# Quantitative Phosphoproteomics Reveals SLP-76 Dependent Regulation of PAG and Src Family Kinases in T Cells

**DOI:** 10.1371/journal.pone.0046725

**Published:** 2012-10-11

**Authors:** Lulu Cao, Yiyuan Ding, Norris Hung, Kebing Yu, Anna Ritz, Benjamin J. Raphael, Arthur R. Salomon

**Affiliations:** 1 Department of Chemistry, Brown University, Providence, Rhode Island, United States of America; 2 Department of Molecular Biology, Cell Biology, and Biochemistry, Brown University, Providence, Rhode Island, United States of America; 3 Department of Computer Science, Brown University, Providence, Rhode Island, United States of America; Wayne State University, United States of America

## Abstract

The SH2-domain-containing leukocyte protein of 76 kDa (SLP-76) plays a critical scaffolding role in T cell receptor (TCR) signaling. As an adaptor protein that contains multiple protein-binding domains, SLP-76 interacts with many signaling molecules and links proximal receptor stimulation to downstream effectors. The function of SLP-76 in TCR signaling has been widely studied using the Jurkat human leukaemic T cell line through protein disruption or site-directed mutagenesis. However, a wide-scale characterization of SLP-76-dependant phosphorylation events is still lacking. Quantitative profiling of over a hundred tyrosine phosphorylation sites revealed new modes of regulation of phosphorylation of PAG, PI3K, and WASP while reconfirming previously established regulation of Itk, PLCγ, and Erk phosphorylation by SLP-76. The absence of SLP-76 also perturbed the phosphorylation of Src family kinases (SFKs) Lck and Fyn, and subsequently a large number of SFK-regulated signaling molecules. Altogether our data suggests unique modes of regulation of positive and negative feedback pathways in T cells by SLP-76, reconfirming its central role in the pathway.

## Introduction

**Figure 1:**
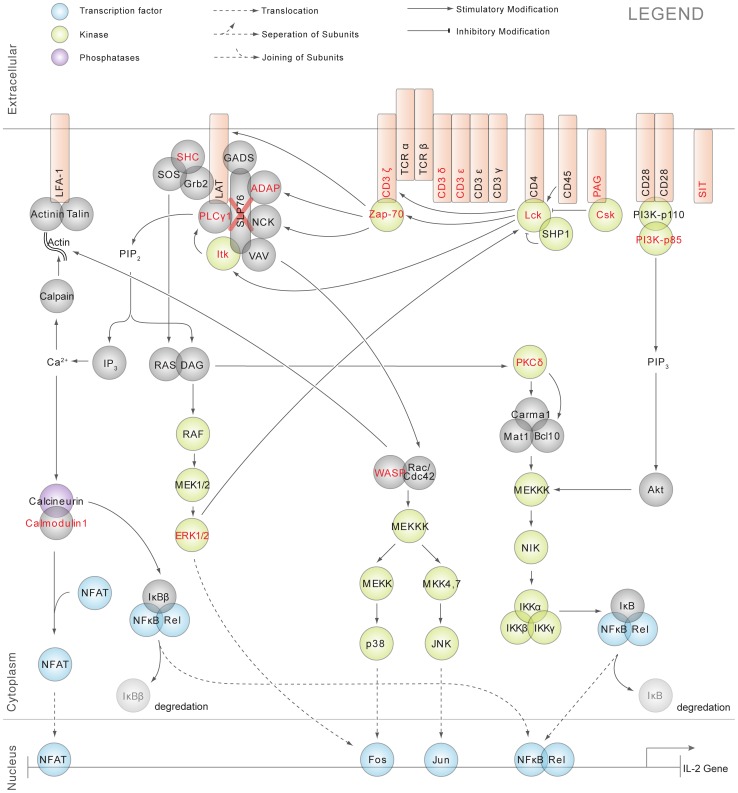
Canonical TCR signaling pathway. Established signaling cascades in activated T cells. Proteins identified in our quantitative phosphoproteomic analysis are highlighted in red and the detailed data regarding these proteins, their phosphorylation kinetics in both SLP-76 reconstituted and deficient Jurkat T cells upon TCR stimulation, were presented later in Figure 3 and Figure S3.

TCR signaling plays an essential role in the regulation of the adaptive immune response, and it has been intensively investigated and described (Figure 1). TCR engagement results in the activation of Src family kinases Lck and Fyn. Active Lck phosphorylates the CD3 and ζ-chain immunoreceptor tyrosine-based acivation motifs (ITAMs) [1], resulting in the recruitment of the Syk-family tyrosine kinase ζ-chain associated protein of 70 kDa (ZAP70) [2]. Lck-activated ZAP70 then phosphorylates a number of downstream proteins, including the key adapter proteins linker for activation of T cells (LAT) and SLP-76, resulting in the assembly of a “signalsome” complex [3].

SLP-76 consists of three domains capable of mediating intermolecular interactions: an N-terminal acidic region containing three tyrosine phosphorylation sites, a central proline-rich region, and a C-terminal SH2 domain [4]. The indispensable role of SLP-76 for T cell development and activation has been demonstrated by various studies in T cell lines and in vivo [5]–[9]. In SLP-76 deficient Jurkat T cells, phosphorylation and activation of phospholipase C-γ1 (PLCγ1) is severely impaired, resulting in defective calcium mobilization, Erk activation, and cytokine gene transcription following TCR ligation [6]. In vivo, SLP-76 deficiency results in a complete block in thymocyte development at the CD4^−^CD8^−^ double-negative stage and the lack of peripheral T cells [7]–[9]. Upon TCR activation, Grb2-related adaptor downstream of Shc (GADS) binds and recruits SLP-76 to the LAT signaling complex [10], where SLP-76 nucleates the interaction of signaling proteins, including PLCγ1, IL-2-inducible T cell kinase (Itk), VAV, NCK, adhesion and degranulation promoting adaptor protein (ADAP) [11], leading to more distal signaling events. PLCγ1 is recruited to the SLP-76 signaling complex by binding to both LAT and SLP-76 [12], [13]. The SH2 and SH3 domains of the Tec-family protein tyrosine kinase Itk binds selectively to N-terminal phosphotyrosyl residue Tyr^145^ and short motifs within the proline-rich domain of SLP-76 [14], [15], allowing the maintenance of Itk in an active conformation [4]. The interaction between SLP-76 and Itk juxtaposes PLCγ1 with the active Itk, resulting in the full activation of PLCγ1 and the subsequent generation of the second messengers inositol 1,4,5-trisphosphate (IP_3_) and diacylglcycerol (DAG) [16]. SLP-76 also acts as a scaffold to coordinate the assembly of a tri-molecular signaling complex with VAV and Nck that regulates cytoskeletion rearrangement [17]. Tyrosine phosphorylation of VAV activates its guanine nucleotide exchange factor (GEF) activity and leads to the activation of Rac and Cdc42 [18]. Activated Rac and Cdc42 then bind and activate the adaptor protein Nck-associated proteins, including p21-activated kinase 1 (Pak1) and Wiskott-Aaldrich syndrome protein (WASP), resulting in the regulation of actin polymerization and IL-2 gene transcription [17], [19], [20]. Additionally, SLP-76 regulates integrin activation through its interaction with the tyrosine-phosphorylated adaptor protein ADAP (previously termed SLAP-130/Fyb) [21].

T cells must discriminate foreign peptide-MHC agonists from a large variety of self peptide-MHC antagonists to appropriately trigger the TCR activation pathway only in the proper context [22]. Biochemically, this discrimination is accomplished through the half-life of the interaction between peptide-MHC and the TCR [23]. To discriminate foreign from self peptides, the T cell signaling pathway utilizes both positive and negative feedback pathways to establish an ultrasensitive, bistable switch [23]. These feedback activation and inhibition pathways are critically important in tuning the sensitivity of TCR activation to self and foreign ligands [23]. While some of the important regulatory proteins involved in positive and negative feedback are beginning to be defined, the pathways leading to the regulation of these molecules are much more obscure. For example, the dynamic equilibrium between Lck mediated CD3 ITAM phosphorylation and phosphatase mediated dephosphorylation of these ITAMs and Lck is only beginning to be understood [23]. Csk, CD45, c-Cbl, and SHP-1 are proteins known to function in negative feedback mechanisms in TCR signaling [24]–[28]. Positive feedback mechanisms have also been proposed in T cells such as ERK phosphorylation of Lck [28]–[30]. In this study, new SLP-76 dependent phosphorylation sites are revealed on a variety of signaling proteins, providing an unprecedented detailed view of the central role of this signaling protein.

## Materials and Methods

### Cell culture, SILAC labeling and T cell stimulation

The SLP-76 deficient Jurkat-derived cell line J14 and its wild-type (WT) SLP-76 reconstituted derivative J14-76-11 [6] were provided by Deborah Yablonski at Technion-Israel Institute of Technology. All cells were initially maintained in RPMI 1640 medium (Sigma, St. Louis, MO) supplemented with 10% fetal bovine serum (Hyclone, Logan, UT), 2 mM L-glutamine, 100 U/ml penicillin G, and 100 µg/ml streptomycin (Invitrogen, Carlsbad, CA) in a humidified incubator with 5% CO_2_ at 37°C. In addition, growth media for J14-76-11, the stable transfectant of J14, was supplemented with 2 mg/ml G418, which was washed out 2 days prior to SILAC labeling of the cells. For SILAC labeling, all cell lines were washed twice with RPMI 1640 medium without Arginine, Lysine, and Leucine (Sigma), and reconstituted in RPMI 1640 medium containing either ^12^C_6_, ^14^N_4_ L-Arginine, and ^12^C_6_, ^14^N_2_ L-Lysine (Sigma) or ^13^C_6_, ^15^N_4_ L-Arginine, ^13^C_6_, ^15^N_2_ L-Lysine (Cambridge Isotope Laboratories, Andover, MA), supplemented with 10% dialyzed fetal bovine serum (Sigma), 0.381 mM L-leucine (Sigma), glutamine, penicillin, and streptomycin for 7 cell doublings. The concentration of L-Arginine and L-Lysine chosen for SILAC labeling of Jurkat cells in experiments described here was 0.383 mM and 0.219 mM, respectively (Method S1).

Anti-CD3 and anti-CD4 (clones OKT3 and OKT4; eBioscience, San Diego, CA) stimulation was performed as described [31]. Briefly, cells were washed once with 4°C phosphate buffer saline (PBS), and reconstituted at a concentration of 1×10^8^ cells/ml in PBS. For each timepoint, 1×10^8^ cells were treated with OKT3 and OKT4 primary antibodies, at a concentration of 2.5 µg/ml of each antibody, for 10 min at 4°C. Cells were then crosslinked with 22 µg/ml of goat anti-mouse IgG (Jackson ImmunoResearch, West Grove, PA) at 37°C for 0, 1, 1.5, 2, 3, 5, or 10 minutes.

### Cell lysis, protein reduction, alkylation, digestion, and peptide immunoprecipitation

To stop the stimulation, cells were lysed with lysis buffer (8 M urea, 1 mM sodium orthovanadate, and 100 mM ammonium bicarbonate, pH 8.0) and incubated for 20 min at 4°C. Lysates were then cleared at 14,000×g for 15 min at 4°C, and protein concentration was measured by the DC Protein Assay (Bio-Rad, Hercules, CA). Cell lysates from J14 and J14-76-11 were combined at a 1∶1 protein concentration ratio and reduced with 10 mM DTT for 1 hr at 56°C, followed by alkylation with 55 mM iodoacetamide for 1 hr at room temperature in the dark. Cell lysates were then diluted 5 fold with 100 mM ammonium bicarbonate, pH 8.9 and digested with sequencing grade modified trypsin (Promega, Madison, WI) at 1∶100 (w/w) trypsin∶protein ratio overnight at room temperature. Tryptic peptides were desalted using C18 Sep-Pak plus cartridges (Waters, Milford, MA), as described [32], and lyophilized in a Speed Vac plus (Thermo Fisher Scientific, Waltham, MA). Dry peptides from each timepoint were reconstituted and immunoprecipitated as previously described [33] except 20 µl of anti-phosphotyrosine resin was used per 1×10^8^ cells and eluted peptides were filtered through a 0.22 µM filter (Millipore, Billerica, MA). 10 pmol synthetic phosphopeptide LIEDAEpYTAK was added to each timepoint prior to peptide immunoprecipitation as a control for label-free quantitation. Results presented in this manuscript represent the average of three total replicate analyses.

### Western blotting for SLP-76 and phospho-PLC-γ1

Total cellular protein from 8 M urea cell lysates was diluted 1∶1 with gel loading buffer containing 4% SDS, 125 mM Tris-HCl (pH 6.8), 20% v/v glycerol, 5% 2-mercaptoethanol, 0.01% bromophenol blue, pH 6.8 from each proteomic sample. Equal amounts of protein (as measured by Lowry DC assay, Bio-Rad) were separated by 4–20% gradient SDS-polyacrylamide gel electrophoresis (Thermo Fisher Scientific), and electroblotted onto an Immobilon transfer membrane (Millipore). SLP-76 expressions in J14-76-11 and J14 Jurkat T cells were probed using rabbit anti-SLP-76 pAb and rabbit anti-β-Actin mAb (Cell signaling Technology, Danvers, MA). Phosphorylation of PLCγ1 (Tyr^783^) and PLCγ1 expression were detected by western blotting analysis using Odyssey Infrared Imaging System (LI-COR Biosciences, Lincoln, NE). Generally, membranes were blocked in Odyssey Blocking Buffer (LI-COR Biosciences) for 1 hour and then incubated overnight at 4°C with rabbit anti-phospho-PLCγ1 (Tyr^783^) pAb and rabbit anti-PLCγ1 pAb (Cell Signaling Technology) respectively in Odyssey Blocking Buffer. After rinsing, membranes were incubated with IRDye 800CW Donkey Anti-Rabbit IgG (H+L) (LI-COR Biosciences) in Odyssey Blocking Buffer for 45 minute in the dark at room temperature. Membranes were then extensively rinsed and bands were visualized using Odyssey infrared imaging system.

### Automated desalt-IMAC/nano-LC/ESI-MS

Tryptic peptides were analyzed by a fully automated phosphoproteomic technology platform incorporating peptide desalting via reversed-phase chromatography, and Fe^3+^ IMAC enrichment of phosphopeptides as previously described [33]. IMAC enriched phosphopeptides were eluted into the mass spectrometer (LTQ-FT; Thermo Fisher Scientific) through an analytical column (360 µm OD×75 µm ID fused silica with 12 cm of 5 µm Monitor C18 particles with an integrated ∼4 µm ESI emitter tip fritted with 3 µm silica; Bangs Laboratories) with a reversed-phase gradient (0–70% solvent B in 30 min). Static peak parking was performed via flow rate reduction from 200 nl/min to ∼20 nl/min when peptides began to elute as judged from a BSA peptide scouting run, as described previously [34]. An electrospray voltage of 1.8 kV was applied in a split flow configuration, as described [34]. Spectra were collected in positive ion mode and in cycles of one full MS scan in the FT (m/z: 400–1800), followed by data-dependent MS/MS scans in the LTQ (∼0.3 s each) sequentially of the five most abundant ions in each MS scan with charge state screening for +1, +2, +3 ions and dynamic exclusion time of 30 s. The automatic gain control was 1,000,000 for the FTMS scan and 10,000 for the ion trap MS (ITMS) scan. The maximum ion time was 100 ms for the ITMS scan and 500 ms for the FTMS full scan. FTMS resolution was set at 100,000.

### Database analysis

MS/MS spectra were searched against the human National Center for Biotechnology Information non-redundant protein database using both the SEQUEST algorithm provided with Bioworks 3.2 (SEQUEST v.27 rev12) [35] and the Mascot algorithm v.2.2.1 provided by Matrix science [36]. Peak lists were generated using extract_msn.exe 07/12/07 using a mass range of 600–4500, precursor ion tolerance (for grouping) of 0.005 AMU, minimum ion count of 5, group scan of 0, minimum group count of 1. The NCBI human database contained 438,778 protein sequence entries (50% forward, 50% reversed). SEQUEST and Mascot were performed with the following parameters: trypsin enzyme specificity, 2 possible missed cleavages, 0.2 Da mass tolerance for precursor ions, 0.5 Da mass tolerance for fragment ions. Search parameters specified a differential modification of phosphorylation (+79.9663 Da) on serine, threonine, and tyrosine residues and a static modification of carbamidomethylation (+57.0215 Da) on cysteine. Search parameters also included a differential modification for arginine (+10.00827 Da) and lysine (+8.01420 Da) amino acids. To provide high confidence phosphopeptide sequence assignments, data was filtered for Xcorr (+1 >1.5; +2 >2.0; +3 >2.5) for SEQUEST results, Mowse score (>10) for Mascot results, and precursor mass error (<20 ppm). In addition, a logistic regression statistical analysis was performed on the non-redundant list of tyrosine phosphopeptides using a newly developed spectral score [37] to achieve a final estimated false discovery rate of 0.5%. After filtering by logistic score to 0.5% FDR, SEQUEST and Mascot results were combined together to generate the whole peptide list. For quantitative measurements, a minimum peak area threshold of 500 for both the SILAC and label free quantitation were required. Additionally, repeat observations of MS/MS spectra of each tyrosine-phosphorylated peptide in minimally 4 of 8 total timepoints were required. False discovery rate (FDR) was estimated with the decoy database approach after final assembly of nonredundant data into heatmaps [38]. To validate the position of the phosphorylation site, the Ascore algorithm [39] was applied to all data and the reported phosphorylation site position reflects the top Ascore prediction. Ascore probabilities are reported in the full data table (Dataset S1). Assigned MS/MS spectra for all reported phosphopeptides in this analysis are also available (Dataset S2).

### Quantitation of relative phosphopeptide abundance

Relative quantification of peptide abundance was performed via calculation of selected ion chromatogram (SIC) peak areas of heavy and light SILAC labeled phosphopeptides. For label free comparison of phosphopeptide abundance in the SLP-76 reconstituted Jurkat cells between different timepoints of TCR stimulation, individual SIC peak areas were normalized to the SIC peak area of the copurified synthetic peptide LIEDAEpYTAK in the same timepoint. The exogenous peptide LIEDAEpYTAK was added to each timepoint at 10 pmol. This exogenous peptide accompanied cellular phosphopeptides through the peptide immunoprecipitation, desalt, IMAC, and reversed-phase elution into the mass spectrometer. Peak areas were calculated by inspection of SICs using recently developed software programmed in Microsoft Visual Basic 6.0 based on Xcalibur Development Kit 2.0 SR2 (Thermo Fisher Scientific). Quantitative data was calculated automatically for every assigned peptide using the ICIS algorithm available in the Xcalibur XDK with the following parameters: multiple resolution of 8, noise tolerance of 0.1, noise window of 40, scans in baseline of 5, include of RefExc peaks False. A minimum SIC peak area equivalent to the typical spectral noise level of 500 was required of all data reported for label free and SILAC quantitation. SIC peak areas for all reported phosphopeptides were then manually validated.

Temporal changes in phosphorylation abundance are represented as heatmaps from 3 replicate experiments. A label free heatmap that represents the change of abundance of phosphopeptides in J14-76-11 across 10 minutes of TCR stimulation and a SILAC heatmap that represents the ratios of abundance of phosphopeptides between J14 and J14-76-11 at each of the TCR stimulation timepoints were generated as previously described [40]. In the label free heatmap representation, the magnitude of change of the heatmap color was calculated through the log of the ratio of peak area of each peptide compared with the geometric mean of peak areas for that peptide across 8 timepoints. Any changes (either an increase or decrease of peptide abundance above the average) greater than 100 fold were displayed as the same color as the 100-fold change. Black represents average abundance of a given phosphopeptide across all timepoints, while yellow (blue) represents levels of phosphorylation above (below) the average. In the SILAC heatmap representation, the magnitude of change of the heatmap color was calculated through the log of the ratio of peak area of each peptide in J14 compared with J14-76-11 at each of the TCR stimulation timepoints. Any changes greater than 50 fold was displayed as the same color as the 50-fold change. Black represents no change in abundance of a given phosphopeptide in response to SLP-76 removal, while green (red) represents elevated (reduced) phosphorylation.

In both label free and SILAC heatmaps, blanks indicate timepoints without a clearly defined SIC peak in any of the replicate analyses. The coefficient of variation (CV) for each heapmap square amongst the three replicate observations was calculated (Dataset S1) and represented as a color bar on the bottom of that heatmap. According to the CV color key, black represents 0% CV and more orange represents larger CV. Note that, according to Human Protein Reference Database (hprd) Release 7, phosphorylation sites discussed in the literature previously are marked with *^P^ if identified using phosphoproteomic method alone or * if identified using traditional approaches such as site-directed mutagenesis. For label free heatmap representation, P-values were calculated for each timepoint compared to the timepoint with the minimal average peak area for that phosphopeptide using unpaired, 2- sided student t-test. Q value has been defined as the measure of the minimum positive false discovery rate (pFDR) at which the test can be called significant [41]. pFDR was calculated for each test based on the determined p-values using the R package QVALUE as previously described [42] (Dataset S1). White dots on label free heapmaps represent timepoints with false discovery rate less than 2% for significant changes in phosphorylation abundance compared to the timepoint with minimal average abundance. For SILAC heatmap representation, P-values were calculated between the SLP-76 deficient and SLP-76 reconstituted replicate measurements for each phosphopeptide and timepoint using paired, 2 sided student t-test. pFDR was calculated for each test based on the determined p-values using the R package QVALUE as described above (Dataset S1). White dots on SILAC heapmaps represent timepoints with false discovery rate less than 2% for significant changes in phosphorylation abundance between J14 and J14-76-11.

## Results

**Figure 2:**
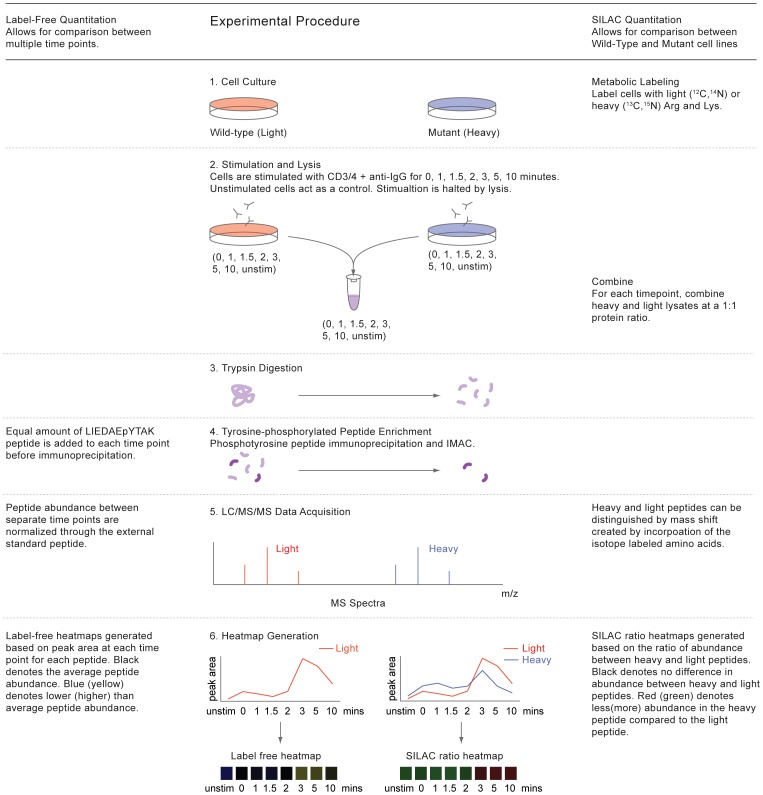
Experimental procedure. Two cell populations of human Jurkat T cell clones (J14-76-11 and J14) were incubated with RPMI 1640 medium containing normal or heavy isotope labeled arginine and lysine amino acids, physically differentiating the two proteomes by a shift in molecular weights. Each cell population was then pre-incubated with OKT3 and OKT4 antibodies for 10 minutes at 4°C and then crosslinked with anti-IgG at 37°C for the times indicated. After cell lysis, light and heavy cell lysates were combined at an equal protein concentration ratio for each timepoint. Proteins were then reduced, alkylated, and trypsin-digested into peptides. Peptides were desalted by Sep-Pak cartridges, enriched by phosphotyrosine peptide immunoprecipitation and Fe^3+^ IMAC, and then subjected to reversed-phase LC-MS/MS analysis. MS shifts introduced by heavy isotope labeling allow for differentiation between light and heavy peptide counterparts in MS spectra. Selected ion chromatogram (SIC) peak areas of light and heavy isotope labeled phosphopeptides were calculated for relative quantification of peptide abundance. Individual SIC peak areas were normalized to the SIC peak area of the copurified synthetic peptide LIEDAEpYTAK in the same timepoint. A label-free heatmap was generated based on peptide abundance for a certain peptide in SLP-76 reconstituted Jurkat cells through a time course of receptor stimulation and SILAC ratio heatmaps were generated based on the ratio of abundance between light (SLP-76 reconstituted) and heavy (SLP-76 deficient) peptide counterparts for each timepoint (SLP-76 deficient in relative to SLP-76 reconstituted).

A quantitative phosphoproteomic analysis of TCR signaling comparing SLP-76 deficient Jurkat T cell line J14 and SLP-76 reconstituted Jurkat T cell line J14-76-11 was performed (Figure 2). A receptor stimulation time course experiment of 8 total timepoints, 3 replicates for each cell line was performed to present a wide-scale view of the temporal changes of tyrosine phosphorylation events following TCR stimulation and SLP-76 perturbation. High-quality sequence assignments were determined using stringent criteria as described above in methods. A total of 260 non-redundant phosphopeptides assigned at a 0.5% false discovery rate for phosphopeptide sequence confidence were identified after all filtering and assembly.

Relative quantification of peptide abundance via calculation of SIC peak areas was performed for each light or heavy SILAC labeled phosphopeptide in each timepoint. SILAC ratios were calculated by comparison of the SIC peak areas of phosphopeptides from SLP-76 deficient cells to their SLP-76 reconstituted counterparts. Label free fold changes were also calculated by comparison of the SIC peak areas of phosphopeptides in SLP-76 reconstituted cells between different timepoints of TCR stimulation. A total of 3 replicate experiments were performed and SIC peak areas were generated from the average values. The complete list of quantitative replicate data, calculated CV, p-values and q-values are available (Dataset S1). SIC peak areas for each identified phosphopeptide from both SLP-76 deficient and SLP-76 reconstituted Jurkat T cells were plotted for direct view of the dynamic phosphorylation changes (Dataset S3).

The reproducibility of SILAC analysis among 3 replicate experiments was assessed by comparisons of three SILAC ratios (R_1_, R_2_, R_3_) at 2 min after TCR stimulation (Figure S1). Difference between SILAC ratios were represented using a scatter plot of observed phosphopeptides (Figure S1A–C) and a histogram (Figure S1D–F) of Log_2_ (R_2_/R_1_), Log_2_ (R_3_/R_1_), and Log_2_ (R_3_/R_2_) for each identified phosphopeptide. In good agreement of the expected value of 0, the measured average value of Log_2_ (R_2_/R_1_), Log_2_ (R_3_/R_1_), and Log_2_ (R_3_/R_2_) was 0.08±0.27, 0.01±0.18, and 0.01±0.46, respectively. Additionally, histogram plots showed that 85.2% (Figure S1D), 82.2% (Figure S1E), and 86.8% (Figure S1F) of identified phosphopeptides displayed Log_2_ (R_2_/R_1_), Log_2_ (R_3_/R_1_), and Log_2_ (R_3_/R_2_) values respectively in the range of −0.6 to 0.6.

### Representation of Label Free and SILAC Quantitation

**Figure 3:**
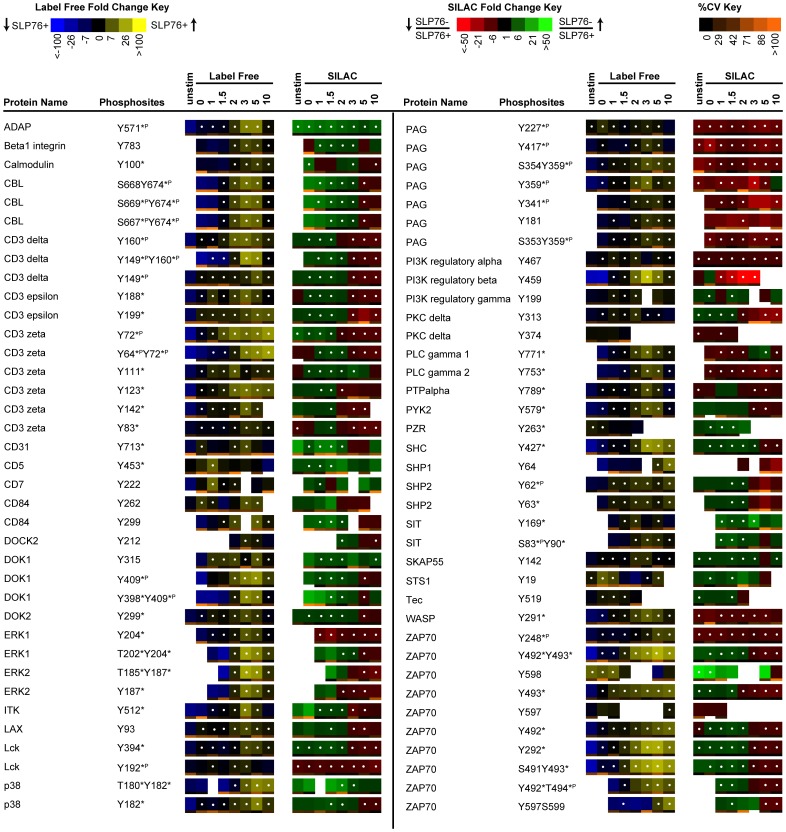
Quantitative phosphoproteomic analysis of known TCR signaling proteins. Listed above is a portion of the data collected, representing proteins previously established to be involved in TCR signaling. Temporal changes in phosphorylation abundance are represented as heatmaps from 3 replicate experiments as described in methods. The label free heatmap represents the change of abundance of phosphopeptides in SLP-76 reconstituted cells across 10 minutes of TCR stimulation while the SILAC heatmap represents the ratios of abundance of phosphopeptides in SLP-76 deficient cells relative to SLP-76 reconstituted cells at each of the TCR stimulation timepoints. In label free heatmaps, black represents average abundance for a certain peptide across all timepoints, while yellow (blue) represents levels of phosphorylation above (below) the average. White dots on heapmaps represent timepoints with false discovery rate less than 2% for significant changes in phosphorylation abundance compared to the timepoint with minimal average abundance. In SILAC heatmaps, black represents no change in phosphorylation abundance in response to SLP-76 removal, while green (red) represents elevated (reduced) phosphorylation. White dots on heapmaps represent timepoints with false discovery rate less than 2% for significant changes in phosphorylation abundance between SLP-76-reconstituted and deficient cells. For all heatmaps, blanks indicate timepoints without a clearly defined SIC peak for that peptide. The coefficient of variation (CV) for each heapmap square amongst the three replicate experiments was calculated and represented as a color bar on the bottom of that heatmap. According to the CV color key, black represents 0% CV and more orange represents larger CV. Note that, according to Human Protein Reference Database (hprd) Release 7, phosphorylation sites discussed in the literature previously are marked with *^P^ if identified using phosphoproteomic method alone or * if identified using traditional approaches such as site-directed mutagenesis.

**Figure 4:**
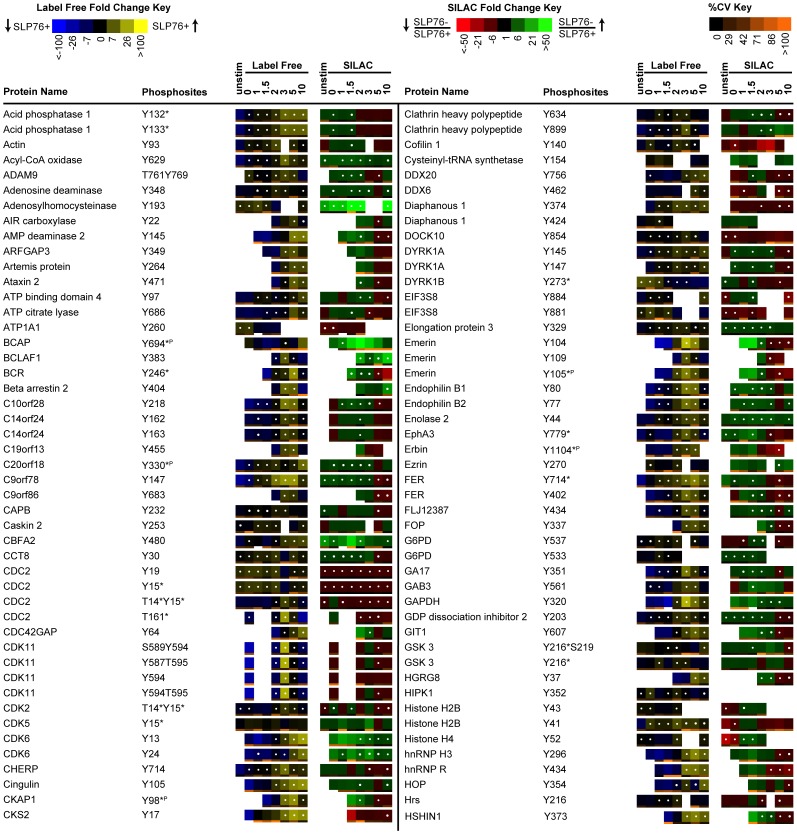
Quantitative phosphoproteomic analysis of proteins not previously known to be associated with TCR signaling. Listed above is a portion of the data collected, representing proteins not previously established to be involved in TCR signaling. Temporal changes in phosphorylation abundance are represented as heatmaps from 3 replicate experiments as described in methods. The label free heatmap represents the change of abundance of phosphopeptides in SLP-76 reconstituted cells across 10 minutes of TCR stimulation while the SILAC heatmap represents the ratios of abundance of phosphopeptides in SLP-76 deficient cells relative to SLP-76 reconstituted cells at each of the TCR stimulation timepoints. The label free and SILAC heatmaps are described in detail as in Figure 3.

**Figure 5:**
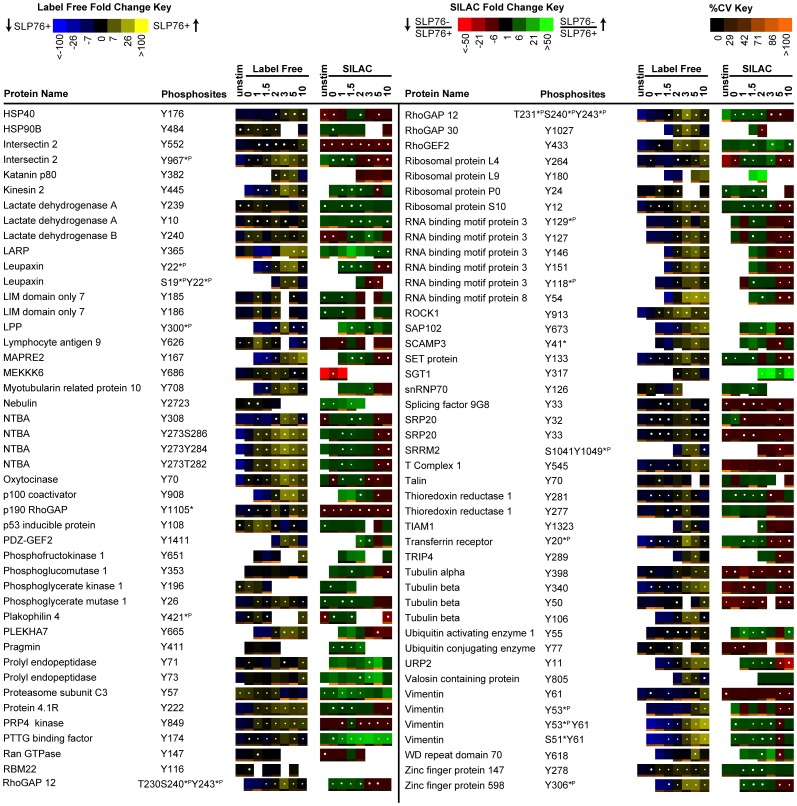
Quantitative phosphoproteomic analysis of proteins not previously known to be associated with TCR signaling (con't). Listed above is a portion of the data collected, representing proteins not previously established to be involved in TCR signaling. Temporal changes in phosphorylation abundance are represented as heatmaps from 3 replicate experiments as described in methods. The label free heatmap represents the change of abundance of phosphopeptides in SLP-76 reconstituted cells across 10 minutes of TCR stimulation while the SILAC heatmap represents the ratios of abundance of phosphopeptides in SLP-76 deficient cells relative to SLP-76 reconstituted cells at each of the TCR stimulation timepoints. The label free and SILAC heatmaps are described in detail as in Figure 3.

Two different visual representations of quantitative data were generated for each sequenced phosphopeptide, either as SILAC heatmaps to reflect the SILAC ratios between J14 and J14-76-11, or as label free heatmaps to reflect the abundance of phosphorylation in J14-76-11 across the timecourse of TCR stimulation, providing information that indicates changes of phosphorylation for each identified phosphopeptide in response to the removal of SLP-76 or TCR stimulation of J14-76-11. Data from 3 replicate experiments are represented in the form of SILAC or label free heatmaps (Figure 3, 4, 5) accordingly as described above in method section.

### Phosphoproteomic profiling of phosphorylation sites identified on canonical T cell signaling proteins

From this analysis, we observed 270 unique tyrosine phosphorylation sites residing on 159 proteins in the SLP-76 reconstituted T cells across 8 timepoints of receptor stimulation (Figure 3, 4, 5). Among the 159 proteins identified, 21% of them (35 proteins with 74 phosphorylation sites) were previously functionally characterized in TCR signaling (Figure 1 and Figure 3) and 79% of them (124 proteins with 196 phosphorylation sites) were not previously known to be involved in TCR signaling (Figure 4 and Figure 5). Established TCR signaling proteins that were identified in our quantitative phosphoproteomic analysis were also represented in the canonical TCR signaling pathway with SILAC heatmaps beside individual proteins to reflect their quantitative SILAC ratios (SLP-76 deficient in relative to SLP-76 reconstituted) (Figure S3). Phosphorylation events were observed on the proximal protein tyrosine kinases and T cell receptor ITAMs (Lck, ZAP70, TCR-CD3 subunits δεζ), SLP-76 related signaling proteins (Itk, PLCγ1, Erk1/2, ADAP, SKAP55, and WASP), TCR signaling negative regulators (CD31, CD5, PZR, PAG, SIT, and two newly described T cell signaling negative regulators linker for activation of X cells LAX [43] and suppressor of T-cell receptor signaling 1 STS-1 [44]), PI3K regulatory subunits, and many other Lck and/or Fyn-regulated signaling proteins (Figure 1 and Figure 3).

**Figure 6:**
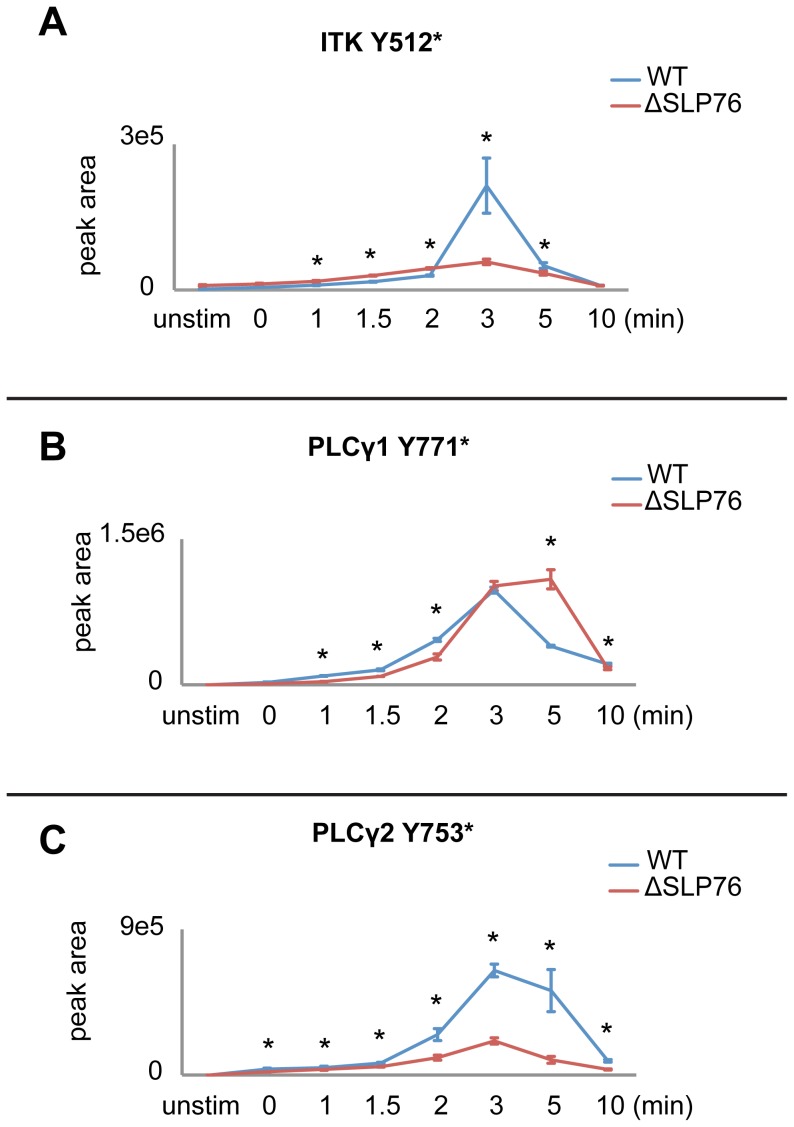
Phosphorylation kinetics of Itk, PLCγ1, and PLCγ2 in SLP-76 reconstituted and deficient cells. Phosphorylation kinetics of A) Itk Y512, B) PLCγ1 Y771, and C) PLCγ2 Y753 in the presence (WT) and absence of SLP-76 (ΔSLP76) are represented for 8 timepoints. Results represent the means of three replicate experiments (error bars indicate SD). “*” represents timepoints with significant changes (less than 2% false discovery rate) in phosphorylation abundance between WT and ΔSLP76 cells.

**Figure 7:**
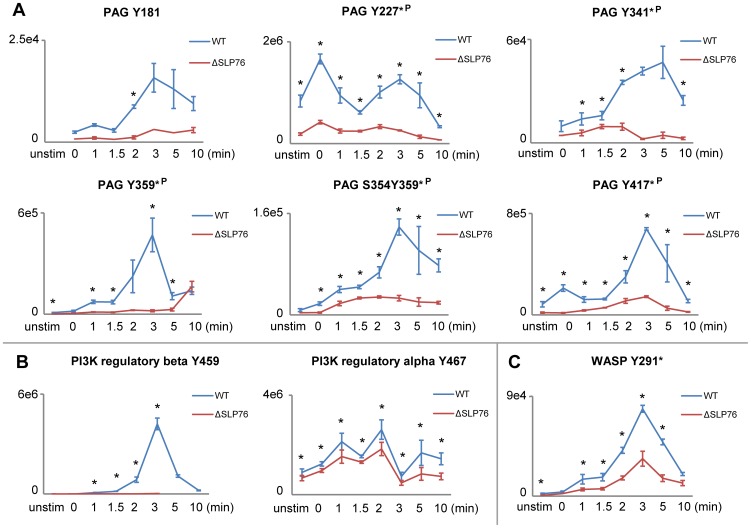
Phosphorylation kinetics of PAG, PI3K, WASP in SLP-76 reconstituted and deficient cells. Phosphorylation kinetics of A) PAG, B) PI3K, and C) WASP in the presence (WT) and absence of SLP-76 (ΔSLP76) are represented for 8 timepoints. Results represent the means of three replicate experiments (error bars indicate SD). “*” represents timepoints with significant changes (less than 2% false discovery rate) in phosphorylation abundance between WT and ΔSLP76 cells.

**Figure 8:**
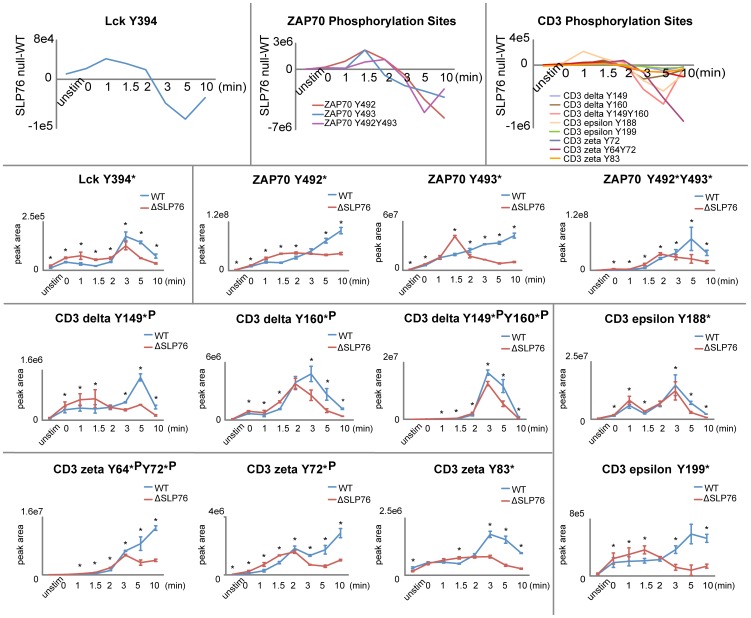
Erk positive feedback on Lck, ZAP70, and CD3 ITAMs. Phosphorylation kinetics of Lck, ZAP70, and CD3 ITAMs from SLP-76 reconstituted (WT) and deficient (ΔSLP76) cells are presented for 8 timepoints. The differences of phosphorylations between WT and ΔSLP76 cells (ΔSLP76–WT) are also presented to show the trend of phosphorylation changes. Results represent the means of three replicate experiments (error bars indicate SD). “*” represents timepoints with significant changes (less than 2% false discovery rate) in phosphorylation abundance between WT and ΔSLP76 cells.

The data revealed changes in phosphorylation abundance when comparing cells with or without SLP-76 expression through a timecourse of TCR-induced tyrosine phosphorylation on hundreds of proteins. Consistent with previous studies in J14 (SLP-76 deficient) Jurkat T cells, significantly reduced phosphorylation of Itk, PLCγ, Erk was observed (Figure 6). As expected, phosphorylation of Itk at its activation site Tyr^512^ reached its peak at 3 min of TCR stimulation and it was significantly reduced in SLP-76 deficient cells compared to reconstituted cells (Figure 6A). Reduced phosphorylation of PLCγ1 at one of its defined SH2-SH3 linker regulatory sites Tyr^783^ in SLP-76 deficient cells was also validated by western blot (Figure S2) [6], [13]. Additionally, our data for the first time revealed that the removal of SLP-76 directly led to significantly reduced phosphorylation of PAG, PI3K, and WASP (Figure 7), suggesting the possible role of SLP-76 in regulating the phosphorylation of these signaling molecules. Phosphorylation of PAG at Tyr^227^, Tyr^417^, Tyr^359^, Tyr^341^, and a previously identified Fyn SH2 domain-interacting tyrosine residue Tyr^181^ (Figure 7A), PI3K at two regulatory subunit tyrosine residues (p85 alpha at Tyr^467^, p85 beta at Tyr^459^)(Figure 7B), and WASP at a Fyn regulated site Tyr^291^ (Figure 7C) [45], were significantly decreased in SLP-76 deficient cells compared to reconstituted cells. Although phosphorylation of the TCR proximal signaling proteins upstream of SLP-76 would be expected to be unaffected by SLP-76 removal, our data revealed that SLP-76 regulates the phosphorylation of Lck, as well as a large number of known Lck regulated signaling molecules. Phosphorylation of Lck at its activation tyrosine residue Tyr^394^, CD3 δ, ε, ζ at their respective ITAMs, ZAP70 at the activating site Tyr^394^ [46] as well as several other tyrosine residues, was significantly elevated at the early timepoints and significantly reduced phosphorylation at later timepoints in the SLP-76 deficient Jurkat T cells (Figure 8). Unfortunately, due to the fact that the sequence of the identified Fyn phosphopeptide at the activation site Tyr^420^ is identical to other Src family kinases (LIEDNEpYTAR), Fyn phosphorylation could not be quantified.

## Discussion

### Phosphorylation of proteins previously established to be downstream of SLP-76

Tyrosine phosphorylation of signaling proteins that are known to be downstream of SLP-76, including Itk, PLCγ, and Erk, were significantly reduced in the SLP-76 deficient Jurkat T cells. Previous studies in Jurkat T cells have demonstrated that SLP-76 is required for TCR-induced tyrosine phosphorylation and activation of Itk and PLCγ1 [4], [6]. Itk is recruited to the cell membrane through the interaction of its PH domain with membrane phophatidylinositol 3,4,5-trisphosphate (PIP_3_), where it is phosphorylated by Lck [47]. Lck then phosphorylates Tyr^512^ in the activation loop of the Itk kinase domain, activating Itk kinase activity [48]. The subsequent interaction between SH2 and SH3 domains of Itk with SLP-76 maintains Itk in an active conformation [16]. As a result, TCR-induced activation of Itk is severely reduced in the absence of SLP-76 [4]. The Itk activation site Tyr^512^ was observed with comparable phosphorylation at the early timepoints and significantly decreased phosphorylation at the later timepoints in SLP-76 deficient cells compared to reconstituted cells (Figure 6A). Upon TCR stimulation, the recruitment of PLCγ1 to TCR signaling complex in lipid rafts requires LAT and the Gads-binding domain of SLP-76 [49]. Also, SLP-76 has been shown to be required for Itk-mediated phosphorylation of PLCγ1 at Tyr^775^ and Tyr^783^, the defined SH2-SH3 linker regulatory sites, leading to the activation of the PLCγ1 signaling pathway [4]. Therefore we expected phosphorylation of PLCγ1 at Tyr^775^ and Tyr^783^ to be significantly reduced in the absence of SLP-76. Unfortunately, phosphorylation of PLCγ1 at Tyr^775^ and Tyr^783^ were not observed in our analysis. In contrast, Tyr^771^ of PLCγ1, another tyrosine residue known to be phosphorylated after TCR engagement, showed an increased and sustained phosphorylation in SLP-76 deficient cells compared to reconstituted cells, indicating a different regulatory mechanism underlying its phosphorylation (Figure 6B). Interestingly, phosphorylation of the PLCγ2 at Tyr^753^ was increased with 18 fold maximal change in the SLP-76 reconstituted cells upon TCR engagement, and it was significantly reduced in the SLP-76 deficient cells (Figure 6C). PLCγ2 plays a crucial role in BCR-dependent calcium mobilization [50] and Tec family kinases were demonstrated to phosphorylate PLCγ2 at Tyr^753^ and Tyr^759^ within the SH2-SH3 linker region, leading to the activation of PLCγ2 phospholipase activity and PLCγ2-mediated calcium signaling [50]. Our observation suggests that PLCγ2 could also be regulated by Itk and may complement the role of PLCγ1 in regulating calcium mobilization in response to TCR engagement.

In the absence of SLP-76, impaired activation of PLCγ leads to reduced production of the second messenger molecules IP3 and DAG. DAG serves as a direct activator for Ras and PKC, leading to the activation of the Ras-Erk-AP1 and NF-κB pathways, respectively [51]. Yablonski *et al.* reported that Erk2 activation is partially reduced in J14 (SLP-76 deficient) cells, indicating an incomplete, but significant defect in TCR-mediated activation of Ras-dependent responses [6]. Consistent with these earlier reports, our SILAC quantification showed significant decreases in phosphorylation on Erk1/2 in the absence of SLP-76 (Erk1 at Tyr^204^, Erk2 at Tyr^187^ and Thr^185^Tyr^187^). Notably, phosphorylation of Erk1 on a peptide containing phosphorylation at both Thr^202^ and Tyr^204^ was significantly increased in the SLP-76 deficient Jurkat T cells. However, the underlying mechanistic explanation for this observation remains obscure.

### Other proteins identified with decreased phosphorylation in J14 (SLP-76 deficient) cells

PAG is exclusively localized to lipid rafts and is known to down-regulate TCR signaling by recruiting C-terminal Src kinase (Csk) via the Fyn-mediated phosphorylation of PAG at Tyr^317^ [52]. Csk phosphorylates the C-terminal inhibitory tyrosine residues of Src-family kinases such as Lck and Fyn, down-regulating their kinase activities. While PAG is constitutively phosphorylated in resting T cells, TCR stimulation has been reported to induce rapid reduction of overall PAG phosphorylation, leading to the dissociation of Csk from lipid rafts and the subsequent activation of Src family kinases [52], [53]. Previous reports measured PAG phosphorylation by western blots using anti-P-Tyr antibodies such as 4G10. The combined average abundance of phosphorylation on many tyrosine residues would be measured by this approach. Information about individual phosphorylation sites are not revealed in a 4G10 blot [52]–[54]. Phosphoproteomic analysis provides detailed quantitative information of phosphorylation on specific sites, allowing for the discrimination between their different roles. PAG phosphorylation at Tyr^227^, Tyr^417^, Tyr^359^, Tyr^341^, and a previously identified Fyn SH2 domain-interacting tyrosine residue Tyr^181^ was significantly decreased in SLP-76 deficient cells (Figure 7A). The significantly reduced phosphorylation of PAG in SLP-76 deficient cells could be due to the regulation of tyrosine phosphatases by SLP-76, such as CD45, which has been indicated as the phosphatase that dephosphorylates PAG upon TCR stimulation [54]. Alternatively, SLP-76 may regulate the upstream tyrosine kinase of PAG. Previous studies have demonstrated that Fyn is predominantly responsible for the phosphorylation of PAG in resting peripheral T cells [55]. Since we did not detect the Fyn-specific phosphopeptides, whether the absence of SLP-76 may impair Fyn activity or the recruitment of Fyn to phosphorylate PAG is unknown. Nevertheless, SLP-76 regulation of PAG phosphorylation has not been previously described and the exact mechanism underlying the connection between PAG phosphorylation and SLP-76 is still not understood. Notably, the expression level of Fyn compared to Lck in Jurkat T cells seems to be controversial, with previous studies showing that expression levels of Fyn and Lck are relatively equal [56], or expression level of Fyn is approximately 30-fold less than that of Lck [57]. Our hypotheses on these putative Fyn-regulated phosphorylation events would need further validation in primary T cells.

PI3K regulates a vast array of signaling pathways in T cells via catalyzing the phosphorylation of PIP2 to yield PIP3, a second messenger molecule that recruits PIP3-specific PH domain-containing signaling proteins to the plasma membrane. Shim *et al*. showed that SLP-76 associates with the N-terminal SH2 domain of p85 in Jurkat T cells after TCR activation, and the TCR-induced PI3K/Akt activation is dependent on membrane translocation and tyrosine phosphorylation of SLP-76 [58], [59]. PI3K phosphorylation was reduced at the regulatory subunits tyrosine residues (p85 alpha at Tyr^467^, p85 beta at Tyr^459^) (Figure 7B). The possible reduced production of PIP3 by membrane-associated p85 in the absence of SLP-76 might also contribute to reduced ITK phosphorylation observed in this analysis. Consistent with these previous studies, phosphorylation of p85 (p85 alpha at Tyr^467^ and p85 beta at Tyr^459^) was significantly reduced in J14 (SLP-76 deficient) cells, supporting the hypothesis that SLP-76 functions to recruit p85 to the membrane via the LAT signaling complex, where PI3K gets phosphorylated and activated.

WASP is a key cytoskeletal regulator in hematopoietic cells. The TCR-induced tyrosine phosphorylation of WASP at Tyr^291^ has been demonstrated to be necessary for WASP effector activities downstream of the T cell receptor [45], [60]. Using Lck-deficient Jurkat cells (JCam-1) and primary T cells deficient for Itk, Lck, or Fyn, Badour *et al.* has identified that Fyn, but not Itk or Lck, is required for WASP phosphorylation at Tyr^291^ after TCR stimulation in both Jurkat and primary T cells [45]. Previous studies suggested that SLP-76, as a scaffolding protein, brings WASP into proximity with Cdc42-GTP, and that this Cdc-GTP binding is required for Fyn-regulated phosphorylation of WASP at Tyr^291^ [61], [62]. We hypothesize that SLP-76 mediates scaffolding of the interaction between WASP and Fyn and therefore the absence of SLP-76 resulted in the significantly reduced phosphorylation of WASP at Tyr^291^ in SLP-76 deficient cells (Figure 7C), but this hypothesis would have to be validated by future experiments.

### Phosphorylation of the proximal signaling proteins upstream of SLP-76

The observation that SLP-76 regulates upstream phosphorylation of Lck, ZAP70, and CD3 δ, ε, ξ ITAMs, and the variation of this effect over time is consistent with a model of SLP-76 regulation of competing positive and negative feedback loops (Figure 1 and Figure 8).

In support of this hypothesis, Stefanova *et al.* have demonstrated the presence of competing ERK positive and SHP-1 negative feedback pathways in T cell signaling regulating Lck activity [28]. The Lck-induced phosphorylation of SHP-1 at Tyr^564^ leads to its association with the SH2 domain of Lck and the recruitment of SHP-1 to the TCR signaling complex. SHP-1 then dephosphorylates Lck within its activation loop at Tyr^394^, resulting in down-regulation of Lck activity and decreased phosphorylation of TCR ITAMs and their associated ZAP70 molecules [30]. Counteracting SHP-1 negative feedback is a positive feedback loop through phosphorylation of Lck by Erk. Previous reports have demonstrated that phosphorylation of Ser^59^ of Lck can be uniquely induced by mitogen-activated protein kinase Erk [63] and that phosphorylation of this site abrogates the binding of SHP-1 [28], [64]. Our data as well as previous reports have shown that the phosphorylation and activation of Erk is reduced in SLP-76 deficient cells [6]. The inhibition of ERK positive feedback on Lck activity in SLP-76 deficient cells would be expected to coincide with the timing of ERK activation, which was observed in our data to occur at 3 min after TCR stimulation. The removal of ERK positive feedback at 3 min and later timepoints in SLP-76 deficient cells could explain the significant reduction in phosphorylation observed in these timepoints on CD3 and ξ-chain ITAMs and the ZAP70 activating site Tyr^493^ (Figure 8) [1], [46], [65], [66]. In contrast, phosphorylation of Lck within the SH2 domain at Tyr^192^, and ZAP70 at a functionally uncharacterized tyrosine residue also within its SH2 domain at Tyr^248^, respond differently to the removal of SLP-76 (constitutively decreased in SLP-76 deficient cells compared to reconstituted cells). However, the unique pathways regulating these particular phosphorylation sites remain obscure.

Constitutively reduced phosphorylation on PAG in SLP-76 deficient cells would be expected to lead to a reduction in recruitment of the negative feedback regulator CSK, leading to constitutive increased phosphorylation of Lck within its activation loop as well as pathway substrates of Lck such as CD3, and ξ-chain ITAMs, and the ZAP70. Before Lck is substantially activated at 3 min, the SLP-76 mediated regulation of PAG and its associated negative regulator CSK could explain the observed significant increased phosphorylation of Lck and its pathway substrates. After 3 min, SLP-76 mediated regulation of ERK positive feedback could predominantly regulate the activity level of Lck. An intricate balance of positive and negative feedback pathways could lead to a fine regulation of the precise levels of activation of the T cell signaling pathway. From this data, the newly appreciated role of SLP-76 in the regulation of feedback pathways provides a new glimpse into the pivotal role of this protein.

### Proteins identified with increased phosphorylation in J14 (SLP-76 deficient) cells

Our analysis also revealed that the removal of SLP-76 led to increased phosphorylation of multiple signaling proteins in J14 (SLP-76 deficient) cells, including the SLP-76 interacting adaptor proteins ADAP (Tyr^571^) and SKAP55 (Tyr^142^), and several negative regulators of TCR signaling, such as the E3 ubiquitin ligase CBL (Ser^667^Tyr^674^, Ser^668^Tyr^674^, Ser^669^Tyr^674^) and the CBL-interacting protein STS-1 (Tyr^19^), membrane glycoprotein CD31 (ITIM motif residue Tyr^713^) and CD5 (ITAM-like motif residue Tyr^453^), and transmembrane adaptor protein PZR (ITIM motif residue Tyr^263^) and SIT (Tyr^199^).

This data also revealed previously undescribed SLP-76 regulation of T cell signaling inhibitory proteins. The phosphorylation of immunoreceptor tyrosine-based inhibitory motif (ITIM) tyrosine residues in the cytoplasmic domain of CD31 (Tyr^690^ and Tyr^713^) and PZR (Tyr^263^), as well as the ITAM-like motif tyrosine residue (Tyr^453^) in CD5 and the tyrosine-based signaling motif Y^199^ASV in SIT, were significantly elevated in SLP-76 deficient cells. The Src family kinase Lck and/or Fyn have been identified as the putative kinases that phosphorylate these tyrosine residues in T cells [67]–[71].

### SLP-76 mediated regulation of Lck substrates

Phosphorylation of many other signaling proteins previously known to be phosphorylated by Src PTKs, including adaptor proteins DOK1 (Tyr^409^) and DOK2 (Tyr^299^) [72]–[74], transmembrane adaptor protein LAX (Tyr^93^) [75], protein serine/threonine kinase PKCδ (Tyr^313^) [76], protein tyrosine phosphatase PTPα (Tyr^789^) [77], proline-rich tyrosine kinase 2 (Pyk2) (Tyr^579^) [78], adaptor protein SHC (Tyr^427^) [79], as well as phosphorylation of protein tyrosine phosphatase SHP2 at Tyr^62^ with a strong scansite motif for Src PTKs, have been observed with SILAC profiles very similar to Lck Tyr^349^ (slightly elevated phosphorylation at the early timepoints and significantly reduced phosphorylation at the later timepoints in the SLP-76 deficient Jurkat T cells compared to reconstituted), supporting the hypothesis that Lck is involved in regulating these sites, either directly or indirectly.

### Phosphorylation of proteins not previously associated with TCR signaling

Many tyrosine phosphorylation sites were also observed on proteins not previously known to be involved in T cell signaling (Figure 4 and Figure 5). The majority of these sites were significantly changed in SLP-76 reconstituted cells through a time course of TCR stimulation as well as when comparing SLP-76 deficient to reconstituted cells. For example, tyrosine phosphorylation of NTBA at Tyr^308^, Tyr^273^, and Tyr^284^ was significantly increased before 3 min and decreased after 3 min. NTBA is an ITIM containing killer Ig-like receptor that has been previously shown to be expressed in all human NK, T, and B-lymphocytes [80], [81]. In NK cells, NTBA has been shown to display inhibitory functions by blocking the ability of NK cells to kill Epstein-Barr virus-infected target cells [80]. Little is known about their role in T cells. Recently, certain KIRs (KIR2DL2 and KLRG1) have been shown to disrupt late T cell receptor-stimulated effector functions such the production of IFN-γ and interleukin-2, respectively [82], [83]. Furthermore, site-directed mutagenesis of specific tyrosine residues in the ITIM motif of KLRG1 demonstrates the importance of tyrosine phosphorylation in the inhibitory process in T cells [83]. In a recently published phosphoproteomic analysis of ZAP70-deficient T cells, NTBA phosphorylation was slightly decreased at Tyr^308^ in ZAP70 deficient cells compared to ZAP70 reconstituted cells, suggesting that this site may be downstream of ZAP70 activation [40]. In the present study, phosphorylation of Tyr^308^ as well as Tyr^273^ and Tyr^284^ on NTBA was significantly elevated before 3 min and significantly decreased after 3 min when comparing SLP-76 deficient to reconstituted cells. This pattern mimics the same pattern observed on Lck Tyr^394^, suggesting that NTBA ITIM phosphorylation could be downstream of Lck and Erk feedback. This observation also colors the interpretation of our previous data on ZAP70 suggesting that ZAP70 mediated regulation of NTBA could either be direct or a consequence of Erk positive feedback inhibition of Lck activity.

In this study, a wide-scale quantitative phosphoproteomic analysis of SLP-76 deficient Jurkat T cells has been performed, not only to elucidate SLP-76 related signaling networks systematically, but also to provide more insights into the molecular mechanisms of TCR signaling. Through the synergistic combination of label-free and SILAC quantification techniques, the subtle fluctuations of cellular signaling networks in response to the removal of SLP-76 were captured and quantified over time. This analysis has provided the most comprehensive view to date of the dynamic tyrosine phosphoproteome after TCR engagement and the role of SLP-76 in regulation of this constellation of phosphorylation. Quantitative, wide-scale phosphoproteomic analysis of protein-disruption or site-directed mutants of canonical T cell signaling proteins can greatly complement traditional biochemical approaches of studying signaling pathways, providing the possibility of mapping the newly discovered phosphorylation sites within the framework of the canonical pathway.

## Supporting Information

Dataset S1 Quantitative and statistic analysis of all identified phosphopeptides. Sequence and phosphorylation site assignment of all identified phosphopeptides with their corresponding SIC peak areas and statistics (standard deviation, p-values and q-values) in both SLP-76 reconstituted and deficient Jurkat T cells.
(XLS)

Dataset S2 Assigned MS/MS spectra for all identified phosphopeptides in this analysis.
(PDF)

Dataset S3 Phosphorylation kinetics of all identified phosphopeptides in SLP-76 reconstituted and deficient Jurkat T cells. The differences of phosphorylations between SLP-76 reconstituted (WT) and deficient (ΔSLP76) cells are also presented as plots of ΔSLP76–WT across 8 timepoints to show the trend of phosphorylation changes. Results represent the means of three replicate experiments (error bars indicate standard deviation). “*” represents timepoints with false discovery rate less than 2% for significant changes in phosphorylation abundance between WT and ΔSLP76 cells.
(PDF)

Figure S1 Variation assessment of SILAC ratios among three replicate experiments. The reproducibility of SILAC analysis among 3 replicate experiments was assessed by comparisons of three SILAC ratios (R_1_, R_2_, R_3_) at 2 min after TCR stimulation. Difference between SILAC ratios were represented using a scatter plot of observed phosphopeptides (A, B, C) and a histogram (D, E, F) of Log_2_ (R_2_/R_1_), Log_2_ (R_3_/R_1_), and Log_2_ (R_3_/R_2_) for each identified phosphopeptide. In good agreement of the expected value of 0, the measured average value of Log_2_ (R_2_/R_1_), Log_2_ (R_3_/R_1_), and Log_2_ (R_3_/R_2_) was 0.08±0.27, 0.01±0.18, and 0.01±0.46, respectively. Additionally, histogram plots showed that D) 85.2%, E) 82.2%, and F) 86.8% identified phosphopeptides displayed Log_2_ (R_2_/R_1_), Log_2_ (R_3_/R_1_), and Log_2_ (R_3_/R_2_) values respectively in the range of −0.6∼0.6.
(TIF)

Figure S2 Disruption of SLP-76 from Jurkat T cells and phosphorylation of PLCγ1. **A**) SLP-76 expression in Jurkat T cells. Protein lysates from J14-76-11 (SLP-76 reconstituted) and J14 (SLP-76 deficient) were separated by SDS-PAGE and immunoblotted with SLP-76 specific antibodies. **B**) Effect of SLP-76 on PLCγ1 phosphorylation in OKT3/OKT4 stimulated Jurkat T cells. Lysates were prepared from J14-76-11 (SLP-76 reconstituted) and J14 (SLP-76 deficient) cells following TCR stimulation for the indicated time periods. Total cell lysates were probed by protein immunoblotting with anti-phospho-PLCγ1 (Y783) (top) and anti-PLCγ1 (bottom).
(TIF)

Figure S3 Canonical TCR signaling pathway. Established signaling cascades in activated T cells with quantitative SILAC ratios (SLP-76 deficient in relative to SLP-76 reconstituted) represented as SILAC heatmaps beside individual proteins. SILAC ratios between J14 and J14-76-11 at each of the TCR stimulation timepoints were represented as SILAC heatmaps as described in methods.
(PDF)

Method S1 Selection of SILAC labeling conditions. SILAC labeling conditions were tested for both Jurkat T cell lines J14-76-11 (SLP-76 reconstituted) and J14 (SLP-76 deficient) to make sure cells grow and function normally.
(PDF)
