# Supplementary figures and images for: Quantitative Phosphoproteomics Reveals SLP-76 Dependent Regulation of PAG and Src Family Kinases in T Cells
Source: PLoS One. 2012 Oct 11;7(10):e46725. doi: 10.1371/journal.pone.0046725 (PMC3469622; doi:10.1371/journal.pone.0046725)

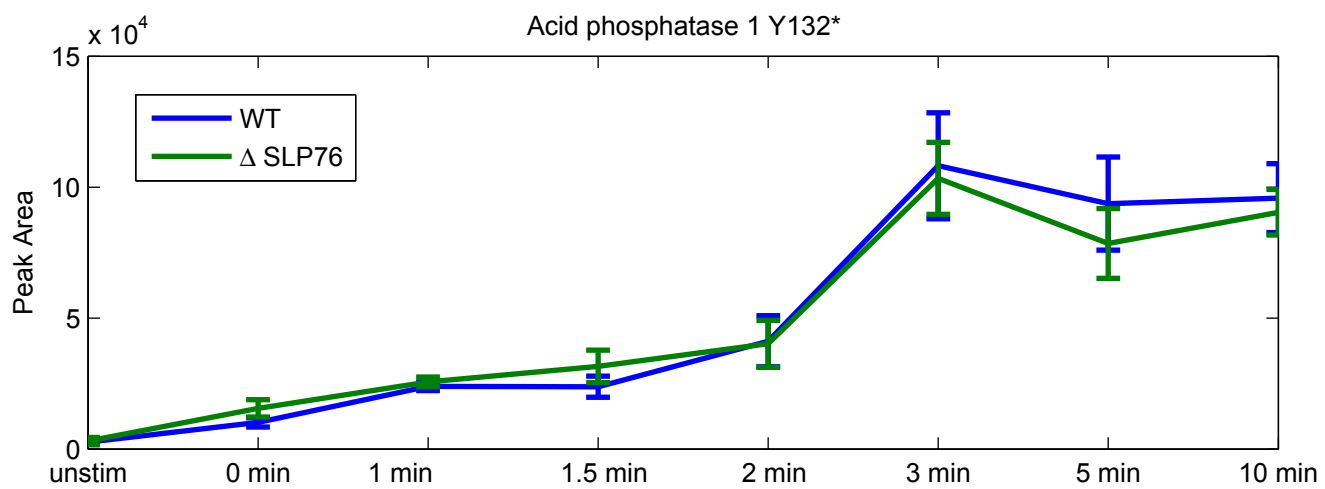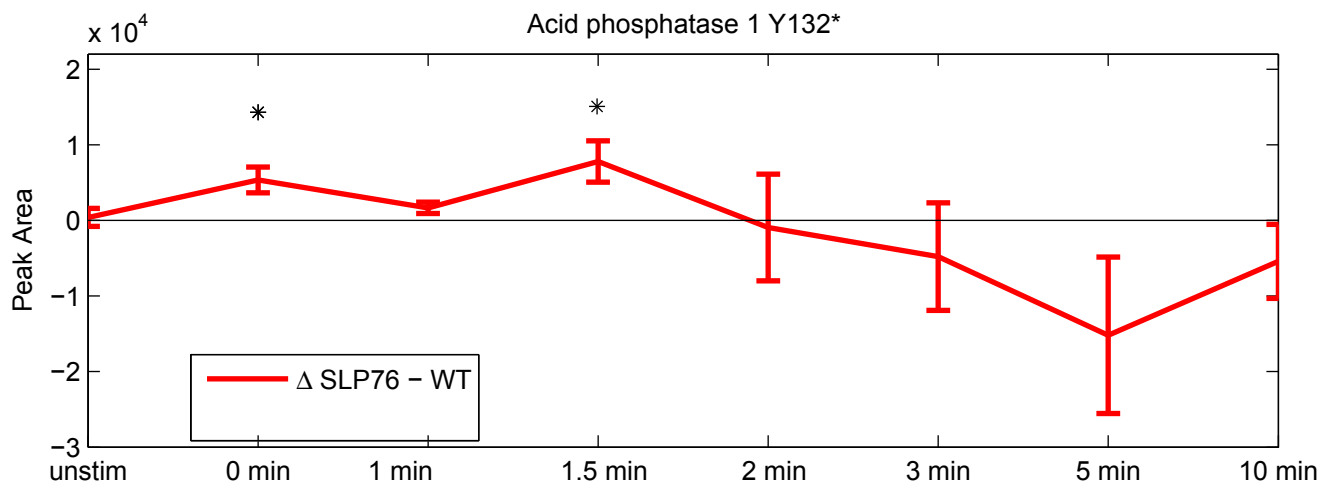

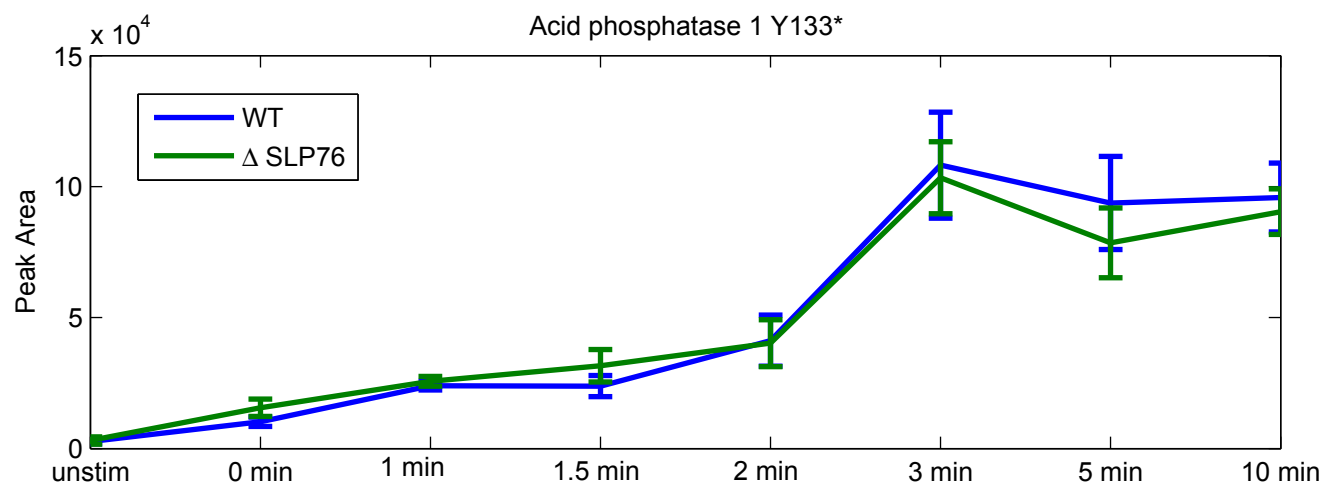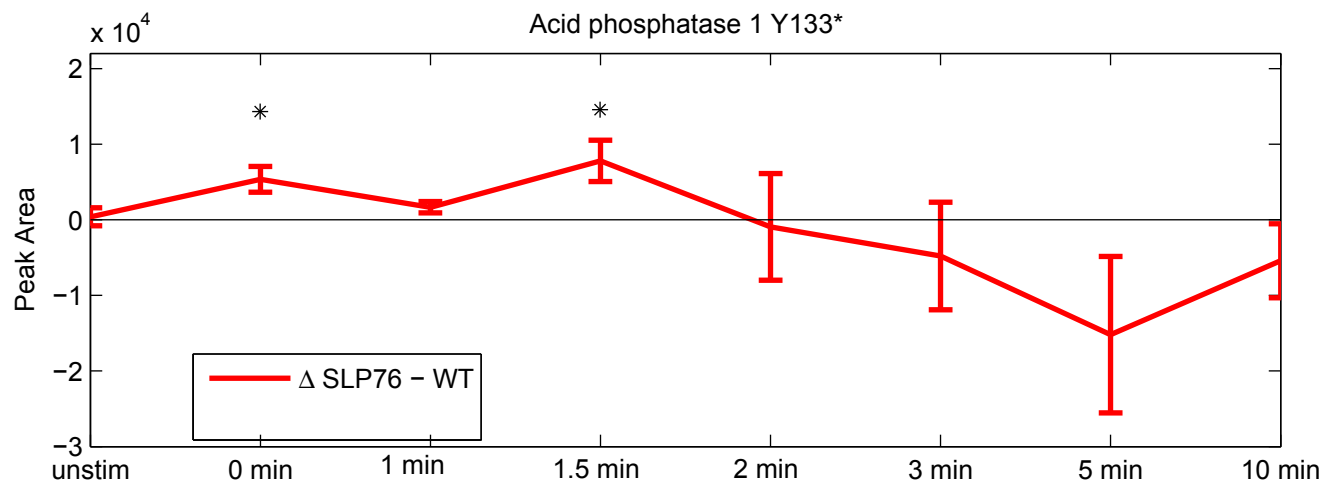

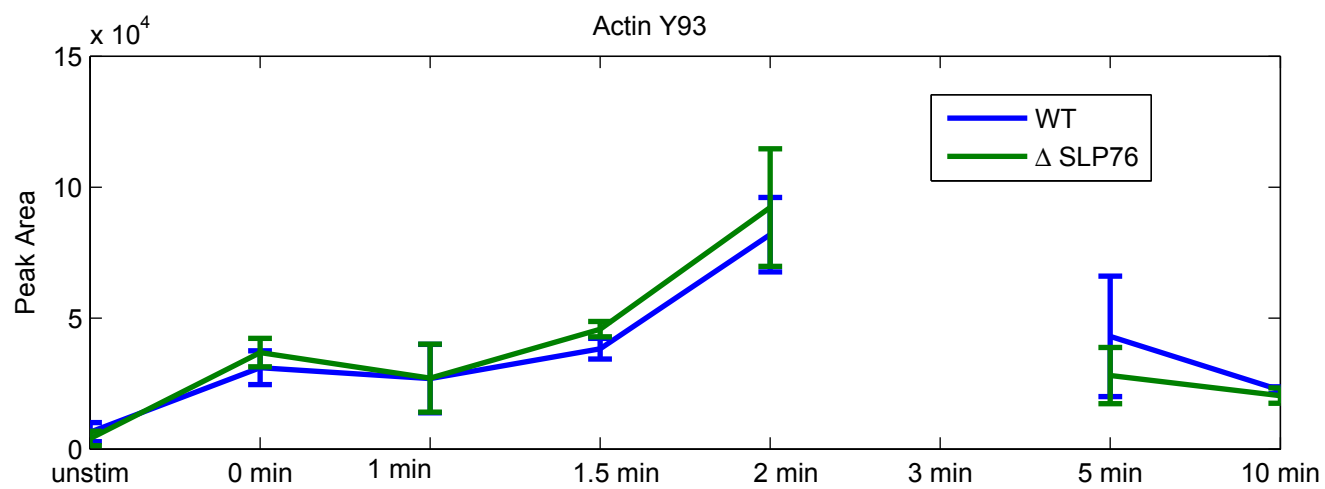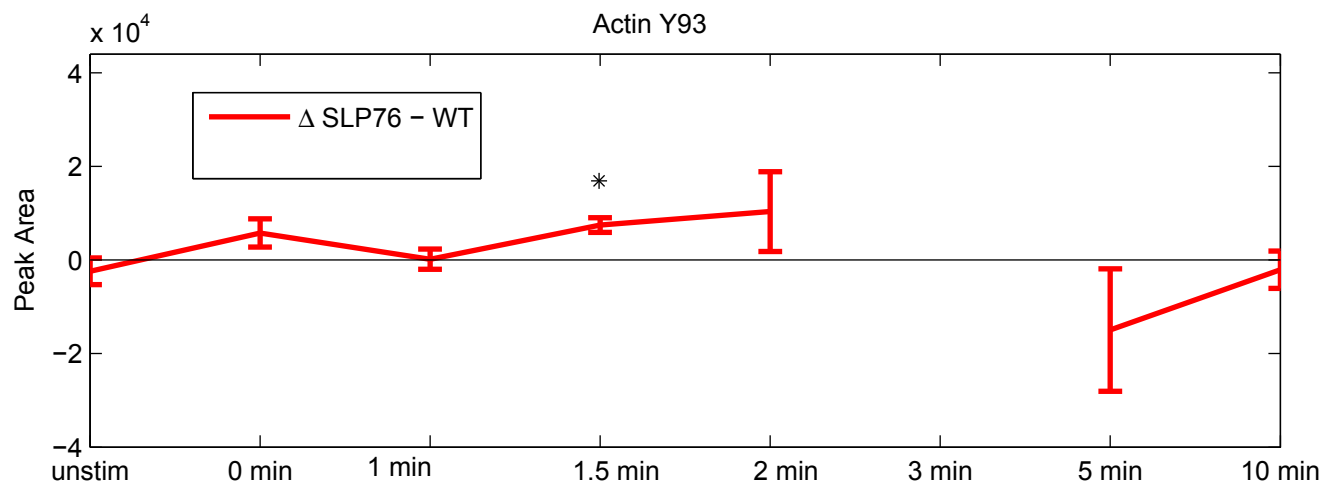

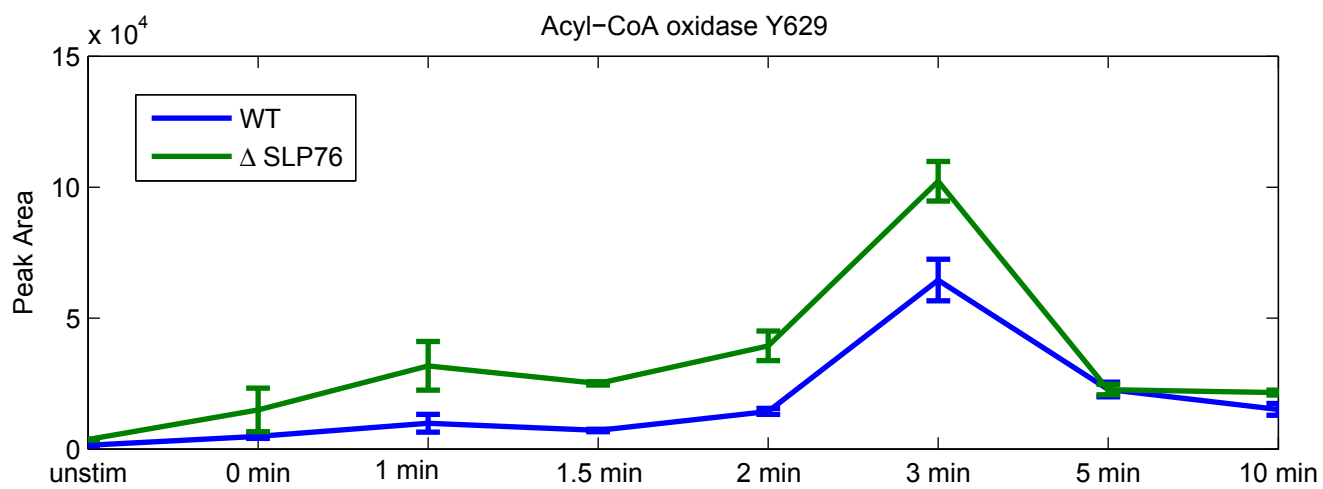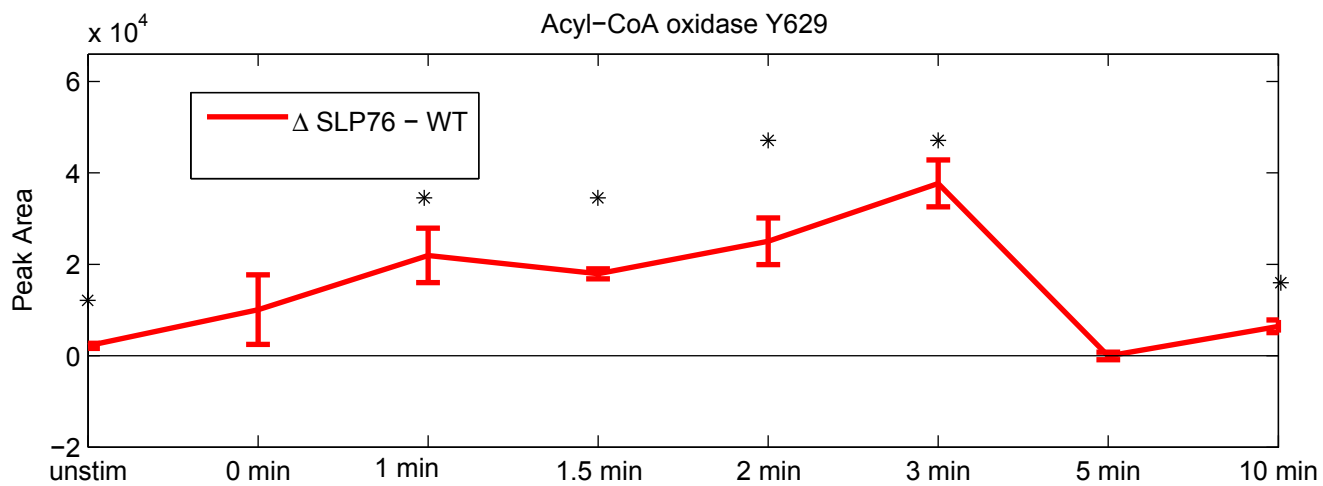

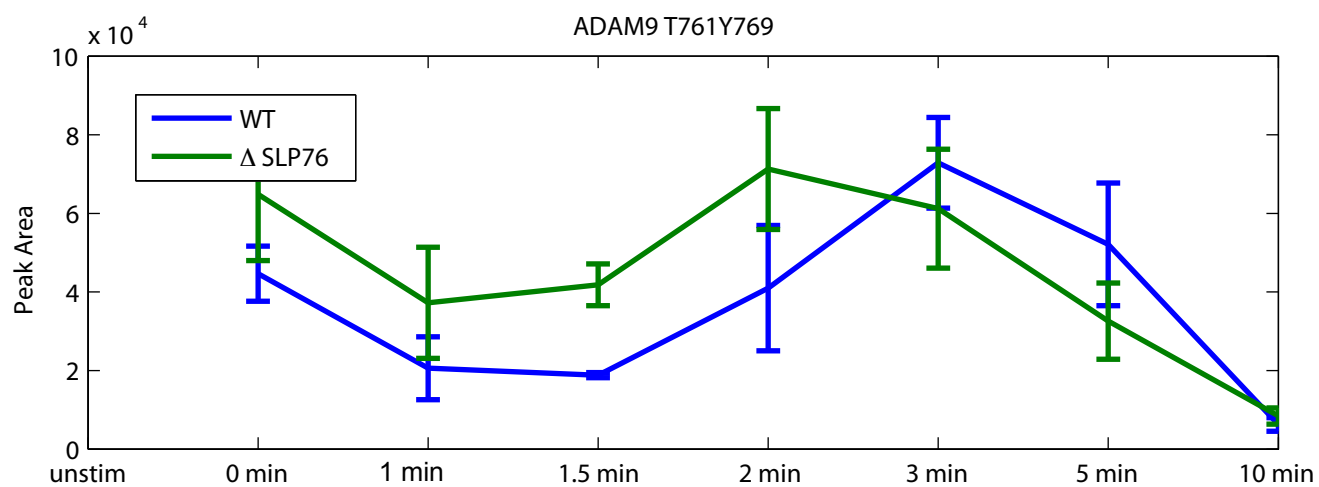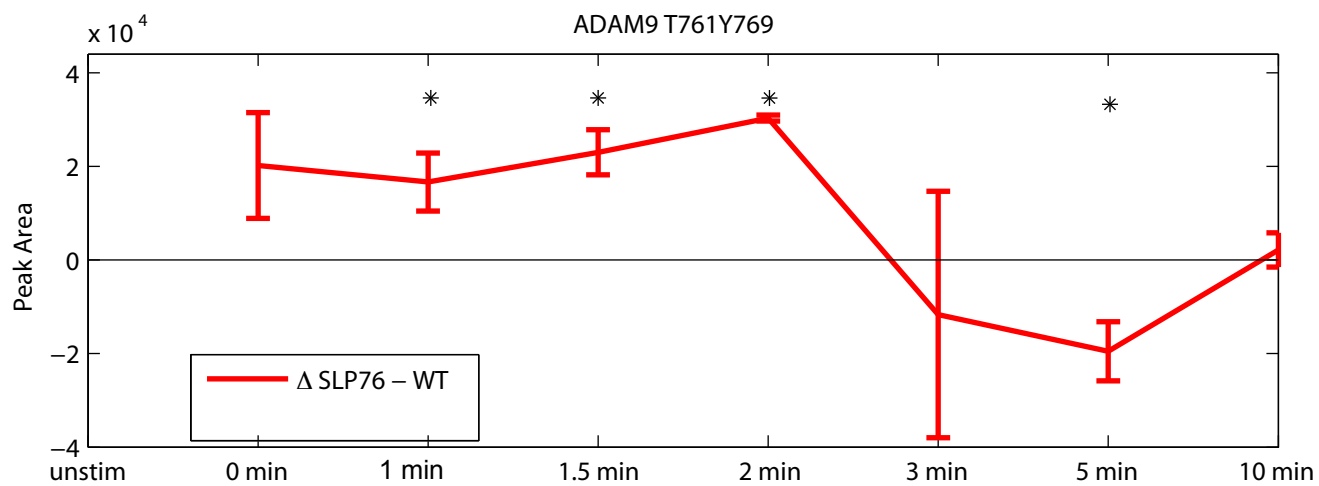

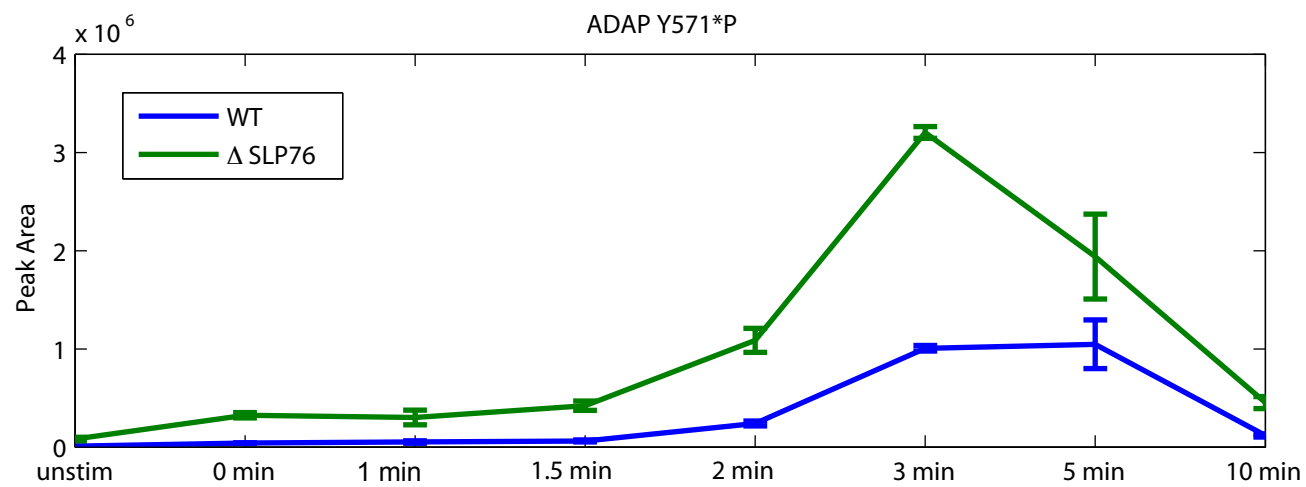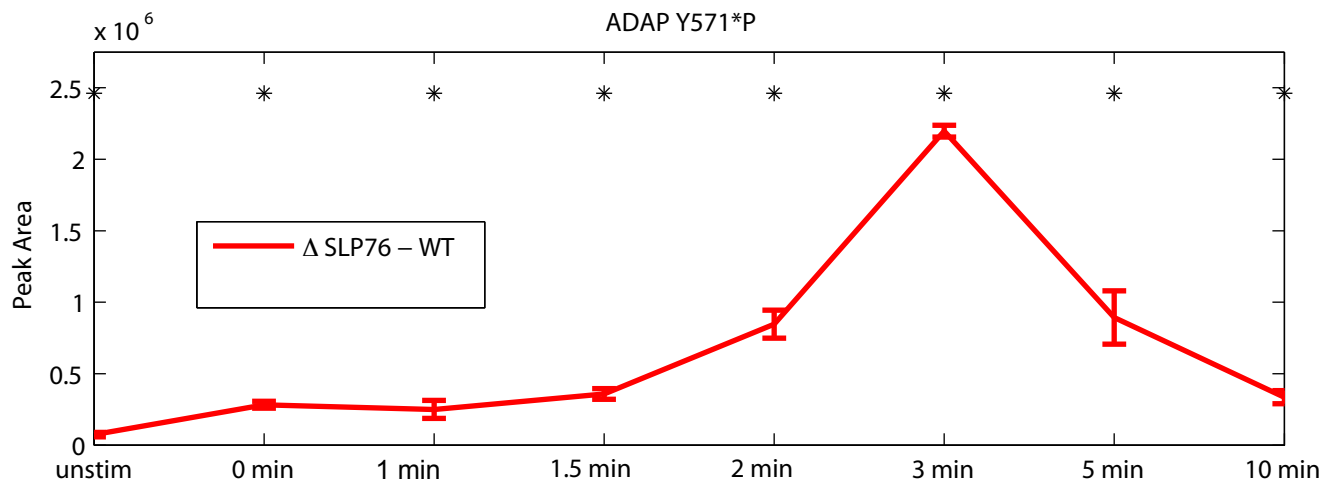

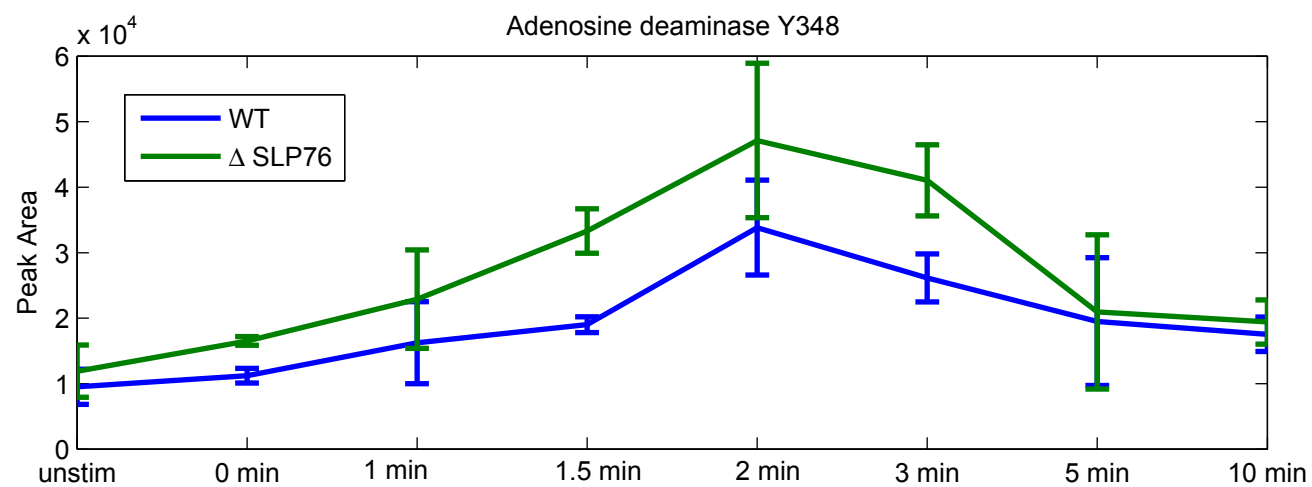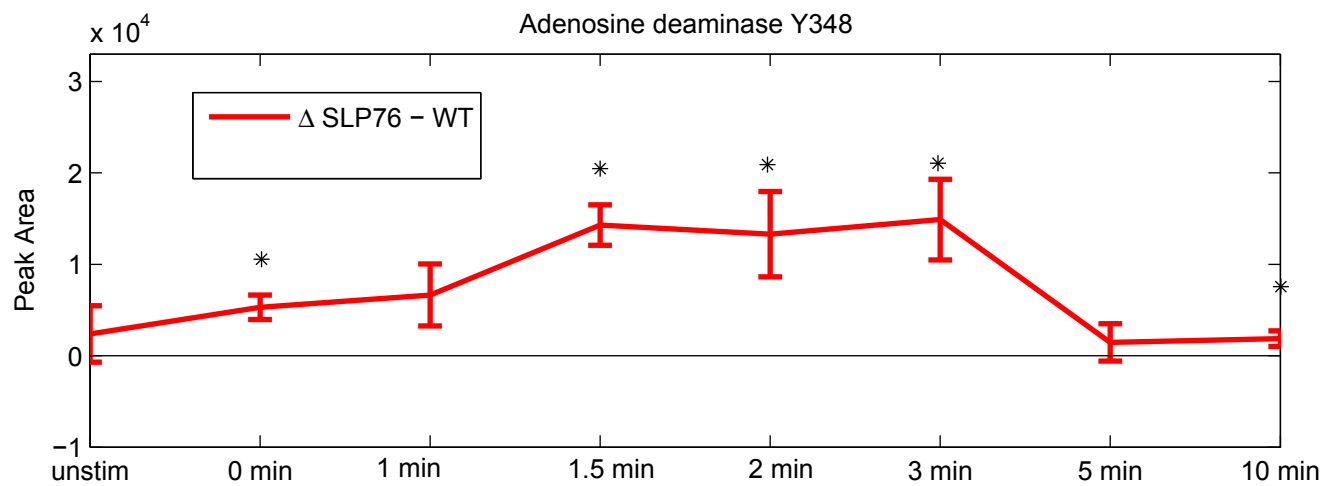

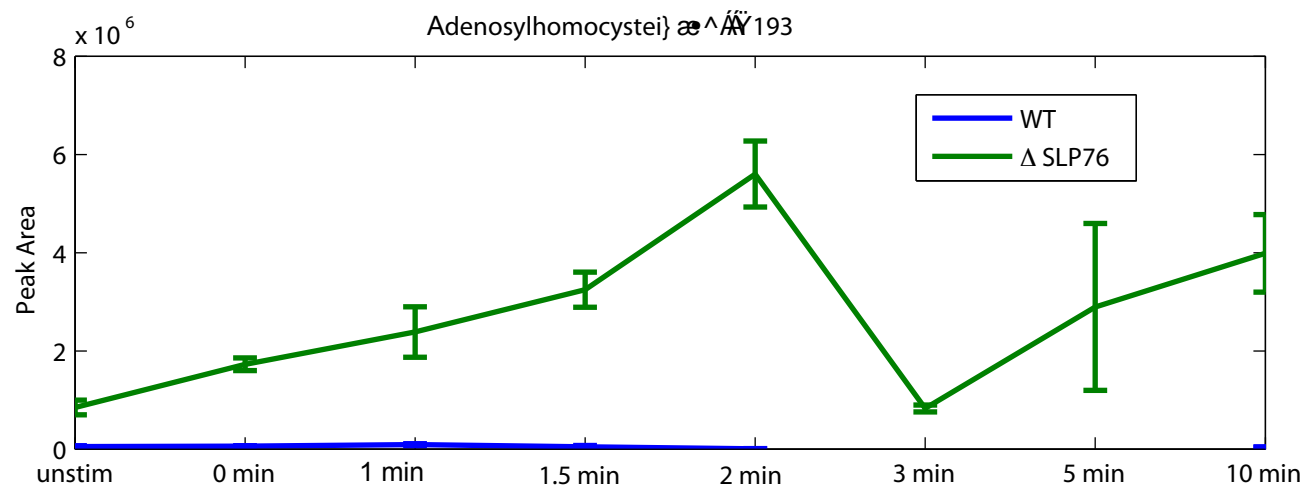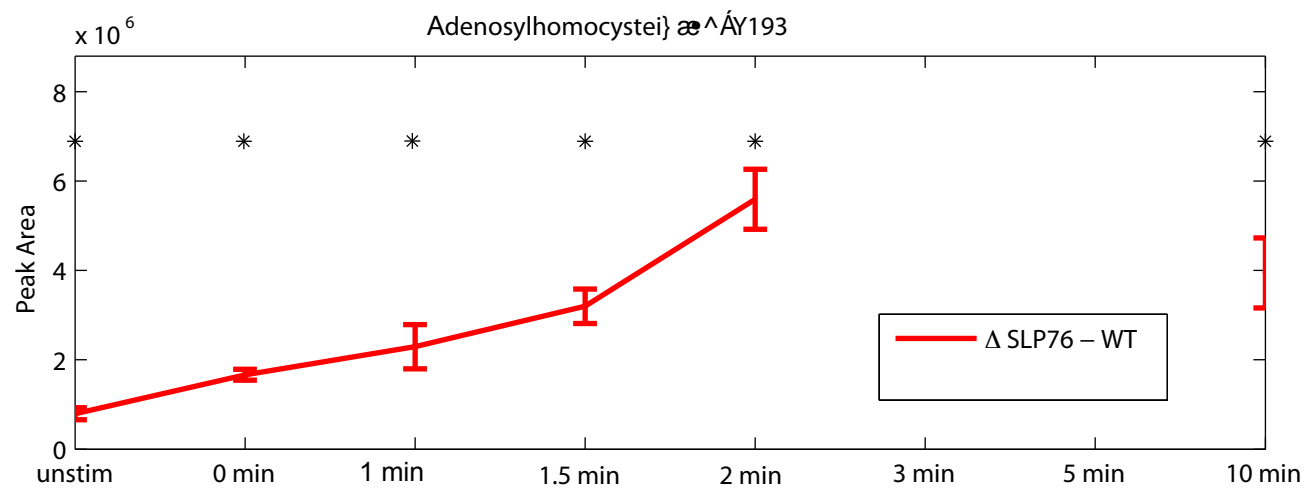

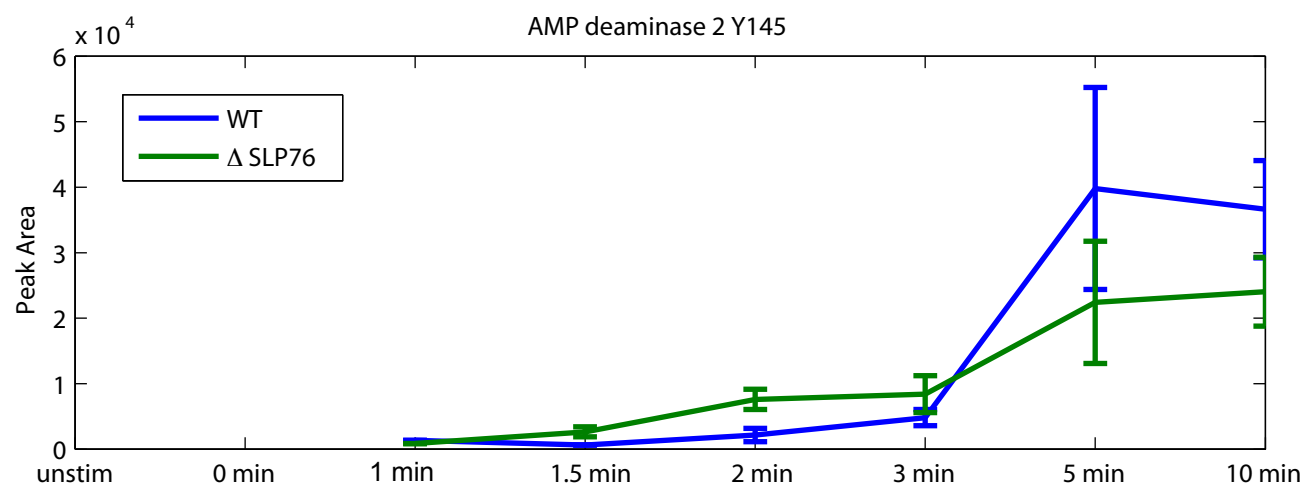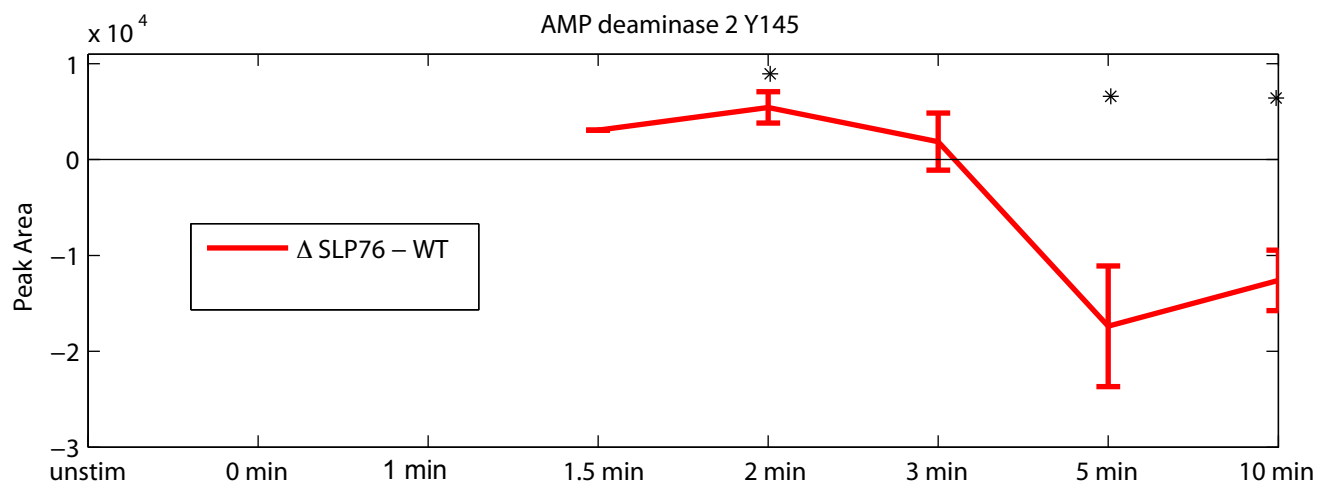

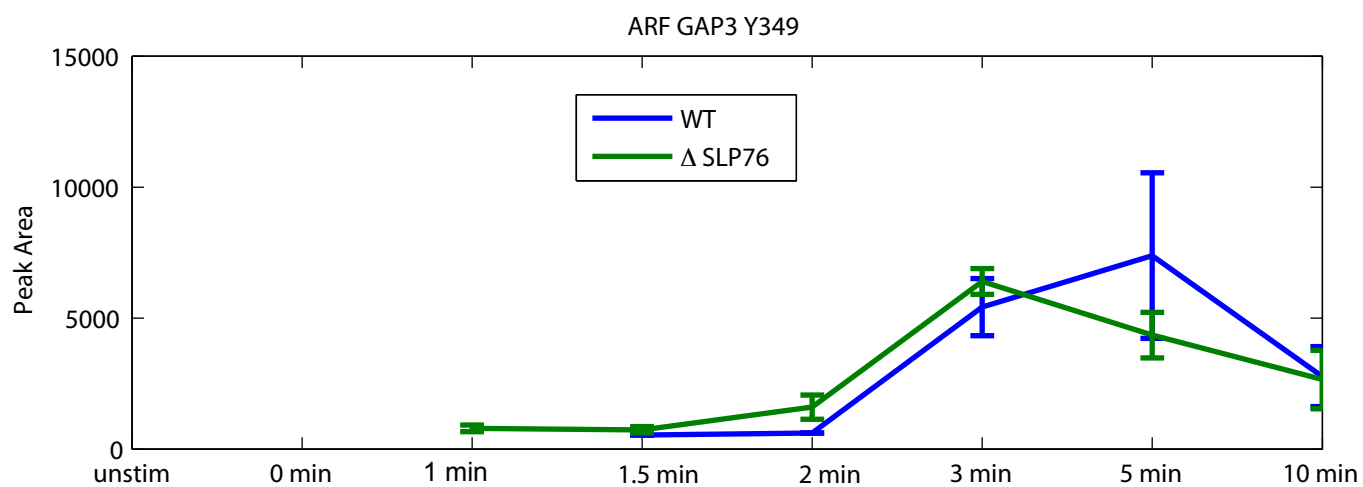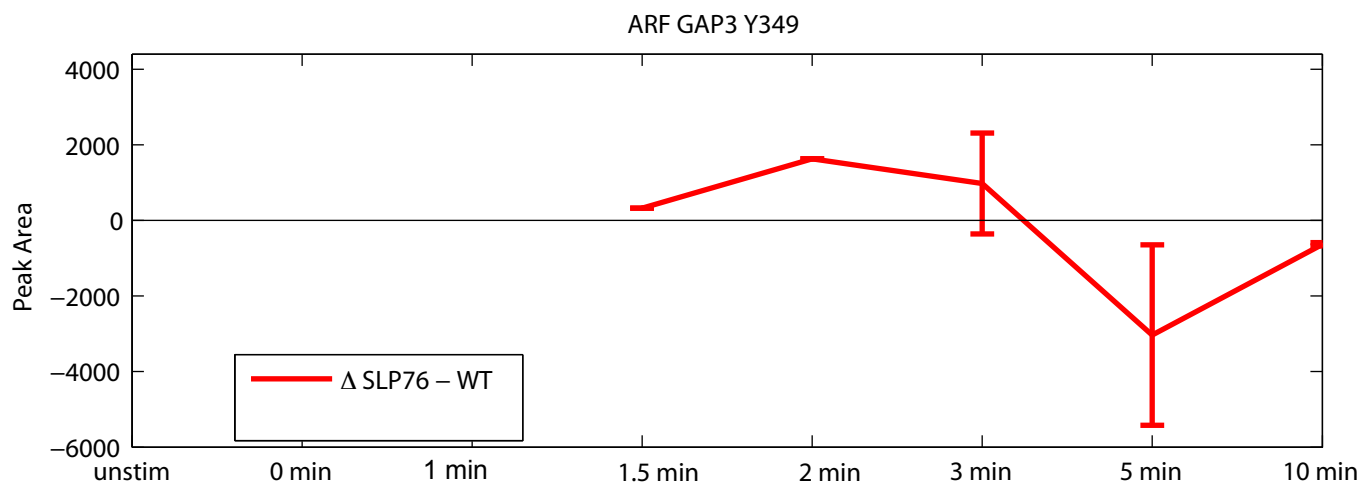

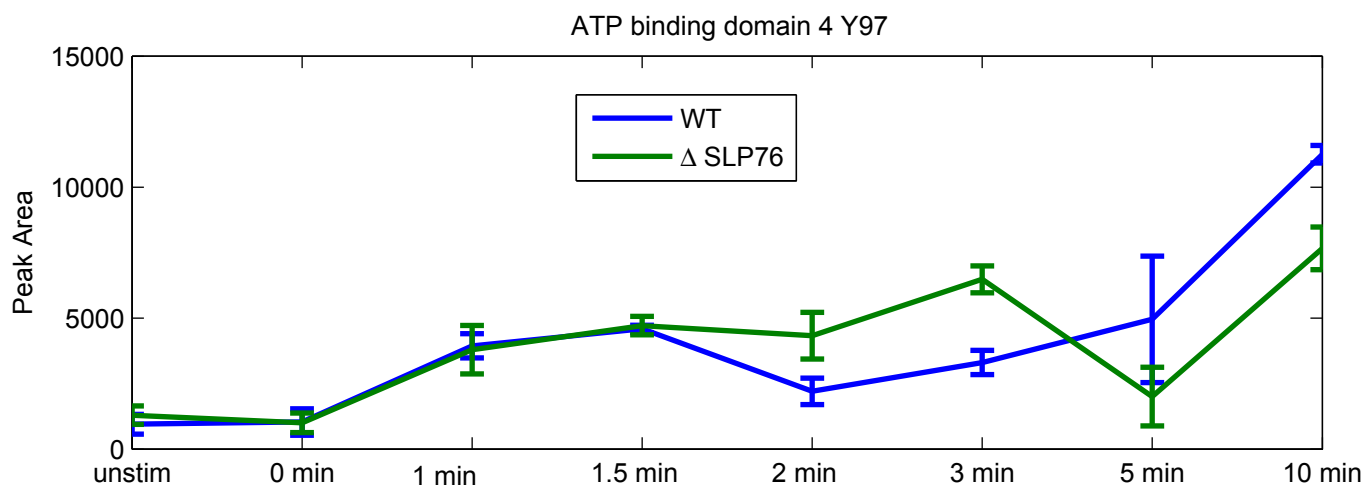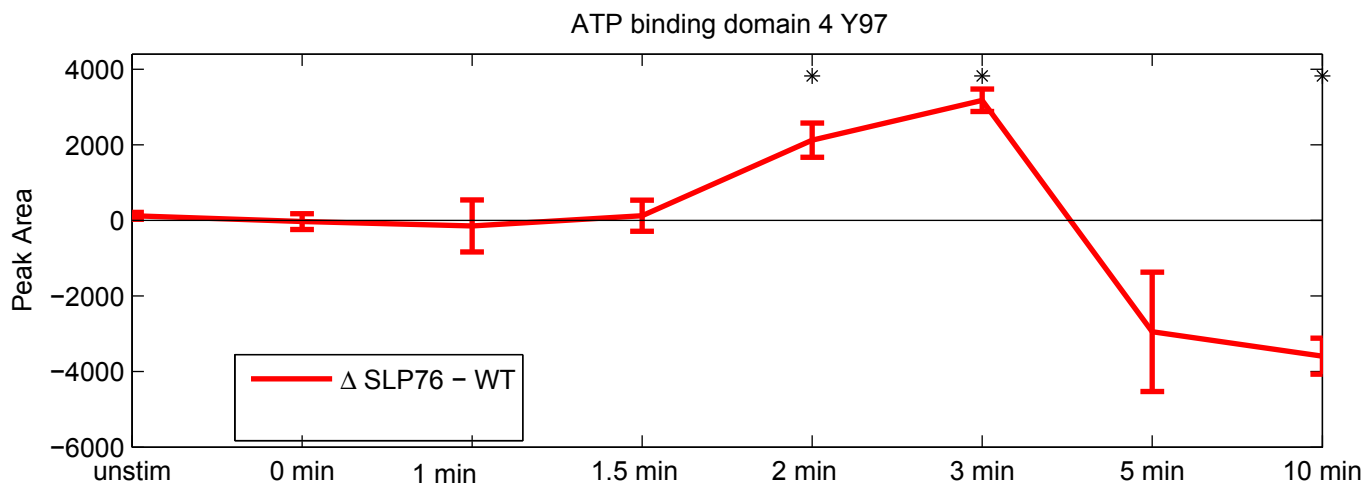

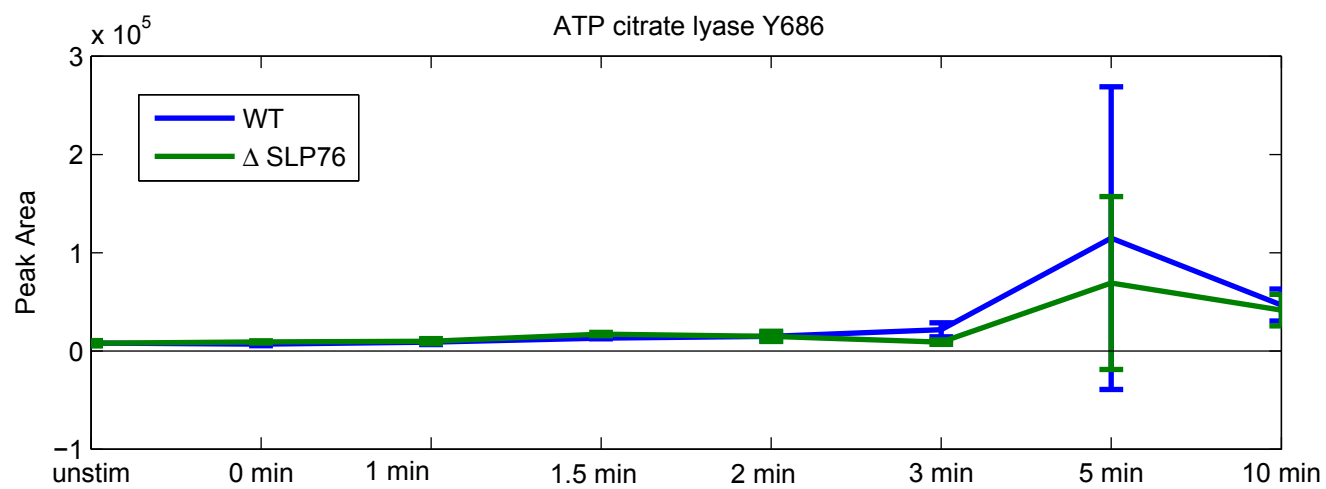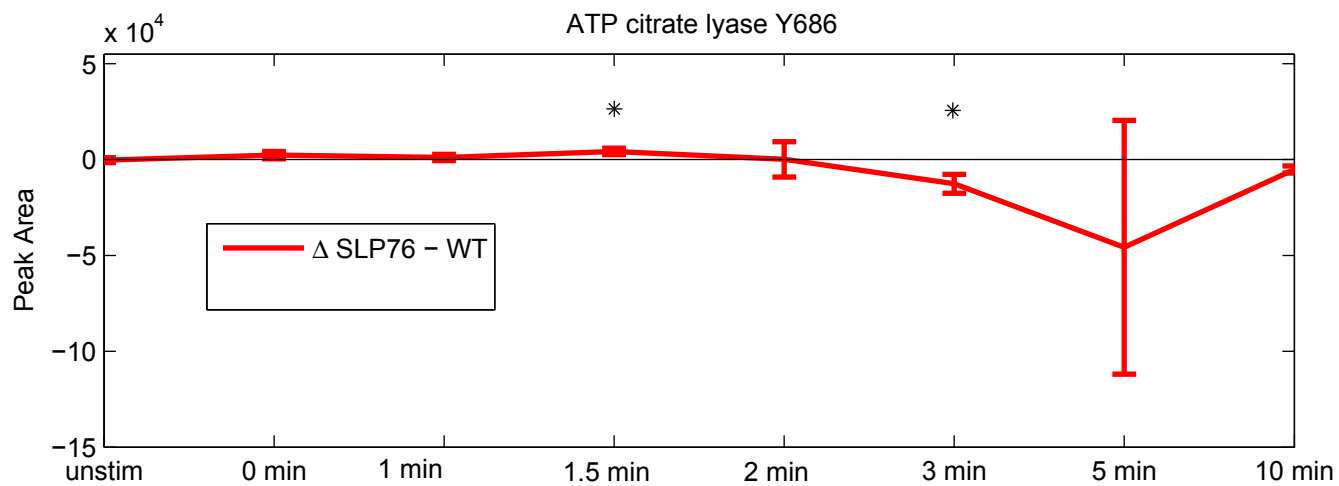

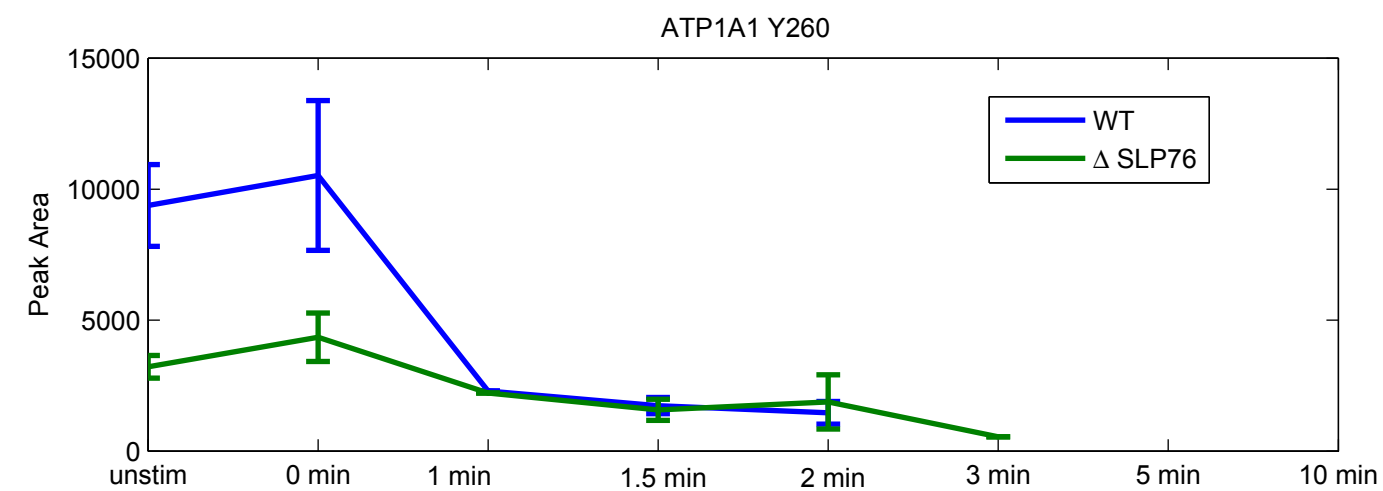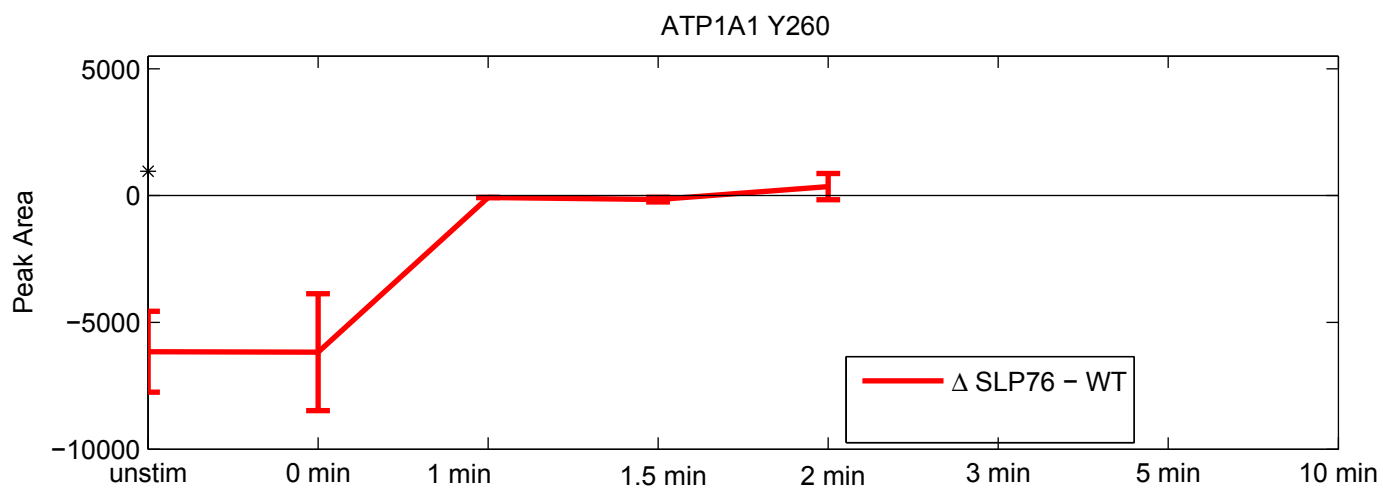

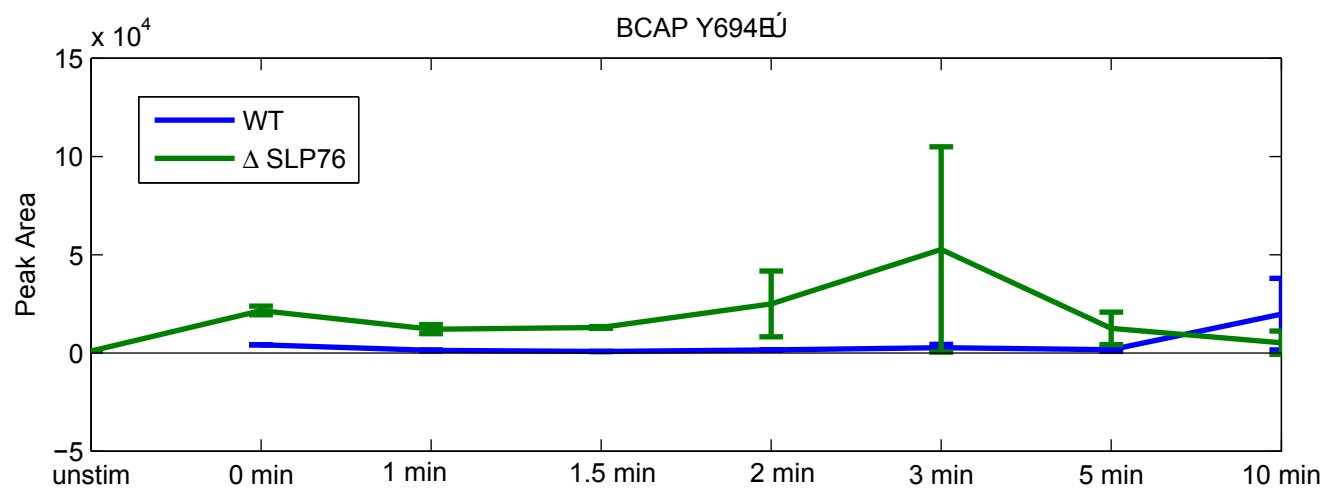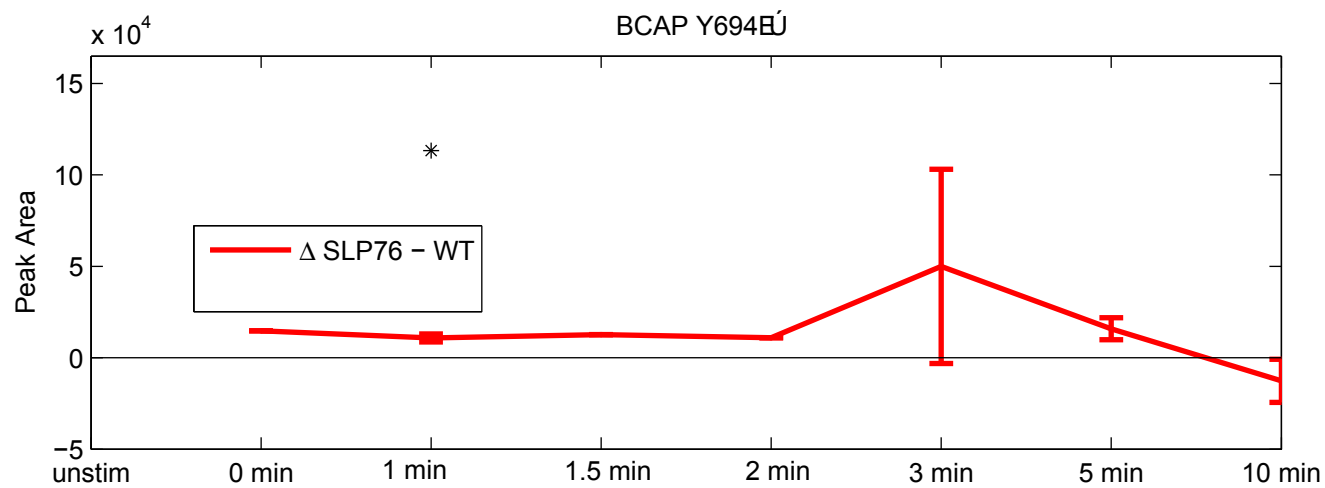

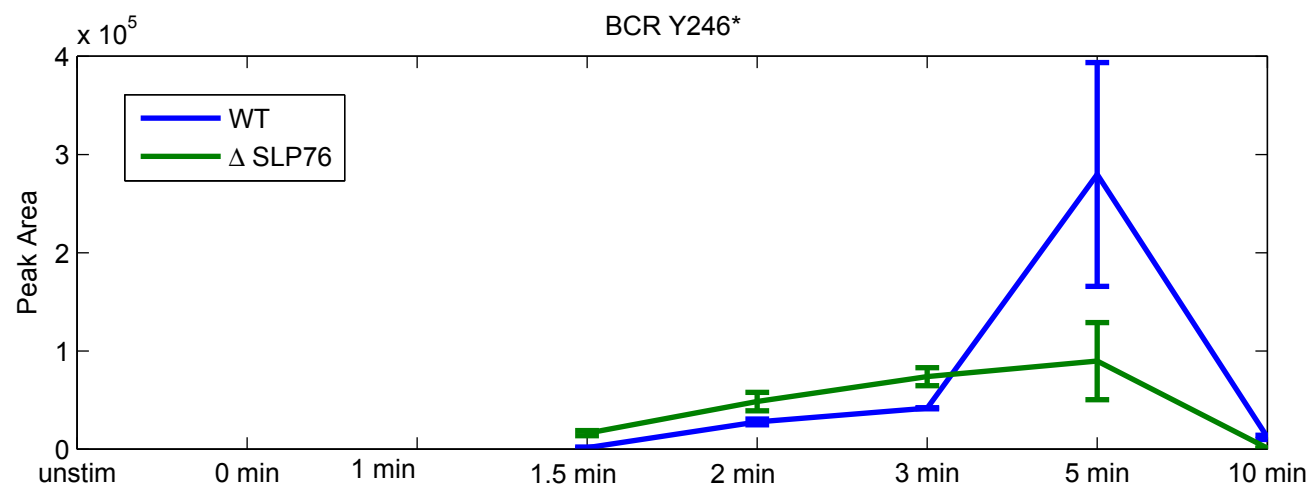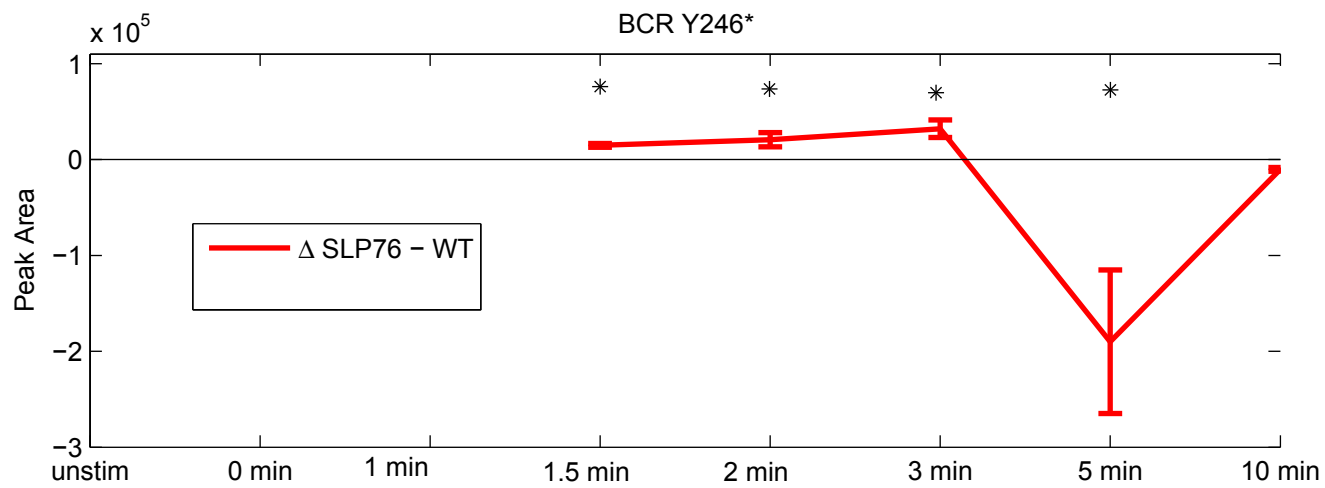

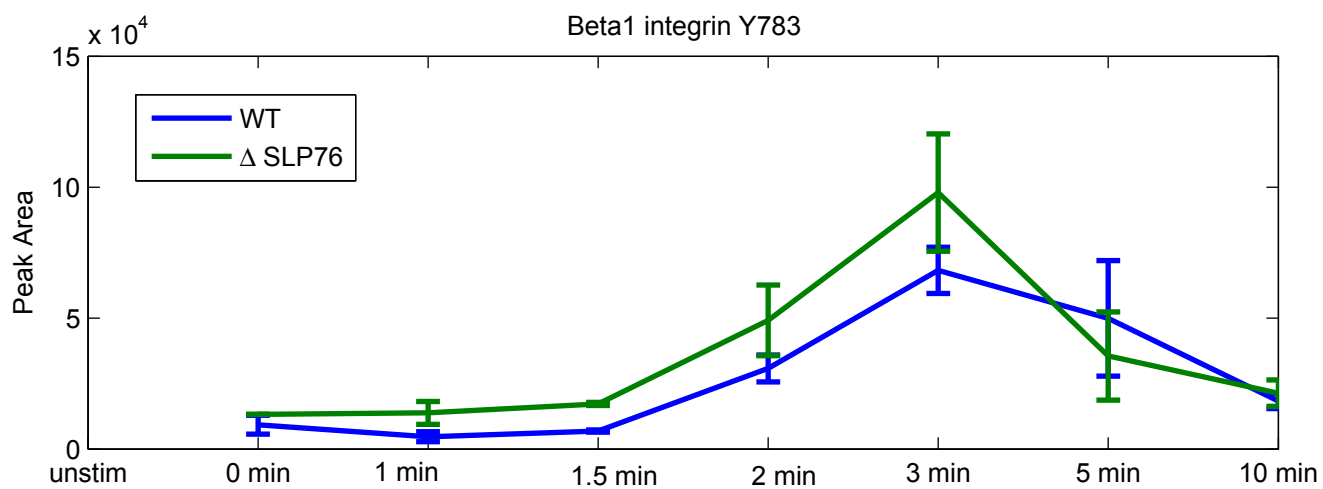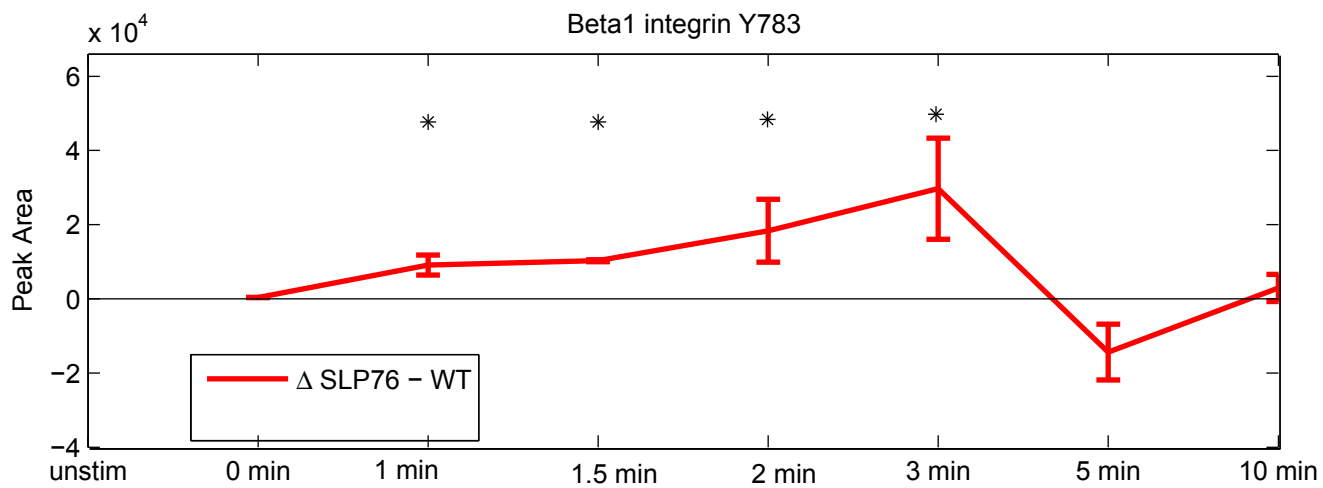

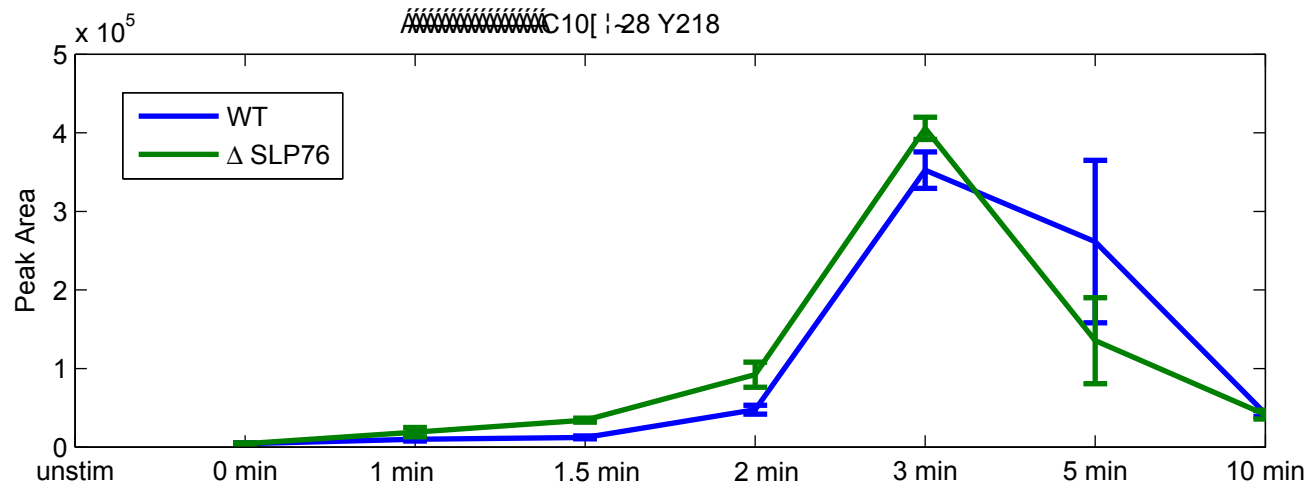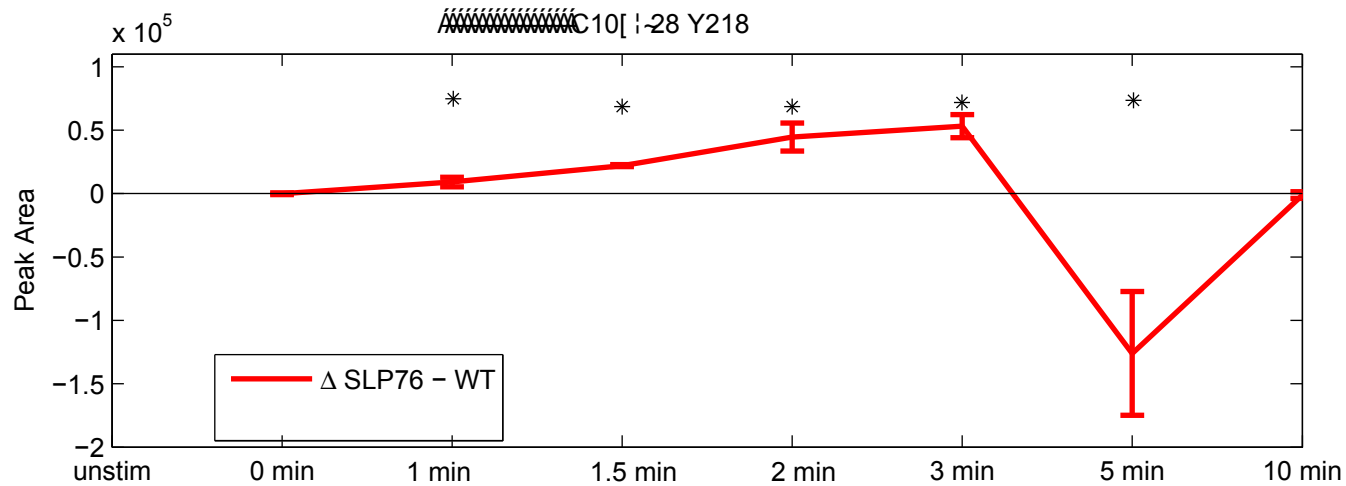

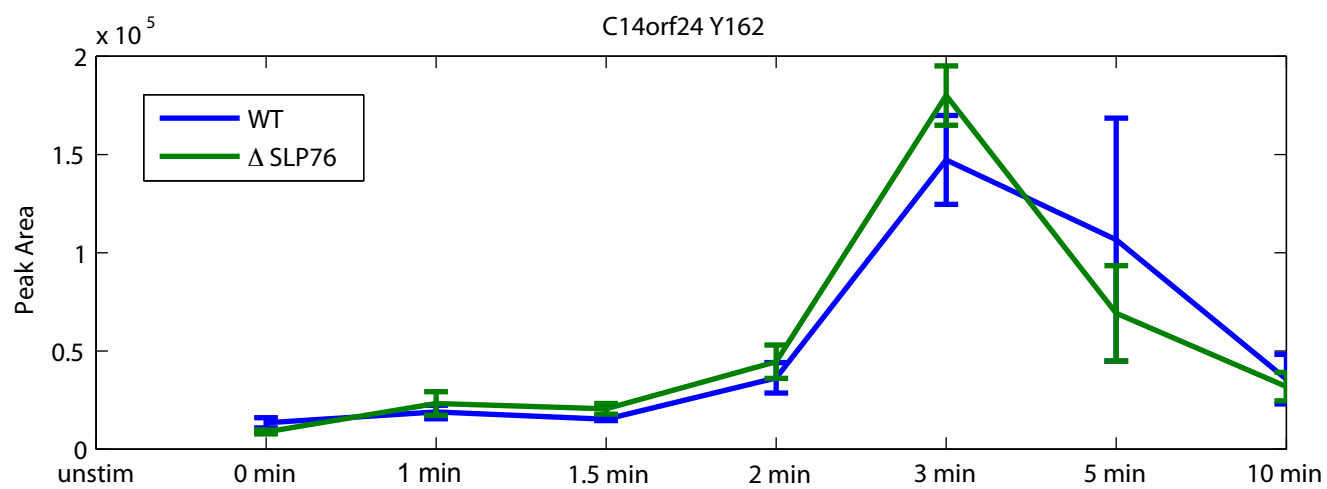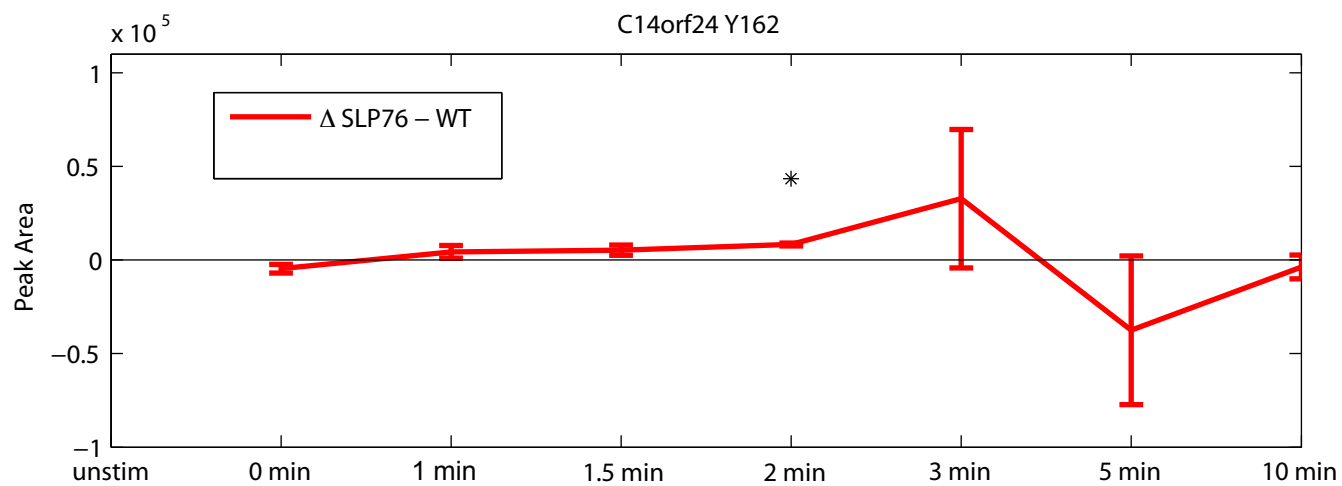

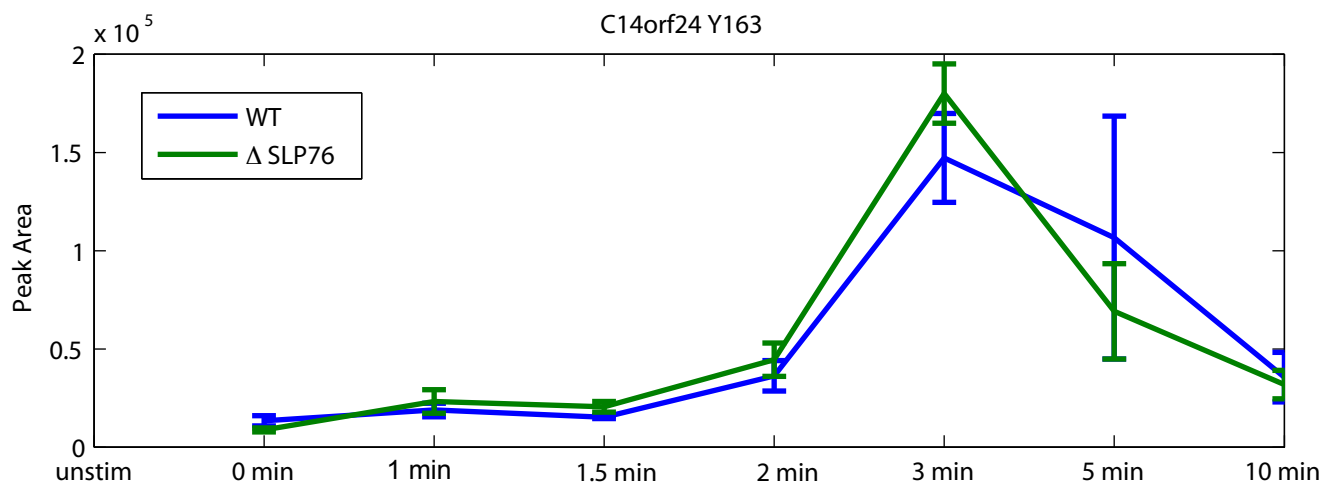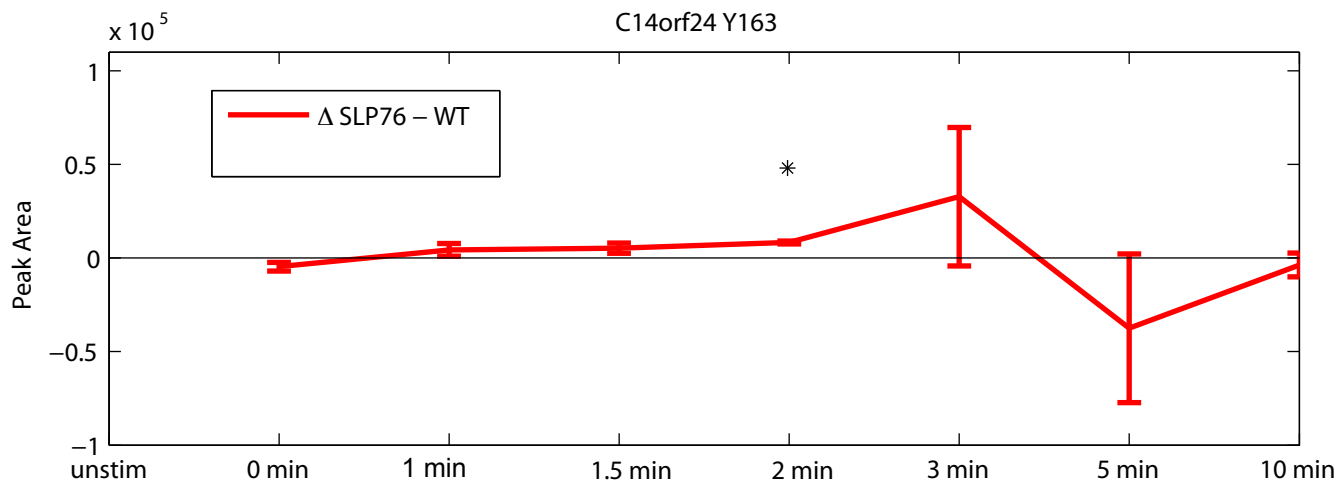

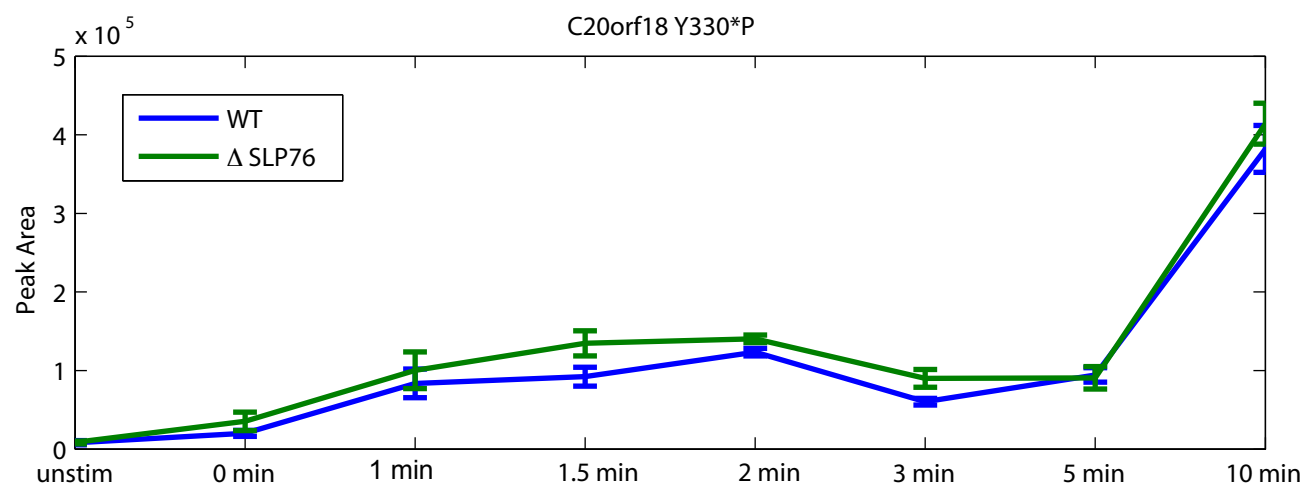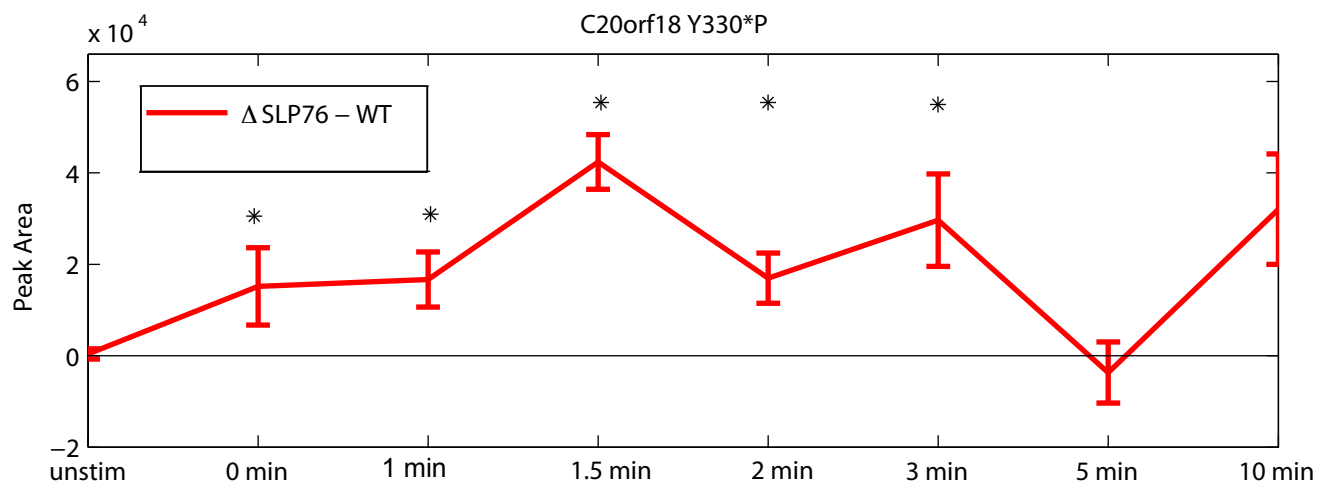

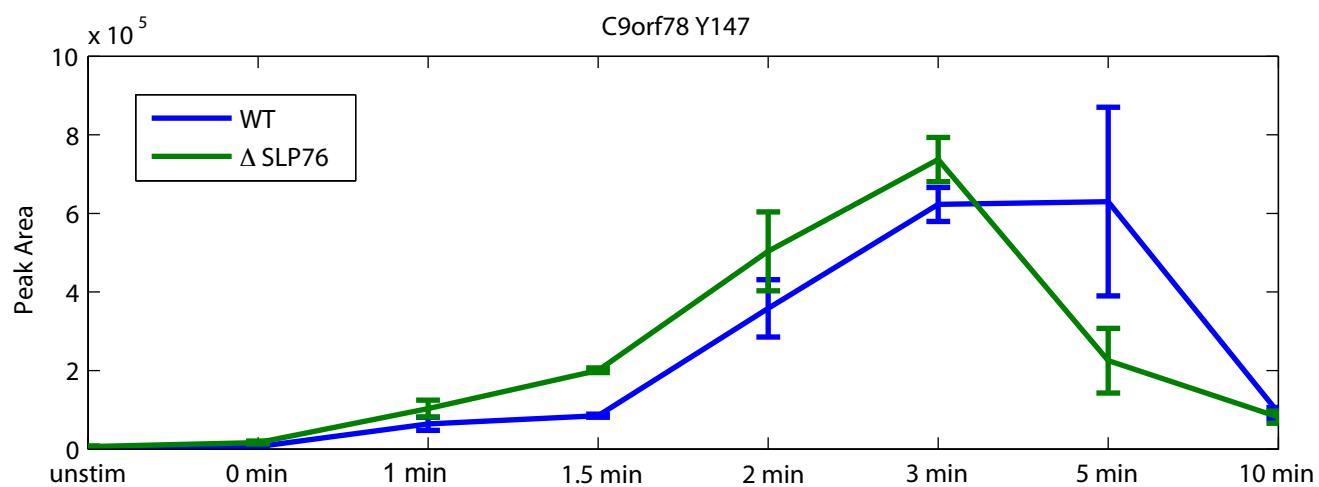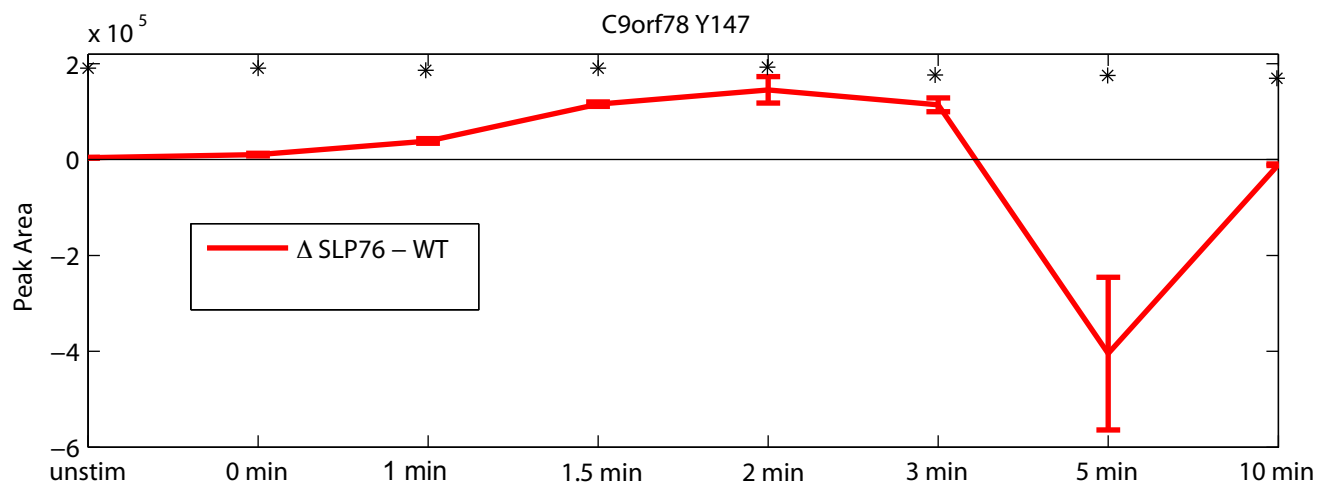

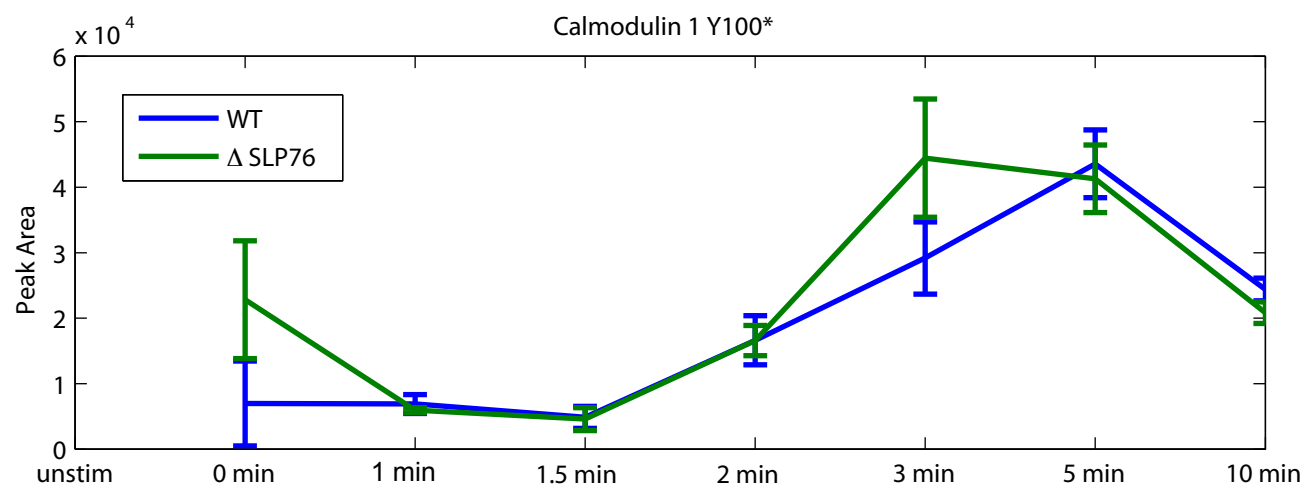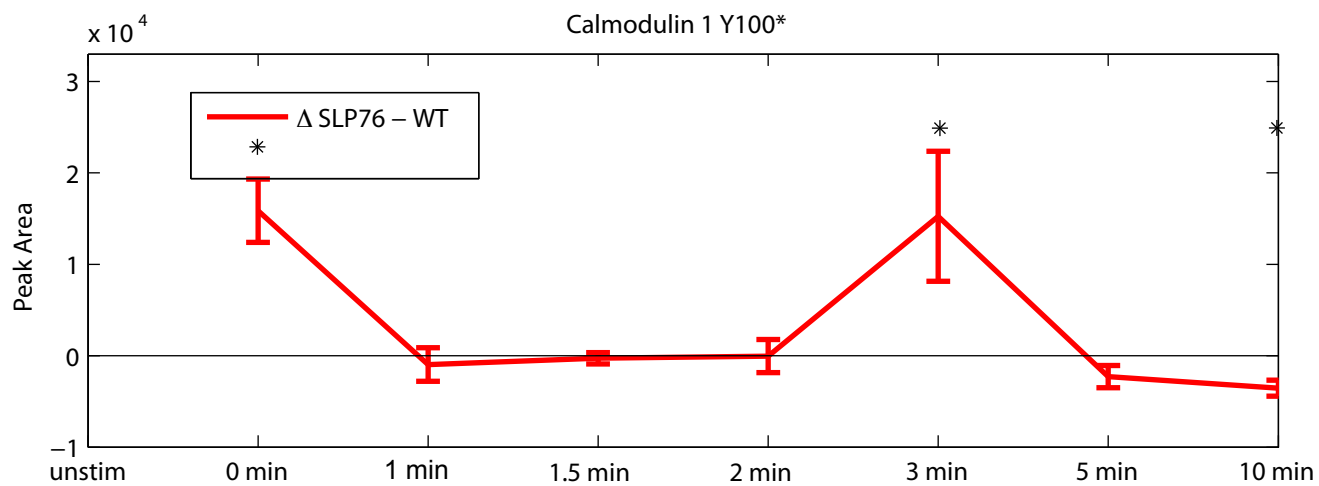

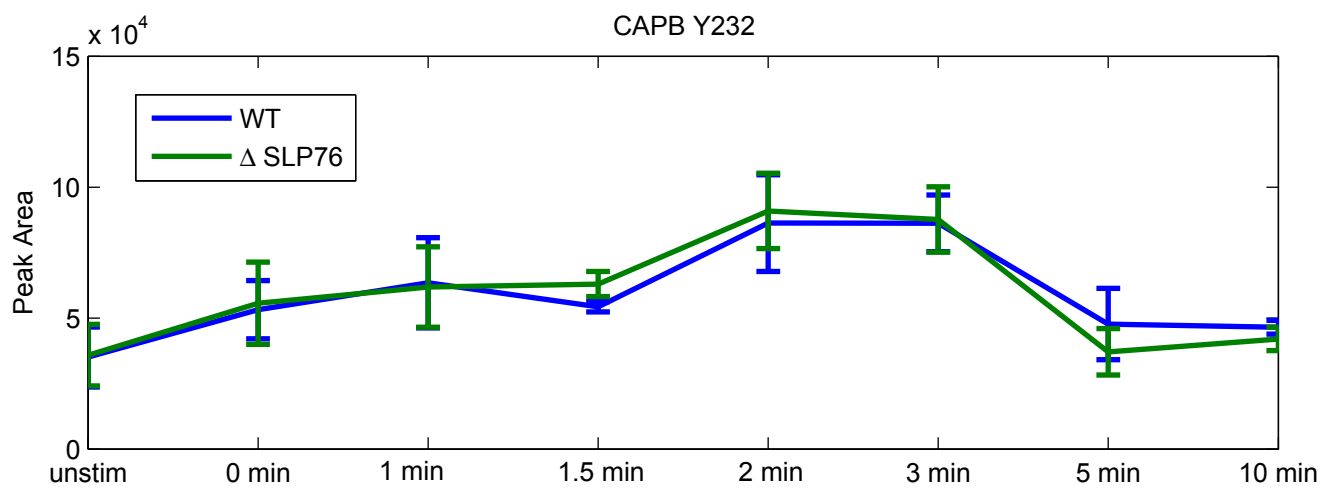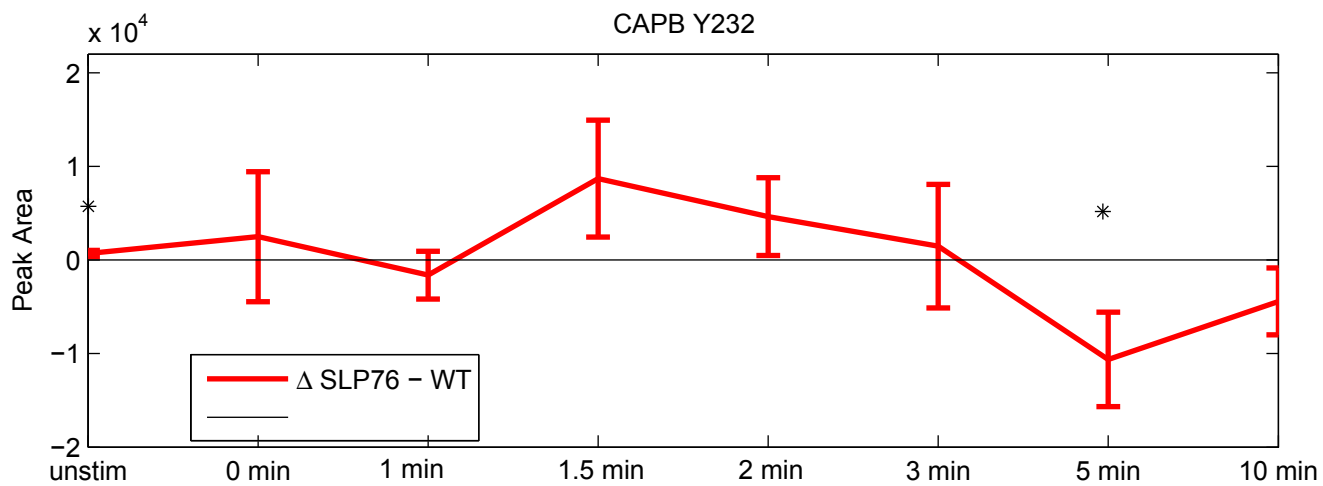

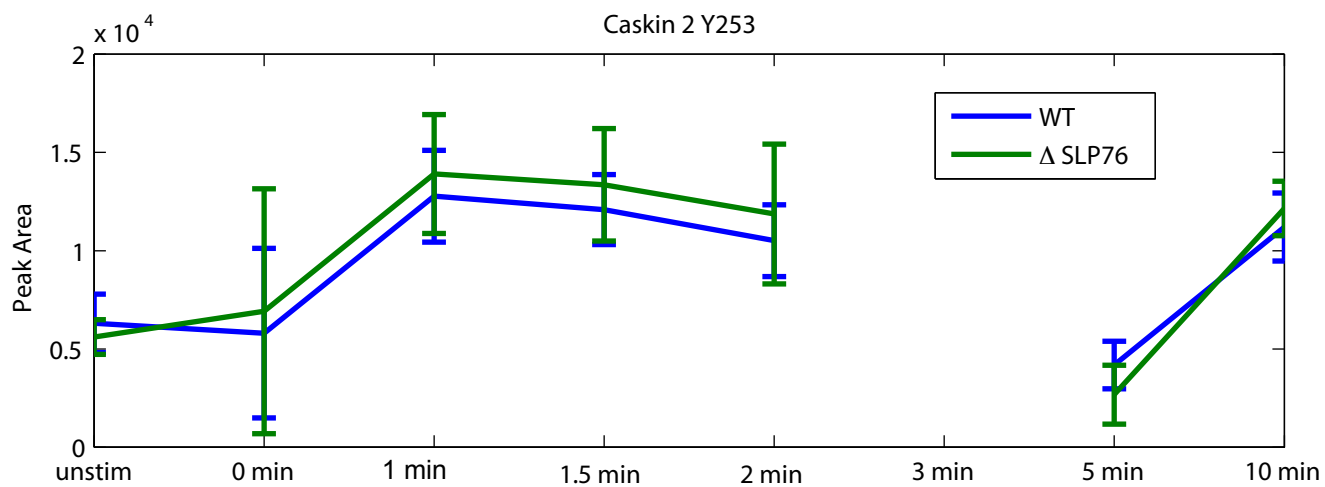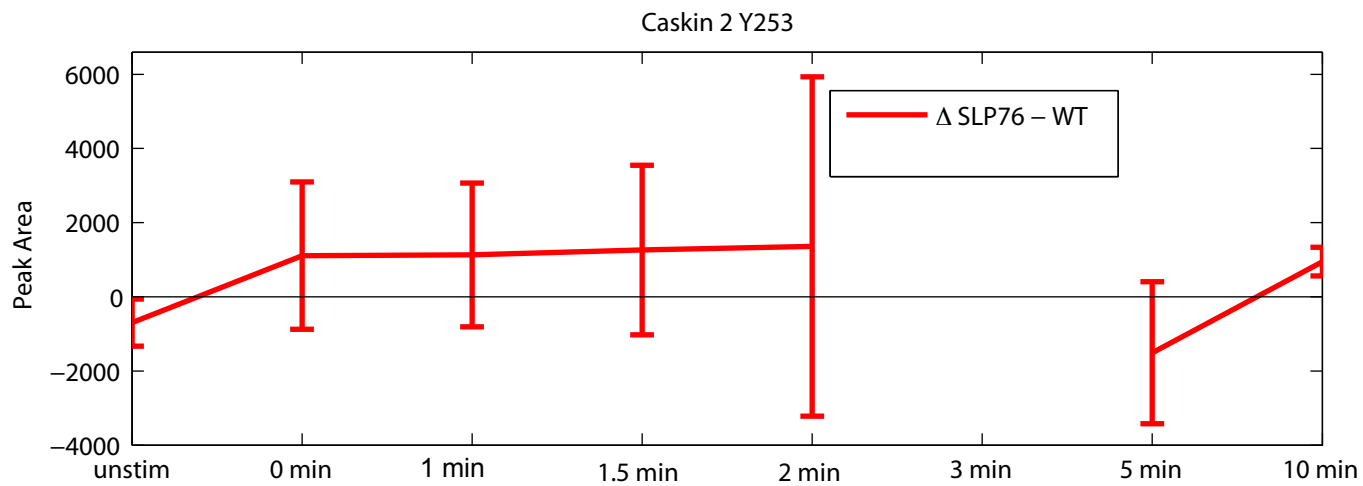

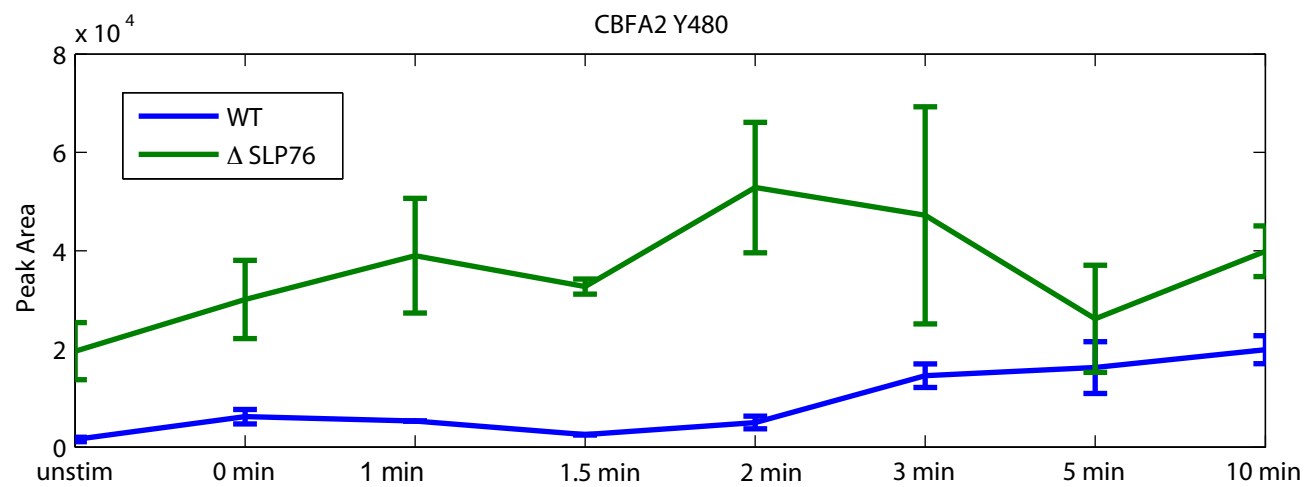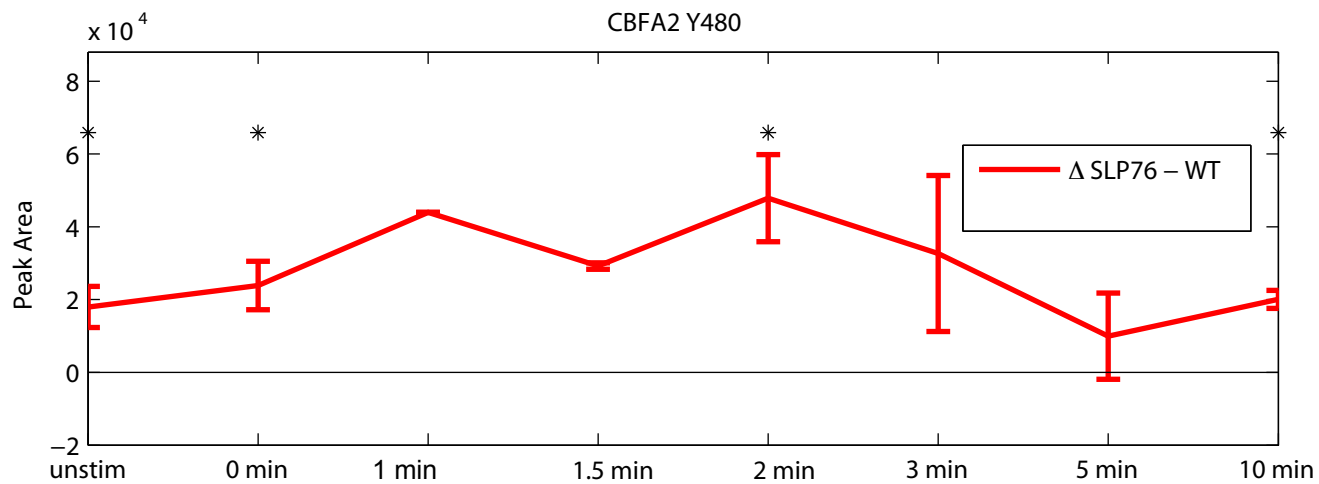

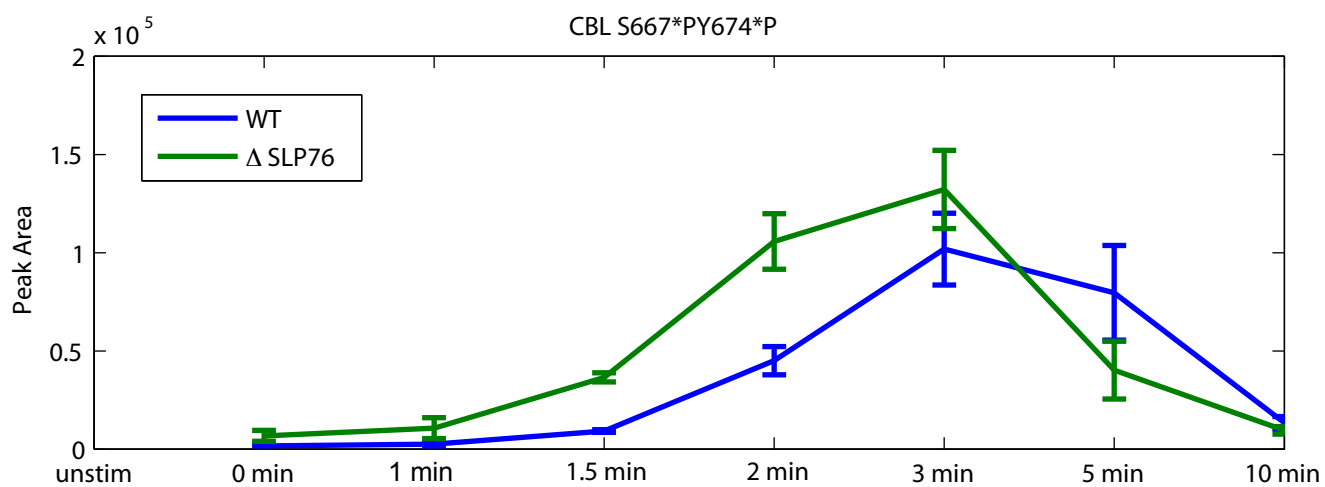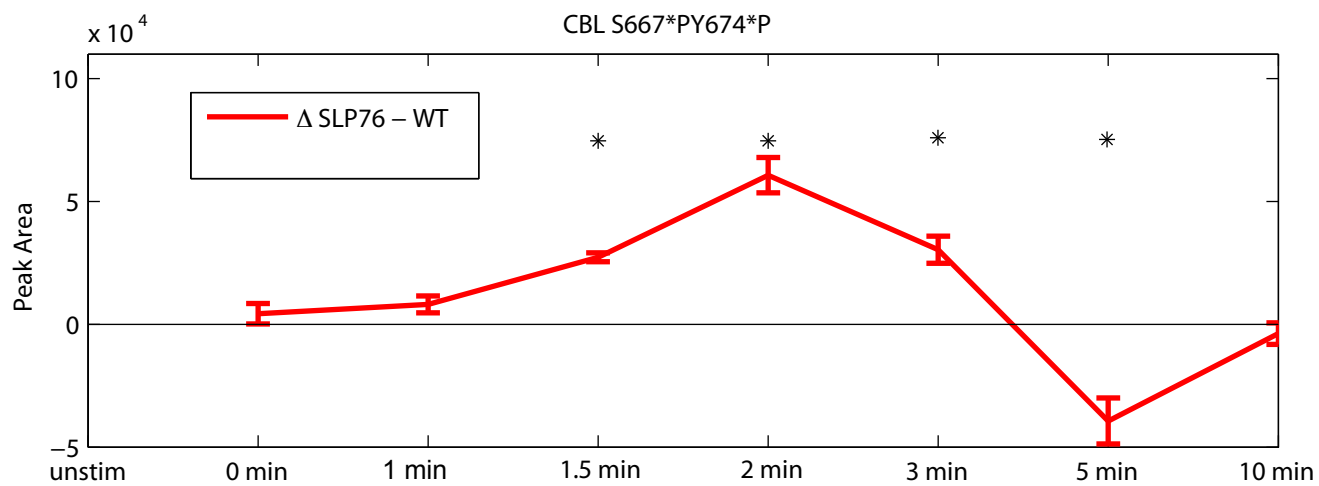

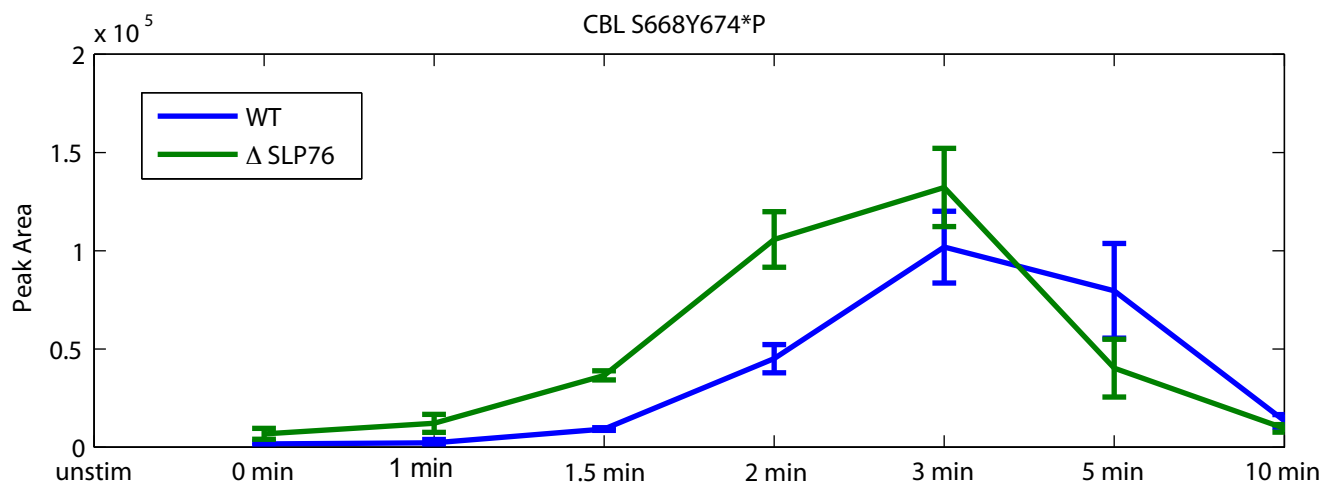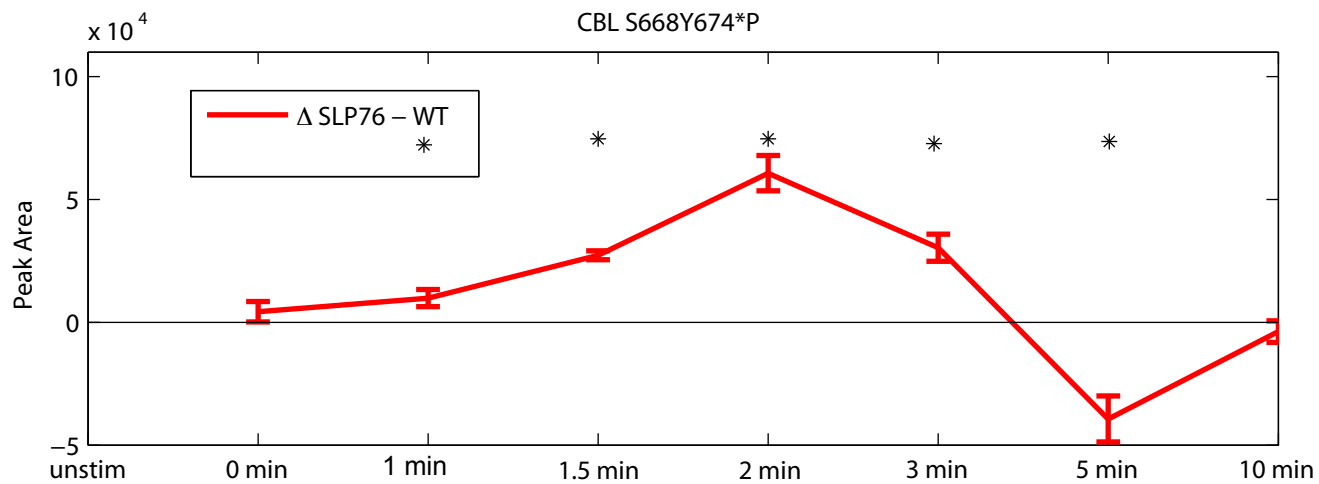

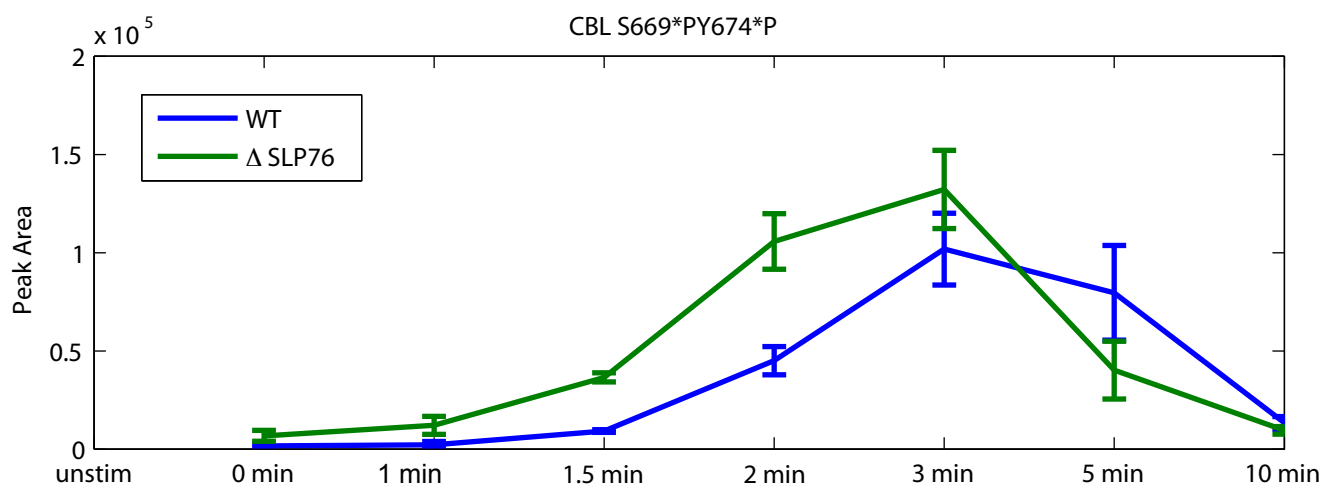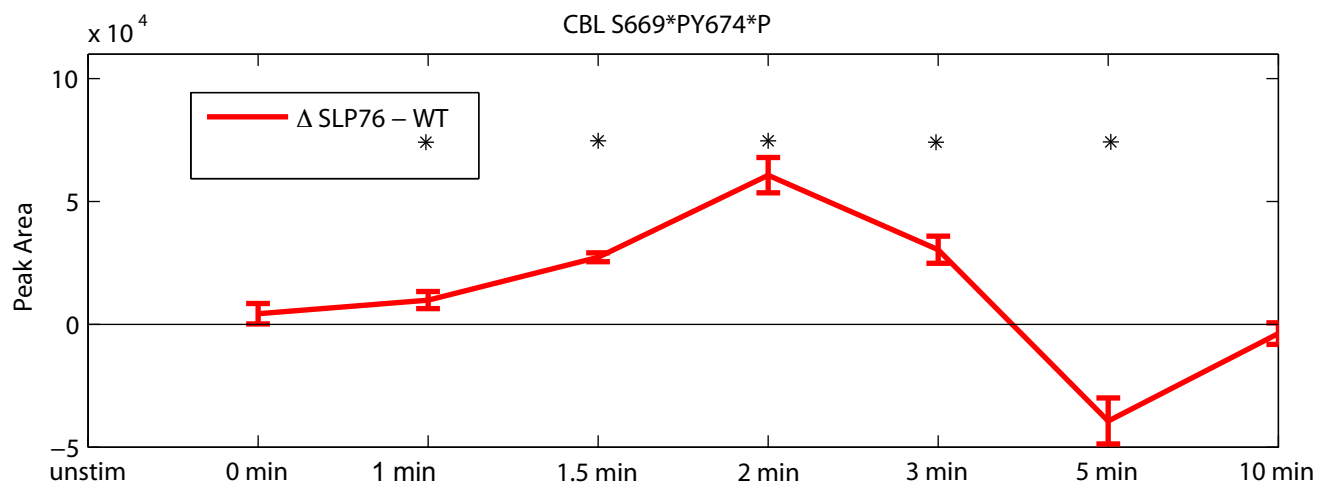

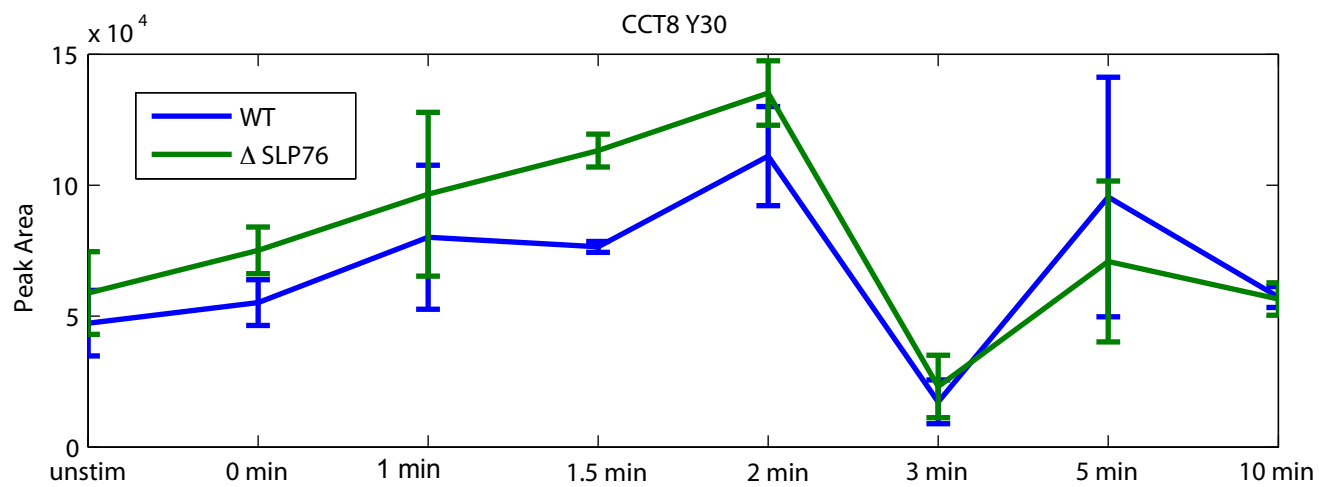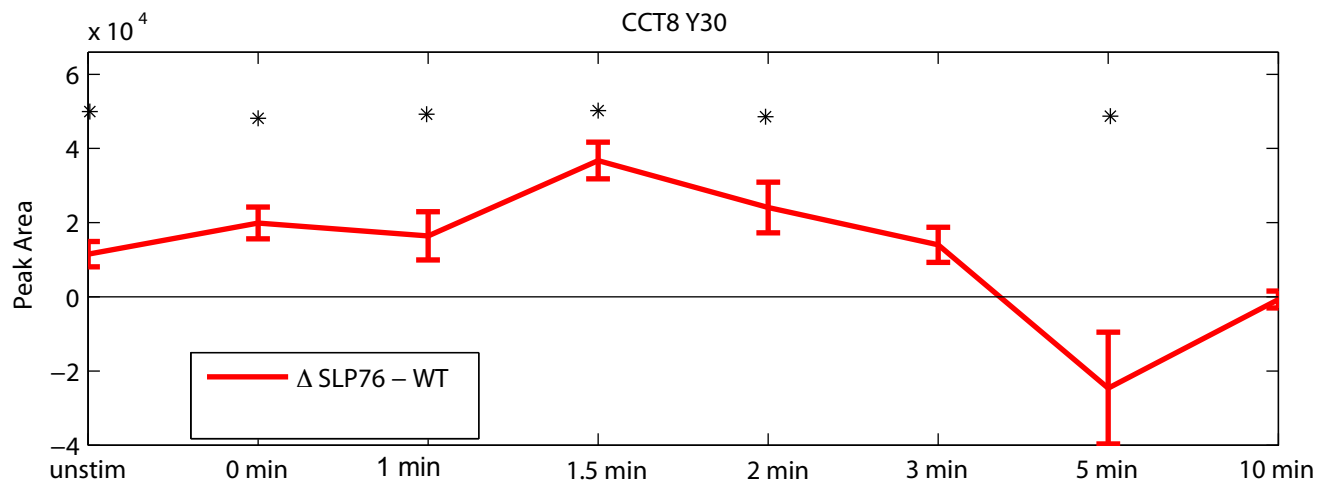

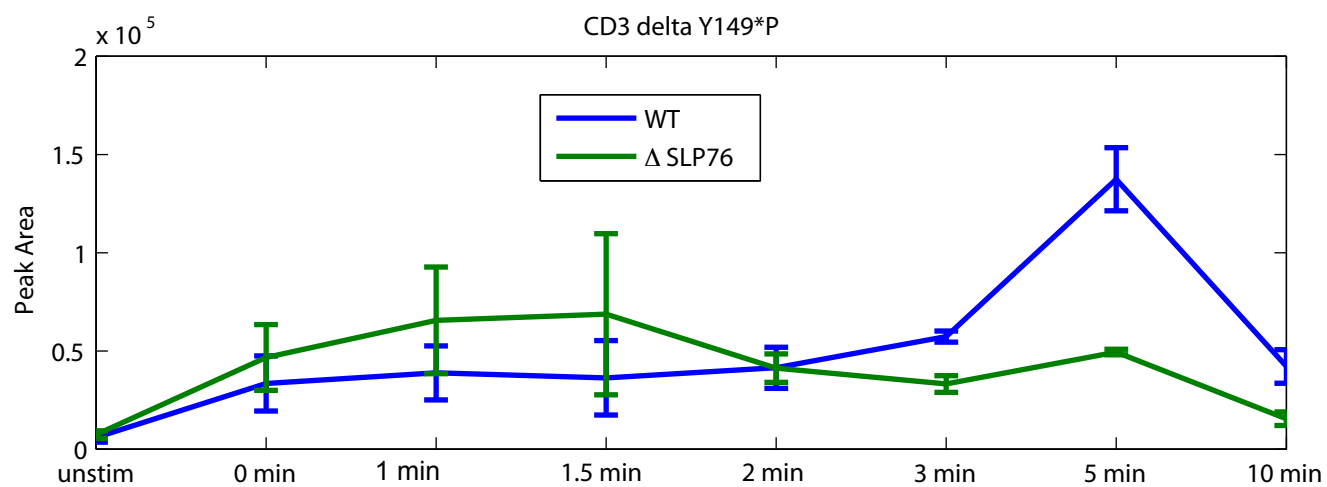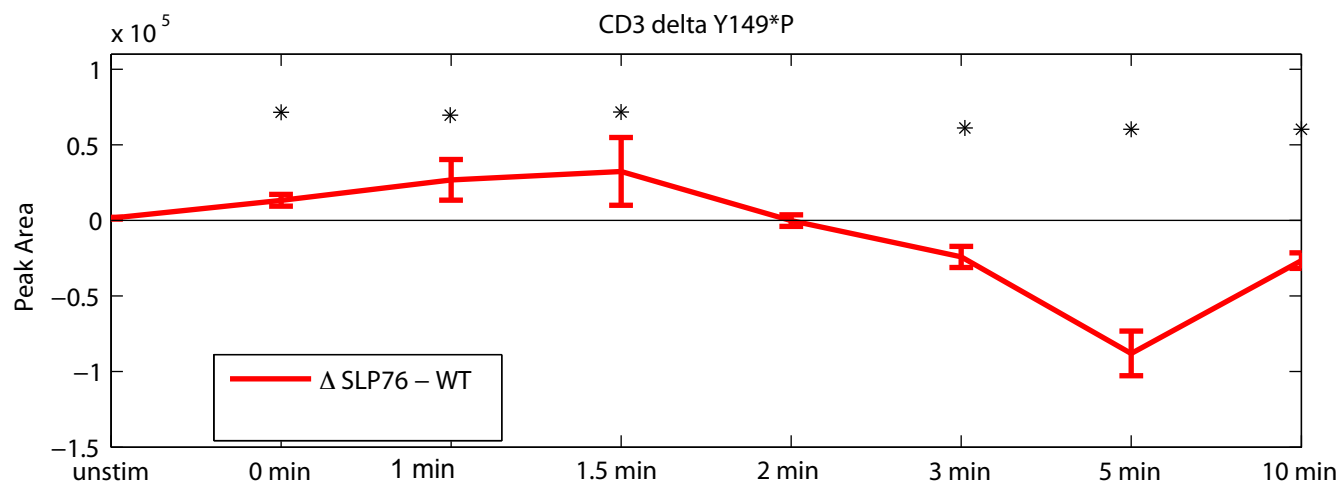

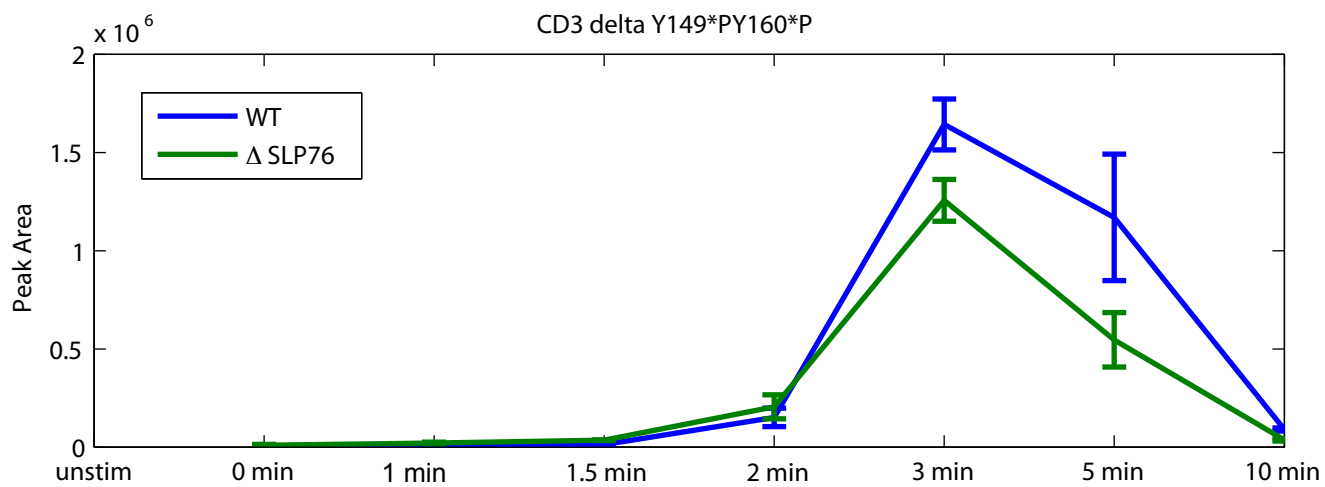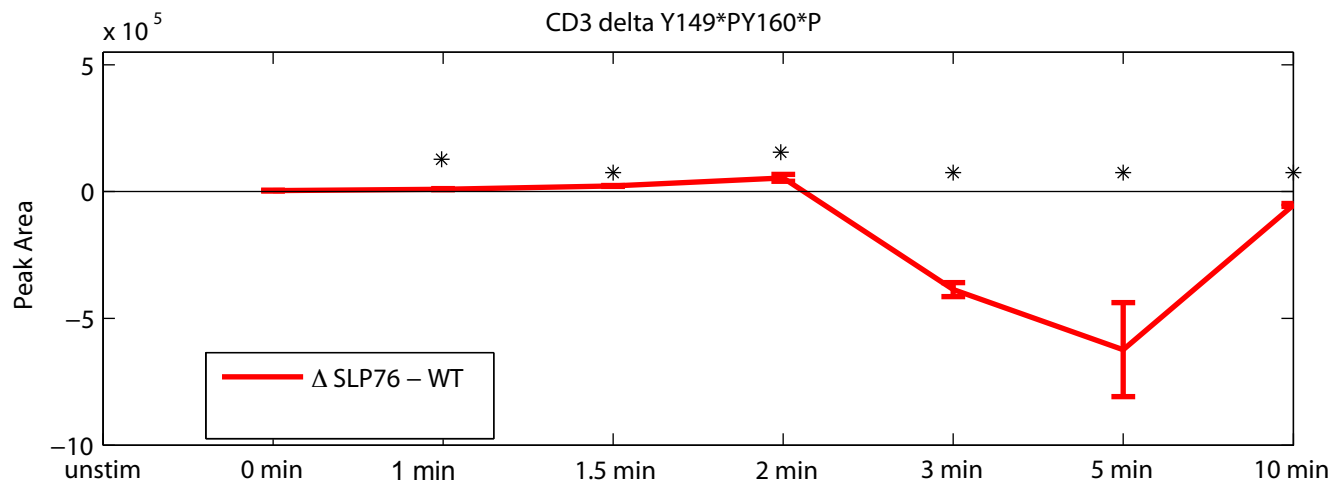

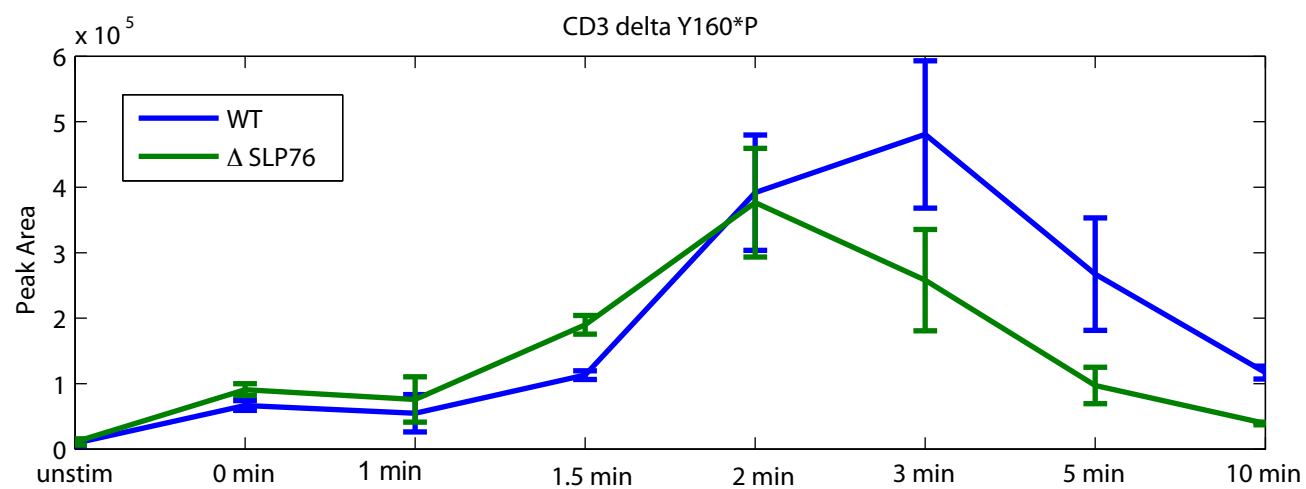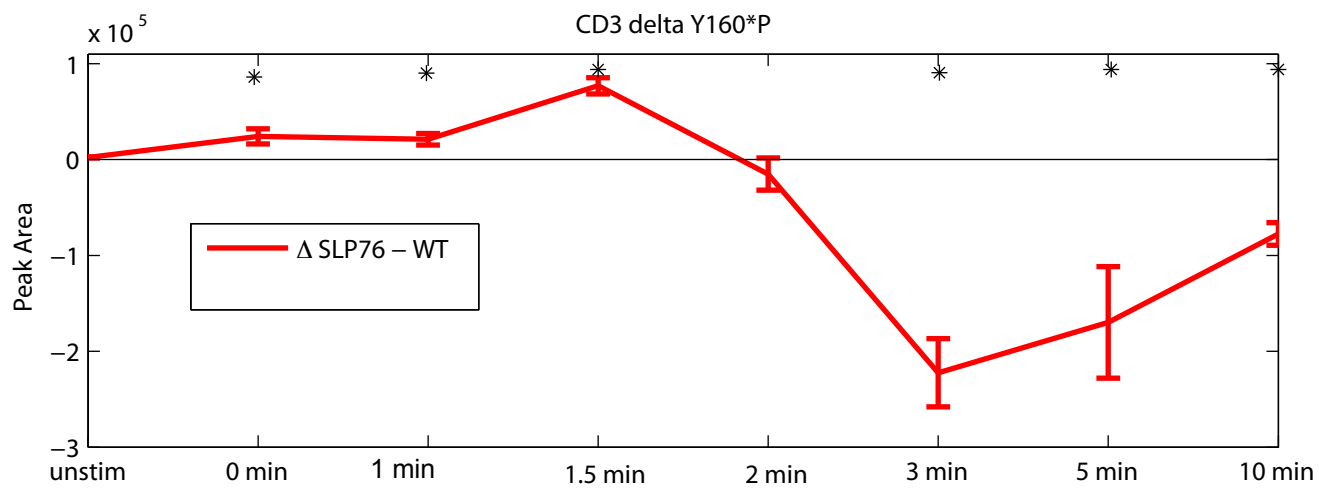

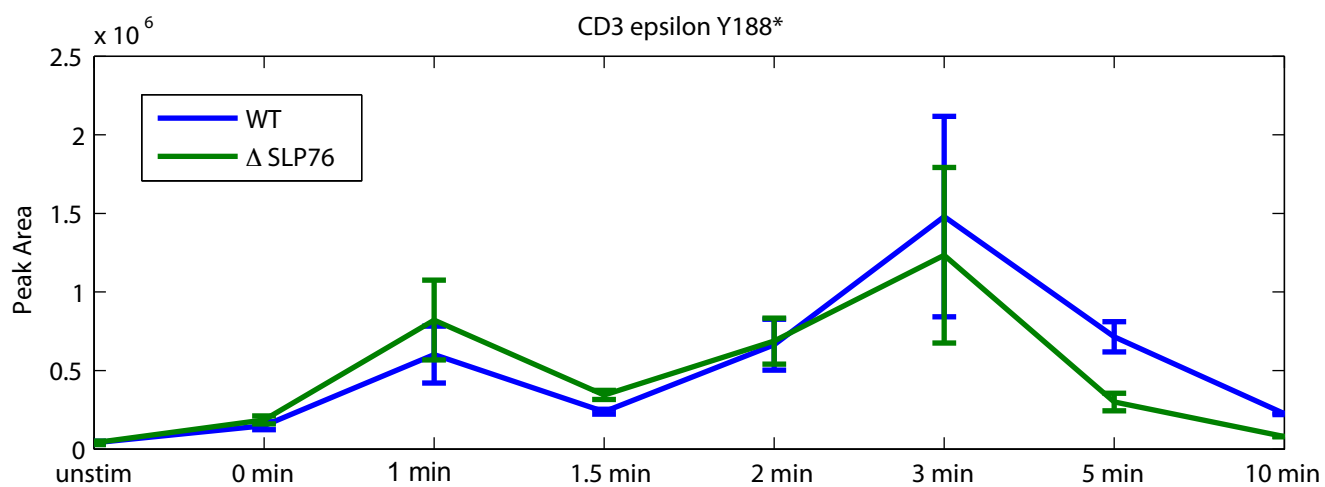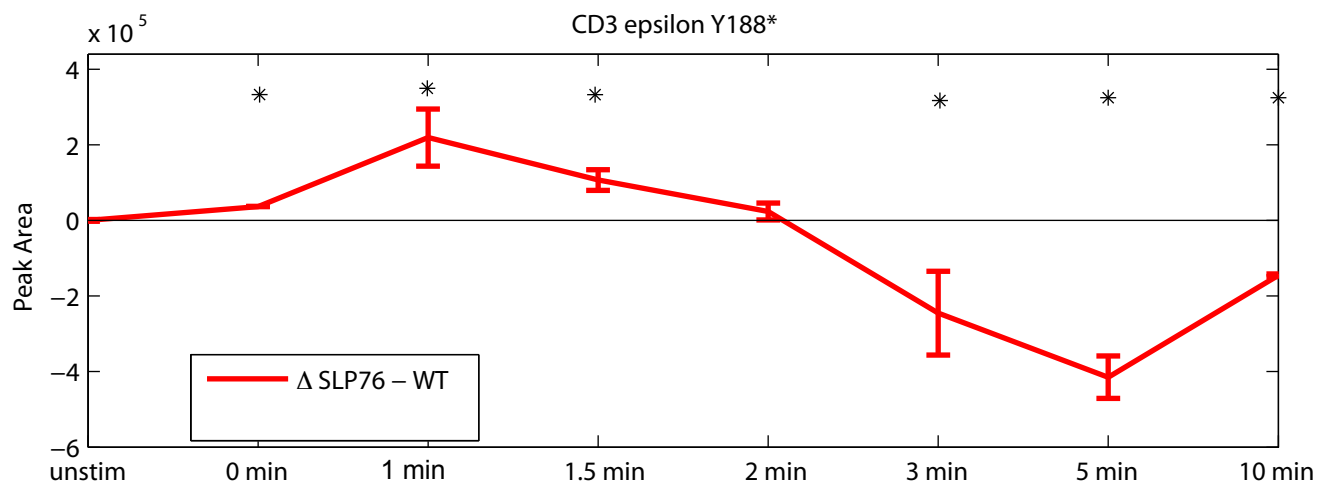

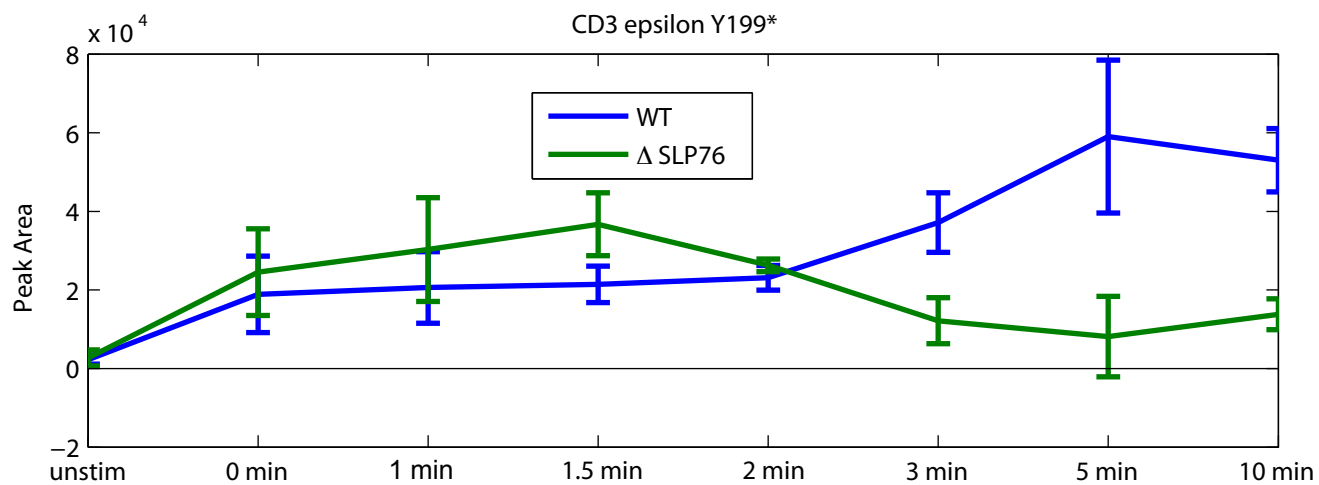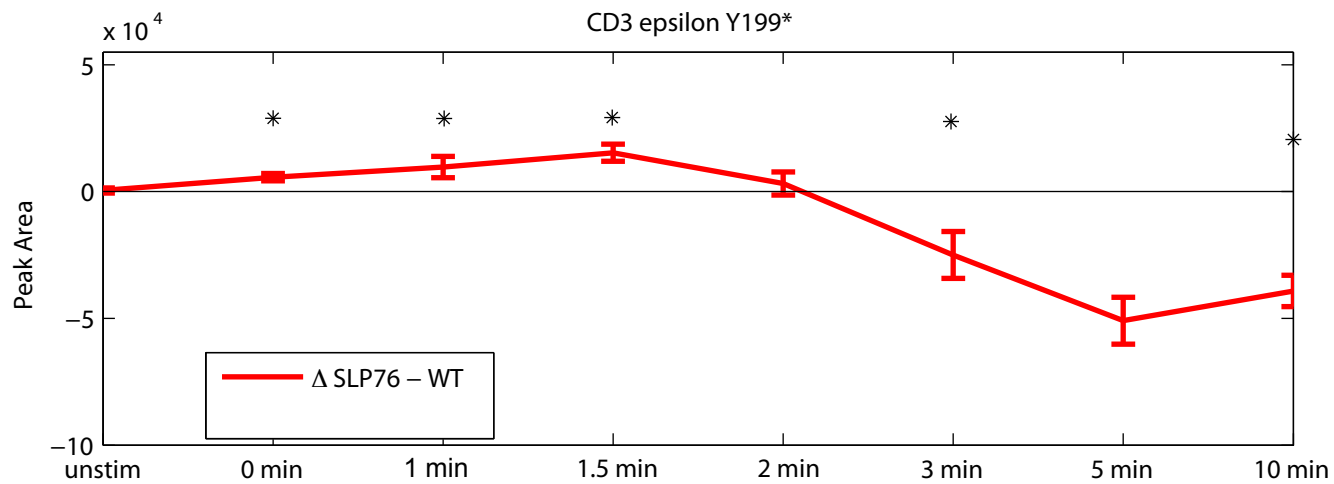

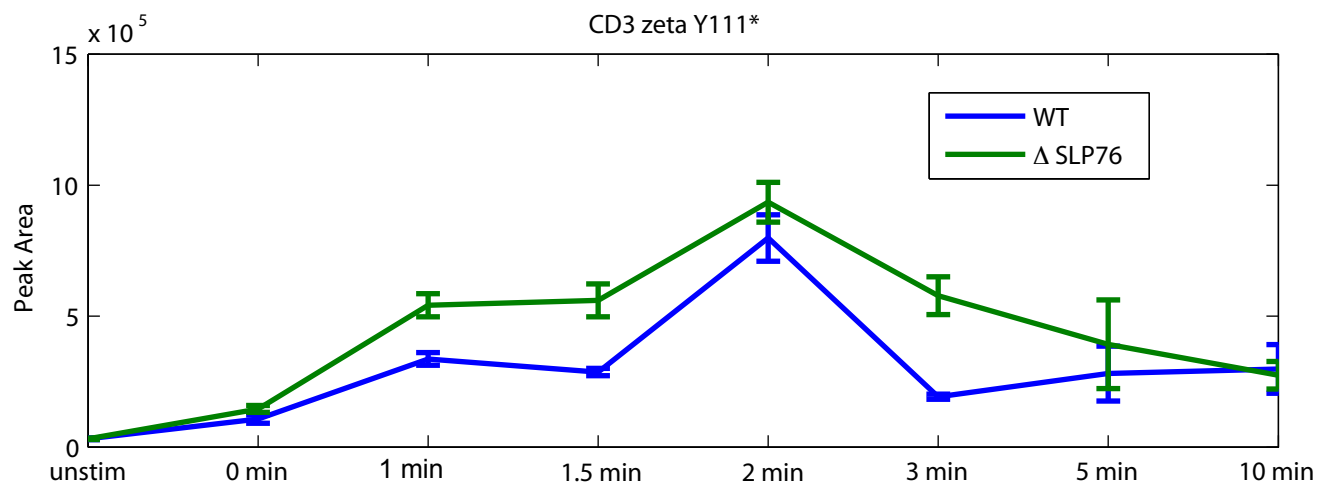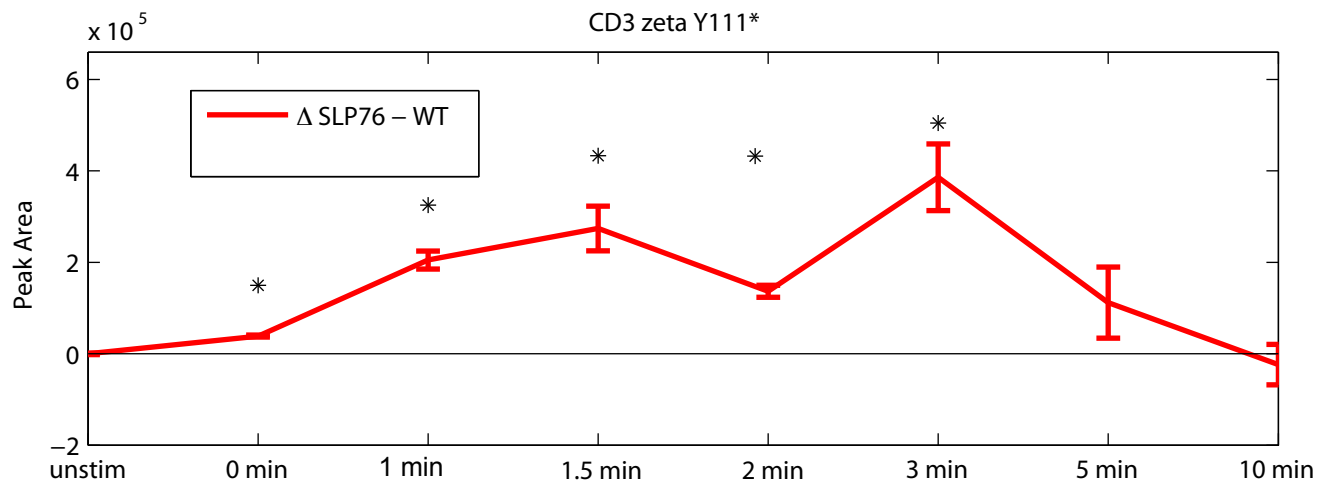

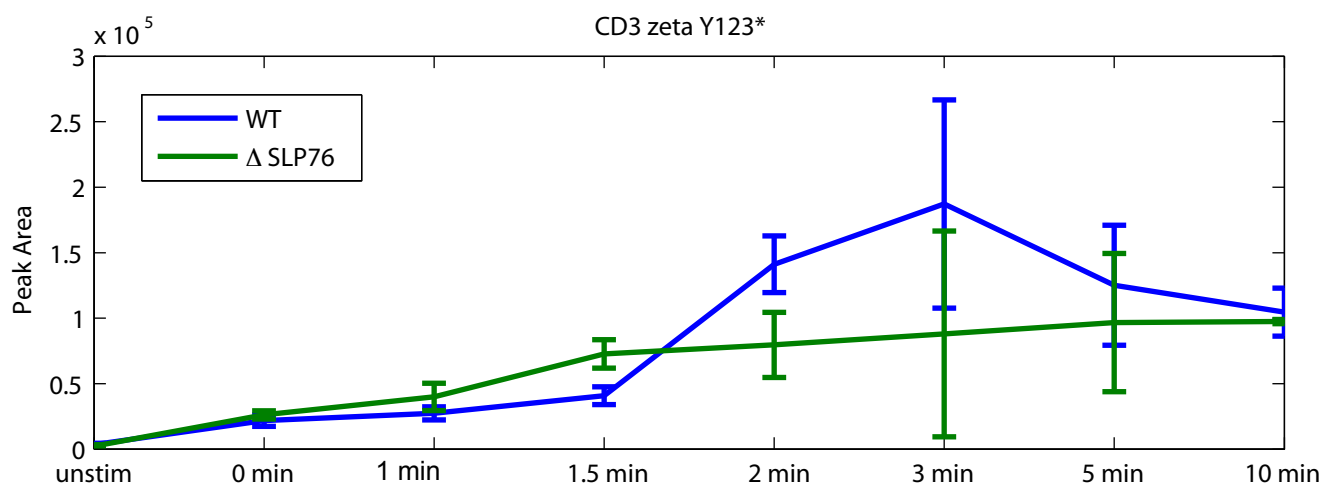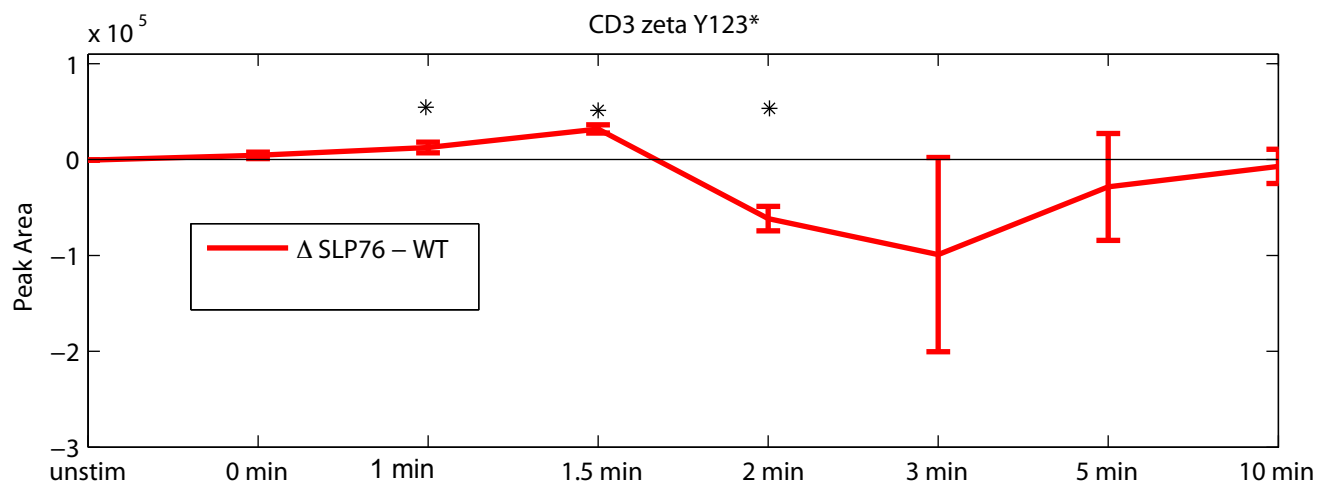

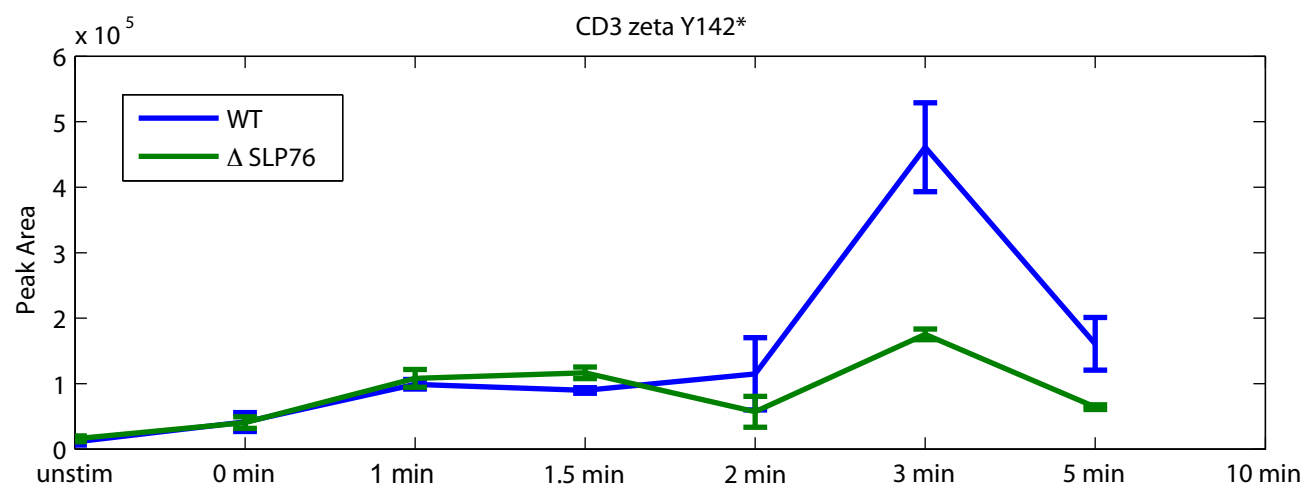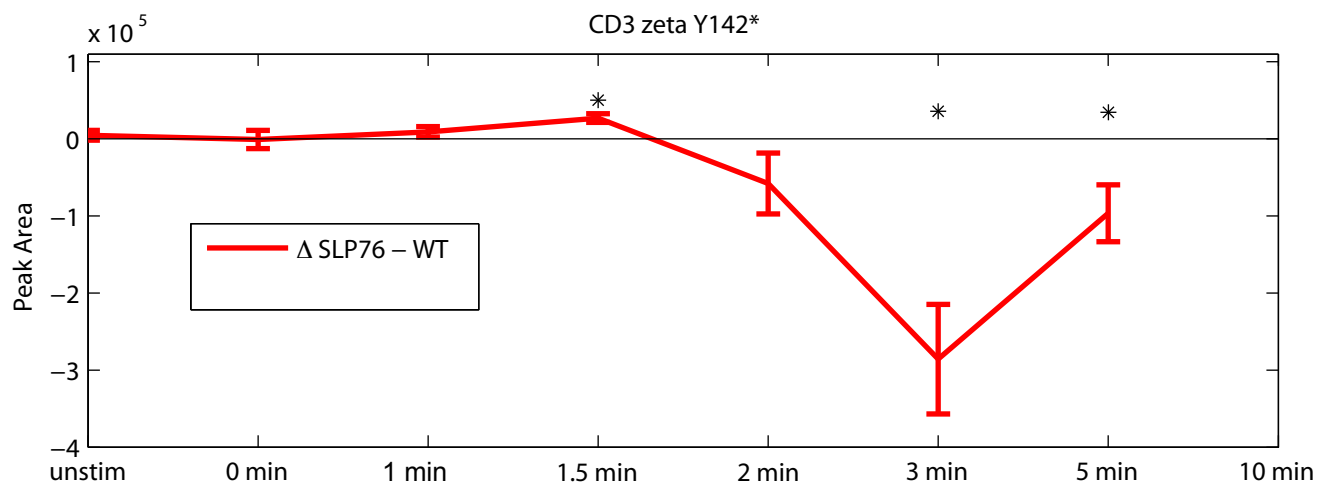

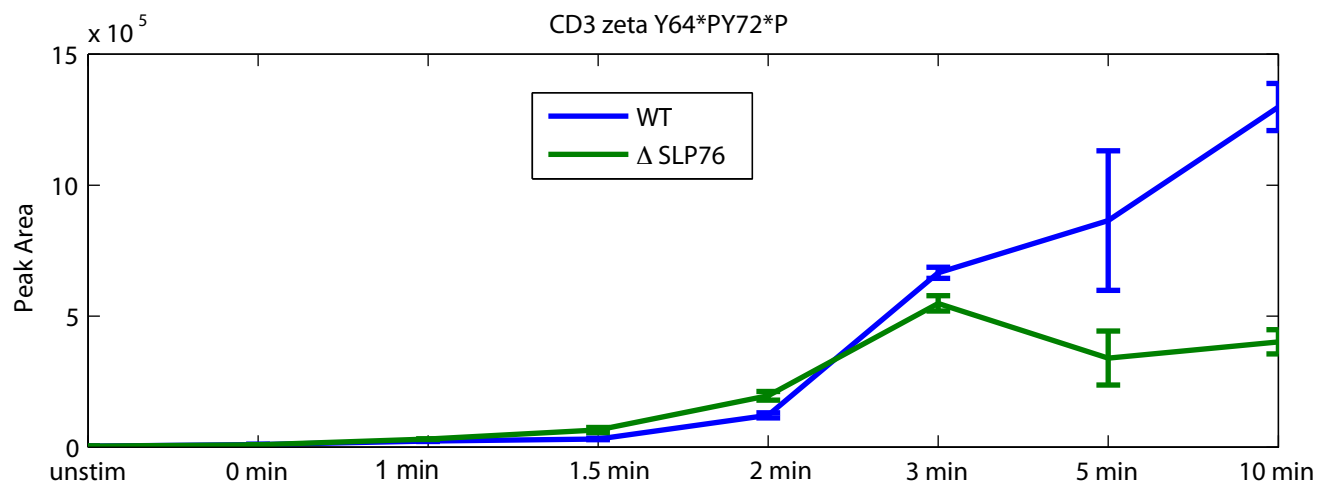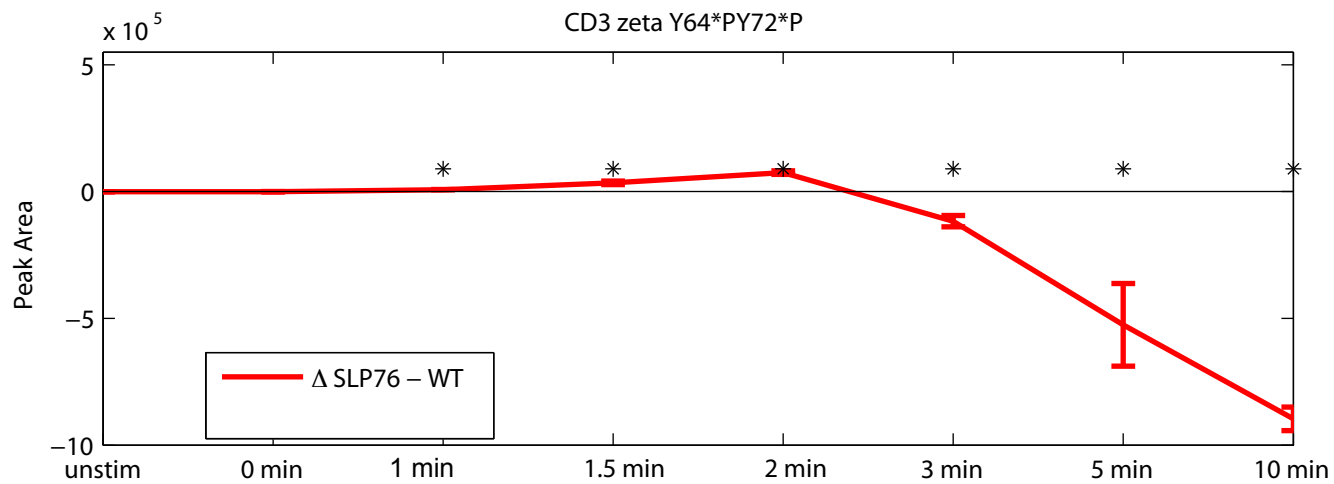

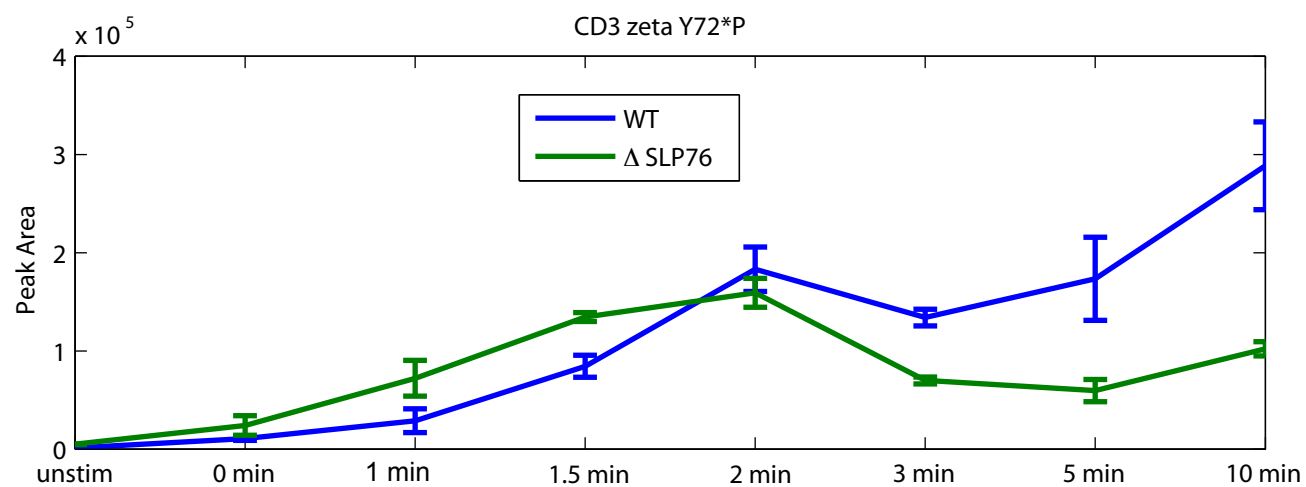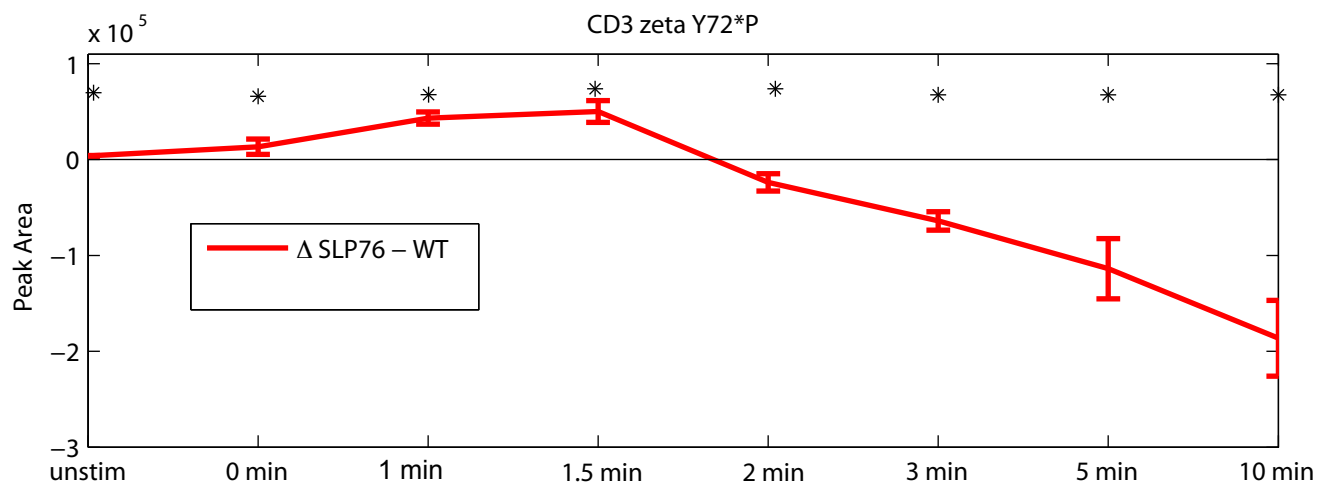

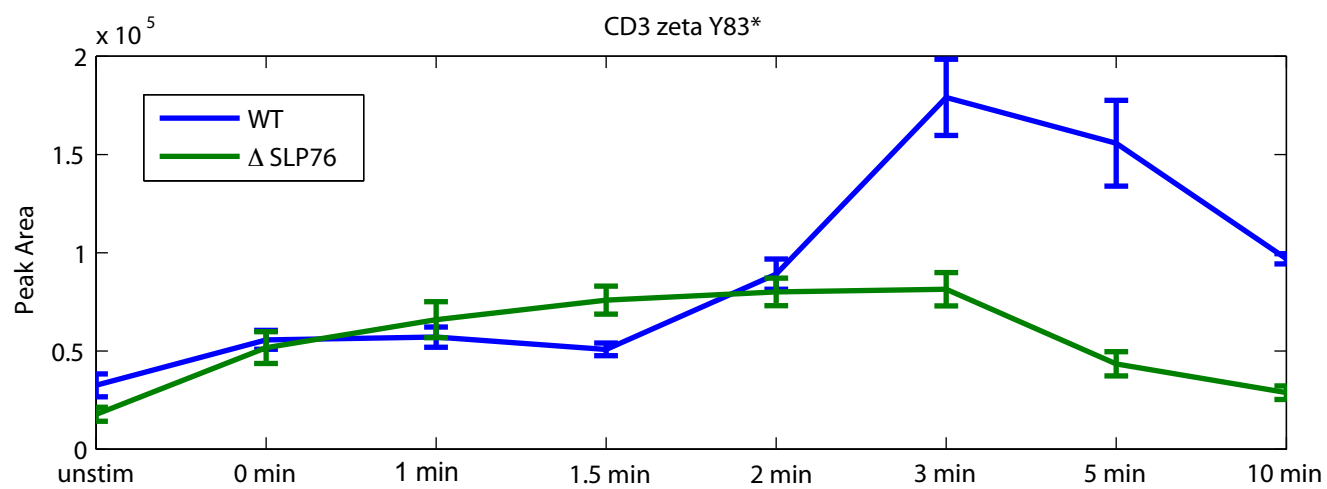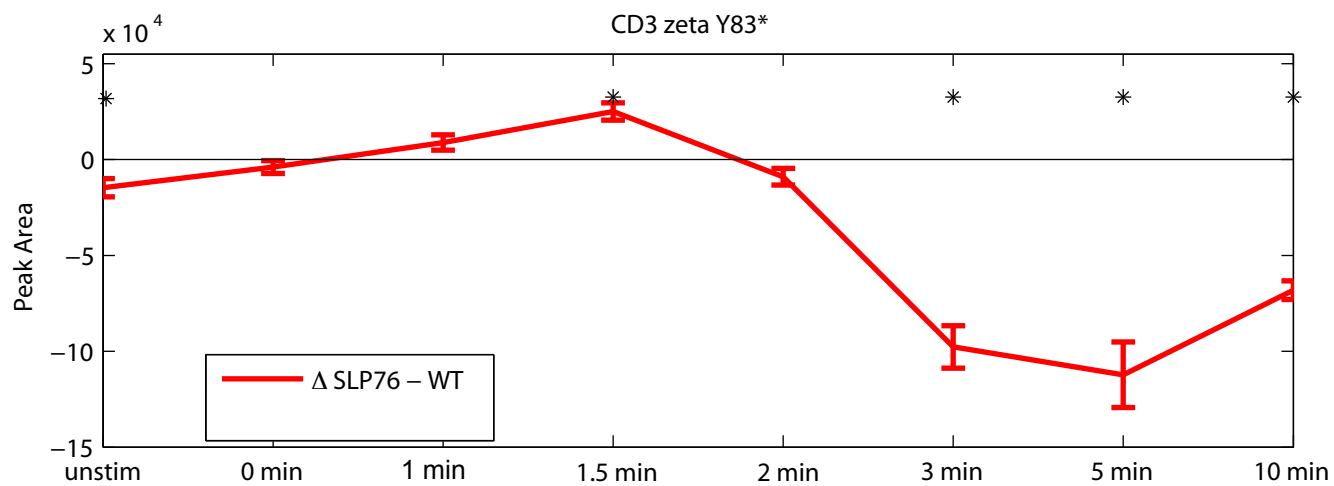

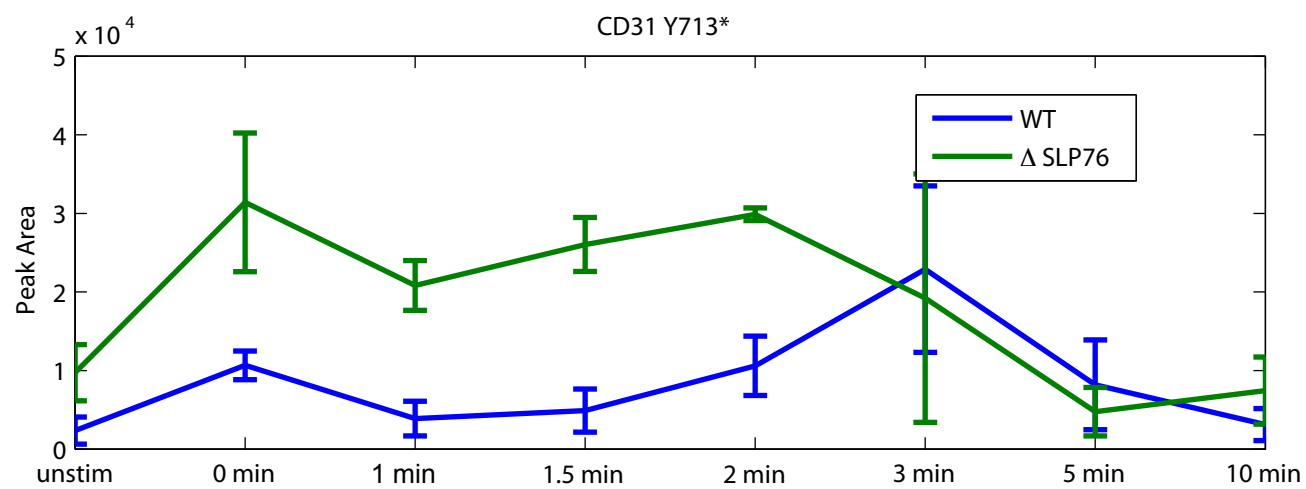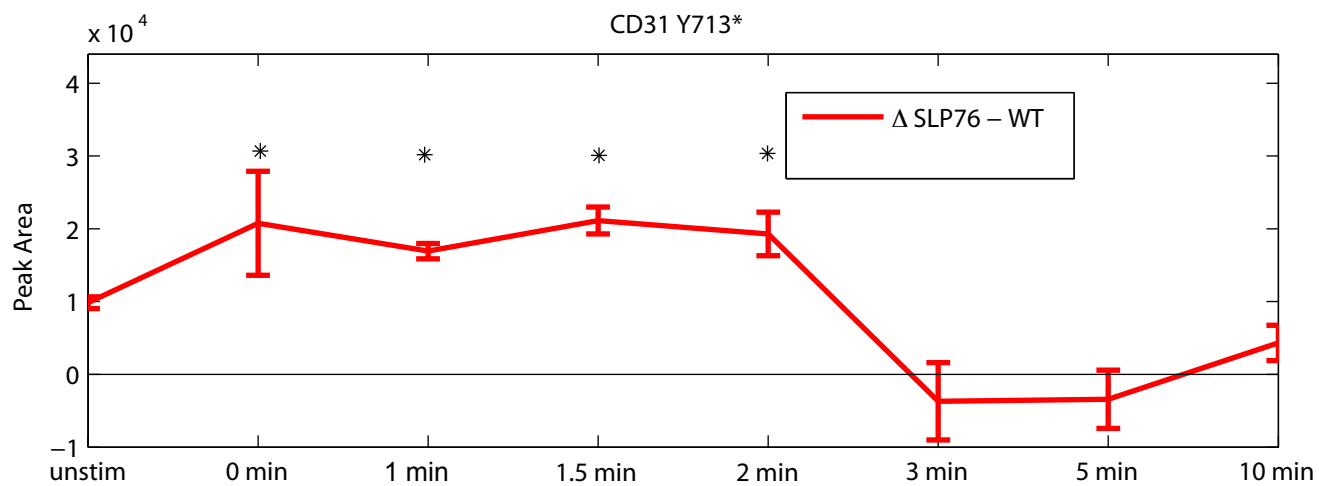

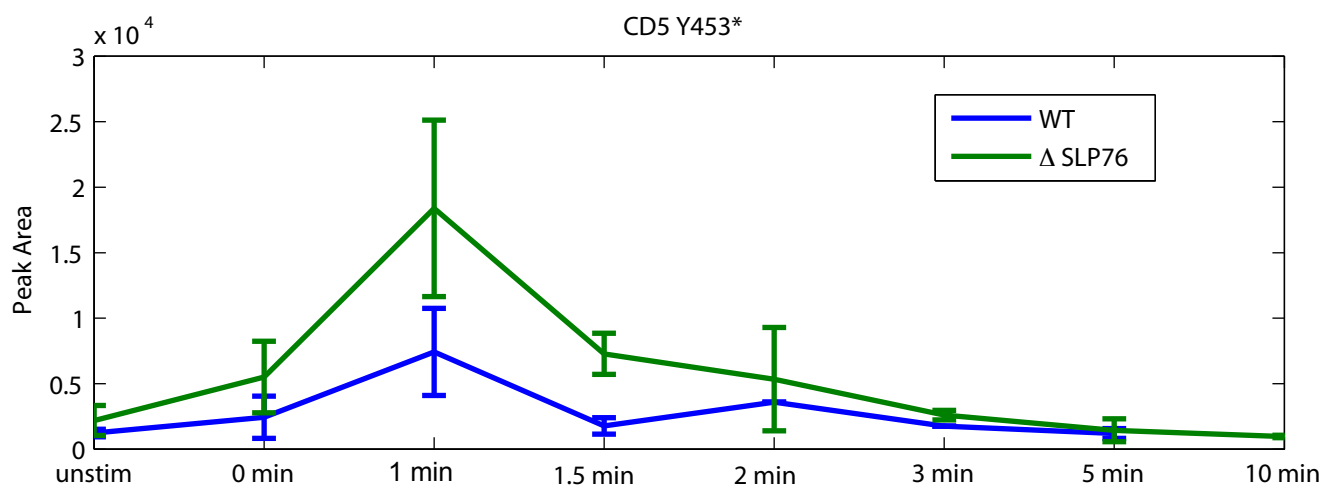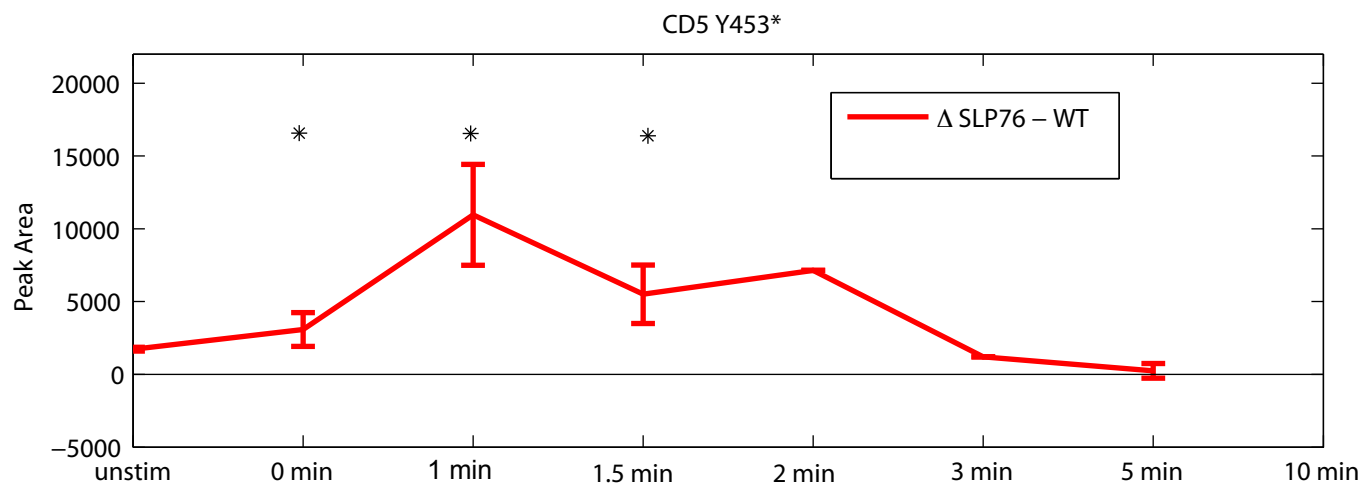

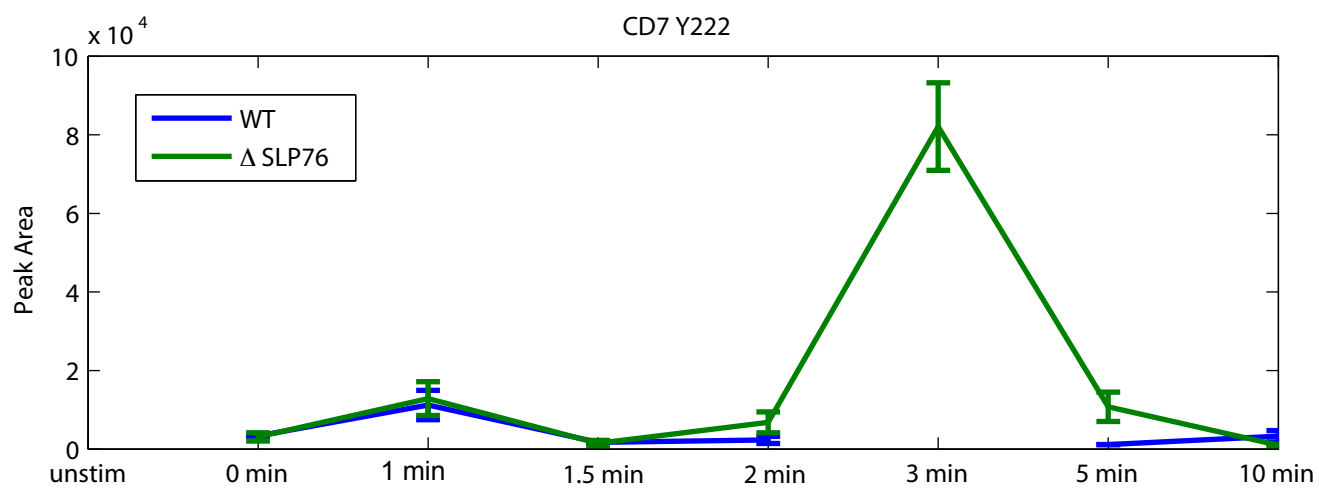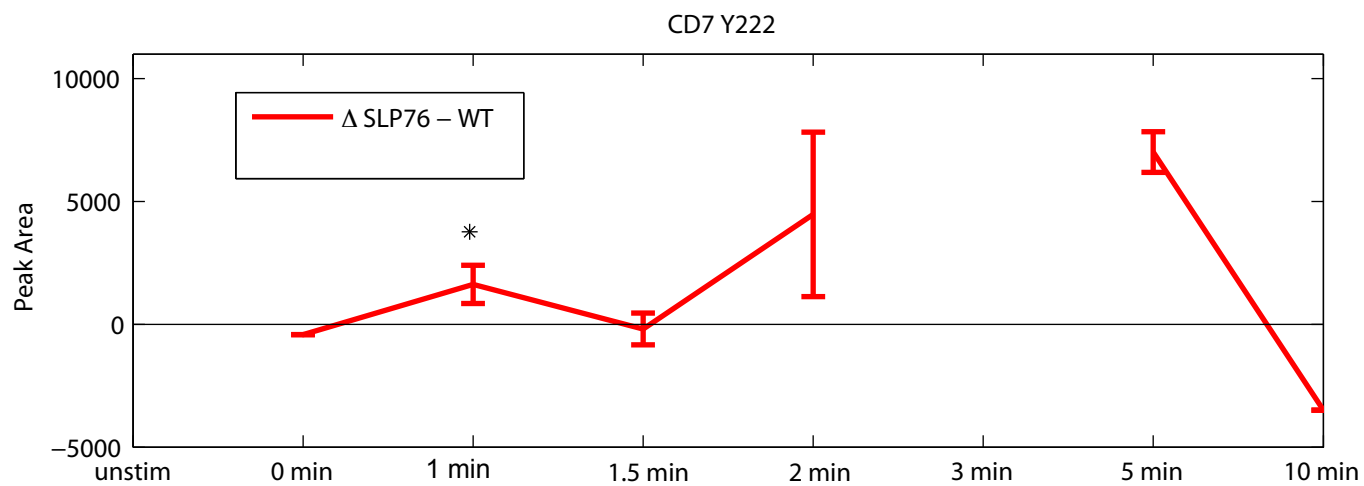

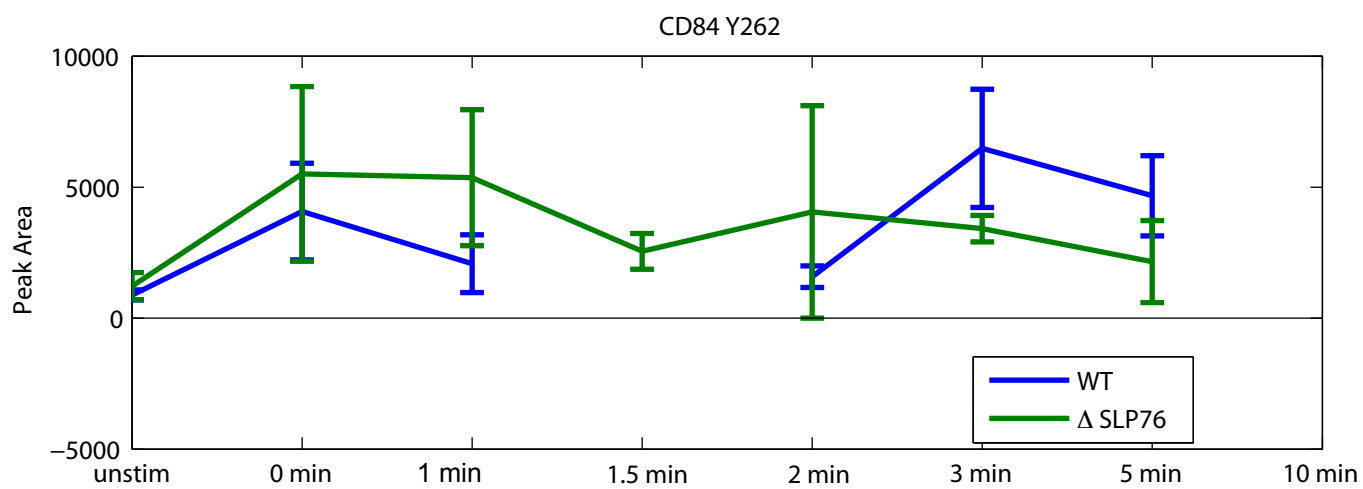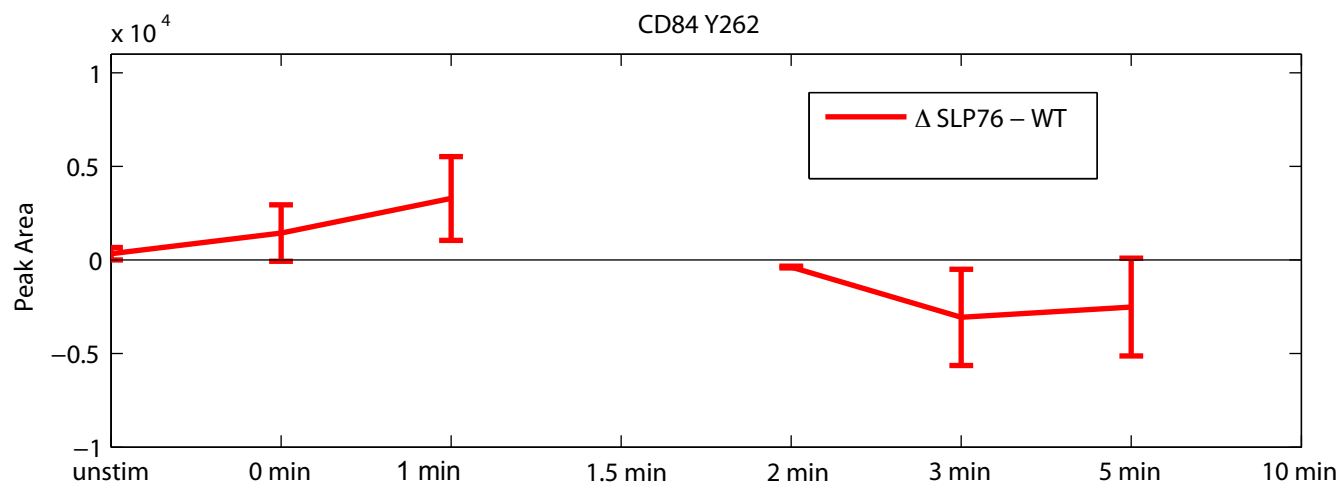

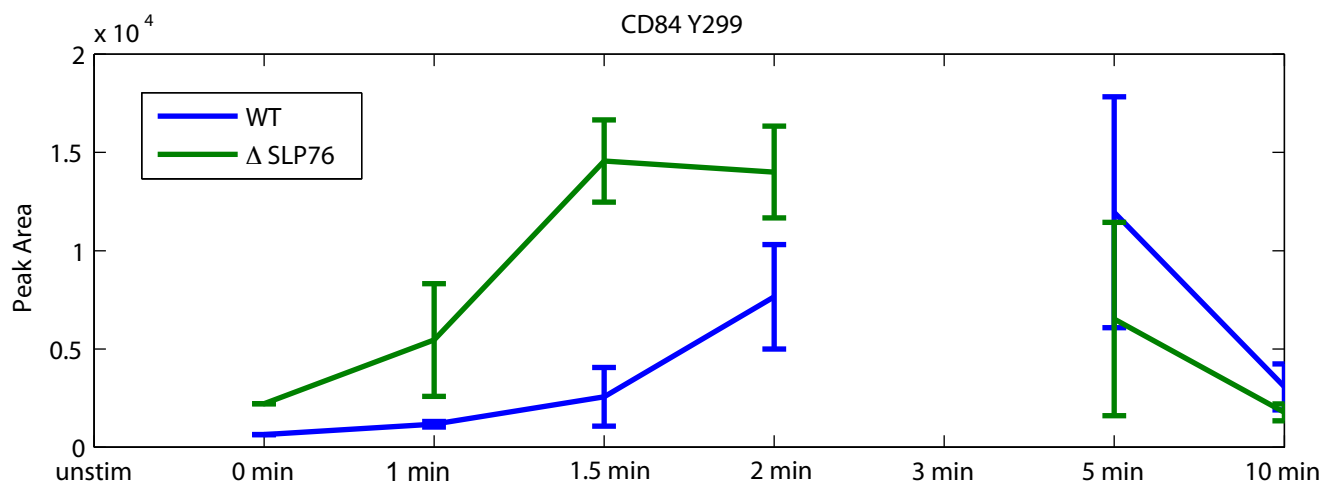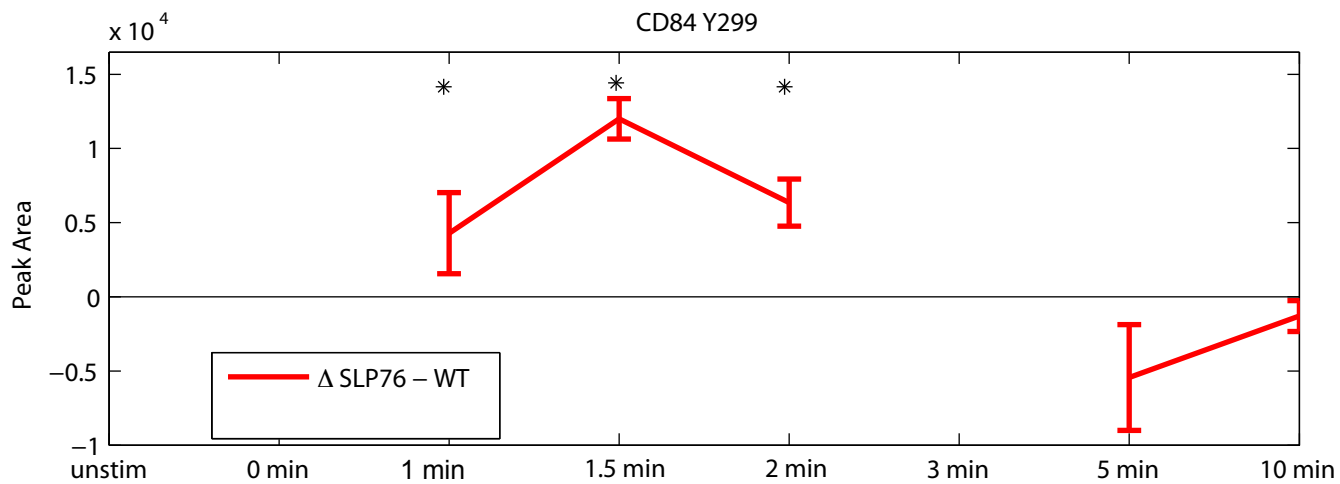

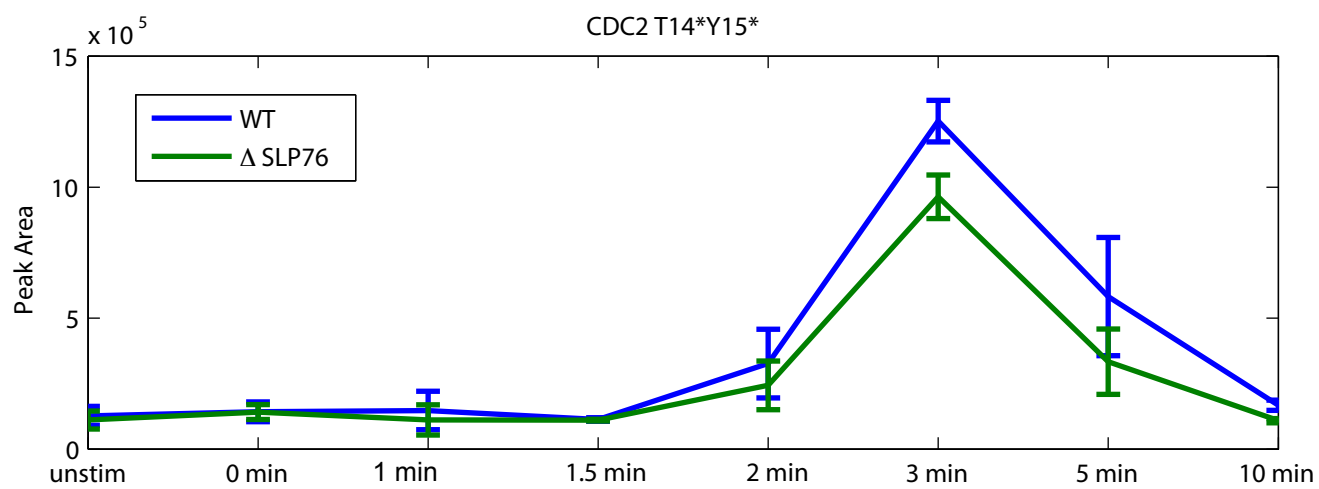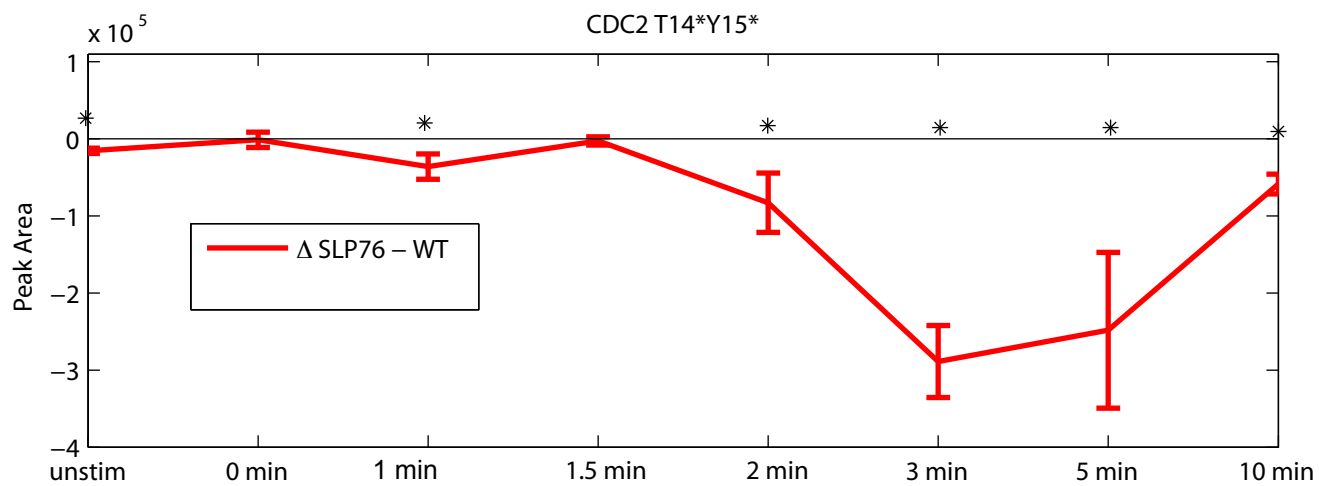

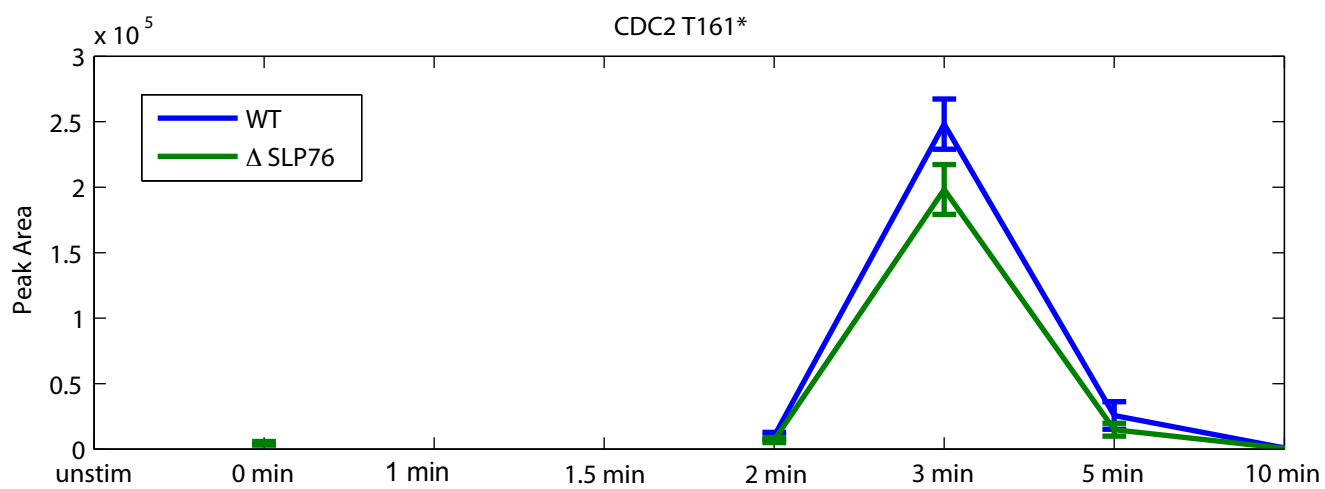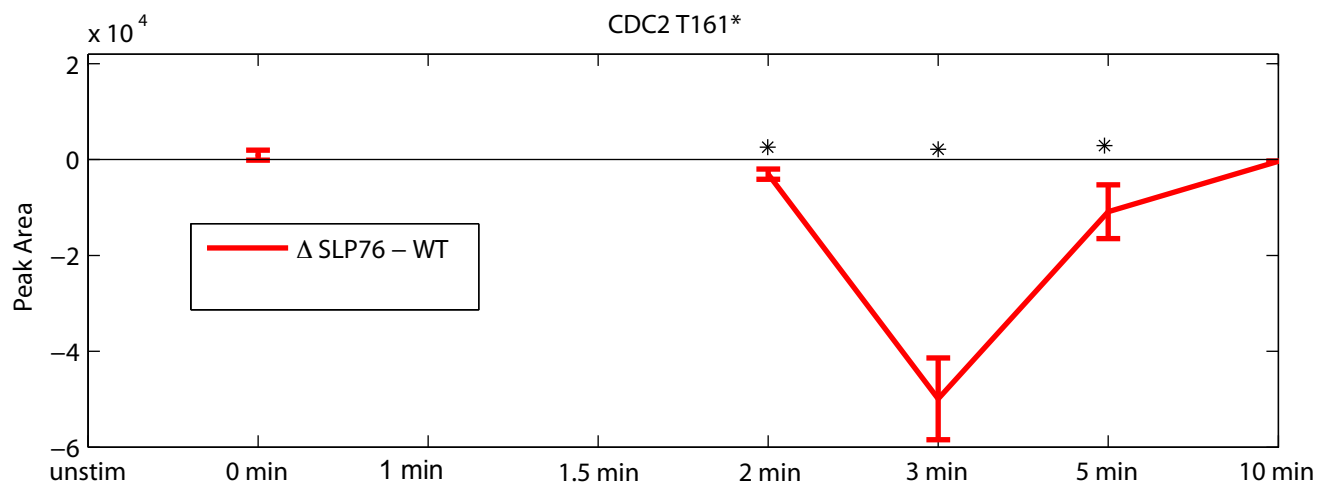

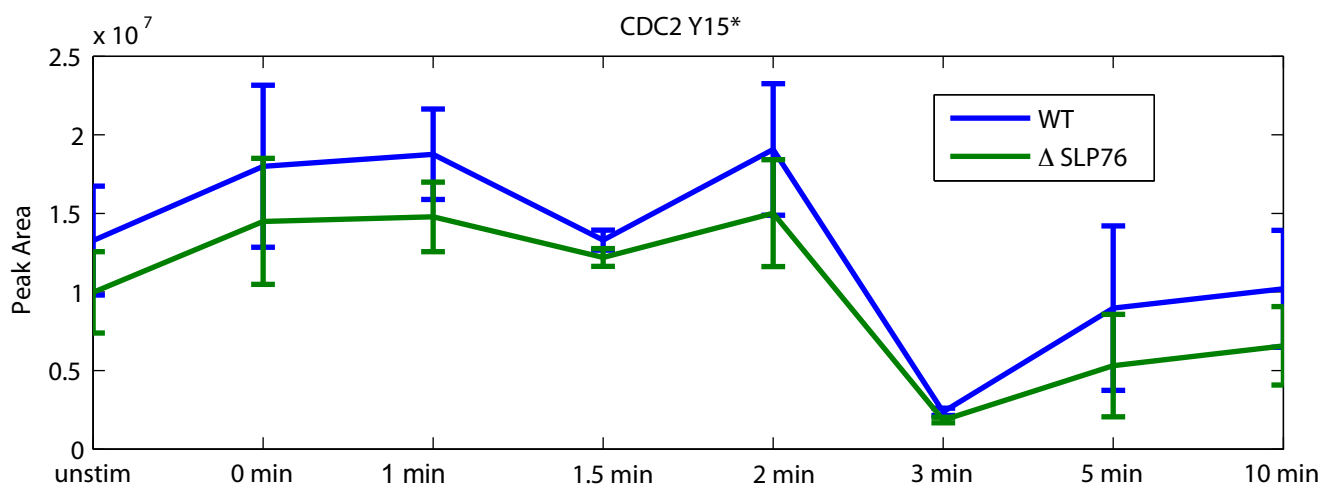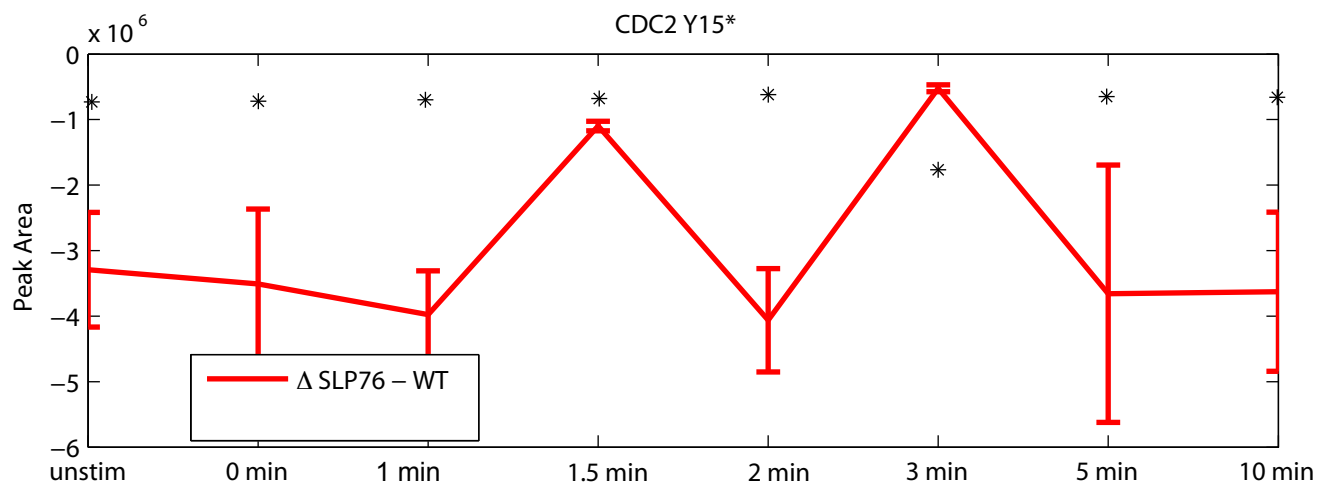

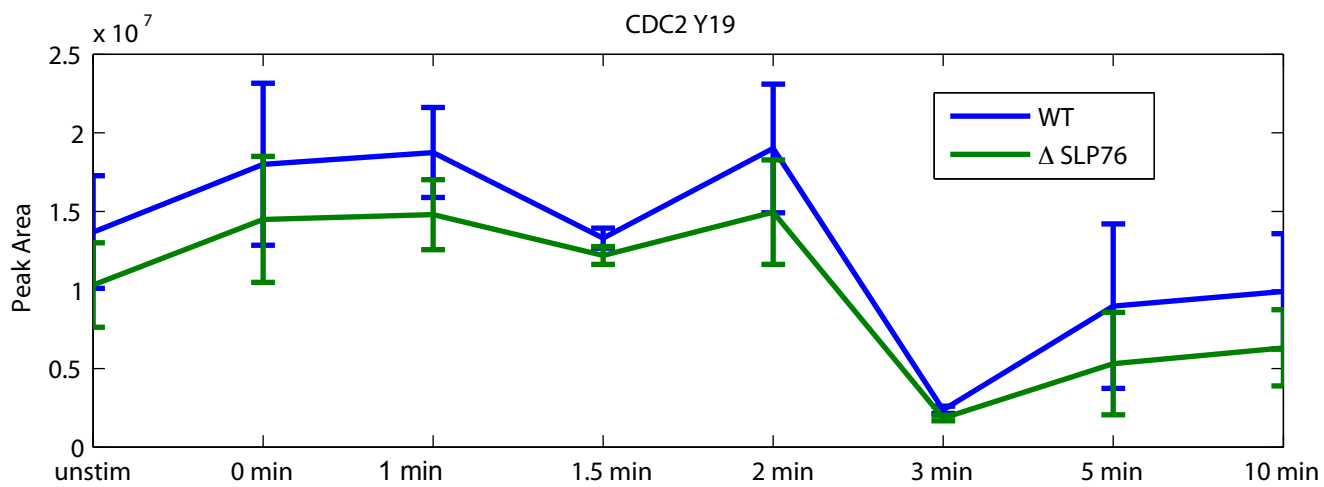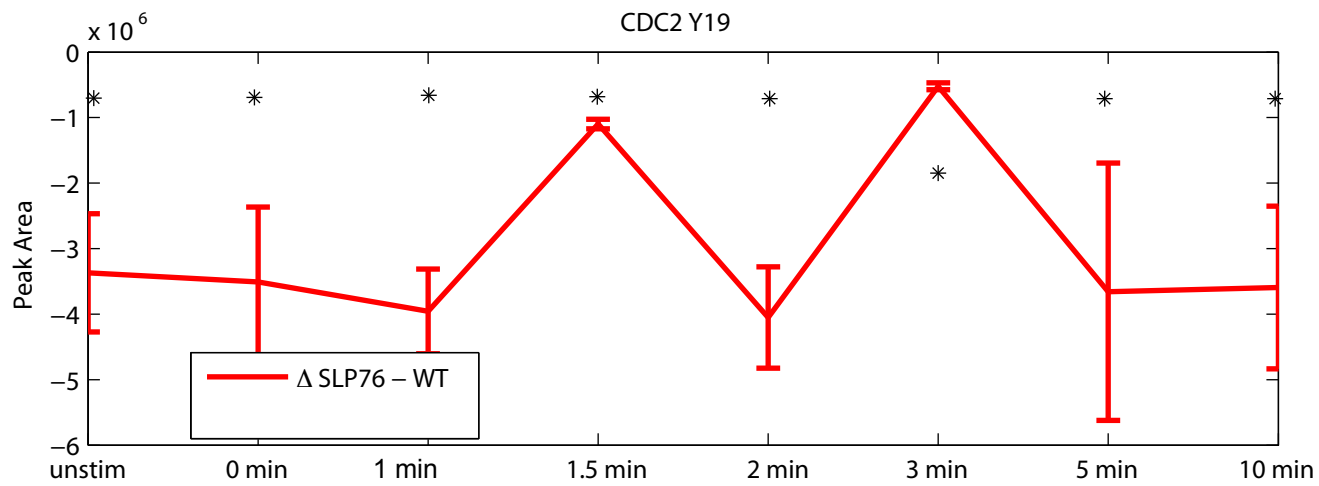

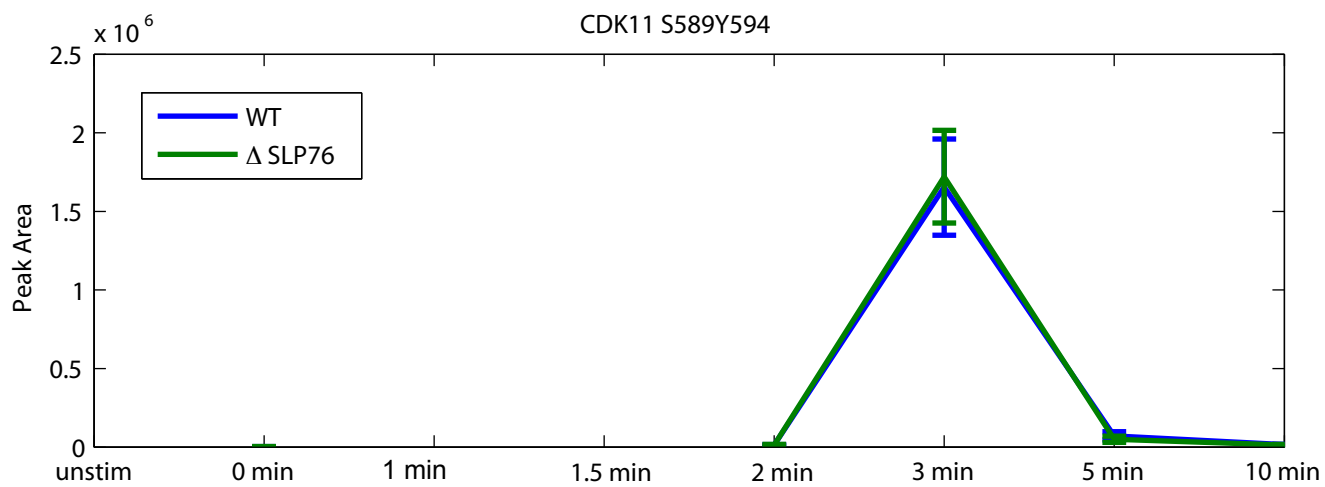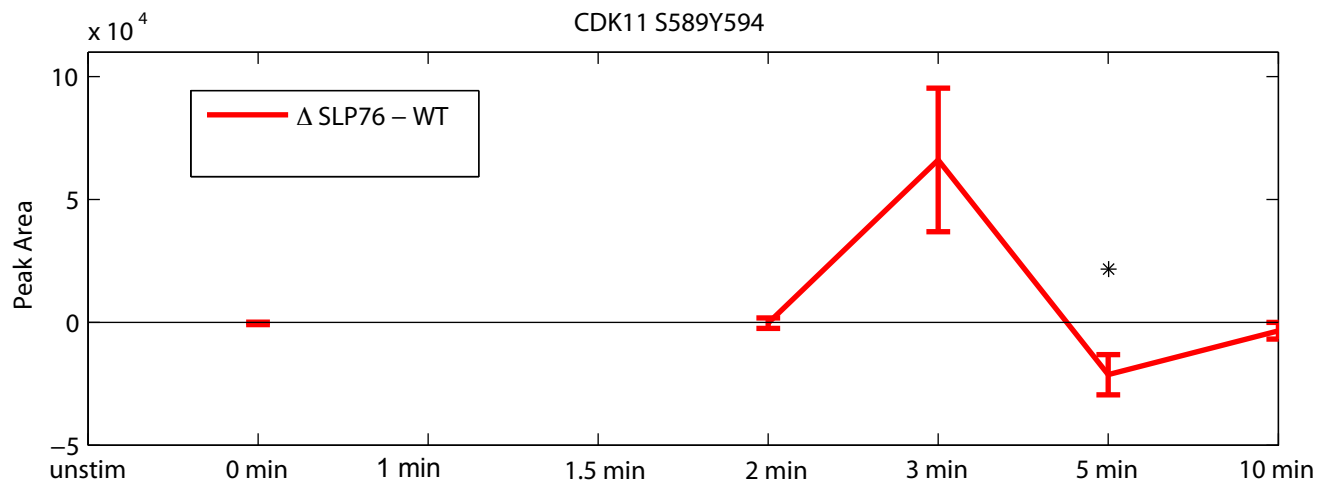

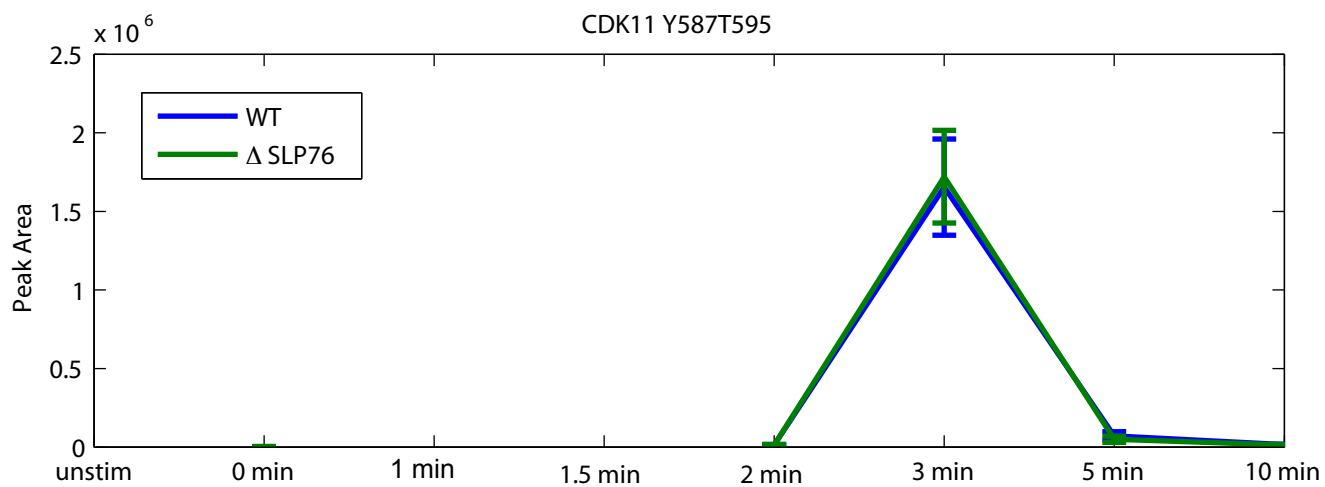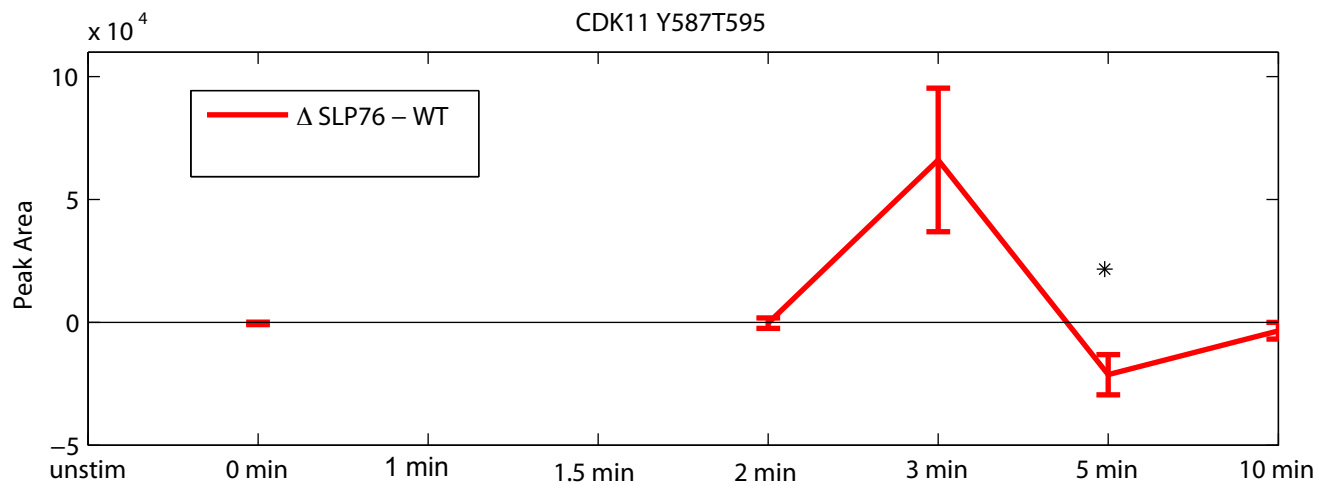

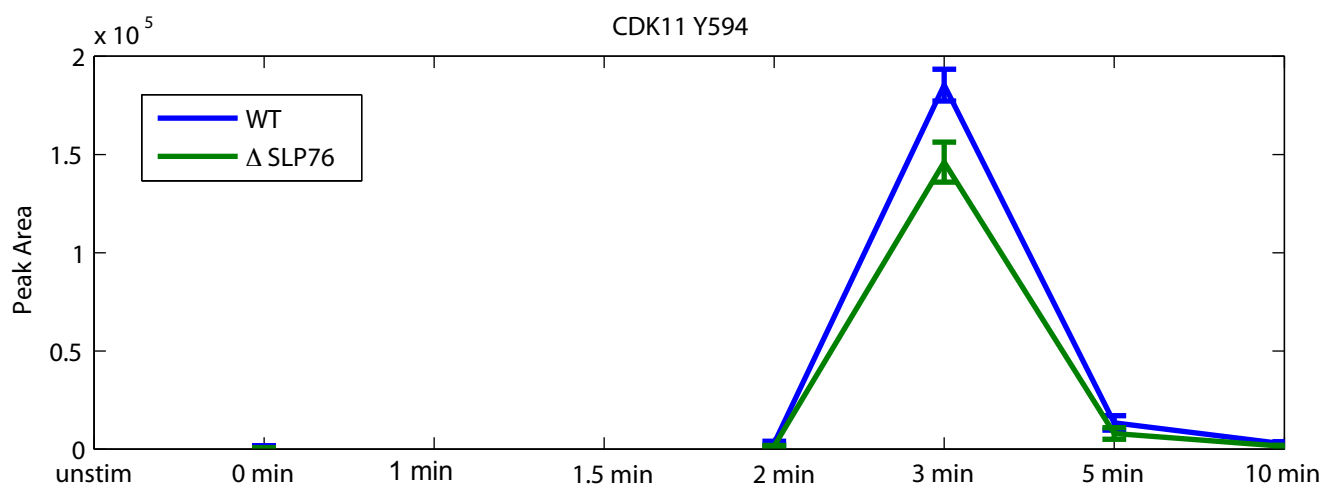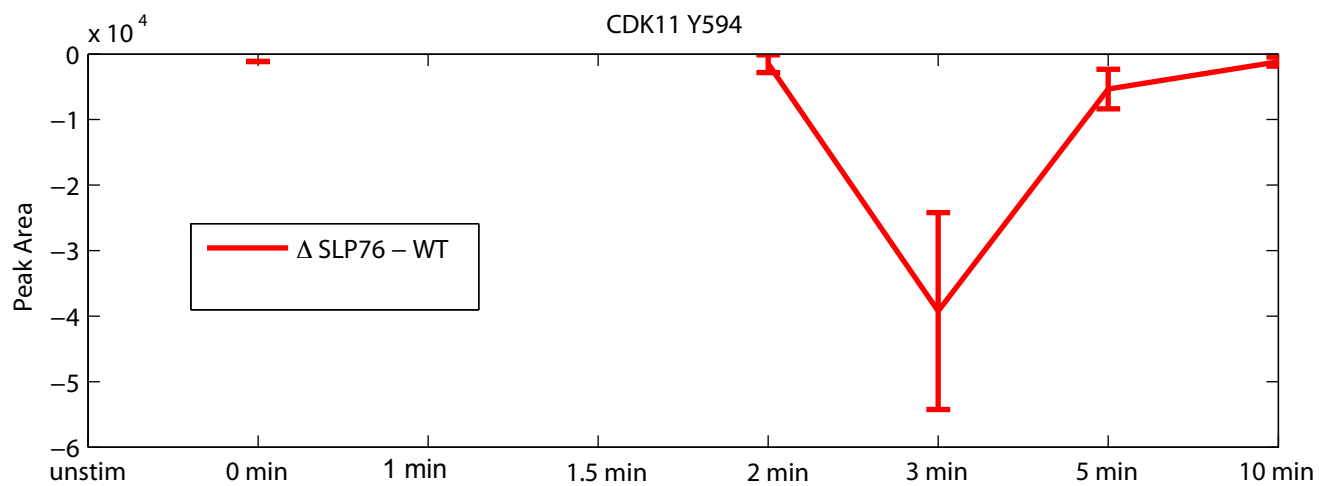

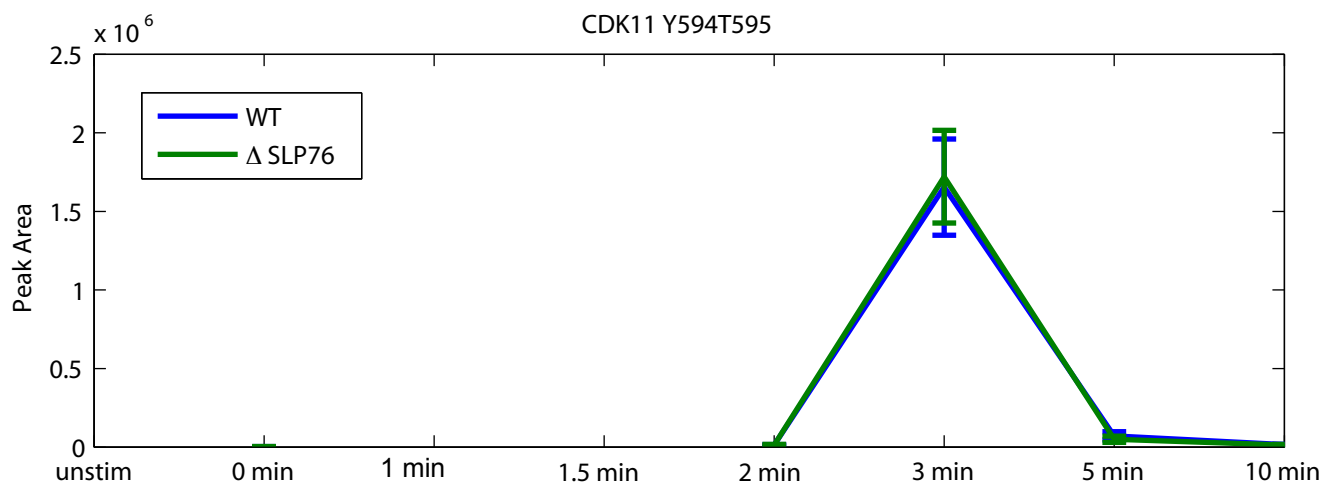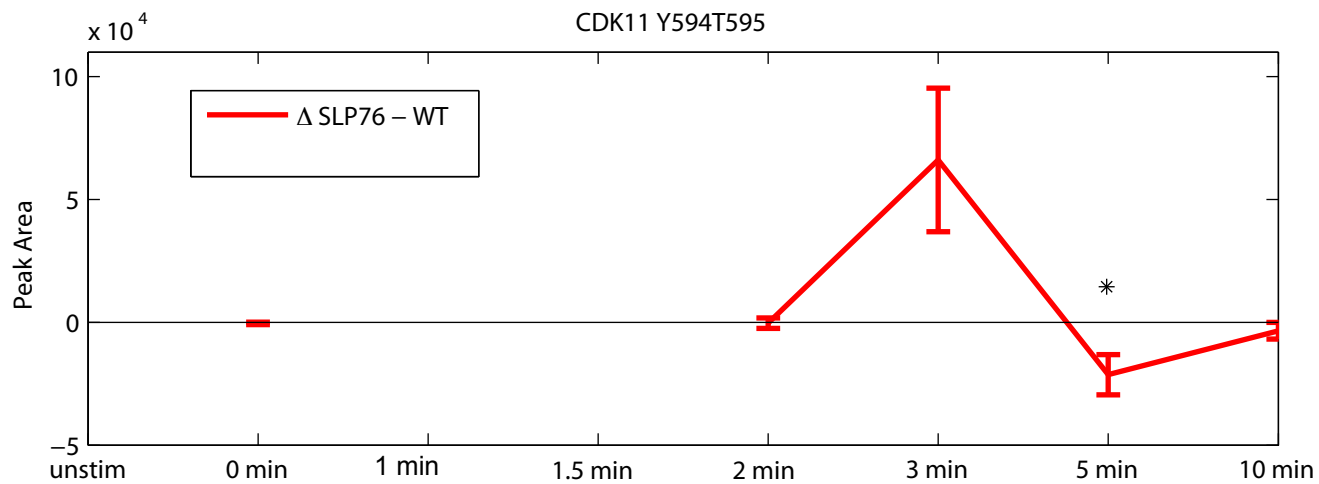

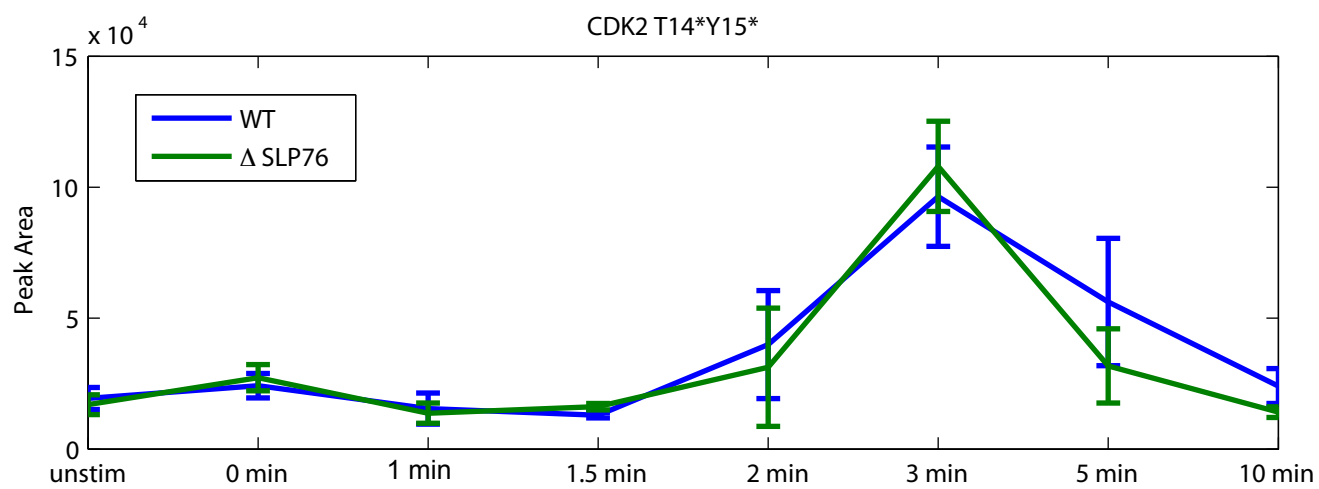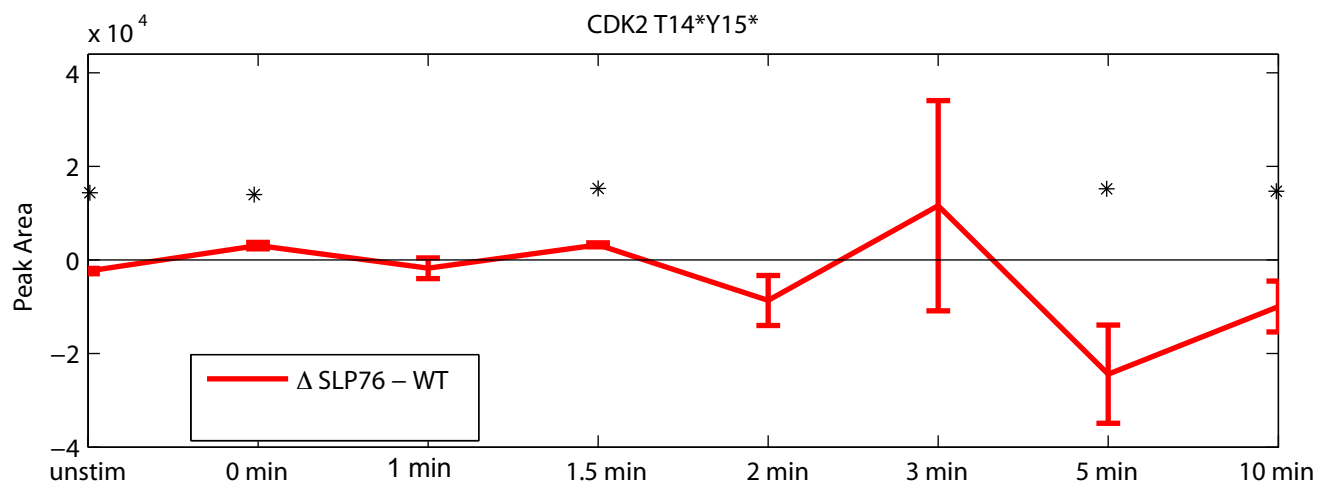

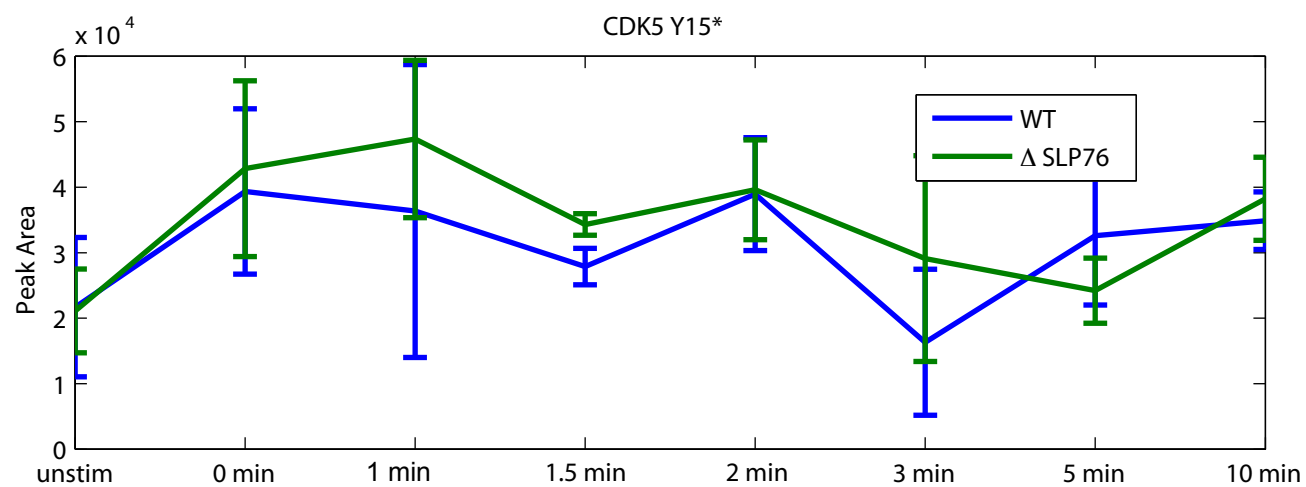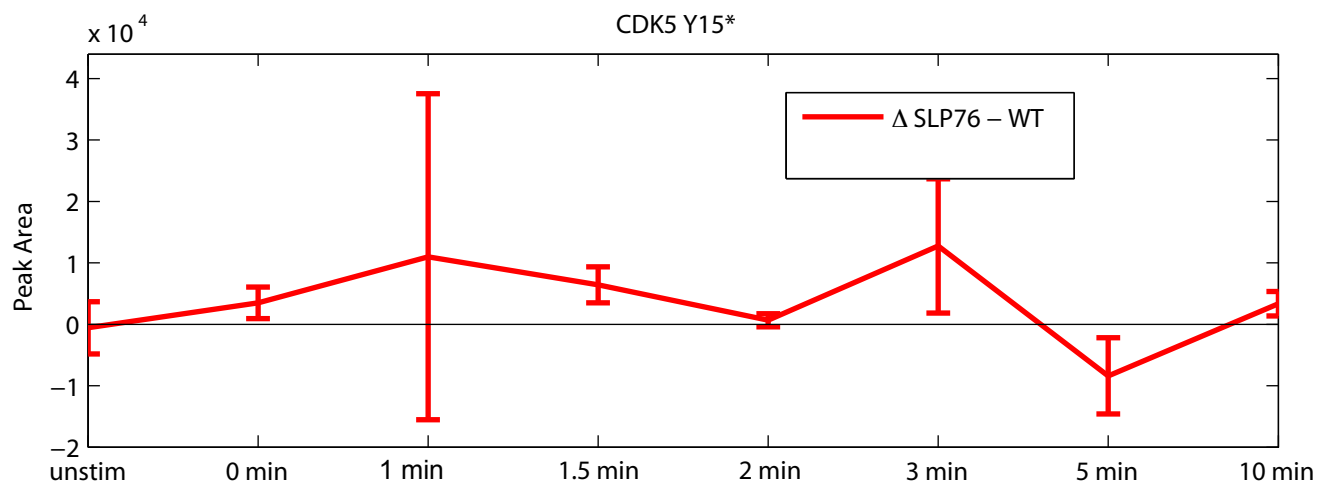

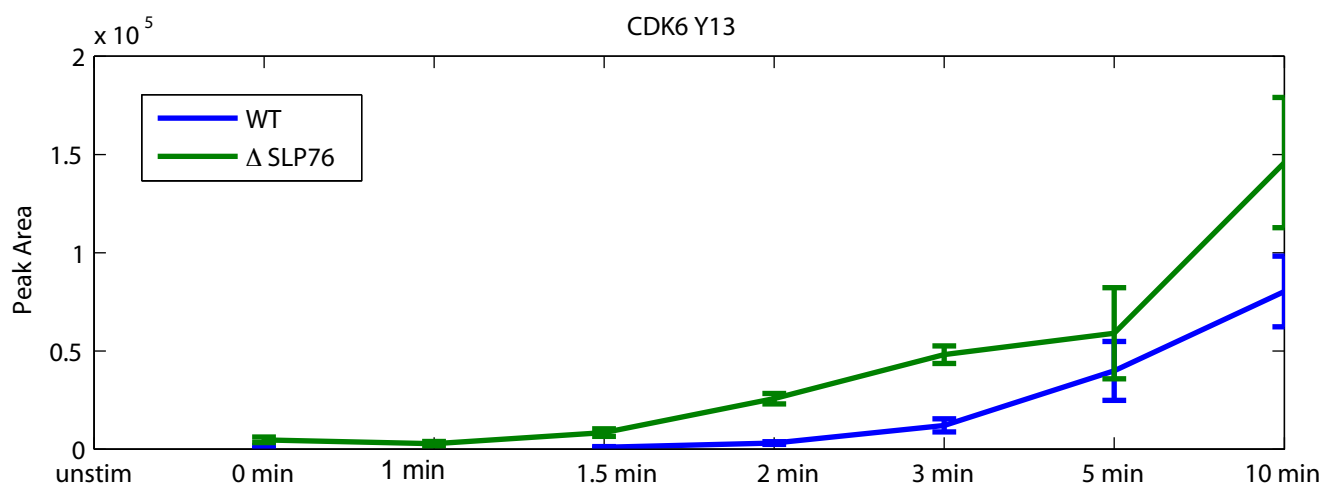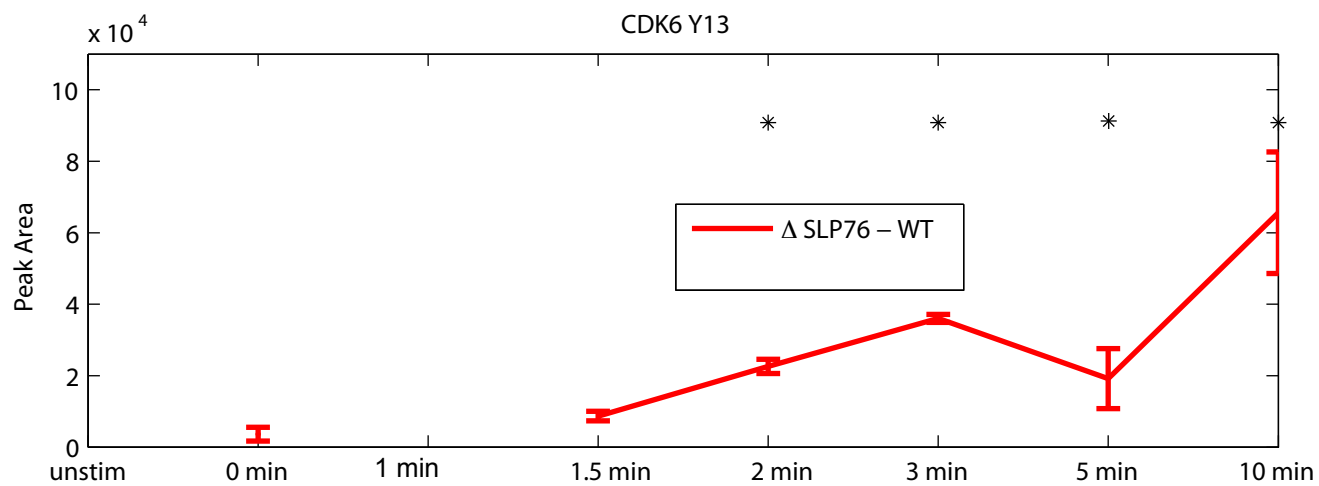

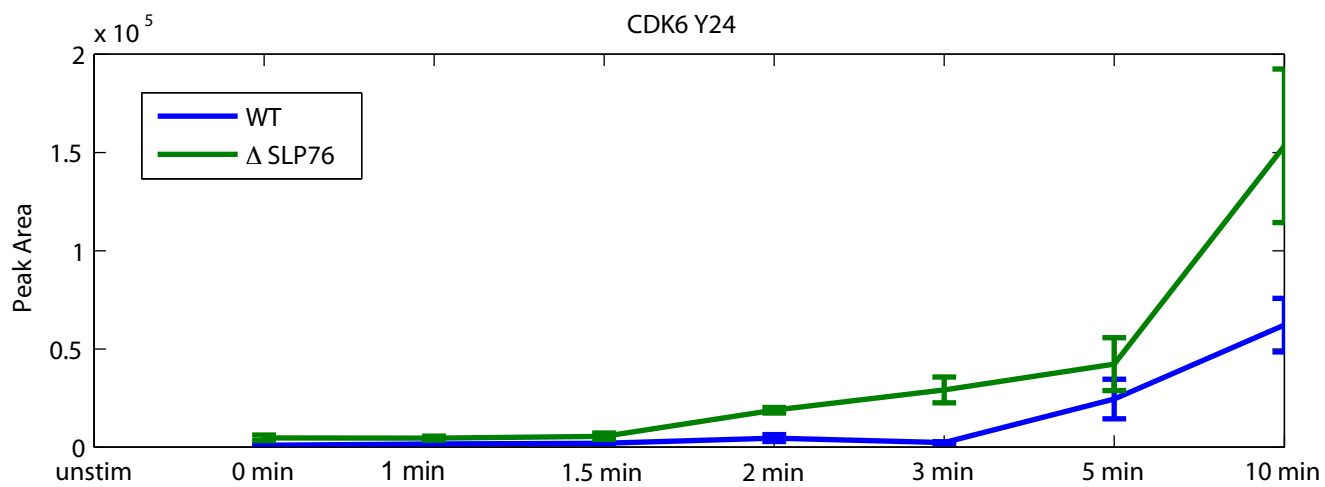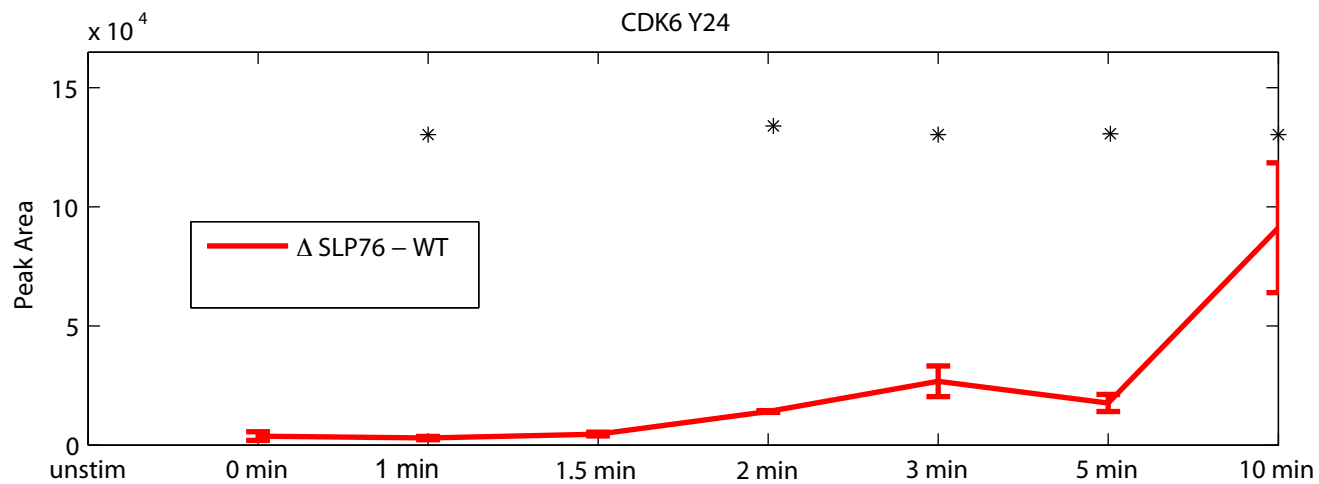

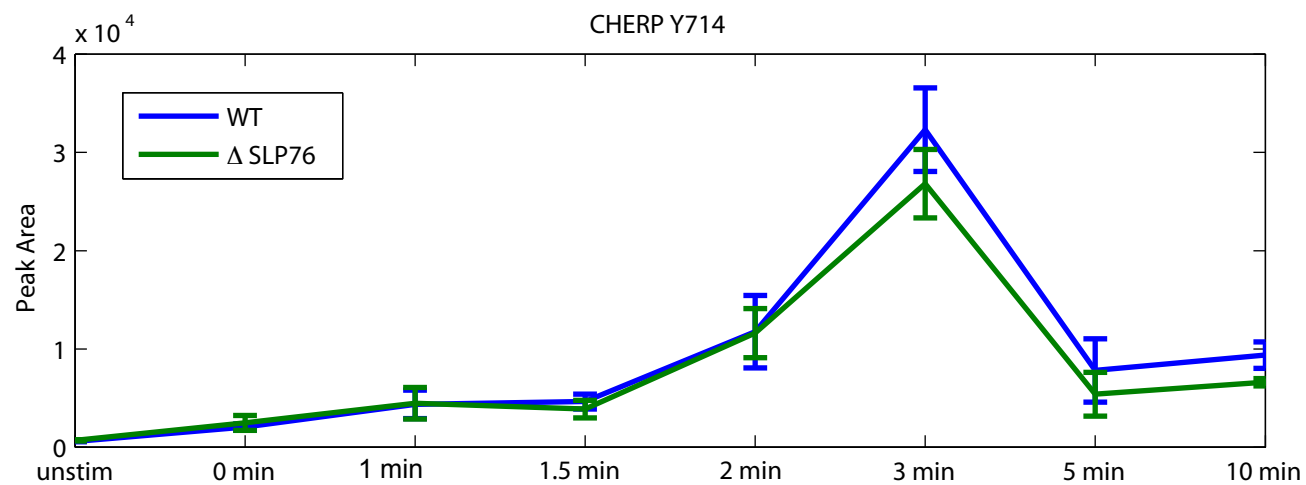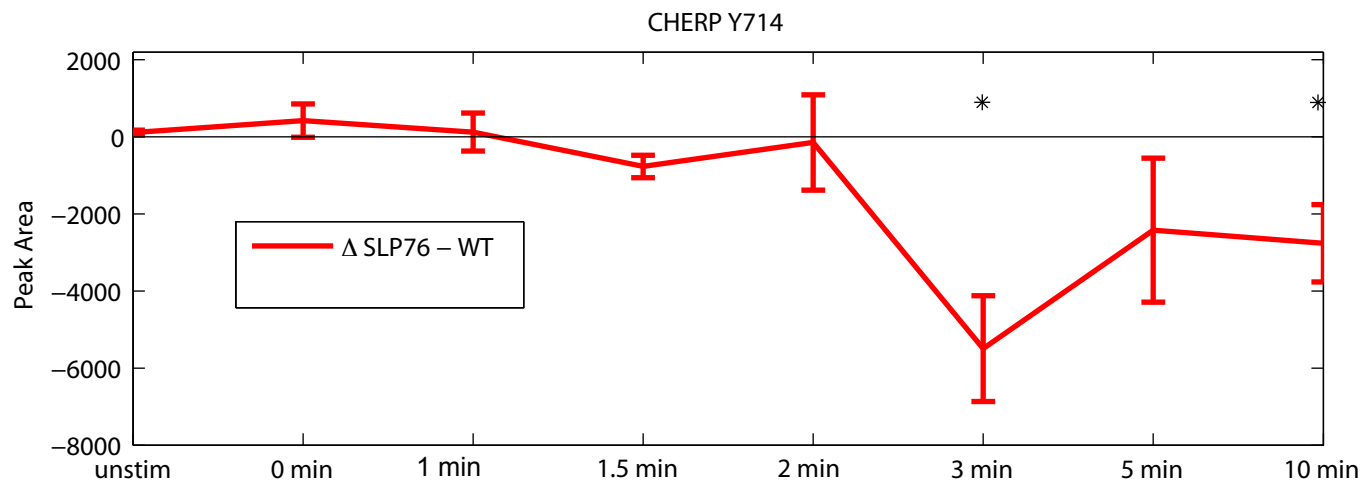

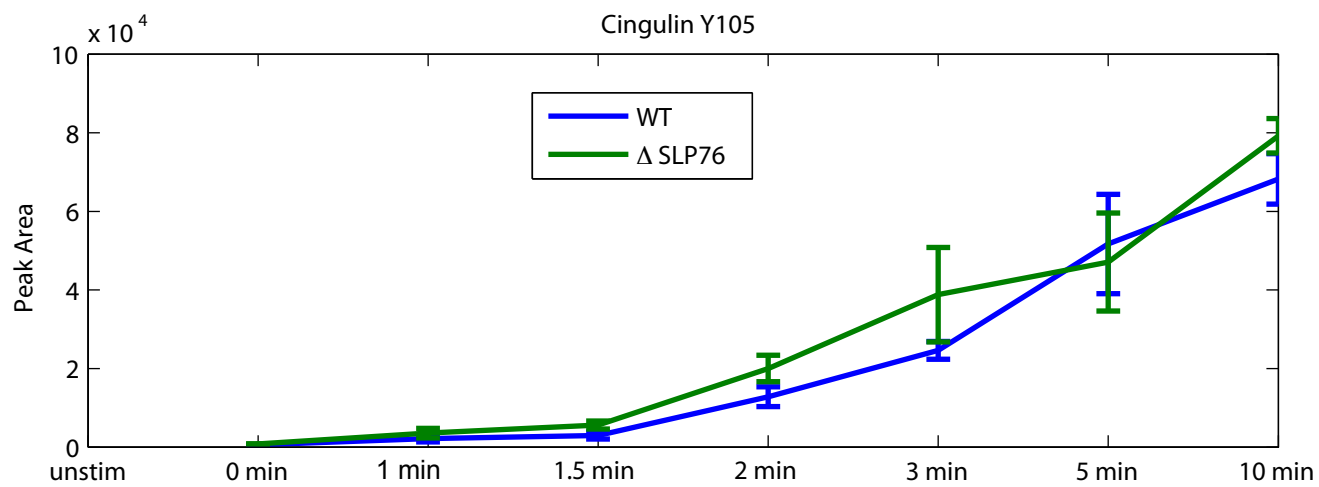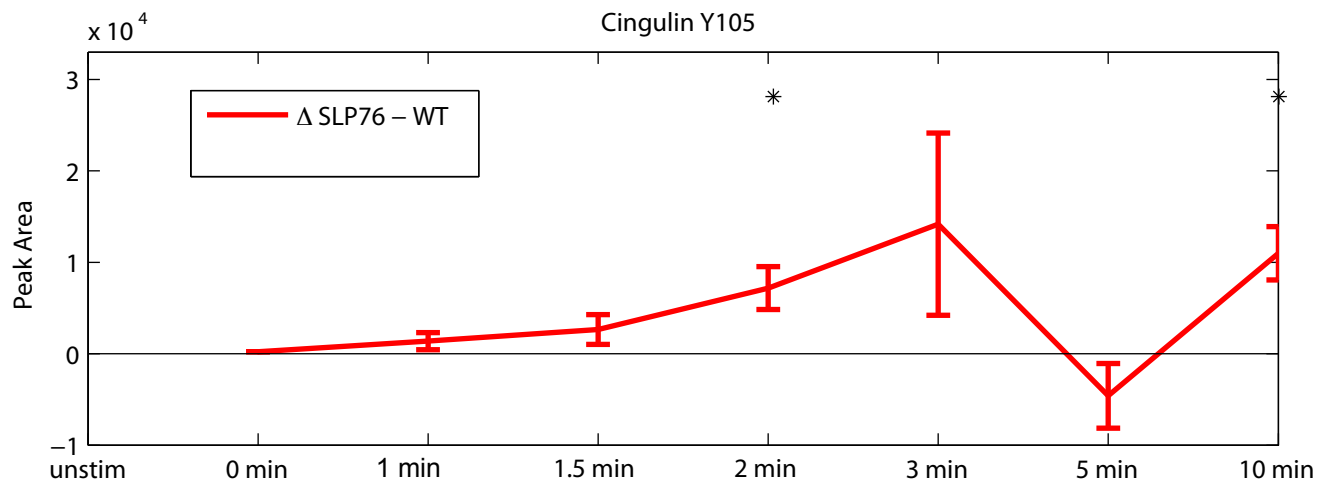

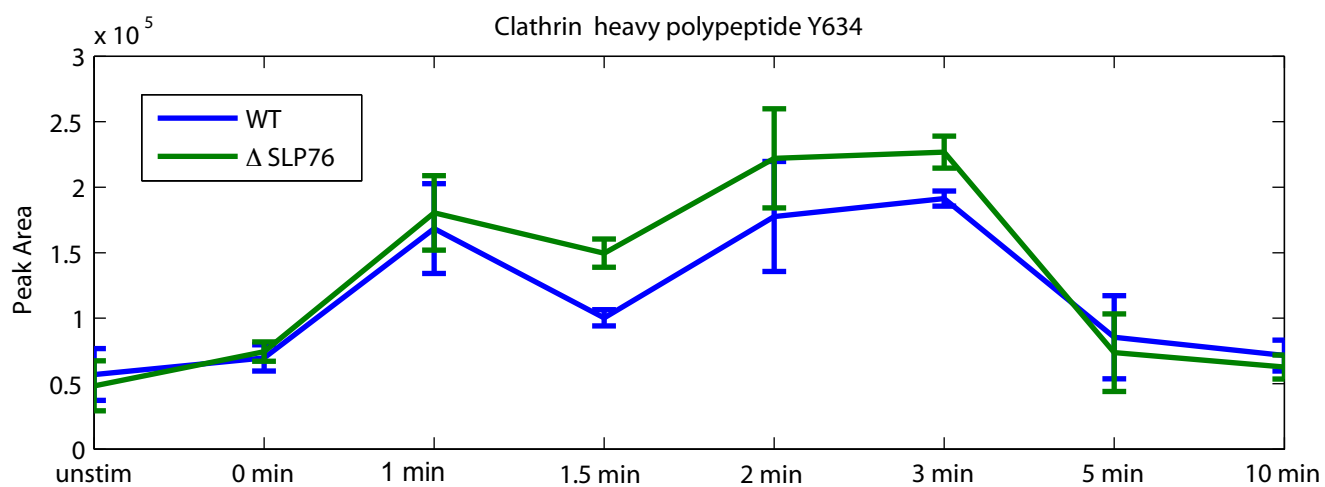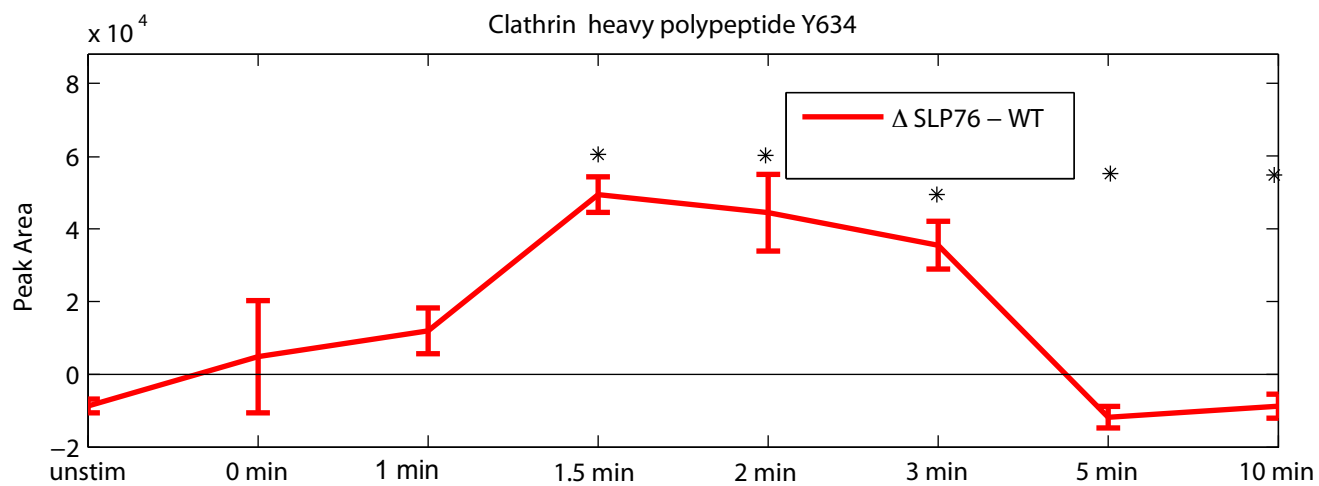

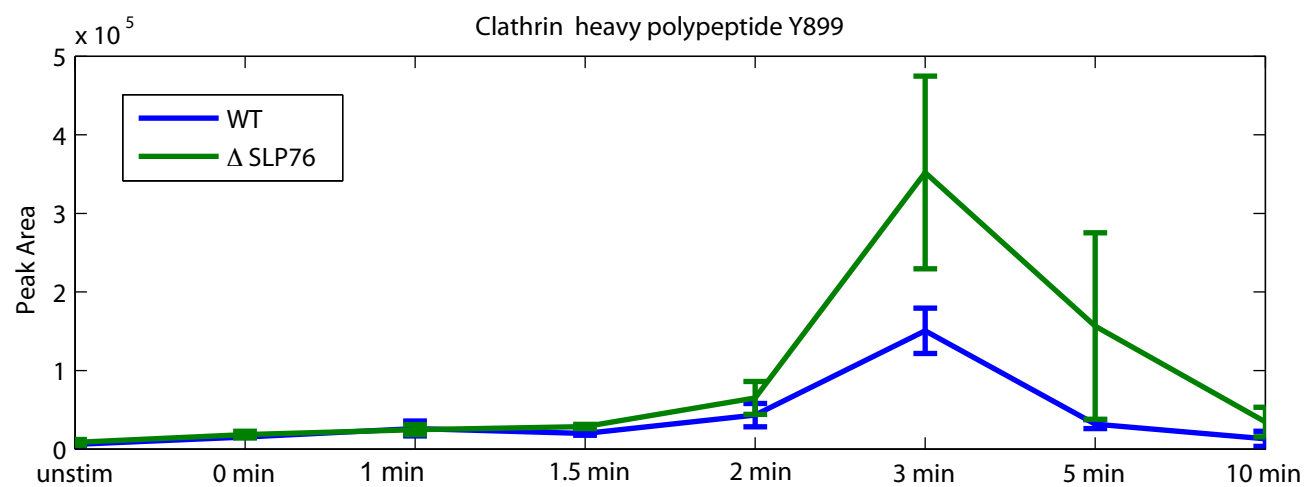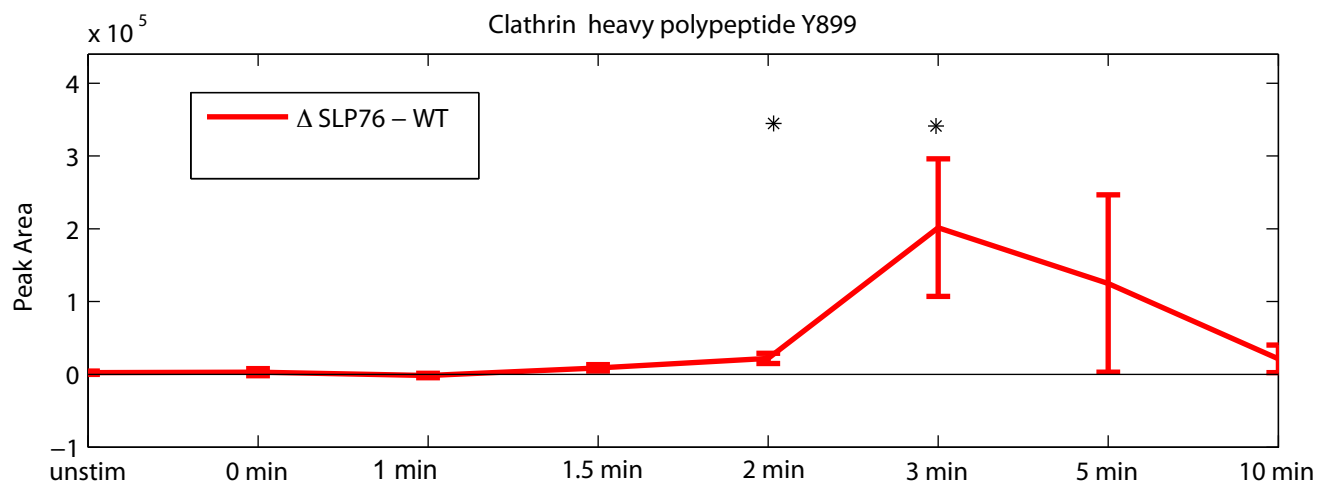

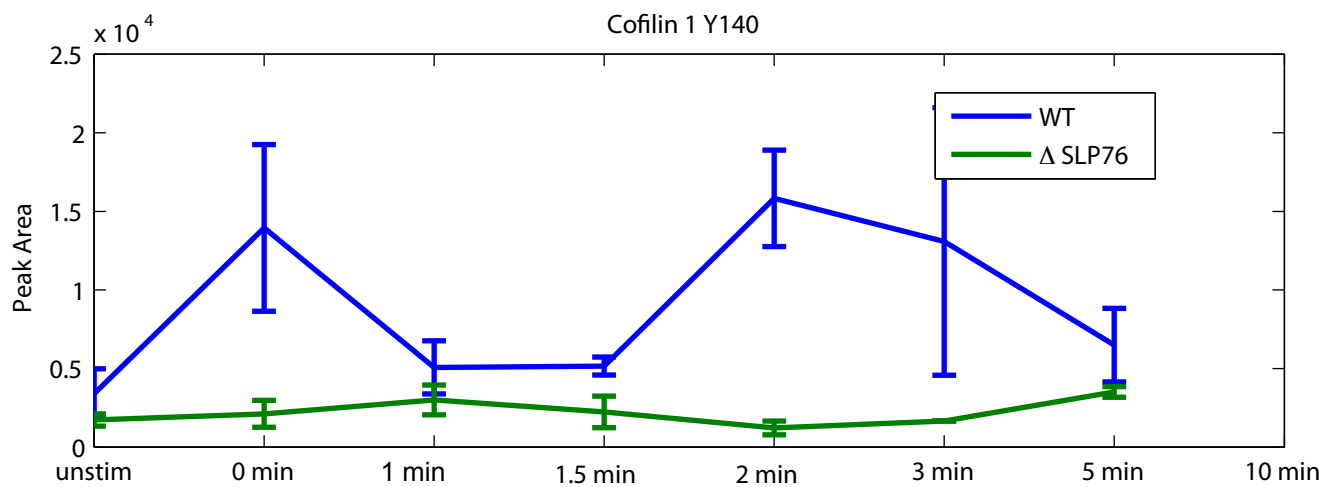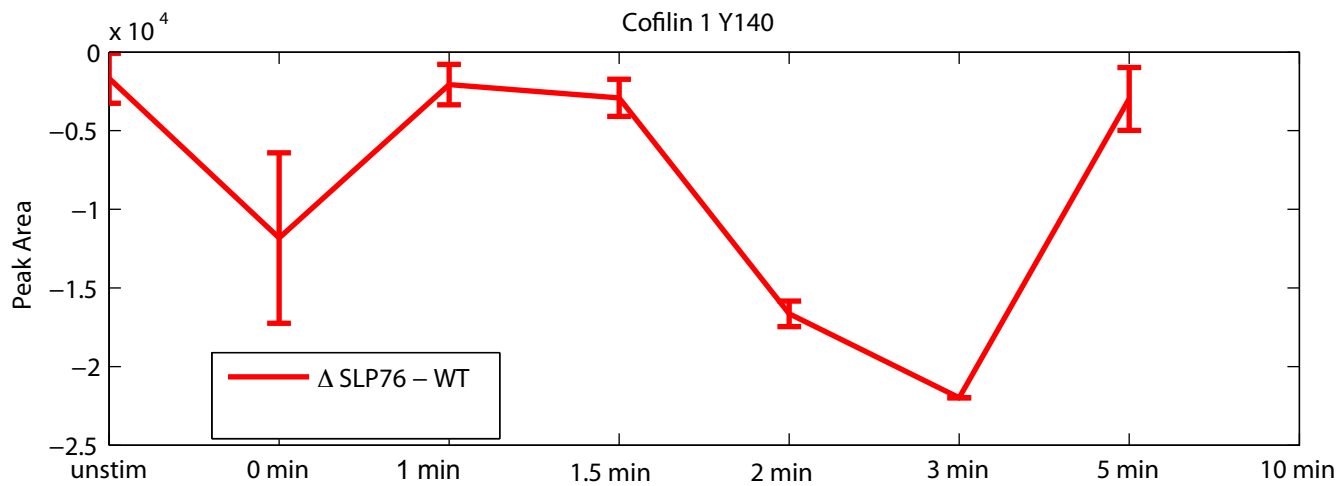

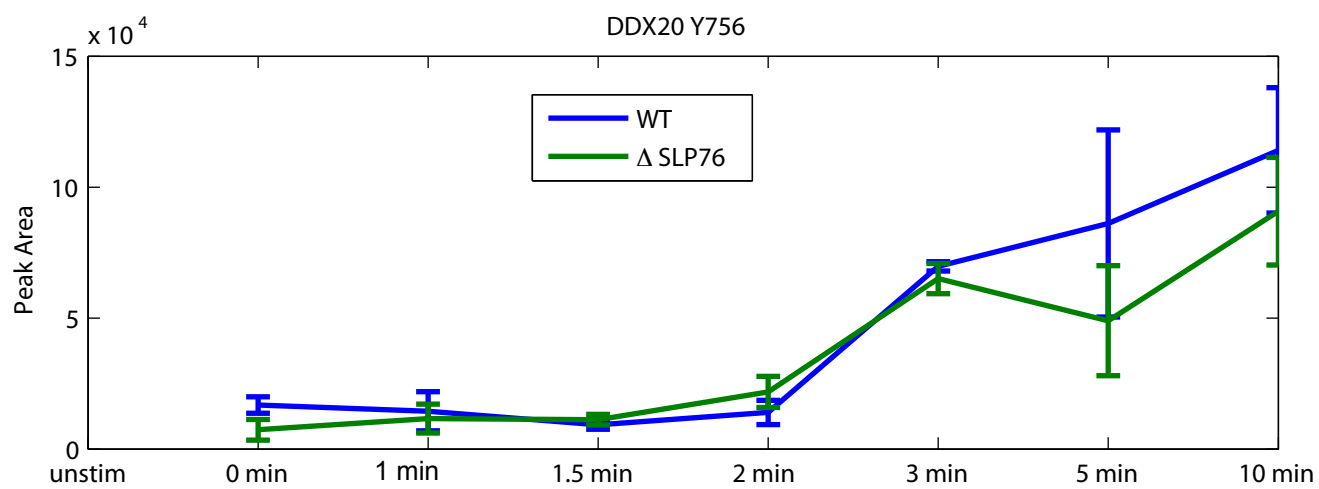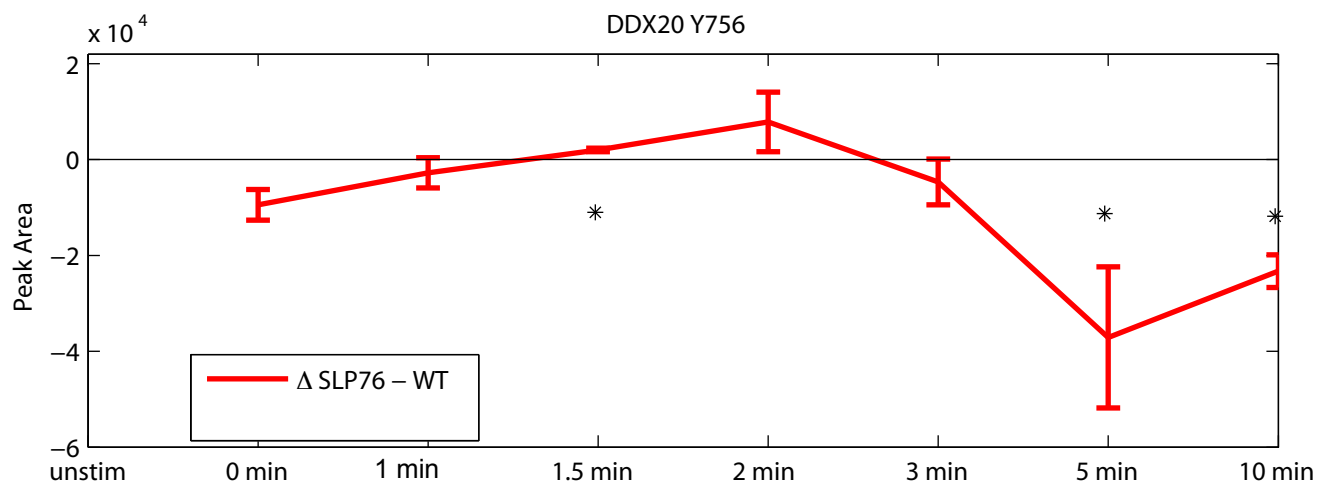

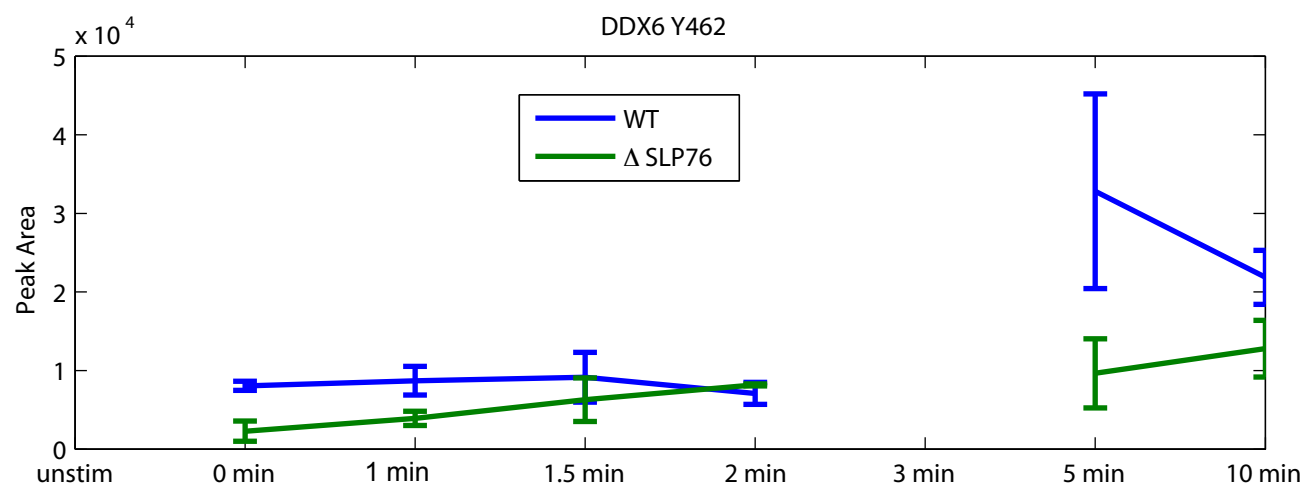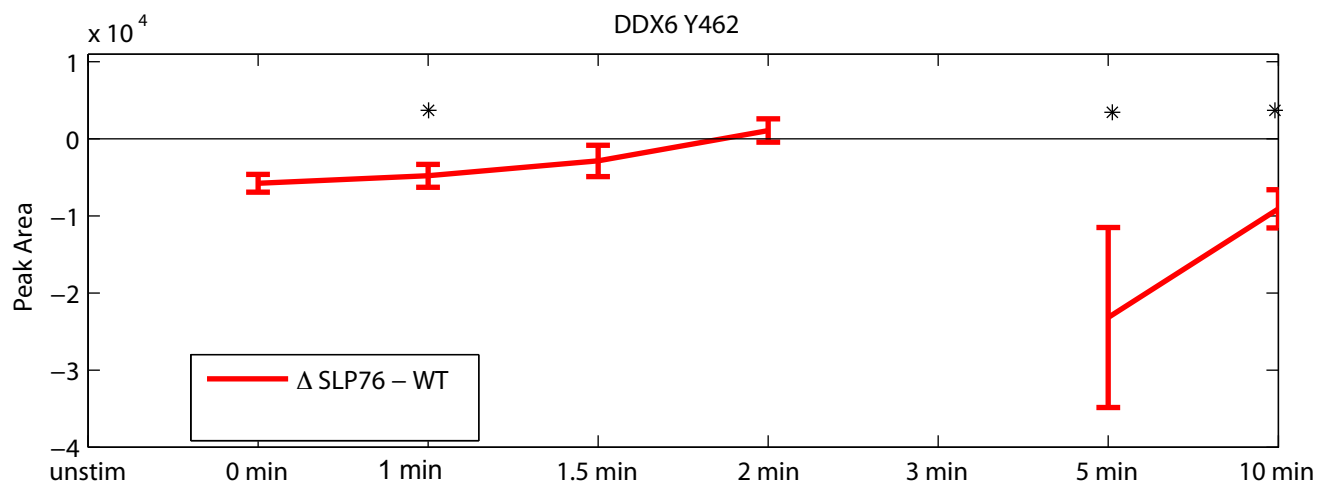

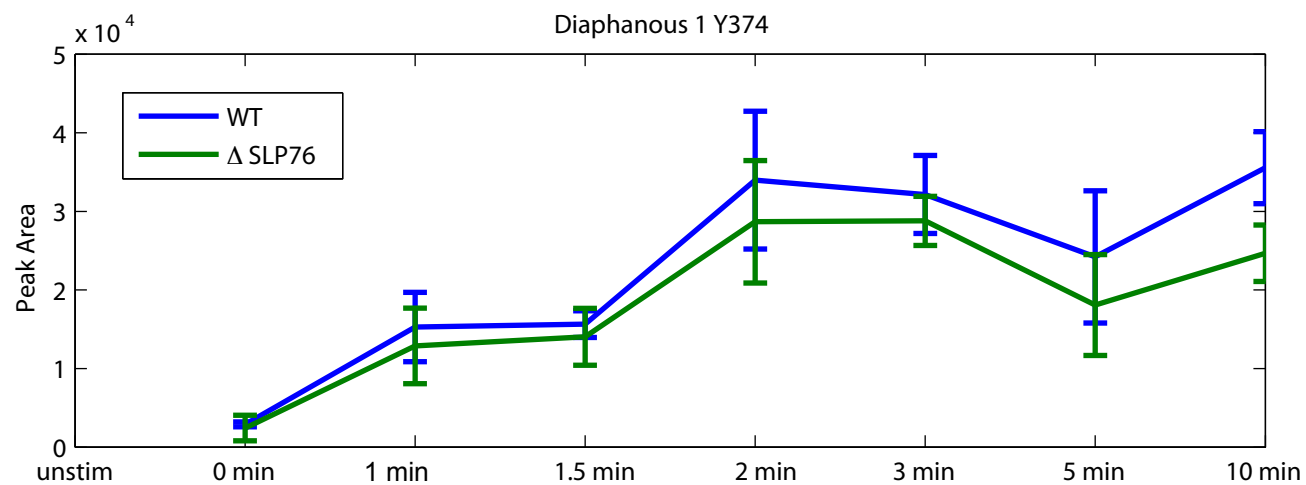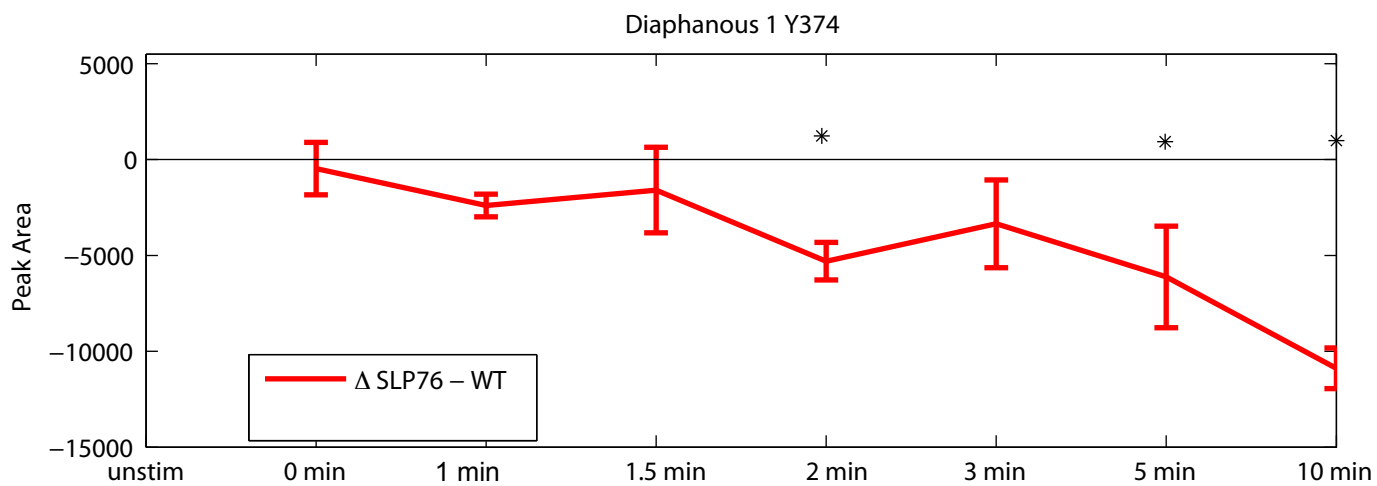

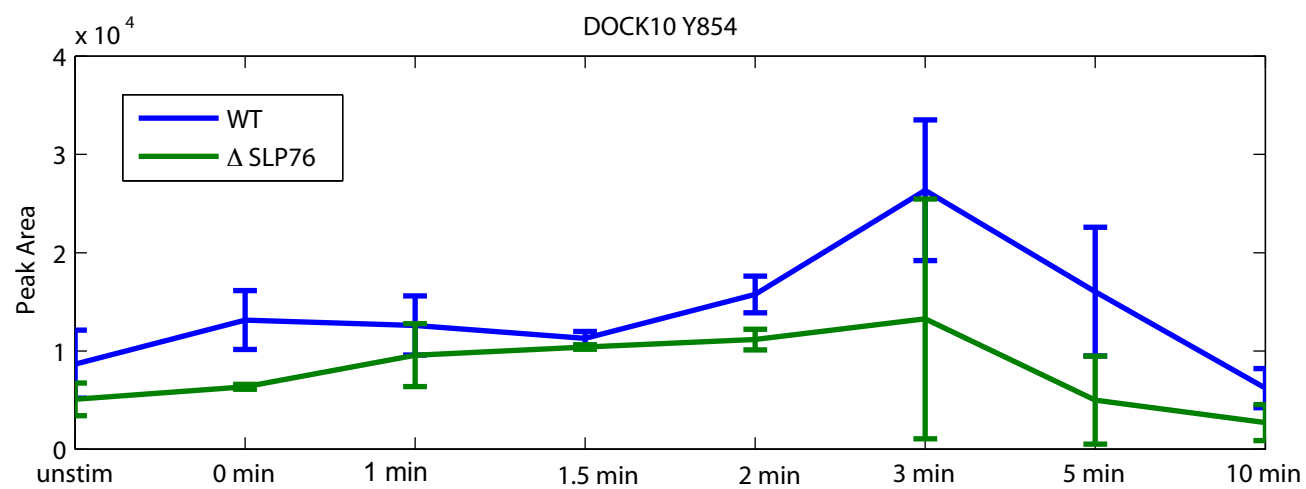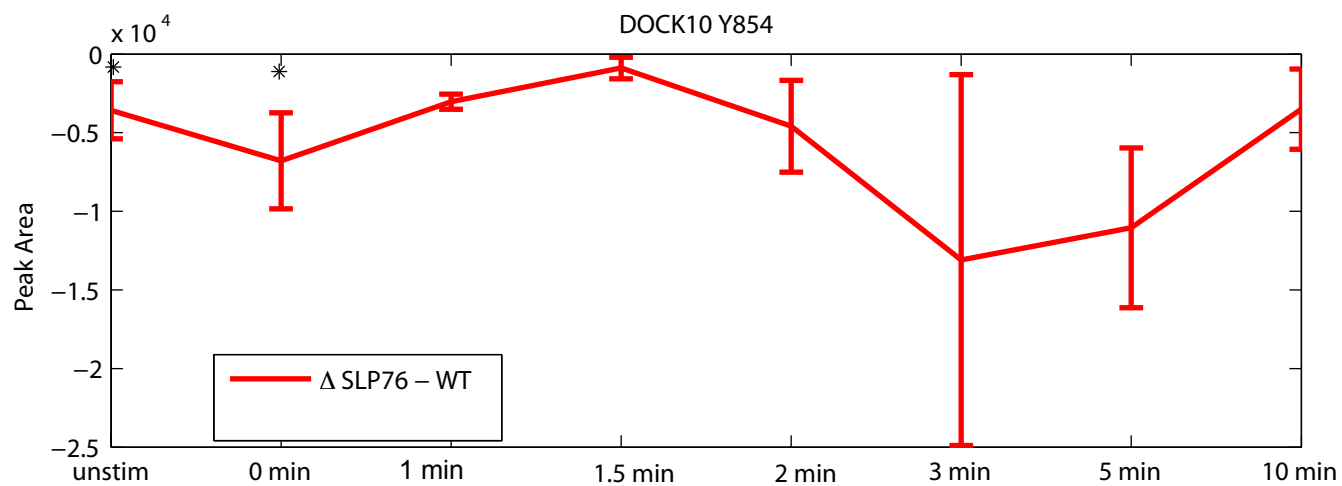

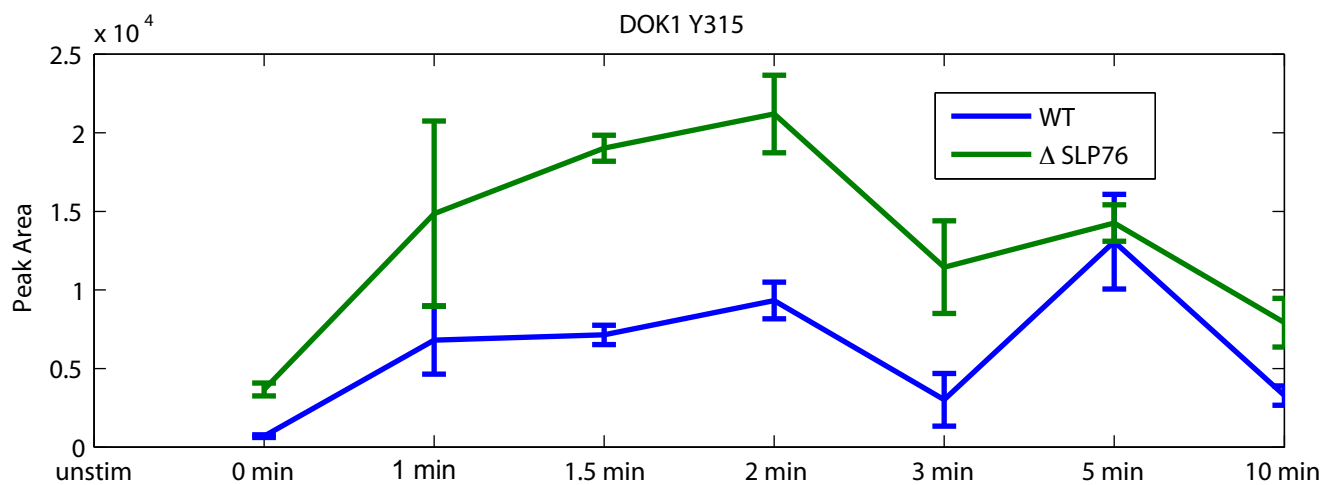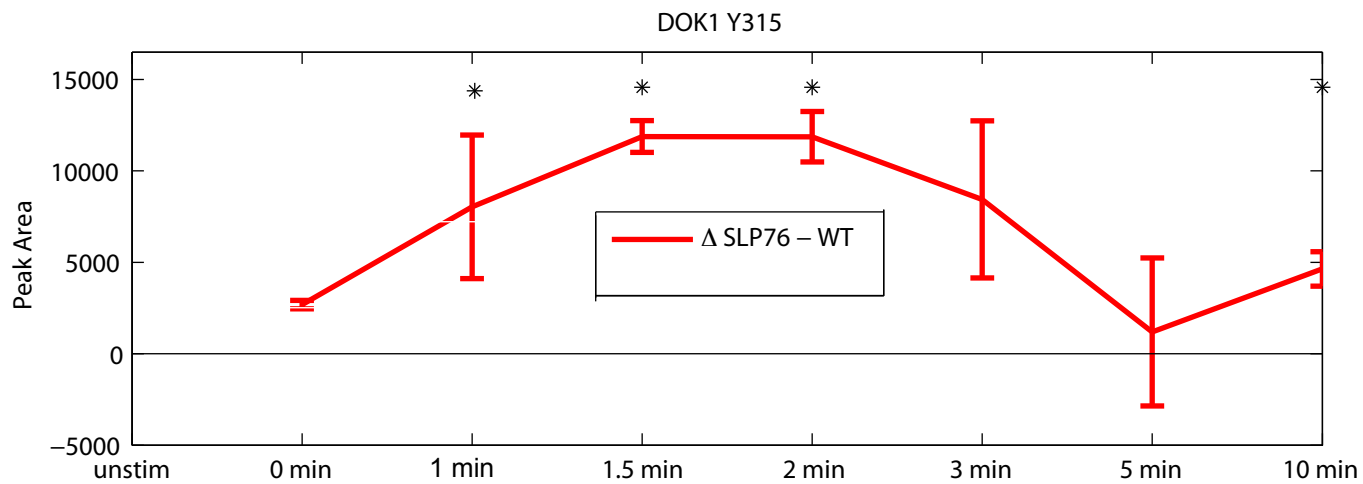

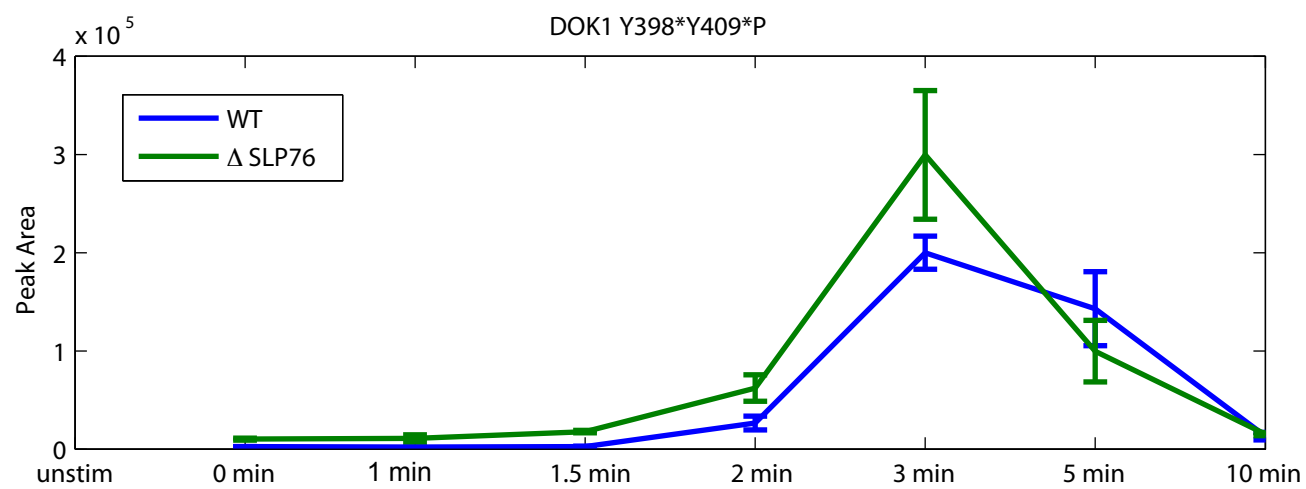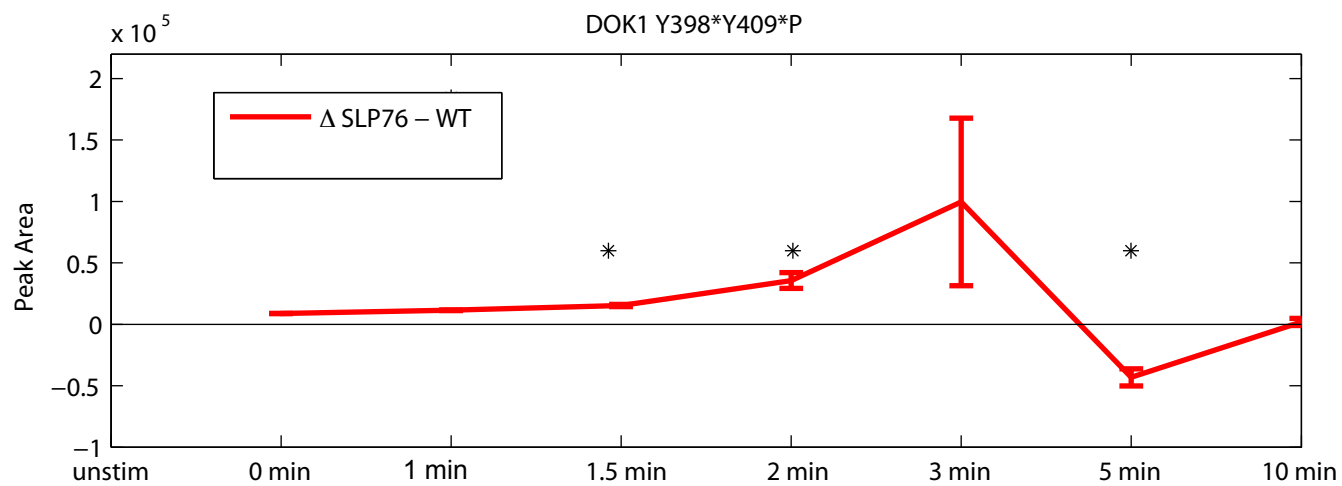

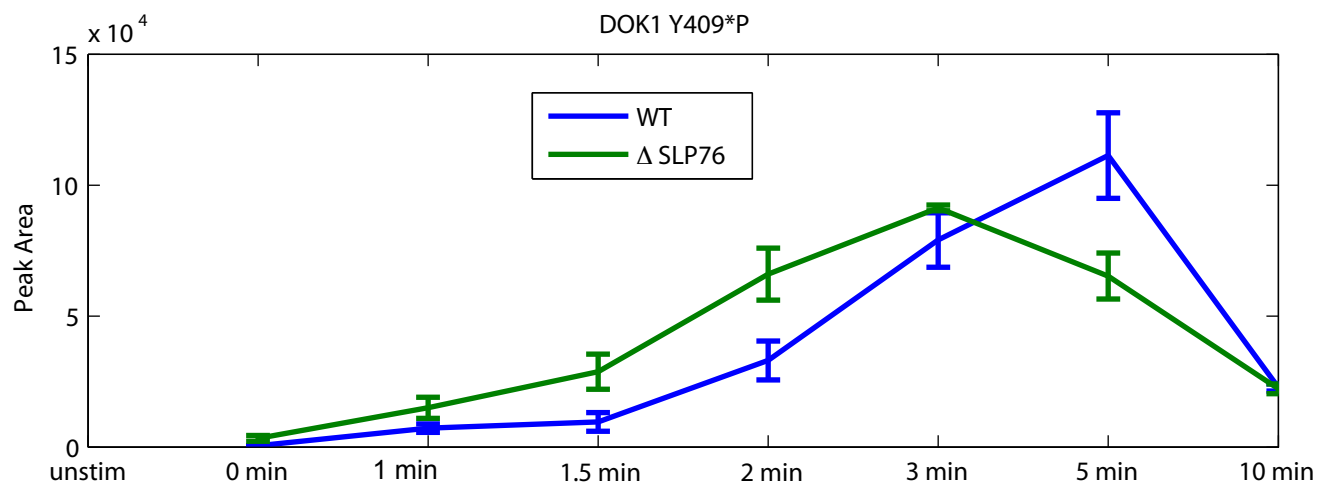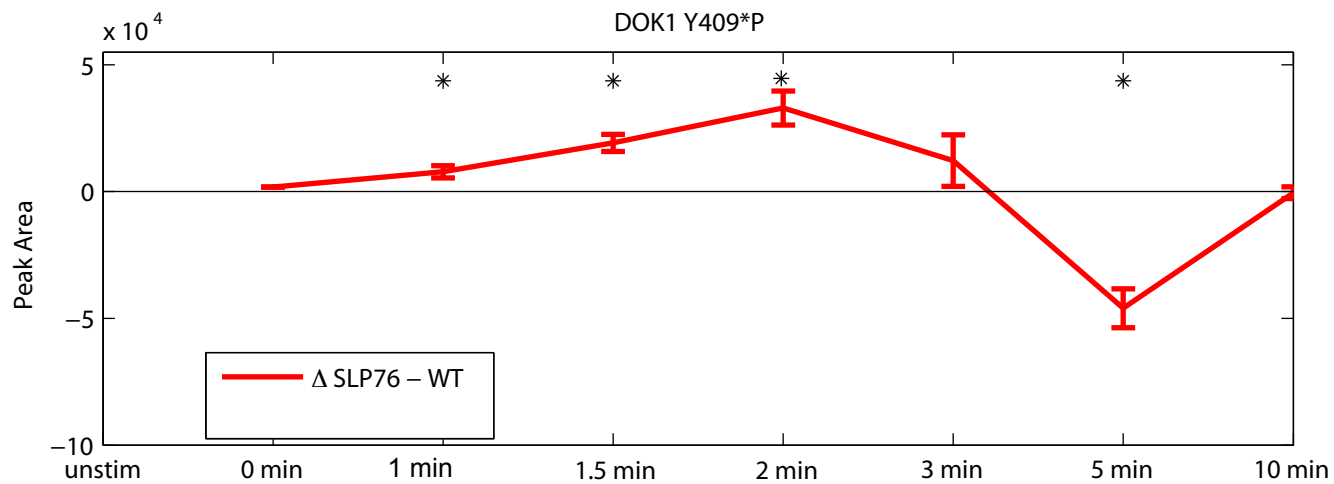

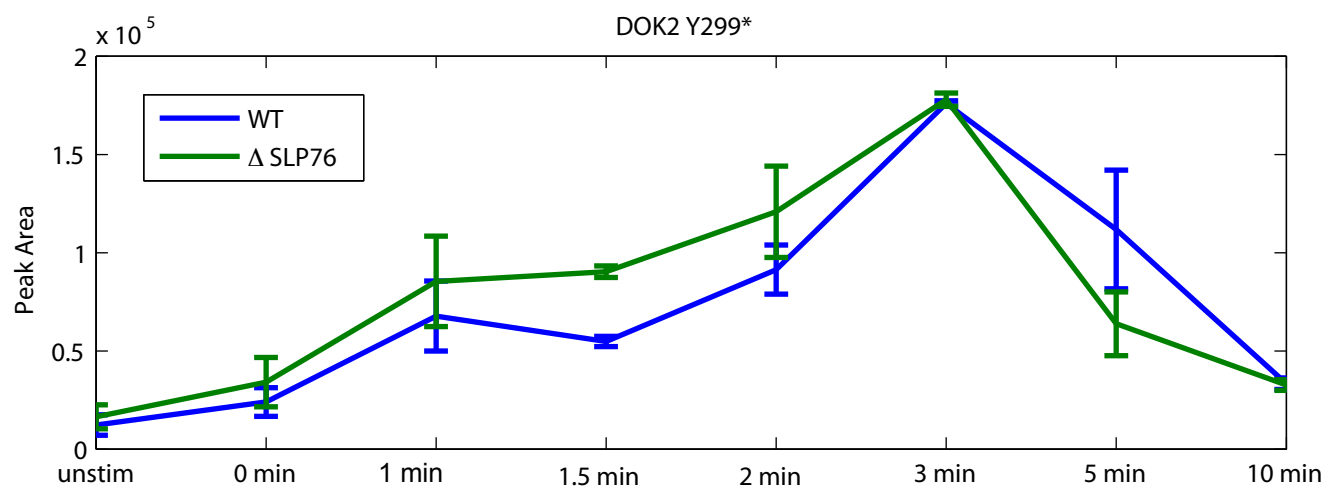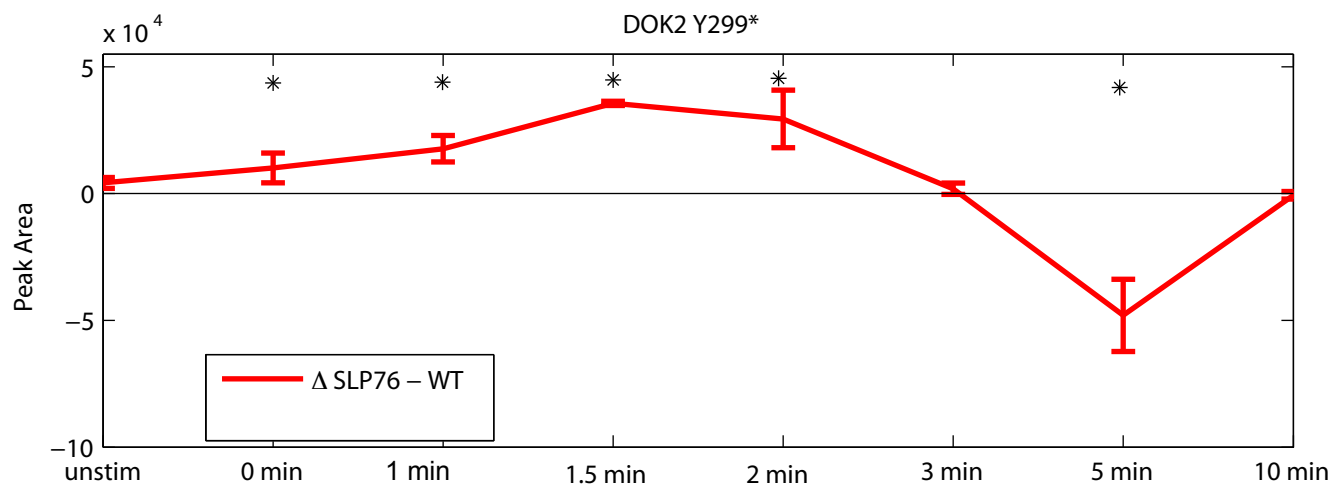

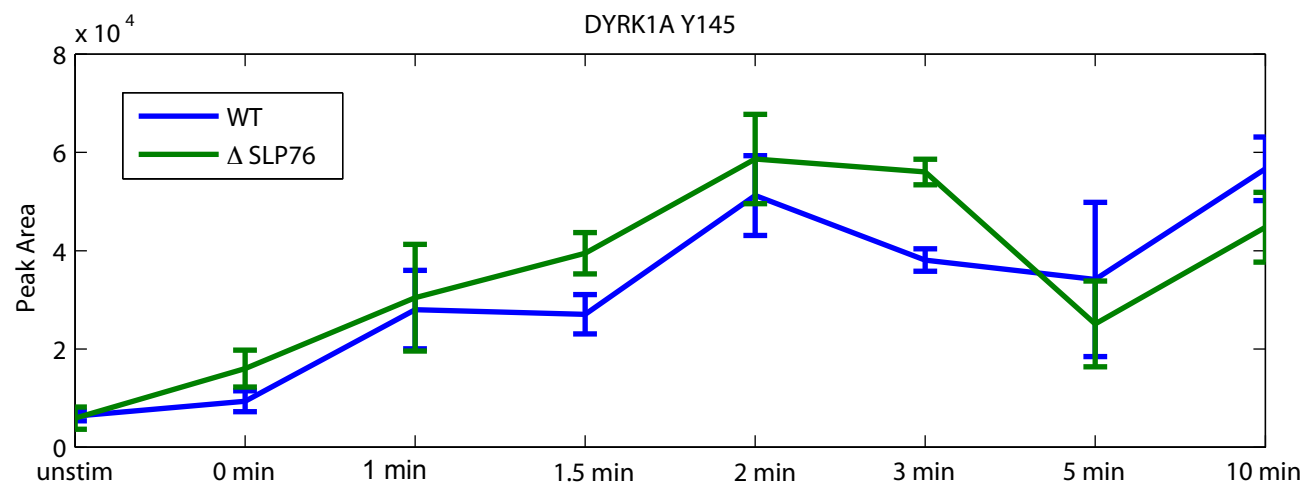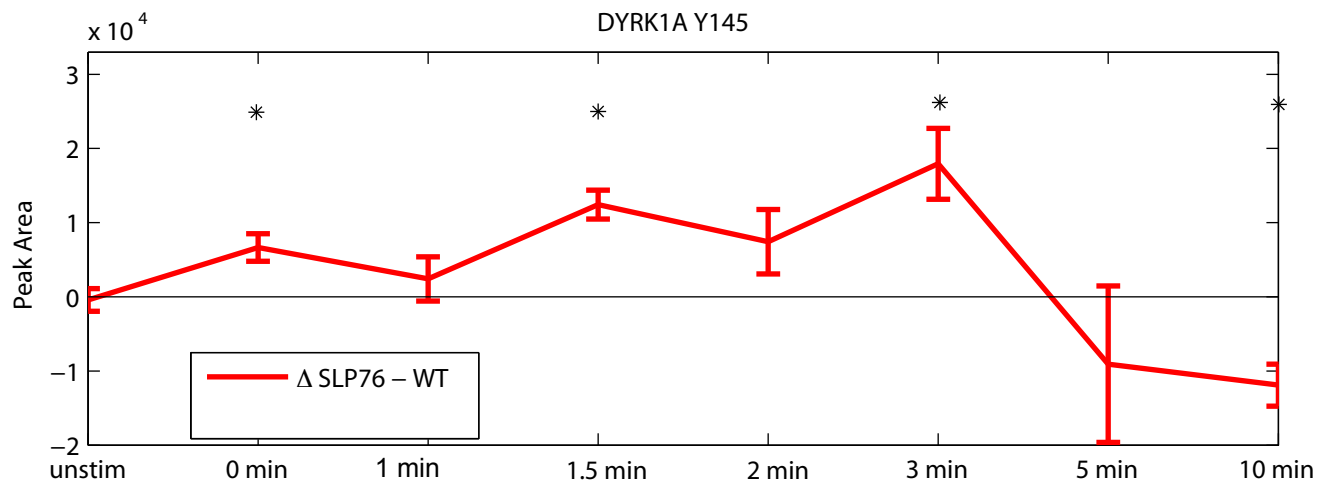

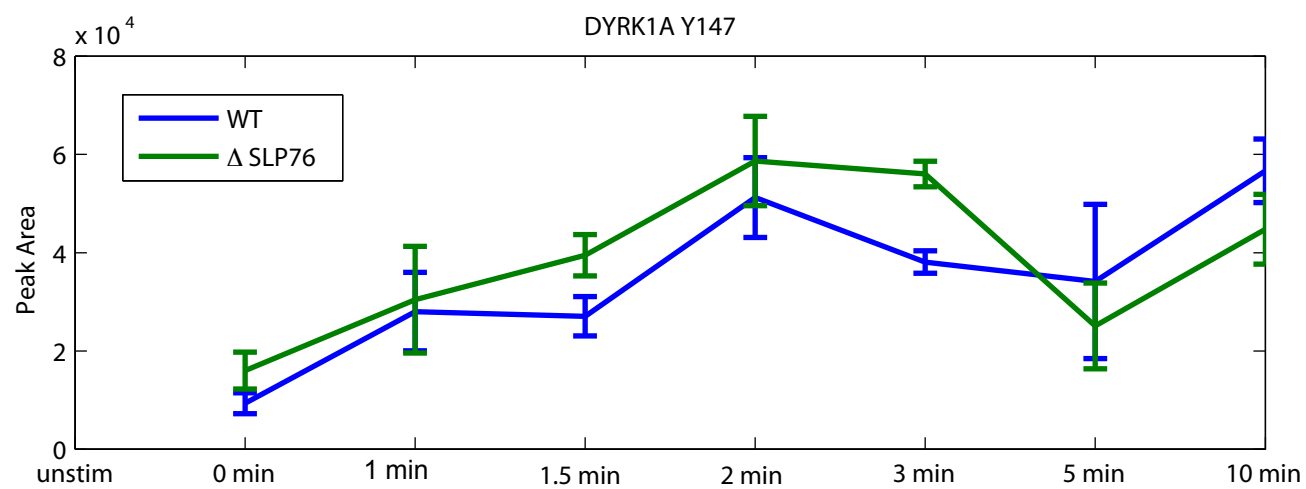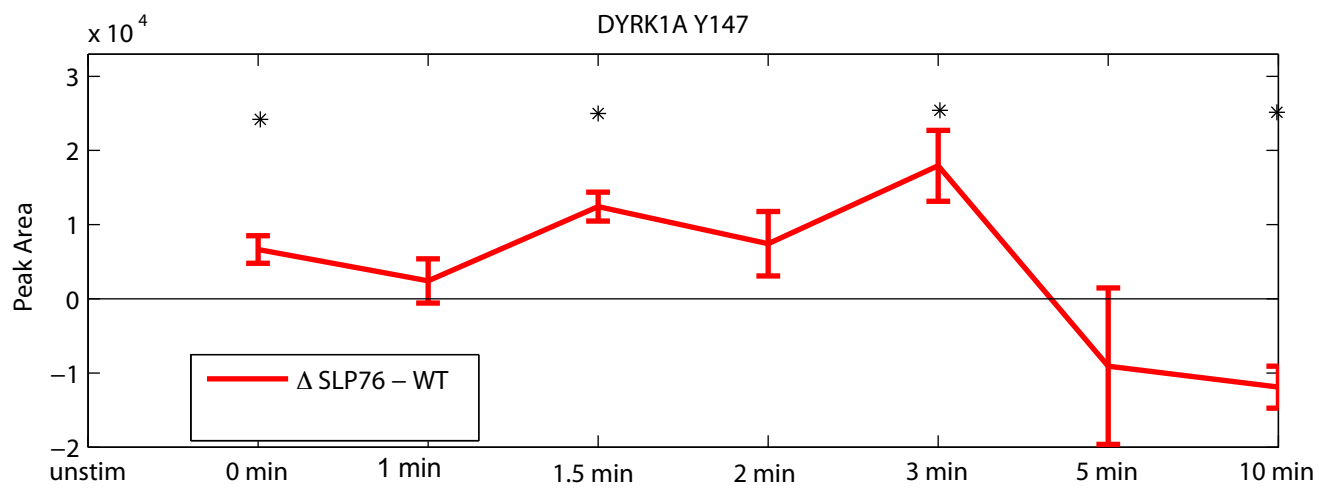

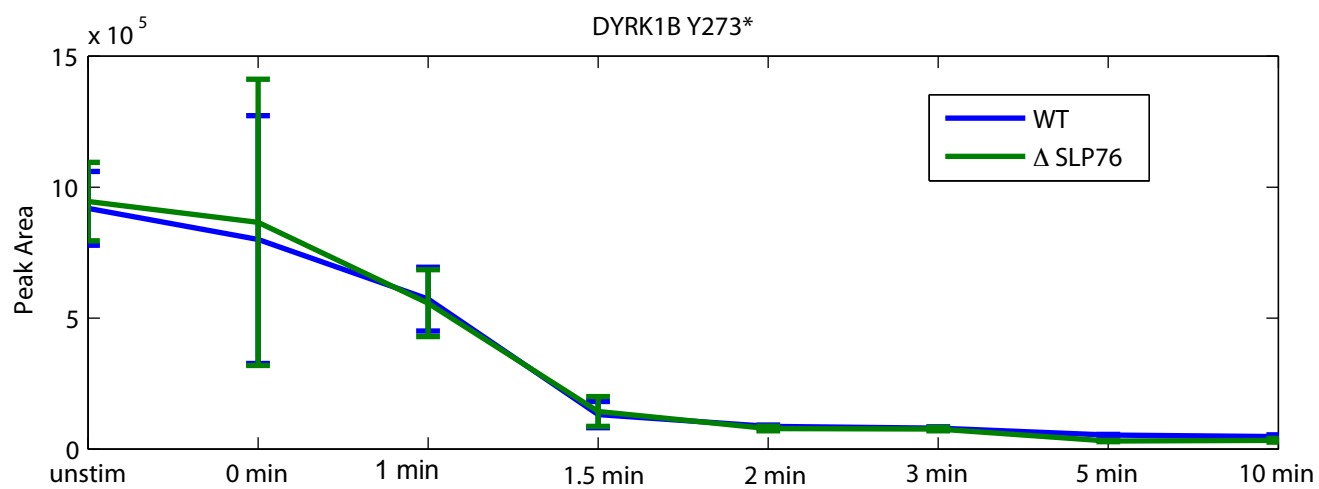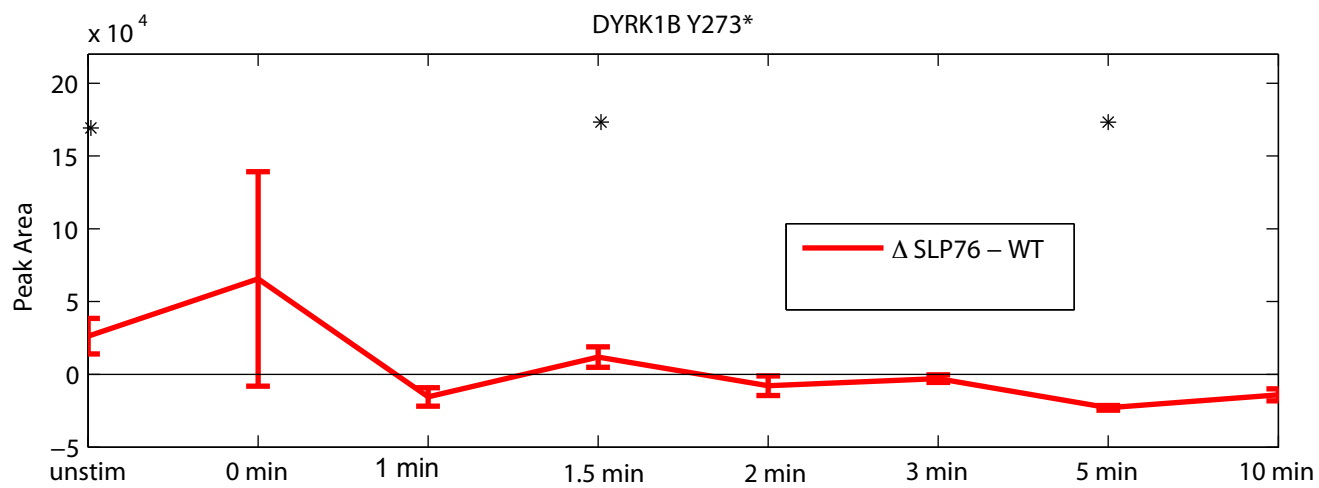

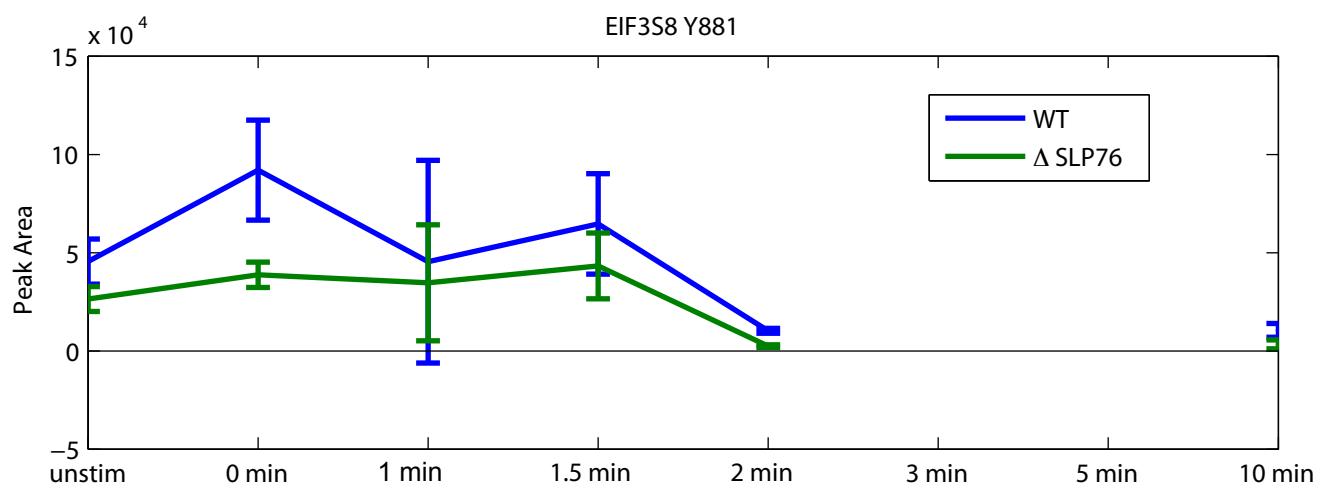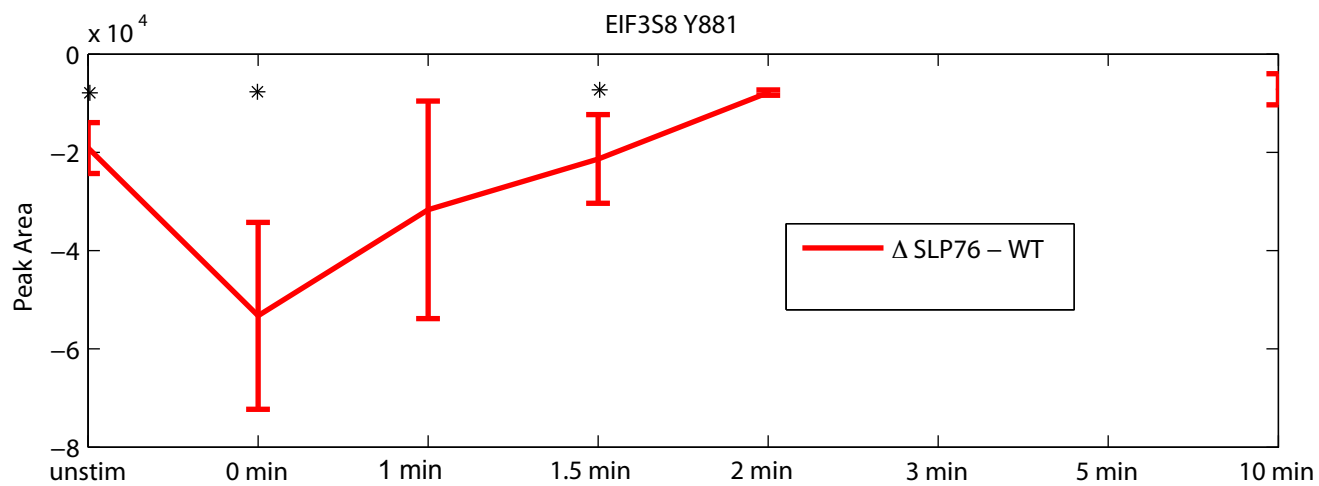

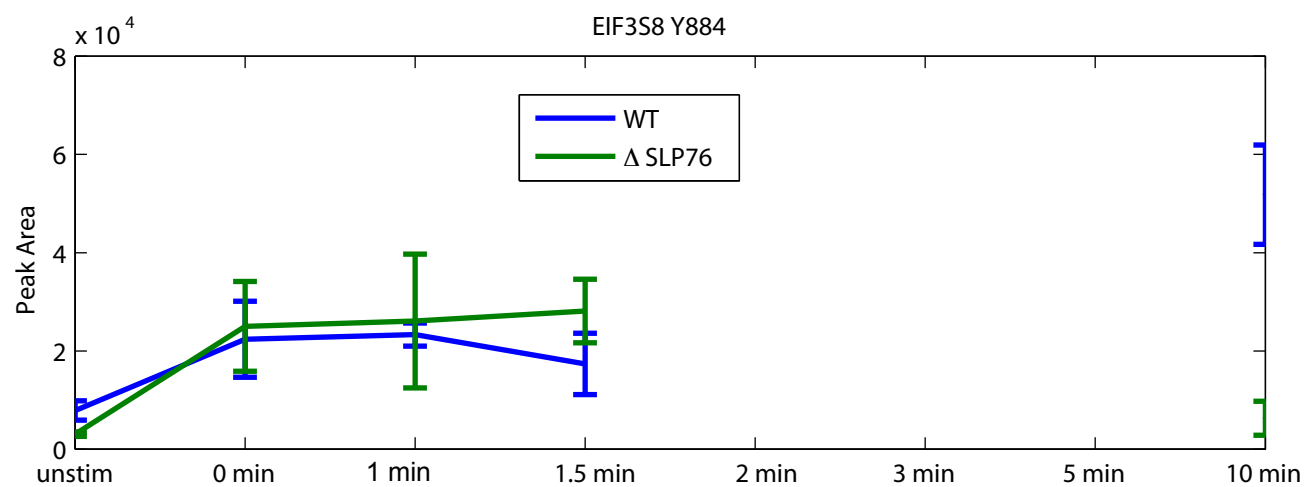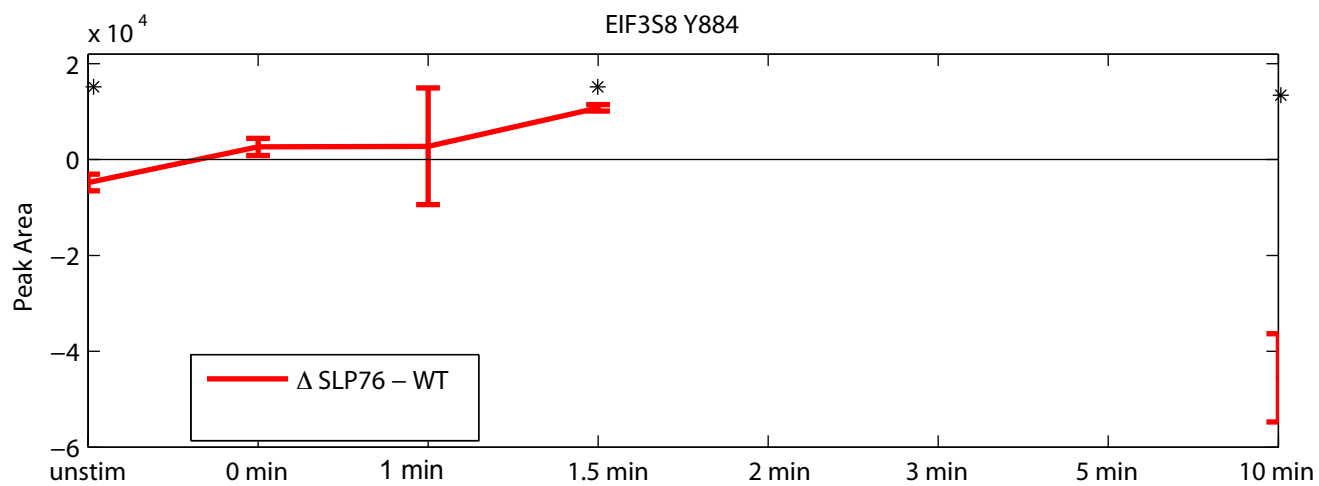

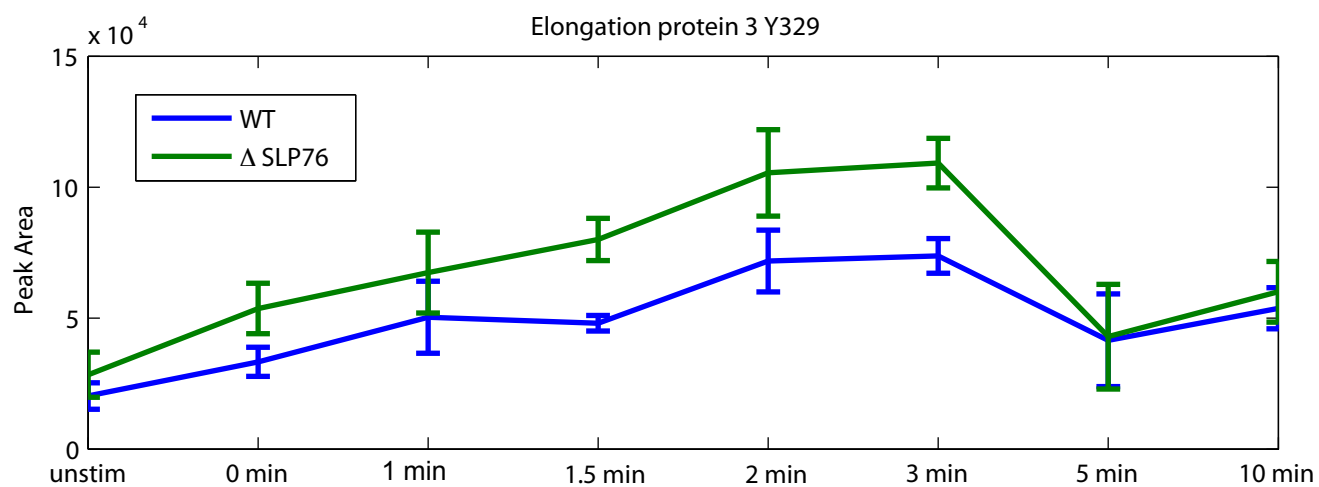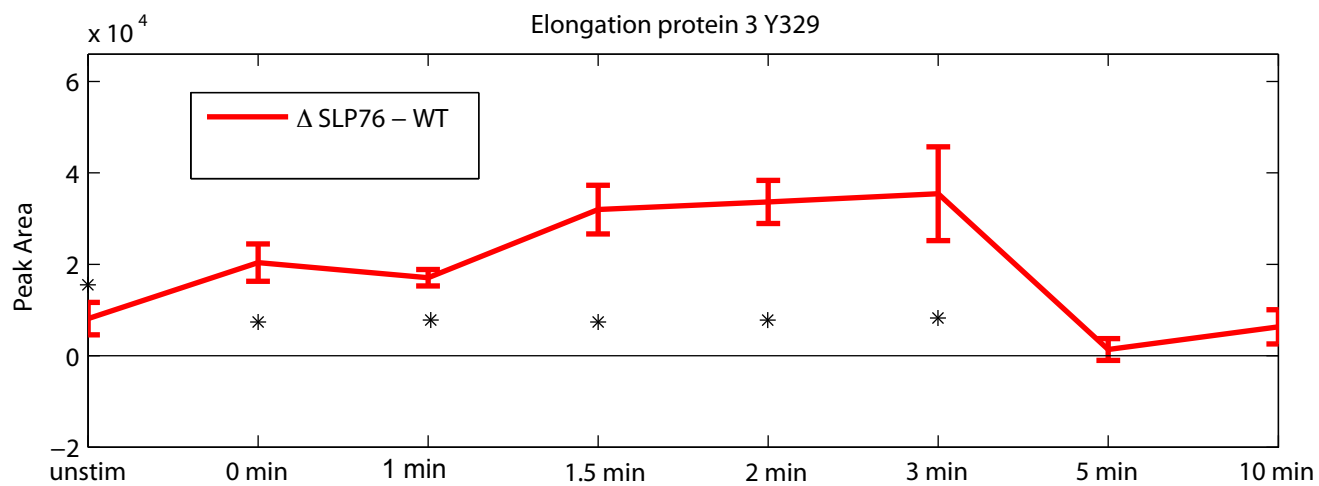

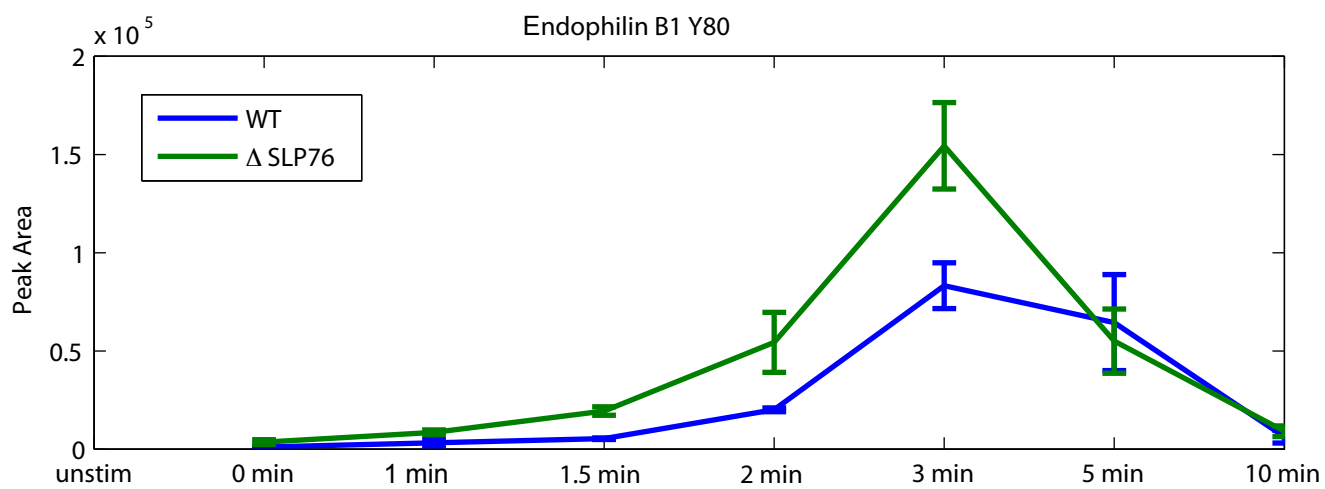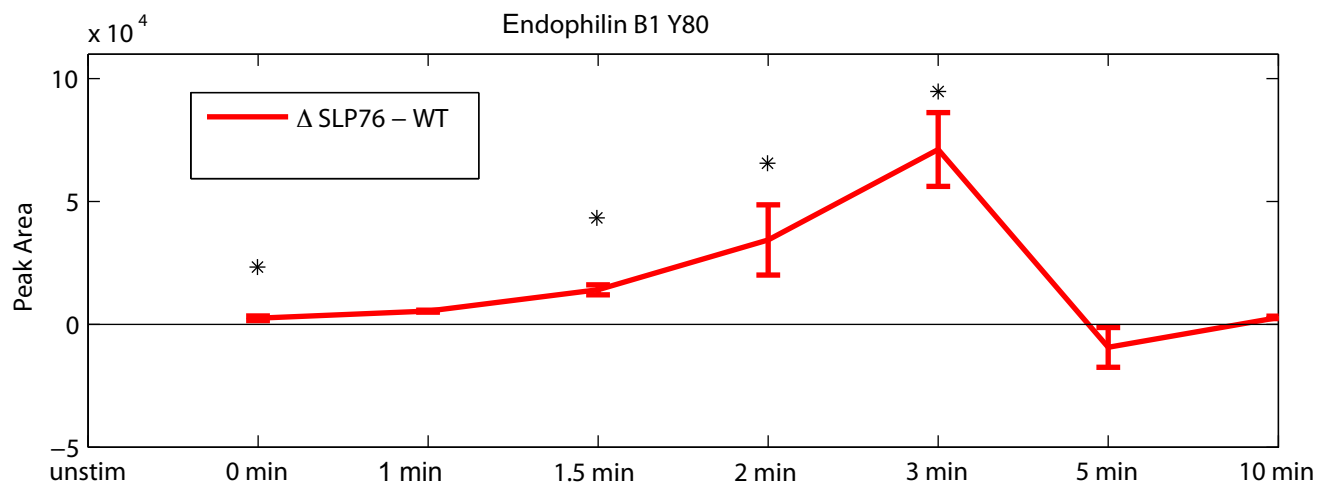

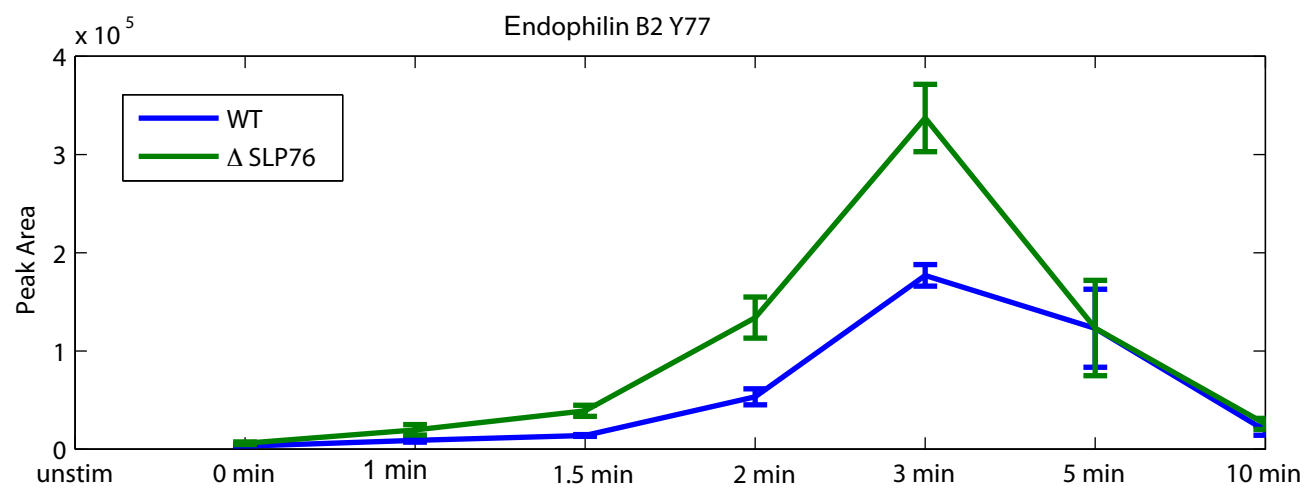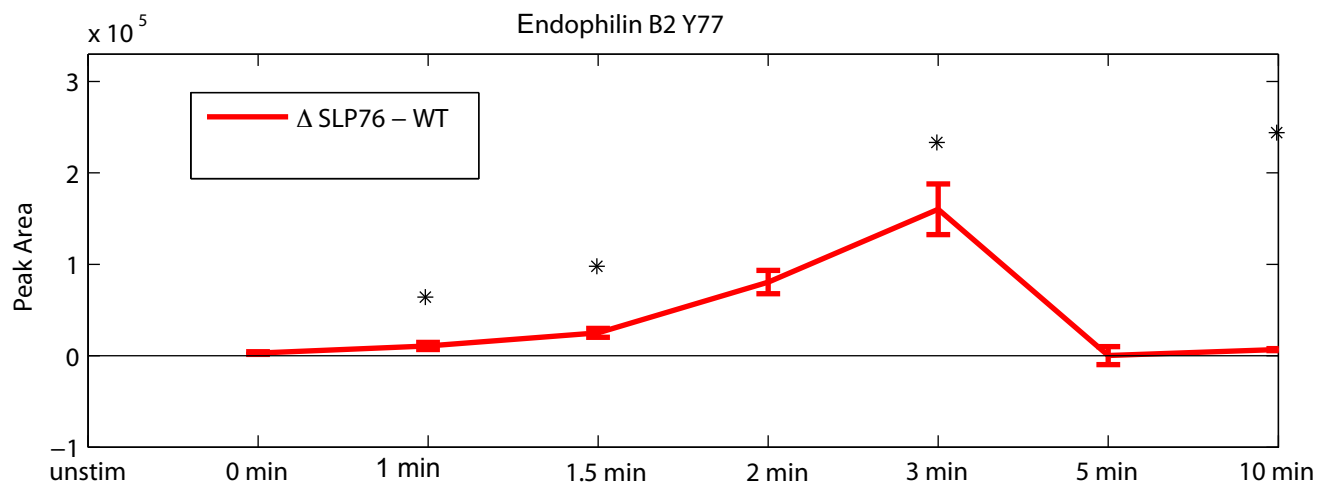

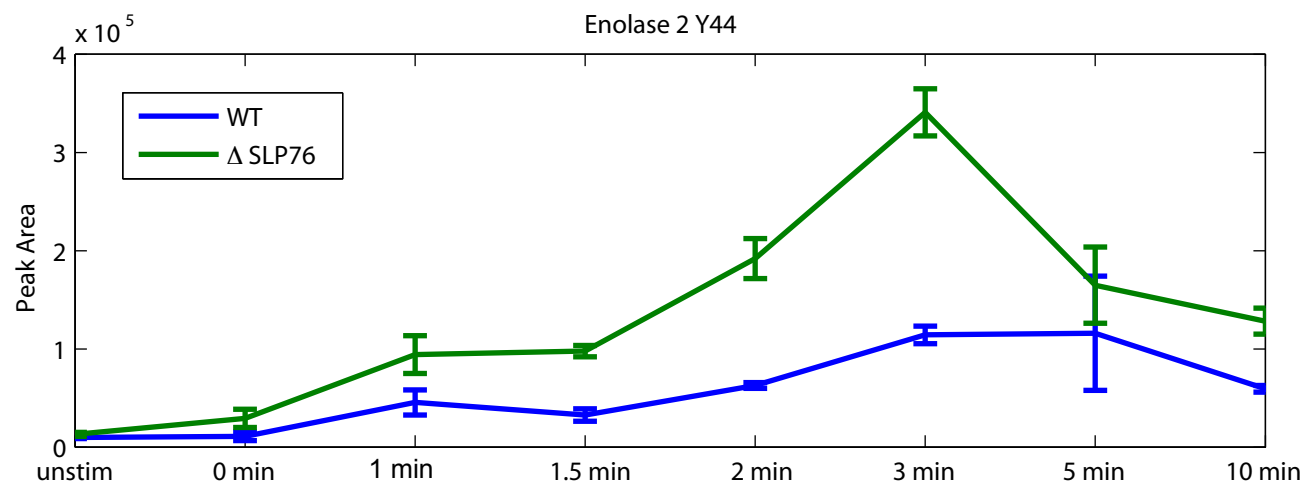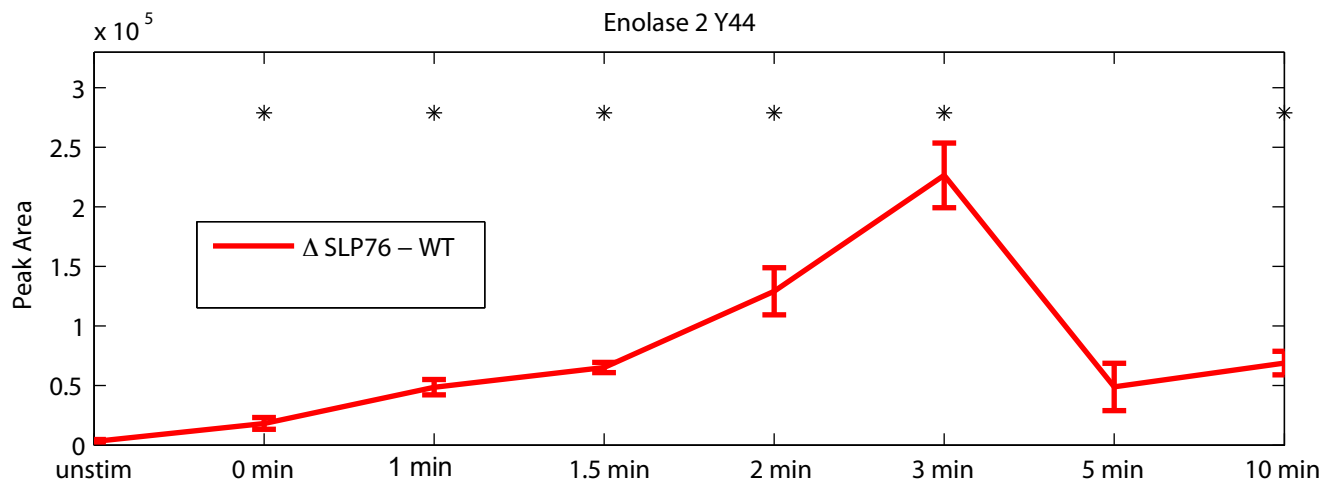

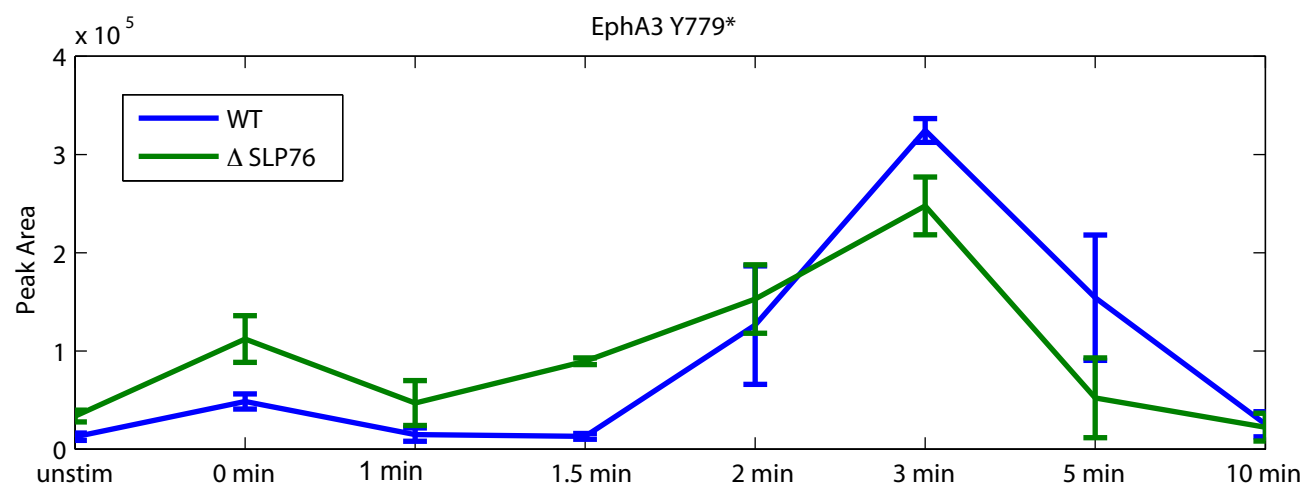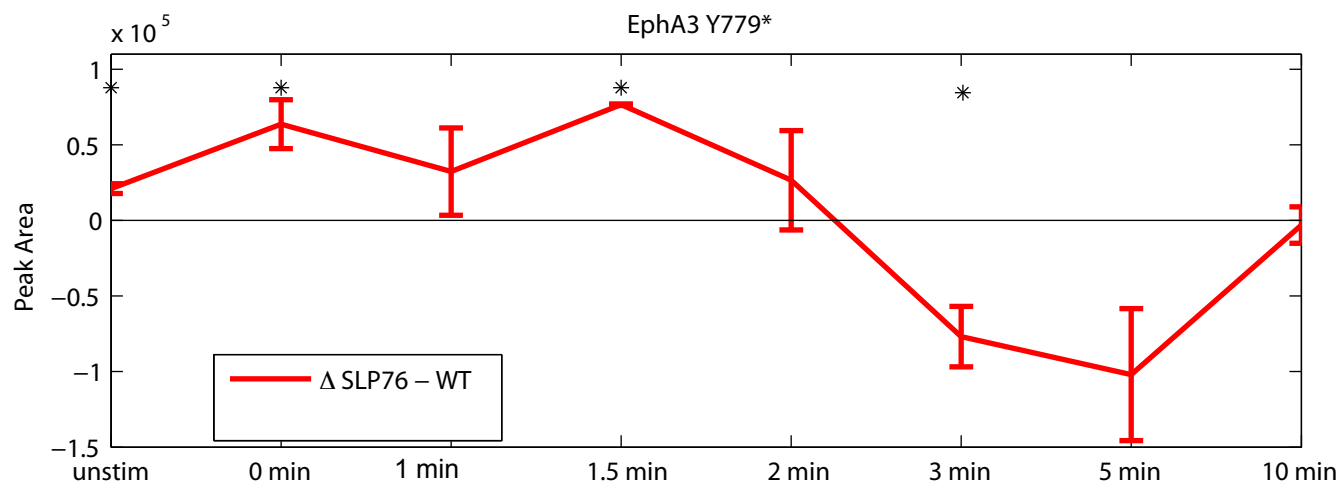

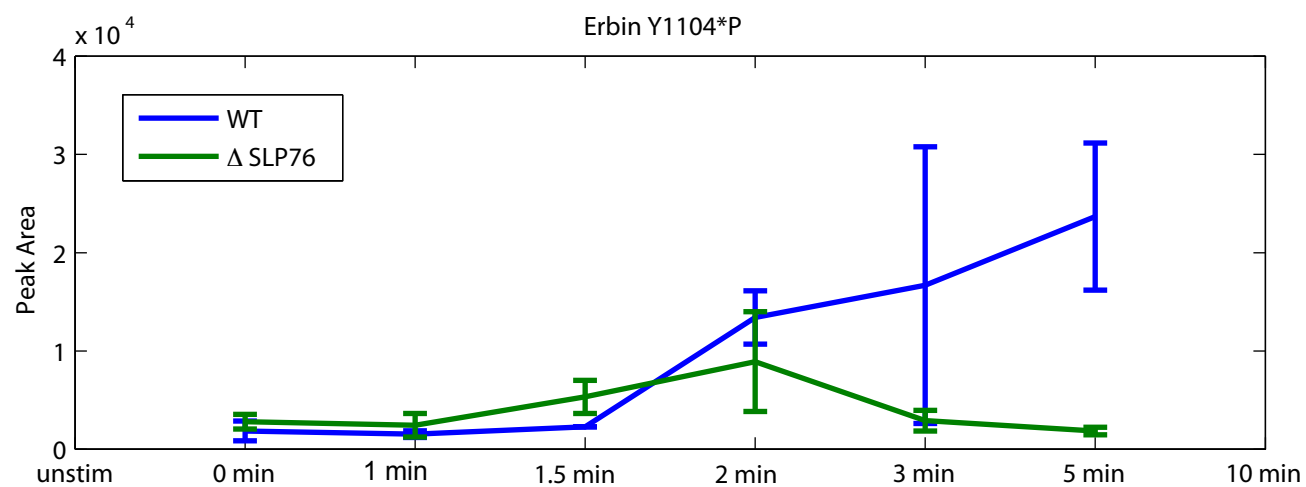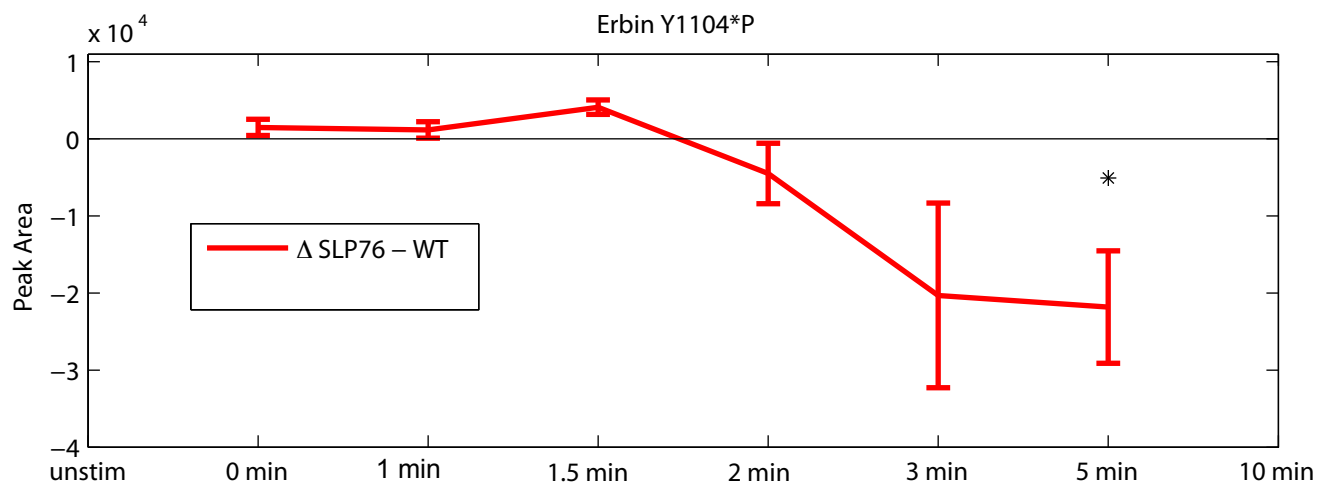

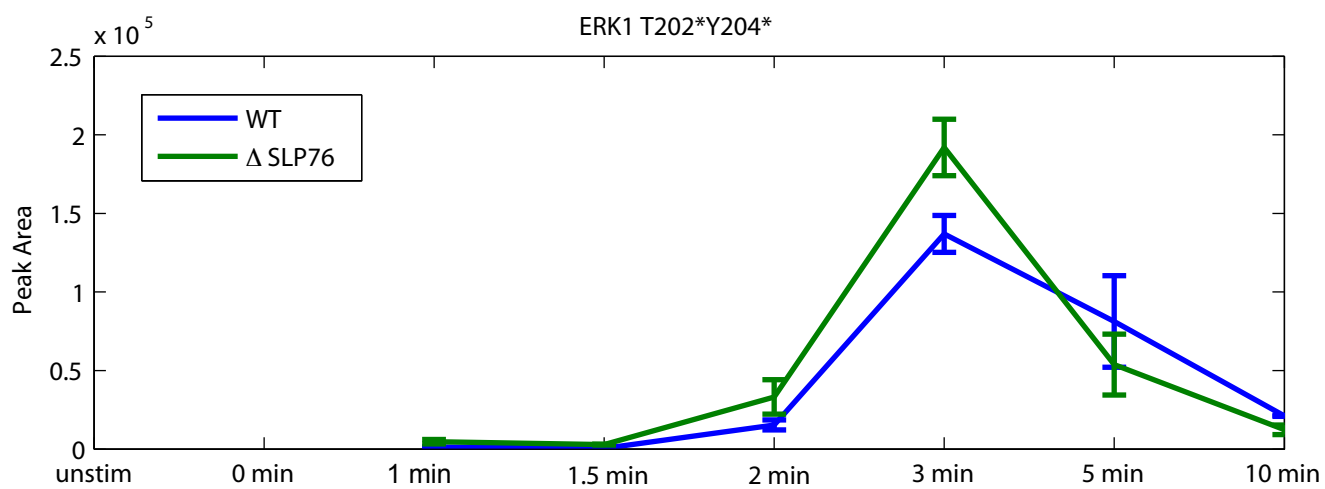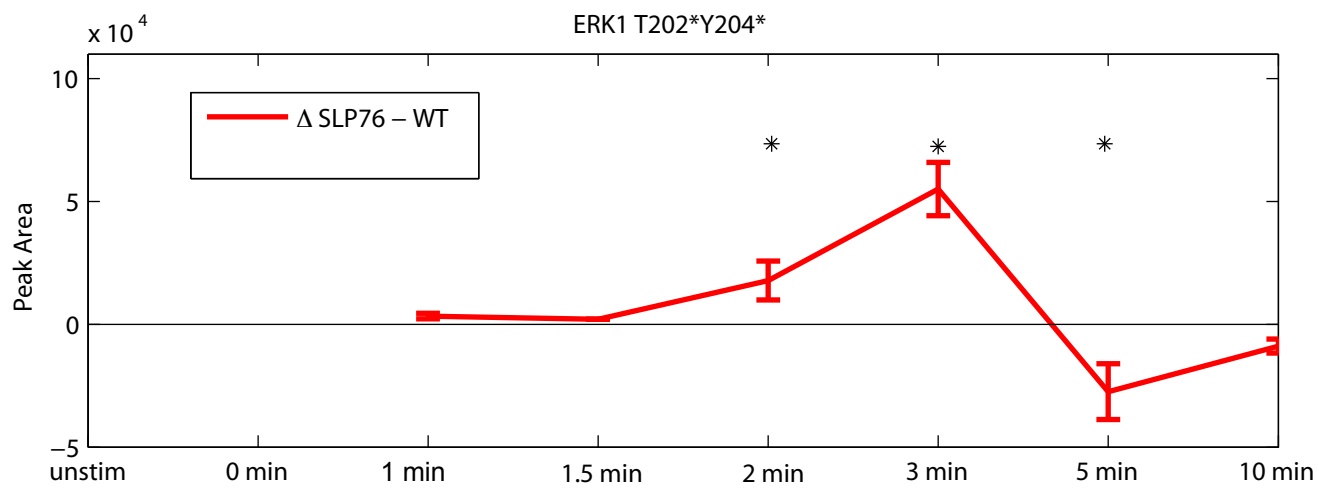

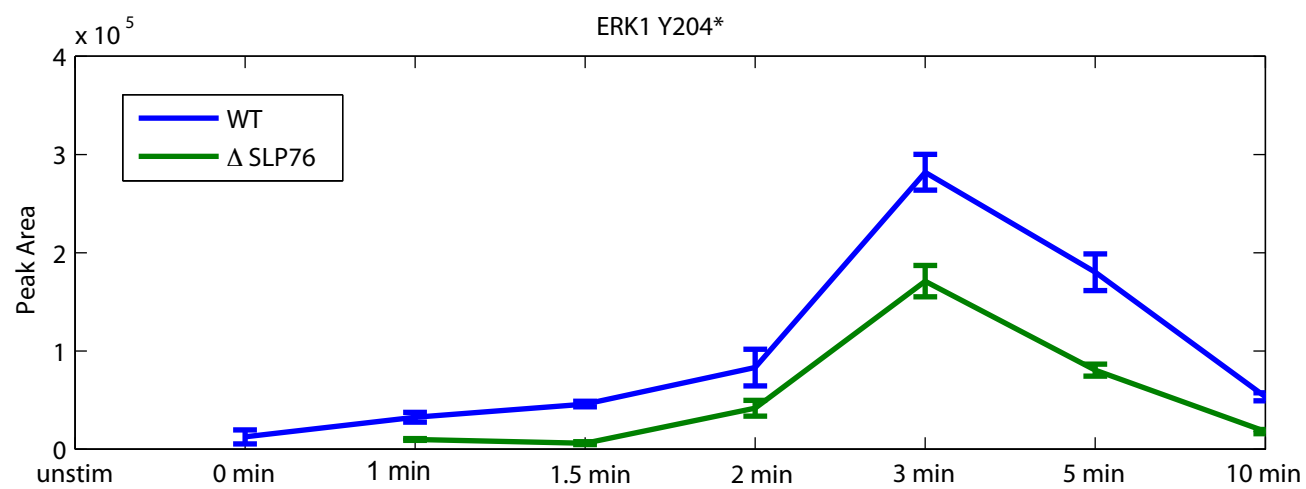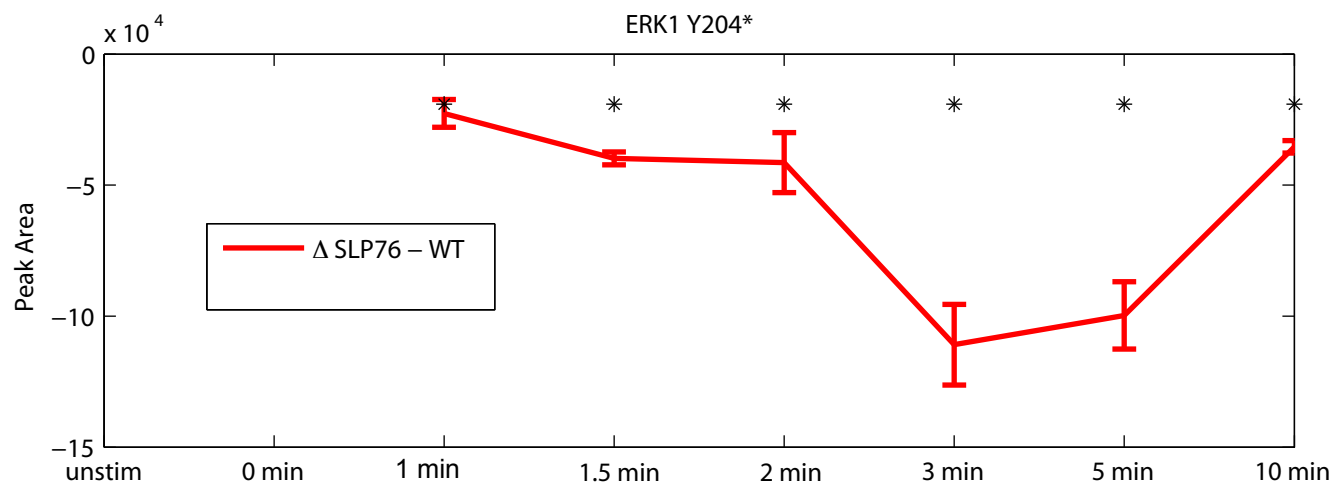

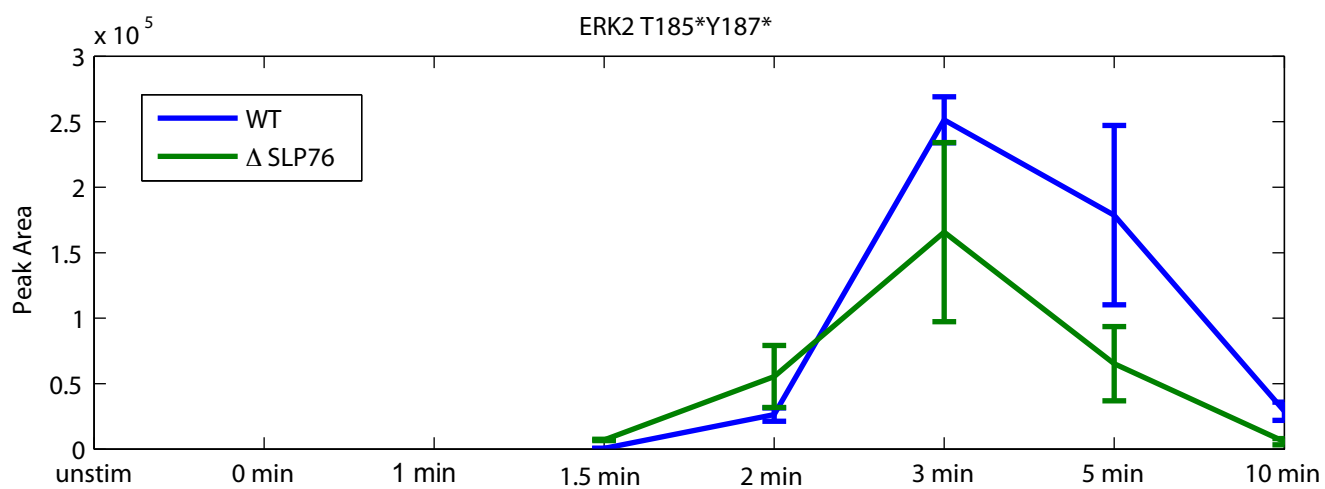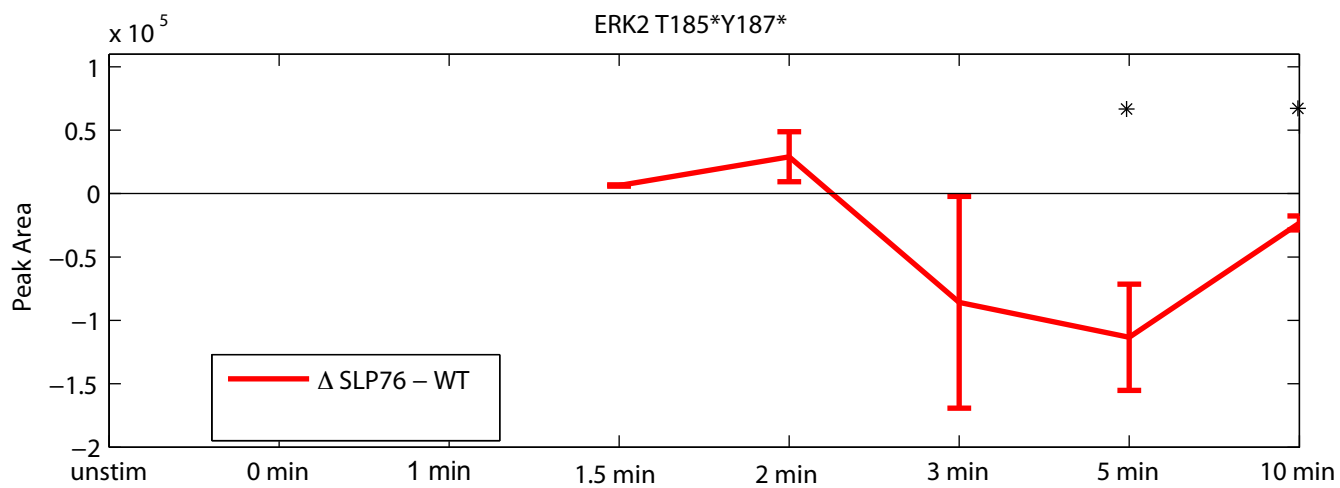

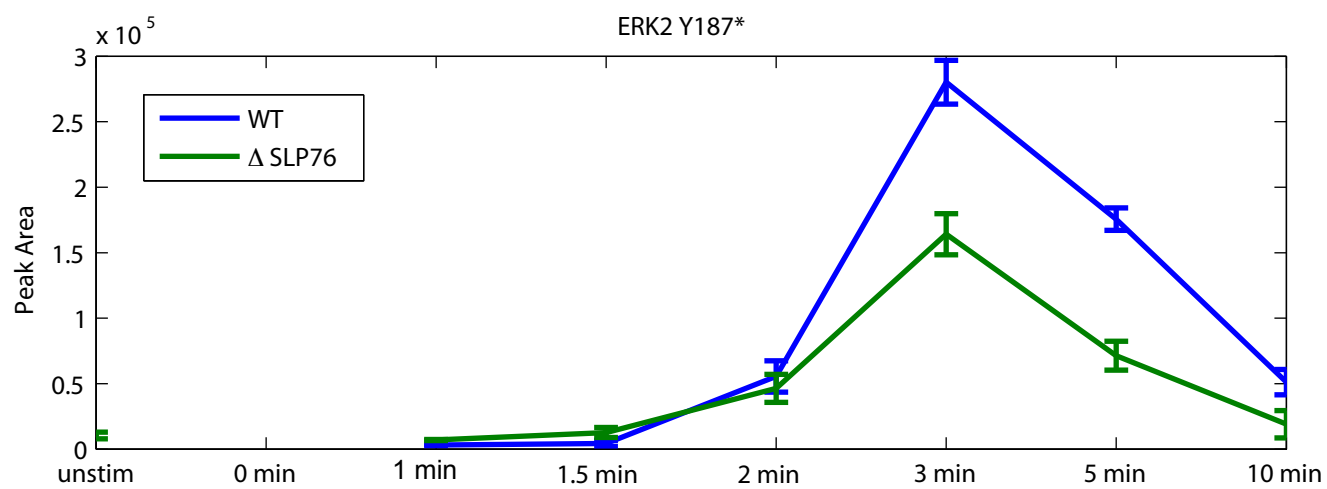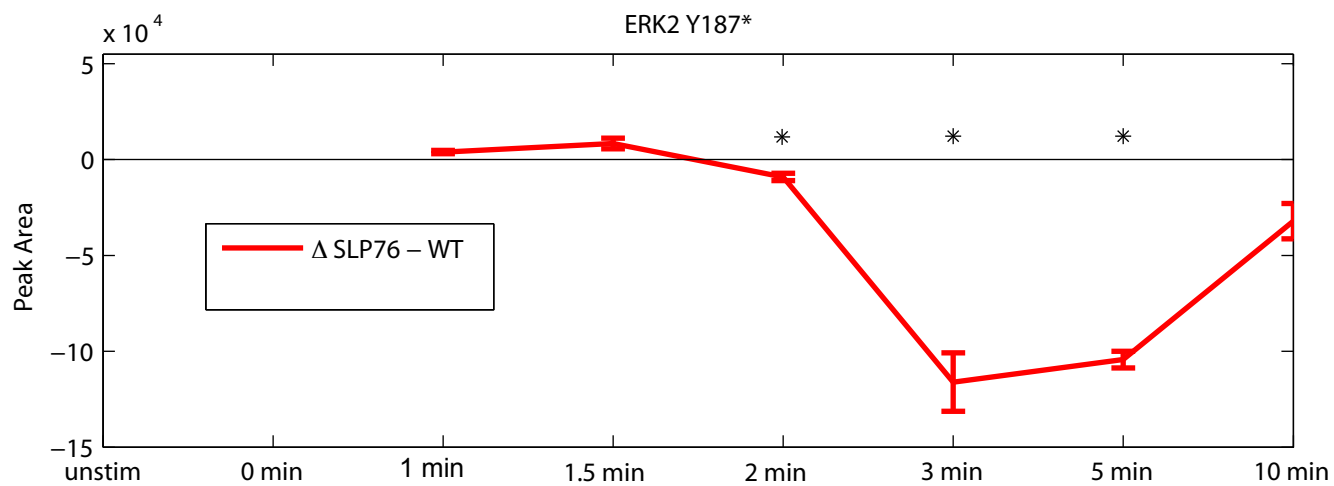

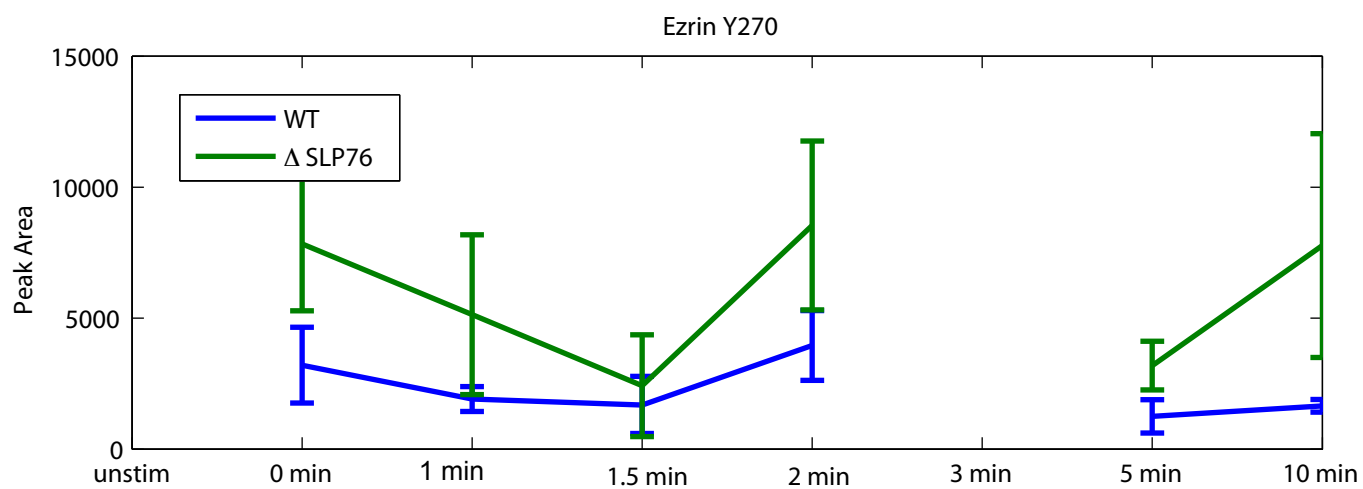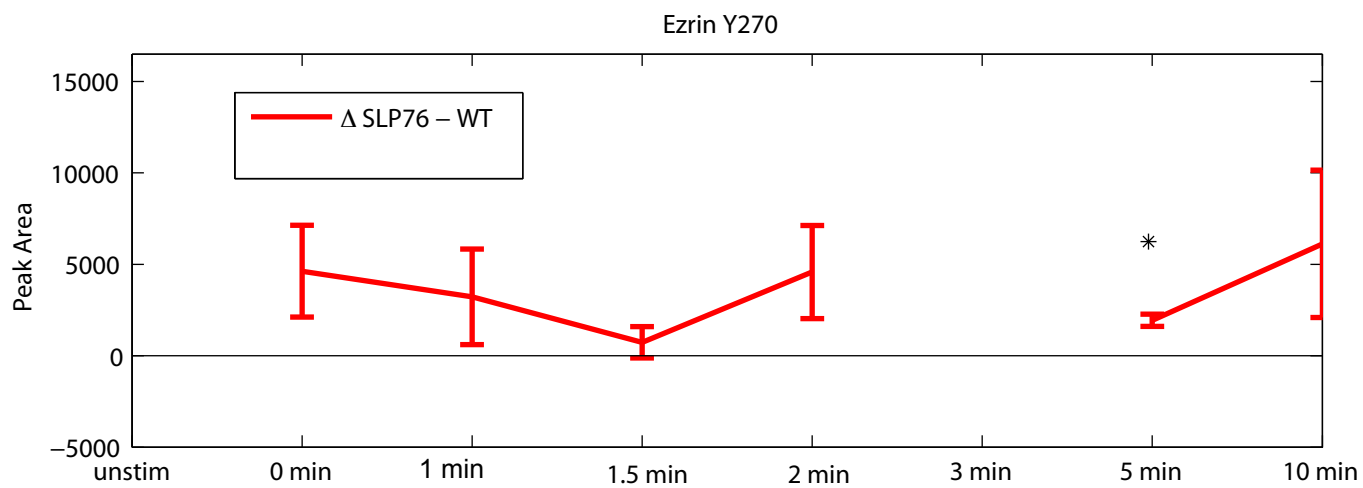

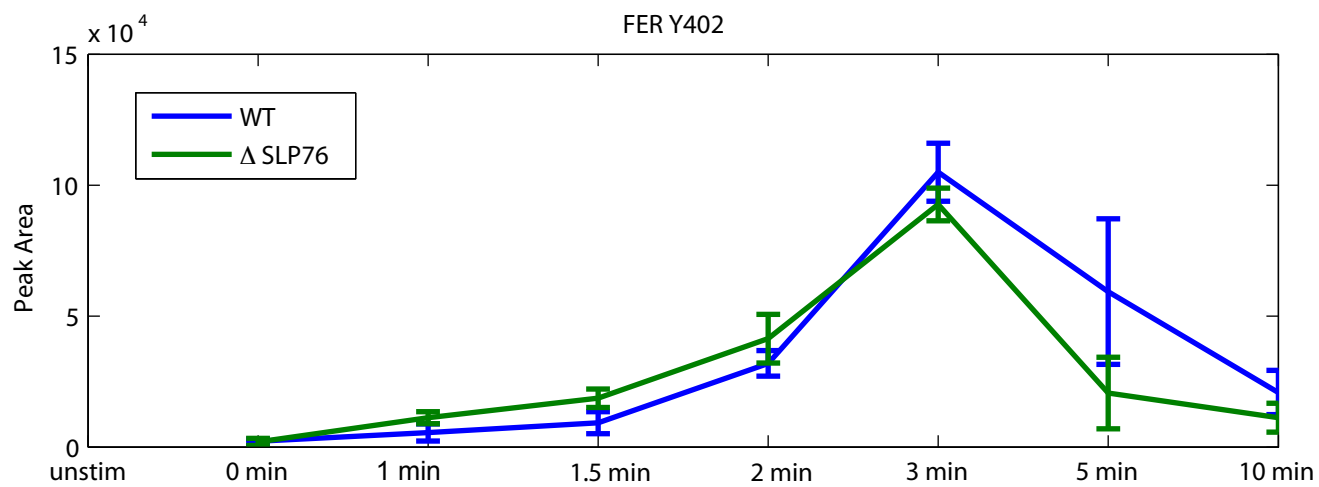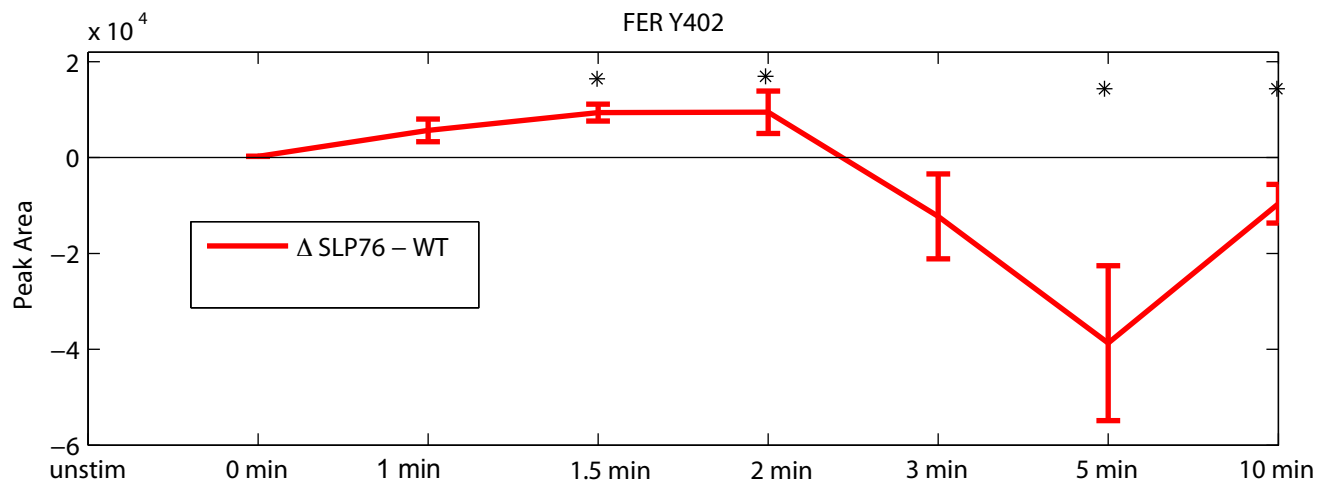

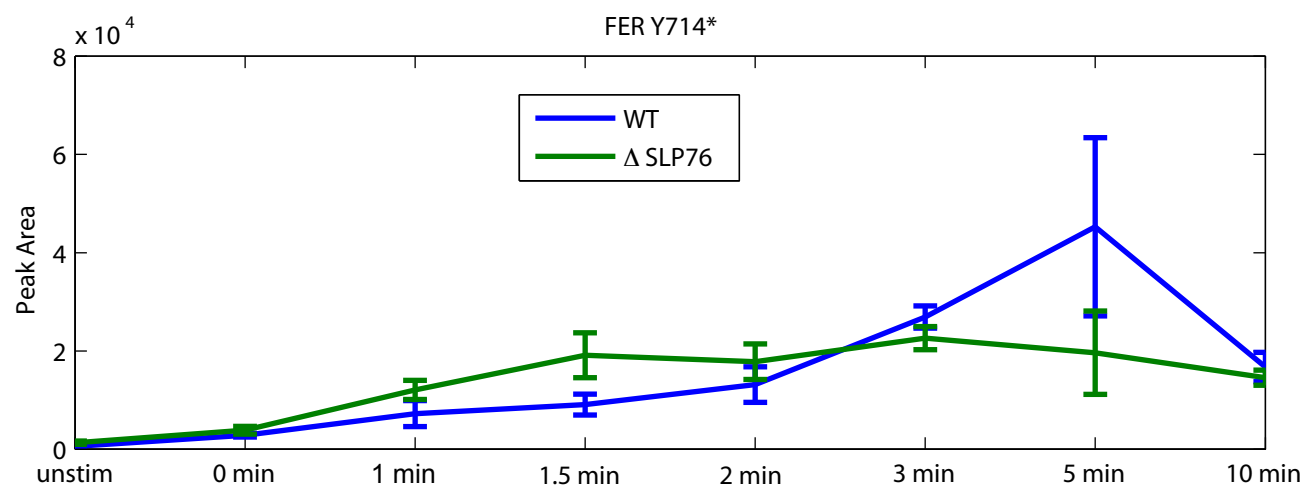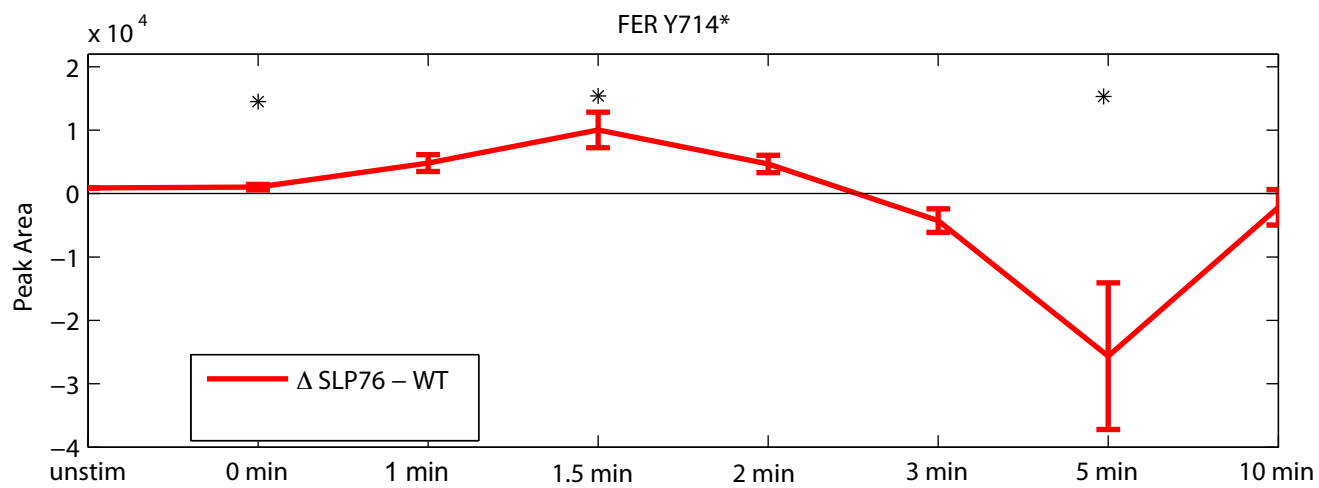

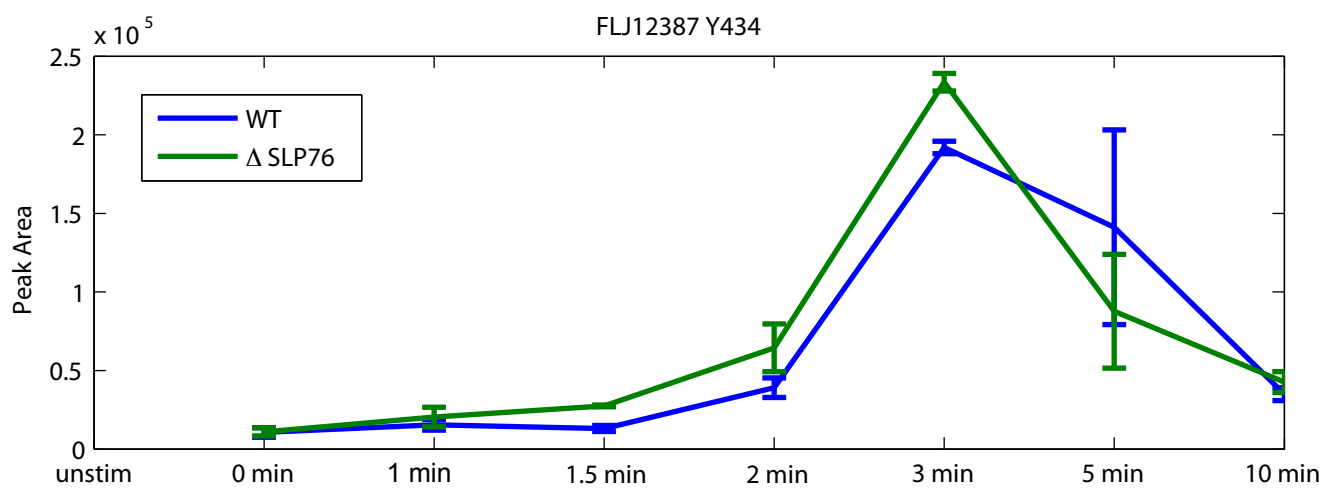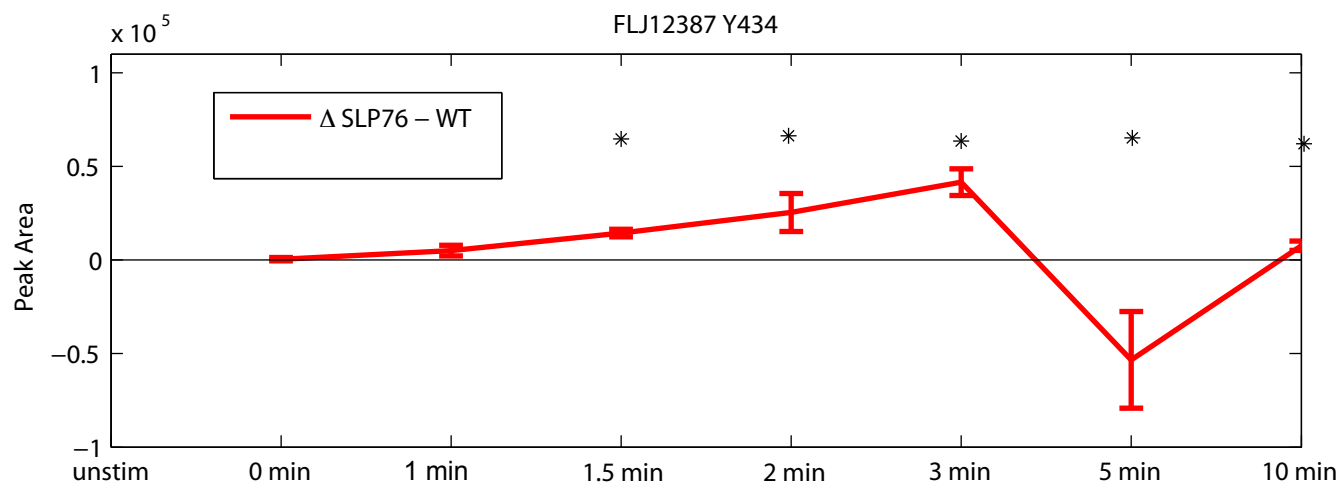

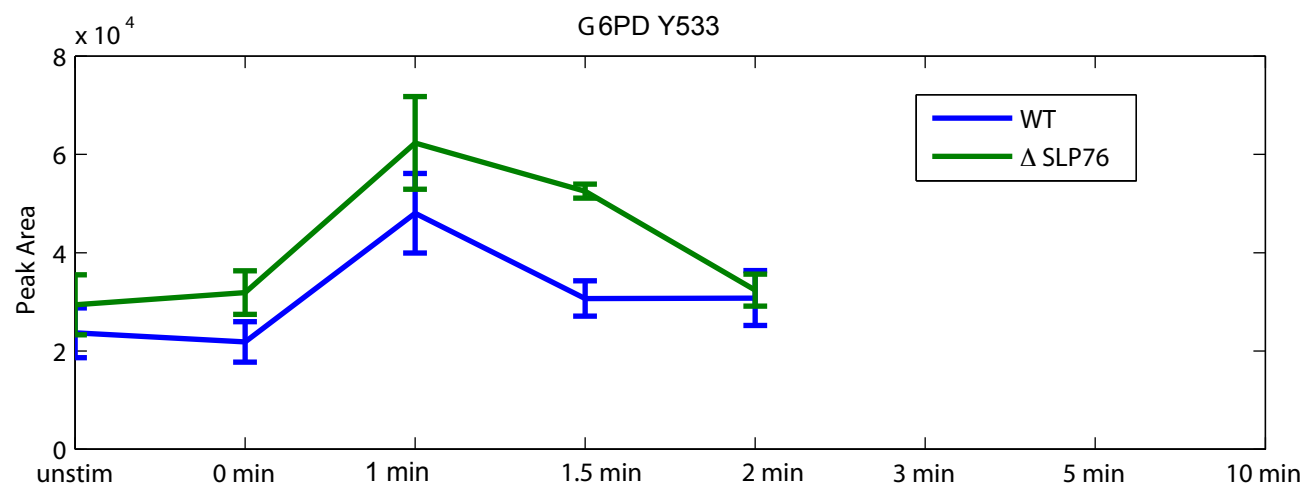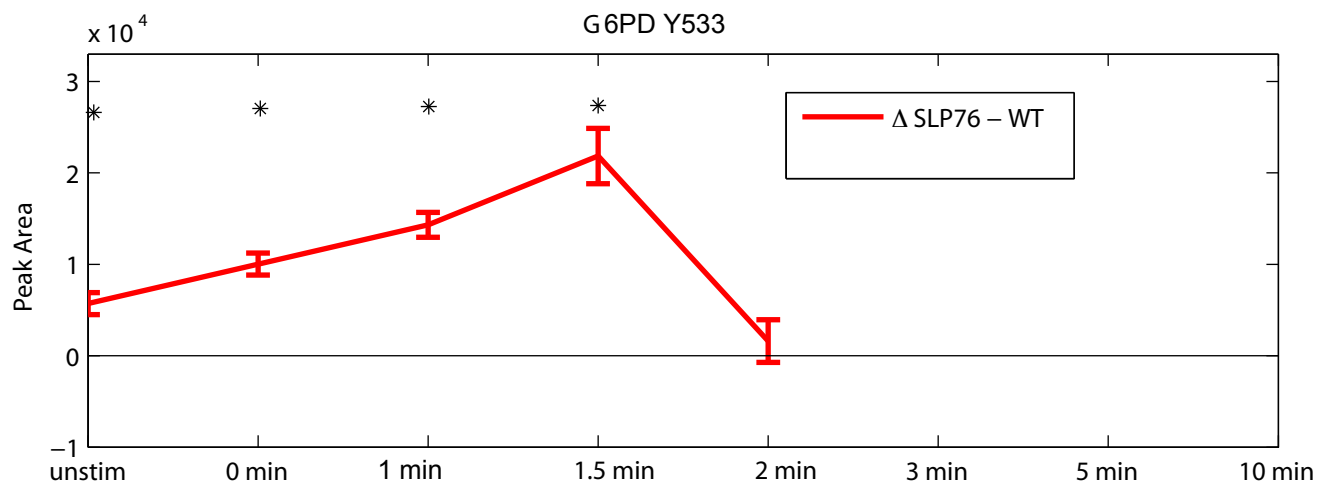

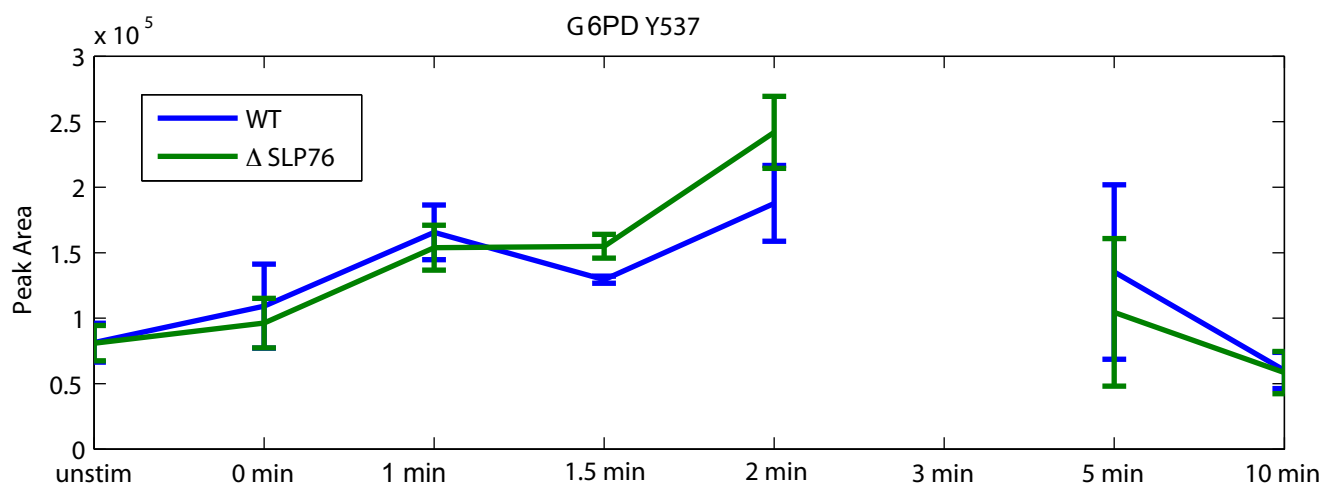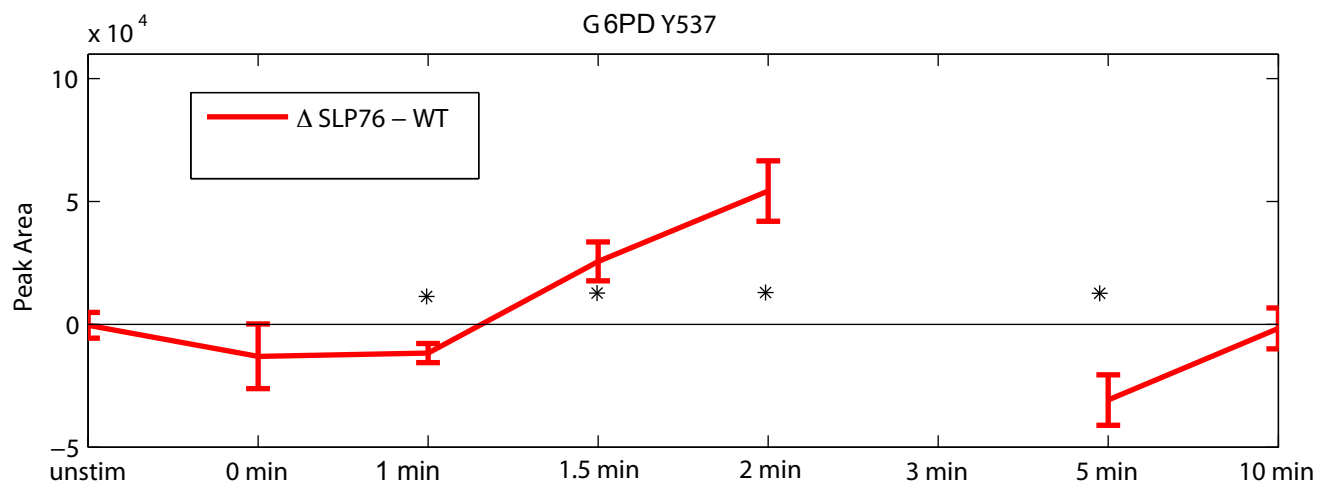

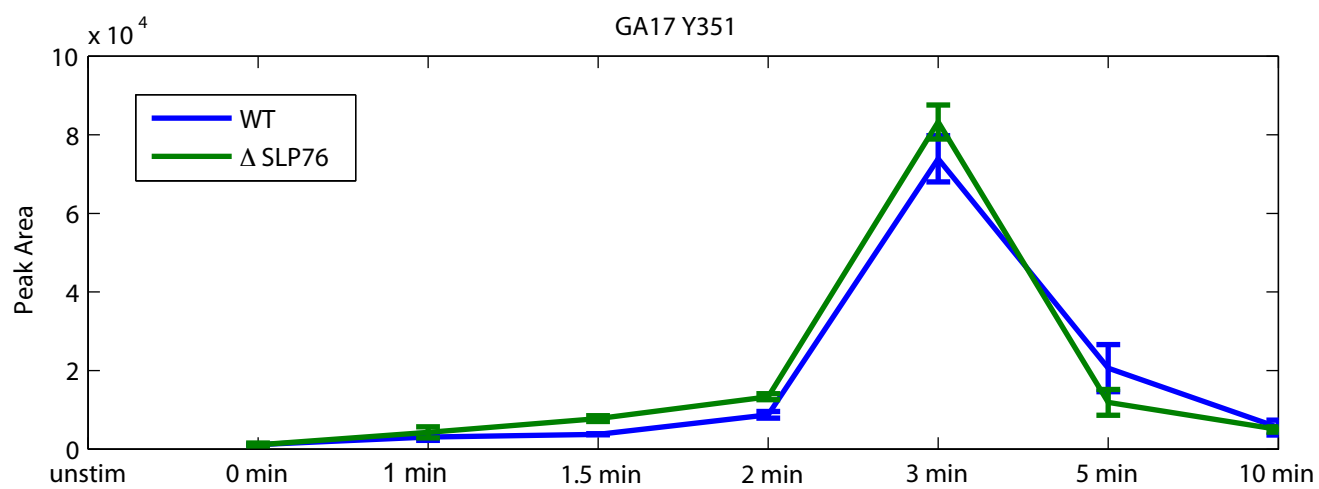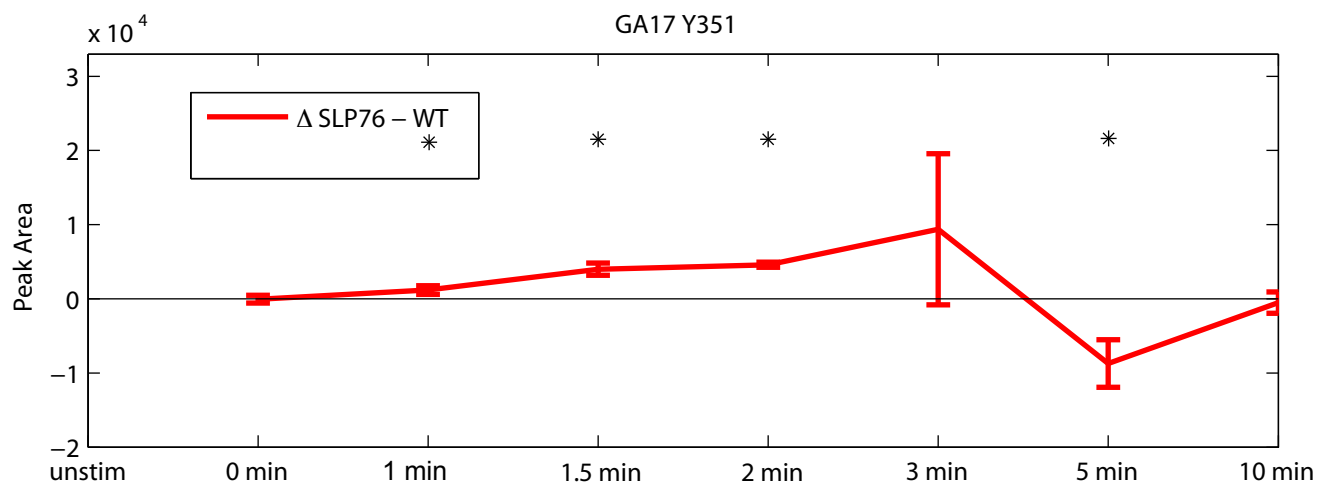

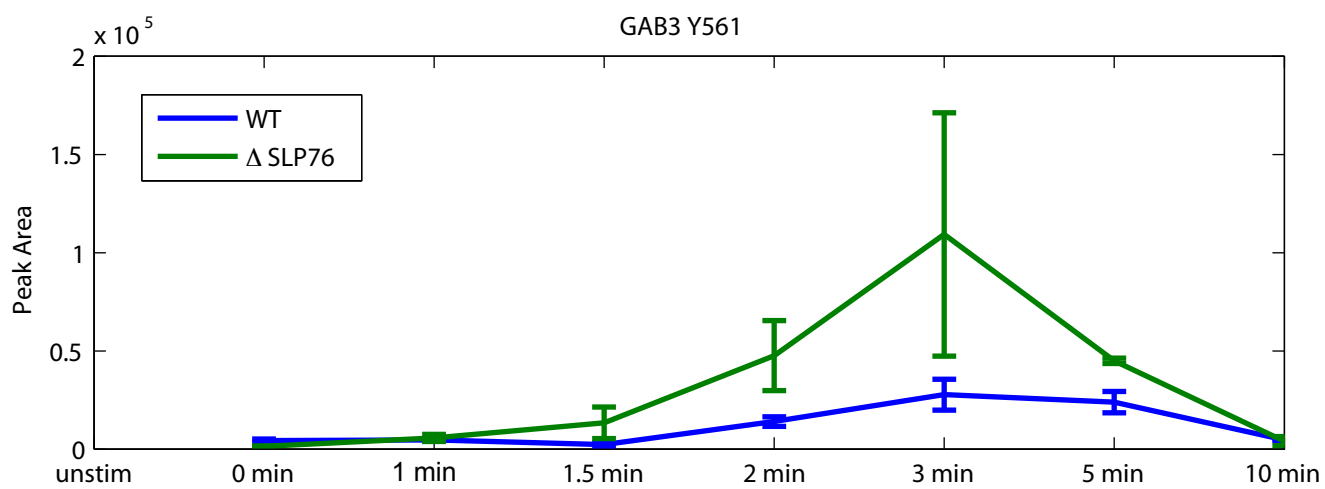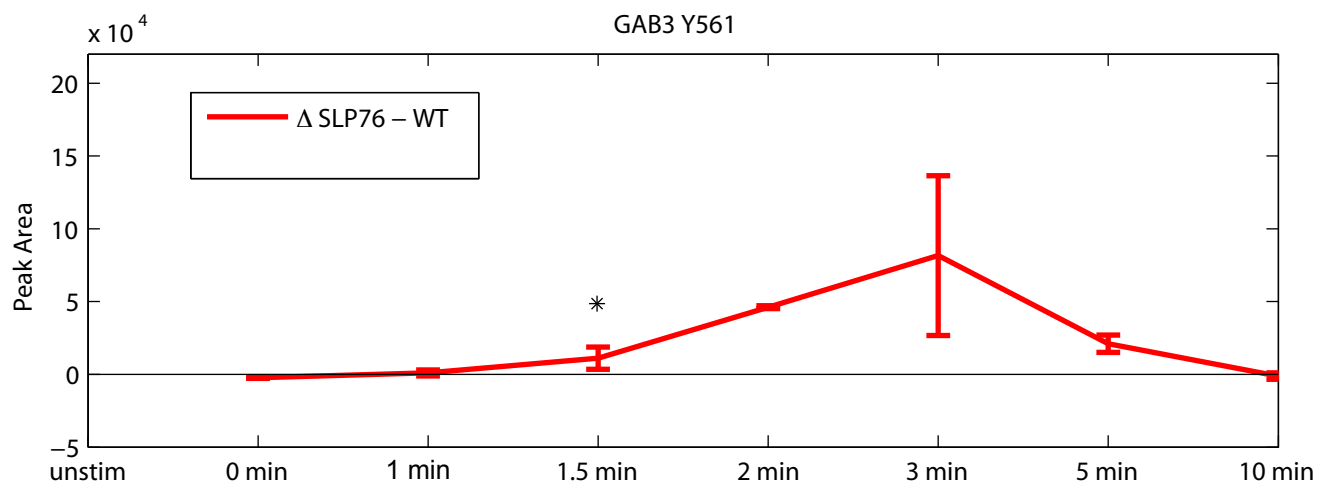

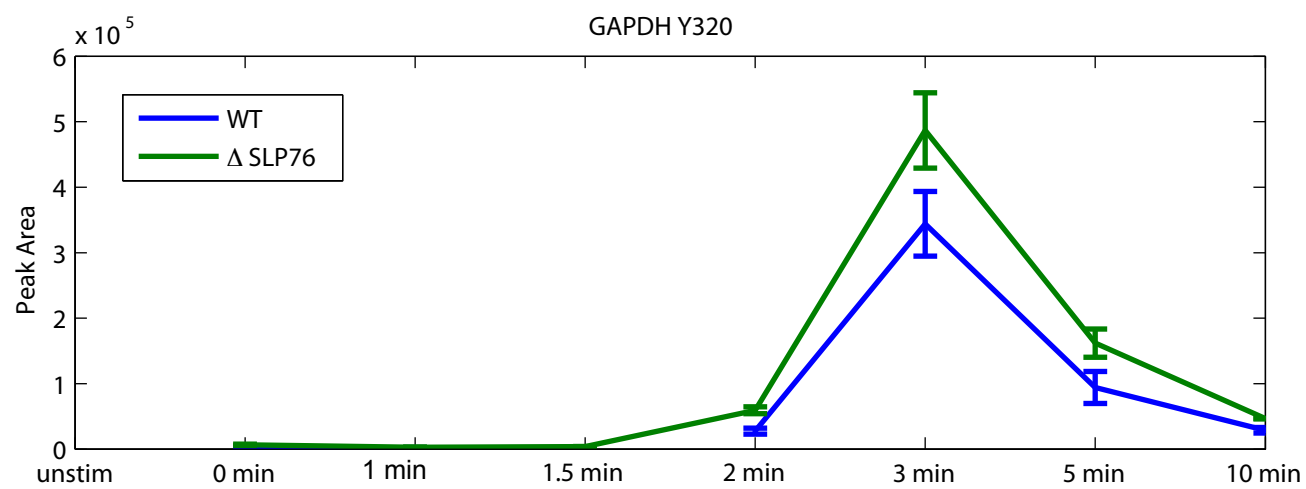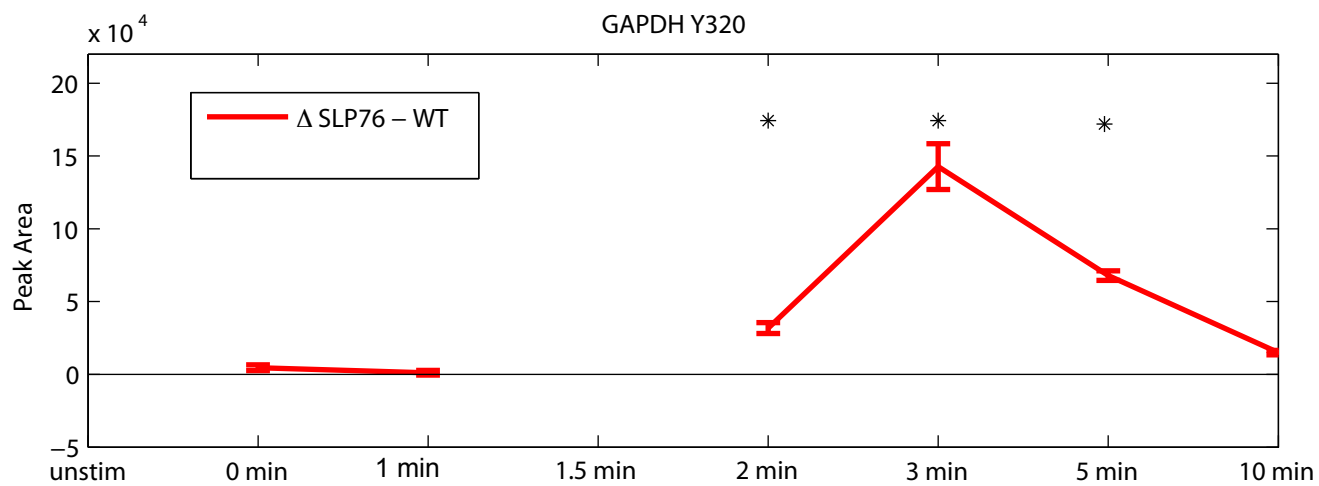

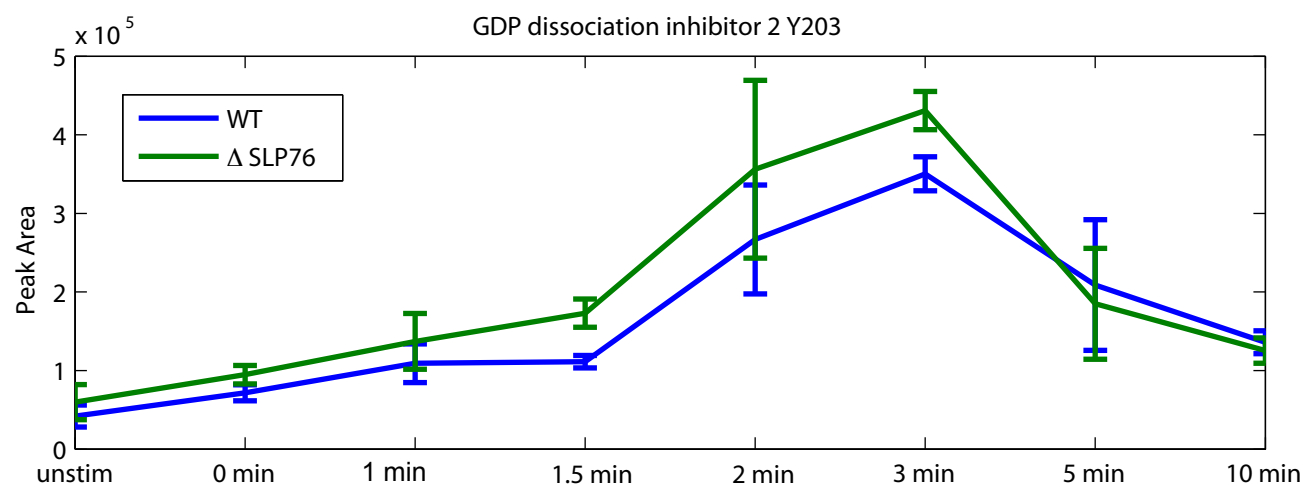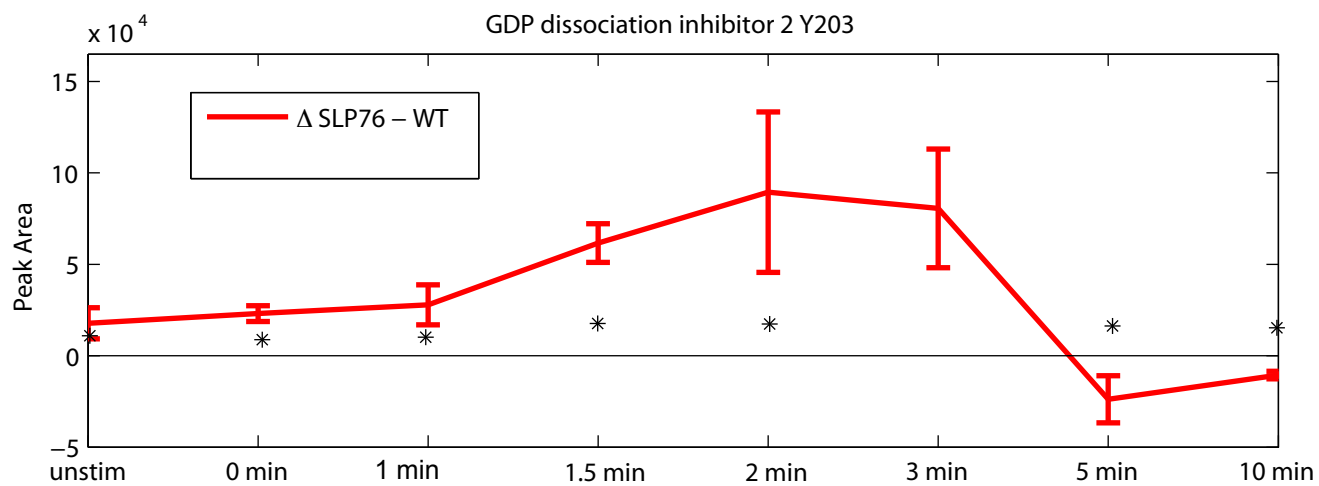

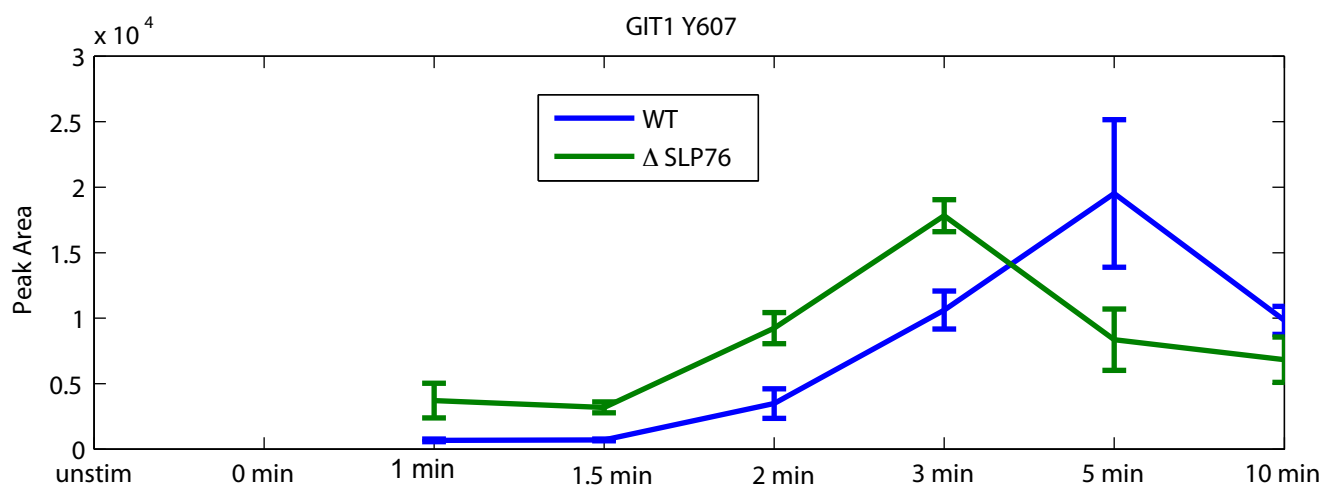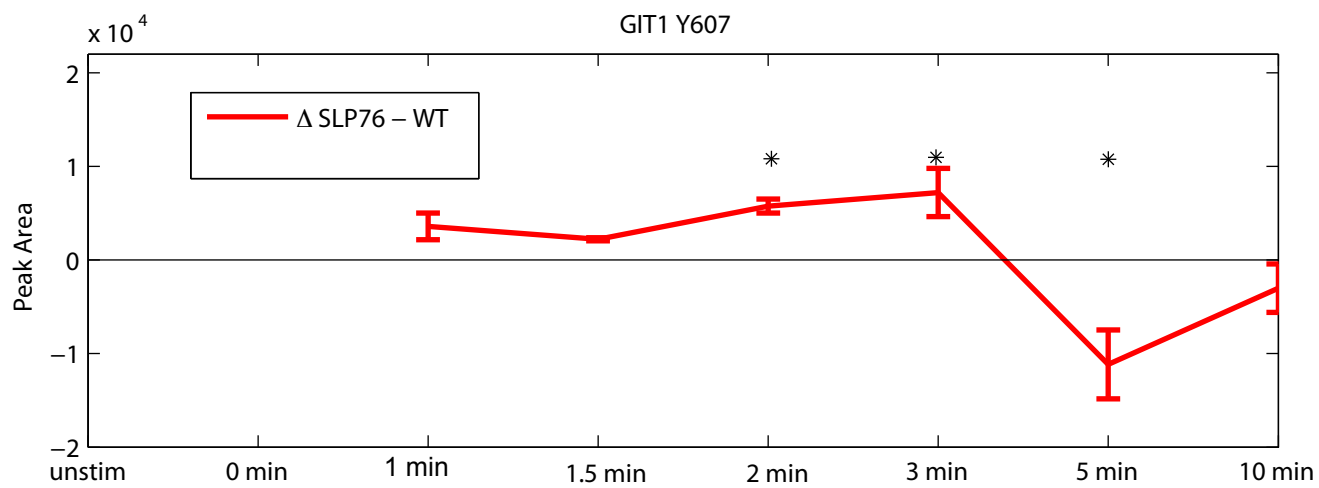

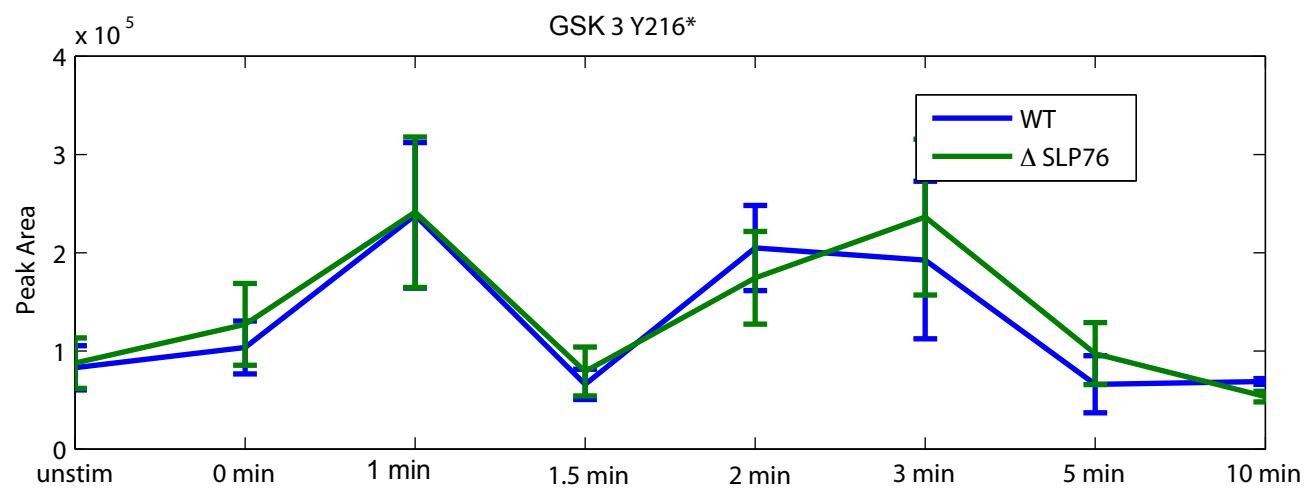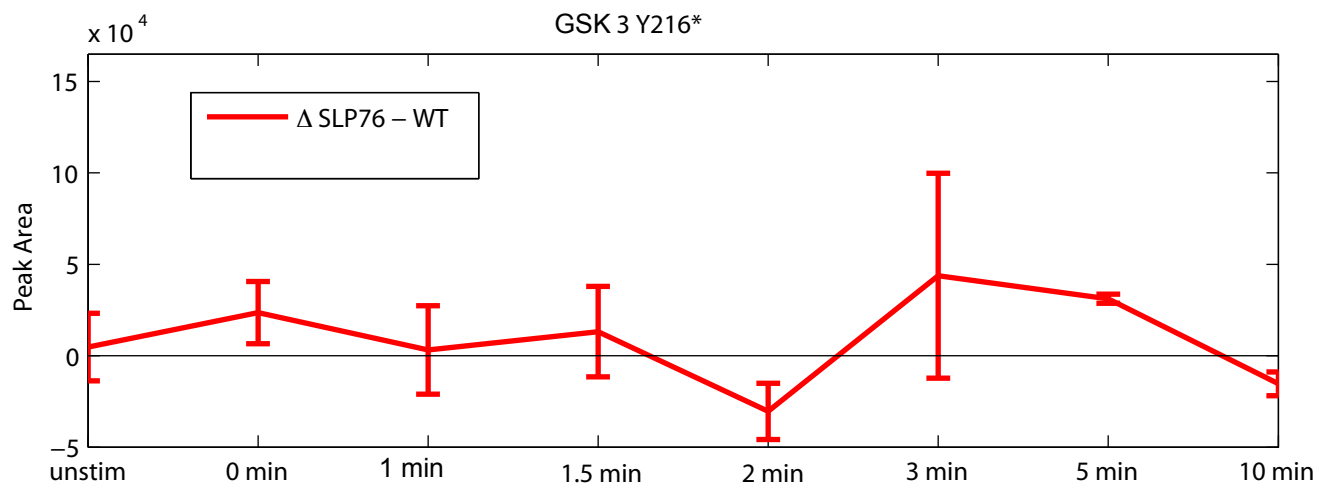

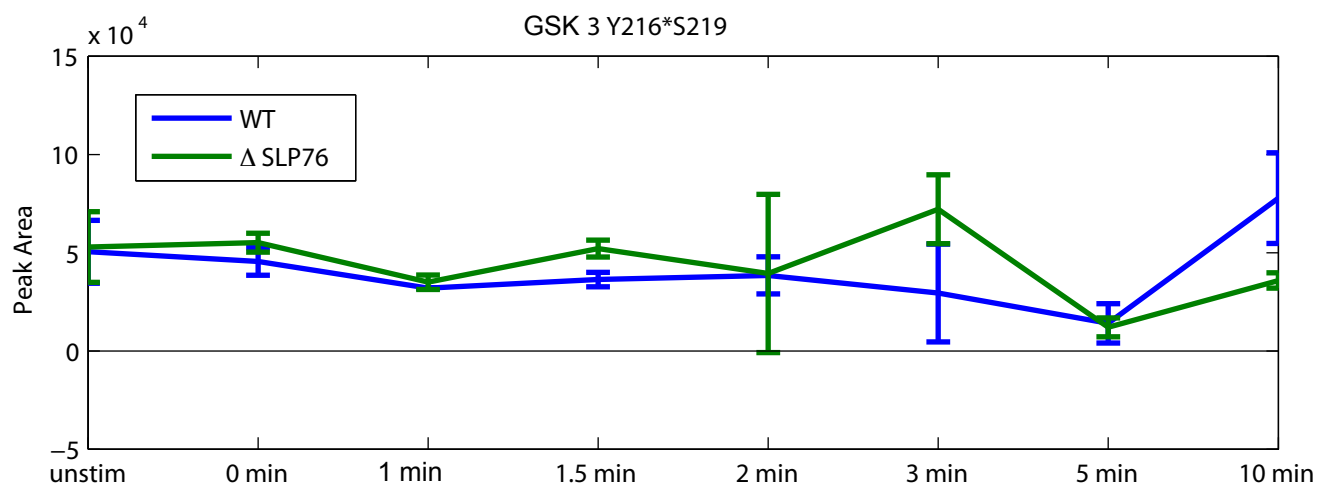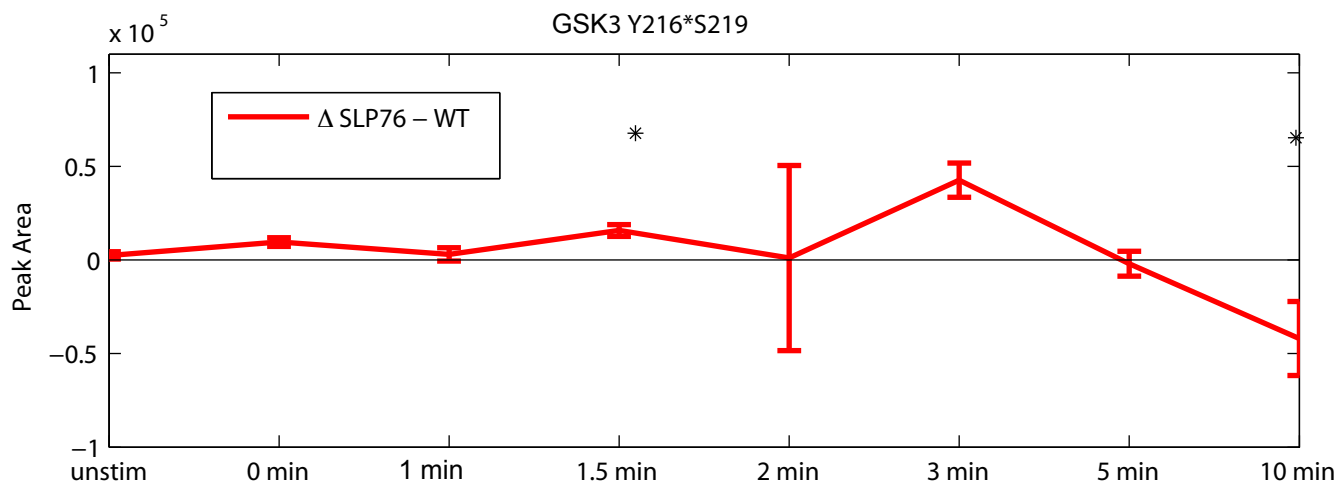

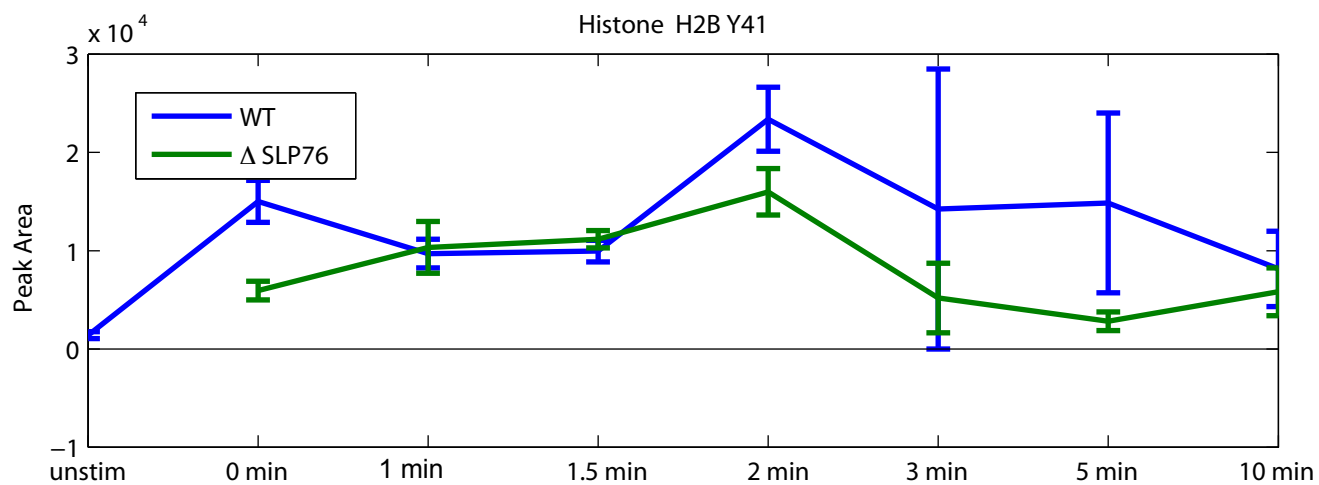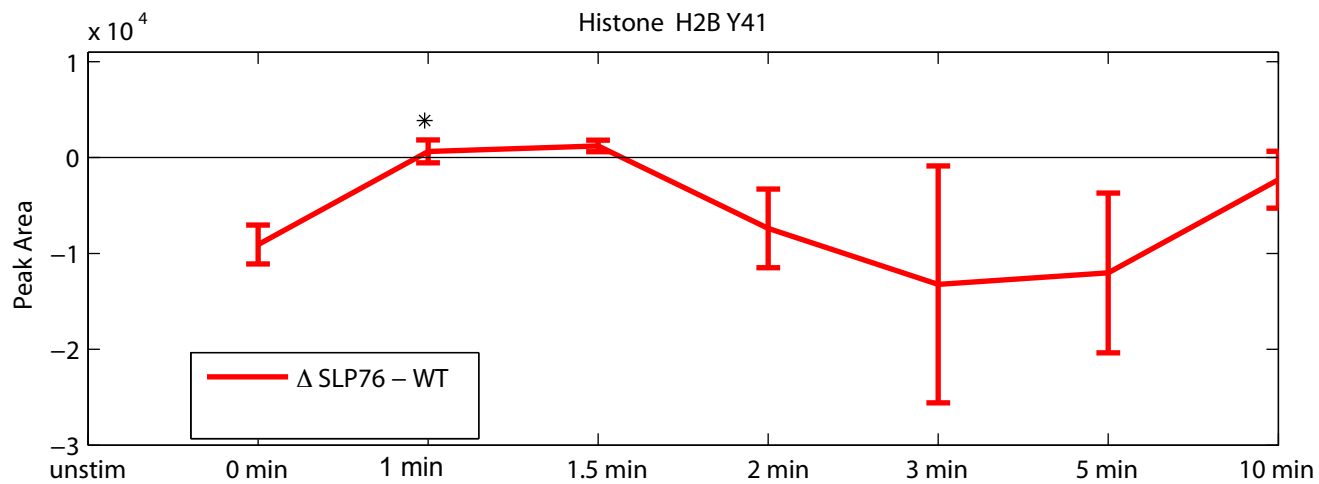

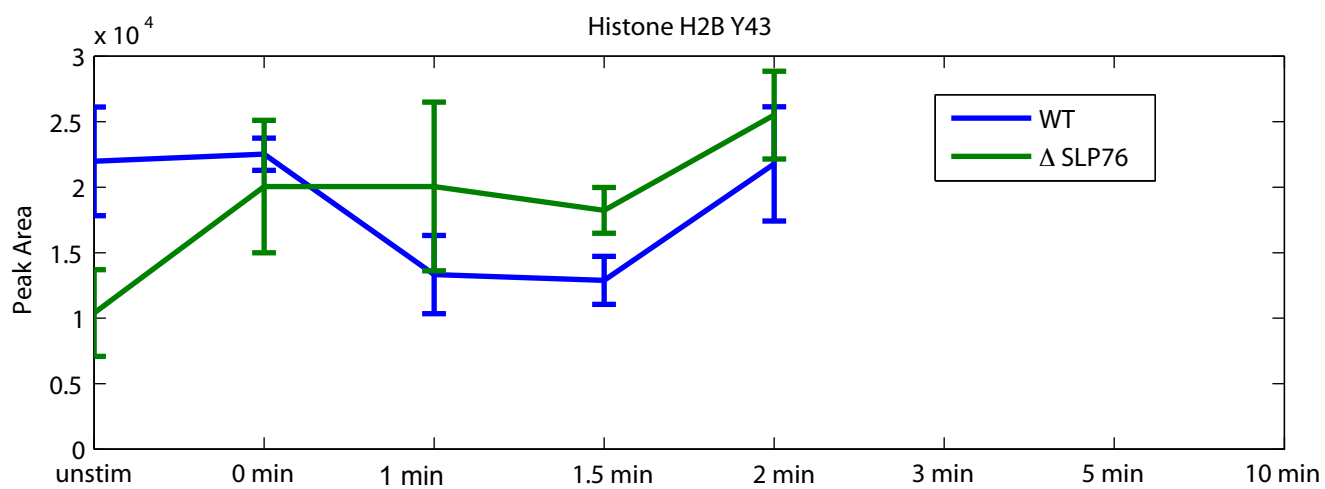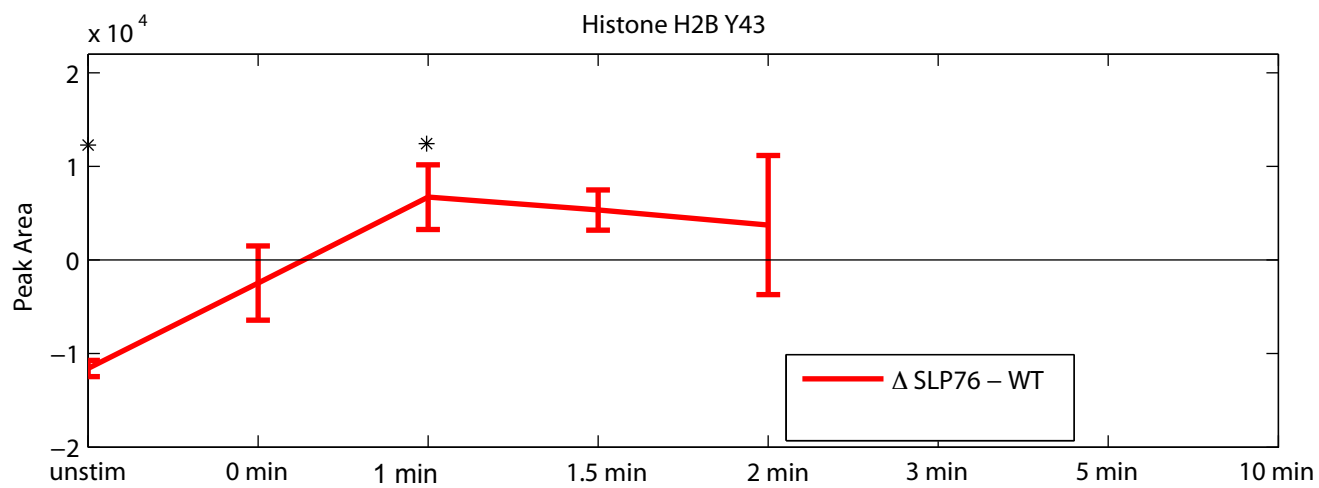

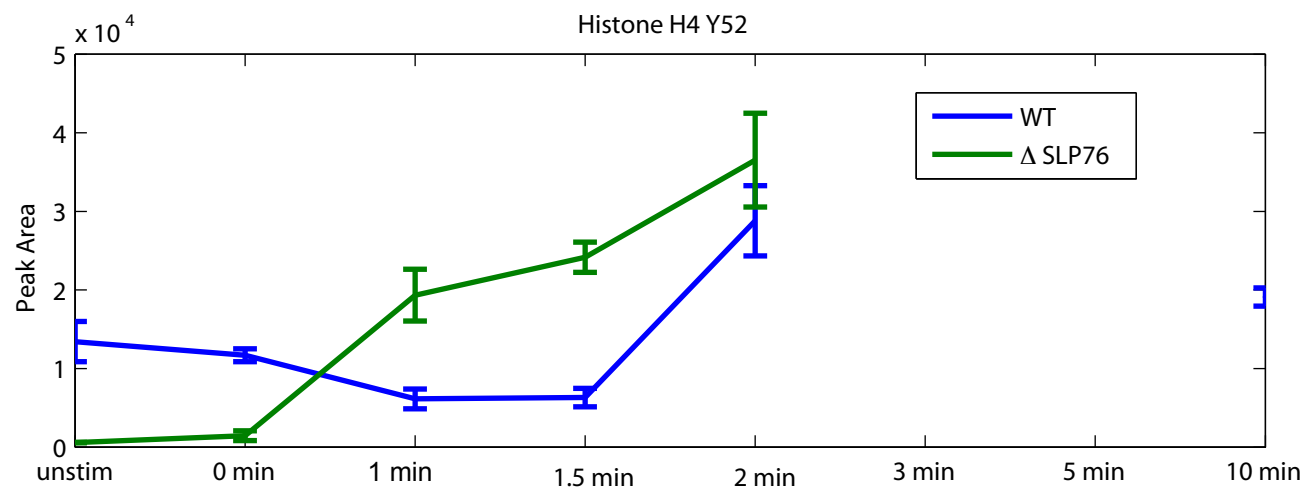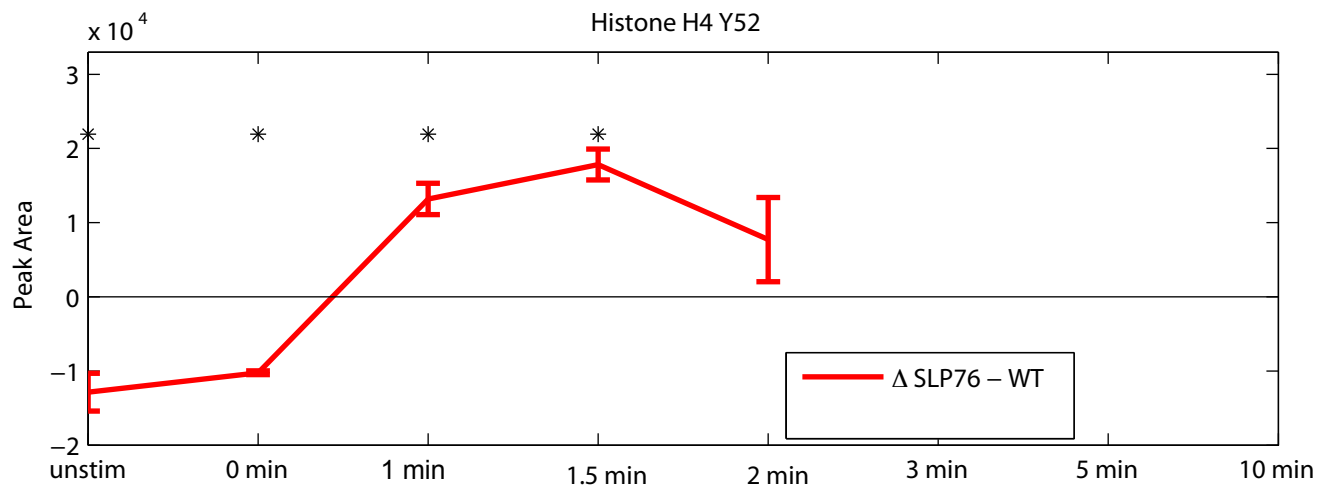

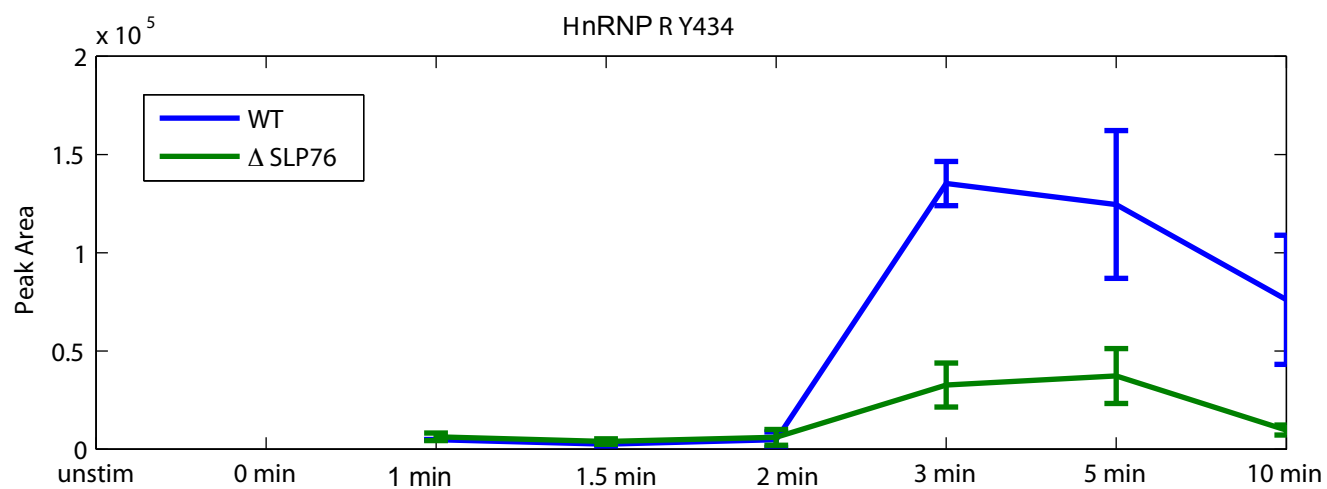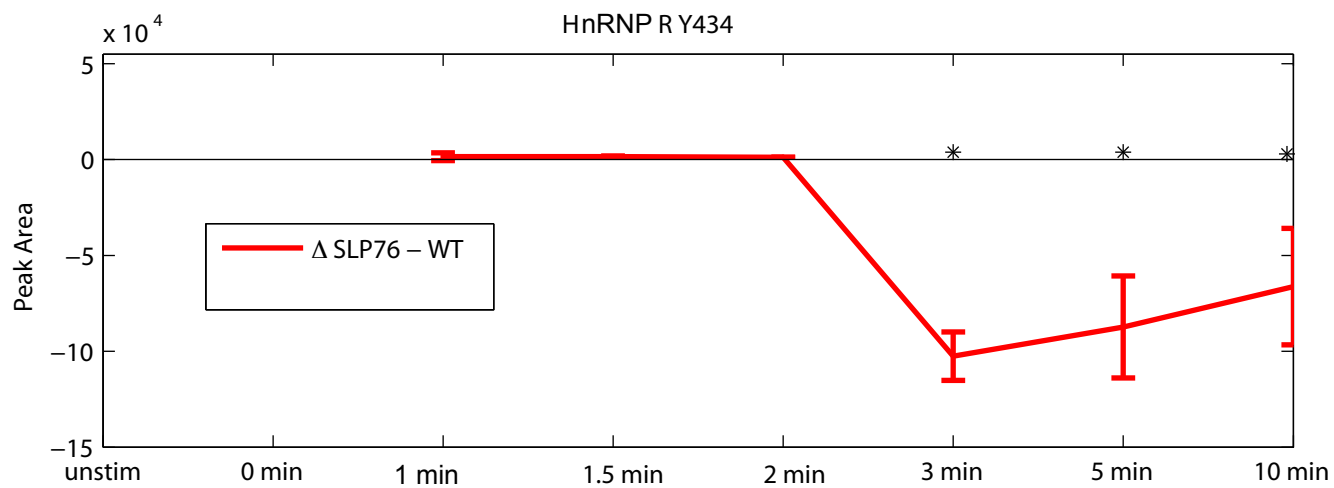

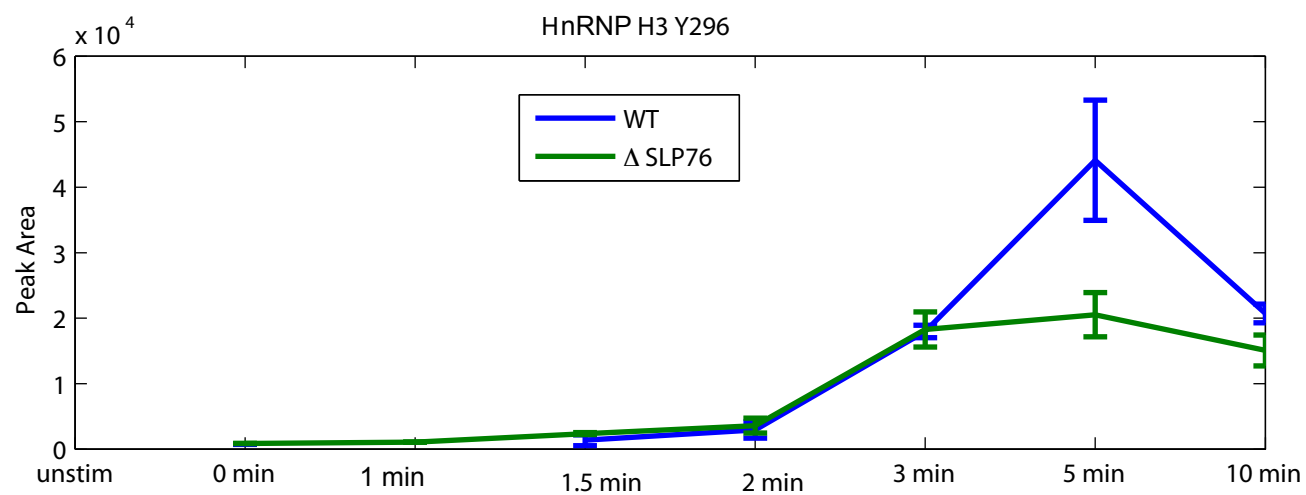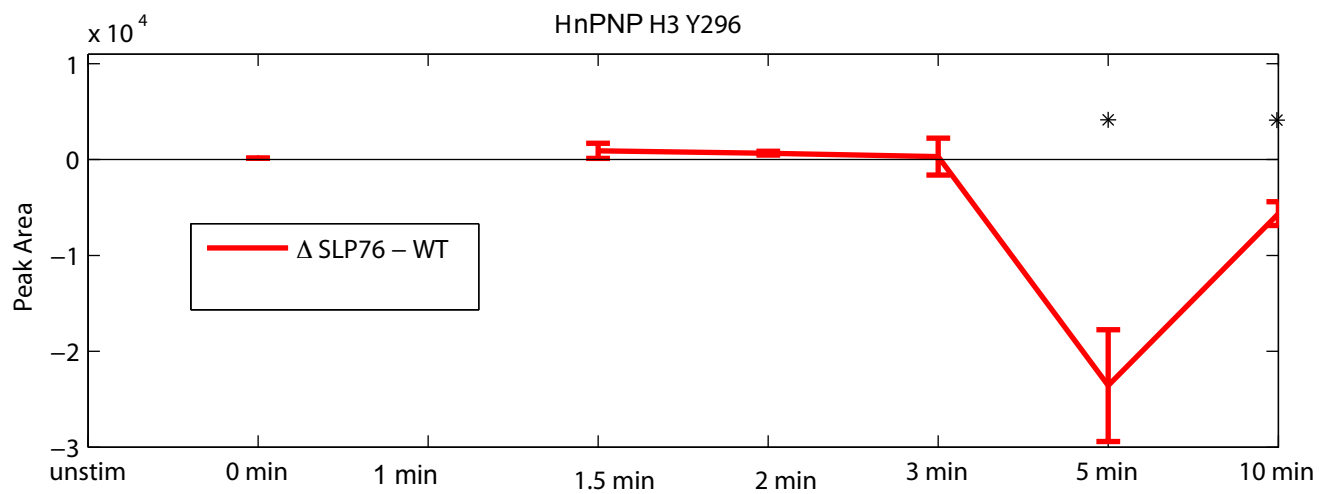

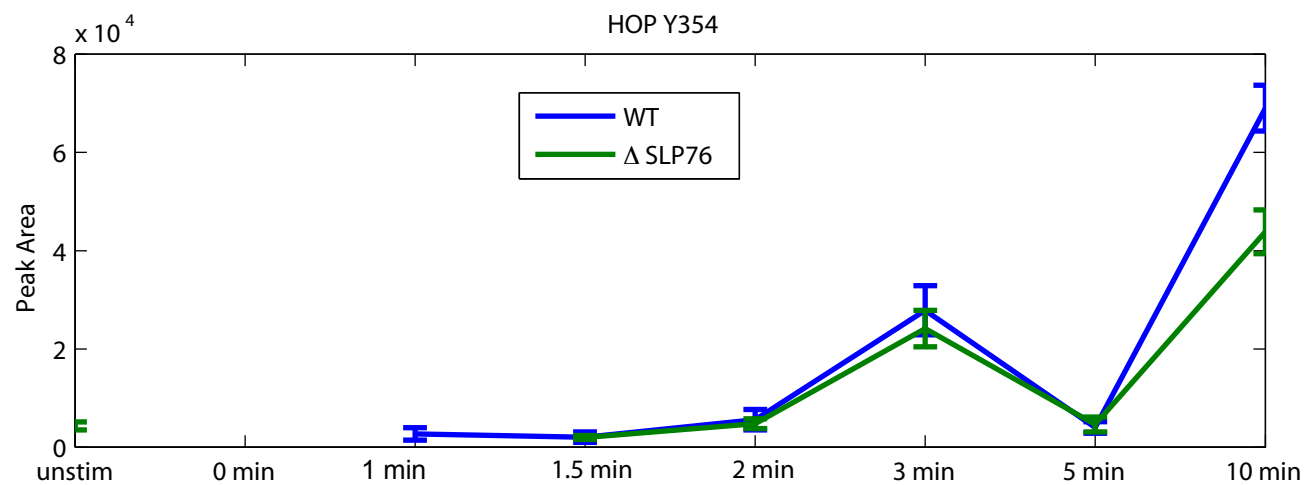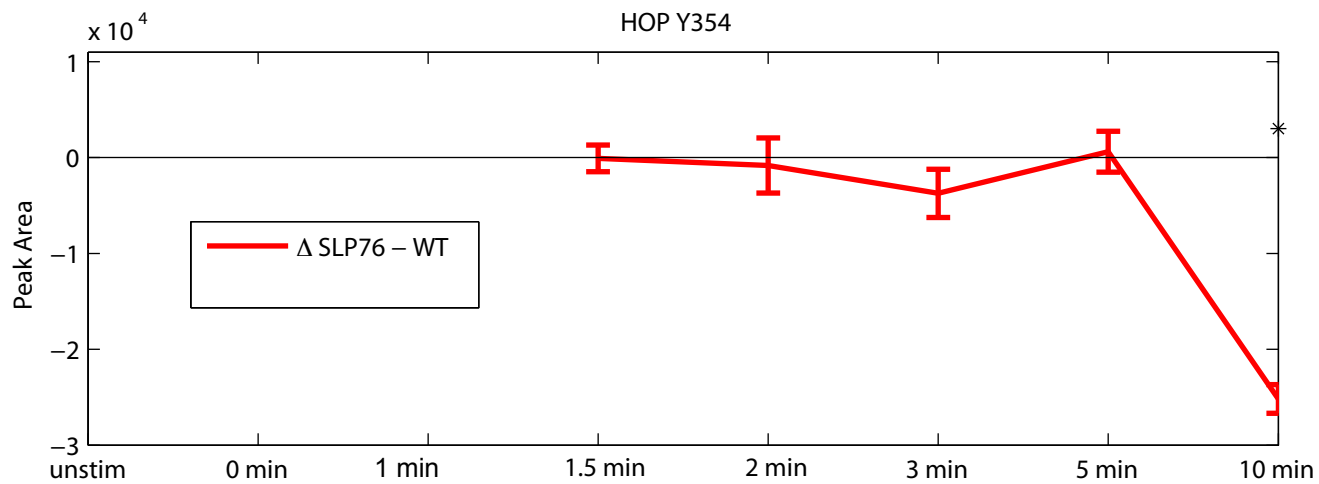

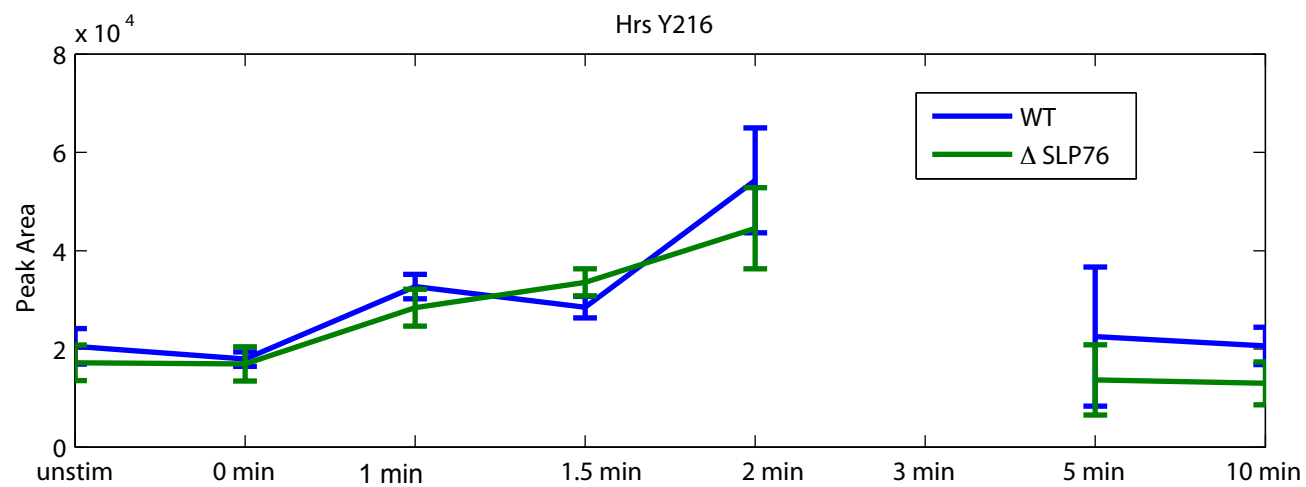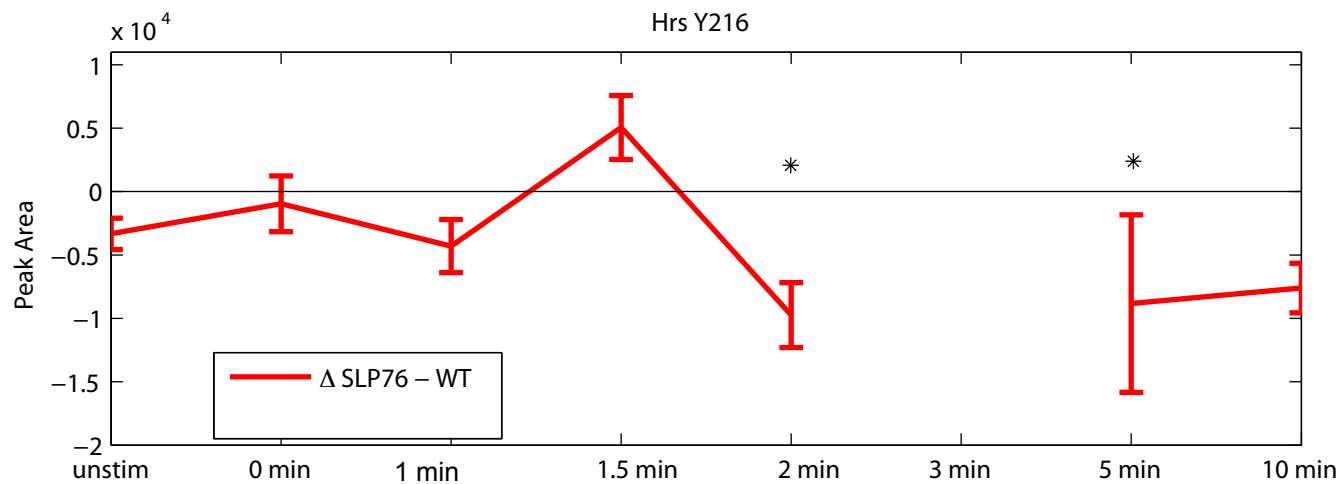

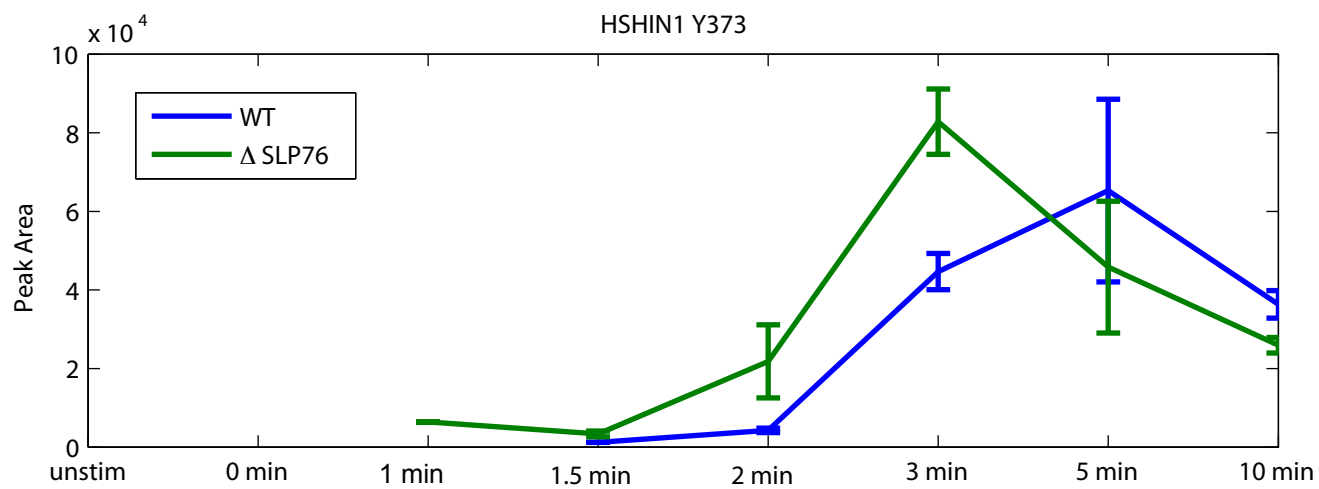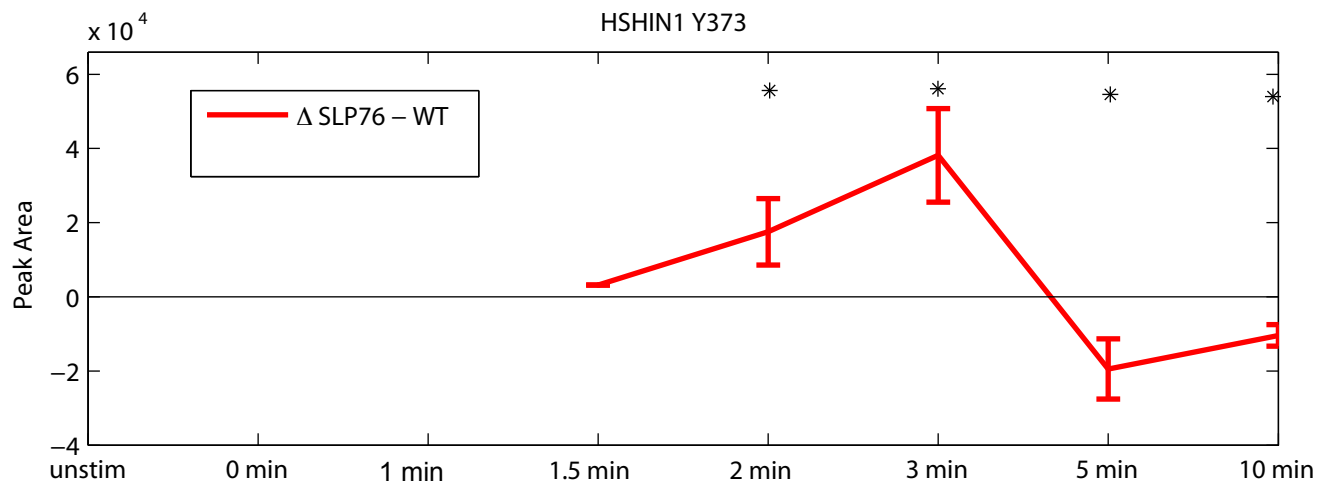

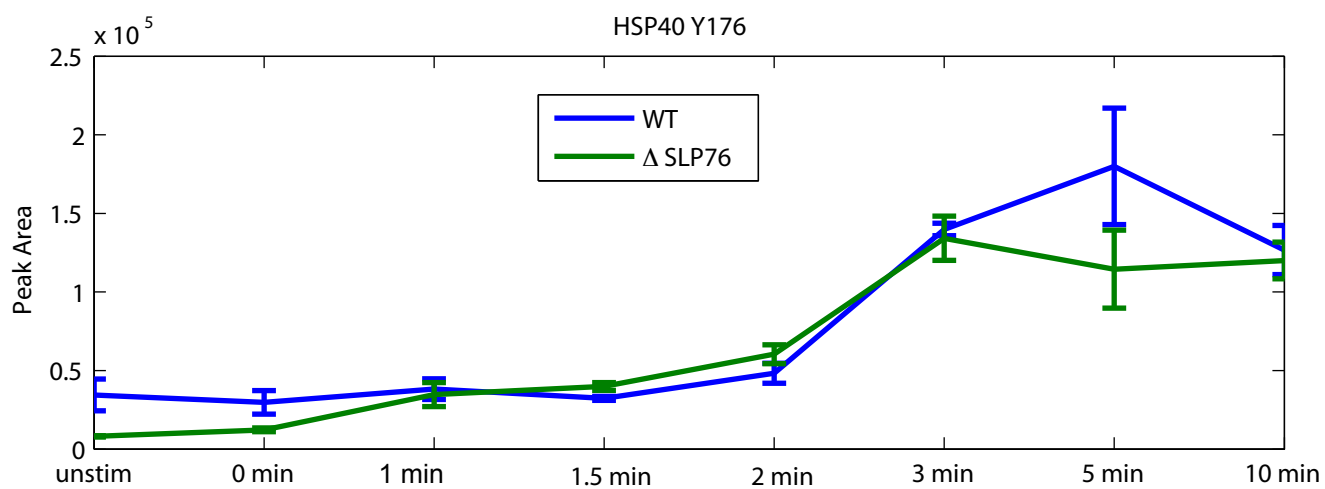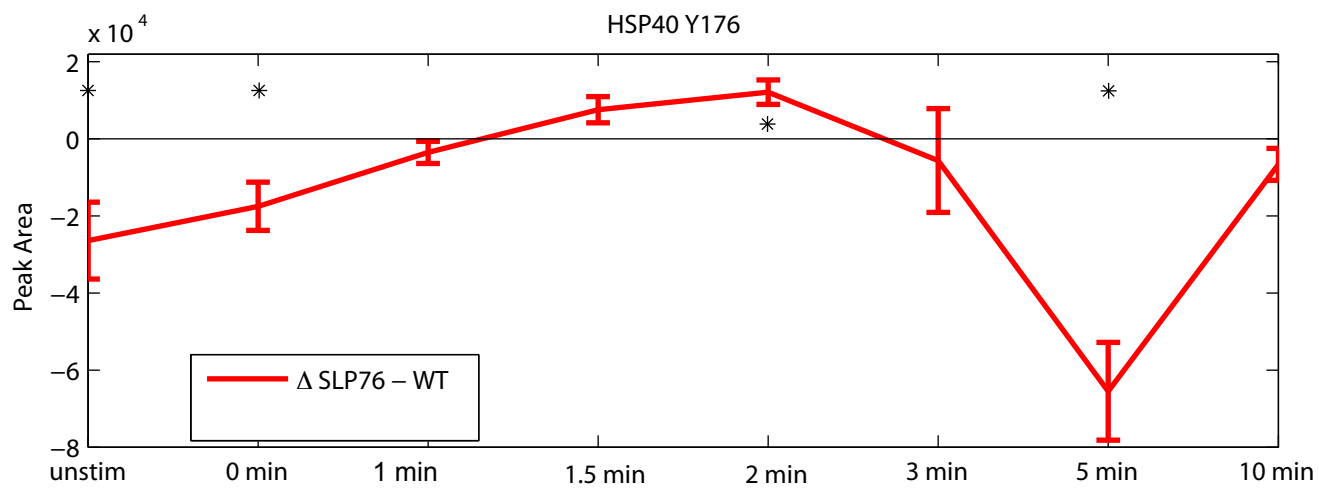

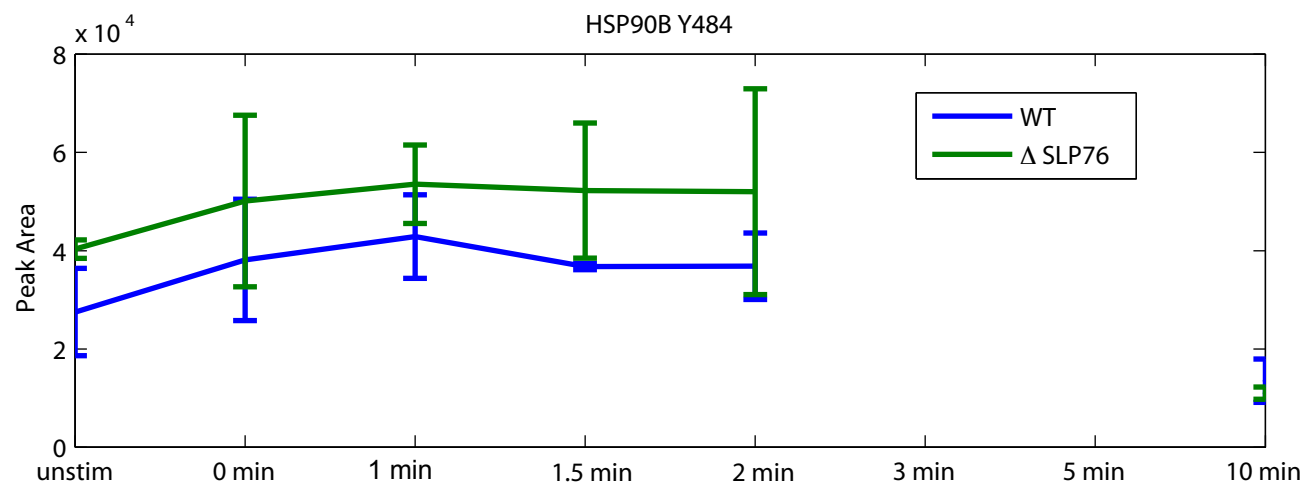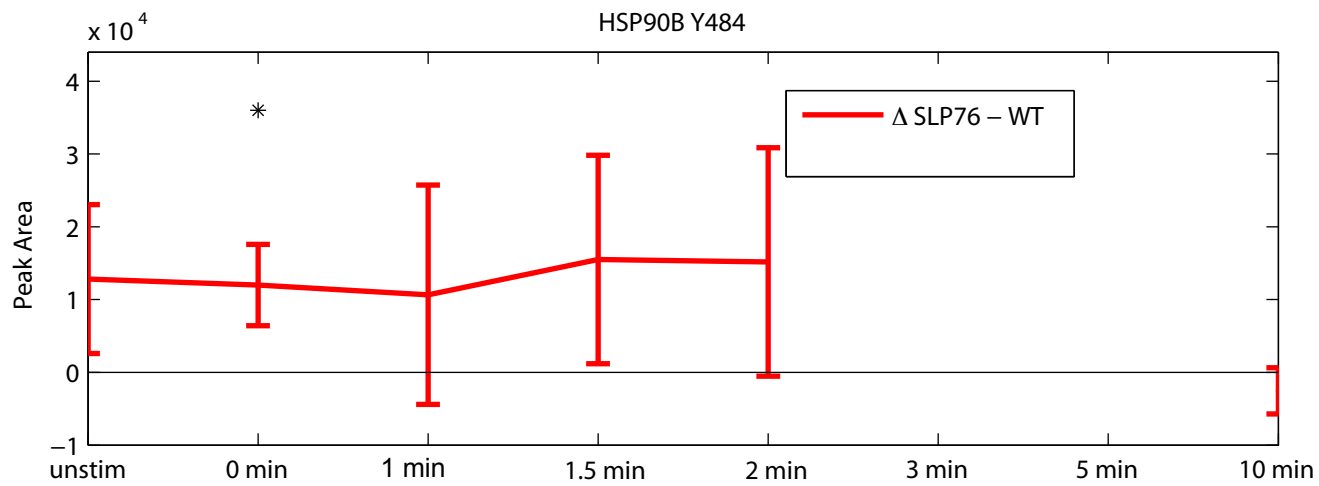

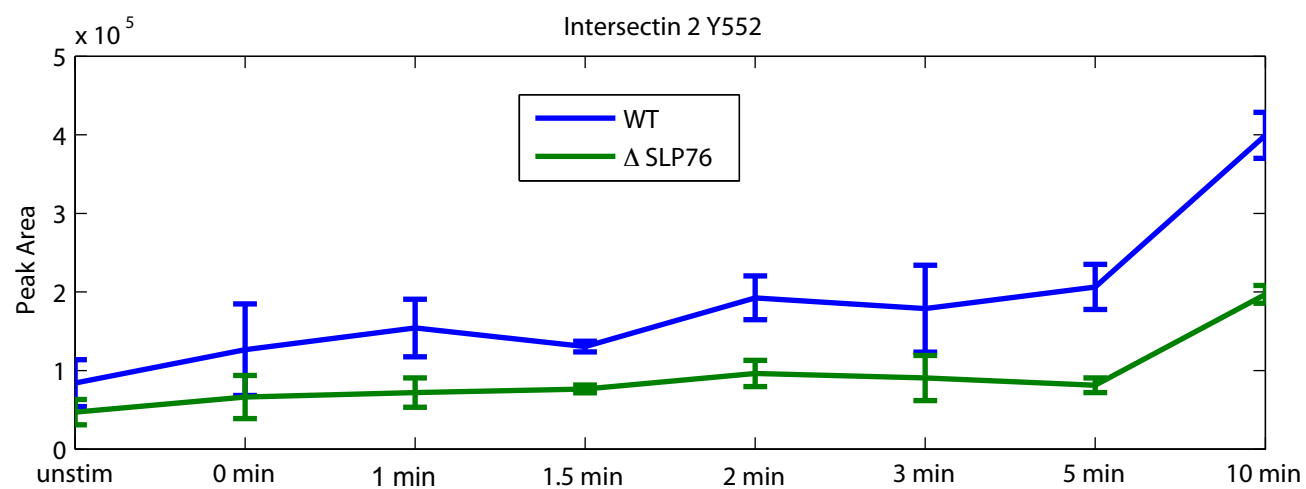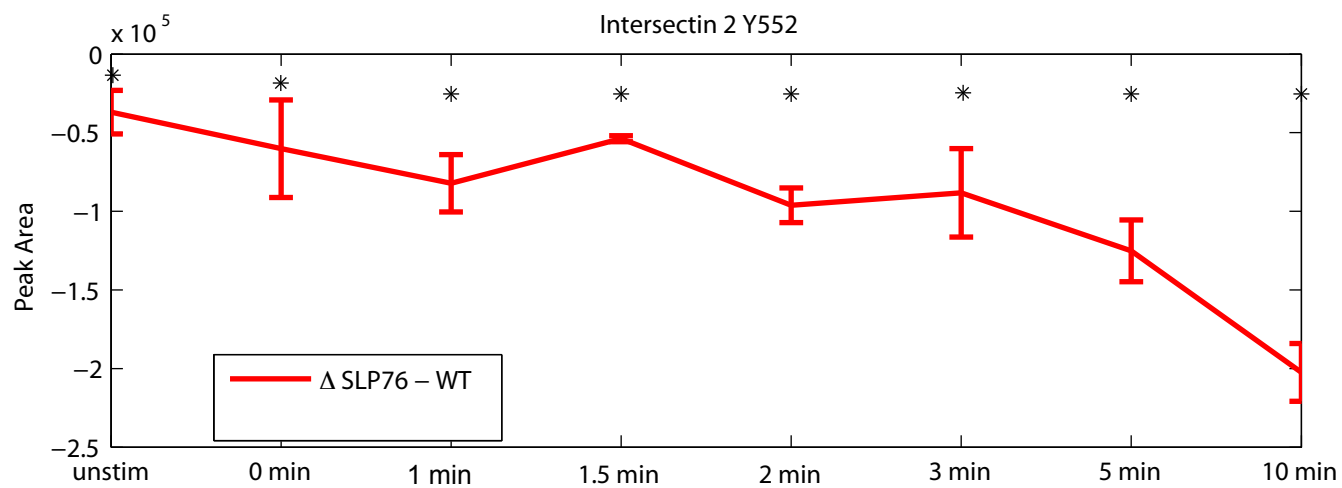

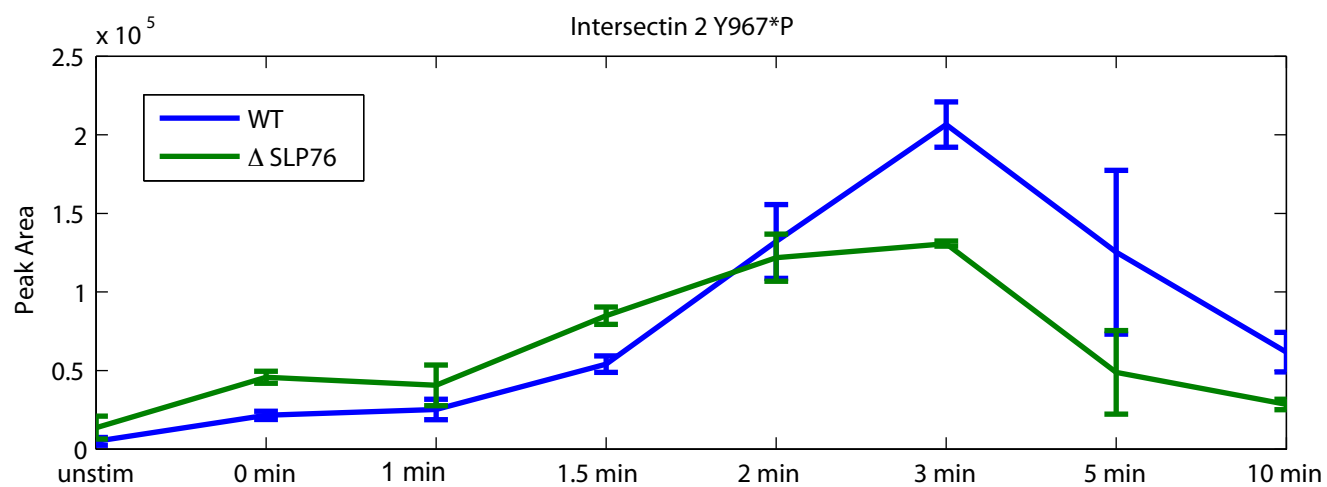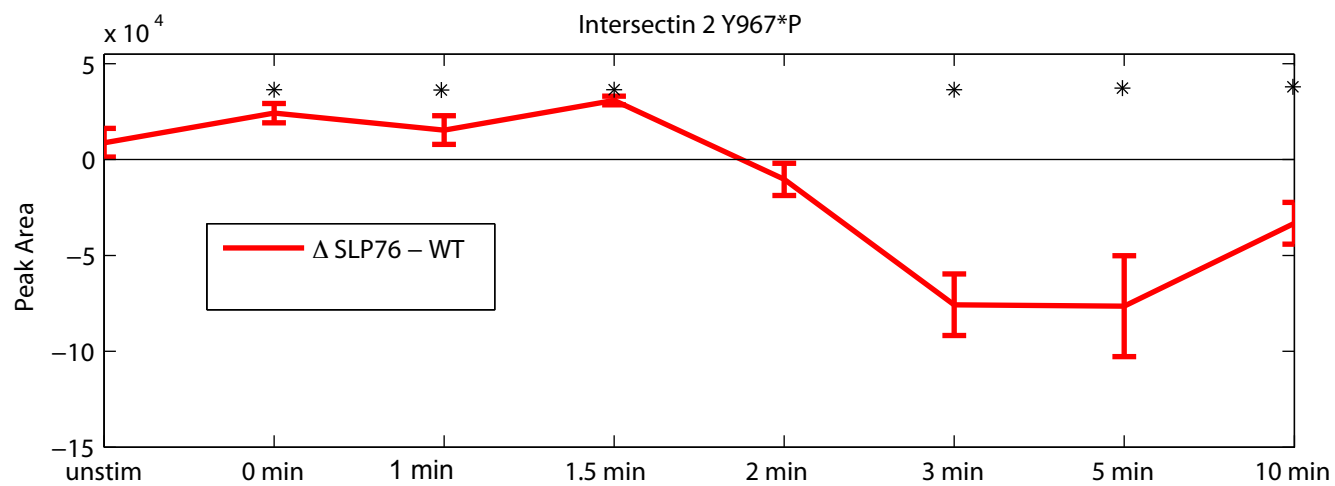

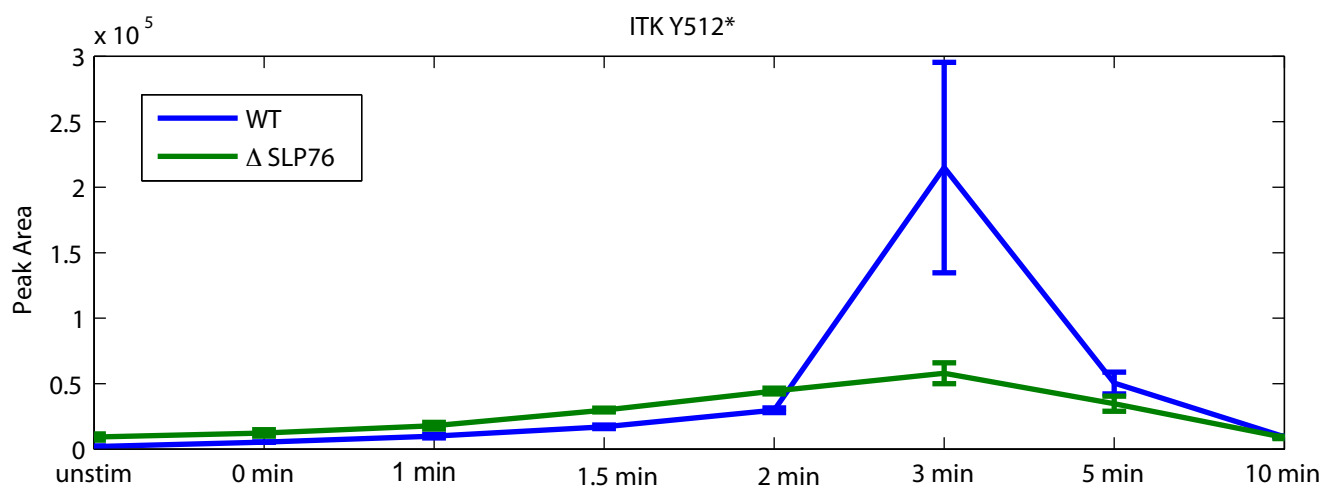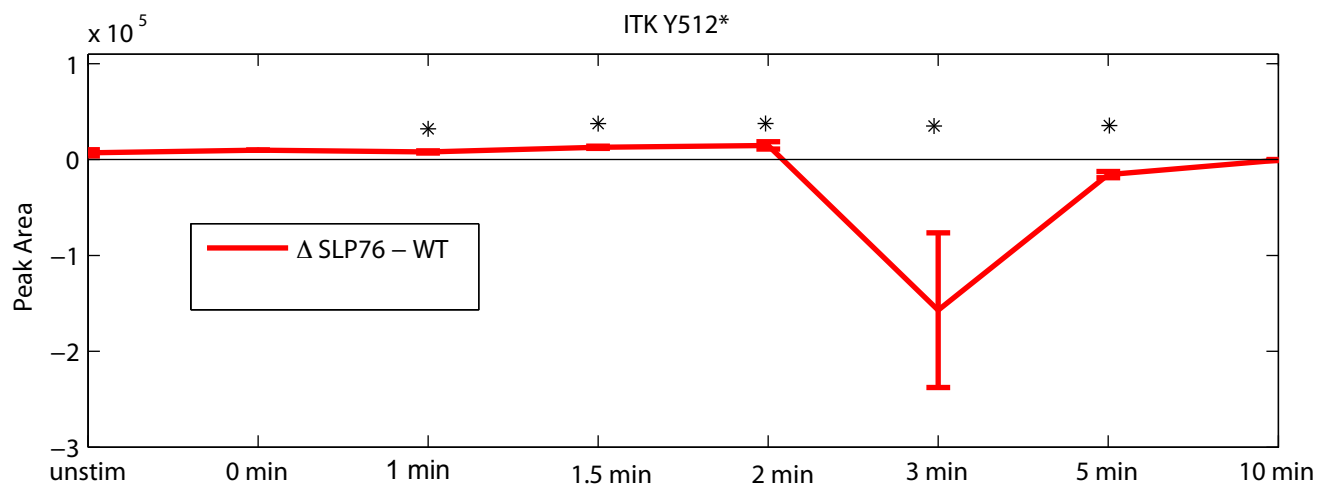

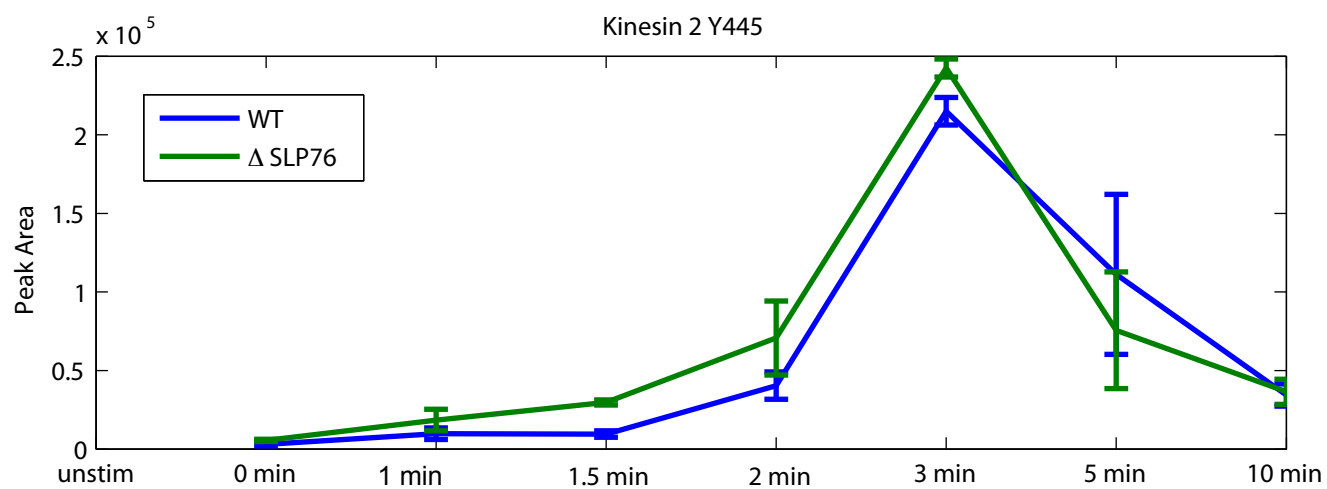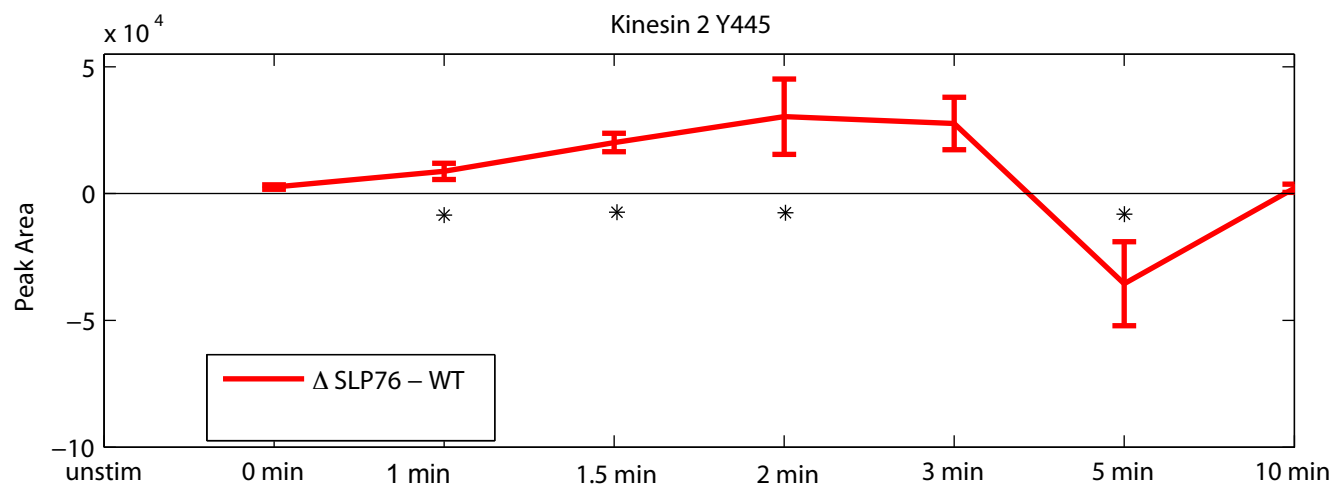

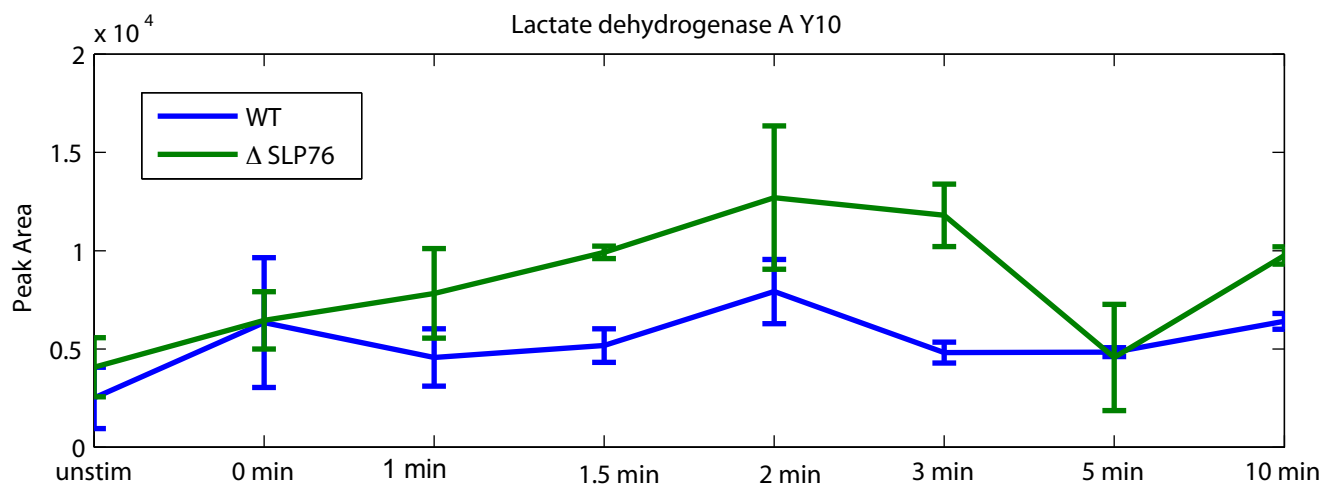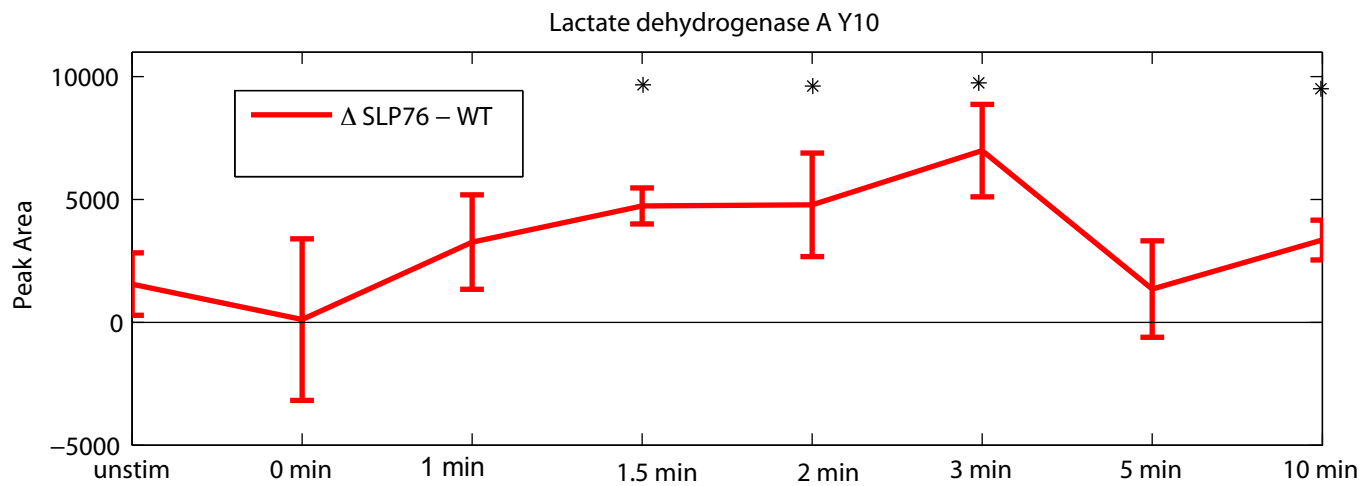

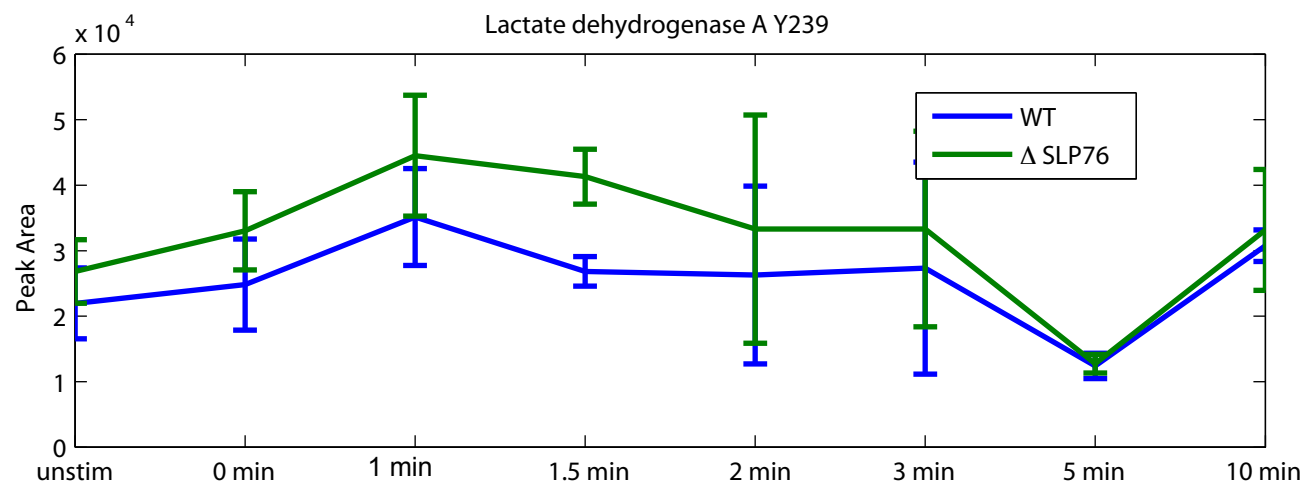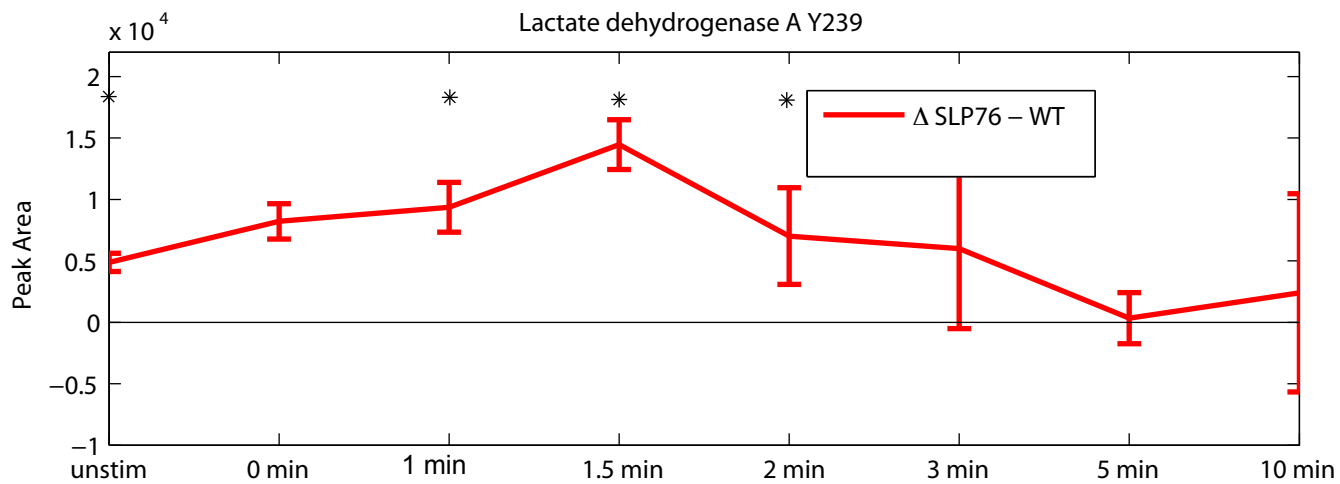

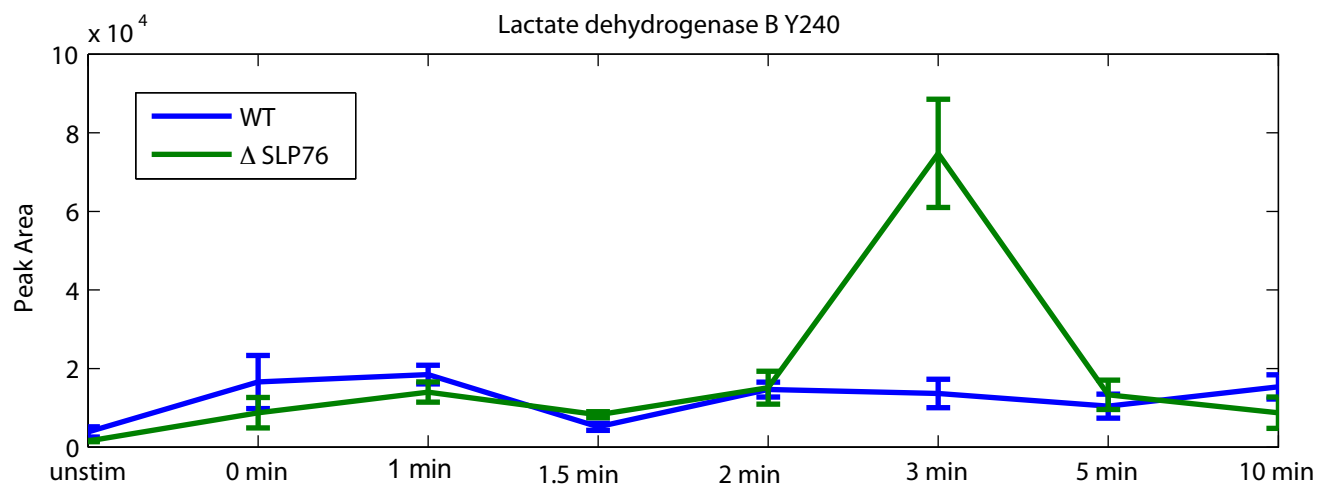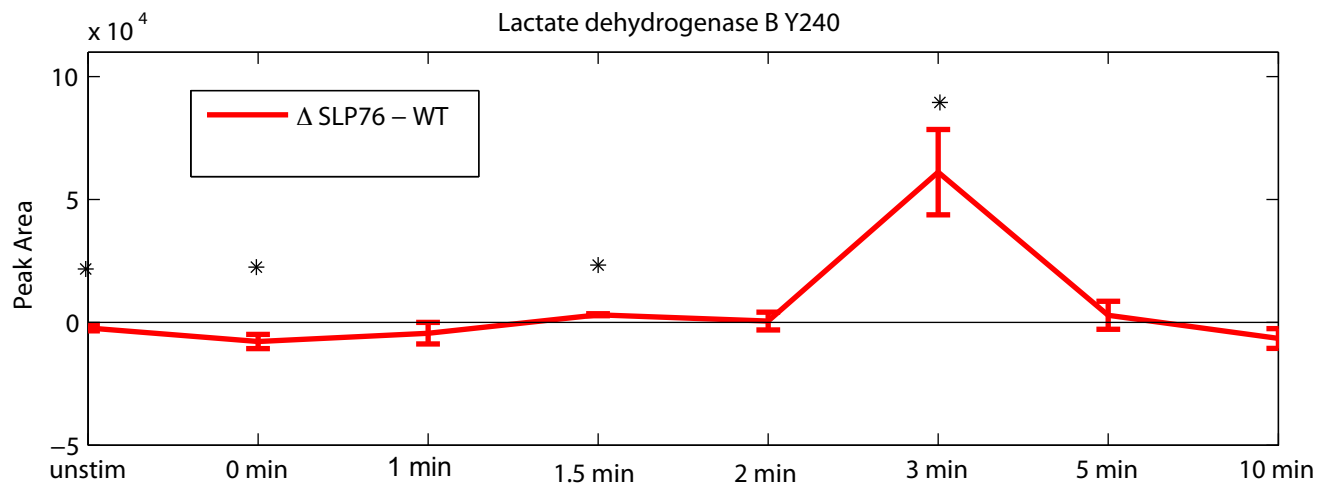

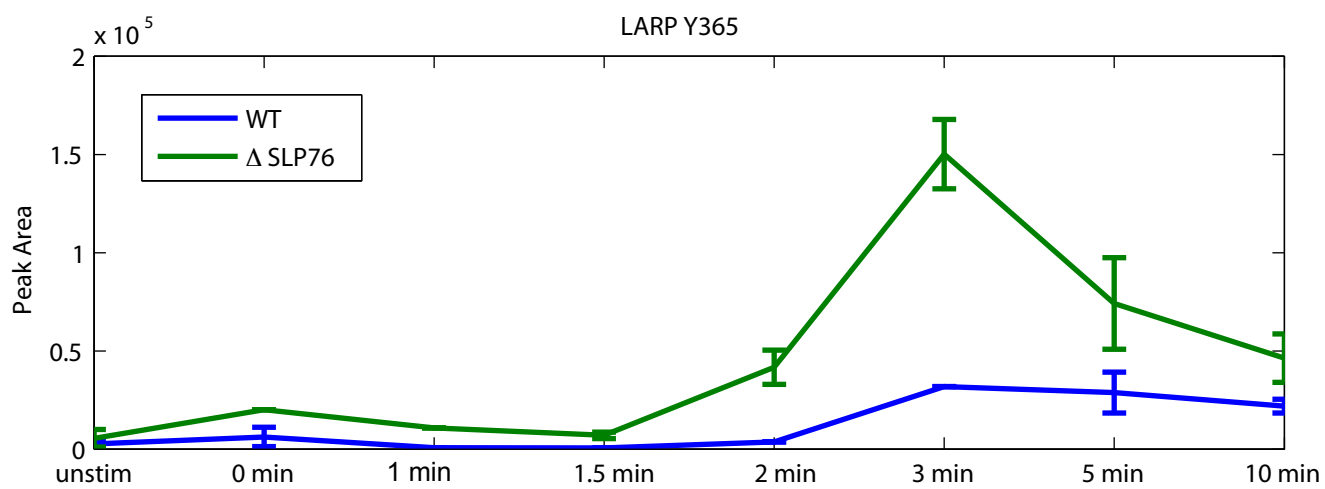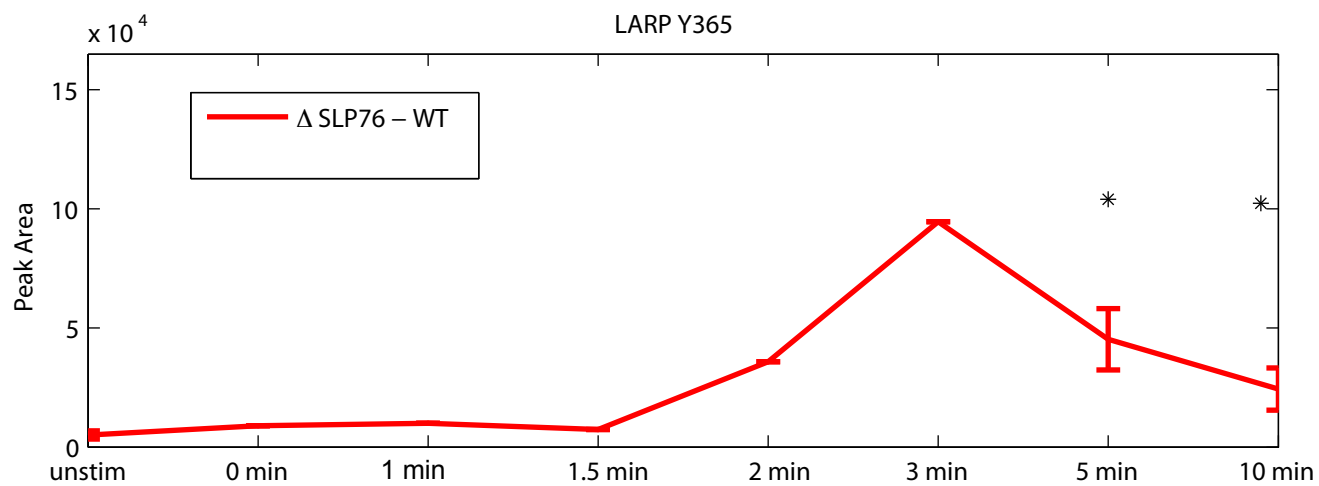

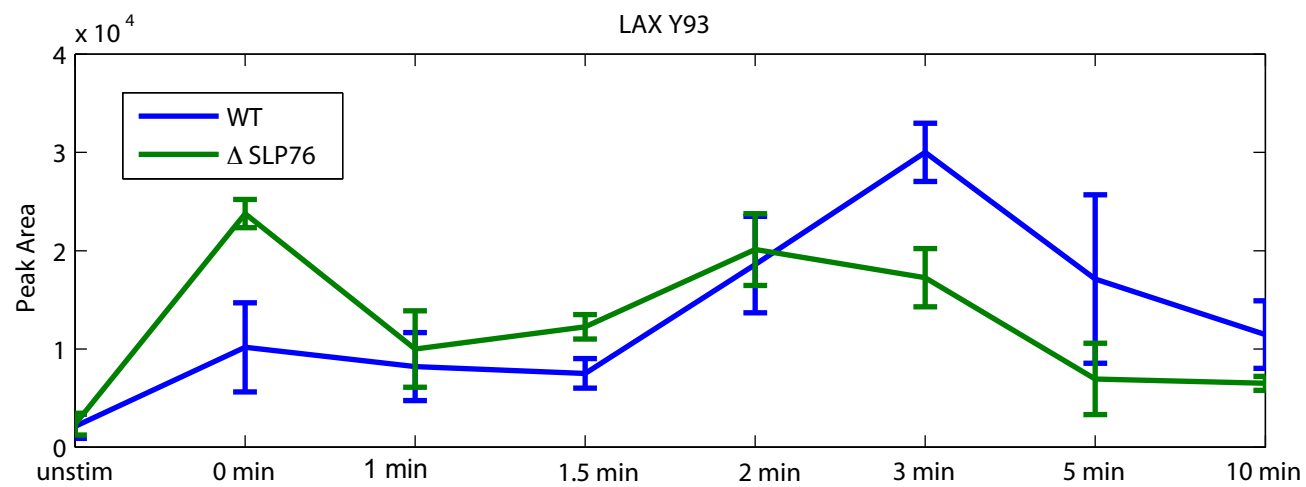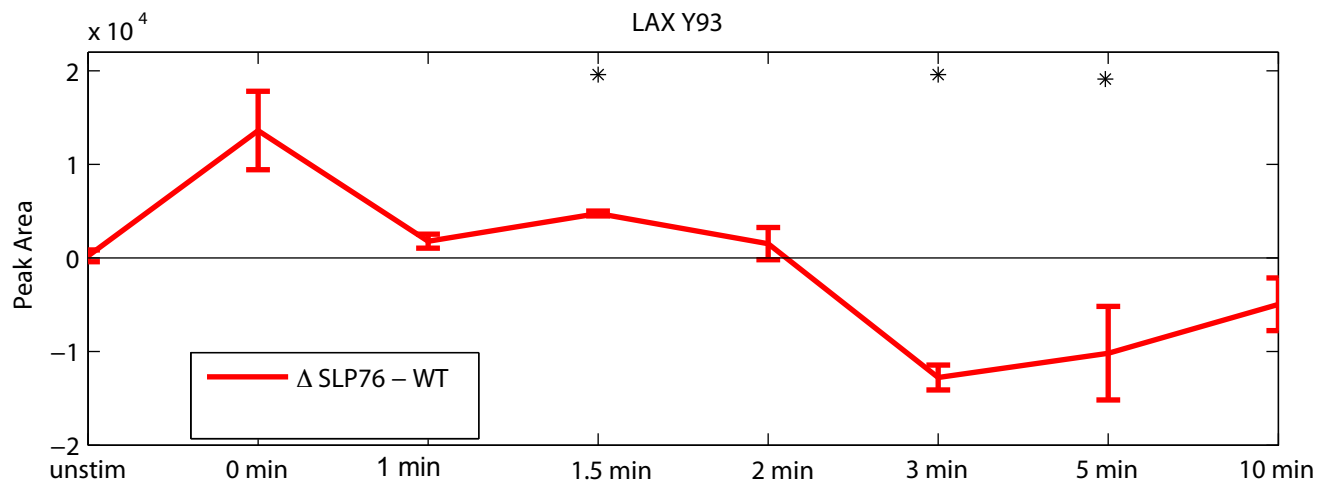

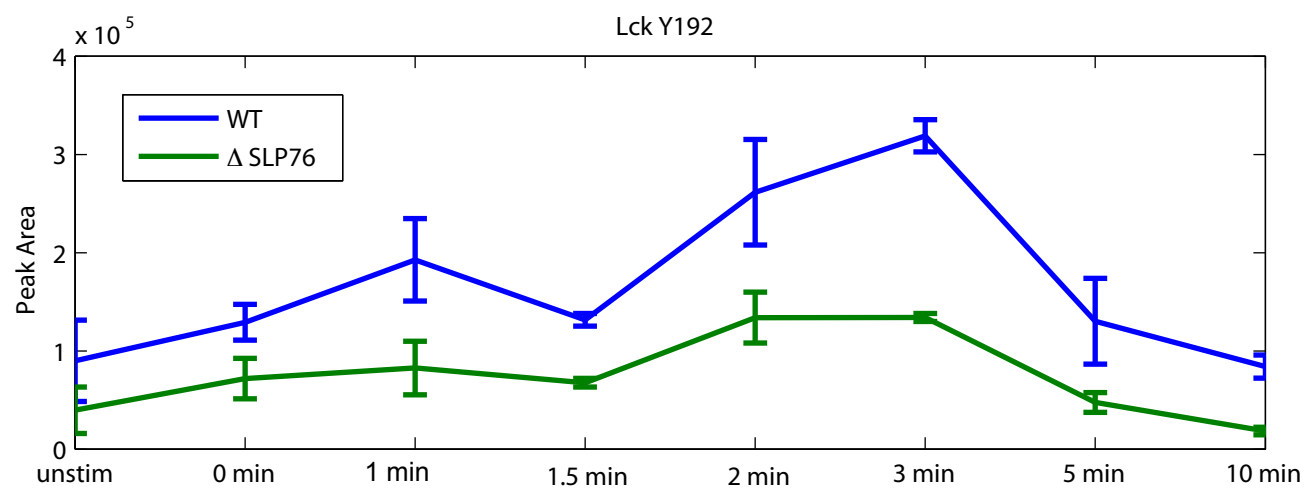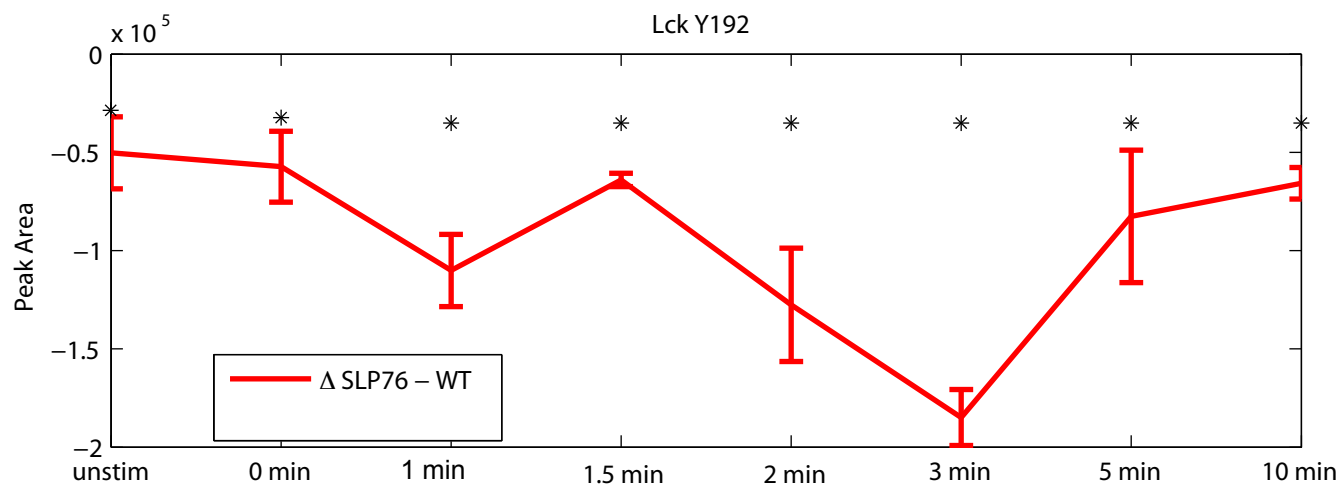

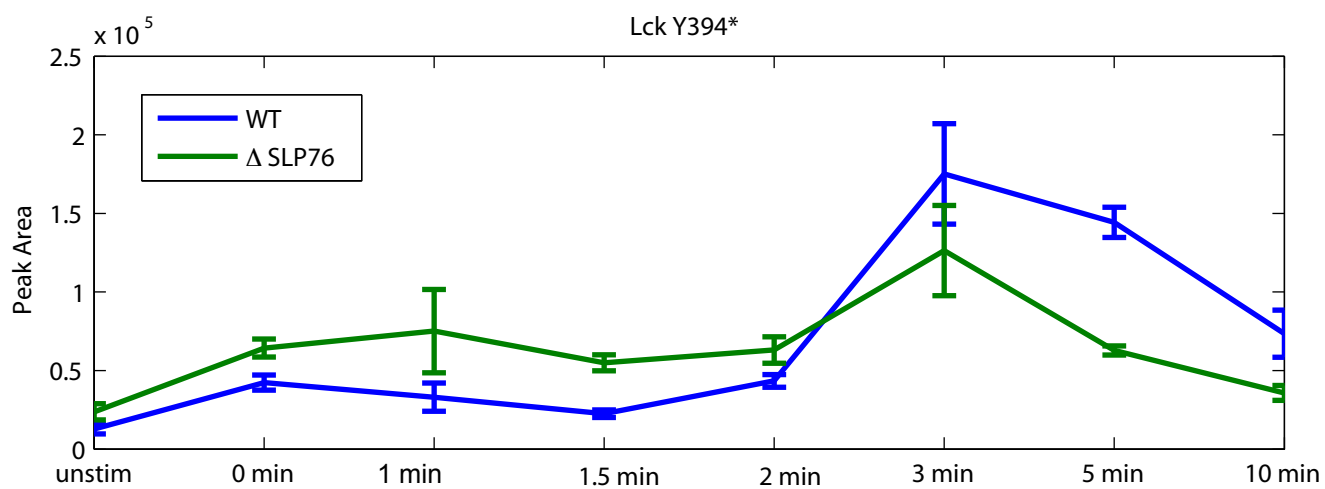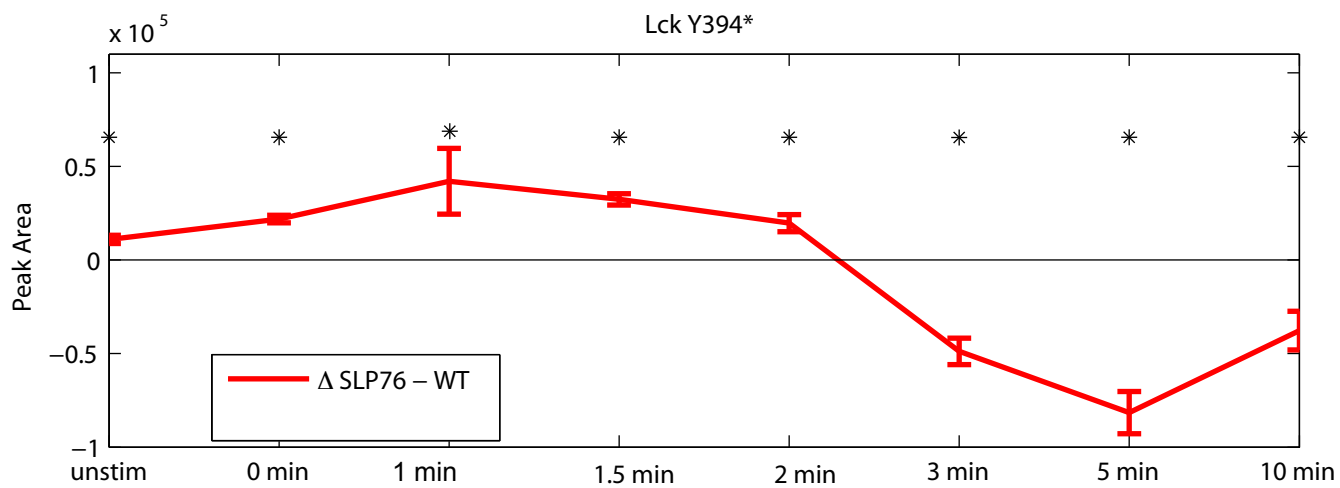

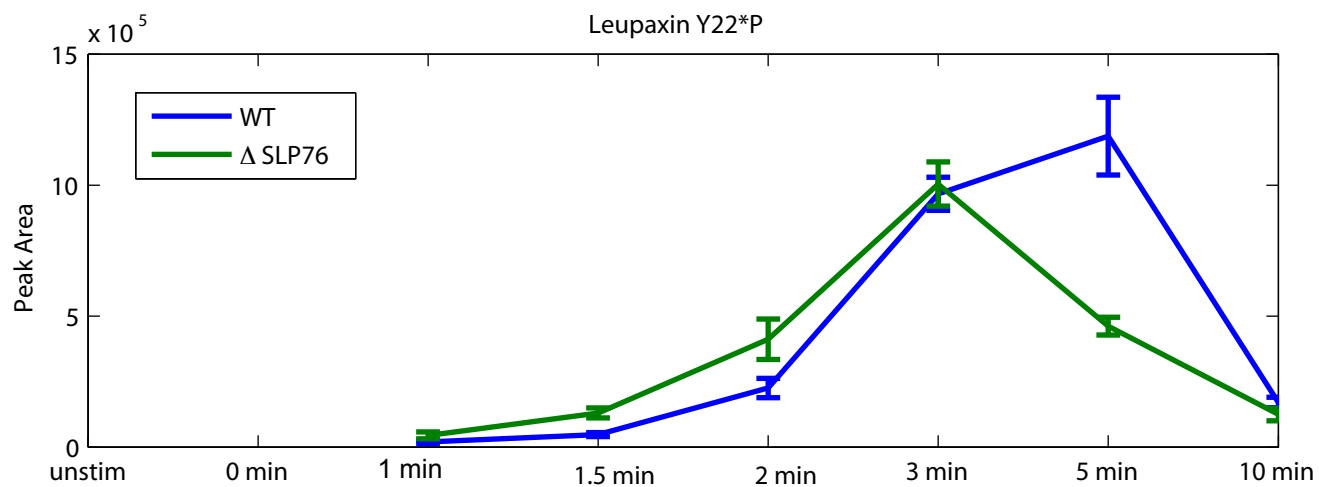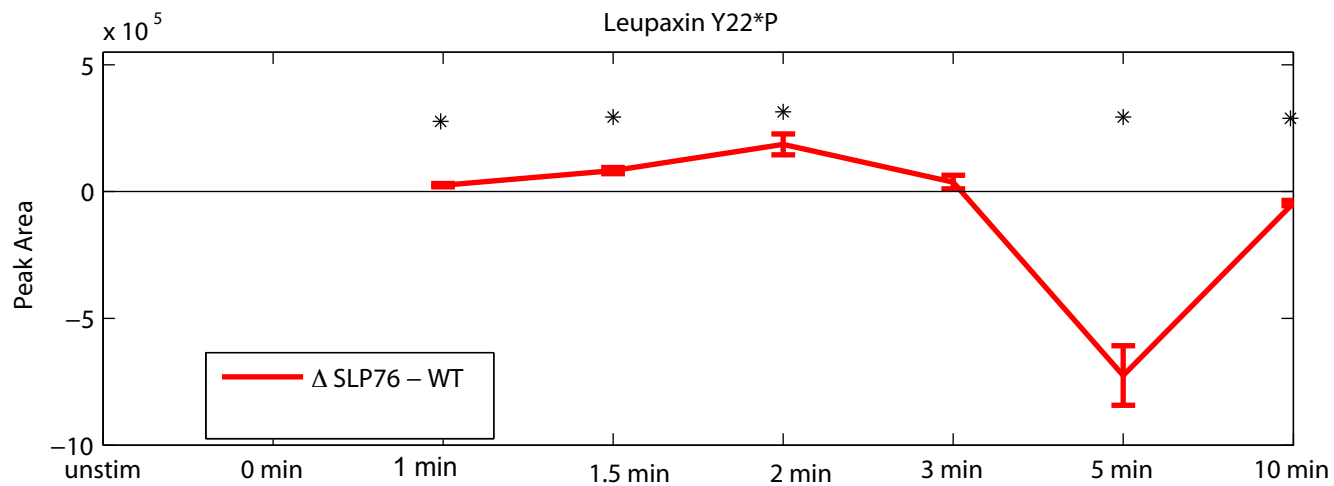

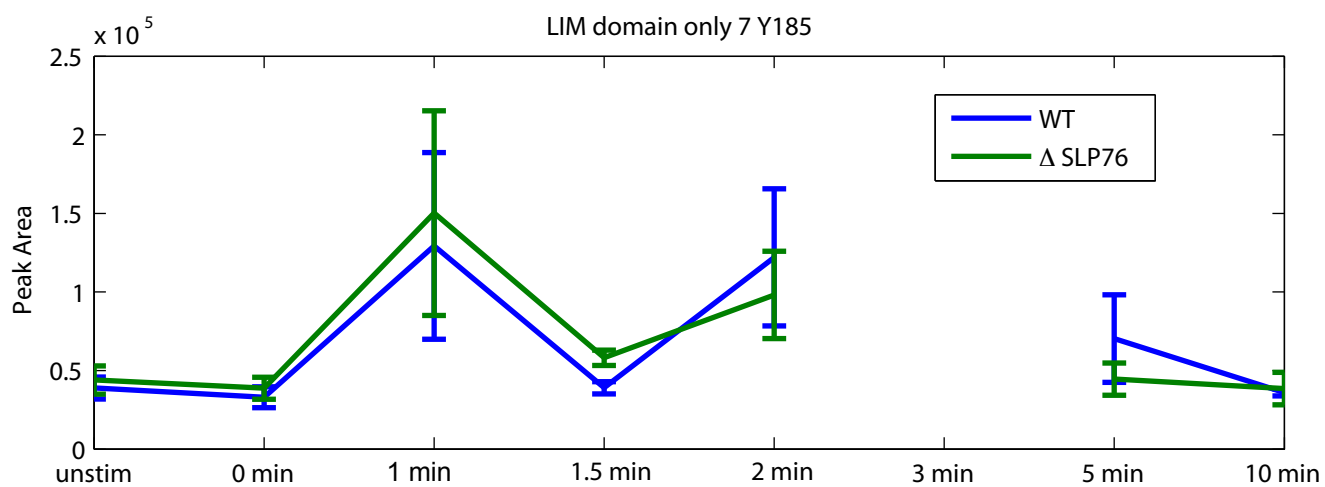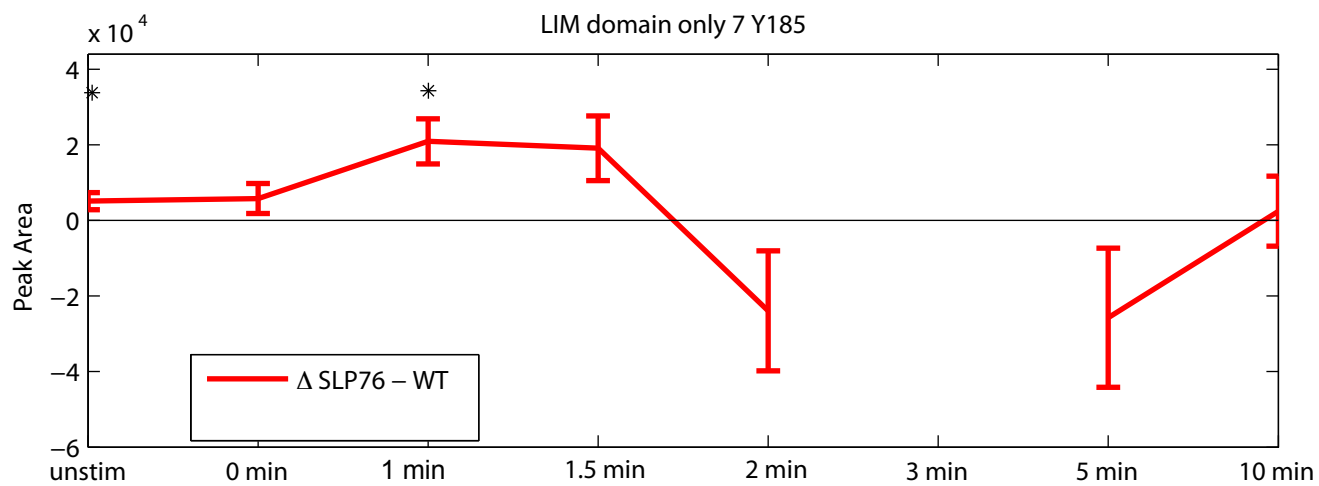

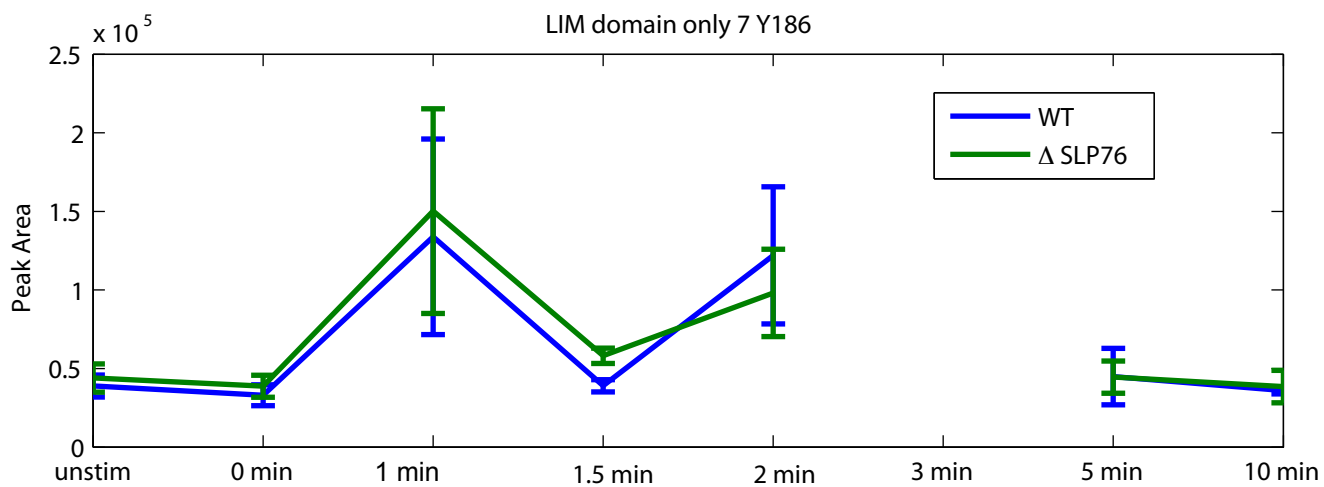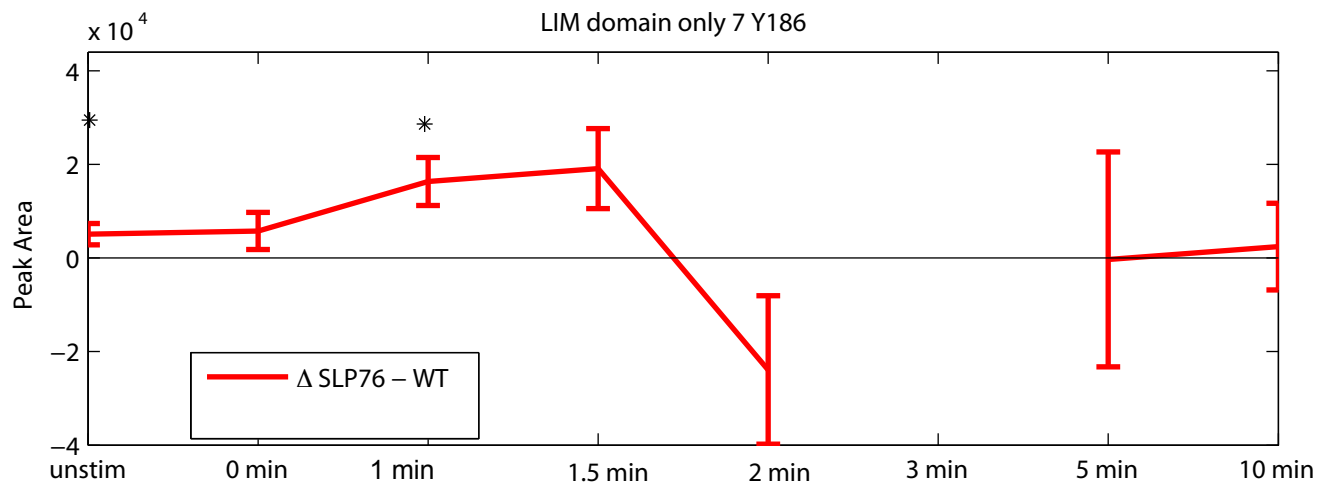

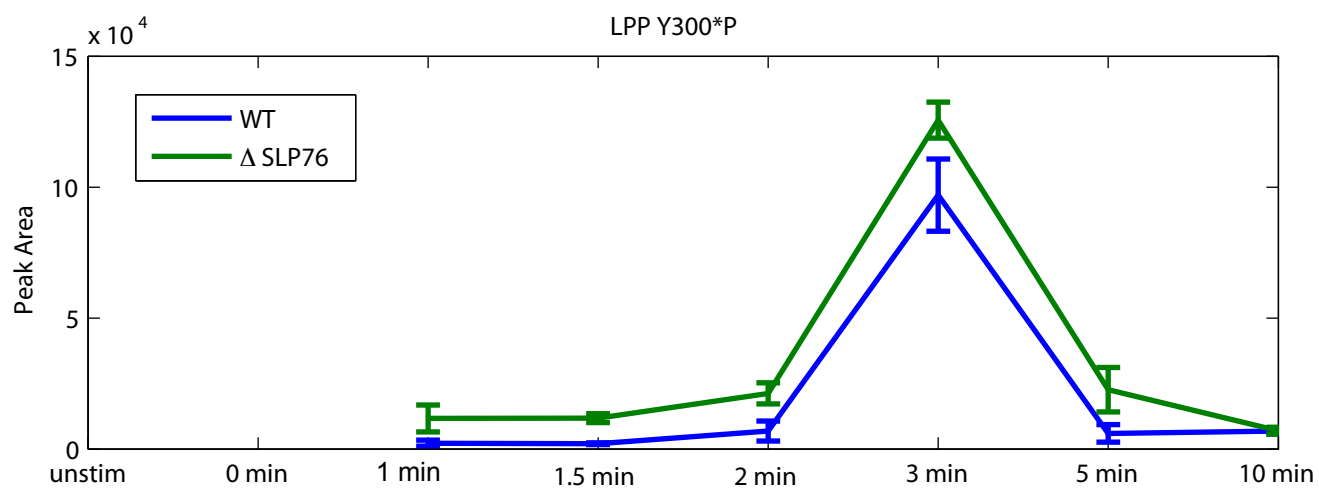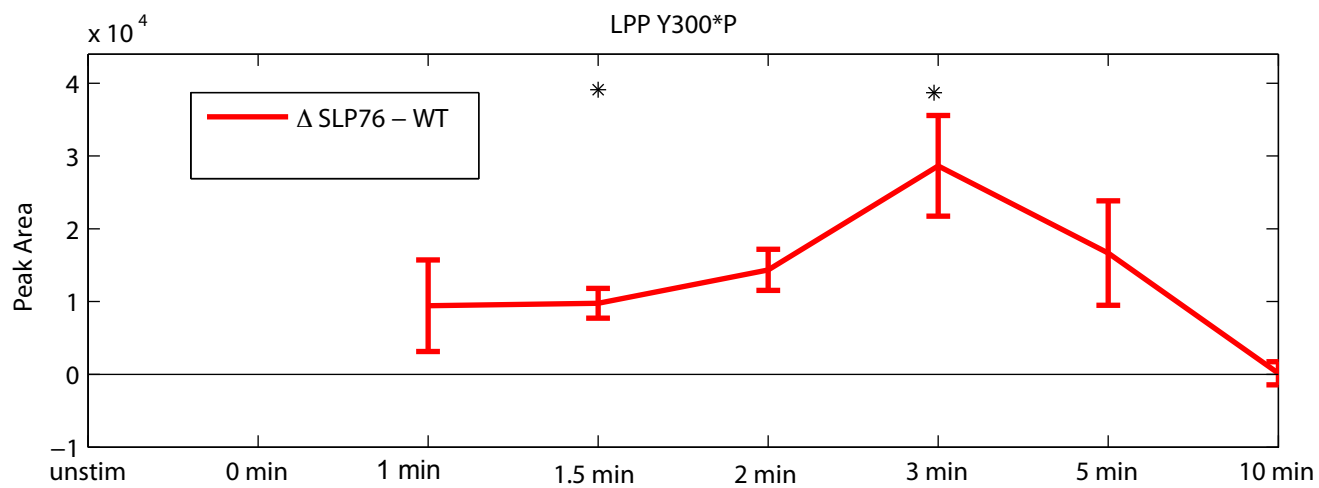

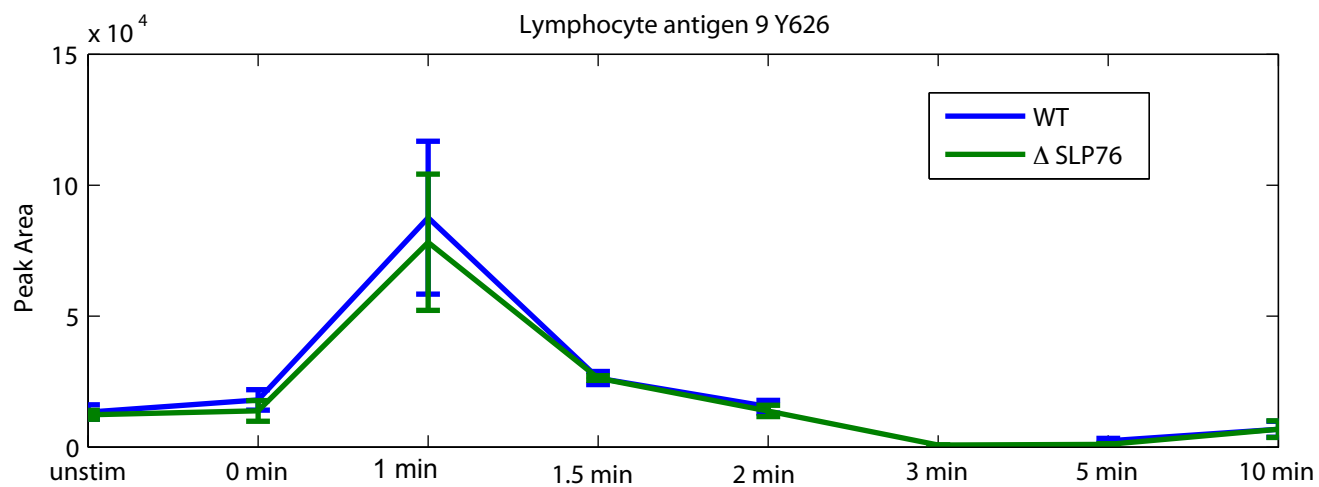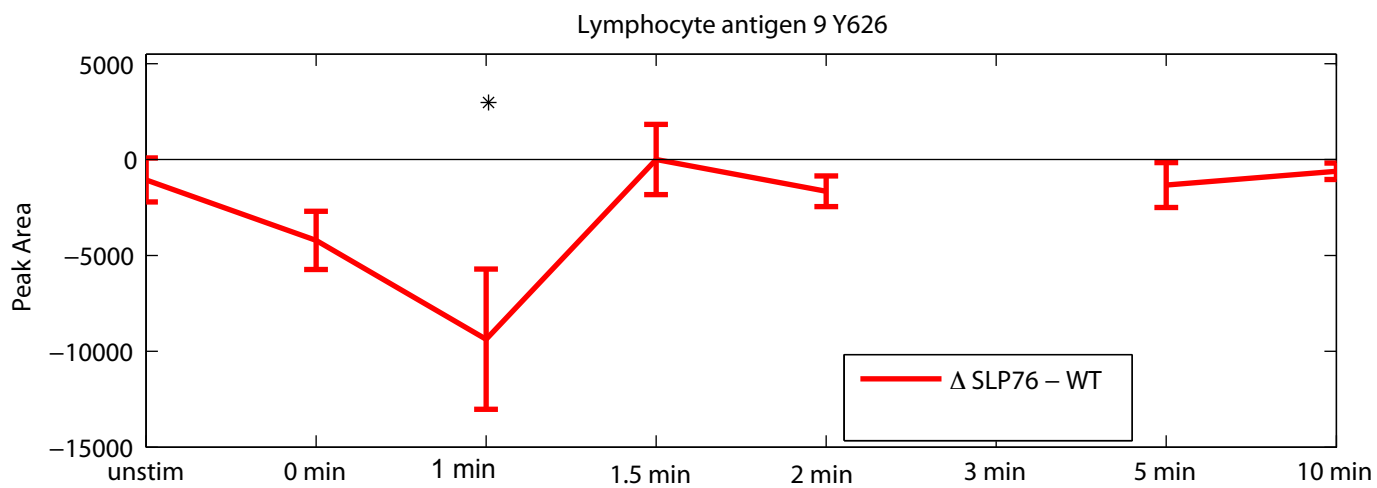

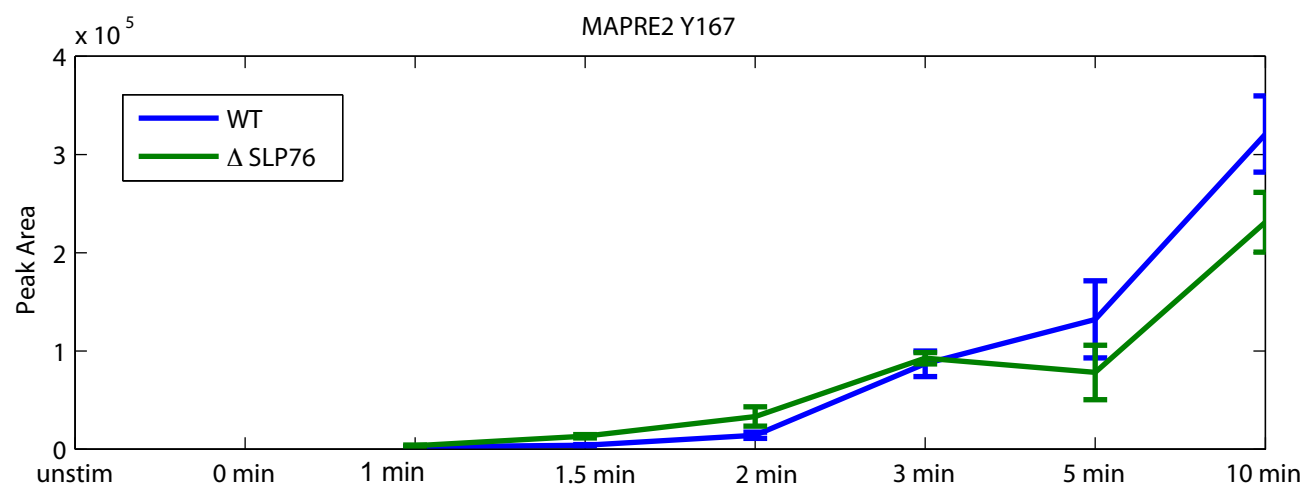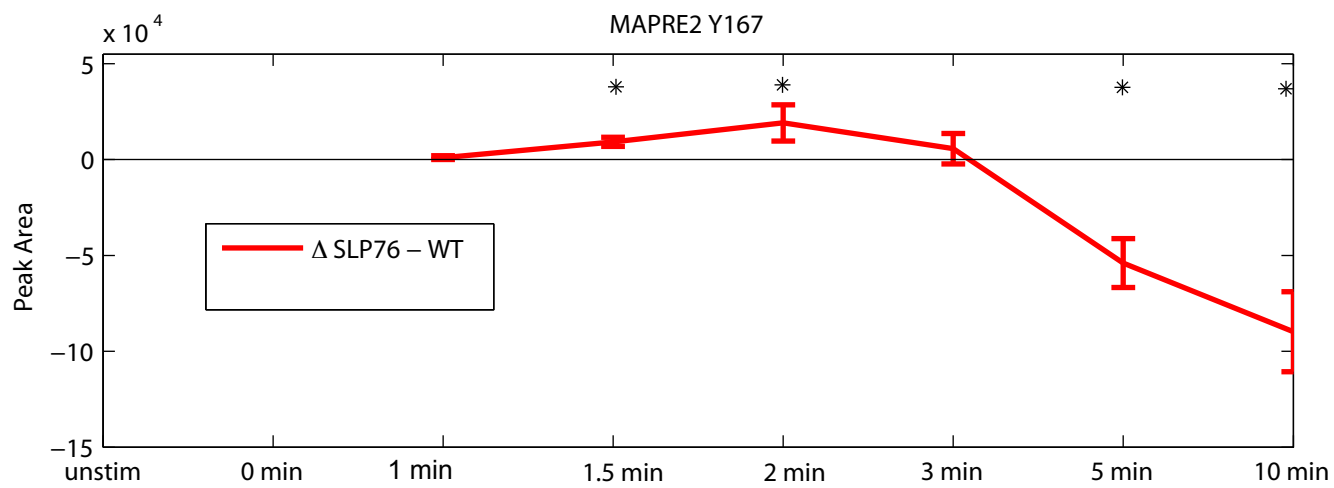

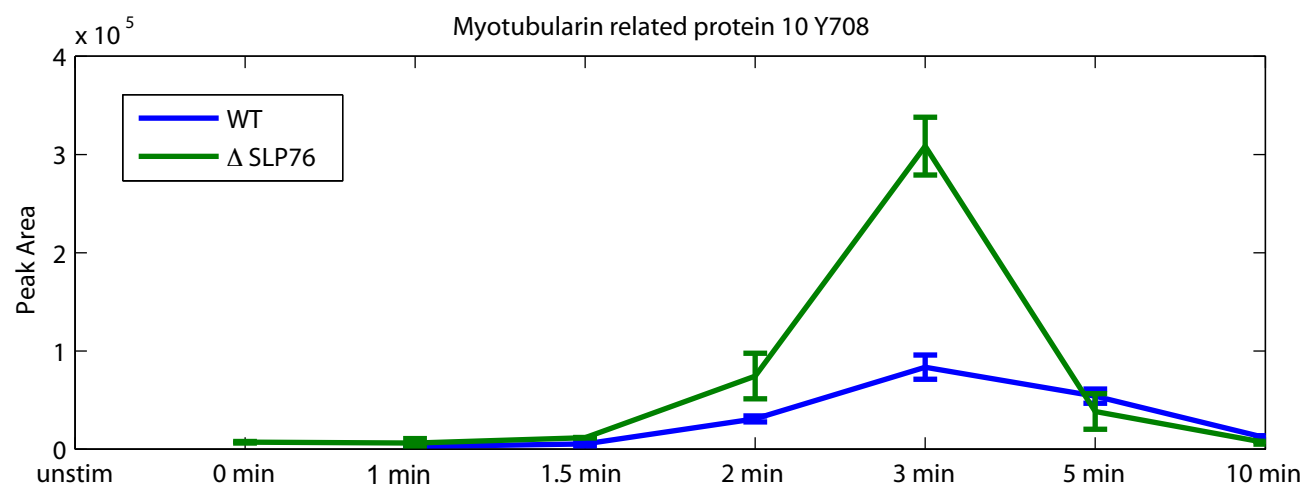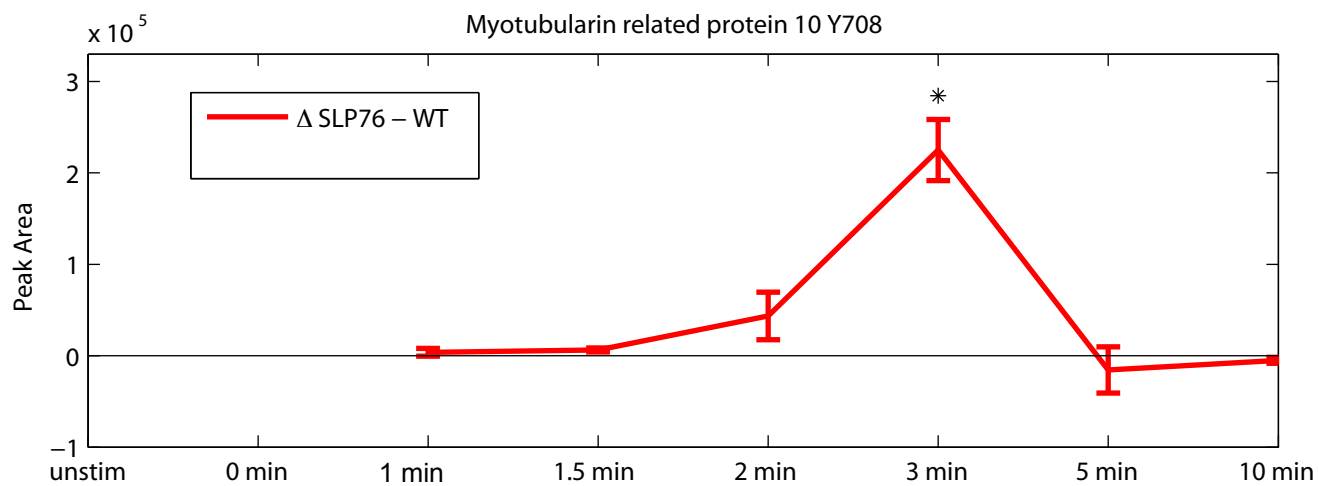

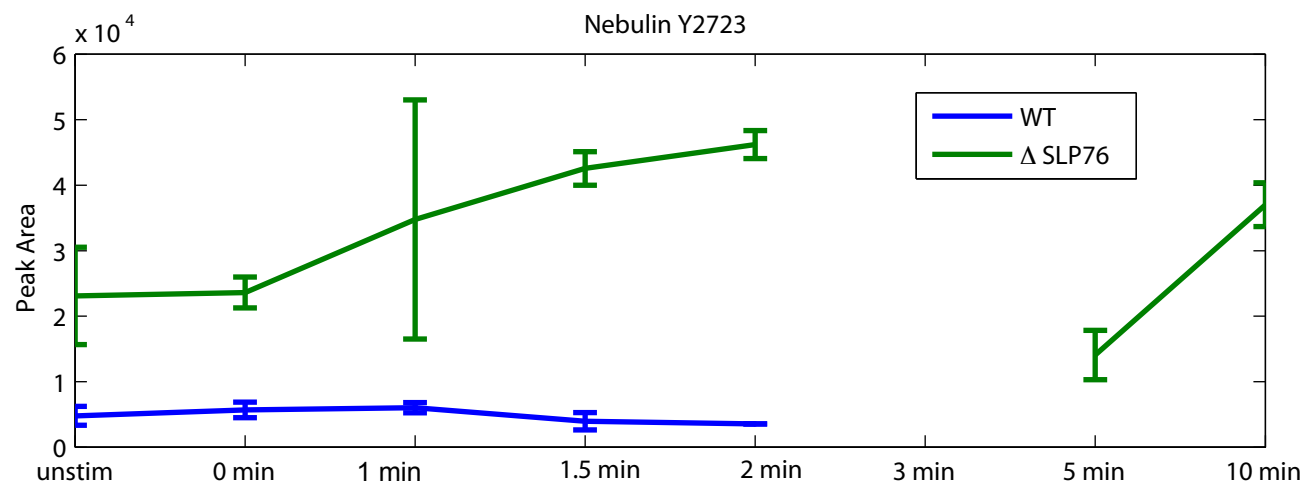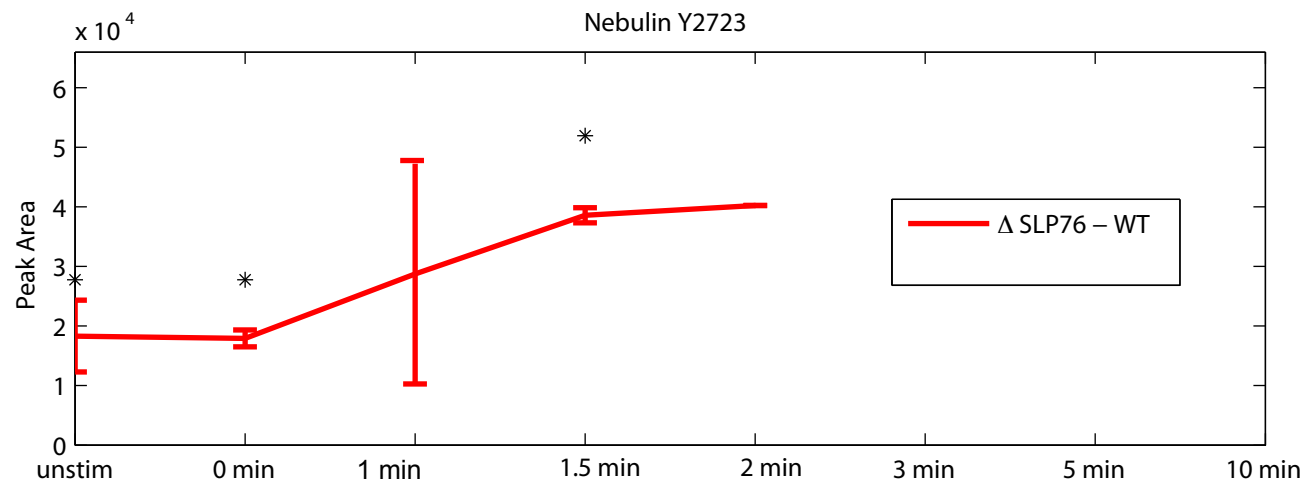

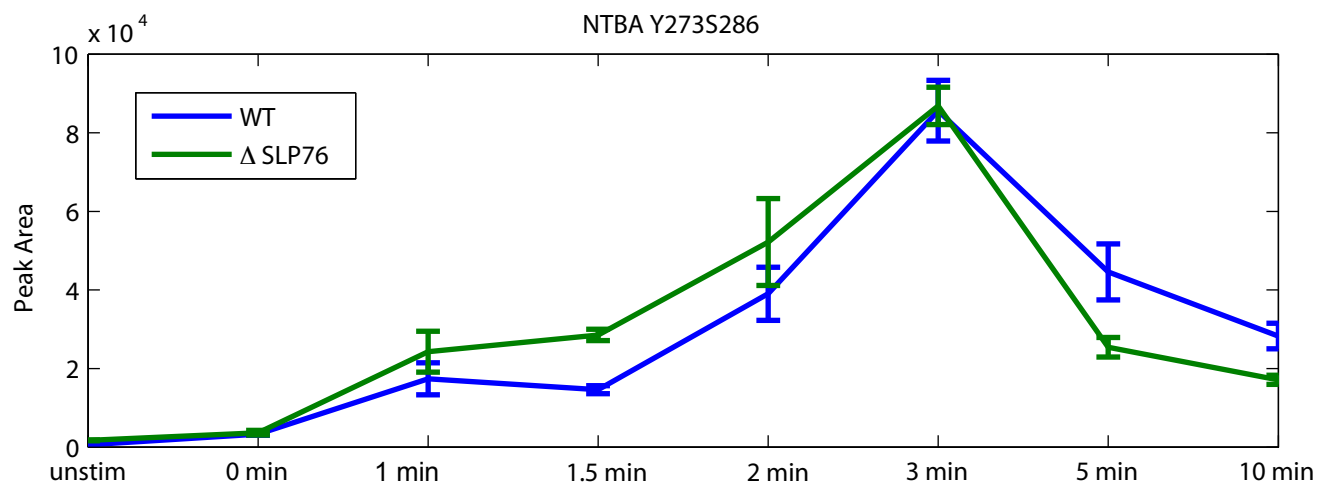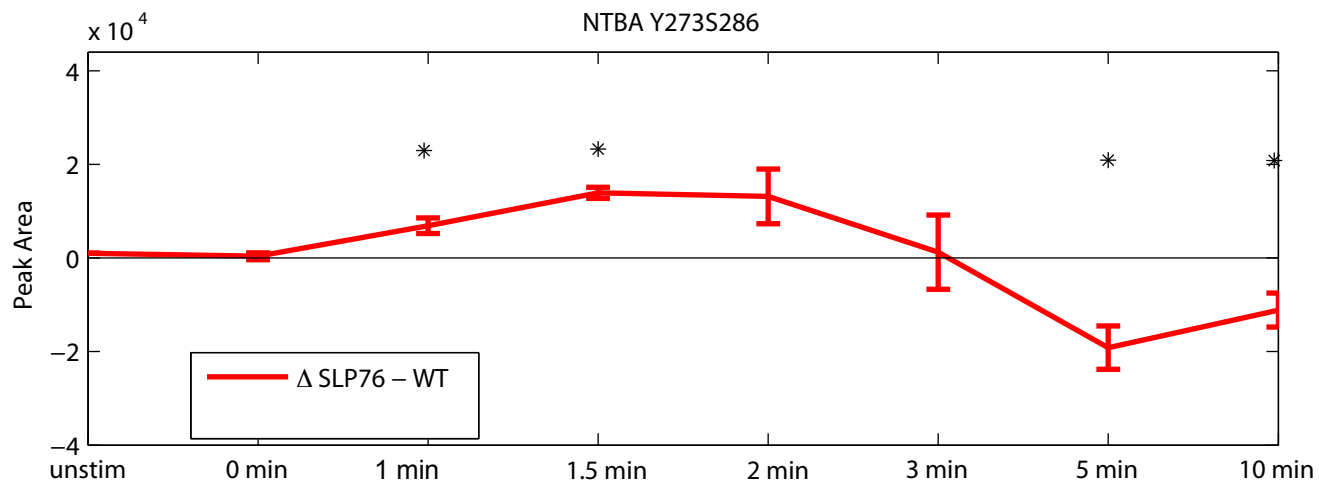

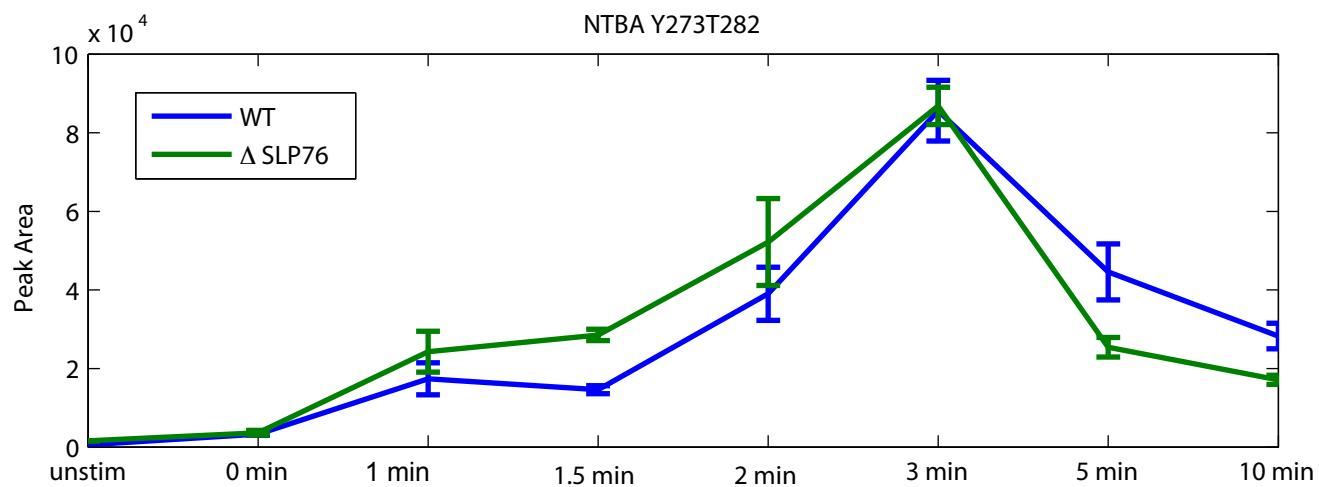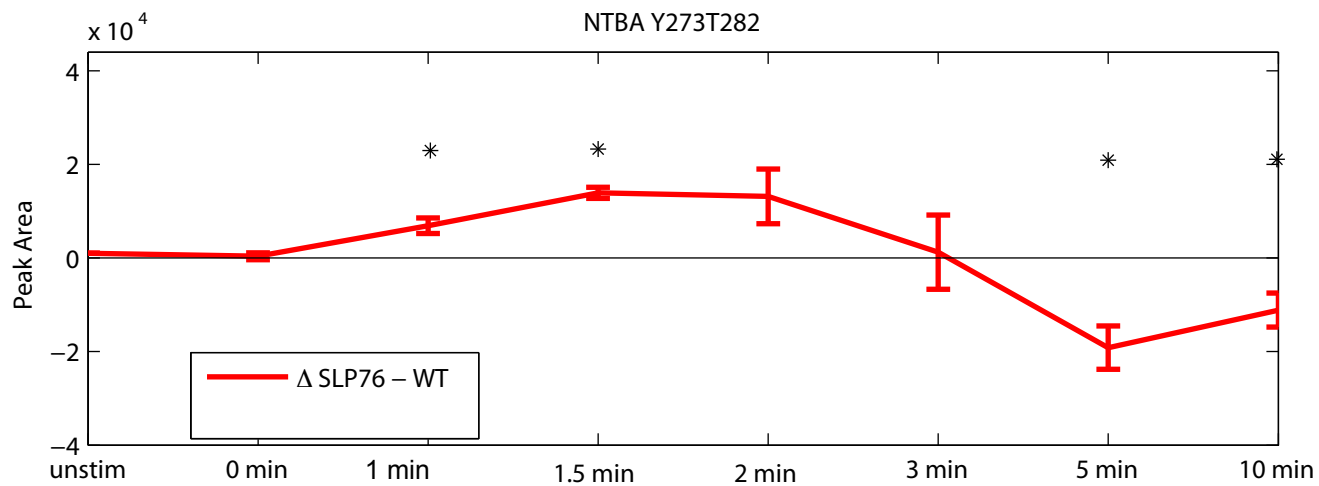

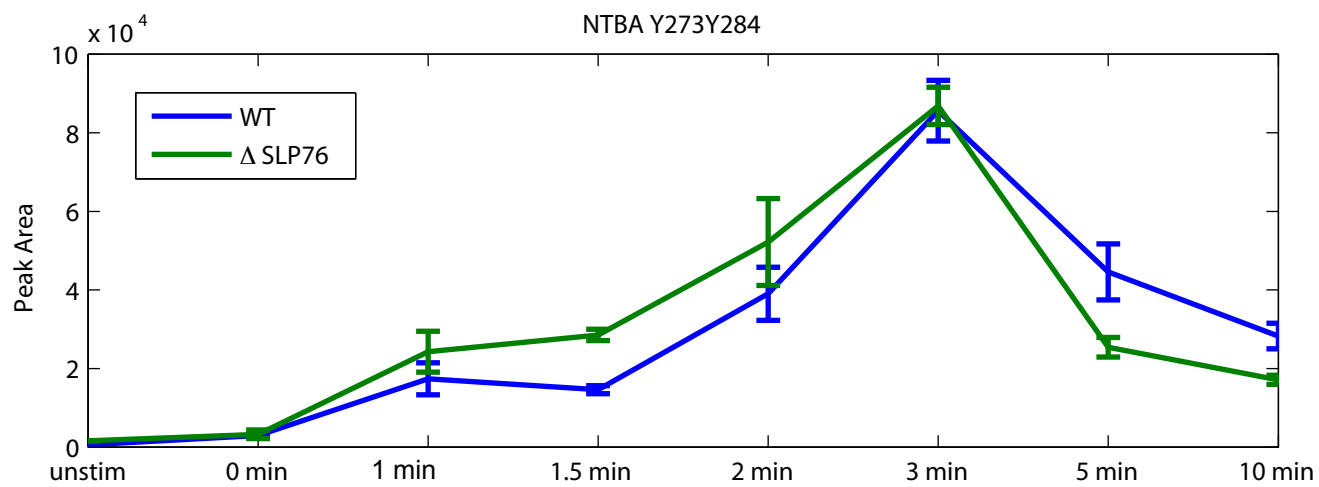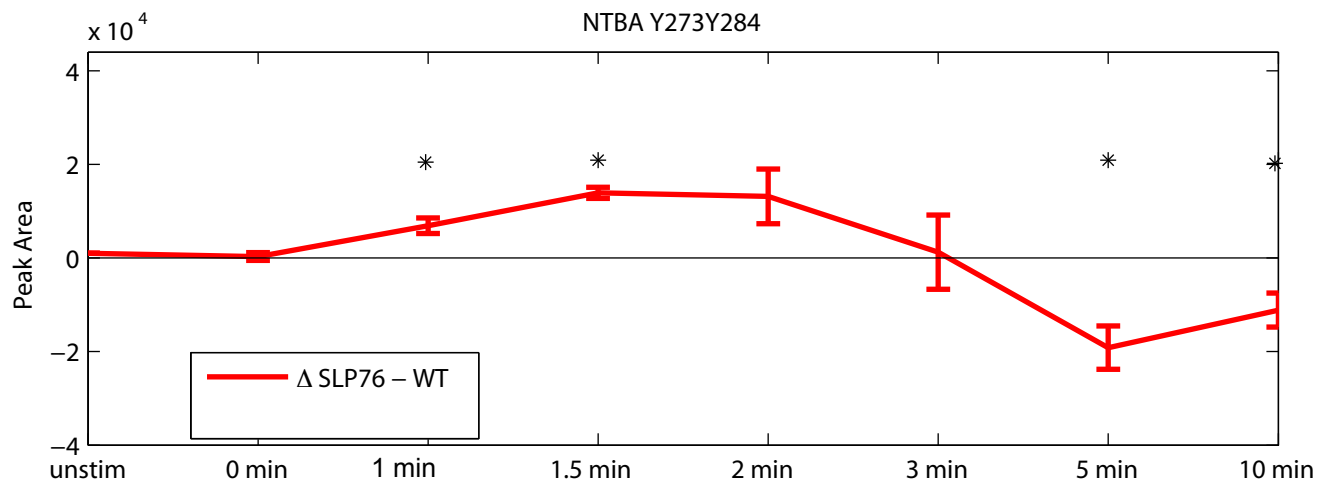

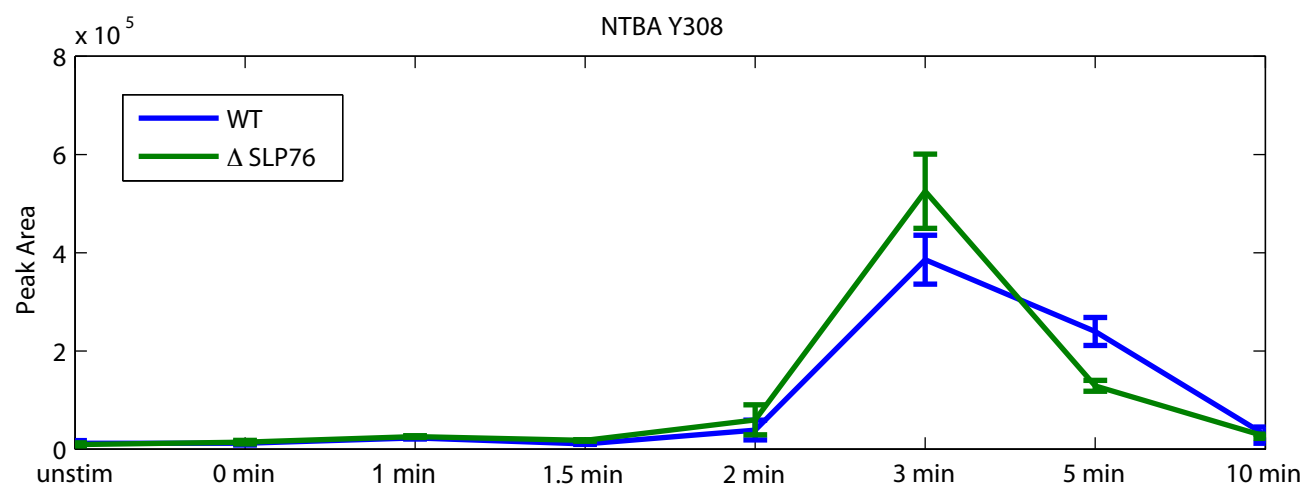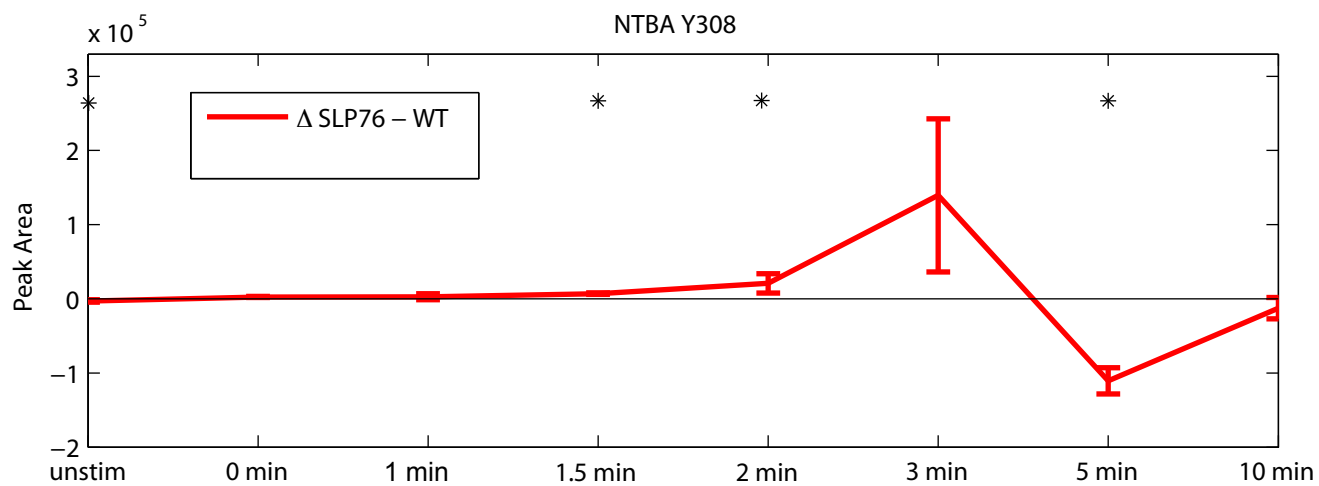

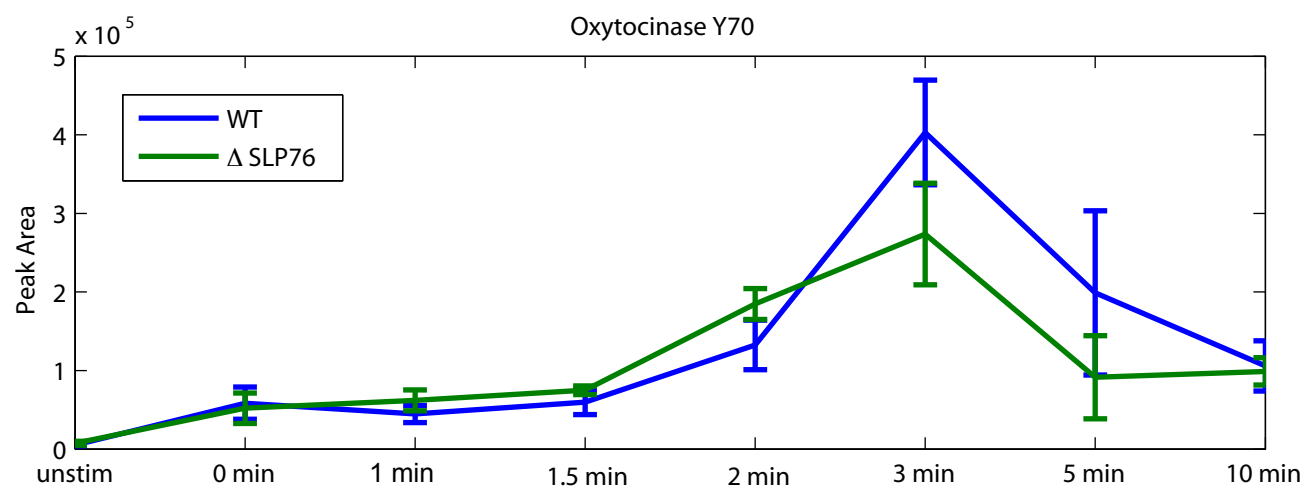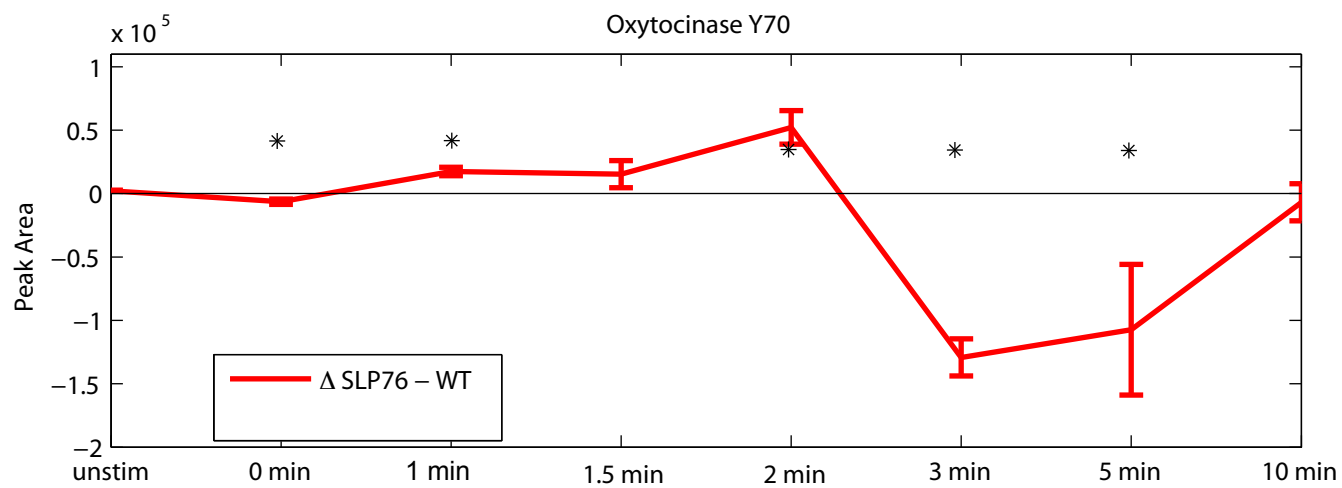

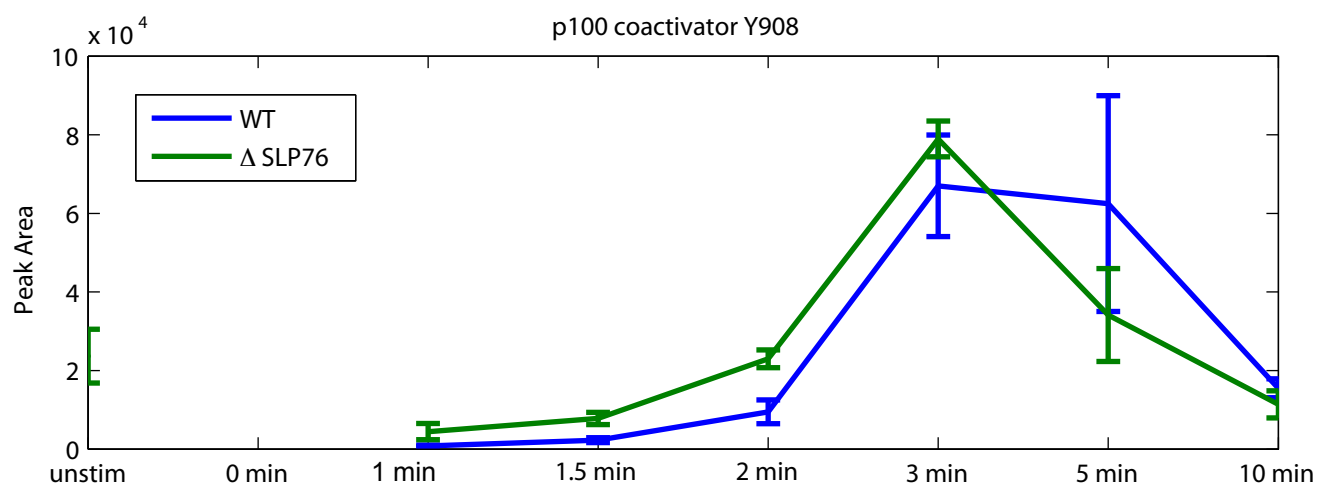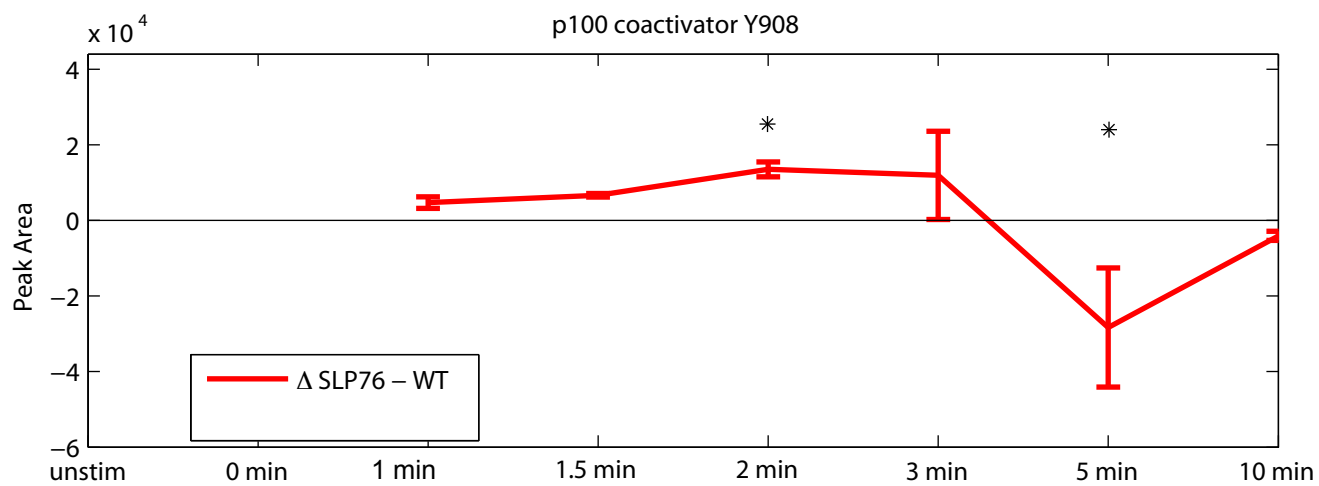

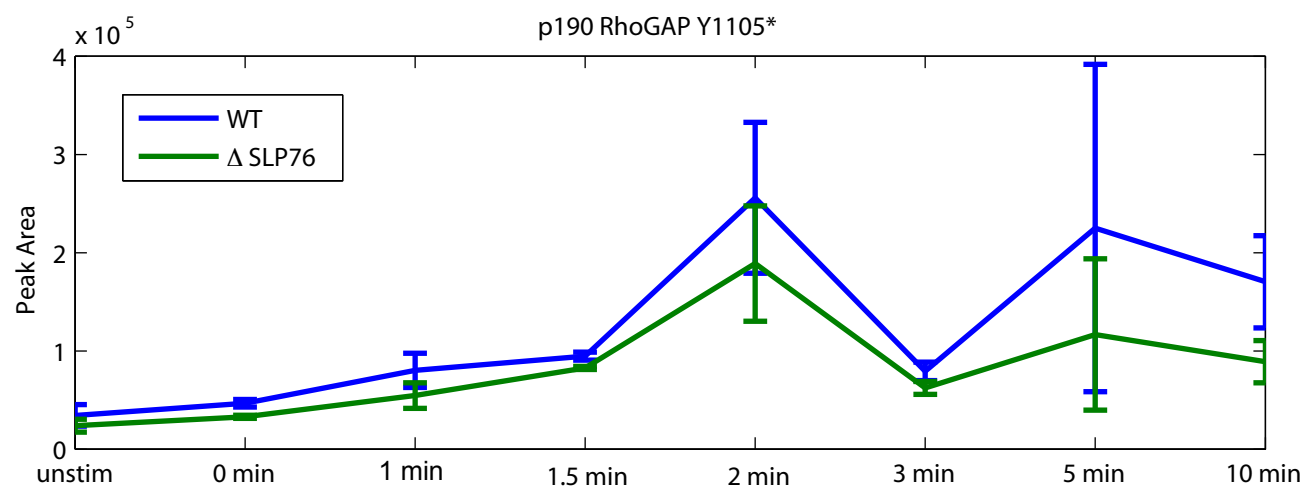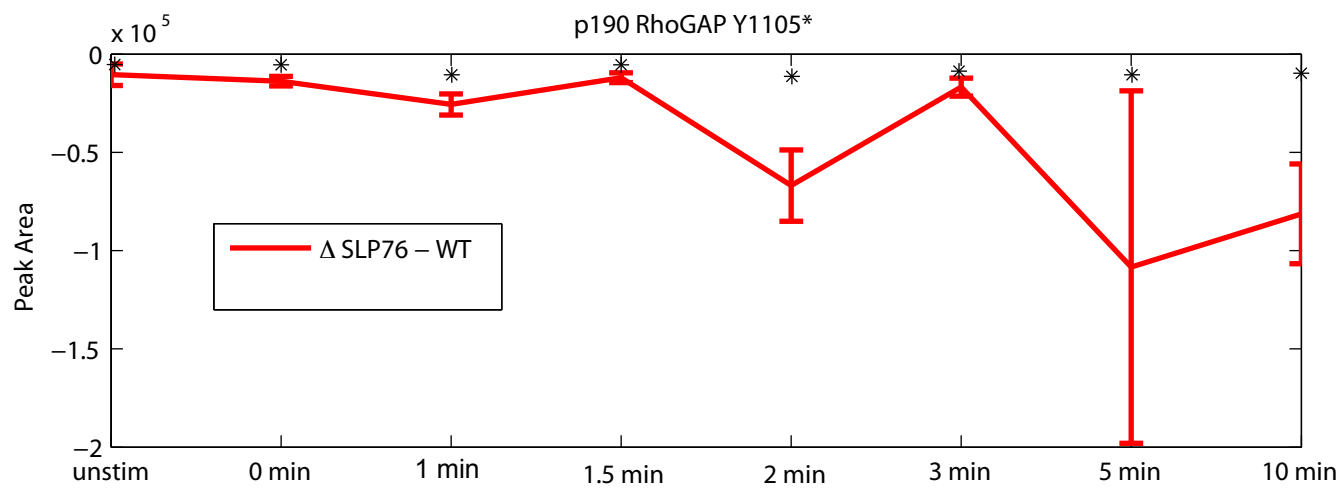

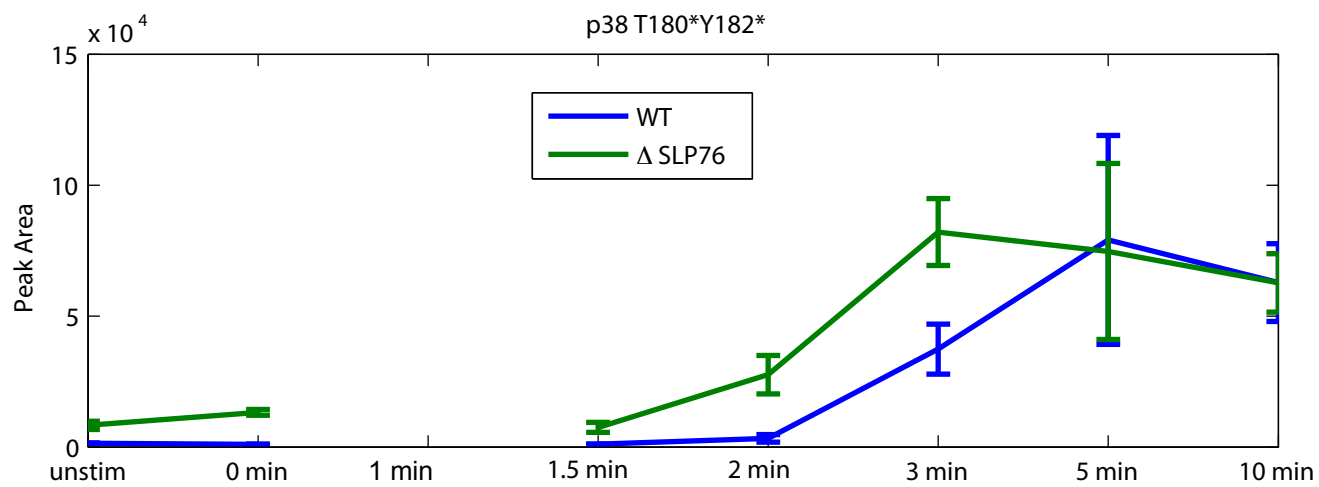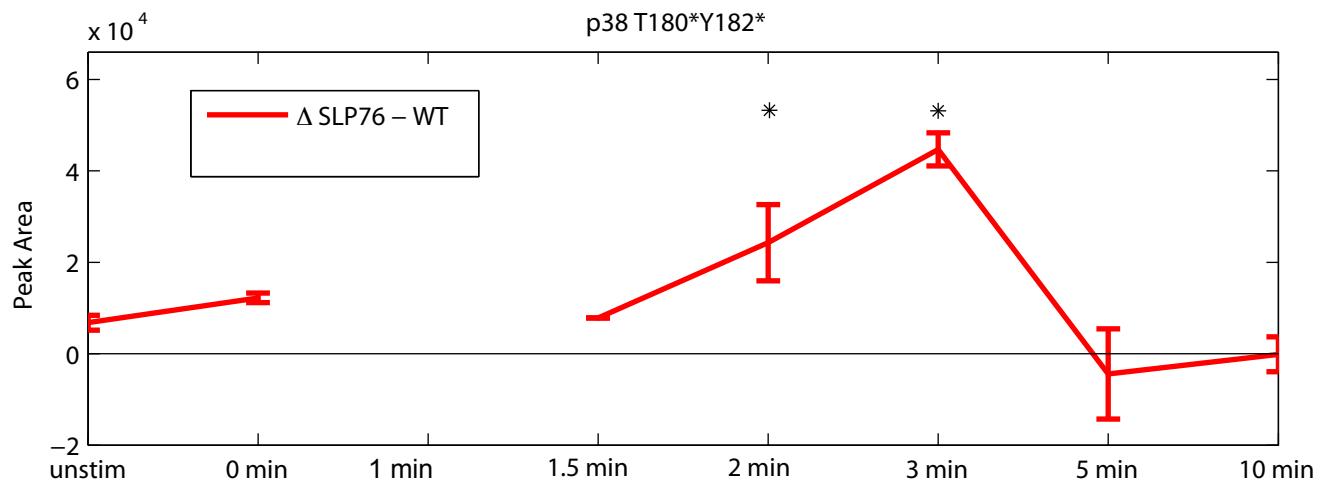

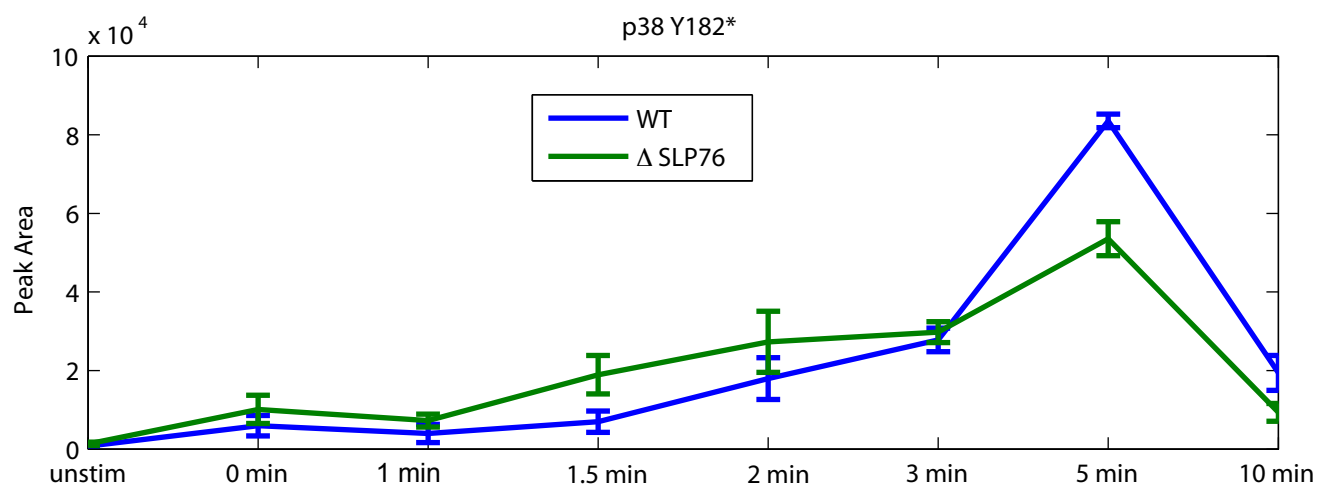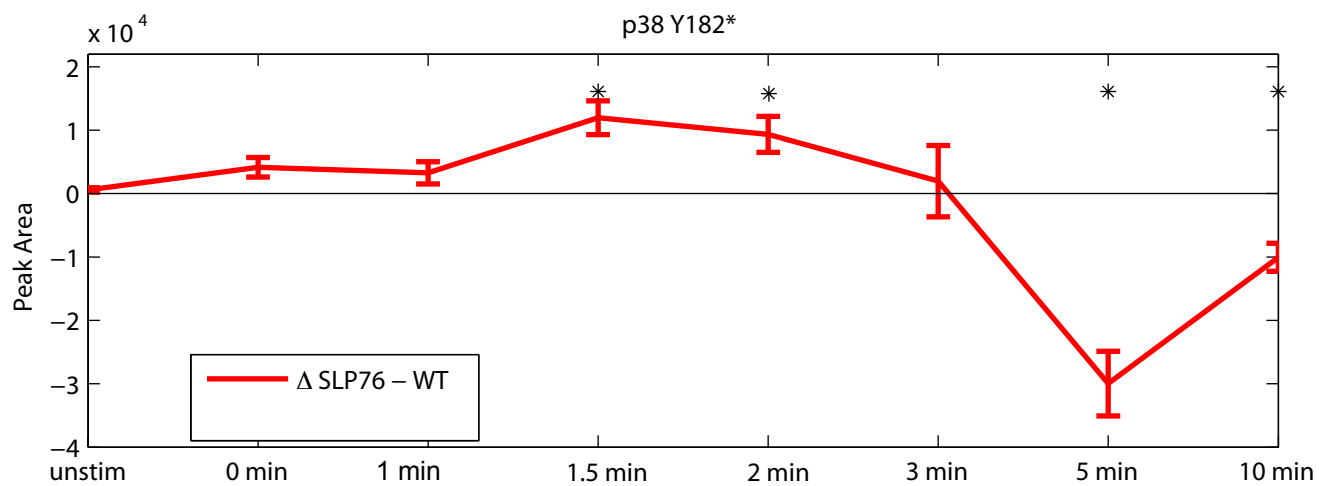

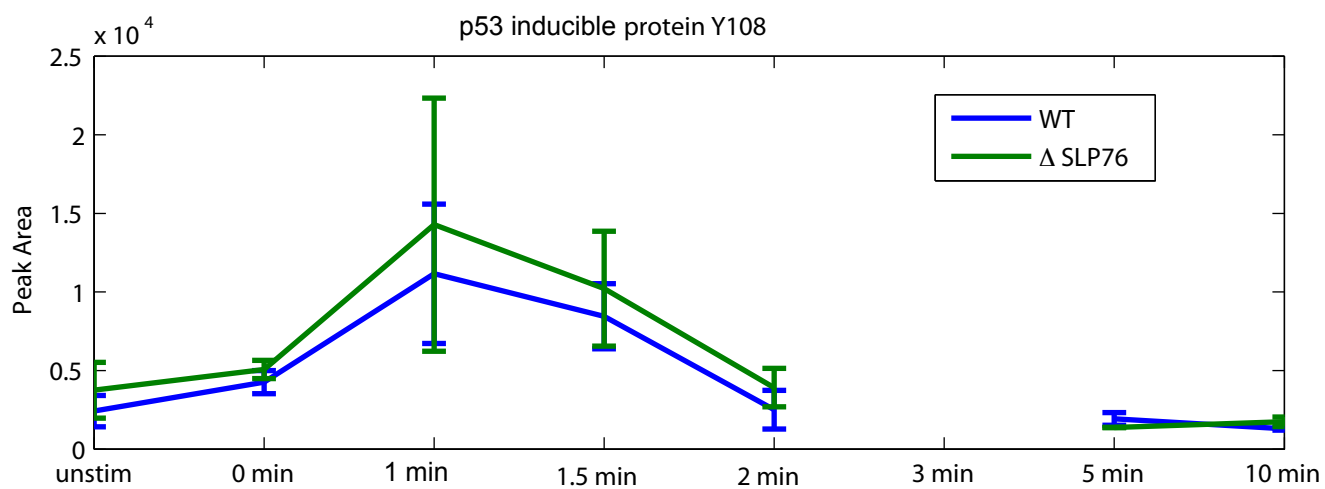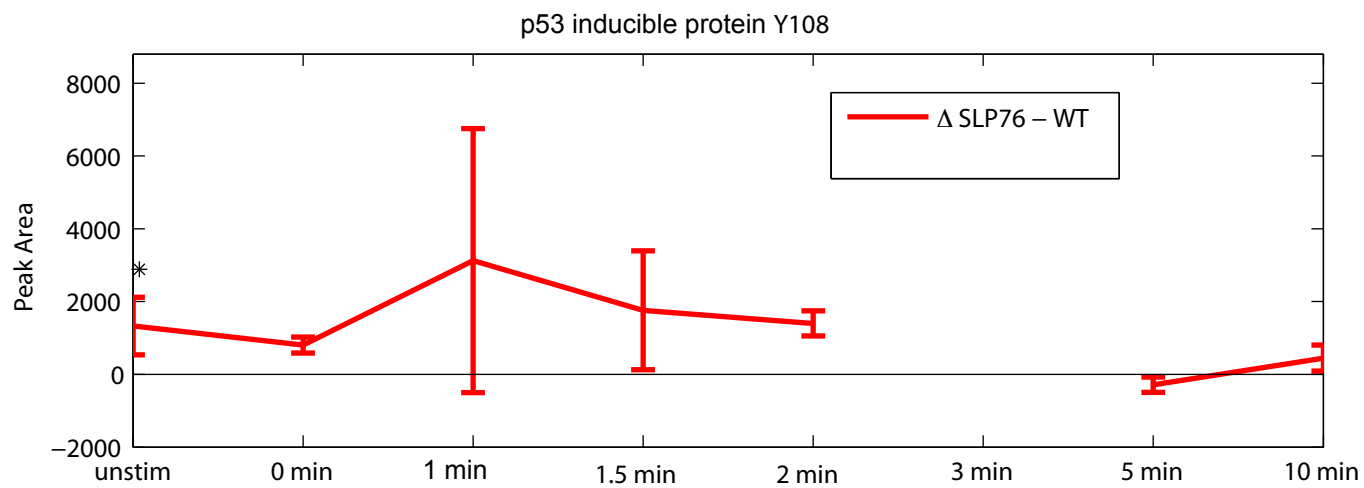

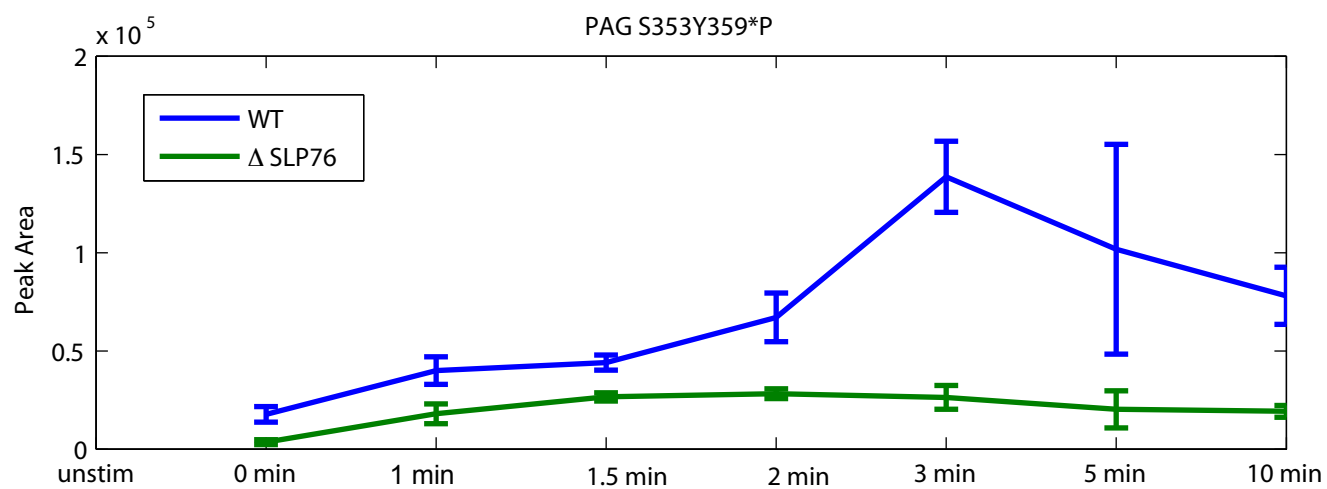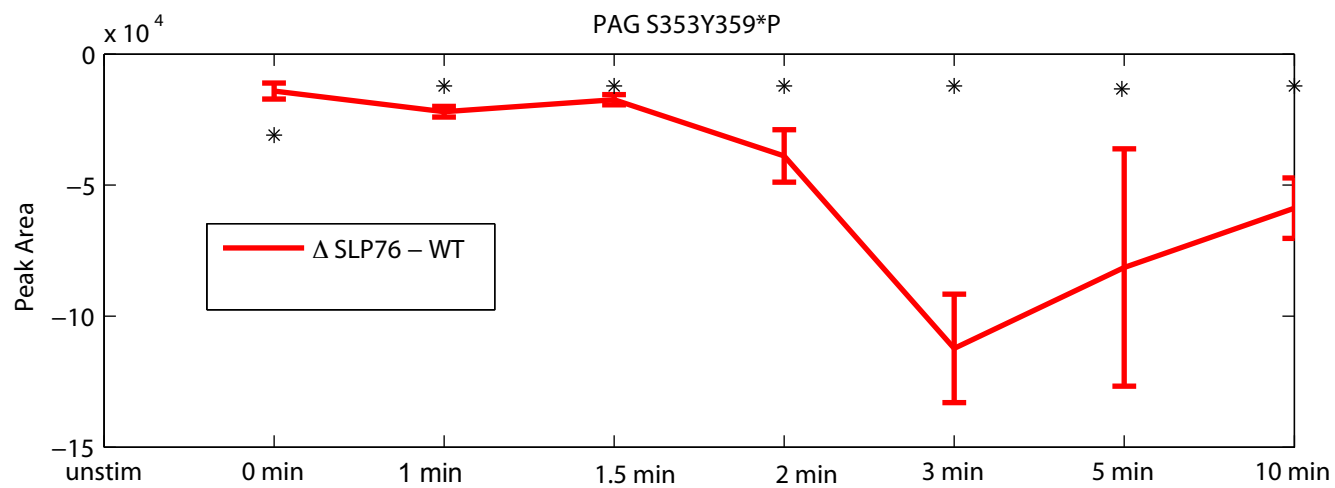

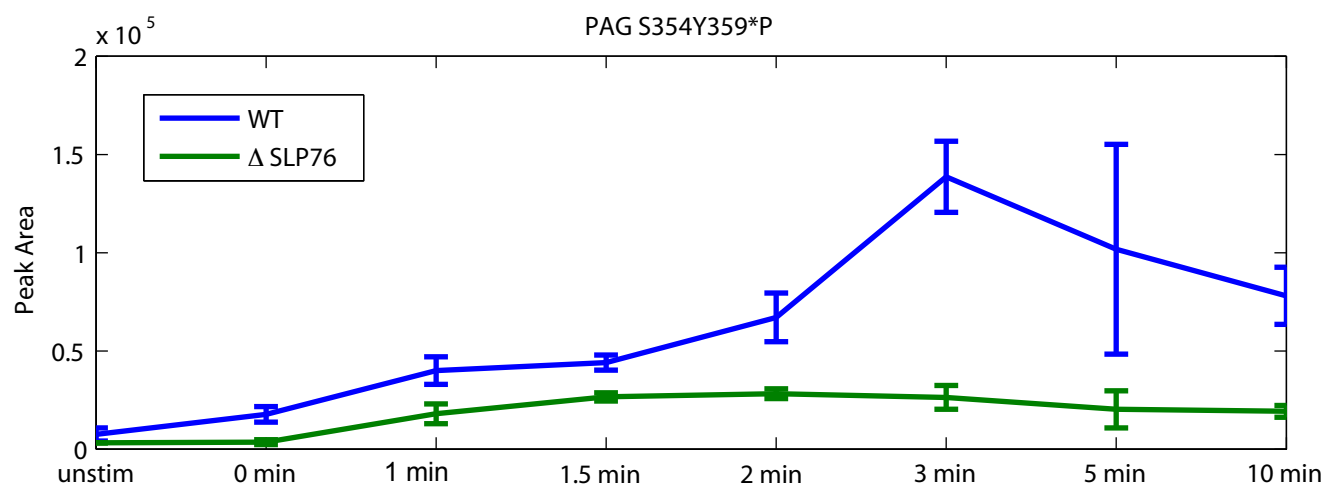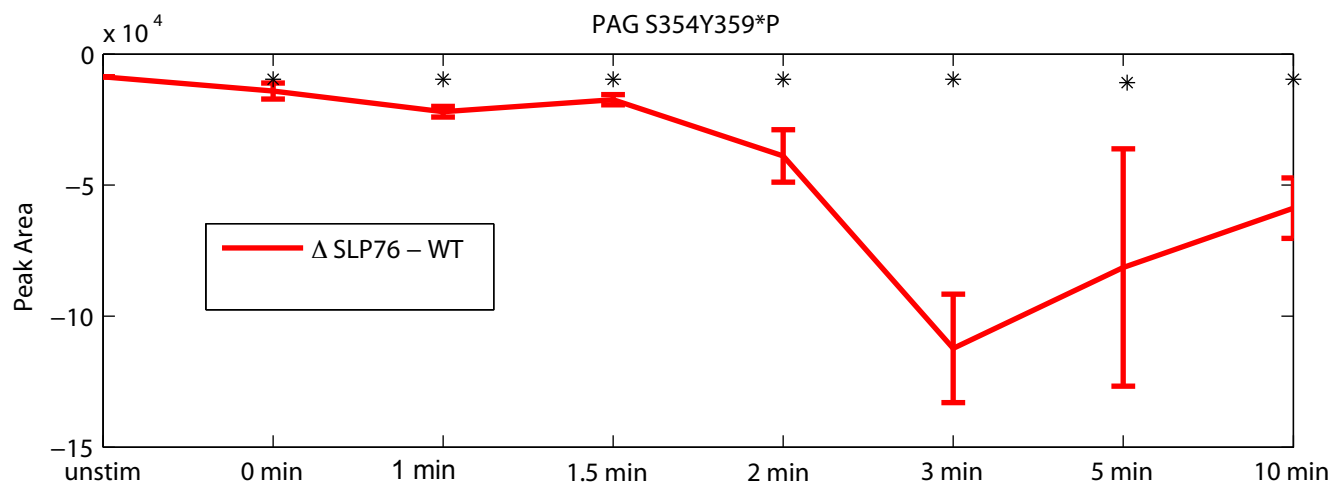

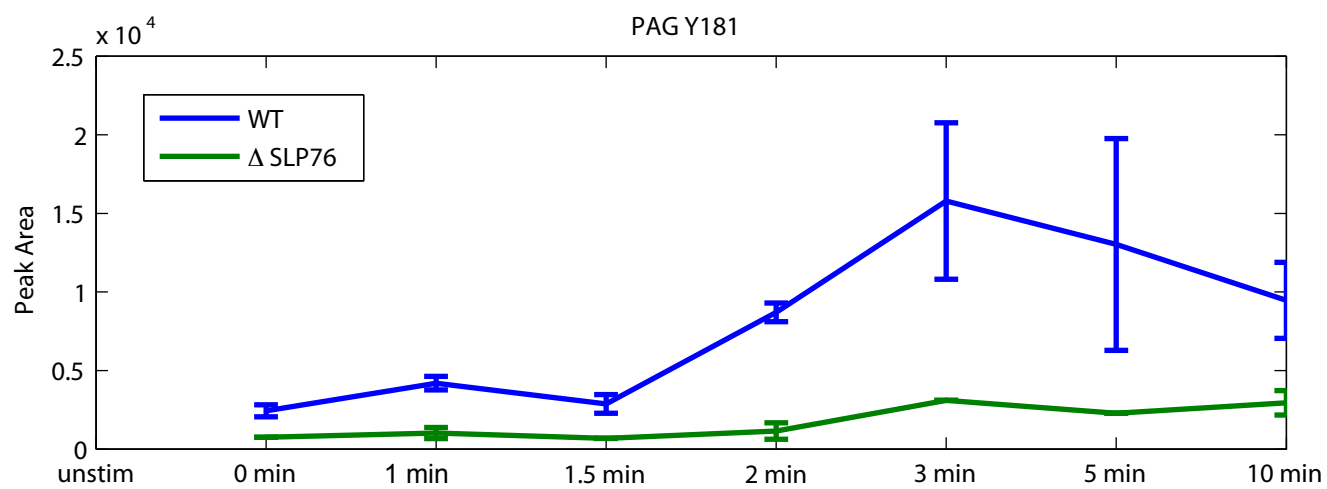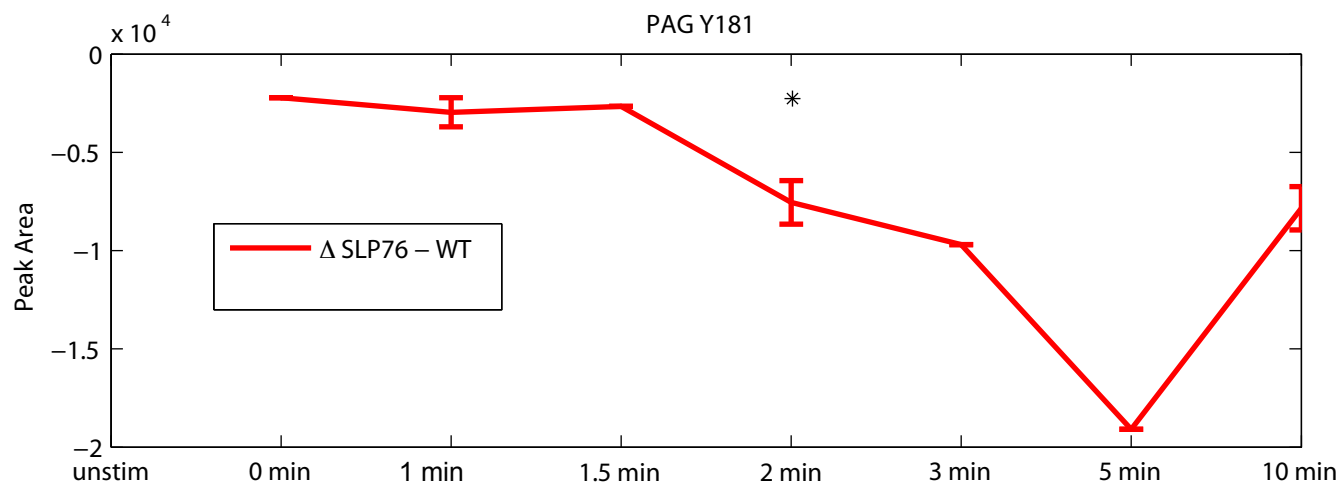

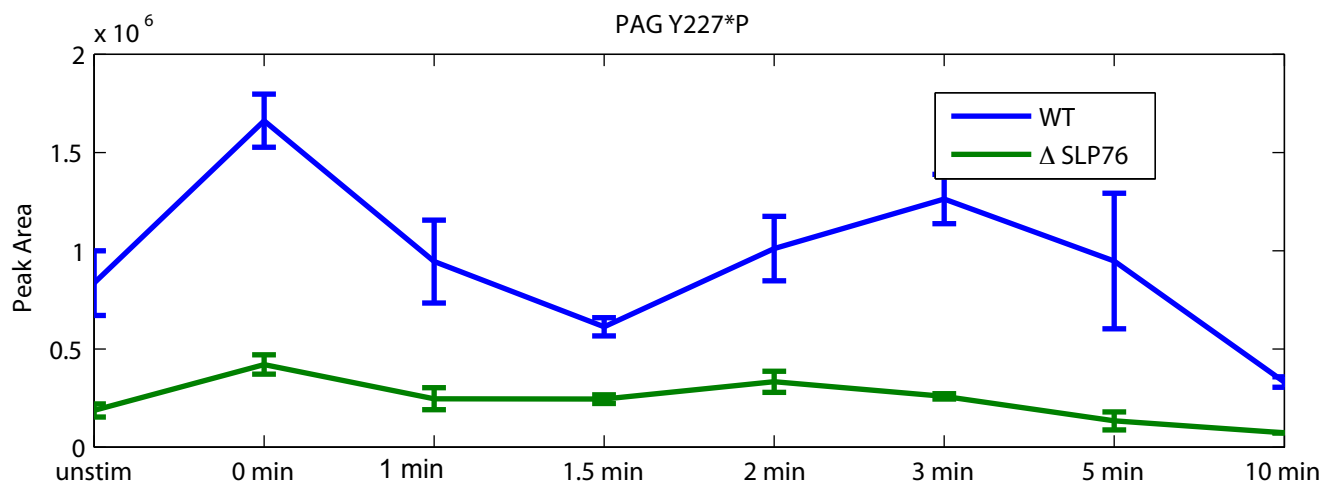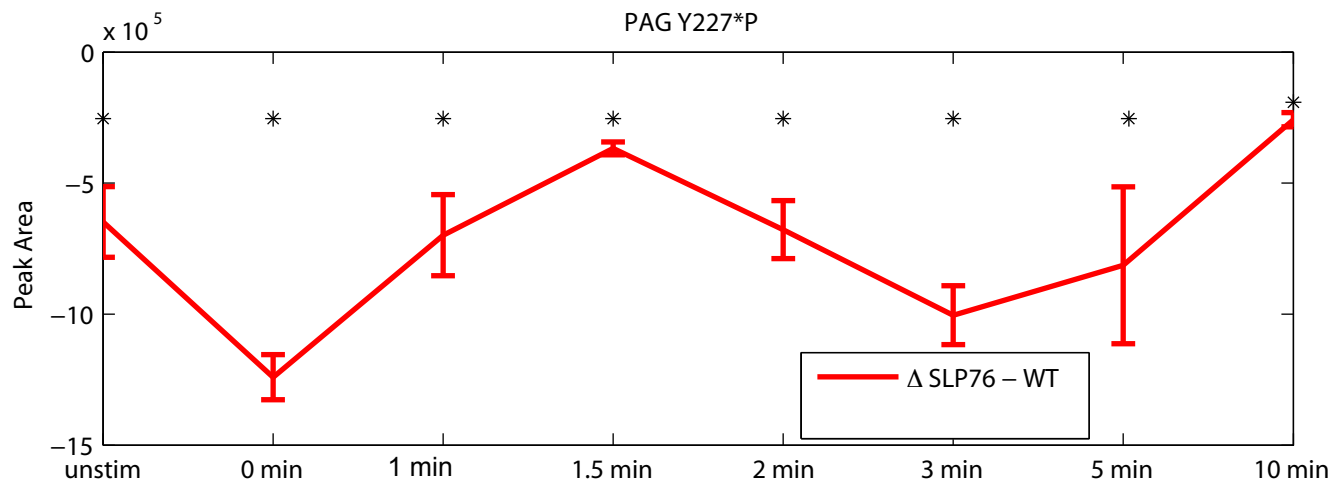

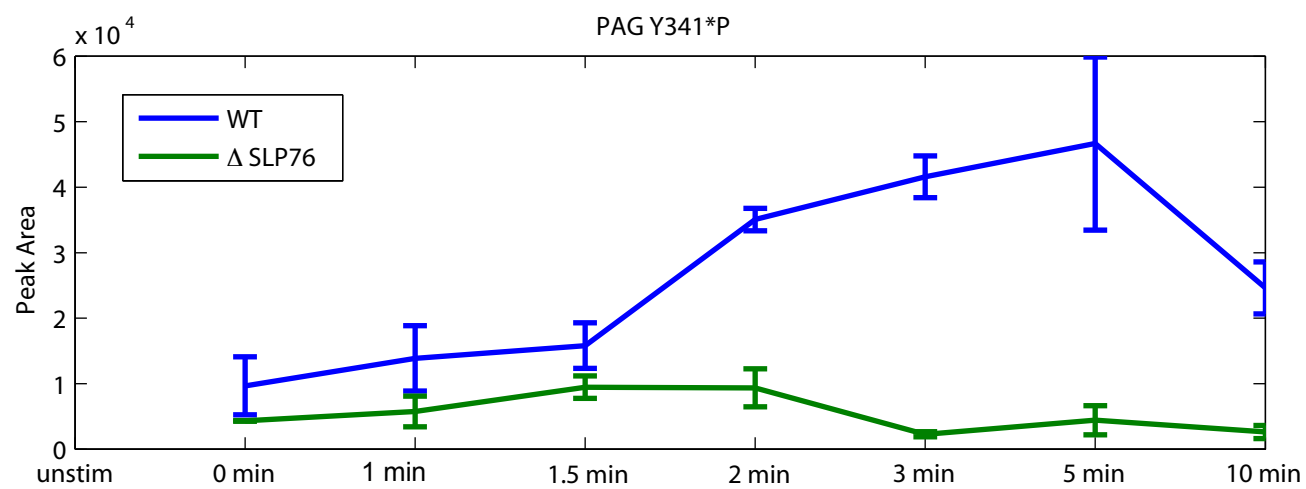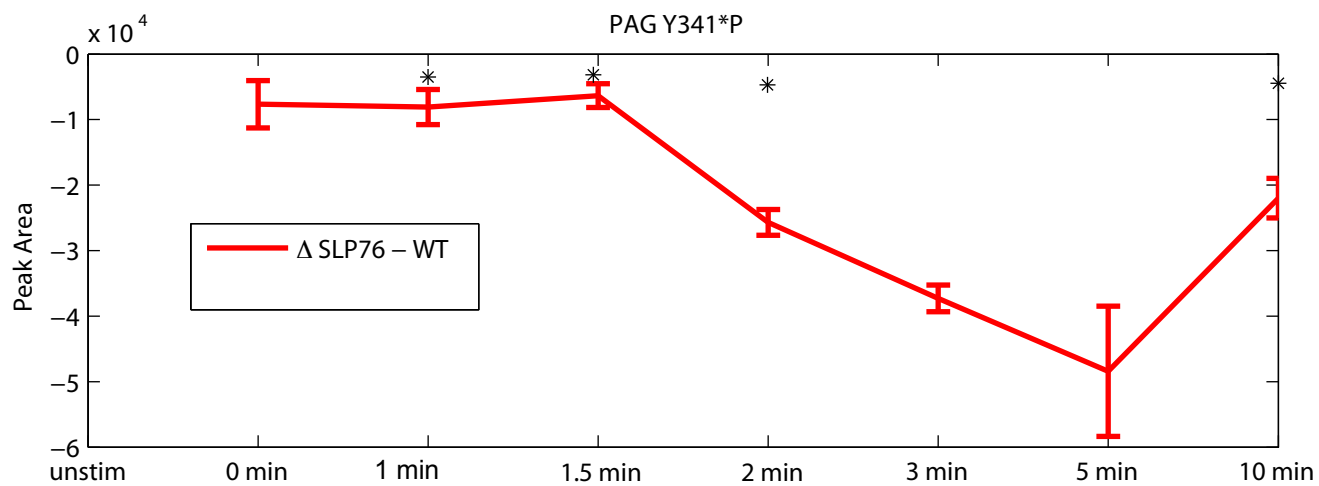

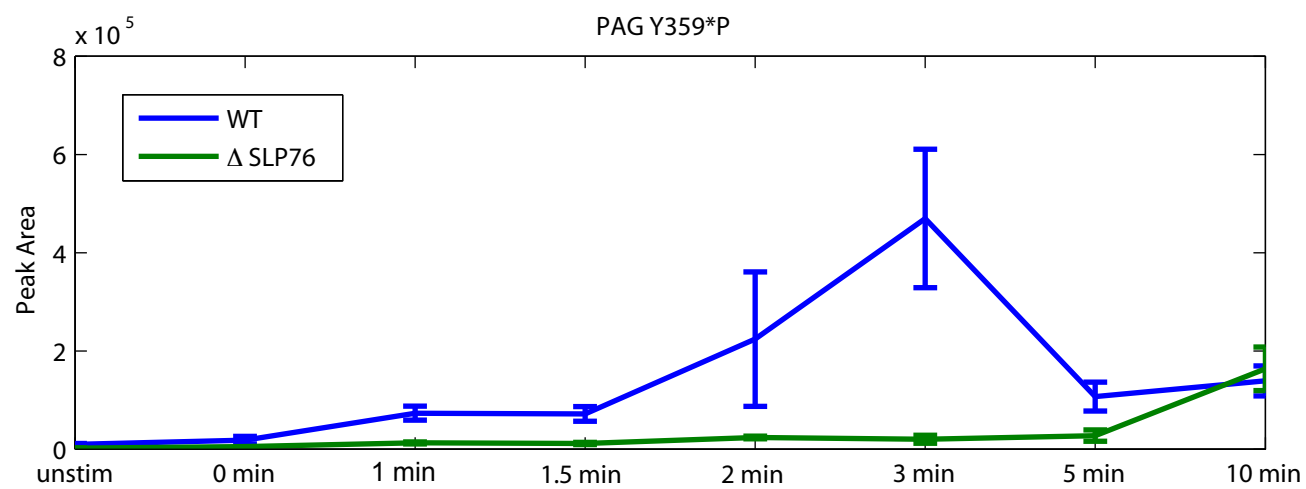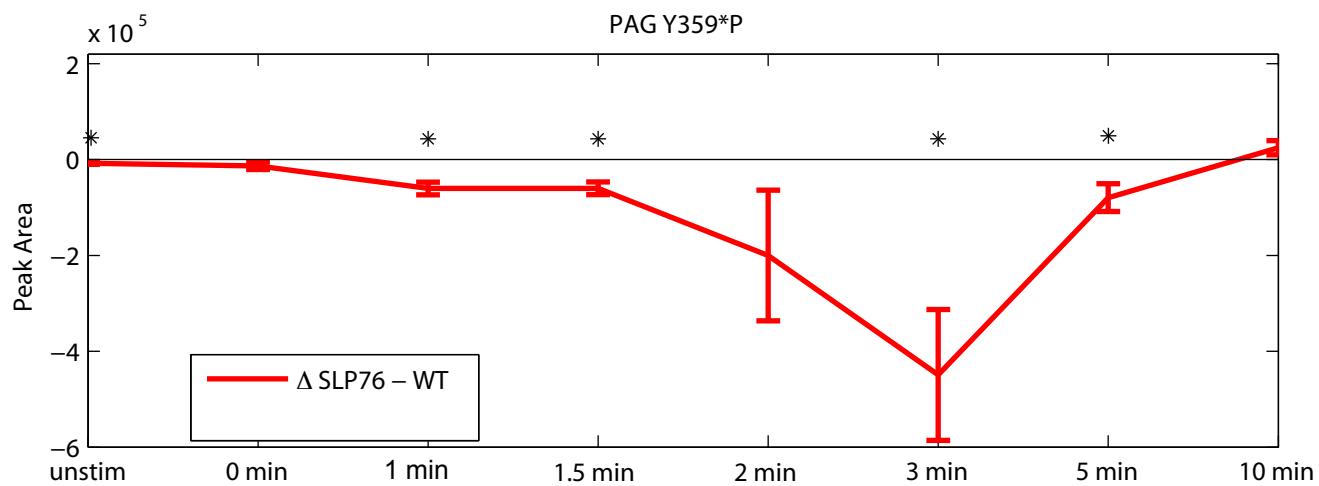

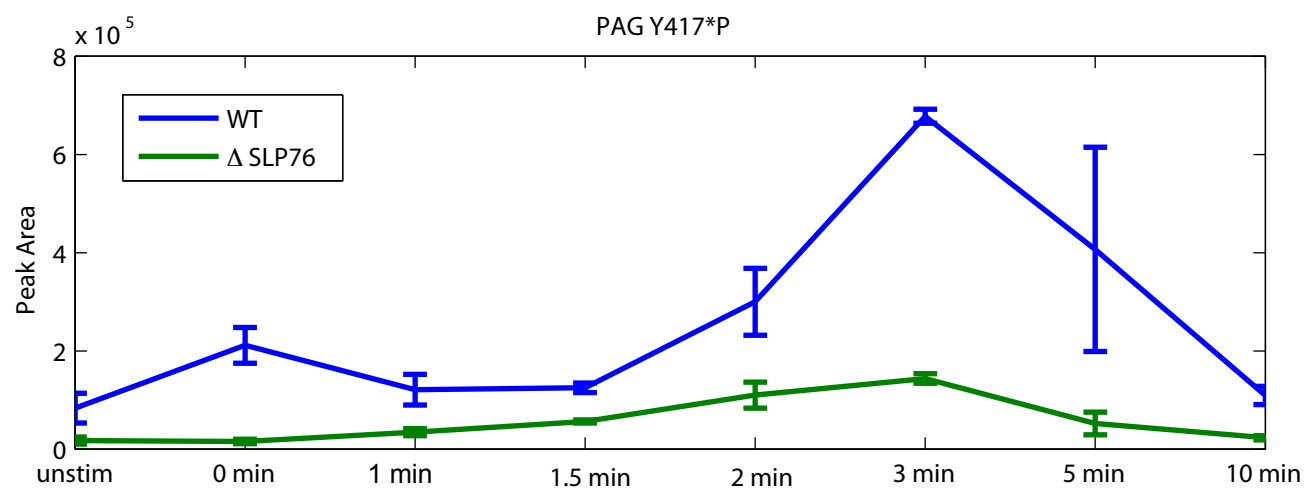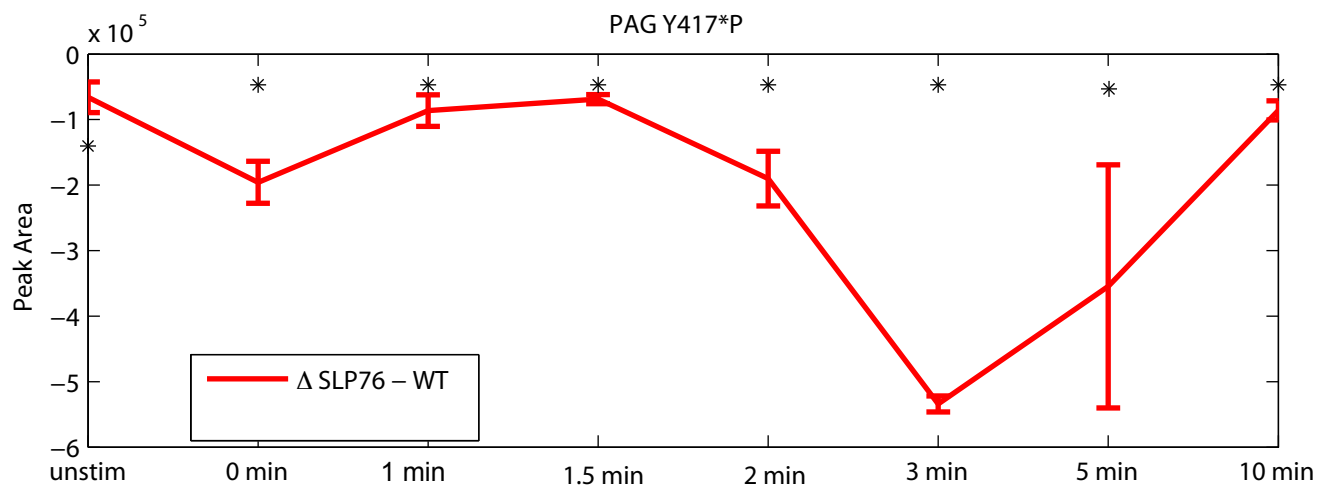

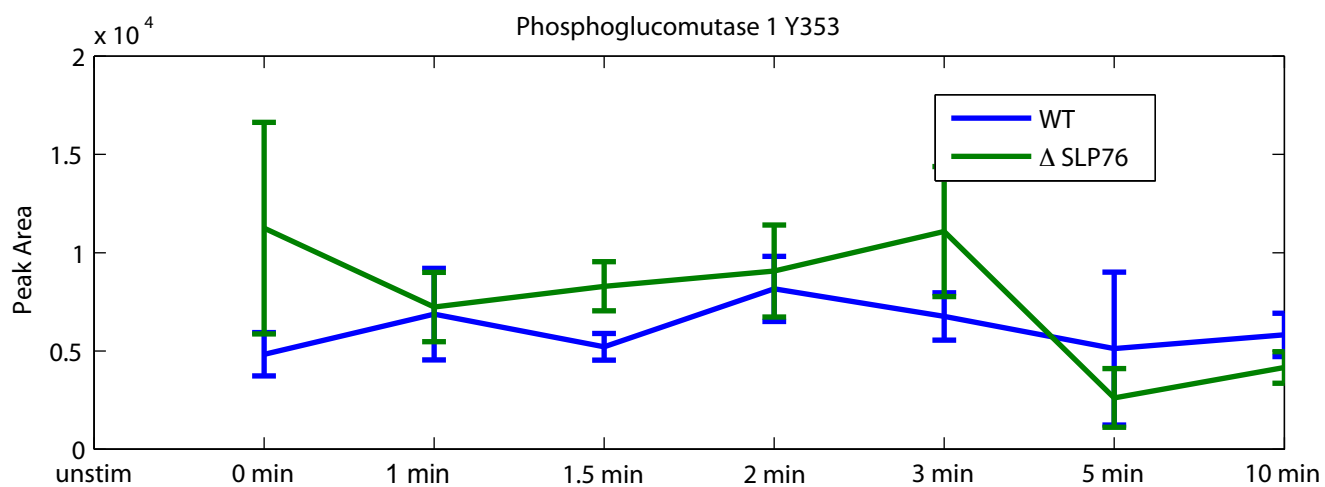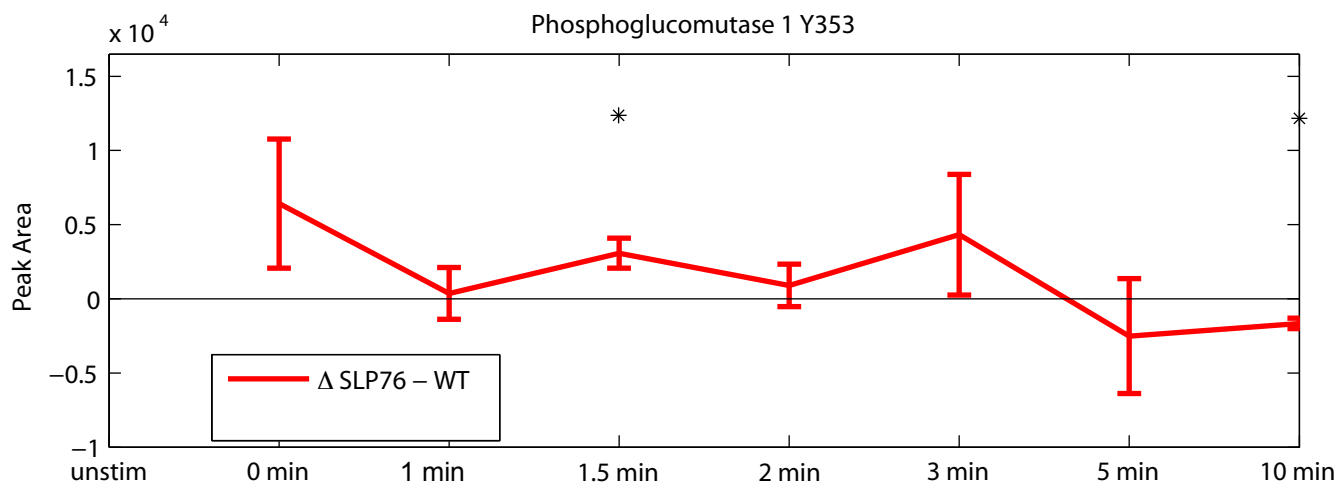

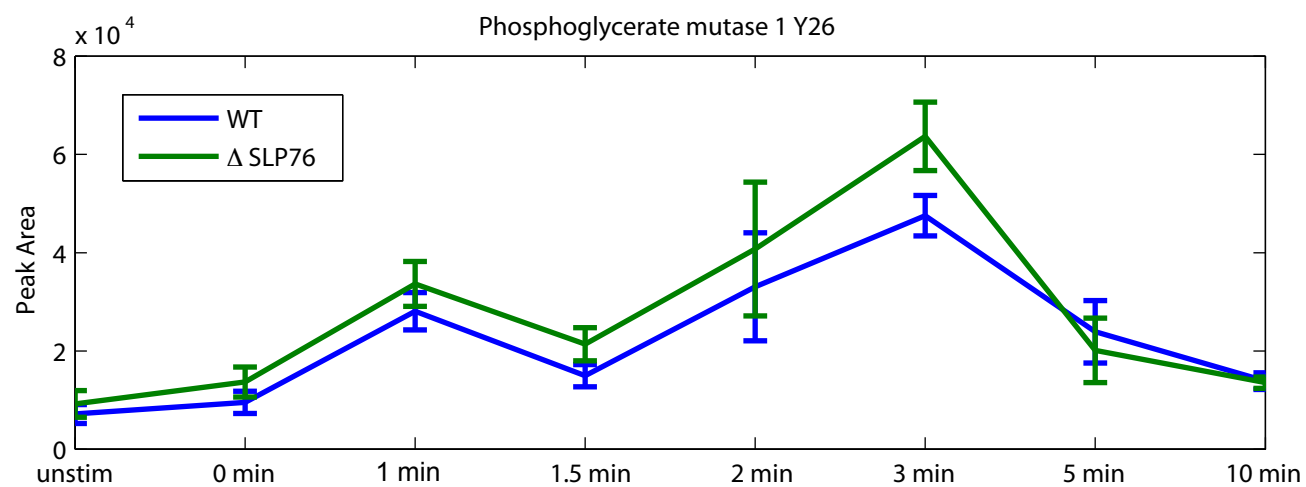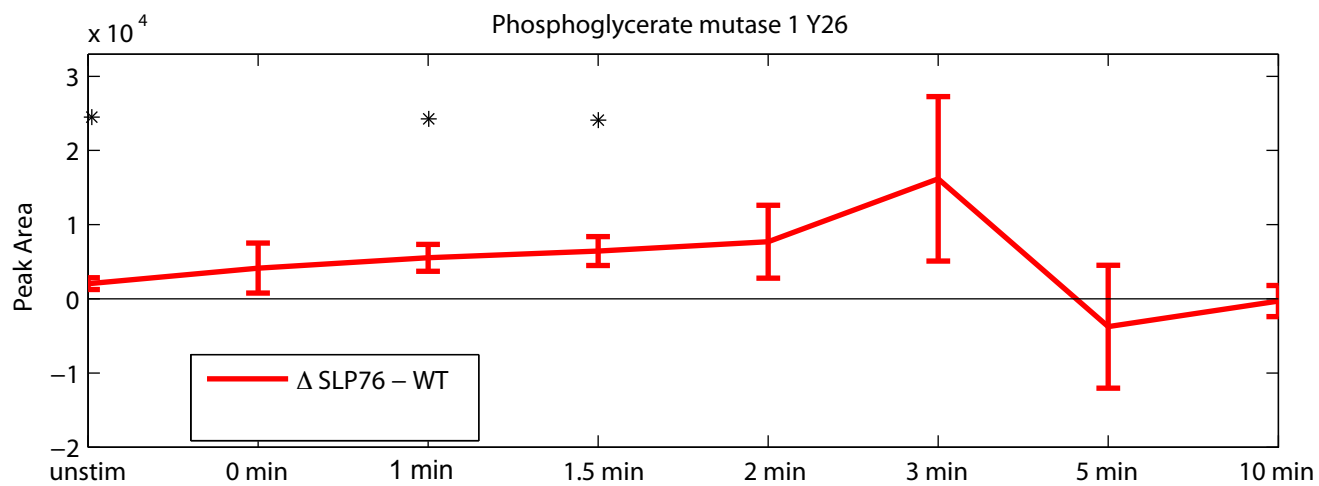

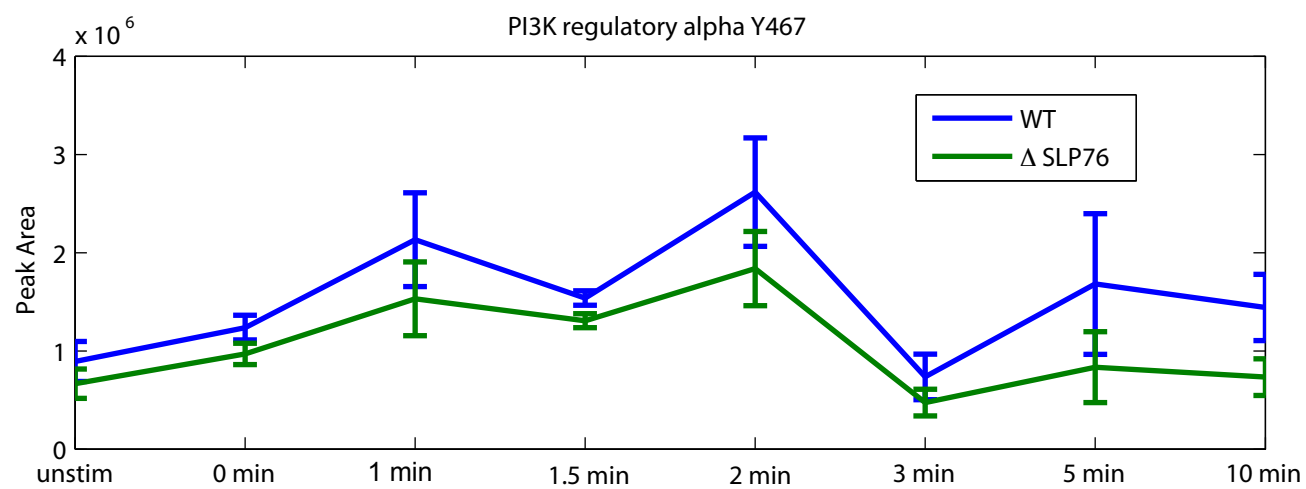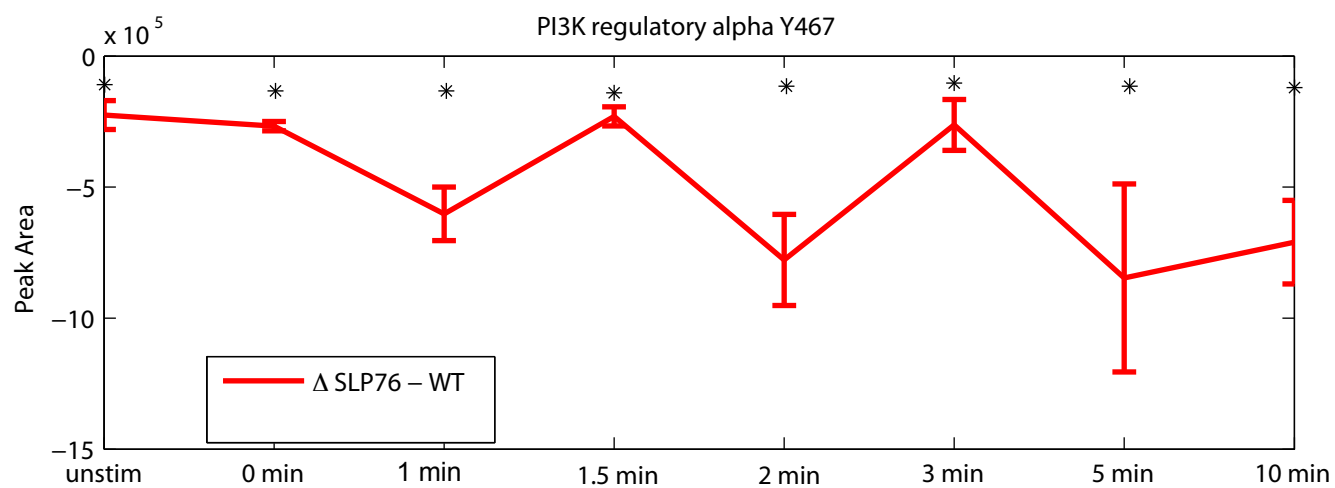

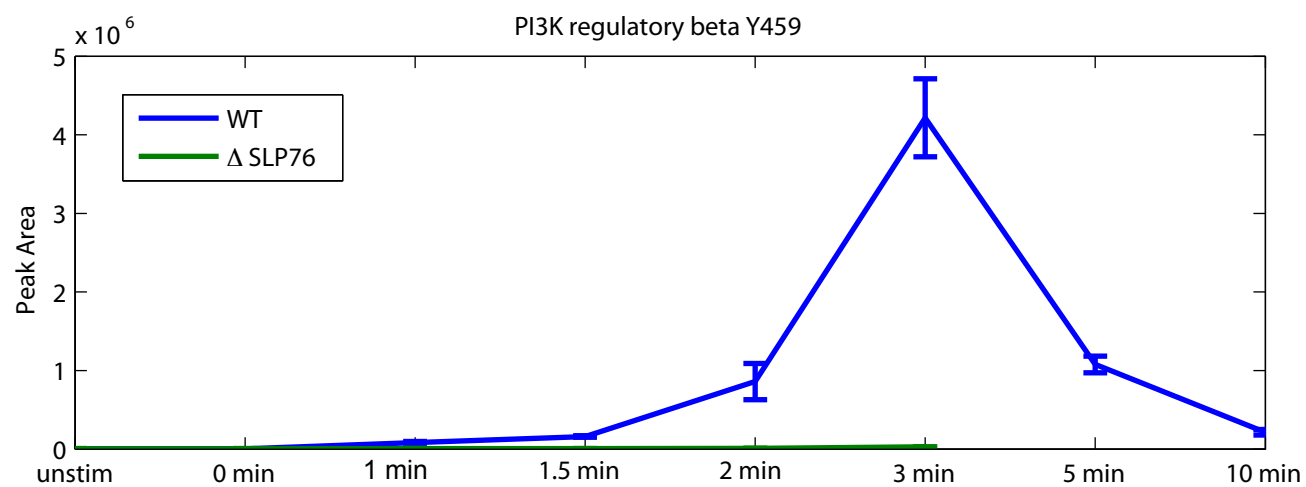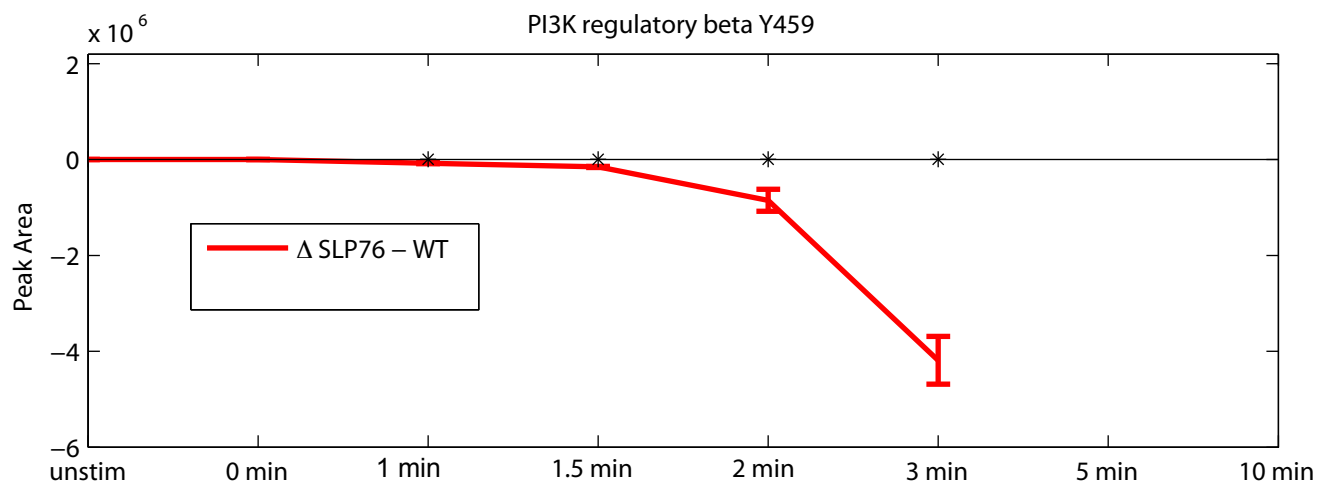

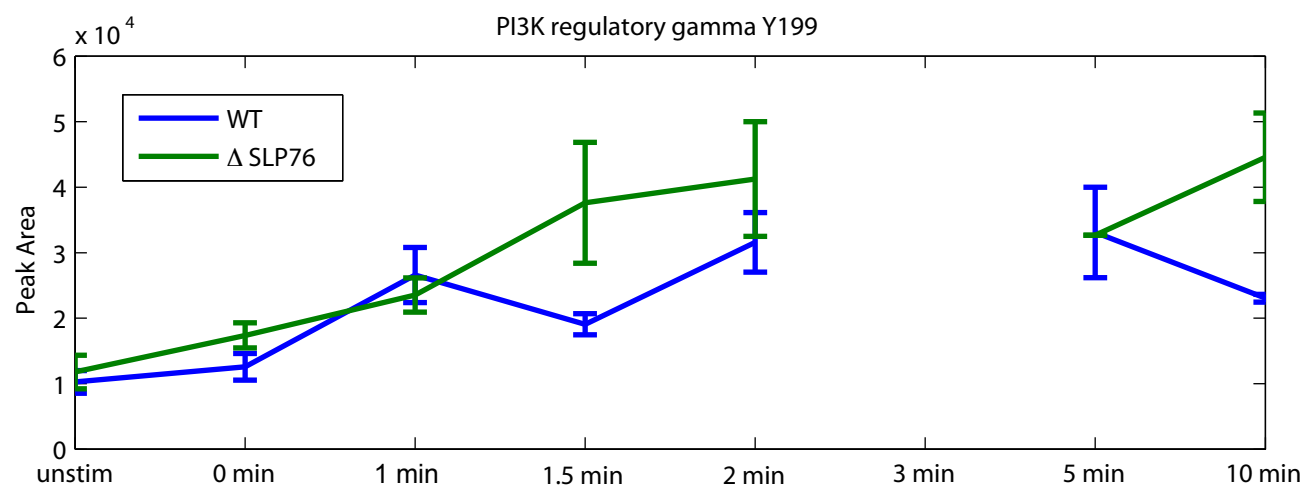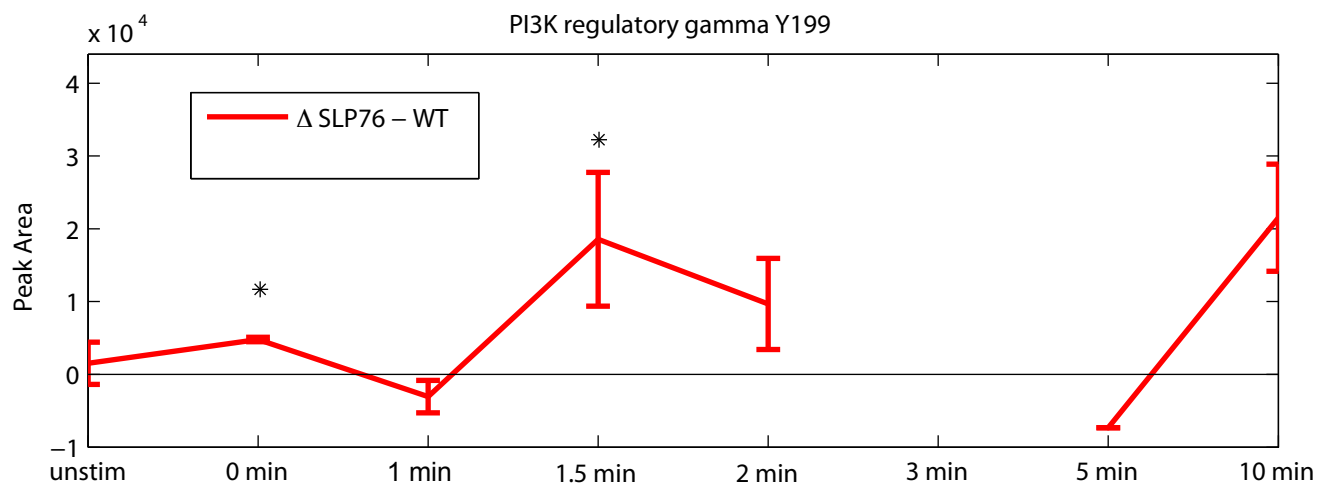

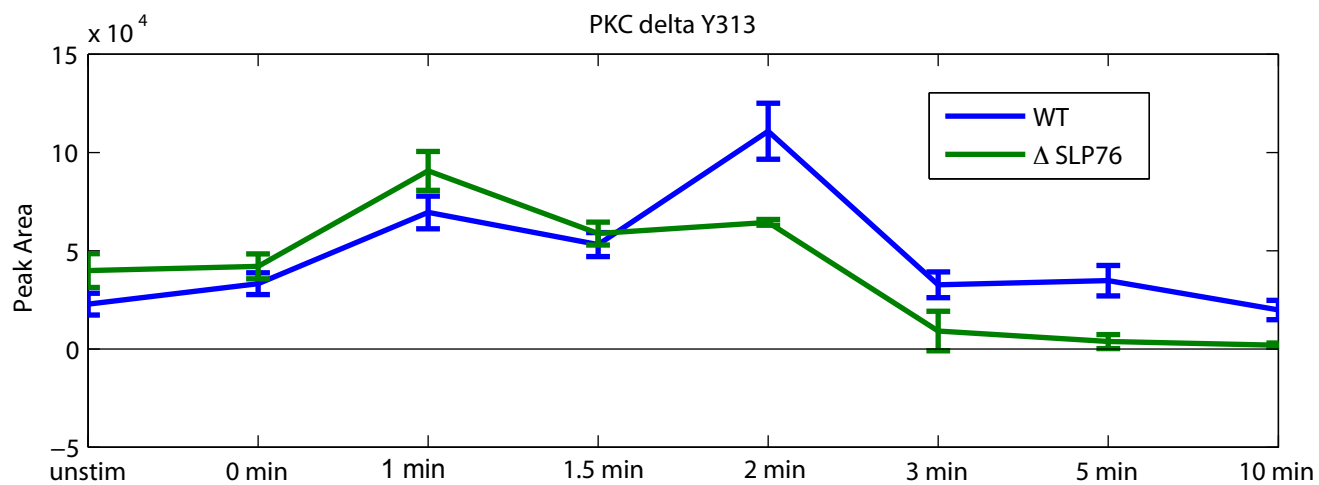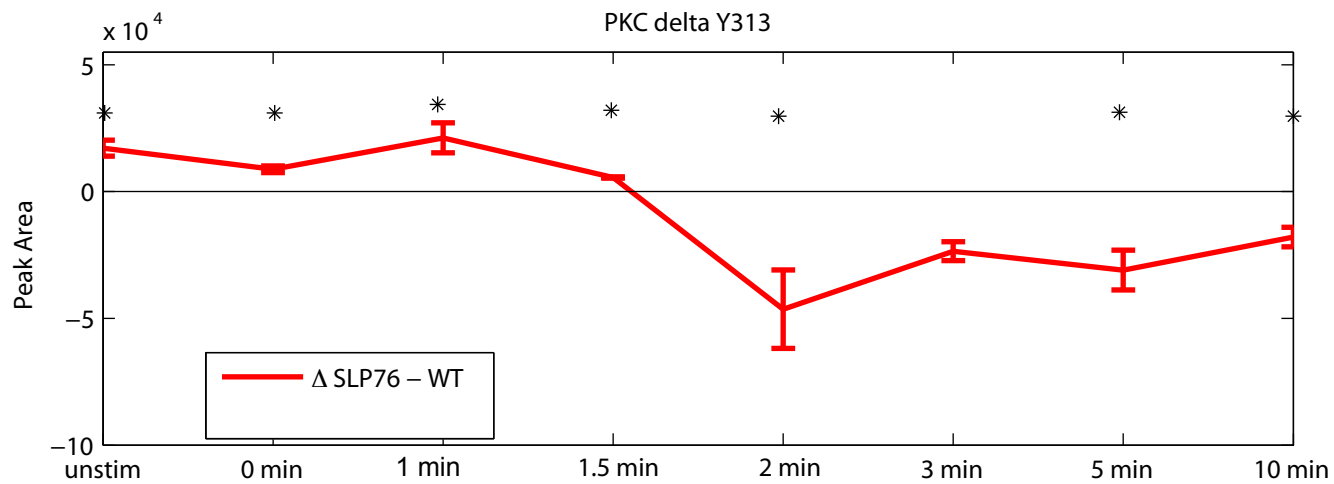

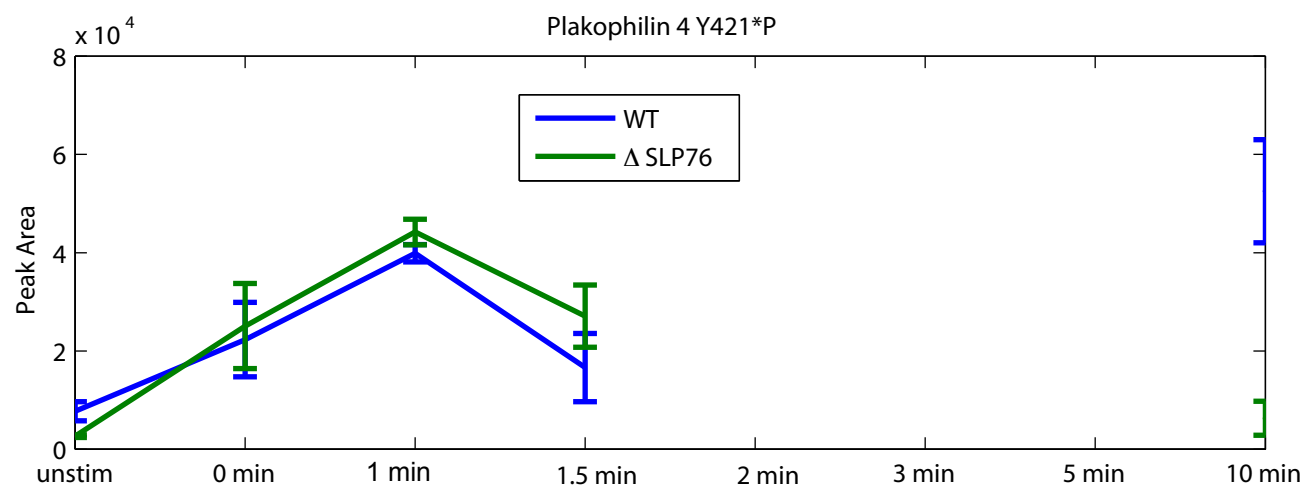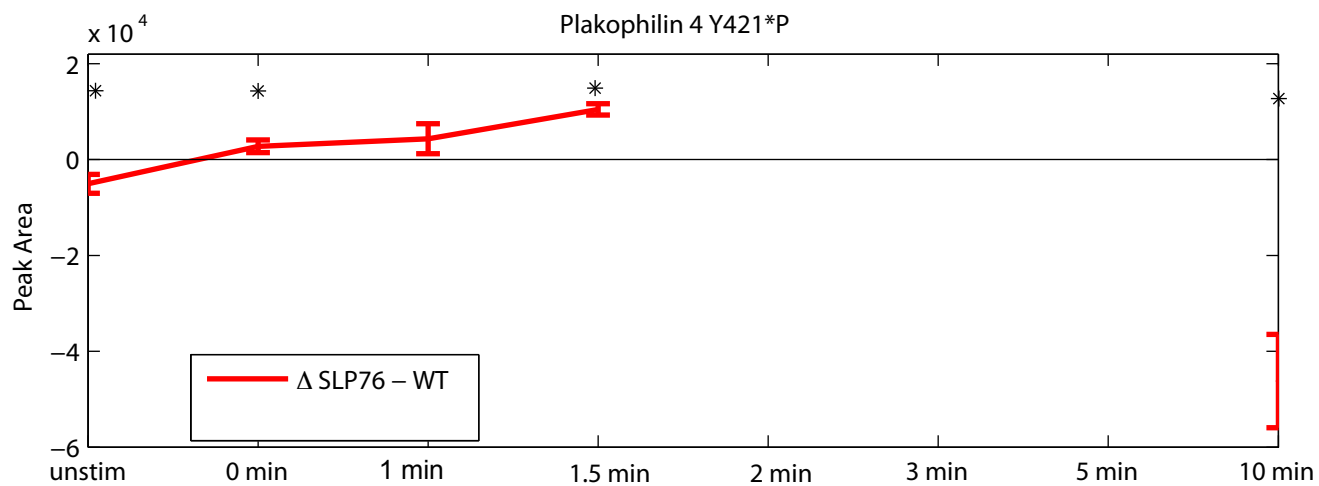

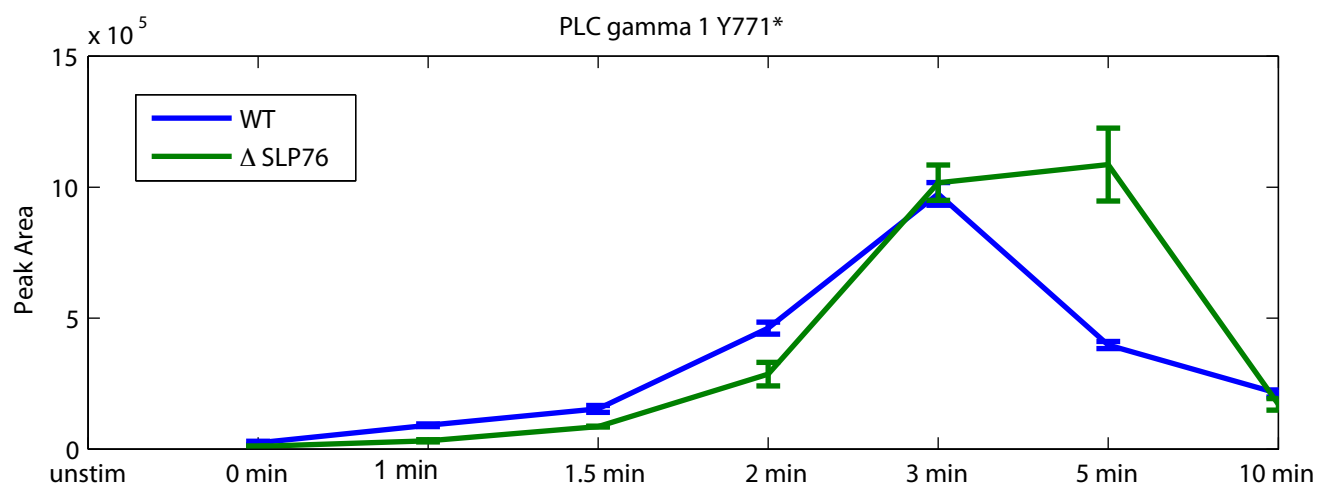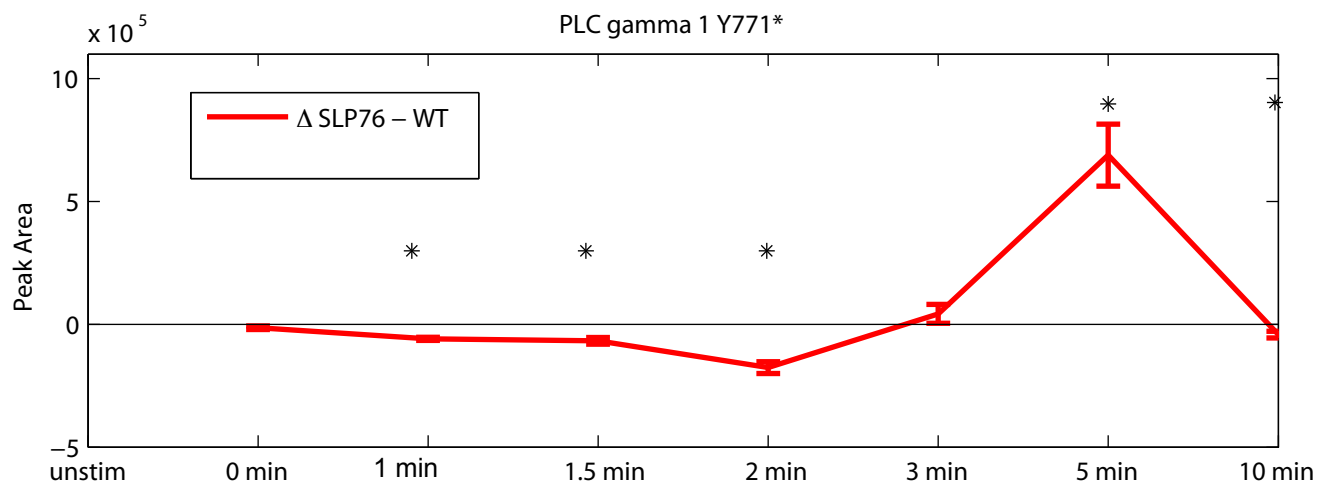

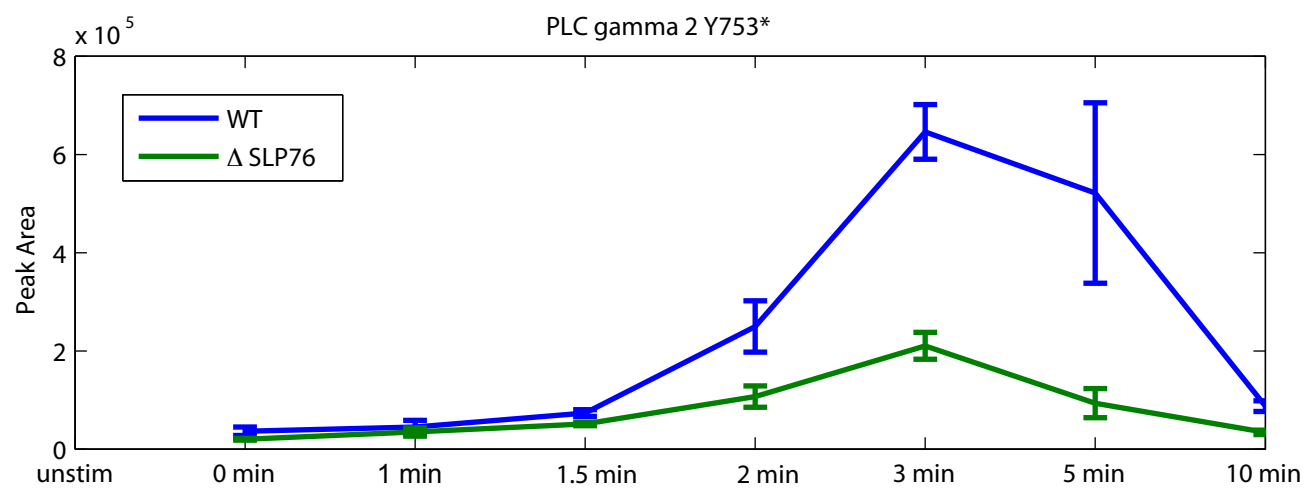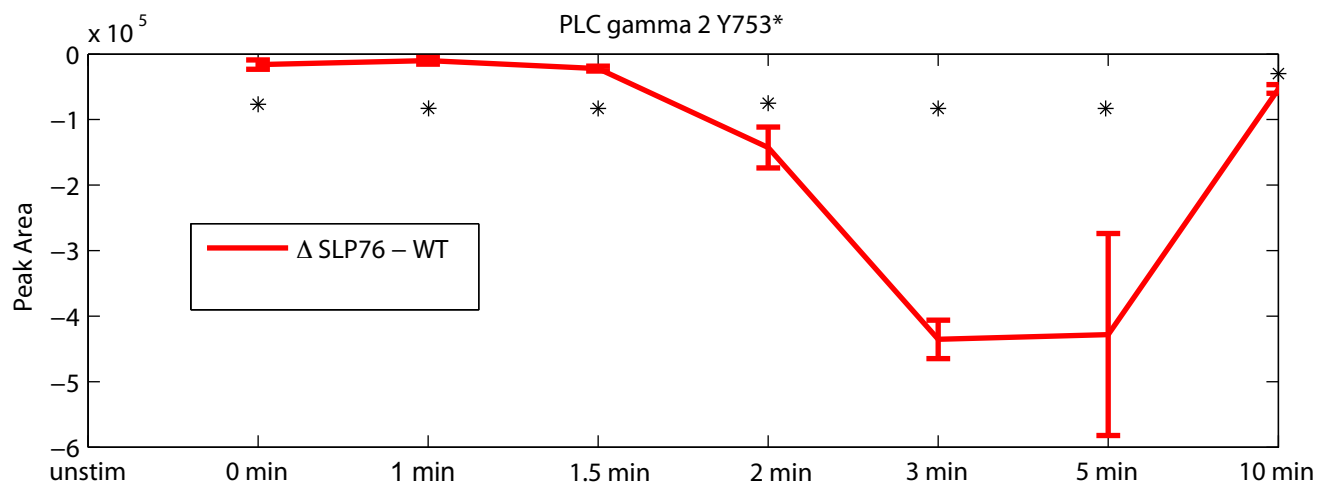

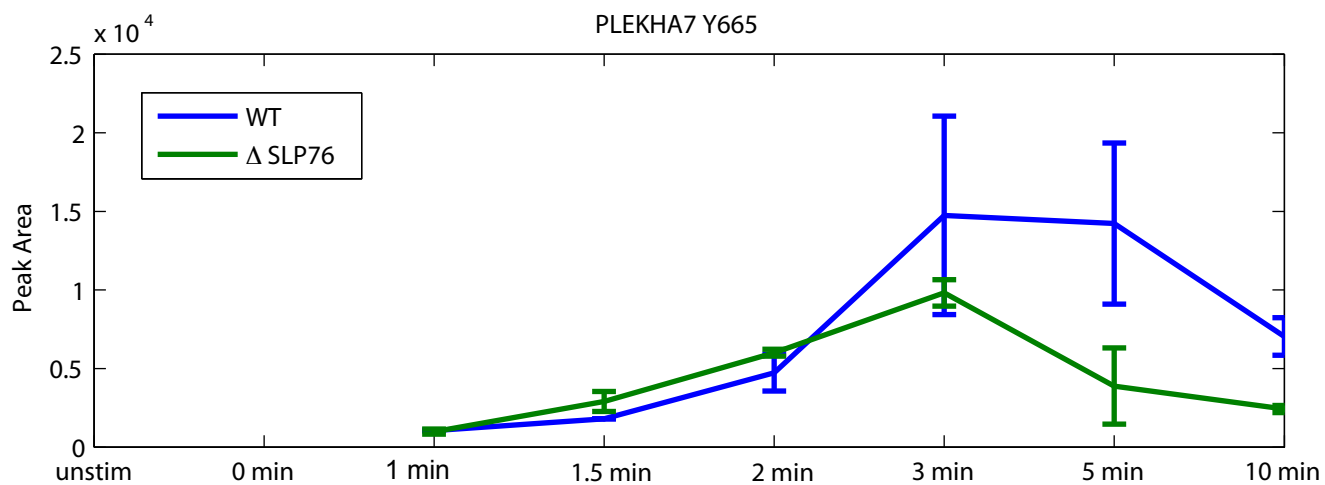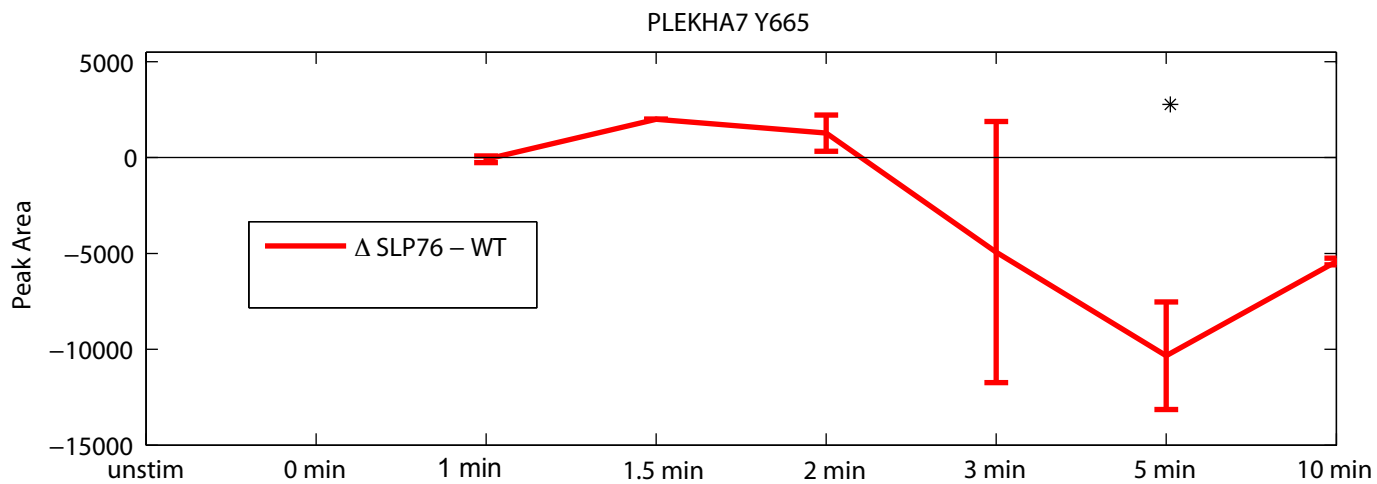

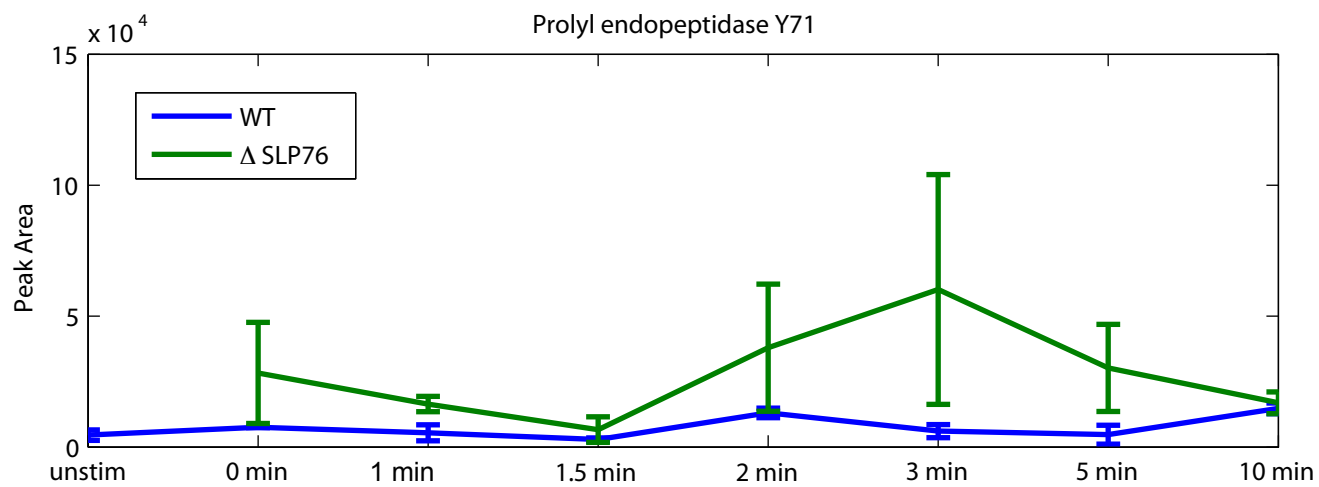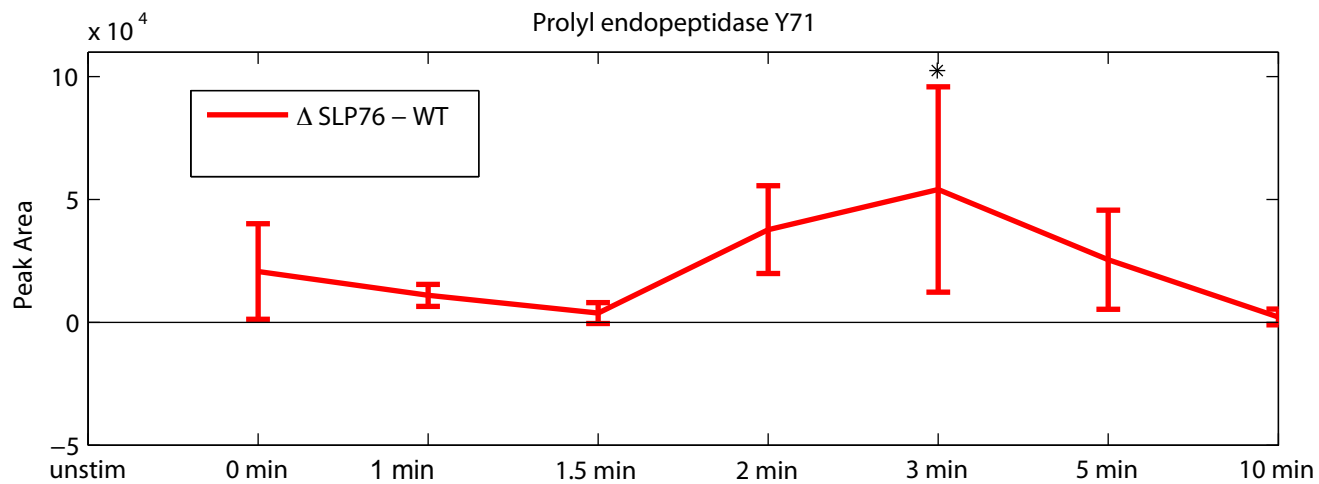

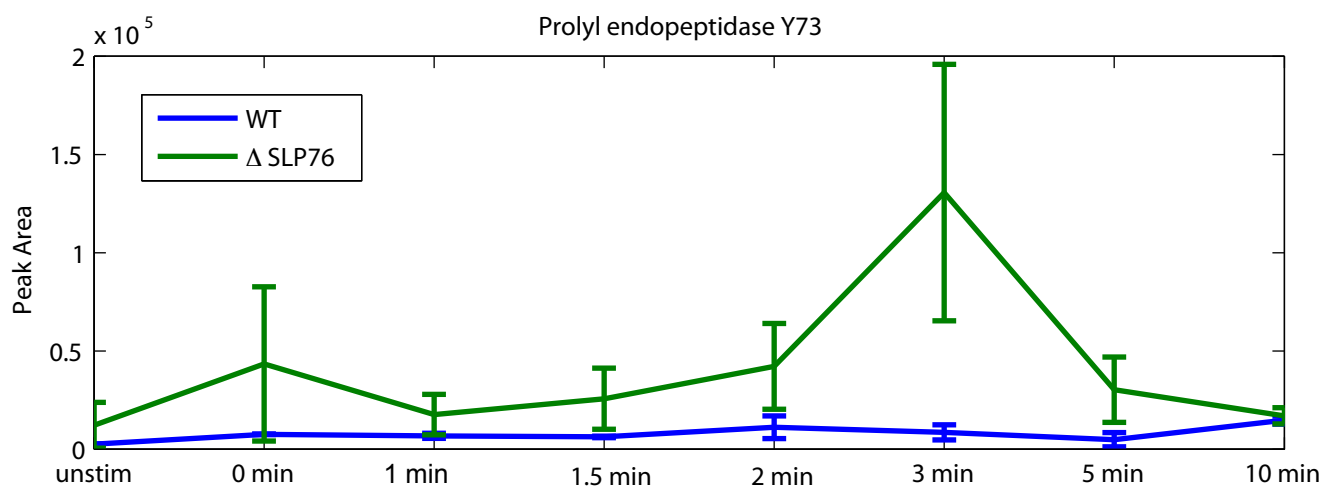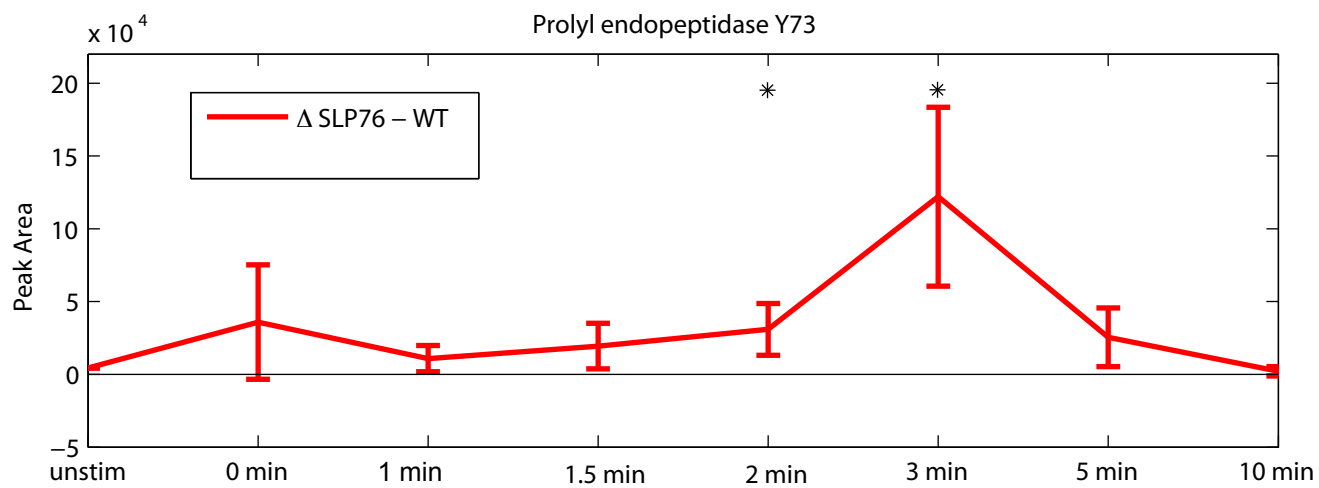

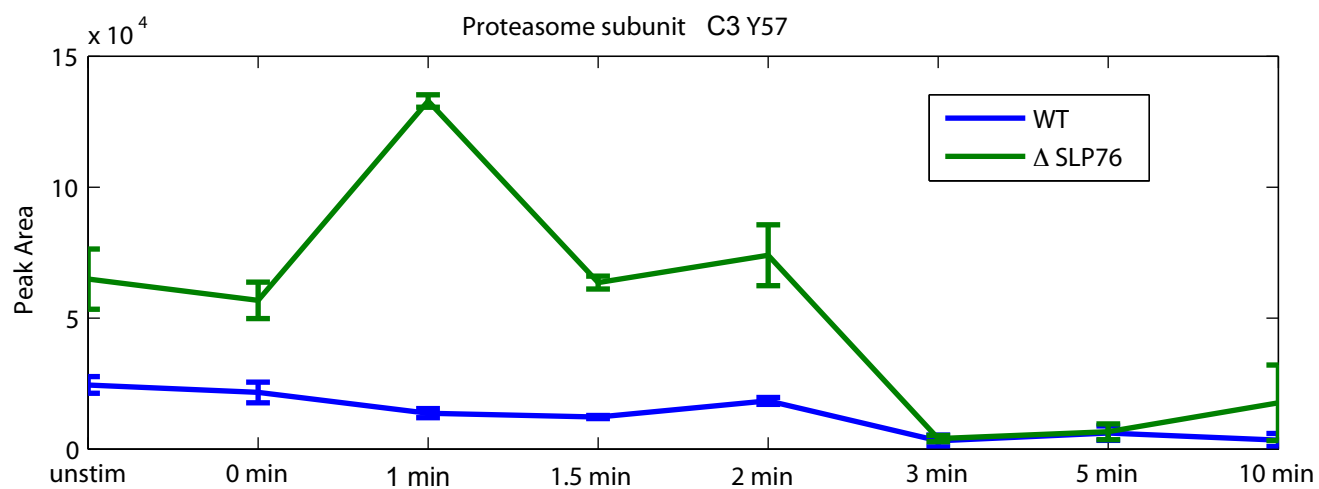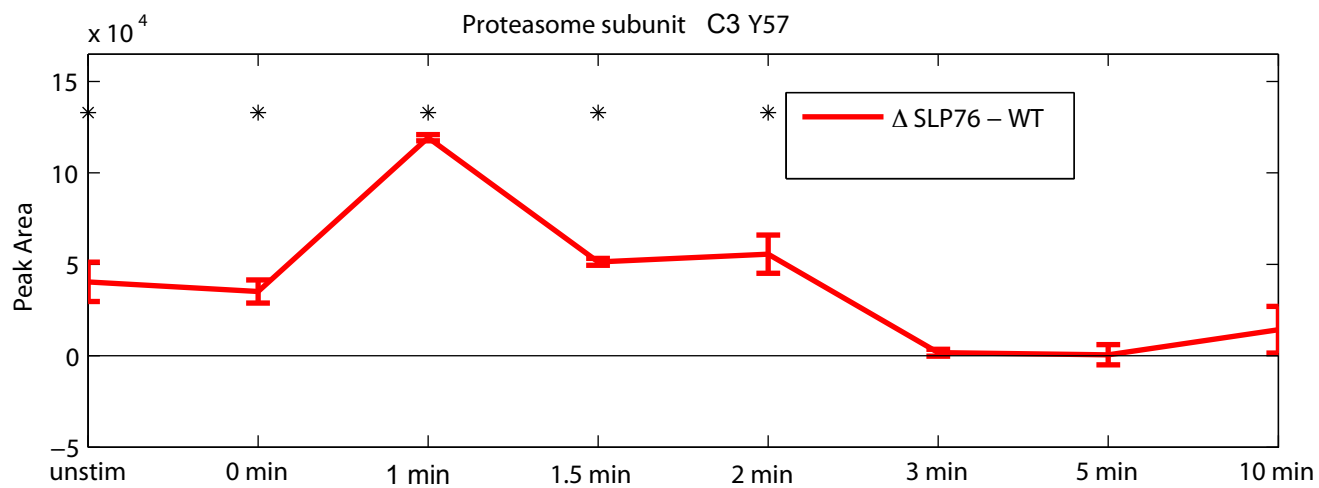

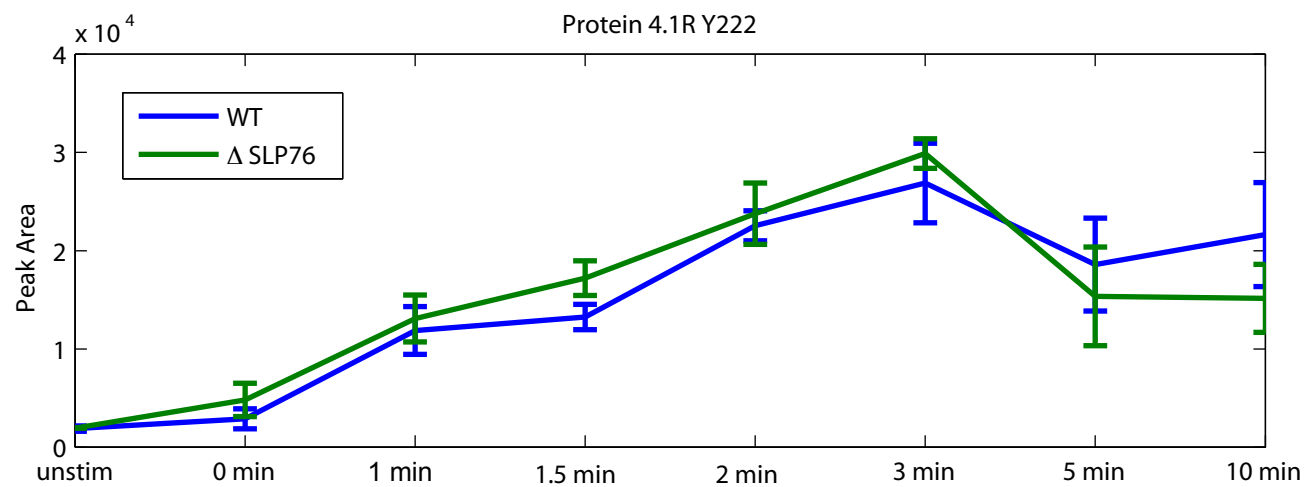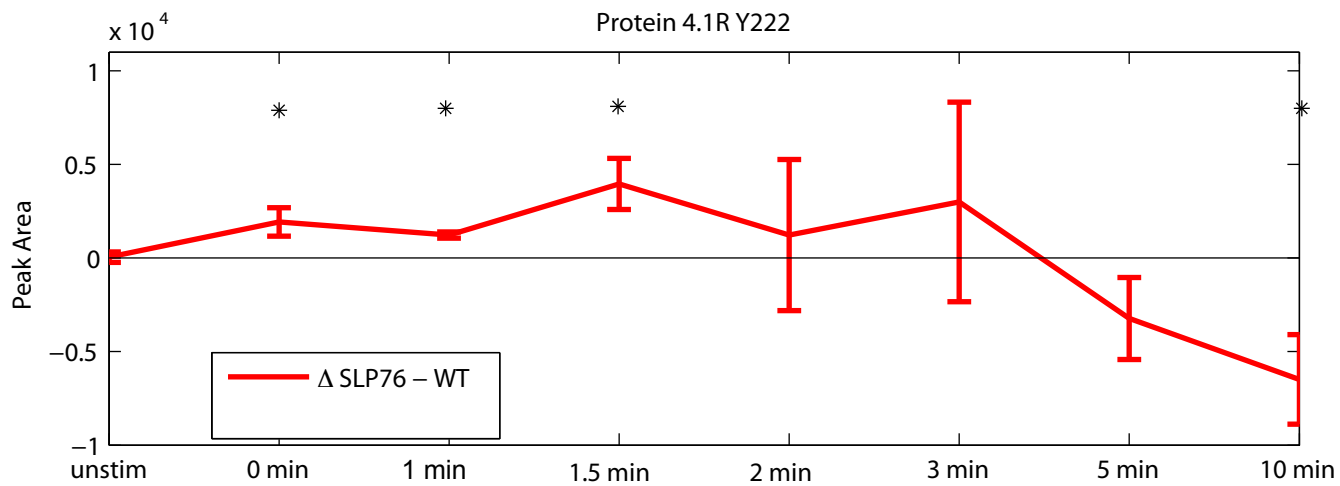

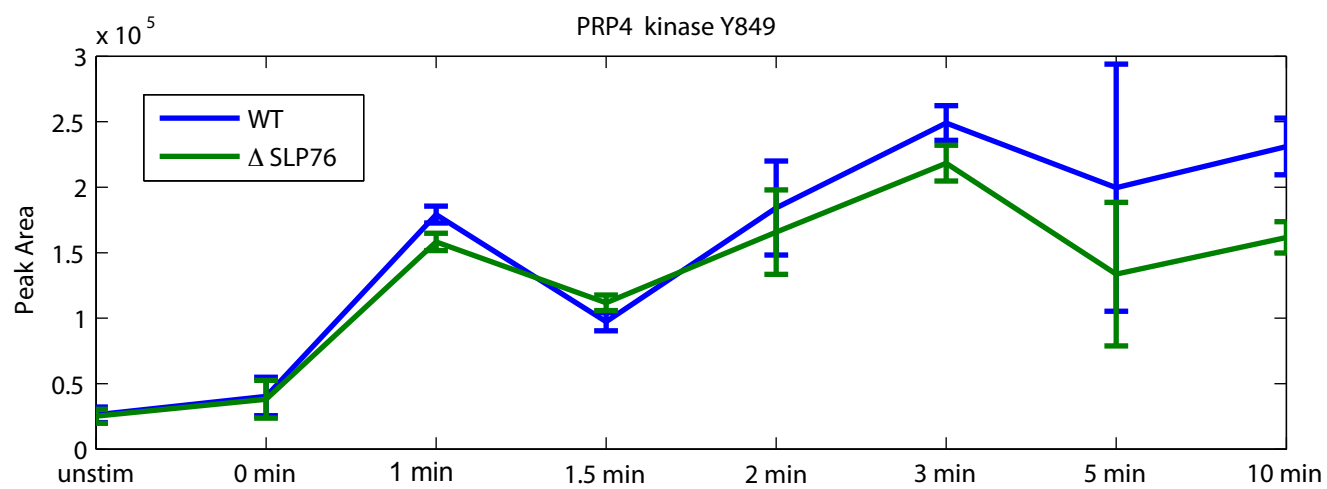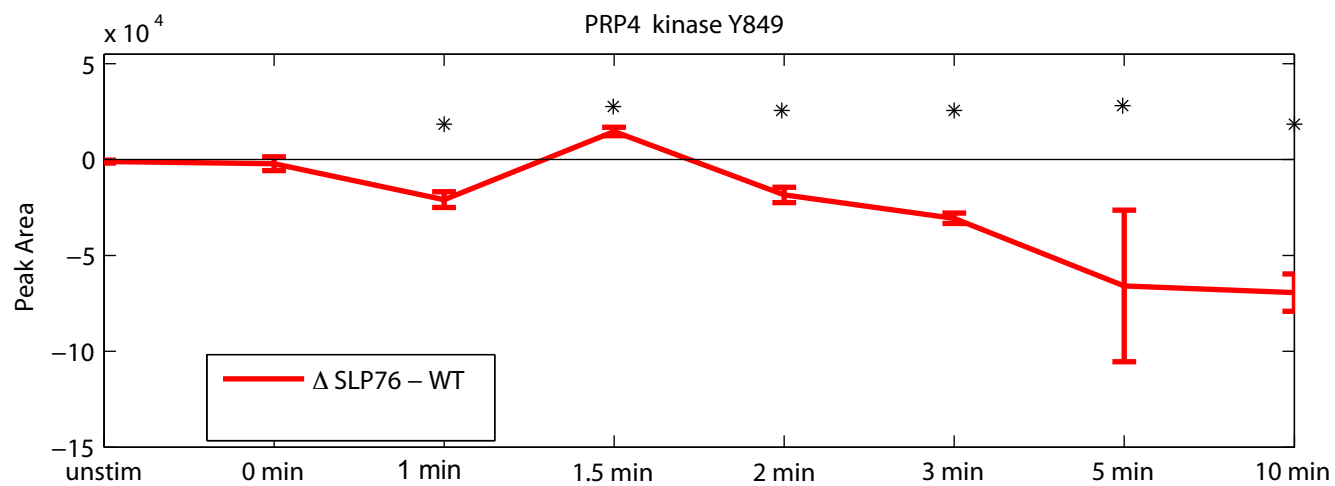

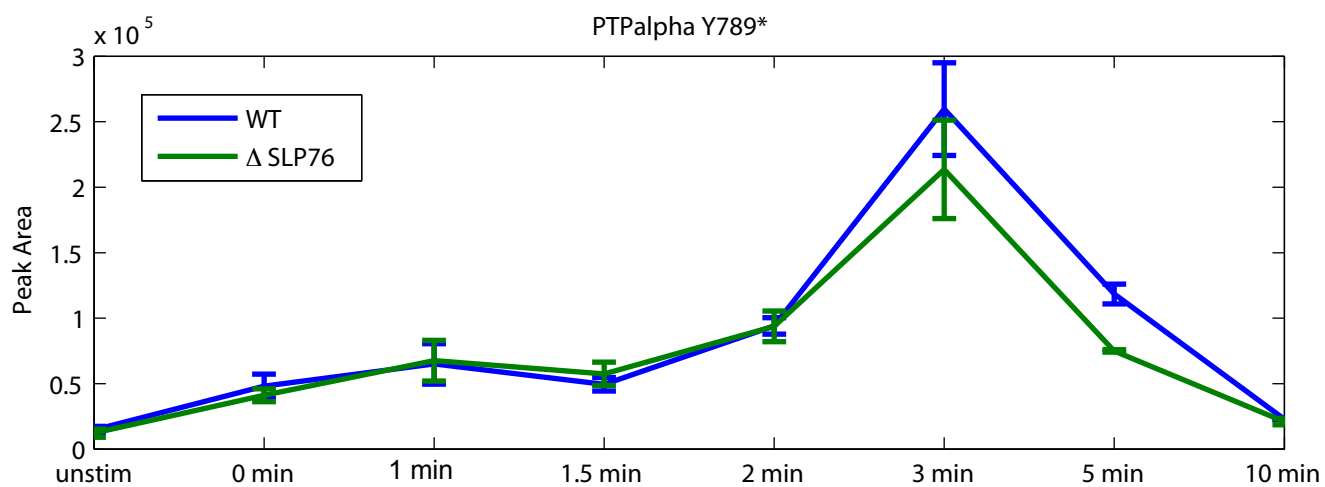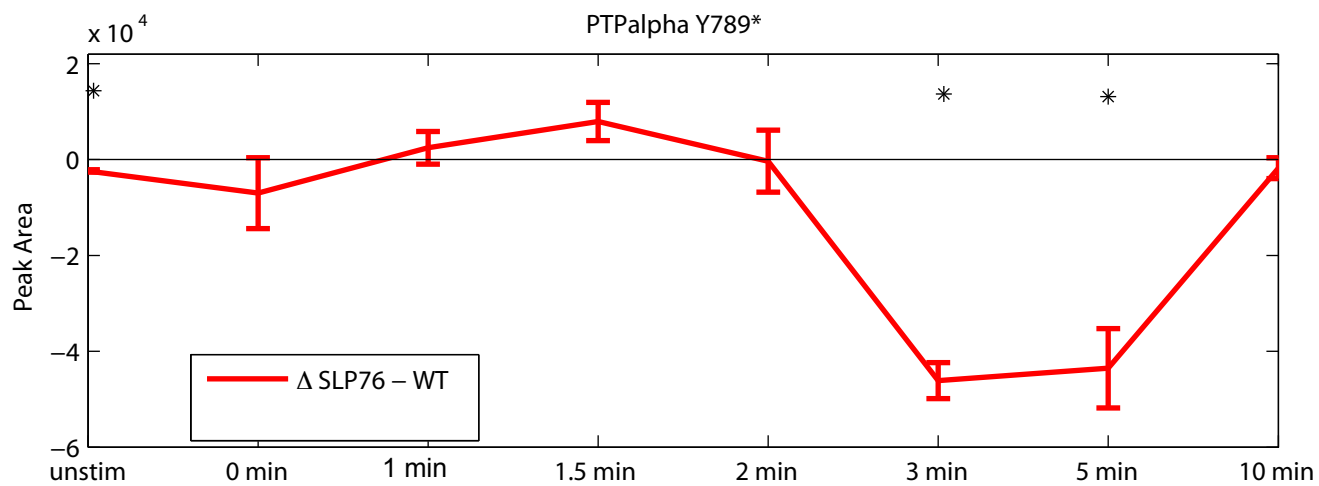

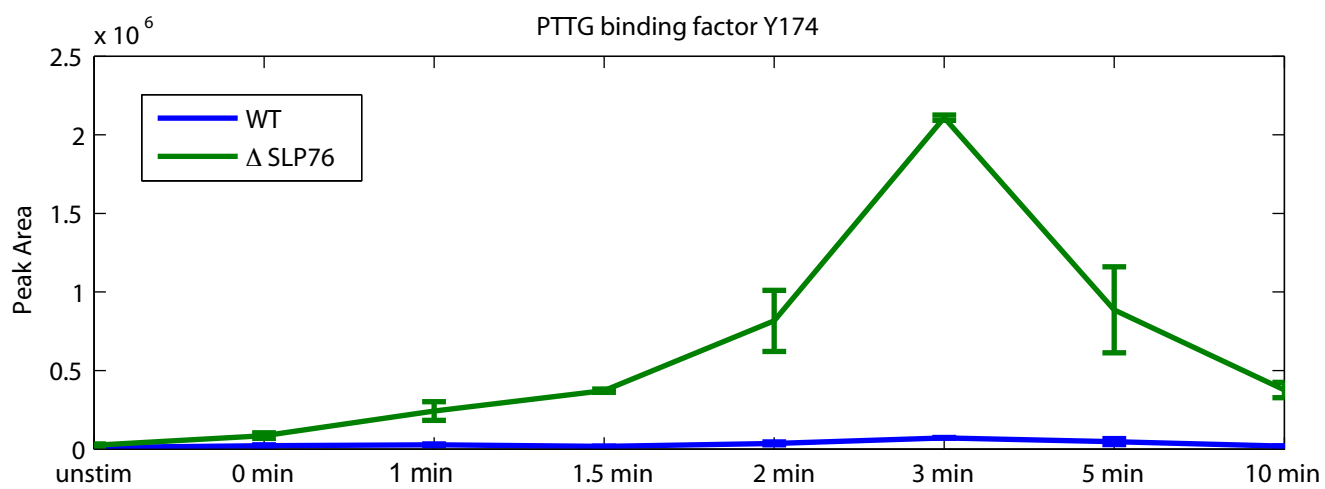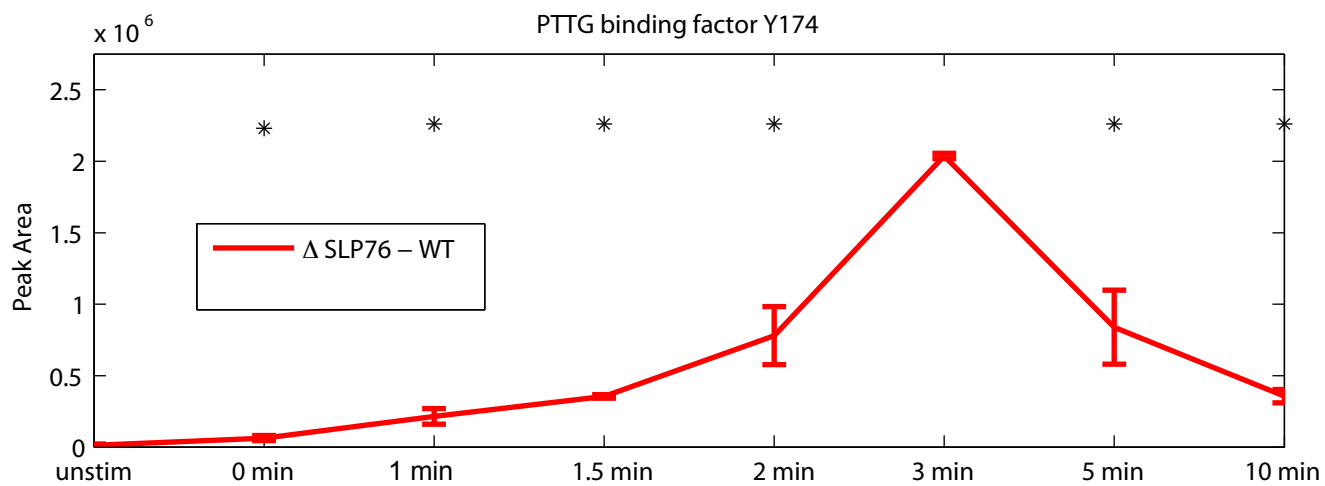

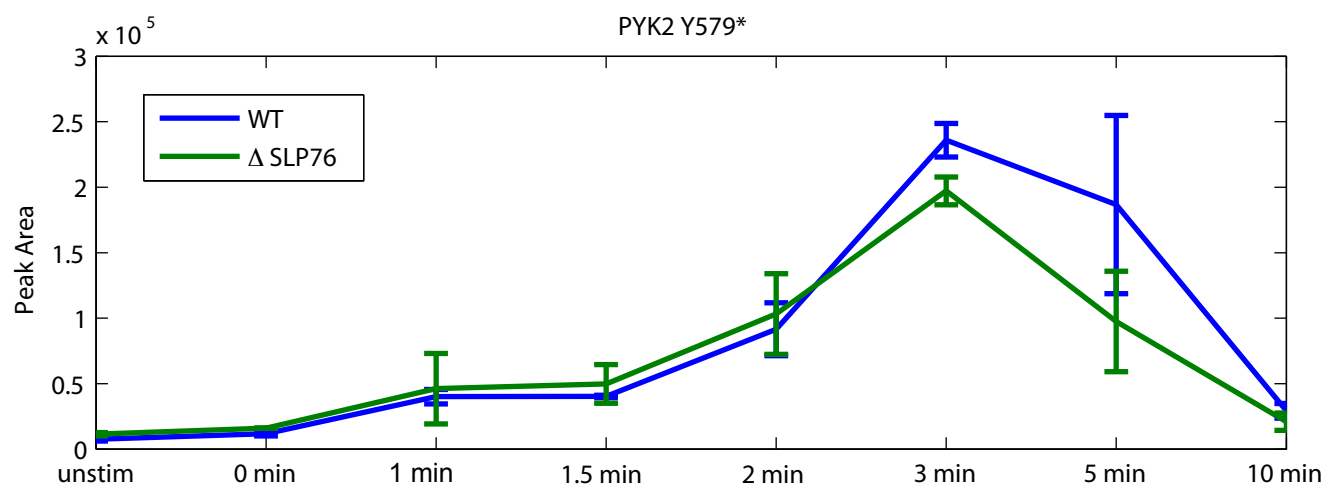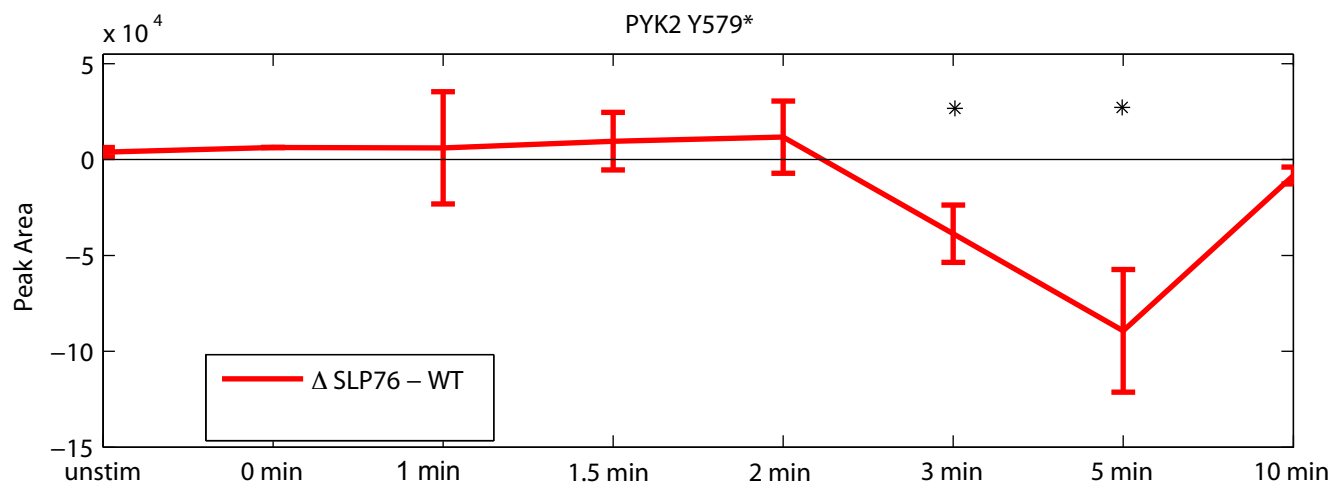

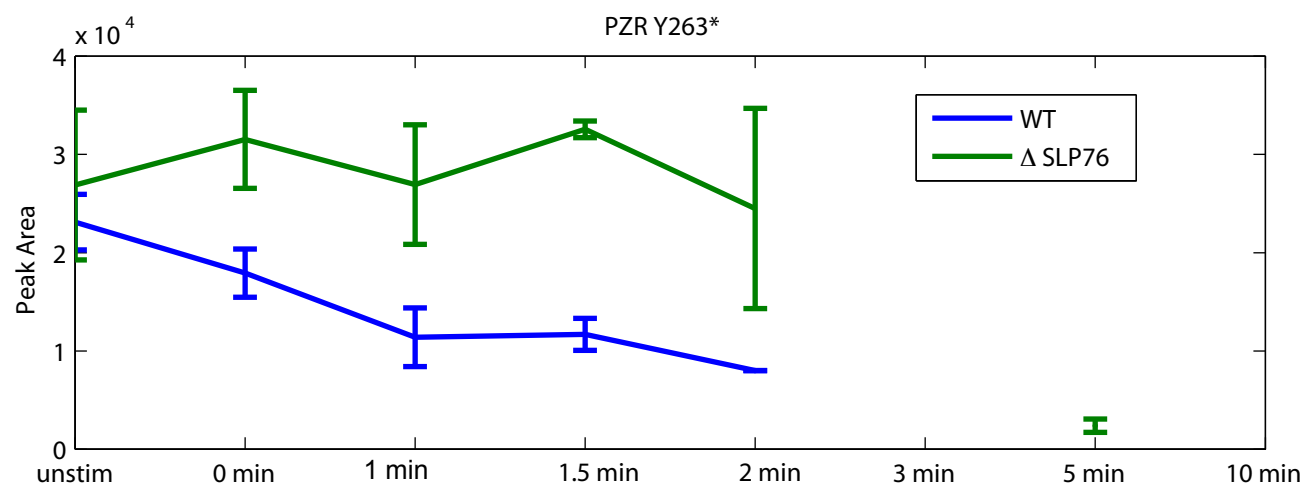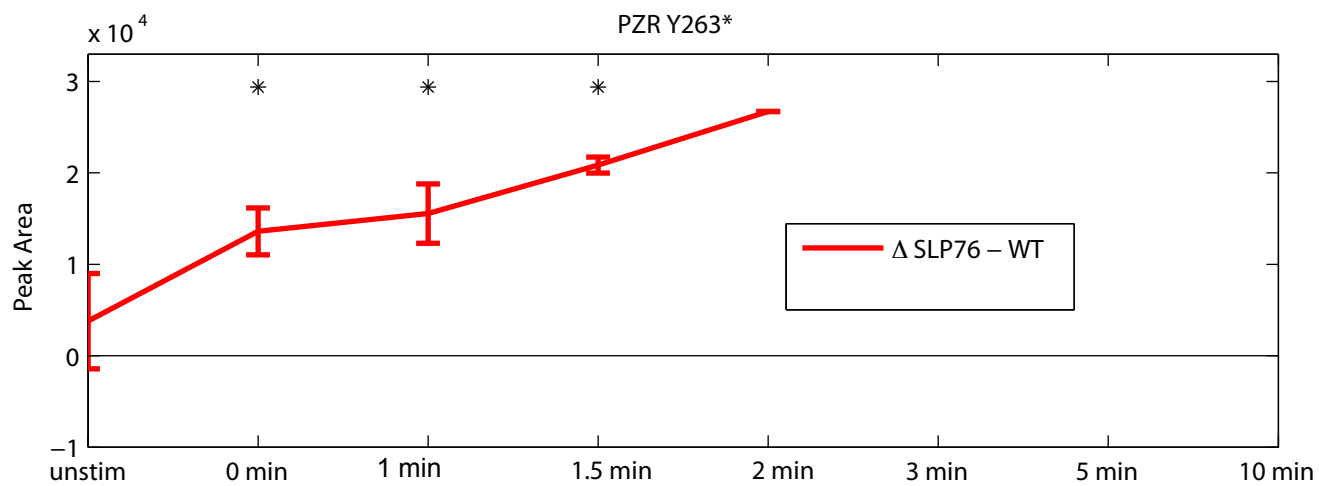

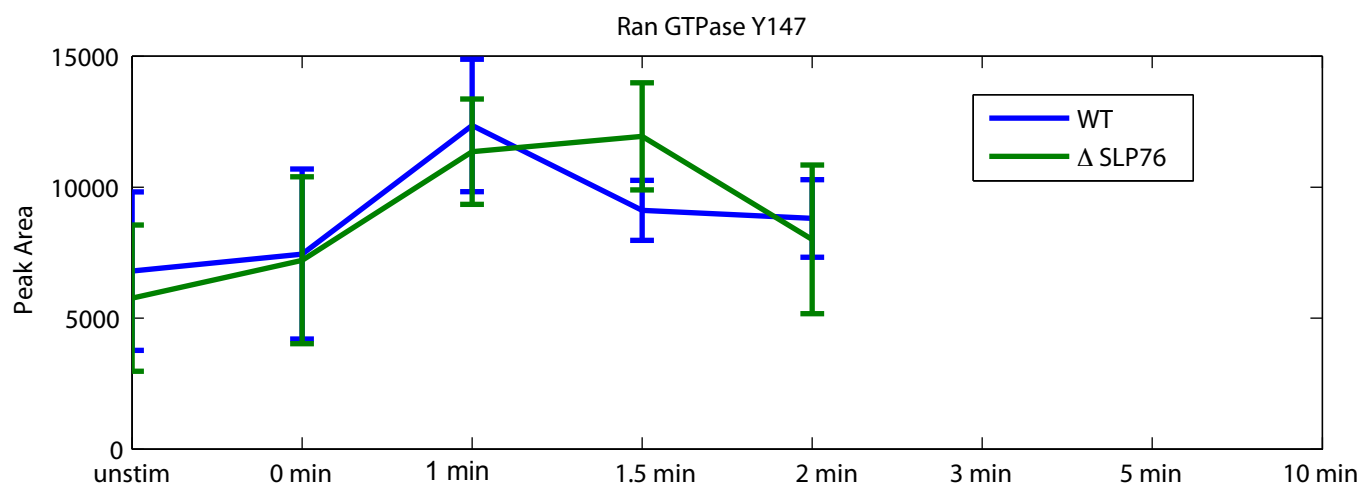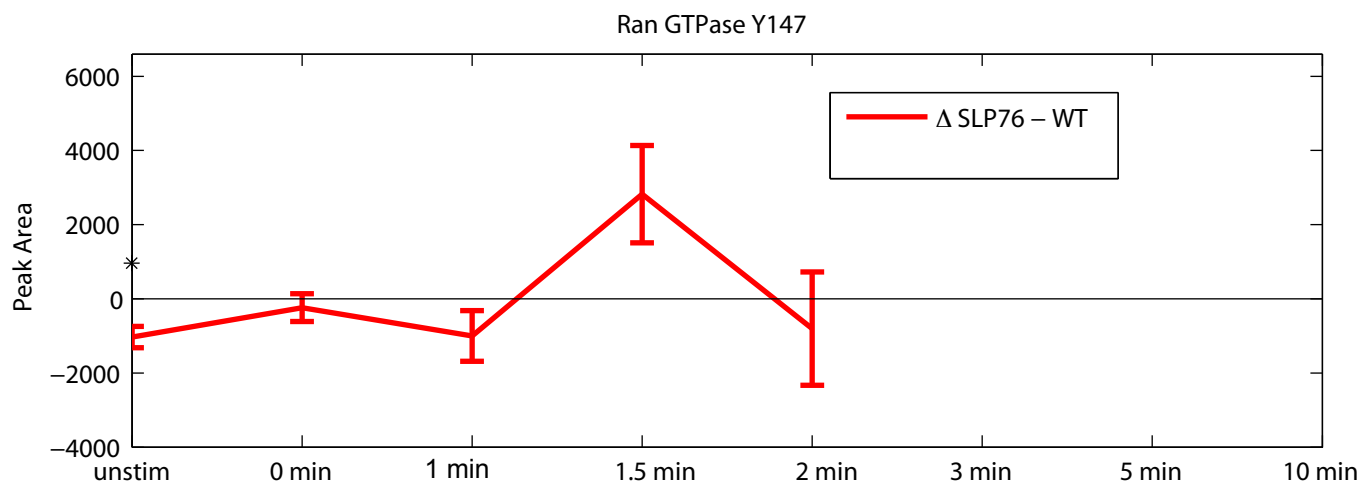

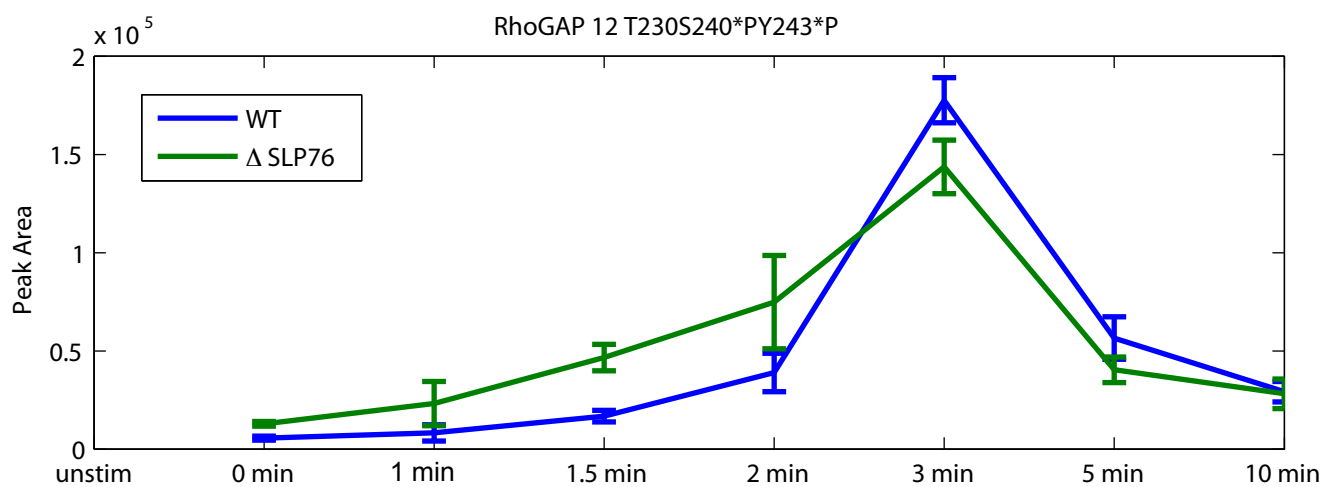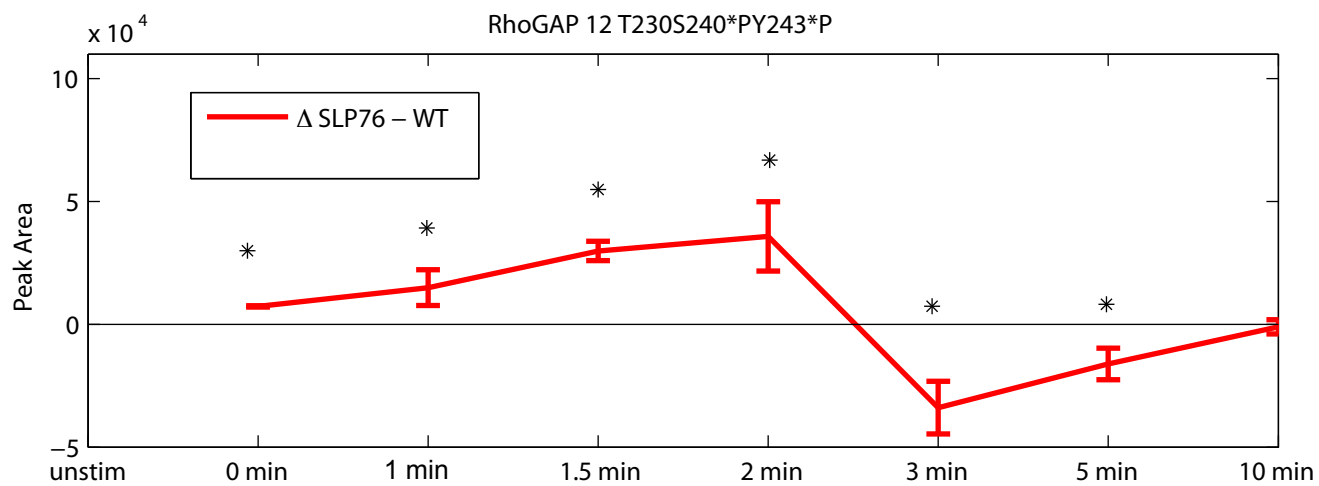

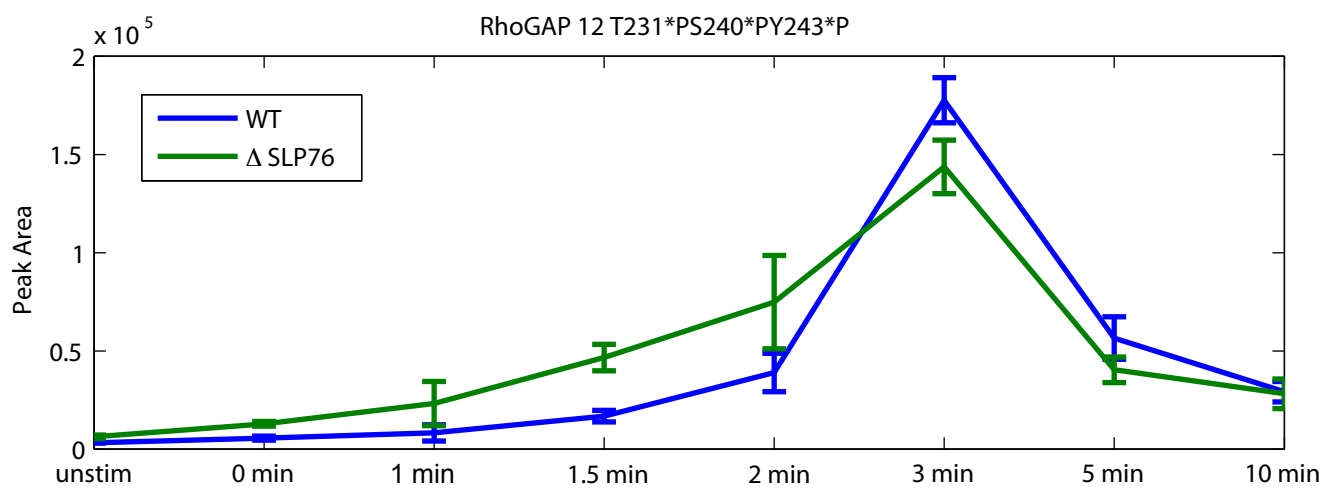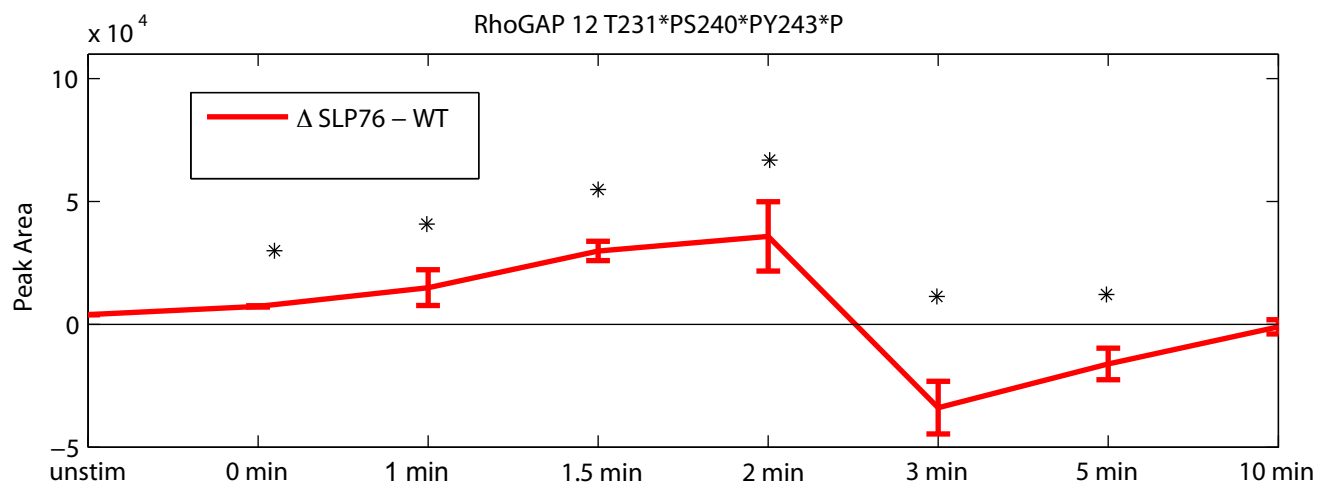

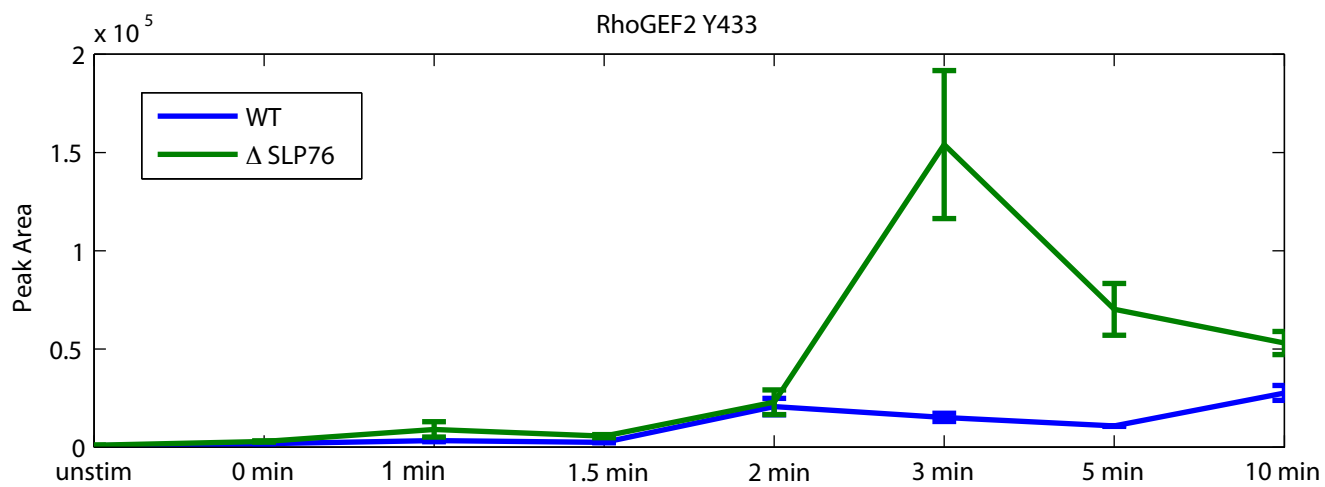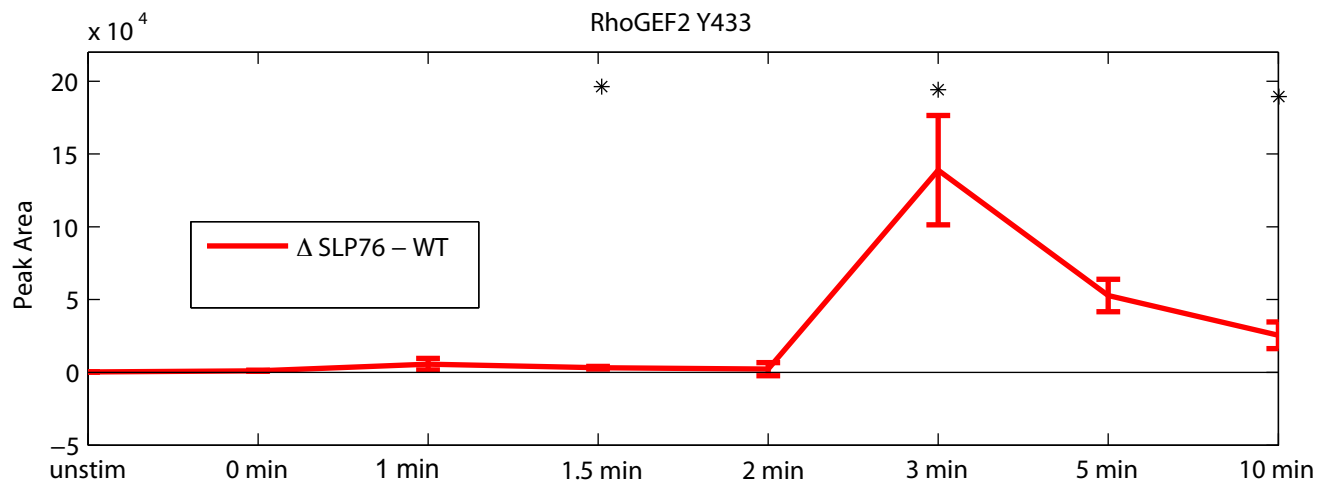

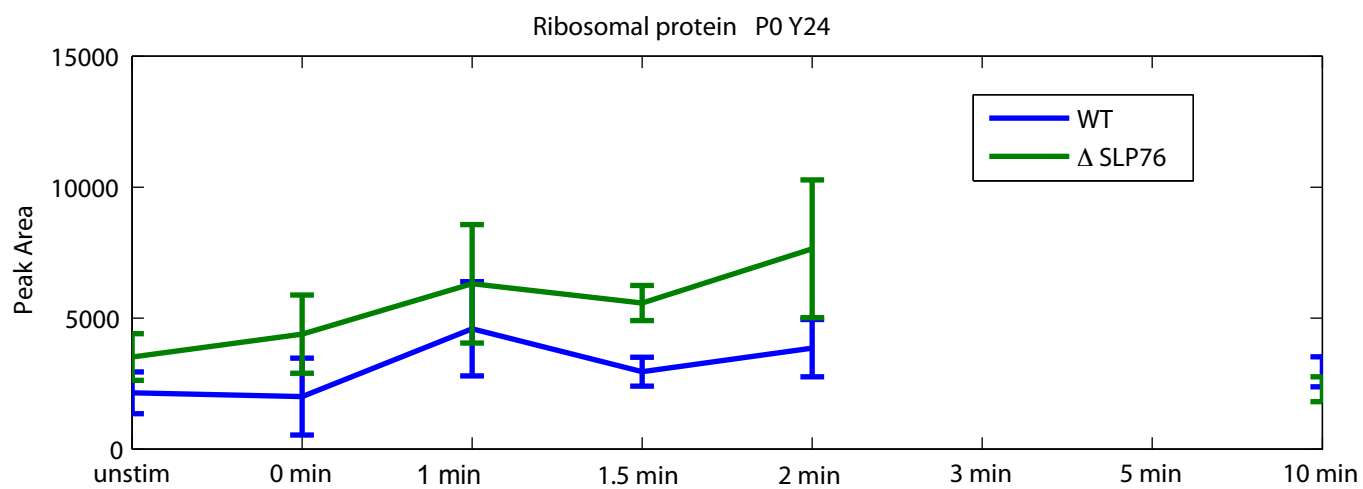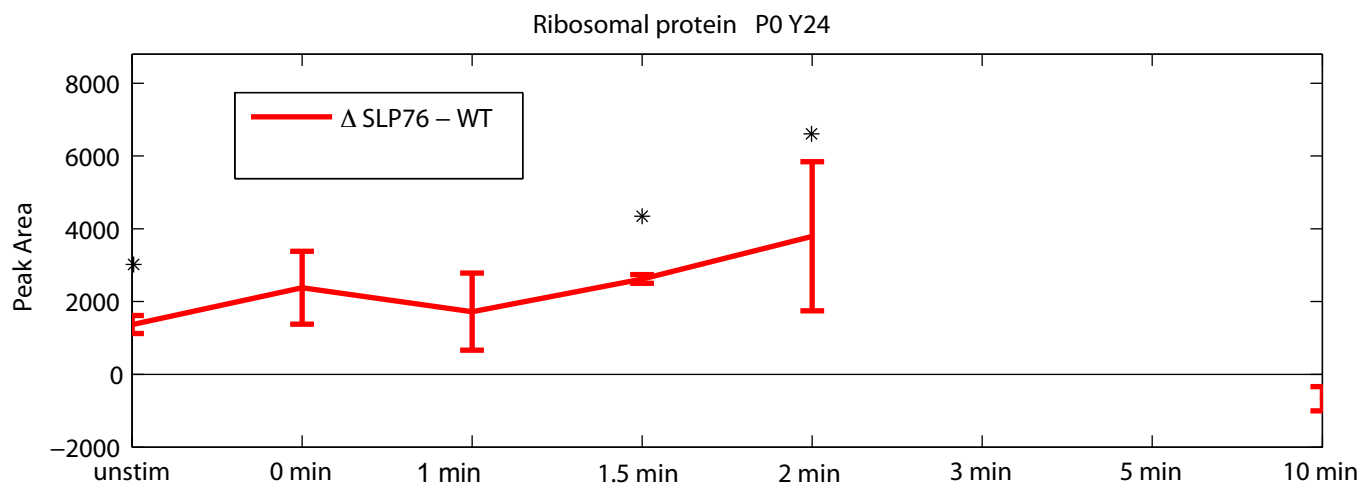

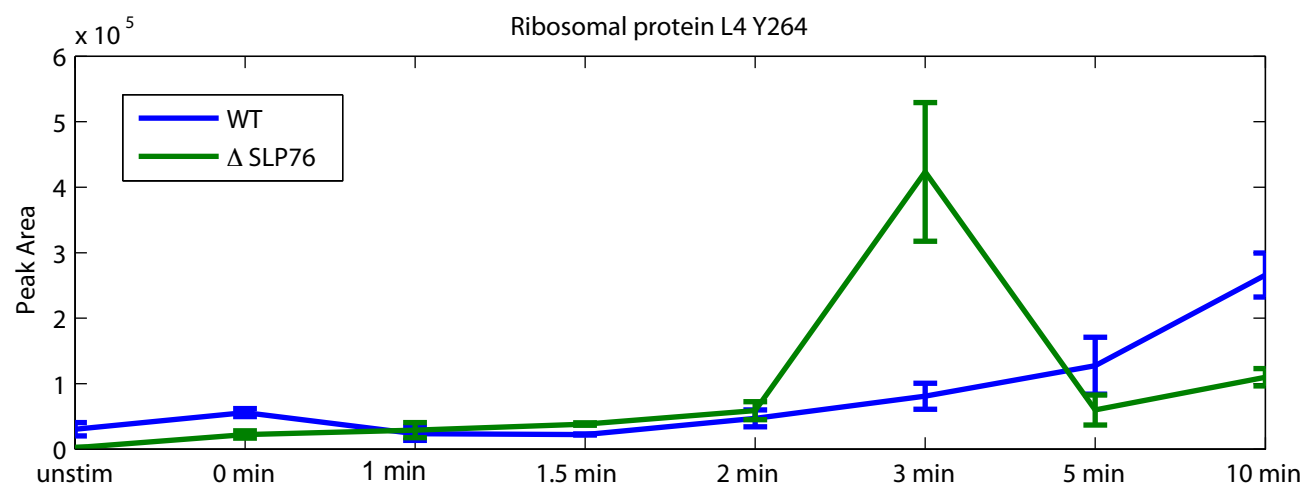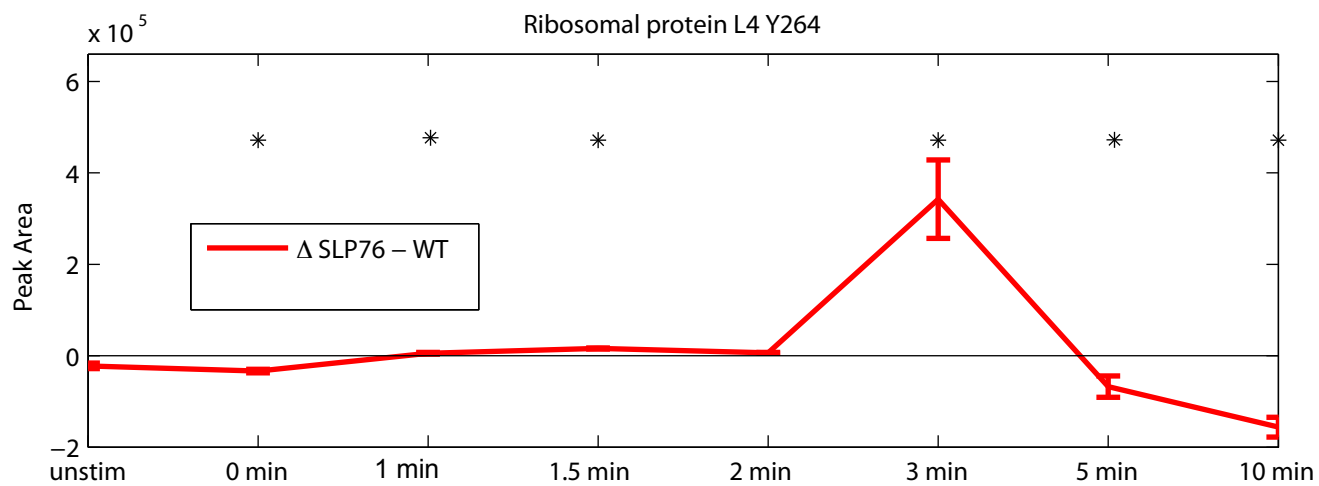

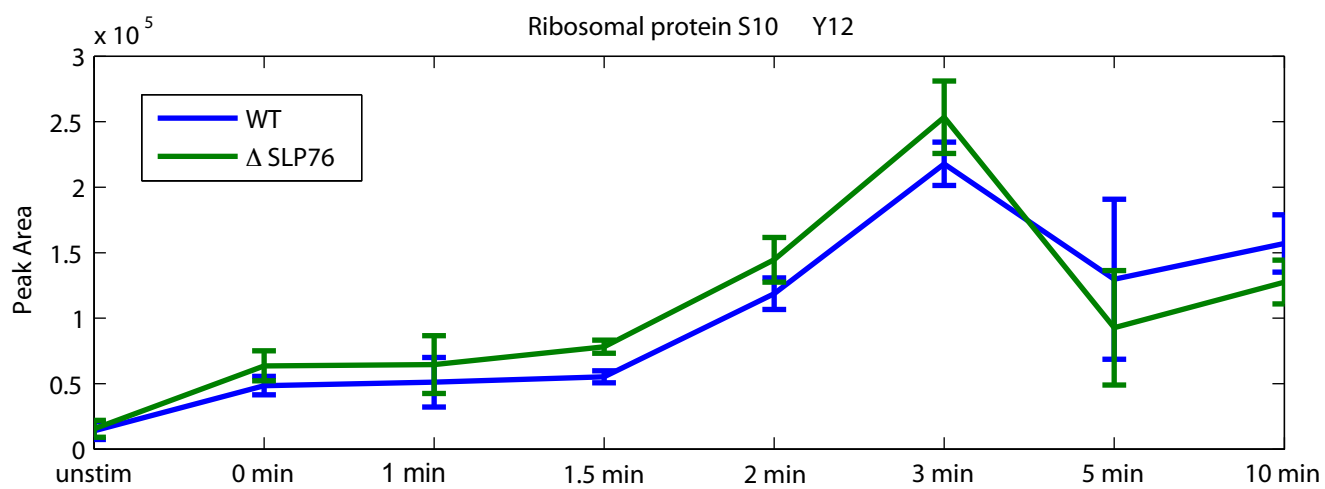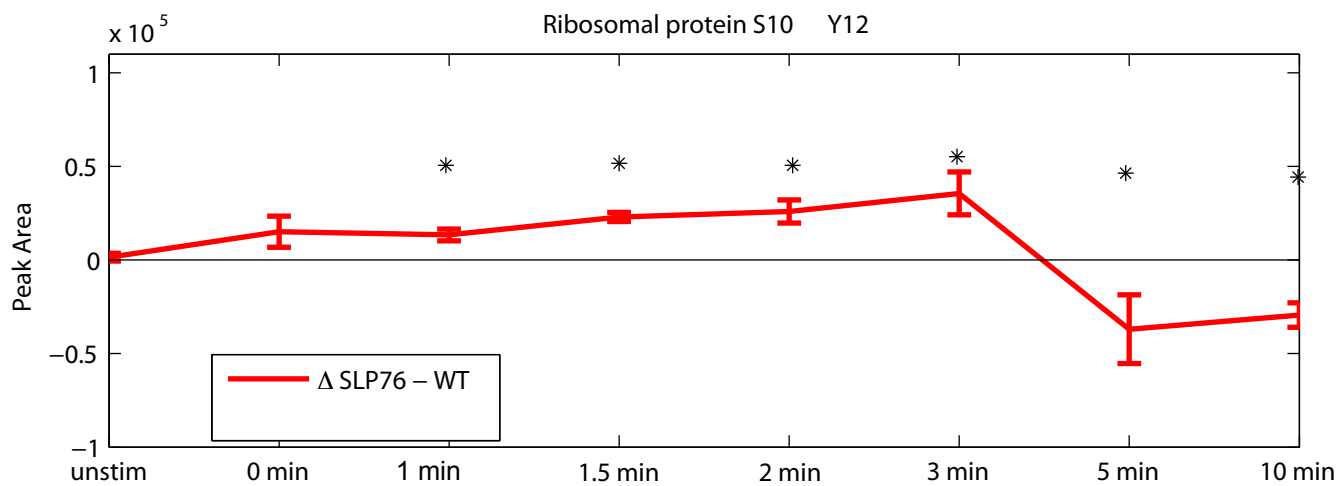

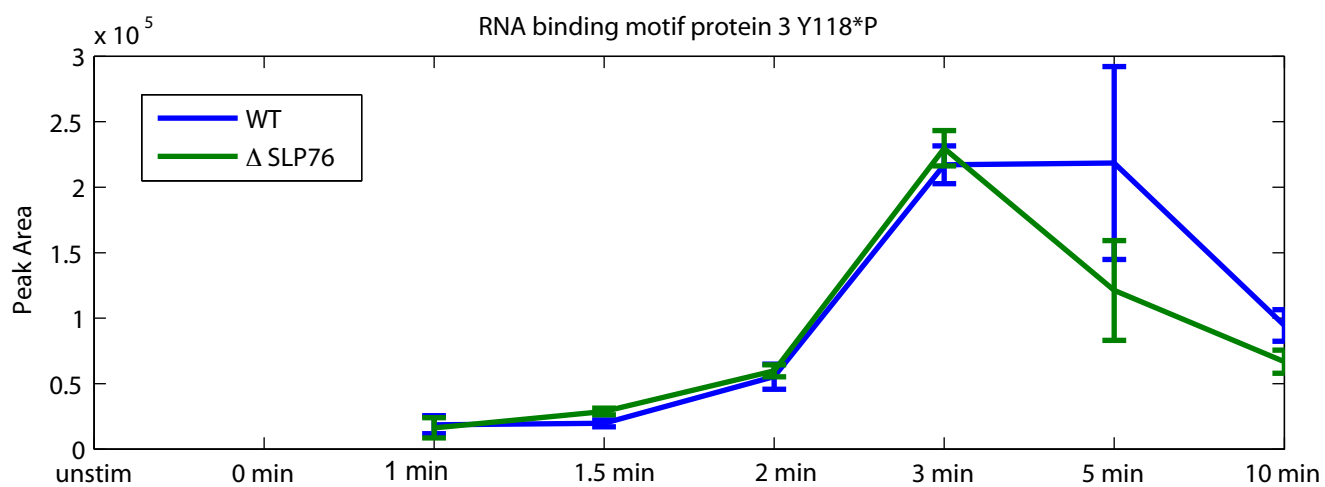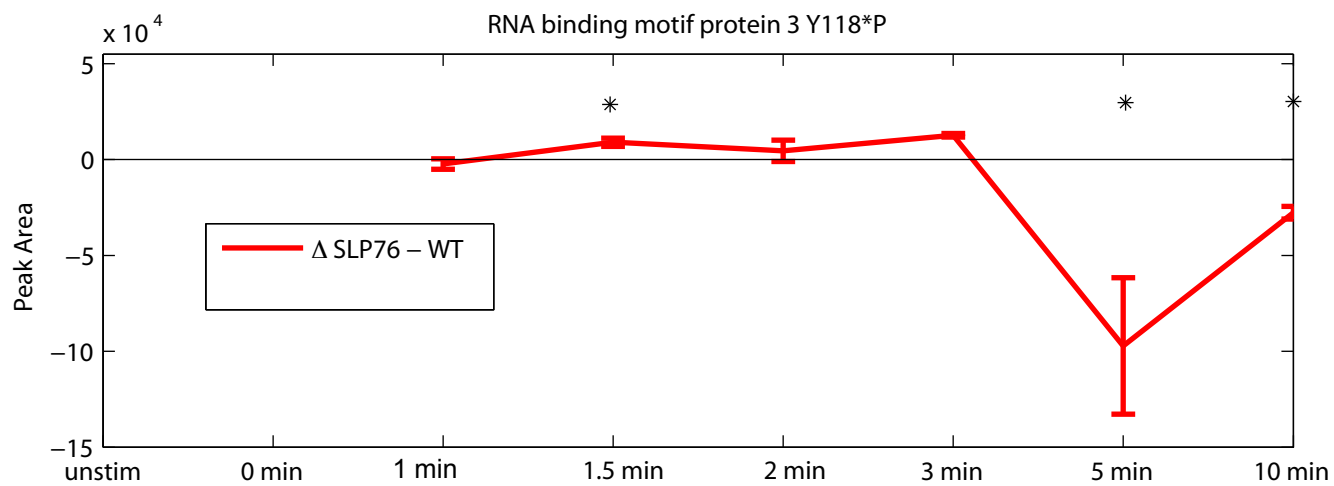

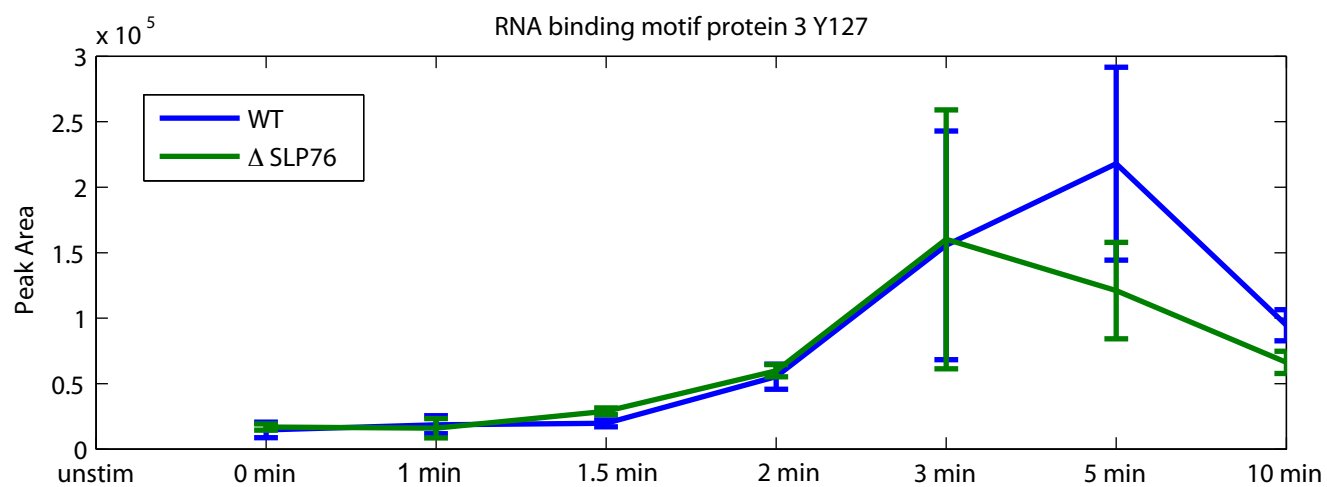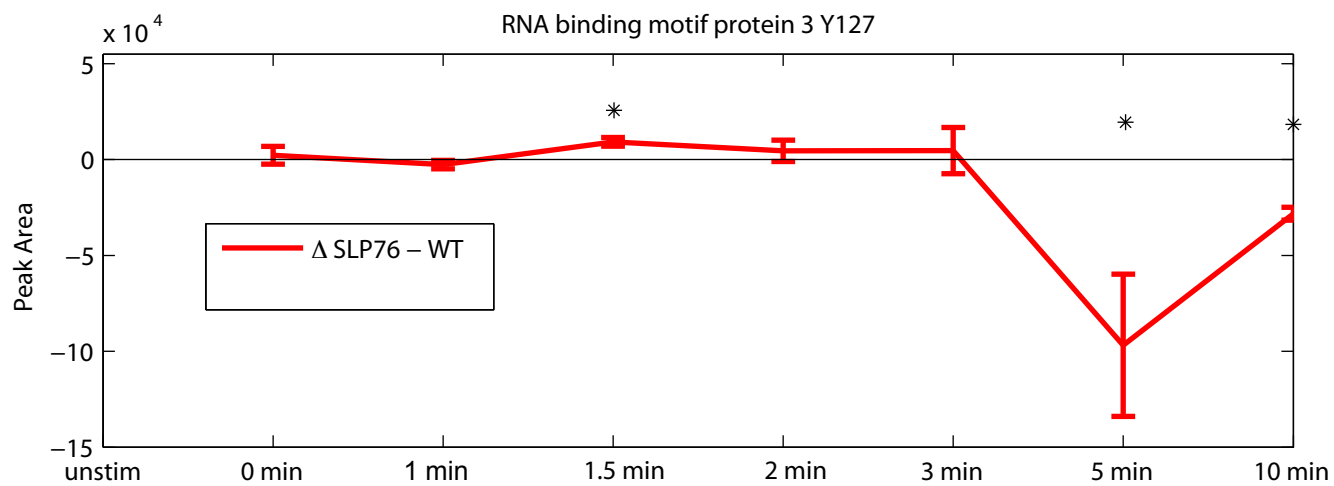

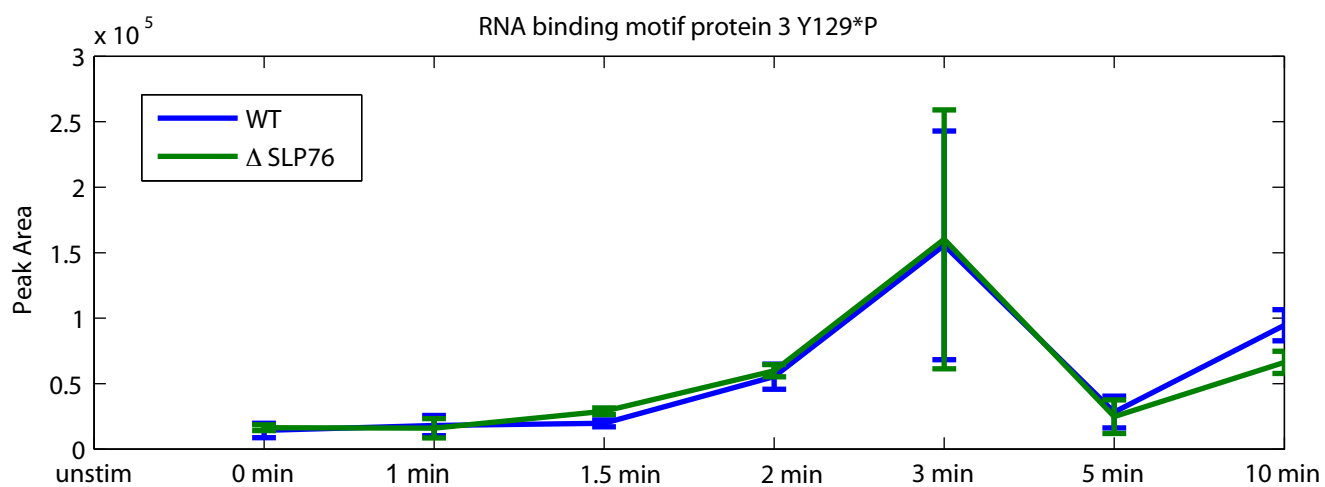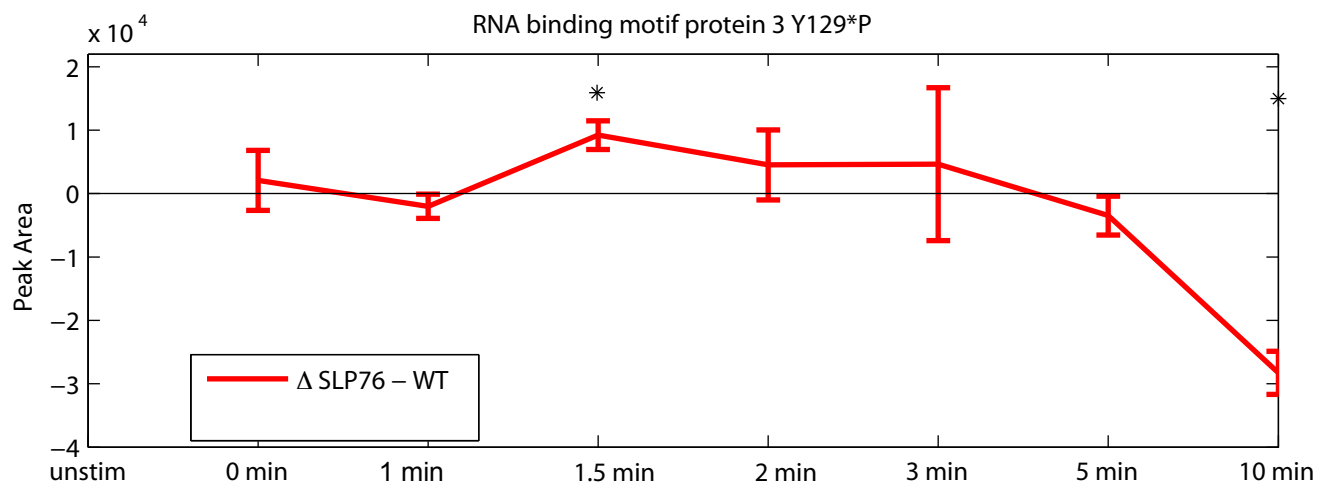

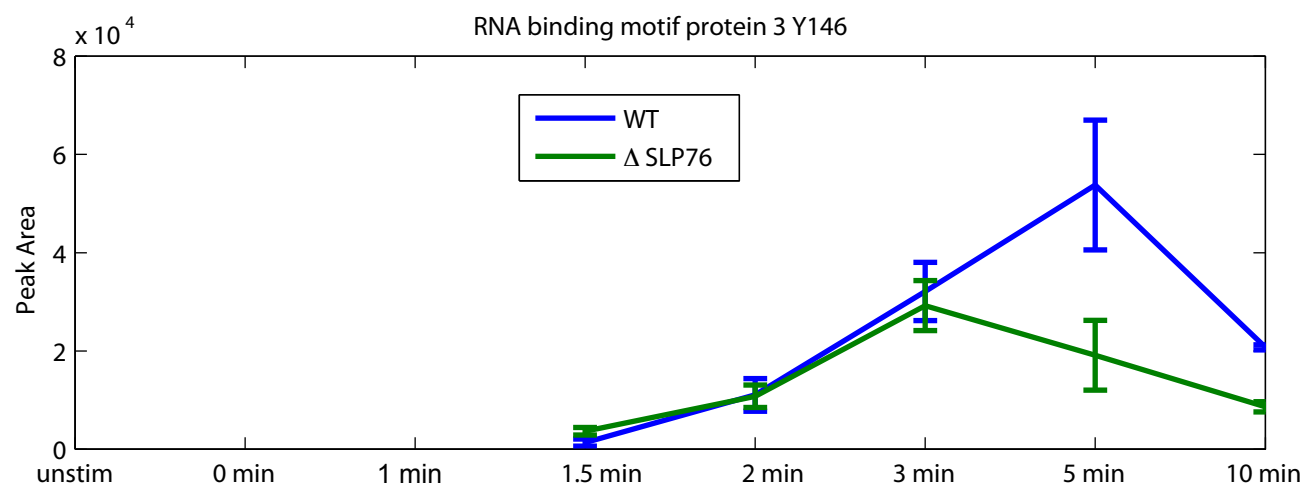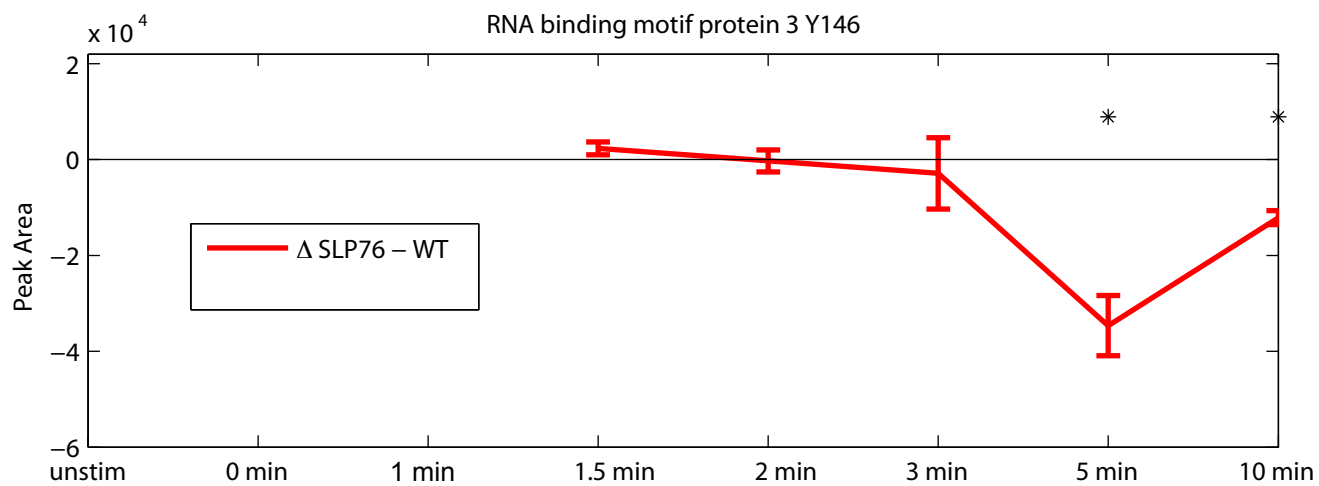

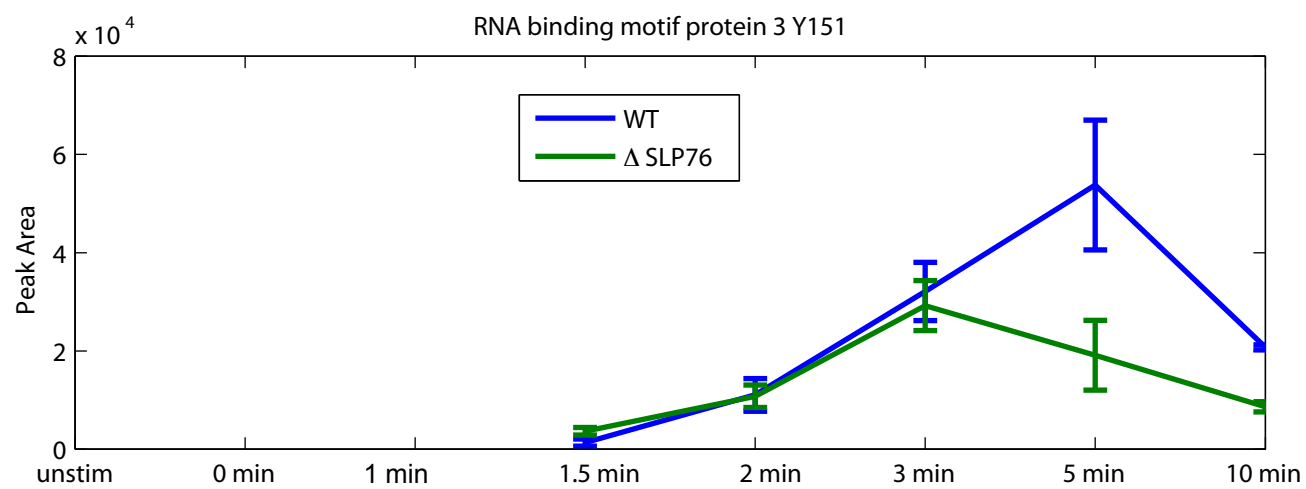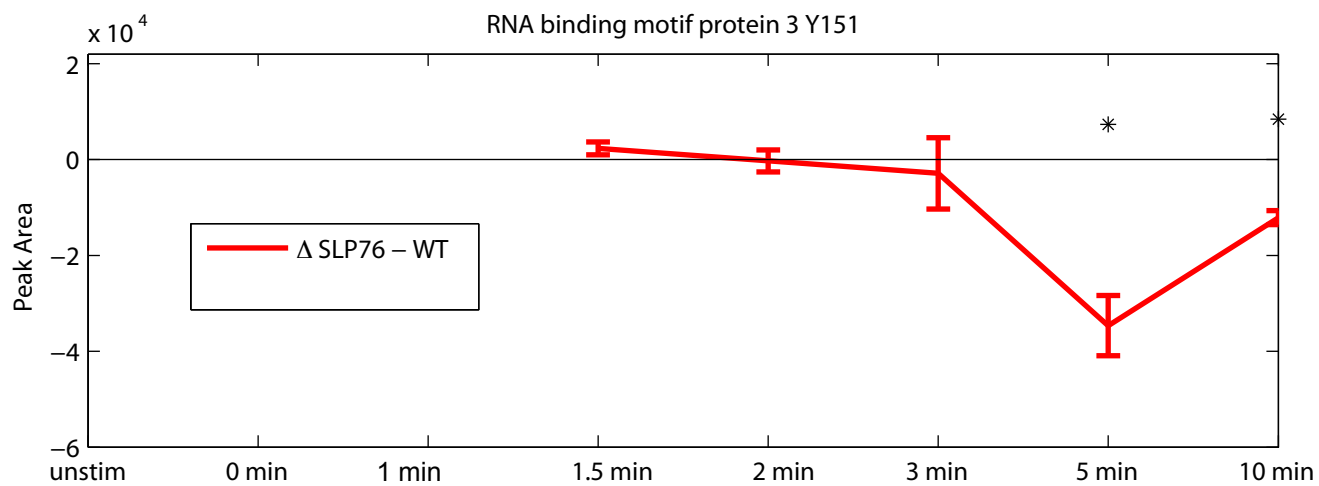

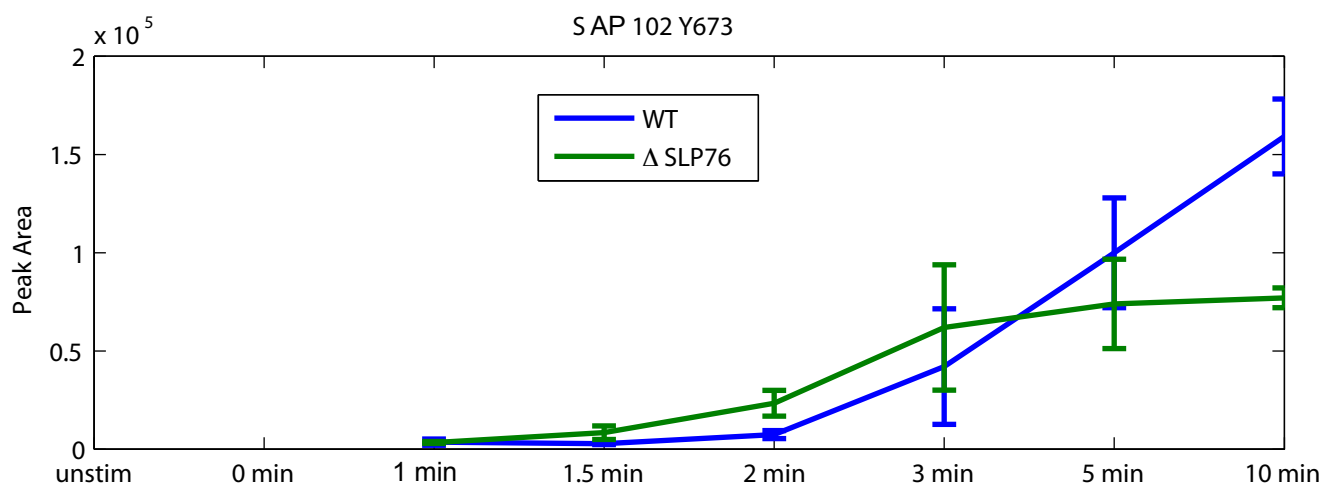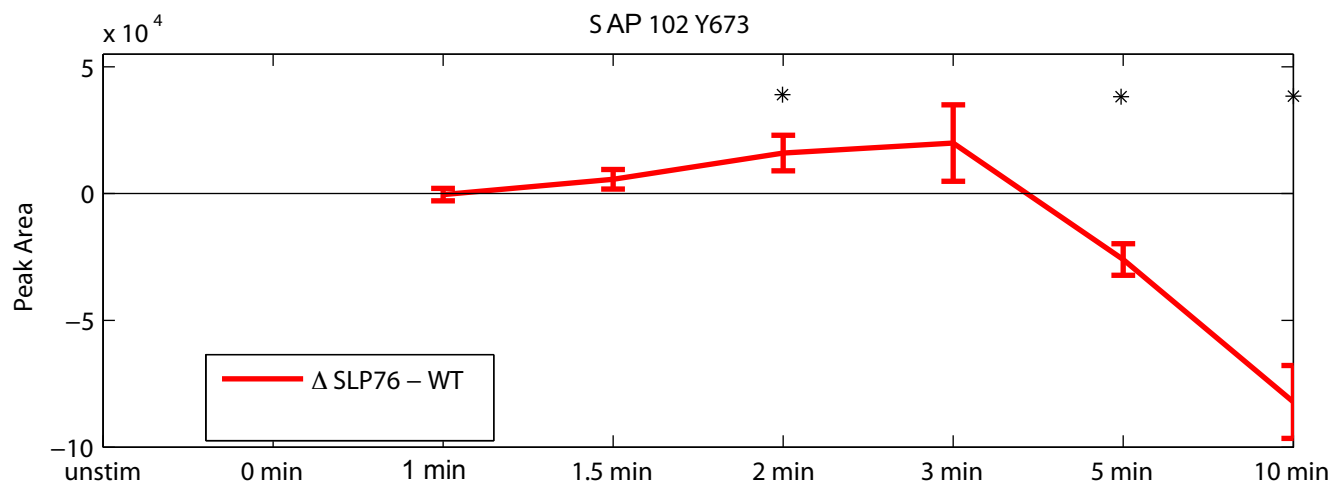

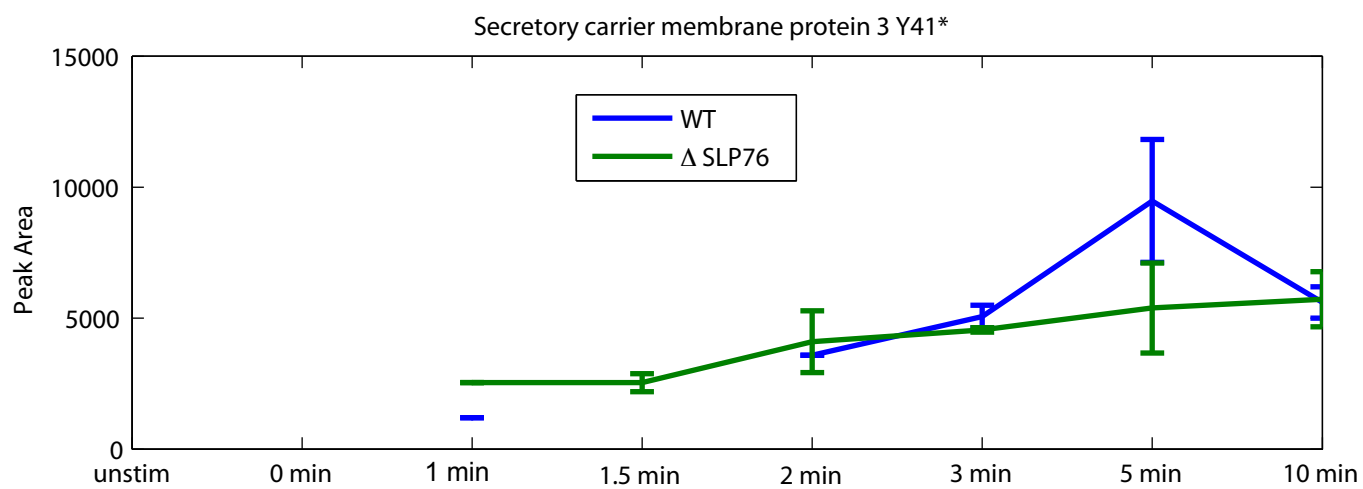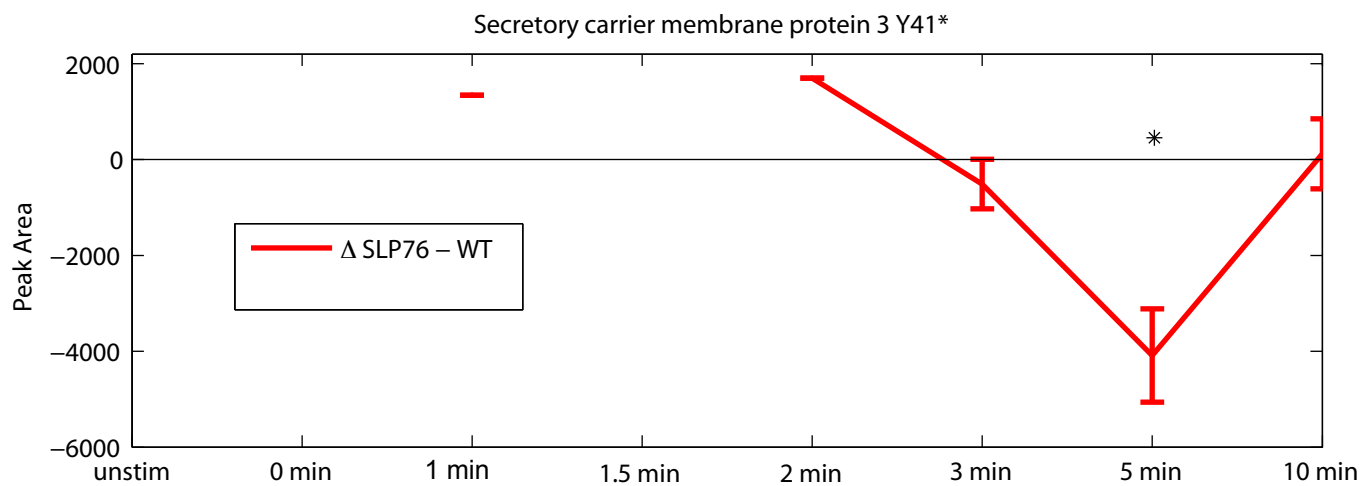

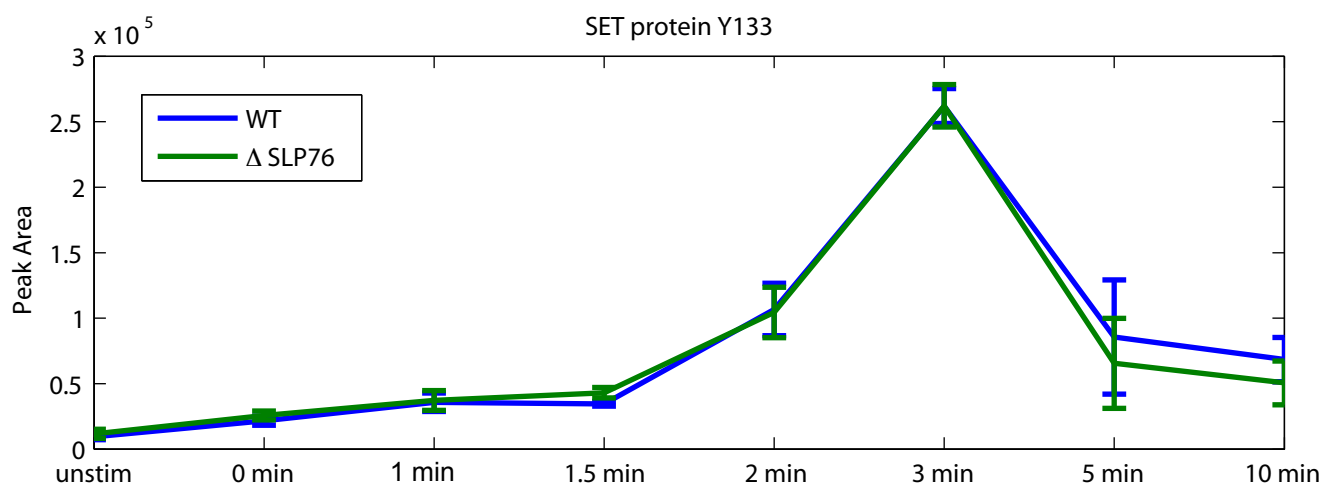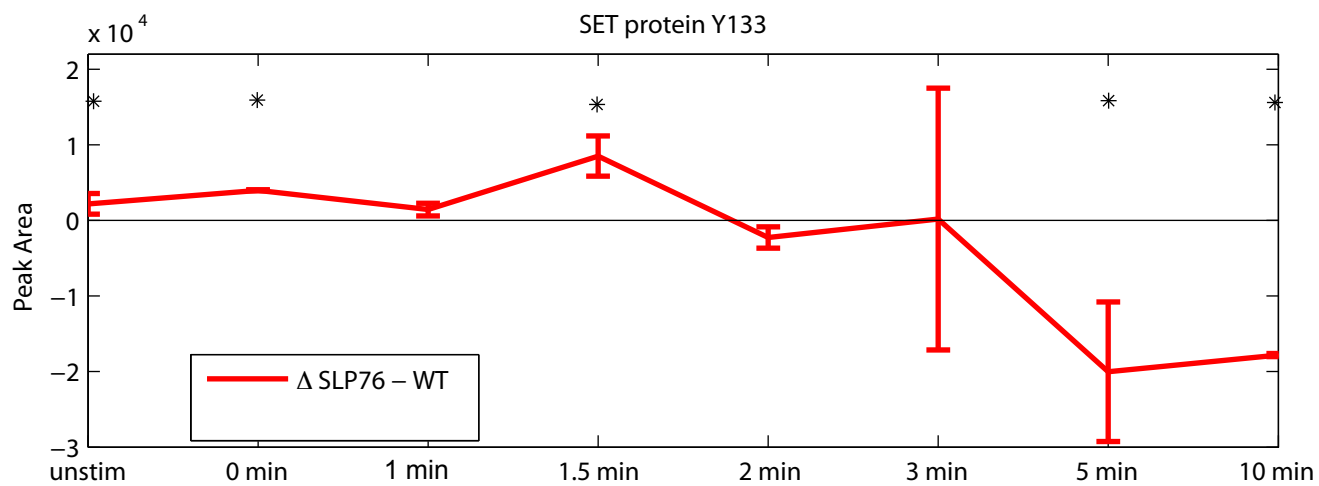

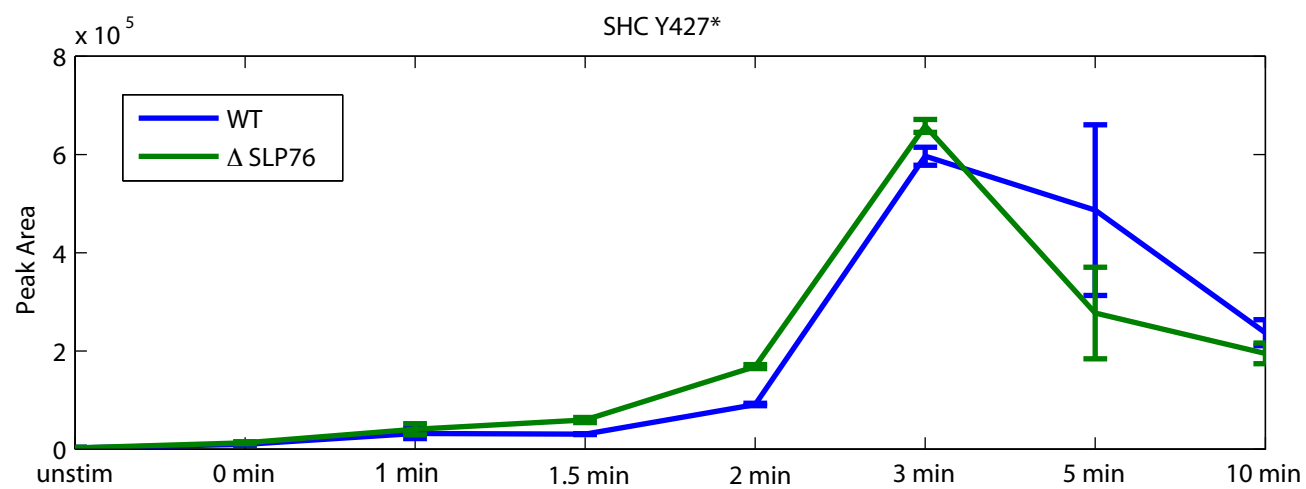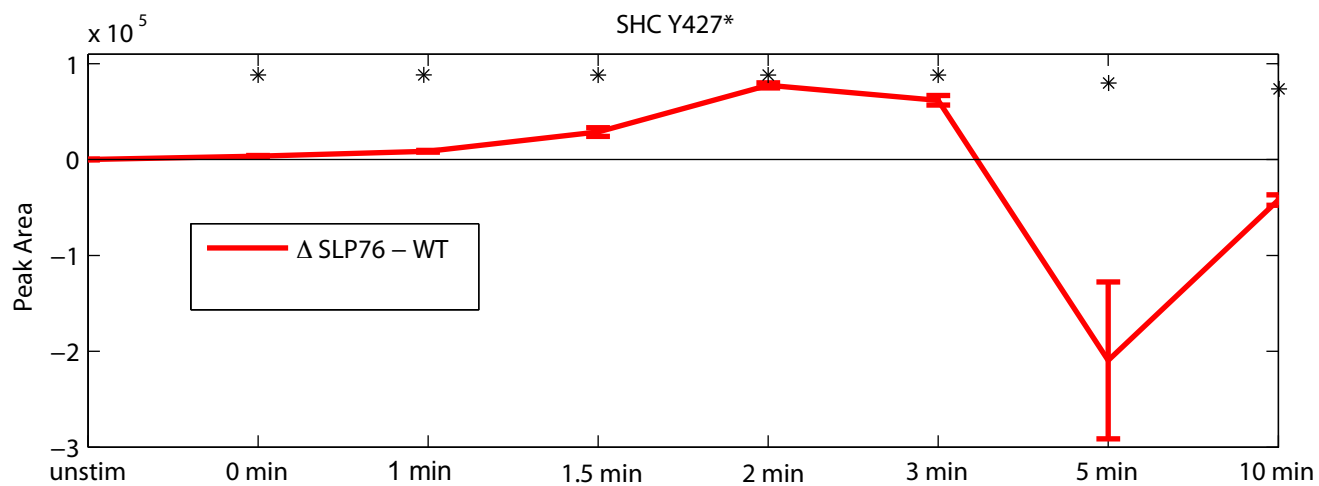

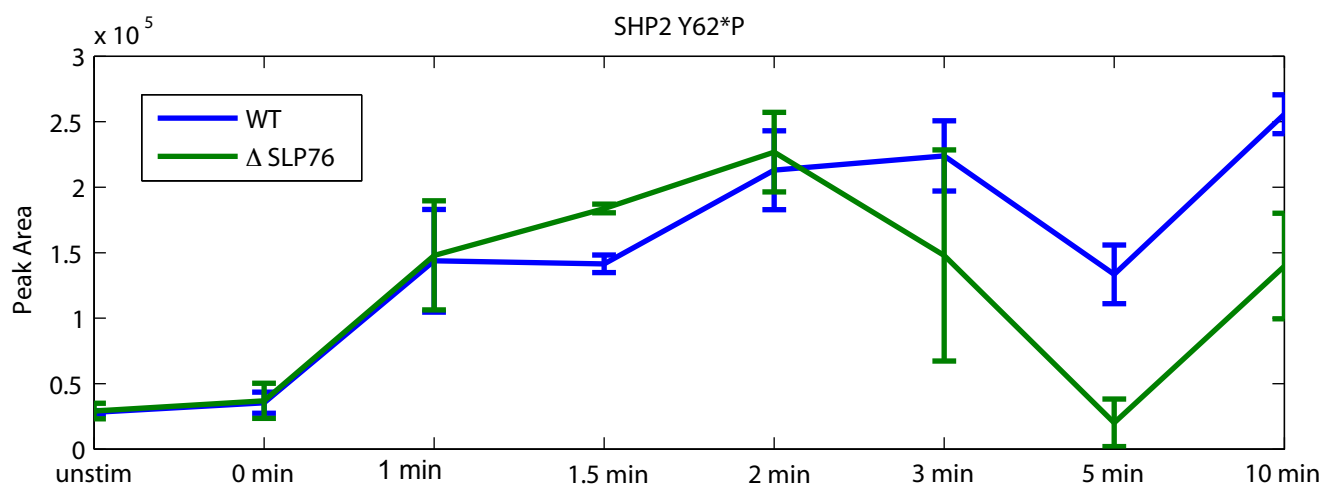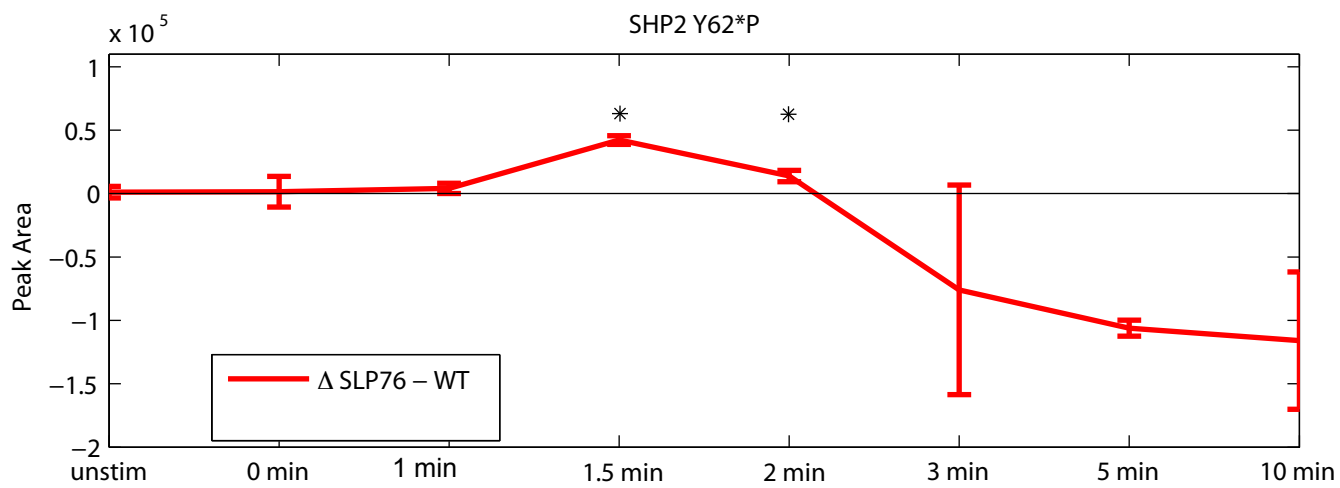

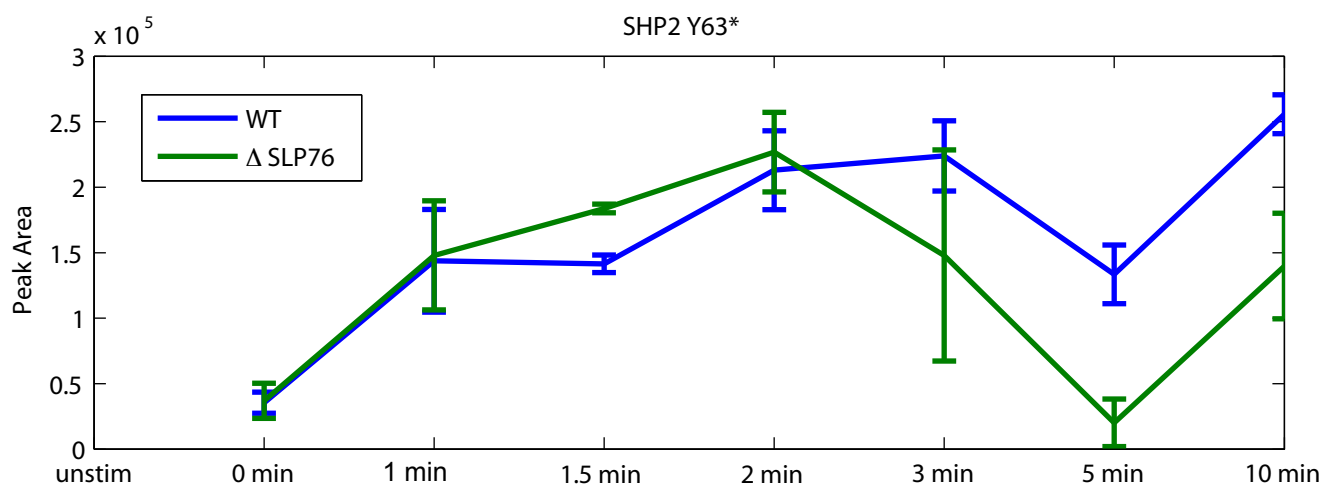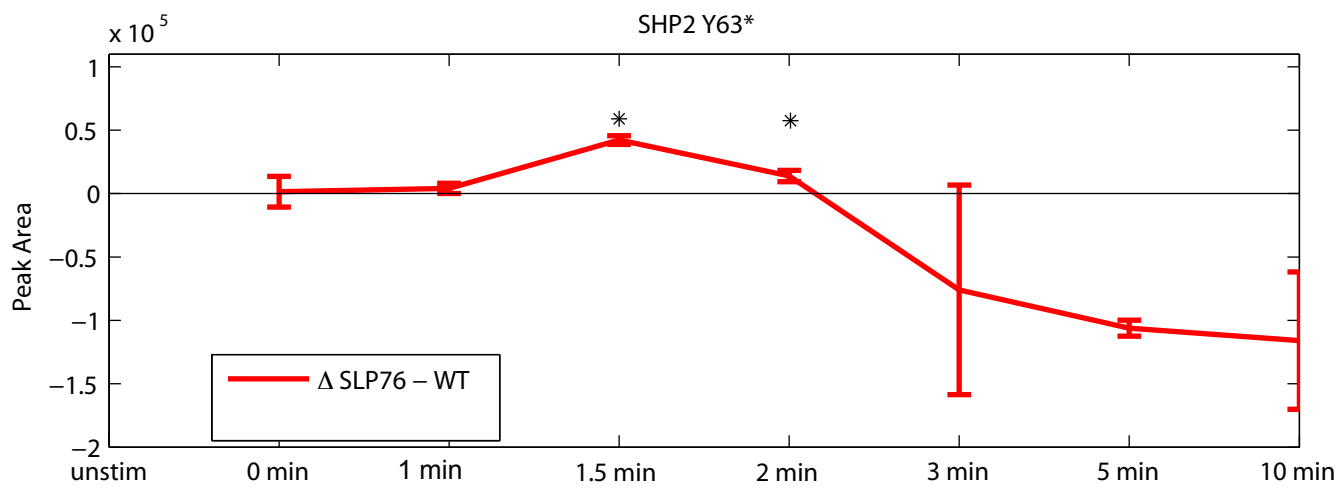

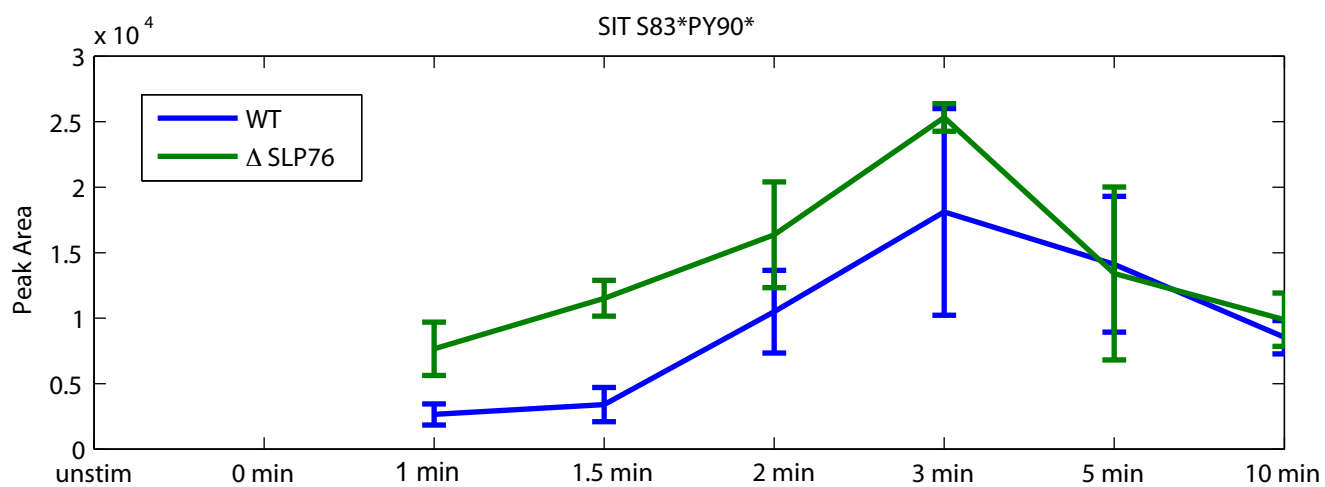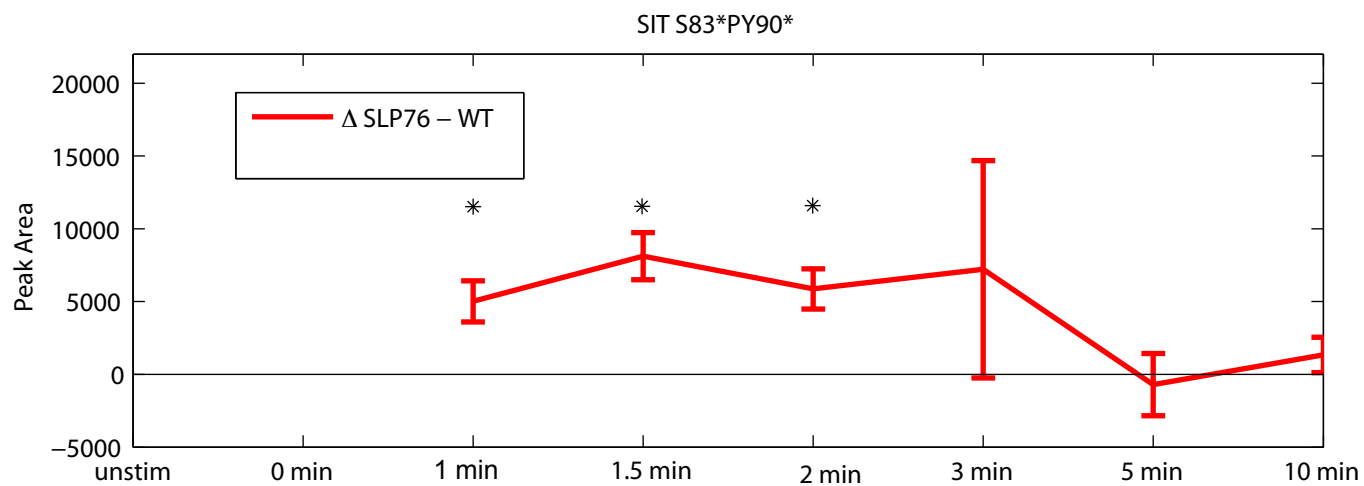

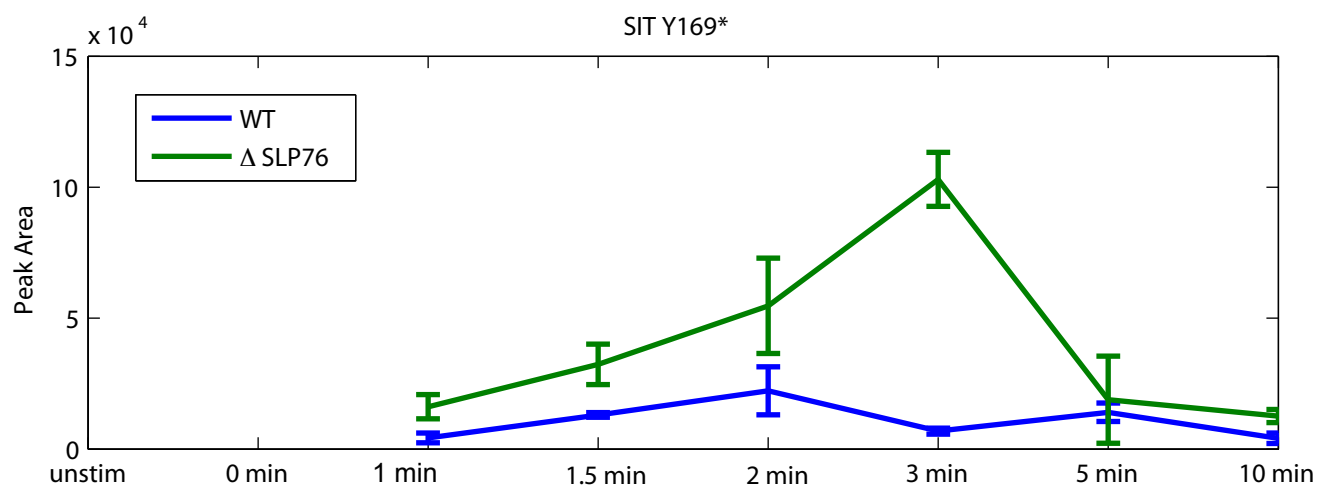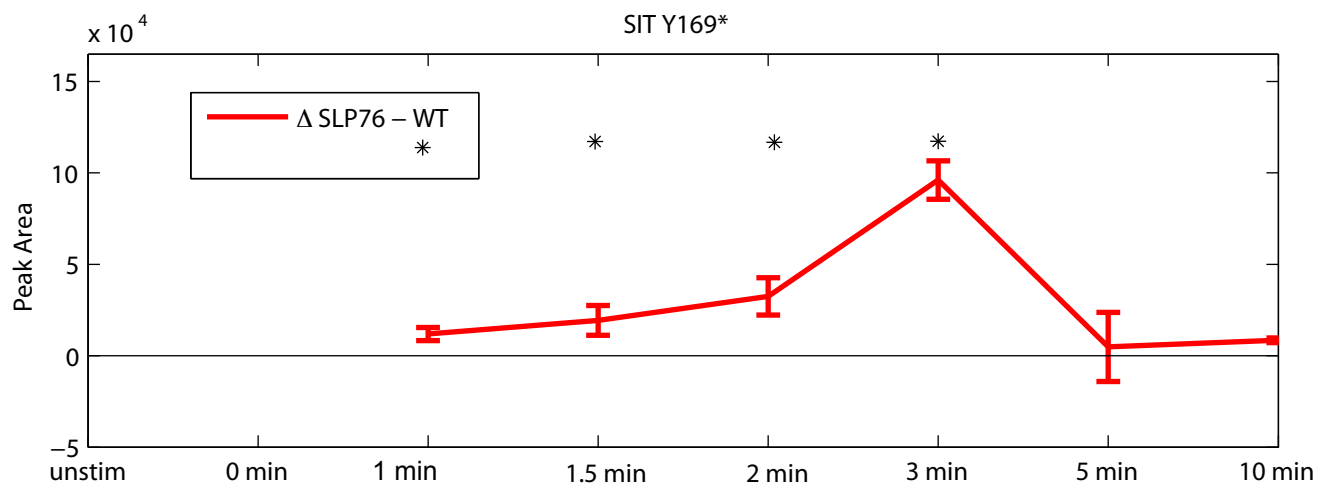

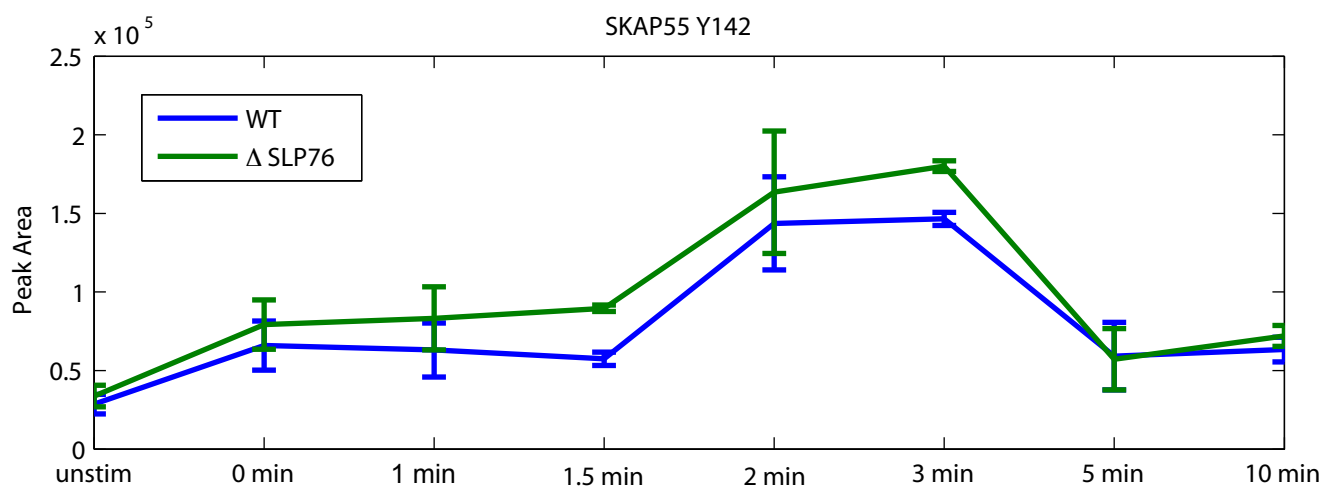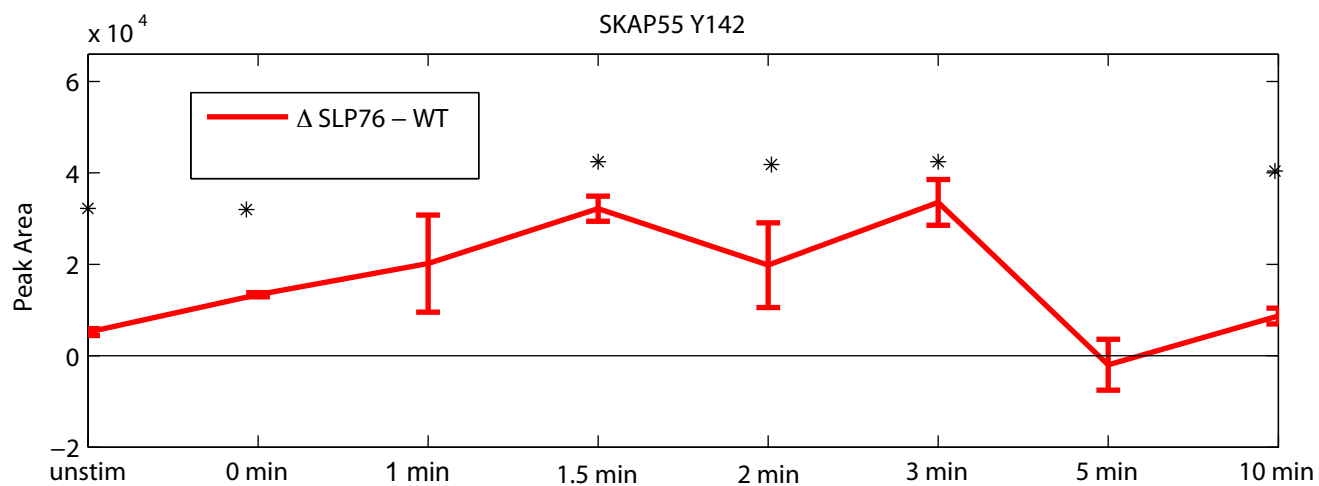

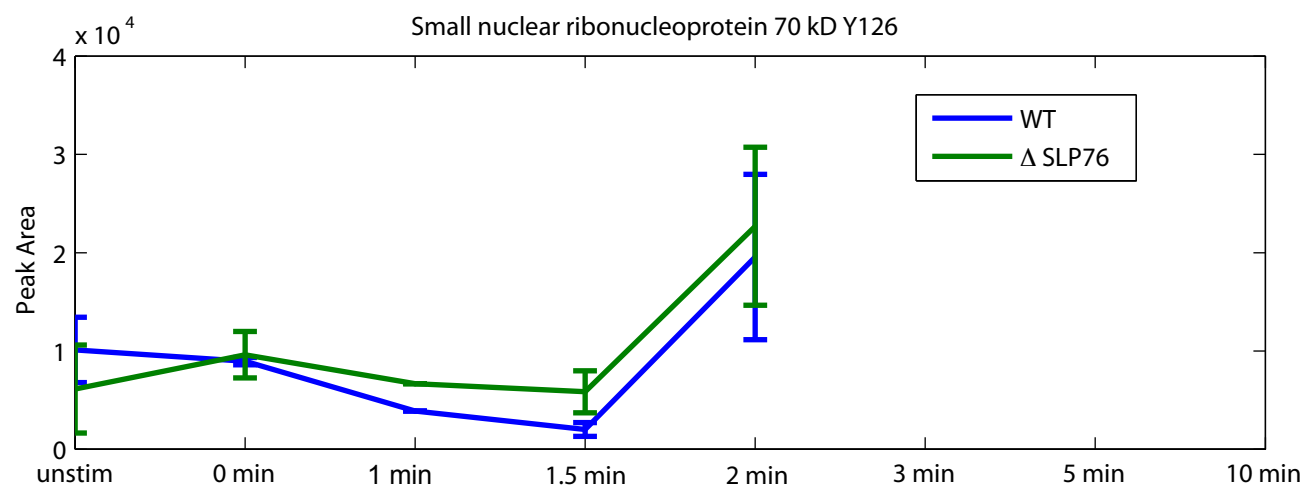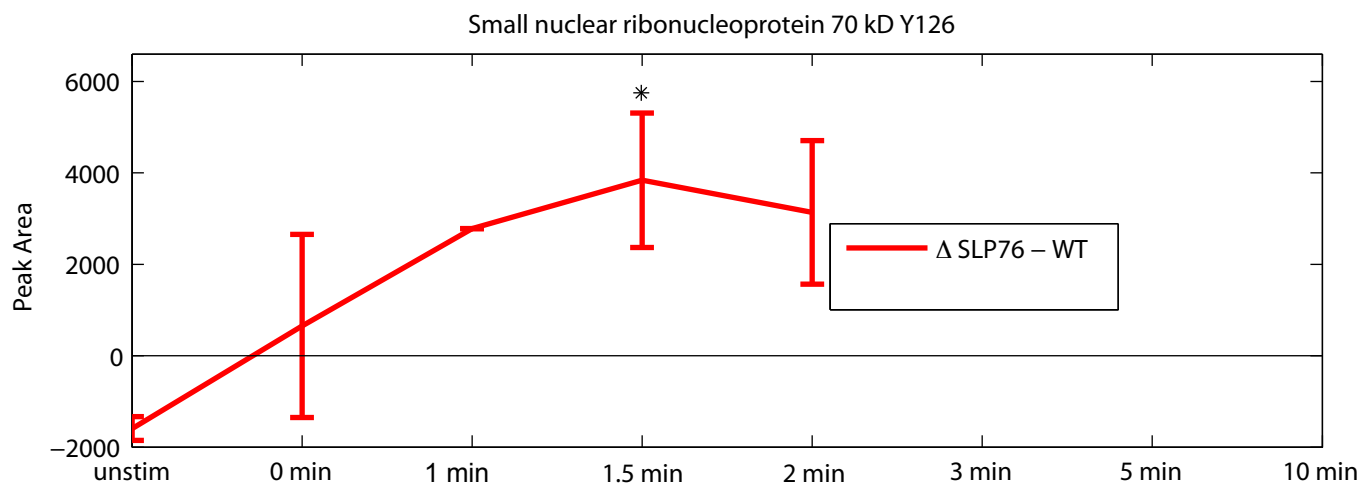

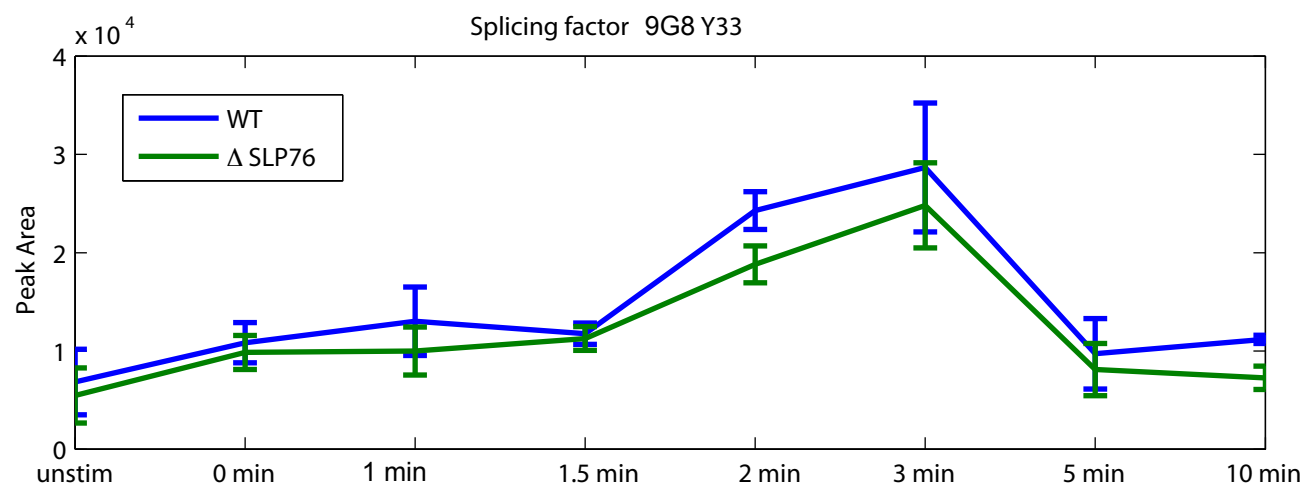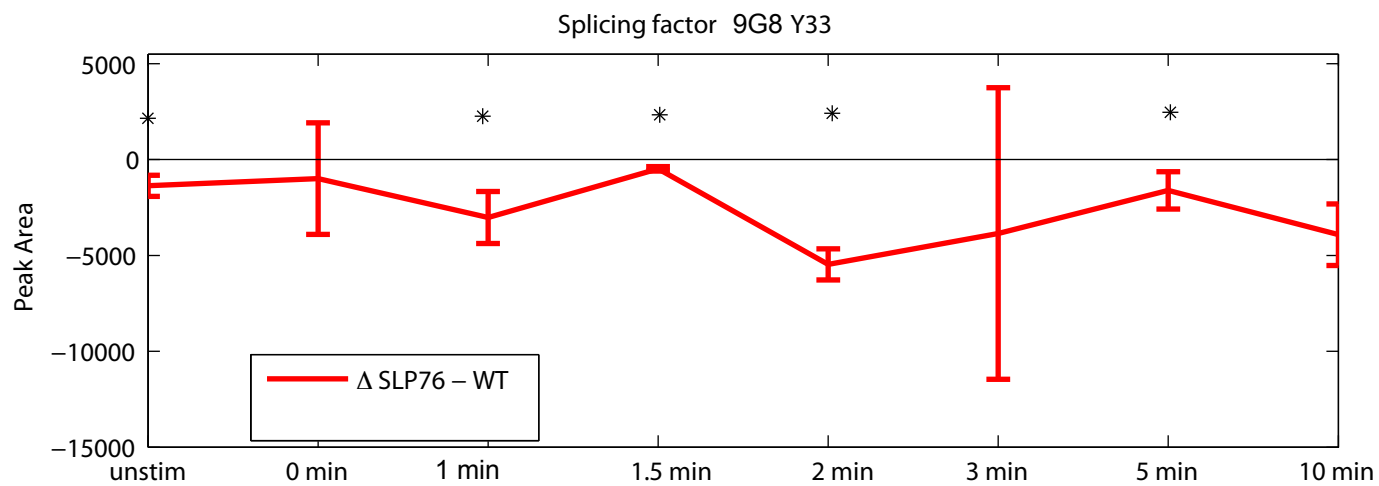

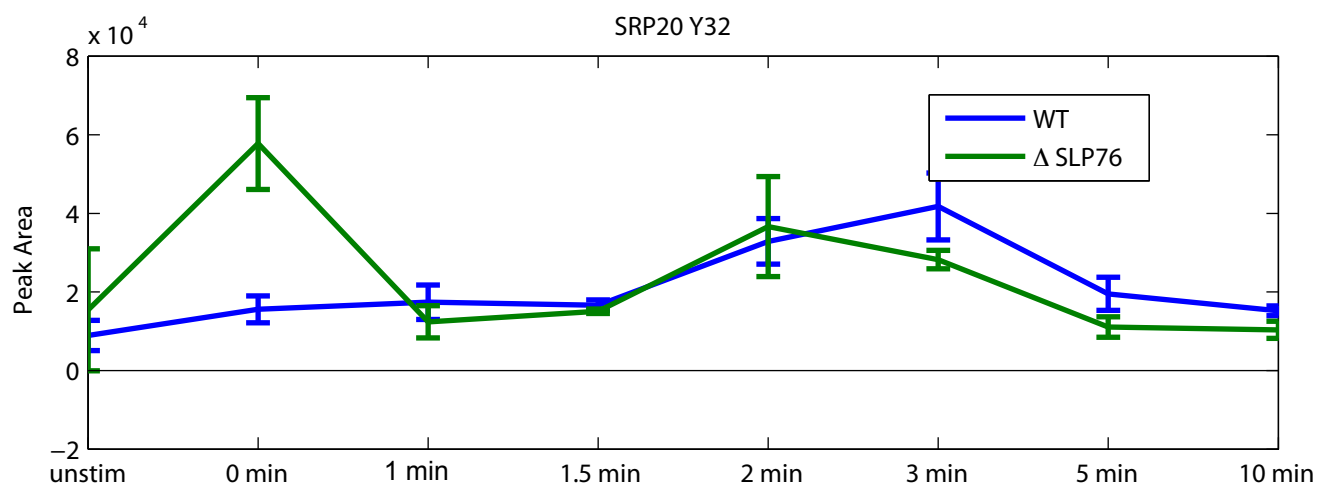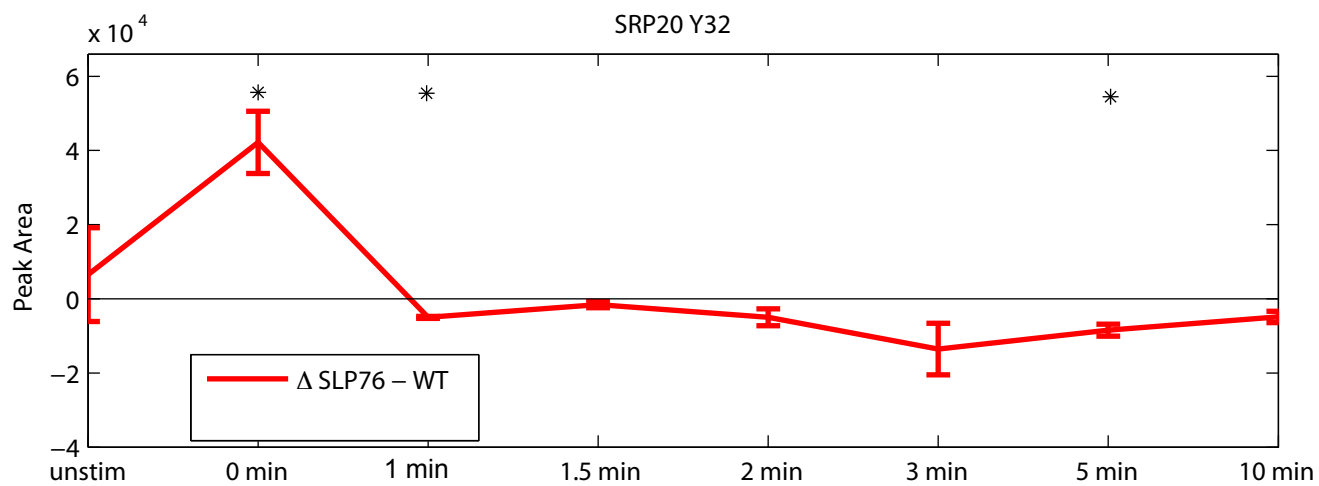

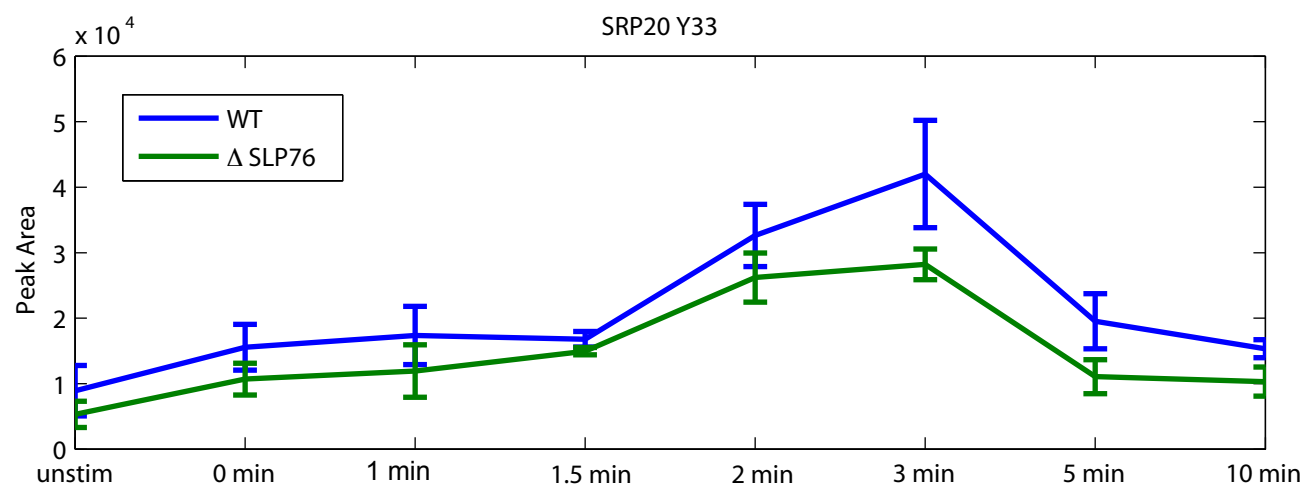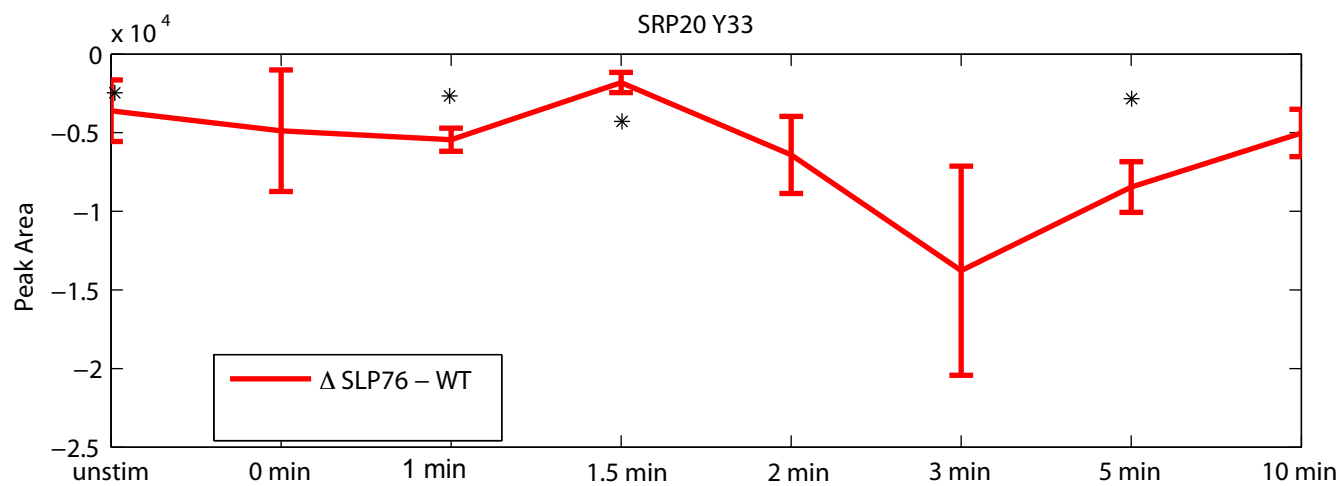

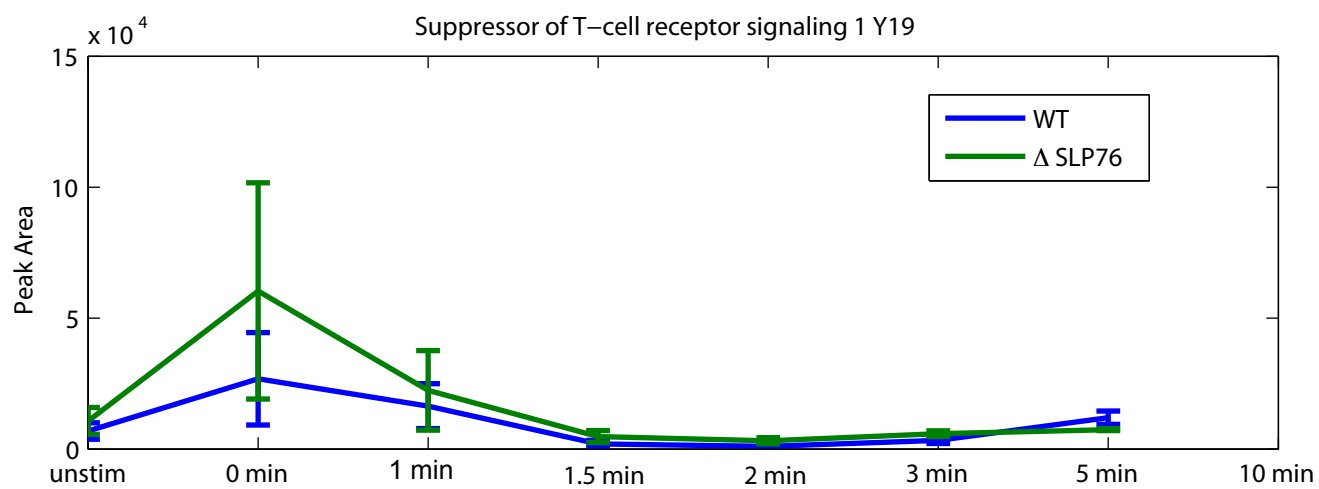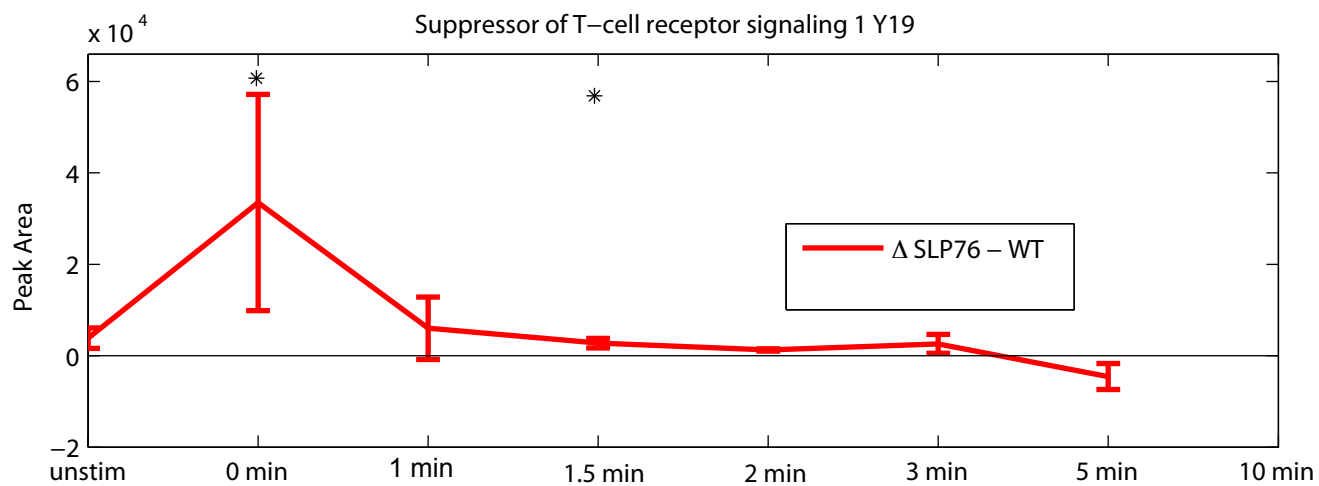

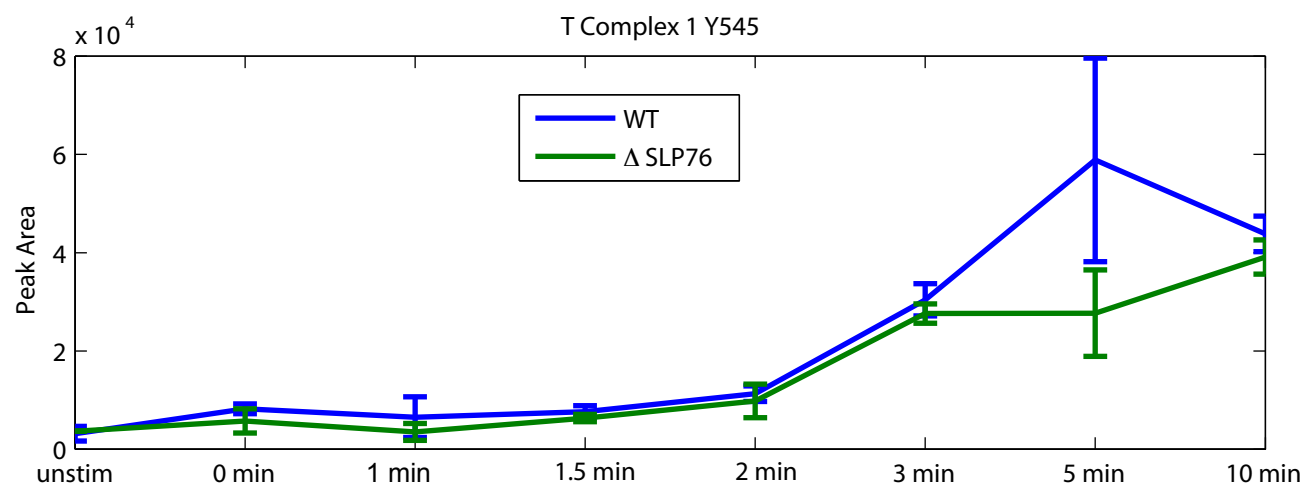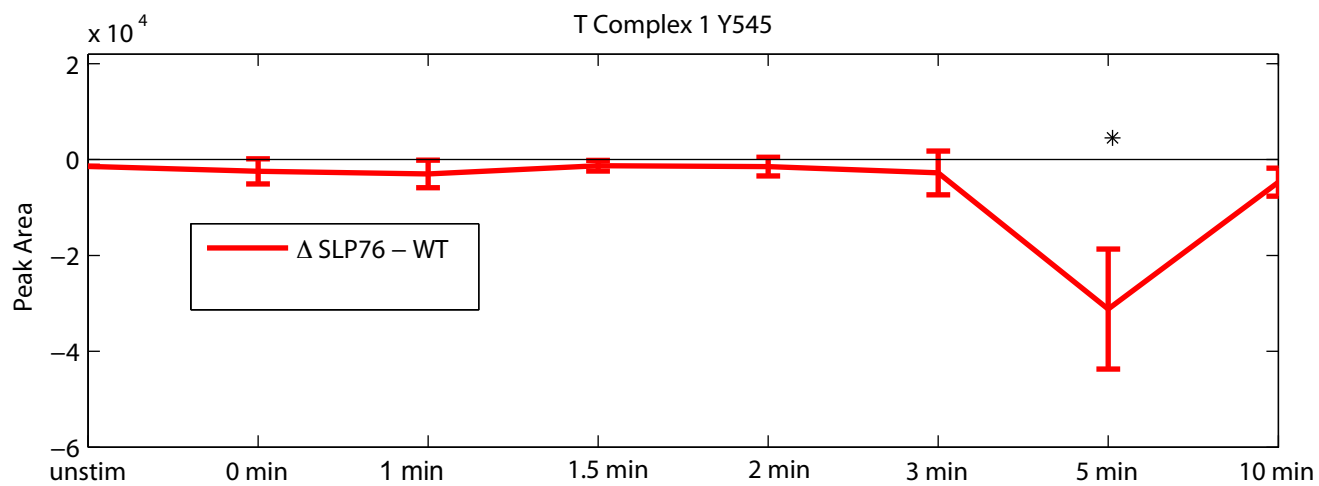

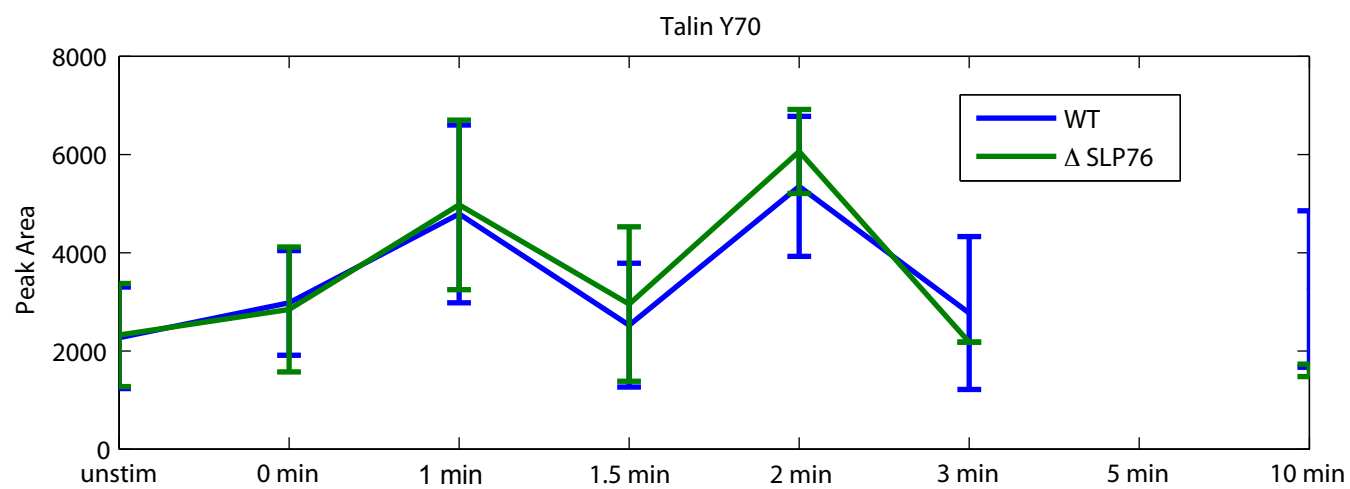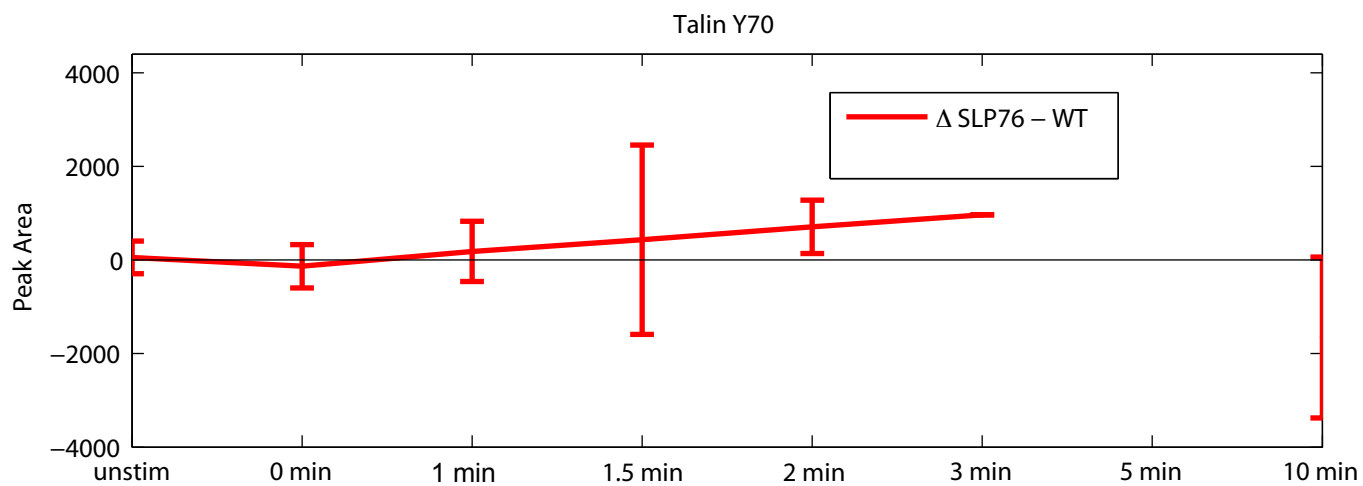

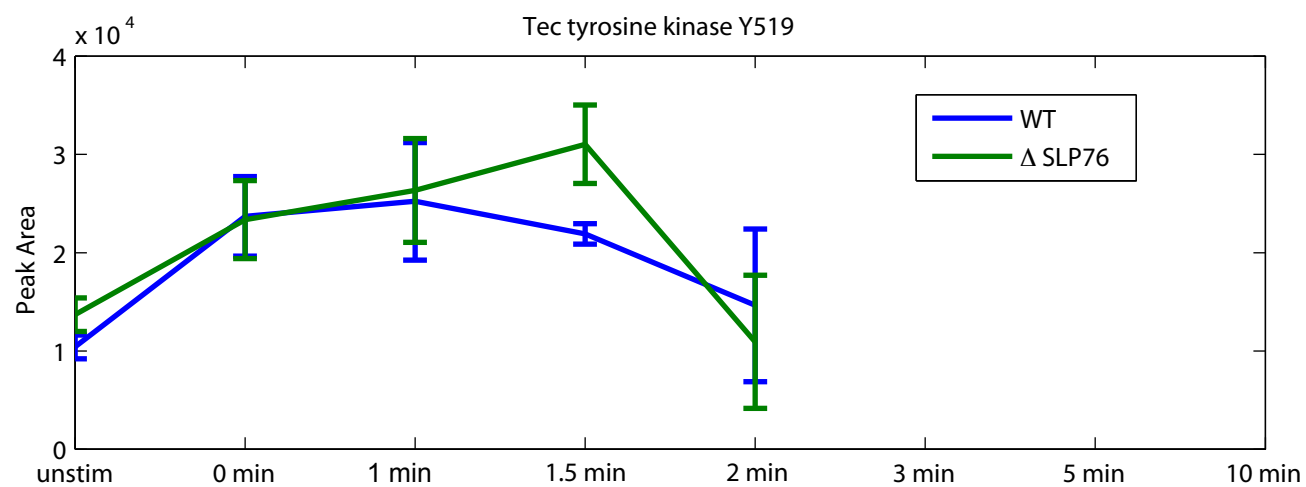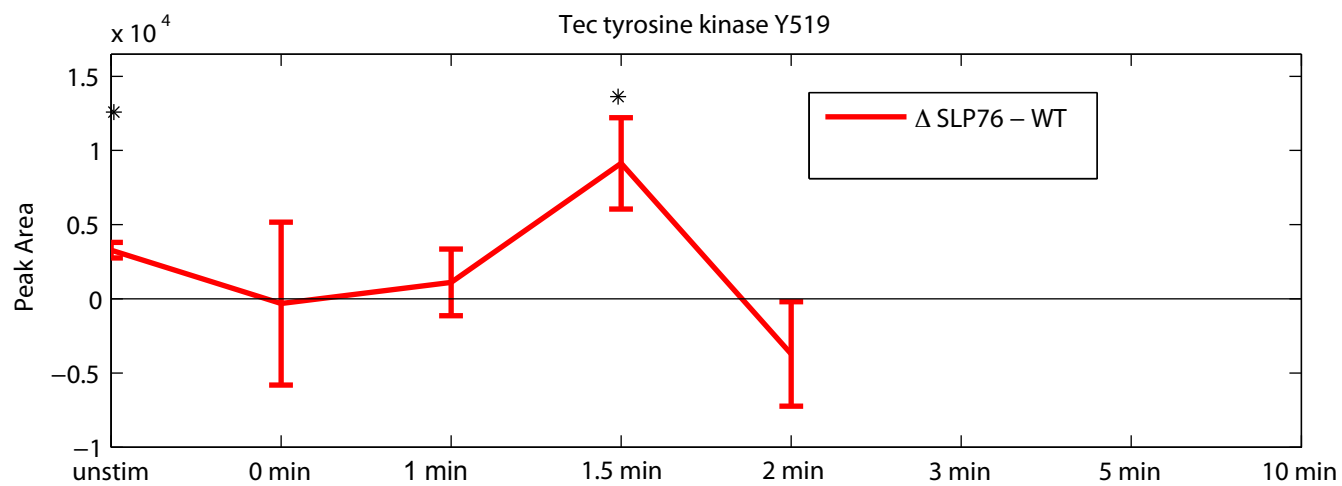

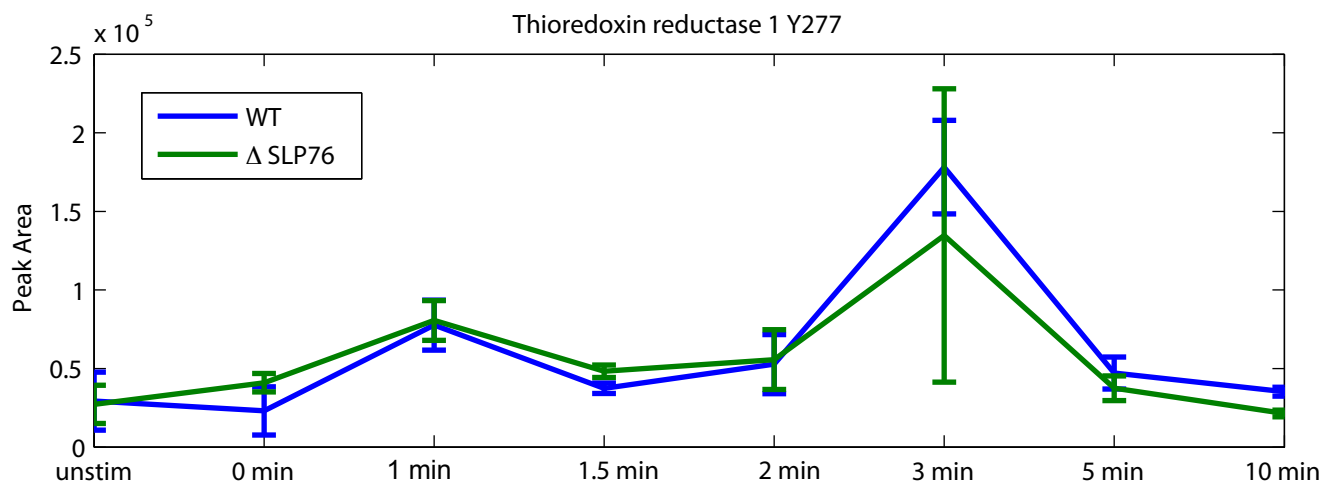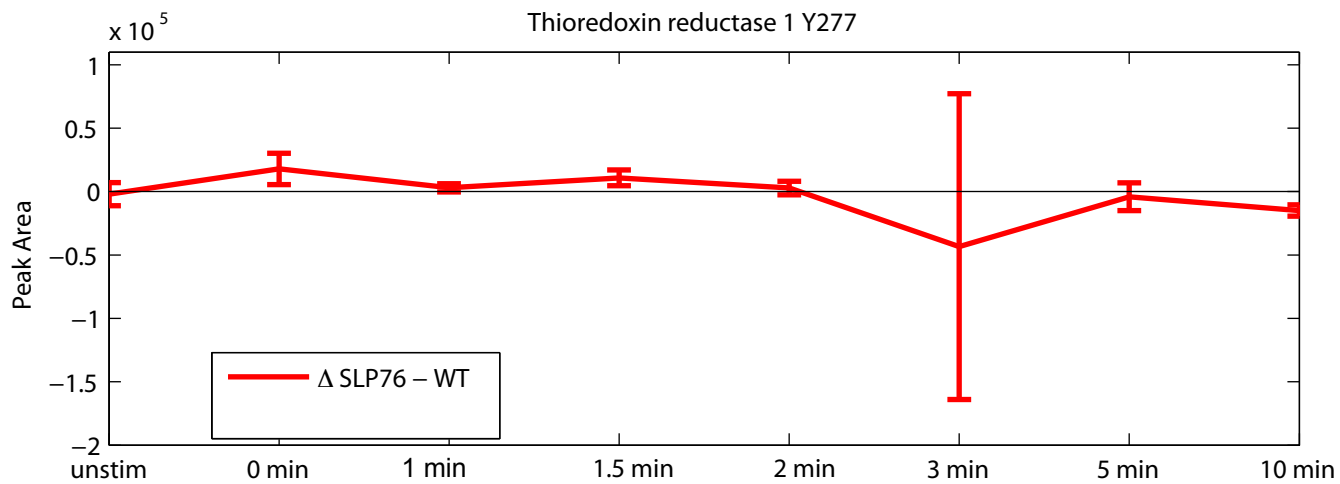

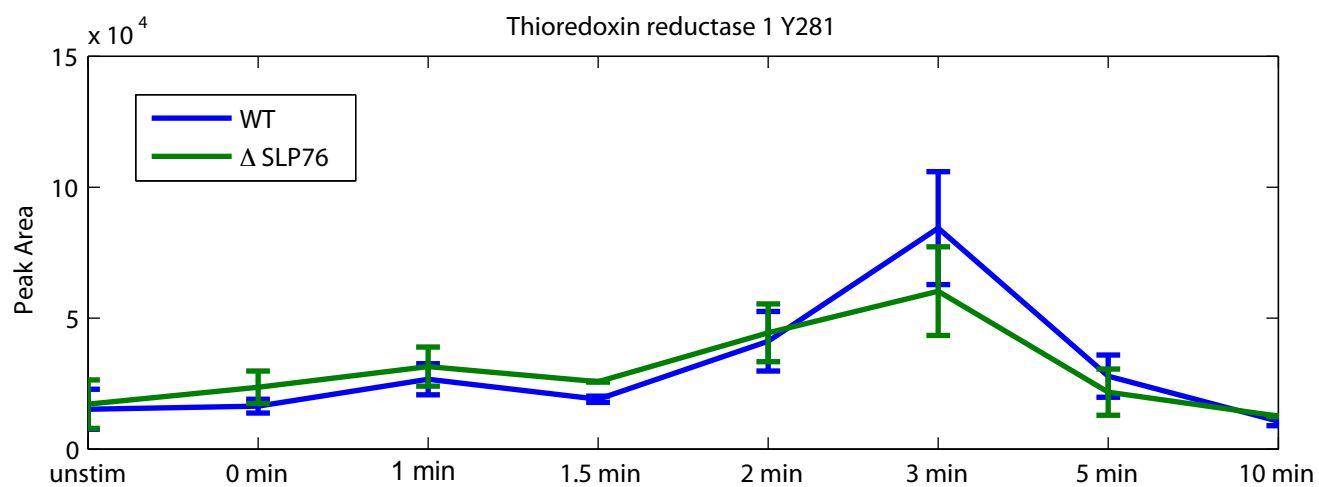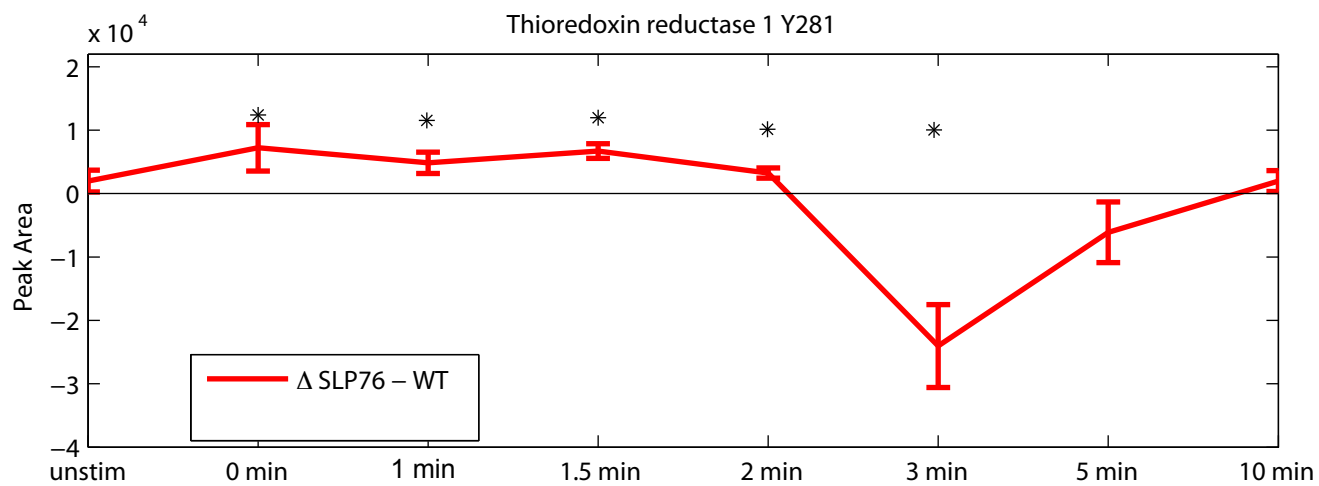

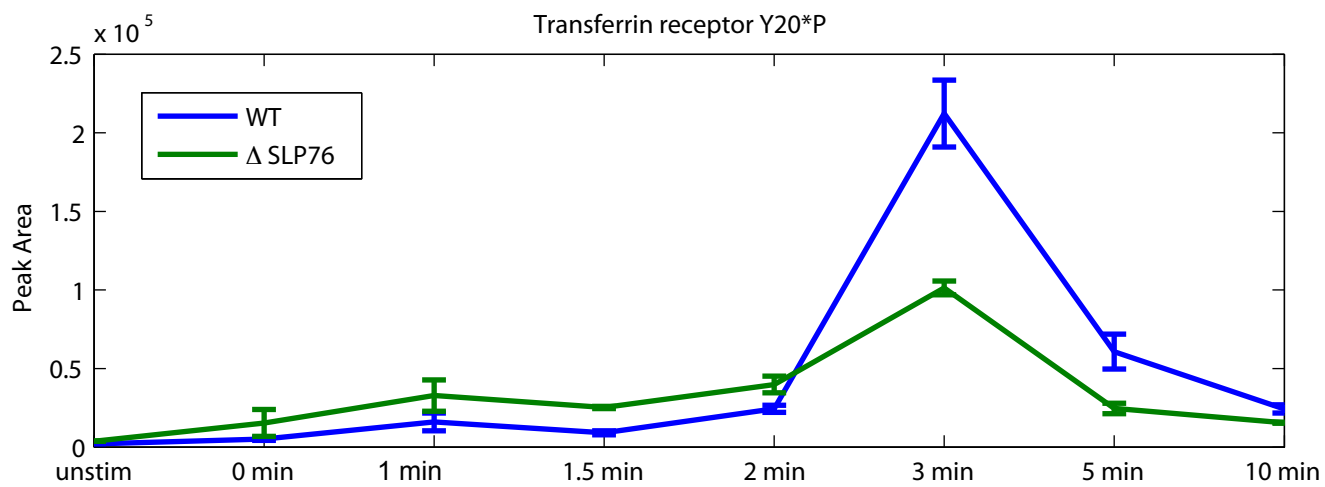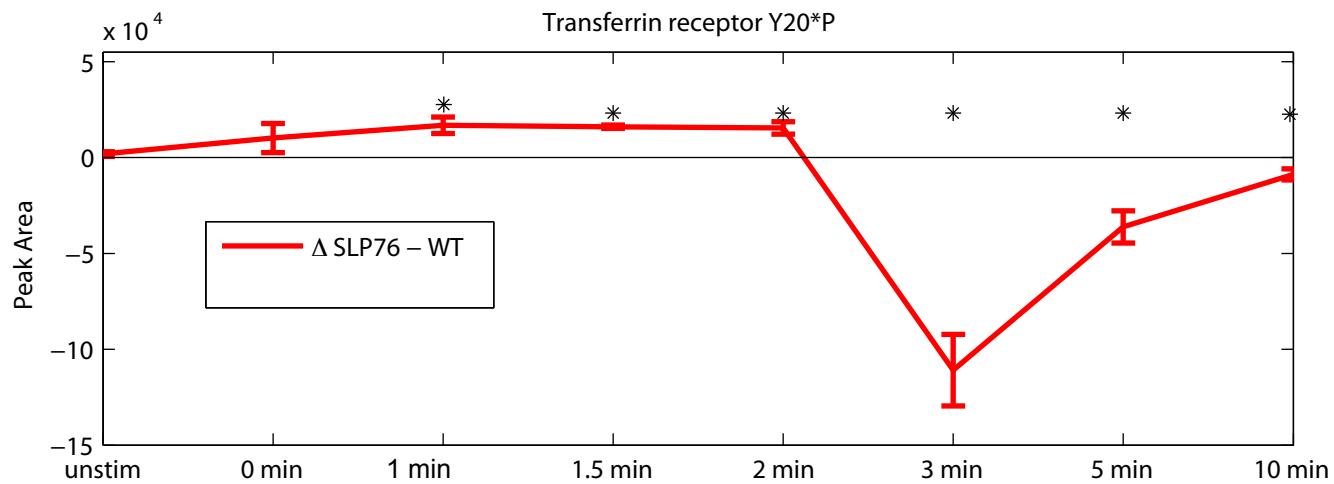

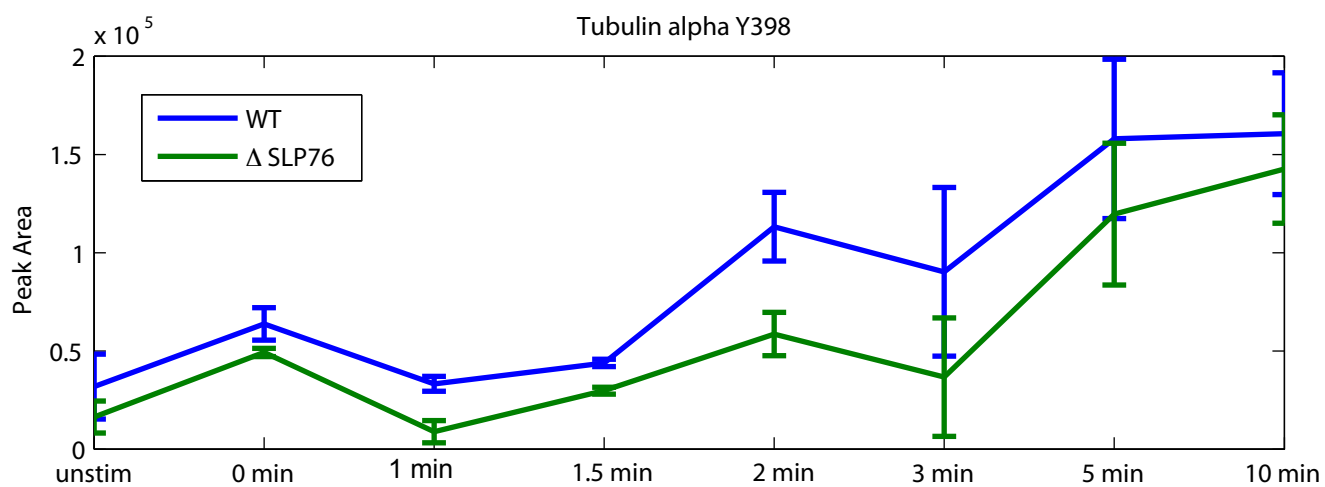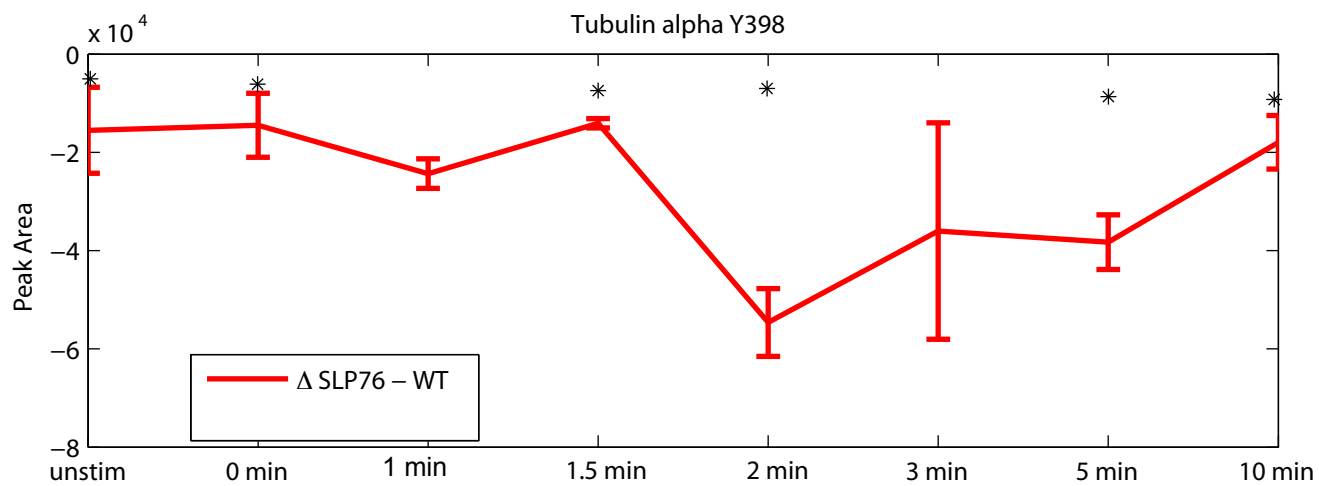

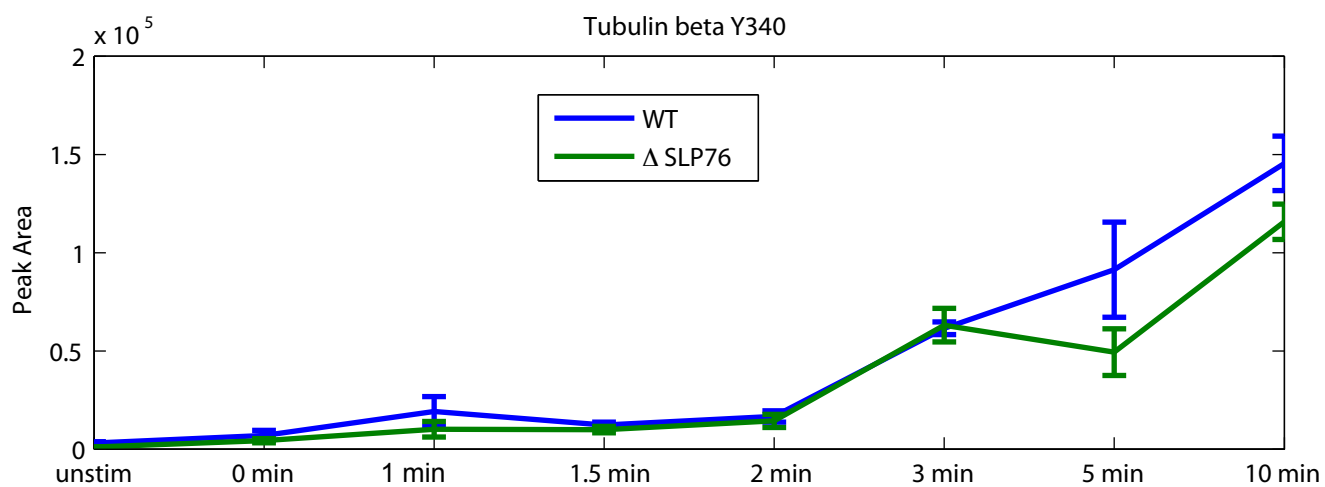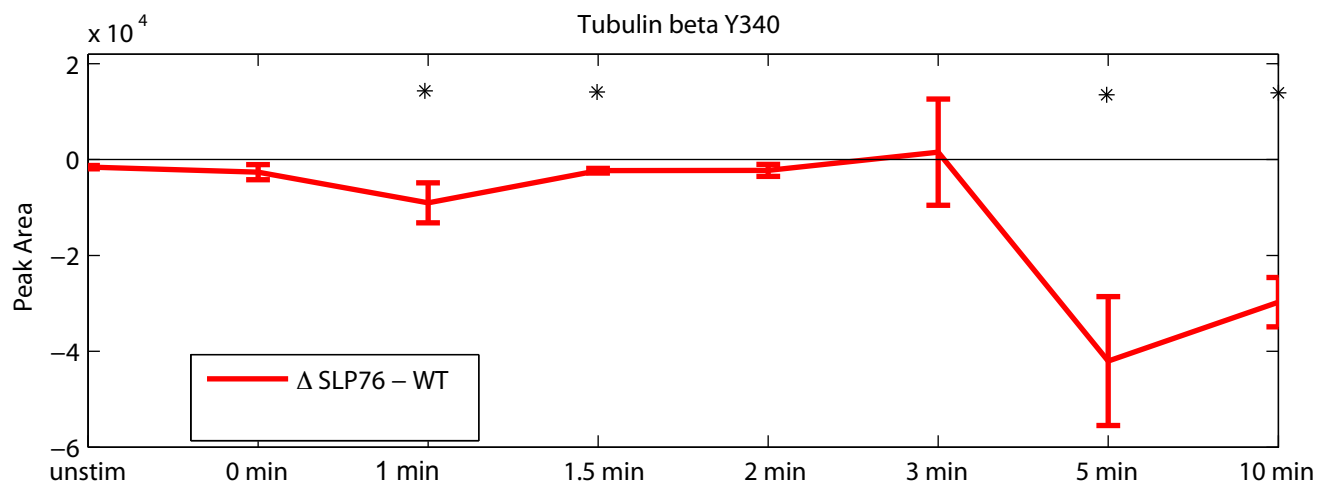

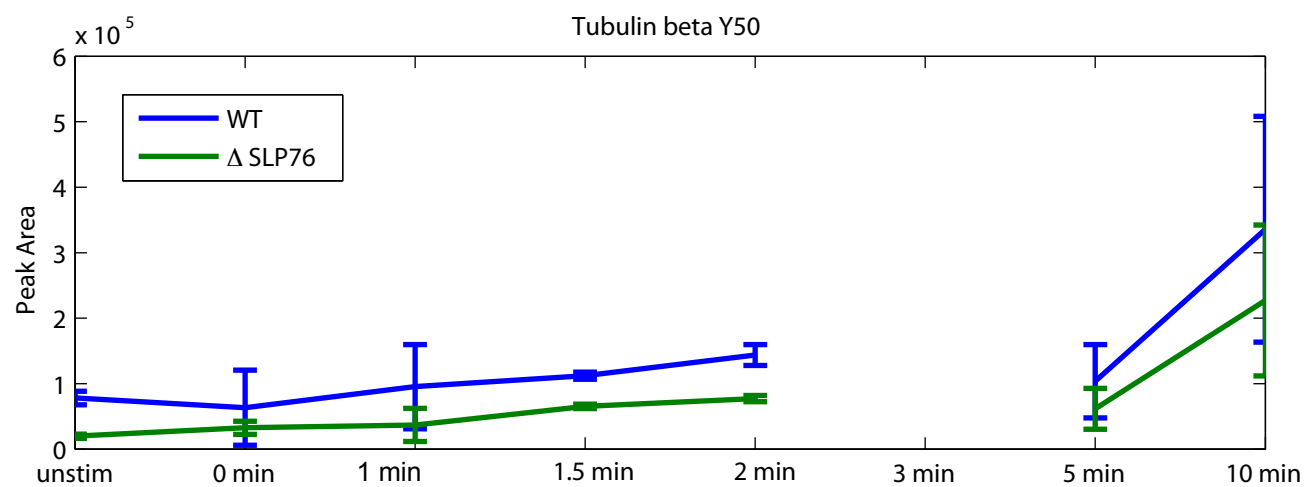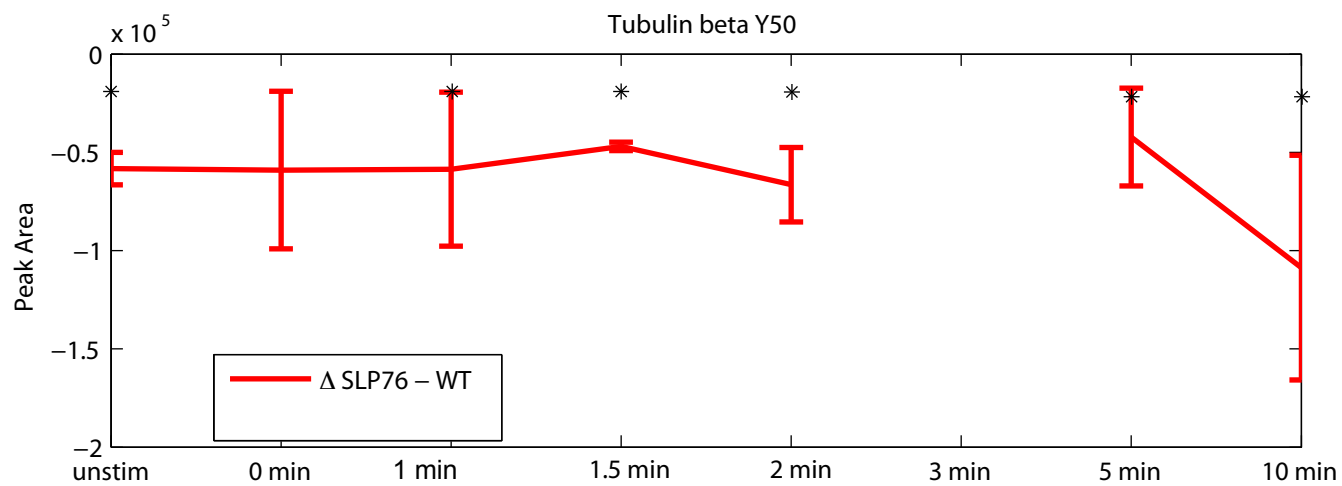

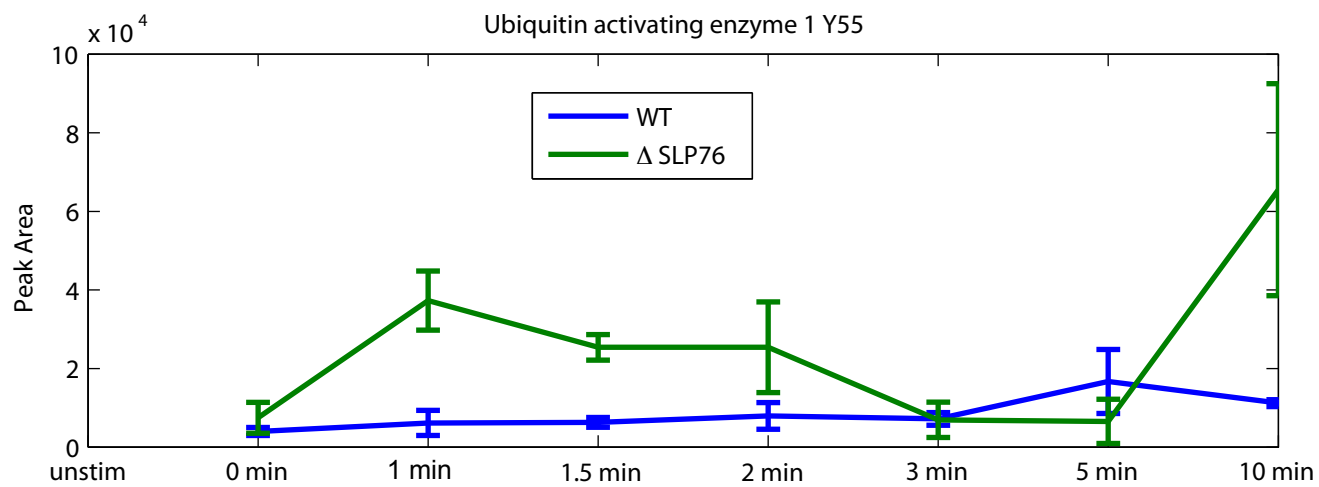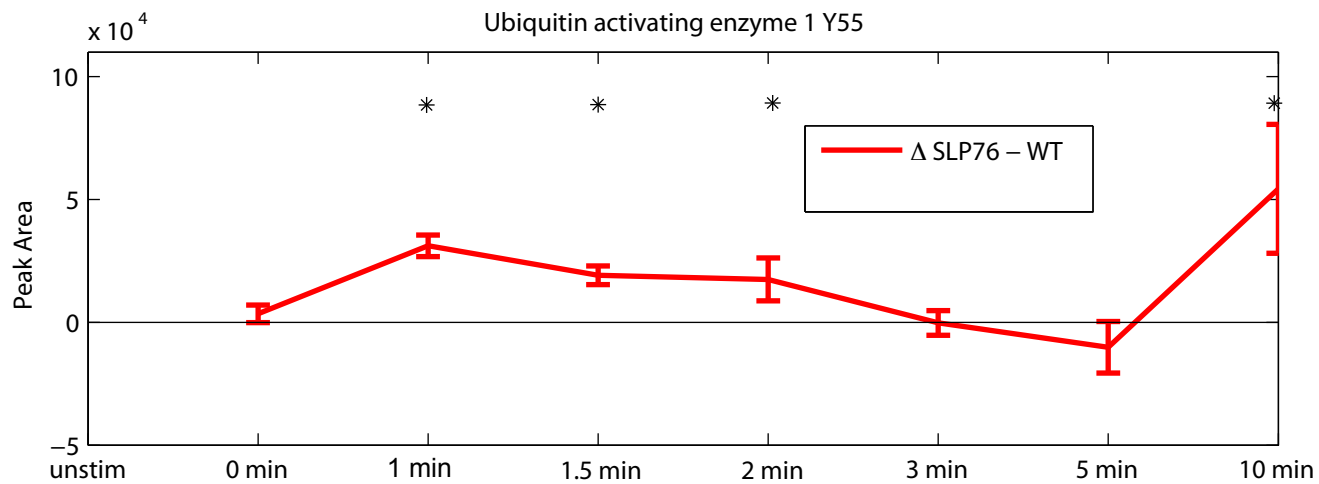

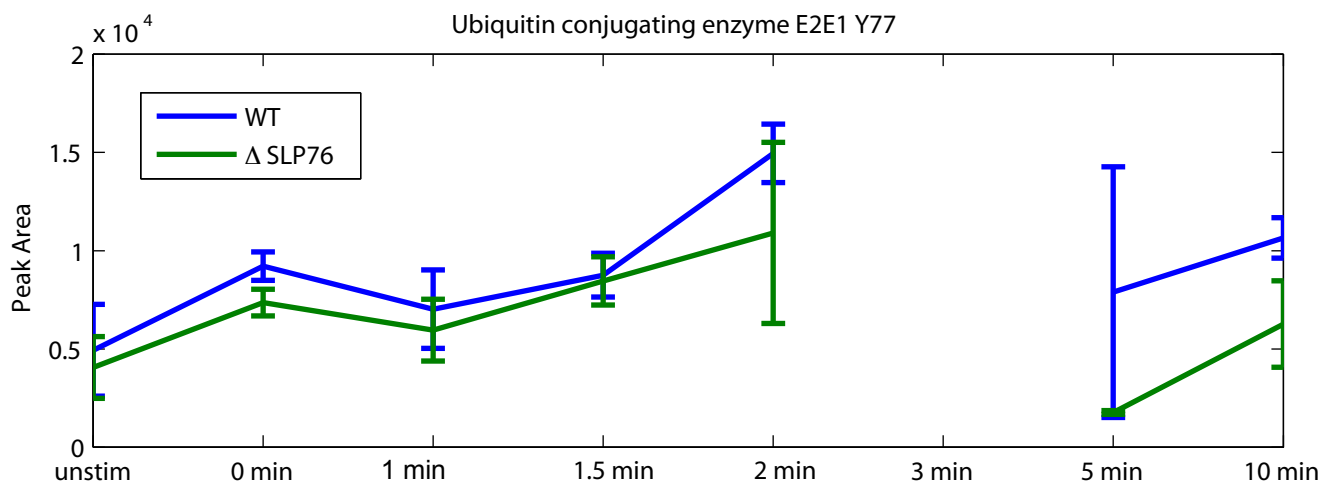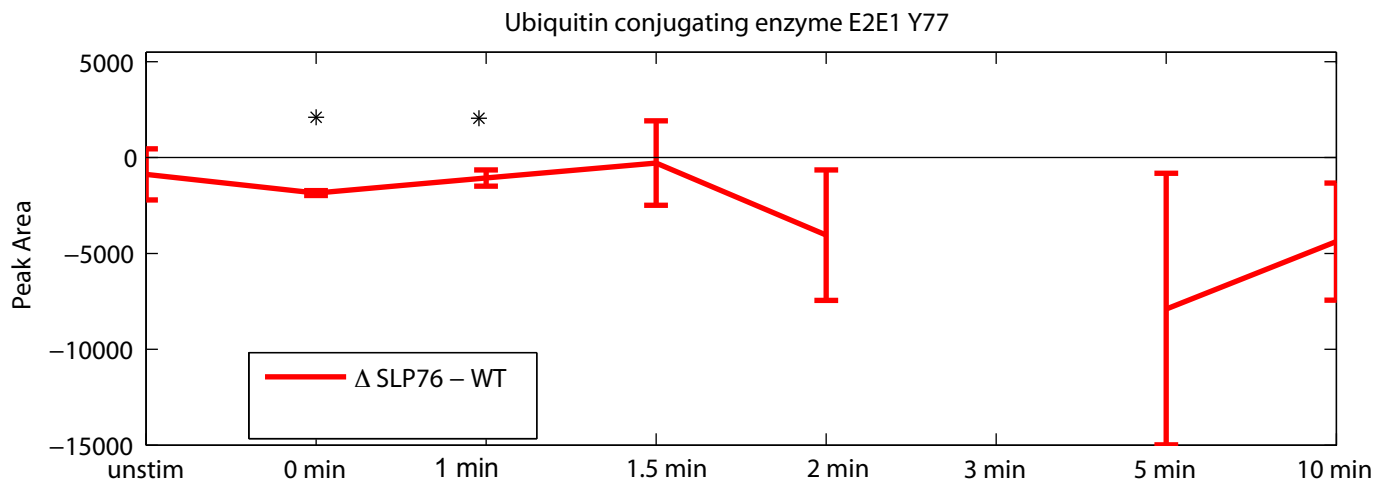

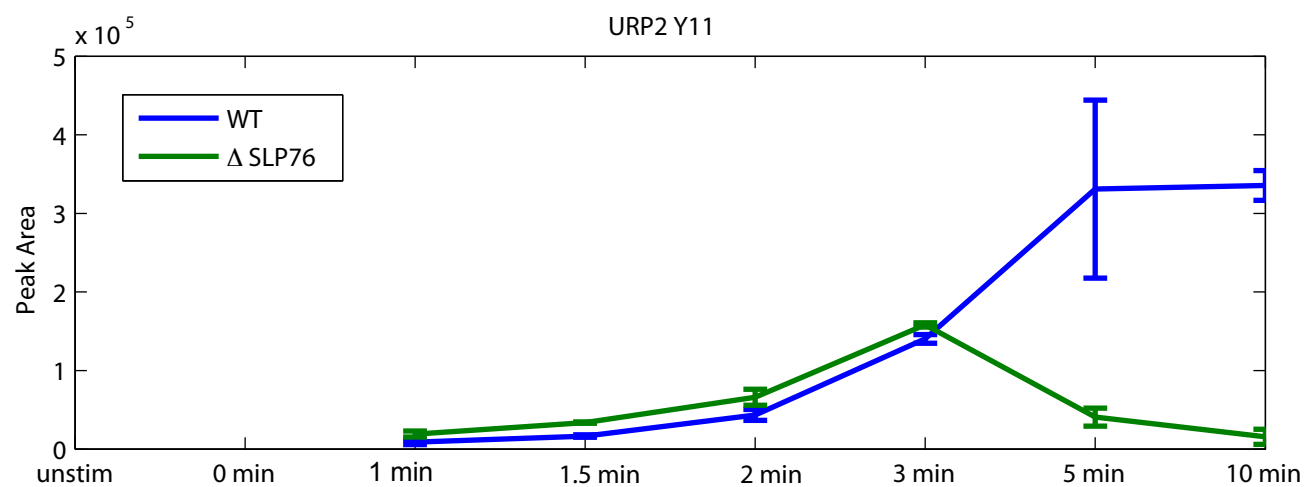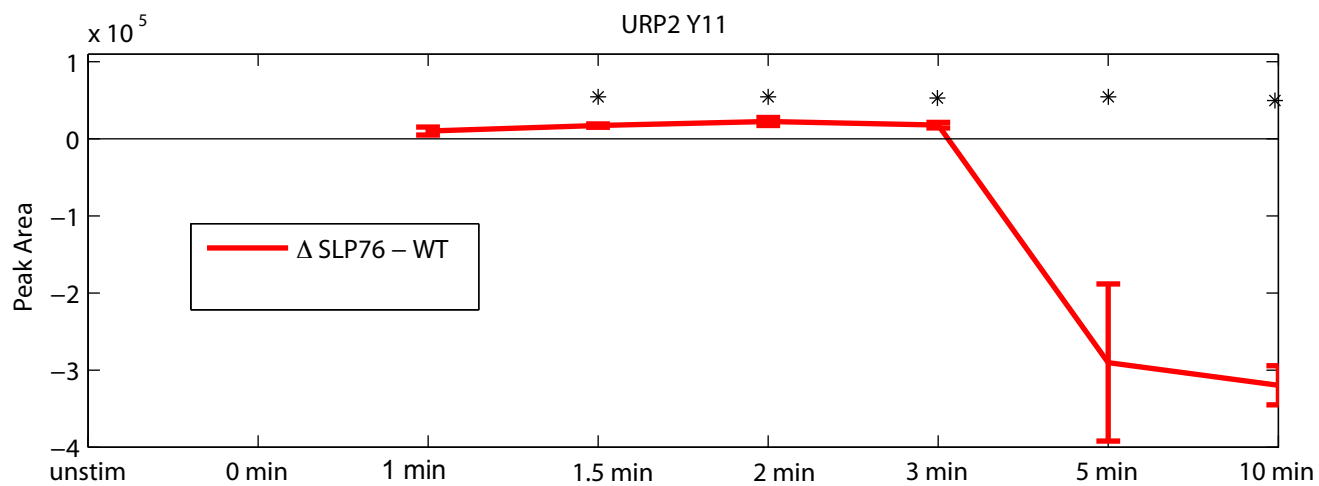

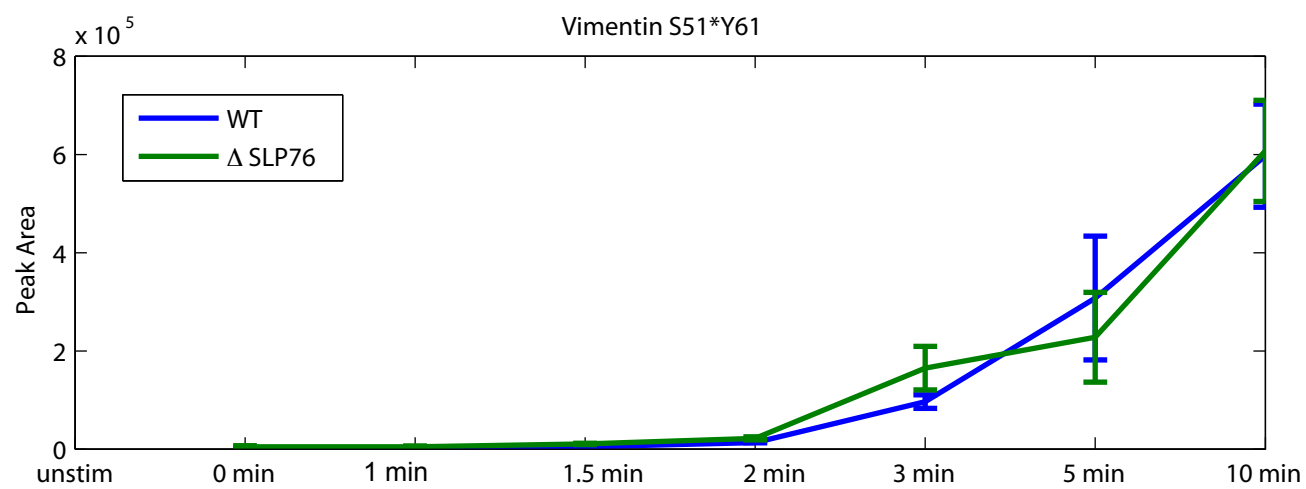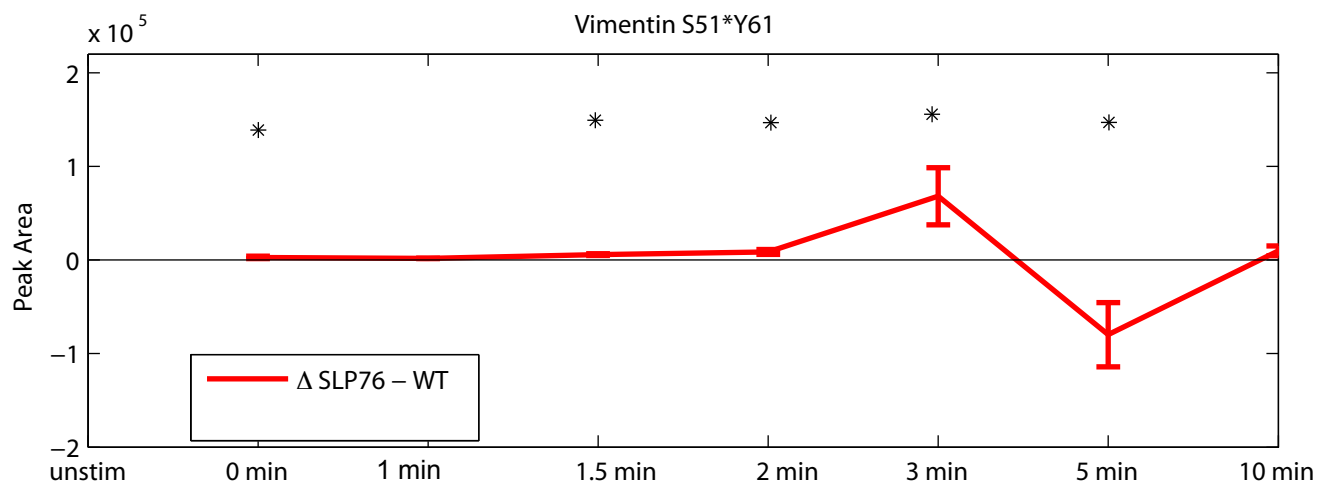

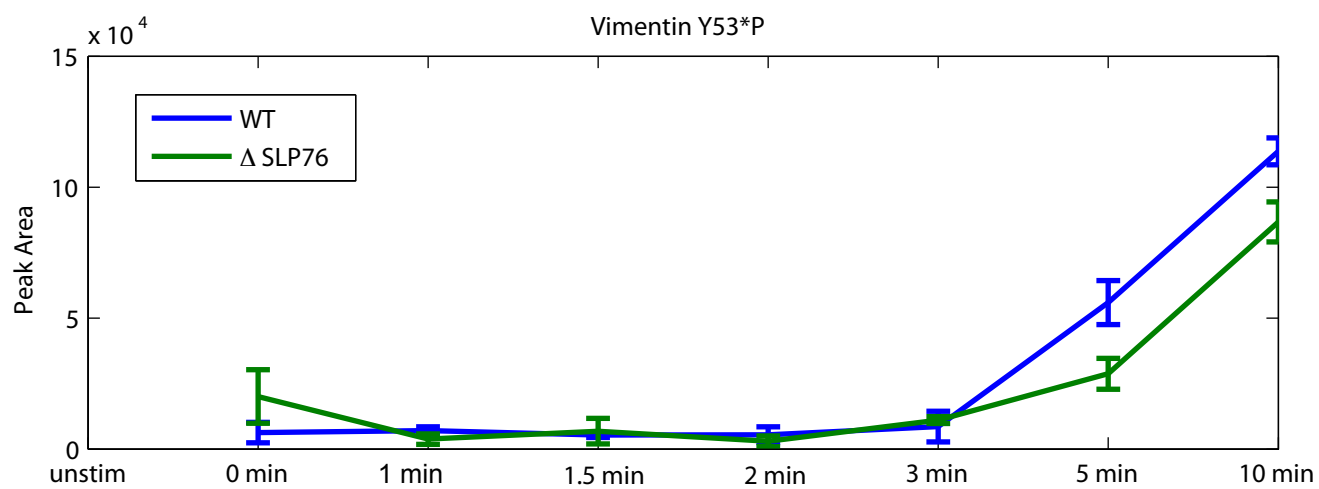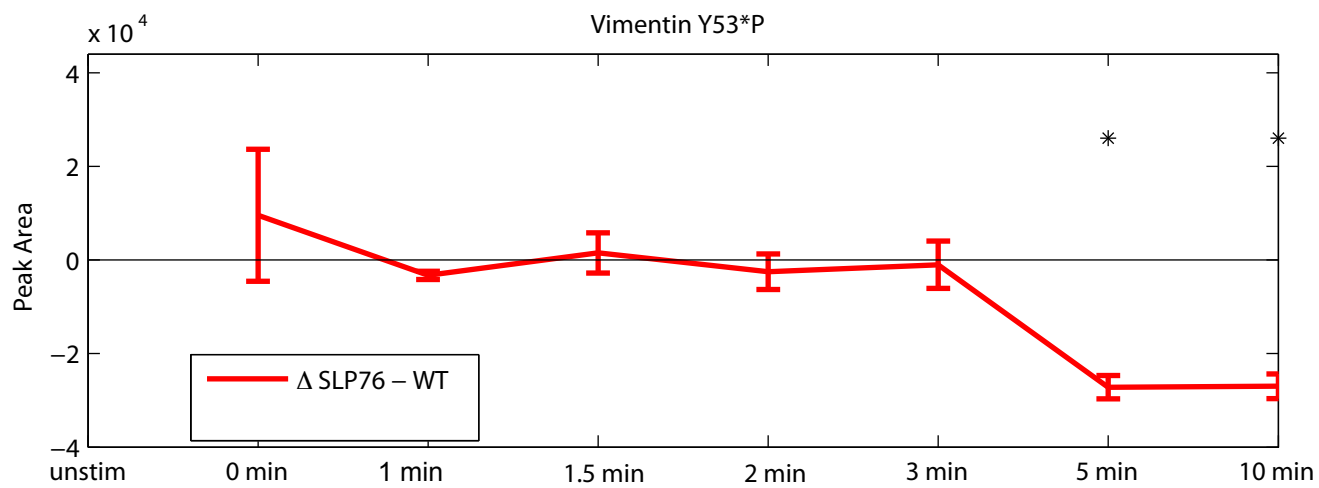

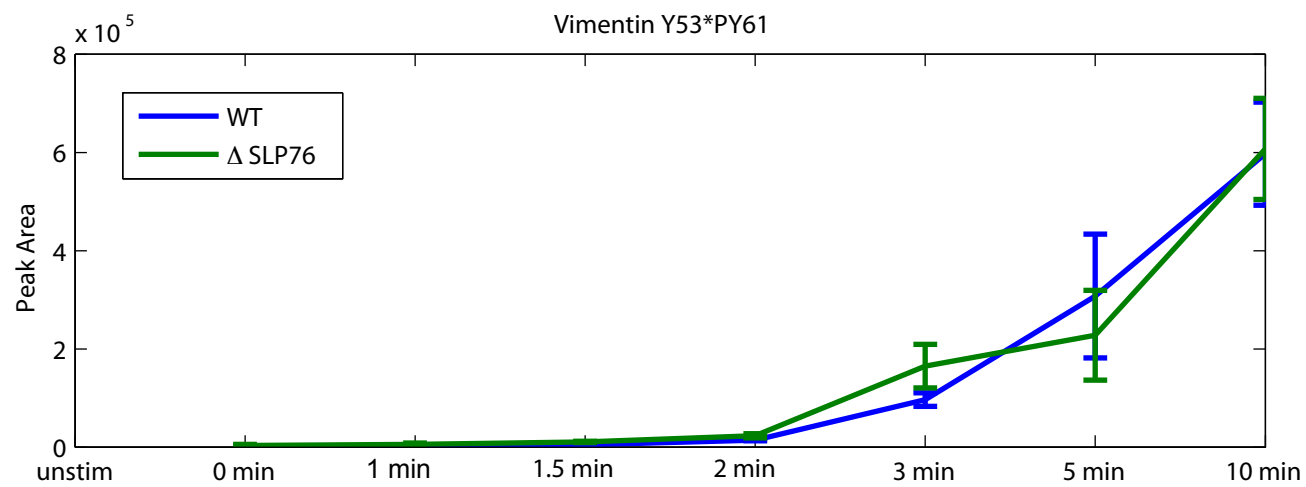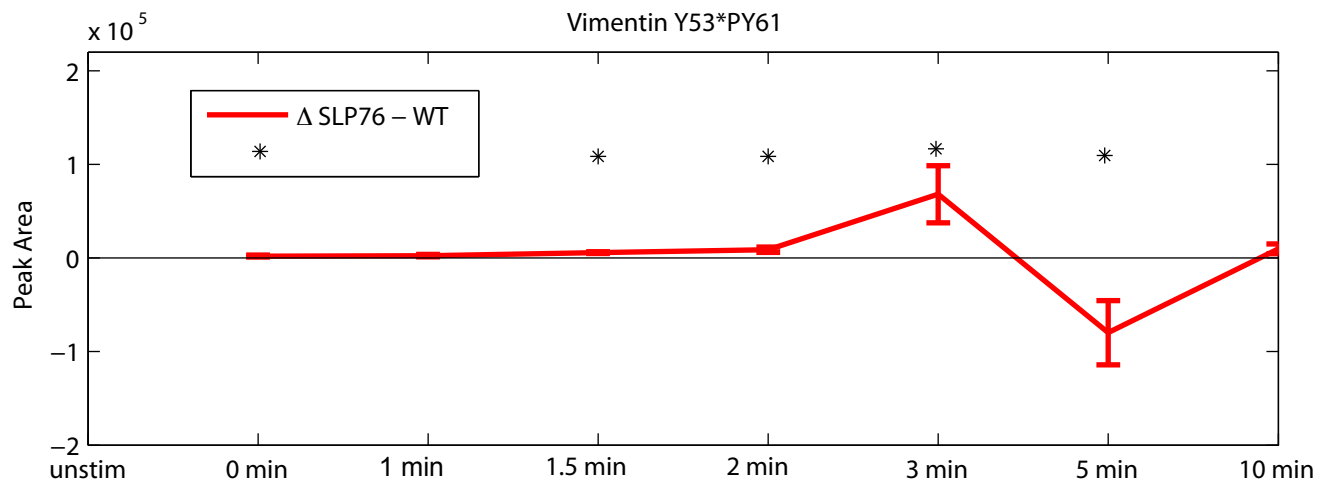

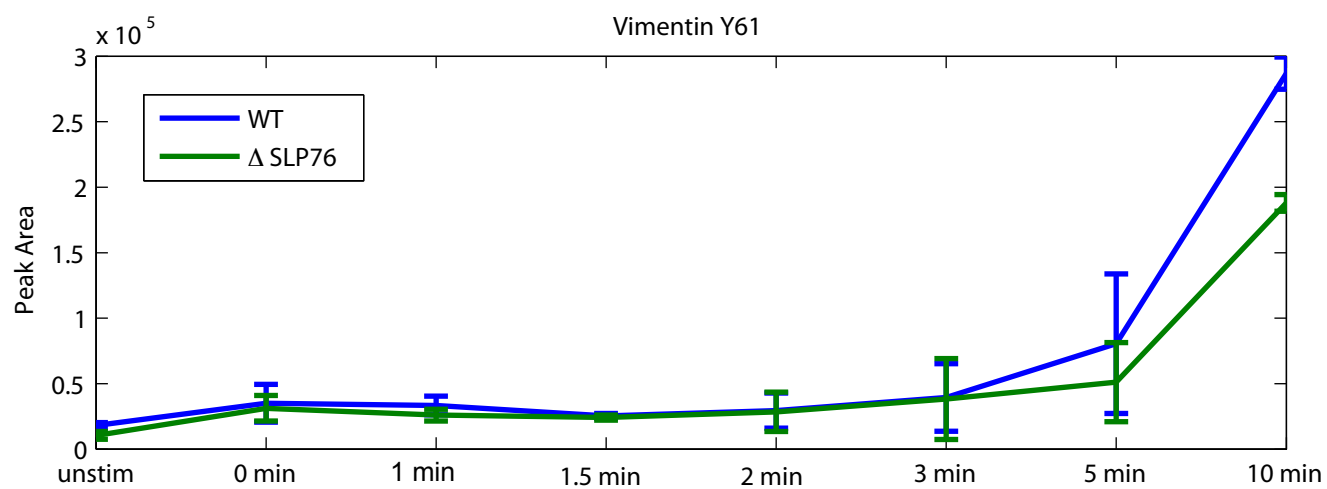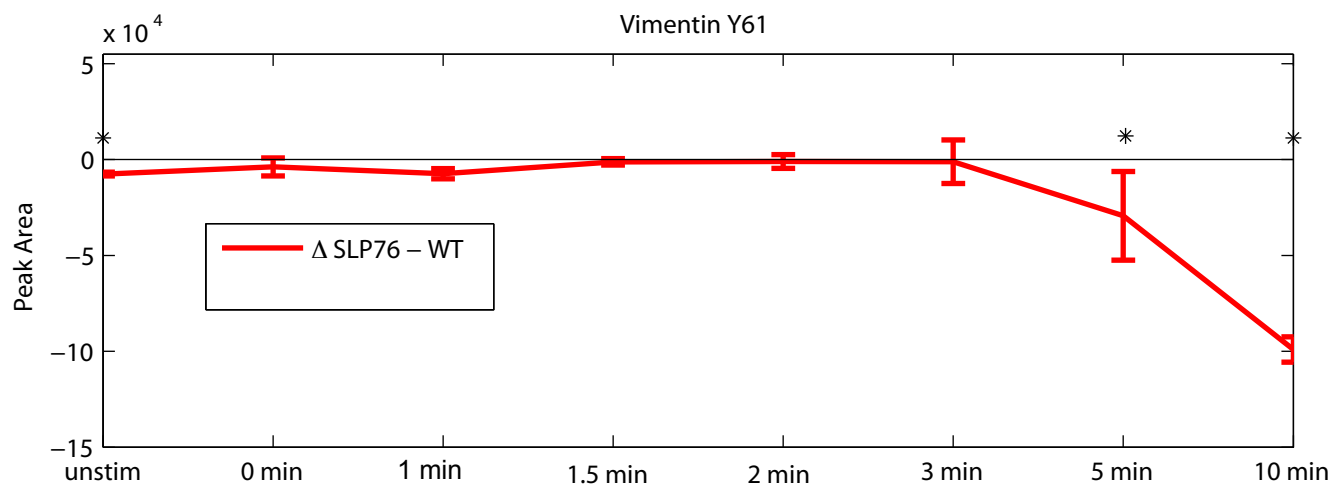

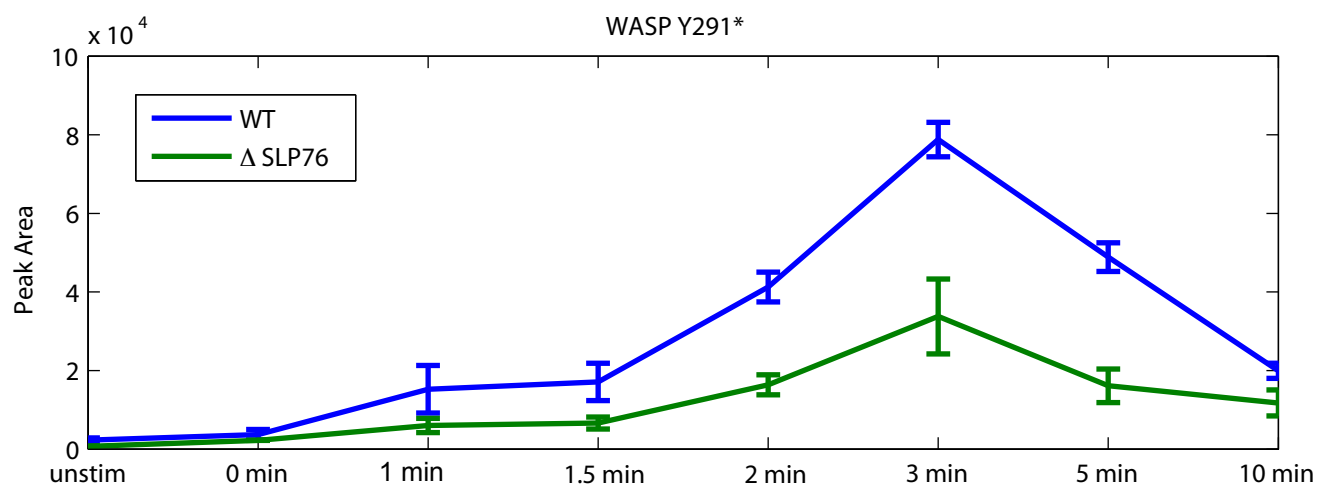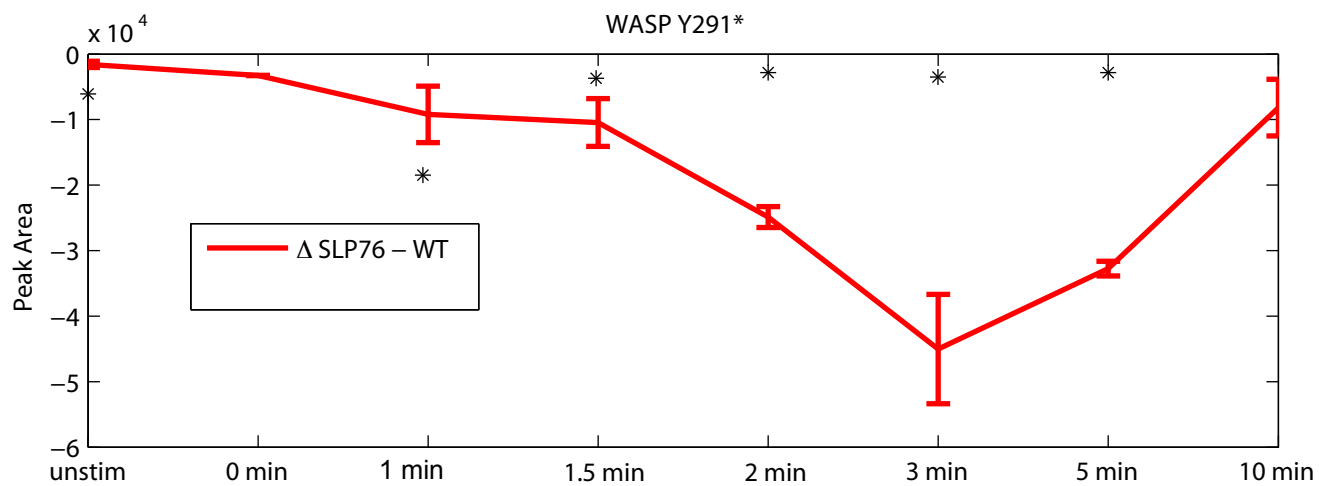

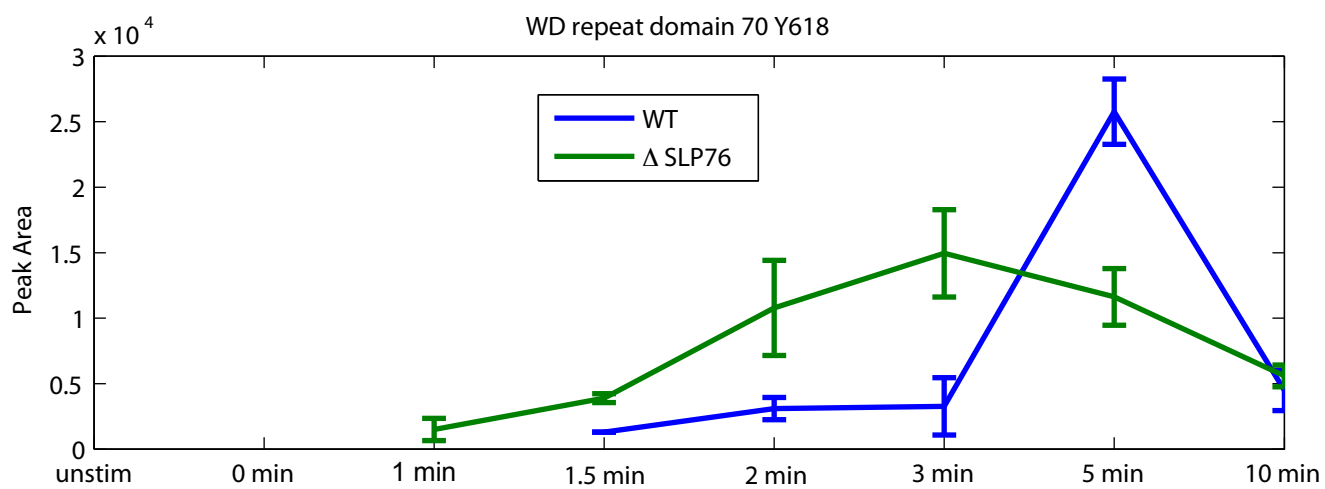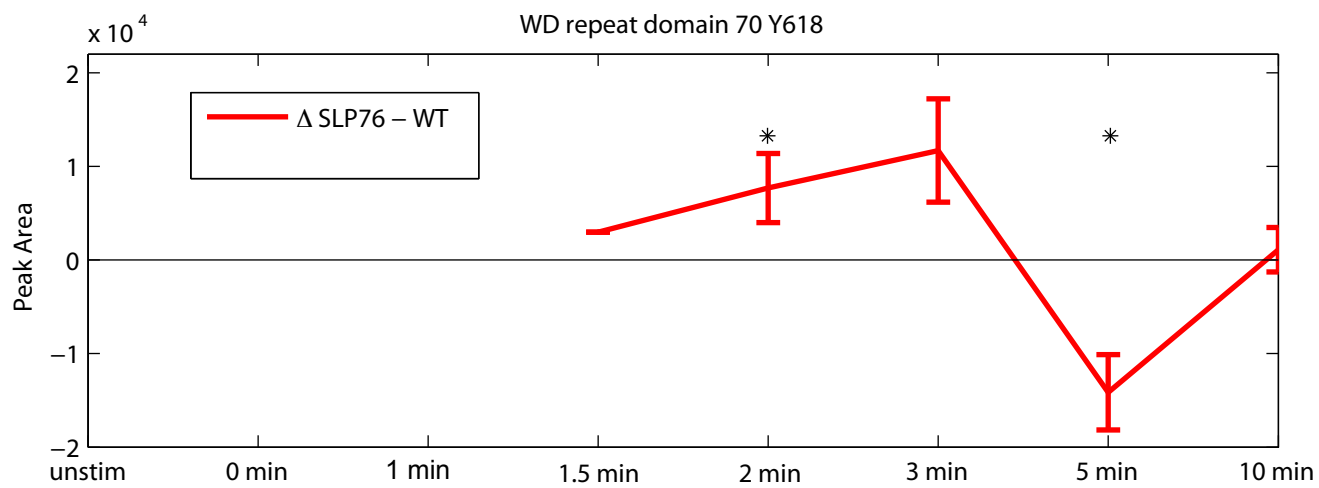

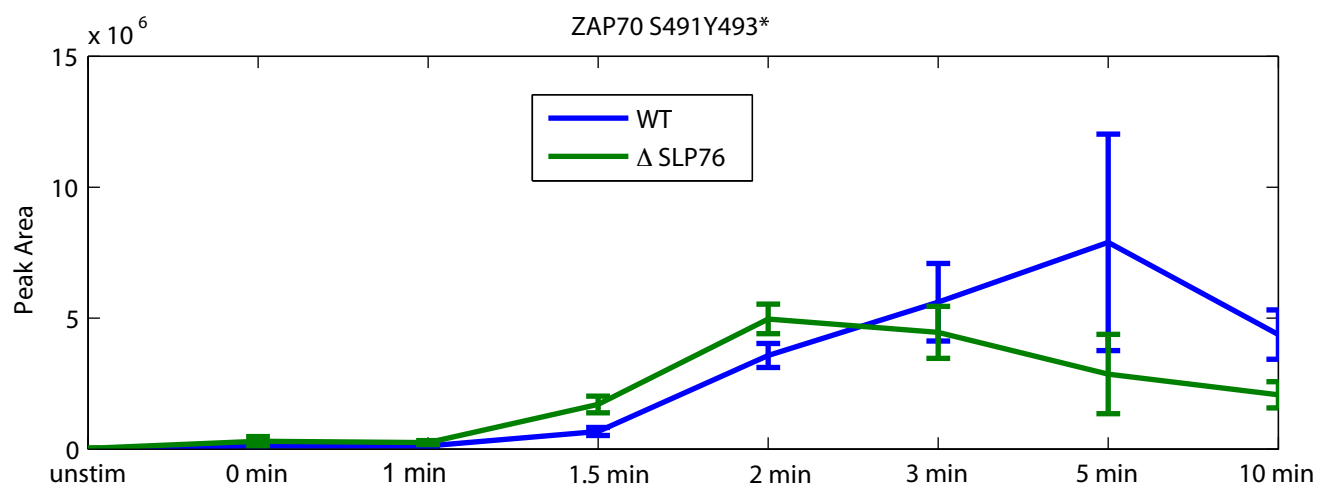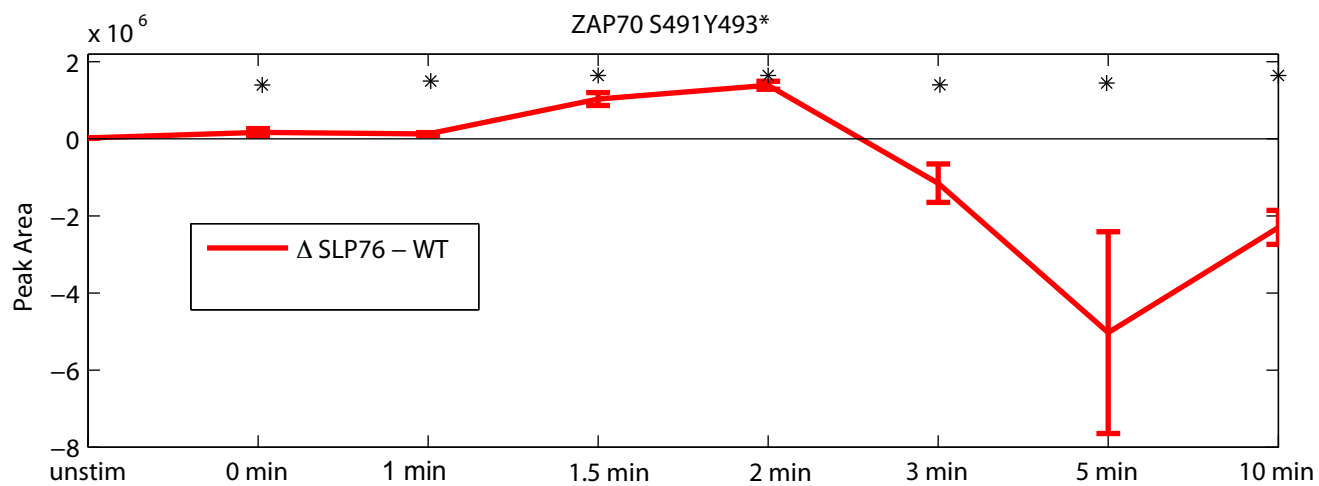

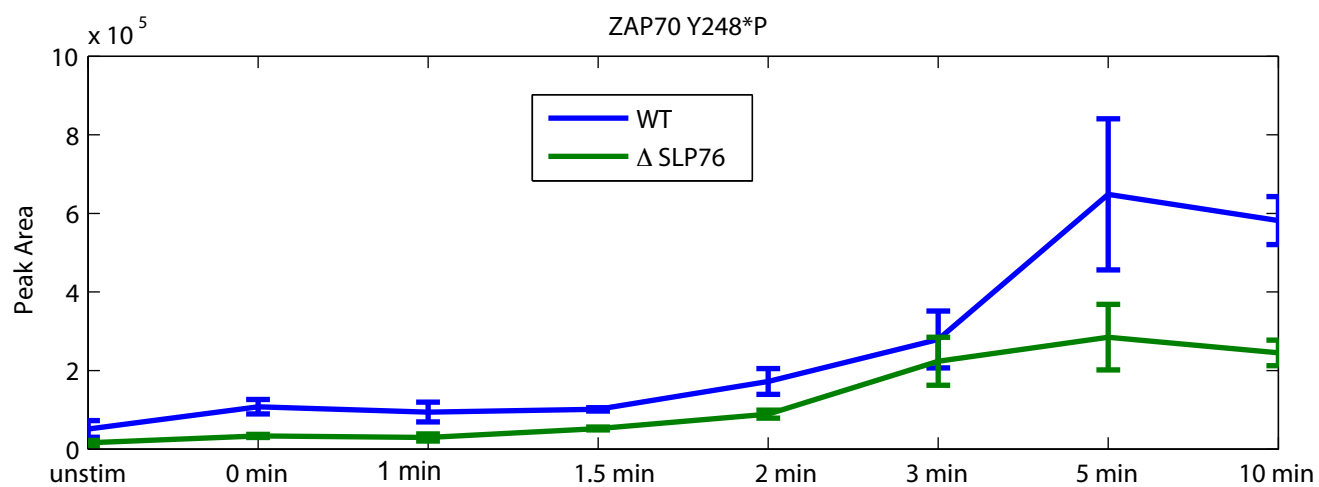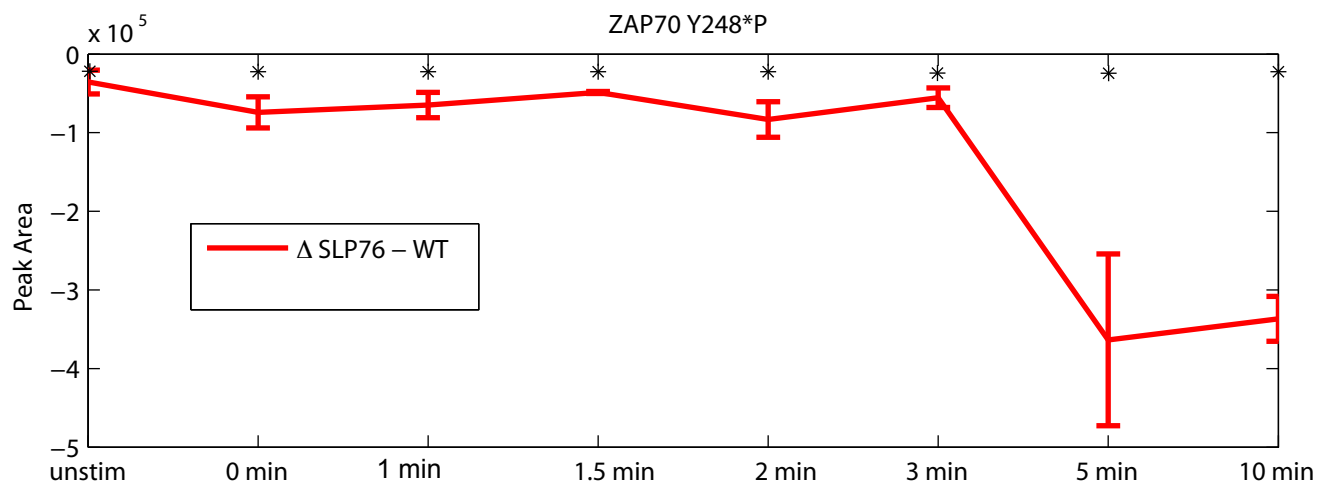

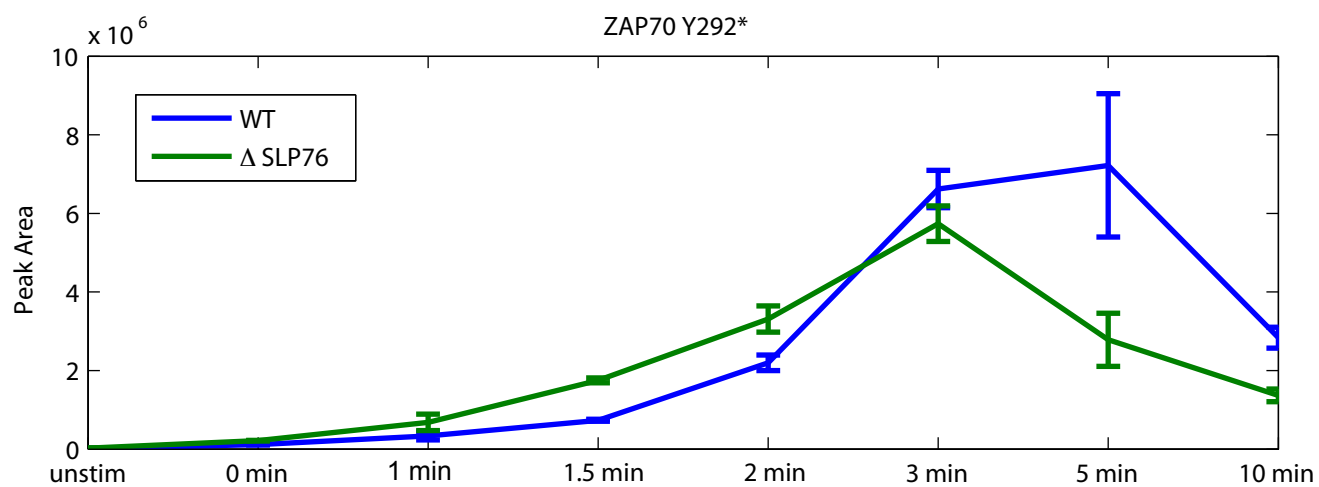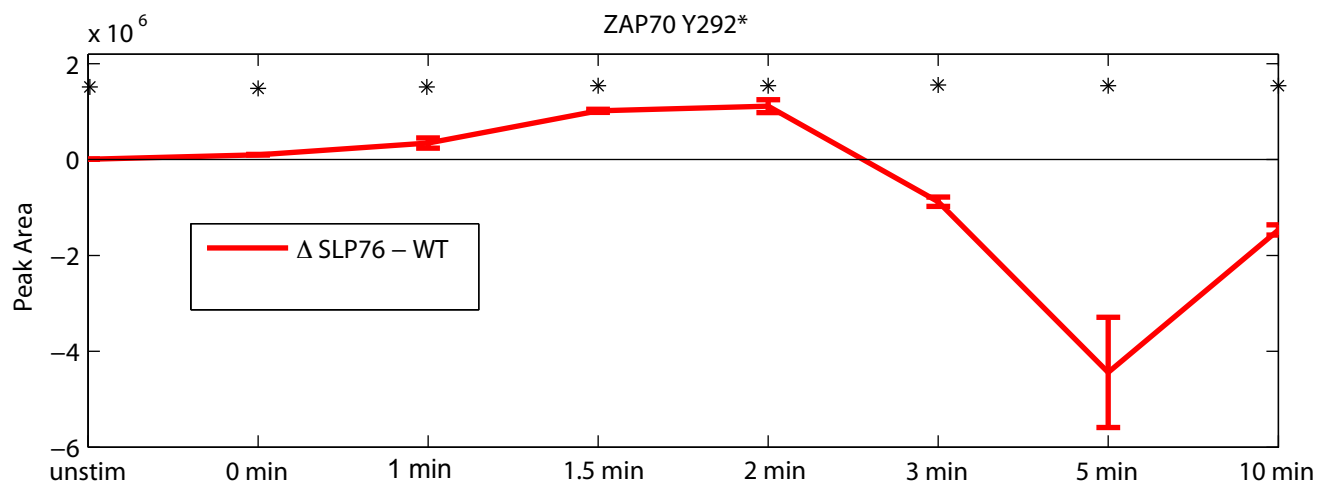

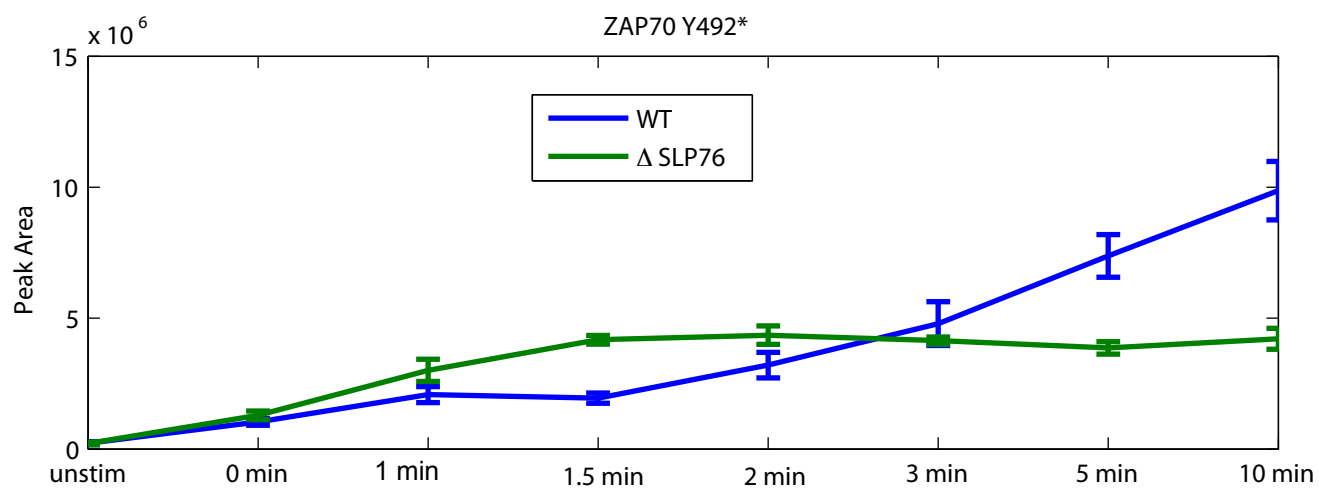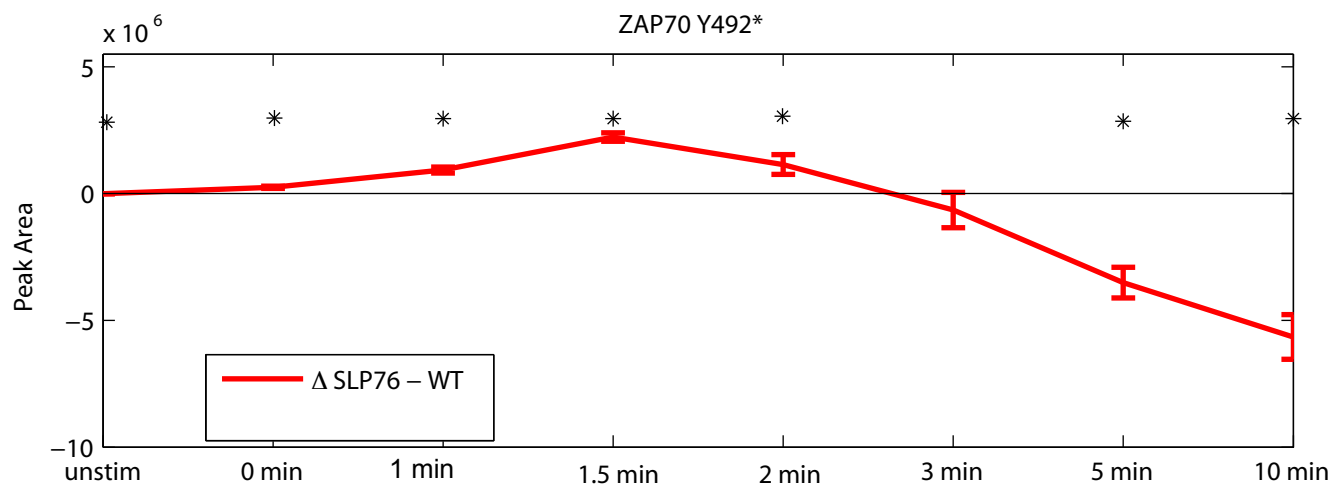

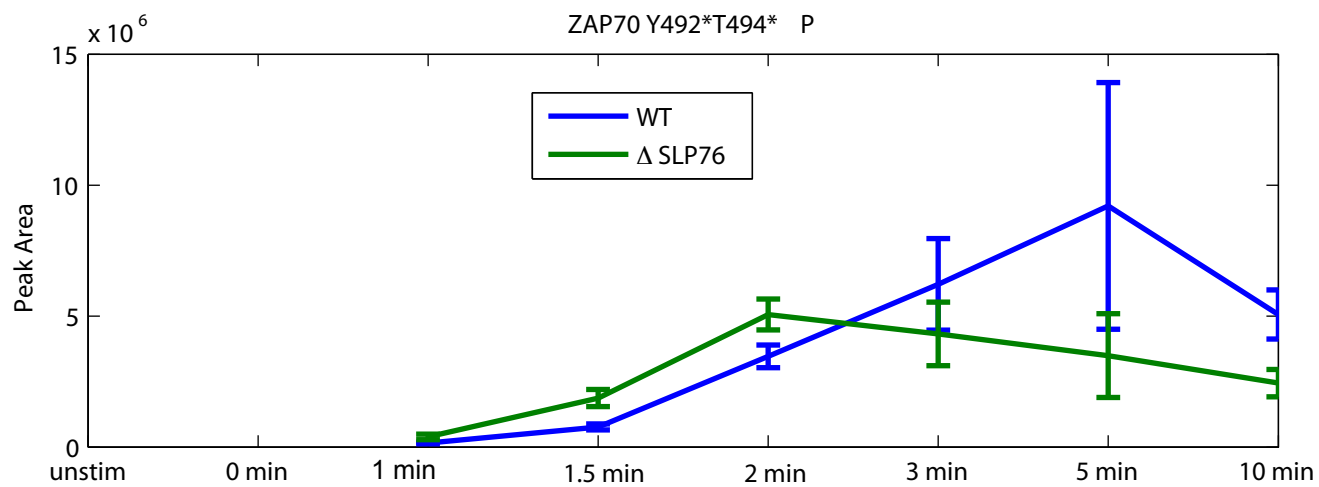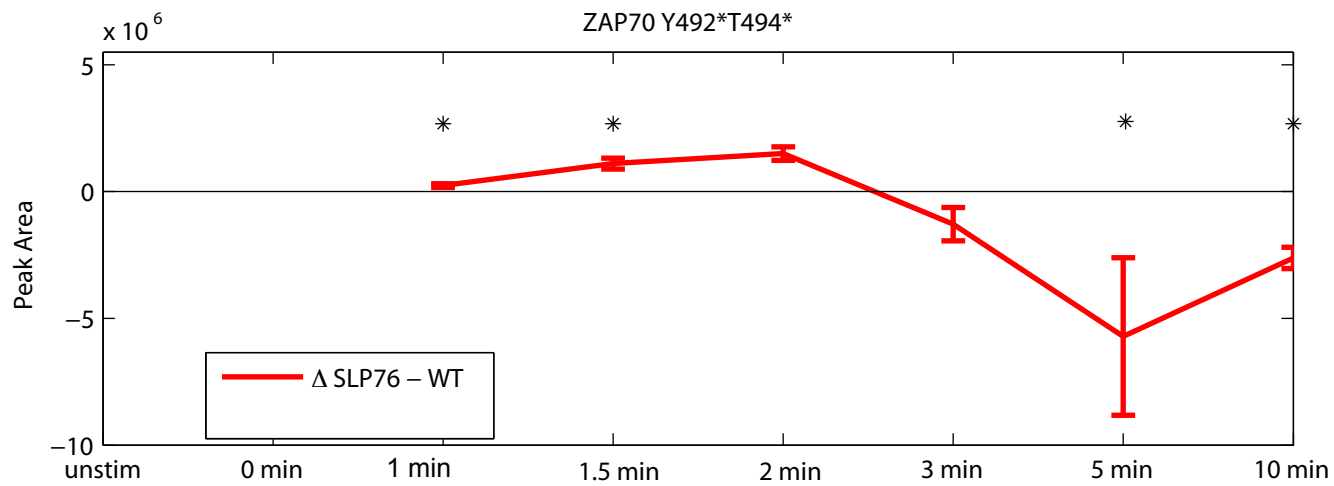

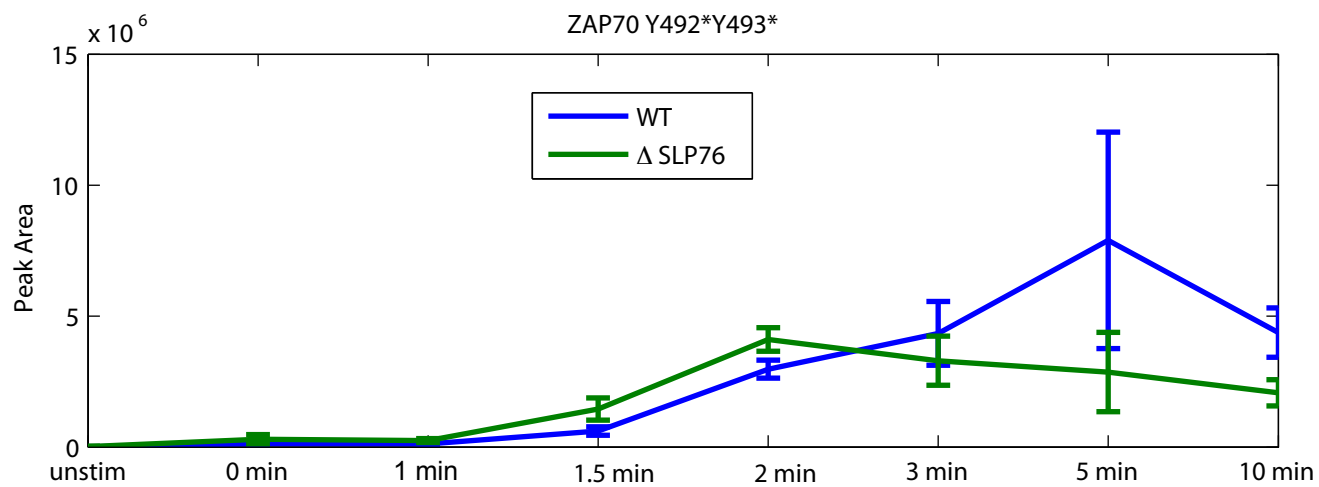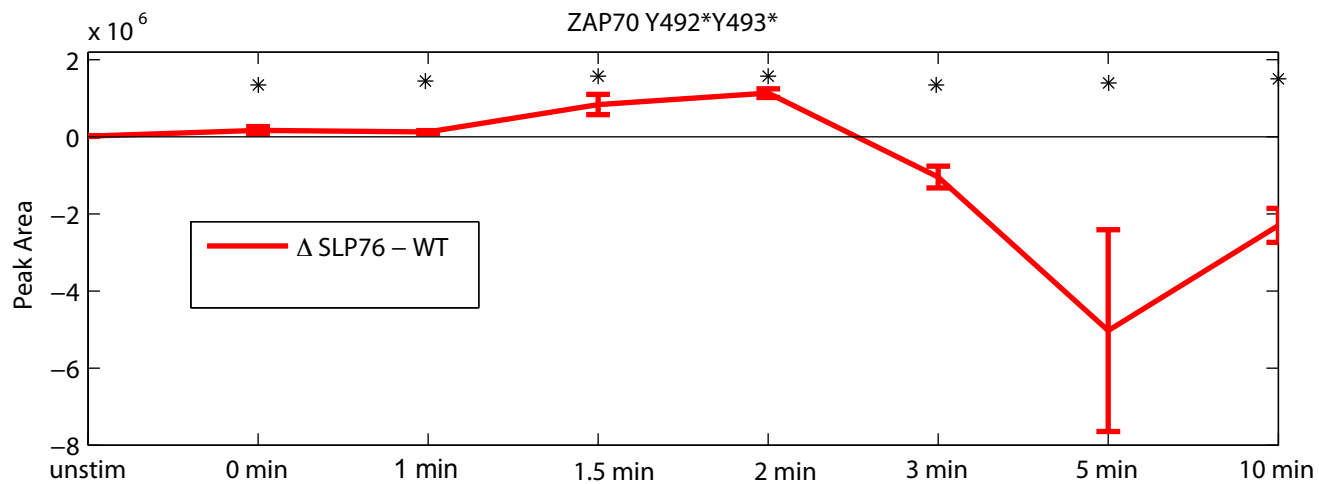

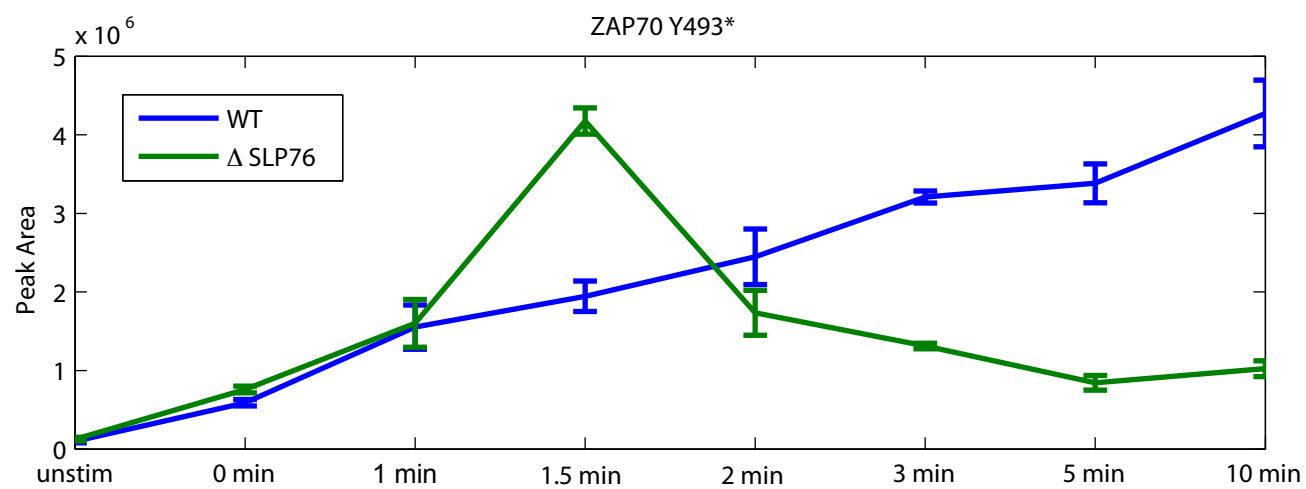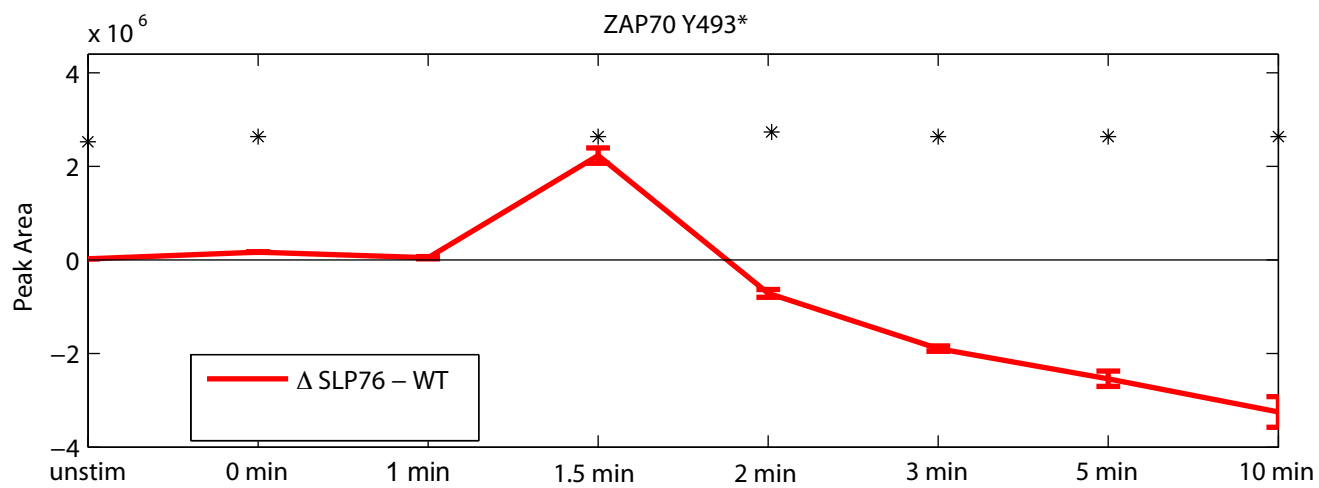

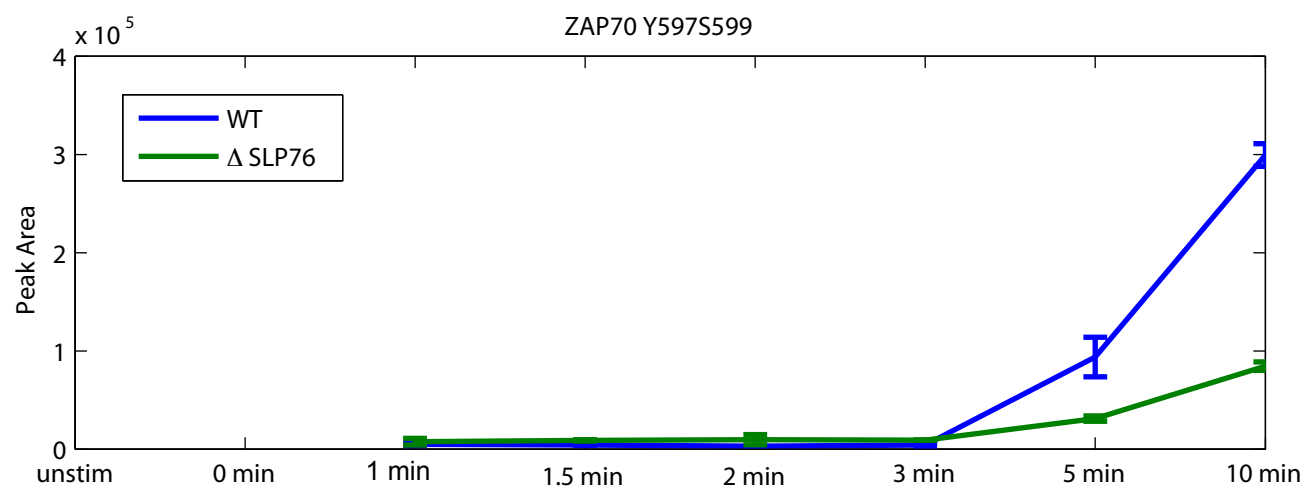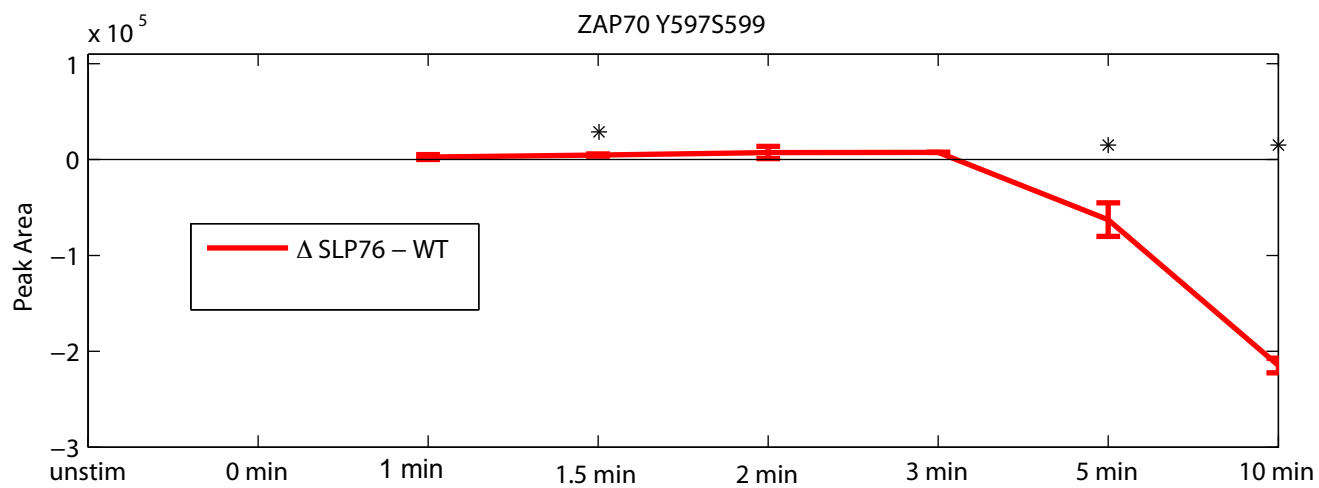

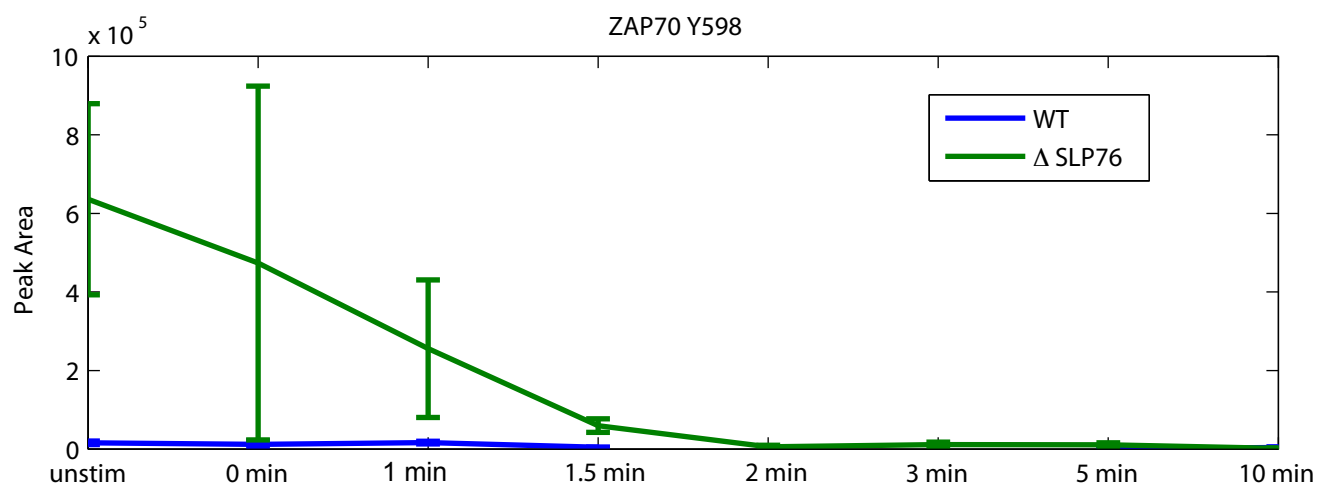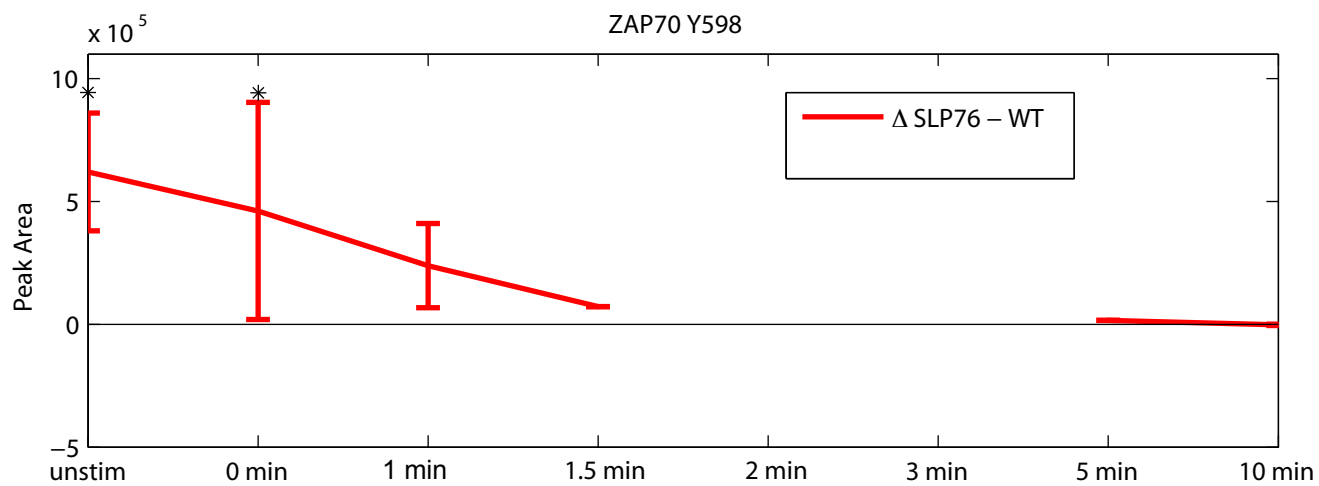

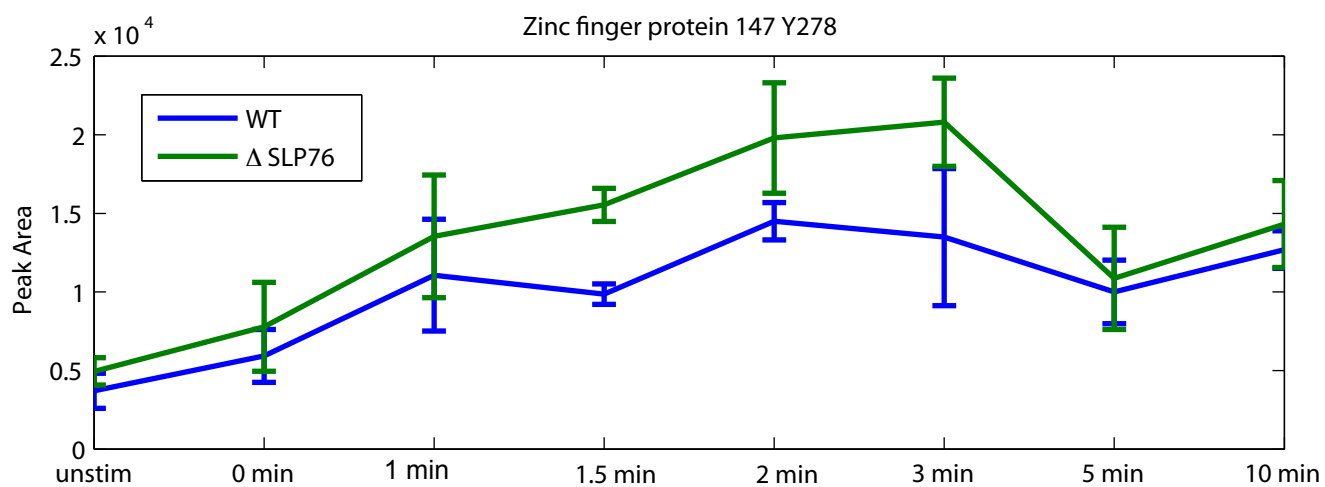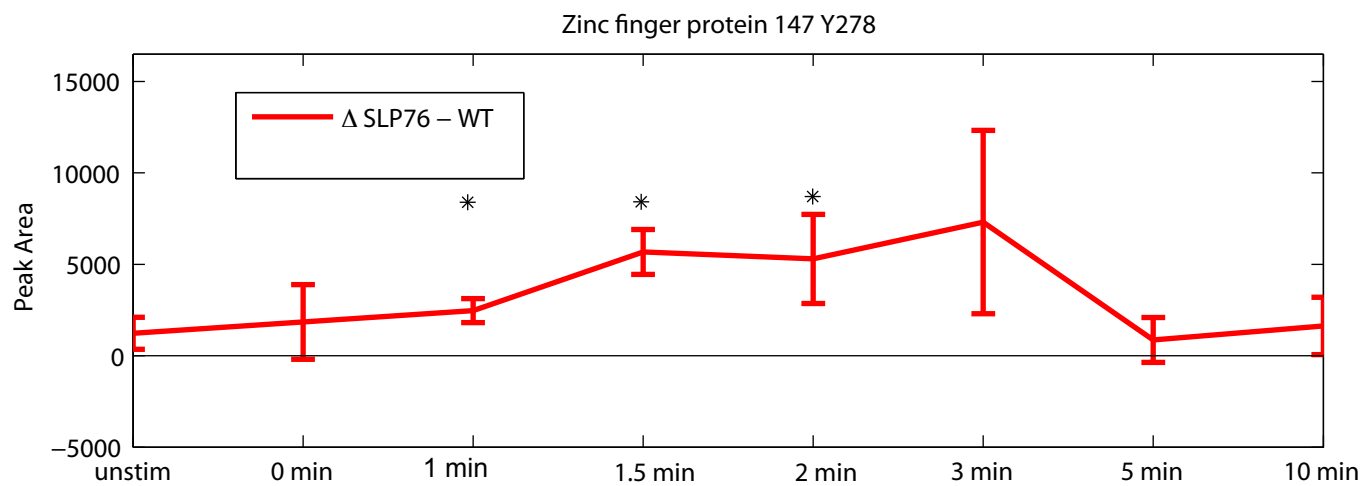

Supplement: Dataset S3 — Phosphorylation kinetics of all identified phosphopeptides in SLP-76 reconstituted and deficient Jurkat T cells. The differences of phosphorylations between SLP-76 reconstituted (WT) and deficient (ΔSLP76) cells are also presented as plots of ΔSLP76–WT across 8 timepoints to show the trend of phosphorylation changes. Results represent the means of three replicate experiments (error bars indicate standard deviation). “*” represents timepoints with false discovery rate less than 2% for significant changes in phosphorylation abundance between WT and ΔSLP76 cells. (PDF) [file pone.0046725.s003.pdf]

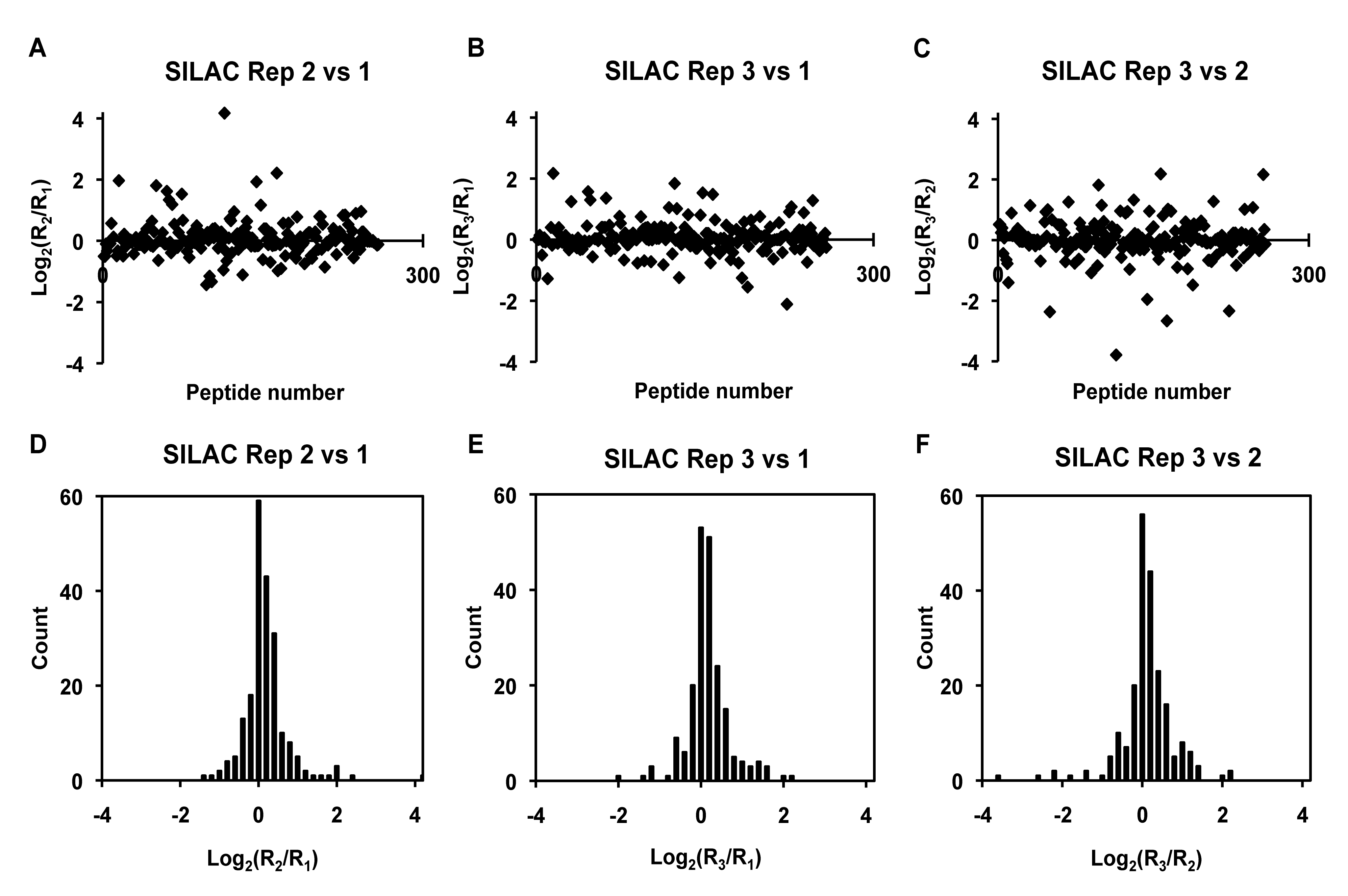

Supplement: Figure S1 — Variation assessment of SILAC ratios among three replicate experiments. The reproducibility of SILAC analysis among 3 replicate experiments was assessed by comparisons of three SILAC ratios (R1, R2, R3) at 2 min after TCR stimulation. Difference between SILAC ratios were represented using a scatter plot of observed phosphopeptides (A, B, C) and a histogram (D, E, F) of Log2 (R2/R1), Log2 (R3/R1), and Log2 (R3/R2) for each identified phosphopeptide. In good agreement of the expected value of 0, the measured average value of Log2 (R2/R1), Log2 (R3/R1), and Log2 (R3/R2) was 0.08±0.27, 0.01±0.18, and 0.01±0.46, respectively. Additionally, histogram plots showed that D) 85.2%, E) 82.2%, and F) 86.8% identified phosphopeptides displayed Log2 (R2/R1), Log2 (R3/R1), and Log2 (R3/R2) values respectively in the range of −0.6∼0.6. (TIF) [file pone.0046725.s004.tif]

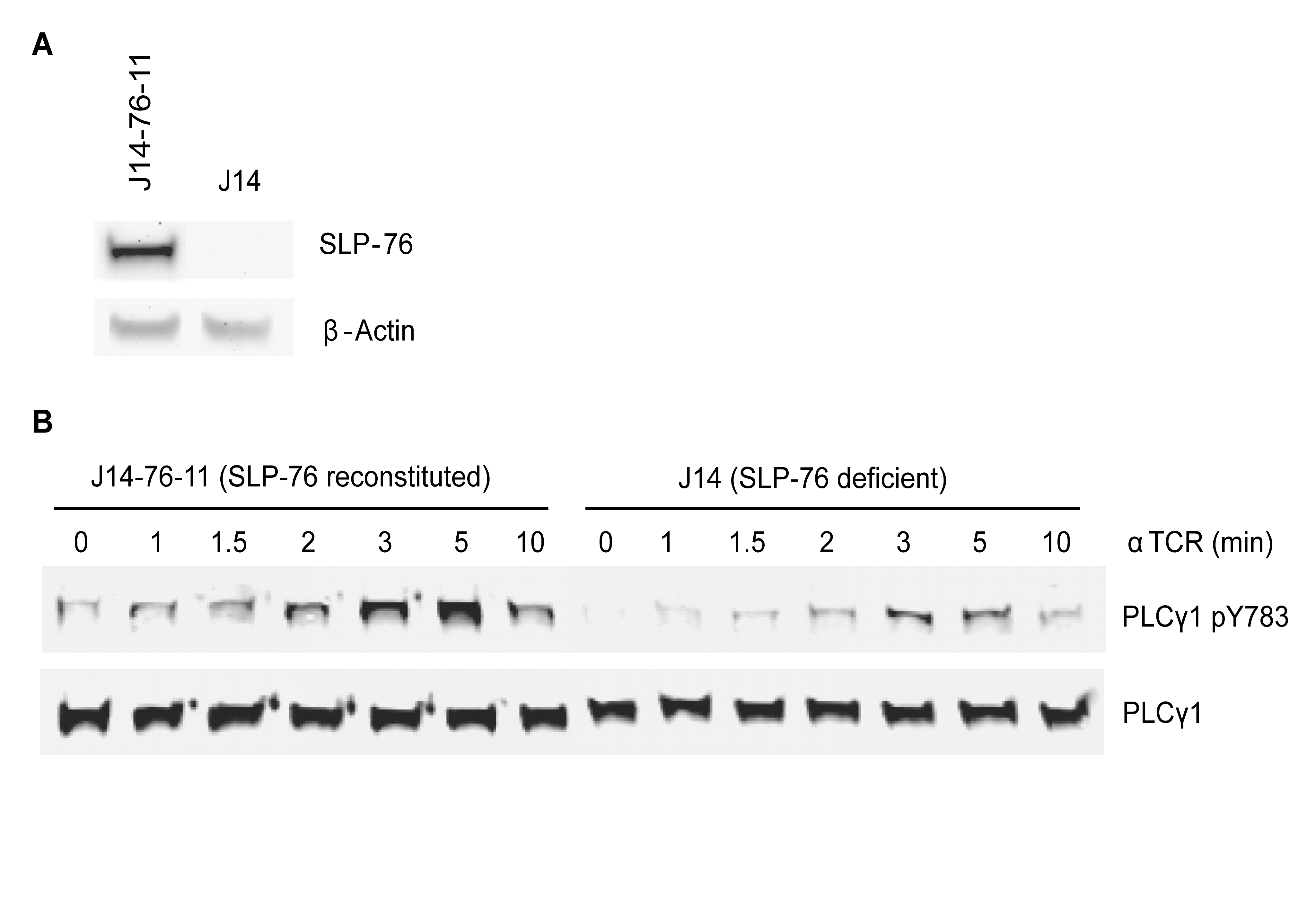

Supplement: Figure S2 — Disruption of SLP-76 from Jurkat T cells and phosphorylation of PLCγ1. A) SLP-76 expression in Jurkat T cells. Protein lysates from J14-76-11 (SLP-76 reconstituted) and J14 (SLP-76 deficient) were separated by SDS-PAGE and immunoblotted with SLP-76 specific antibodies. B) Effect of SLP-76 on PLCγ1 phosphorylation in OKT3/OKT4 stimulated Jurkat T cells. Lysates were prepared from J14-76-11 (SLP-76 reconstituted) and J14 (SLP-76 deficient) cells following TCR stimulation for the indicated time periods. Total cell lysates were probed by protein immunoblotting with anti-phospho-PLCγ1 (Y783) (top) and anti-PLCγ1 (bottom). (TIF) [file pone.0046725.s005.tif]

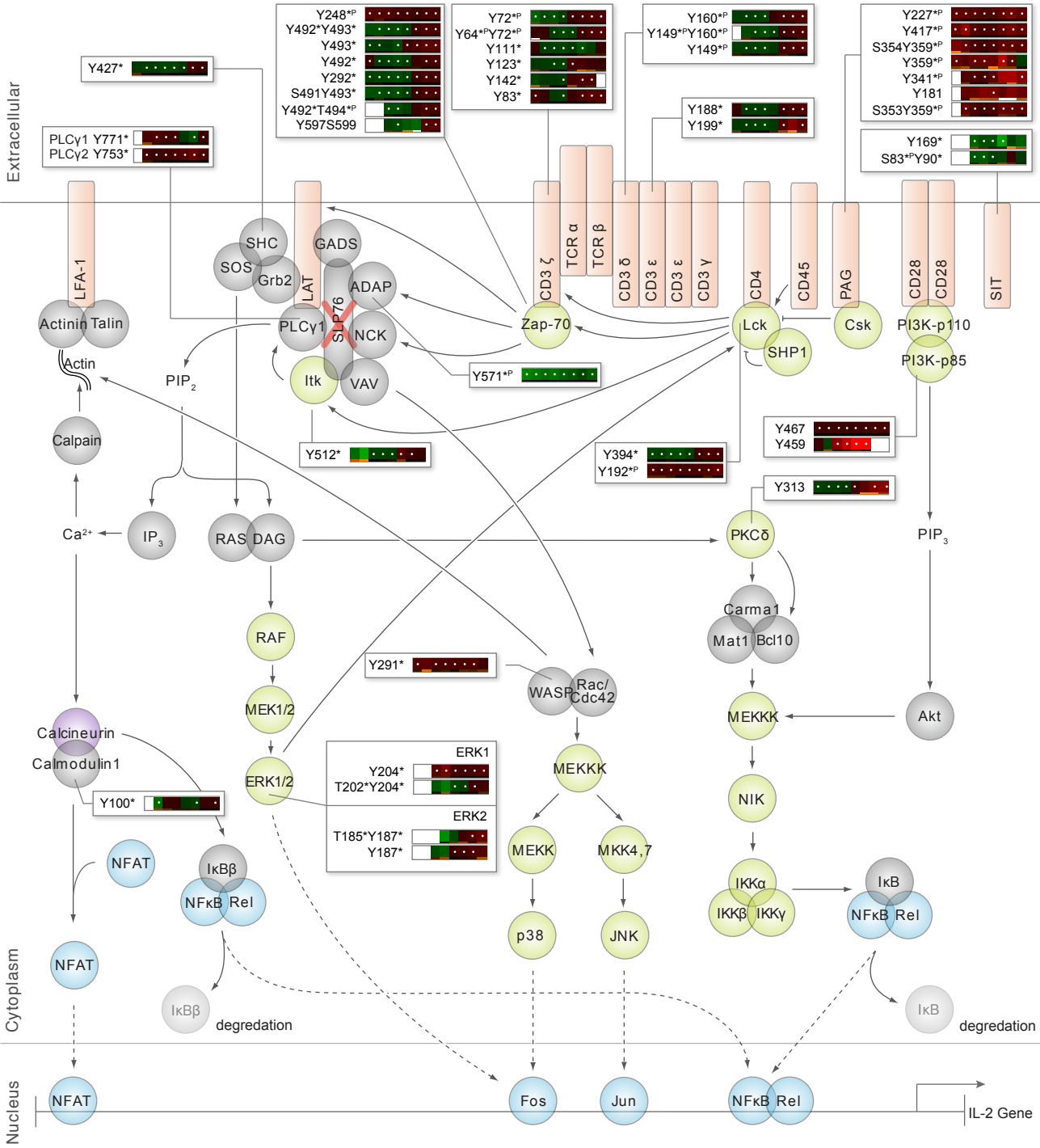

Supplement: Figure S3 — Canonical TCR signaling pathway. Established signaling cascades in activated T cells with quantitative SILAC ratios (SLP-76 deficient in relative to SLP-76 reconstituted) represented as SILAC heatmaps beside individual proteins. SILAC ratios between J14 and J14-76-11 at each of the TCR stimulation timepoints were represented as SILAC heatmaps as described in methods. (PDF) [file pone.0046725.s006.pdf]
