# Supplementary material for: Quantitative Phosphoproteomics Reveals SLP-76 Dependent Regulation of PAG and Src Family Kinases in T Cells
Source: PLoS One. 2012 Oct 11;7(10):e46725. doi: 10.1371/journal.pone.0046725 (PMC3469622; doi:10.1371/journal.pone.0046725)
Supplement: Dataset S2 — Assigned MS/MS spectra for all identified phosphopeptides in this analysis. (PDF) [file pone.0046725.s002.pdf]

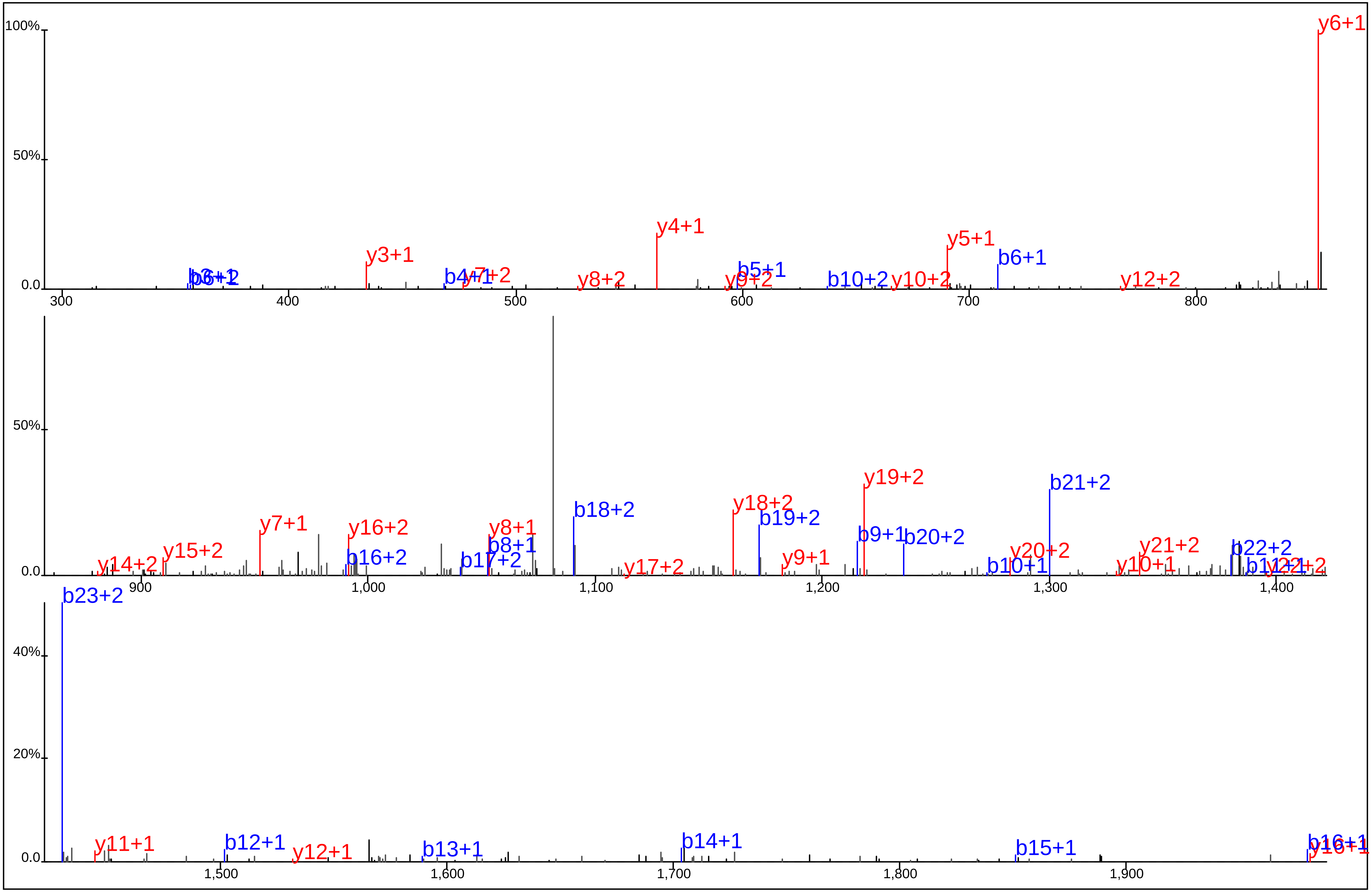

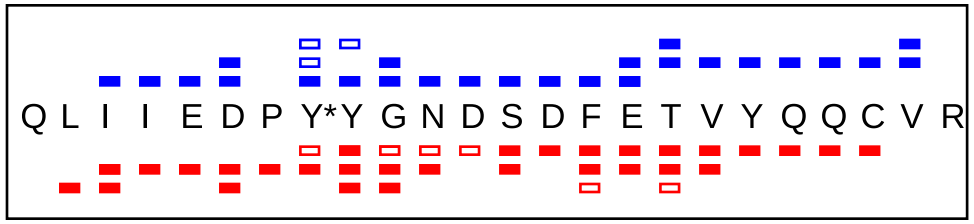

Protein Name: Acid phosphatase 1
gi Accession: 4757714
Precursor Mass: 1,011.4417+3
Delta Mass: 2.786 ppm
XCorr: 60.25

1 - Acidphosphatase1_3433823.pdf

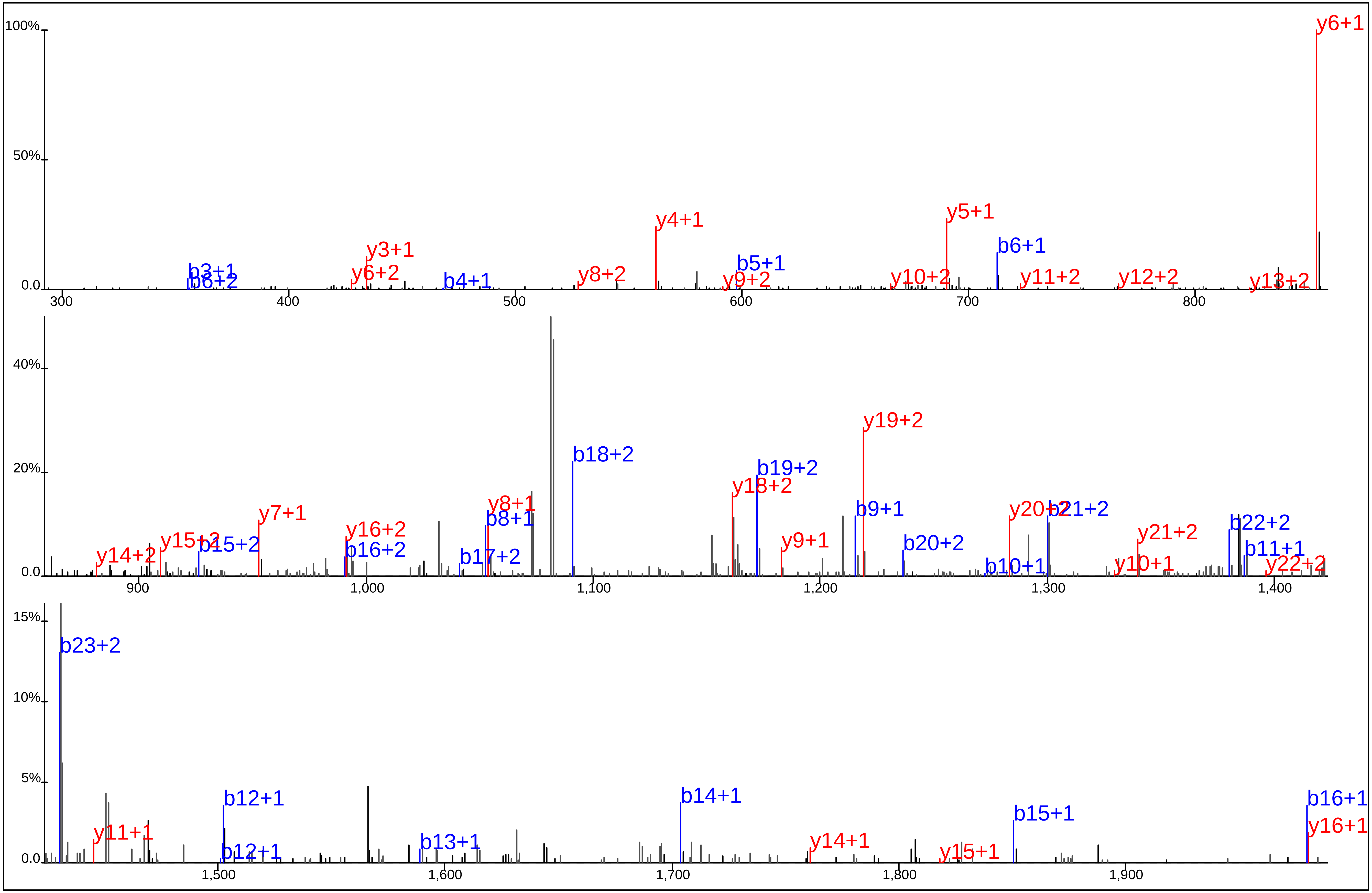

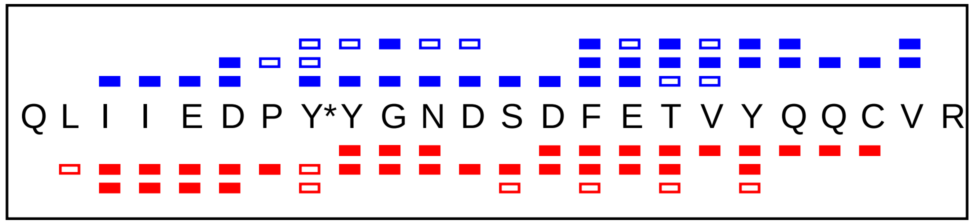

Protein Name: Acid phosphatase 1
gi Accession: 4757714
Precursor Mass: 1,011.4408+3
Delta Mass: 1.938 ppm
XCorr: 61.27

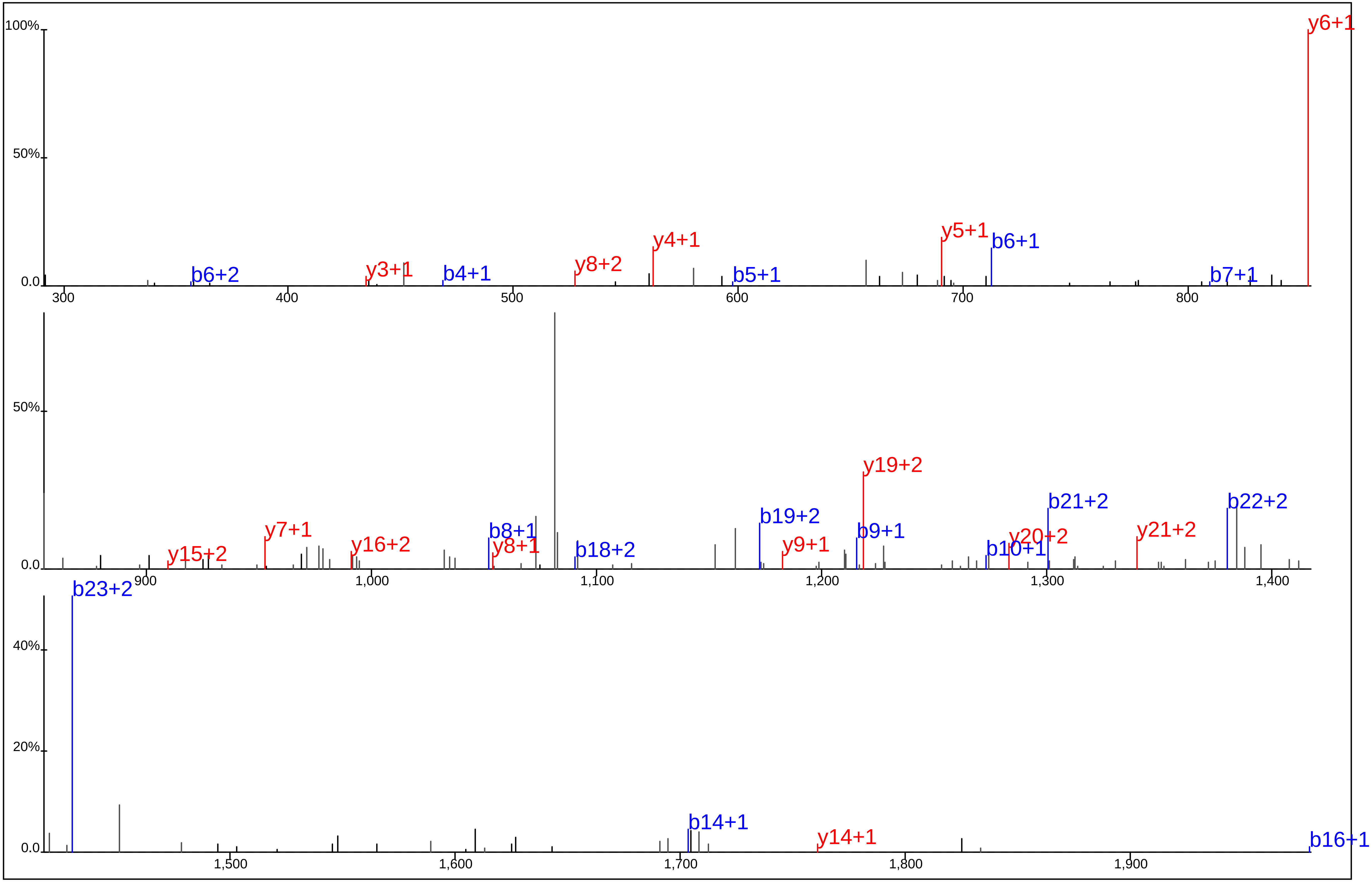

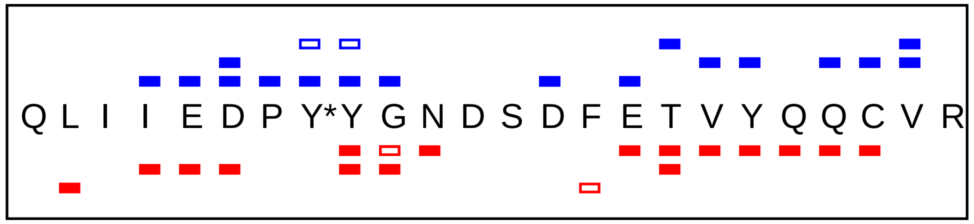

Protein Name: Acid phosphatase 1
gi Accession: 4757714
Precursor Mass: 1,011.4439+3
Delta Mass: 5.018 ppm
XCorr: 36.5

3 - Acidphosphatase1_3439454.pdf

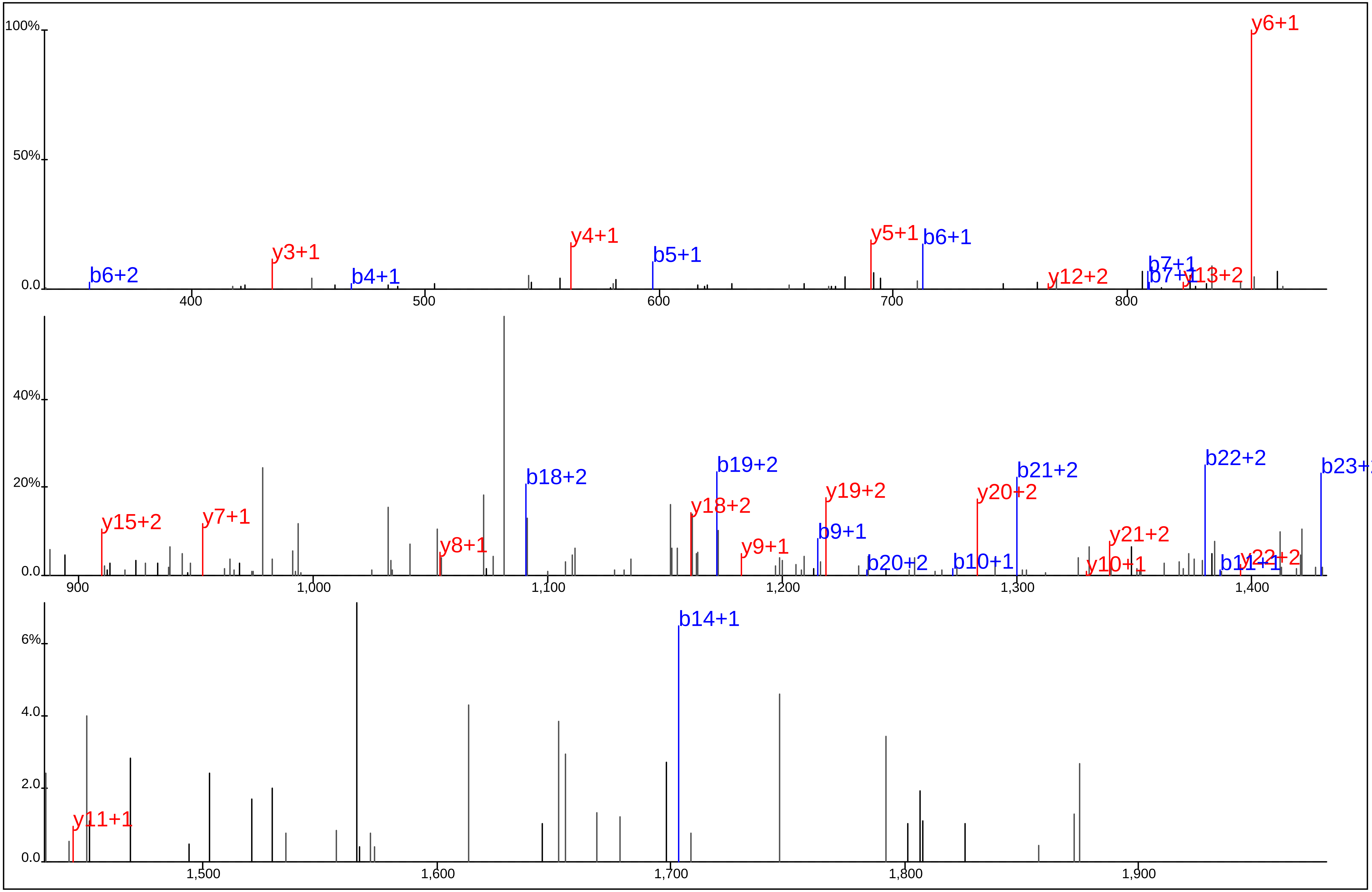

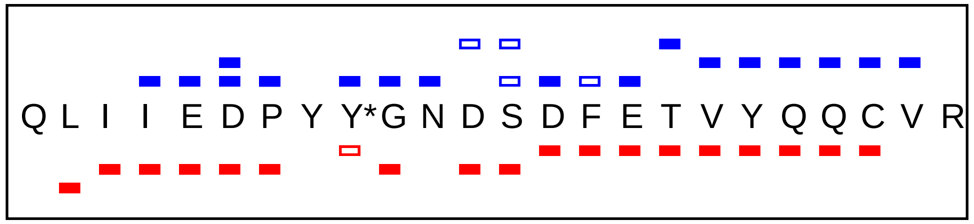

Protein Name:	Acid phosphatase 1
gi Accession:	4757714
Precursor Mass:	1,011.4426+3
Delta Mass:	3.752 ppm
XCorr:	43.2

4 - Acidphosphatase1_3441941.pdf

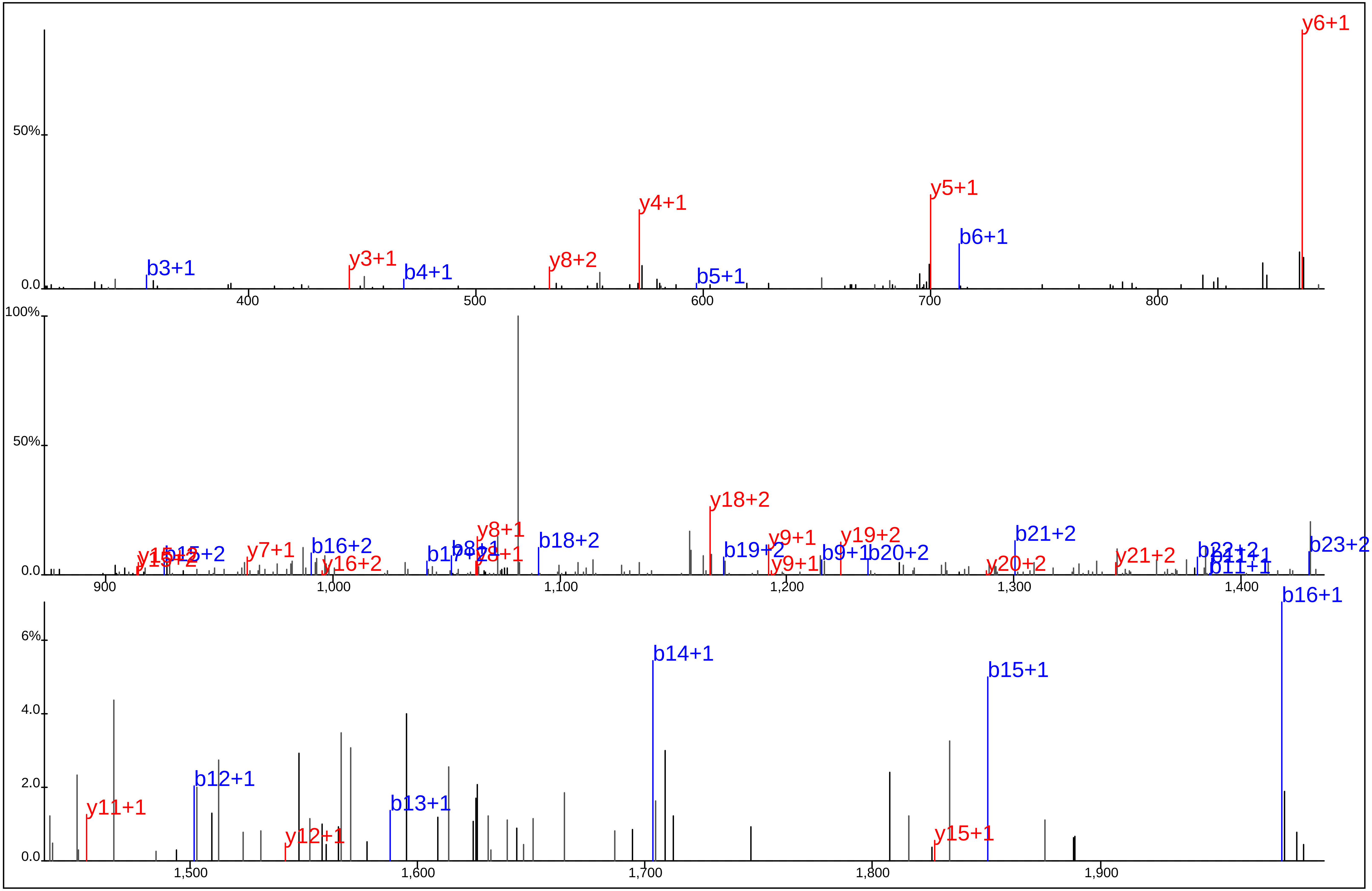

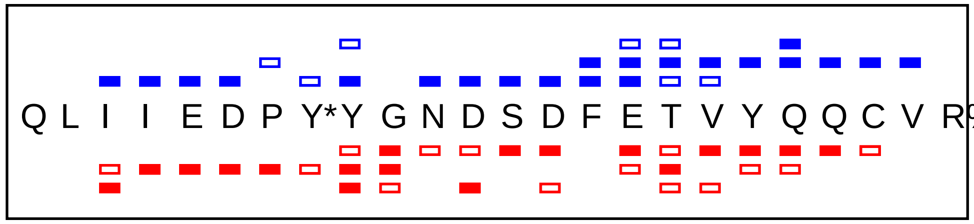

Protein Name:	Acid phosphatase 1
gi Accession:	4757714
Precursor Mass:	1,014.7791+3
Delta Mass:	4.069 ppm
XCorr:	45.41

5 - Acidphosphatase1_3442525.pdf

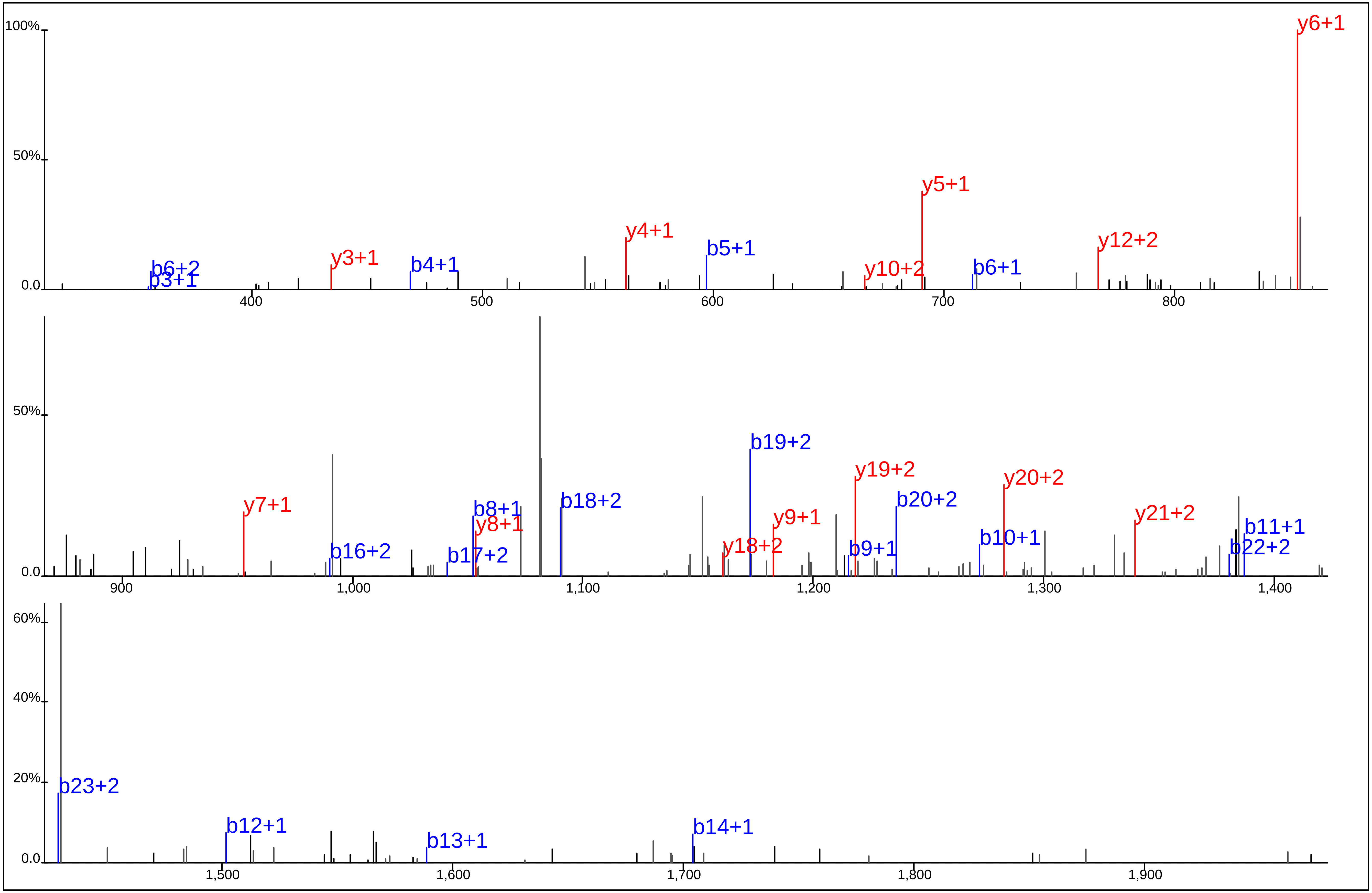

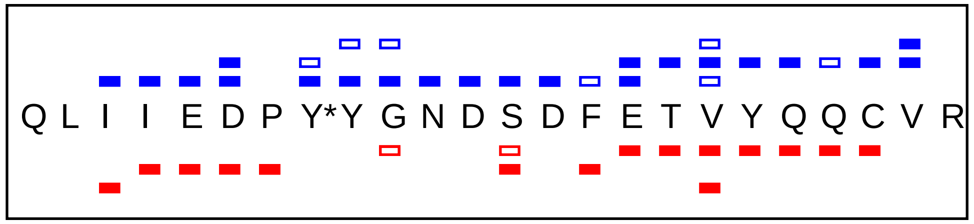

Protein Name: Acid phosphatase 1
gi Accession: 4757714
Precursor Mass: 1,011.4423+3
Delta Mass: 3.449 ppm
XCorr: 49.01

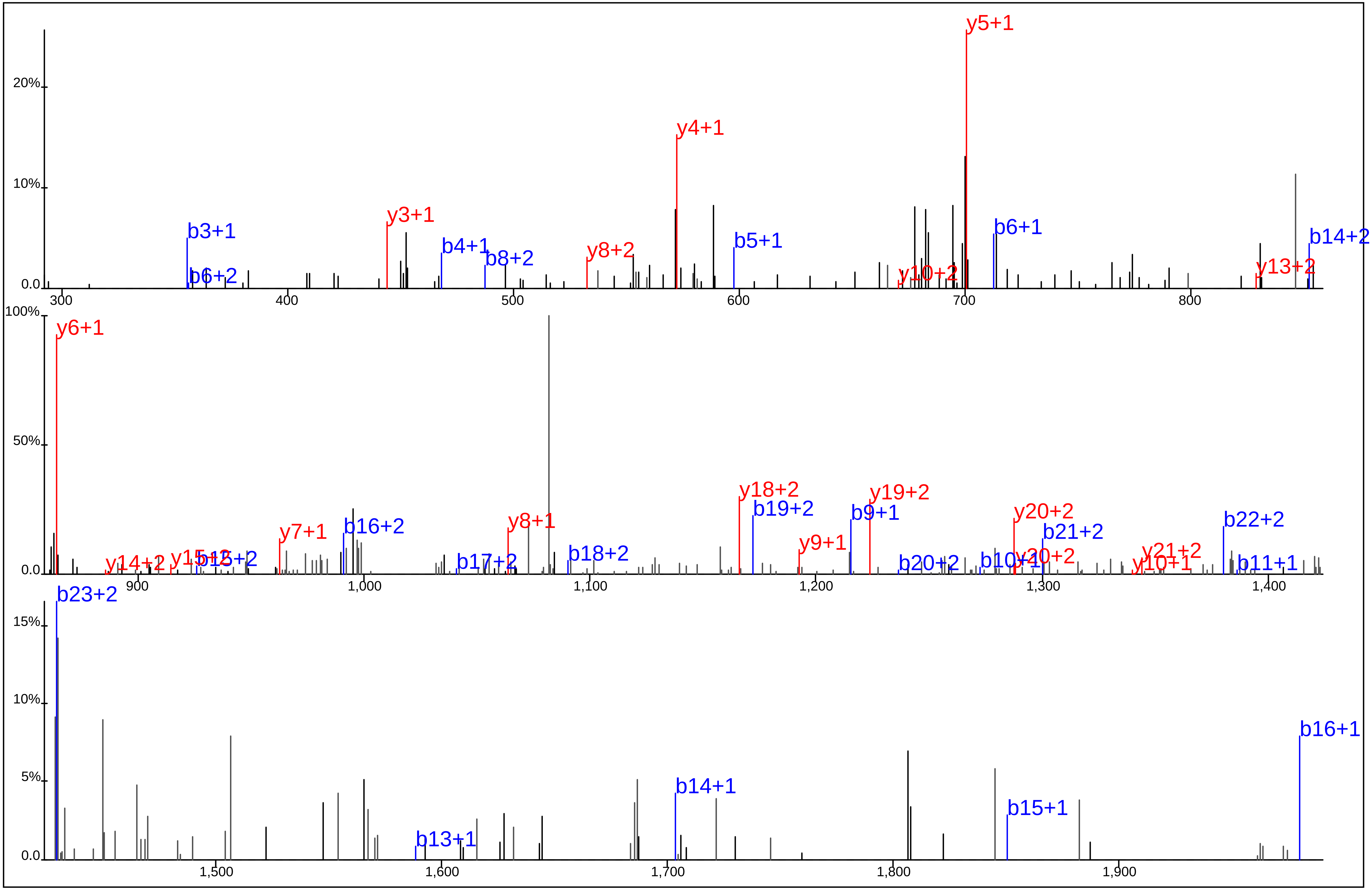

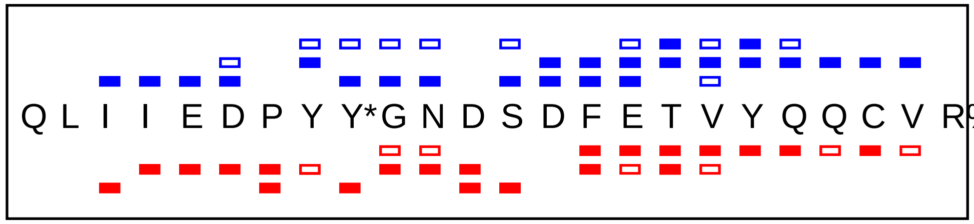

Protein Name:	Acid phosphatase 1
gi Accession:	4757714
Precursor Mass:	1,014.7775+3
Delta Mass:	2.566 ppm
XCorr:	34.77

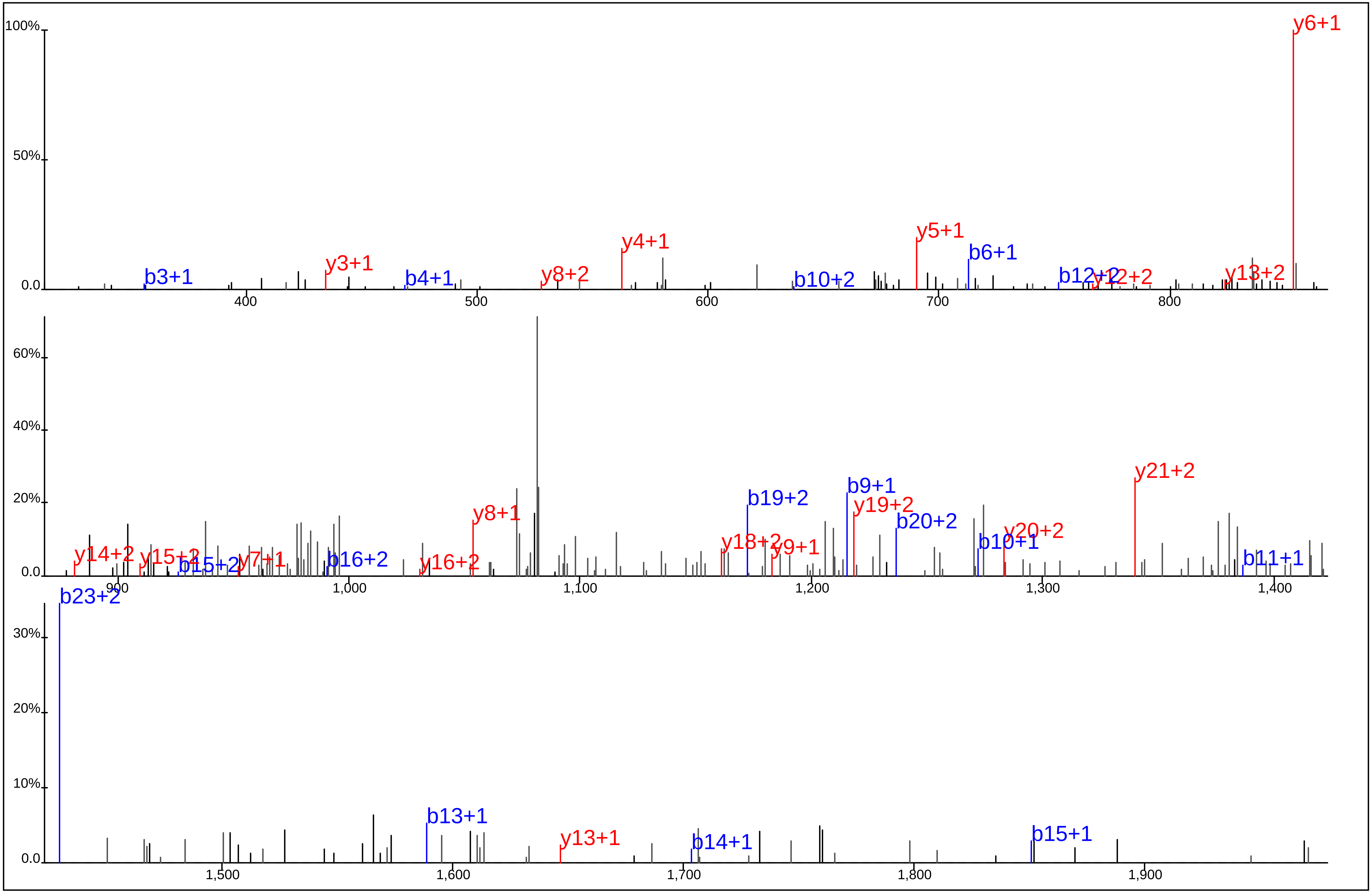

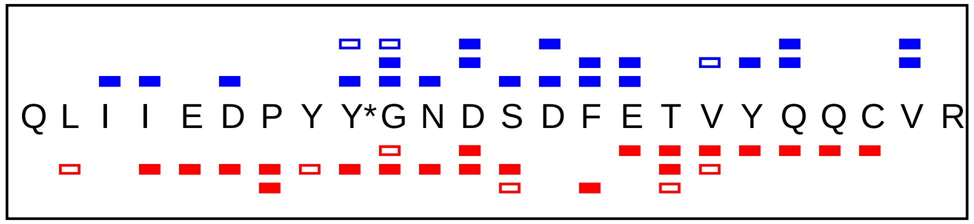

Protein Name: Acid phosphatase 1
gi Accession: 4757714
Precursor Mass: 1,011.4414+3
Delta Mass: 2.545 ppm
XCorr: 16.4

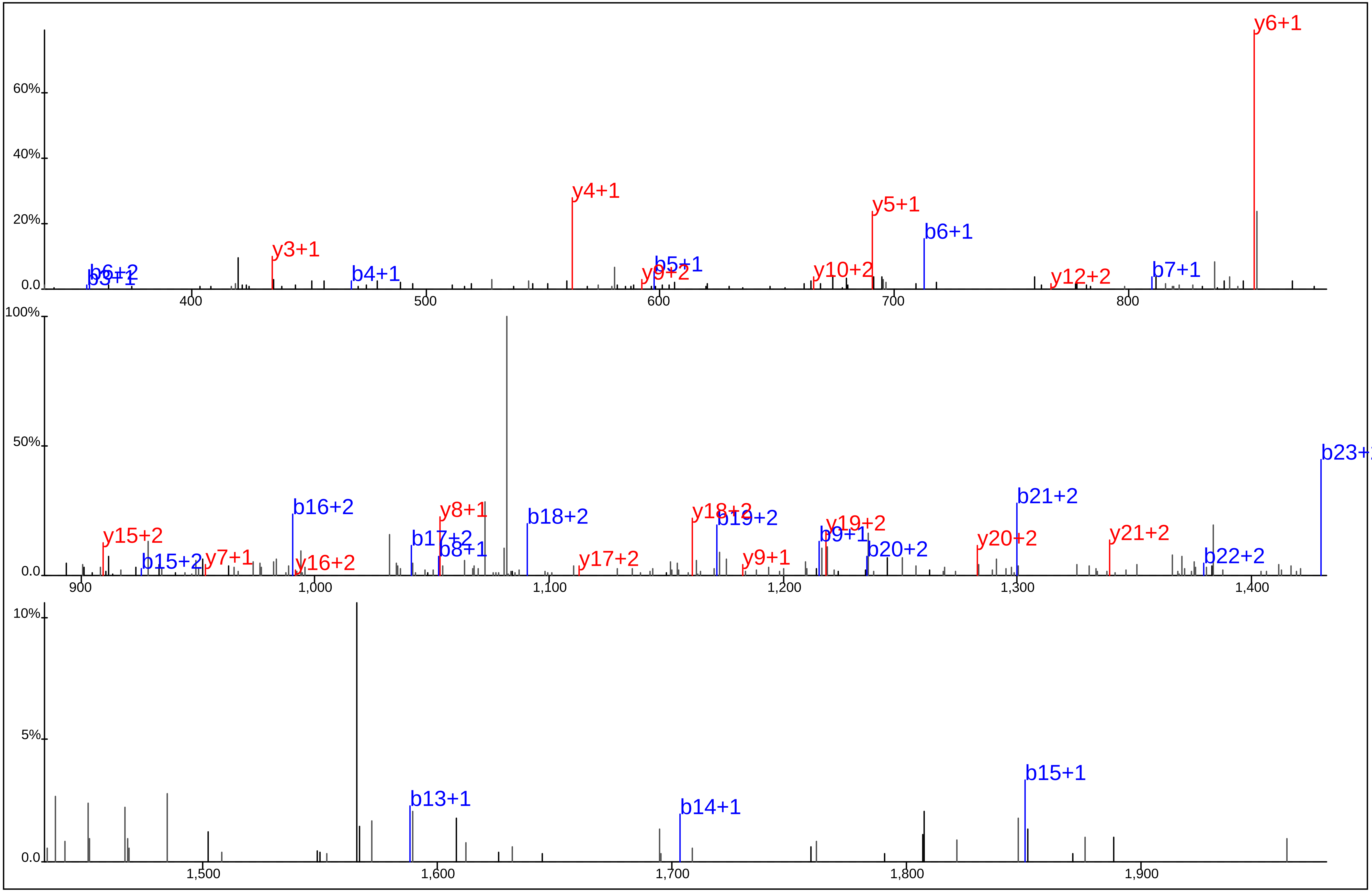

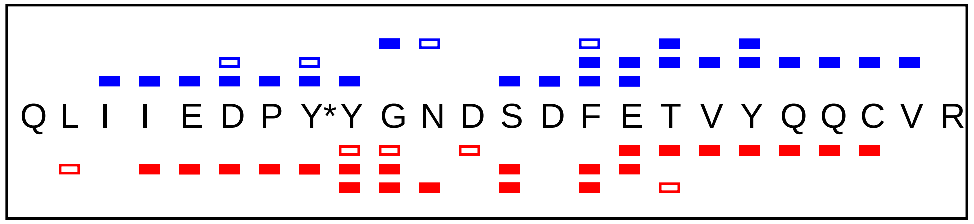

Protein Name:	Acid phosphatase 1
gi Accession:	4757714
Precursor Mass:	1,011.4465+3
Delta Mass:	7.617 ppm
XCorr:	45.31

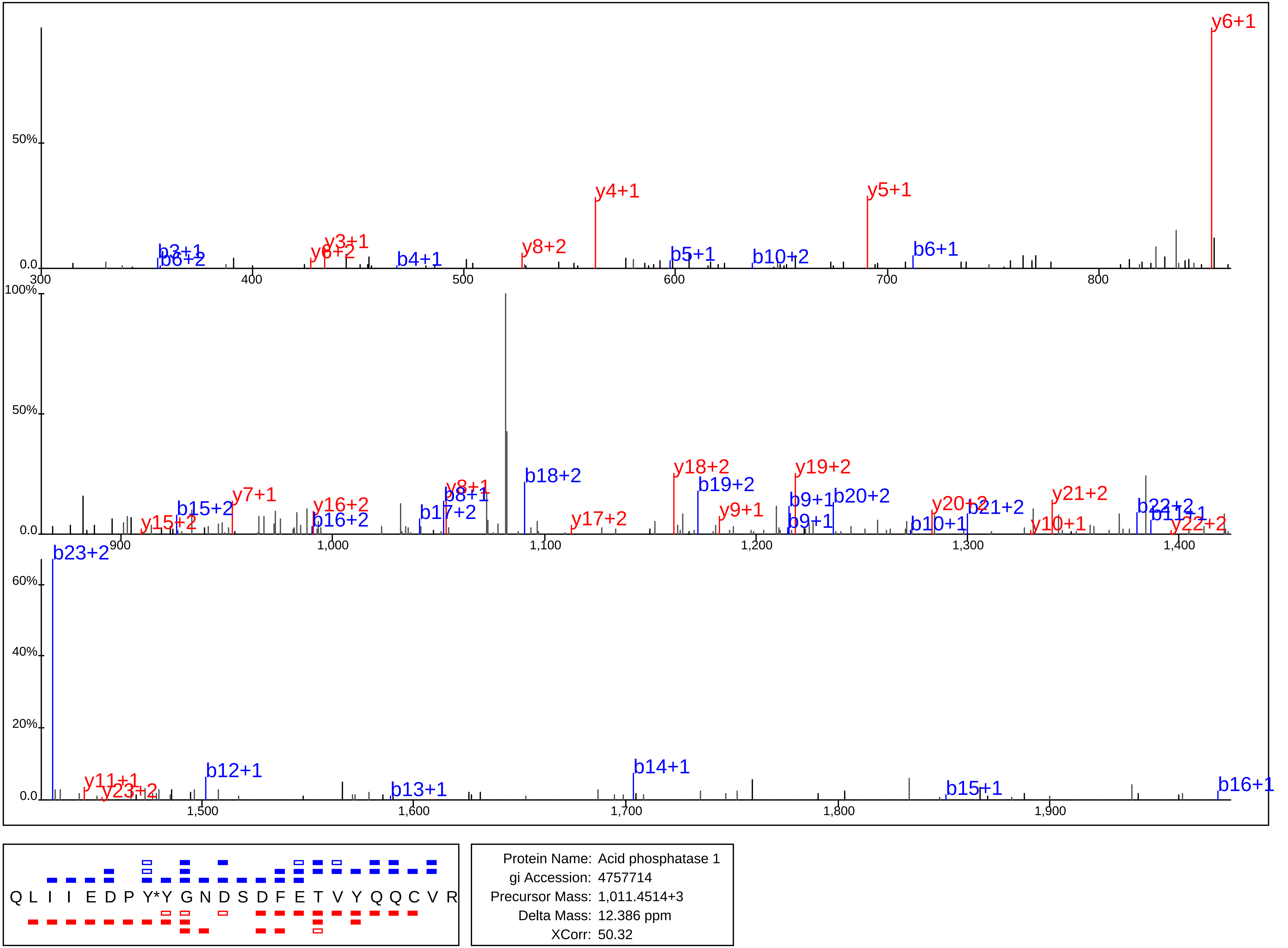

10 - Acidphosphatase1_3453068.pdf

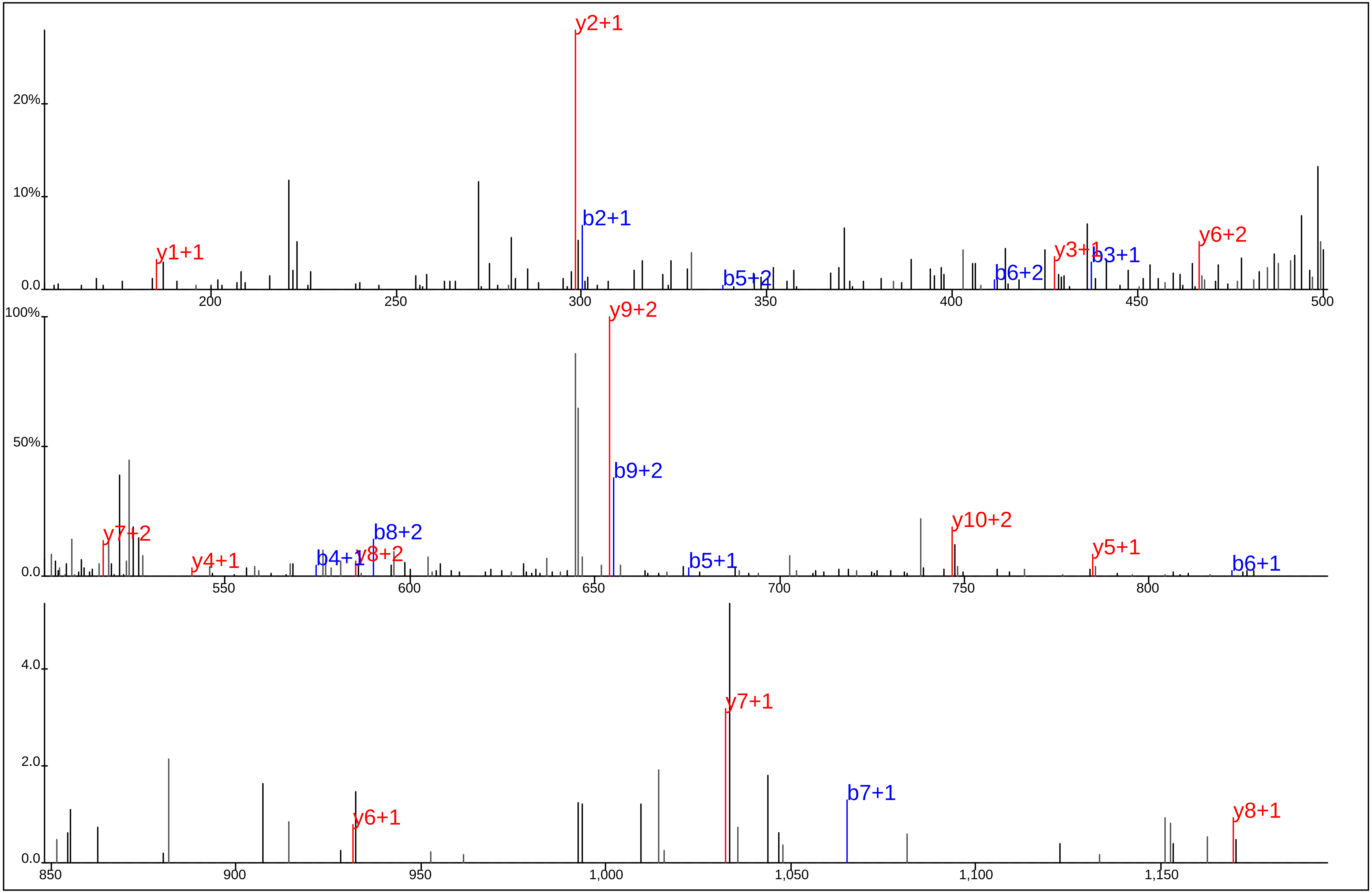

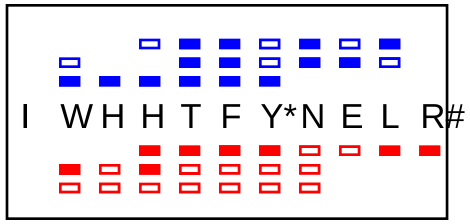

Protein Name: Actin
gi Accession: 7245526
Precursor Mass: 535.9132+3
Delta Mass: 0.816 ppm
XCorr: 3.1026

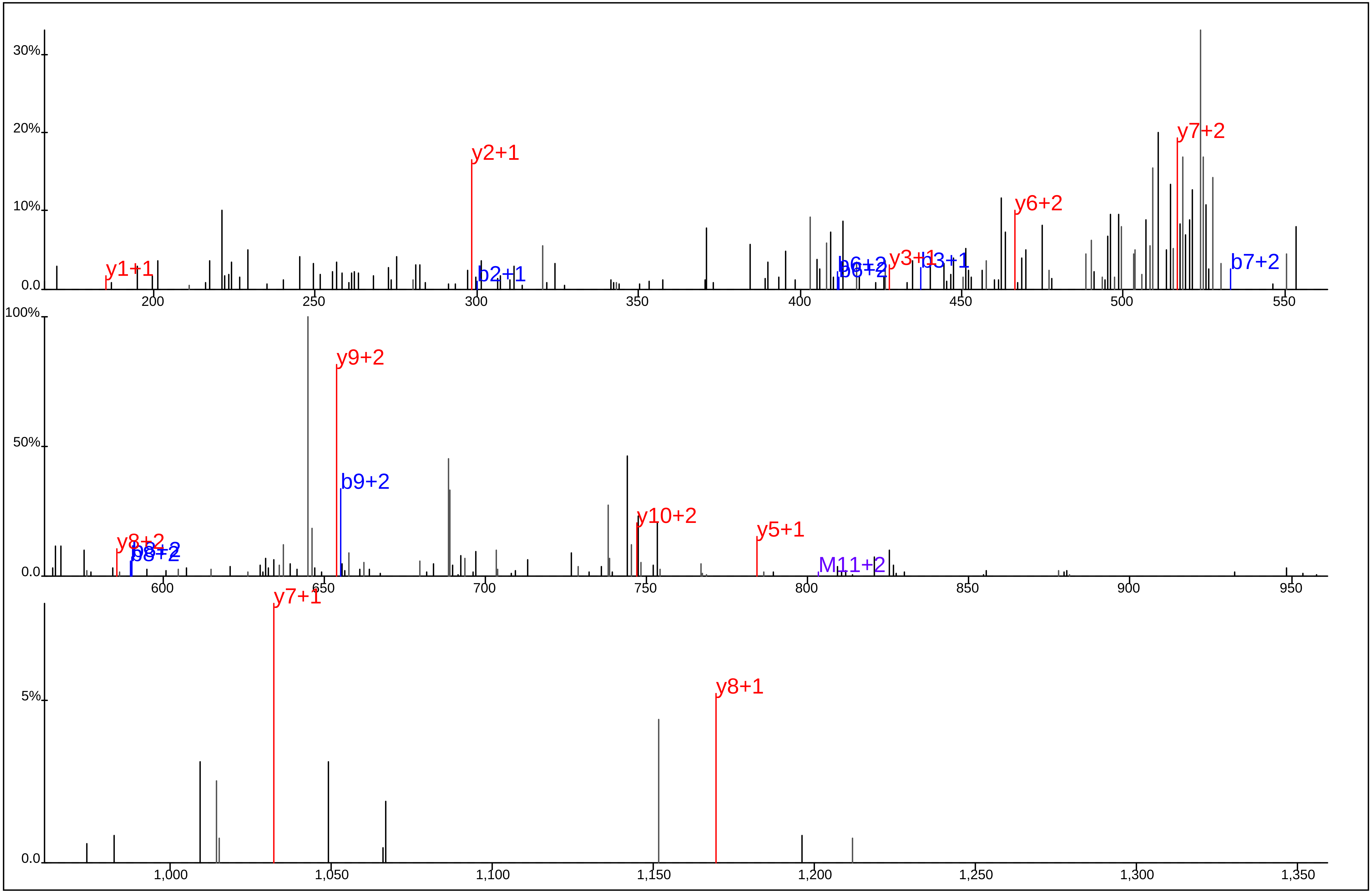

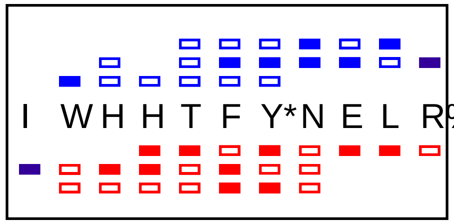

Protein Name: Actin
gi Accession: 7245526
Precursor Mass: 535.9150+3
Delta Mass: 4.245 ppm
XCorr: 16.37

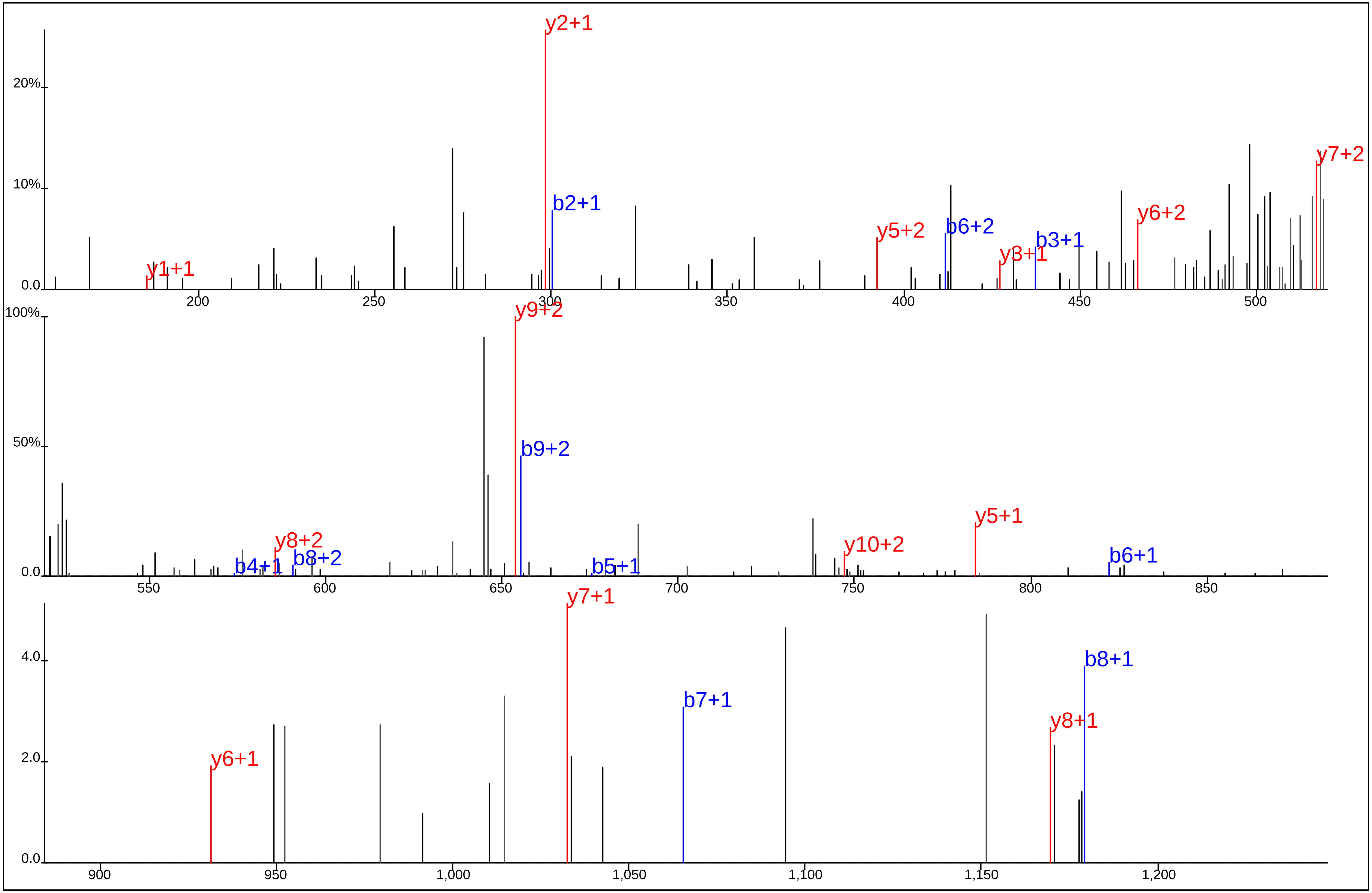

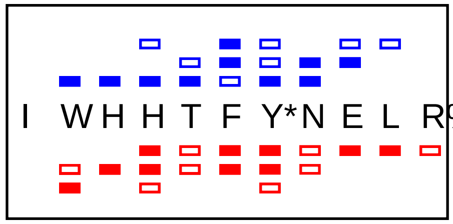

Protein Name: Actin
gi Accession: 7245526
Precursor Mass: 535.9147+3
Delta Mass: 3.679 ppm
XCorr: 20.89

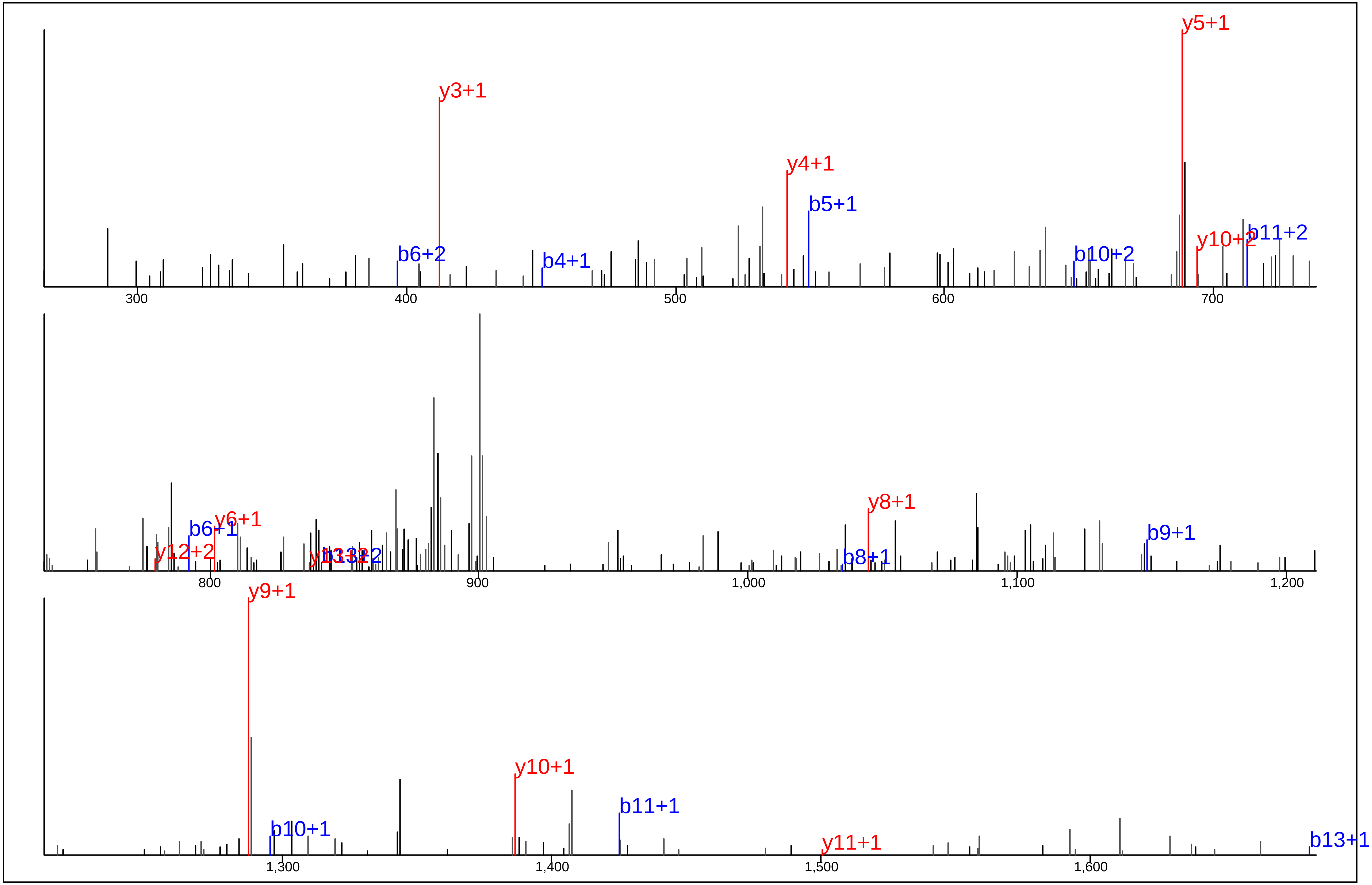

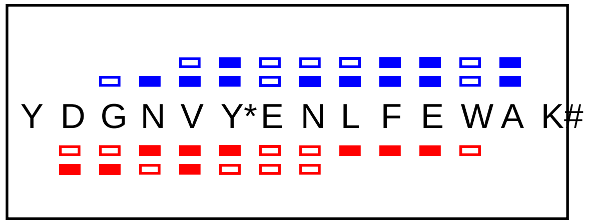

Protein Name:	Acyl-CoA oxidase
gi Accession:	119609755
Precursor Mass:	918.3974+2
Delta Mass:	5.877 ppm
XCorr:	24.12

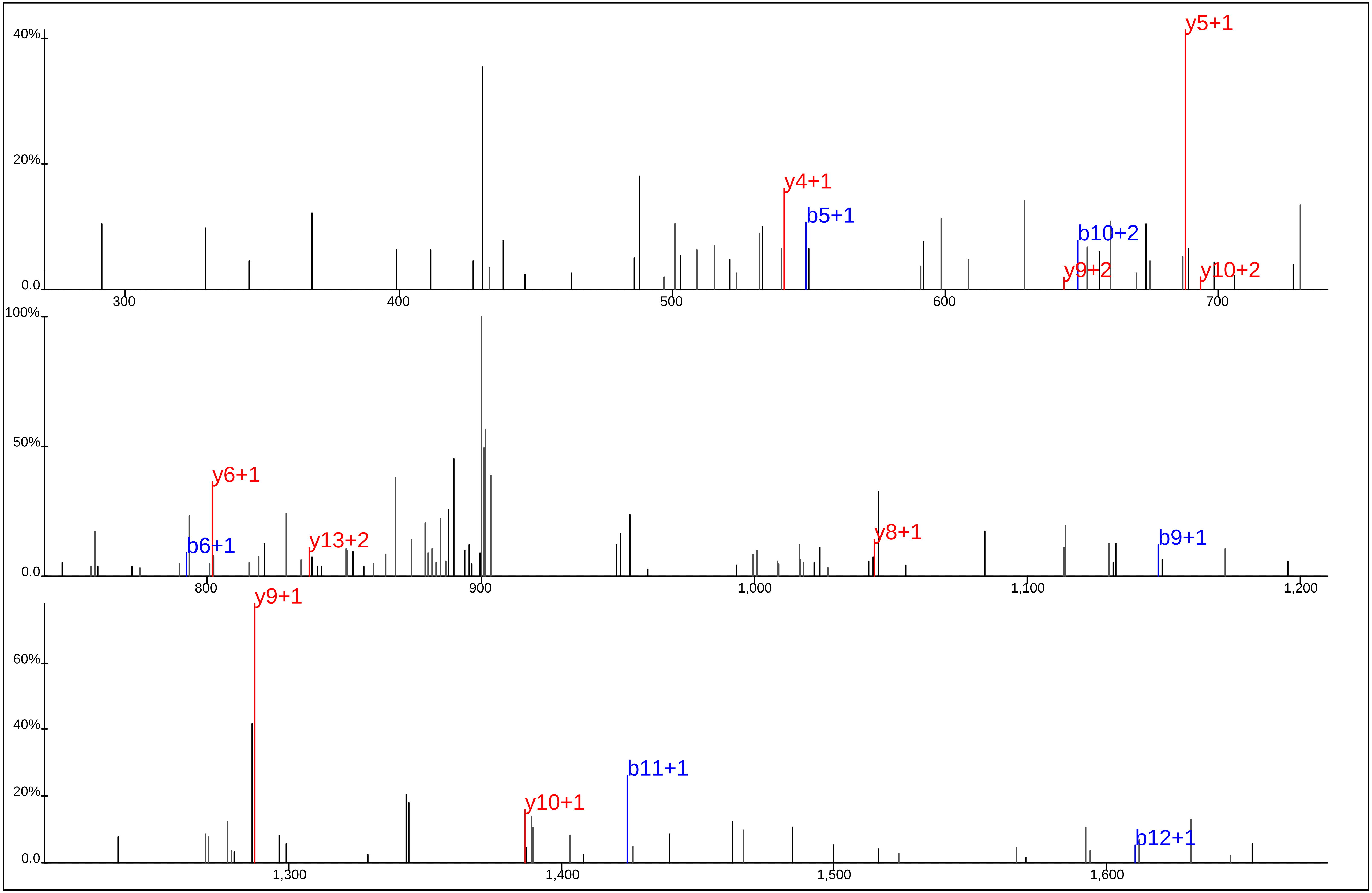

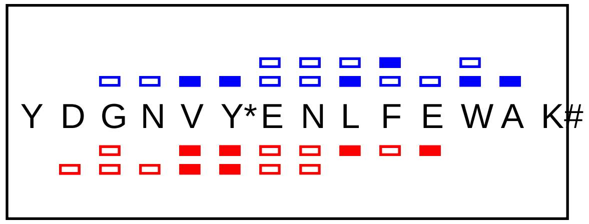

Protein Name:	Acyl-CoA oxidase
gi Accession:	119609755
Precursor Mass:	918.3952+2
Delta Mass:	3.485 ppm
XCorr:	16.39

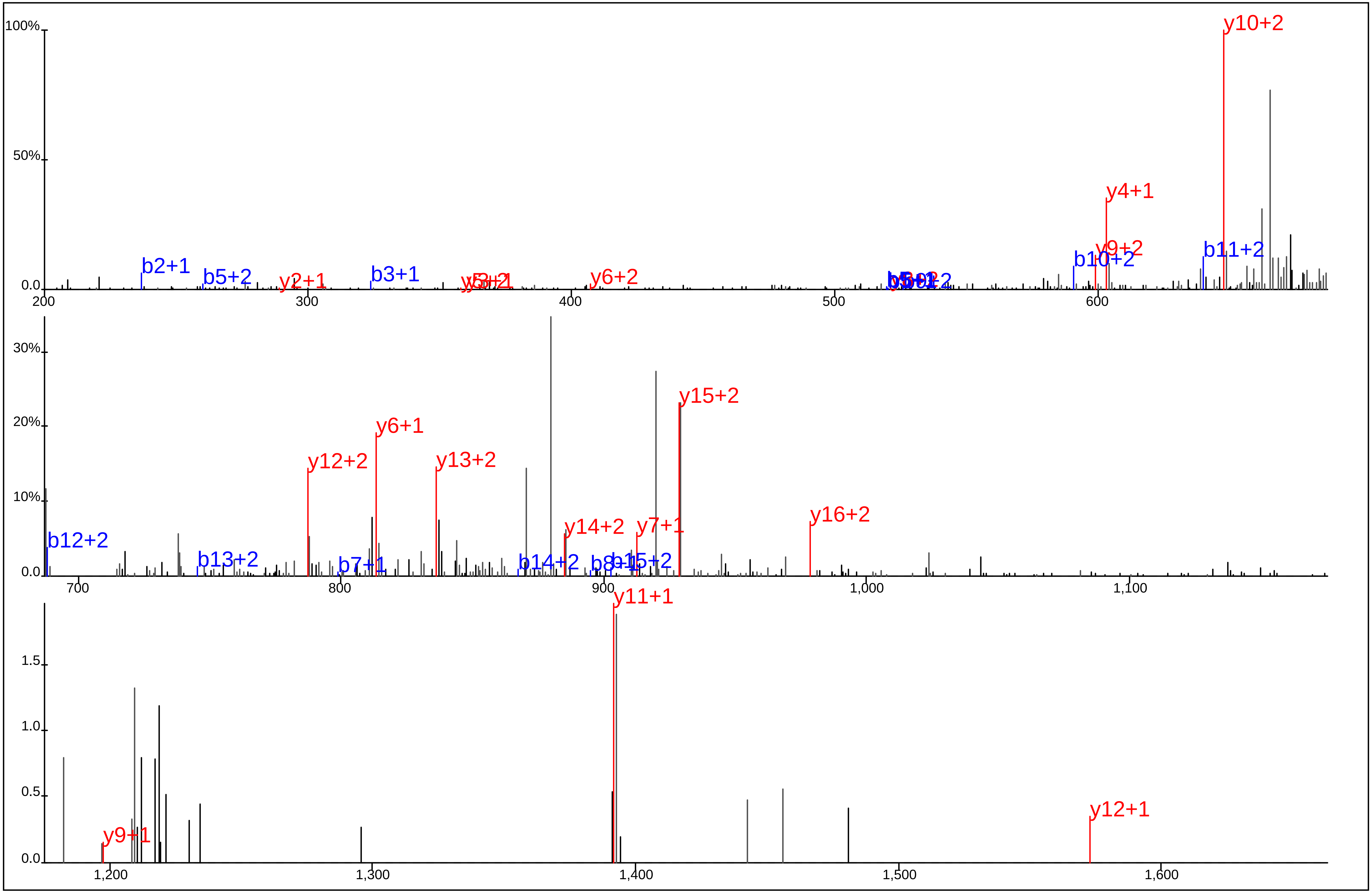

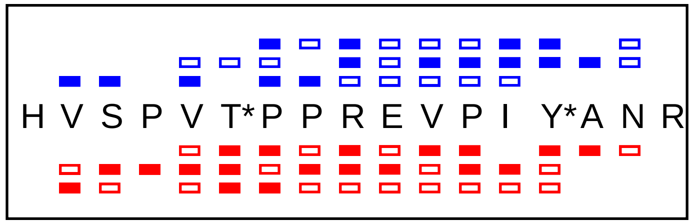

Protein Name: ADAM9
gi Accession: 6630618
Precursor Mass: 697.9979+3
Delta Mass: 0.668 ppm
XCorr: 35.29

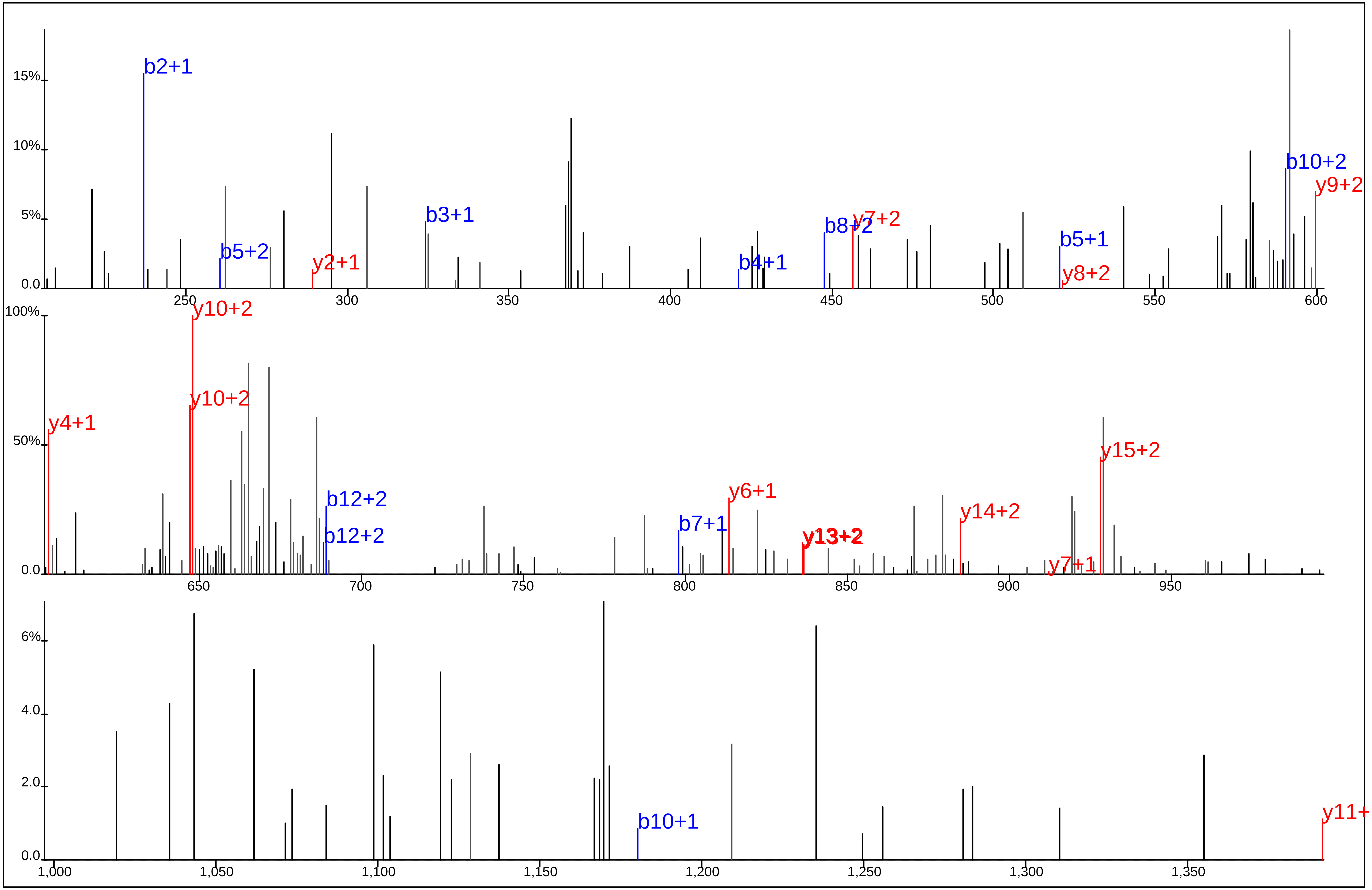

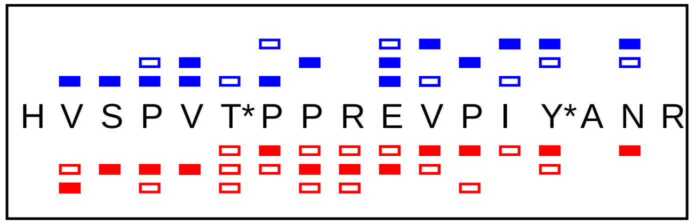

Protein Name:	ADAM9
gi Accession:	6630618
Precursor Mass:	697.9991+3
Delta Mass:	2.504 ppm
XCorr:	20.77

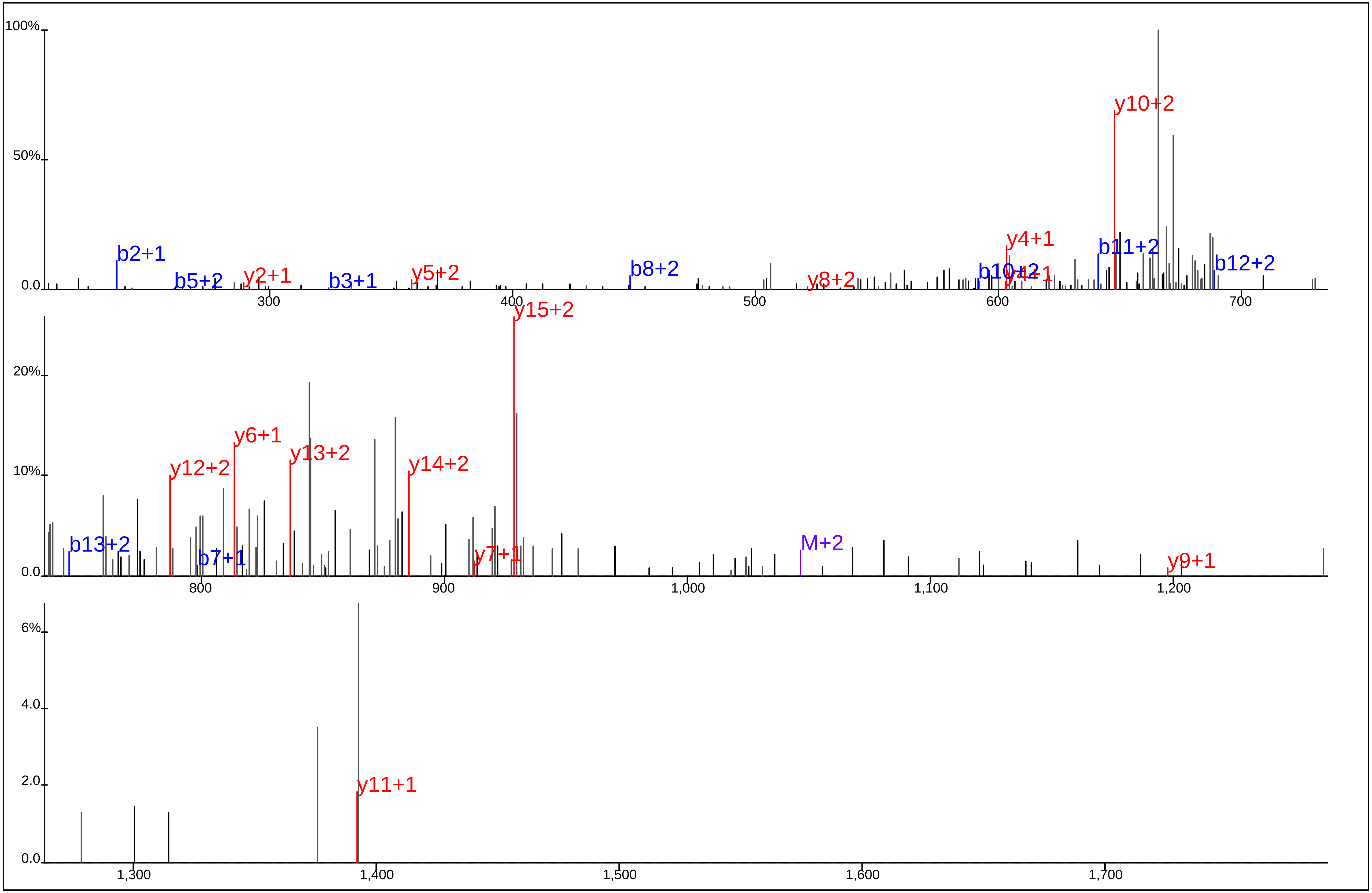

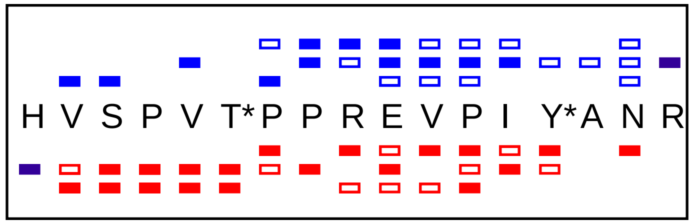

Protein Name:	ADAM9
gi Accession:	6630618
Precursor Mass:	697.9998+3
Delta Mass:	3.379 ppm
XCorr:	15.08

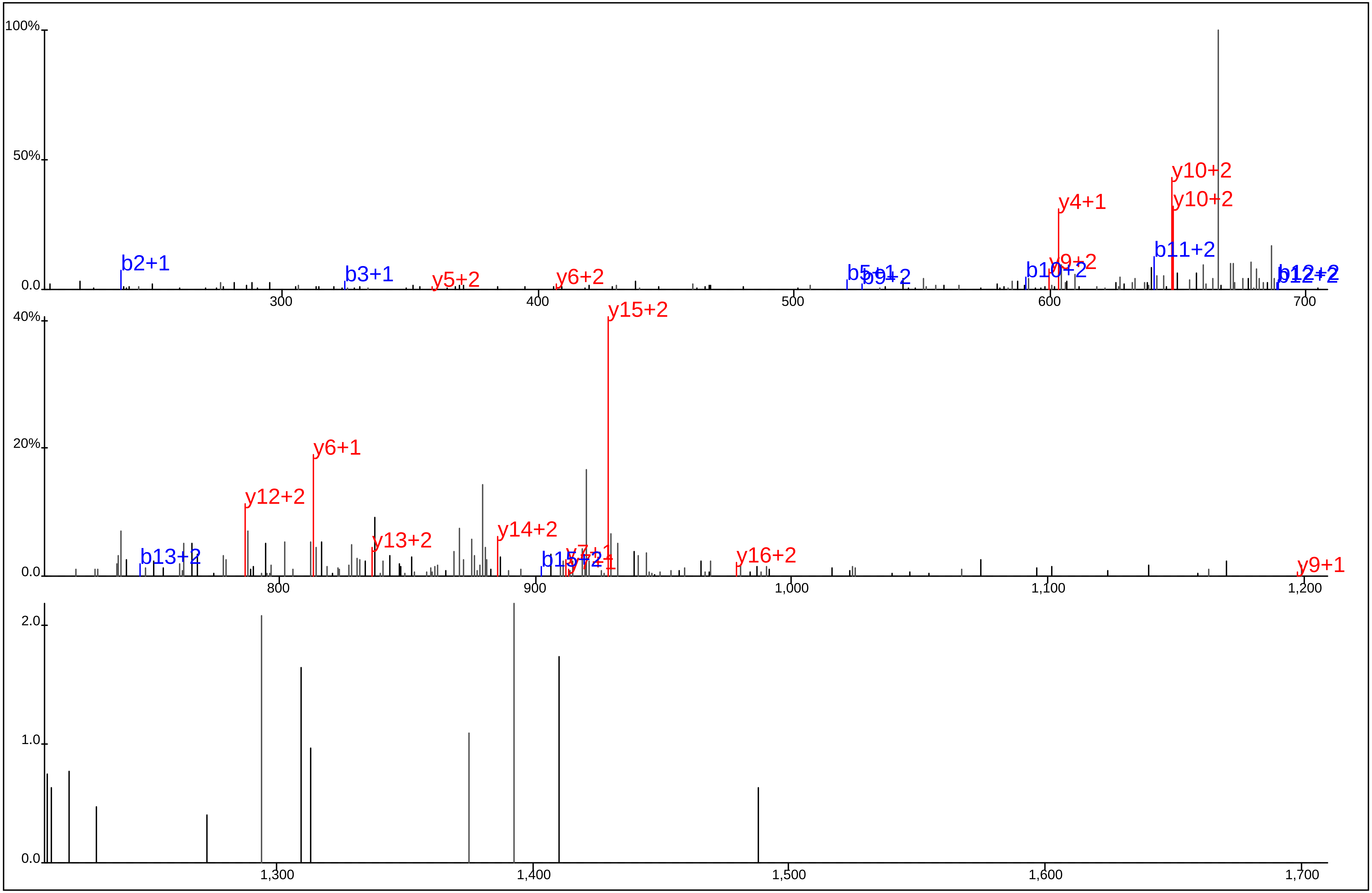

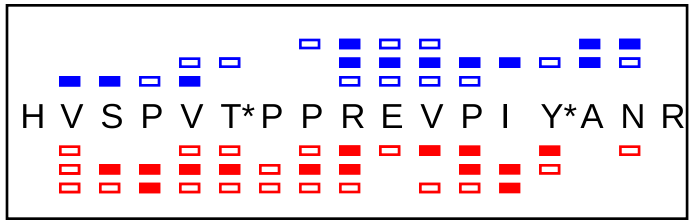

Protein Name: ADAM9
gi Accession: 6630618
Precursor Mass: 698.0002+3
Delta Mass: 4.081 ppm
XCorr: 23.88

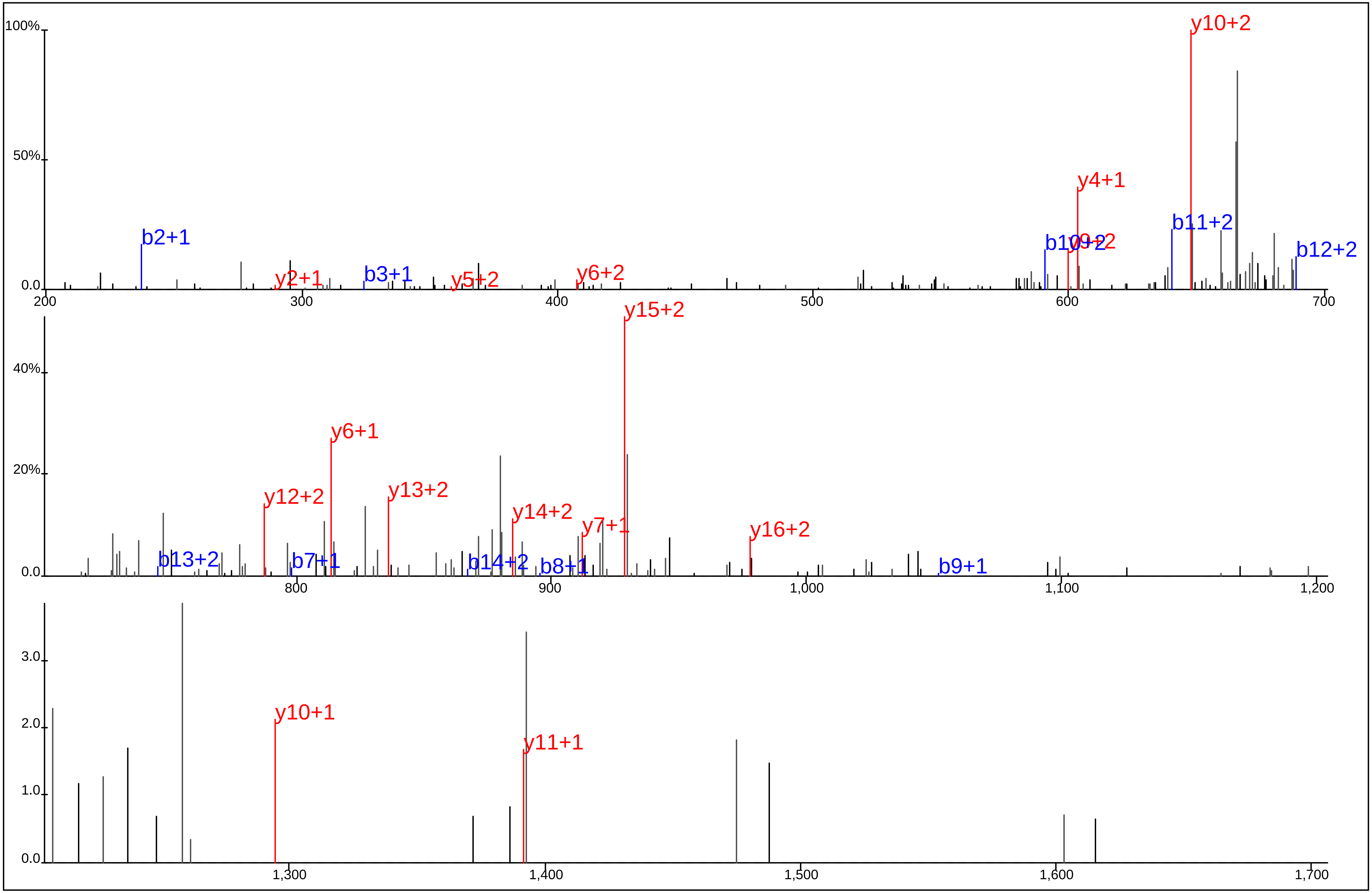

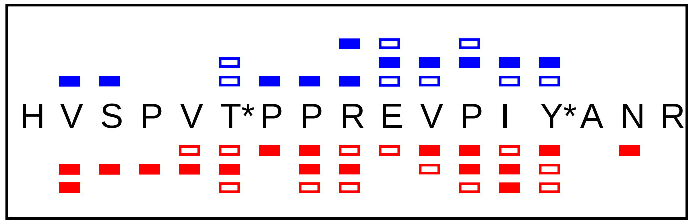

Protein Name: ADAM9
gi Accession: 6630618
Precursor Mass: 697.9998+3
Delta Mass: 3.379 ppm
XCorr: 23.34

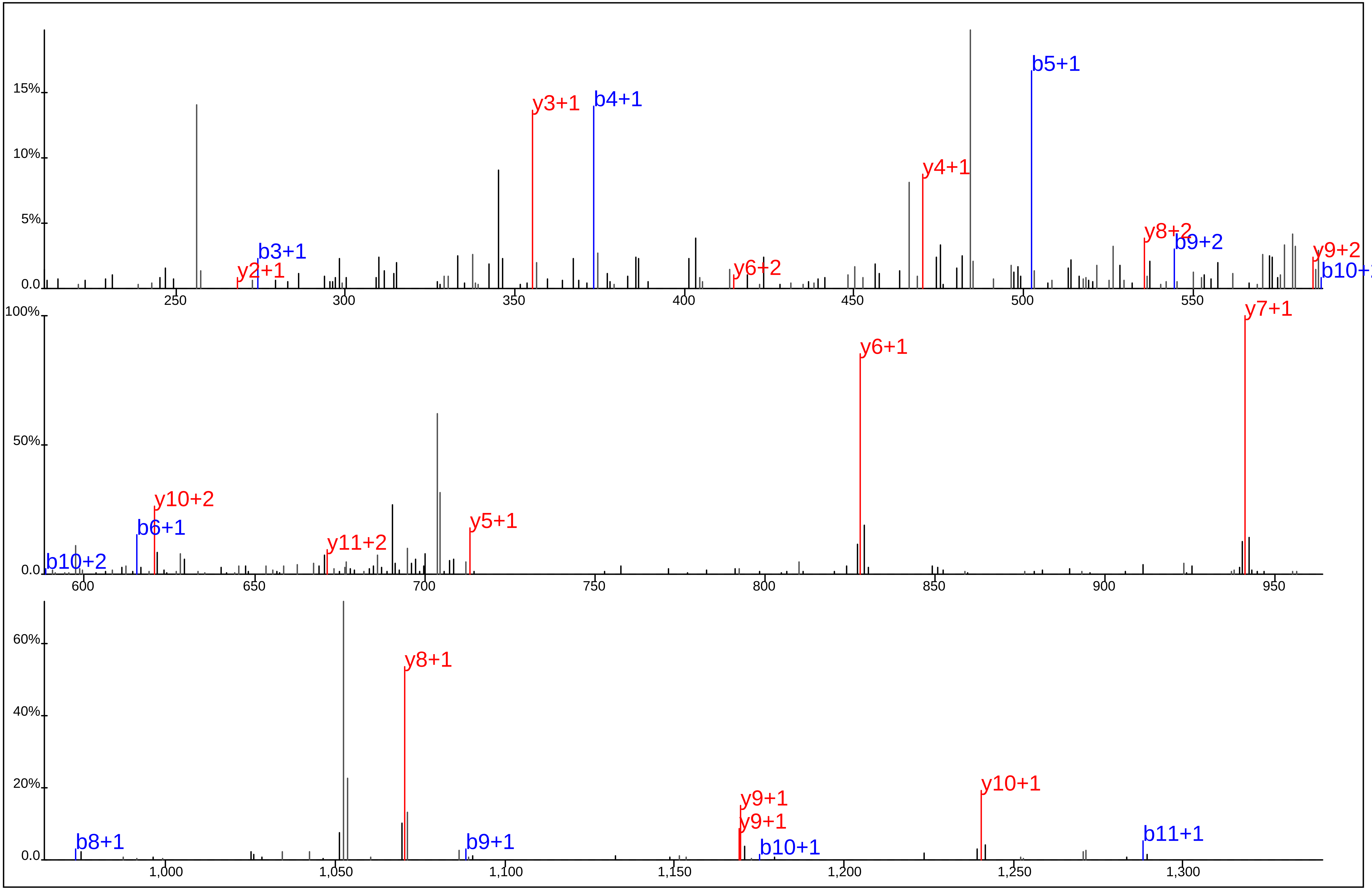

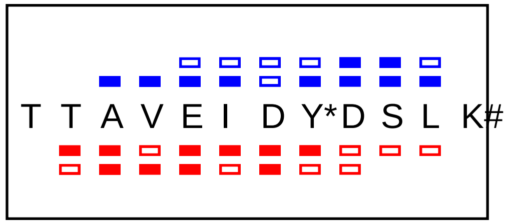

Protein Name:	ADAP
gi Accession:	6166197
Precursor Mass:	721.8338+2
Delta Mass:	4.269 ppm
XCorr:	73.14

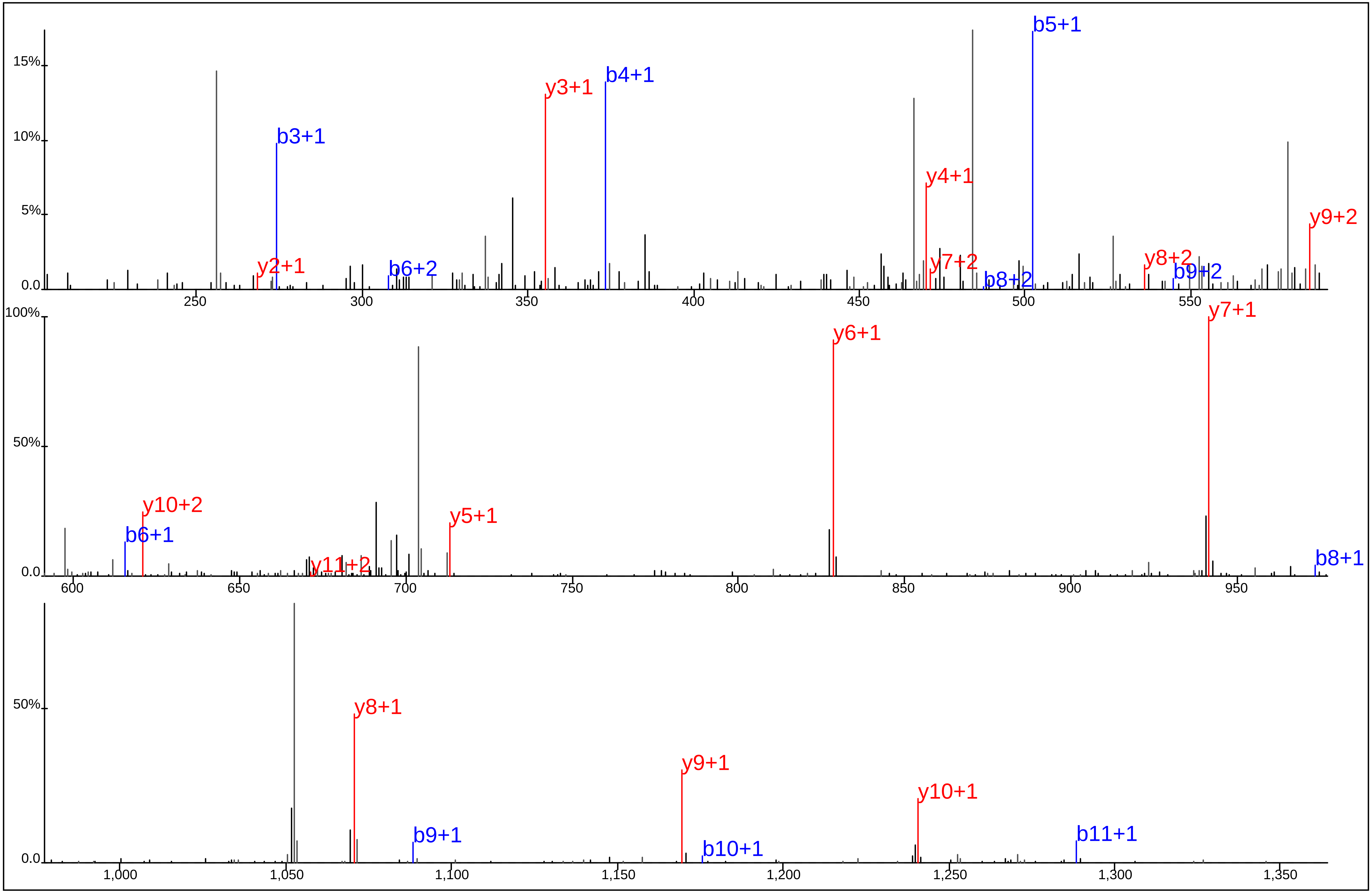

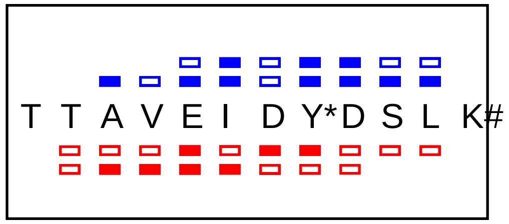

Protein Name: ADAP
gi Accession: 6166197
Precursor Mass: 721.8325+2
Delta Mass: 2.487 ppm
XCorr: 53.32

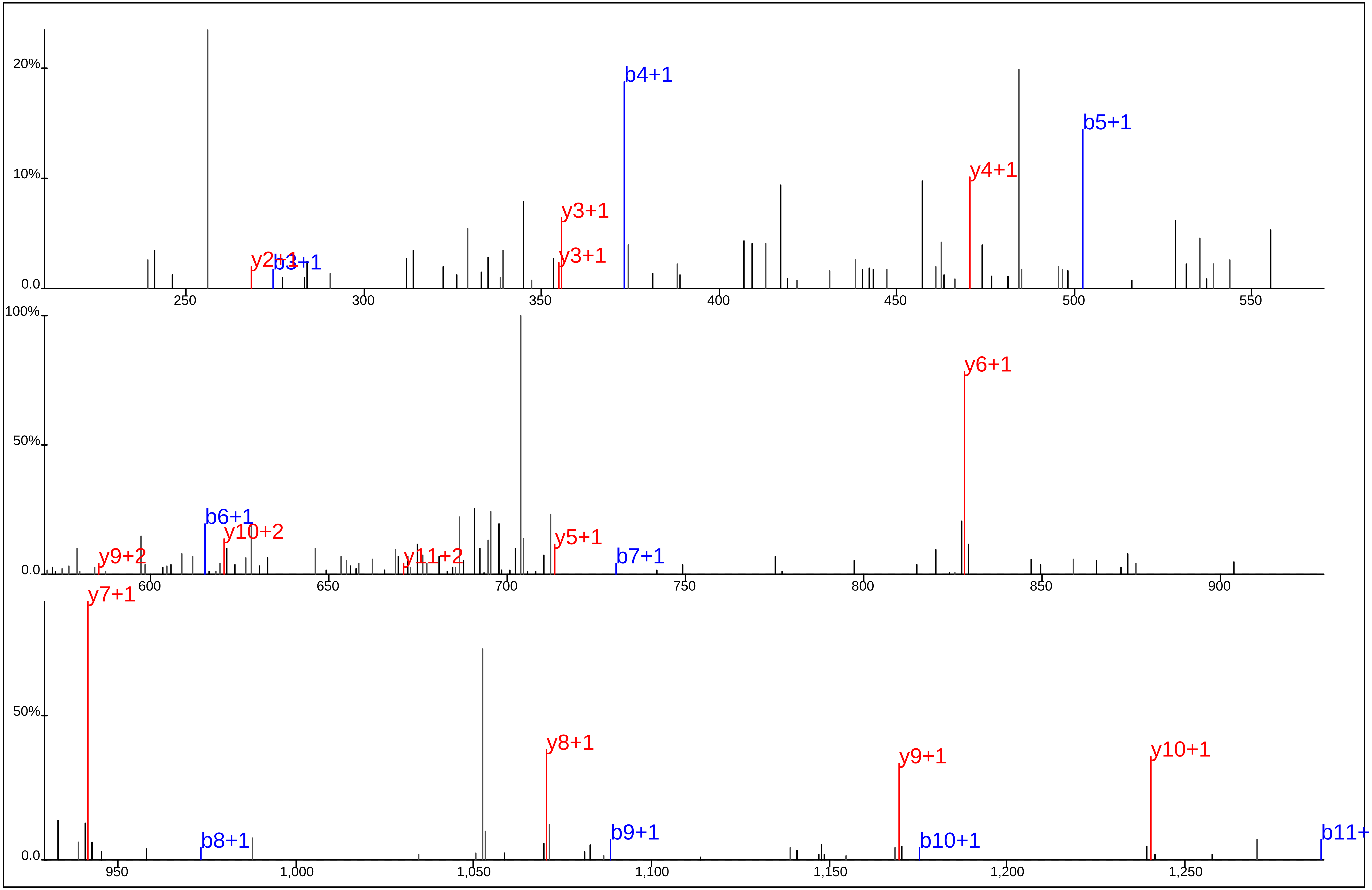

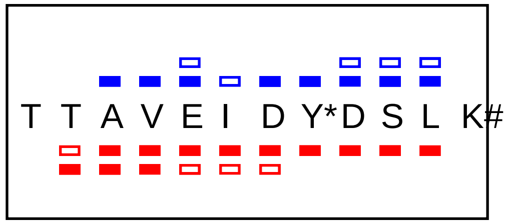

Protein Name: ADAP
gi Accession: 6166197
Precursor Mass: 721.8323+2
Delta Mass: 2.147 ppm
XCorr: 52.61

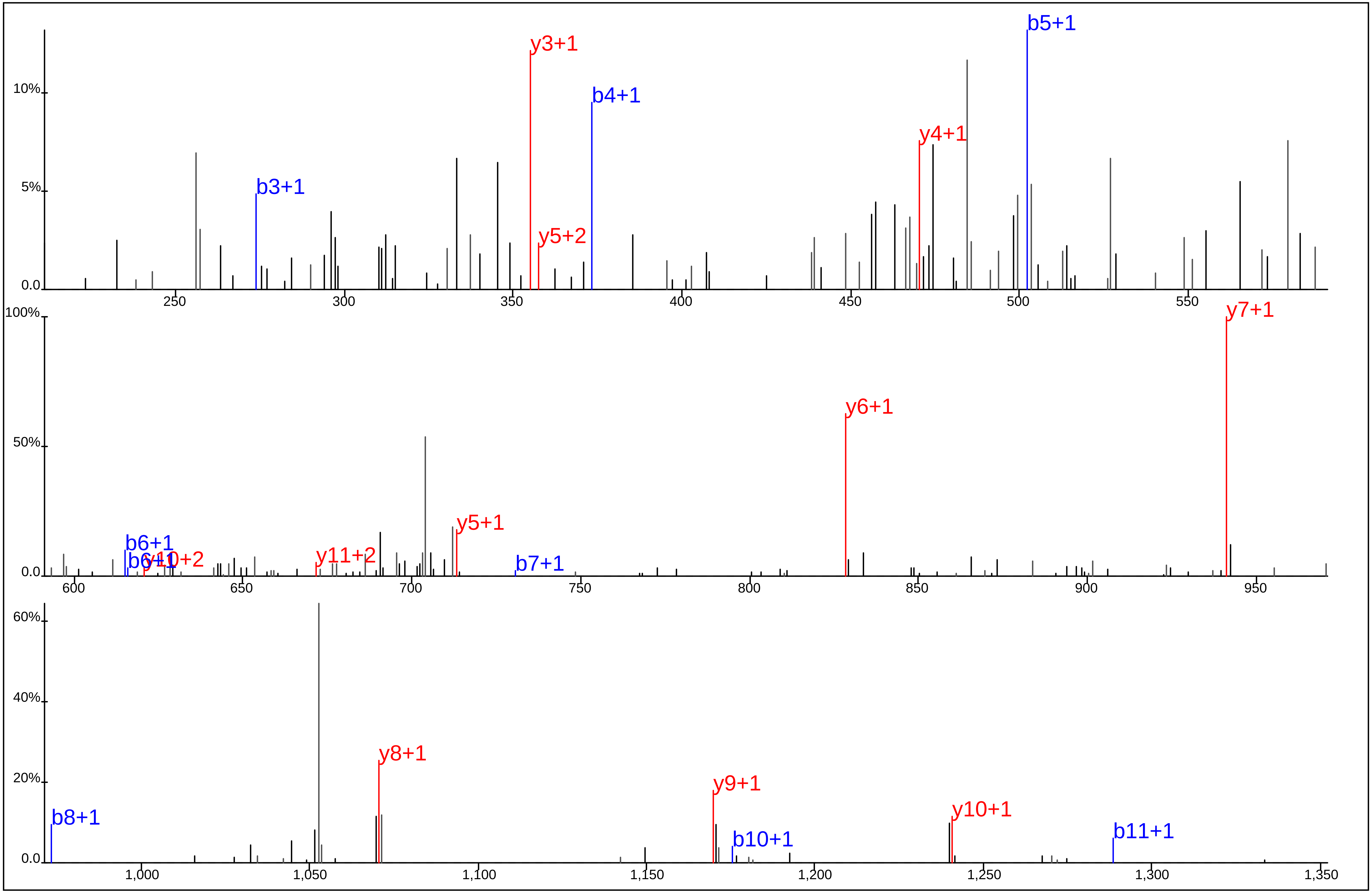

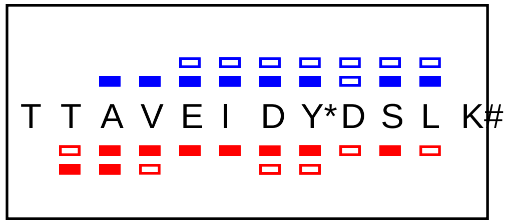

Protein Name: ADAP
gi Accession: 6166197
Precursor Mass: 721.8324+2
Delta Mass: 2.321 ppm
XCorr: 60.66

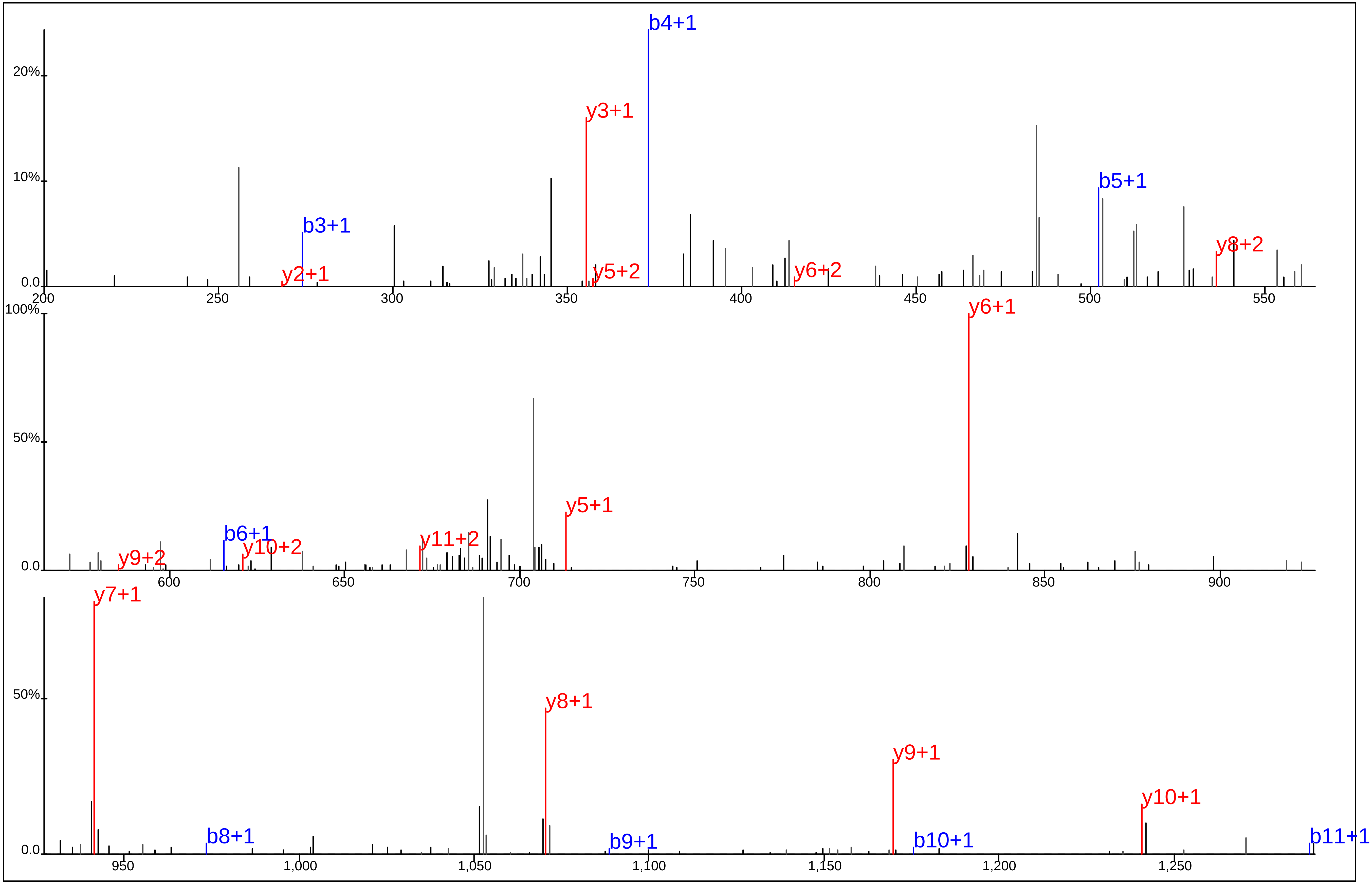

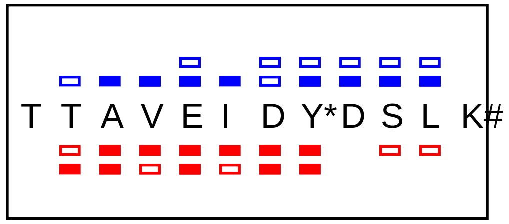

Protein Name: ADAP
gi Accession: 6166197
Precursor Mass: 721.8333+2
Delta Mass: 3.589 ppm
XCorr: 49.9

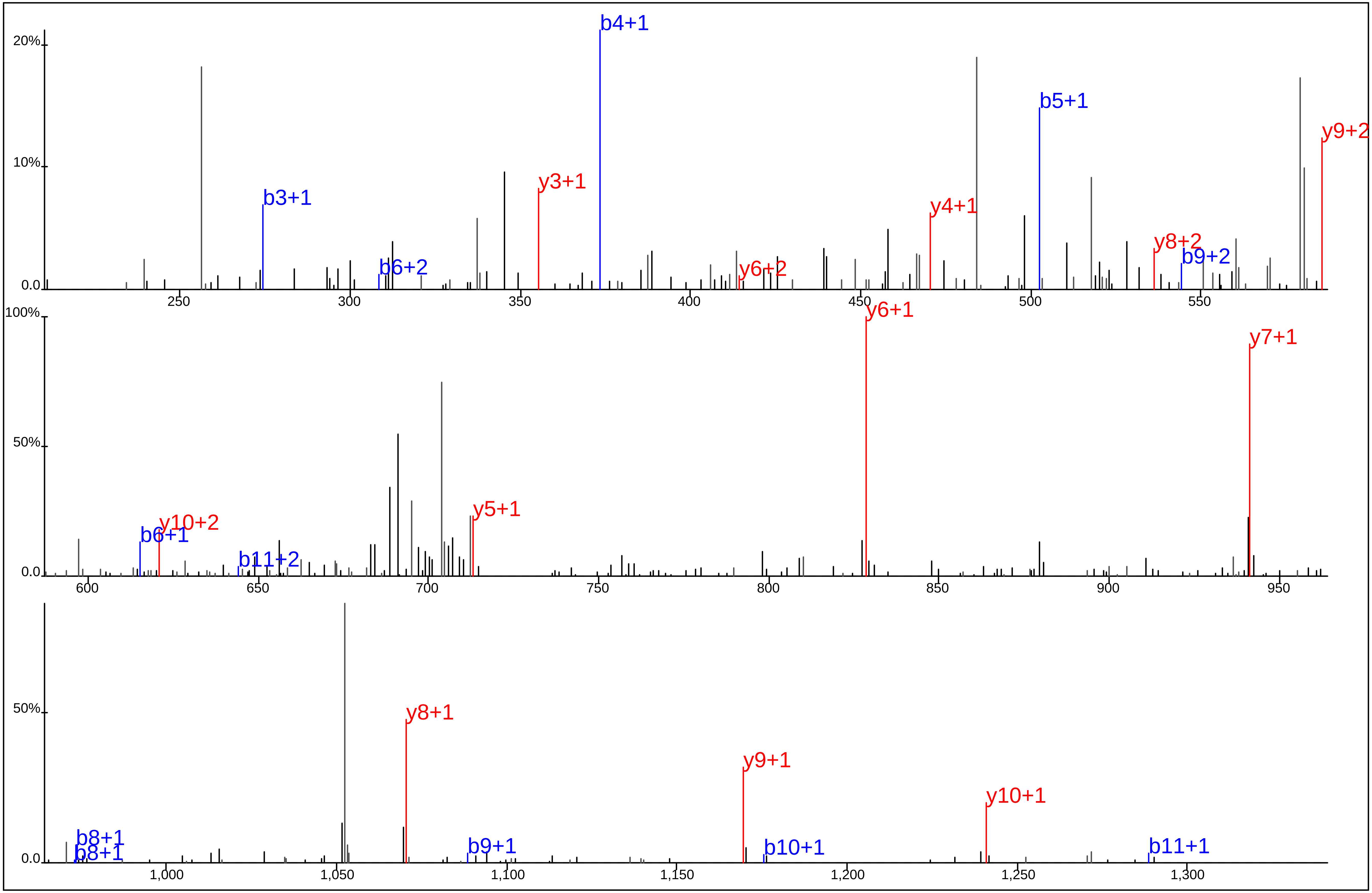

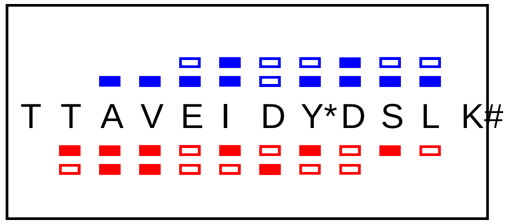

Protein Name: ADAP
gi Accession: 6166197
Precursor Mass: 721.8328+2
Delta Mass: 2.910 ppm
XCorr: 61.91

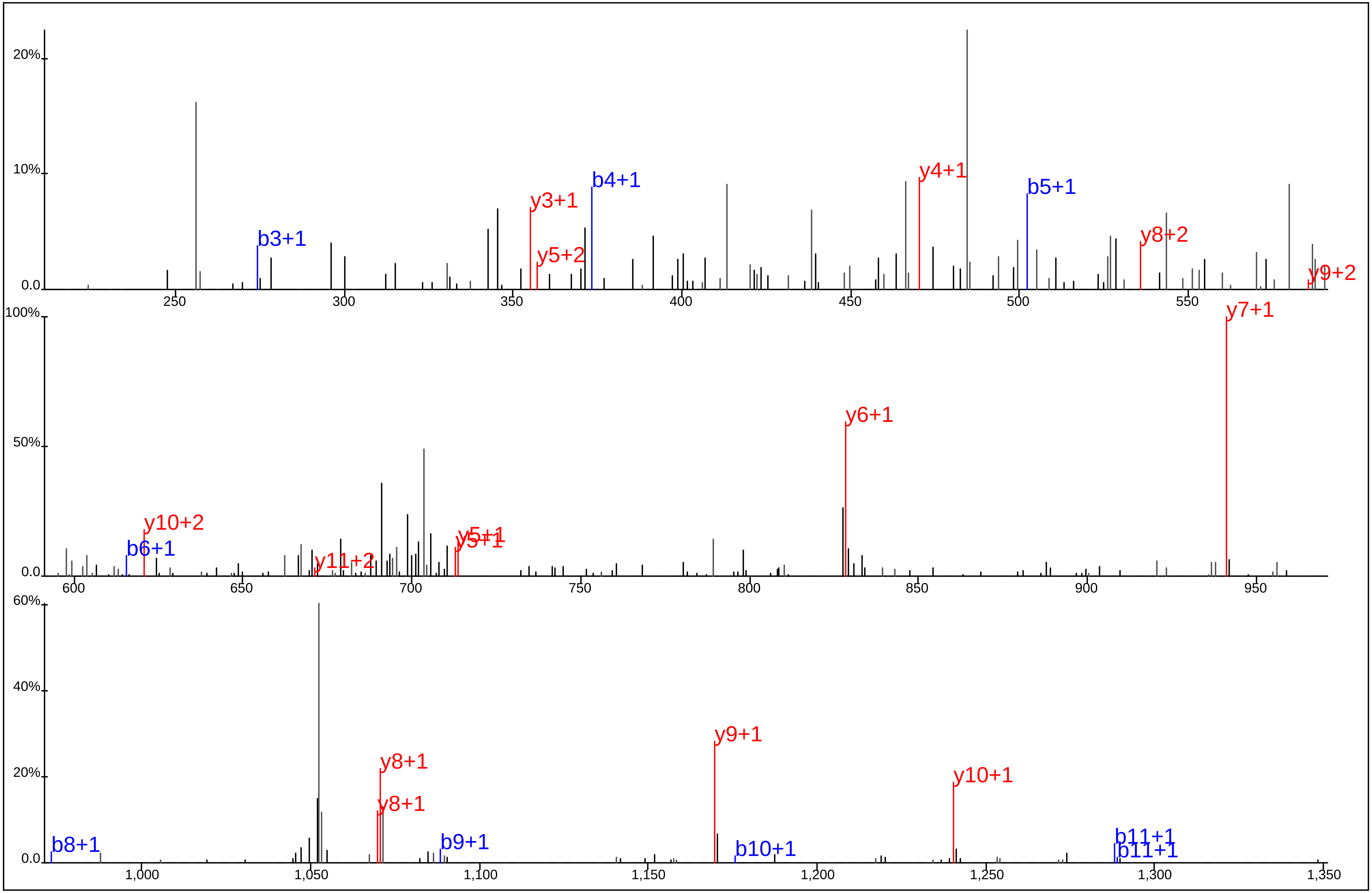

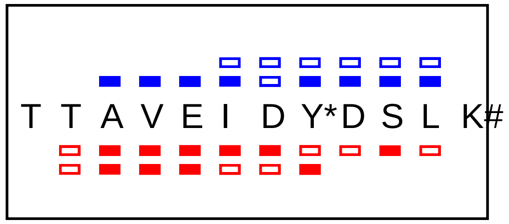

Protein Name: ADAP
gi Accession: 6166197
Precursor Mass: 721.8354+2
Delta Mass: 6.466 ppm
XCorr: 56.2

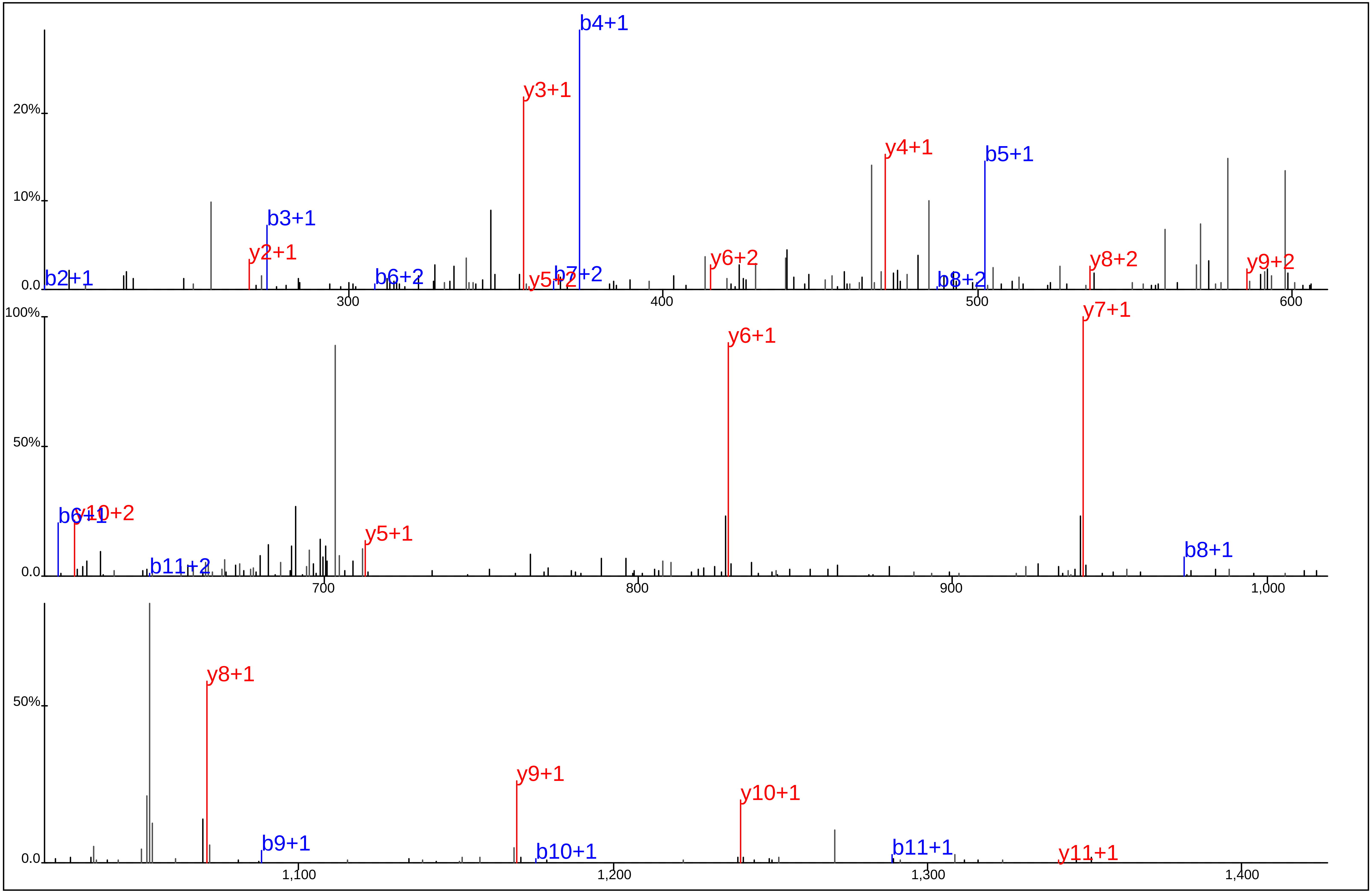

T T A V E I D Y*D S L K#

Protein Name: ADAP
gi Accession: 6166197
Precursor Mass: 721.8339+2
Delta Mass: 4.435 ppm
XCorr: 61.71

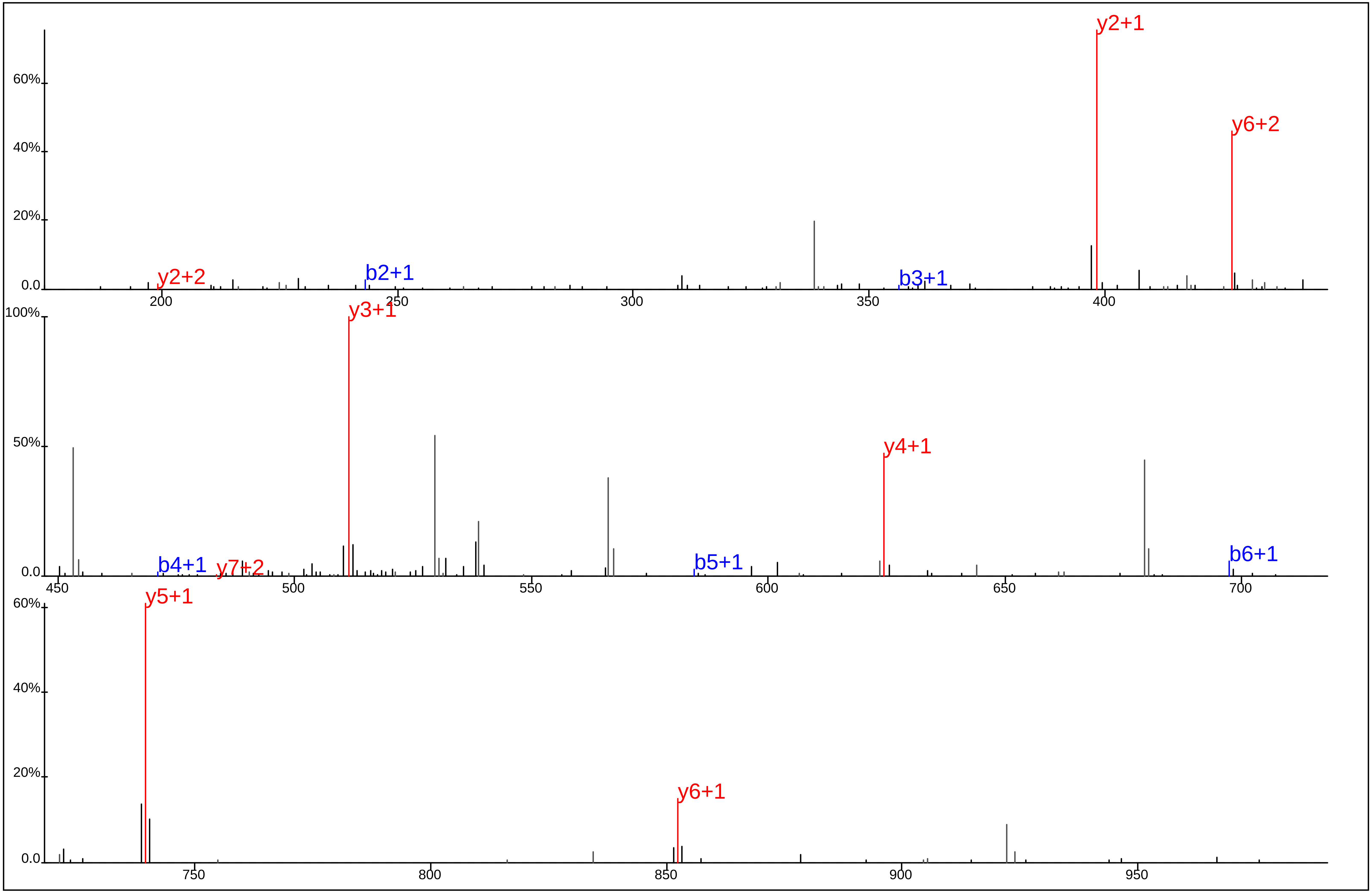

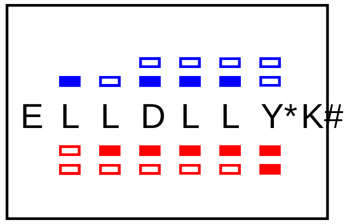

Protein Name: Adenosine deaminase
gi Accession: 1197210
Precursor Mass: 547.7863+2
Delta Mass: 2.684 ppm
XCorr: 33.4

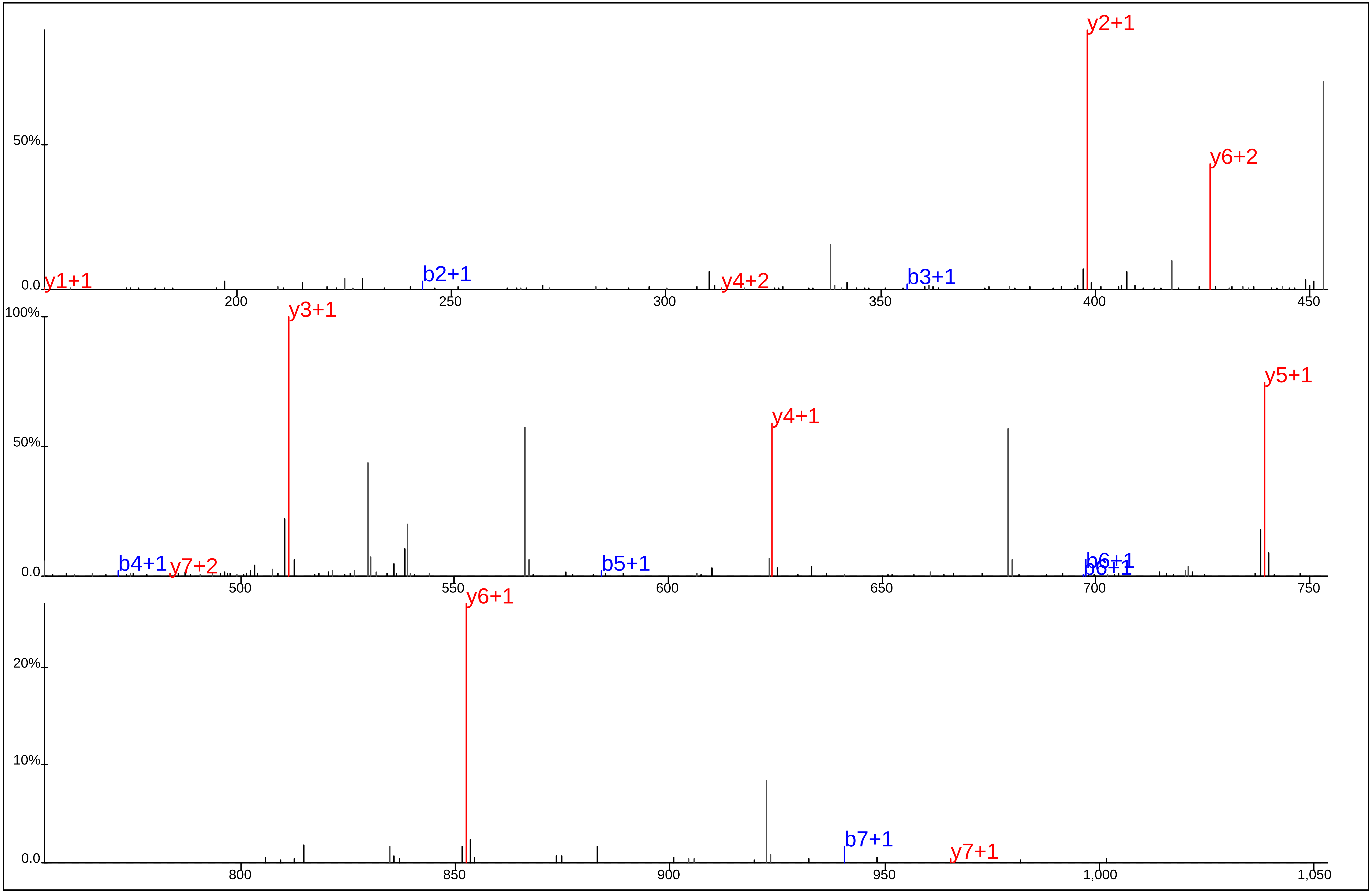

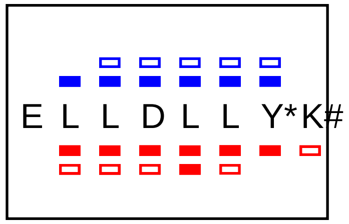

Protein Name: Adenosine deaminase
gi Accession: 1197210
Precursor Mass: 547.7874+2
Delta Mass: 4.575 ppm
XCorr: 48.49

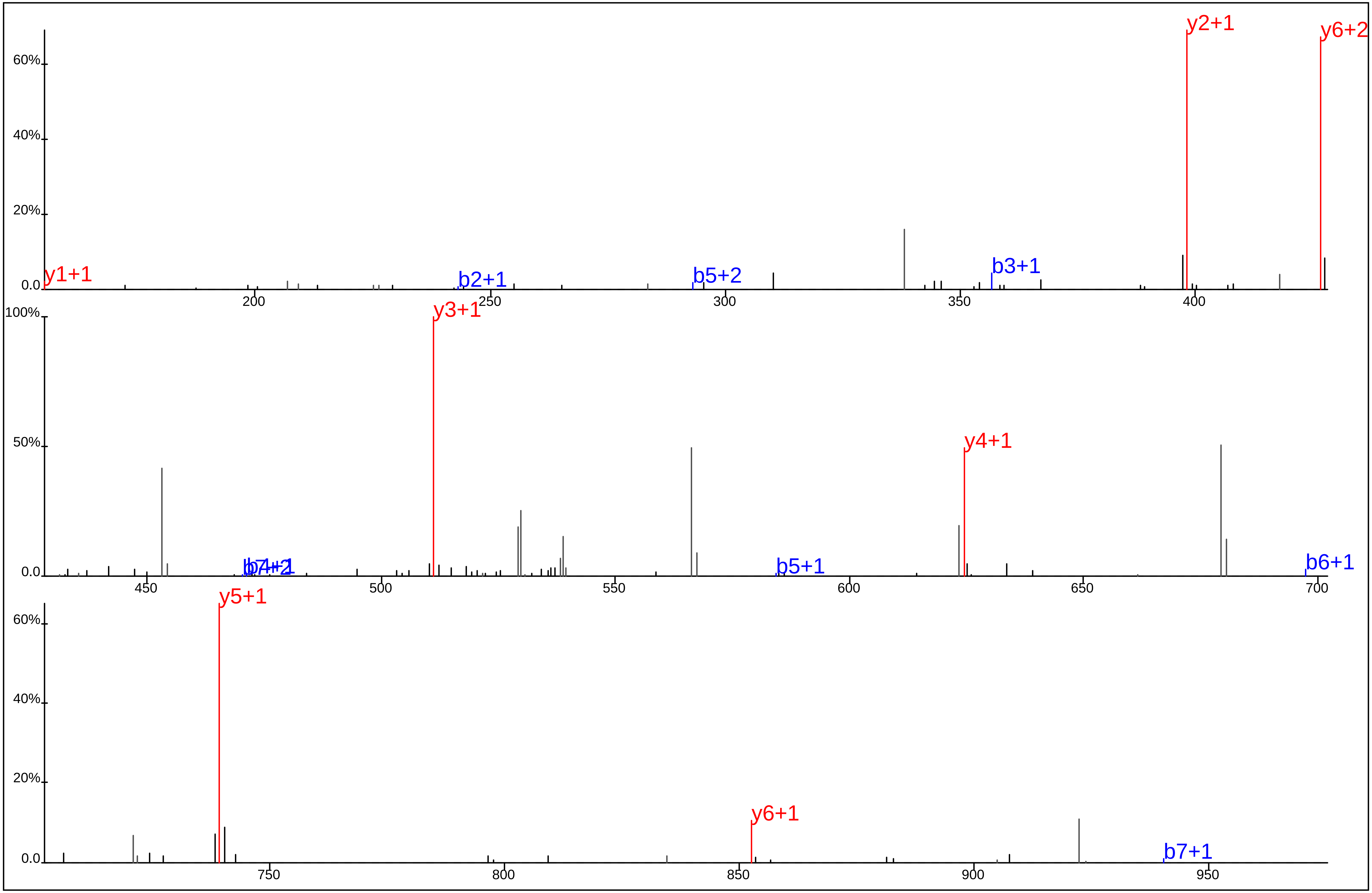

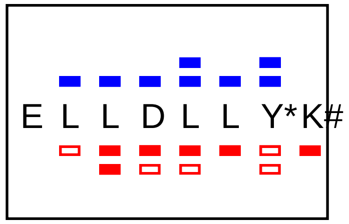

Protein Name: Adenosine deaminase
gi Accession: 1197210
Precursor Mass: 547.7864+2
Delta Mass: 2.794 ppm
XCorr: 48.36

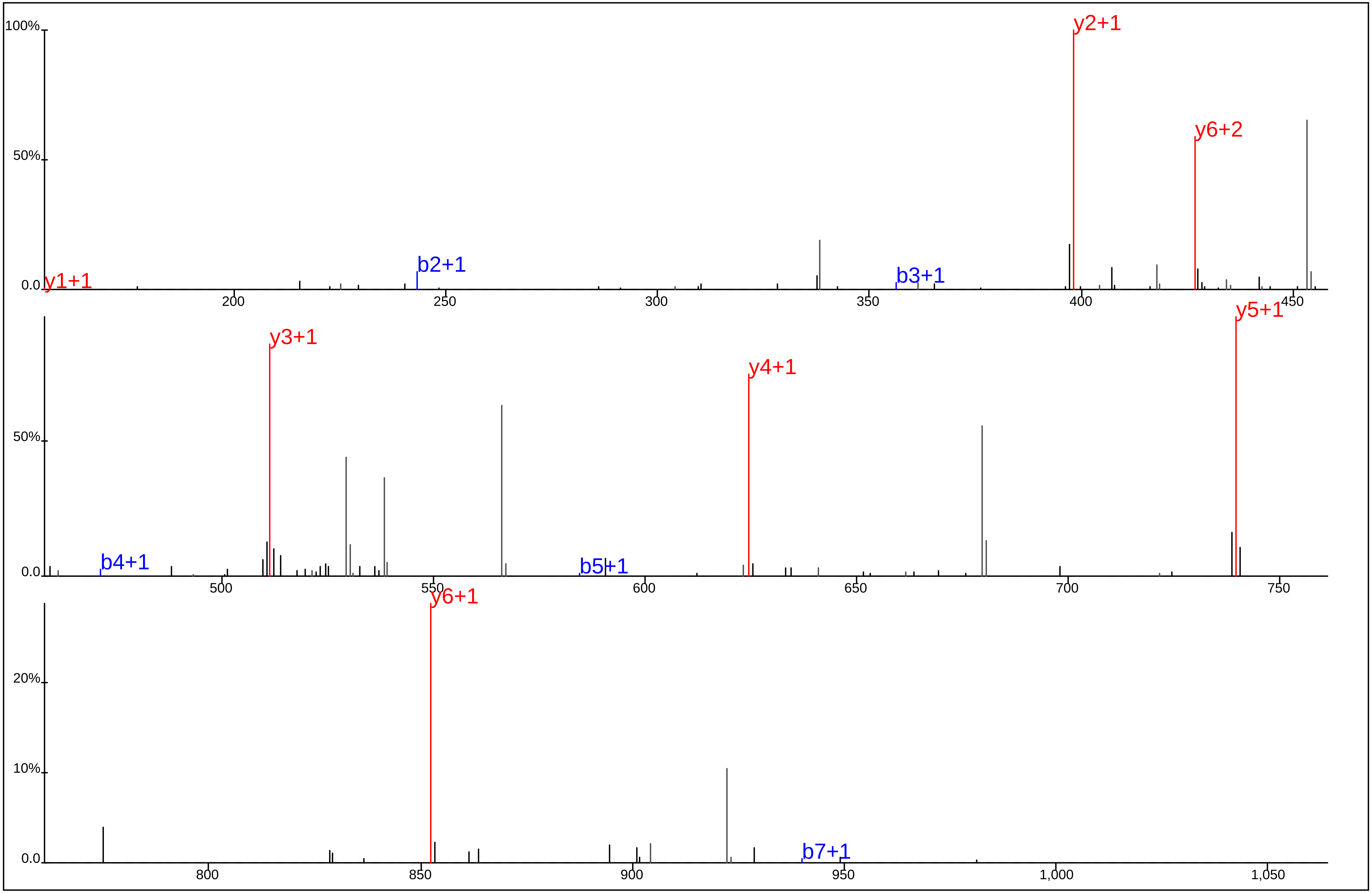

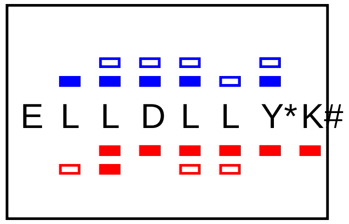

Protein Name:	Adenosine deaminase
gi Accession:	1197210
Precursor Mass:	547.7867+2
Delta Mass:	3.351 ppm
XCorr:	30.93

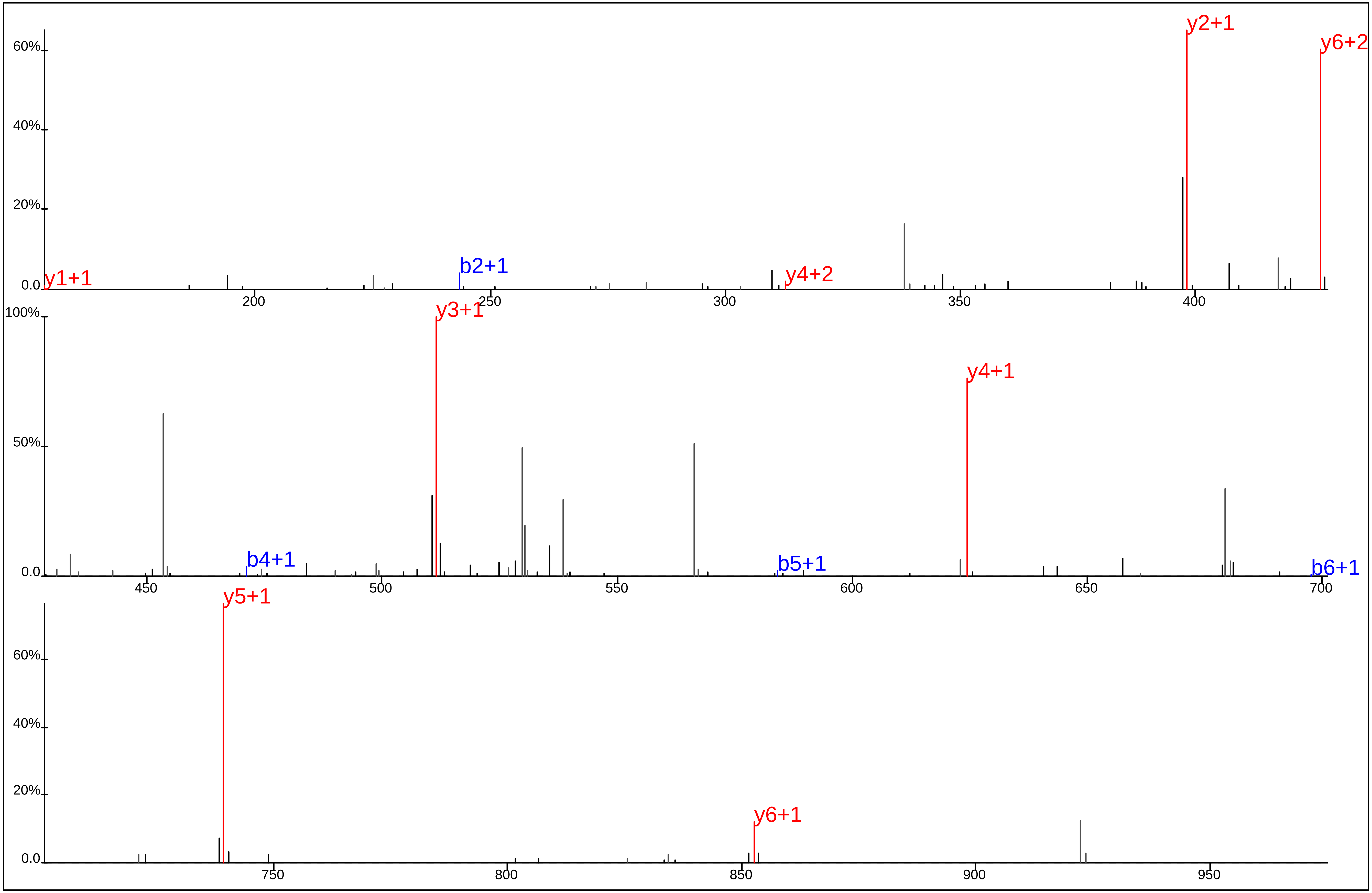

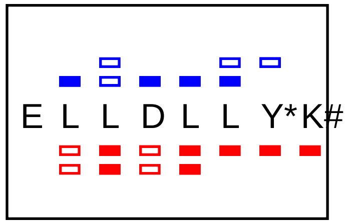

Protein Name: Adenosine deaminase
gi Accession: 1197210
Precursor Mass: 547.7863+2
Delta Mass: 2.565 ppm
XCorr: 33.02

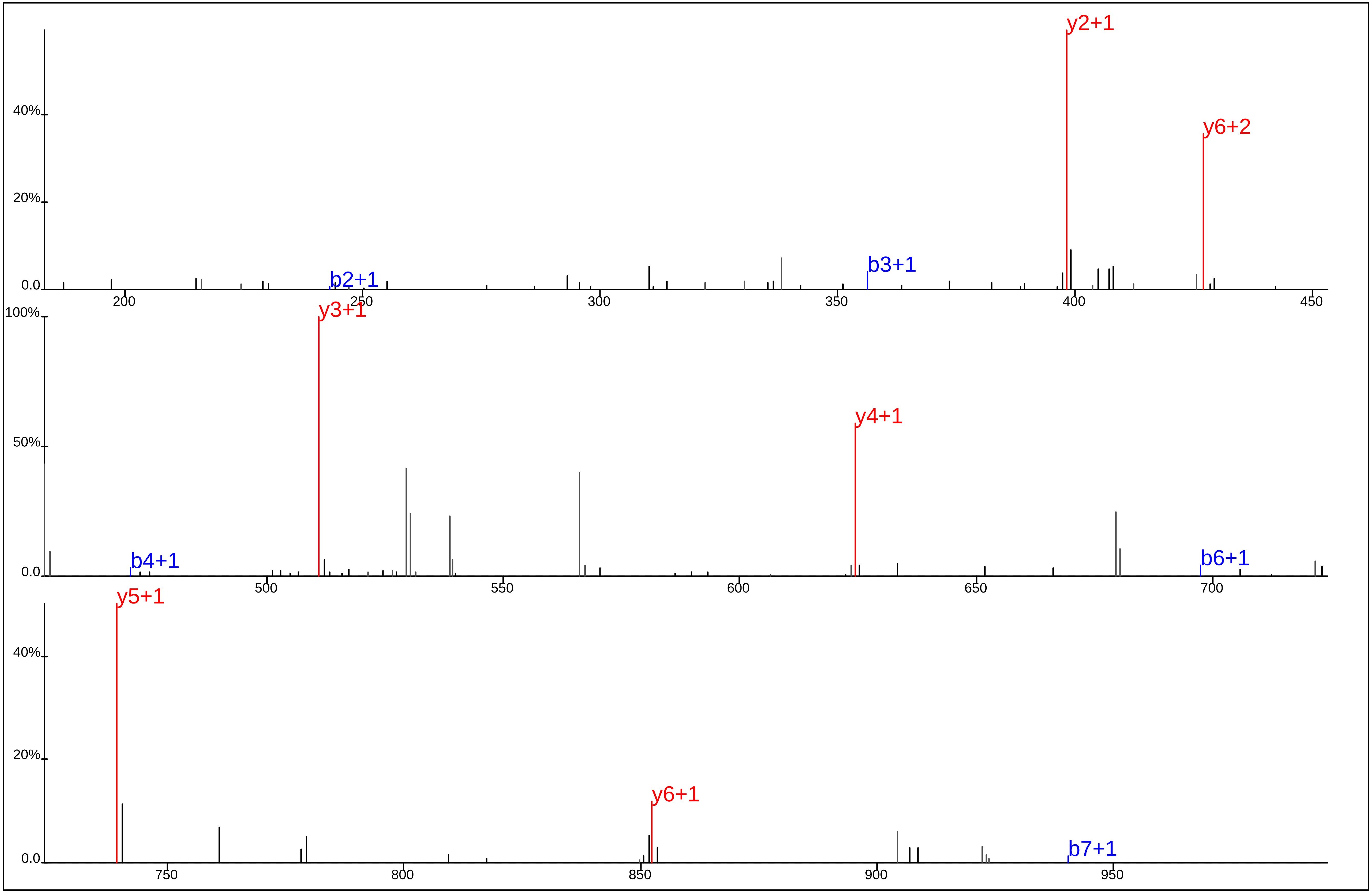

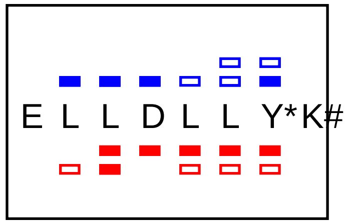

Protein Name: Adenosine deaminase
gi Accession: 1197210
Precursor Mass: 547.7873+2
Delta Mass: 4.466 ppm
XCorr: 33.33

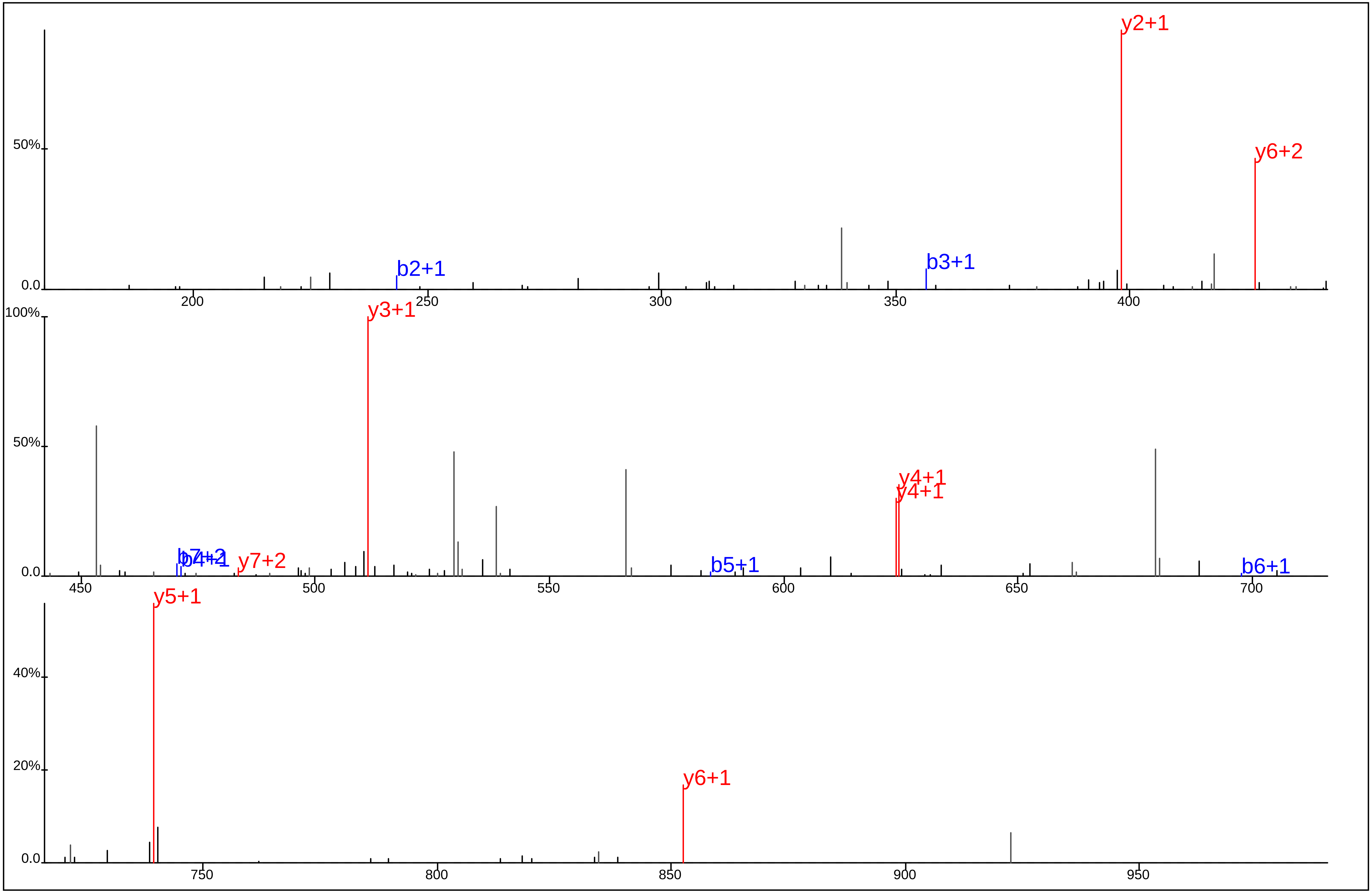

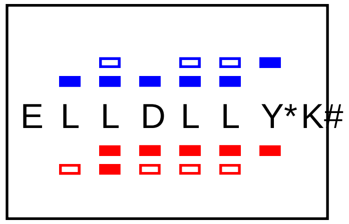

Protein Name:	Adenosine deaminase
gi Accession:	1197210
Precursor Mass:	547.7859+2
Delta Mass:	2.008 ppm
XCorr:	43.52

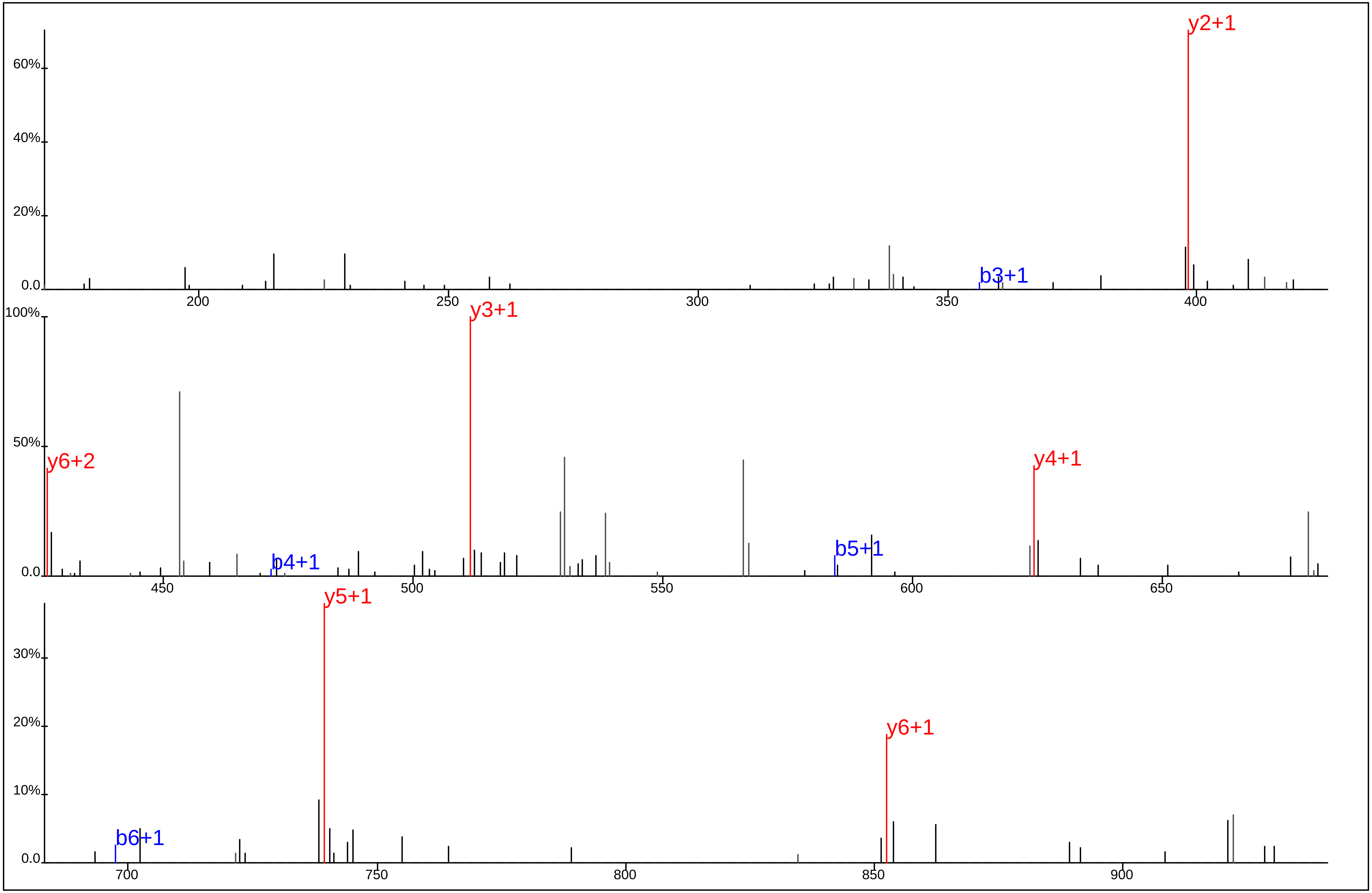

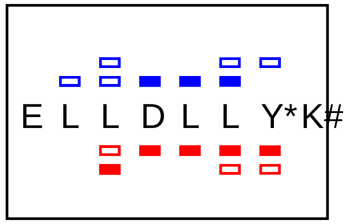

Protein Name:	Adenosine deaminase
gi Accession:	1197210
Precursor Mass:	547.7850+2
Delta Mass:	0.227 ppm
XCorr:	20.26

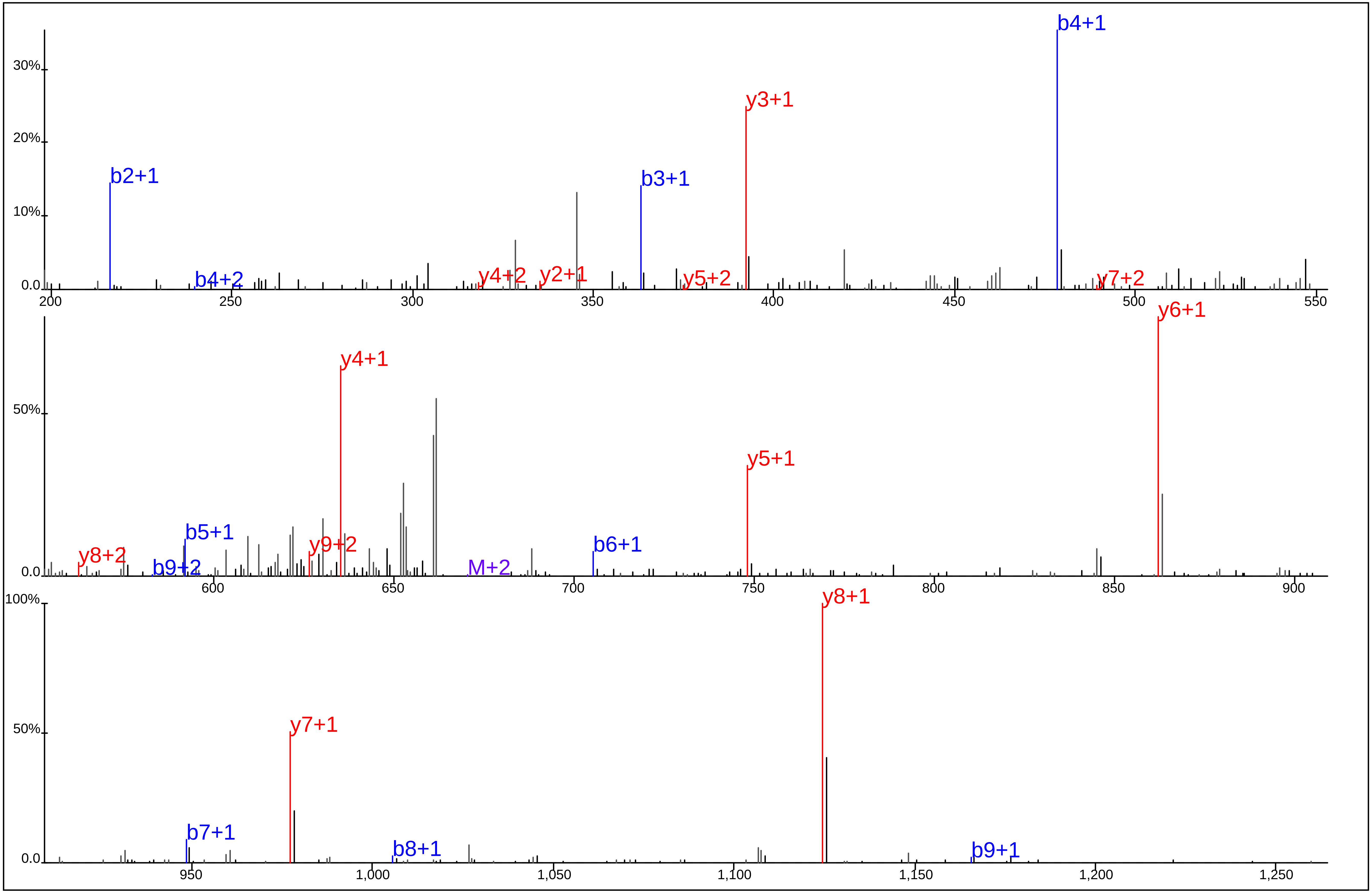

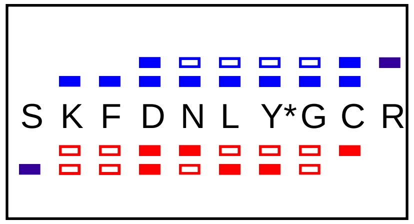

Protein Name:	Adenosylhomocysteinase
gi Accession:	9951915
Precursor Mass:	670.2791+2
Delta Mass:	0.588 ppm
XCorr:	49.11

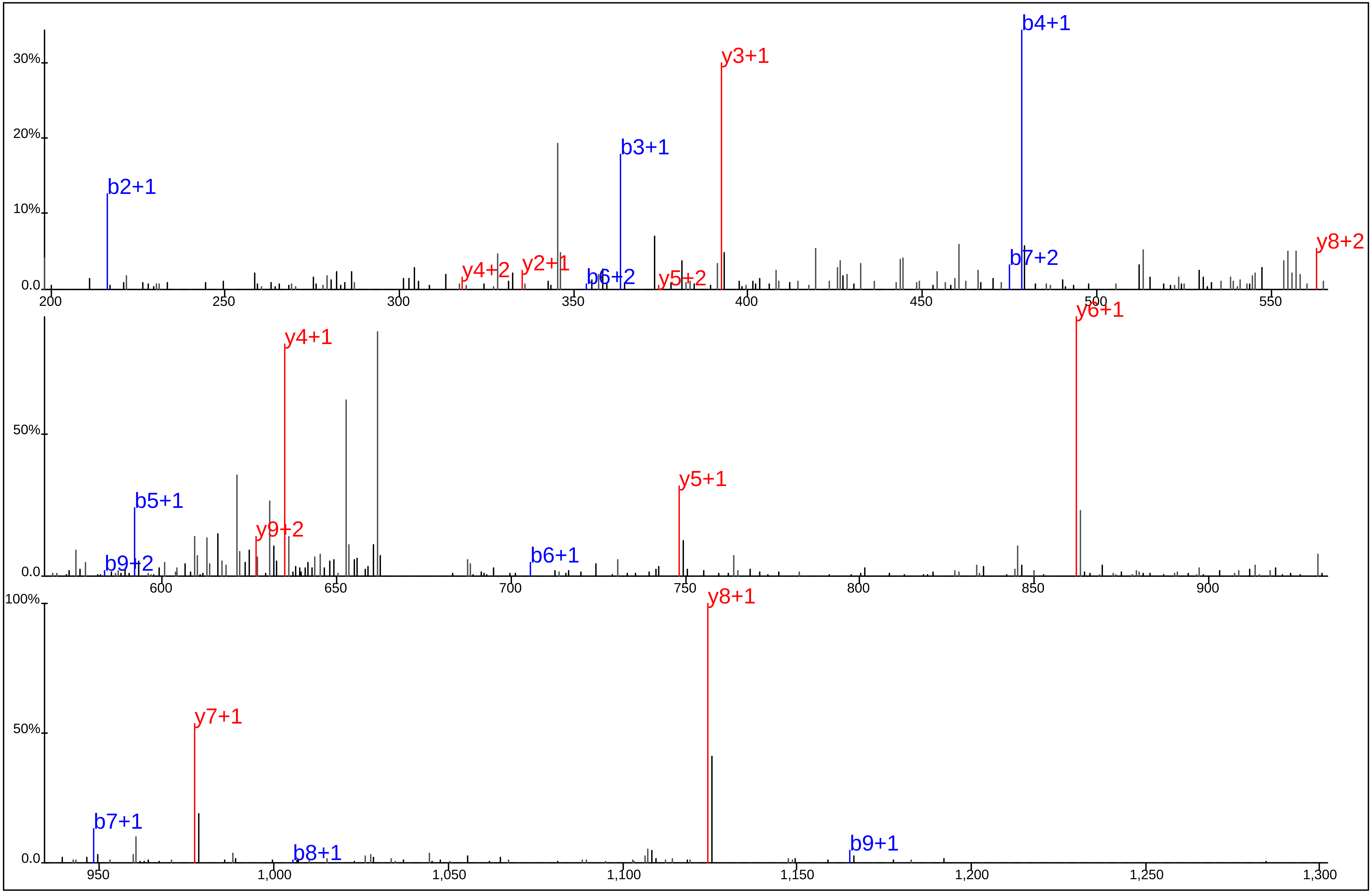

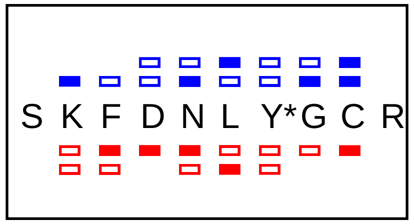

Protein Name: Adenosylhomocysteinase
gi Accession: 9951915
Precursor Mass: 670.2816+2
Delta Mass: 4.321 ppm
XCorr: 37.4

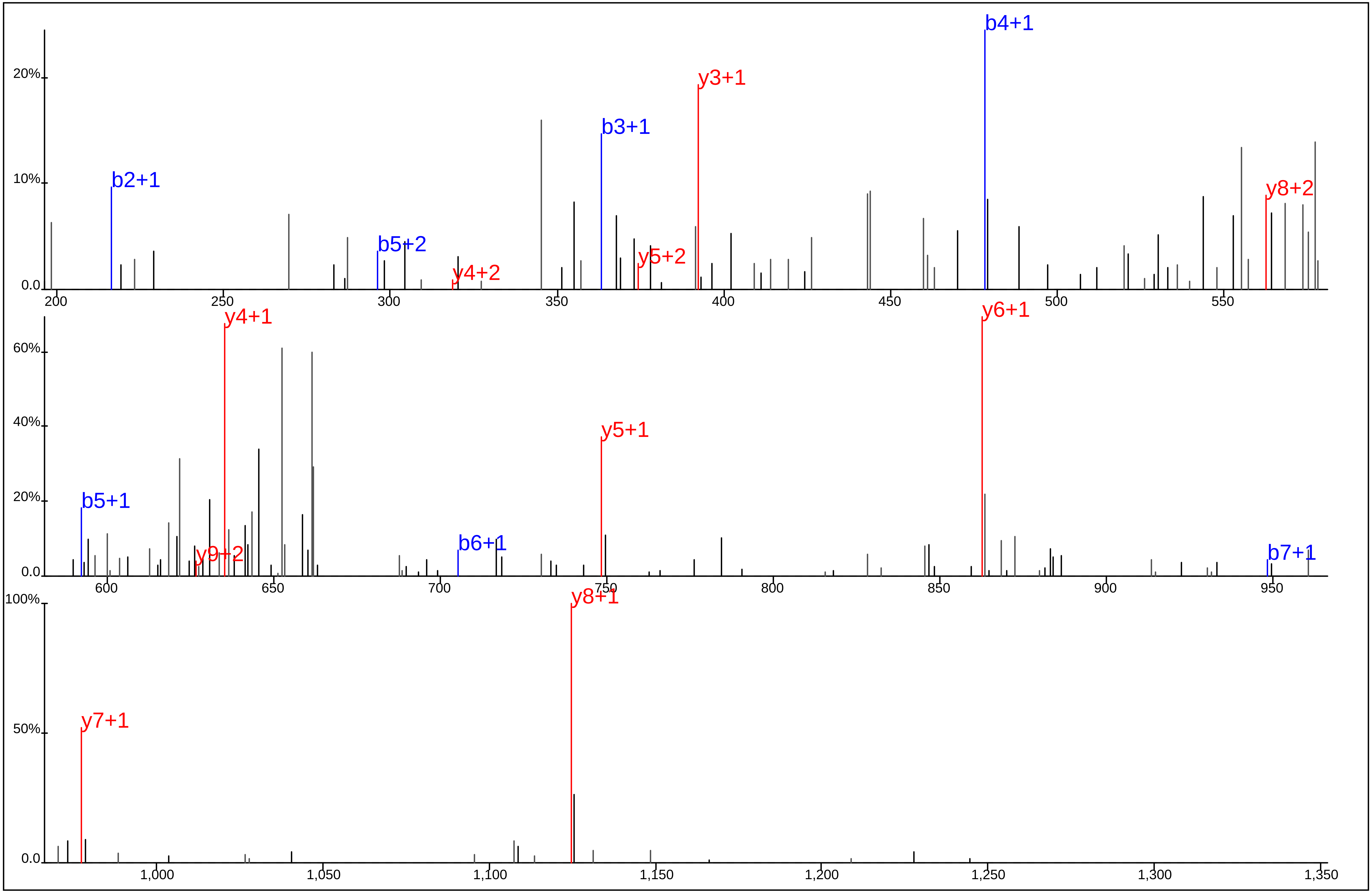

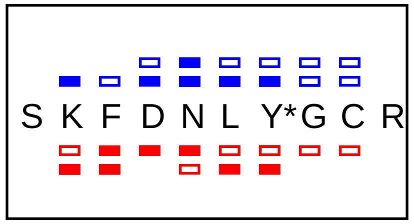

Protein Name: Adenosylhomocysteinase
gi Accession: 9951915
Precursor Mass: 670.2802+2
Delta Mass: 2.231 ppm
XCorr: 48.09

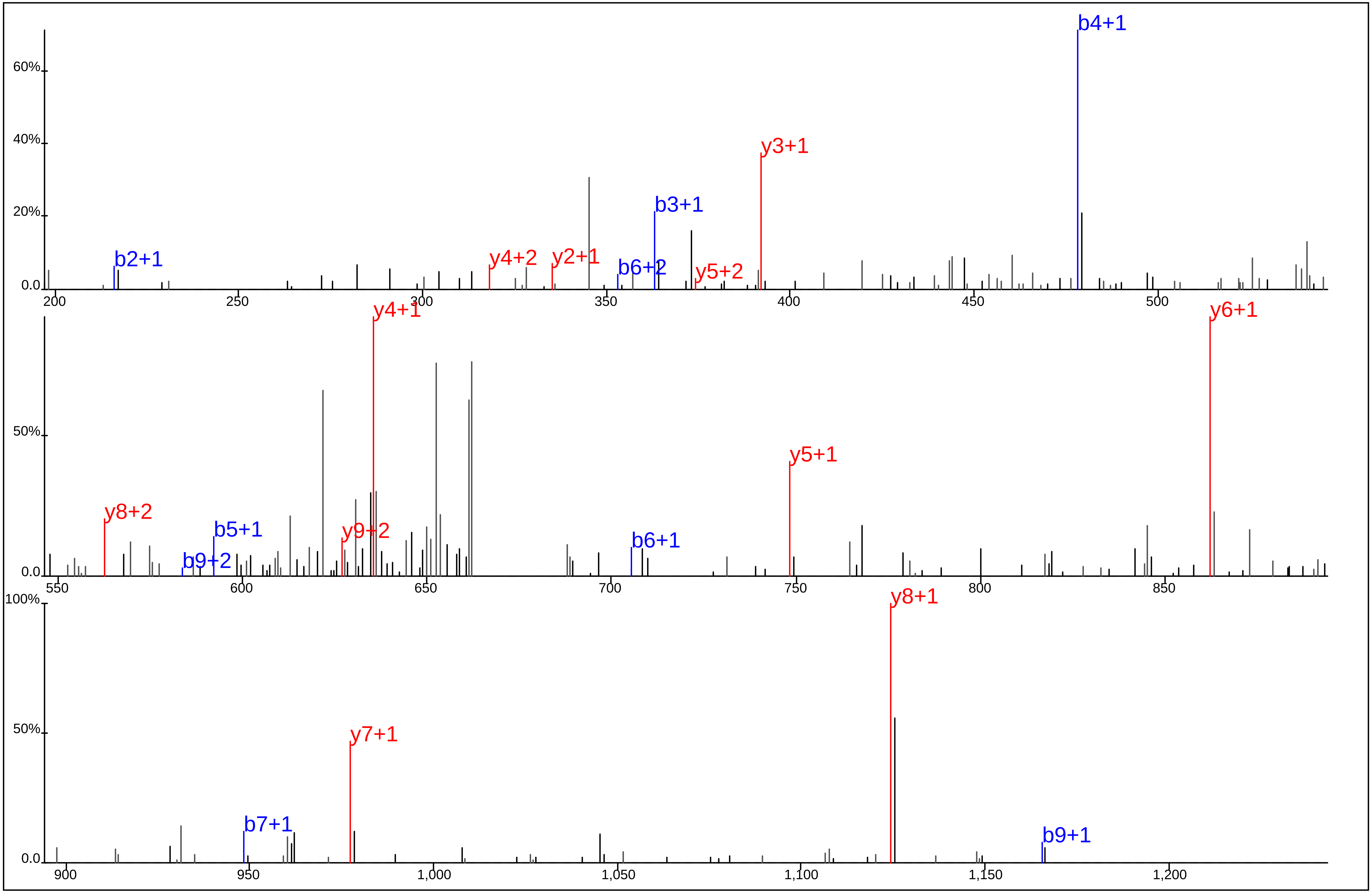

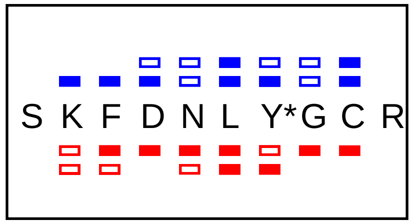

Protein Name: Adenosylhomocysteinase
gi Accession: 9951915
Precursor Mass: 670.2789+2
Delta Mass: 0.402 ppm
XCorr: 37.24

40 - Adenosylhomocysteinase_3453698.pdf

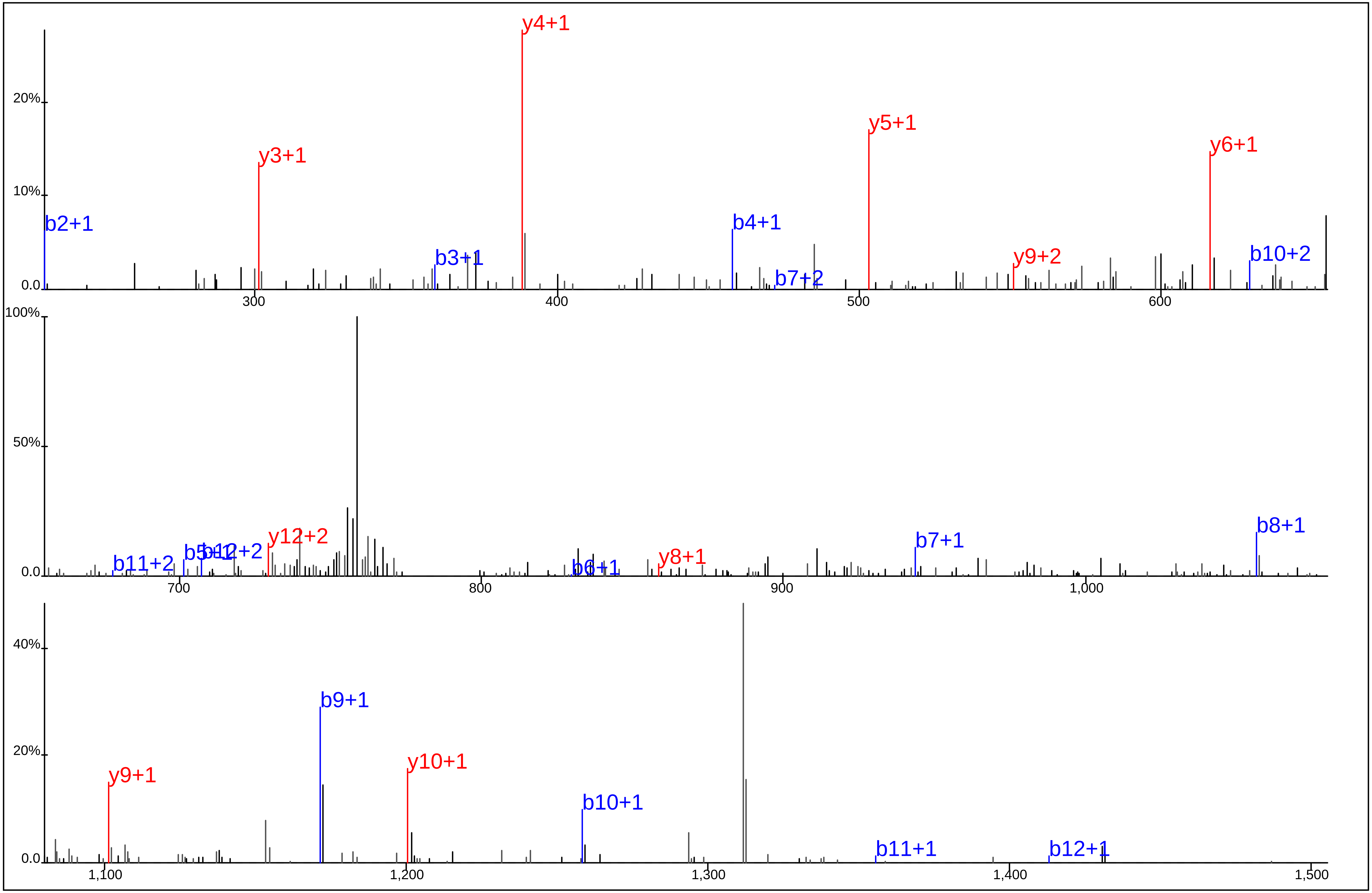

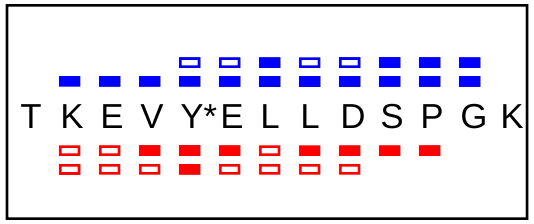

Protein Name: AIR carboxylase
gi Accession: 5453539
Precursor Mass: 779.8774+2
Delta Mass: 4.797 ppm
XCorr: 49.03

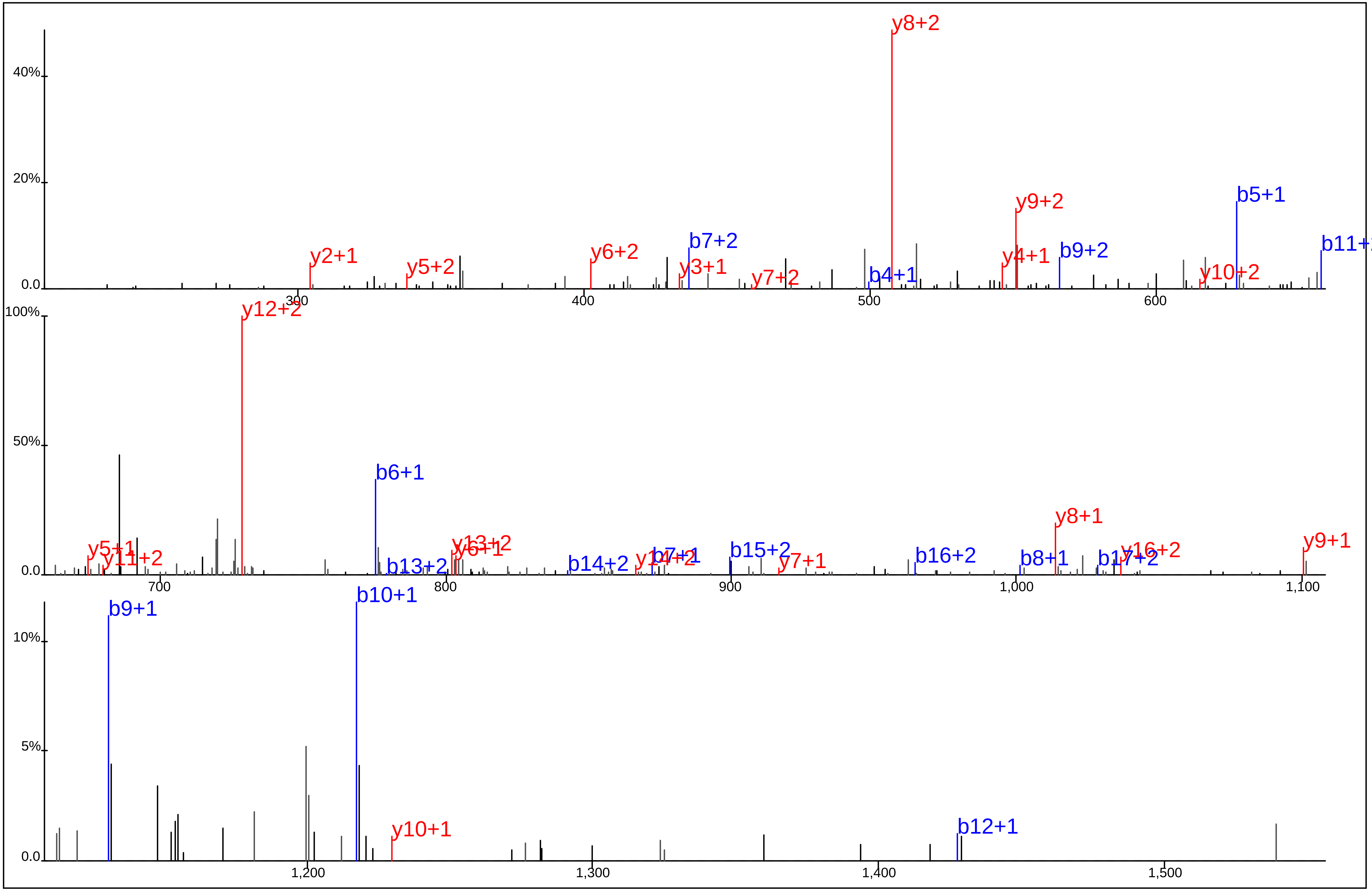

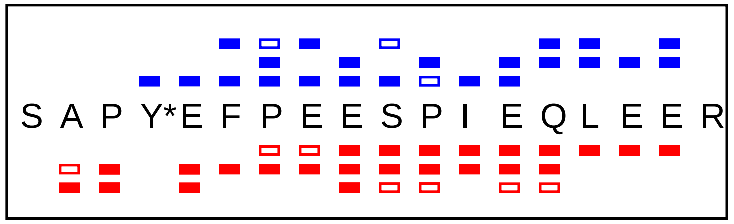

Protein Name: AMP deaminase 2
gi Accession: 21264318
Precursor Mass: 743.9952+3
Delta Mass: 5.510 ppm
XCorr: 38.67

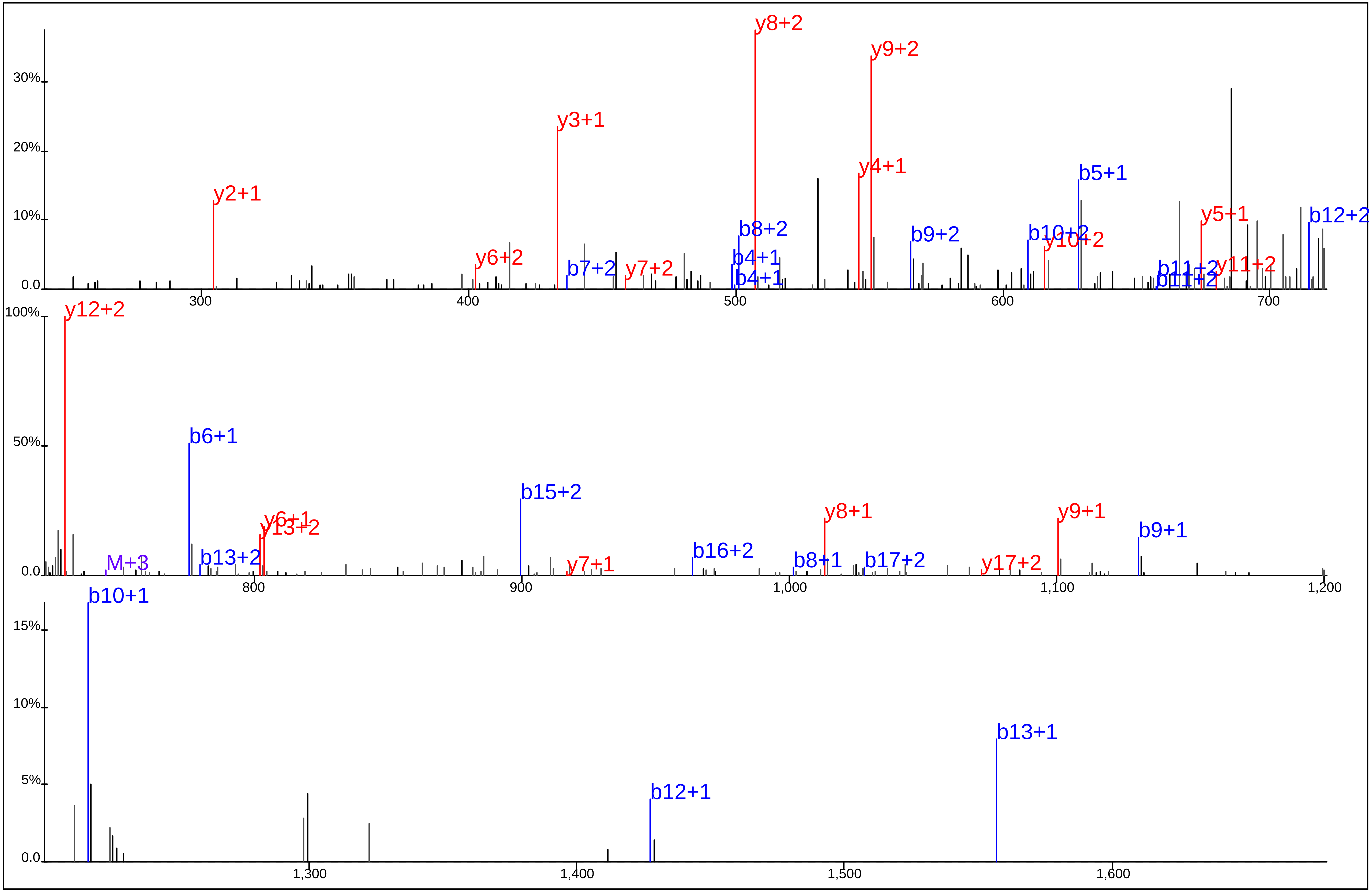

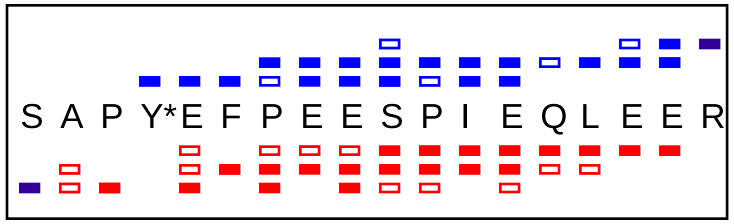

Protein Name:	AMP deaminase 2
gi Accession:	21264318
Precursor Mass:	743.9942+3
Delta Mass:	4.197 ppm
XCorr:	36.04

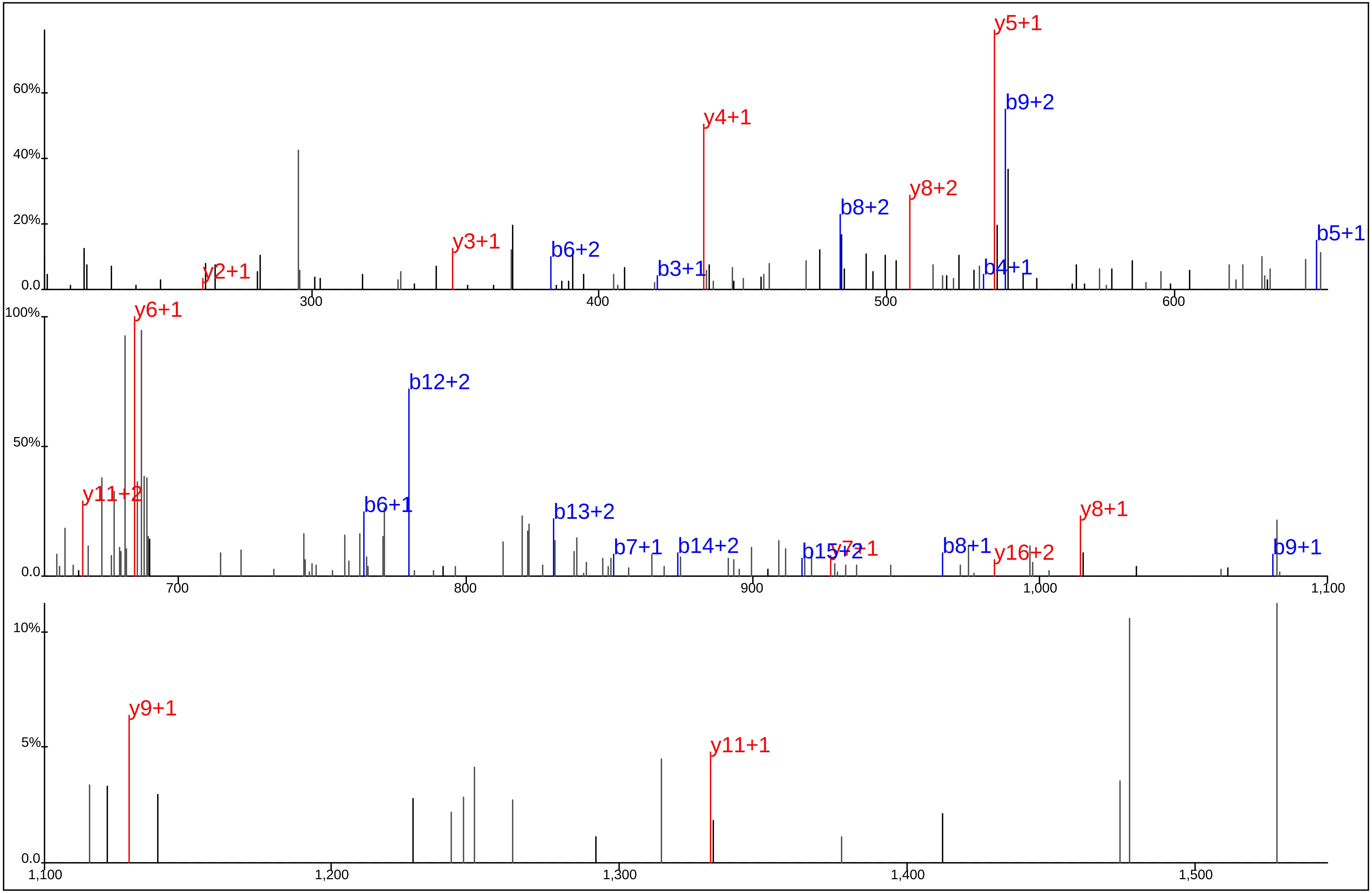

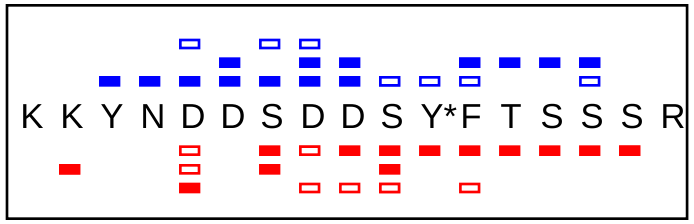

Protein Name: ARF GAP3
gi Accession: 10434365
Precursor Mass: 698.9518+3
Delta Mass: 5.733 ppm
XCorr: 30.2

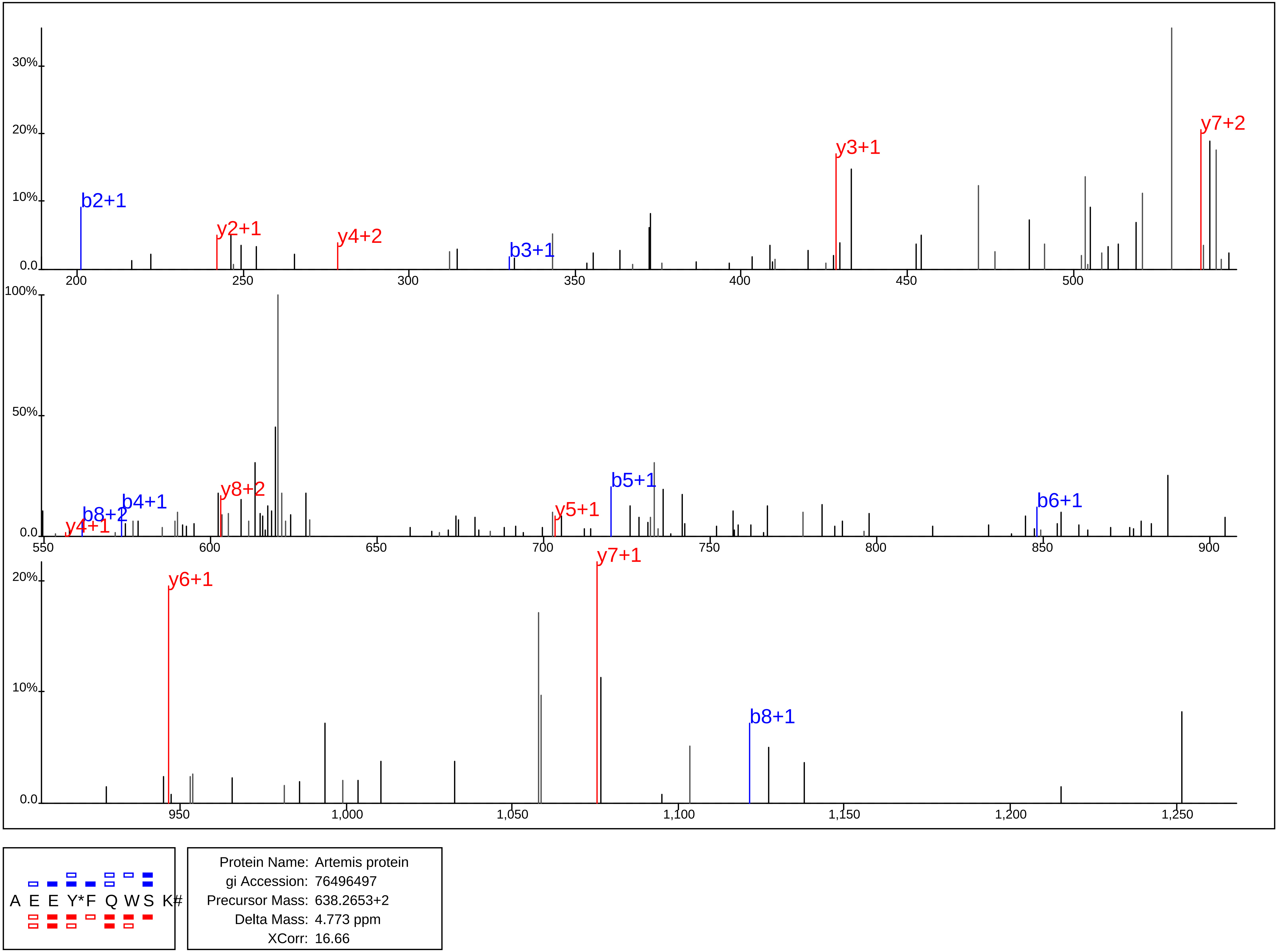

Protein Name: Artemis protein
gi Accession: 76496497
Precursor Mass: 638.2653+2
Delta Mass: 4.773 ppm
XCorr: 16.66

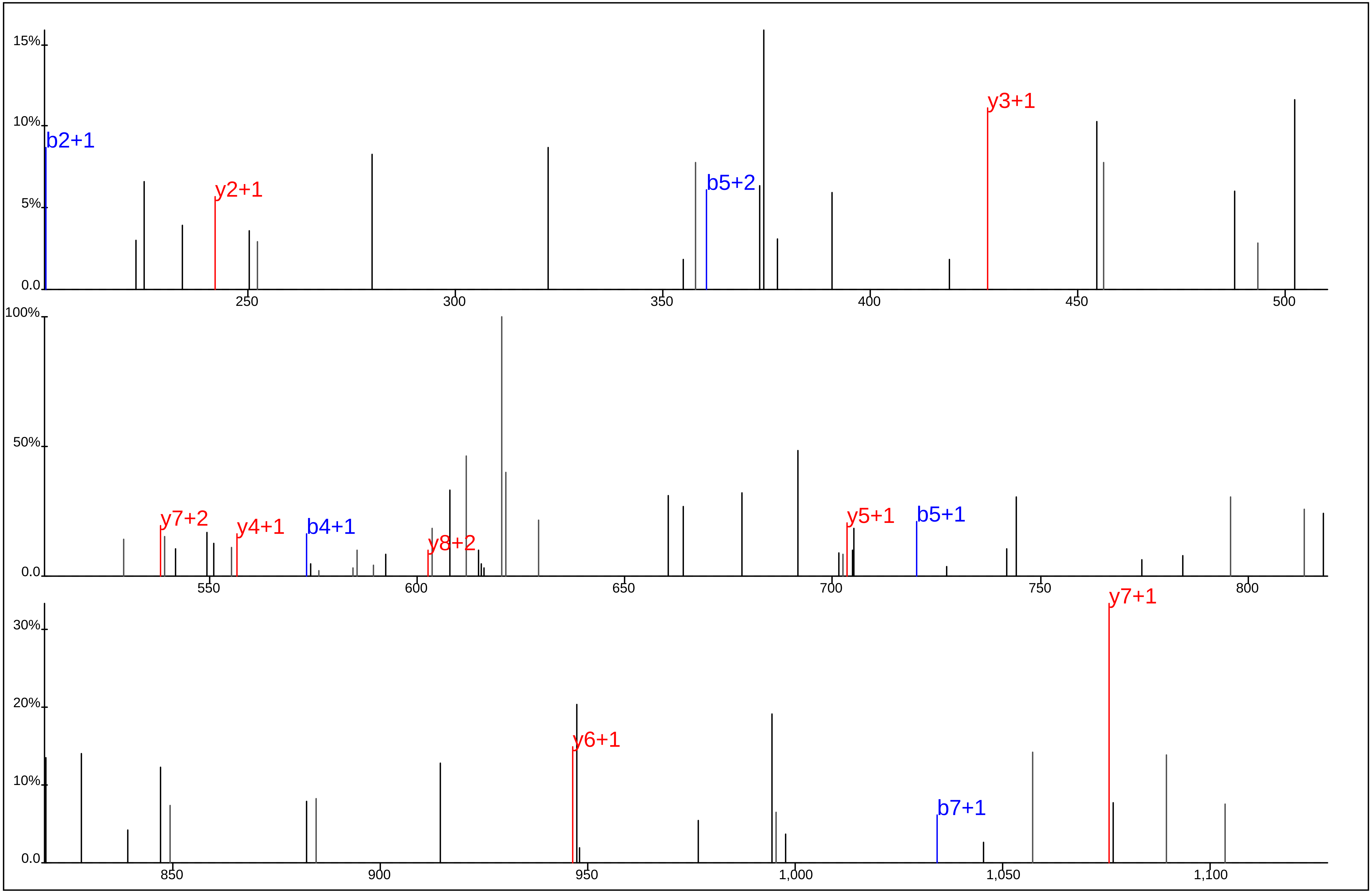

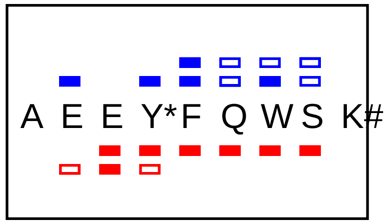

Protein Name: Artemis protein
gi Accession: 76496497
Precursor Mass: 638.2633+2
Delta Mass: 1.613 ppm
XCorr: 19.76

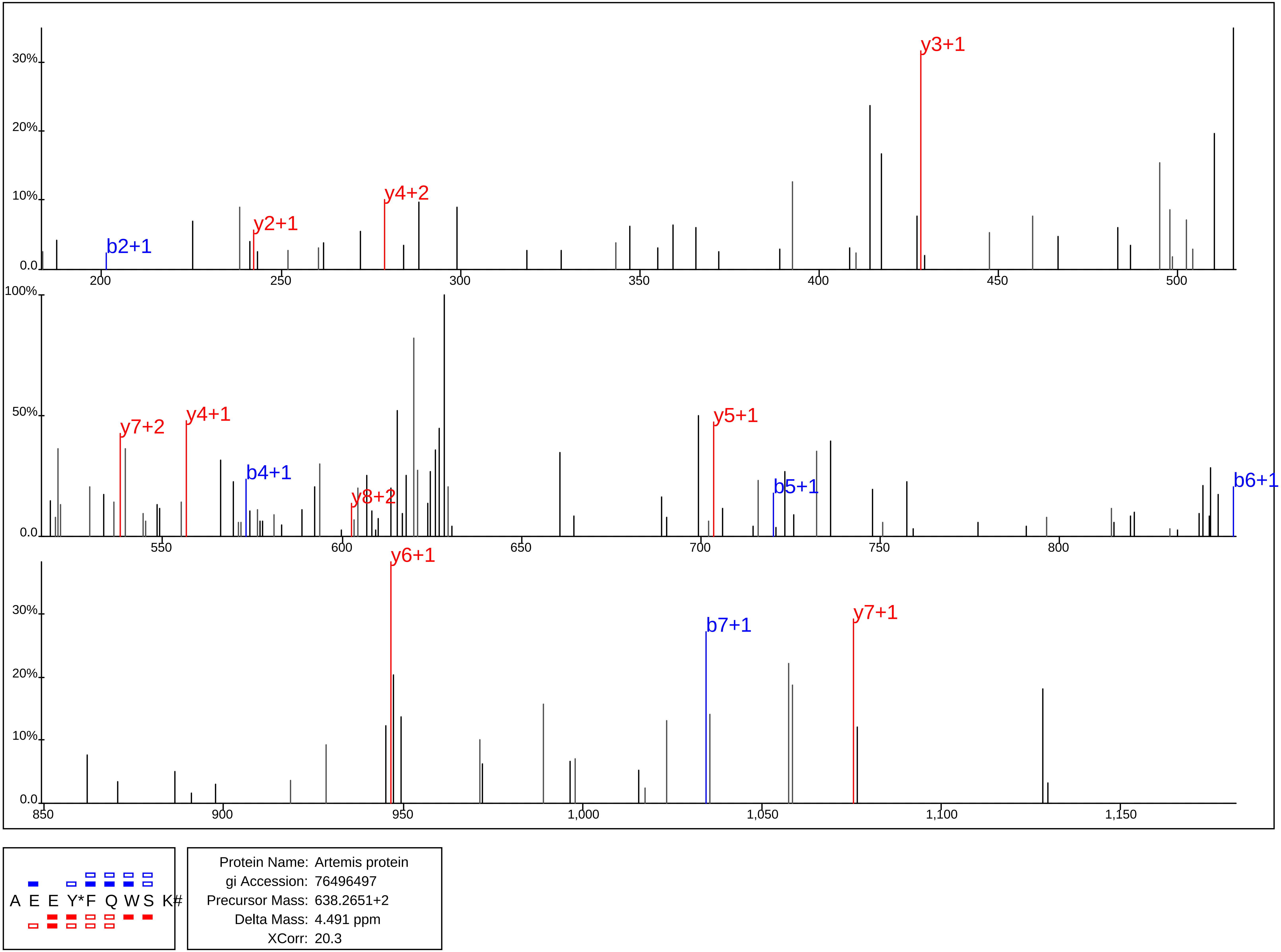

Protein Name: Artemis protein
gi Accession: 76496497
Precursor Mass: 638.2651+2
Delta Mass: 4.491 ppm
XCorr: 20.3

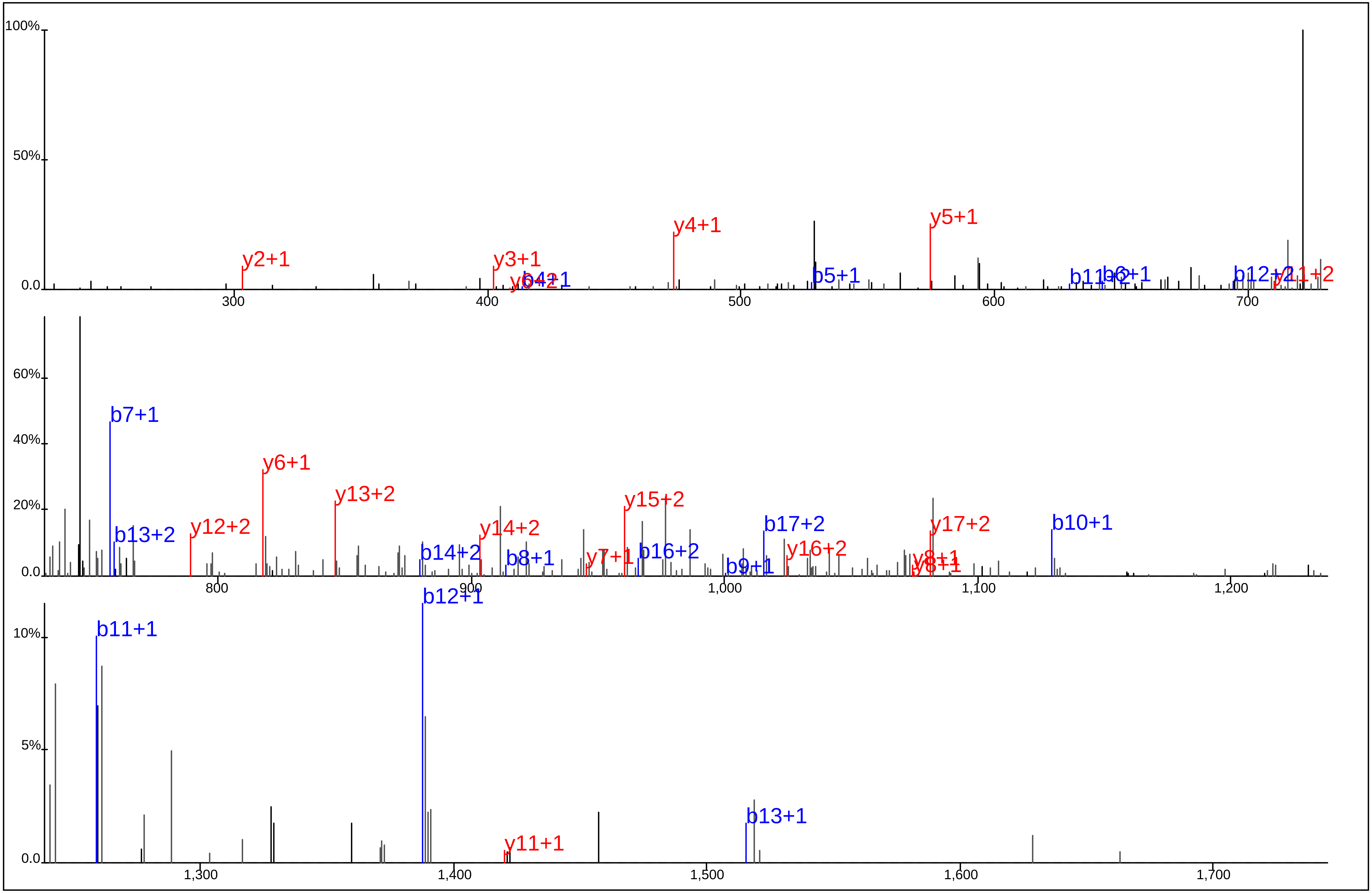

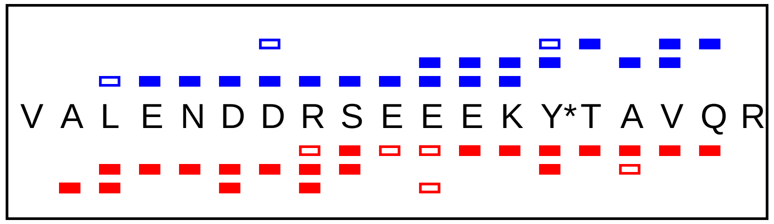

Protein Name: Ataxin 2
gi Accession: 52000732
Precursor Mass: 778.0243+3
Delta Mass: 5.617 ppm
XCorr: 21.22

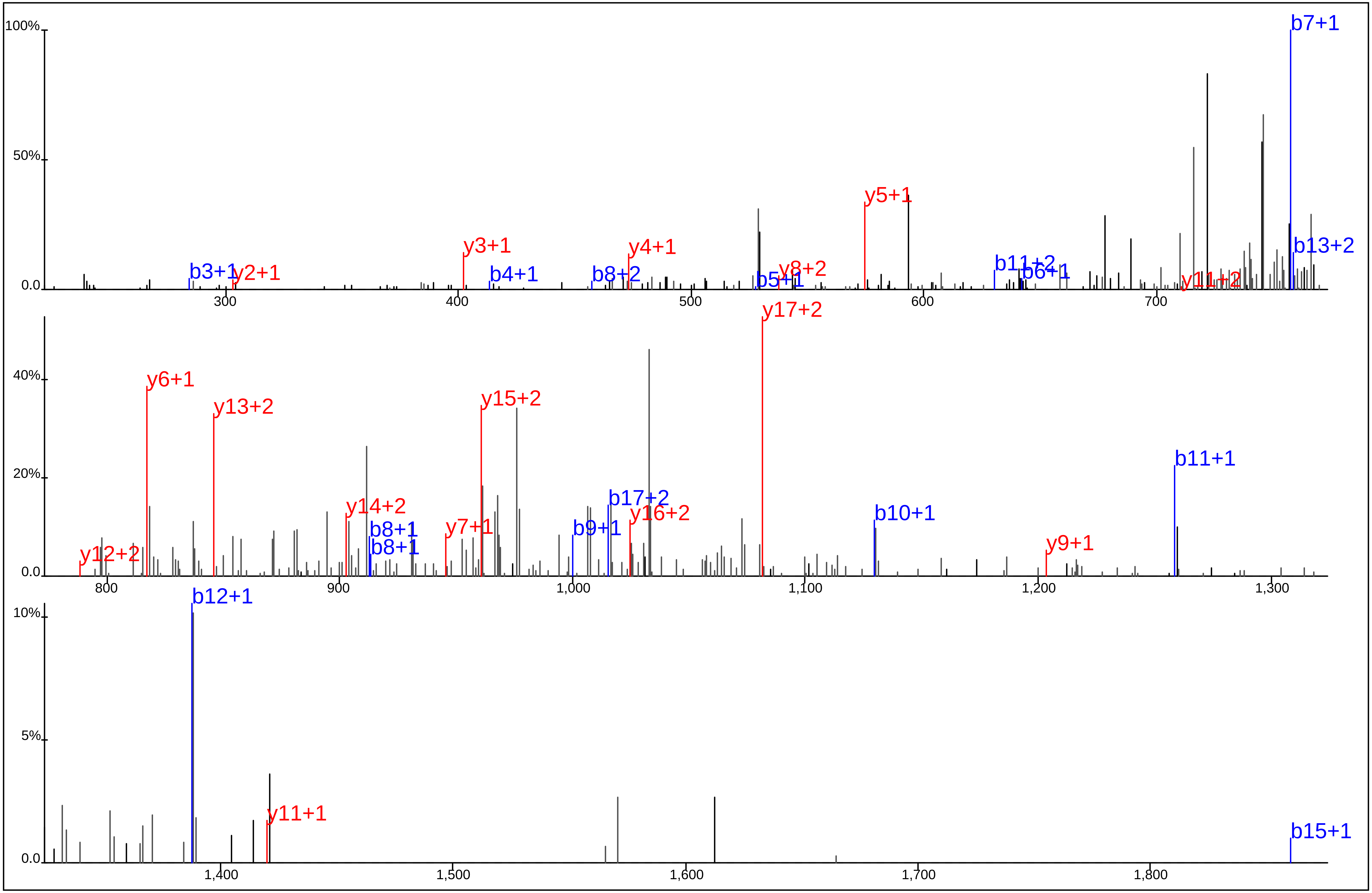

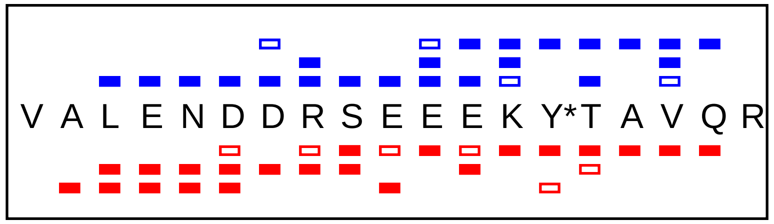

Protein Name: Ataxin 2
gi Accession: 52000732
Precursor Mass: 778.0253+3
Delta Mass: 6.955 ppm
XCorr: 33.98

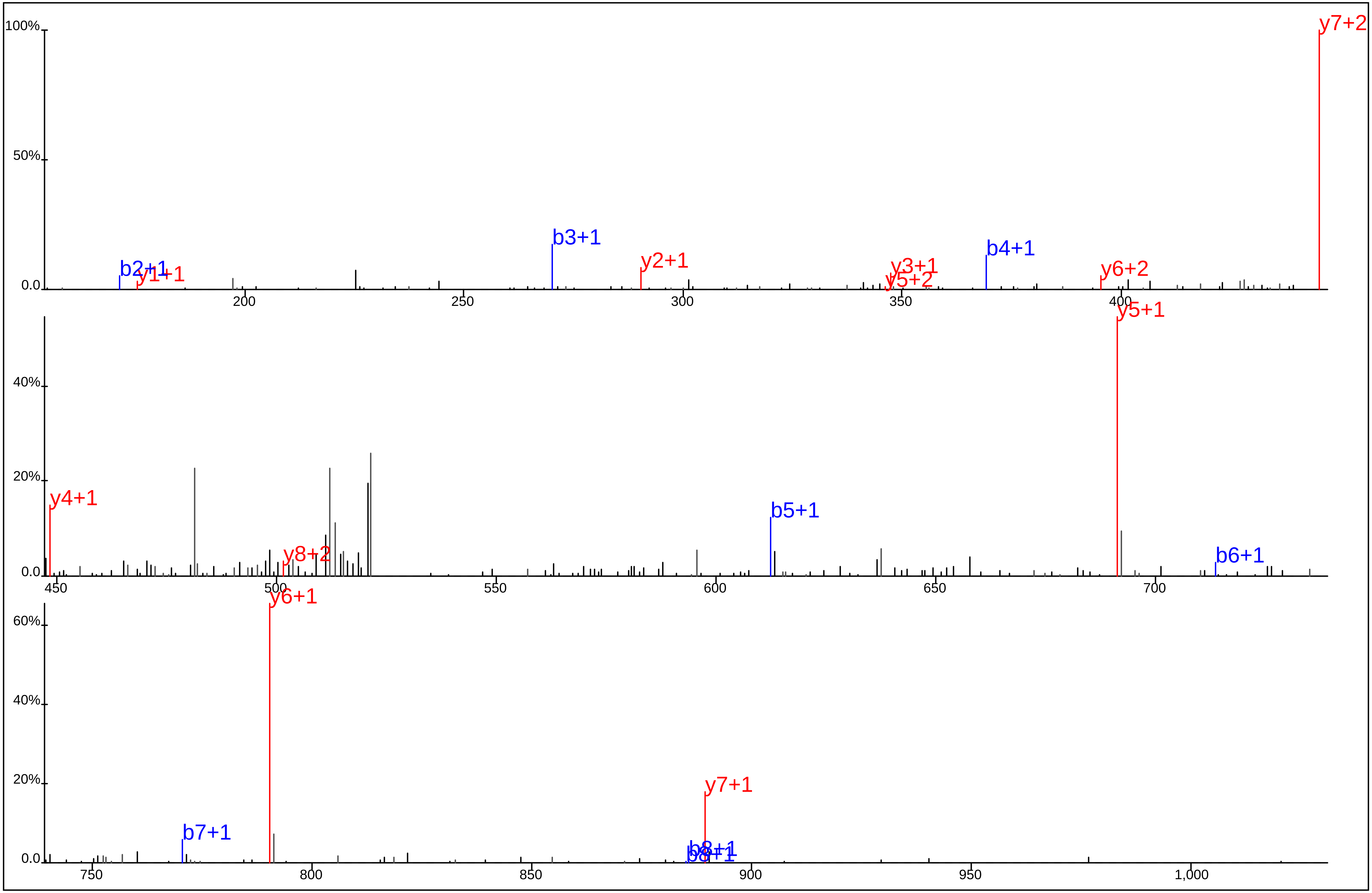

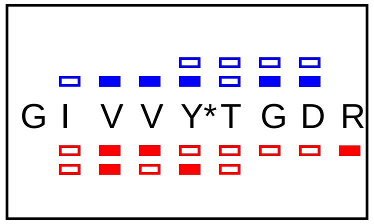

Protein Name: ATP1A1
gi Accession: 21361181
Precursor Mass: 530.2483+2
Delta Mass: 2.122 ppm
XCorr: 43.07

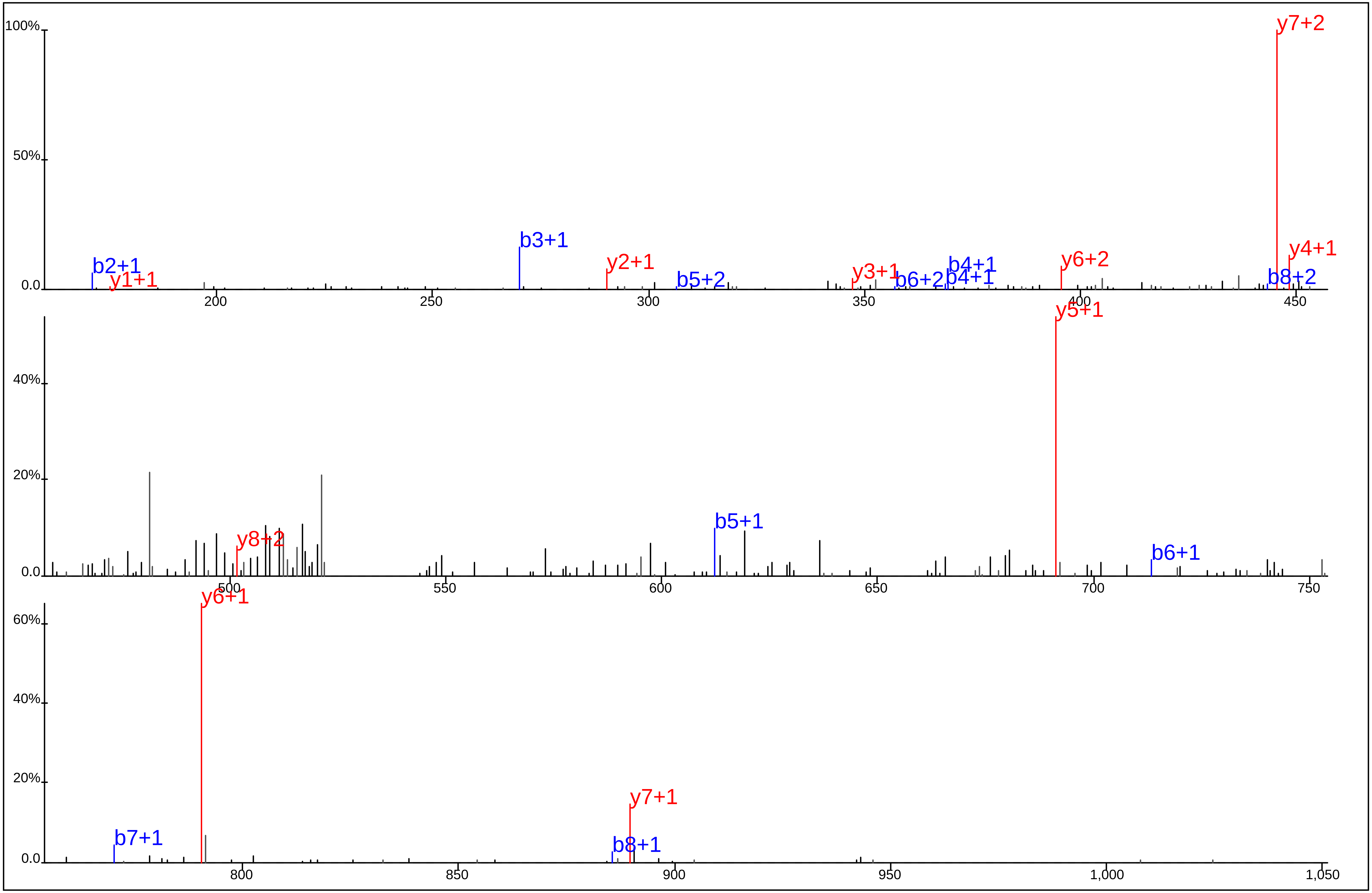

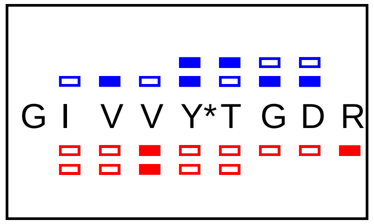

Protein Name:	ATP1A1
gi Accession:	21361181
Precursor Mass:	530.2488+2
Delta Mass:	3.037 ppm
XCorr:	37.7

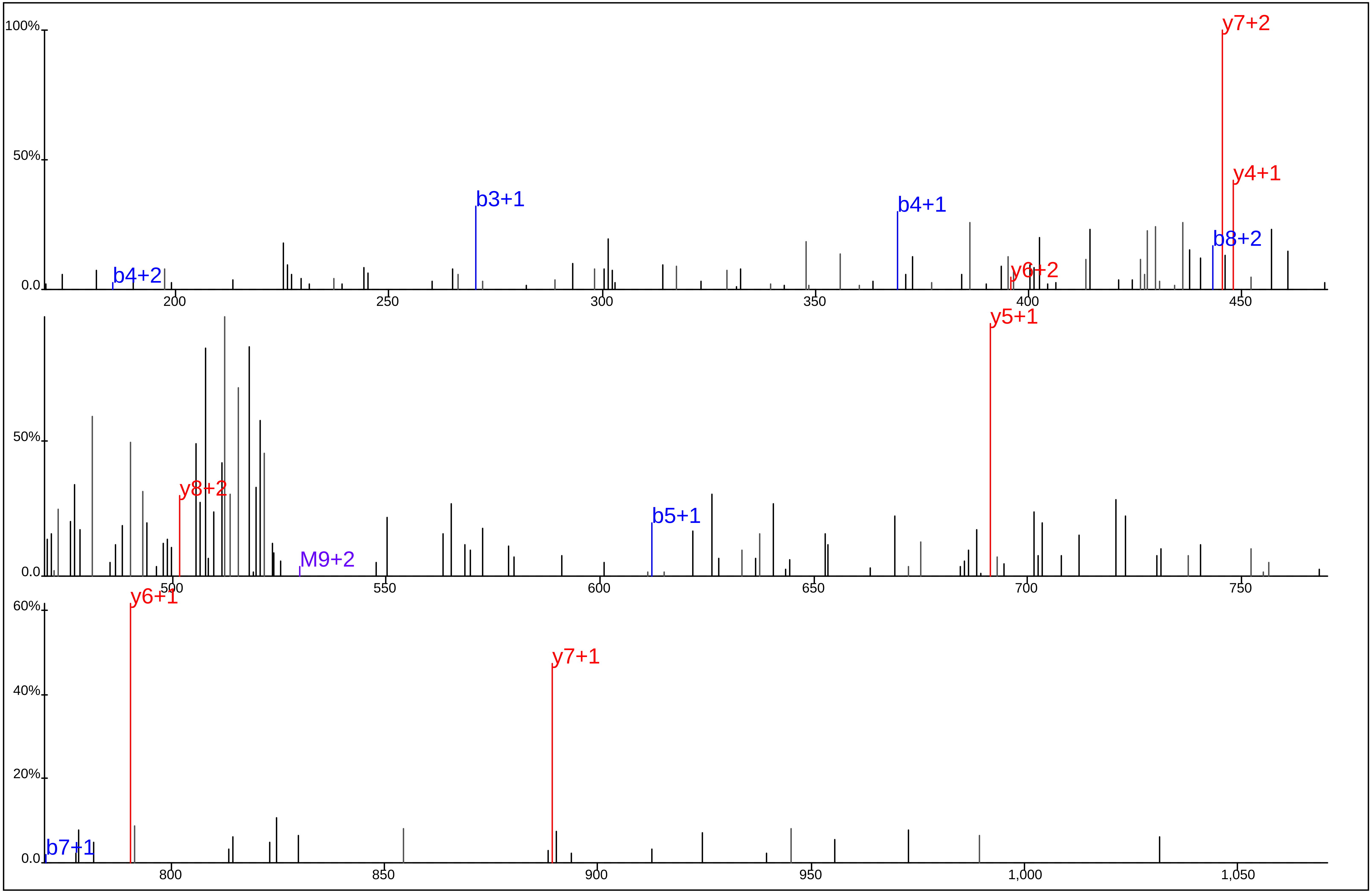

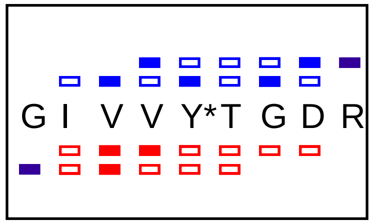

Protein Name: ATP1A1
gi Accession: 21361181
Precursor Mass: 530.2484+2
Delta Mass: 2.348 ppm
XCorr: 13.66

52 - ATP1A1_3453759.pdf

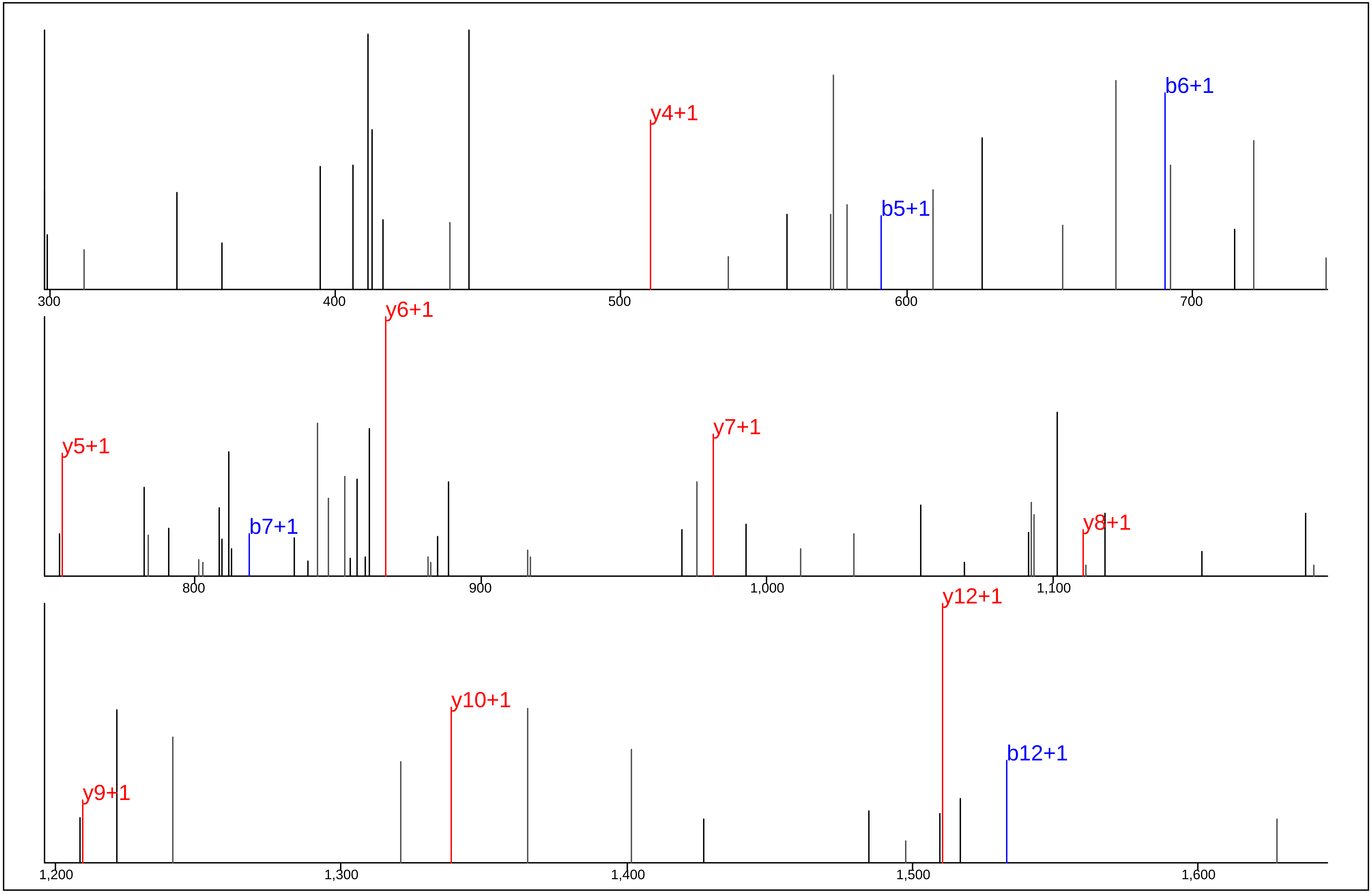

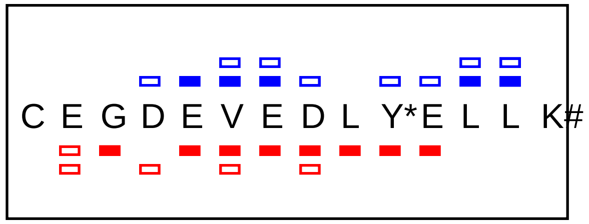

Protein Name:	ATP binding domain 4
gi Accession:	18087809
Precursor Mass:	900.3814+2
Delta Mass:	1.071 ppm
XCorr:	24.26

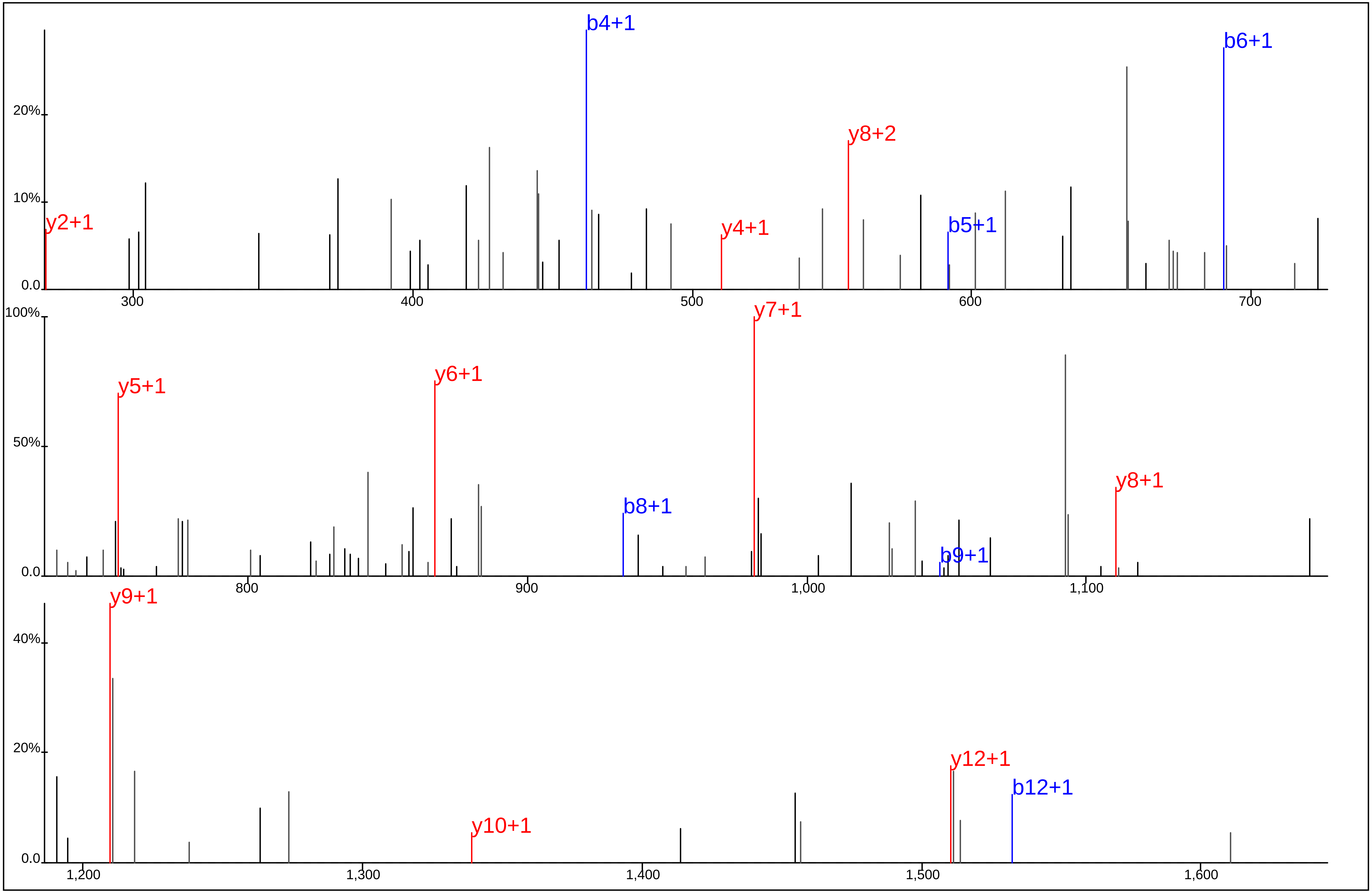

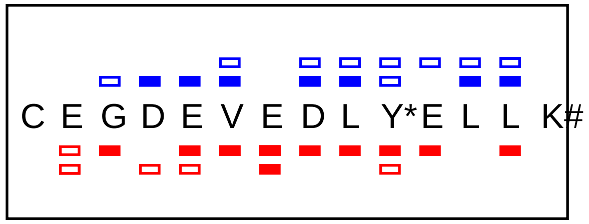

Protein Name:	ATP binding domain 4
gi Accession:	18087809
Precursor Mass:	900.3907+2
Delta Mass:	11.384 ppm
XCorr:	41.34

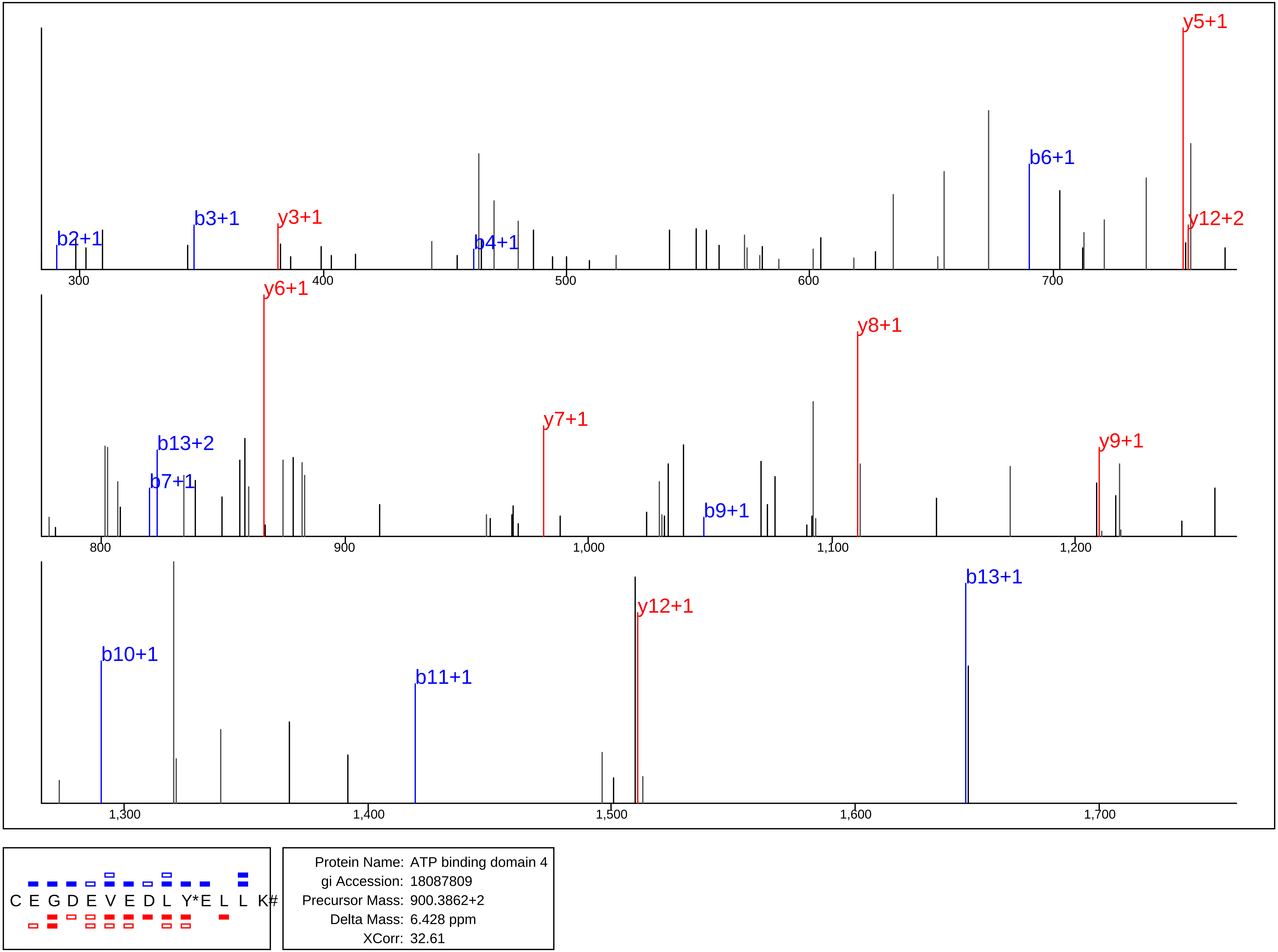

Protein Name:	ATP binding domain 4
gi Accession:	18087809
Precursor Mass:	900.3862+2
Delta Mass:	6.428 ppm
XCorr:	32.61

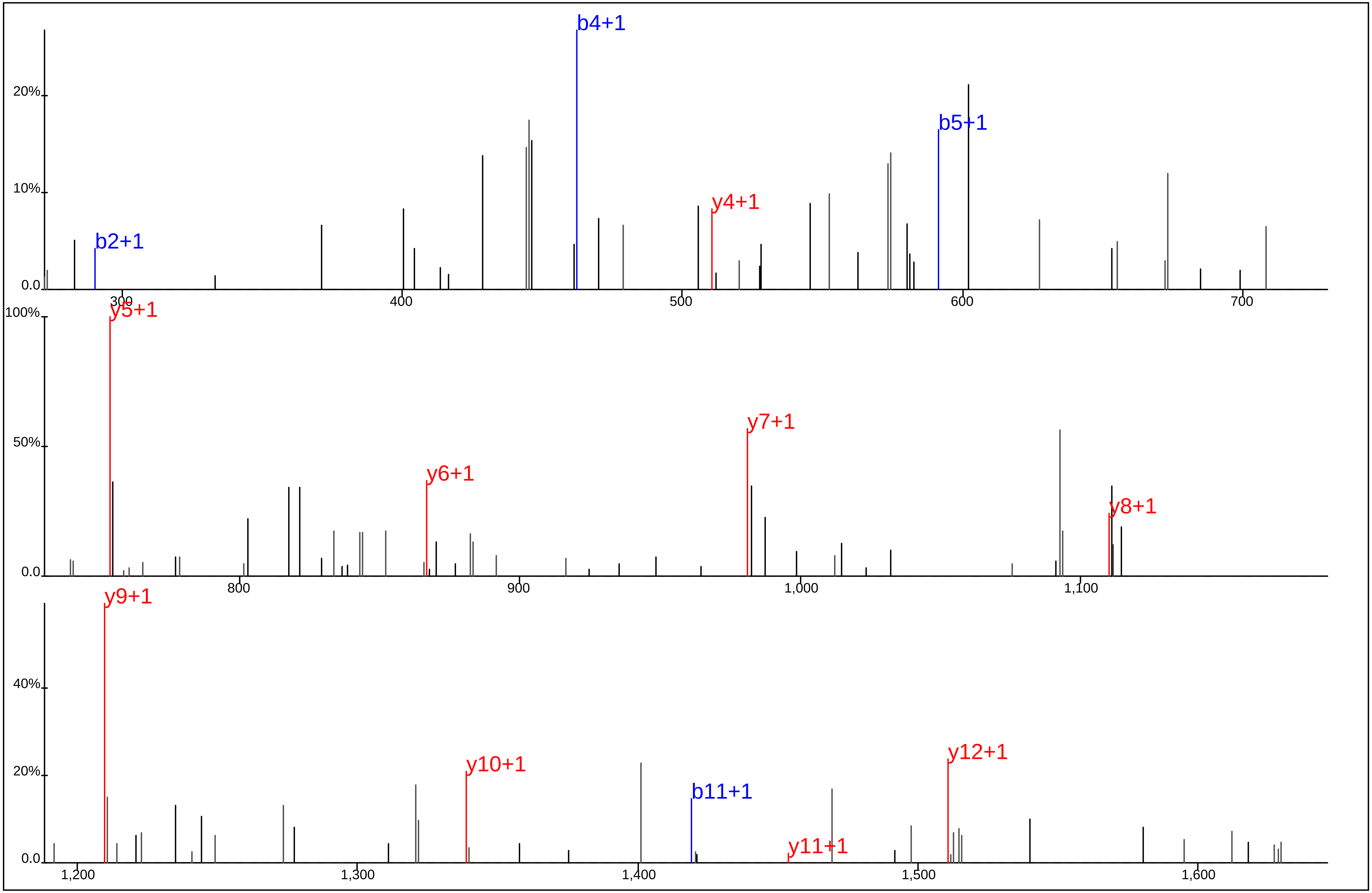

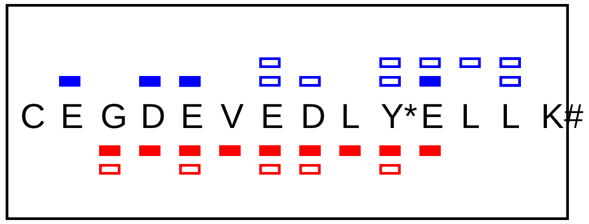

Protein Name: ATP binding domain 4
gi Accession: 18087809
Precursor Mass: 900.3807+2
Delta Mass: 0.327 ppm
XCorr: 29.93

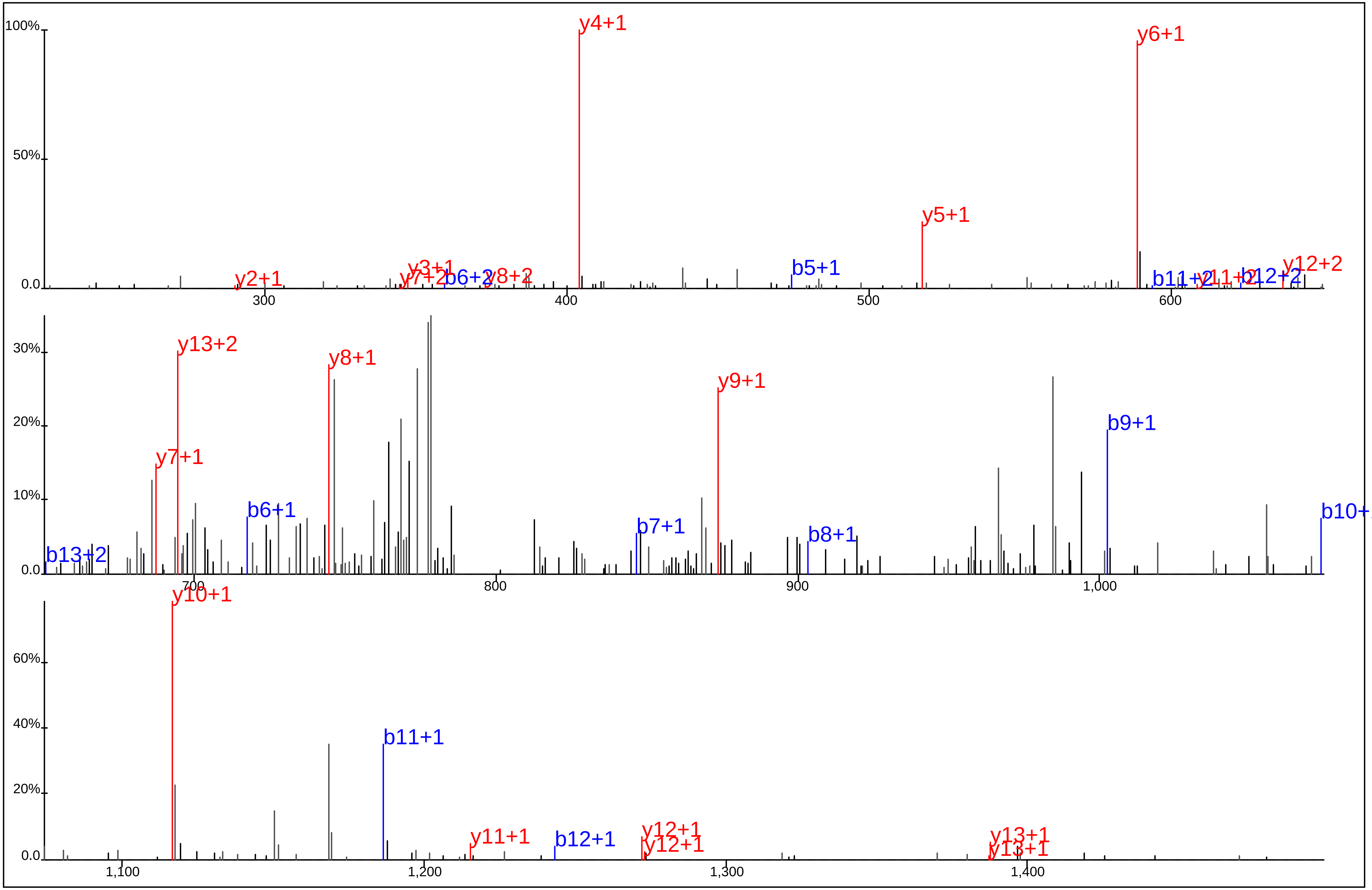

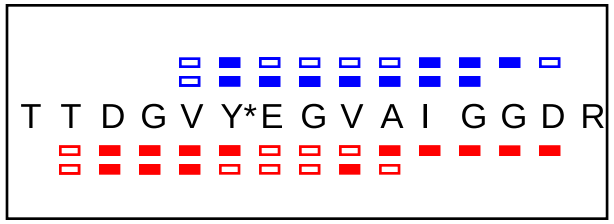

Protein Name: ATP citrate lyase
gi Accession: 68533125
Precursor Mass: 795.3471+2
Delta Mass: 1.647 ppm
XCorr: 74.69

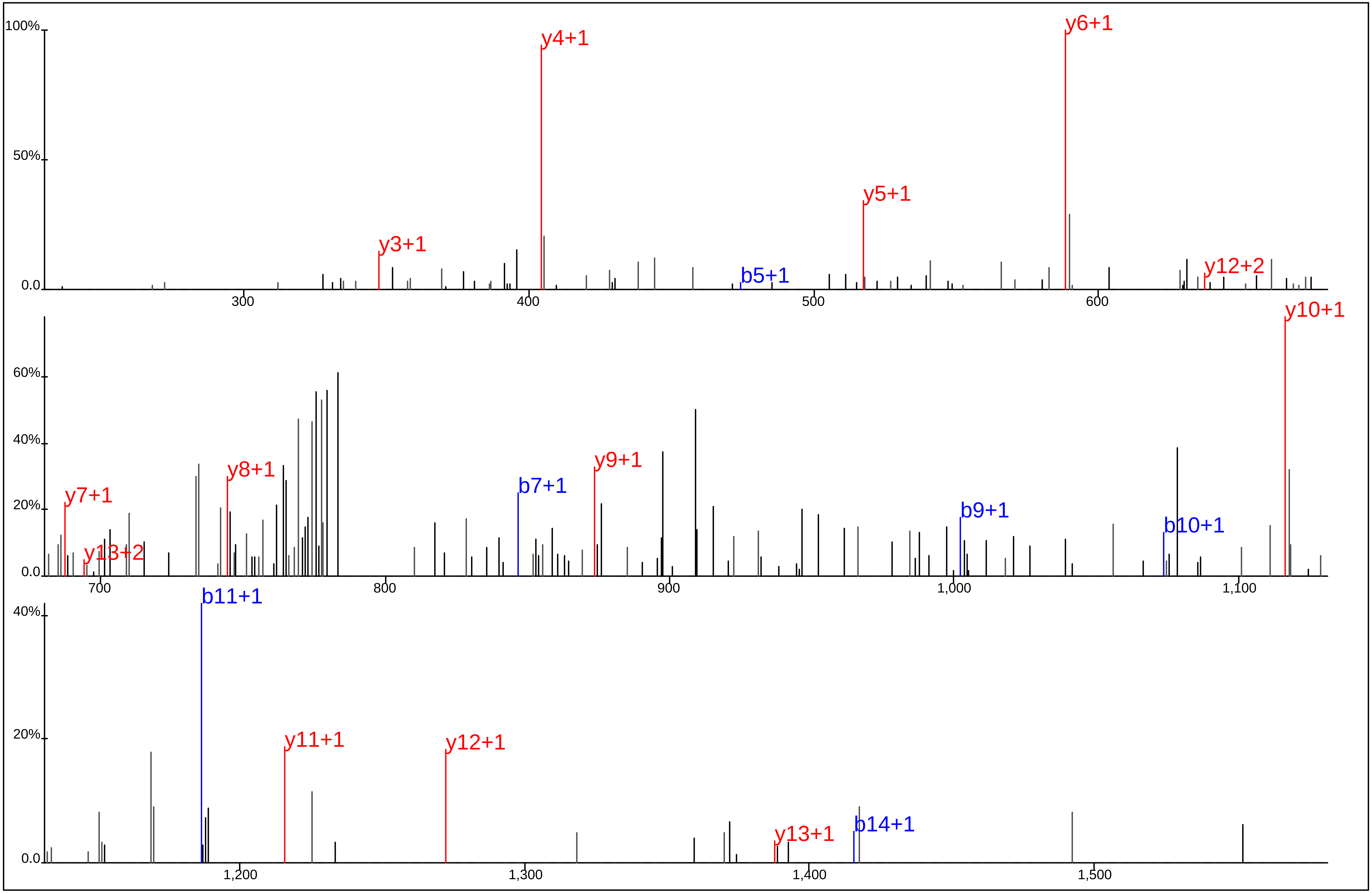

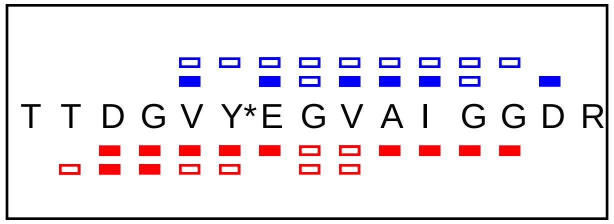

Protein Name: ATP citrate lyase
gi Accession: 68533125
Precursor Mass: 795.3469+2
Delta Mass: 1.339 ppm
XCorr: 38.76

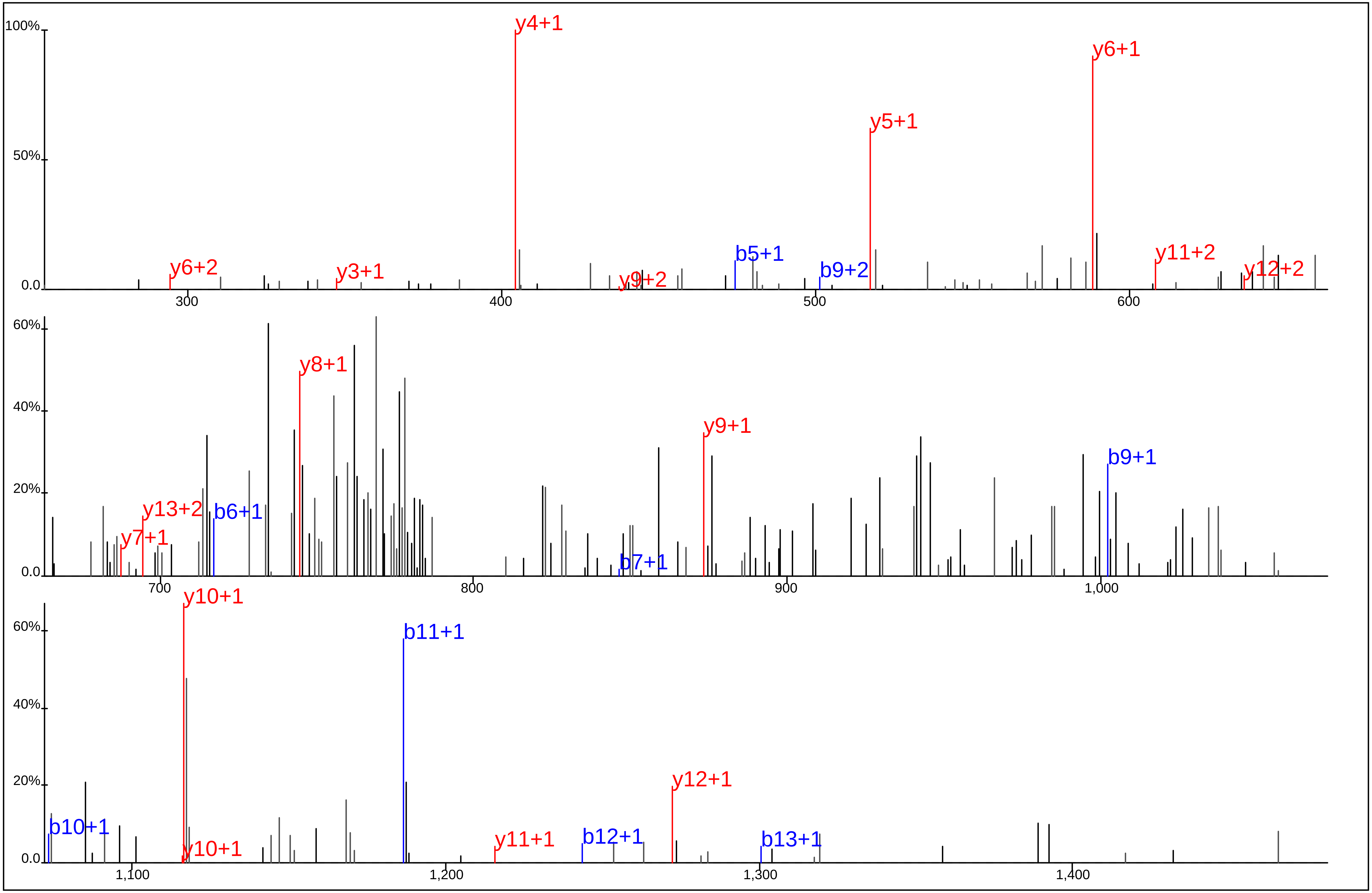

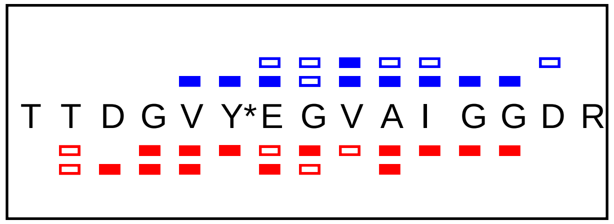

Protein Name: ATP citrate lyase
gi Accession: 68533125
Precursor Mass: 795.3500+2
Delta Mass: 5.333 ppm
XCorr: 29.12

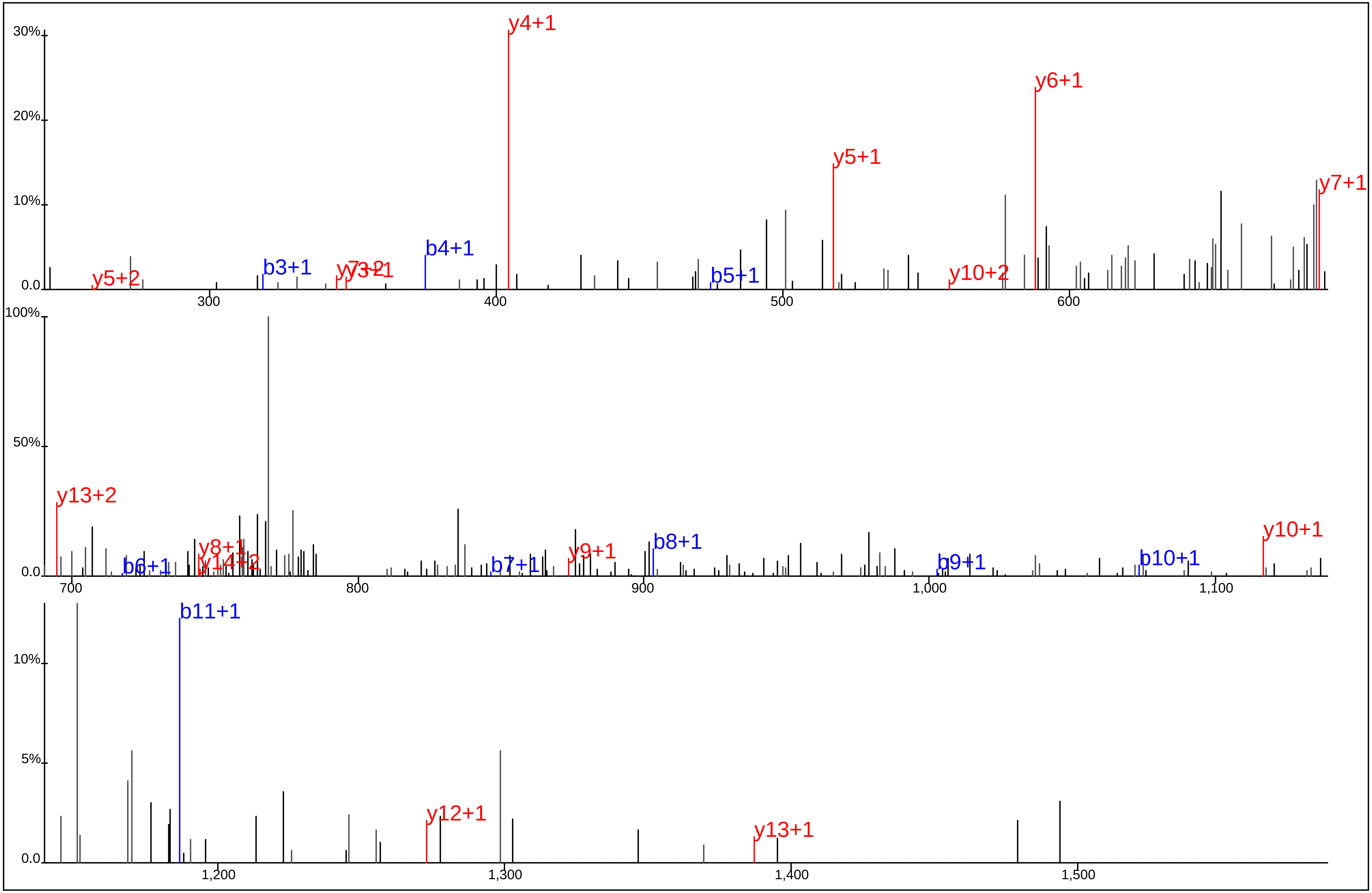

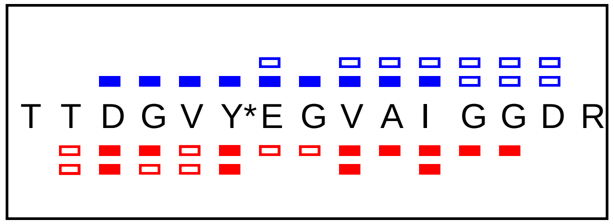

Protein Name:	ATP citrate lyase
gi Accession:	68533125
Precursor Mass:	795.3479+2
Delta Mass:	2.641 ppm
XCorr:	12.16

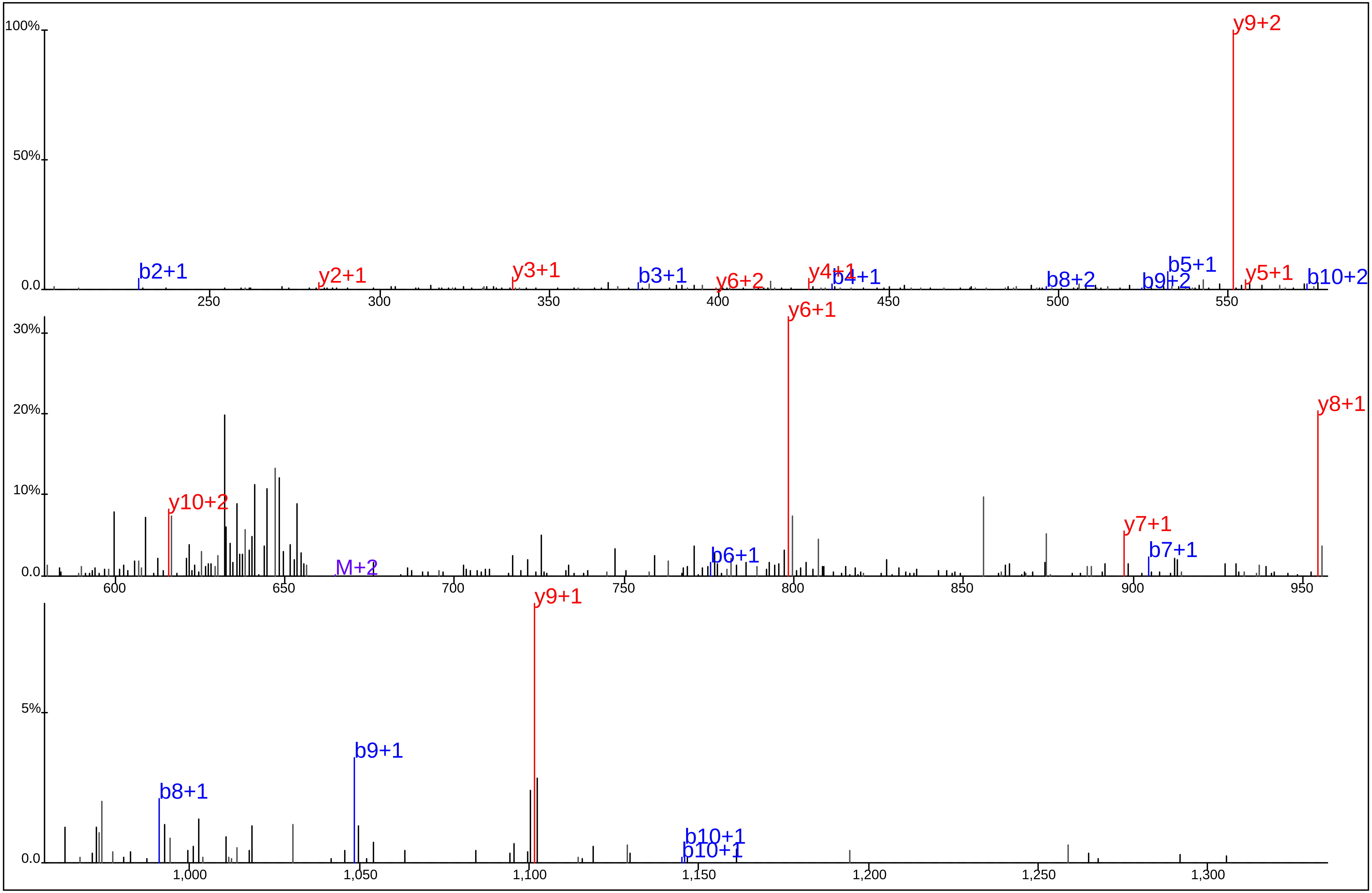

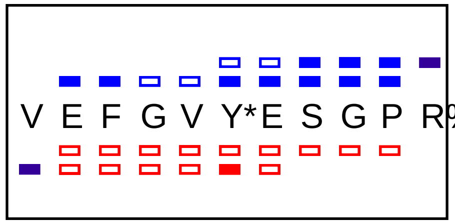

Protein Name: BCAP
gi Accession: 28273124
Precursor Mass: 665.2935+2
Delta Mass: 3.564 ppm
XCorr: 43.13

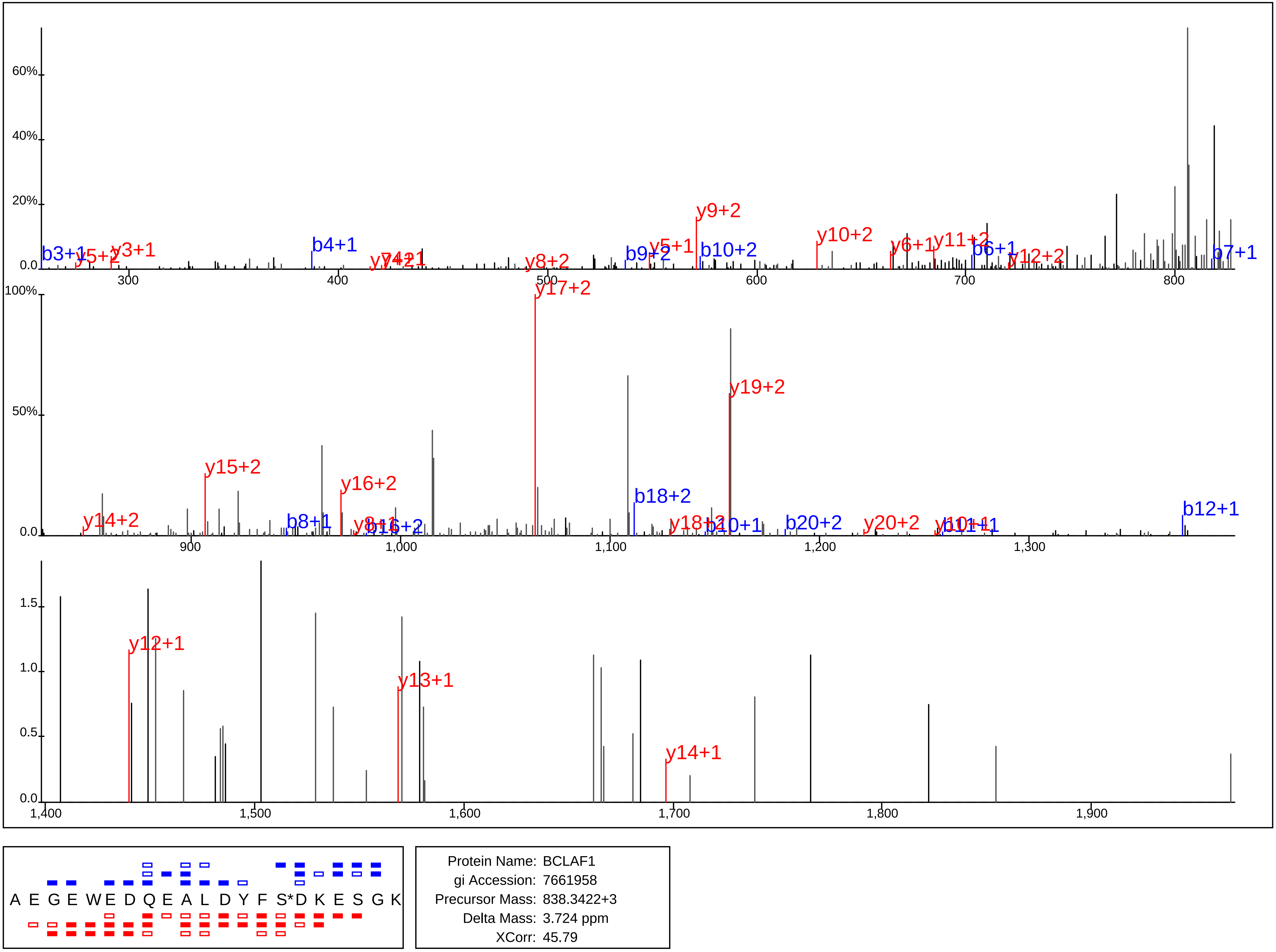

Protein Name: BCLAF1
gi Accession: 7661958
Precursor Mass: 838.3422+3
Delta Mass: 3.724 ppm
XCorr: 45.79

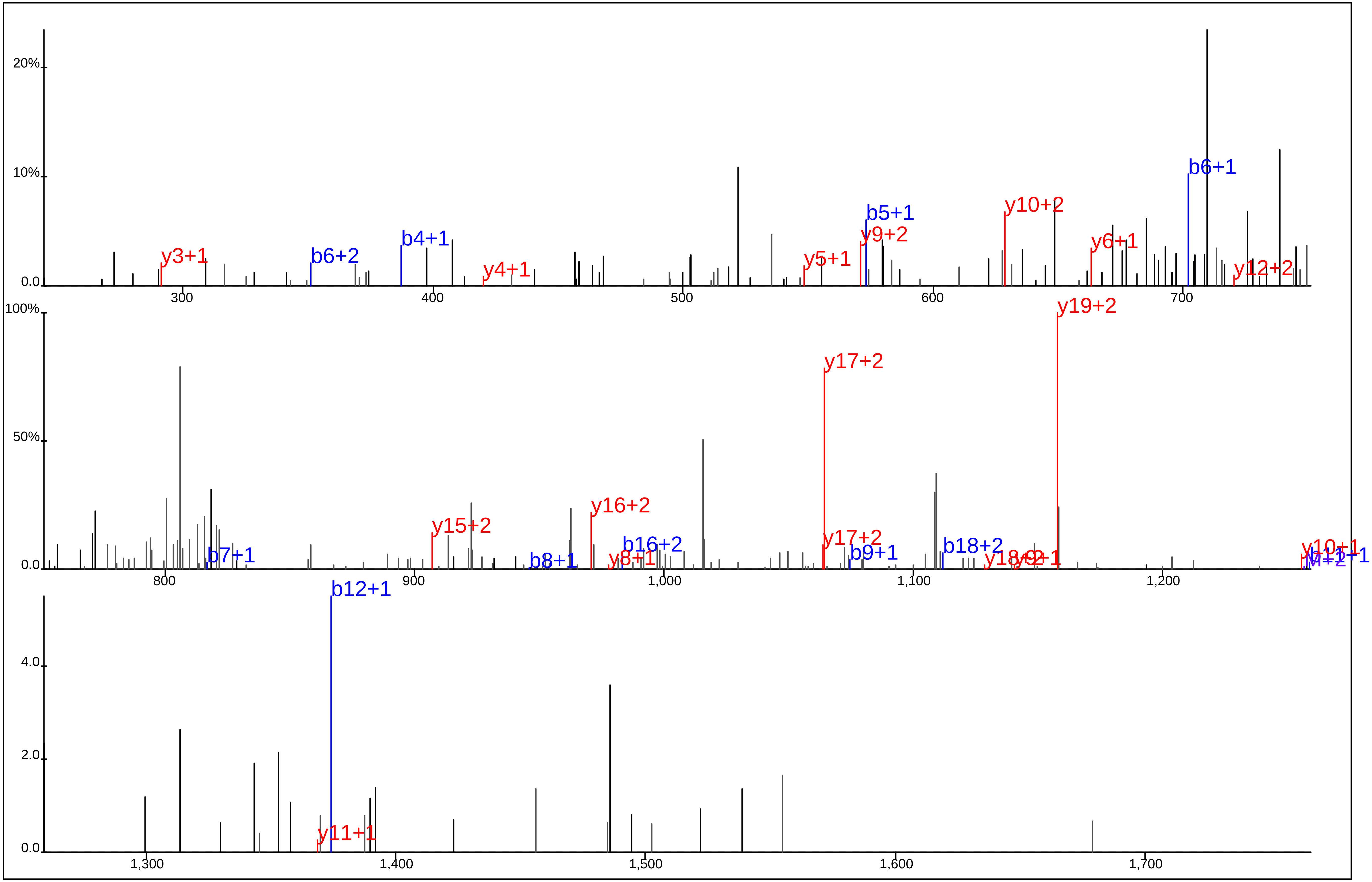

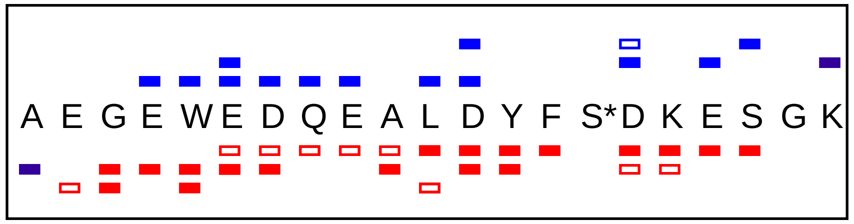

Protein Name: BCLAF1
gi Accession: 7661958
Precursor Mass: 838.3437+3
Delta Mass: 5.470 ppm
XCorr: 13.42

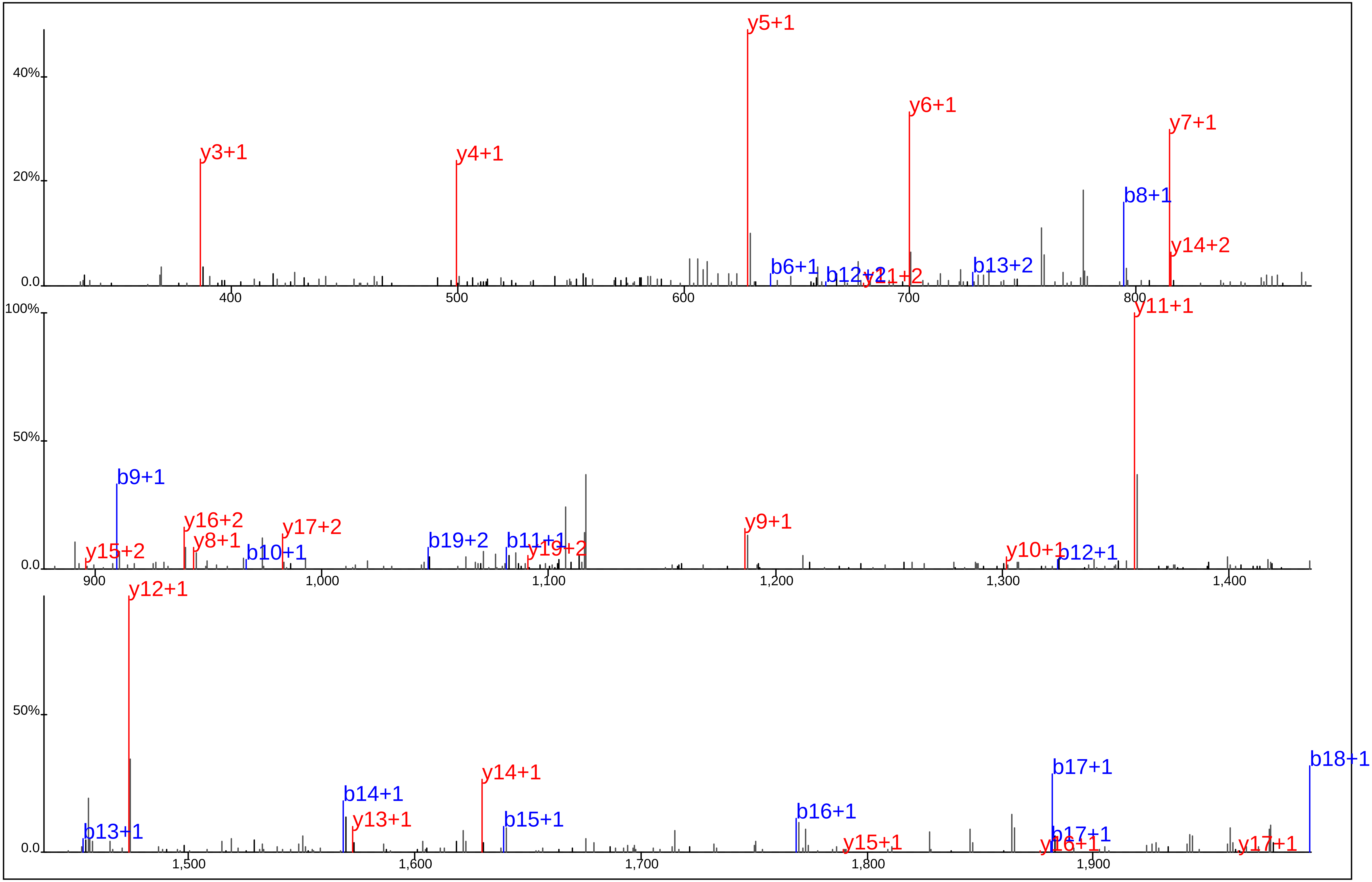

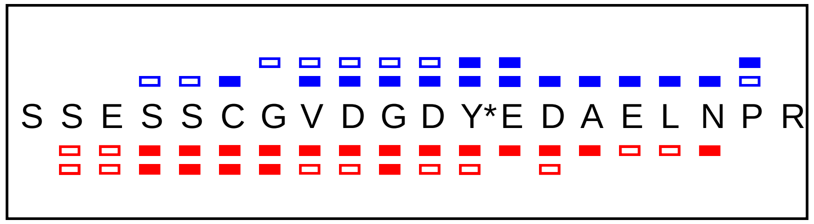

Protein Name: BCR
gi Accession: 28629
Precursor Mass: 1,133.9270+2
Delta Mass: 1.128 ppm
XCorr: 75.74

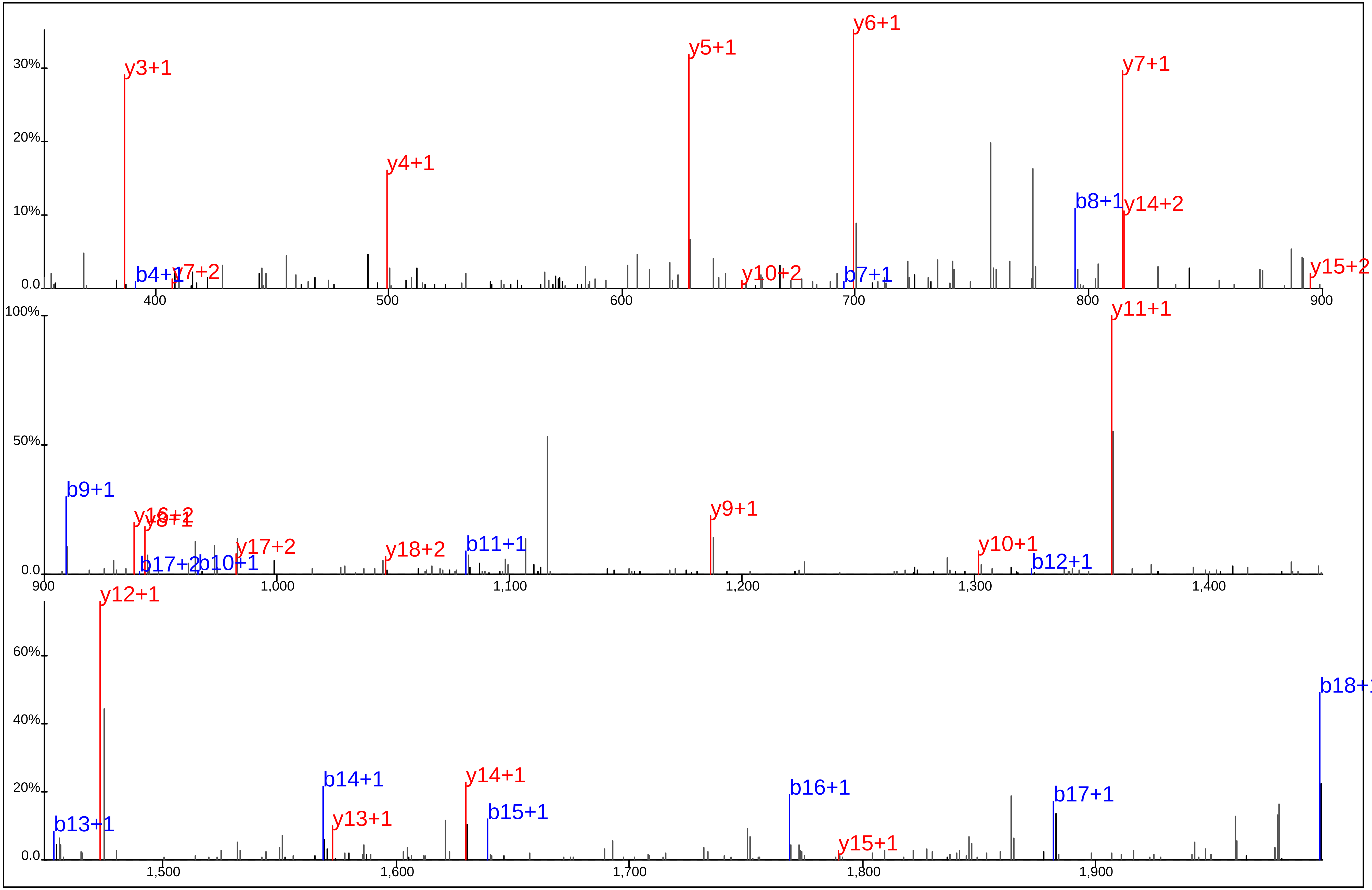

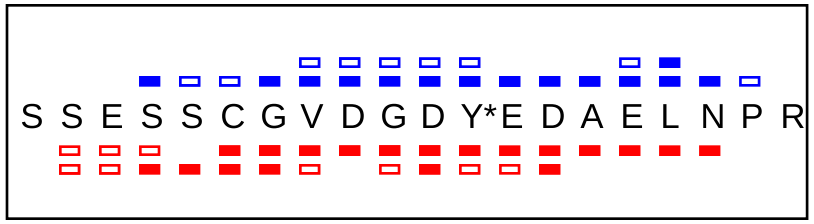

Protein Name: BCR
gi Accession: 28629
Precursor Mass: 1,133.9359+2
Delta Mass: 8.990 ppm
XCorr: 92.49

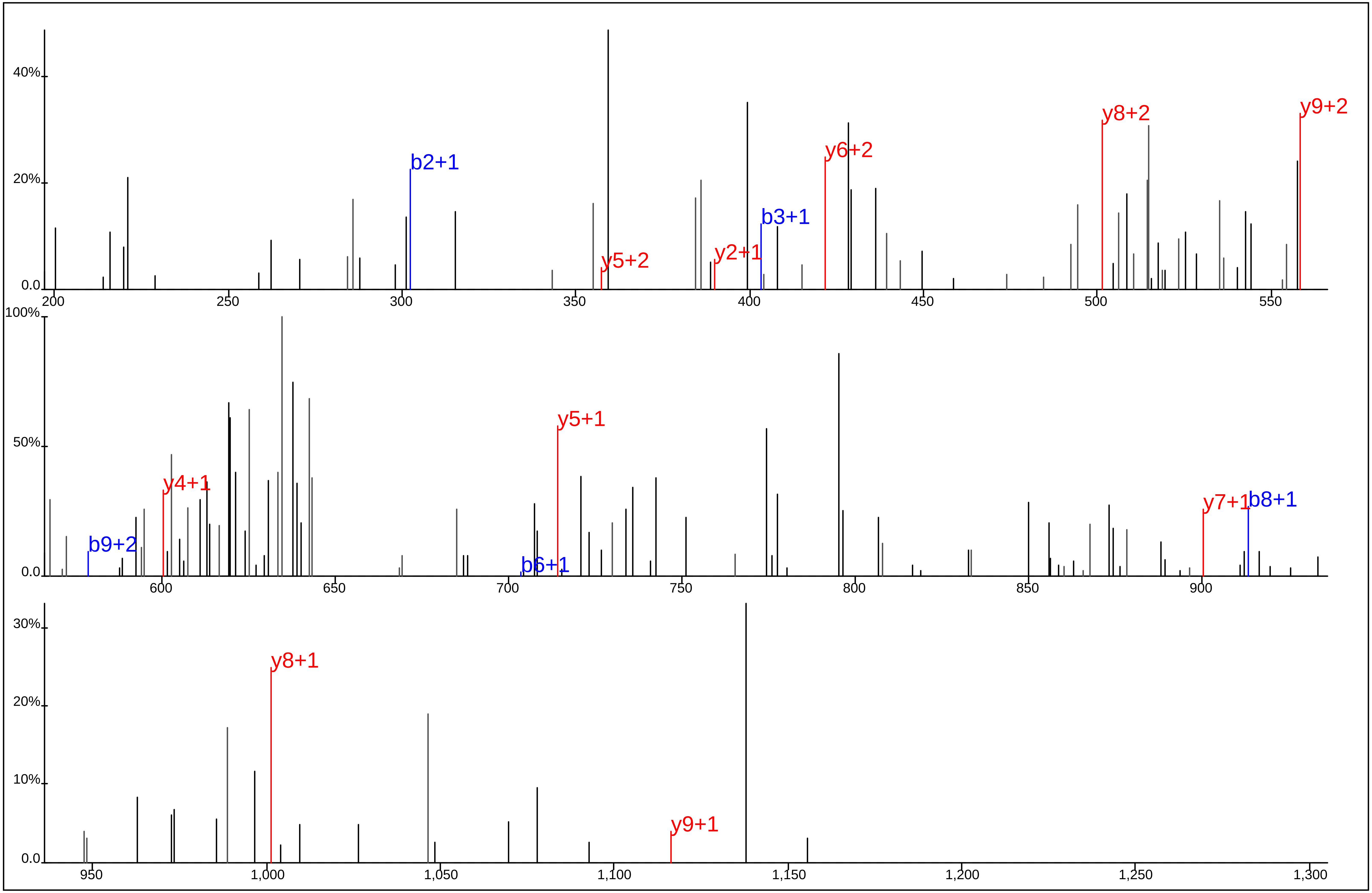

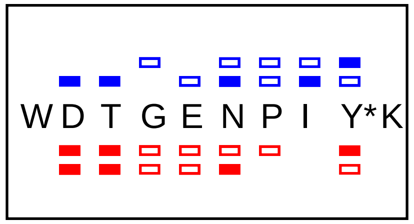

Protein Name:	Beta1 integrin
gi Accession:	124963
Precursor Mass:	651.7760+2
Delta Mass:	3.484 ppm
XCorr:	12.28

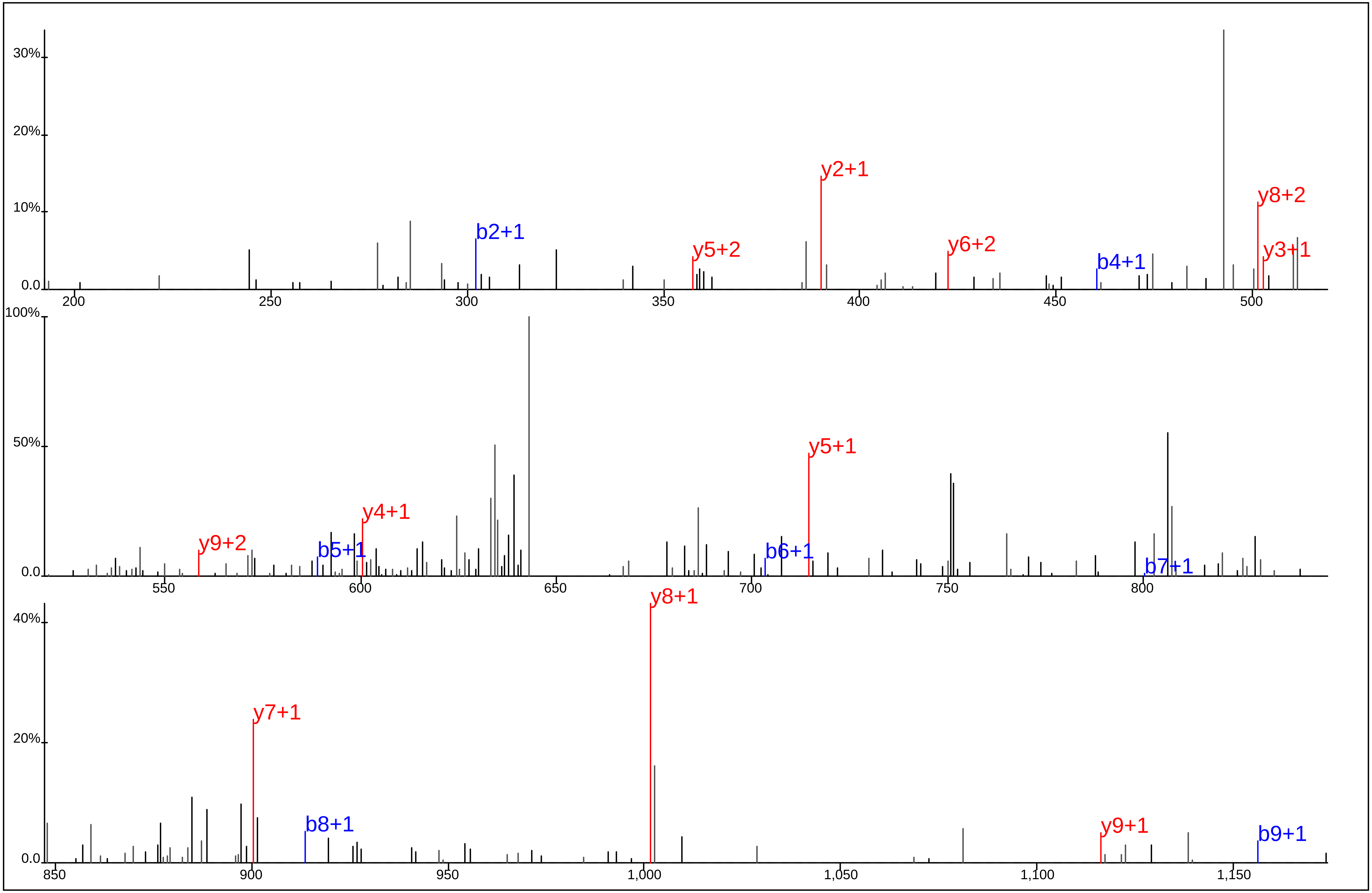

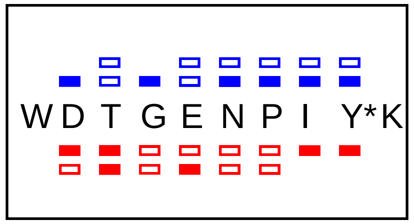

Protein Name:	Beta1 integrin
gi Accession:	124963
Precursor Mass:	651.7756+2
Delta Mass:	2.824 ppm
XCorr:	33.08

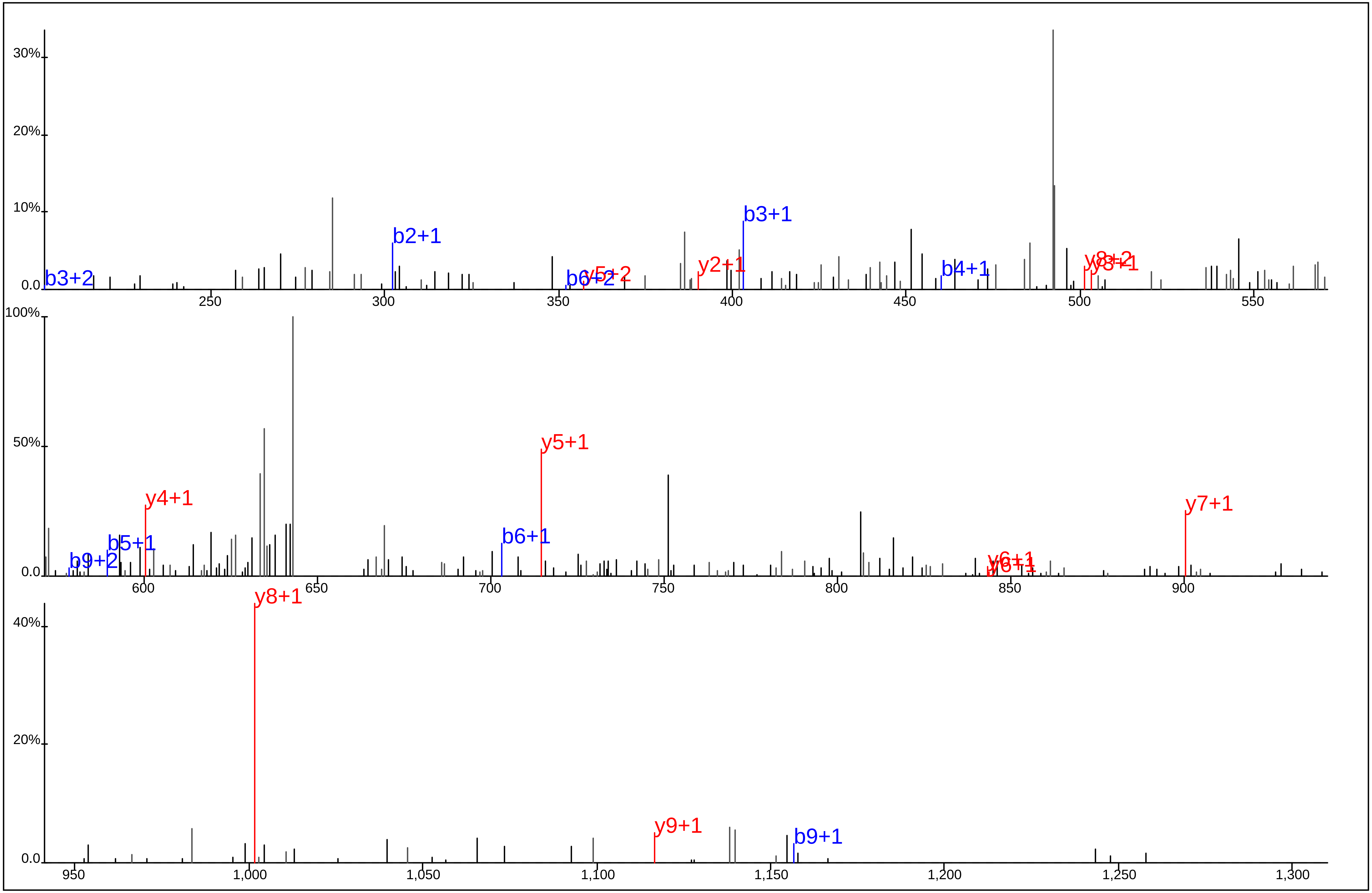

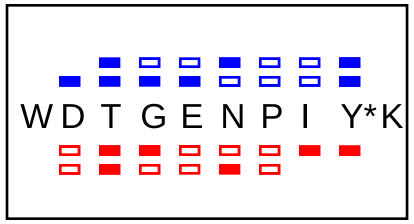

Protein Name:	Beta1 integrin
gi Accession:	124963
Precursor Mass:	651.7782+2
Delta Mass:	6.854 ppm
XCorr:	21.71

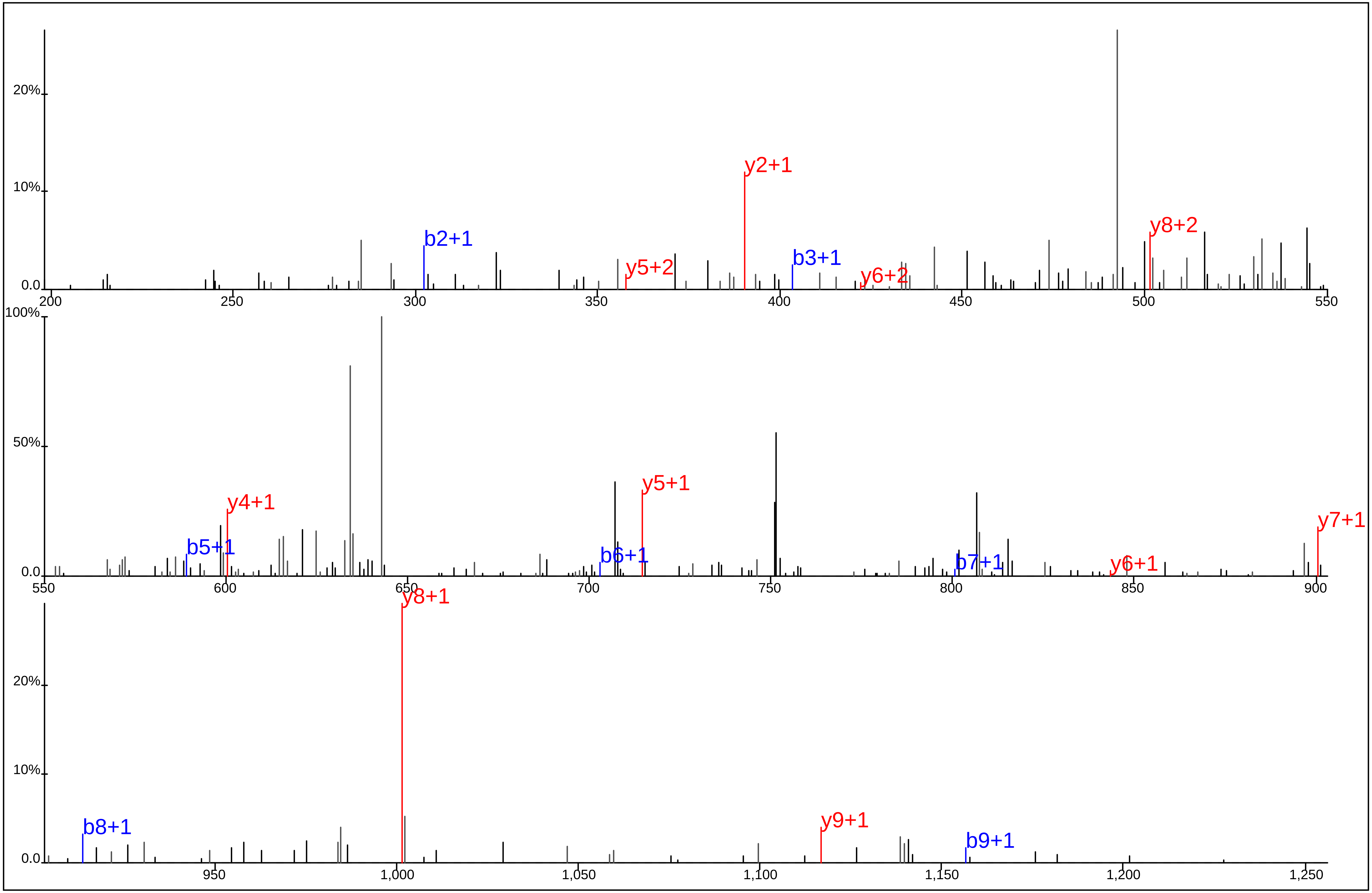

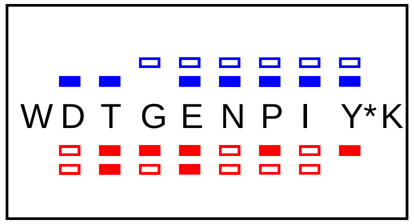

Protein Name: Beta1 integrin
gi Accession: 124963
Precursor Mass: 651.7761+2
Delta Mass: 3.576 ppm
XCorr: 25.12

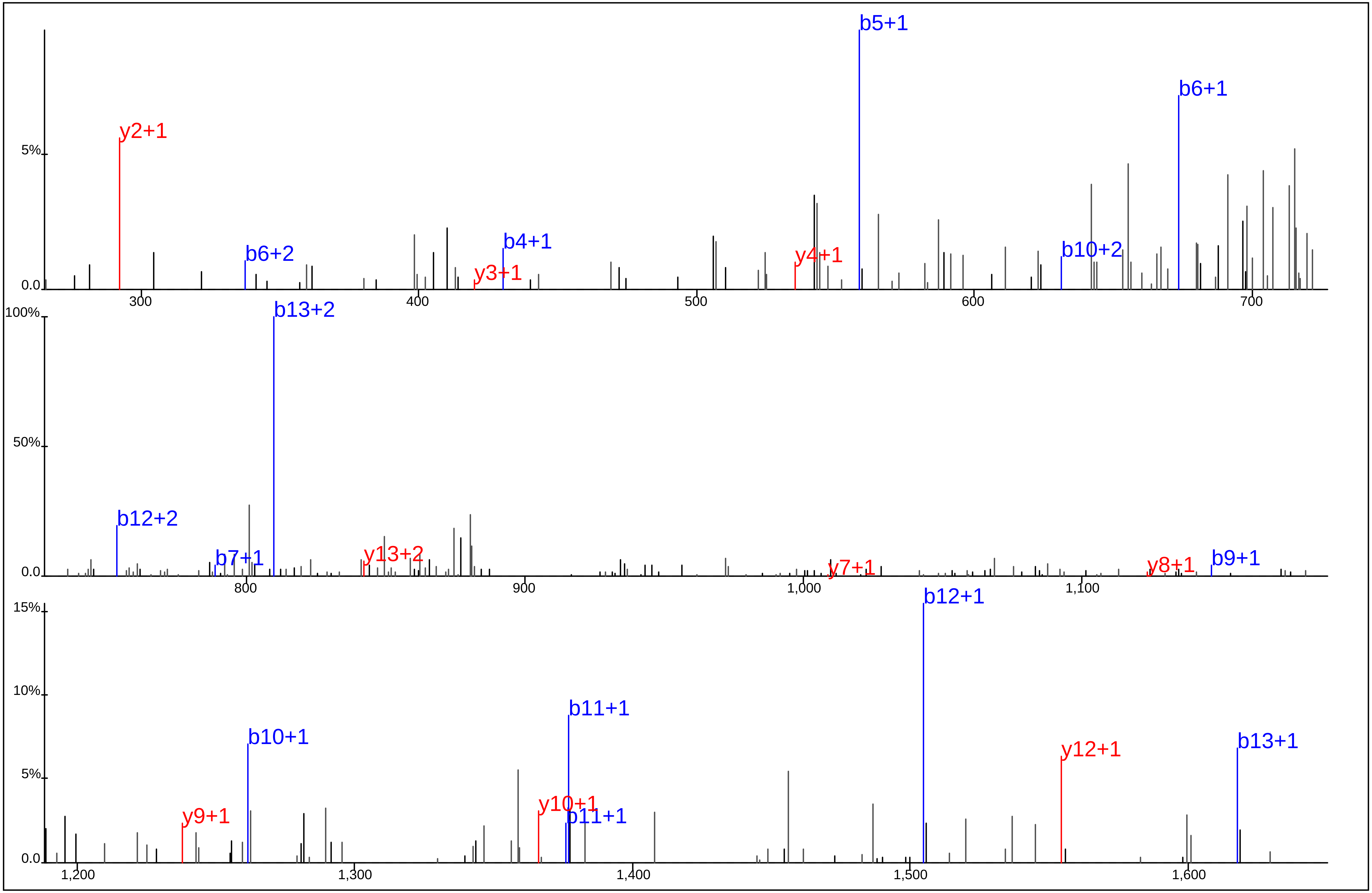

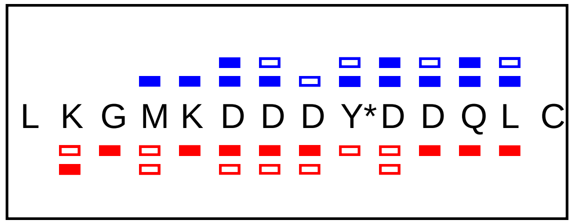

Protein Name:	Beta arrestin 2
gi Accession:	21757343
Precursor Mass:	898.3524+2
Delta Mass:	3.413 ppm
XCorr:	35.93

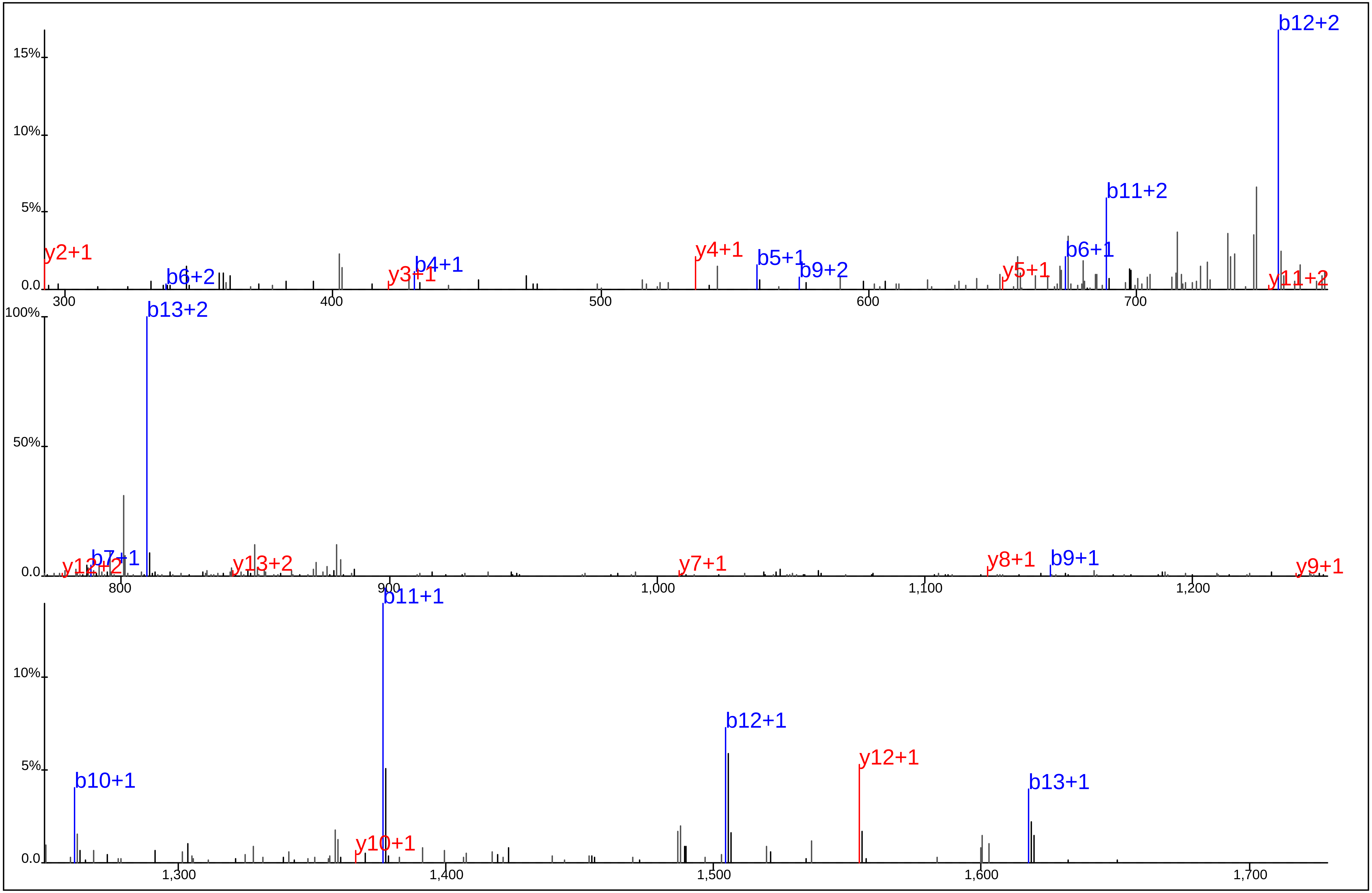

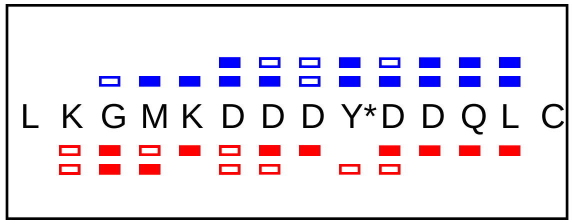

Protein Name:	Beta arrestin 2
gi Accession:	21757343
Precursor Mass:	898.3492+2
Delta Mass:	0.190 ppm
XCorr:	44.68

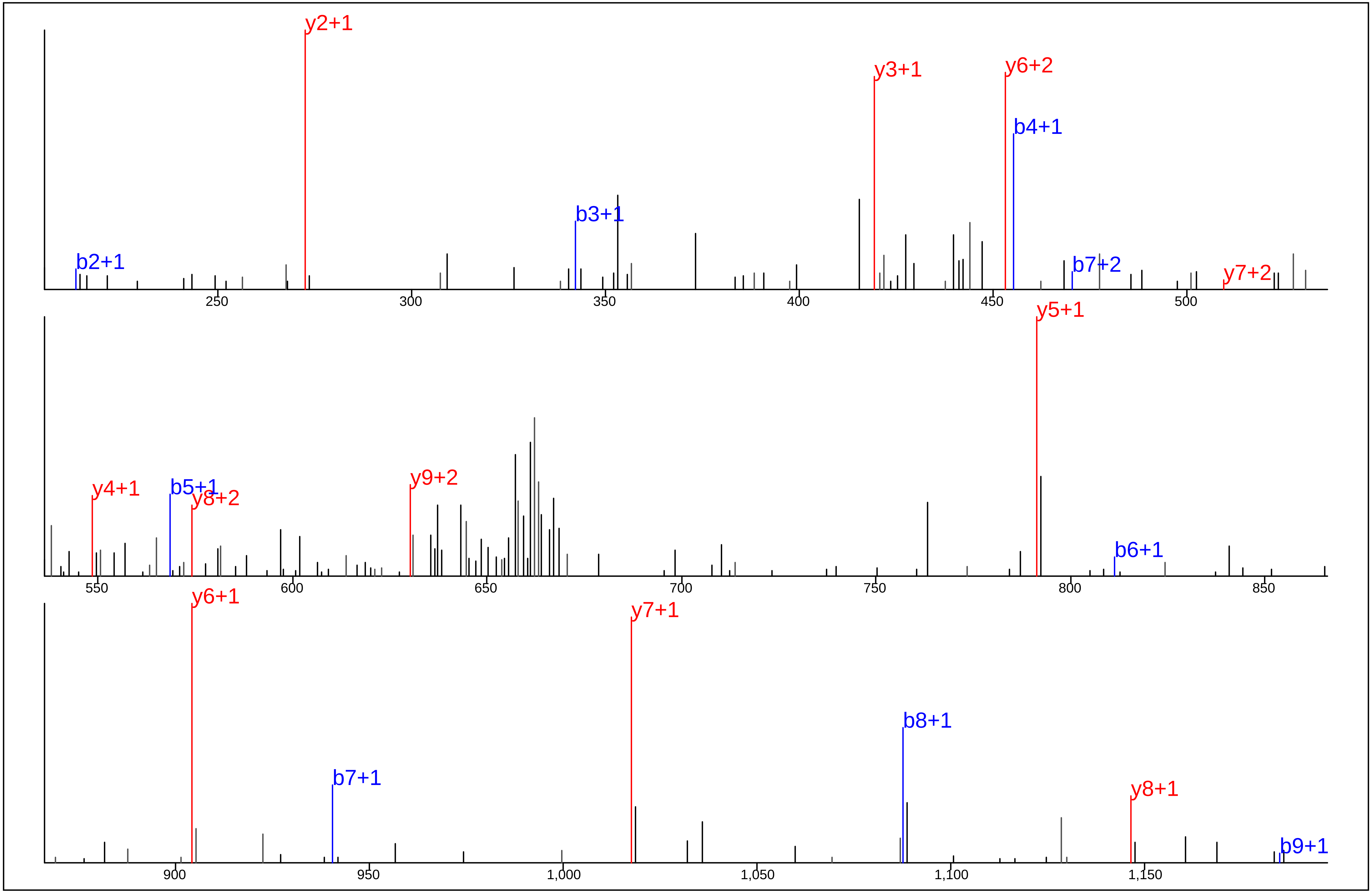

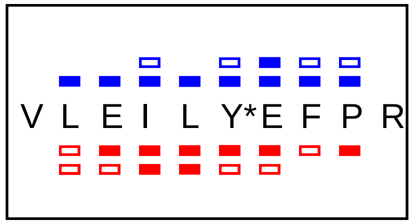

Protein Name:	C10orf28
gi Accession:	71648680
Precursor Mass:	679.8438+2
Delta Mass:	3.436 ppm
XCorr:	48.18

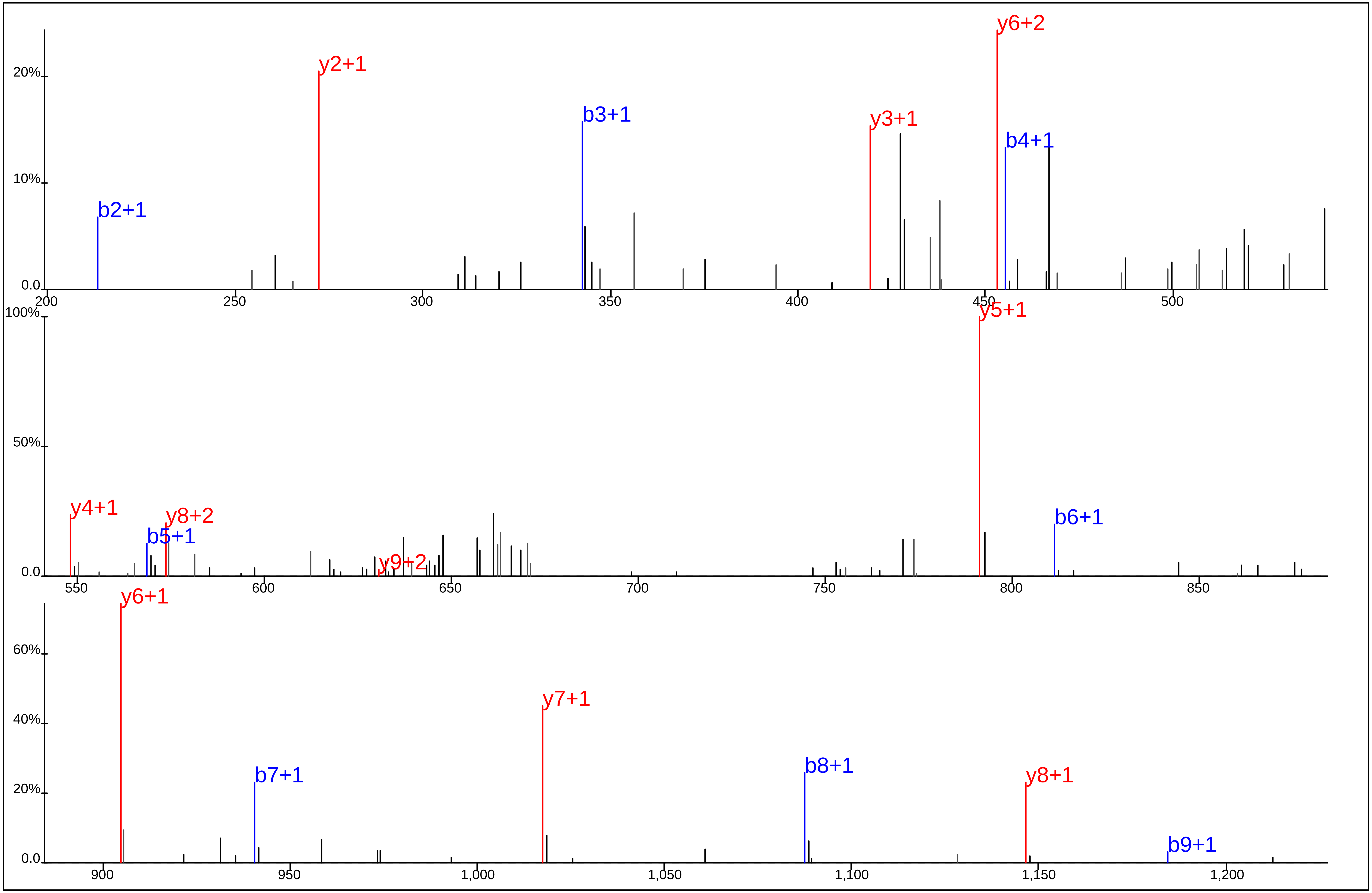

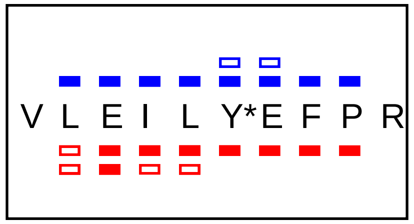

Protein Name:	C10orf28
gi Accession:	71648680
Precursor Mass:	679.8434+2
Delta Mass:	2.898 ppm
XCorr:	51.75

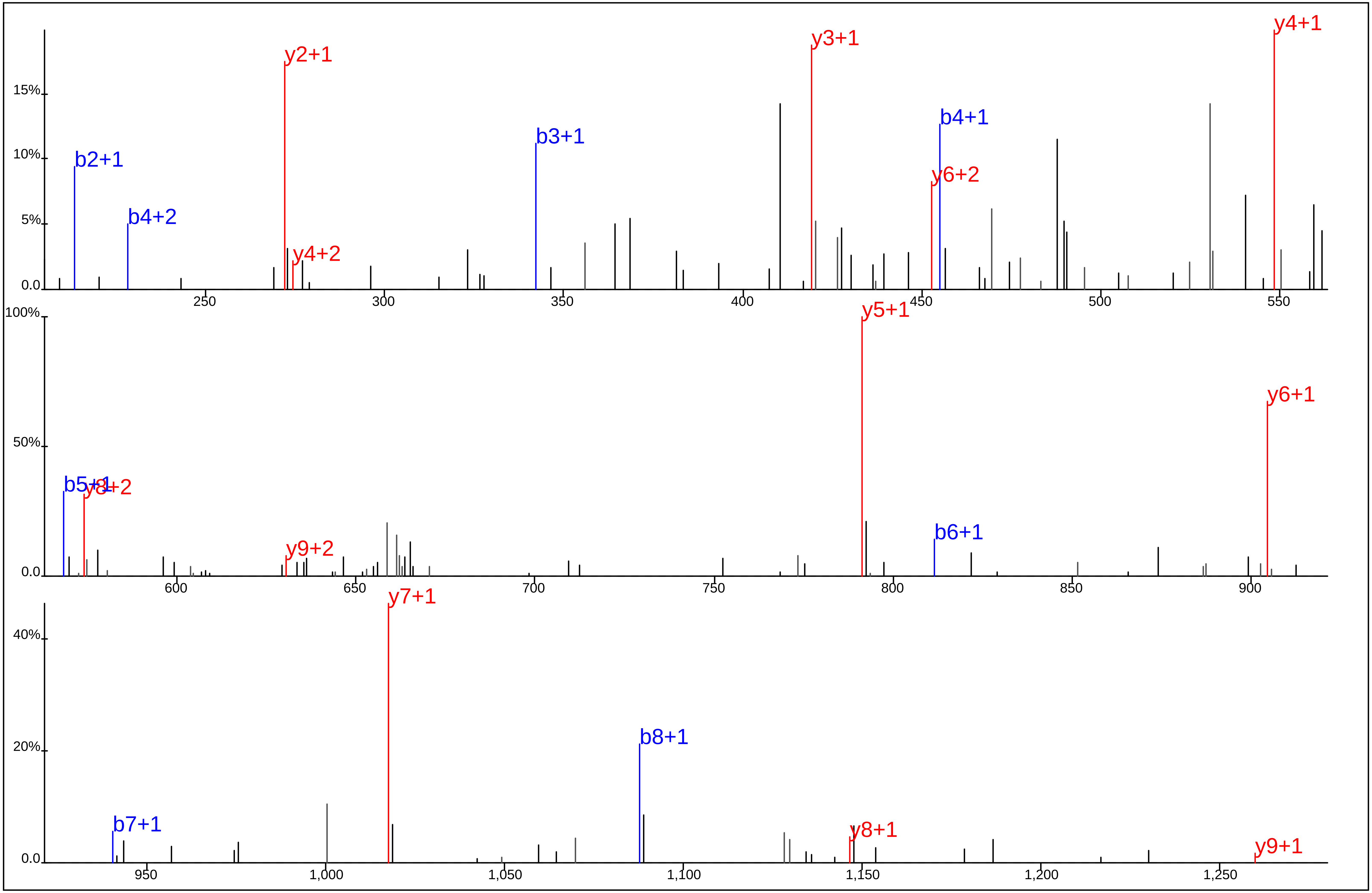

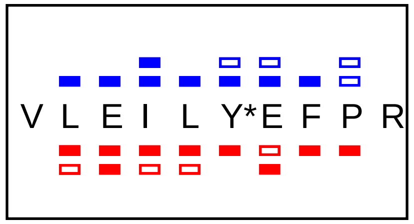

Protein Name: C10orf28
gi Accession: 71648680
Precursor Mass: 679.8449+2
Delta Mass: 5.143 ppm
XCorr: 50.7

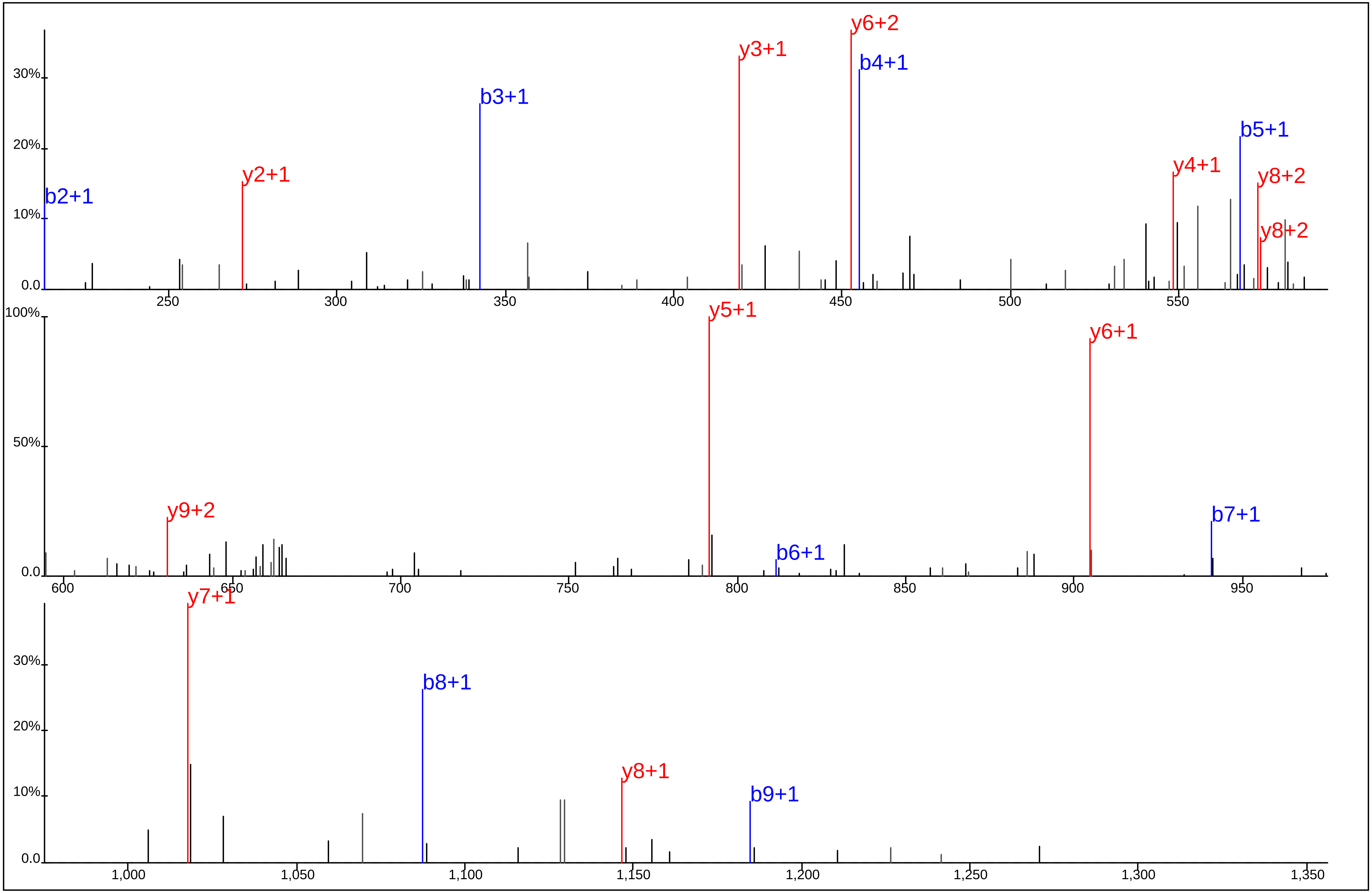

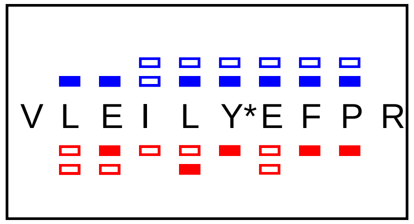

Protein Name:	C10orf28
gi Accession:	71648680
Precursor Mass:	679.8450+2
Delta Mass:	5.232 ppm
XCorr:	50.13

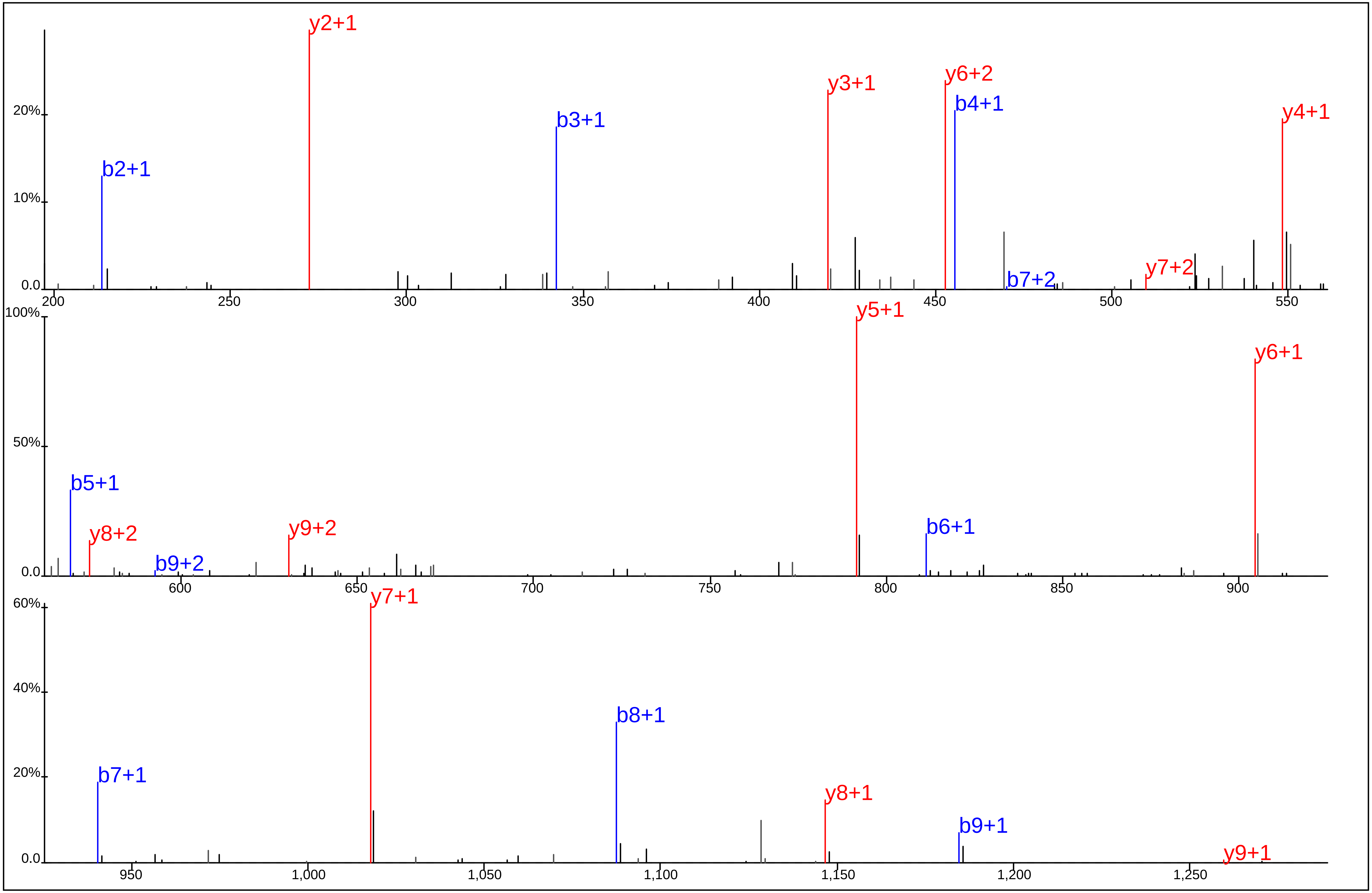

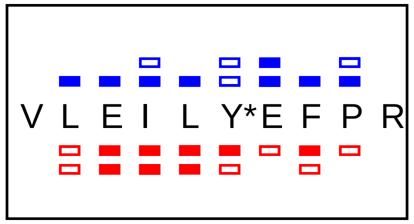

Protein Name: C10orf28
gi Accession: 71648680
Precursor Mass: 679.8459+2
Delta Mass: 6.586 ppm
XCorr: 67.43

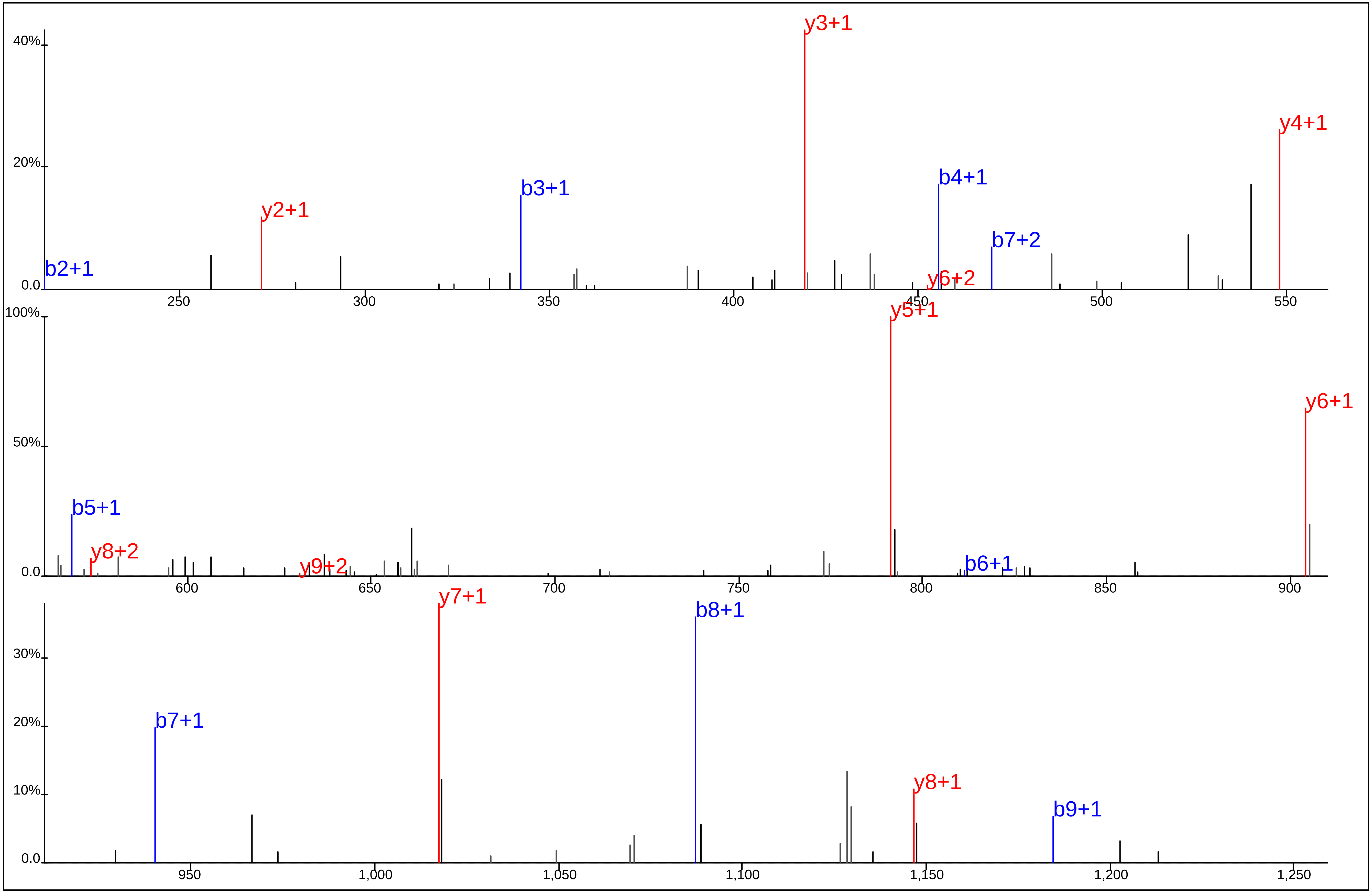

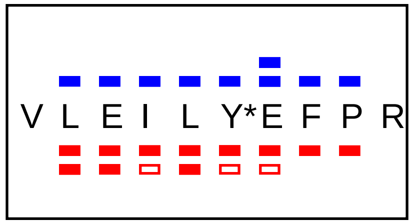

Protein Name: C10orf28
gi Accession: 71648680
Precursor Mass: 679.8455+2
Delta Mass: 6.041 ppm
XCorr: 65.71

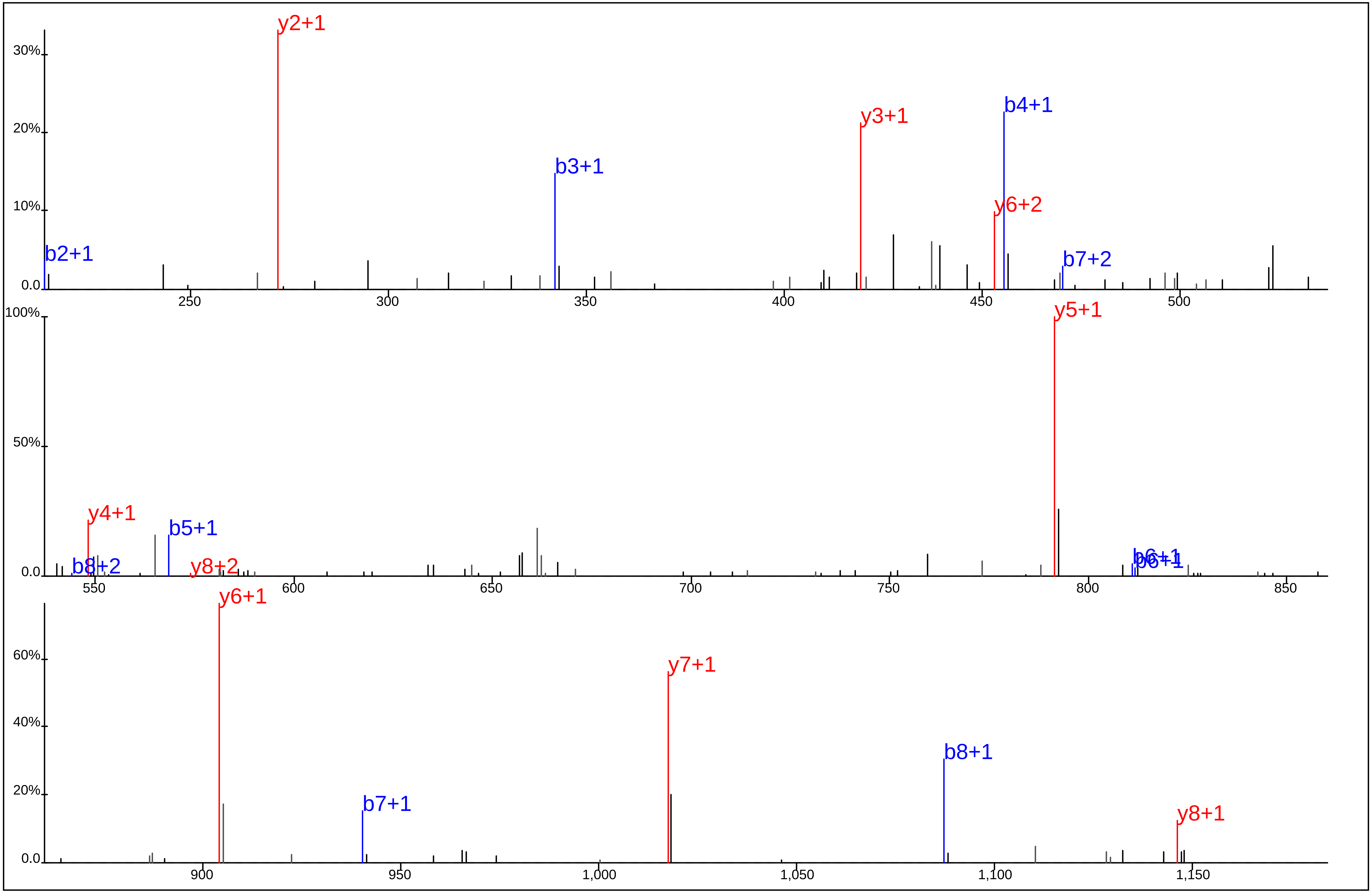

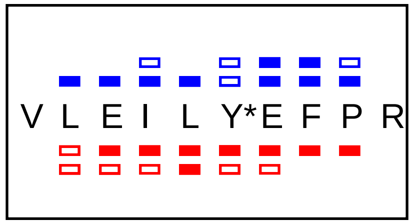

Protein Name: C10orf28
gi Accession: 71648680
Precursor Mass: 679.8445+2
Delta Mass: 4.606 ppm
XCorr: 59.13

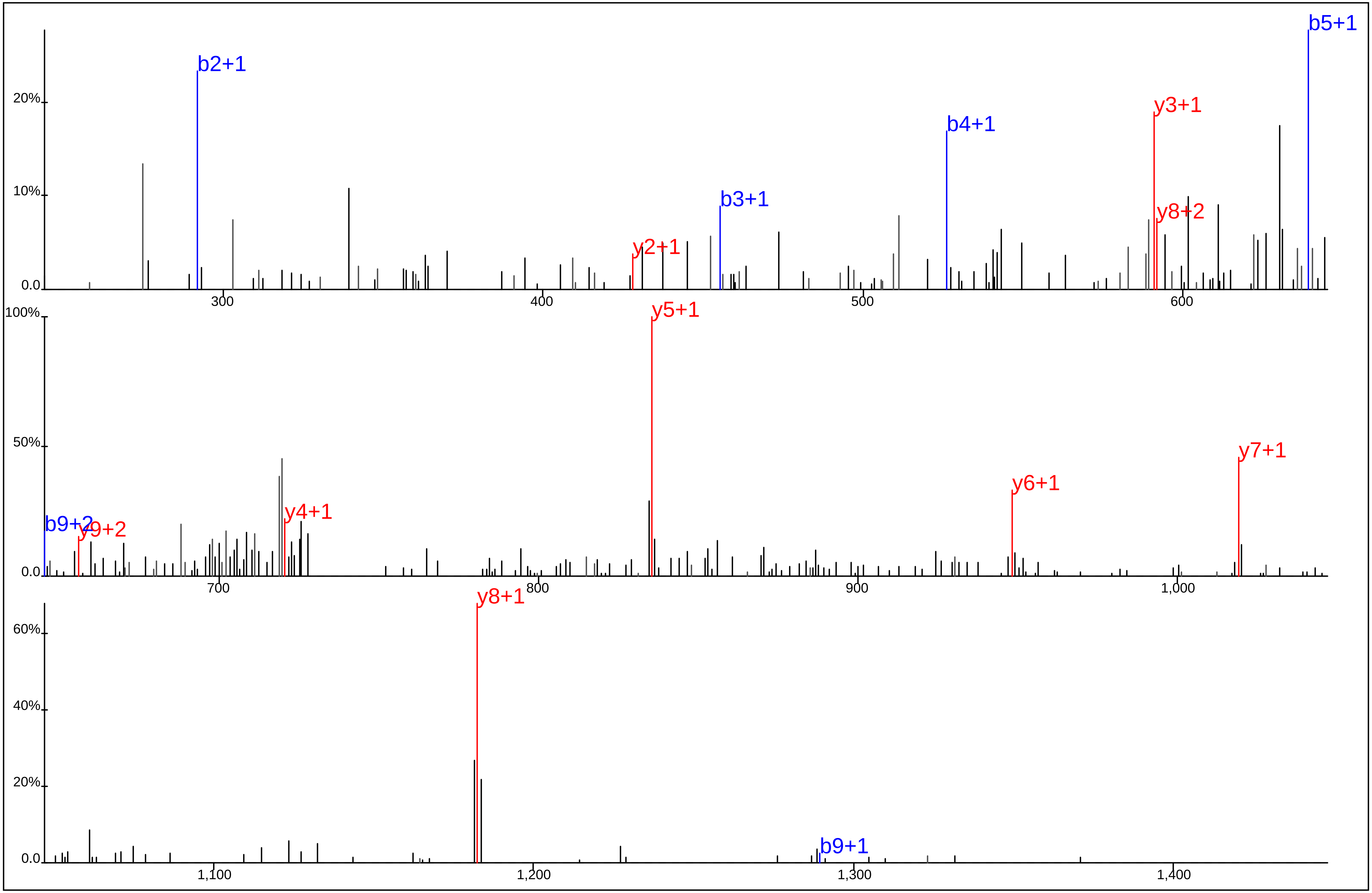

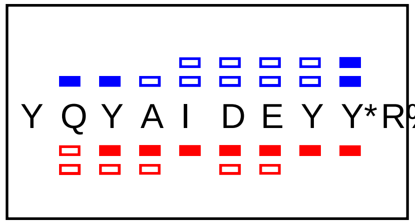

Protein Name: C14orf24
gi Accession: 118498366
Precursor Mass: 737.3041+2
Delta Mass: 3.317 ppm
XCorr: 26.6

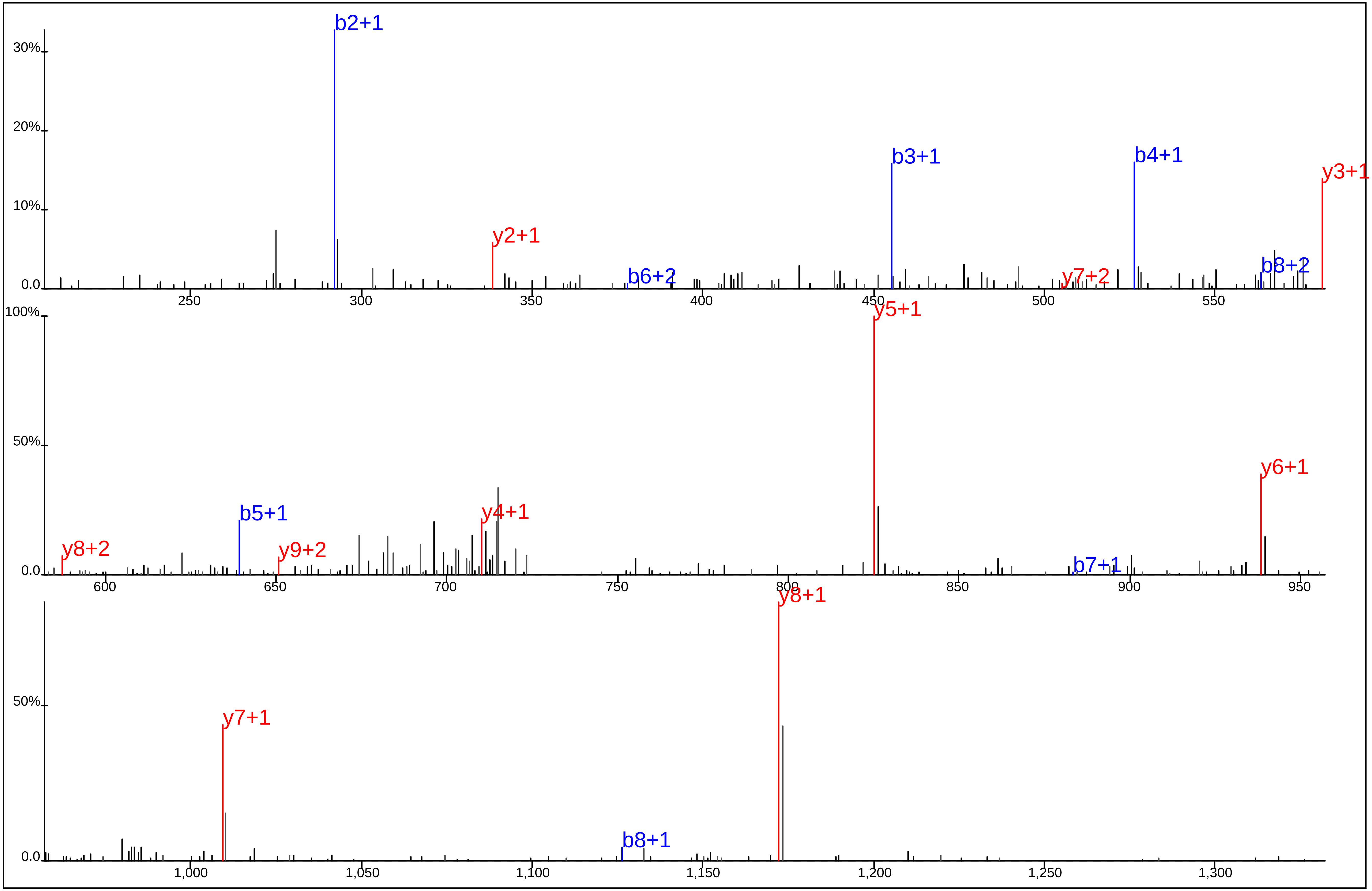

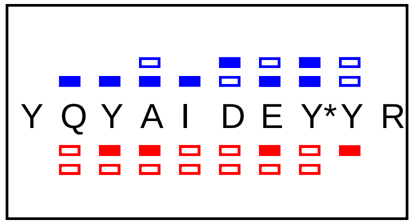

Protein Name: C14orf24
gi Accession: 118498366
Precursor Mass: 732.3010+2
Delta Mass: 4.734 ppm
XCorr: 40.56

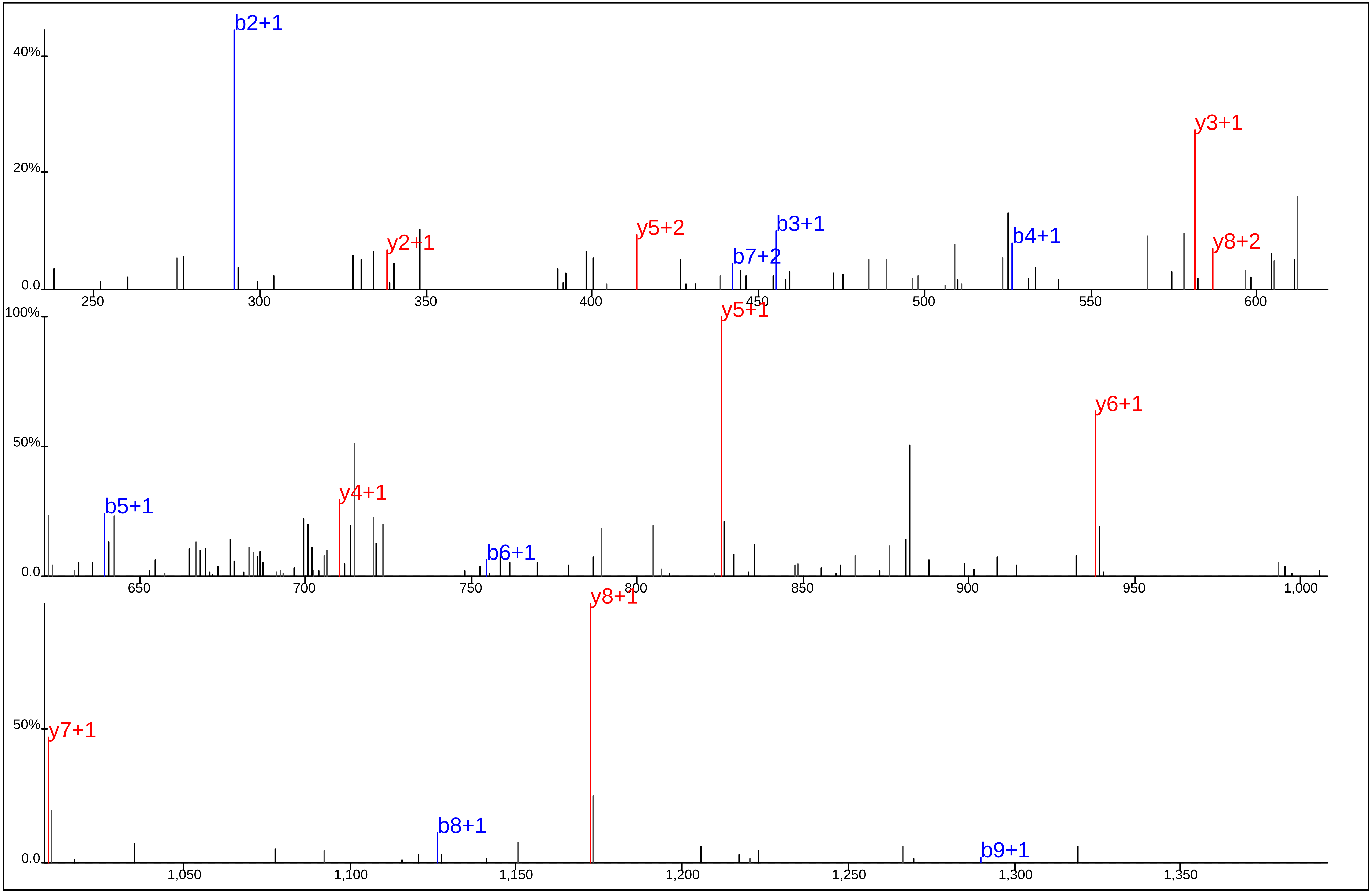

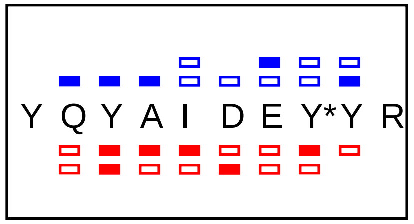

Protein Name: C14orf24
gi Accession: 118498366
Precursor Mass: 732.3017+2
Delta Mass: 5.656 ppm
XCorr: 33.05

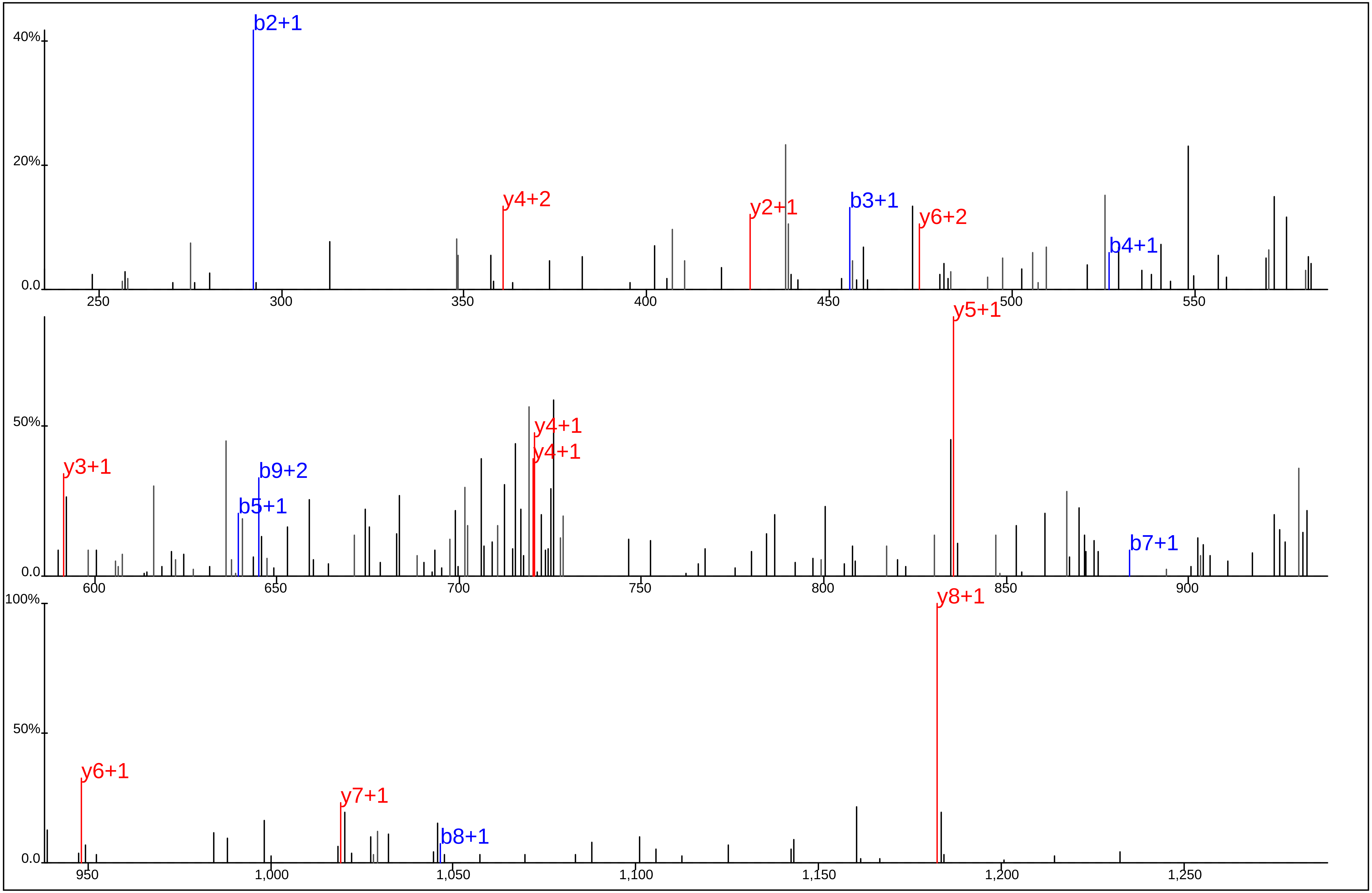

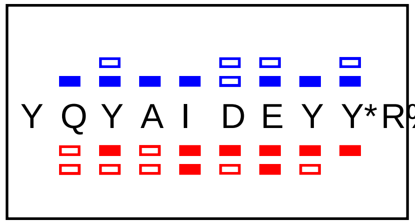

Protein Name: C14orf24
gi Accession: 118498366
Precursor Mass: 737.3040+2
Delta Mass: 3.066 ppm
XCorr: 33.54

82 - C14orf24_3440800.pdf

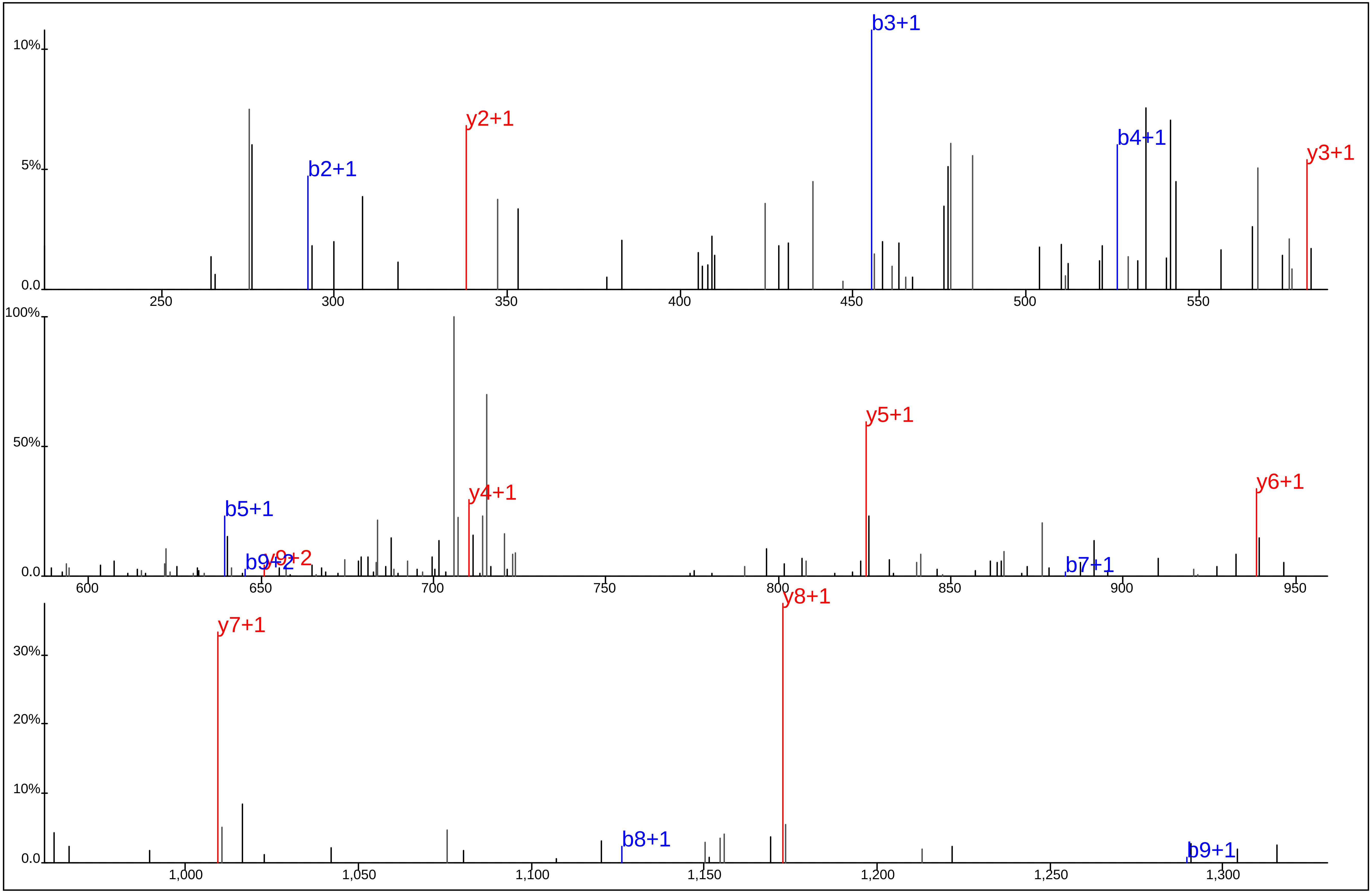

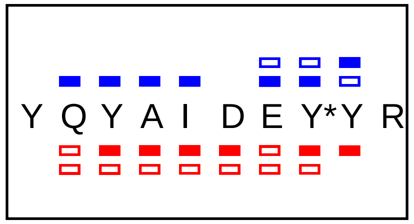

Protein Name: C14orf24
gi Accession: 118498366
Precursor Mass: 732.3018+2
Delta Mass: 5.820 ppm
XCorr: 24.21

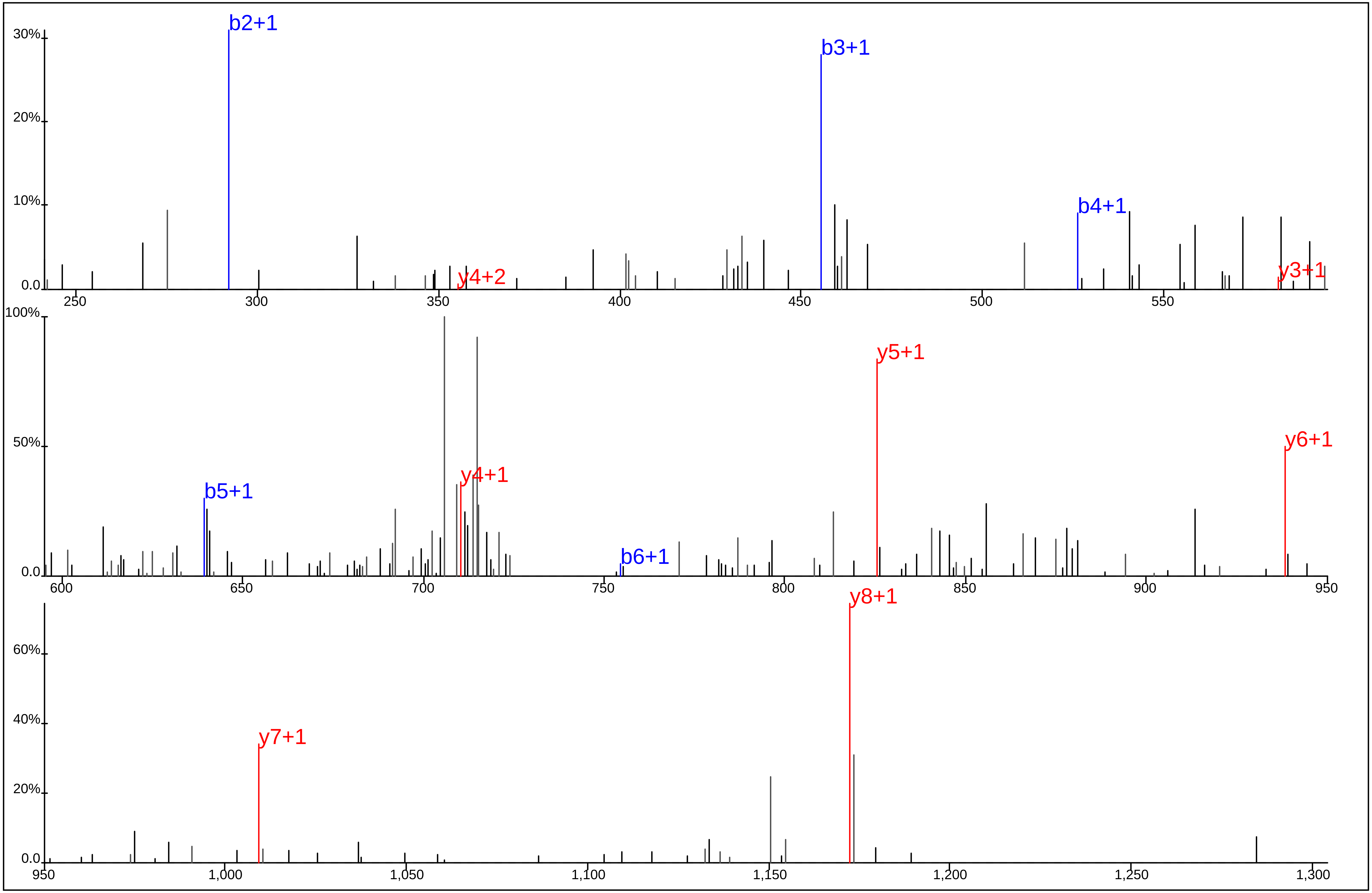

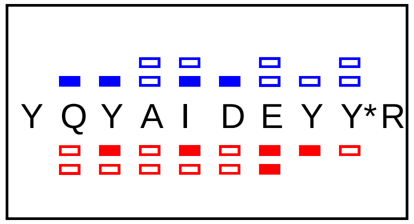

Protein Name: C14orf24
gi Accession: 118498366
Precursor Mass: 732.3000+2
Delta Mass: 3.319 ppm
XCorr: 21.92

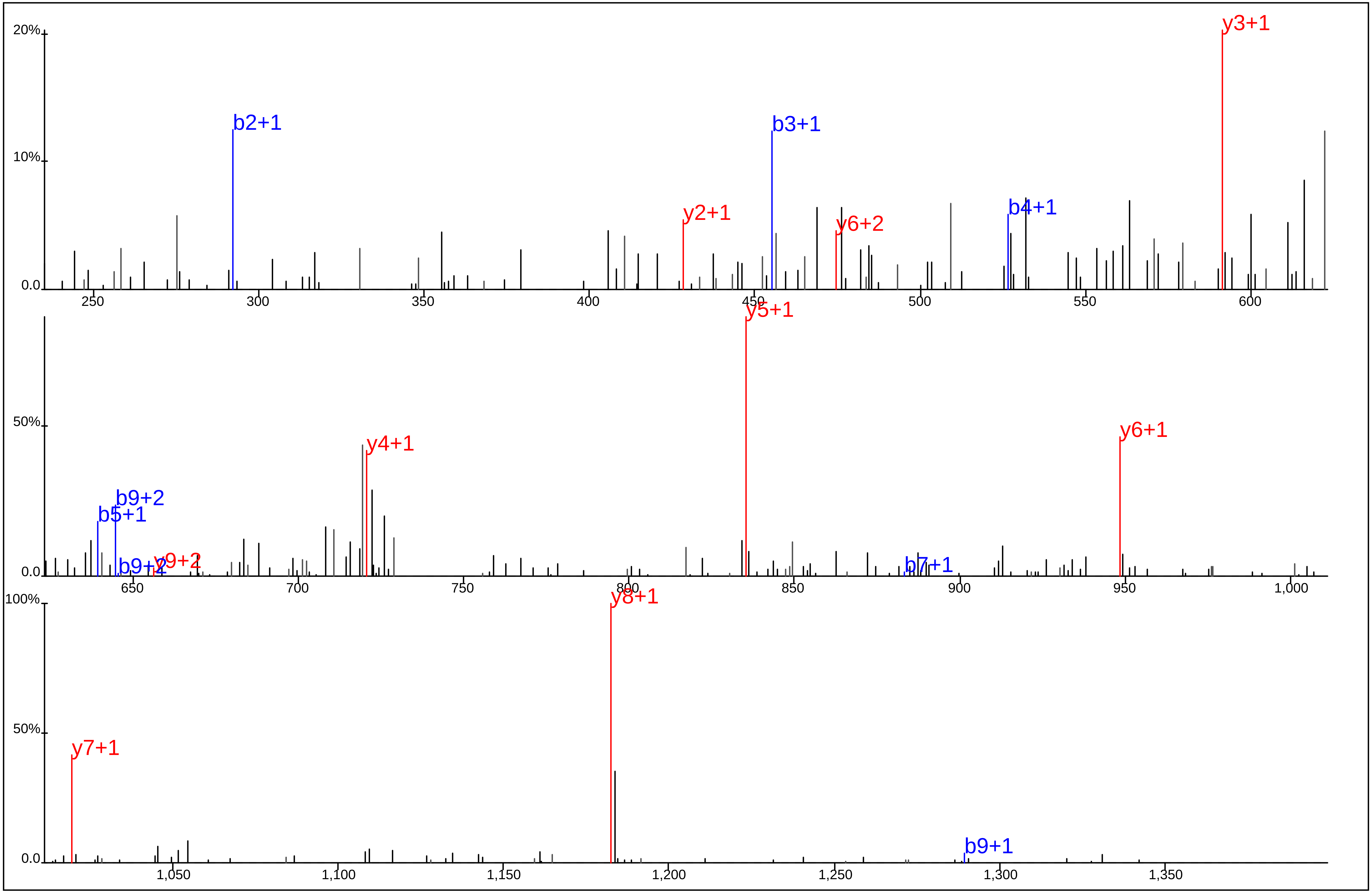

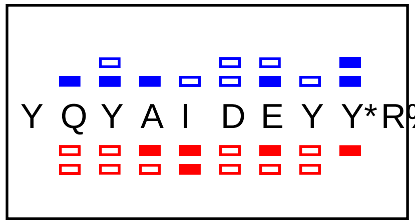

Protein Name: C14orf24
gi Accession: 118498366
Precursor Mass: 737.3048+2
Delta Mass: 4.226 ppm
XCorr: 42.32

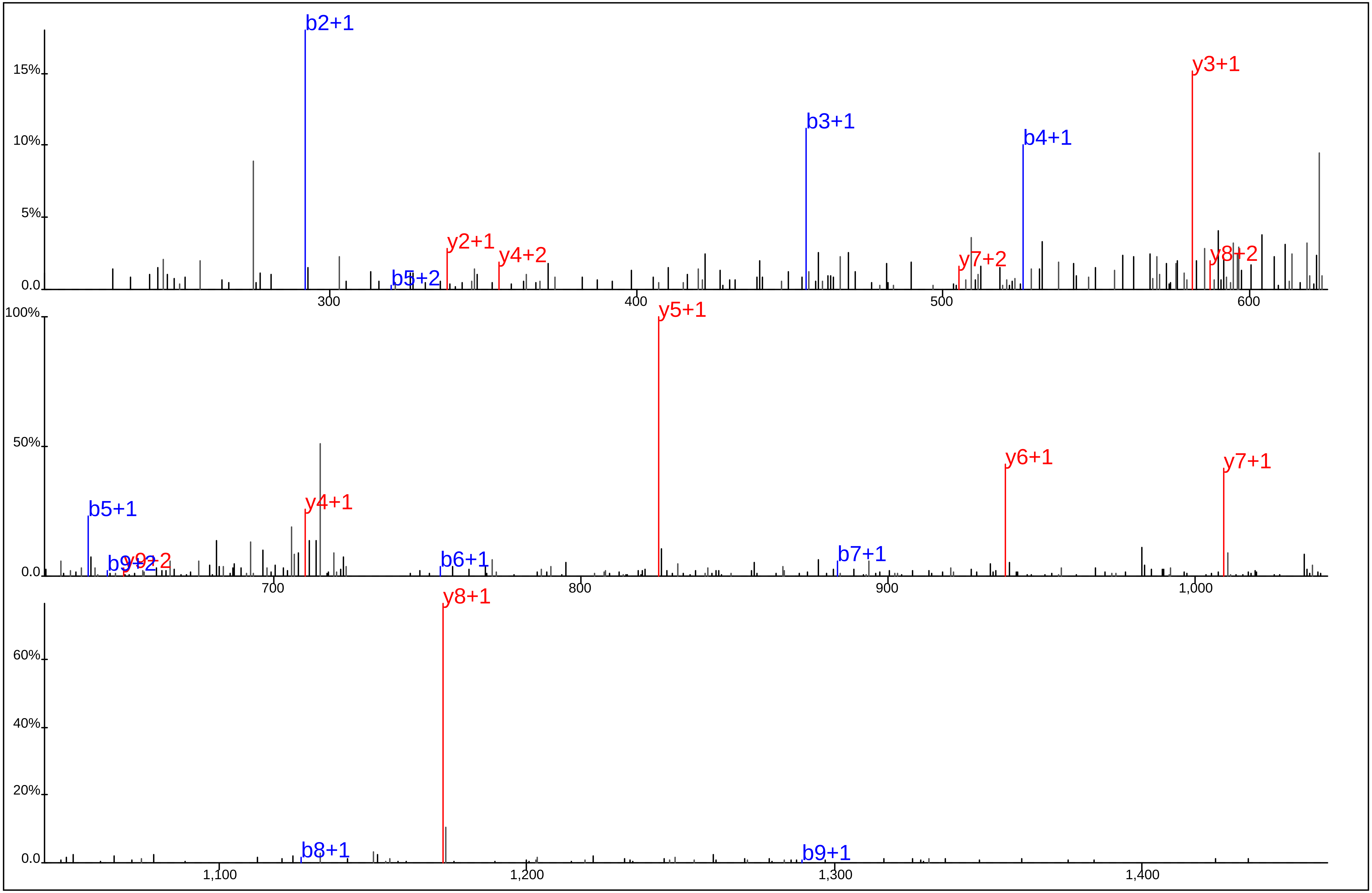

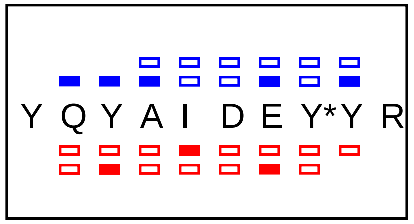

Protein Name:	C14orf24
gi Accession:	118498366
Precursor Mass:	732.2985+2
Delta Mass:	1.317 ppm
XCorr:	45.44

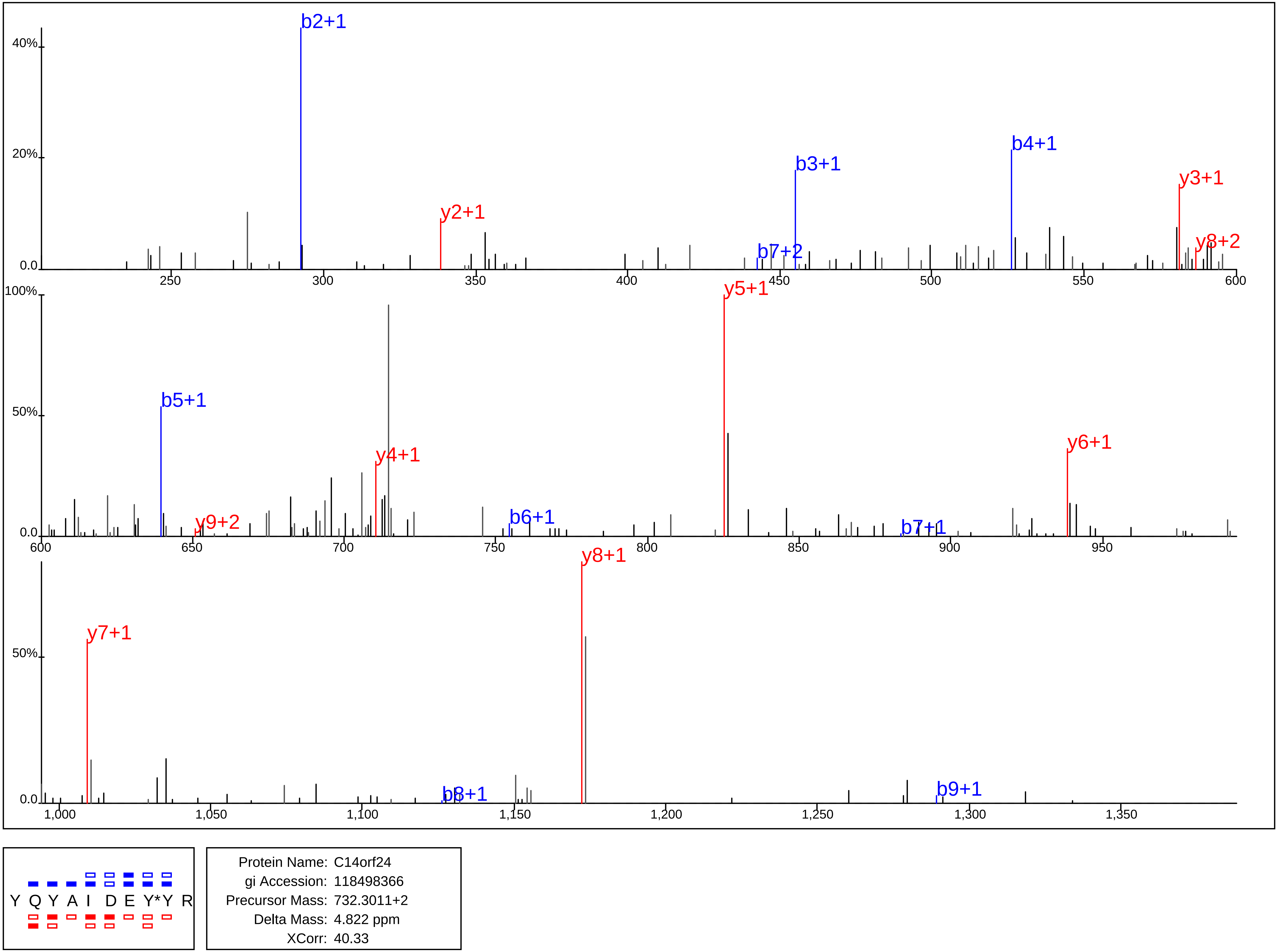

Protein Name: C14orf24
gi Accession: 118498366
Precursor Mass: 732.3011+2
Delta Mass: 4.822 ppm
XCorr: 40.33

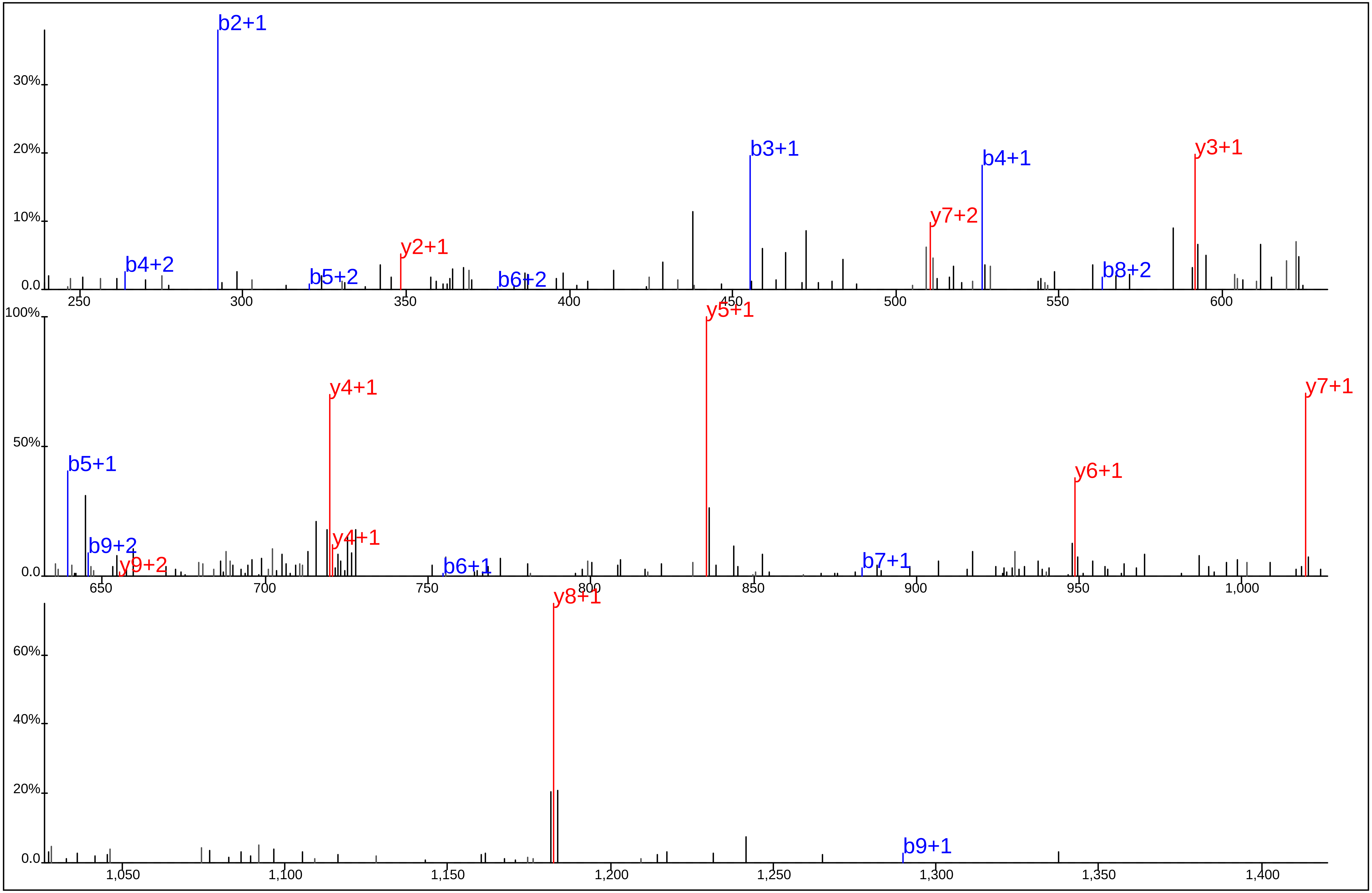

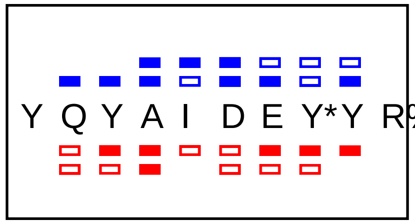

Protein Name: C14orf24
gi Accession: 118498366
Precursor Mass: 737.3066+2
Delta Mass: 6.710 ppm
XCorr: 40.02

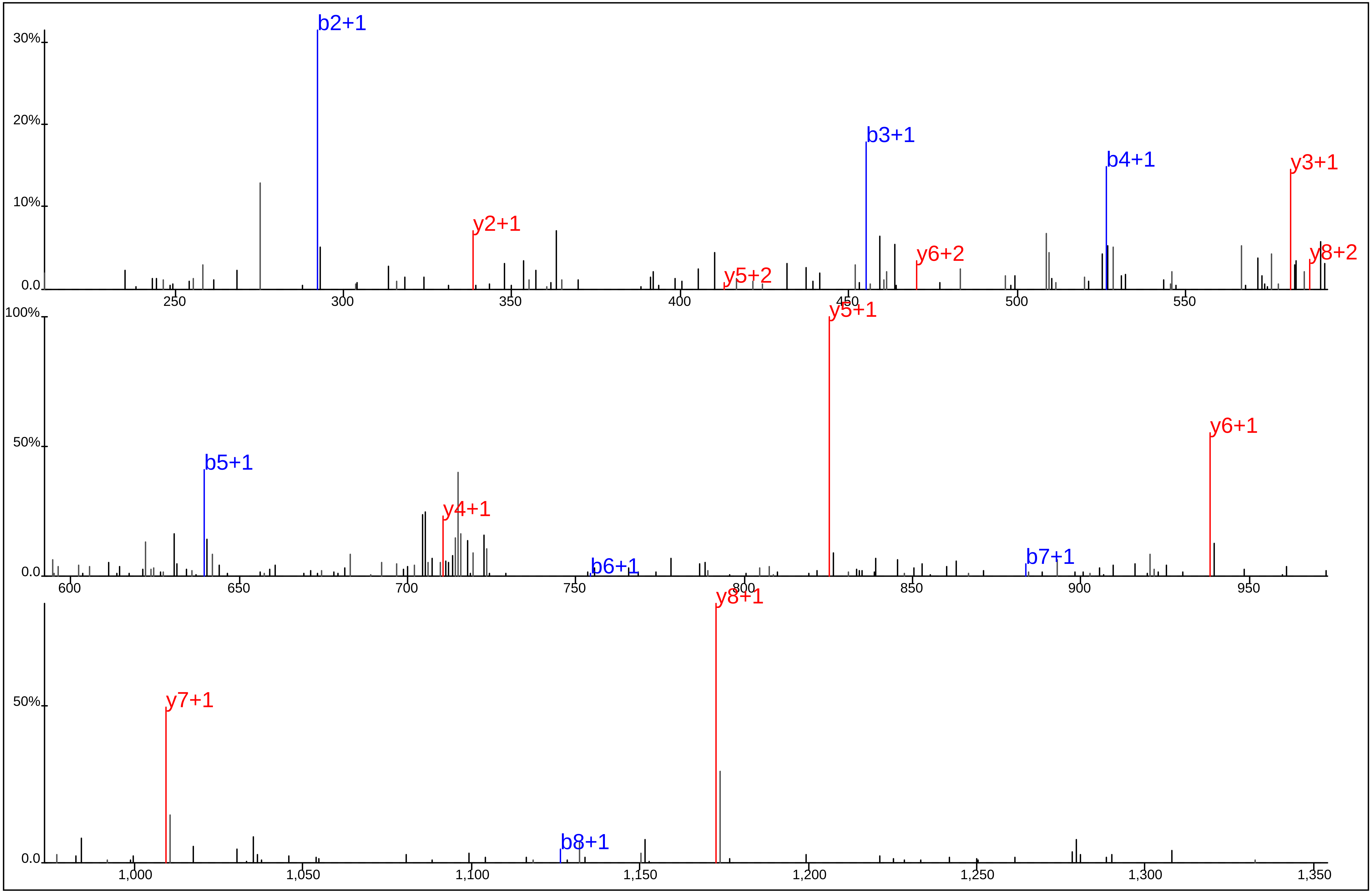

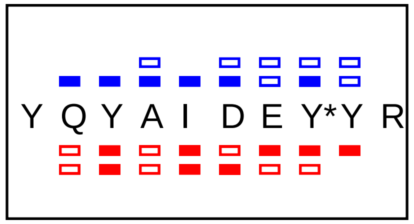

Protein Name: C14orf24
gi Accession: 118498366
Precursor Mass: 732.3017+2
Delta Mass: 5.656 ppm
XCorr: 31.31

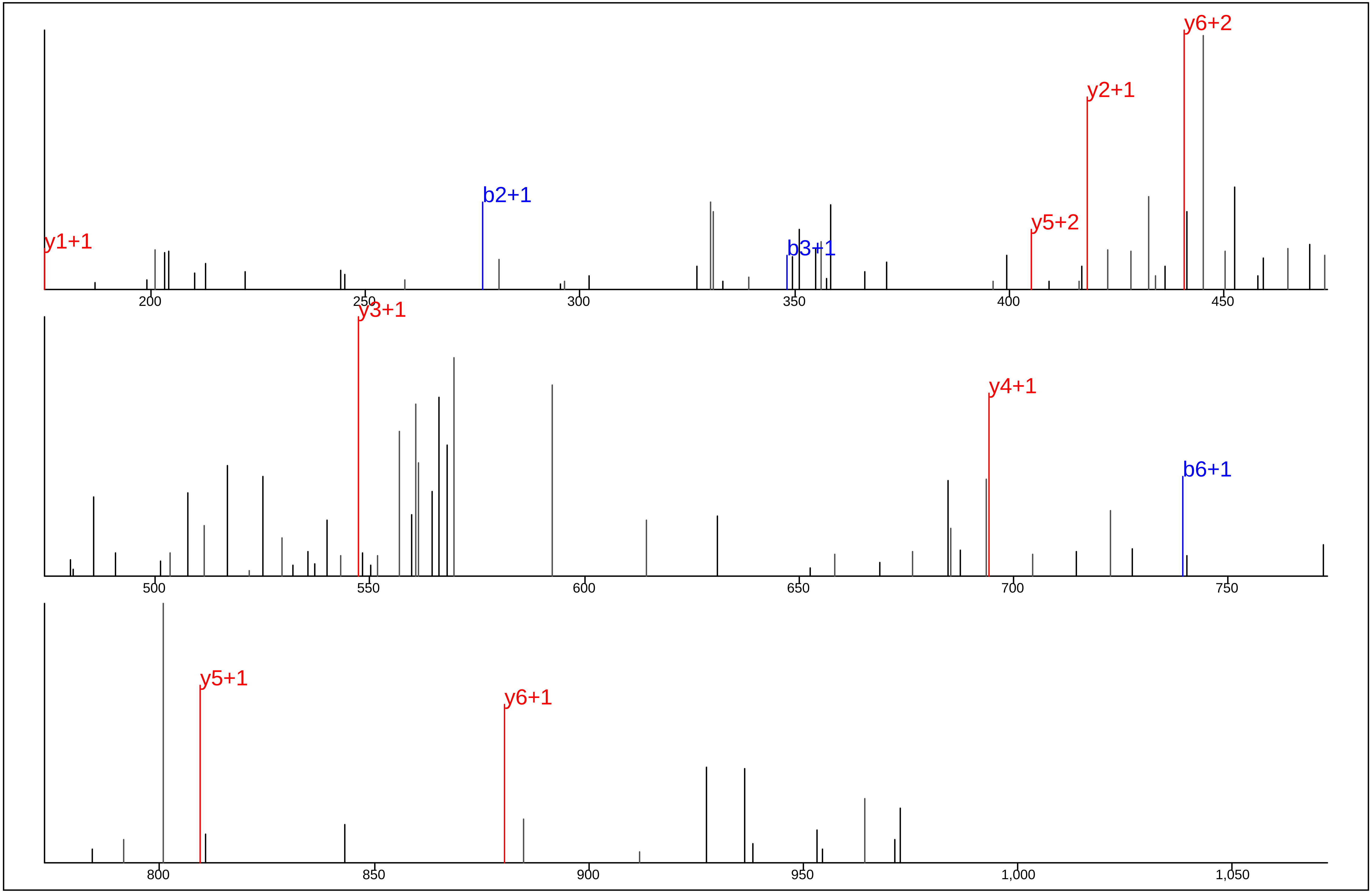

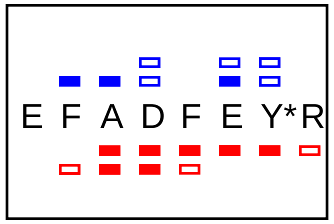

Protein Name: C19orf13
gi Accession: 14041999
Precursor Mass: 578.7244+2
Delta Mass: 5.870 ppm
XCorr: 32.84

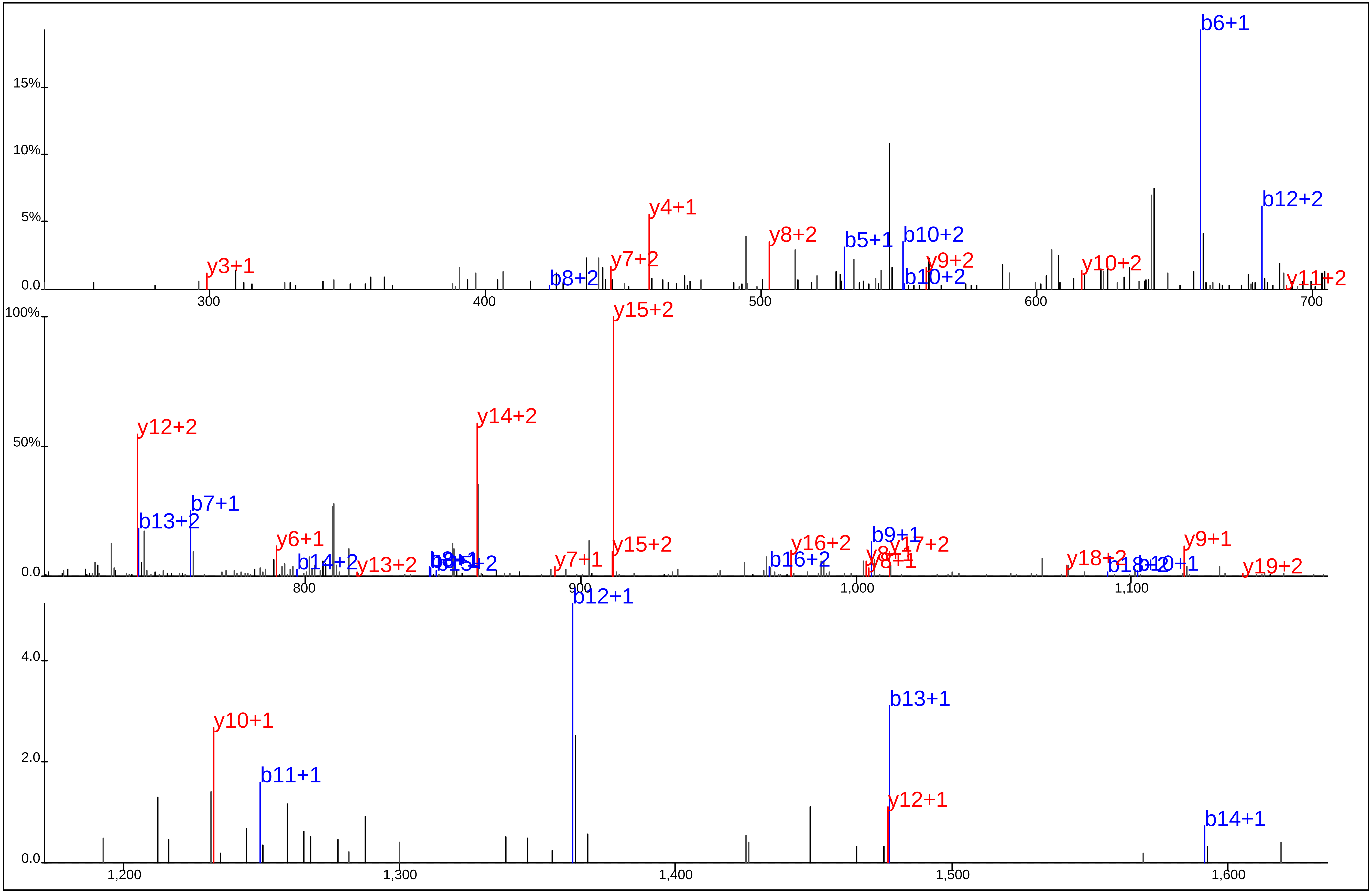

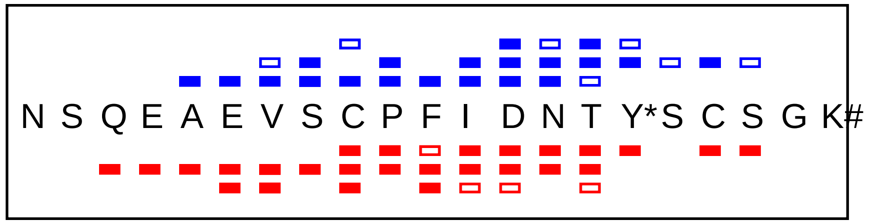

Protein Name:	C20orf18
gi Accession:	34189561
Precursor Mass:	827.6694+3
Delta Mass:	4.518 ppm
XCorr:	62.99

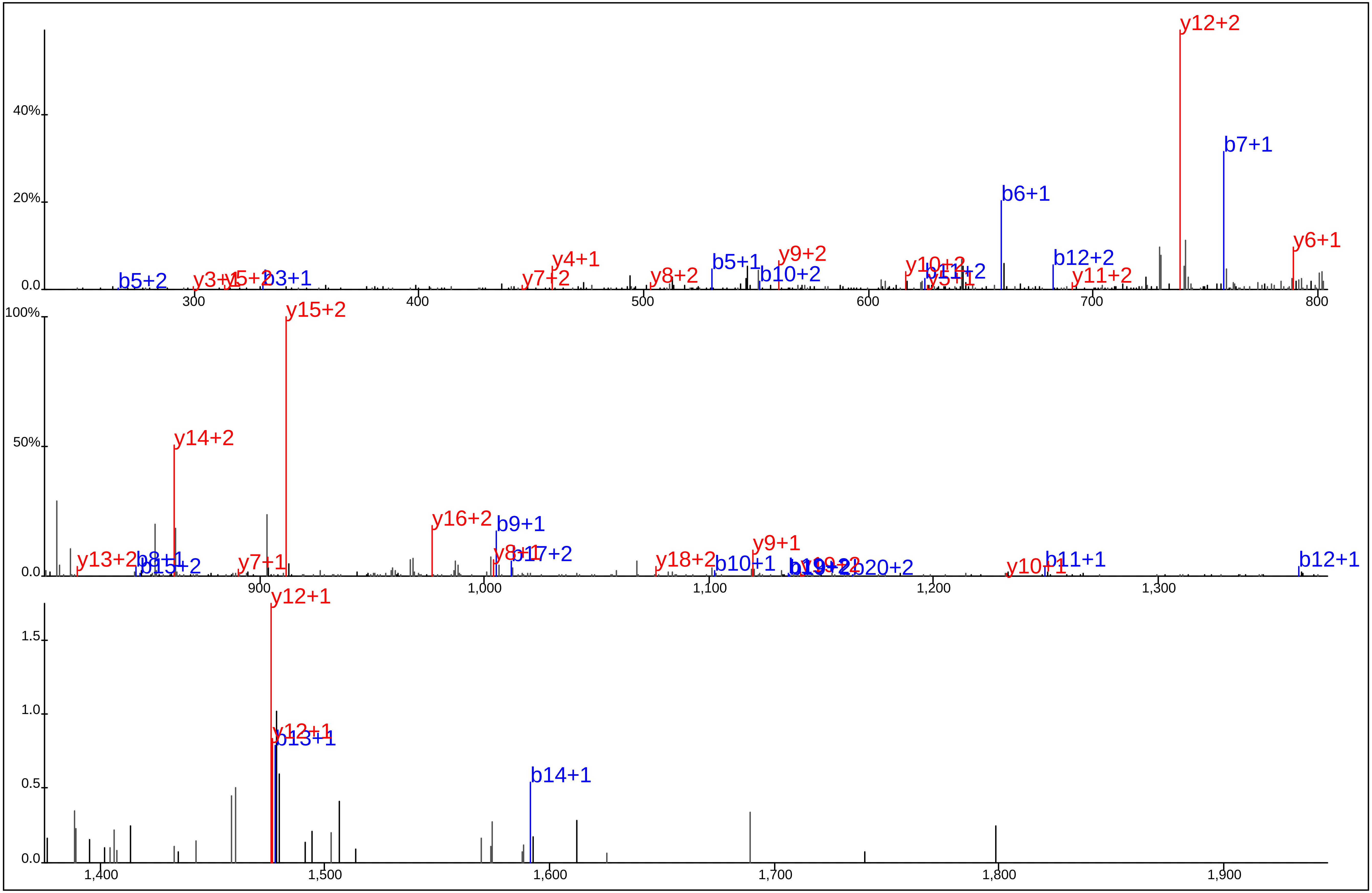

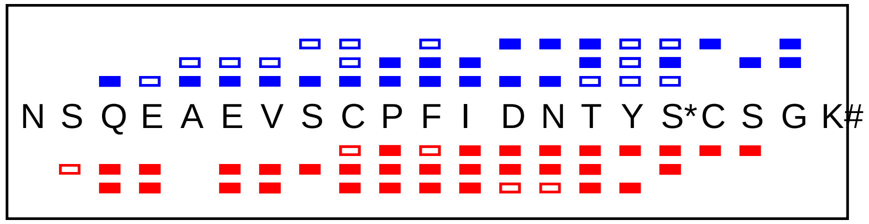

Protein Name: C20orf18
gi Accession: 34189561
Precursor Mass: 827.6711+3
Delta Mass: 6.582 ppm
XCorr: 63.66

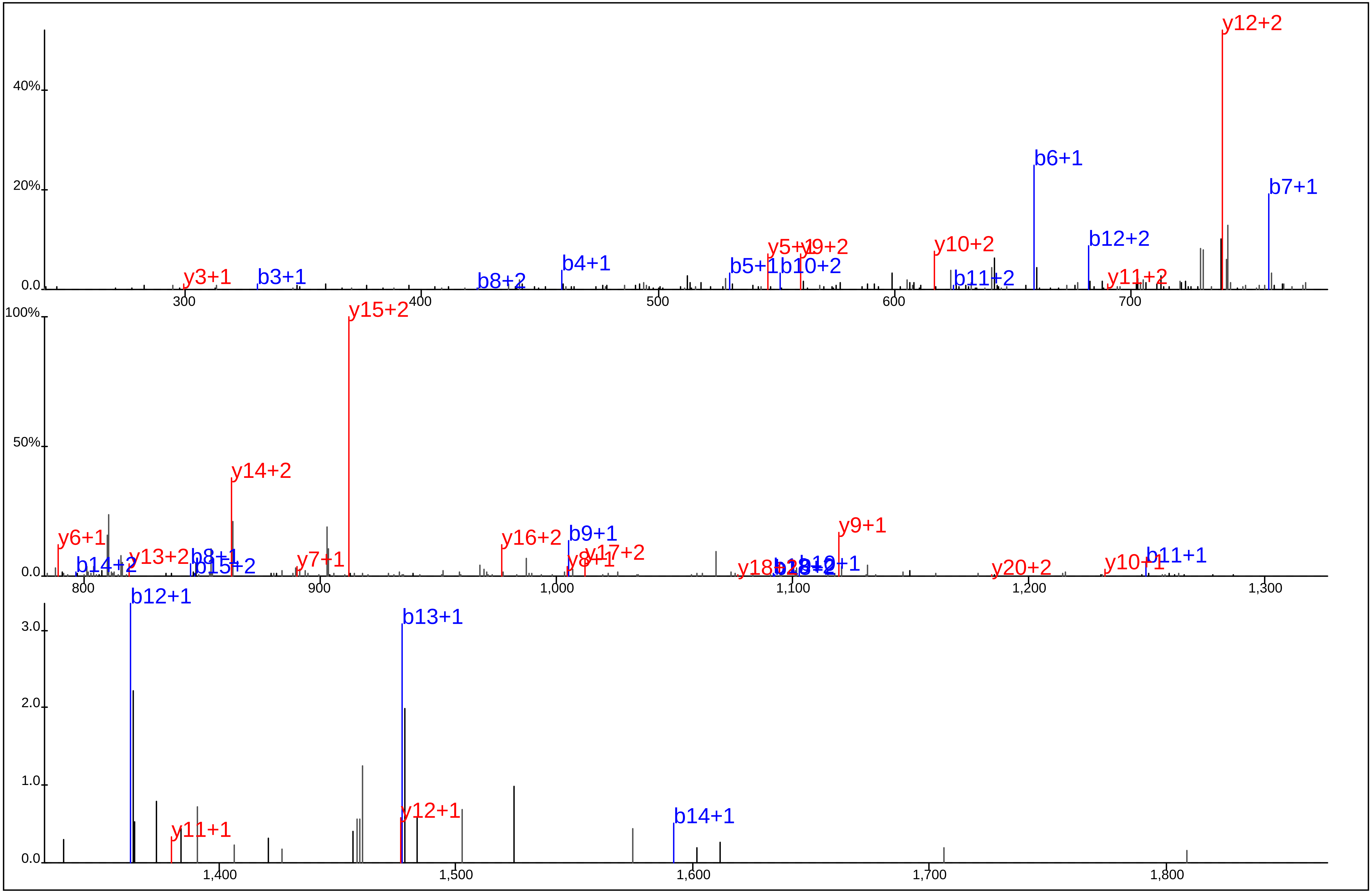

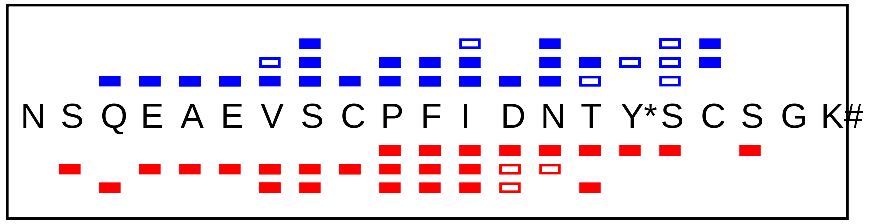

Protein Name: C20orf18
gi Accession: 34189561
Precursor Mass: 827.6680+3
Delta Mass: 2.821 ppm
XCorr: 72.07

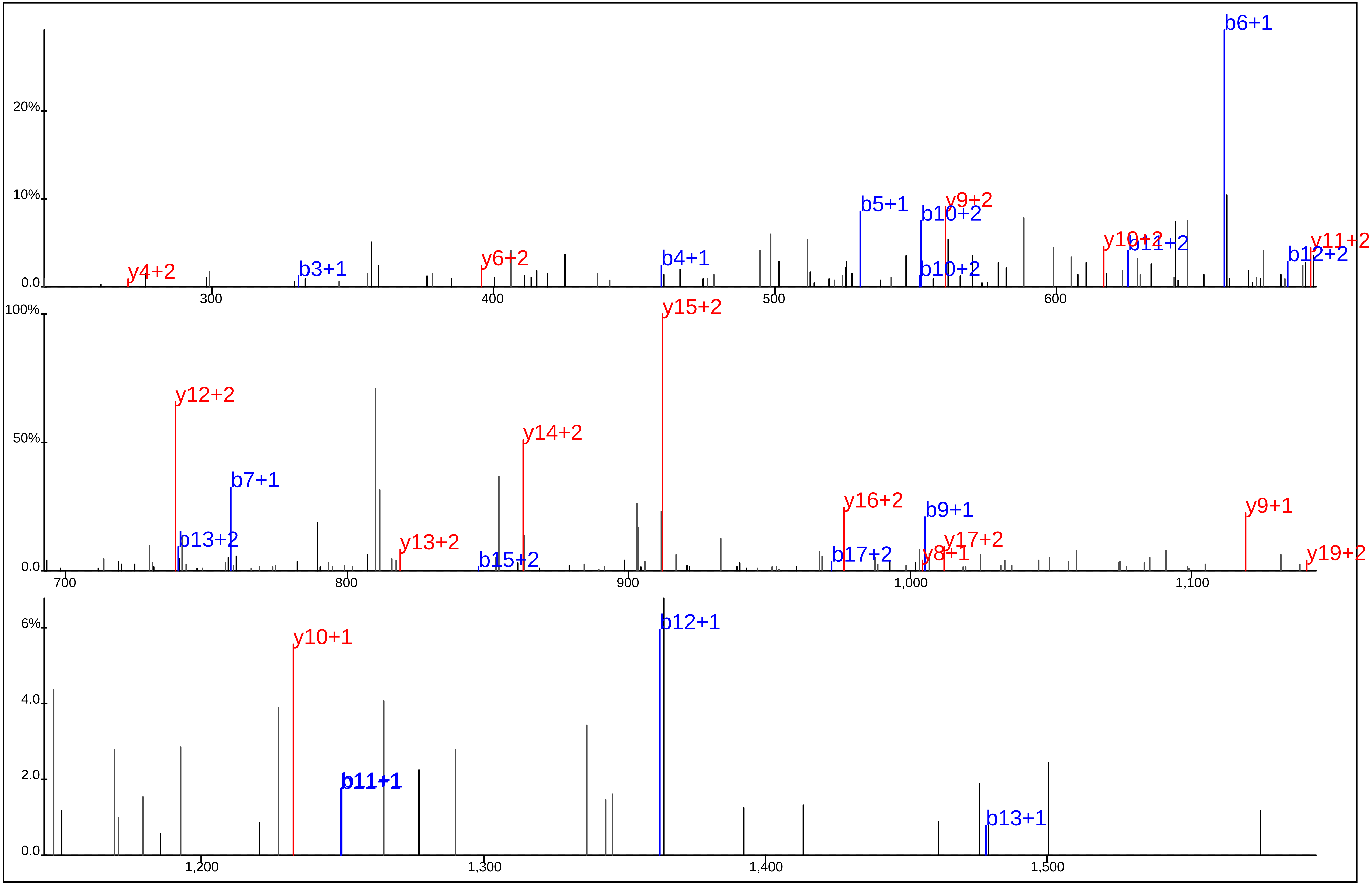

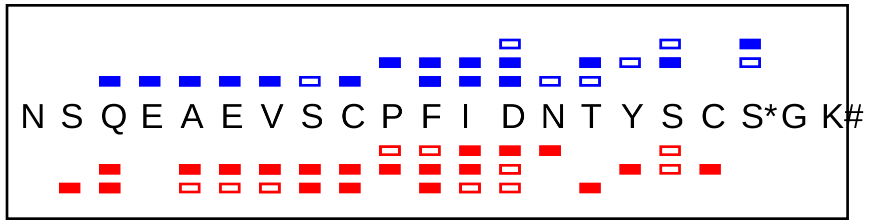

Protein Name: C20orf18
gi Accession: 34189561
Precursor Mass: 827.6586+3
Delta Mass: 8.545 ppm
XCorr: 41.81

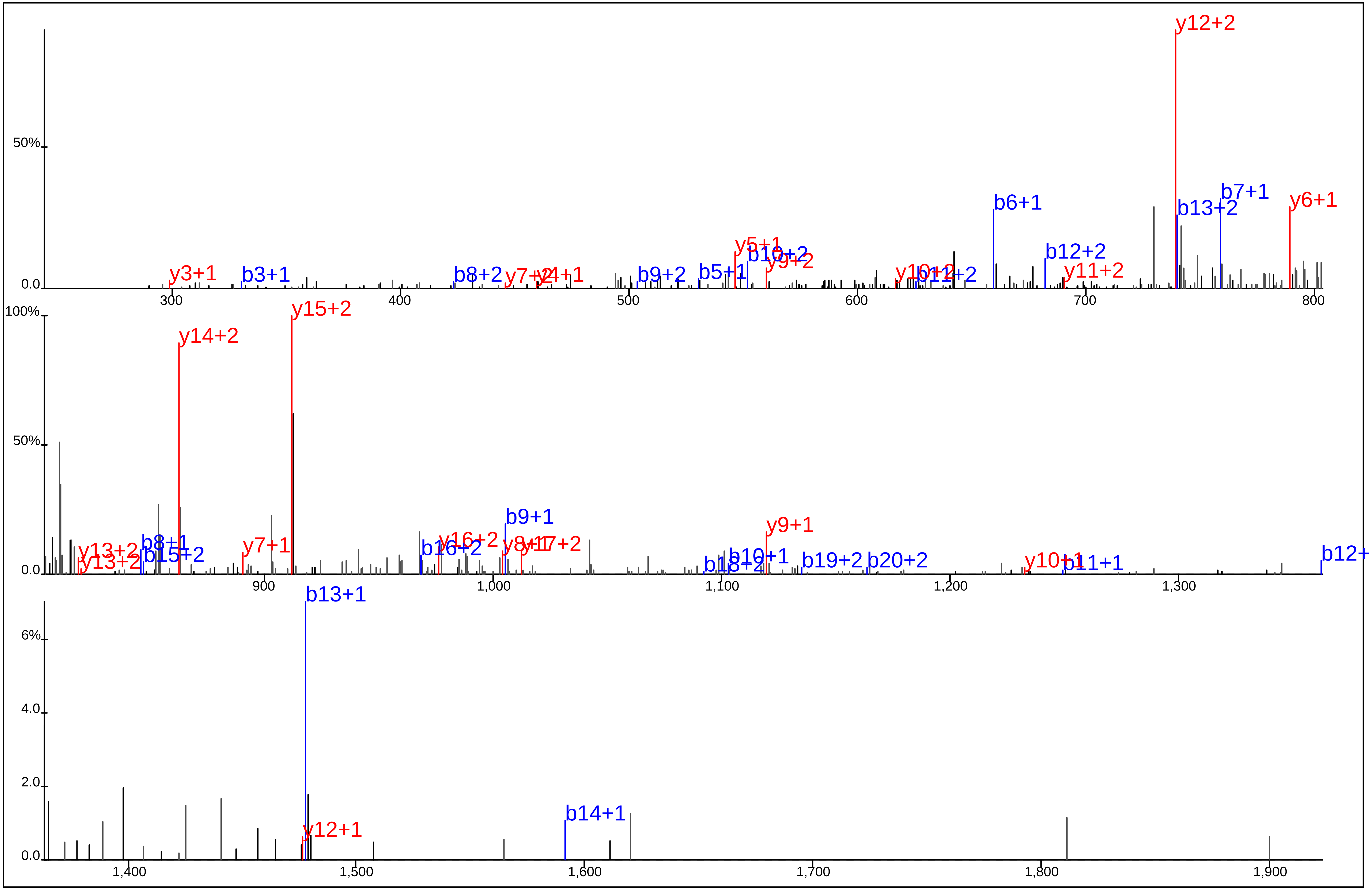

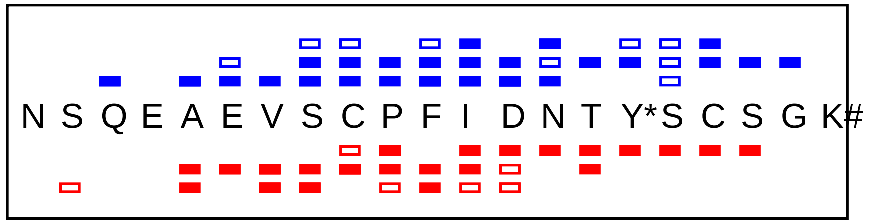

Protein Name: C20orf18
gi Accession: 34189561
Precursor Mass: 827.6707+3
Delta Mass: 6.065 ppm
XCorr: 40.0

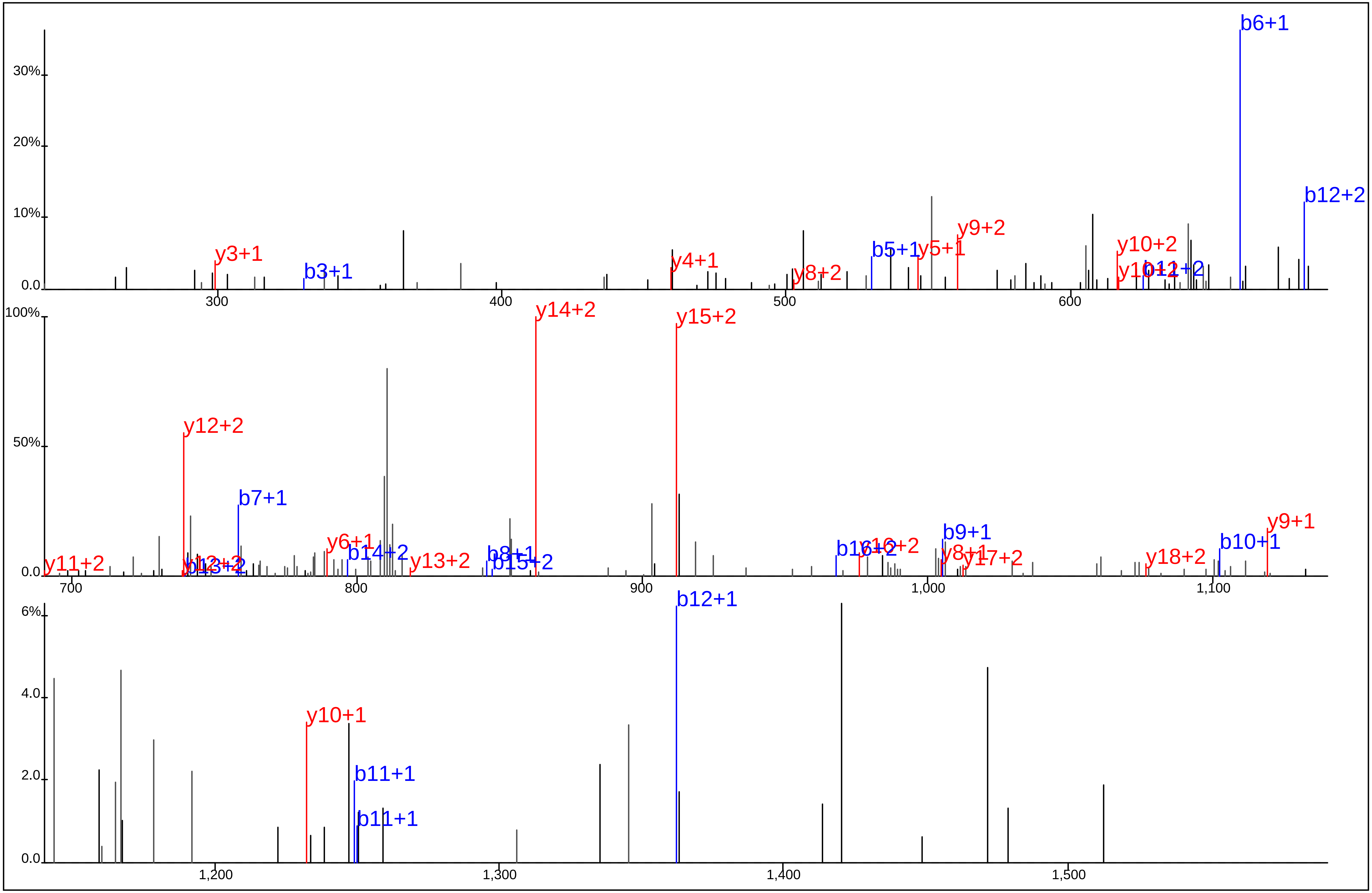

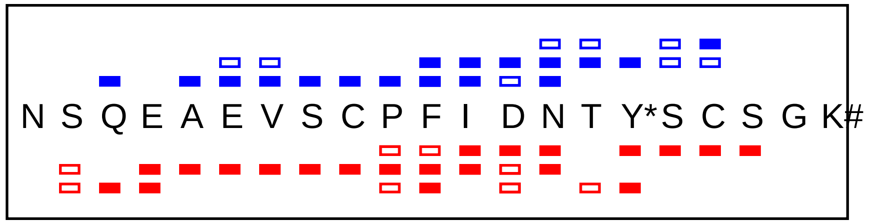

Protein Name:	C20orf18
gi Accession:	34189561
Precursor Mass:	827.6722+3
Delta Mass:	7.912 ppm
XCorr:	38.62

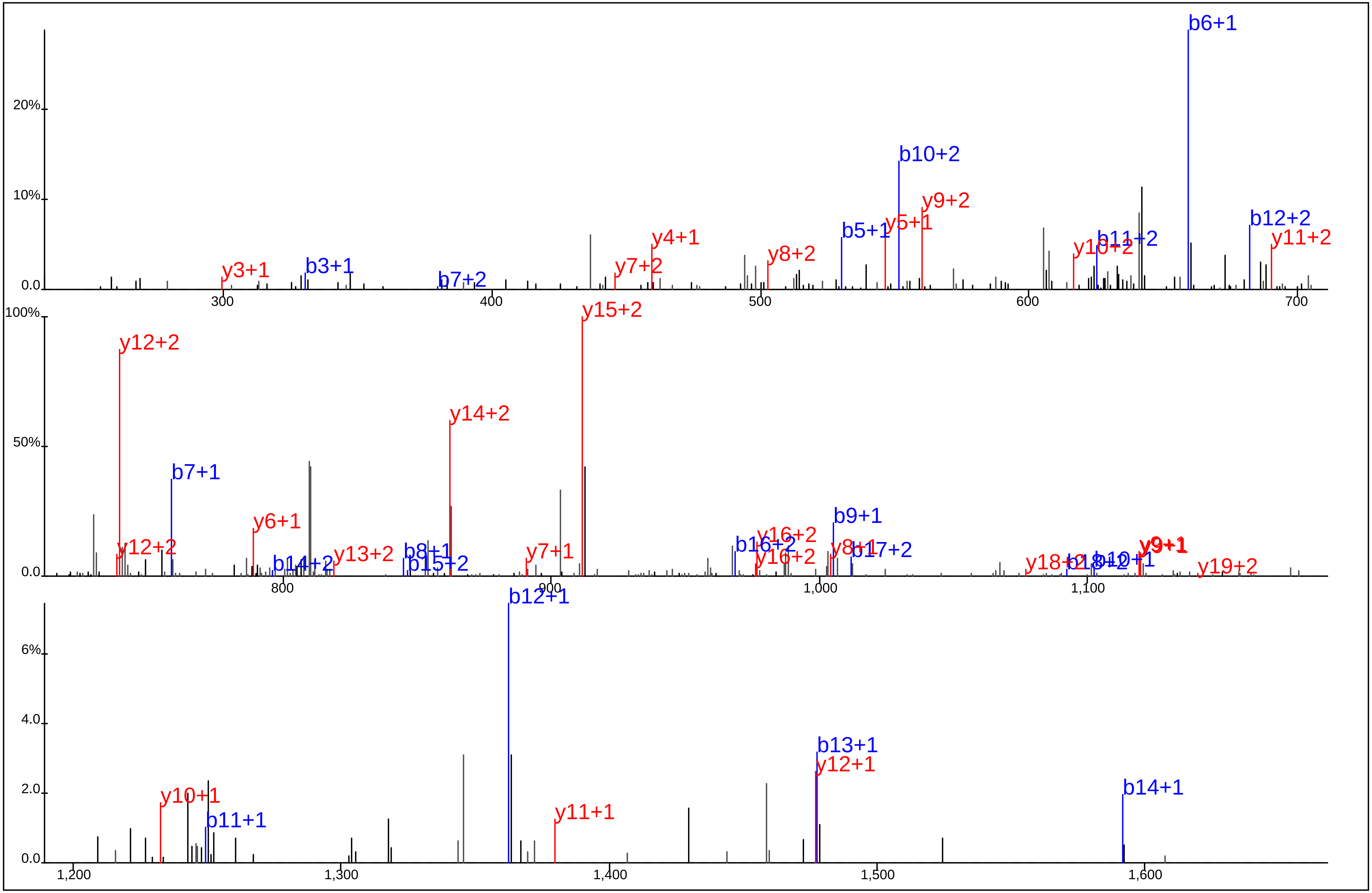

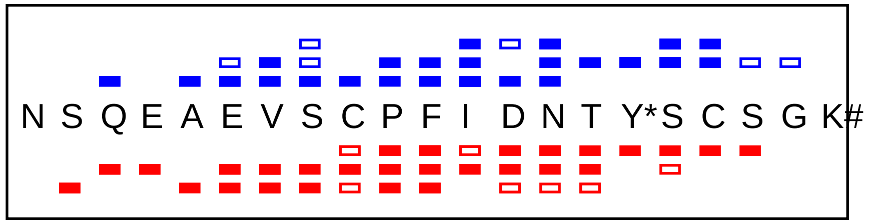

Protein Name: C20orf18
gi Accession: 34189561
Precursor Mass: 827.6749+3
Delta Mass: 11.084 ppm
XCorr: 56.08

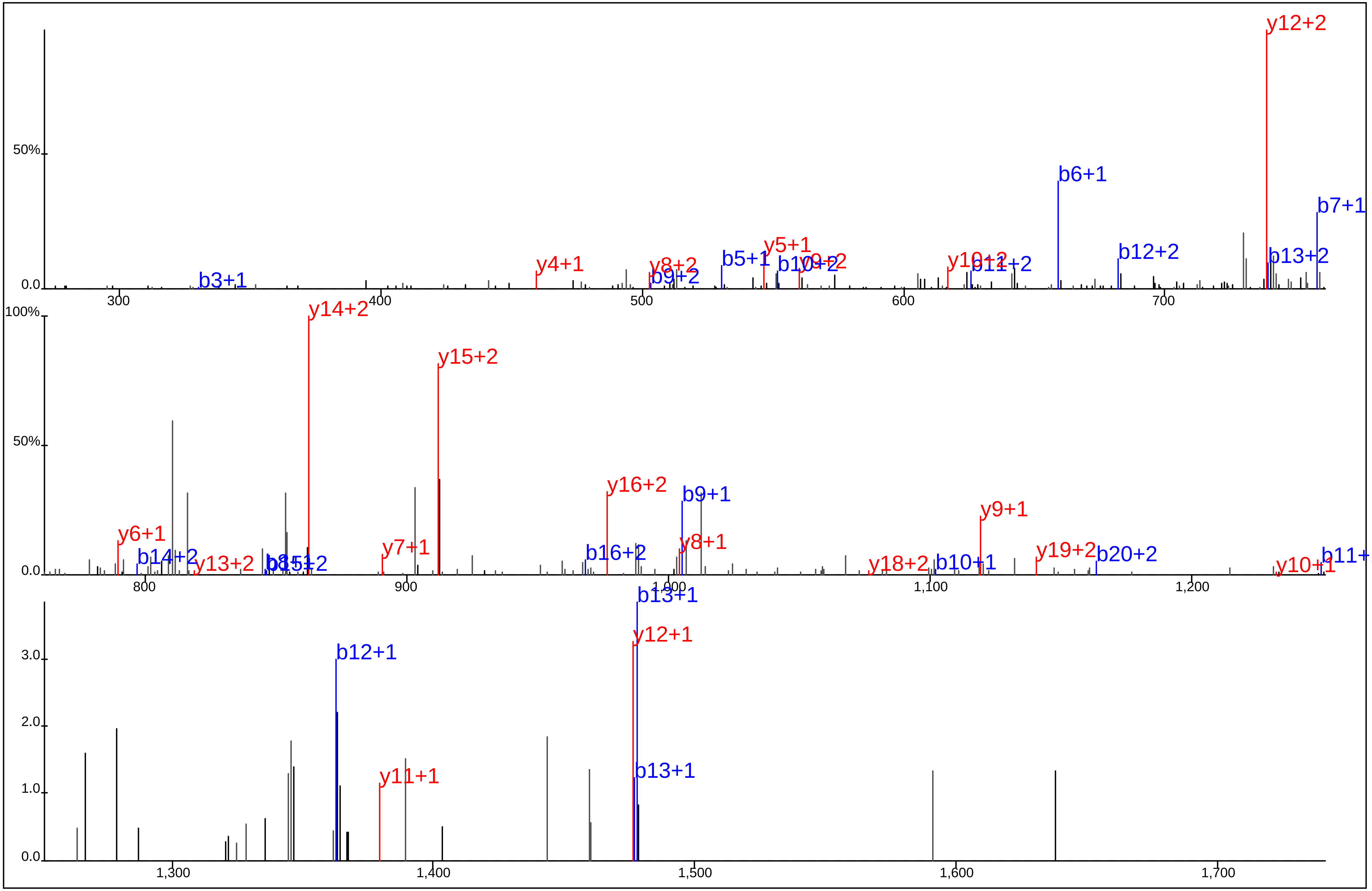

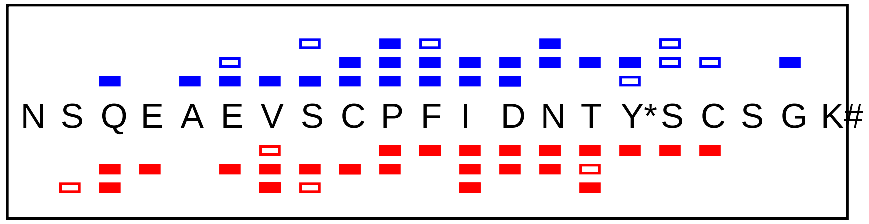

Protein Name: C20orf18
gi Accession: 34189561
Precursor Mass: 827.6700+3
Delta Mass: 5.183 ppm
XCorr: 46.05

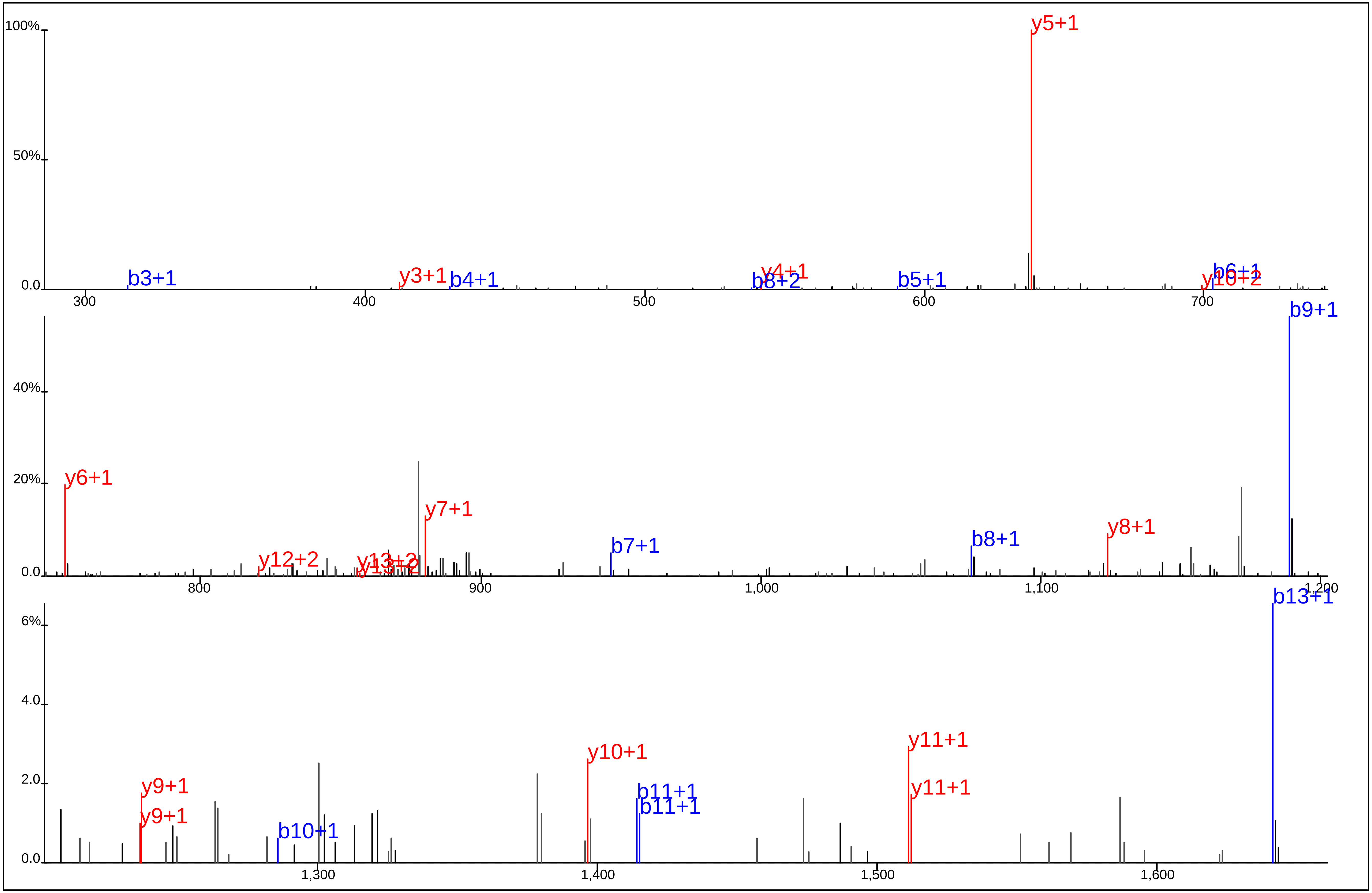

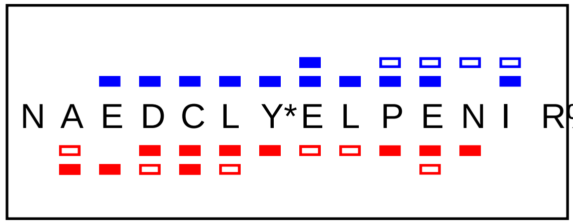

Protein Name: C9orf78
gi Accession: 6808233
Precursor Mass: 913.3892+2
Delta Mass: 0.574 ppm
XCorr: 50.94

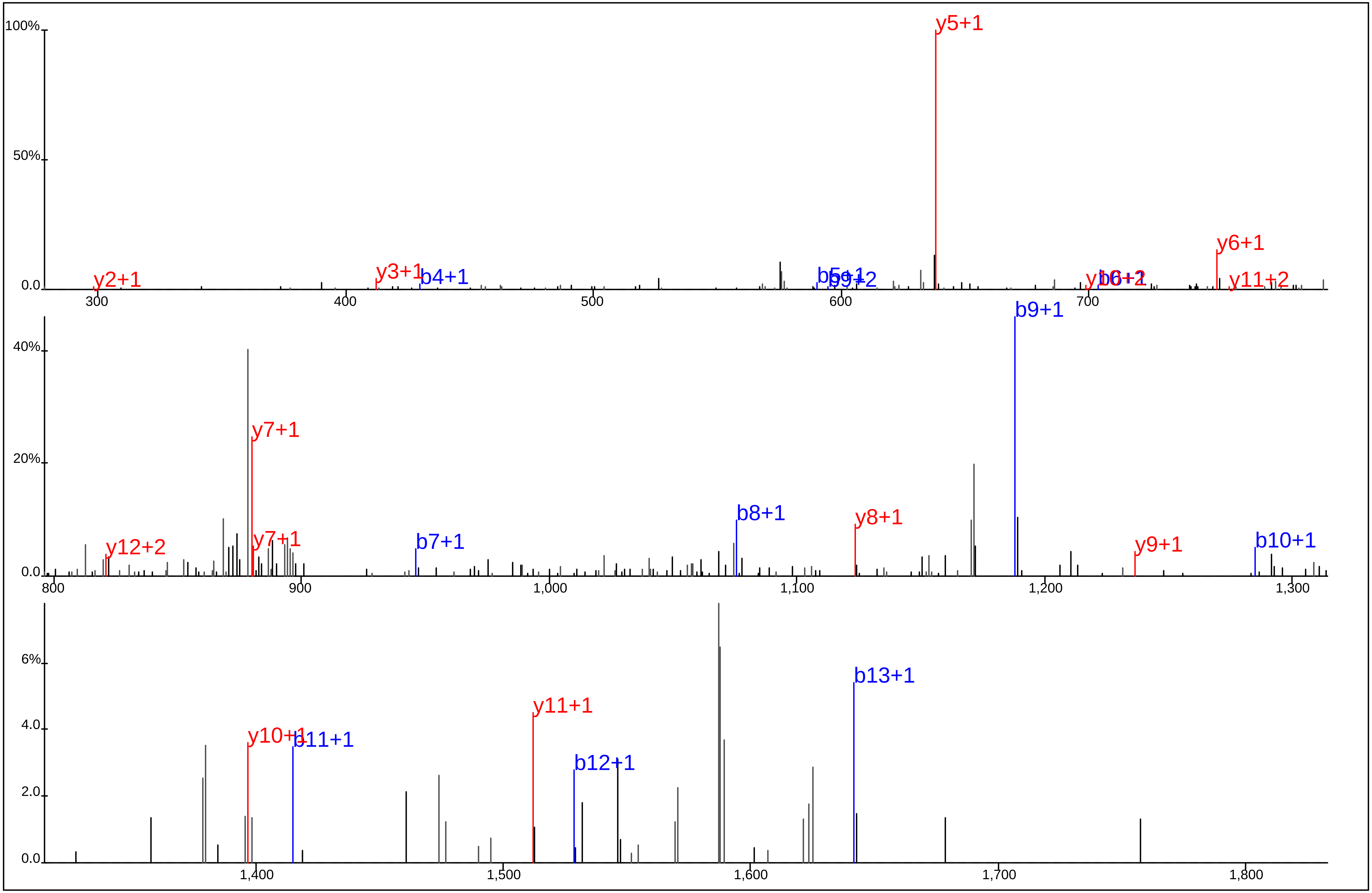

N A E D C L Y*E L P E N I R%

Protein Name:	C9orf78
gi Accession:	6808233
Precursor Mass:	913.3901+2
Delta Mass:	1.576 ppm
XCorr:	49.44

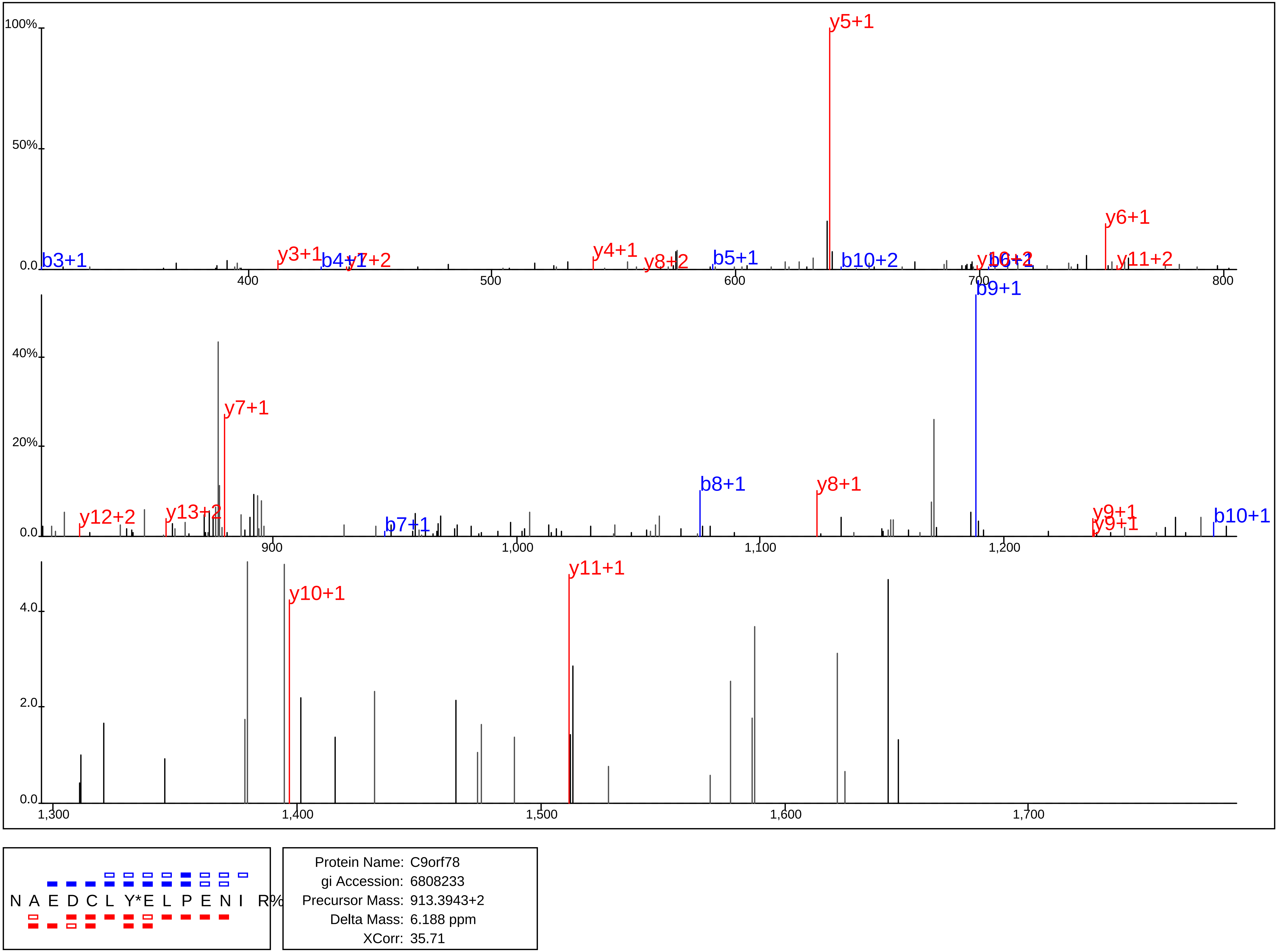

Protein Name: C9orf78
gi Accession: 6808233
Precursor Mass: 913.3943+2
Delta Mass: 6.188 ppm
XCorr: 35.71

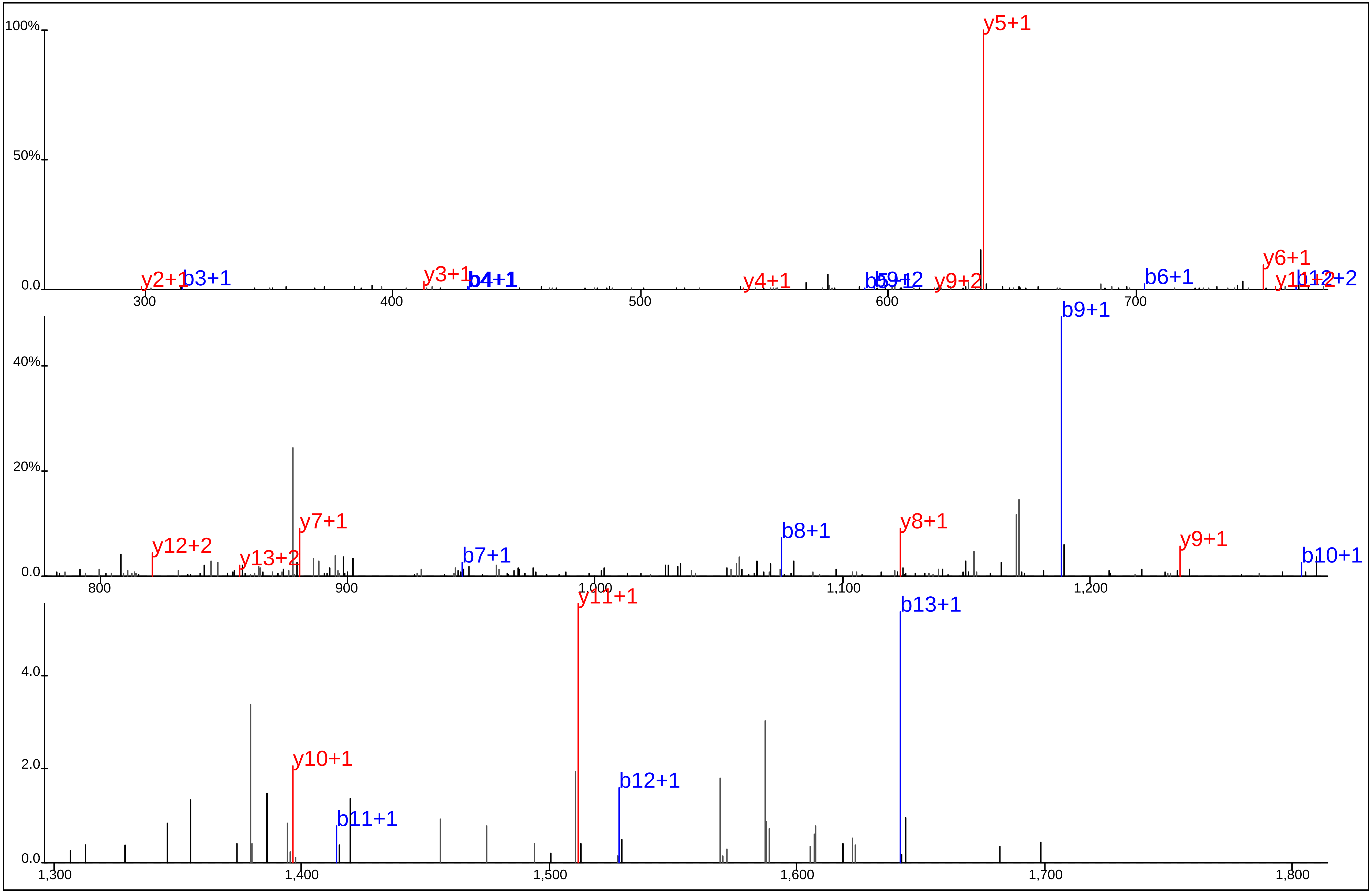

N A E D C L Y*E L P E N I R%

Protein Name: C9orf78
gi Accession: 6808233
Precursor Mass: 913.3908+2
Delta Mass: 2.310 ppm
XCorr: 44.52

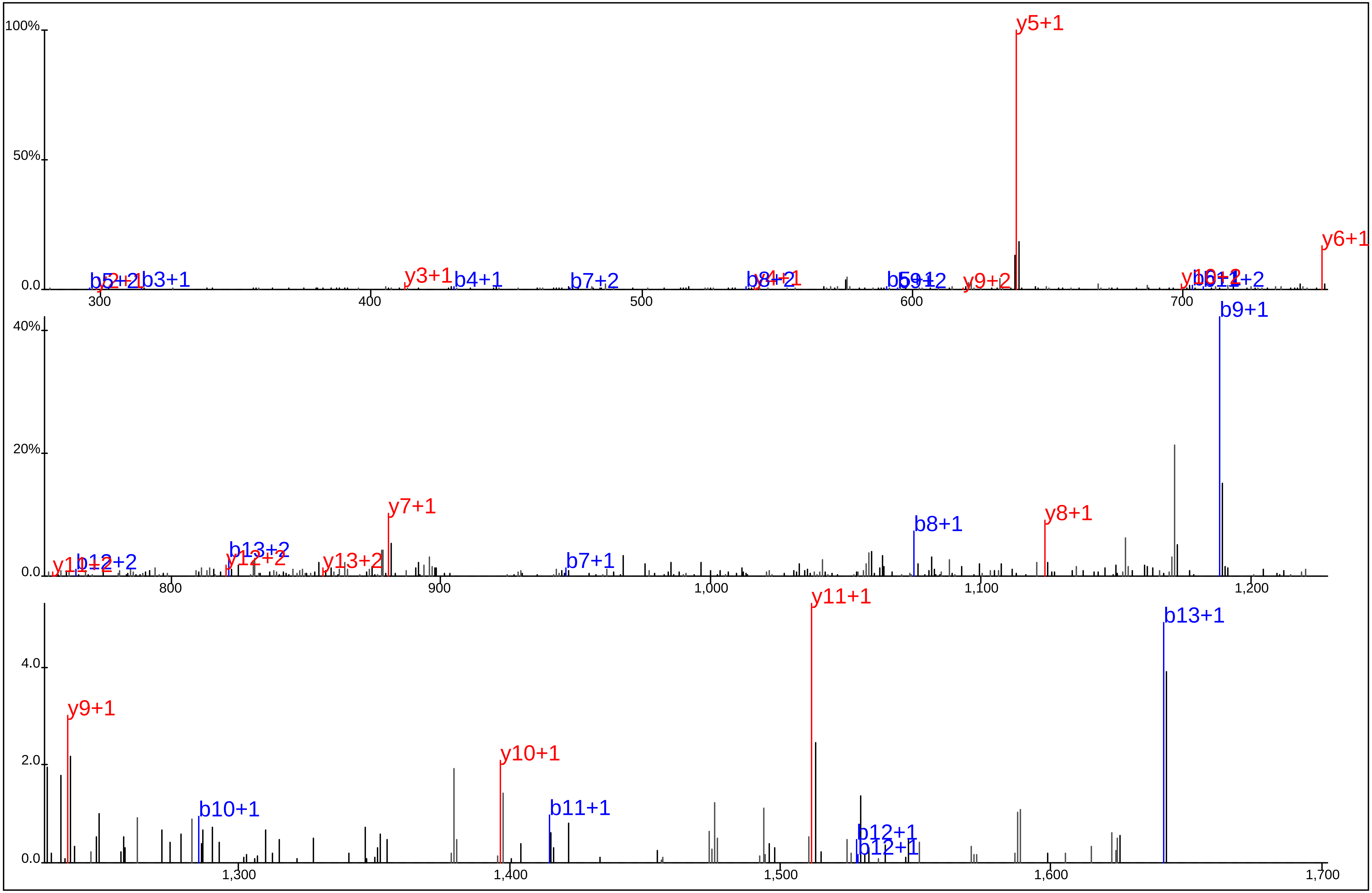

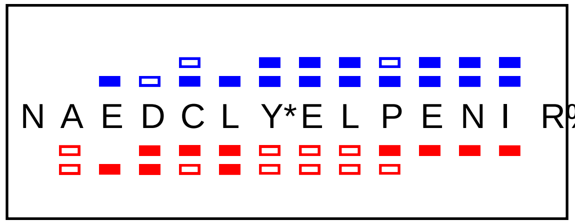

Protein Name: C9orf78
gi Accession: 6808233
Precursor Mass: 913.3917+2
Delta Mass: 3.313 ppm
XCorr: 50.53

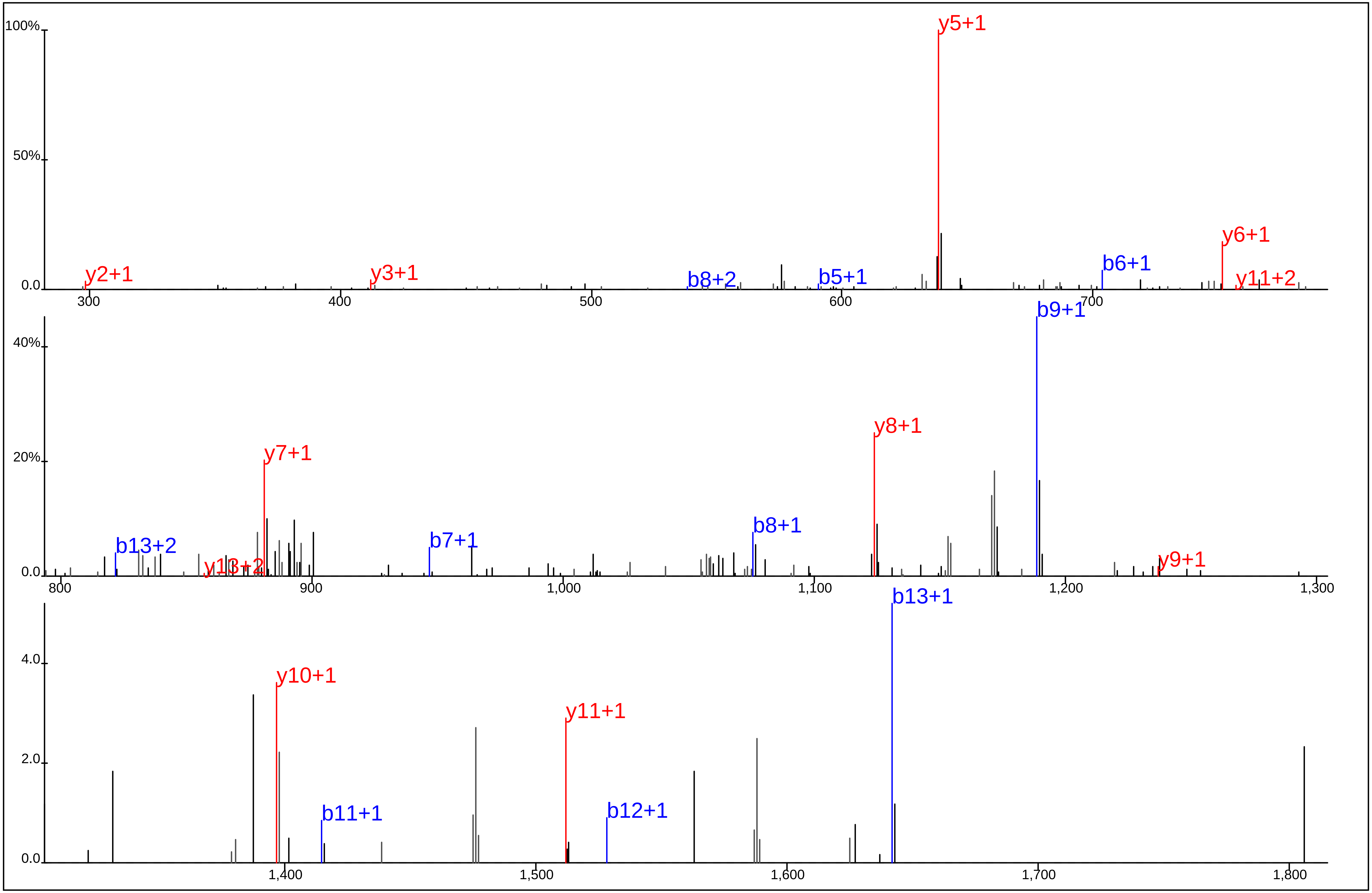

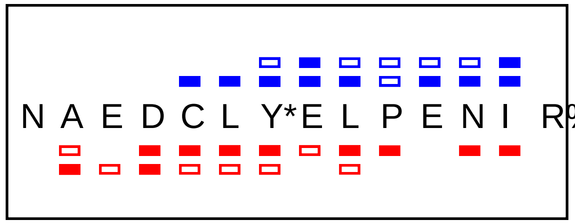

Protein Name: C9orf78
gi Accession: 6808233
Precursor Mass: 913.3918+2
Delta Mass: 3.378 ppm
XCorr: 60.38

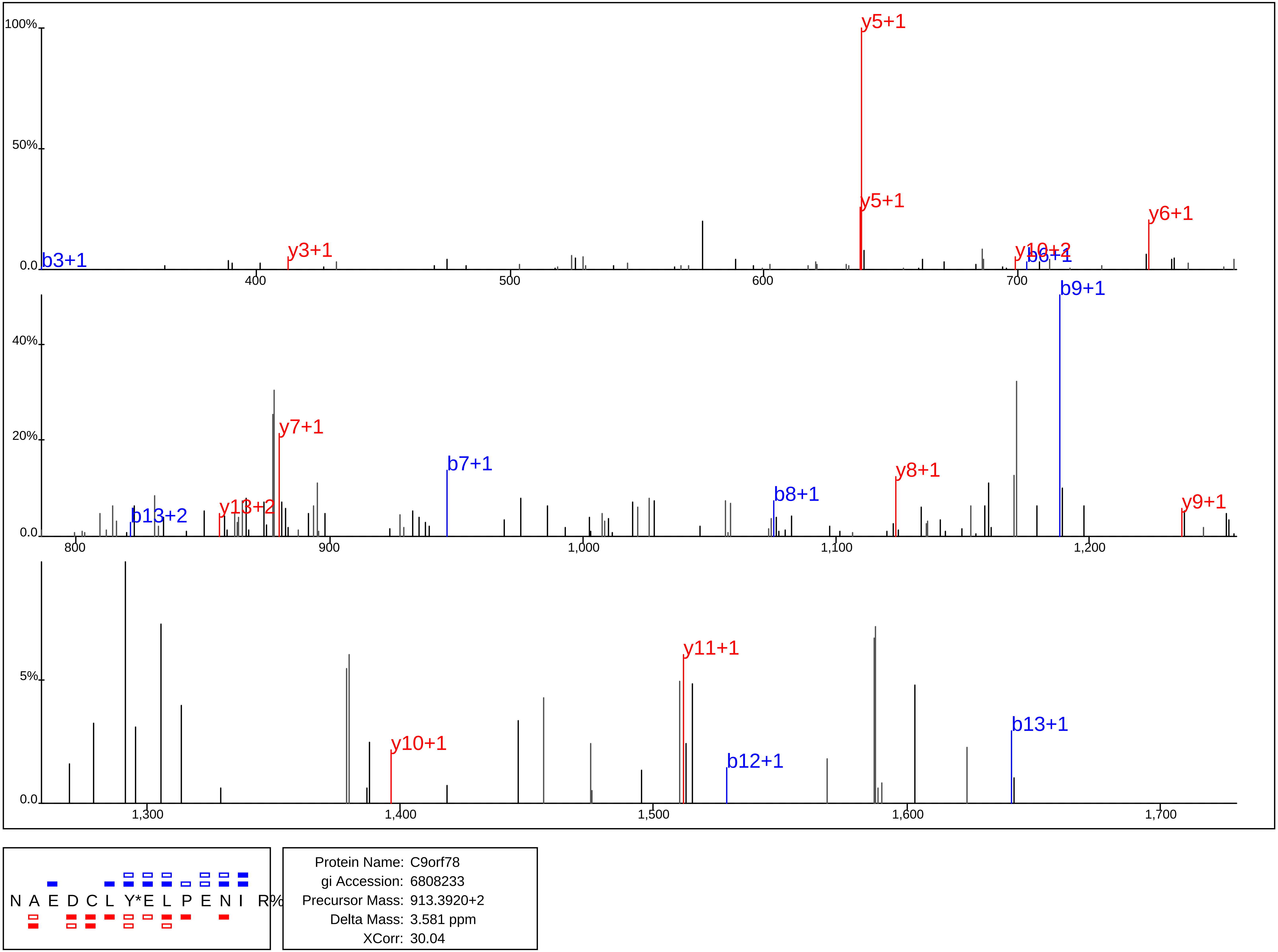

Protein Name: C9orf78
gi Accession: 6808233
Precursor Mass: 913.3920+2
Delta Mass: 3.581 ppm
XCorr: 30.04

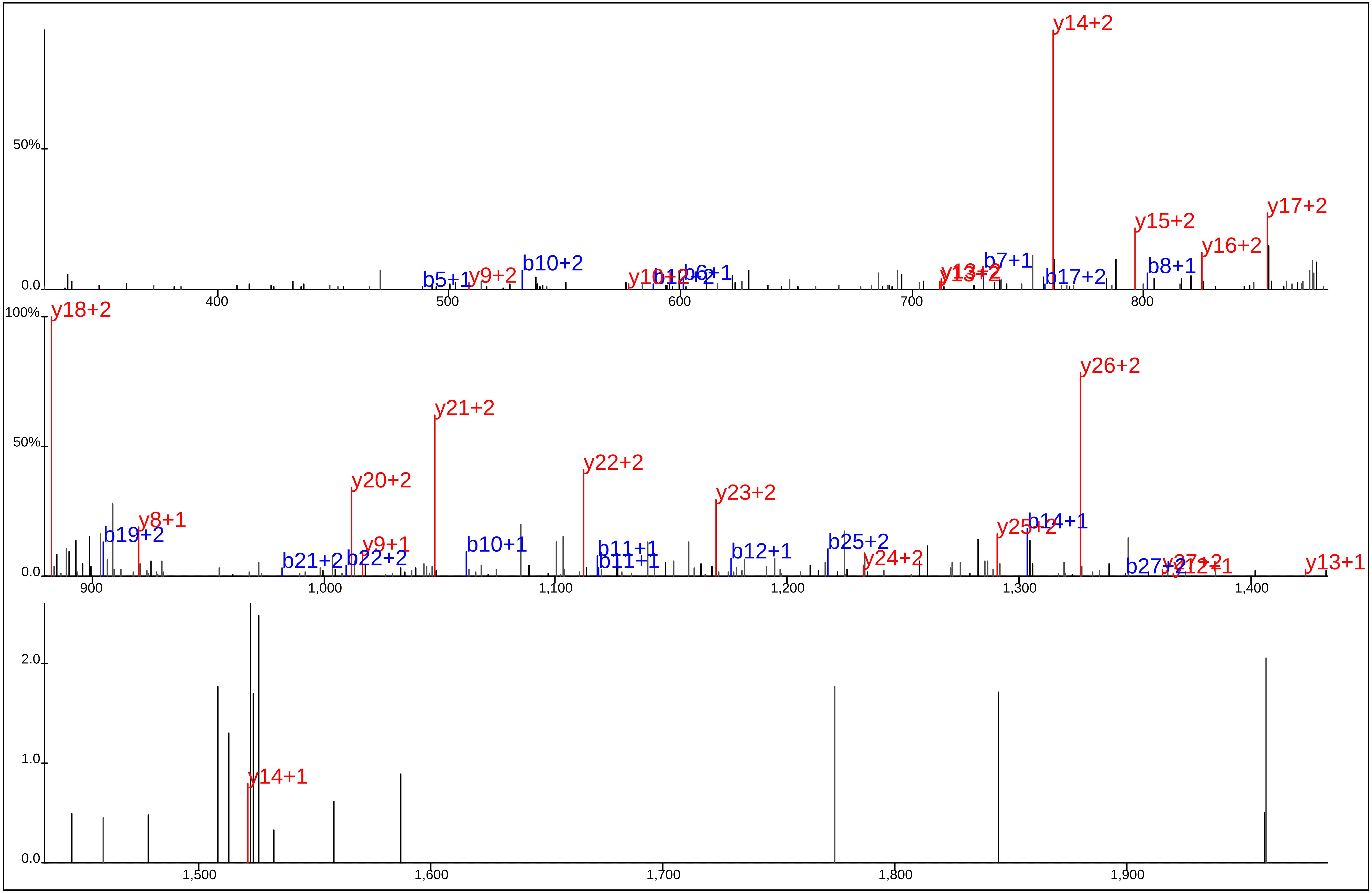

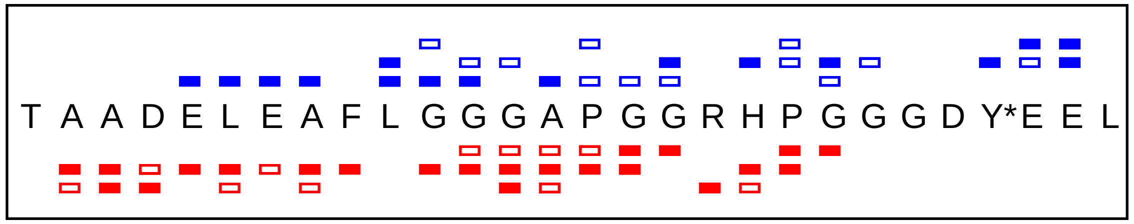

Protein Name:	C9orf86
gi Accession:	14042363
Precursor Mass:	941.7533+3
Delta Mass:	6.935 ppm
XCorr:	53.19

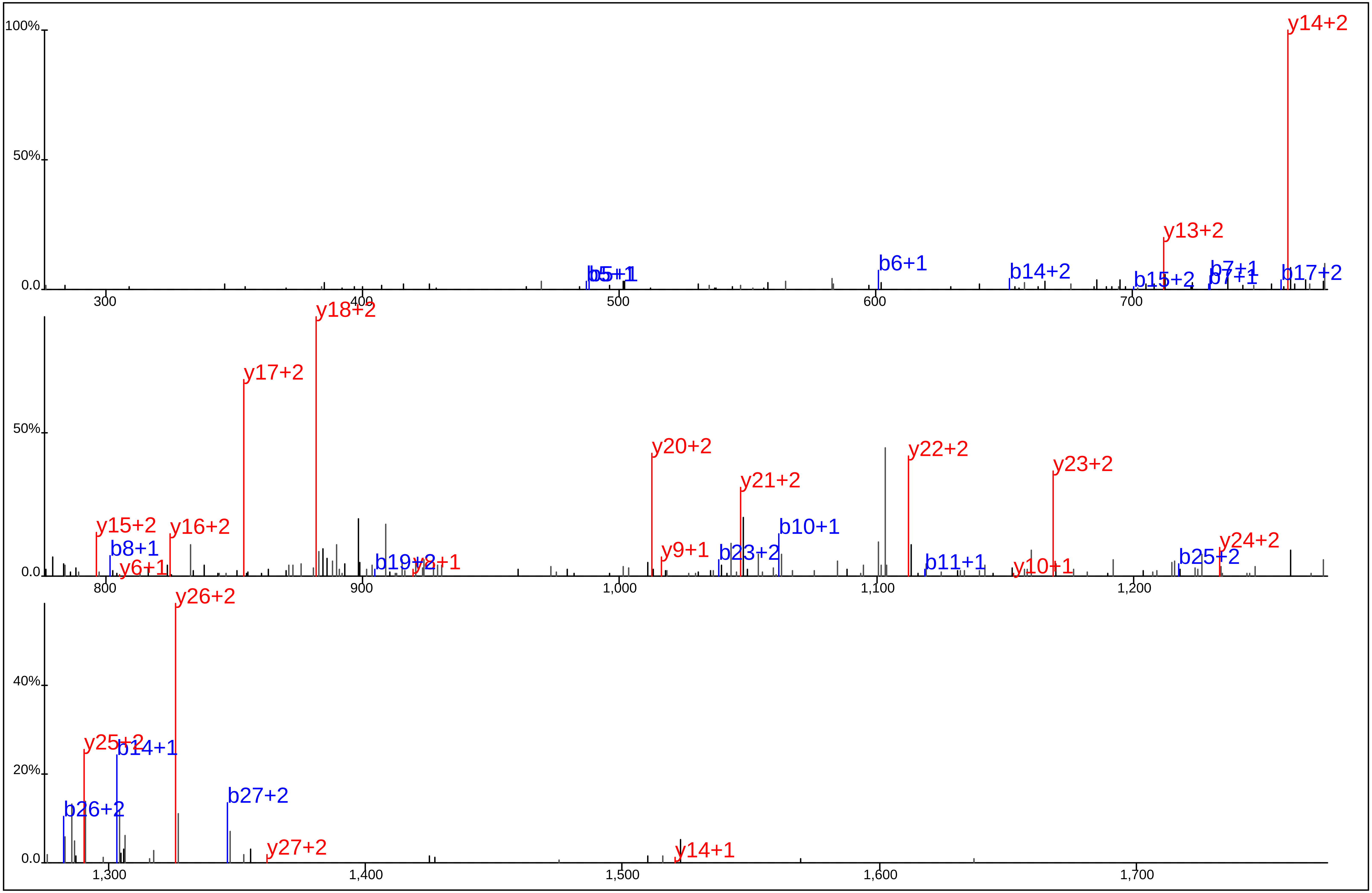

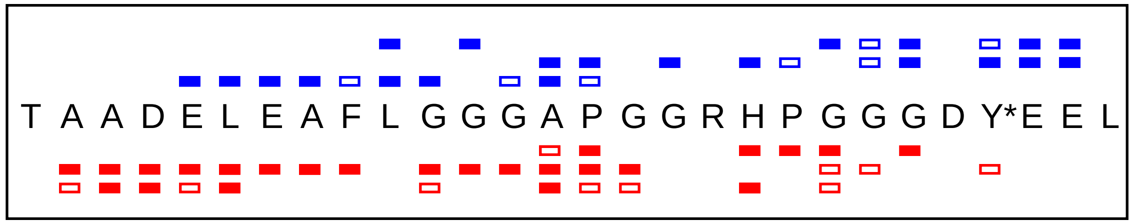

Protein Name:	C9orf86
gi Accession:	14042363
Precursor Mass:	941.7481+3
Delta Mass:	1.423 ppm
XCorr:	64.15

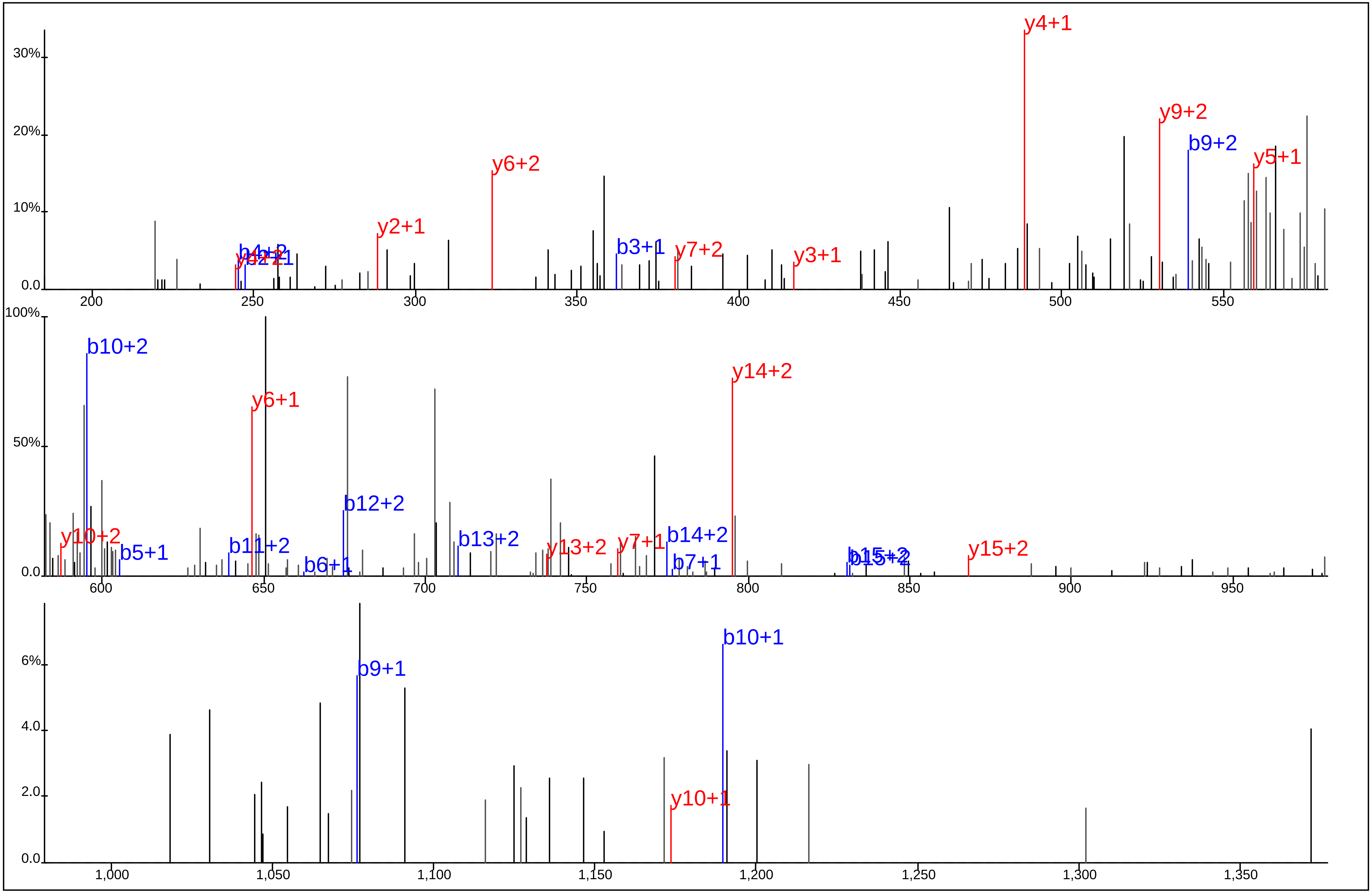

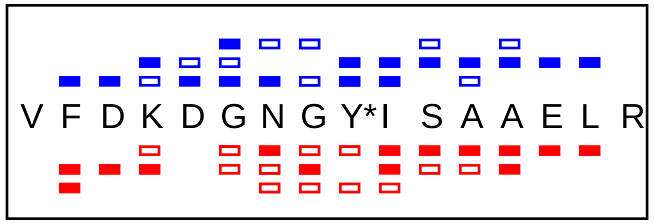

Protein Name: Calmodulin
gi Accession: 825635
Precursor Mass: 612.2878+3
Delta Mass: 6.474 ppm
XCorr: 14.15

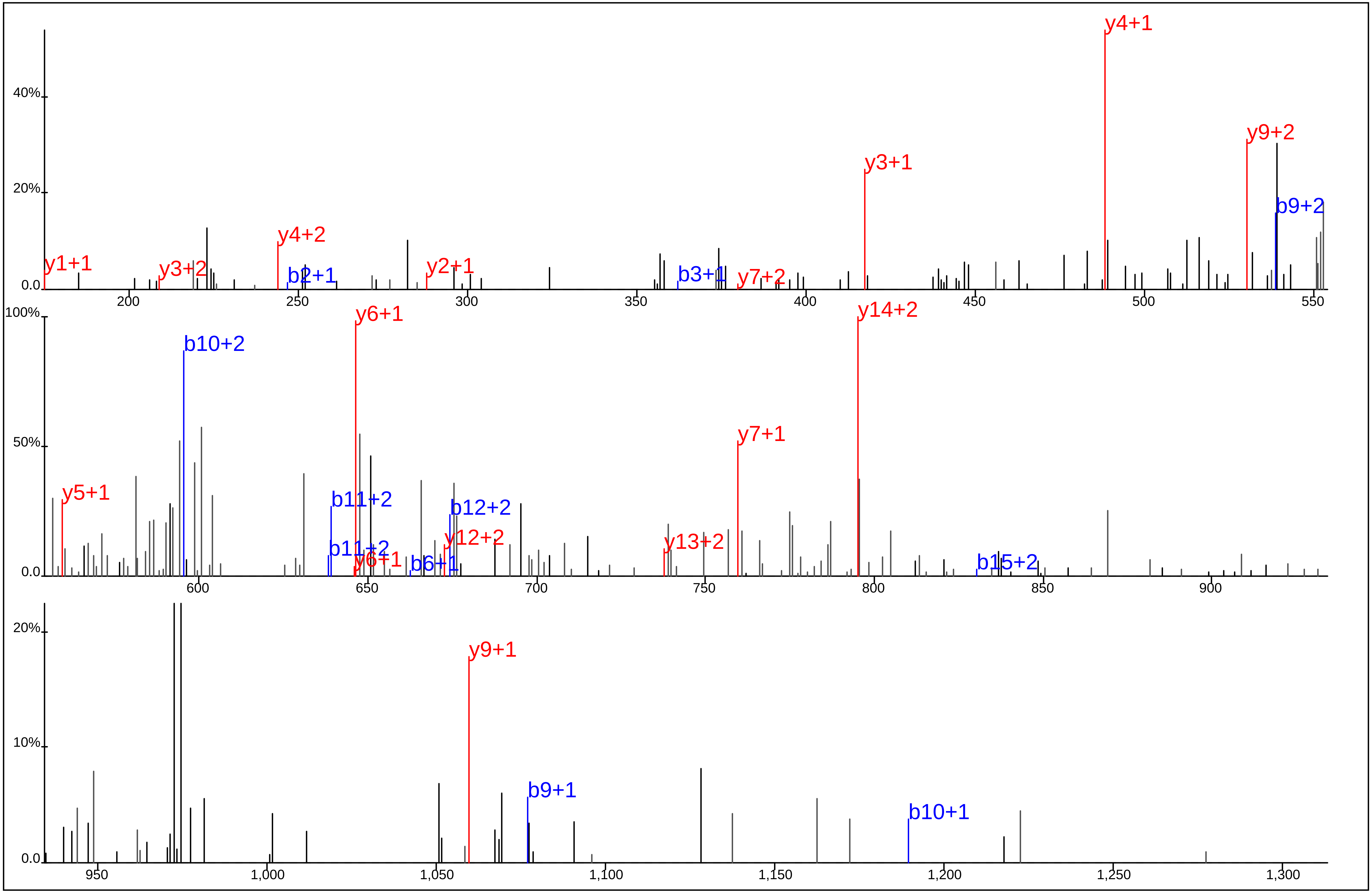

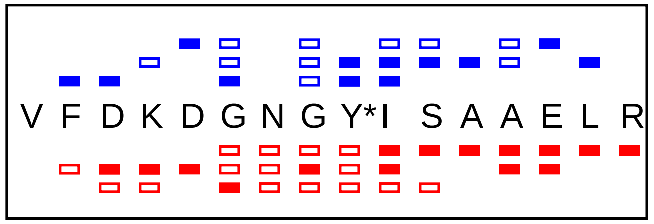

Protein Name: Calmodulin
gi Accession: 825635
Precursor Mass: 612.2853+3
Delta Mass: 2.282 ppm
XCorr: 19.11

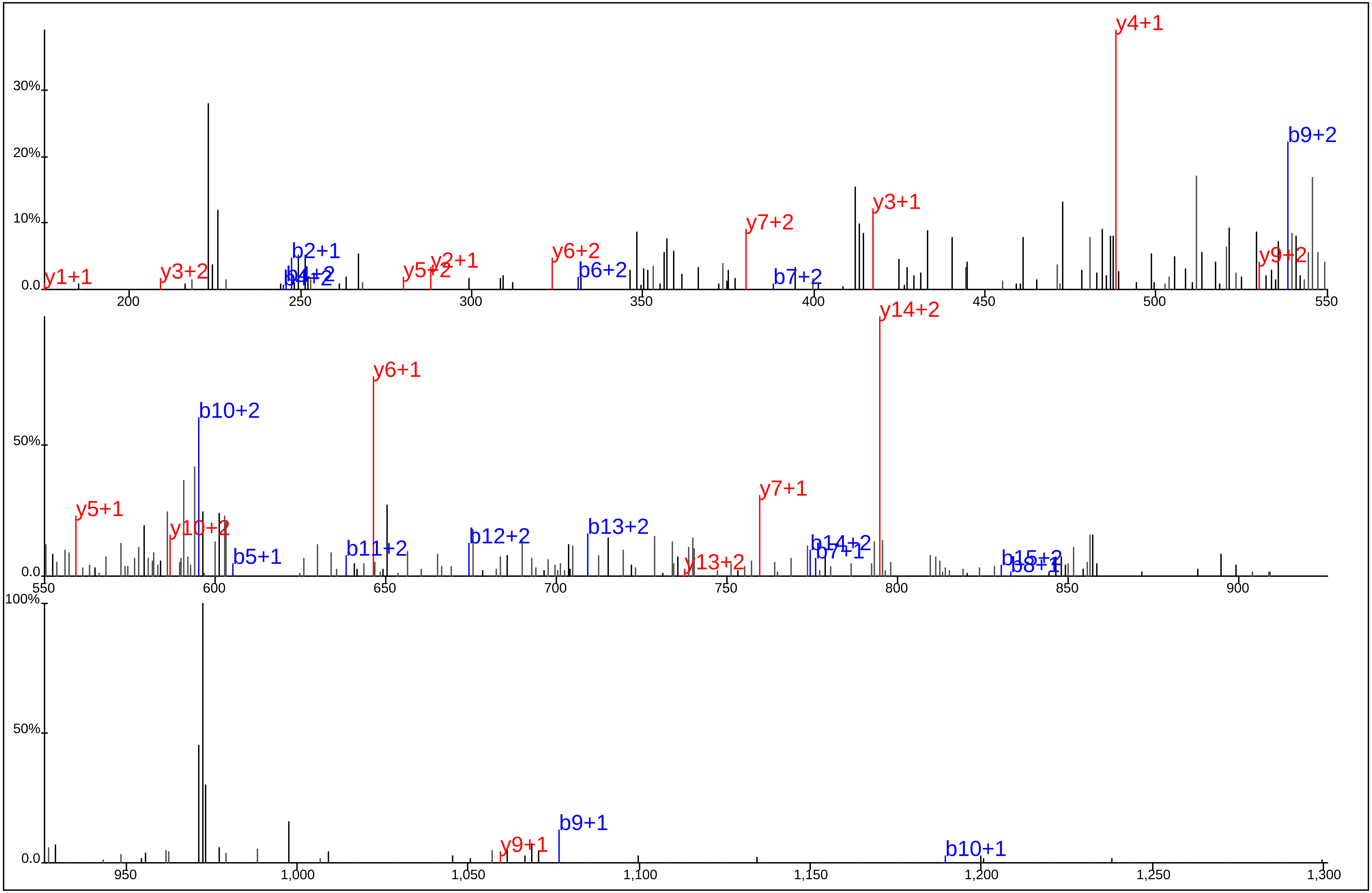

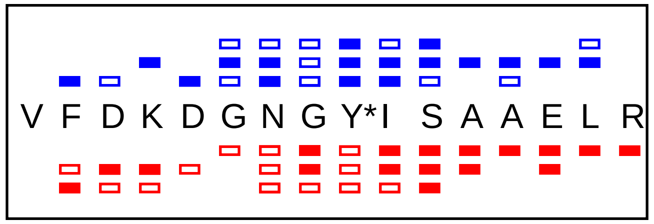

Protein Name: Calmodulin
gi Accession: 825635
Precursor Mass: 612.2867+3
Delta Mass: 4.578 ppm
XCorr: 10.82

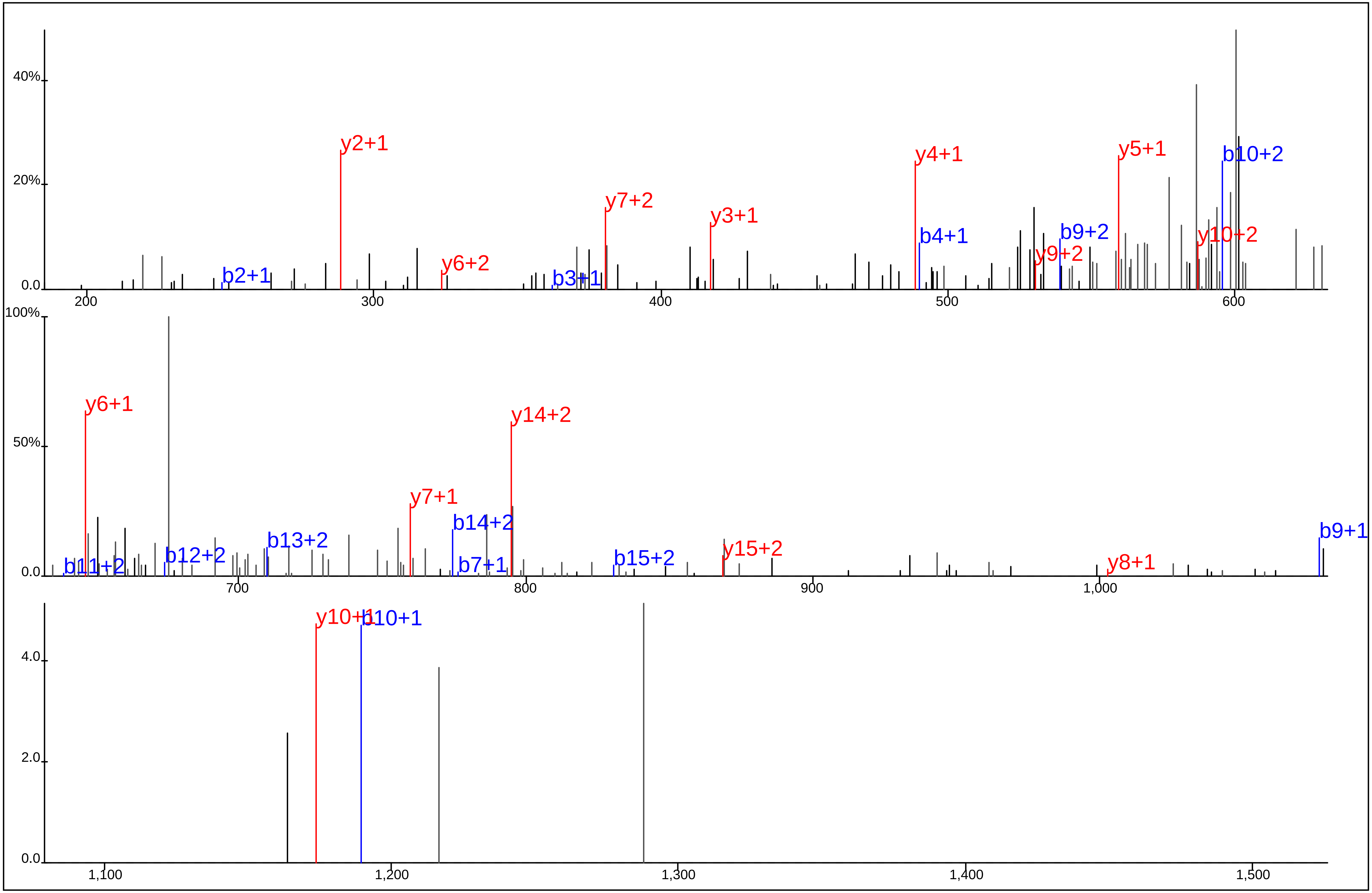

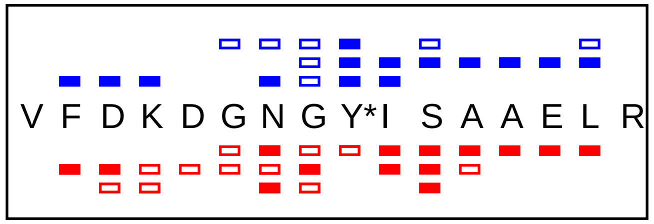

Protein Name: Calmodulin
gi Accession: 825635
Precursor Mass: 612.2861+3
Delta Mass: 3.683 ppm
XCorr: 30.78

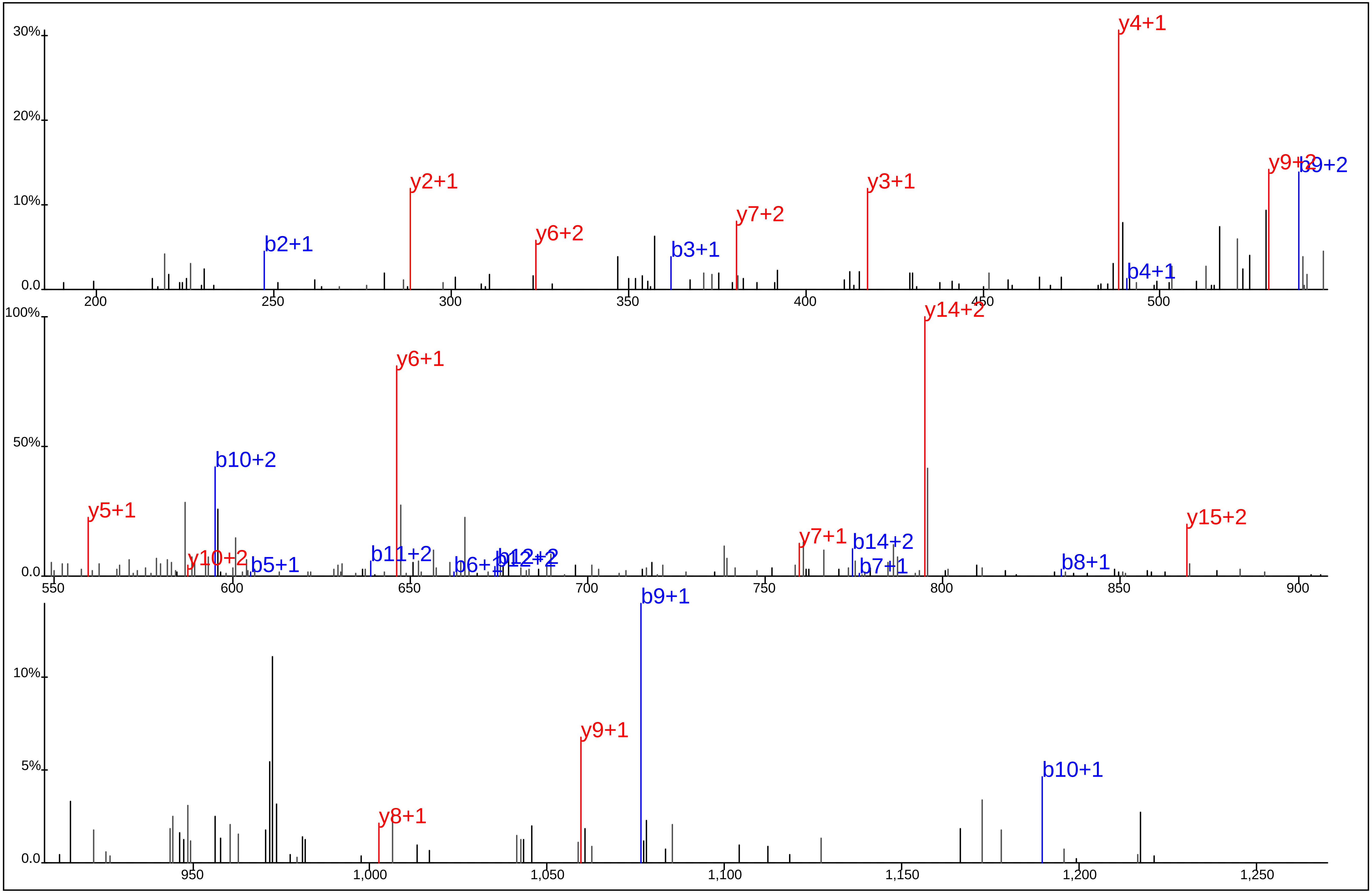

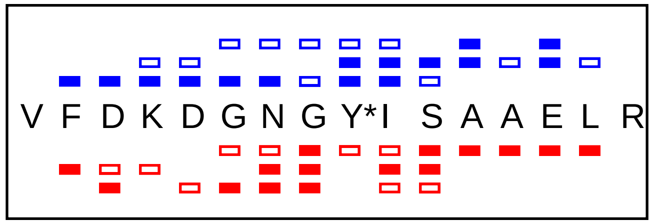

Protein Name: Calmodulin
gi Accession: 825635
Precursor Mass: 612.2847+3
Delta Mass: 1.383 ppm
XCorr: 42.07

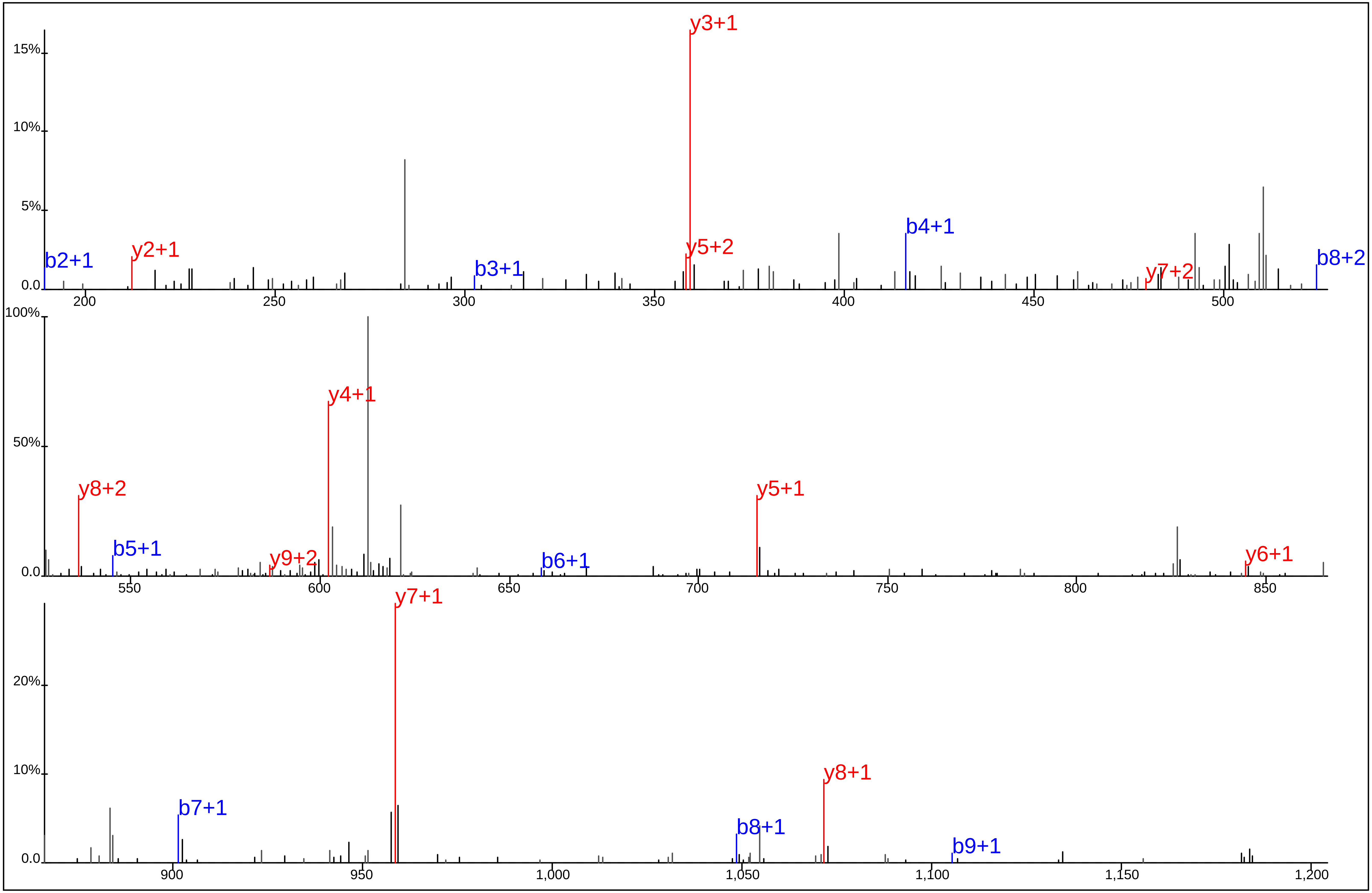

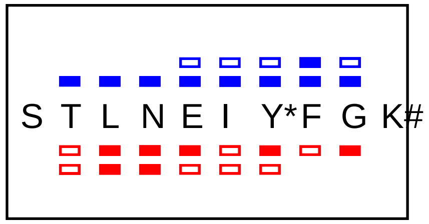

Protein Name:	CAPB
gi Accession:	13124696
Precursor Mass:	630.2958+2
Delta Mass:	3.587 ppm
XCorr:	41.61

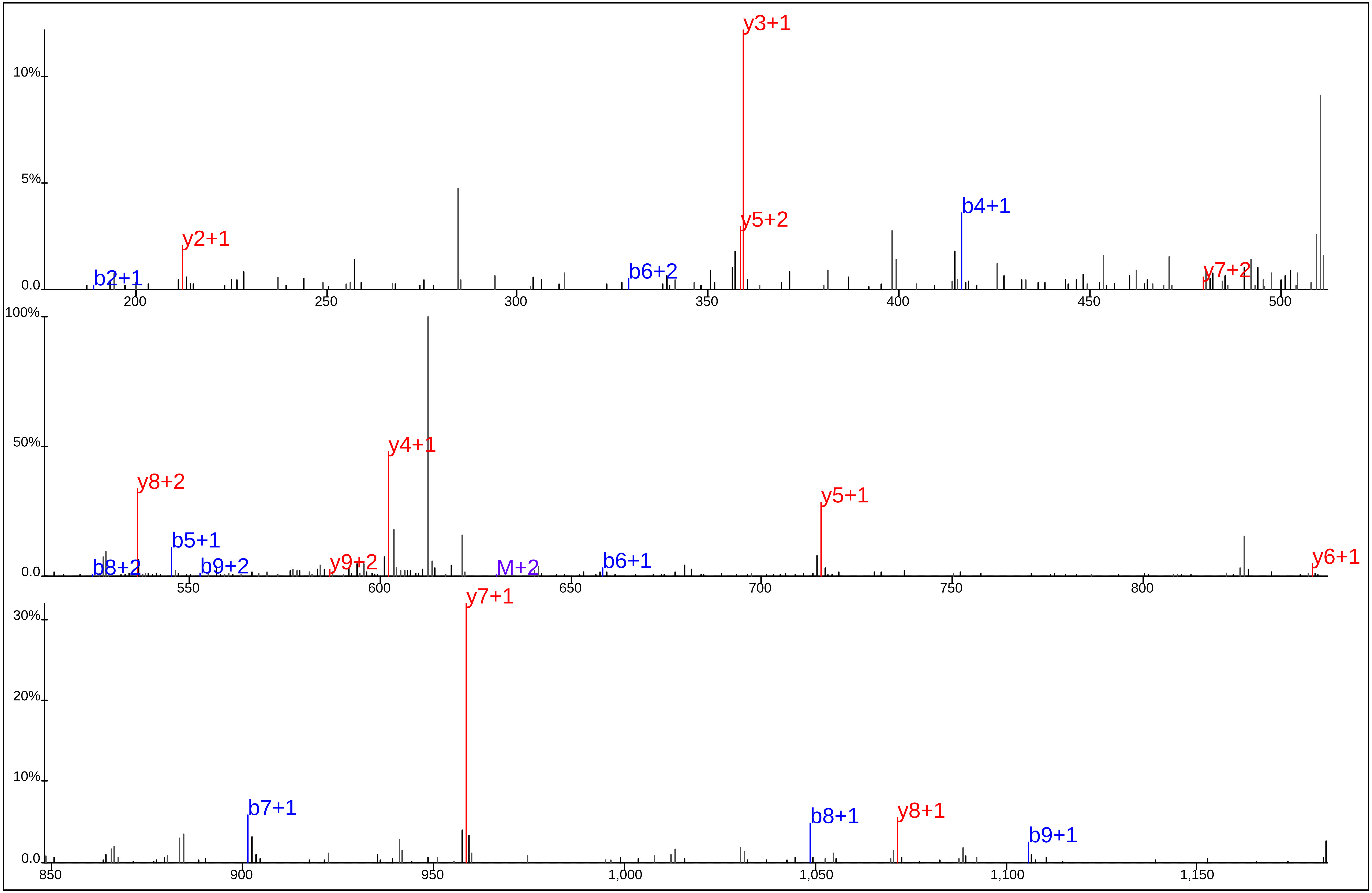

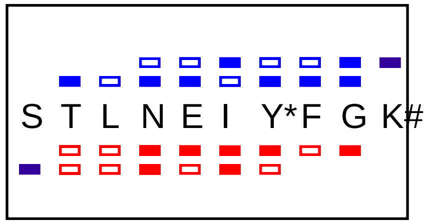

Protein Name: CAPB
gi Accession: 13124696
Precursor Mass: 630.2954+2
Delta Mass: 2.904 ppm
XCorr: 56.85

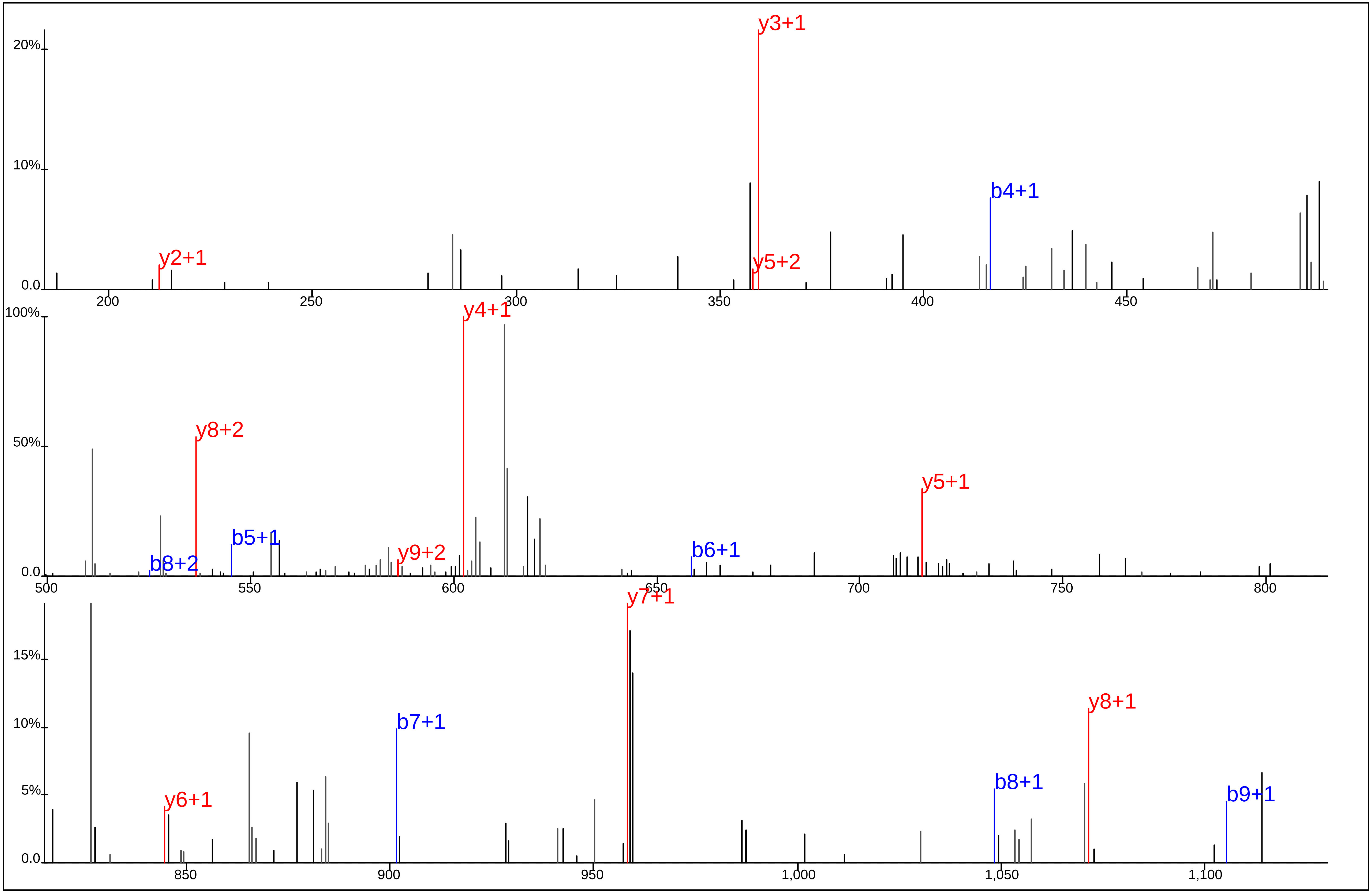

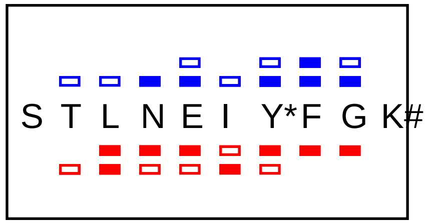

Protein Name: CAPB
gi Accession: 13124696
Precursor Mass: 630.2946+2
Delta Mass: 1.650 ppm
XCorr: 44.37

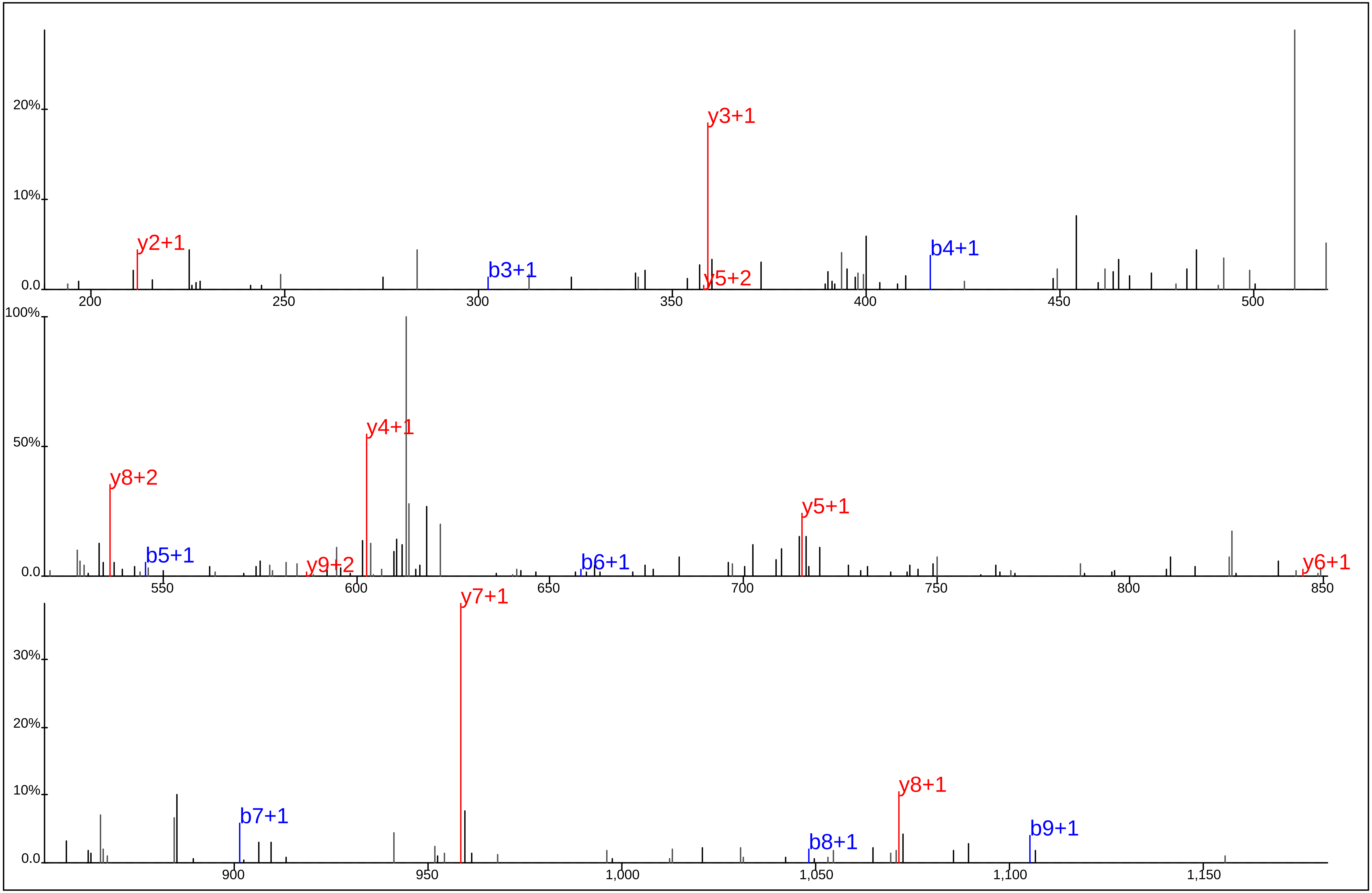

S T L N E I Y*F G K#

Protein Name: CAPB
gi Accession: 13124696
Precursor Mass: 630.2961+2
Delta Mass: 4.071 ppm
XCorr: 30.97

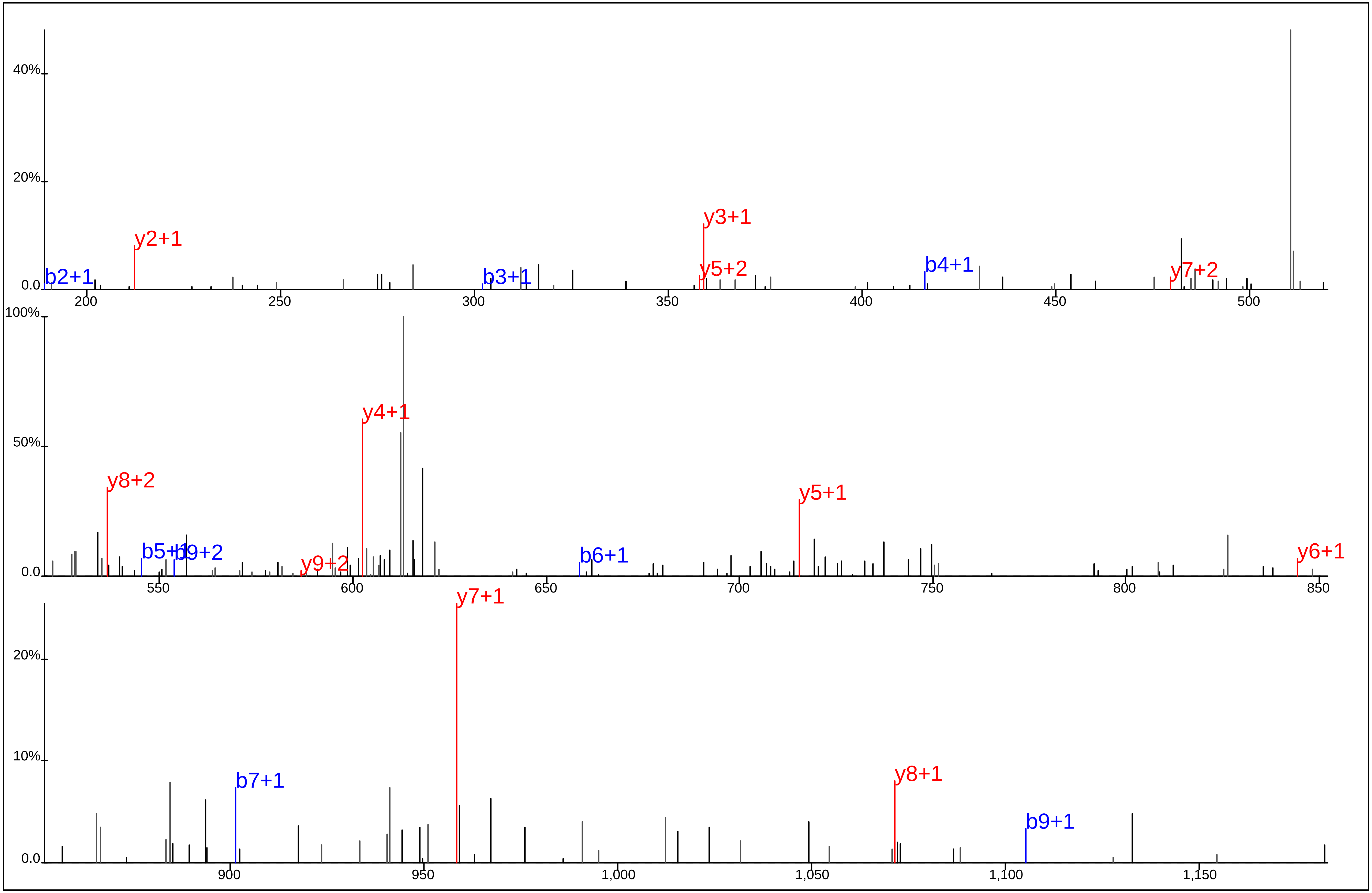

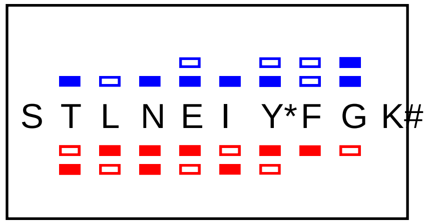

Protein Name: CAPB
gi Accession: 13124696
Precursor Mass: 630.2975+2
Delta Mass: 6.199 ppm
XCorr: 38.67

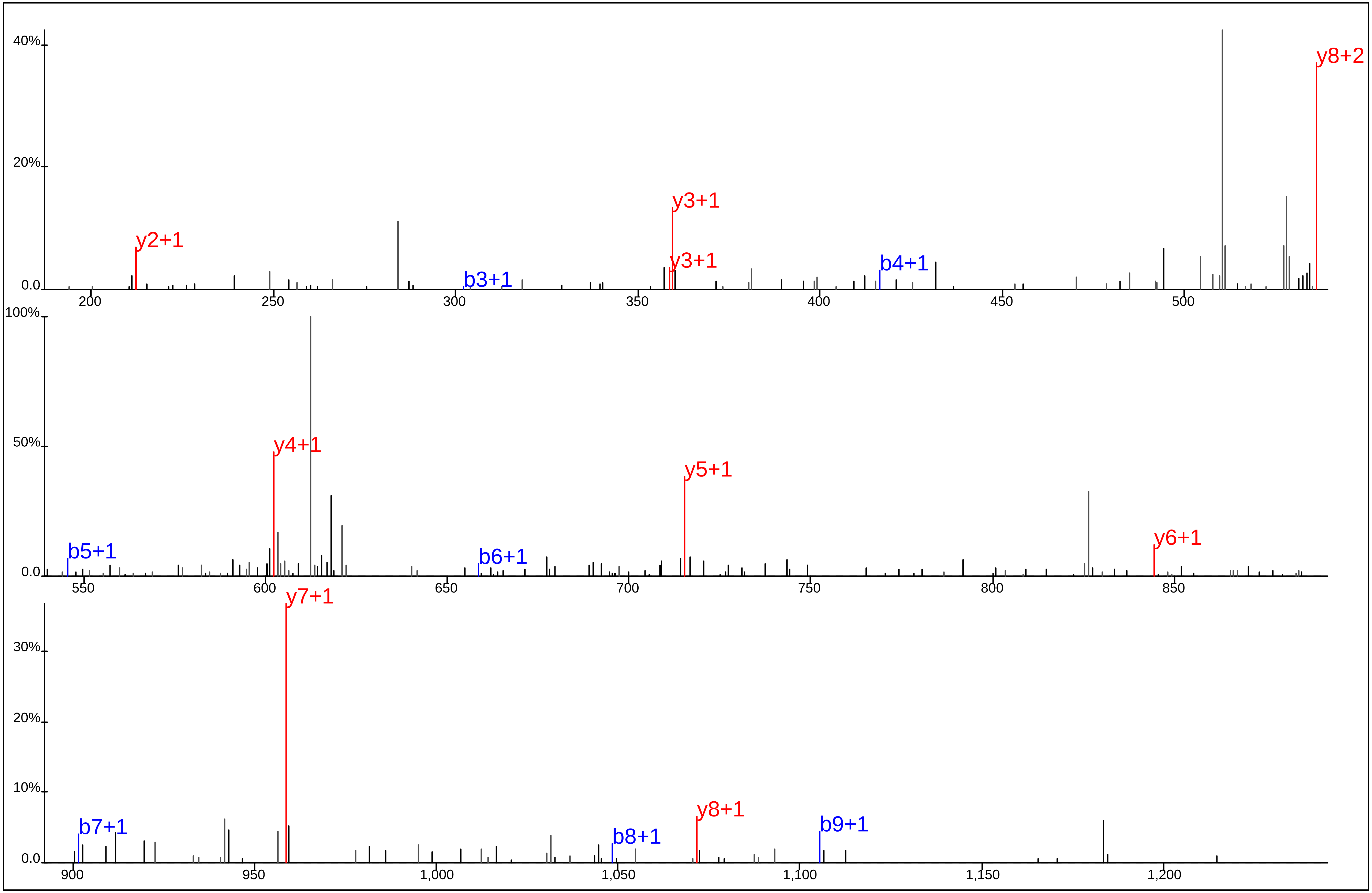

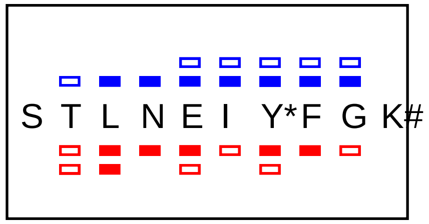

Protein Name: CAPB
gi Accession: 13124696
Precursor Mass: 630.2958+2
Delta Mass: 3.587 ppm
XCorr: 48.74

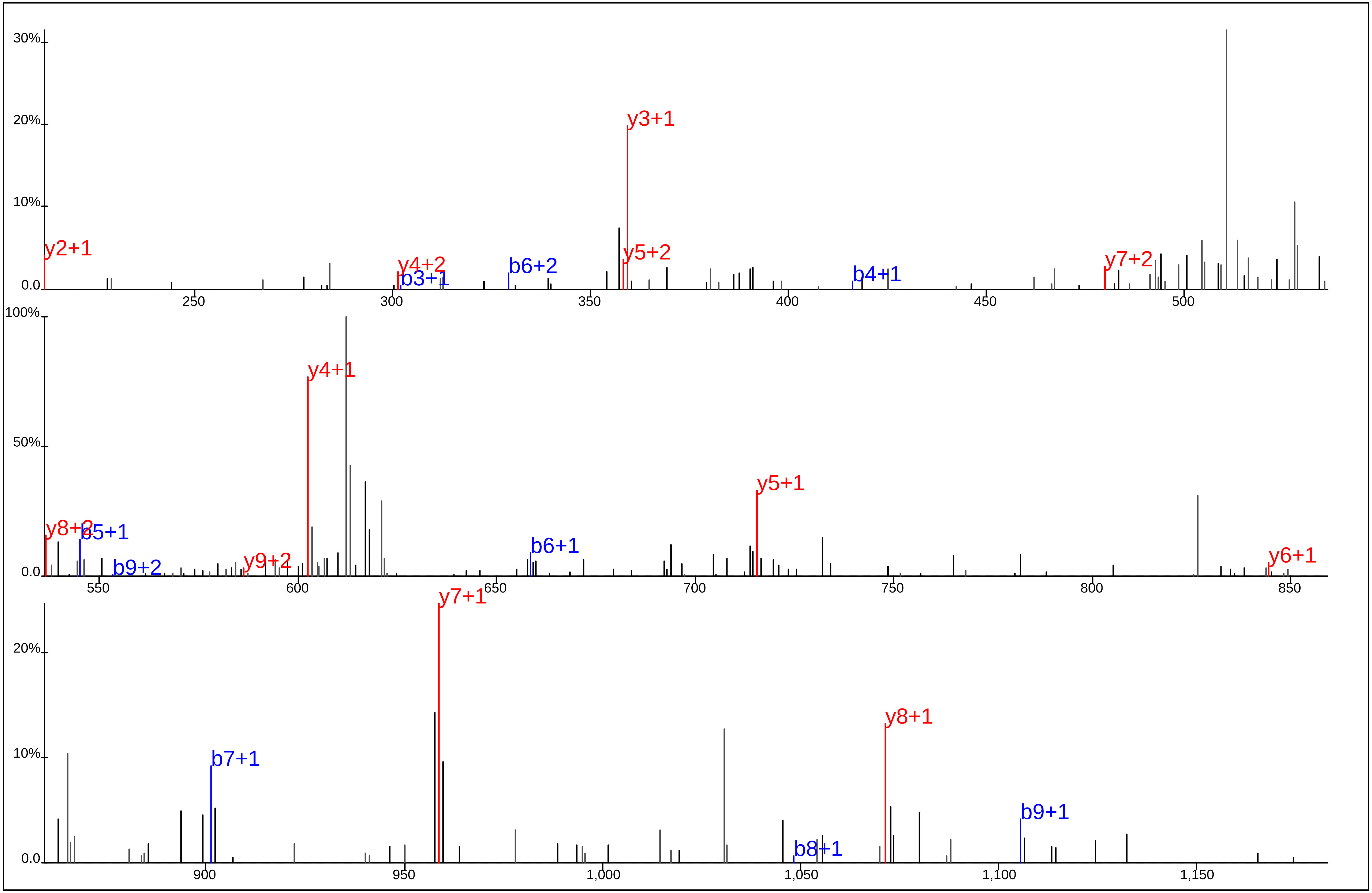

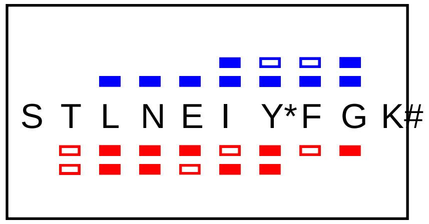

Protein Name: CAPB
gi Accession: 13124696
Precursor Mass: 630.2967+2
Delta Mass: 4.945 ppm
XCorr: 45.21

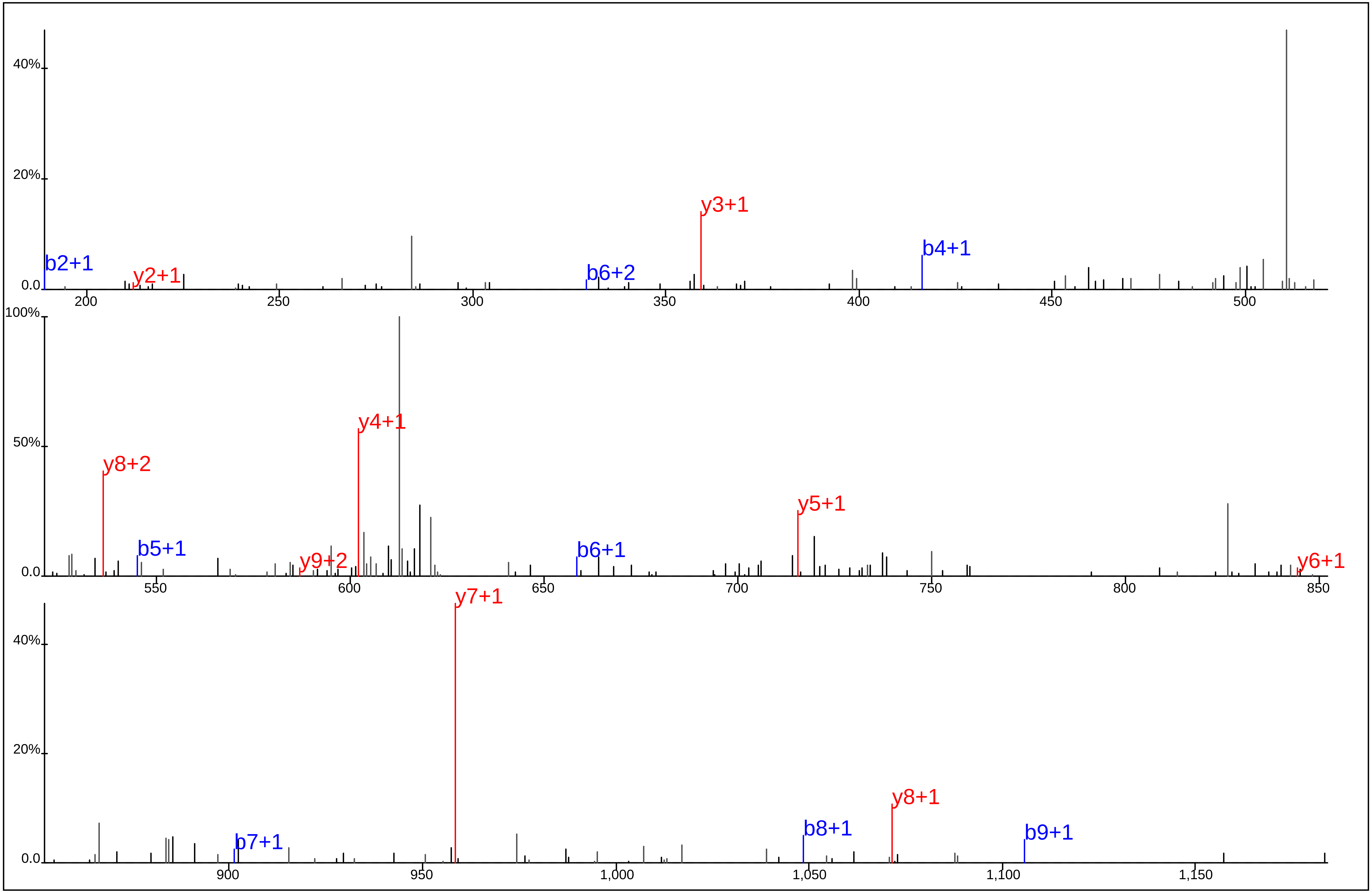

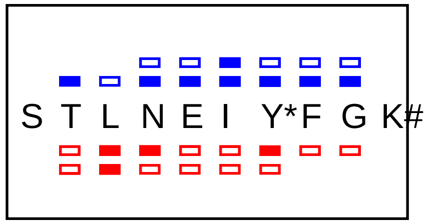

Protein Name: CAPB
gi Accession: 13124696
Precursor Mass: 630.3030+2
Delta Mass: 14.924 ppm
XCorr: 37.41

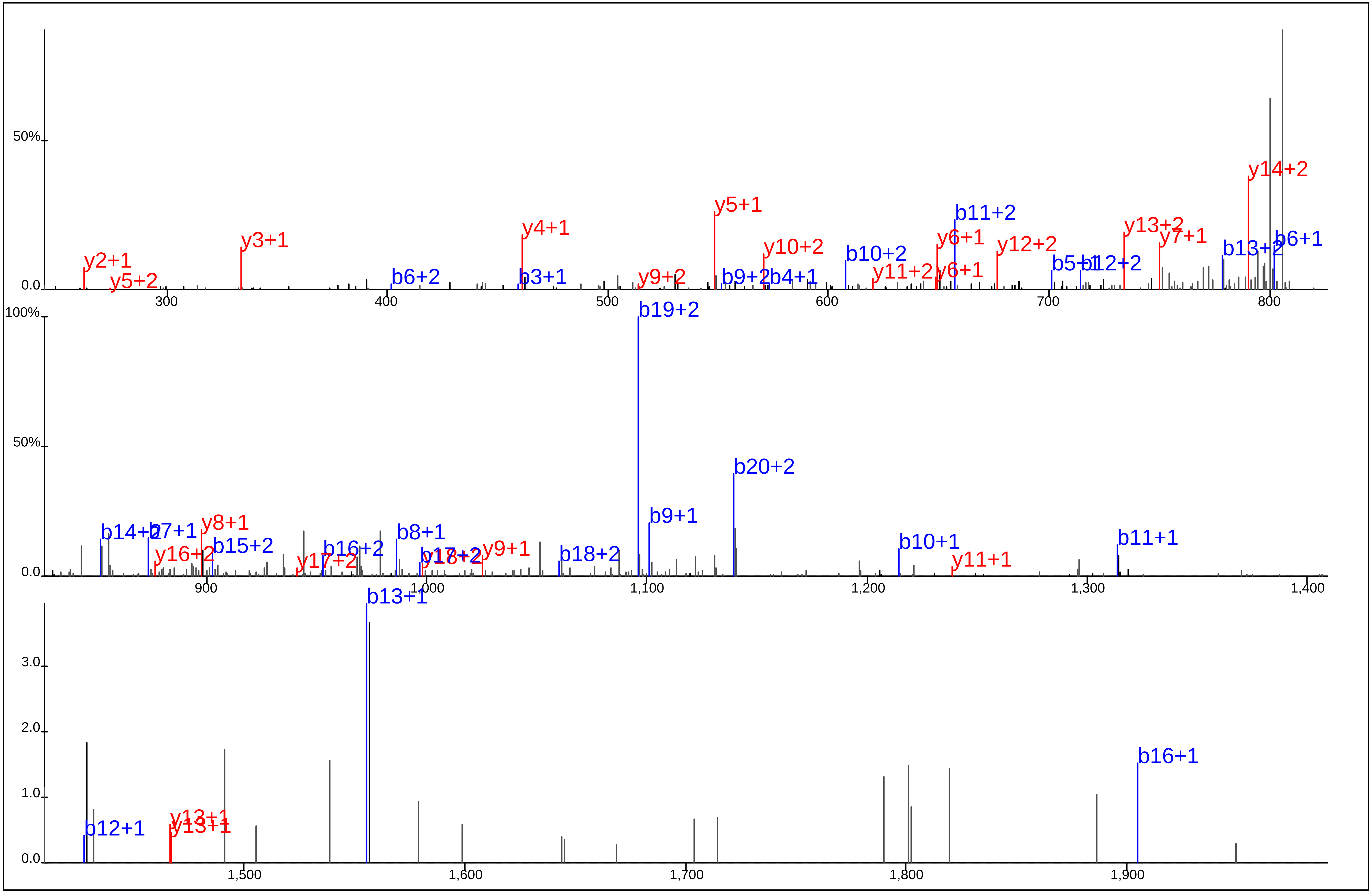

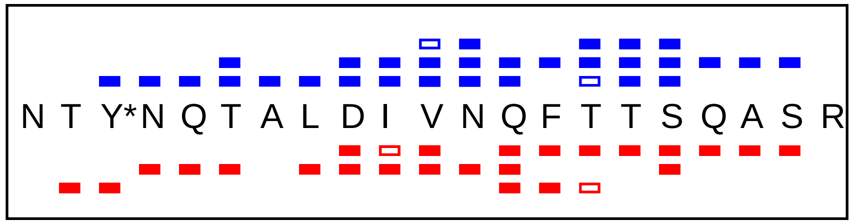

Protein Name:	Caskin 2
gi Accession:	24638431
Precursor Mass:	818.0461+3
Delta Mass:	3.947 ppm
XCorr:	61.73

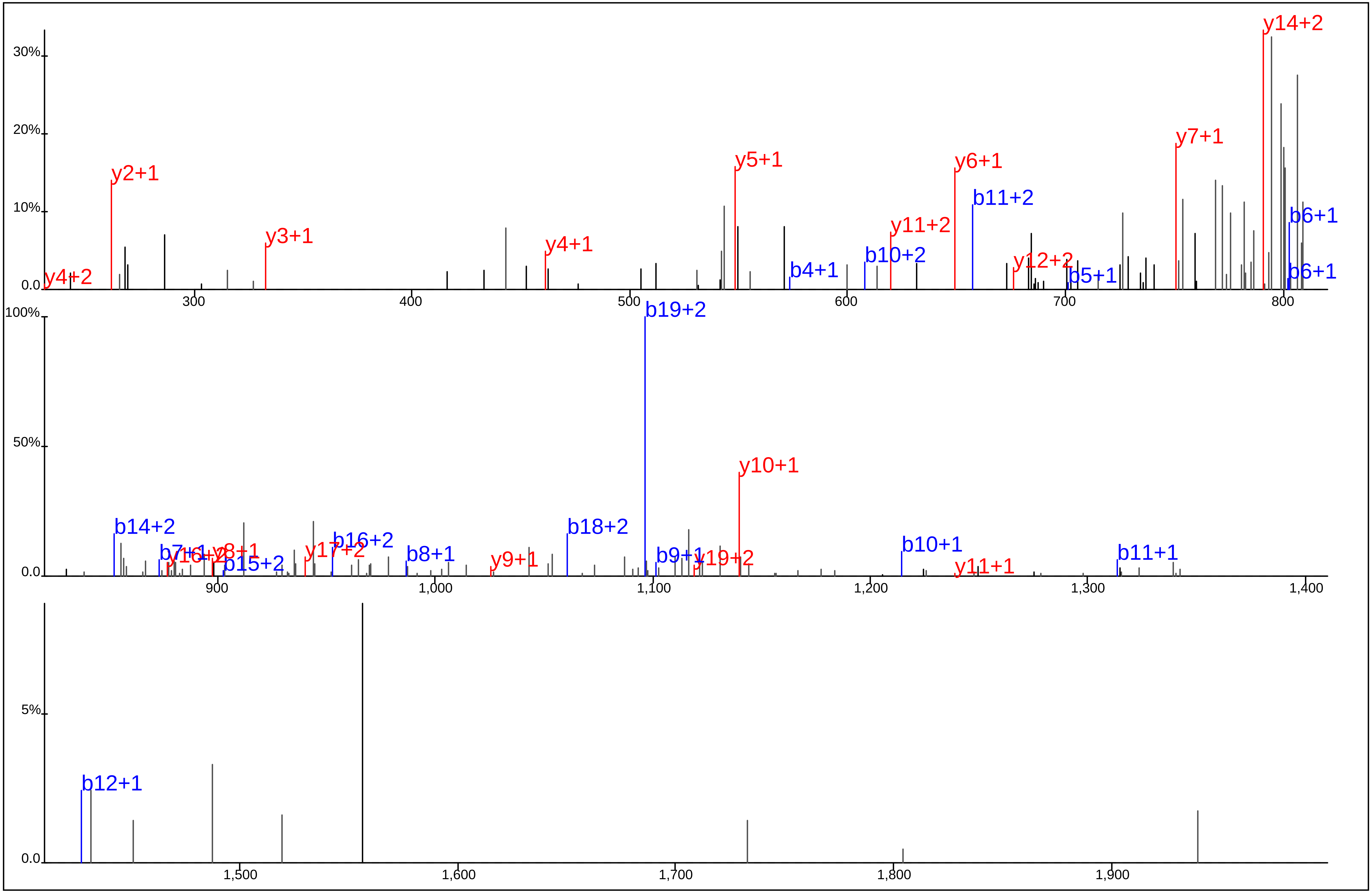

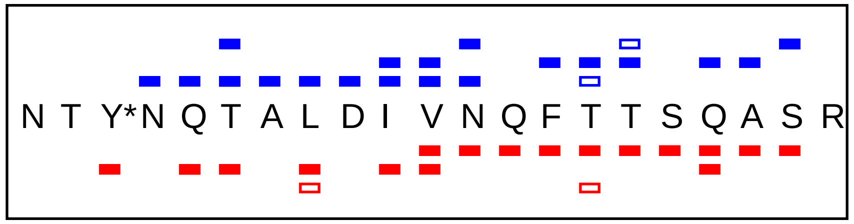

Protein Name: Caskin 2
gi Accession: 24638431
Precursor Mass: 818.0475+3
Delta Mass: 5.741 ppm
XCorr: 26.61

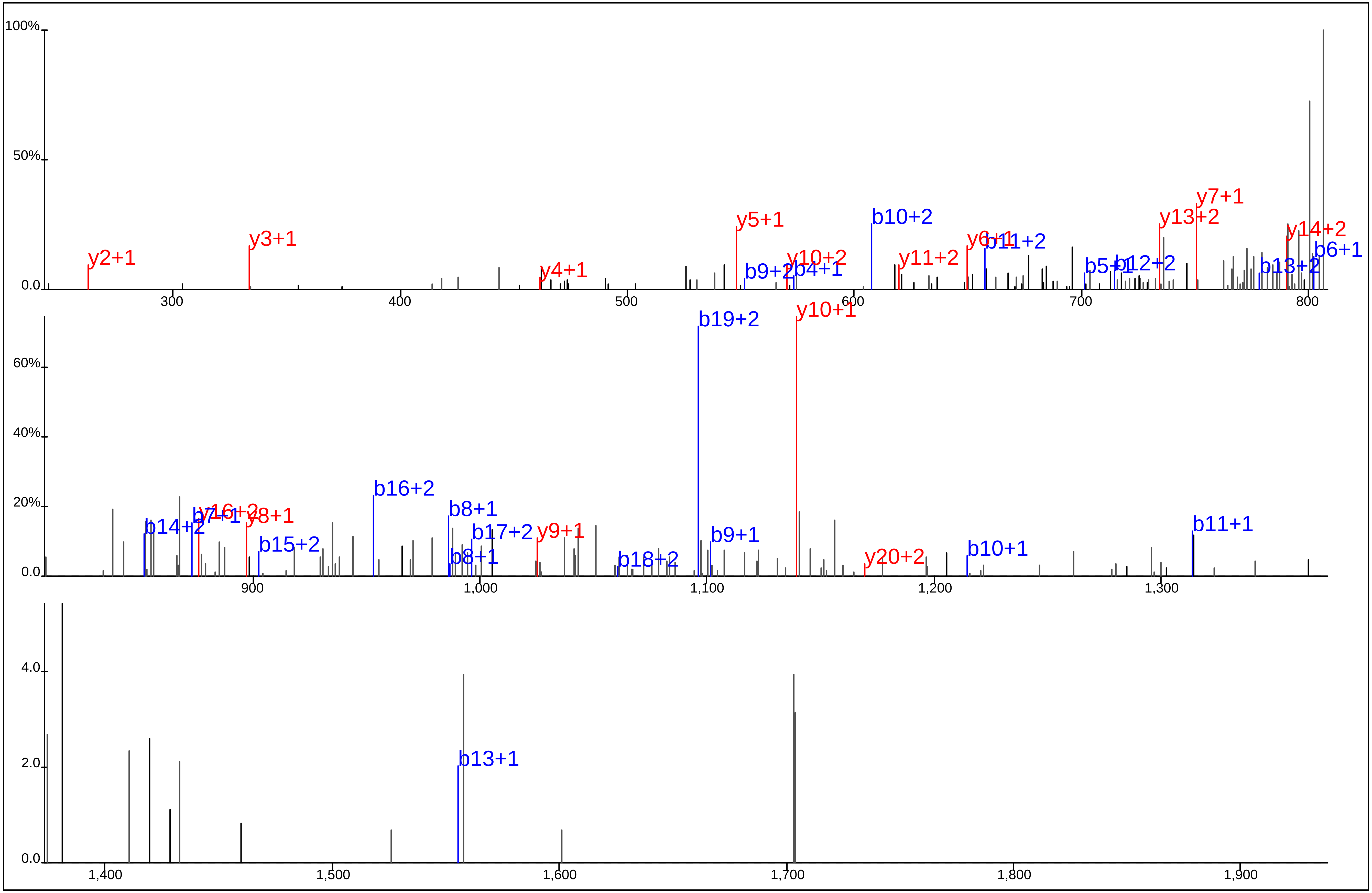

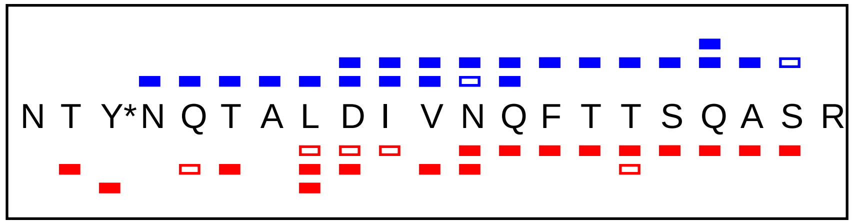

Protein Name: Caskin 2
gi Accession: 24638431
Precursor Mass: 818.0450+3
Delta Mass: 2.679 ppm
XCorr: 33.39

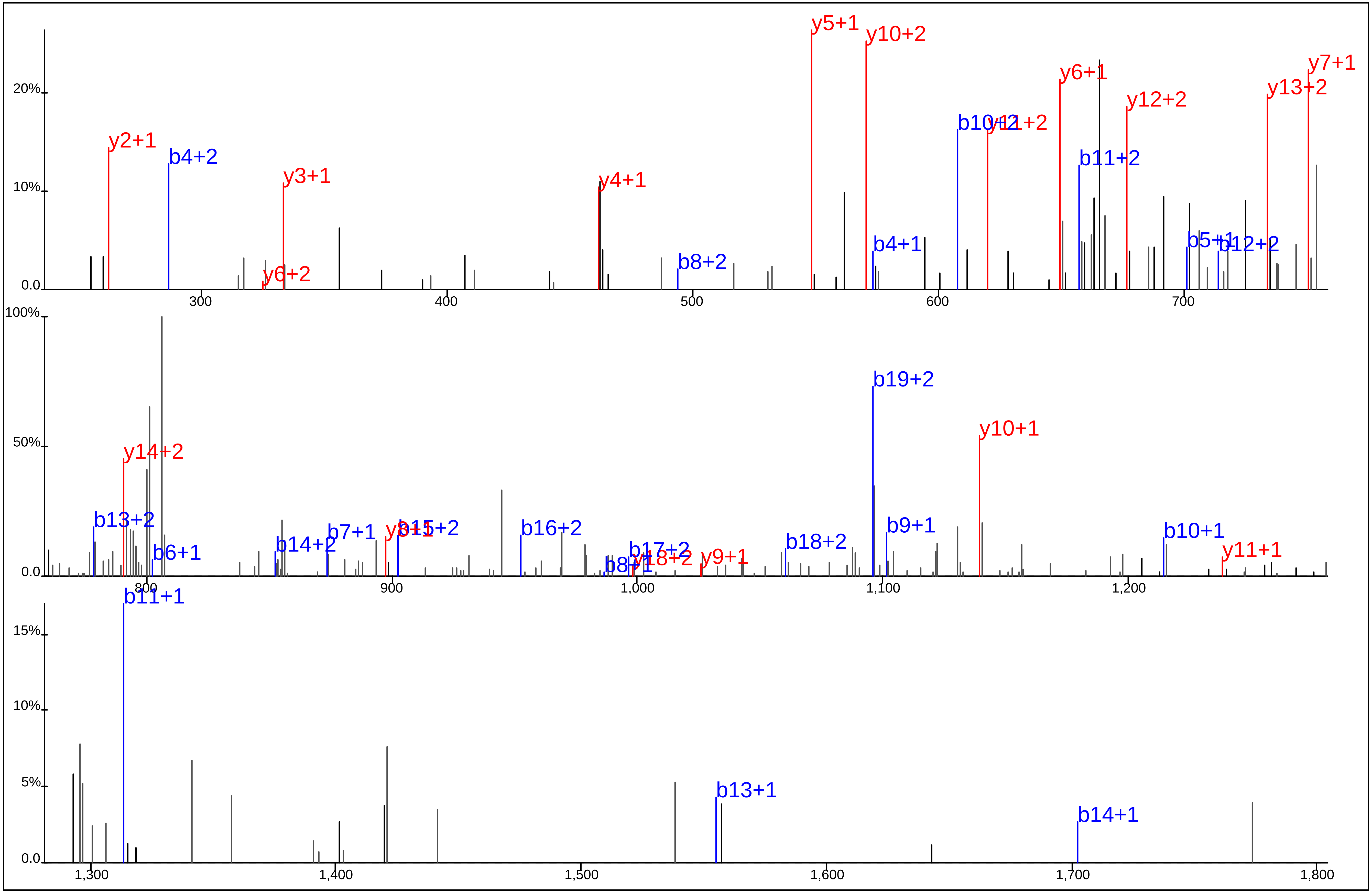

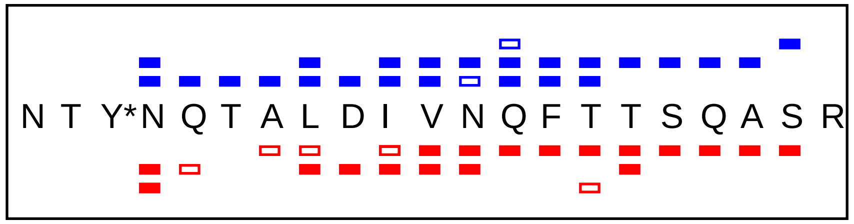

Protein Name: Caskin 2
gi Accession: 24638431
Precursor Mass: 818.0468+3
Delta Mass: 4.844 ppm
XCorr: 44.52

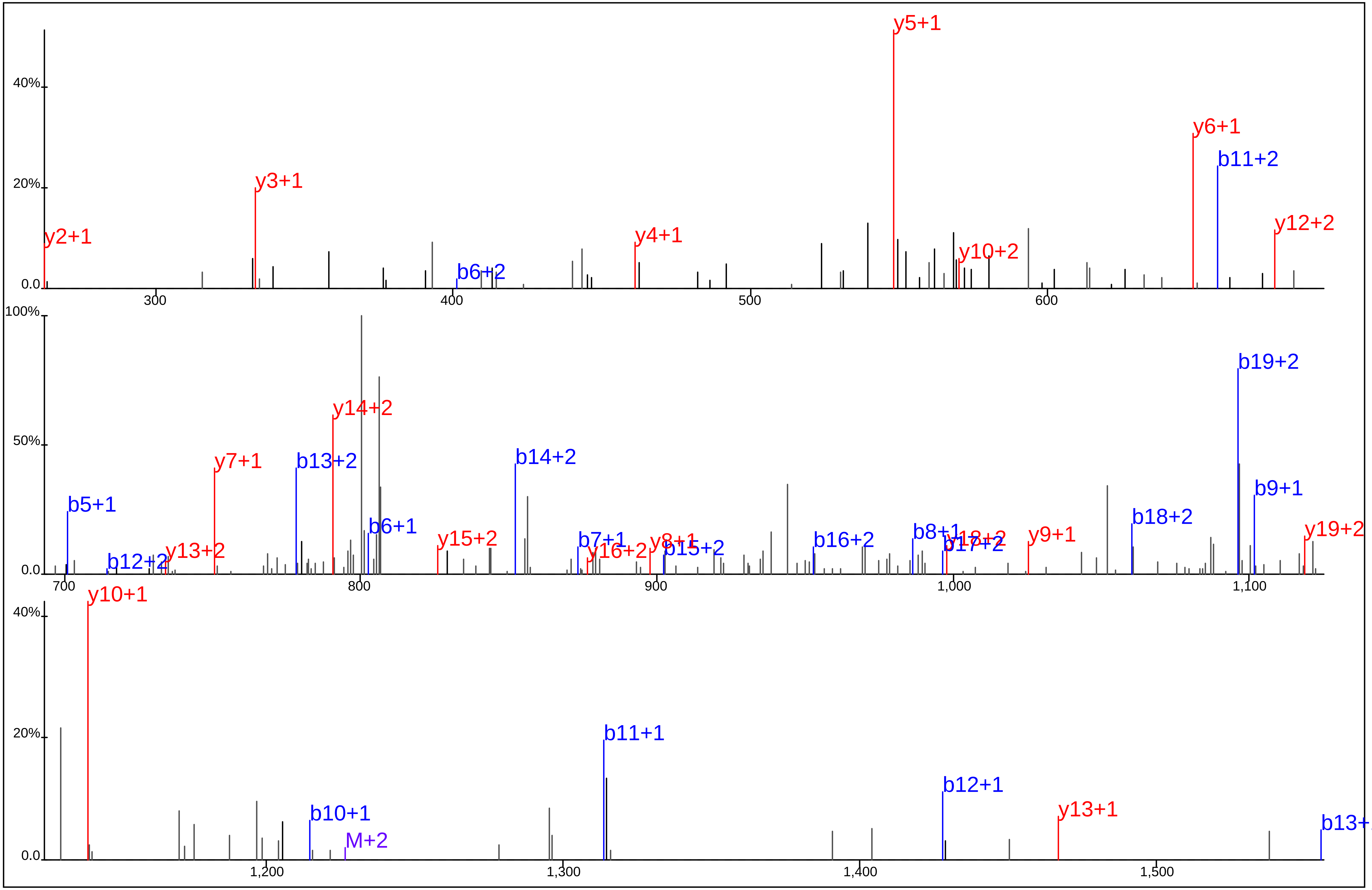

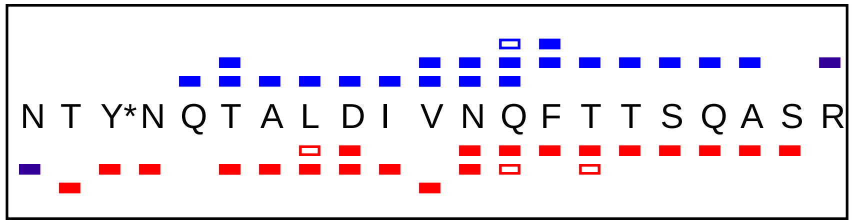

Protein Name: Caskin 2
gi Accession: 24638431
Precursor Mass: 818.0455+3
Delta Mass: 3.201 ppm
XCorr: 59.93

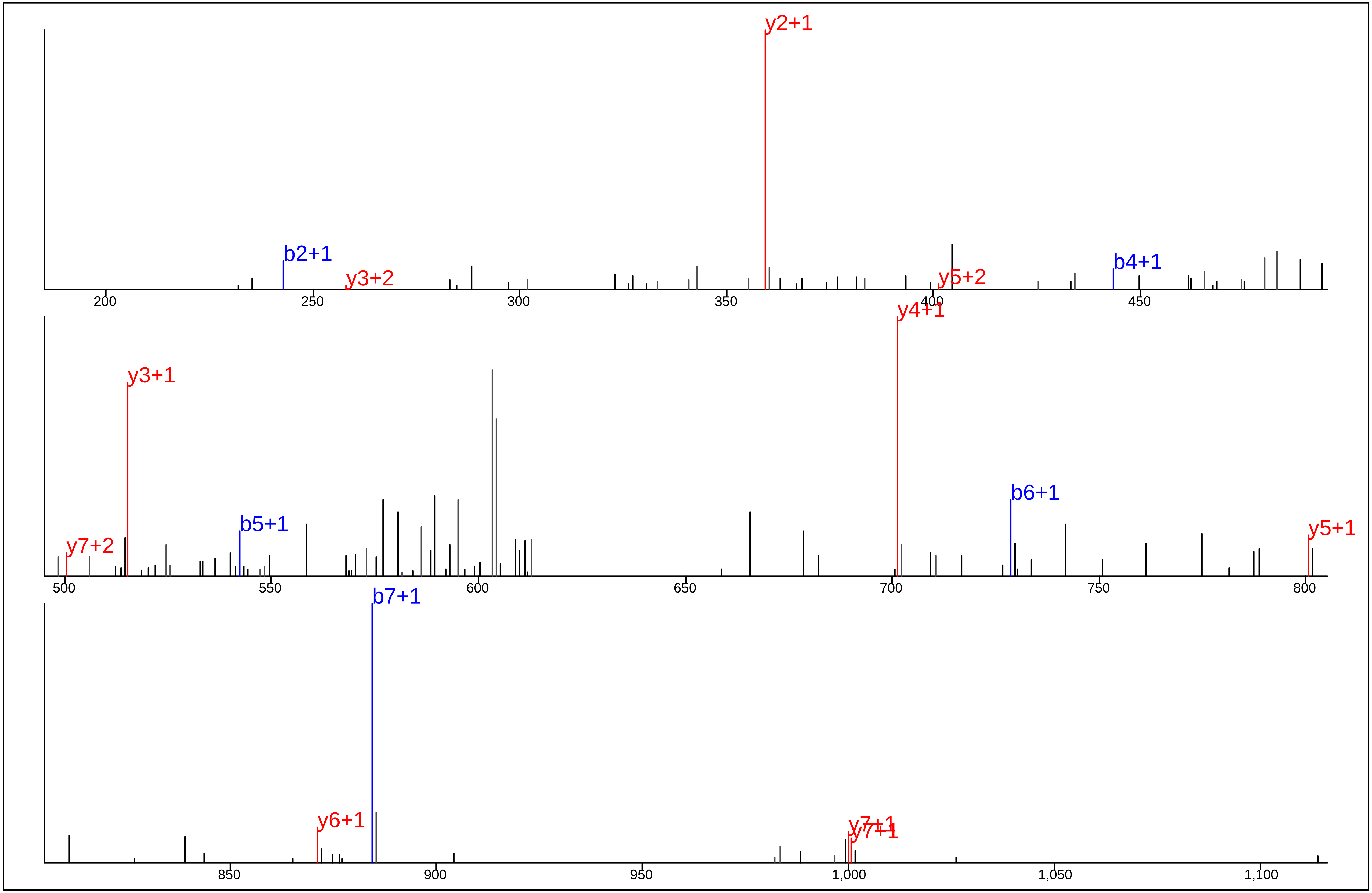

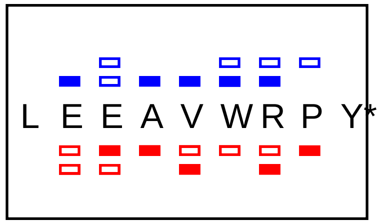

Protein Name: CBFA2
gi Accession: 2506974
Precursor Mass: 621.7822+2
Delta Mass: 1.302 ppm
XCorr: 29.28

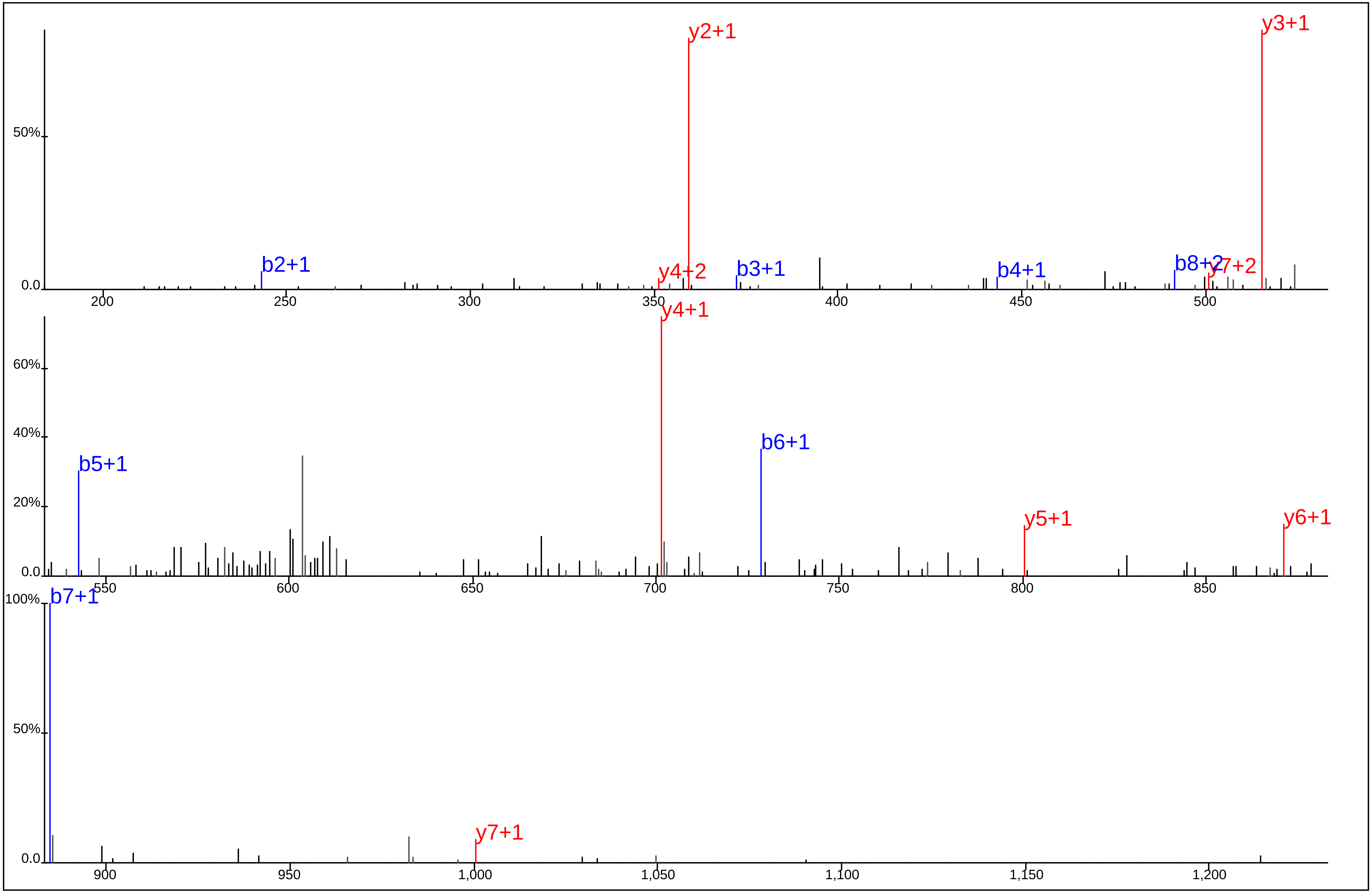

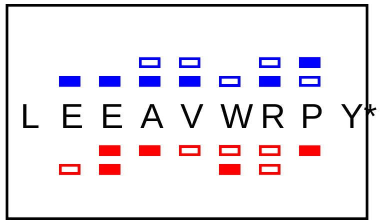

Protein Name: CBFA2
gi Accession: 2506974
Precursor Mass: 621.7828+2
Delta Mass: 2.389 ppm
XCorr: 36.99

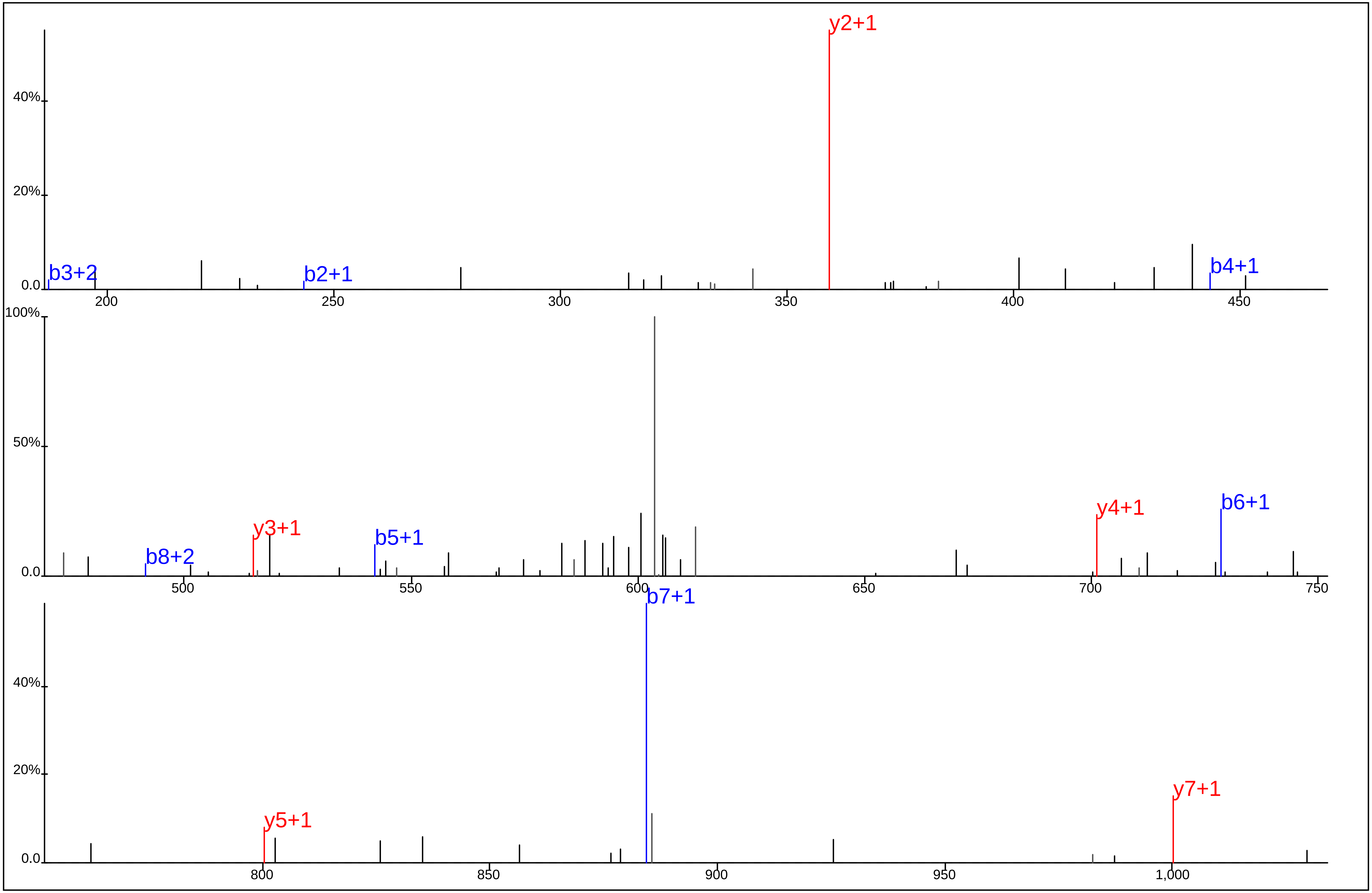

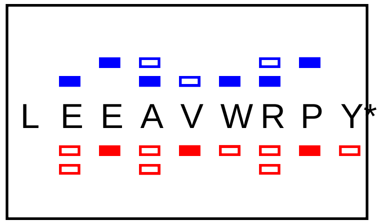

Protein Name:	CBFA2
gi Accession:	2506974
Precursor Mass:	621.7844+2
Delta Mass:	4.843 ppm
XCorr:	20.3

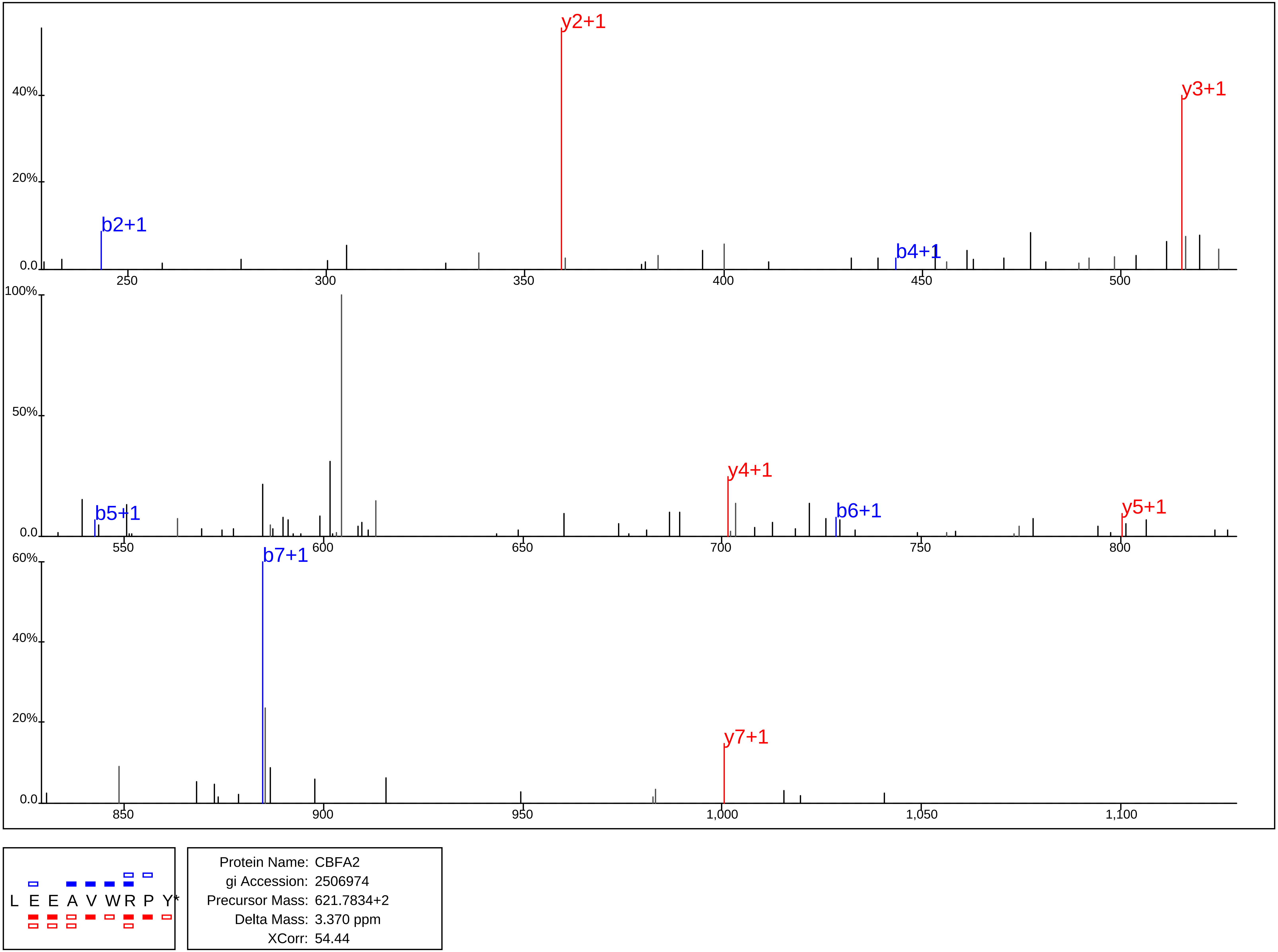

Protein Name: CBFA2
gi Accession: 2506974
Precursor Mass: 621.7834+2
Delta Mass: 3.370 ppm
XCorr: 54.44

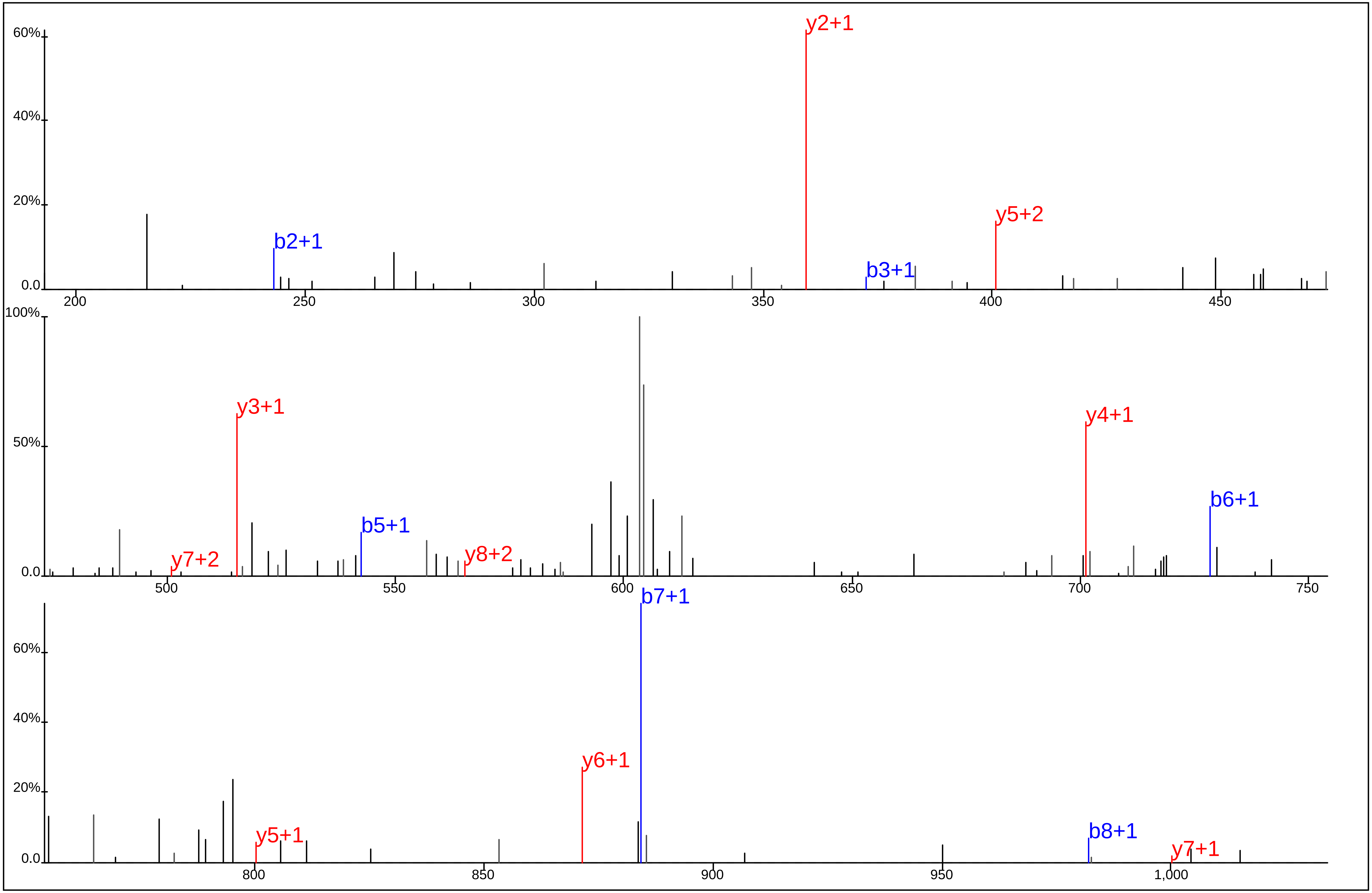

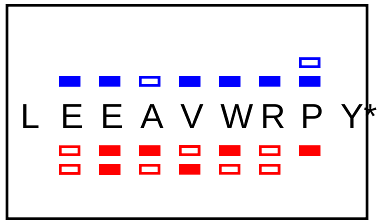

Protein Name: CBFA2
gi Accession: 2506974
Precursor Mass: 621.7847+2
Delta Mass: 5.334 ppm
XCorr: 26.23

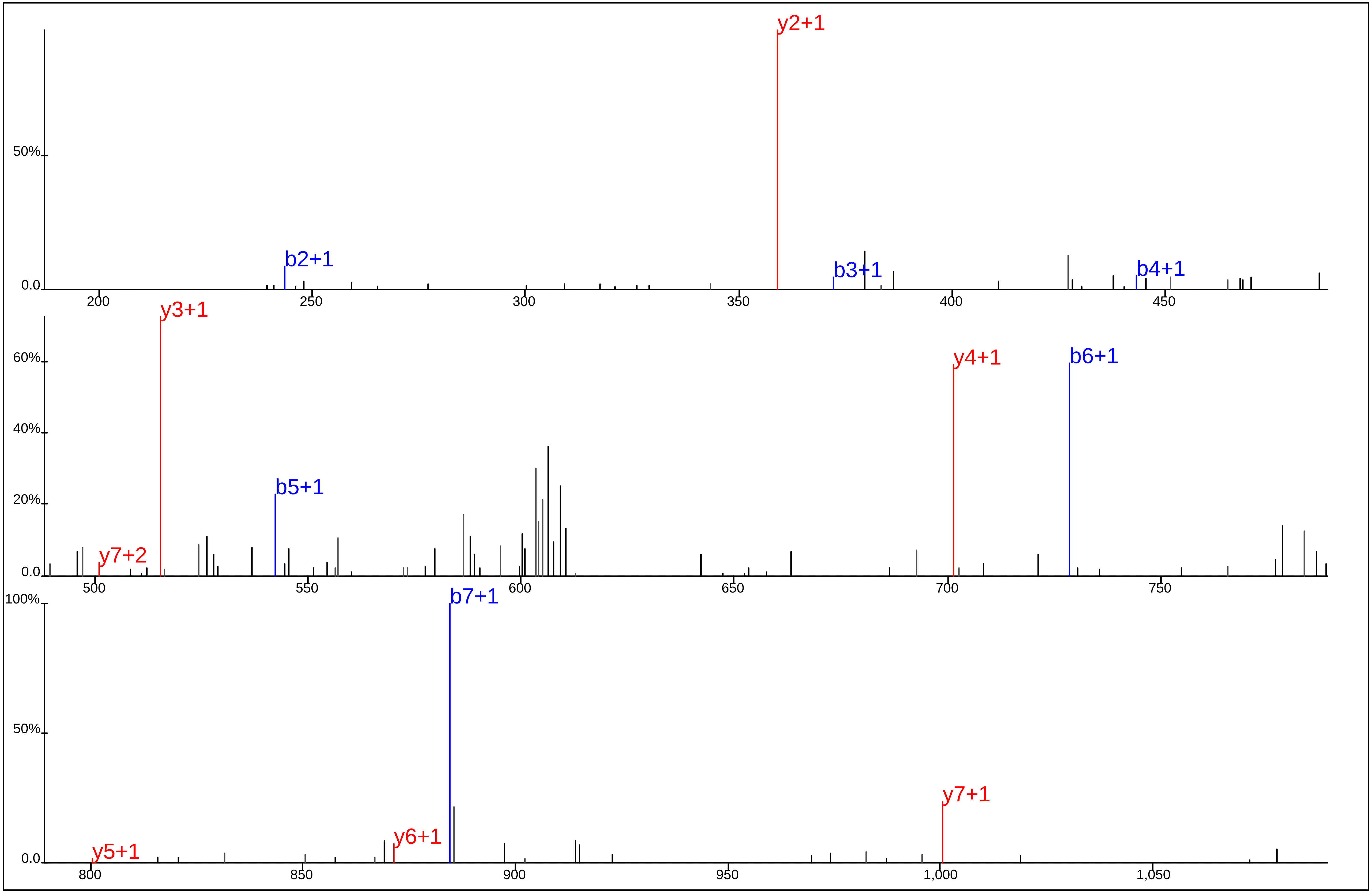

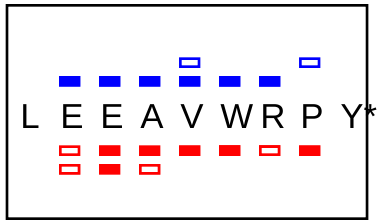

Protein Name: CBFA2
gi Accession: 2506974
Precursor Mass: 621.7798+2
Delta Mass: 2.424 ppm
XCorr: 24.74

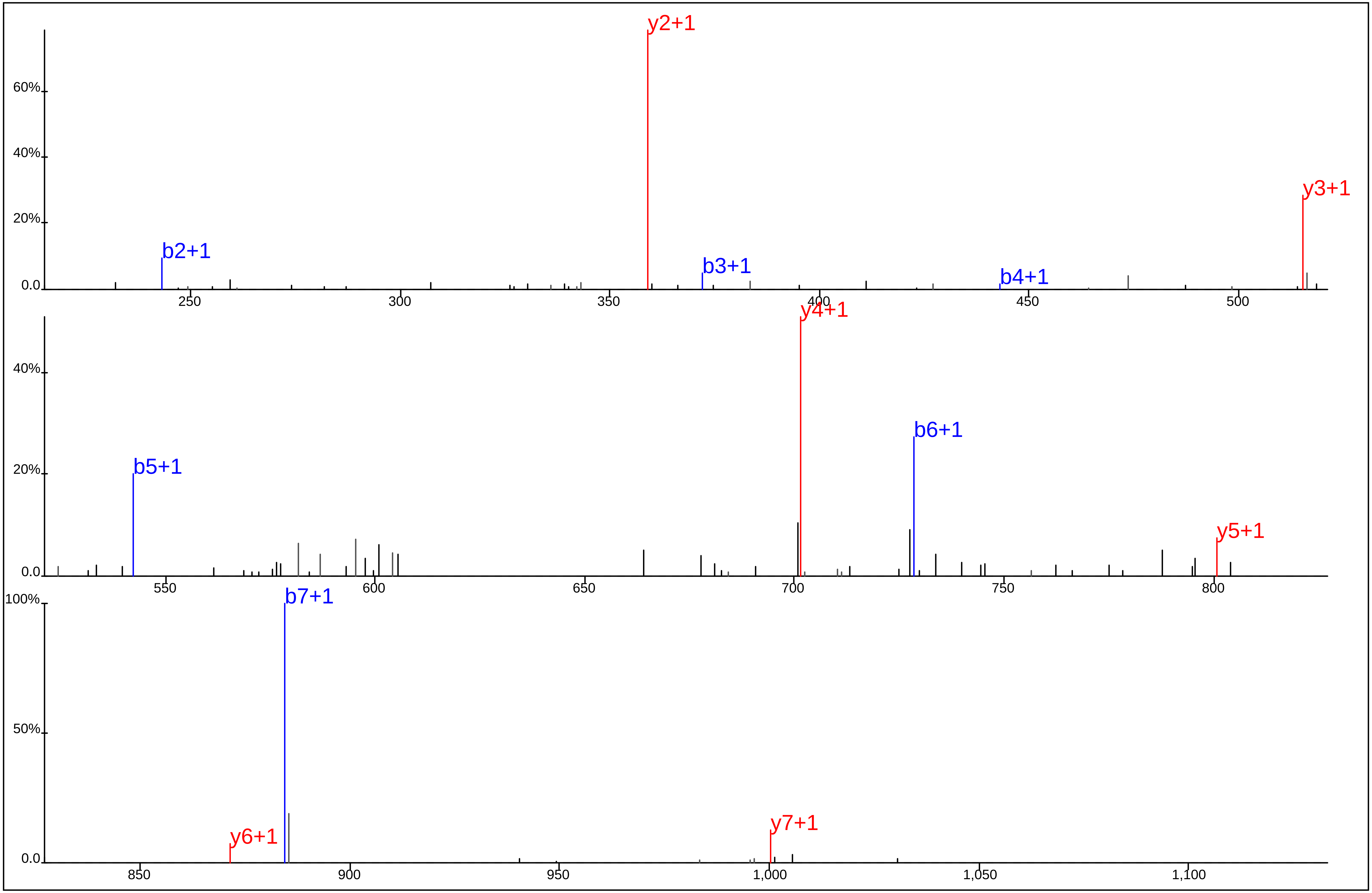

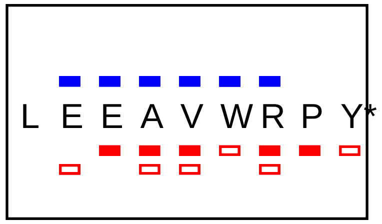

Protein Name: CBFA2
gi Accession: 2506974
Precursor Mass: 621.7823+2
Delta Mass: 1.503 ppm
XCorr: 32.75

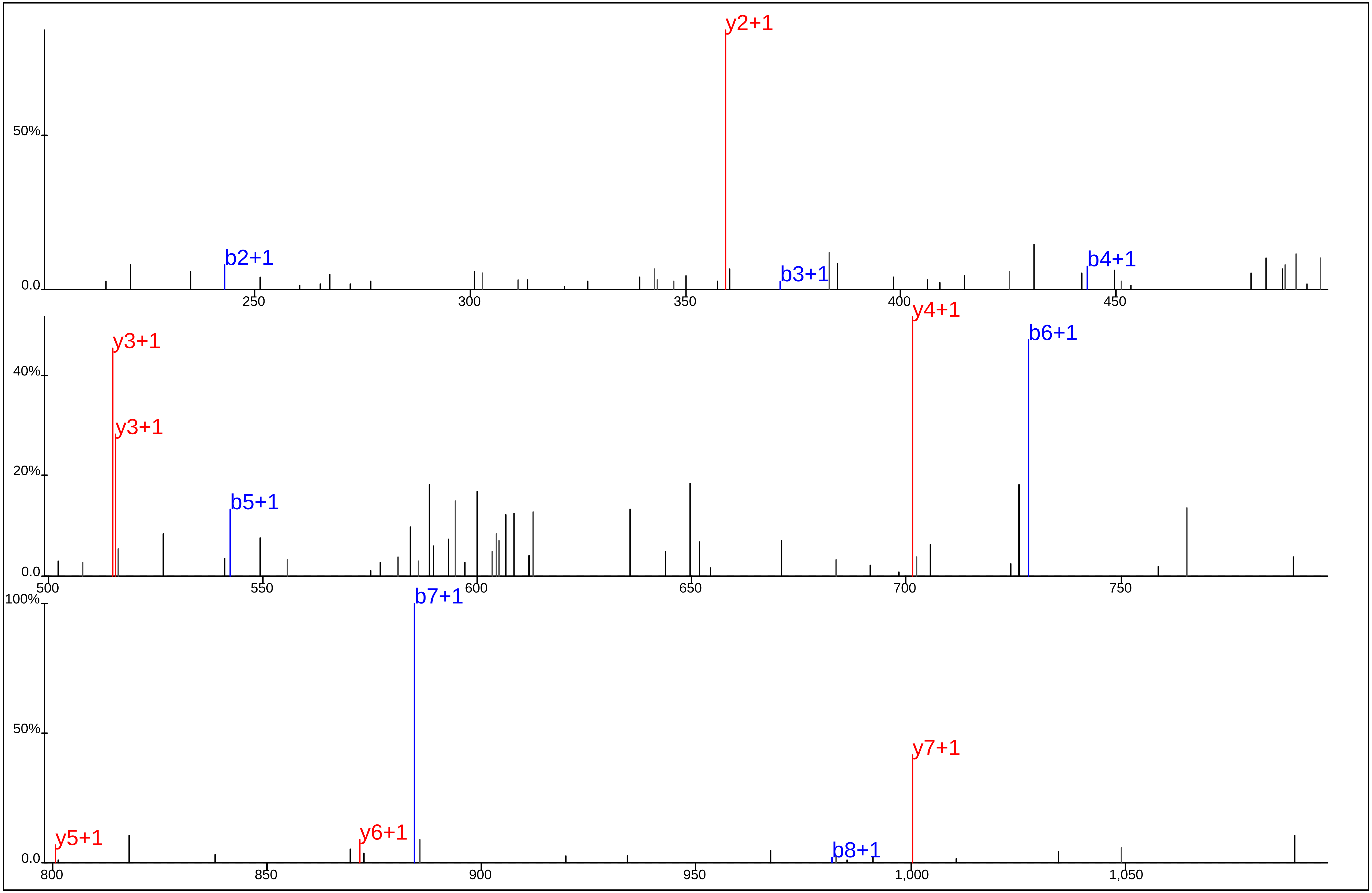

L E E A V W R P Y*

Protein Name:	CBFA2
gi Accession:	2506974
Precursor Mass:	621.7841+2
Delta Mass:	4.449 ppm
XCorr:	24.36

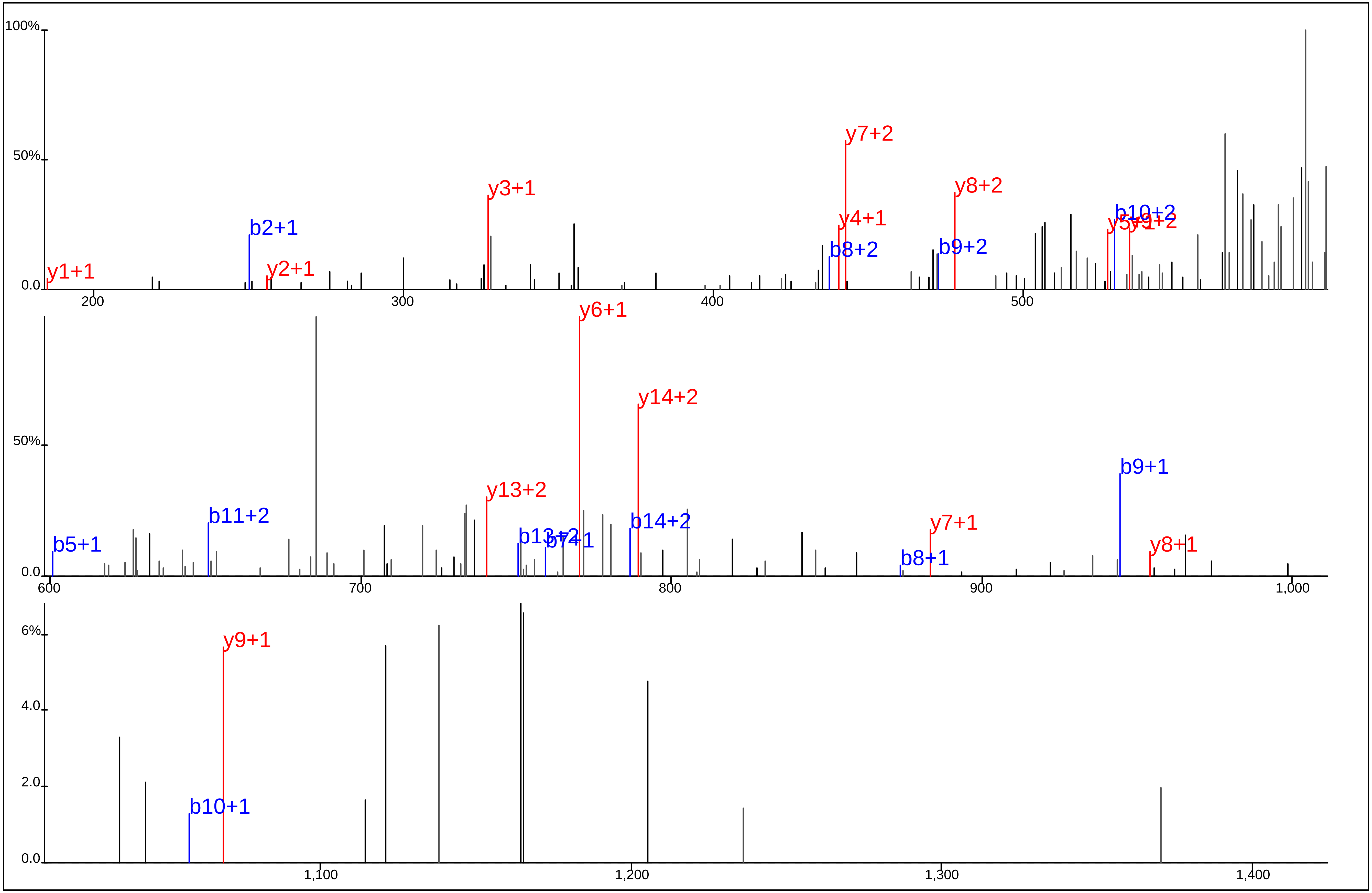

I K@P S S*S A N A I Y*S L A A R#

Protein Name: CBL
gi Accession: 115855
Precursor Mass: 609.6208+3
Delta Mass: 4.932 ppm
XCorr: 3.1716

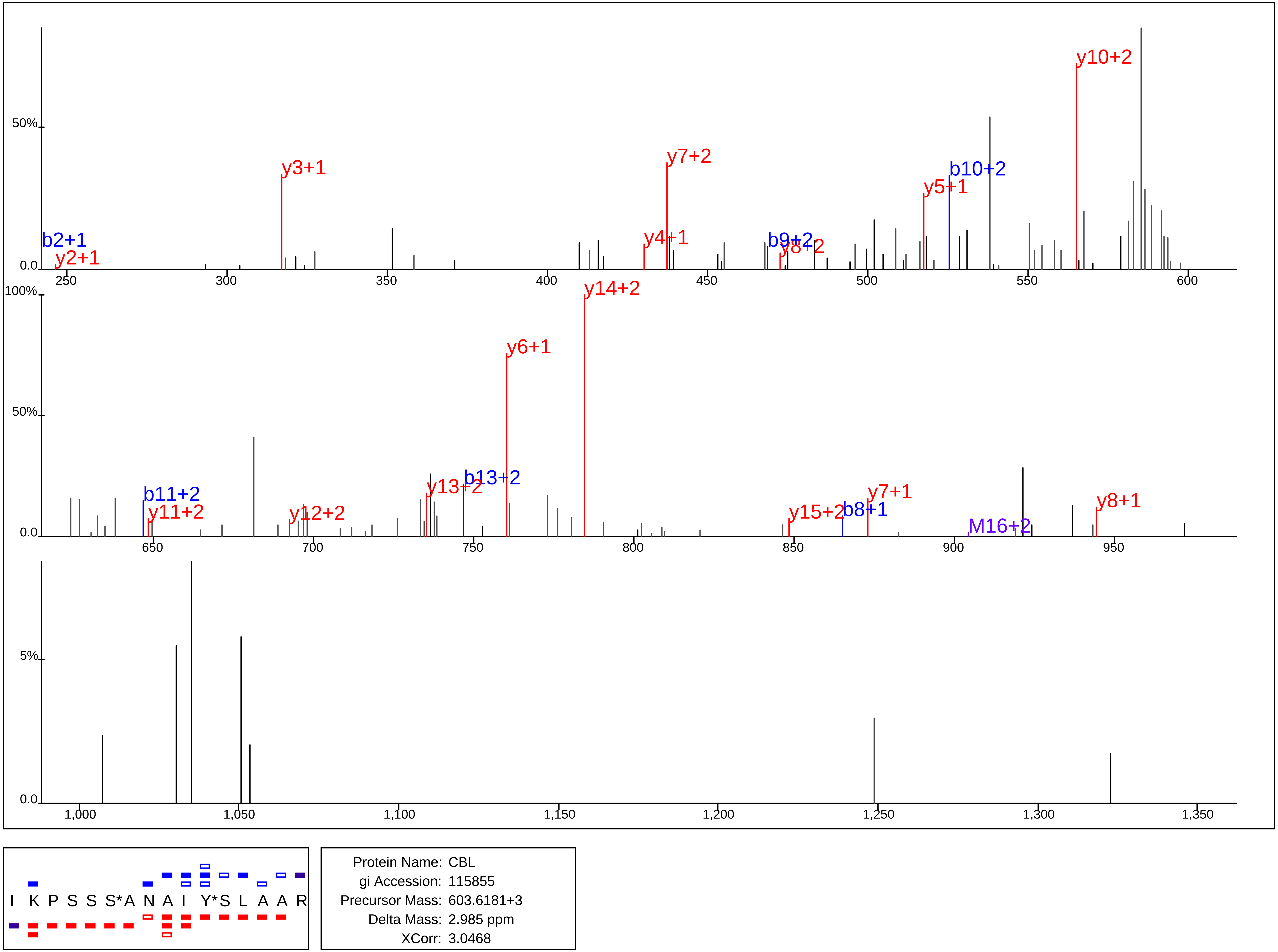

Protein Name: CBL
gi Accession: 115855
Precursor Mass: 603.6181+3
Delta Mass: 2.985 ppm
XCorr: 3.0468

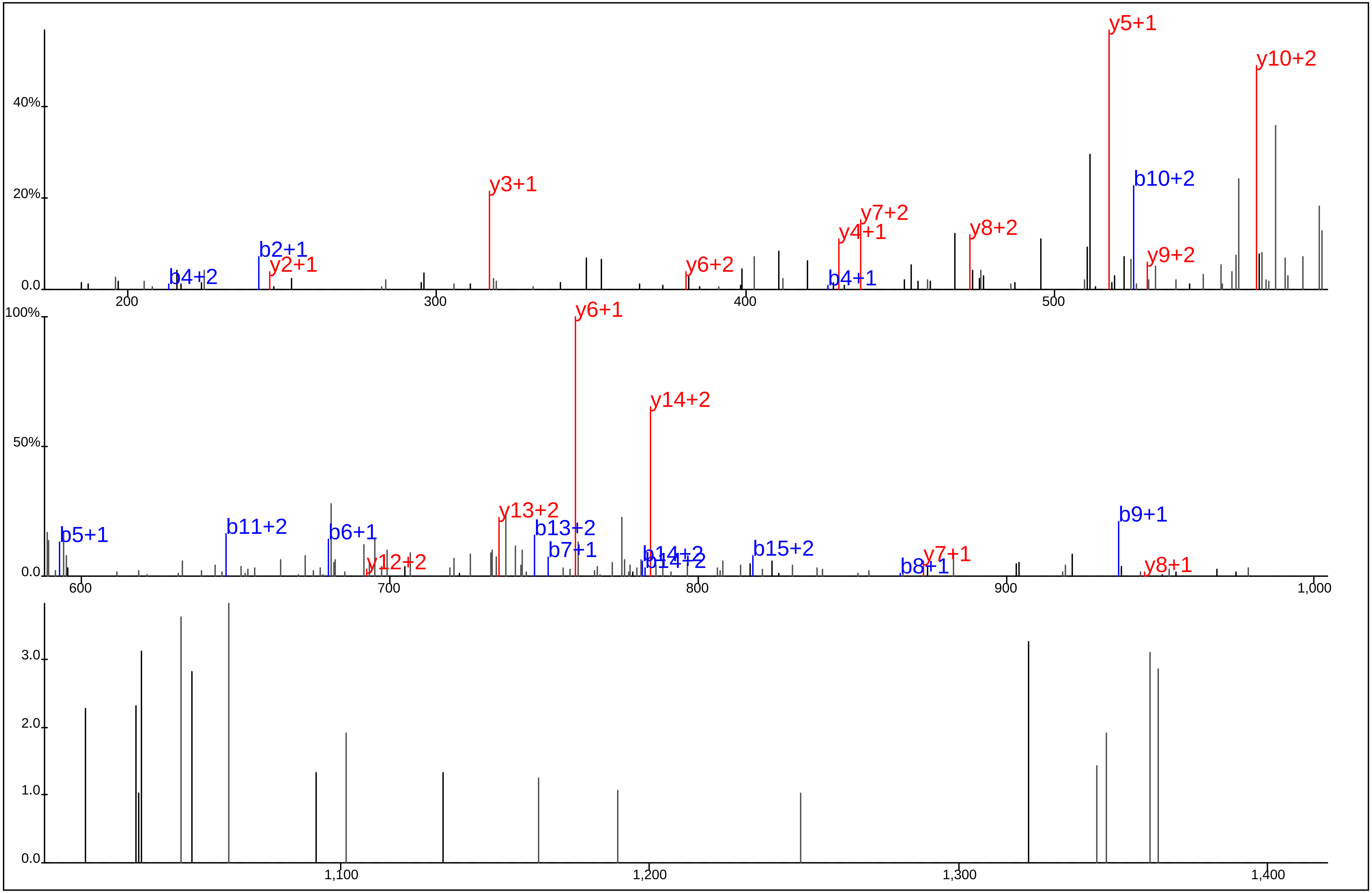

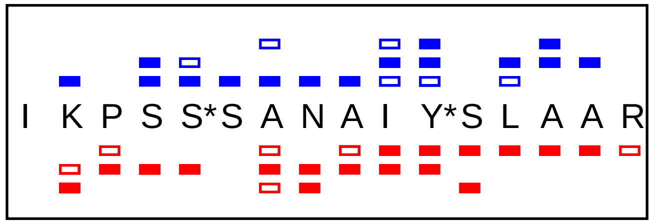

Protein Name: CBL
gi Accession: 115855
Precursor Mass: 603.6175+3
Delta Mass: 1.968 ppm
XCorr: 3.6091

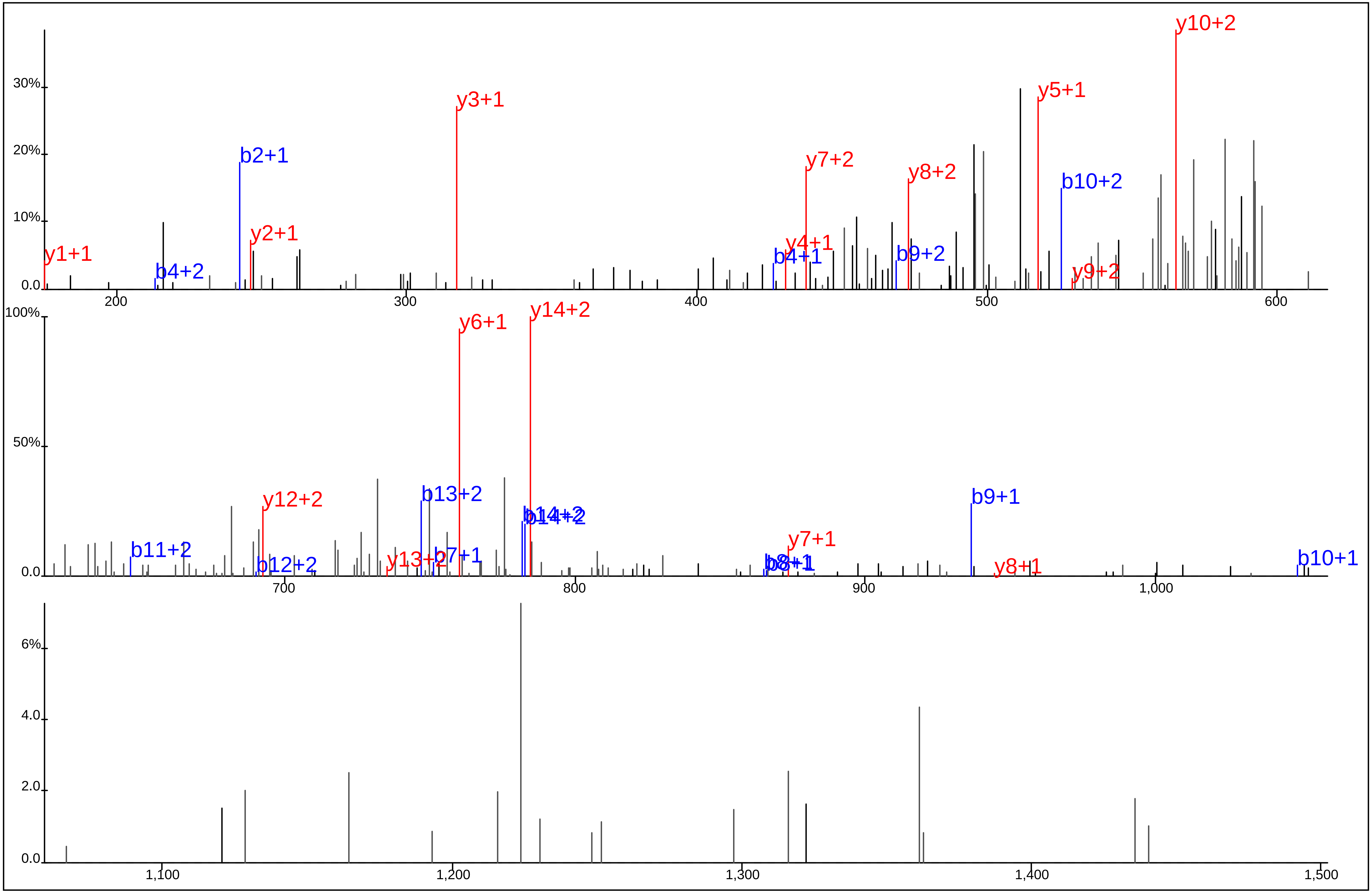

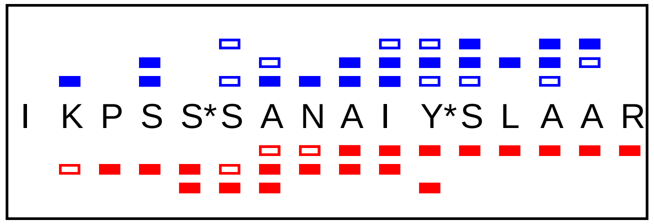

Protein Name: CBL
gi Accession: 115855
Precursor Mass: 603.6166+3
Delta Mass: 0.553 ppm
XCorr: 3.0698

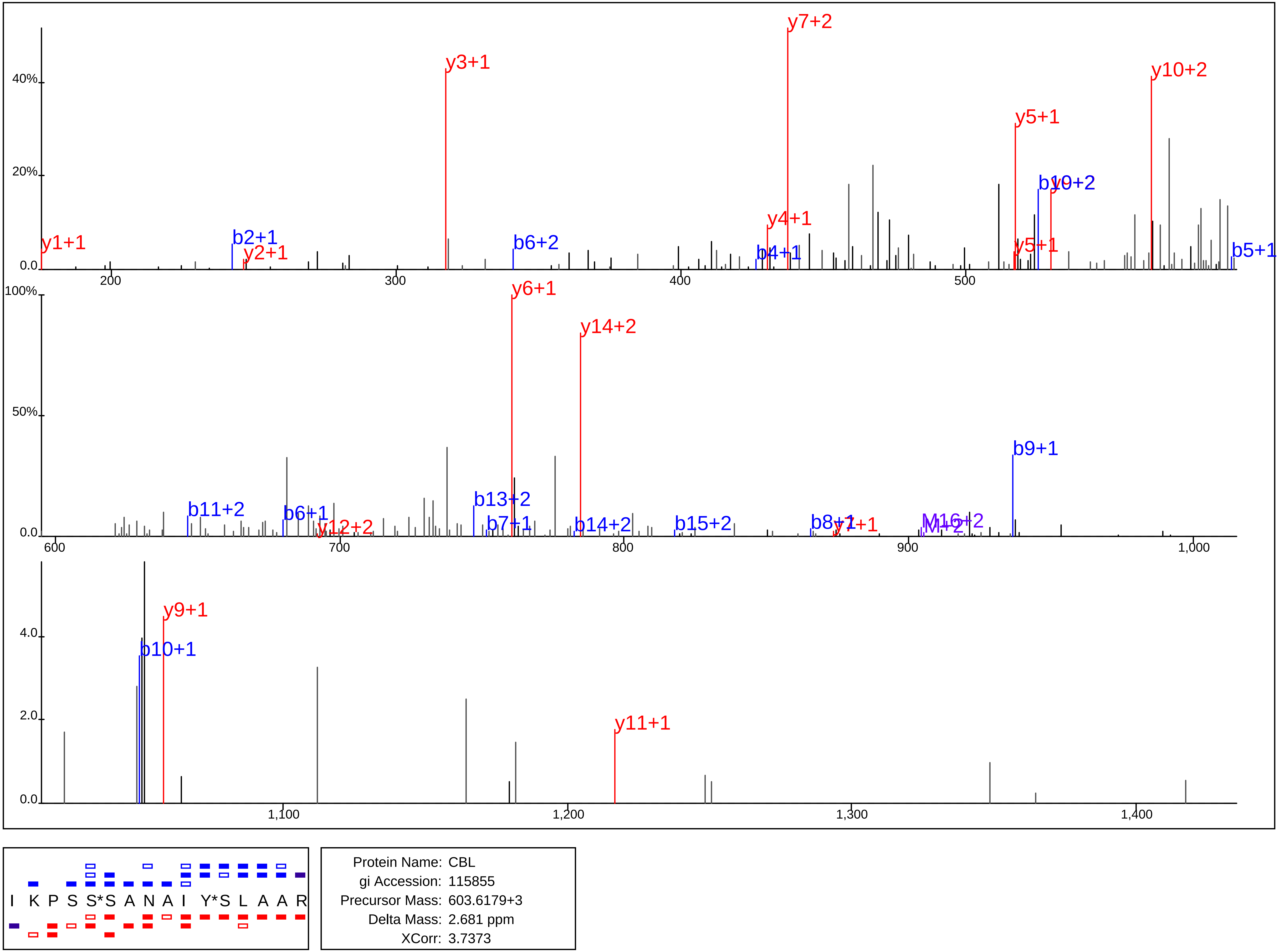

Protein Name: CBL
gi Accession: 115855
Precursor Mass: 603.6179+3
Delta Mass: 2.681 ppm
XCorr: 3.7373

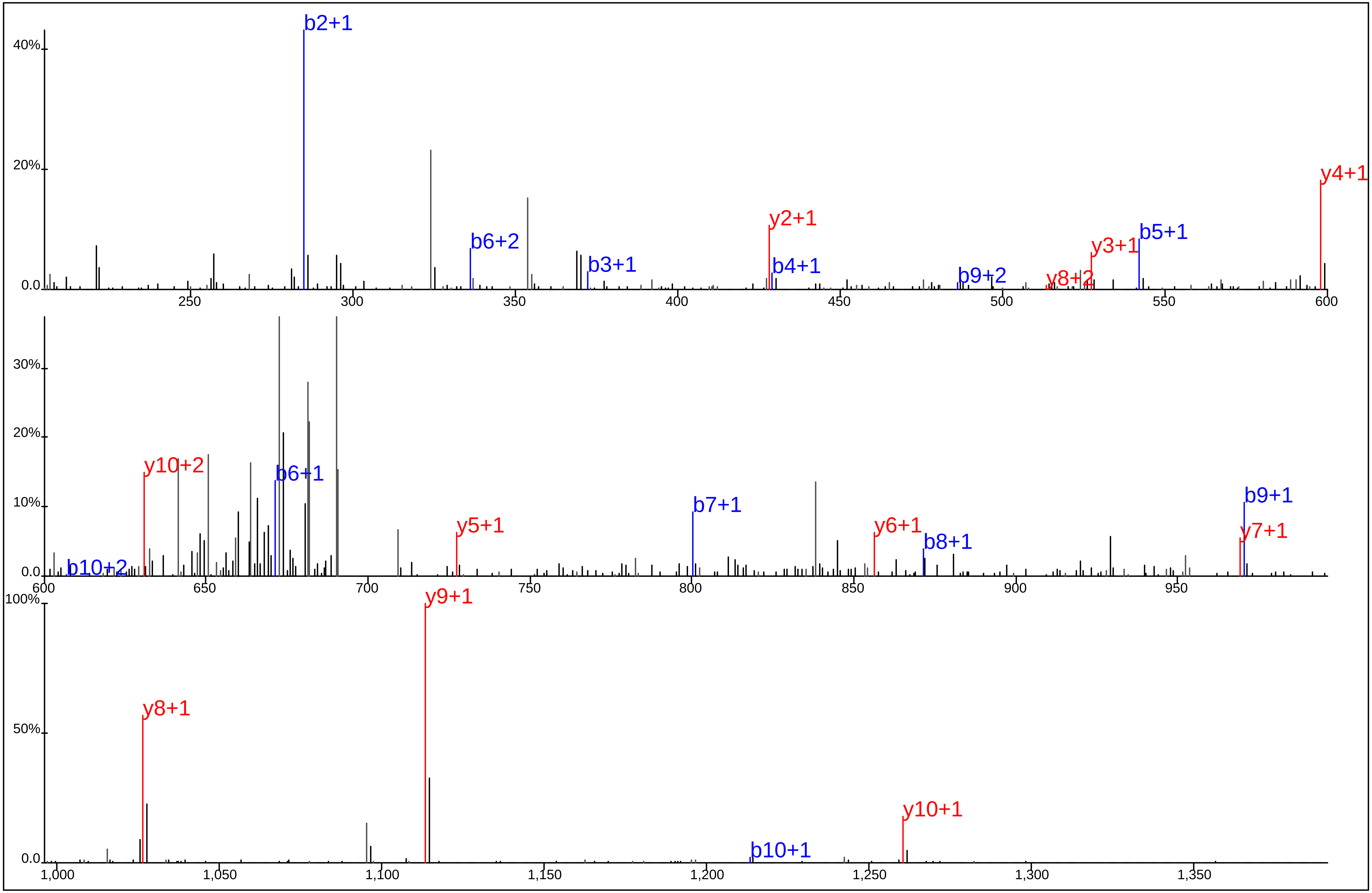

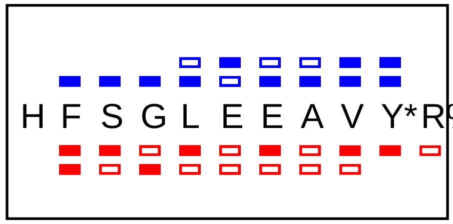

Protein Name: CCT8
gi Accession: 48762932
Precursor Mass: 699.3123+2
Delta Mass: 3.426 ppm
XCorr: 60.15

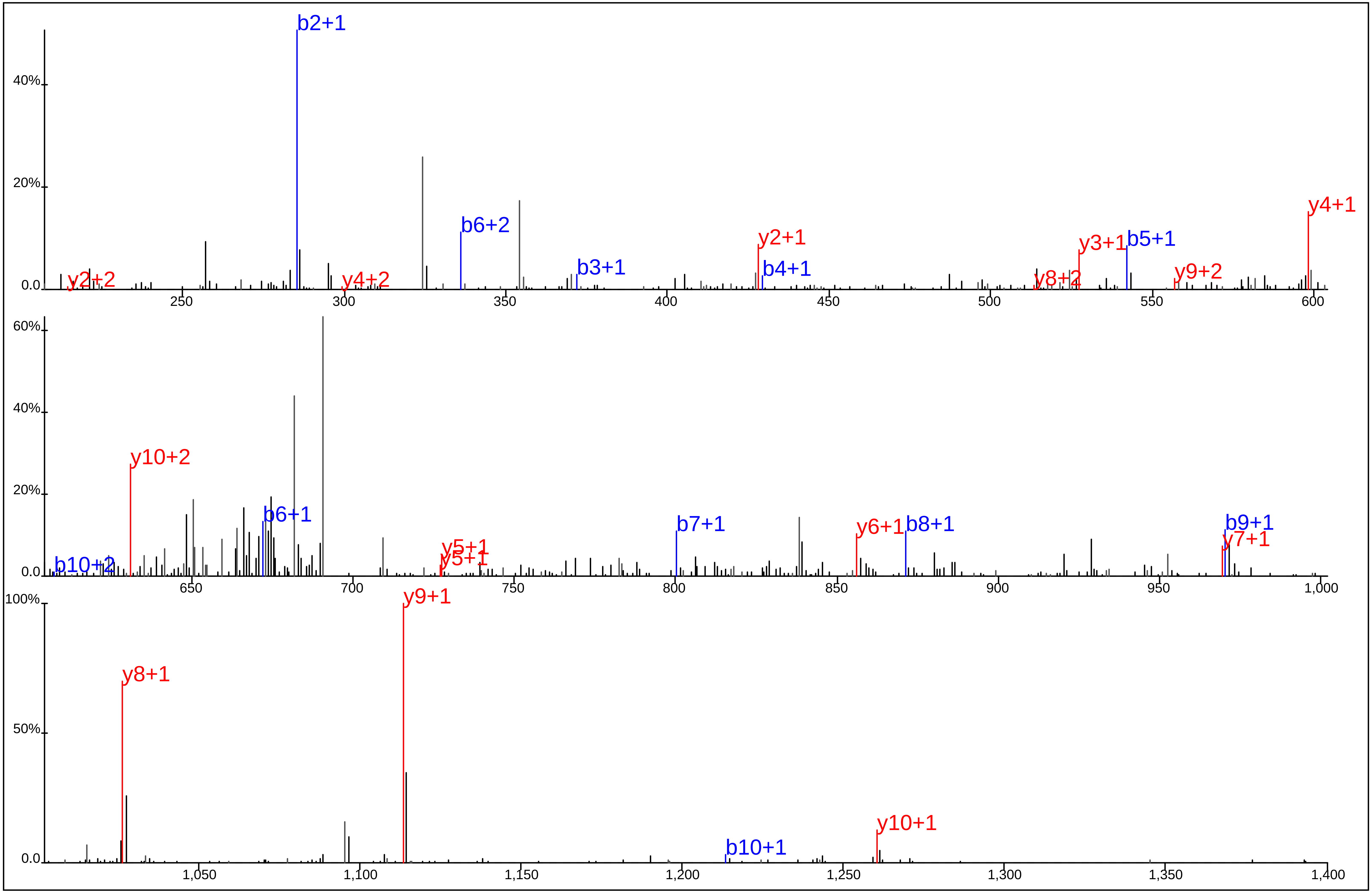

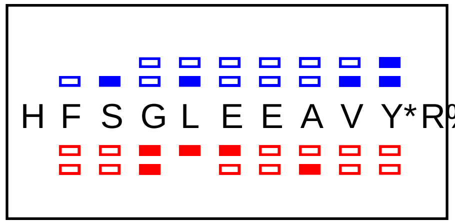

Protein Name: CCT8
gi Accession: 48762932
Precursor Mass: 699.3113+2
Delta Mass: 2.109 ppm
XCorr: 54.61

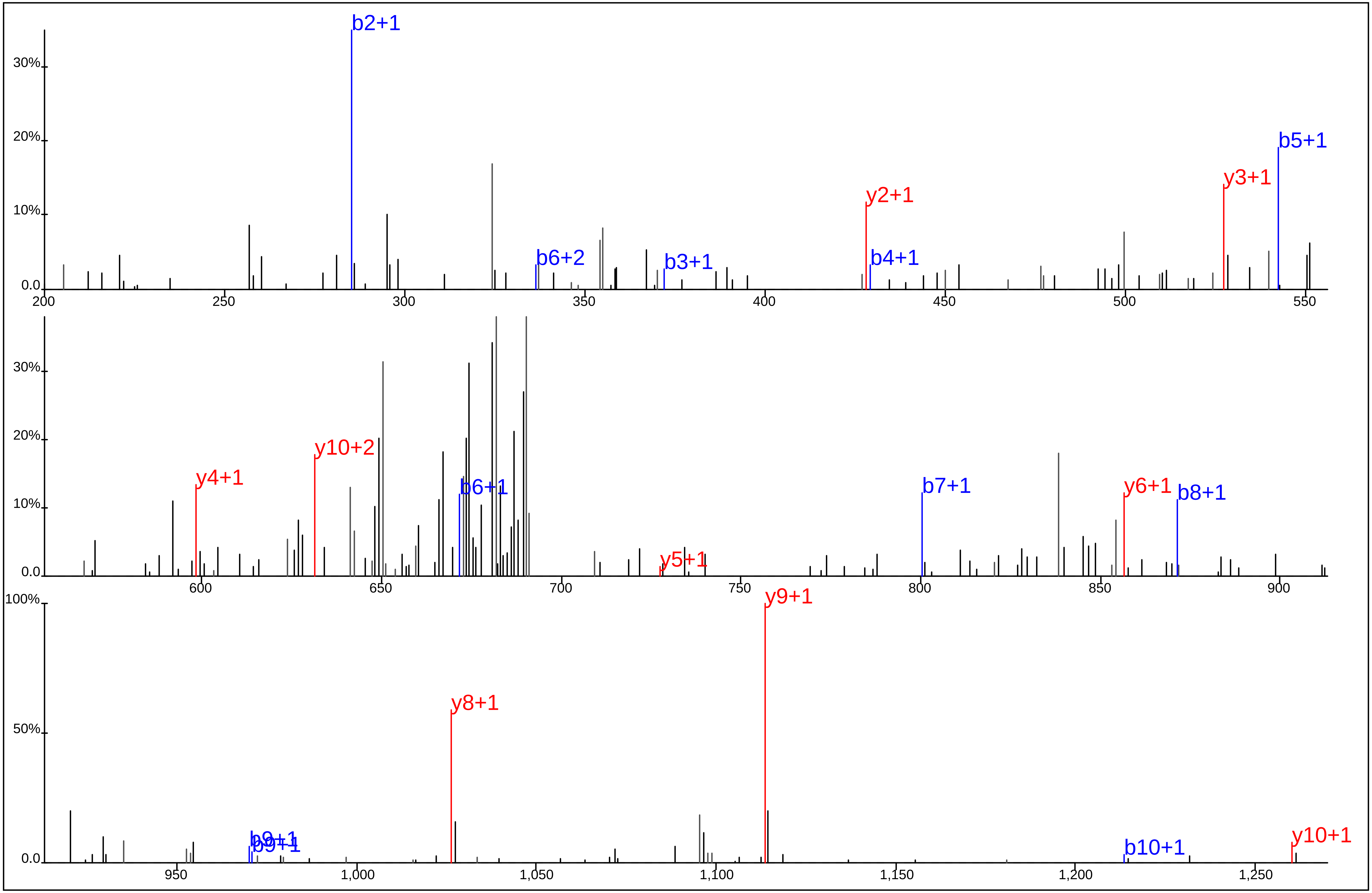

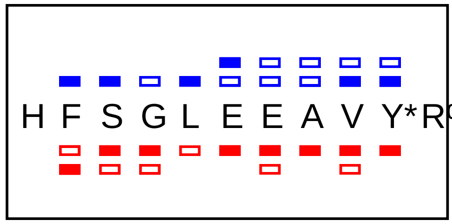

Protein Name: CCT8
gi Accession: 48762932
Precursor Mass: 699.3124+2
Delta Mass: 3.598 ppm
XCorr: 38.23

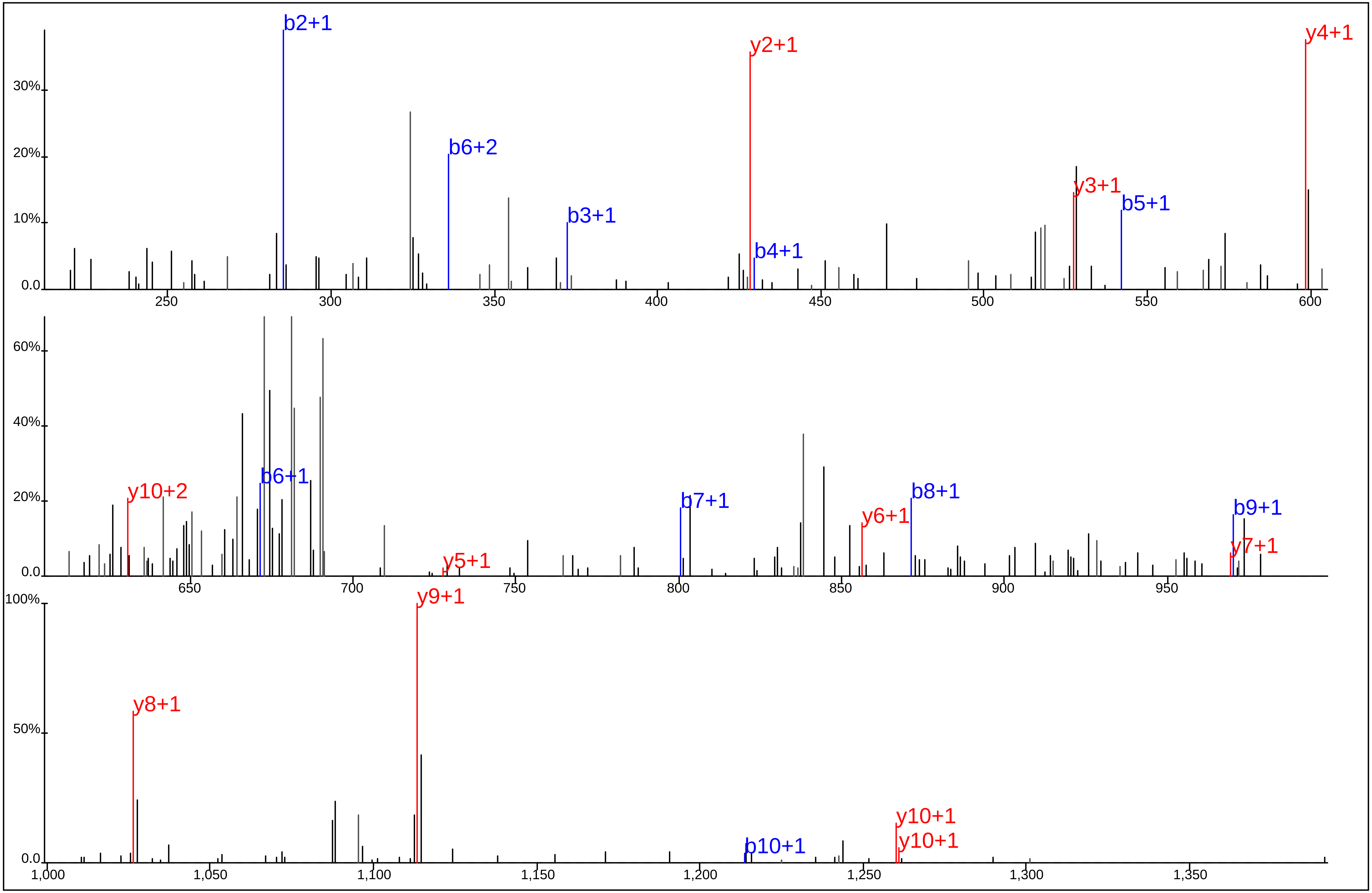

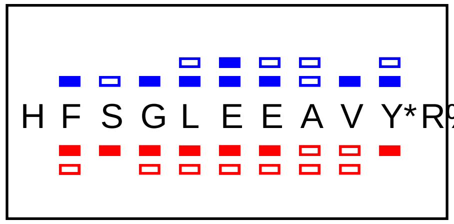

Protein Name: CCT8
gi Accession: 48762932
Precursor Mass: 699.3121+2
Delta Mass: 3.161 ppm
XCorr: 29.71

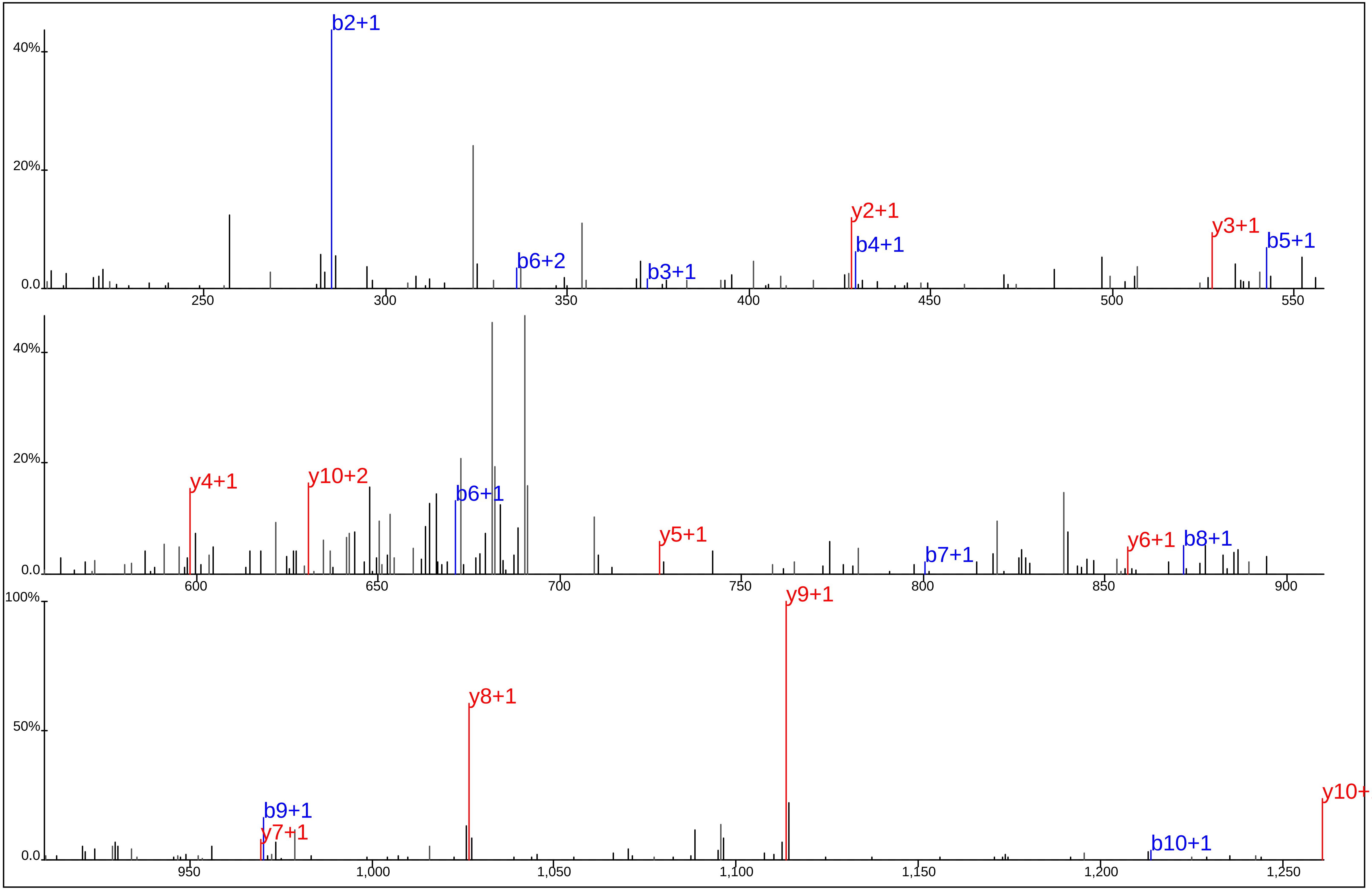

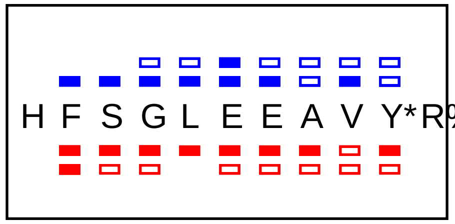

Protein Name: CCT8
gi Accession: 48762932
Precursor Mass: 699.3116+2
Delta Mass: 2.460 ppm
XCorr: 51.58

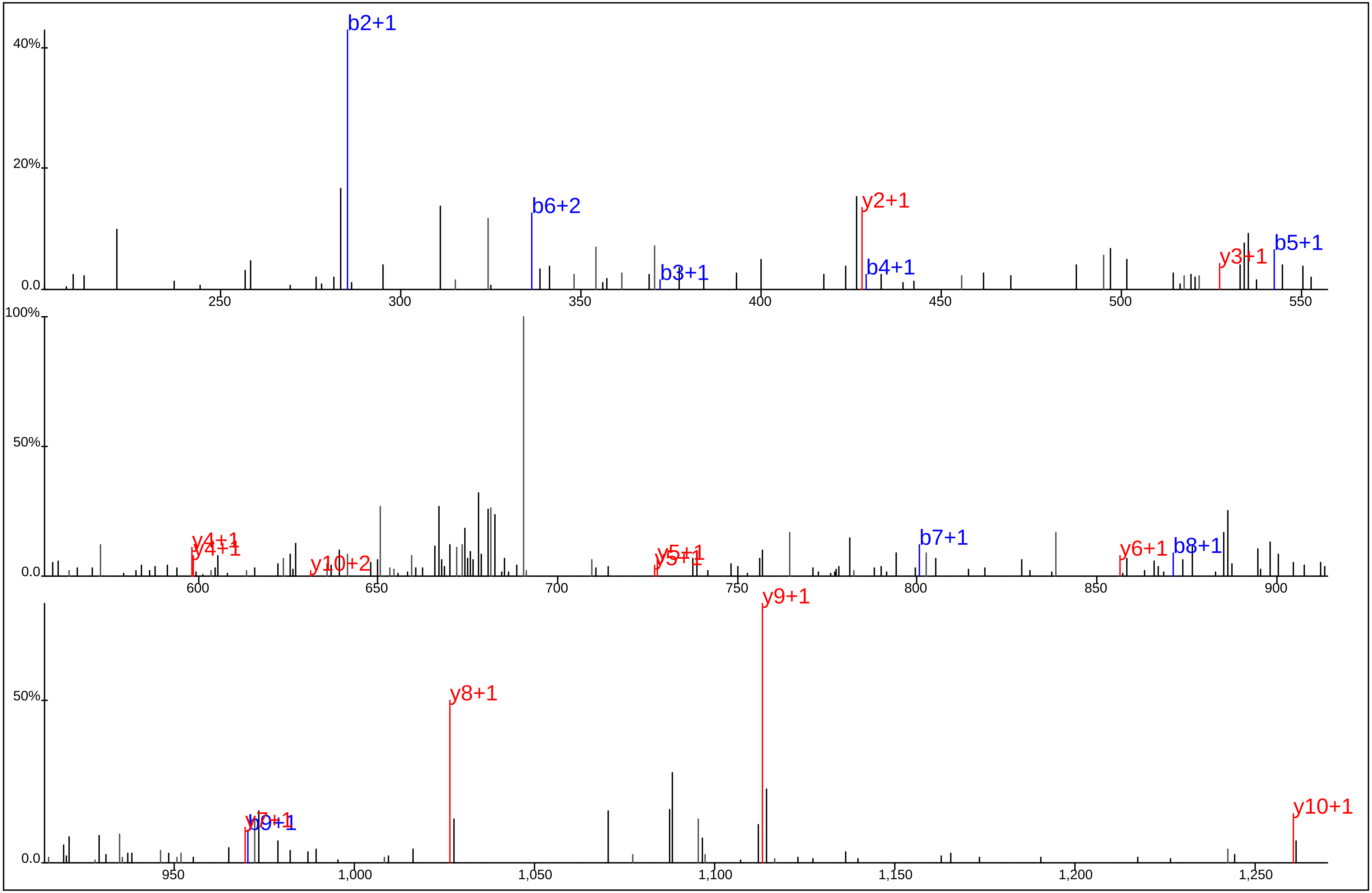

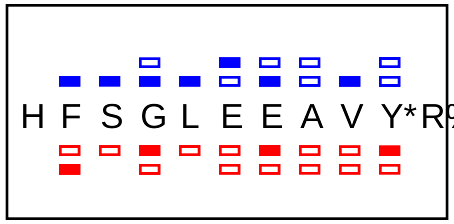

Protein Name: CCT8
gi Accession: 48762932
Precursor Mass: 699.3122+2
Delta Mass: 3.333 ppm
XCorr: 20.89

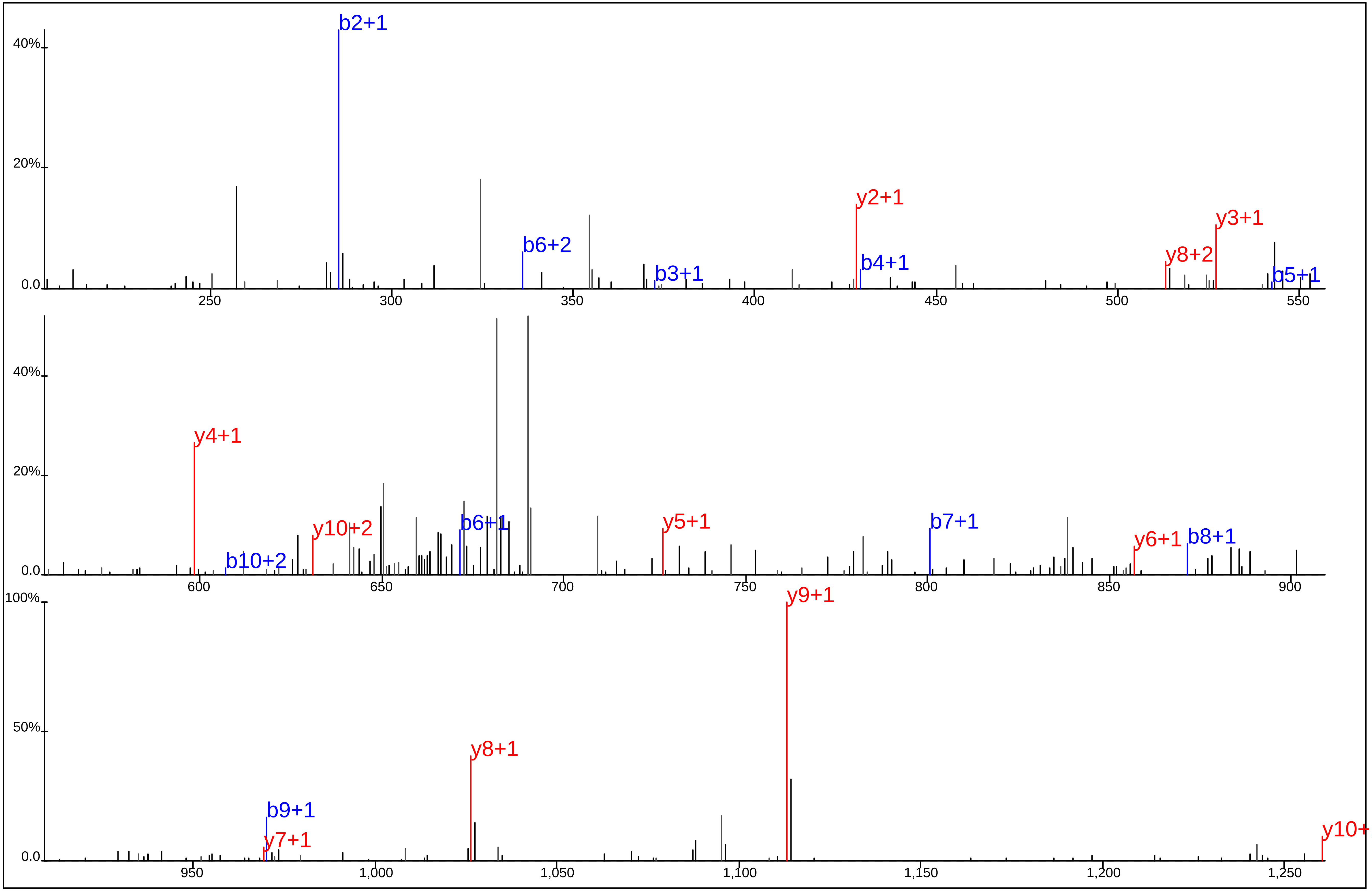

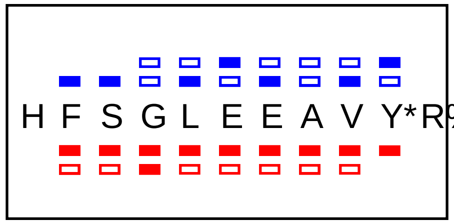

Protein Name: CCT8
gi Accession: 48762932
Precursor Mass: 699.3110+2
Delta Mass: 1.587 ppm
XCorr: 63.31

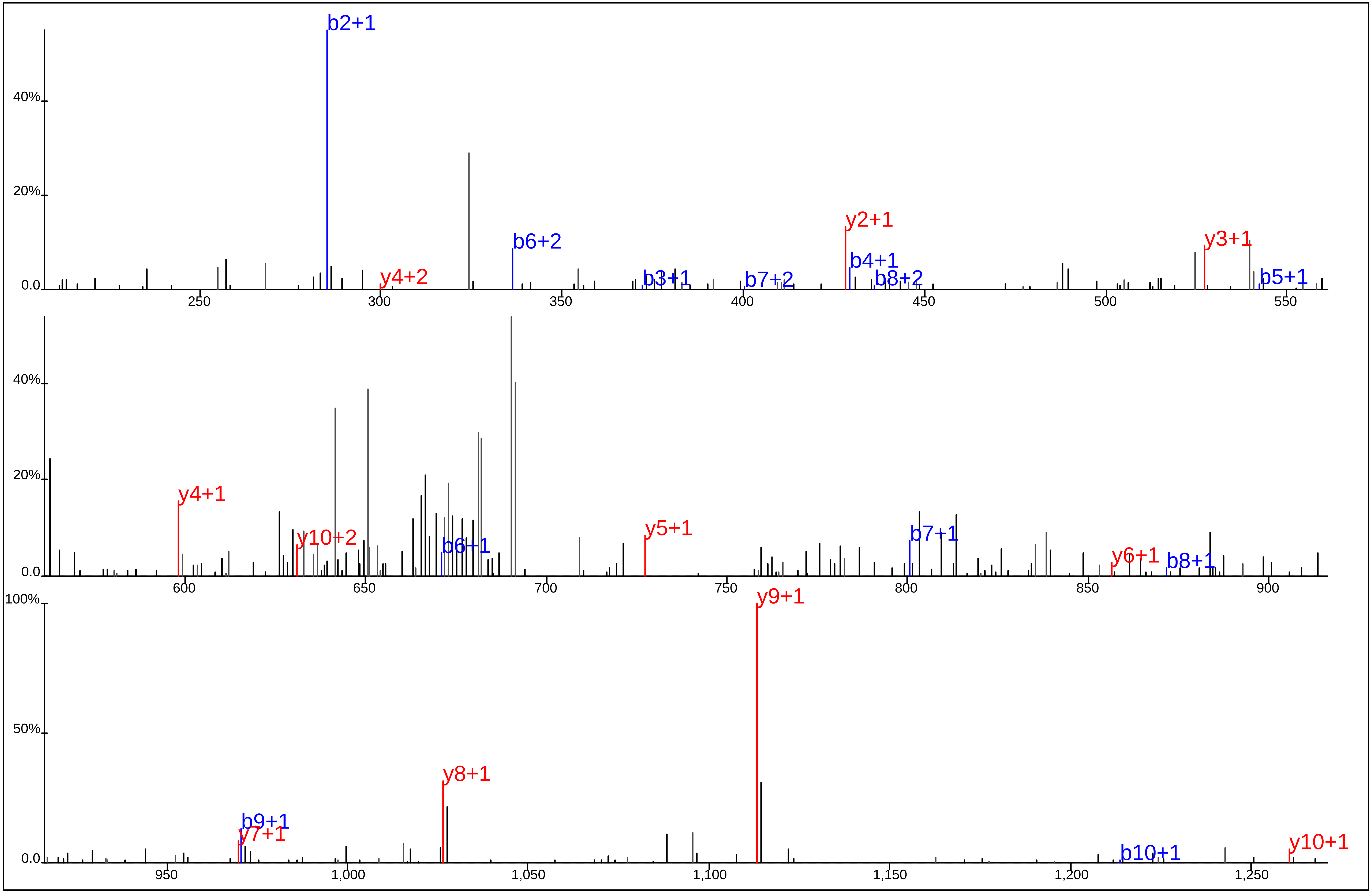

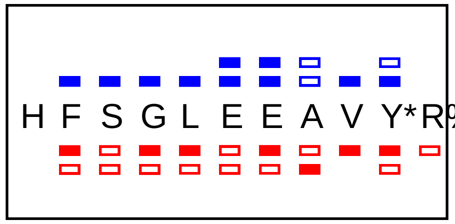

Protein Name:	CCT8
gi Accession:	48762932
Precursor Mass:	699.3132+2
Delta Mass:	4.821 ppm
XCorr:	41.09

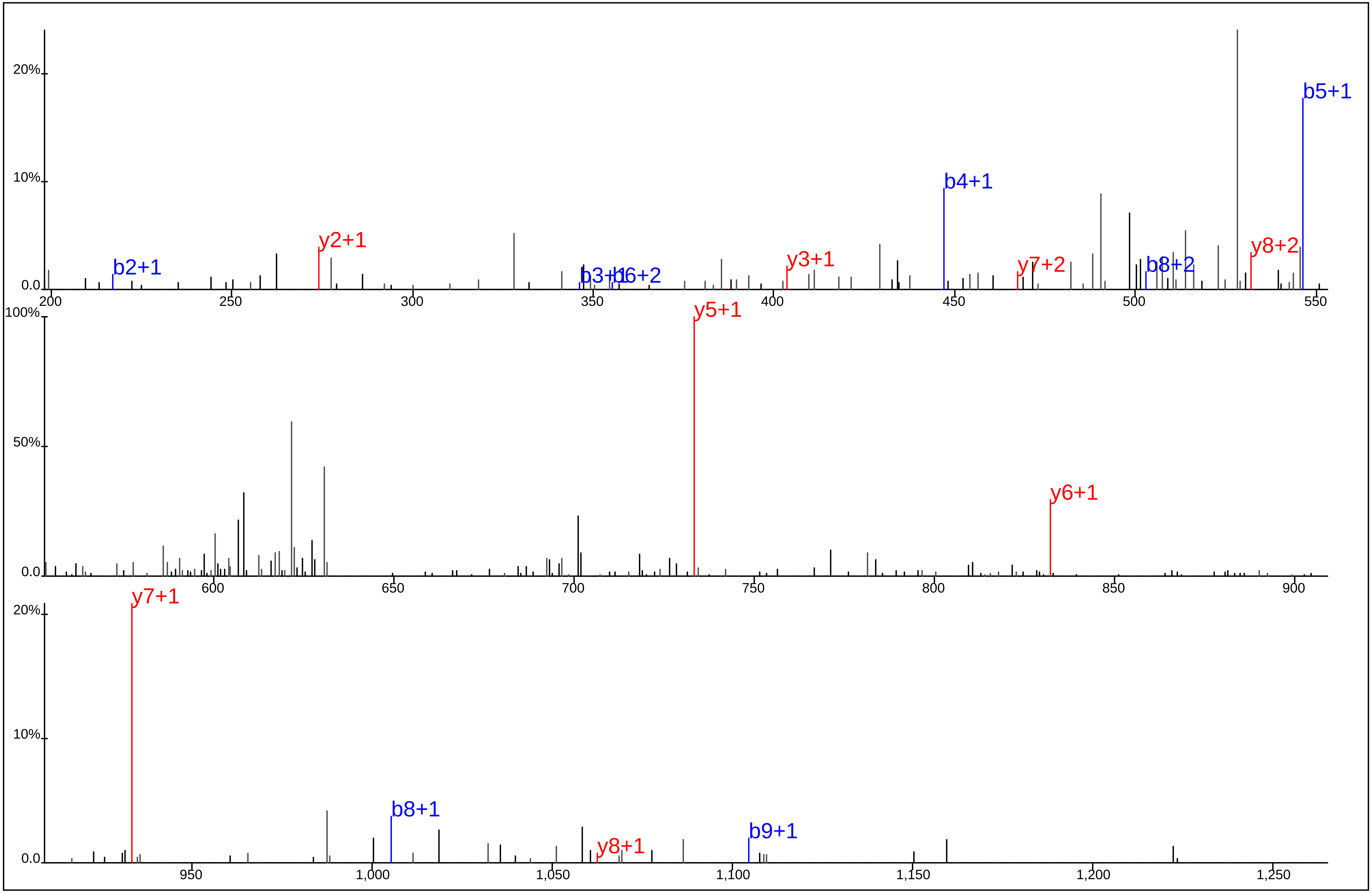

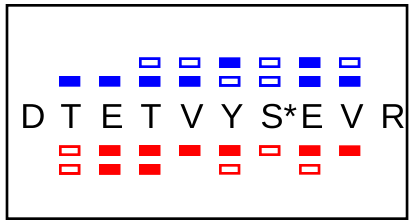

Protein Name: CD31
gi Accession: 110347451
Precursor Mass: 639.7679+2
Delta Mass: 2.775 ppm
XCorr: 33.6

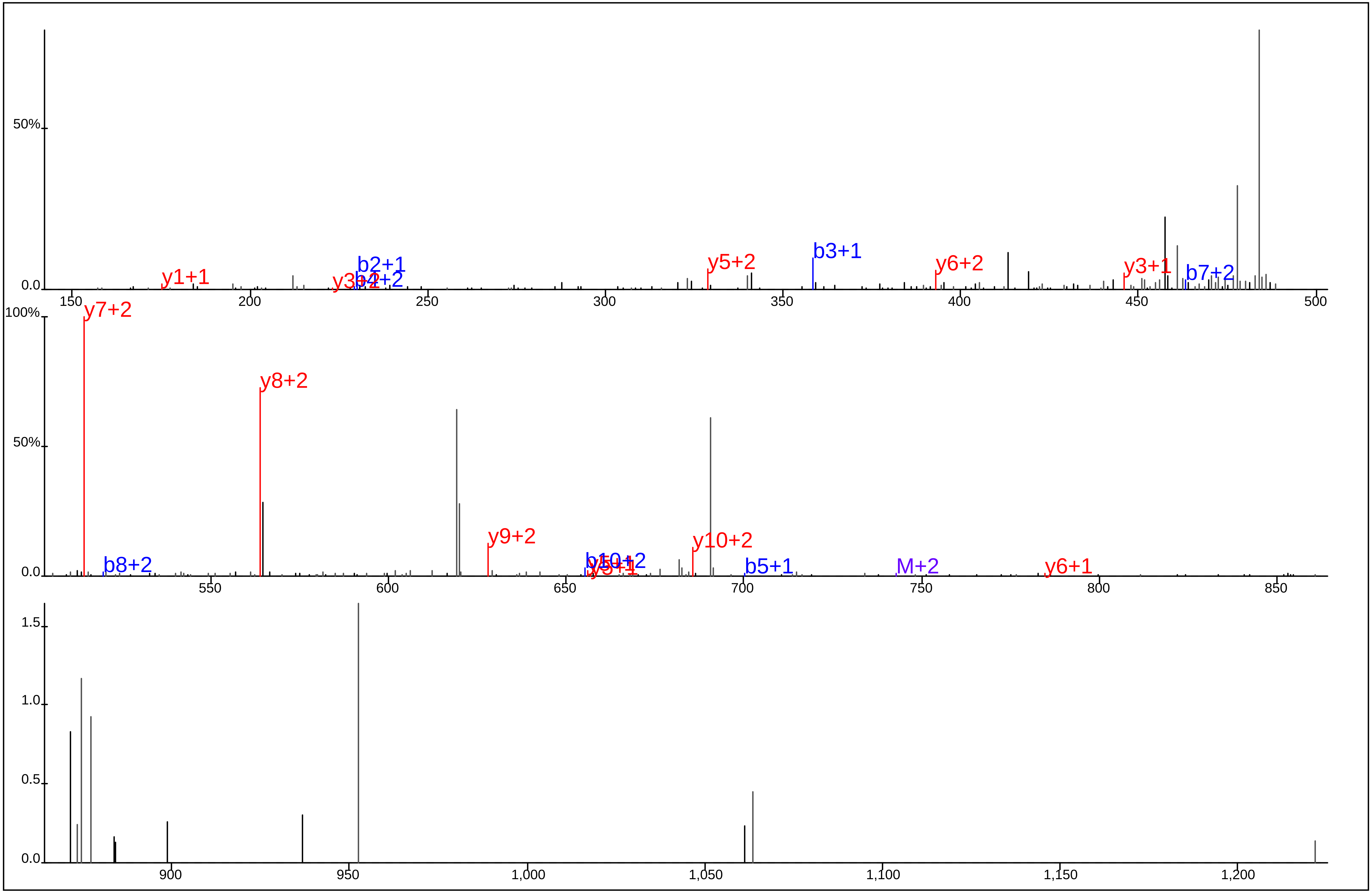

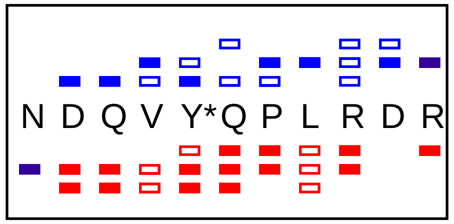

Protein Name: CD3 delta
gi Accession: 4502669
Precursor Mass: 495.2290+3
Delta Mass: 2.391 ppm
XCorr: 19.85

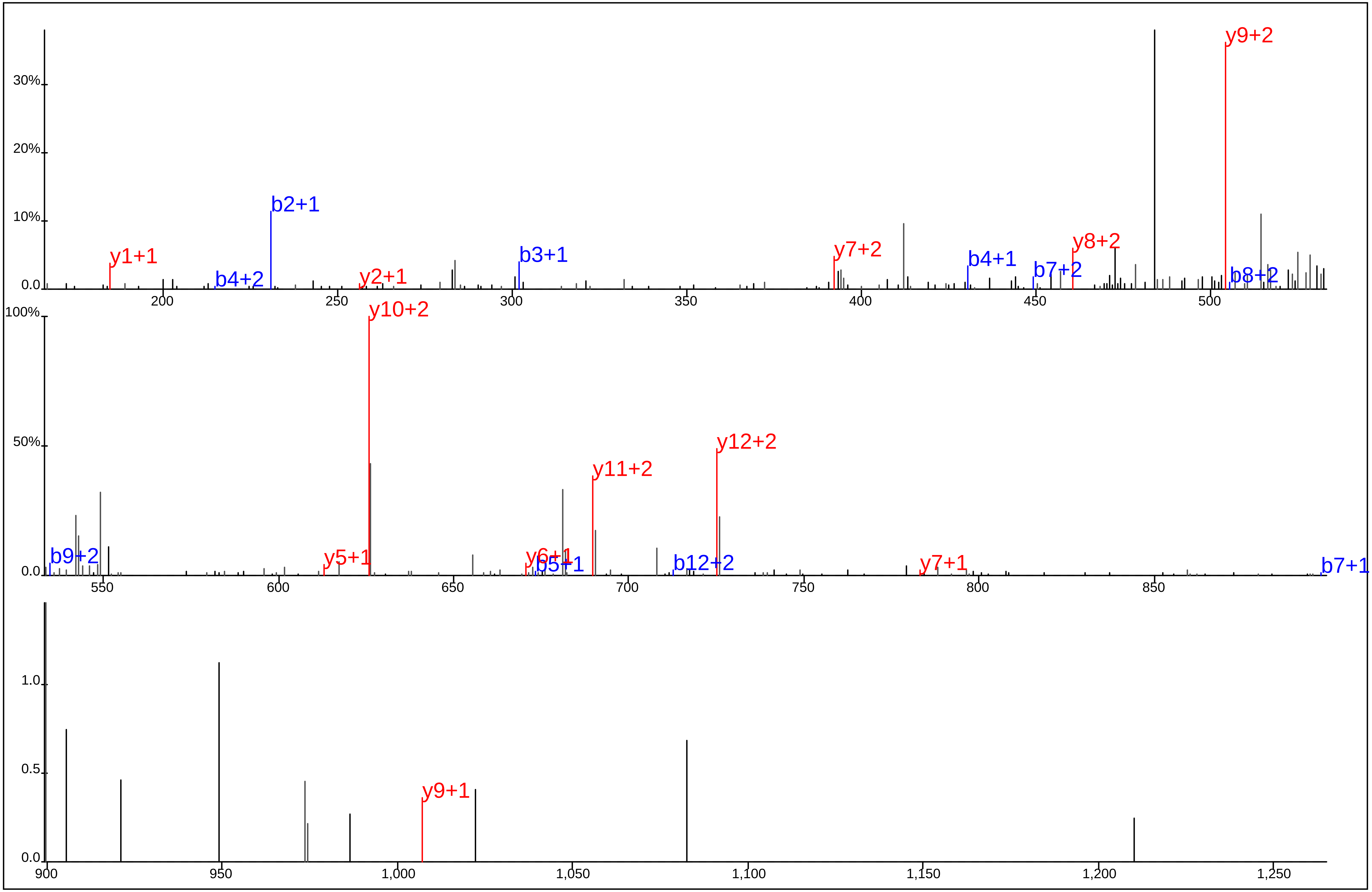

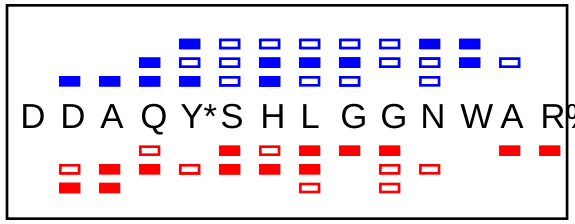

Protein Name: CD3 delta
gi Accession: 4502669
Precursor Mass: 560.5684+3
Delta Mass: 4.155 ppm
XCorr: 29.77

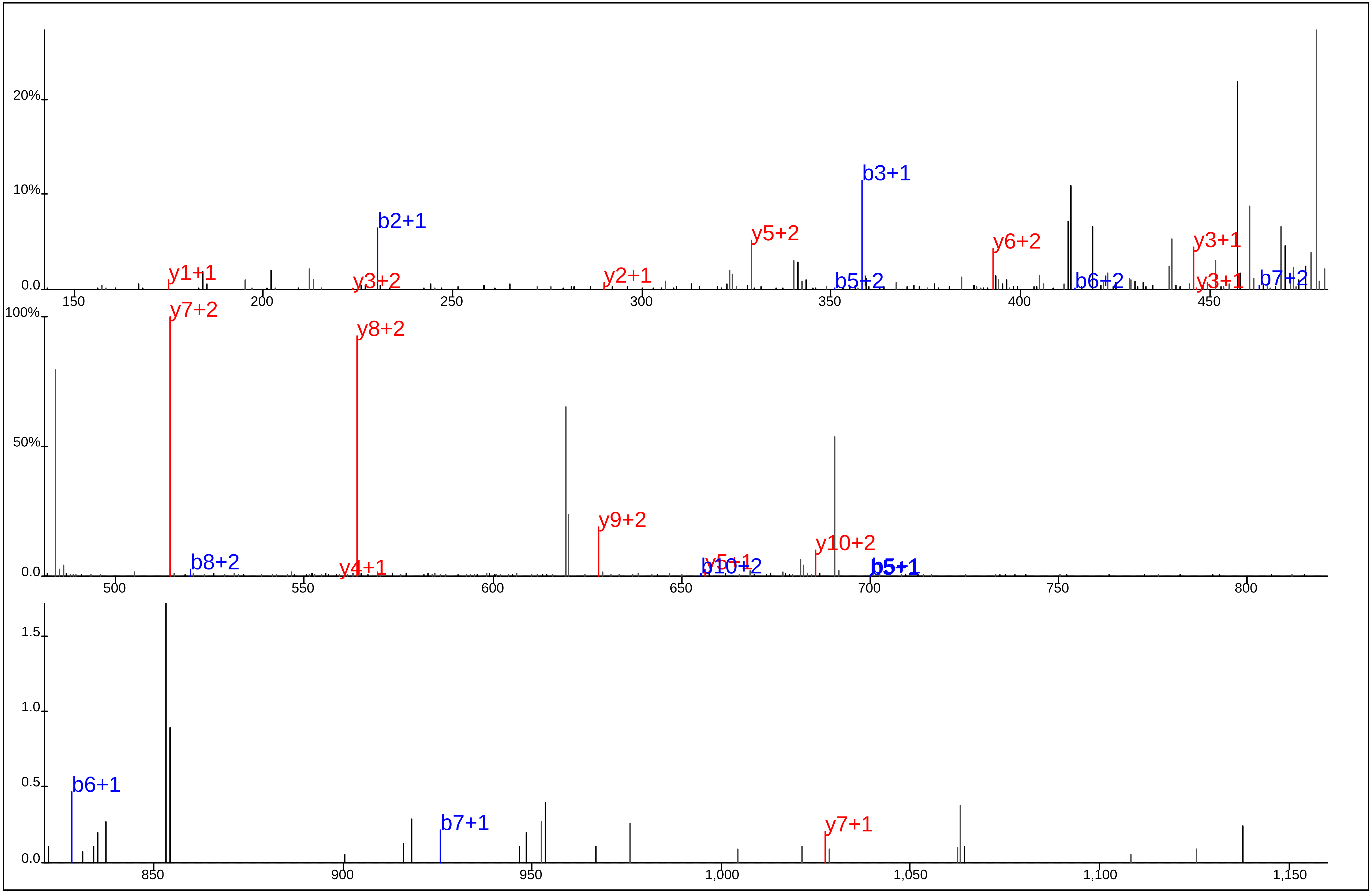

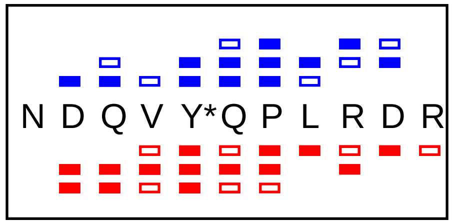

Protein Name: CD3 delta
gi Accession: 4502669
Precursor Mass: 495.2291+3
Delta Mass: 2.573 ppm
XCorr: 21.59

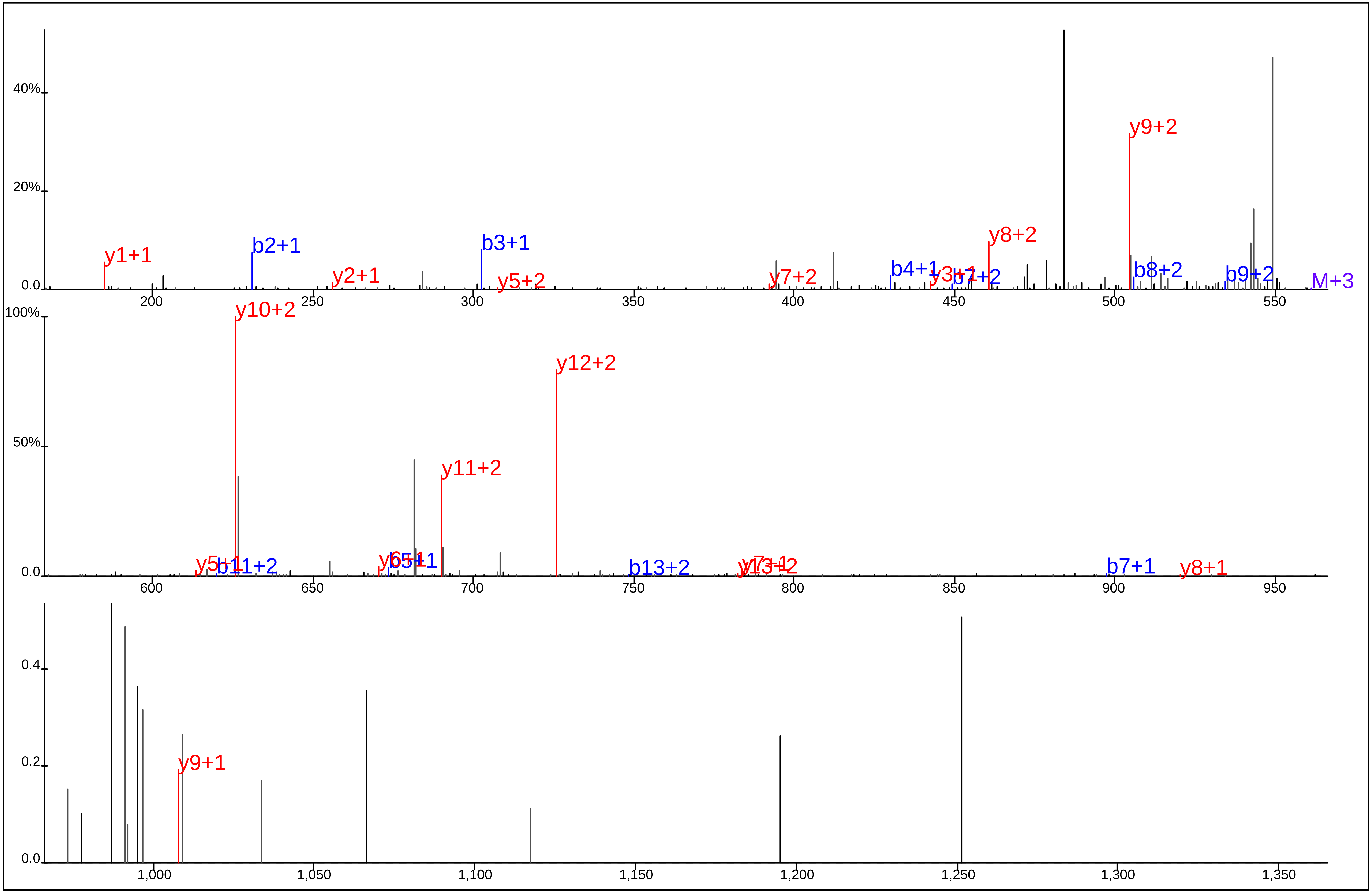

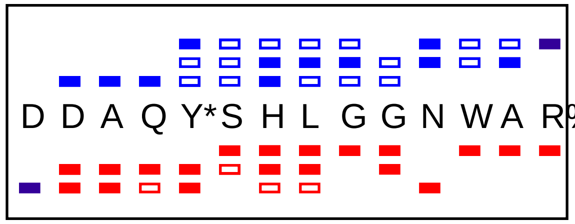

Protein Name: CD3 delta
gi Accession: 4502669
Precursor Mass: 560.5678+3
Delta Mass: 3.065 ppm
XCorr: 30.44

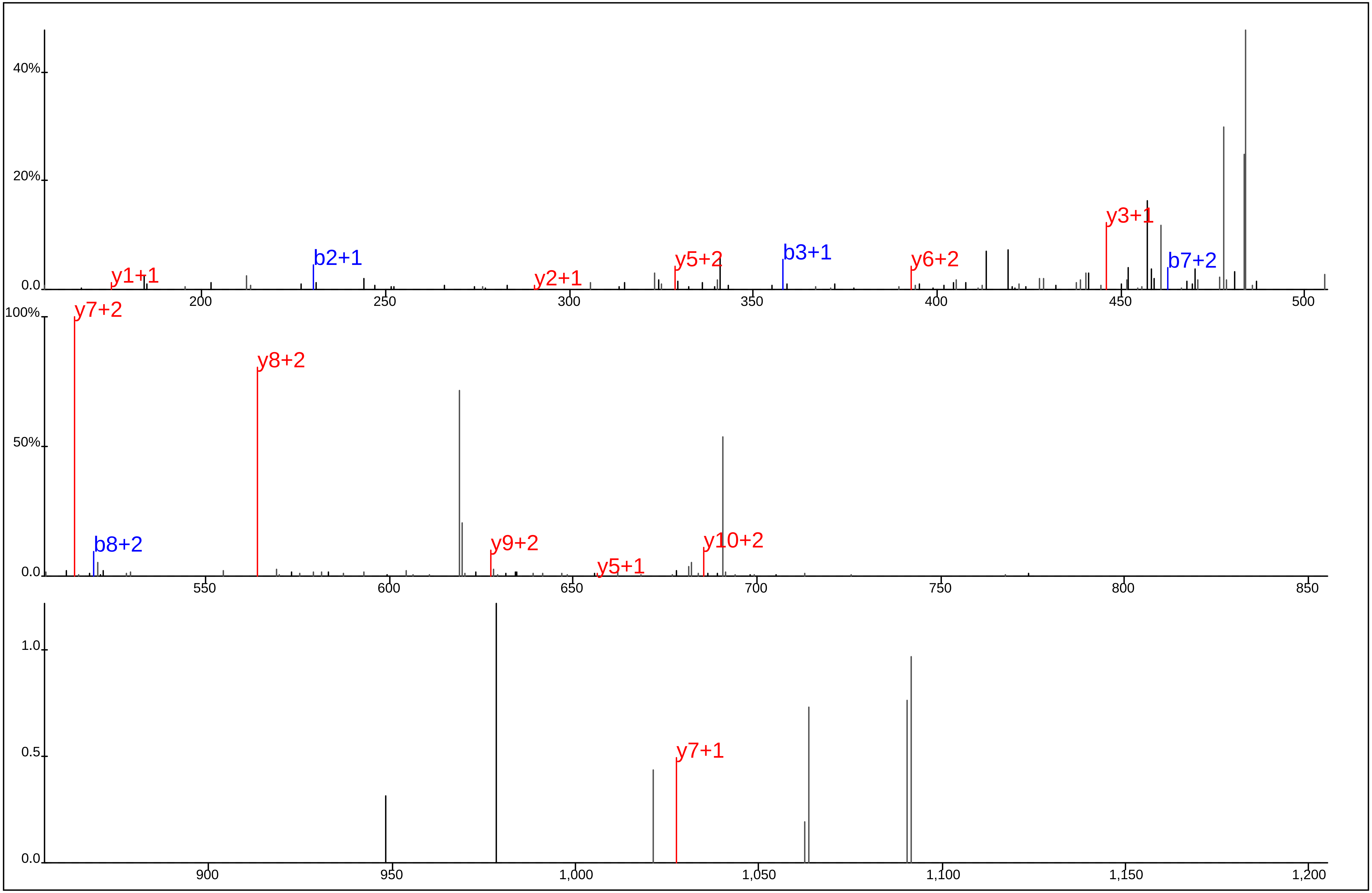

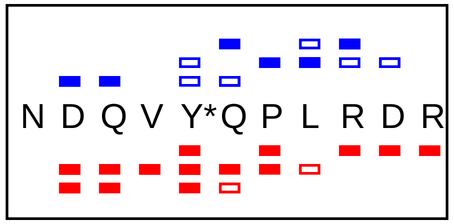

Protein Name: CD3 delta
gi Accession: 4502669
Precursor Mass: 495.2285+3
Delta Mass: 1.467 ppm
XCorr: 23.23

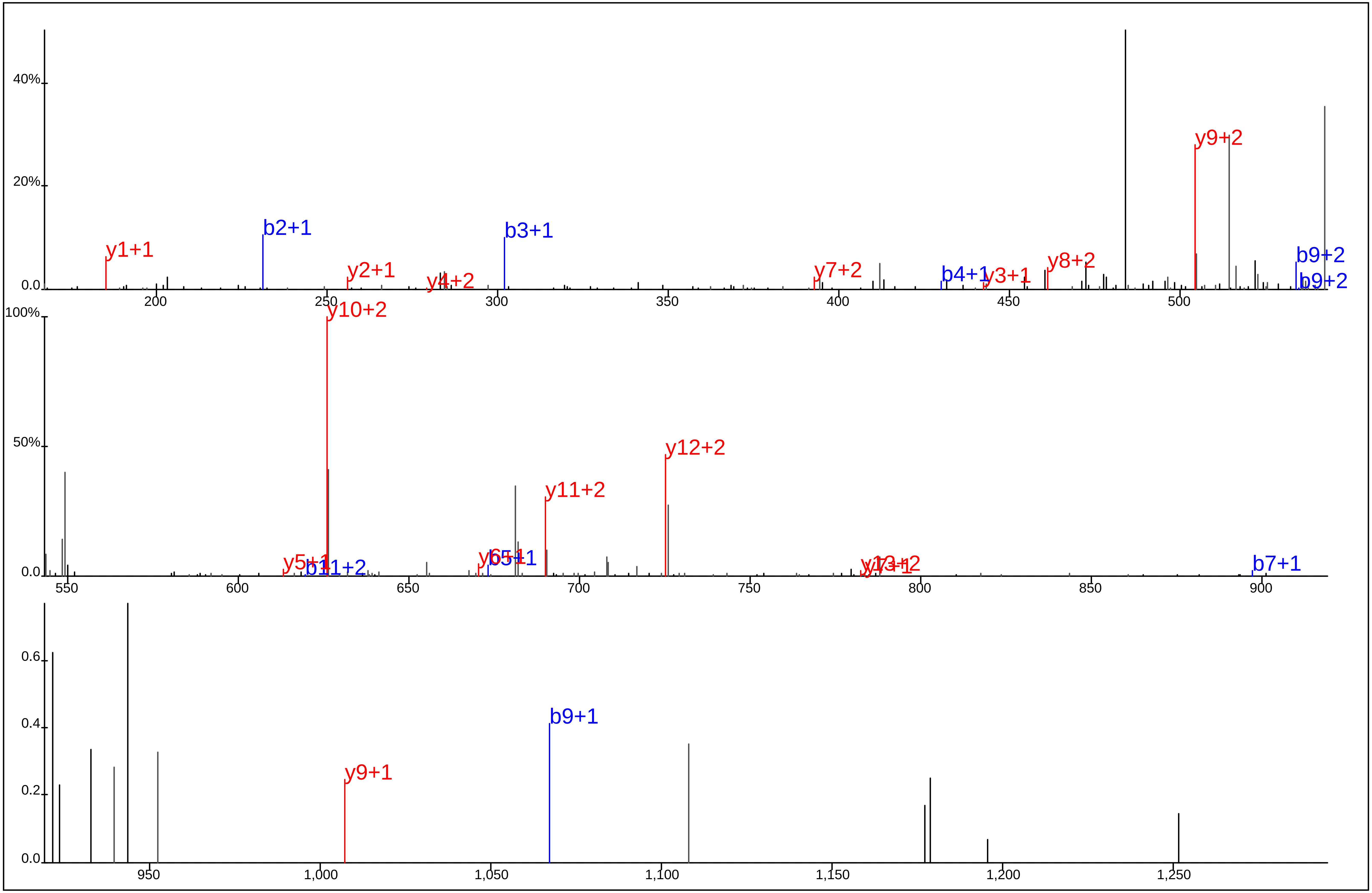

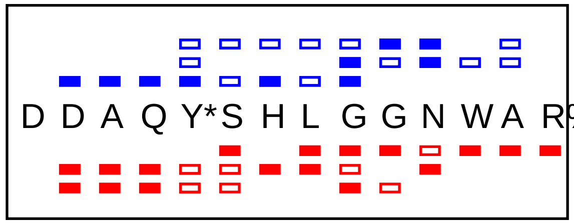

Protein Name:	CD3 delta
gi Accession:	4502669
Precursor Mass:	560.5673+3
Delta Mass:	2.083 ppm
XCorr:	30.91

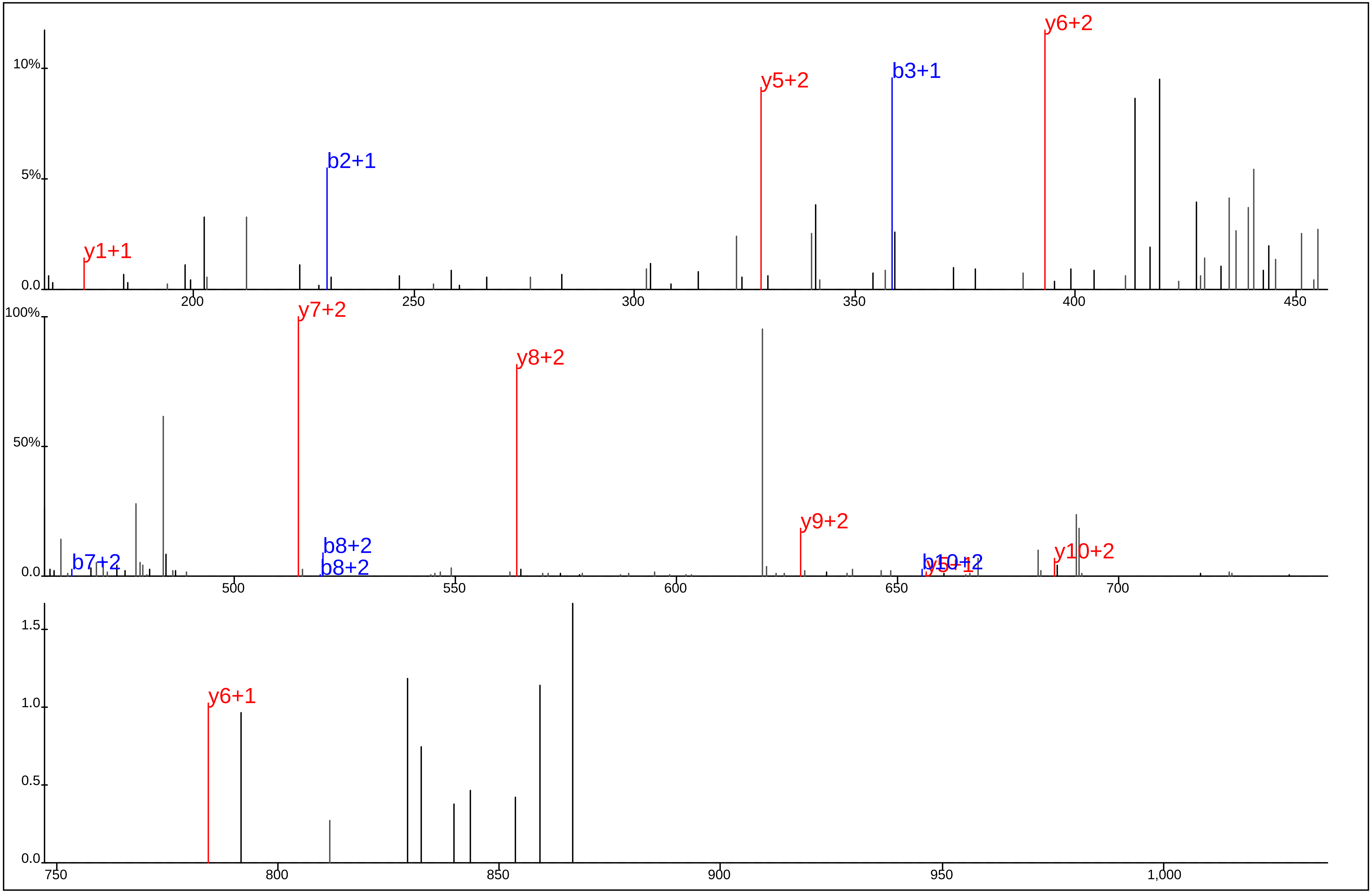

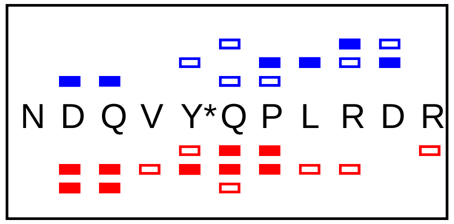

Protein Name: CD3 delta
gi Accession: 4502669
Precursor Mass: 495.2290+3
Delta Mass: 2.391 ppm
XCorr: 27.18

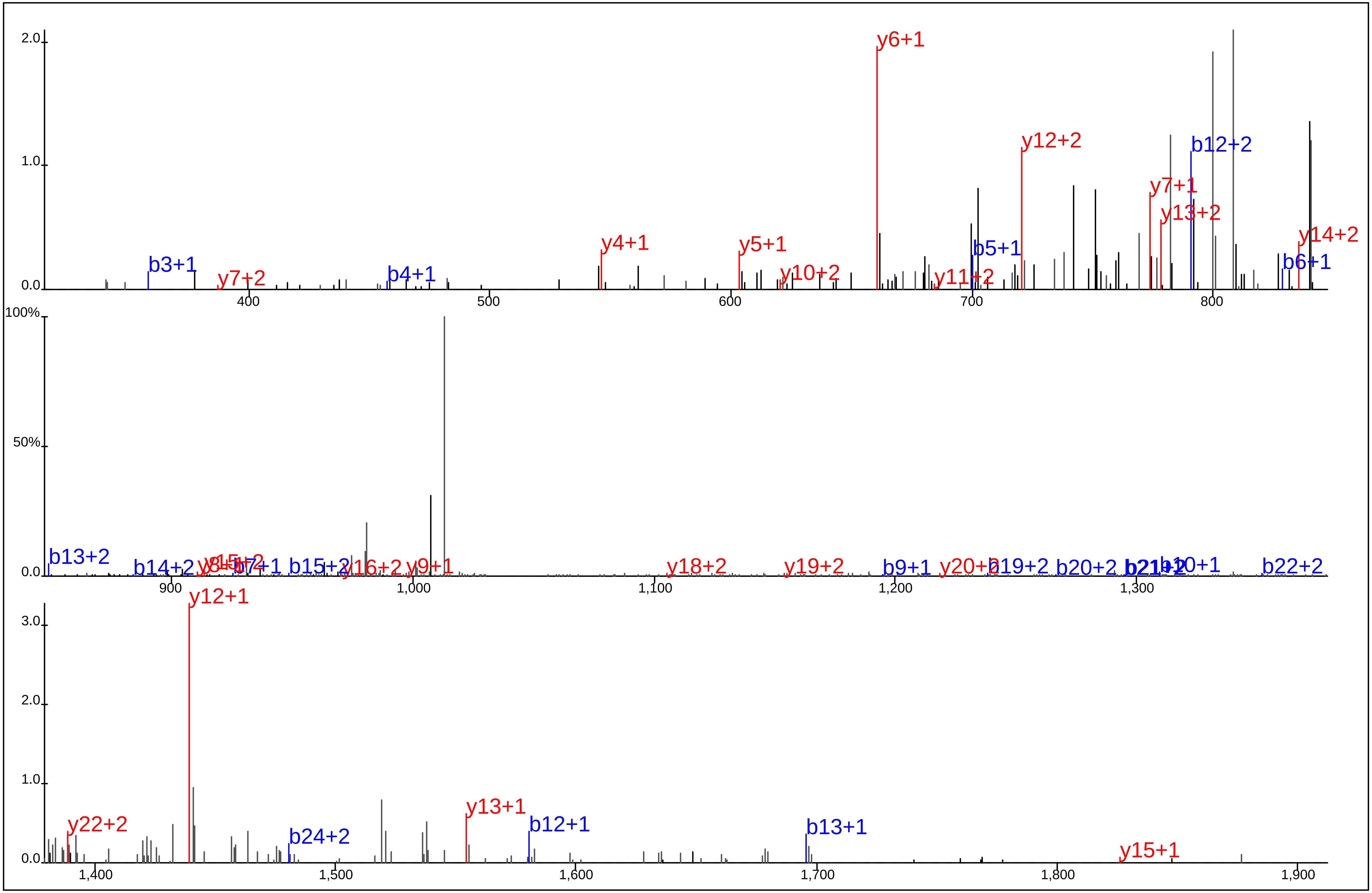

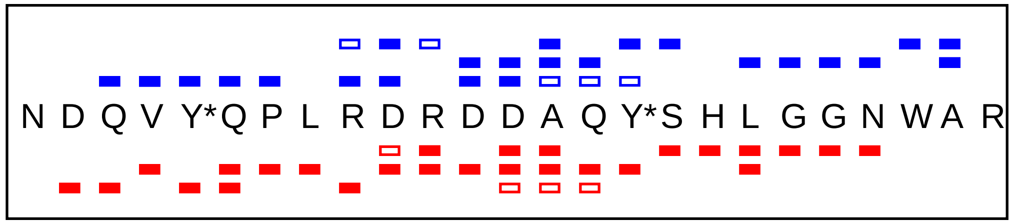

Protein Name: CD3 delta
gi Accession: 4502669
Precursor Mass: 1,045.4508+3
Delta Mass: 3.614 ppm
XCorr: 20.08

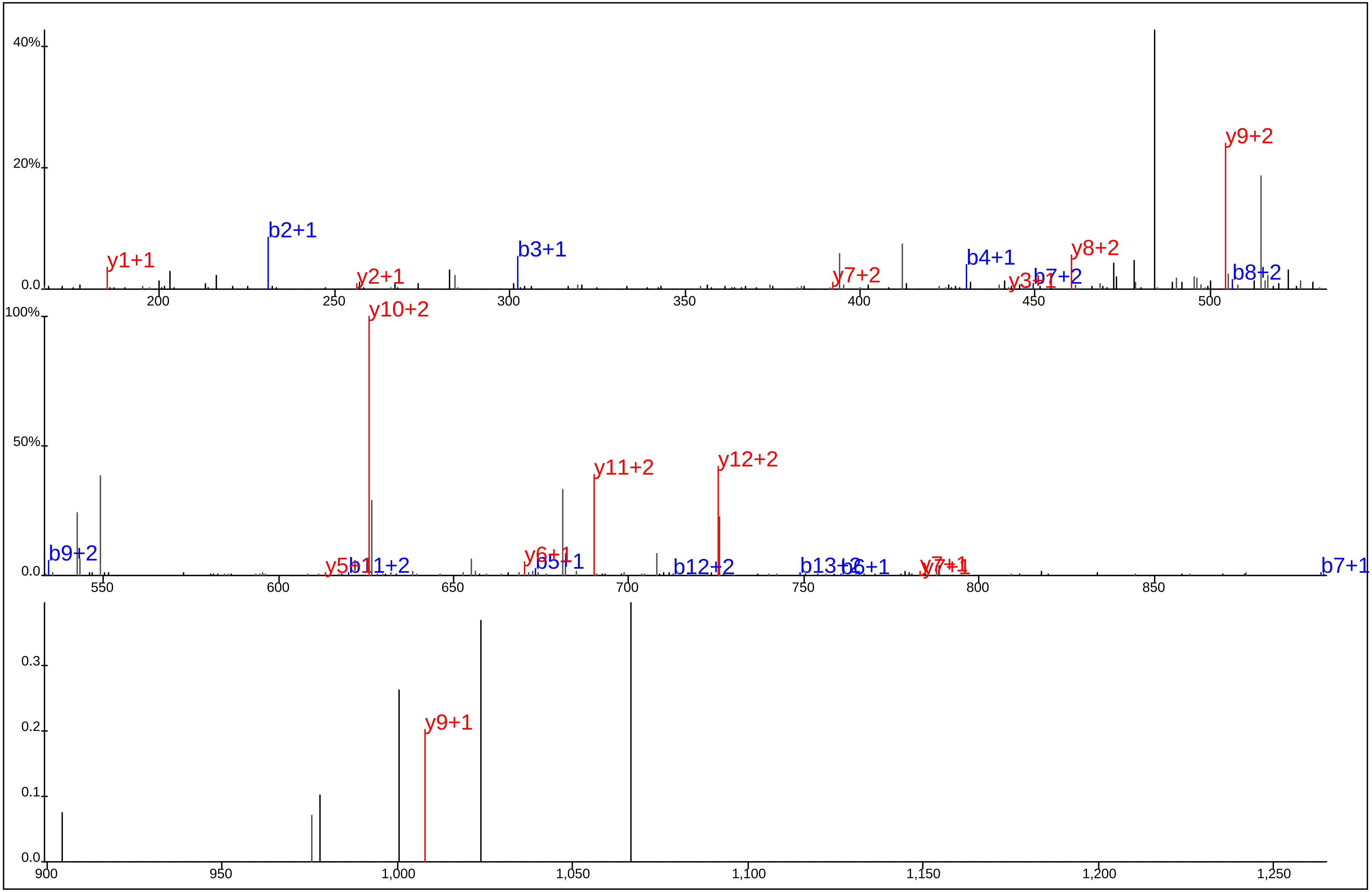

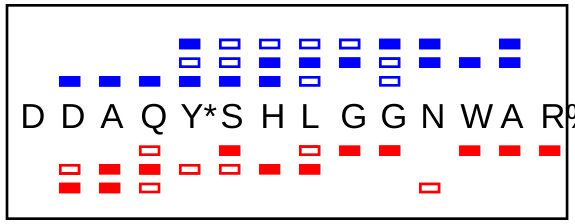

Protein Name:	CD3 delta
gi Accession:	4502669
Precursor Mass:	560.5669+3
Delta Mass:	1.428 ppm
XCorr:	29.13

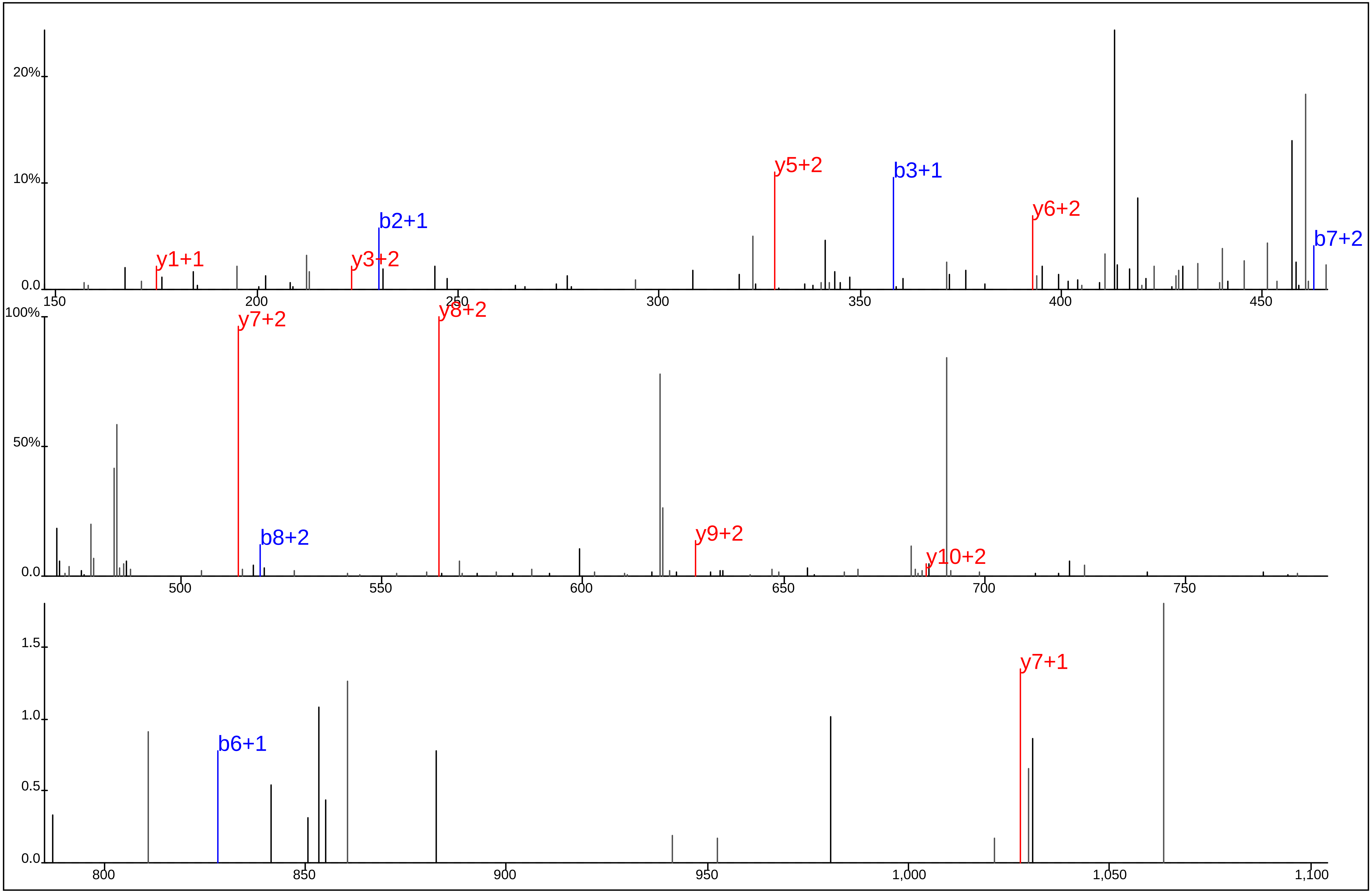

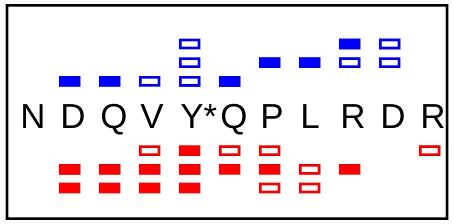

Protein Name: CD3 delta
gi Accession: 4502669
Precursor Mass: 495.2296+3
Delta Mass: 3.564 ppm
XCorr: 24.43

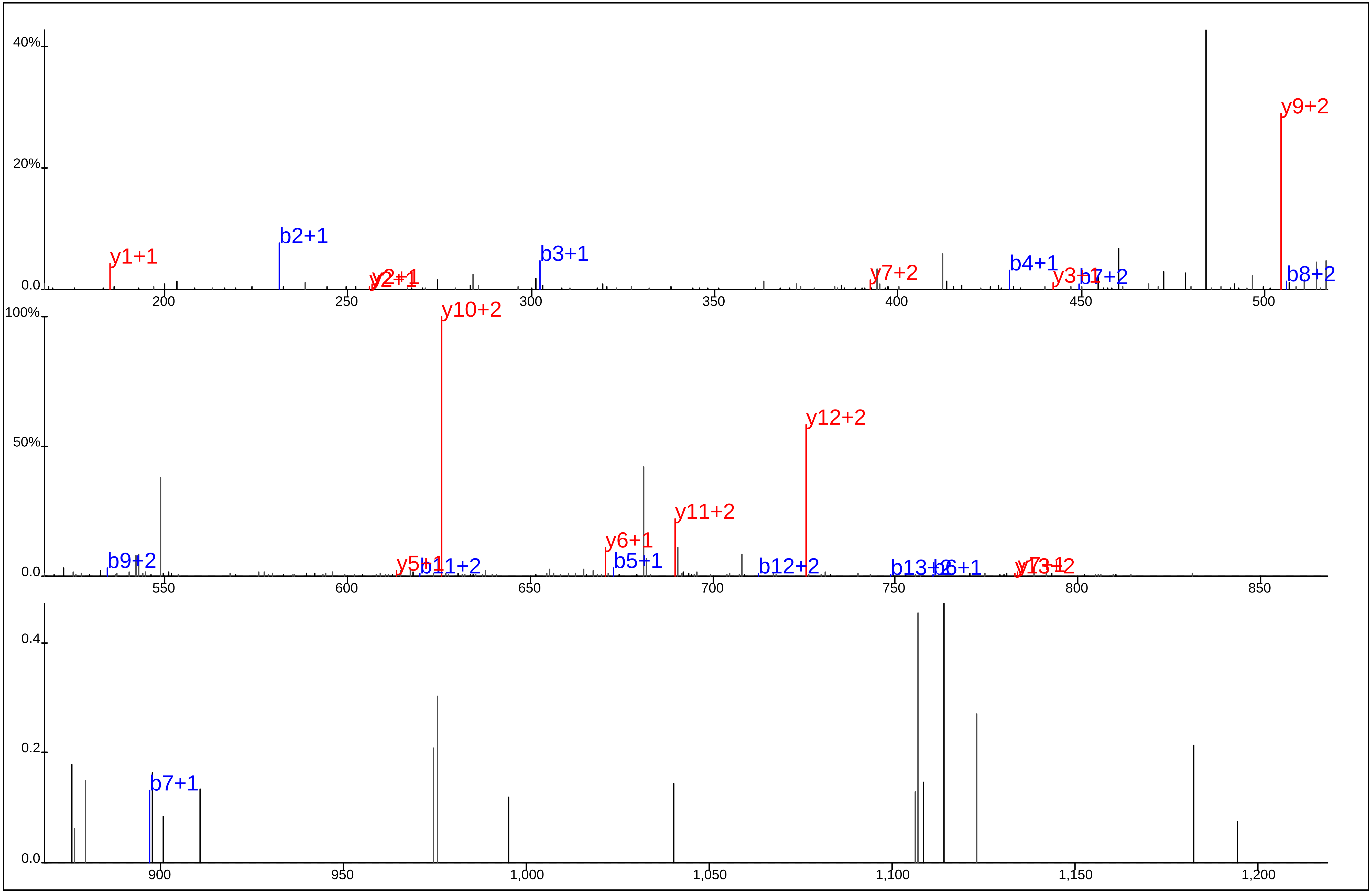

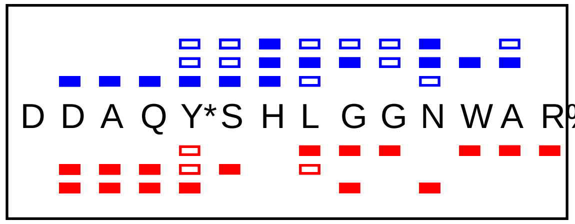

Protein Name:	CD3 delta
gi Accession:	4502669
Precursor Mass:	560.5677+3
Delta Mass:	2.844 ppm
XCorr:	35.41

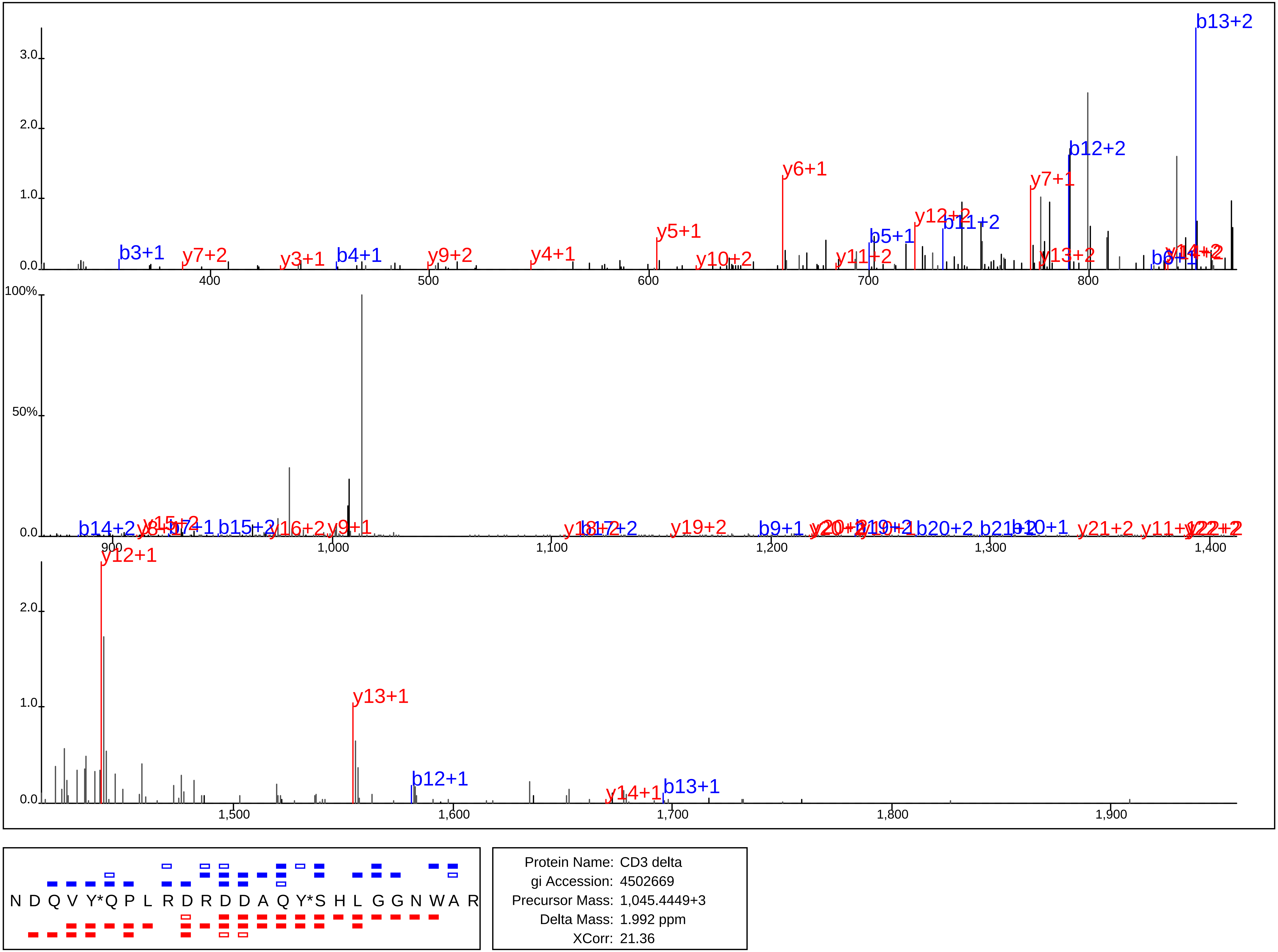

Protein Name: CD3 delta
gi Accession: 4502669
Precursor Mass: 1,045.4449+3
Delta Mass: 1.992 ppm
XCorr: 21.36

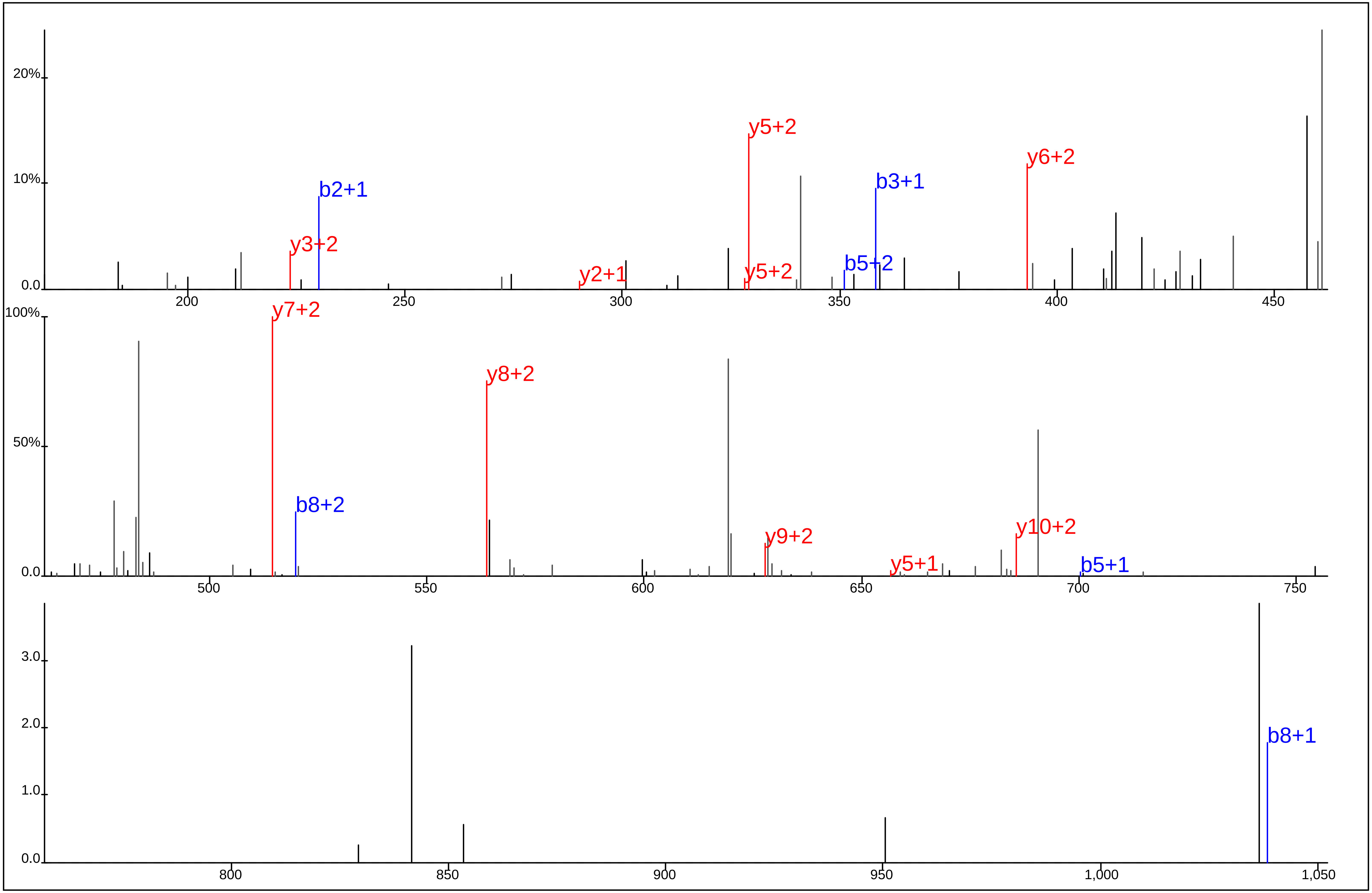

N D Q V Y*Q P L R D R

Protein Name: CD3 delta
gi Accession: 4502669
Precursor Mass: 495.2290+3
Delta Mass: 2.331 ppm
XCorr: 32.43

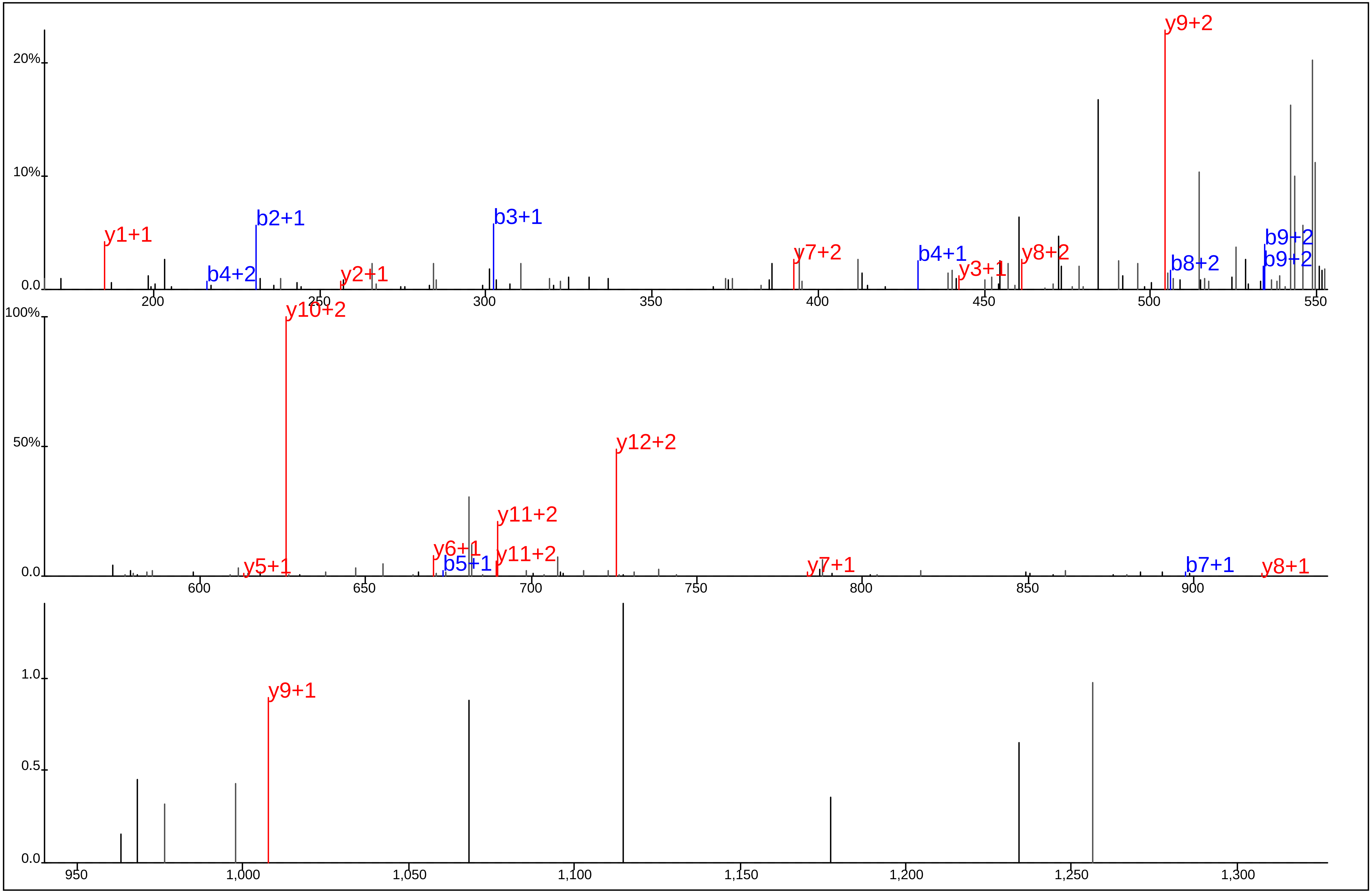

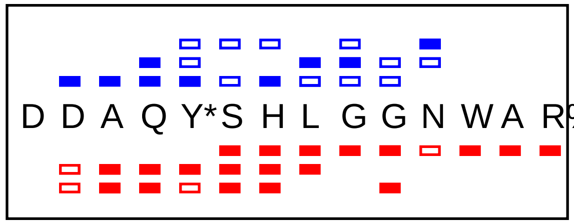

Protein Name:	CD3 delta
gi Accession:	4502669
Precursor Mass:	560.5682+3
Delta Mass:	3.719 ppm
XCorr:	23.66

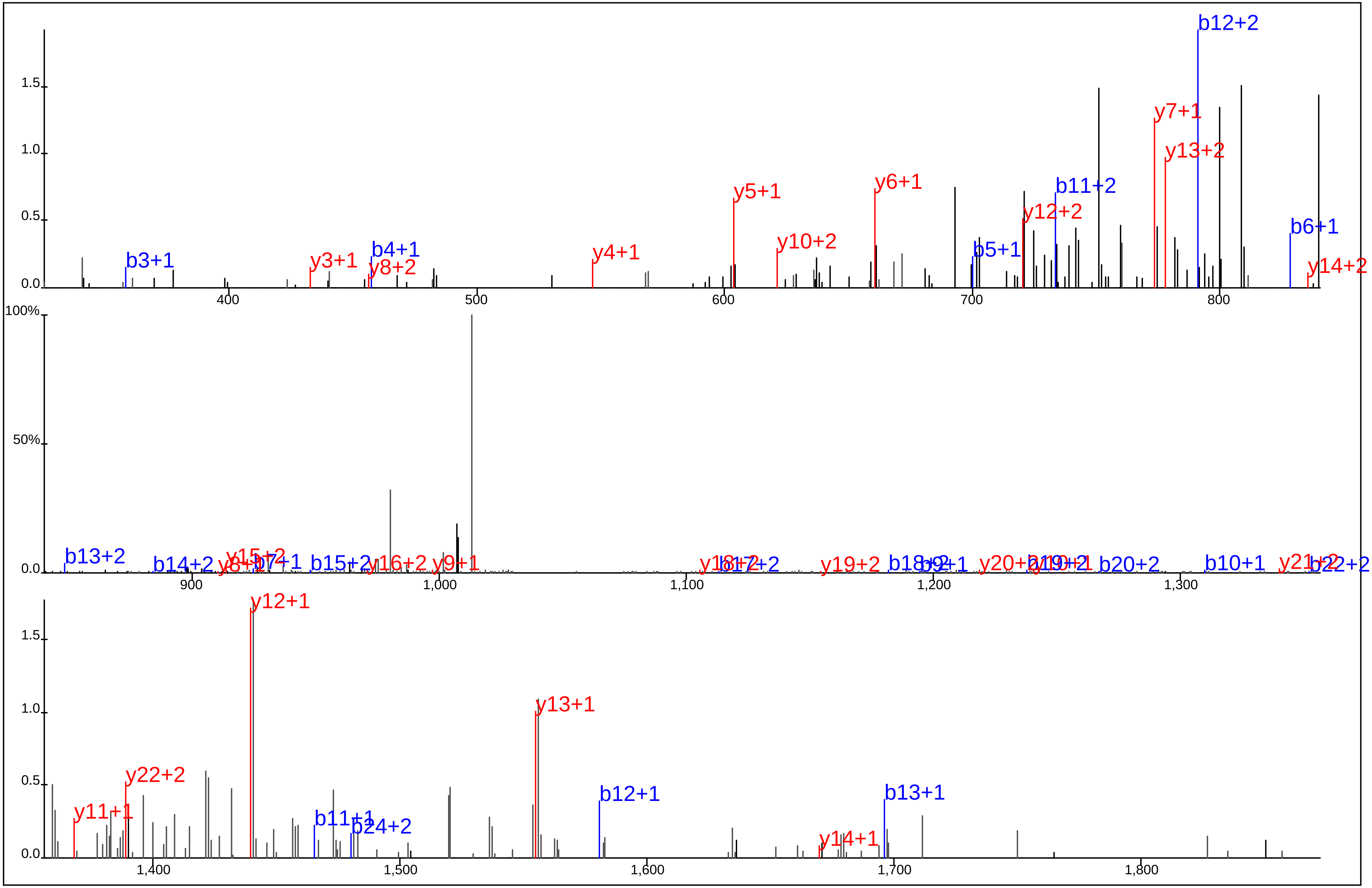

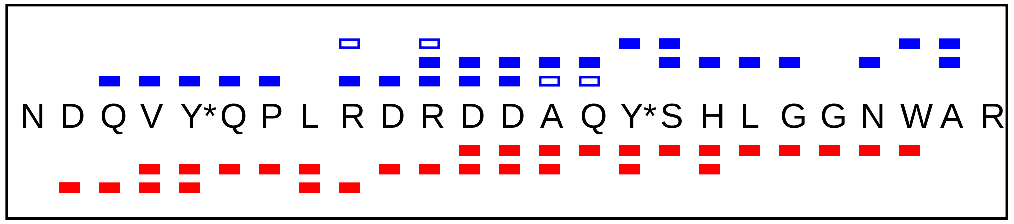

Protein Name: CD3 delta
gi Accession: 4502669
Precursor Mass: 1,045.4462+3
Delta Mass: 0.824 ppm
XCorr: 35.88

162 - CD3delta_3448741.pdf

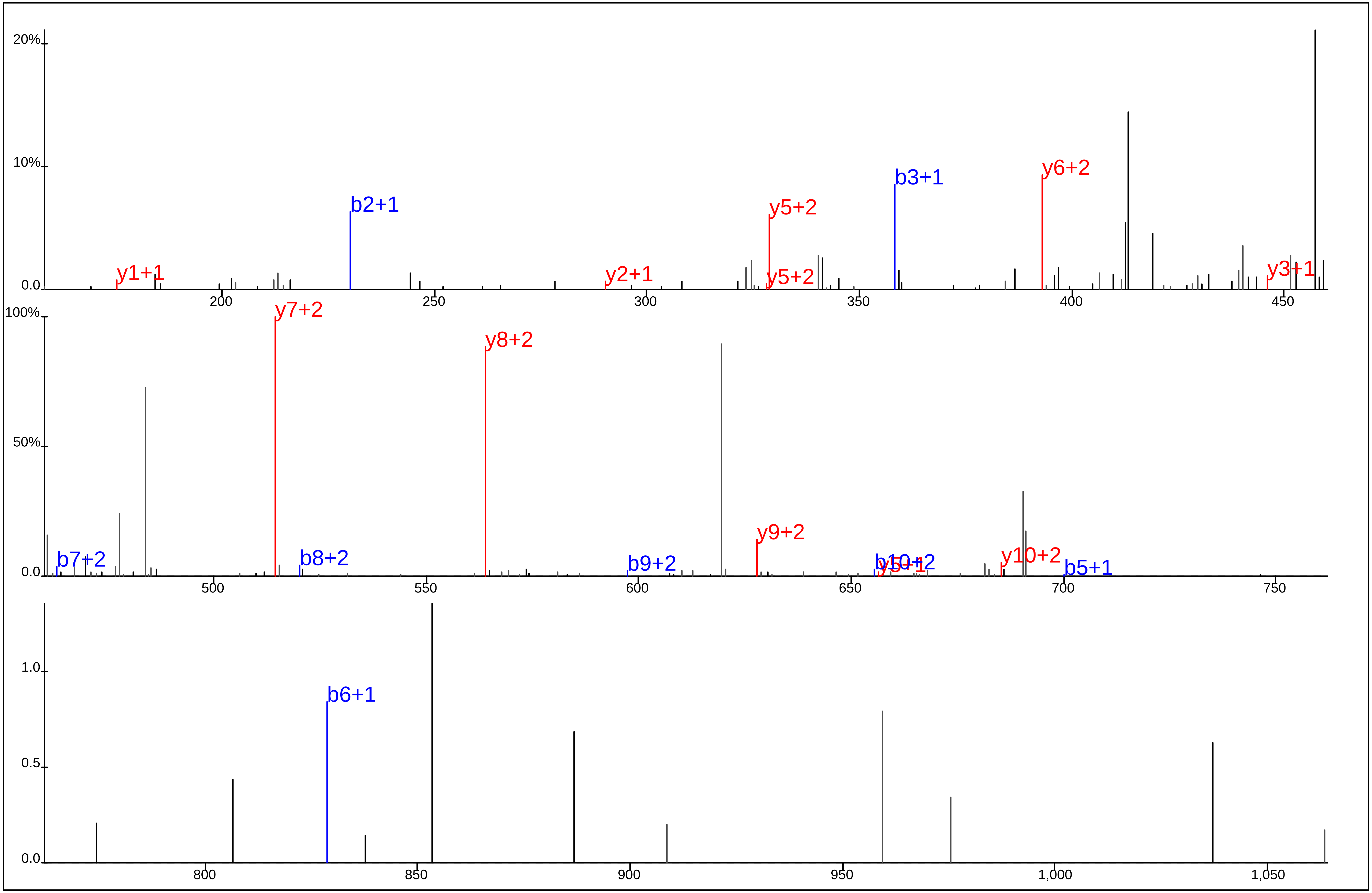

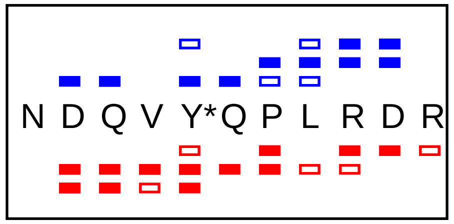

Protein Name: CD3 delta
gi Accession: 4502669
Precursor Mass: 495.2290+3
Delta Mass: 2.391 ppm
XCorr: 26.46

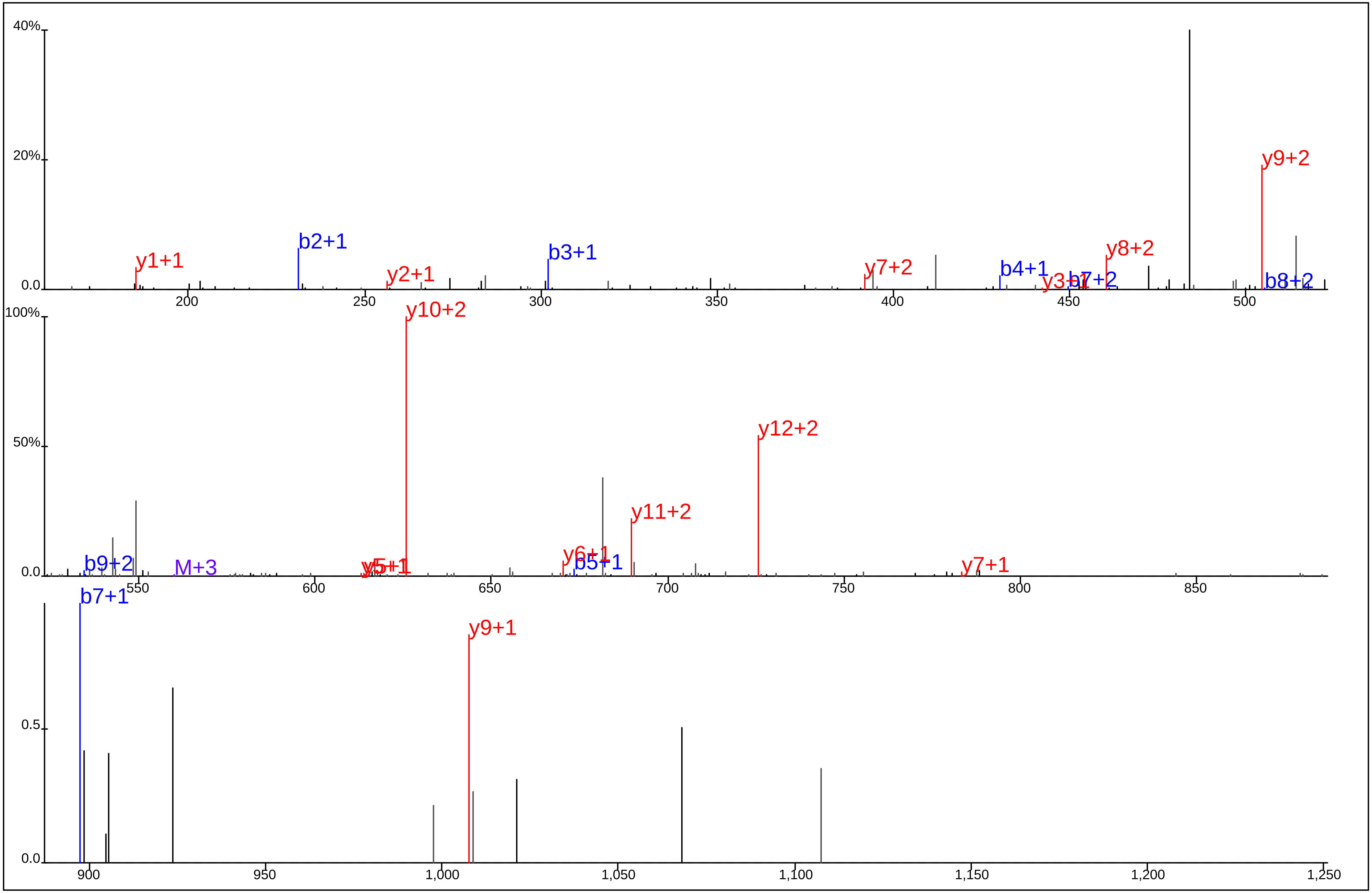

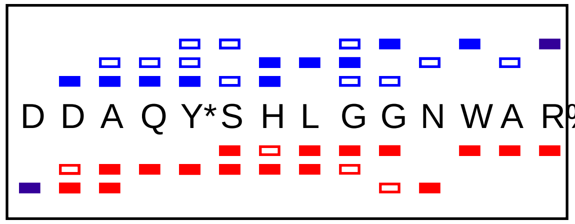

Protein Name: CD3 delta
gi Accession: 4502669
Precursor Mass: 560.5666+3
Delta Mass: 0.879 ppm
XCorr: 29.46

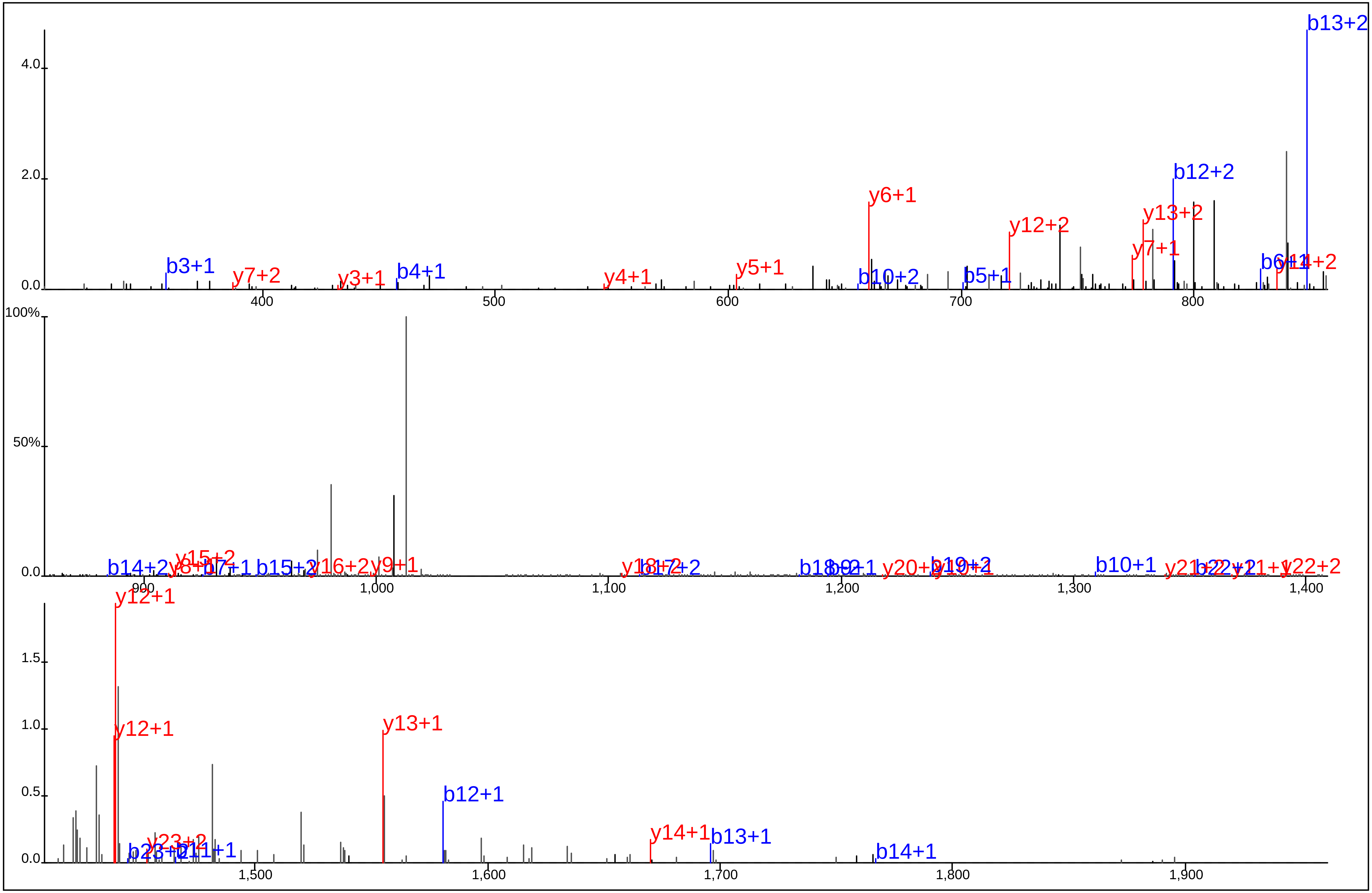

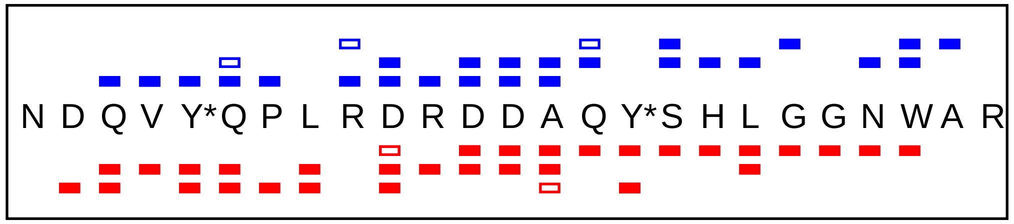

Protein Name: CD3 delta
gi Accession: 4502669
Precursor Mass: 1,045.4479+3
Delta Mass: 0.810 ppm
XCorr: 20.66

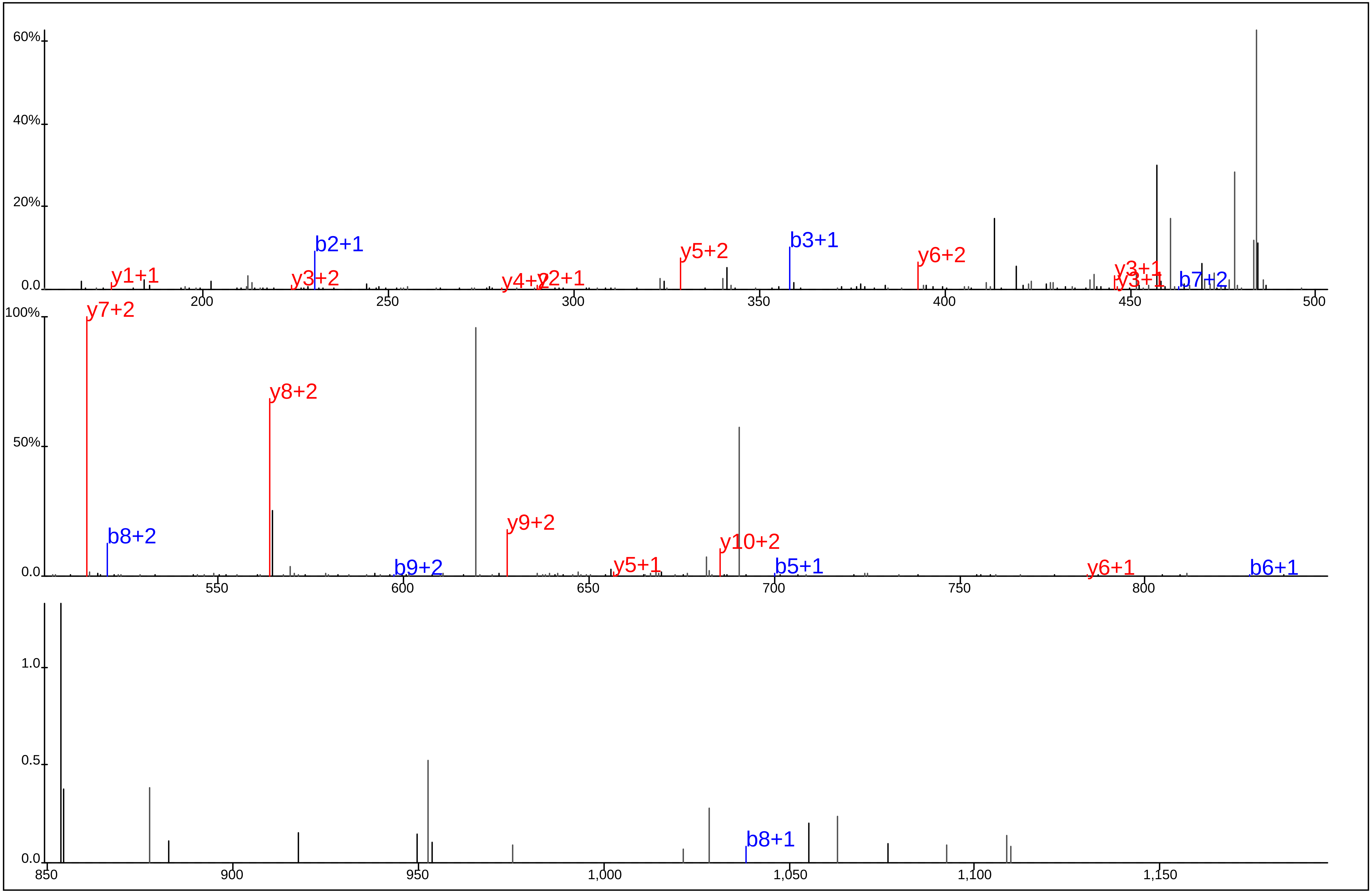

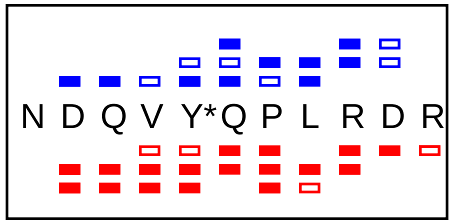

Protein Name: CD3 delta
gi Accession: 4502669
Precursor Mass: 495.2288+3
Delta Mass: 2.021 ppm
XCorr: 25.57

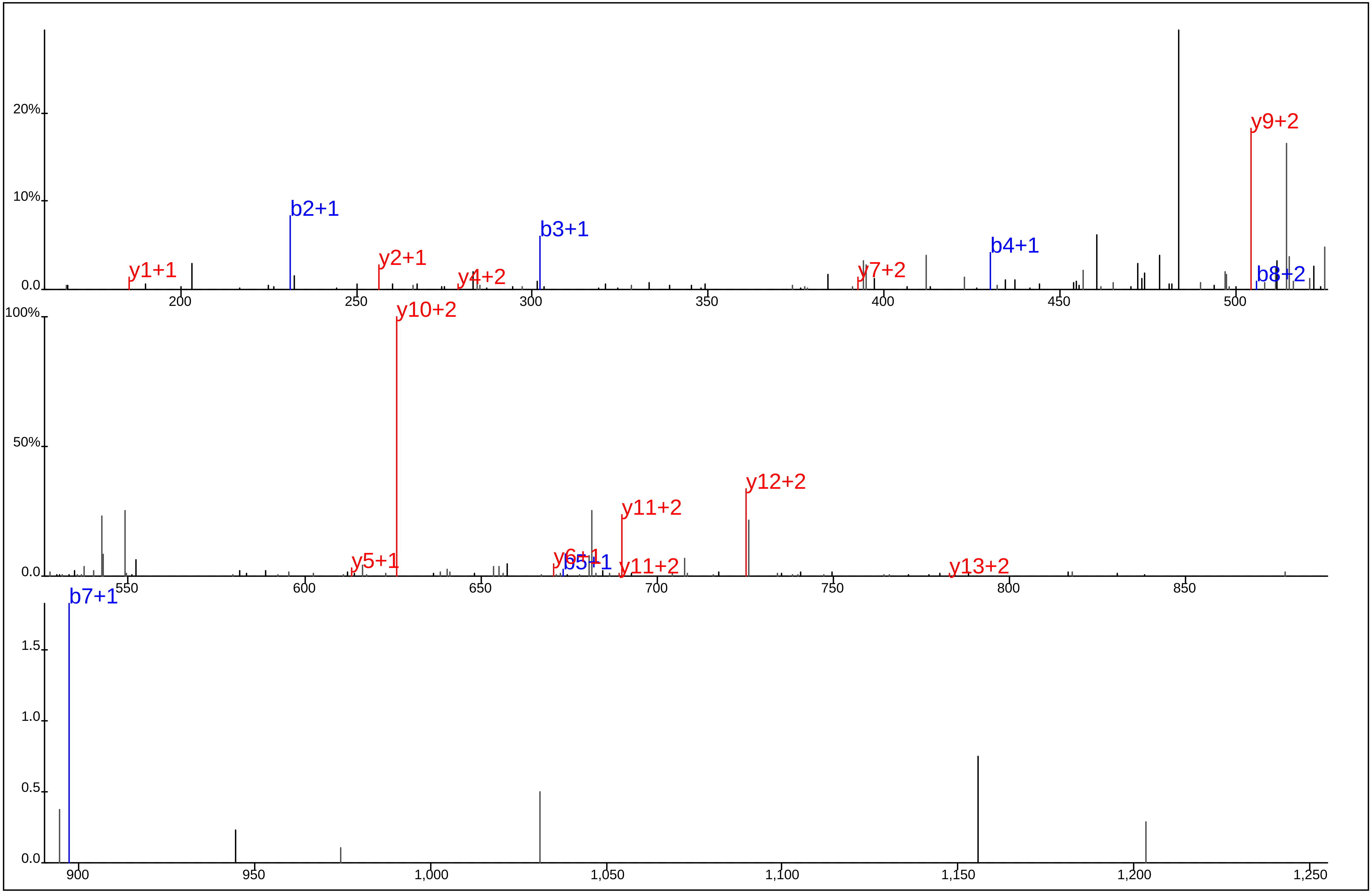

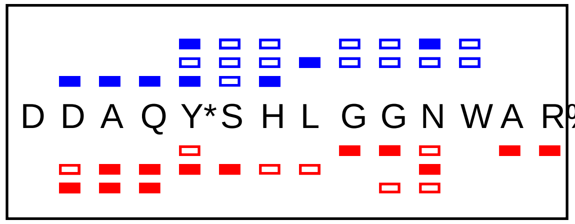

Protein Name: CD3 delta
gi Accession: 4502669
Precursor Mass: 560.5682+3
Delta Mass: 3.719 ppm
XCorr: 36.96

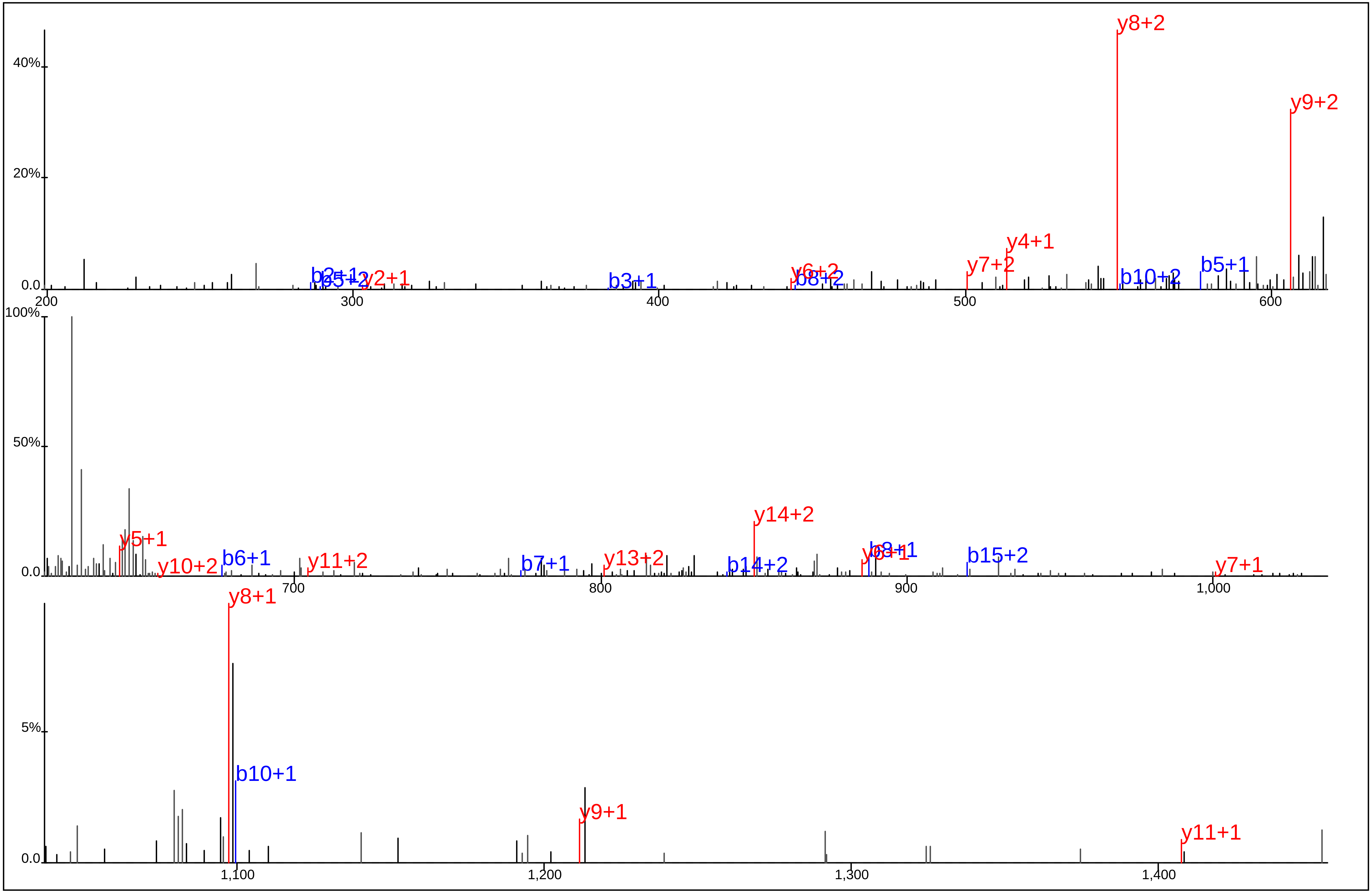

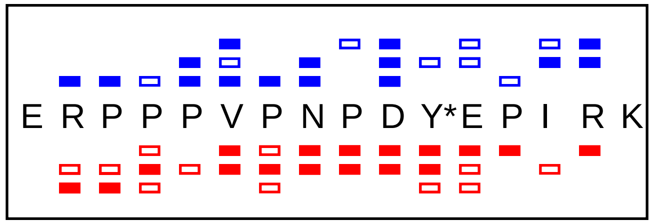

Protein Name: CD3 epsilon
gi Accession: 4502671
Precursor Mass: 661.9945+3
Delta Mass: 0.086 ppm
XCorr: 2.5629

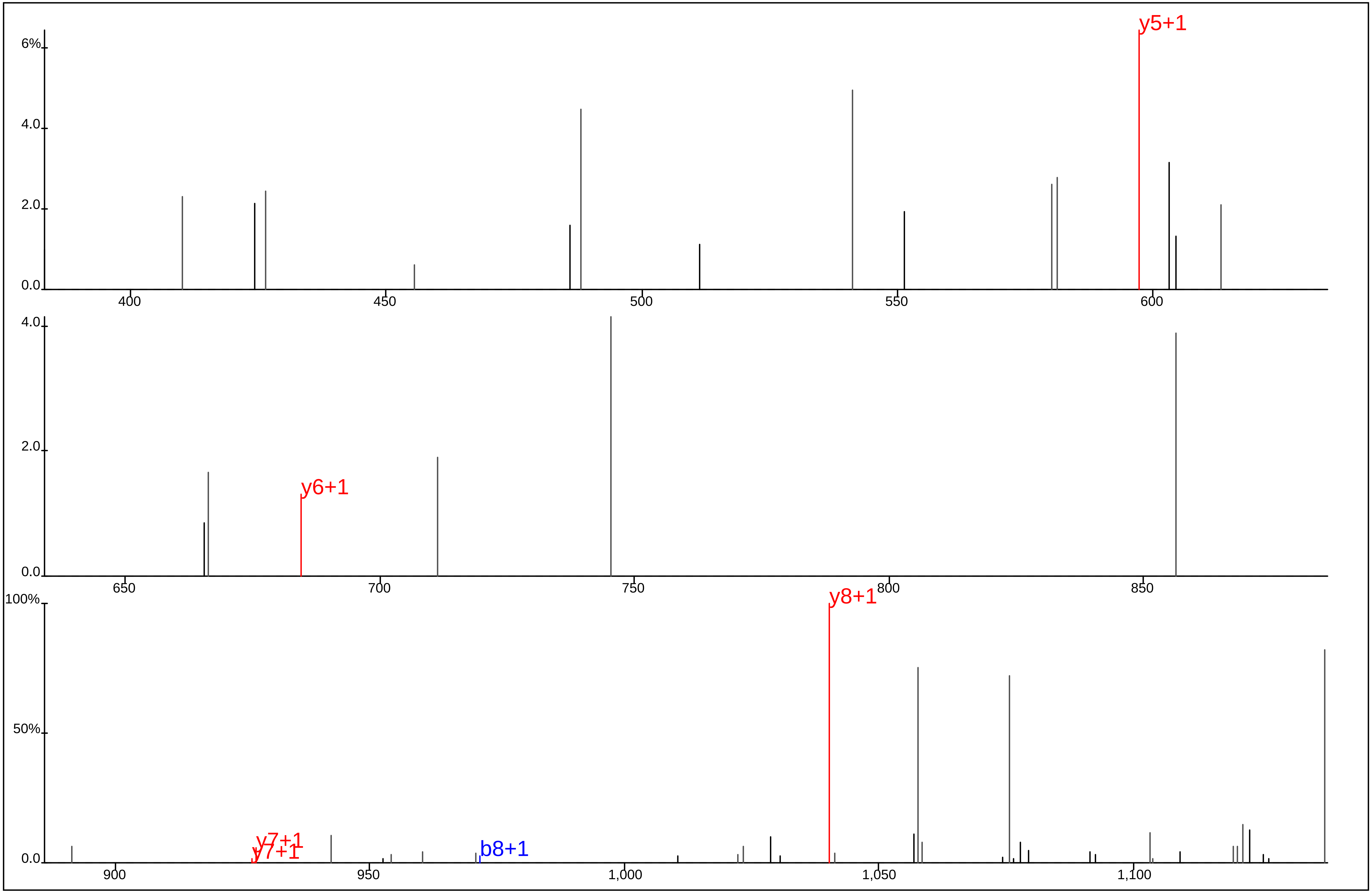

D L Y*S G L N Q R#

Protein Name: CD3 epsilon
gi Accession: 4502671
Precursor Mass: 1,155.4916+1
Delta Mass: 13.302 ppm
XCorr: 1.7836

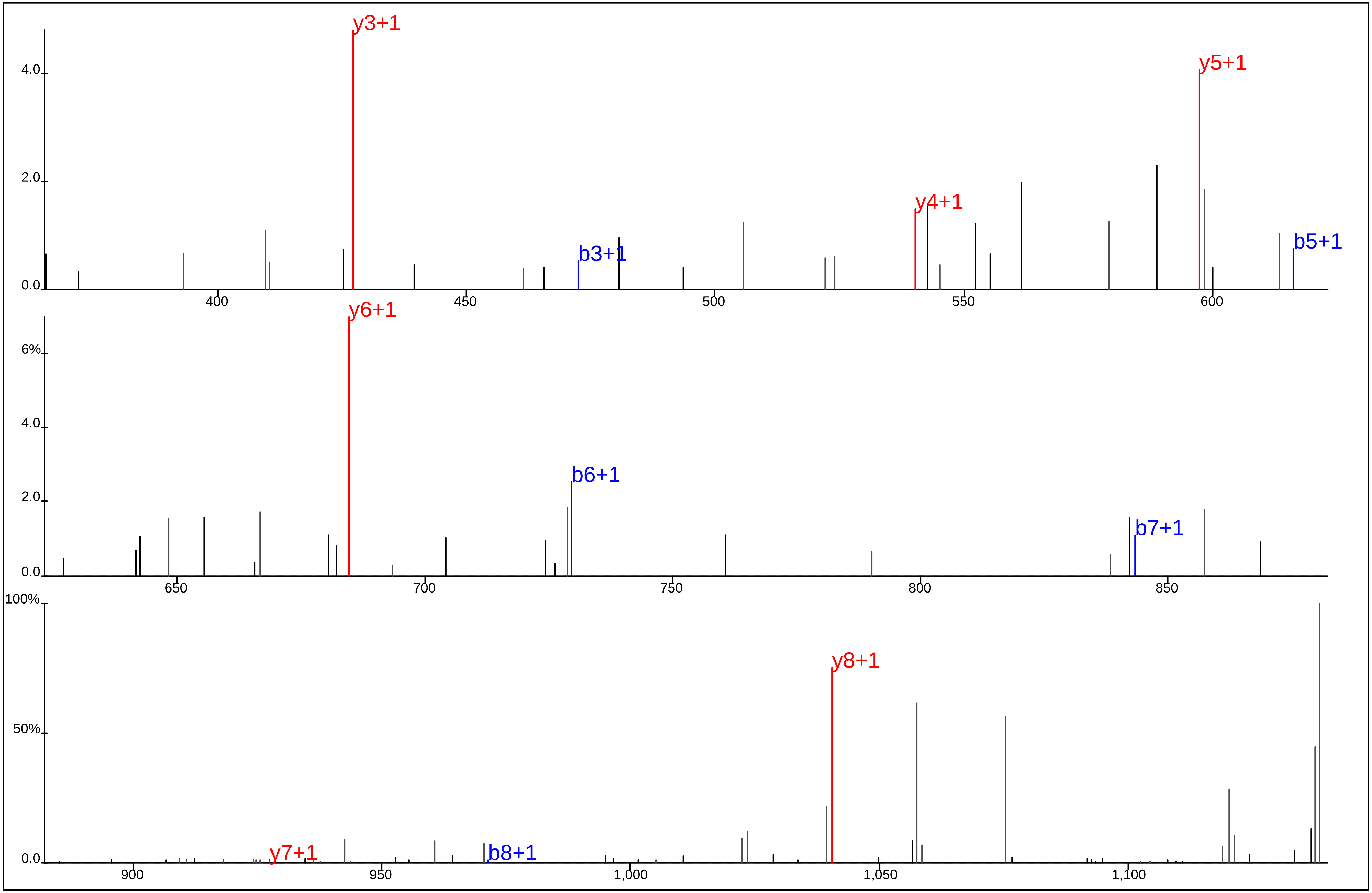

D L Y*S G L N Q R%

Protein Name: CD3 epsilon
gi Accession: 4502671
Precursor Mass: 1,155.5138+1
Delta Mass: 5.944 ppm
XCorr: 24.3

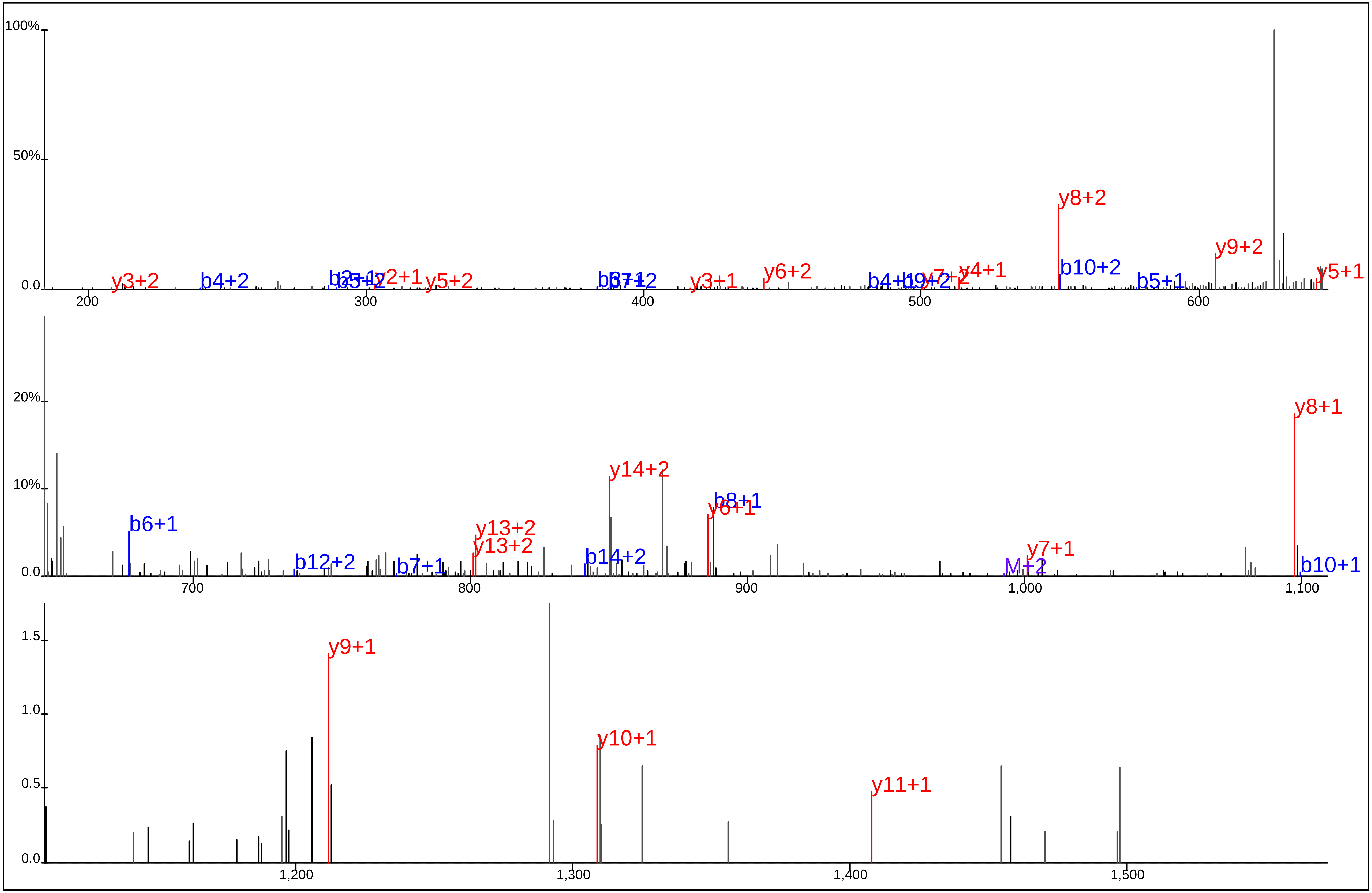

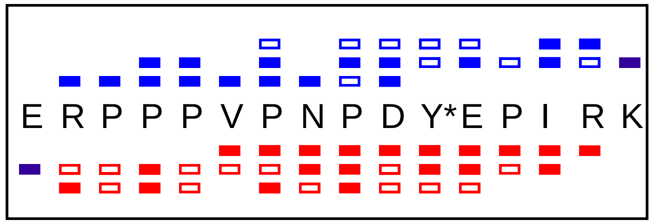

Protein Name: CD3 epsilon
gi Accession: 4502671
Precursor Mass: 661.9969+3
Delta Mass: 3.794 ppm
XCorr: 36.43

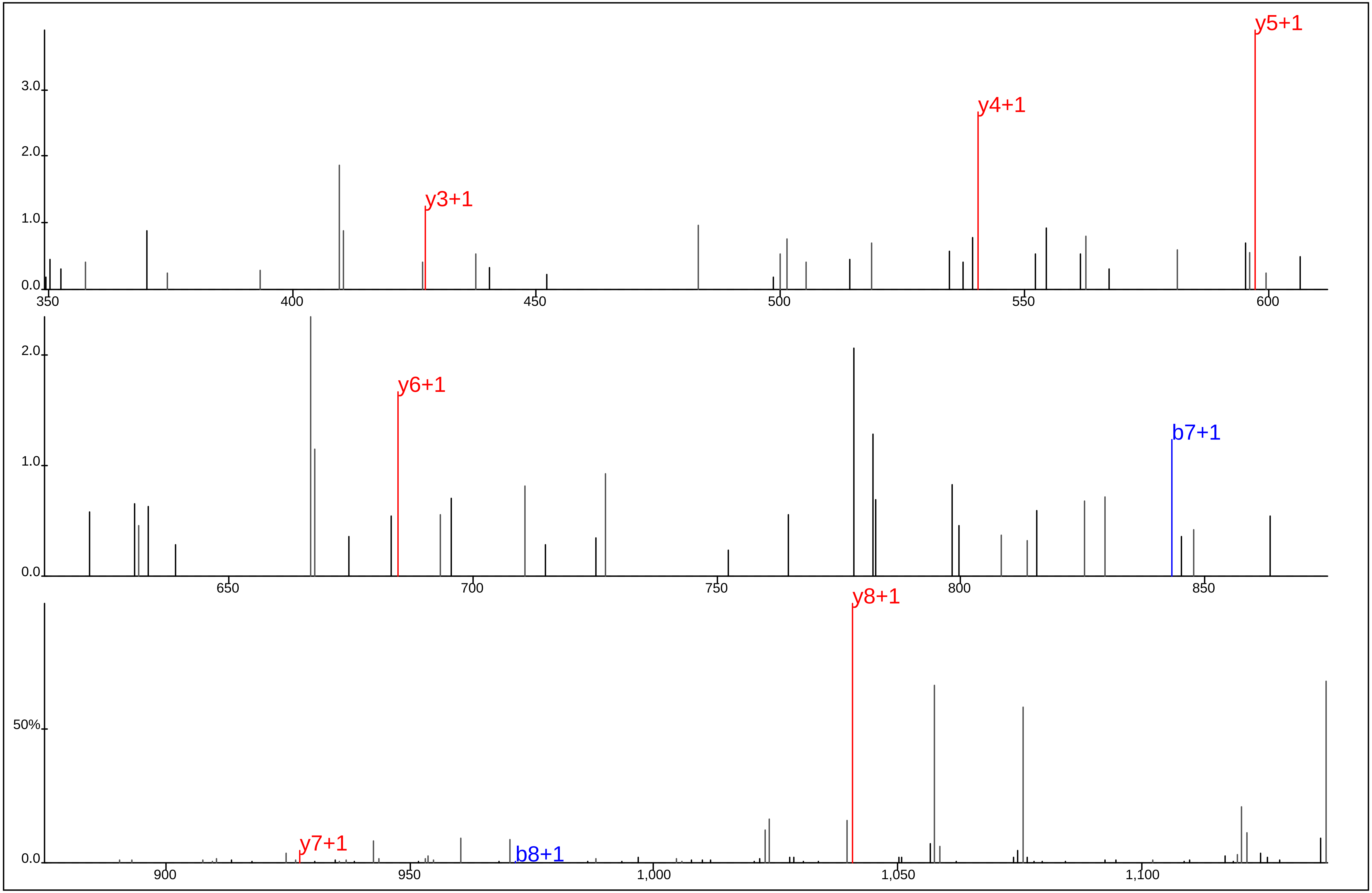

D L Y*S G L N Q R%

Protein Name:	CD3 epsilon
gi Accession:	4502671
Precursor Mass:	1,155.5055+1
Delta Mass:	1.239 ppm
XCorr:	42.06

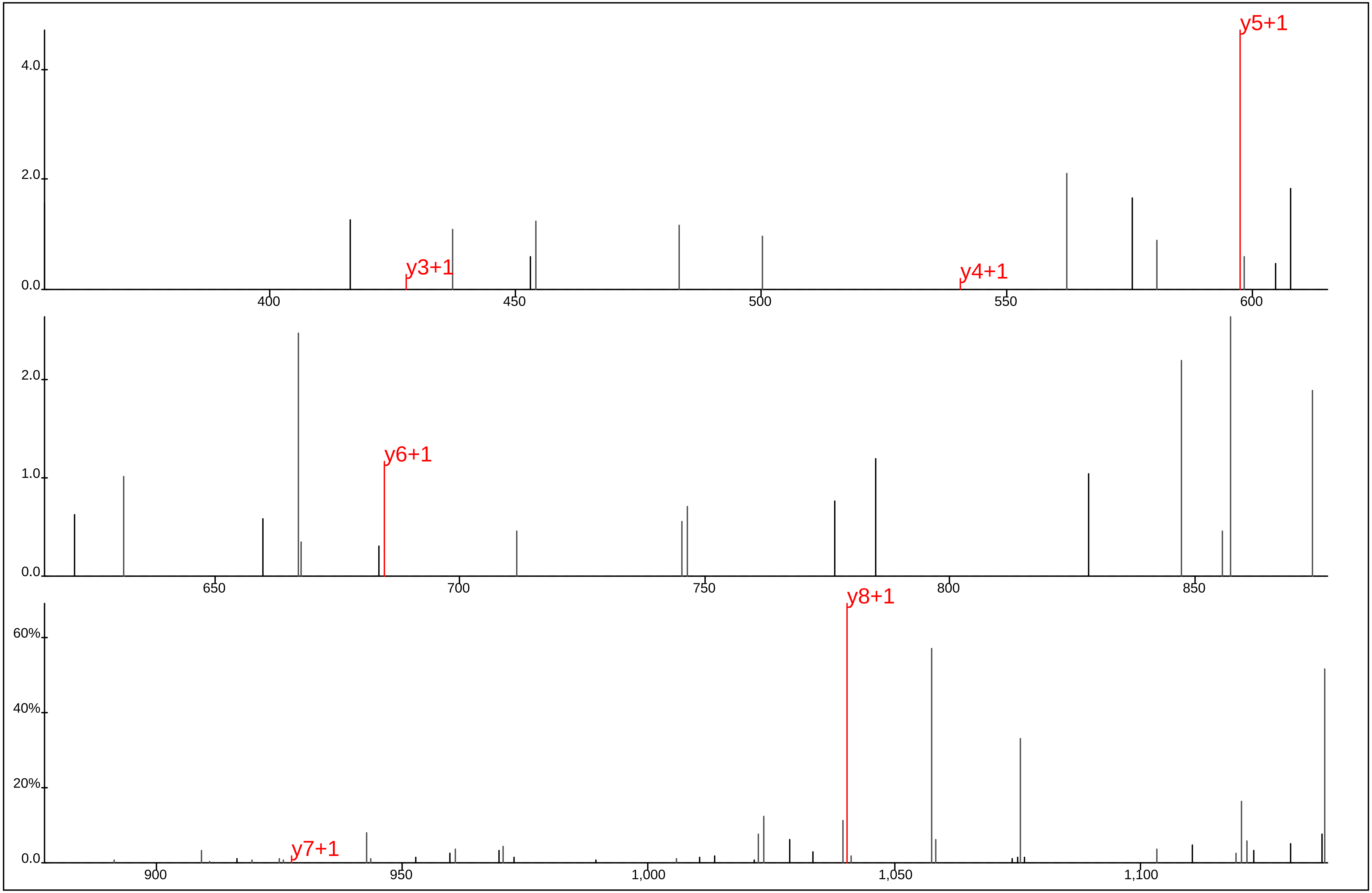

D L Y*S G L N Q R%

Protein Name: CD3 epsilon
gi Accession: 4502671
Precursor Mass: 1,155.5094+1
Delta Mass: 2.145 ppm
XCorr: 20.08

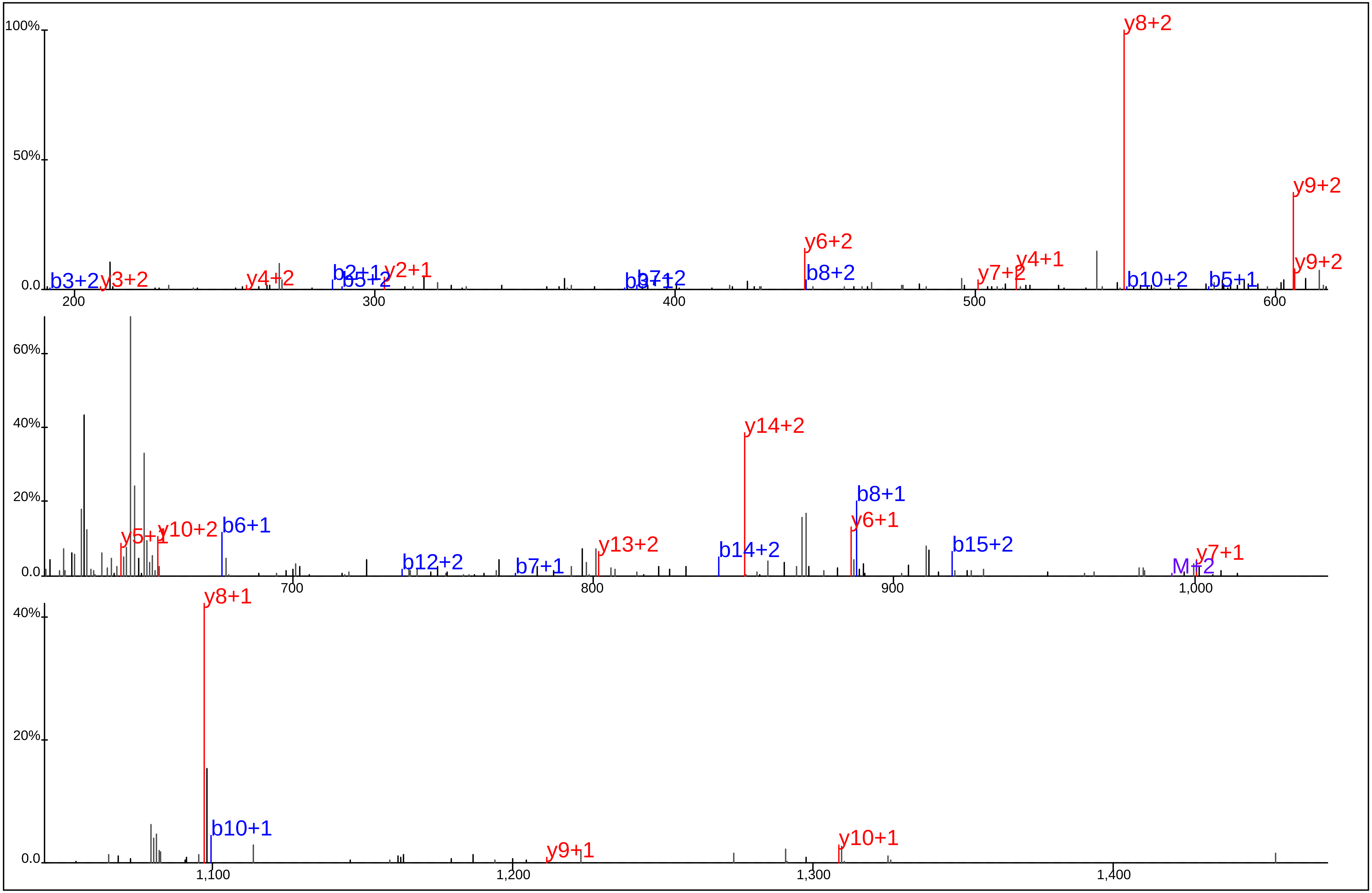

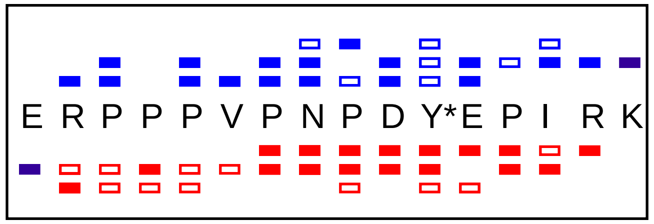

Protein Name: CD3 epsilon
gi Accession: 4502671
Precursor Mass: 661.9952+3
Delta Mass: 1.117 ppm
XCorr: 31.59

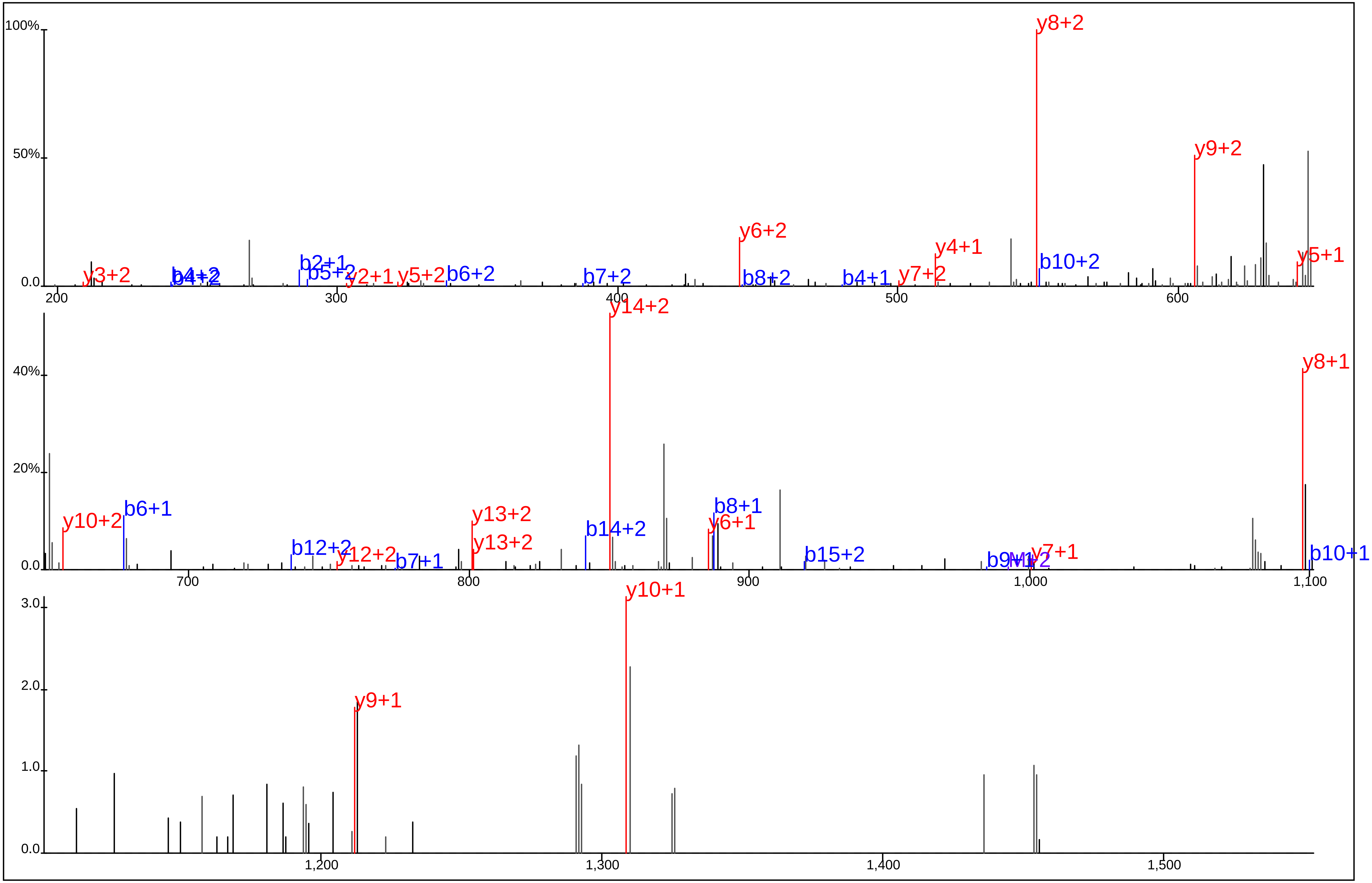

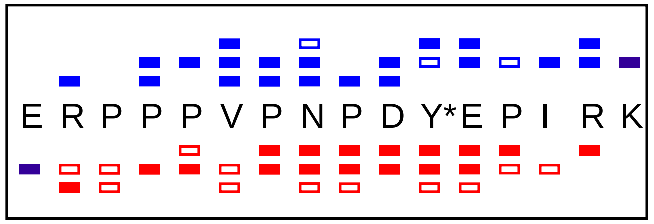

Protein Name: CD3 epsilon
gi Accession: 4502671
Precursor Mass: 661.9977+3
Delta Mass: 4.995 ppm
XCorr: 36.38

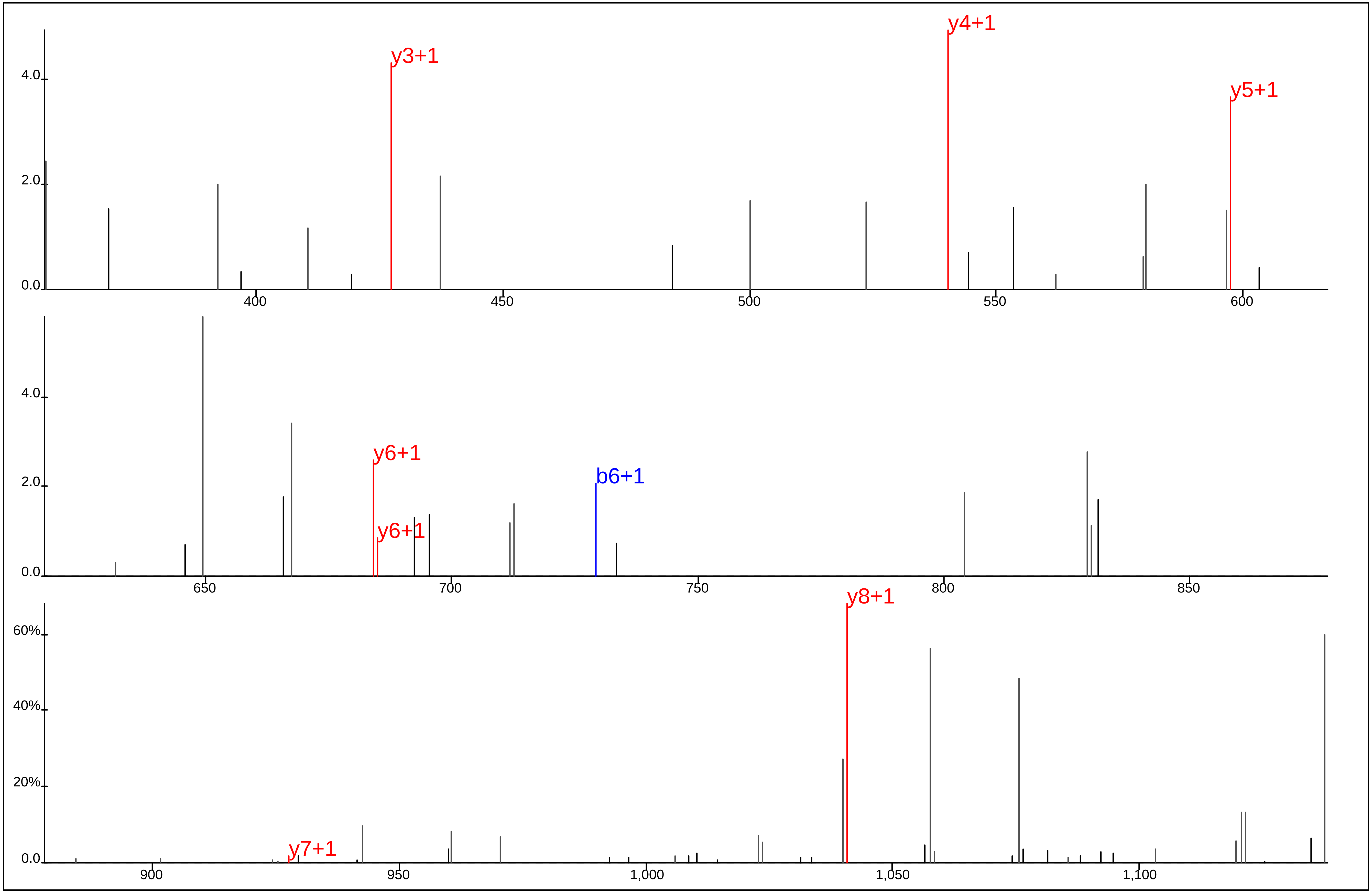

D L Y*S G L N Q R%

Protein Name:	CD3 epsilon
gi Accession:	4502671
Precursor Mass:	1,155.5034+1
Delta Mass:	3.031 ppm
XCorr:	39.24

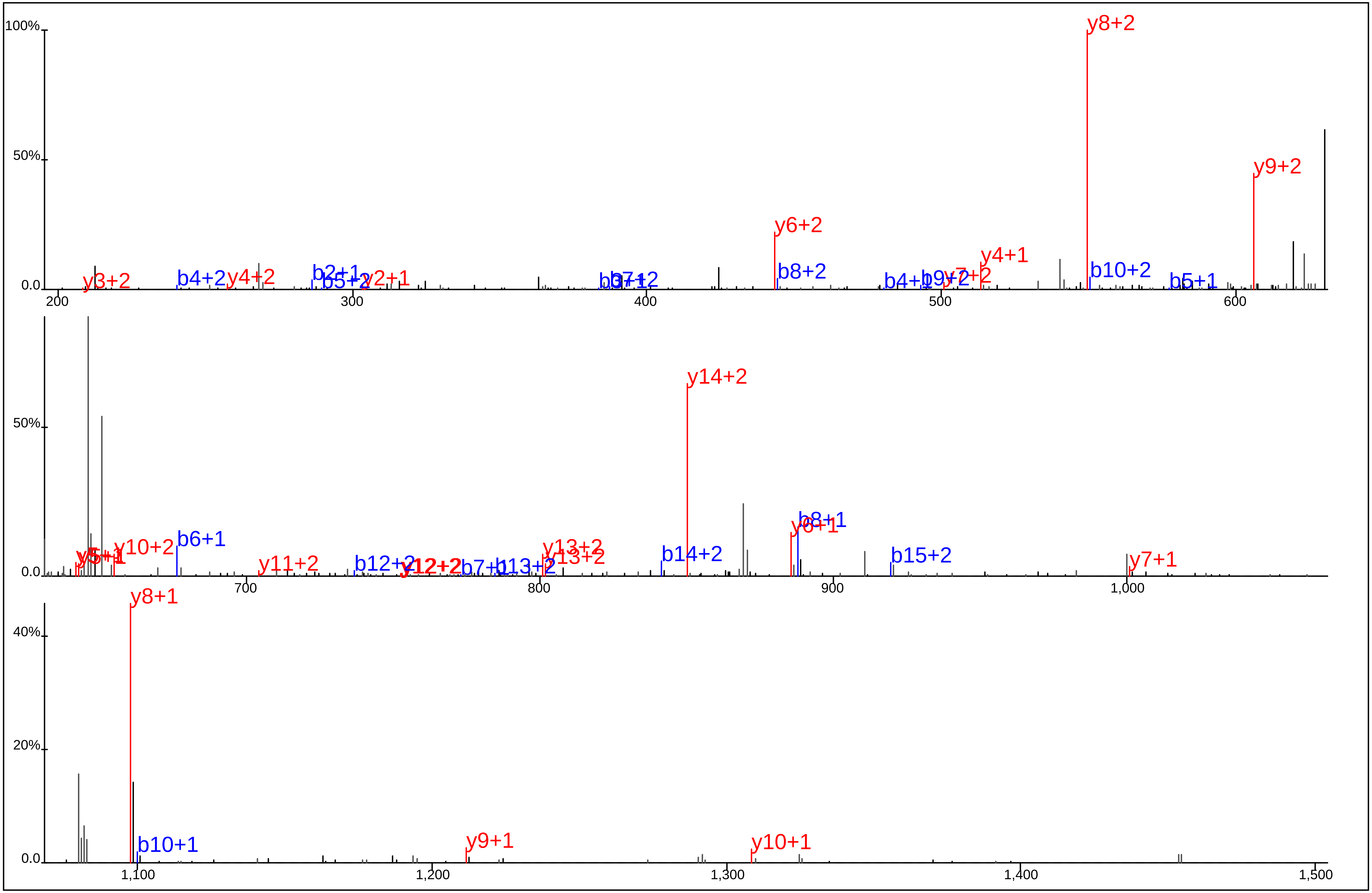

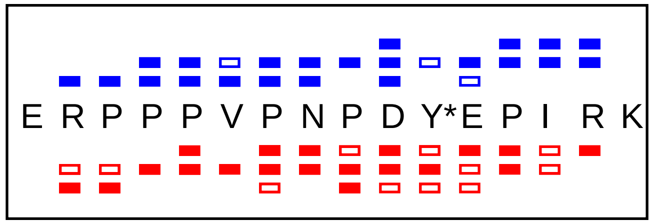

Protein Name: CD3 epsilon
gi Accession: 4502671
Precursor Mass: 661.9951+3
Delta Mass: 0.936 ppm
XCorr: 34.66

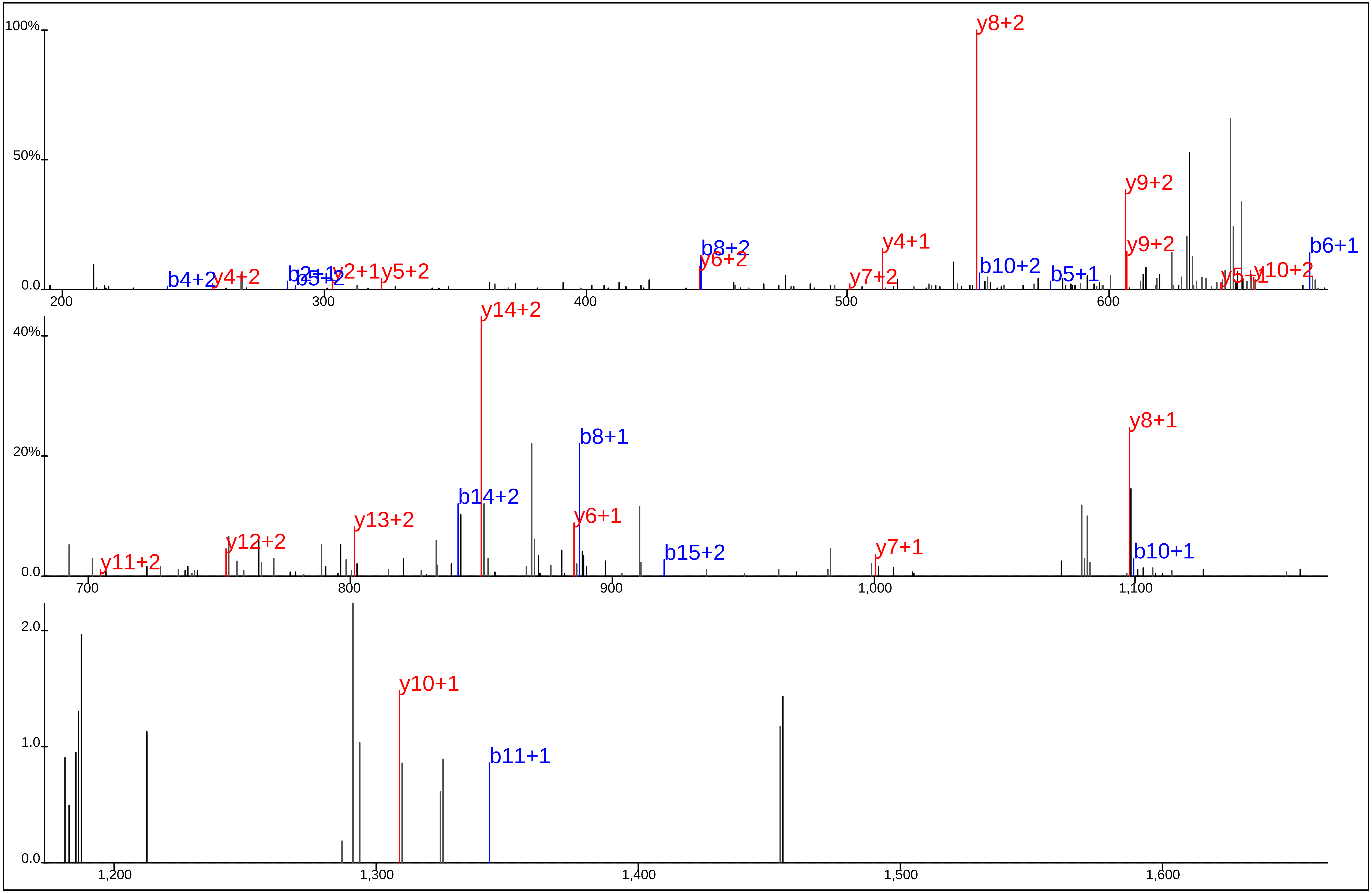

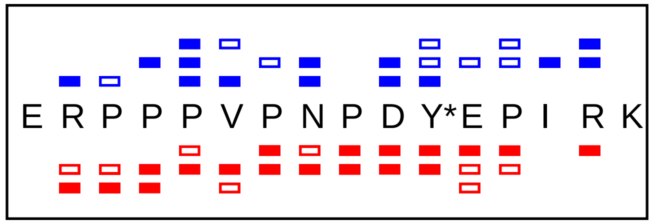

Protein Name: CD3 epsilon
gi Accession: 4502671
Precursor Mass: 661.9974+3
Delta Mass: 4.535 ppm
XCorr: 37.51

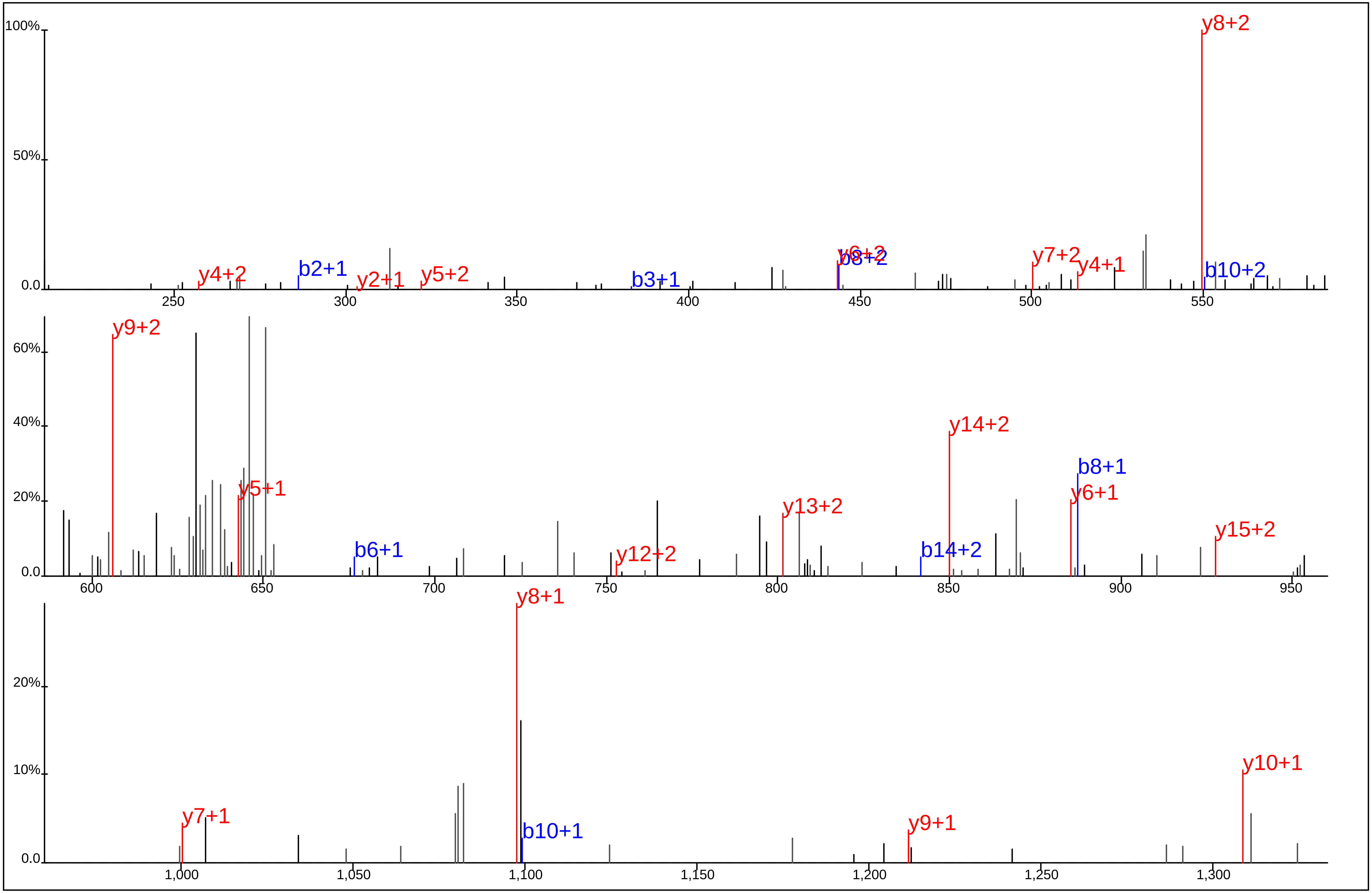

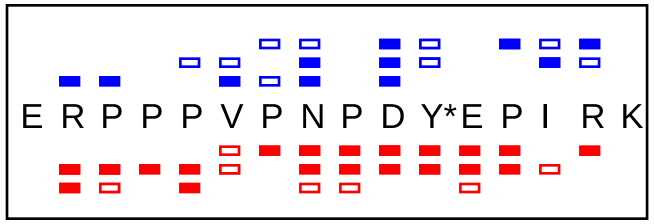

Protein Name:	CD3 epsilon
gi Accession:	4502671
Precursor Mass:	661.9993+3
Delta Mass:	7.393 ppm
XCorr:	40.15

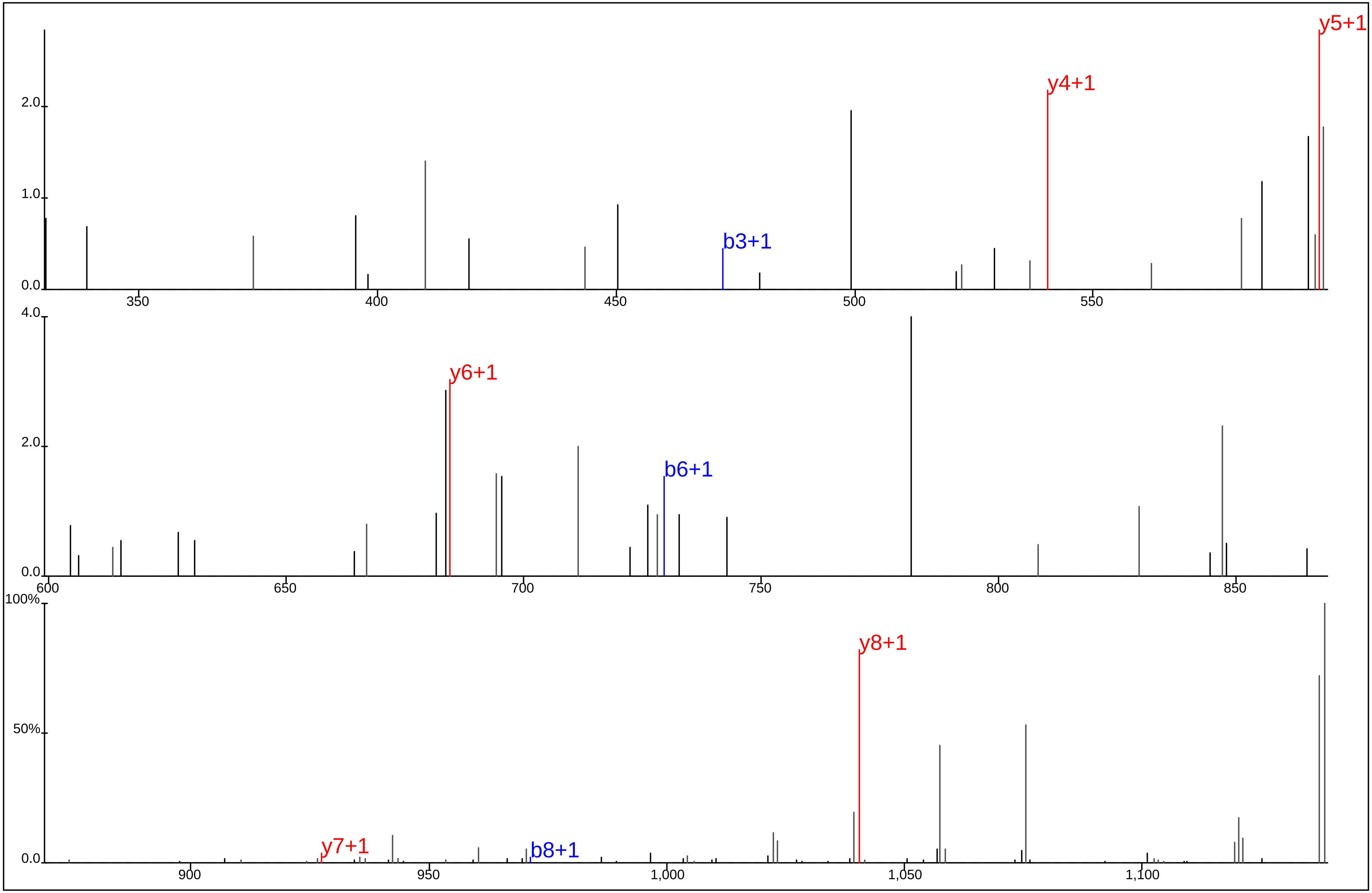

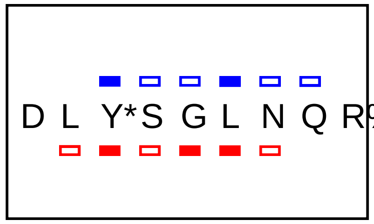

Protein Name: CD3 epsilon
gi Accession: 4502671
Precursor Mass: 1,155.5107+1
Delta Mass: 3.304 ppm
XCorr: 28.21

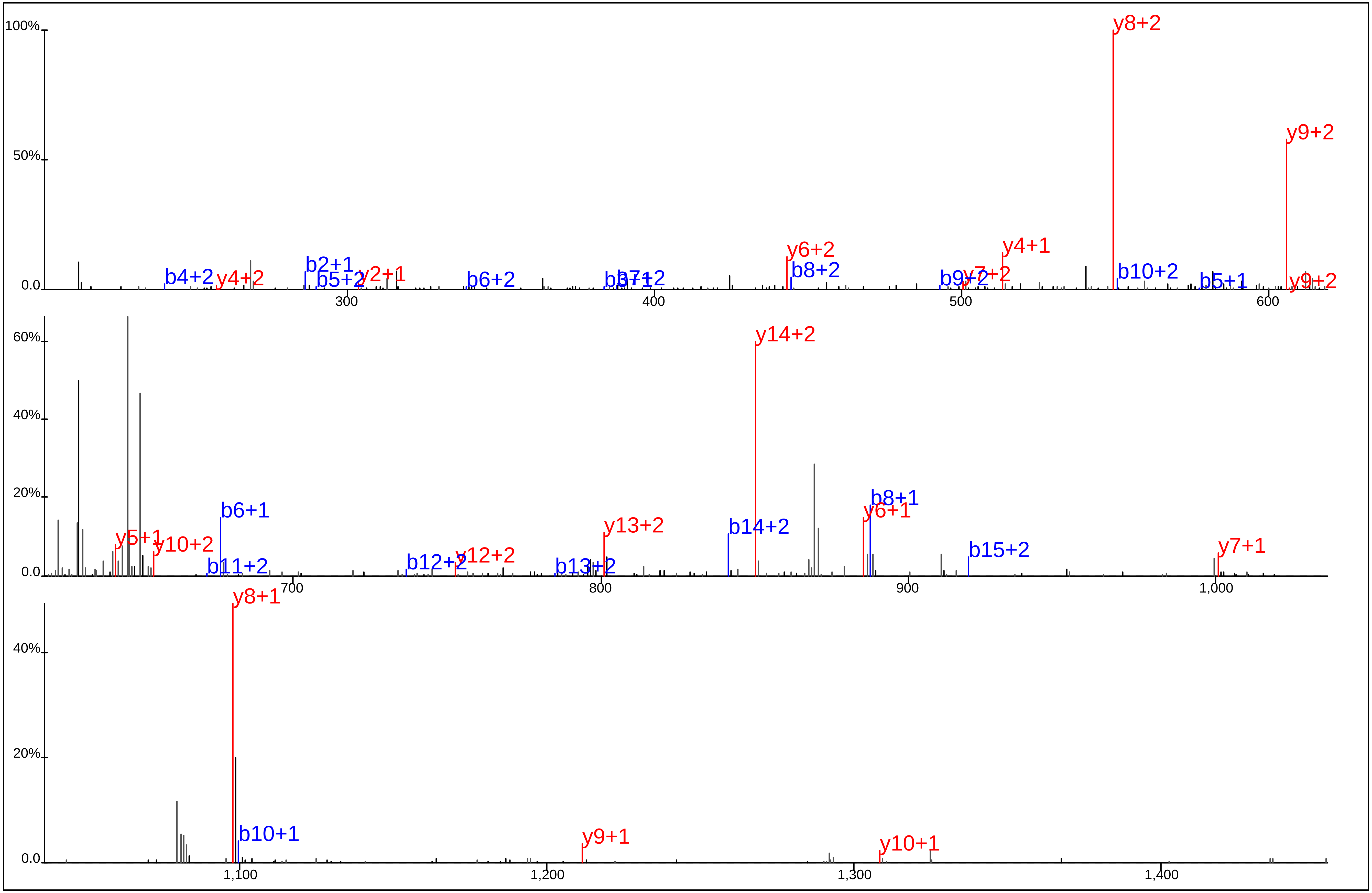

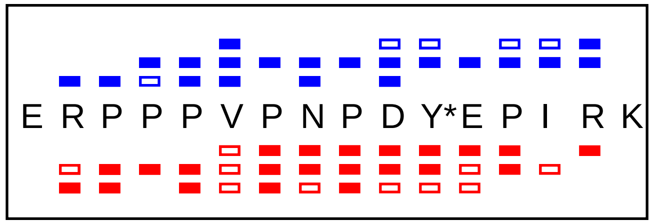

Protein Name:	CD3 epsilon
gi Accession:	4502671
Precursor Mass:	661.9959+3
Delta Mass:	2.227 ppm
XCorr:	38.54

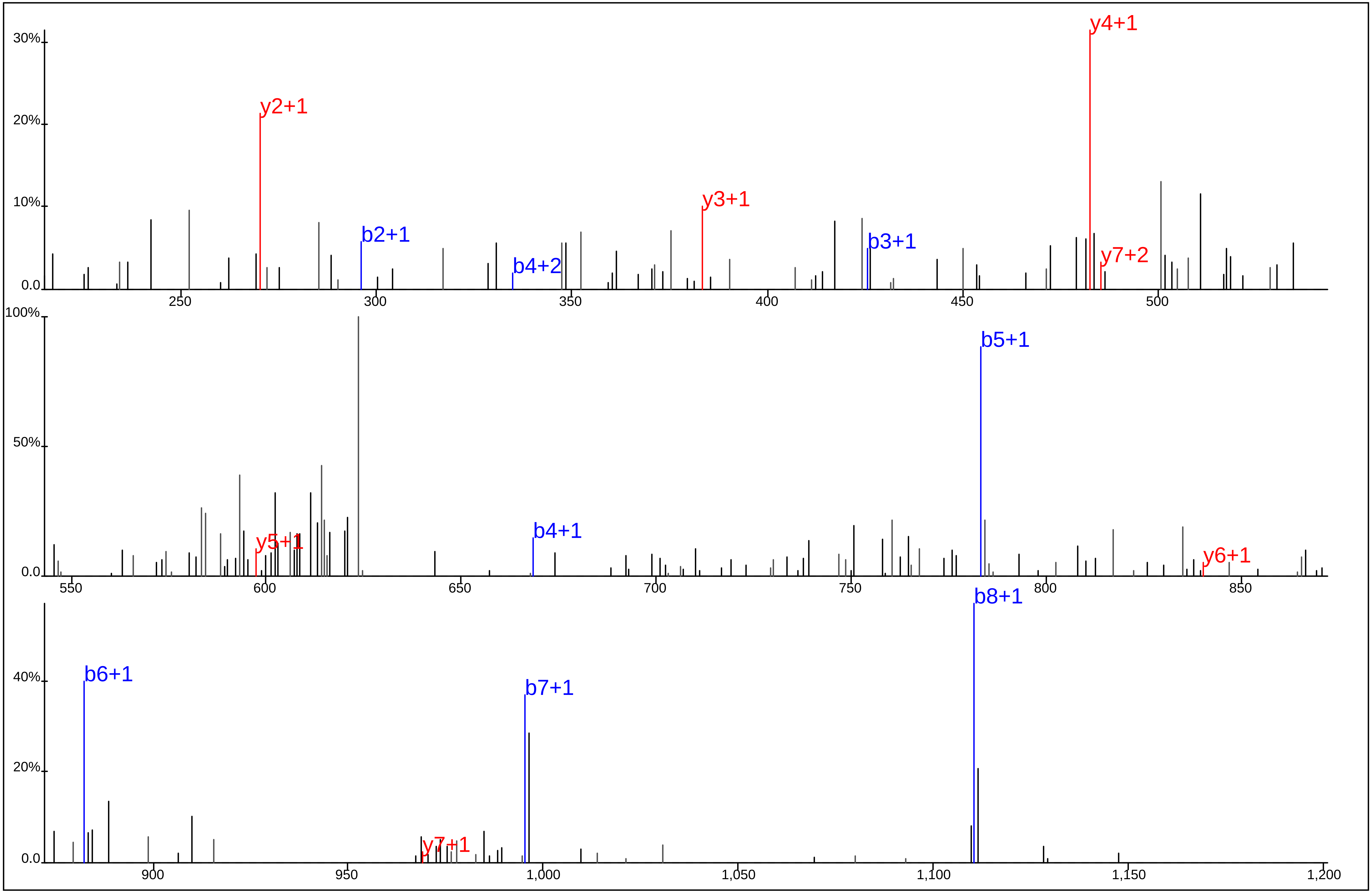

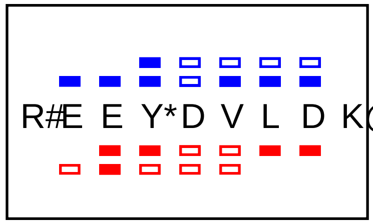

Protein Name:	CD3 zeta
gi Accession:	37595565
Precursor Mass:	632.7835+2
Delta Mass:	1.700 ppm
XCorr:	2.8817

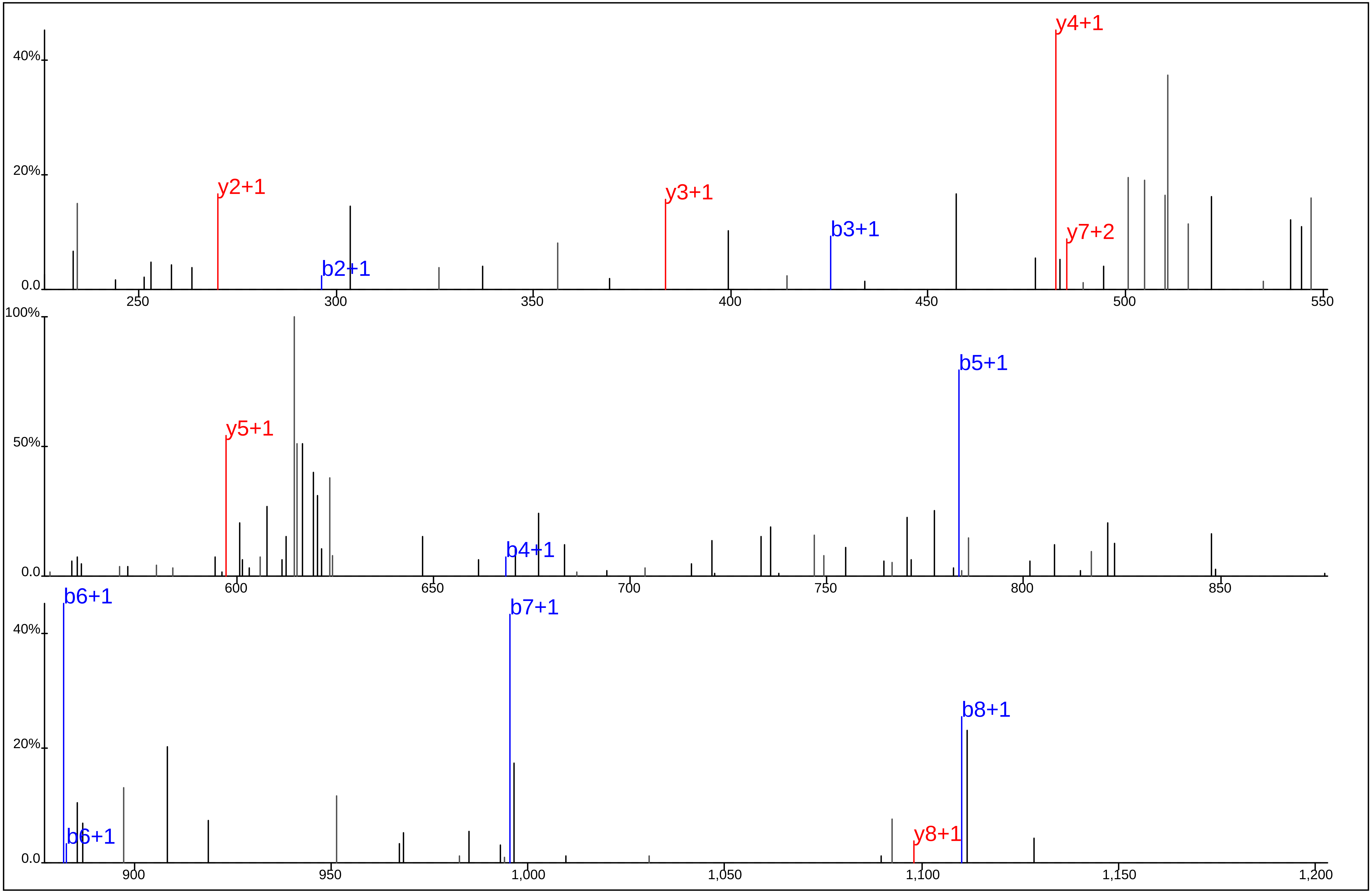

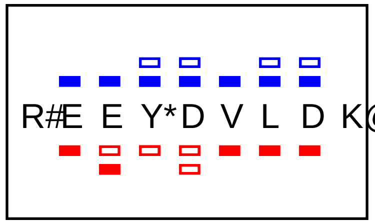

Protein Name: CD3 zeta
gi Accession: 37595565
Precursor Mass: 632.7896+2
Delta Mass: 11.356 ppm
XCorr: 2.0799

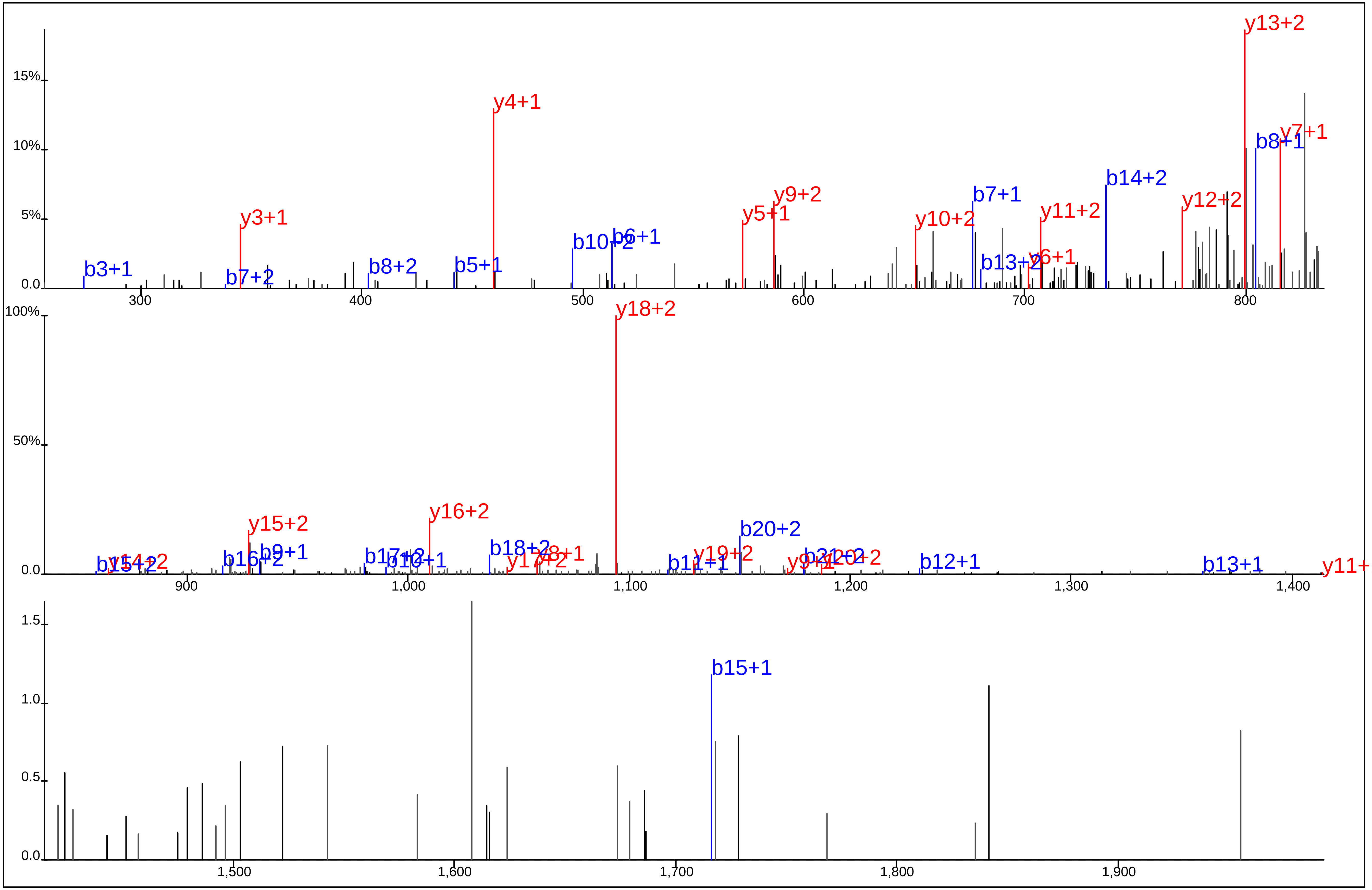

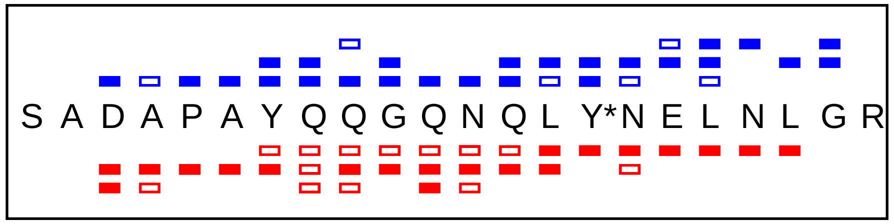

Protein Name: CD3 zeta
gi Accession: 37595565
Precursor Mass: 844.0528+3
Delta Mass: 3.185 ppm
XCorr: 56.68

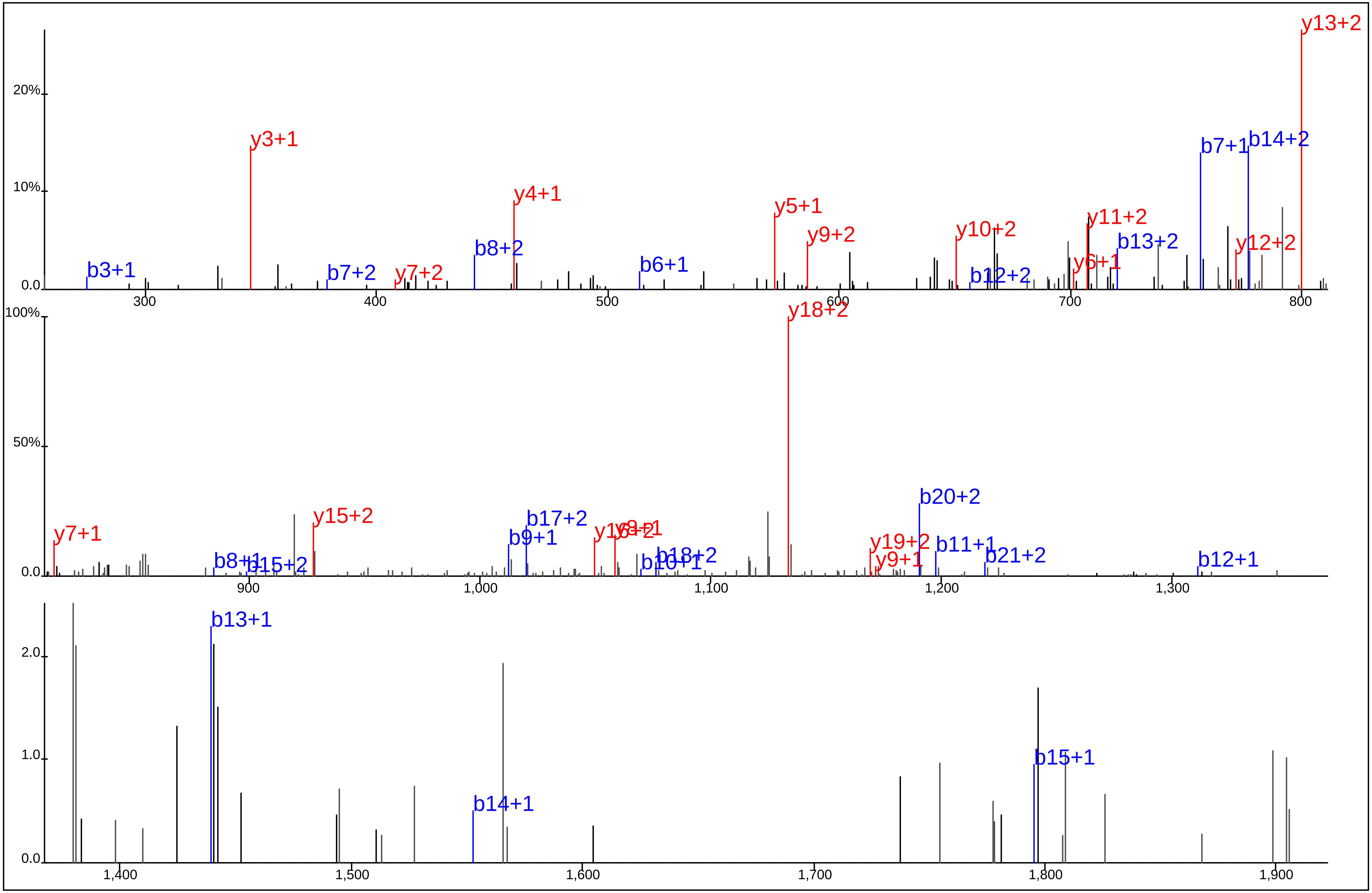

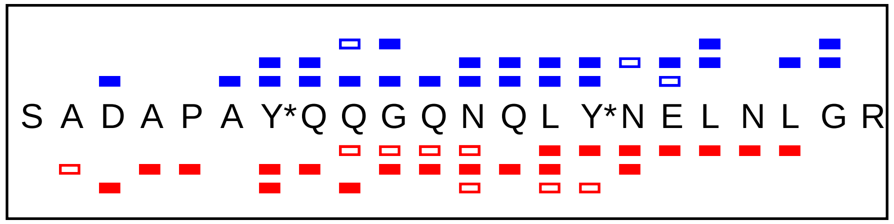

Protein Name:	CD3 zeta
gi Accession:	37595565
Precursor Mass:	870.7092+3
Delta Mass:	4.160 ppm
XCorr:	50.64

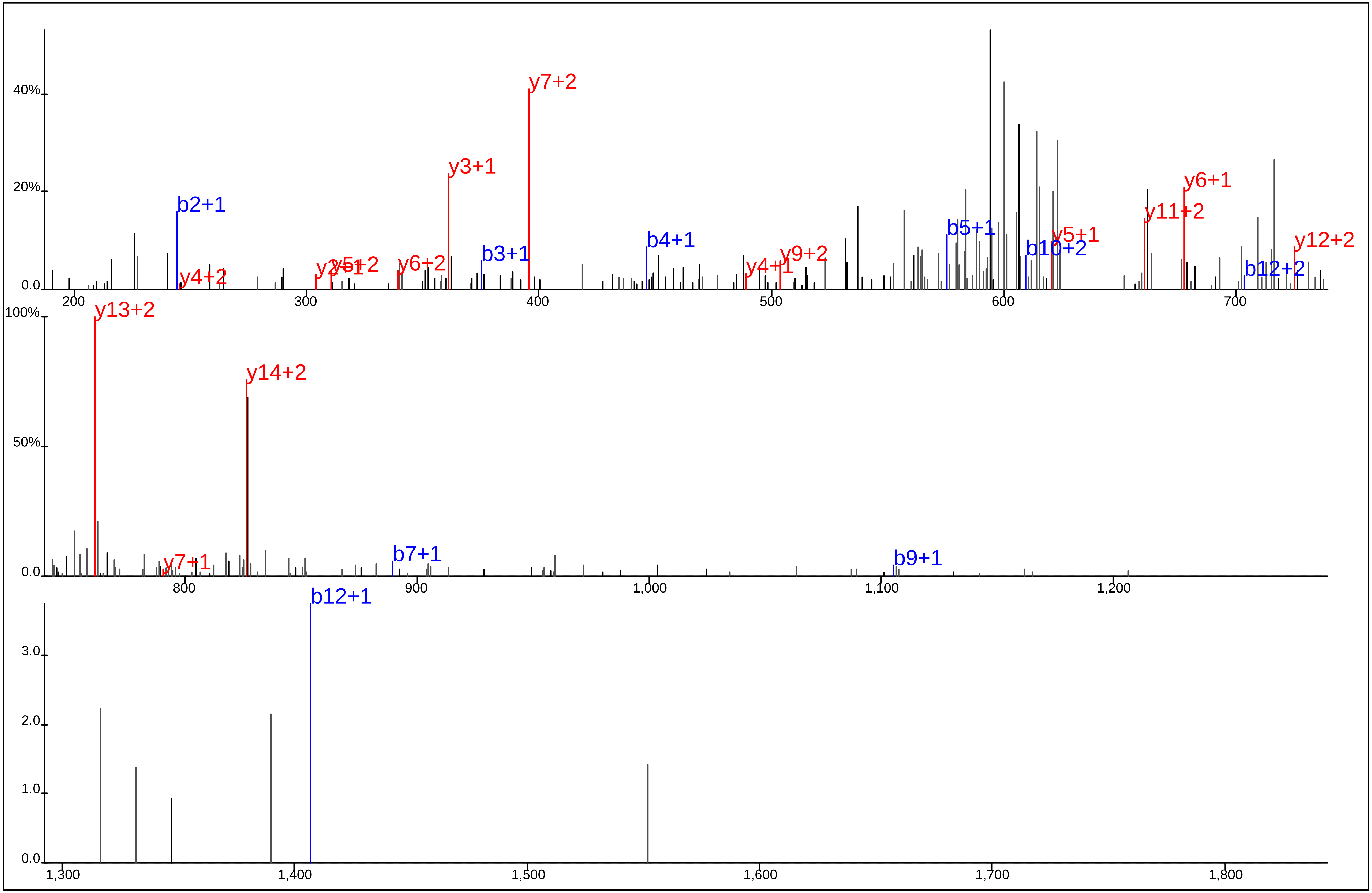

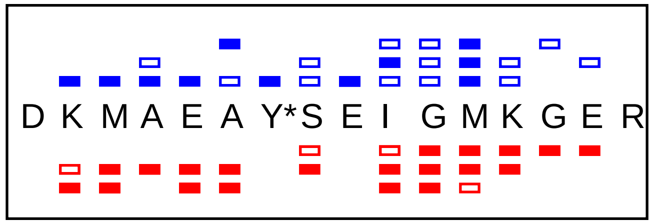

Protein Name:	CD3 zeta
gi Accession:	37595565
Precursor Mass:	632.2758+3
Delta Mass:	2.896 ppm
XCorr:	17.07

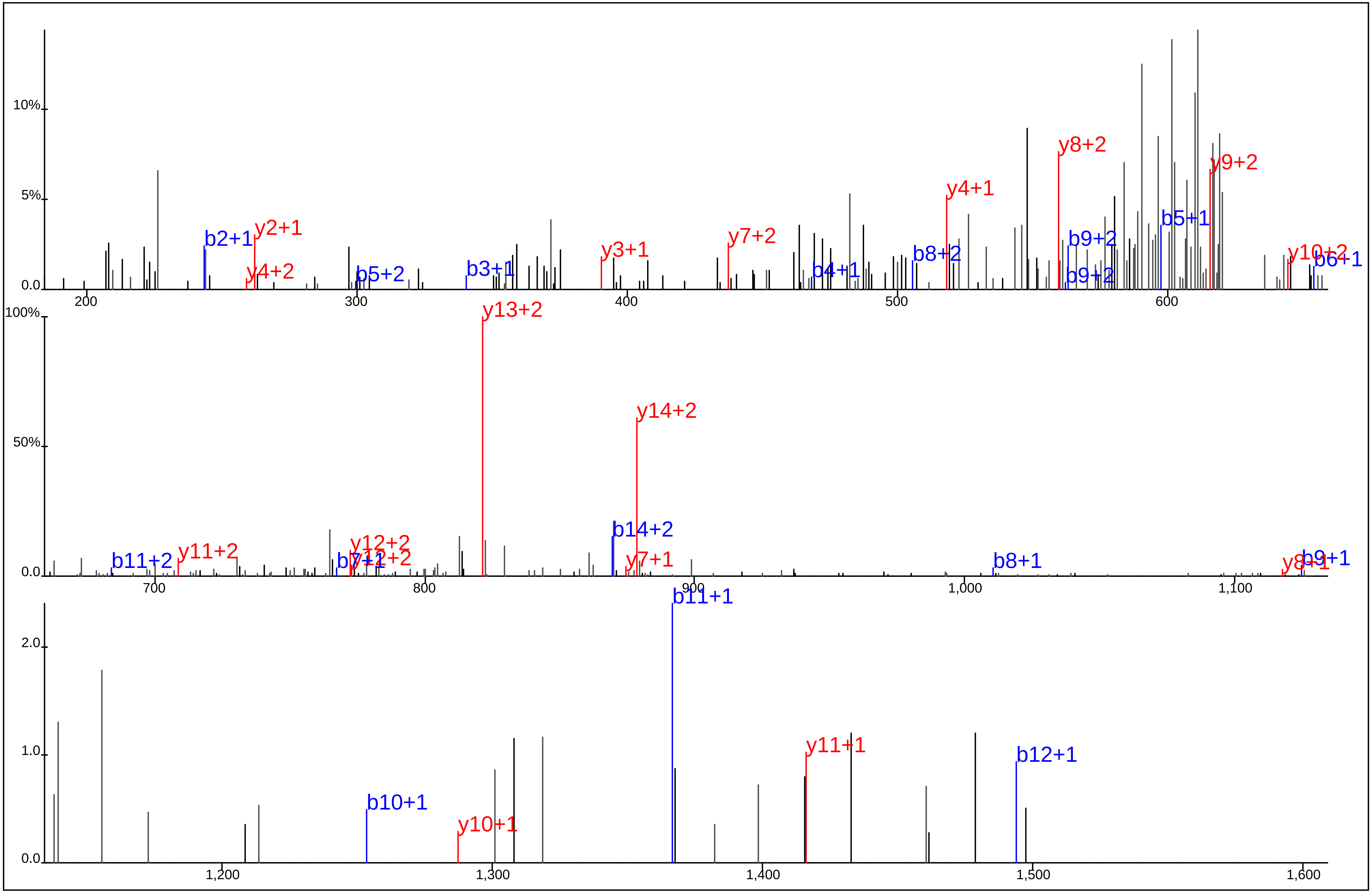

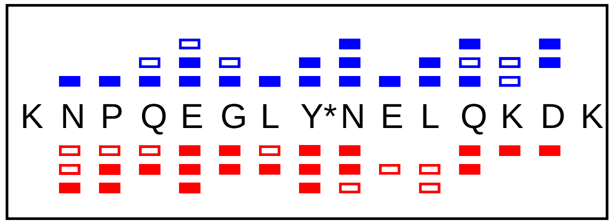

Protein Name:	CD3 zeta
gi Accession:	37595565
Precursor Mass:	628.6352+3
Delta Mass:	0.636 ppm
XCorr:	29.66

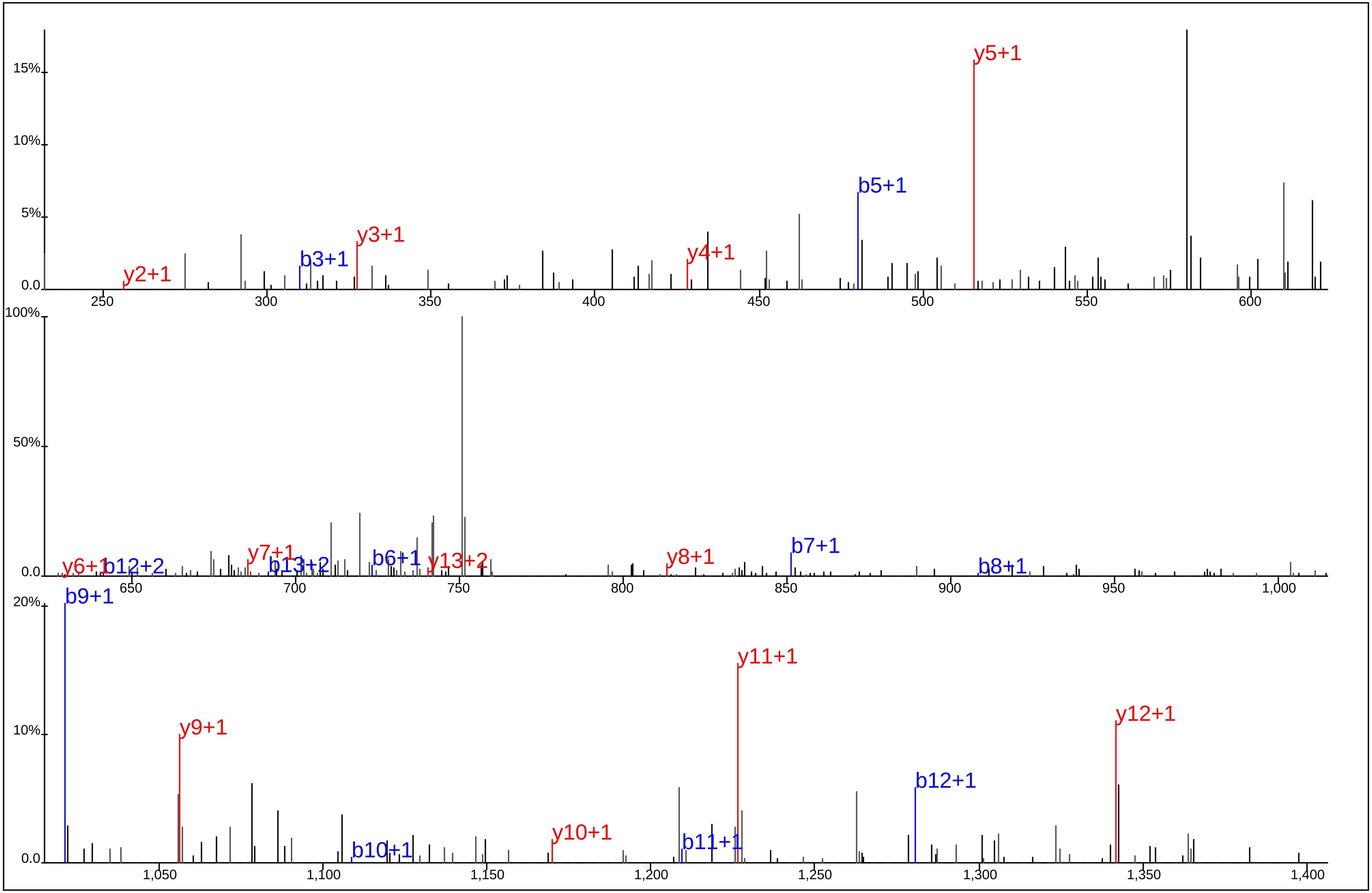

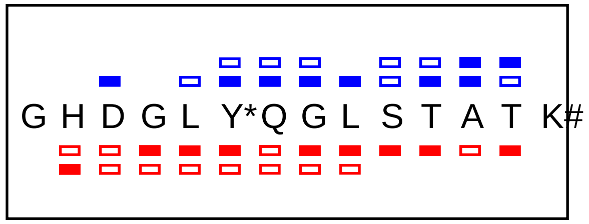

Protein Name: CD3 zeta
gi Accession: 37595565
Precursor Mass: 768.3516+2
Delta Mass: 1.401 ppm
XCorr: 29.54

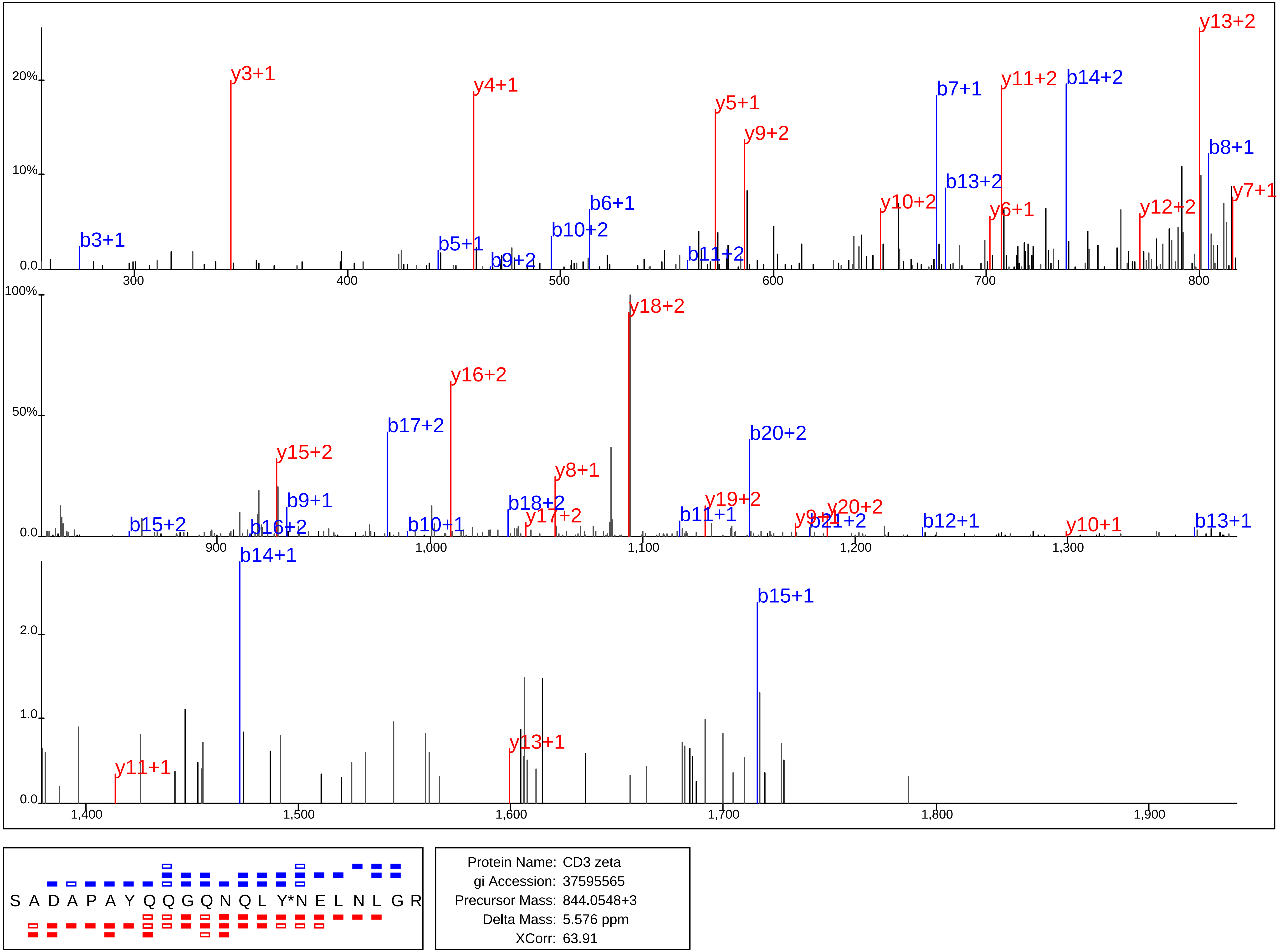

Protein Name: CD3 zeta
gi Accession: 37595565
Precursor Mass: 844.0548+3
Delta Mass: 5.576 ppm
XCorr: 63.91

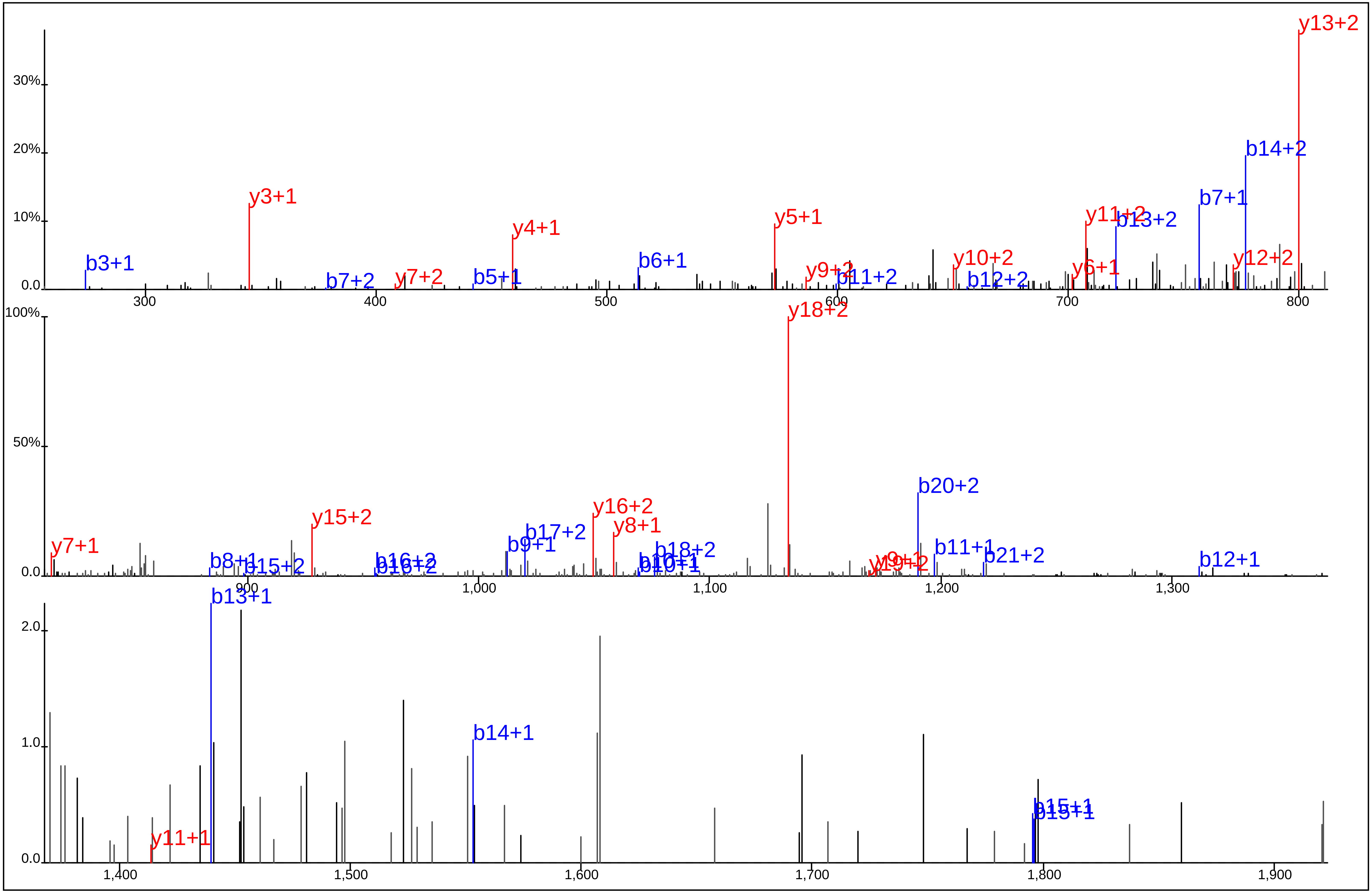

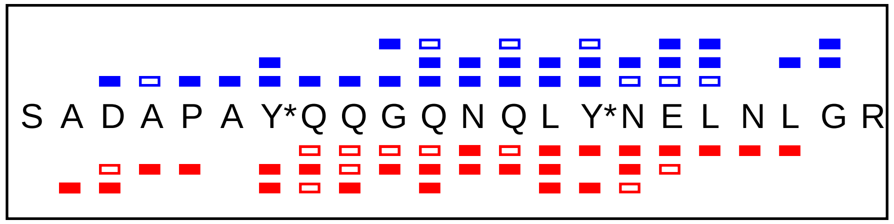

Protein Name:	CD3 zeta
gi Accession:	37595565
Precursor Mass:	870.7111+3
Delta Mass:	6.405 ppm
XCorr:	53.97

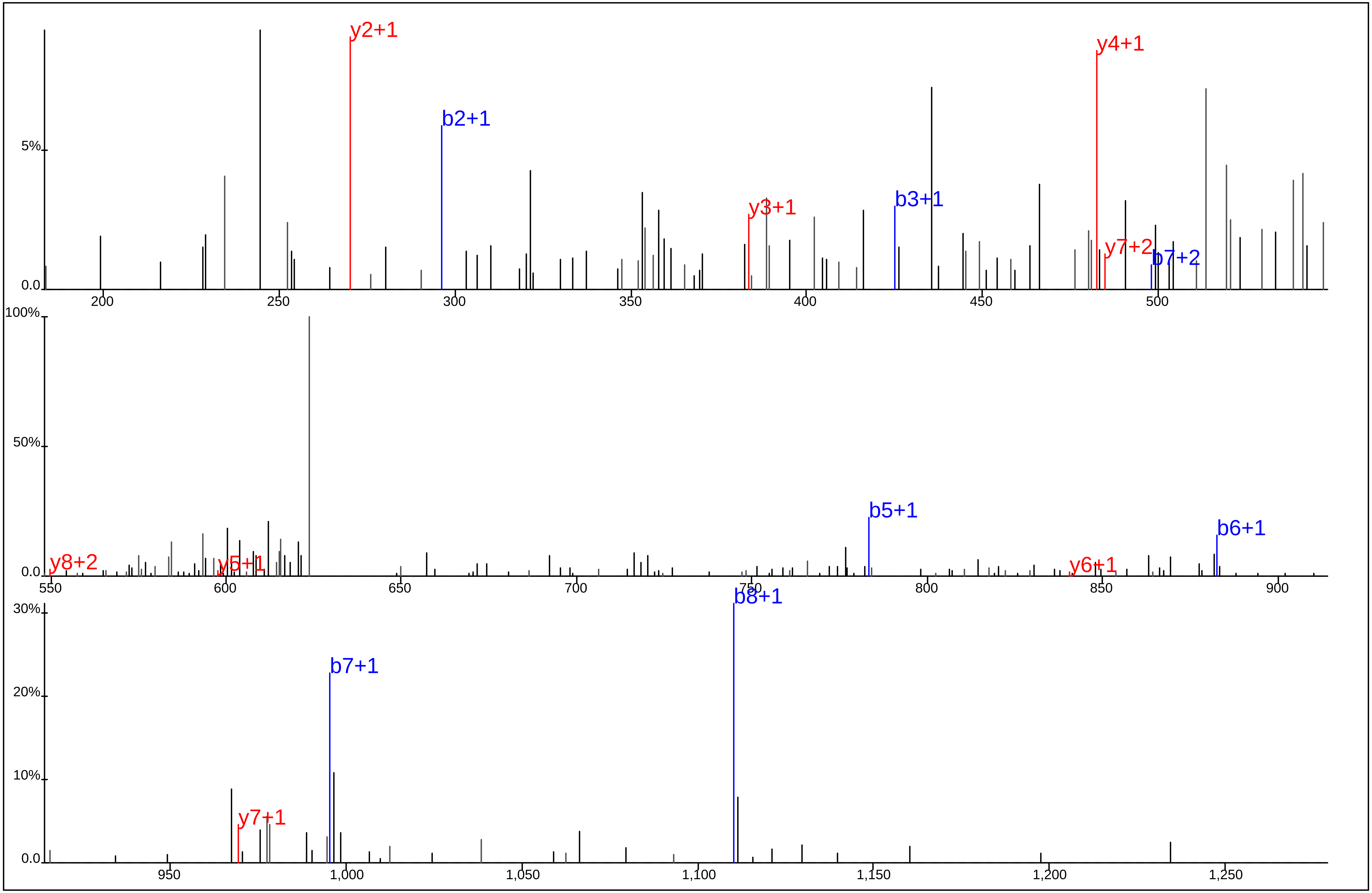

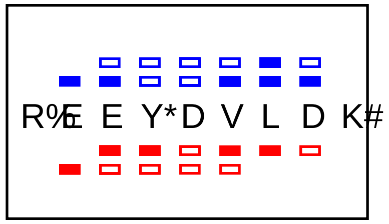

Protein Name: CD3 zeta
gi Accession: 37595565
Precursor Mass: 632.7861+2
Delta Mass: 5.771 ppm
XCorr: 25.79

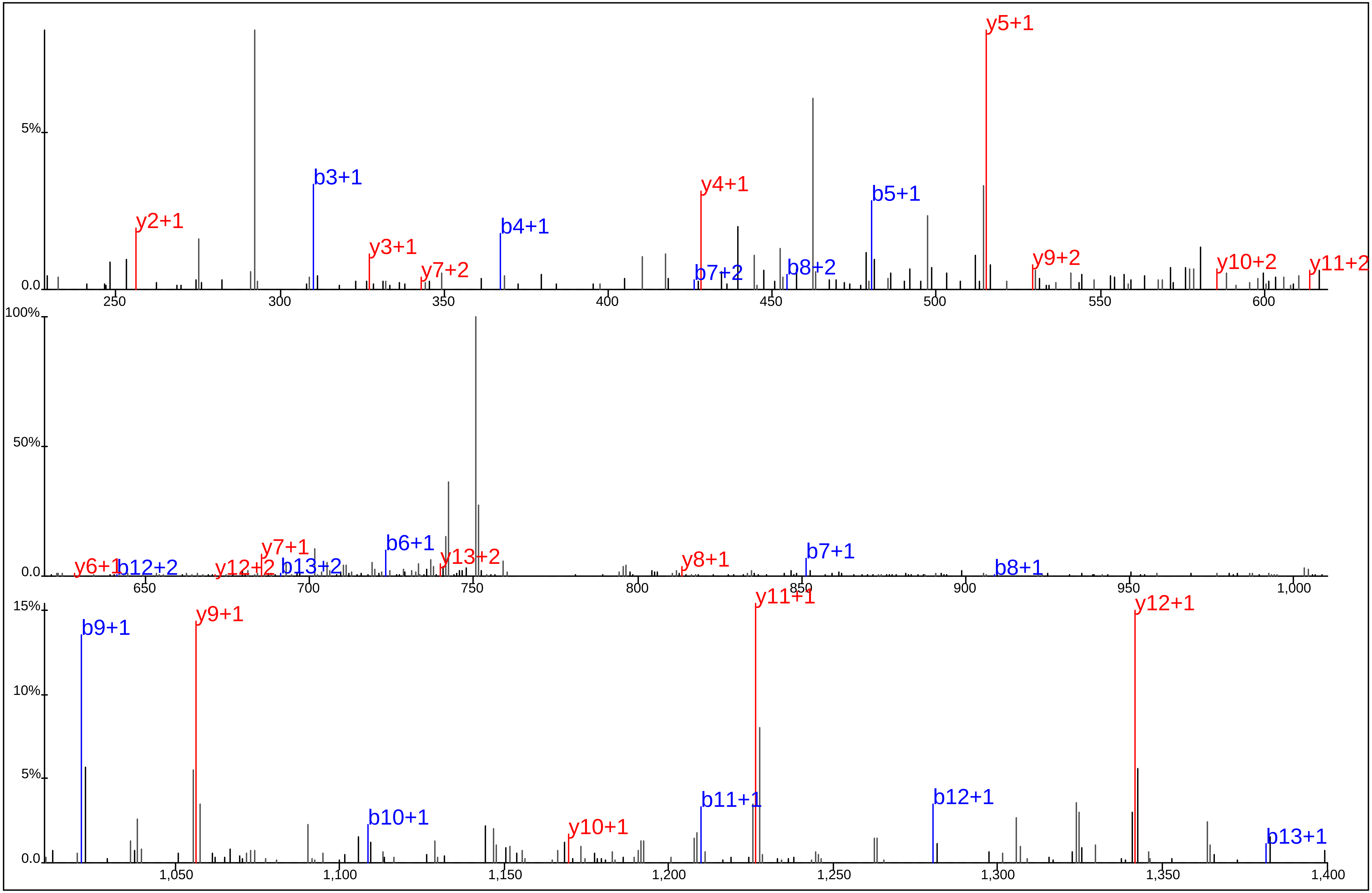

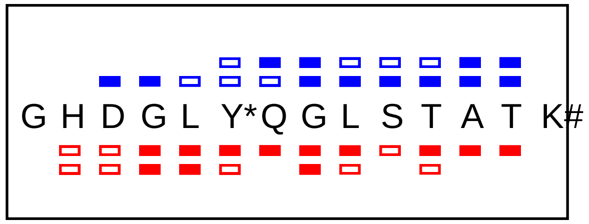

Protein Name: CD3 zeta
gi Accession: 37595565
Precursor Mass: 768.3546+2
Delta Mass: 2.415 ppm
XCorr: 54.59

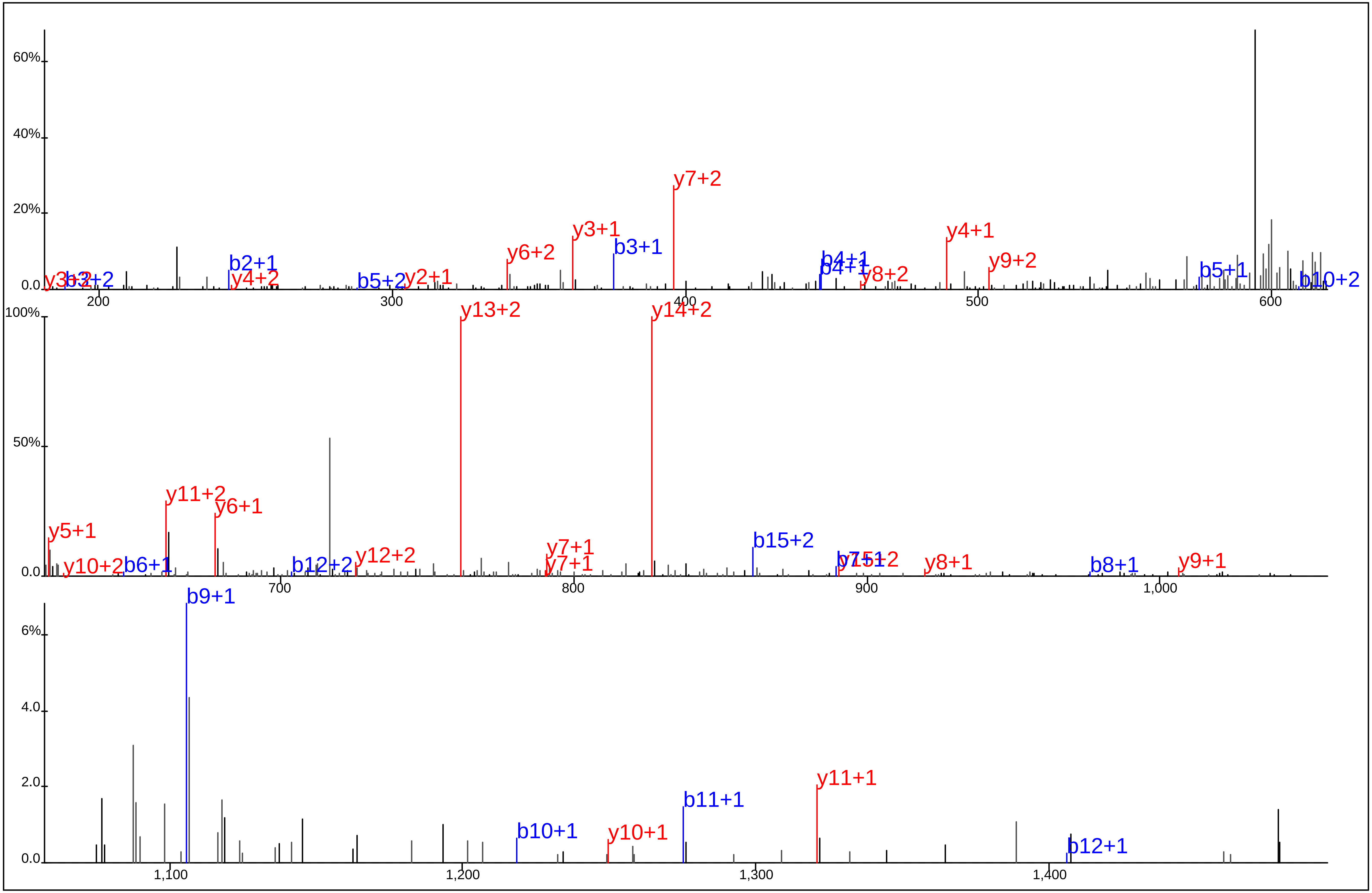

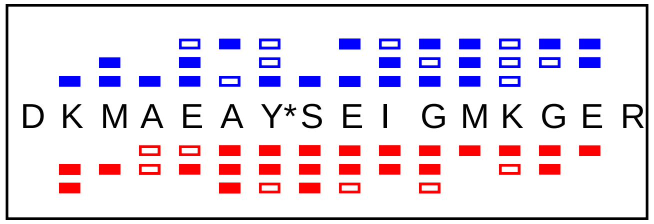

Protein Name: CD3 zeta
gi Accession: 37595565
Precursor Mass: 632.2749+3
Delta Mass: 1.446 ppm
XCorr: 51.3

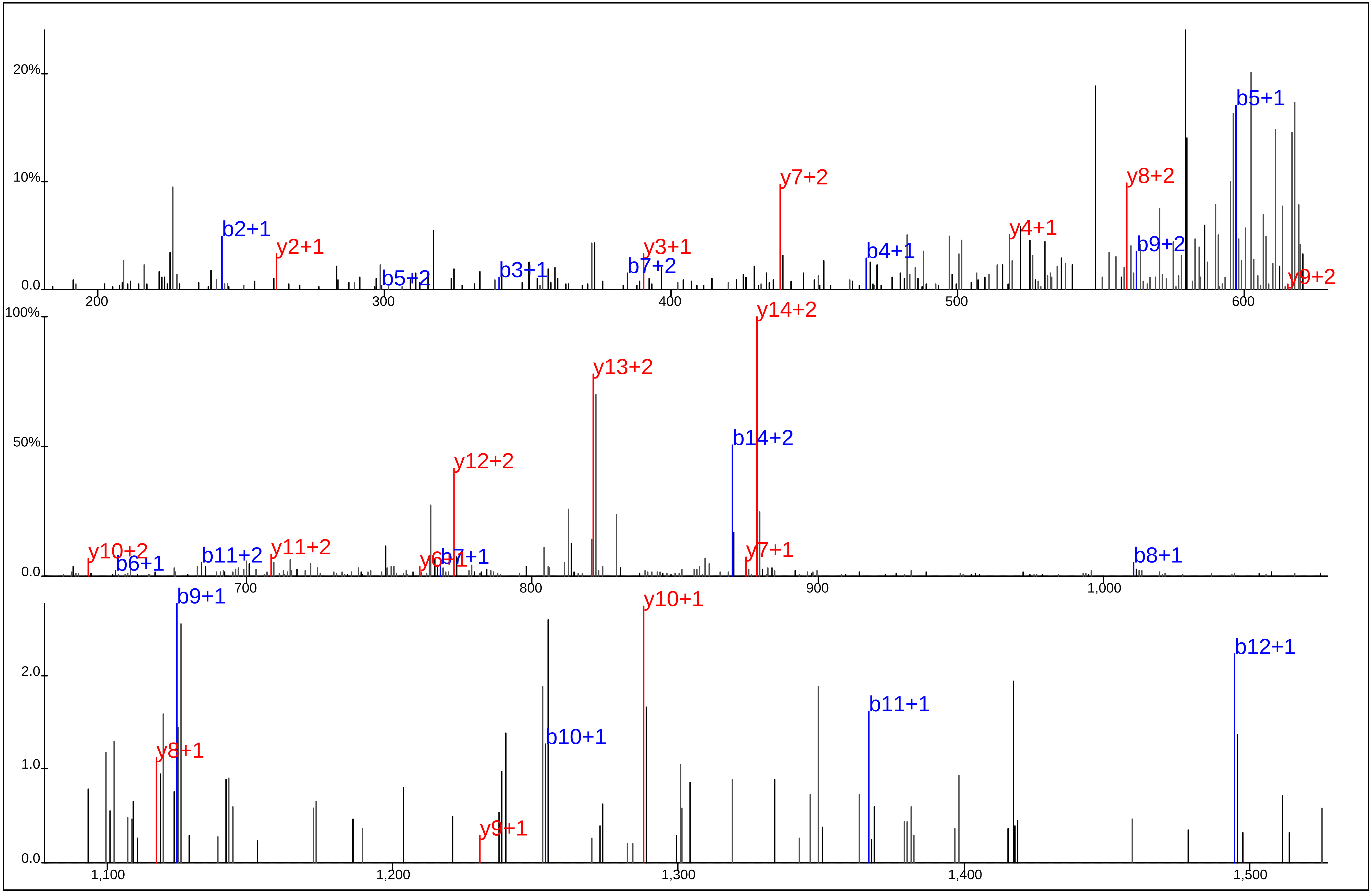

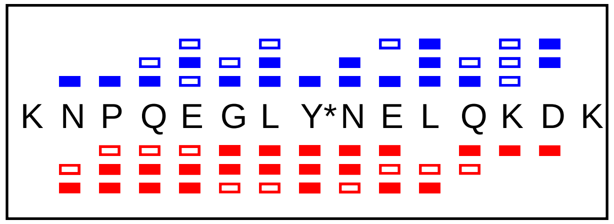

Protein Name: CD3 zeta
gi Accession: 37595565
Precursor Mass: 628.6361+3
Delta Mass: 2.090 ppm
XCorr: 38.67

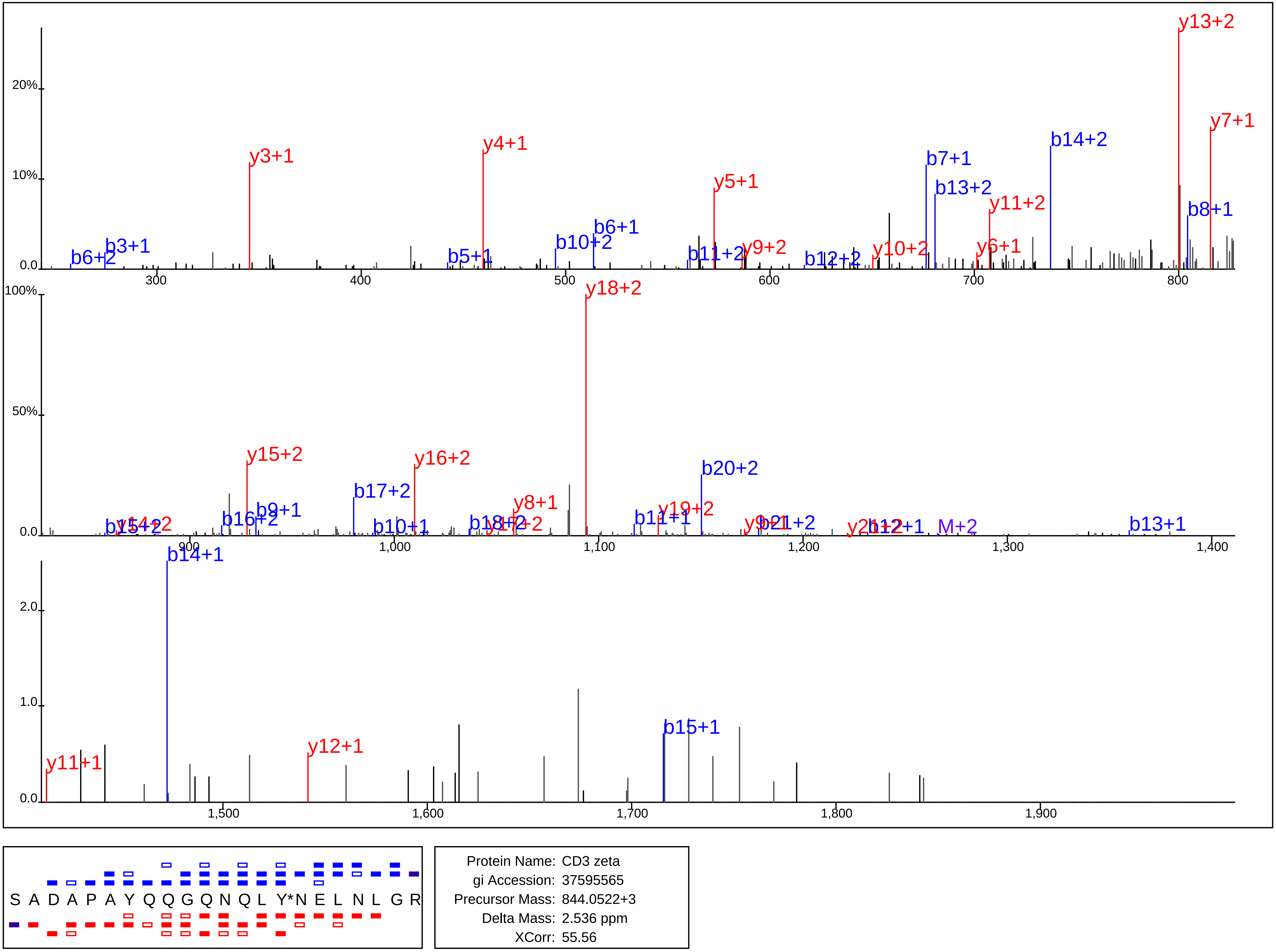

Protein Name:	CD3 zeta
gi Accession:	37595565
Precursor Mass:	844.0522+3
Delta Mass:	2.536 ppm
XCorr:	55.56

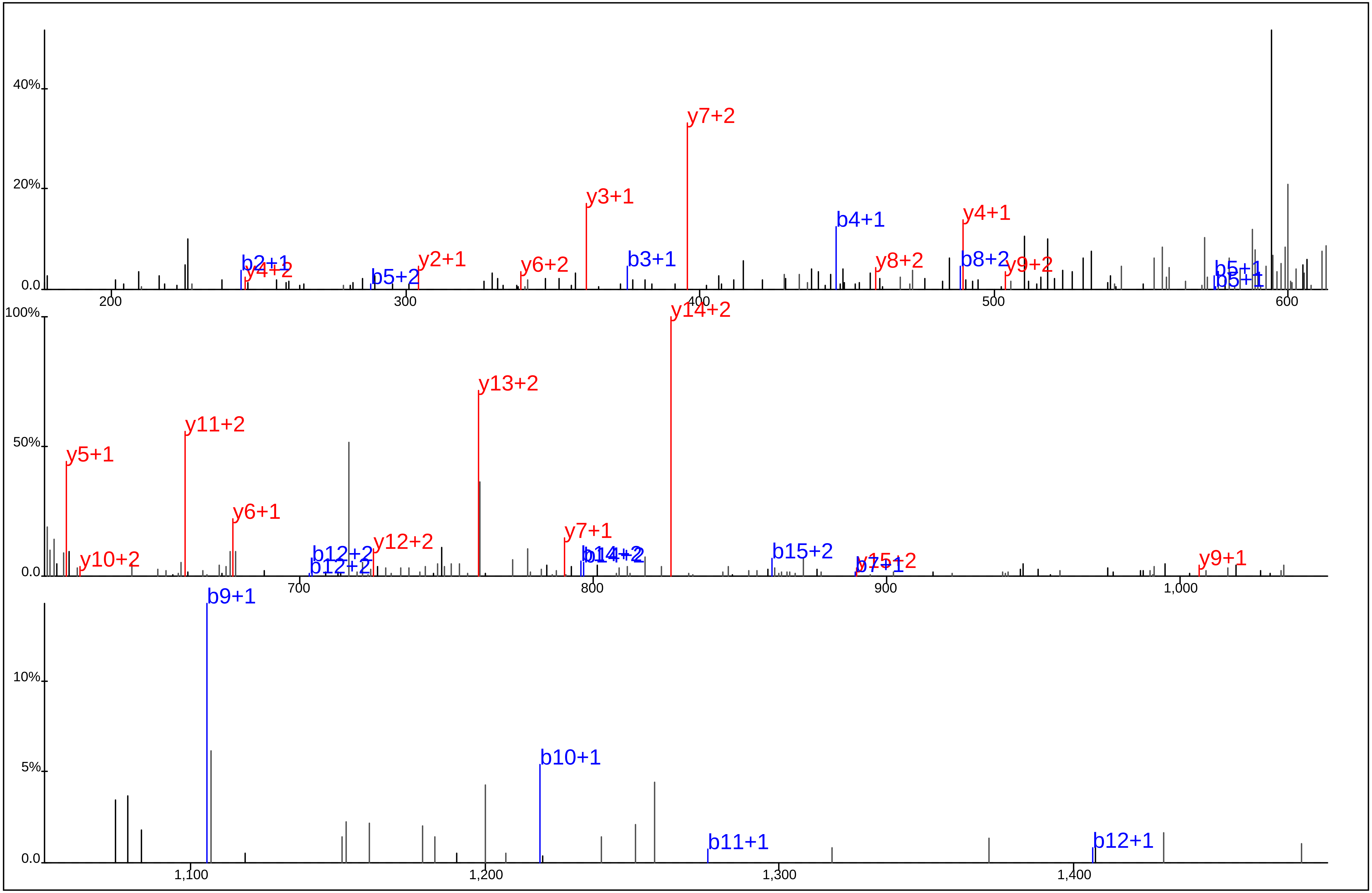

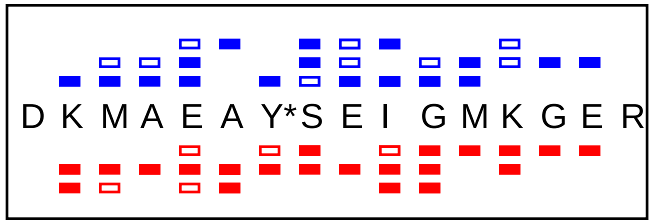

Protein Name: CD3 zeta
gi Accession: 37595565
Precursor Mass: 632.2767+3
Delta Mass: 4.348 ppm
XCorr: 43.32

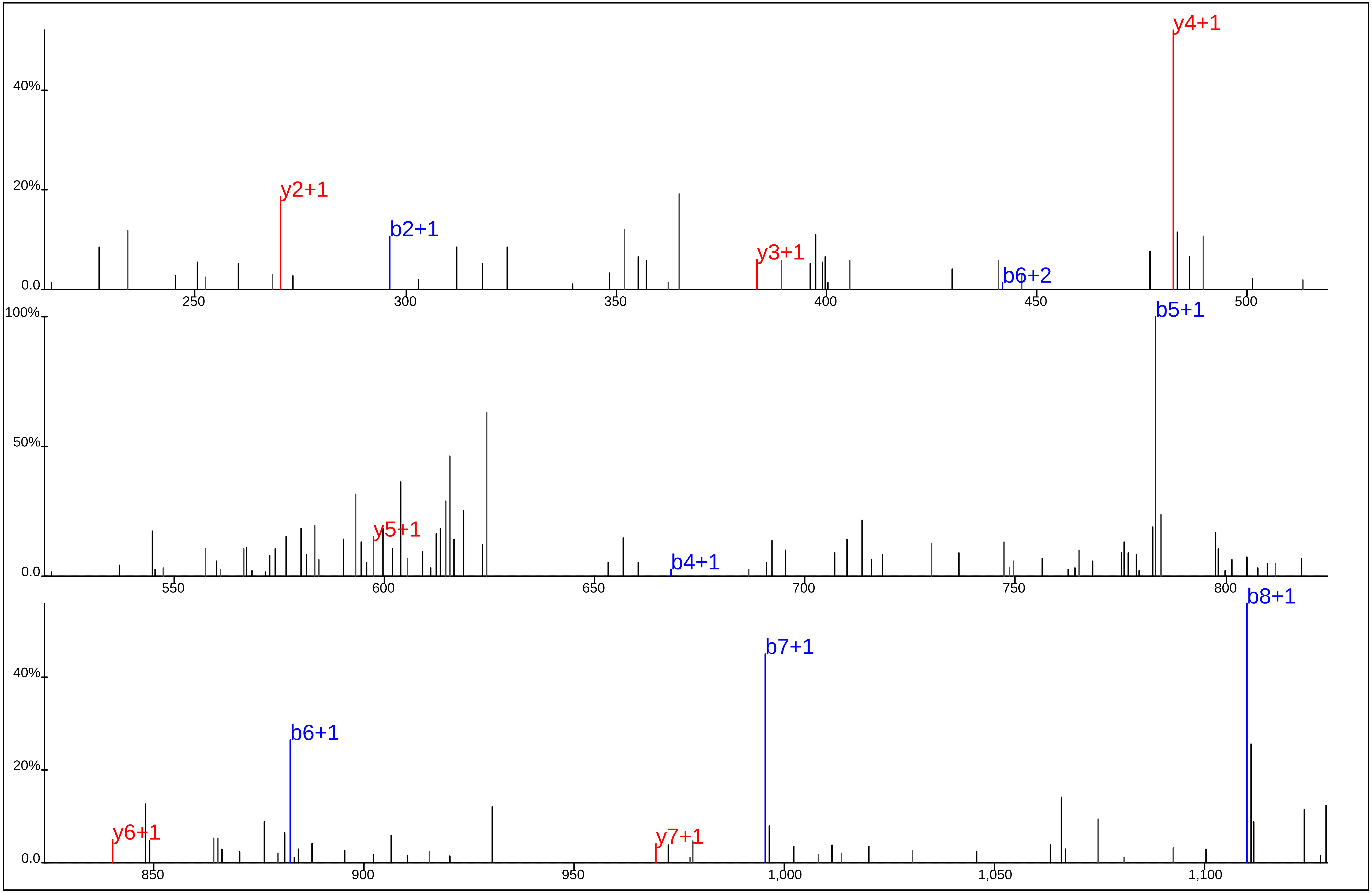

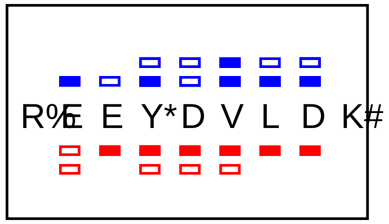

Protein Name:	CD3 zeta
gi Accession:	37595565
Precursor Mass:	632.7834+2
Delta Mass:	1.620 ppm
XCorr:	26.56

197 - CD3zeta_3441009.pdf

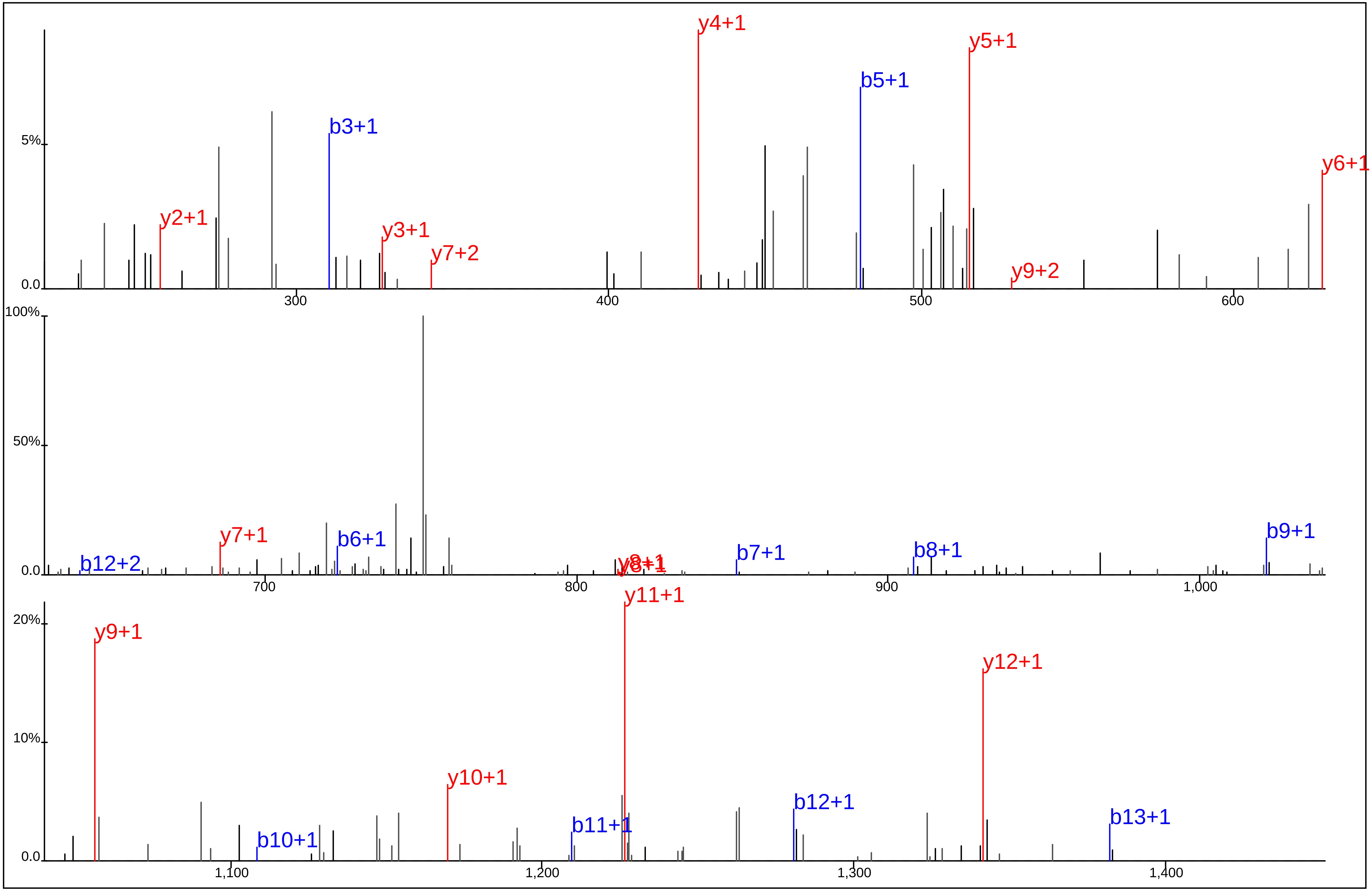

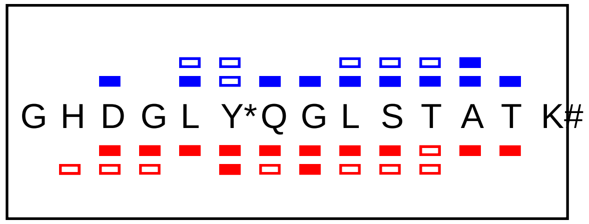

Protein Name:	CD3 zeta
gi Accession:	37595565
Precursor Mass:	768.3528+2
Delta Mass:	0.109 ppm
XCorr:	52.14

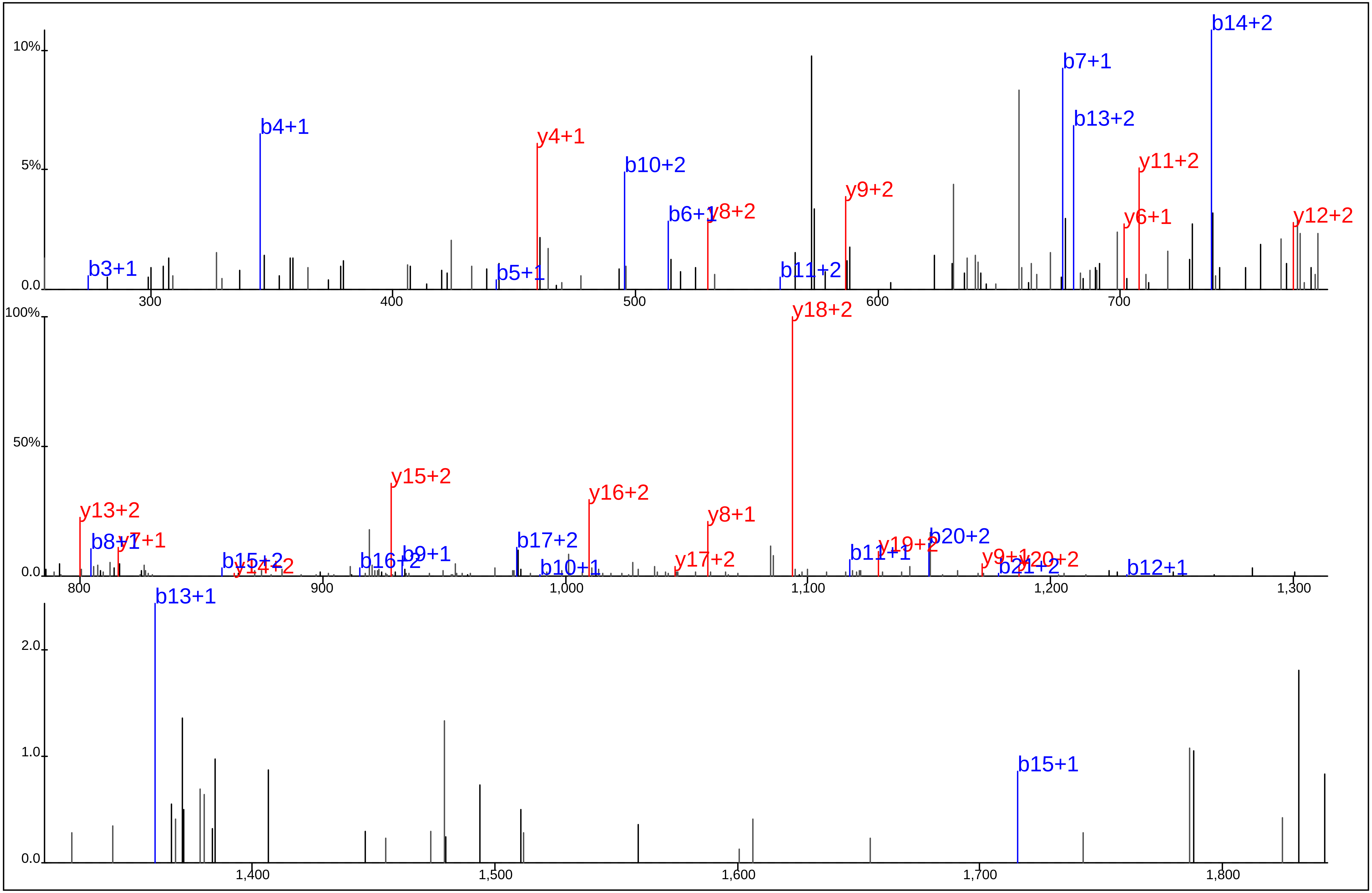

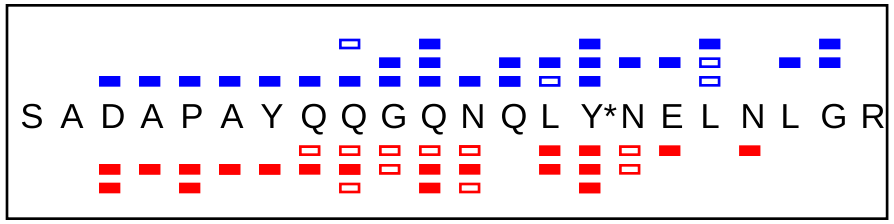

Protein Name: CD3 zeta
gi Accession: 37595565
Precursor Mass: 844.0534+3
Delta Mass: 3.908 ppm
XCorr: 42.06

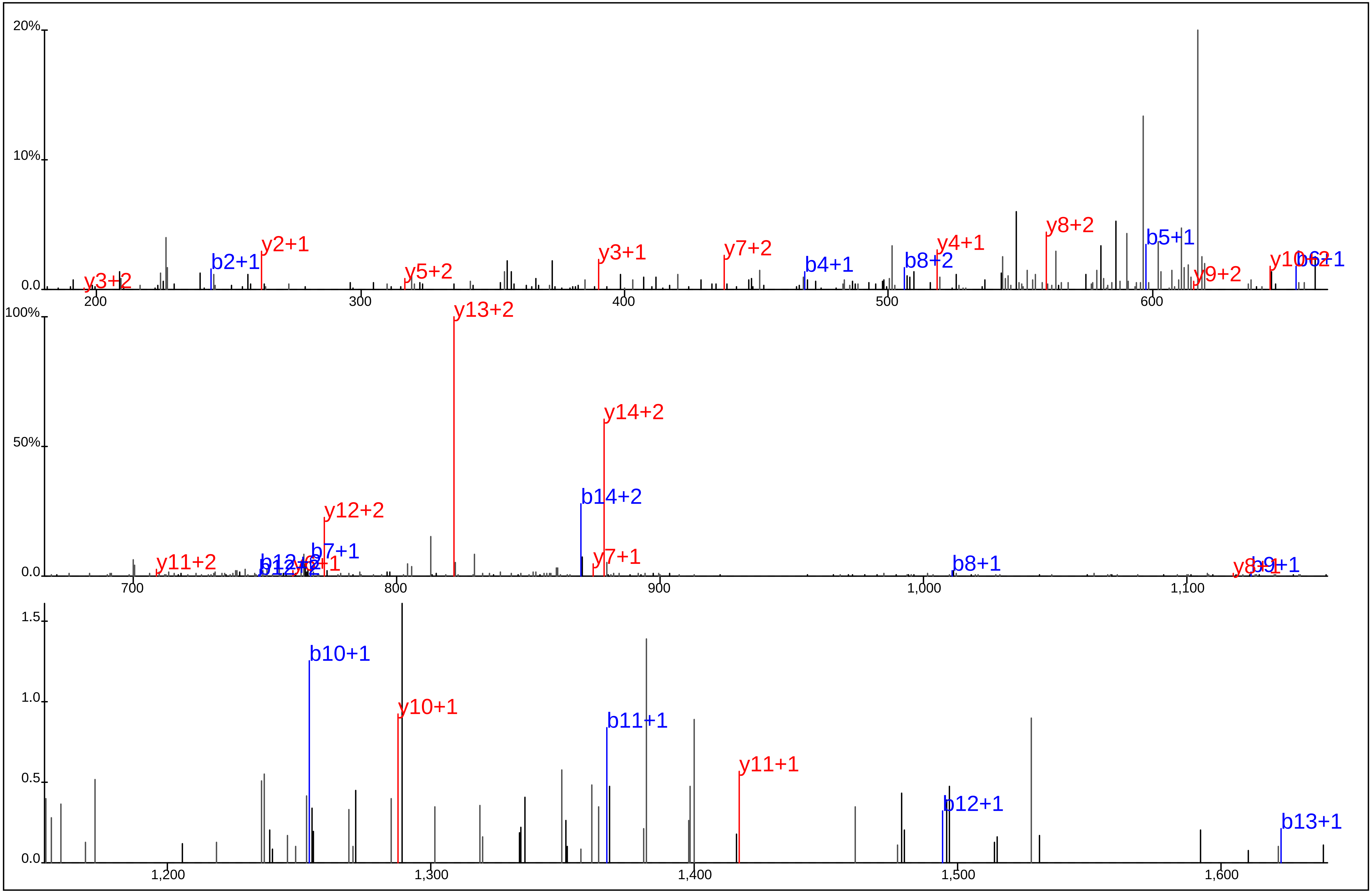

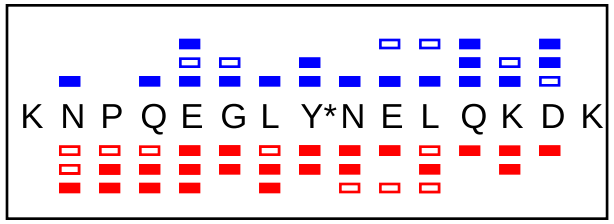

Protein Name:	CD3 zeta
gi Accession:	37595565
Precursor Mass:	628.6342+3
Delta Mass:	1.020 ppm
XCorr:	47.64

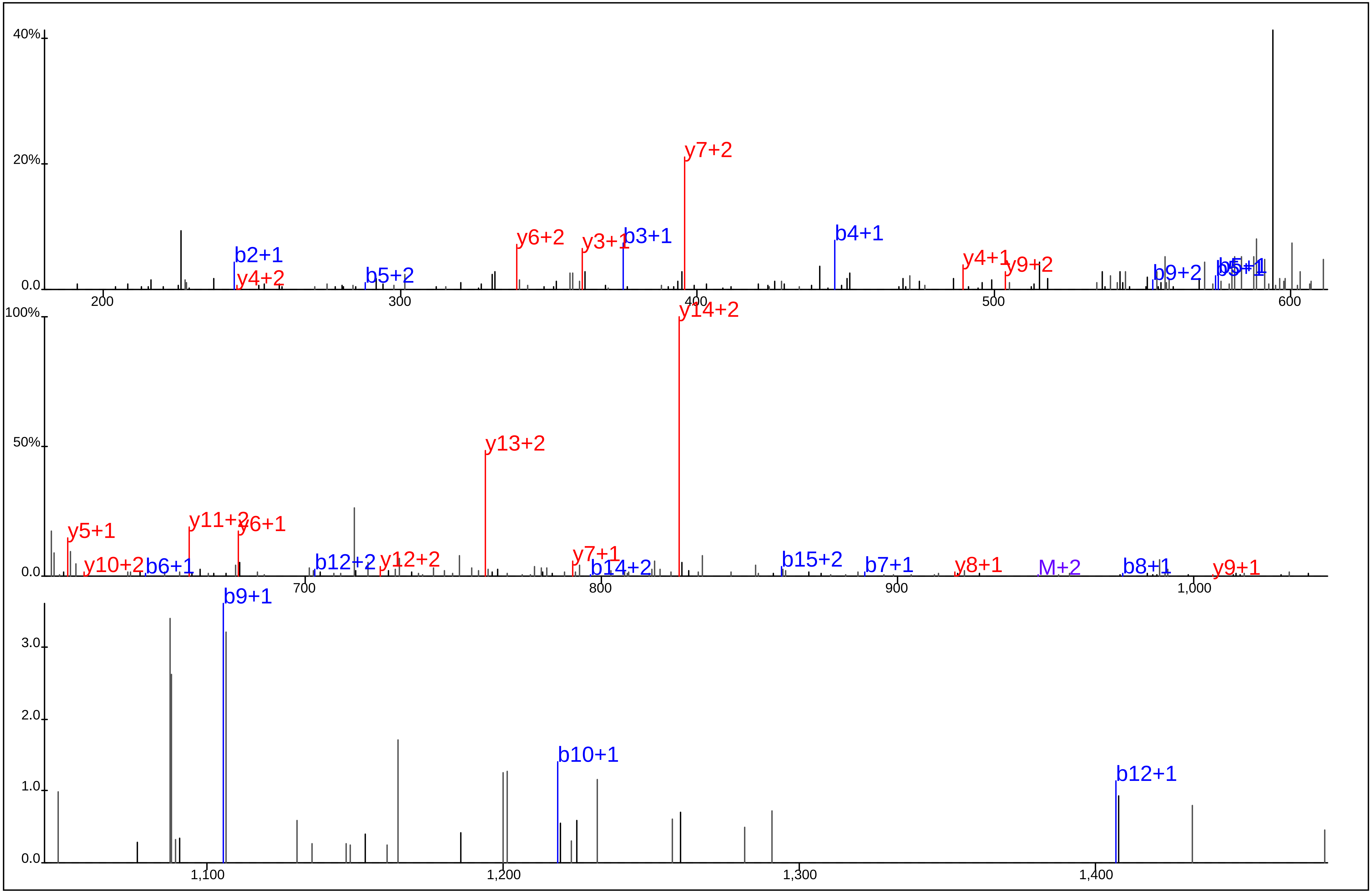

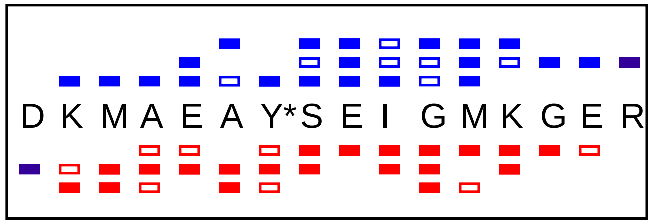

Protein Name: CD3 zeta
gi Accession: 37595565
Precursor Mass: 632.2751+3
Delta Mass: 1.735 ppm
XCorr: 36.14

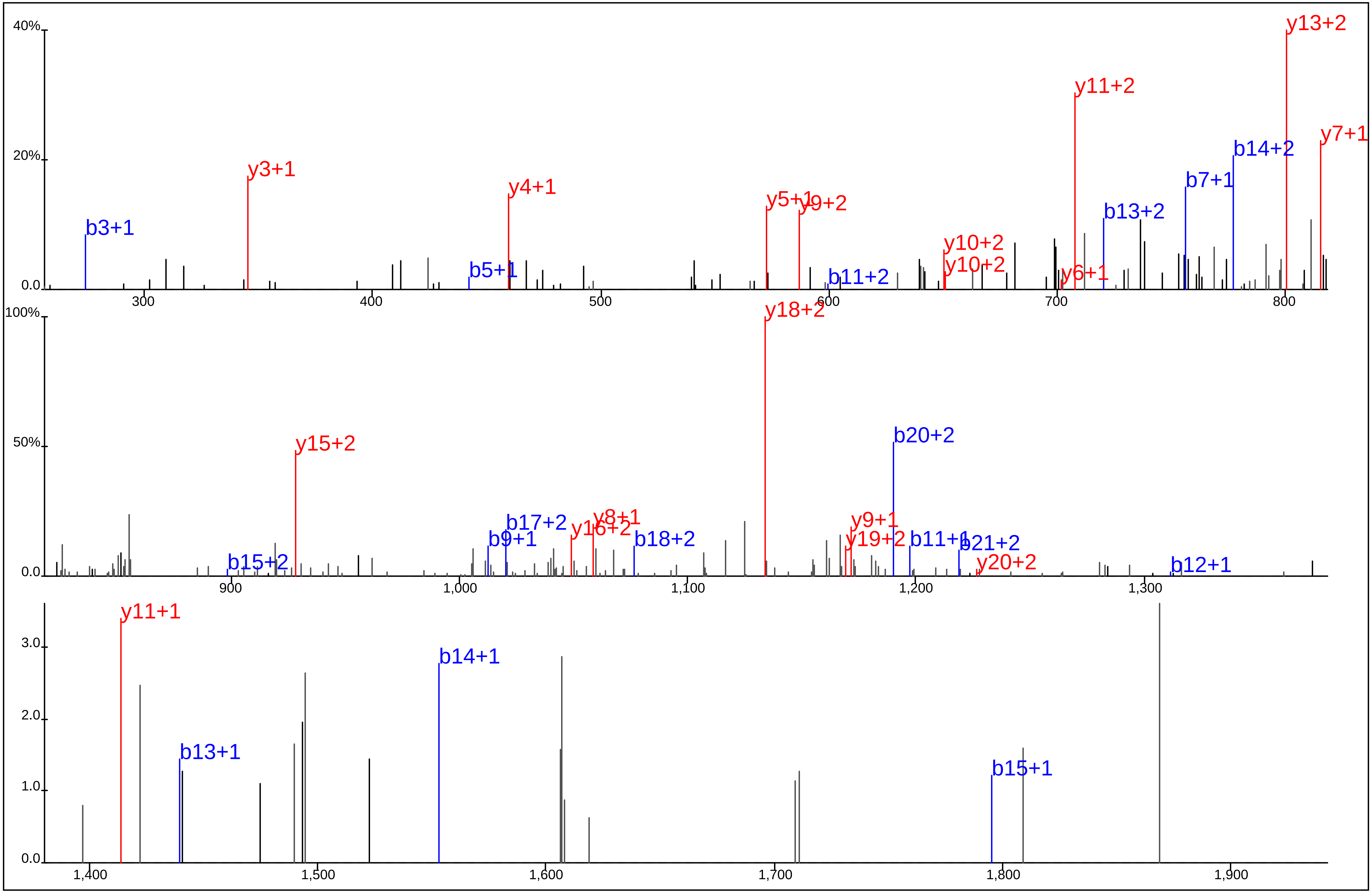

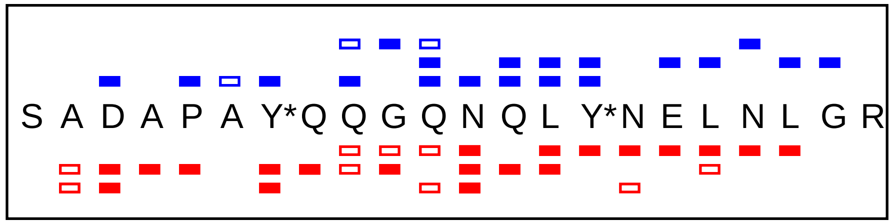

Protein Name: CD3 zeta
gi Accession: 37595565
Precursor Mass: 870.7101+3
Delta Mass: 5.214 ppm
XCorr: 45.45

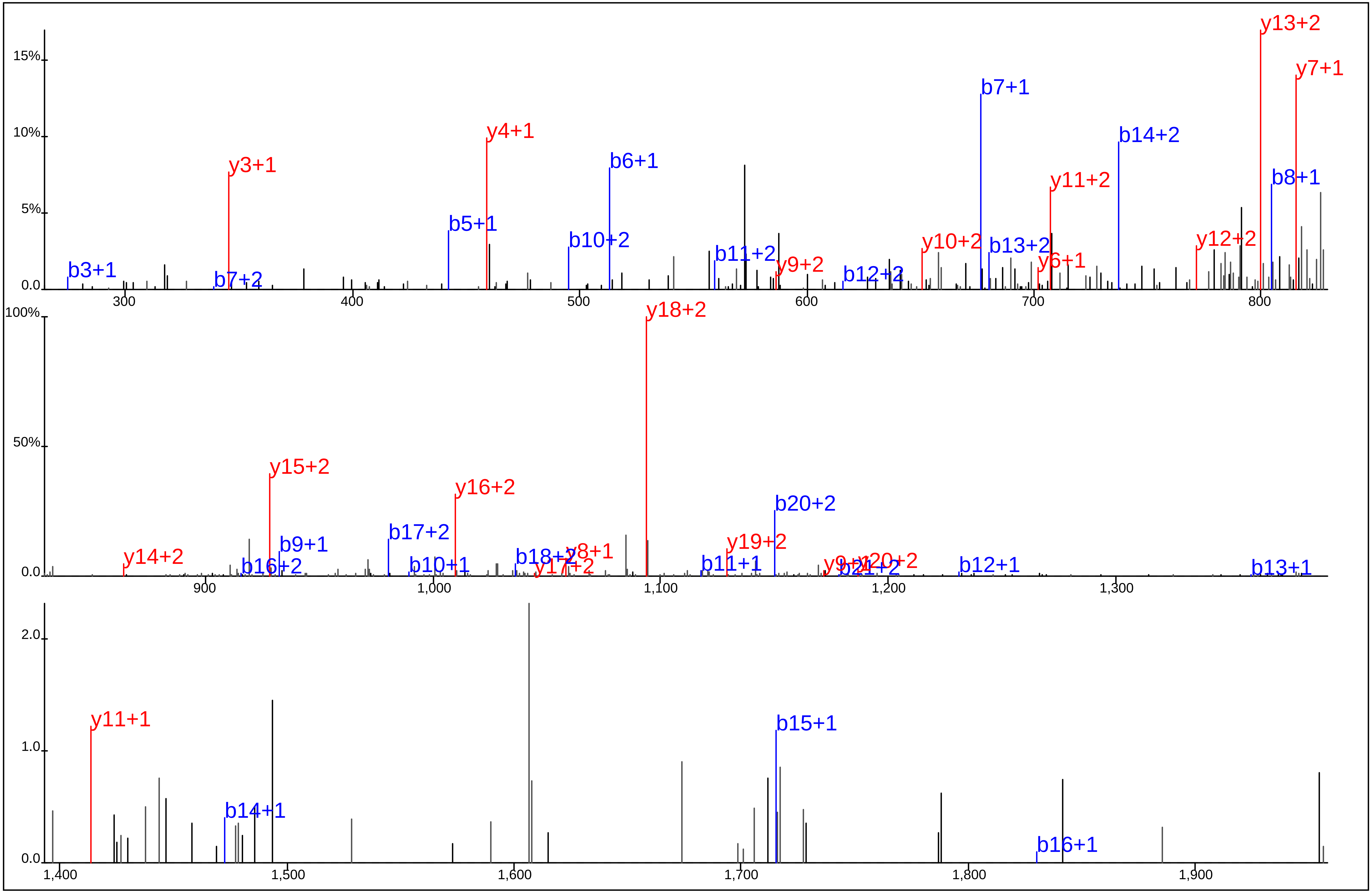

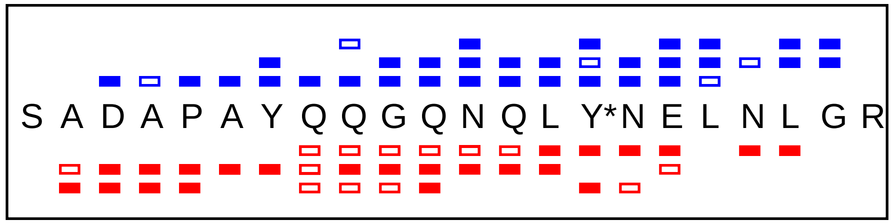

Protein Name: CD3 zeta
gi Accession: 37595565
Precursor Mass: 844.0536+3
Delta Mass: 4.201 ppm
XCorr: 65.48

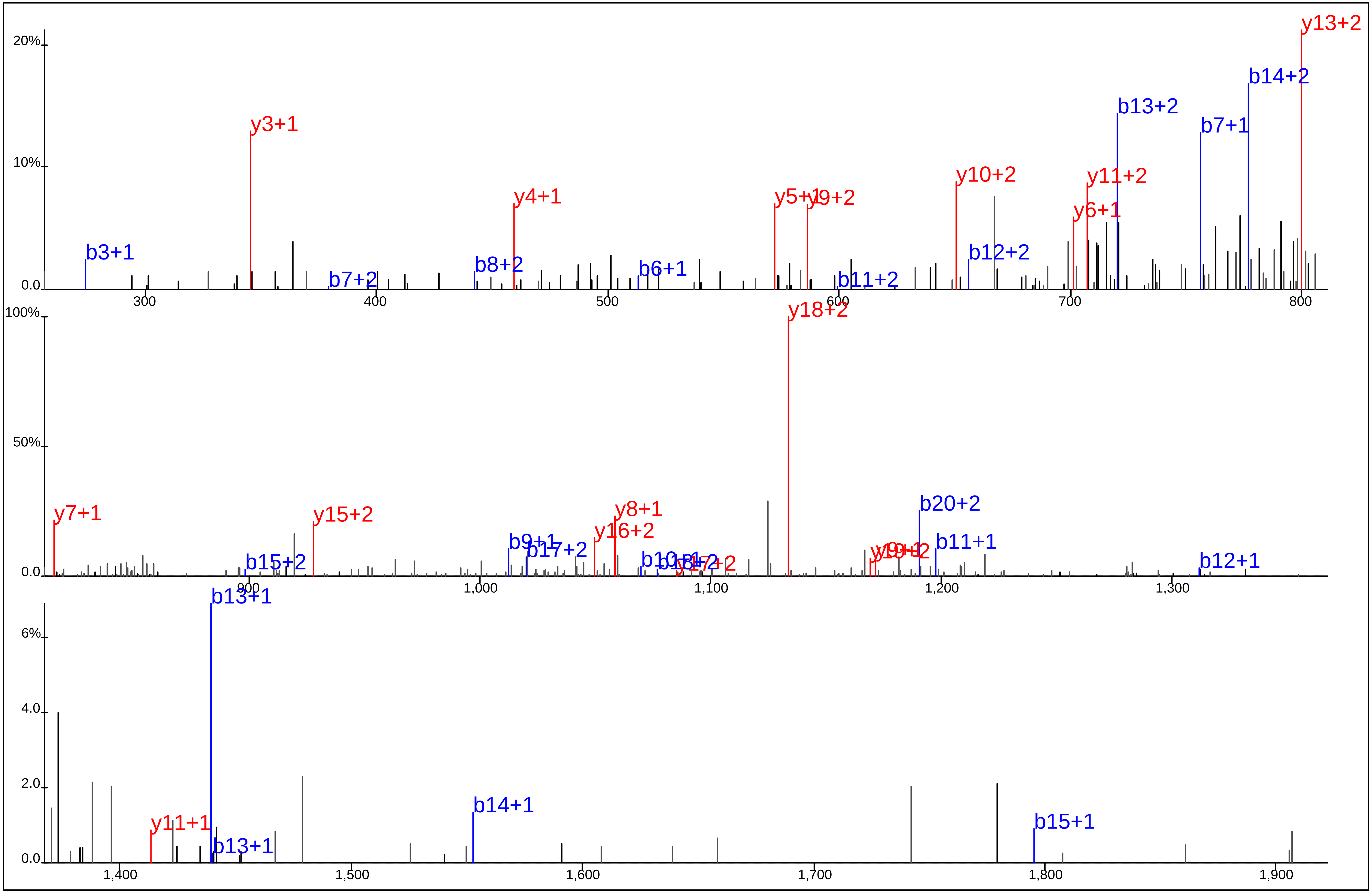

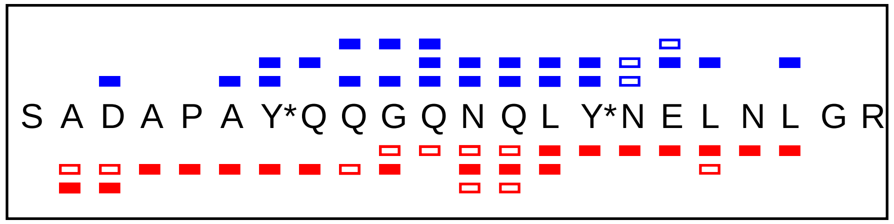

Protein Name: CD3 zeta
gi Accession: 37595565
Precursor Mass: 870.7094+3
Delta Mass: 4.371 ppm
XCorr: 48.42

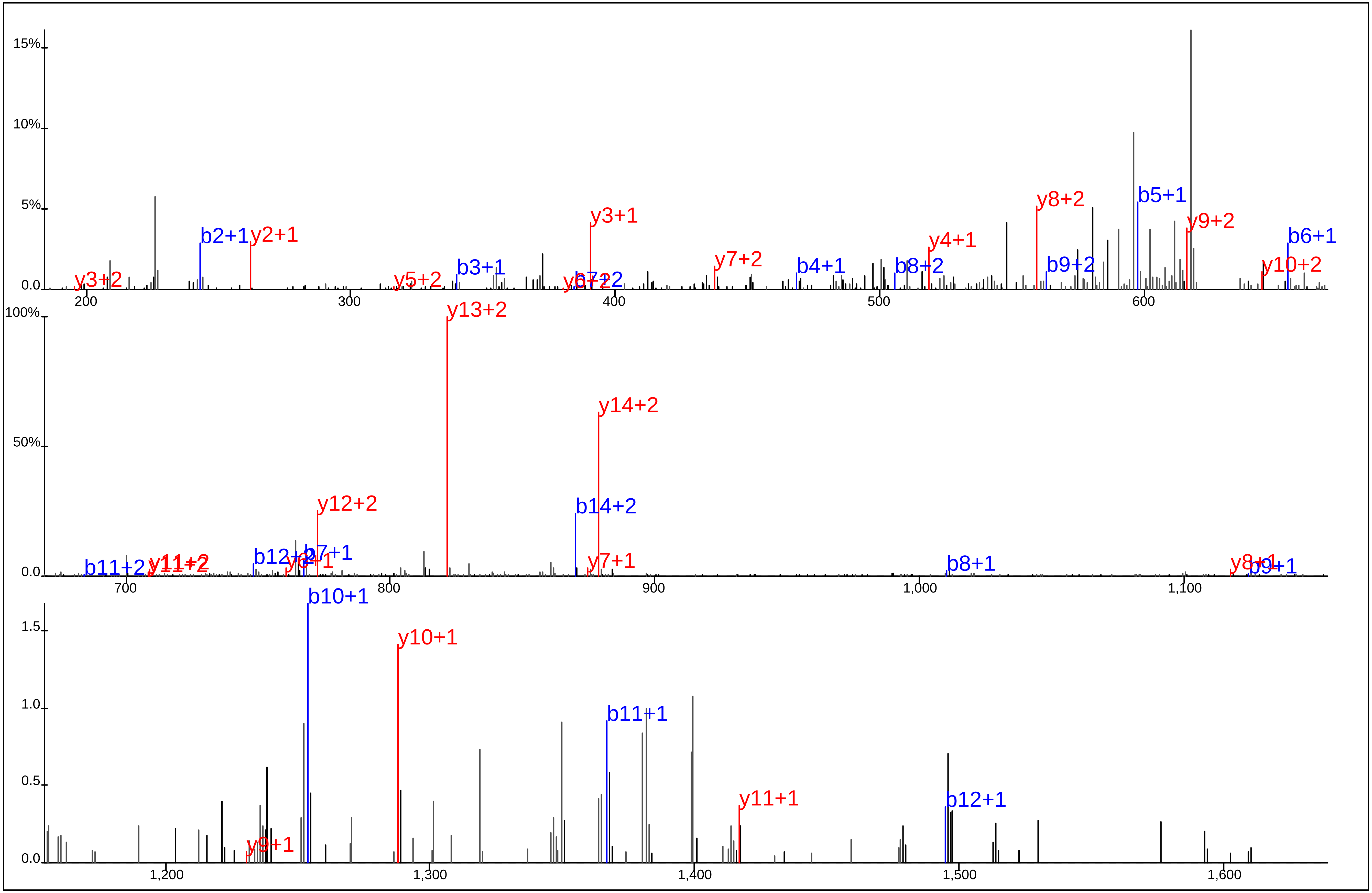

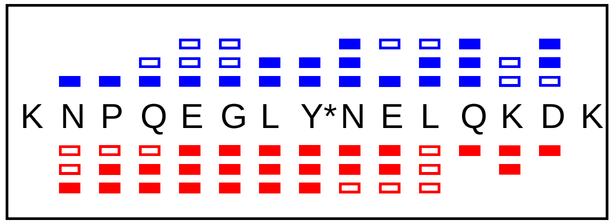

Protein Name: CD3 zeta
gi Accession: 37595565
Precursor Mass: 628.6359+3
Delta Mass: 1.703 ppm
XCorr: 46.93

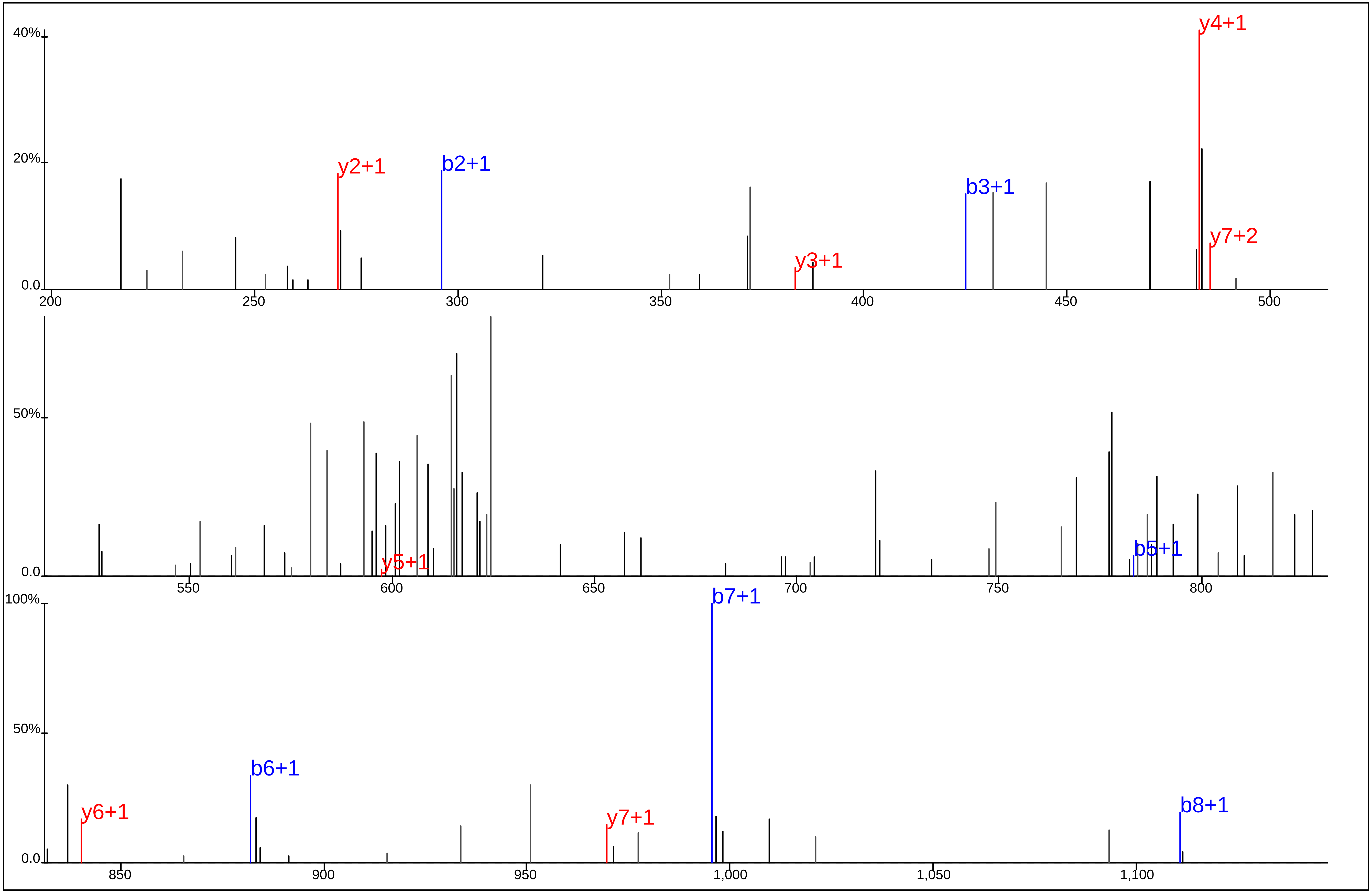

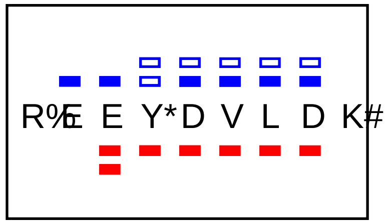

Protein Name: CD3 zeta
gi Accession: 37595565
Precursor Mass: 632.7827+2
Delta Mass: 0.457 ppm
XCorr: 14.03

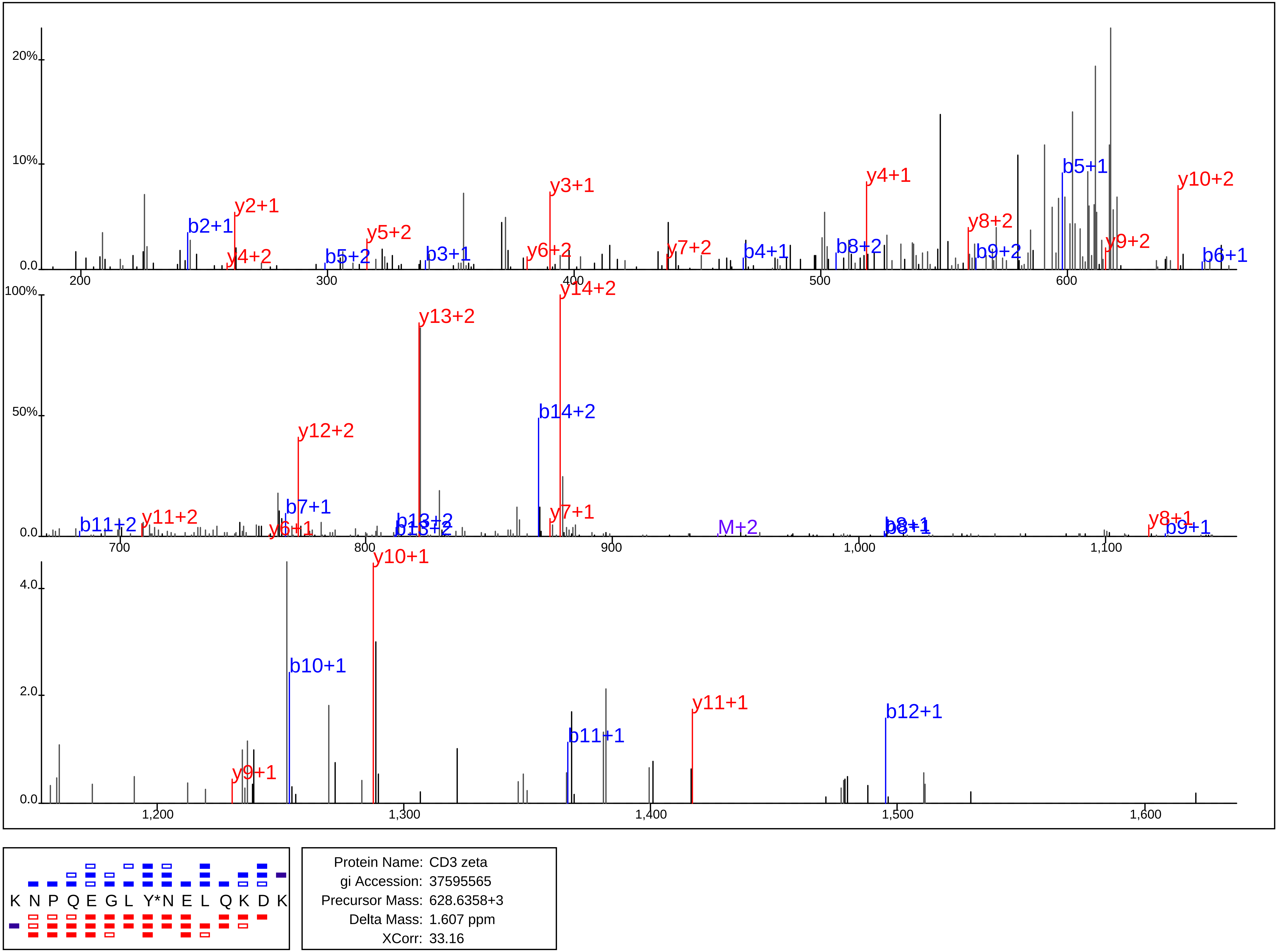

Protein Name:	CD3 zeta
gi Accession:	37595565
Precursor Mass:	628.6358+3
Delta Mass:	1.607 ppm
XCorr:	33.16

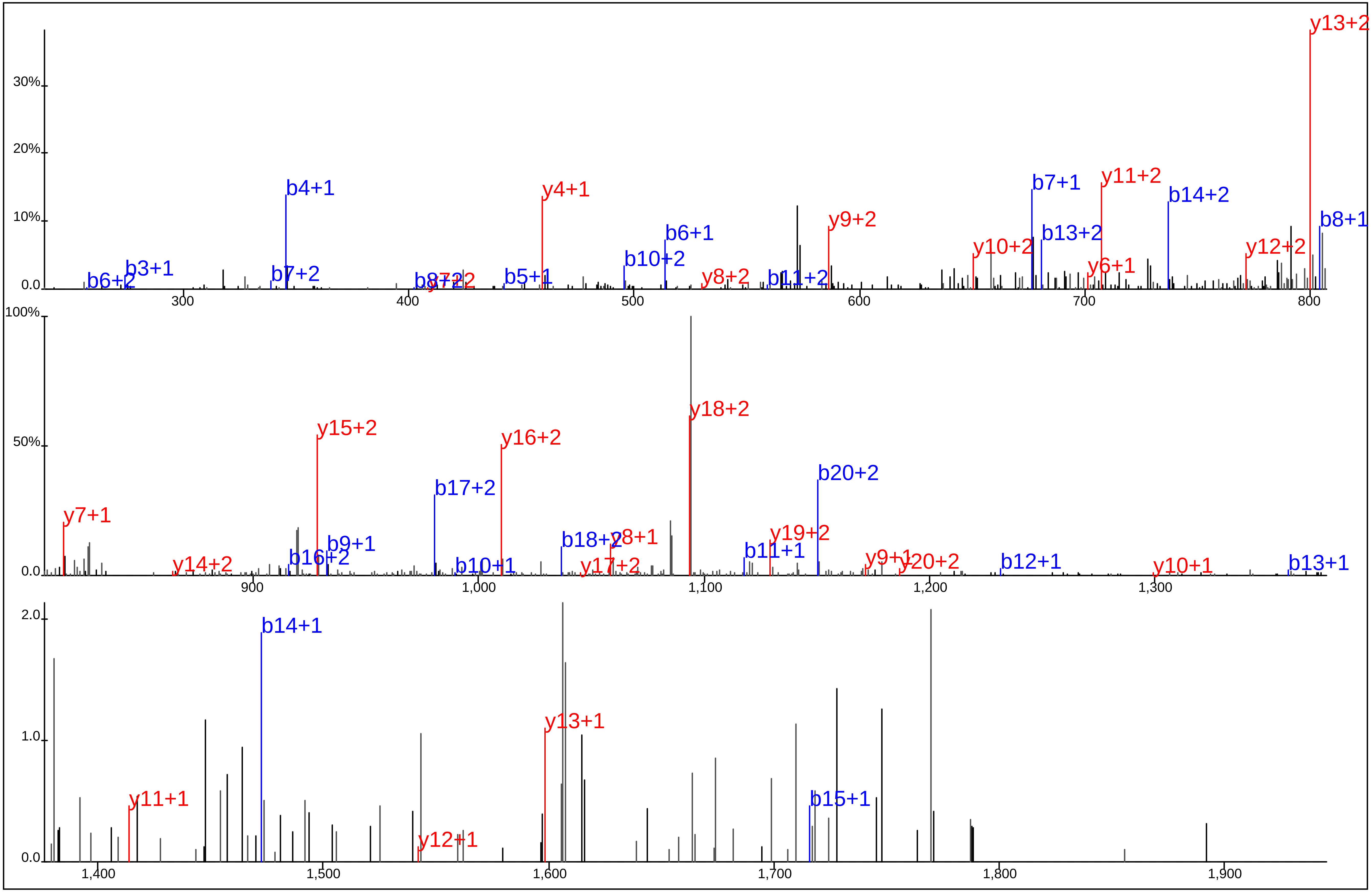

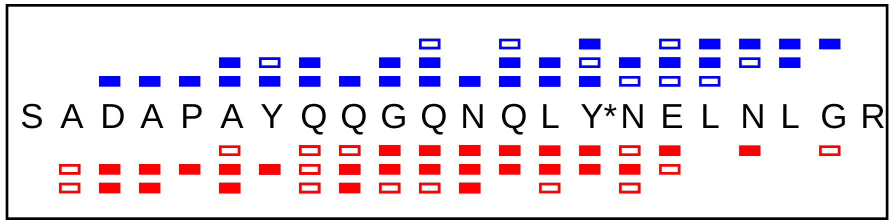

Protein Name:	CD3 zeta
gi Accession:	37595565
Precursor Mass:	844.0529+3
Delta Mass:	3.331 ppm
XCorr:	50.18

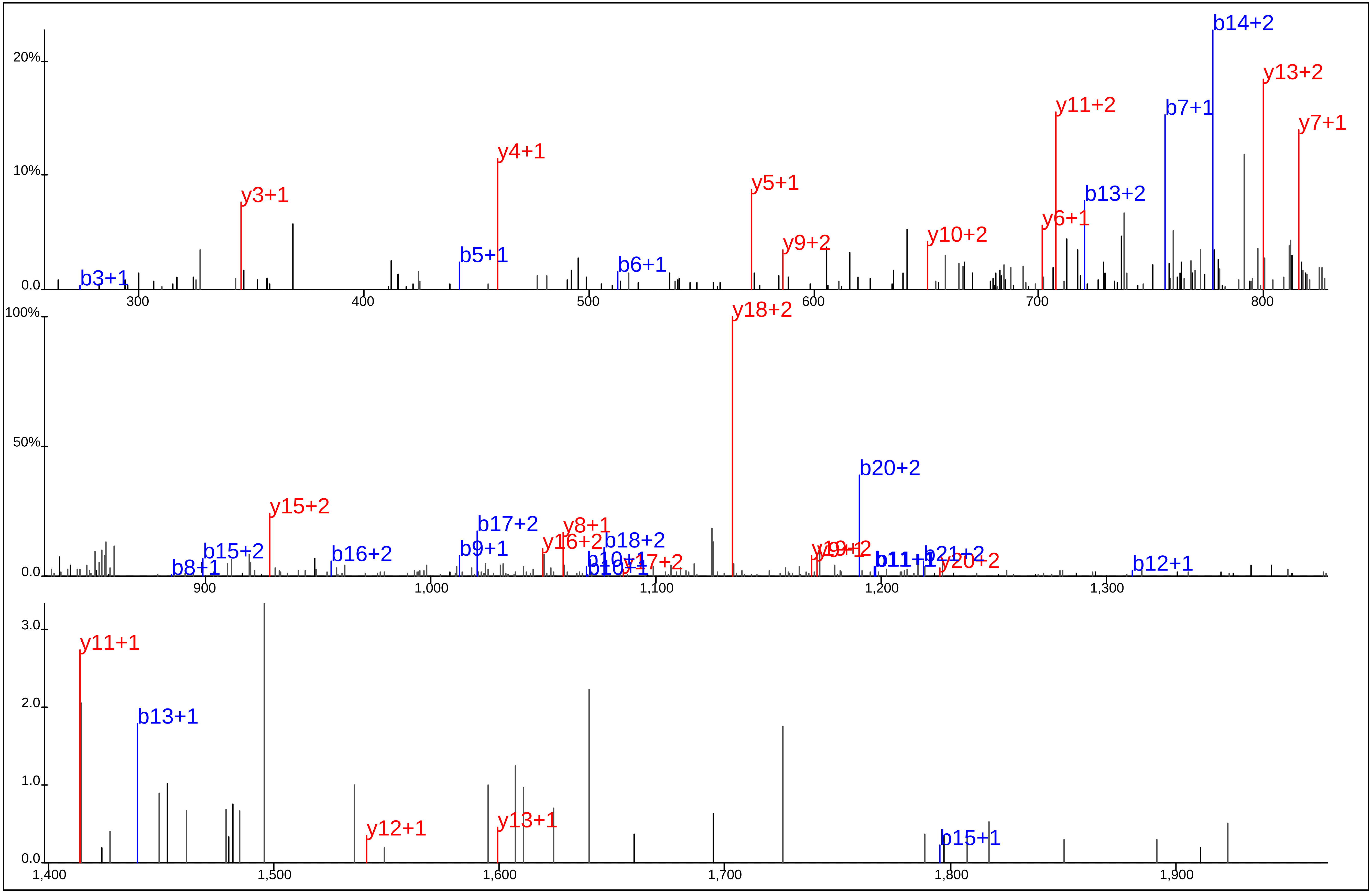

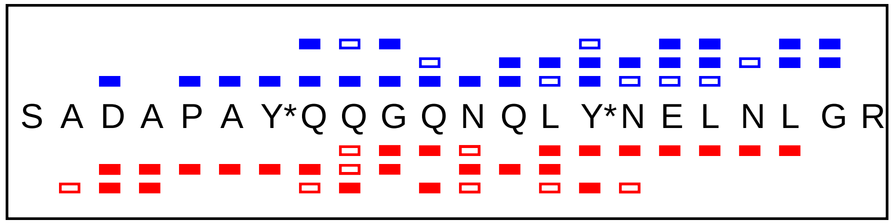

Protein Name: CD3 zeta
gi Accession: 37595565
Precursor Mass: 870.7053+3
Delta Mass: 0.330 ppm
XCorr: 42.5

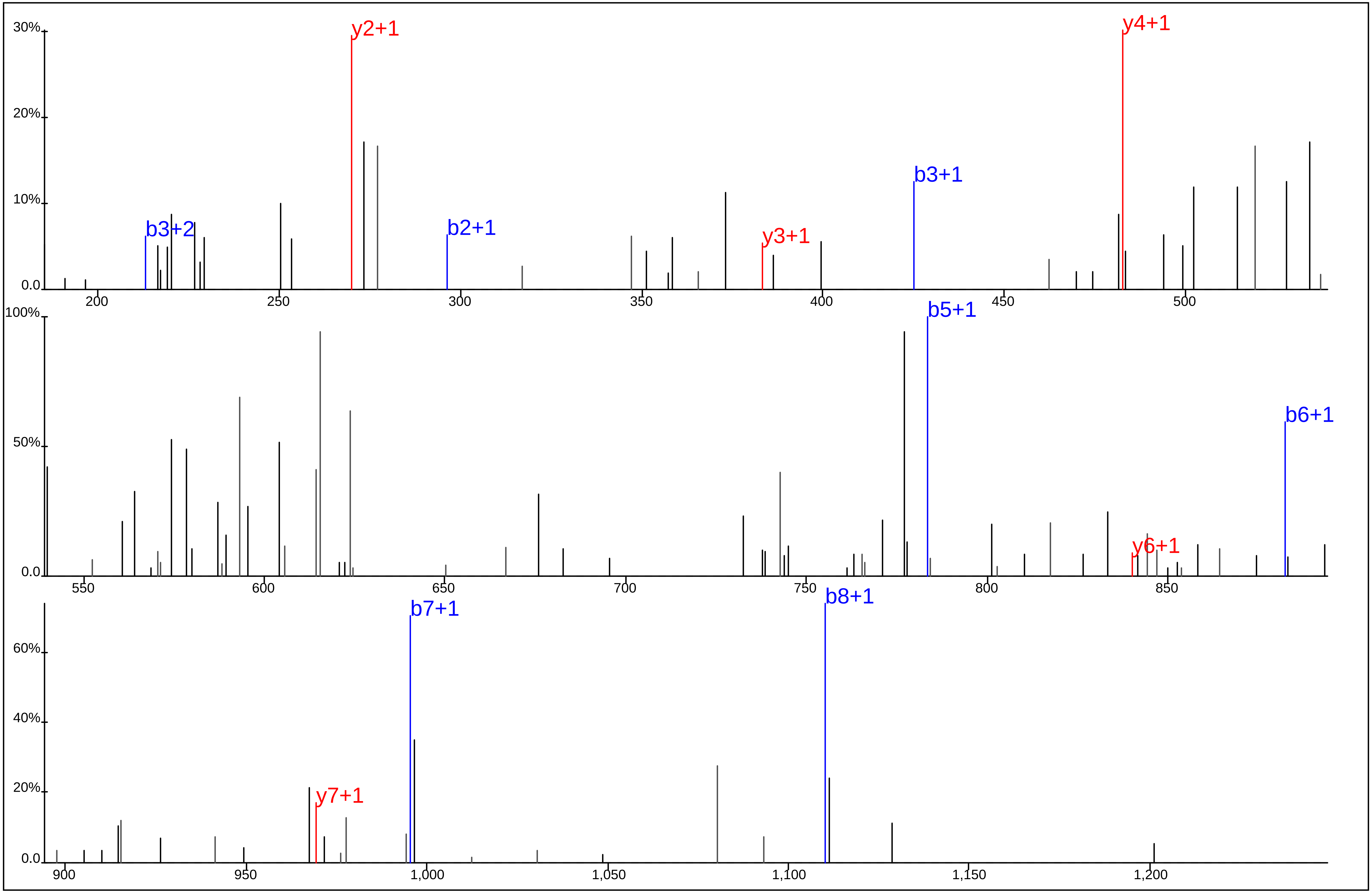

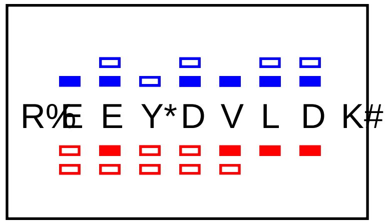

Protein Name: CD3 zeta
gi Accession: 37595565
Precursor Mass: 632.7826+2
Delta Mass: 0.267 ppm
XCorr: 28.42

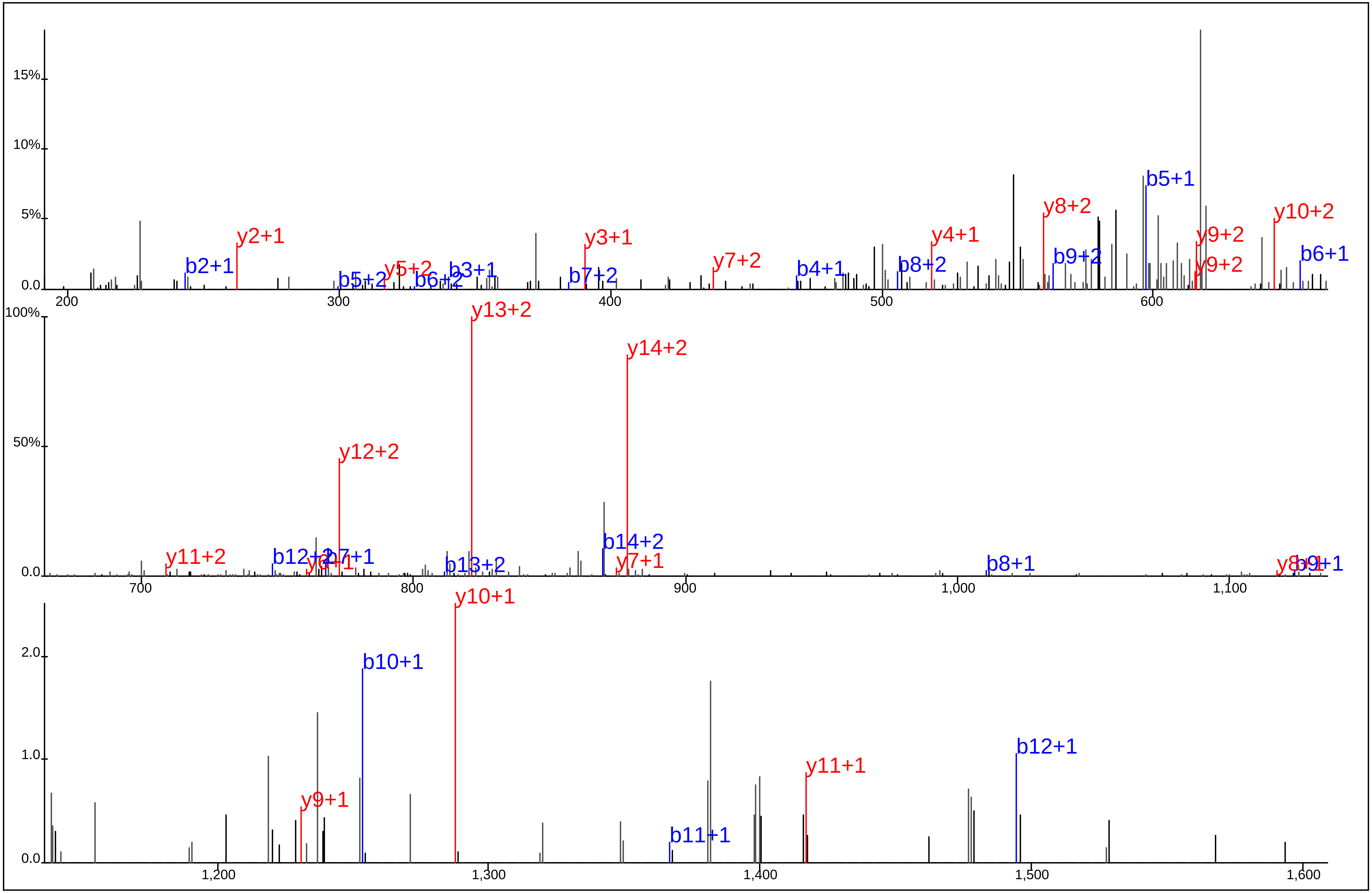

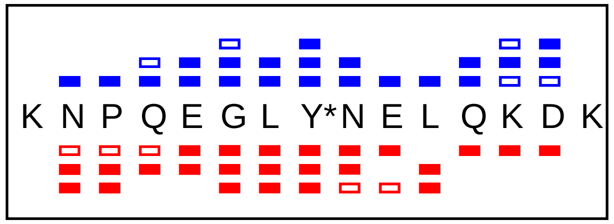

Protein Name: CD3 zeta
gi Accession: 37595565
Precursor Mass: 628.6362+3
Delta Mass: 2.192 ppm
XCorr: 40.94

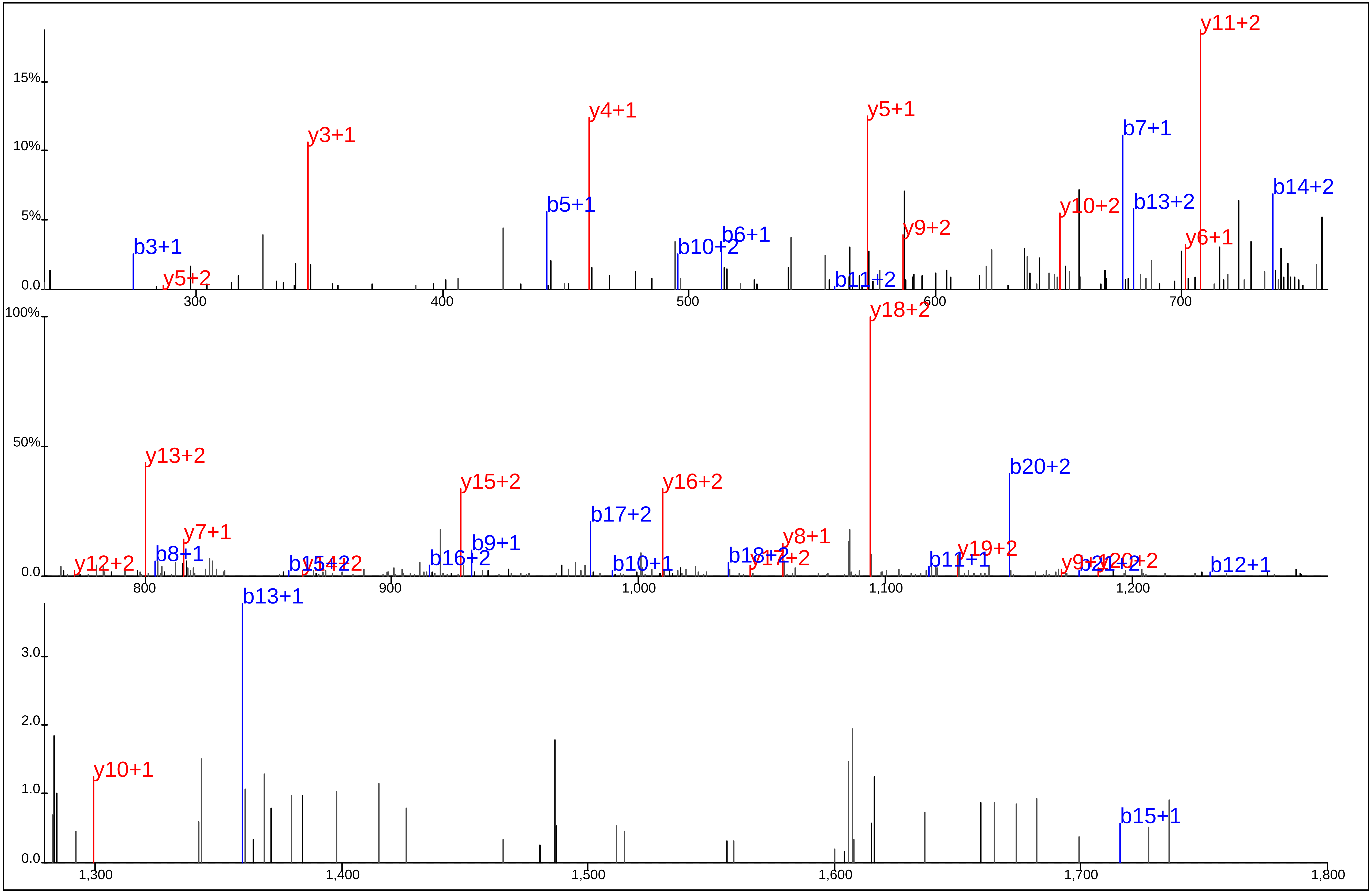

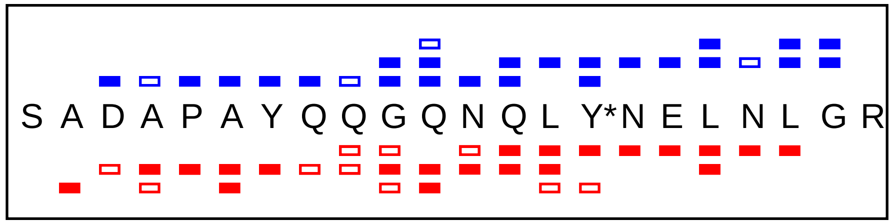

Protein Name: CD3 zeta
gi Accession: 37595565
Precursor Mass: 844.0544+3
Delta Mass: 5.067 ppm
XCorr: 45.22

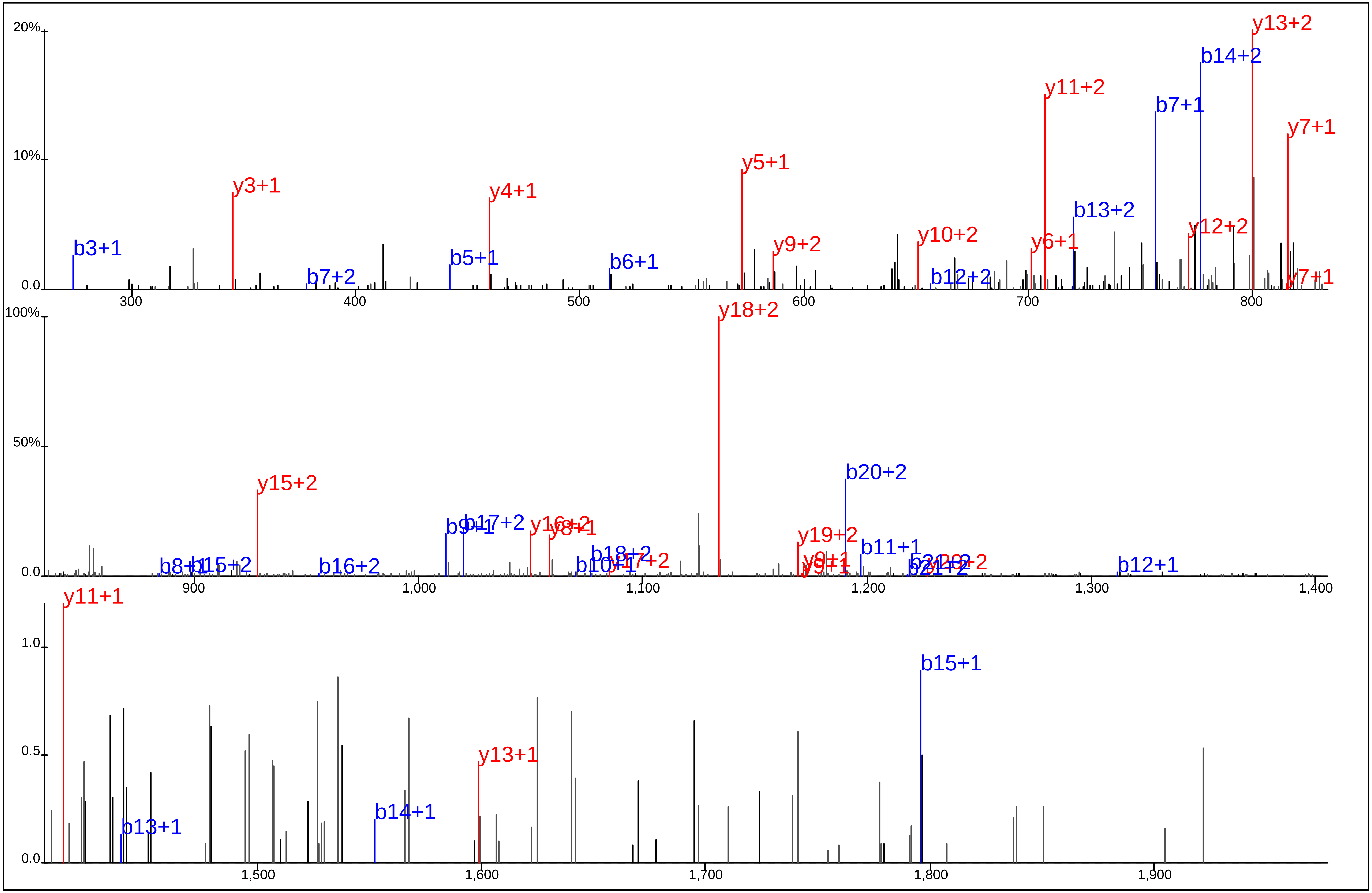

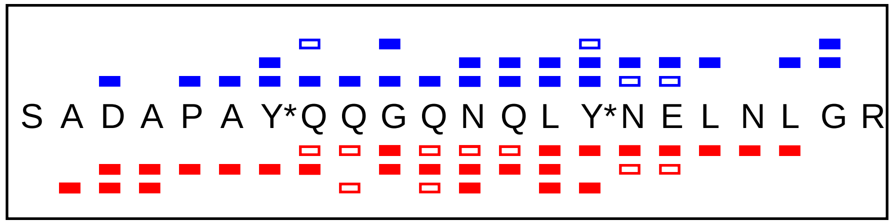

Protein Name: CD3 zeta
gi Accession: 37595565
Precursor Mass: 870.7090+3
Delta Mass: 4.018 ppm
XCorr: 48.53

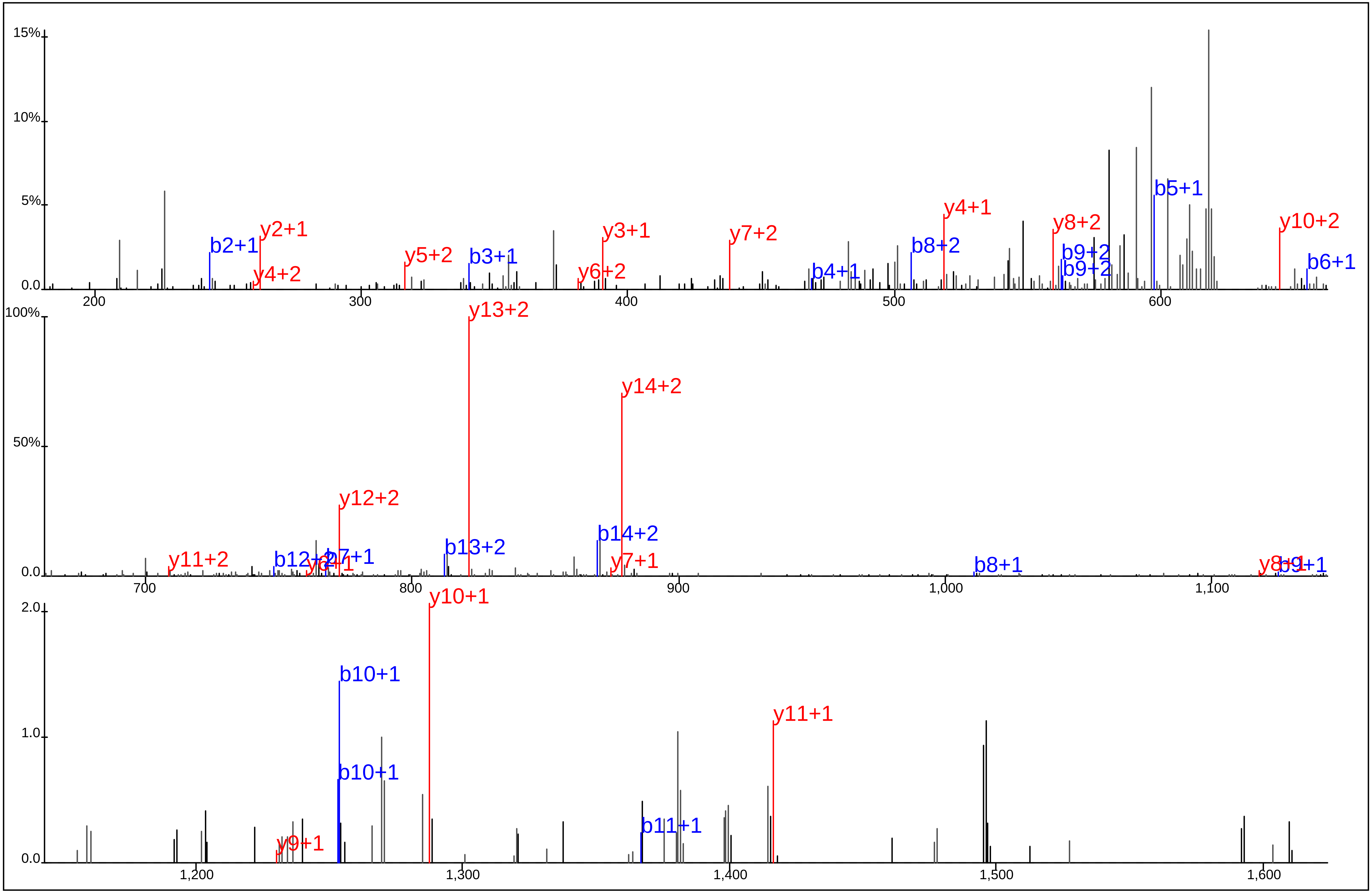

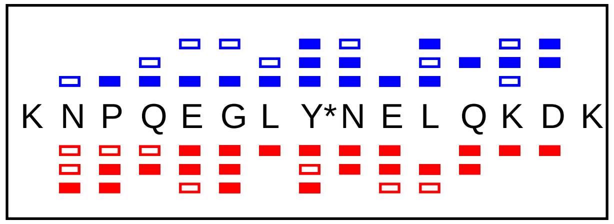

Protein Name: CD3 zeta
gi Accession: 37595565
Precursor Mass: 628.6366+3
Delta Mass: 2.870 ppm
XCorr: 40.79

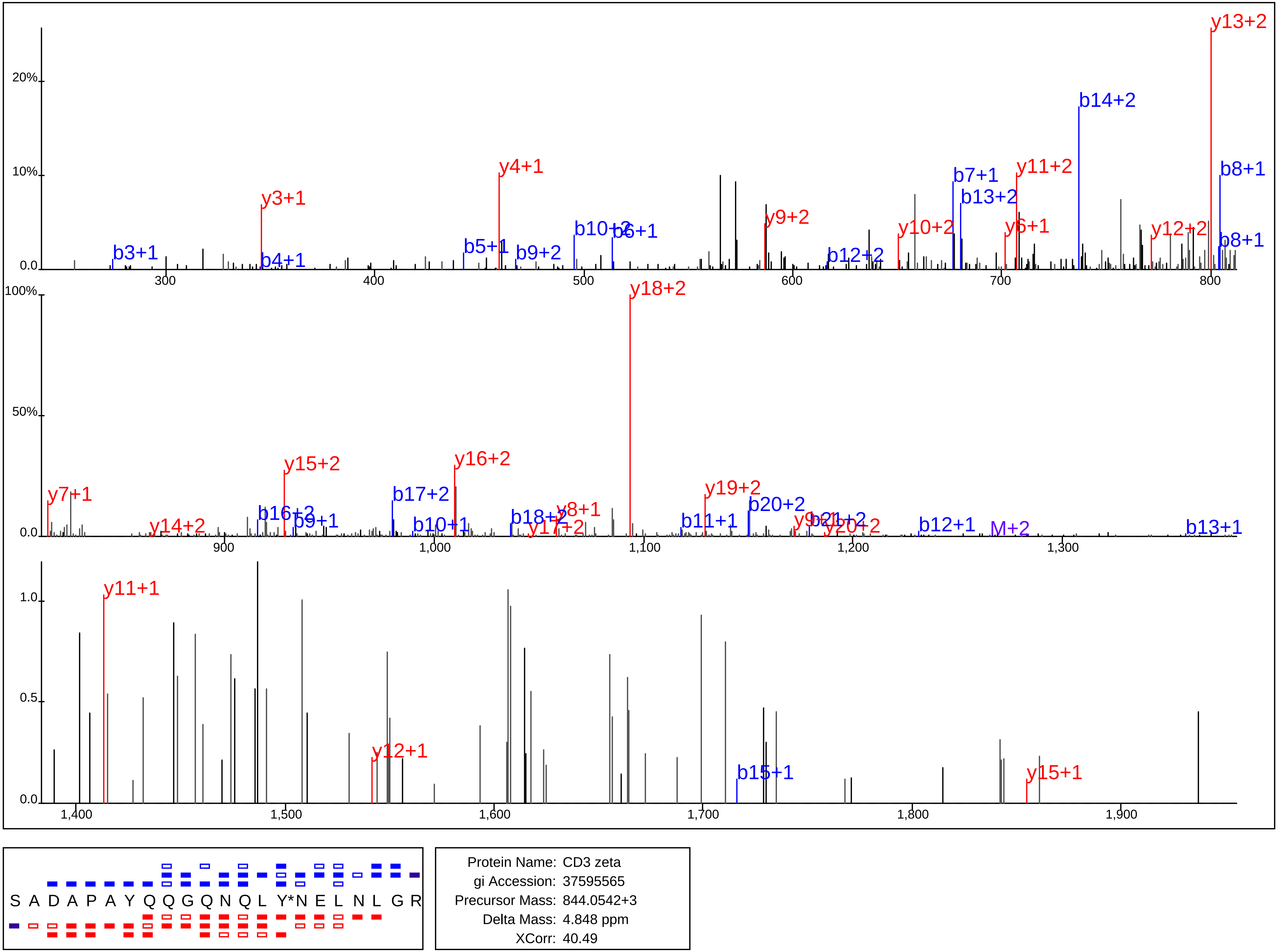

Protein Name:	CD3 zeta
gi Accession:	37595565
Precursor Mass:	844.0542+3
Delta Mass:	4.848 ppm
XCorr:	40.49

215 - CD3zeta_3452280.pdf

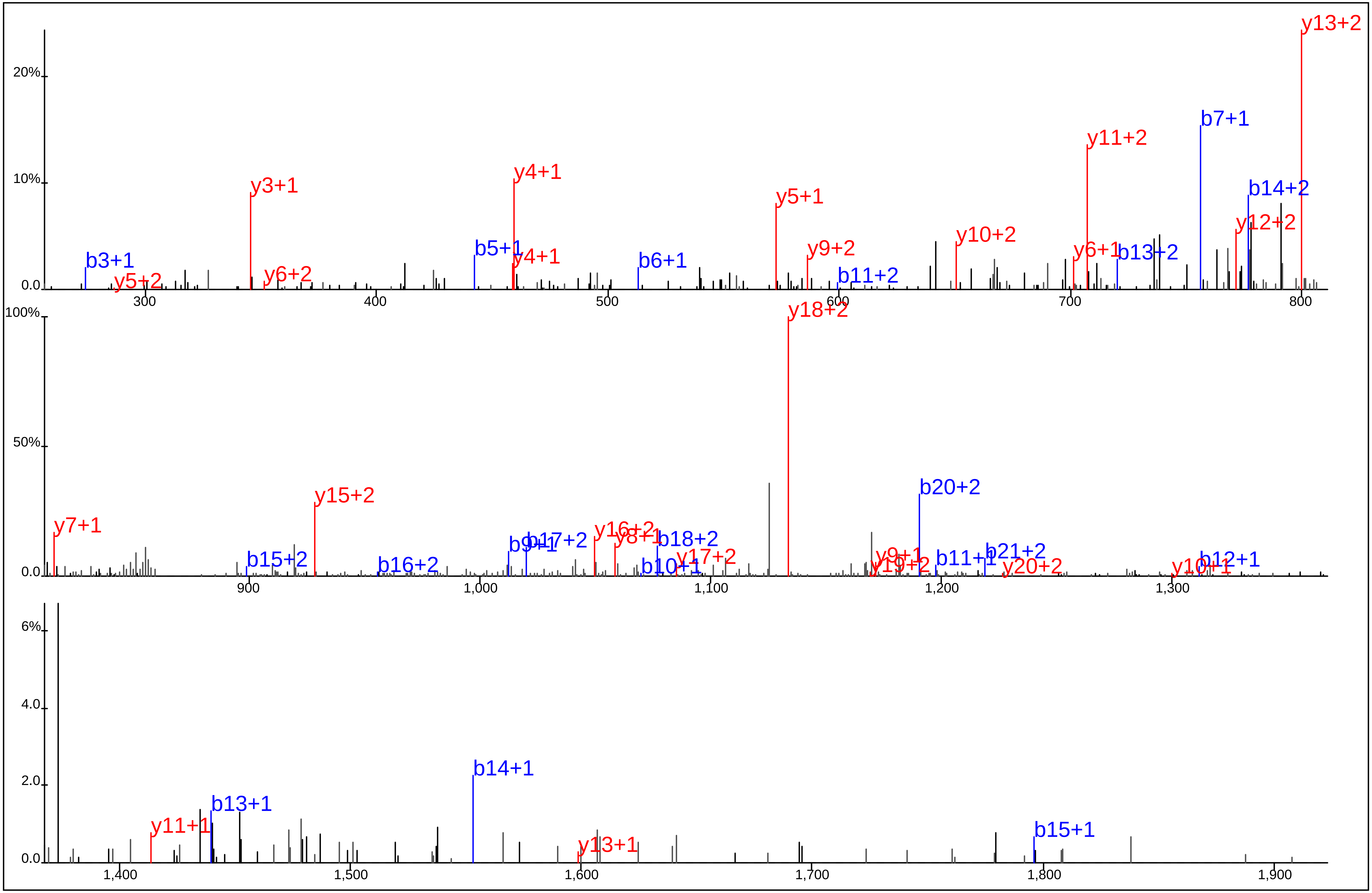

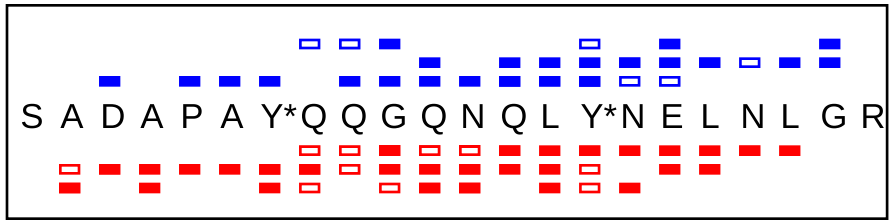

Protein Name: CD3 zeta
gi Accession: 37595565
Precursor Mass: 870.7144+3
Delta Mass: 10.121 ppm
XCorr: 53.49

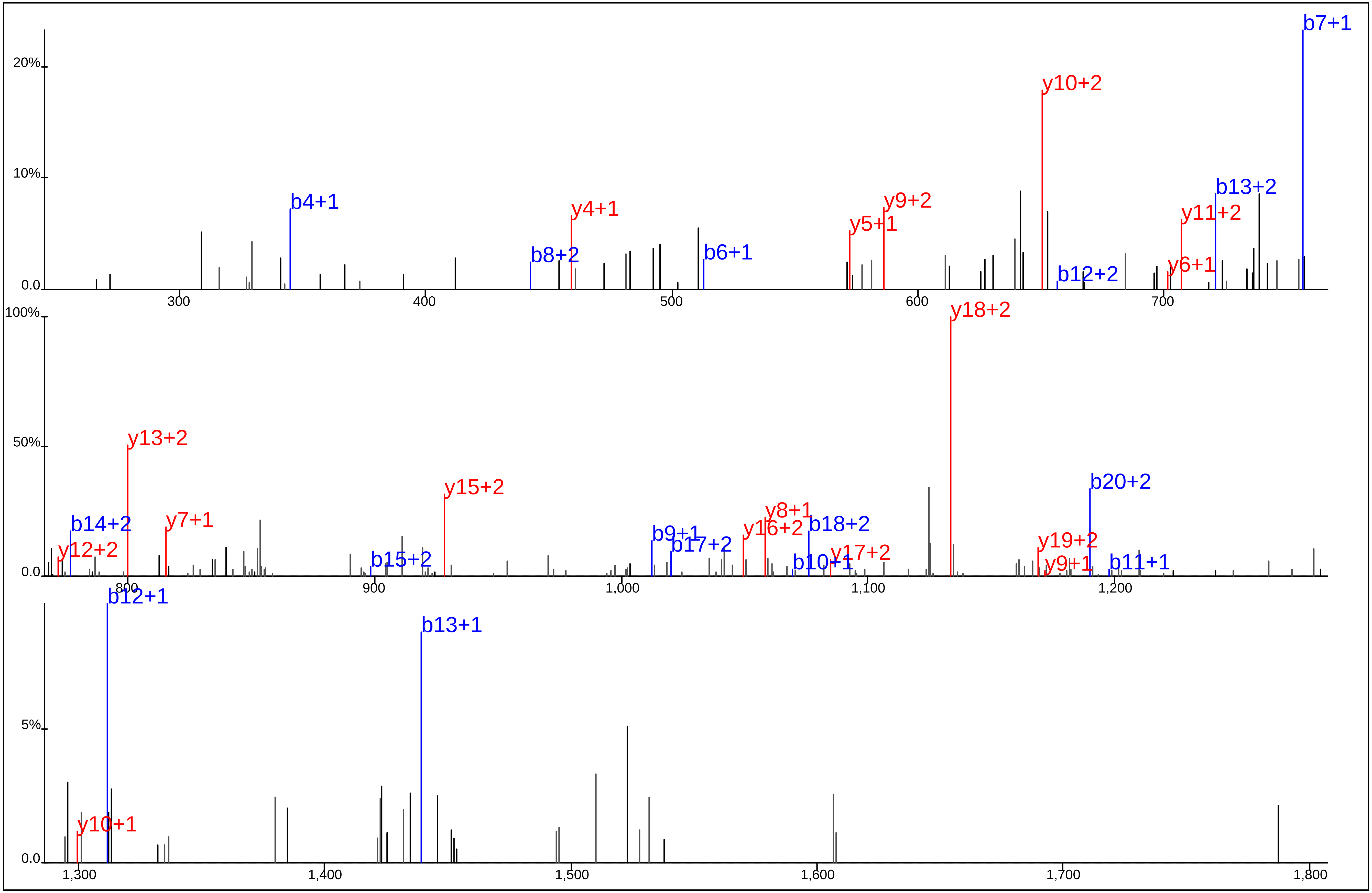

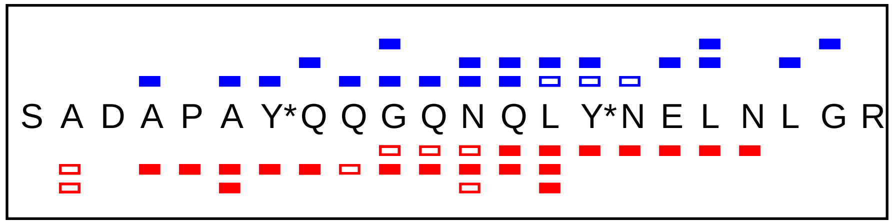

Protein Name: CD3 zeta
gi Accession: 37595565
Precursor Mass: 870.7115+3
Delta Mass: 6.895 ppm
XCorr: 34.19

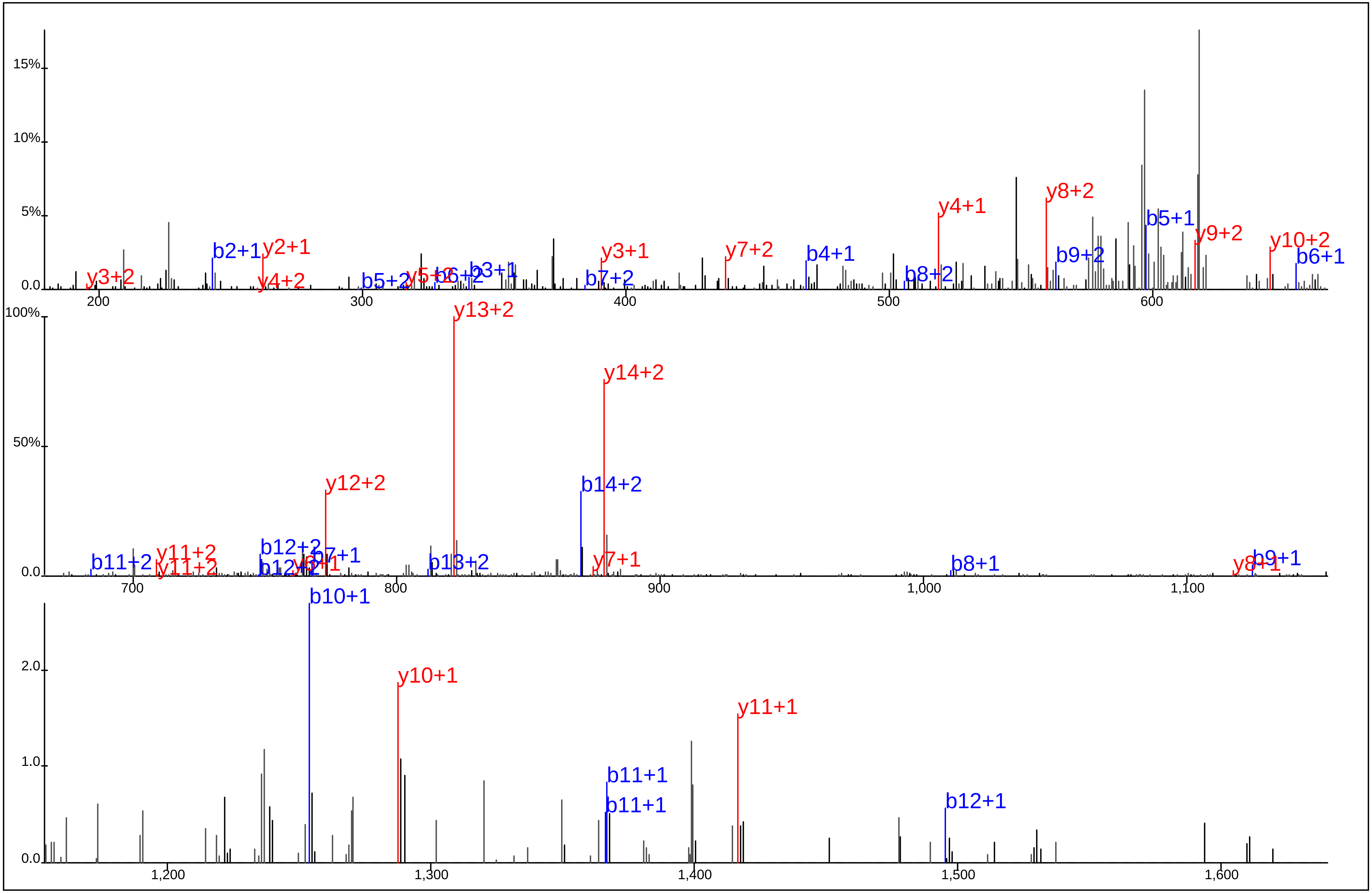

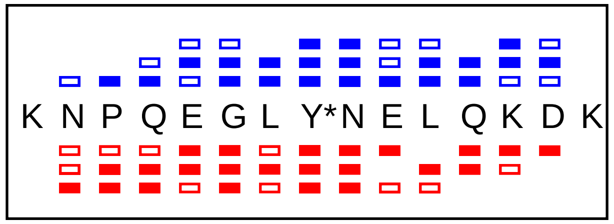

Protein Name: CD3 zeta
gi Accession: 37595565
Precursor Mass: 628.6354+3
Delta Mass: 0.923 ppm
XCorr: 37.02

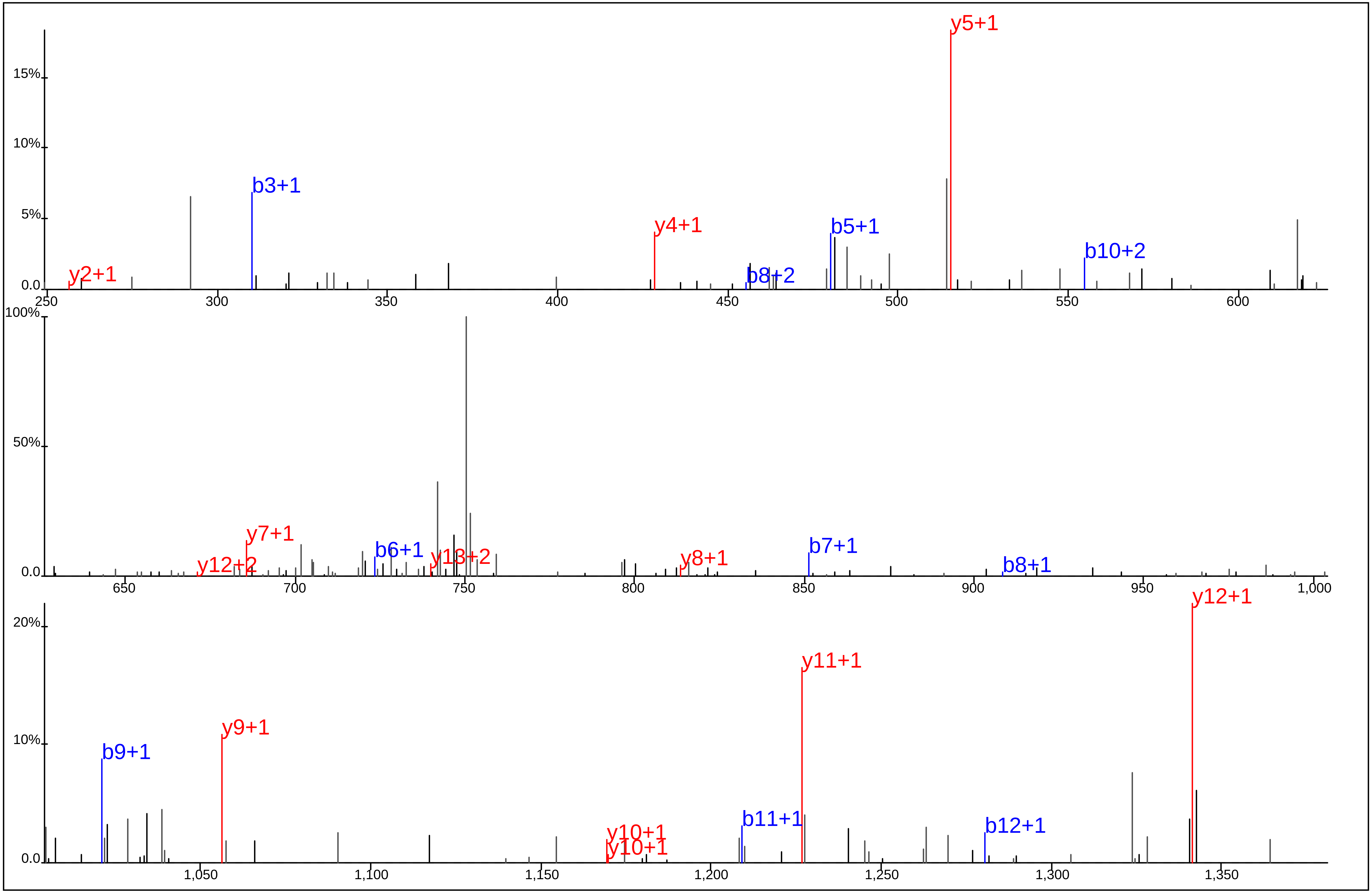

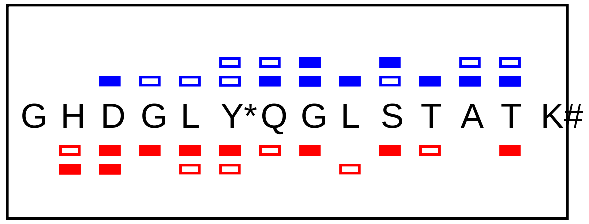

Protein Name:	CD3 zeta
gi Accession:	37595565
Precursor Mass:	768.3522+2
Delta Mass:	0.685 ppm
XCorr:	36.51

219 - CD3zeta_3453717.pdf

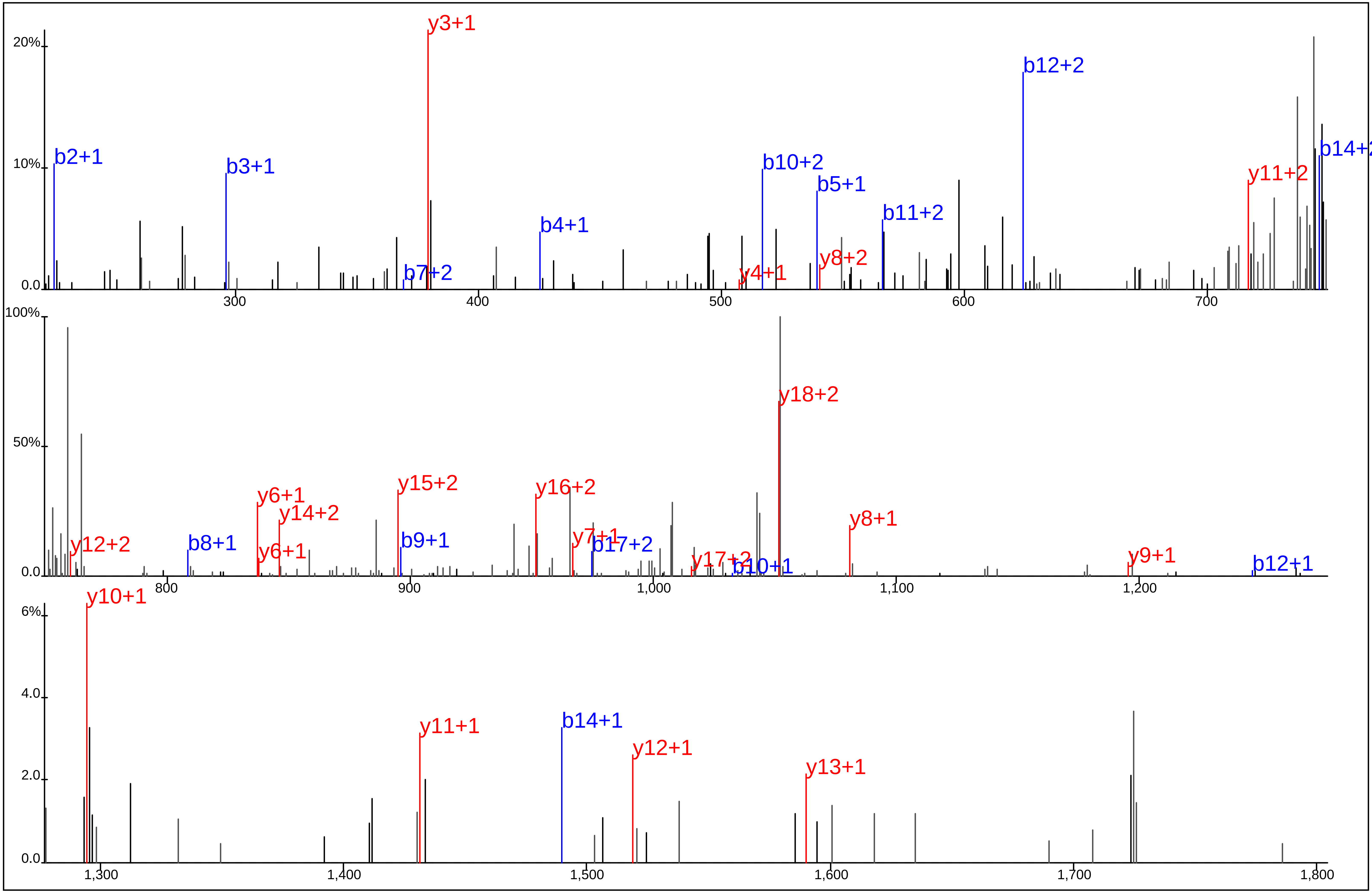

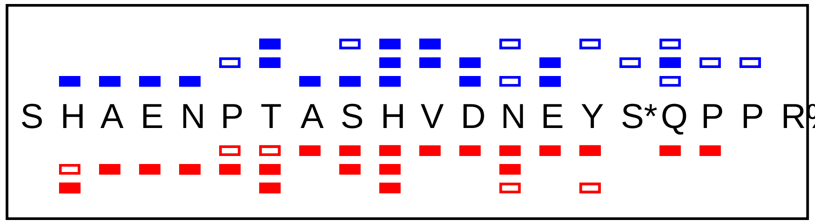

Protein Name: CD5
gi Accession: 116024
Precursor Mass: 775.9984+3
Delta Mass: 2.046 ppm
XCorr: 34.07

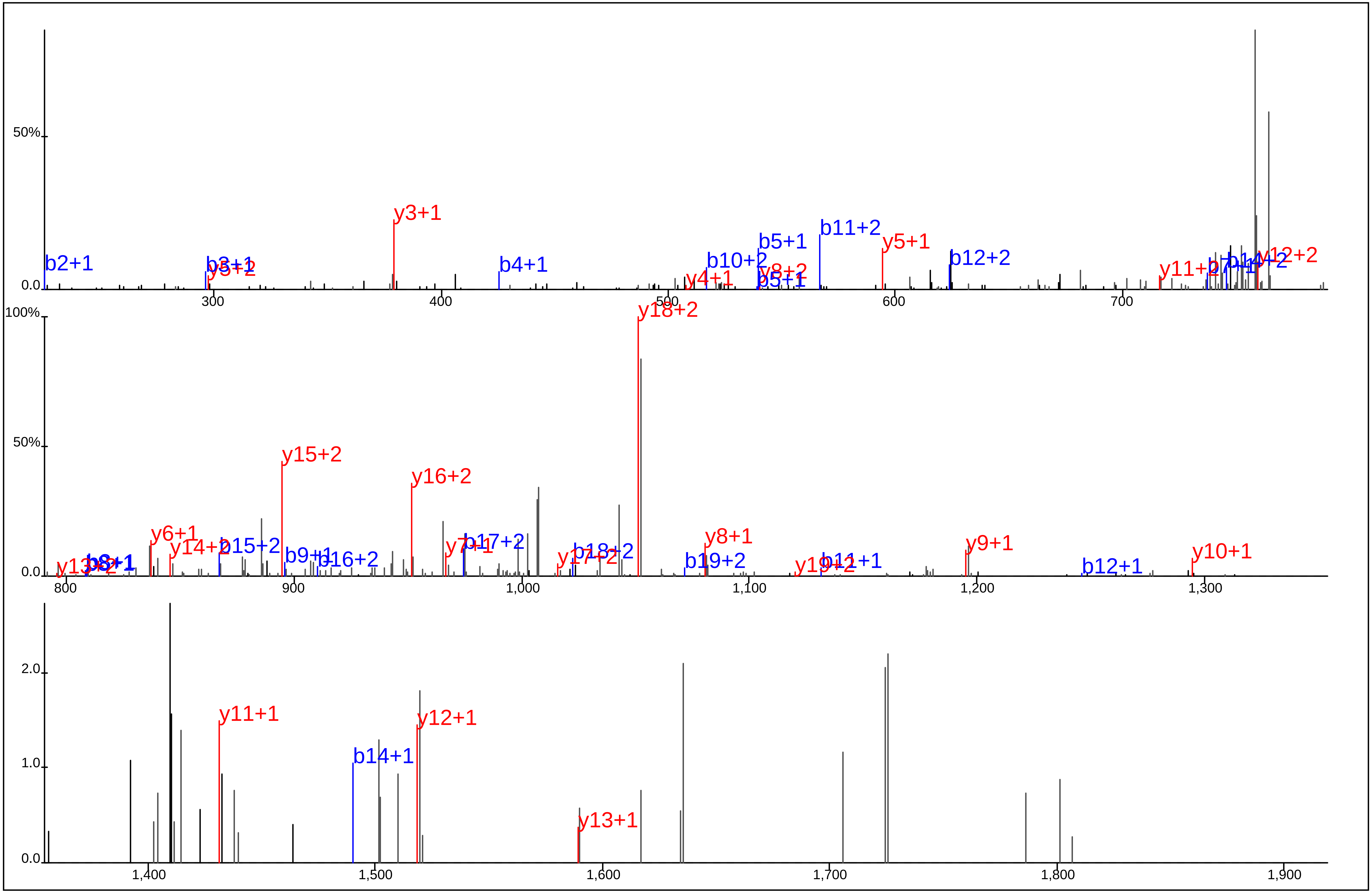

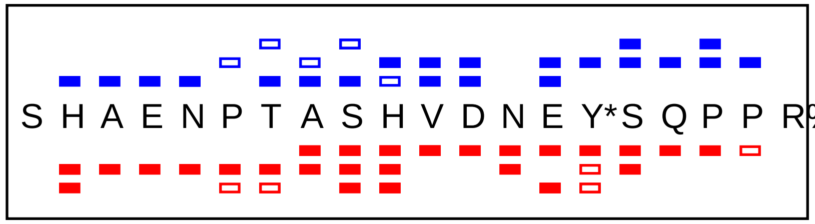

Protein Name: CD5
gi Accession: 116024
Precursor Mass: 776.0000+3
Delta Mass: 4.092 ppm
XCorr: 48.14

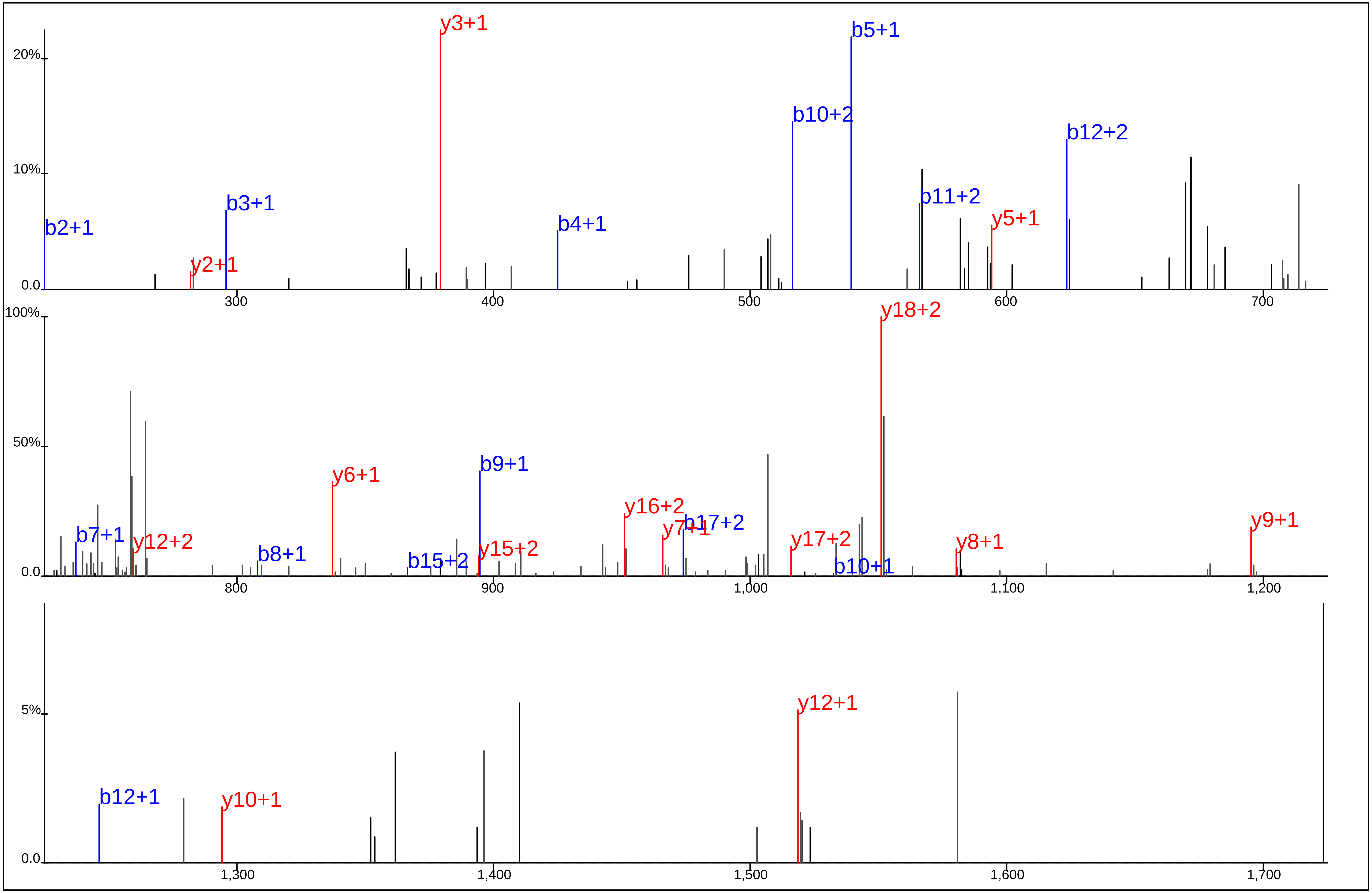

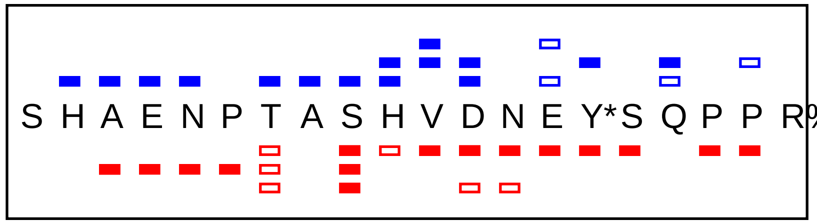

Protein Name: CD5
gi Accession: 116024
Precursor Mass: 775.9993+3
Delta Mass: 3.146 ppm
XCorr: 48.37

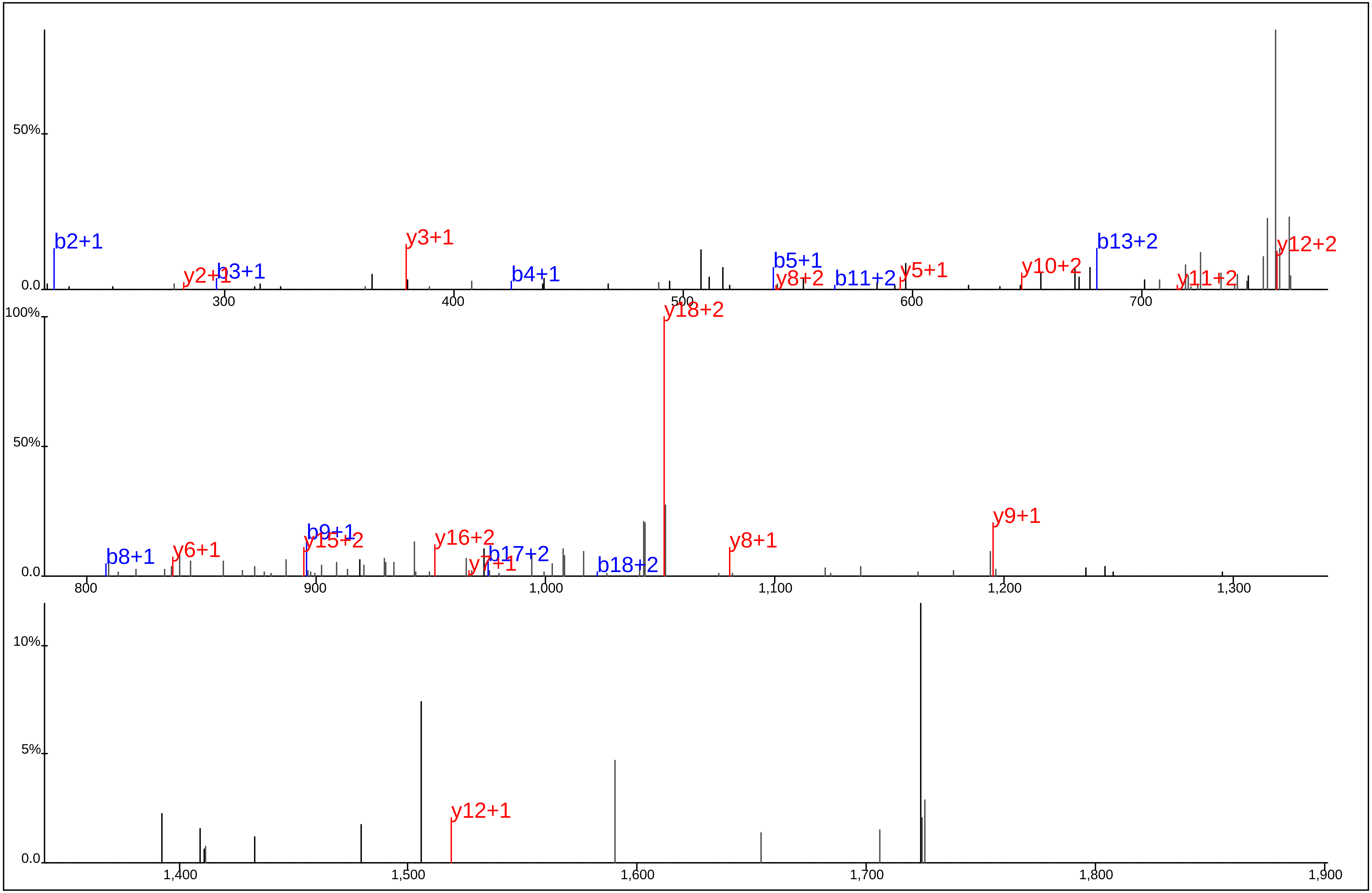

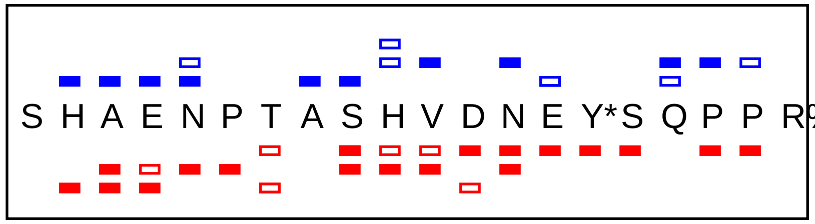

Protein Name: CD5
gi Accession: 116024
Precursor Mass: 776.0038+3
Delta Mass: 8.972 ppm
XCorr: 23.83

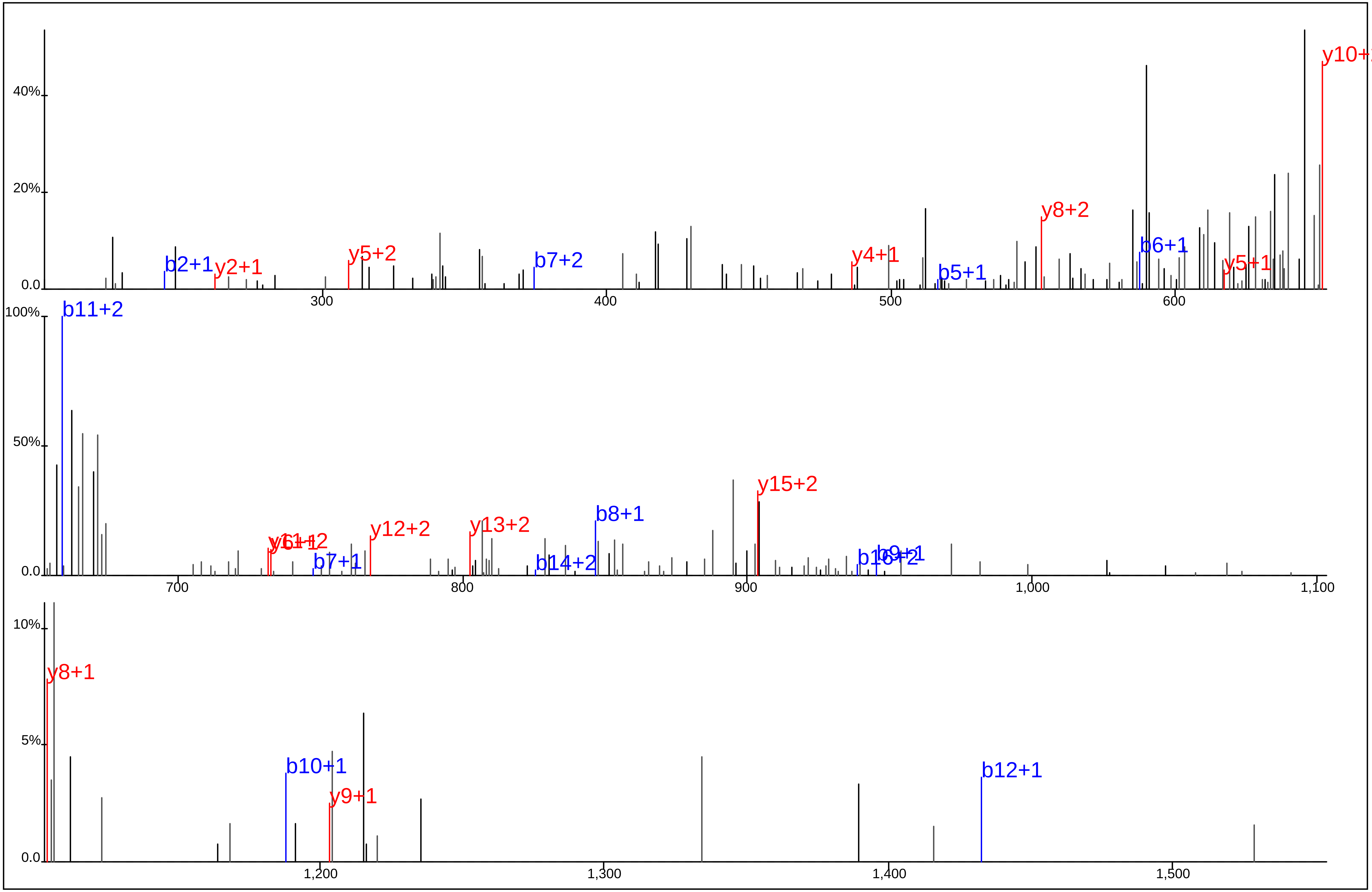

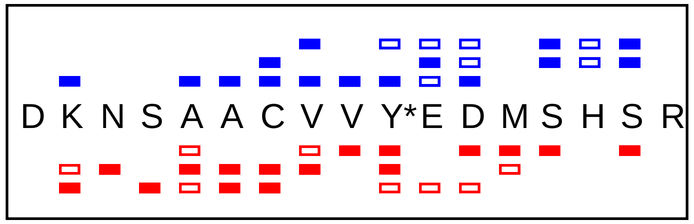

Protein Name: CD7
gi Accession: 5453613
Precursor Mass: 683.6118+3
Delta Mass: 0.277 ppm
XCorr: 21.79

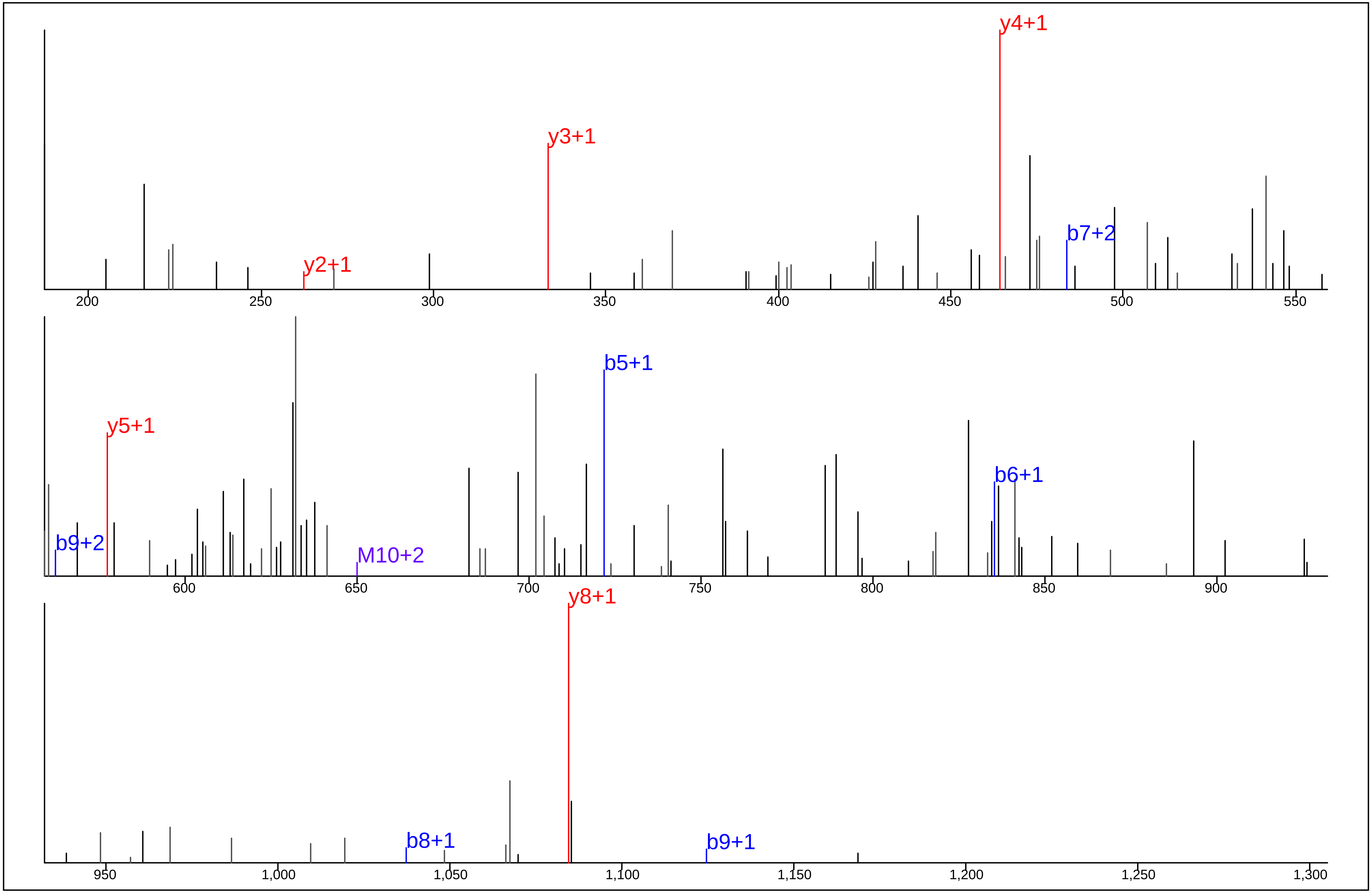

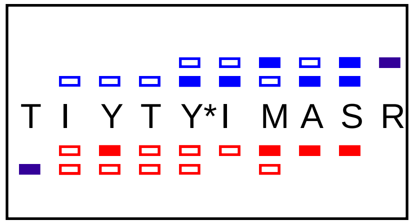

Protein Name: CD84
gi Accession: 74762772
Precursor Mass: 649.7964+2
Delta Mass: 0.368 ppm
XCorr: 22.68

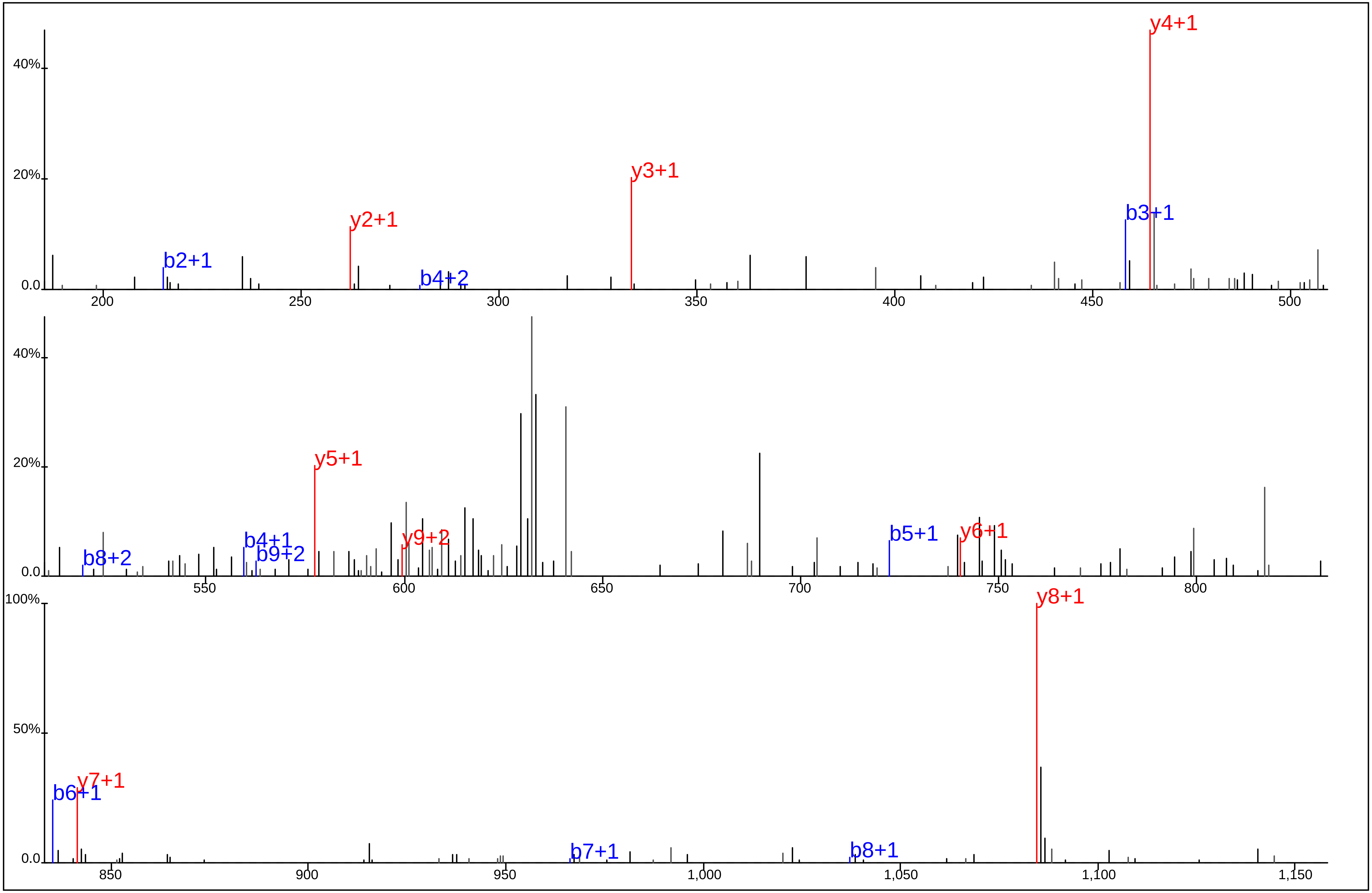

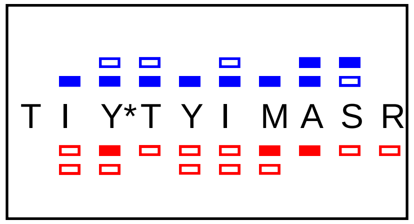

Protein Name: CD84
gi Accession: 74762772
Precursor Mass: 649.7988+2
Delta Mass: 4.034 ppm
XCorr: 41.17

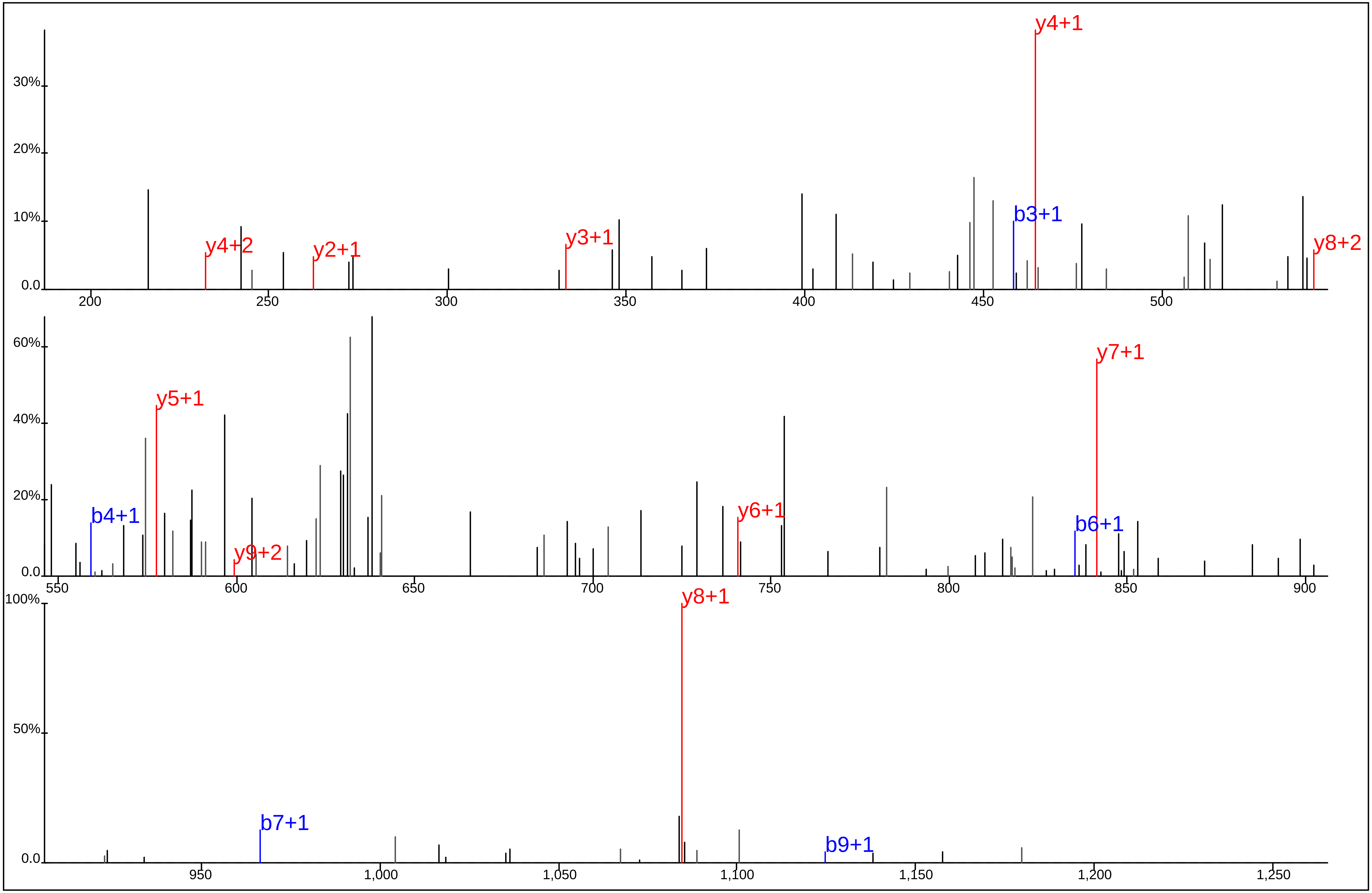

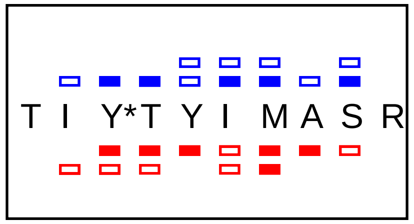

Protein Name: CD84
gi Accession: 74762772
Precursor Mass: 649.7974+2
Delta Mass: 1.870 ppm
XCorr: 17.88

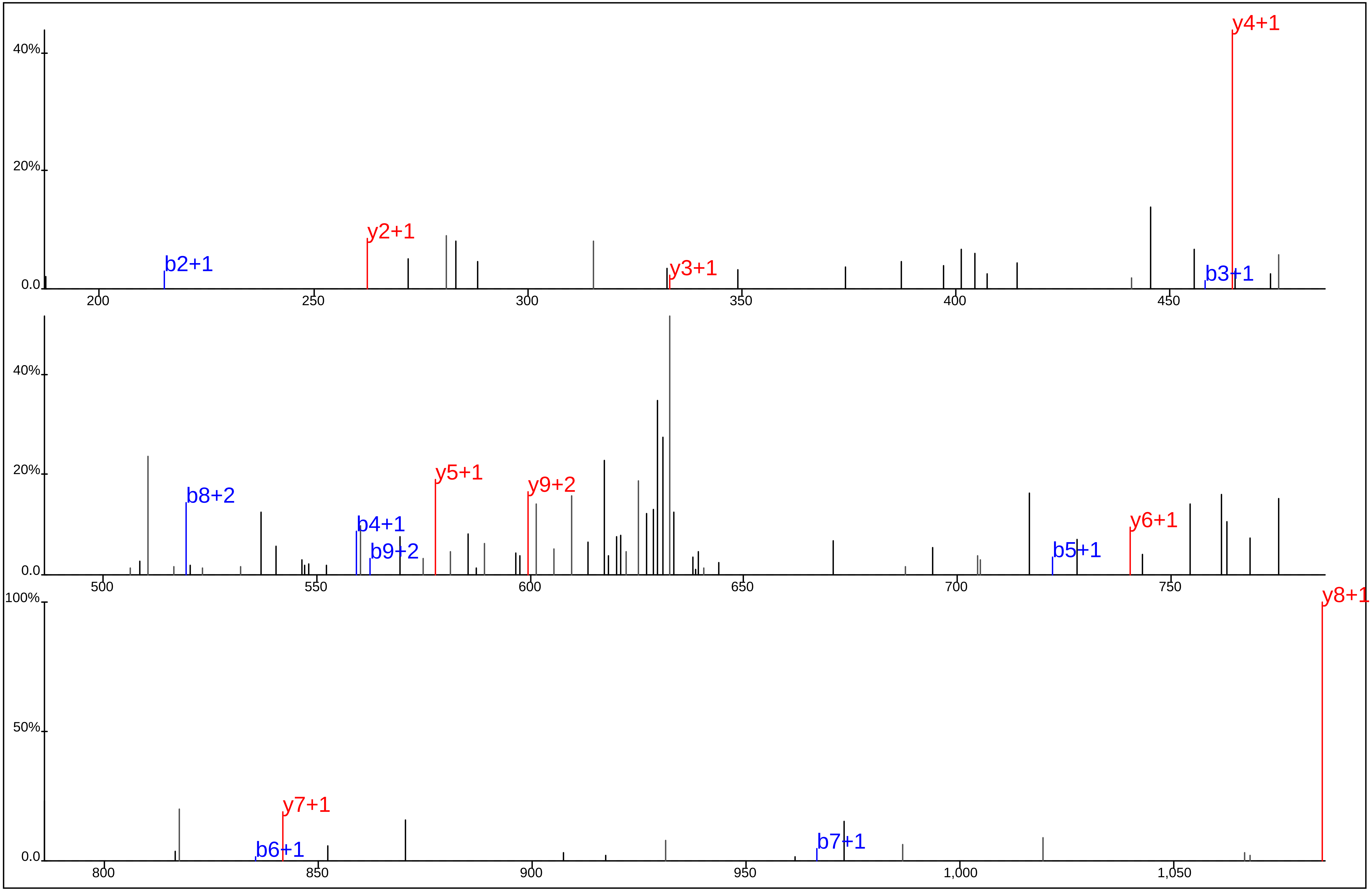

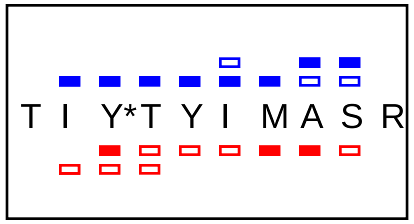

Protein Name: CD84
gi Accession: 74762772
Precursor Mass: 649.8002+2
Delta Mass: 6.198 ppm
XCorr: 23.63

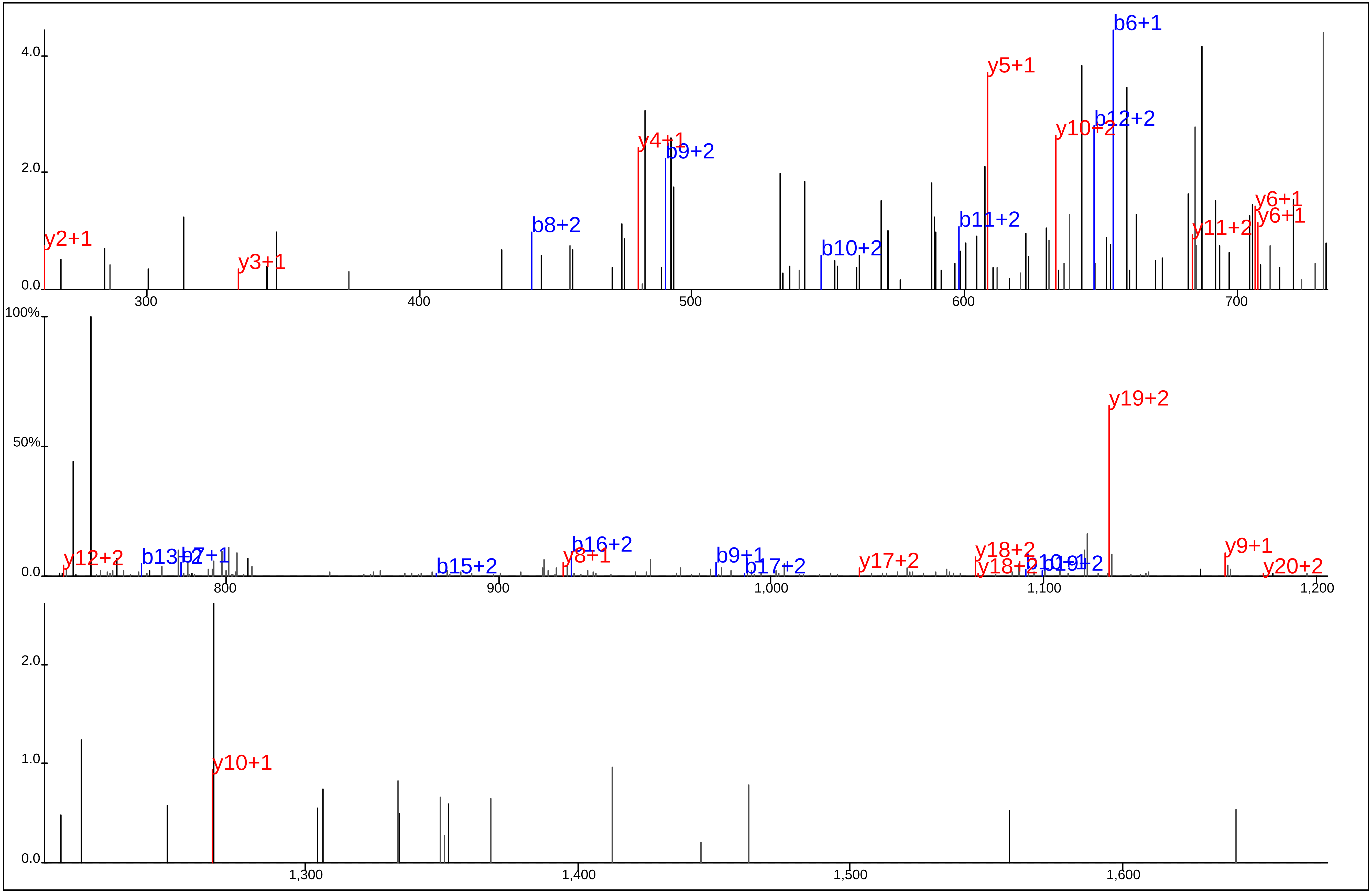

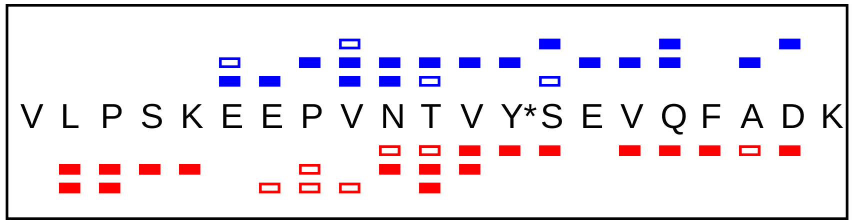

Protein Name: CD84
gi Accession: 74762772
Precursor Mass: 820.3973+3
Delta Mass: 1.349 ppm
XCorr: 18.58

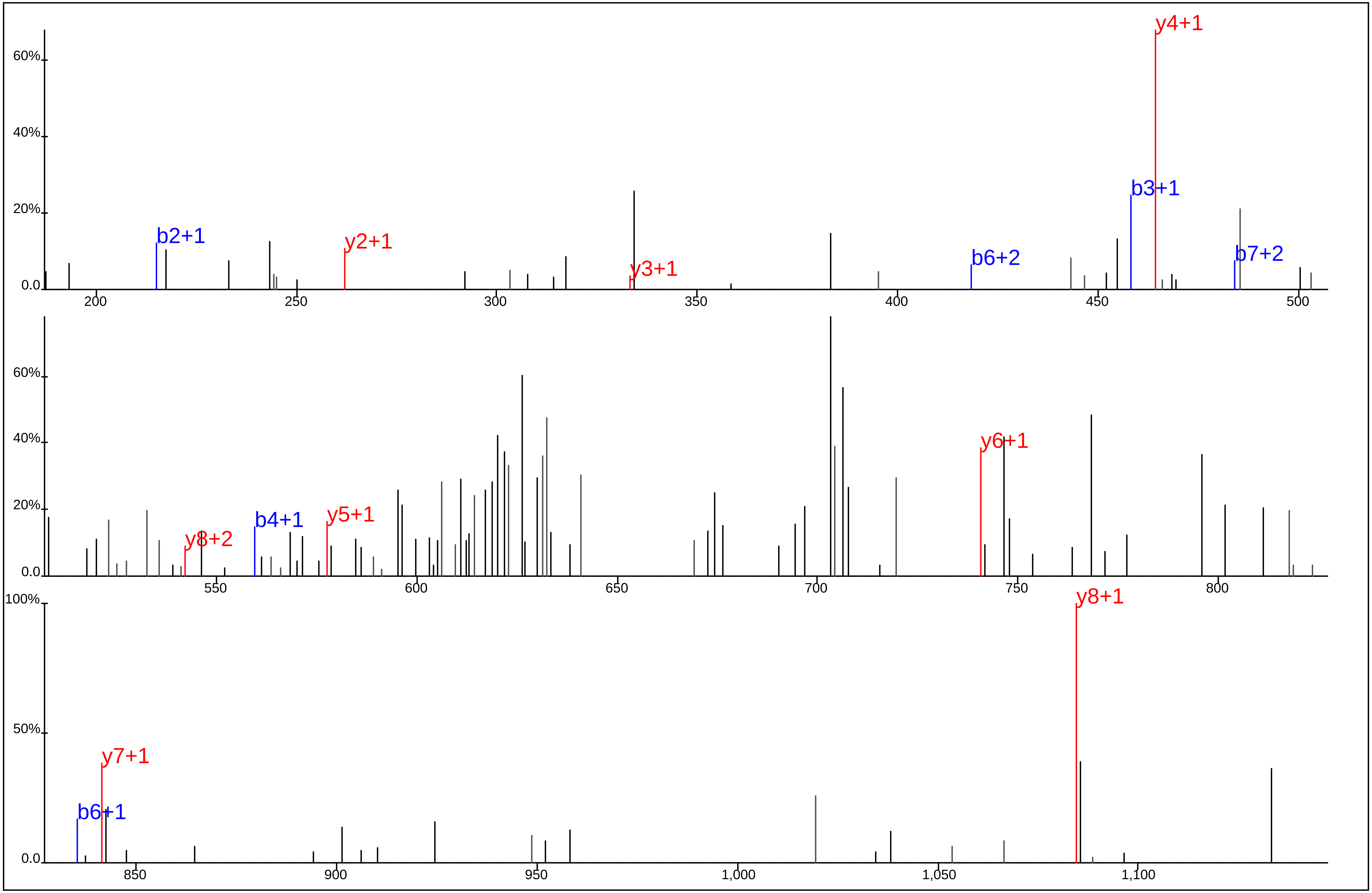

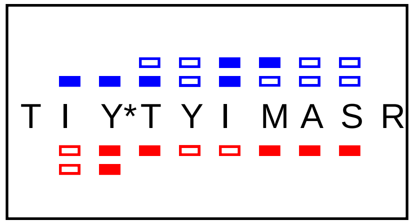

Protein Name: CD84
gi Accession: 74762772
Precursor Mass: 649.7984+2
Delta Mass: 3.471 ppm
XCorr: 18.2

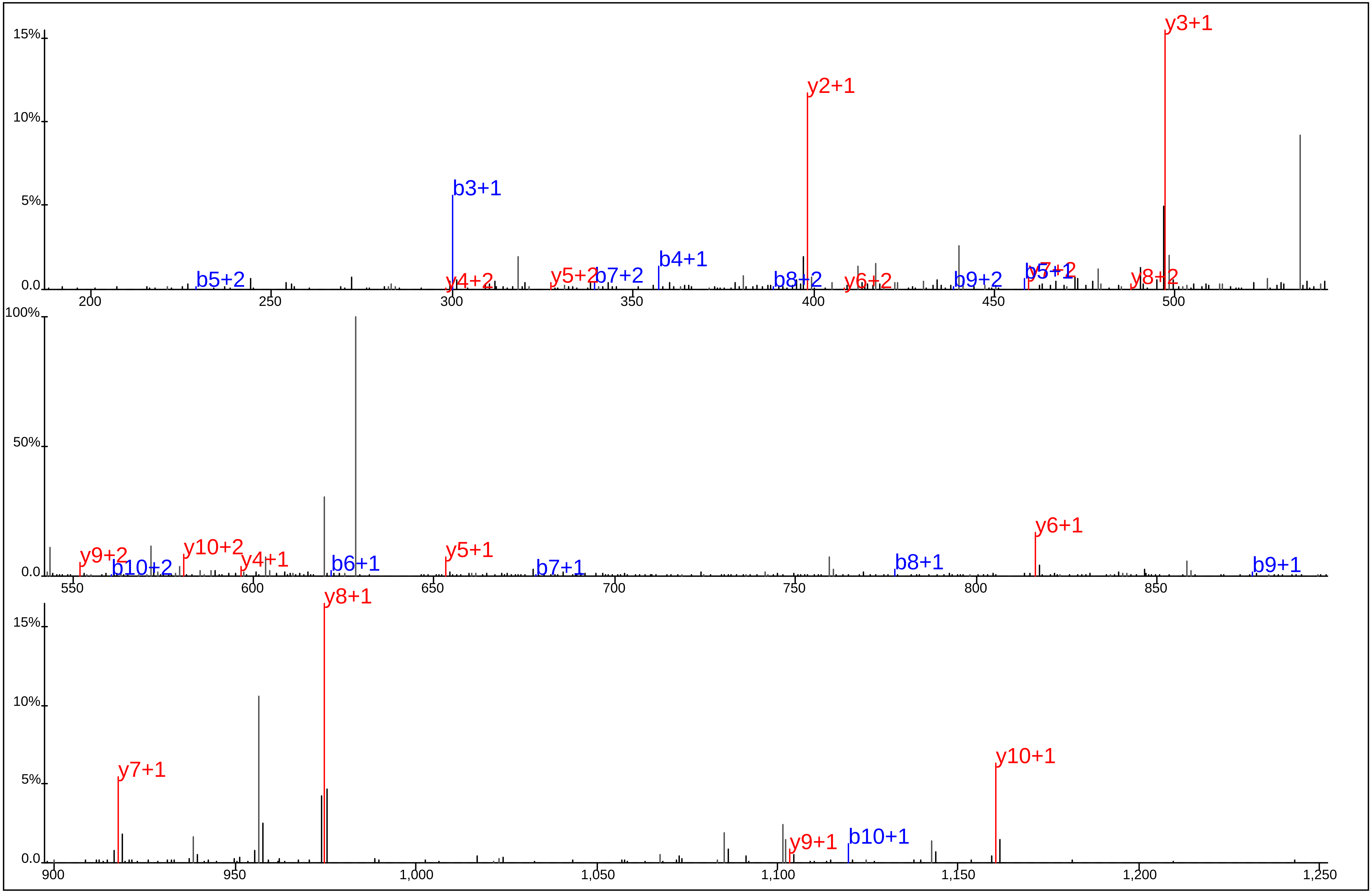

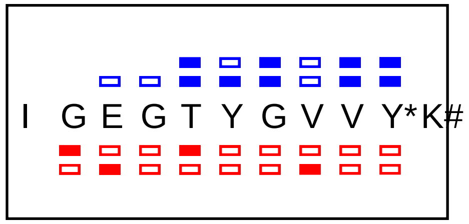

Protein Name:	CDC2
gi Accession:	24158643
Precursor Mass:	637.3041+2
Delta Mass:	4.278 ppm
XCorr:	47.92

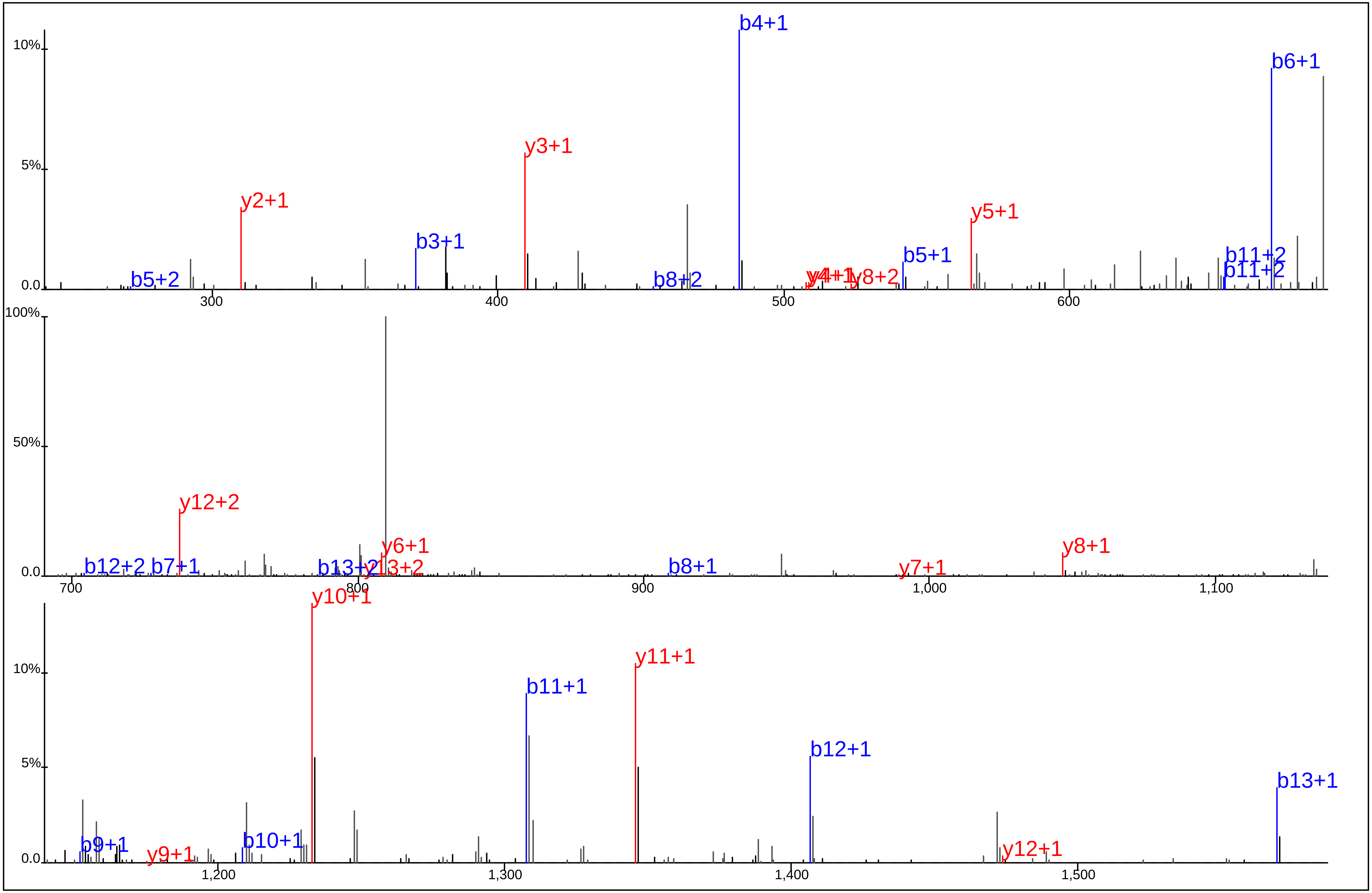

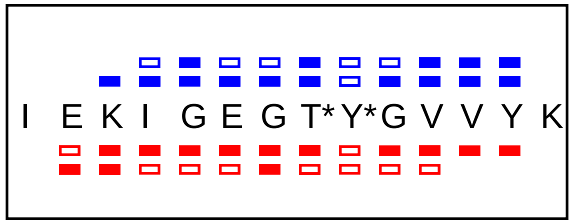

Protein Name: CDC2
gi Accession: 16306492
Precursor Mass: 858.3904+2
Delta Mass: 2.458 ppm
XCorr: 48.24

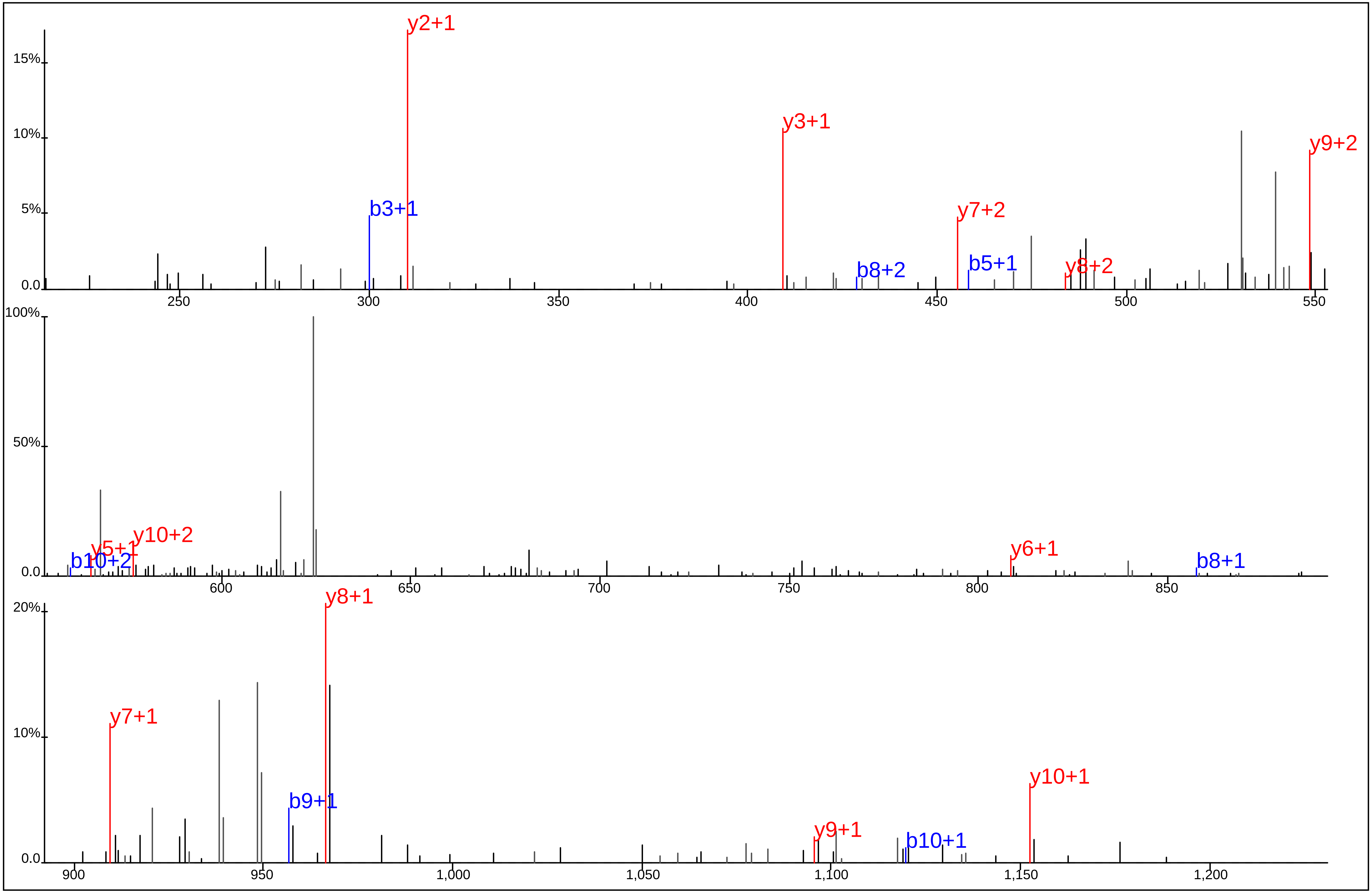

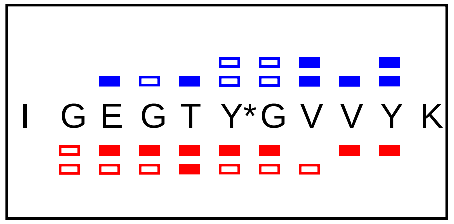

Protein Name: CDC2
gi Accession: 24158643
Precursor Mass: 633.2957+2
Delta Mass: 2.219 ppm
XCorr: 48.85

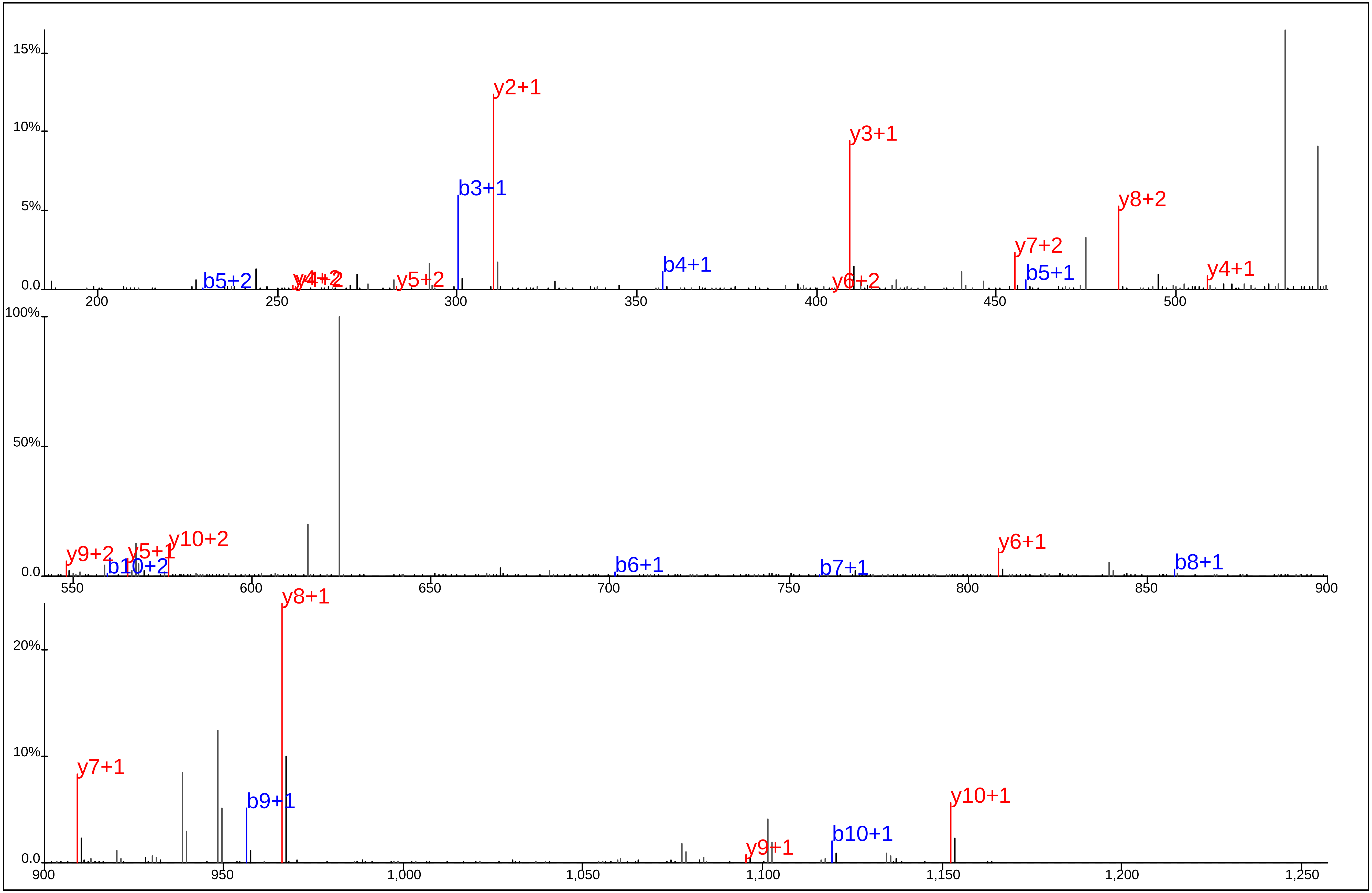

I G E G T Y*G V V Y K

Protein Name:	CDC2
gi Accession:	24158643
Precursor Mass:	633.2950+2
Delta Mass:	1.160 ppm
XCorr:	42.98

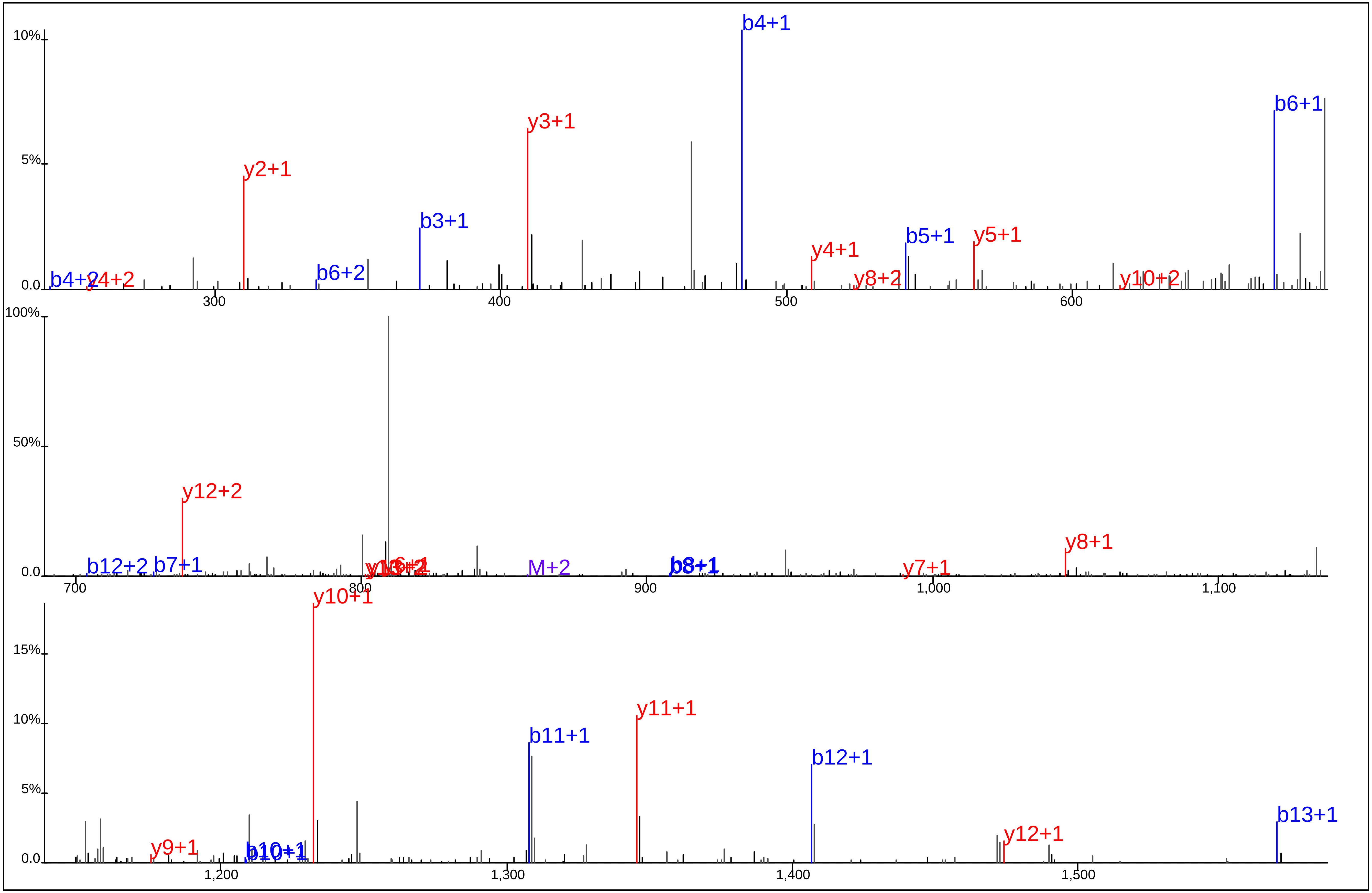

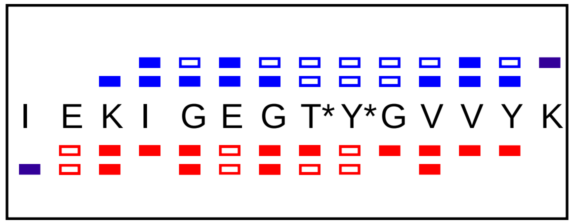

Protein Name: CDC2
gi Accession: 16306492
Precursor Mass: 858.3882+2
Delta Mass: 0.036 ppm
XCorr: 45.78

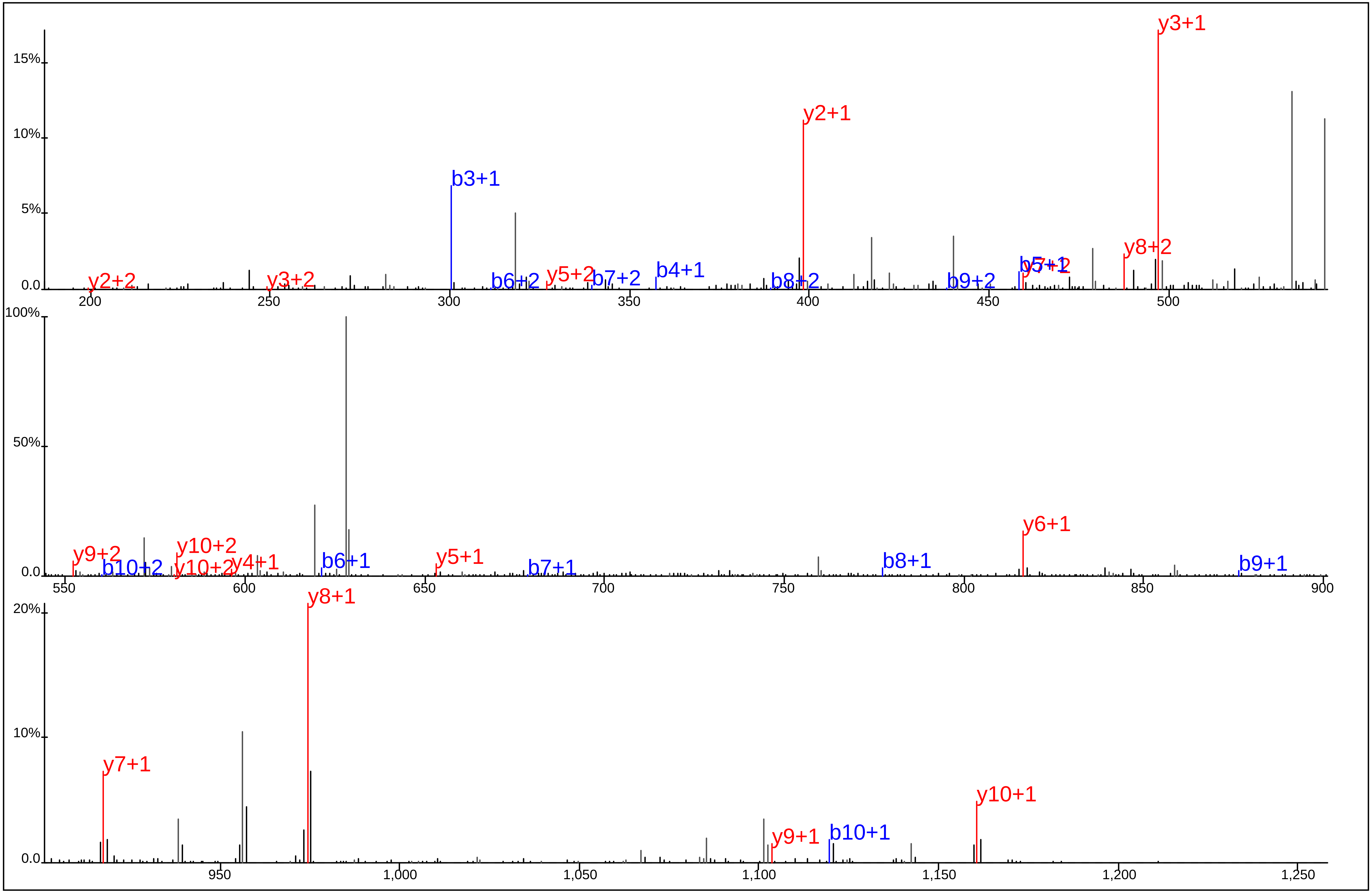

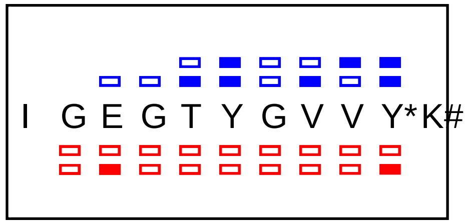

Protein Name: CDC2
gi Accession: 24158643
Precursor Mass: 637.3022+2
Delta Mass: 1.310 ppm
XCorr: 47.75

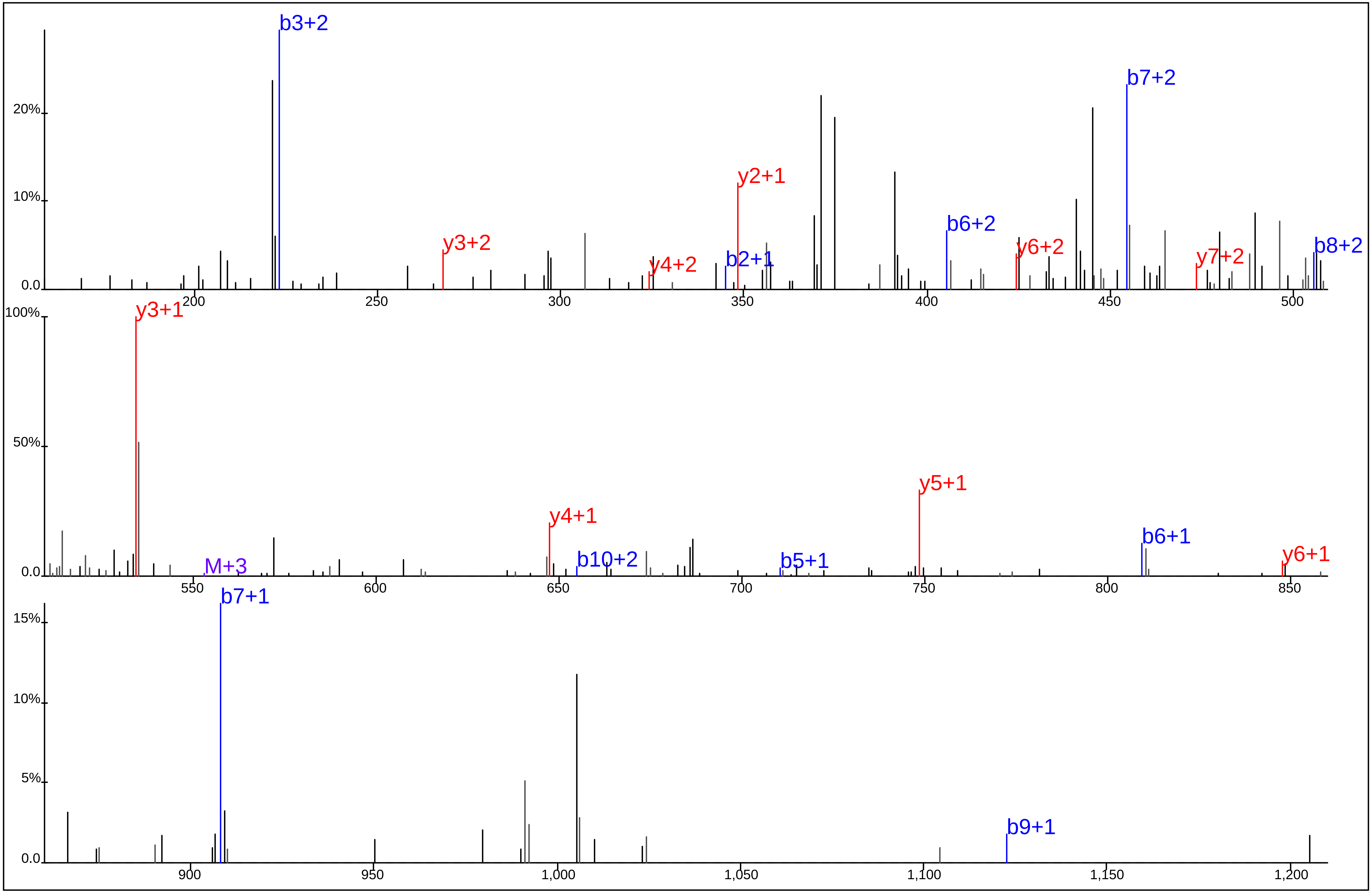

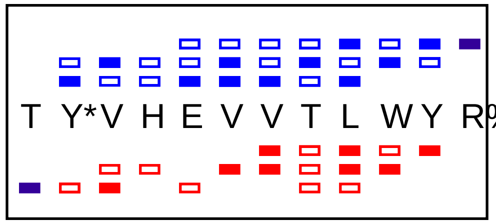

Protein Name:	CDC2
gi Accession:	24158643
Precursor Mass:	552.6034+3
Delta Mass:	6.002 ppm
XCorr:	27.89

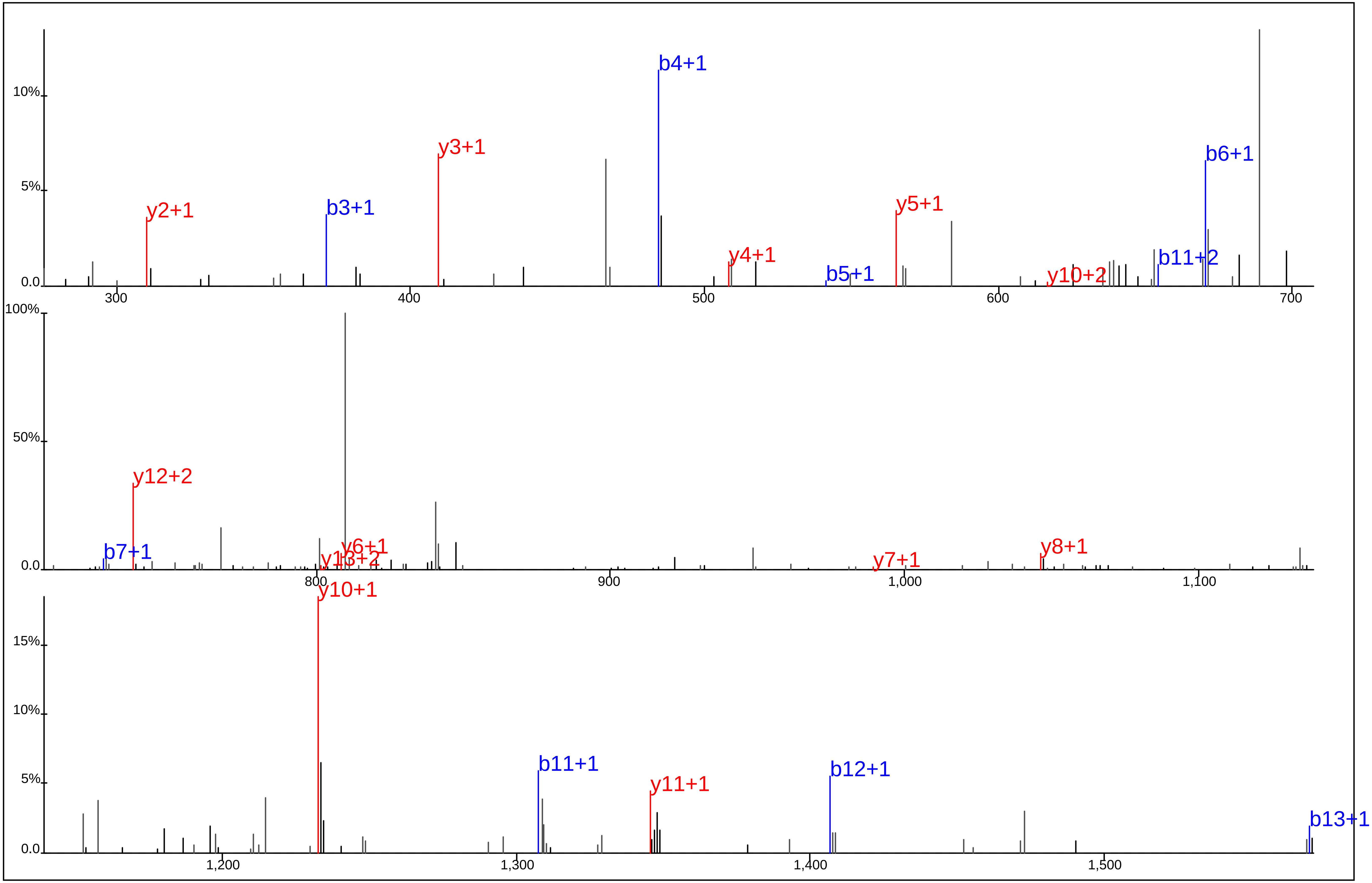

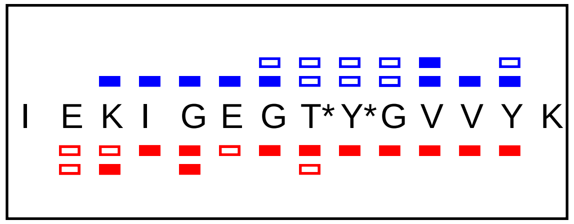

Protein Name:	CDC2
gi Accession:	16306492
Precursor Mass:	858.3895+2
Delta Mass:	1.462 ppm
XCorr:	43.68

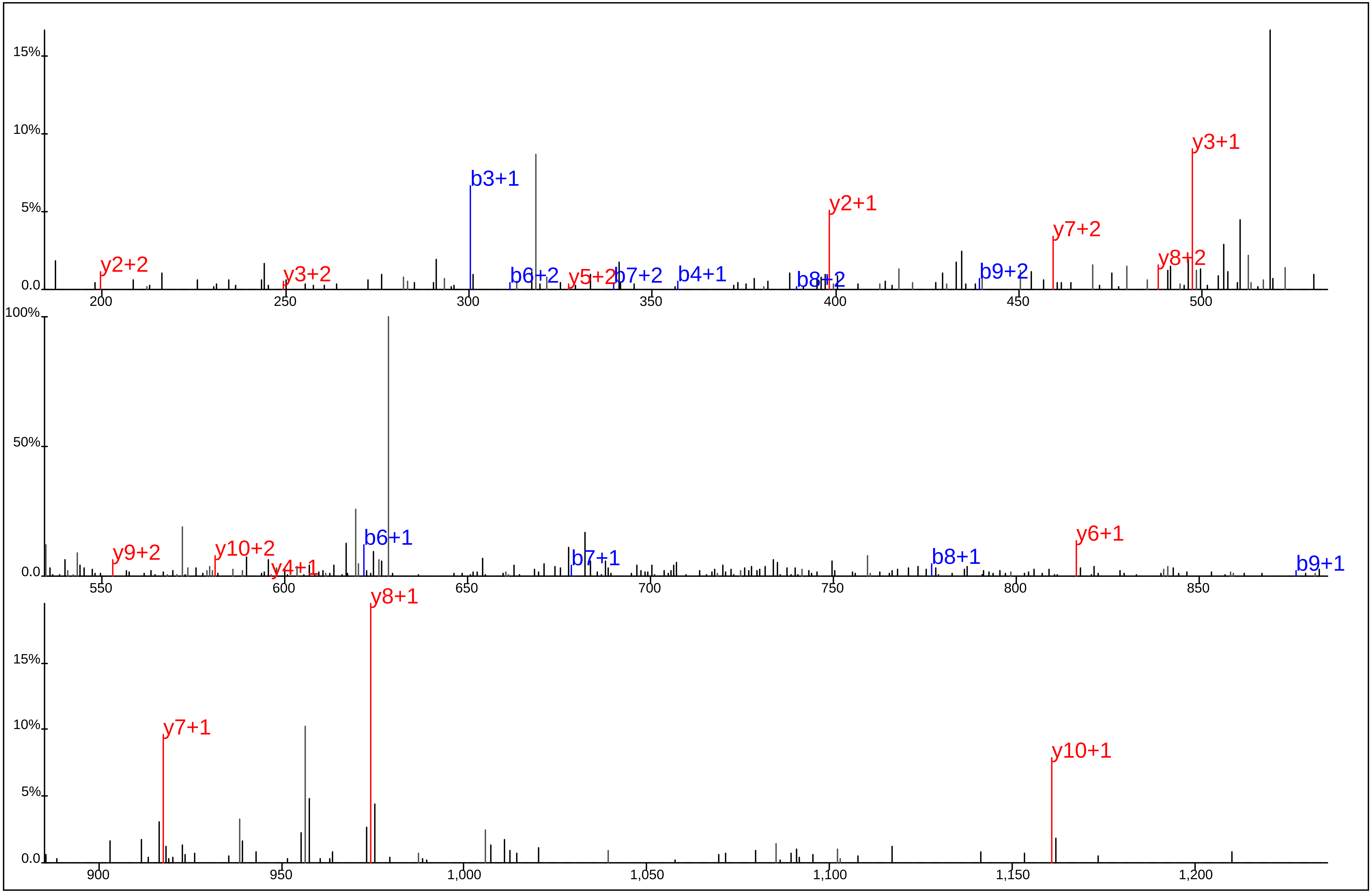

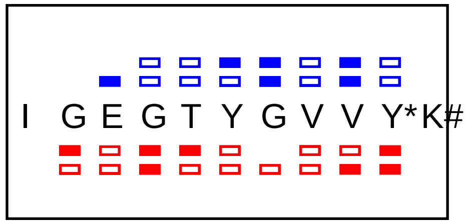

Protein Name: CDC2
gi Accession: 24158643
Precursor Mass: 637.3025+2
Delta Mass: 1.687 ppm
XCorr: 22.34

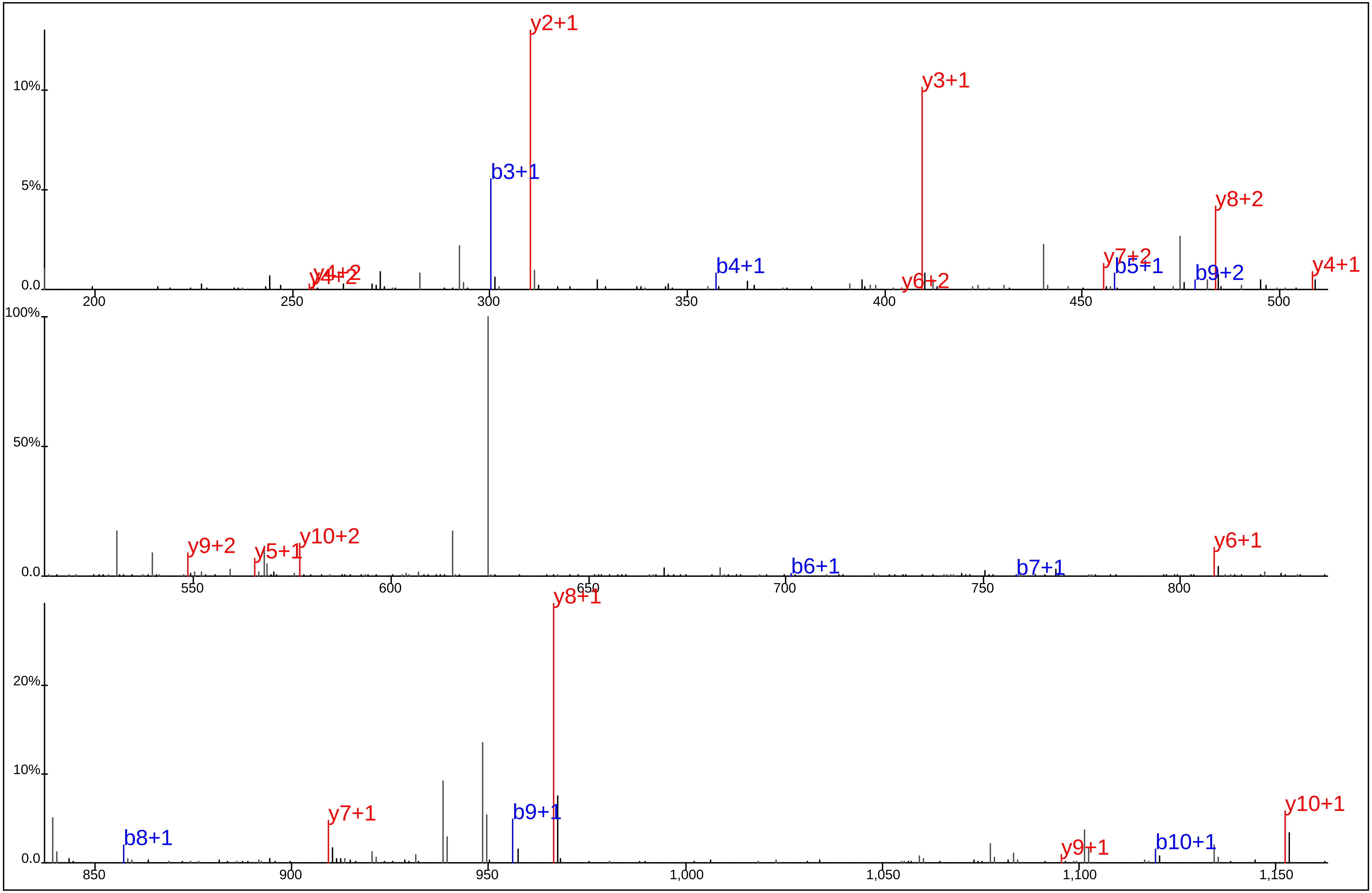

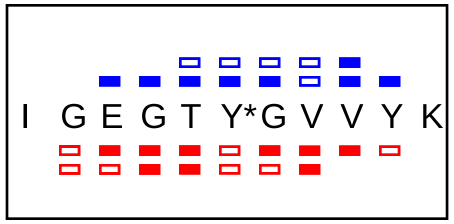

Protein Name: CDC2
gi Accession: 24158643
Precursor Mass: 633.2935+2
Delta Mass: 1.250 ppm
XCorr: 48.84

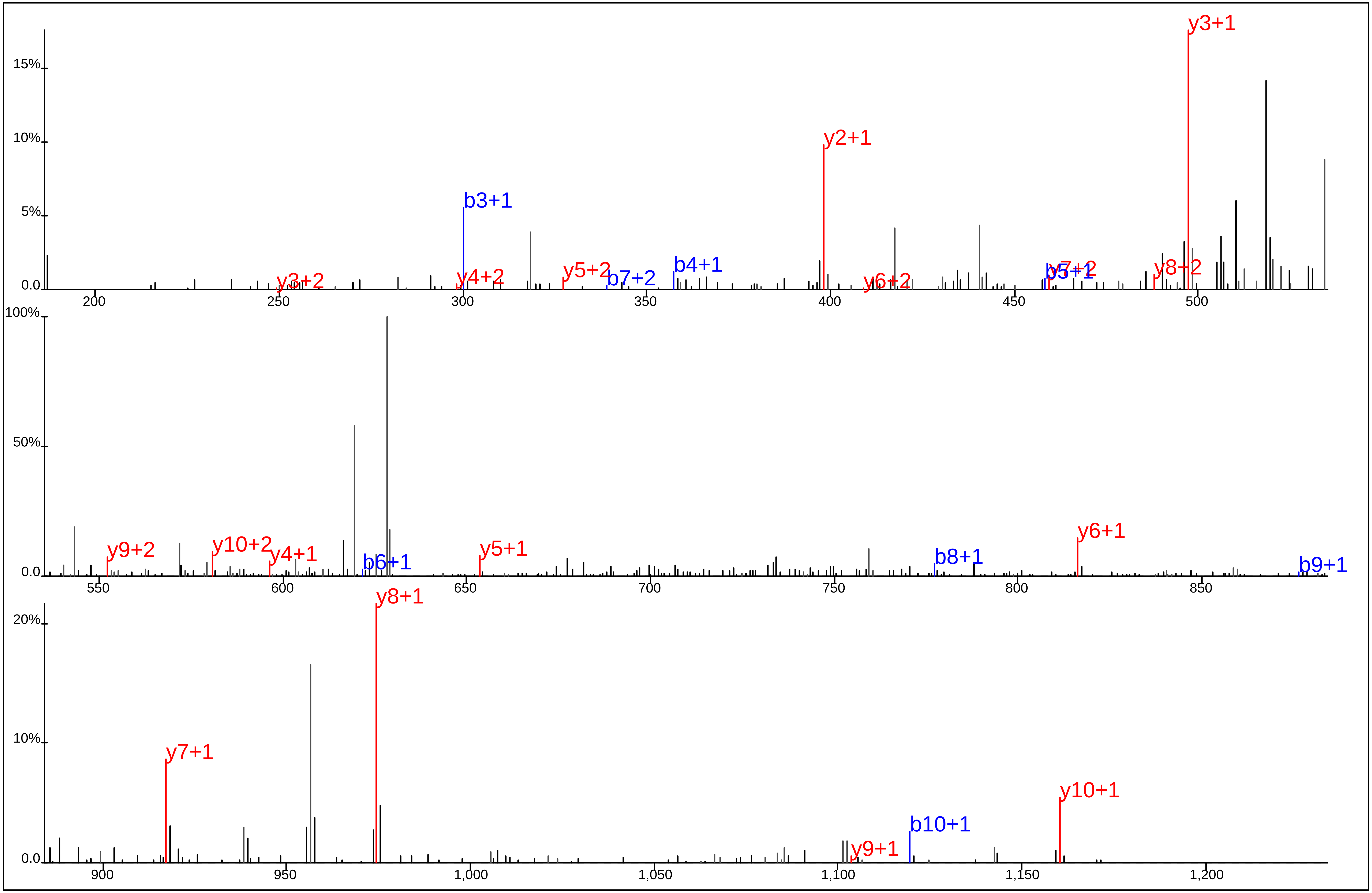

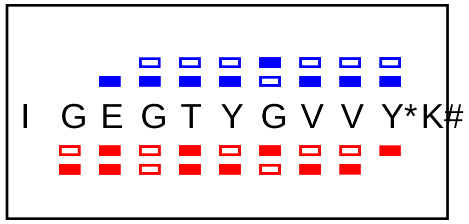

Protein Name: CDC2
gi Accession: 24158643
Precursor Mass: 637.3022+2
Delta Mass: 1.310 ppm
XCorr: 36.17

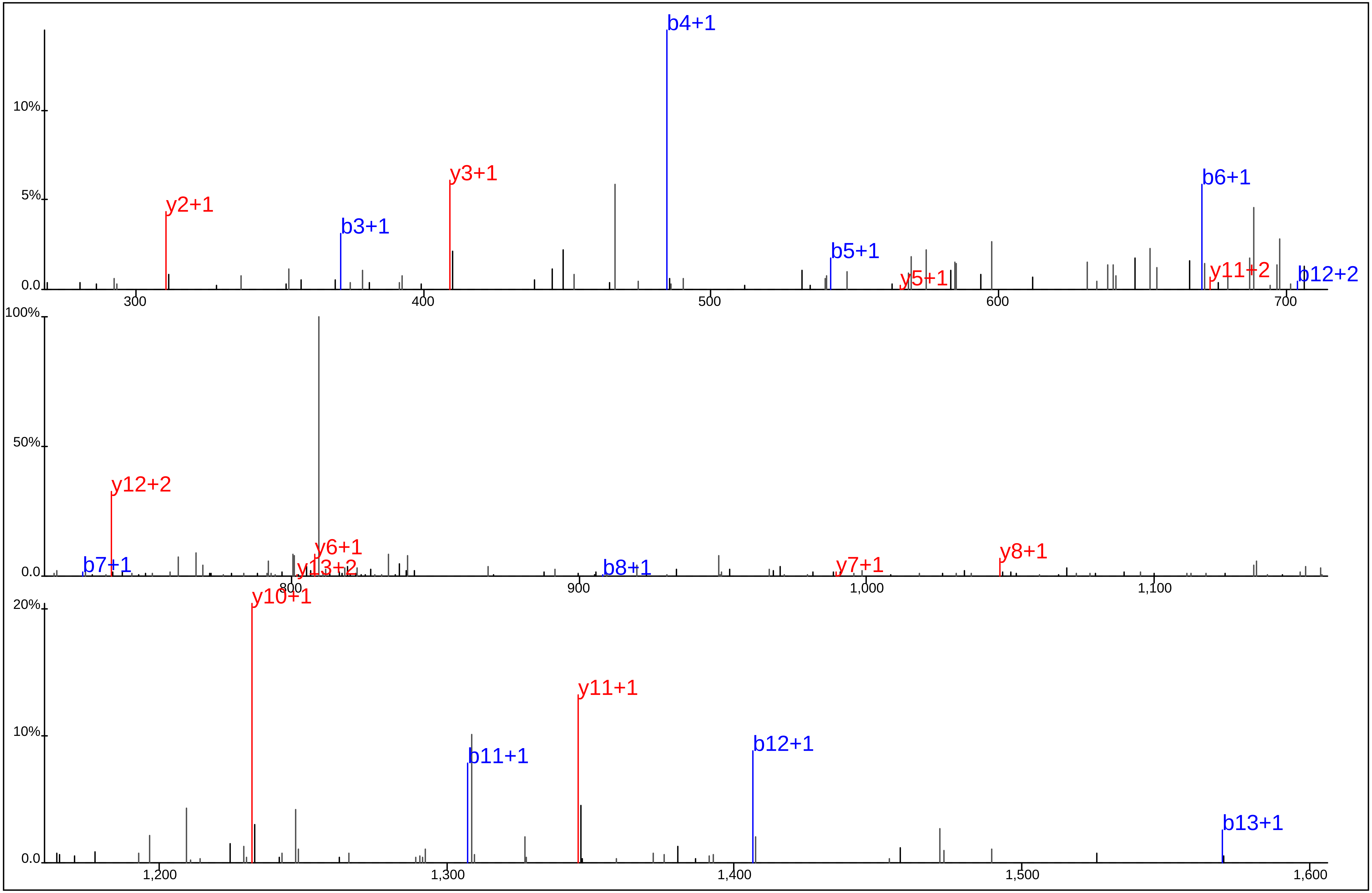

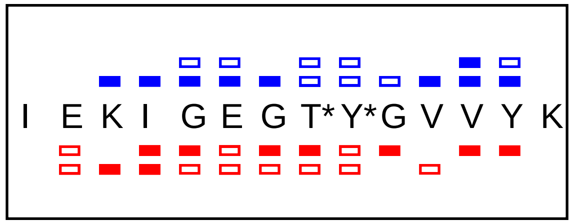

Protein Name: CDC2
gi Accession: 16306492
Precursor Mass: 858.3910+2
Delta Mass: 3.169 ppm
XCorr: 32.29

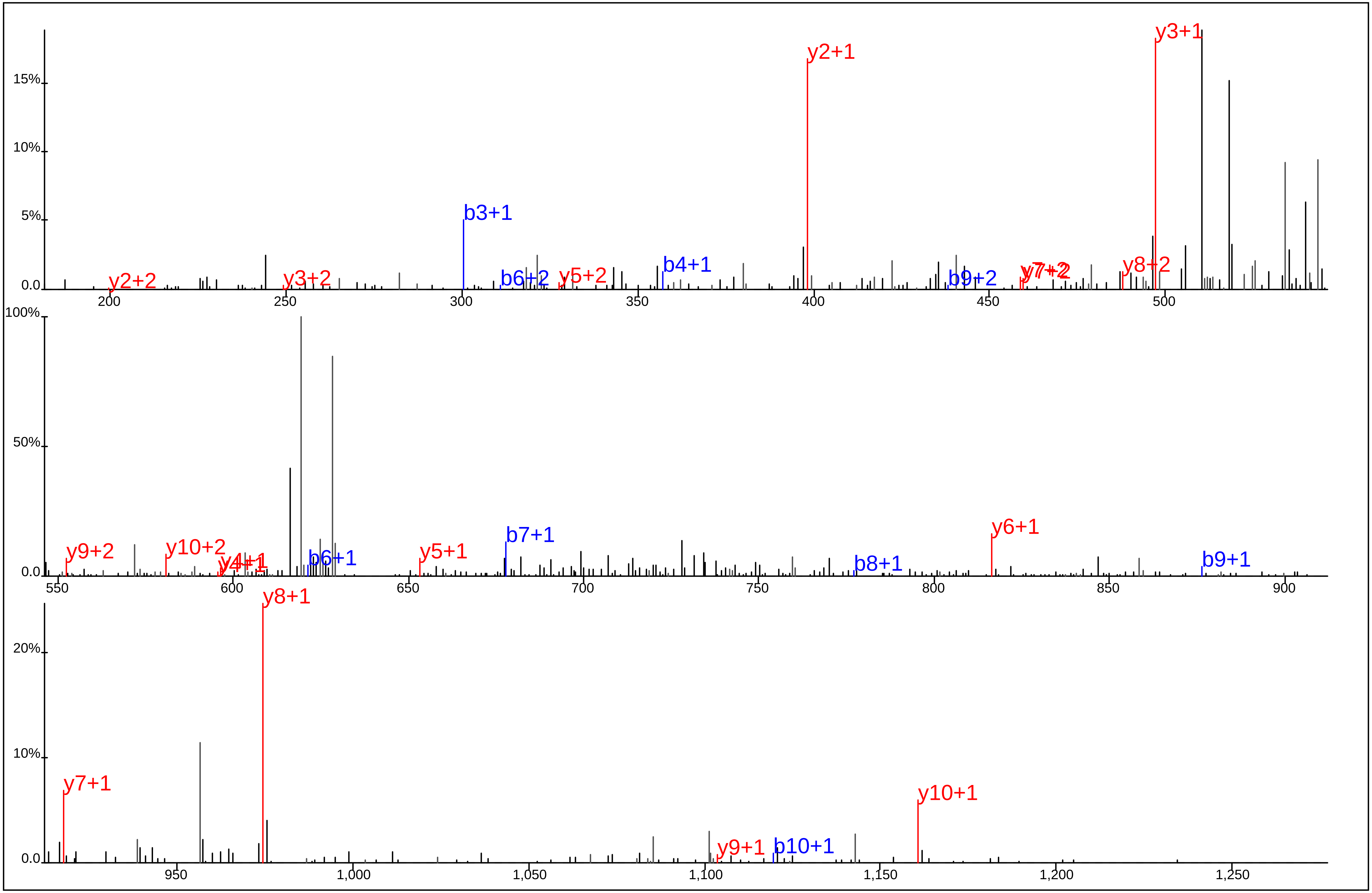

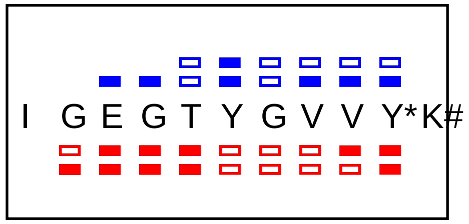

Protein Name: CDC2
gi Accession: 24158643
Precursor Mass: 637.3030+2
Delta Mass: 2.550 ppm
XCorr: 24.24

243 - CDC2_3444211.pdf

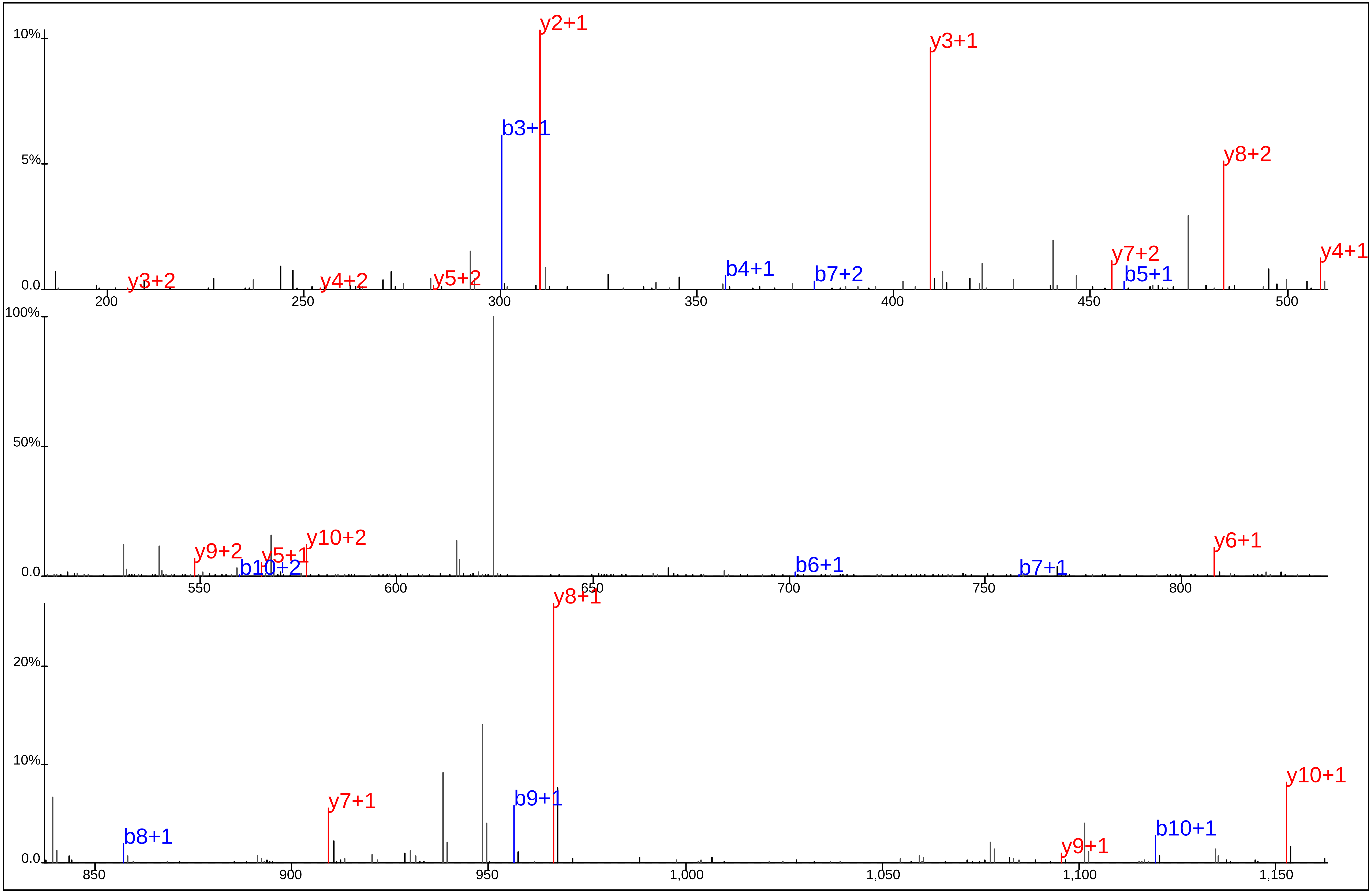

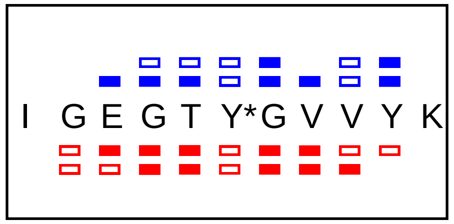

Protein Name: CDC2
gi Accession: 24158643
Precursor Mass: 633.2947+2
Delta Mass: 0.583 ppm
XCorr: 41.96

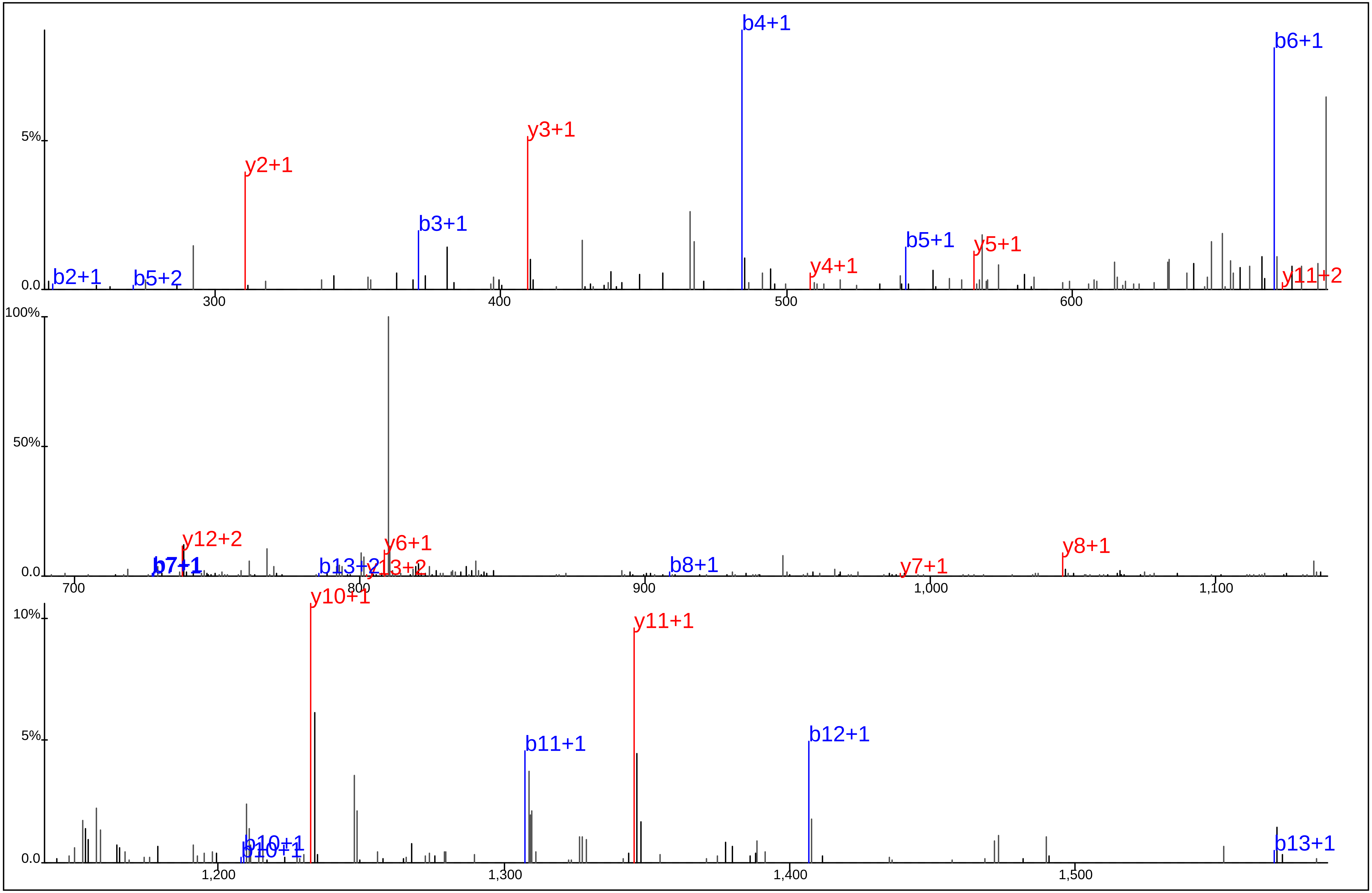

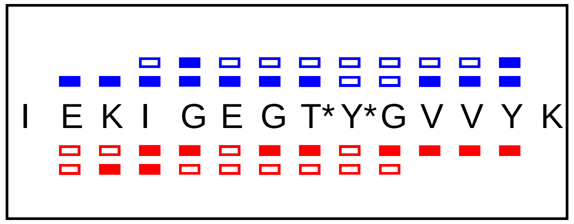

Protein Name: CDC2
gi Accession: 16306492
Precursor Mass: 858.3903+2
Delta Mass: 2.383 ppm
XCorr: 41.99

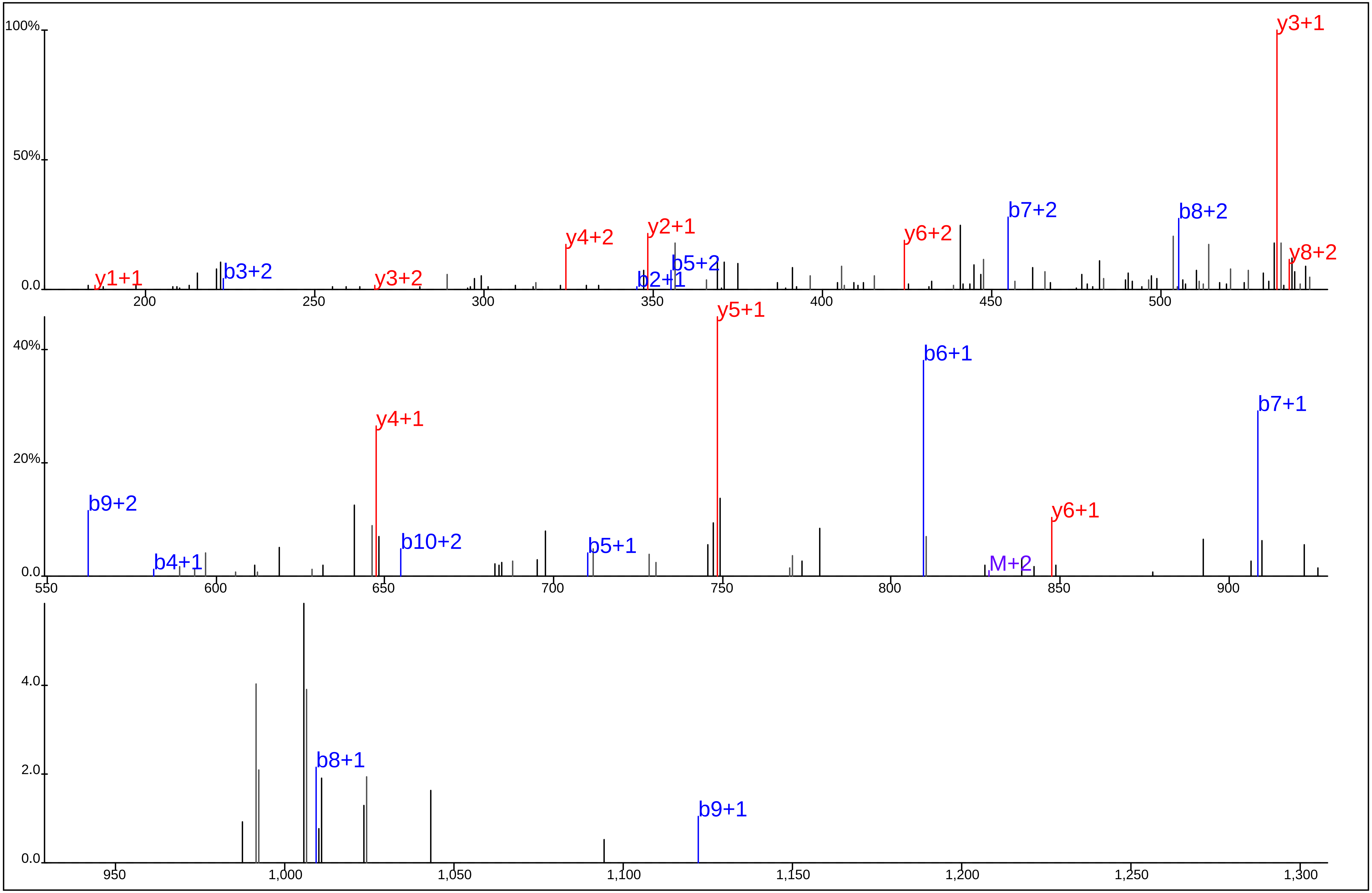

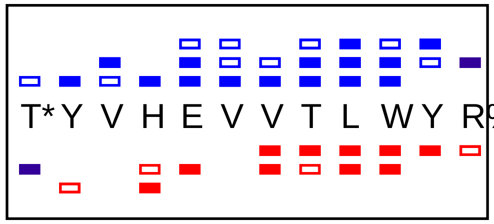

Protein Name: CDC2
gi Accession: 24158643
Precursor Mass: 552.5987+3
Delta Mass: 2.514 ppm
XCorr: 31.77

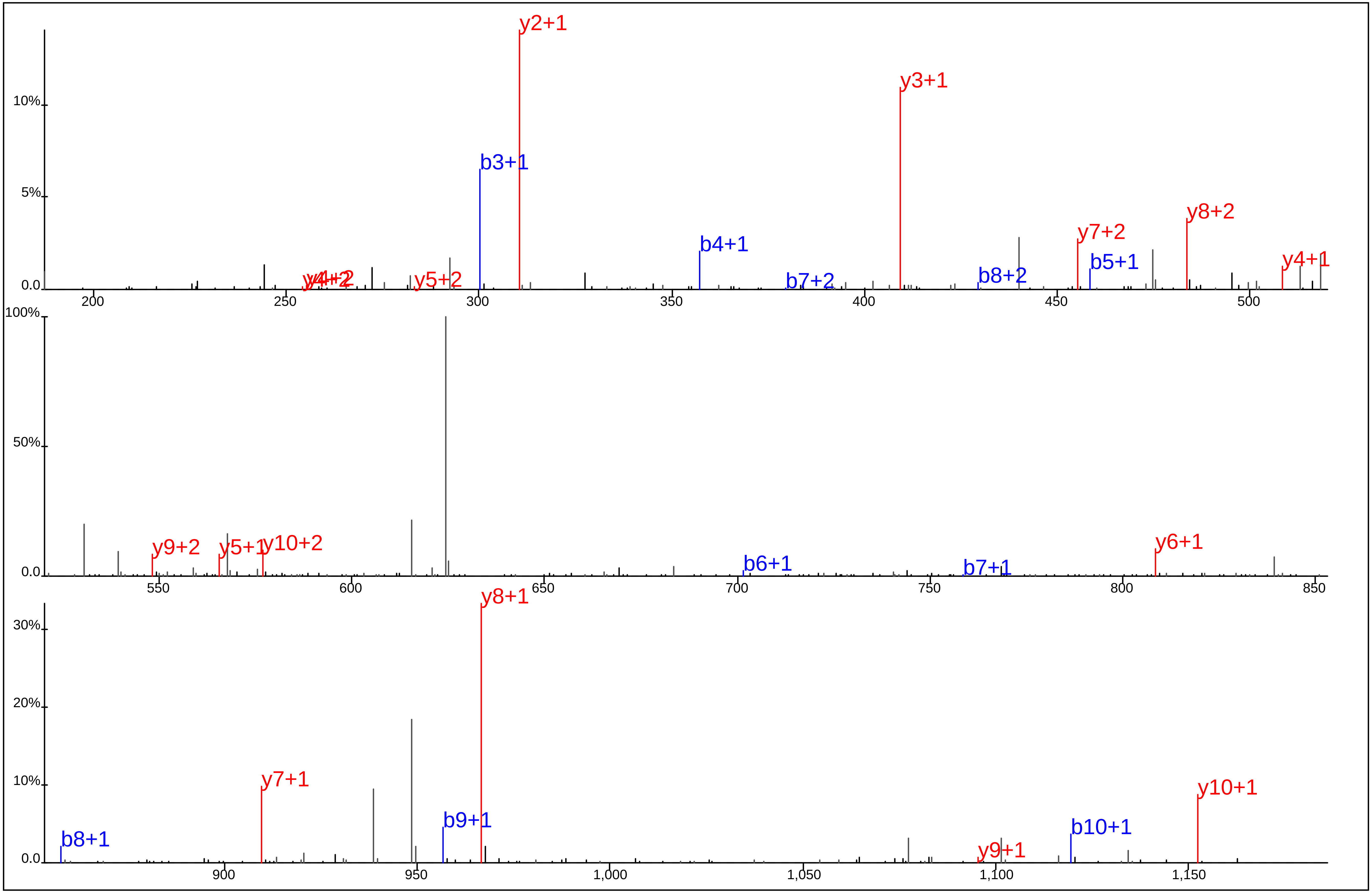

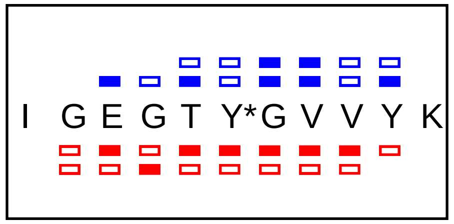

Protein Name: CDC2
gi Accession: 24158643
Precursor Mass: 633.2961+2
Delta Mass: 2.898 ppm
XCorr: 47.73

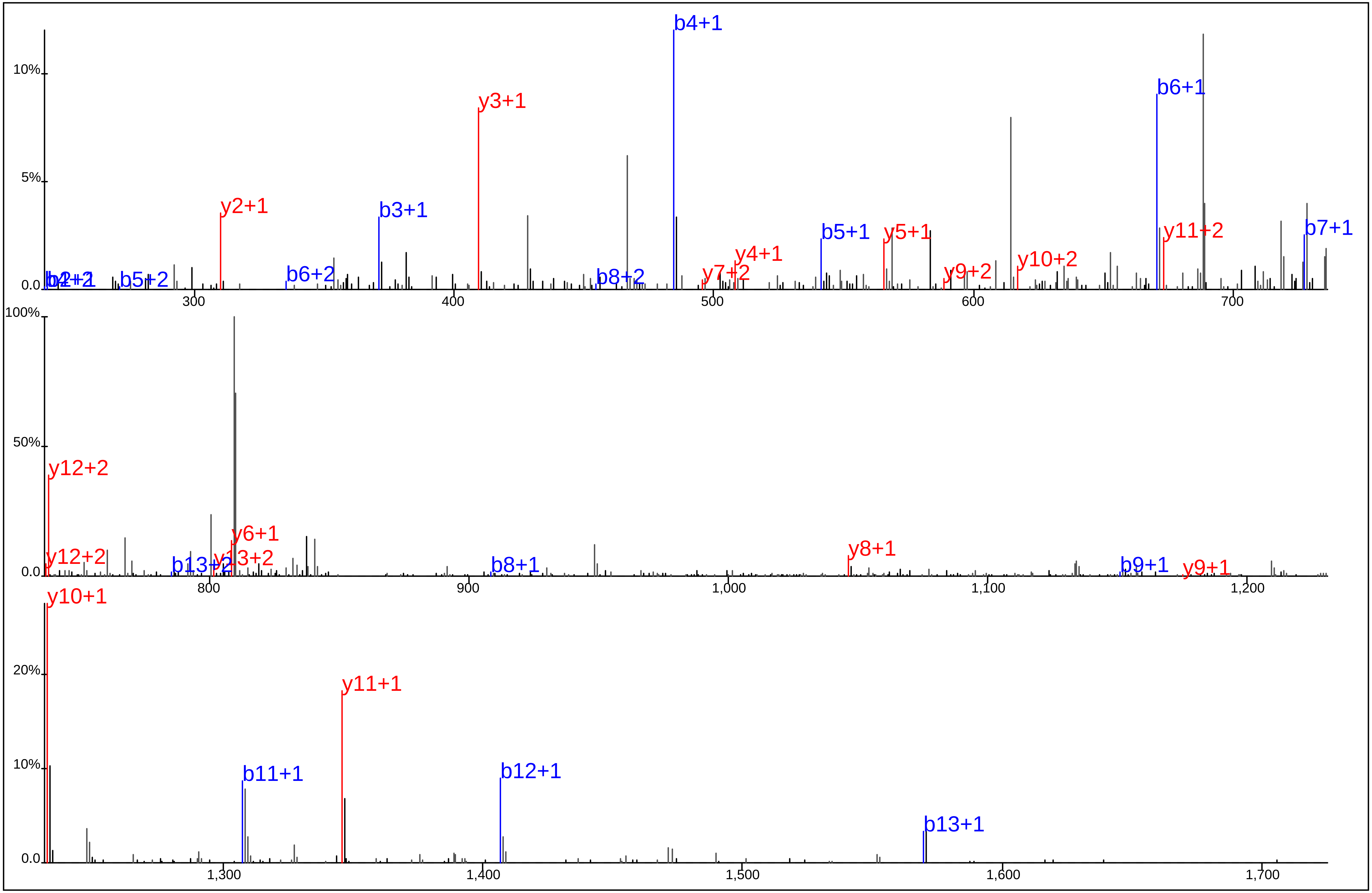

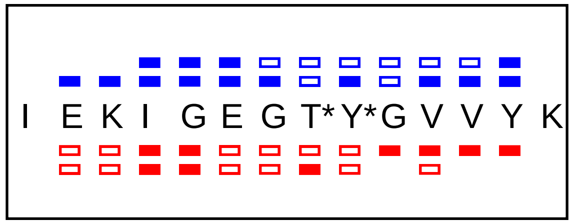

Protein Name:	CDC2
gi Accession:	16306492
Precursor Mass:	858.3878+2
Delta Mass:	0.532 ppm
XCorr:	42.94

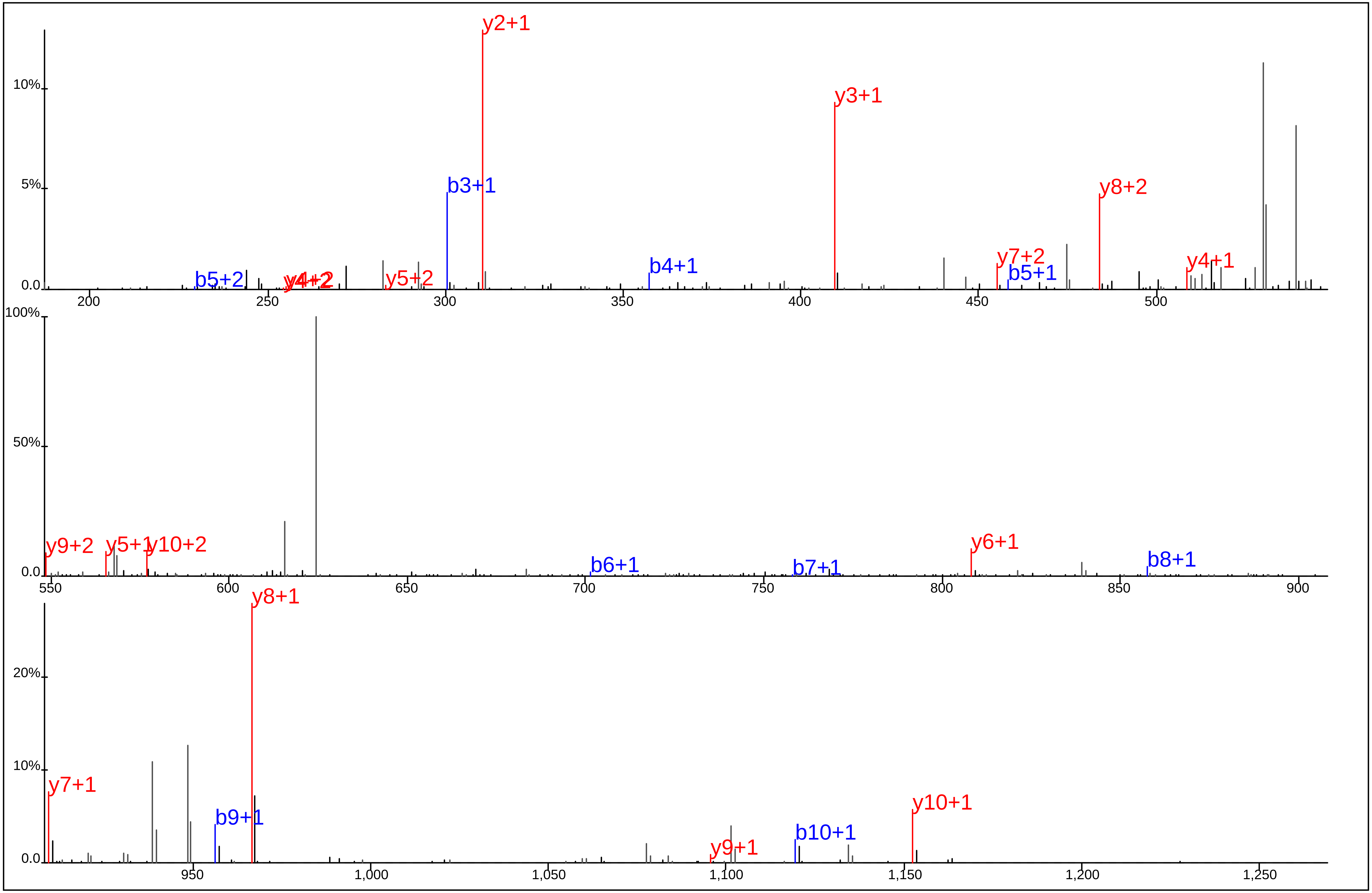

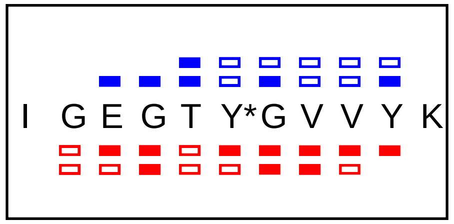

Protein Name: CDC2
gi Accession: 24158643
Precursor Mass: 633.2955+2
Delta Mass: 1.839 ppm
XCorr: 45.38

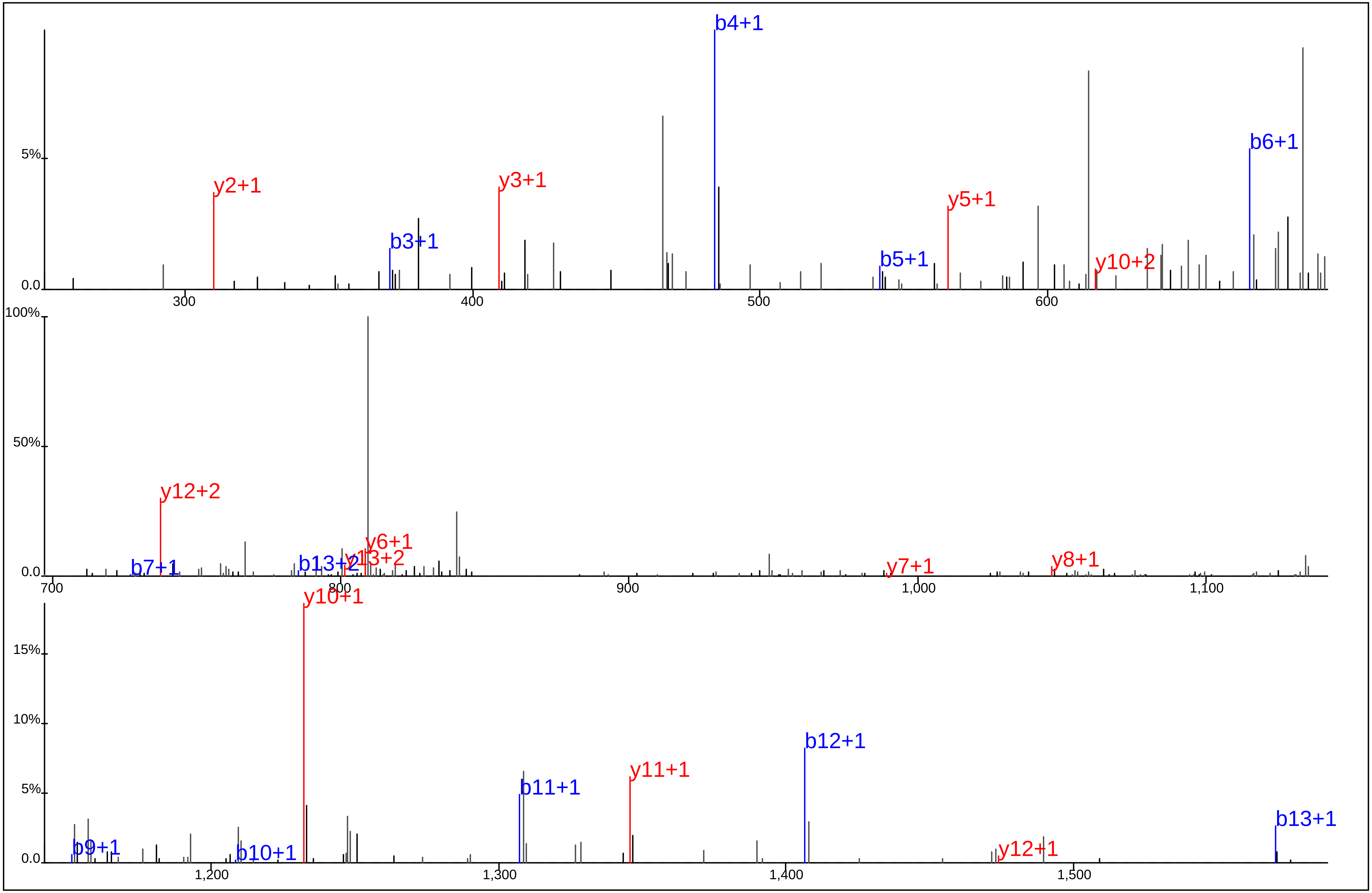

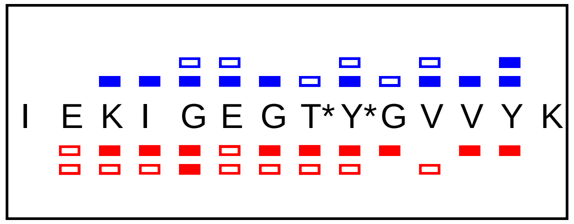

Protein Name: CDC2
gi Accession: 16306492
Precursor Mass: 858.3896+2
Delta Mass: 1.602 ppm
XCorr: 27.78

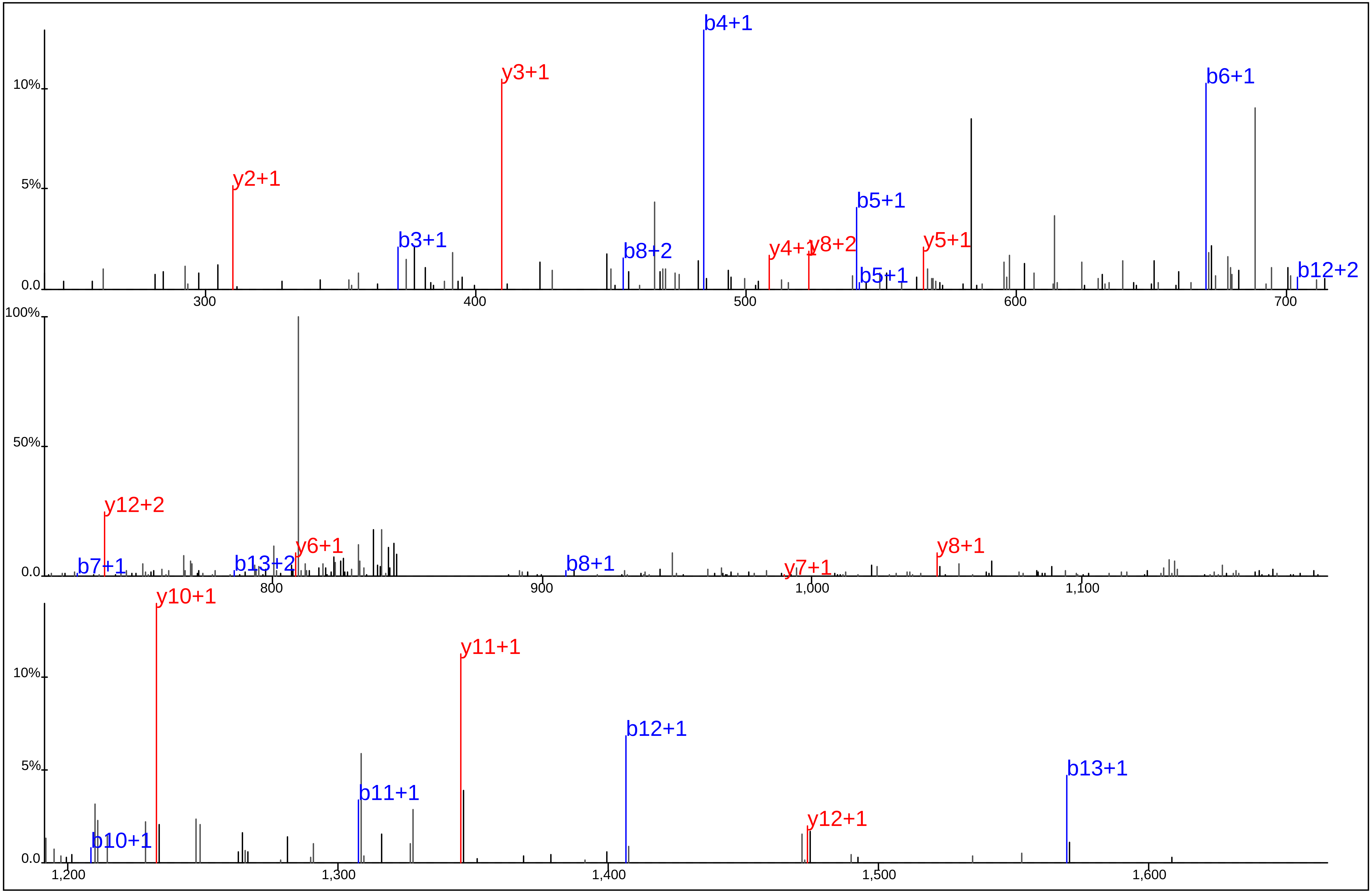

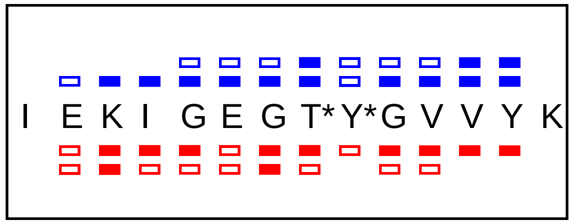

Protein Name: CDC2
gi Accession: 16306492
Precursor Mass: 858.3919+2
Delta Mass: 4.236 ppm
XCorr: 36.08

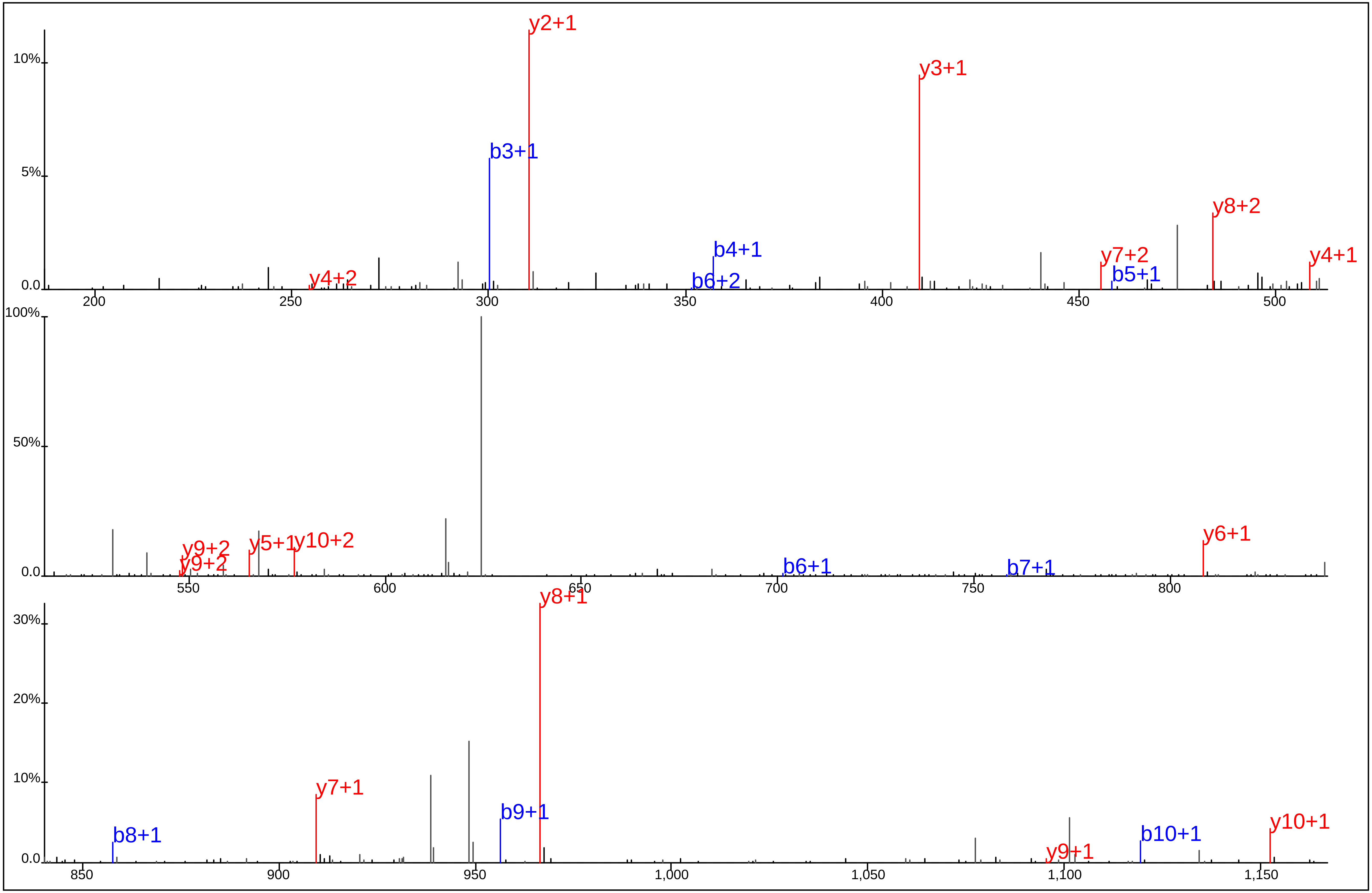

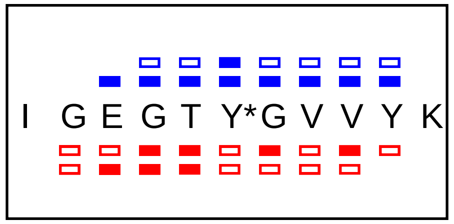

Protein Name: CDC2
gi Accession: 24158643
Precursor Mass: 633.2942+2
Delta Mass: 0.191 ppm
XCorr: 44.16

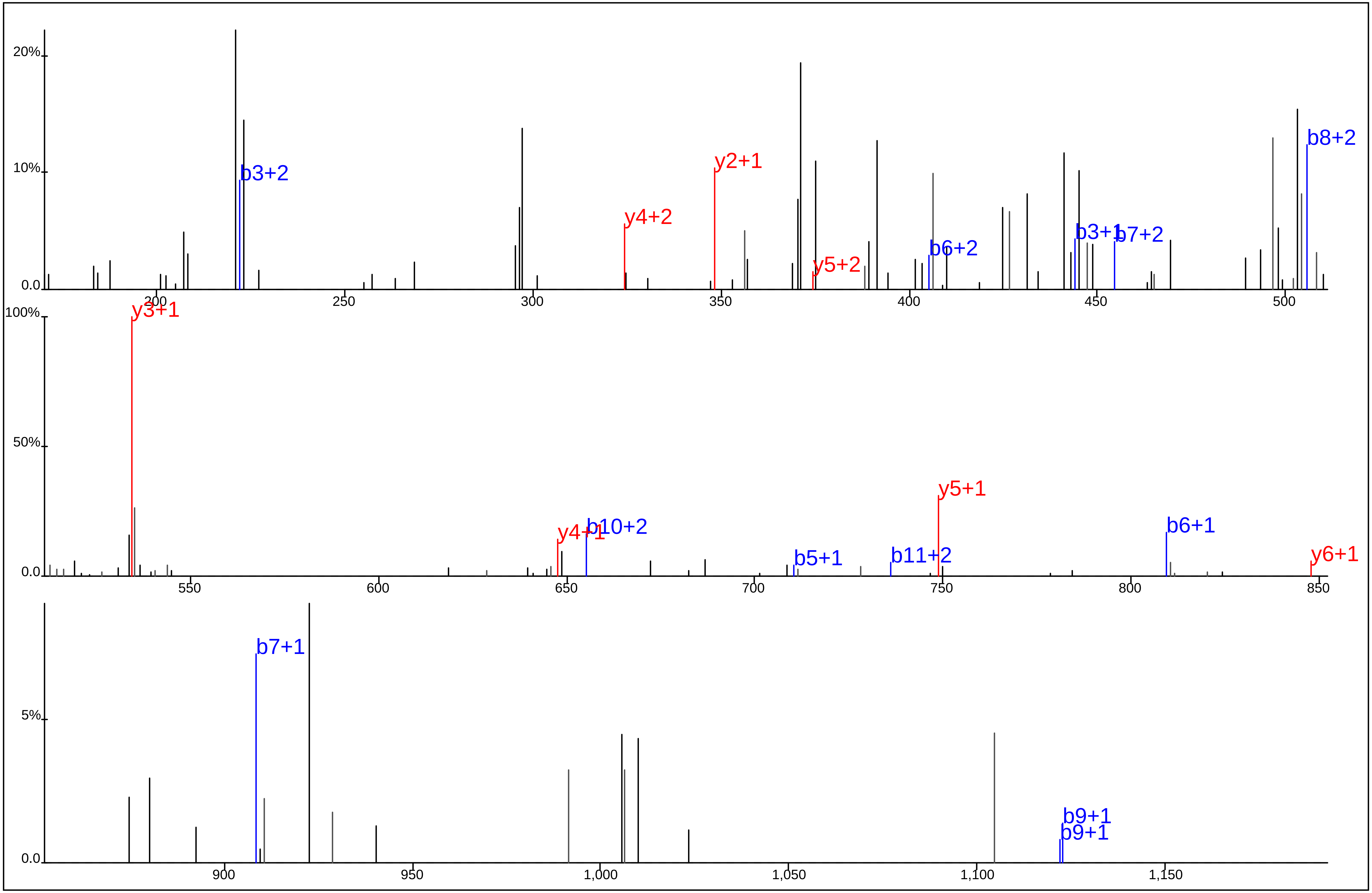

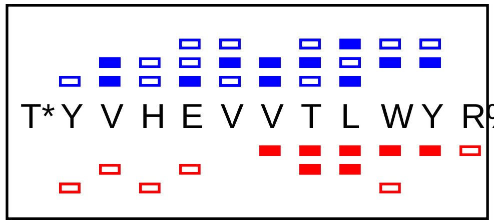

Protein Name:	CDC2
gi Accession:	24158643
Precursor Mass:	552.5995+3
Delta Mass:	0.968 ppm
XCorr:	19.88

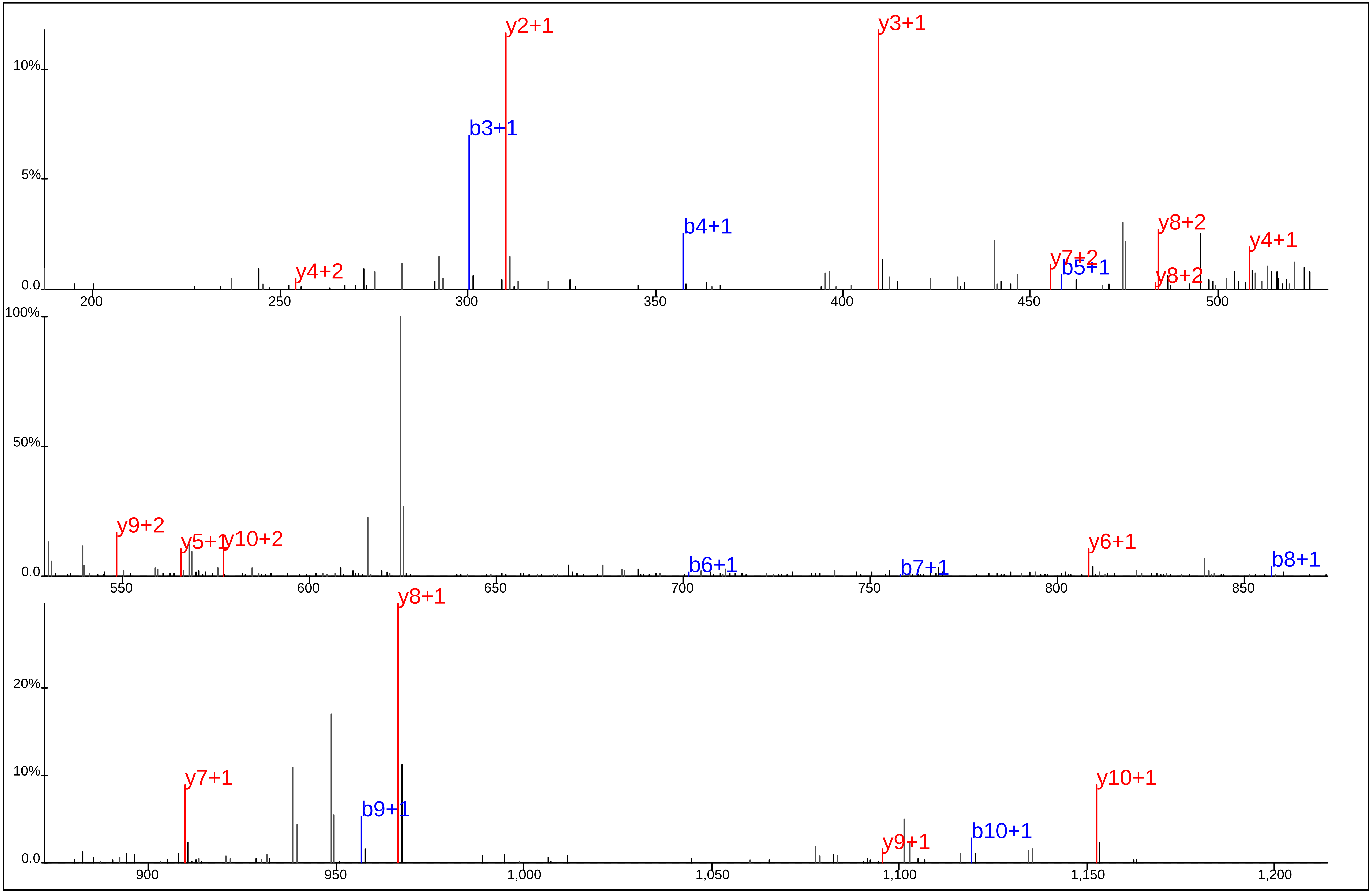

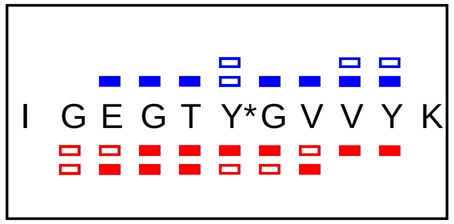

Protein Name:	CDC2
gi Accession:	24158643
Precursor Mass:	633.2962+2
Delta Mass:	2.993 ppm
XCorr:	43.55

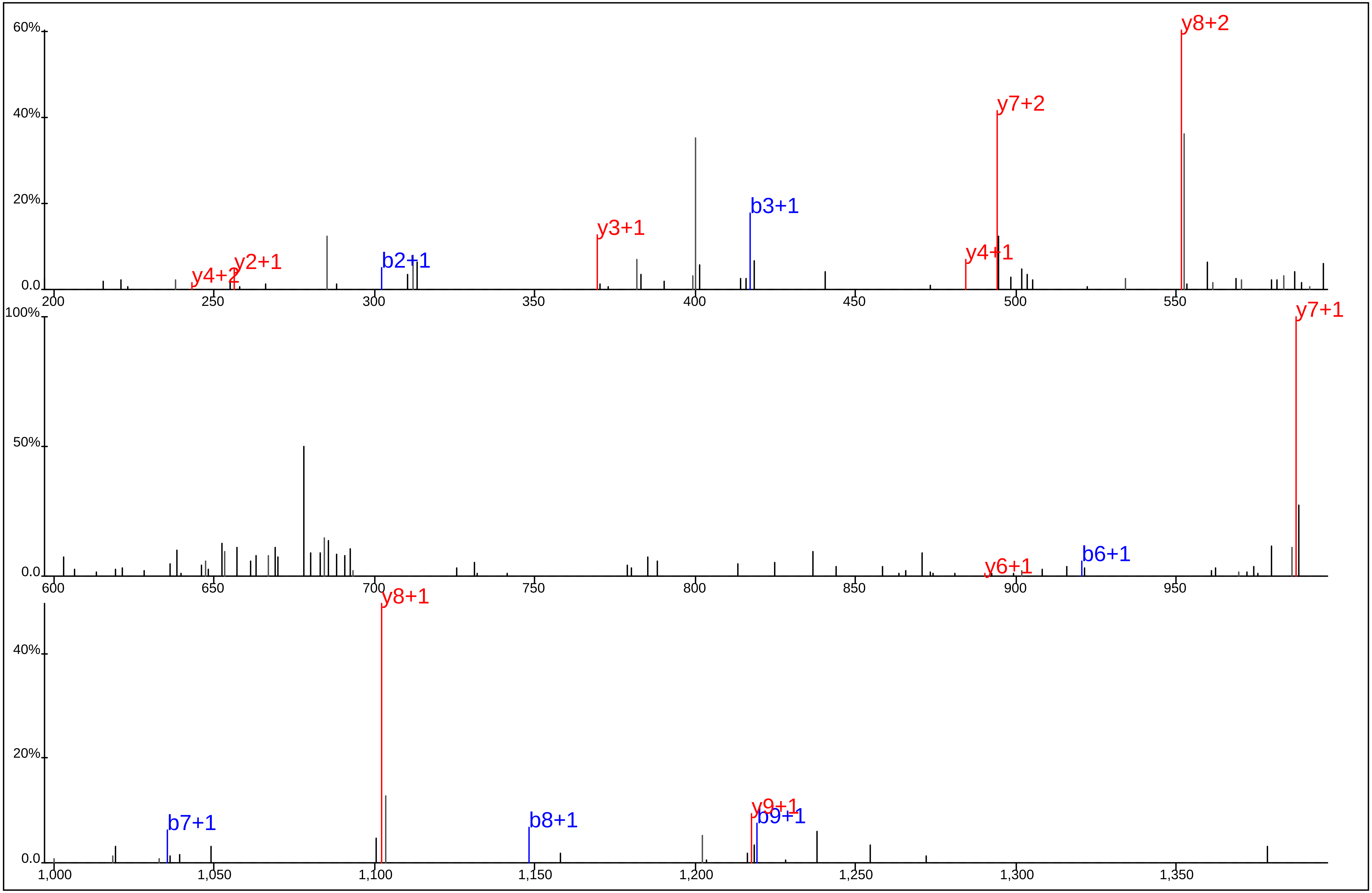

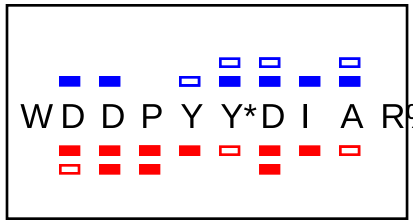

Protein Name: CDC42GAP
gi Accession: 4757766
Precursor Mass: 702.2848+2
Delta Mass: 5.720 ppm
XCorr: 18.66

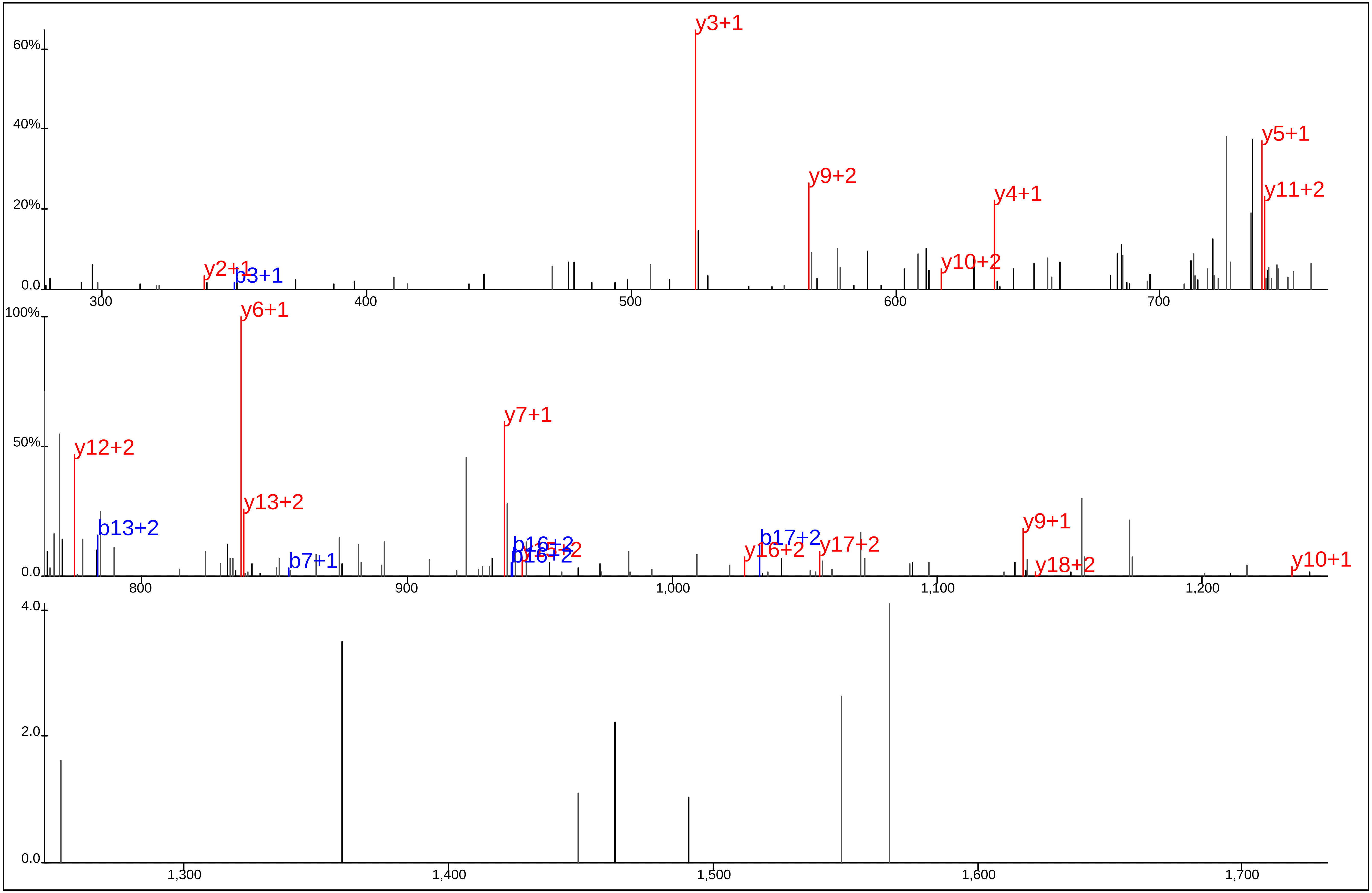

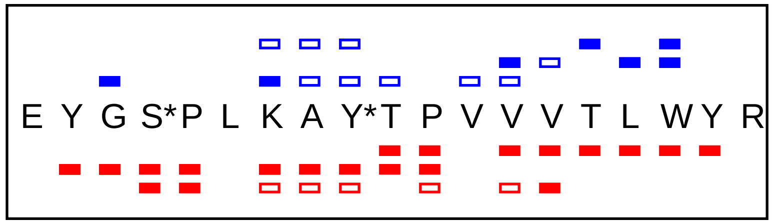

Protein Name: CDK11
gi Accession: 34978359
Precursor Mass: 801.3840+3
Delta Mass: 5.931 ppm
XCorr: 27.21

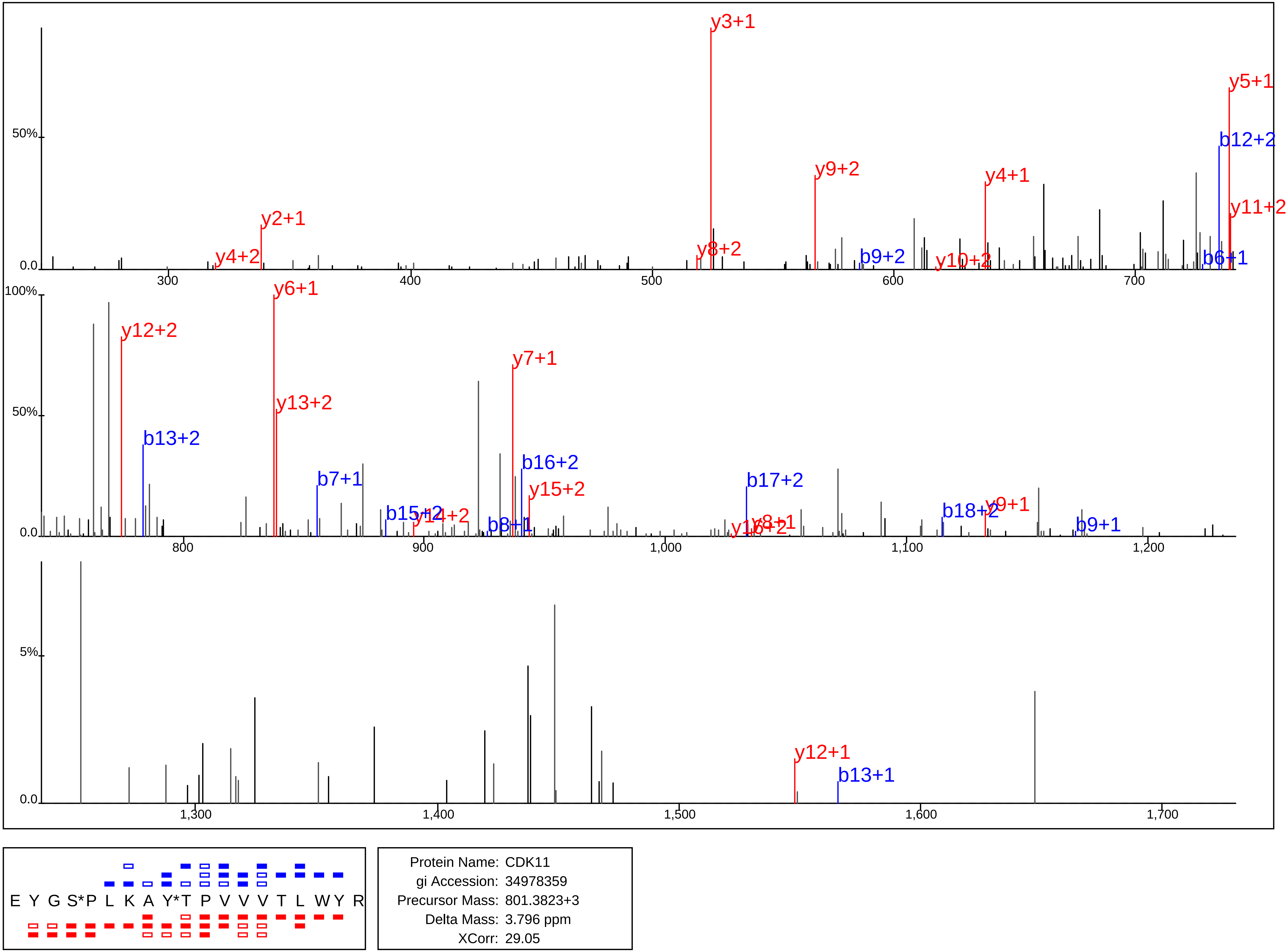

Protein Name: CDK11
gi Accession: 34978359
Precursor Mass: 801.3823+3
Delta Mass: 3.796 ppm
XCorr: 29.05

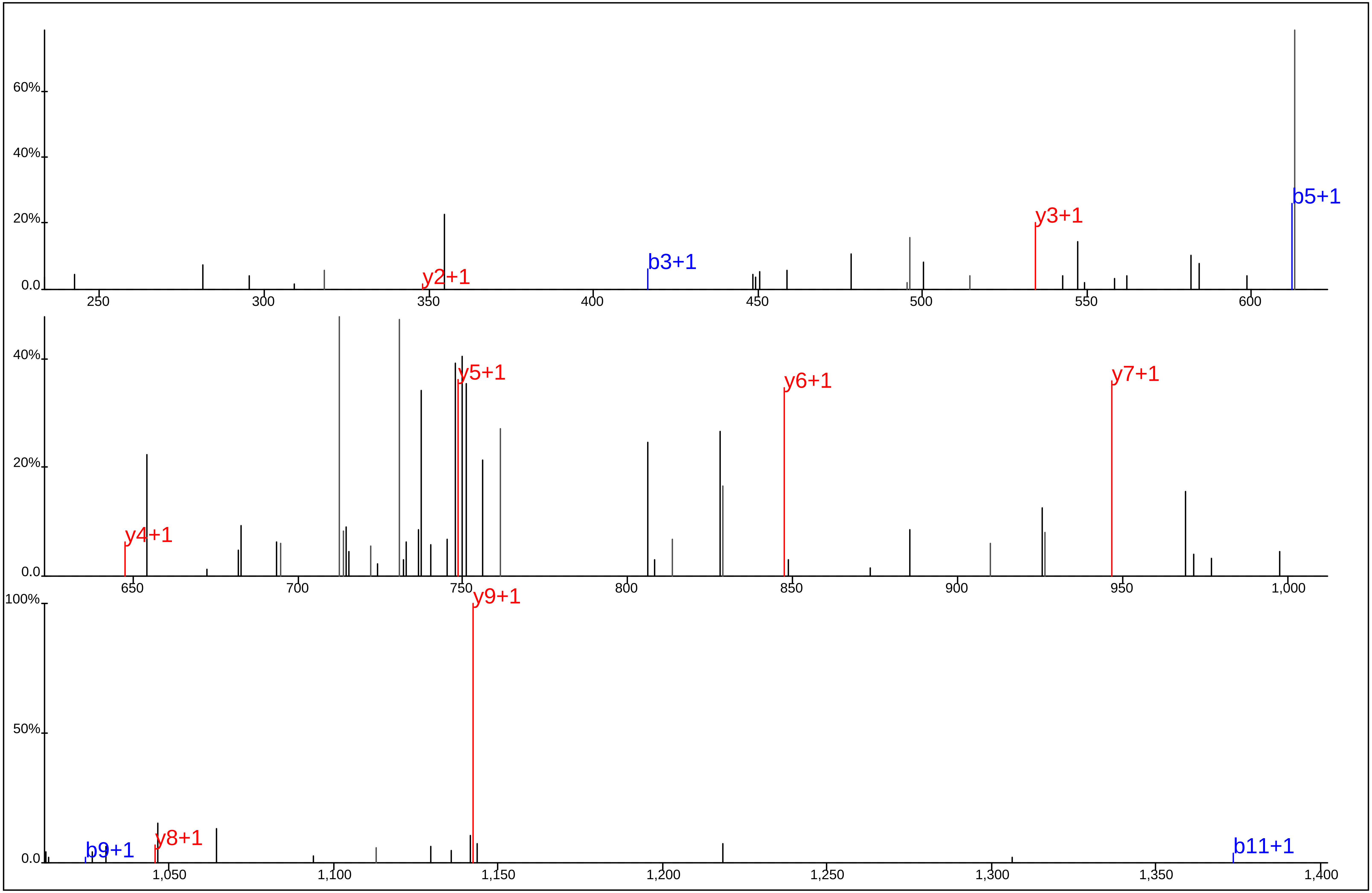

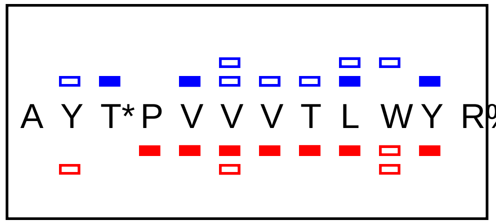

Protein Name: CDK11
gi Accession: 34978359
Precursor Mass: 779.4008+2
Delta Mass: 12.953 ppm
XCorr: 31.52

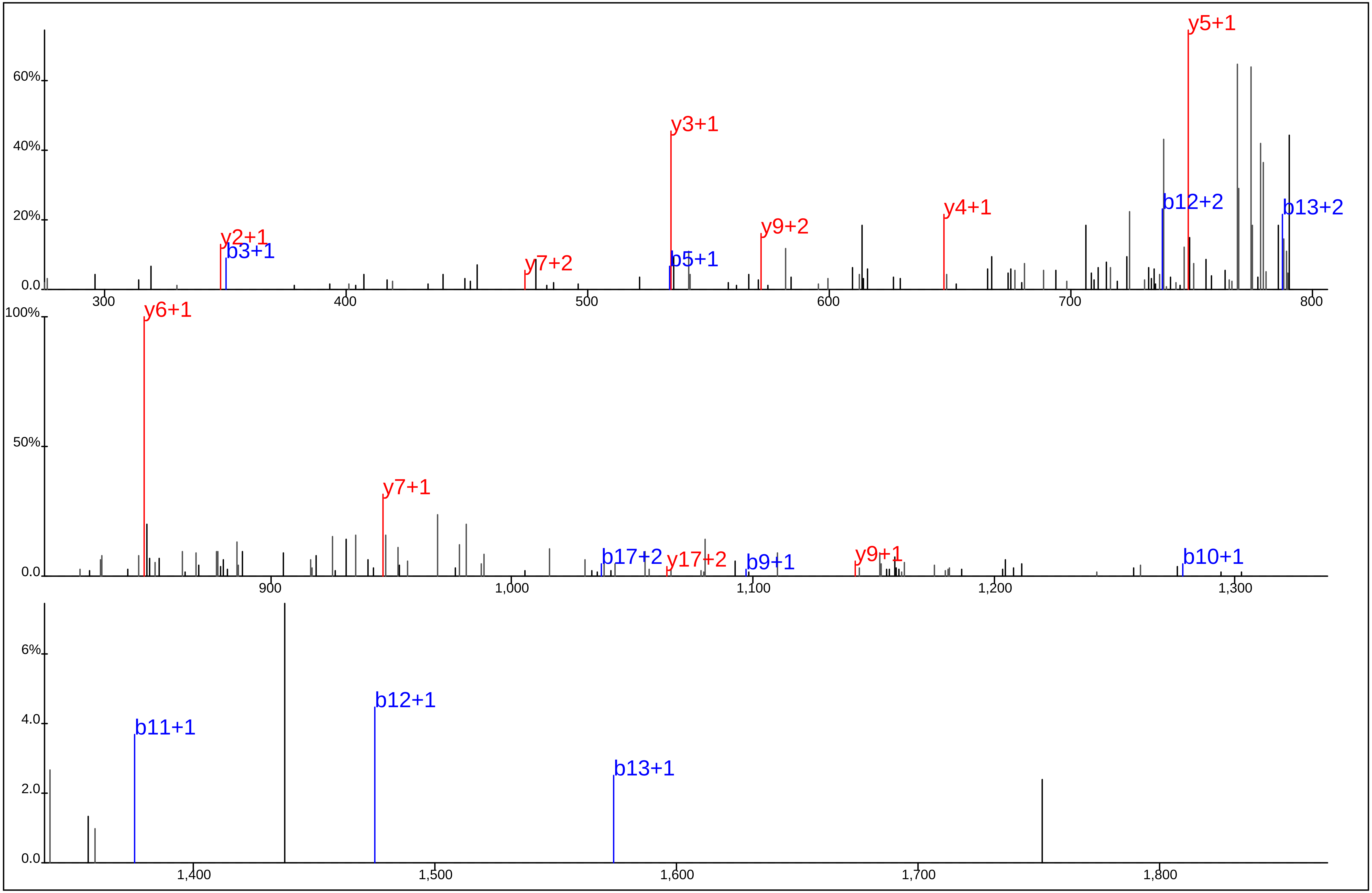

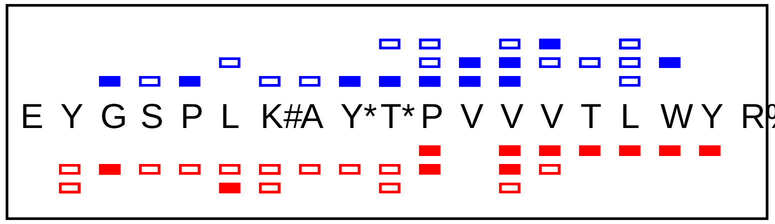

Protein Name: CDK11
gi Accession: 34978359
Precursor Mass: 807.3903+3
Delta Mass: 4.313 ppm
XCorr: 31.98

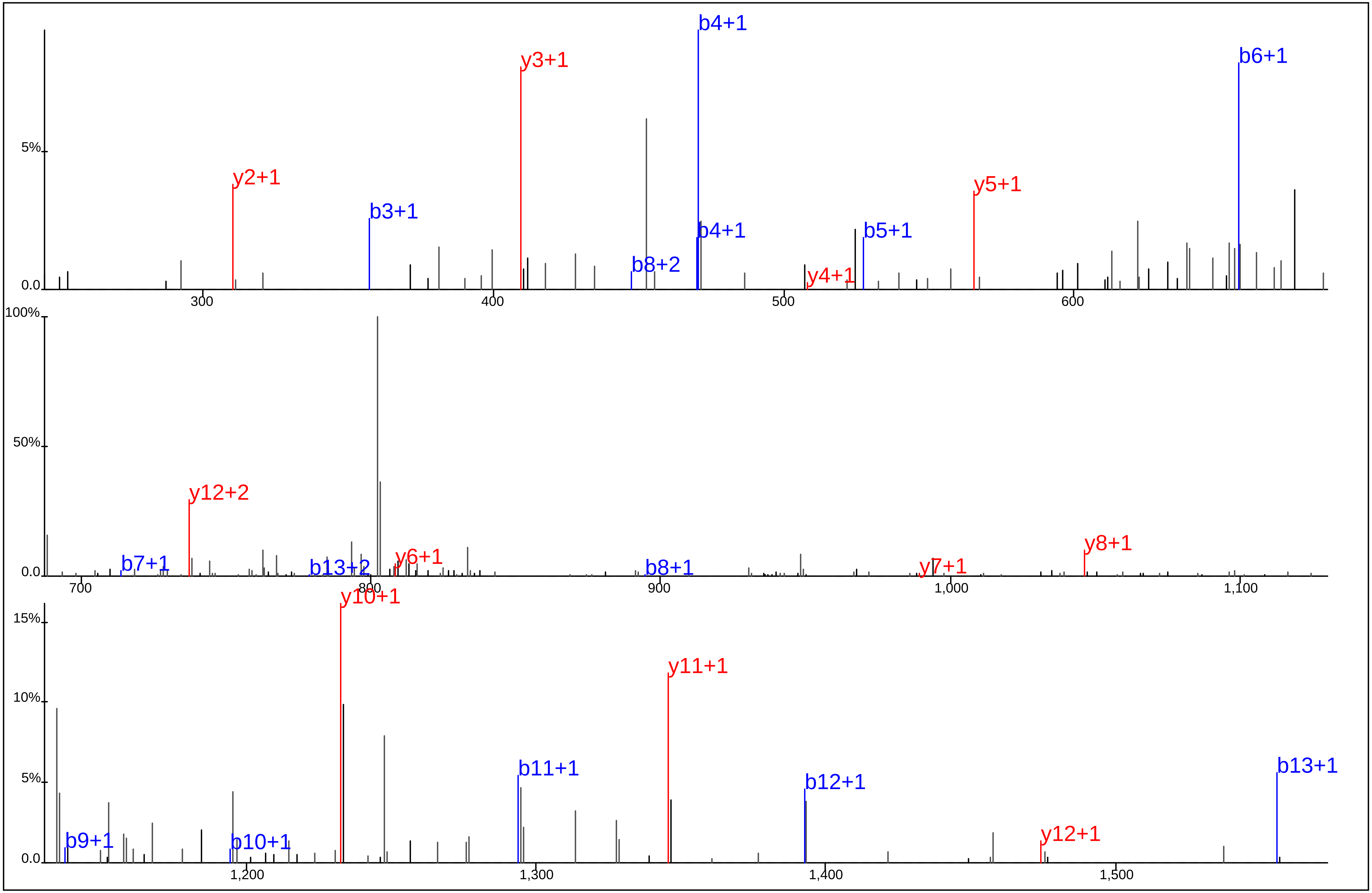

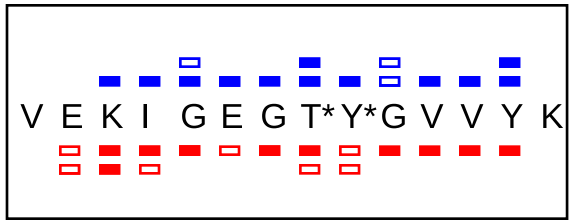

Protein Name: CDK2
gi Accession: 24158643
Precursor Mass: 851.3840+2
Delta Mass: 4.136 ppm
XCorr: 45.43

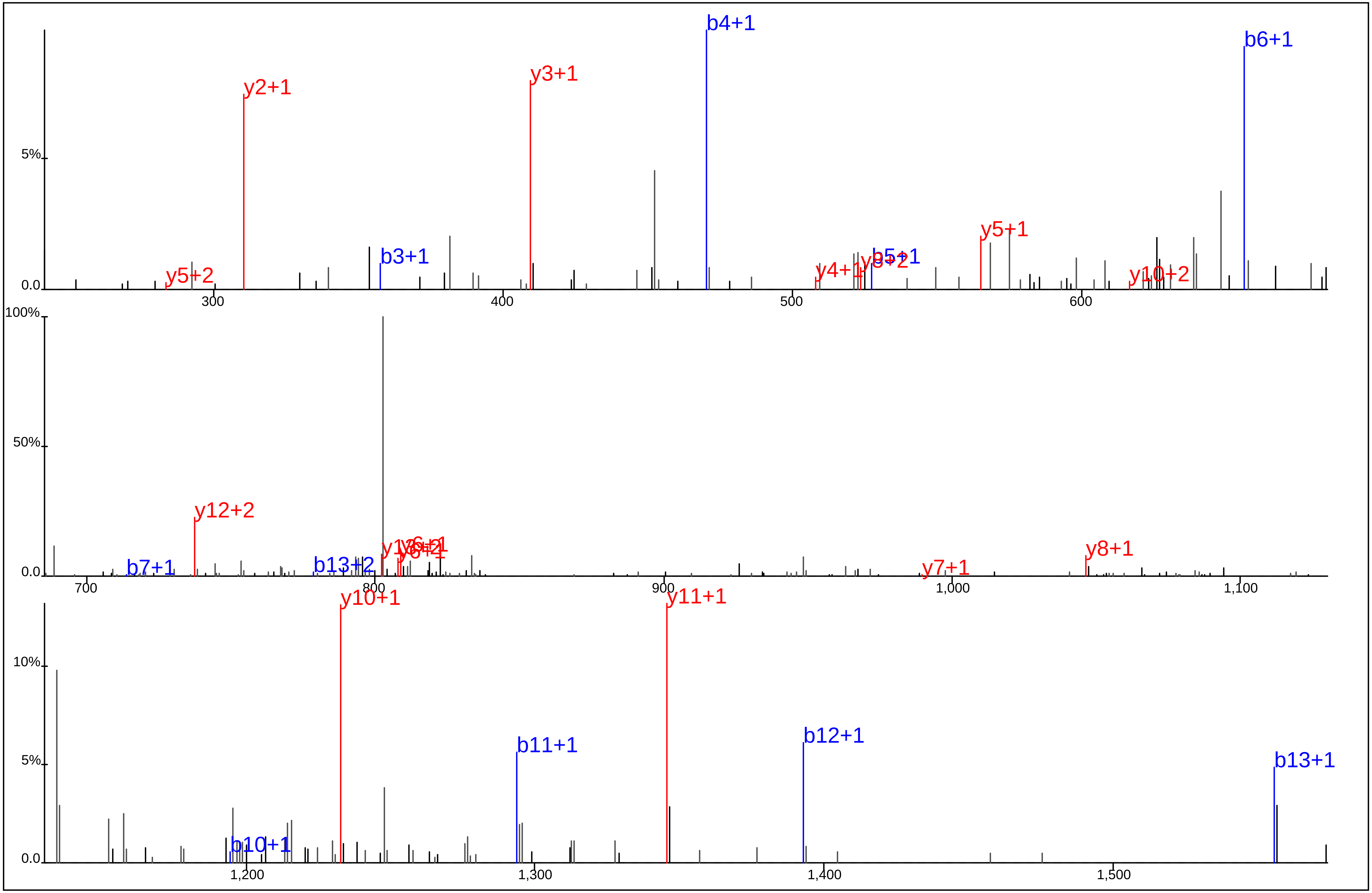

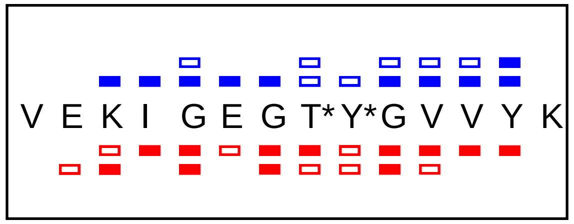

Protein Name: CDK2
gi Accession: 24158643
Precursor Mass: 851.3781+2
Delta Mass: 2.822 ppm
XCorr: 42.33

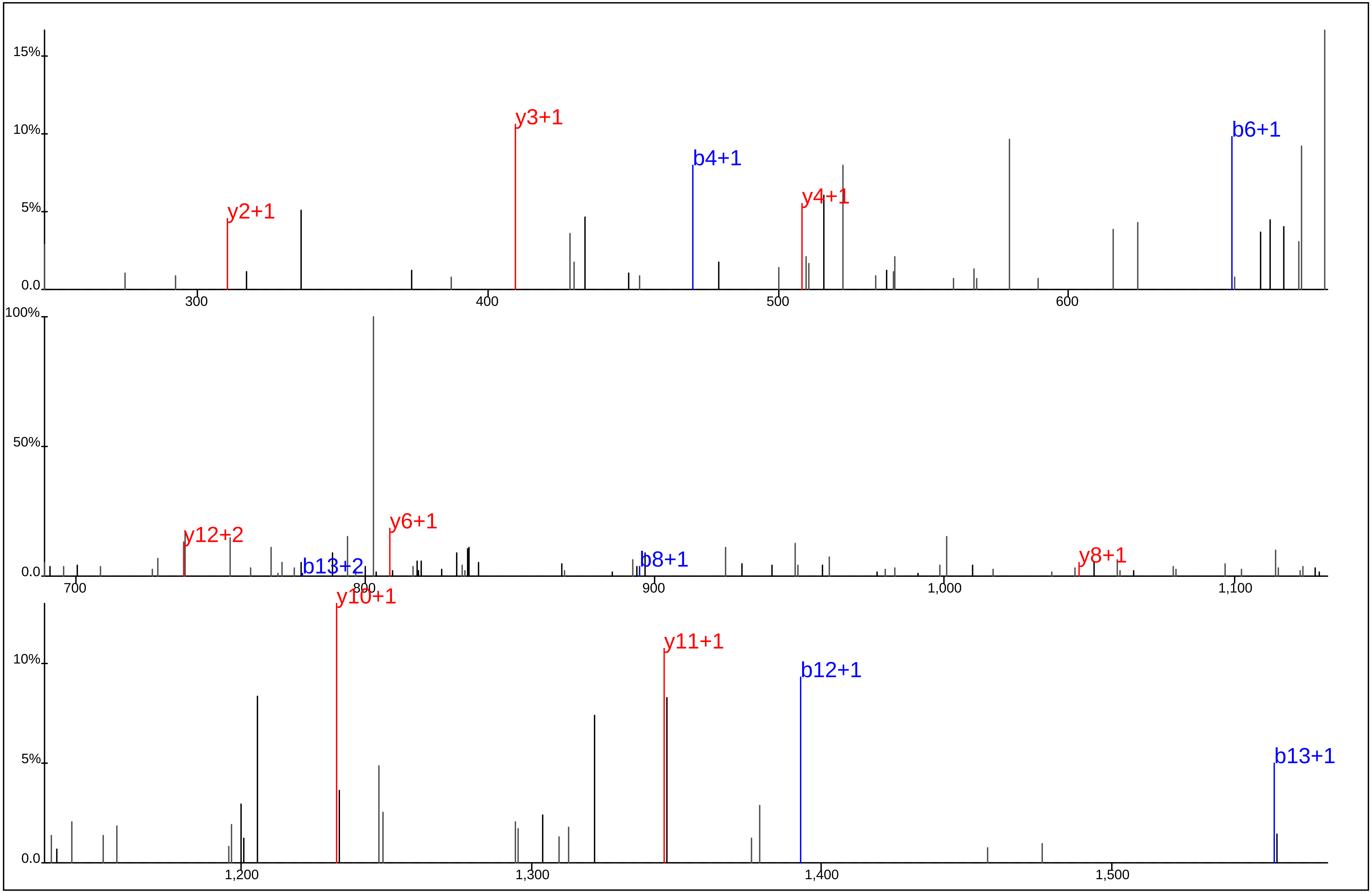

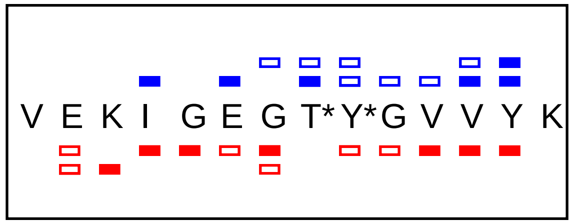

Protein Name: CDK2
gi Accession: 24158643
Precursor Mass: 851.3876+2
Delta Mass: 8.367 ppm
XCorr: 25.39

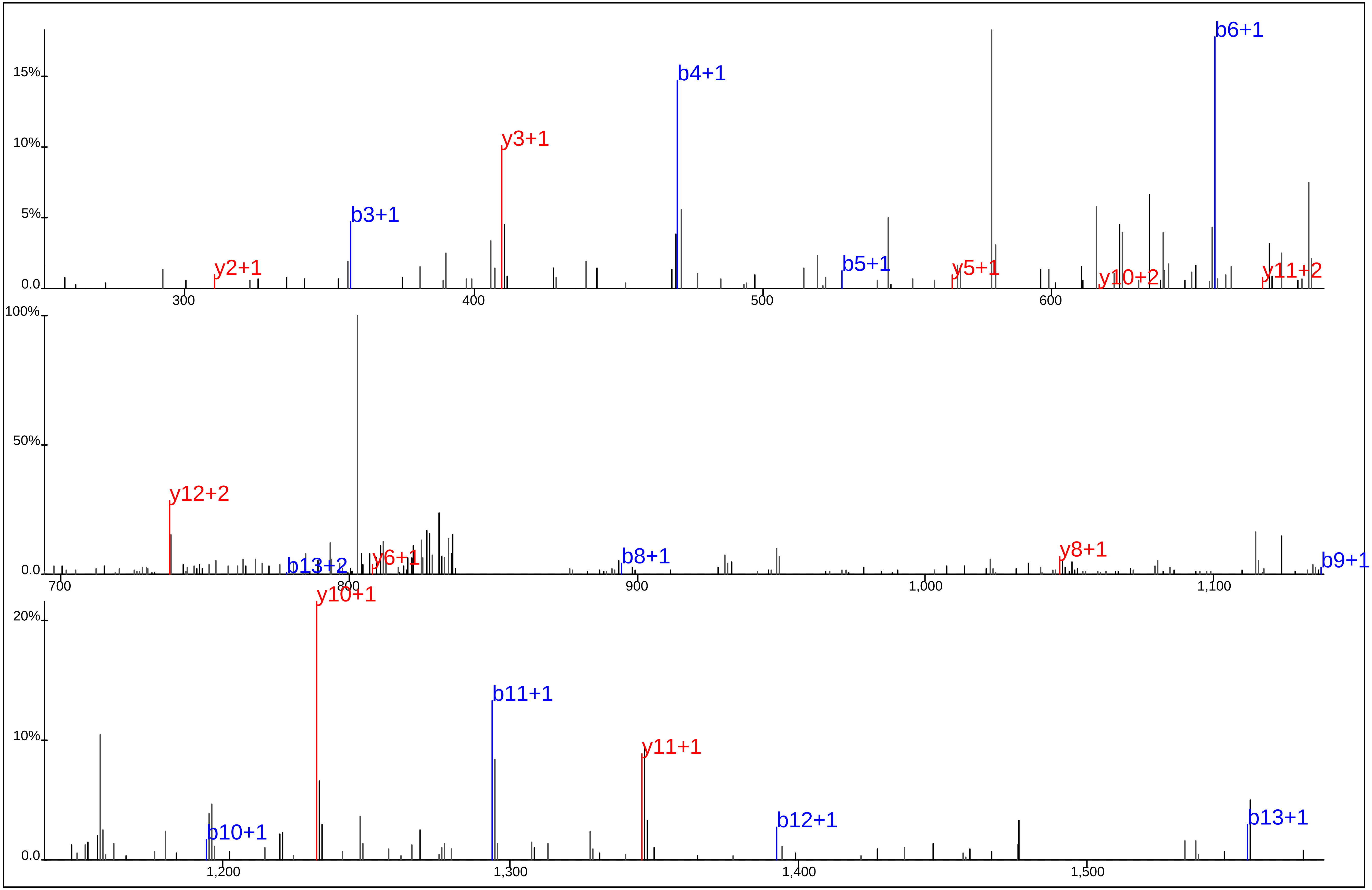

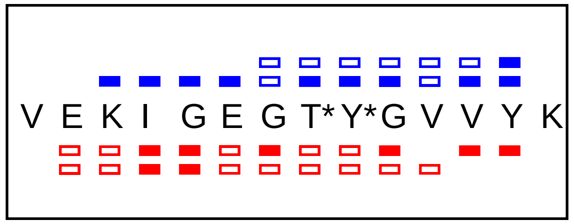

Protein Name: CDK2
gi Accession: 24158643
Precursor Mass: 851.3804+2
Delta Mass: 0.025 ppm
XCorr: 20.43

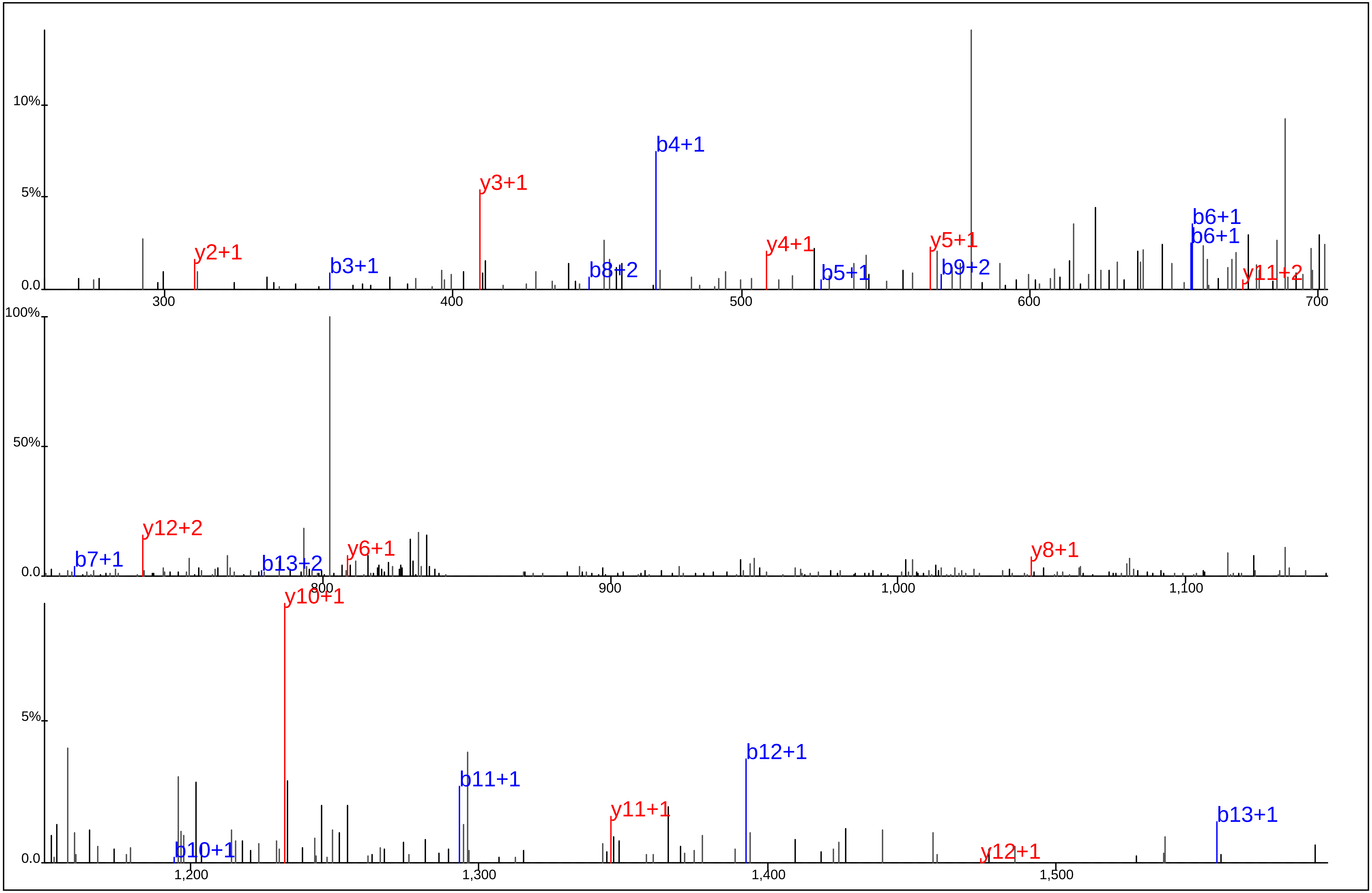

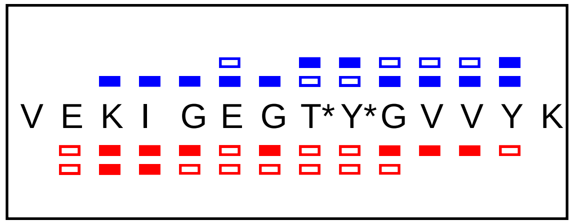

Protein Name: CDK2
gi Accession: 24158643
Precursor Mass: 851.3798+2
Delta Mass: 0.742 ppm
XCorr: 19.66

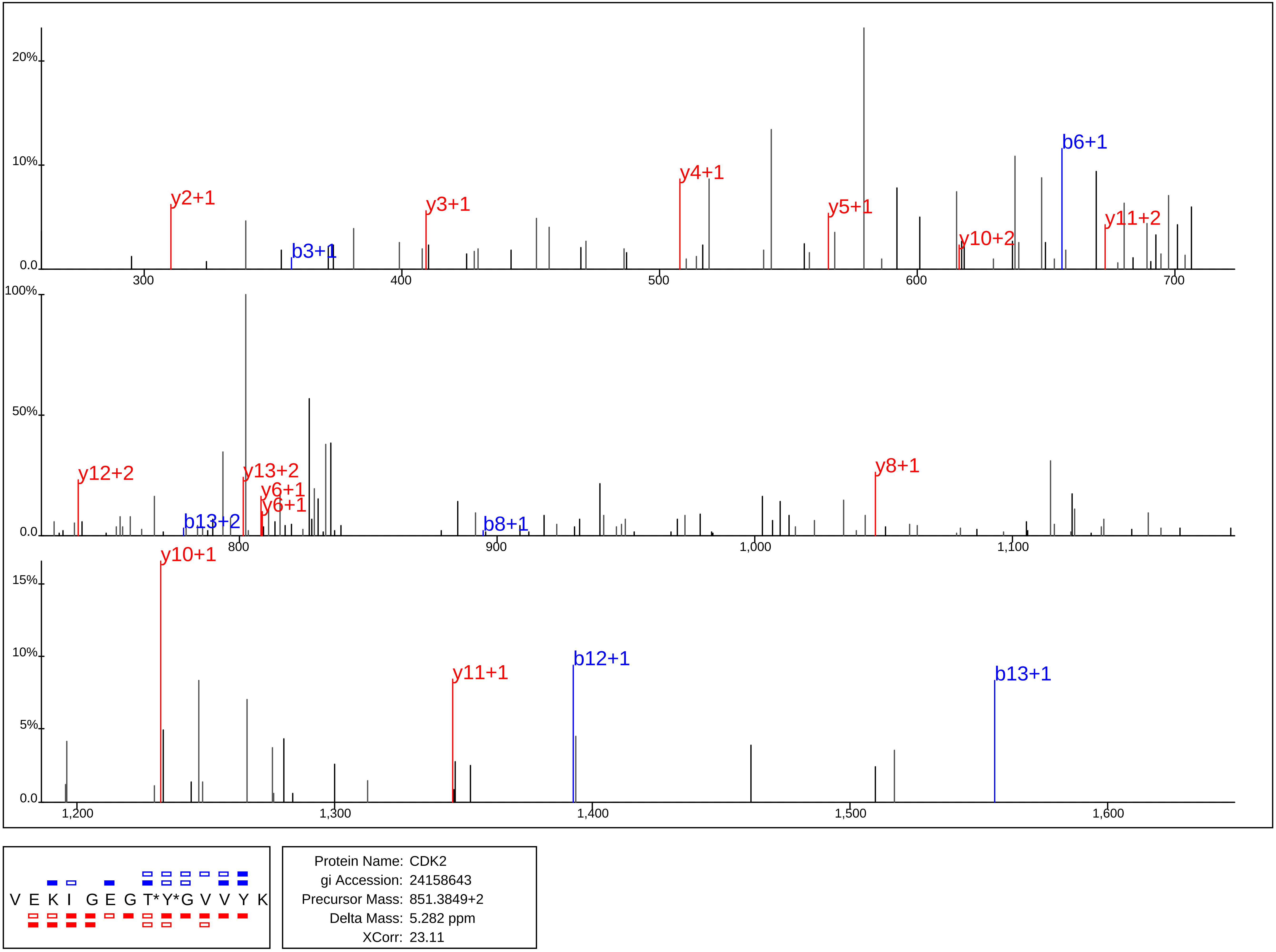

Protein Name:	CDK2
gi Accession:	24158643
Precursor Mass:	851.3849+2
Delta Mass:	5.282 ppm
XCorr:	23.11

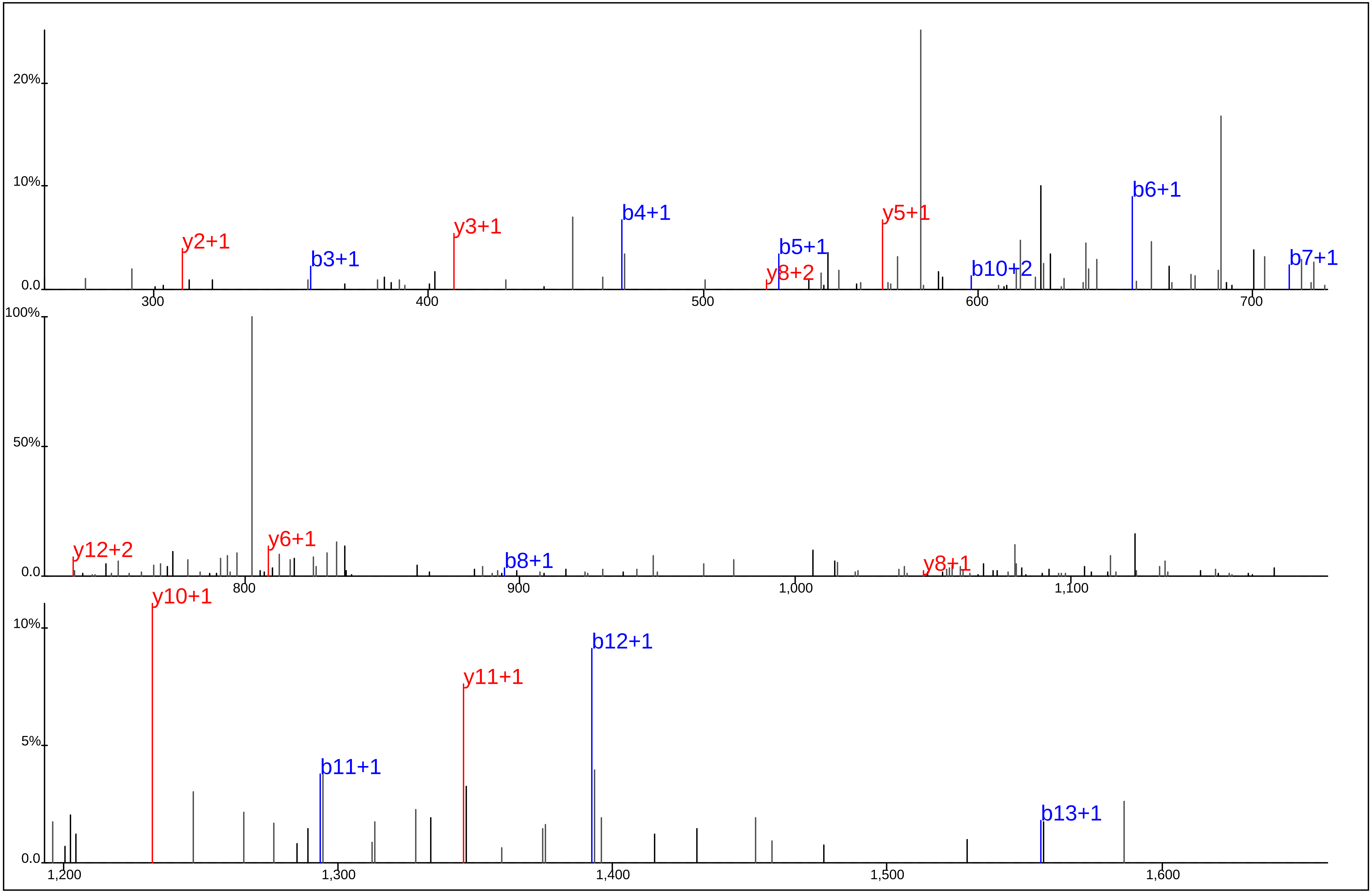

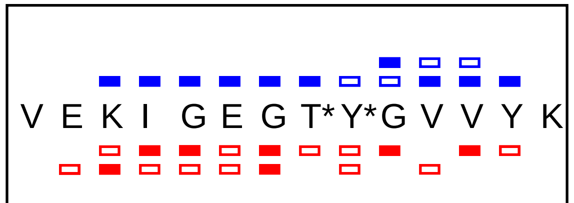

Protein Name: CDK2
gi Accession: 24158643
Precursor Mass: 851.3770+2
Delta Mass: 4.115 ppm
XCorr: 27.27

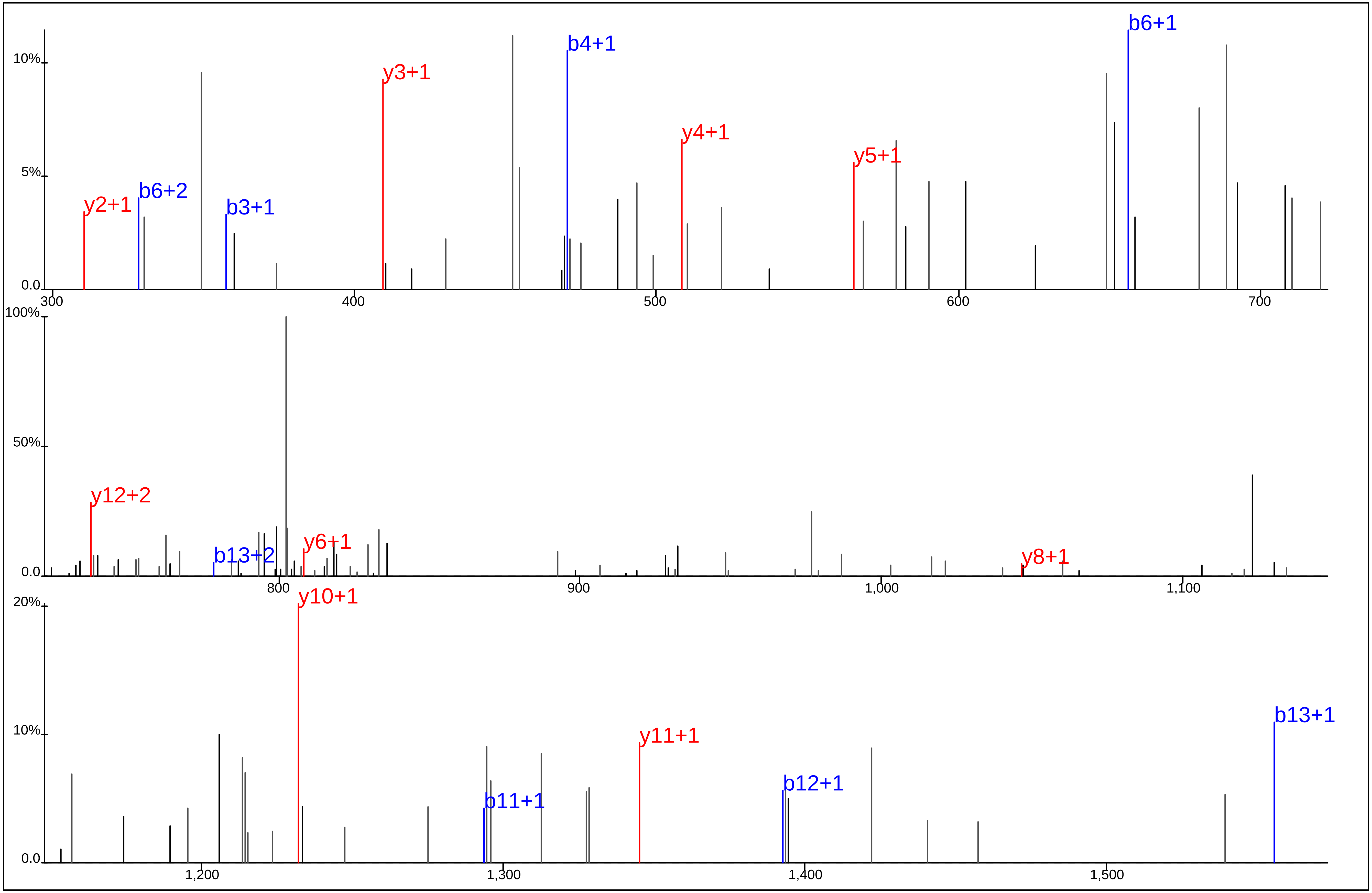

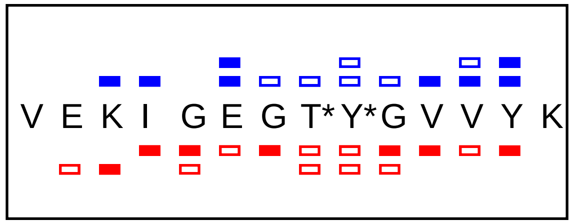

Protein Name: CDK2
gi Accession: 24158643
Precursor Mass: 851.3826+2
Delta Mass: 2.484 ppm
XCorr: 23.45

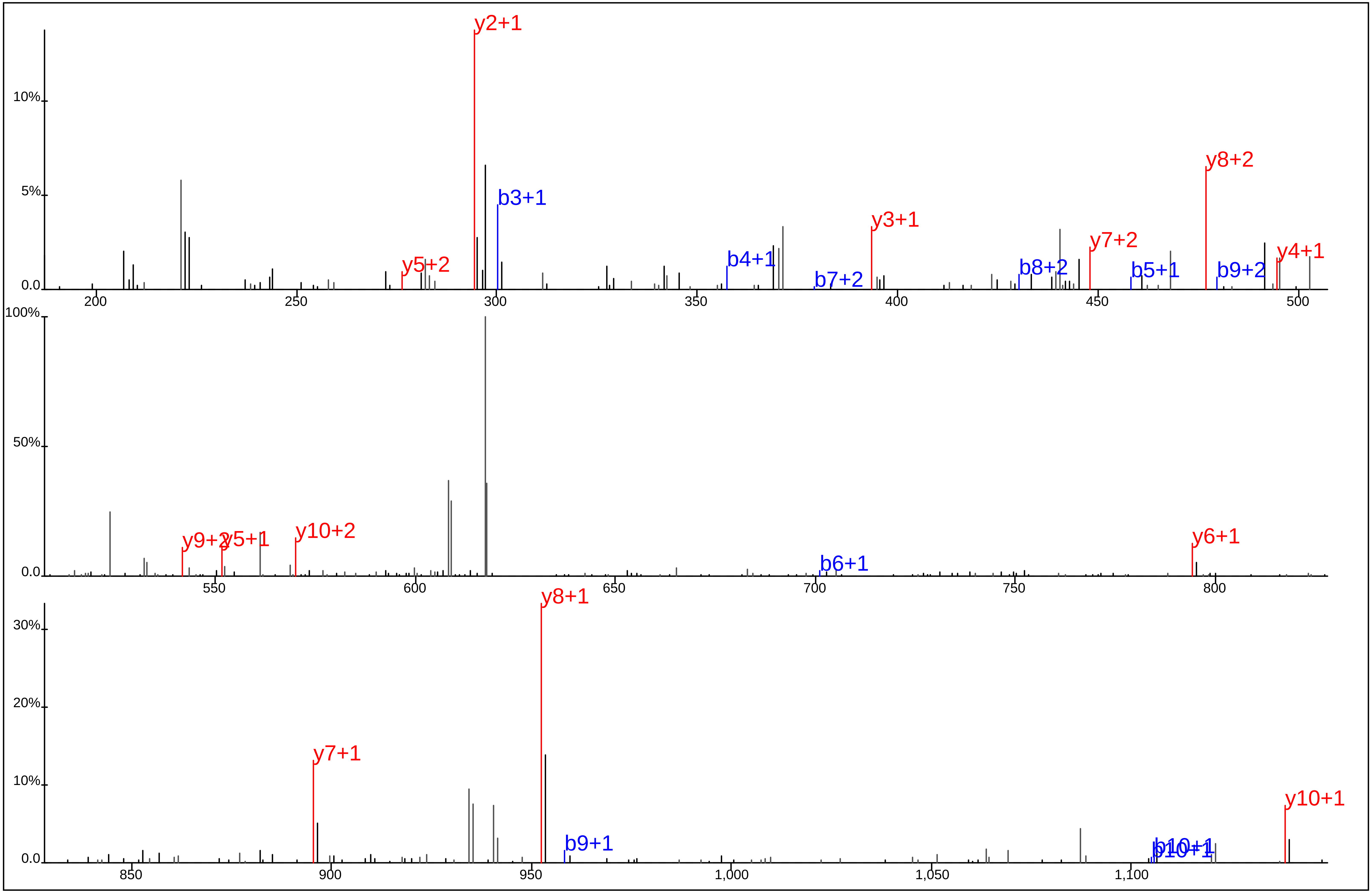

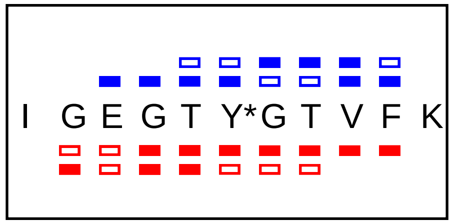

Protein Name: CDK5
gi Accession: 4826675
Precursor Mass: 626.2895+2
Delta Mass: 4.792 ppm
XCorr: 39.78

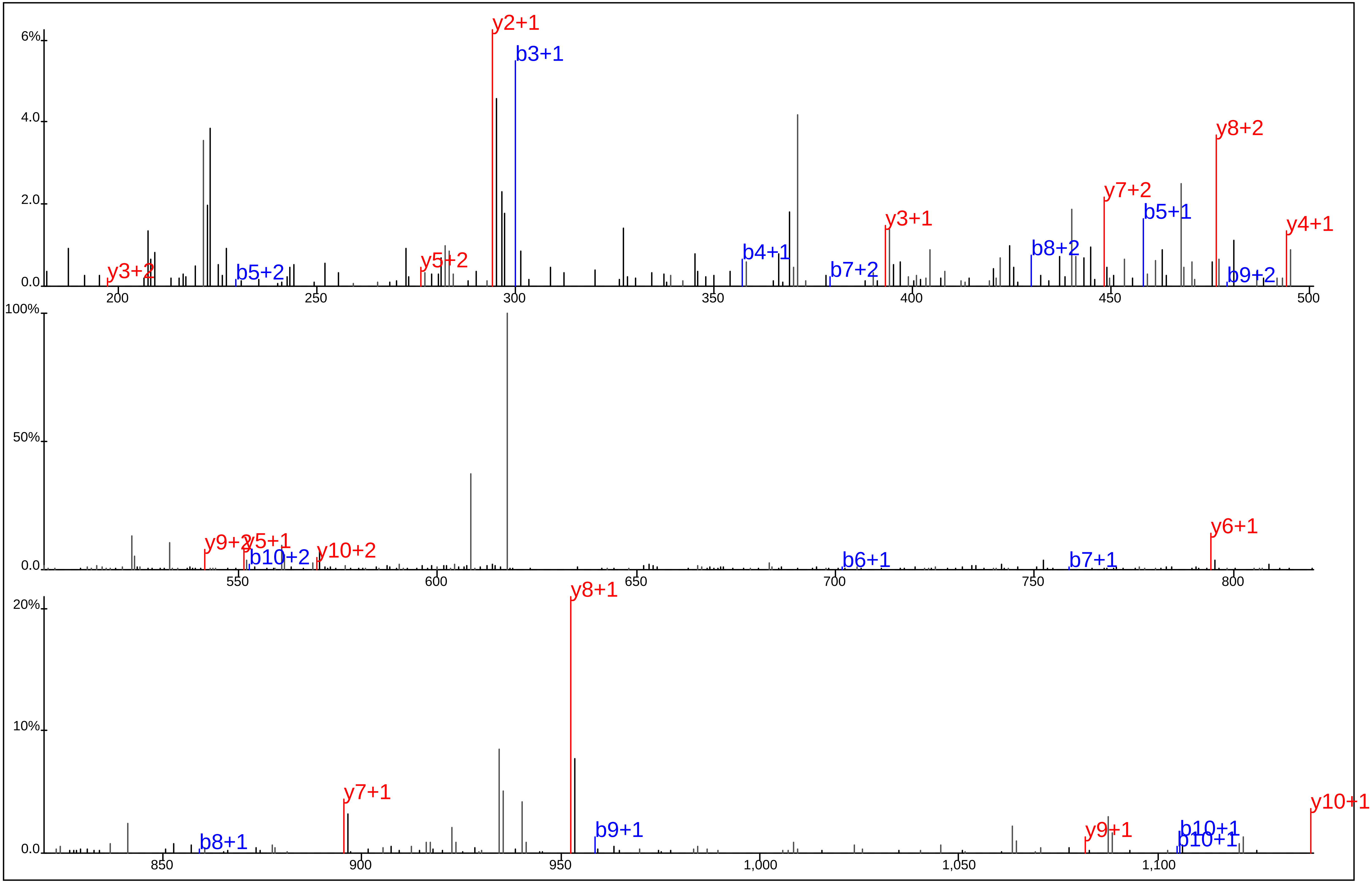

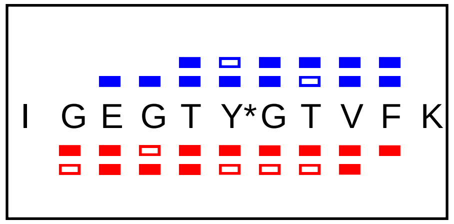

Protein Name: CDK5
gi Accession: 4826675
Precursor Mass: 626.2901+2
Delta Mass: 5.767 ppm
XCorr: 42.16

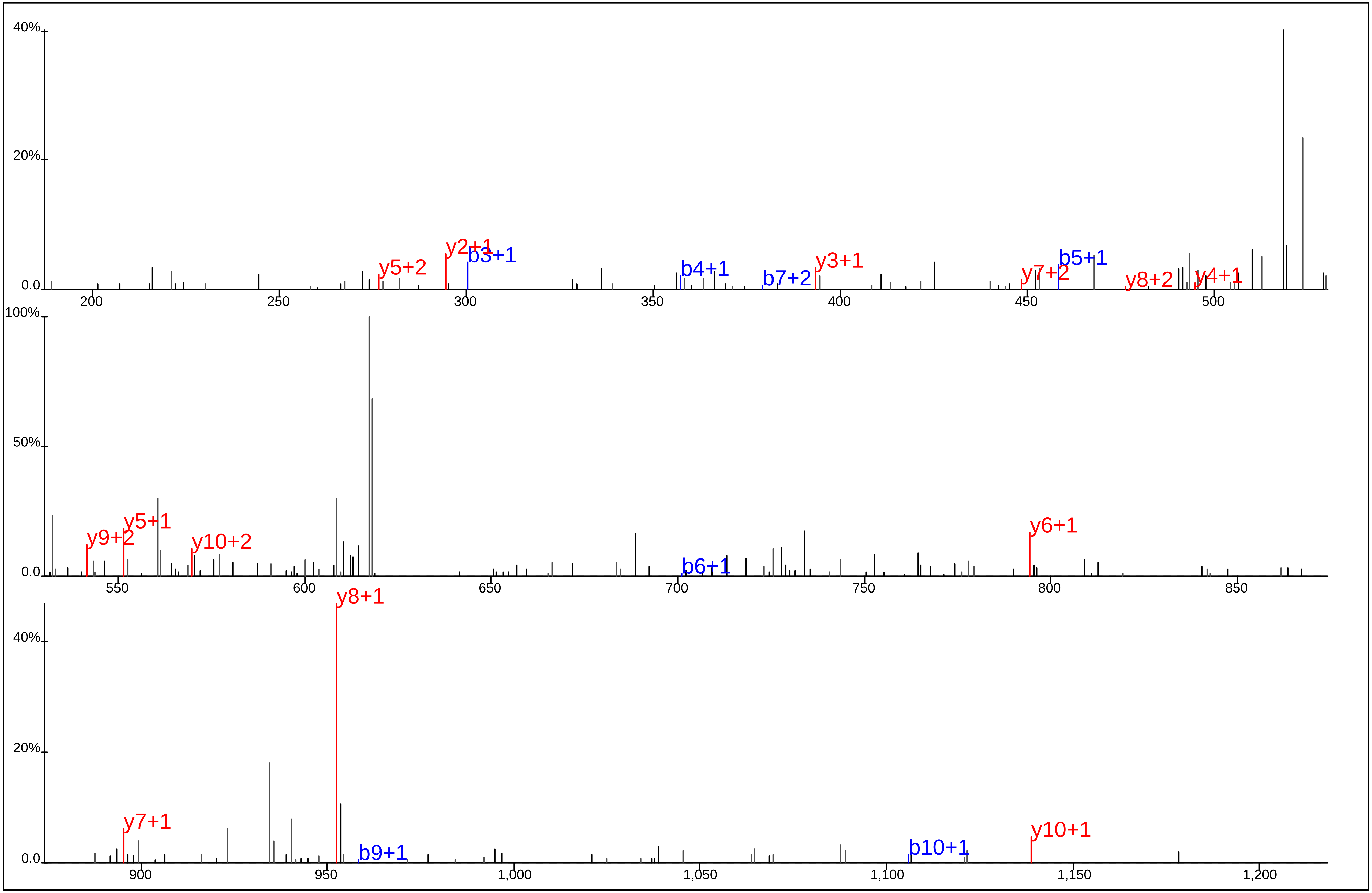

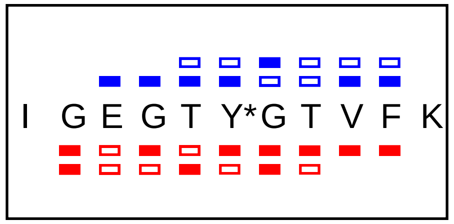

Protein Name: CDK5
gi Accession: 4826675
Precursor Mass: 626.2889+2
Delta Mass: 3.913 ppm
XCorr: 25.65

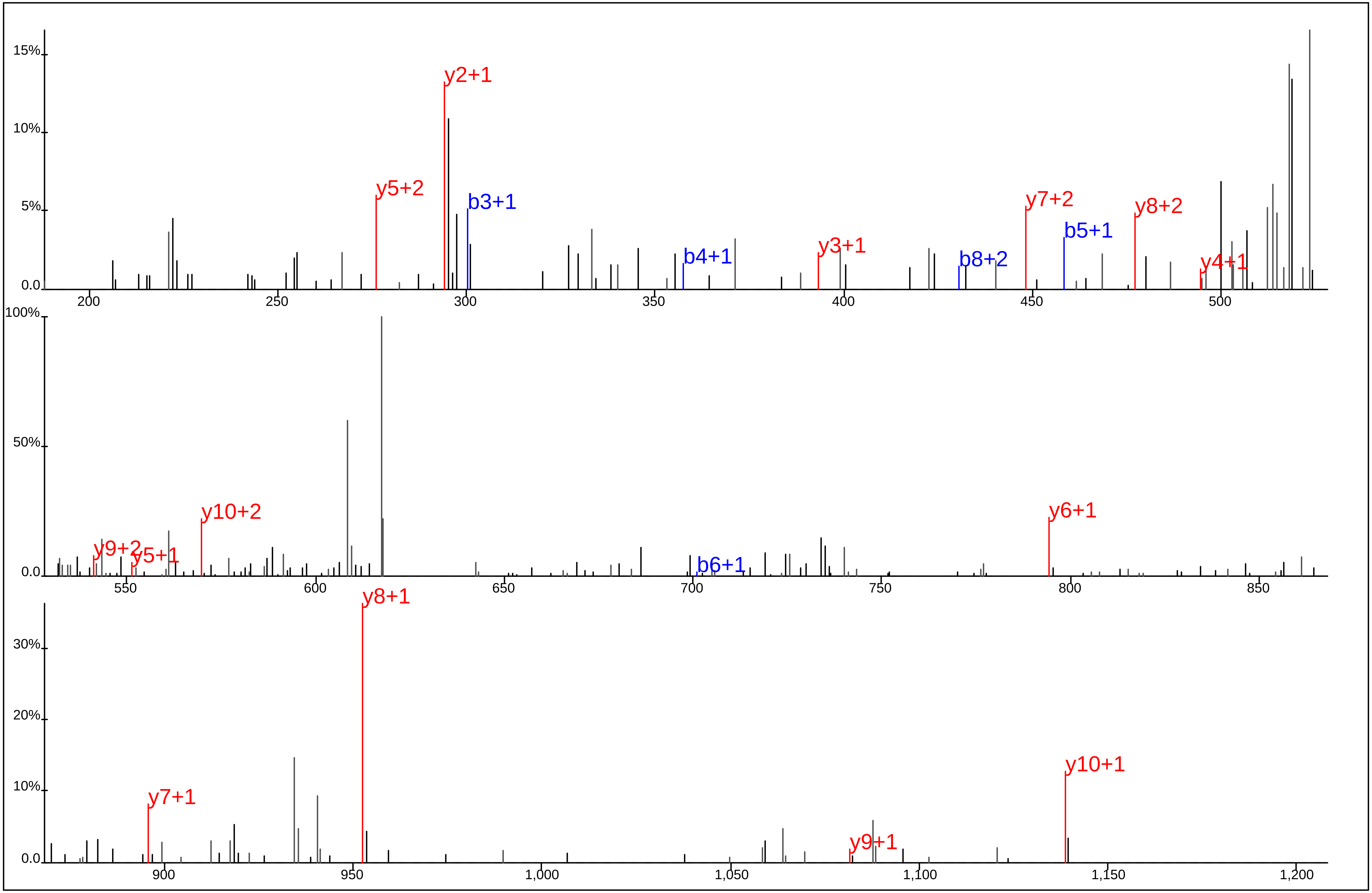

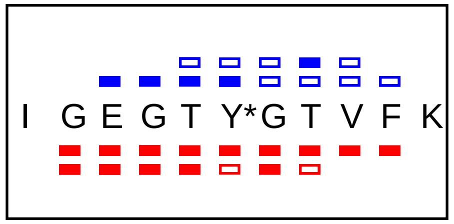

Protein Name: CDK5
gi Accession: 4826675
Precursor Mass: 626.2878+2
Delta Mass: 2.060 ppm
XCorr: 29.77

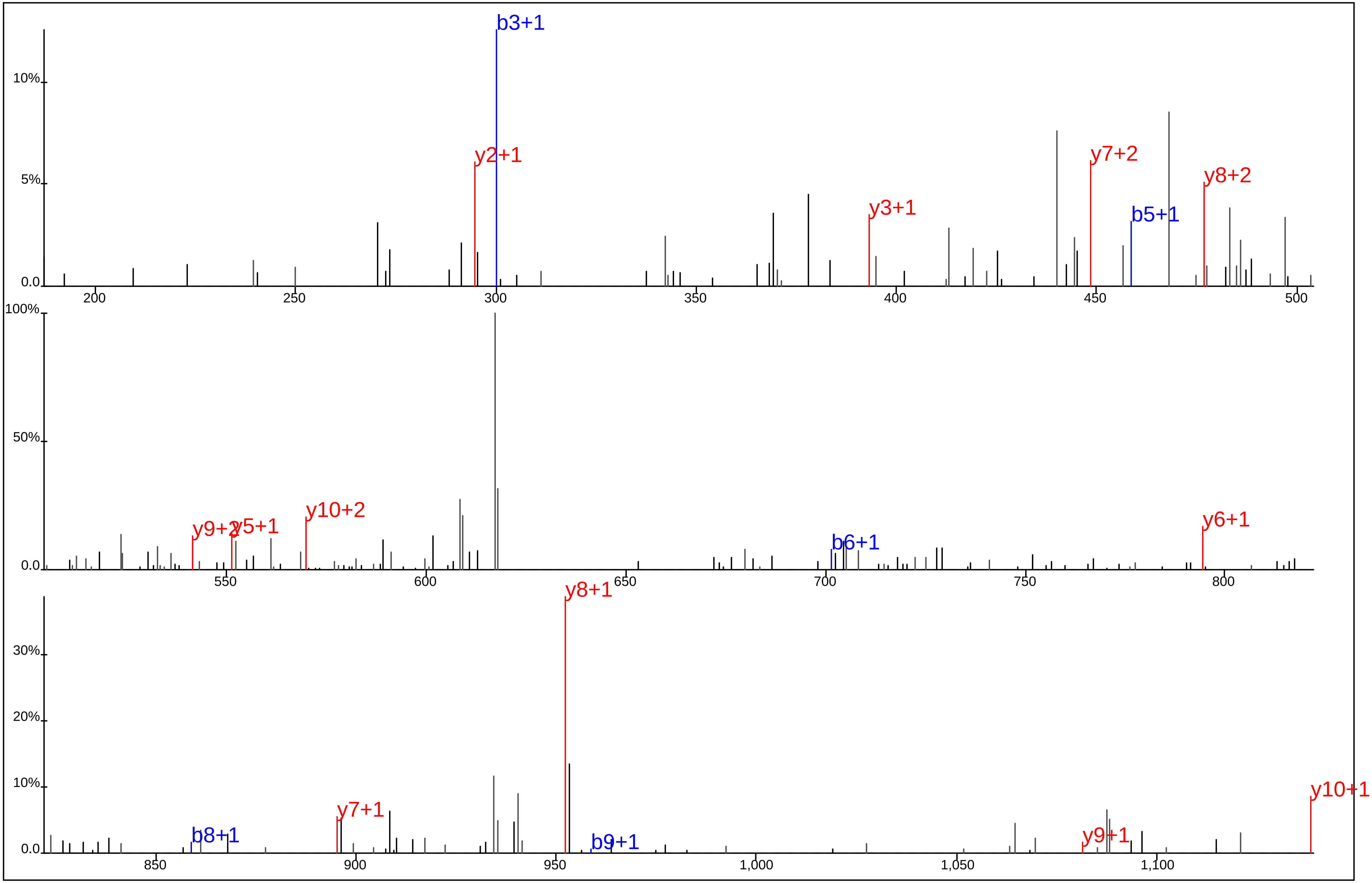

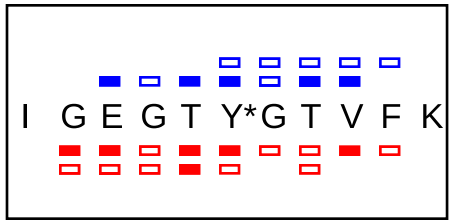

Protein Name: CDK5
gi Accession: 4826675
Precursor Mass: 626.2889+2
Delta Mass: 3.913 ppm
XCorr: 36.68

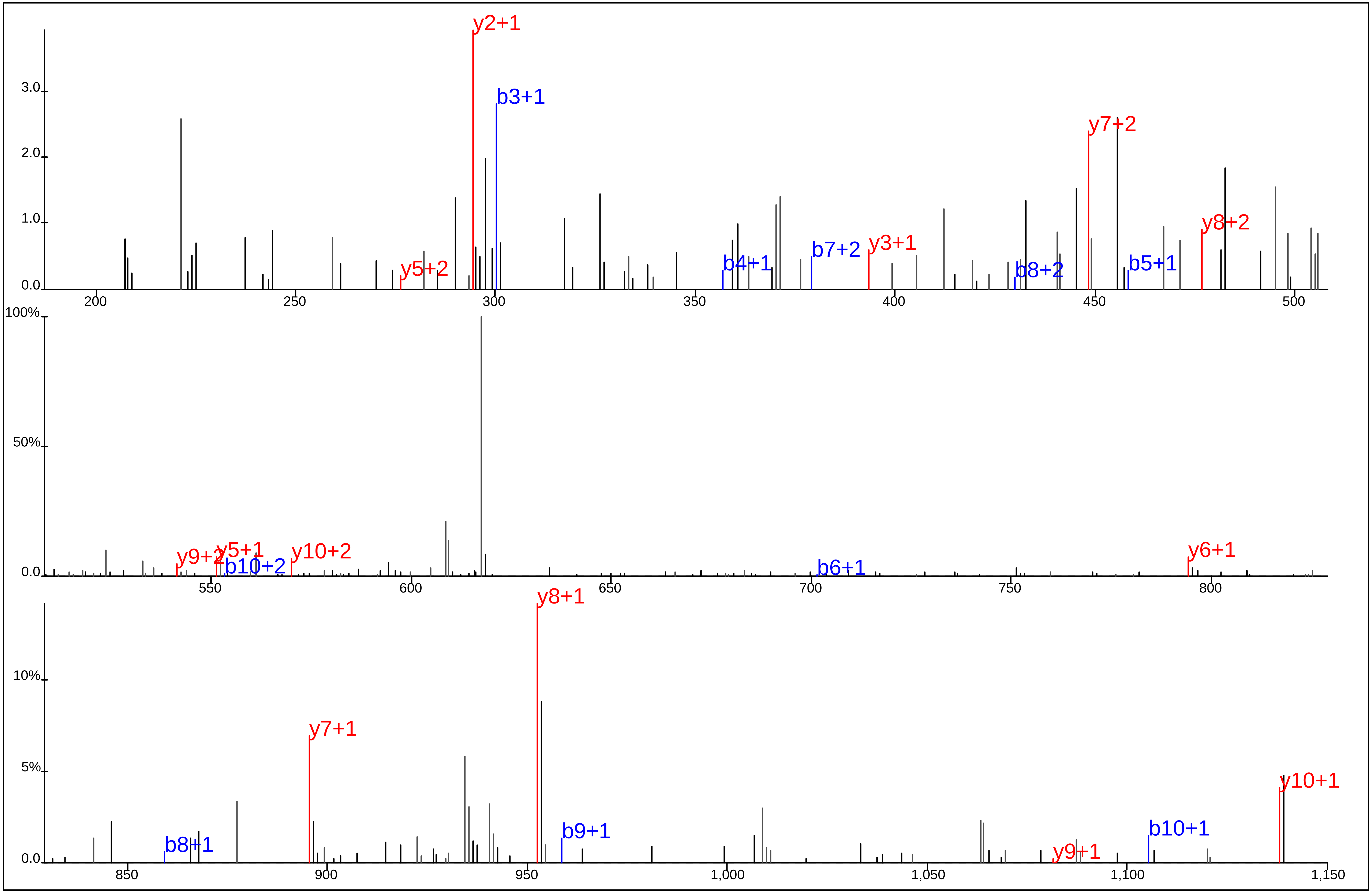

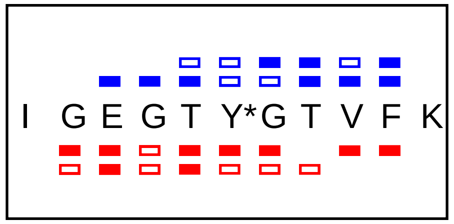

Protein Name: CDK5
gi Accession: 4826675
Precursor Mass: 626.2870+2
Delta Mass: 0.797 ppm
XCorr: 28.86

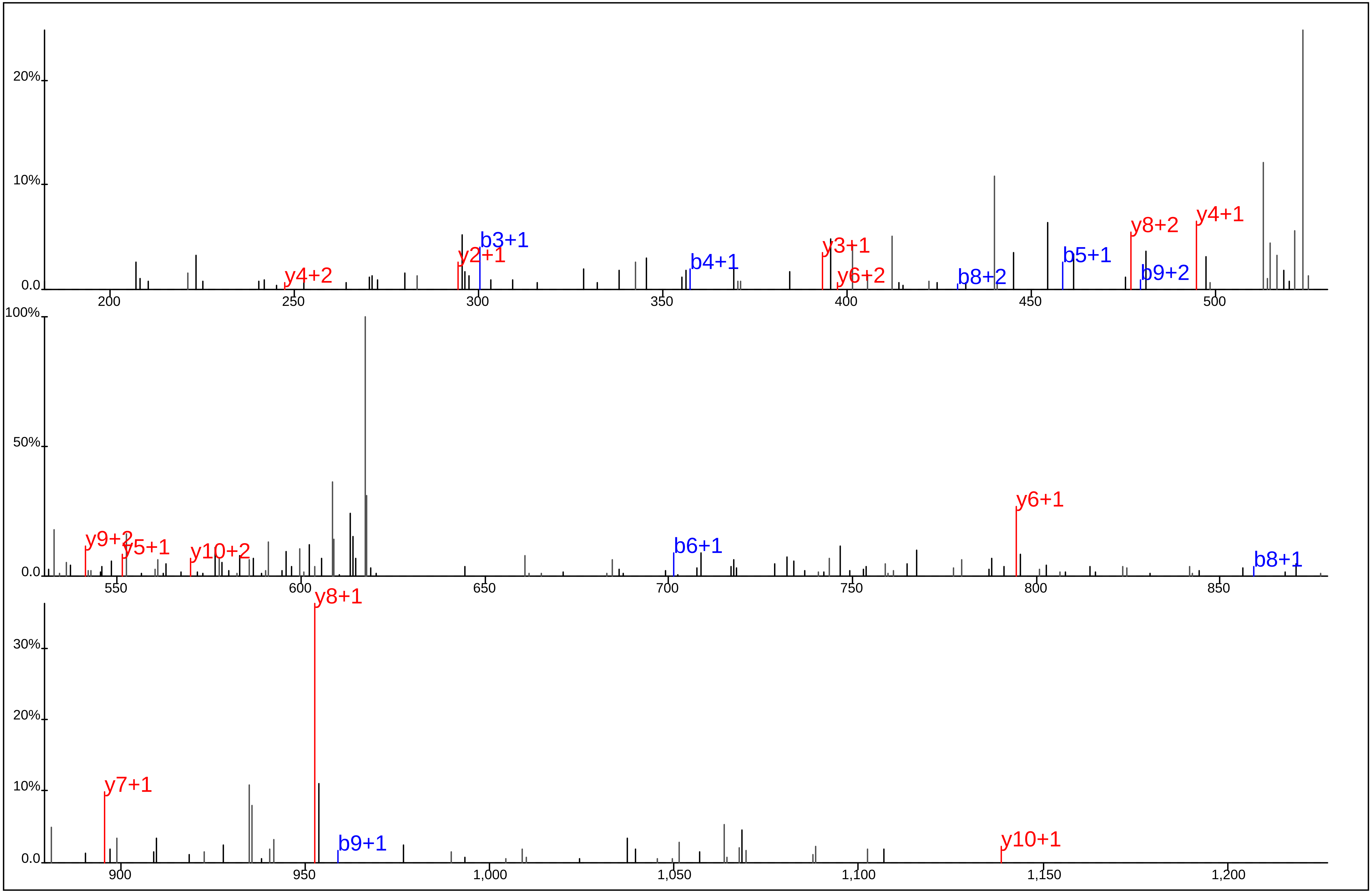

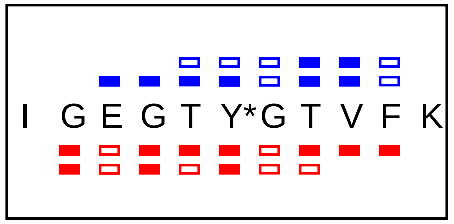

Protein Name: CDK5
gi Accession: 4826675
Precursor Mass: 626.2893+2
Delta Mass: 4.505 ppm
XCorr: 24.15

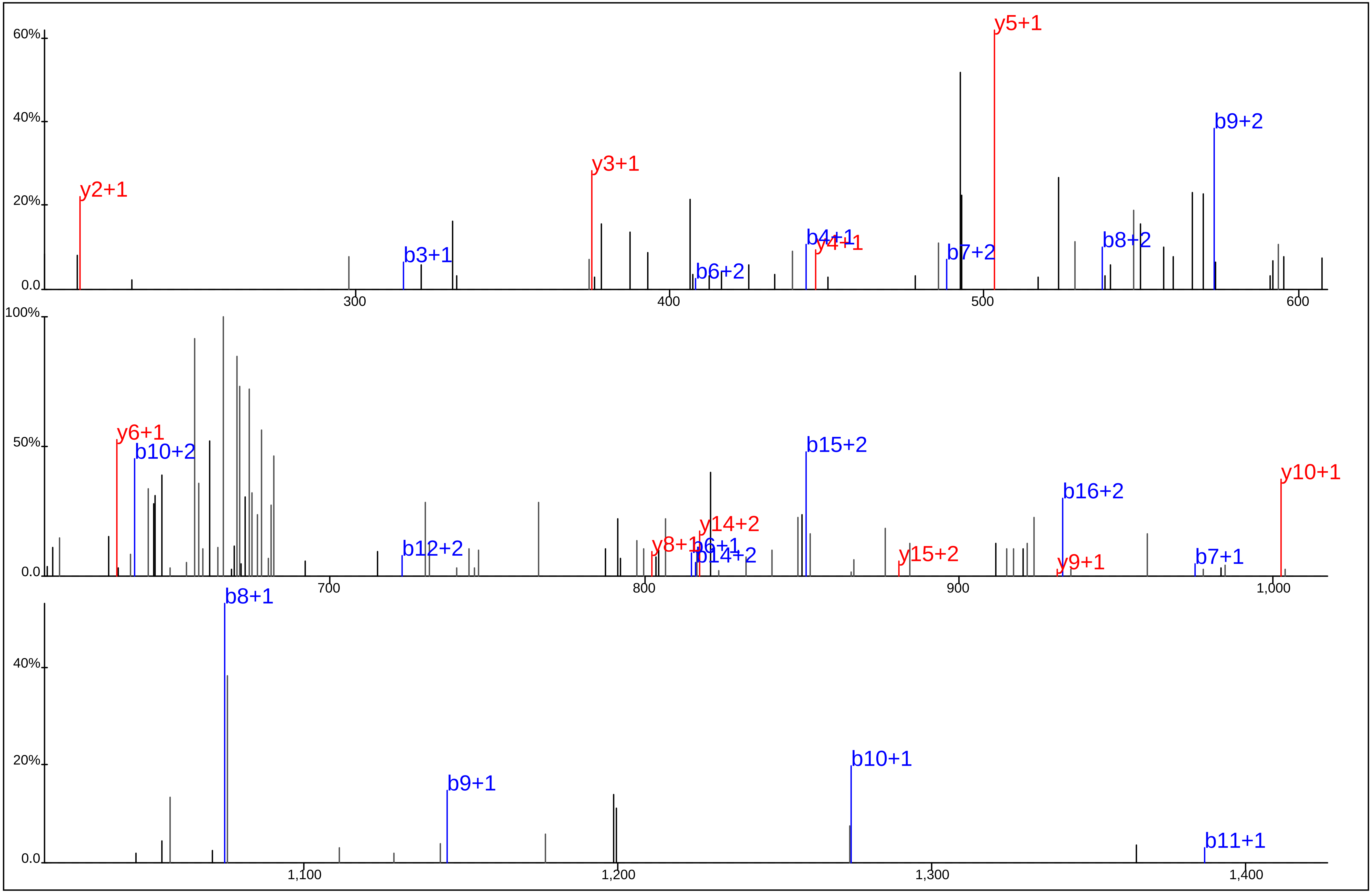

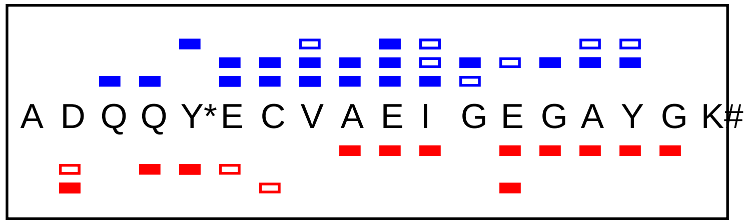

Protein Name: CDK6
gi Accession: 4502741
Precursor Mass: 692.6265+3
Delta Mass: 6.897 ppm
XCorr: 23.83

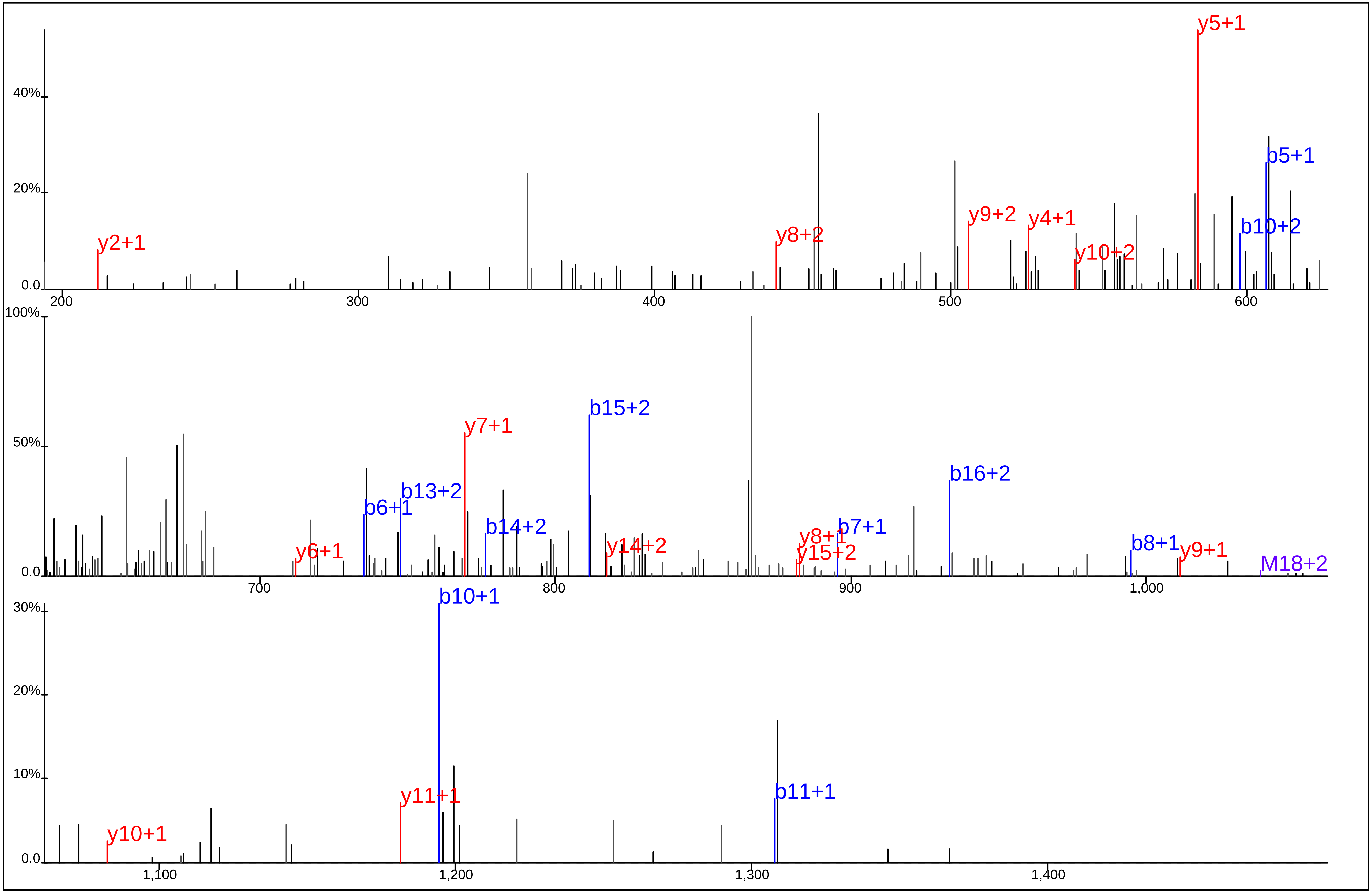

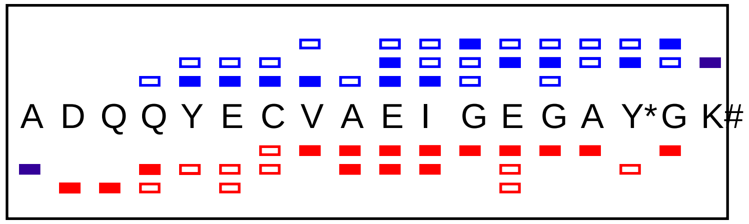

Protein Name: CDK6
gi Accession: 4502741
Precursor Mass: 692.6183+3
Delta Mass: 4.920 ppm
XCorr: 22.87

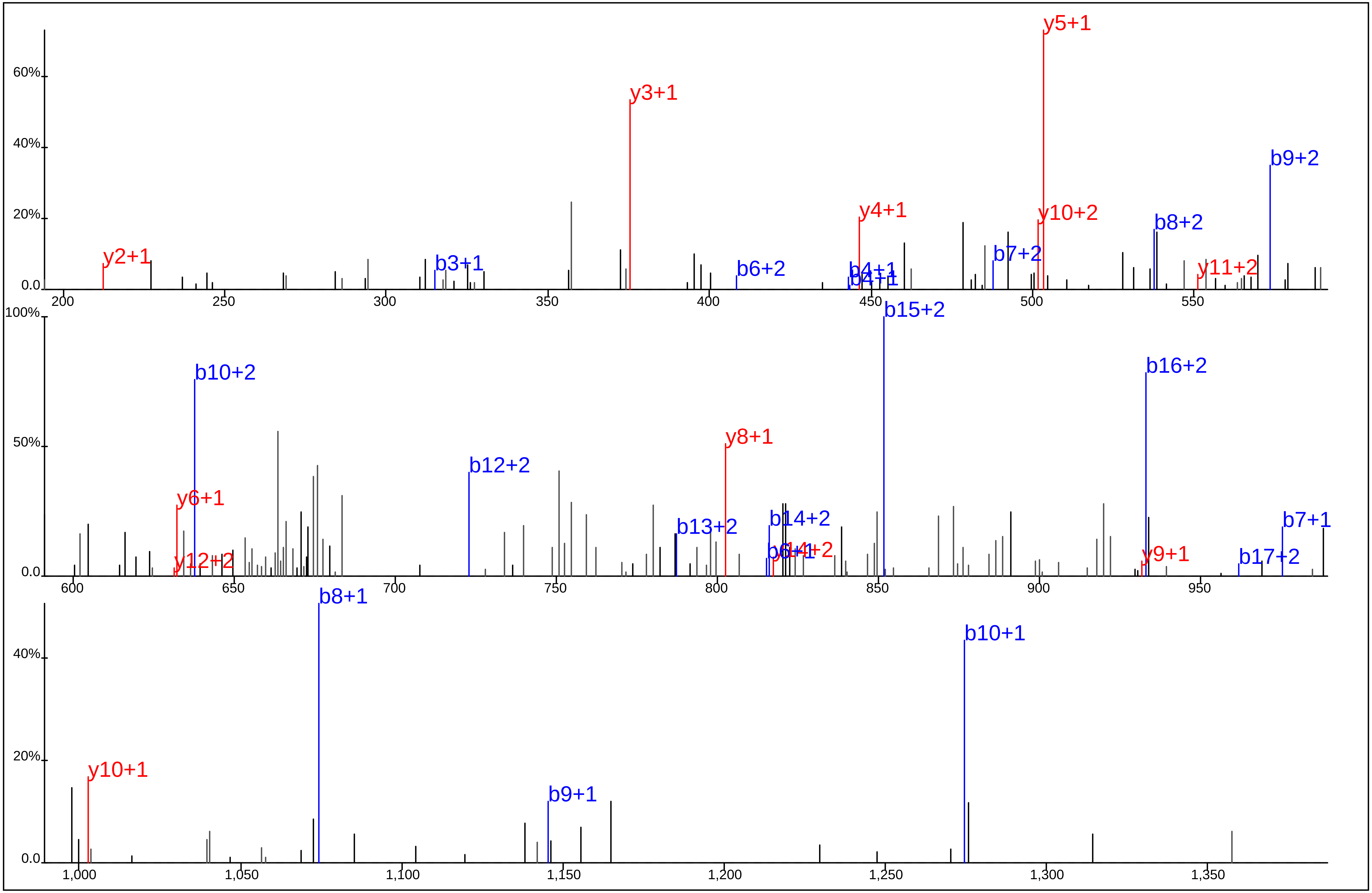

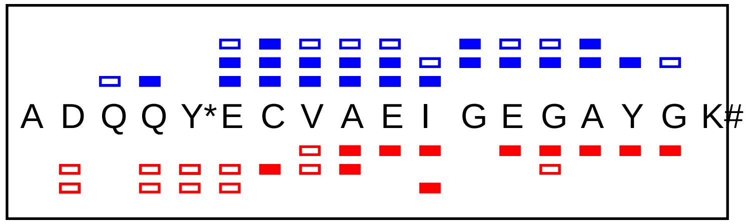

Protein Name: CDK6
gi Accession: 4502741
Precursor Mass: 692.6249+3
Delta Mass: 4.604 ppm
XCorr: 33.19

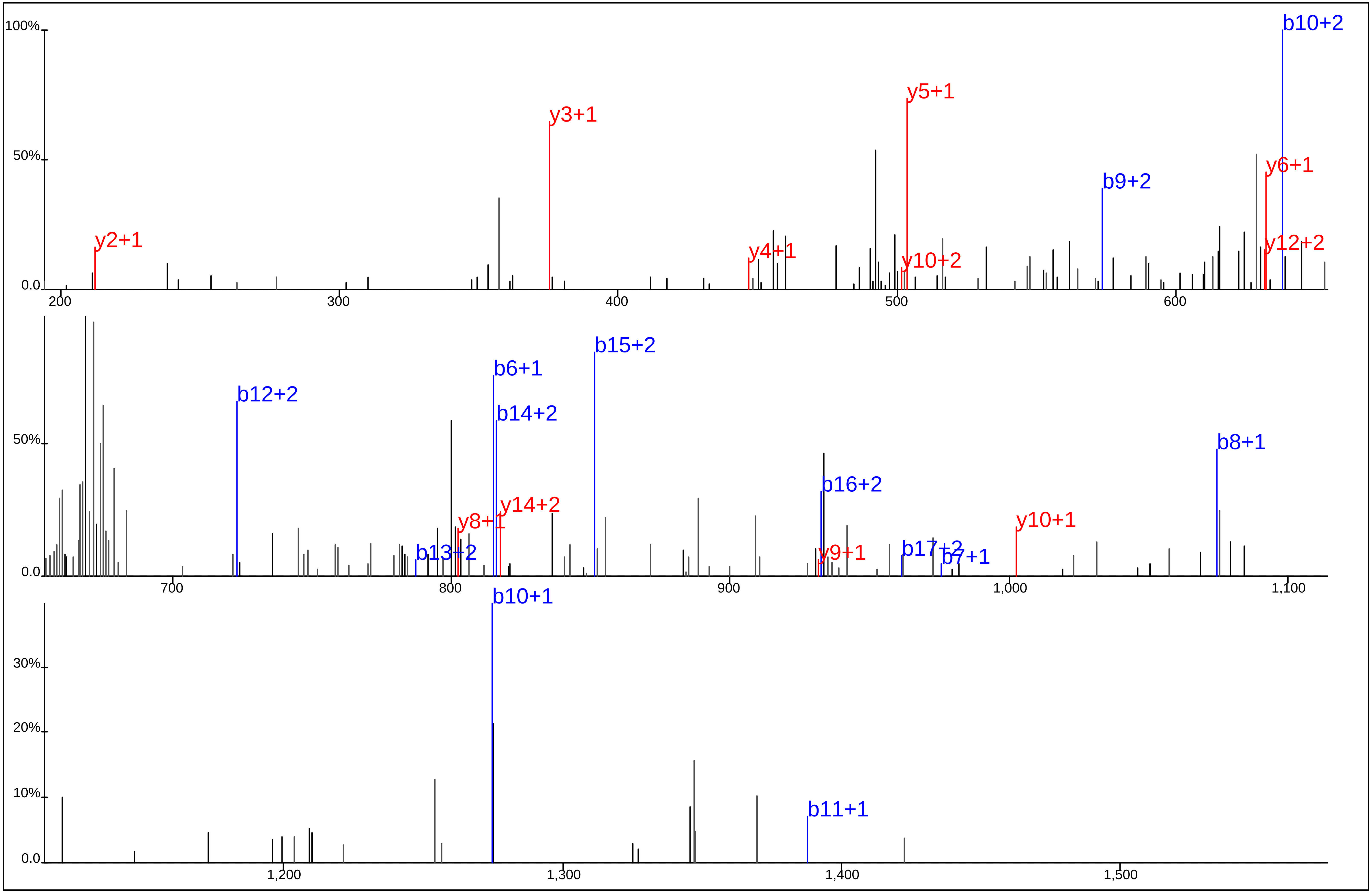

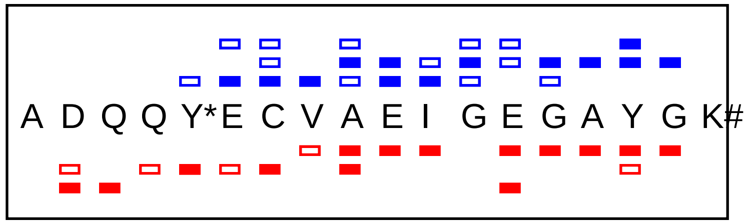

Protein Name: CDK6
gi Accession: 4502741
Precursor Mass: 692.6242+3
Delta Mass: 3.549 ppm
XCorr: 21.46

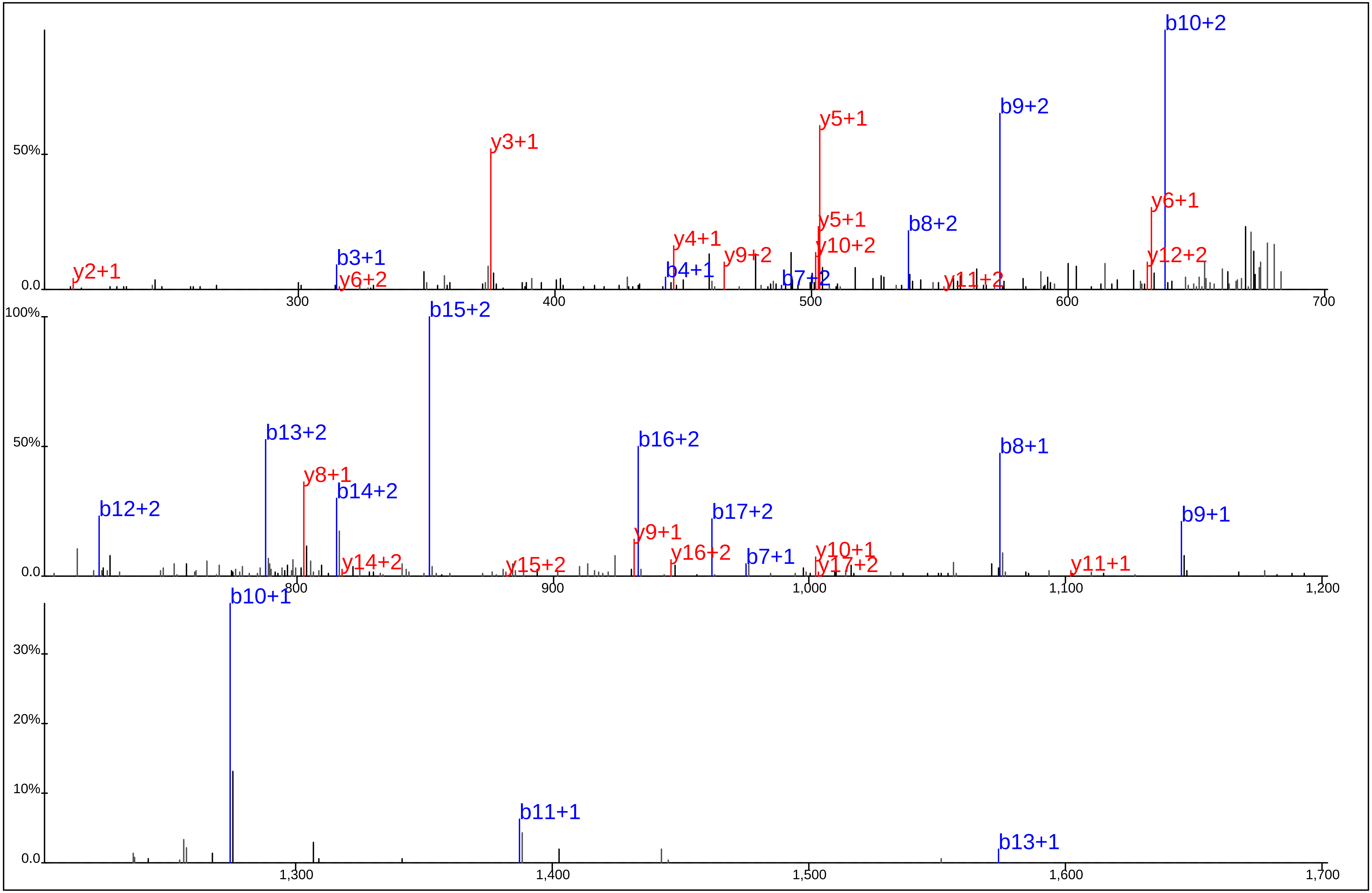

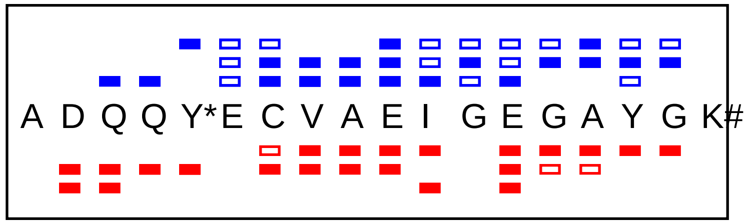

Protein Name: CDK6
gi Accession: 4502741
Precursor Mass: 692.6228+3
Delta Mass: 1.517 ppm
XCorr: 62.63

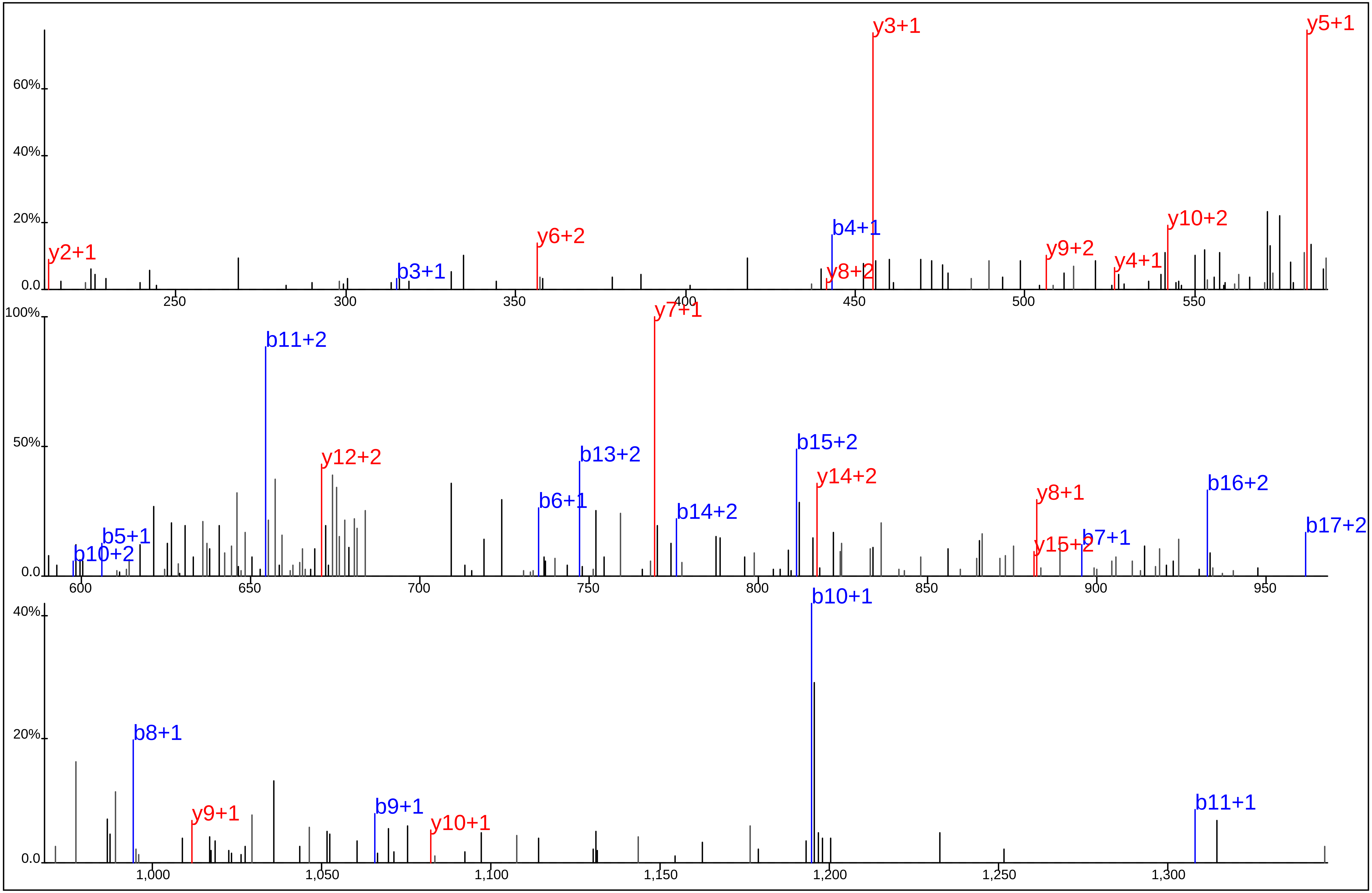

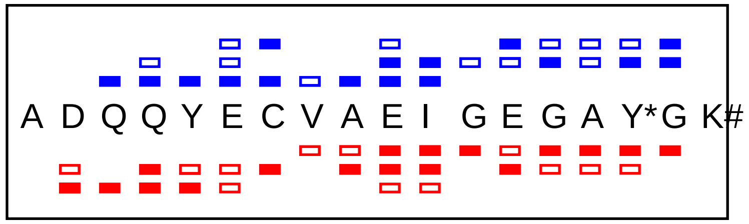

Protein Name: CDK6
gi Accession: 4502741
Precursor Mass: 692.6248+3
Delta Mass: 4.339 ppm
XCorr: 38.54

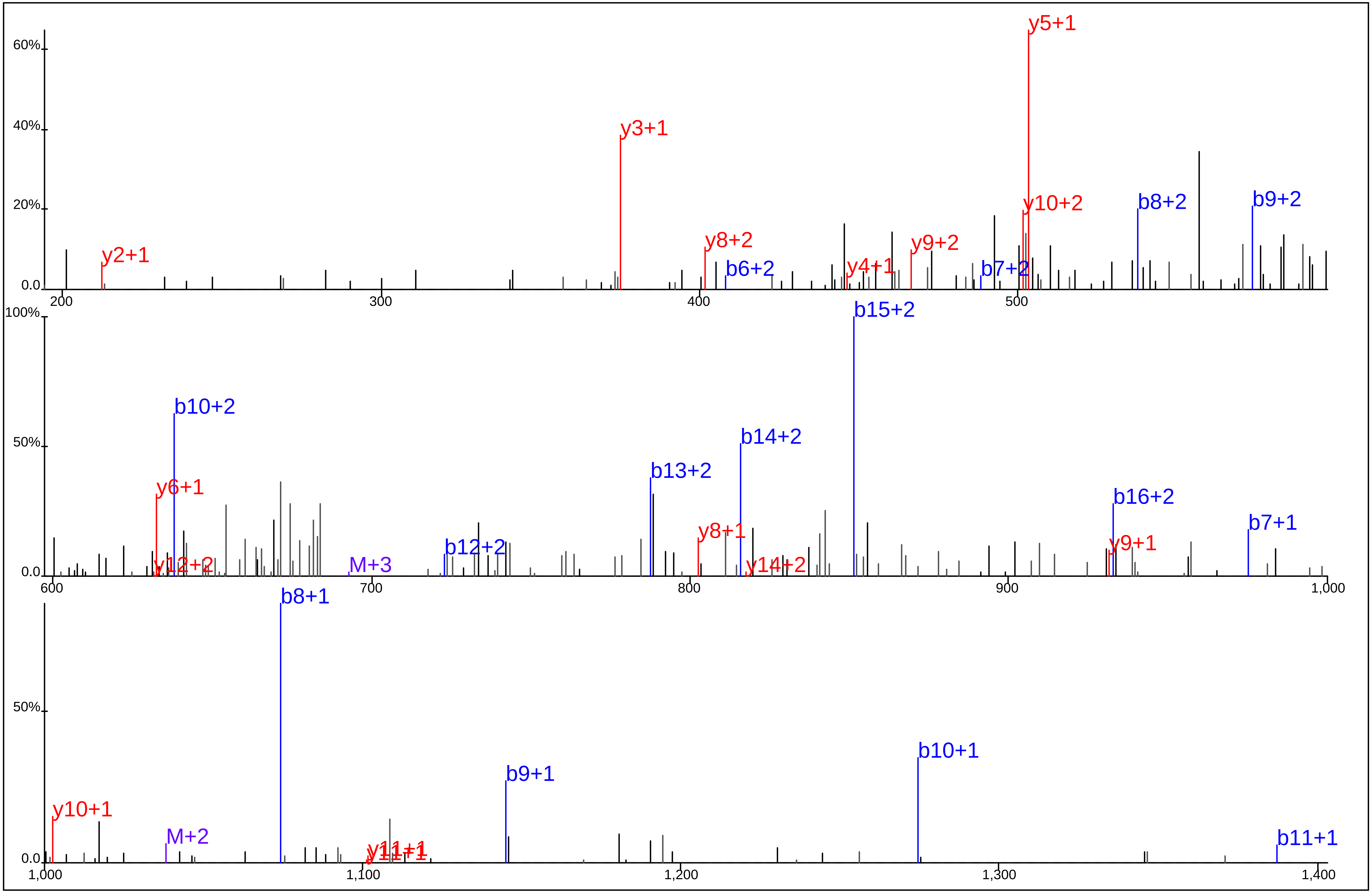

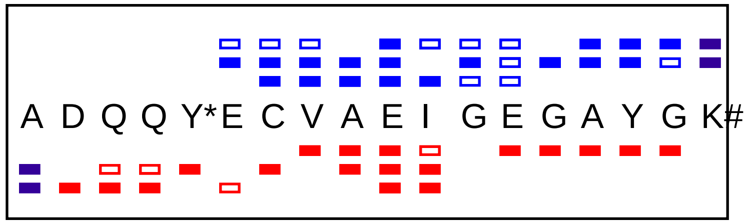

Protein Name: CDK6
gi Accession: 4502741
Precursor Mass: 692.6238+3
Delta Mass: 3.020 ppm
XCorr: 39.06

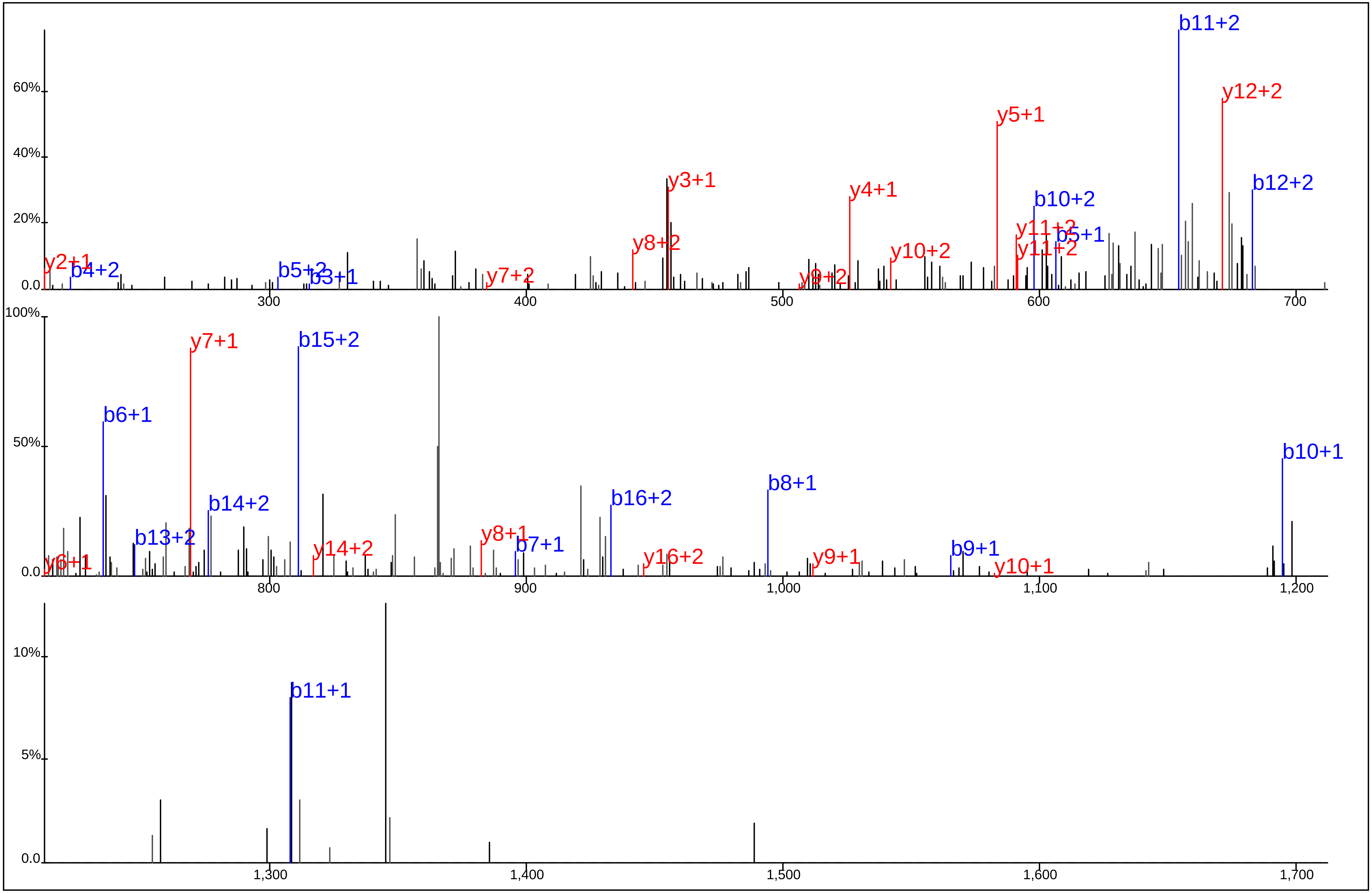

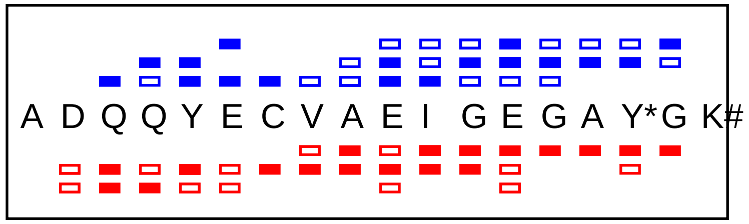

Protein Name: CDK6
gi Accession: 4502741
Precursor Mass: 692.6289+3
Delta Mass: 10.341 ppm
XCorr: 26.65

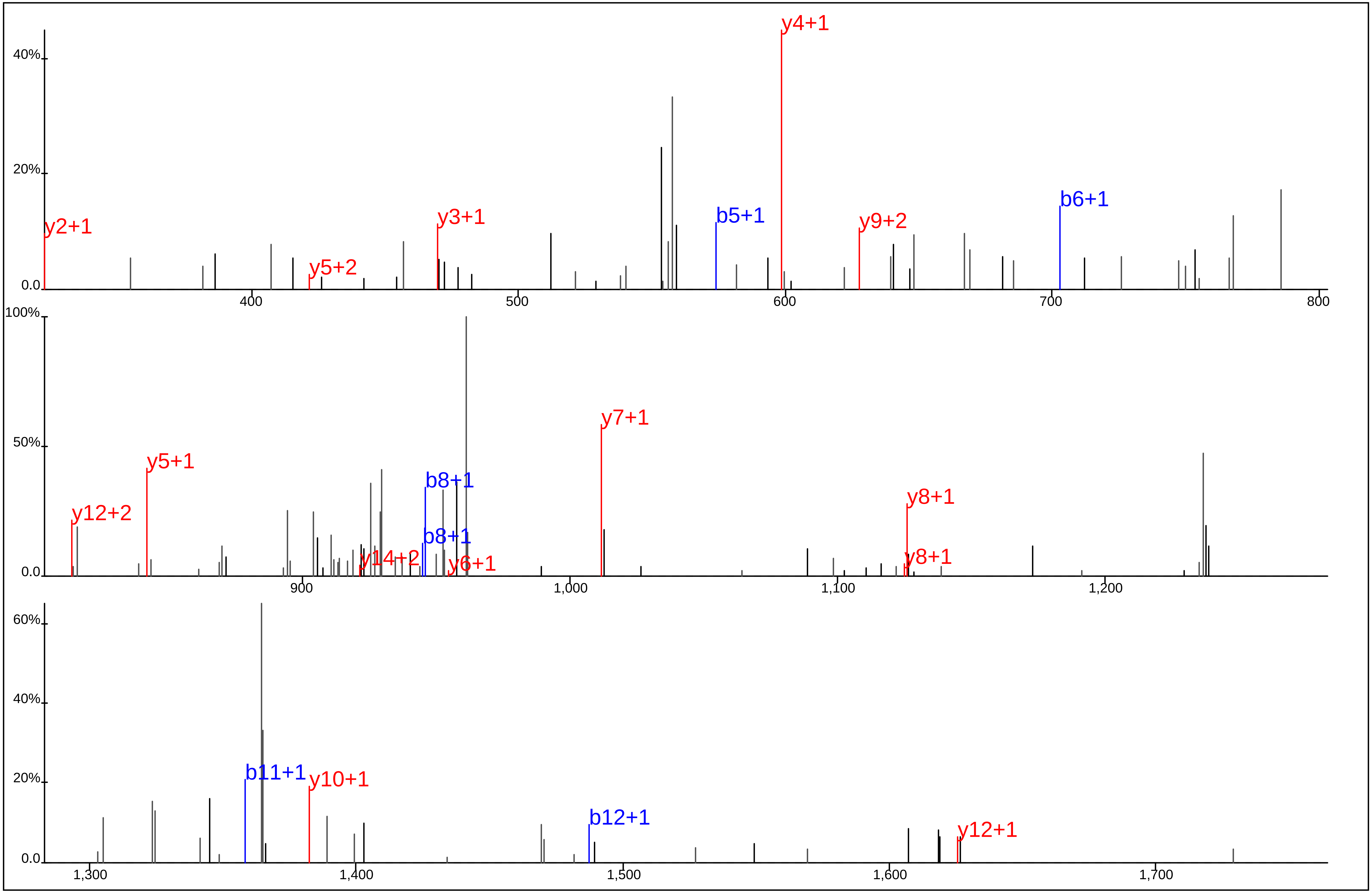

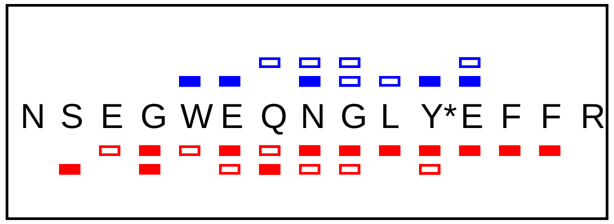

Protein Name: CHERP
gi Accession: 25992622
Precursor Mass: 978.4077+2
Delta Mass: 6.170 ppm
XCorr: 39.3

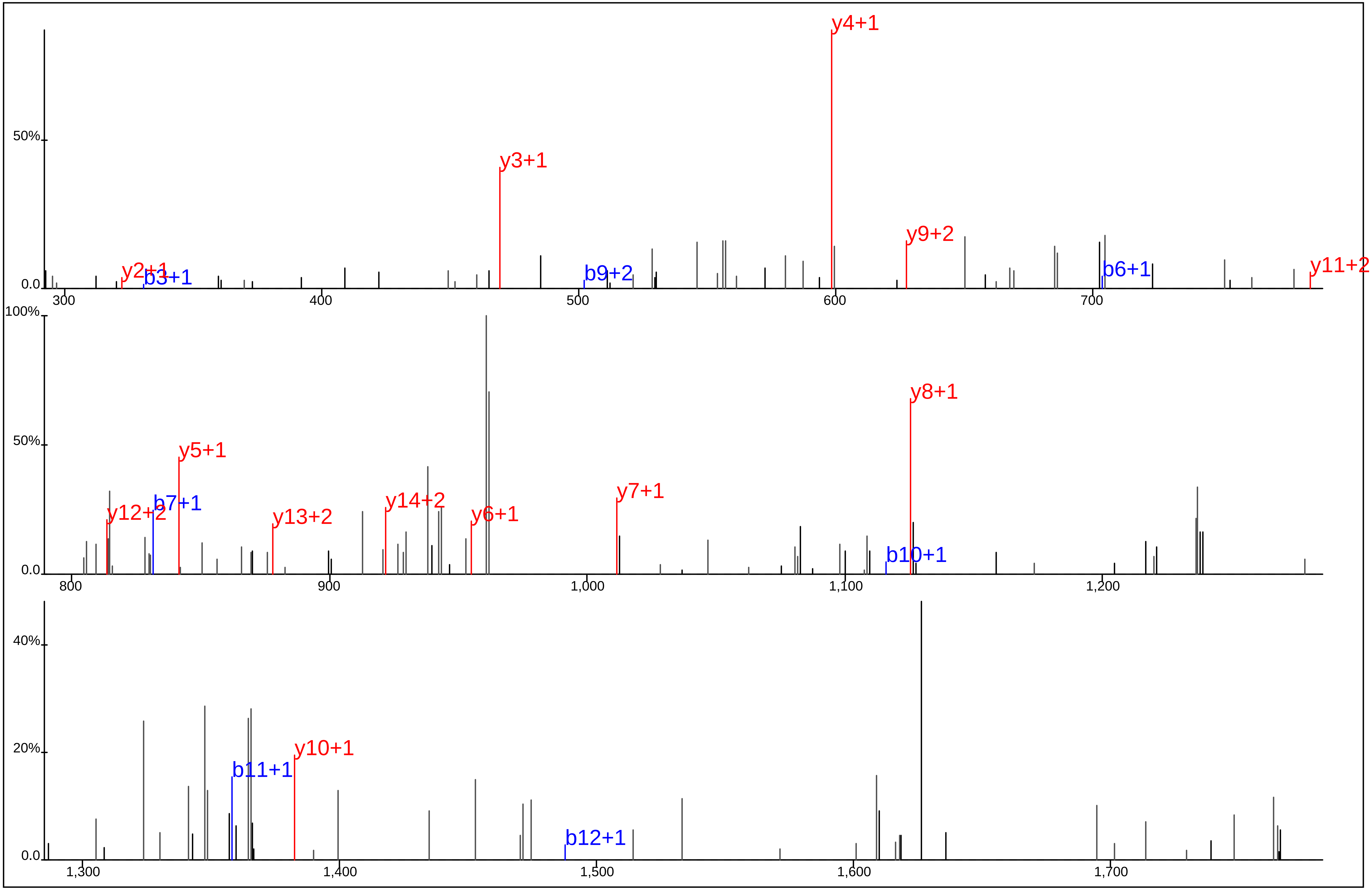

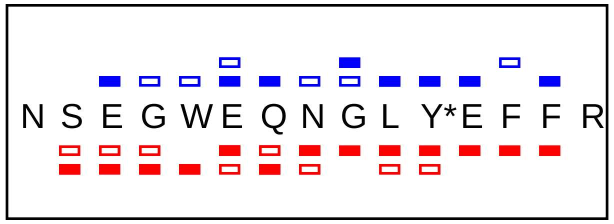

Protein Name:	CHERP
gi Accession:	25992622
Precursor Mass:	978.4067+2
Delta Mass:	5.173 ppm
XCorr:	30.38

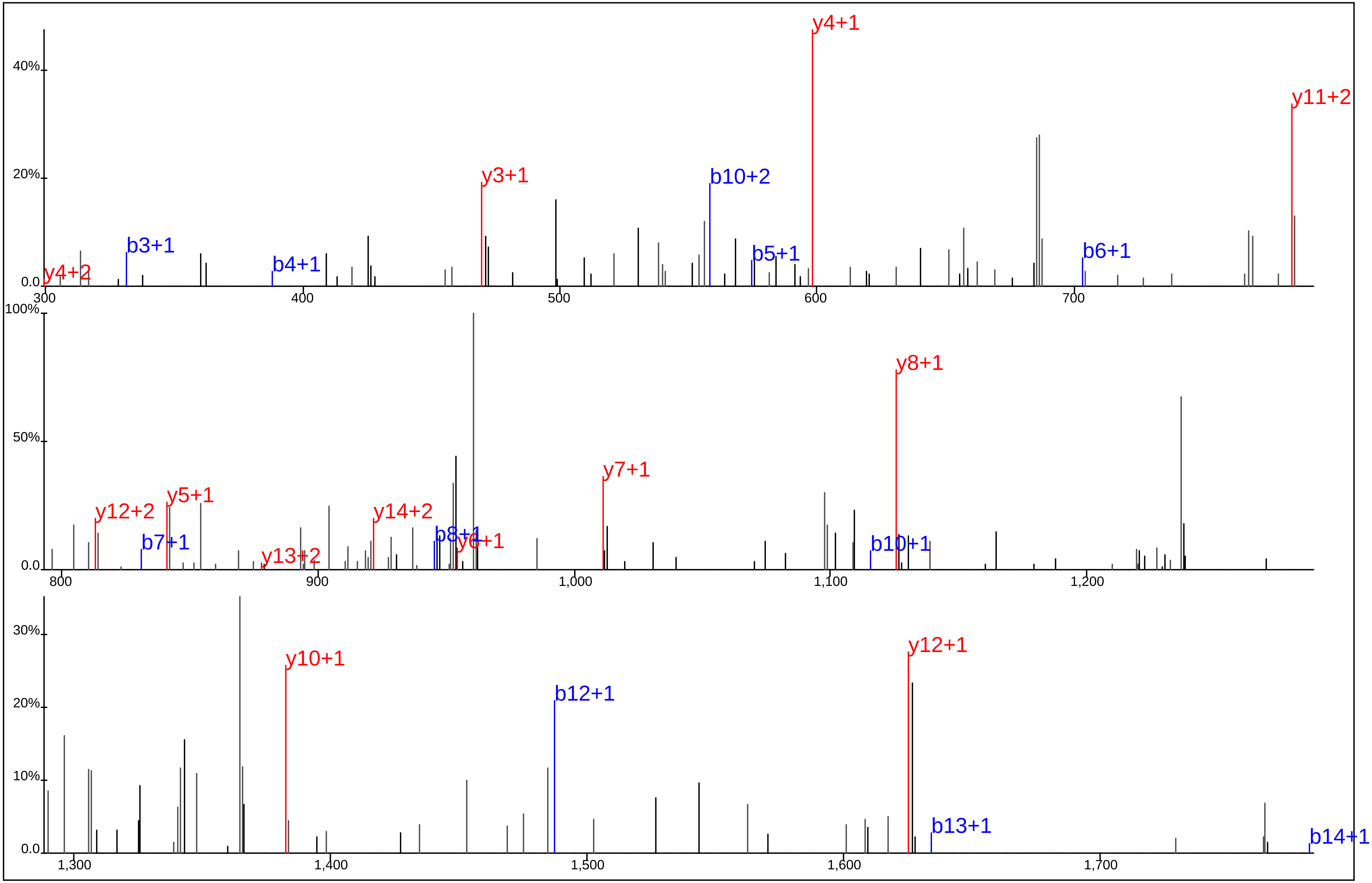

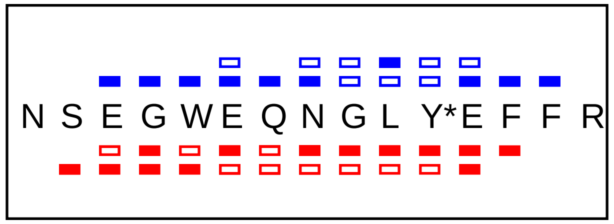

Protein Name: CHERP
gi Accession: 25992622
Precursor Mass: 978.4062+2
Delta Mass: 4.734 ppm
XCorr: 34.43

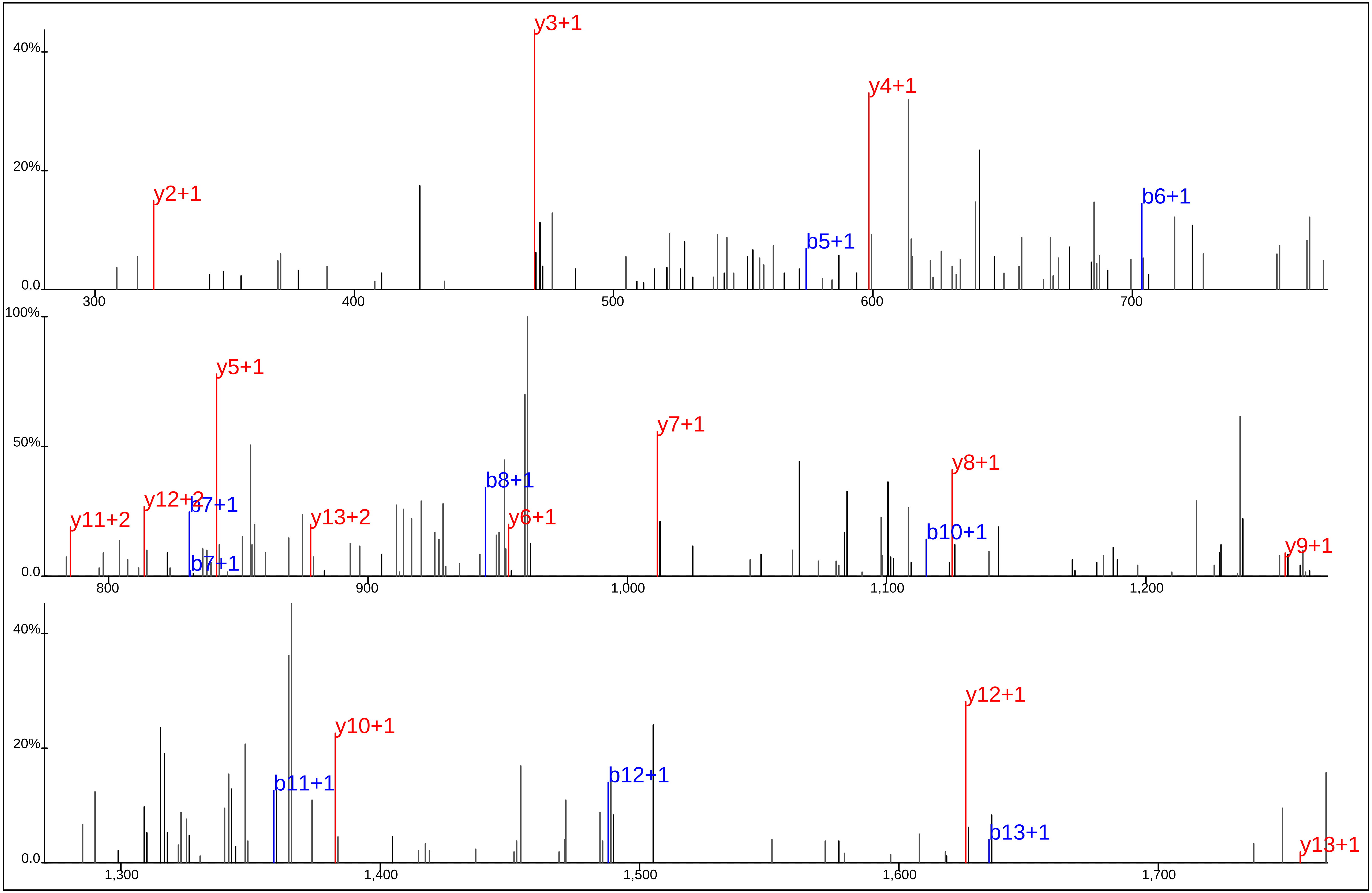

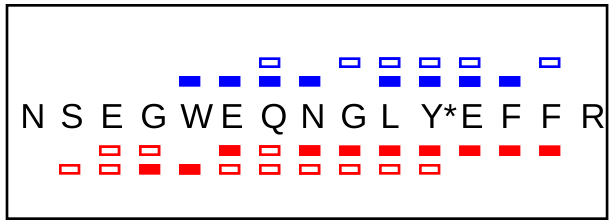

Protein Name: CHERP
gi Accession: 25992622
Precursor Mass: 978.4061+2
Delta Mass: 4.611 ppm
XCorr: 53.17

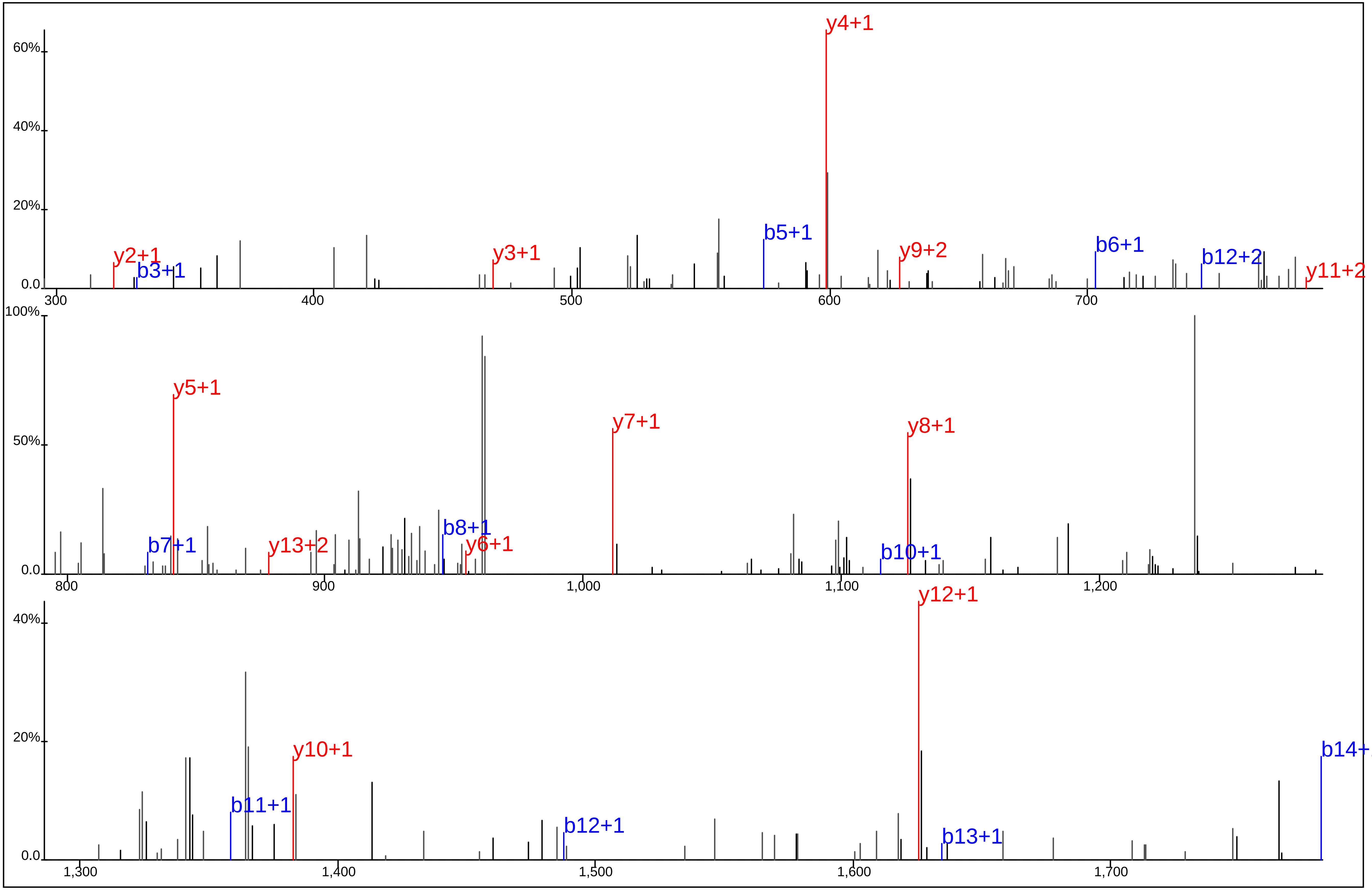

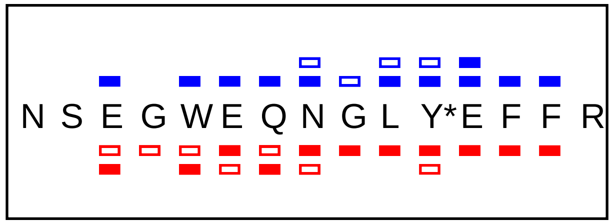

Protein Name: CHERP
gi Accession: 25992622
Precursor Mass: 978.4073+2
Delta Mass: 5.797 ppm
XCorr: 35.74

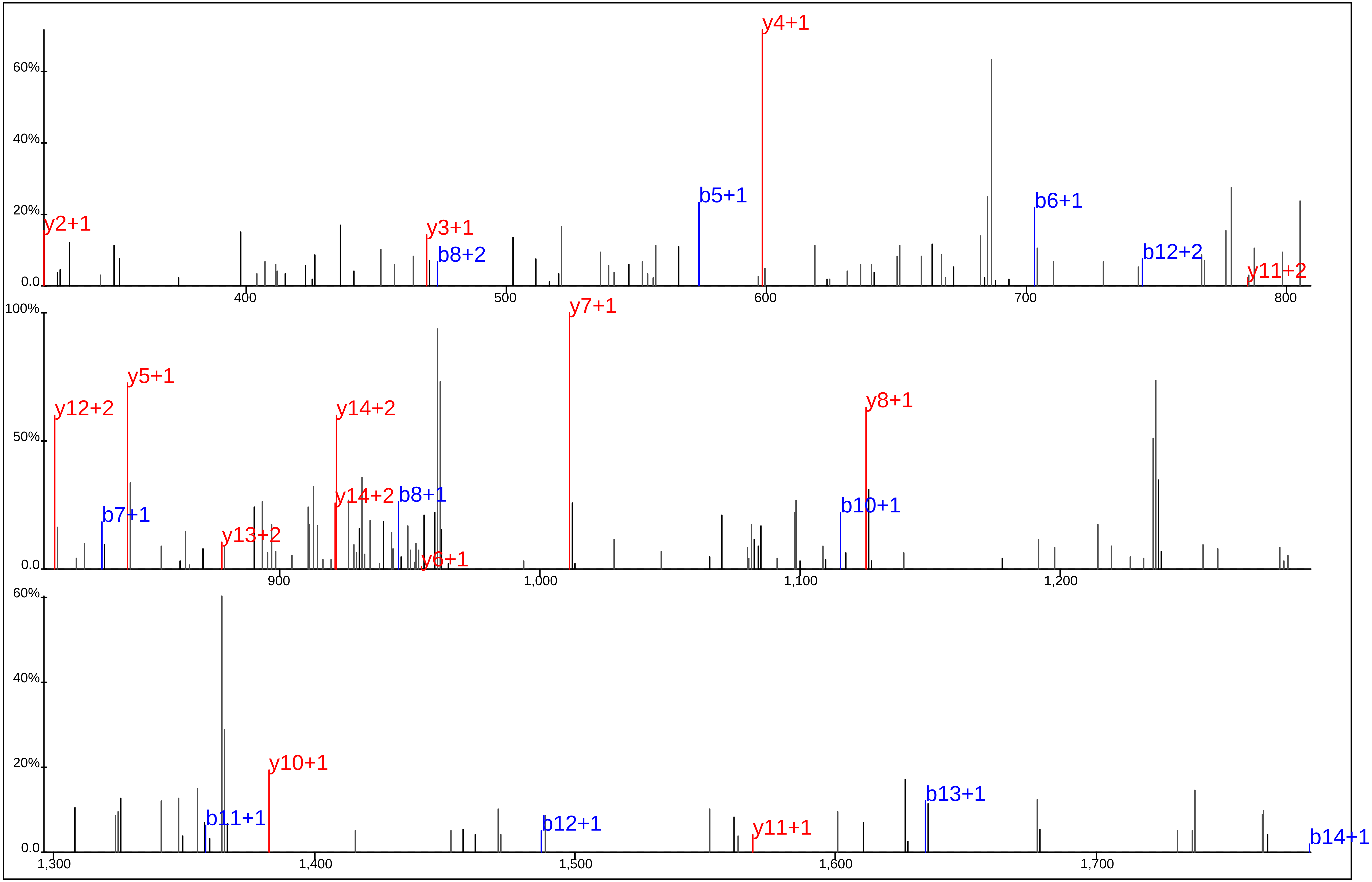

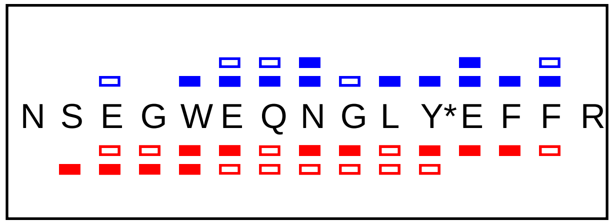

Protein Name: CHERP
gi Accession: 25992622
Precursor Mass: 978.4031+2
Delta Mass: 1.553 ppm
XCorr: 32.54

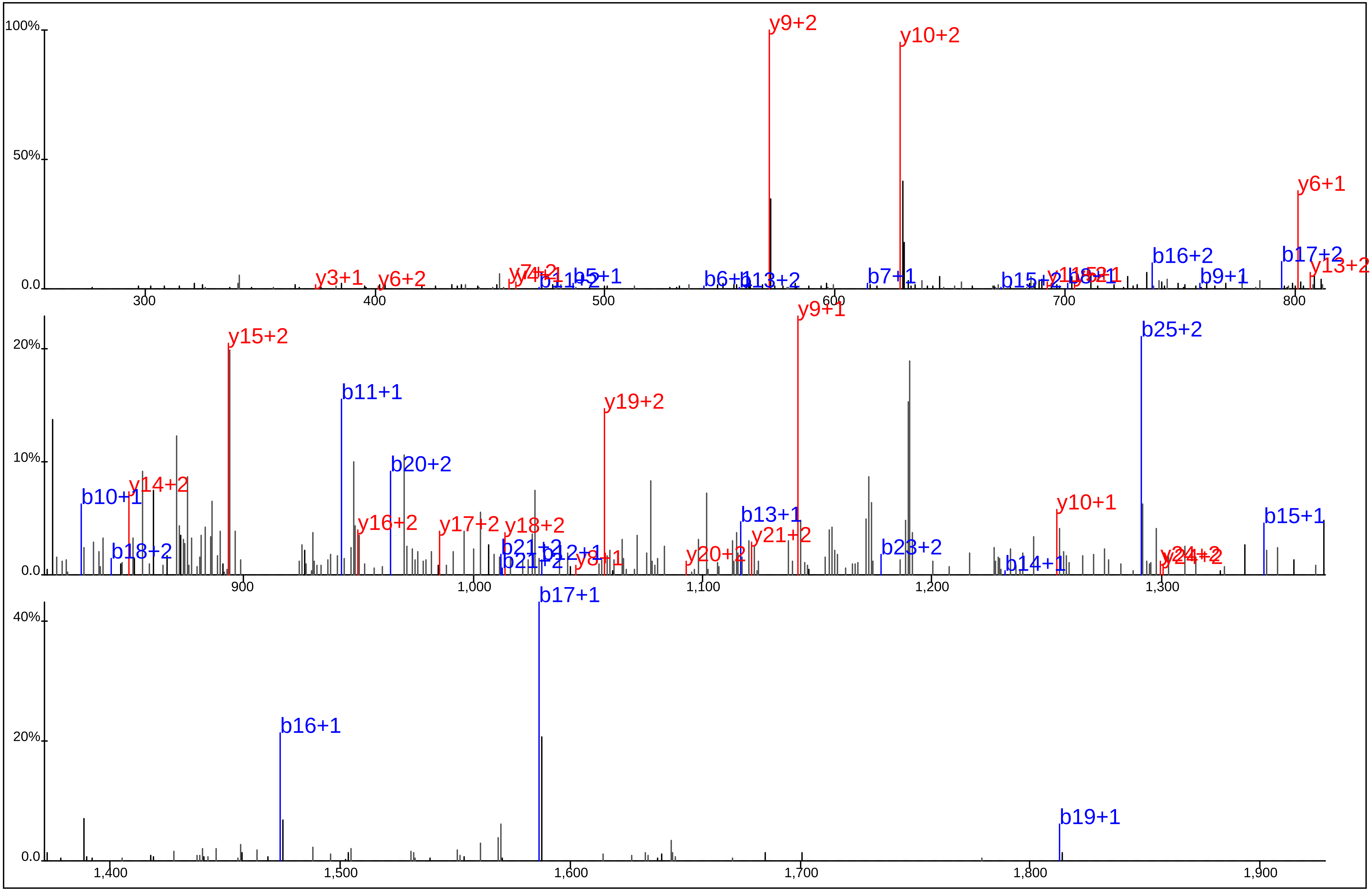

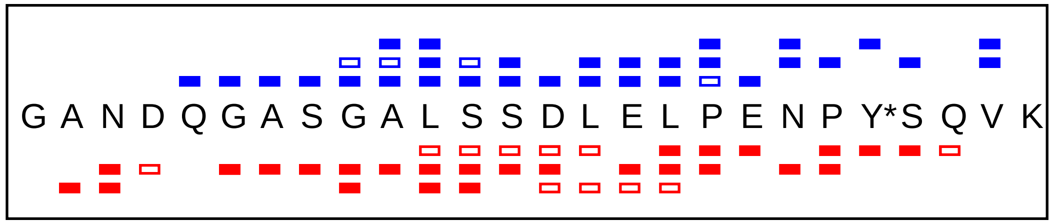

Protein Name:	Cingulin
gi Accession:	16262452
Precursor Mass:	909.7468+3
Delta Mass:	4.194 ppm
XCorr:	23.18

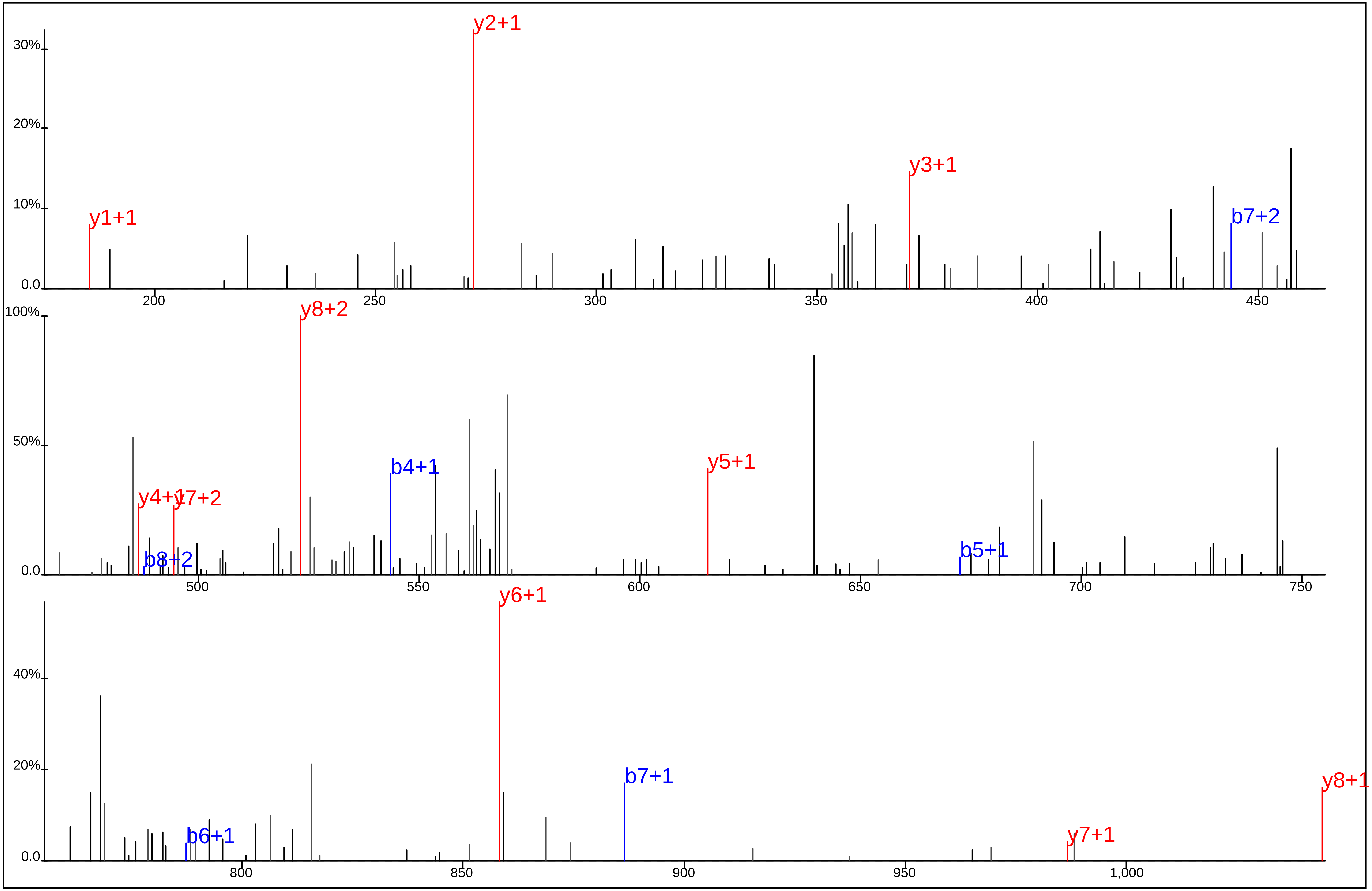

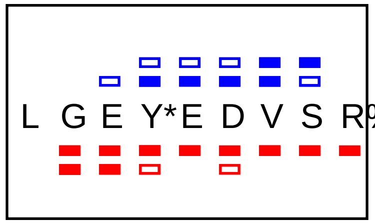

Protein Name: CKAP1
gi Accession: 736704
Precursor Mass: 579.2377+2
Delta Mass: 5.851 ppm
XCorr: 31.92

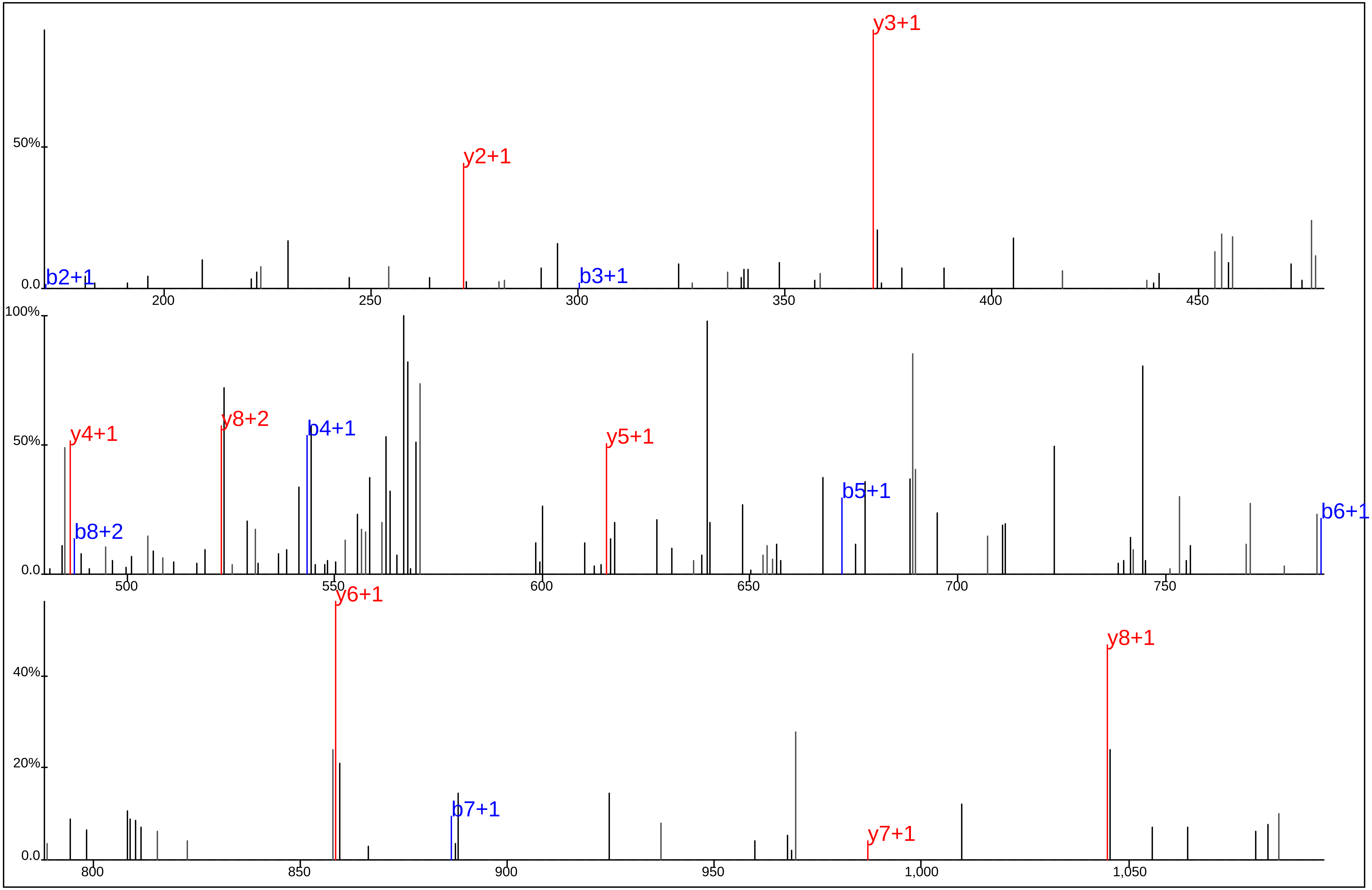

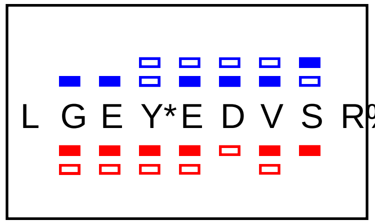

Protein Name: CKAP1
gi Accession: 736704
Precursor Mass: 579.2431+2
Delta Mass: 3.428 ppm
XCorr: 13.56

291 - CKAP1_3442613.pdf

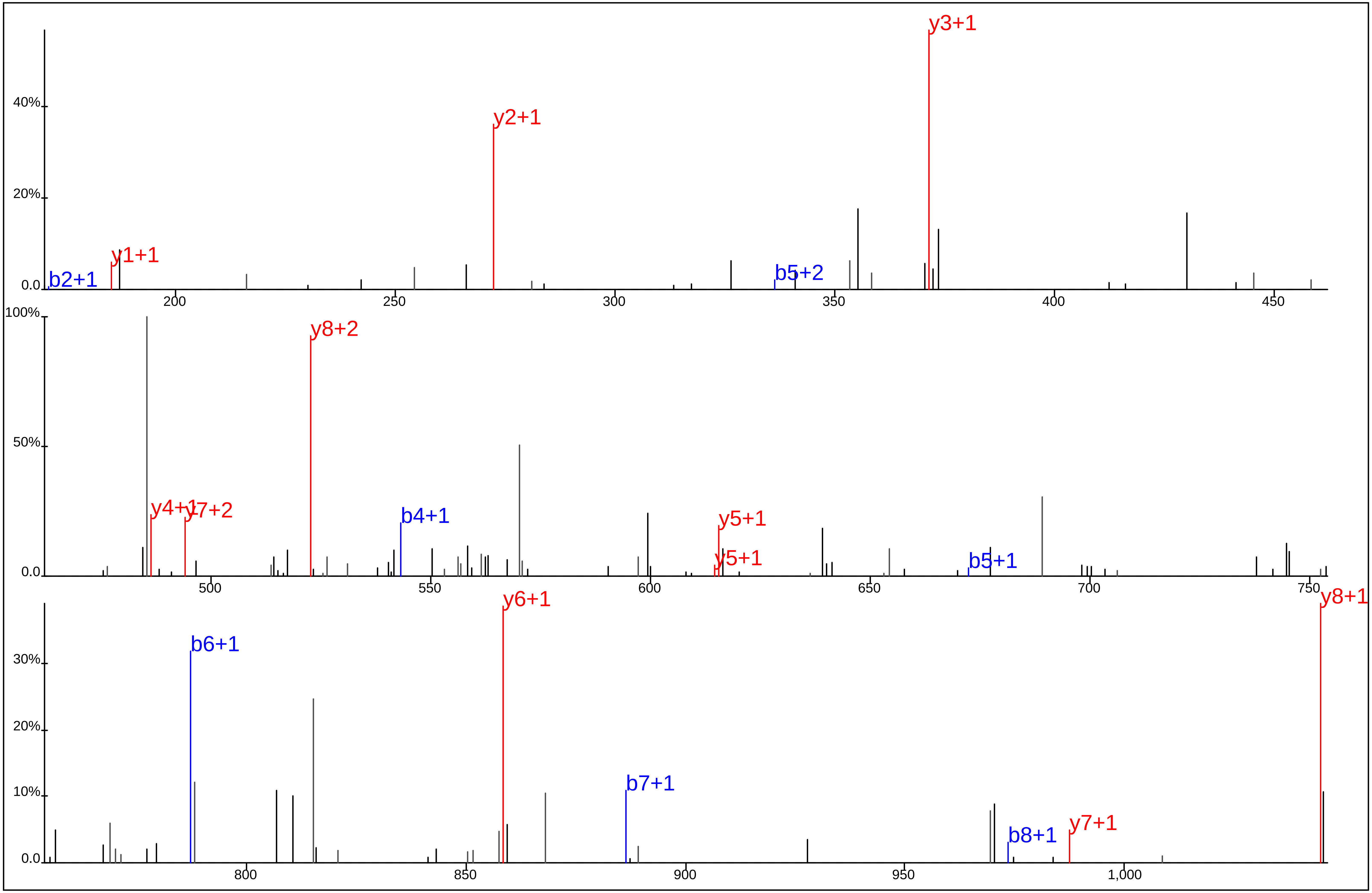

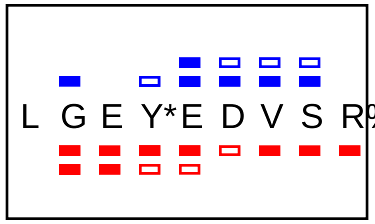

Protein Name: CKAP1
gi Accession: 736704
Precursor Mass: 579.2421+2
Delta Mass: 1.743 ppm
XCorr: 49.57

292 - CKAP1_3447360.pdf

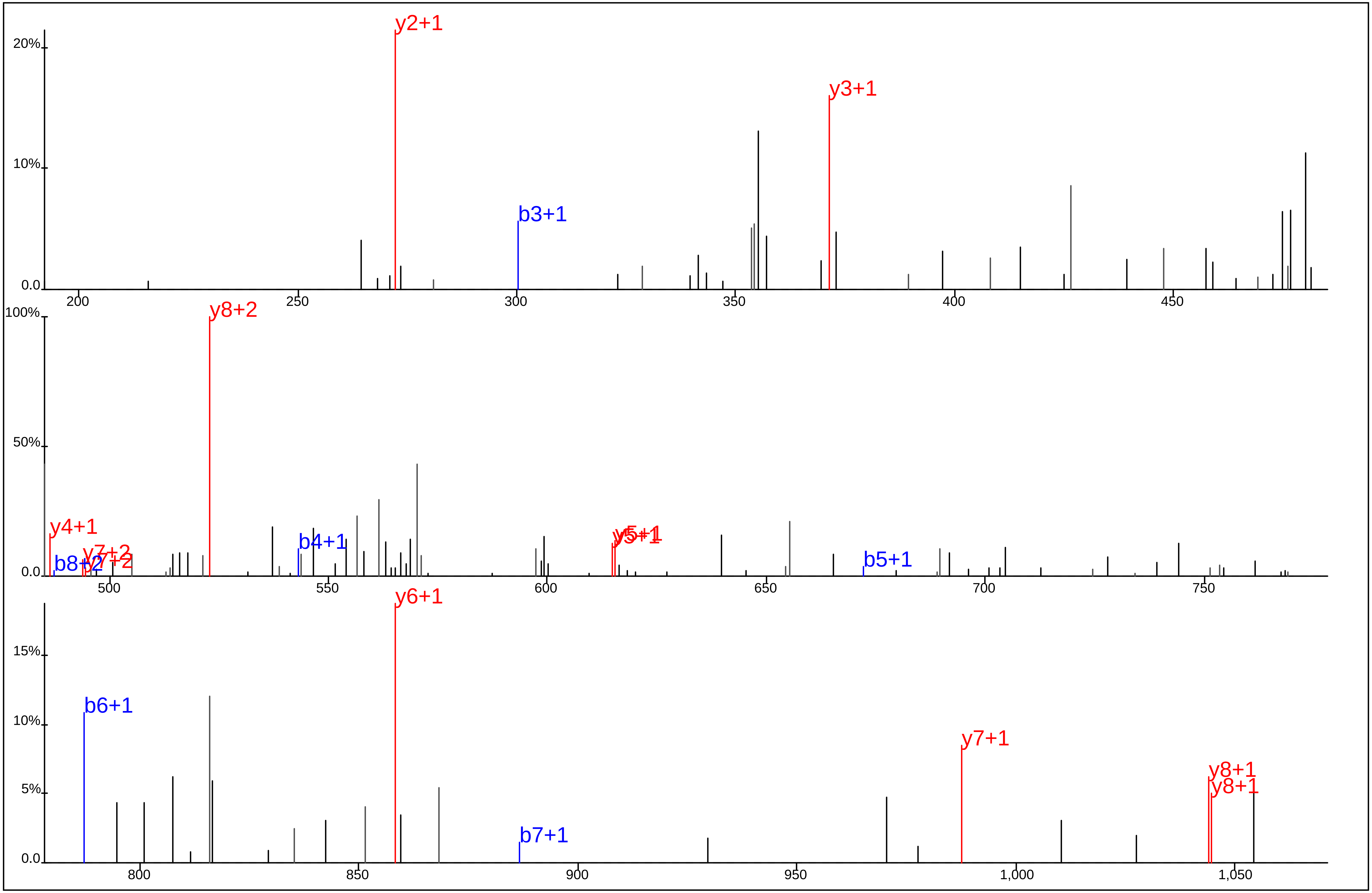

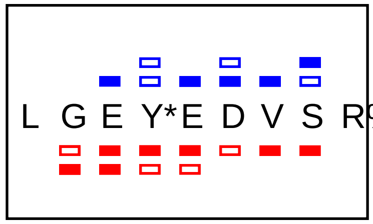

Protein Name: CKAP1
gi Accession: 736704
Precursor Mass: 579.2441+2
Delta Mass: 5.217 ppm
XCorr: 34.7

293 - CKAP1_3449090.pdf

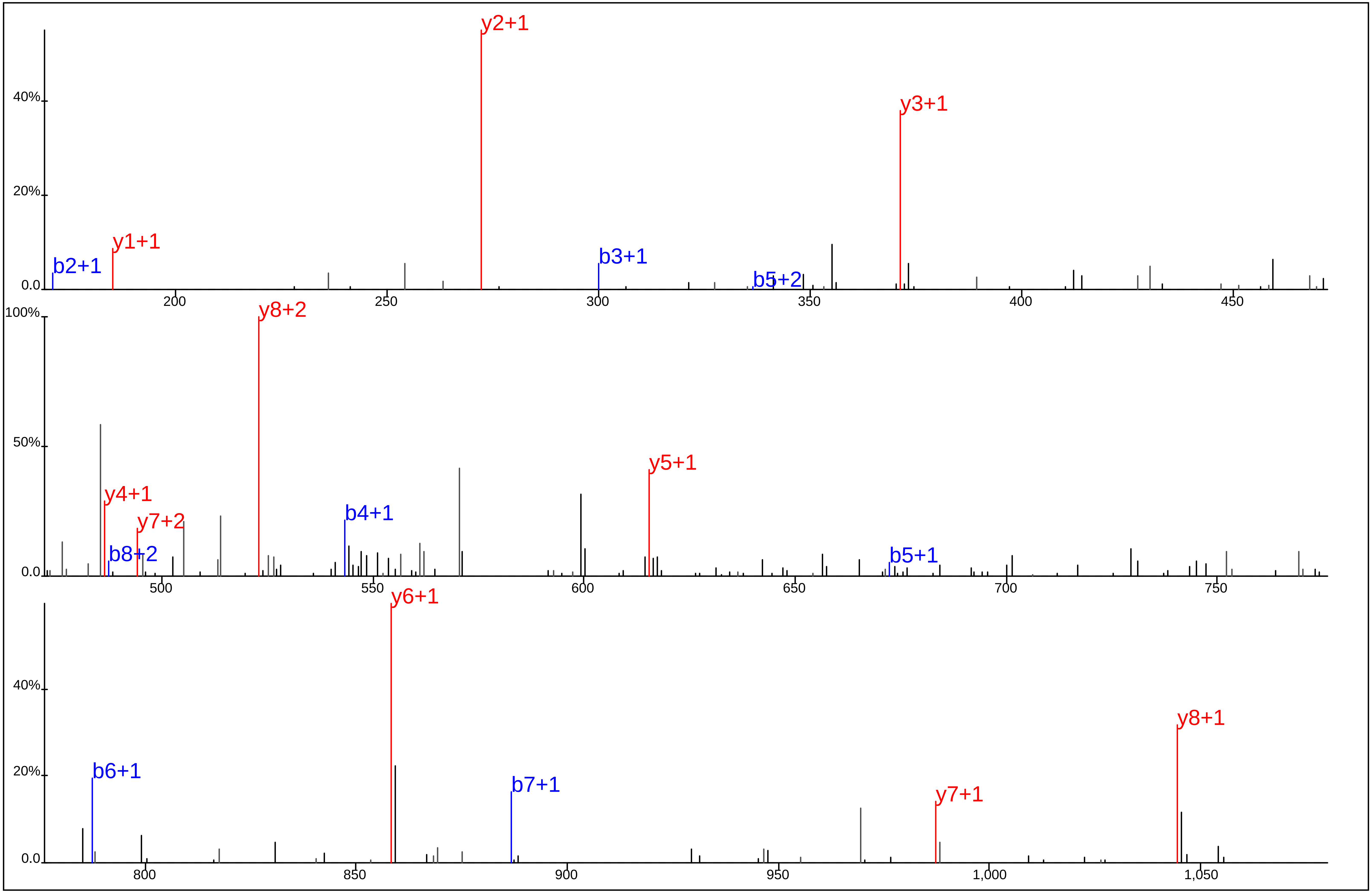

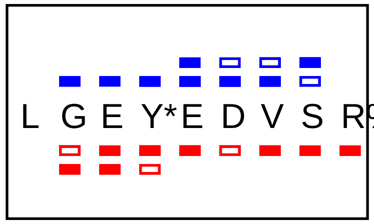

Protein Name: CKAP1
gi Accession: 736704
Precursor Mass: 579.2435+2
Delta Mass: 4.059 ppm
XCorr: 54.62

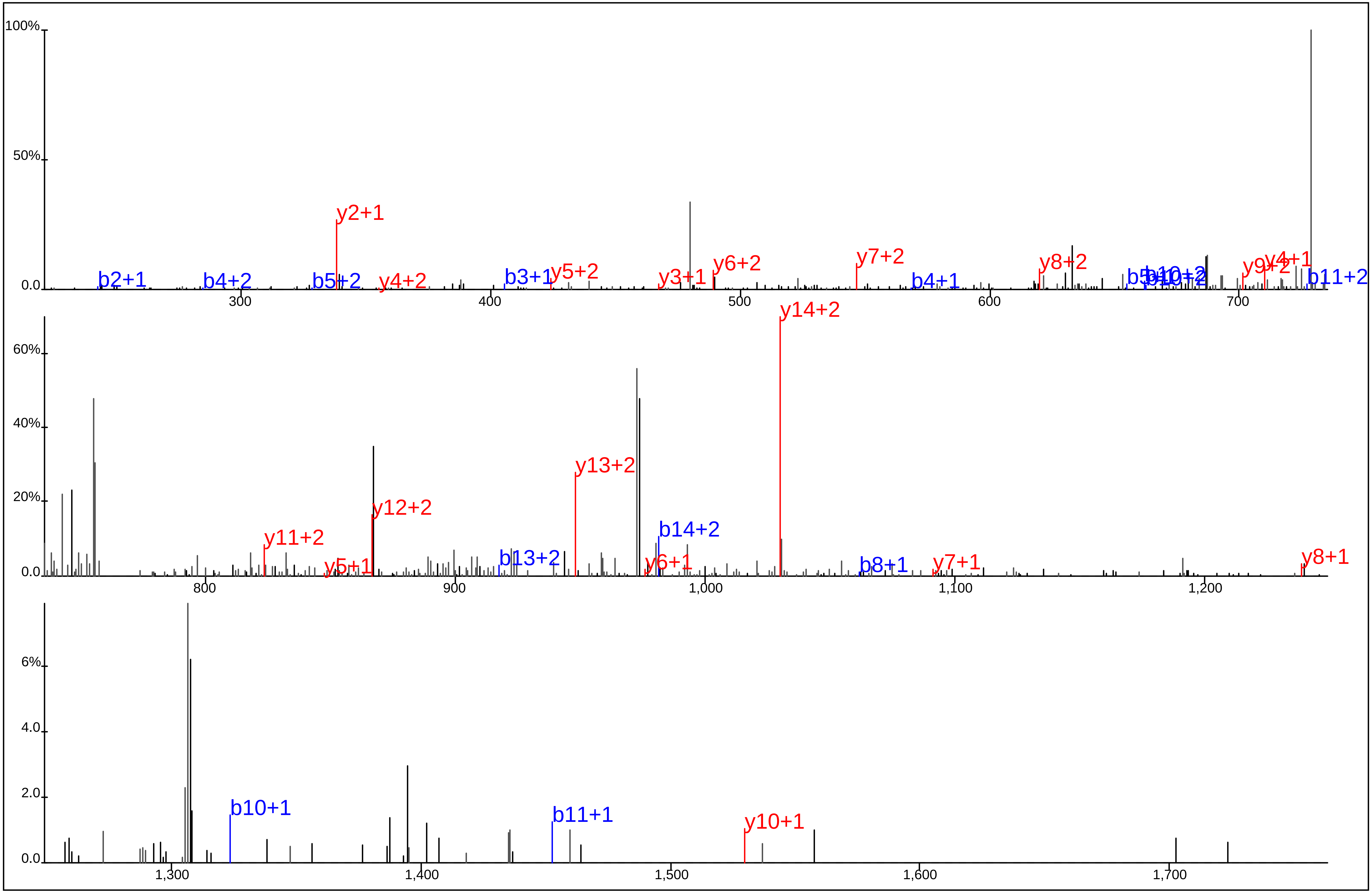

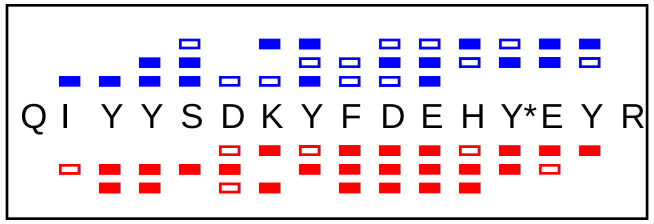

Protein Name: CKS2
gi Accession: 4502859
Precursor Mass: 766.9894+3
Delta Mass: 6.820 ppm
XCorr: 24.08

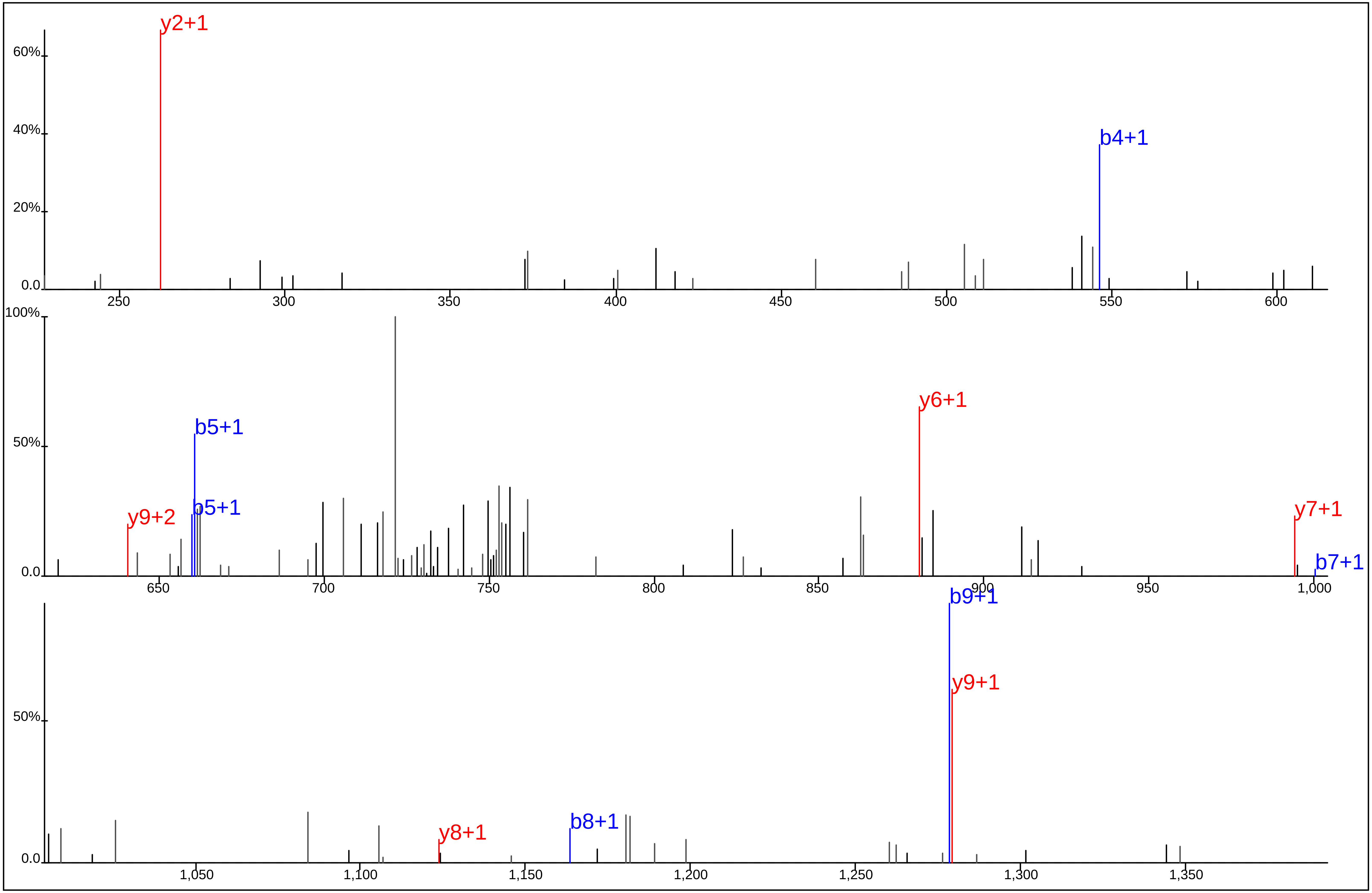

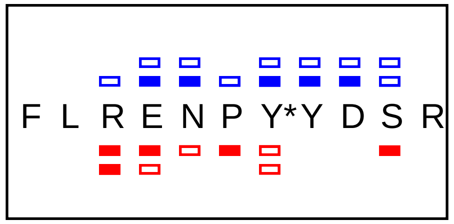

Protein Name: Clathrin, heavy polypeptide
gi Accession: 4758012
Precursor Mass: 770.3329+2
Delta Mass: 2.786 ppm
XCorr: 2.8871

296 - Clathrinheavypolypeptide_3414851.pdf

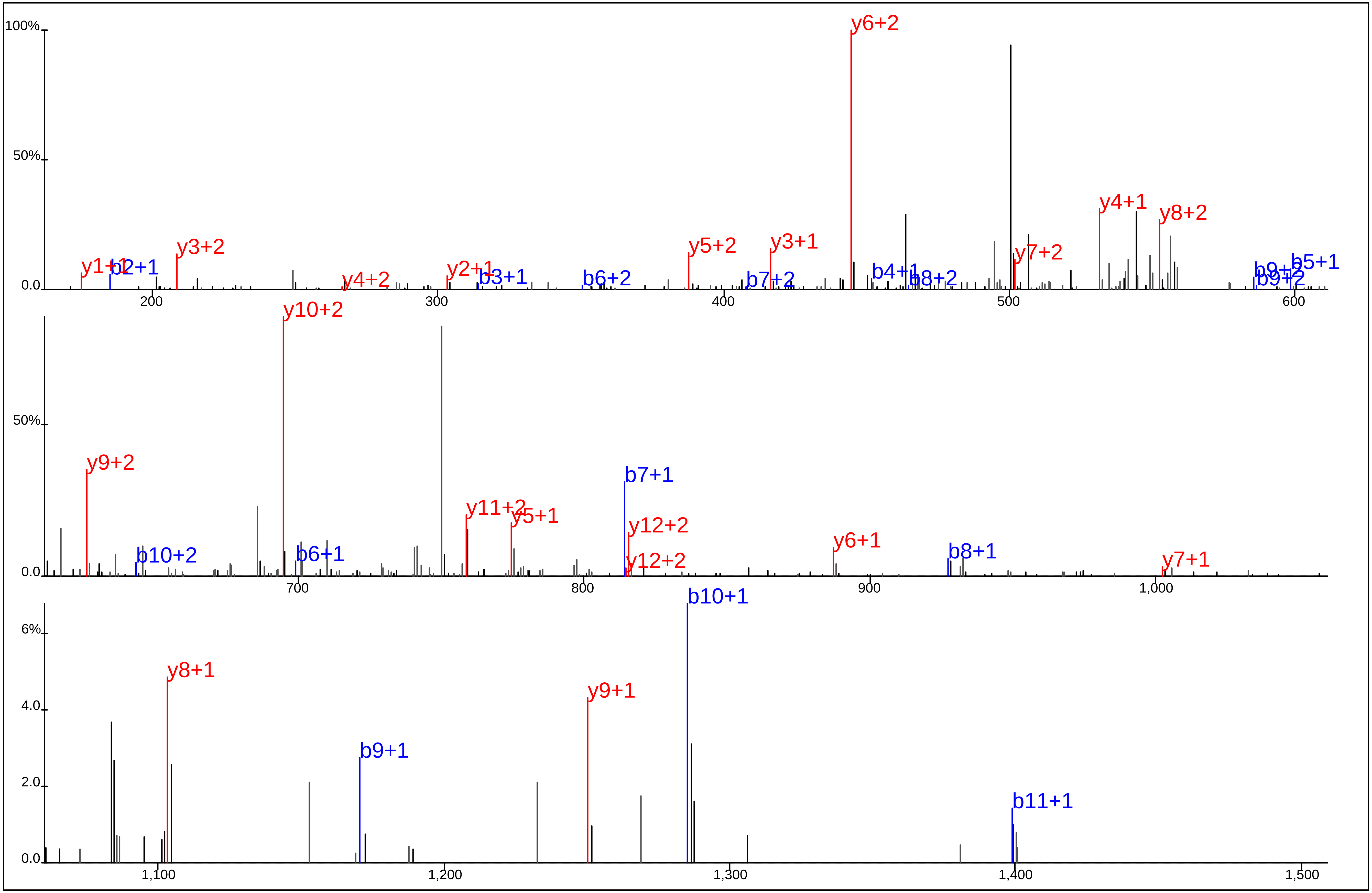

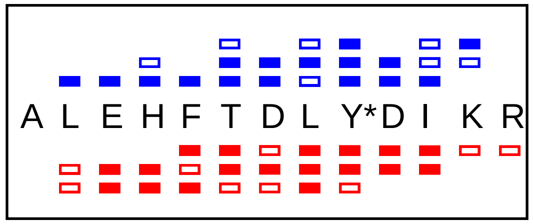

Protein Name: Clathrin, heavy polypeptide
gi Accession: 4758012
Precursor Mass: 567.6088+3
Delta Mass: 4.356 ppm
XCorr: 58.09

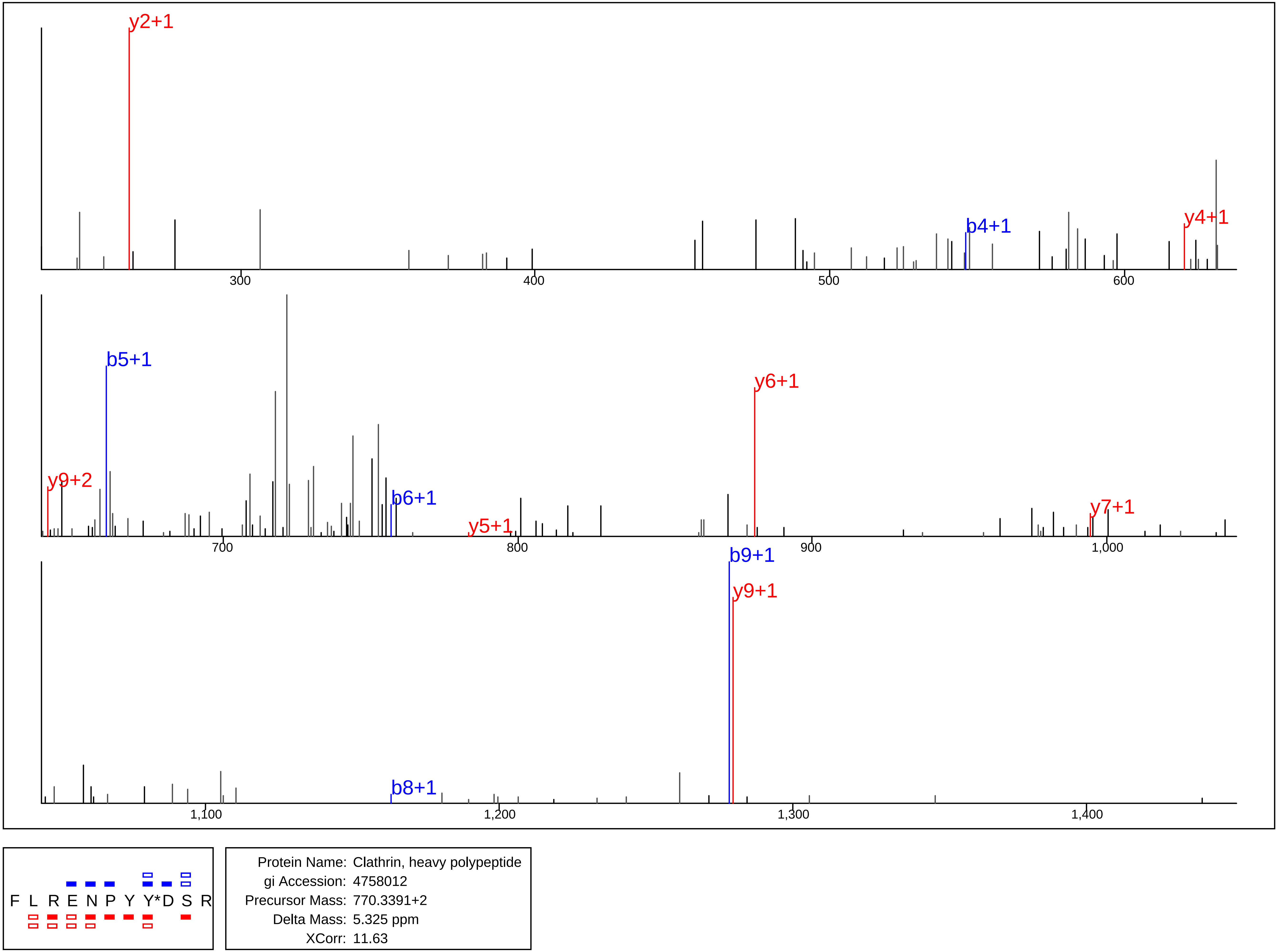

Protein Name:	Clathrin, heavy polypeptide
gi Accession:	4758012
Precursor Mass:	770.3391+2
Delta Mass:	5.325 ppm
XCorr:	11.63

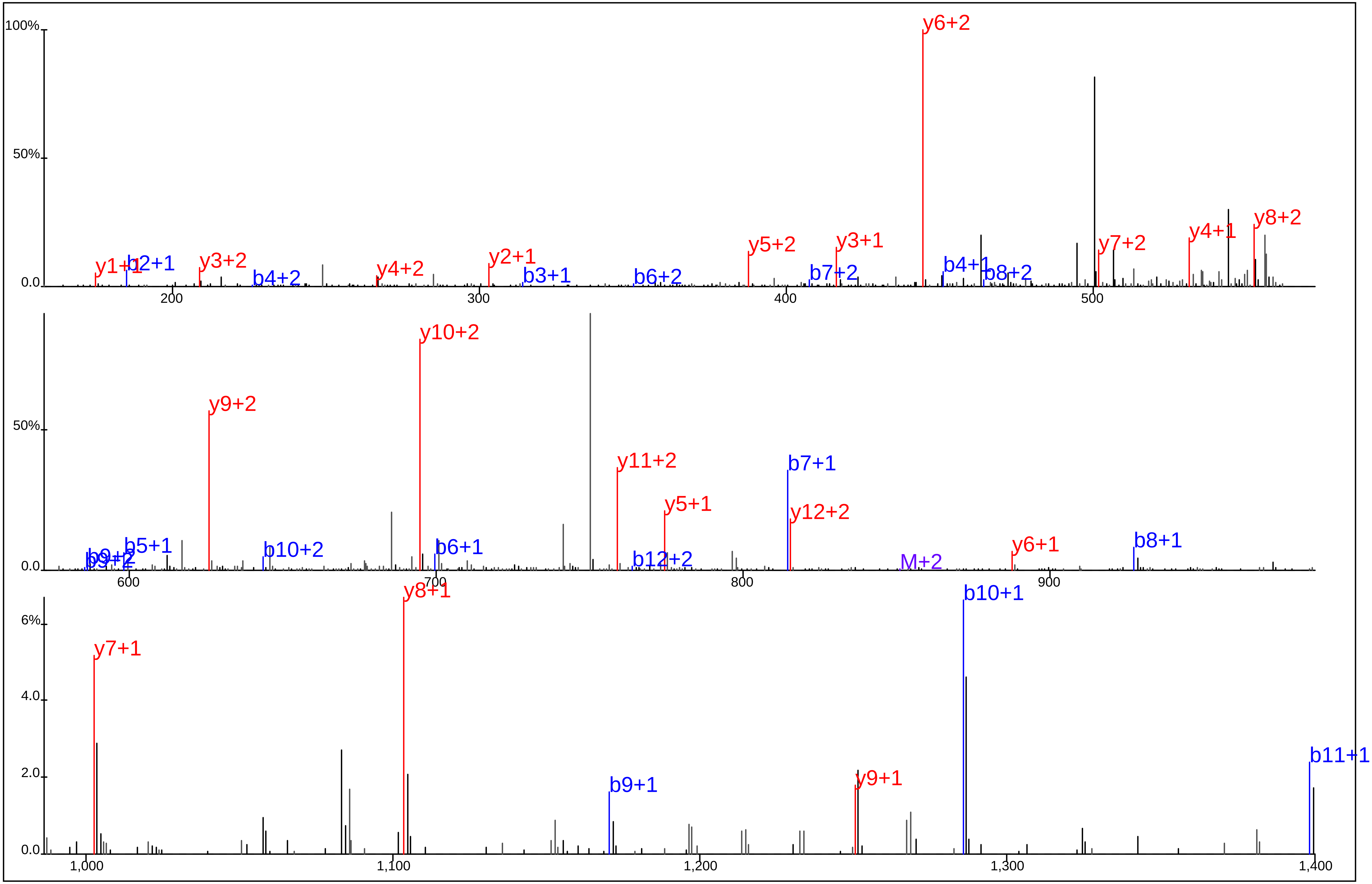

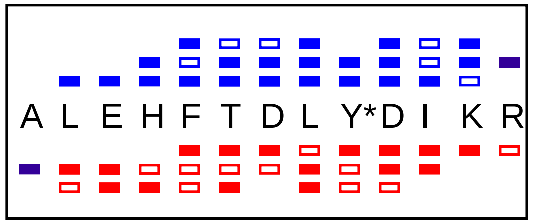

Protein Name:	Clathrin, heavy polypeptide
gi Accession:	4758012
Precursor Mass:	567.6085+3
Delta Mass:	3.927 ppm
XCorr:	60.88

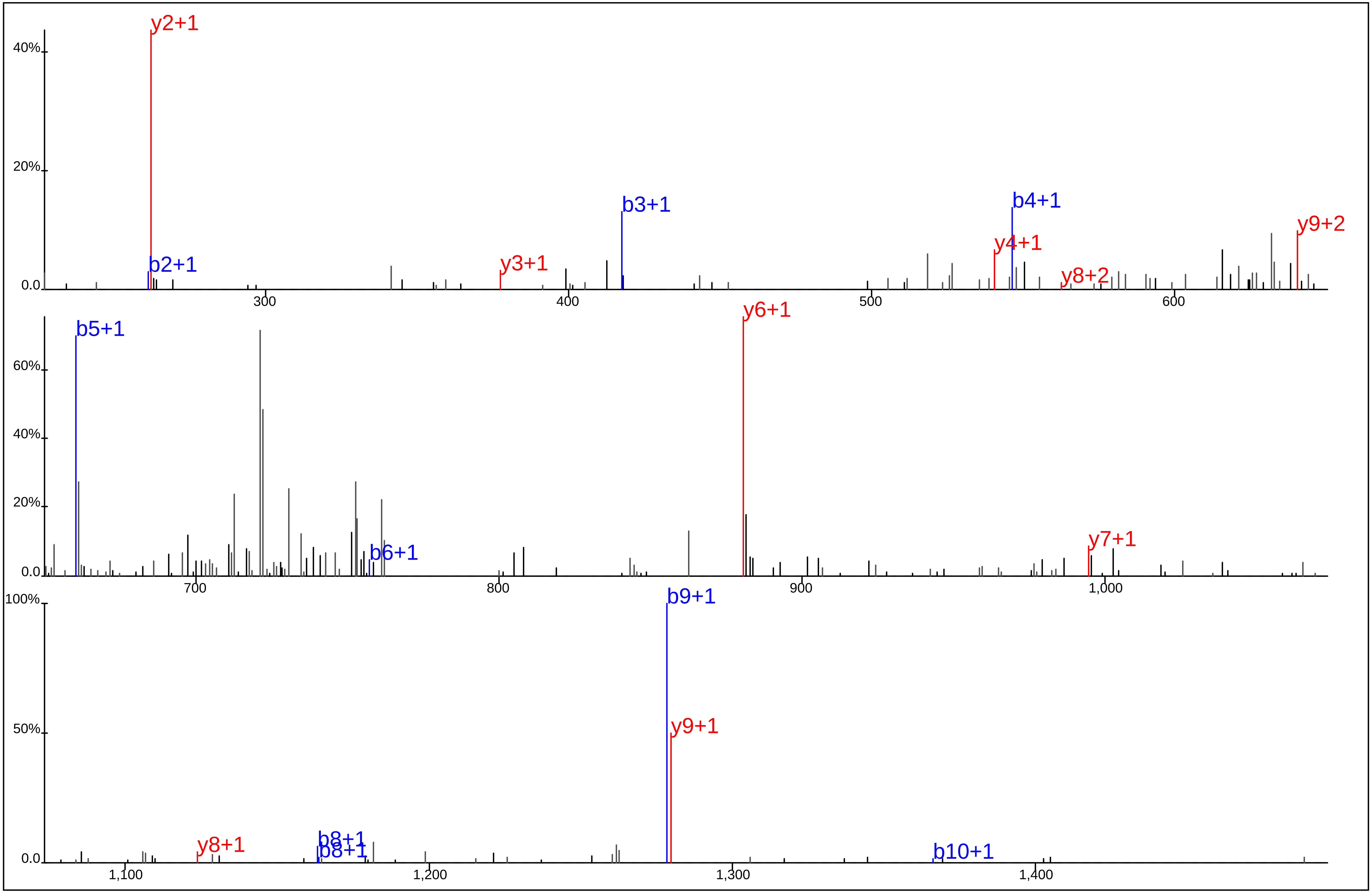

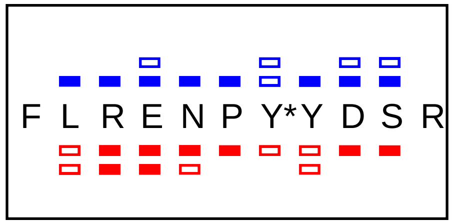

Protein Name:	Clathrin, heavy polypeptide
gi Accession:	4758012
Precursor Mass:	770.3395+2
Delta Mass:	5.799 ppm
XCorr:	34.38

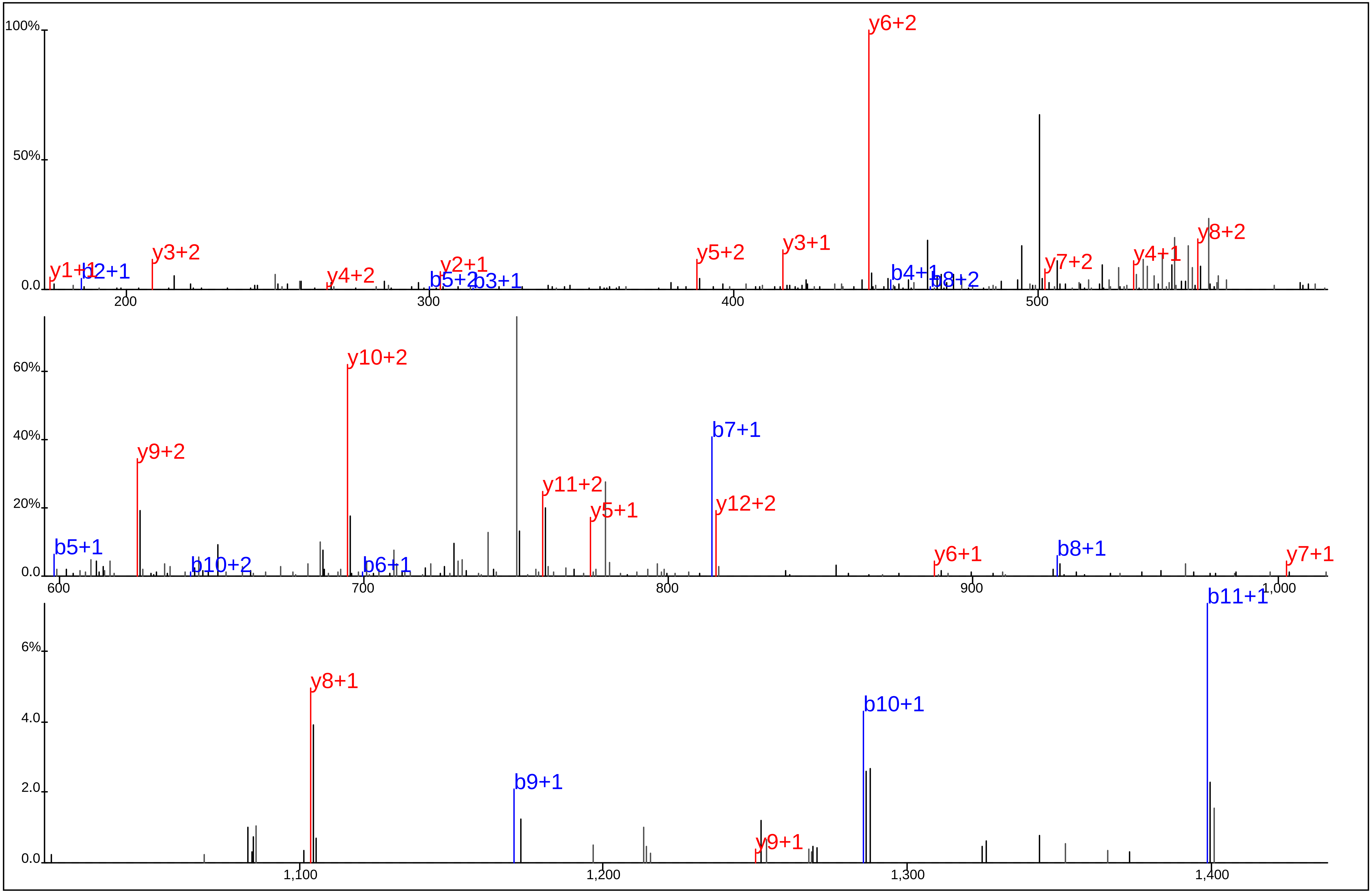

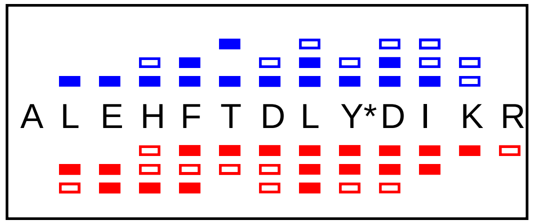

Protein Name:	Clathrin, heavy polypeptide
gi Accession:	4758012
Precursor Mass:	567.6075+3
Delta Mass:	2.098 ppm
XCorr:	41.95

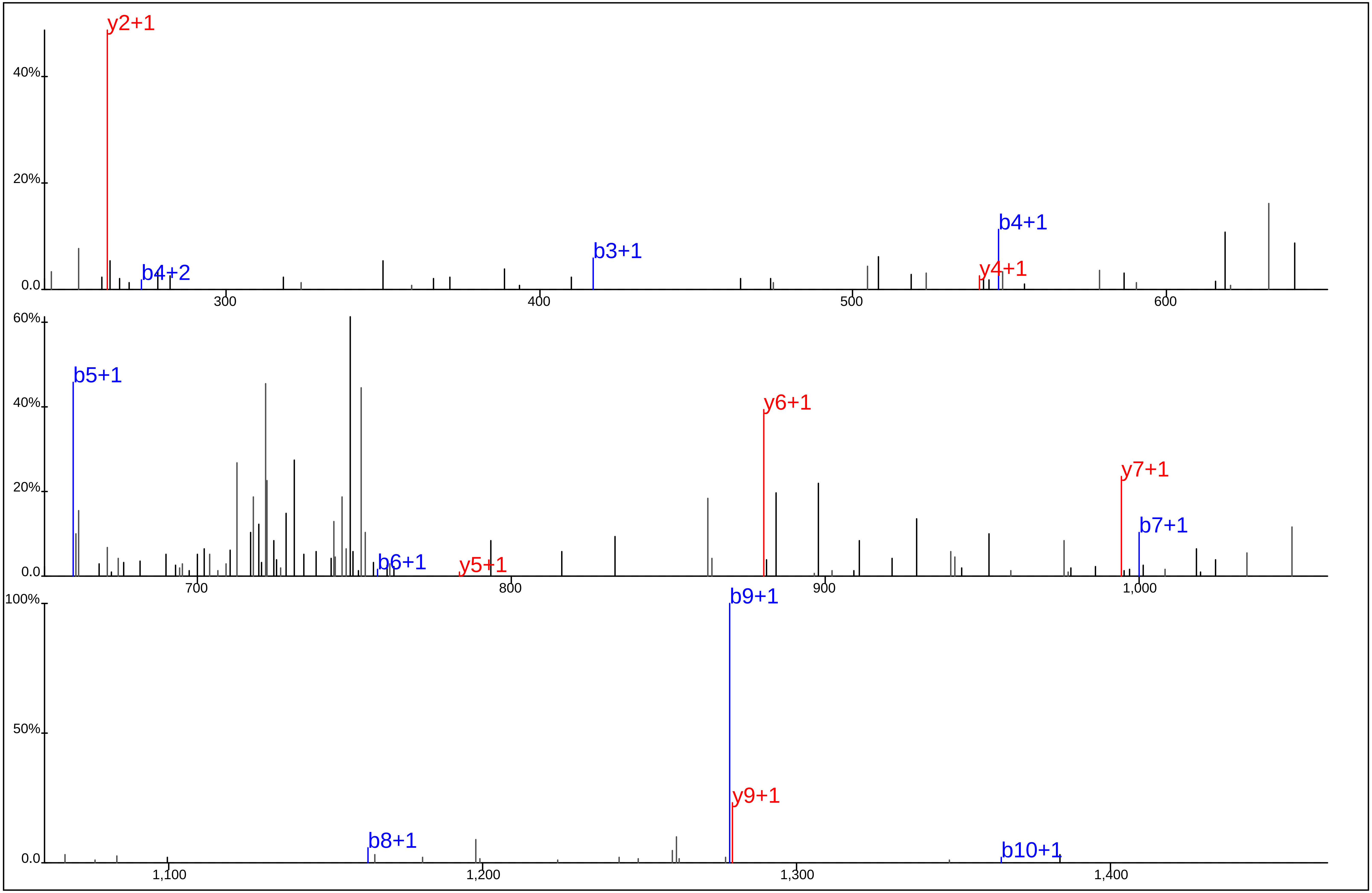

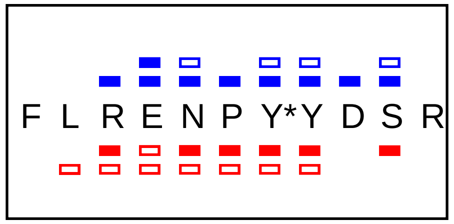

Protein Name: Clathrin, heavy polypeptide
gi Accession: 4758012
Precursor Mass: 770.3392+2
Delta Mass: 5.480 ppm
XCorr: 35.98

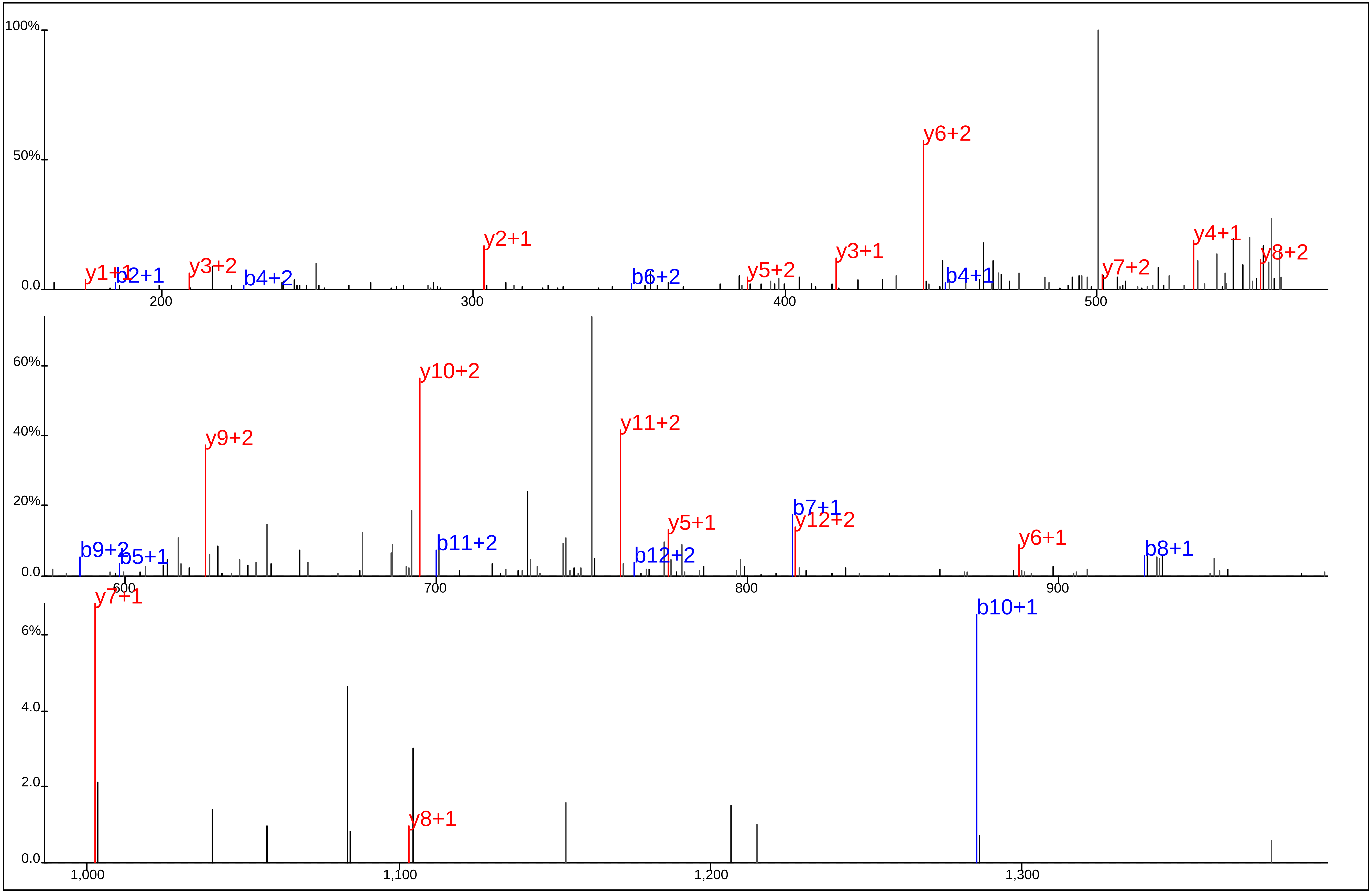

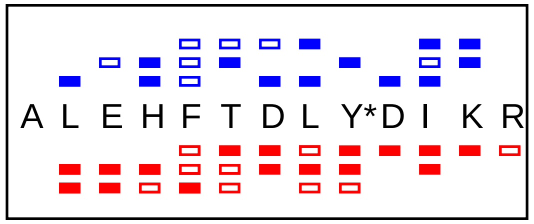

Protein Name:	Clathrin, heavy polypeptide
gi Accession:	4758012
Precursor Mass:	567.6055+3
Delta Mass:	1.347 ppm
XCorr:	40.83

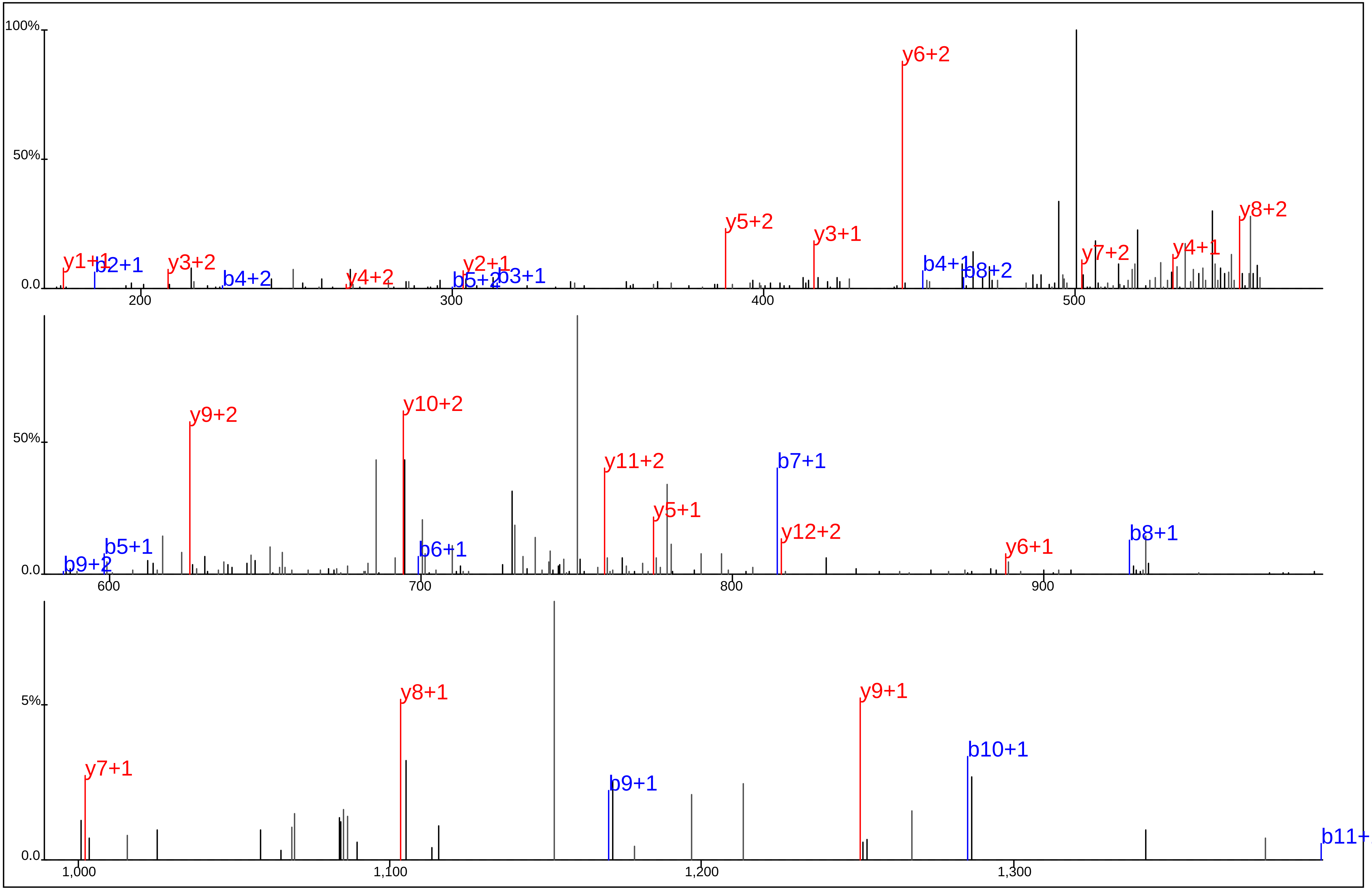

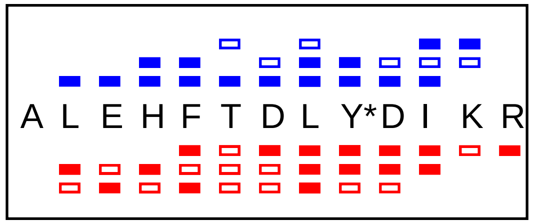

Protein Name: Clathrin, heavy polypeptide
gi Accession: 4758012
Precursor Mass: 567.6069+3
Delta Mass: 1.128 ppm
XCorr: 42.44

304 - Clathrinheavypolypeptide_3444311.pdf

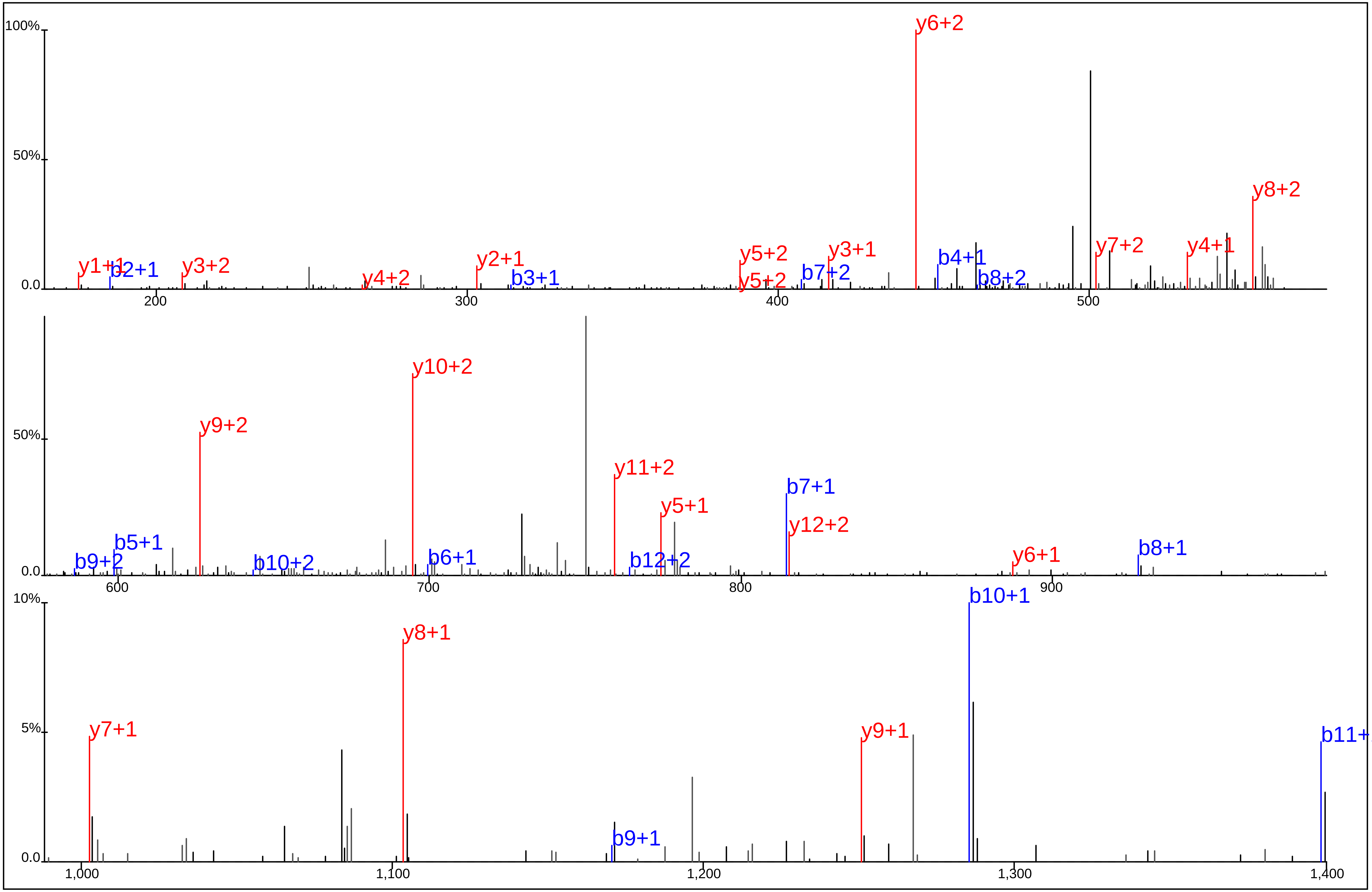

A L E H F T D L Y*D I K R

Protein Name: Clathrin, heavy polypeptide
gi Accession: 4758012
Precursor Mass: 567.6070+3
Delta Mass: 1.234 ppm
XCorr: 49.54

305 - Clathrinheavypolypeptide_3446888.pdf

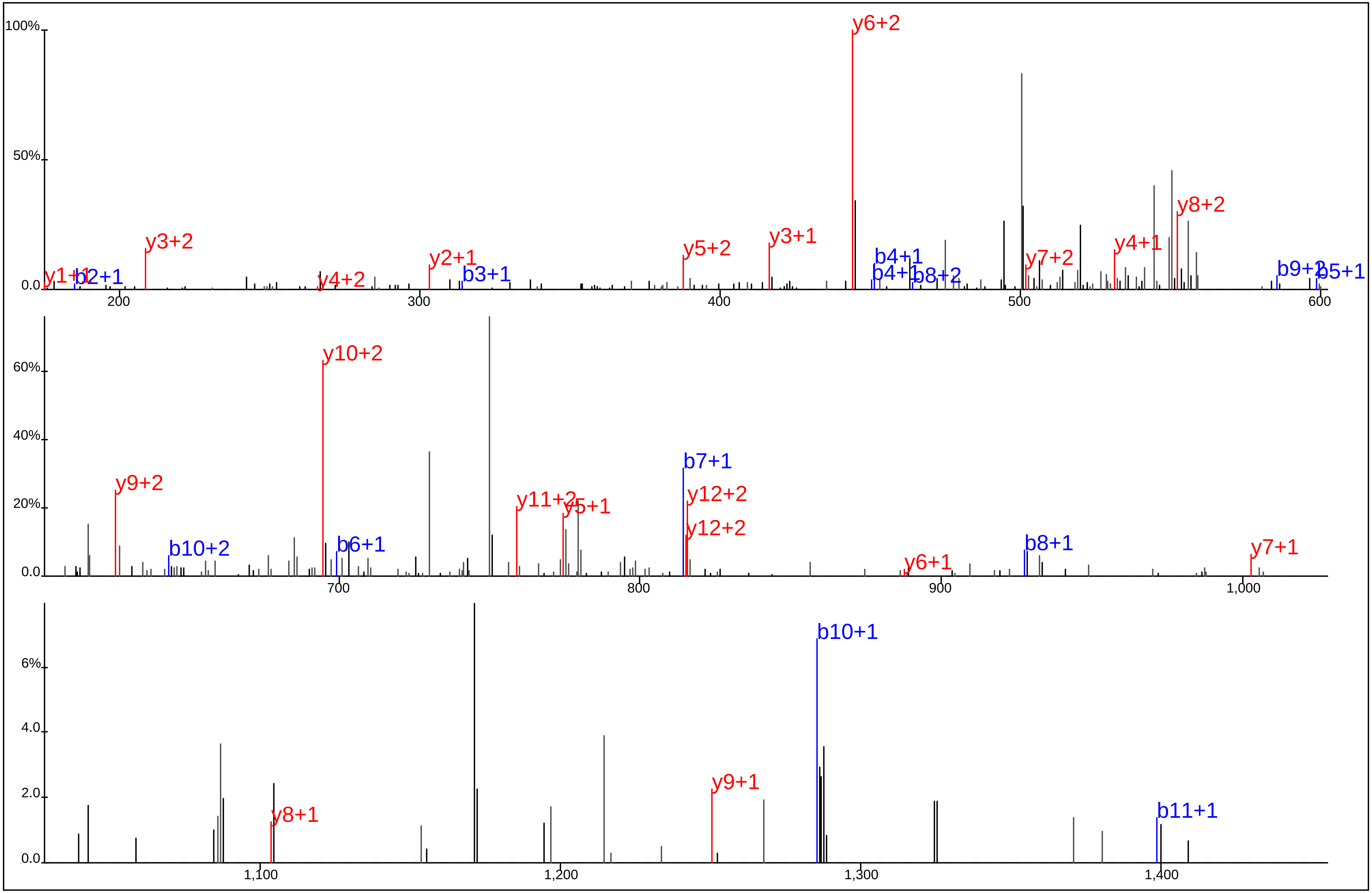

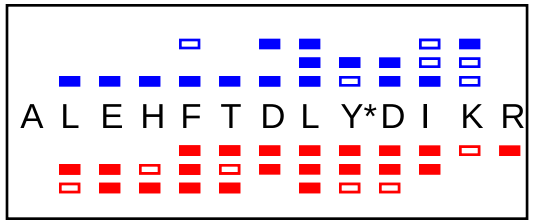

Protein Name: Clathrin, heavy polypeptide
gi Accession: 4758012
Precursor Mass: 567.6086+3
Delta Mass: 4.033 ppm
XCorr: 29.64

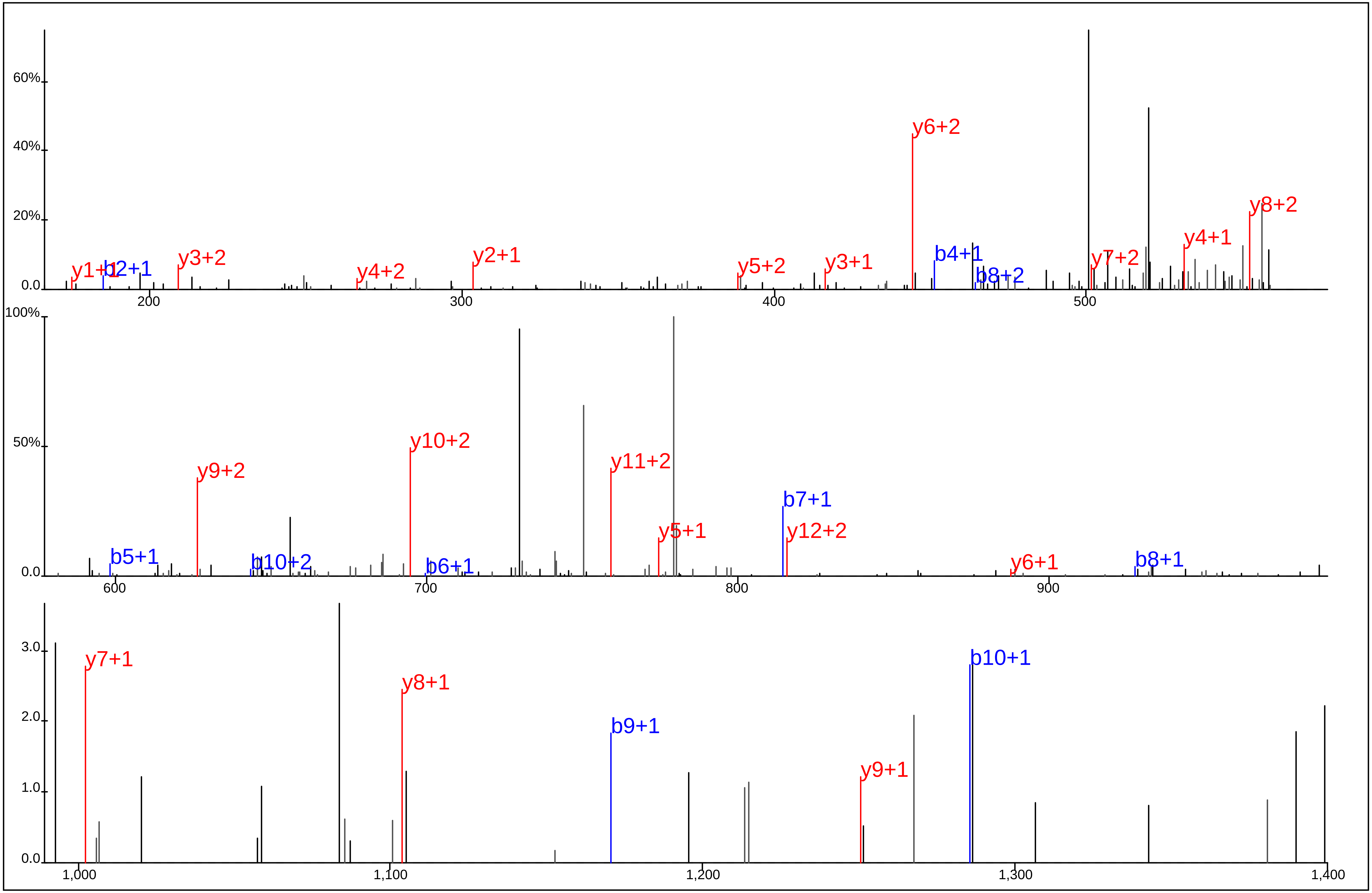

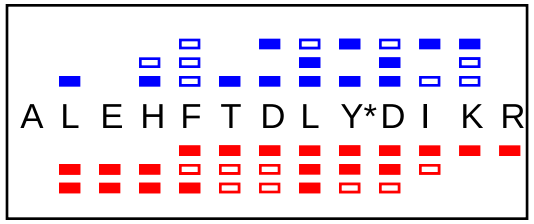

Protein Name:	Clathrin, heavy polypeptide
gi Accession:	4758012
Precursor Mass:	567.6093+3
Delta Mass:	5.326 ppm
XCorr:	44.4

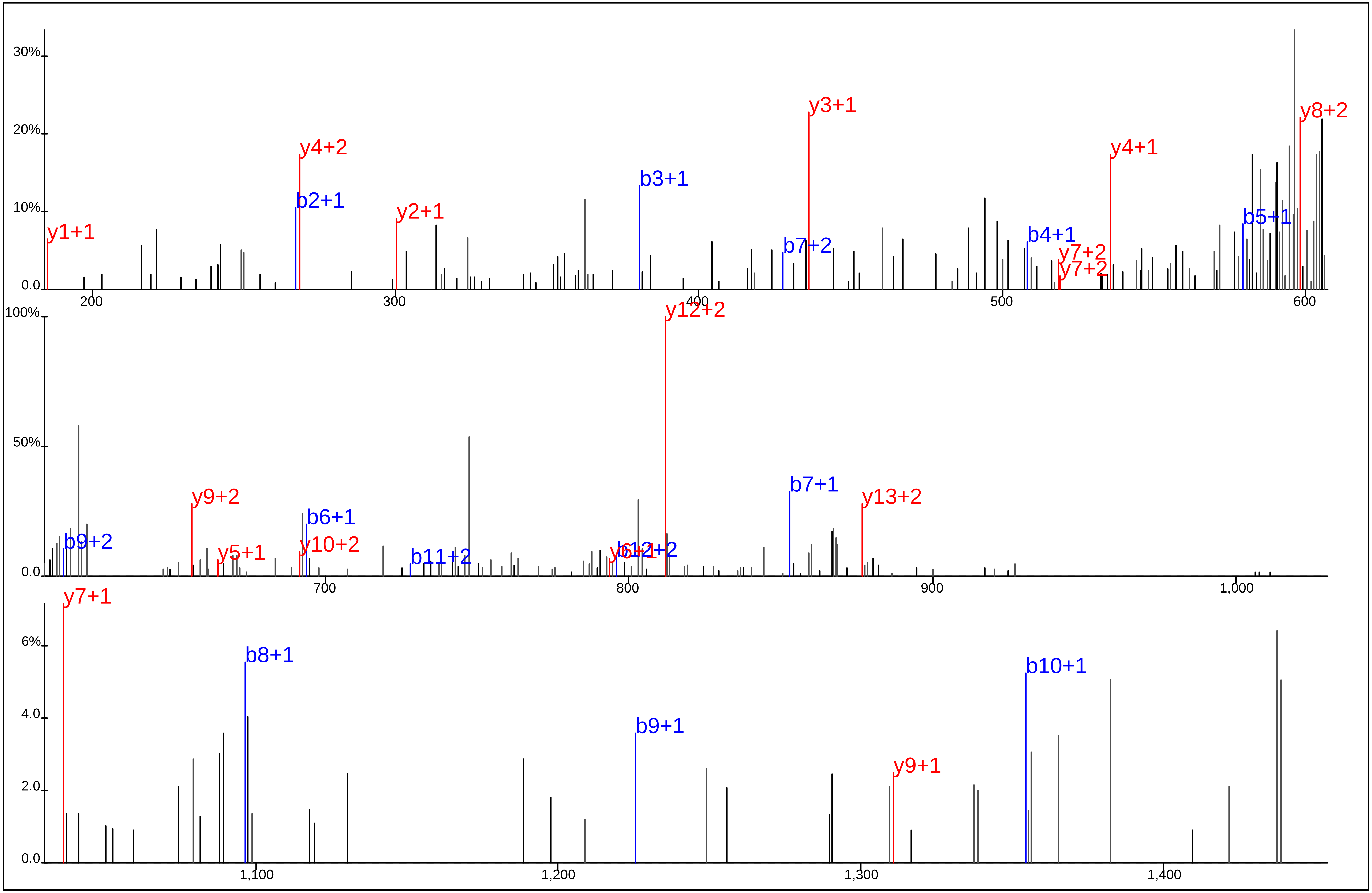

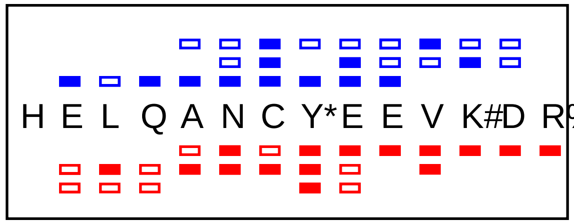

Protein Name: Cofilin 1
gi Accession: 5031635
Precursor Mass: 630.2734+3
Delta Mass: 2.217 ppm
XCorr: 31.45

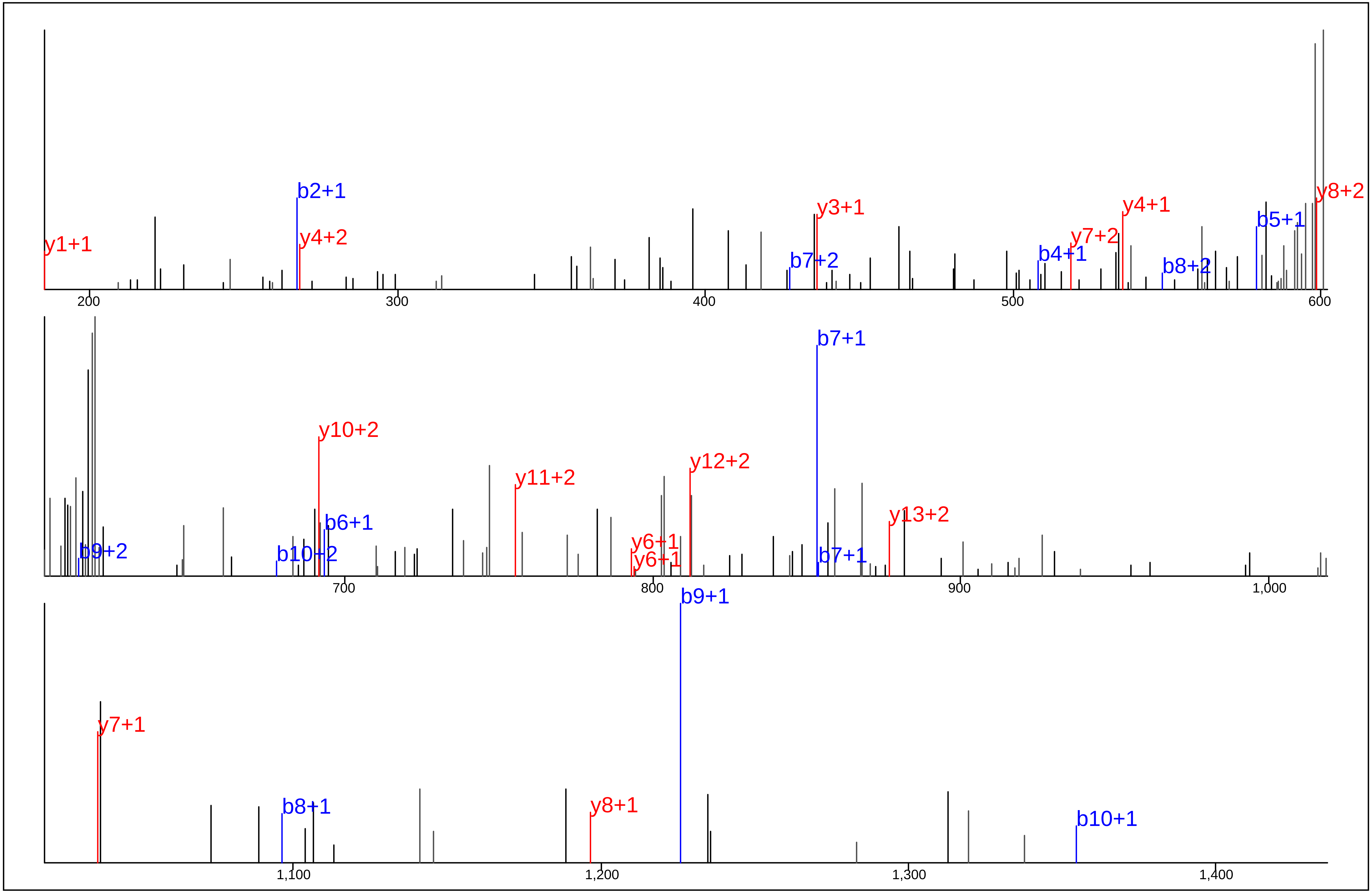

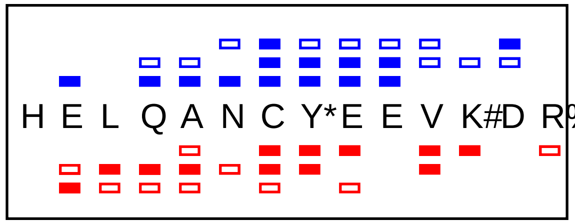

Protein Name:	Cofilin 1
gi Accession:	5031635
Precursor Mass:	630.2743+3
Delta Mass:	3.668 ppm
XCorr:	24.5

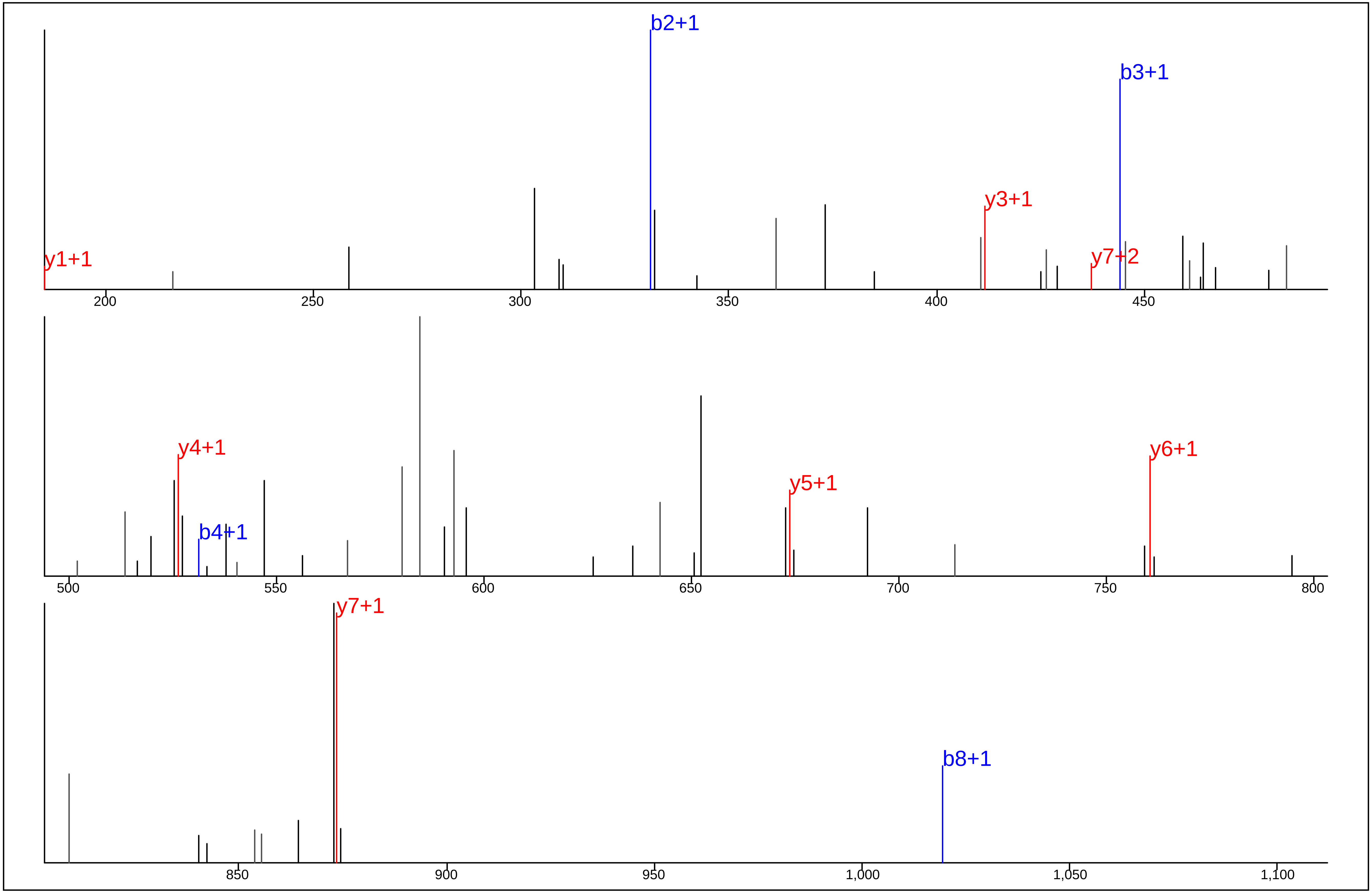

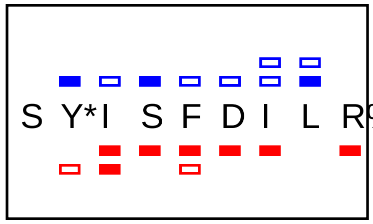

Protein Name:	Cysteinyl-tRNA synthetase
gi Accession:	10835051
Precursor Mass:	602.2893+2
Delta Mass:	2.391 ppm
XCorr:	31.78

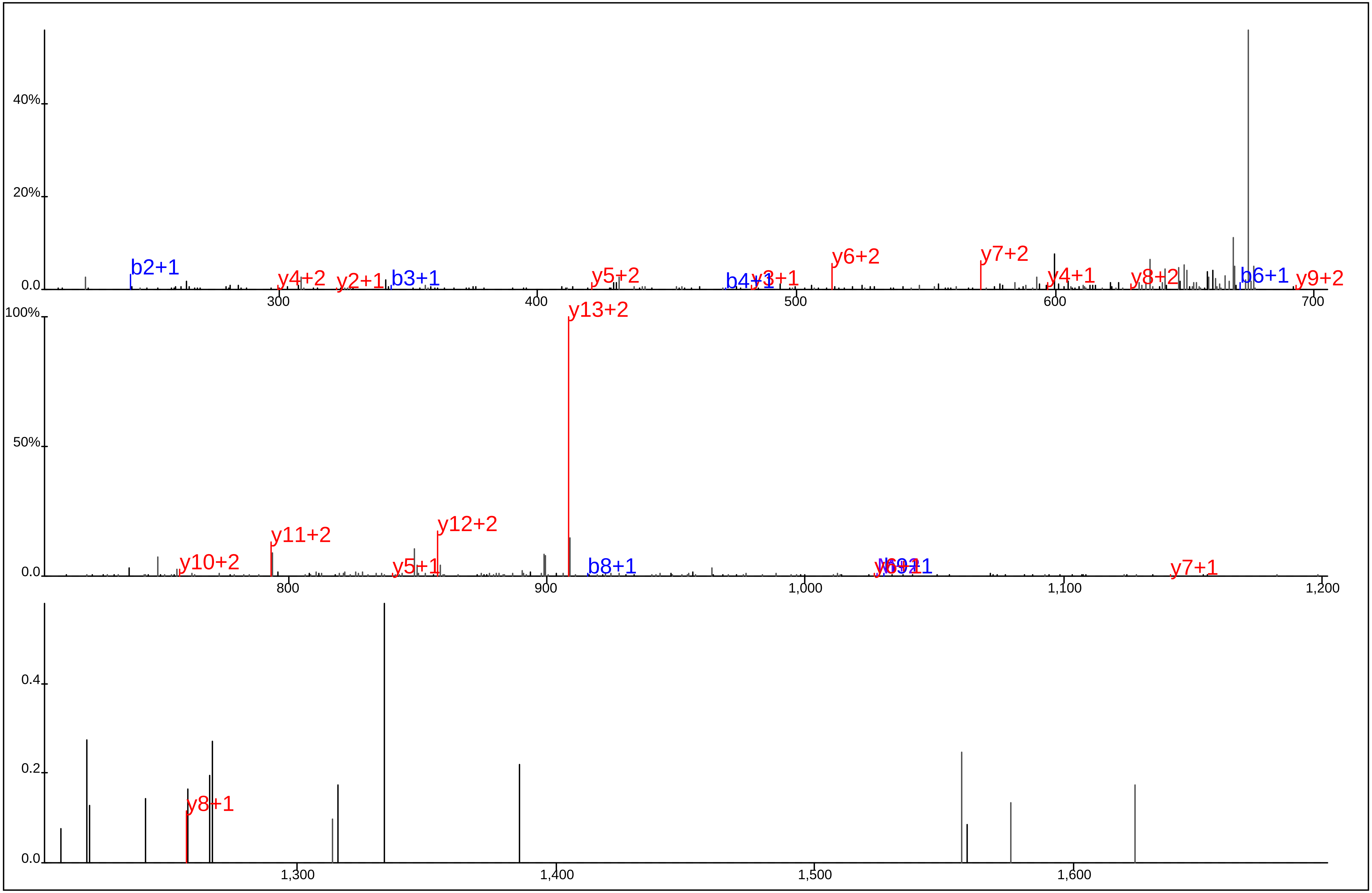

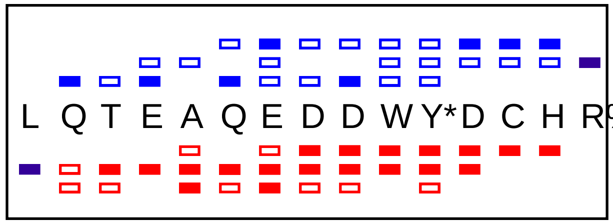

Protein Name: DDX20
gi Accession: 12643886
Precursor Mass: 685.9323+3
Delta Mass: 2.154 ppm
XCorr: 18.95

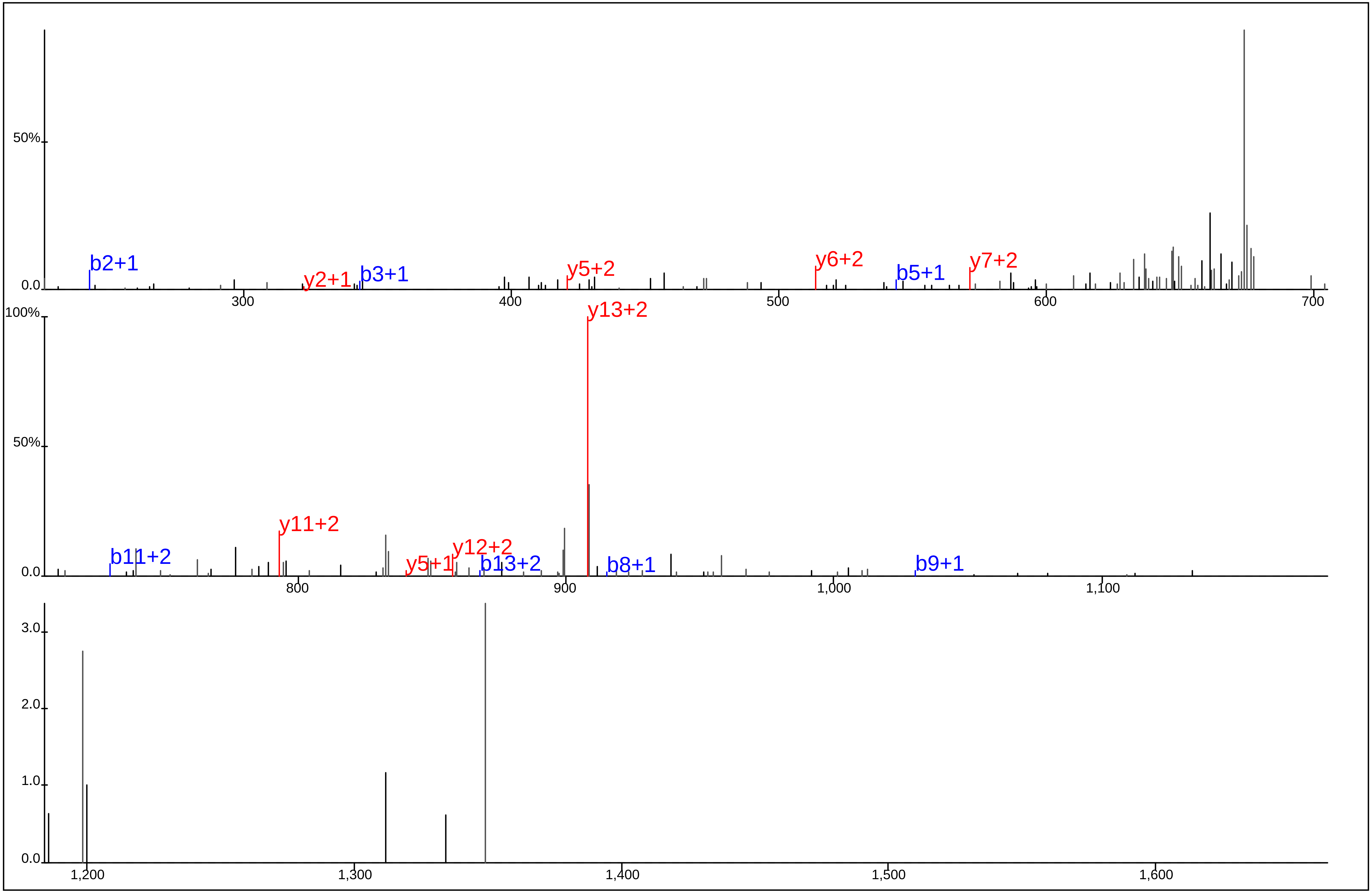

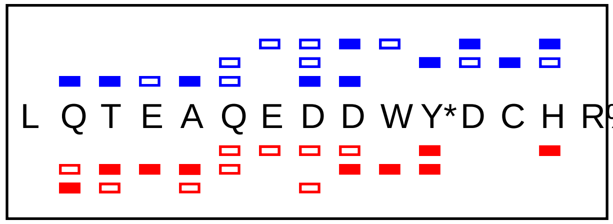

Protein Name: DDX20
gi Accession: 12643886
Precursor Mass: 685.9350+3
Delta Mass: 6.162 ppm
XCorr: 23.62

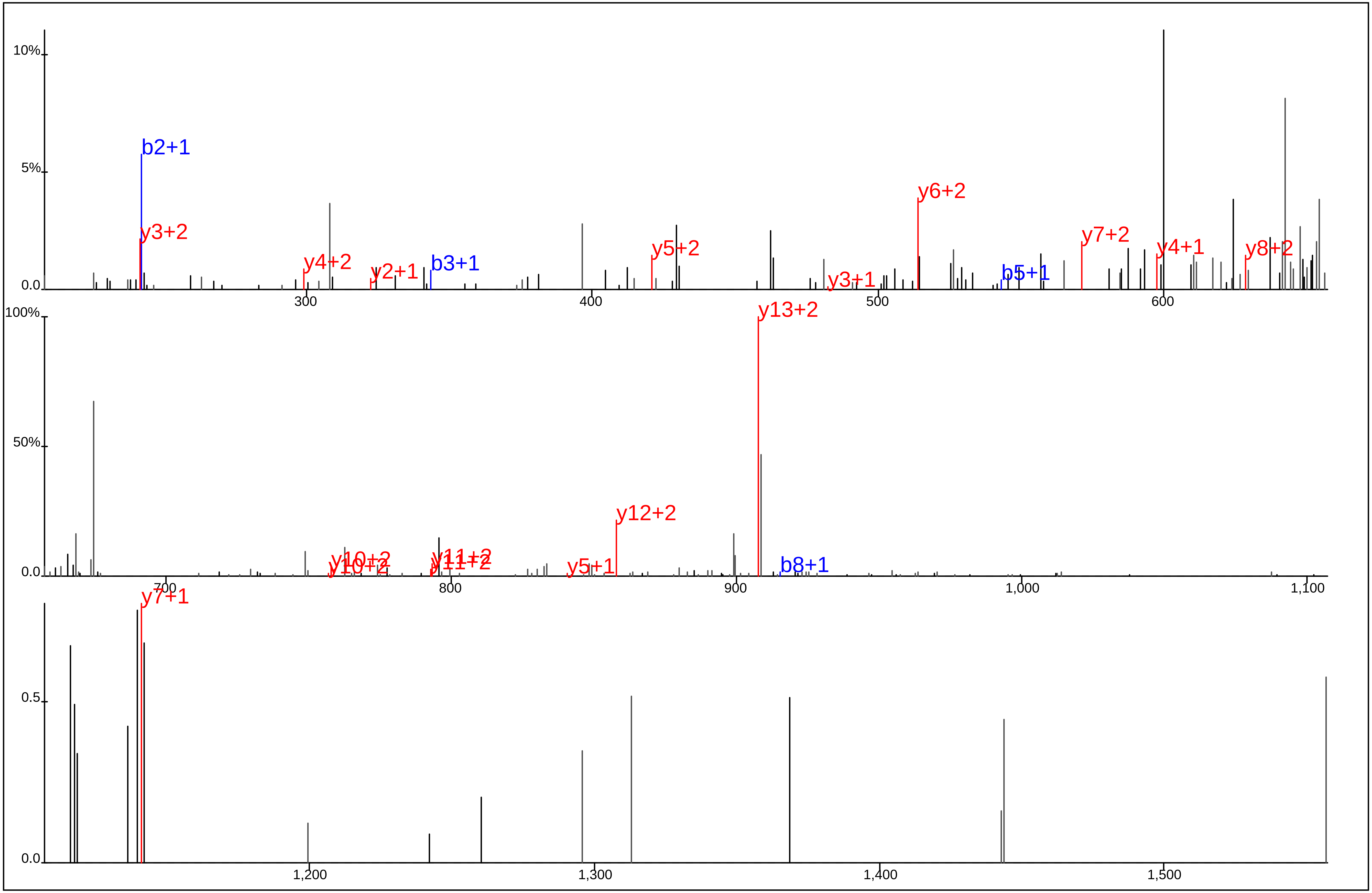

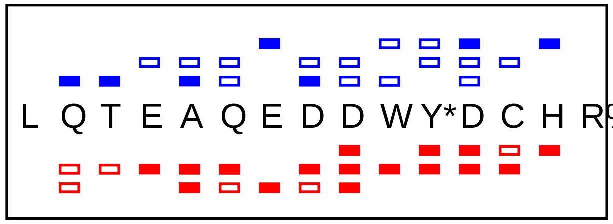

Protein Name: DDX20
gi Accession: 12643886
Precursor Mass: 685.9316+3
Delta Mass: 1.177 ppm
XCorr: 19.19

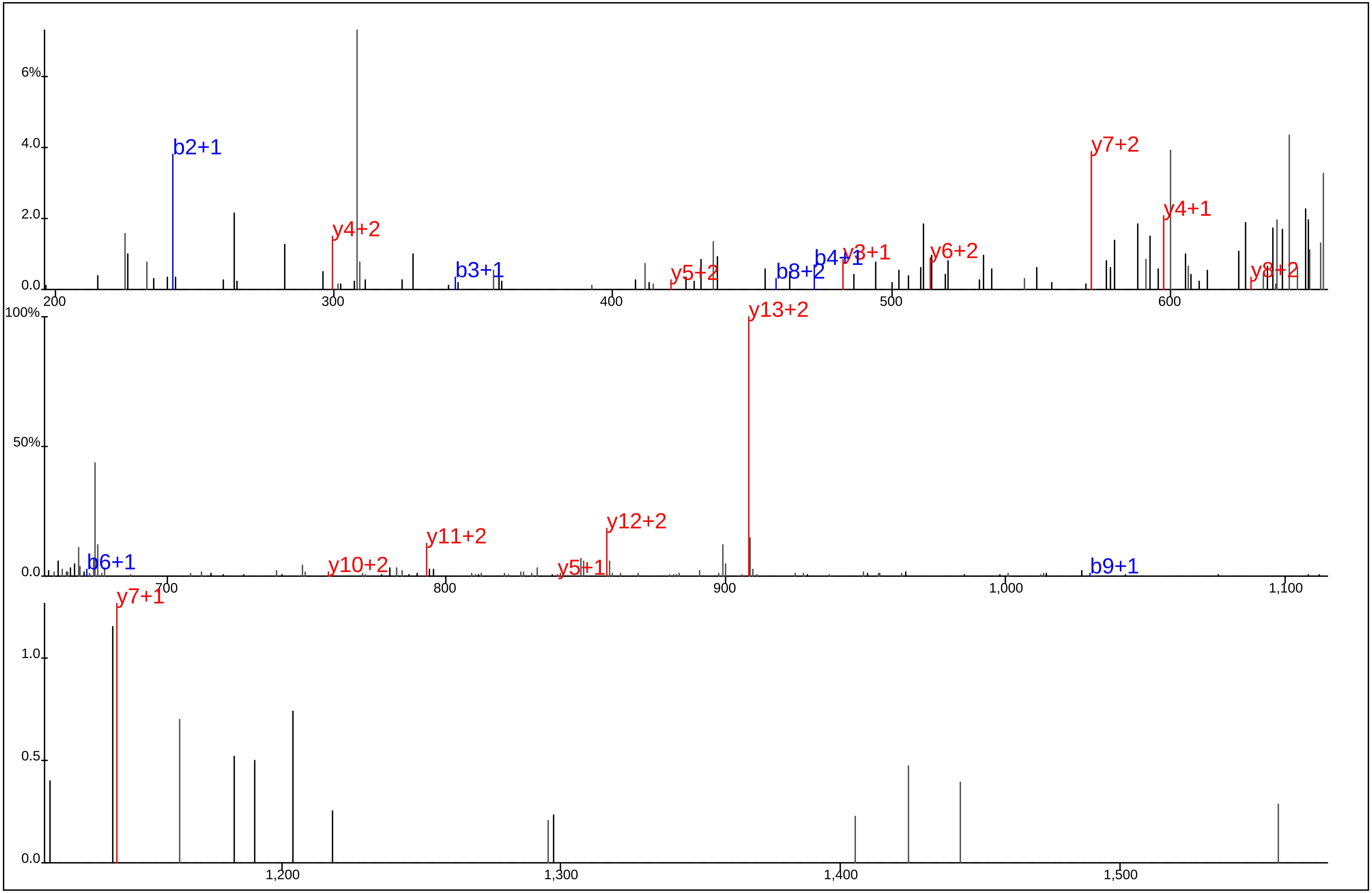

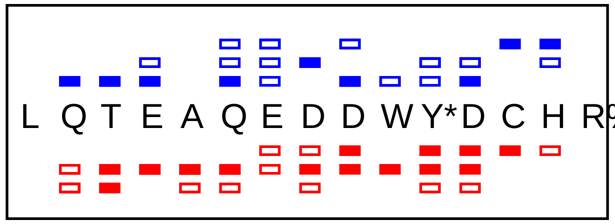

Protein Name: DDX20
gi Accession: 12643886
Precursor Mass: 685.9318+3
Delta Mass: 1.531 ppm
XCorr: 13.73

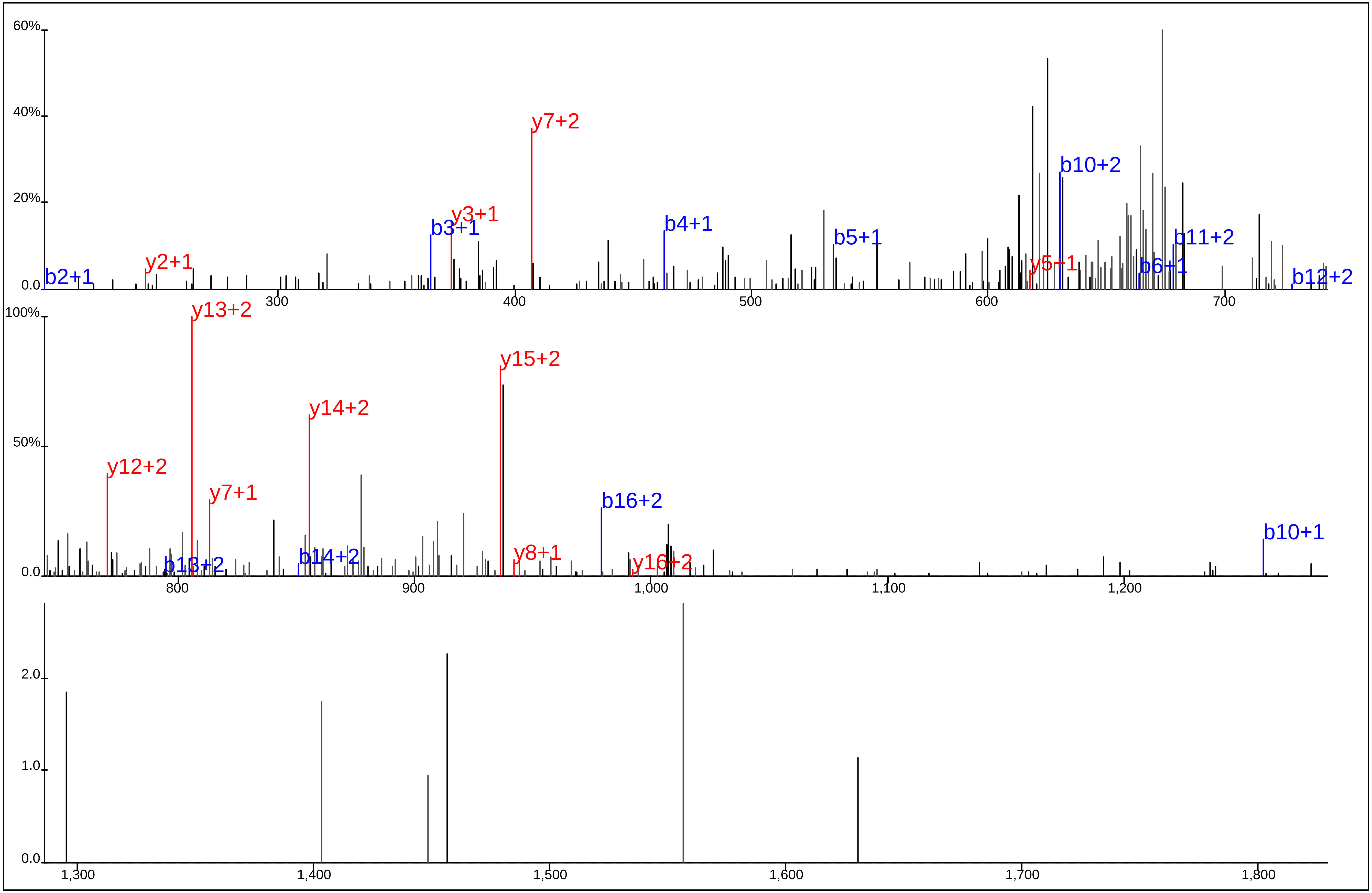

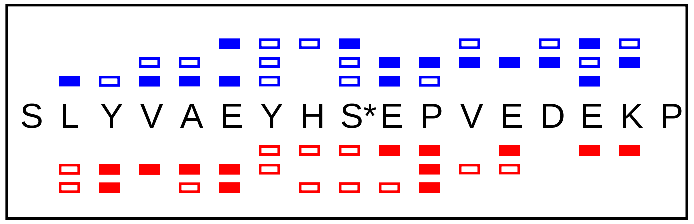

Protein Name:	DDX6
gi Accession:	458727
Precursor Mass:	691.3044+3
Delta Mass:	4.353 ppm
XCorr:	14.26

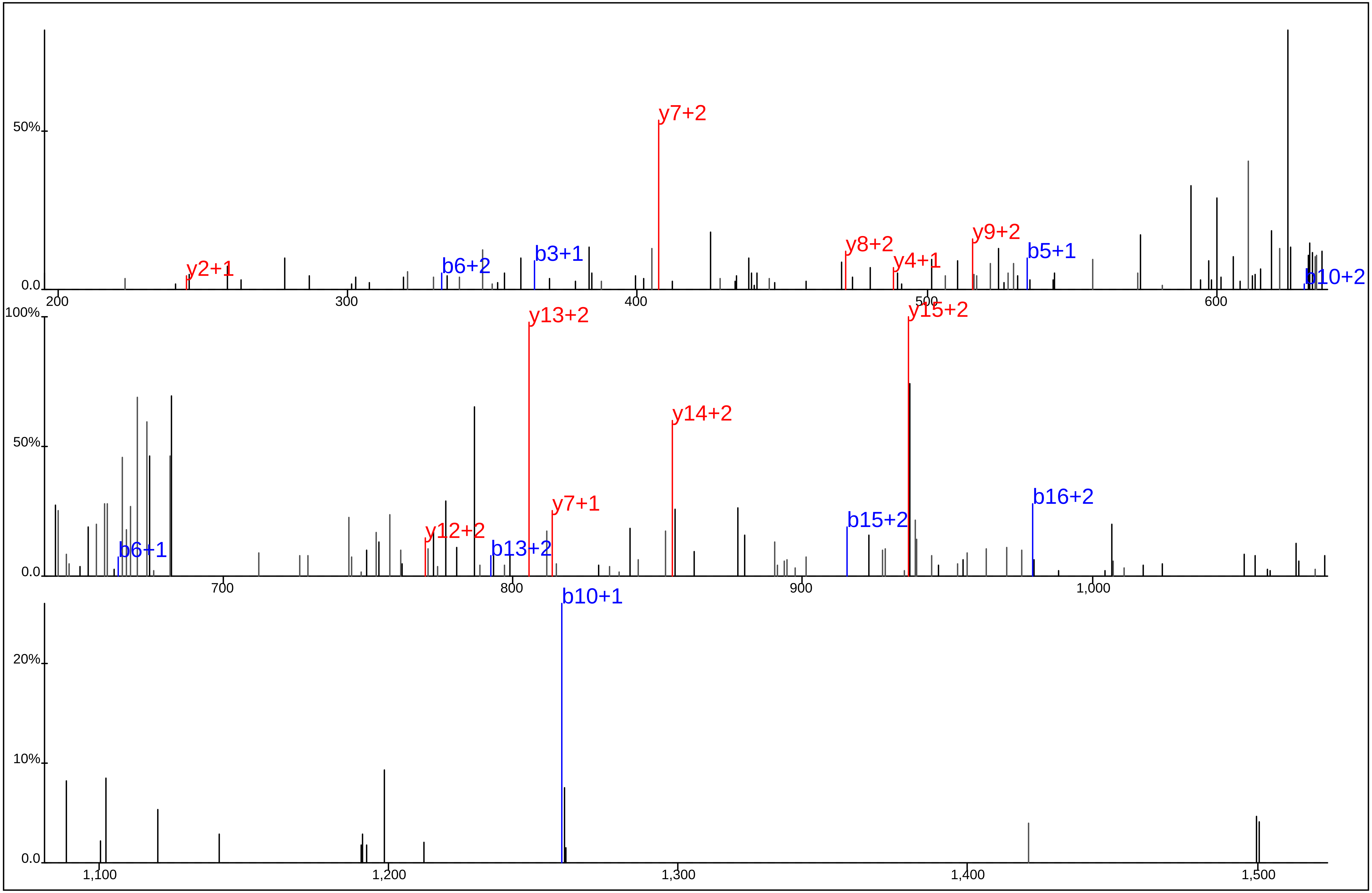

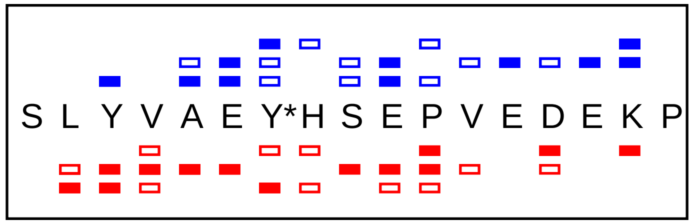

Protein Name: DDX6
gi Accession: 458727
Precursor Mass: 691.3016+3
Delta Mass: 0.289 ppm
XCorr: 12.0

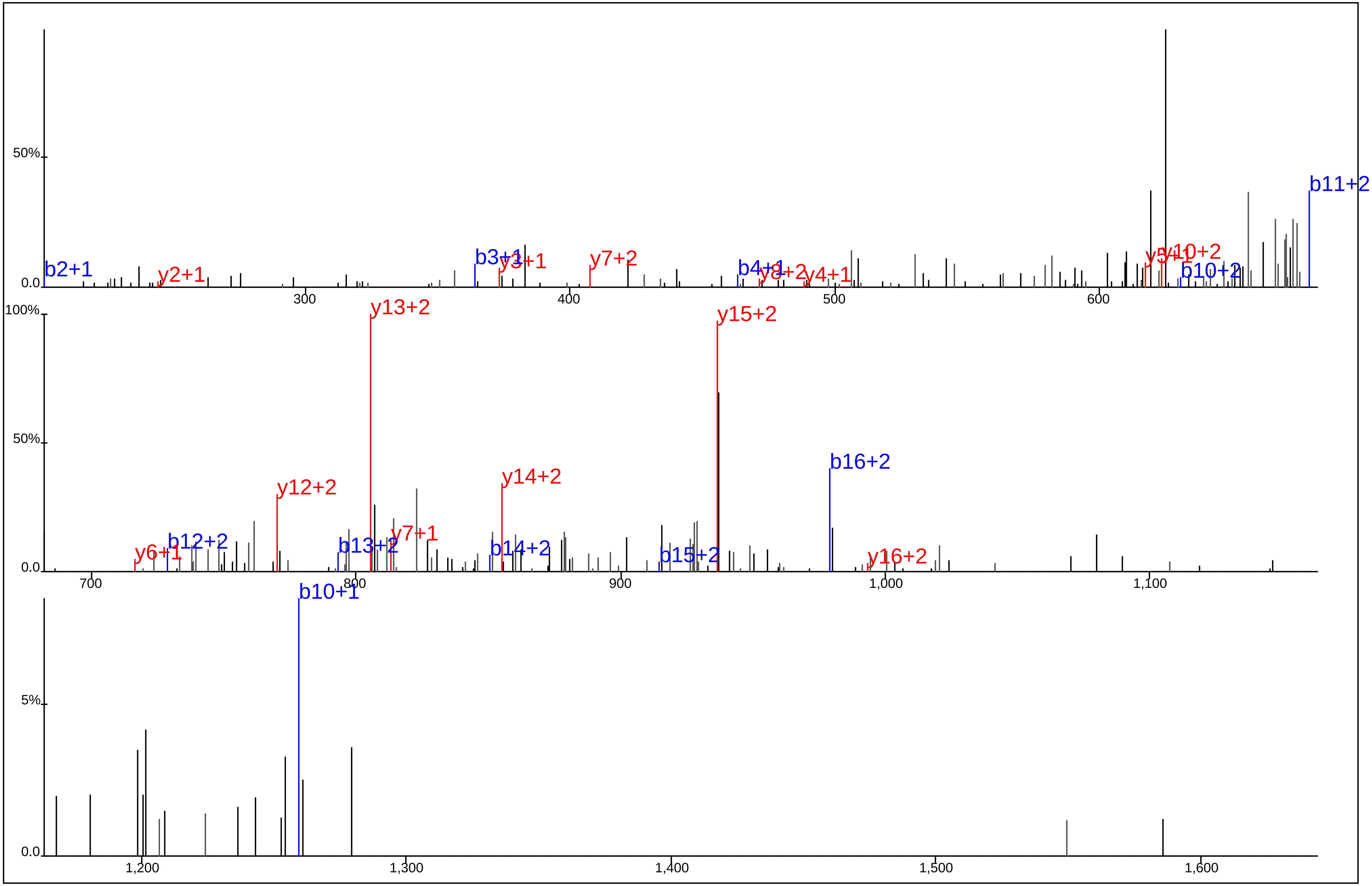

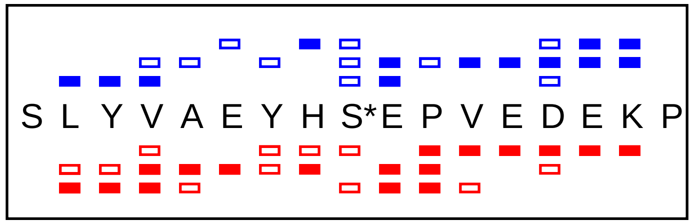

Protein Name: DDX6
gi Accession: 458727
Precursor Mass: 691.3028+3
Delta Mass: 2.055 ppm
XCorr: 10.65

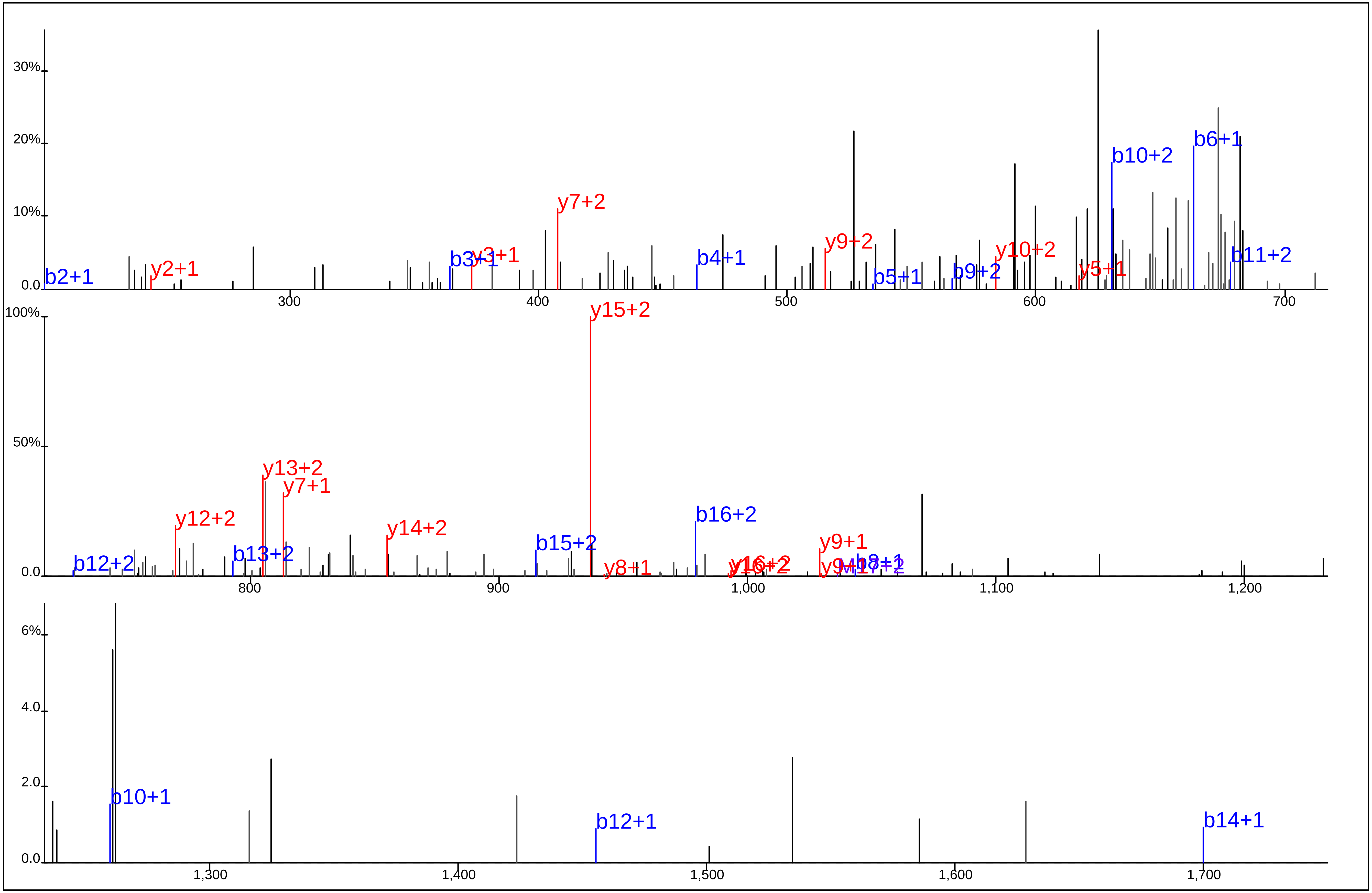

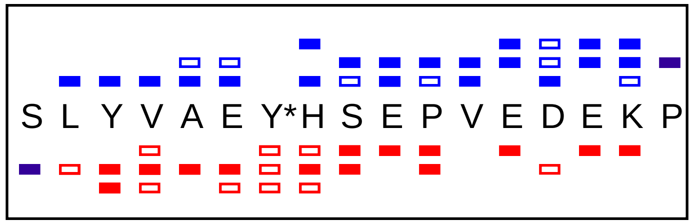

Protein Name: DDX6
gi Accession: 458727
Precursor Mass: 691.3049+3
Delta Mass: 5.149 ppm
XCorr: 14.5

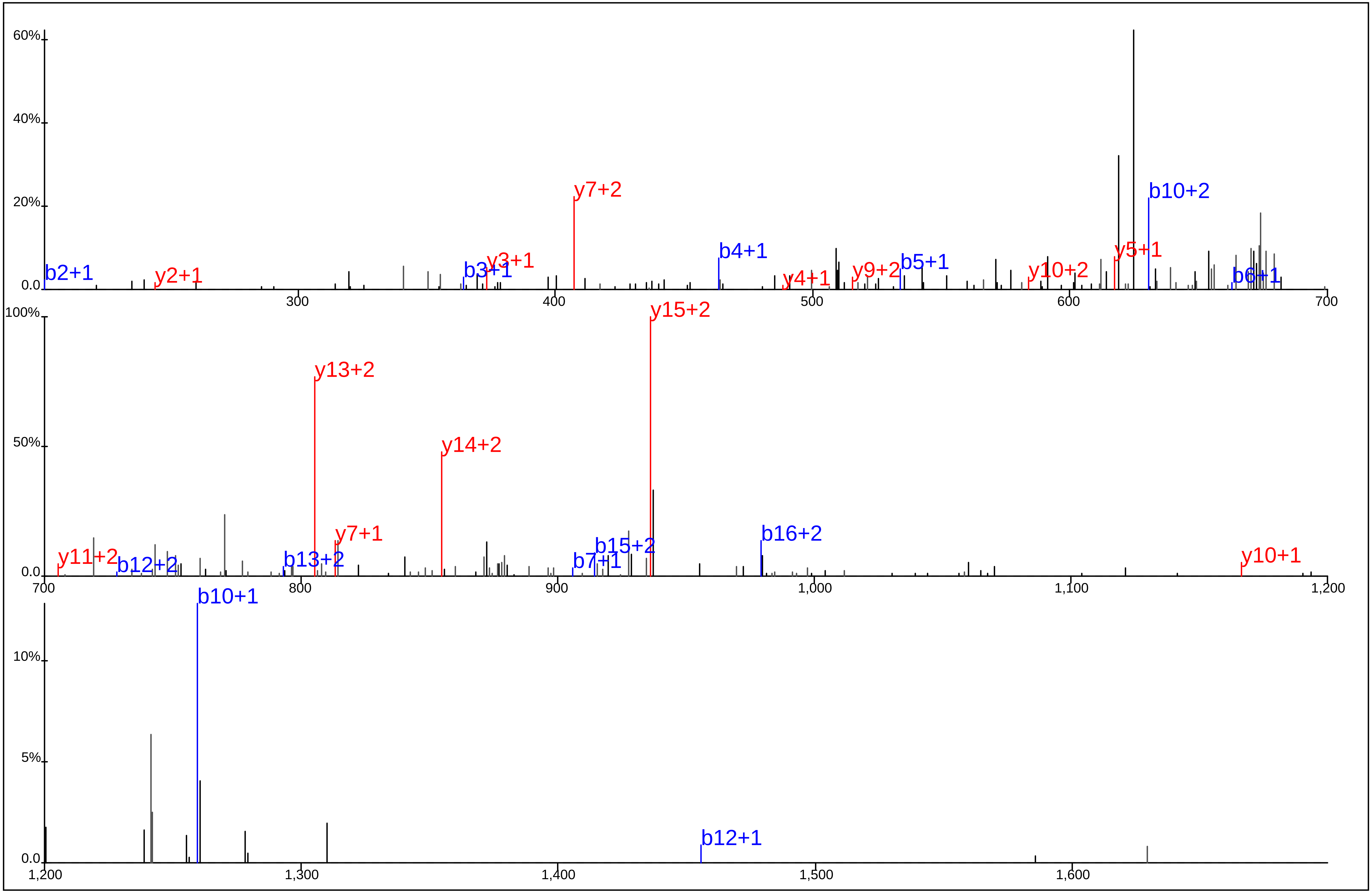

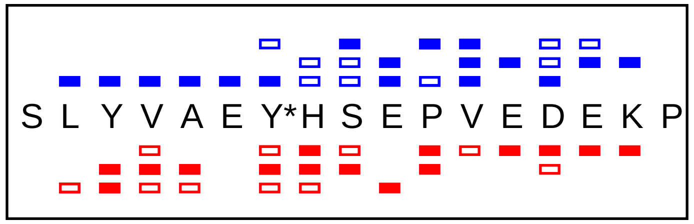

Protein Name: DDX6
gi Accession: 458727
Precursor Mass: 691.3055+3
Delta Mass: 6.033 ppm
XCorr: 17.85

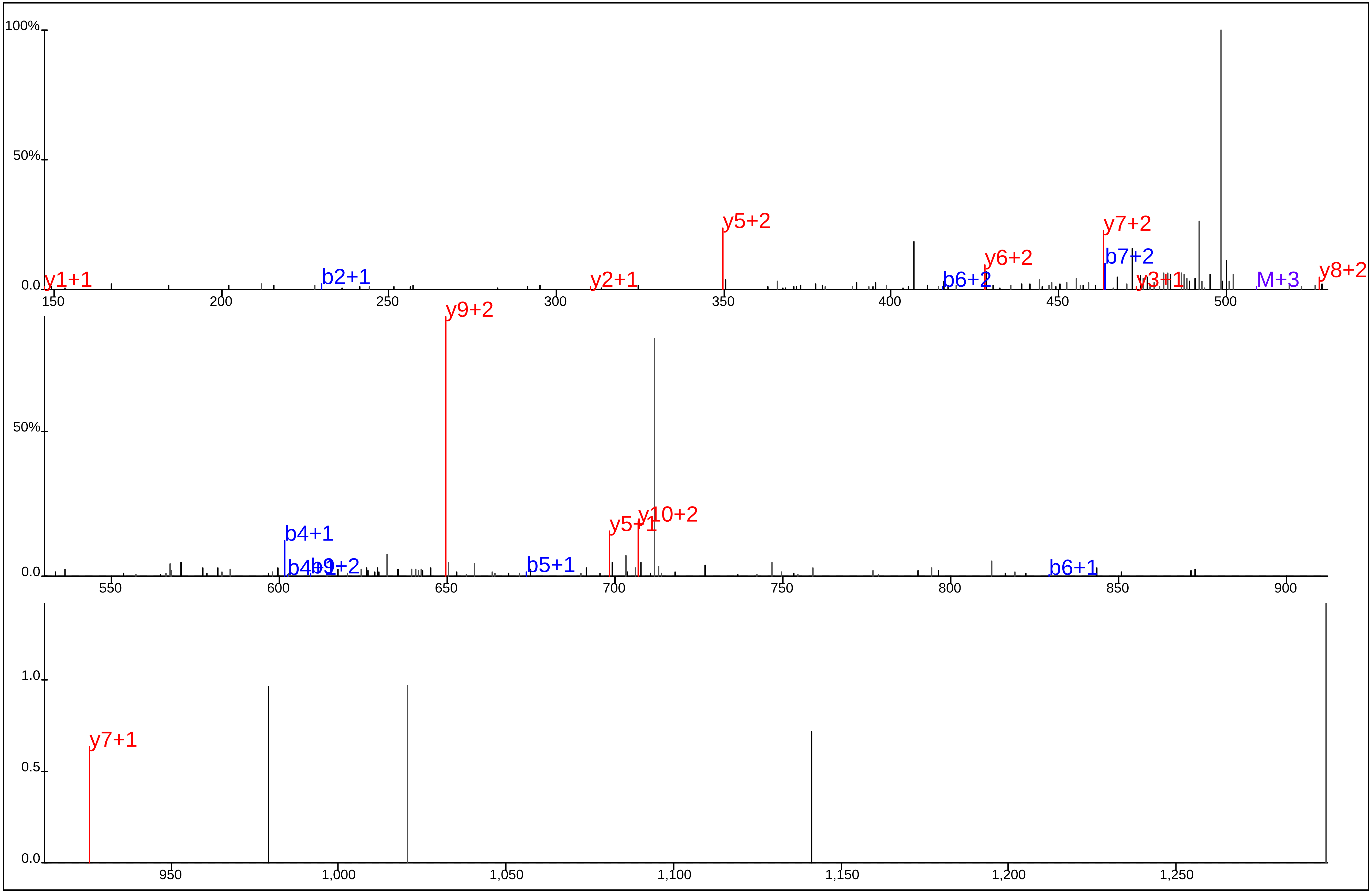

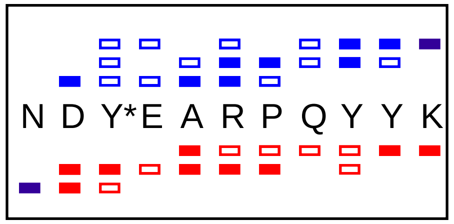

Protein Name:	Diaphanous 1
gi Accession:	10435239
Precursor Mass:	509.5492+3
Delta Mass:	1.322 ppm
XCorr:	13.94

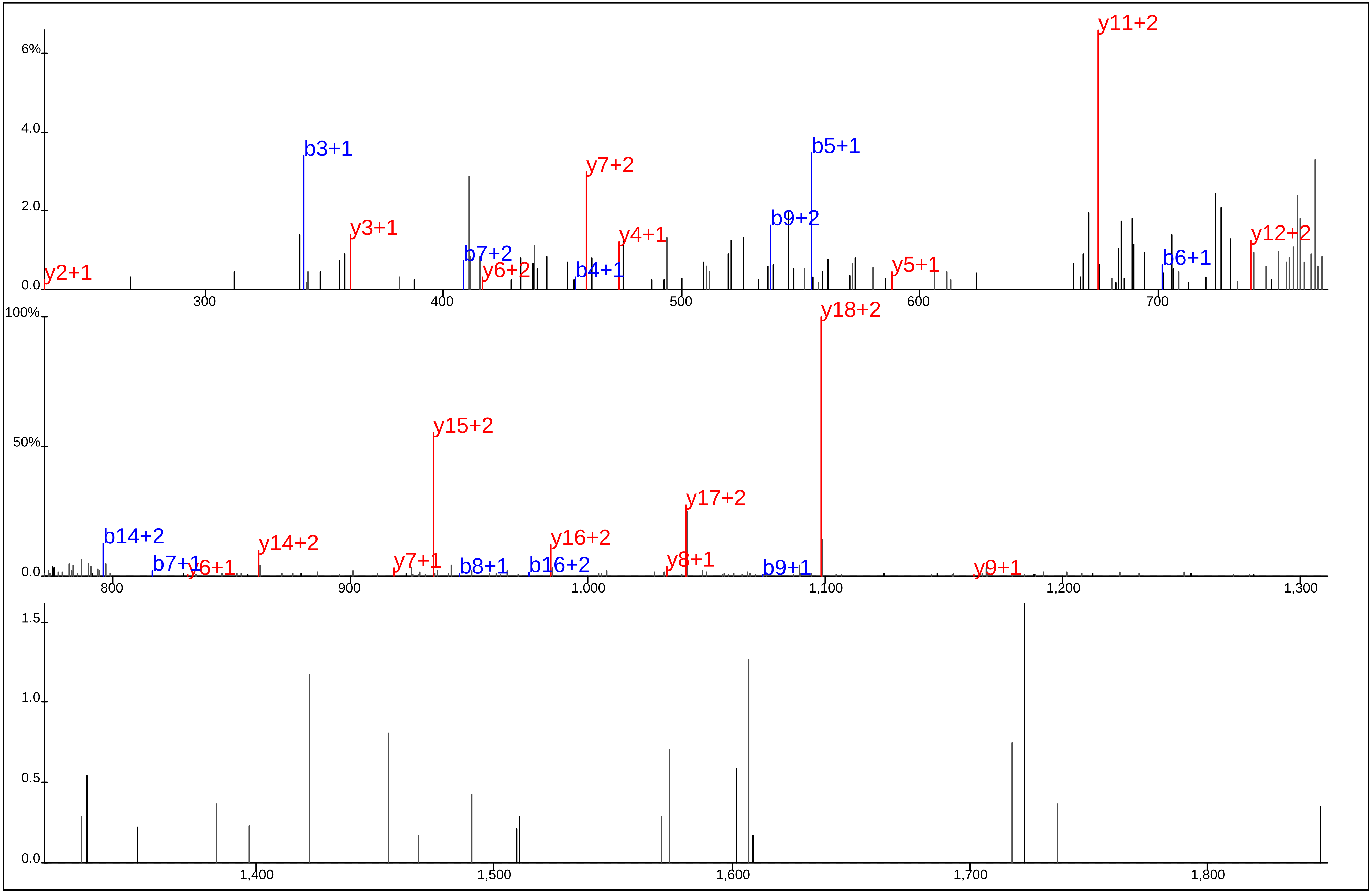

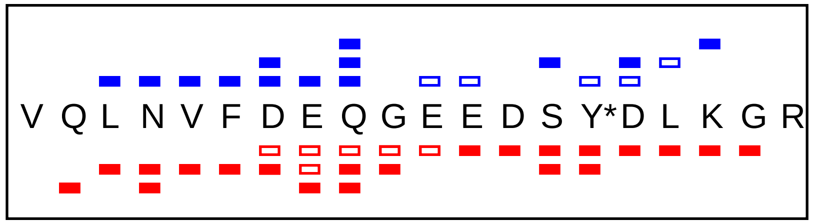

Protein Name:	Diaphanous 1
gi Accession:	10435239
Precursor Mass:	807.6954+3
Delta Mass:	4.617 ppm
XCorr:	22.17

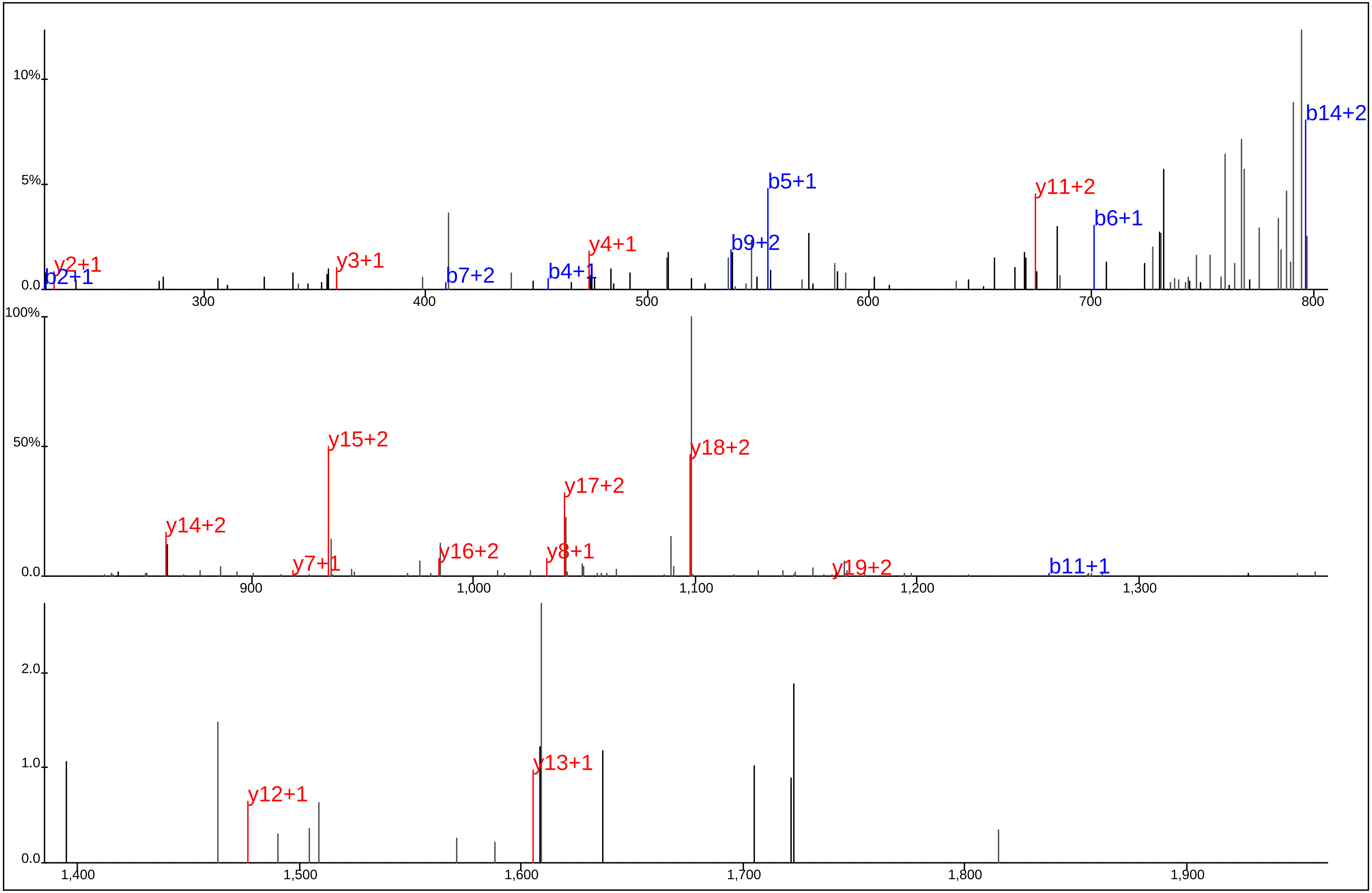

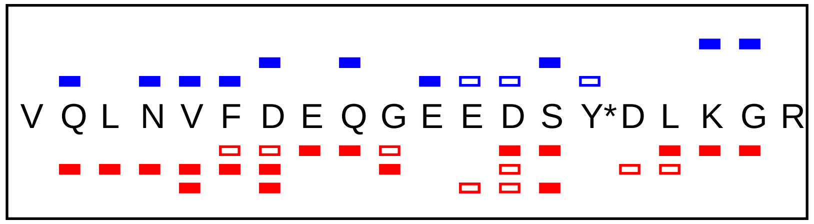

Protein Name:	Diaphanous 1
gi Accession:	10435239
Precursor Mass:	807.6942+3
Delta Mass:	3.105 ppm
XCorr:	21.84

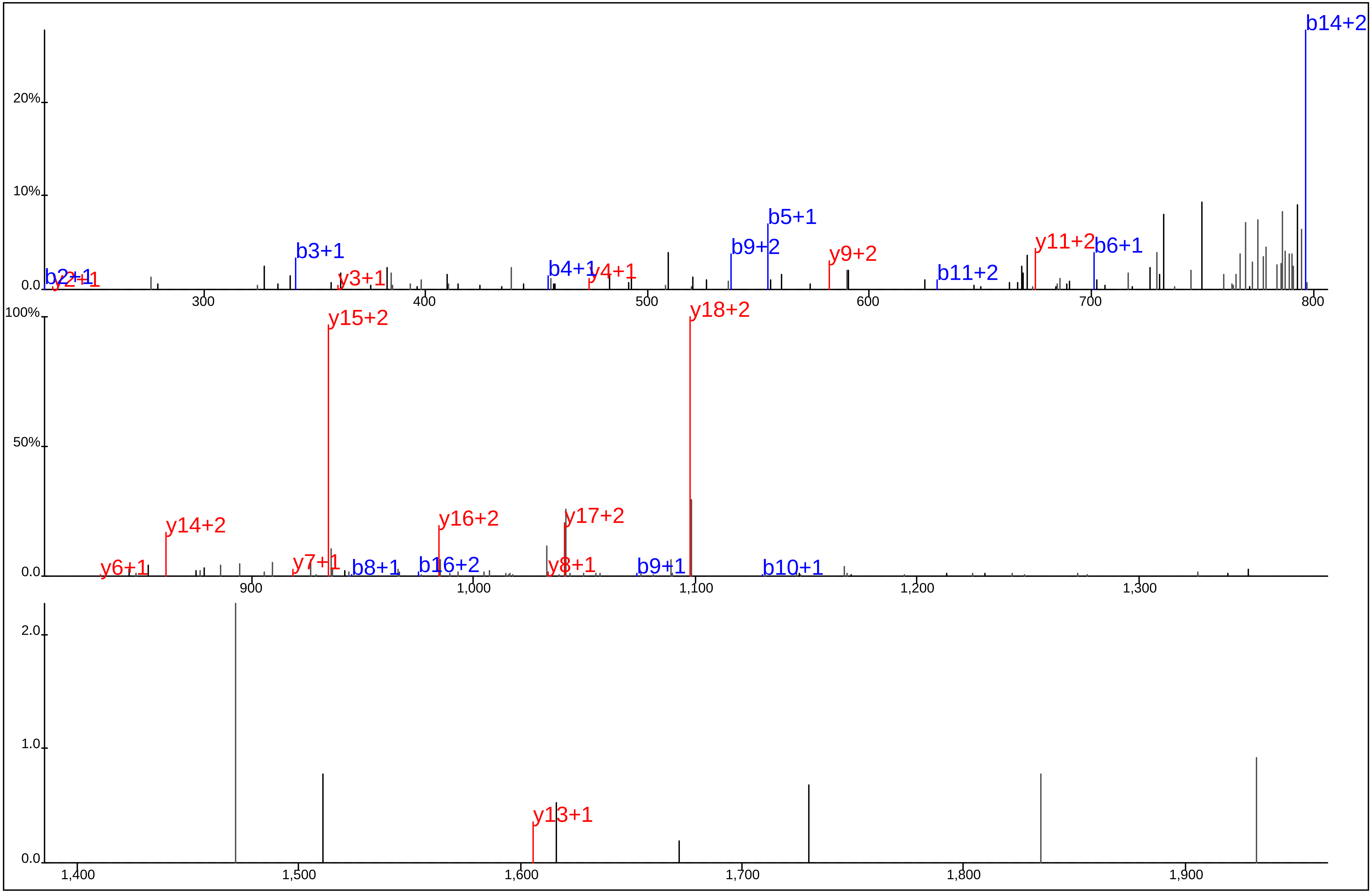

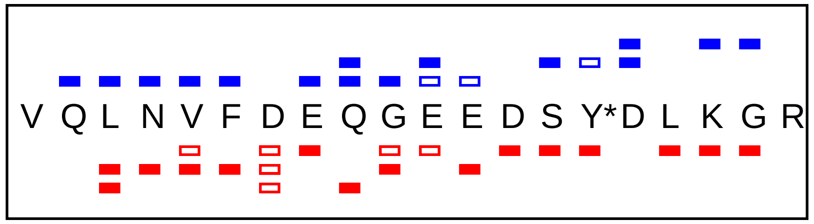

Protein Name: Diaphanous 1
gi Accession: 10435239
Precursor Mass: 807.6951+3
Delta Mass: 4.237 ppm
XCorr: 15.35

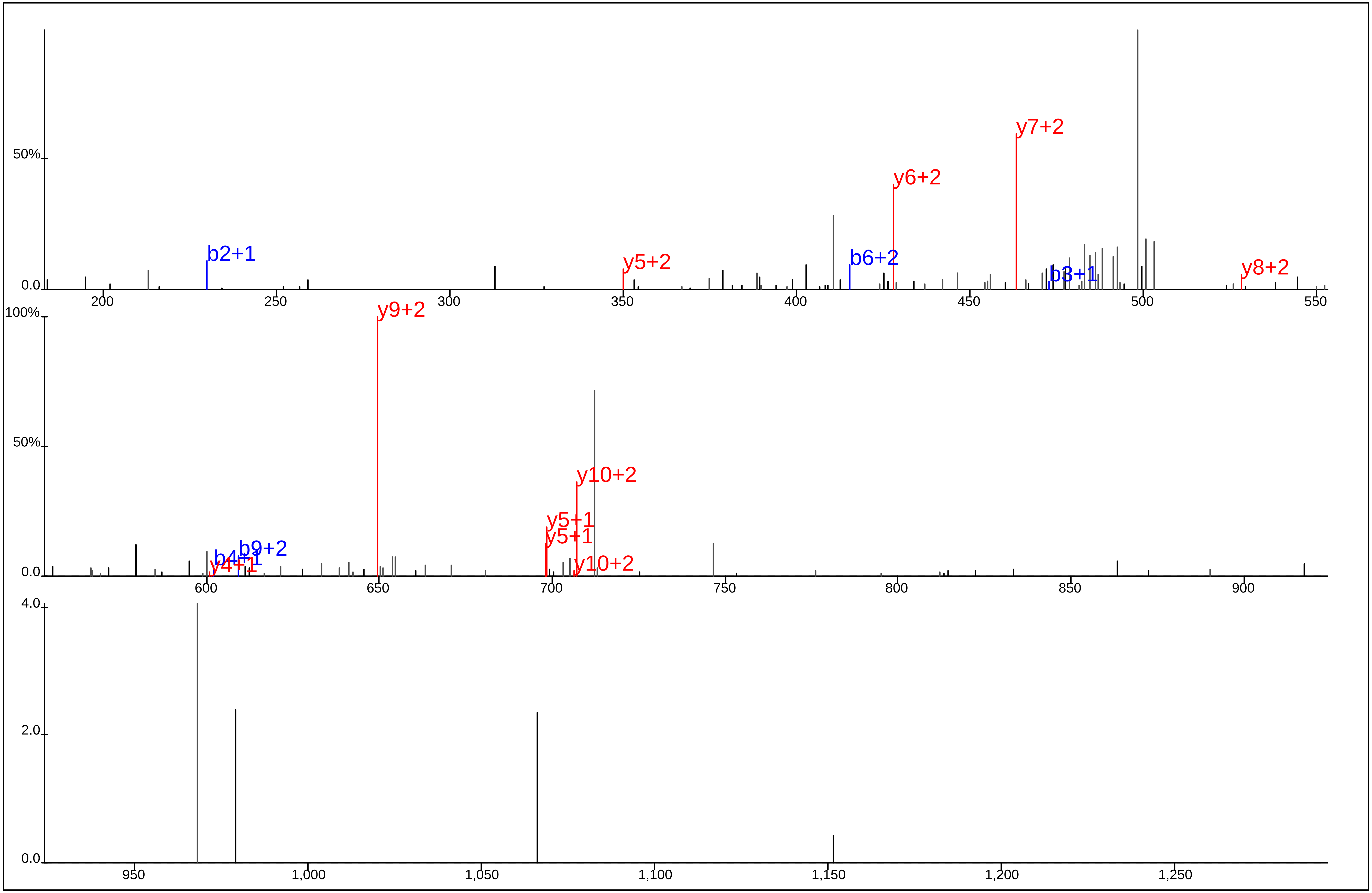

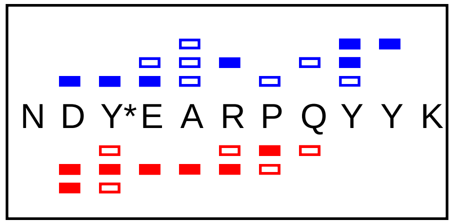

Protein Name: Diaphanous 1
gi Accession: 10435239
Precursor Mass: 509.5495+3
Delta Mass: 1.866 ppm
XCorr: 13.86

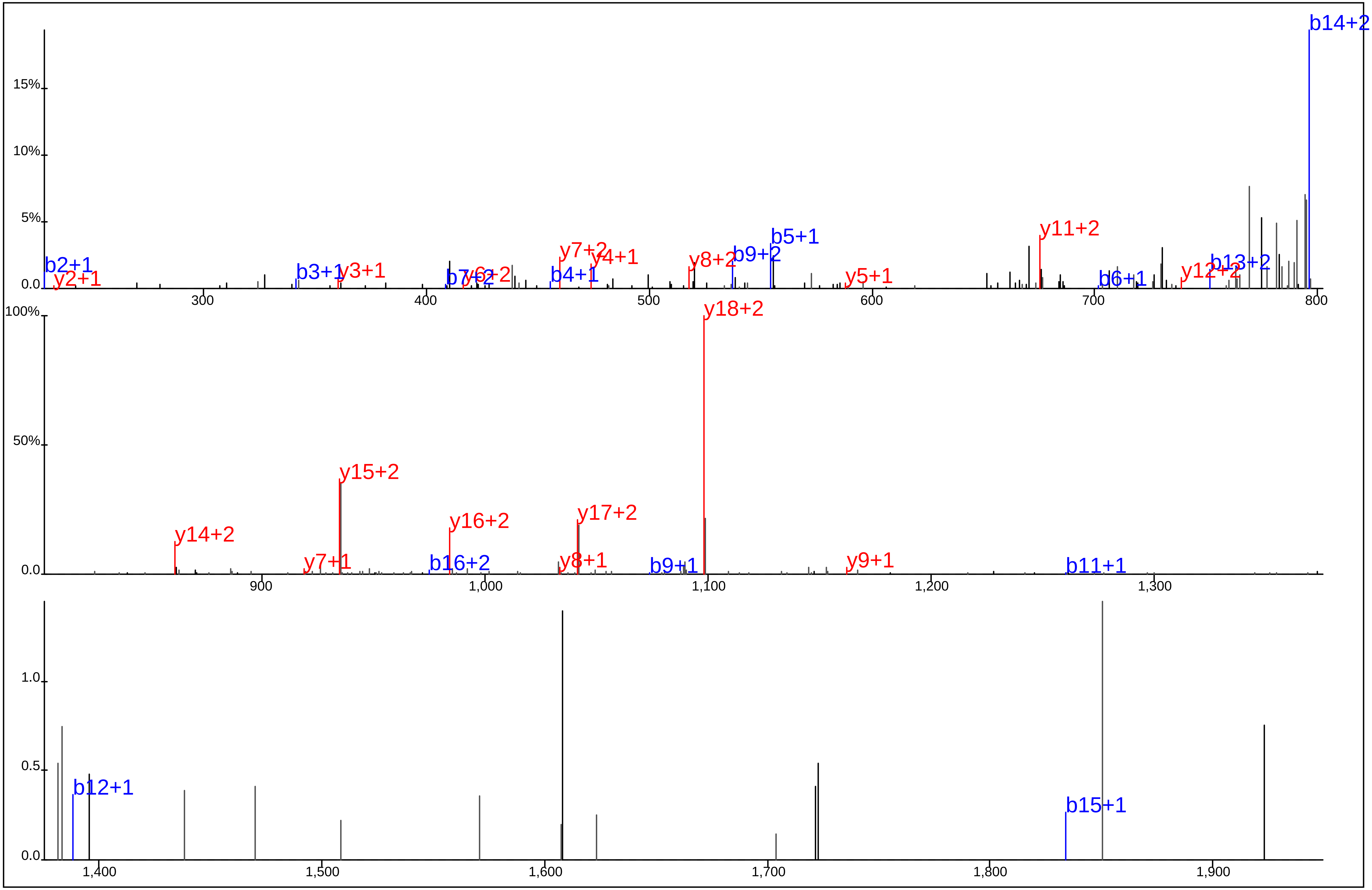

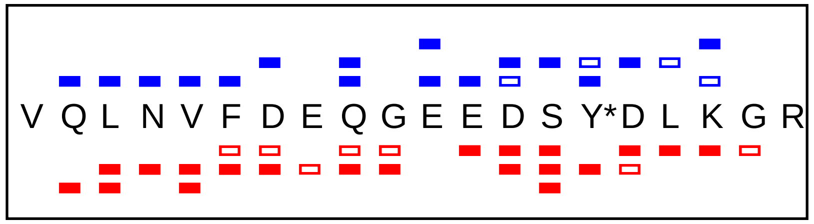

Protein Name:	Diaphanous 1
gi Accession:	10435239
Precursor Mass:	807.6920+3
Delta Mass:	0.383 ppm
XCorr:	30.1

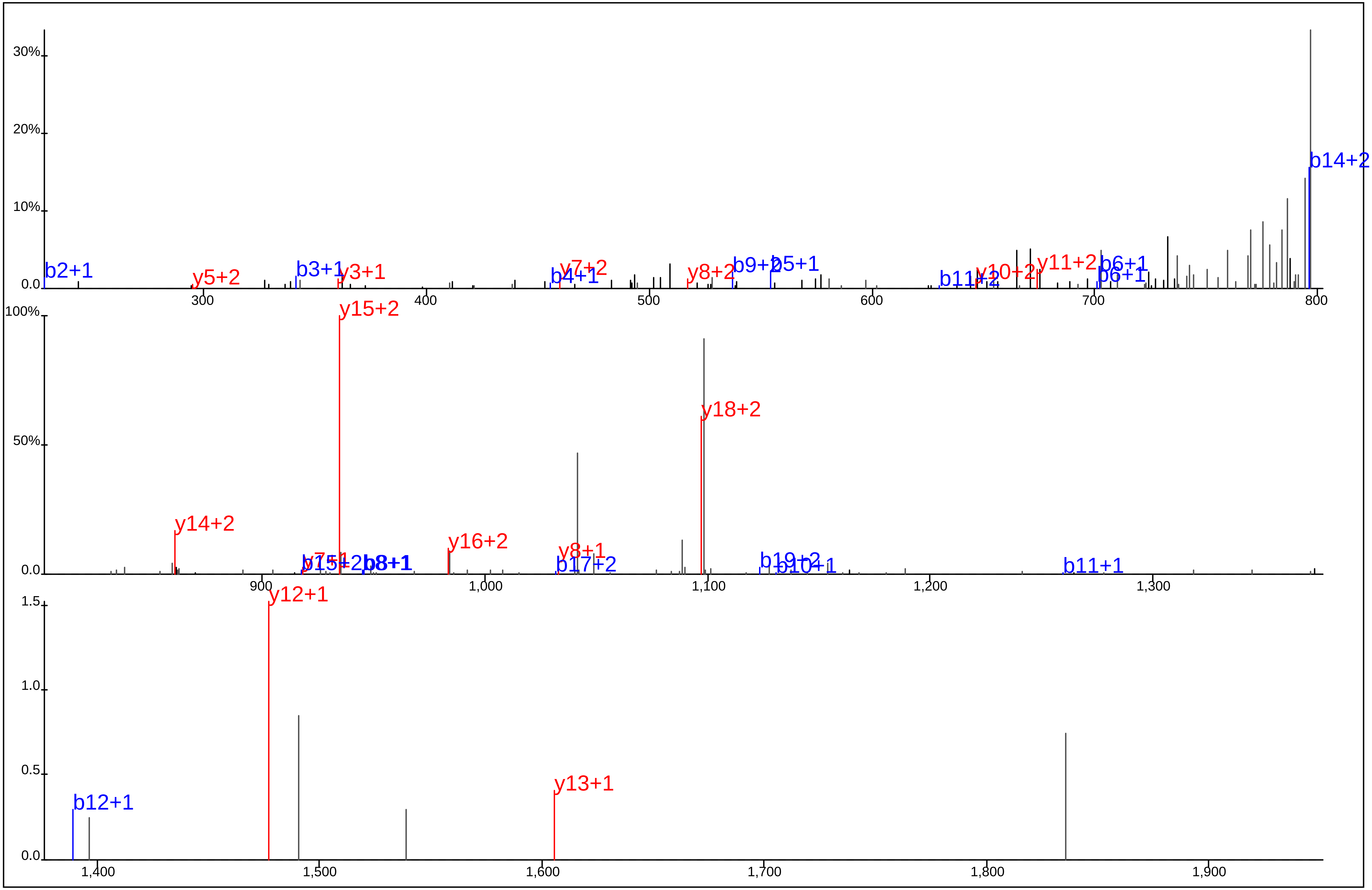

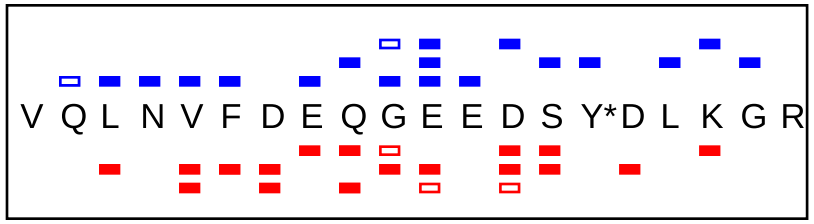

Protein Name: Diaphanous 1
gi Accession: 10435239
Precursor Mass: 807.6925+3
Delta Mass: 0.986 ppm
XCorr: 23.67

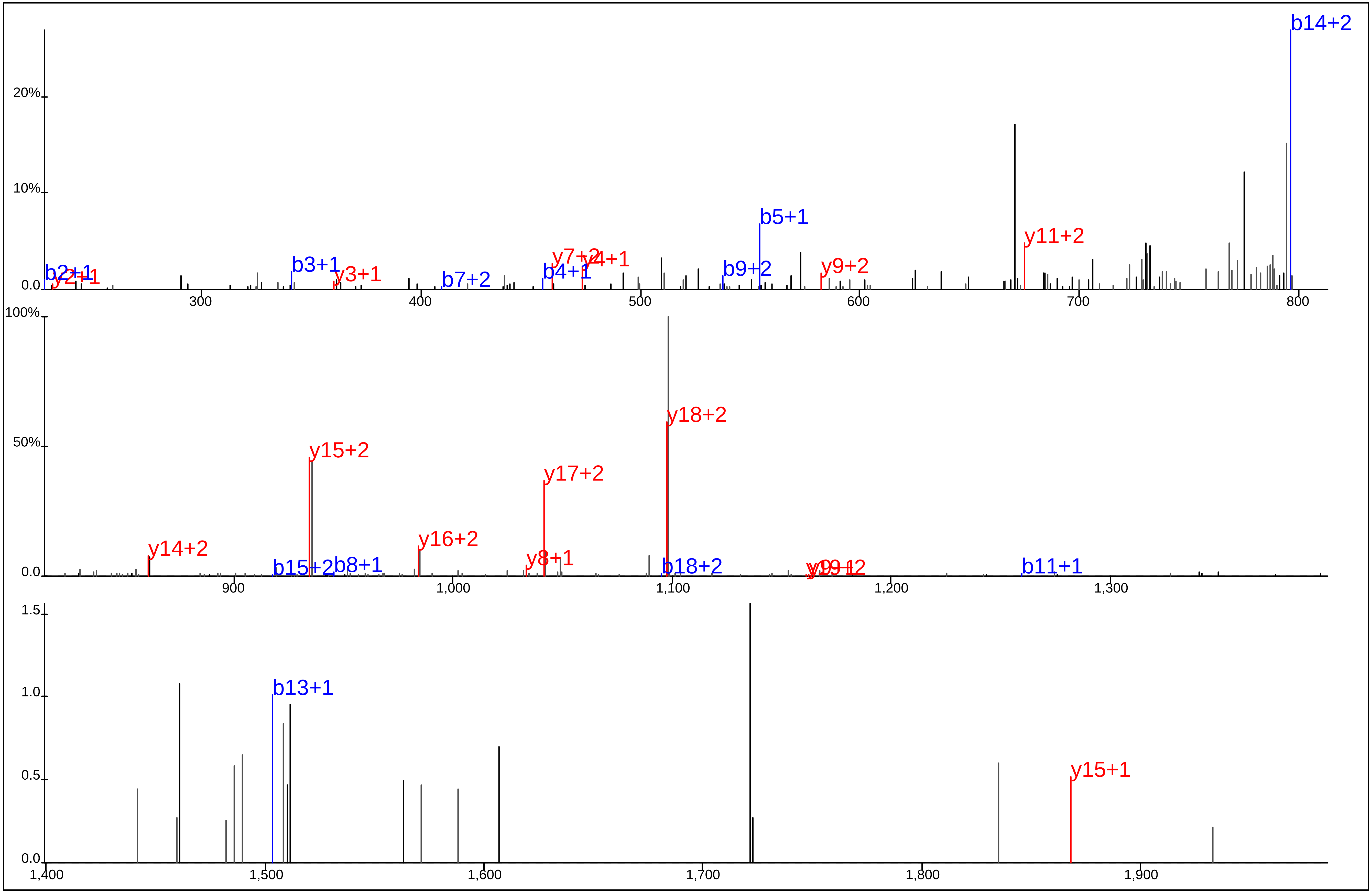

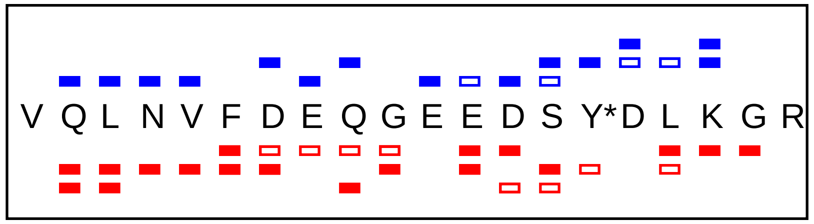

Protein Name:	Diaphanous 1
gi Accession:	10435239
Precursor Mass:	807.6961+3
Delta Mass:	5.447 ppm
XCorr:	22.11

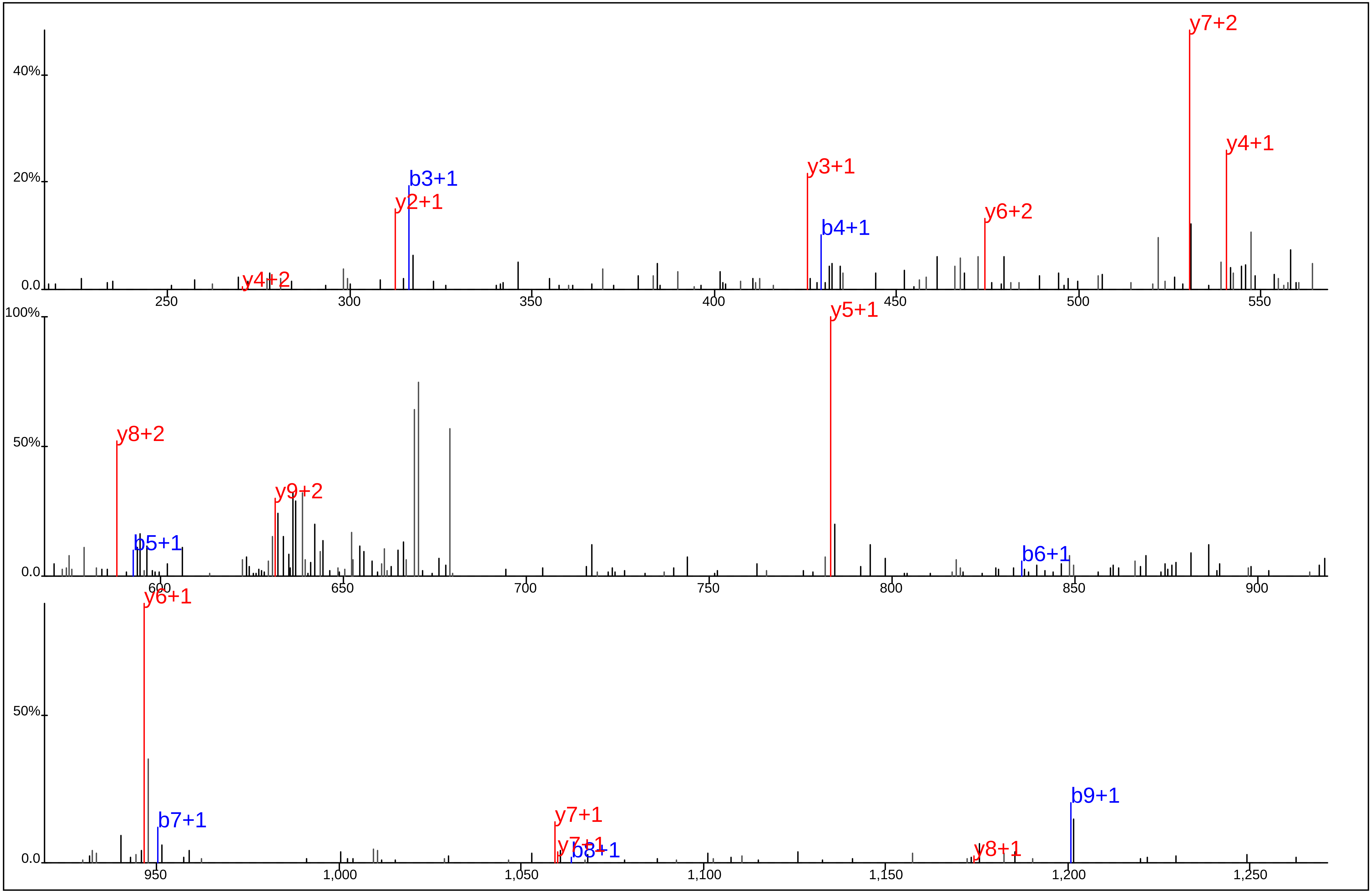

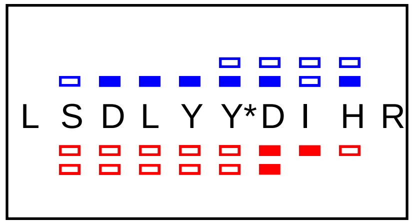

Protein Name: DOCK10
gi Accession: 7020173
Precursor Mass: 687.8108+2
Delta Mass: 3.912 ppm
XCorr: 33.56

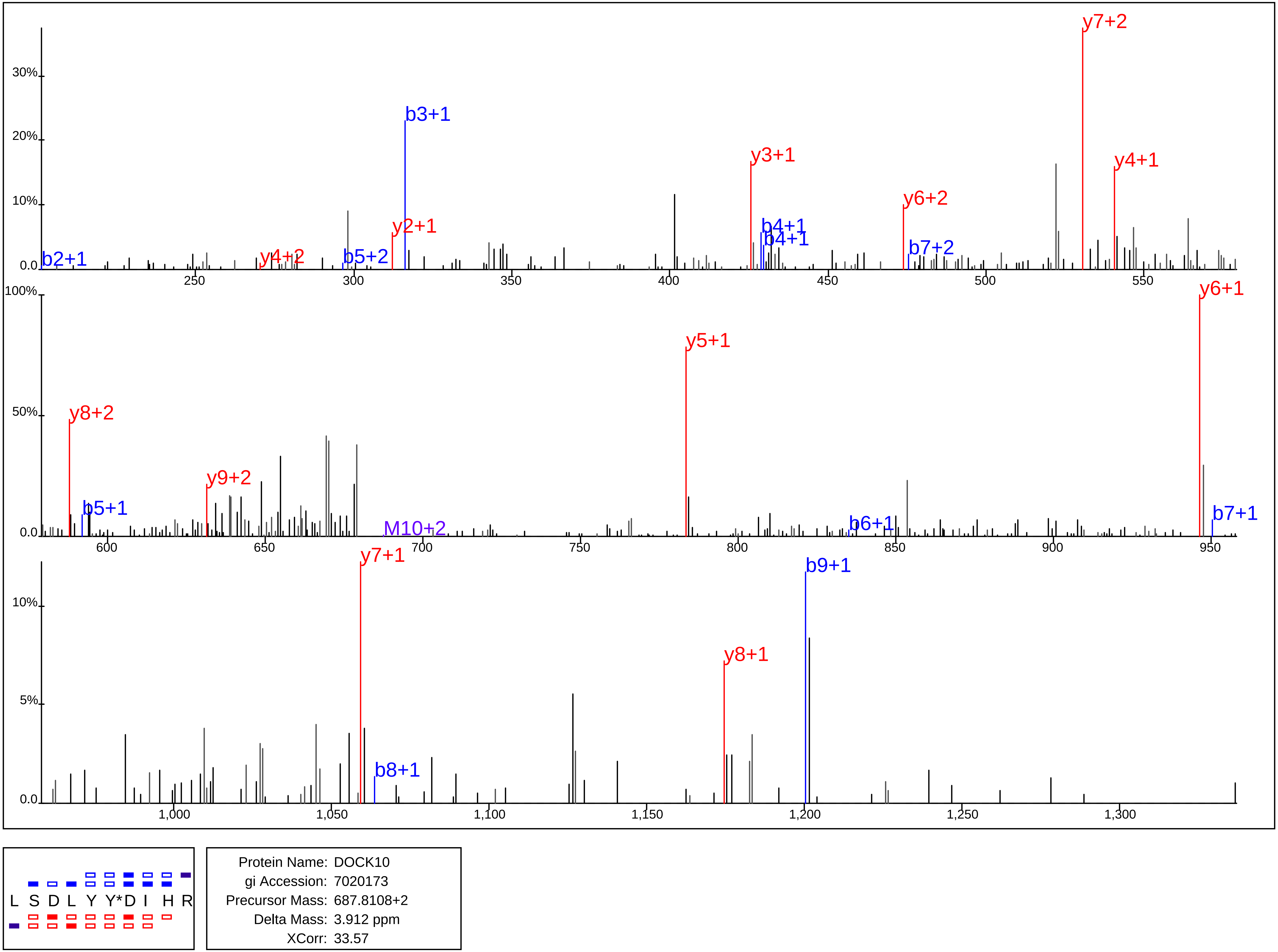

Protein Name: DOCK10
gi Accession: 7020173
Precursor Mass: 687.8108+2
Delta Mass: 3.912 ppm
XCorr: 33.57

329 - DOCK10_3435401.pdf

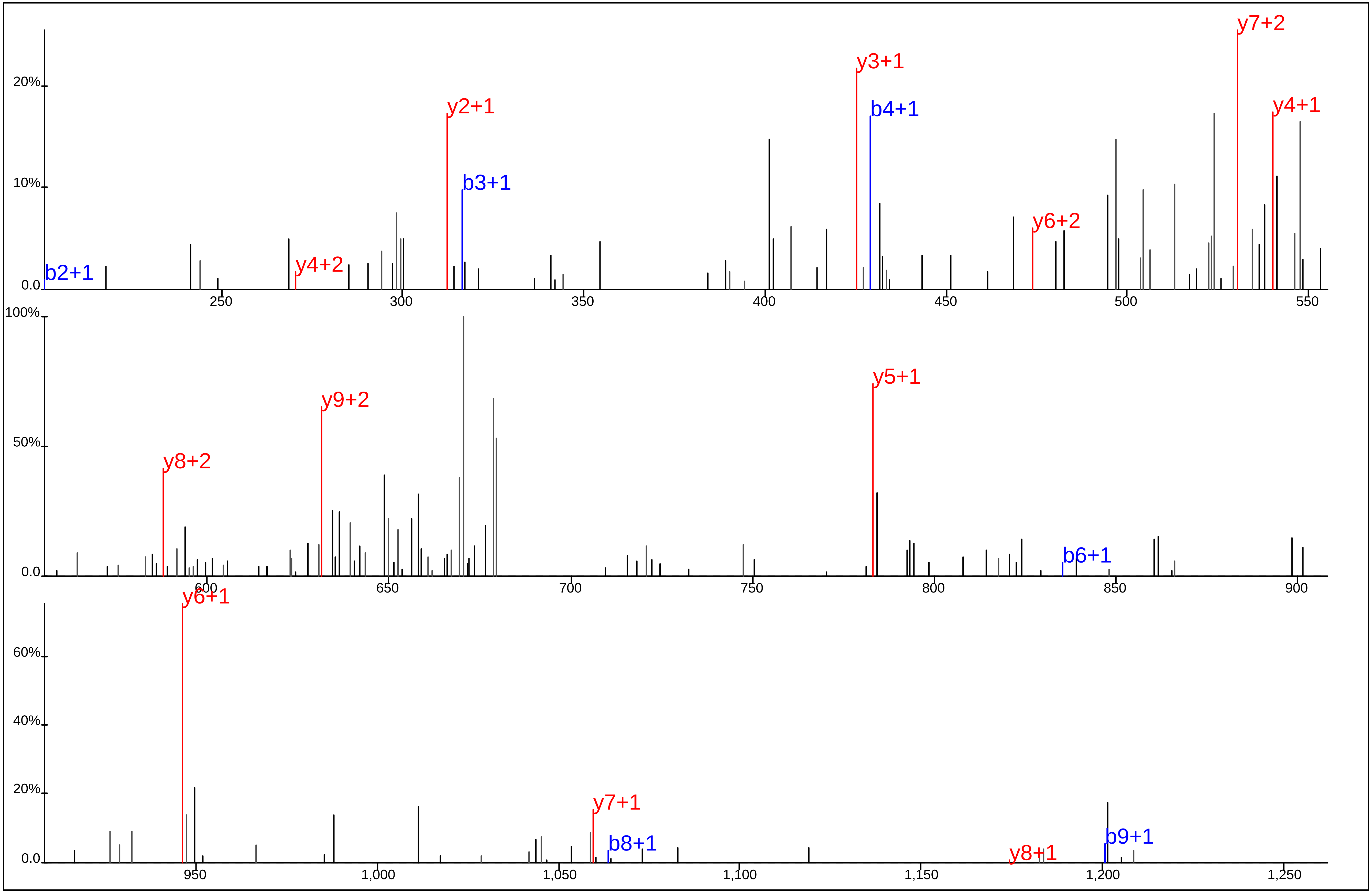

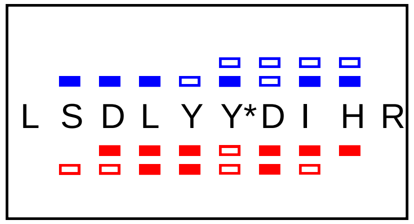

Protein Name: DOCK10
gi Accession: 7020173
Precursor Mass: 687.8111+2
Delta Mass: 4.356 ppm
XCorr: 24.82

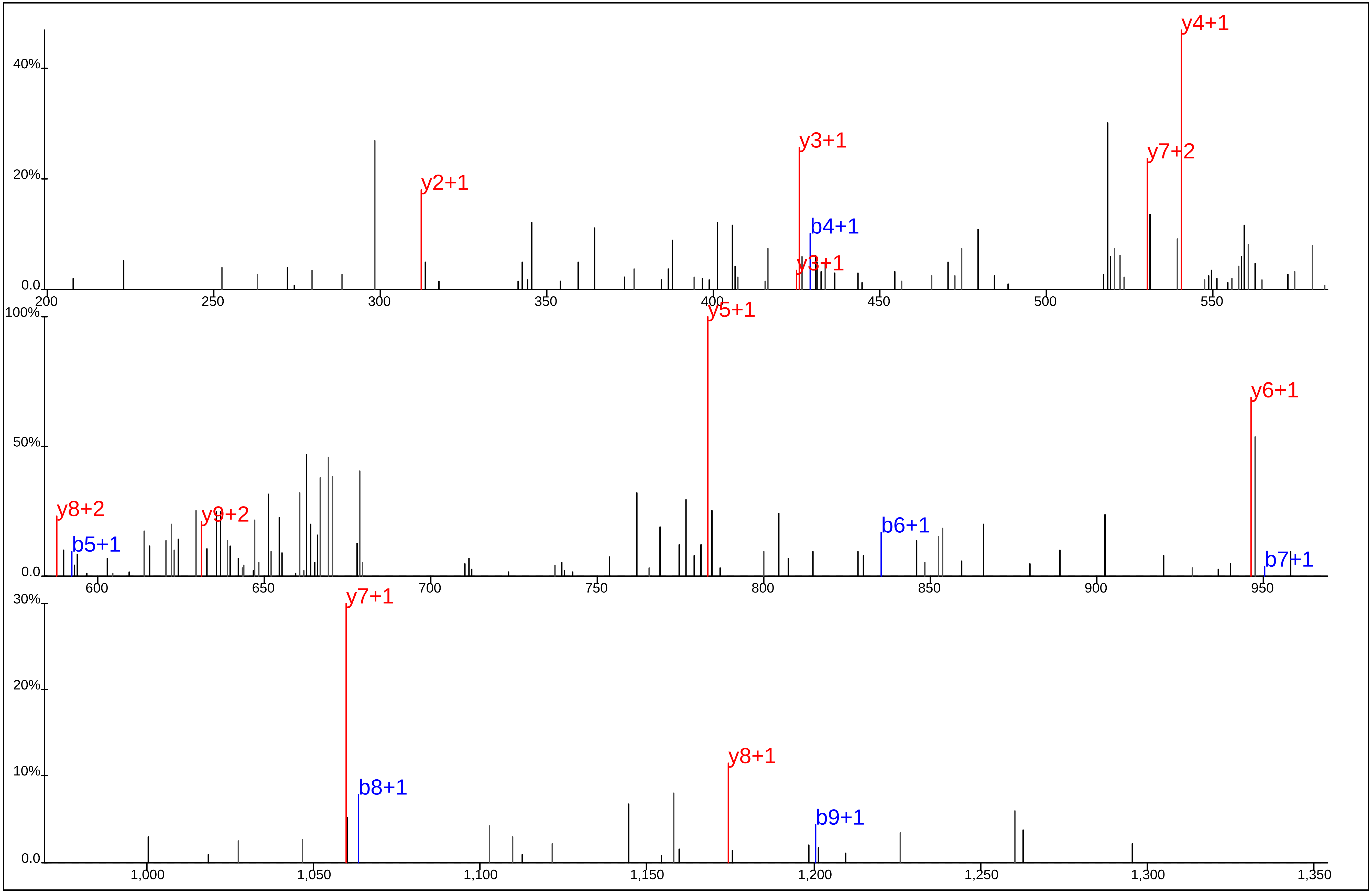

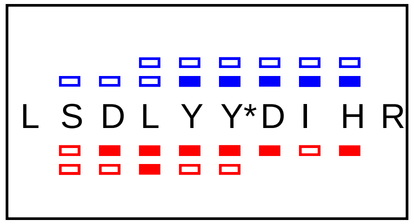

Protein Name: DOCK10
gi Accession: 7020173
Precursor Mass: 687.8099+2
Delta Mass: 2.668 ppm
XCorr: 47.03

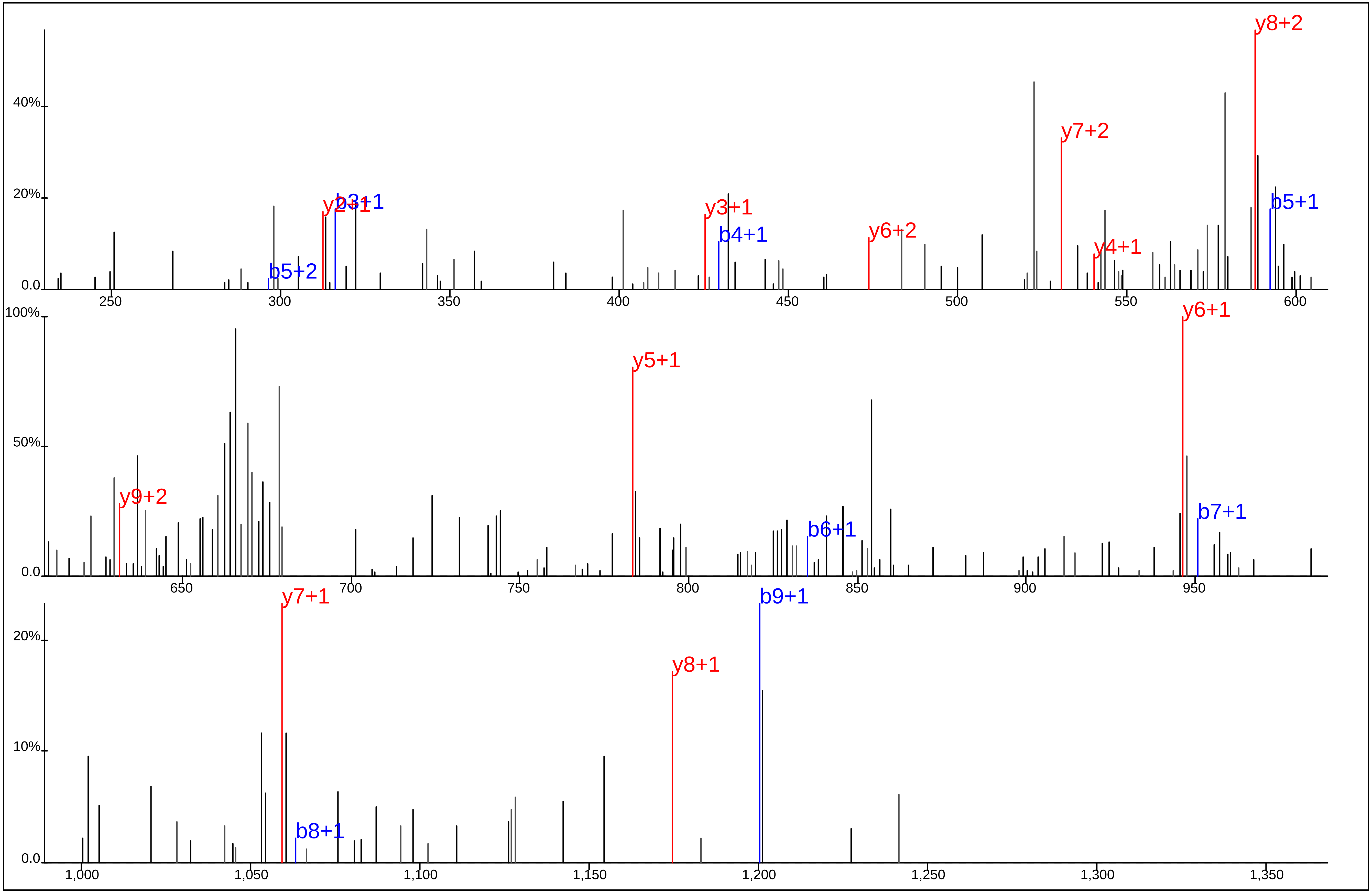

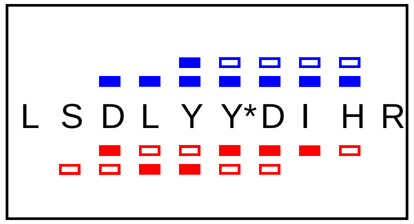

Protein Name: DOCK10
gi Accession: 7020173
Precursor Mass: 687.8090+2
Delta Mass: 1.337 ppm
XCorr: 20.15

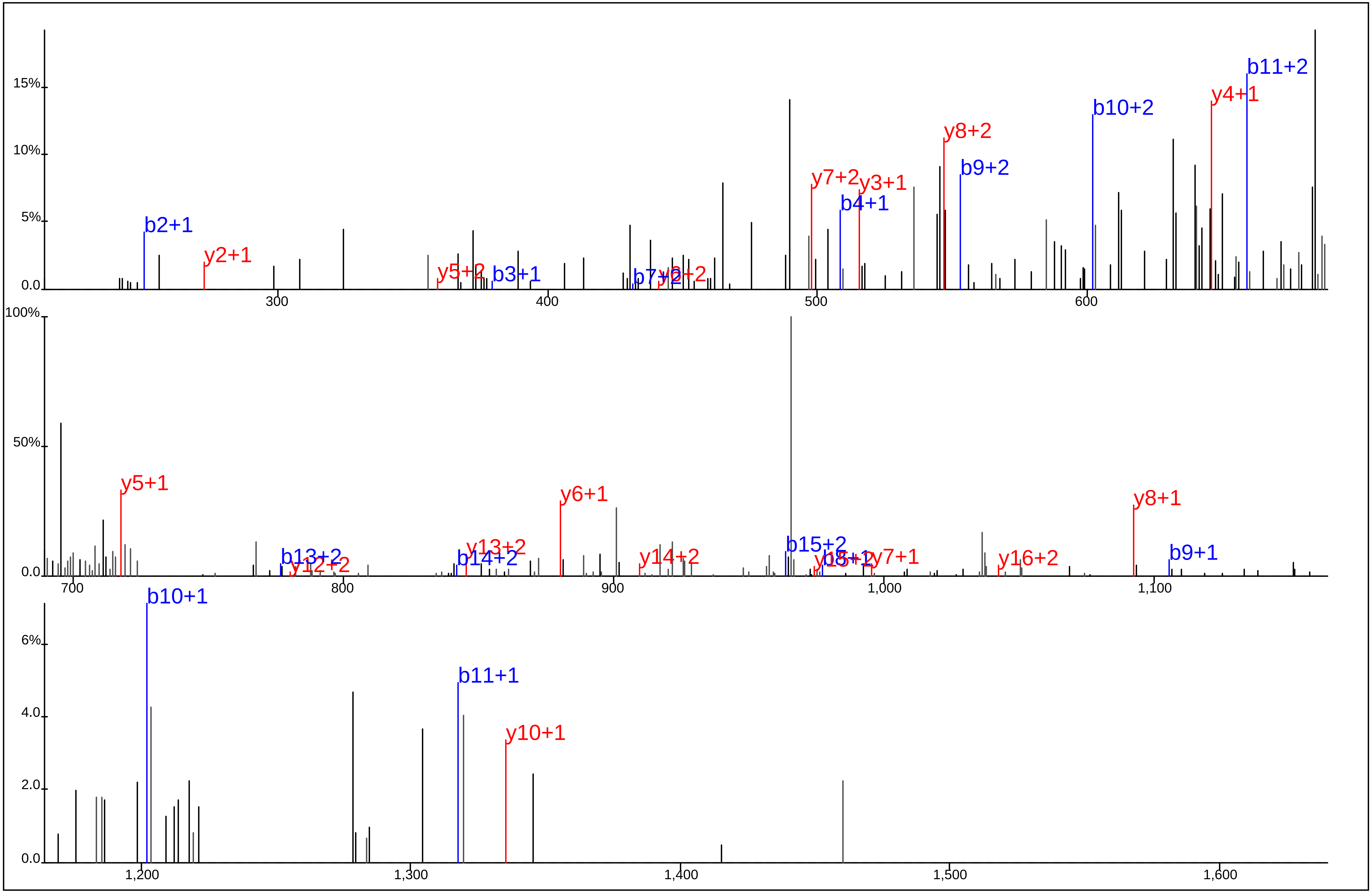

I K#E E M S K#D Q P D Y A M Y*S R%

Protein Name: DOCK2
gi Accession: 1504002
Precursor Mass: 732.9909+3
Delta Mass: 1.392 ppm
XCorr: 19.25

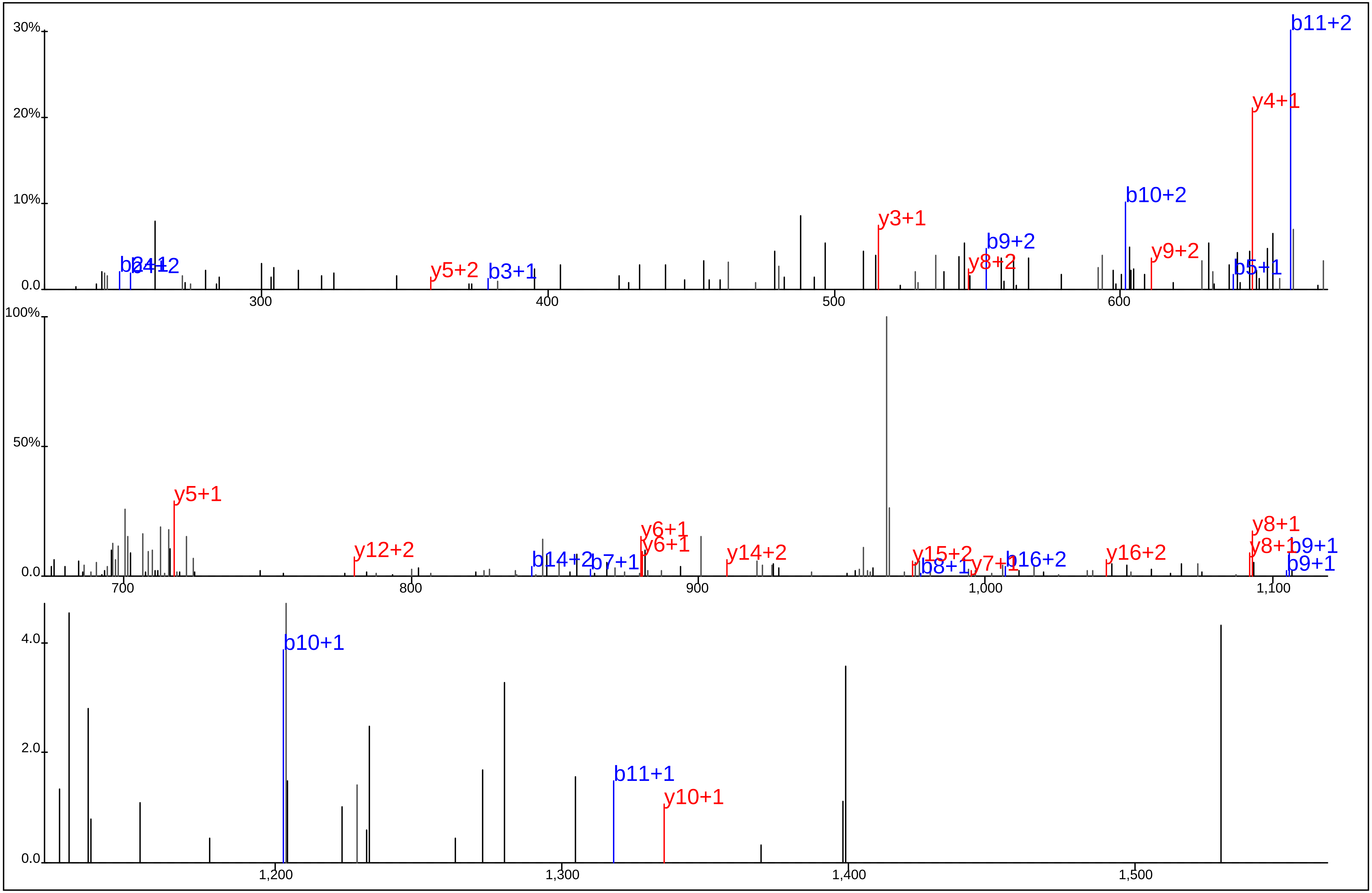

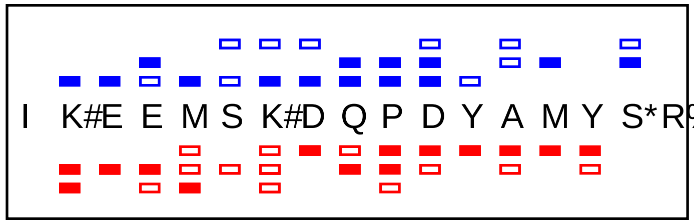

Protein Name:	DOCK2
gi Accession:	1504002
Precursor Mass:	732.9904+3
Delta Mass:	0.727 ppm
XCorr:	18.41

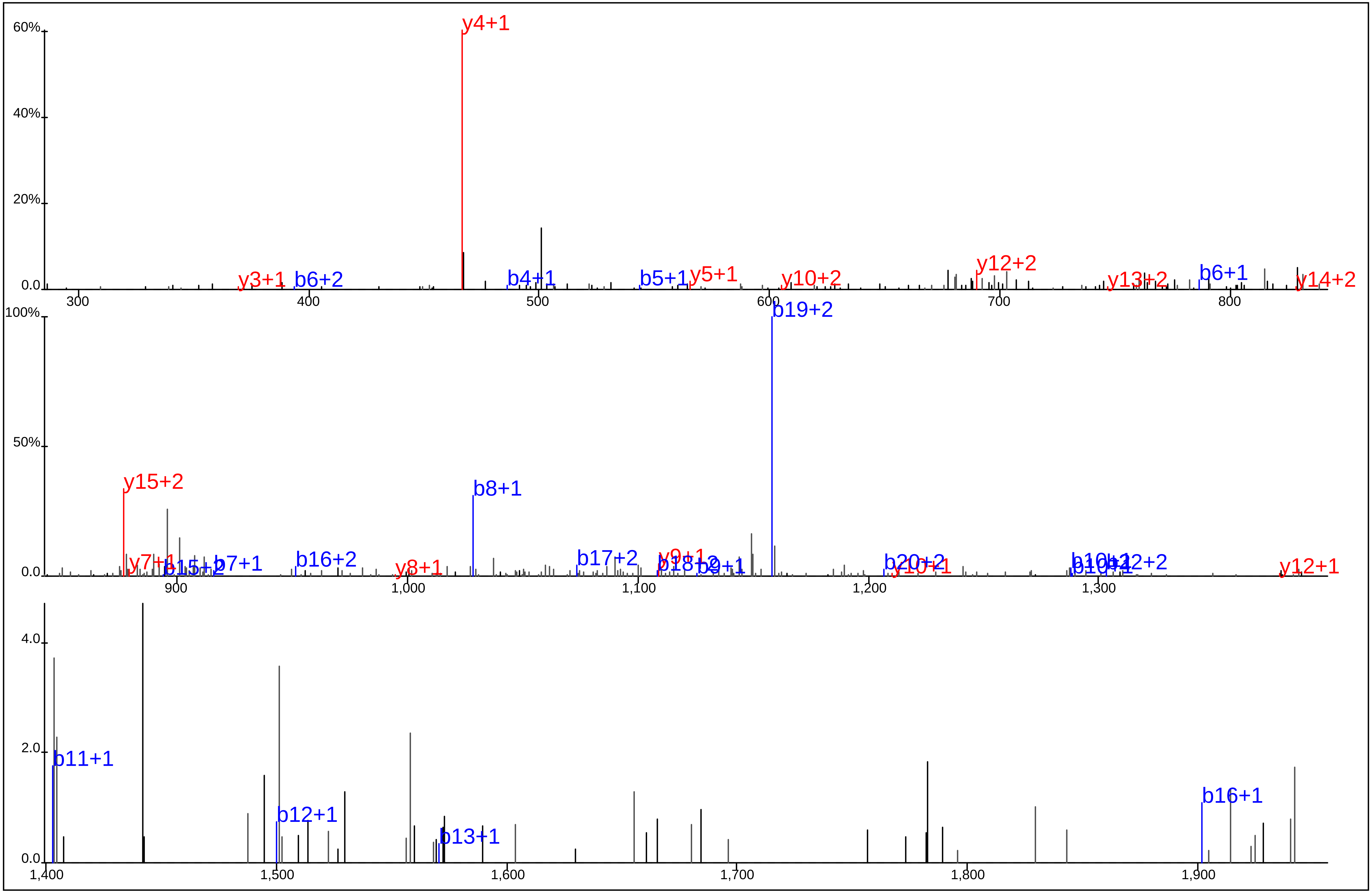

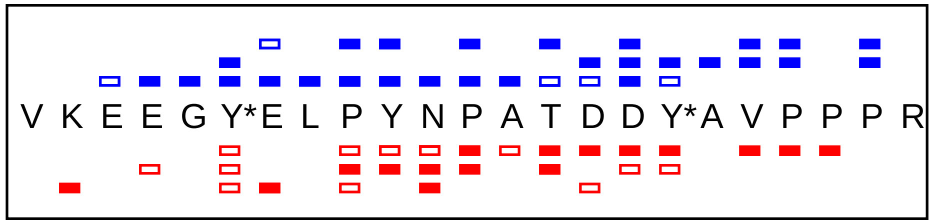

Protein Name: DOK1
gi Accession: 4503357
Precursor Mass: 927.3994+3
Delta Mass: 1.993 ppm
XCorr: 2.6606

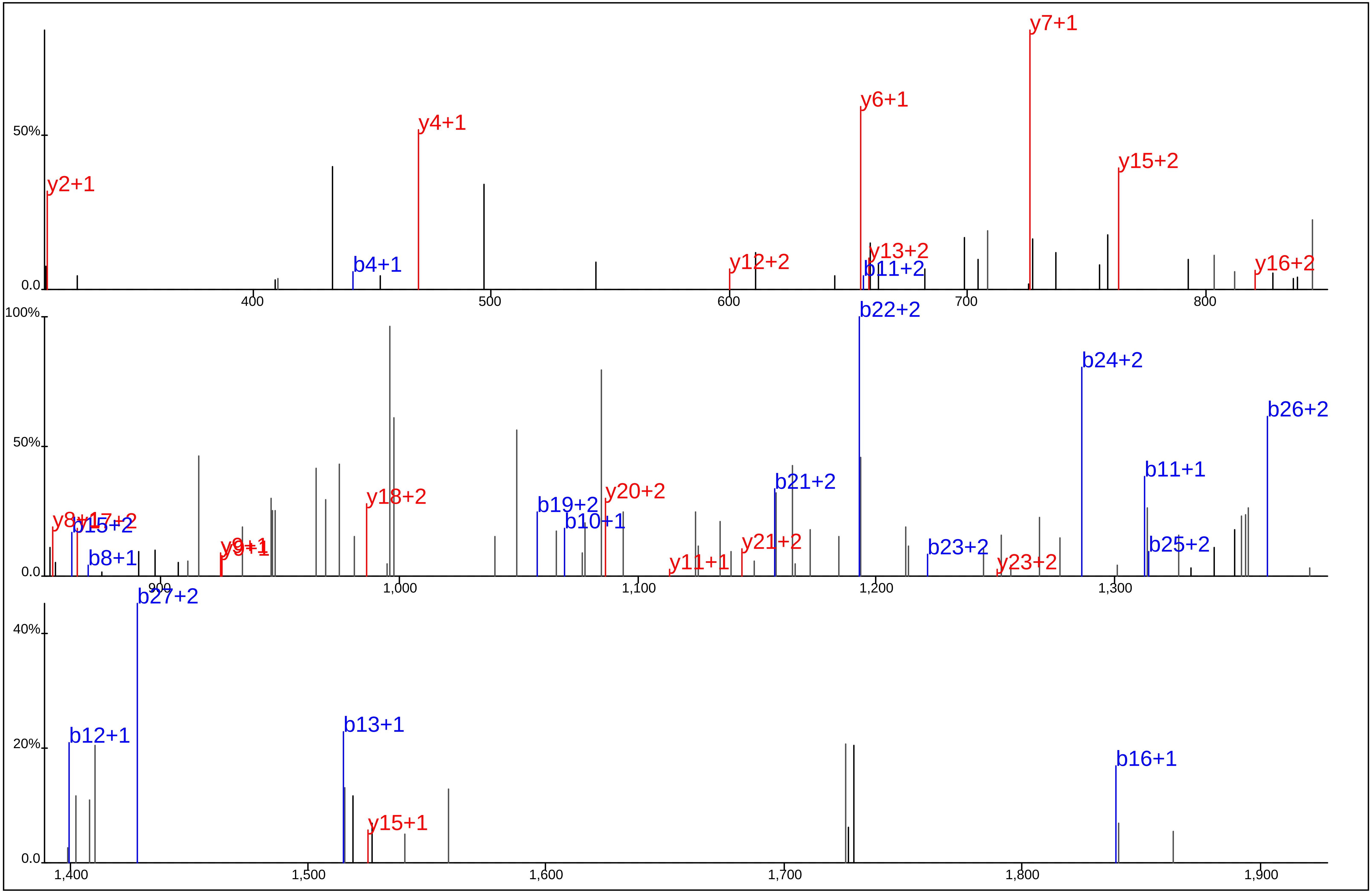

I A P C P S Q D S L Y*S D P L D S T S A Q A G E G V Q R%

Protein Name:	DOK1
gi Accession:	4503357
Precursor Mass:	1,013.7863+3
Delta Mass:	5.504 ppm
XCorr:	32.61

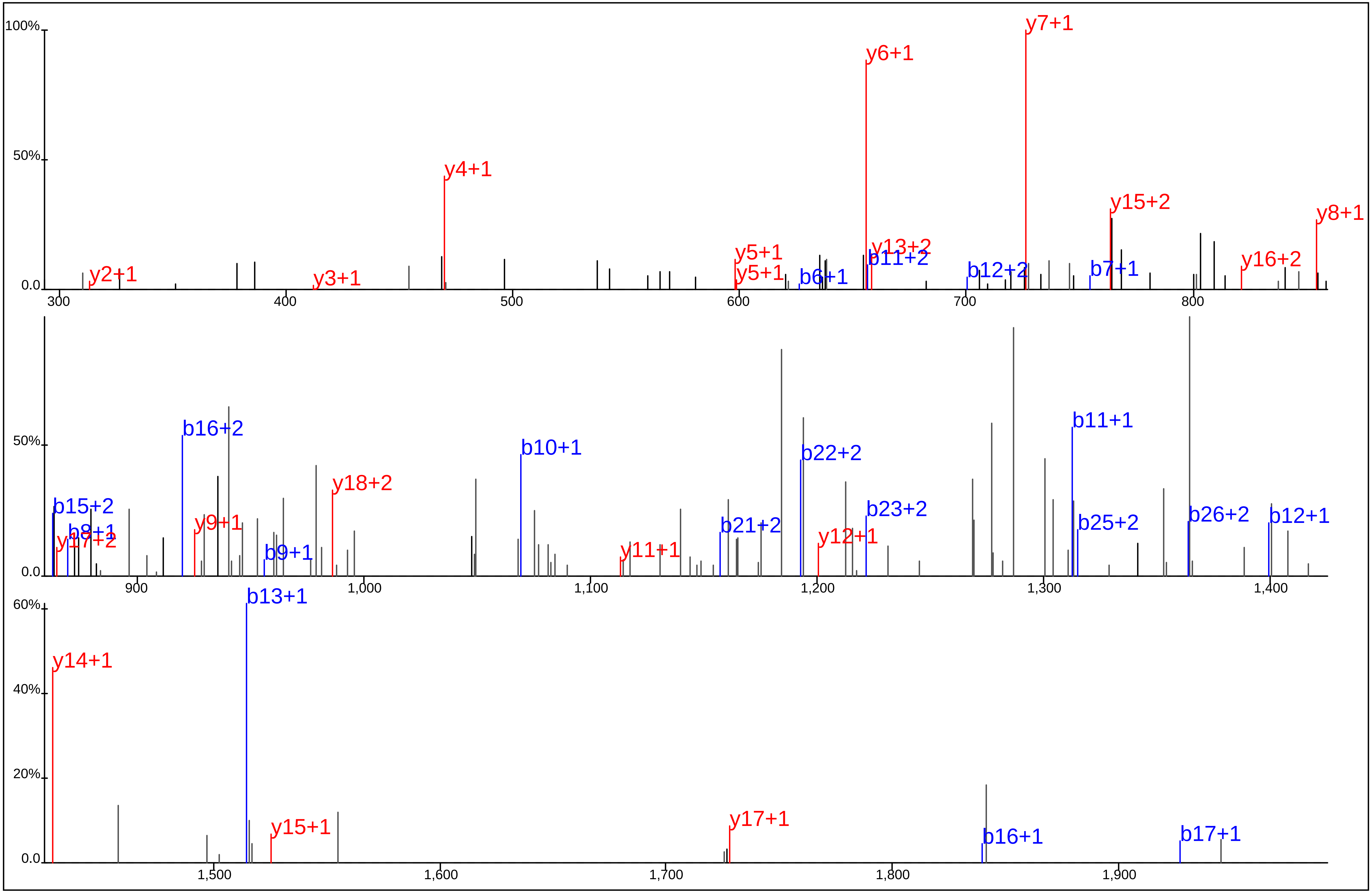

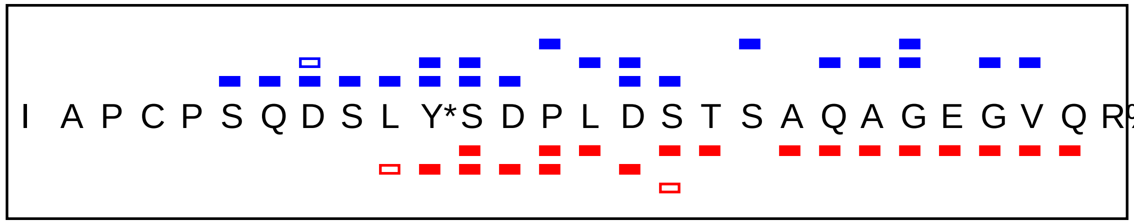

Protein Name:	DOK1
gi Accession:	4503357
Precursor Mass:	1,013.7837+3
Delta Mass:	2.915 ppm
XCorr:	30.4

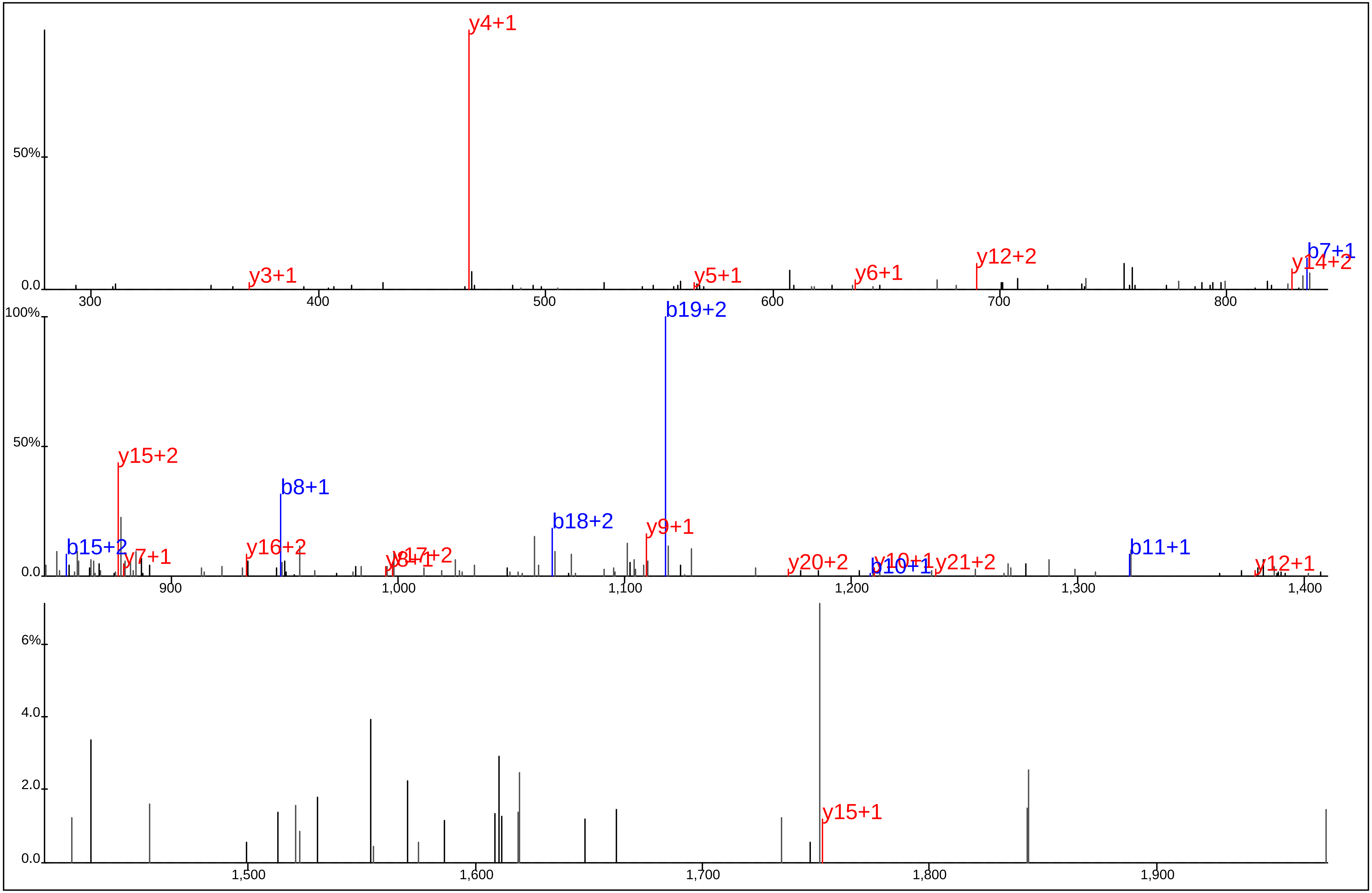

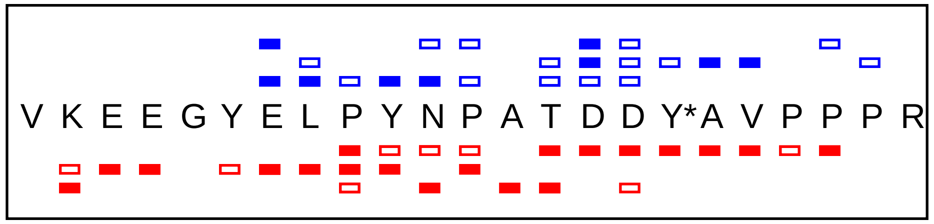

Protein Name: DOK1
gi Accession: 4503357
Precursor Mass: 900.7465+3
Delta Mass: 0.796 ppm
XCorr: 12.24

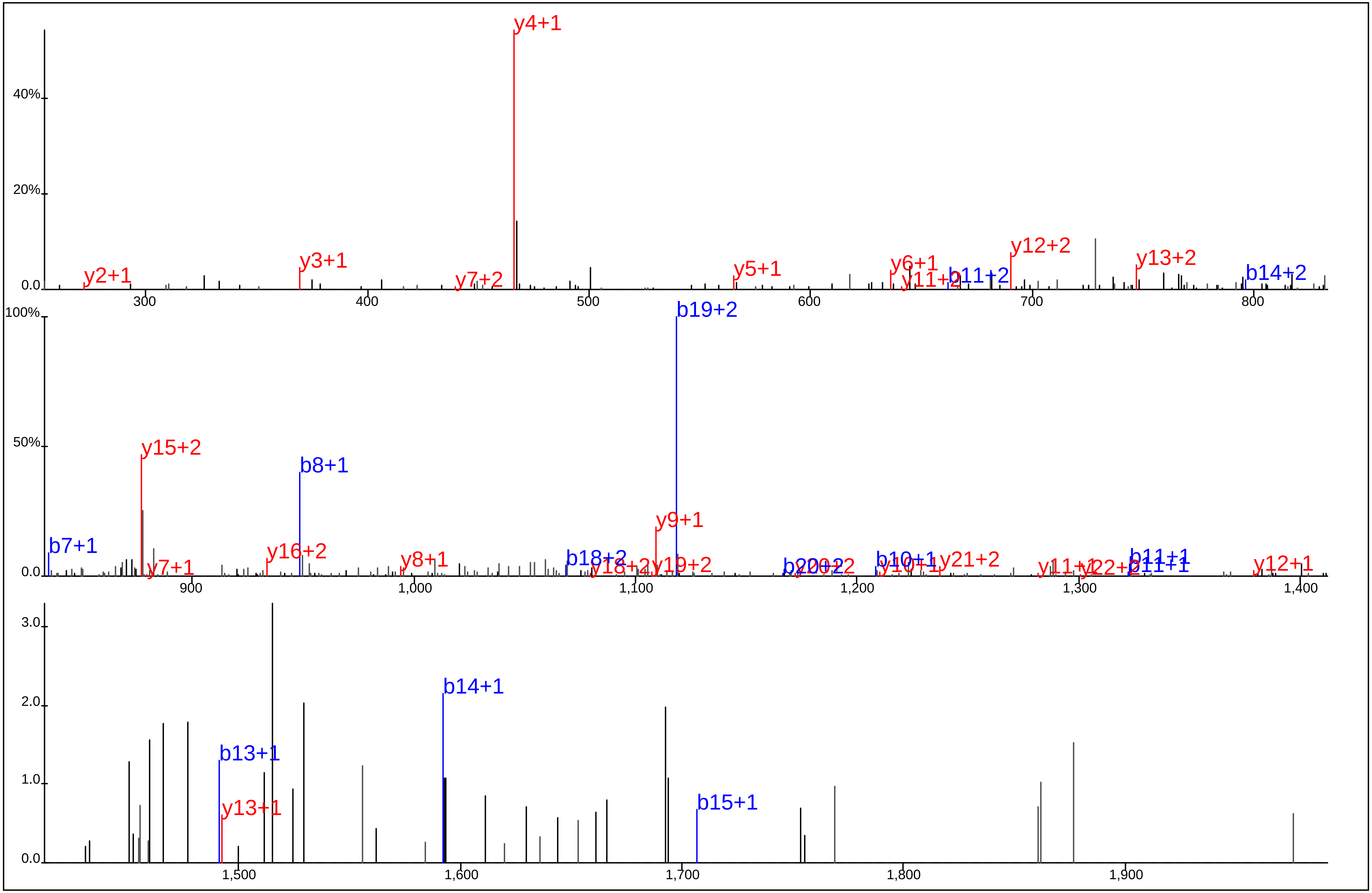

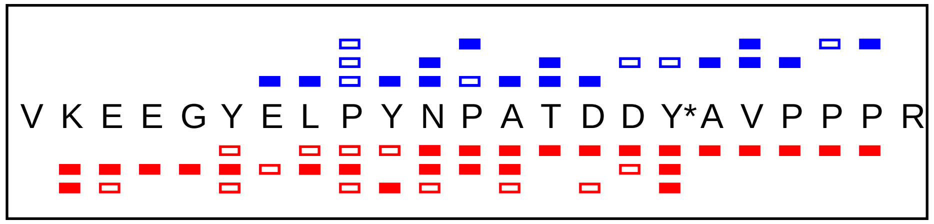

Protein Name: DOK1
gi Accession: 4503357
Precursor Mass: 900.7410+3
Delta Mass: 5.308 ppm
XCorr: 16.7

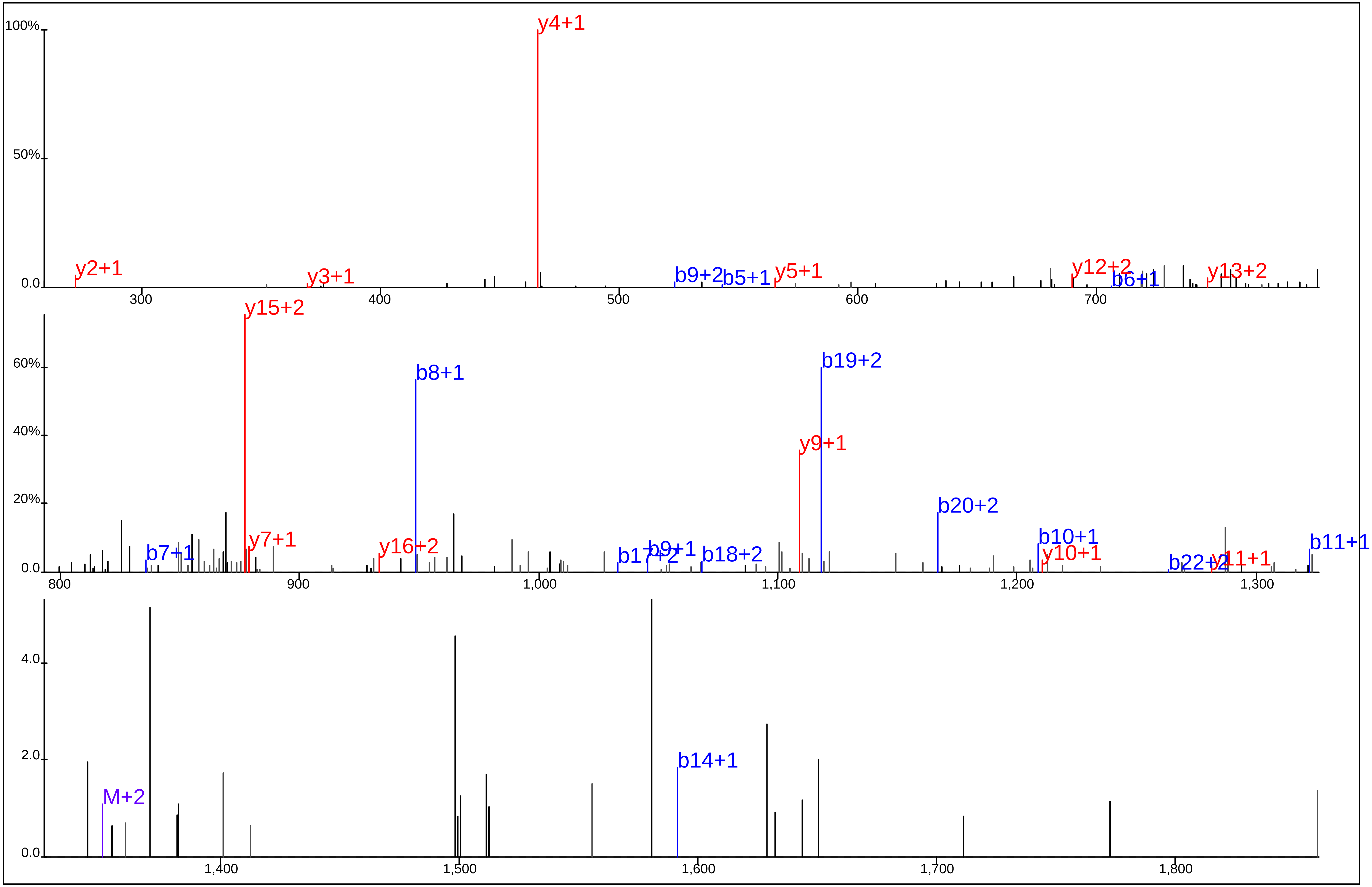

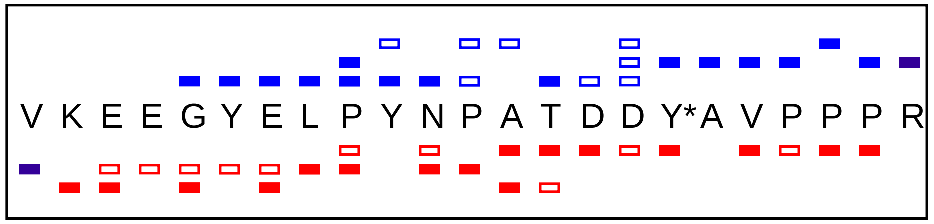

Protein Name:	DOK1
gi Accession:	4503357
Precursor Mass:	900.7521+3
Delta Mass:	6.965 ppm
XCorr:	17.44

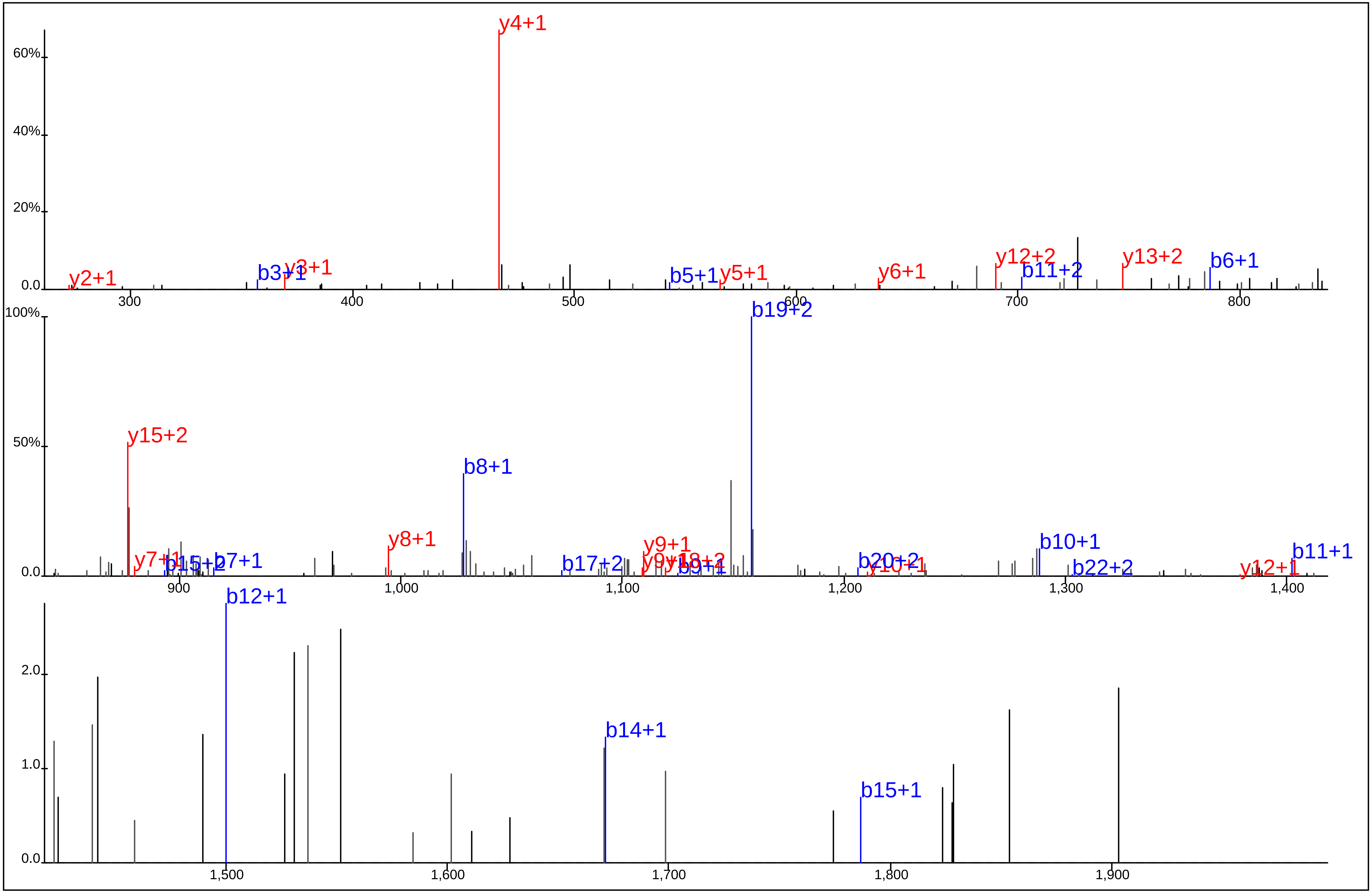

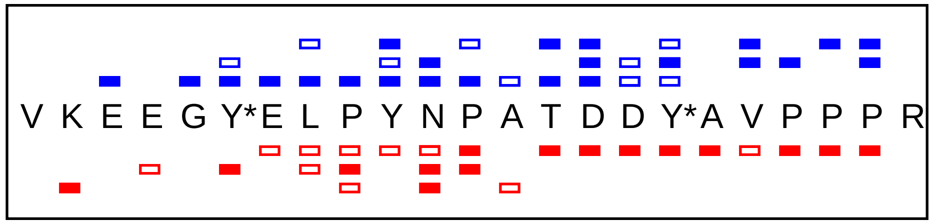

Protein Name: DOK1
gi Accession: 4503357
Precursor Mass: 927.4031+3
Delta Mass: 1.974 ppm
XCorr: 27.12

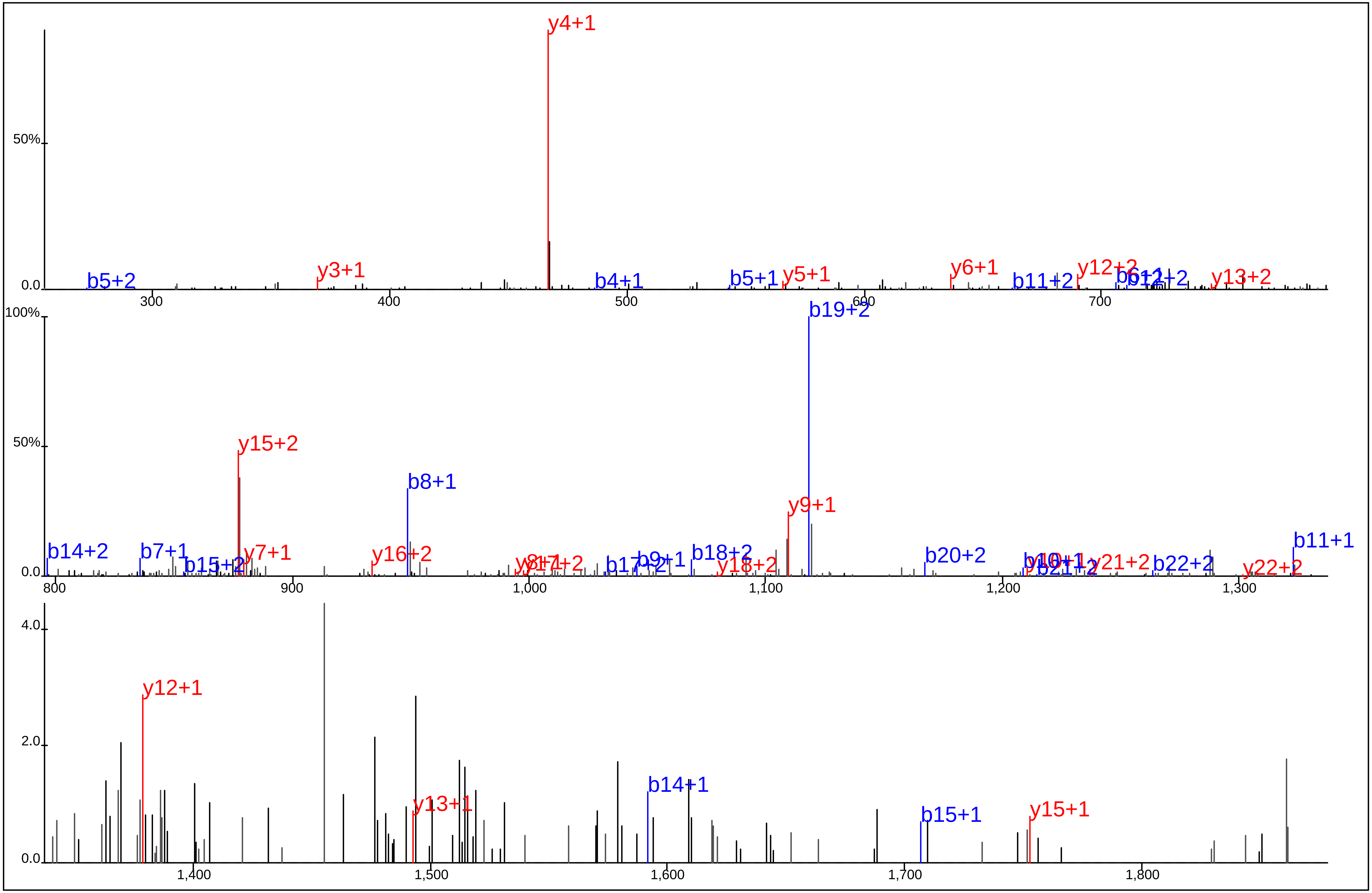

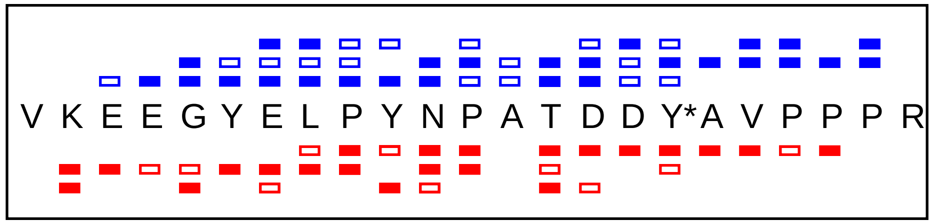

Protein Name: DOK1
gi Accession: 4503357
Precursor Mass: 900.7444+3
Delta Mass: 1.512 ppm
XCorr: 31.74

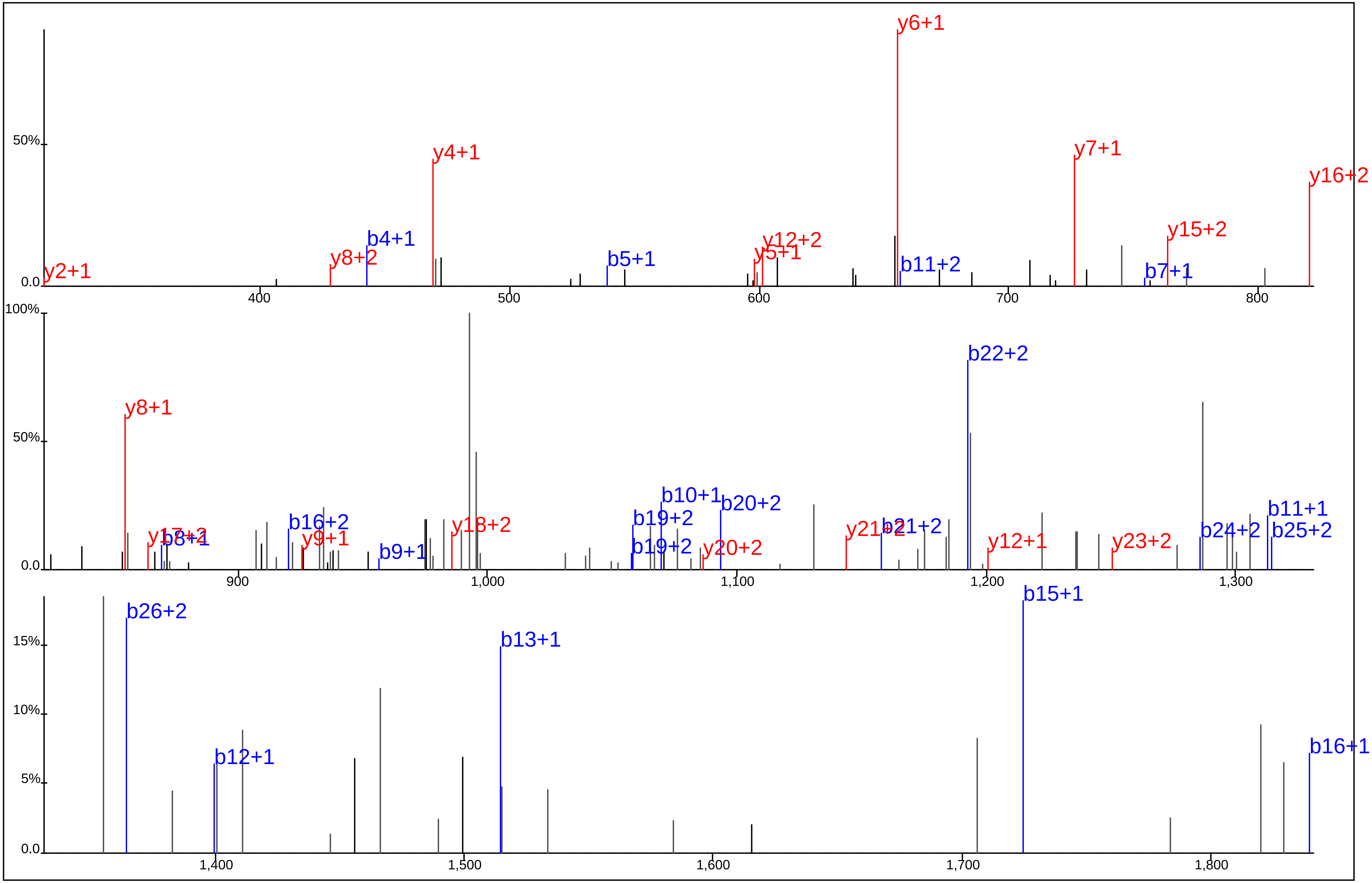

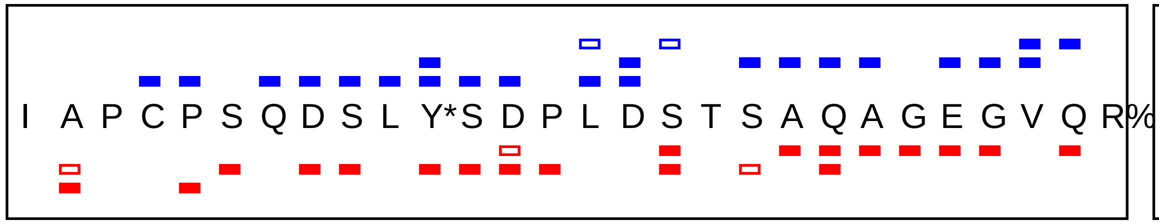

Protein Name:	DOK1
gi Accession:	4503357
Precursor Mass:	1,013.7812+3
Delta Mass:	0.443 ppm
XCorr:	46.63

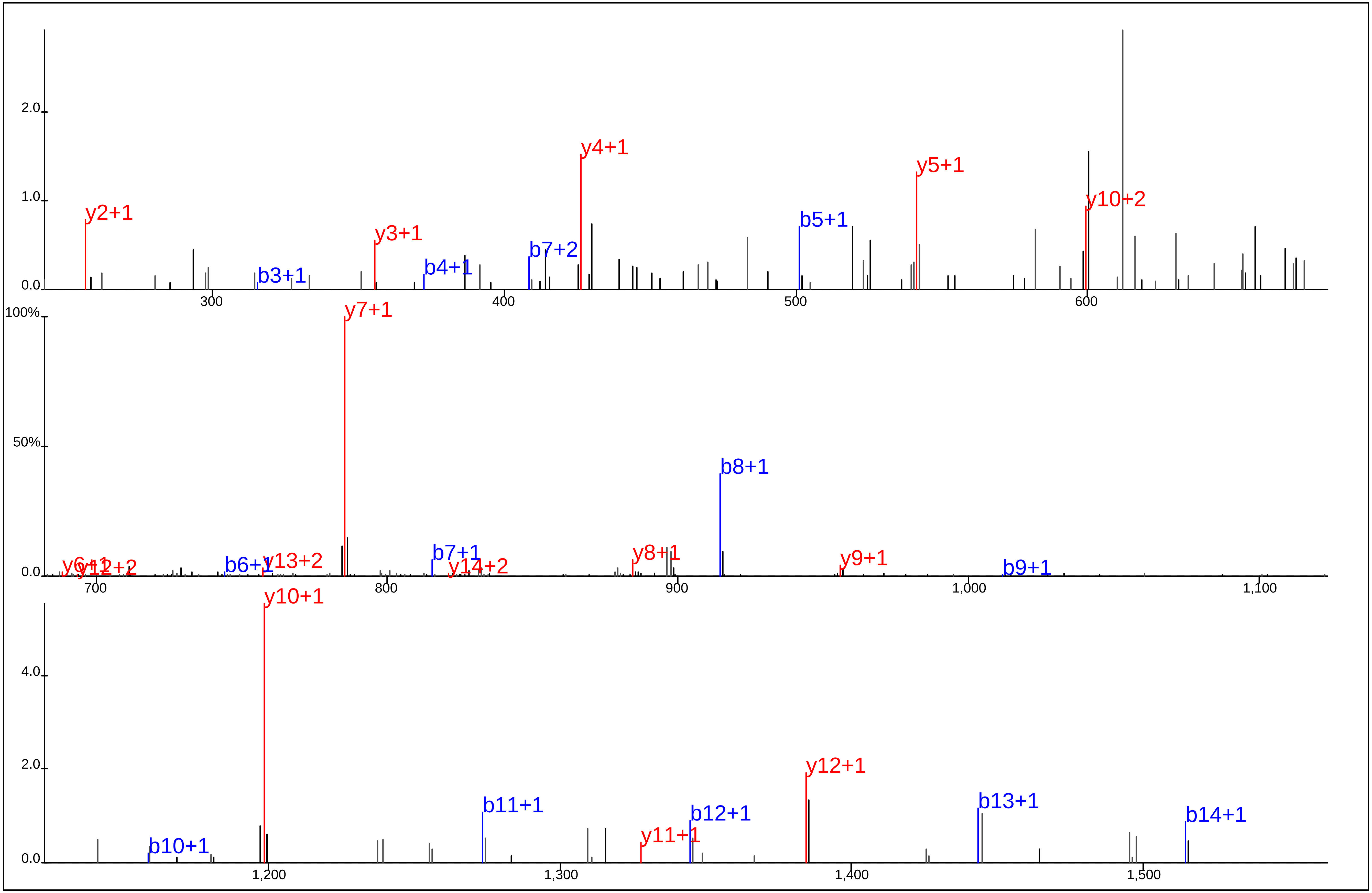

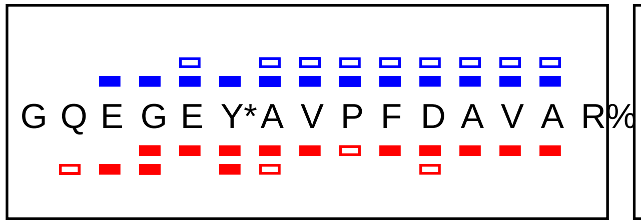

Protein Name: DOK2
gi Accession: 3043919
Precursor Mass: 849.8810+2
Delta Mass: 8.805 ppm
XCorr: 56.52

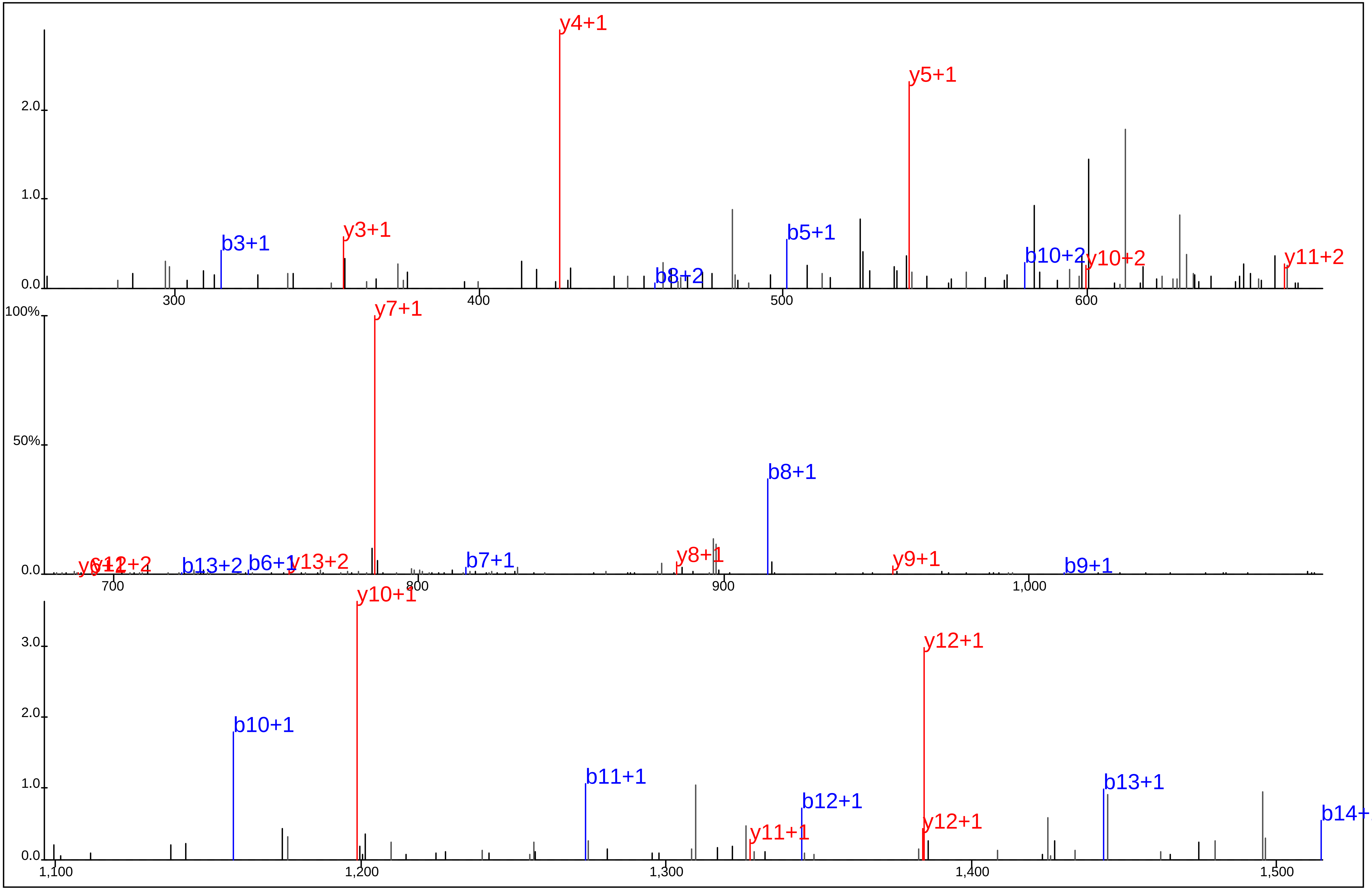

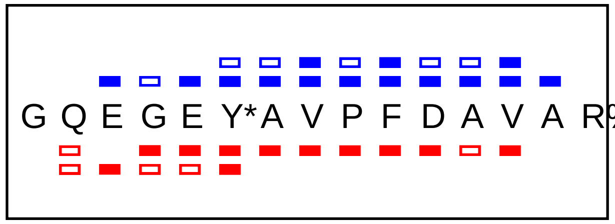

Protein Name:	DOK2
gi Accession:	3043919
Precursor Mass:	849.8753+2
Delta Mass:	2.047 ppm
XCorr:	61.46

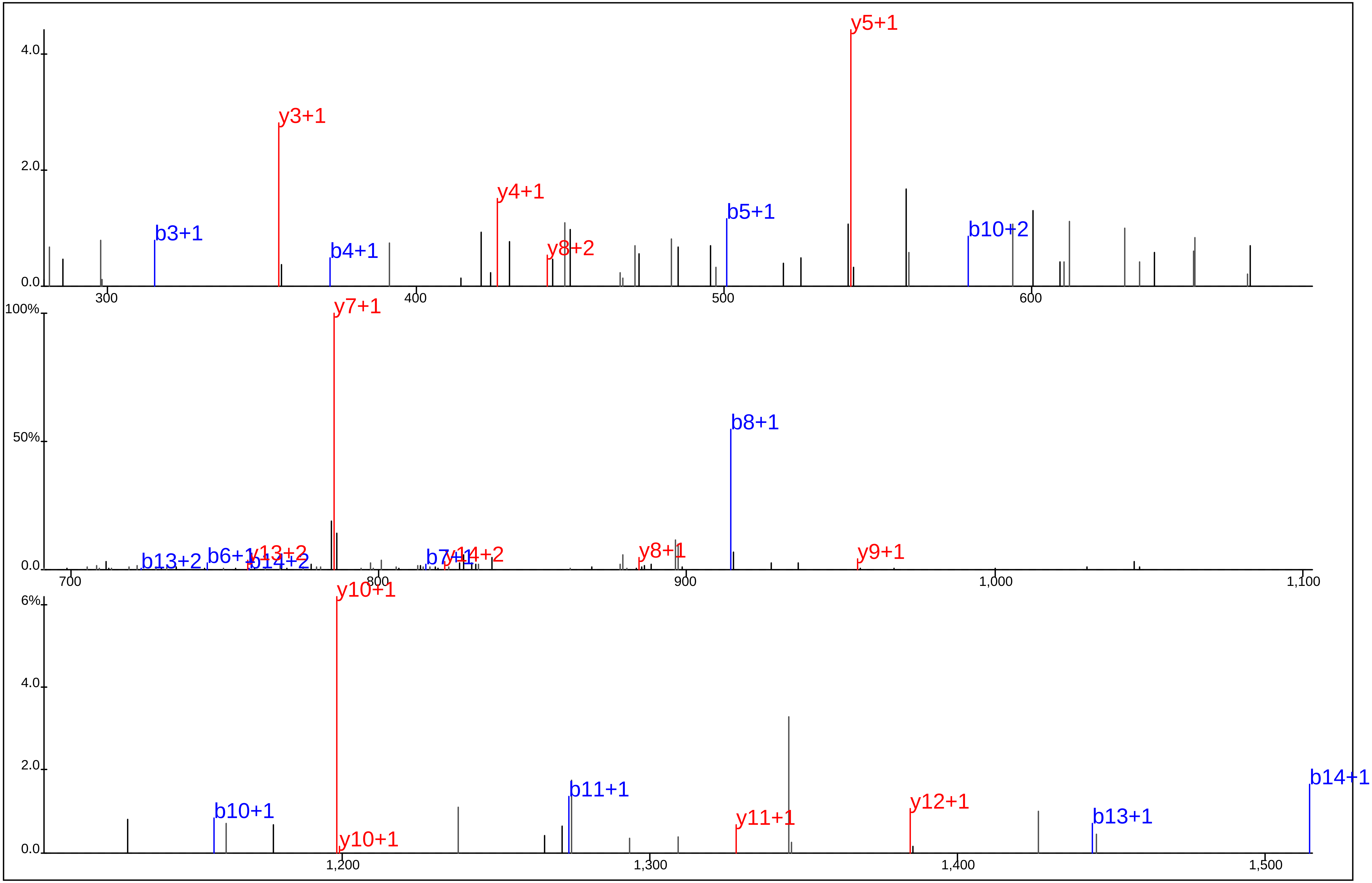

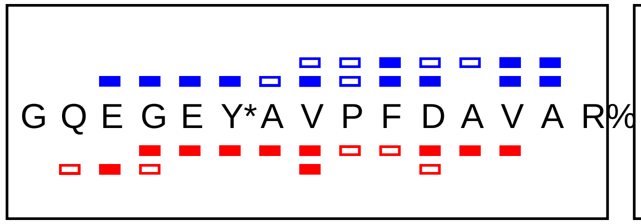

Protein Name: DOK2
gi Accession: 3043919
Precursor Mass: 849.8783+2
Delta Mass: 5.574 ppm
XCorr: 49.23

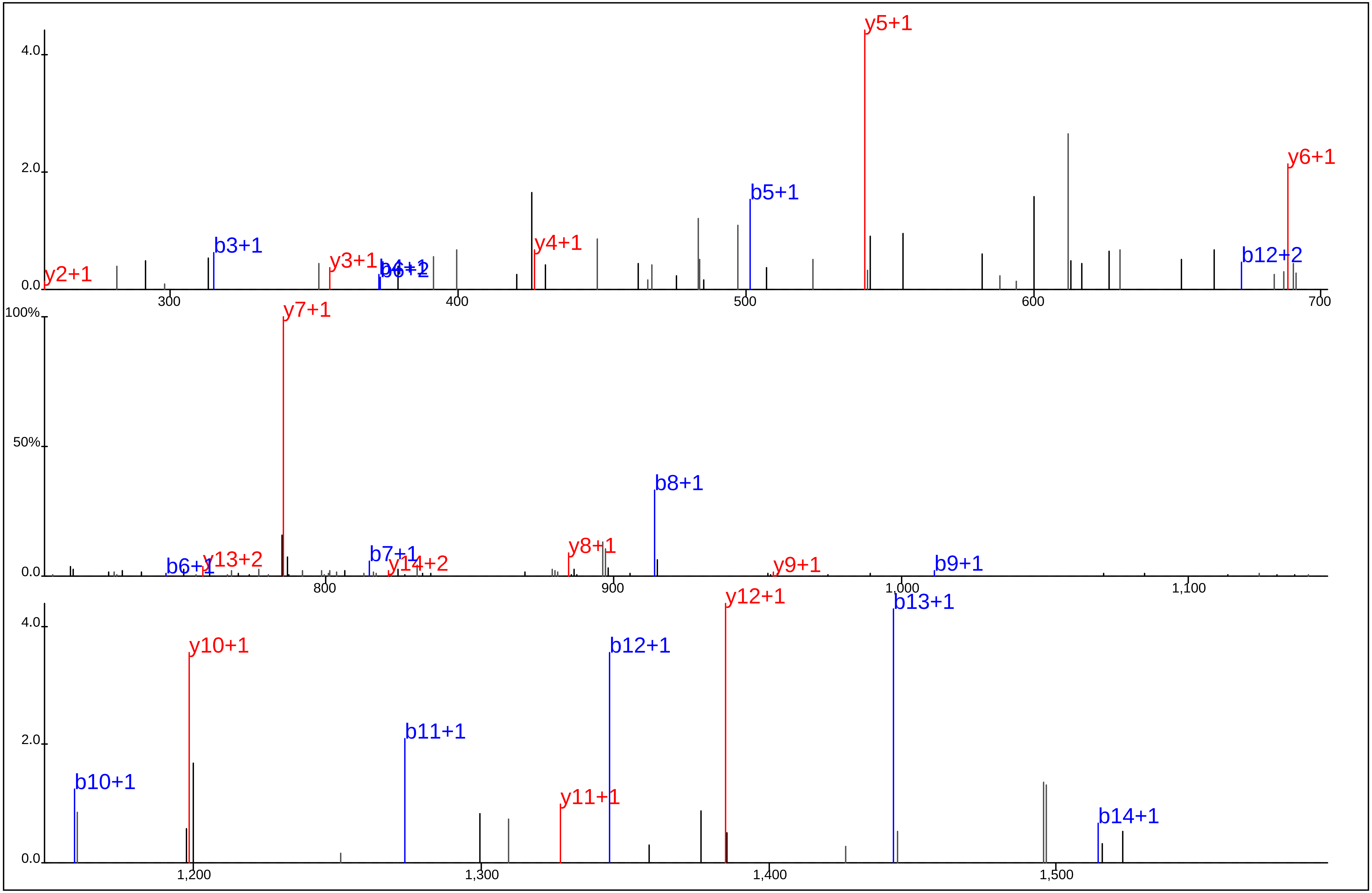

G Q E G E Y*A V P F D A V A R%

Protein Name: DOK2
gi Accession: 3043919
Precursor Mass: 849.8795+2
Delta Mass: 7.010 ppm
XCorr: 56.42

347 - DOK2_3441375.pdf

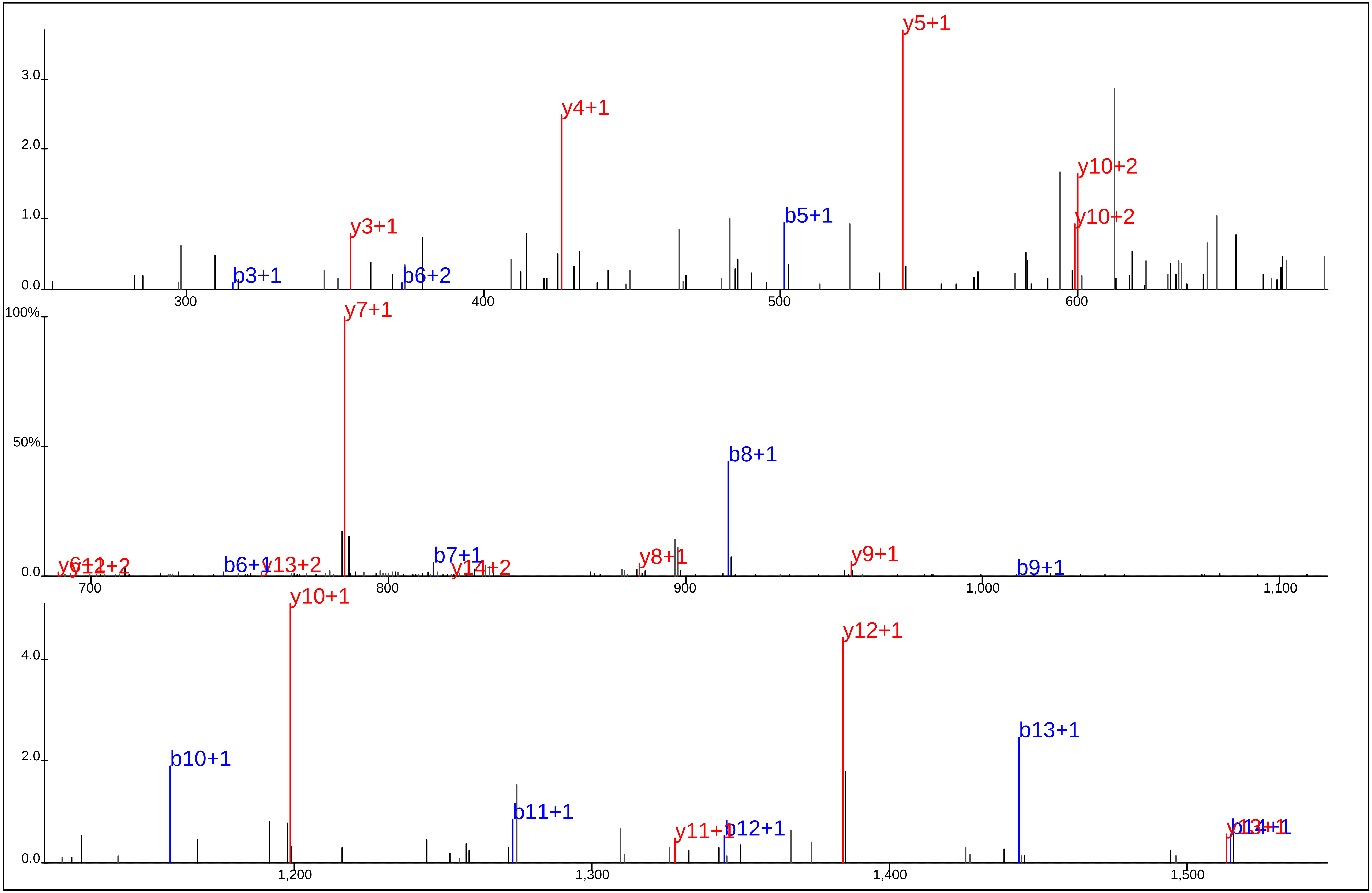

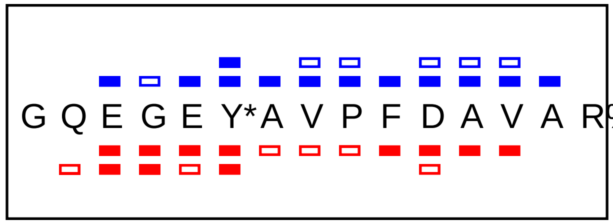

Protein Name:	DOK2
gi Accession:	3043919
Precursor Mass:	849.8755+2
Delta Mass:	2.265 ppm
XCorr:	57.09

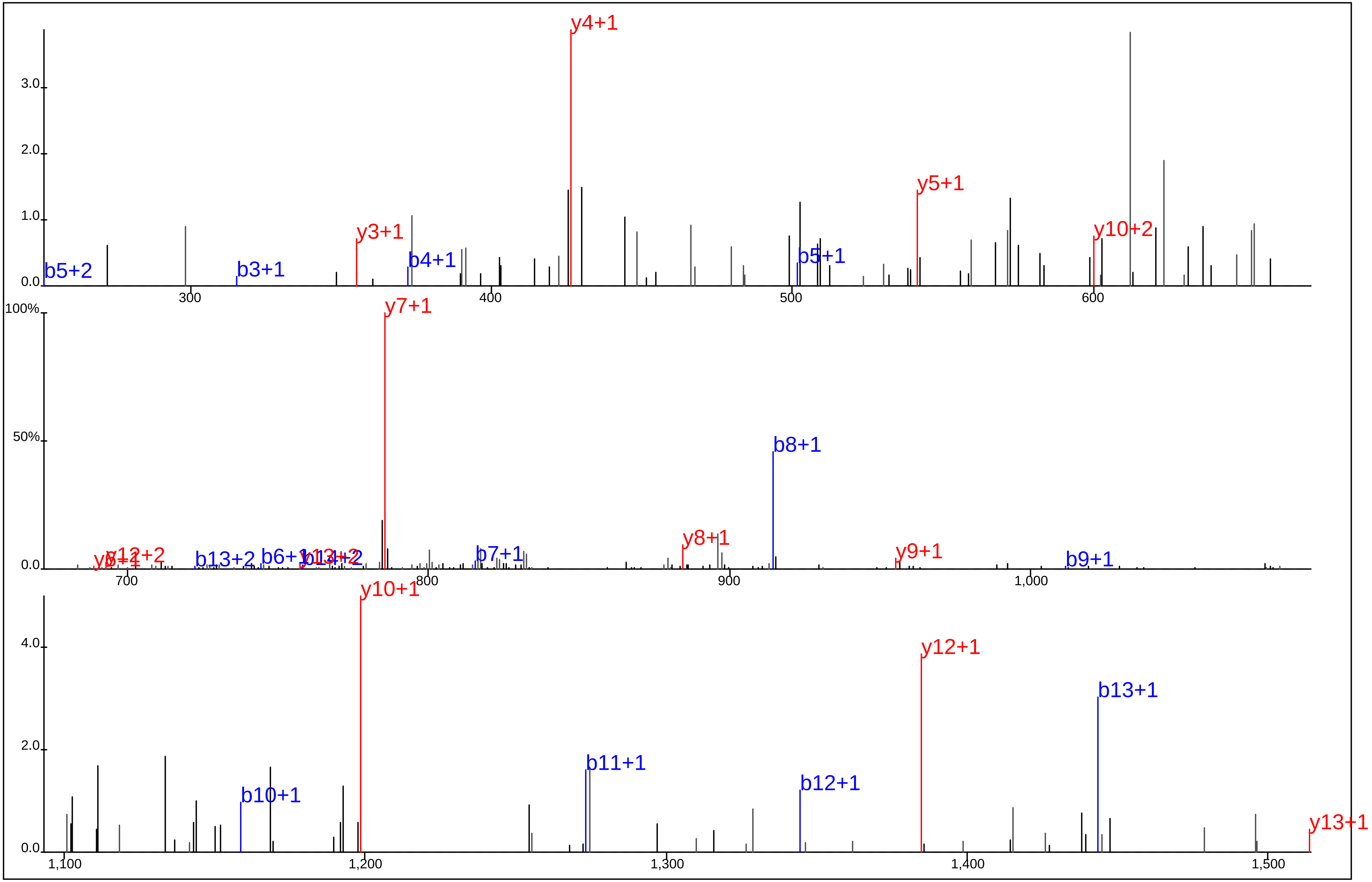

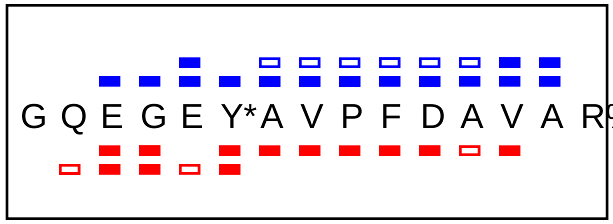

Protein Name: DOK2
gi Accession: 3043919
Precursor Mass: 849.8807+2
Delta Mass: 8.376 ppm
XCorr: 40.49

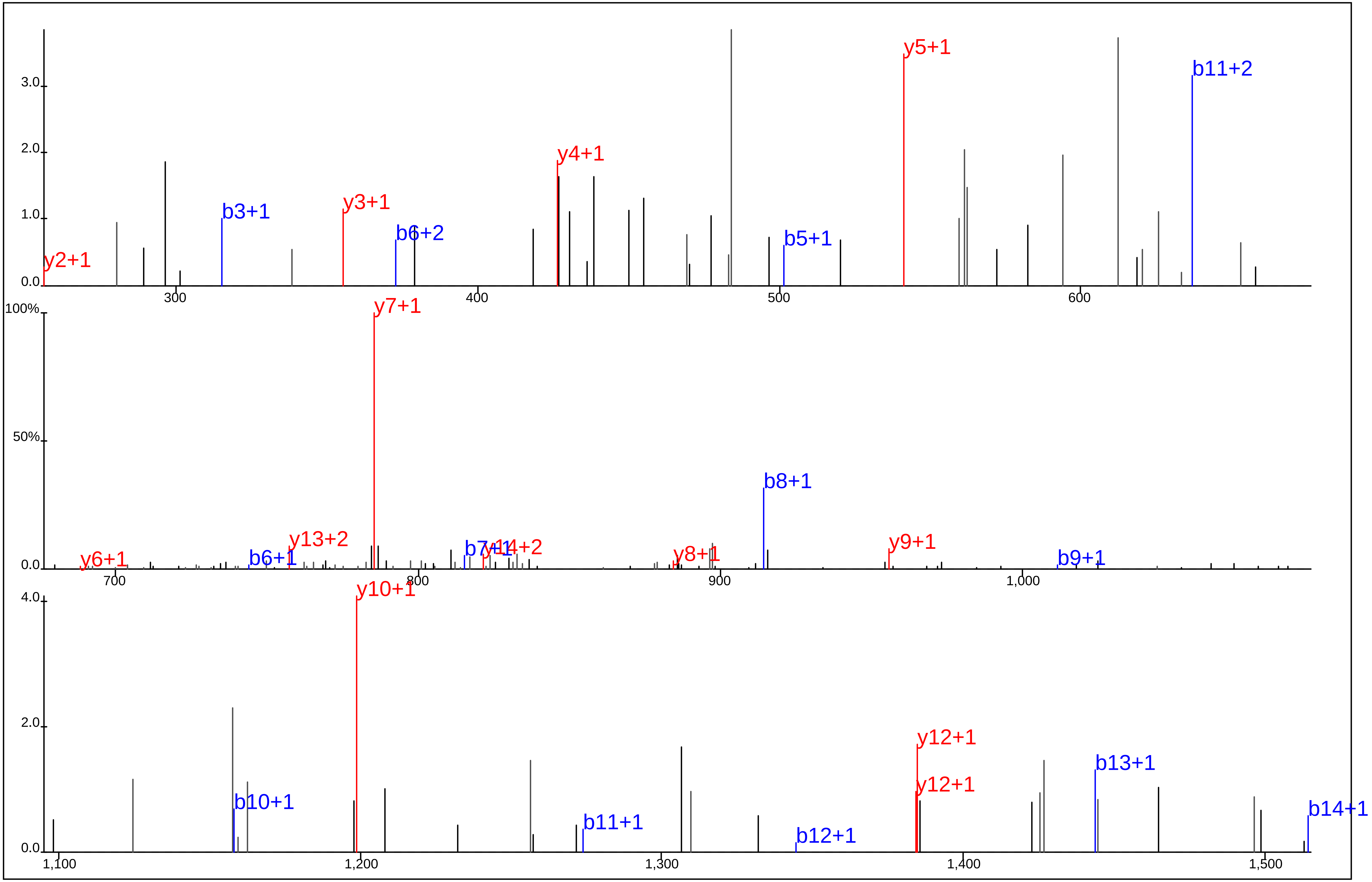

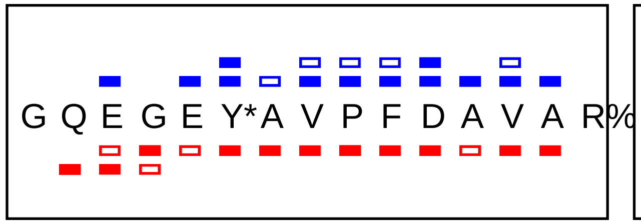

Protein Name: DOK2
gi Accession: 3043919
Precursor Mass: 849.8831+2
Delta Mass: 11.248 ppm
XCorr: 41.97

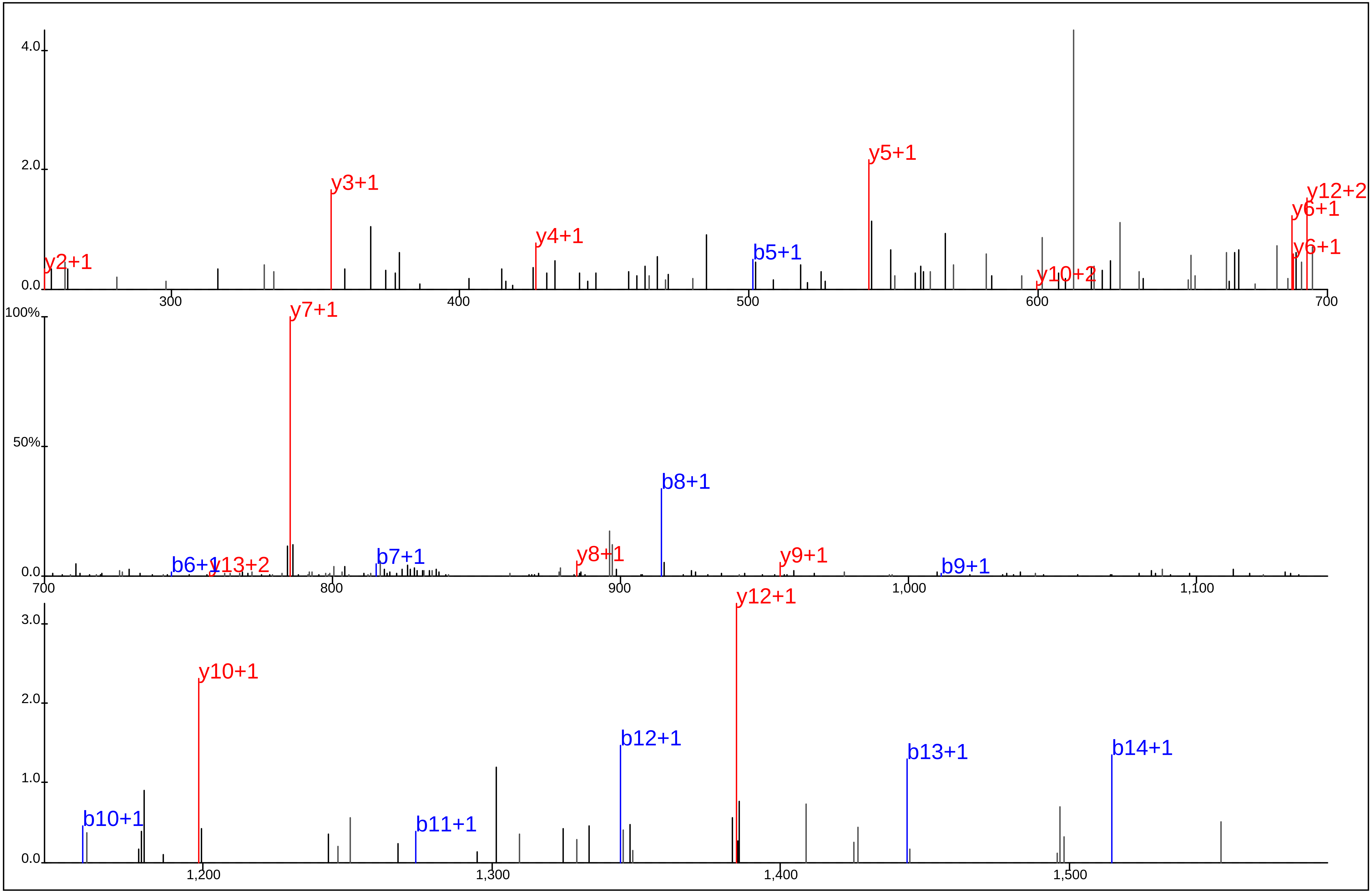

G Q E G E Y*A V P F D A V A R%

Protein Name: DOK2
gi Accession: 3043919
Precursor Mass: 849.8763+2
Delta Mass: 3.201 ppm
XCorr: 35.51

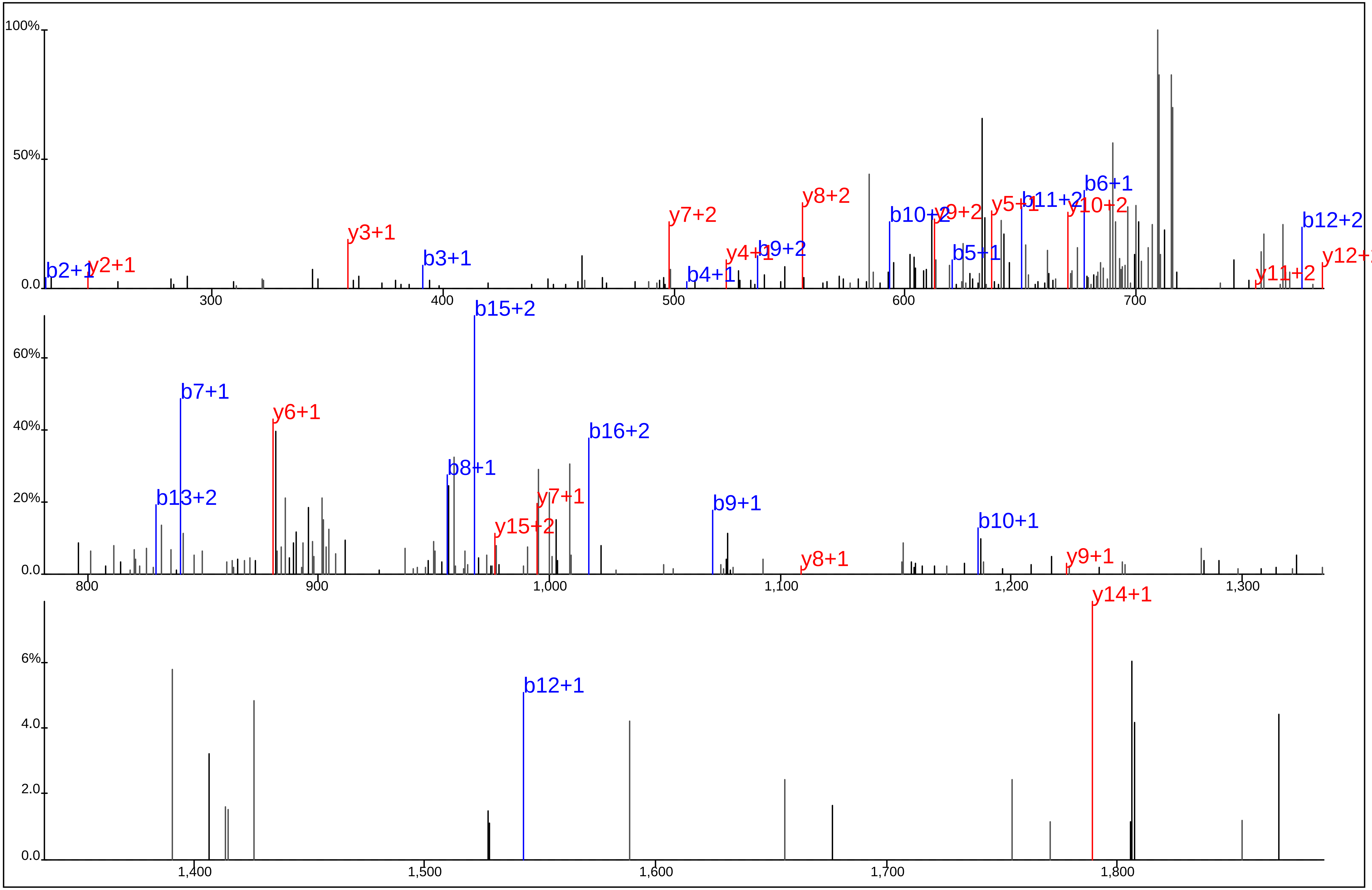

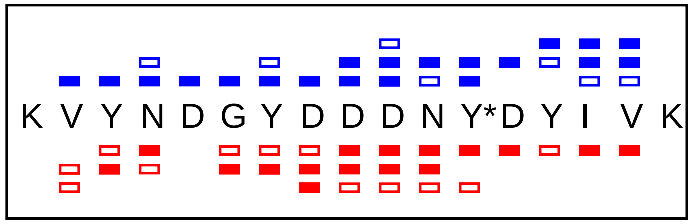

Protein Name: DYRK1A
gi Accession: 18765752
Precursor Mass: 726.9736+3
Delta Mass: 7.269 ppm
XCorr: 25.95

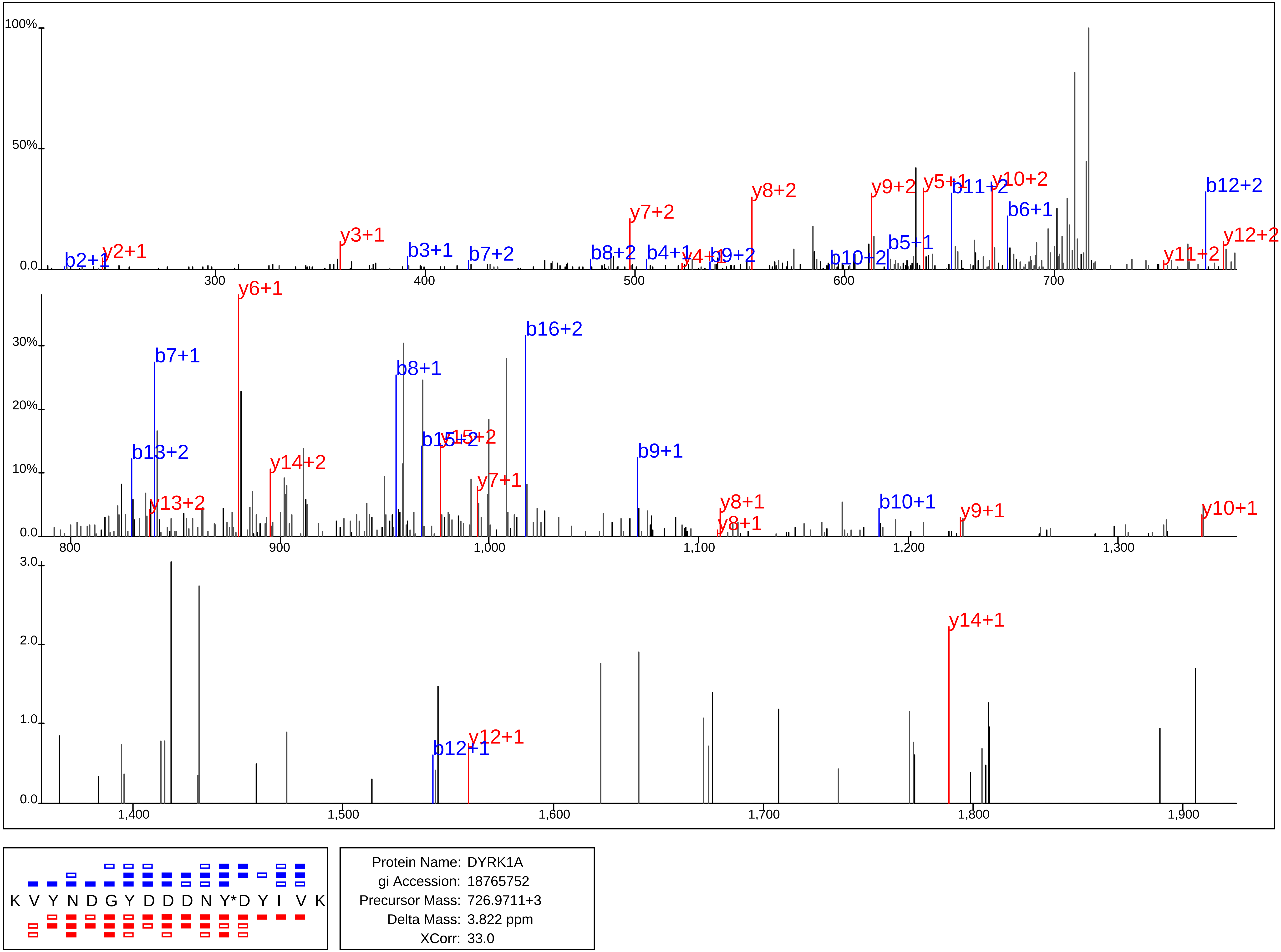

Protein Name: DYRK1A
gi Accession: 18765752
Precursor Mass: 726.9711+3
Delta Mass: 3.822 ppm
XCorr: 33.0

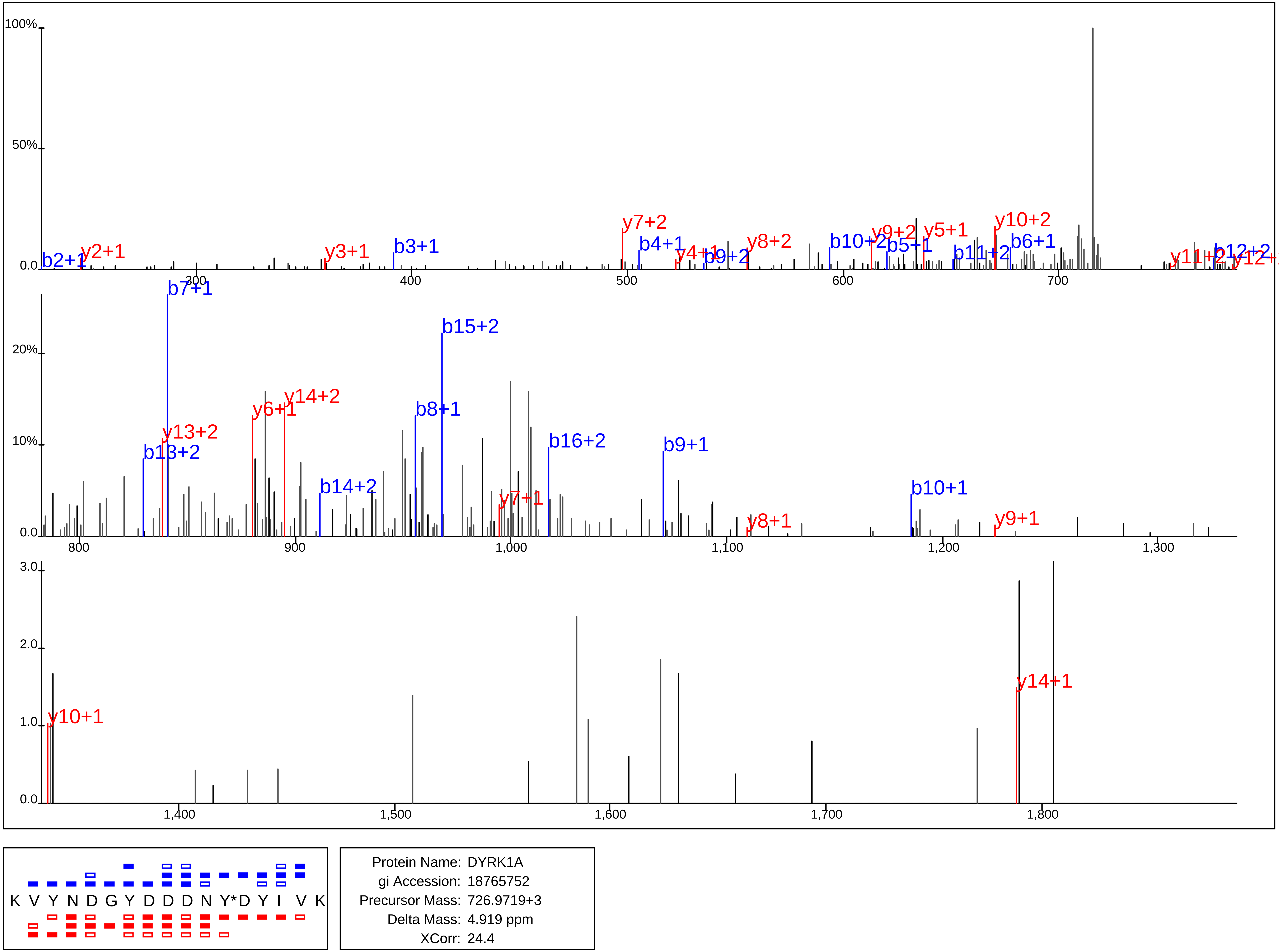

Protein Name: DYRK1A
gi Accession: 18765752
Precursor Mass: 726.9719+3
Delta Mass: 4.919 ppm
XCorr: 24.4

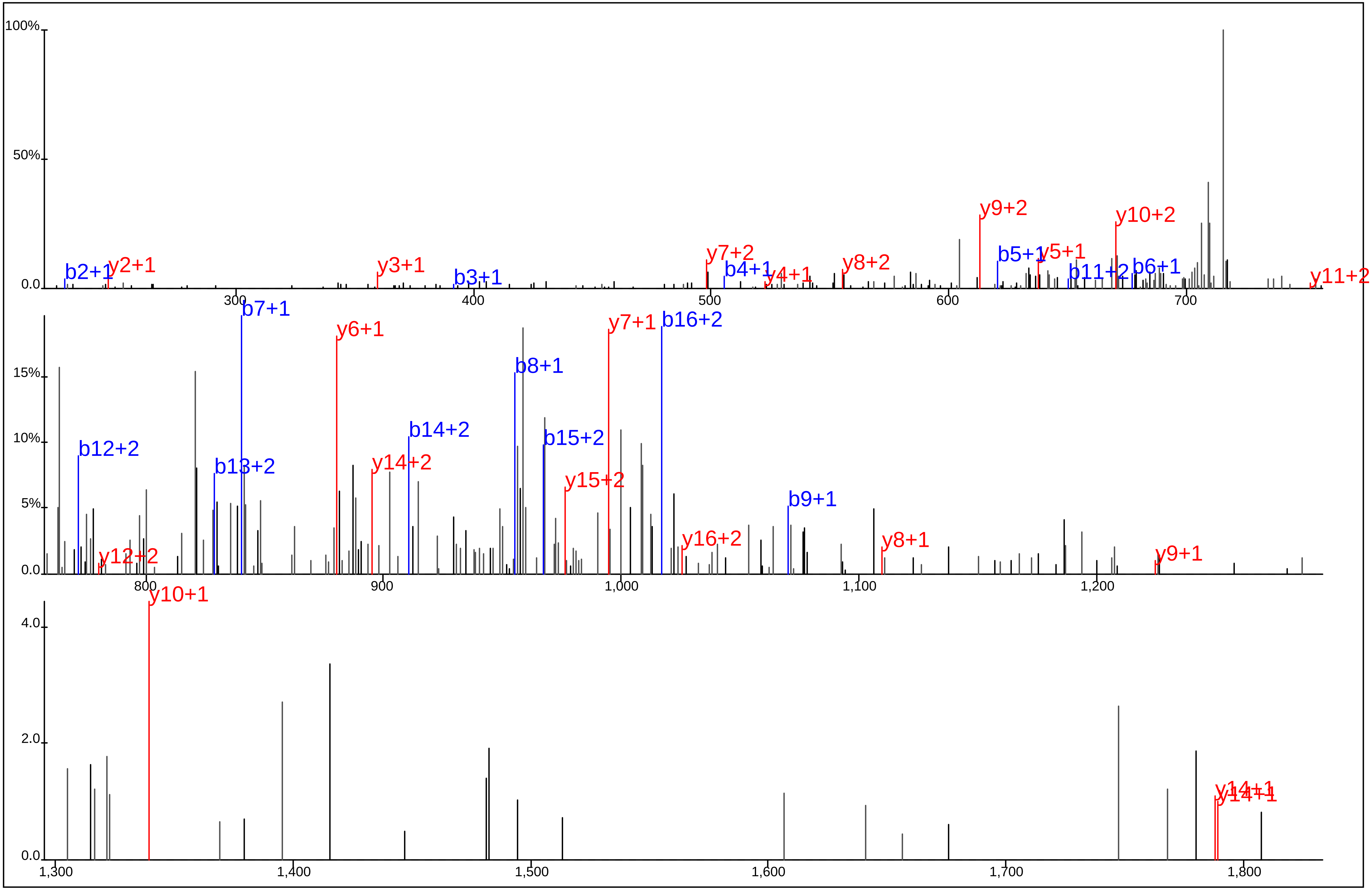

K V Y N D G Y D D D N Y*D Y I V K

Protein Name: DYRK1A
gi Accession: 18765752
Precursor Mass: 726.9721+3
Delta Mass: 5.254 ppm
XCorr: 23.19

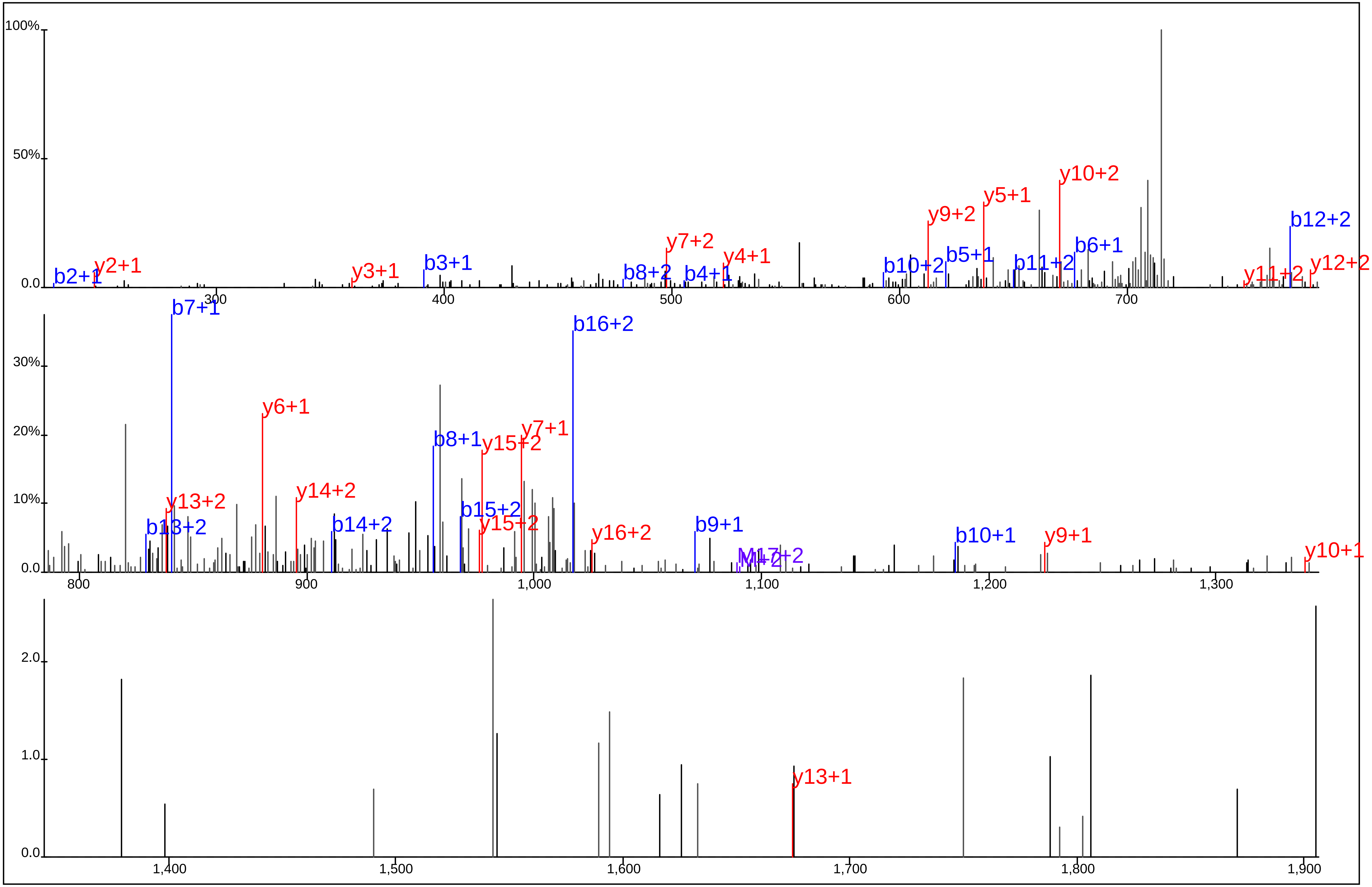

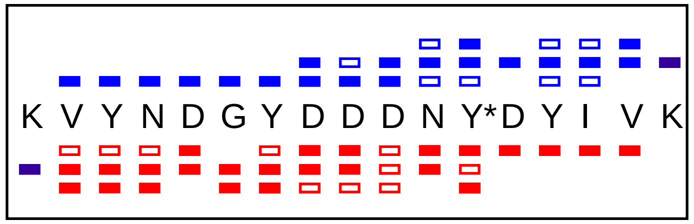

Protein Name: DYRK1A
gi Accession: 18765752
Precursor Mass: 726.9728+3
Delta Mass: 6.176 ppm
XCorr: 45.47

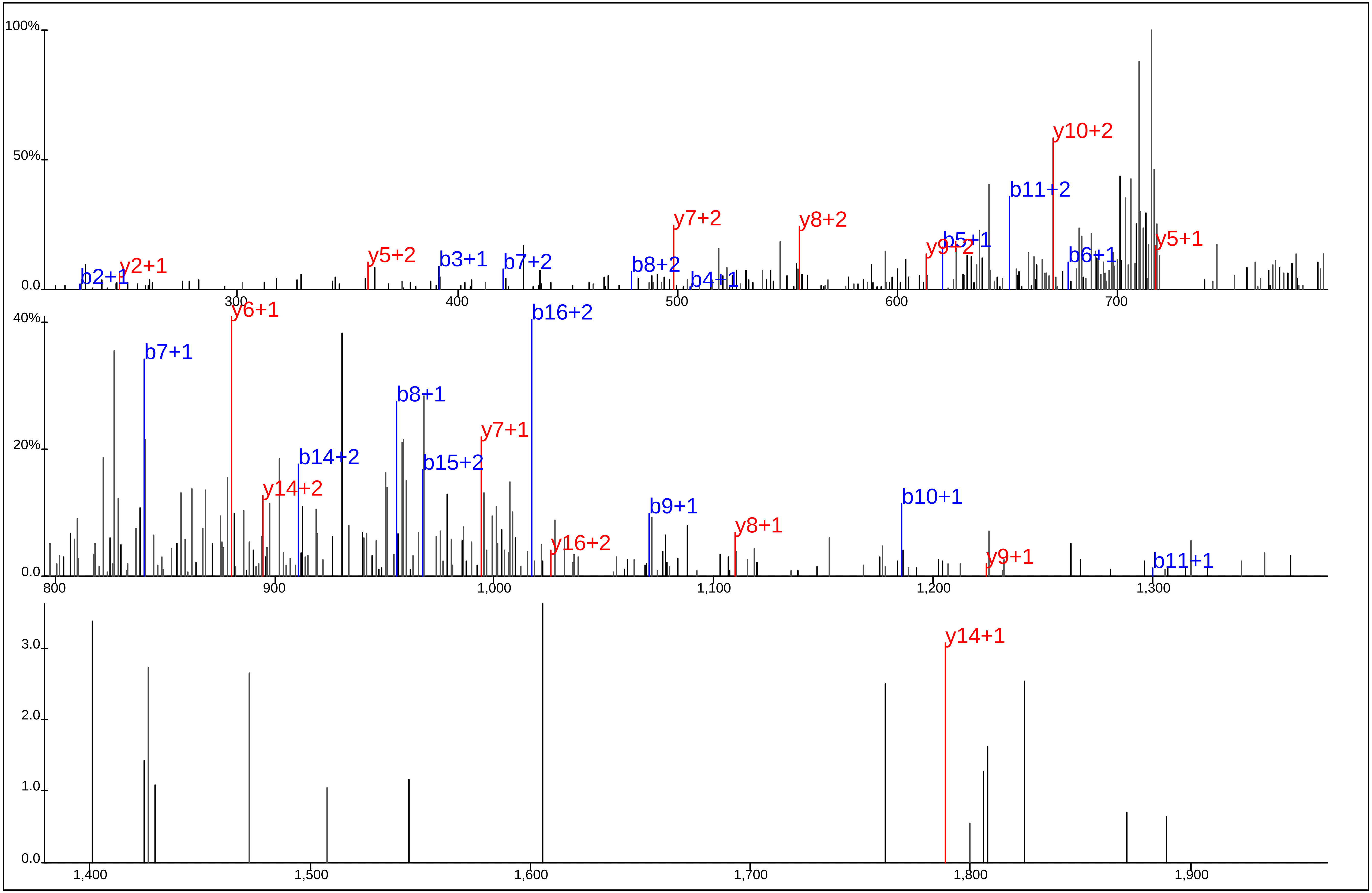

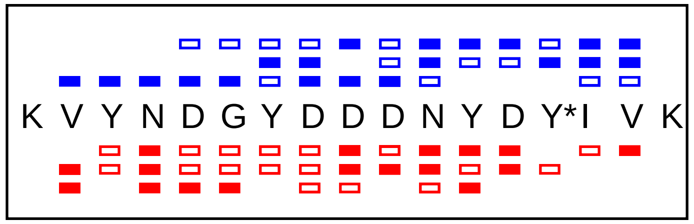

Protein Name:	DYRK1A
gi Accession:	18765752
Precursor Mass:	726.9701+3
Delta Mass:	2.482 ppm
XCorr:	17.11

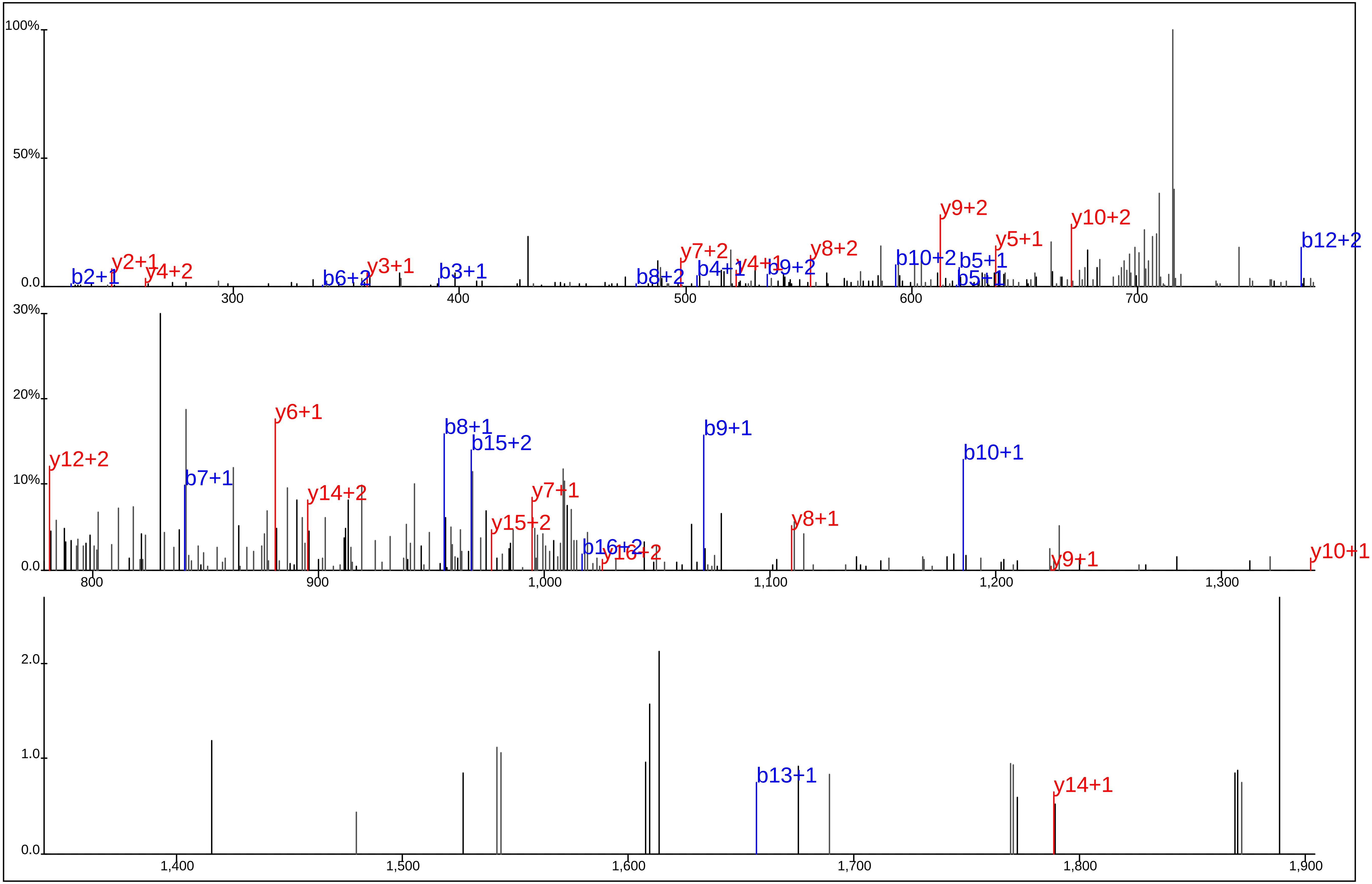

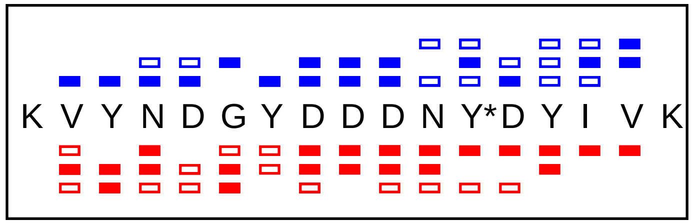

Protein Name:	DYRK1A
gi Accession:	18765752
Precursor Mass:	726.9711+3
Delta Mass:	3.822 ppm
XCorr:	22.69

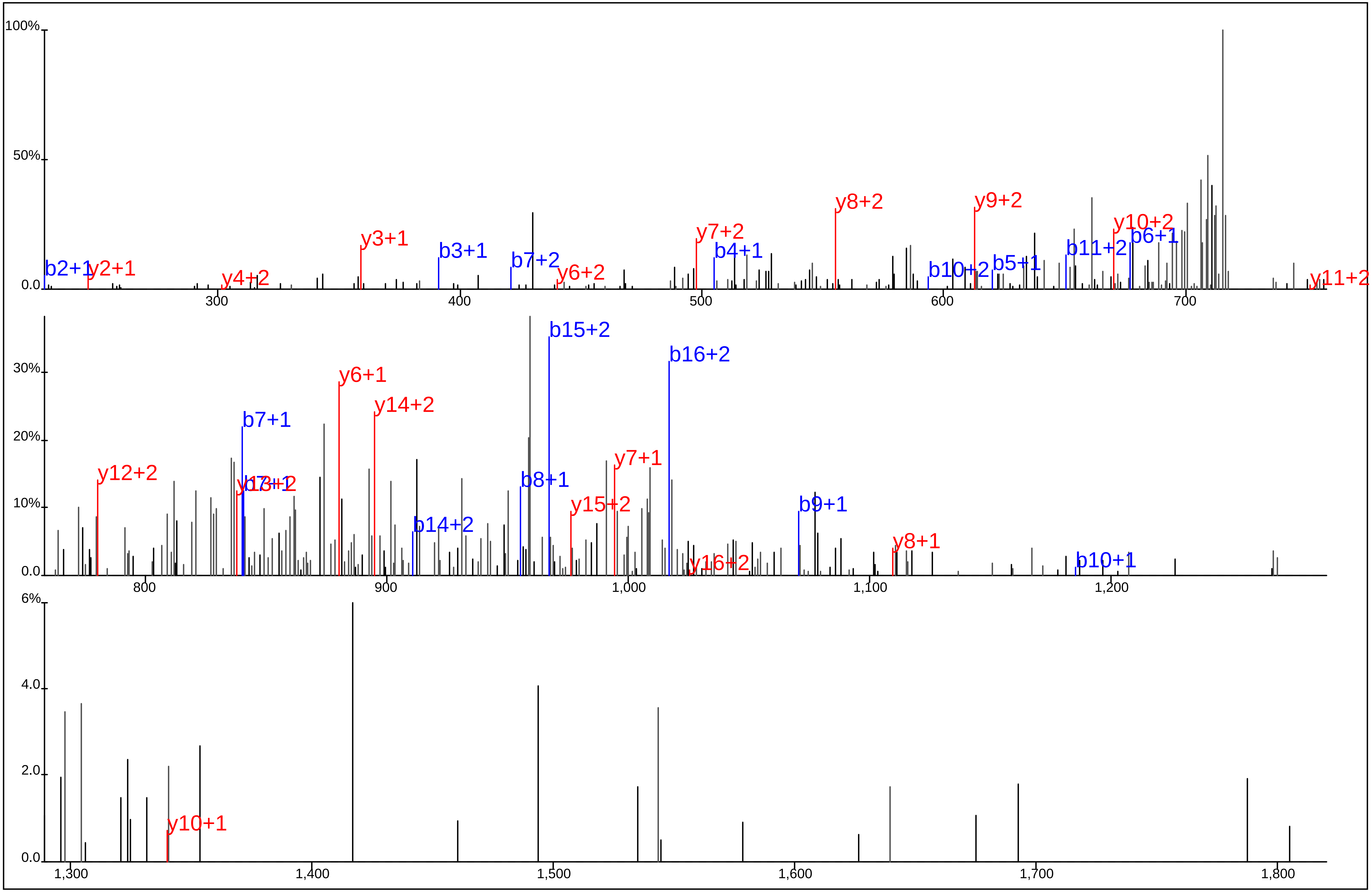

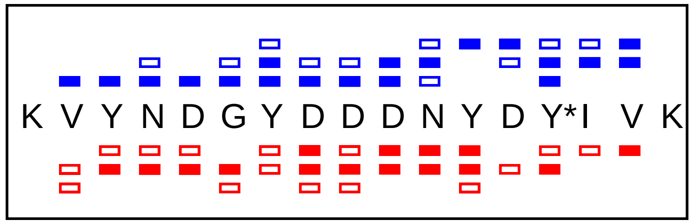

Protein Name: DYRK1A
gi Accession: 18765752
Precursor Mass: 726.9680+3
Delta Mass: 0.460 ppm
XCorr: 21.61

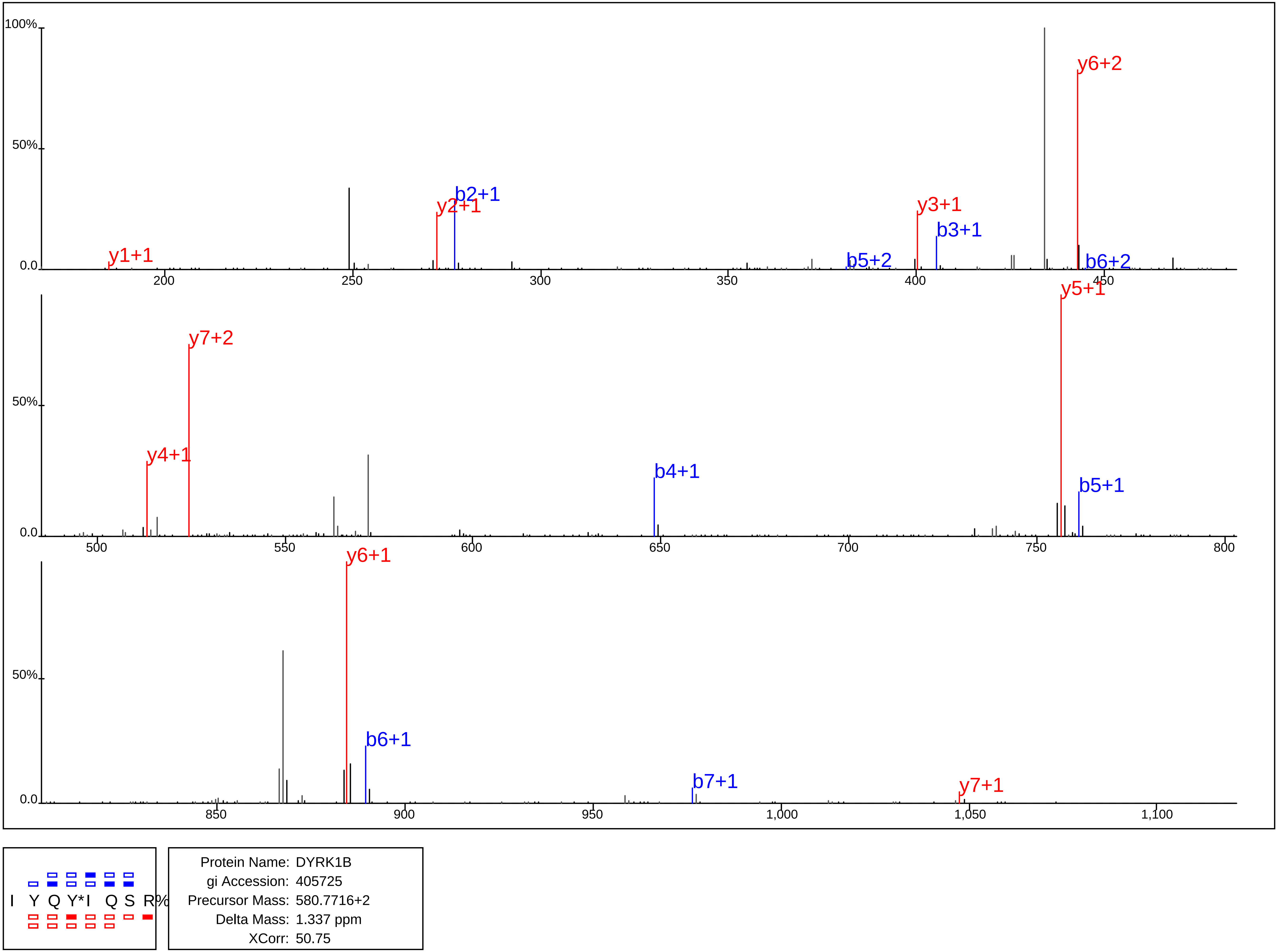

Protein Name:	DYRK1B
gi Accession:	405725
Precursor Mass:	580.7716+2
Delta Mass:	1.337 ppm
XCorr:	50.75

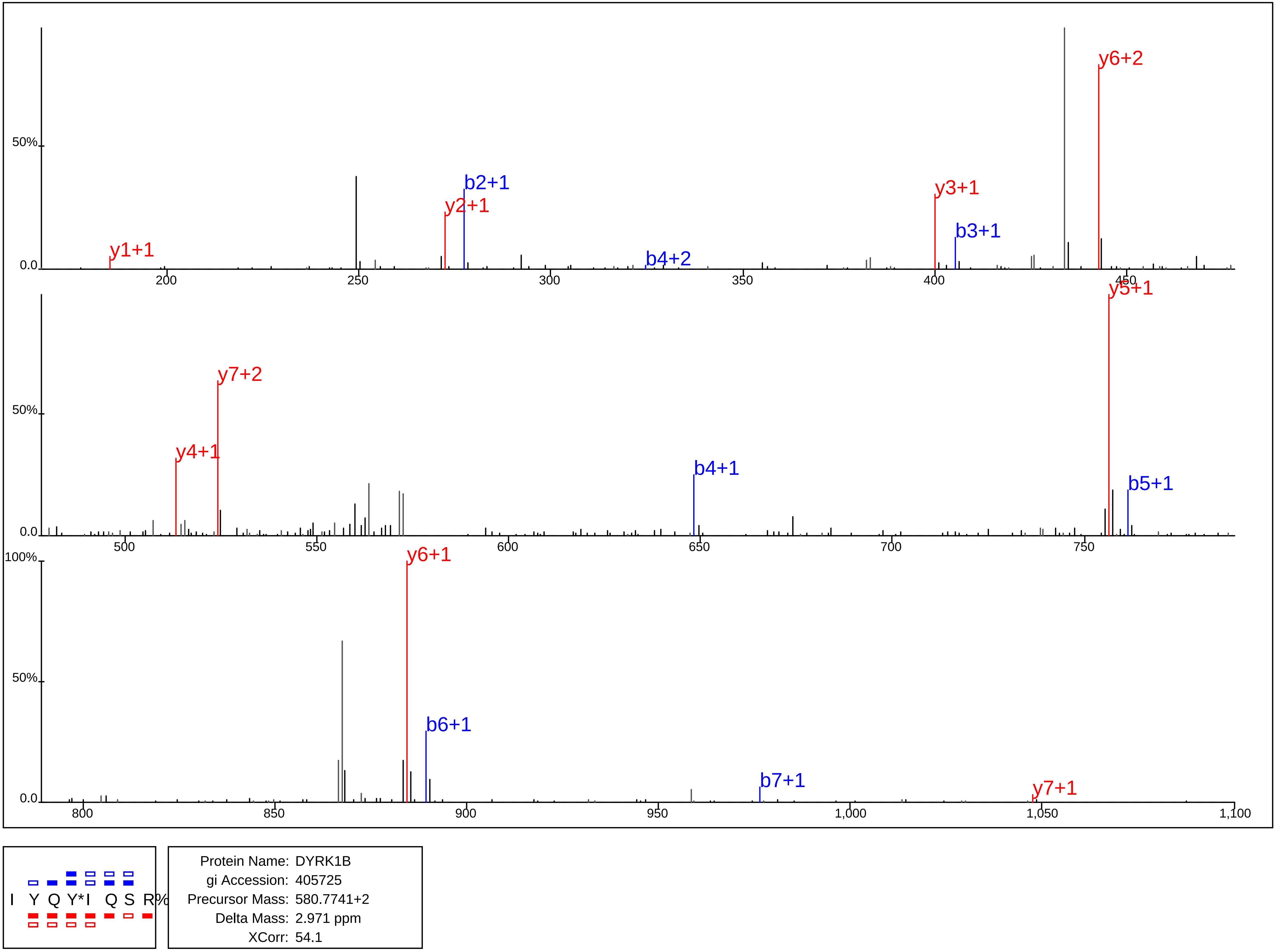

Protein Name: DYRK1B
gi Accession: 405725
Precursor Mass: 580.7741+2
Delta Mass: 2.971 ppm
XCorr: 54.1

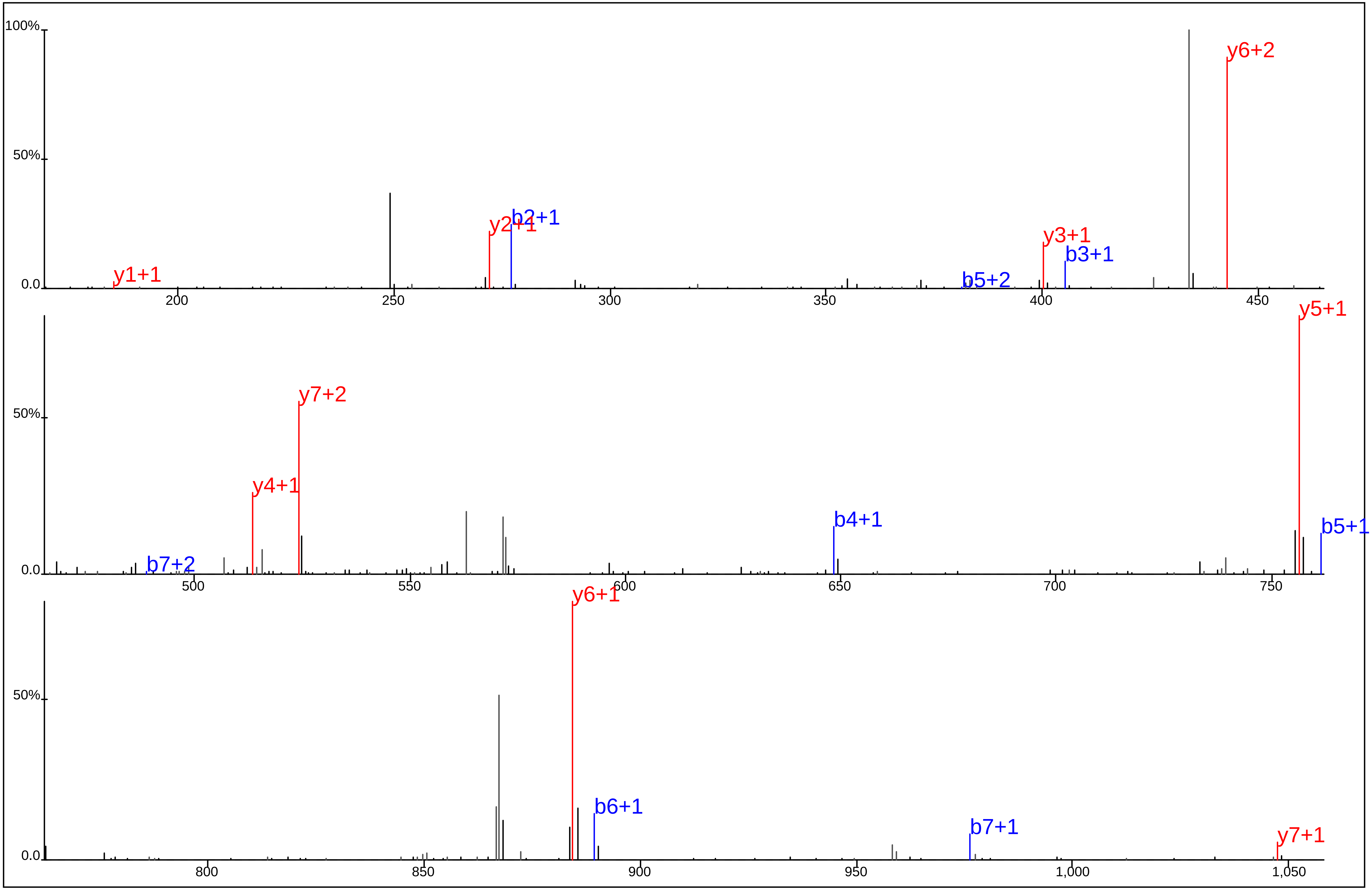

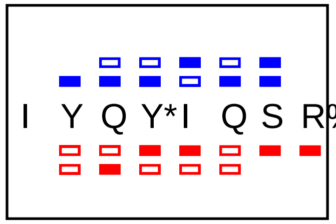

Protein Name: DYRK1B
gi Accession: 405725
Precursor Mass: 580.7729+2
Delta Mass: 0.972 ppm
XCorr: 57.9

362 - DYRK1B_3439034.pdf

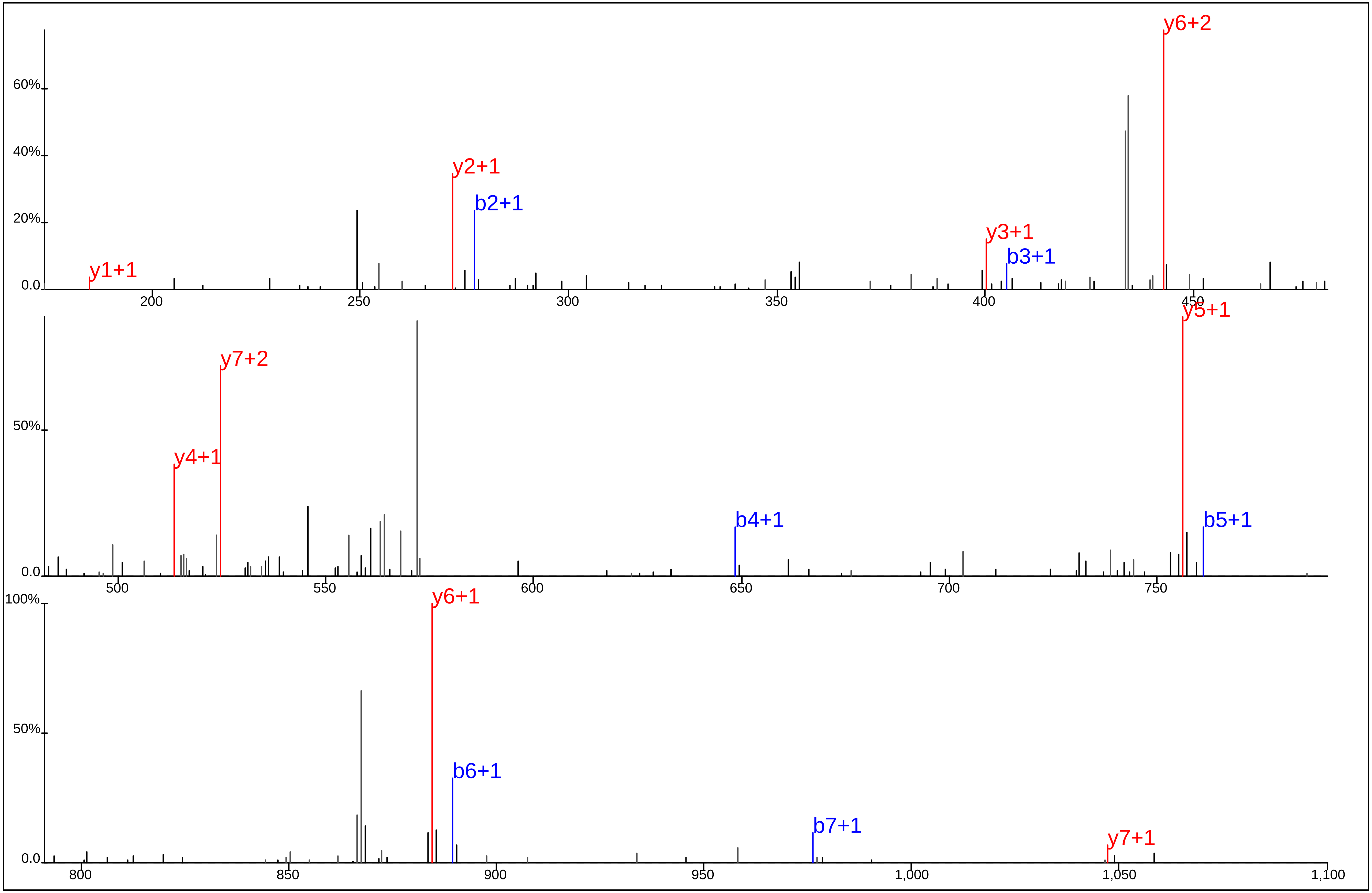

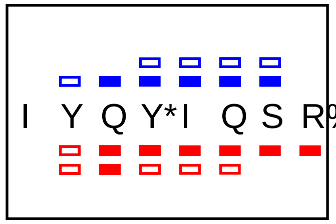

Protein Name:	DYRK1B
gi Accession:	405725
Precursor Mass:	580.7732+2
Delta Mass:	1.394 ppm
XCorr:	44.69

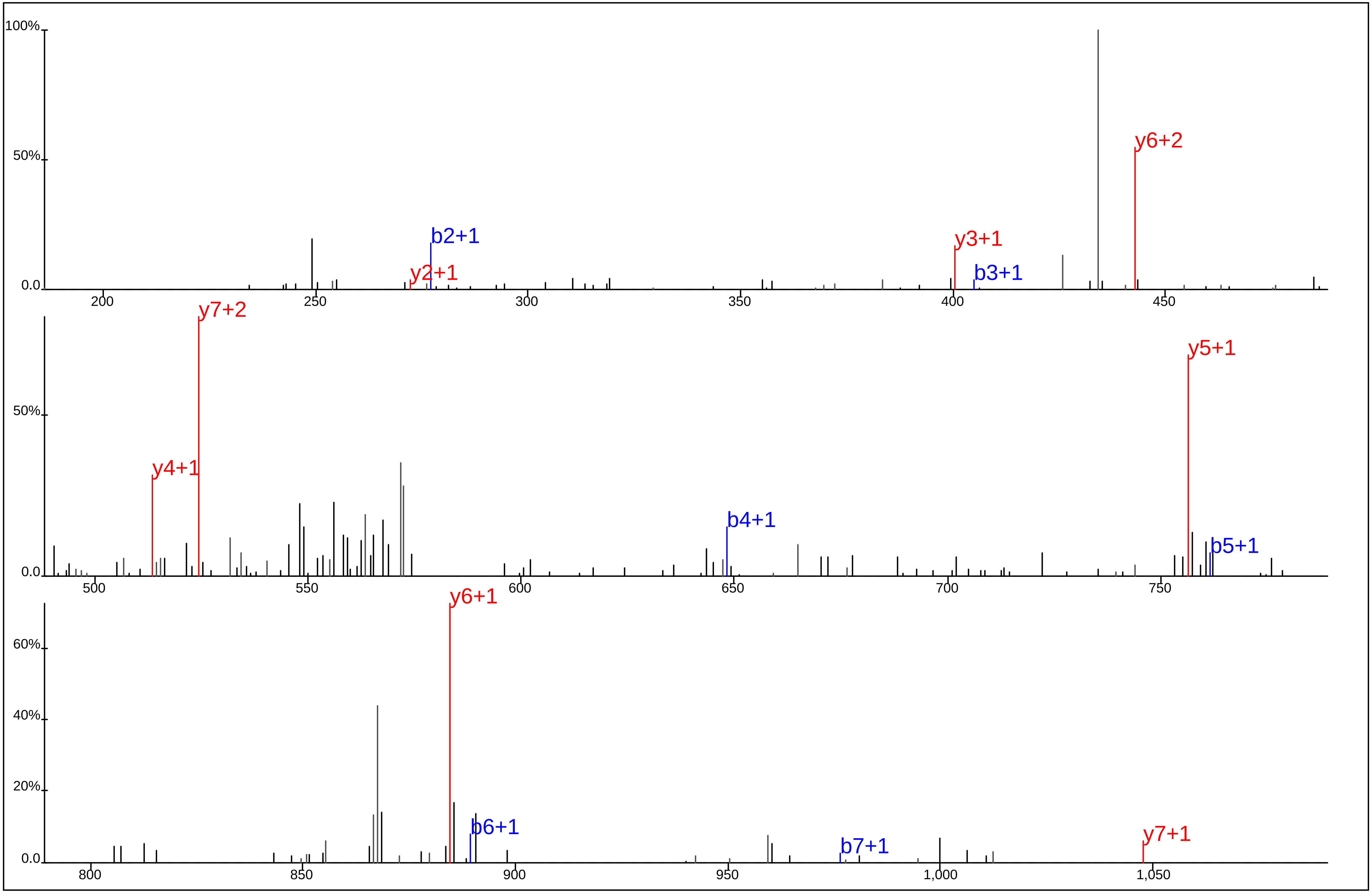

I Y Q Y*I Q S R%

Protein Name: DYRK1B
gi Accession: 405725
Precursor Mass: 580.7750+2
Delta Mass: 4.548 ppm
XCorr: 35.43

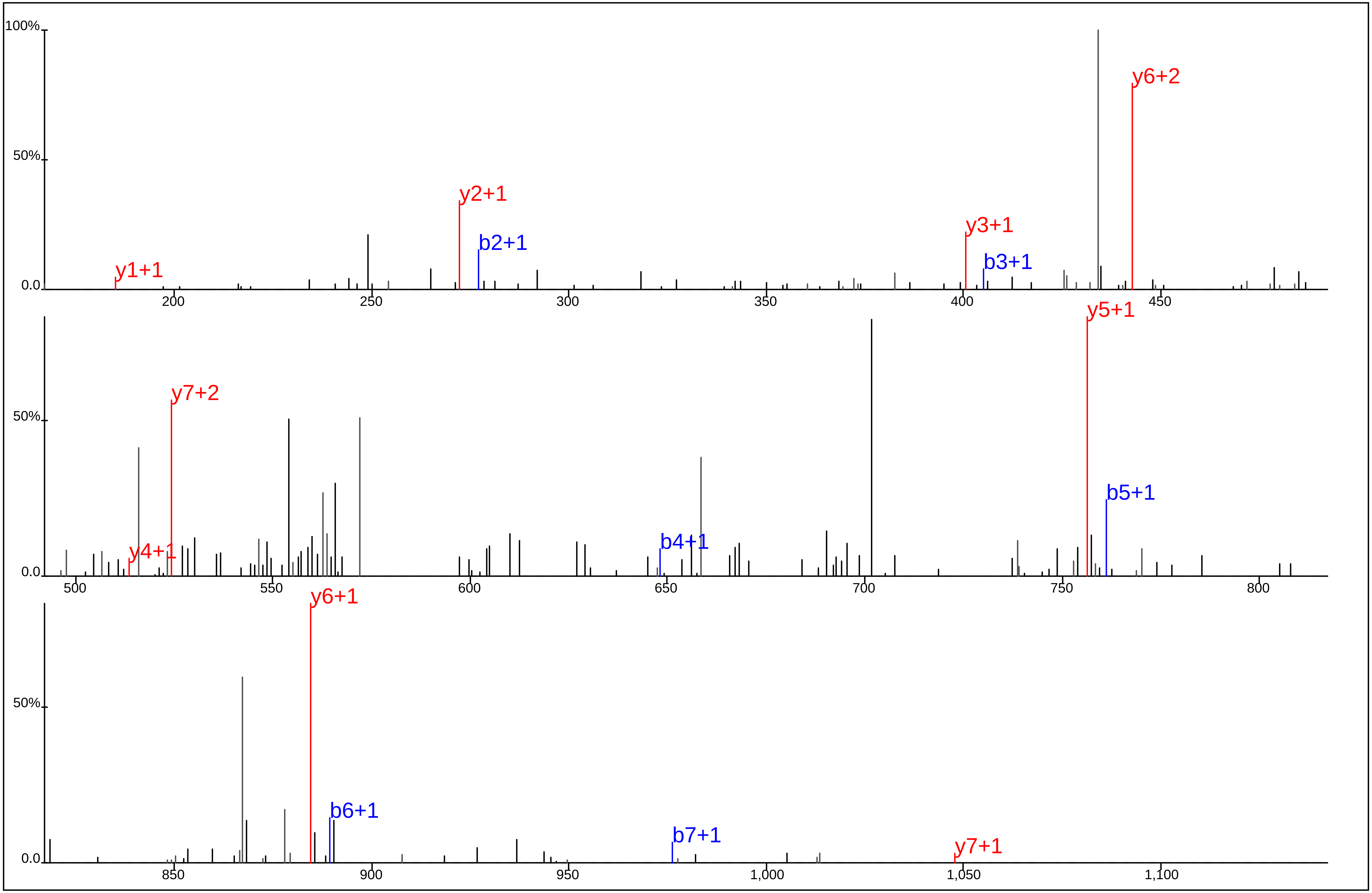

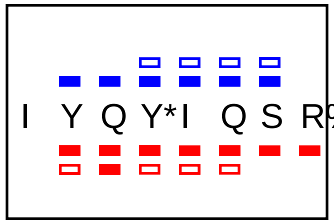

Protein Name: DYRK1B
gi Accession: 405725
Precursor Mass: 580.7734+2
Delta Mass: 1.816 ppm
XCorr: 34.9

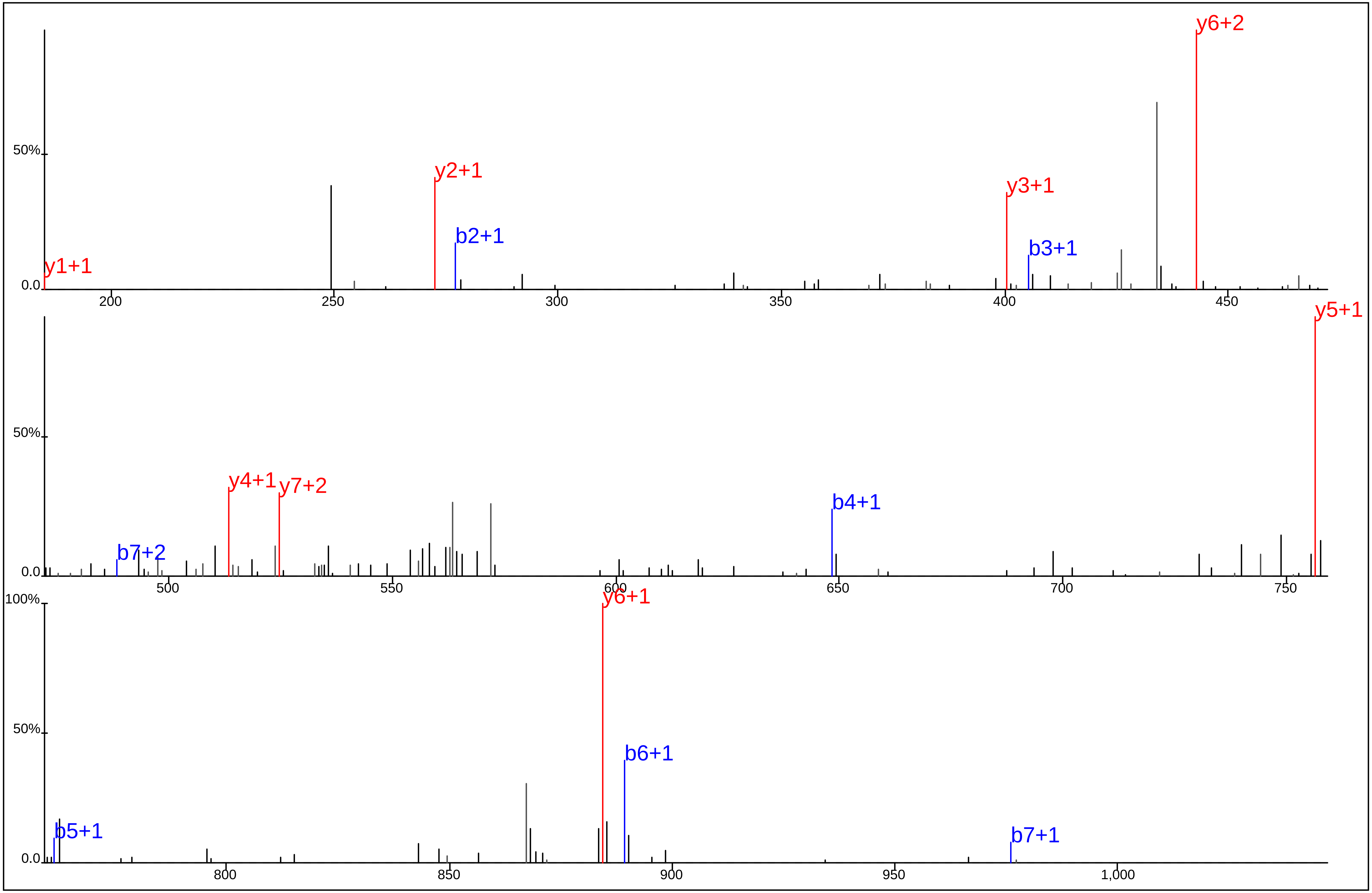

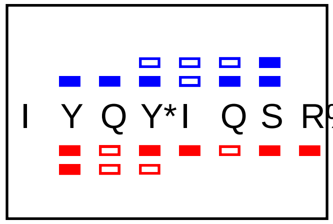

Protein Name: DYRK1B
gi Accession: 405725
Precursor Mass: 580.7738+2
Delta Mass: 2.445 ppm
XCorr: 41.61

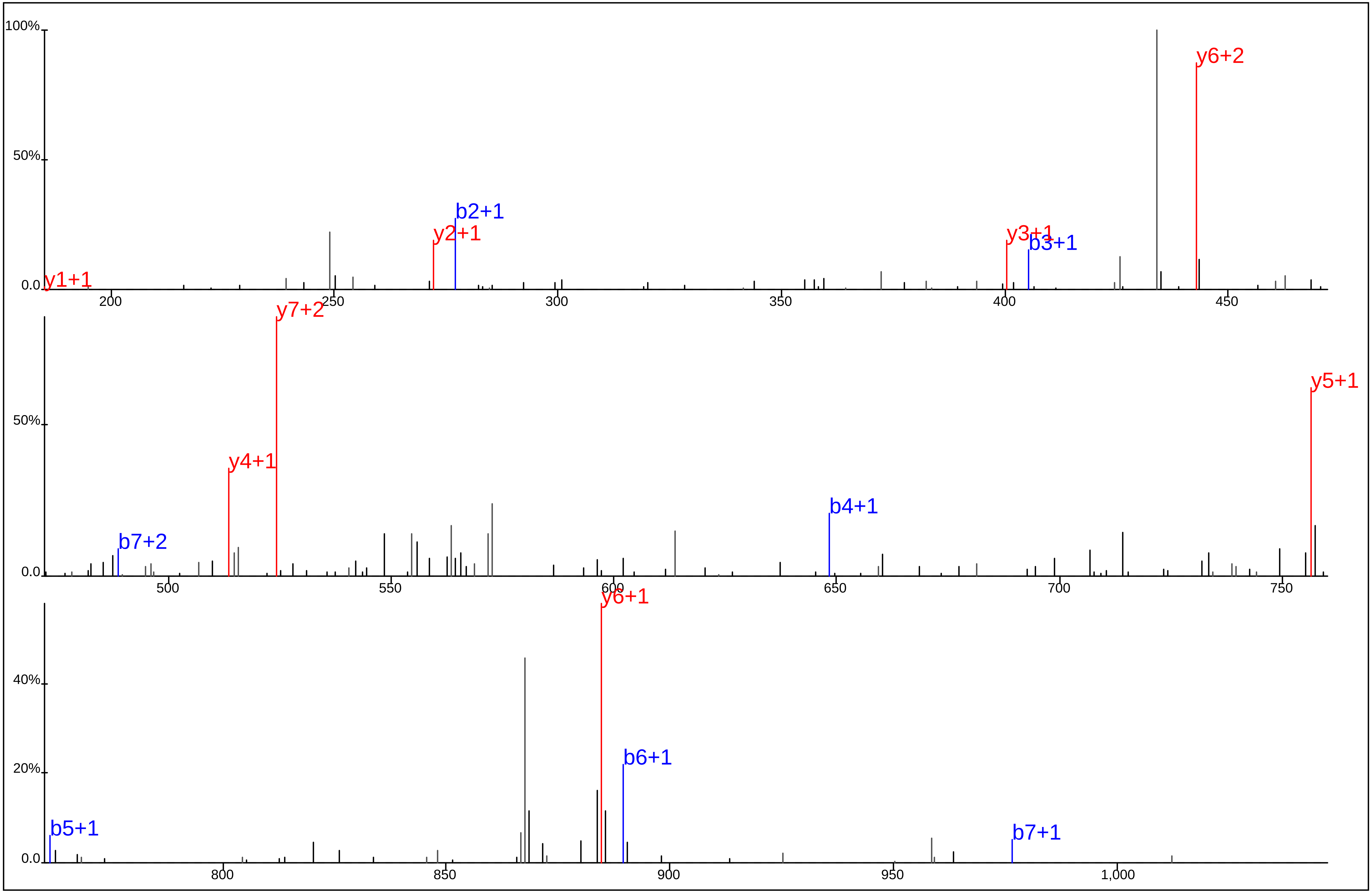

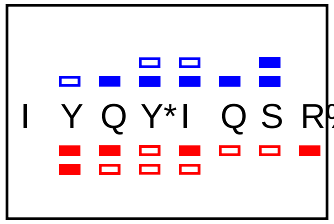

Protein Name:	DYRK1B
gi Accession:	405725
Precursor Mass:	580.7761+2
Delta Mass:	6.444 ppm
XCorr:	40.06

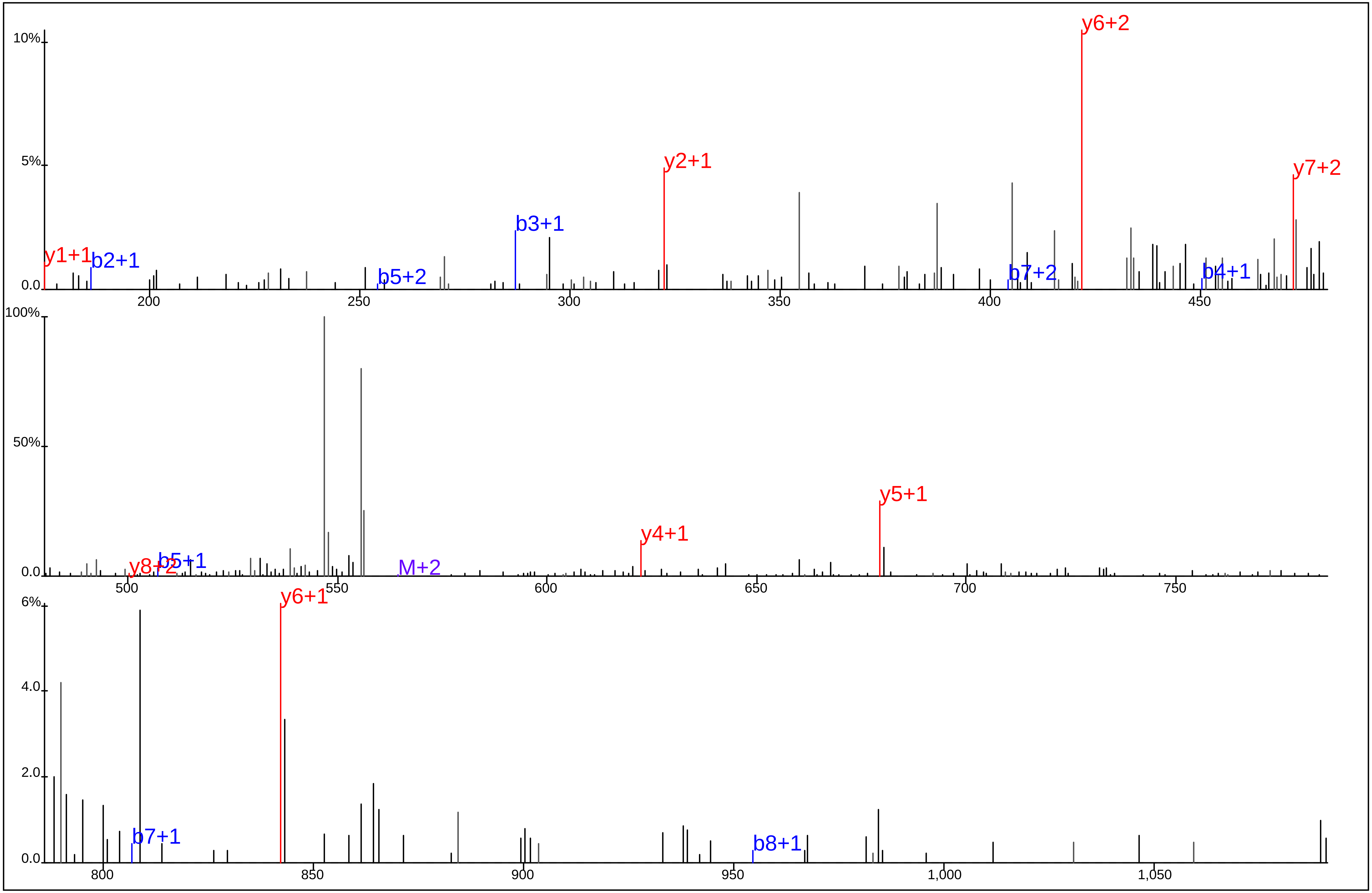

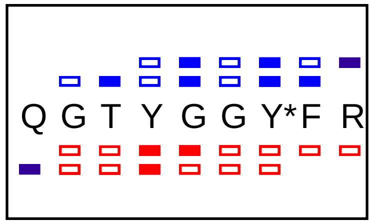

Protein Name: EIF3S8
gi Accession: 4503525
Precursor Mass: 564.7303+2
Delta Mass: 2.028 ppm
XCorr: 22.81

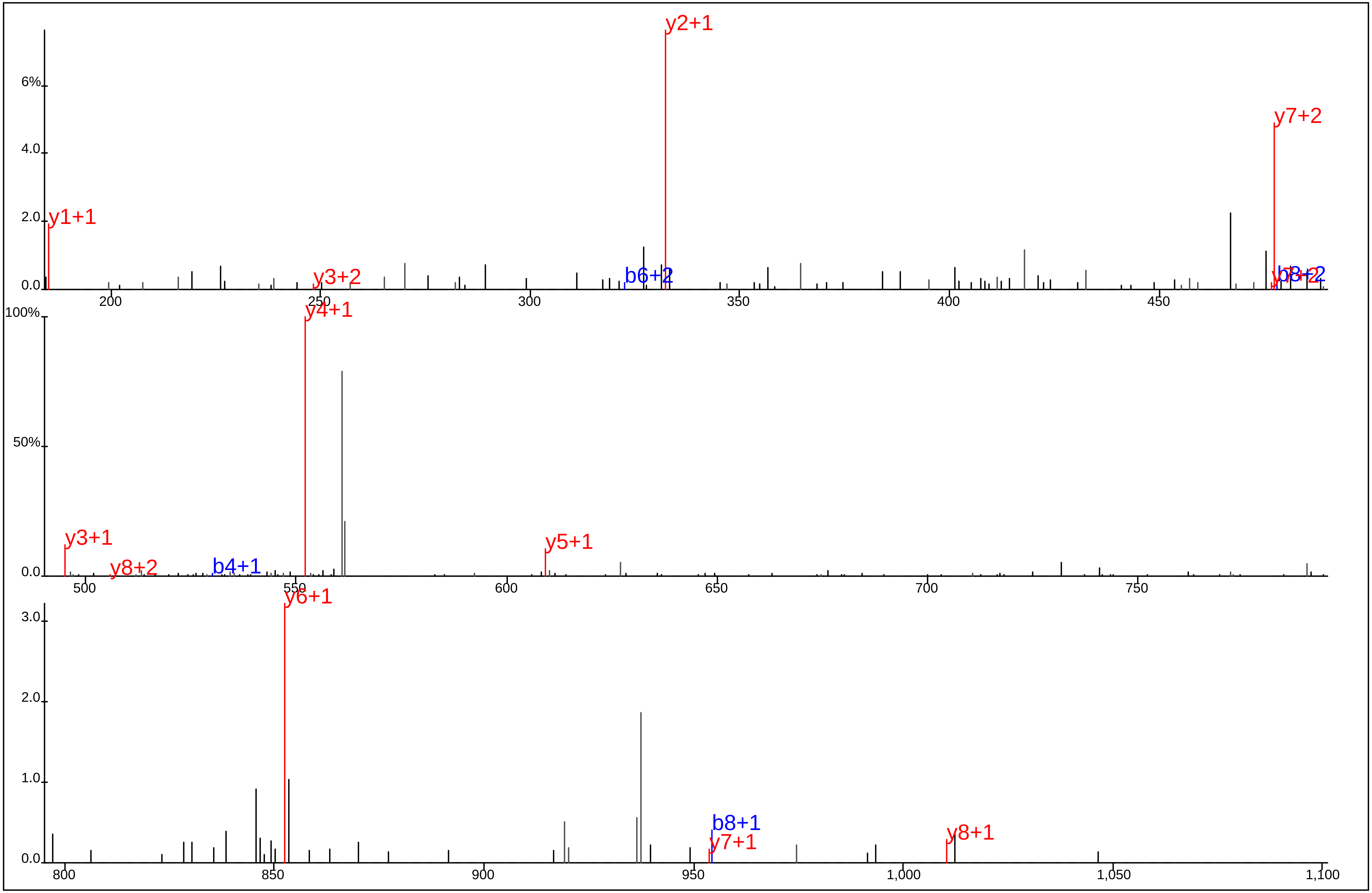

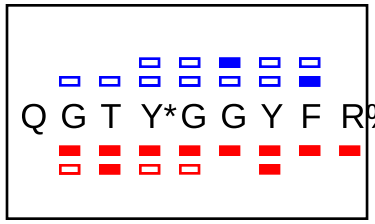

Protein Name: EIF3S8
gi Accession: 4503525
Precursor Mass: 569.7360+2
Delta Mass: 4.829 ppm
XCorr: 33.46

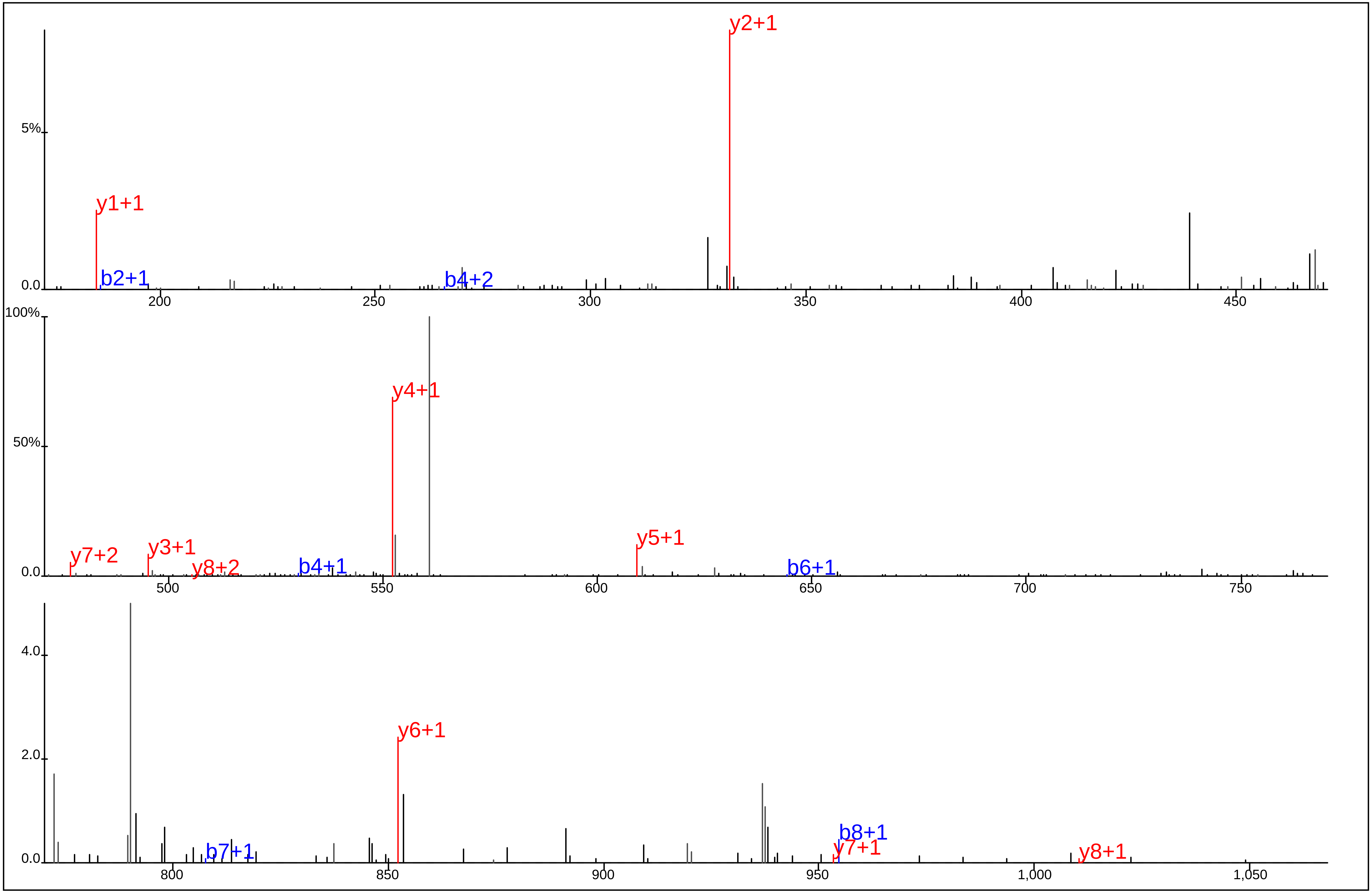

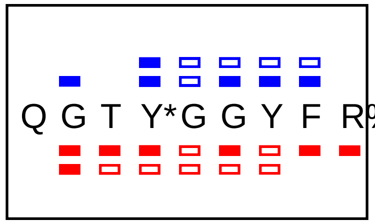

Protein Name:	EIF3S8
gi Accession:	4503525
Precursor Mass:	569.7360+2
Delta Mass:	4.724 ppm
XCorr:	24.37

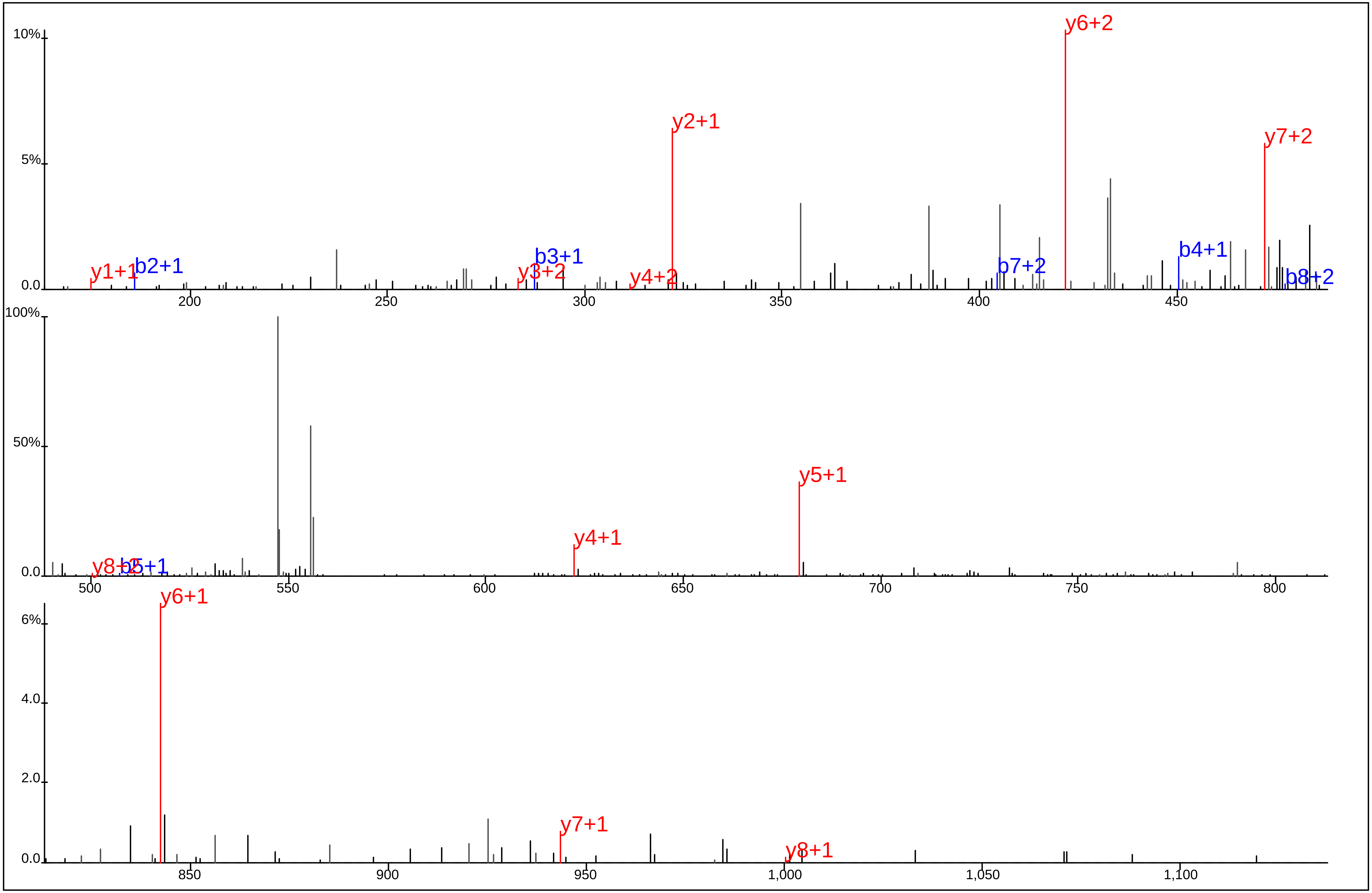

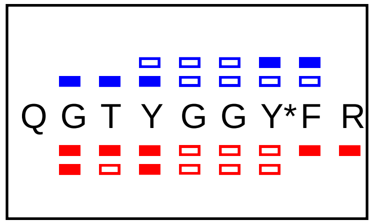

Protein Name: EIF3S8
gi Accession: 4503525
Precursor Mass: 564.7313+2
Delta Mass: 3.871 ppm
XCorr: 26.44

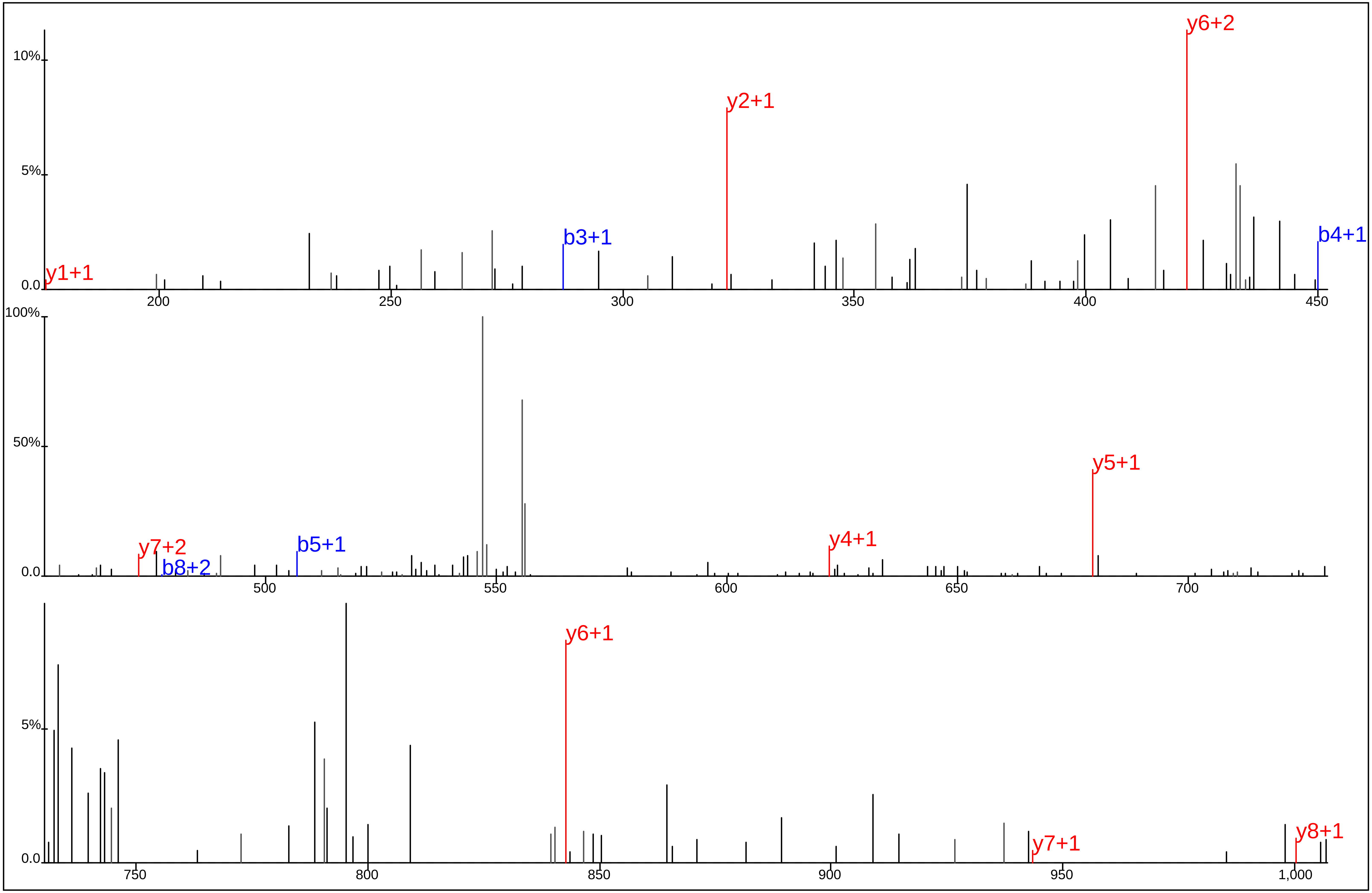

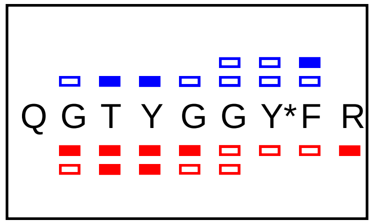

Protein Name: EIF3S8
gi Accession: 4503525
Precursor Mass: 564.7306+2
Delta Mass: 2.577 ppm
XCorr: 22.57

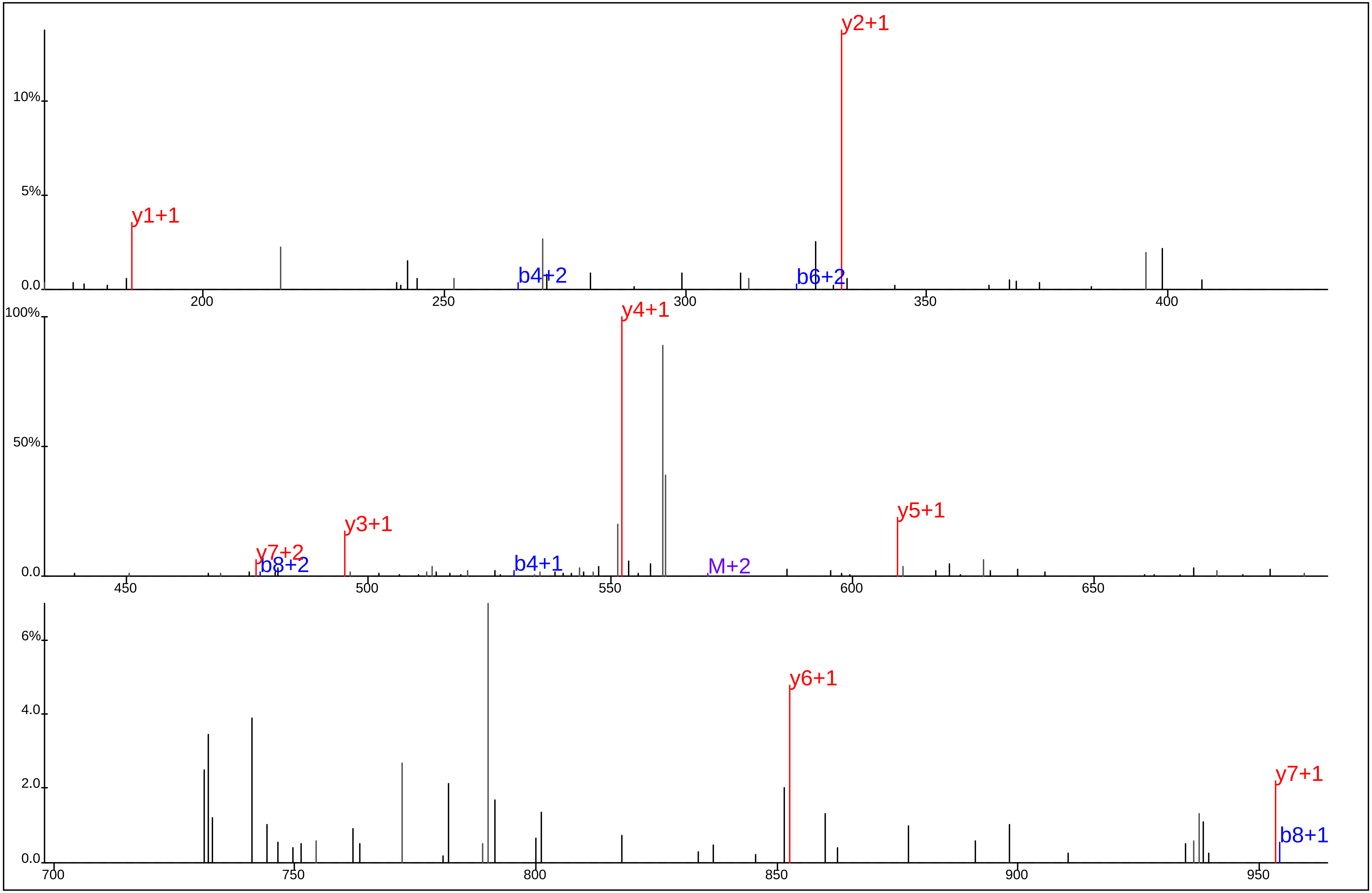

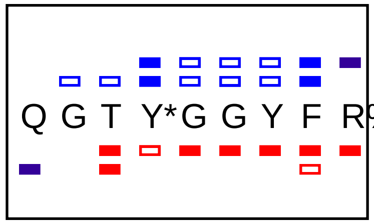

Protein Name: EIF3S8
gi Accession: 4503525
Precursor Mass: 569.7353+2
Delta Mass: 3.538 ppm
XCorr: 34.78

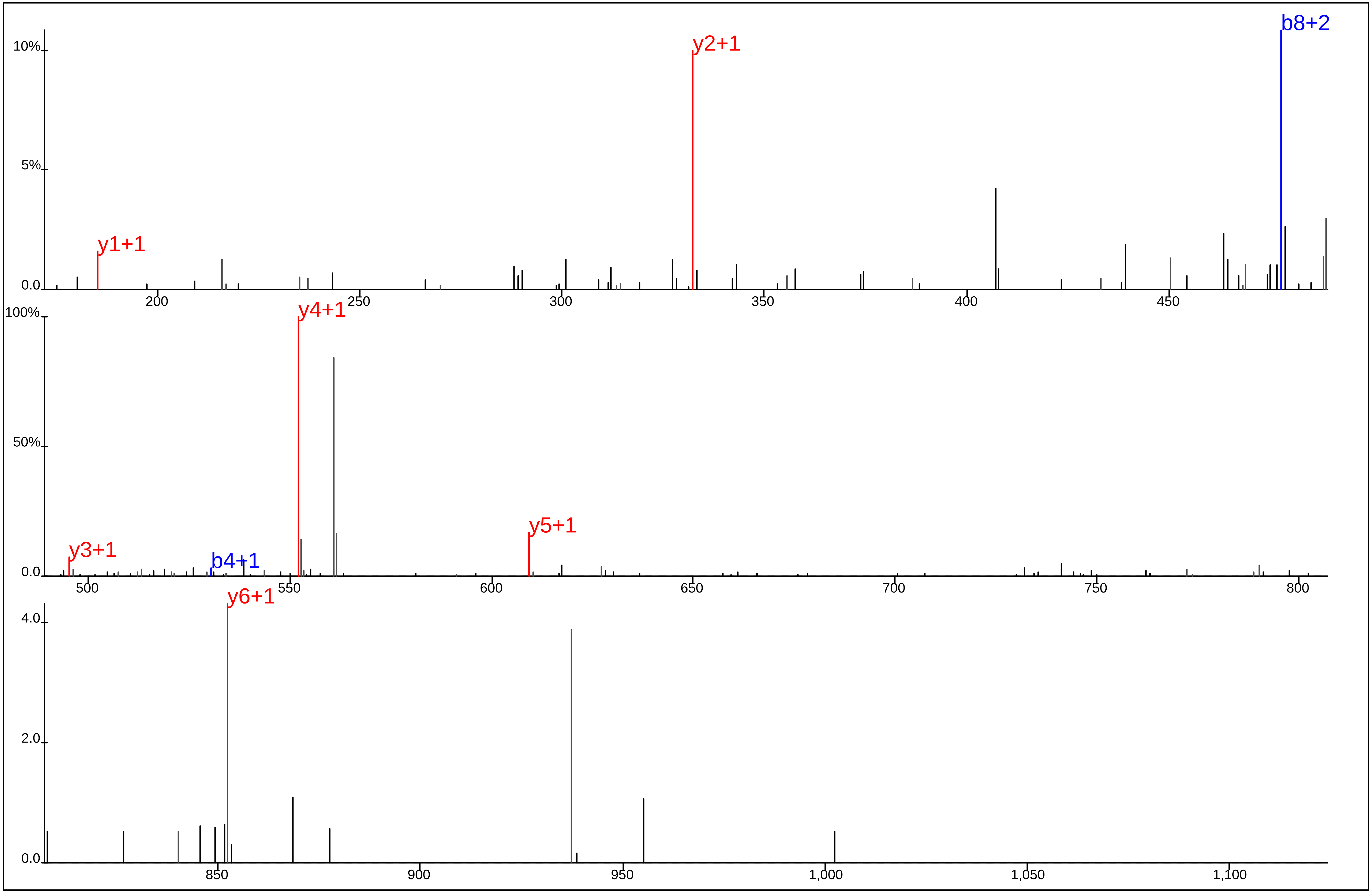

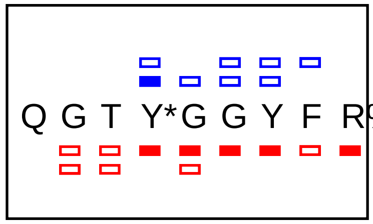

Protein Name: EIF3S8
gi Accession: 4503525
Precursor Mass: 569.7352+2
Delta Mass: 3.433 ppm
XCorr: 29.46

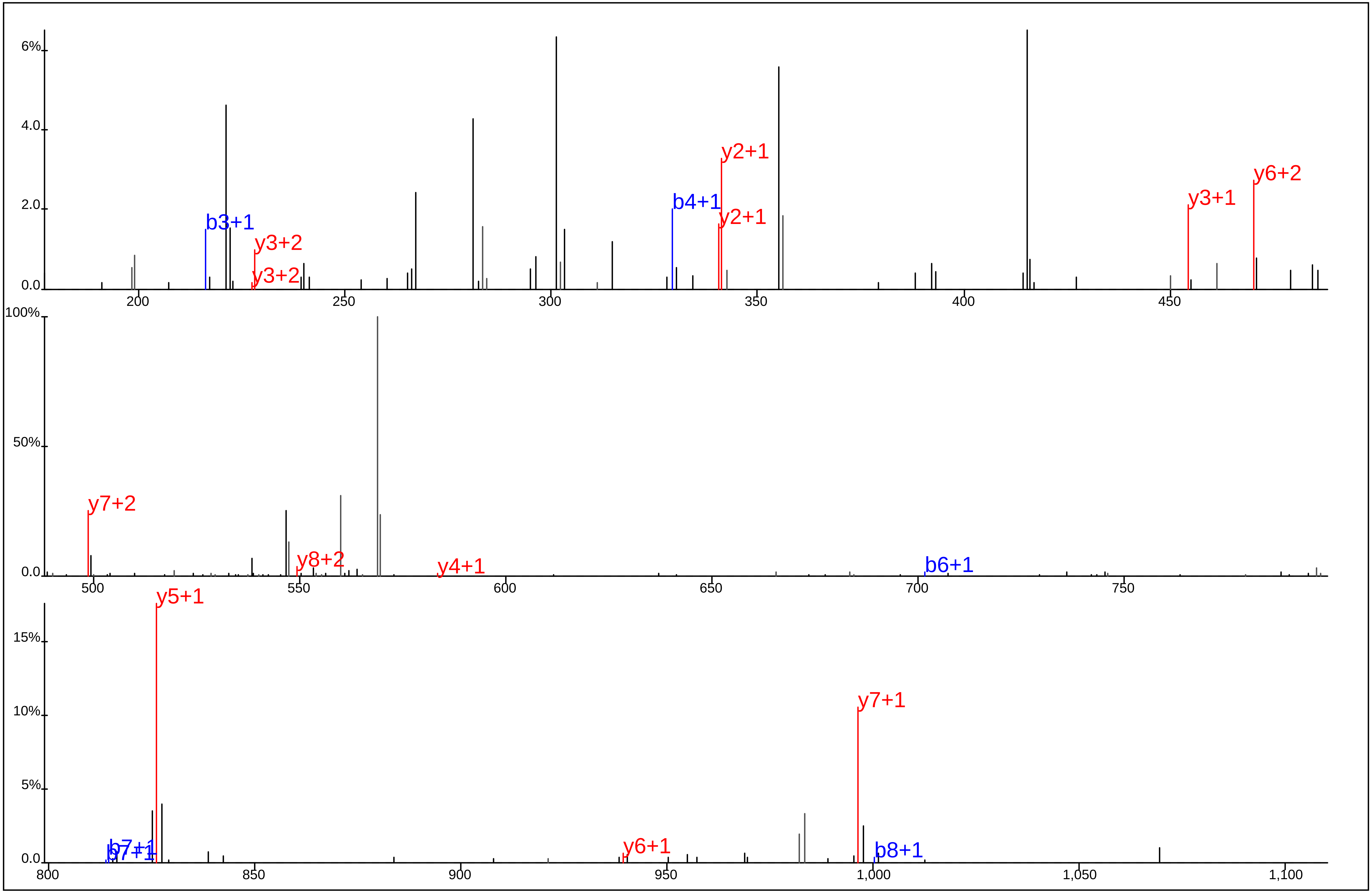

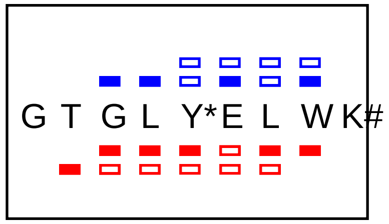

Protein Name: Elongation protein 3
gi Accession: 23510283
Precursor Mass: 577.7748+2
Delta Mass: 4.433 ppm
XCorr: 26.04

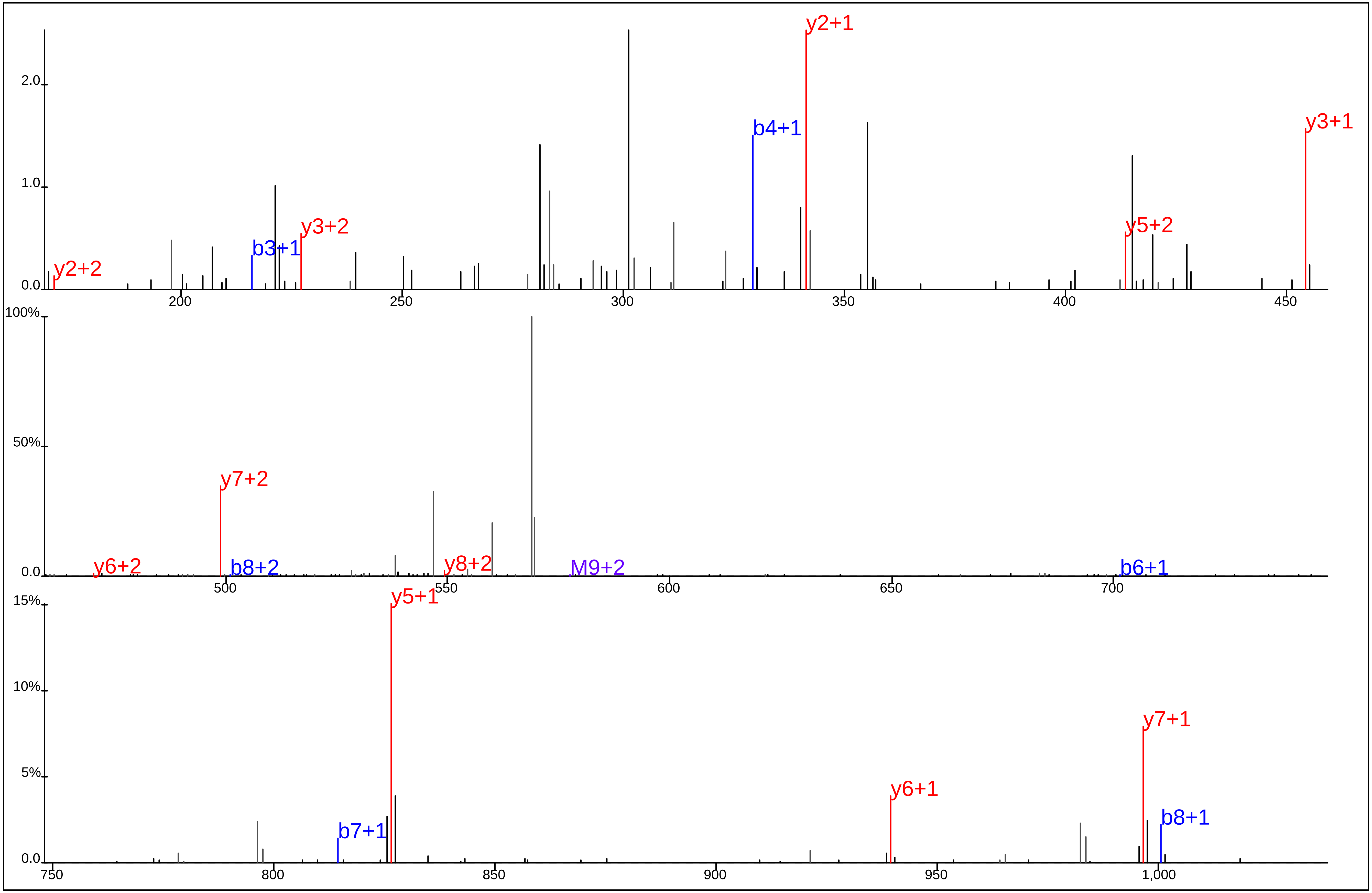

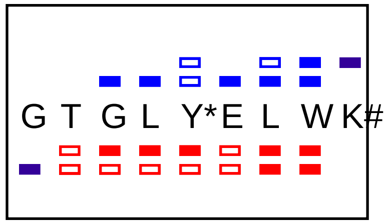

Protein Name:	Elongation protein 3
gi Accession:	23510283
Precursor Mass:	577.7744+2
Delta Mass:	3.584 ppm
XCorr:	40.12

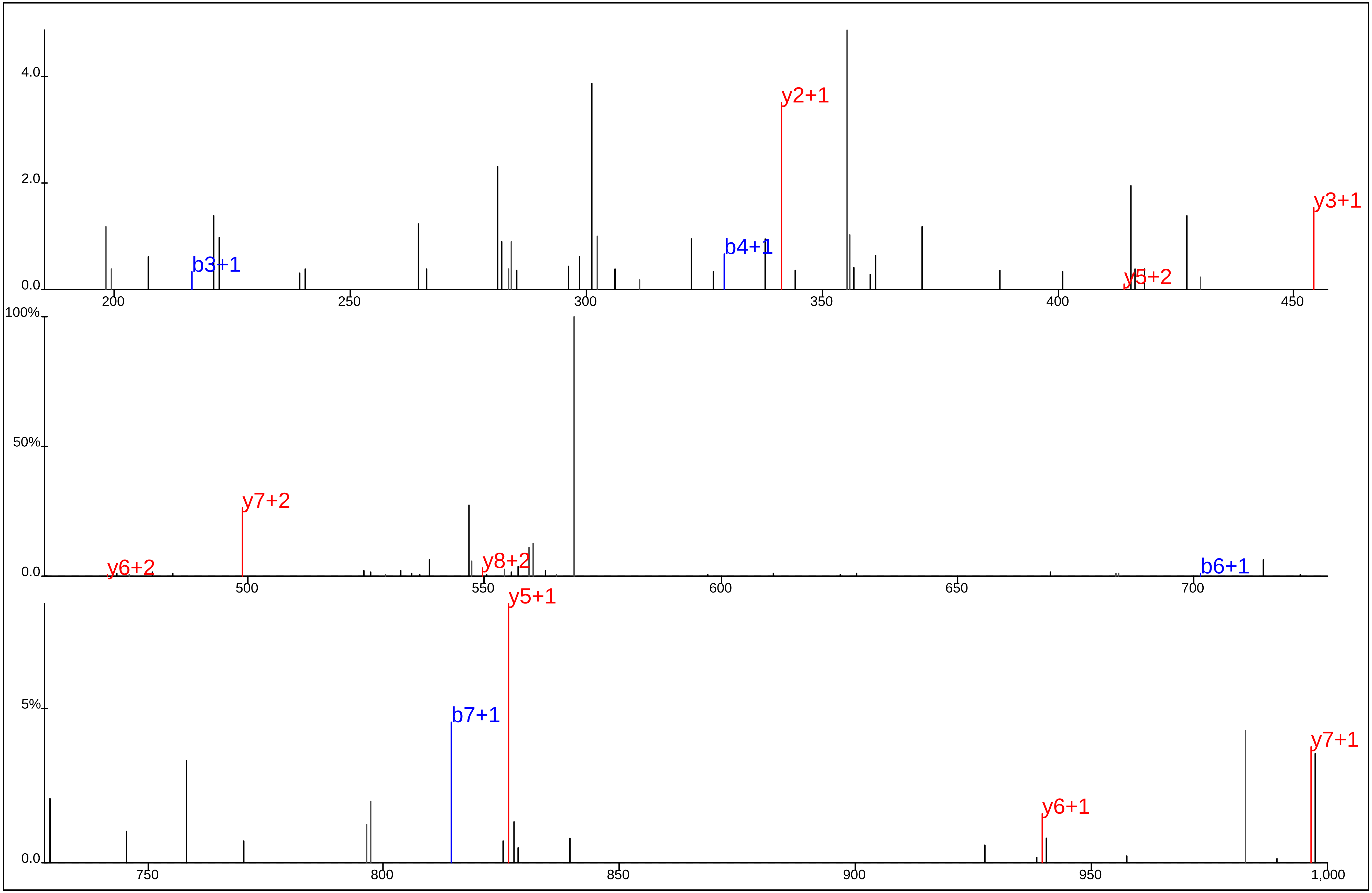

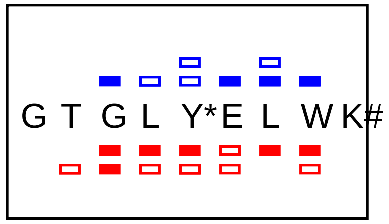

Protein Name: Elongation protein 3
gi Accession: 23510283
Precursor Mass: 577.7738+2
Delta Mass: 2.631 ppm
XCorr: 18.29

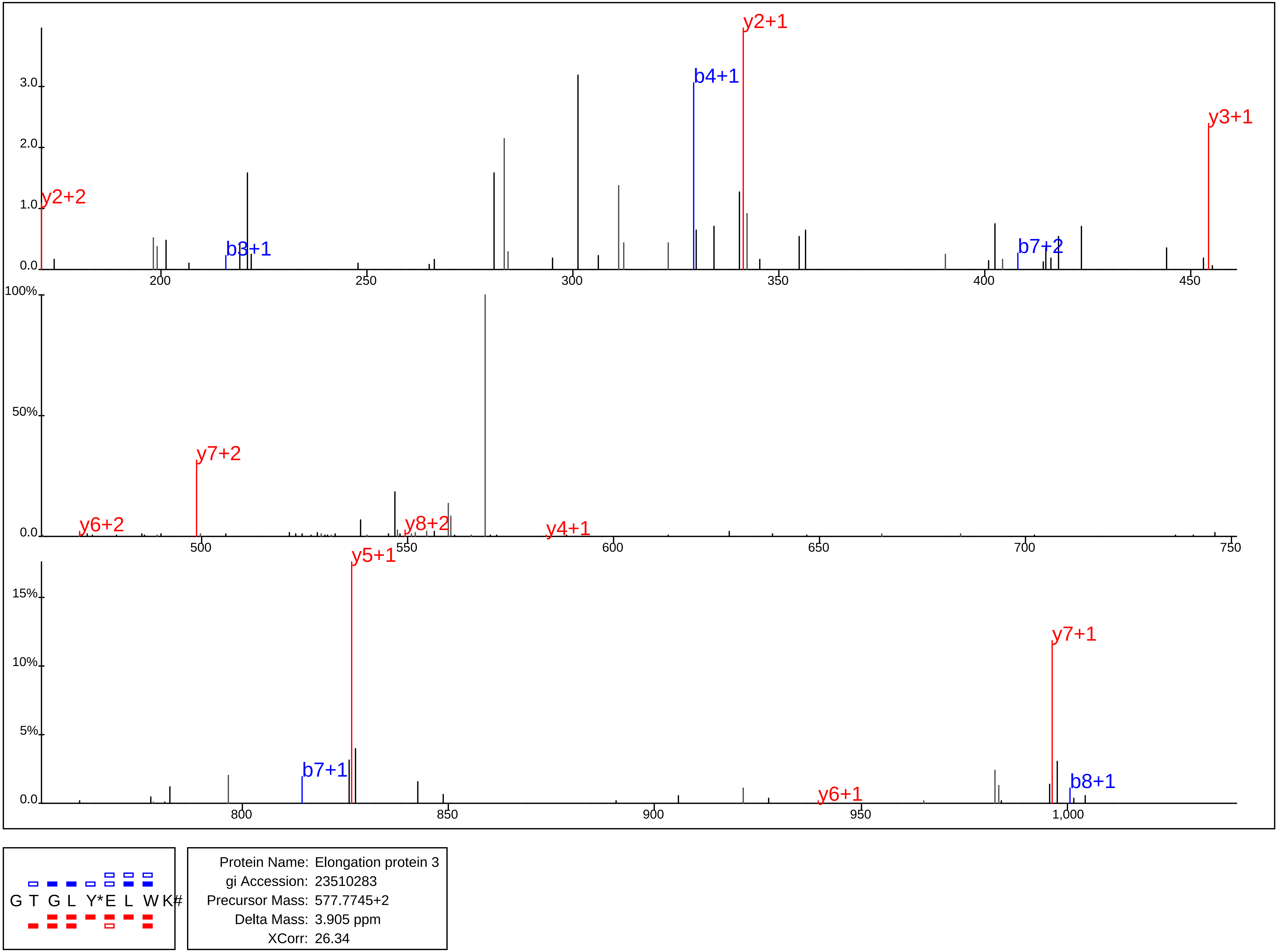

Protein Name: Elongation protein 3
gi Accession: 23510283
Precursor Mass: 577.7745+2
Delta Mass: 3.905 ppm
XCorr: 26.34

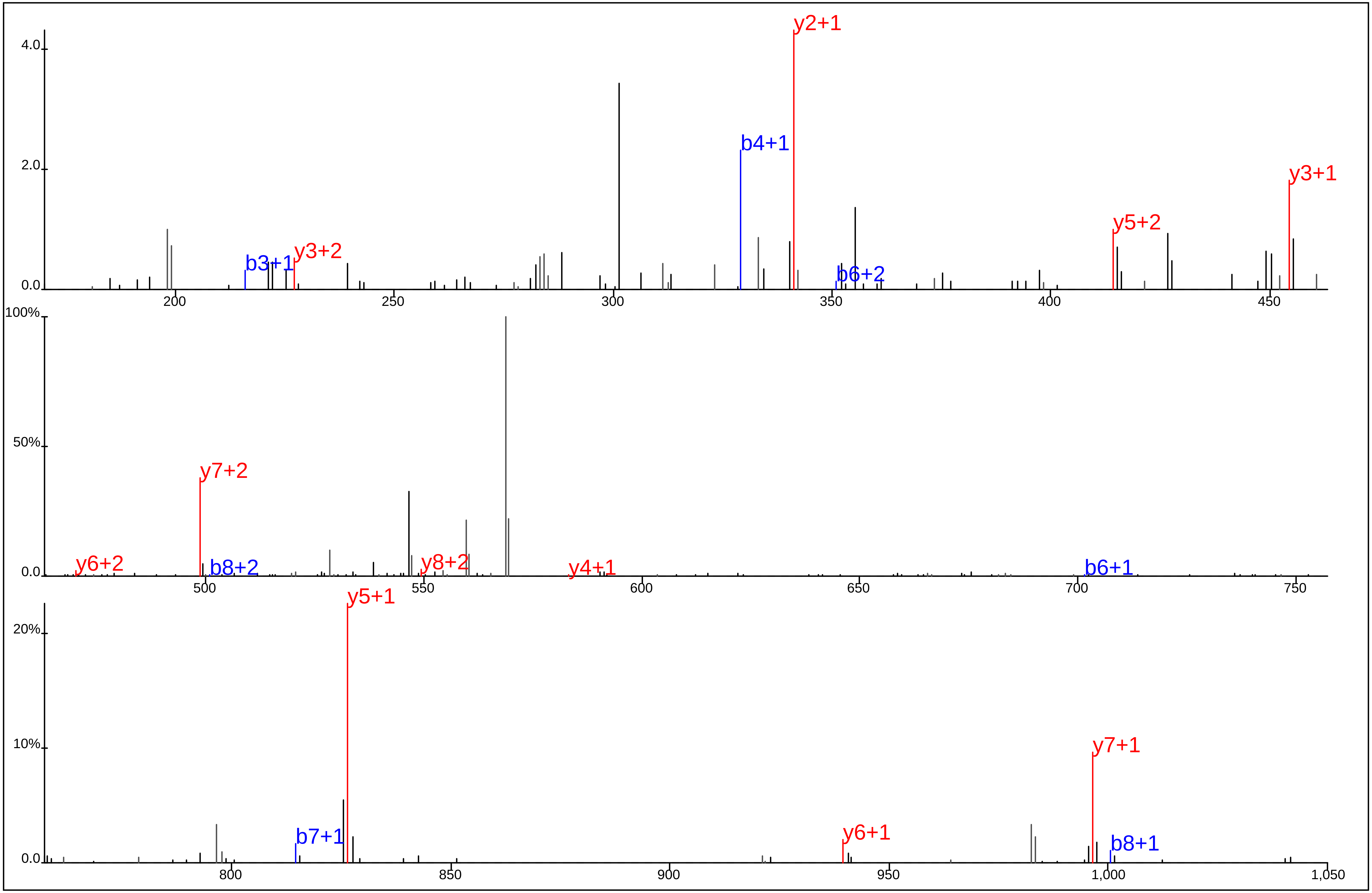

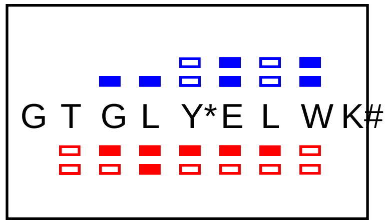

Protein Name:	Elongation protein 3
gi Accession:	23510283
Precursor Mass:	577.7756+2
Delta Mass:	5.698 ppm
XCorr:	35.63

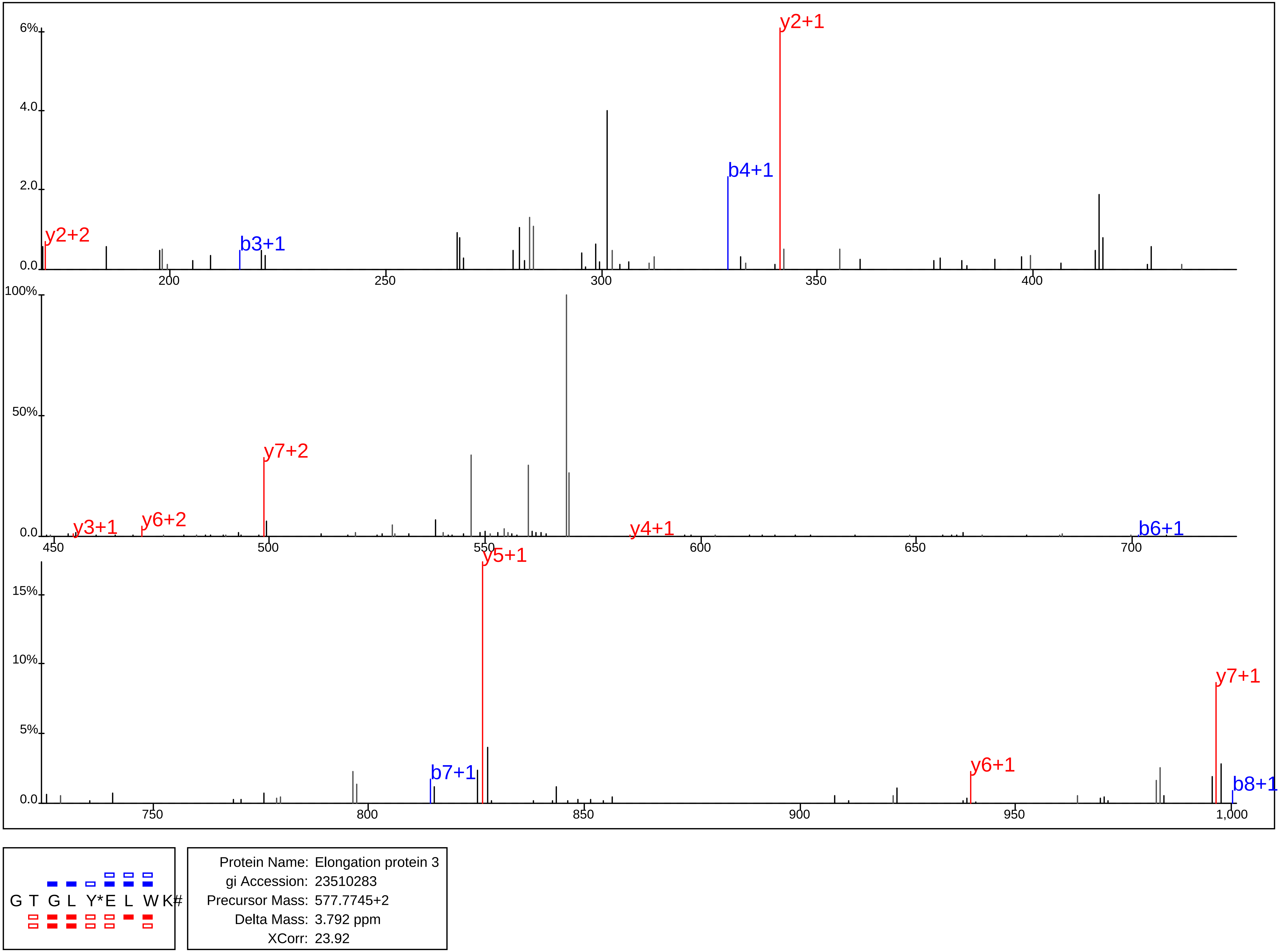

Protein Name: Elongation protein 3
gi Accession: 23510283
Precursor Mass: 577.7745+2
Delta Mass: 3.792 ppm
XCorr: 23.92

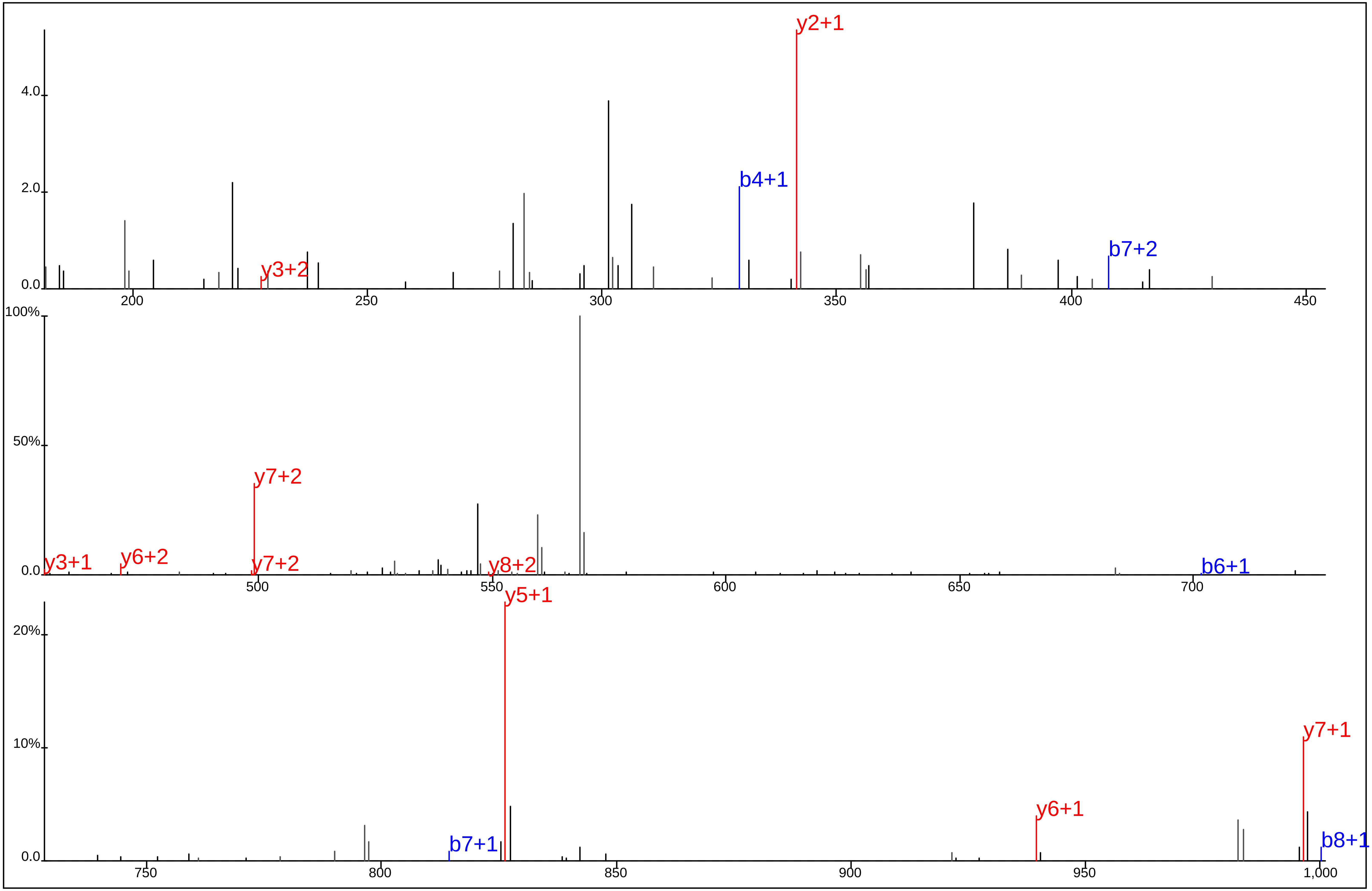

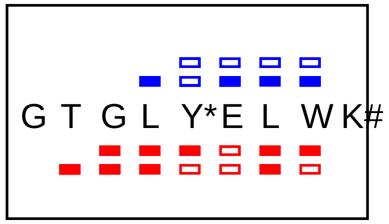

Protein Name:	Elongation protein 3
gi Accession:	23510283
Precursor Mass:	577.7755+2
Delta Mass:	5.594 ppm
XCorr:	27.11

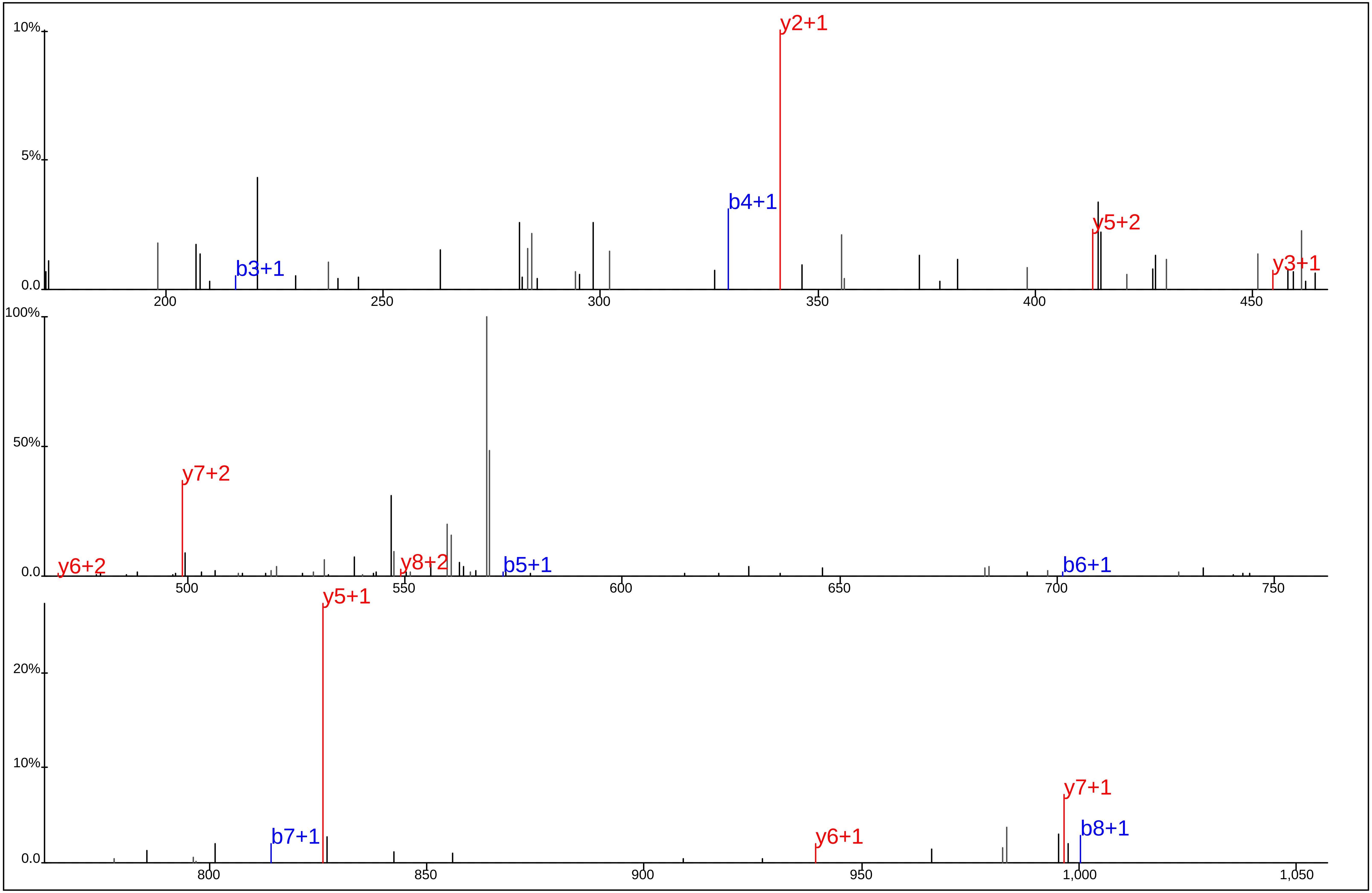

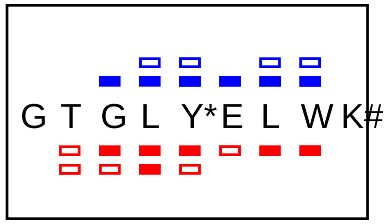

Protein Name: Elongation protein 3
gi Accession: 23510283
Precursor Mass: 577.7762+2
Delta Mass: 6.858 ppm
XCorr: 22.08

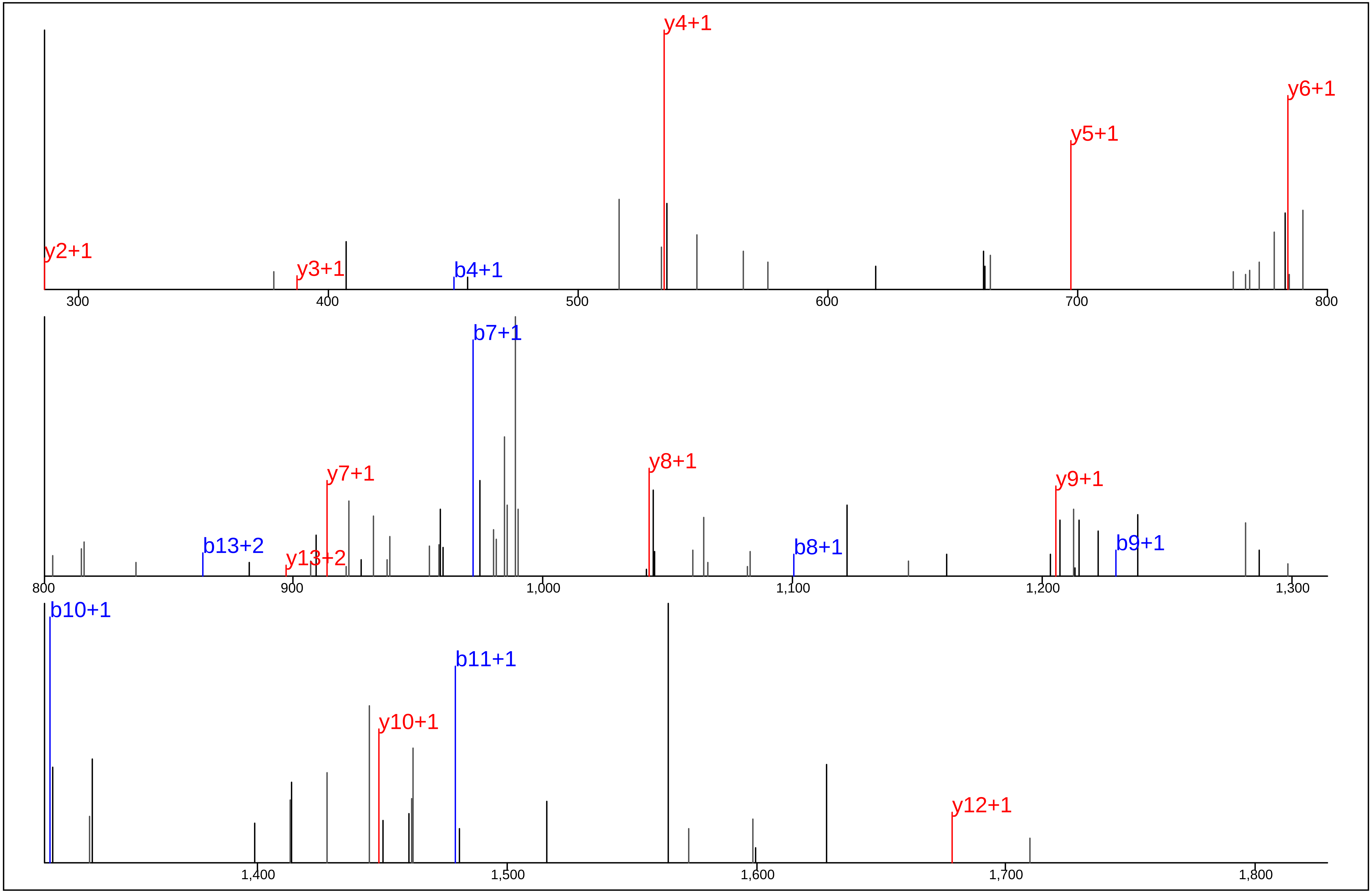

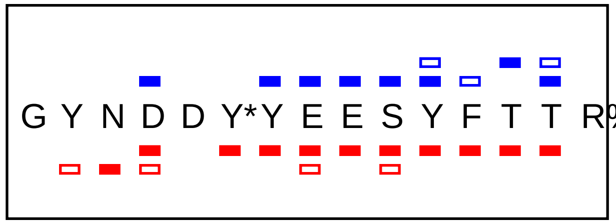

Protein Name: Emerin
gi Accession: 4557553
Precursor Mass: 1,006.8837+2
Delta Mass: 6.994 ppm
XCorr: 44.28

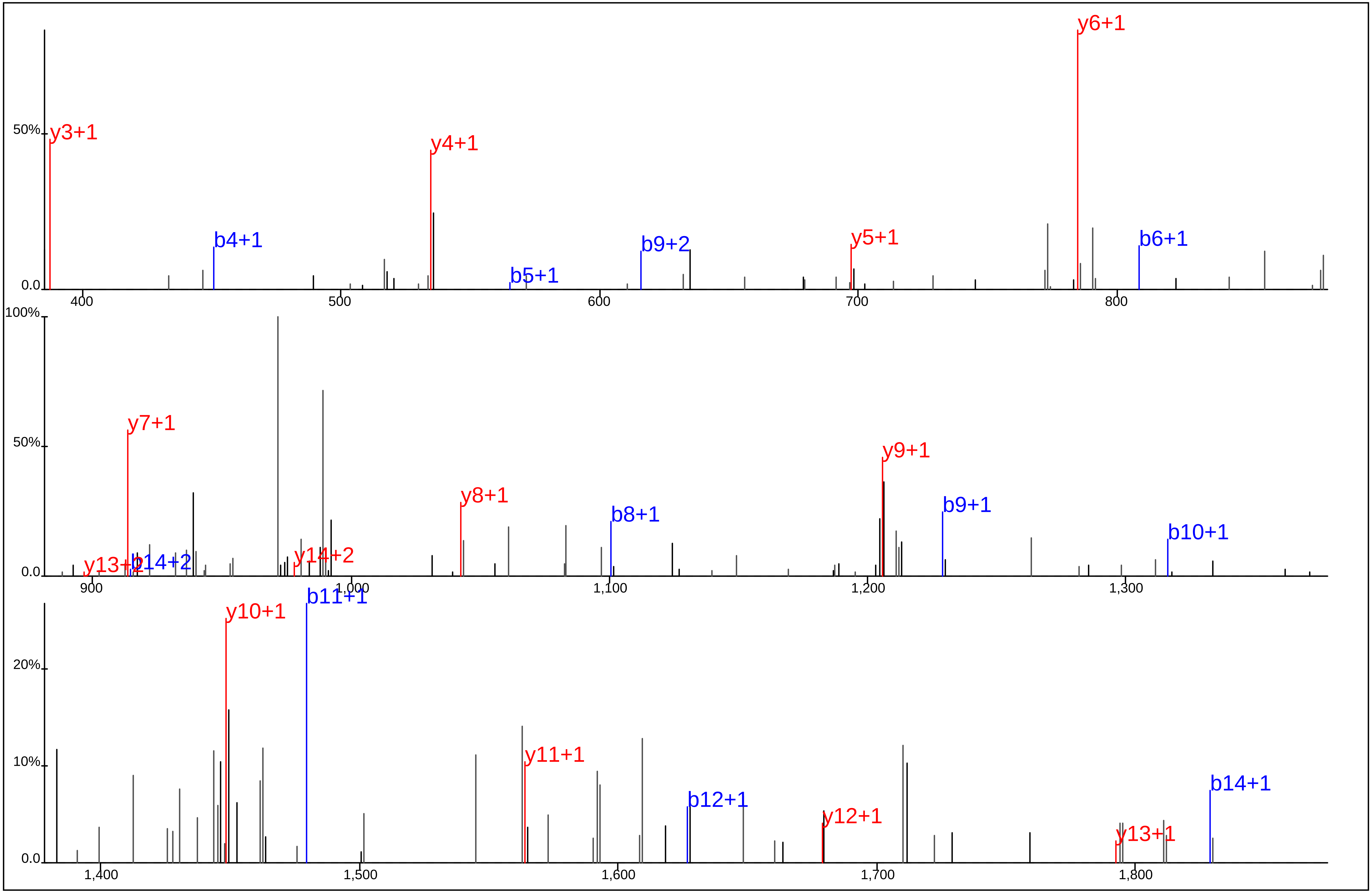

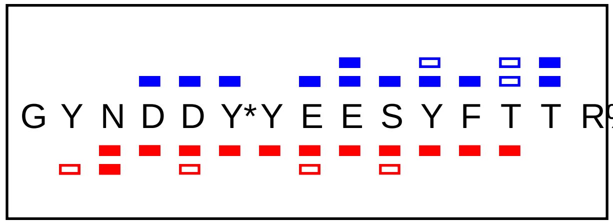

Protein Name: Emerin
gi Accession: 4557553
Precursor Mass: 1,006.8843+2
Delta Mass: 7.601 ppm
XCorr: 44.07

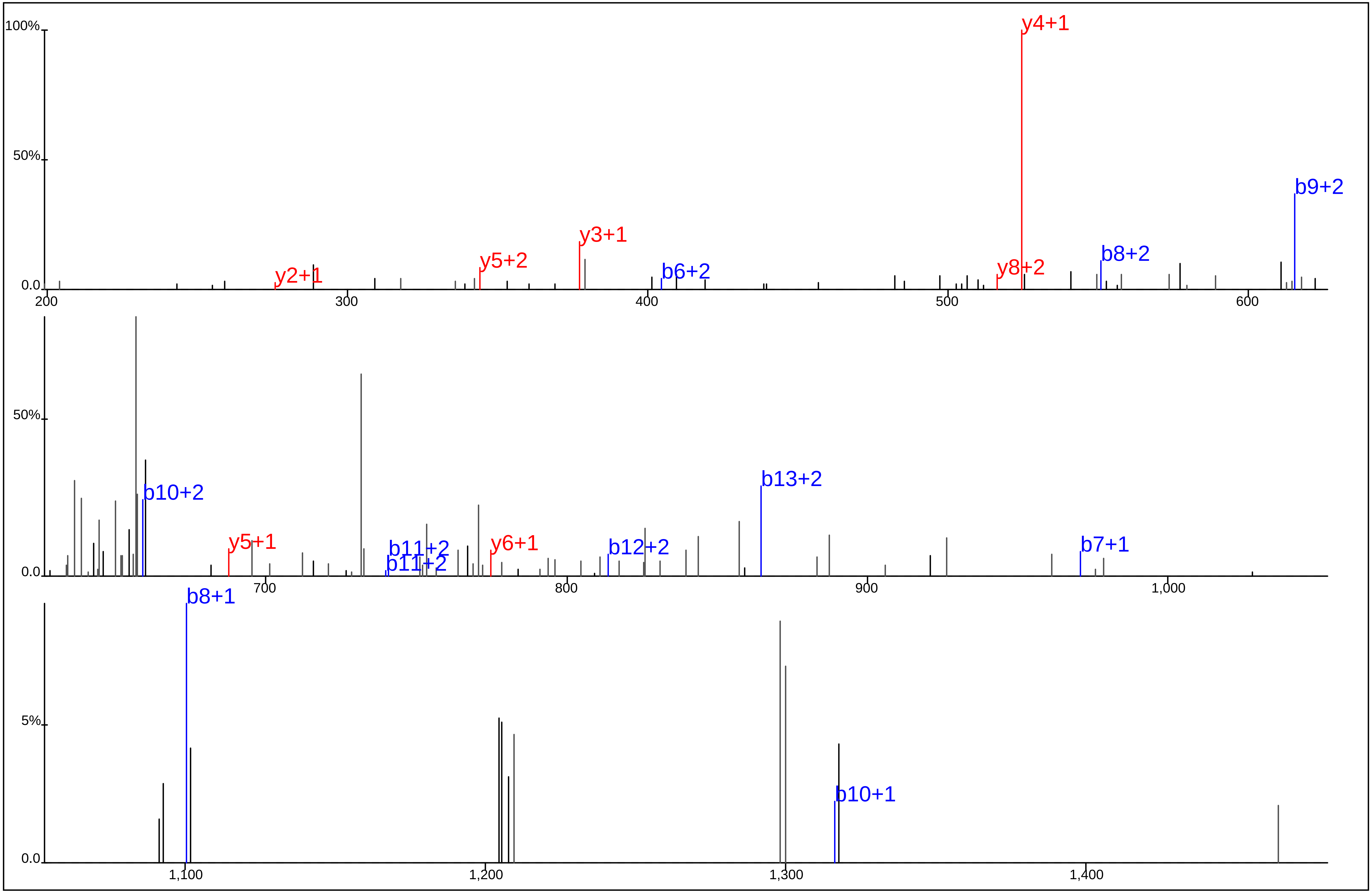

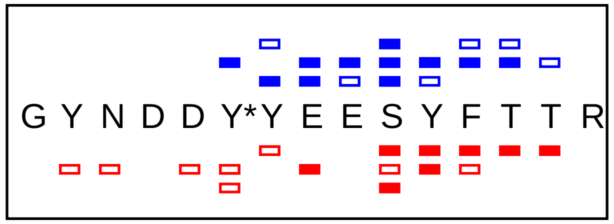

Protein Name:	Emerin
gi Accession:	4557553
Precursor Mass:	668.2517+3
Delta Mass:	1.367 ppm
XCorr:	12.6

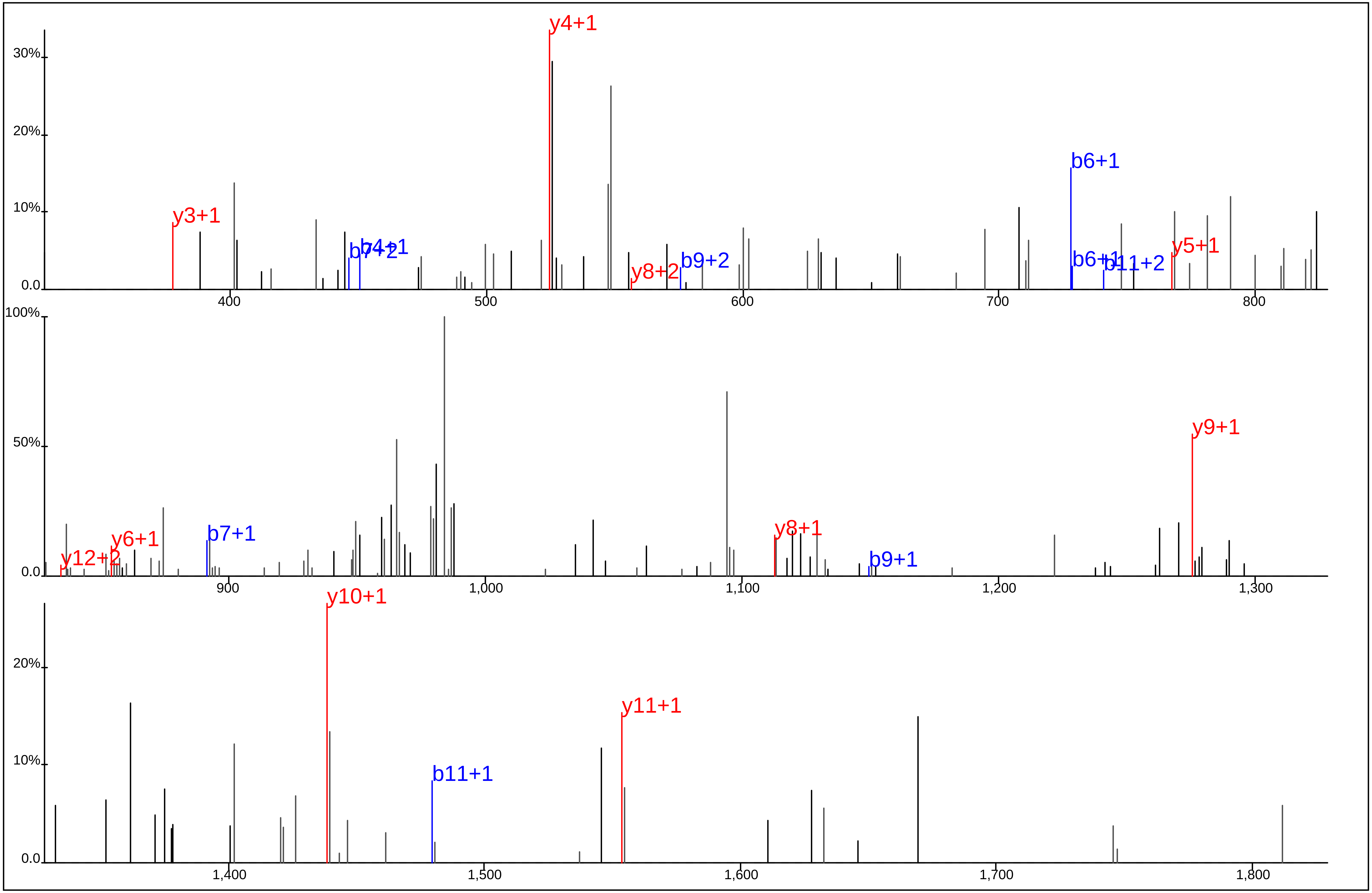

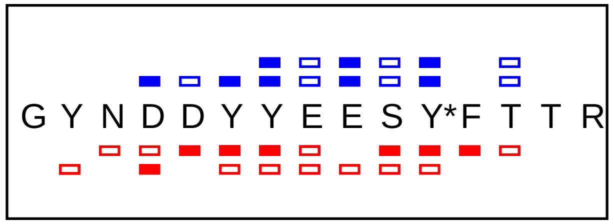

Protein Name: Emerin
gi Accession: 4557553
Precursor Mass: 1,001.8753+2
Delta Mass: 2.745 ppm
XCorr: 14.62

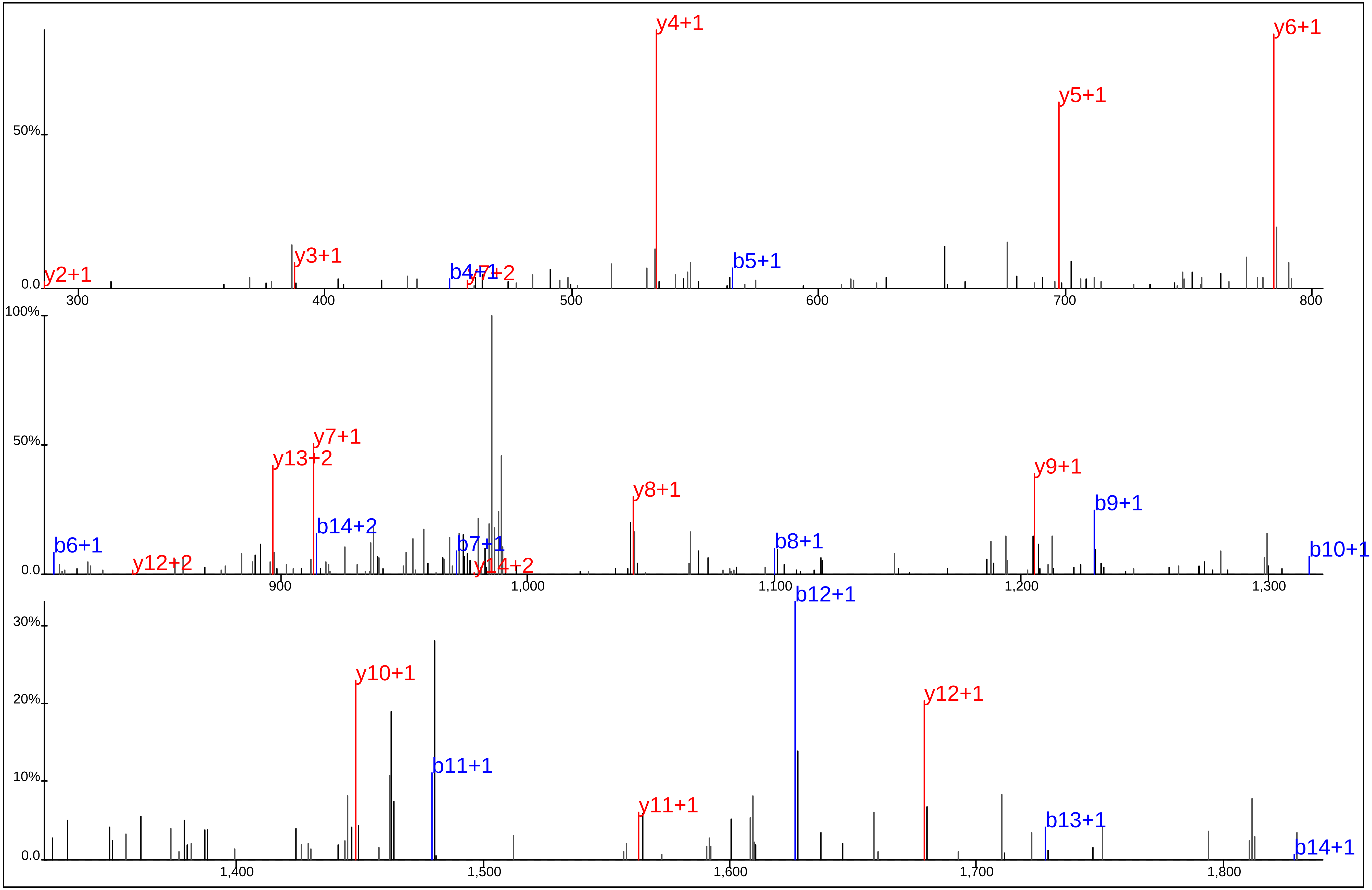

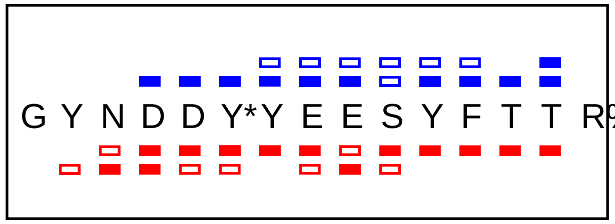

Protein Name:	Emerin
gi Accession:	4557553
Precursor Mass:	1,006.8769+2
Delta Mass:	0.203 ppm
XCorr:	79.39

387 - Emerin_3446370.pdf

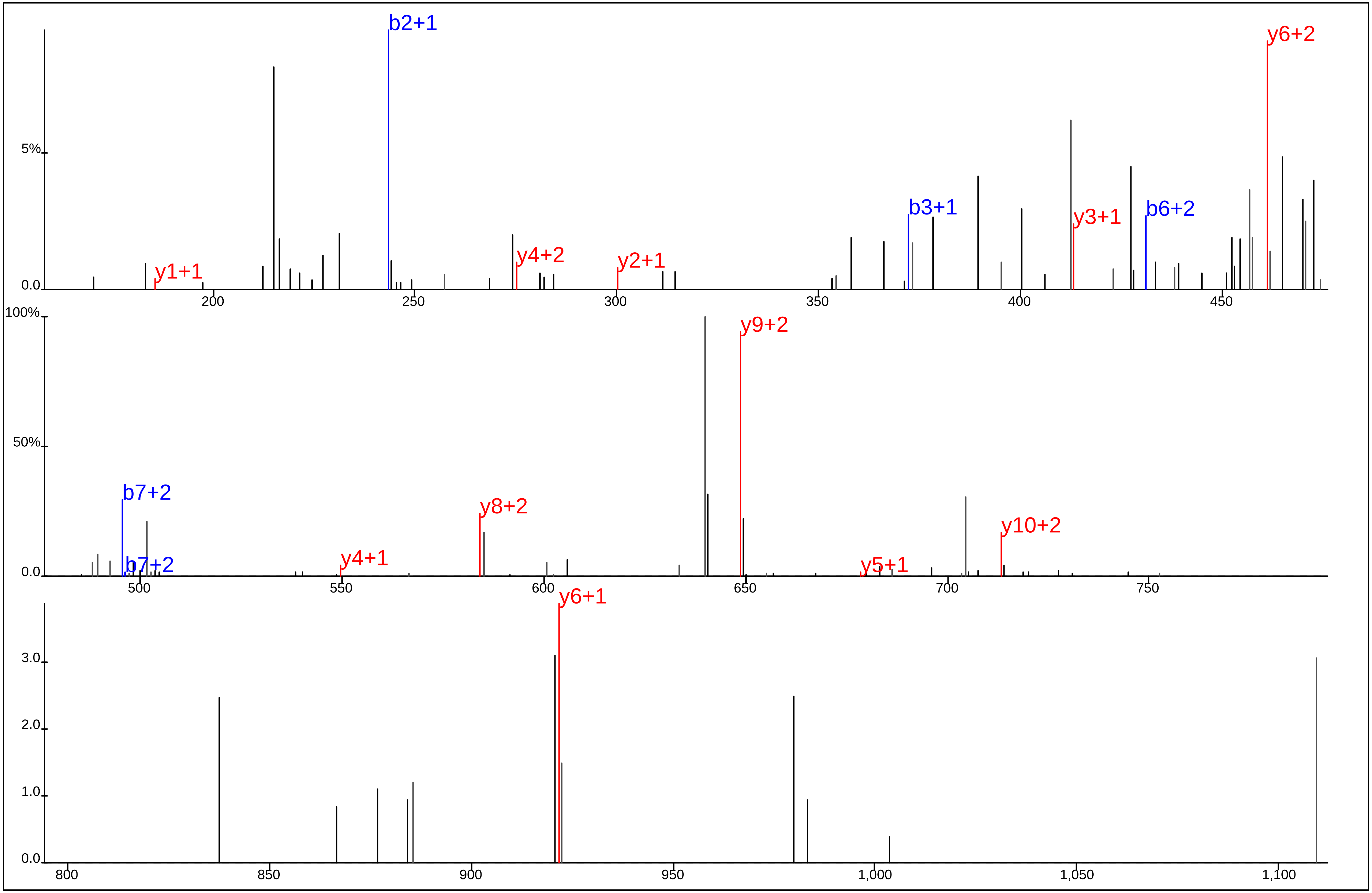

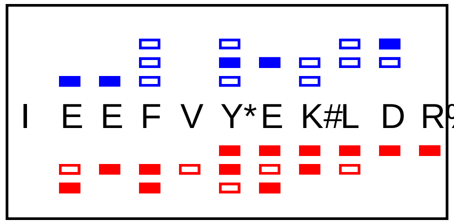

Protein Name: Endophilin B1
gi Accession: 8118530
Precursor Mass: 513.5831+3
Delta Mass: 5.946 ppm
XCorr: 16.11

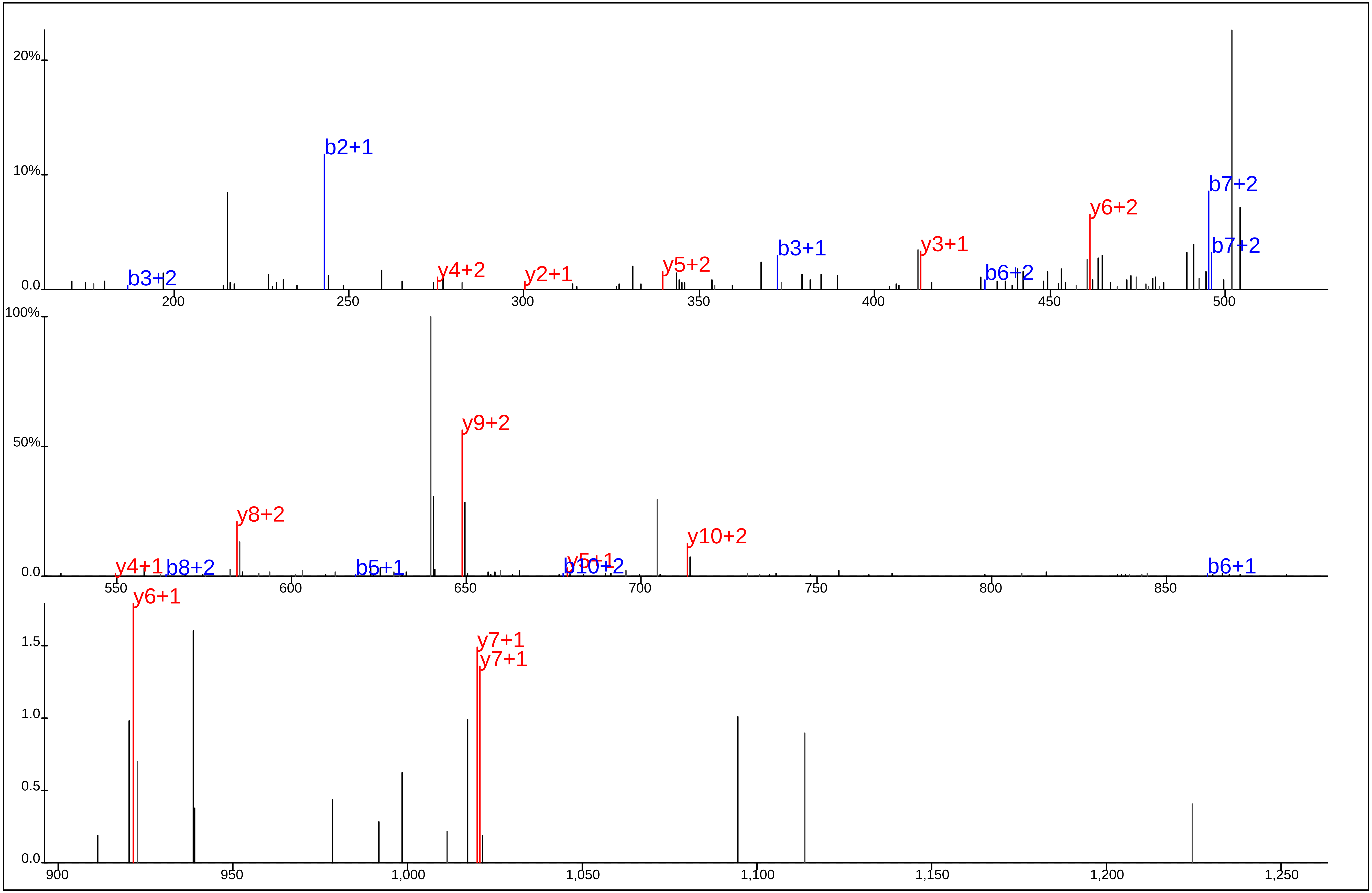

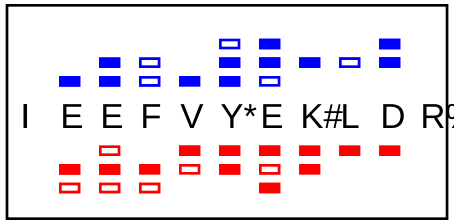

Protein Name: Endophilin B1
gi Accession: 8118530
Precursor Mass: 513.5812+3
Delta Mass: 2.136 ppm
XCorr: 22.69

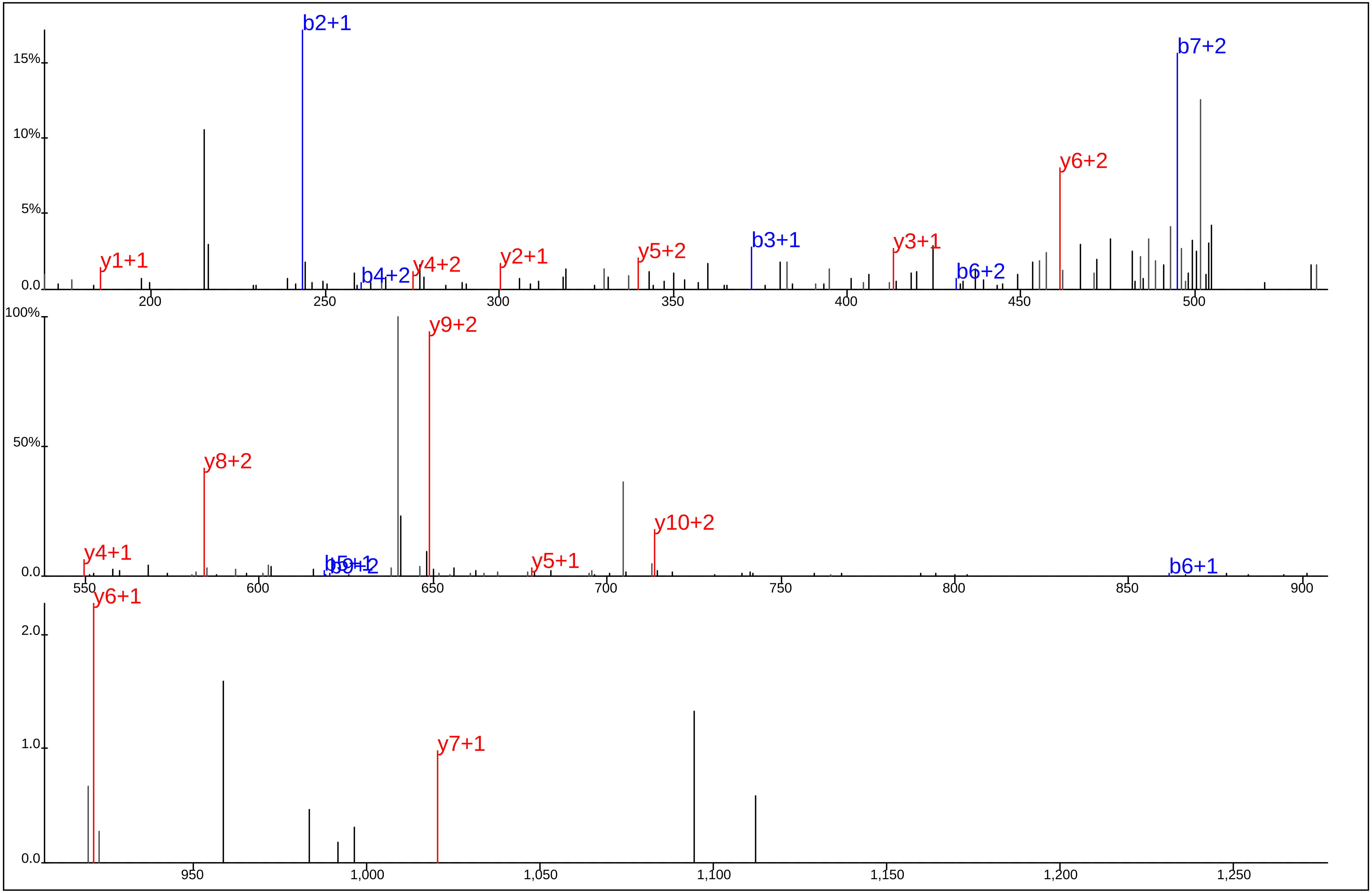

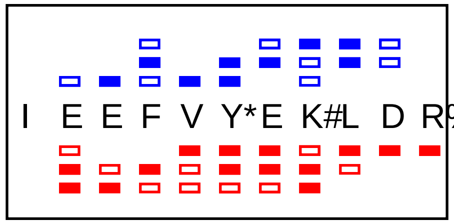

Protein Name: Endophilin B1
gi Accession: 8118530
Precursor Mass: 513.5810+3
Delta Mass: 1.779 ppm
XCorr: 36.07

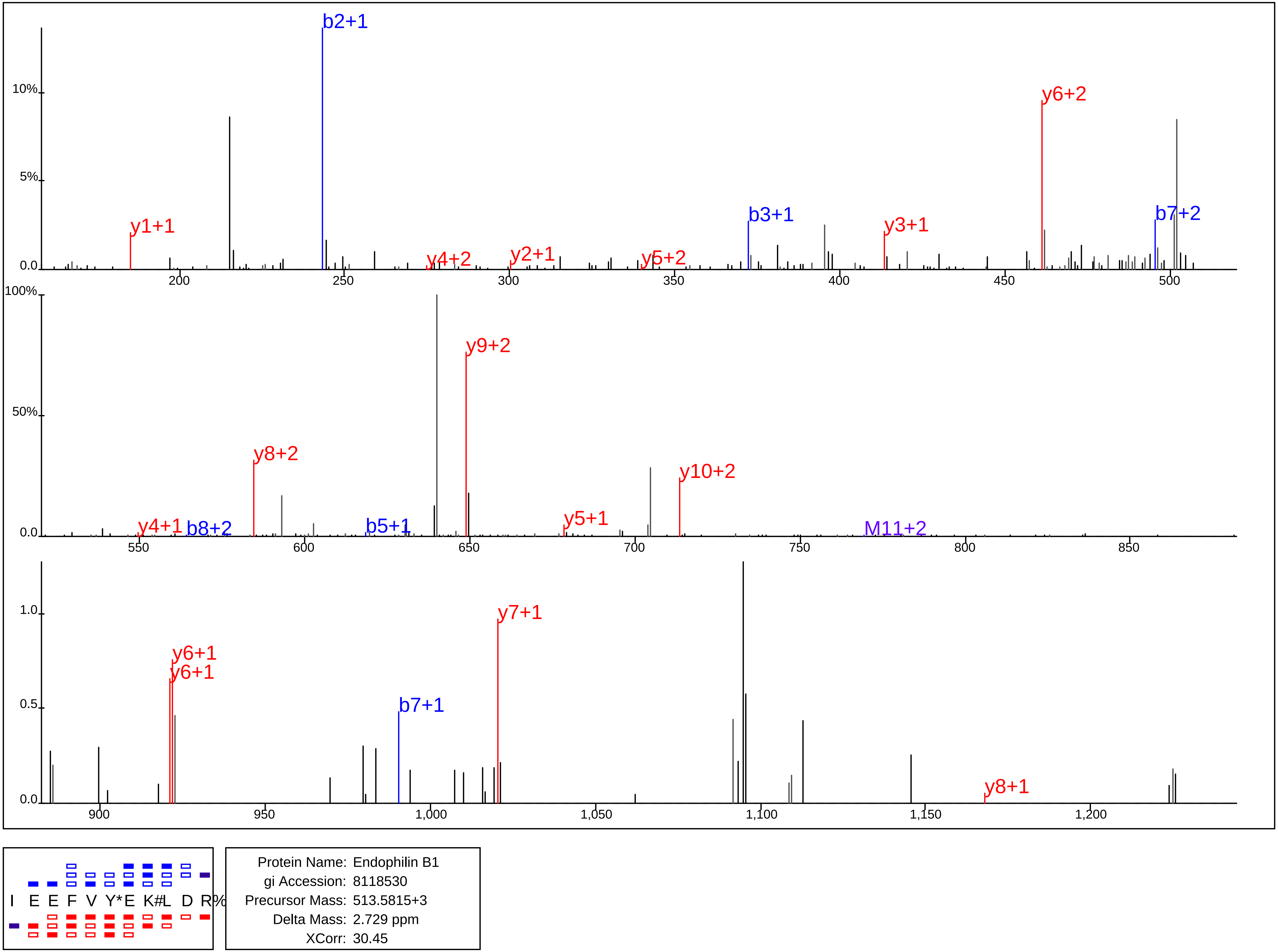

Protein Name:	Endophilin B1
gi Accession:	8118530
Precursor Mass:	513.5815+3
Delta Mass:	2.729 ppm
XCorr:	30.45

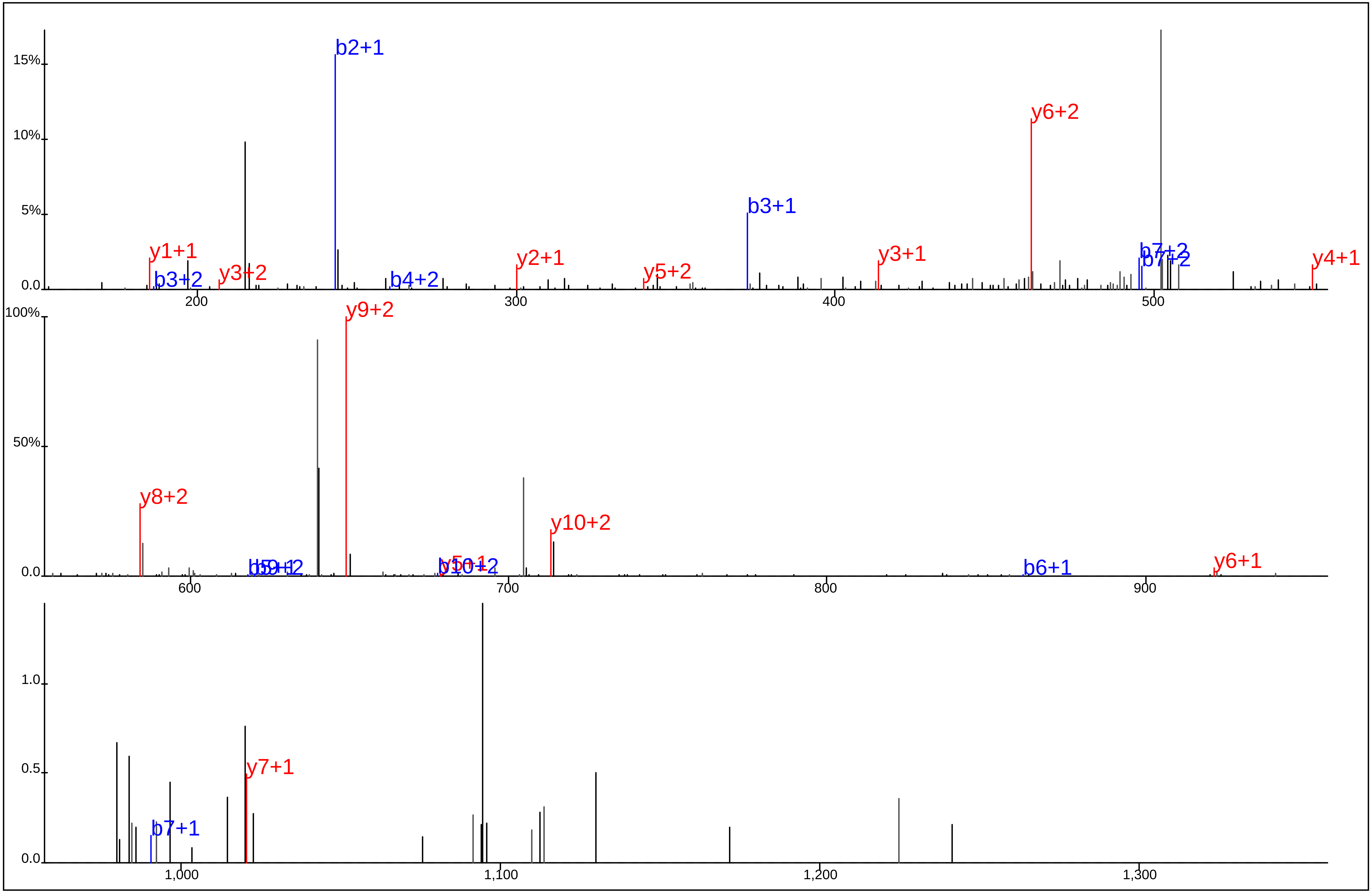

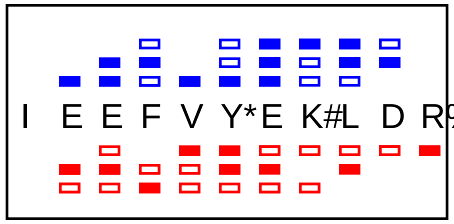

Protein Name:	Endophilin B1
gi Accession:	8118530
Precursor Mass:	513.5807+3
Delta Mass:	1.306 ppm
XCorr:	25.1

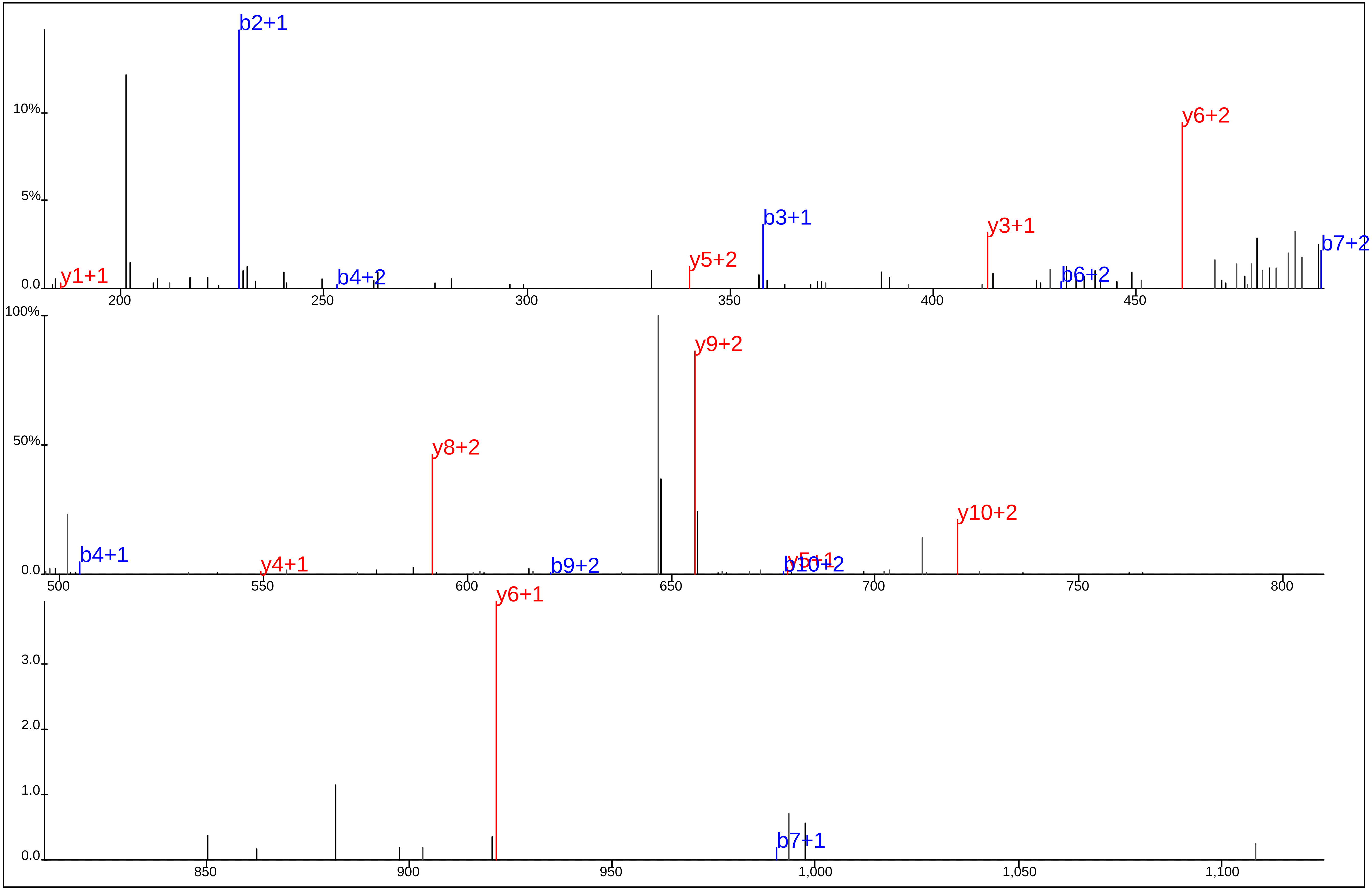

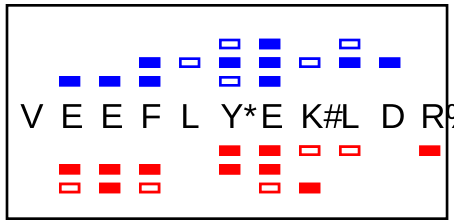

Protein Name: Endophilin B2
gi Accession: 55961144
Precursor Mass: 513.5814+3
Delta Mass: 2.612 ppm
XCorr: 27.77

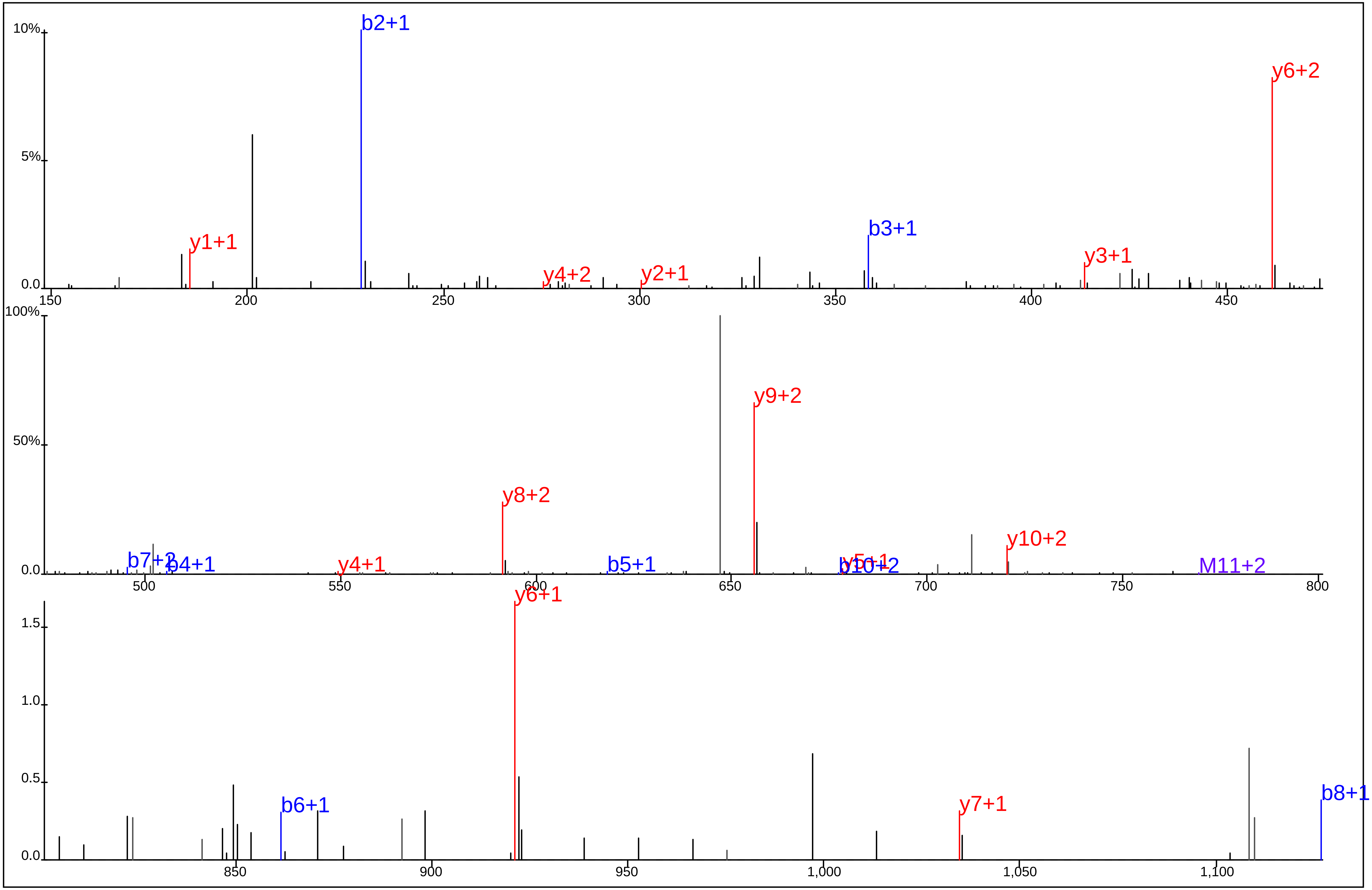

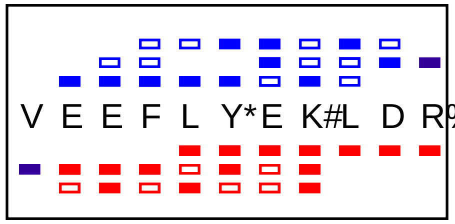

Protein Name: Endophilin B2
gi Accession: 55961144
Precursor Mass: 513.5809+3
Delta Mass: 1.540 ppm
XCorr: 24.56

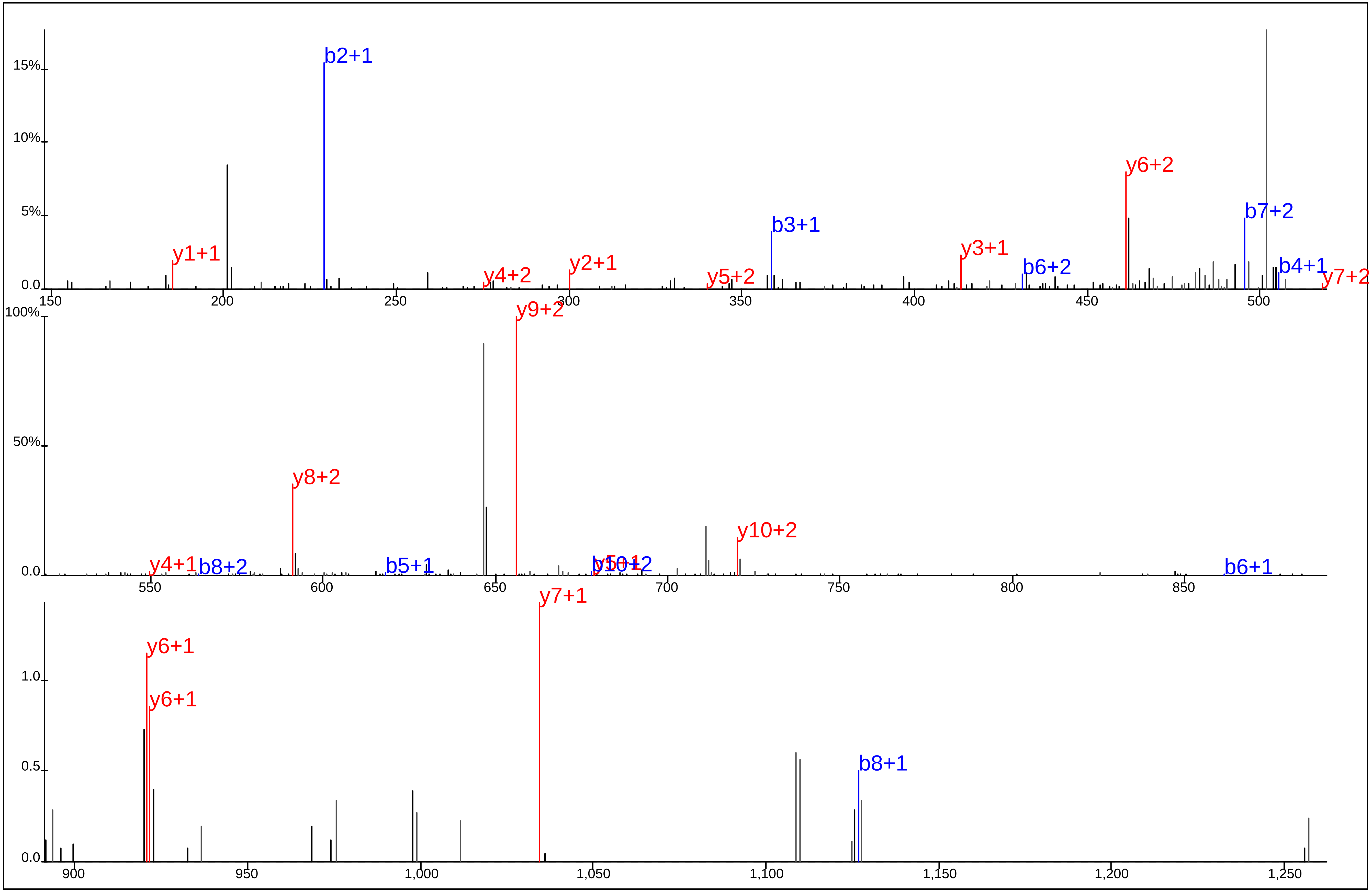

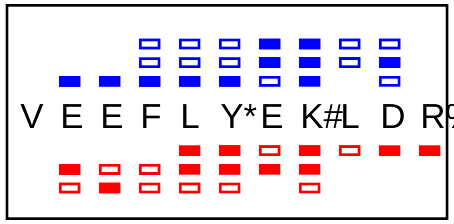

Protein Name: Endophilin B2
gi Accession: 55961144
Precursor Mass: 513.5803+3
Delta Mass: 0.350 ppm
XCorr: 31.77

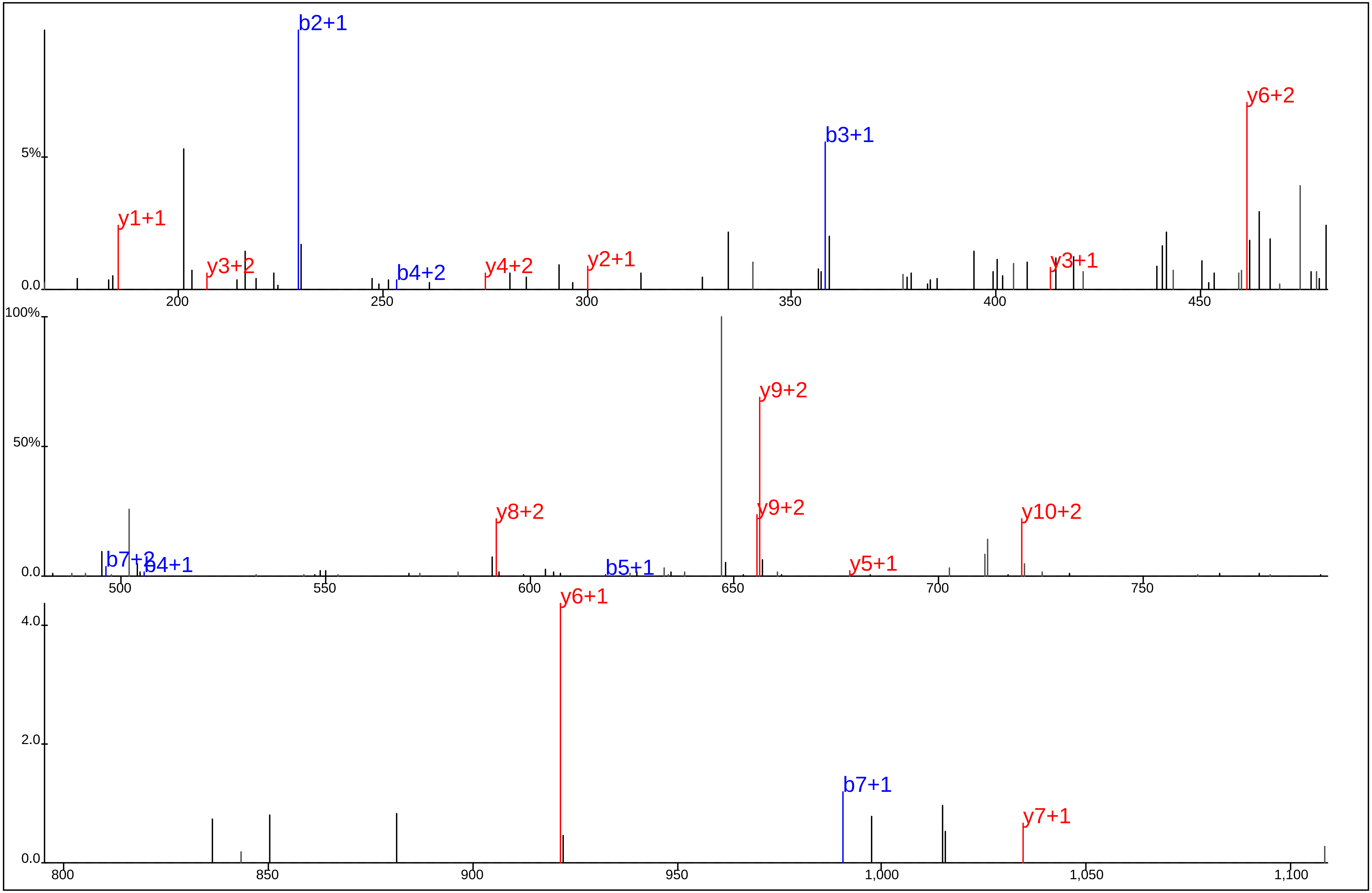

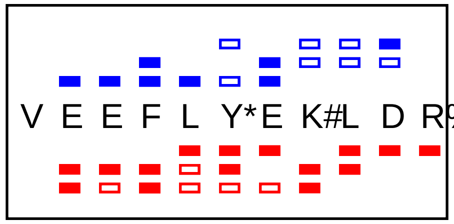

Protein Name: Endophilin B2
gi Accession: 55961144
Precursor Mass: 513.5810+3
Delta Mass: 1.779 ppm
XCorr: 22.81

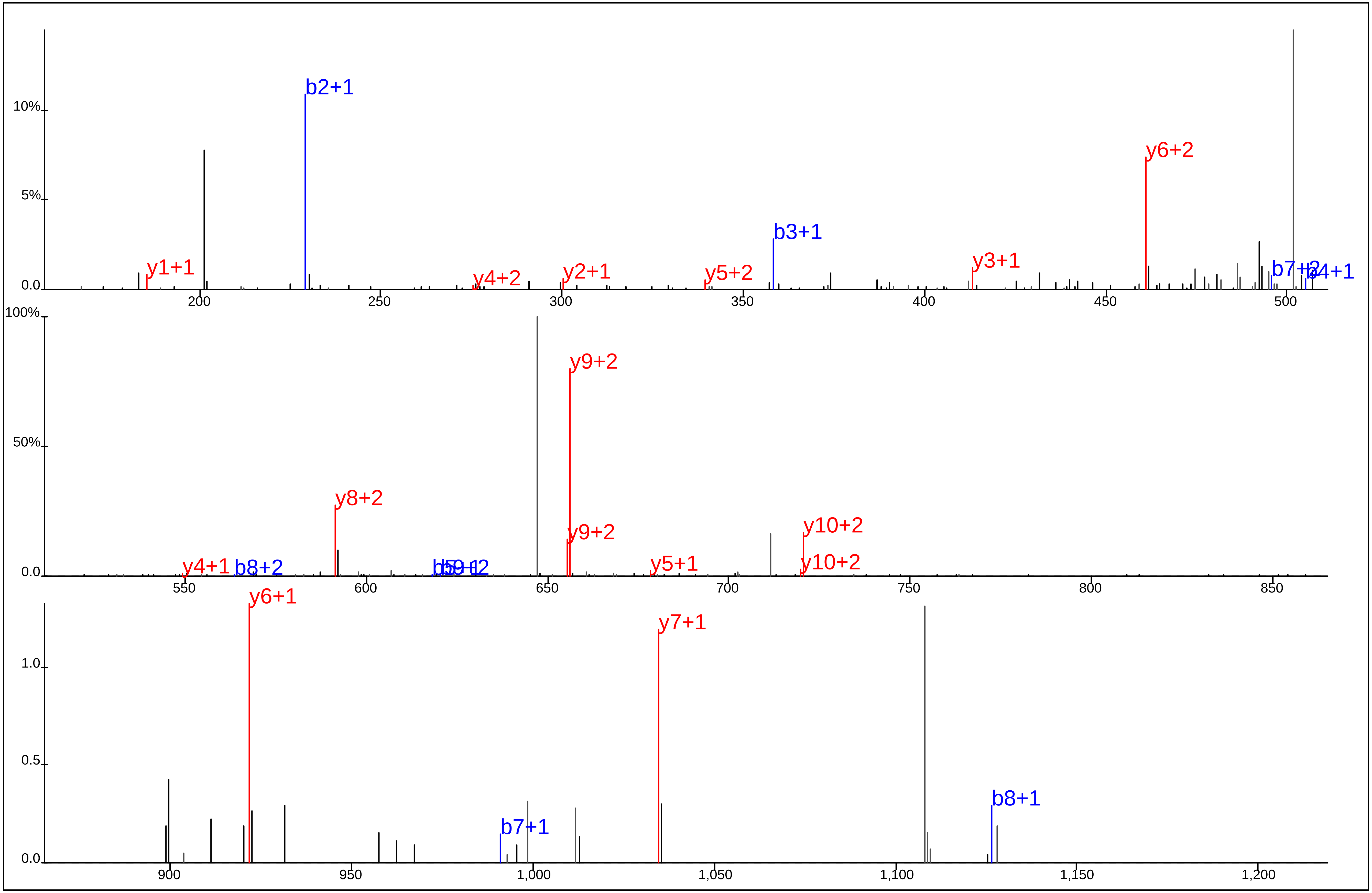

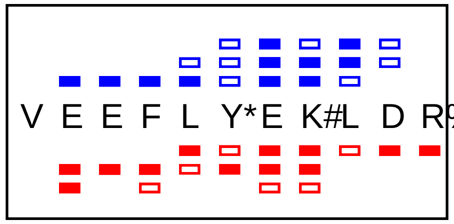

Protein Name: Endophilin B2
gi Accession: 55961144
Precursor Mass: 513.5808+3
Delta Mass: 1.423 ppm
XCorr: 32.48

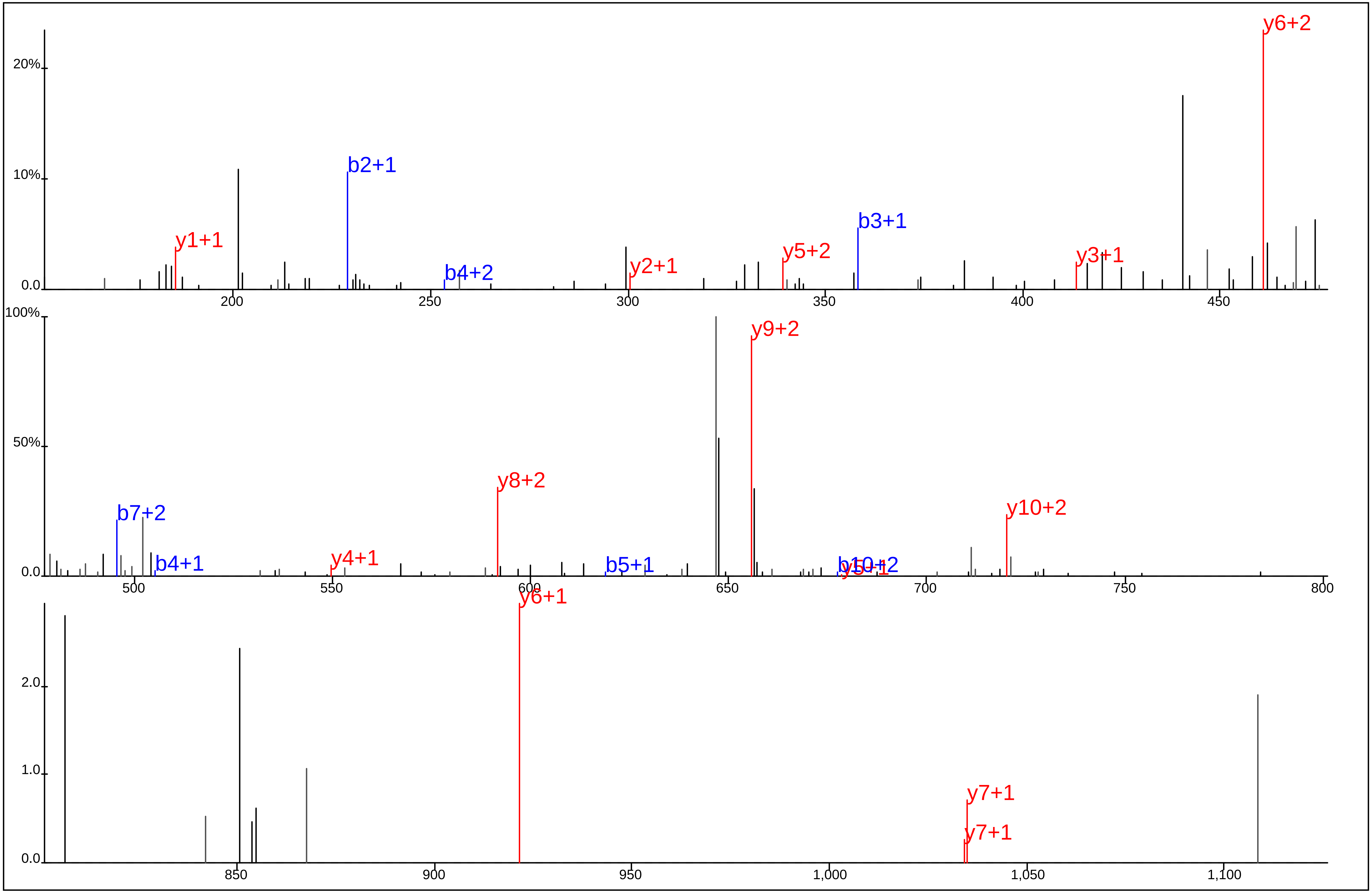

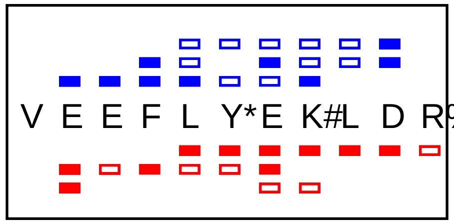

Protein Name: Endophilin B2
gi Accession: 55961144
Precursor Mass: 513.5810+3
Delta Mass: 1.779 ppm
XCorr: 25.06

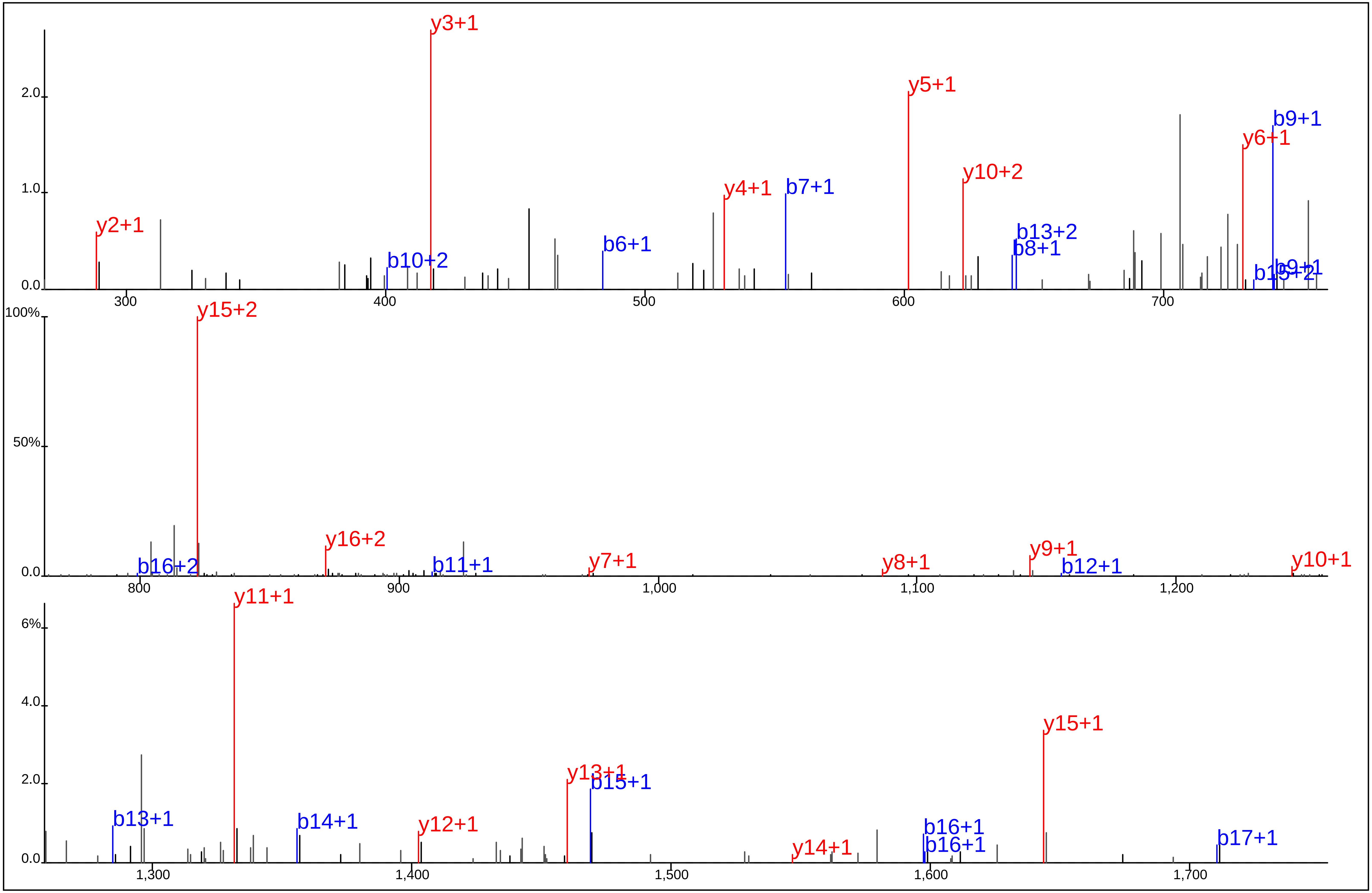

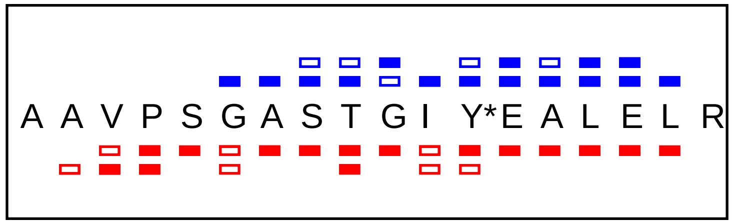

Protein Name: Enolase 2
gi Accession: 114665857
Precursor Mass: 942.9620+2
Delta Mass: 3.479 ppm
XCorr: 100.46

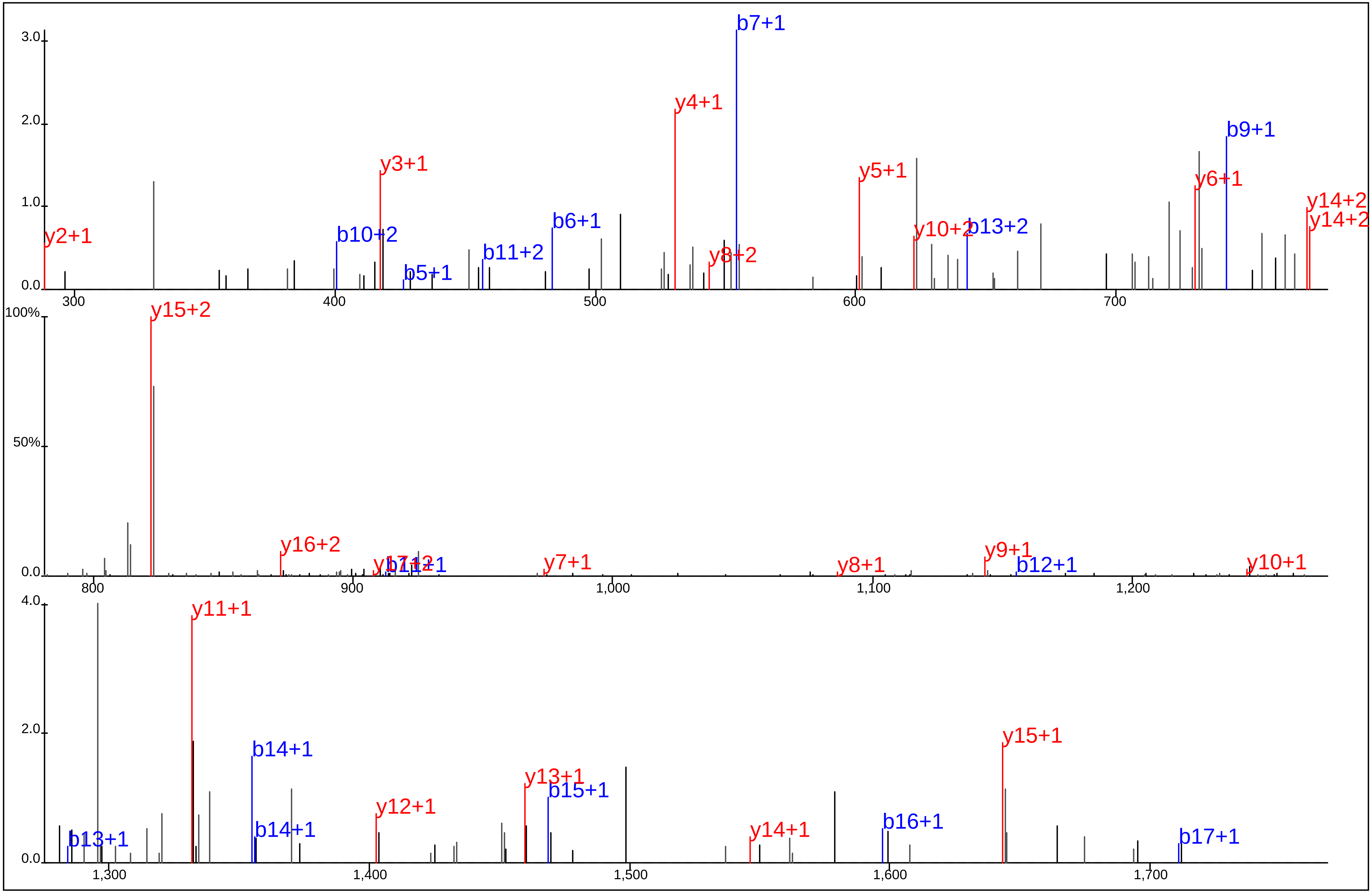

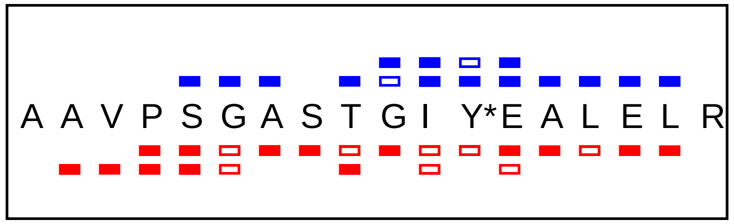

Protein Name: Enolase 2
gi Accession: 114665857
Precursor Mass: 942.9605+2
Delta Mass: 1.856 ppm
XCorr: 77.72

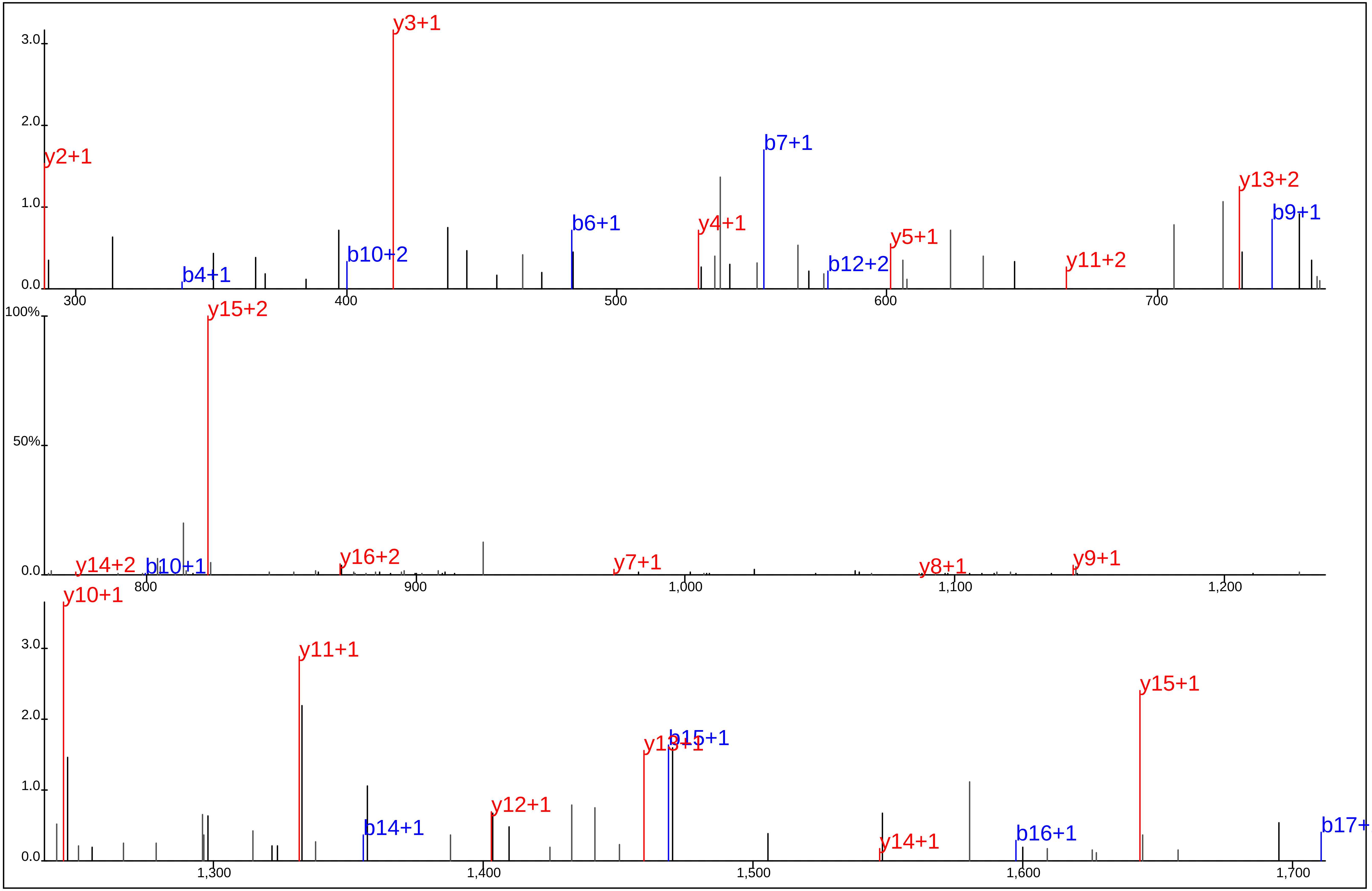

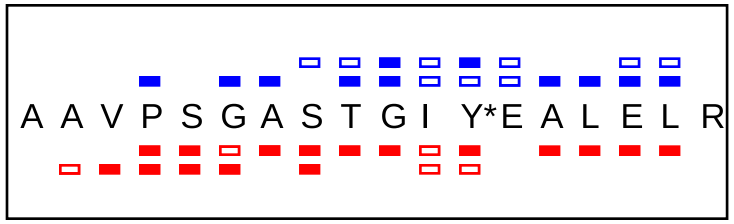

Protein Name: Enolase 2
gi Accession: 114665857
Precursor Mass: 942.9613+2
Delta Mass: 2.699 ppm
XCorr: 67.13

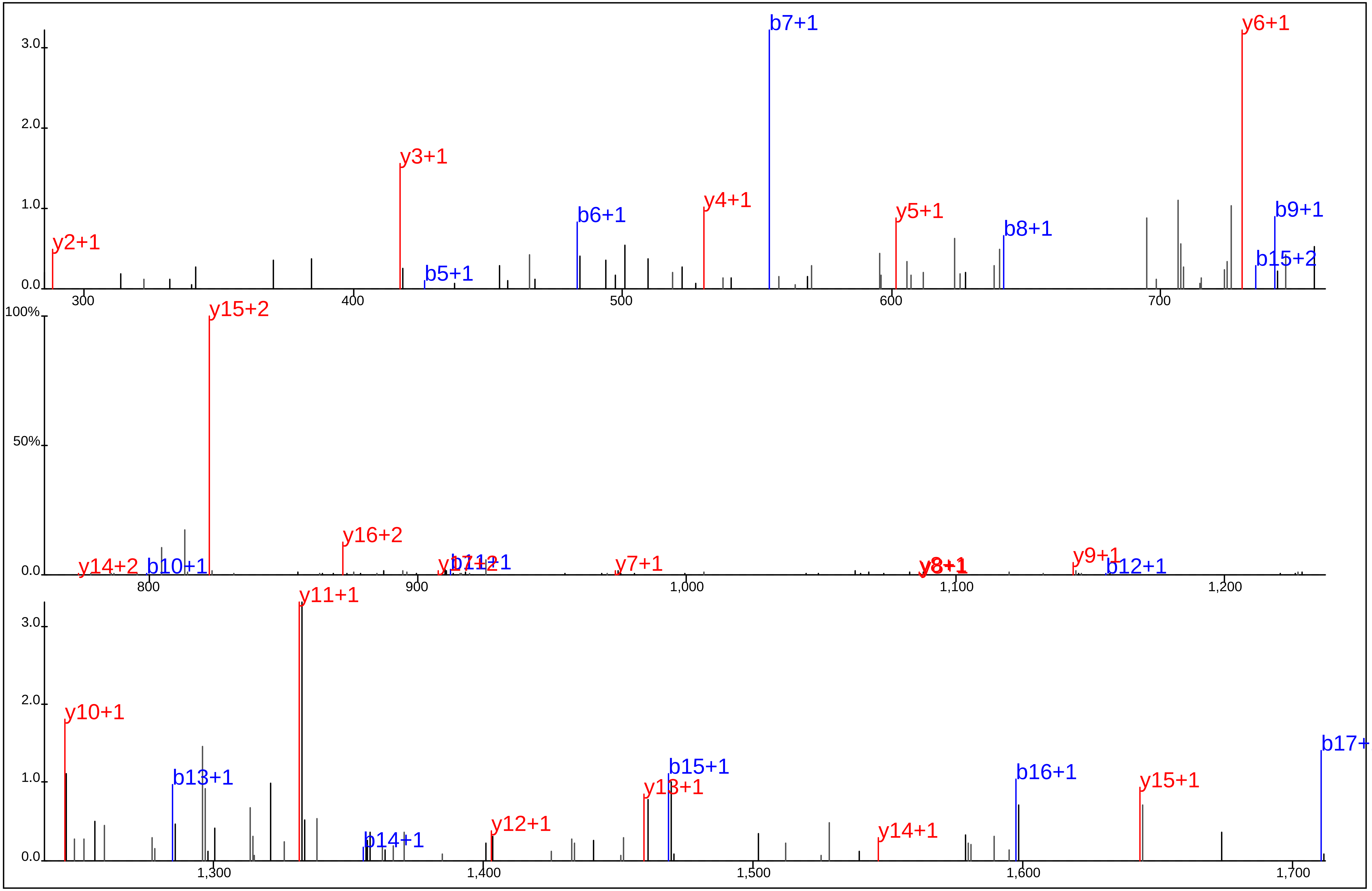

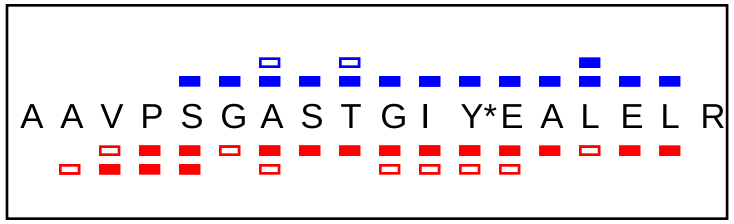

Protein Name: Enolase 2
gi Accession: 114665857
Precursor Mass: 942.9613+2
Delta Mass: 2.699 ppm
XCorr: 70.99

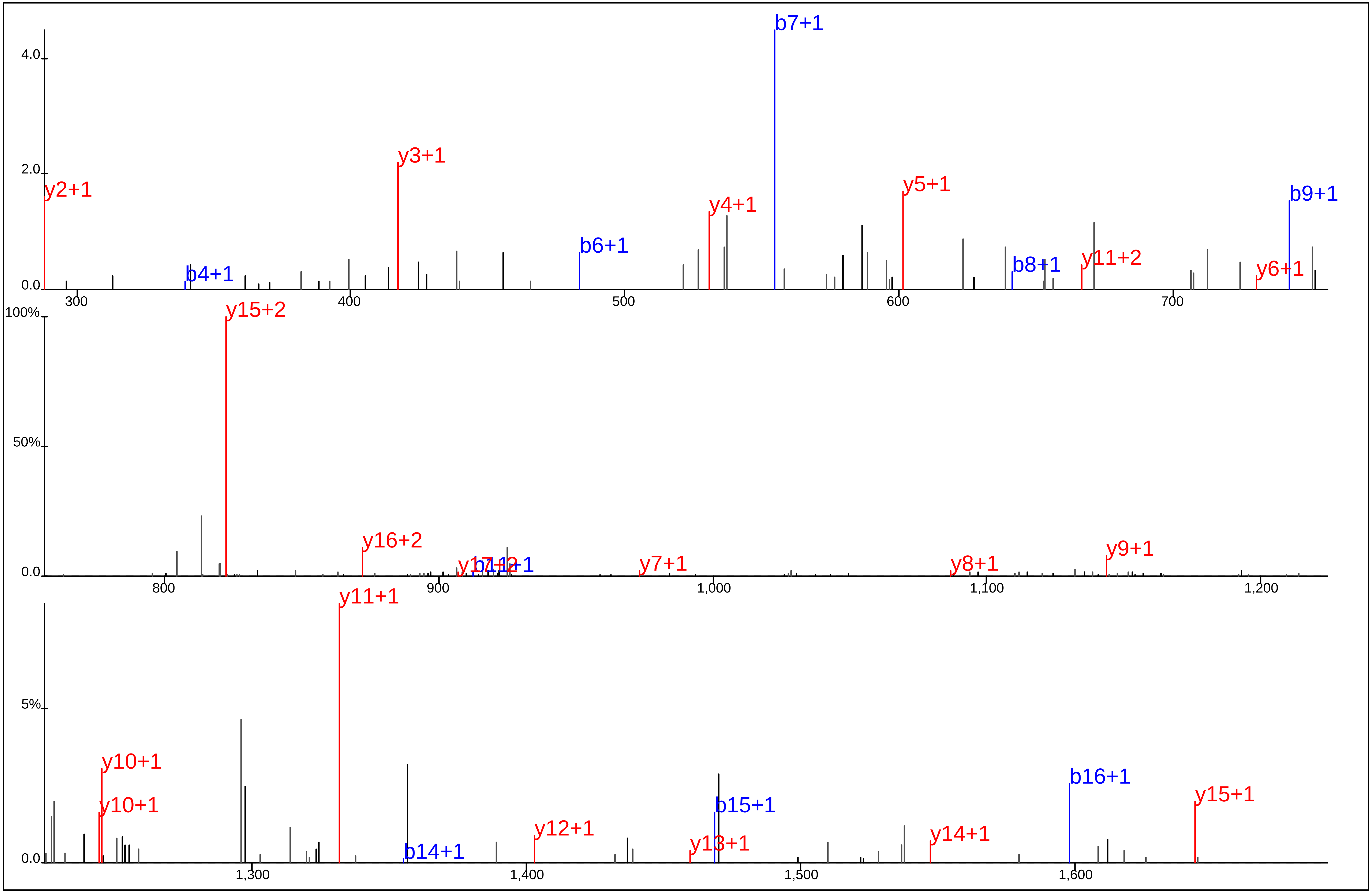

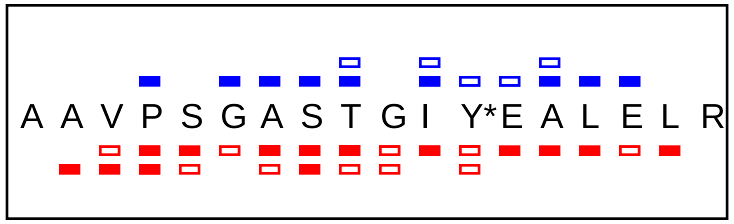

Protein Name:	Enolase 2
gi Accession:	114665857
Precursor Mass:	942.9591+2
Delta Mass:	0.370 ppm
XCorr:	68.9

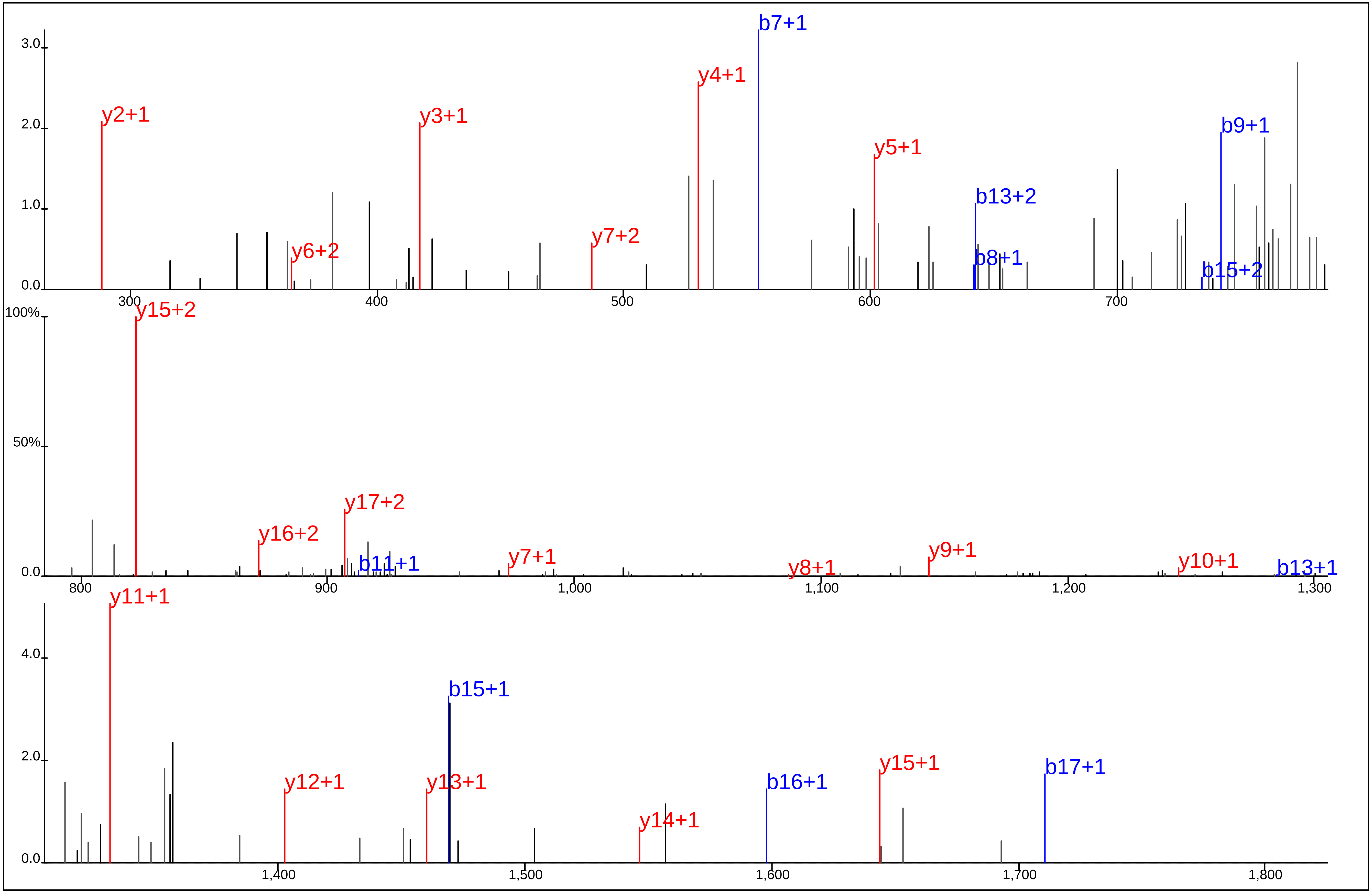

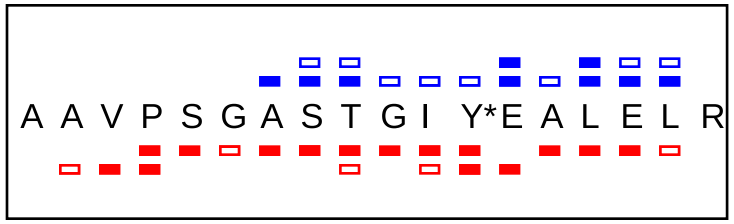

Protein Name:	Enolase 2
gi Accession:	114665857
Precursor Mass:	942.9606+2
Delta Mass:	1.988 ppm
XCorr:	84.57

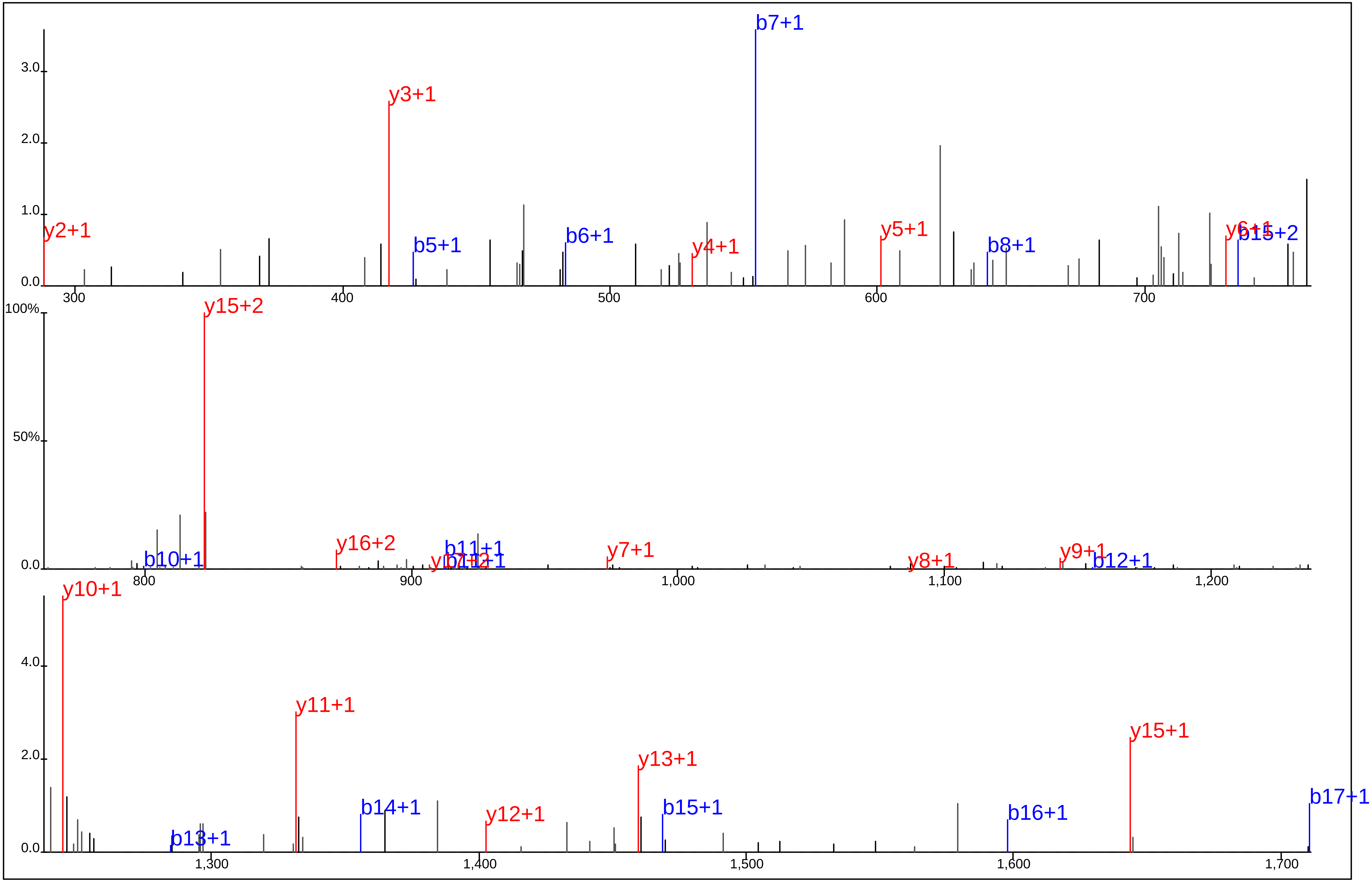

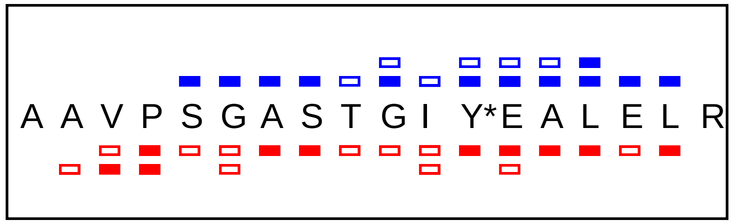

Protein Name: Enolase 2
gi Accession: 114665857
Precursor Mass: 942.9592+2
Delta Mass: 0.498 ppm
XCorr: 52.8

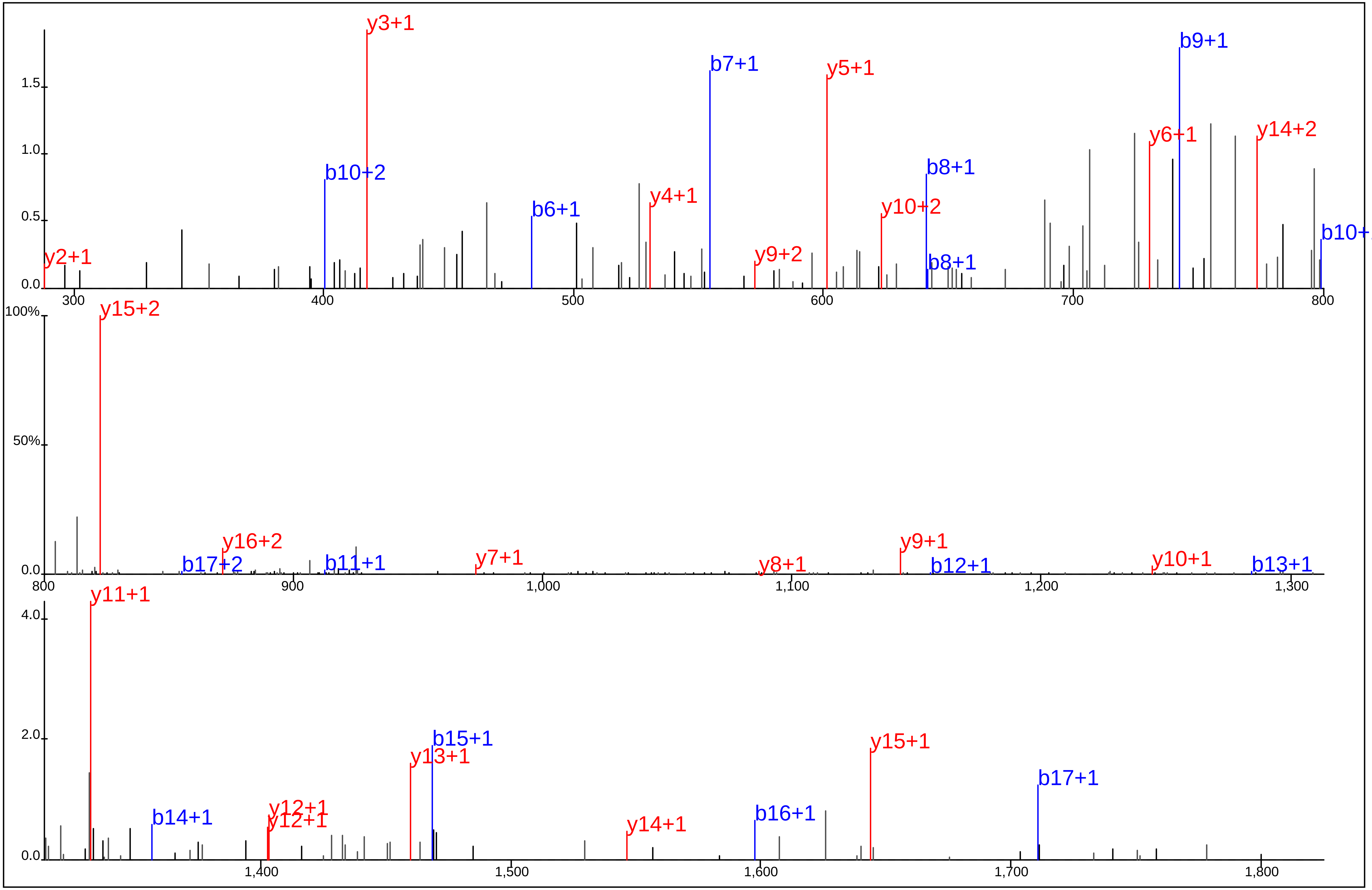

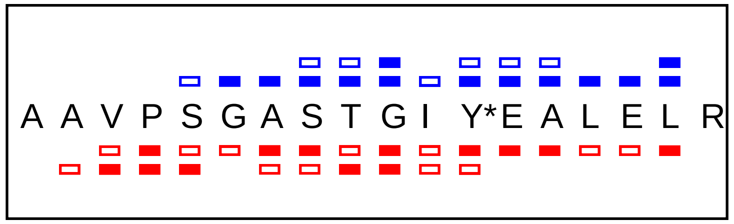

Protein Name: Enolase 2
gi Accession: 114665857
Precursor Mass: 942.9608+2
Delta Mass: 2.116 ppm
XCorr: 94.61

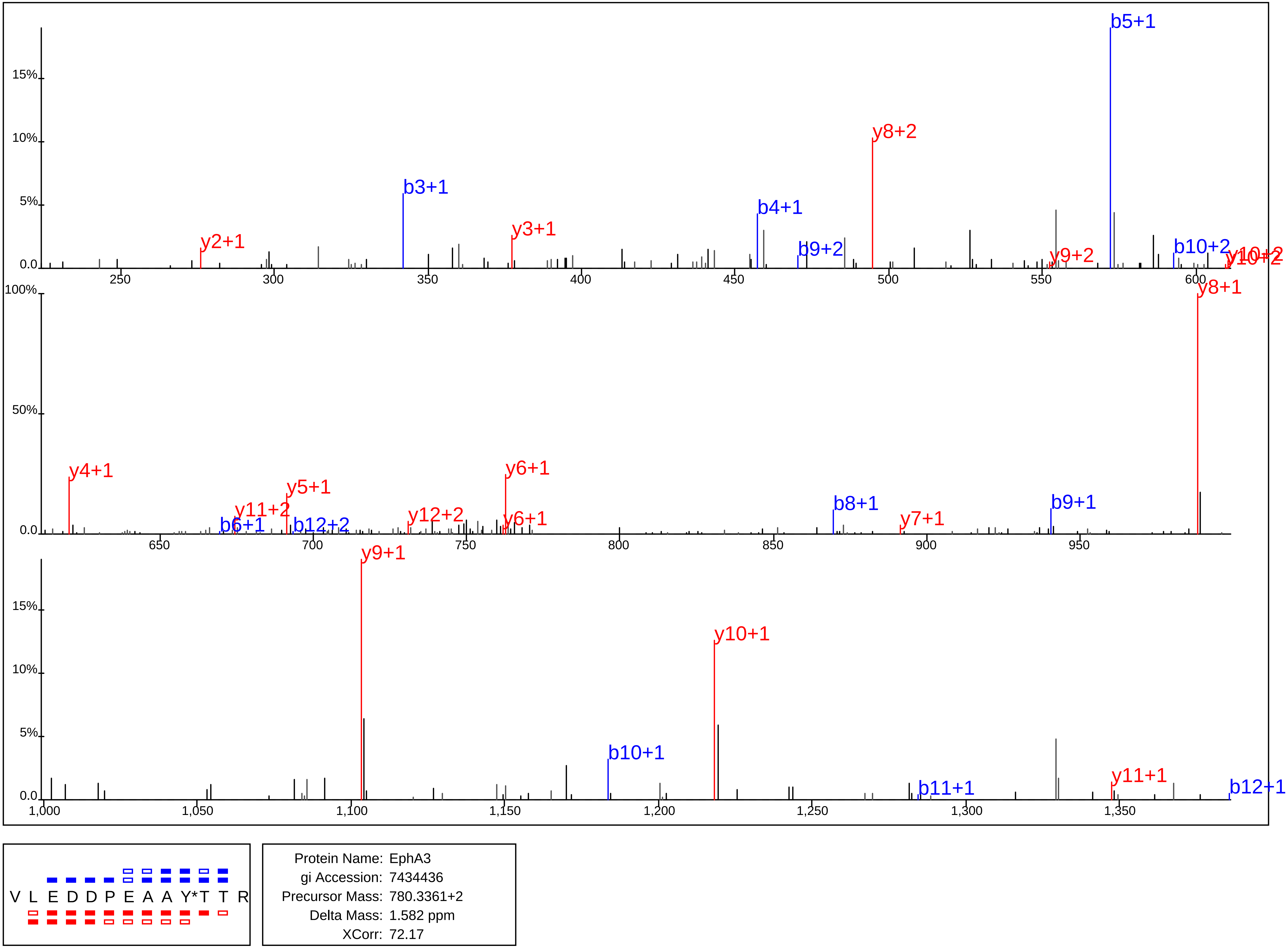

Protein Name: EphA3
gi Accession: 7434436
Precursor Mass: 780.3361+2
Delta Mass: 1.582 ppm
XCorr: 72.17

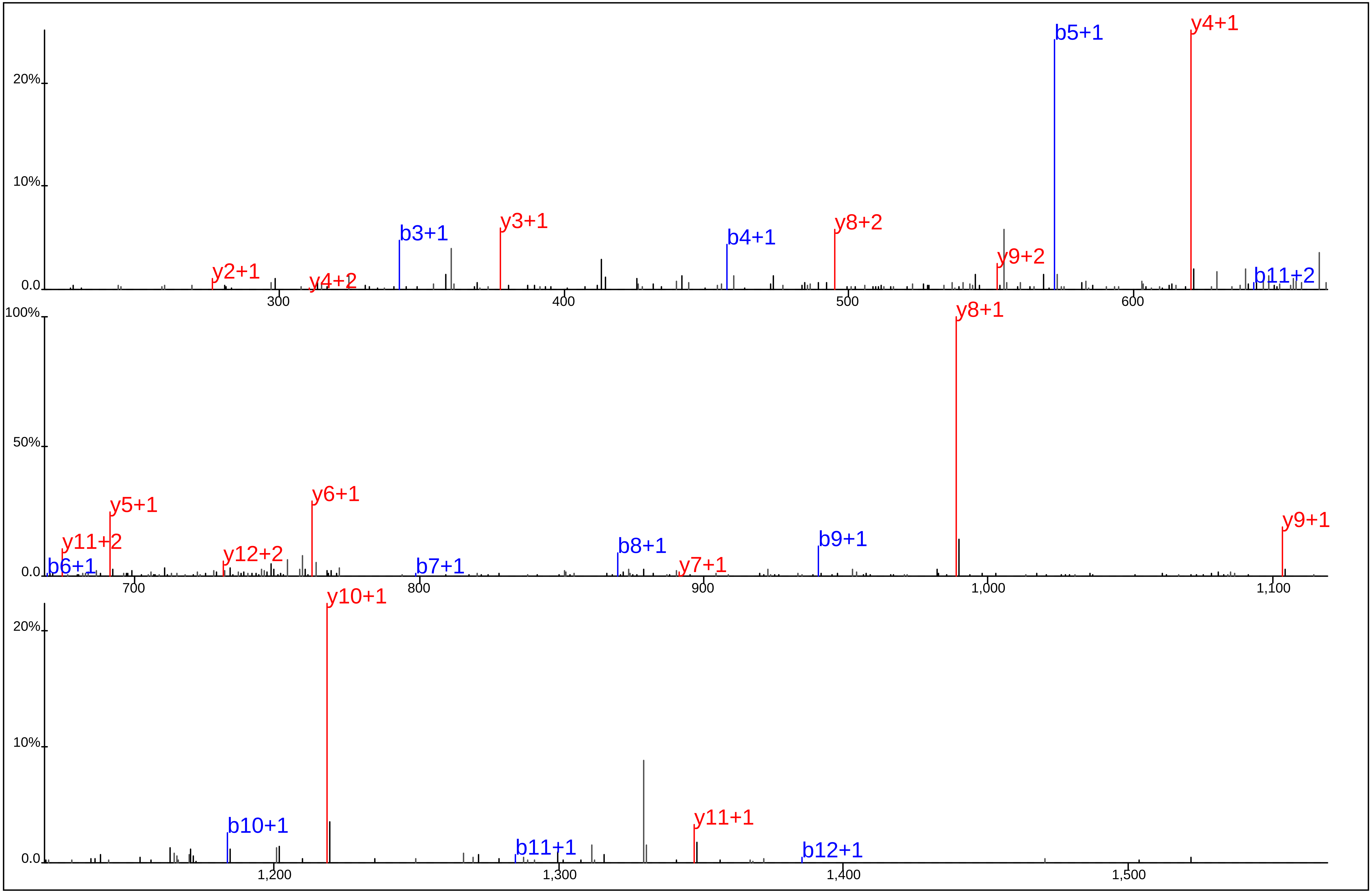

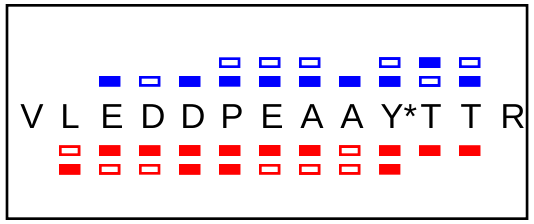

Protein Name: EphA3
gi Accession: 7434436
Precursor Mass: 780.3369+2
Delta Mass: 2.525 ppm
XCorr: 61.97

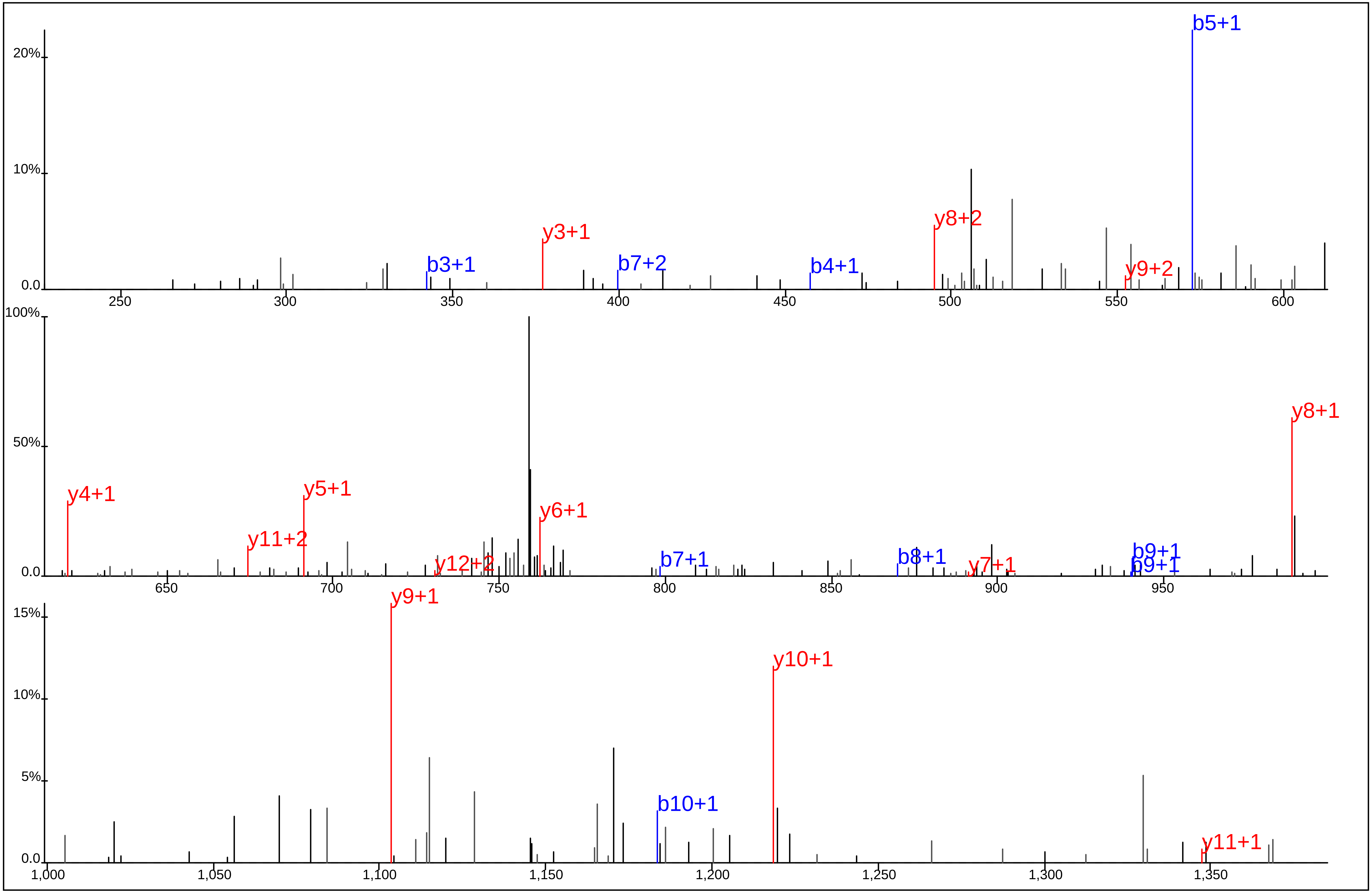

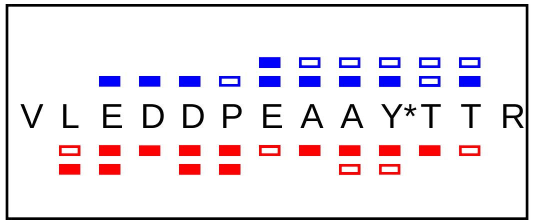

Protein Name: EphA3
gi Accession: 7434436
Precursor Mass: 780.3383+2
Delta Mass: 4.327 ppm
XCorr: 34.49

409 - EphA3_3441605.pdf

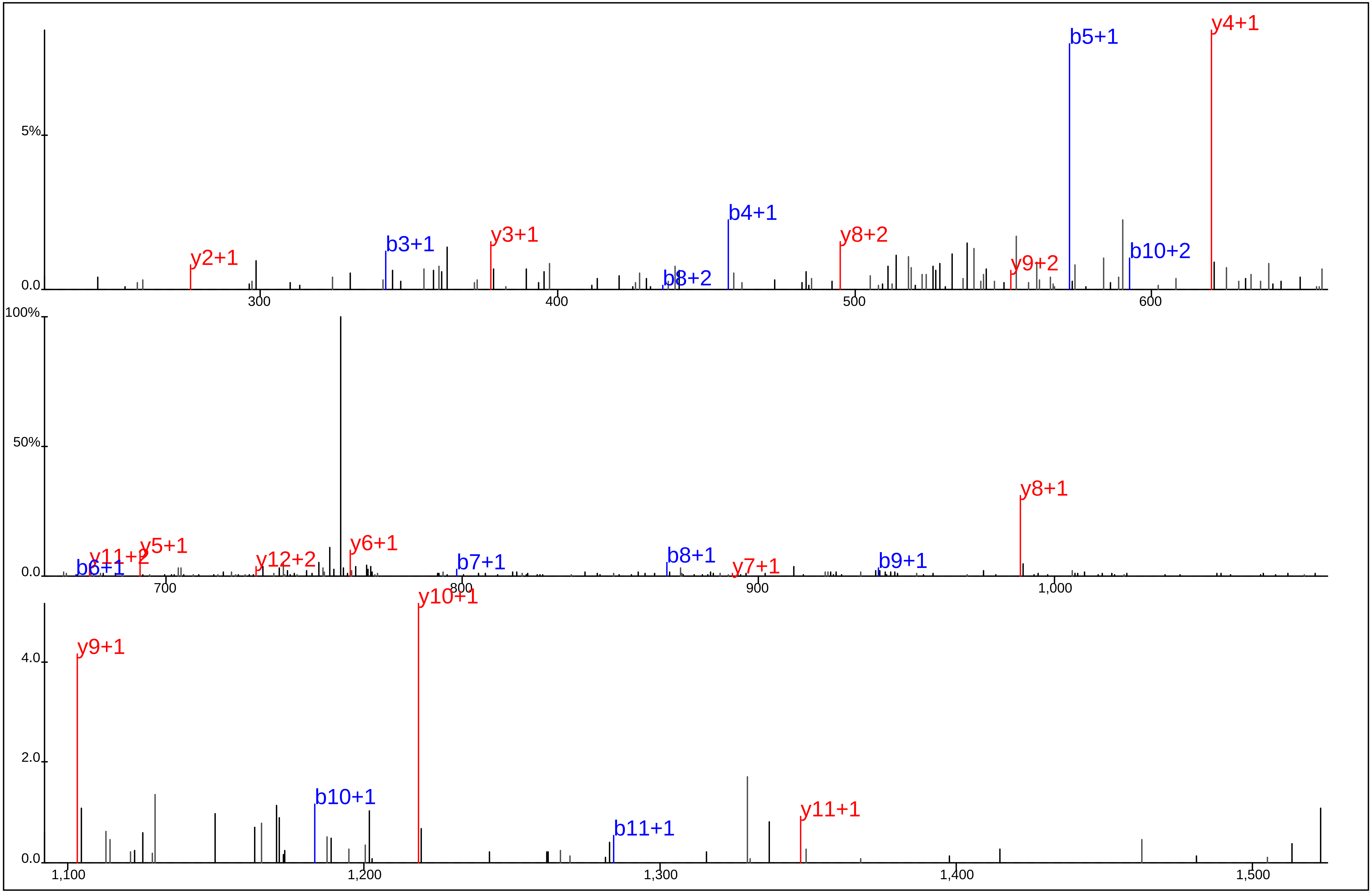

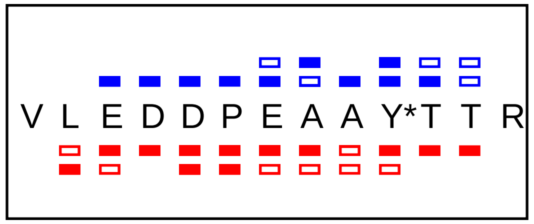

Protein Name: EphA3
gi Accession: 7434436
Precursor Mass: 780.3367+2
Delta Mass: 2.371 ppm
XCorr: 50.8

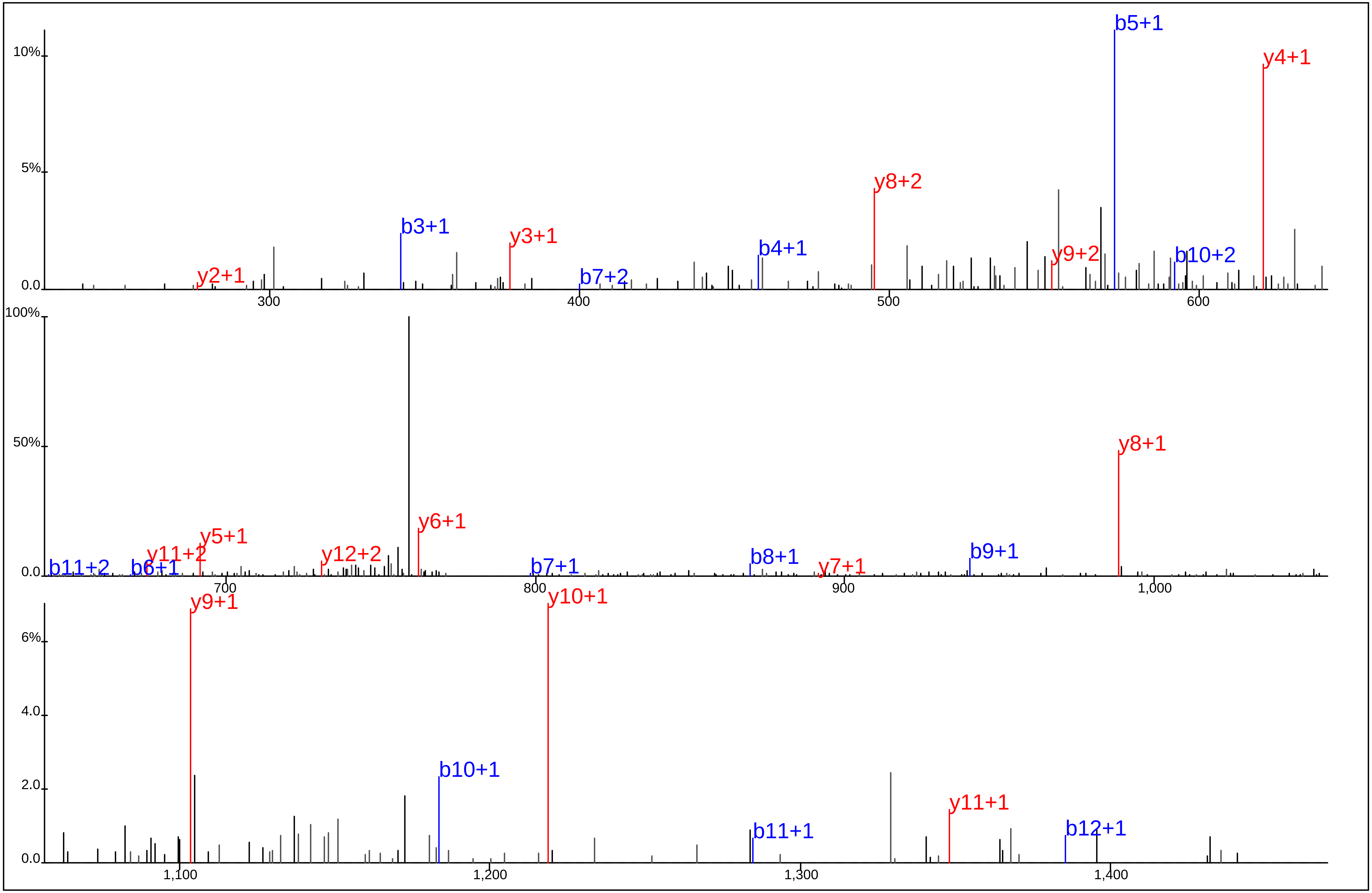

V L E D D P E A A Y*T T R

Protein Name: EphA3
gi Accession: 7434436
Precursor Mass: 780.3342+2
Delta Mass: 0.918 ppm
XCorr: 57.6

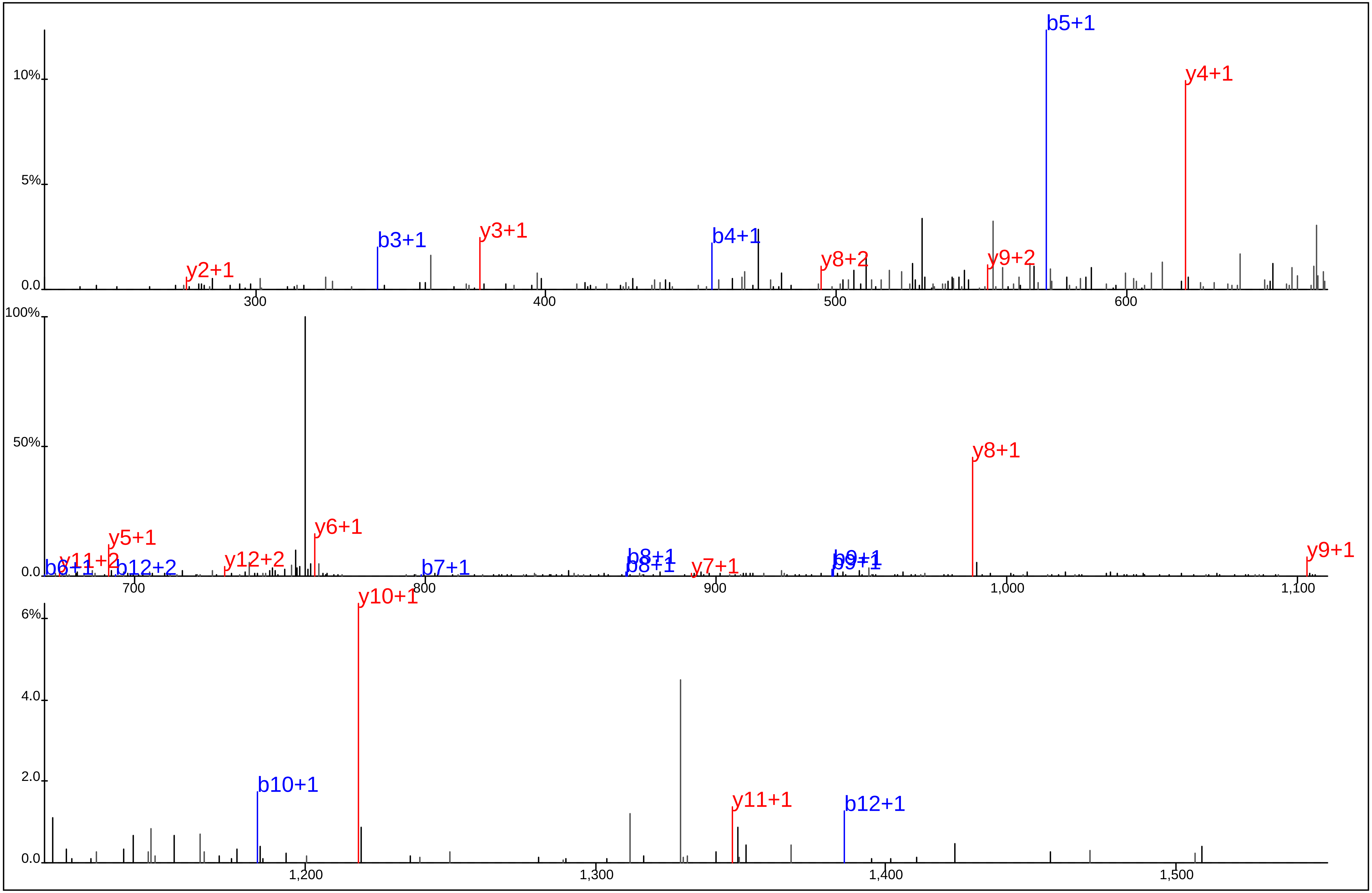

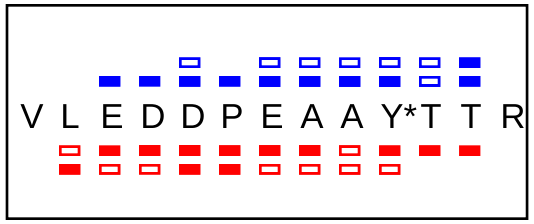

Protein Name:	EphA3
gi Accession:	7434436
Precursor Mass:	780.3357+2
Delta Mass:	1.037 ppm
XCorr:	52.18

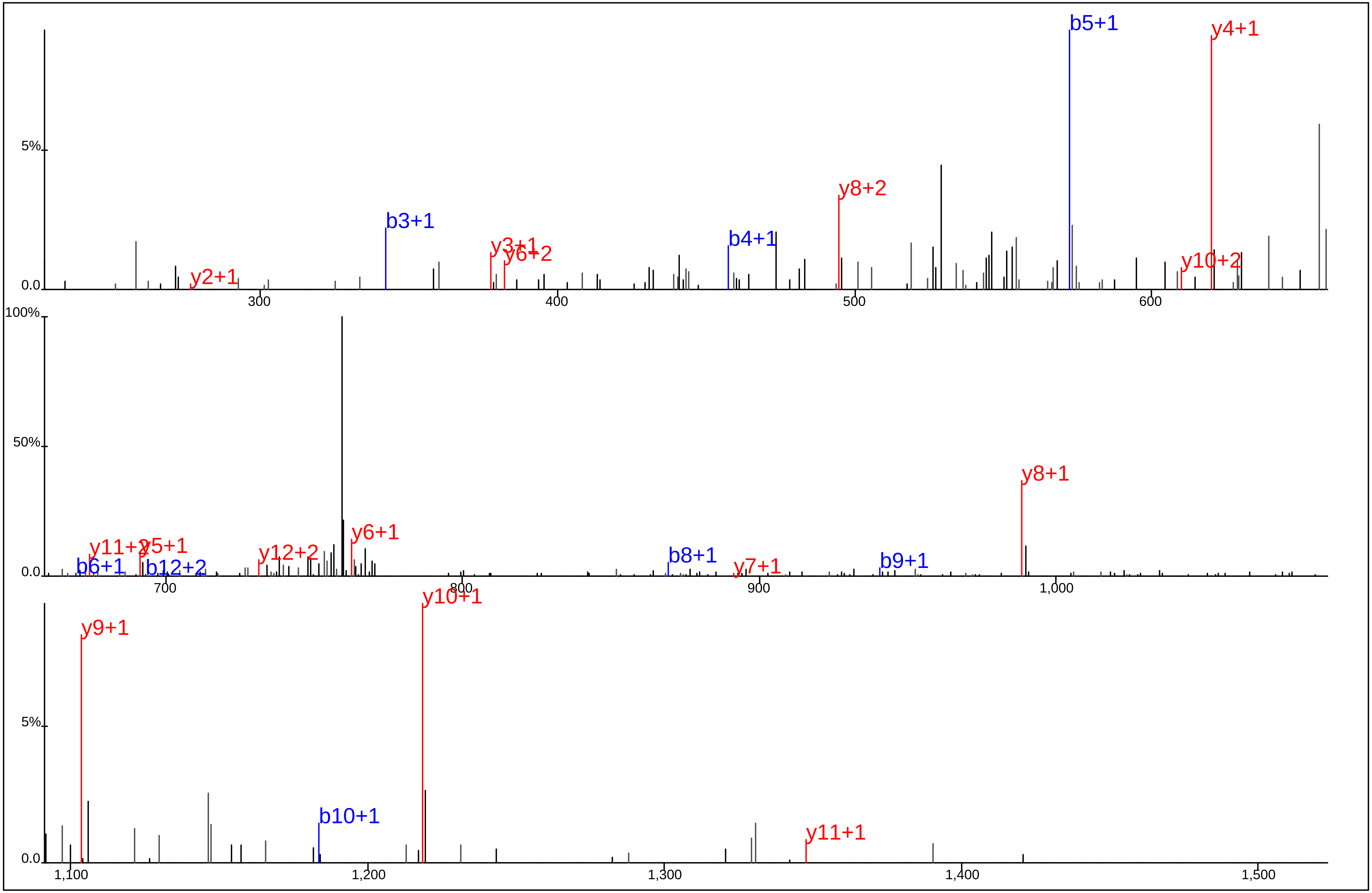

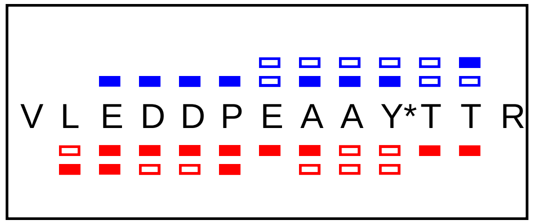

Protein Name: EphA3
gi Accession: 7434436
Precursor Mass: 780.3395+2
Delta Mass: 5.891 ppm
XCorr: 40.13

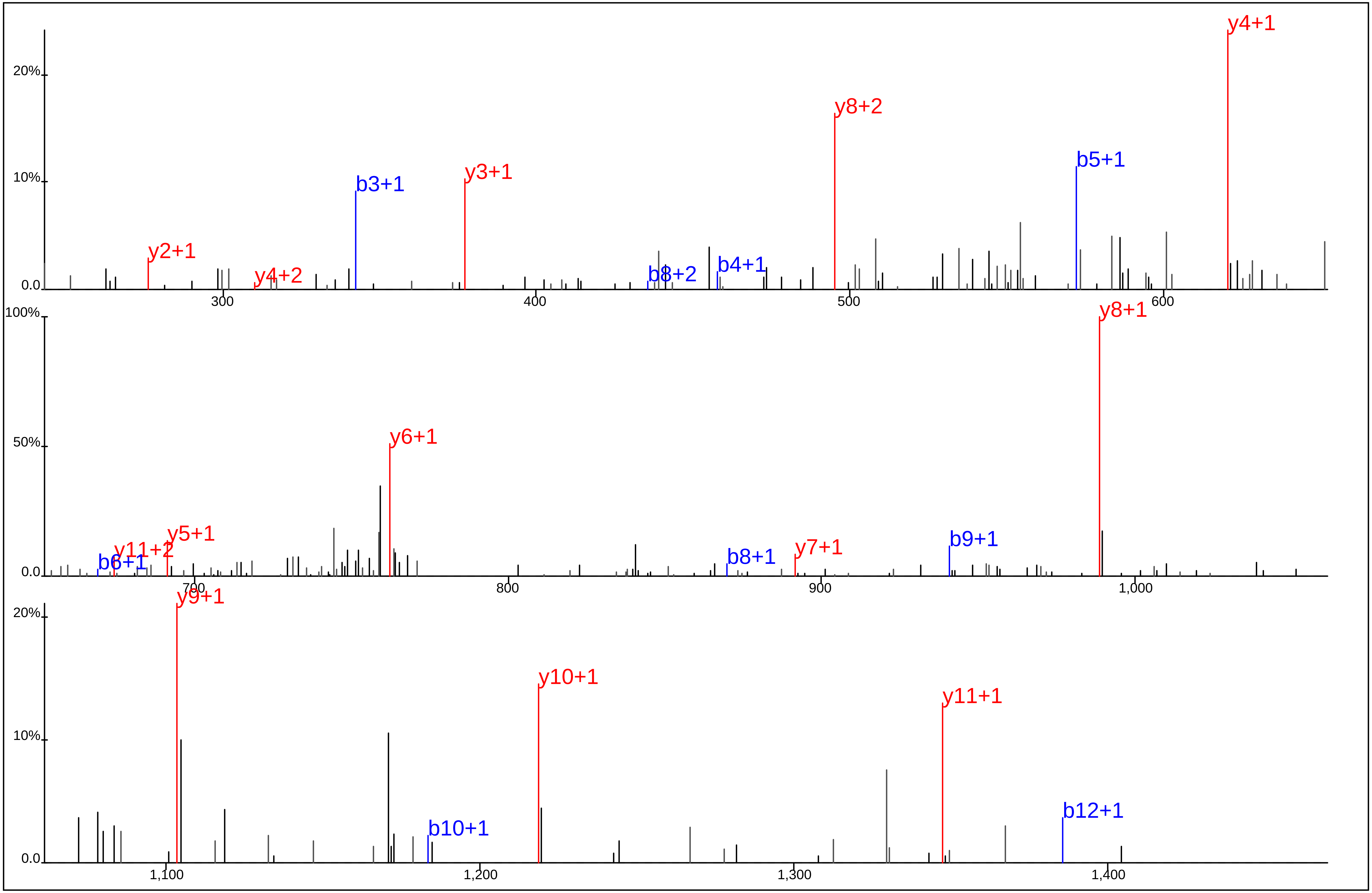

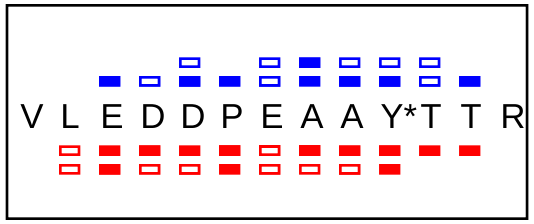

Protein Name: EphA3
gi Accession: 7434436
Precursor Mass: 780.3355+2
Delta Mass: 0.800 ppm
XCorr: 84.5

414 - EphA3_3453776.pdf

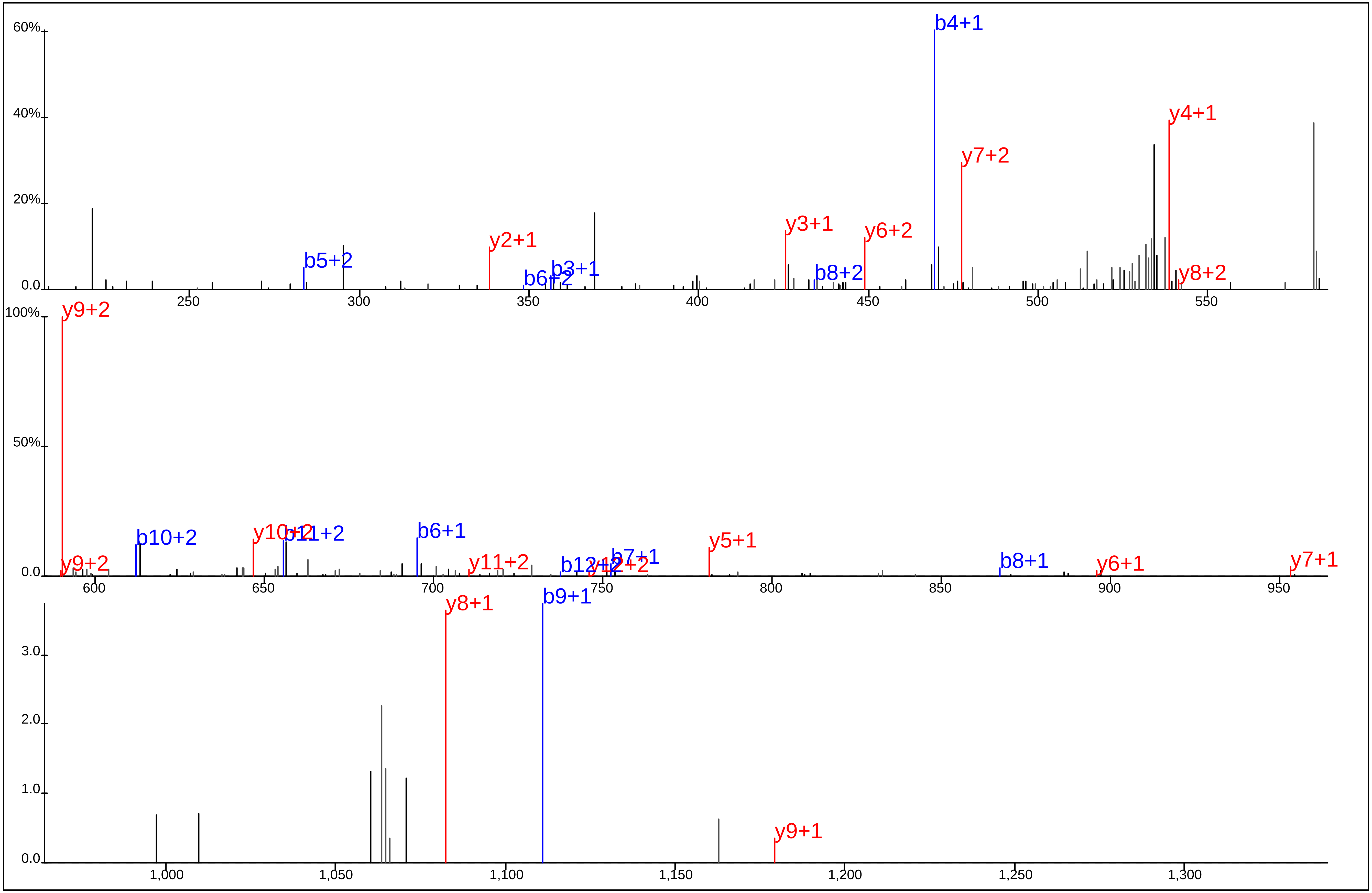

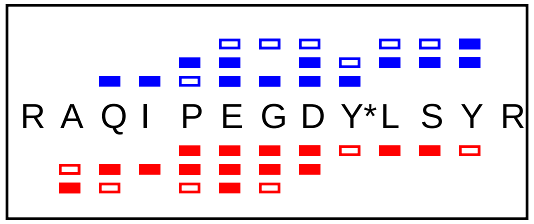

Protein Name: Erbin
gi Accession: 7022273
Precursor Mass: 549.9238+3
Delta Mass: 2.505 ppm
XCorr: 51.74

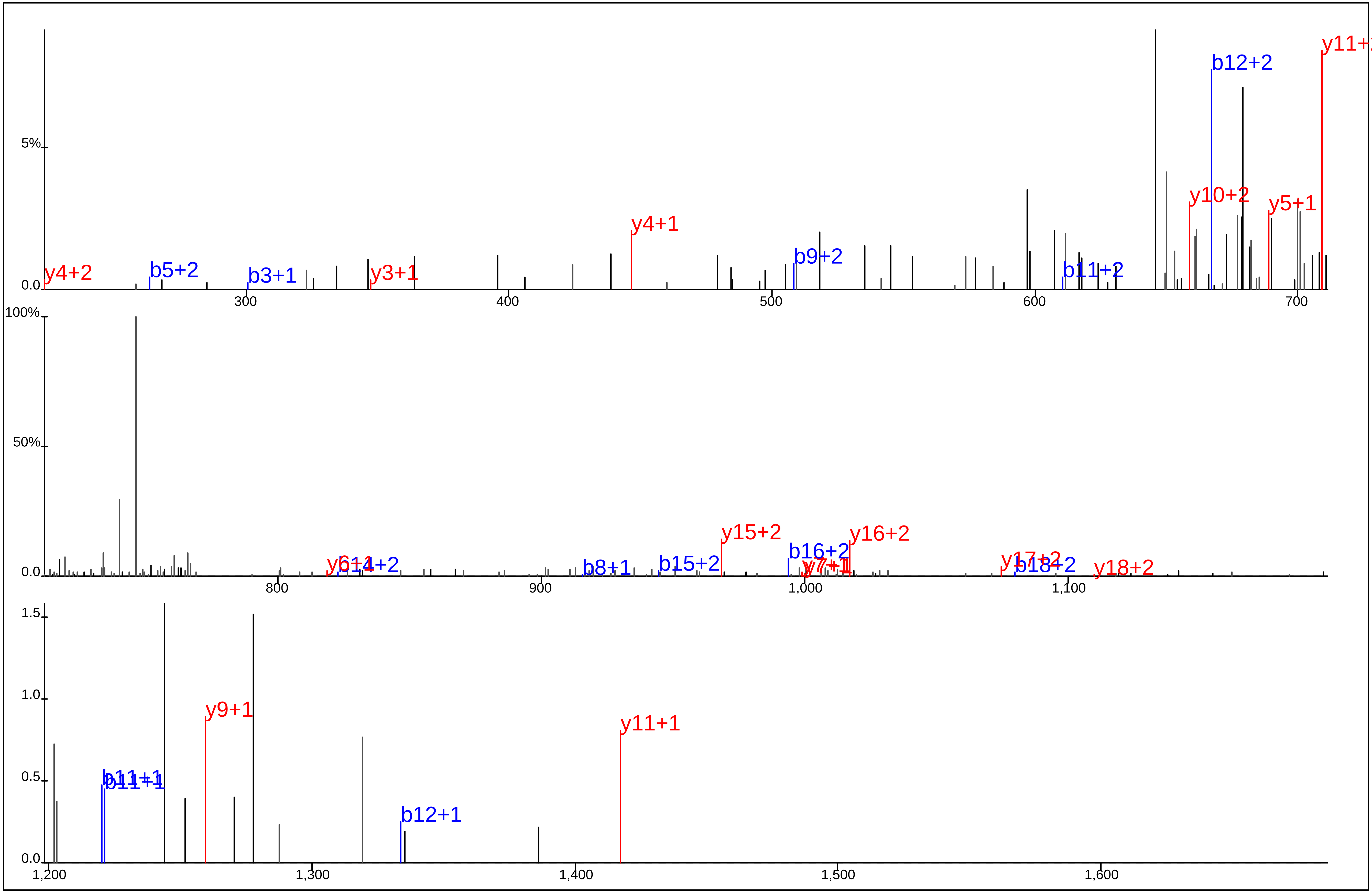

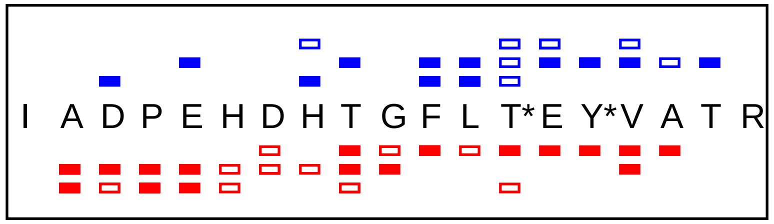

Protein Name: ERK1
gi Accession: 31221
Precursor Mass: 778.0002+3
Delta Mass: 7.599 ppm
XCorr: 2.5865

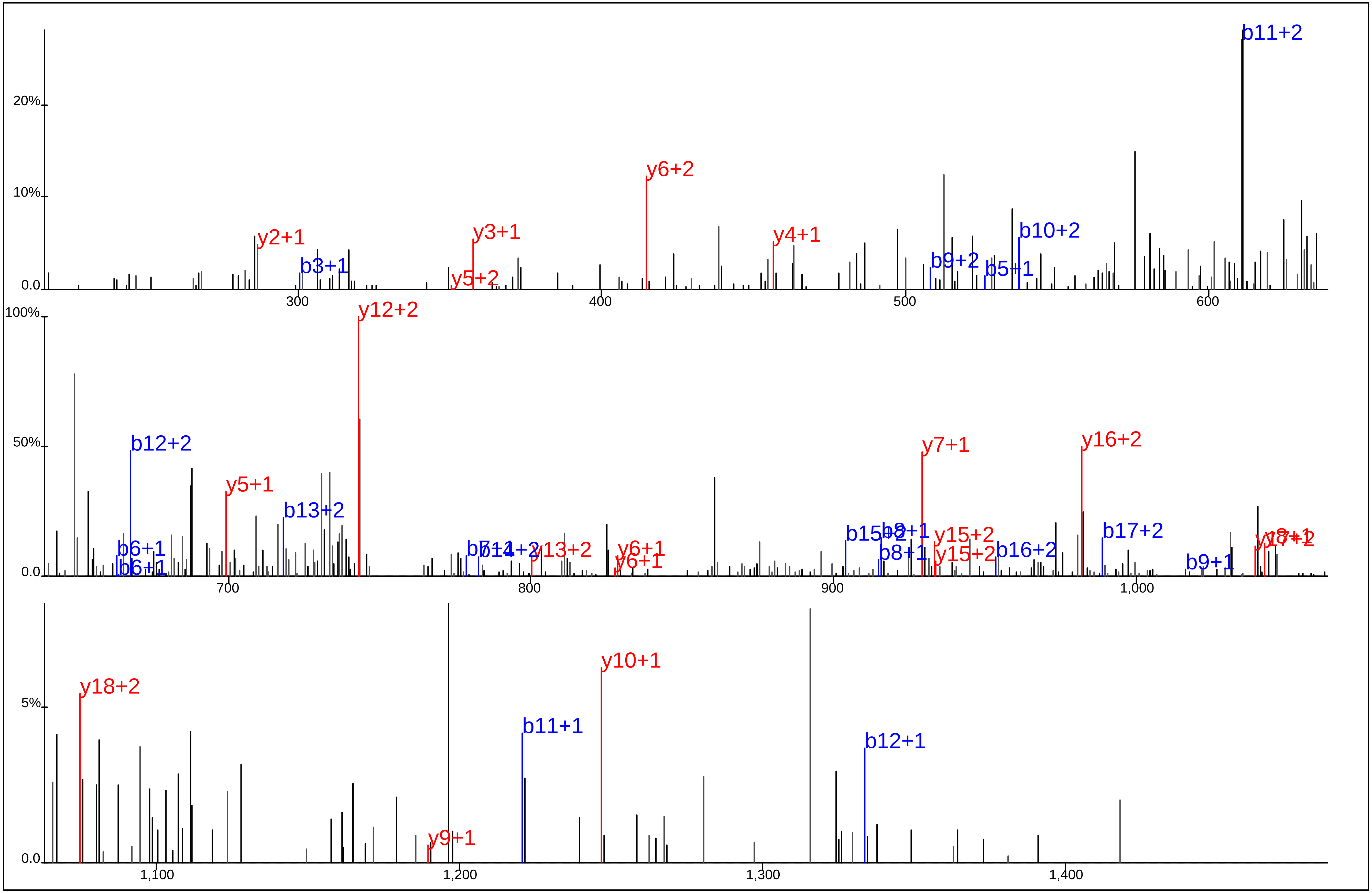

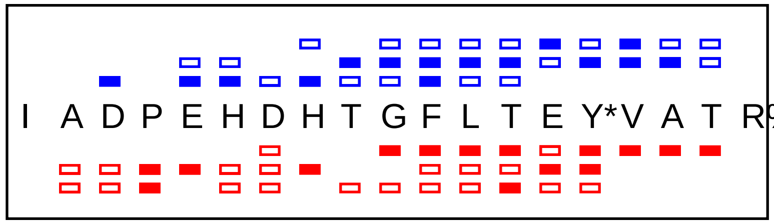

Protein Name: ERK1
gi Accession: 31221
Precursor Mass: 754.6790+3
Delta Mass: 5.427 ppm
XCorr: 26.07

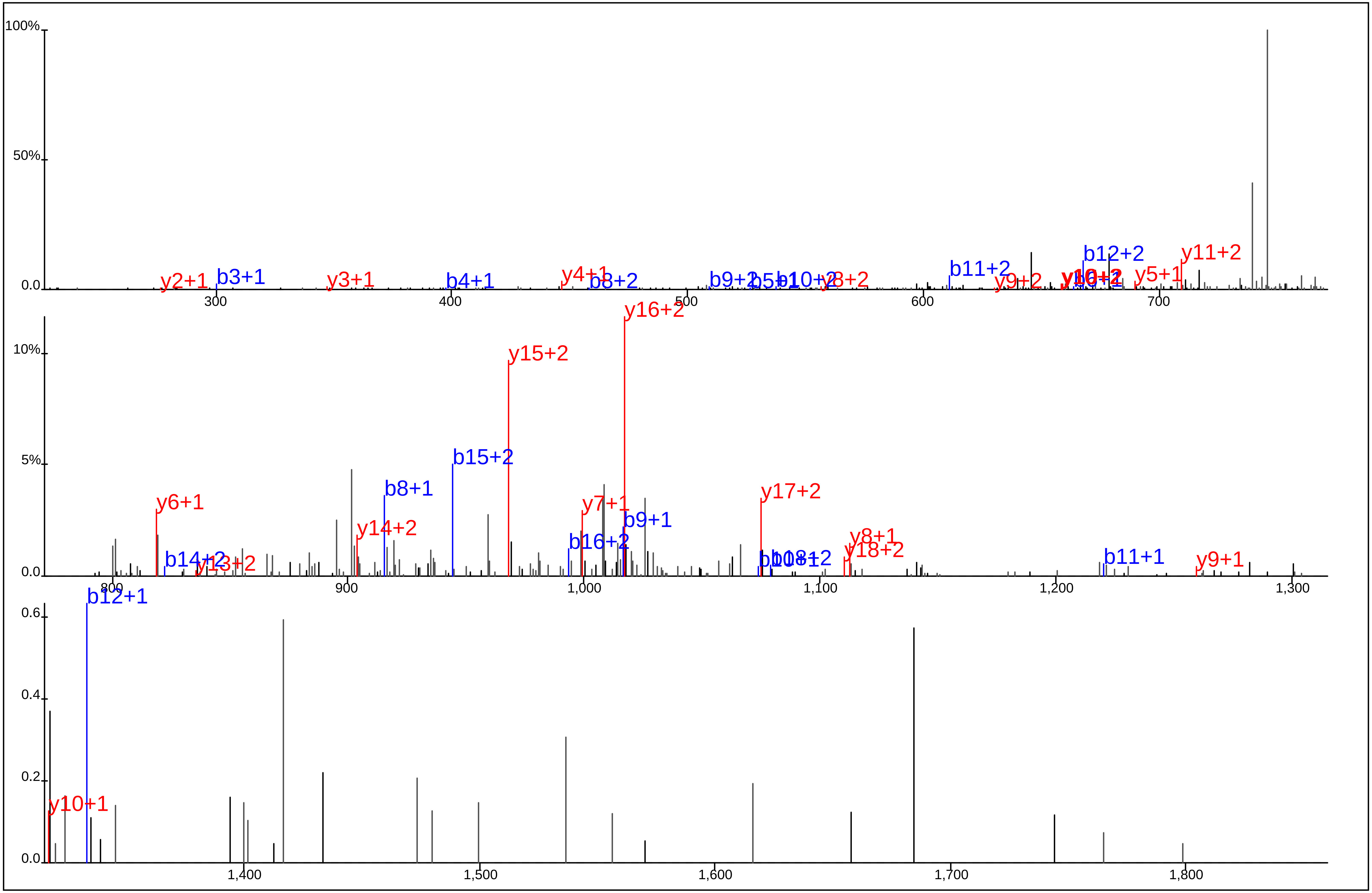

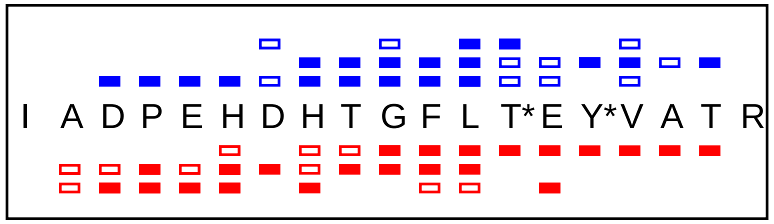

Protein Name: ERK1
gi Accession: 31221
Precursor Mass: 777.9960+3
Delta Mass: 2.182 ppm
XCorr: 21.61

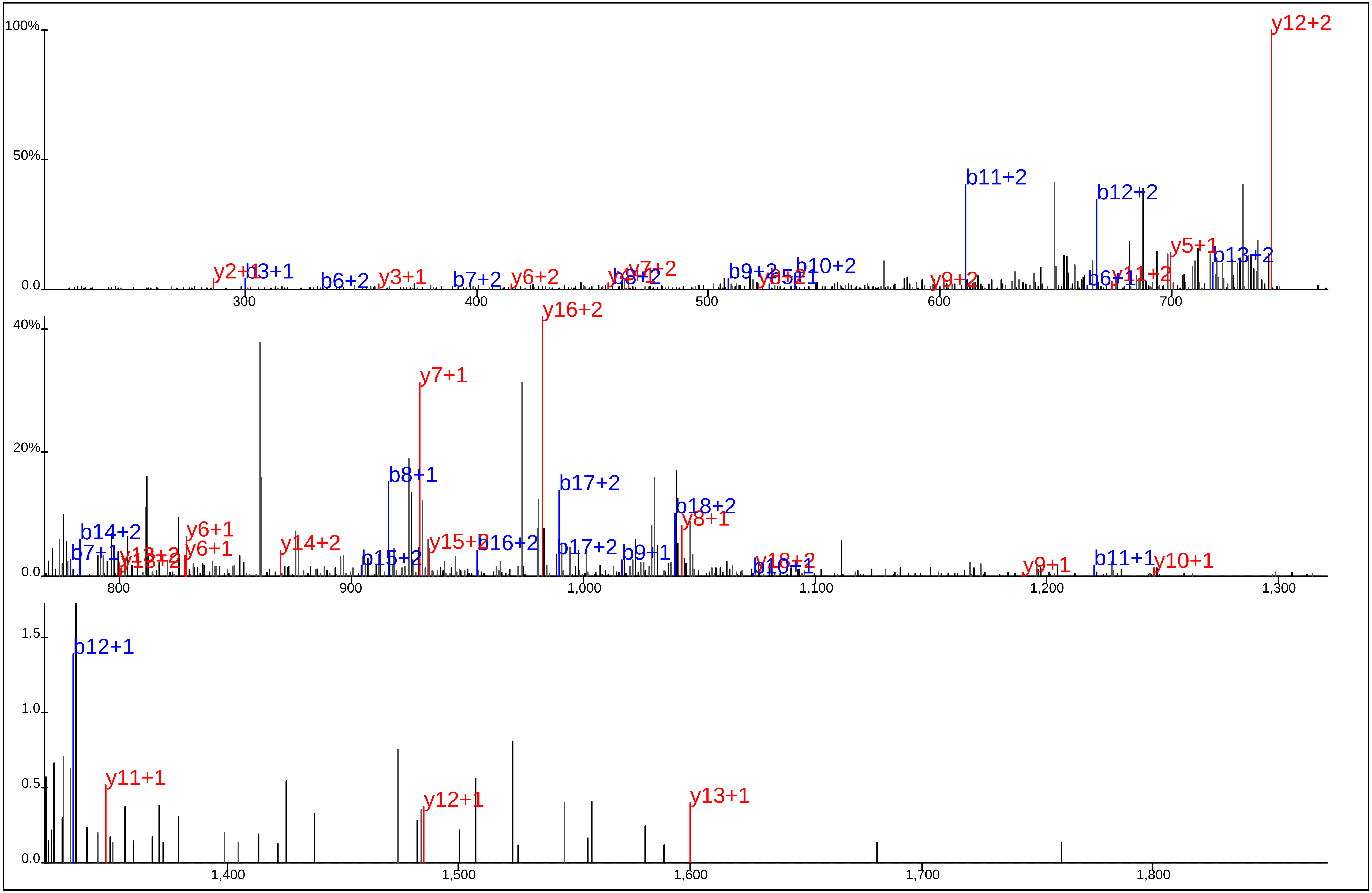

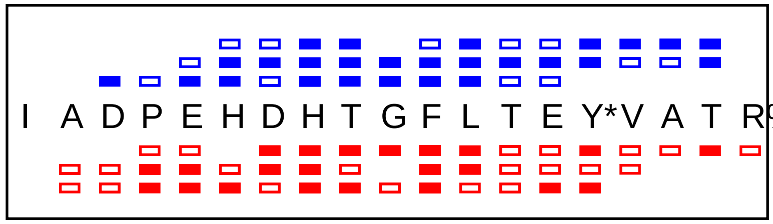

Protein Name: ERK1
gi Accession: 31221
Precursor Mass: 754.6741+3
Delta Mass: 1.132 ppm
XCorr: 36.13

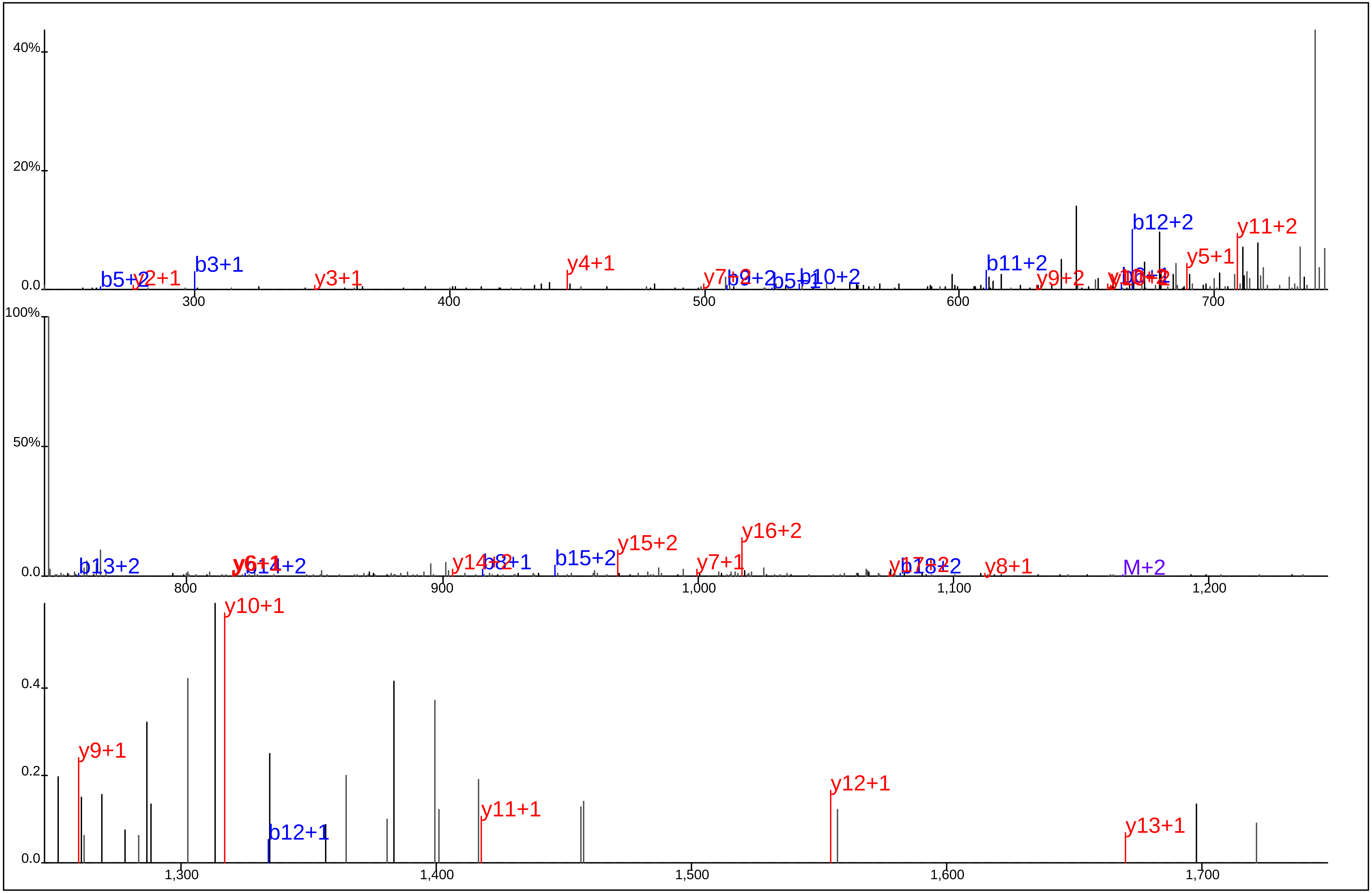

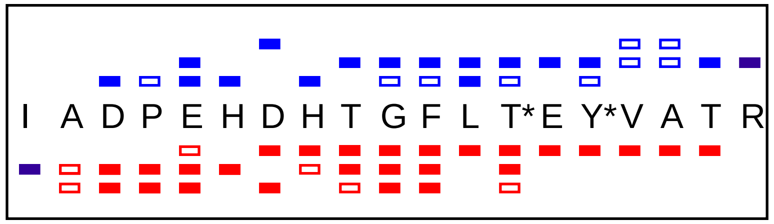

Protein Name: ERK1
gi Accession: 31221
Precursor Mass: 777.9987+3
Delta Mass: 5.638 ppm
XCorr: 28.75

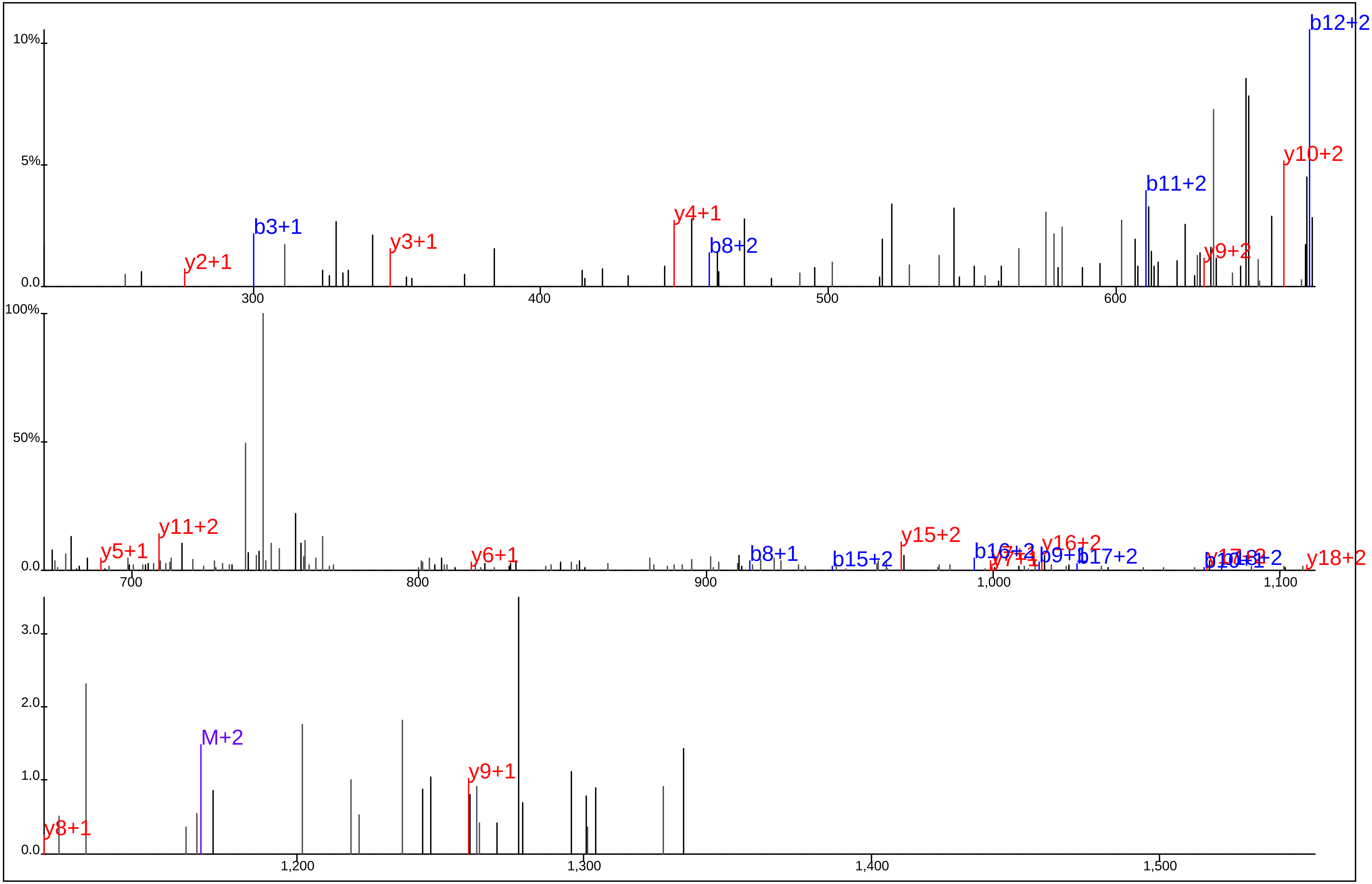

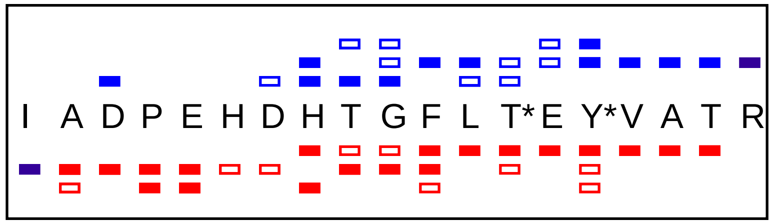

Protein Name: ERK1
gi Accession: 31221
Precursor Mass: 777.9967+3
Delta Mass: 3.125 ppm
XCorr: 13.37

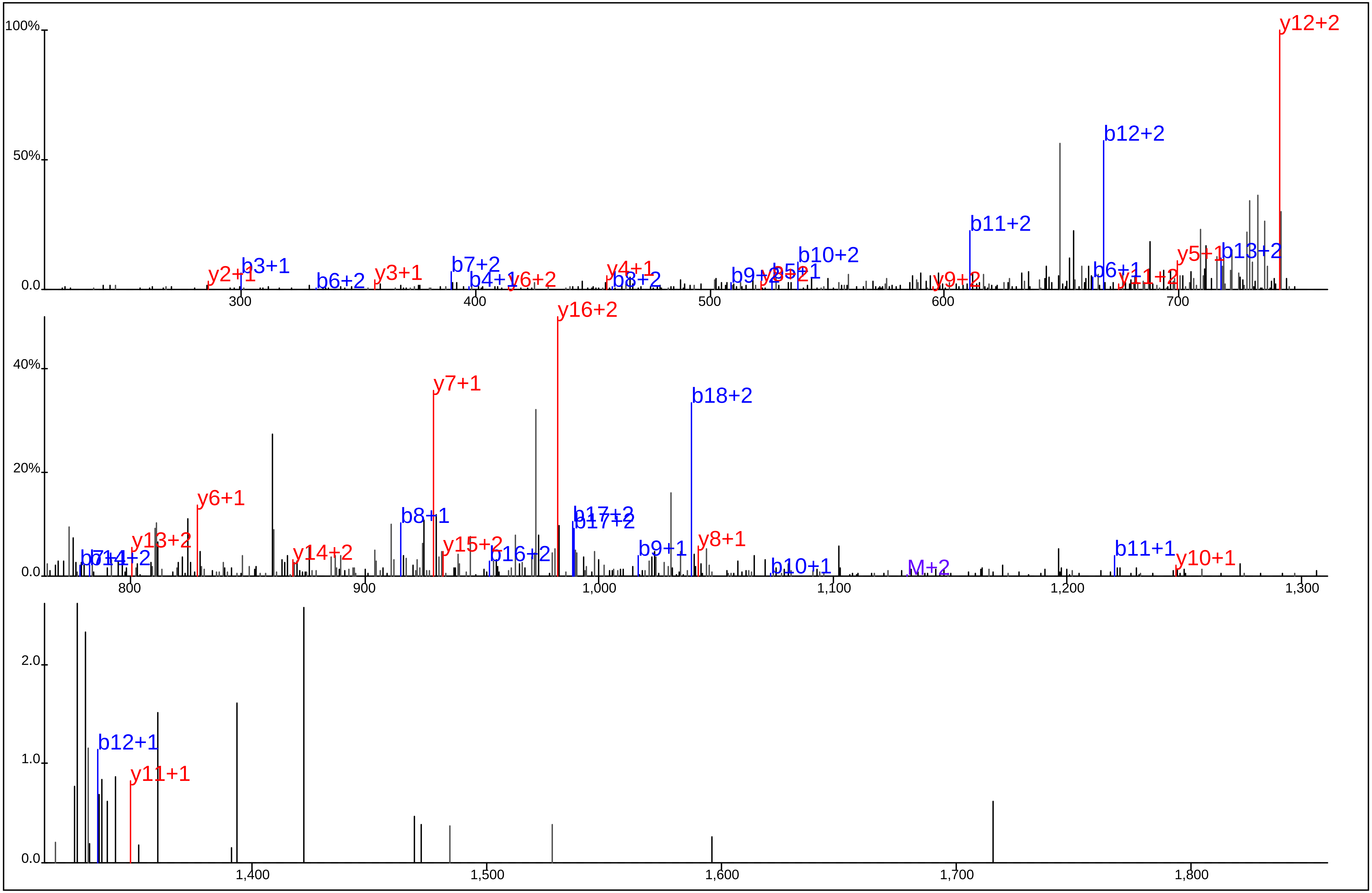

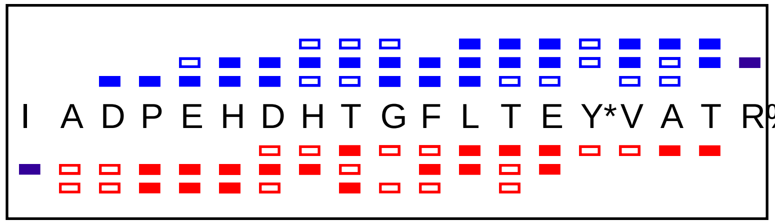

Protein Name: ERK1
gi Accession: 31221
Precursor Mass: 754.6797+3
Delta Mass: 6.396 ppm
XCorr: 28.37

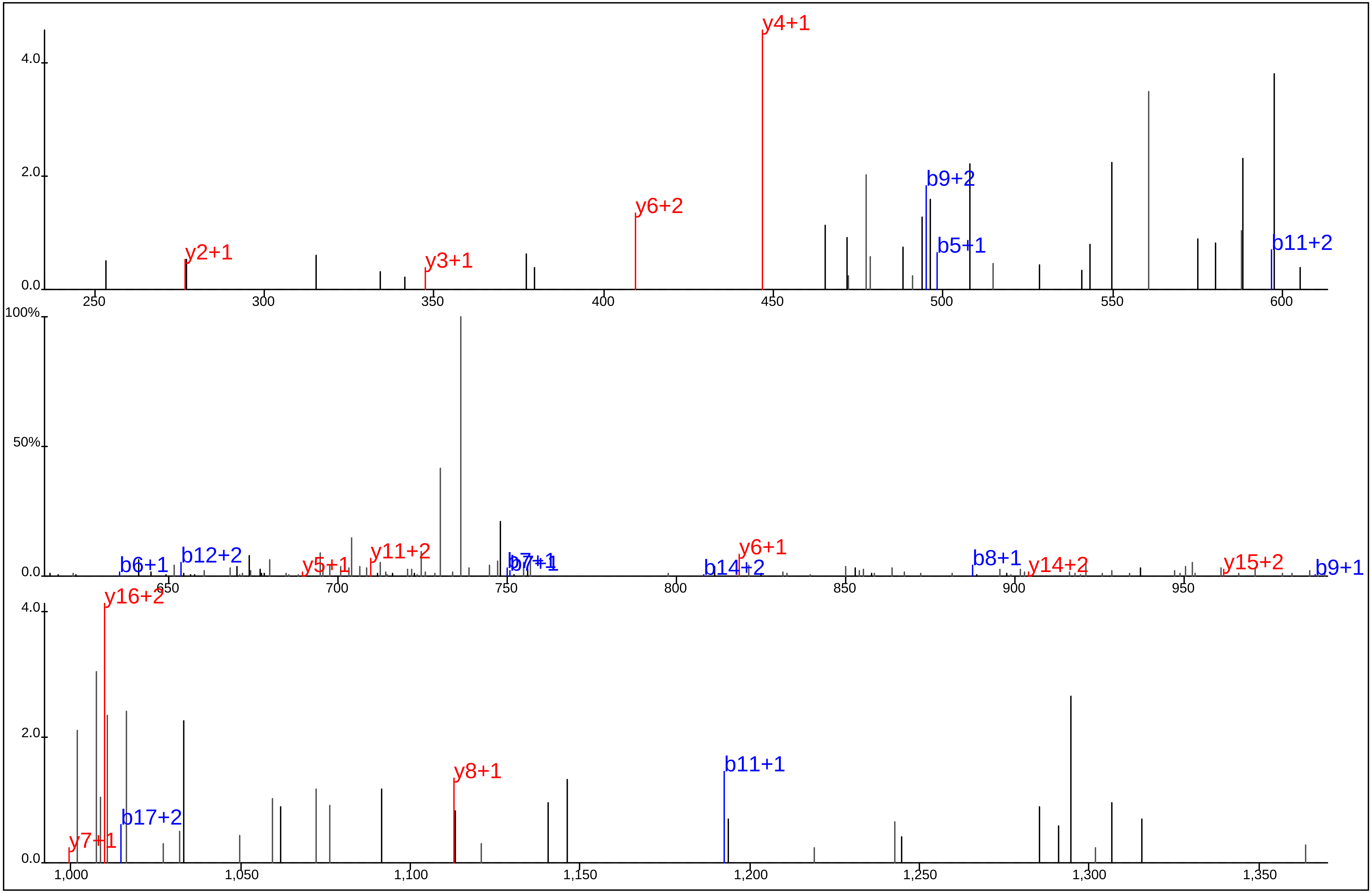

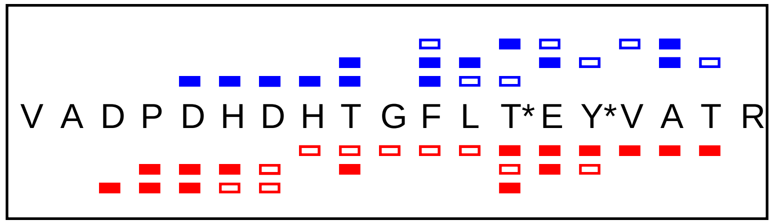

Protein Name: ERK2
gi Accession: 23881
Precursor Mass: 768.6531+3
Delta Mass: 3.342 ppm
XCorr: 2.7

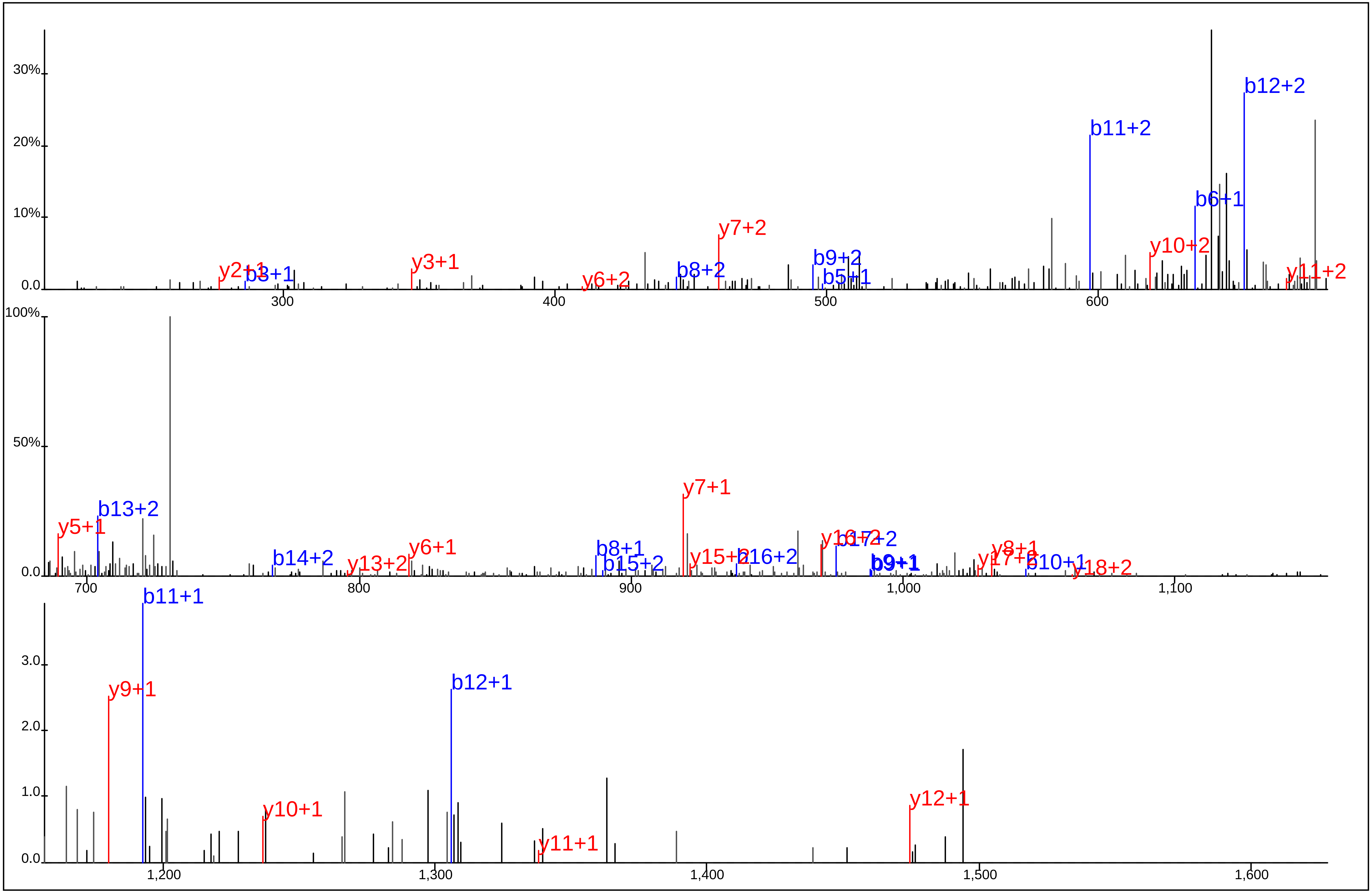

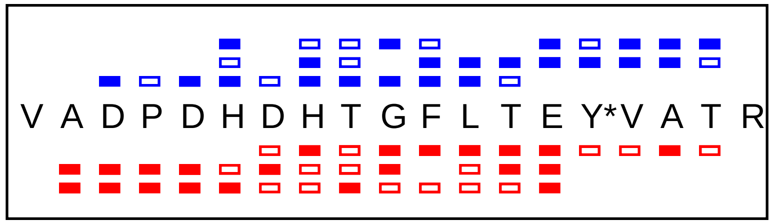

Protein Name: ERK2
gi Accession: 23881
Precursor Mass: 741.9973+3
Delta Mass: 2.940 ppm
XCorr: 35.2

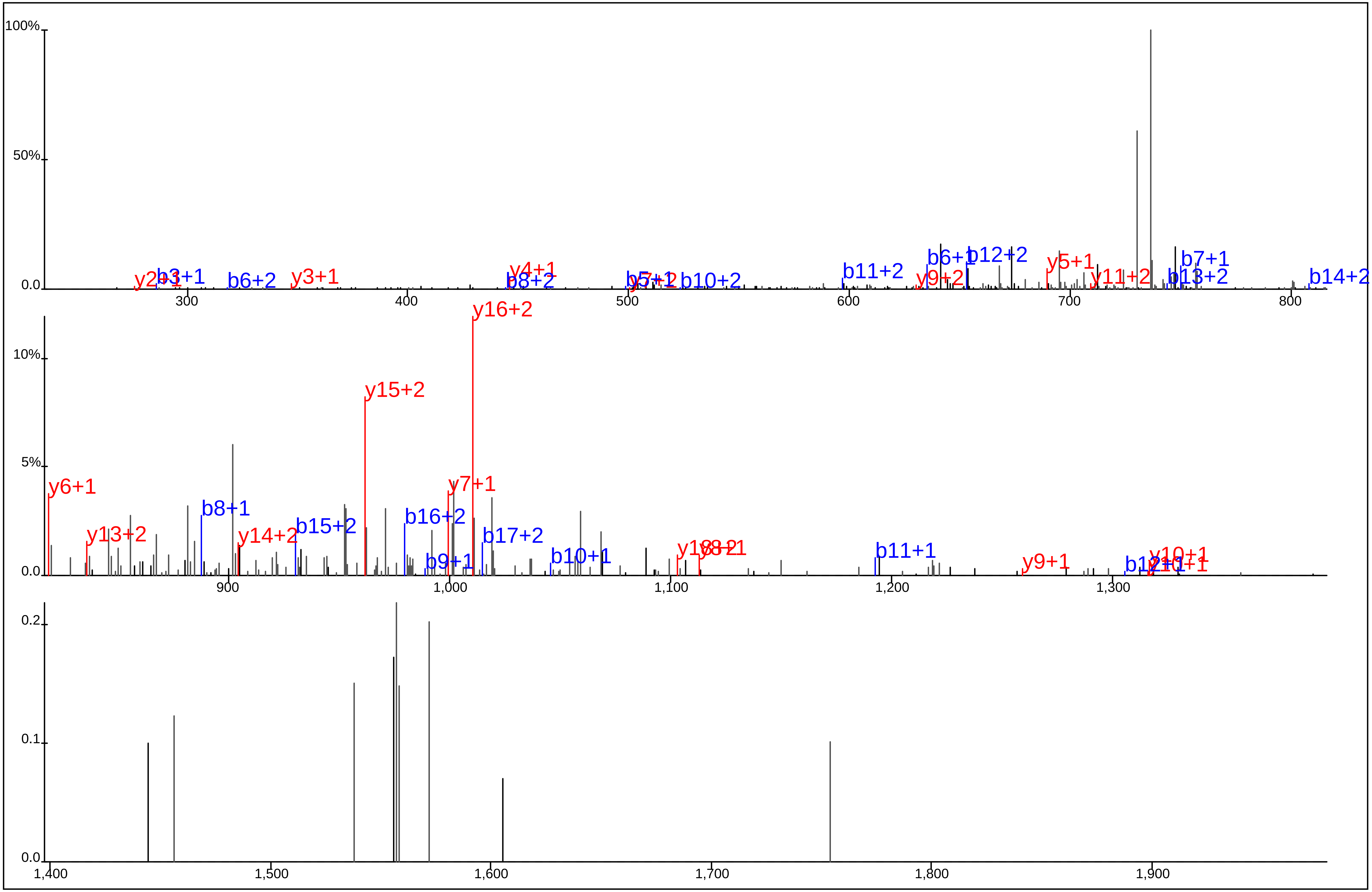

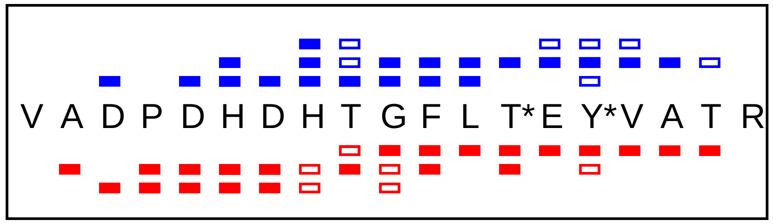

Protein Name: ERK2
gi Accession: 23881
Precursor Mass: 768.6525+3
Delta Mass: 2.538 ppm
XCorr: 35.75

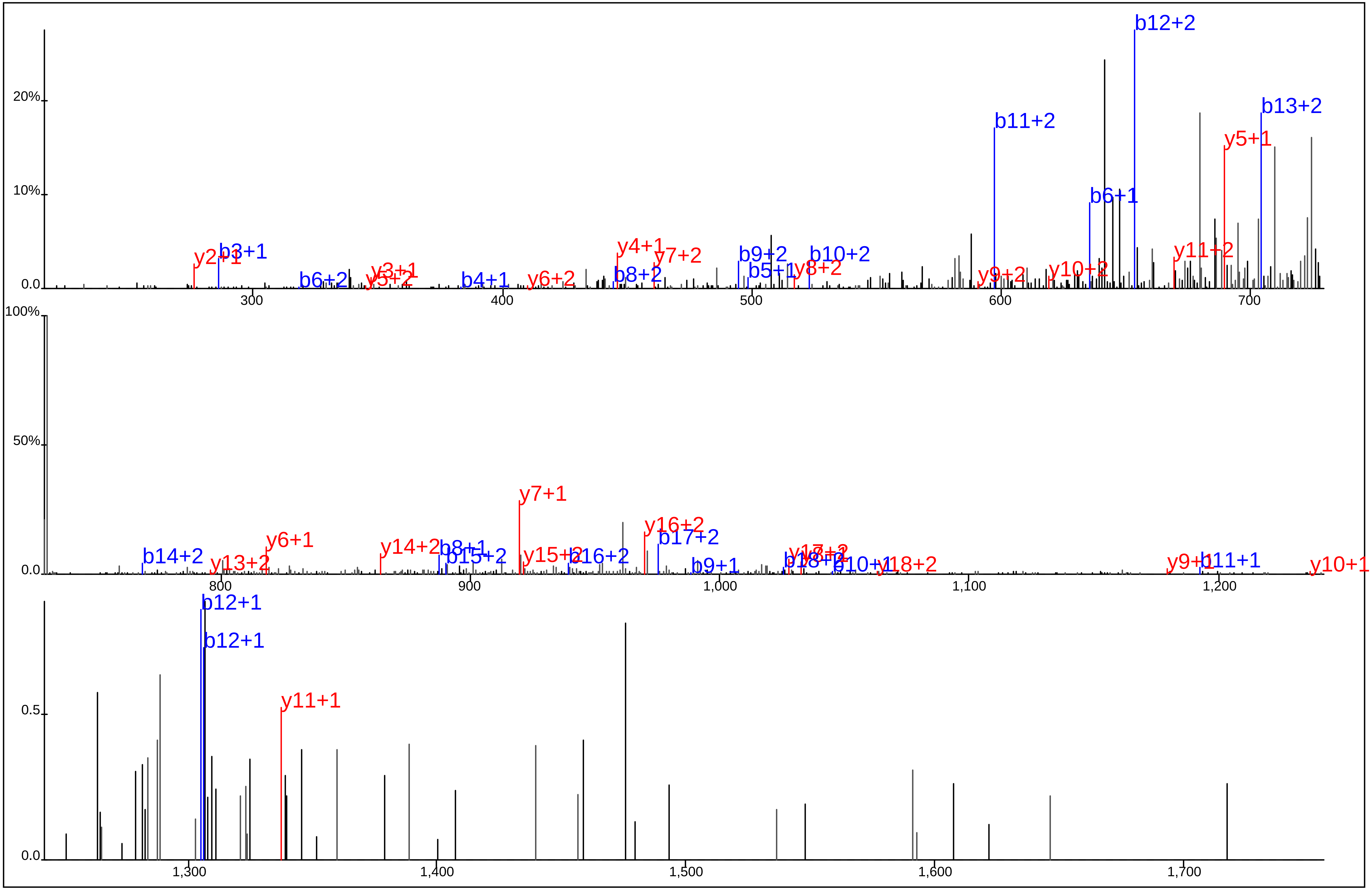

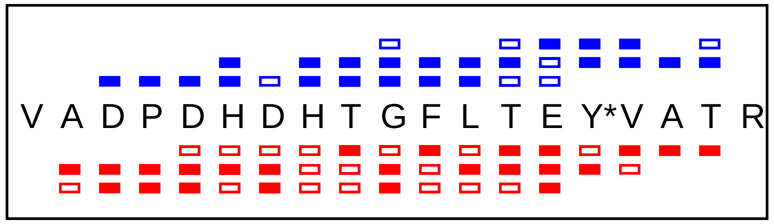

Protein Name: ERK2
gi Accession: 23881
Precursor Mass: 741.9971+3
Delta Mass: 2.774 ppm
XCorr: 41.46

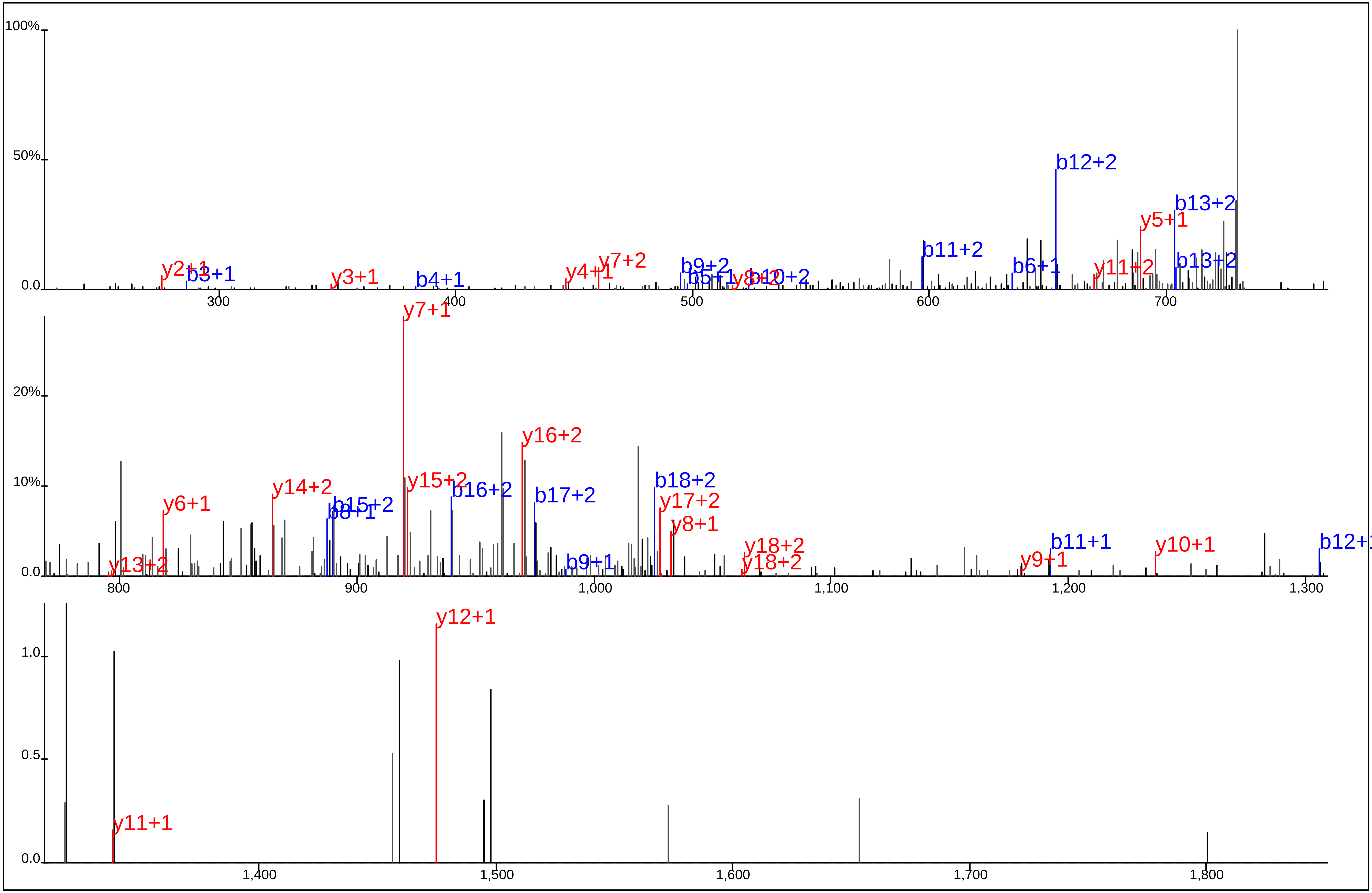

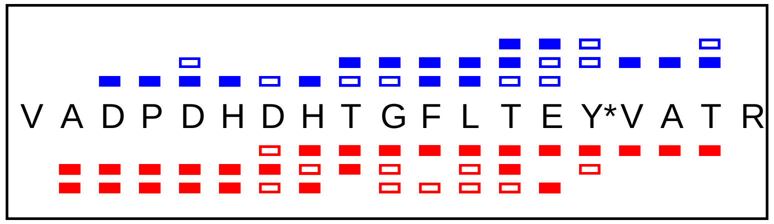

Protein Name: ERK2
gi Accession: 23881
Precursor Mass: 741.9982+3
Delta Mass: 4.258 ppm
XCorr: 47.39

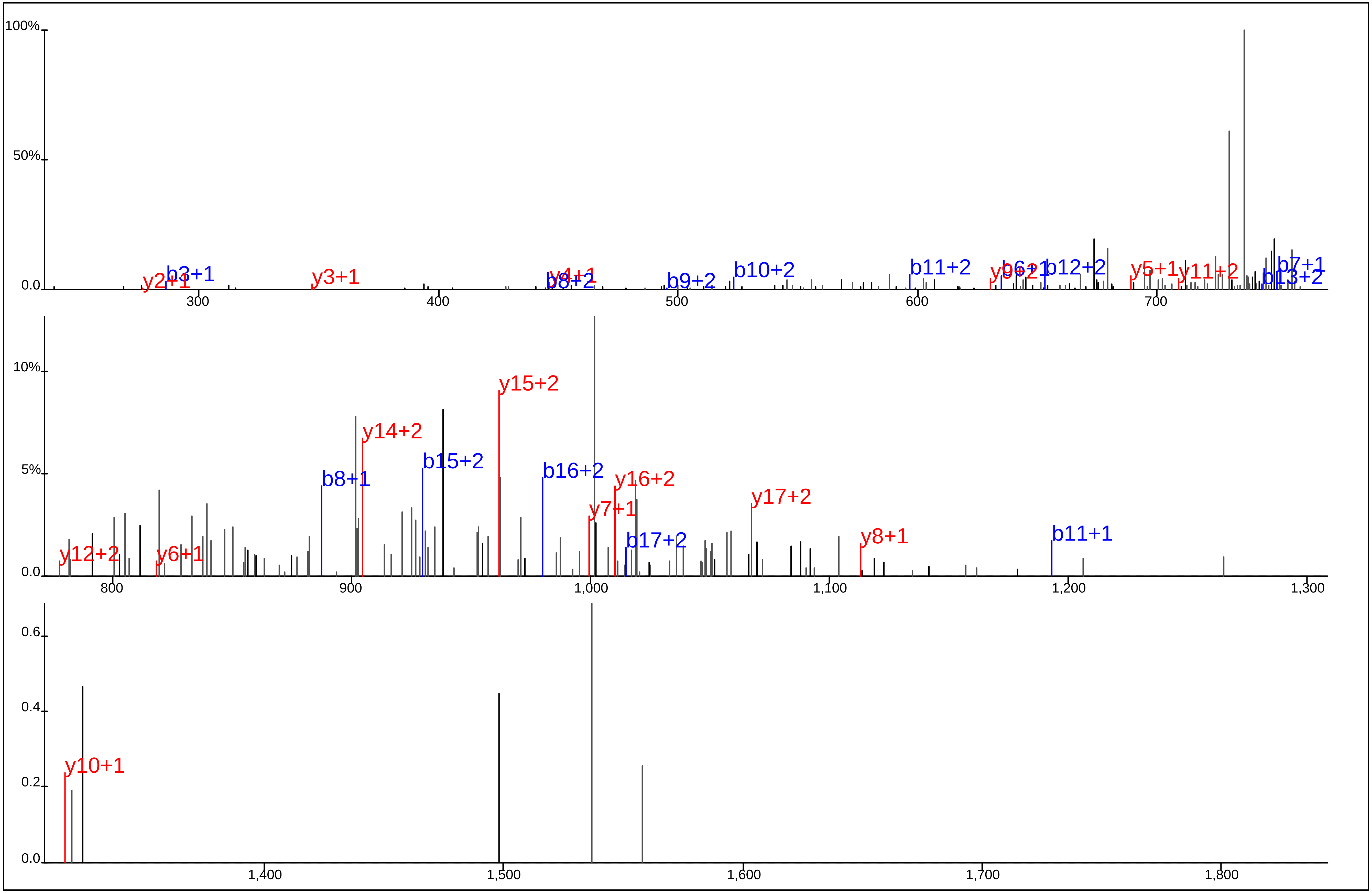

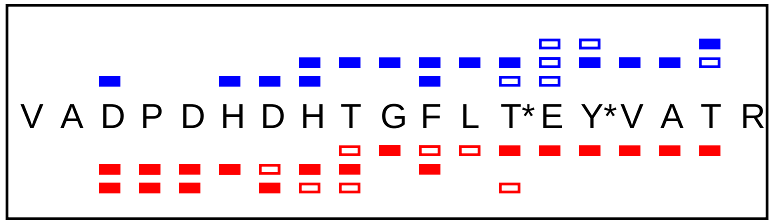

Protein Name:	ERK2
gi Accession:	23881
Precursor Mass:	768.6541+3
Delta Mass:	4.605 ppm
XCorr:	16.96

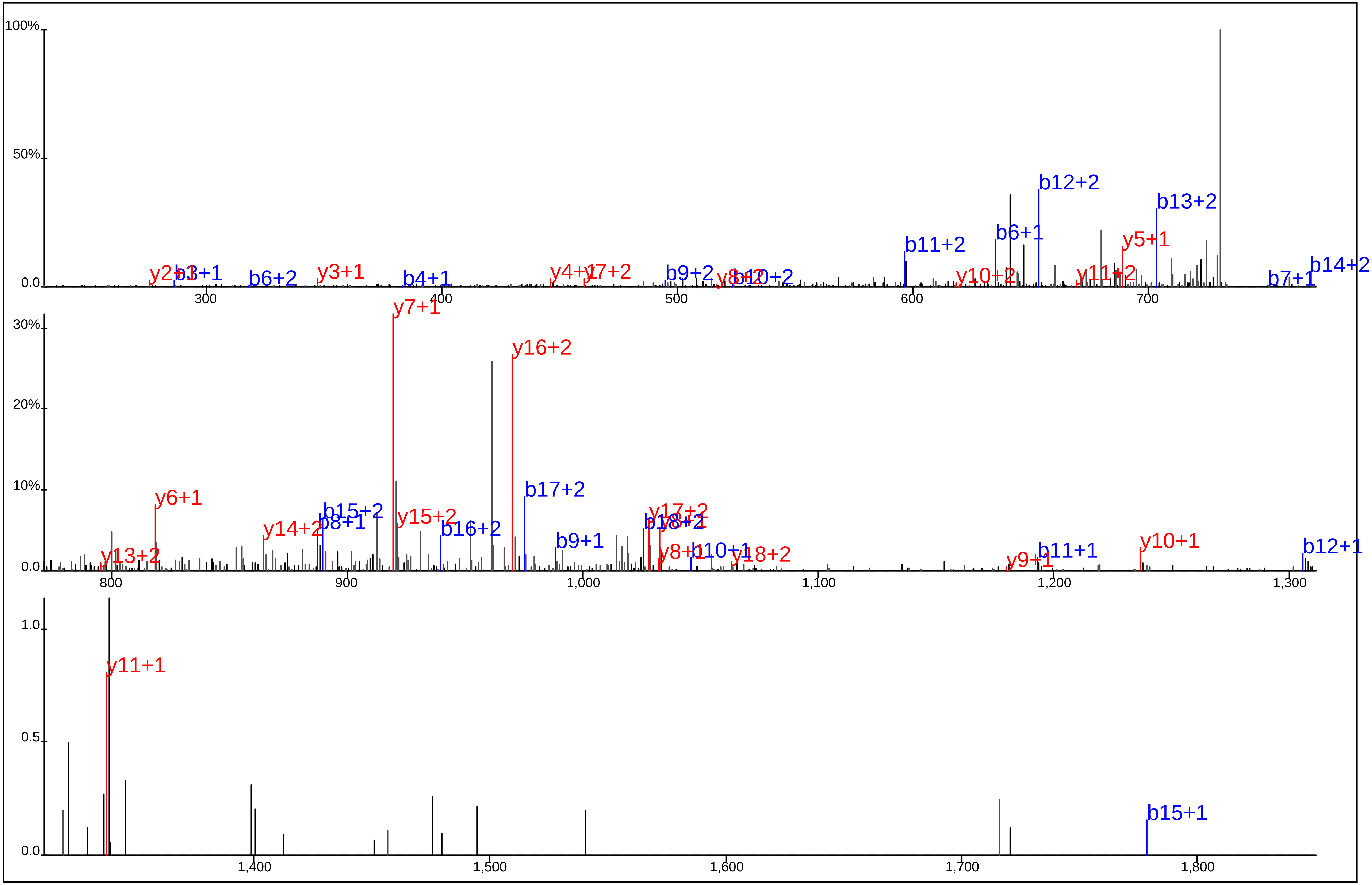

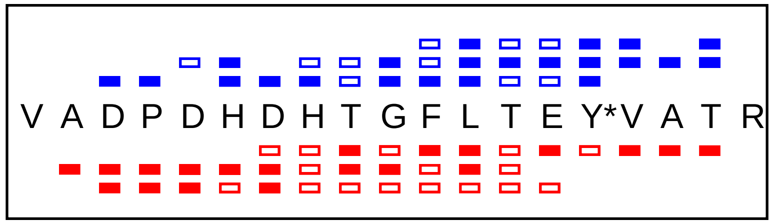

Protein Name: ERK2
gi Accession: 23881
Precursor Mass: 741.9996+3
Delta Mass: 6.069 ppm
XCorr: 40.39

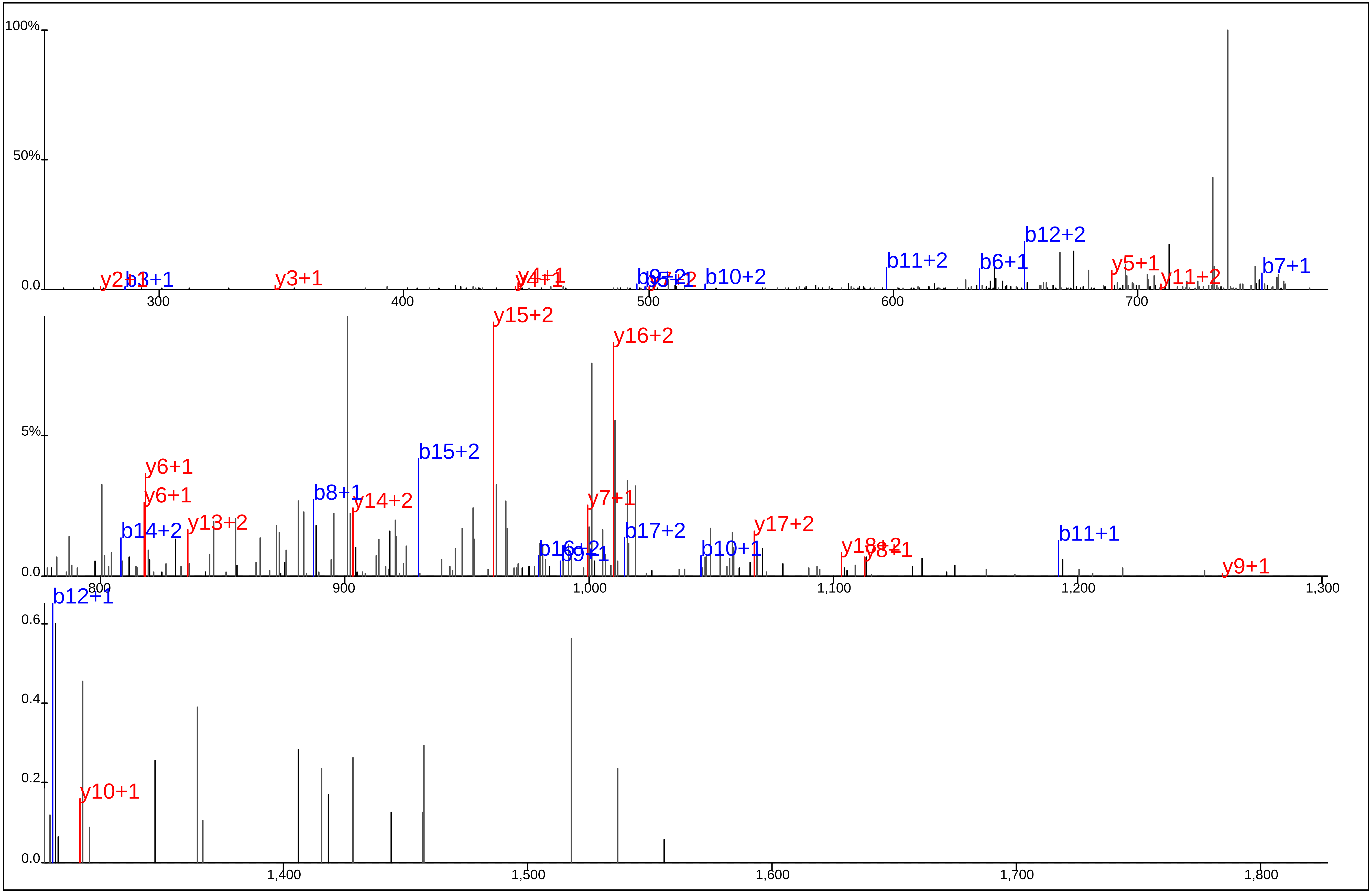

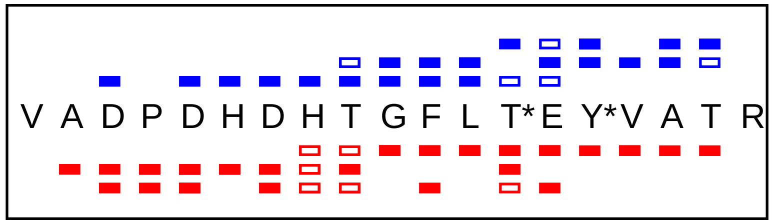

Protein Name: ERK2
gi Accession: 23881
Precursor Mass: 768.6506+3
Delta Mass: 0.077 ppm
XCorr: 33.21

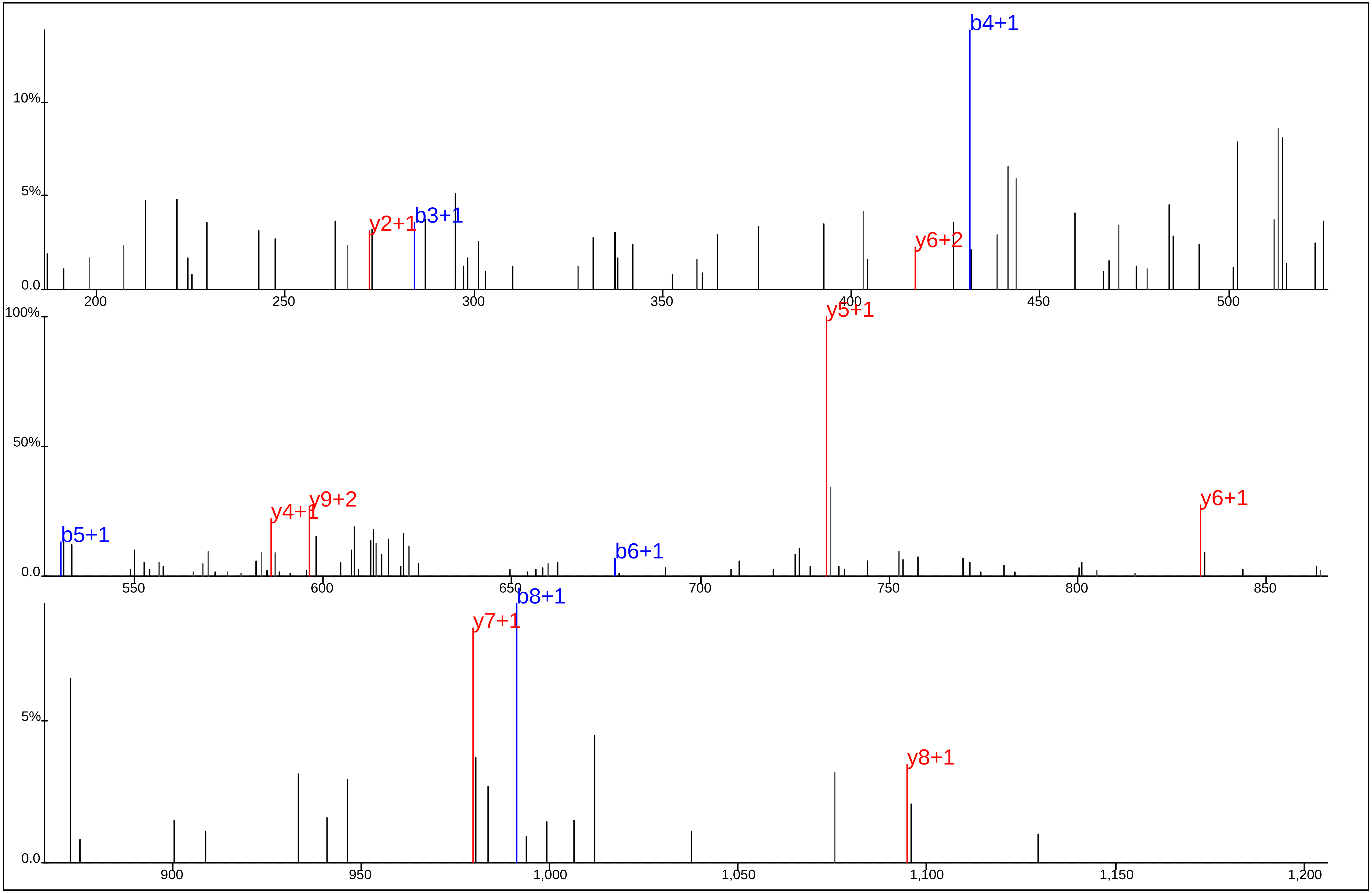

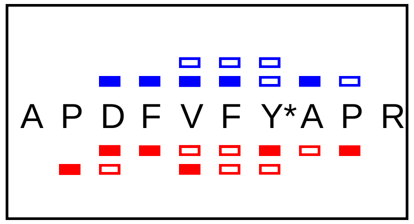

Protein Name: Ezrin
gi Accession: 31283
Precursor Mass: 631.7859+2
Delta Mass: 3.230 ppm
XCorr: 31.34

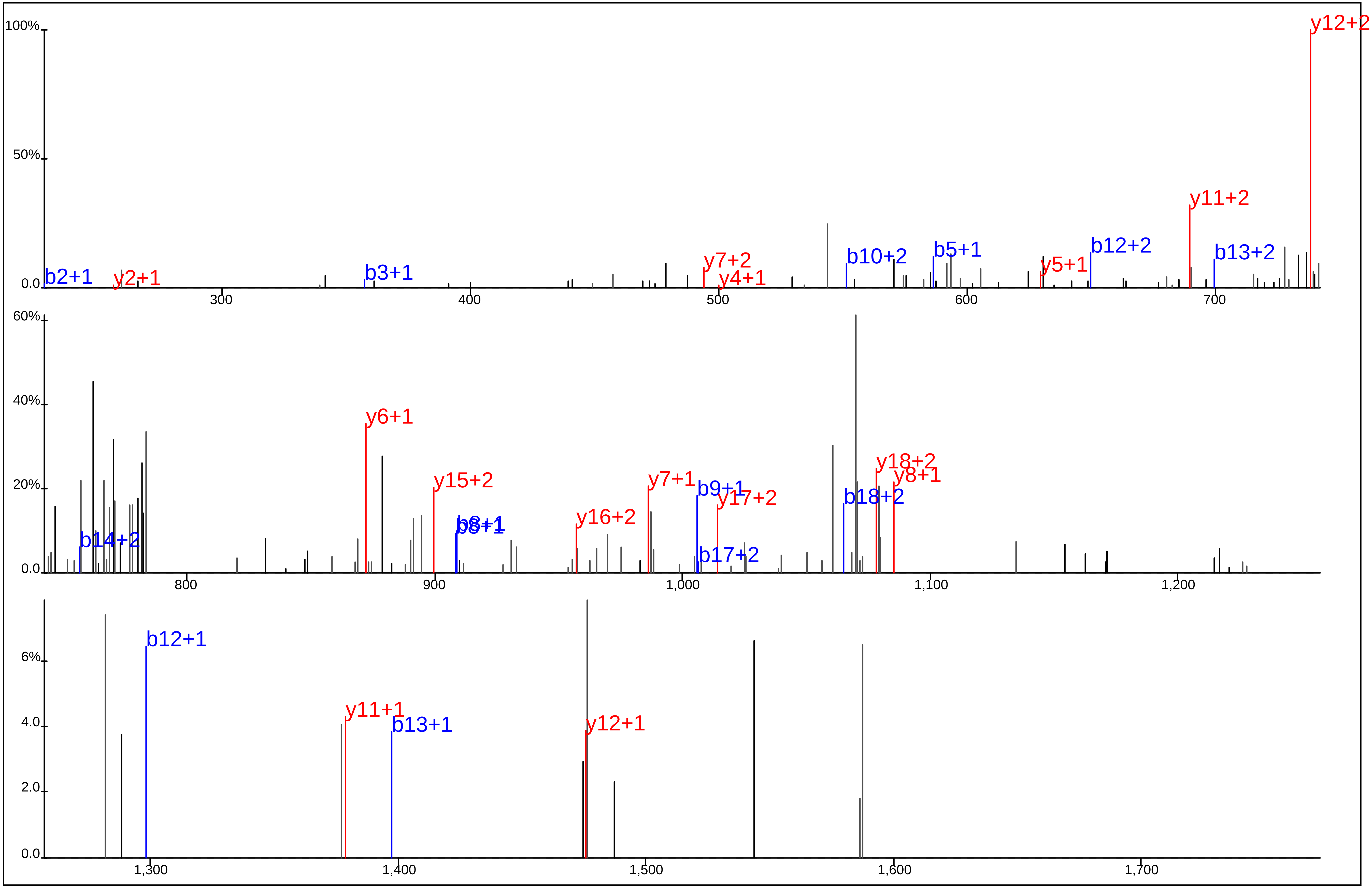

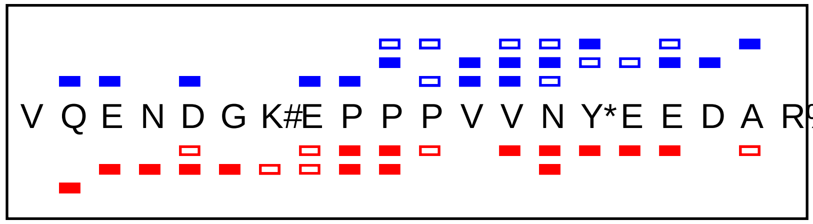

Protein Name: FER
gi Accession: 119964721
Precursor Mass: 795.0258+3
Delta Mass: 2.550 ppm
XCorr: 28.88

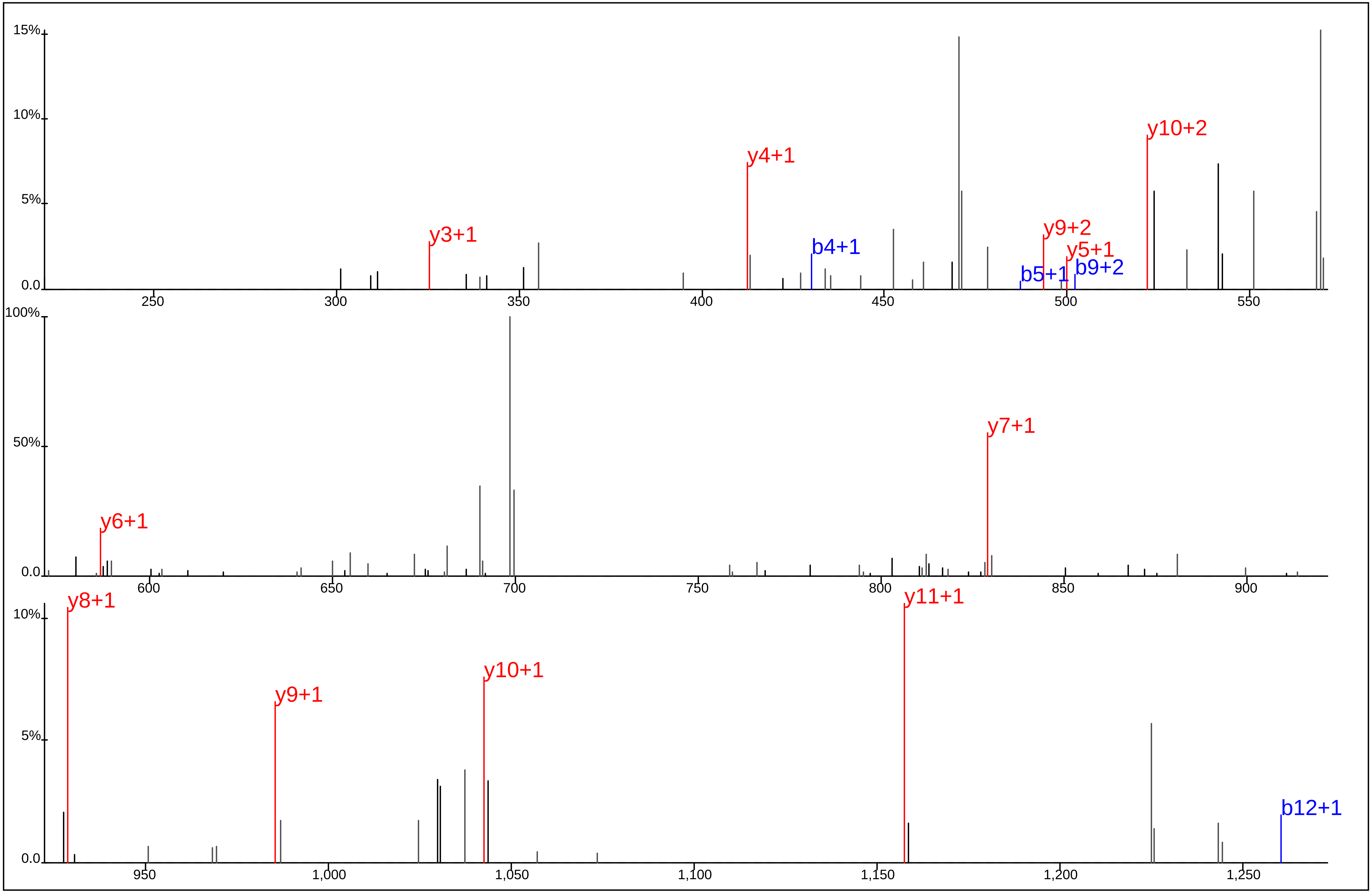

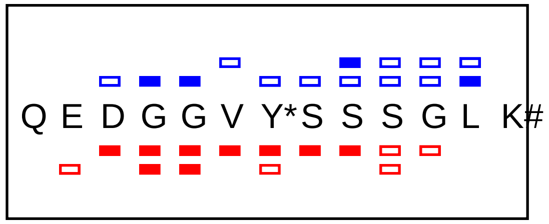

Protein Name: FER
gi Accession: 119964721
Precursor Mass: 707.8046+2
Delta Mass: 3.003 ppm
XCorr: 58.53

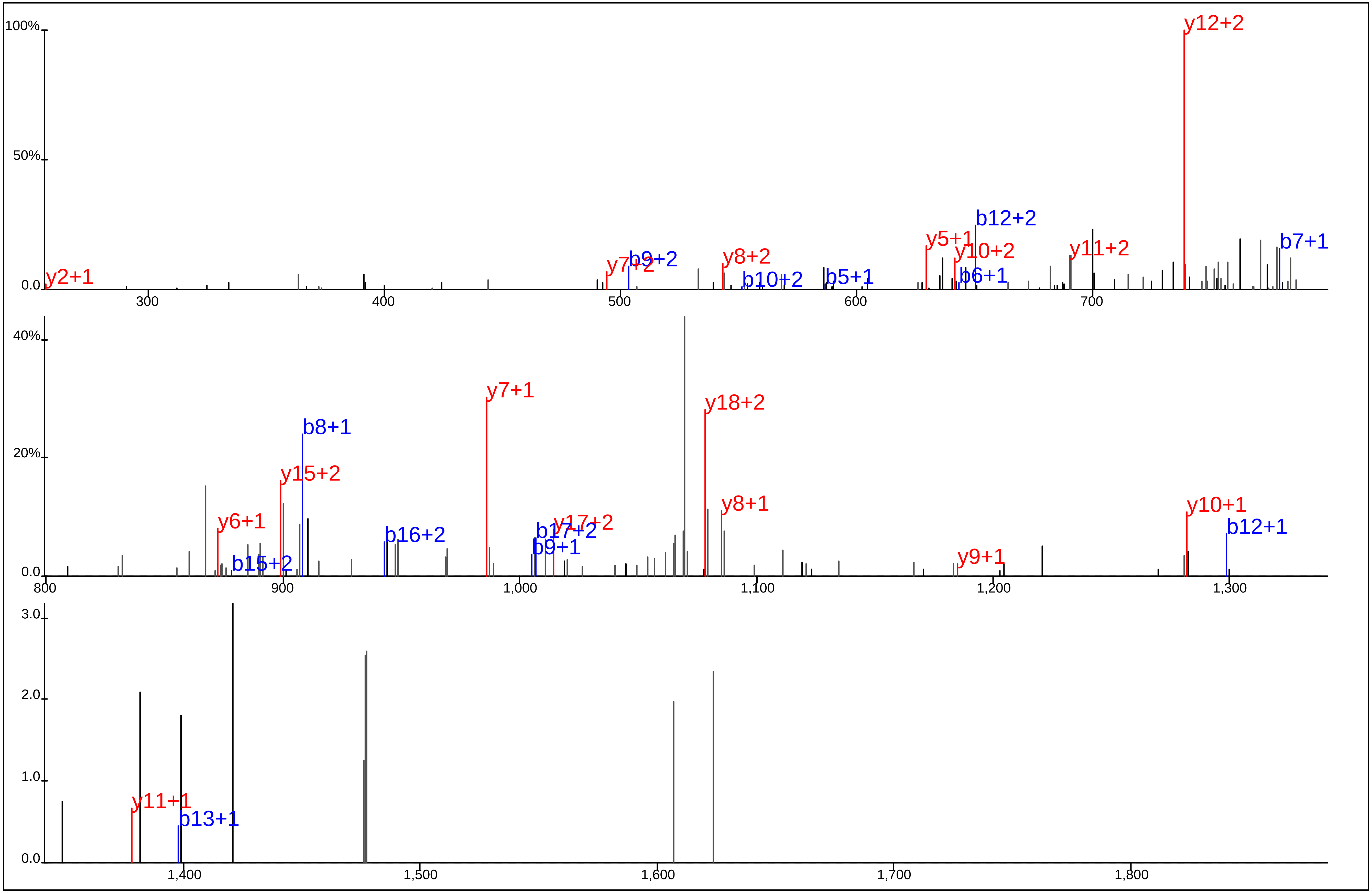

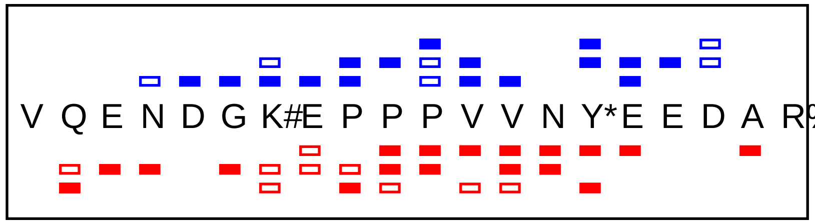

Protein Name:	FER
gi Accession:	119964721
Precursor Mass:	795.0248+3
Delta Mass:	1.246 ppm
XCorr:	32.78

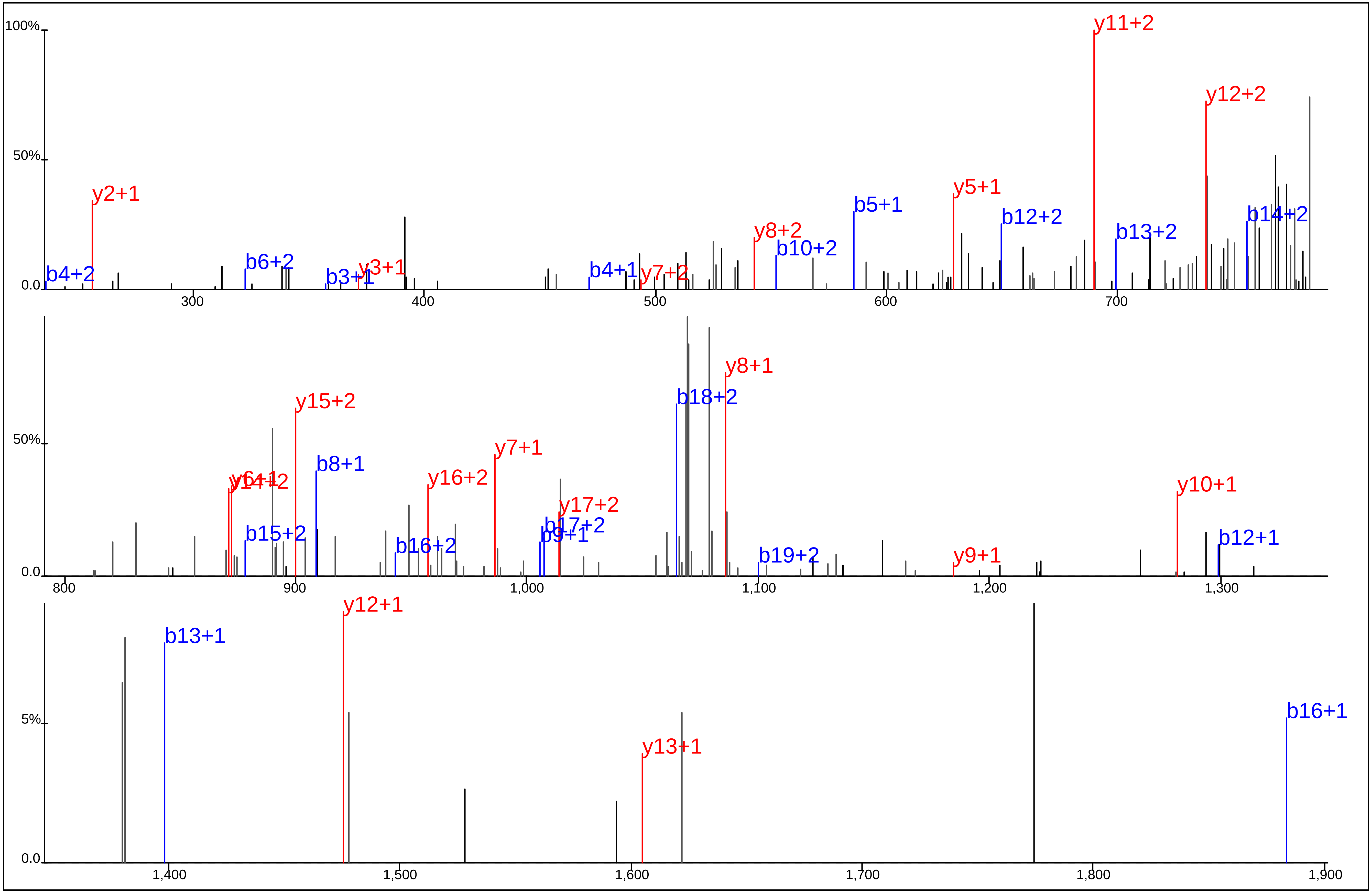

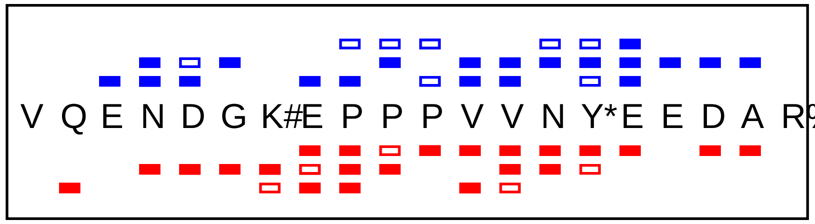

Protein Name:	FER
gi Accession:	119964721
Precursor Mass:	795.0276+3
Delta Mass:	4.778 ppm
XCorr:	49.89

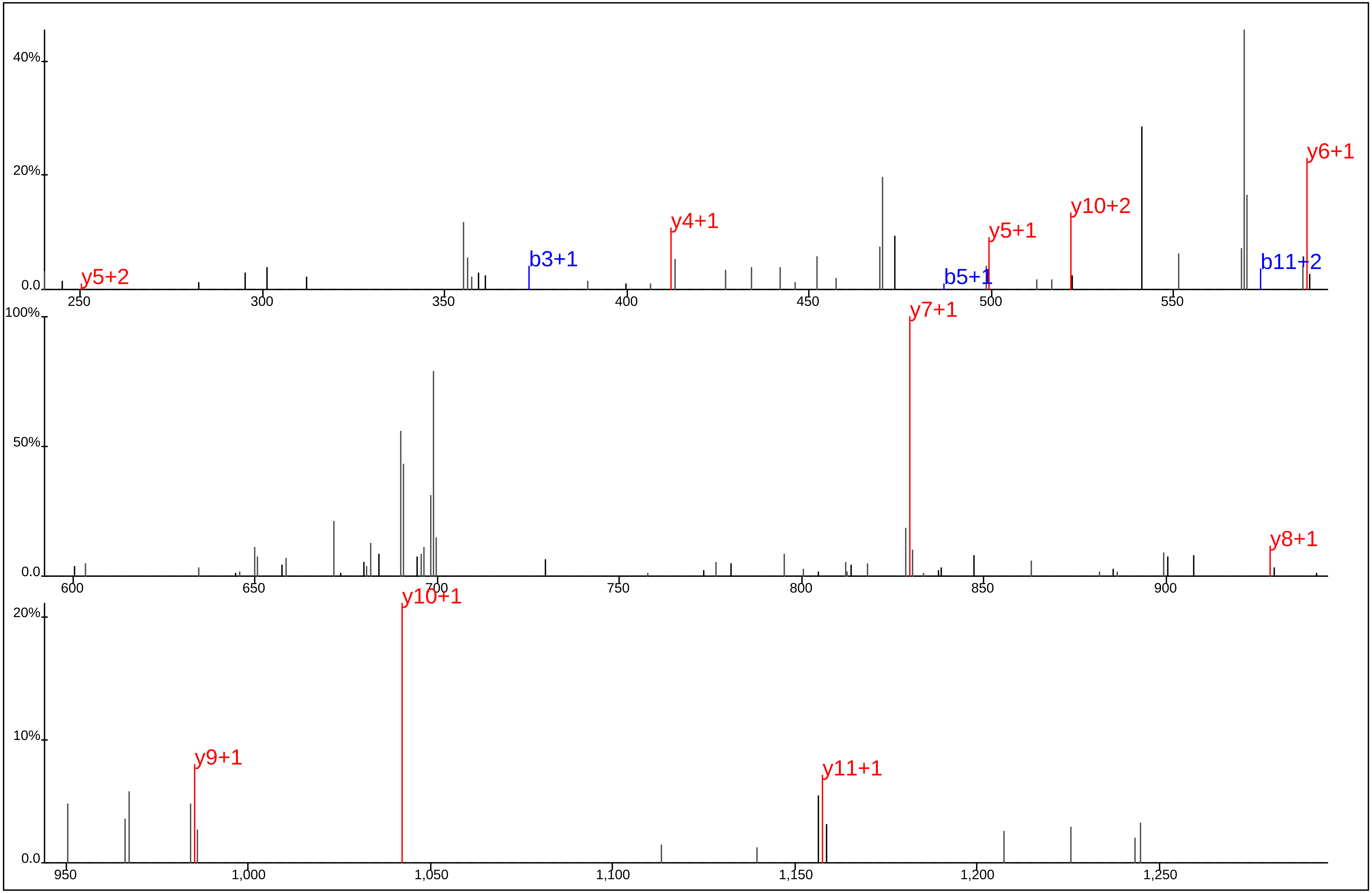

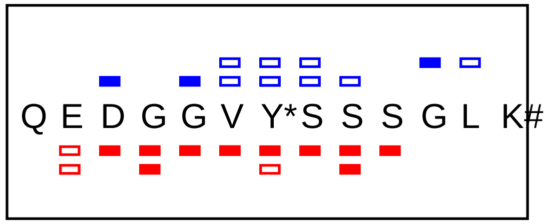

Protein Name: FER
gi Accession: 119964721
Precursor Mass: 707.8057+2
Delta Mass: 4.466 ppm
XCorr: 46.9

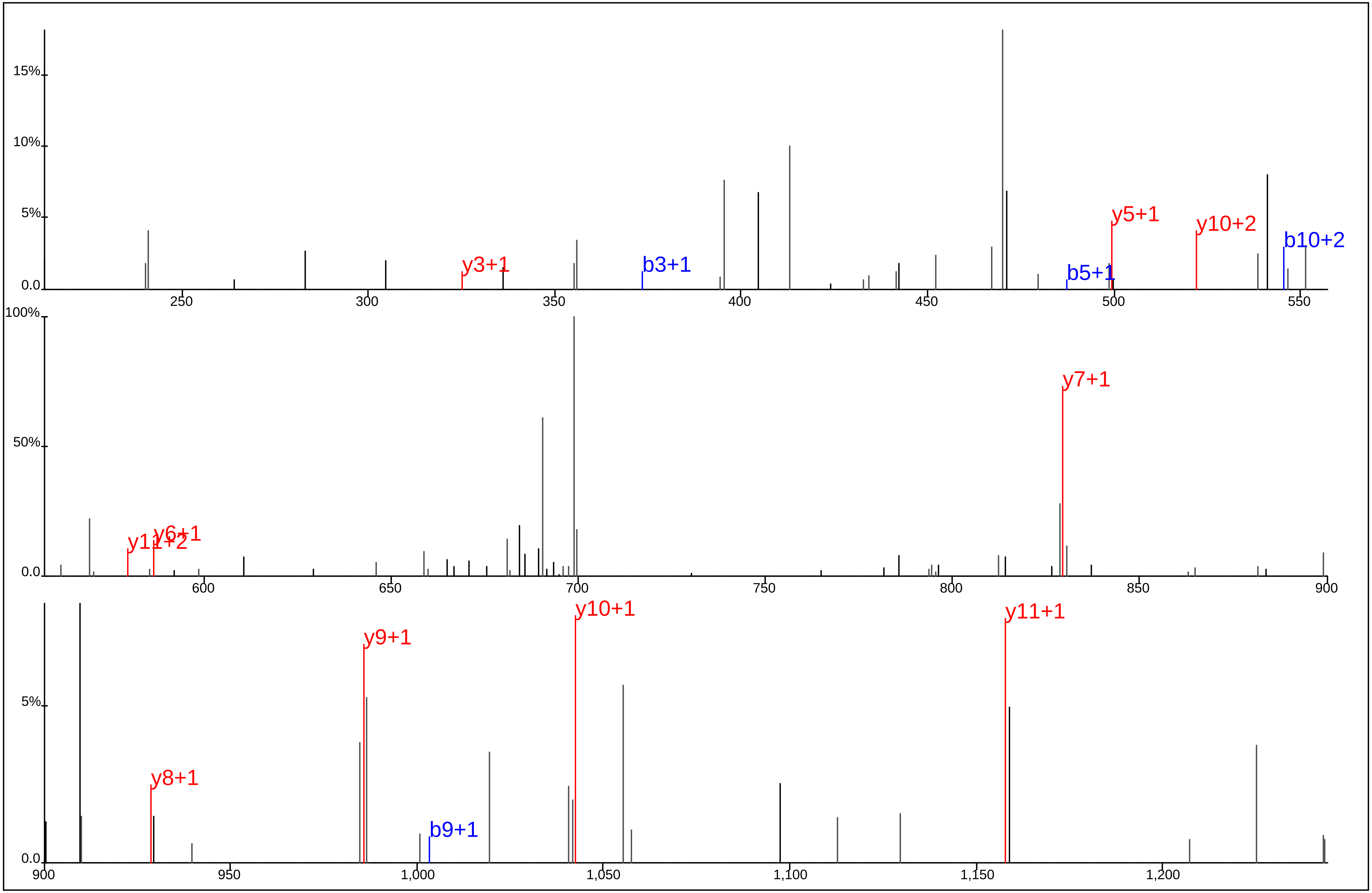

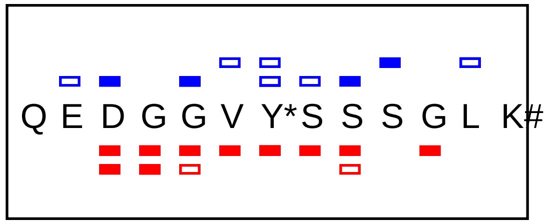

Protein Name: FER
gi Accession: 119964721
Precursor Mass: 707.8062+2
Delta Mass: 5.244 ppm
XCorr: 30.78

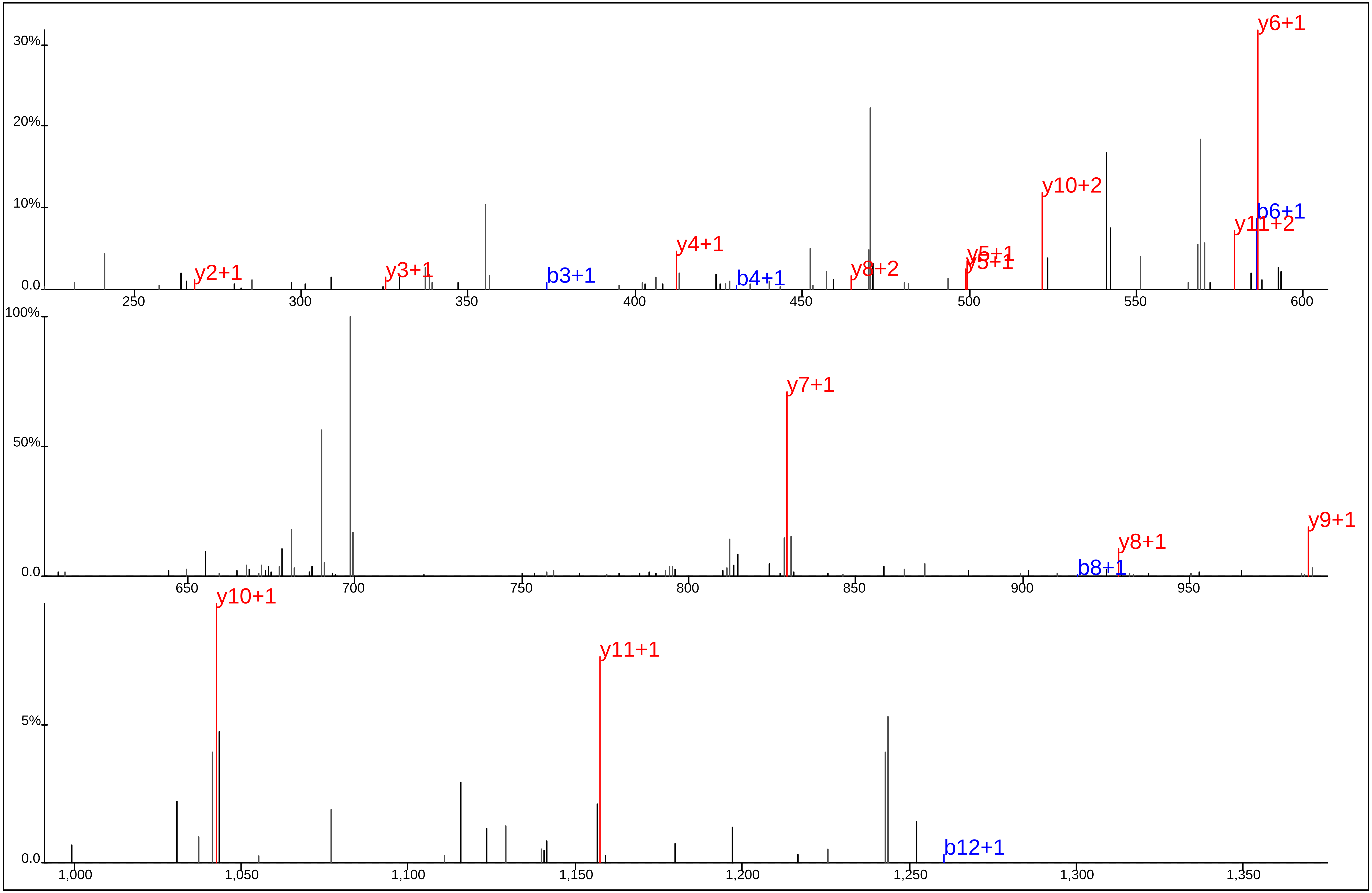

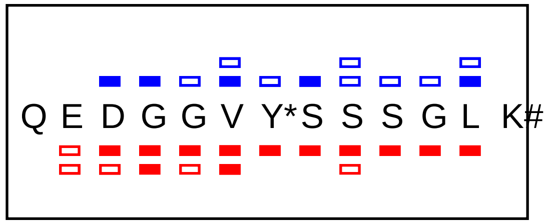

Protein Name: FER
gi Accession: 119964721
Precursor Mass: 707.8089+2
Delta Mass: 9.040 ppm
XCorr: 50.61

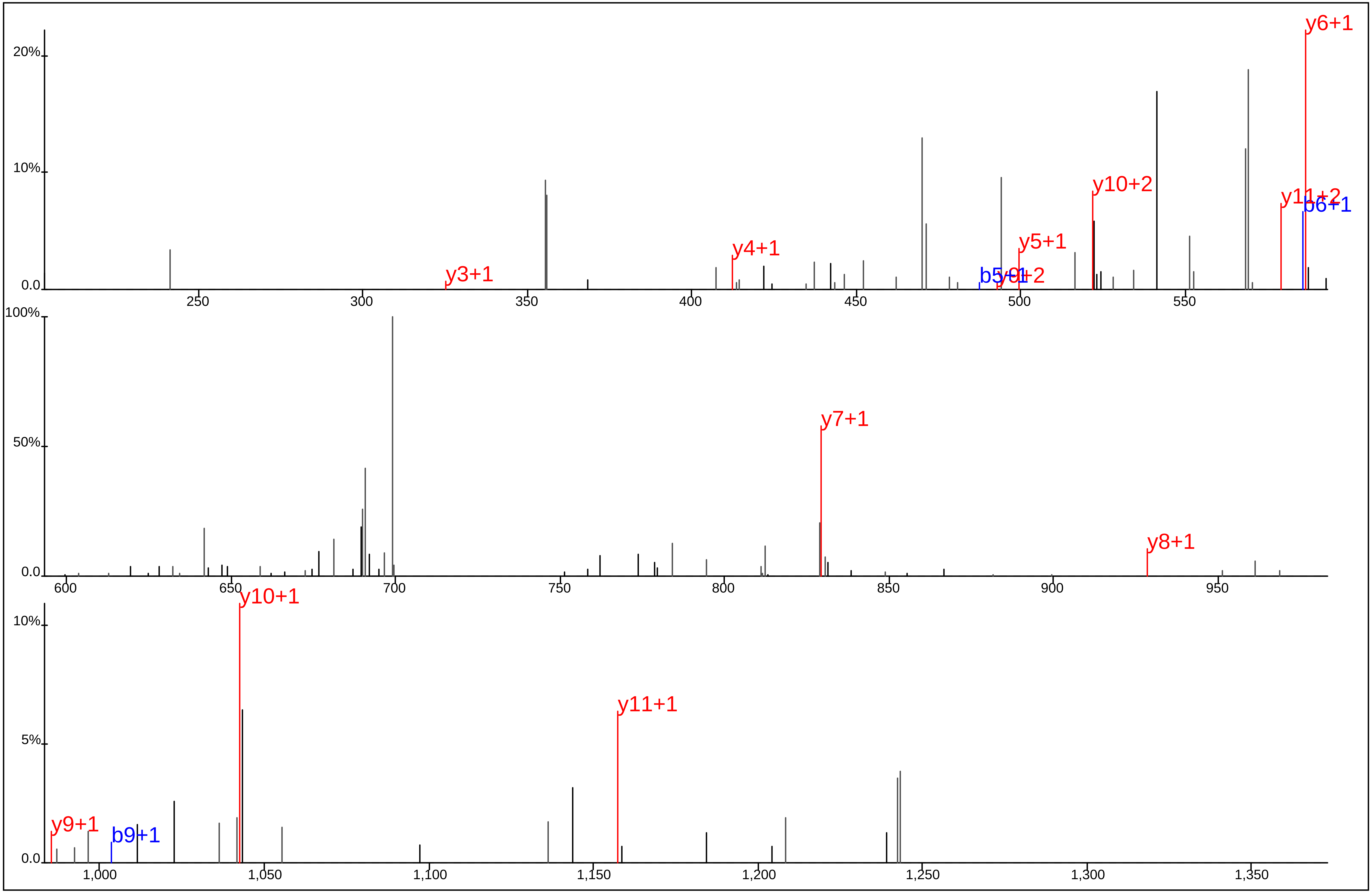

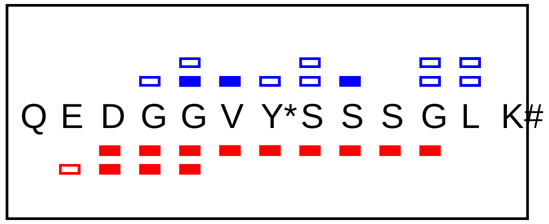

Protein Name: FER
gi Accession: 119964721
Precursor Mass: 707.8065+2
Delta Mass: 5.590 ppm
XCorr: 38.14

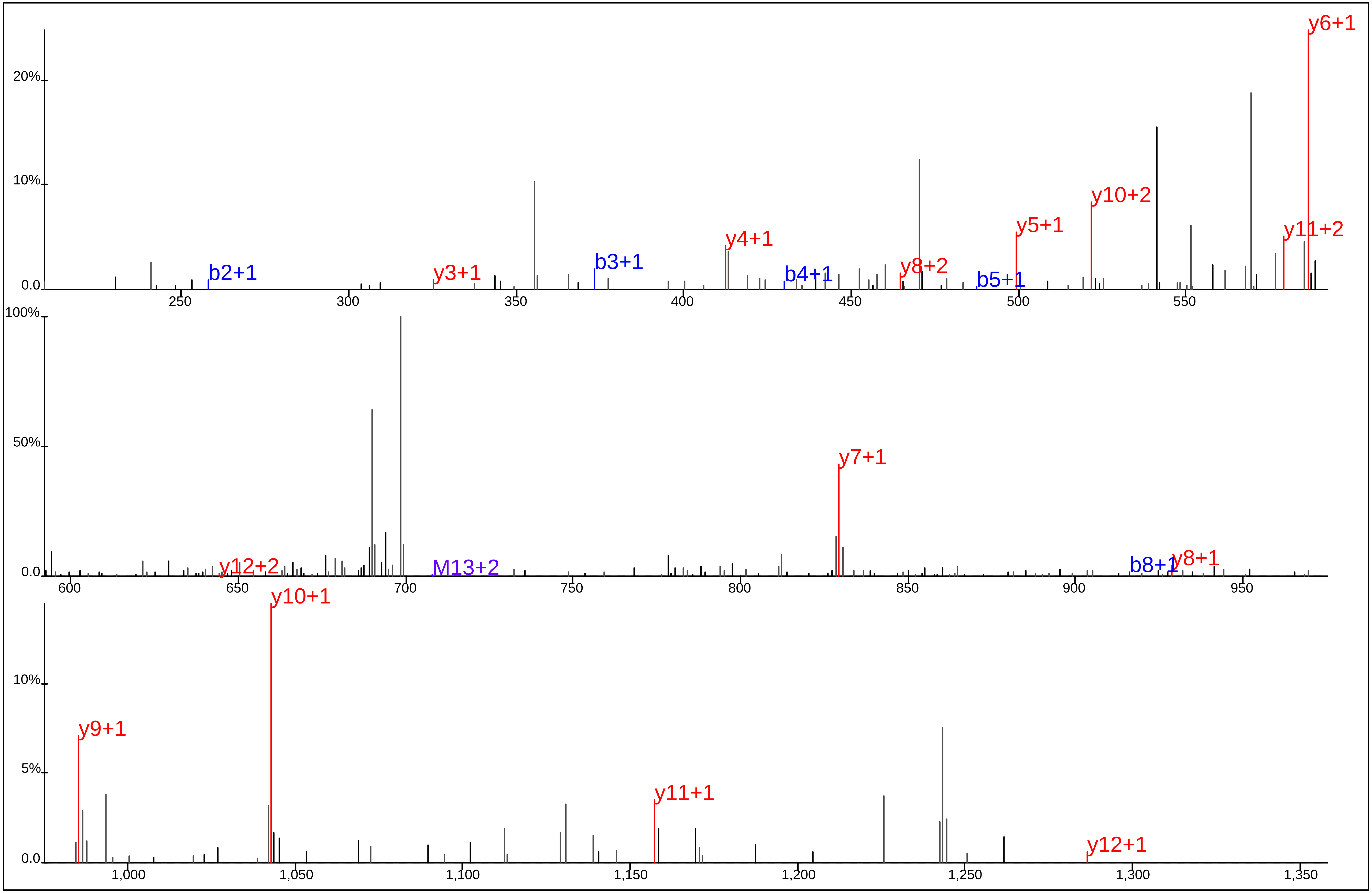

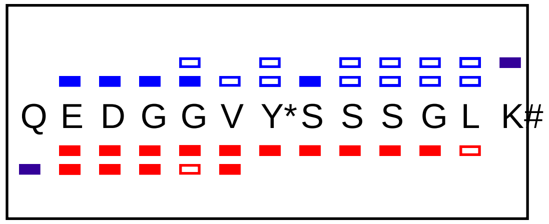

Protein Name: FER
gi Accession: 119964721
Precursor Mass: 707.8035+2
Delta Mass: 1.448 ppm
XCorr: 50.13

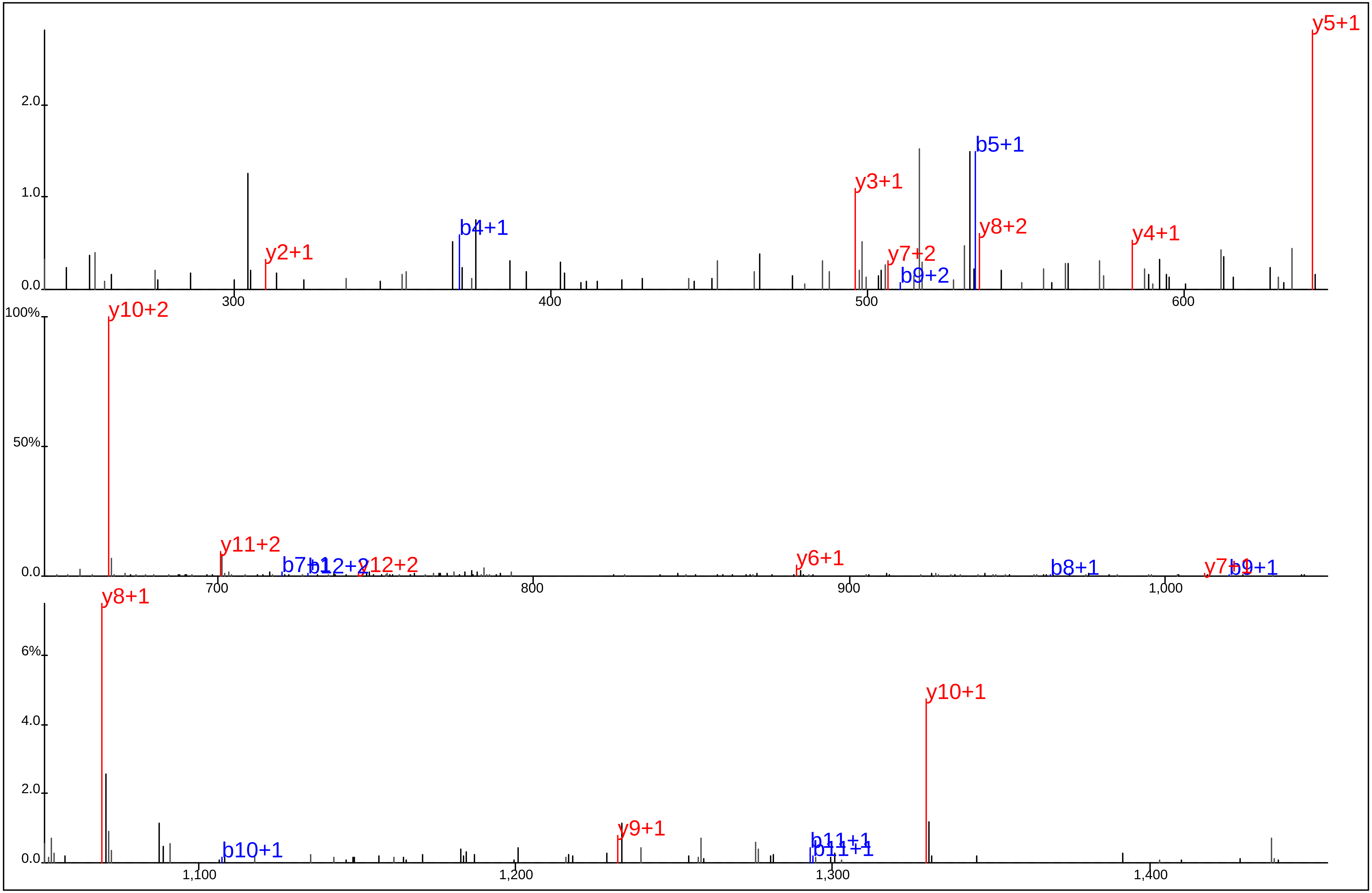

D S A P Y G E Y*G S W Y K

Protein Name: FLJ12387
gi Accession: 10433849
Precursor Mass: 801.8164+2
Delta Mass: 6.700 ppm
XCorr: 37.07

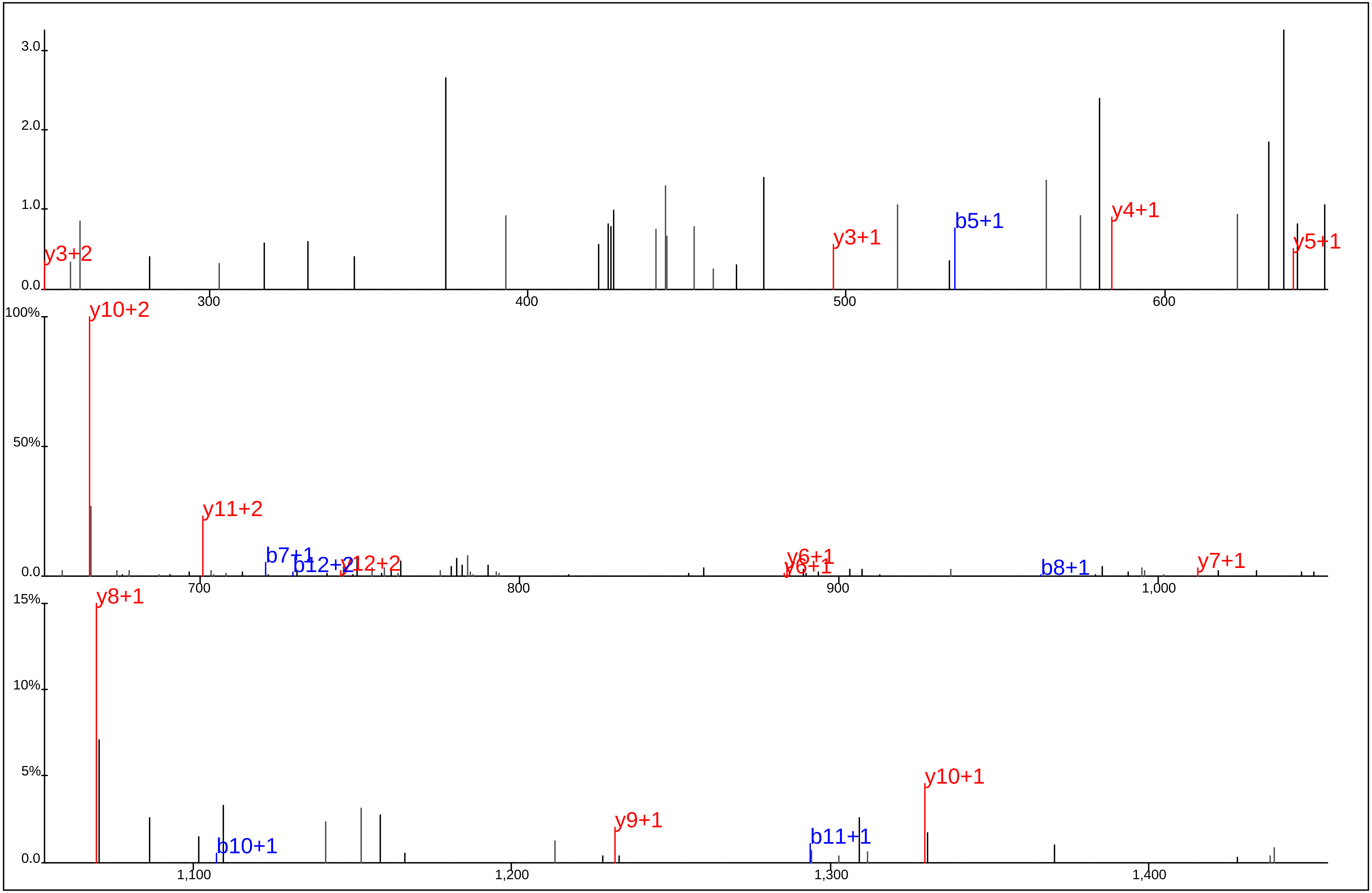

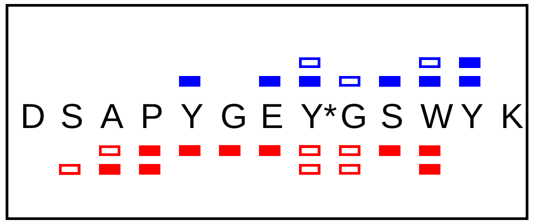

Protein Name:	FLJ12387
gi Accession:	10433849
Precursor Mass:	801.8150+2
Delta Mass:	4.947 ppm
XCorr:	19.69

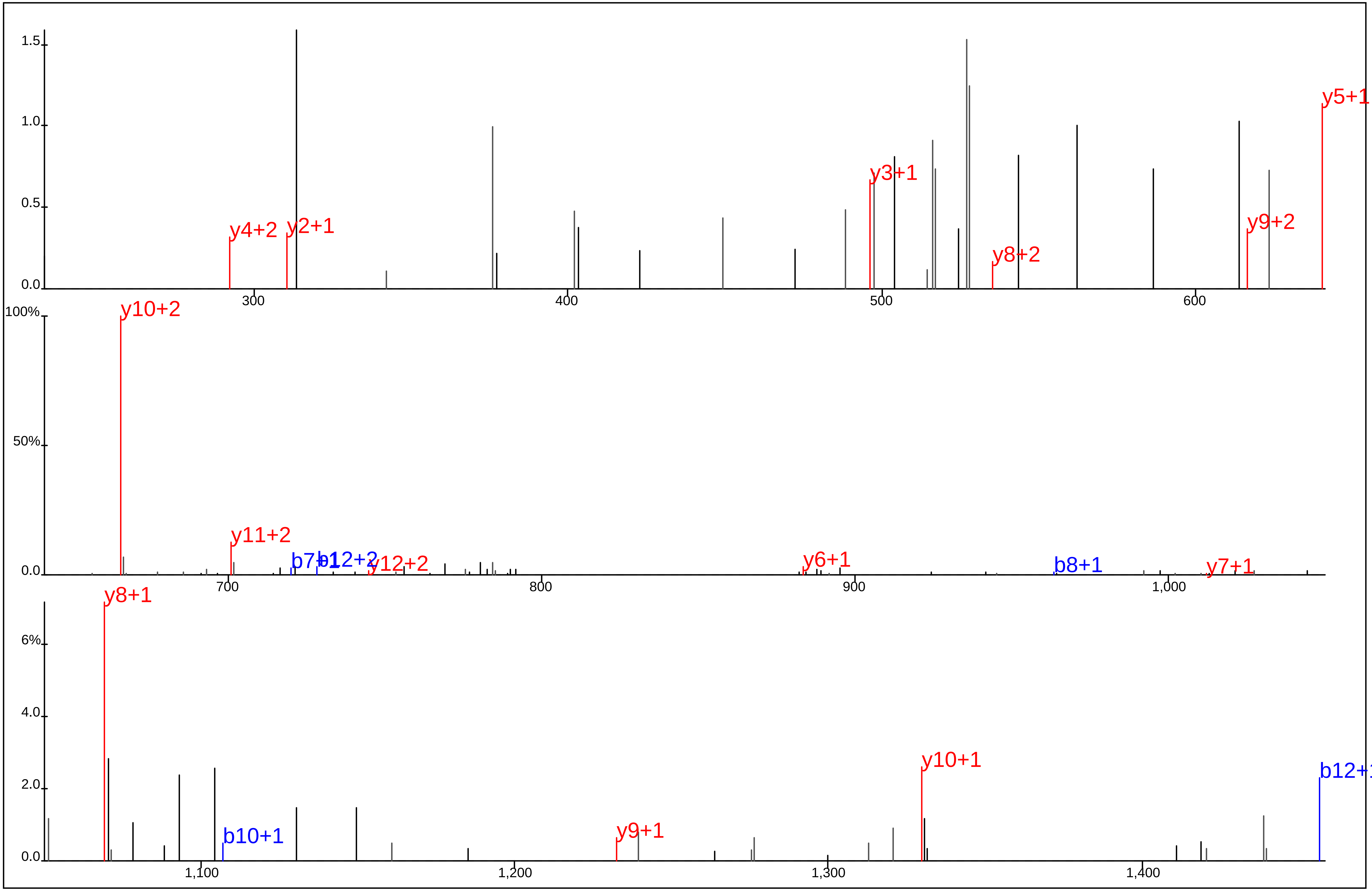

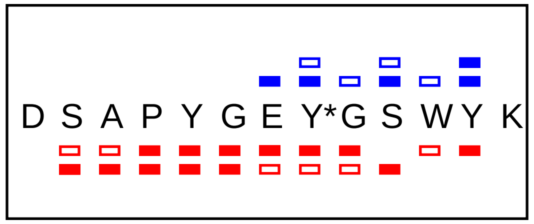

Protein Name: FLJ12387
gi Accession: 10433849
Precursor Mass: 801.8148+2
Delta Mass: 4.641 ppm
XCorr: 26.84

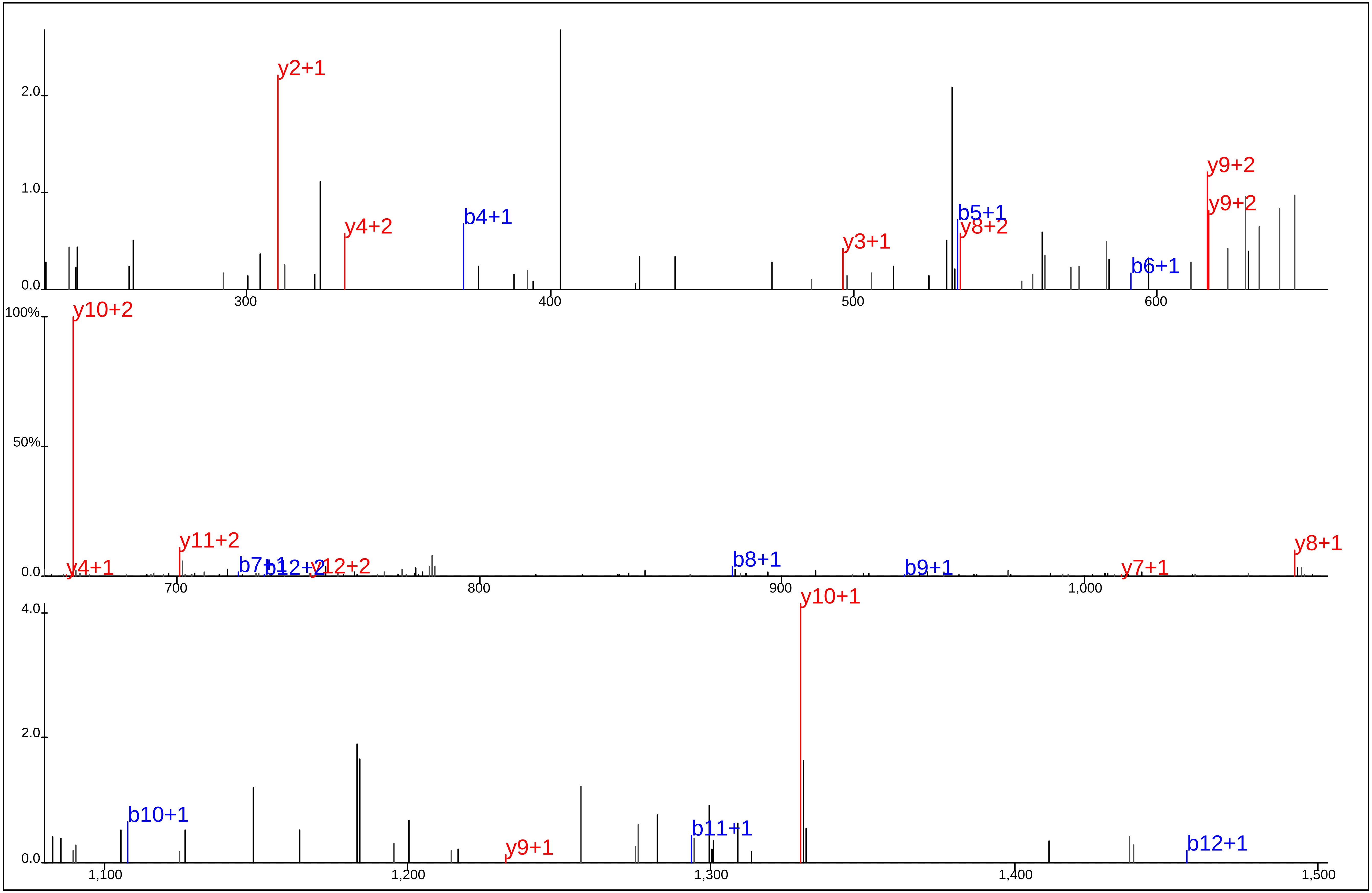

D S A P Y G E Y G S*W Y K

Protein Name: FLJ12387
gi Accession: 10433849
Precursor Mass: 801.8138+2
Delta Mass: 3.499 ppm
XCorr: 22.42

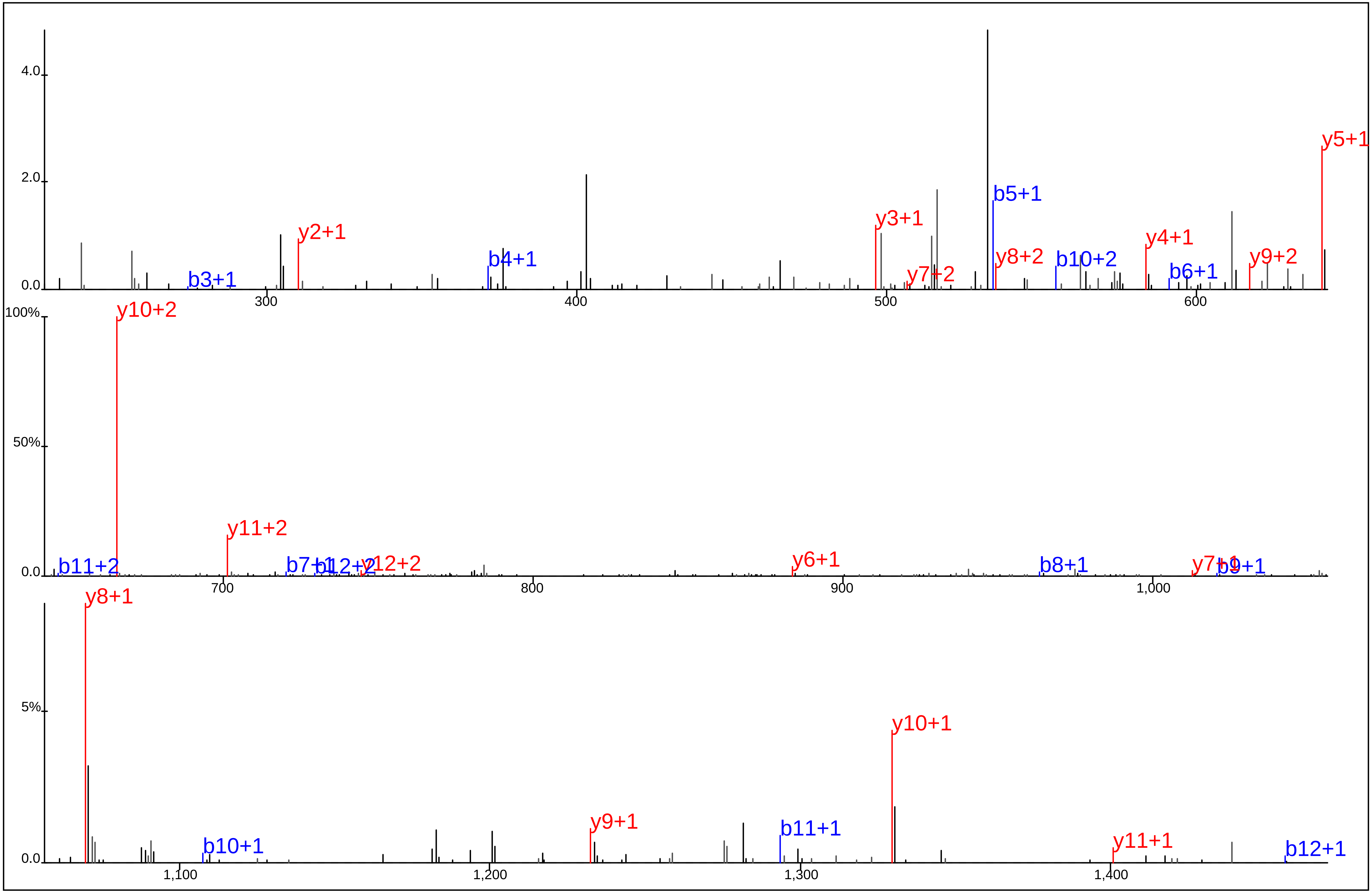

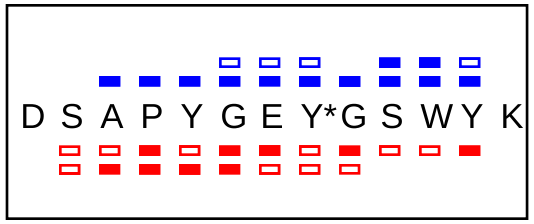

Protein Name: FLJ12387
gi Accession: 10433849
Precursor Mass: 801.8165+2
Delta Mass: 6.775 ppm
XCorr: 55.99

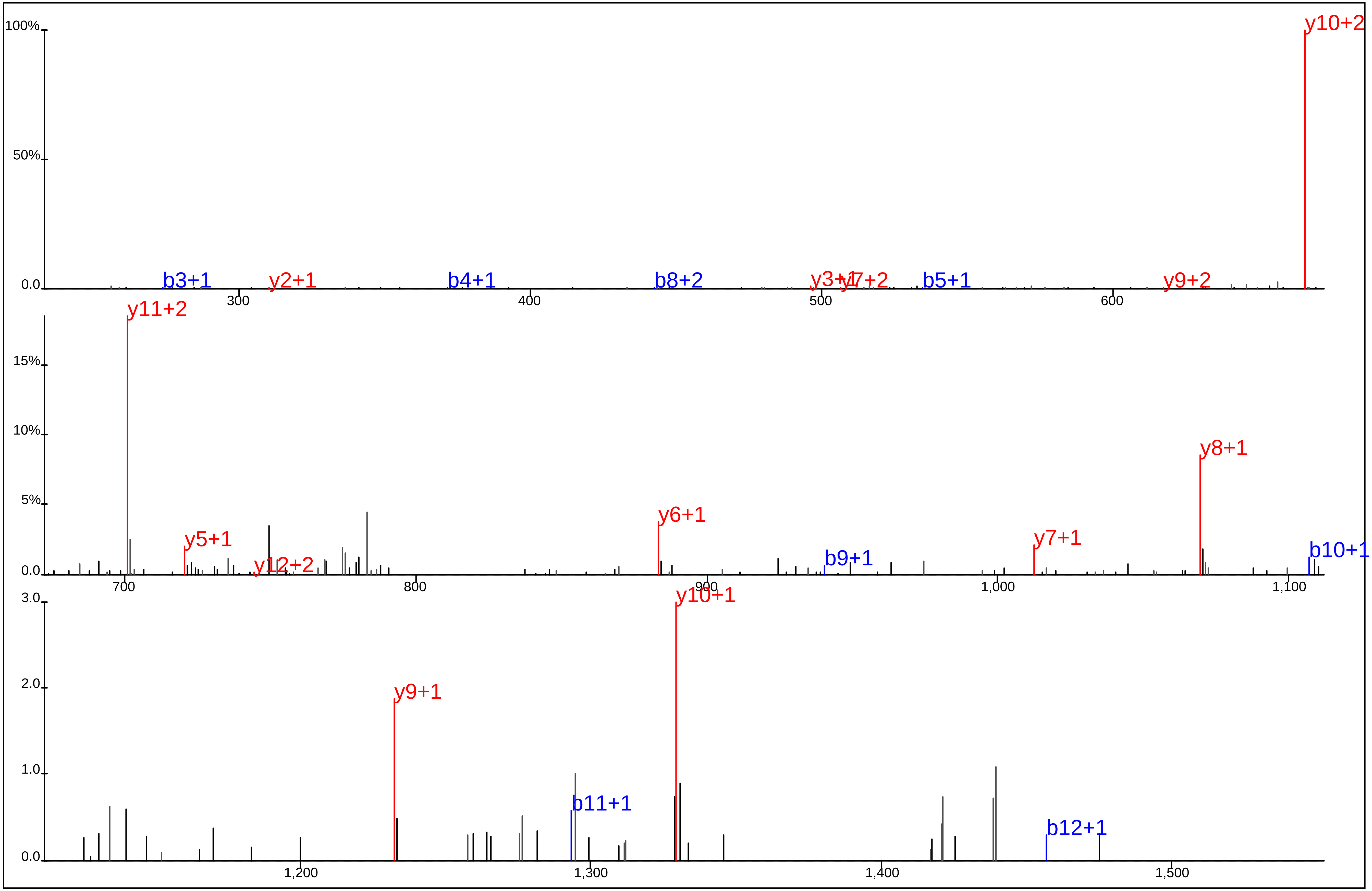

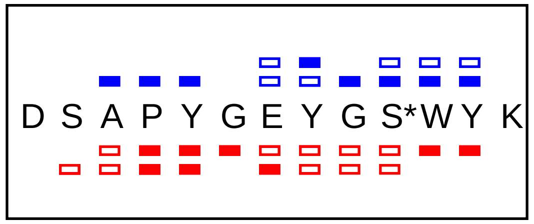

Protein Name: FLJ12387
gi Accession: 10433849
Precursor Mass: 801.8151+2
Delta Mass: 5.097 ppm
XCorr: 33.39

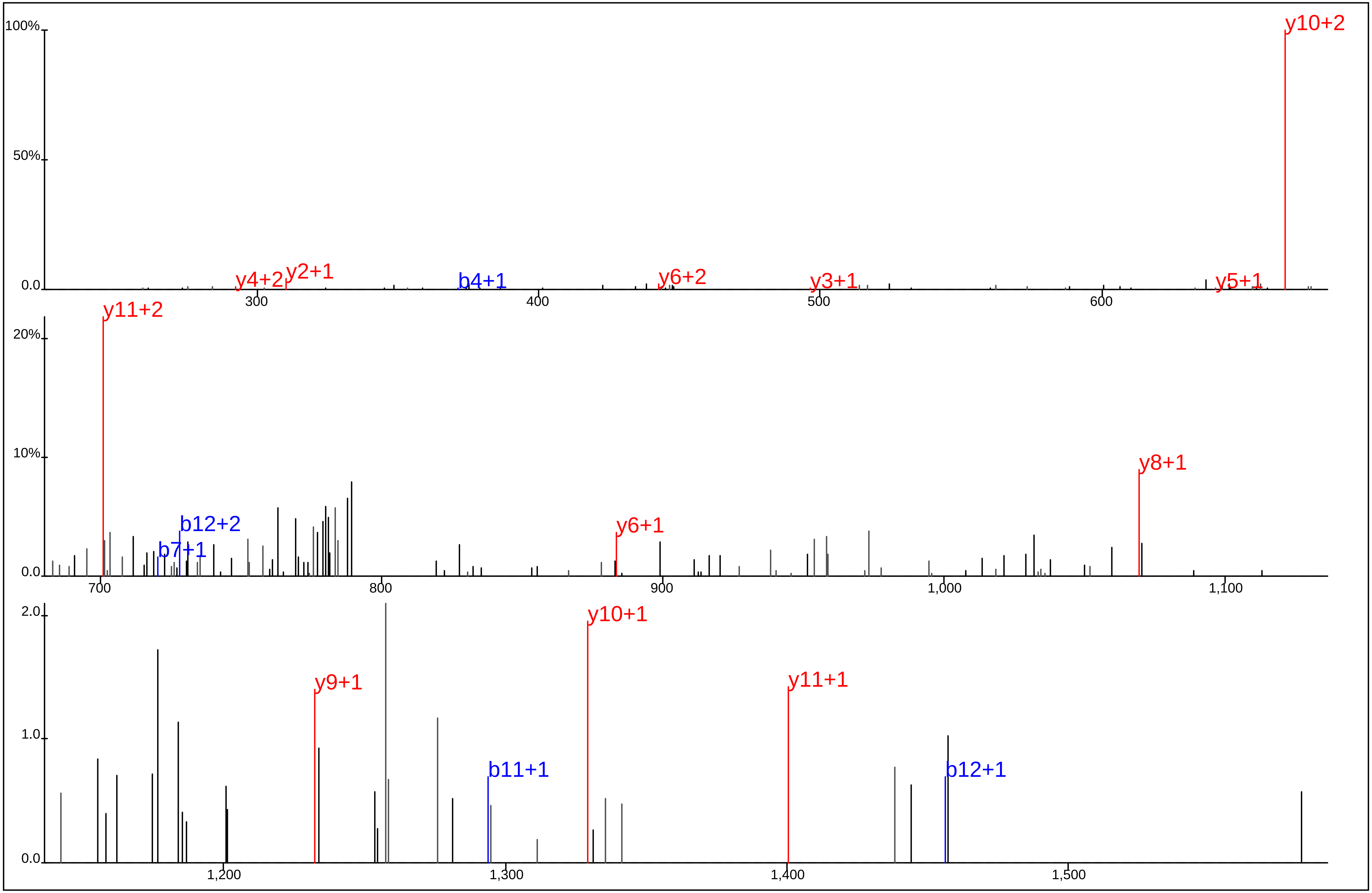

D S A P Y G E Y*G S W Y K

Protein Name: FLJ12387
gi Accession: 10433849
Precursor Mass: 801.8156+2
Delta Mass: 5.633 ppm
XCorr: 21.75

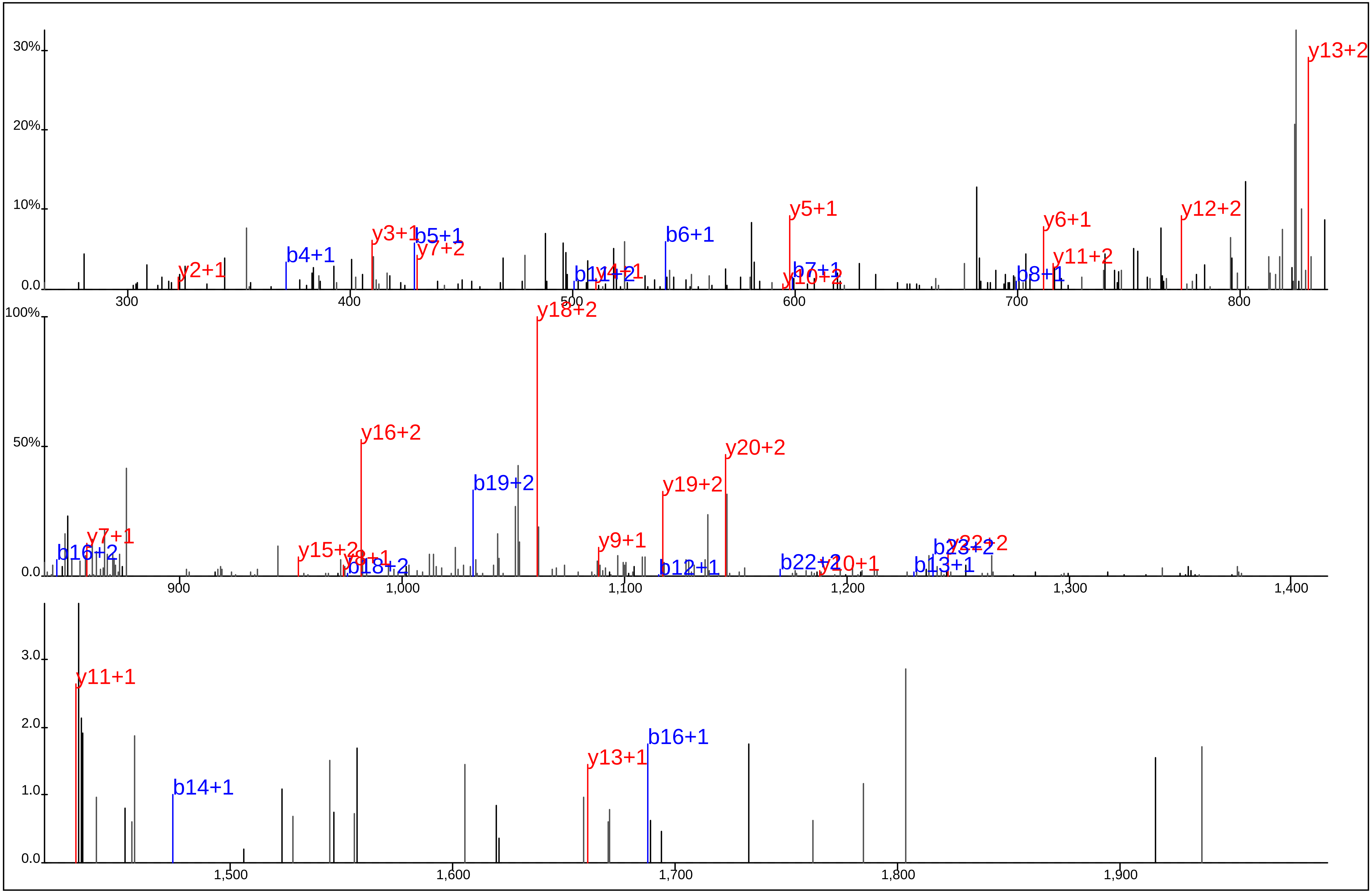

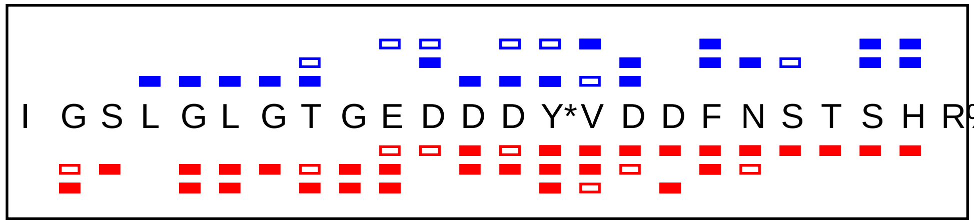

Protein Name: FOP
gi Accession: 5901954
Precursor Mass: 887.3713+3
Delta Mass: 1.163 ppm
XCorr: 34.34

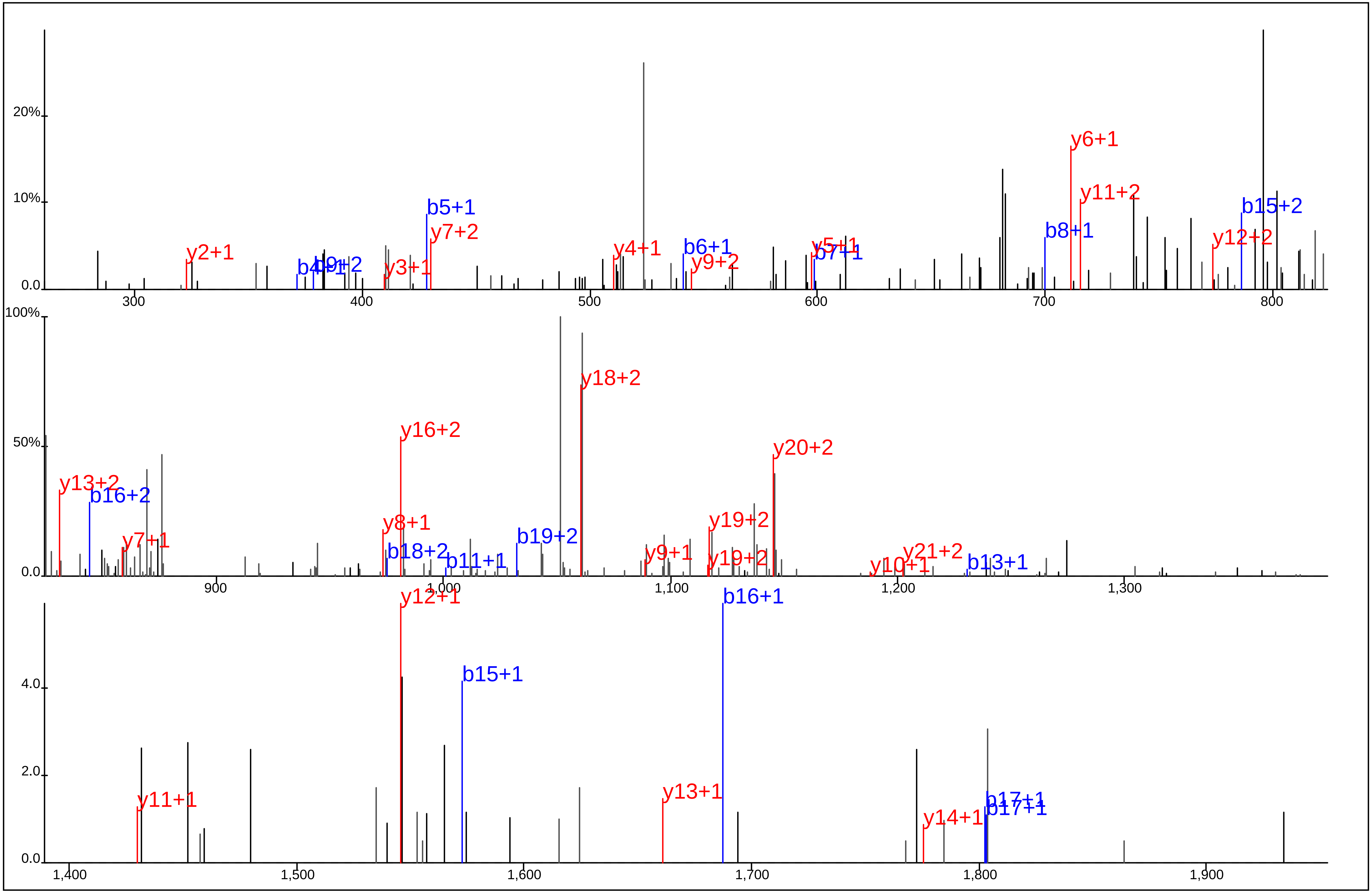

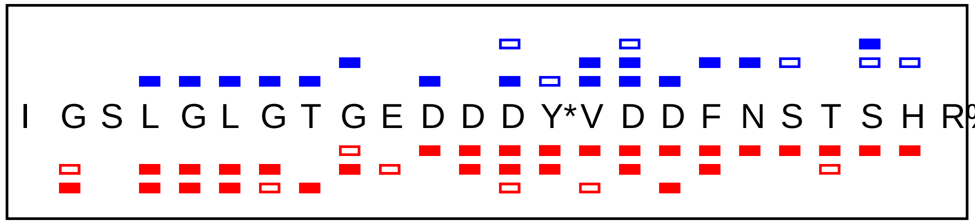

Protein Name: FOP
gi Accession: 5901954
Precursor Mass: 887.3731+3
Delta Mass: 0.901 ppm
XCorr: 39.77

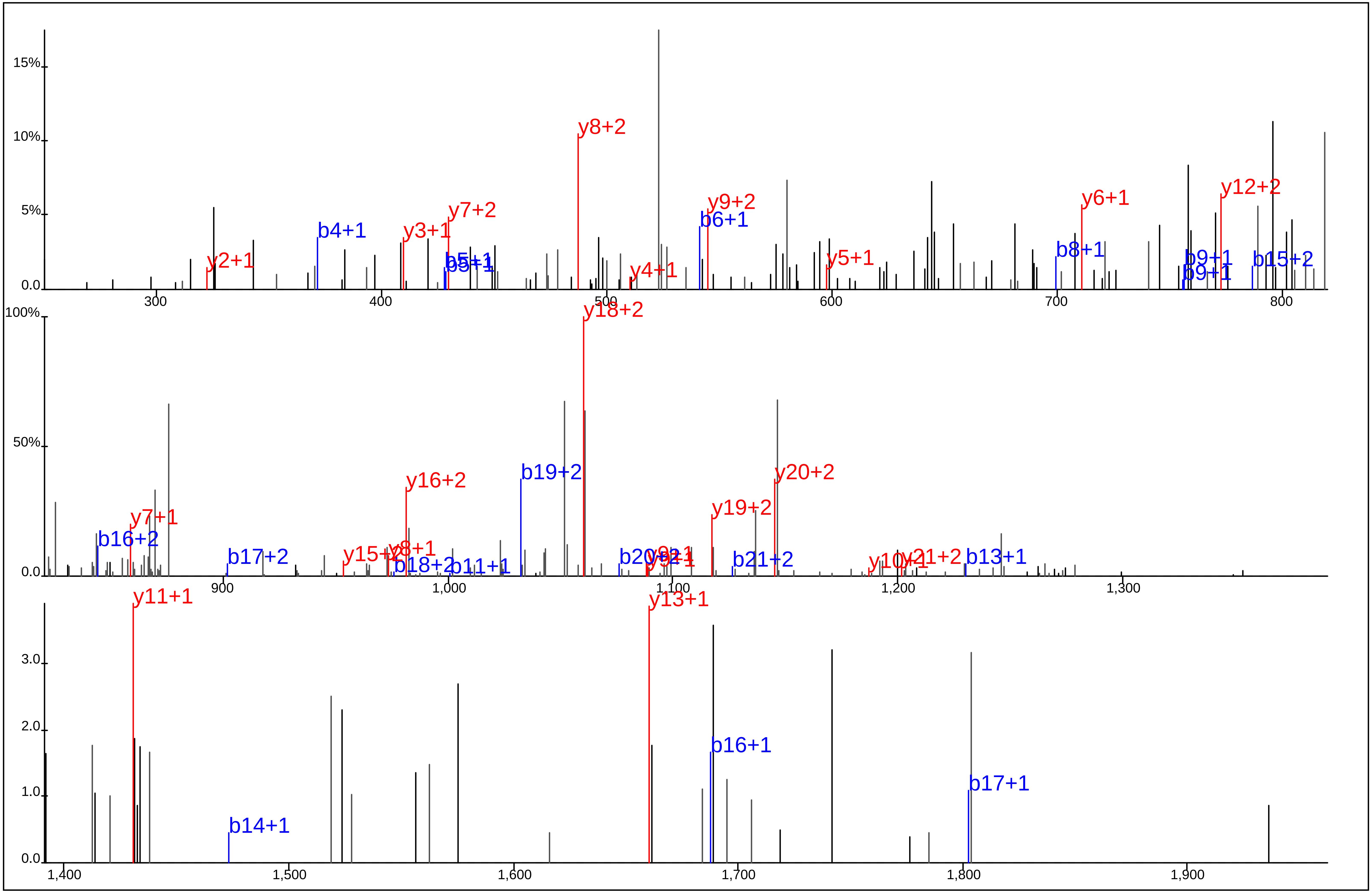

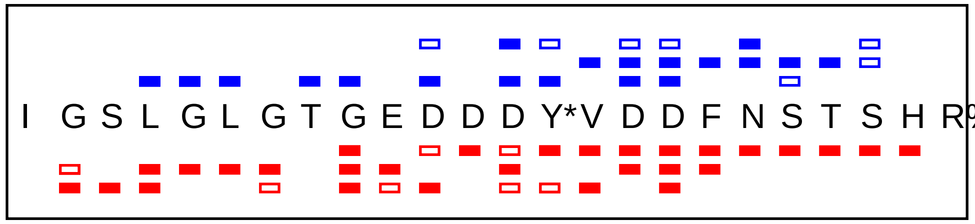

Protein Name: FOP
gi Accession: 5901954
Precursor Mass: 887.3735+3
Delta Mass: 1.383 ppm
XCorr: 30.85

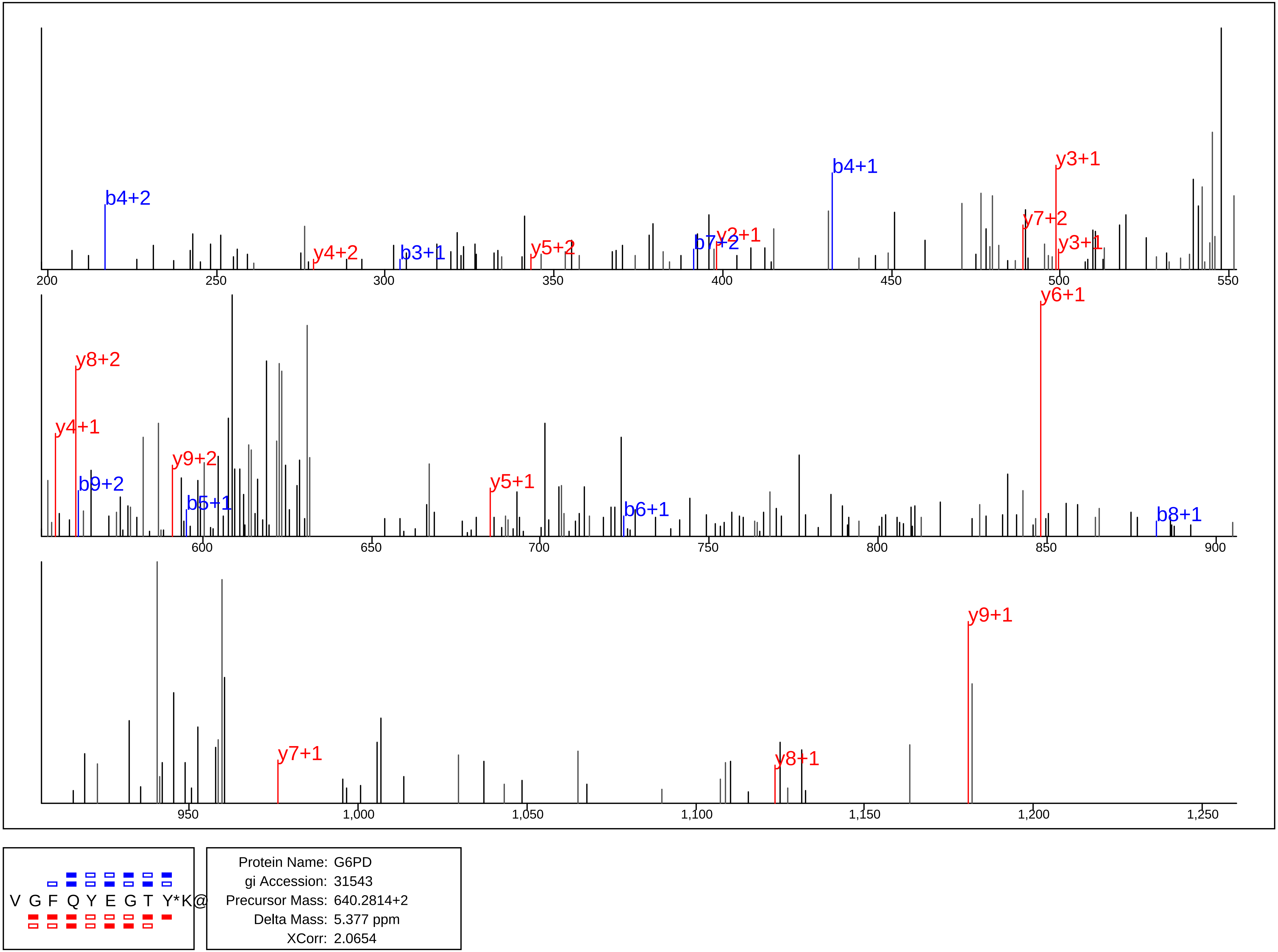

Protein Name: G6PD
gi Accession: 31543
Precursor Mass: 640.2814+2
Delta Mass: 5.377 ppm
XCorr: 2.0654

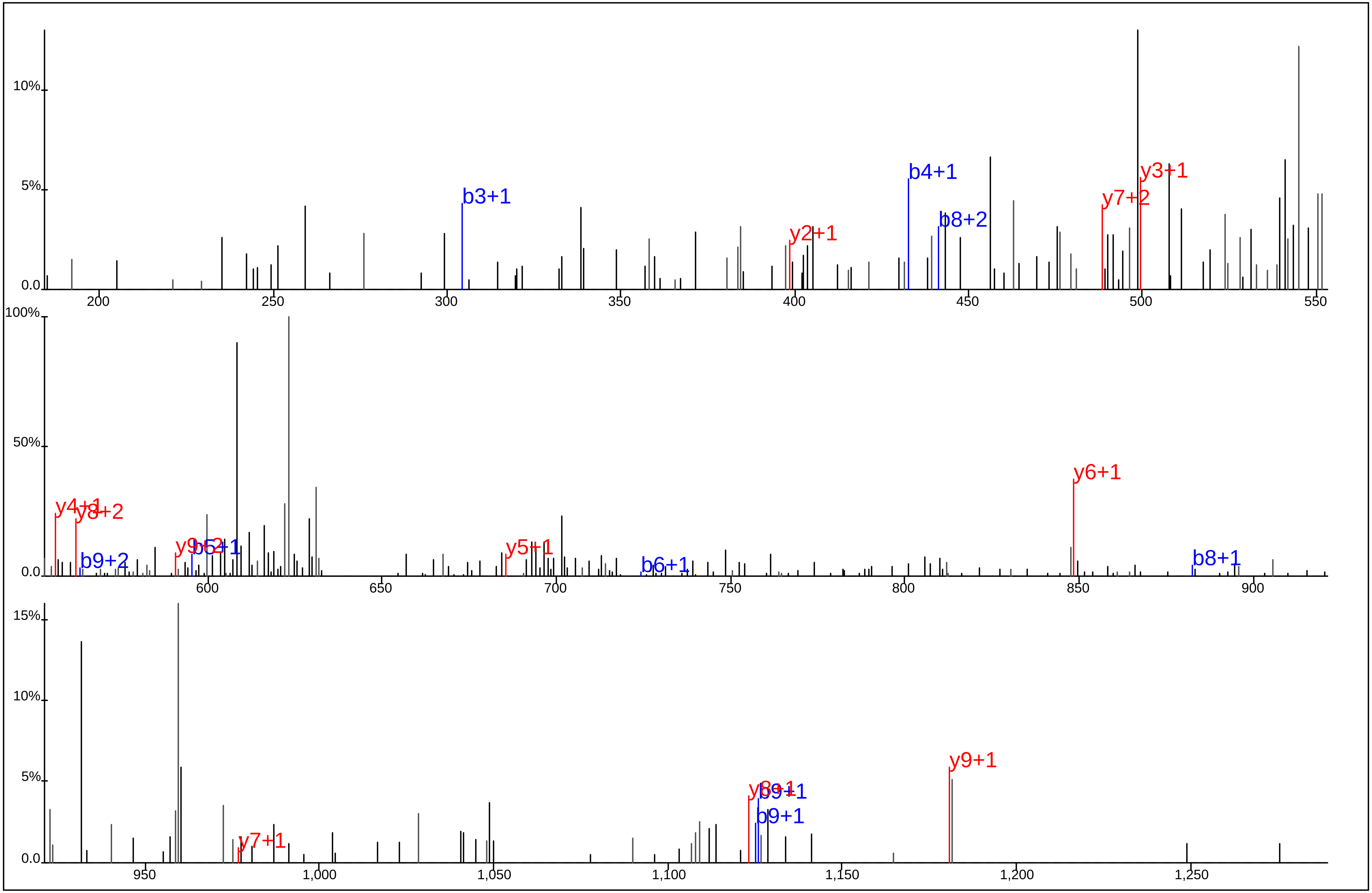

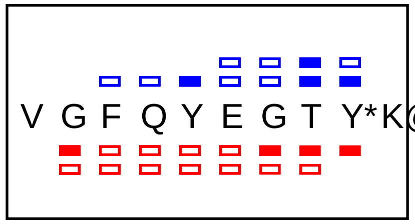

Protein Name: G6PD
gi Accession: 31543
Precursor Mass: 640.2830+2
Delta Mass: 7.854 ppm
XCorr: 2.3201

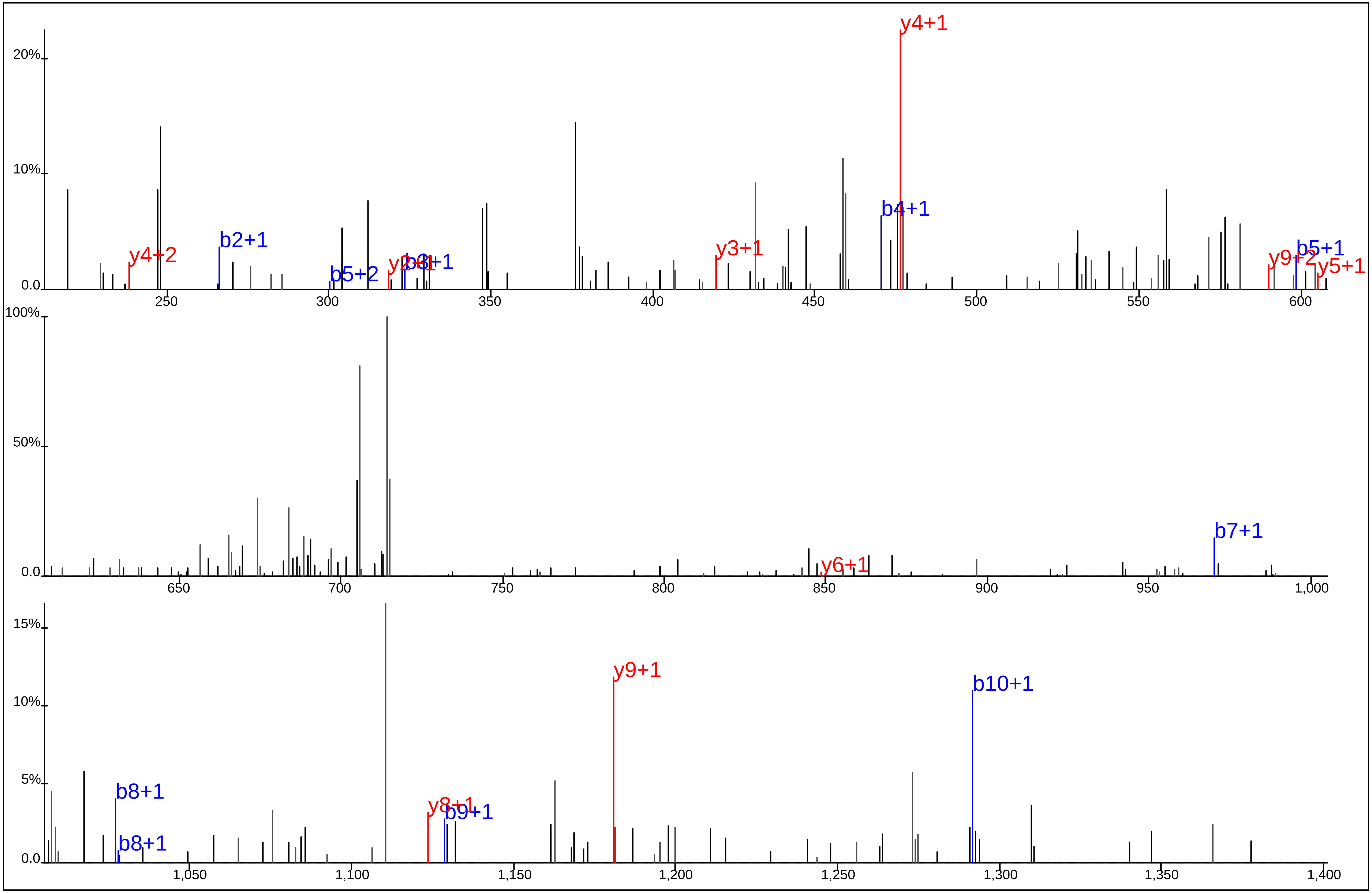

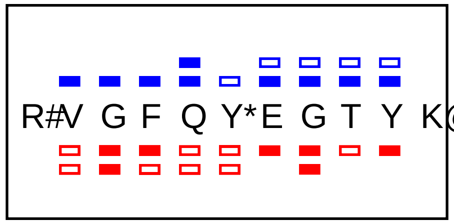

Protein Name: G6PD
gi Accession: 31543
Precursor Mass: 723.3350+2
Delta Mass: 3.237 ppm
XCorr: 2.3645

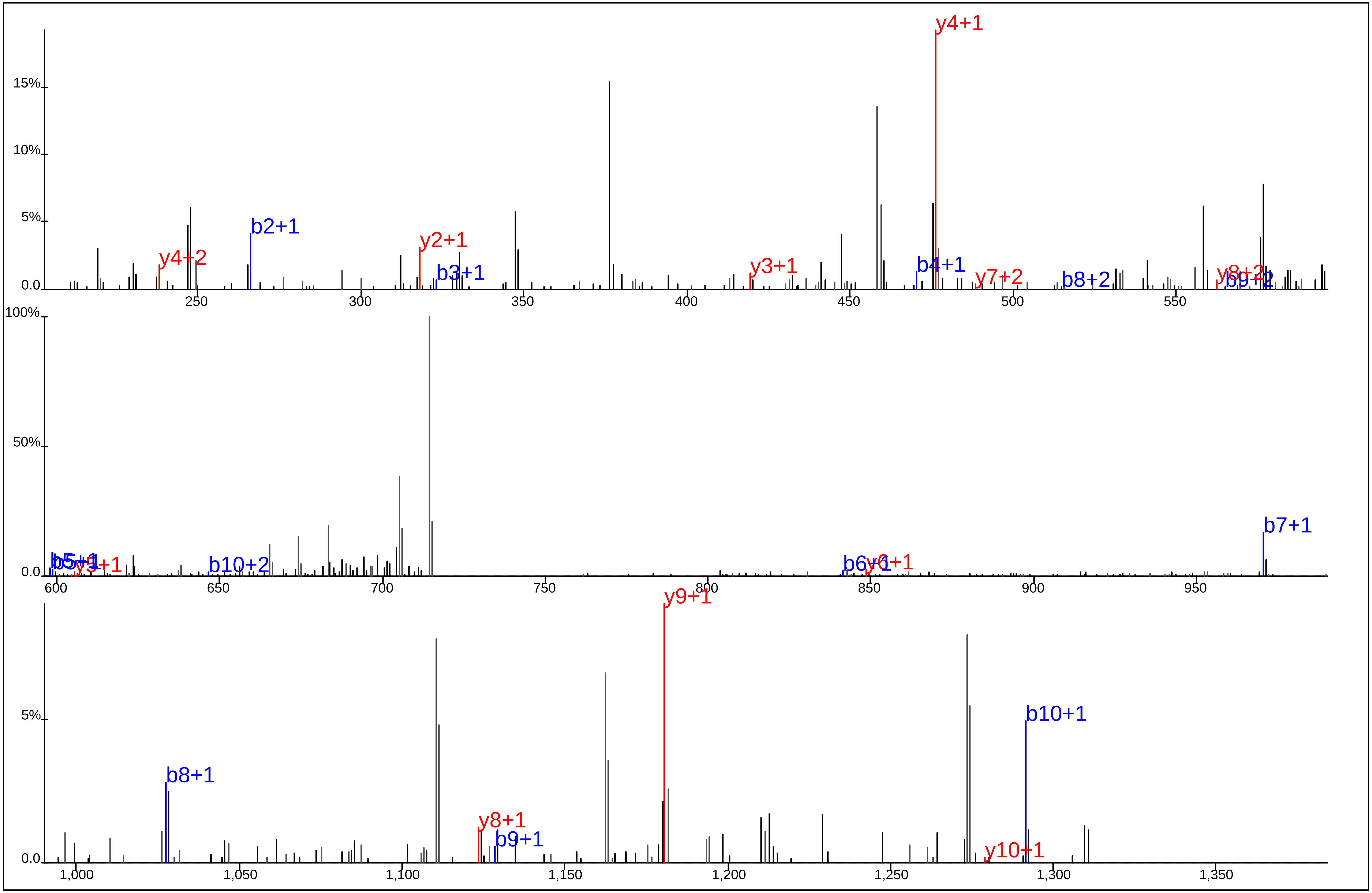

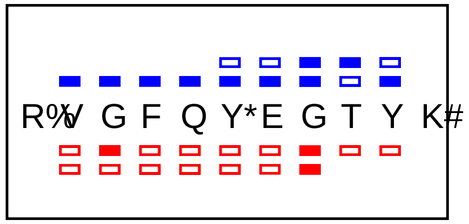

Protein Name: G6PD
gi Accession: 31543
Precursor Mass: 723.3333+2
Delta Mass: 0.870 ppm
XCorr: 24.14

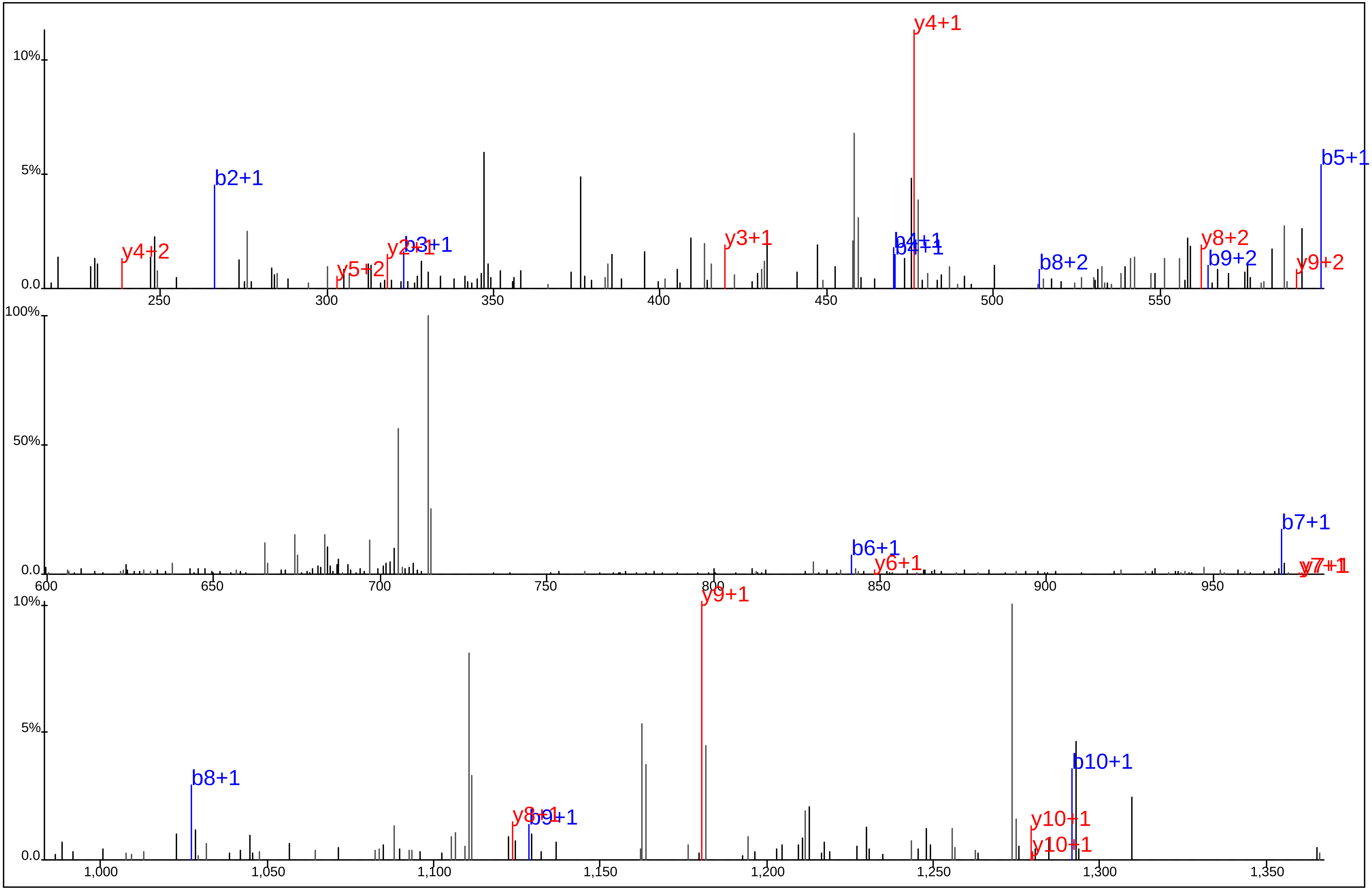

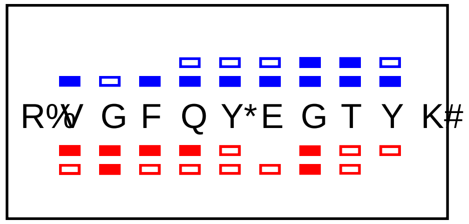

Protein Name: G6PD
gi Accession: 31543
Precursor Mass: 723.3364+2
Delta Mass: 5.173 ppm
XCorr: 26.9

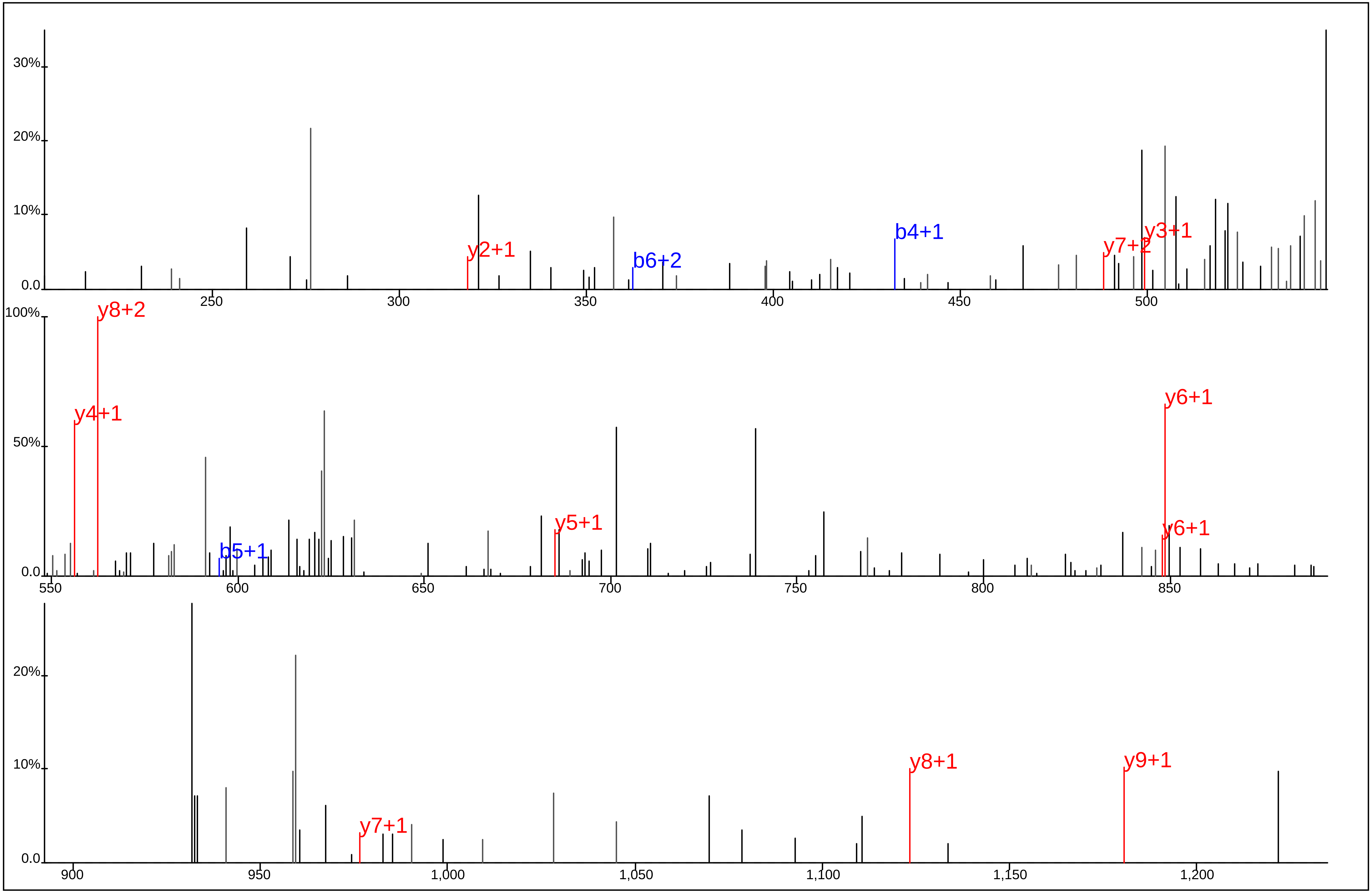

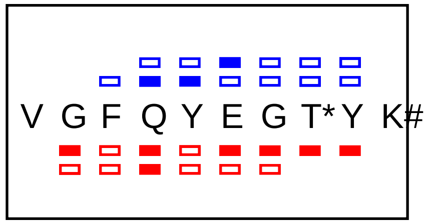

Protein Name: G6PD
gi Accession: 31543
Precursor Mass: 640.2791+2
Delta Mass: 1.741 ppm
XCorr: 15.4

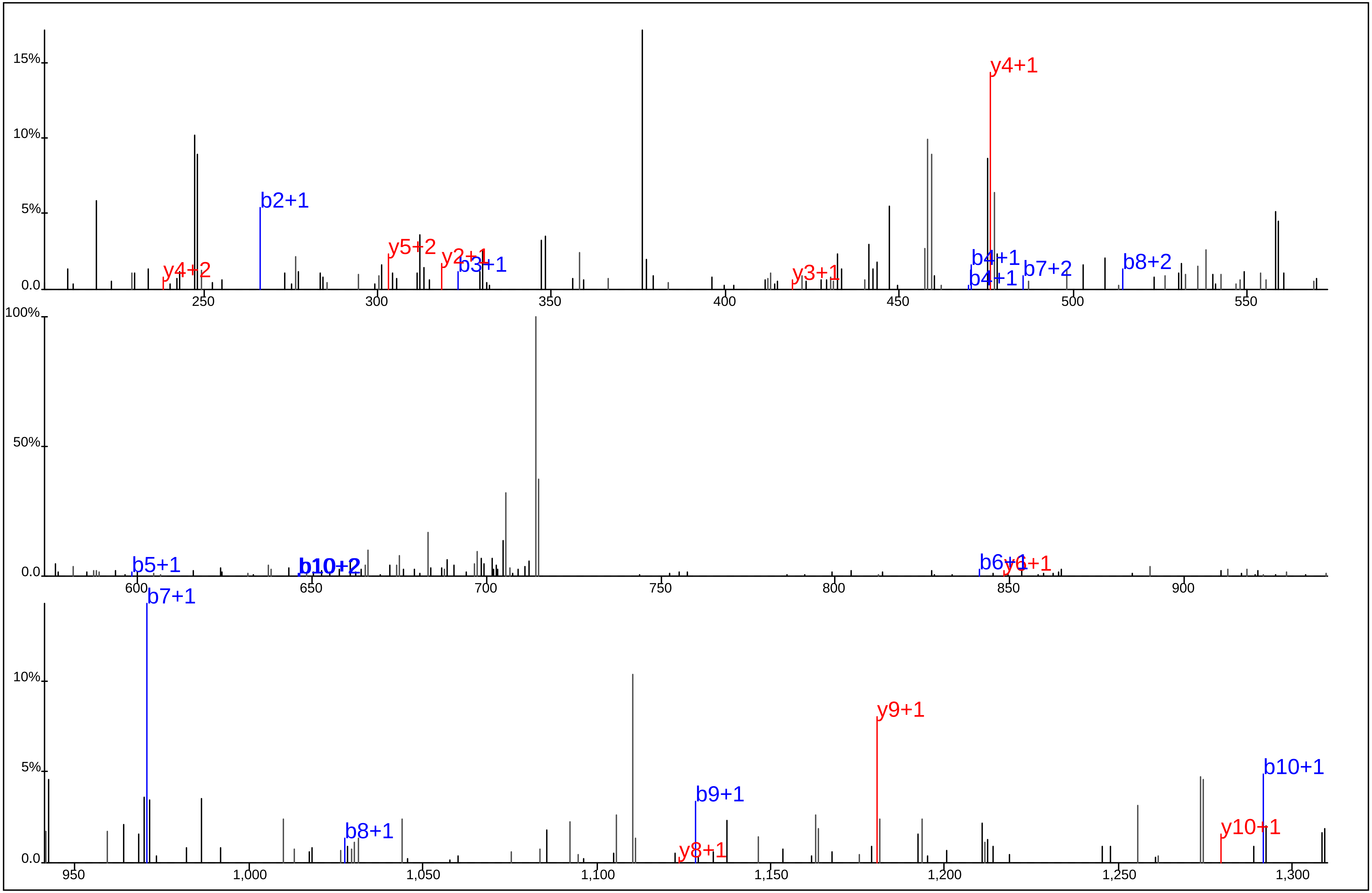

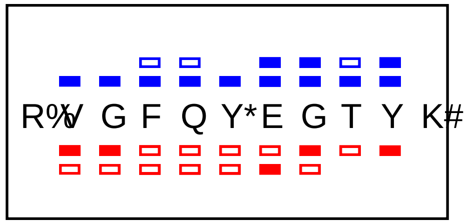

Protein Name: G6PD
gi Accession: 31543
Precursor Mass: 723.3342+2
Delta Mass: 2.136 ppm
XCorr: 17.43

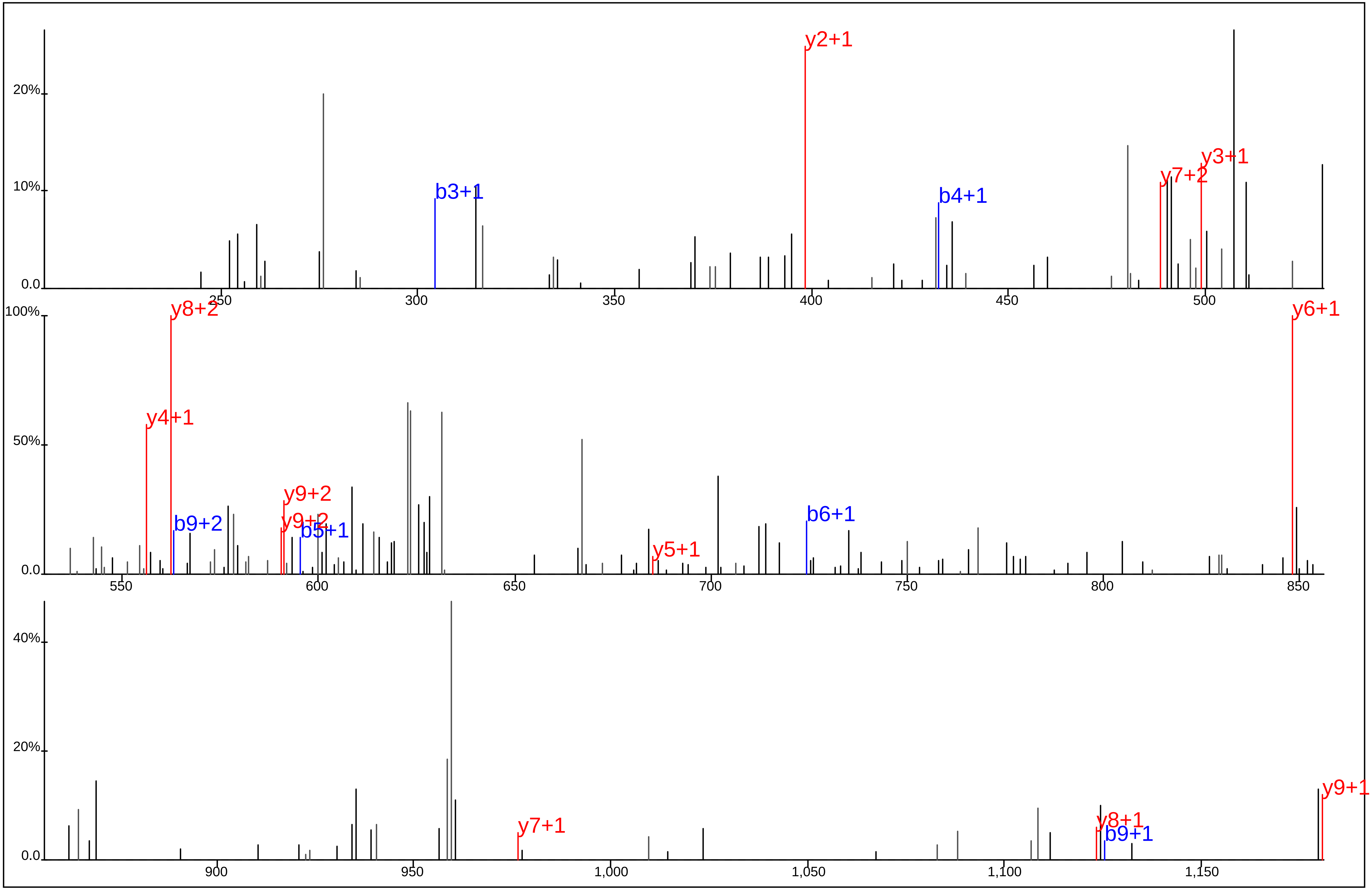

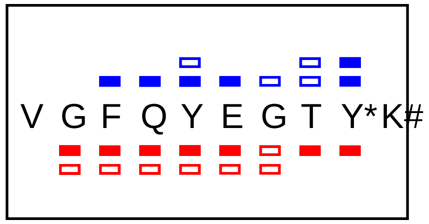

Protein Name:	G6PD
gi Accession:	31543
Precursor Mass:	640.2803+2
Delta Mass:	3.648 ppm
XCorr:	19.72

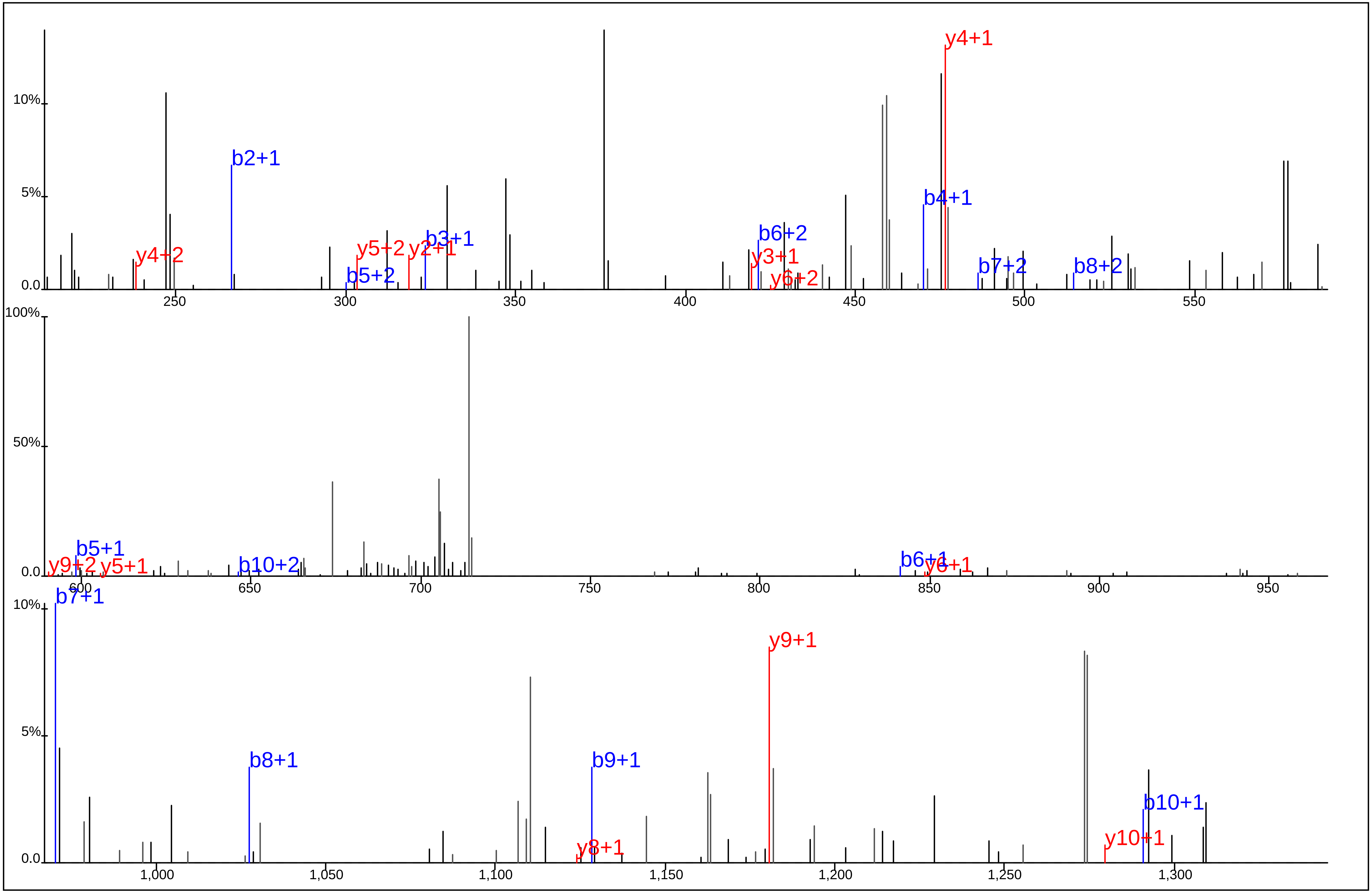

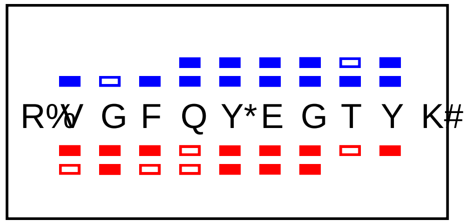

Protein Name: G6PD
gi Accession: 31543
Precursor Mass: 723.3335+2
Delta Mass: 1.209 ppm
XCorr: 33.76

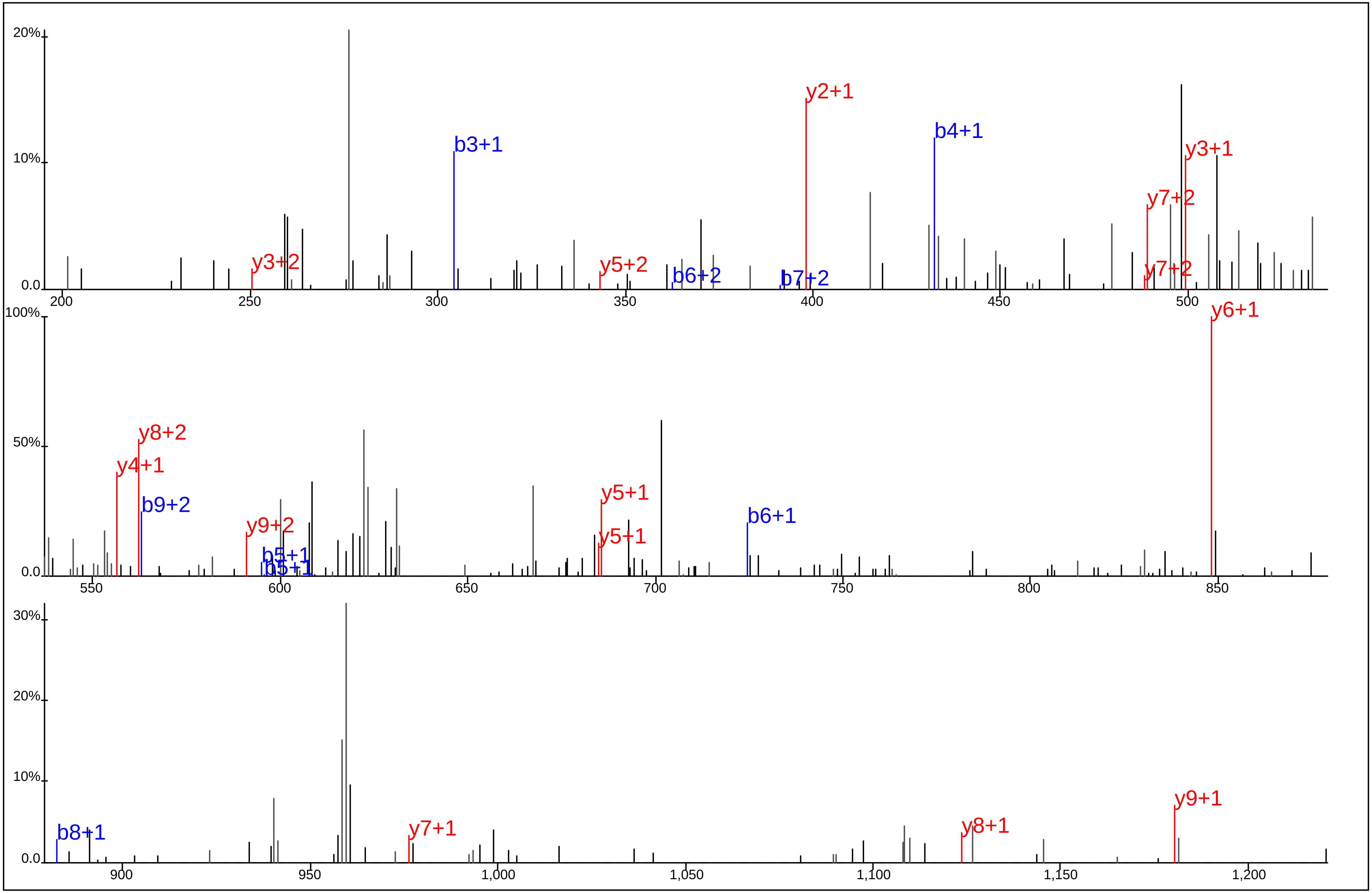

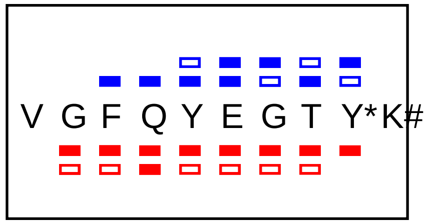

Protein Name: G6PD
gi Accession: 31543
Precursor Mass: 640.2794+2
Delta Mass: 2.218 ppm
XCorr: 25.32

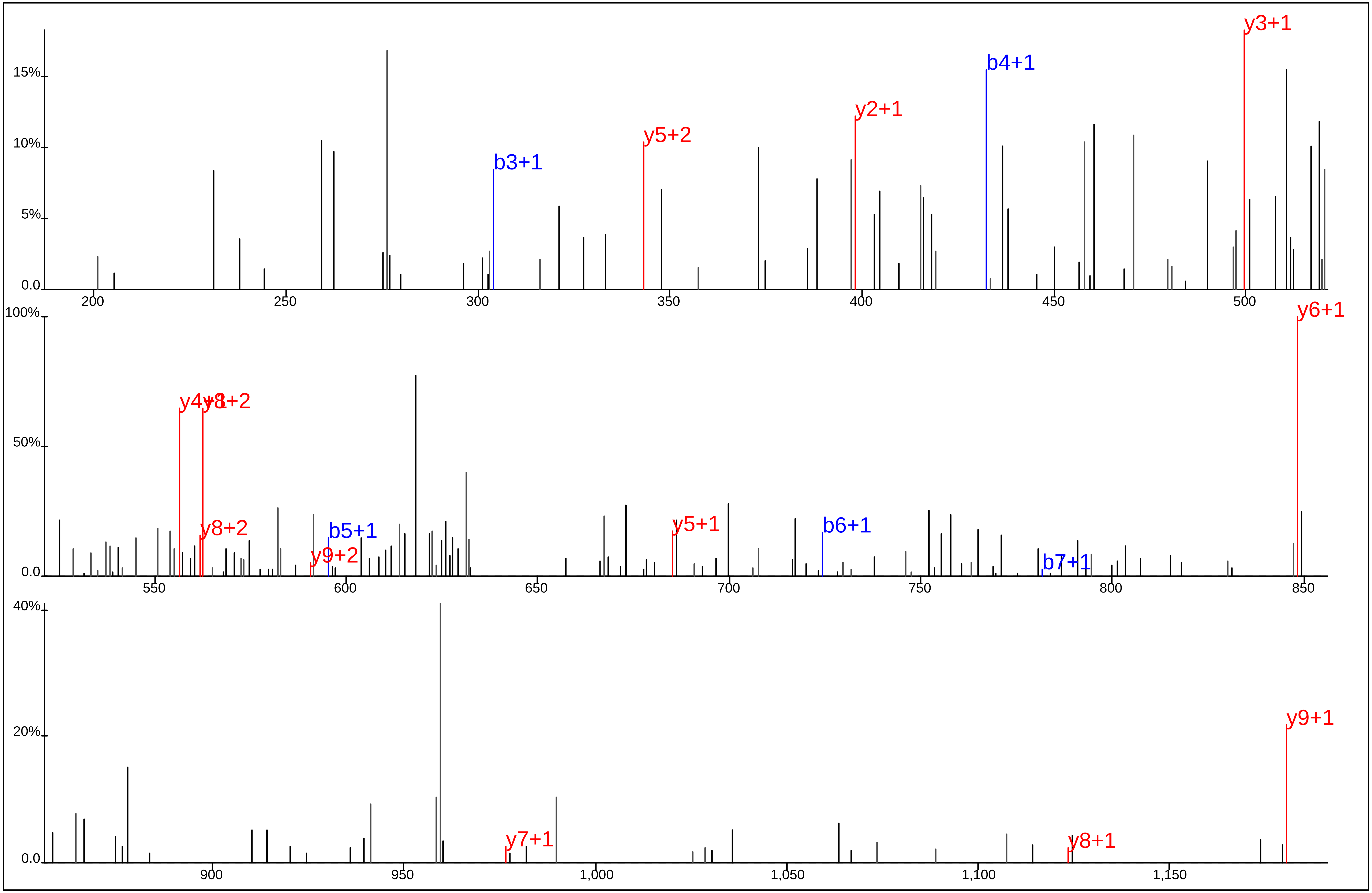

V G F Q Y E G T Y*K#

Protein Name: G6PD
gi Accession: 31543
Precursor Mass: 640.2805+2
Delta Mass: 3.937 ppm
XCorr: 18.66

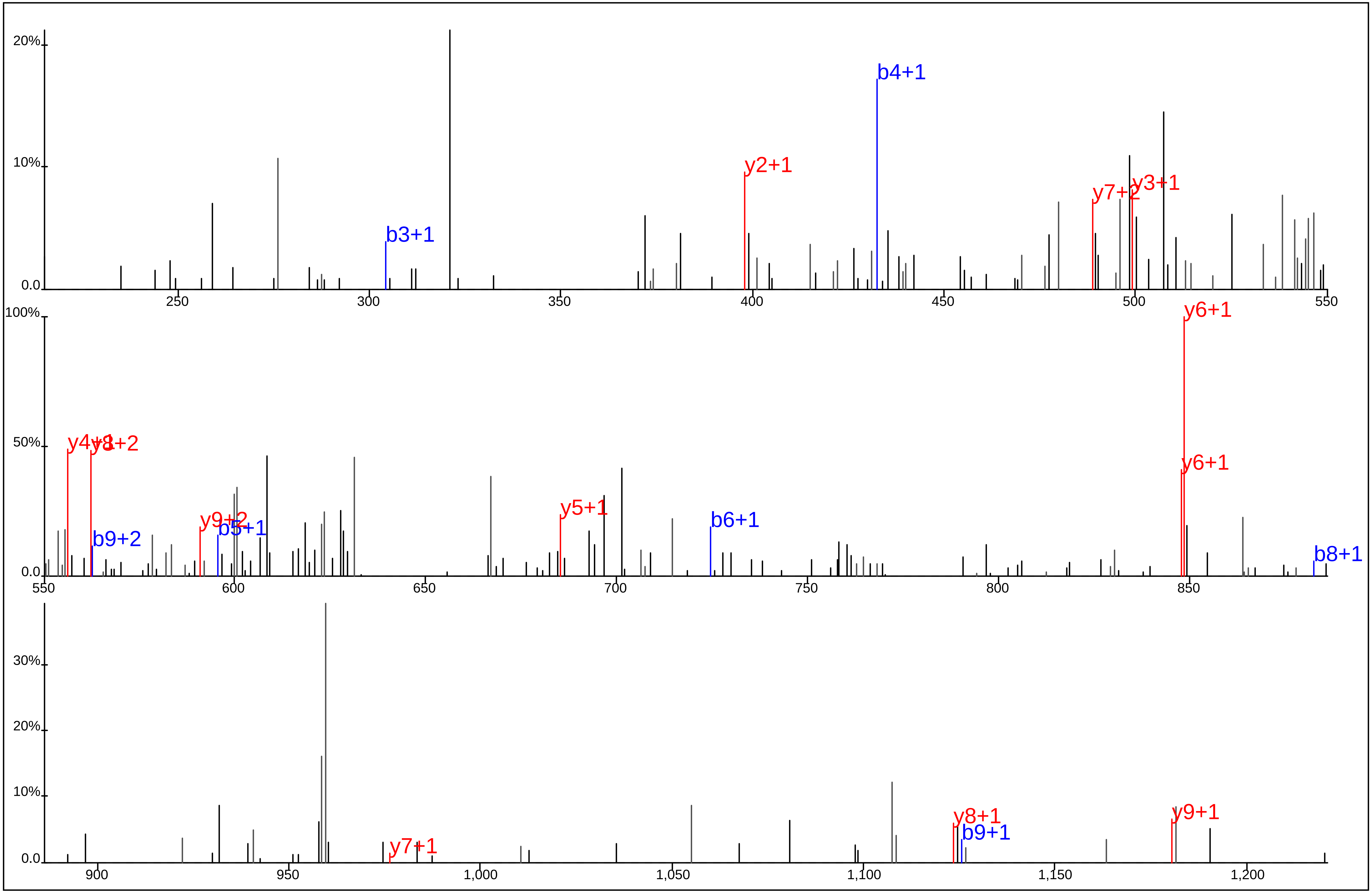

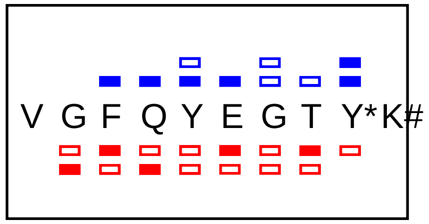

Protein Name: G6PD
gi Accession: 31543
Precursor Mass: 640.2787+2
Delta Mass: 1.265 ppm
XCorr: 24.8

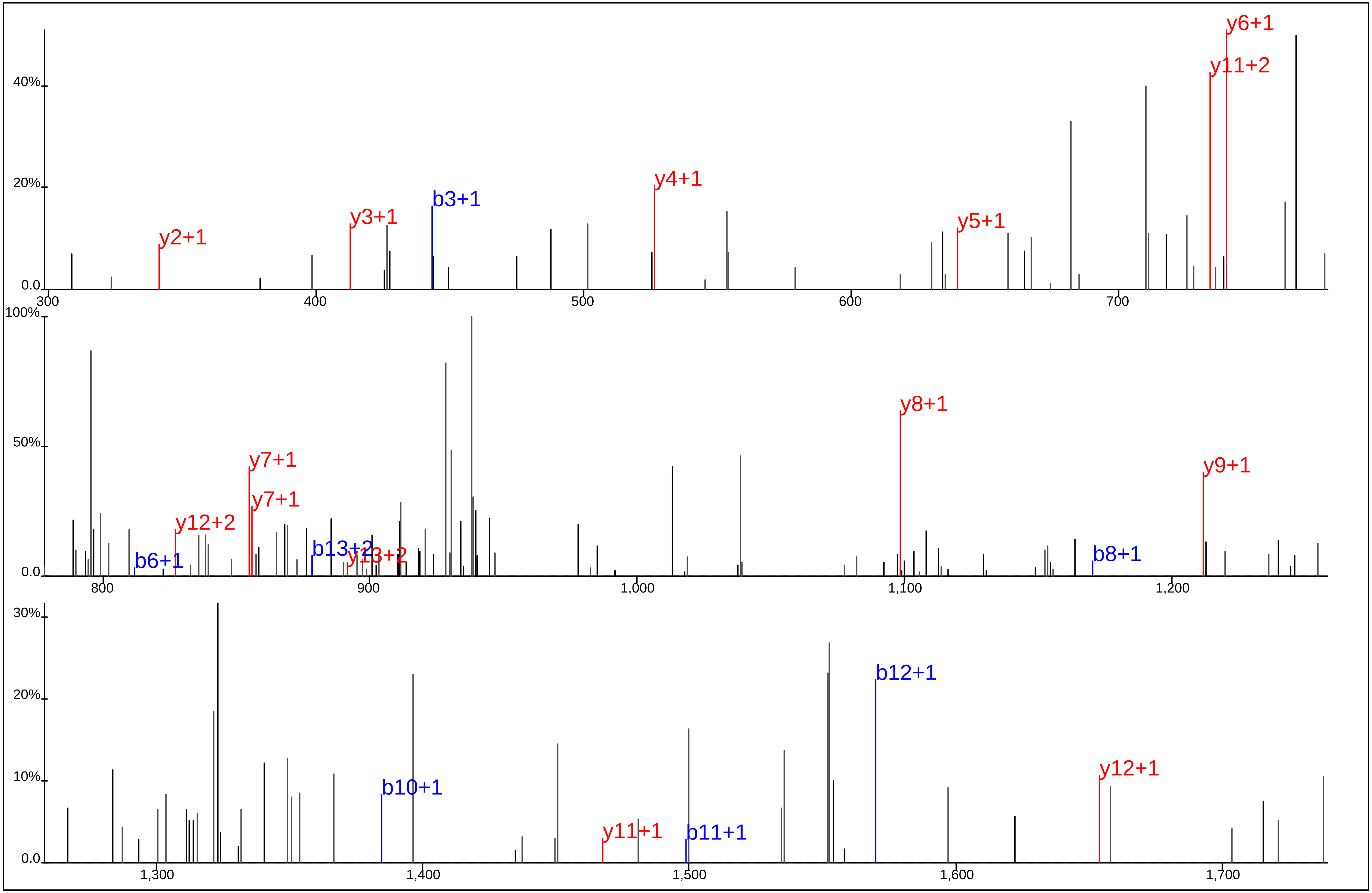

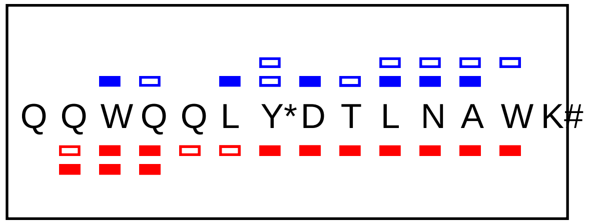

Protein Name: GA17
gi Accession: 23397429
Precursor Mass: 955.4443+2
Delta Mass: 4.727 ppm
XCorr: 39.47

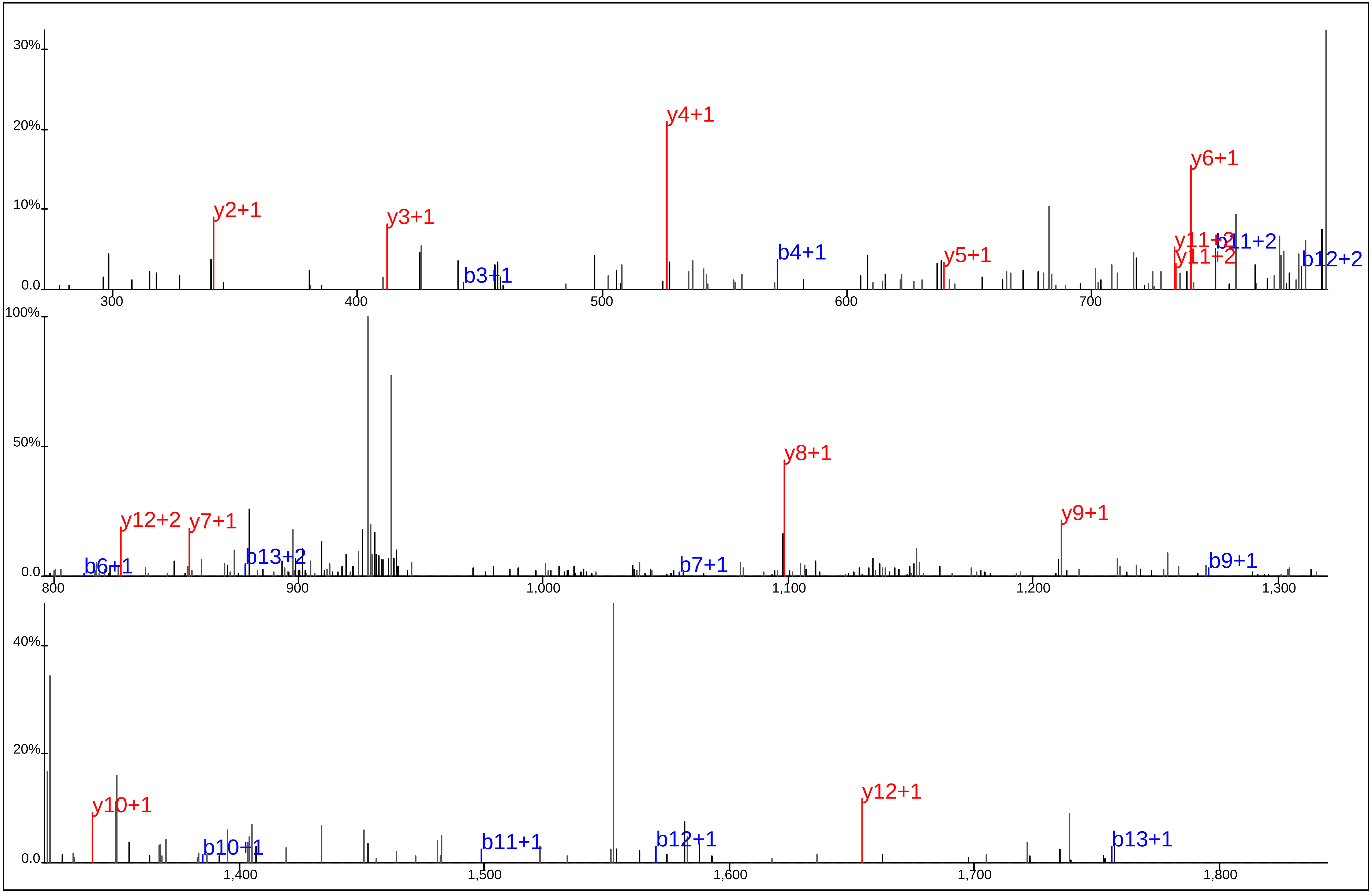

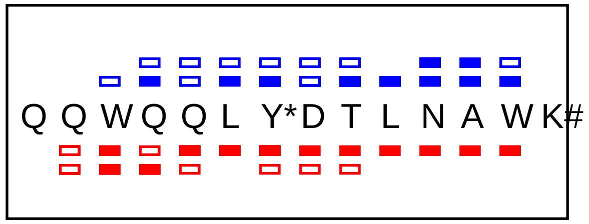

Protein Name: GA17
gi Accession: 23397429
Precursor Mass: 955.4433+2
Delta Mass: 3.638 ppm
XCorr: 35.59

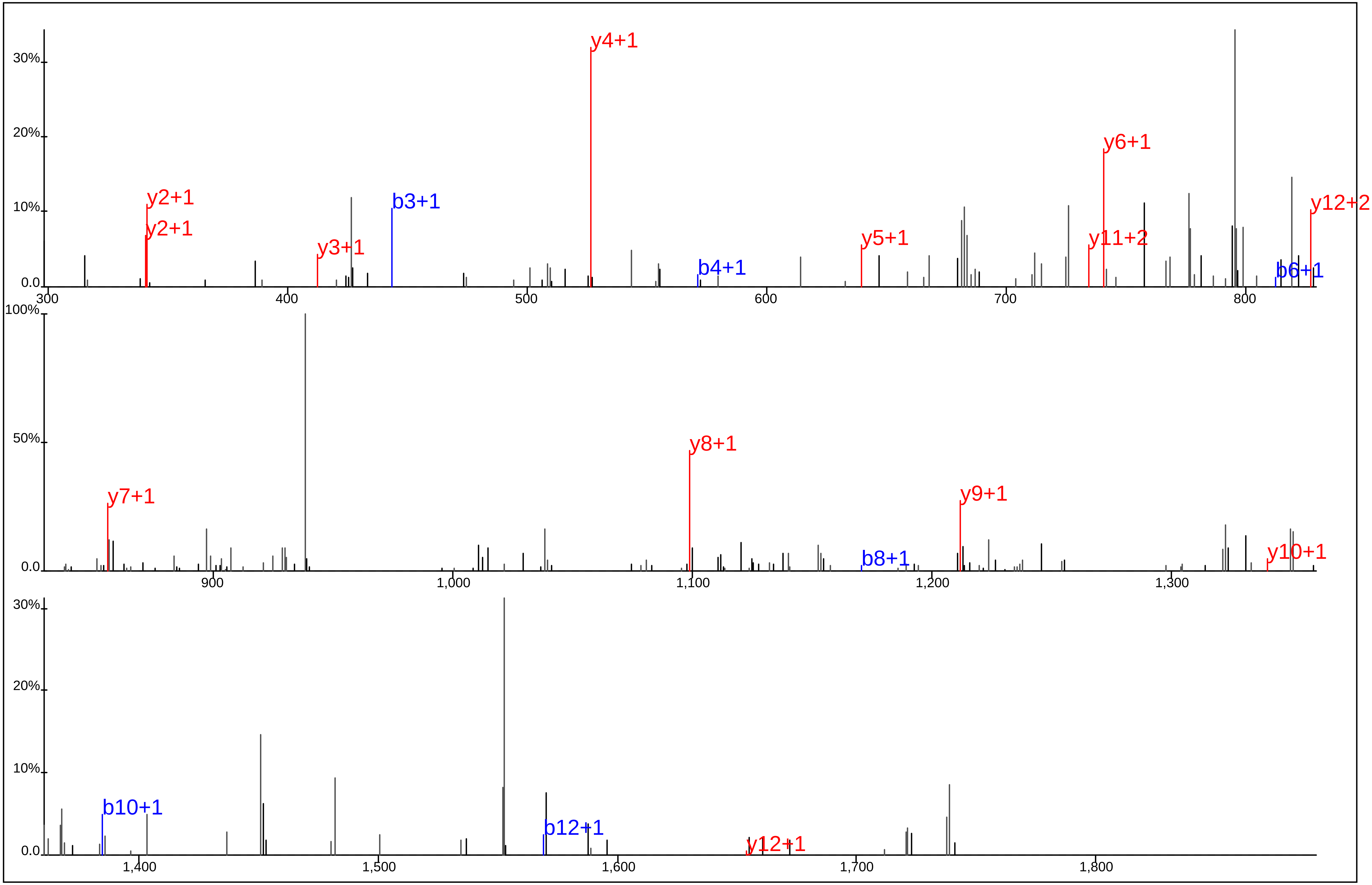

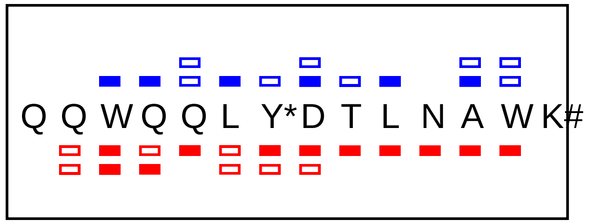

Protein Name:	GA17
gi Accession:	23397429
Precursor Mass:	955.4456+2
Delta Mass:	6.005 ppm
XCorr:	27.71

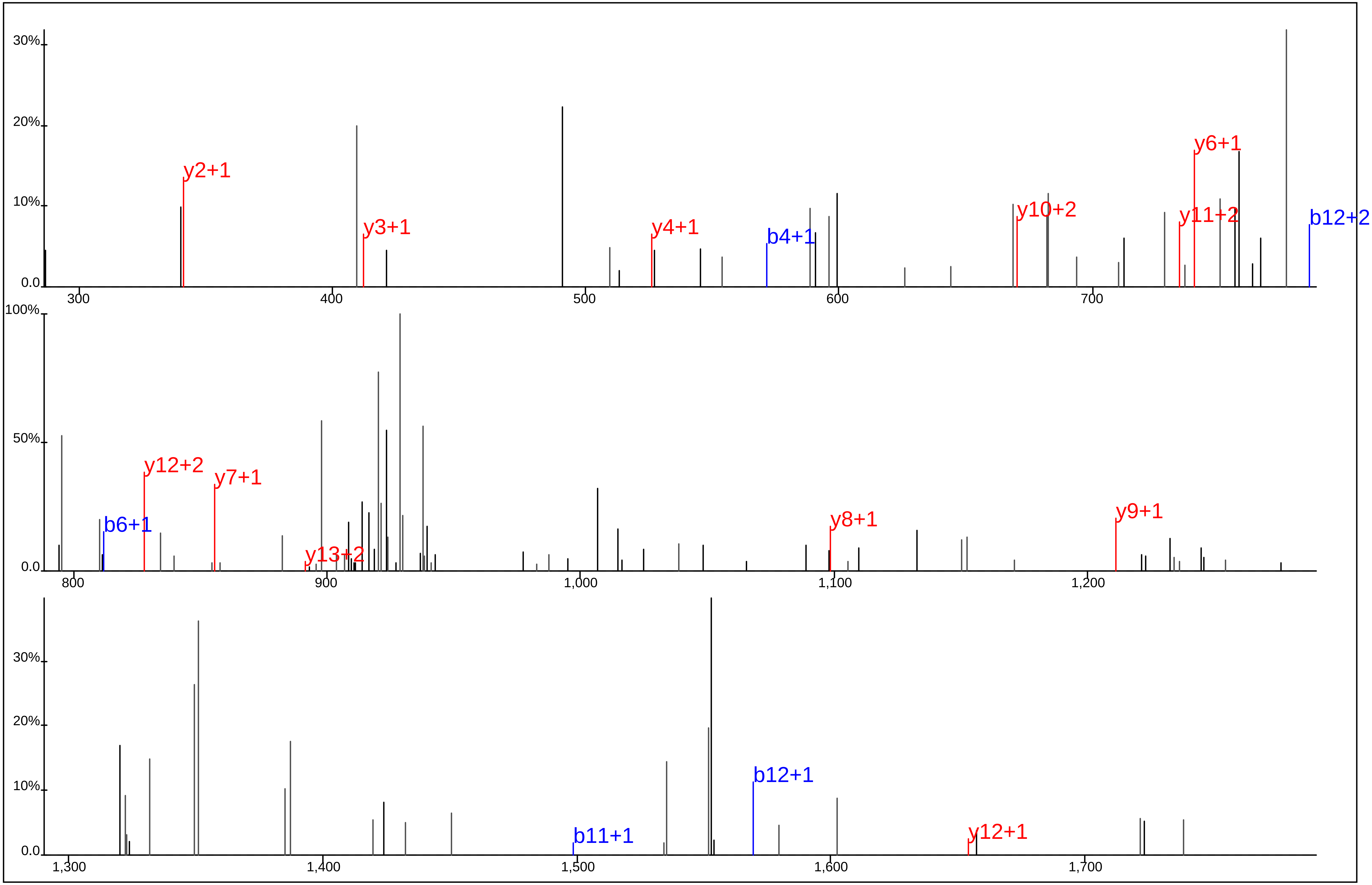

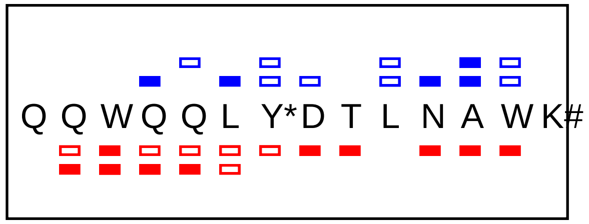

Protein Name: GA17
gi Accession: 23397429
Precursor Mass: 955.4420+2
Delta Mass: 2.298 ppm
XCorr: 18.78

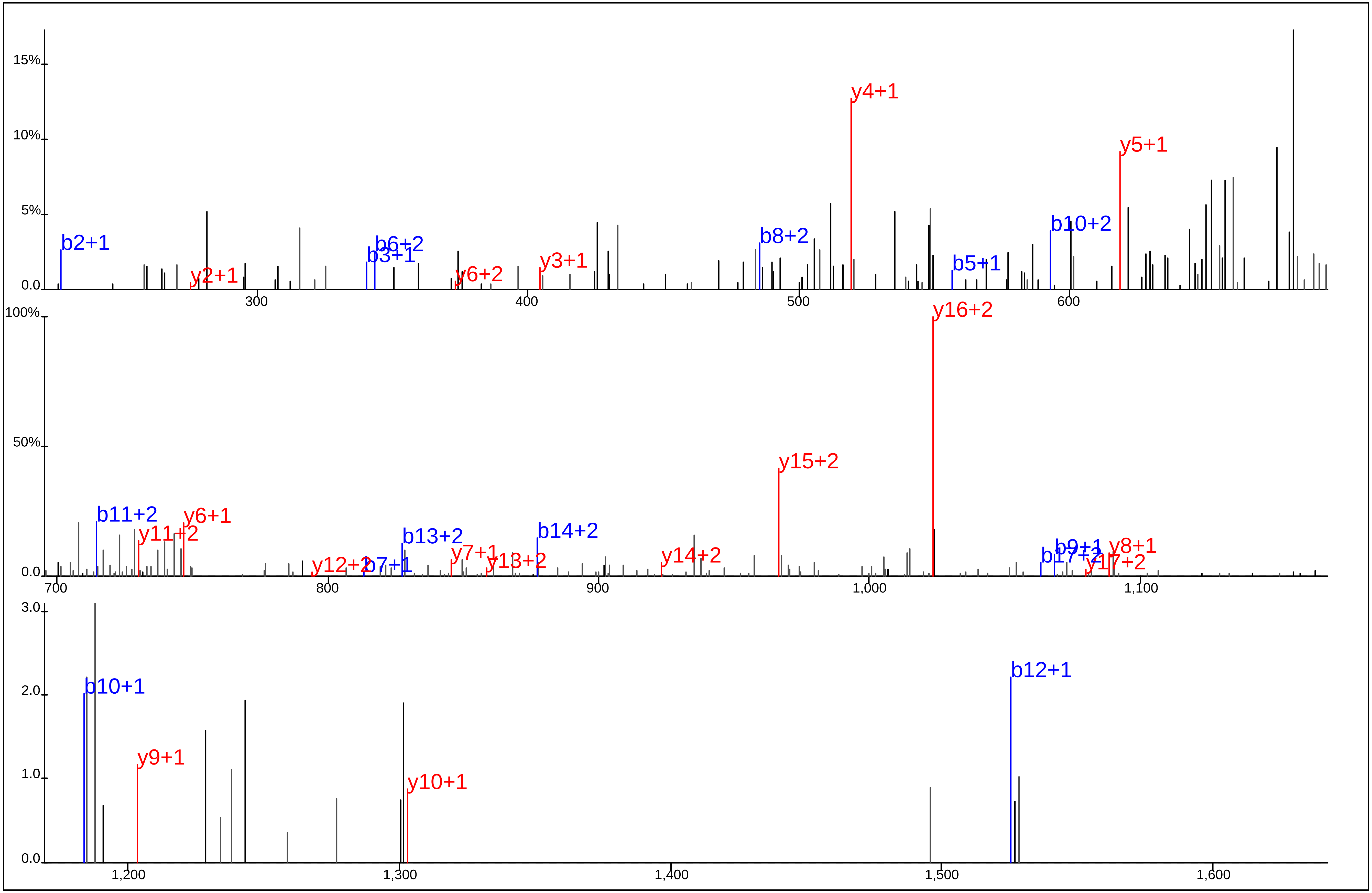

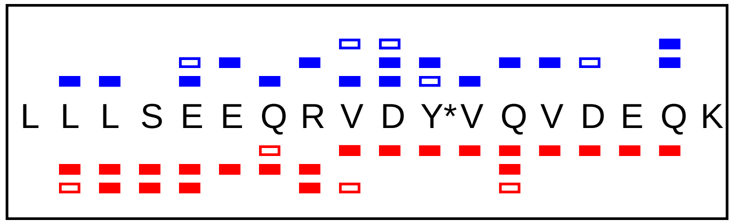

Protein Name: GAB3
gi Accession: 18079323
Precursor Mass: 757.7050+3
Delta Mass: 4.446 ppm
XCorr: 25.68

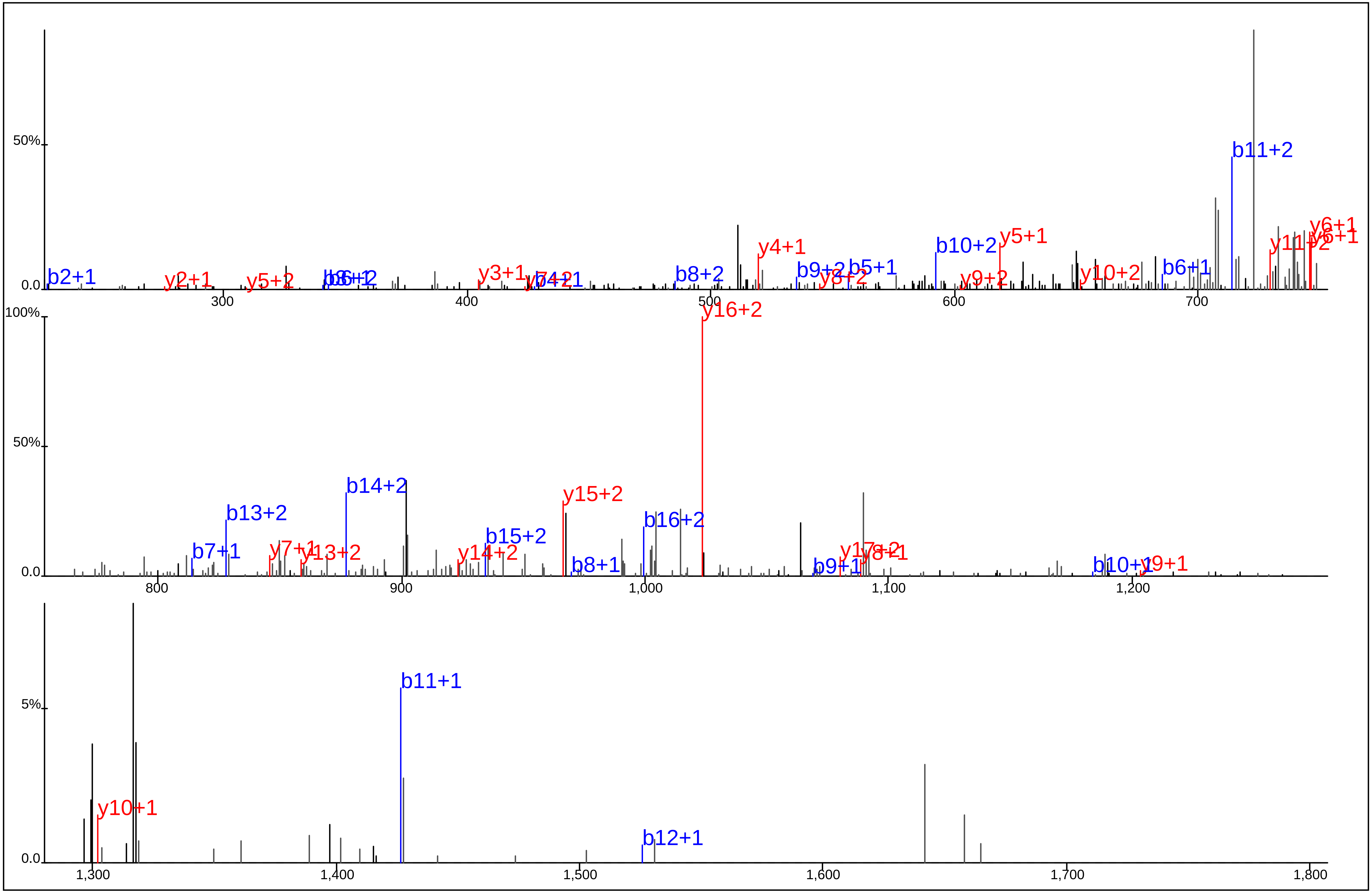

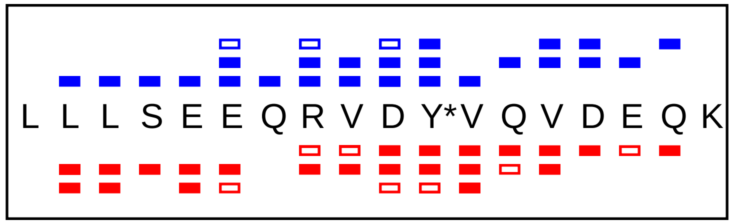

Protein Name:	GAB3
gi Accession:	18079323
Precursor Mass:	757.7081+3
Delta Mass:	8.475 ppm
XCorr:	11.43

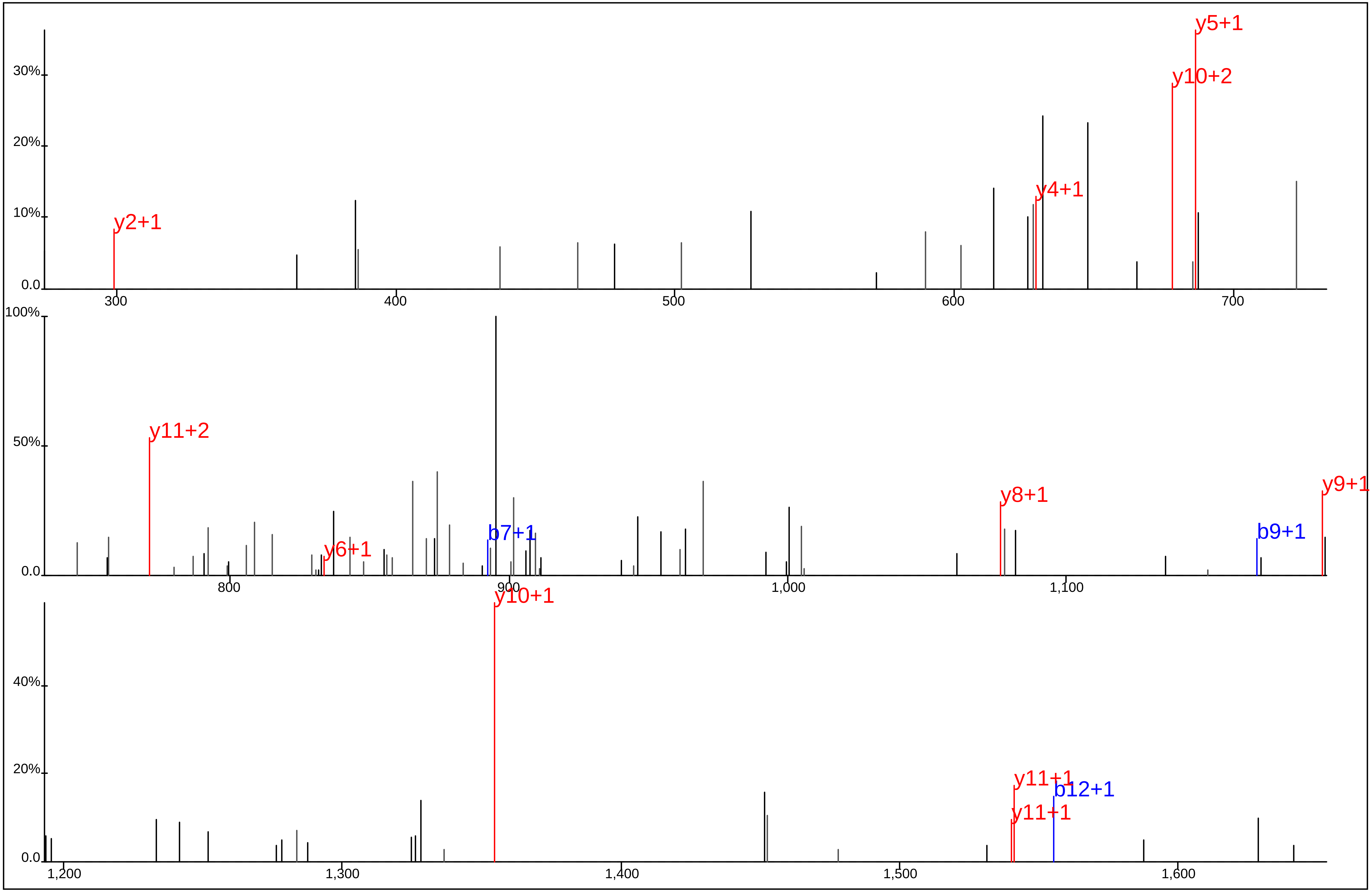

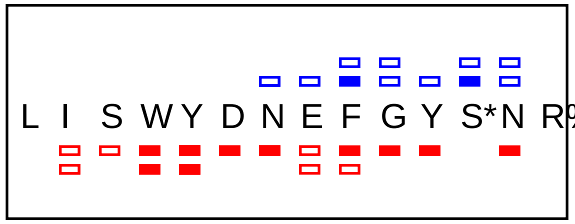

Protein Name: GAPDH
gi Accession: 7669492
Precursor Mass: 927.3979+2
Delta Mass: 6.300 ppm
XCorr: 16.97

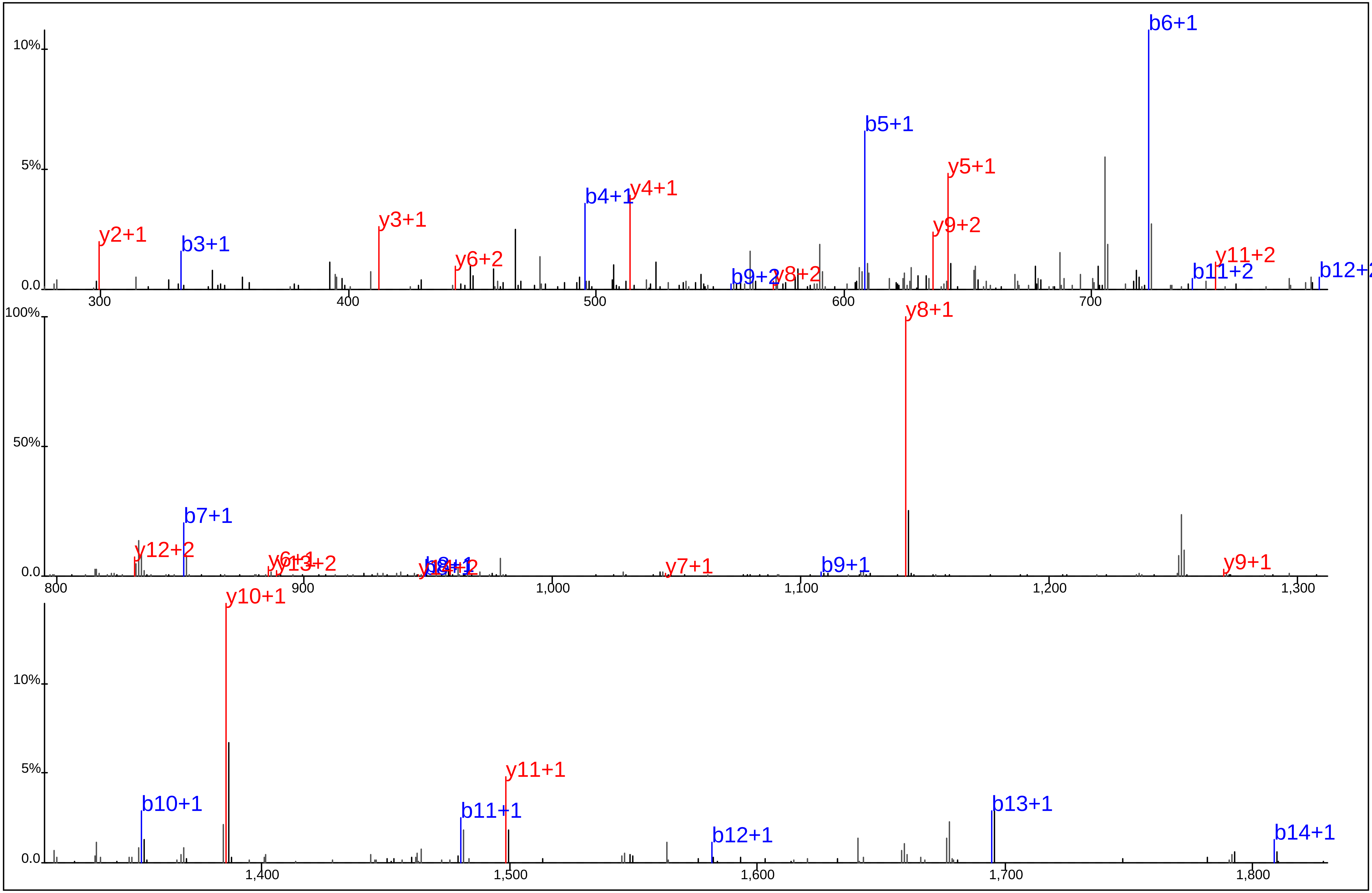

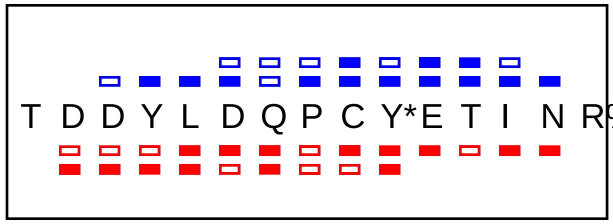

Protein Name:	GDP dissociation inhibitor 2
gi Accession:	6598323
Precursor Mass:	996.9056+2
Delta Mass:	5.950 ppm
XCorr:	70.46

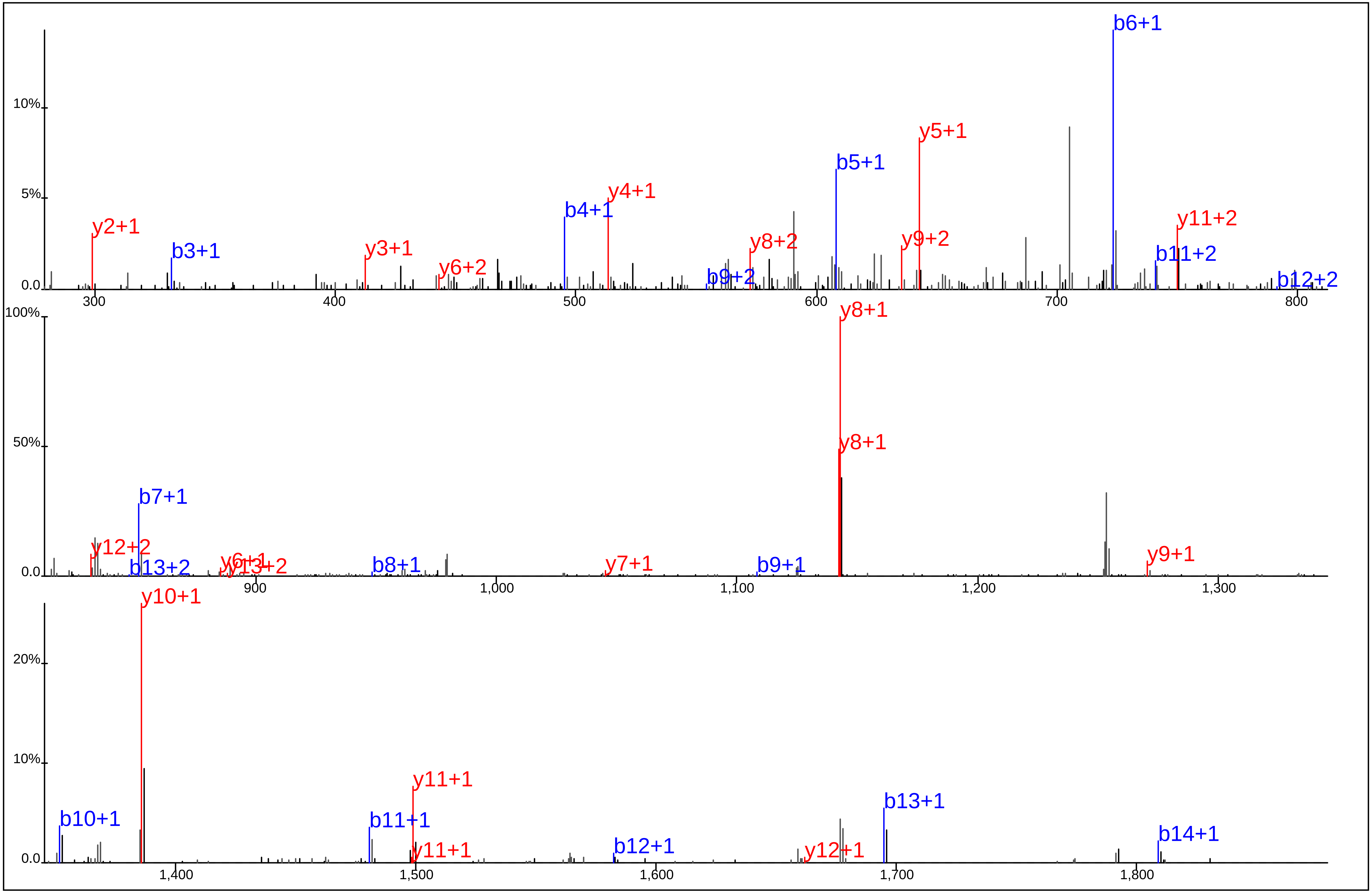

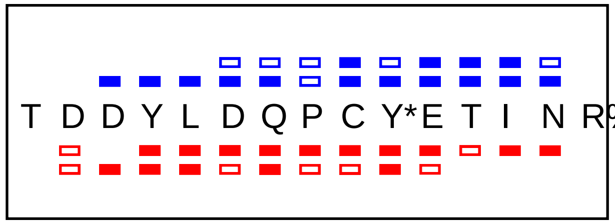

Protein Name: GDP dissociation inhibitor 2
gi Accession: 6598323
Precursor Mass: 996.9064+2
Delta Mass: 6.809 ppm
XCorr: 77.89

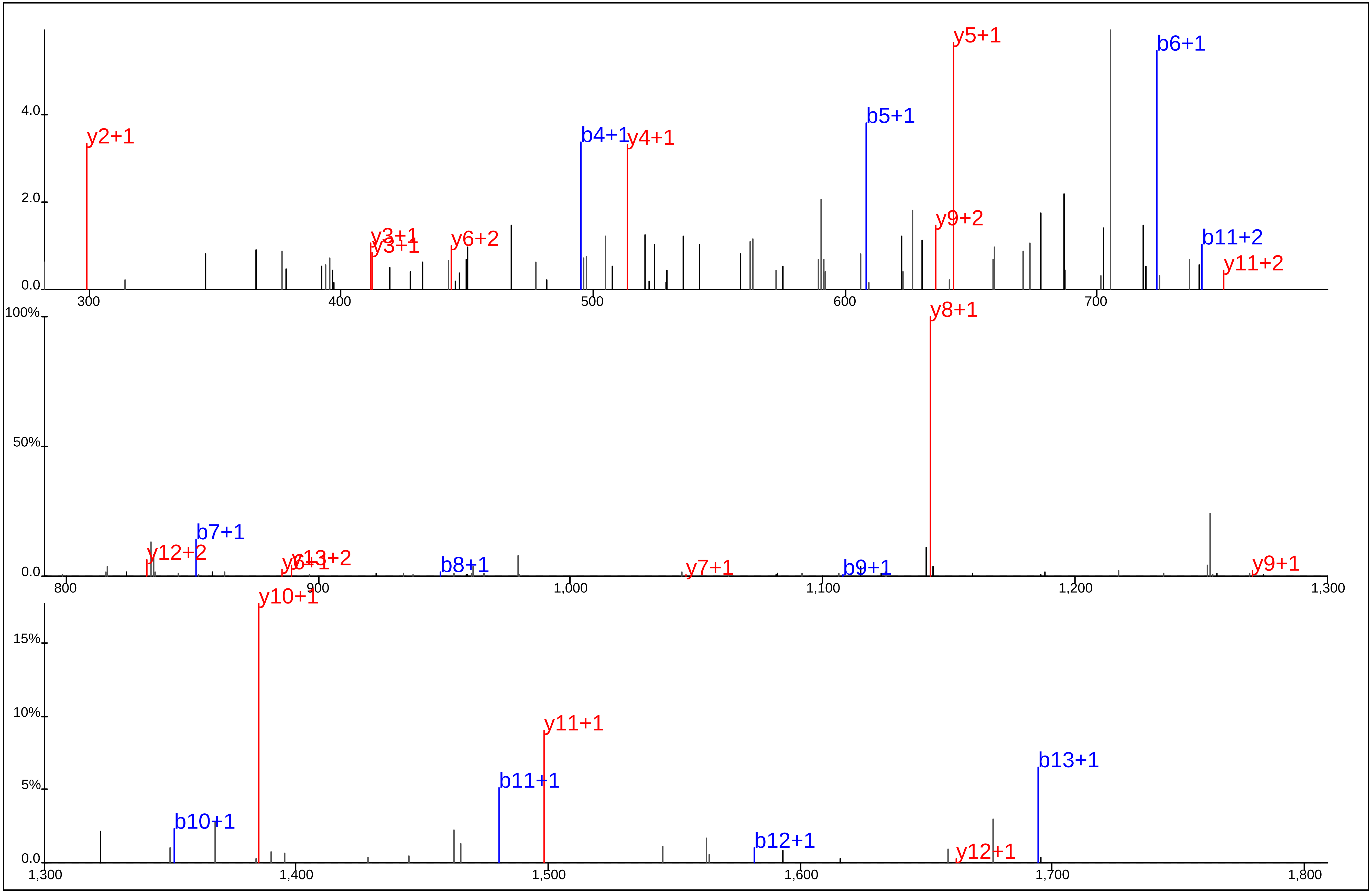

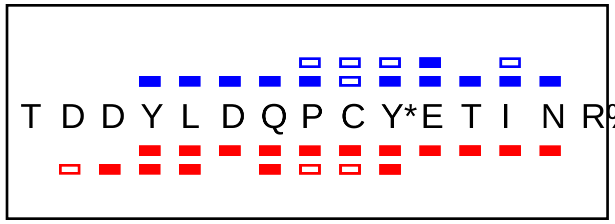

Protein Name: GDP dissociation inhibitor 2
gi Accession: 6598323
Precursor Mass: 996.9006+2
Delta Mass: 0.988 ppm
XCorr: 67.31

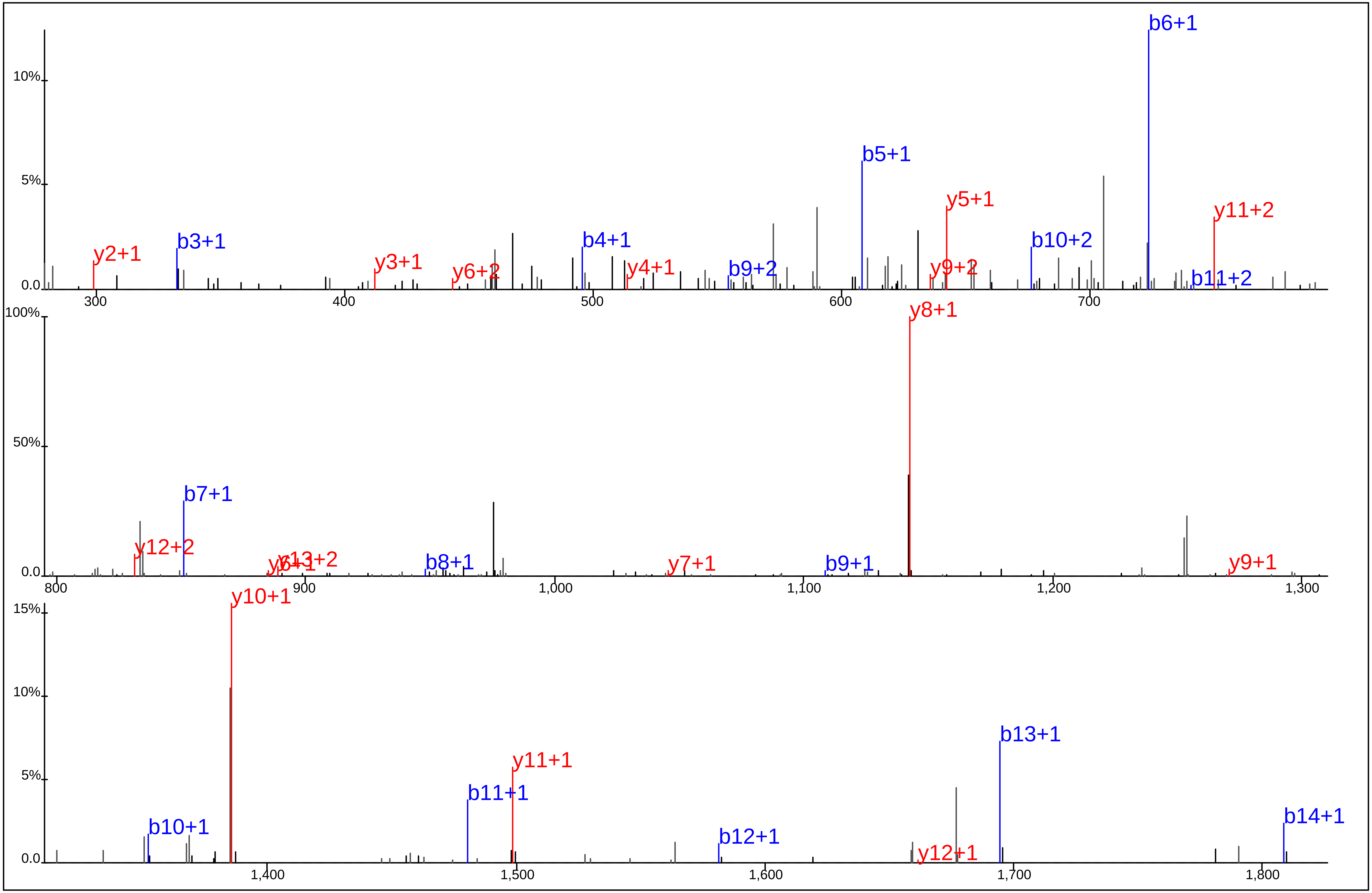

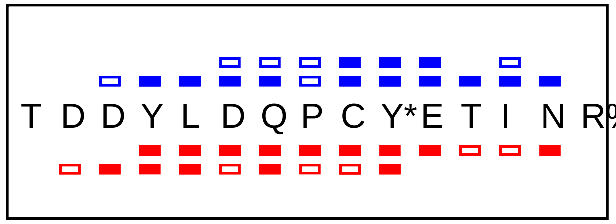

Protein Name: GDP dissociation inhibitor 2
gi Accession: 6598323
Precursor Mass: 996.9018+2
Delta Mass: 2.152 ppm
XCorr: 60.83

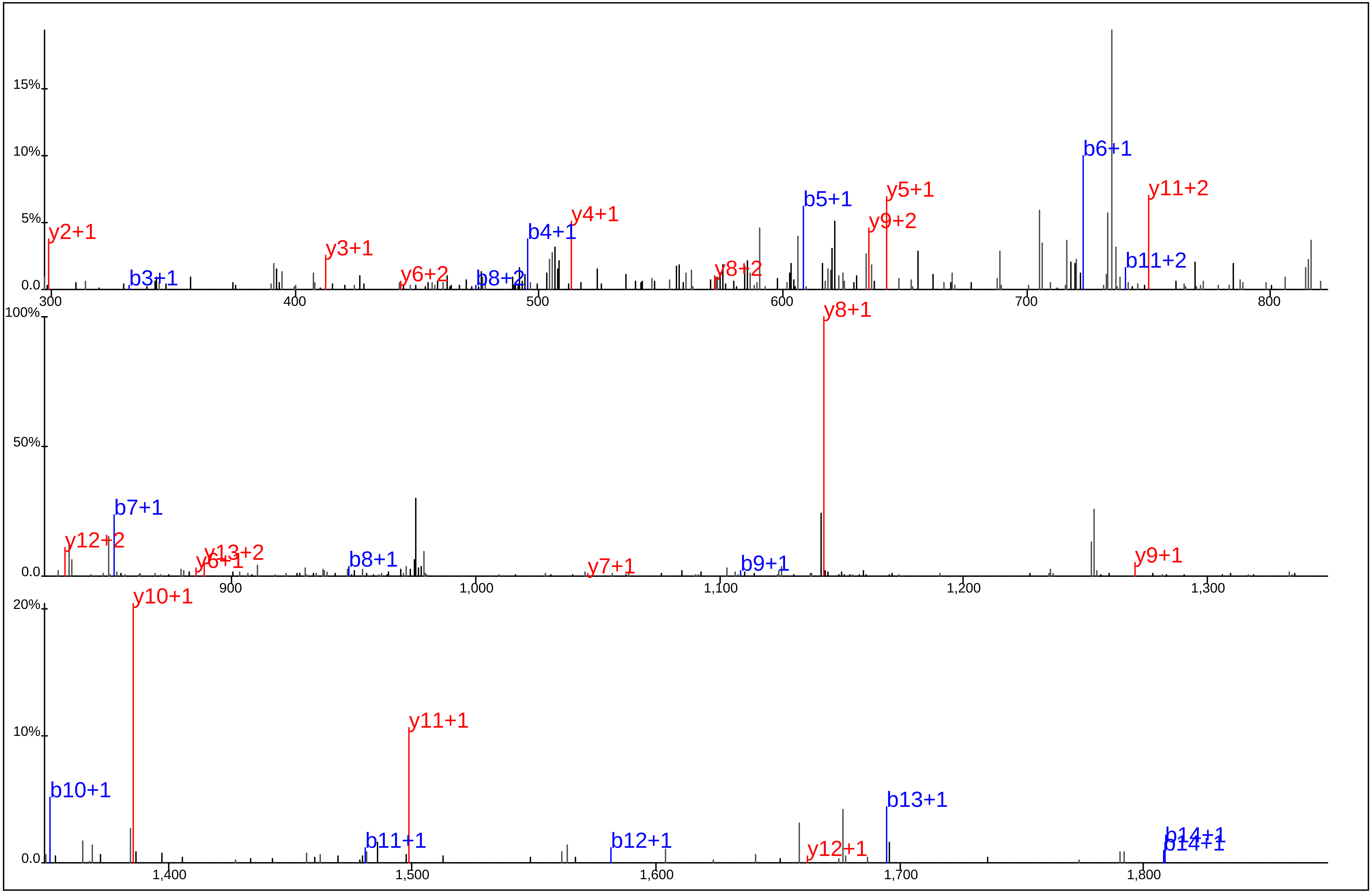

T D D Y L D Q P C Y*E T I N R%

Protein Name:	GDP dissociation inhibitor 2
gi Accession:	6598323
Precursor Mass:	996.9050+2
Delta Mass:	5.398 ppm
XCorr:	56.41

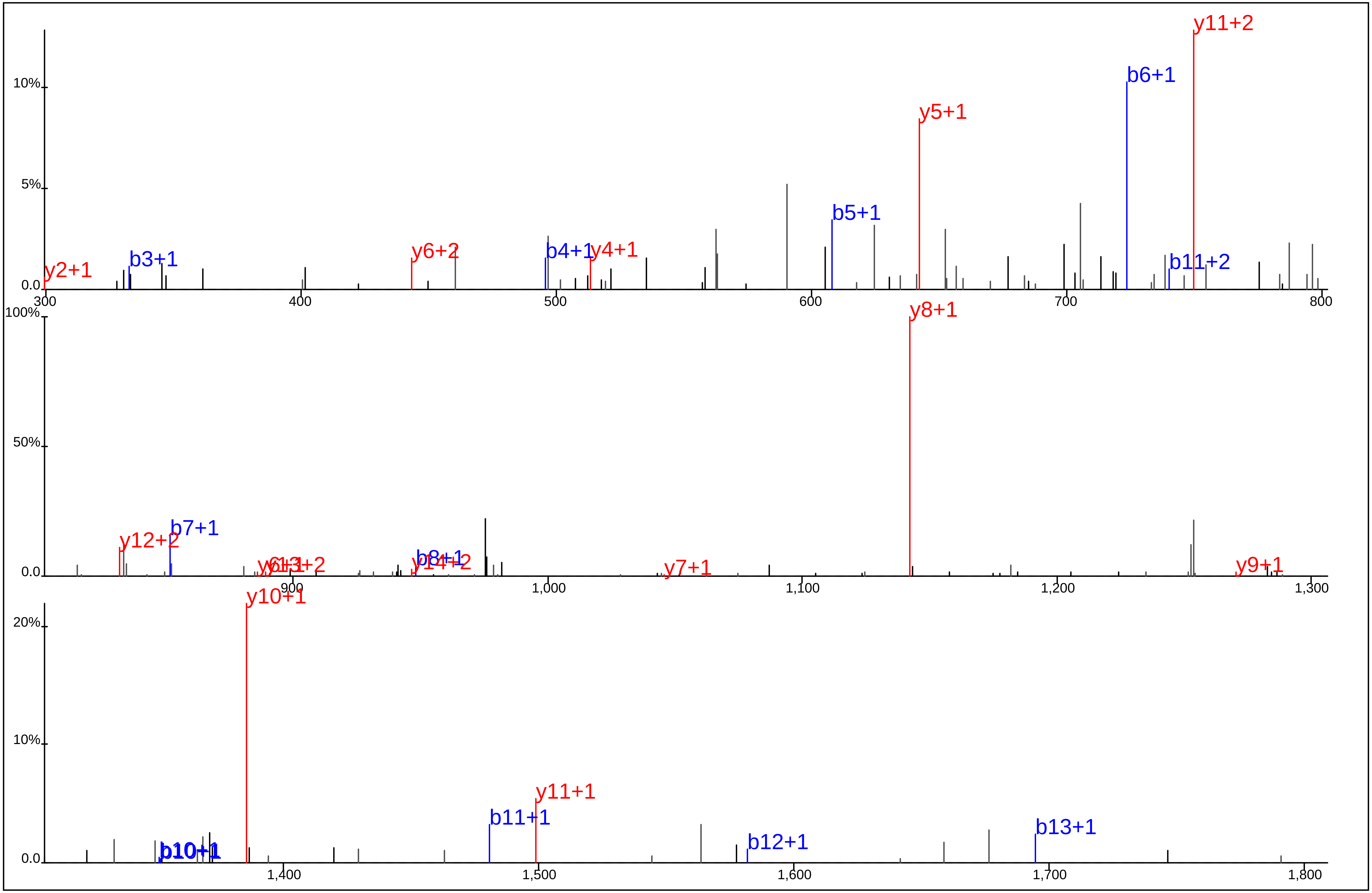

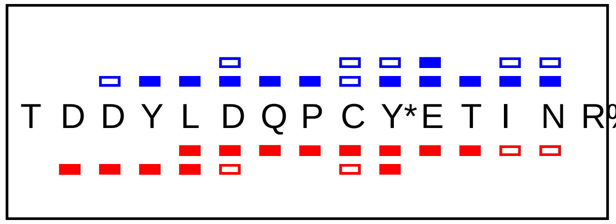

Protein Name: GDP dissociation inhibitor 2
gi Accession: 6598323
Precursor Mass: 996.9023+2
Delta Mass: 2.704 ppm
XCorr: 54.55

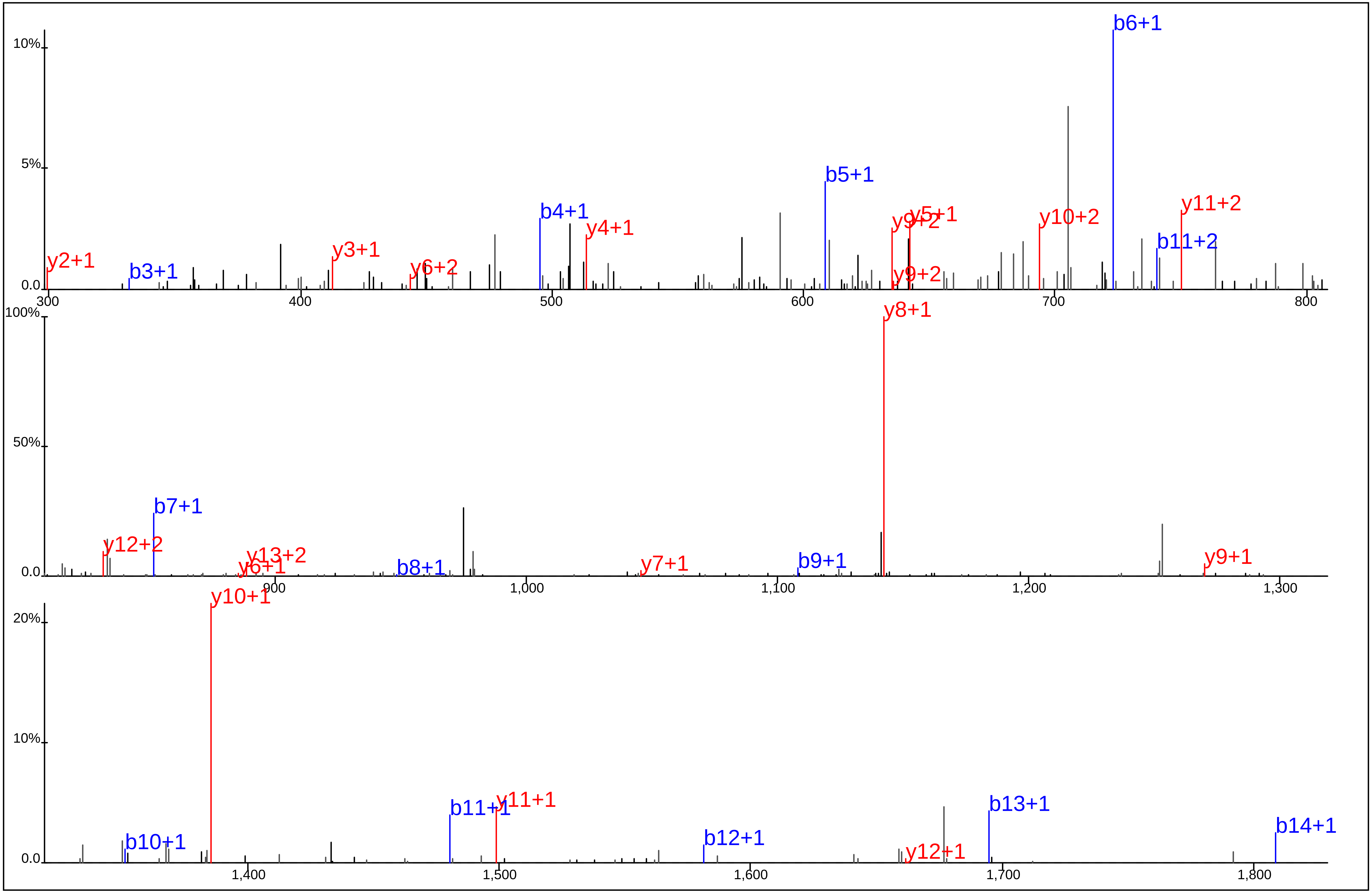

T D D Y L D Q P C Y*E T I N R%

Protein Name:	GDP dissociation inhibitor 2
gi Accession:	6598323
Precursor Mass:	996.9010+2
Delta Mass:	1.359 ppm
XCorr:	65.78

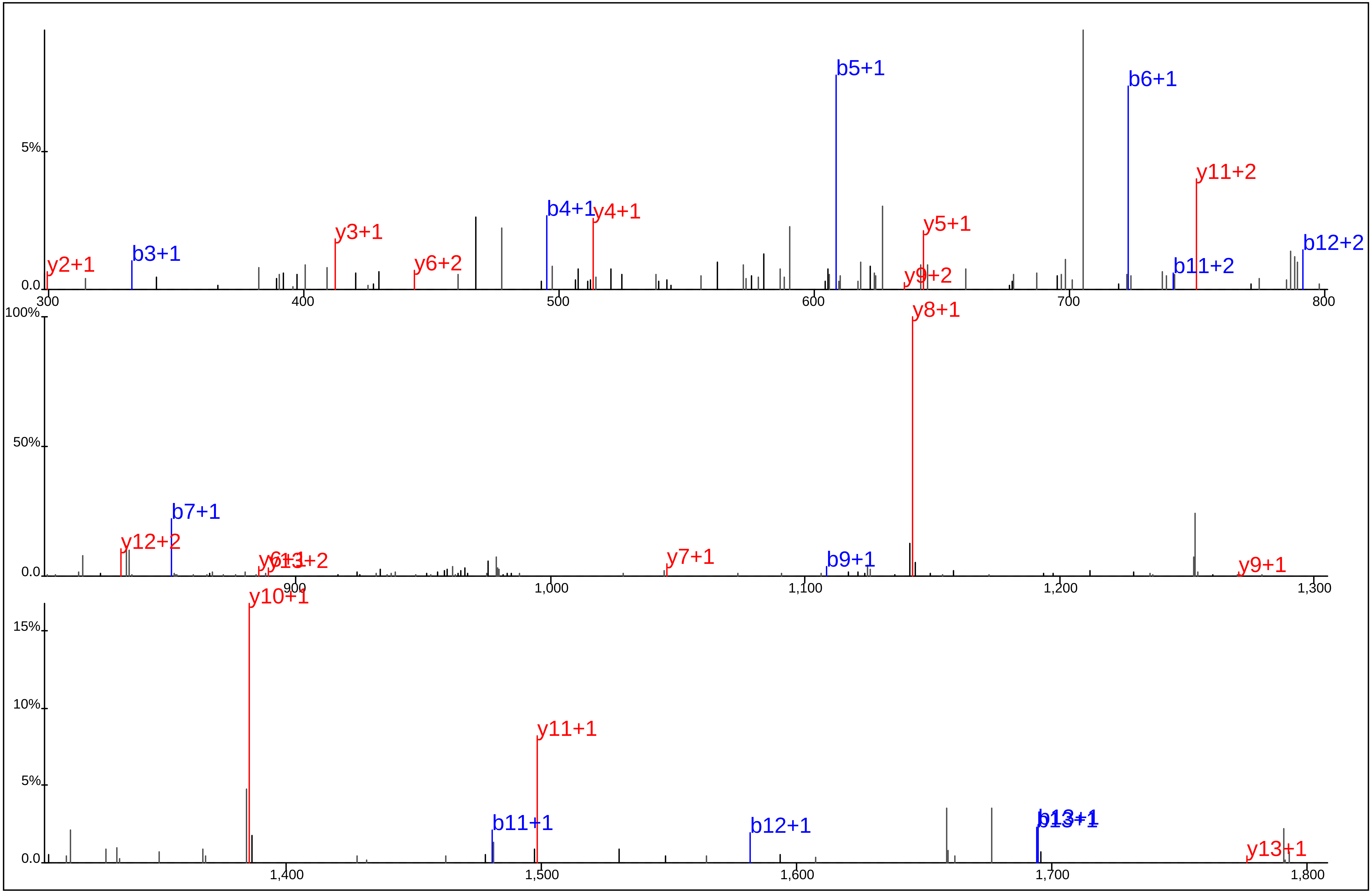

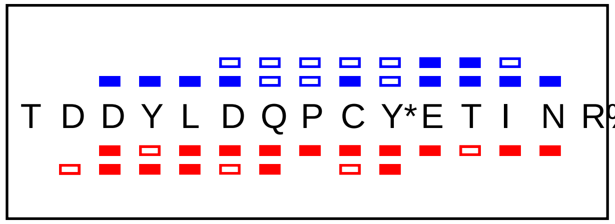

Protein Name: GDP dissociation inhibitor 2
gi Accession: 6598323
Precursor Mass: 996.9021+2
Delta Mass: 2.458 ppm
XCorr: 62.43

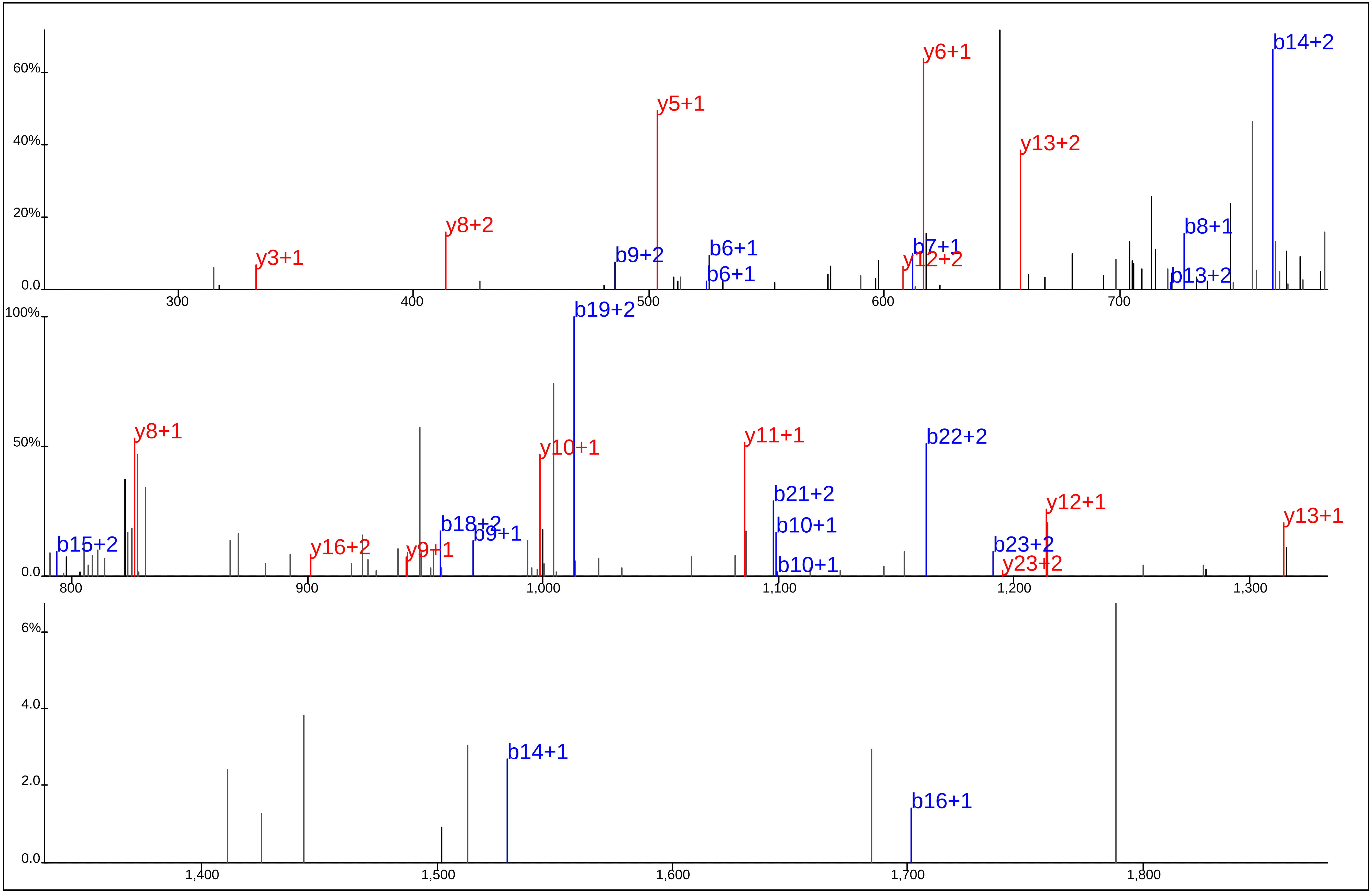

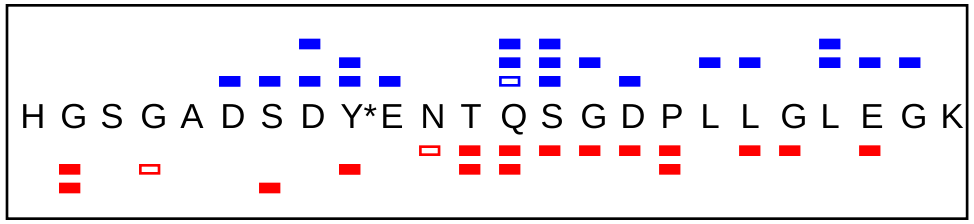

Protein Name: GIT1
gi Accession: 41393573
Precursor Mass: 843.0291+3
Delta Mass: 4.178 ppm
XCorr: 55.68

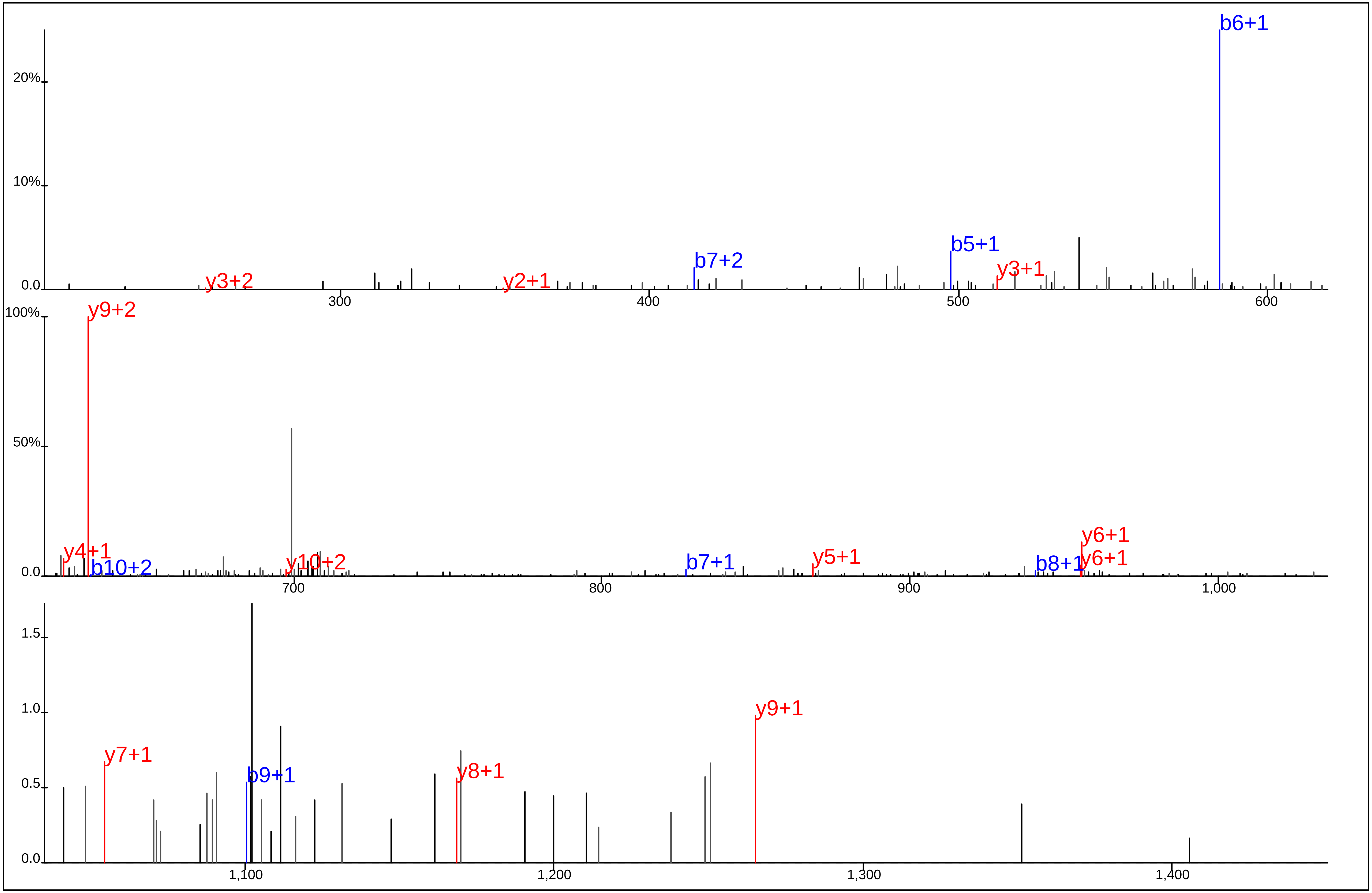

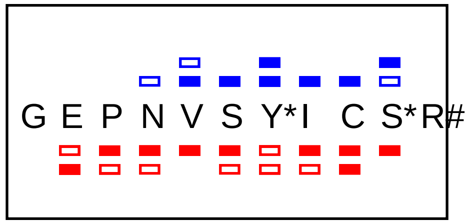

Protein Name:	GSK 3
gi Accession:	49574532
Precursor Mass:	726.2708+2
Delta Mass:	2.900 ppm
XCorr:	2.8991

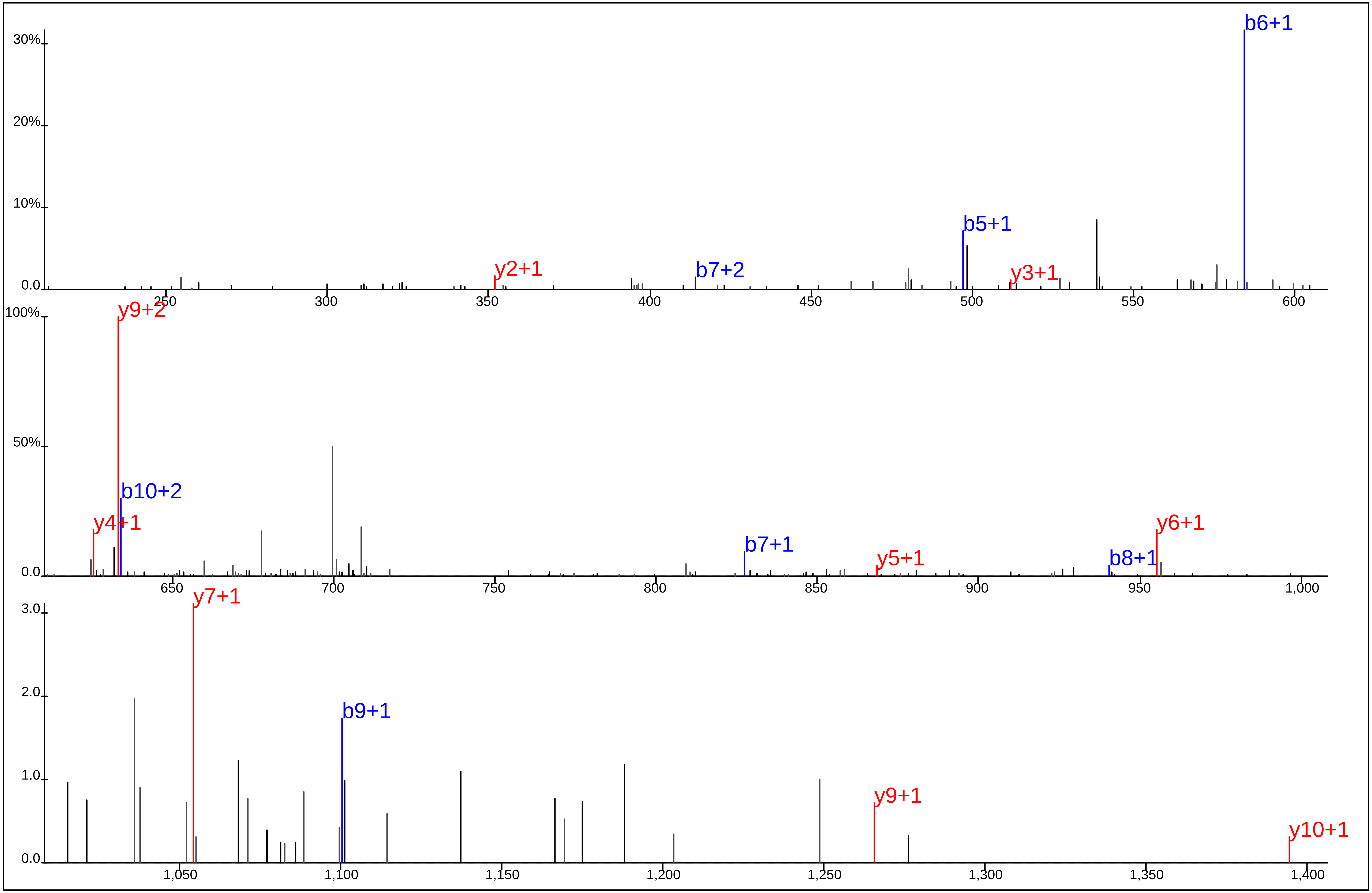

G E P N V S Y*I C S*R%

Protein Name: GSK 3
gi Accession: 49574532
Precursor Mass: 726.2732+2
Delta Mass: 6.192 ppm
XCorr: 34.61

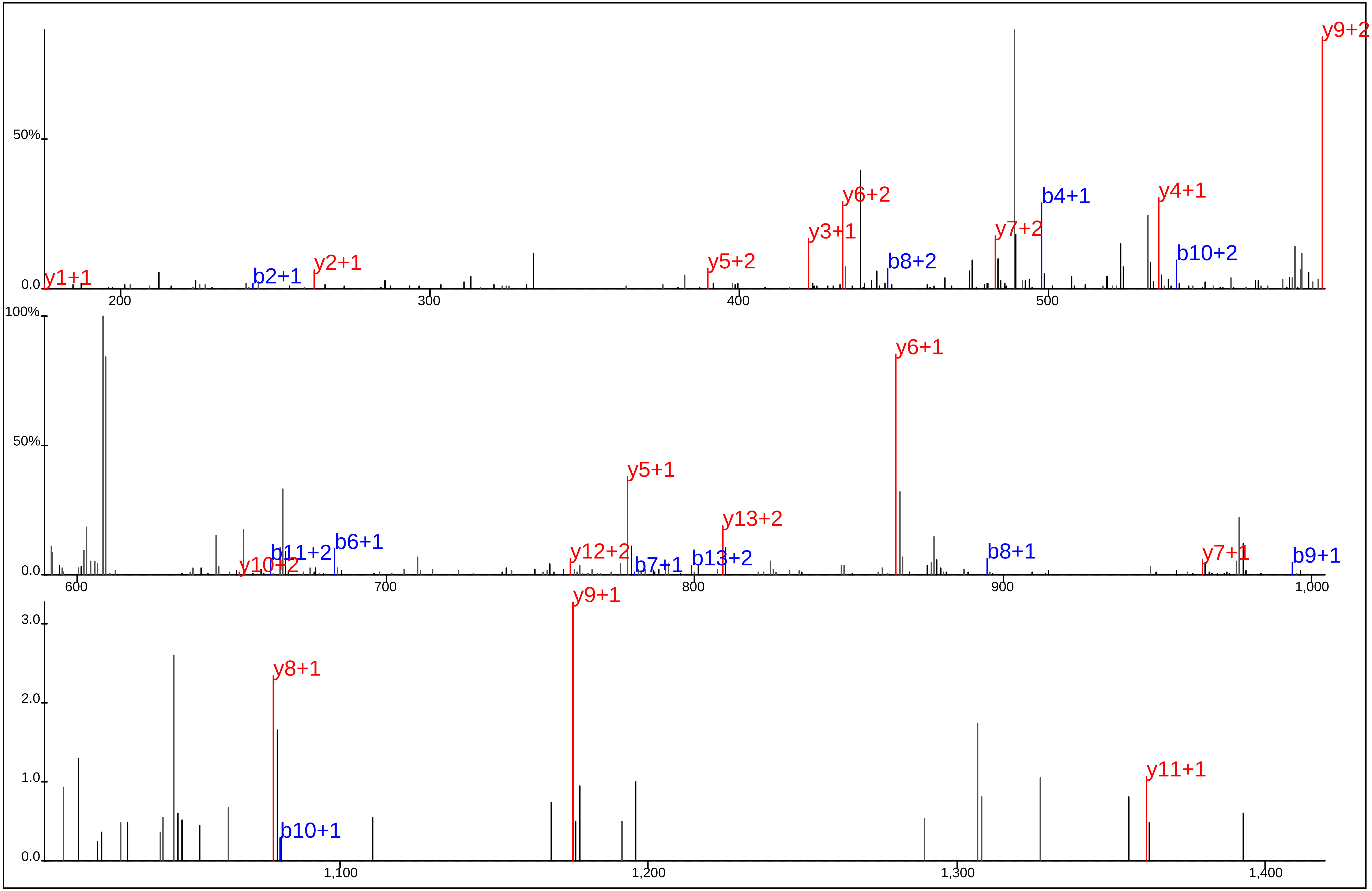

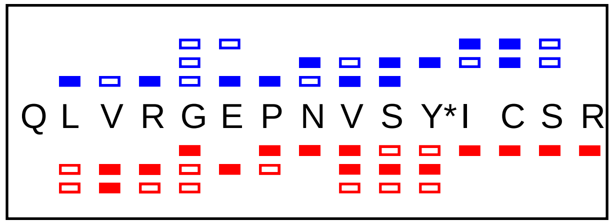

Protein Name:	GSK 3
gi Accession:	49574532
Precursor Mass:	619.9626+3
Delta Mass:	2.965 ppm
XCorr:	55.58

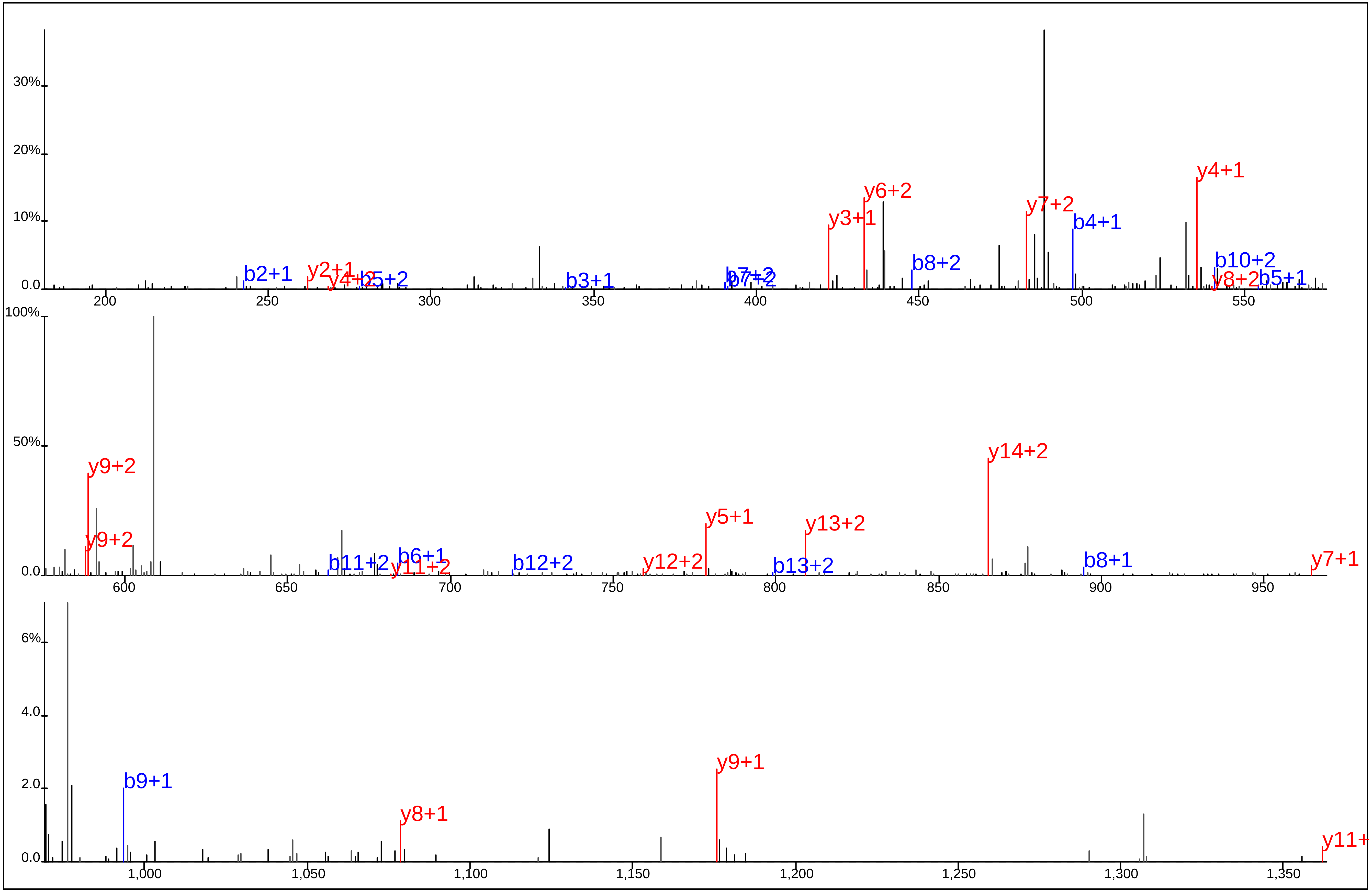

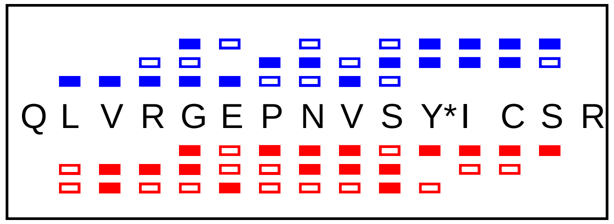

Protein Name: GSK 3
gi Accession: 49574532
Precursor Mass: 619.9625+3
Delta Mass: 2.868 ppm
XCorr: 64.45

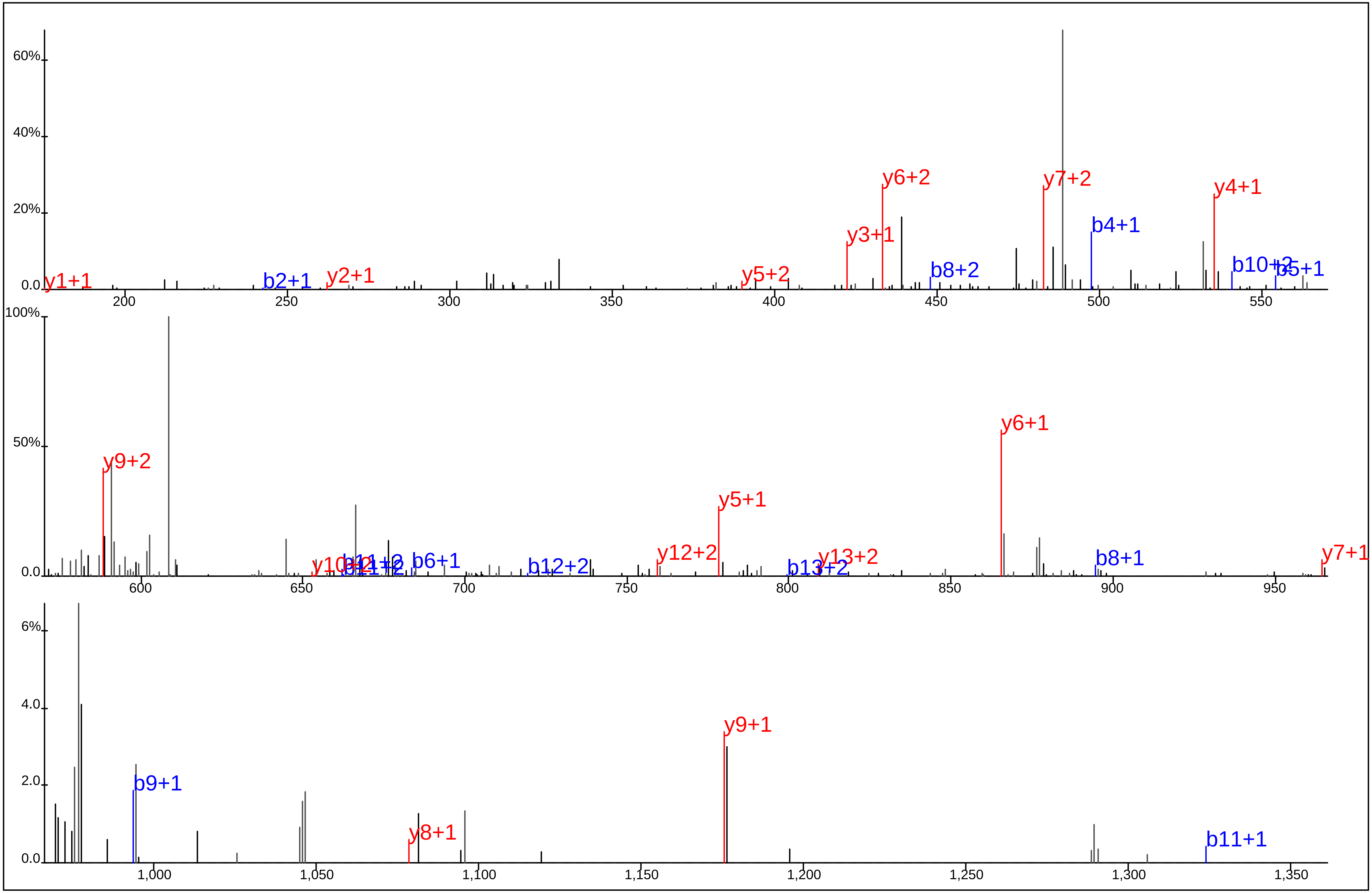

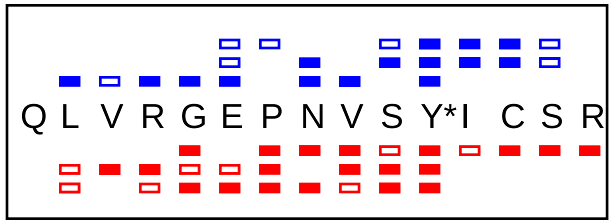

Protein Name: GSK 3
gi Accession: 49574532
Precursor Mass: 619.9621+3
Delta Mass: 2.178 ppm
XCorr: 42.22

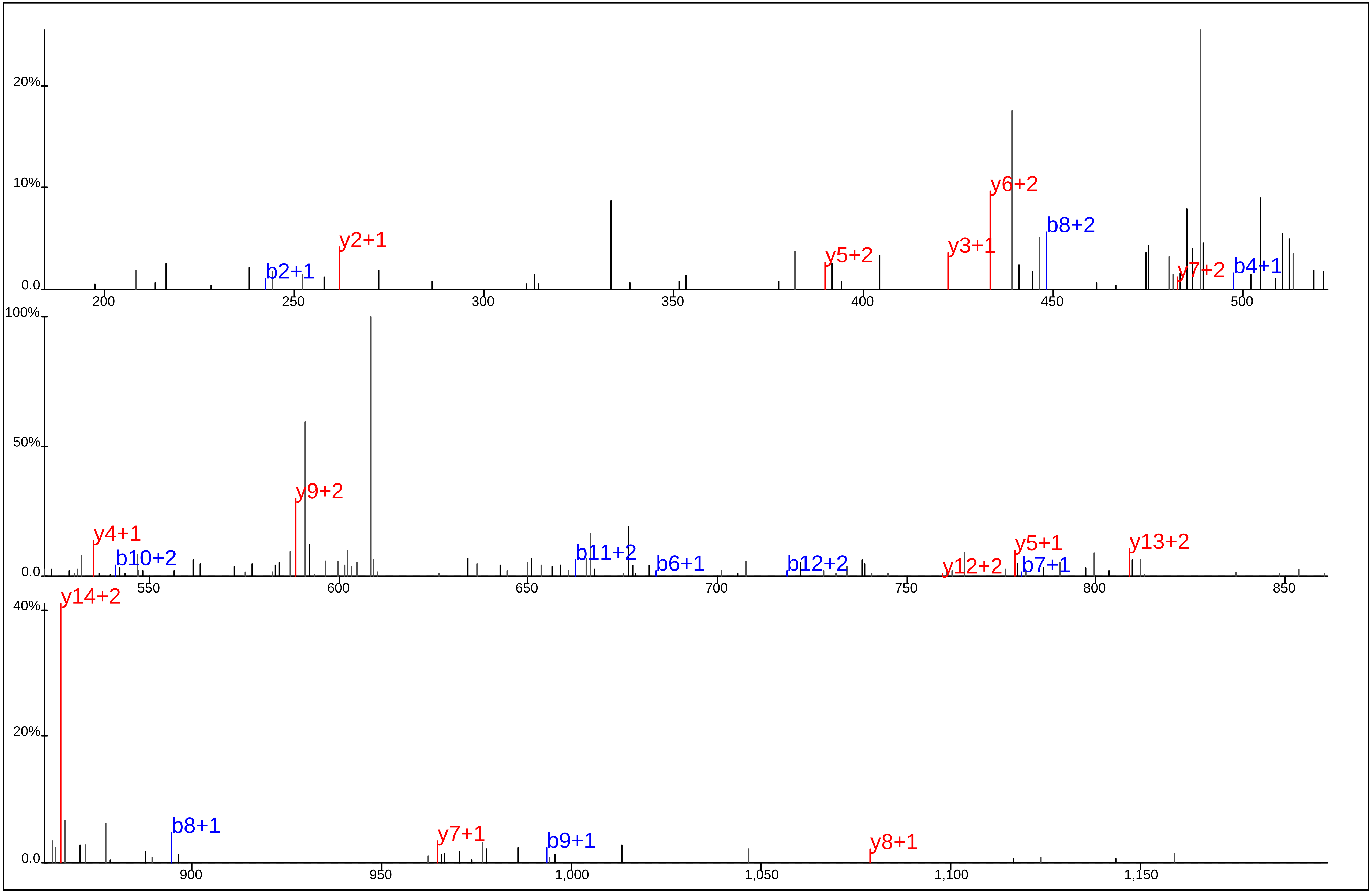

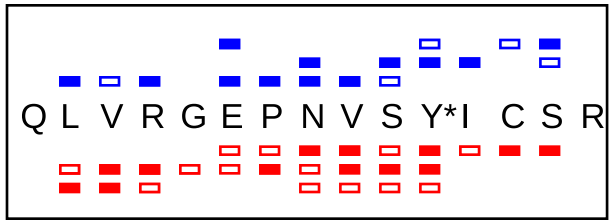

Protein Name: GSK 3
gi Accession: 49574532
Precursor Mass: 619.9625+3
Delta Mass: 2.771 ppm
XCorr: 23.54

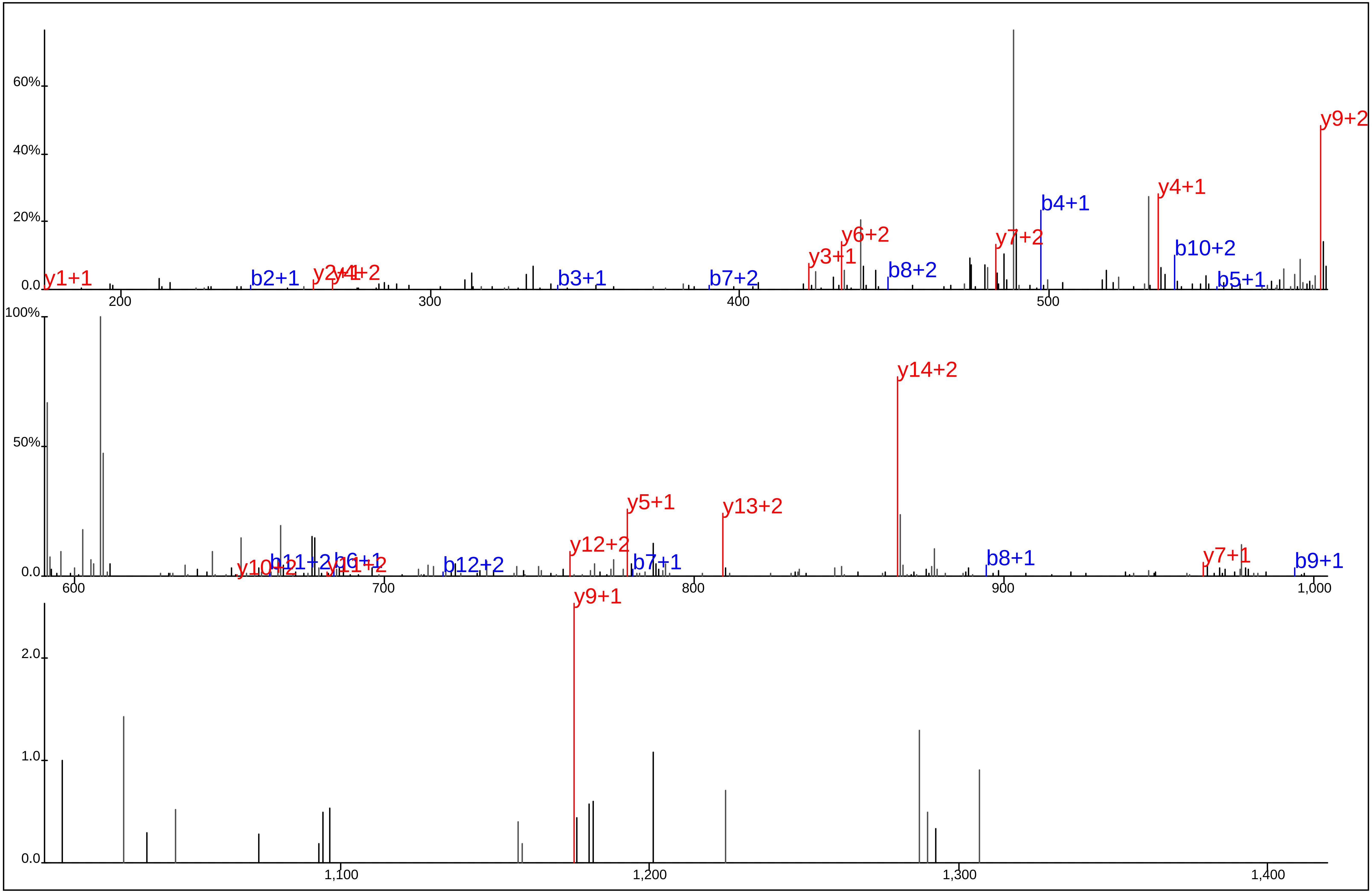

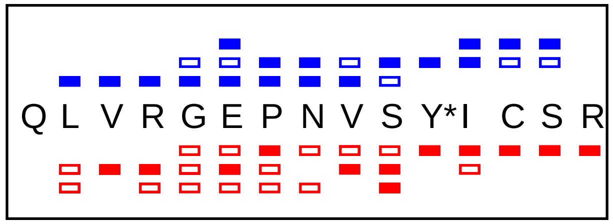

Protein Name: GSK 3
gi Accession: 49574532
Precursor Mass: 619.9621+3
Delta Mass: 2.178 ppm
XCorr: 36.15

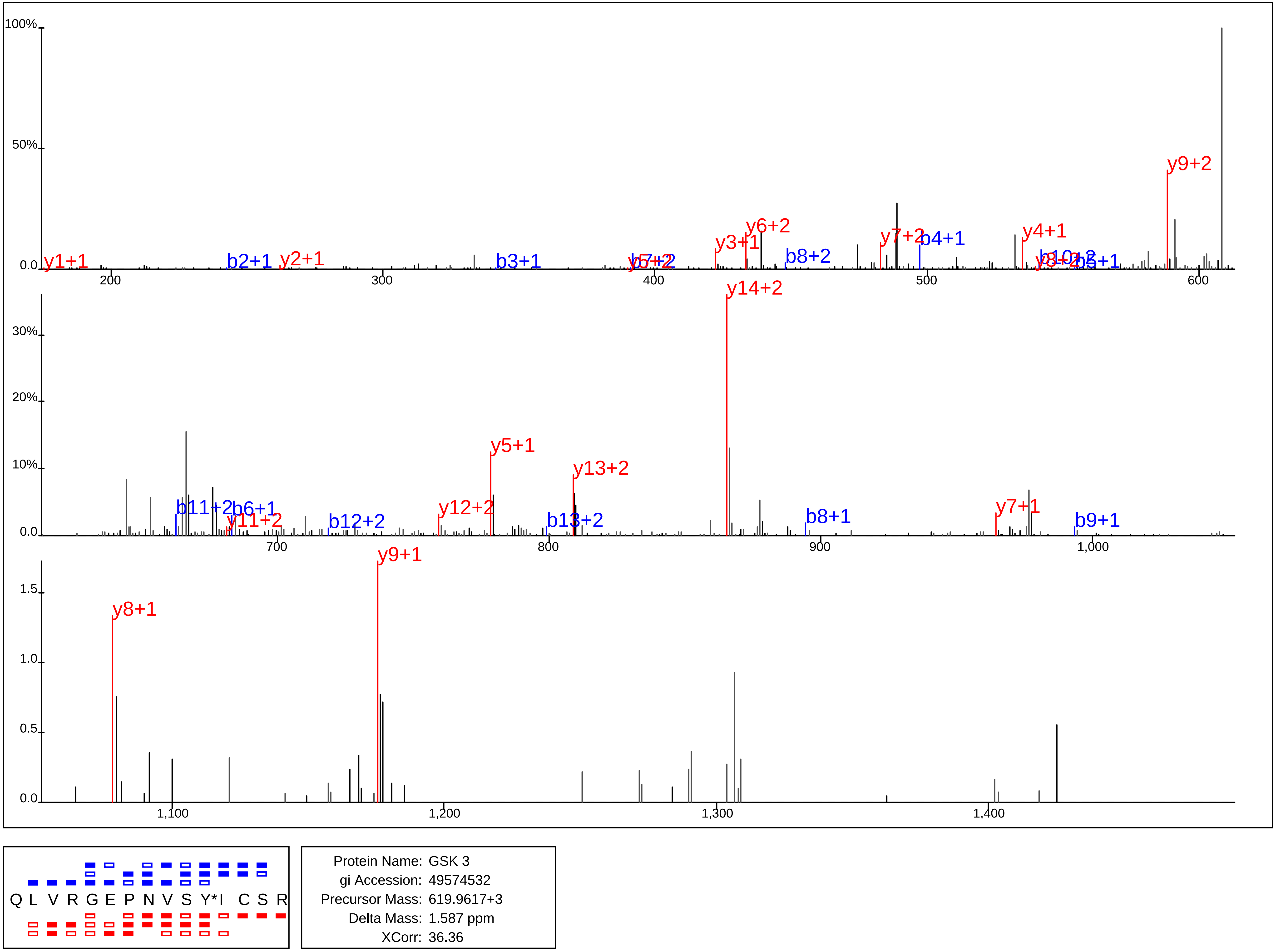

Protein Name: GSK 3
gi Accession: 49574532
Precursor Mass: 619.9617+3
Delta Mass: 1.587 ppm
XCorr: 36.36

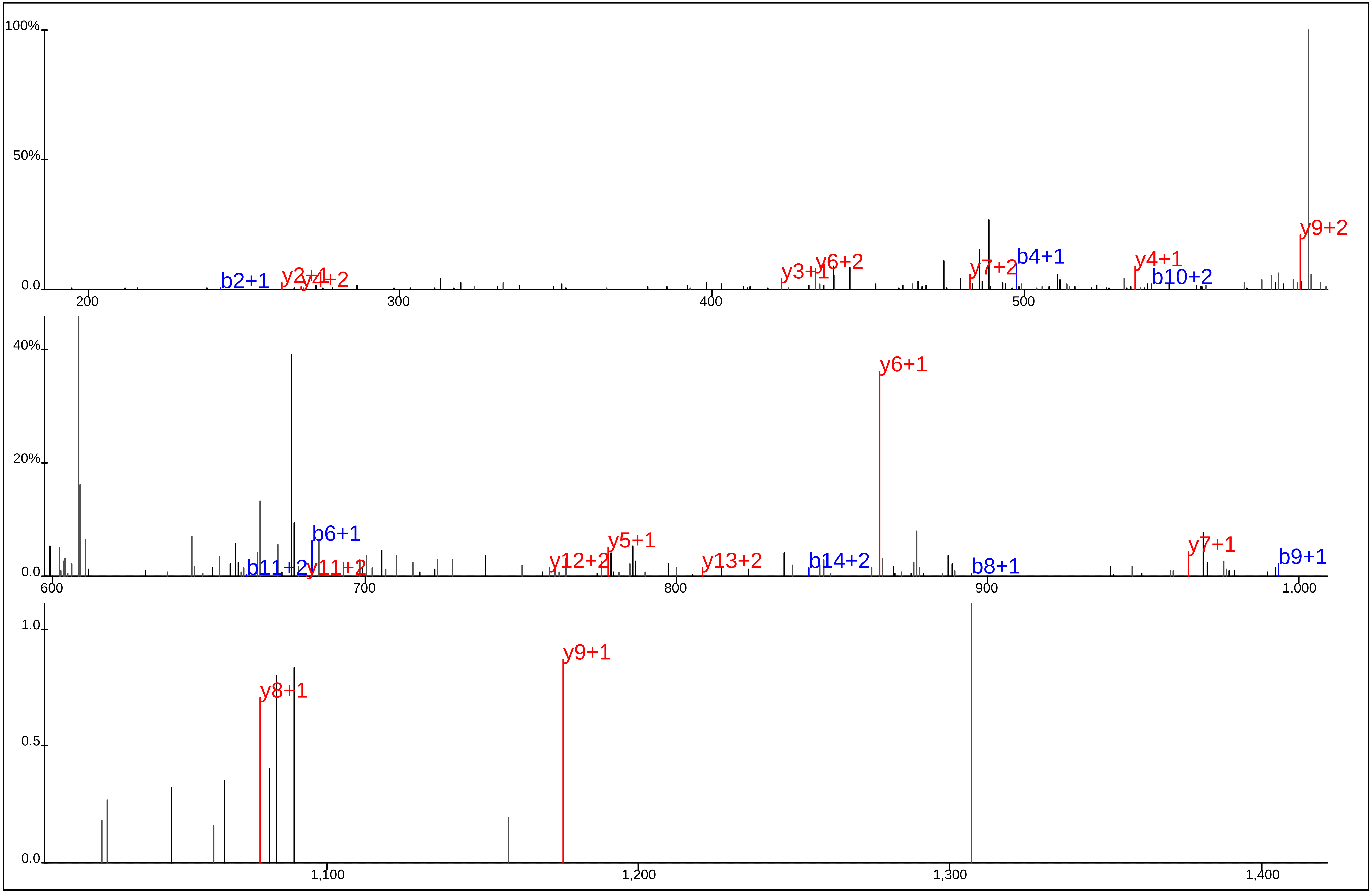

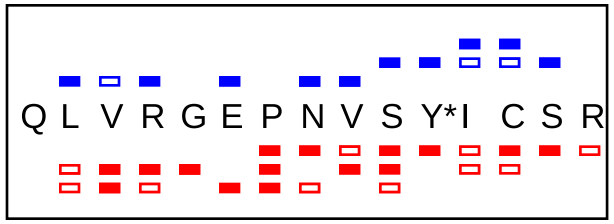

Protein Name:	GSK 3
gi Accession:	49574532
Precursor Mass:	619.9646+3
Delta Mass:	6.222 ppm
XCorr:	26.86

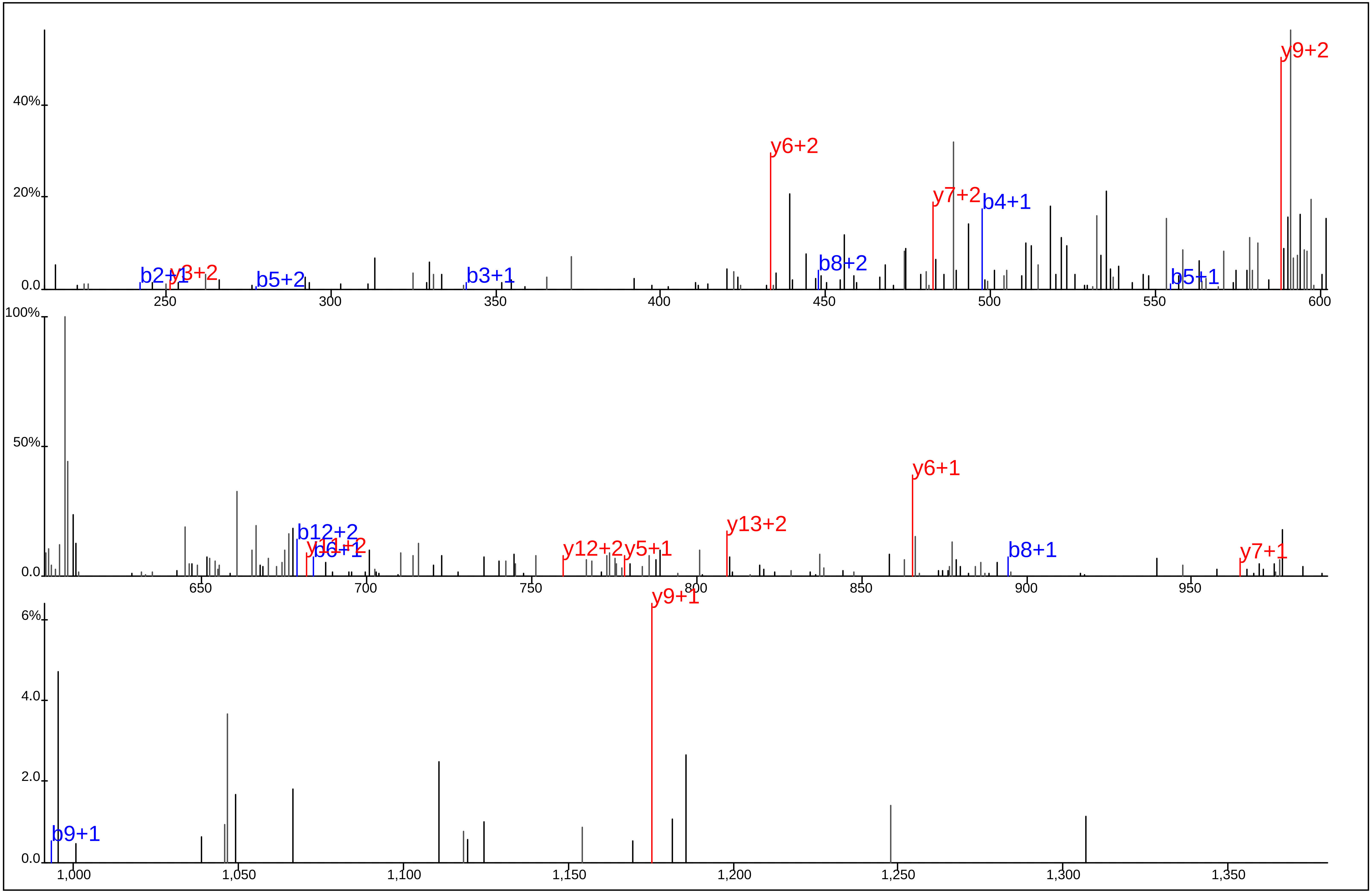

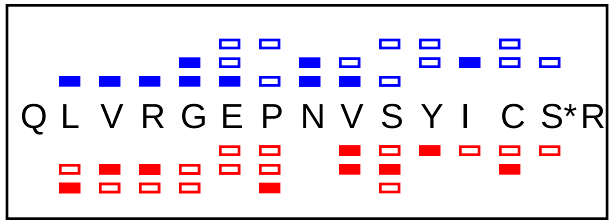

Protein Name: GSK 3
gi Accession: 49574532
Precursor Mass: 619.9624+3
Delta Mass: 2.669 ppm
XCorr: 11.56

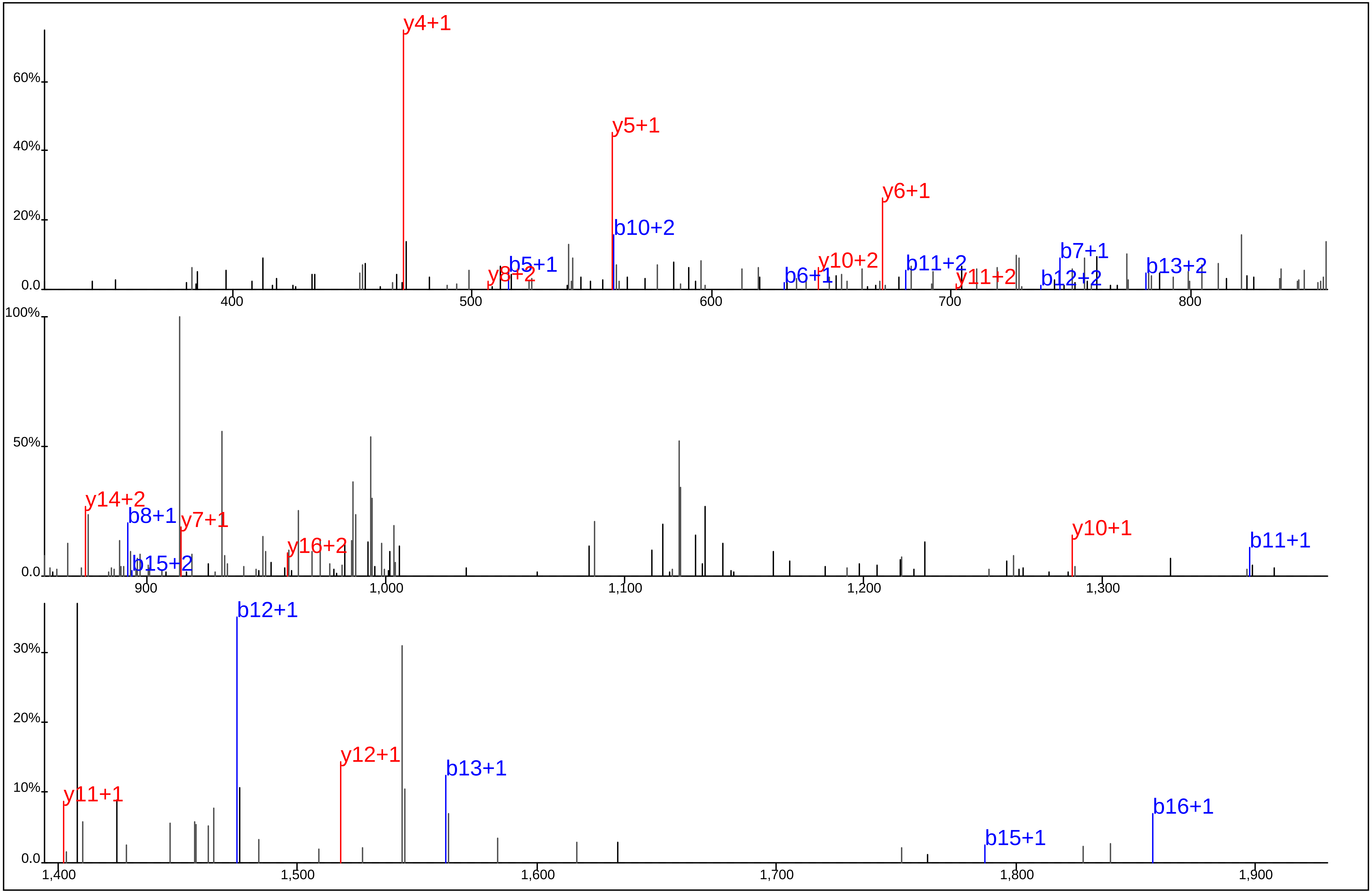

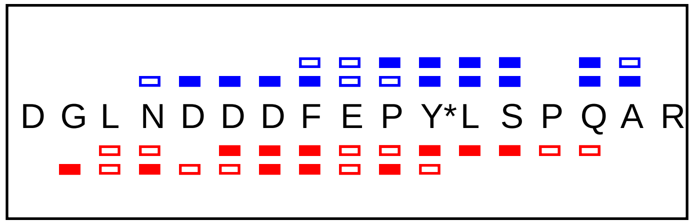

Protein Name: HGRG8
gi Accession: 116812575
Precursor Mass: 1,016.4171+2
Delta Mass: 3.115 ppm
XCorr: 3.0193

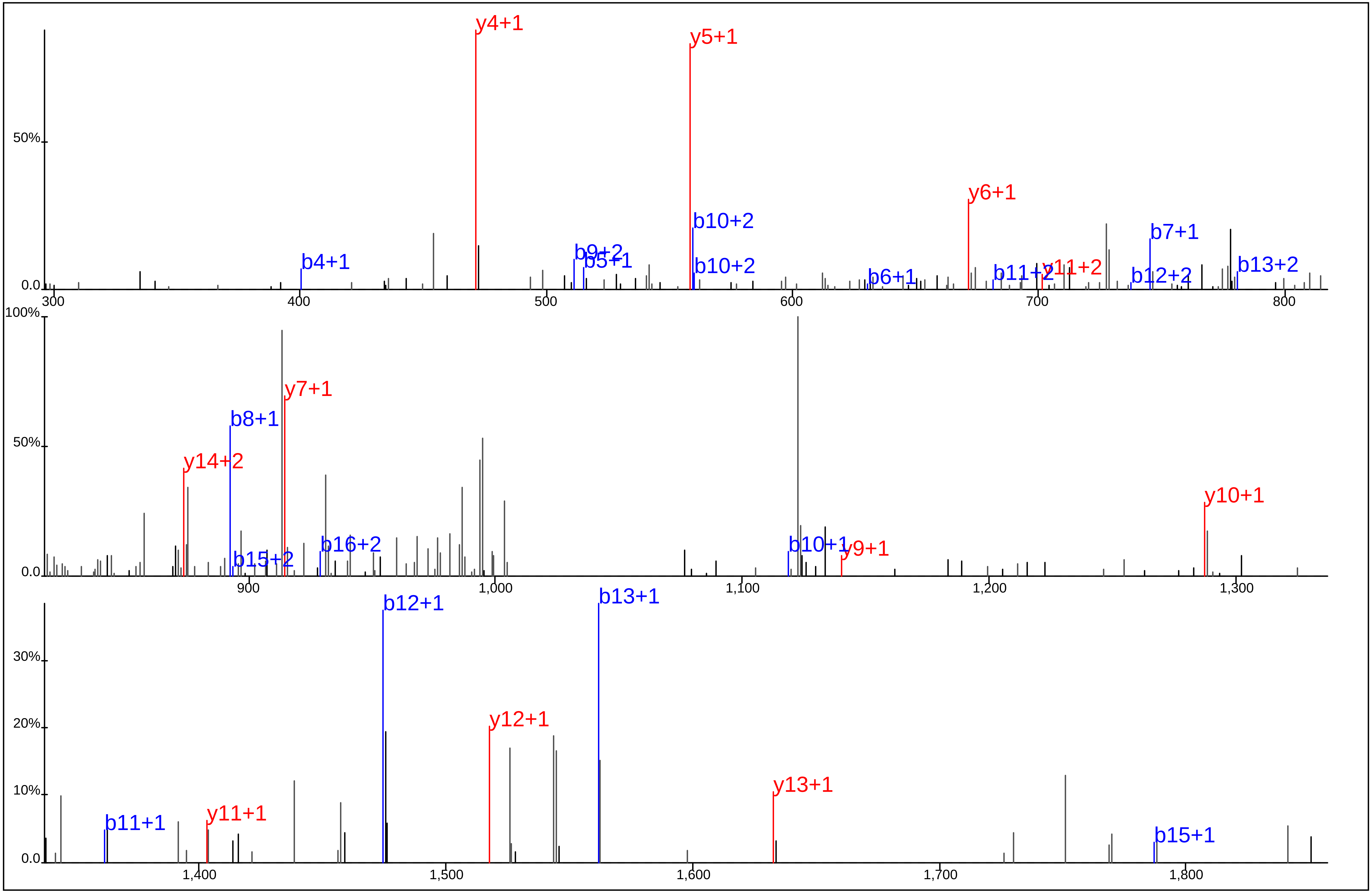

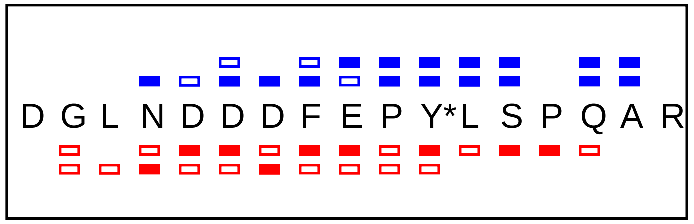

Protein Name: HGRG8
gi Accession: 116812575
Precursor Mass: 1,016.4301+2
Delta Mass: 9.681 ppm
XCorr: 3.6512

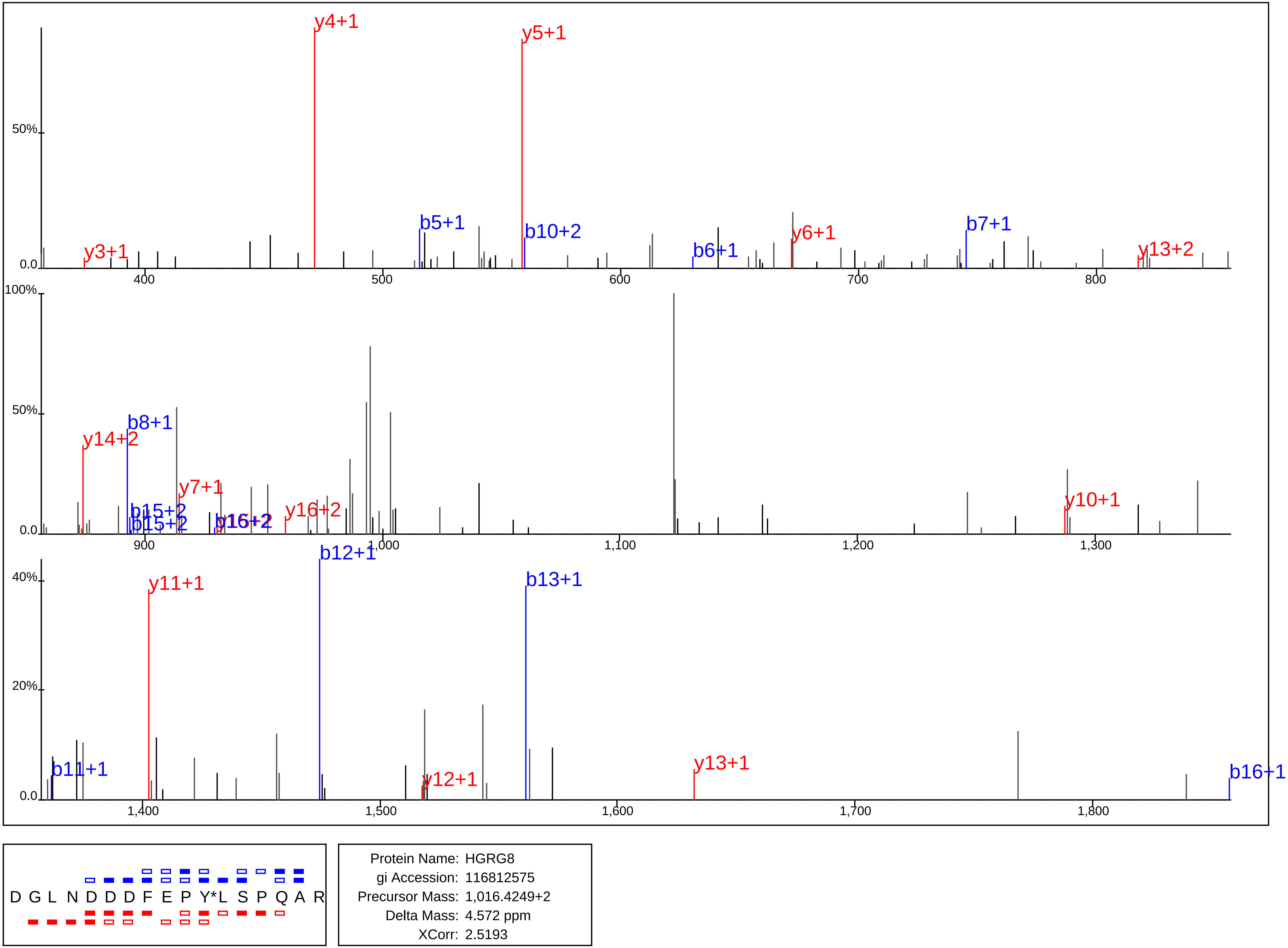

Protein Name: HGRG8
gi Accession: 116812575
Precursor Mass: 1,016.4249+2
Delta Mass: 4.572 ppm
XCorr: 2.5193

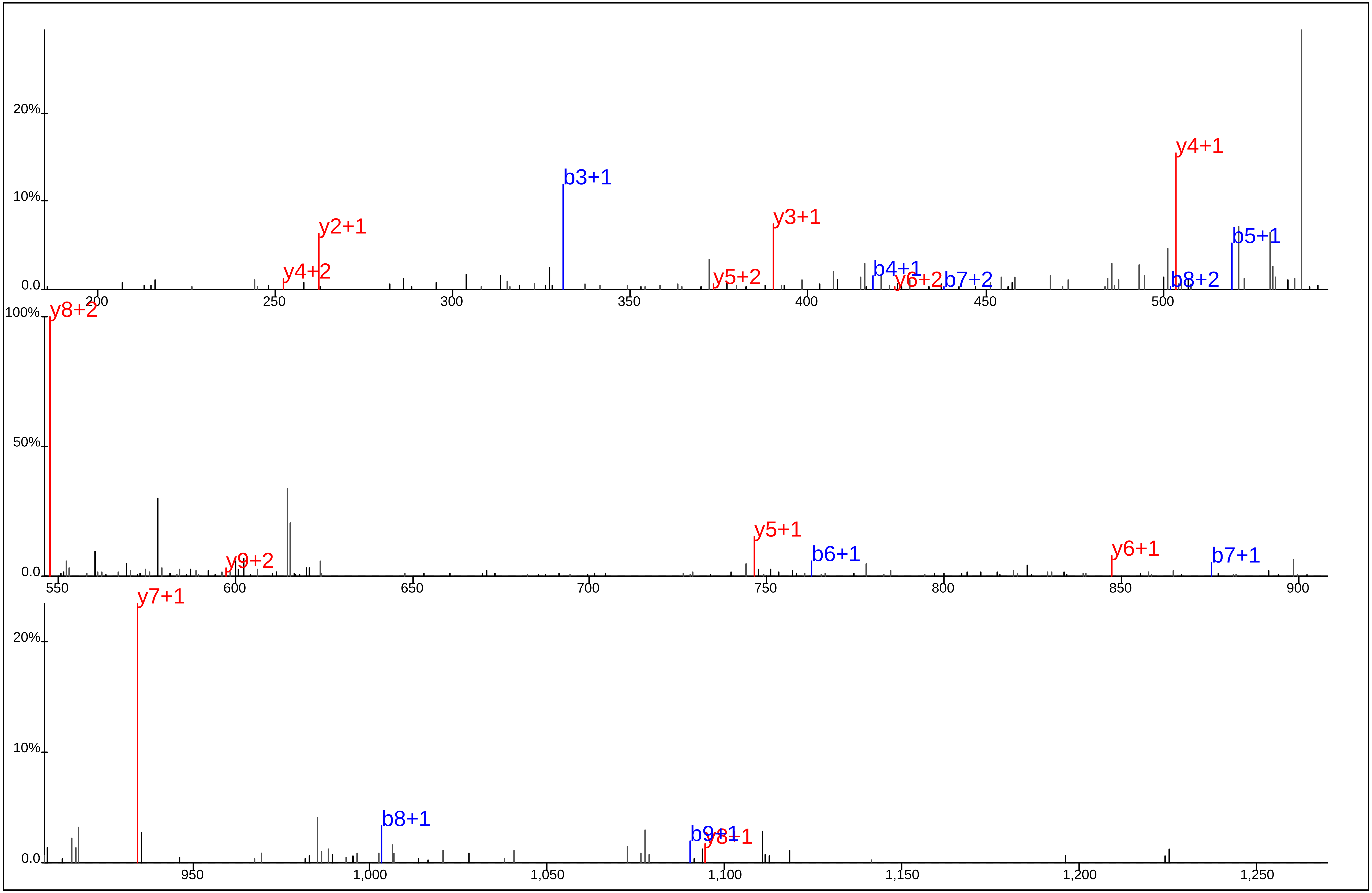

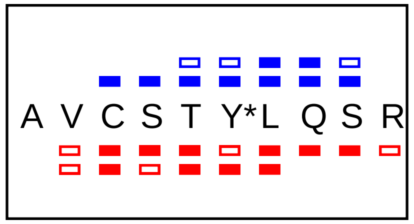

Protein Name: HIPK1
gi Accession: 38201640
Precursor Mass: 632.7764+2
Delta Mass: 5.083 ppm
XCorr: 43.34

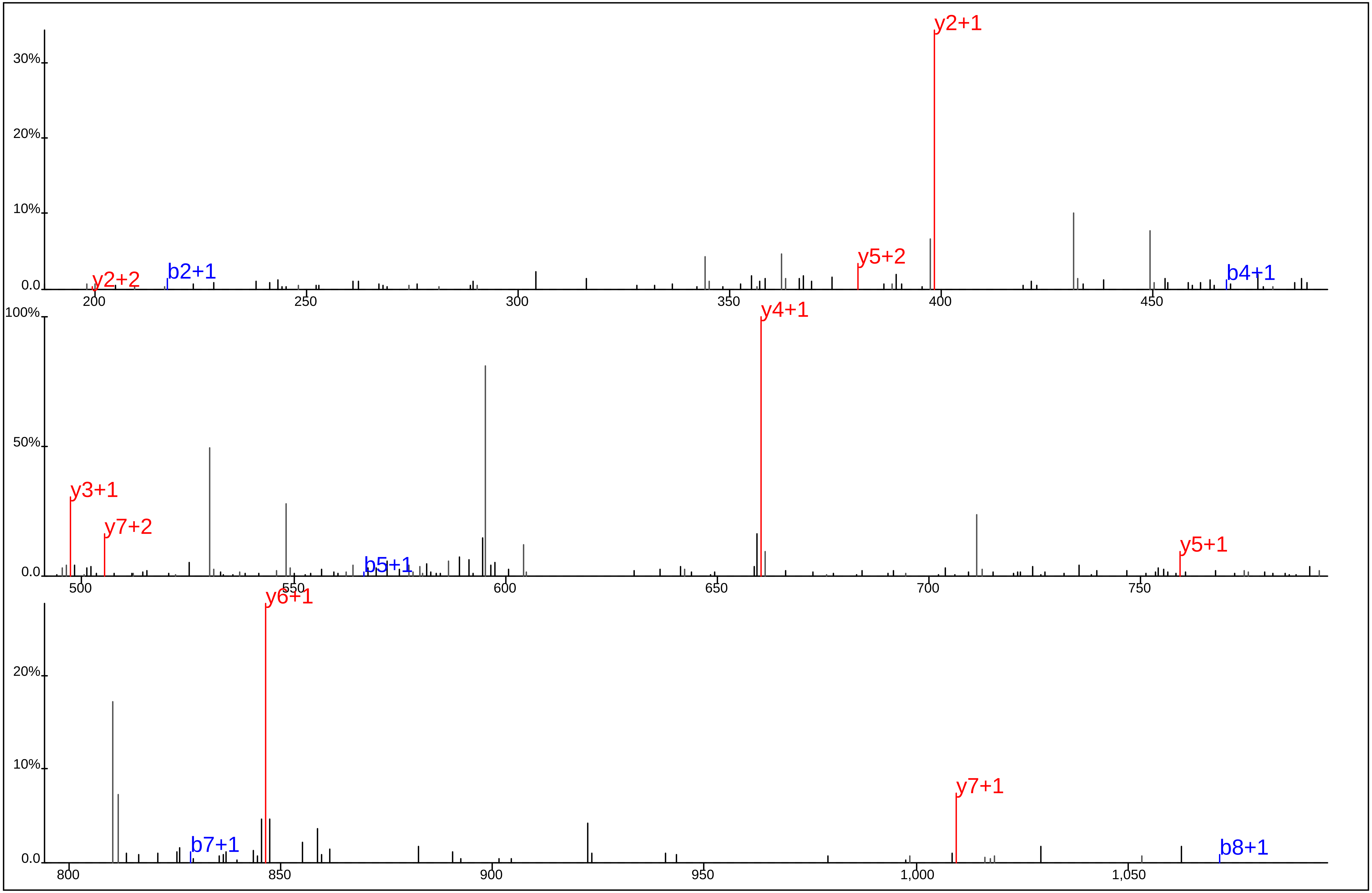

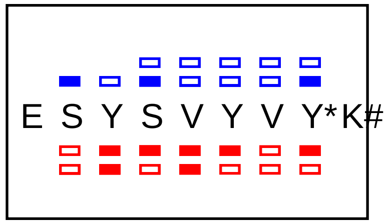

Protein Name: Histone H2B
gi Accession: 1568557
Precursor Mass: 613.2697+2
Delta Mass: 4.380 ppm
XCorr: 31.37

493 - HistoneH2B_3433639.pdf

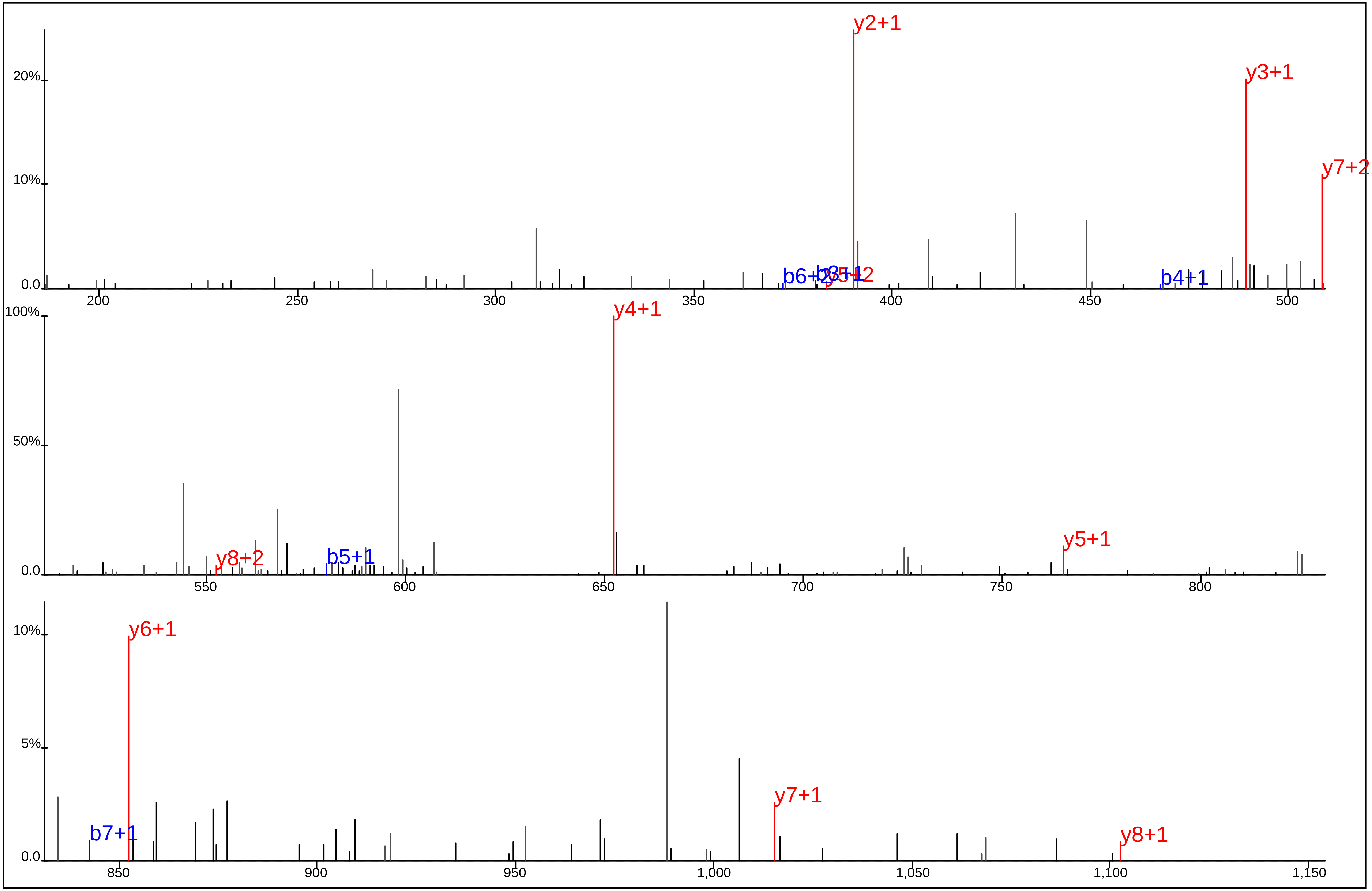

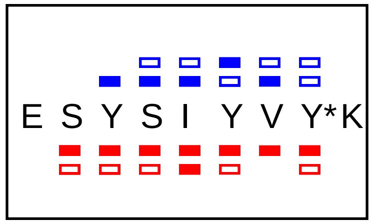

Protein Name: Histone H2B
gi Accession: 31979
Precursor Mass: 616.2711+2
Delta Mass: 5.479 ppm
XCorr: 26.9

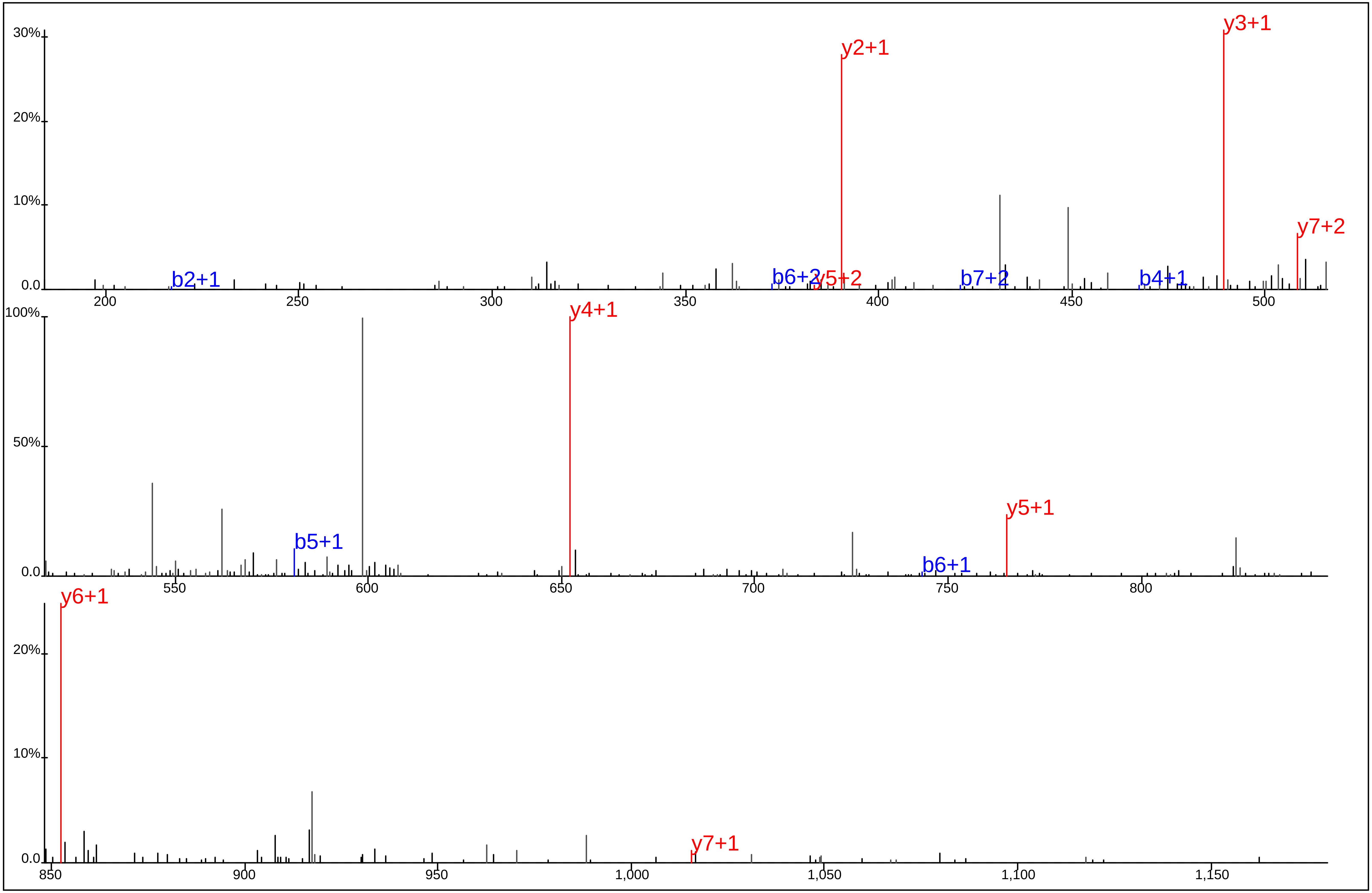

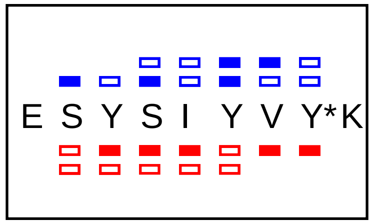

Protein Name: Histone H2B
gi Accession: 31979
Precursor Mass: 616.2651+2
Delta Mass: 4.232 ppm
XCorr: 23.53

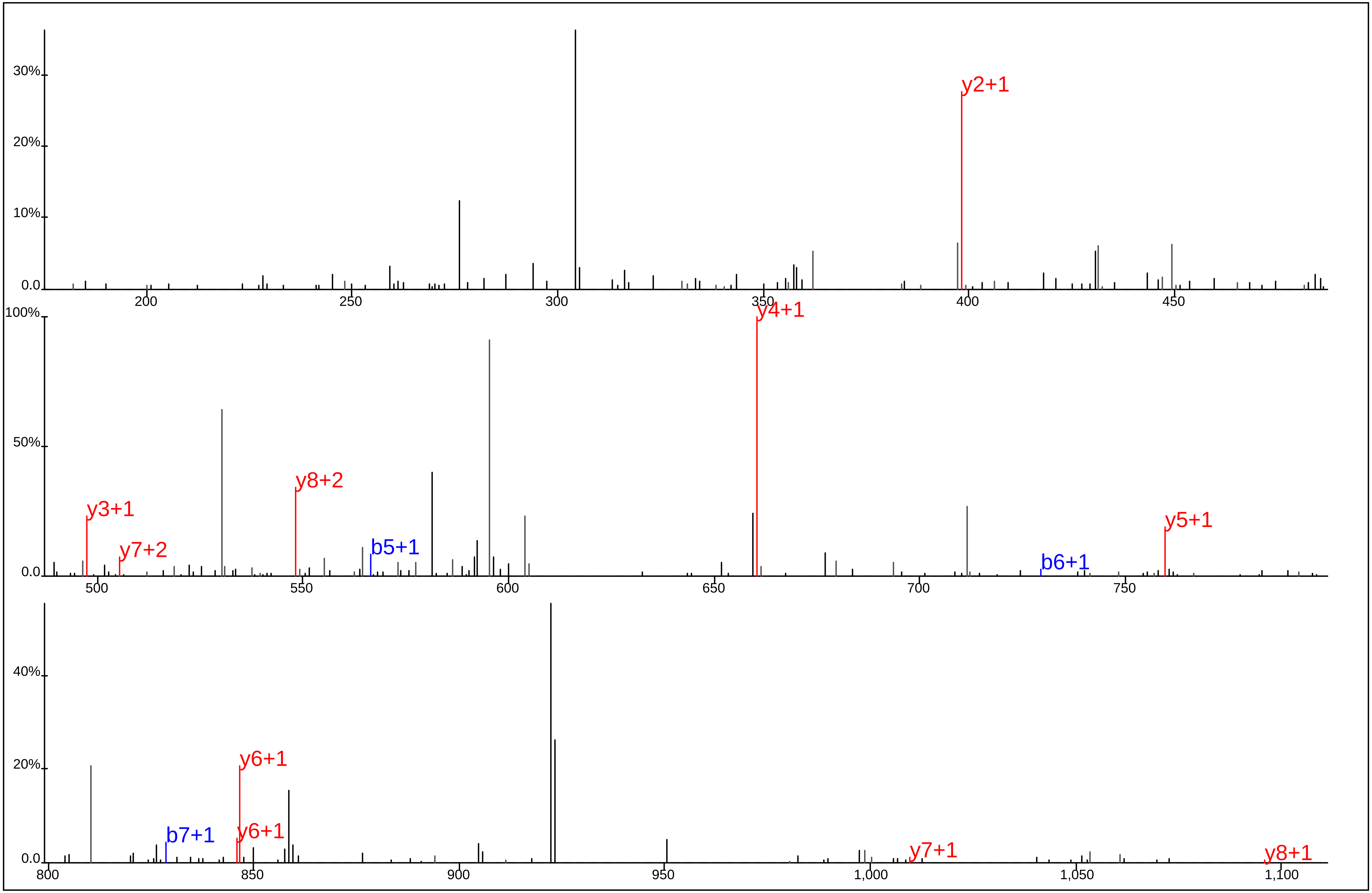

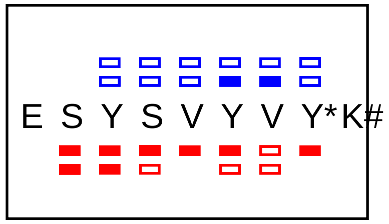

Protein Name: Histone H2B
gi Accession: 1568557
Precursor Mass: 613.2664+2
Delta Mass: 0.997 ppm
XCorr: 18.97

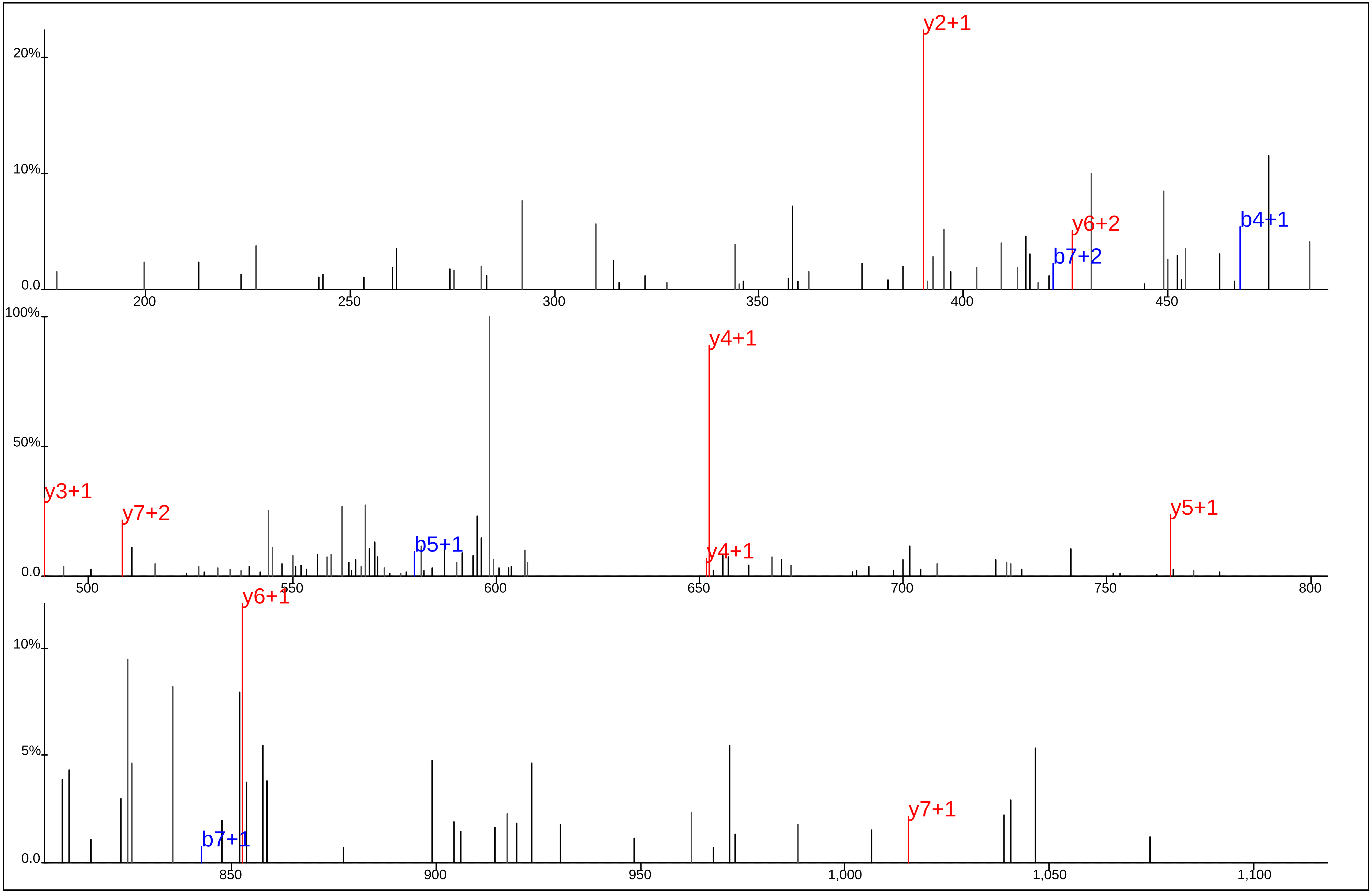

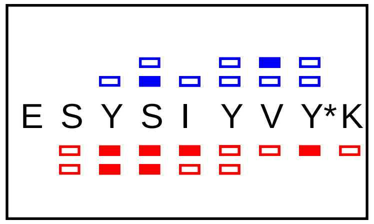

Protein Name: Histone H2B
gi Accession: 31979
Precursor Mass: 616.2698+2
Delta Mass: 3.295 ppm
XCorr: 13.72

497 - HistoneH2B_3438760.pdf

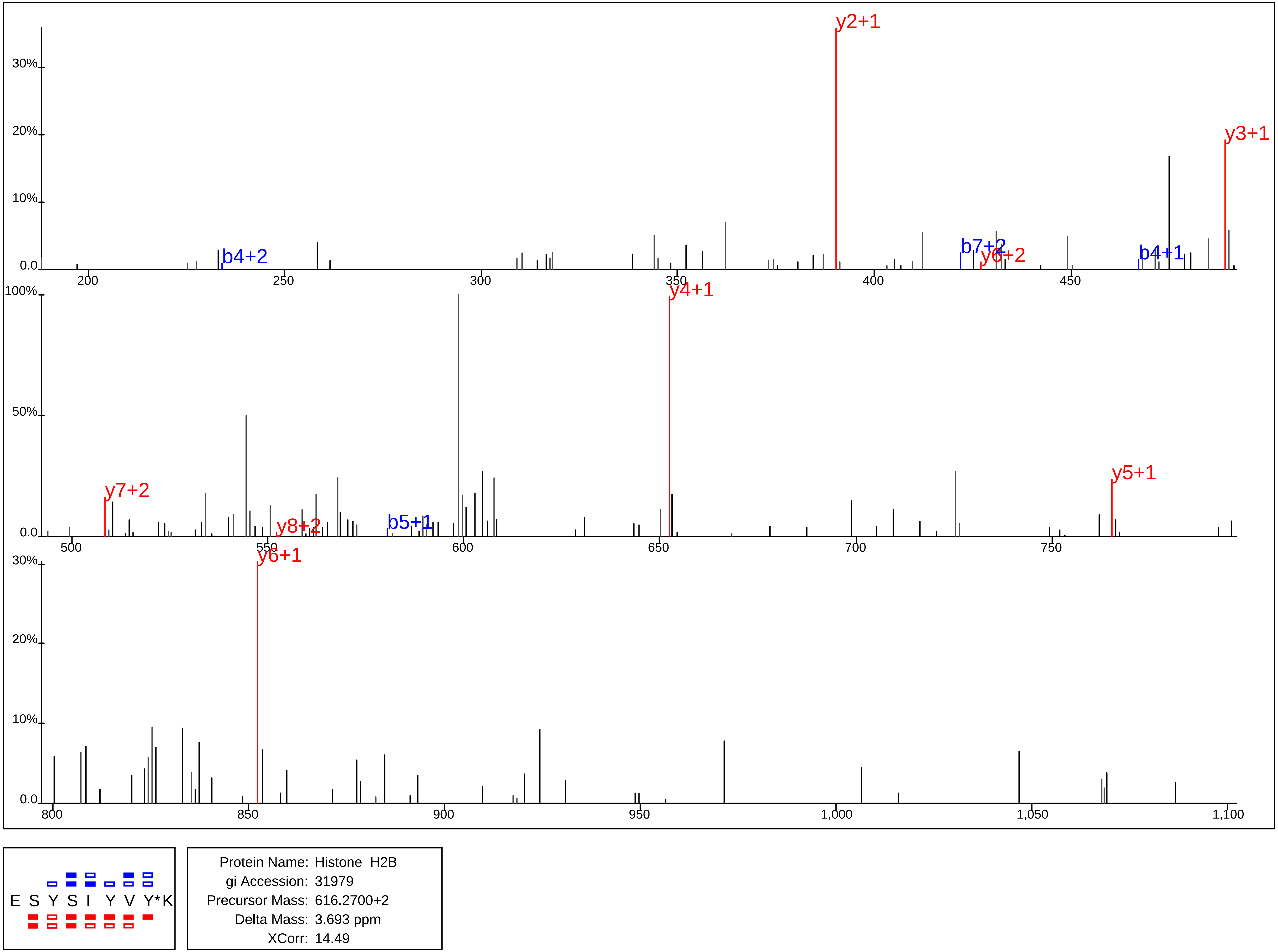

Protein Name: Histone H2B
gi Accession: 31979
Precursor Mass: 616.2700+2
Delta Mass: 3.693 ppm
XCorr: 14.49

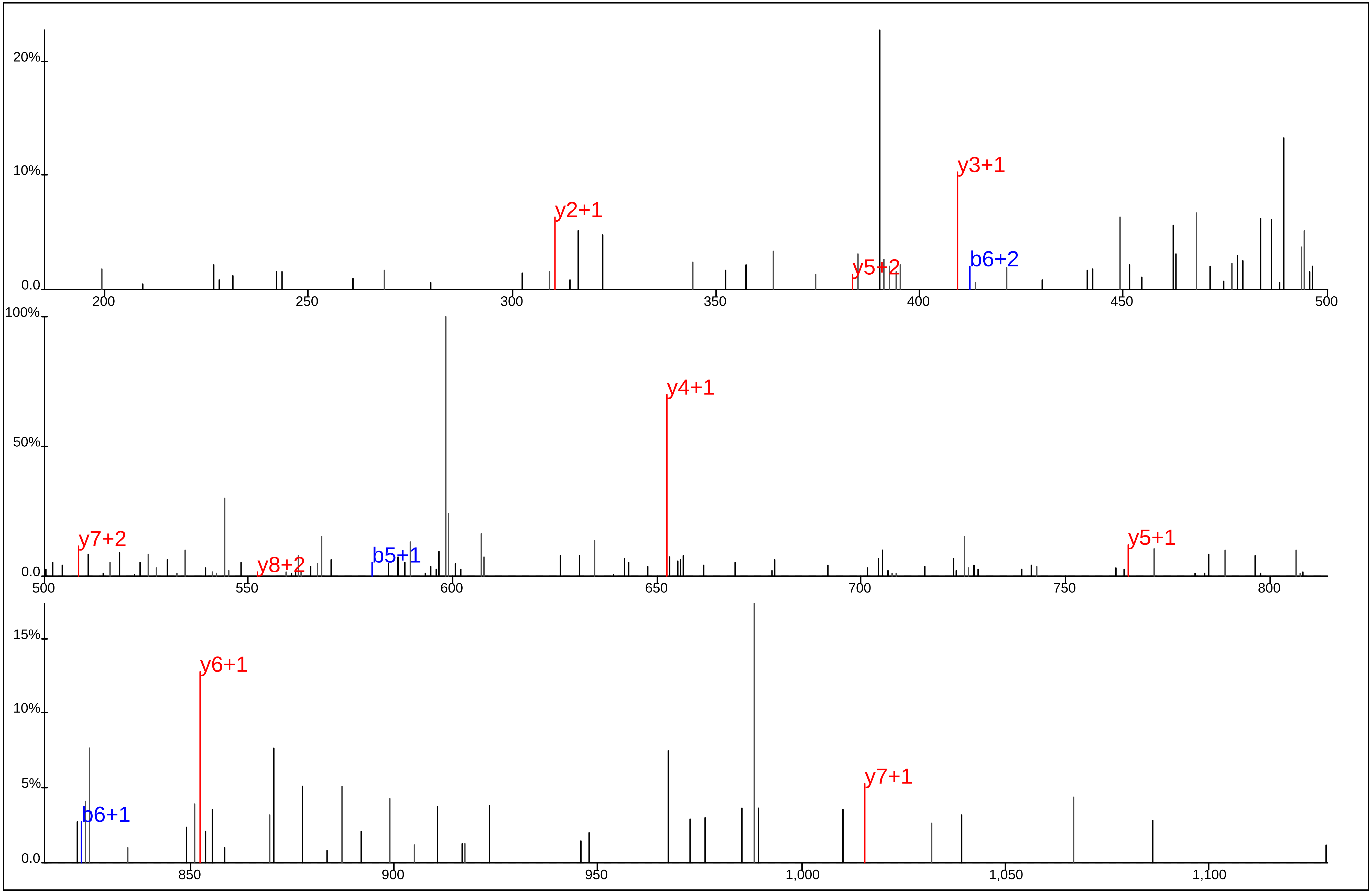

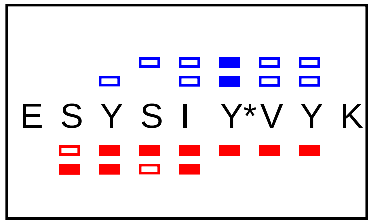

Protein Name: Histone H2B
gi Accession: 31979
Precursor Mass: 616.2690+2
Delta Mass: 2.110 ppm
XCorr: 20.2

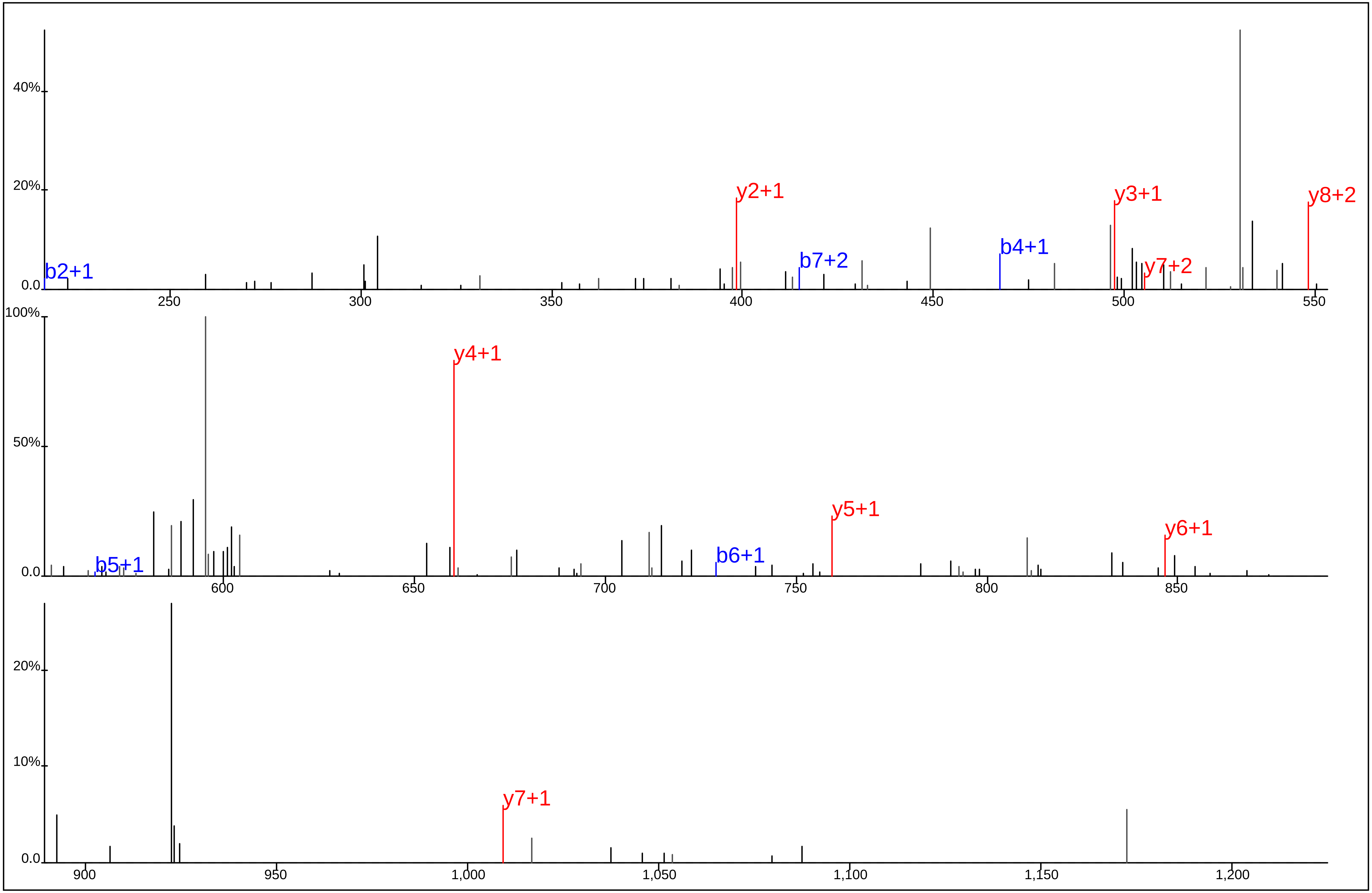

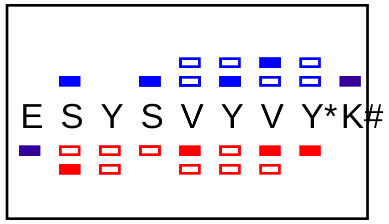

Protein Name: Histone H2B
gi Accession: 1568557
Precursor Mass: 613.2651+2
Delta Mass: 3.086 ppm
XCorr: 27.73

500 - HistoneH2B_3442207.pdf

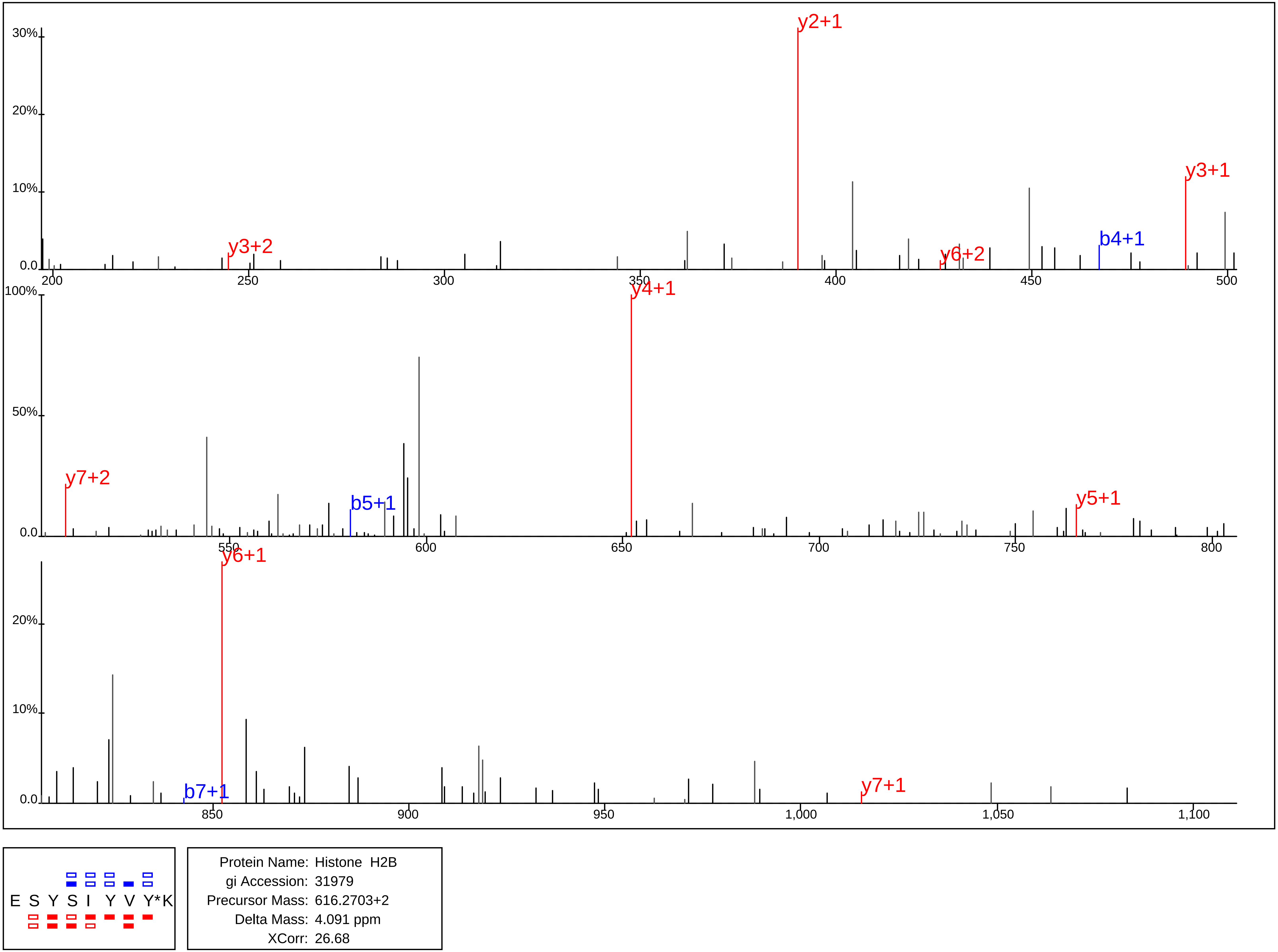

Protein Name: Histone H2B
gi Accession: 31979
Precursor Mass: 616.2703+2
Delta Mass: 4.091 ppm
XCorr: 26.68

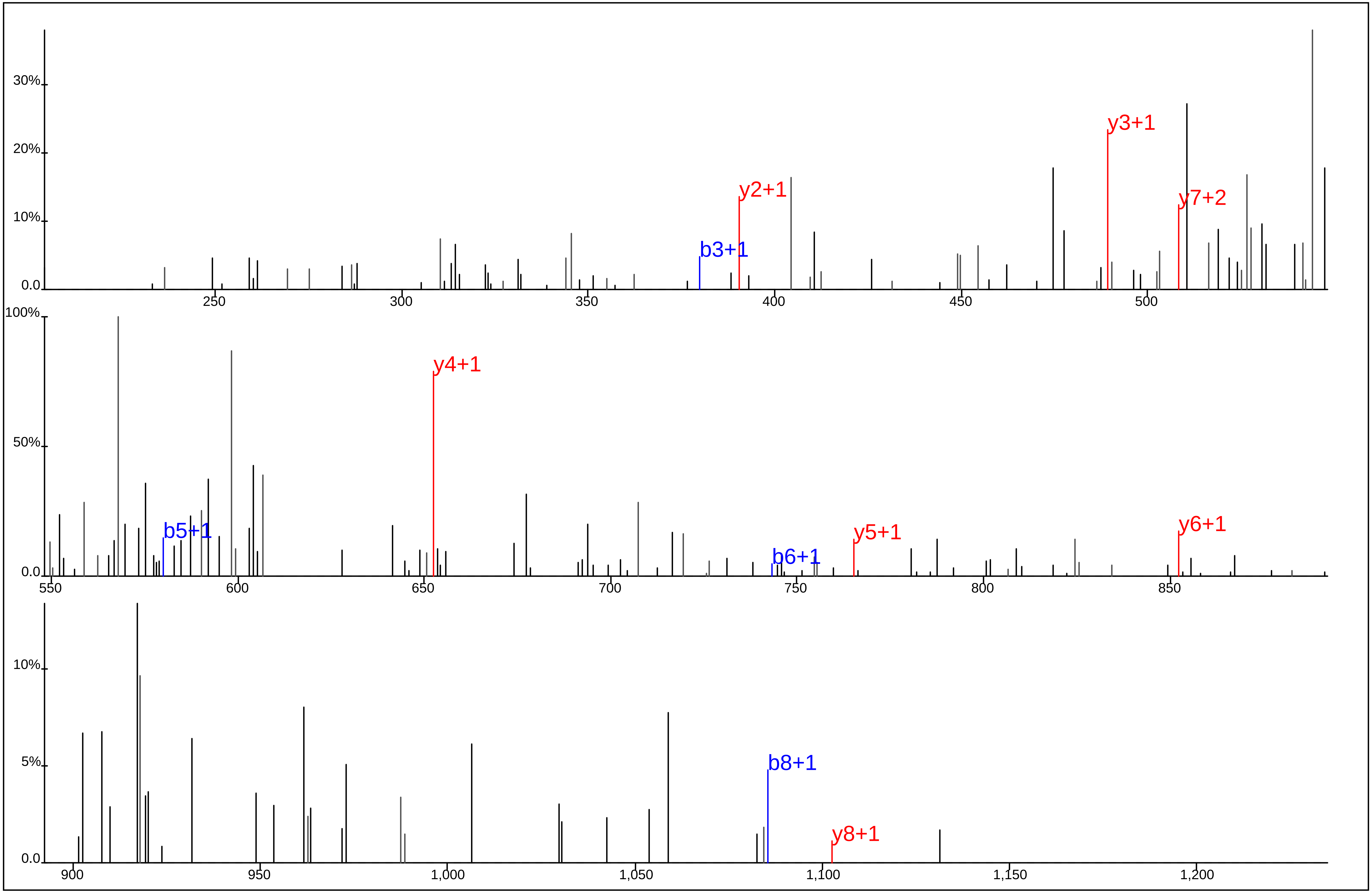

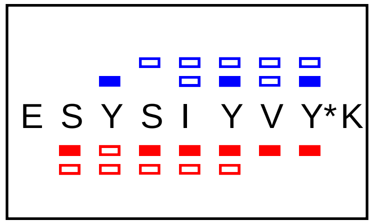

Protein Name: Histone H2B
gi Accession: 31979
Precursor Mass: 616.2706+2
Delta Mass: 4.684 ppm
XCorr: 12.23

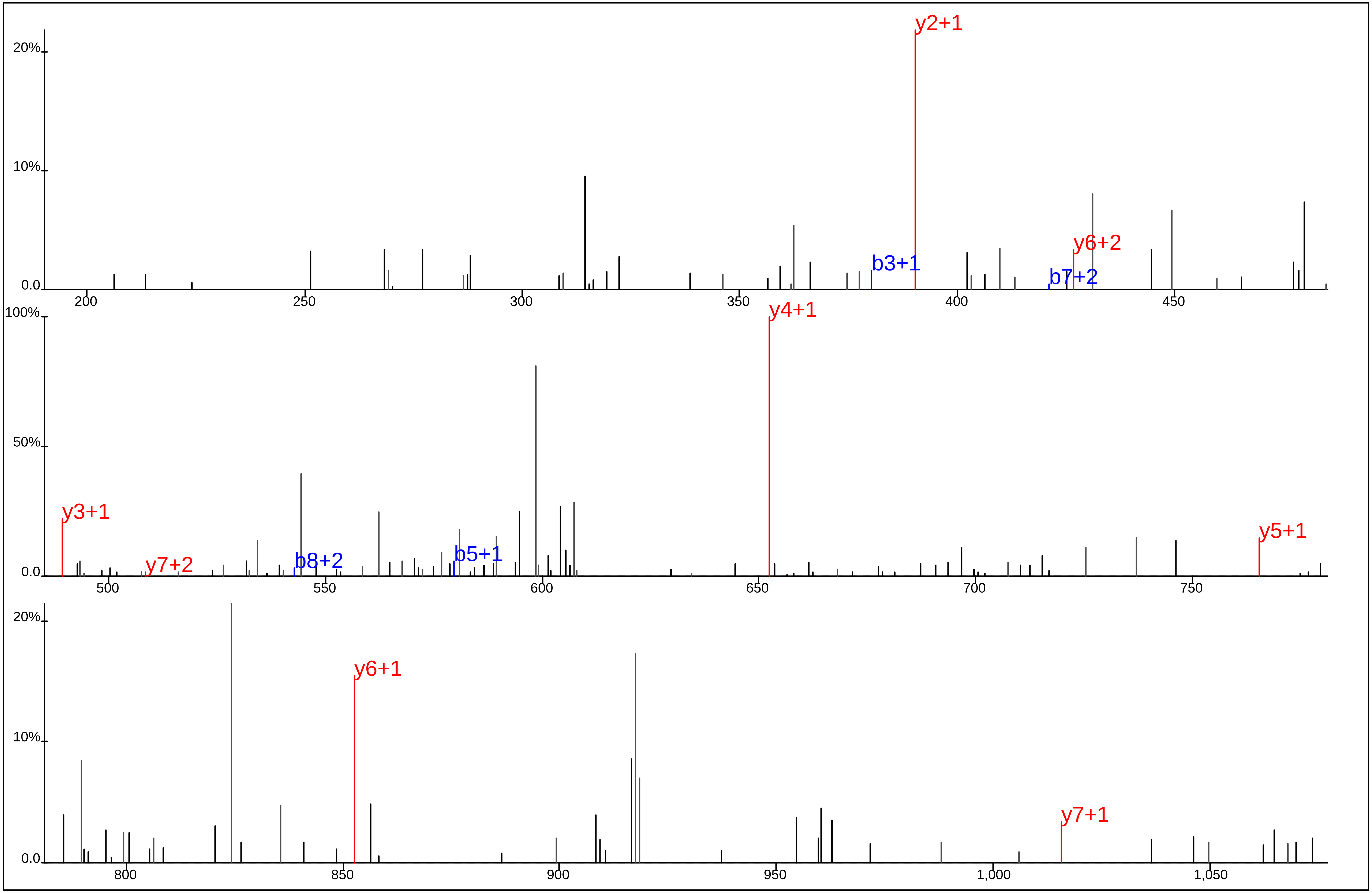

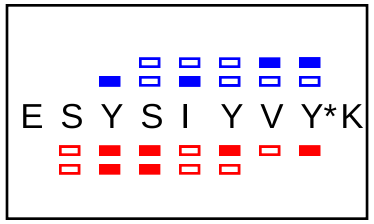

Protein Name: Histone H2B
gi Accession: 31979
Precursor Mass: 616.2724+2
Delta Mass: 7.558 ppm
XCorr: 24.18

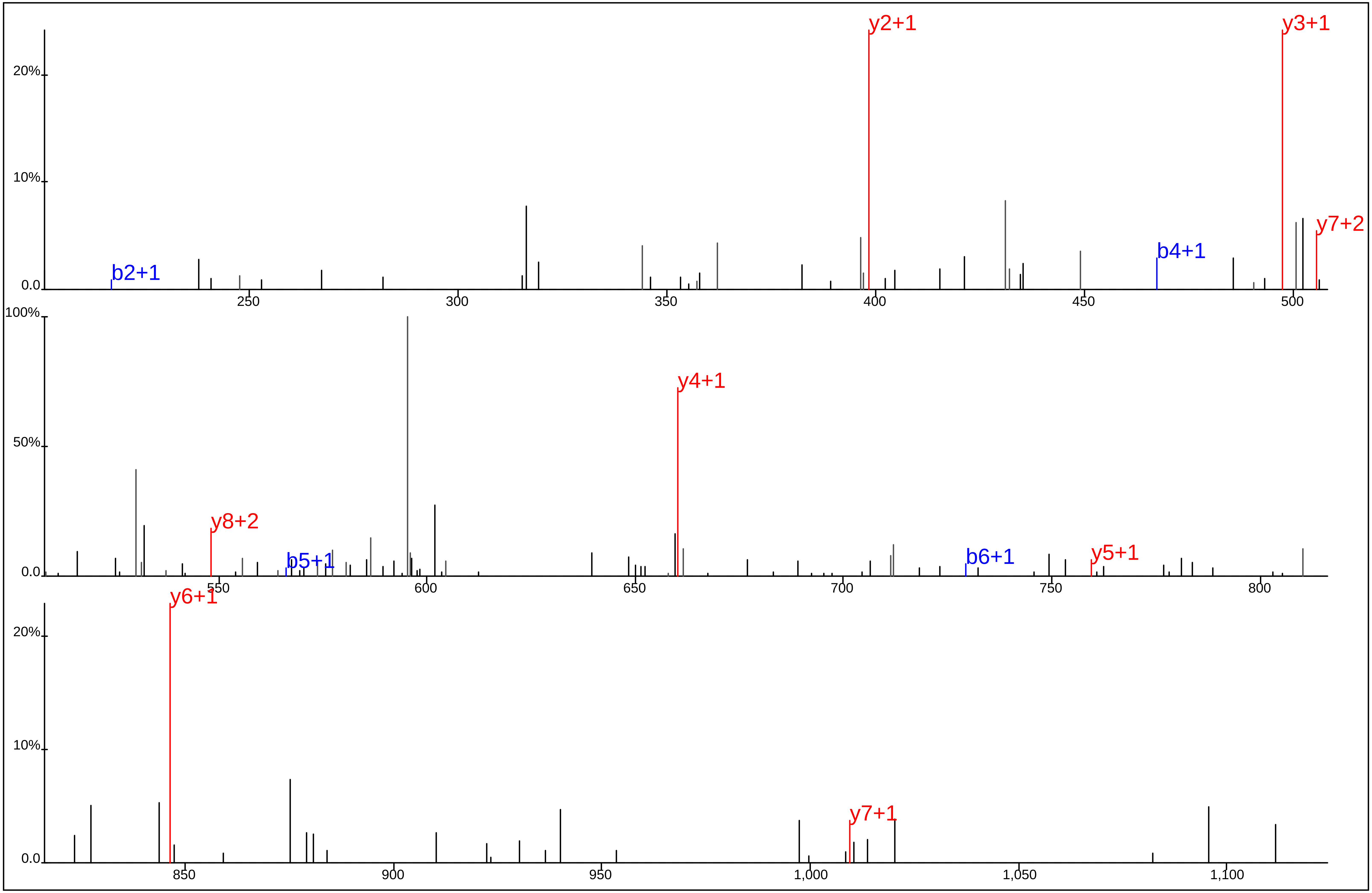

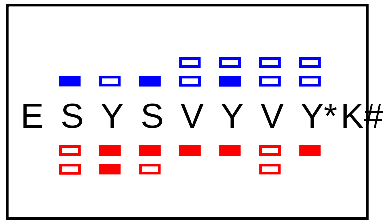

Protein Name: Histone H2B
gi Accession: 1568557
Precursor Mass: 613.2678+2
Delta Mass: 1.296 ppm
XCorr: 17.28

504 - HistoneH2B_3453384.pdf

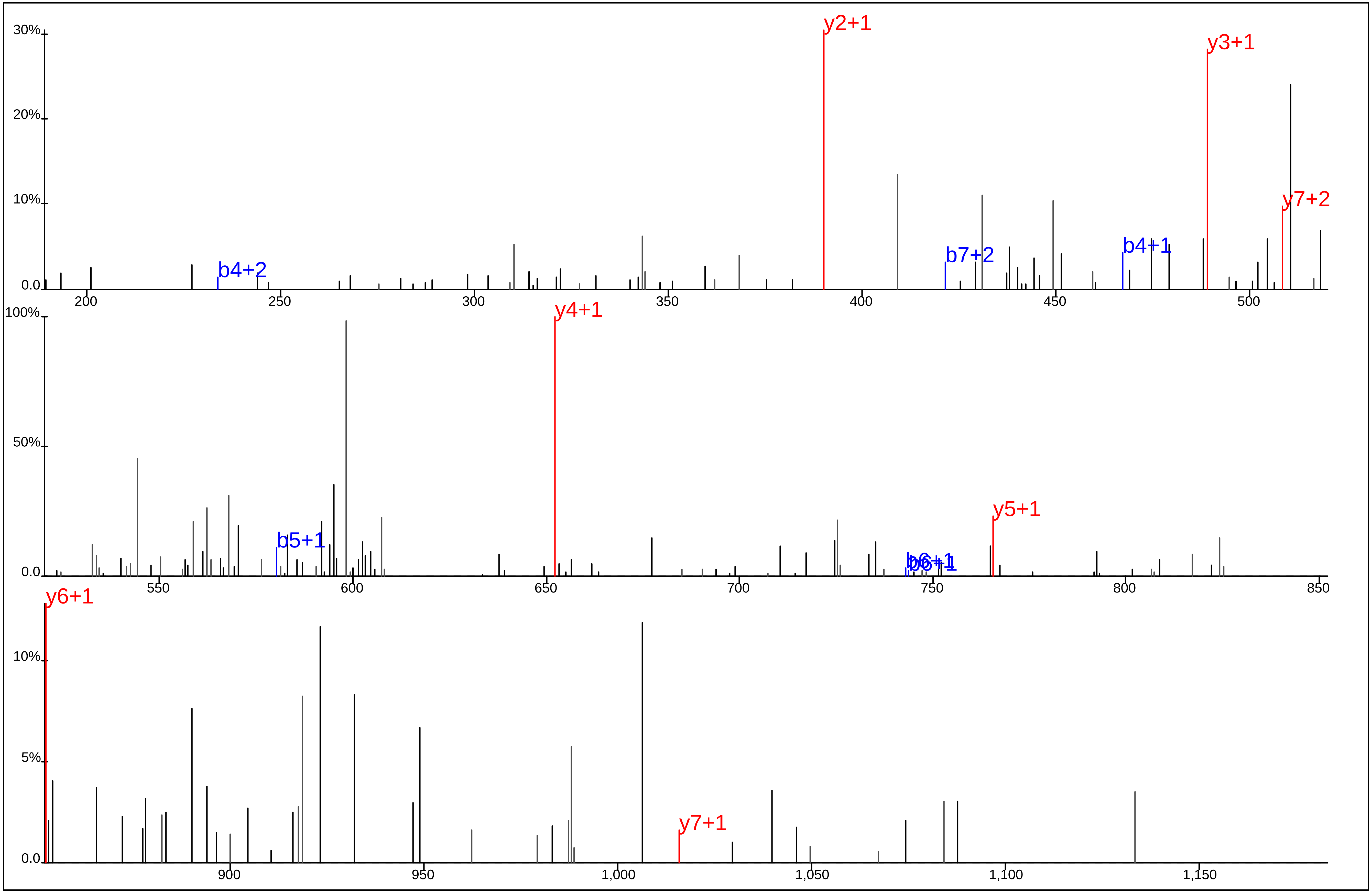

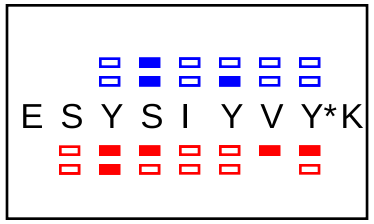

Protein Name: Histone H2B
gi Accession: 31979
Precursor Mass: 616.2685+2
Delta Mass: 1.216 ppm
XCorr: 12.27

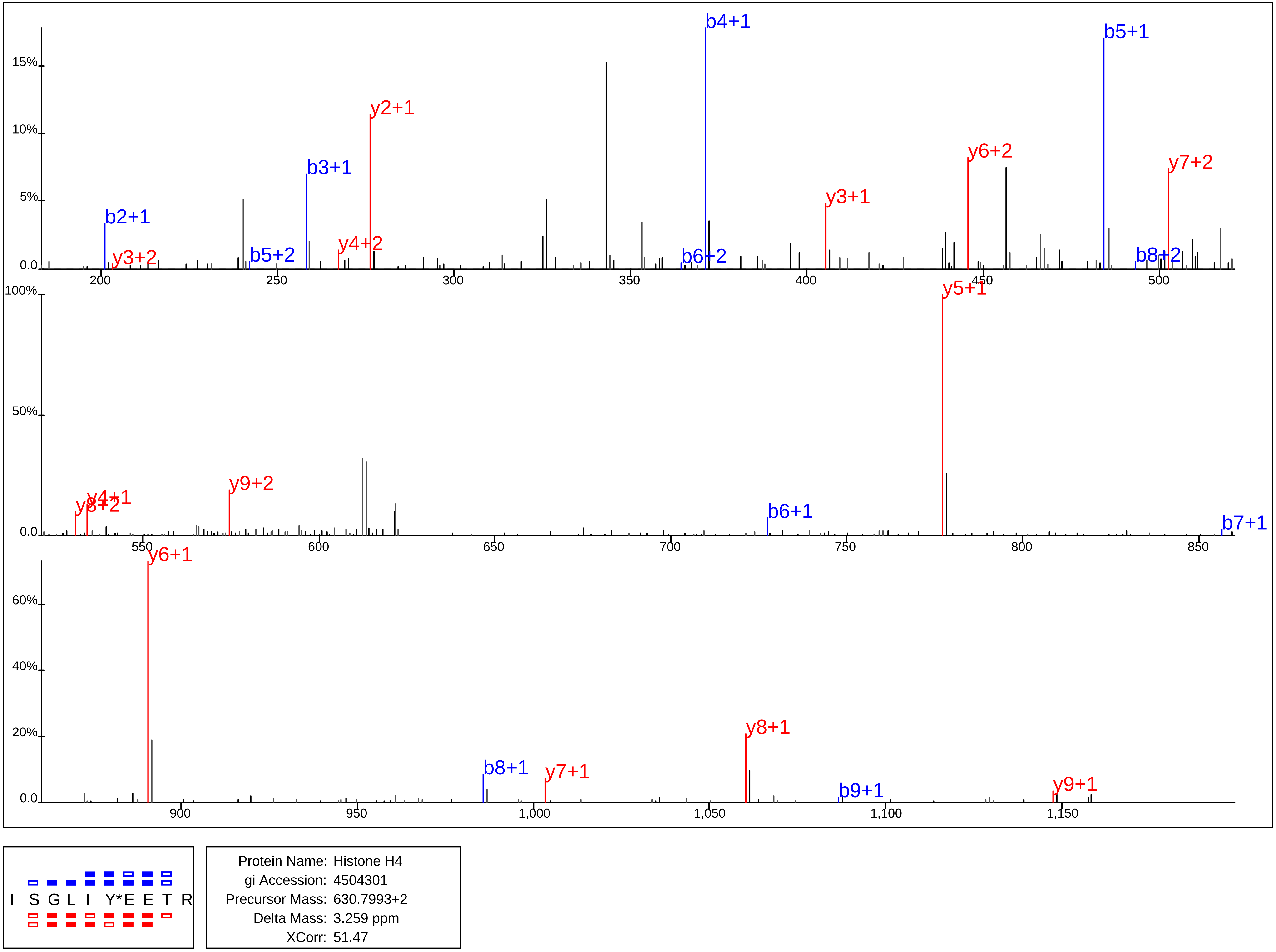

Protein Name: Histone H4
gi Accession: 4504301
Precursor Mass: 630.7993+2
Delta Mass: 3.259 ppm
XCorr: 51.47

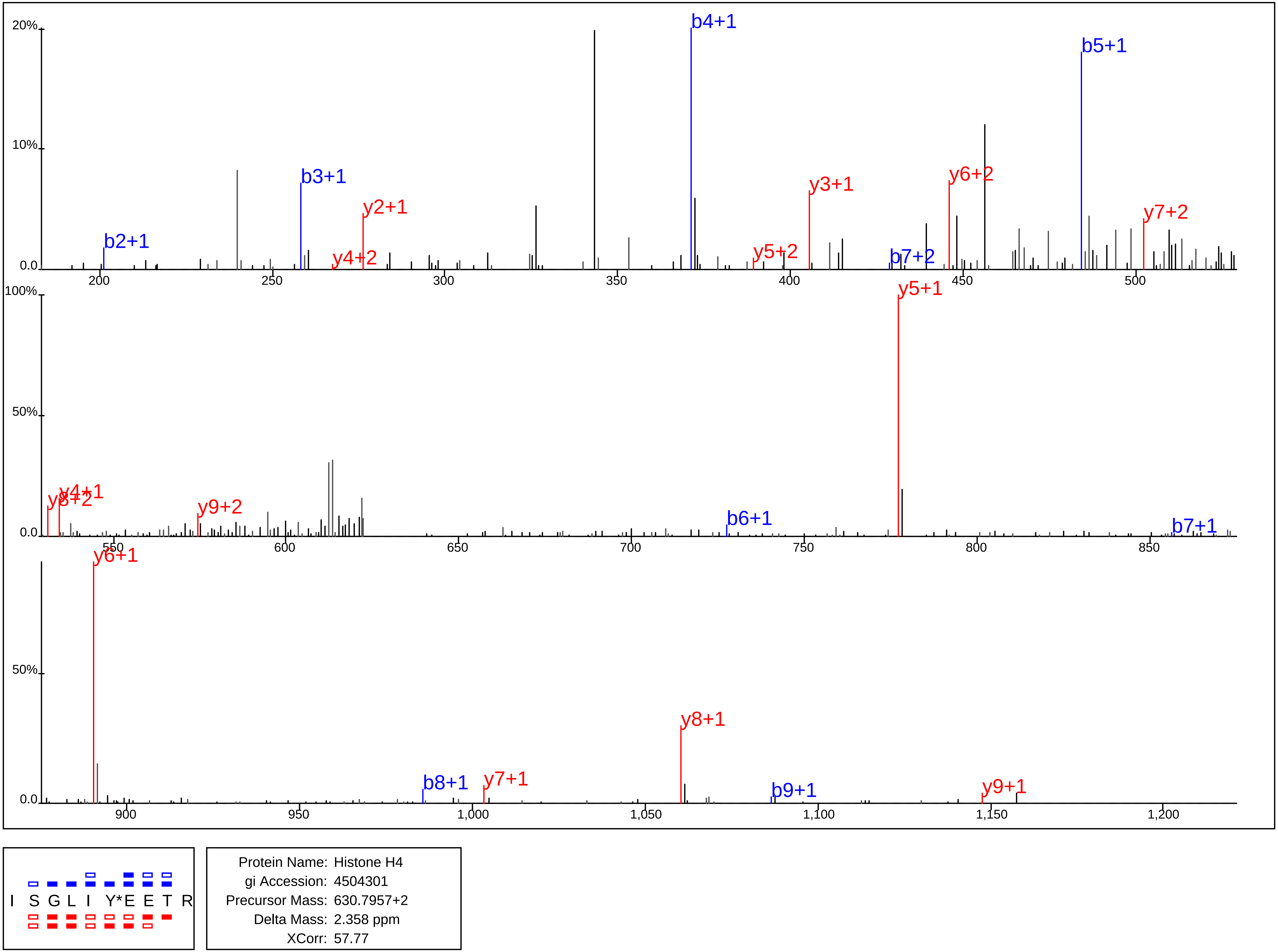

Protein Name: Histone H4
gi Accession: 4504301
Precursor Mass: 630.7957+2
Delta Mass: 2.358 ppm
XCorr: 57.77

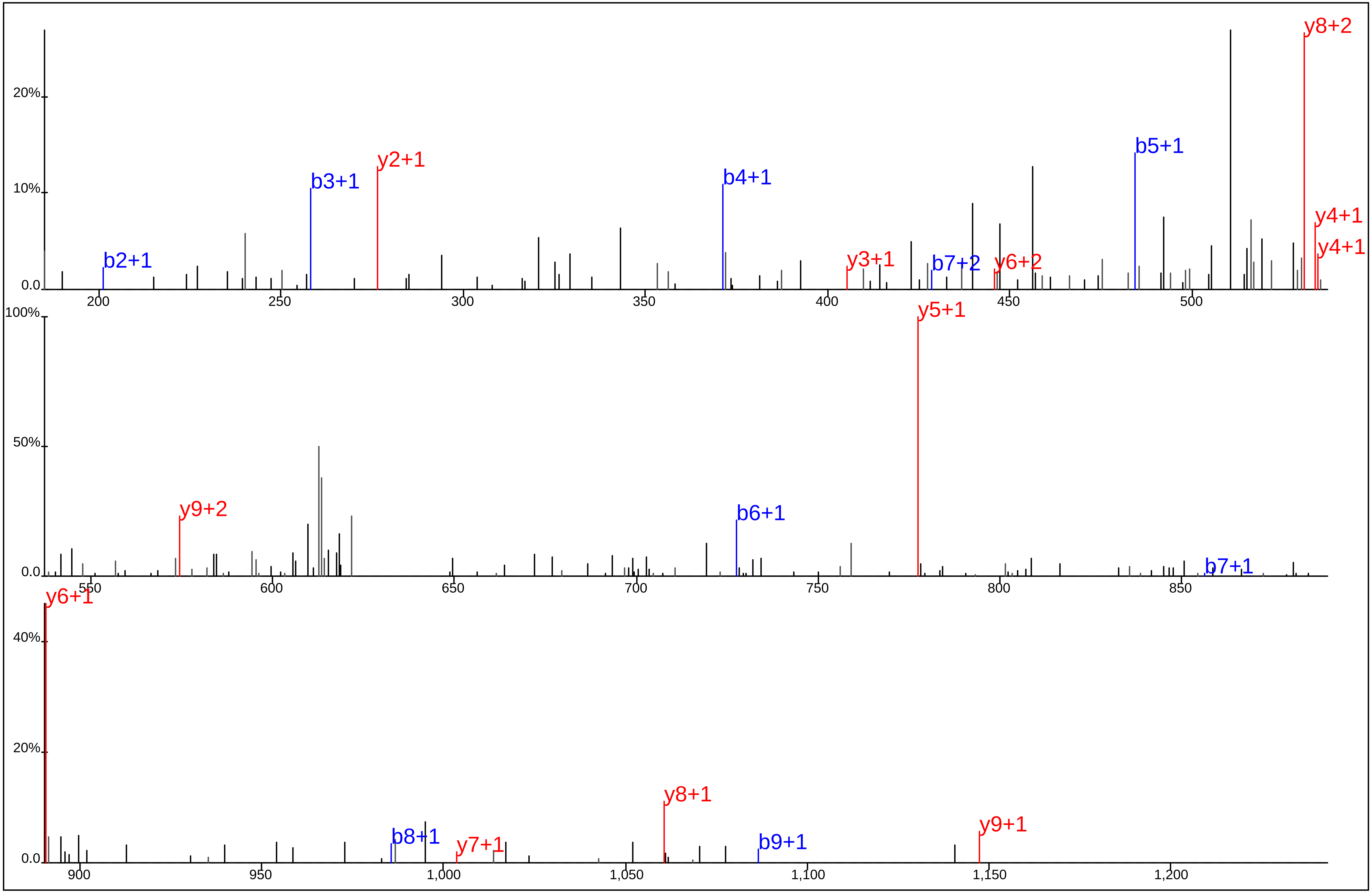

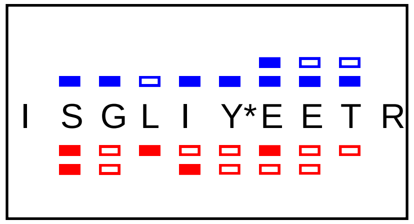

Protein Name: Histone H4
gi Accession: 4504301
Precursor Mass: 630.7954+2
Delta Mass: 2.842 ppm
XCorr: 37.44

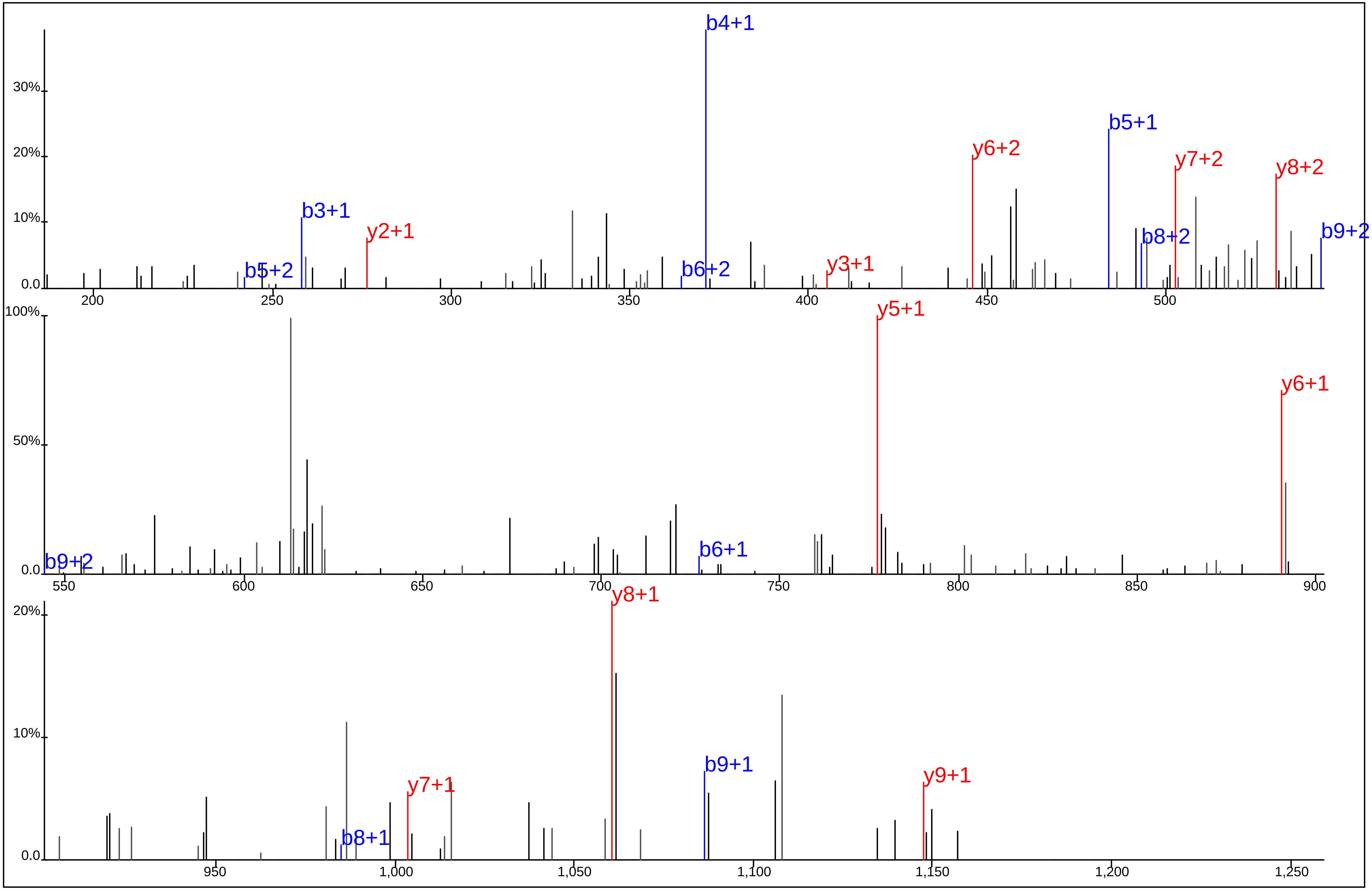

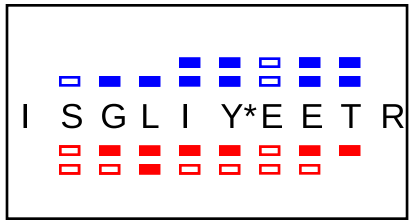

Protein Name: Histone H4
gi Accession: 4504301
Precursor Mass: 630.8008+2
Delta Mass: 5.686 ppm
XCorr: 26.68

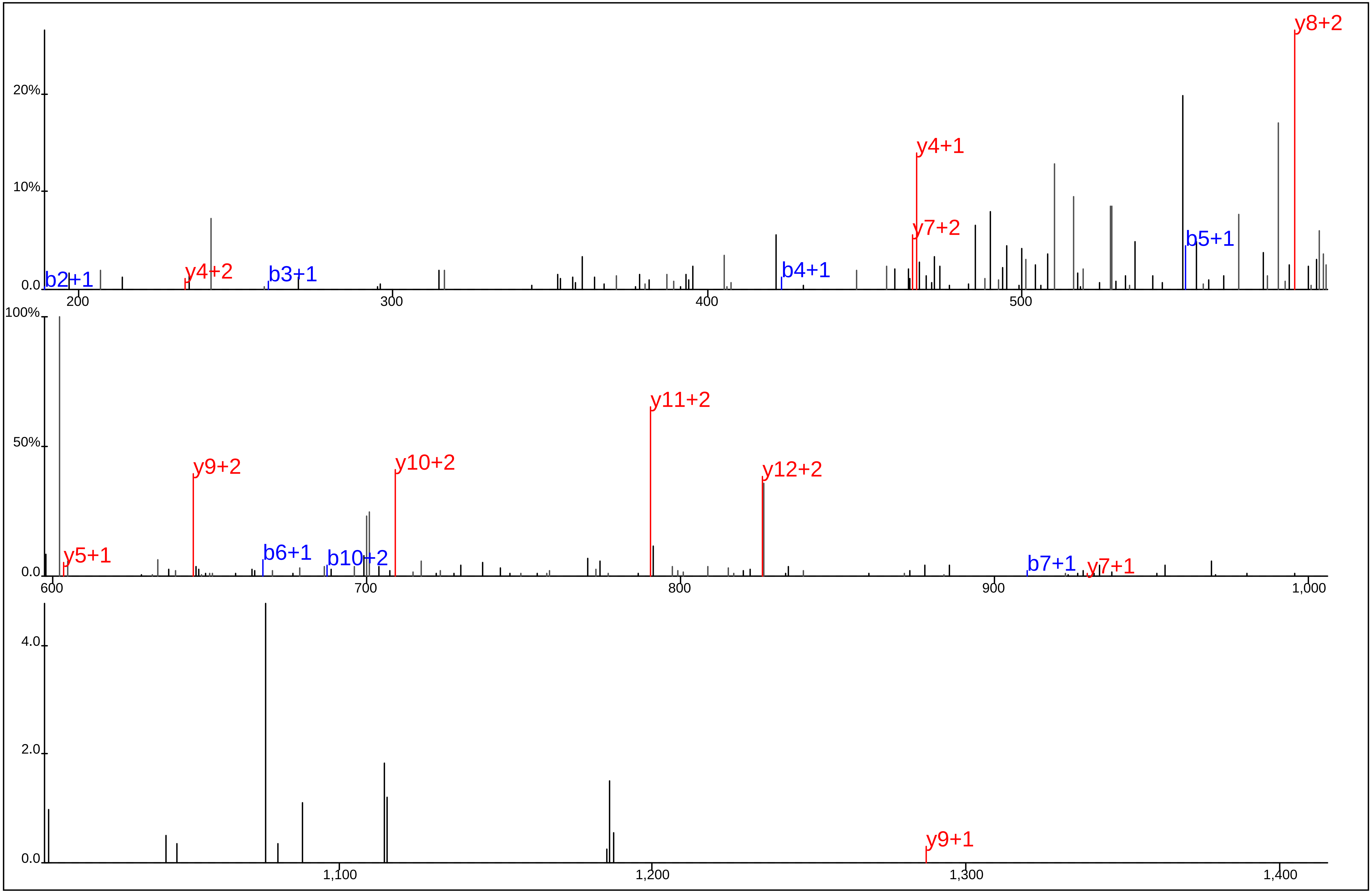

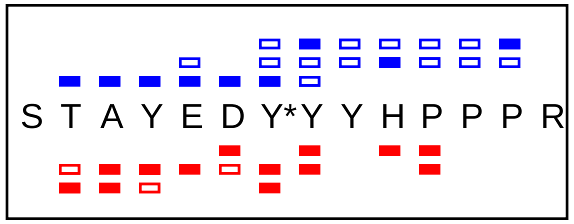

Protein Name:	hnRNP H3
gi Accession:	5031755
Precursor Mass:	613.5892+3
Delta Mass:	5.998 ppm
XCorr:	16.83

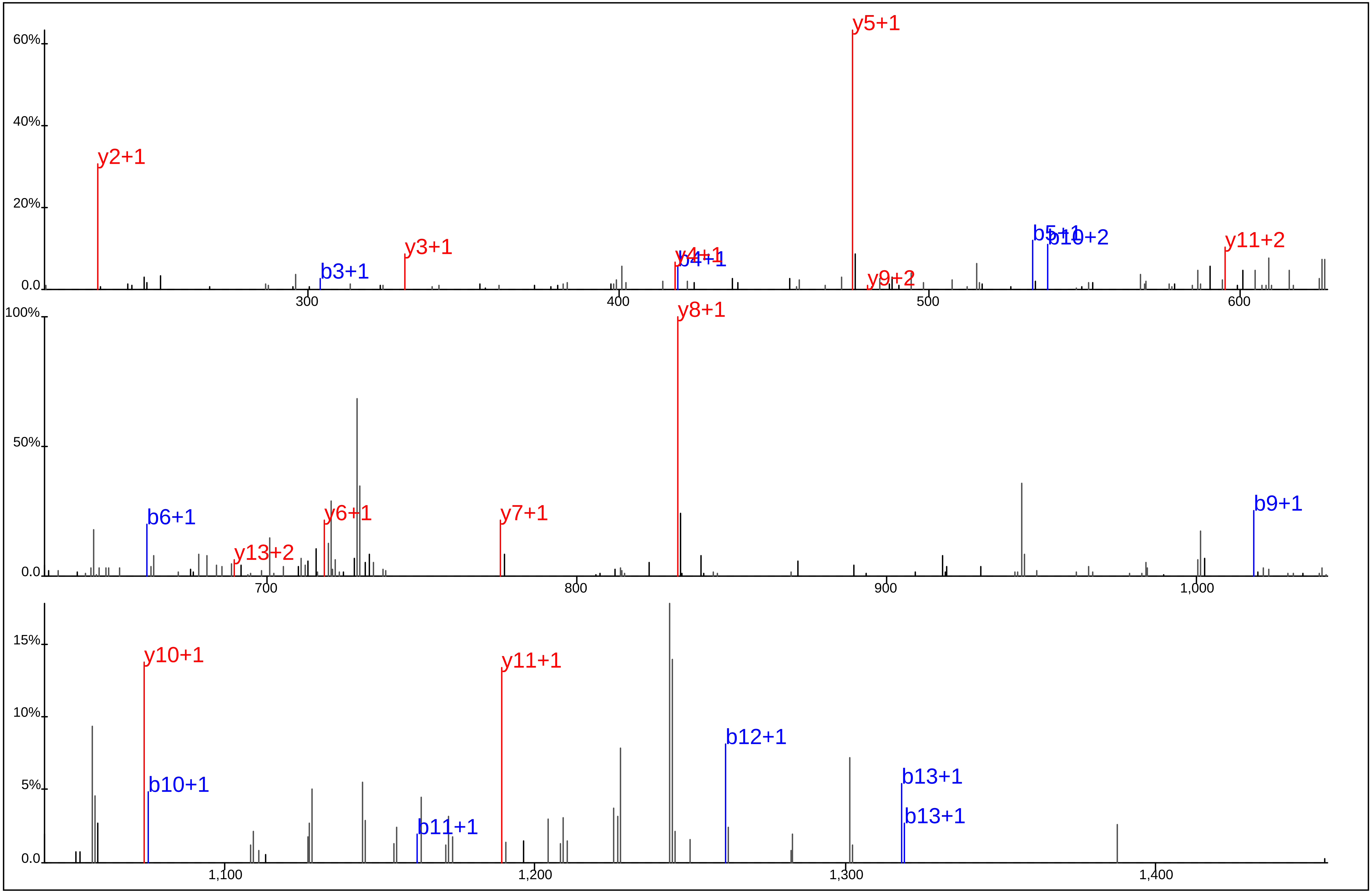

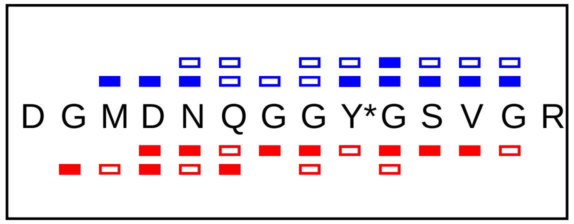

Protein Name: hnRNP H3
gi Accession: 73952775
Precursor Mass: 746.7856+2
Delta Mass: 7.791 ppm
XCorr: 61.63

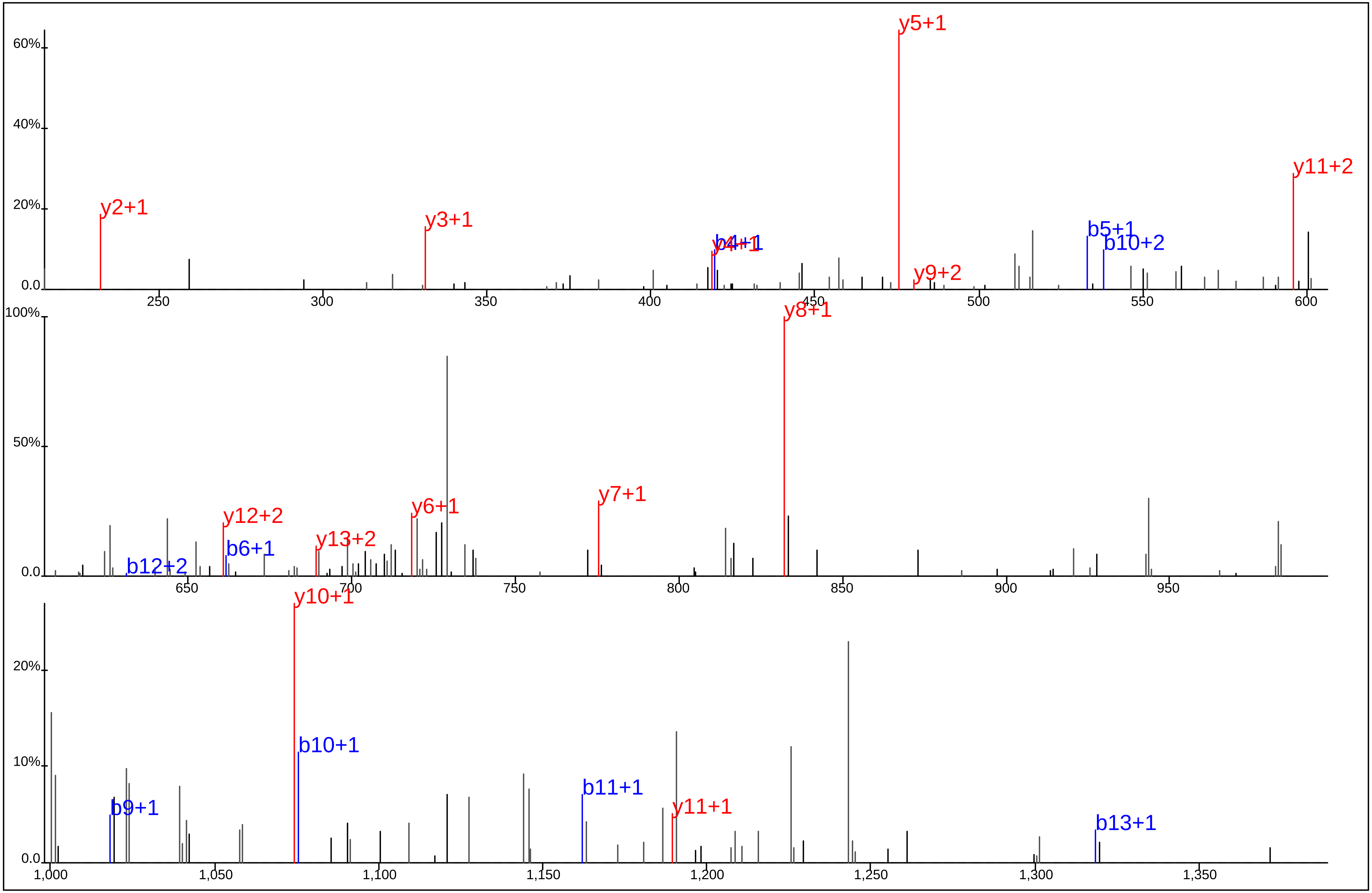

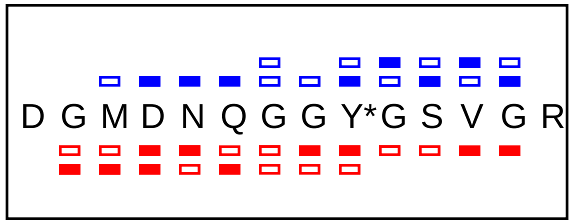

Protein Name: hnRNP H3
gi Accession: 73952775
Precursor Mass: 746.7826+2
Delta Mass: 3.784 ppm
XCorr: 51.19

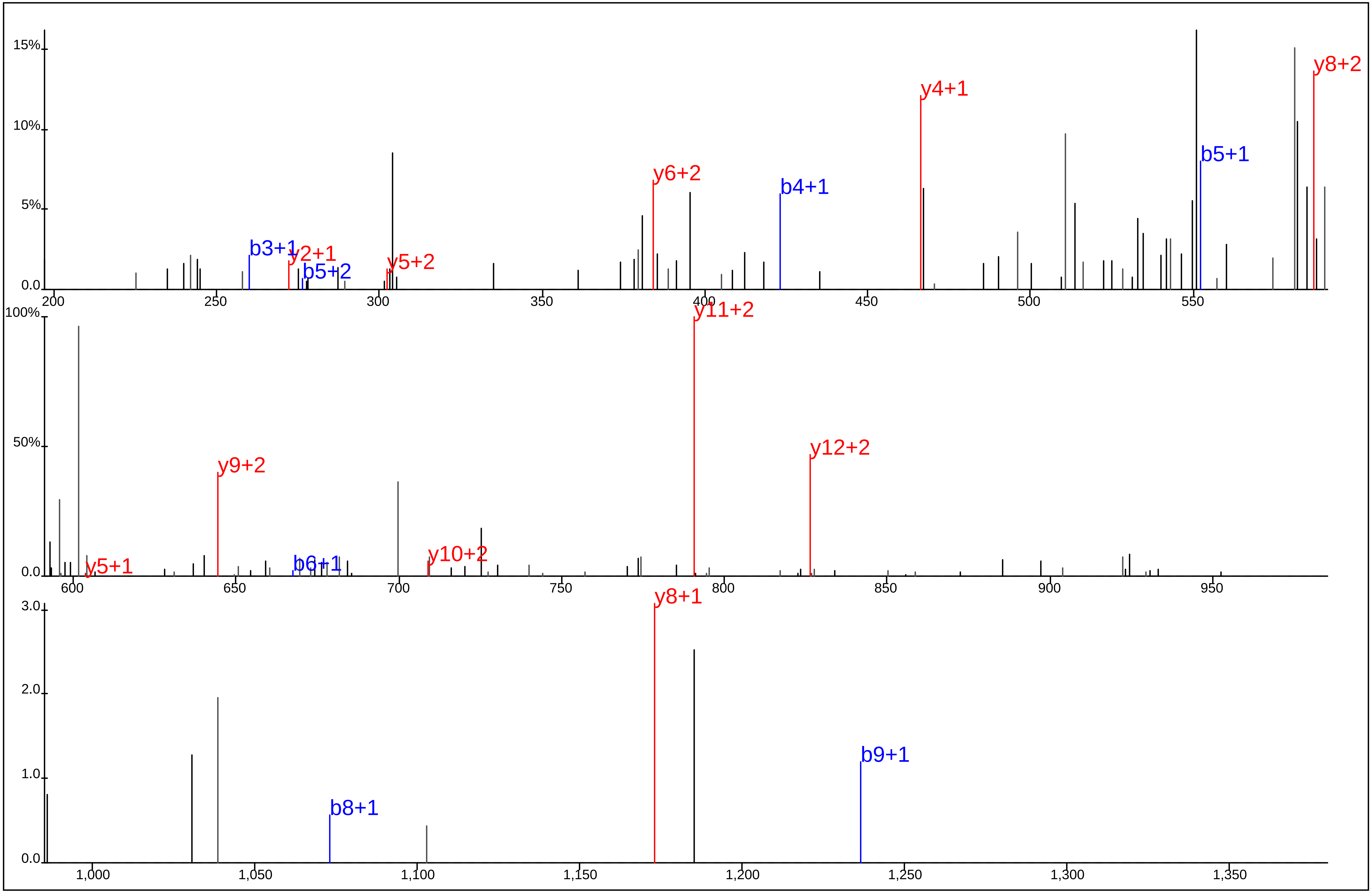

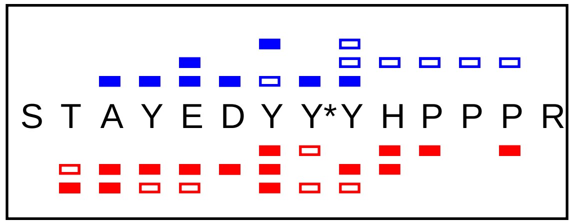

Protein Name: hnRNP H3
gi Accession: 5031755
Precursor Mass: 613.5883+3
Delta Mass: 4.501 ppm
XCorr: 13.04

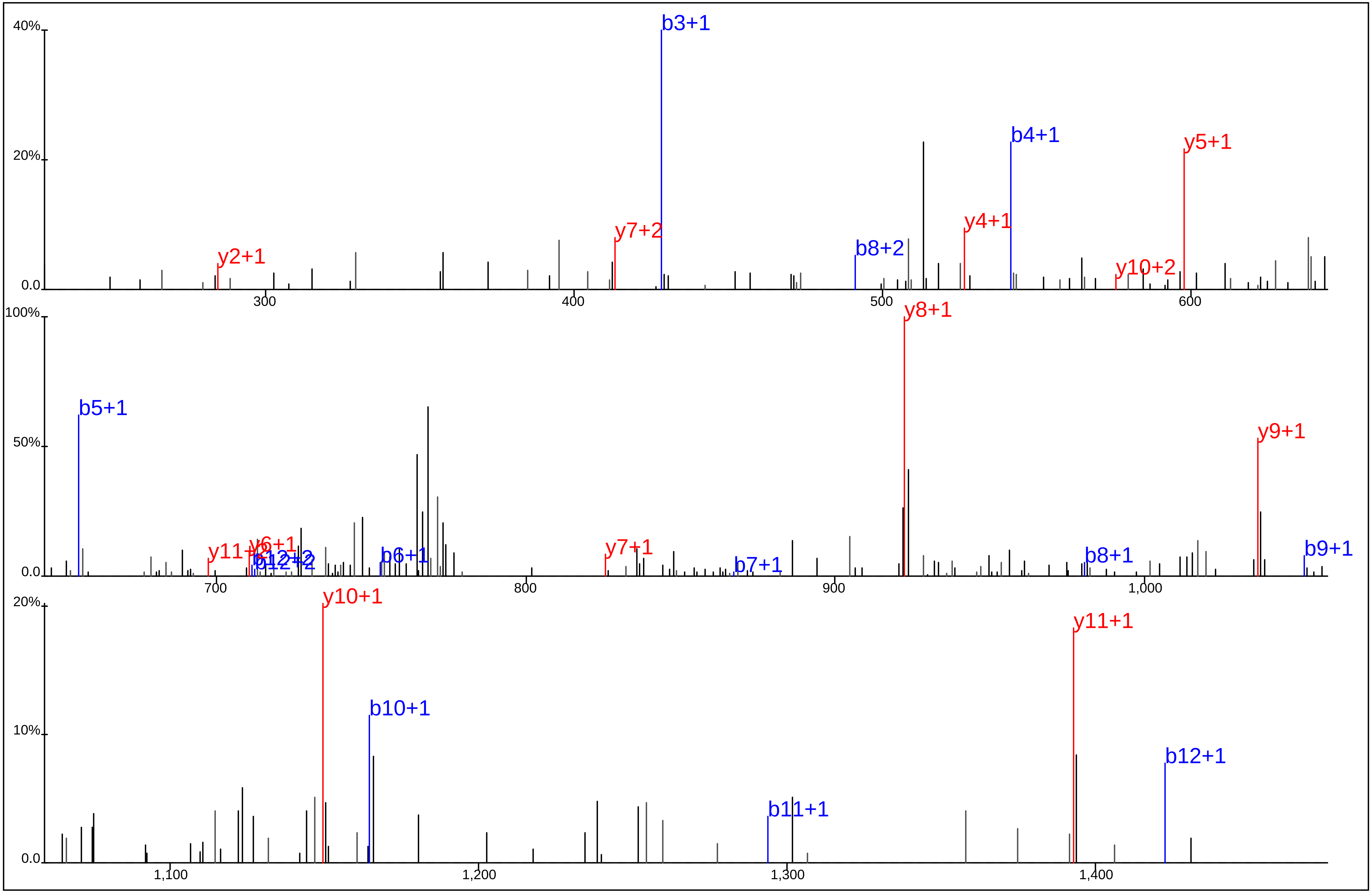

L A Y*I N P D L A L E E K#

Protein Name:	HOP
gi Accession:	5803181
Precursor Mass:	788.8940+2
Delta Mass:	3.664 ppm
XCorr:	41.66

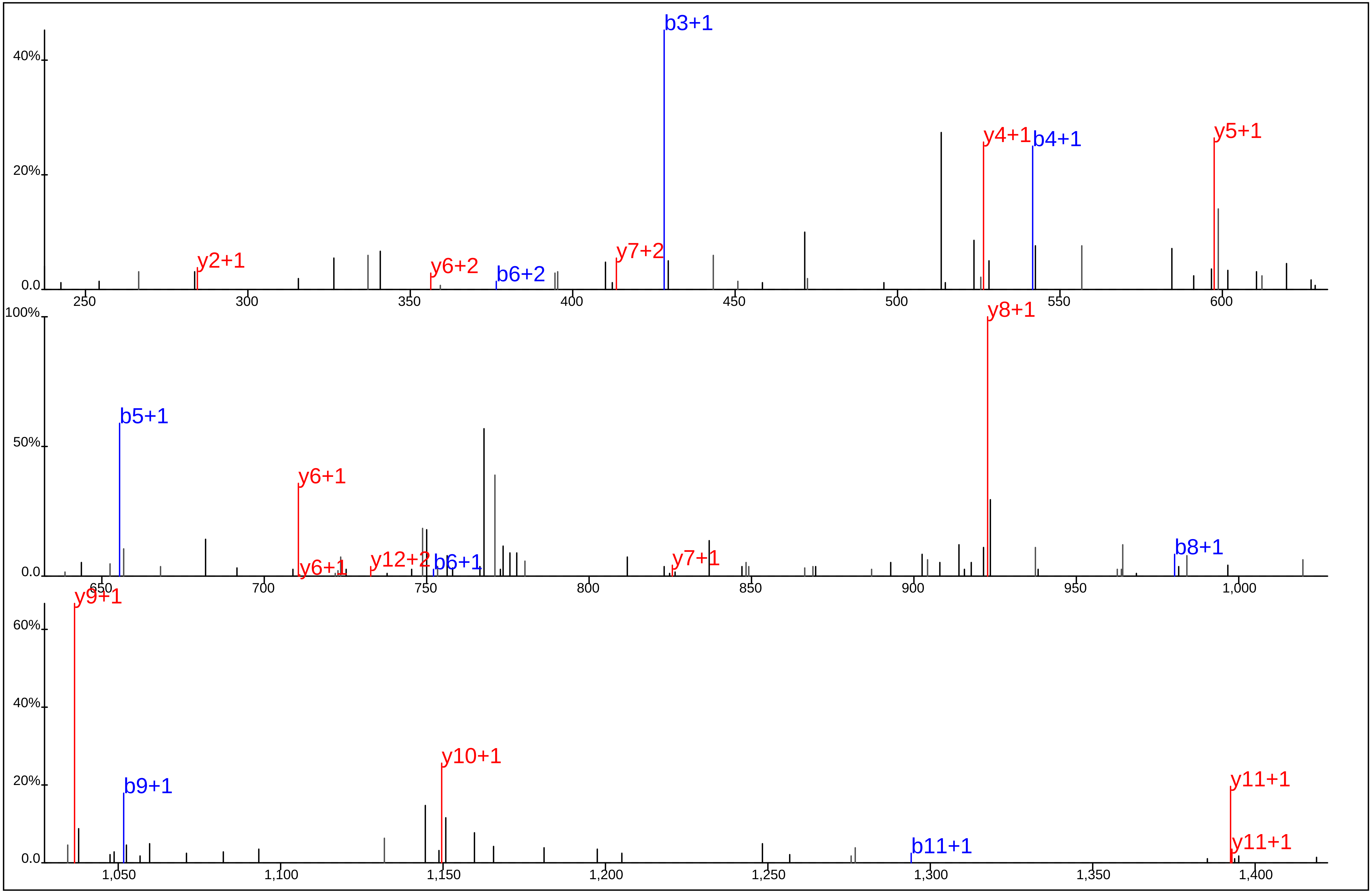

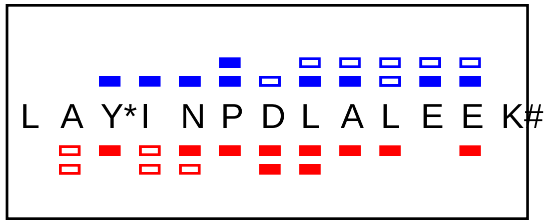

Protein Name:	HOP
gi Accession:	5803181
Precursor Mass:	788.8946+2
Delta Mass:	4.438 ppm
XCorr:	48.39

515 - HOP_3448416.pdf

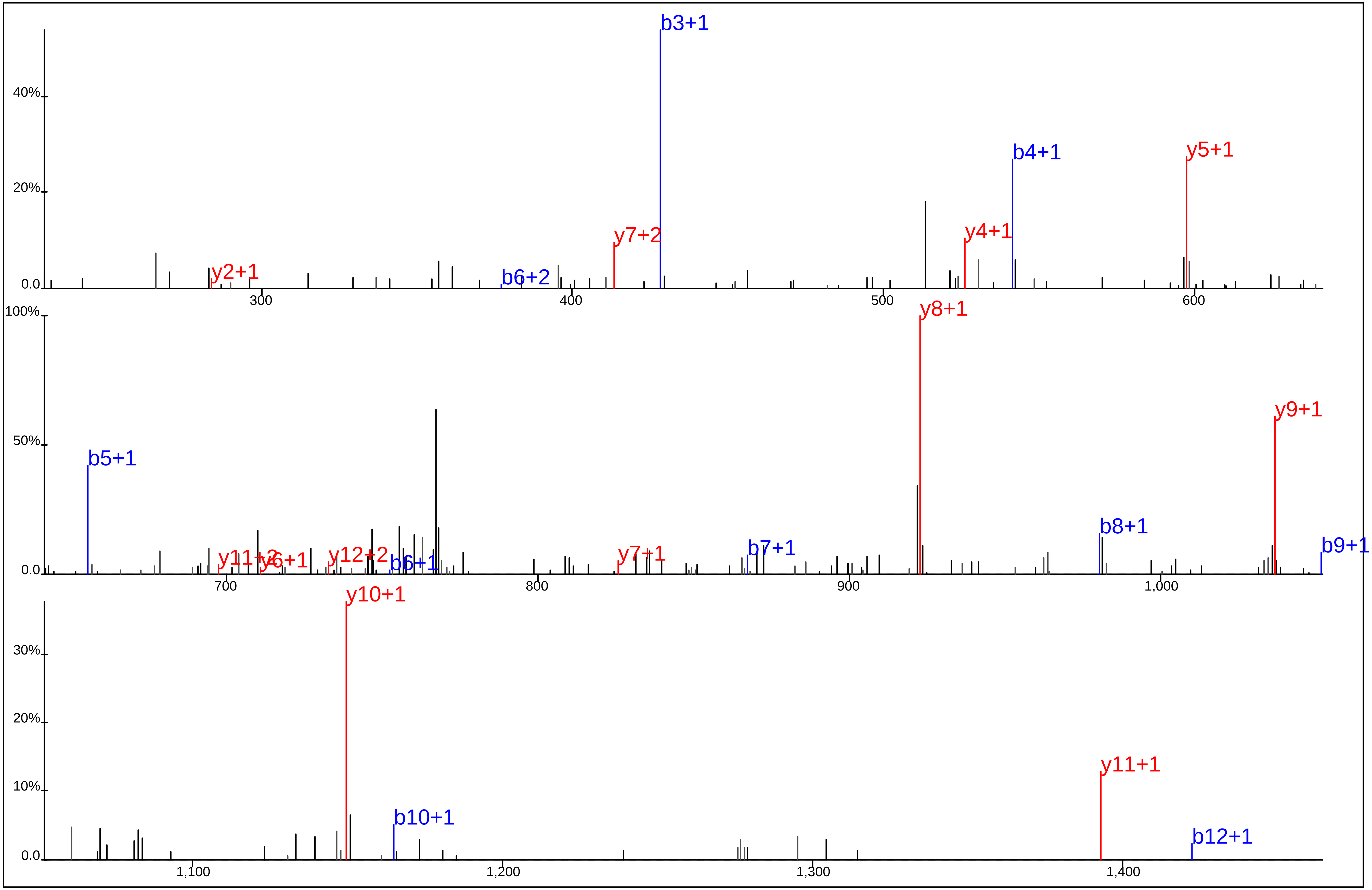

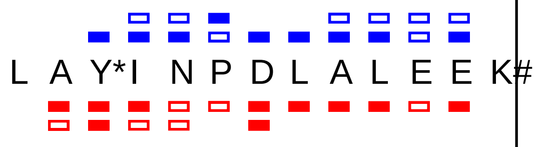

Protein Name:	HOP
gi Accession:	5803181
Precursor Mass:	788.8952+2
Delta Mass:	5.212 ppm
XCorr:	48.71

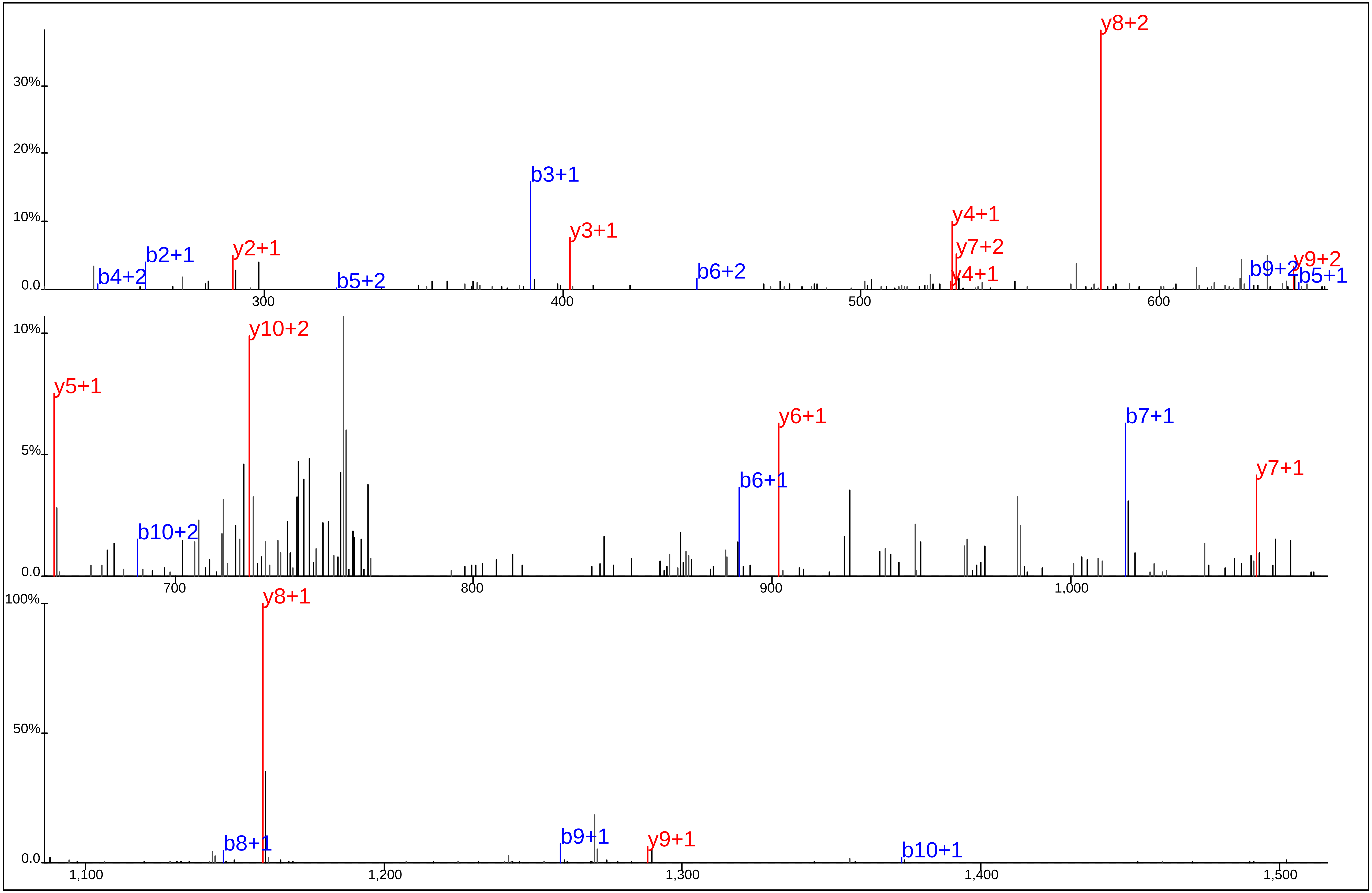

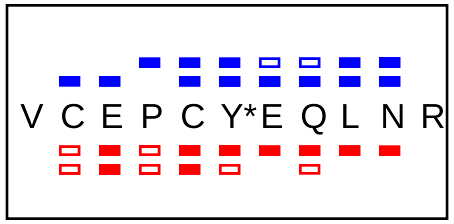

Protein Name: Hrs
gi Accession: 4758528
Precursor Mass: 774.3078+2
Delta Mass: 4.199 ppm
XCorr: 56.14

517 - Hrs_3433929.pdf

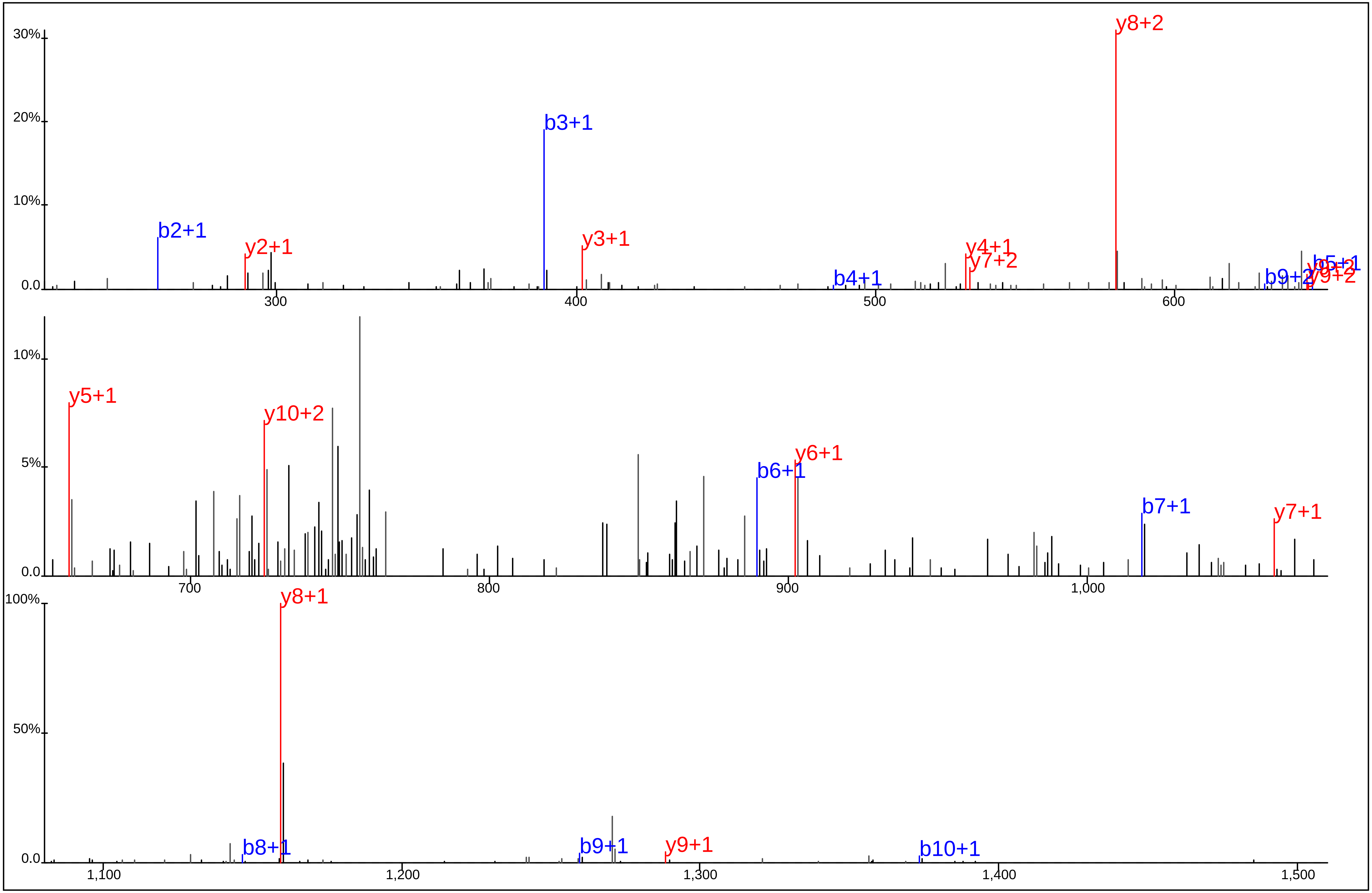

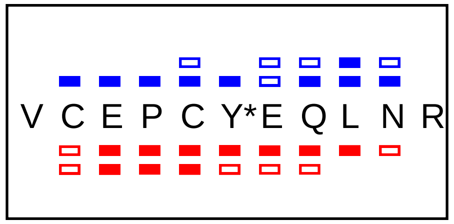

Protein Name: Hrs
gi Accession: 4758528
Precursor Mass: 774.3096+2
Delta Mass: 6.486 ppm
XCorr: 45.98

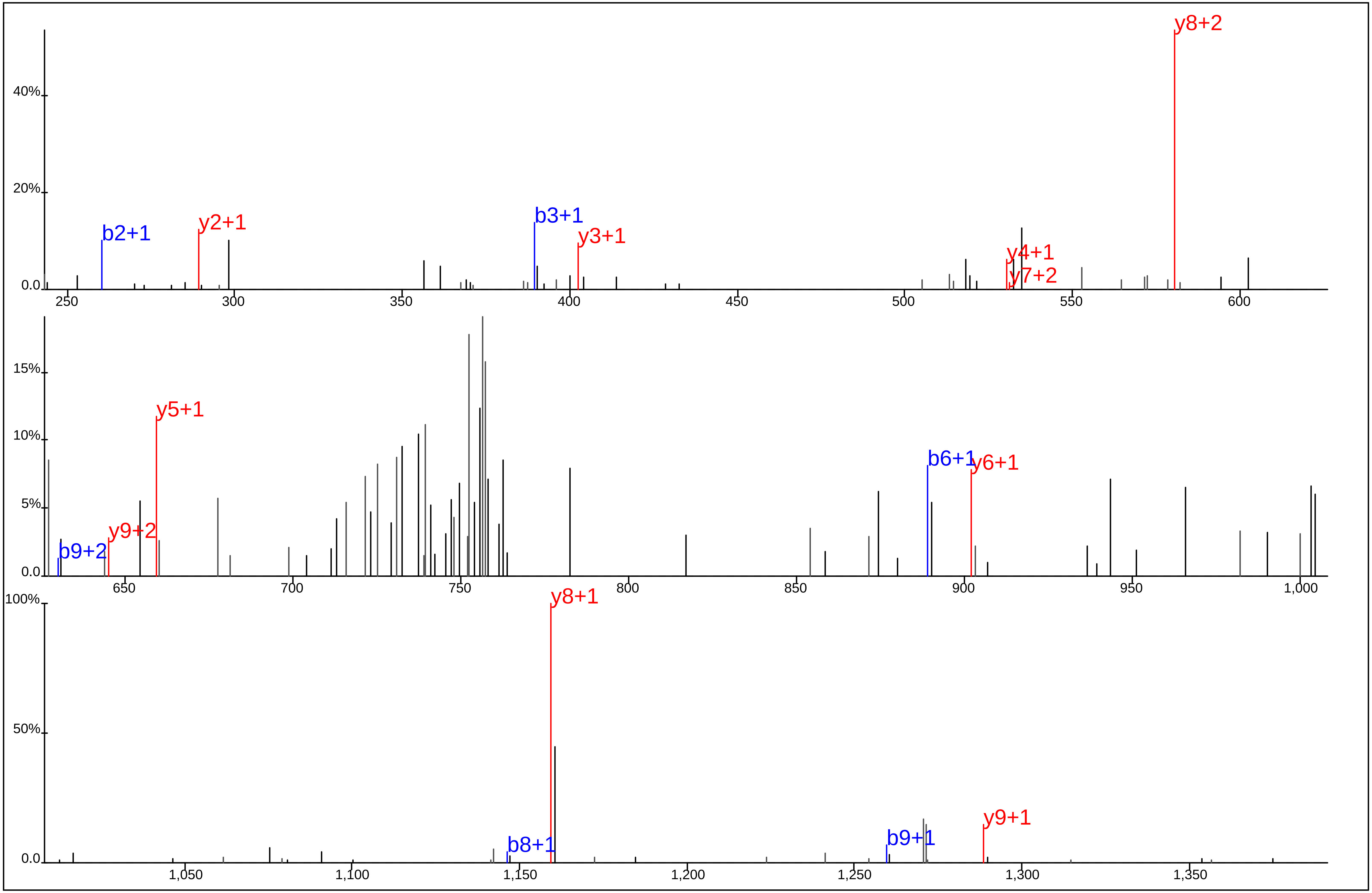

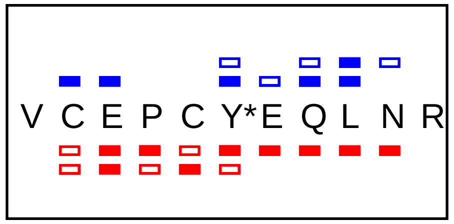

Protein Name: Hrs
gi Accession: 4758528
Precursor Mass: 774.3051+2
Delta Mass: 0.651 ppm
XCorr: 27.09

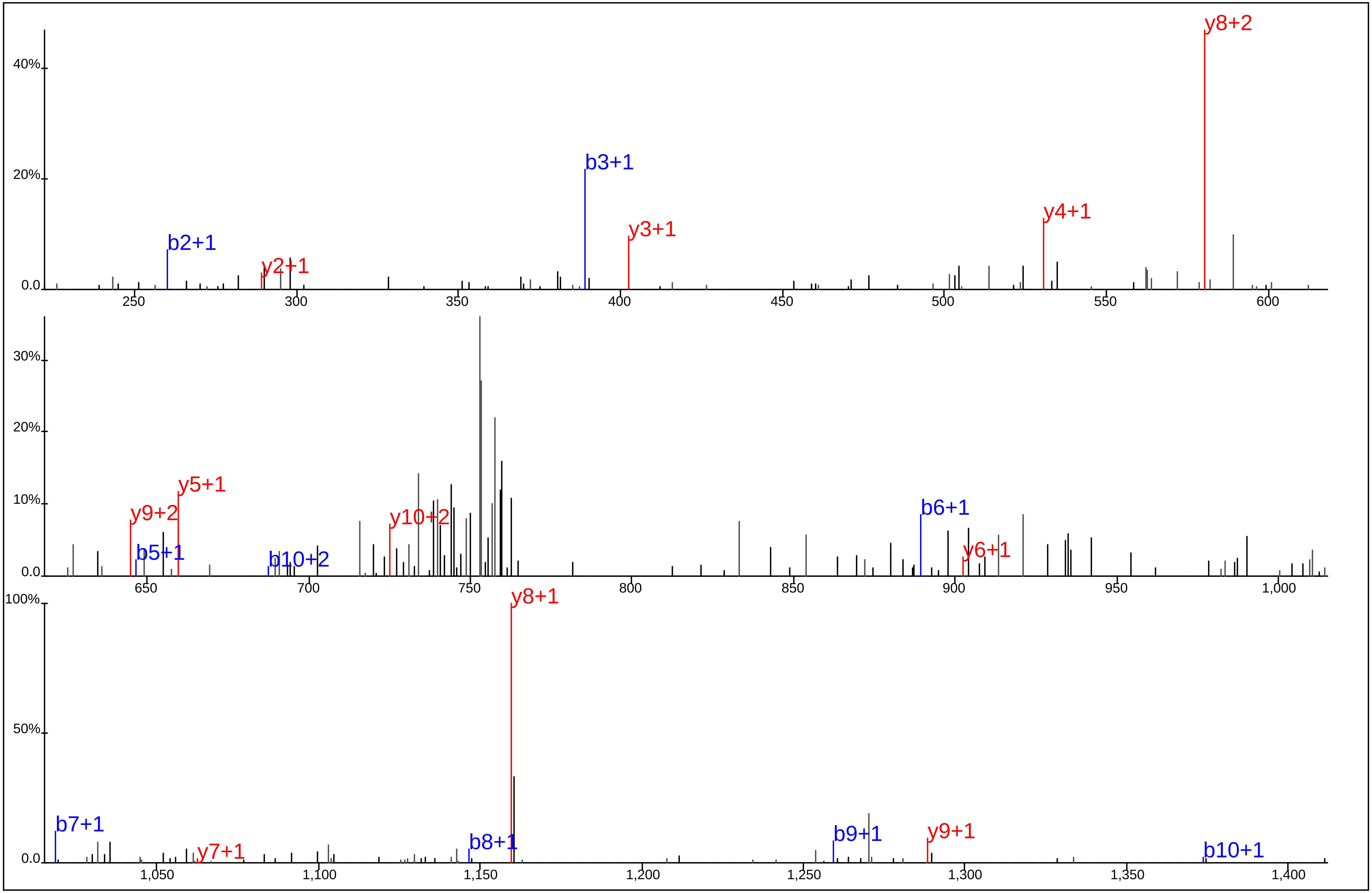

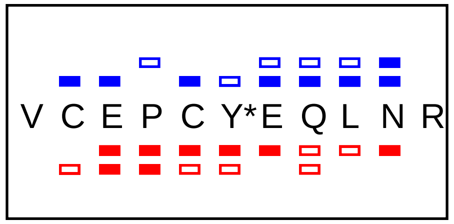

Protein Name: Hrs
gi Accession: 4758528
Precursor Mass: 774.3082+2
Delta Mass: 4.677 ppm
XCorr: 27.5

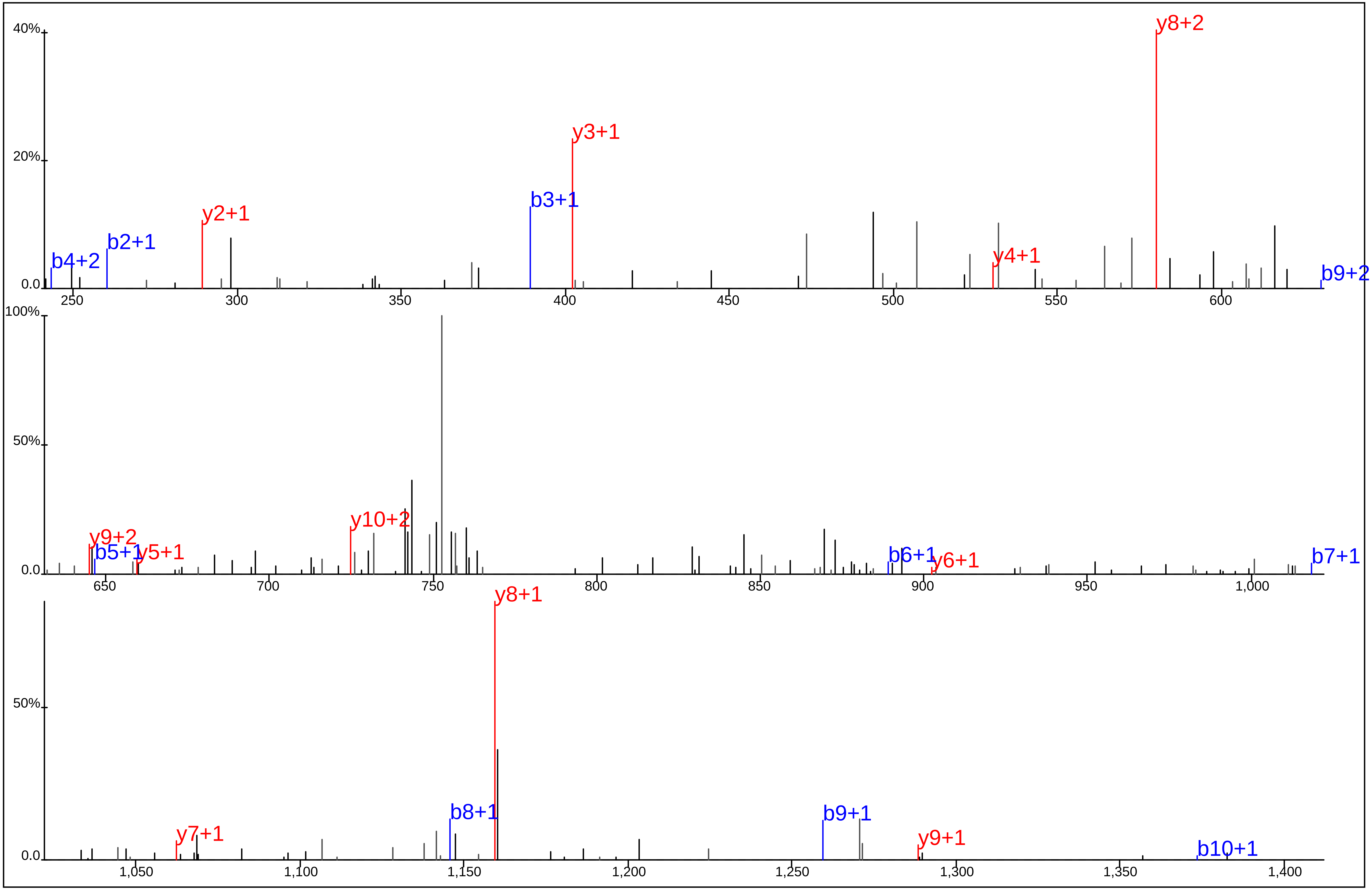

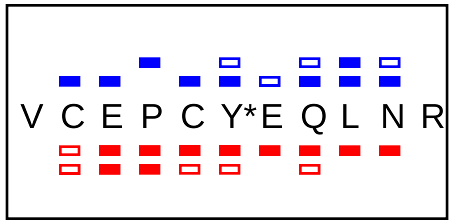

Protein Name: Hrs
gi Accession: 4758528
Precursor Mass: 774.3052+2
Delta Mass: 0.806 ppm
XCorr: 18.08

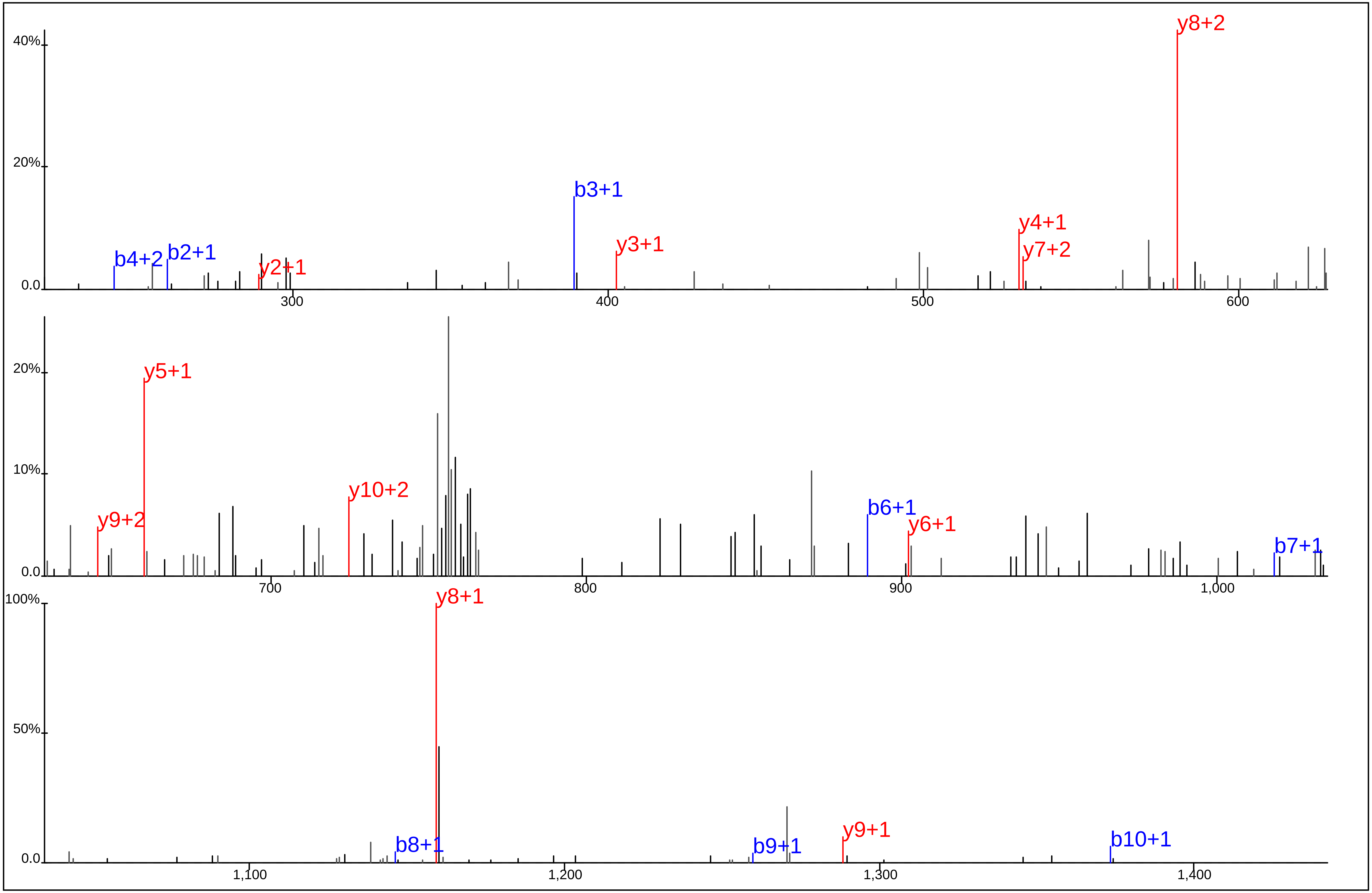

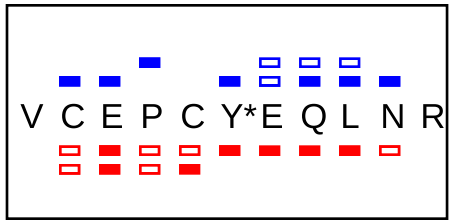

Protein Name: Hrs
gi Accession: 4758528
Precursor Mass: 774.3076+2
Delta Mass: 3.889 ppm
XCorr: 20.73

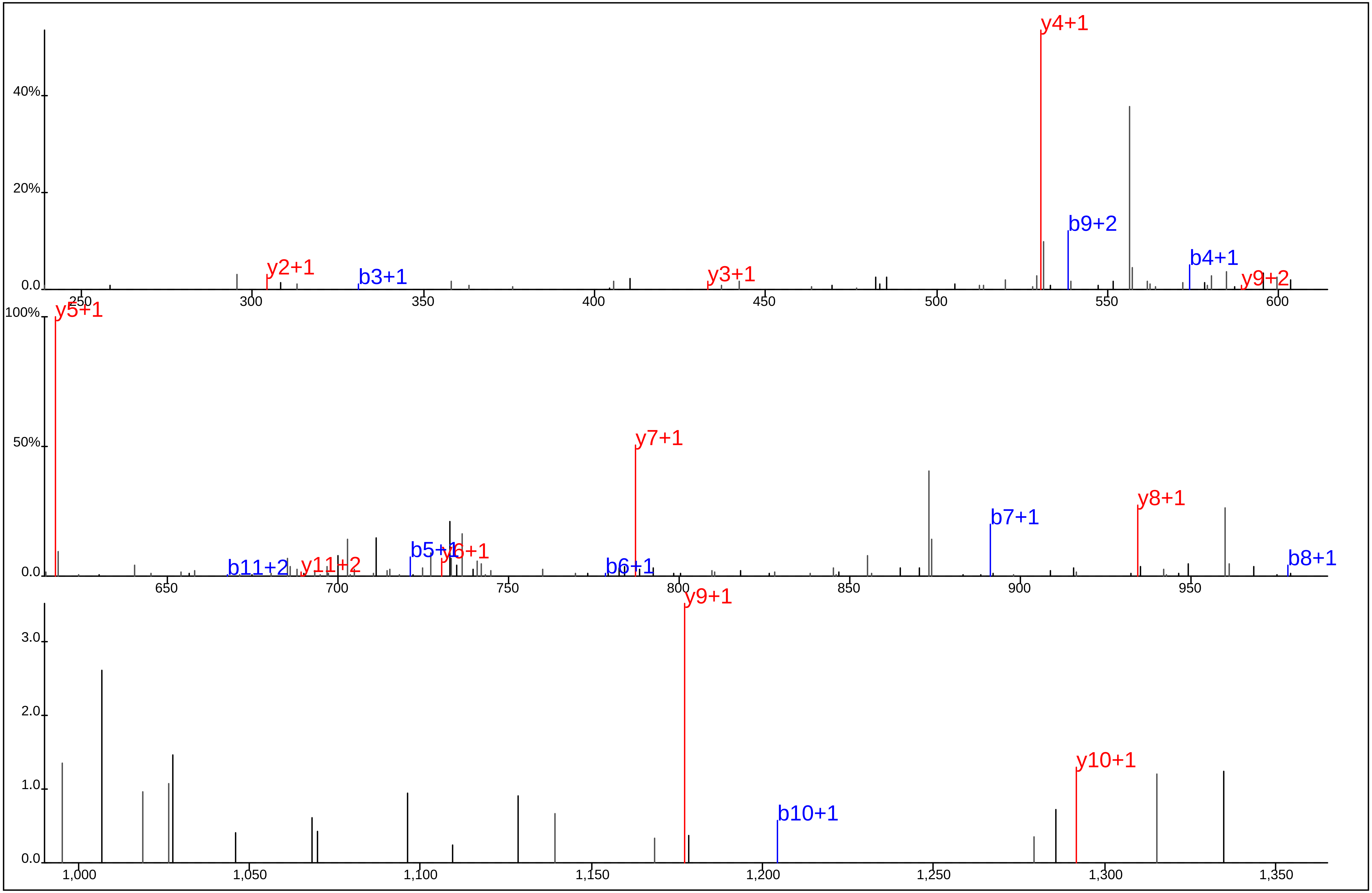

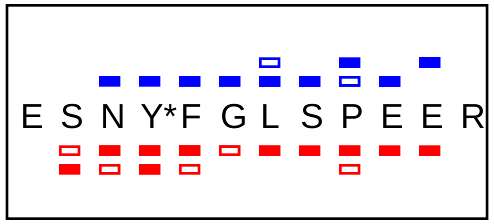

Protein Name: HSHIN1
gi Accession: 16552046
Precursor Mass: 754.3130+2
Delta Mass: 5.743 ppm
XCorr: 43.45

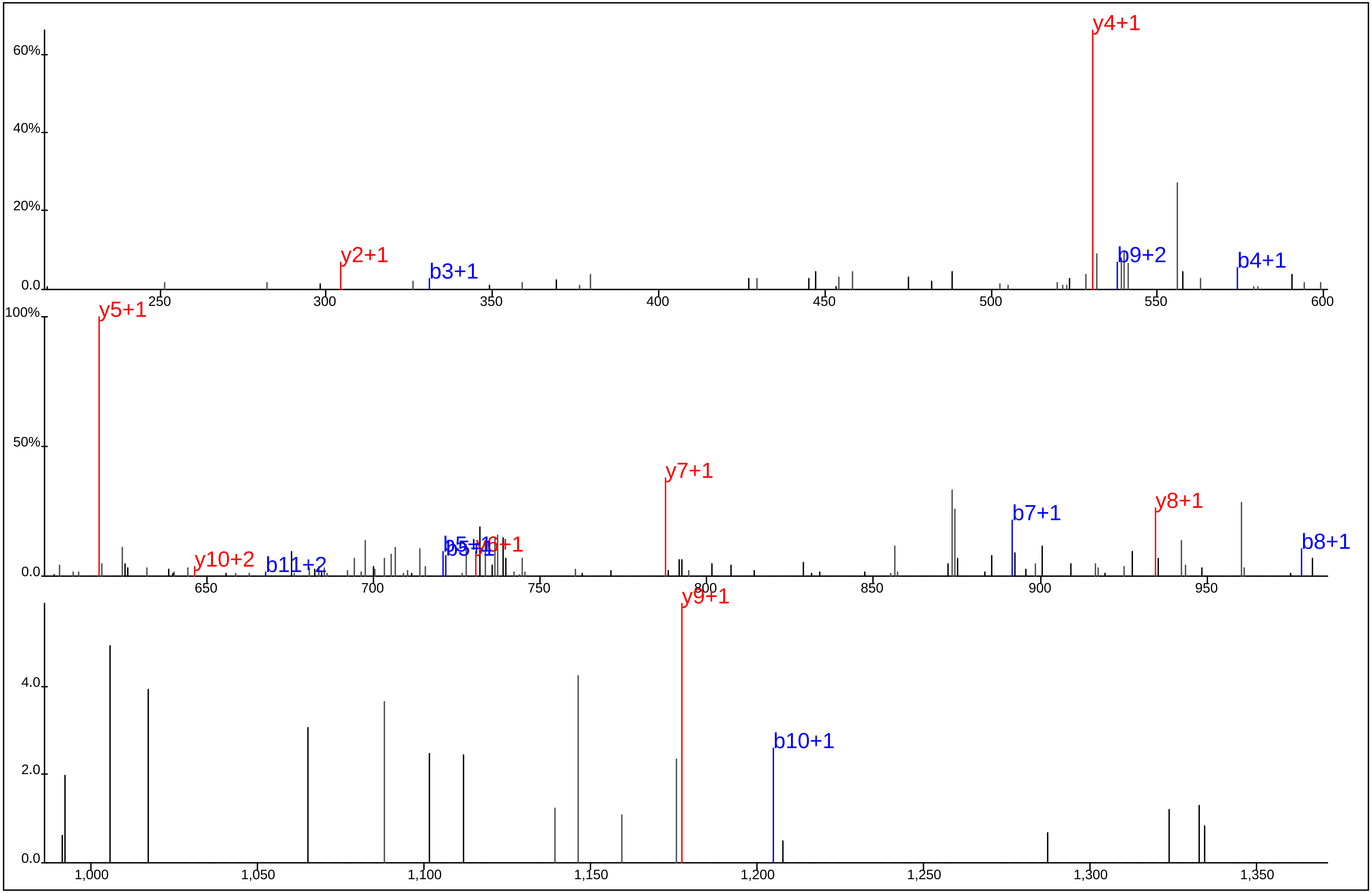

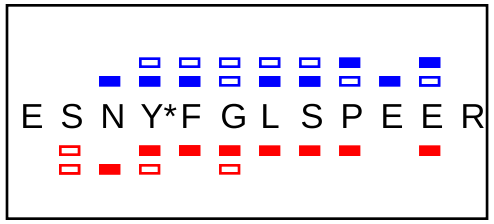

Protein Name: HSHIN1
gi Accession: 16552046
Precursor Mass: 754.3110+2
Delta Mass: 3.070 ppm
XCorr: 25.47

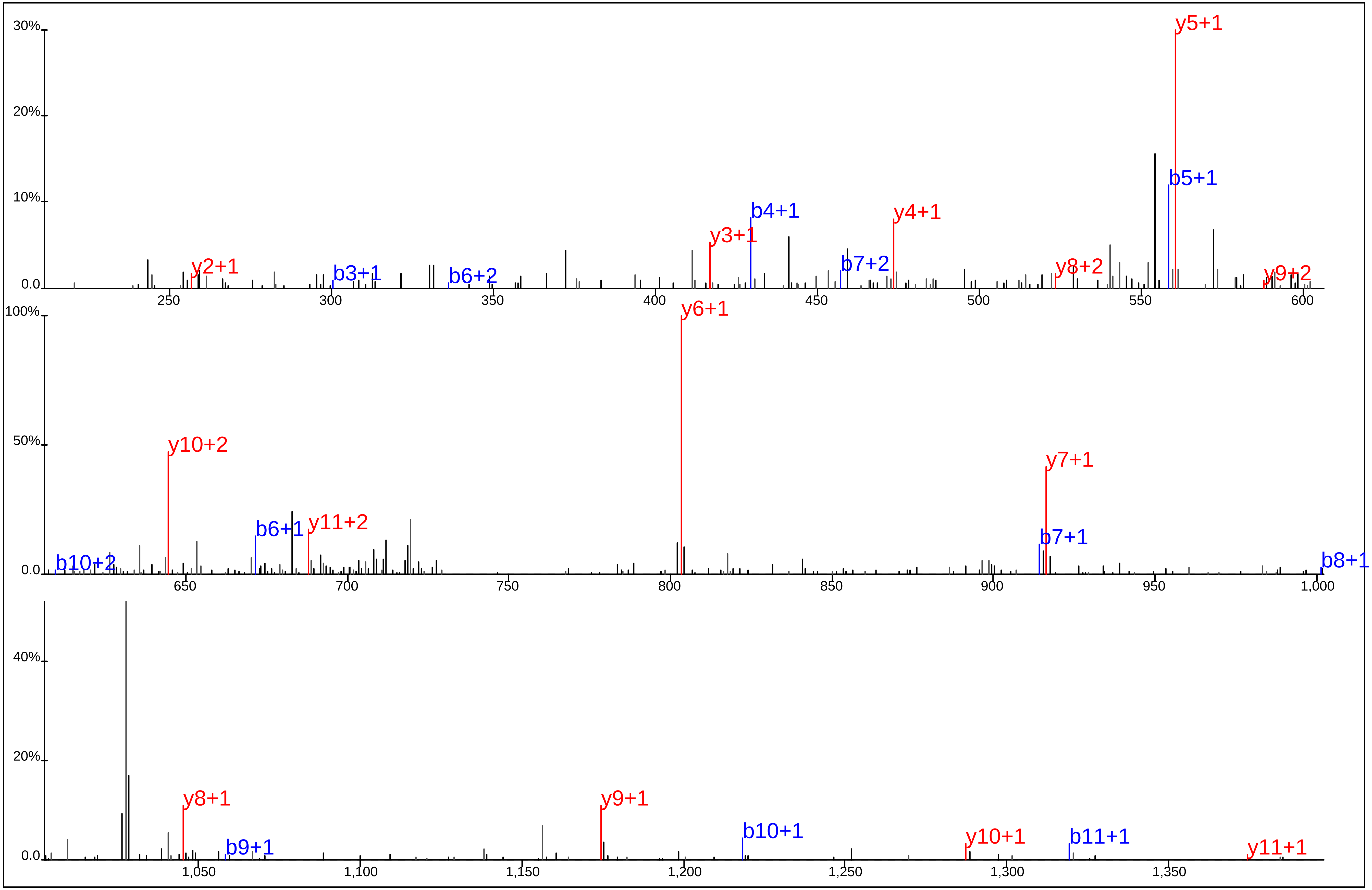

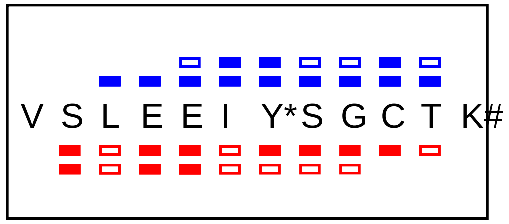

Protein Name: HSP40

gi Accession: 5453690

Precursor Mass: 737.3297+2

Delta Mass: 6.656 ppm

XCorr: 53.15

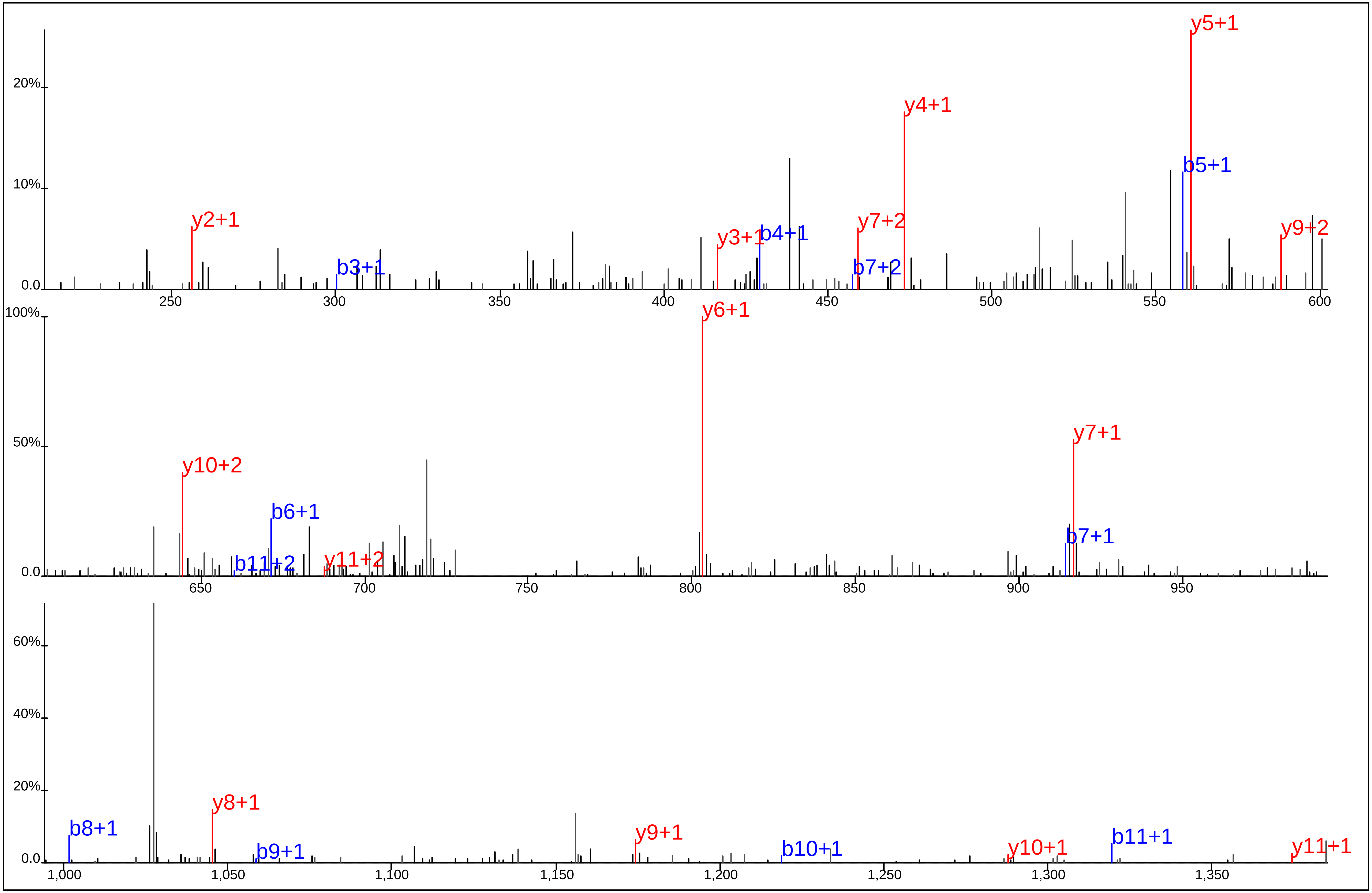

V S L E E I Y*S G C T K#

Protein Name: HSP40
gi Accession: 5453690
Precursor Mass: 737.3281+2
Delta Mass: 4.498 ppm
XCorr: 54.6

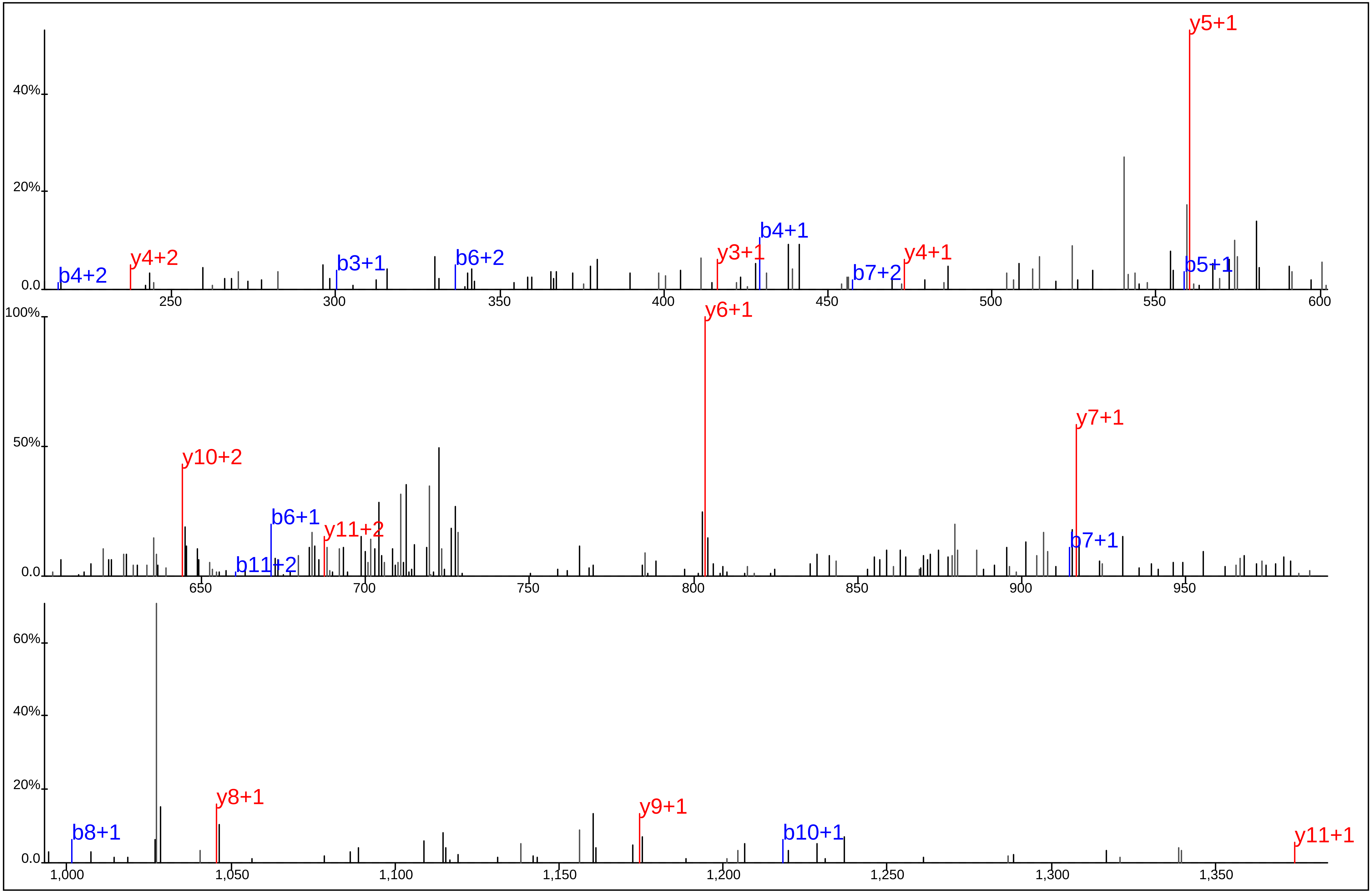

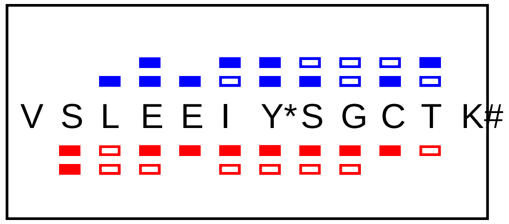

Protein Name: HSP40
gi Accession: 5453690
Precursor Mass: 737.3303+2
Delta Mass: 7.483 ppm
XCorr: 29.57

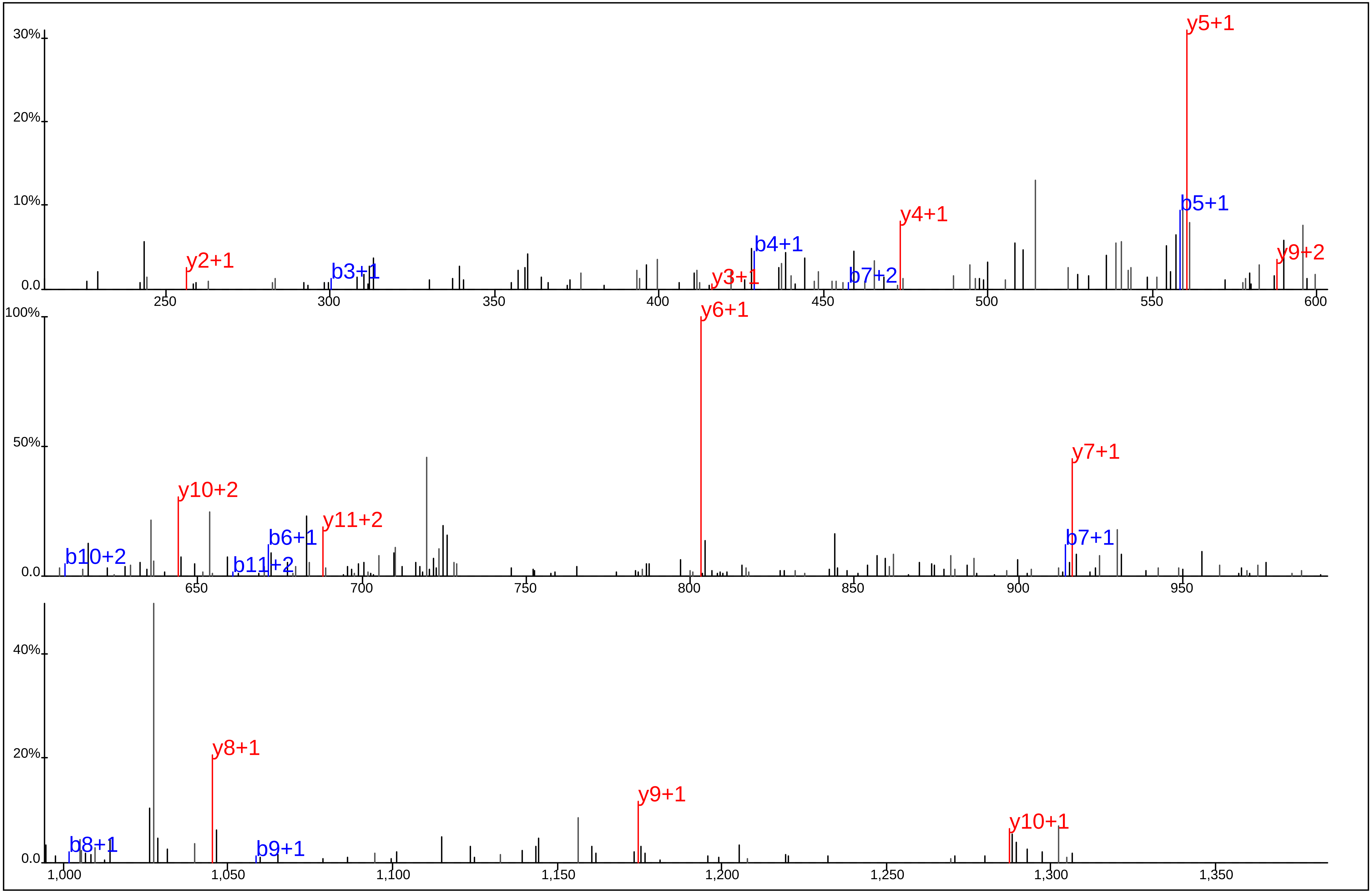

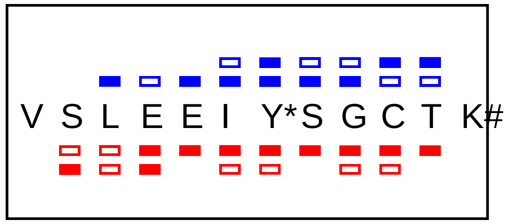

Protein Name: HSP40
gi Accession: 5453690
Precursor Mass: 737.3285+2
Delta Mass: 5.081 ppm
XCorr: 35.2

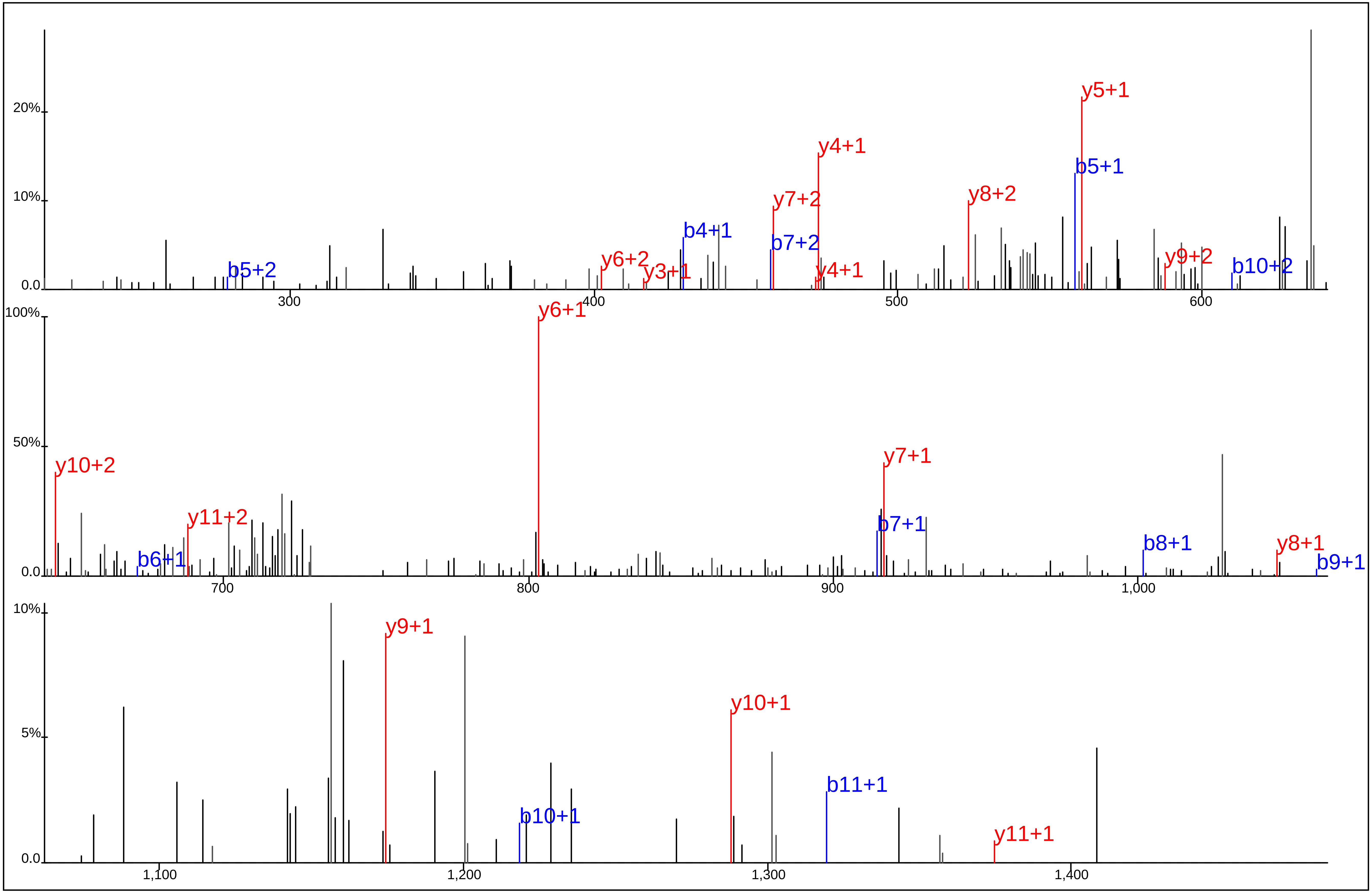

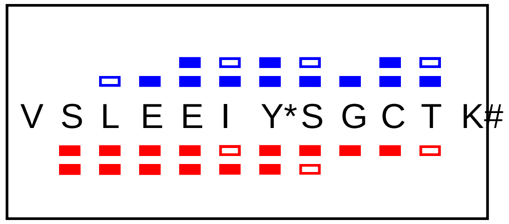

Protein Name: HSP40
gi Accession: 5453690
Precursor Mass: 737.3279+2
Delta Mass: 4.253 ppm
XCorr: 47.7

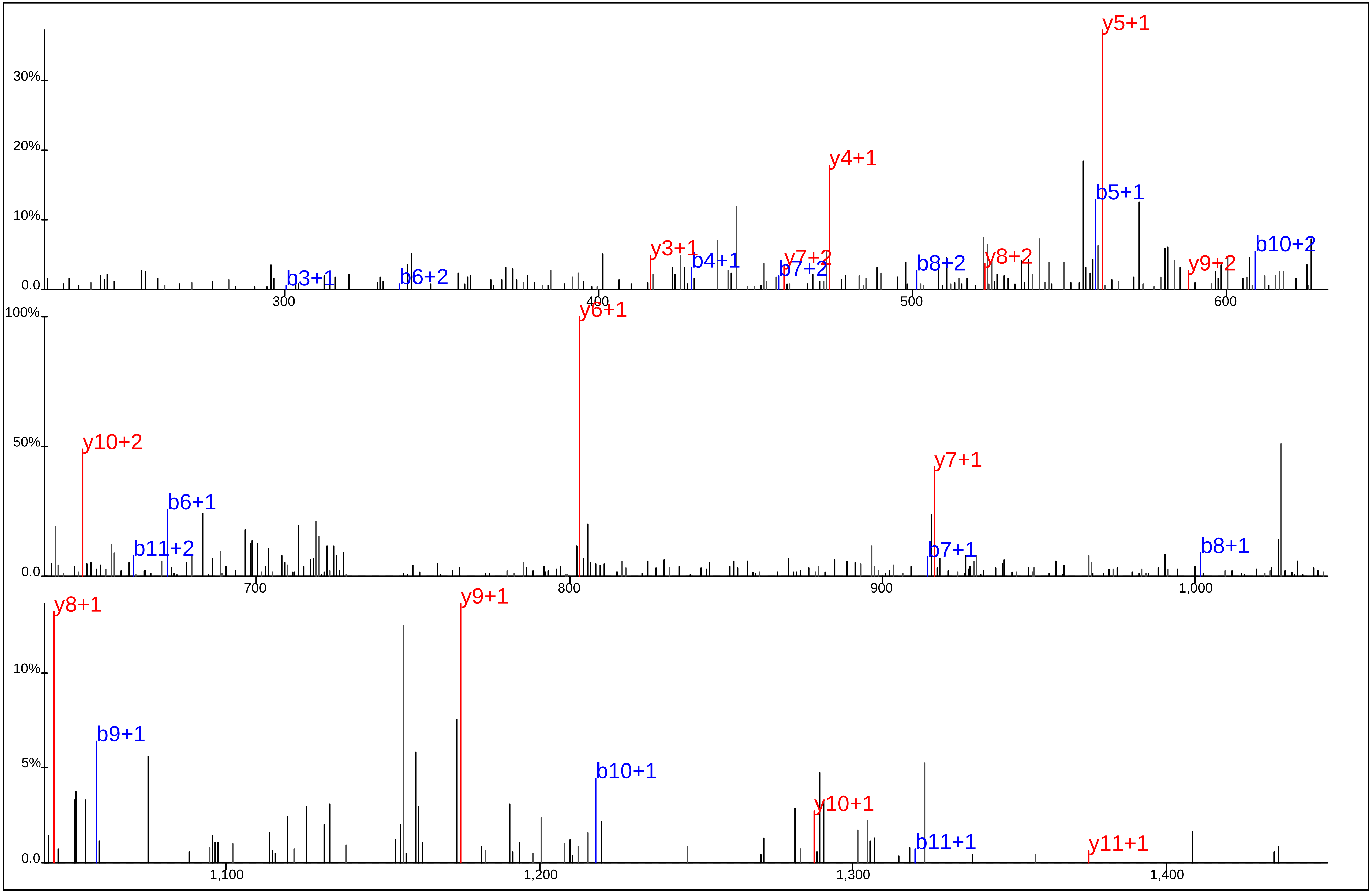

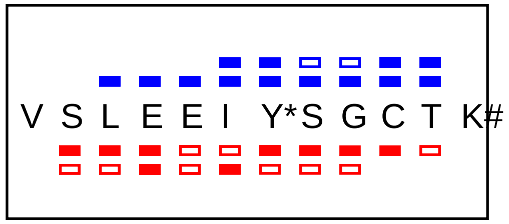

Protein Name: HSP40
gi Accession: 5453690
Precursor Mass: 737.3278+2
Delta Mass: 4.165 ppm
XCorr: 34.91

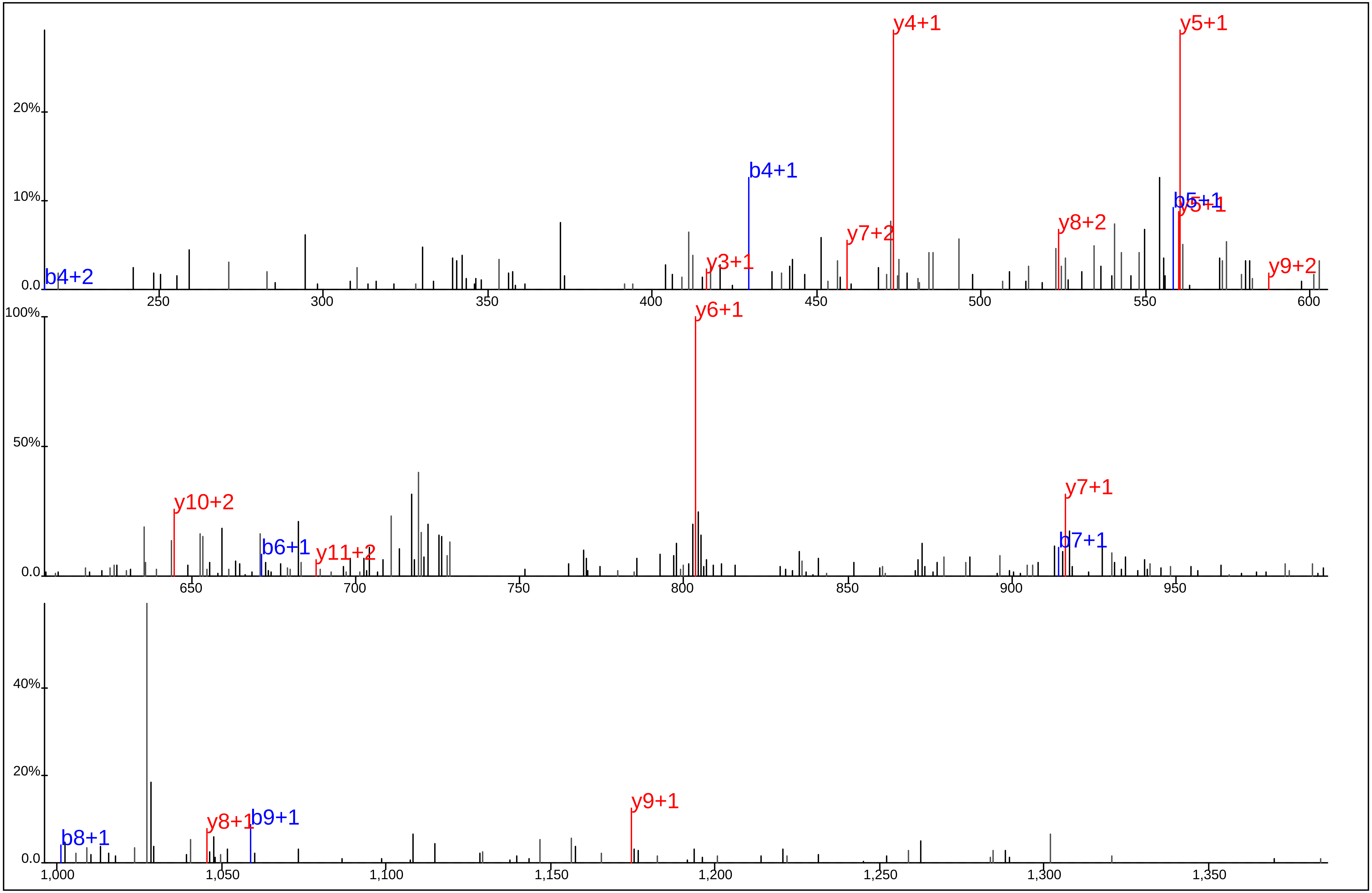

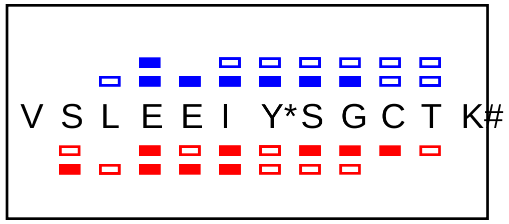

Protein Name: HSP40
gi Accession: 5453690
Precursor Mass: 737.3281+2
Delta Mass: 4.579 ppm
XCorr: 24.28

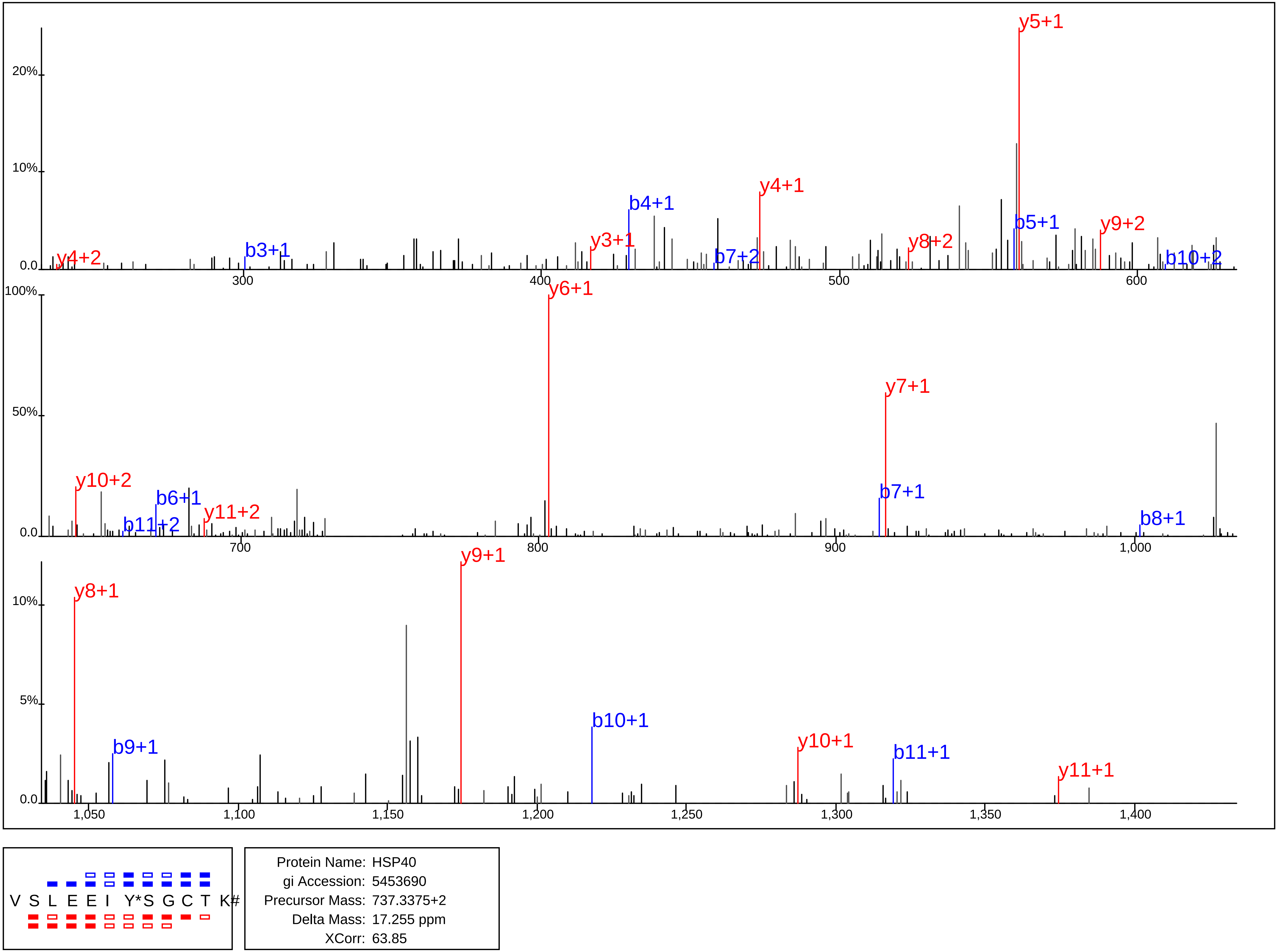

Protein Name:	HSP40
gi Accession:	5453690
Precursor Mass:	737.3375+2
Delta Mass:	17.255 ppm
XCorr:	63.85

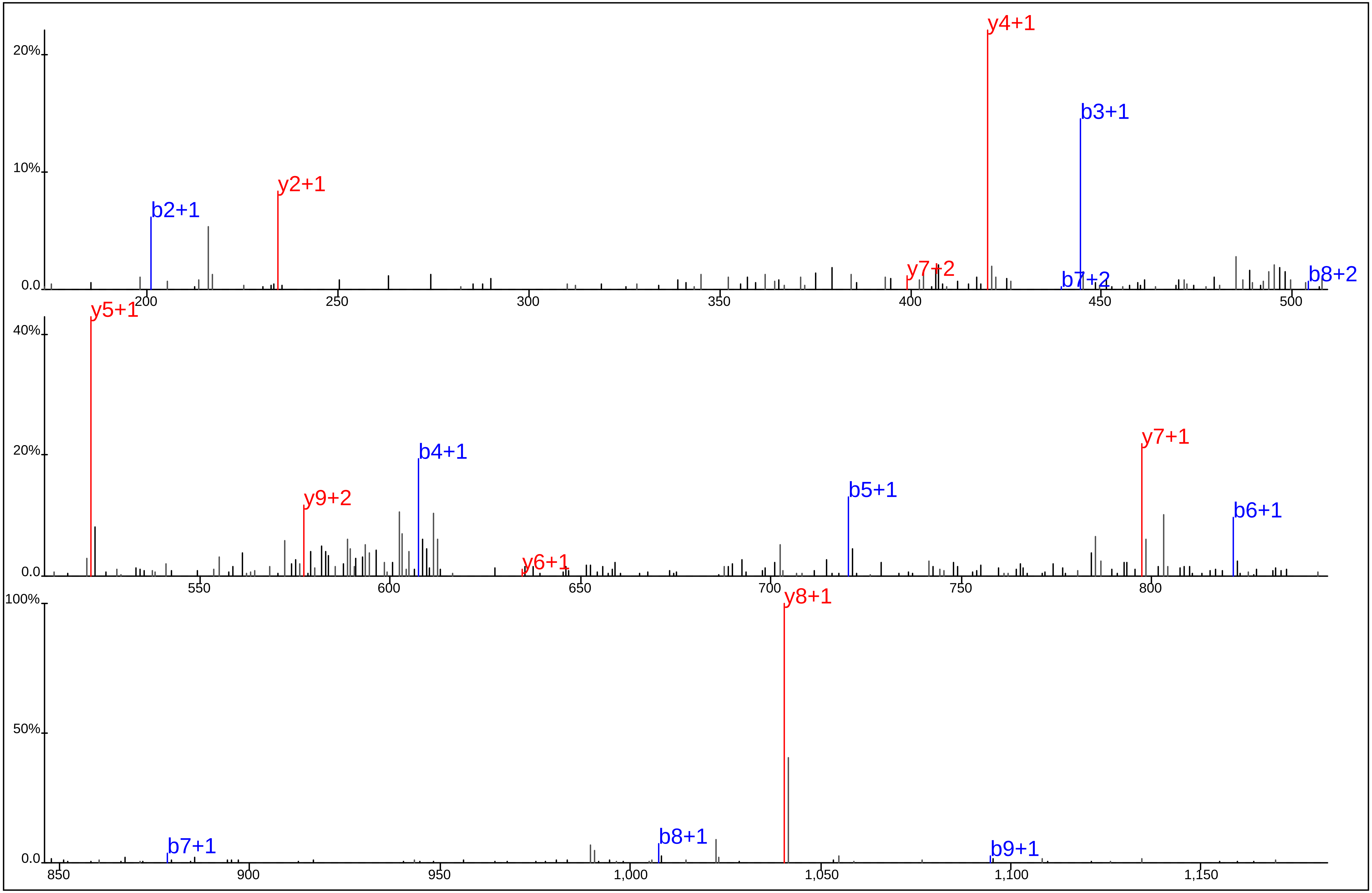

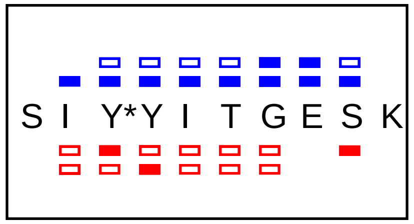

Protein Name: HSP90B
gi Accession: 20149594
Precursor Mass: 620.7792+2
Delta Mass: 1.224 ppm
XCorr: 50.67

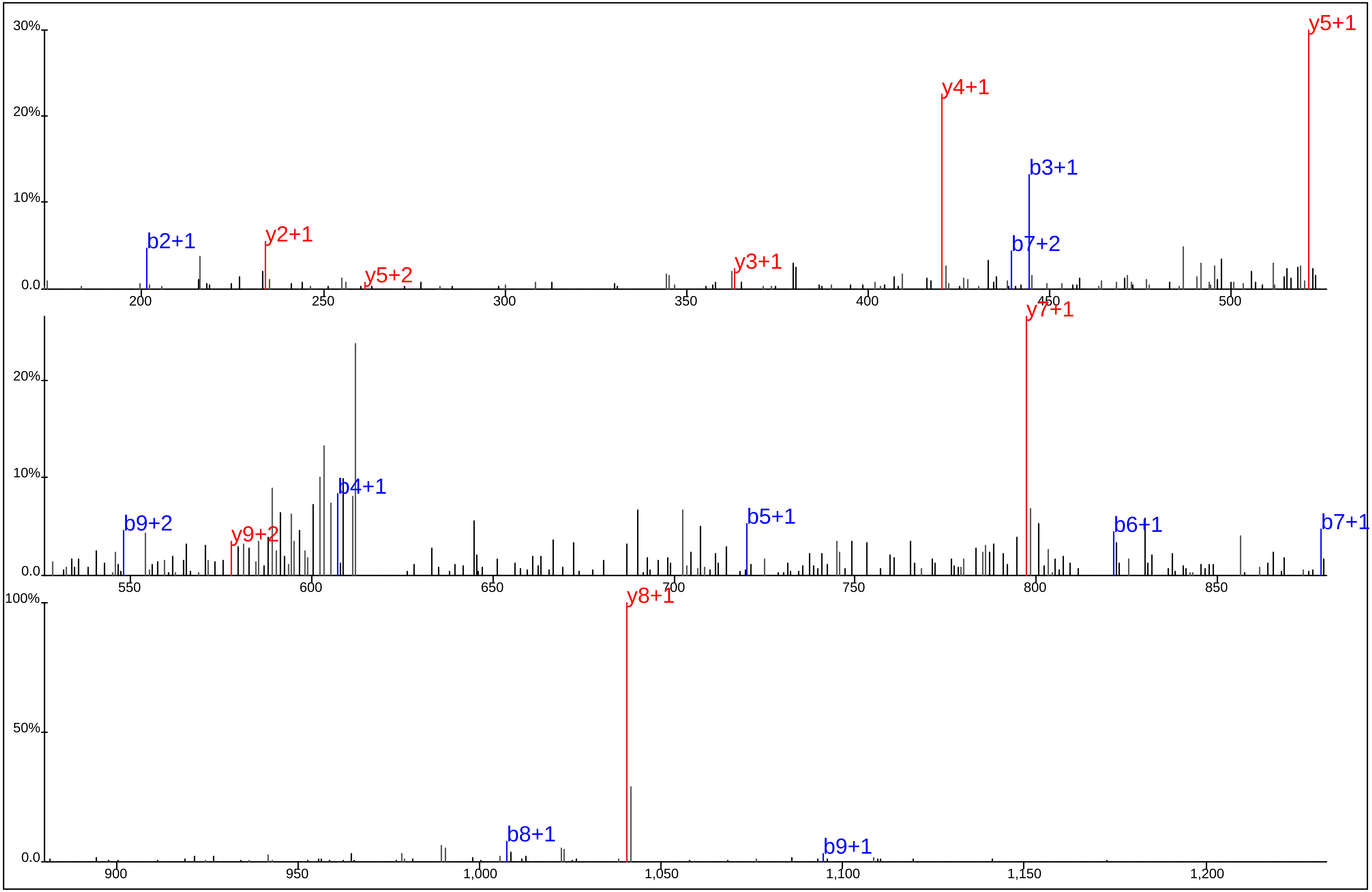

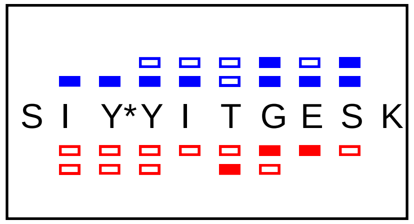

Protein Name: HSP90B
gi Accession: 20149594
Precursor Mass: 620.7800+2
Delta Mass: 2.401 ppm
XCorr: 54.27

534 - HSP90B_3434838.pdf

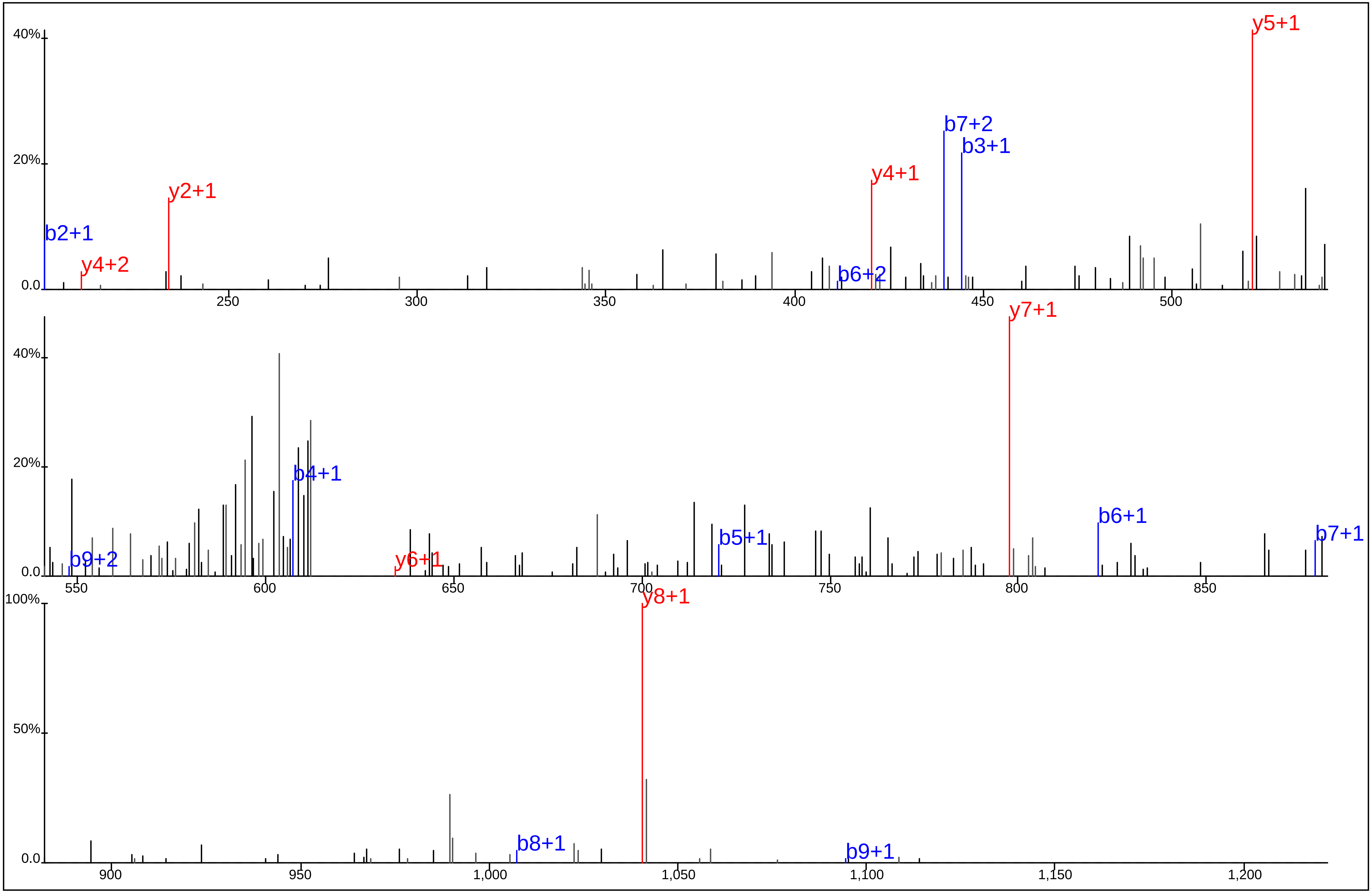

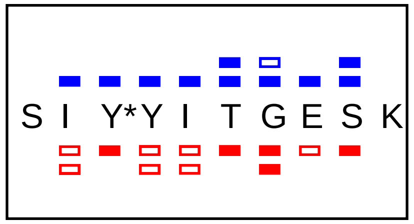

Protein Name:	HSP90B
gi Accession:	20149594
Precursor Mass:	620.7798+2
Delta Mass:	2.207 ppm
XCorr:	18.77

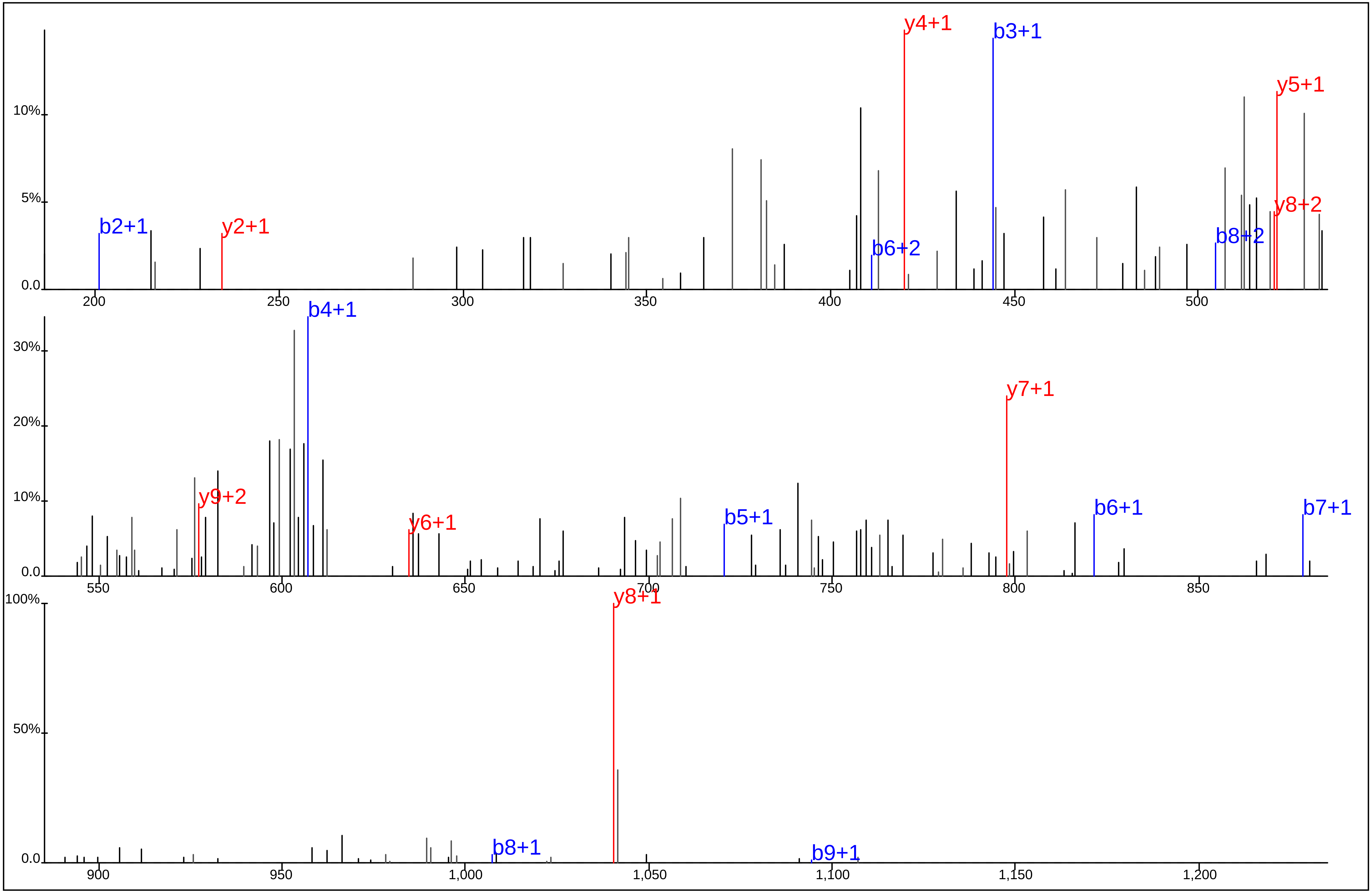

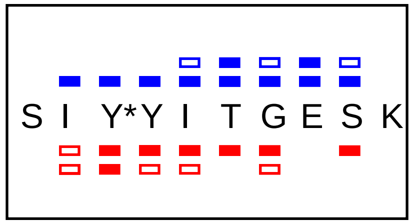

Protein Name: HSP90B
gi Accession: 20149594
Precursor Mass: 620.7740+2
Delta Mass: 7.144 ppm
XCorr: 29.49

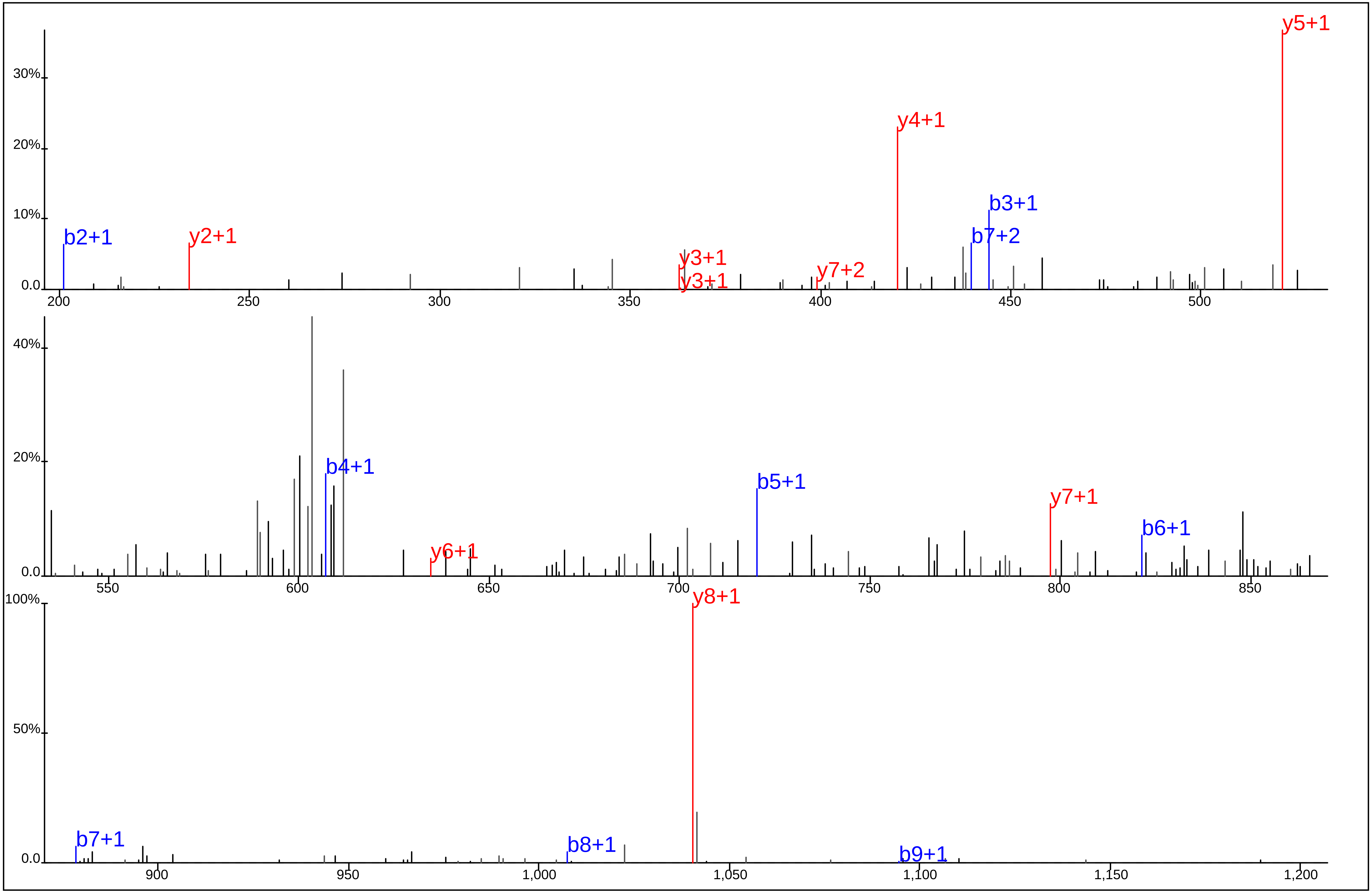

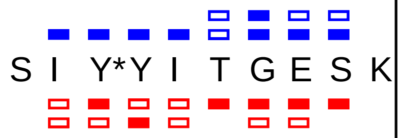

Protein Name: HSP90B
gi Accession: 20149594
Precursor Mass: 620.7787+2
Delta Mass: 0.337 ppm
XCorr: 30.45

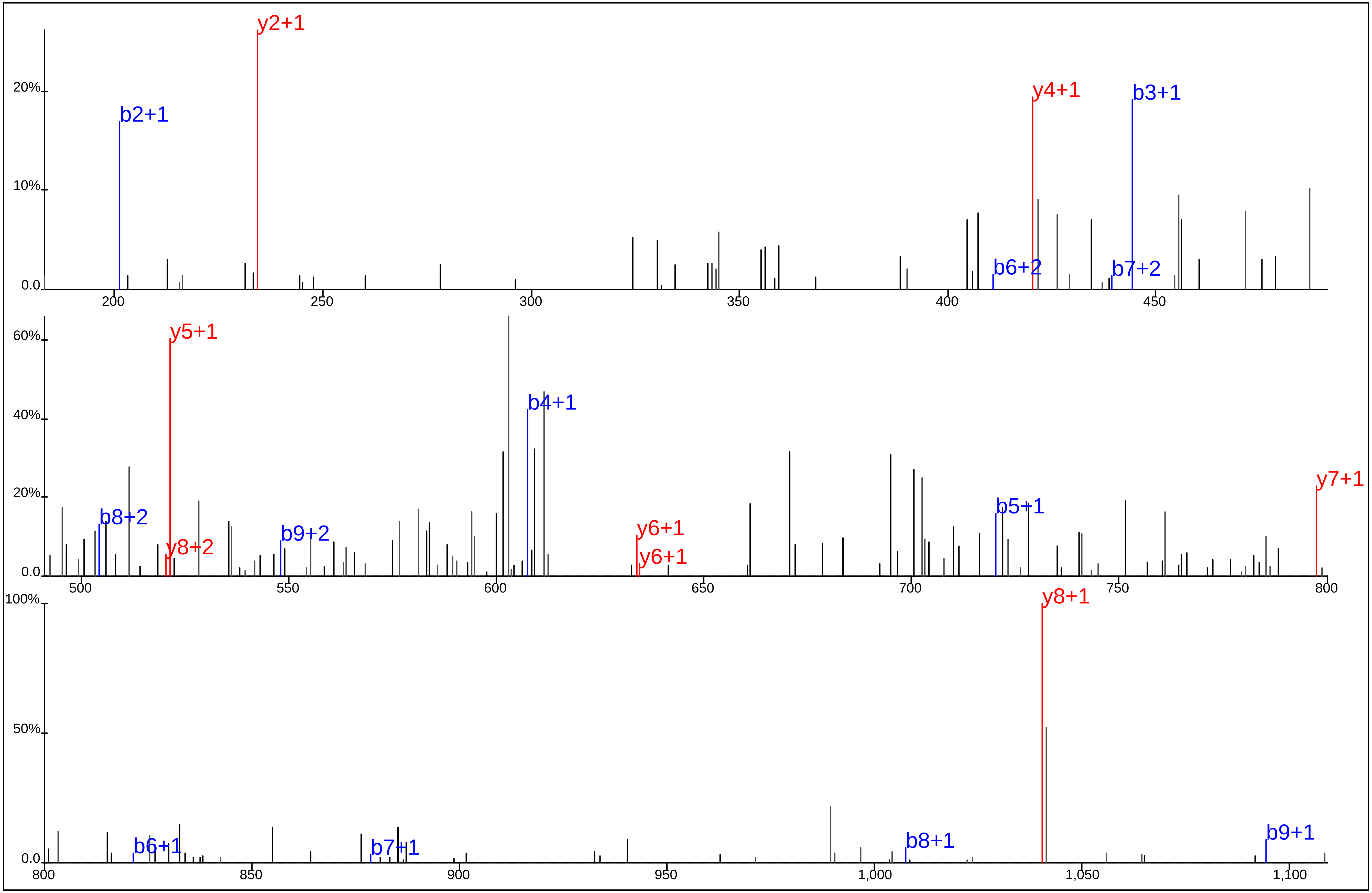

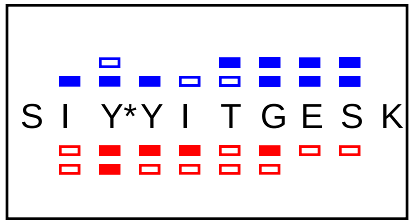

Protein Name: HSP90B
gi Accession: 20149594
Precursor Mass: 620.7789+2
Delta Mass: 0.627 ppm
XCorr: 29.74

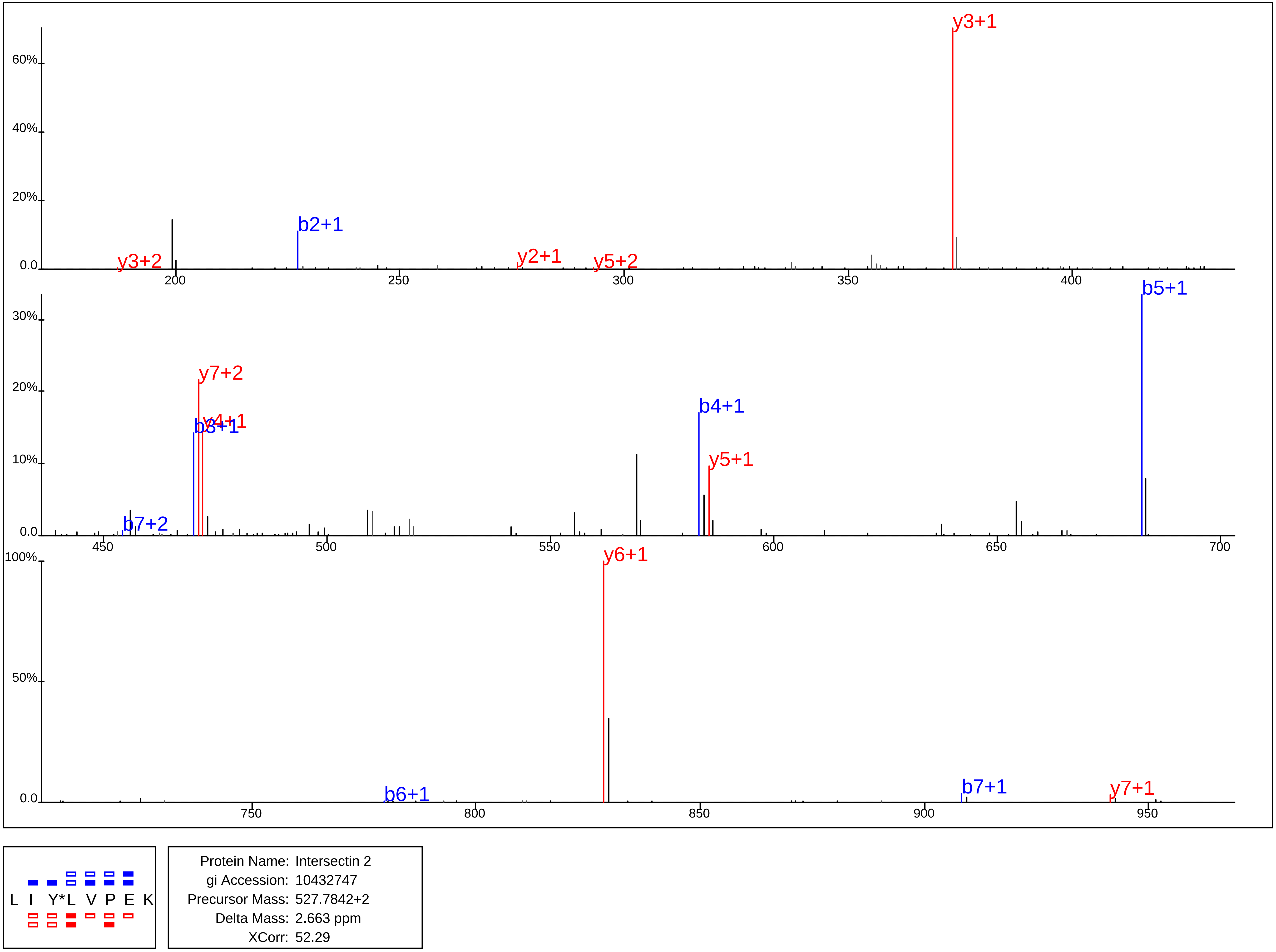

Protein Name: Intersectin 2
gi Accession: 10432747
Precursor Mass: 527.7842+2
Delta Mass: 2.663 ppm
XCorr: 52.29

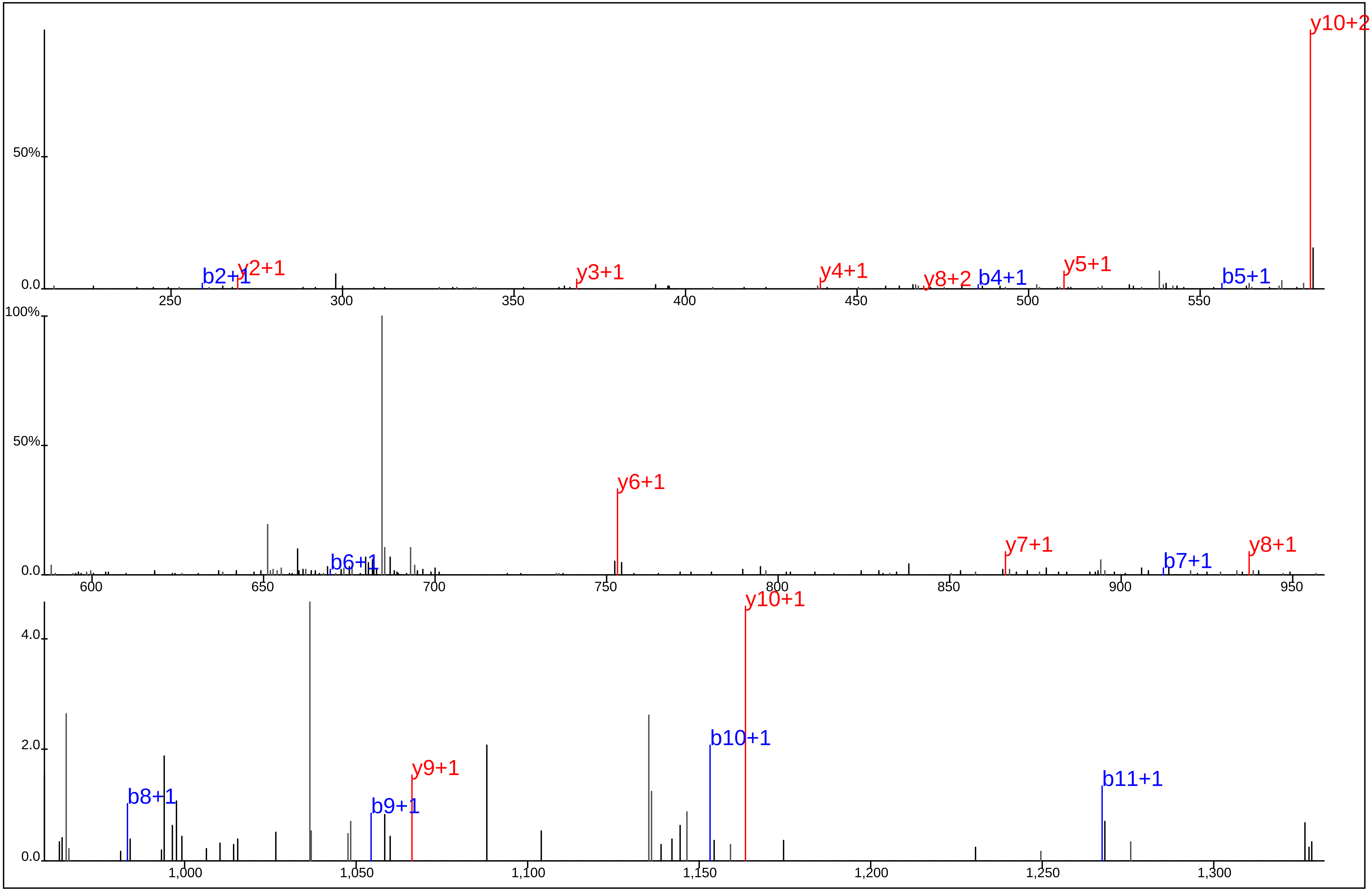

E E P E A L Y*A A V N K#

Protein Name: Intersectin 2
gi Accession: 6330911
Precursor Mass: 711.3283+2
Delta Mass: 3.825 ppm
XCorr: 56.54

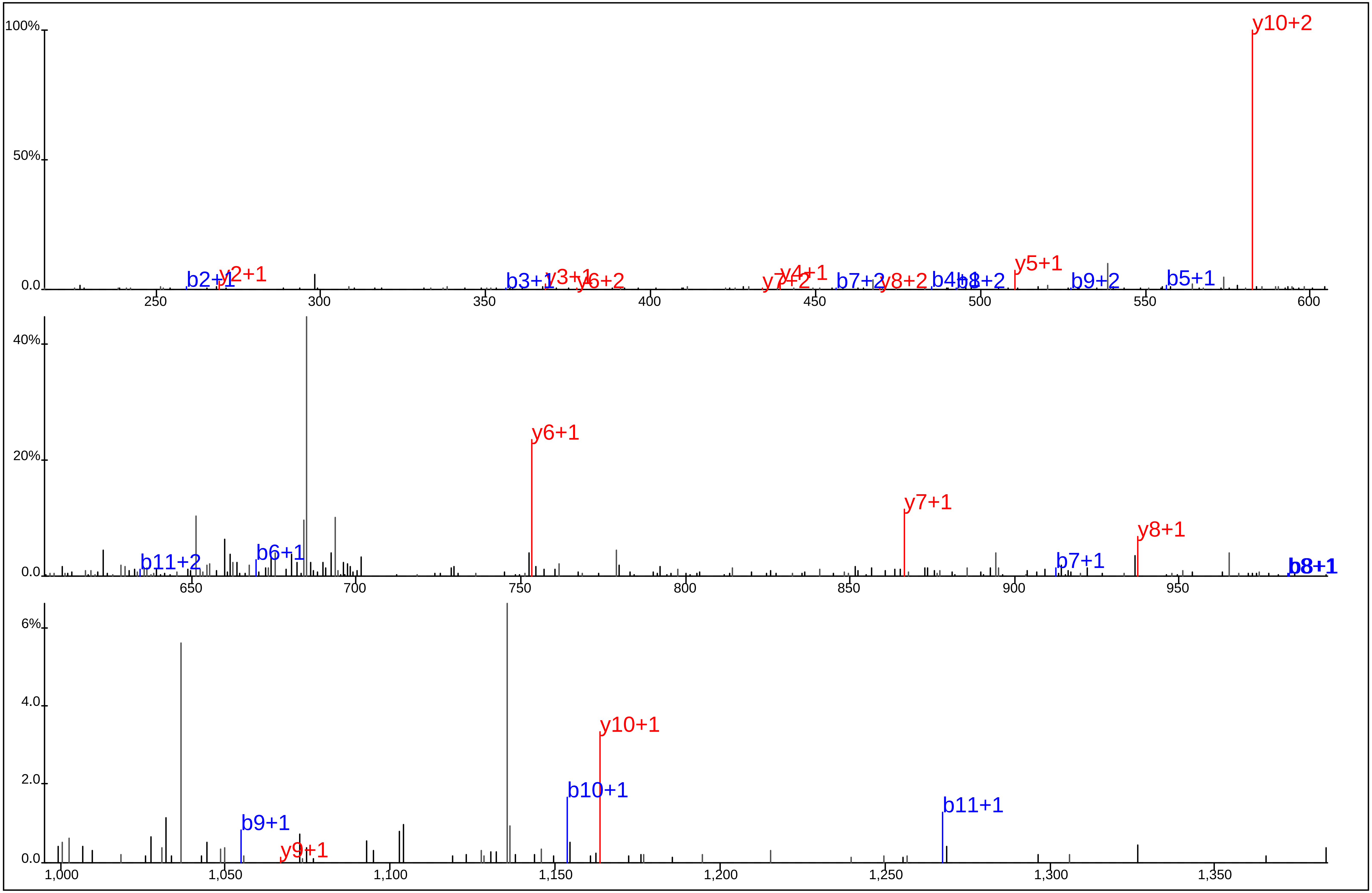

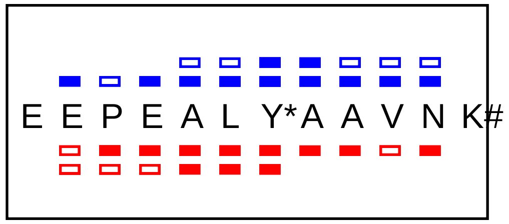

Protein Name: Intersectin 2
gi Accession: 6330911
Precursor Mass: 711.3293+2
Delta Mass: 5.197 ppm
XCorr: 47.36

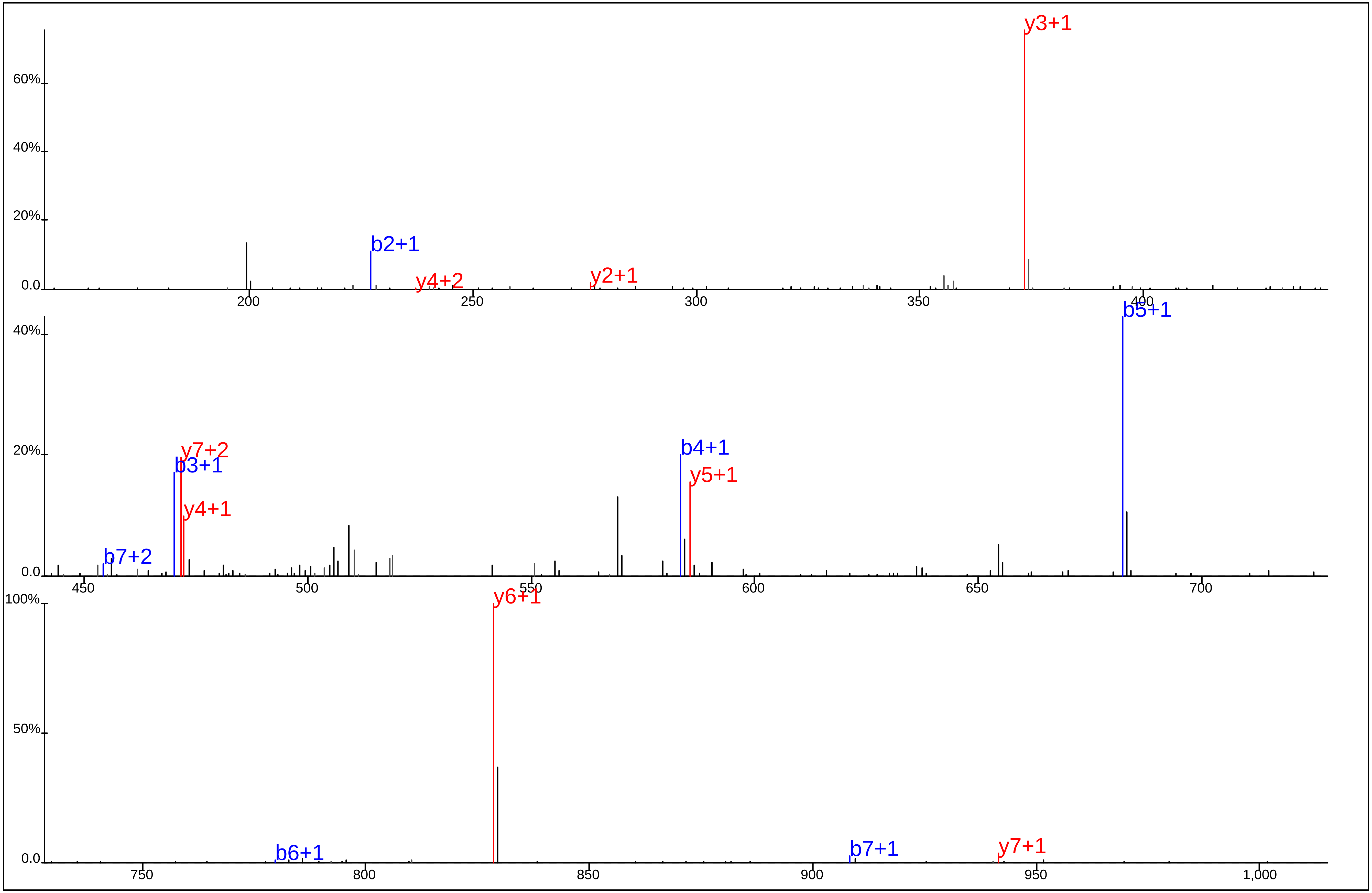

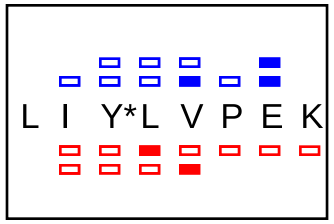

Protein Name: Intersectin 2
gi Accession: 10432747
Precursor Mass: 527.7842+2
Delta Mass: 2.549 ppm
XCorr: 39.28

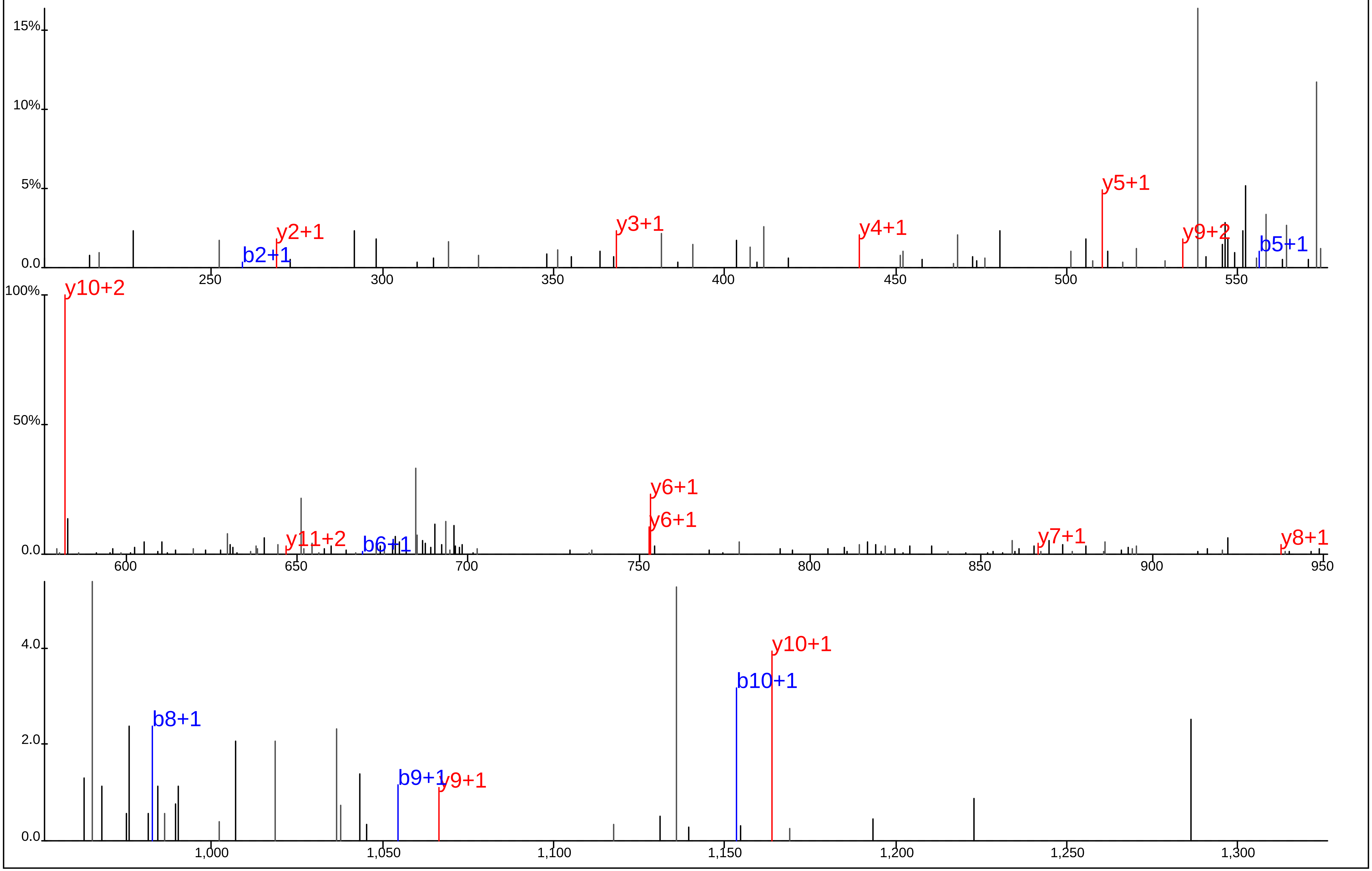

E E P E A L Y*A A V N K#

Protein Name: Intersectin 2
gi Accession: 6330911
Precursor Mass: 711.3286+2
Delta Mass: 4.254 ppm
XCorr: 38.92

543 - Intersectin2_3438749.pdf

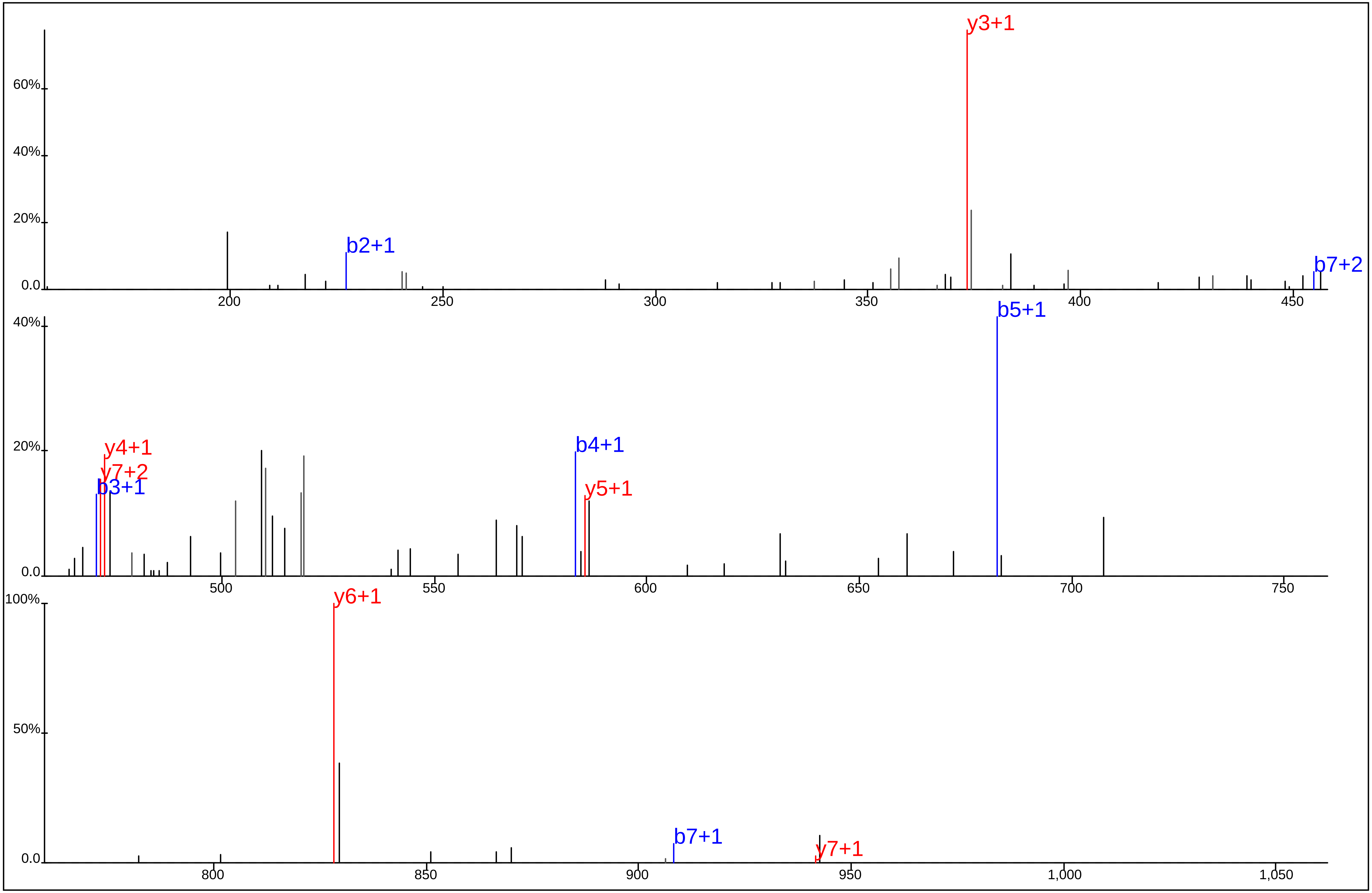

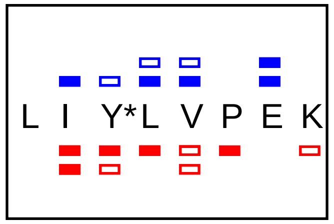

Protein Name: Intersectin 2
gi Accession: 10432747
Precursor Mass: 527.7849+2
Delta Mass: 3.829 ppm
XCorr: 25.14

544 - Intersectin2_3439401.pdf

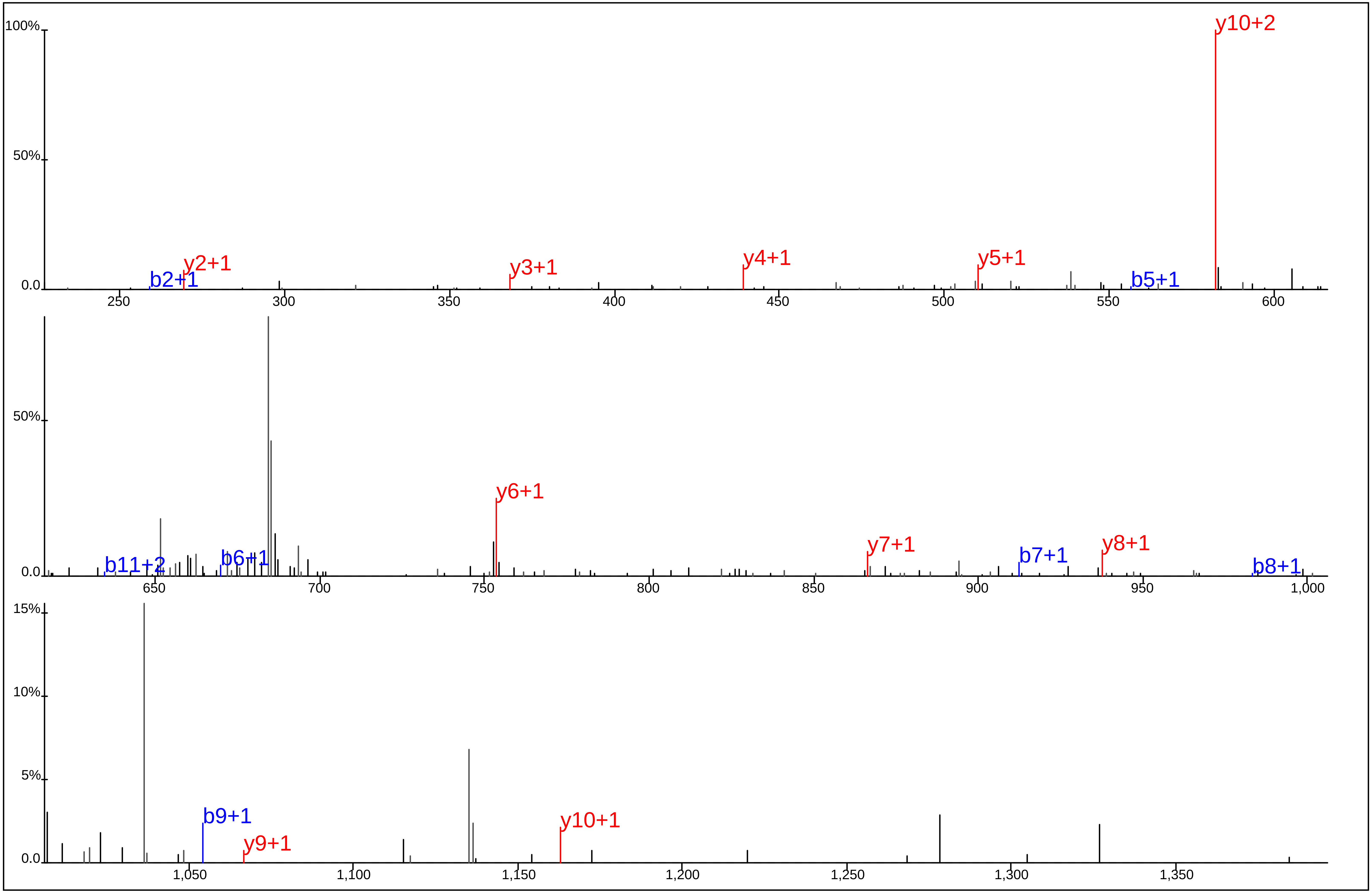

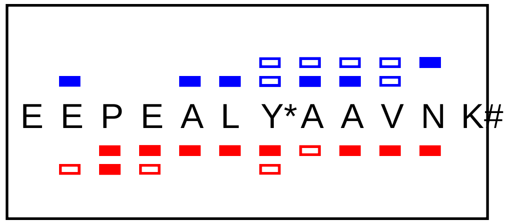

Protein Name: Intersectin 2
gi Accession: 6330911
Precursor Mass: 711.3289+2
Delta Mass: 4.683 ppm
XCorr: 38.84

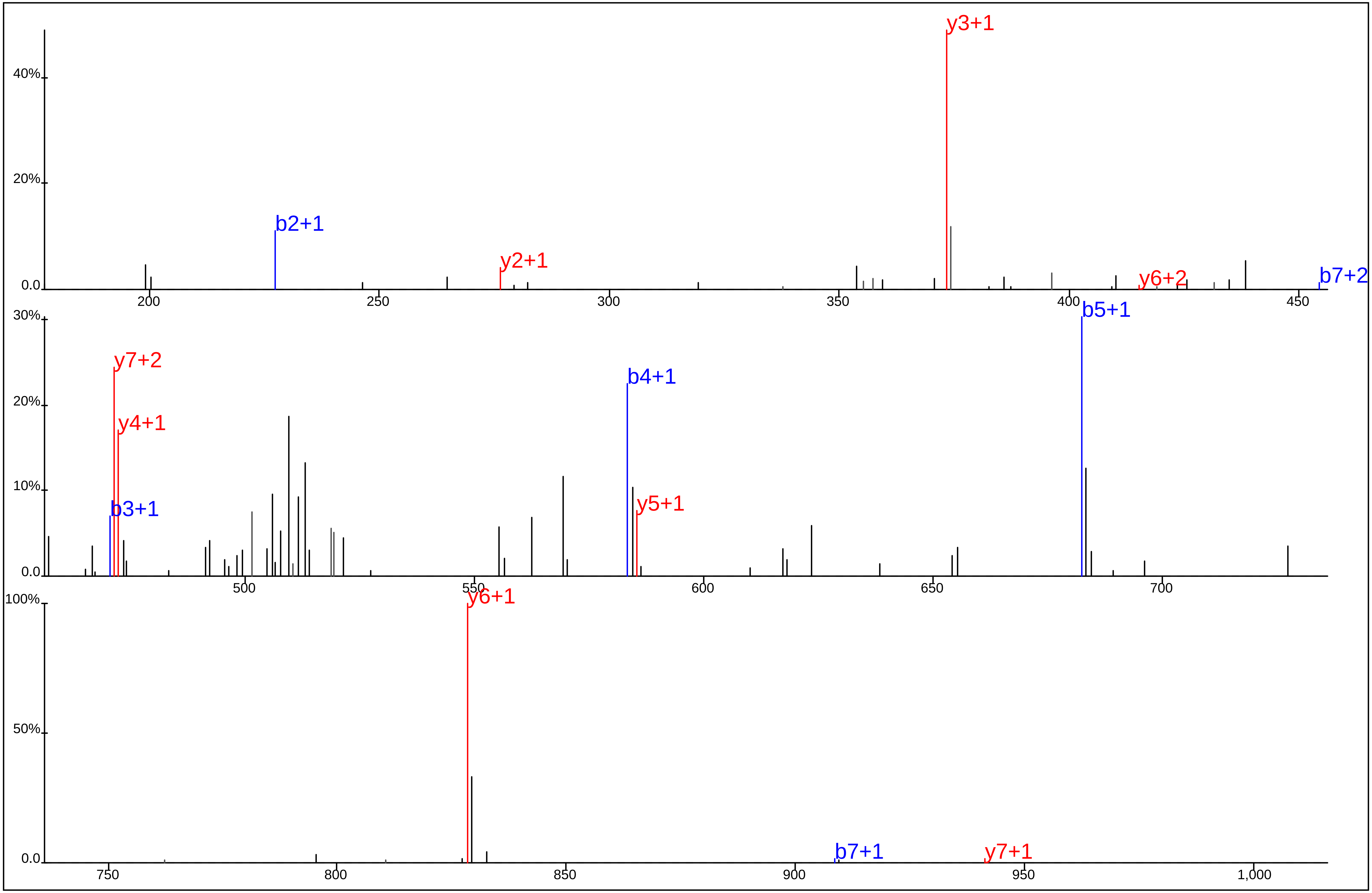

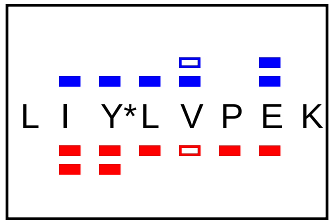

Protein Name: Intersectin 2
gi Accession: 10432747
Precursor Mass: 527.7840+2
Delta Mass: 2.208 ppm
XCorr: 35.31

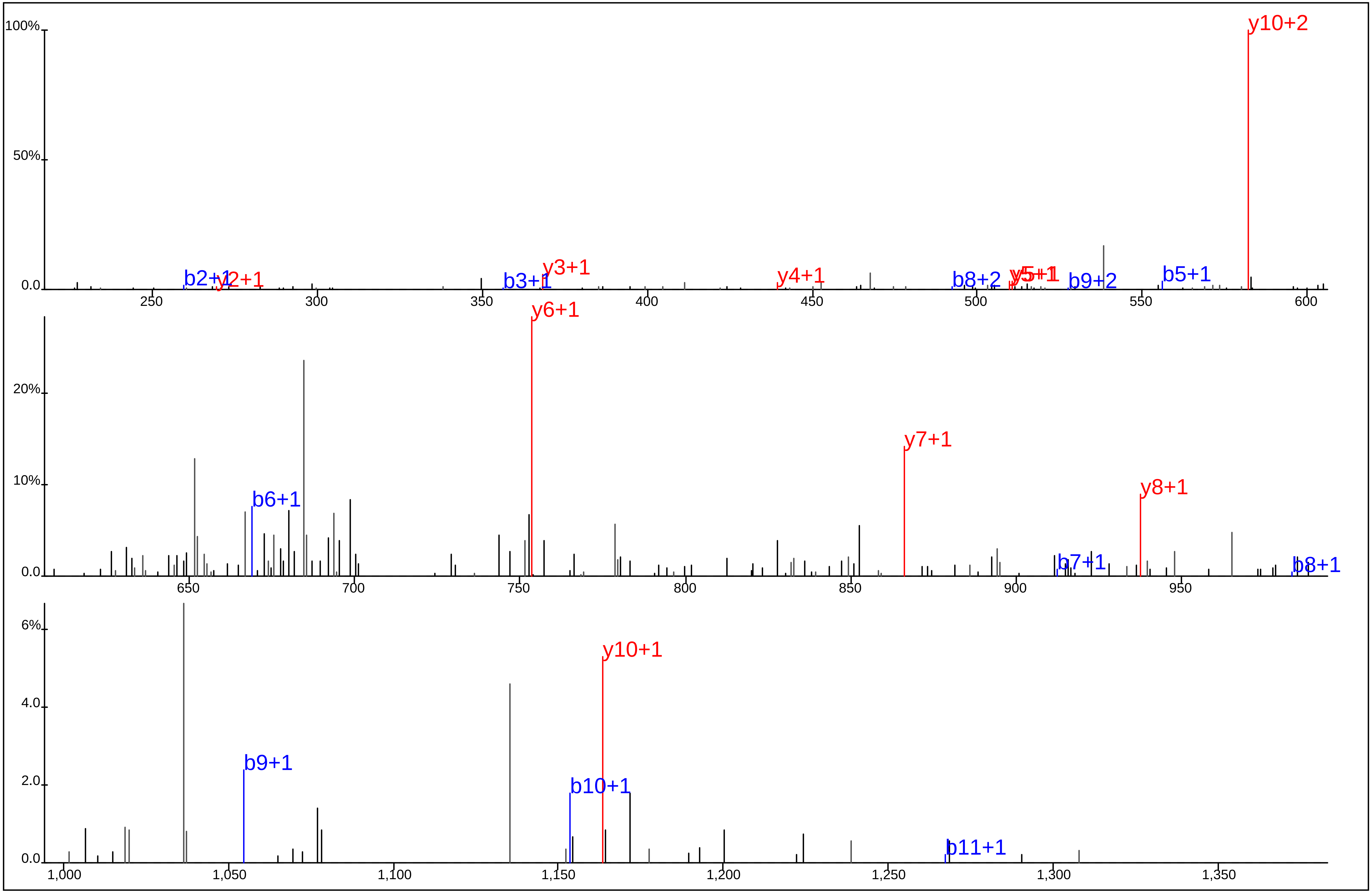

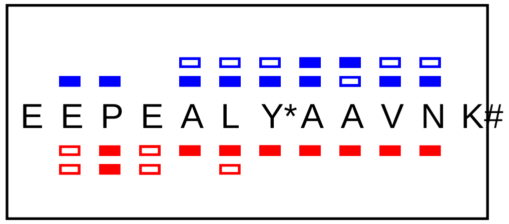

Protein Name: Intersectin 2
gi Accession: 6330911
Precursor Mass: 711.3284+2
Delta Mass: 3.994 ppm
XCorr: 41.08

547 - Intersectin2_3444299.pdf

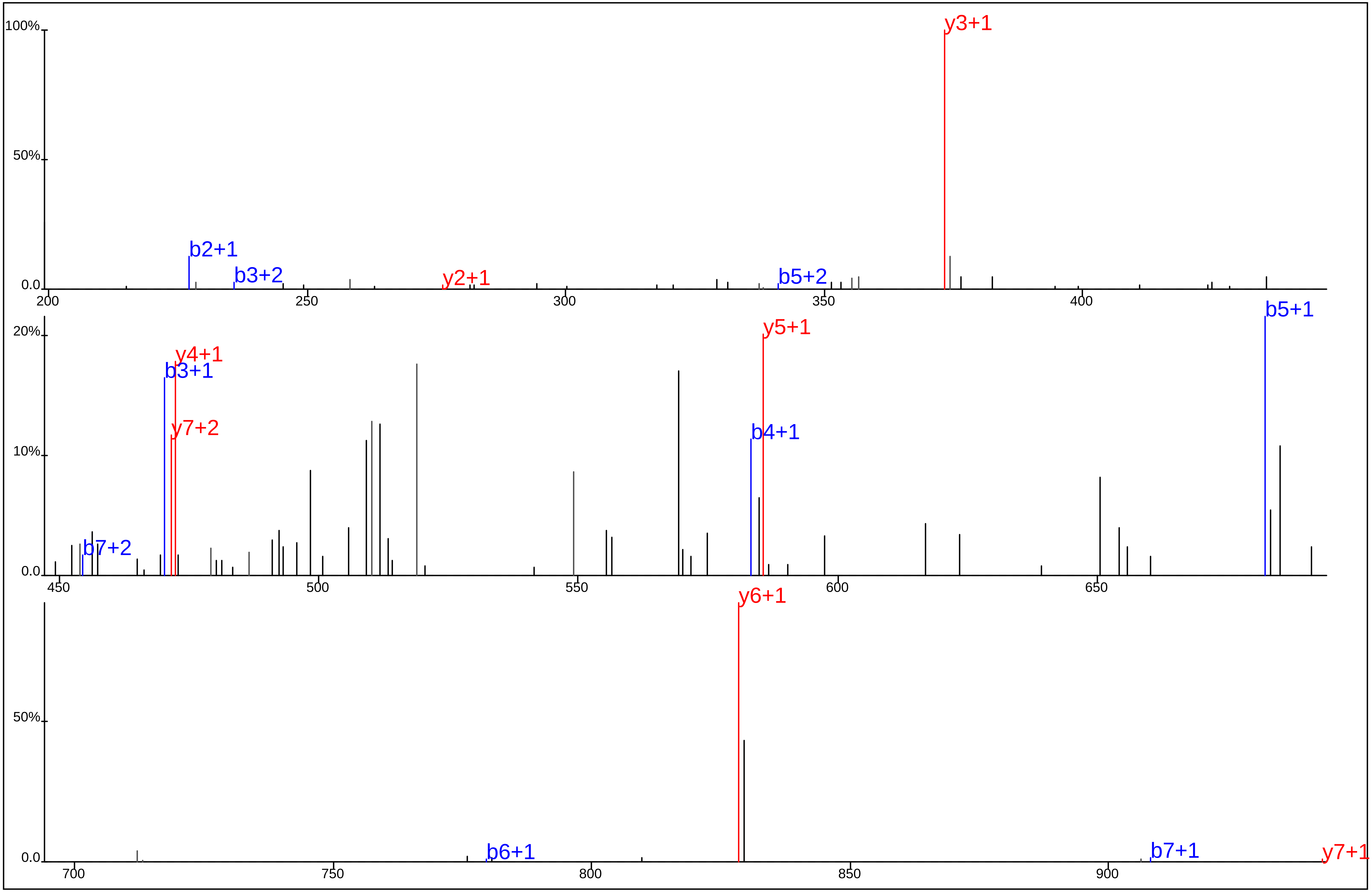

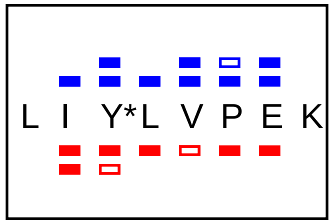

Protein Name: Intersectin 2
gi Accession: 10432747
Precursor Mass: 527.7855+2
Delta Mass: 5.100 ppm
XCorr: 34.64

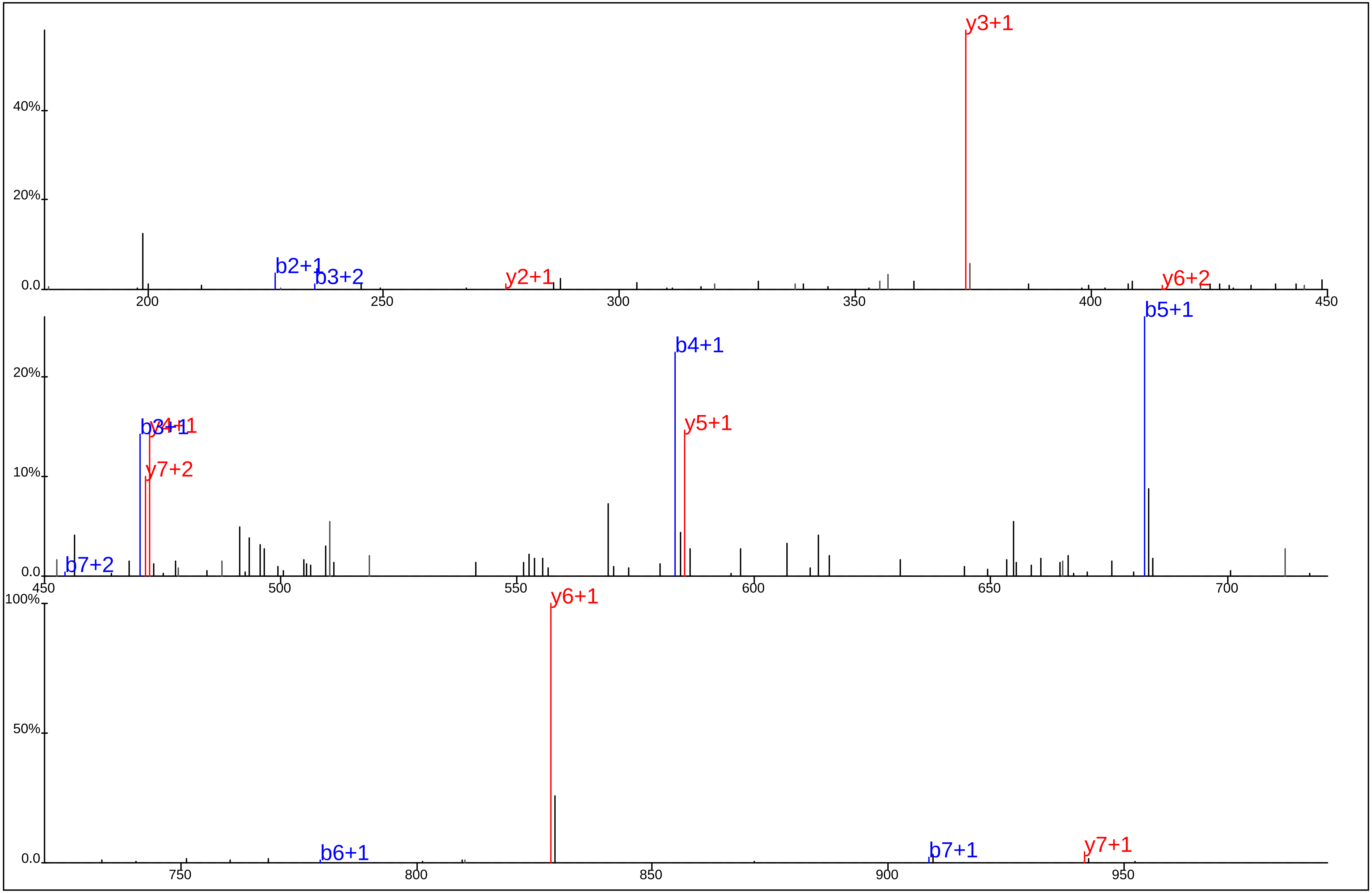

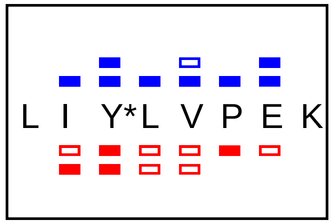

Protein Name: Intersectin 2
gi Accession: 10432747
Precursor Mass: 527.7848+2
Delta Mass: 3.706 ppm
XCorr: 35.88

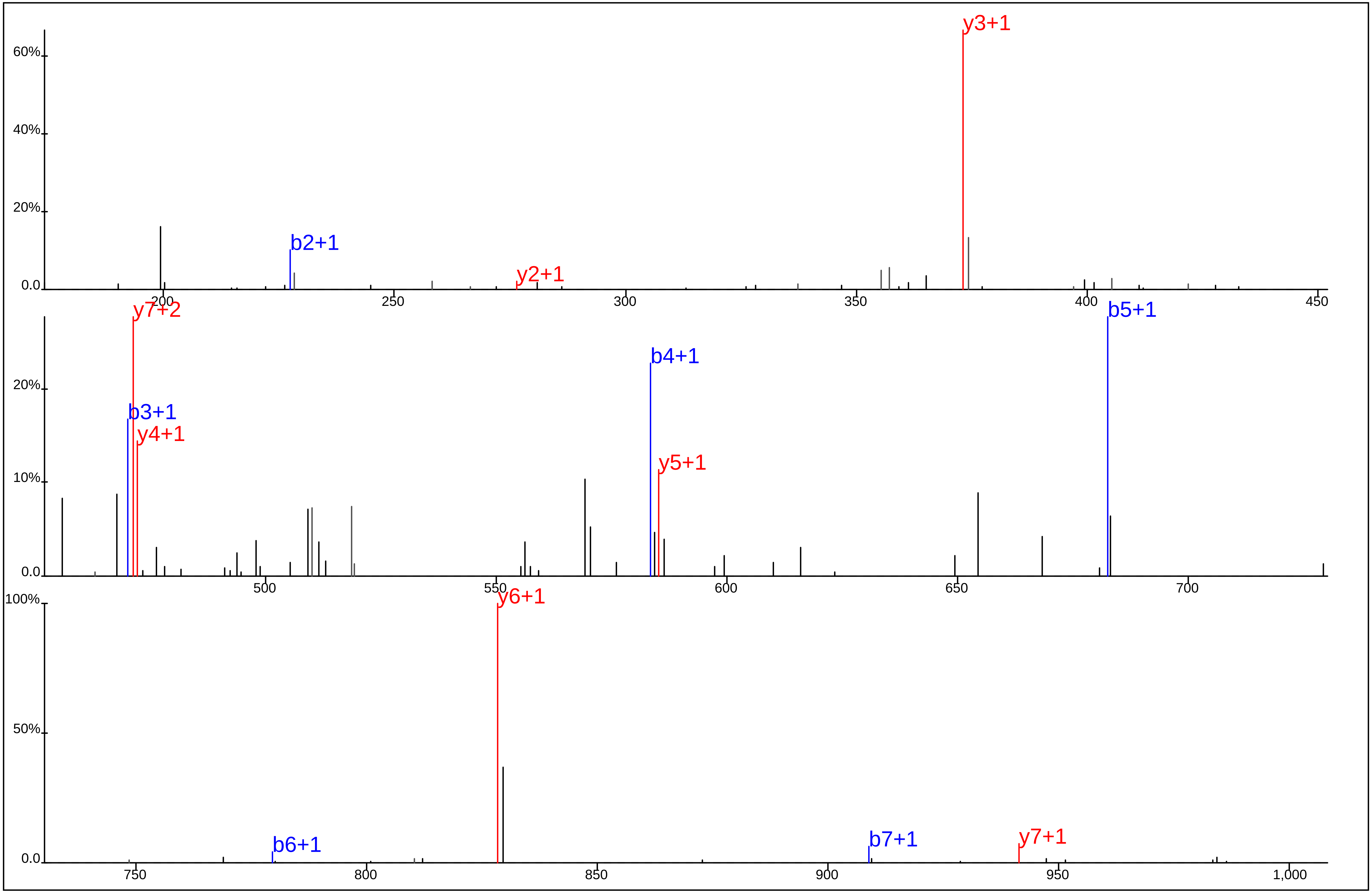

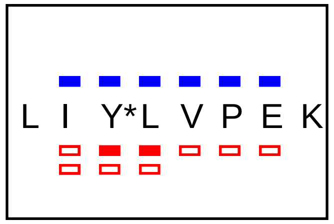

Protein Name: Intersectin 2
gi Accession: 10432747
Precursor Mass: 527.7848+2
Delta Mass: 3.706 ppm
XCorr: 38.04

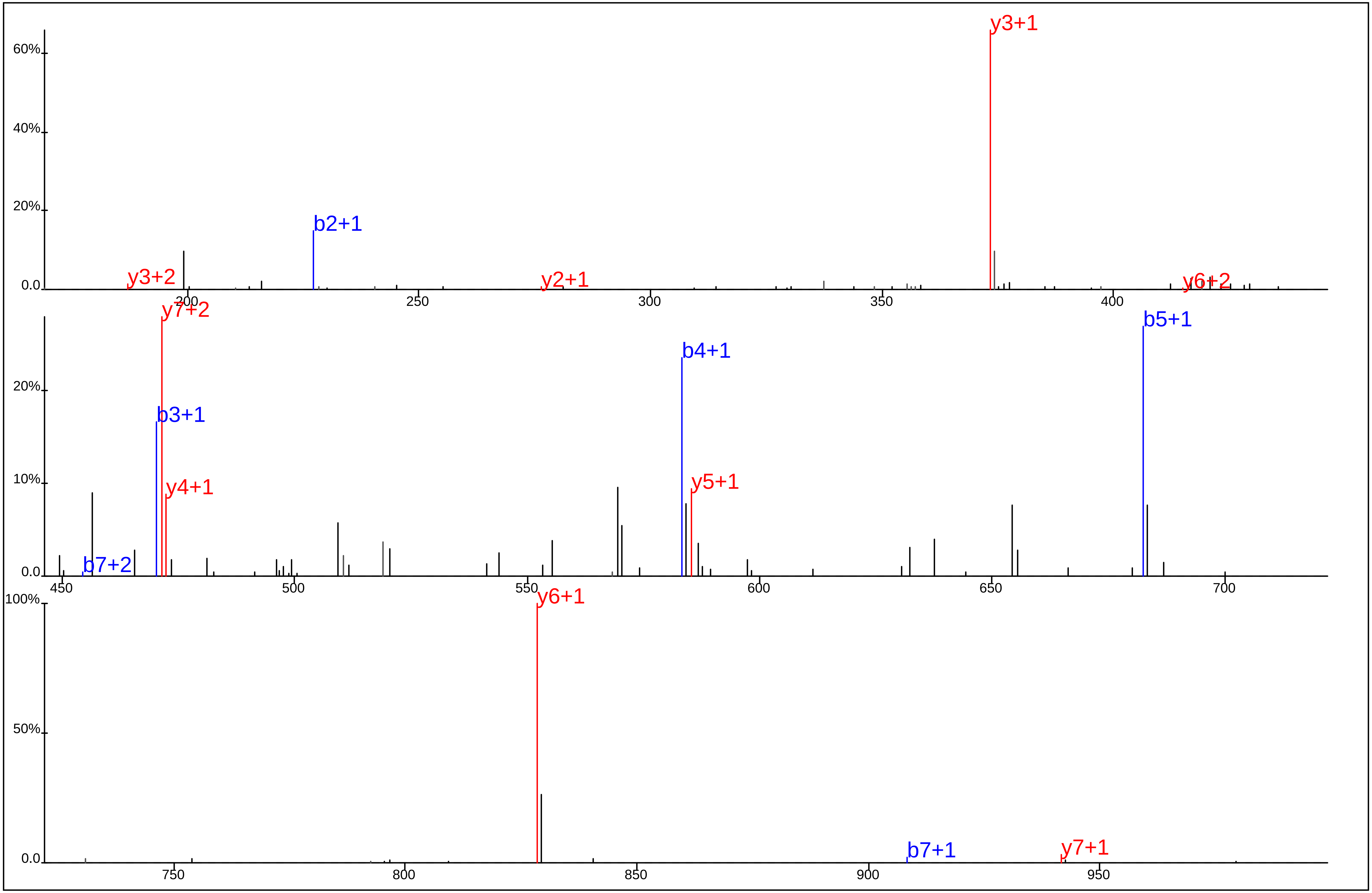

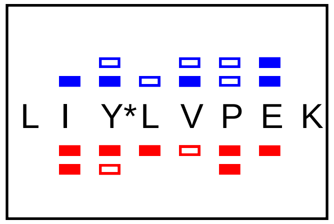

Protein Name: Intersectin 2
gi Accession: 10432747
Precursor Mass: 527.7850+2
Delta Mass: 4.170 ppm
XCorr: 28.67

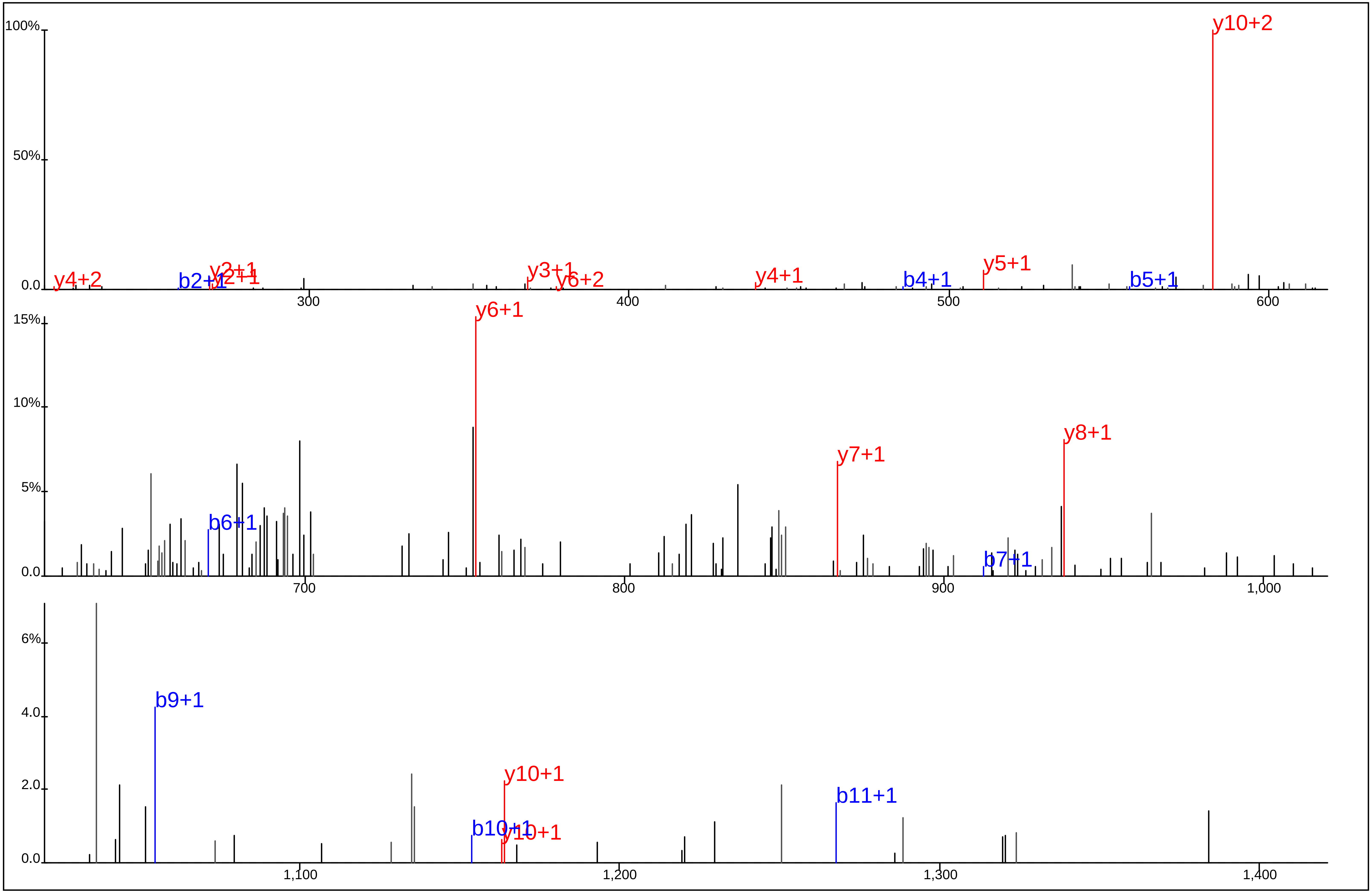

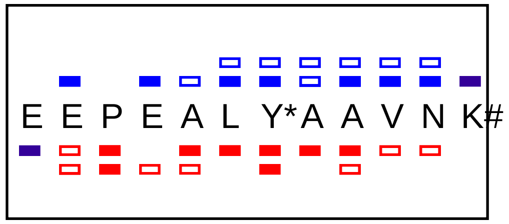

Protein Name: Intersectin 2
gi Accession: 6330911
Precursor Mass: 711.3305+2
Delta Mass: 6.913 ppm
XCorr: 54.31

552 - Intersectin2_3452767.pdf

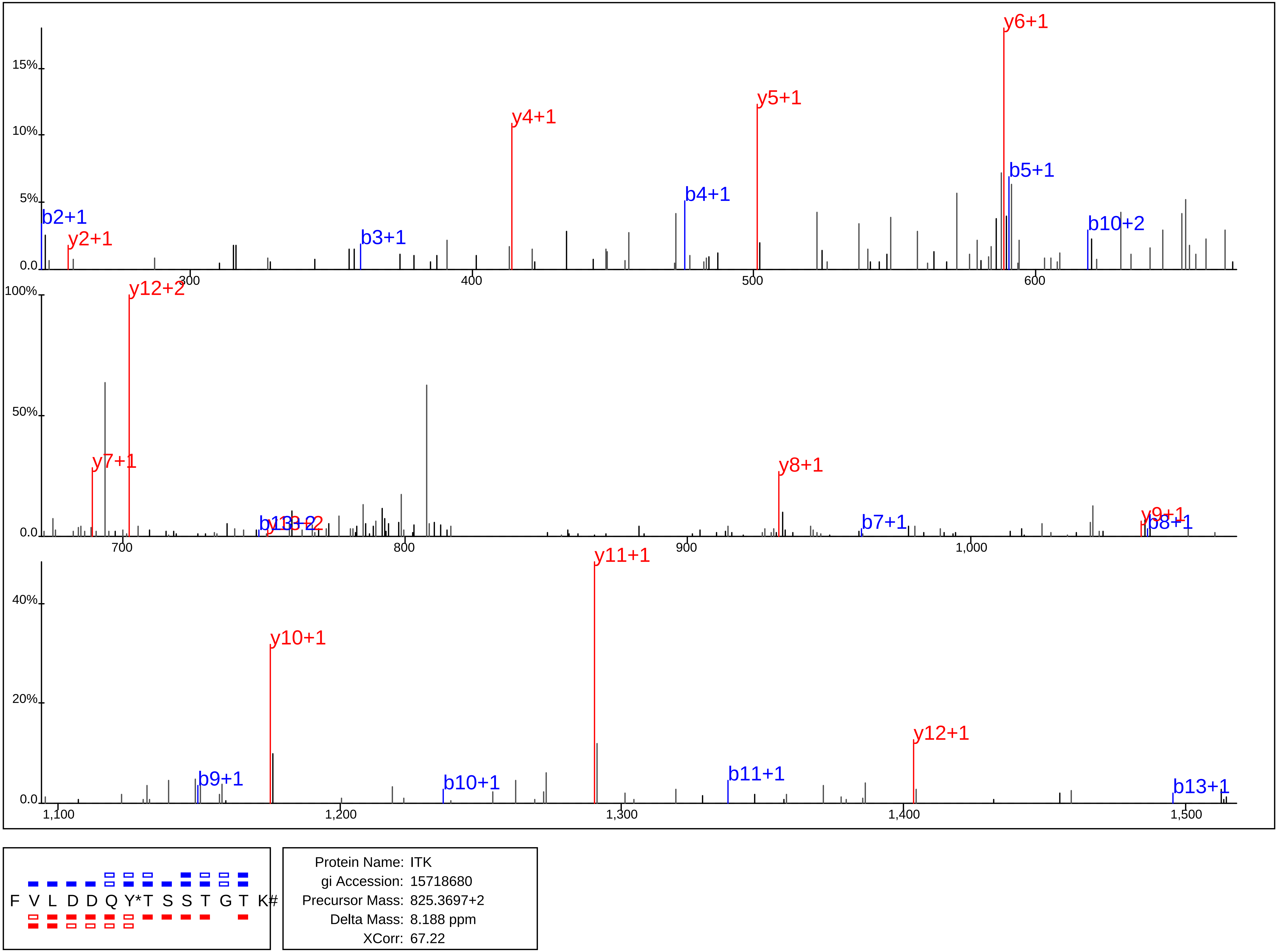

Protein Name: ITK
gi Accession: 15718680
Precursor Mass: 825.3697+2
Delta Mass: 8.188 ppm
XCorr: 67.22

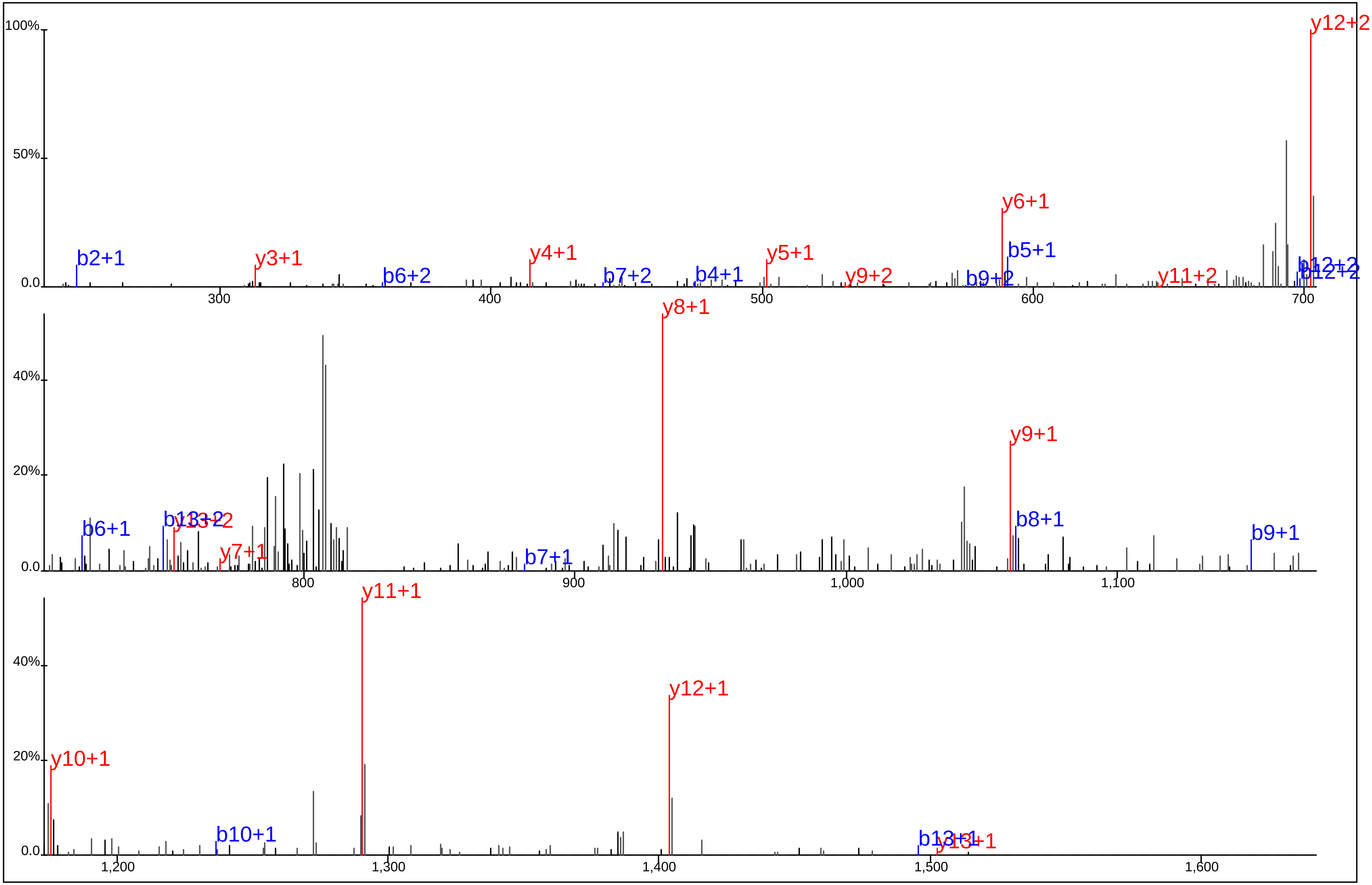

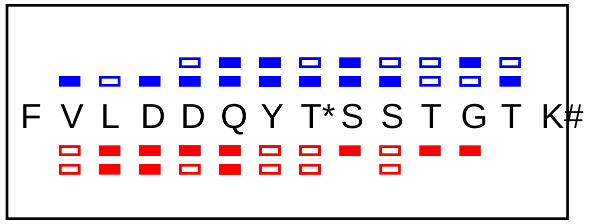

Protein Name:	ITK
gi Accession:	15718680
Precursor Mass:	825.3649+2
Delta Mass:	2.417 ppm
XCorr:	73.66

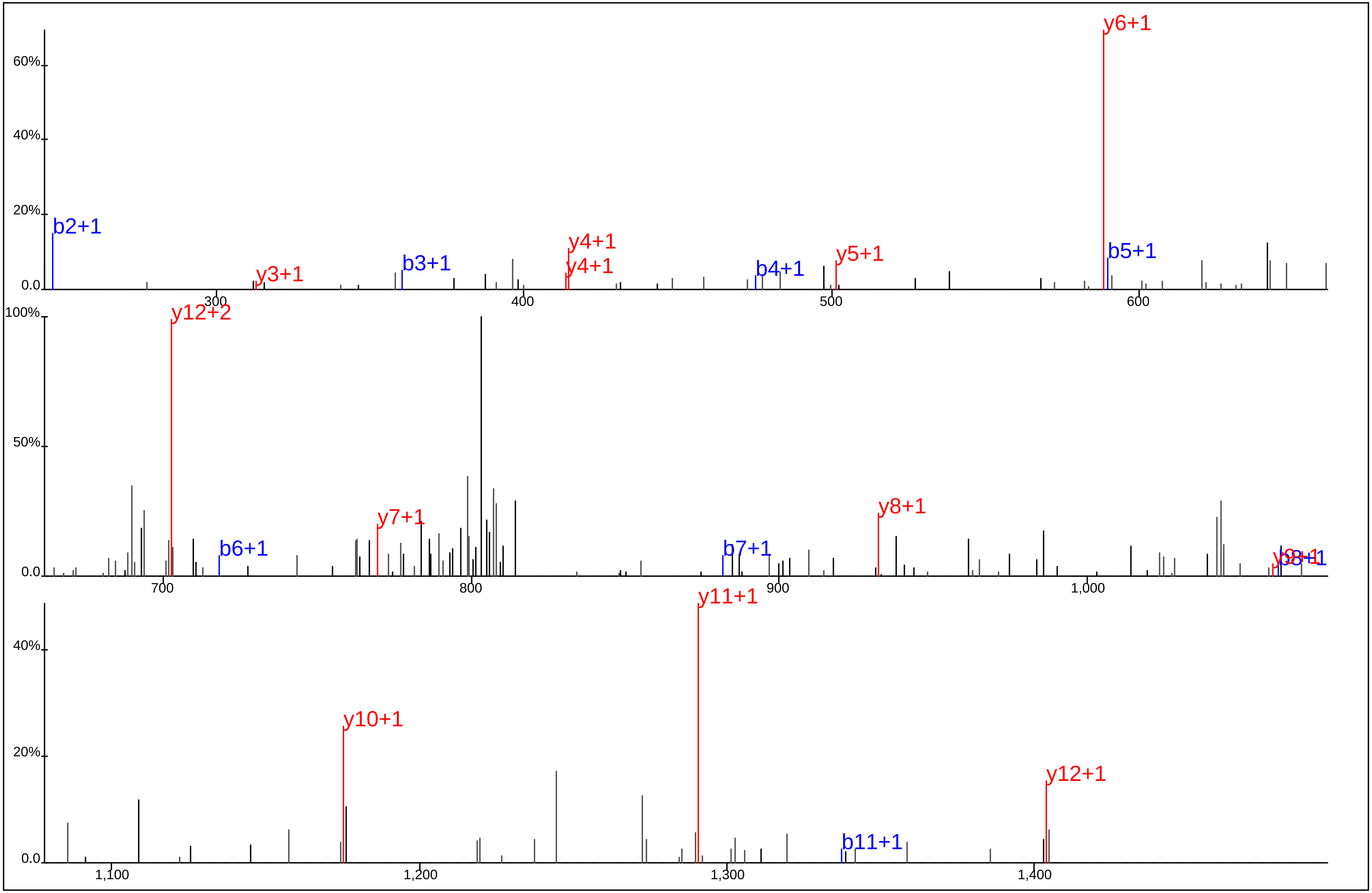

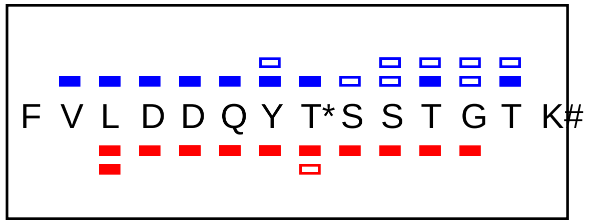

Protein Name:	ITK
gi Accession:	15718680
Precursor Mass:	825.3656+2
Delta Mass:	3.230 ppm
XCorr:	61.35

555 - ITK_3438724.pdf

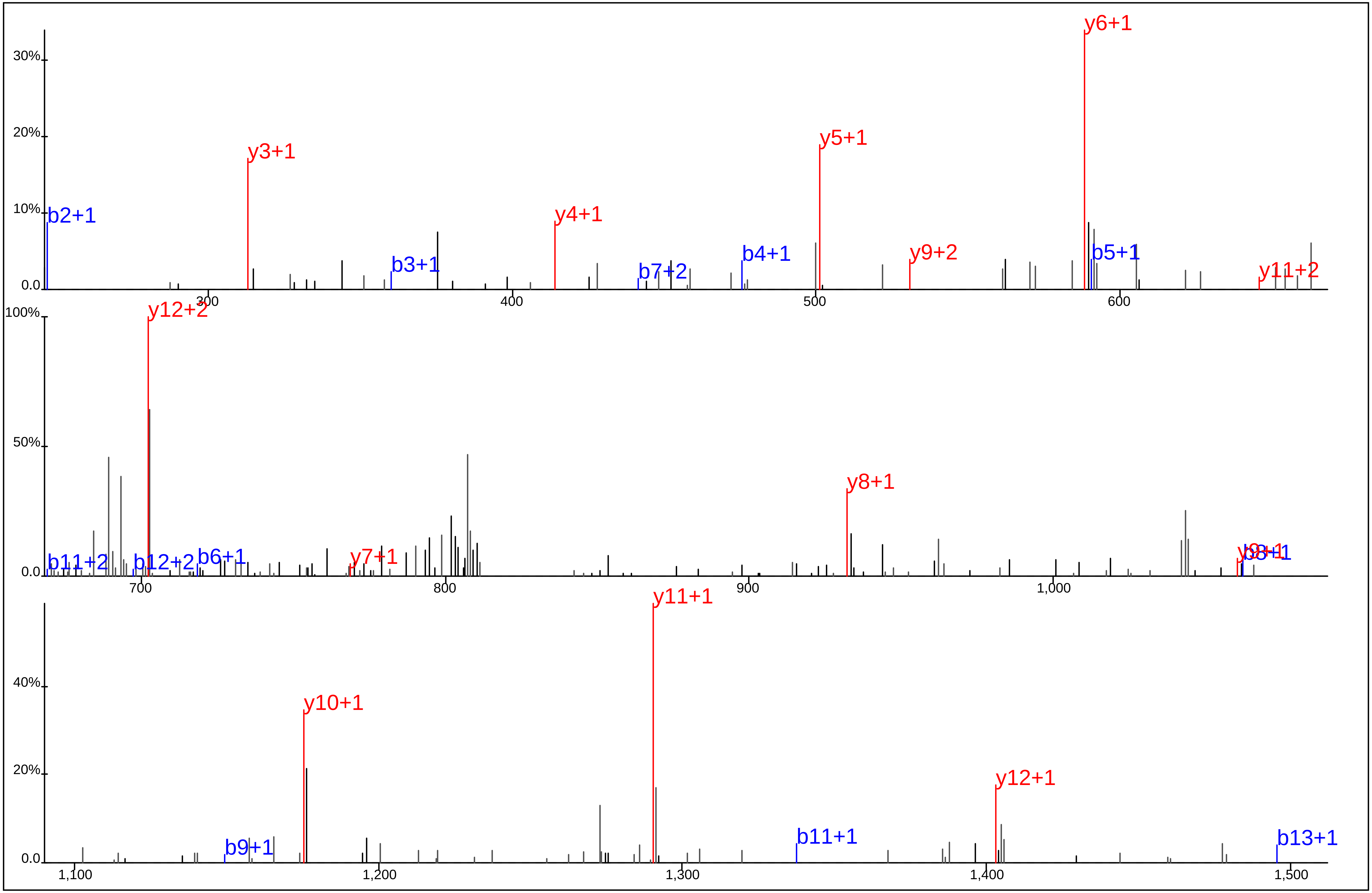

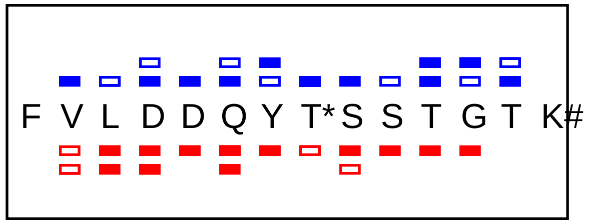

Protein Name: ITK
gi Accession: 15718680
Precursor Mass: 825.3668+2
Delta Mass: 4.715 ppm
XCorr: 82.47

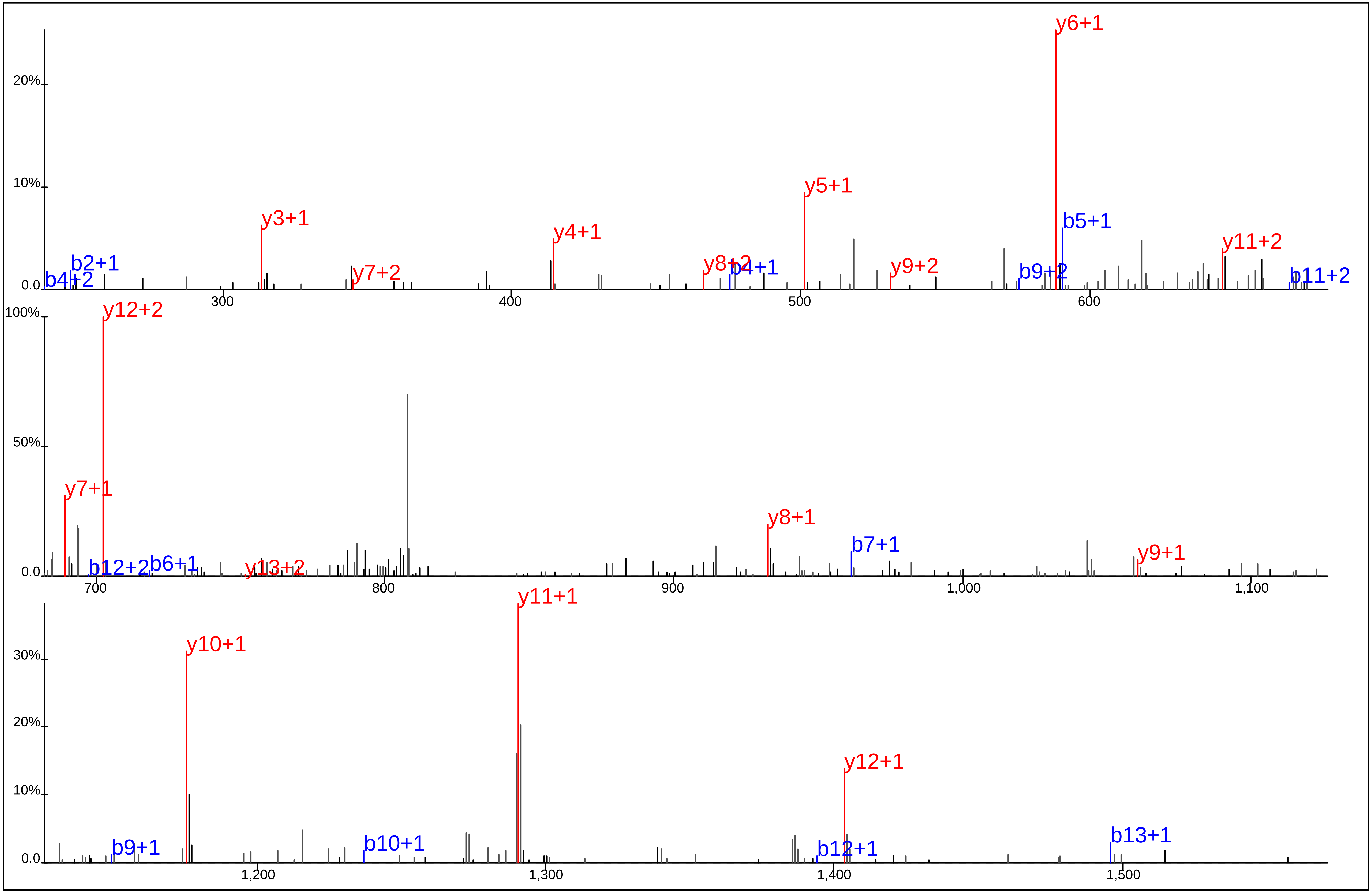

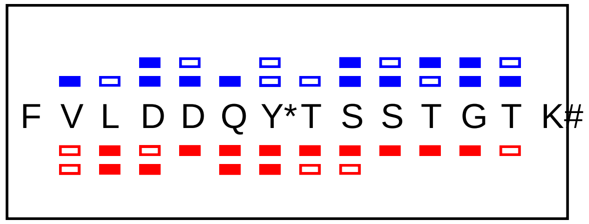

Protein Name:	ITK
gi Accession:	15718680
Precursor Mass:	825.3655+2
Delta Mass:	3.157 ppm
XCorr:	75.54

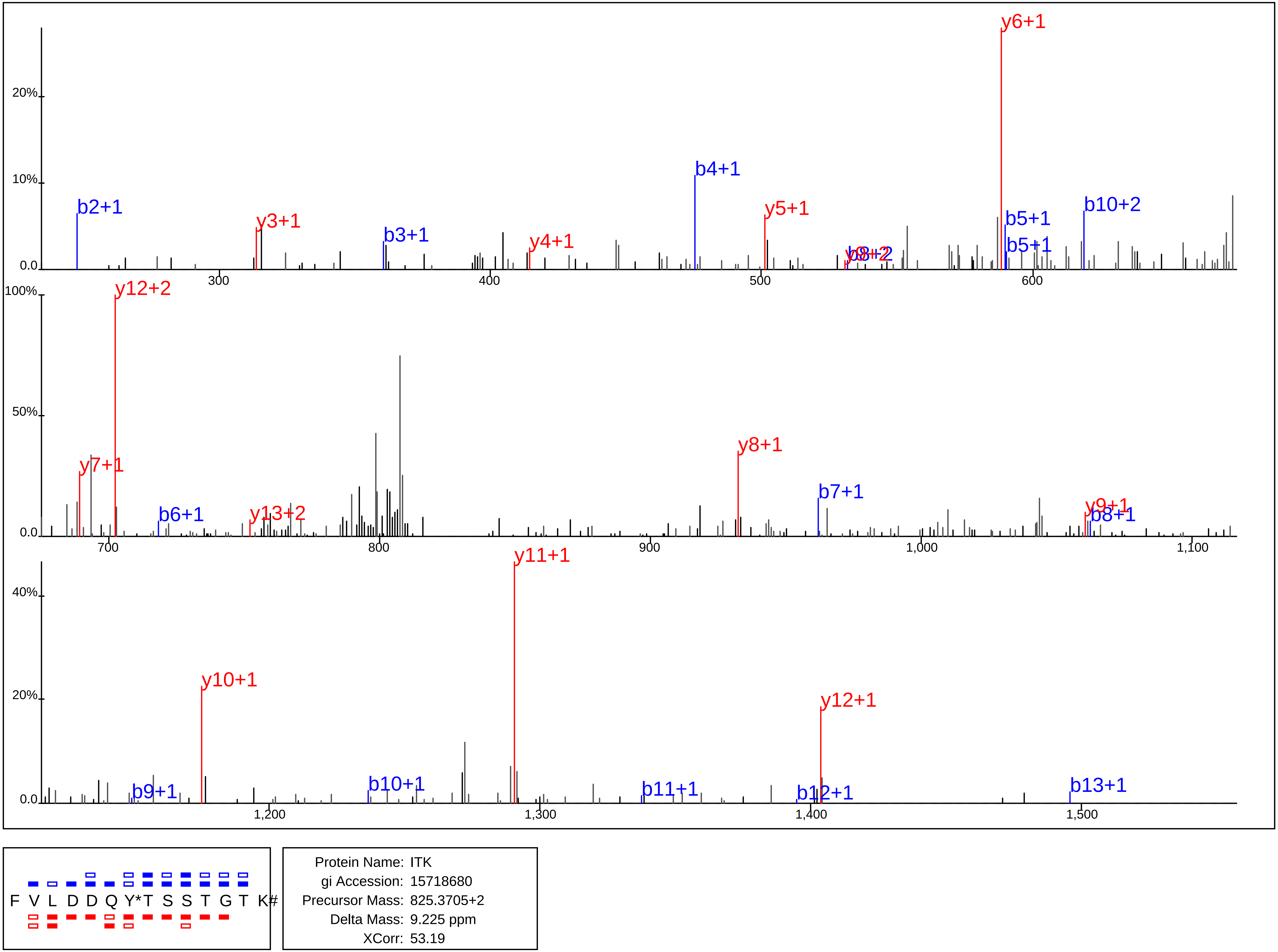

Protein Name:	ITK
gi Accession:	15718680
Precursor Mass:	825.3705+2
Delta Mass:	9.225 ppm
XCorr:	53.19

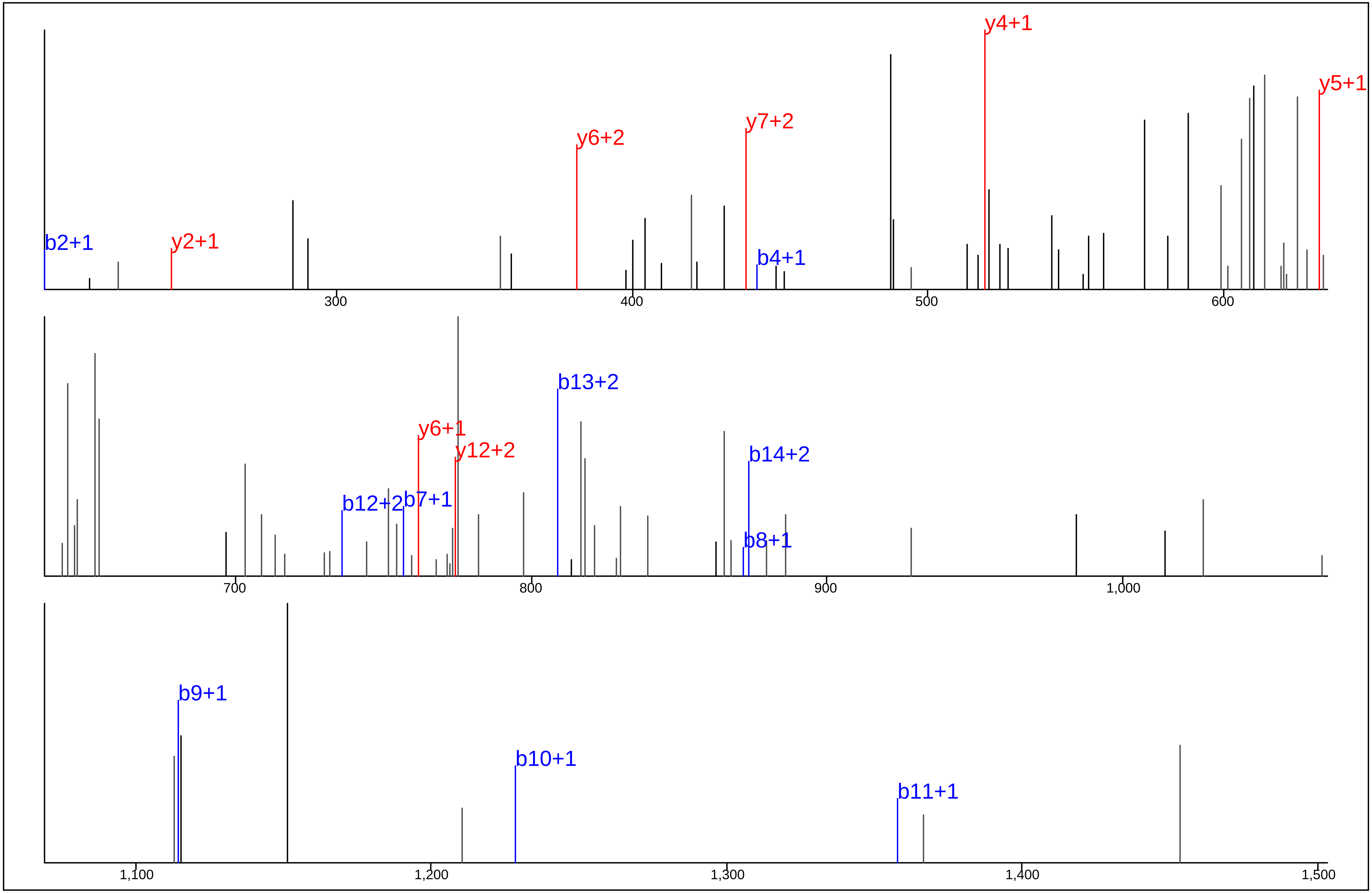

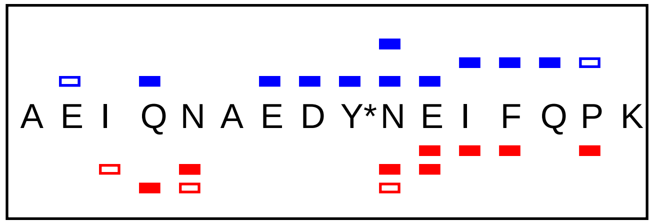

Protein Name: Katanin p80
gi Accession: 83779014
Precursor Mass: 663.6298+3
Delta Mass: 5.651 ppm
XCorr: 13.38

559 - Kataninp80_3452917.pdf

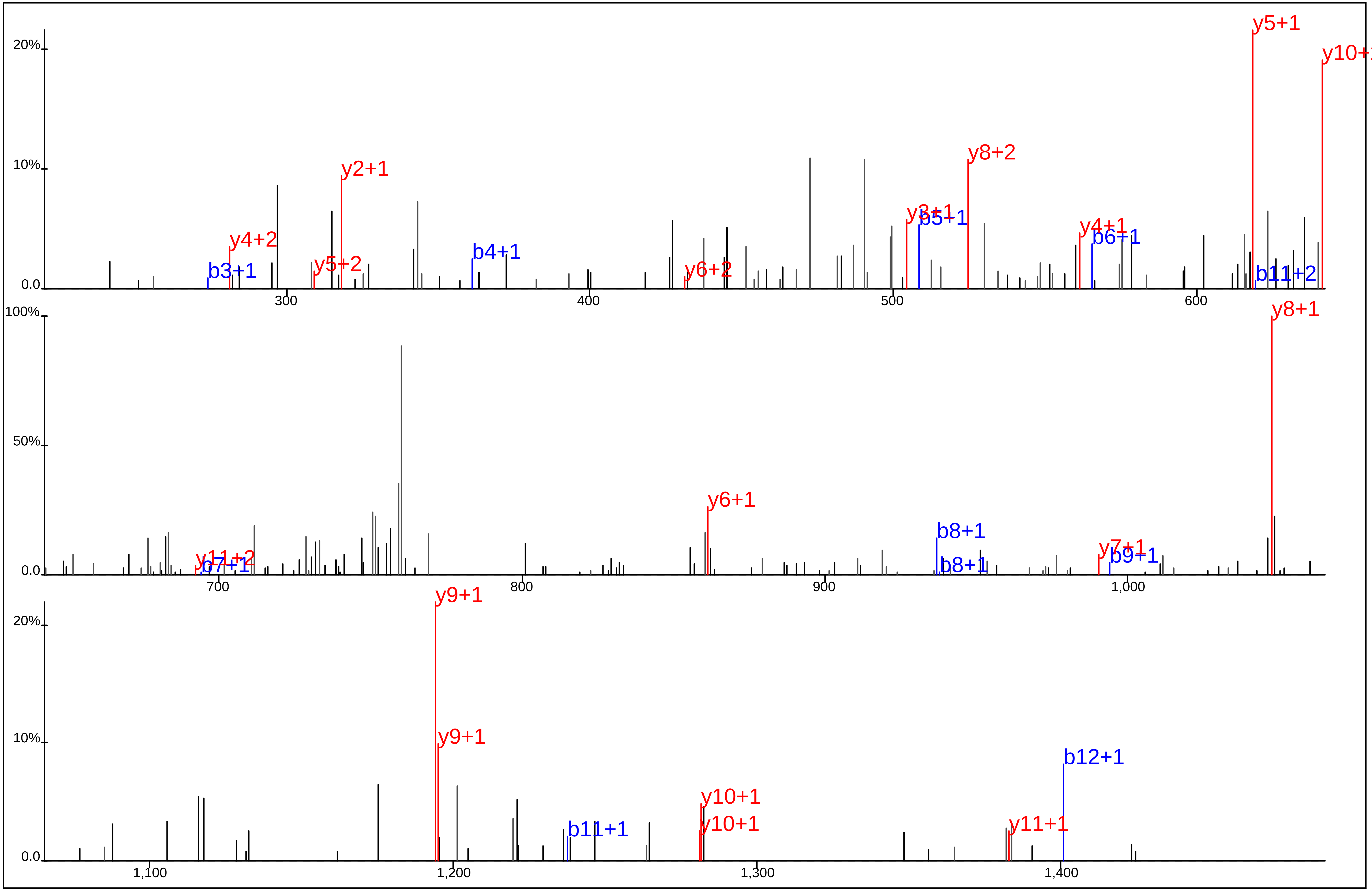

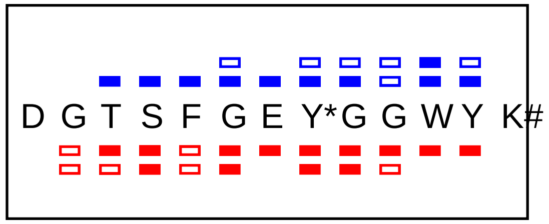

Protein Name: Kinesin 2
gi Accession: 585363
Precursor Mass: 777.8082+2
Delta Mass: 4.090 ppm
XCorr: 55.88

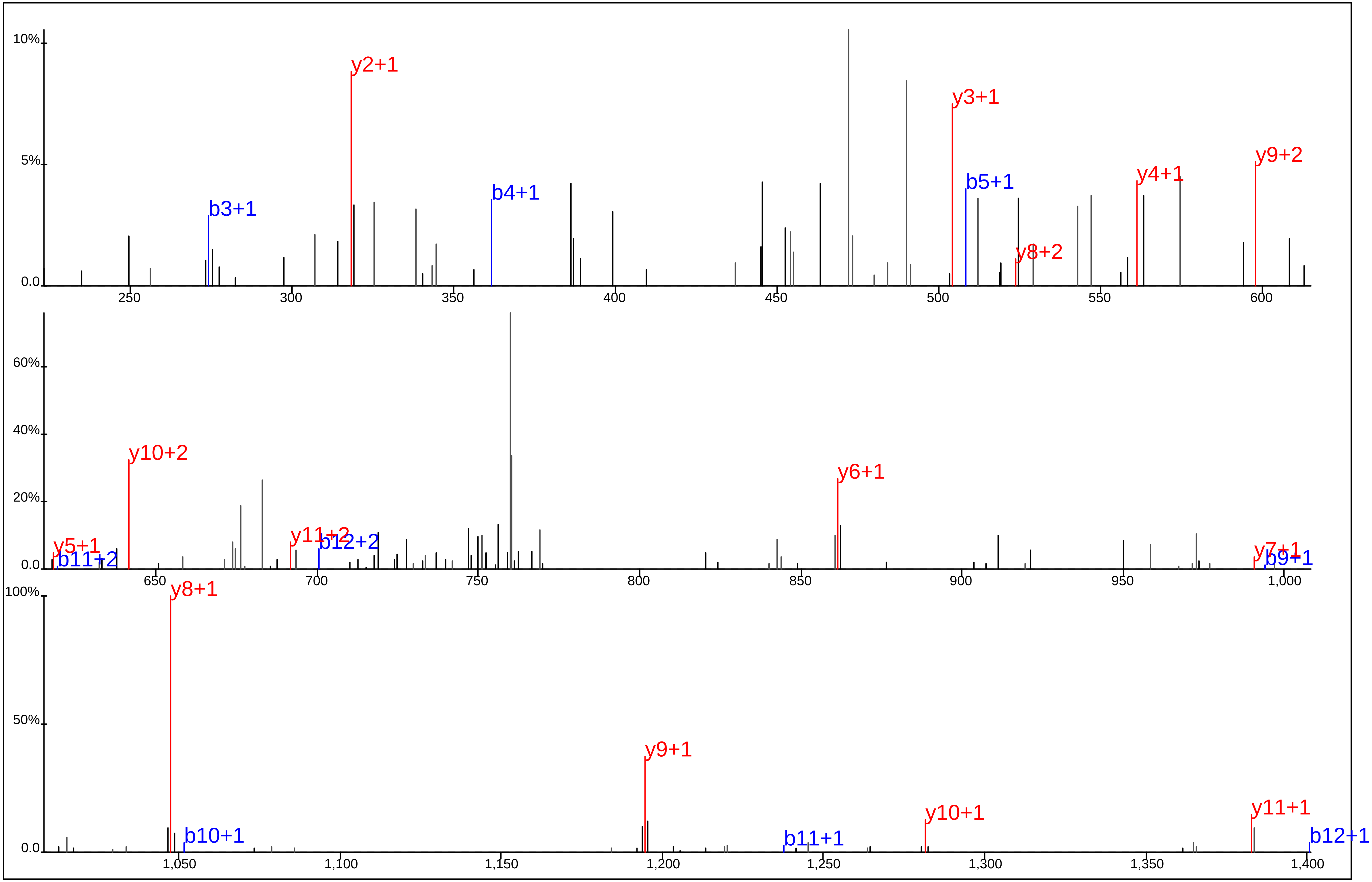

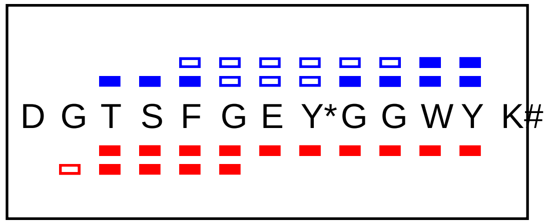

Protein Name: Kinesin 2
gi Accession: 585363
Precursor Mass: 777.8070+2
Delta Mass: 2.520 ppm
XCorr: 55.42

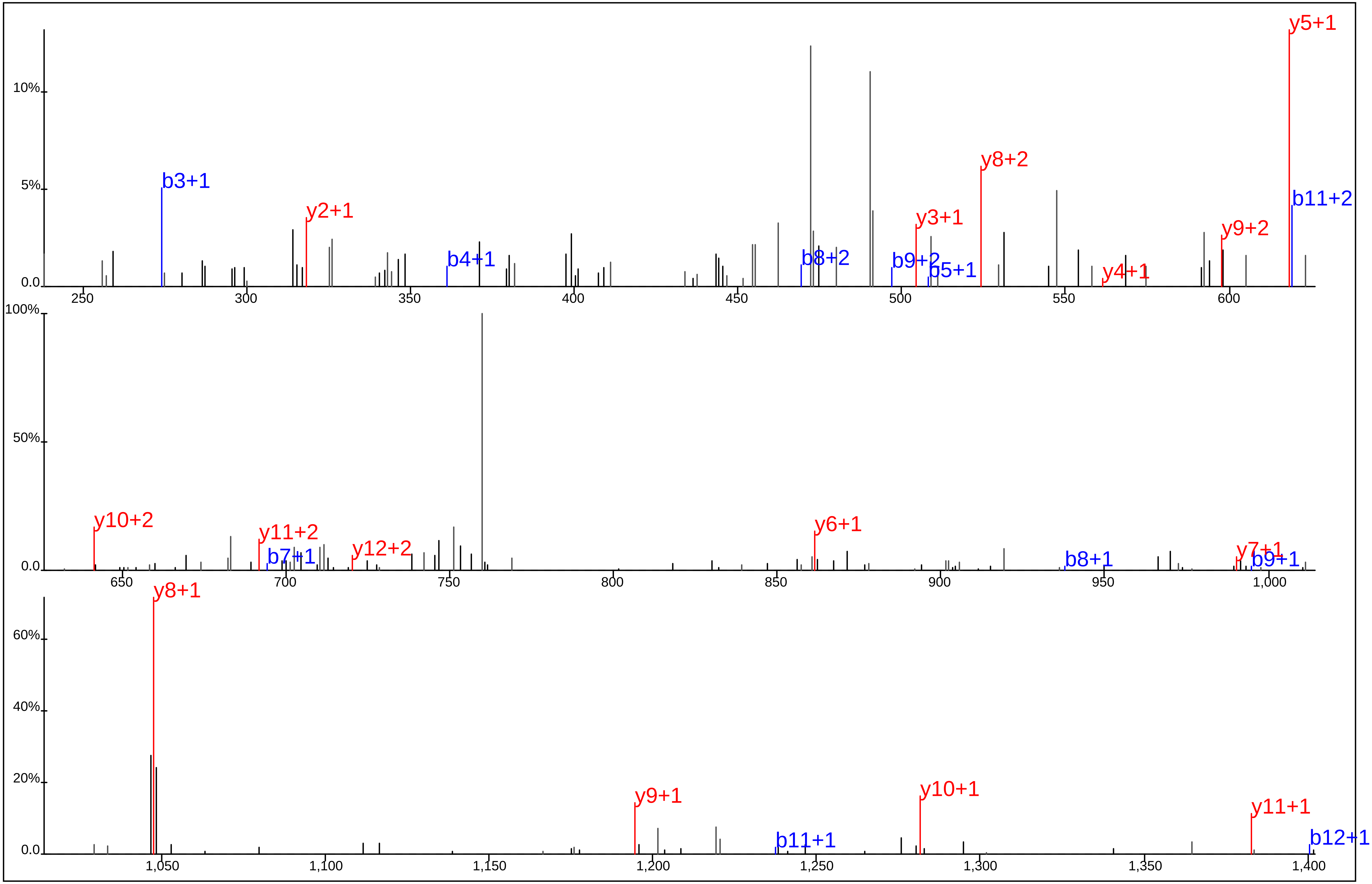

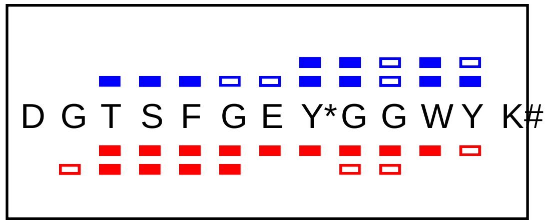

Protein Name:	Kinesin 2
gi Accession:	585363
Precursor Mass:	777.8090+2
Delta Mass:	5.029 ppm
XCorr:	48.1

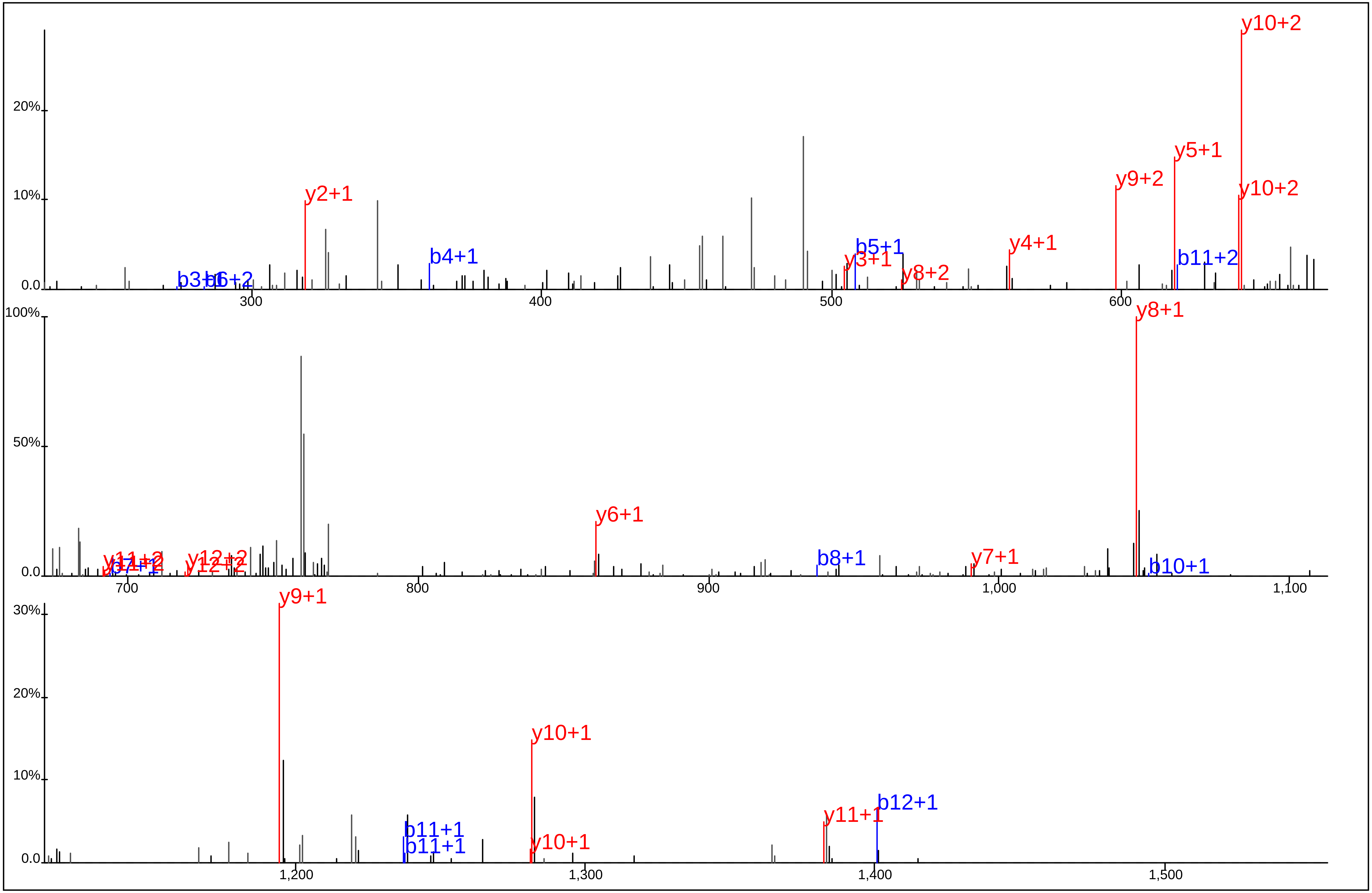

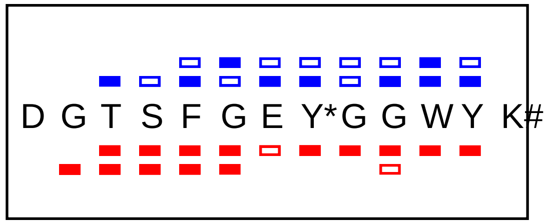

Protein Name:	Kinesin 2
gi Accession:	585363
Precursor Mass:	777.8073+2
Delta Mass:	2.835 ppm
XCorr:	60.55

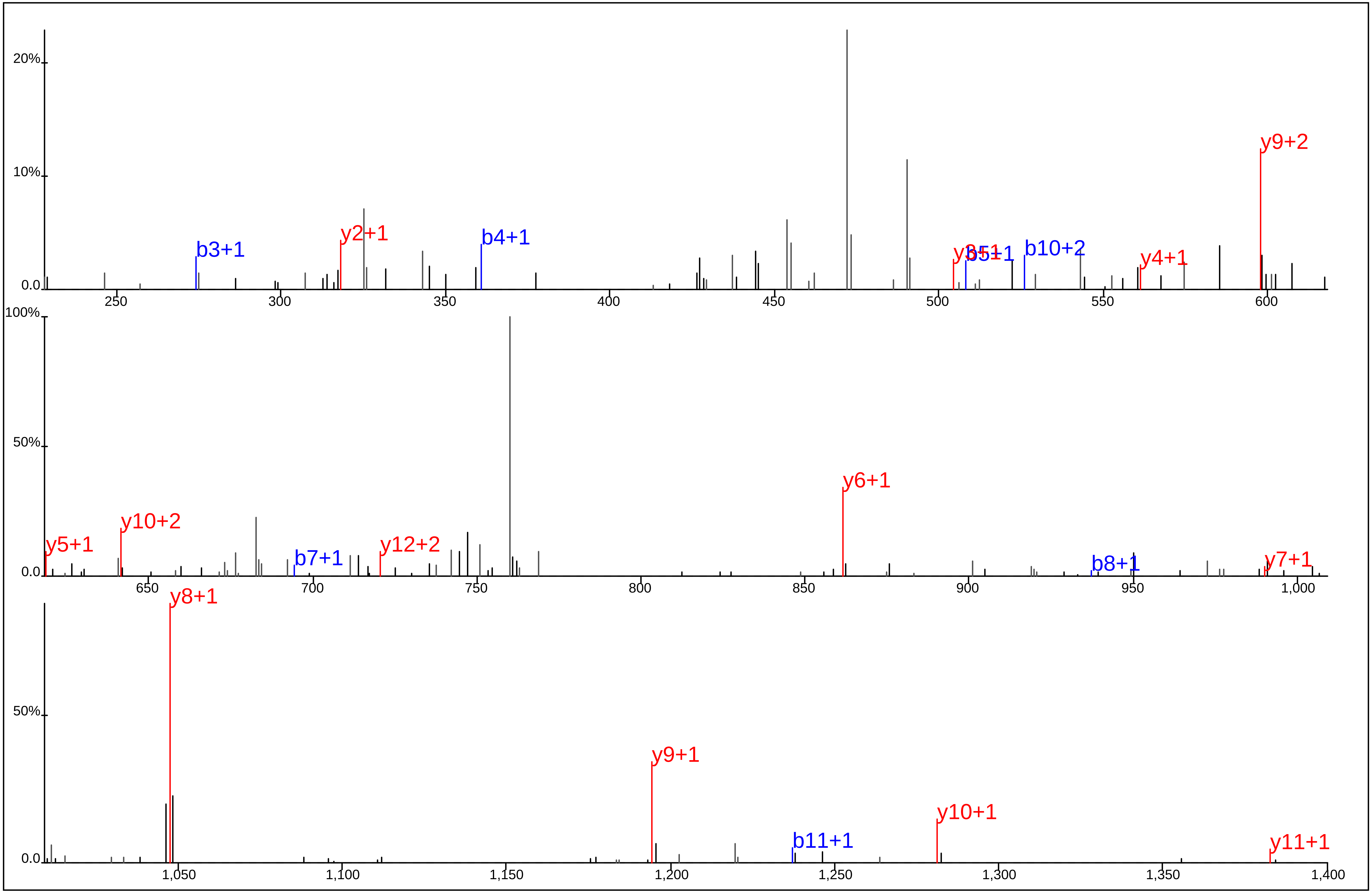

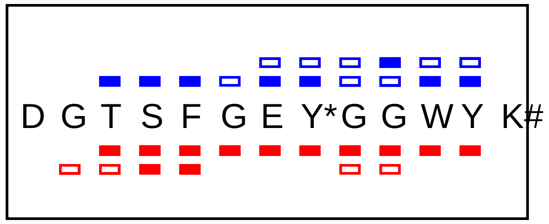

Protein Name: Kinesin 2
gi Accession: 585363
Precursor Mass: 777.8087+2
Delta Mass: 4.720 ppm
XCorr: 46.69

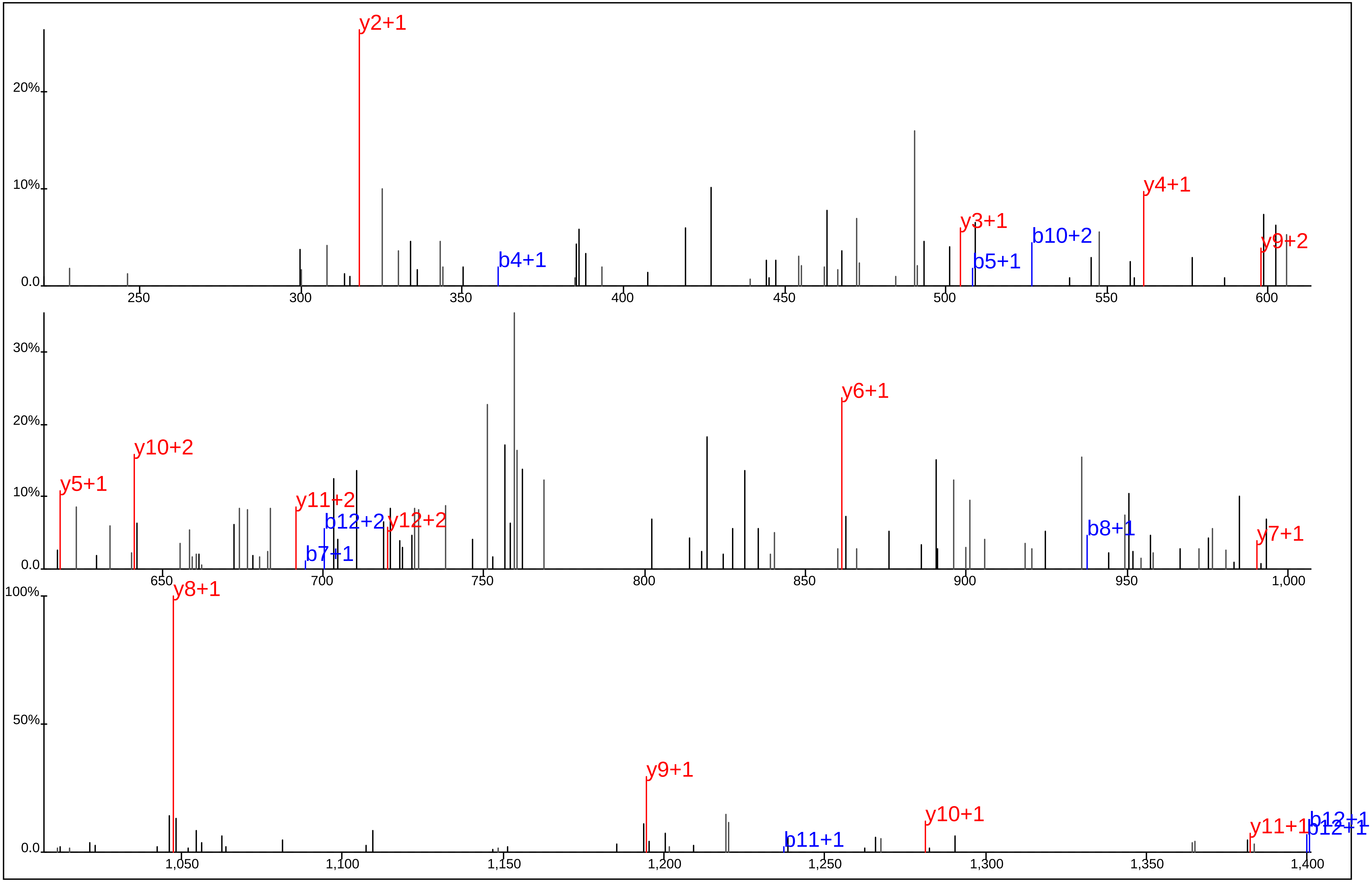

D G T S F G E Y*G G W Y K#

Protein Name:	Kinesin 2
gi Accession:	585363
Precursor Mass:	777.8084+2
Delta Mass:	4.321 ppm
XCorr:	36.71

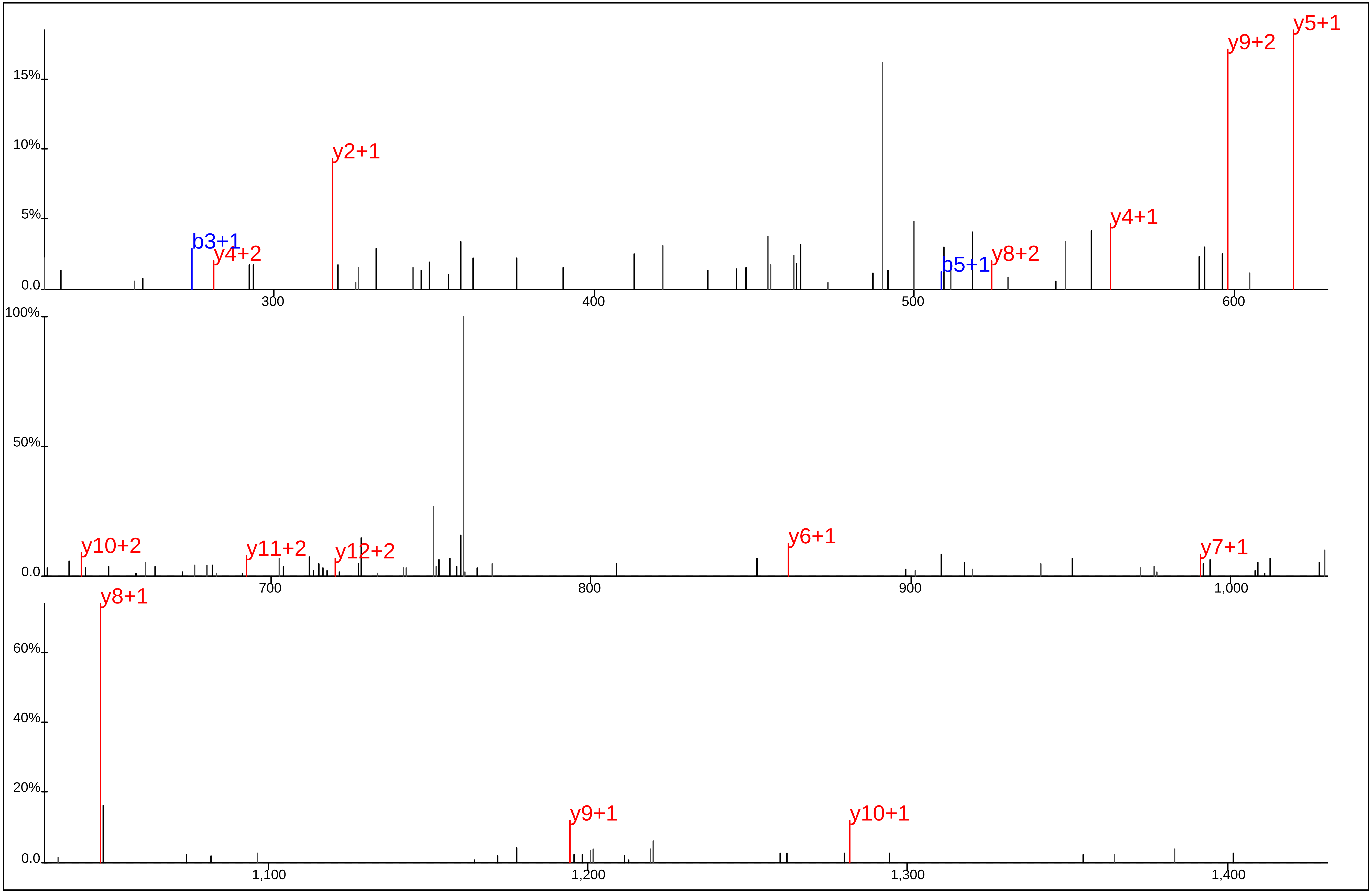

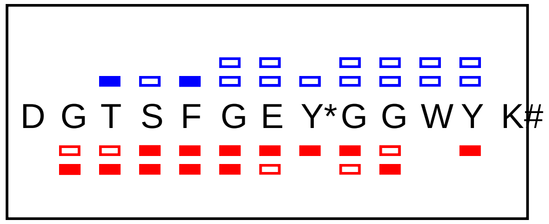

Protein Name:	Kinesin 2
gi Accession:	585363
Precursor Mass:	777.8083+2
Delta Mass:	4.167 ppm
XCorr:	42.92

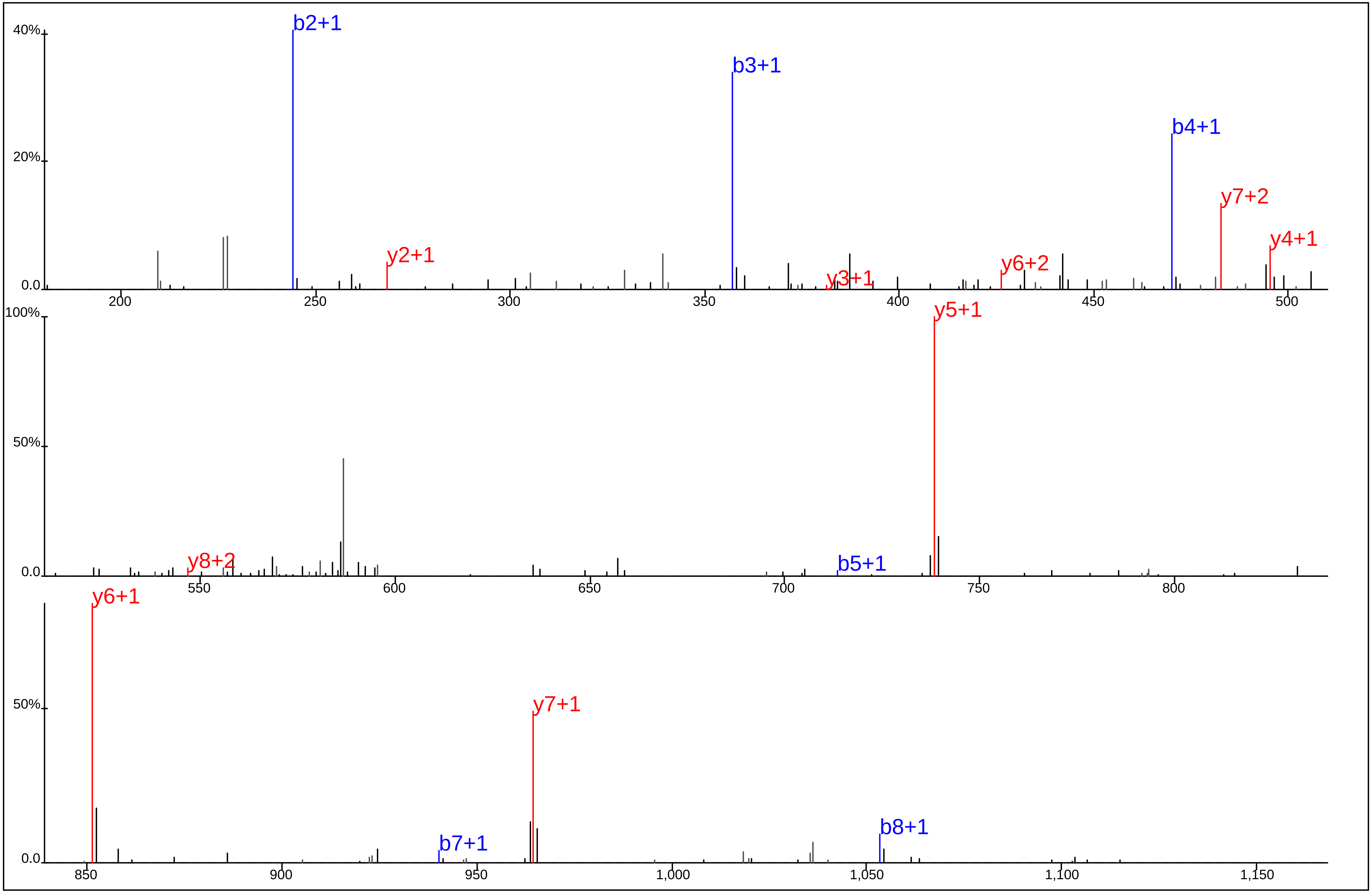

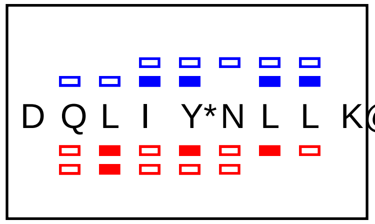

Protein Name:	Lactate dehydrogenase A
gi Accession:	13786849
Precursor Mass:	604.3151+2
Delta Mass:	1.325 ppm
XCorr:	2.4272

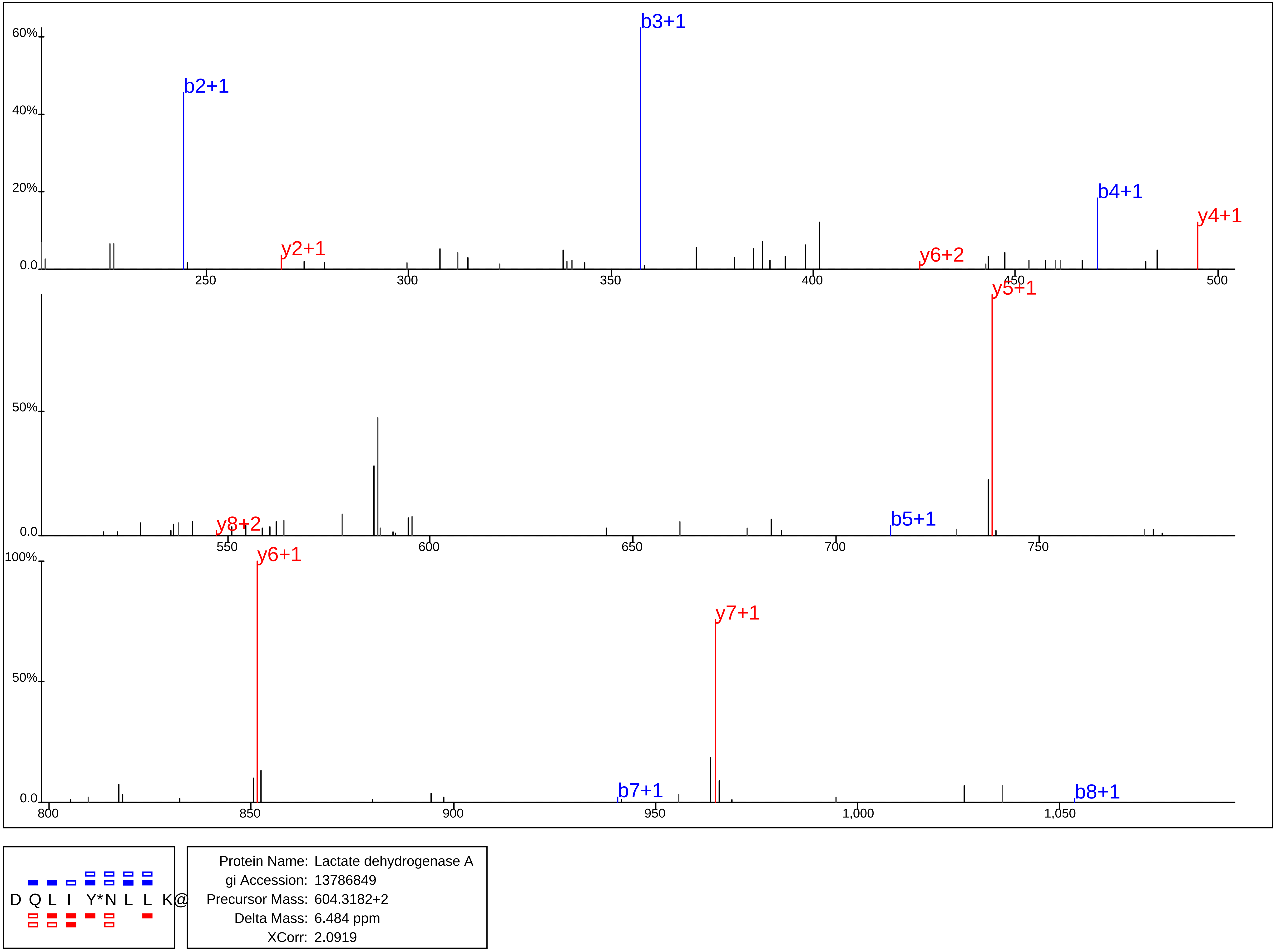

Protein Name:	Lactate dehydrogenase A
gi Accession:	13786849
Precursor Mass:	604.3182+2
Delta Mass:	6.484 ppm
XCorr:	2.0919

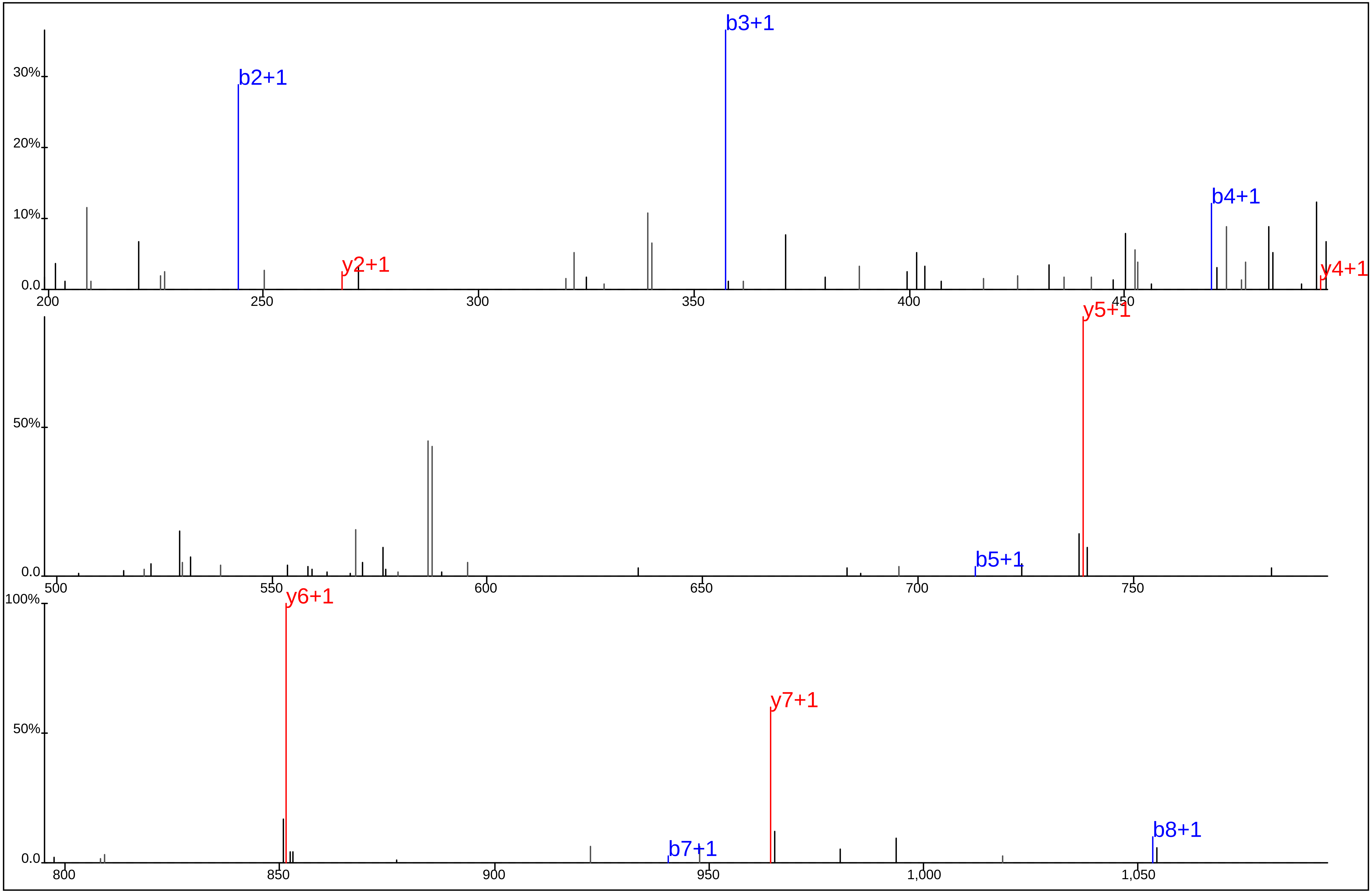

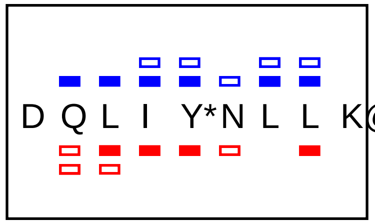

Protein Name:	Lactate dehydrogenase A
gi Accession:	13786849
Precursor Mass:	604.3164+2
Delta Mass:	3.453 ppm
XCorr:	2.3977

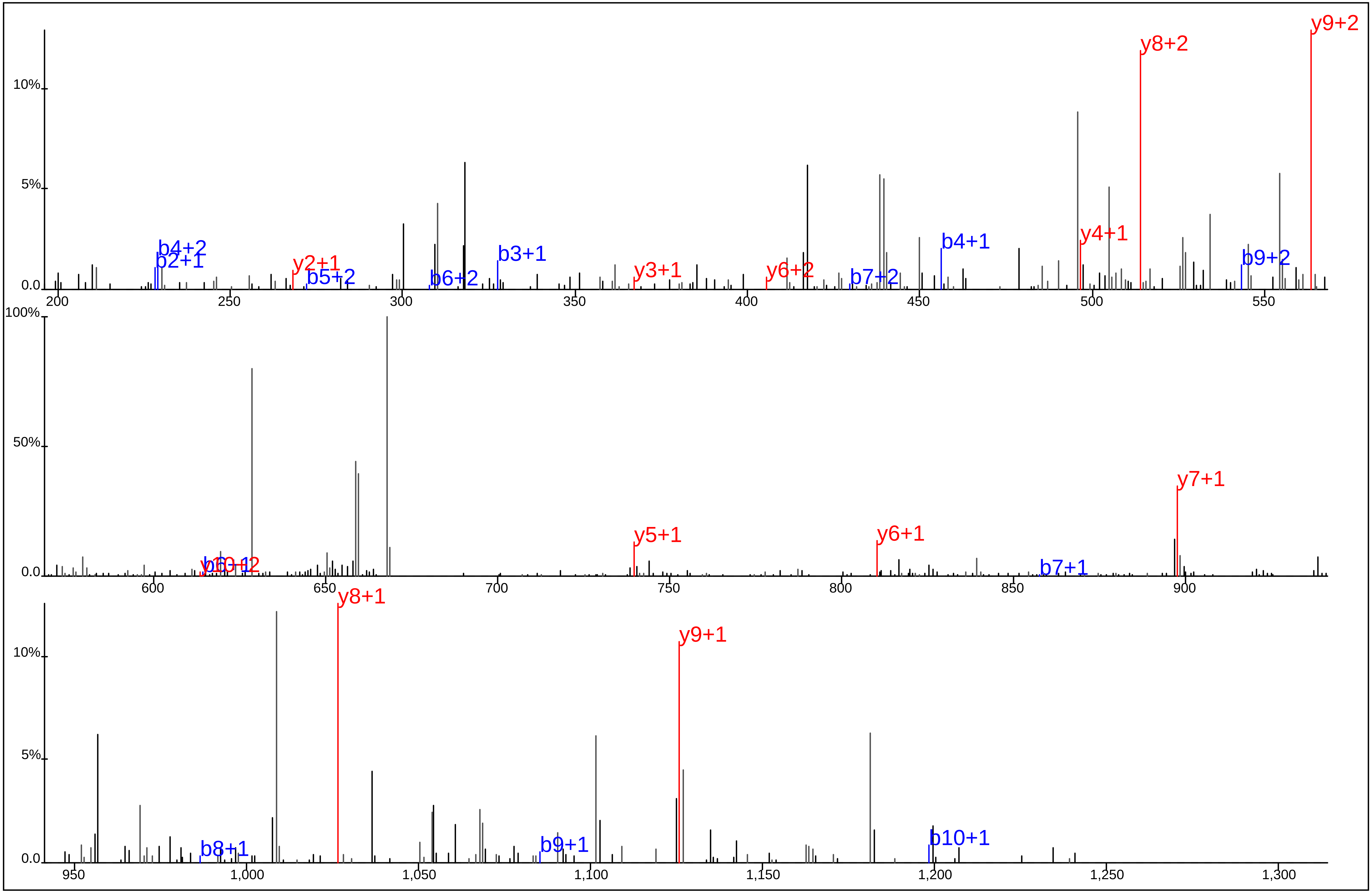

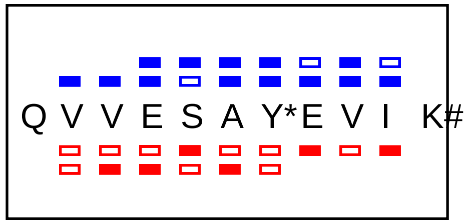

Protein Name:	Lactate dehydrogenase A
gi Accession:	13786849
Precursor Mass:	676.8355+2
Delta Mass:	3.606 ppm
XCorr:	25.49

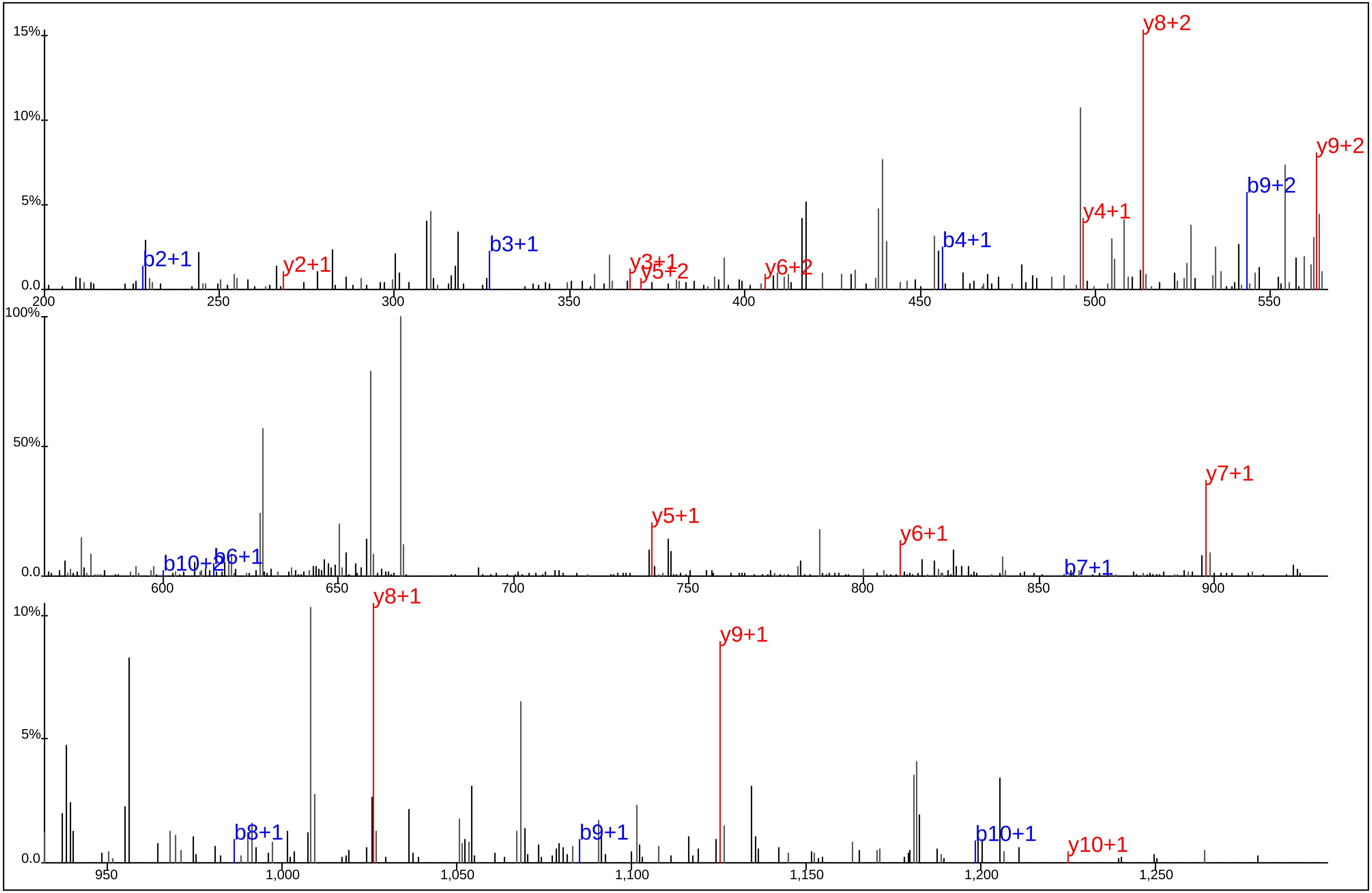

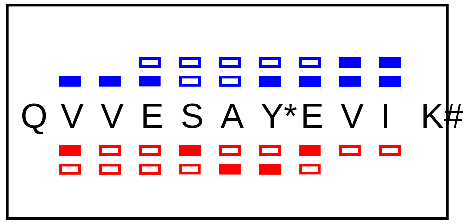

Protein Name: Lactate dehydrogenase A
gi Accession: 13786849
Precursor Mass: 676.8353+2
Delta Mass: 3.251 ppm
XCorr: 31.22

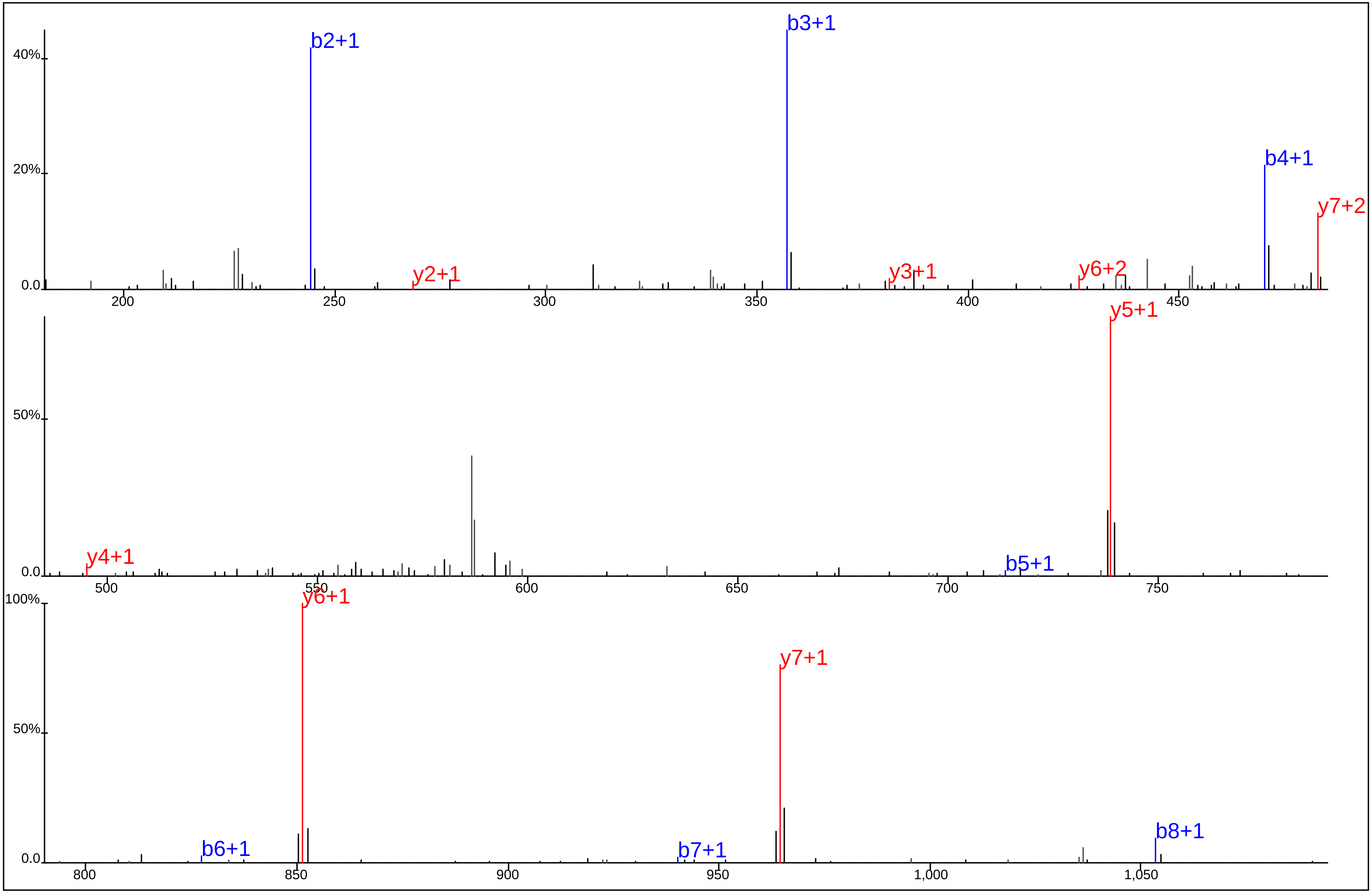

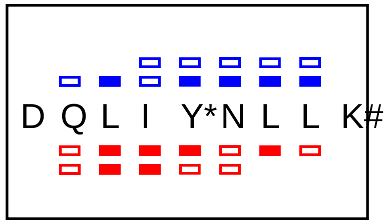

Protein Name: Lactate dehydrogenase A
gi Accession: 13786849
Precursor Mass: 604.3173+2
Delta Mass: 5.000 ppm
XCorr: 33.69

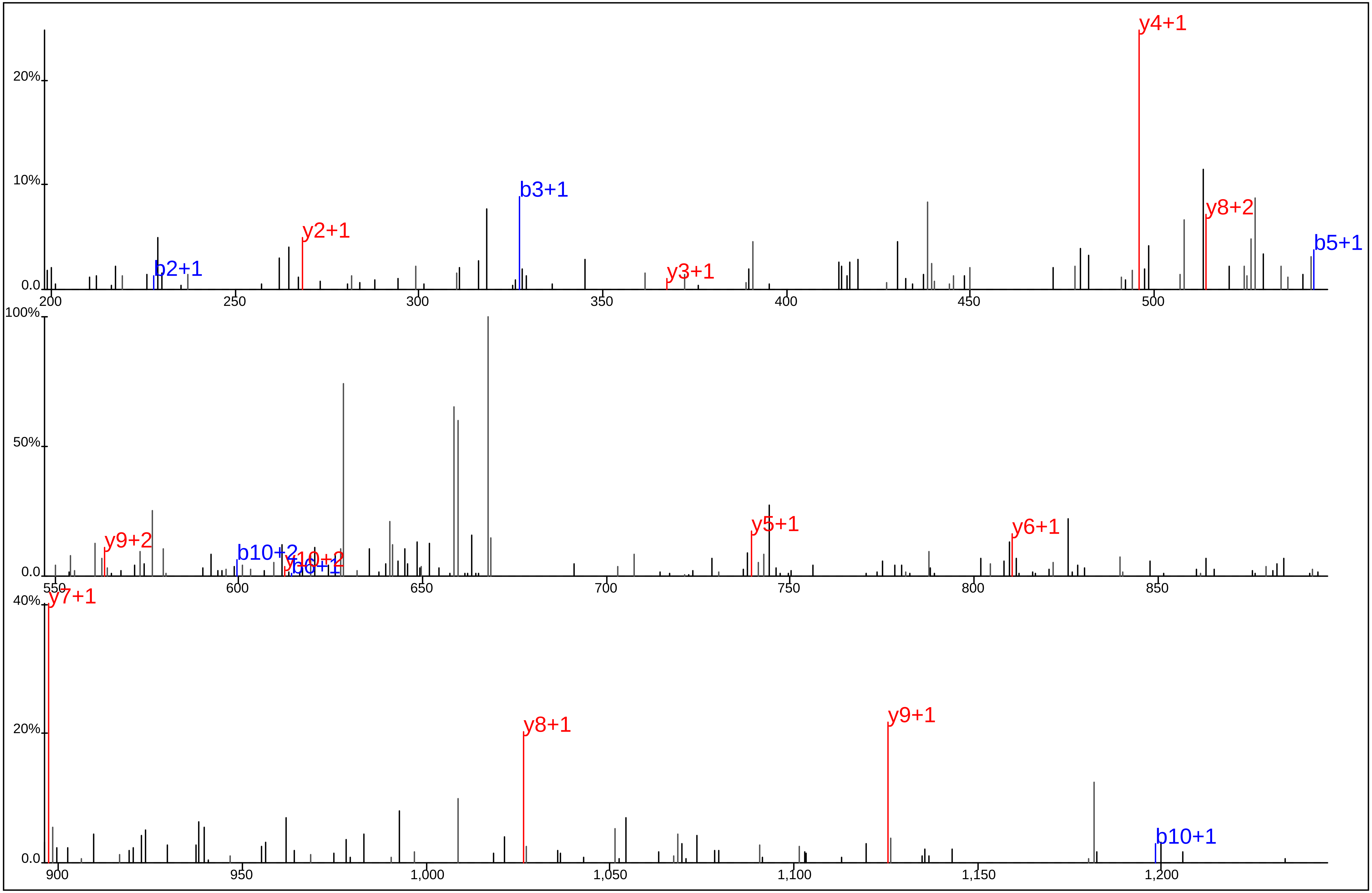

Q V V E S A Y*E V I K#

Protein Name: Lactate dehydrogenase A
gi Accession: 13786849
Precursor Mass: 676.8362+2
Delta Mass: 4.693 ppm
XCorr: 23.07

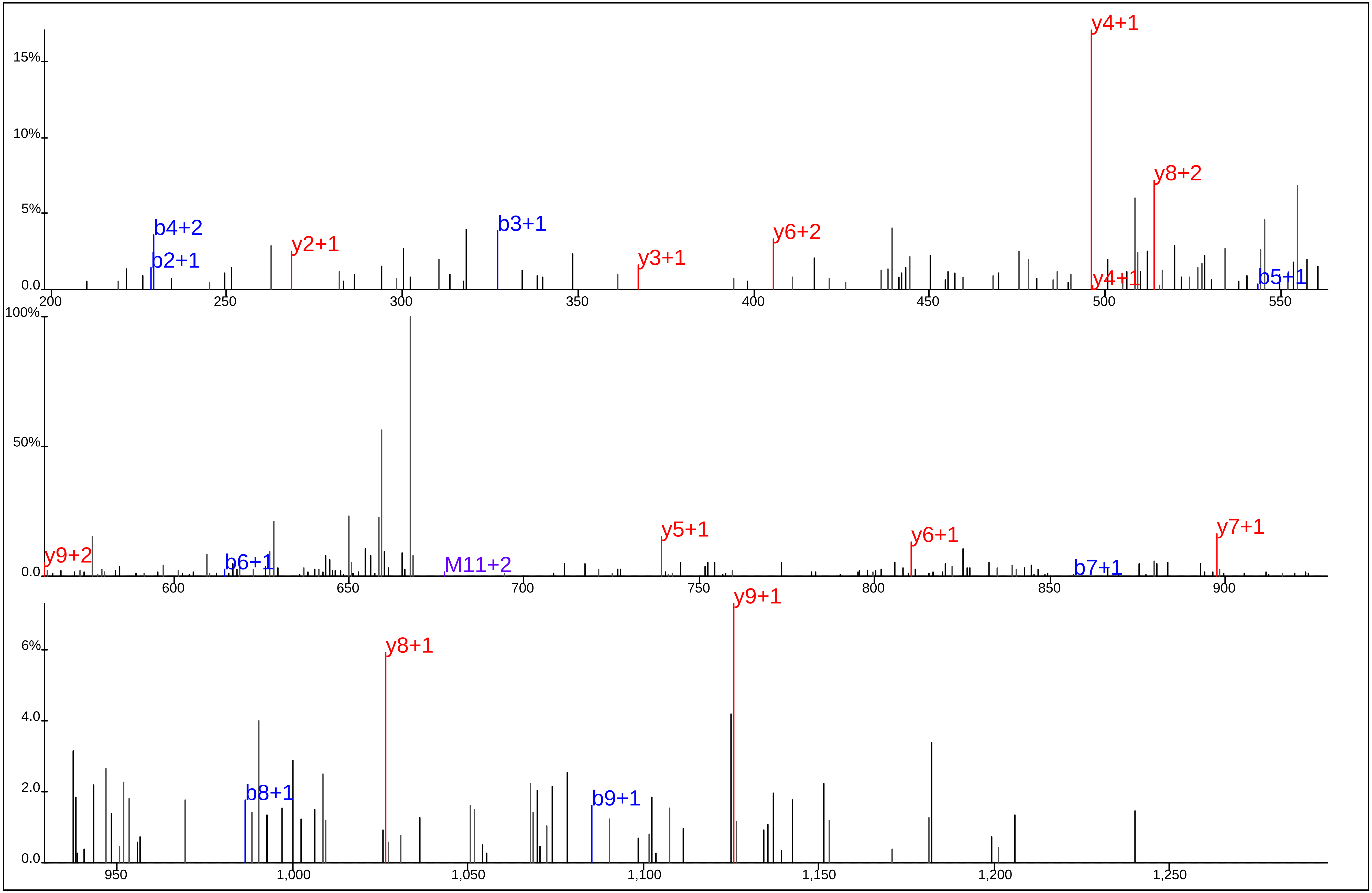

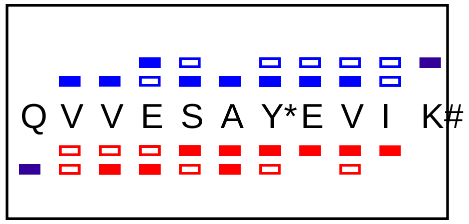

Protein Name: Lactate dehydrogenase A
gi Accession: 13786849
Precursor Mass: 676.8363+2
Delta Mass: 4.782 ppm
XCorr: 27.79

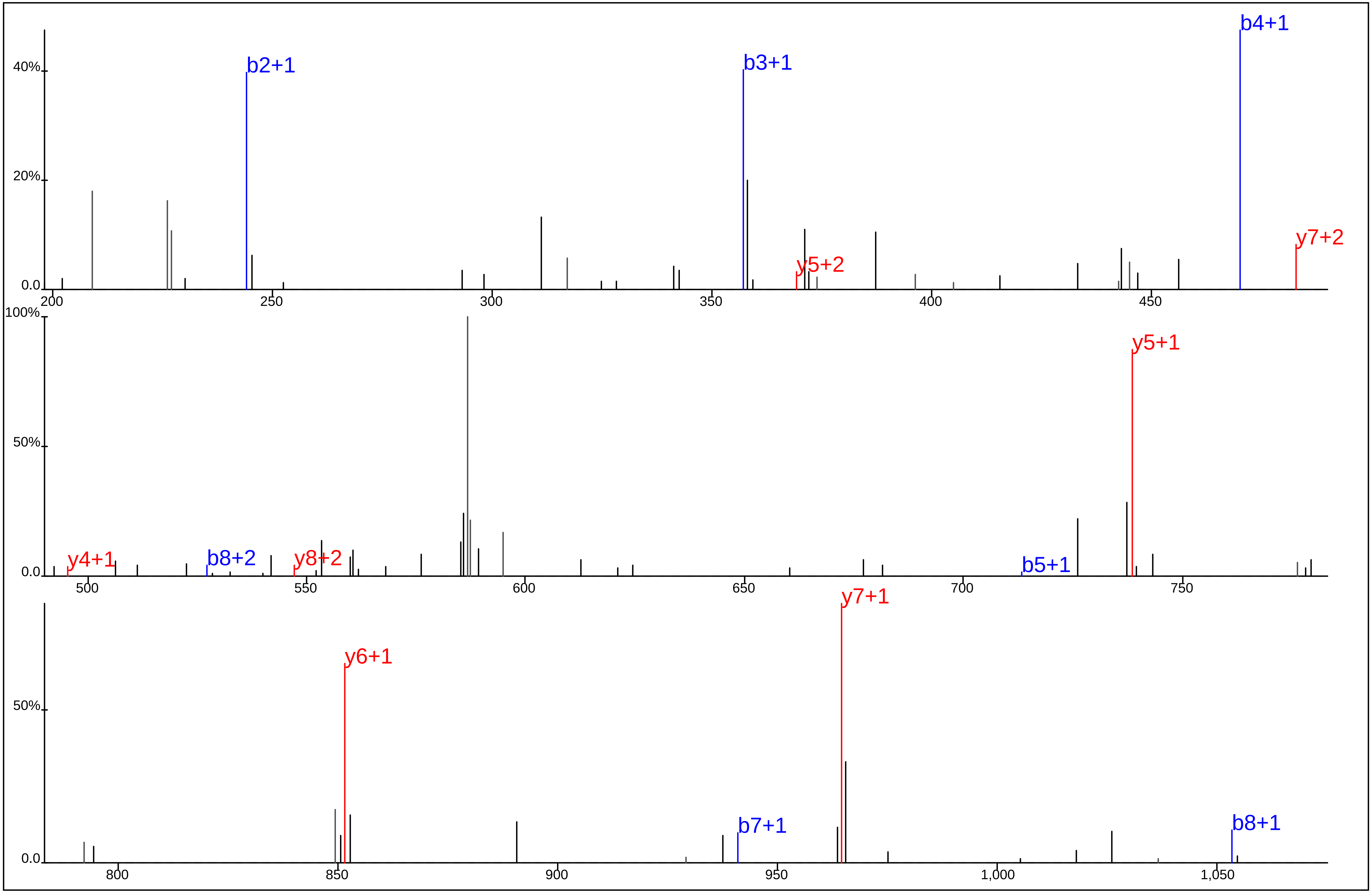

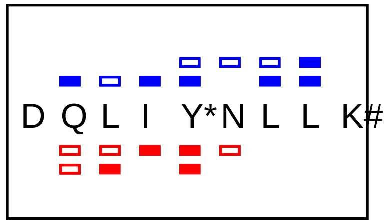

Protein Name: Lactate dehydrogenase A
gi Accession: 13786849
Precursor Mass: 604.3160+2
Delta Mass: 2.772 ppm
XCorr: 32.29

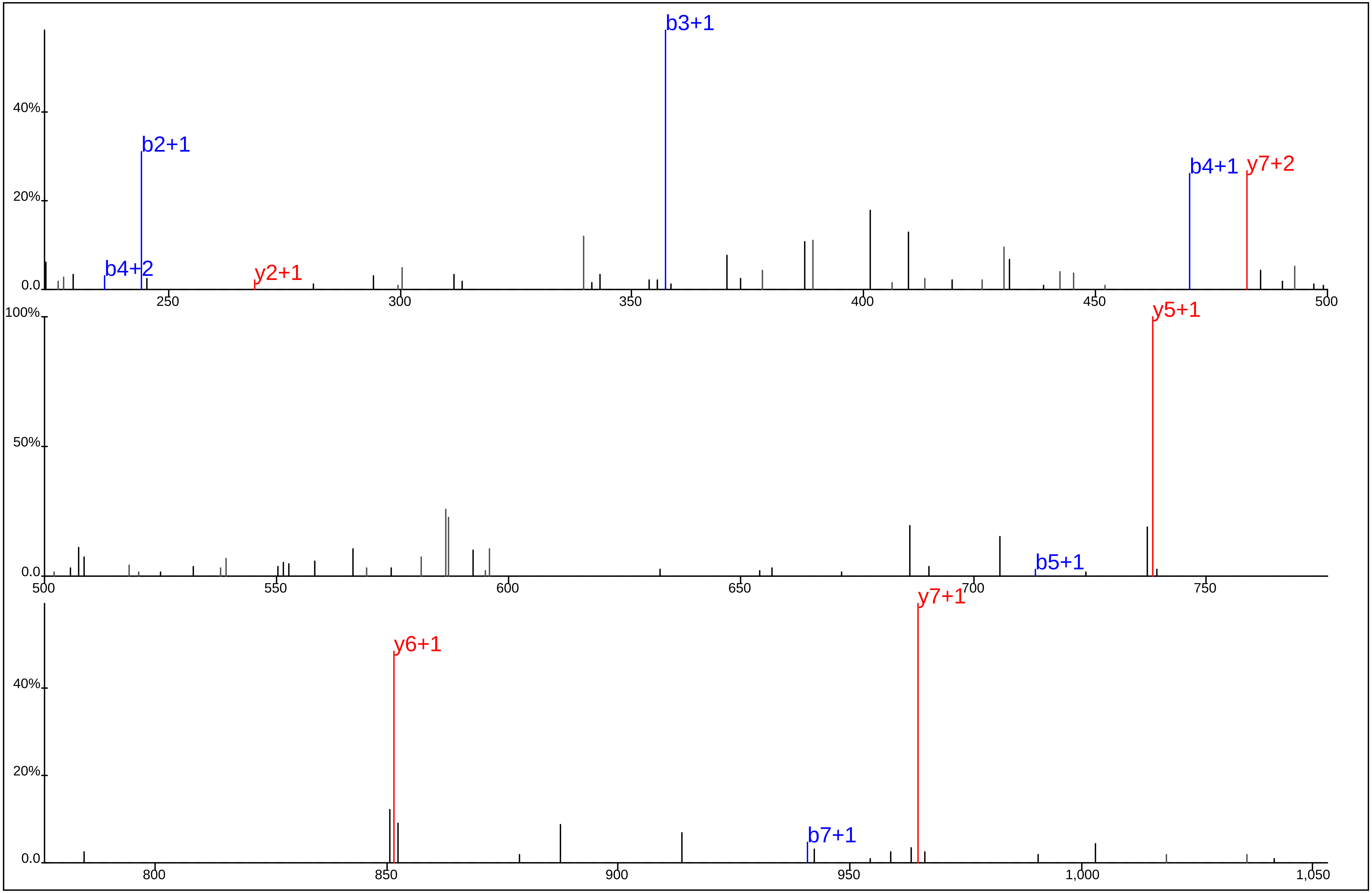

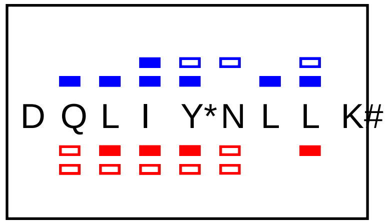

Protein Name:	Lactate dehydrogenase A
gi Accession:	13786849
Precursor Mass:	604.3247+2
Delta Mass:	17.231 ppm
XCorr:	30.76

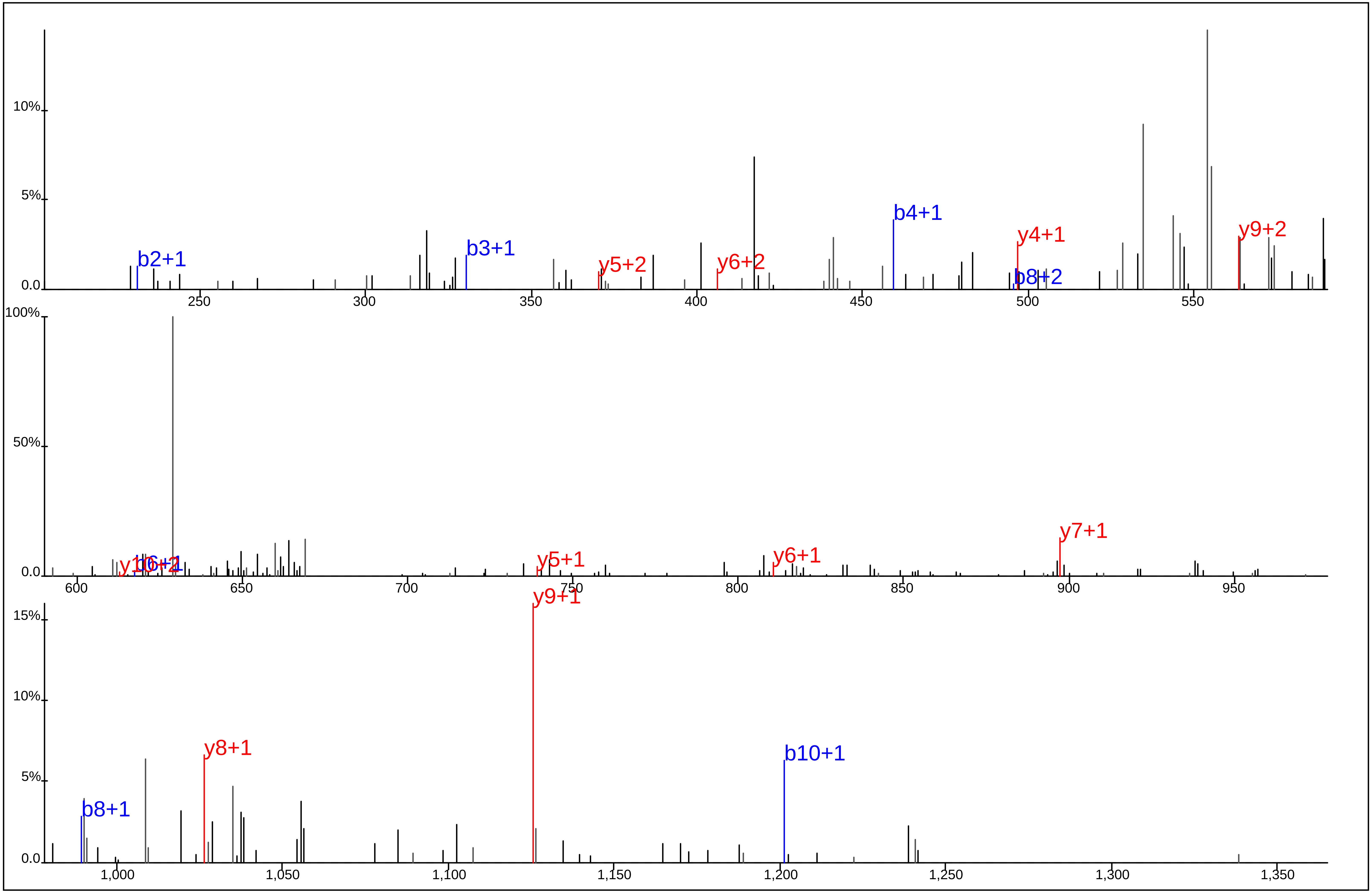

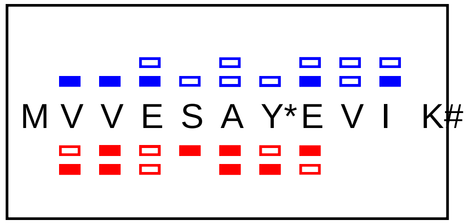

Protein Name: Lactate dehydrogenase B
gi Accession: 13786847
Precursor Mass: 678.3270+2
Delta Mass: 4.439 ppm
XCorr: 17.5

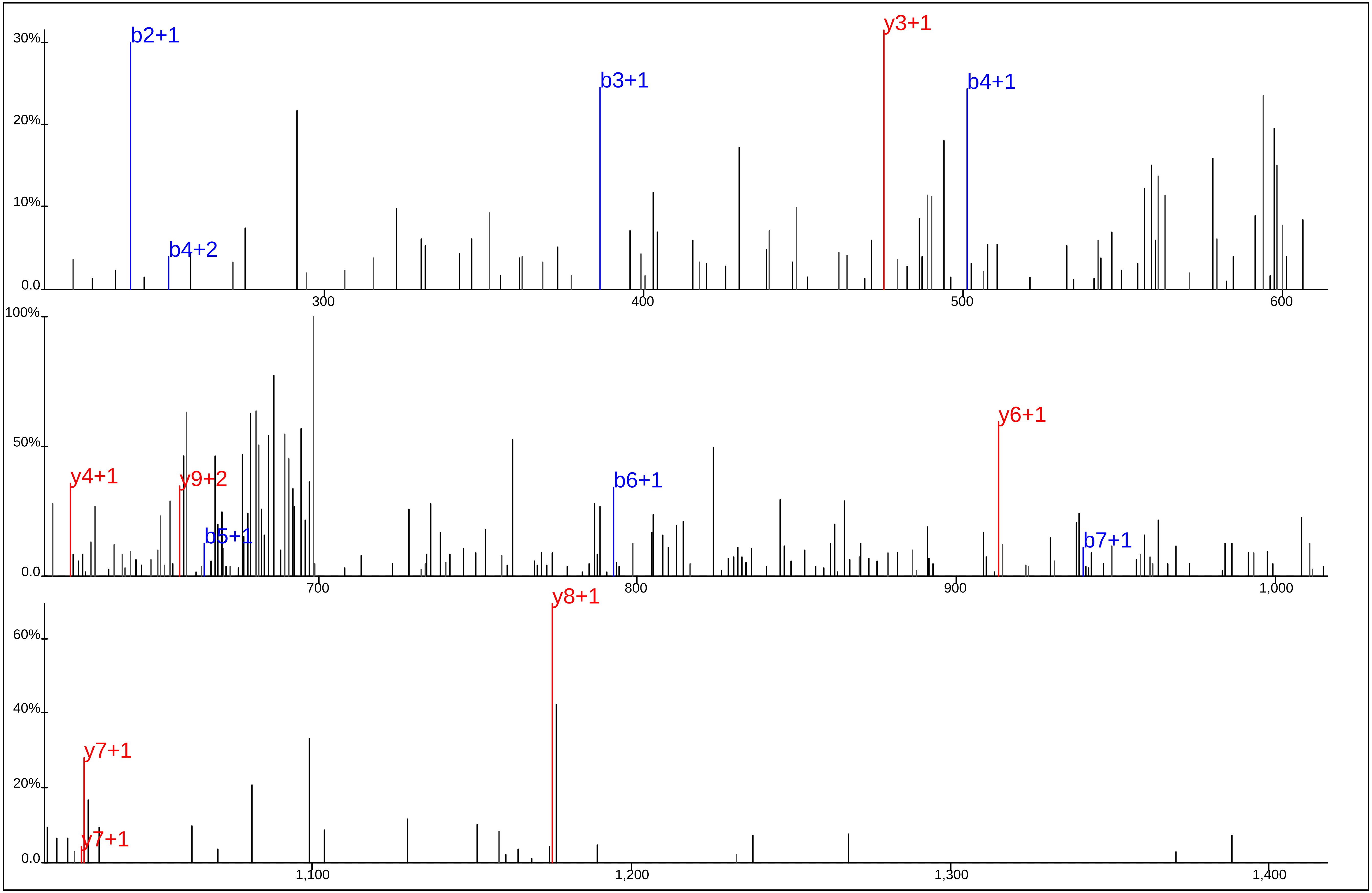

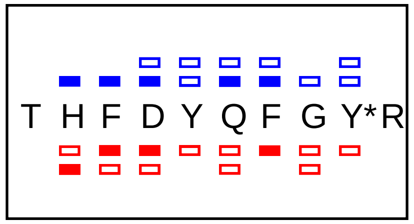

Protein Name: LARP
gi Accession: 3882183
Precursor Mass: 707.2899+2
Delta Mass: 7.229 ppm
XCorr: 11.39

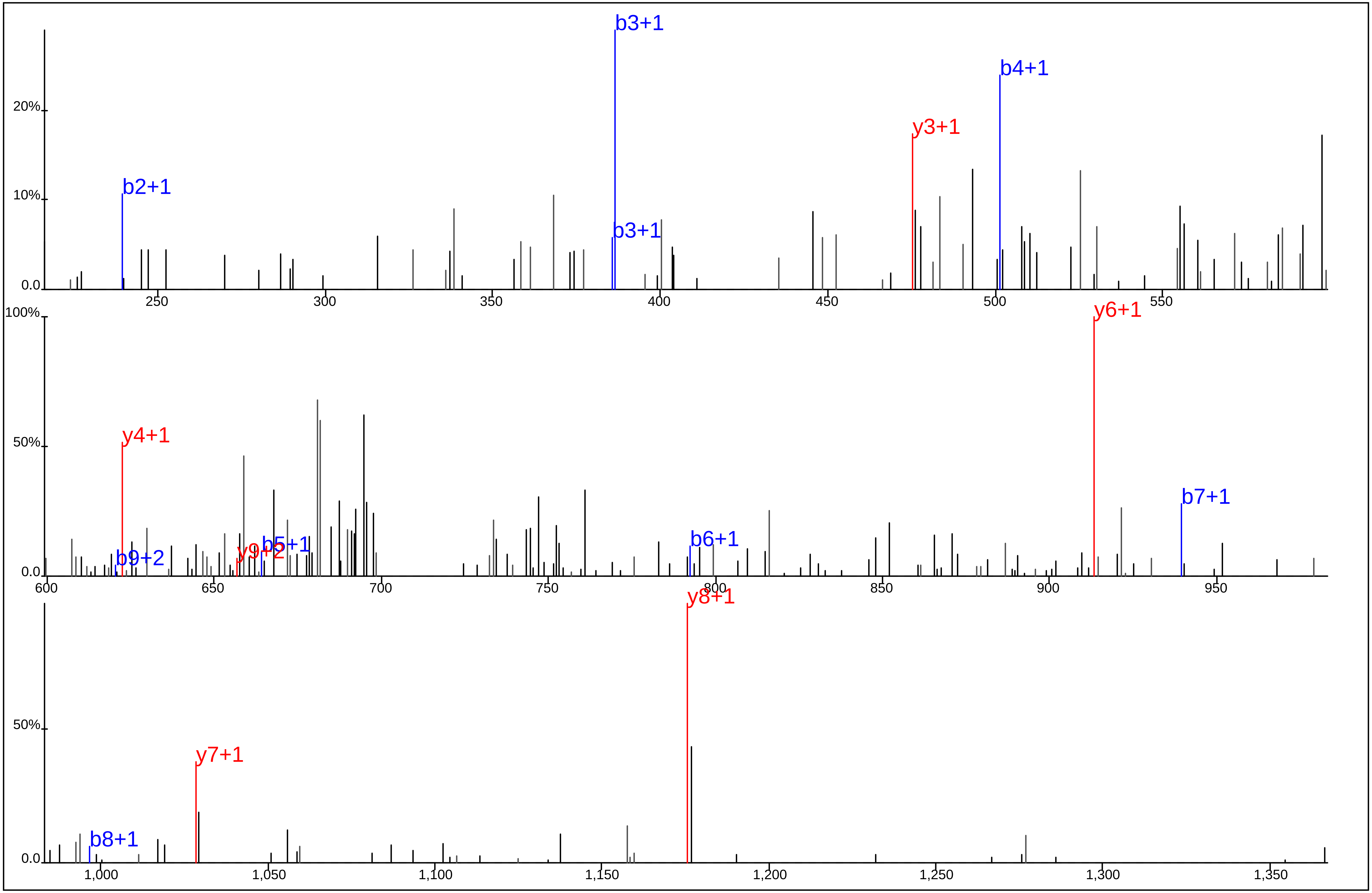

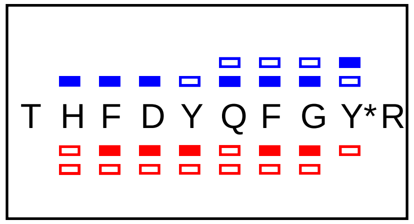

Protein Name:	LARP
gi Accession:	3882183
Precursor Mass:	707.2882+2
Delta Mass:	4.809 ppm
XCorr:	17.74

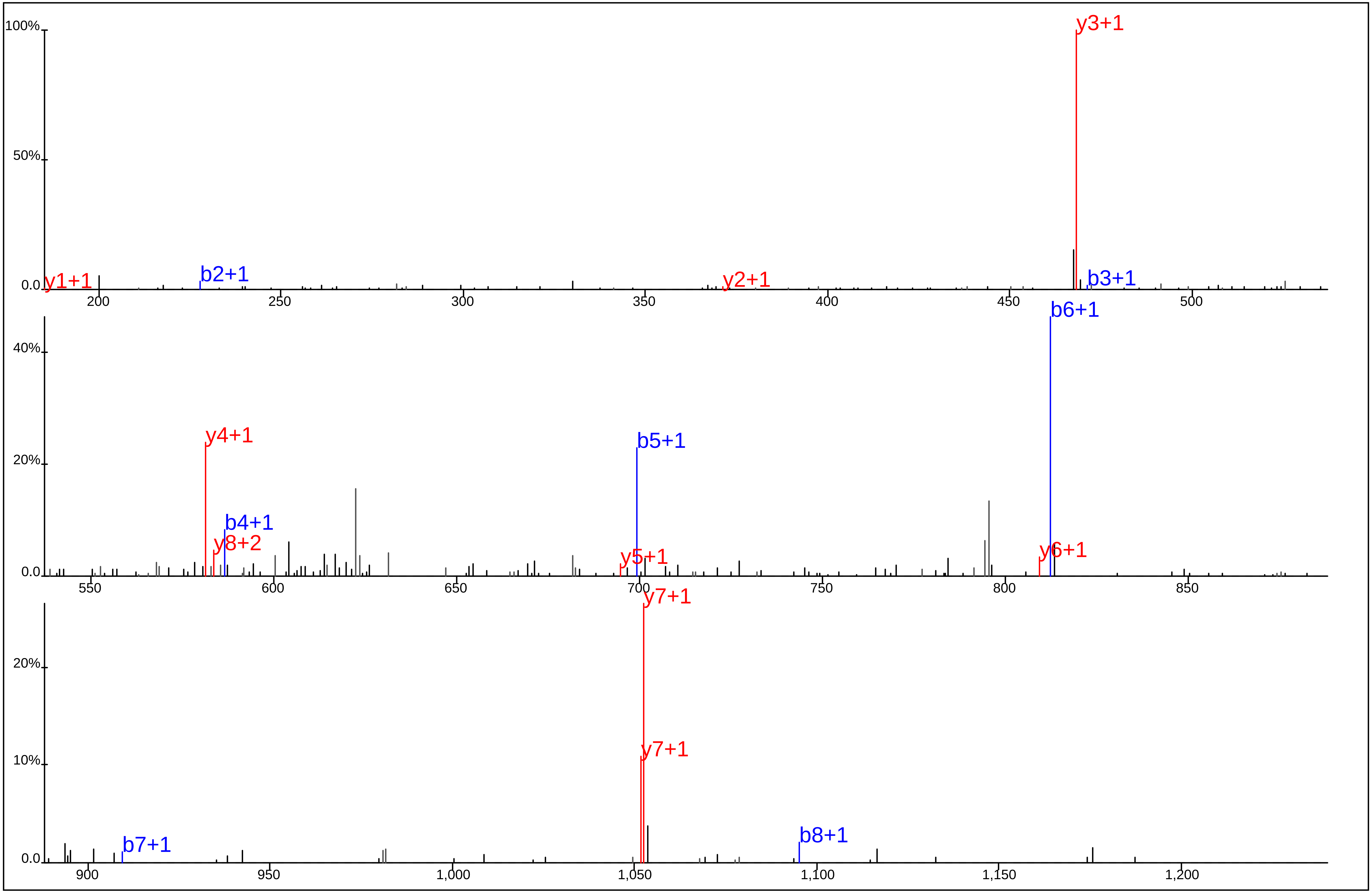

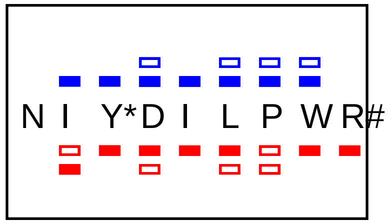

Protein Name: LAX
gi Accession: 7020372
Precursor Mass: 640.3111+2
Delta Mass: 3.048 ppm
XCorr: 2.3089

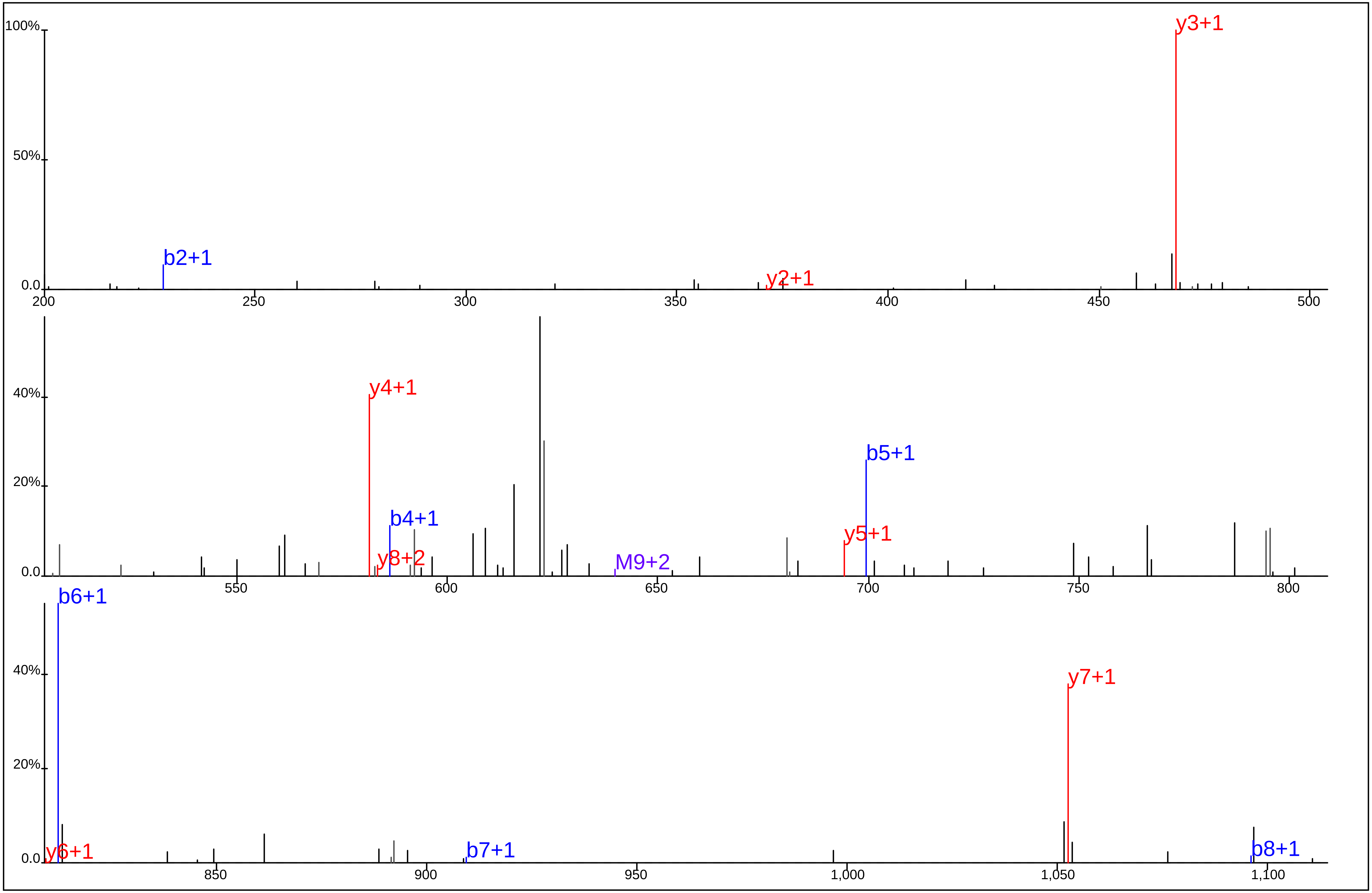

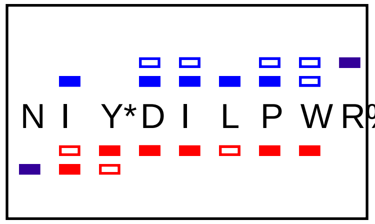

Protein Name: LAX
gi Accession: 7020372
Precursor Mass: 640.3109+2
Delta Mass: 2.781 ppm
XCorr: 19.94

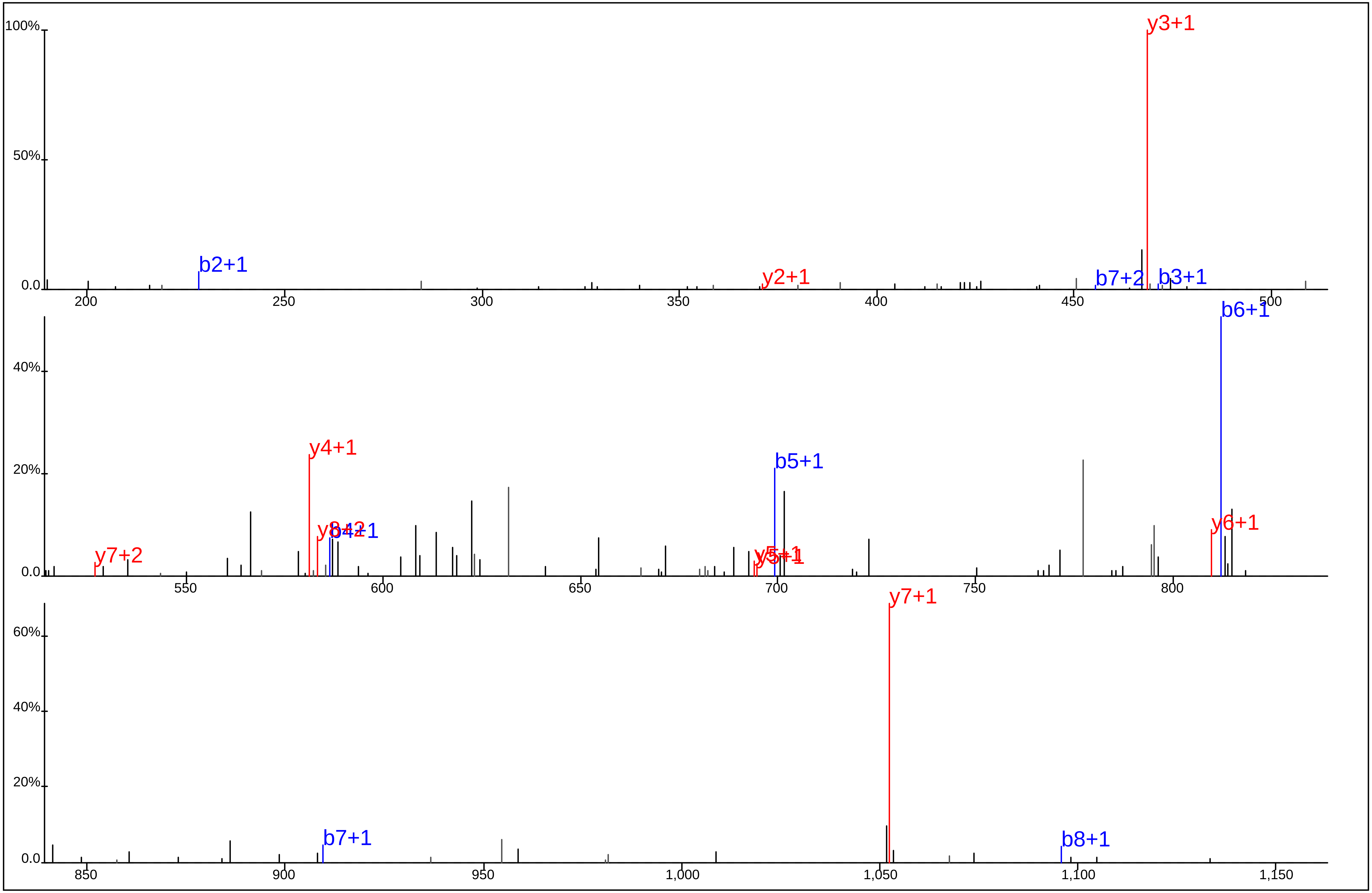

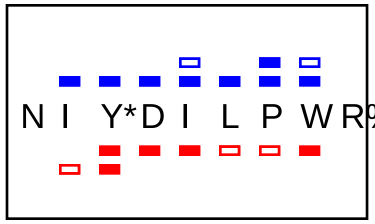

Protein Name:	LAX
gi Accession:	7020372
Precursor Mass:	640.3125+2
Delta Mass:	5.258 ppm
XCorr:	26.54

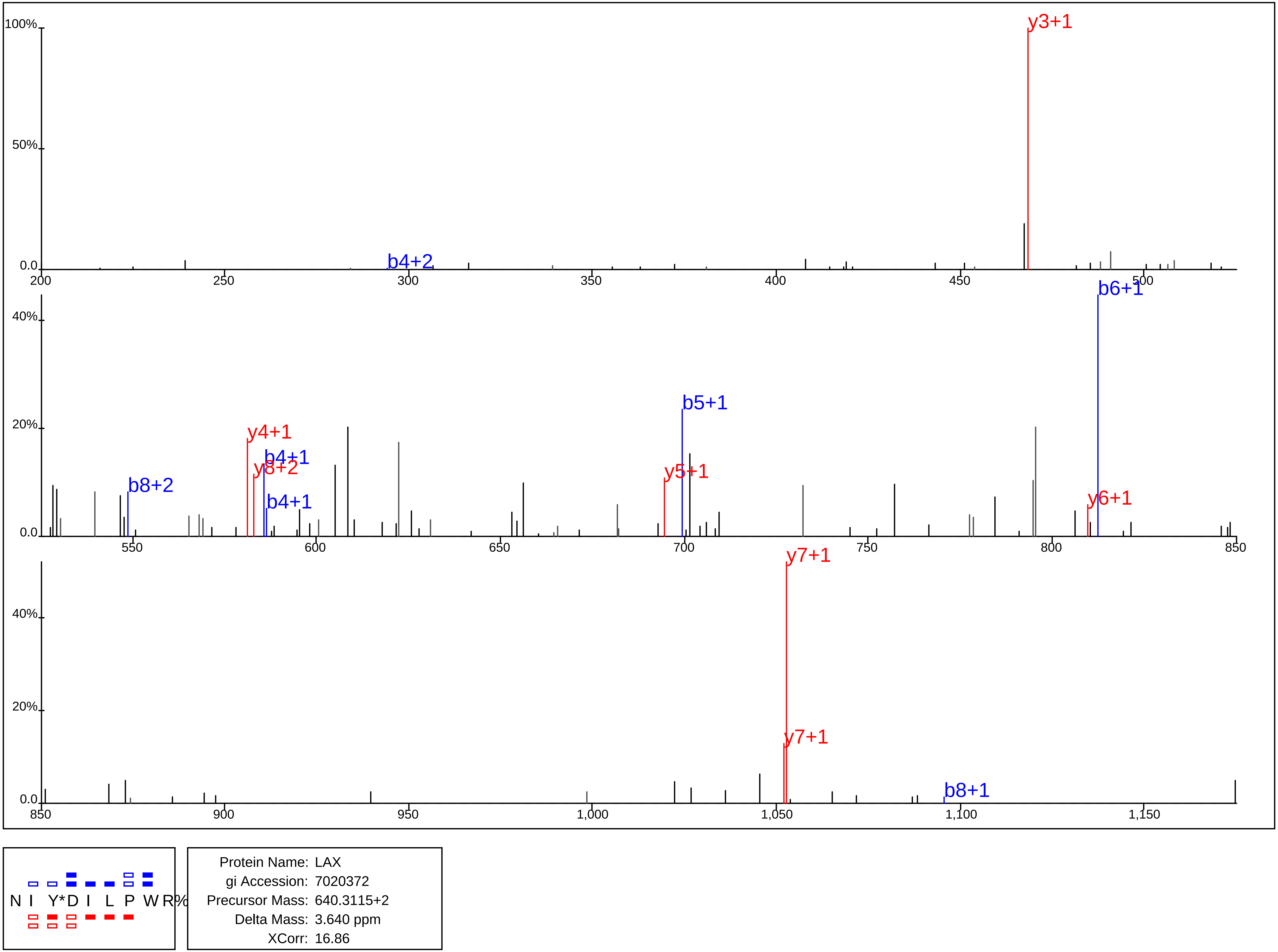

Protein Name: LAX
gi Accession: 7020372
Precursor Mass: 640.3115+2
Delta Mass: 3.640 ppm
XCorr: 16.86

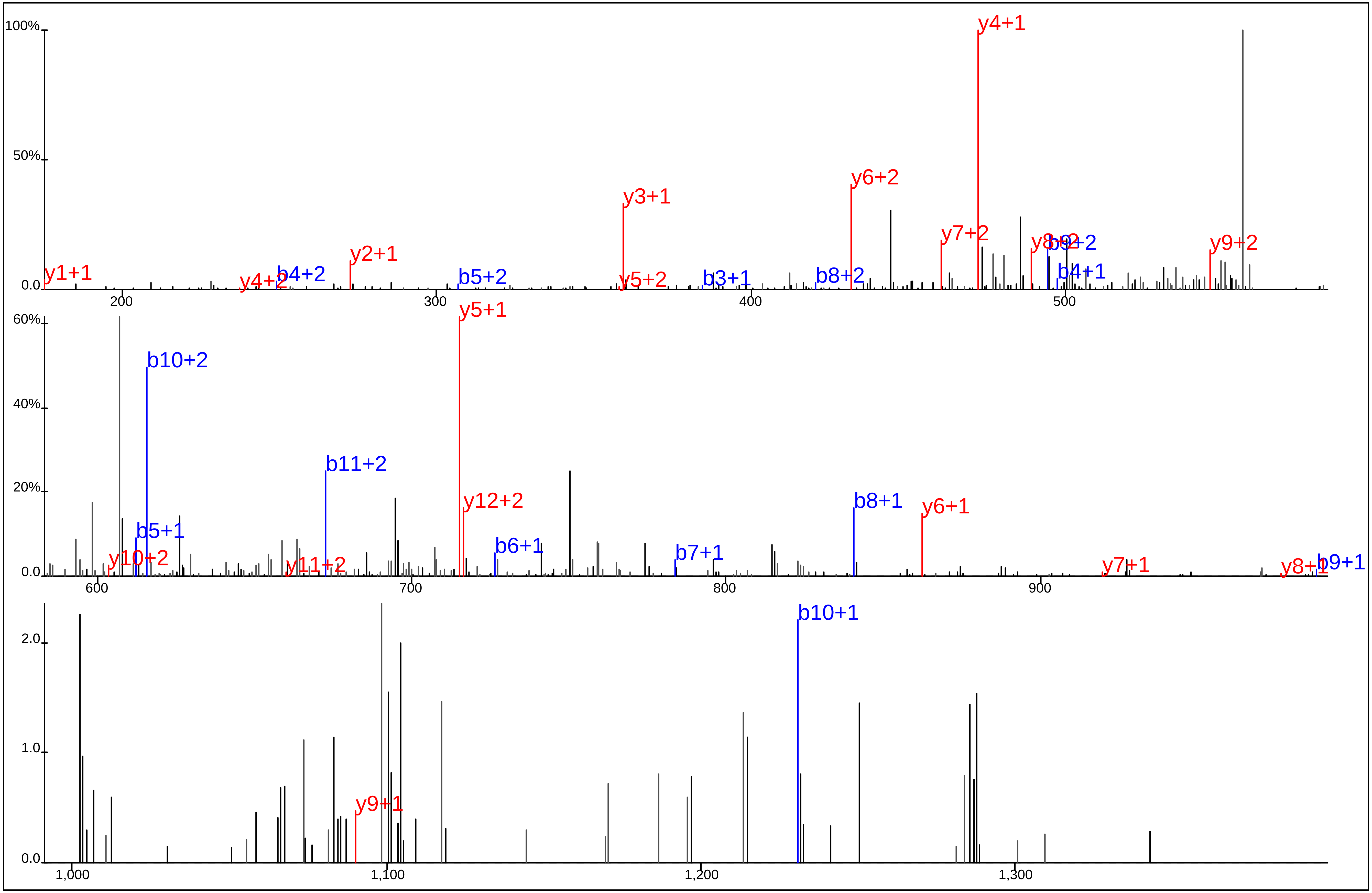

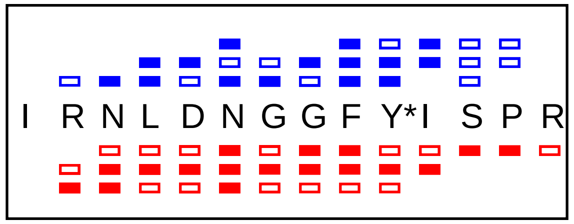

Protein Name:	Lck
gi Accession:	6984209
Precursor Mass:	567.9431+3
Delta Mass:	2.226 ppm
XCorr:	26.35

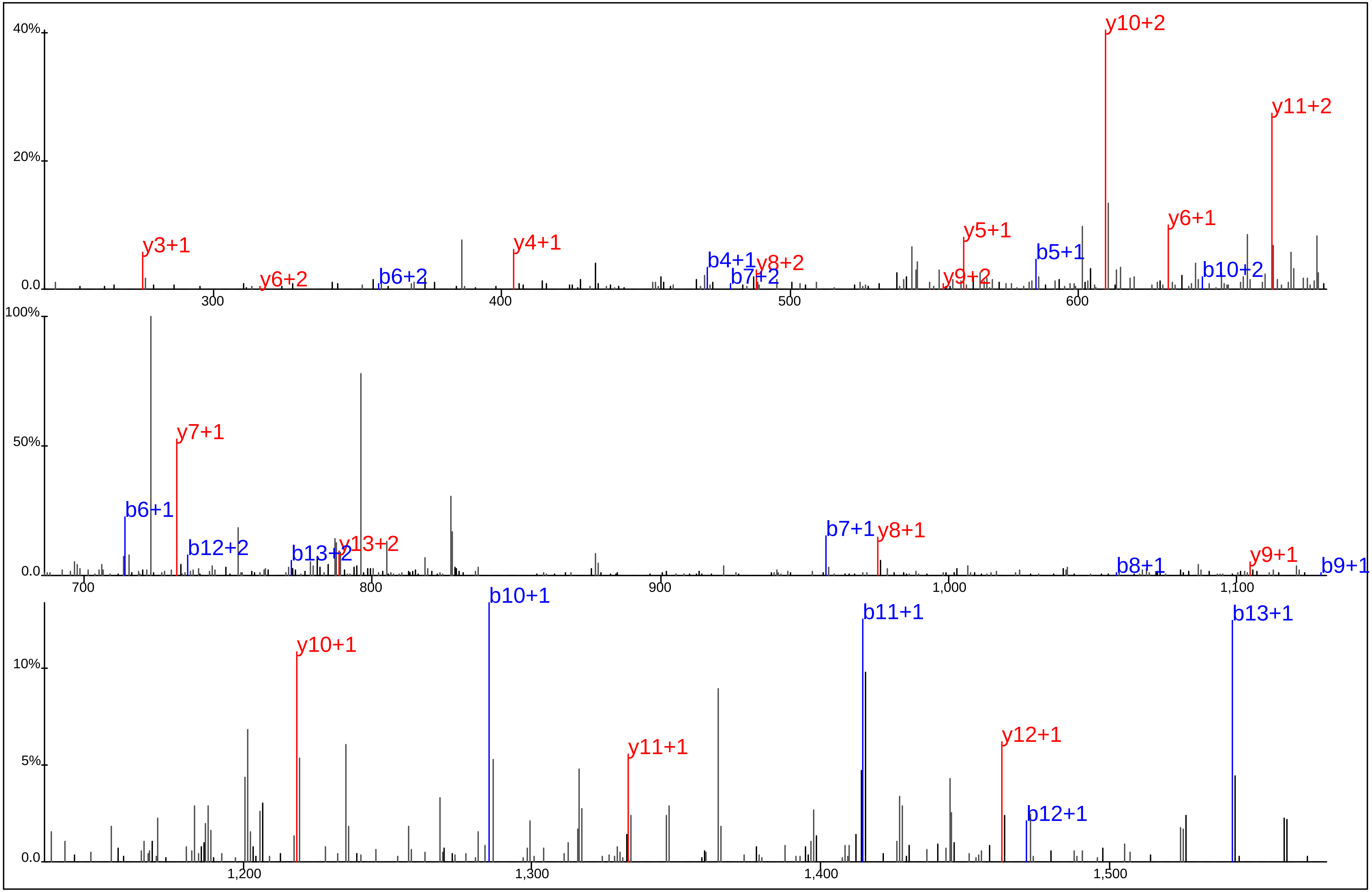

L I E D N E Y*T A R E G A K

Protein Name:	Lck
gi Accession:	6984209
Precursor Mass:	844.8851+2
Delta Mass:	6.033 ppm
XCorr:	56.9

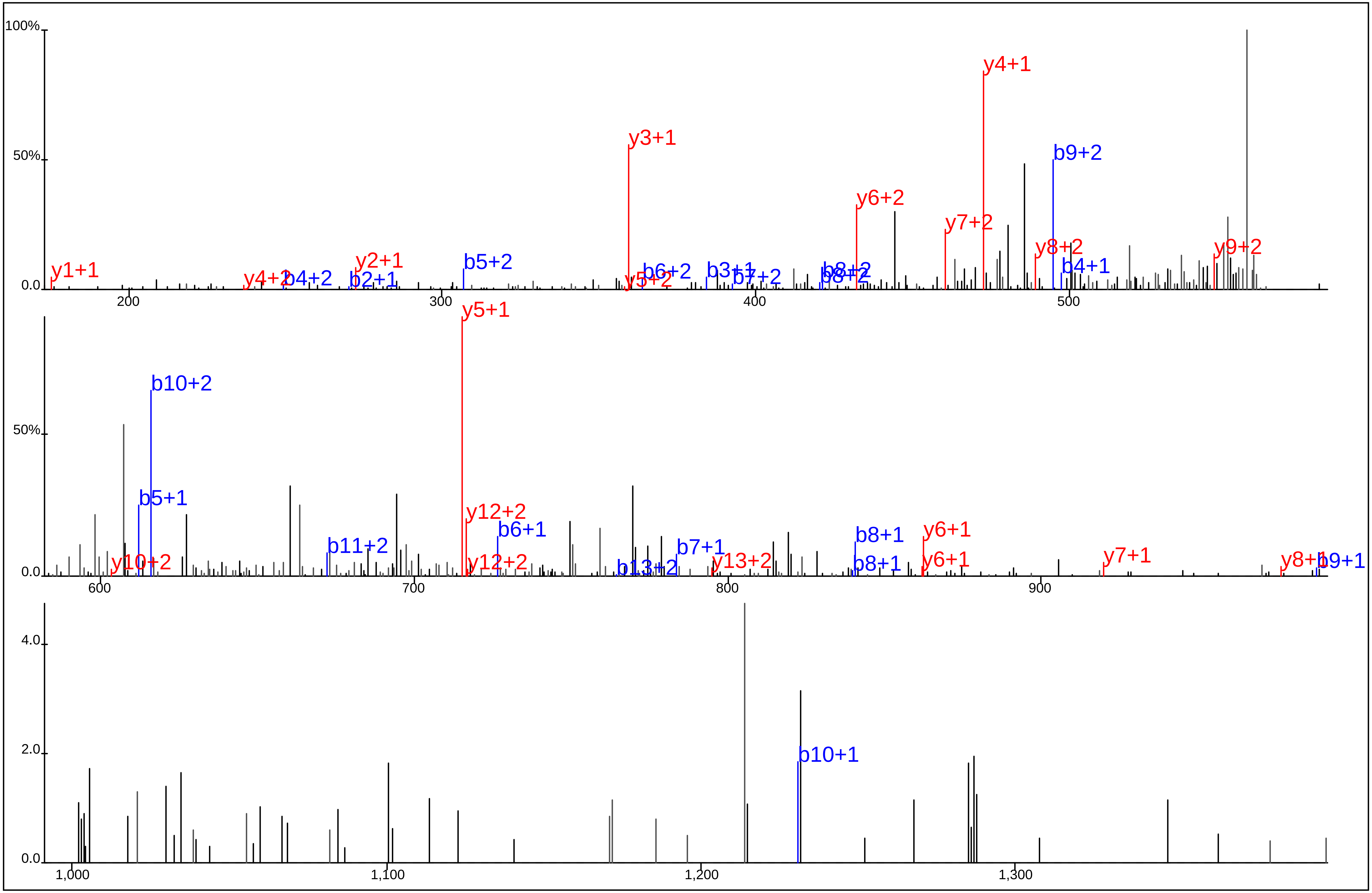

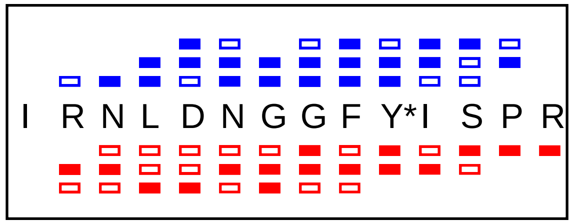

Protein Name: Lck
gi Accession: 6984209
Precursor Mass: 567.9437+3
Delta Mass: 3.301 ppm
XCorr: 38.94

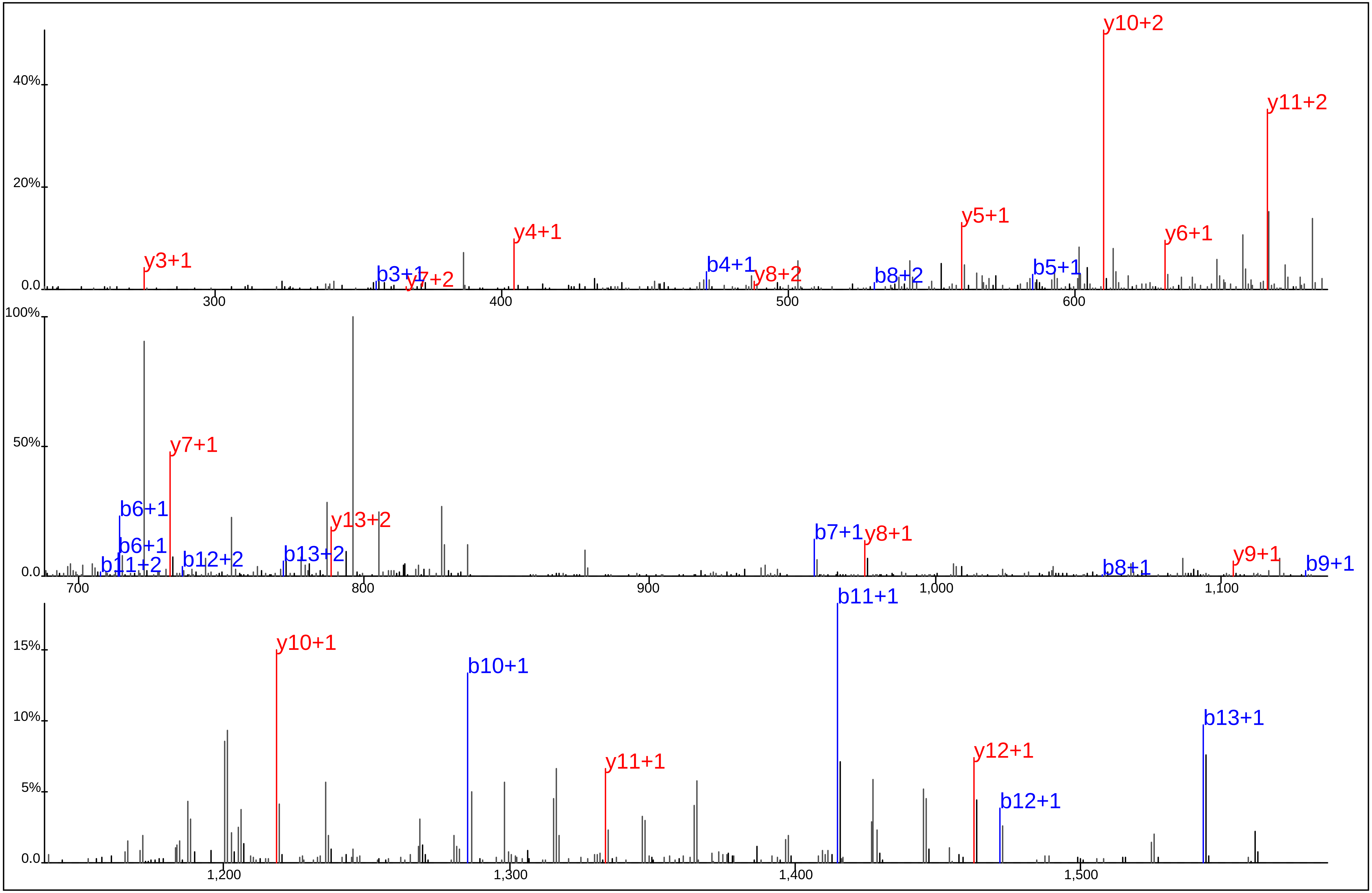

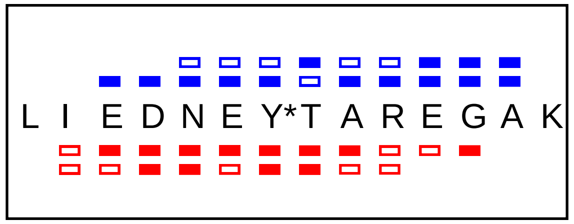

Protein Name: Lck
gi Accession: 6984209
Precursor Mass: 844.8835+2
Delta Mass: 4.156 ppm
XCorr: 68.6

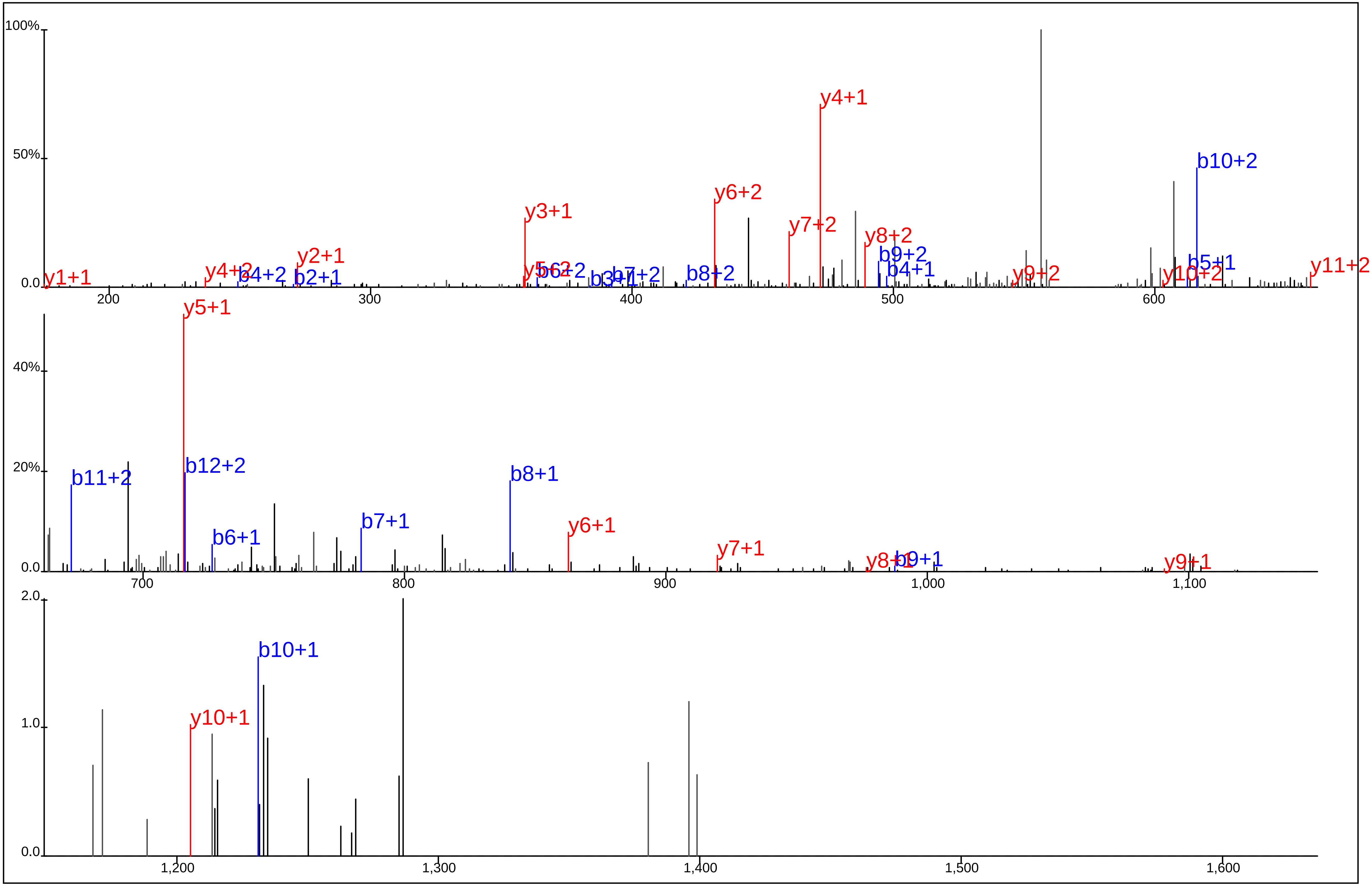

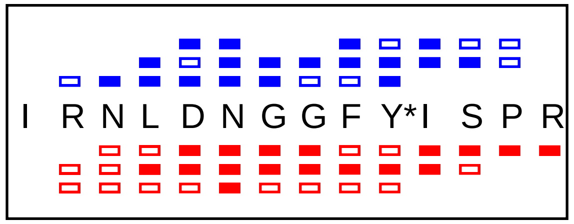

Protein Name:	Lck
gi Accession:	6984209
Precursor Mass:	567.9431+3
Delta Mass:	2.226 ppm
XCorr:	32.9

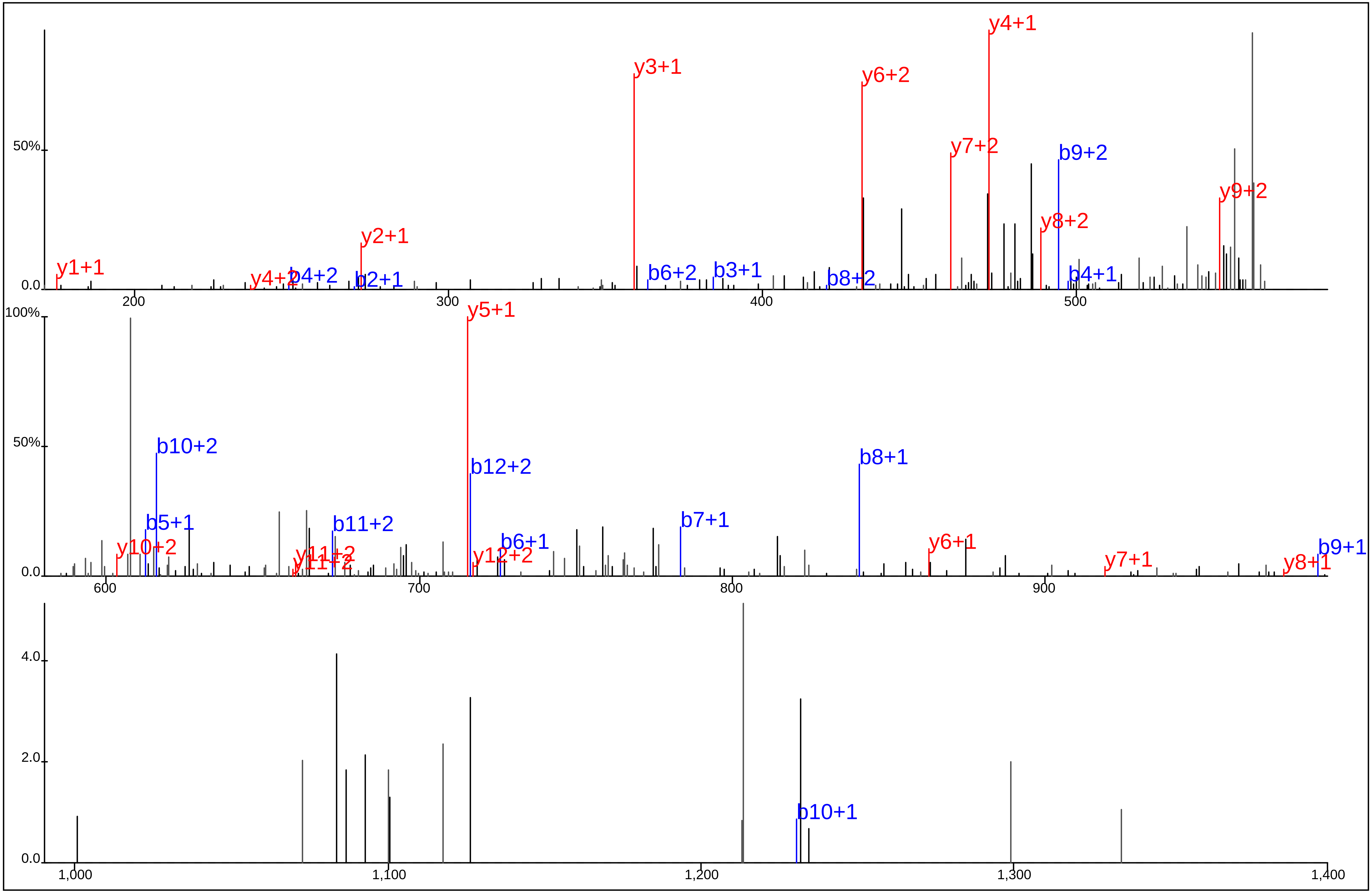

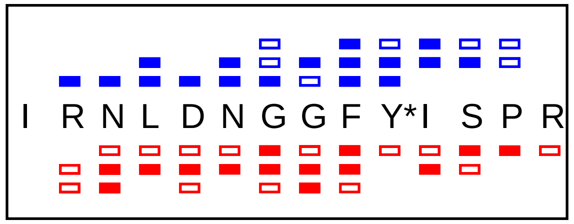

Protein Name:	Lck
gi Accession:	6984209
Precursor Mass:	567.9438+3
Delta Mass:	3.624 ppm
XCorr:	31.23

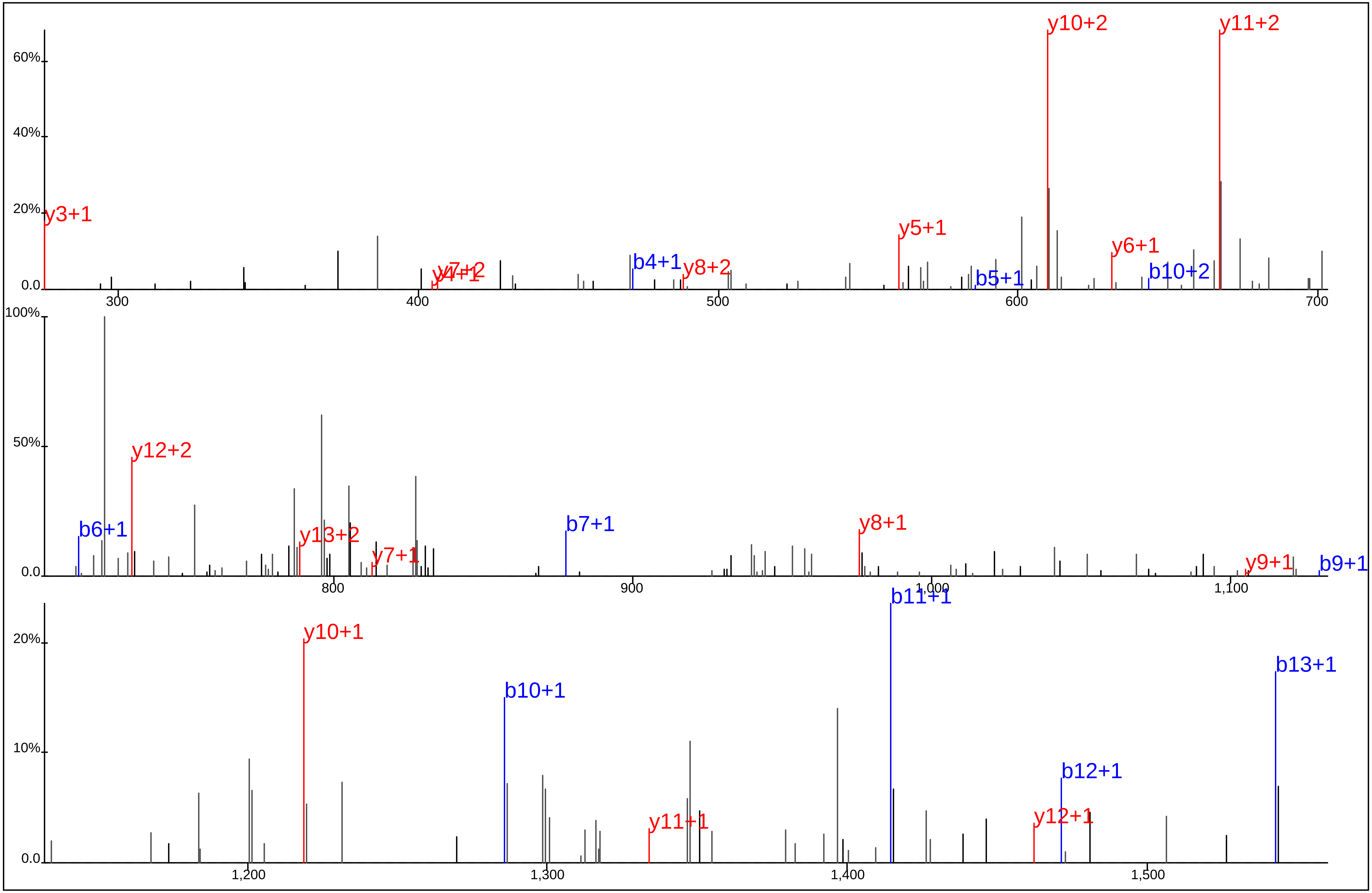

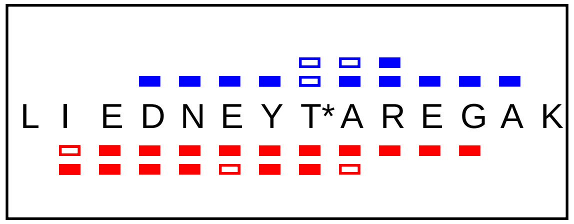

Protein Name:	Lck
gi Accession:	6984209
Precursor Mass:	844.8839+2
Delta Mass:	4.588 ppm
XCorr:	36.51

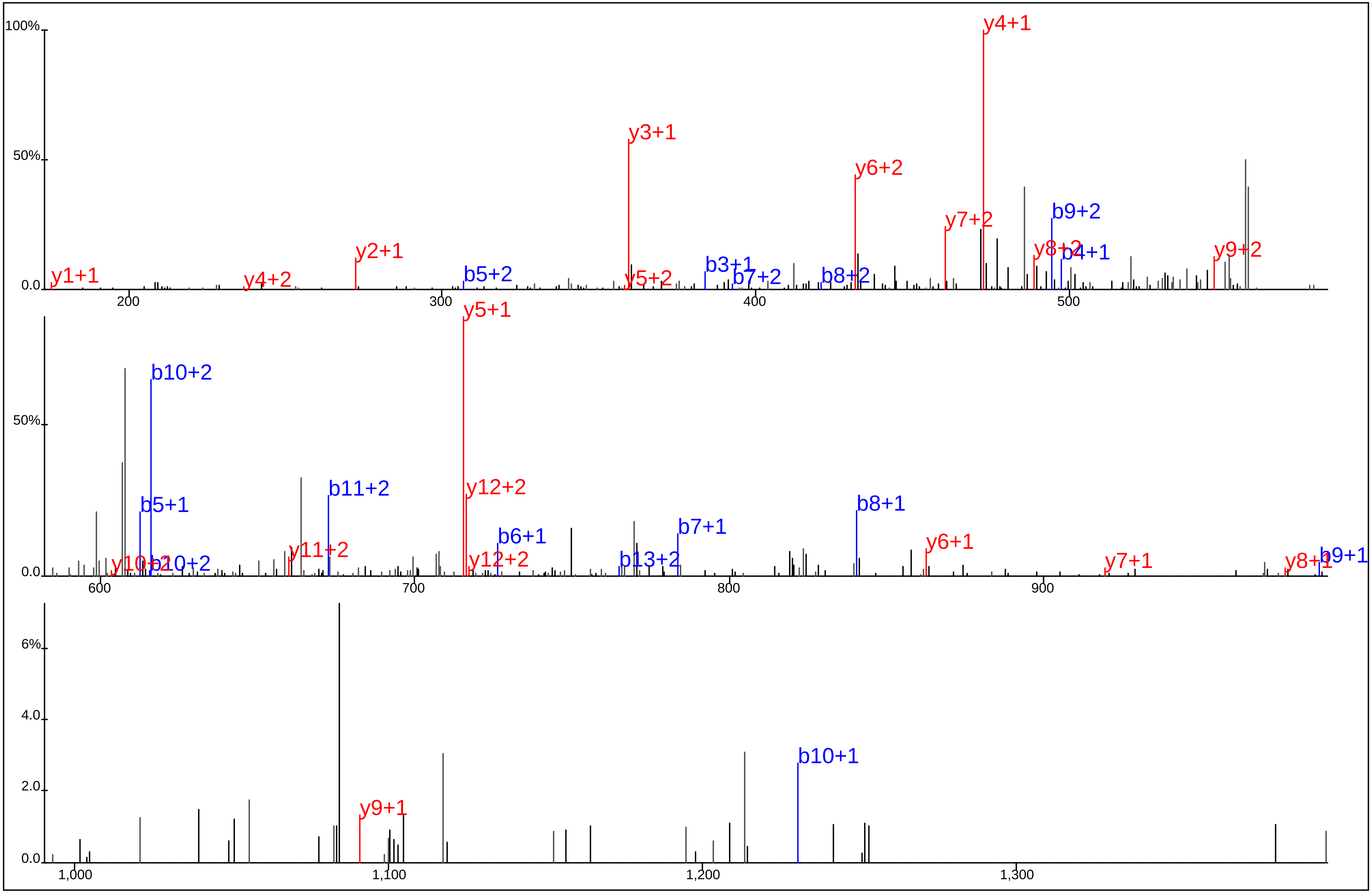

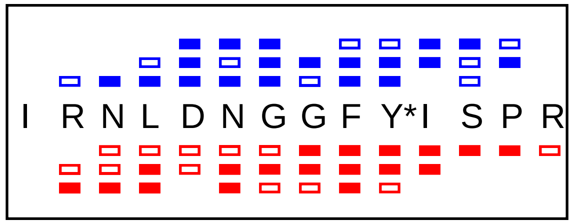

Protein Name:	Lck
gi Accession:	6984209
Precursor Mass:	567.9427+3
Delta Mass:	1.579 ppm
XCorr:	50.18

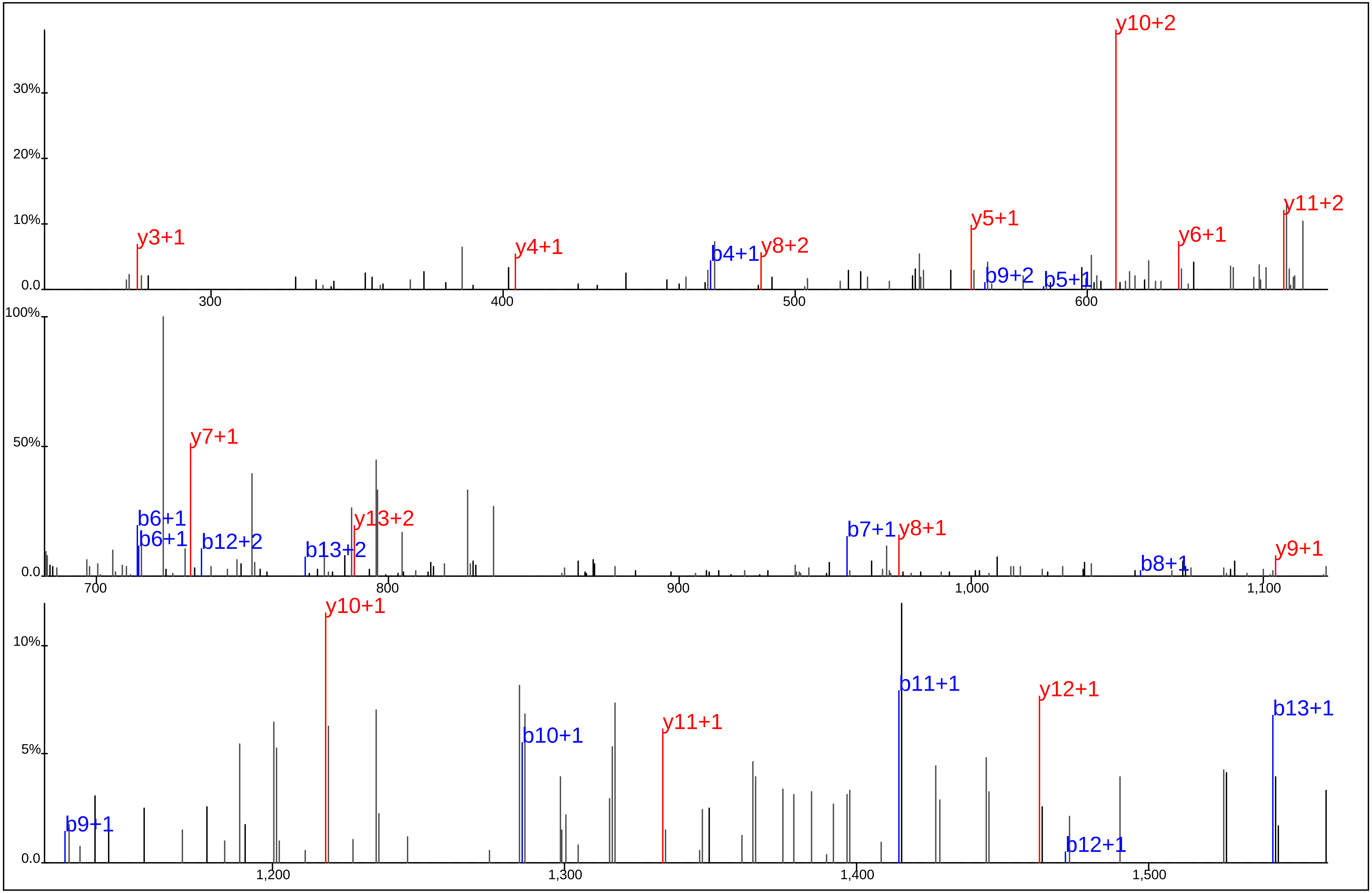

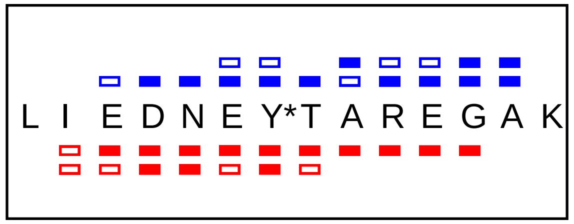

Protein Name: Lck
gi Accession: 6984209
Precursor Mass: 844.8841+2
Delta Mass: 4.878 ppm
XCorr: 49.52

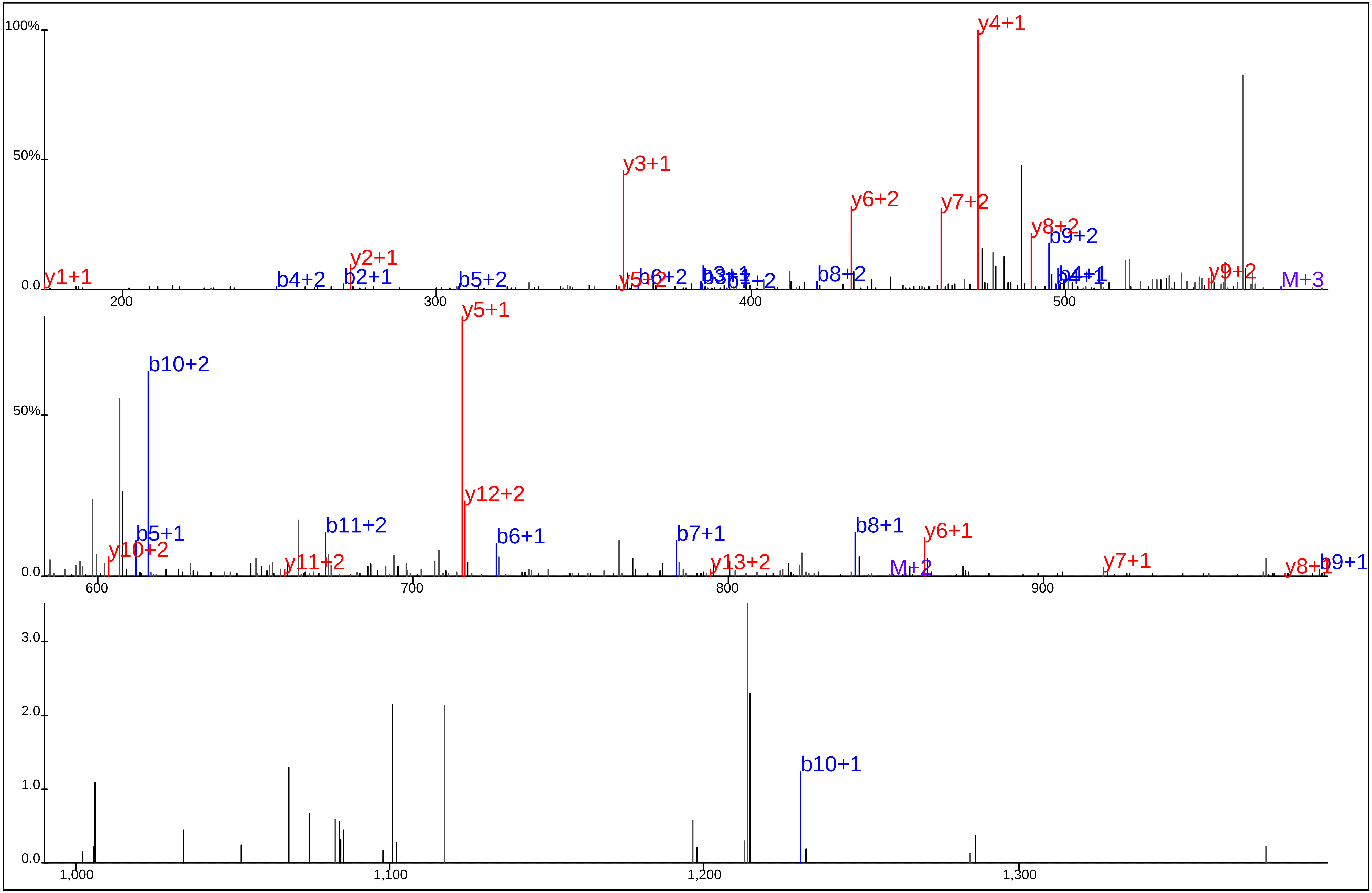

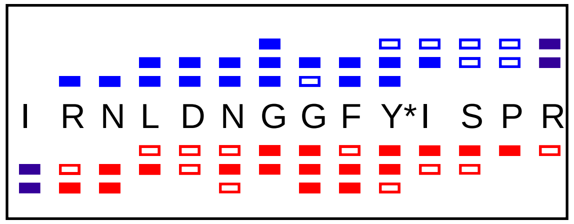

Protein Name: Lck
gi Accession: 6984209
Precursor Mass: 567.9429+3
Delta Mass: 1.903 ppm
XCorr: 41.14

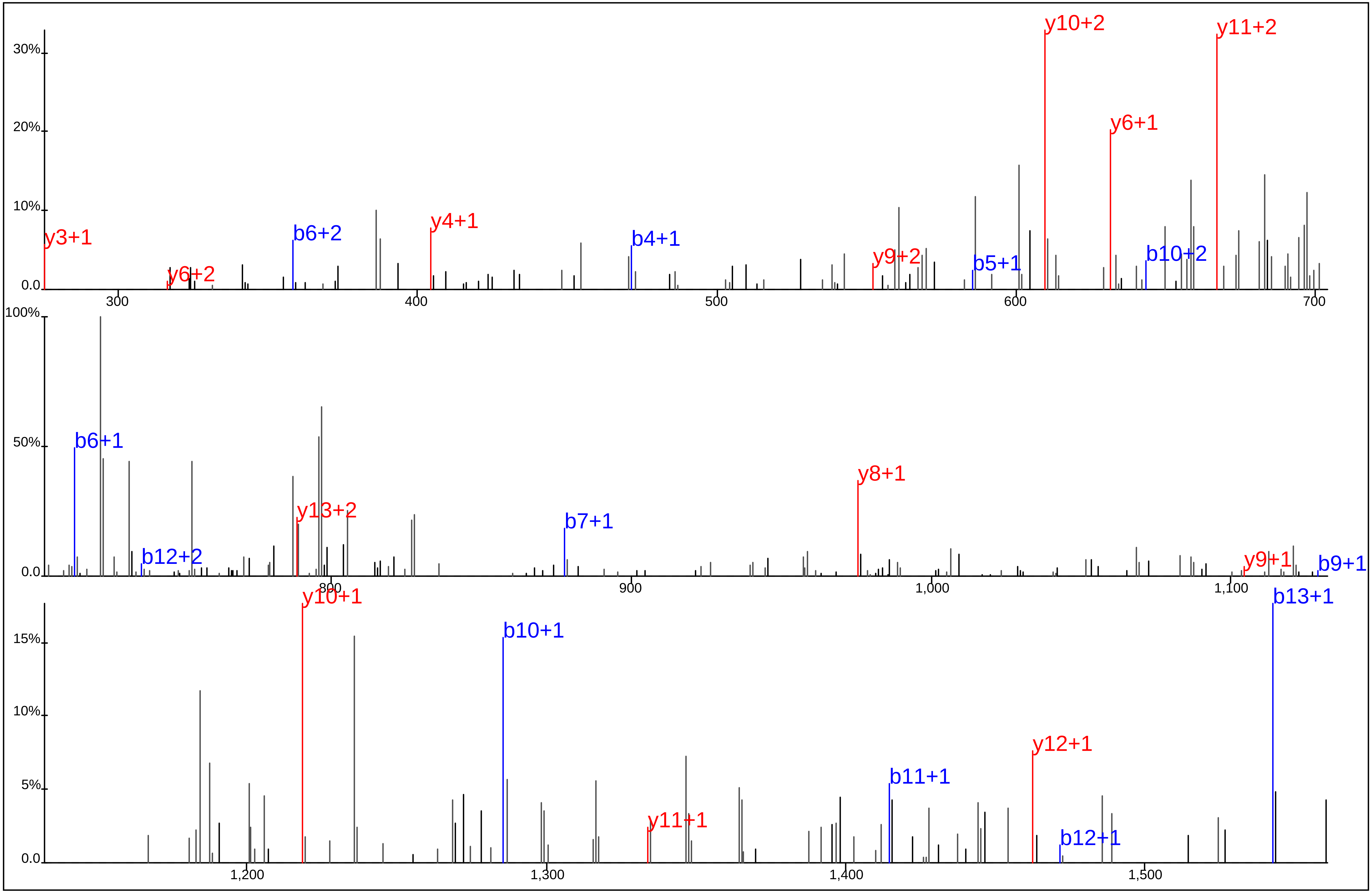

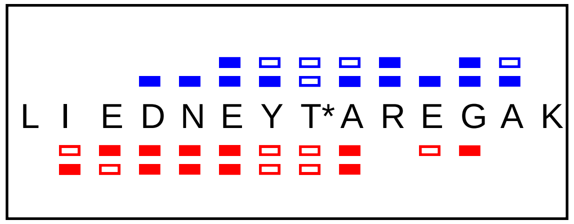

Protein Name: Lck
gi Accession: 6984209
Precursor Mass: 844.8842+2
Delta Mass: 5.026 ppm
XCorr: 45.27

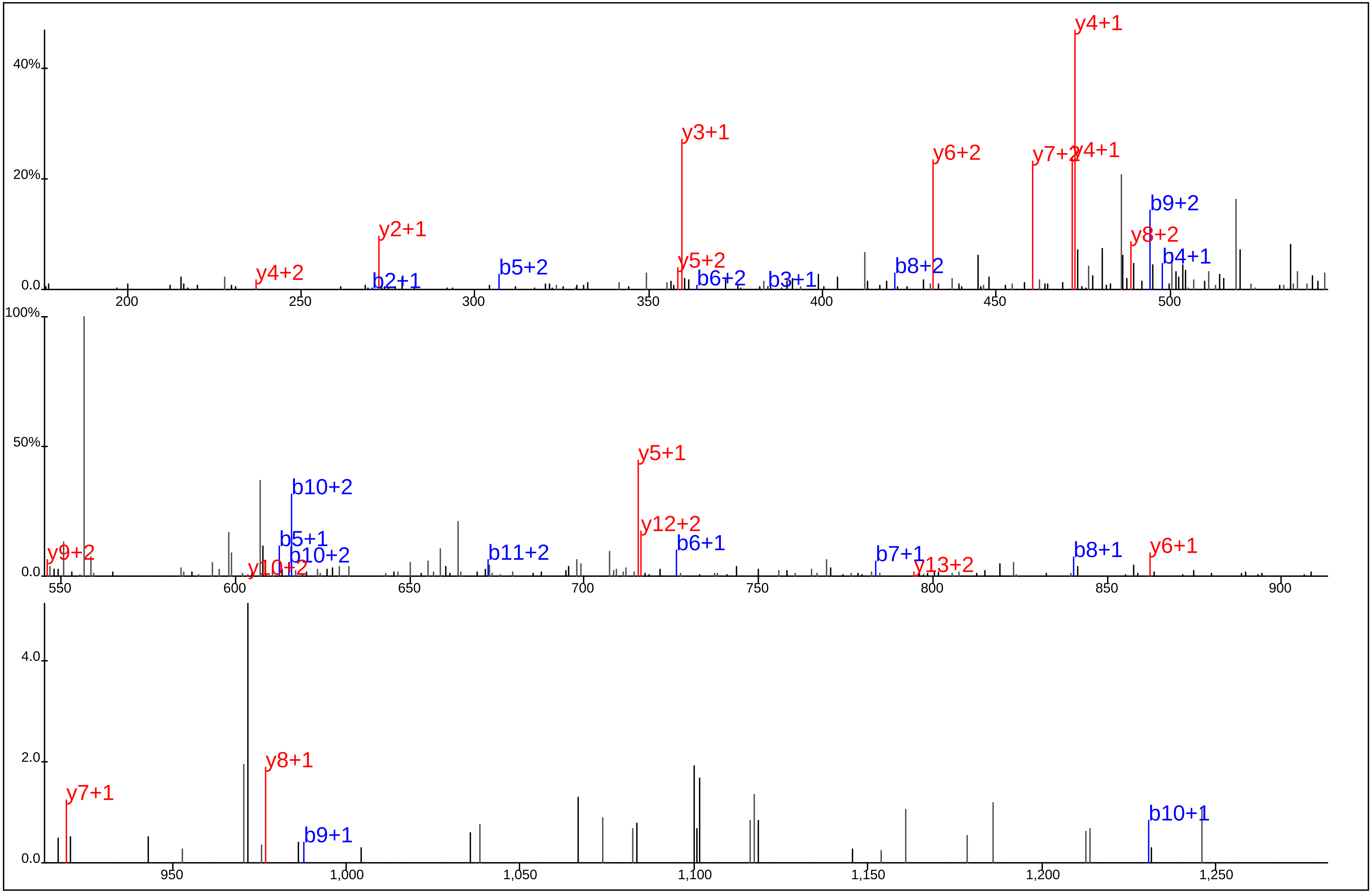

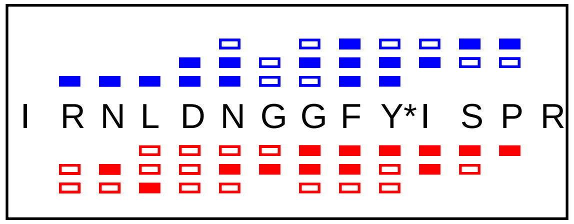

Protein Name: Lck
gi Accession: 6984209
Precursor Mass: 567.9418+3
Delta Mass: 0.036 ppm
XCorr: 41.23

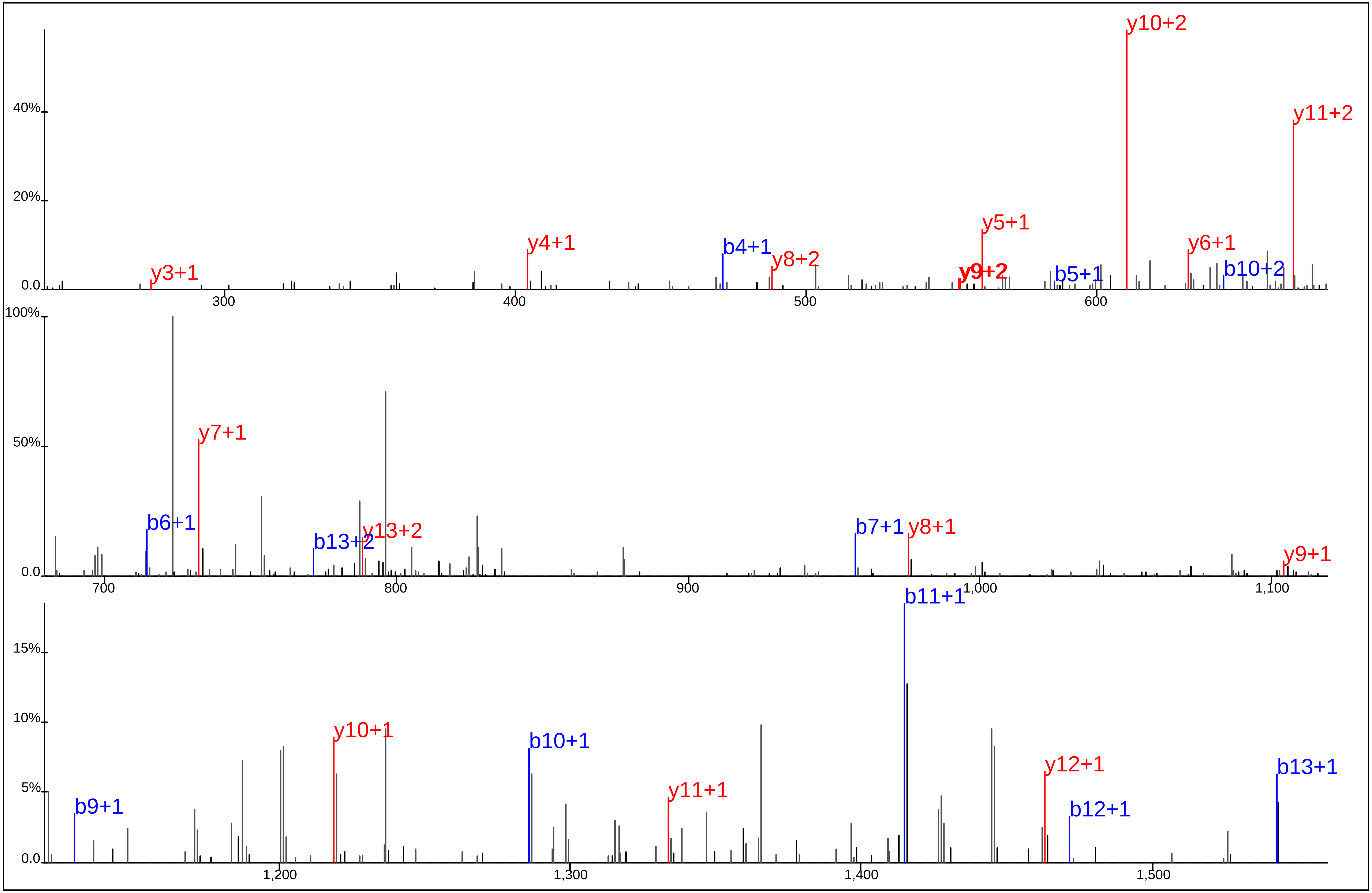

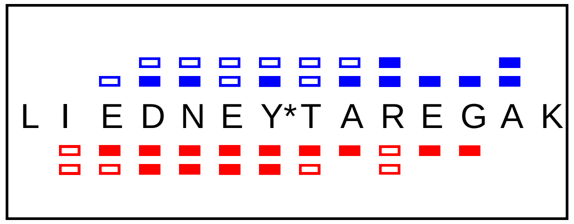

Protein Name: Lck
gi Accession: 6984209
Precursor Mass: 844.8848+2
Delta Mass: 5.672 ppm
XCorr: 63.36

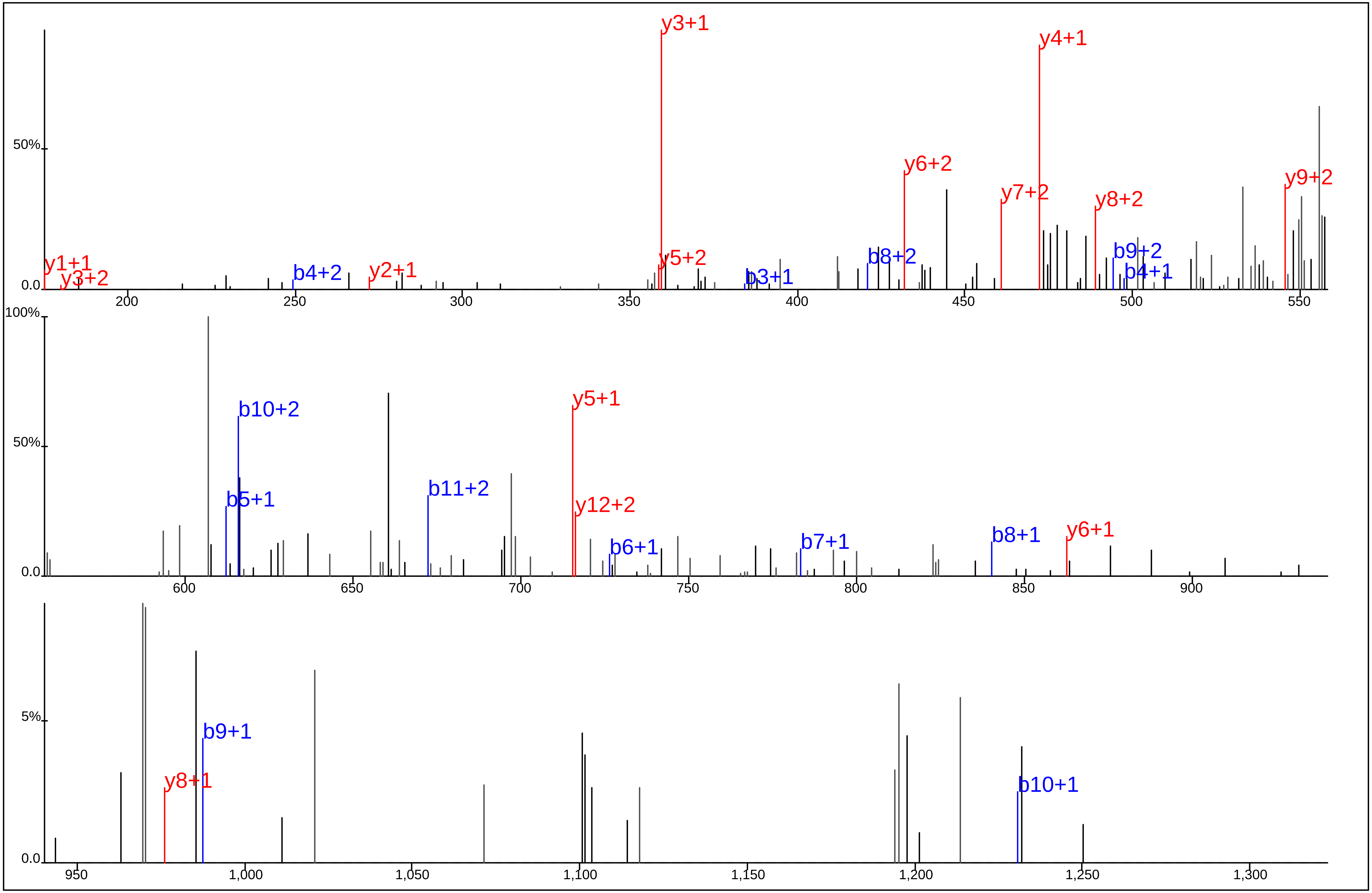

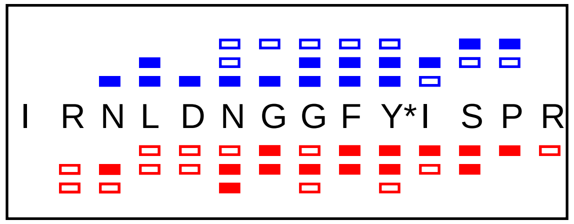

Protein Name: Lck
gi Accession: 6984209
Precursor Mass: 567.9468+3
Delta Mass: 8.895 ppm
XCorr: 31.05

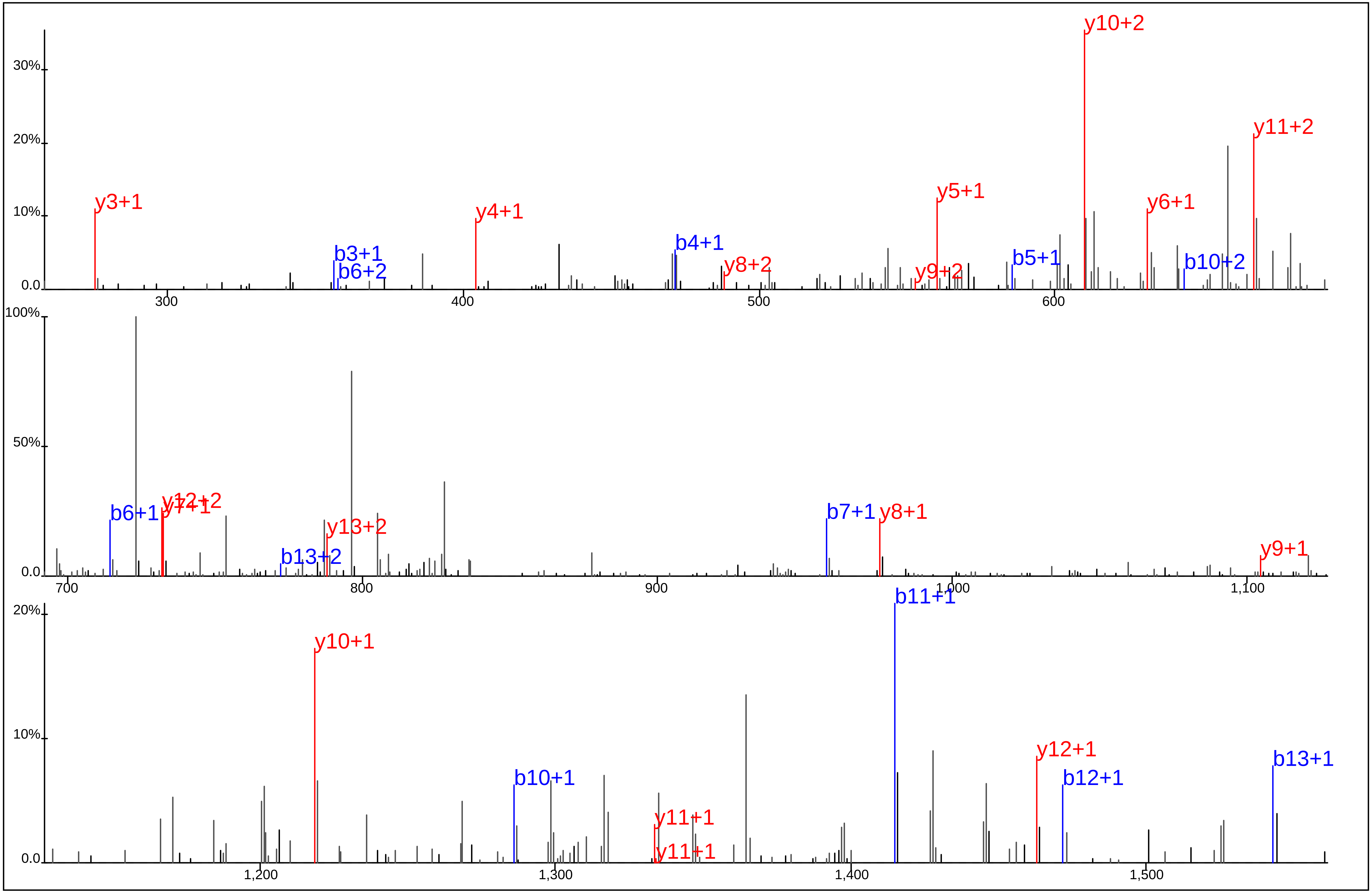

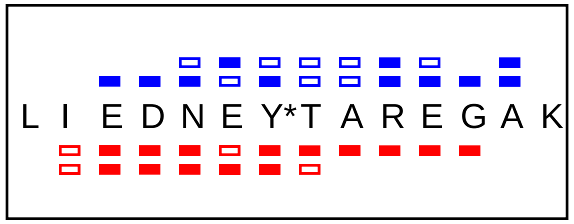

Protein Name:	Lck
gi Accession:	6984209
Precursor Mass:	844.8872+2
Delta Mass:	8.567 ppm
XCorr:	64.86

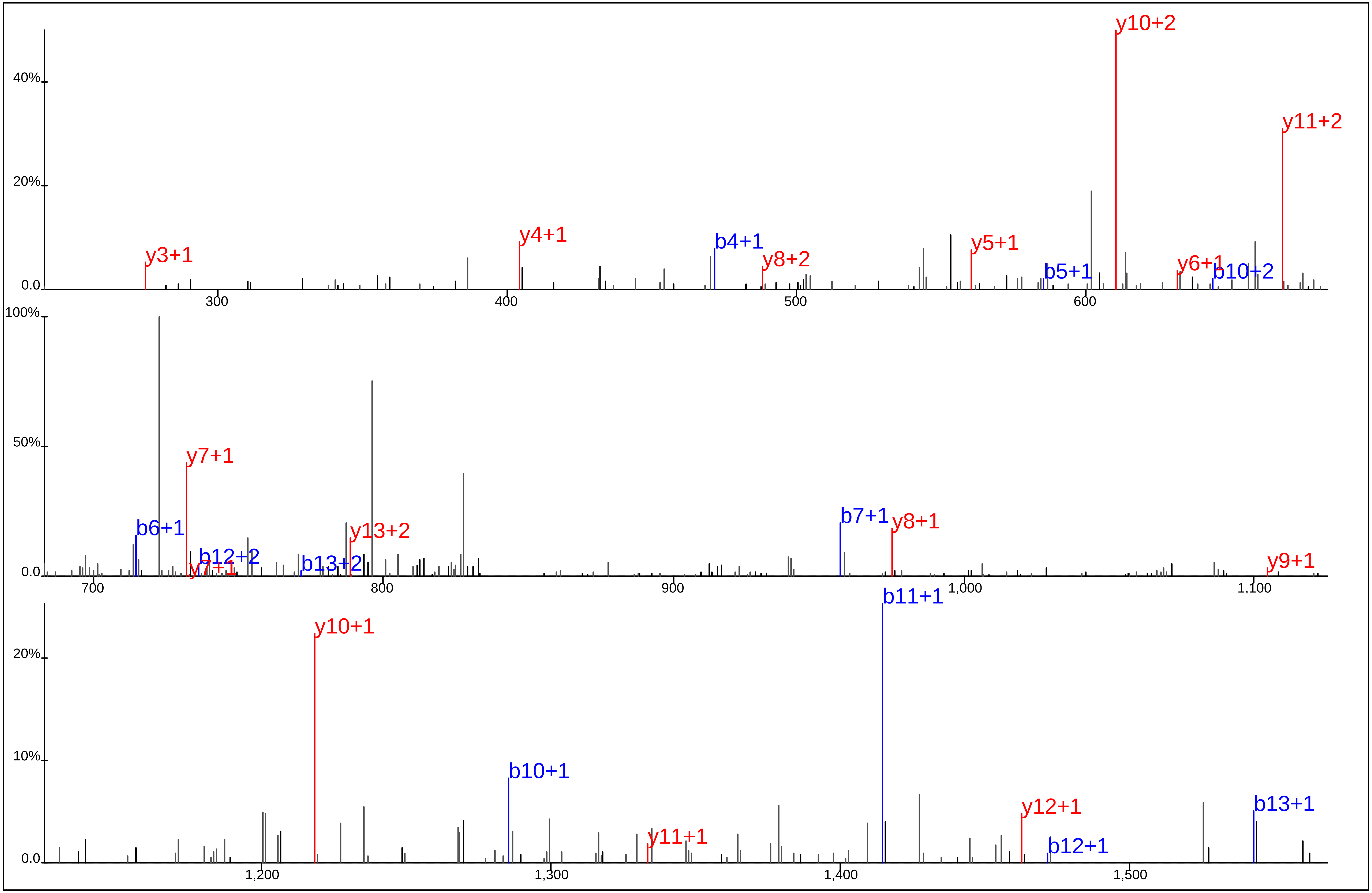

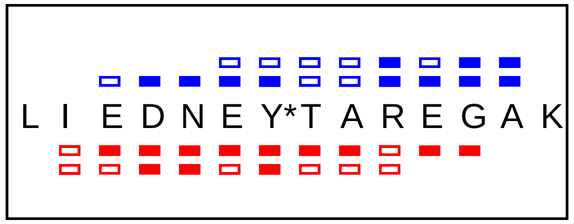

Protein Name: Lck
gi Accession: 6984209
Precursor Mass: 844.8790+2
Delta Mass: 1.120 ppm
XCorr: 37.23

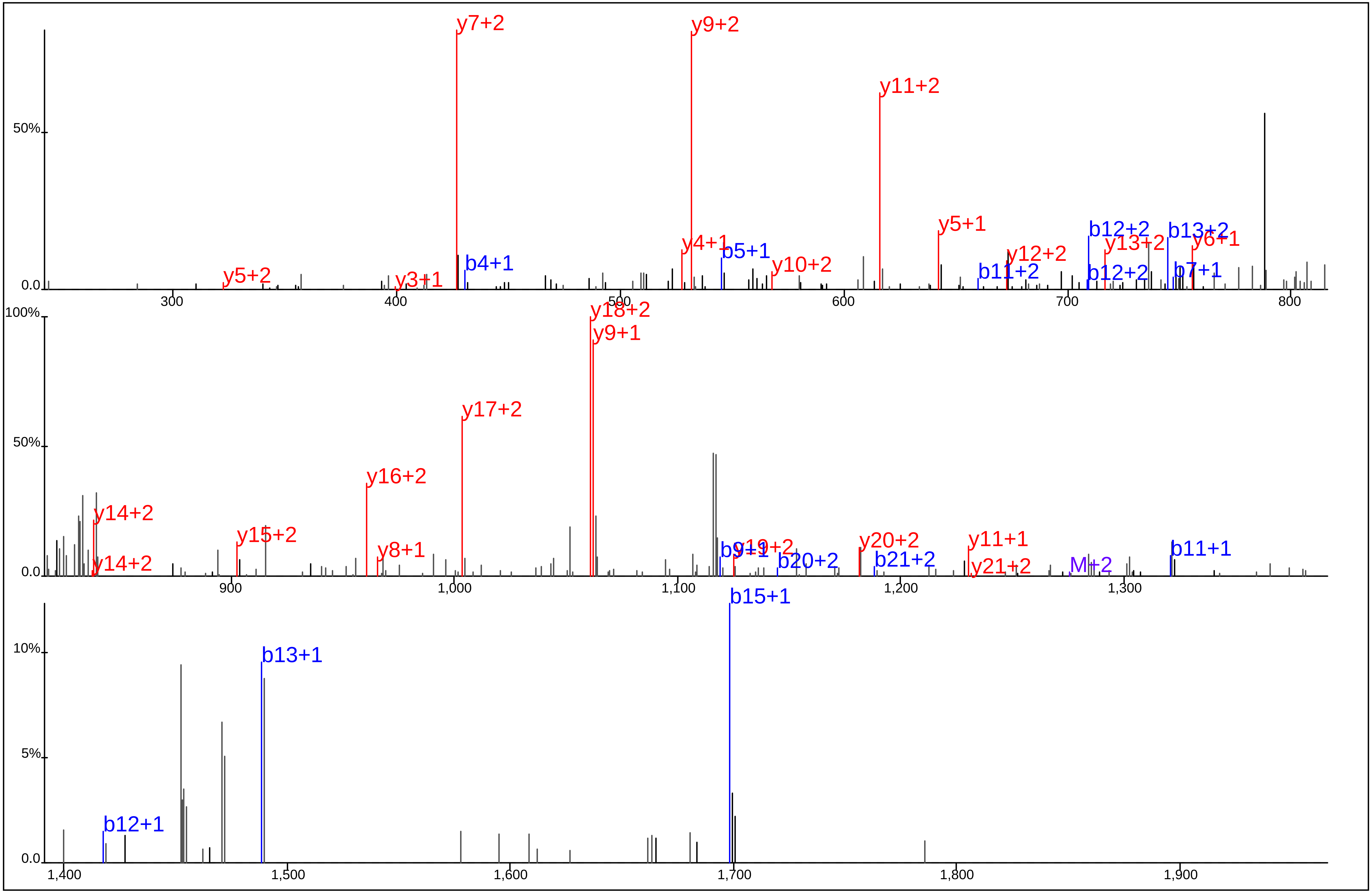

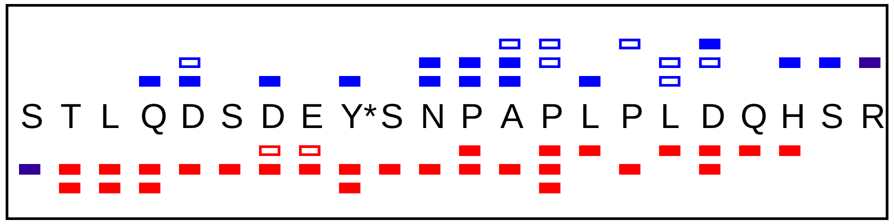

Protein Name:	Leupaxin
gi Accession:	4758670
Precursor Mass:	850.7117+3
Delta Mass:	2.380 ppm
XCorr:	57.24

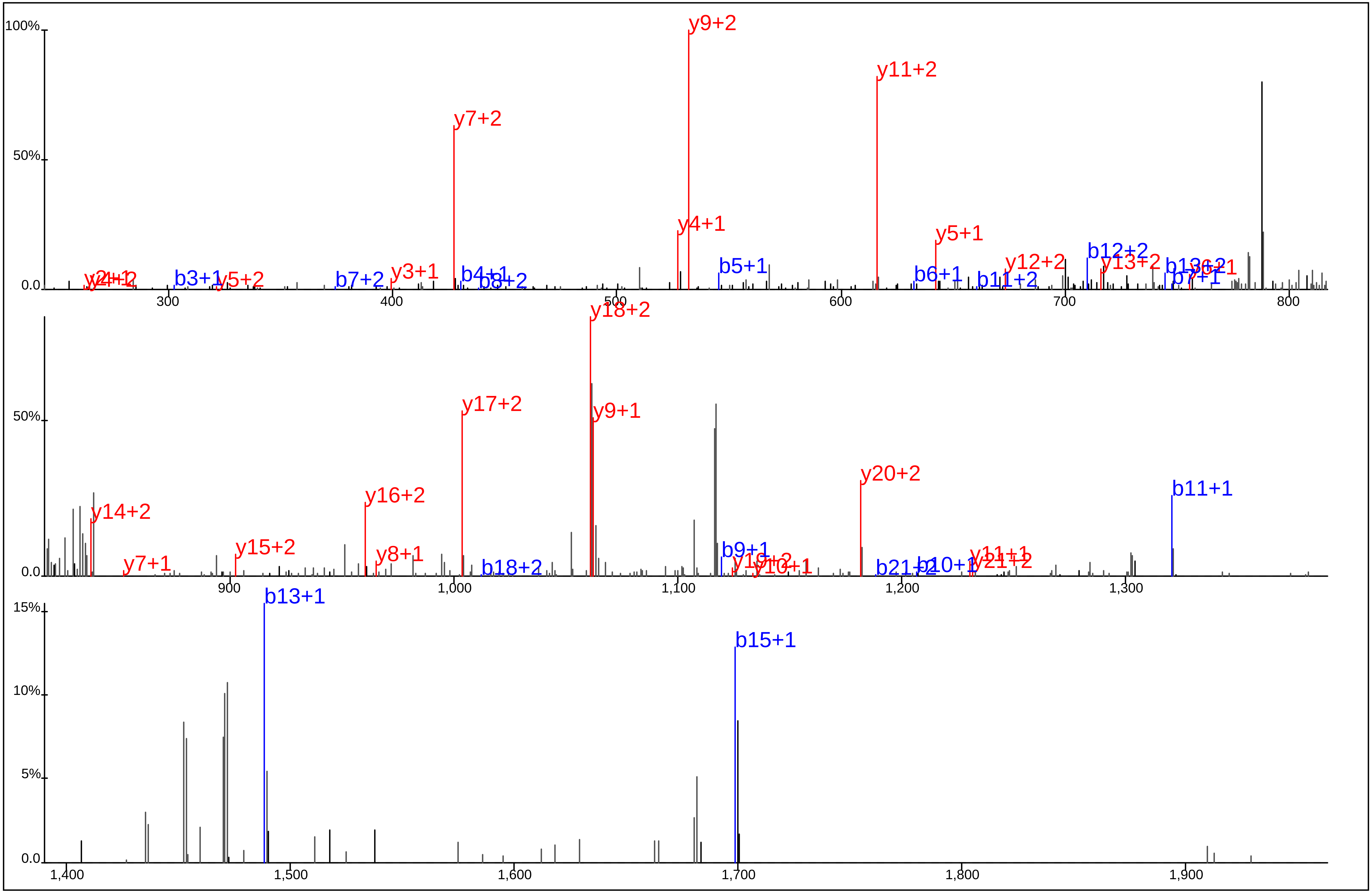

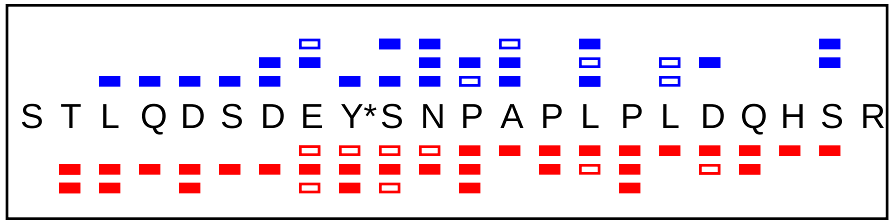

Protein Name: Leupaxin
gi Accession: 4758670
Precursor Mass: 850.7110+3
Delta Mass: 1.587 ppm
XCorr: 52.91

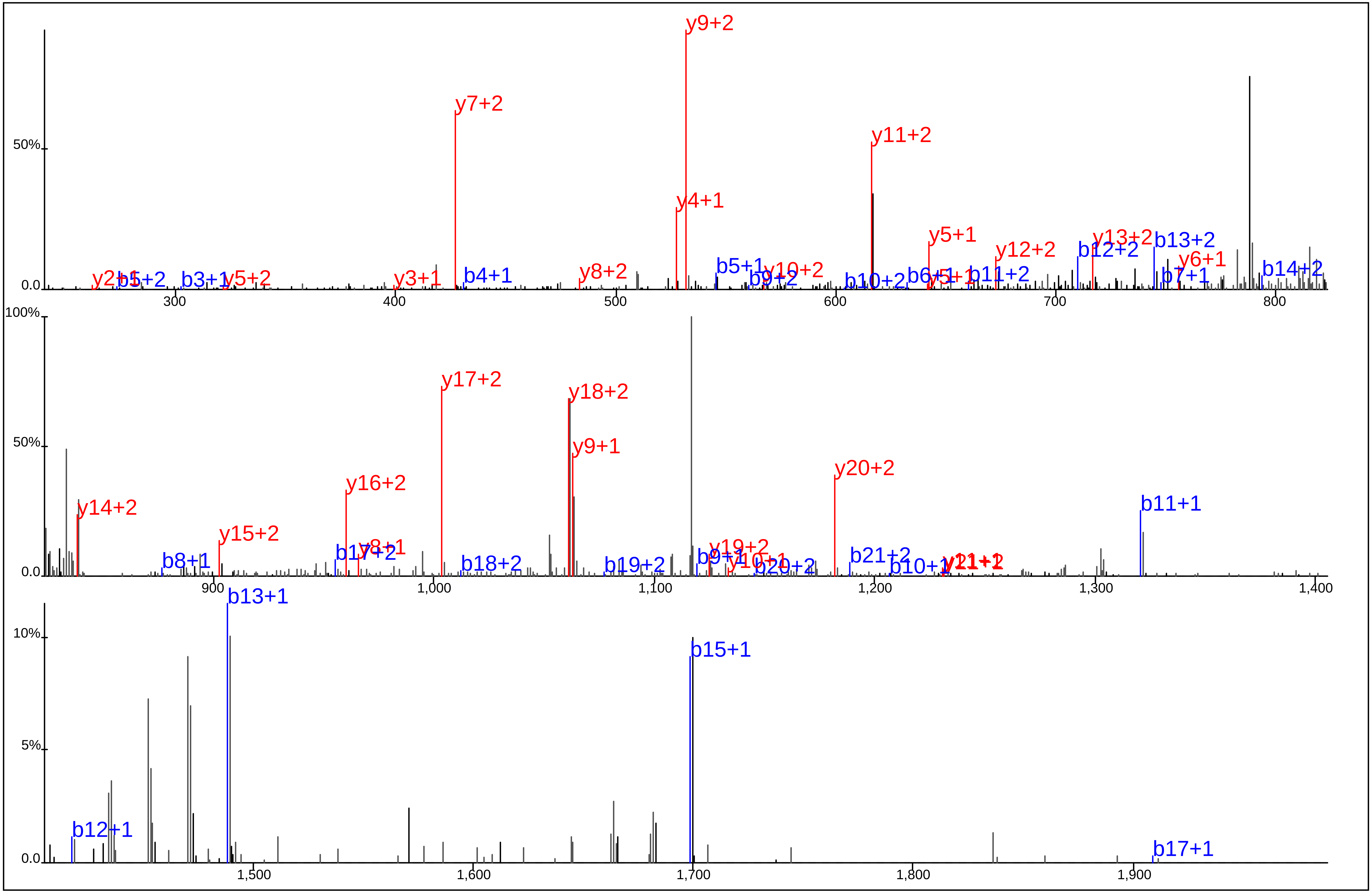

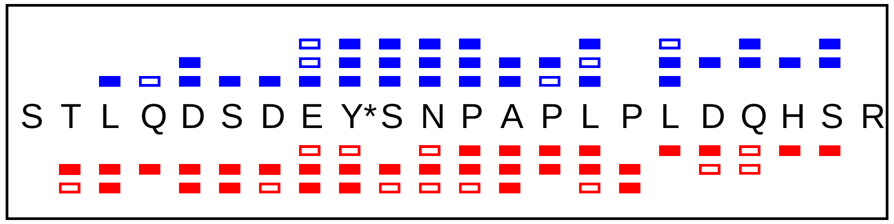

Protein Name: Leupaxin
gi Accession: 4758670
Precursor Mass: 850.7090+3
Delta Mass: 0.711 ppm
XCorr: 79.51

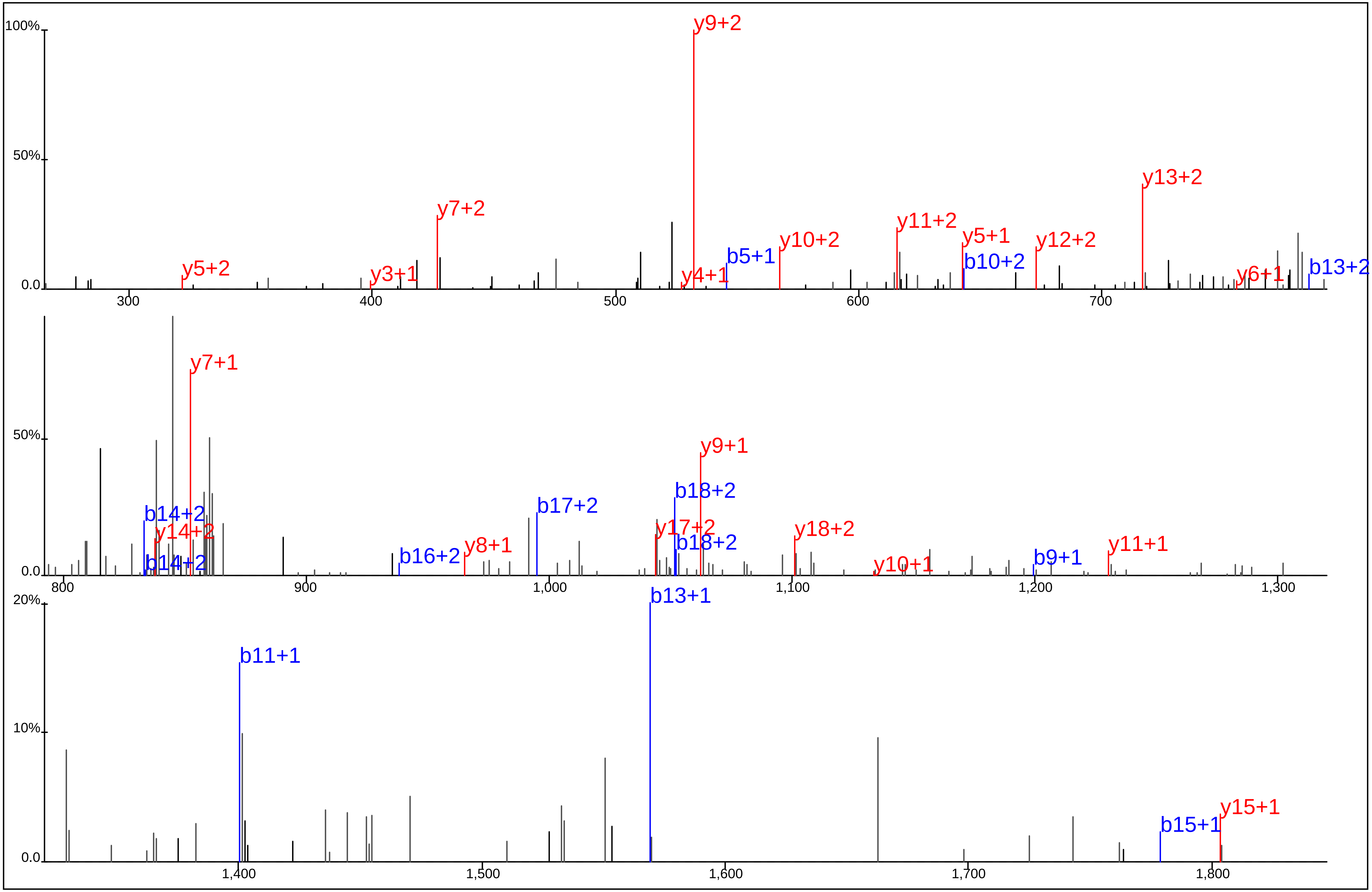

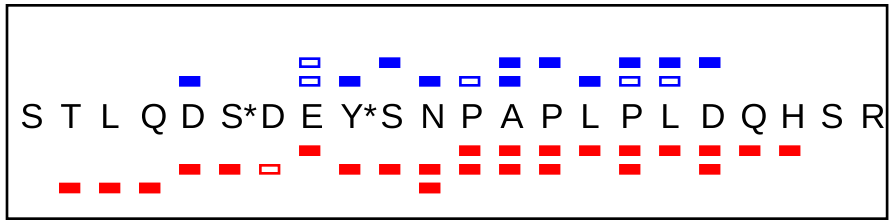

Protein Name: Leupaxin
gi Accession: 4758670
Precursor Mass: 877.3809+3
Delta Mass: 18.056 ppm
XCorr: 41.89

603 - Leupaxin_3447719.pdf

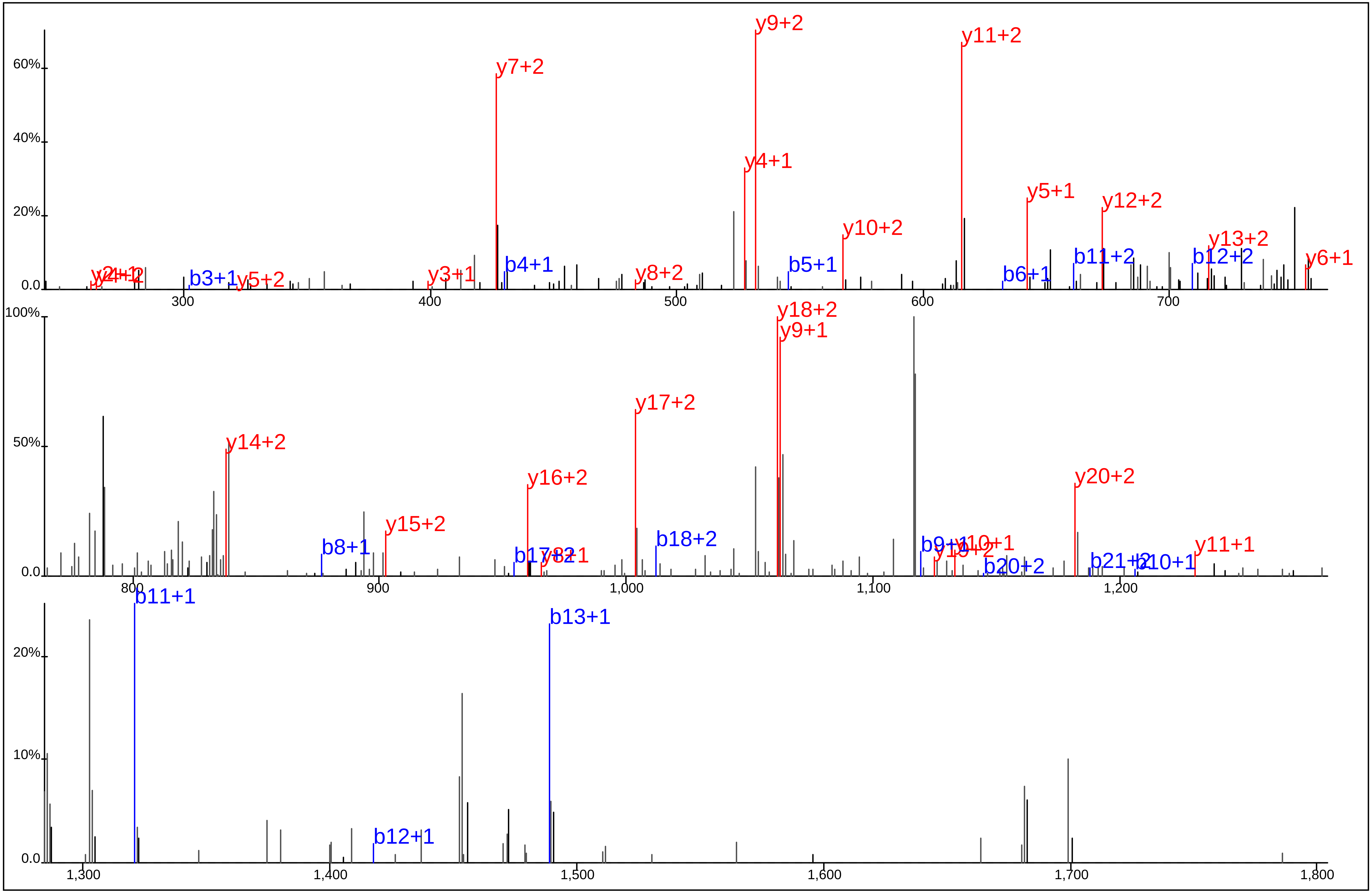

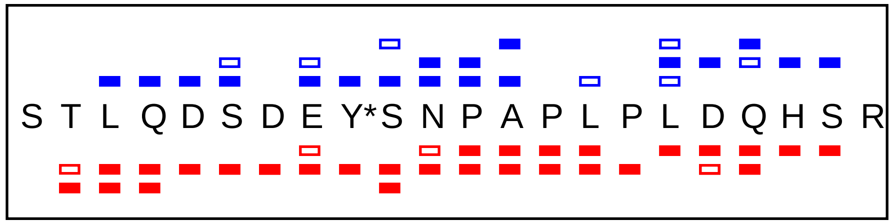

Protein Name: Leupaxin
gi Accession: 4758670
Precursor Mass: 850.7125+3
Delta Mass: 3.383 ppm
XCorr: 59.34

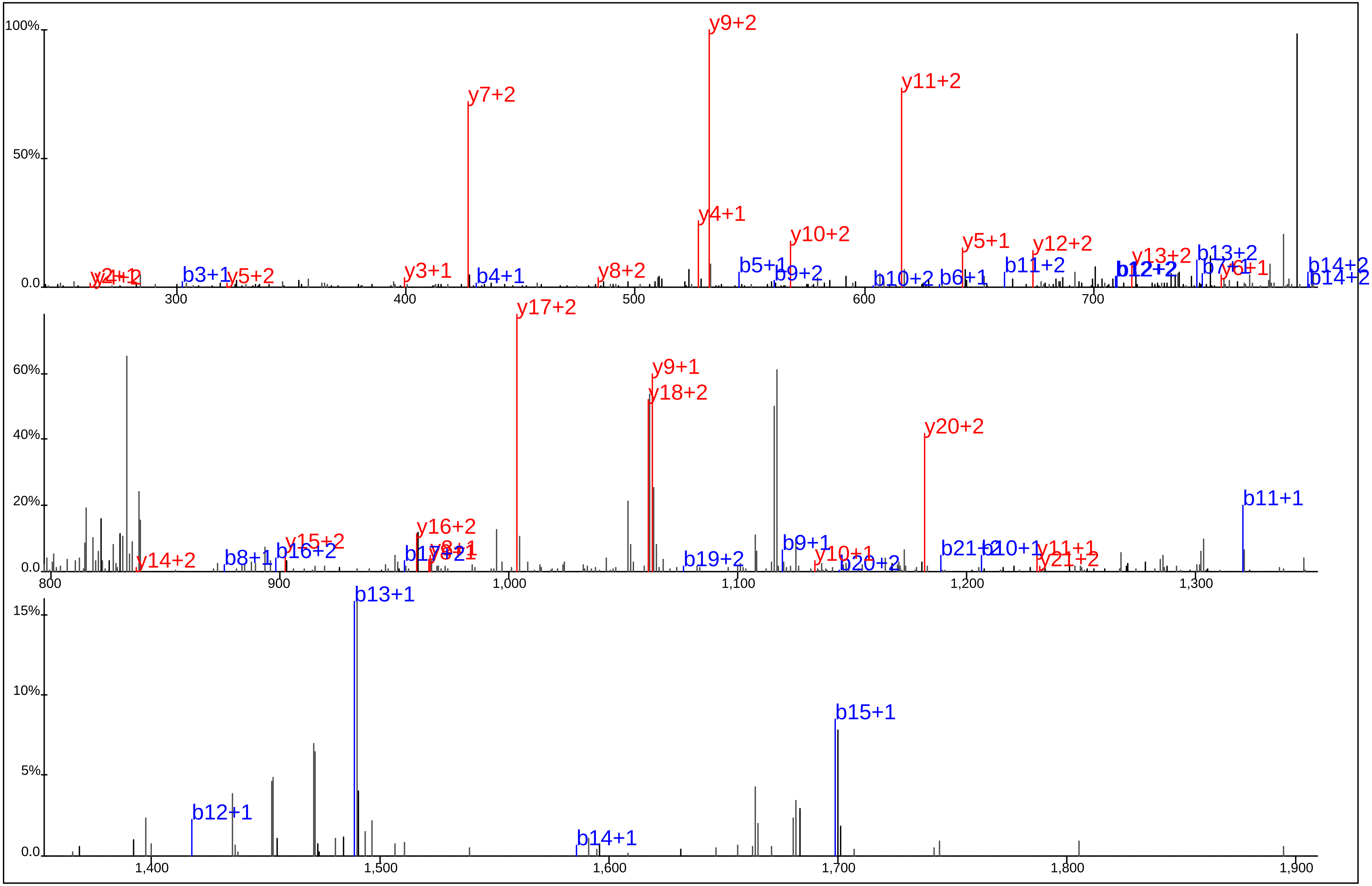

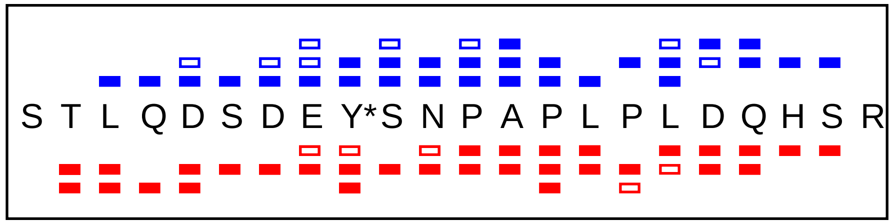

Protein Name: Leupaxin
gi Accession: 4758670
Precursor Mass: 850.7141+3
Delta Mass: 5.180 ppm
XCorr: 58.69

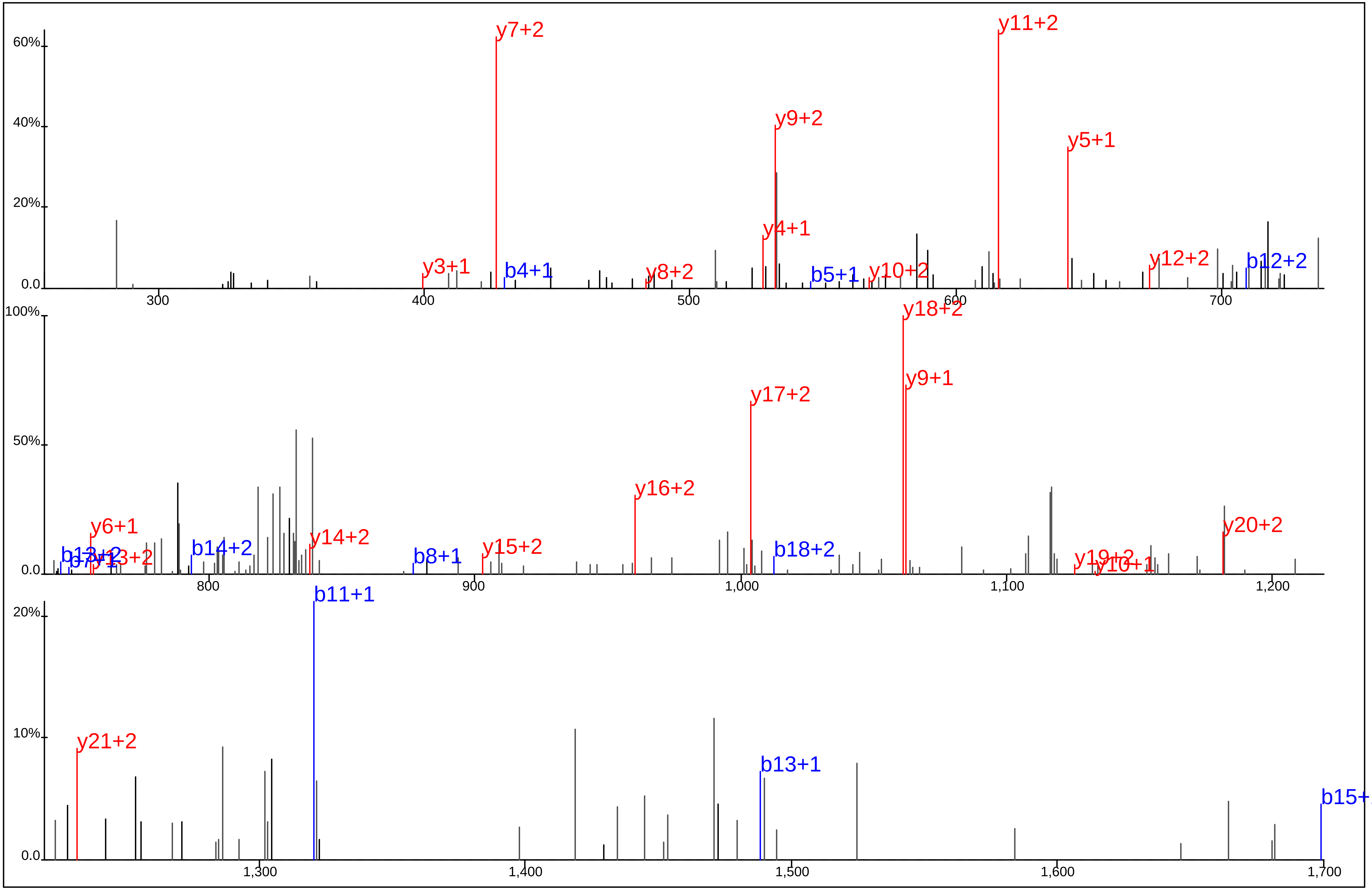

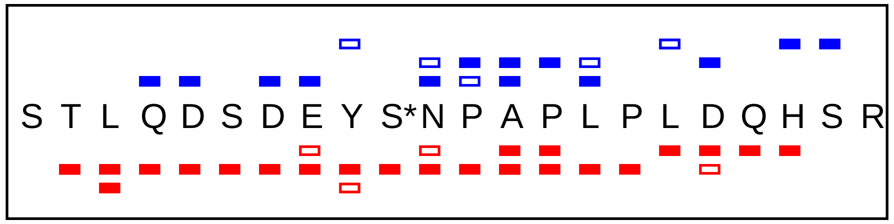

Protein Name:	Leupaxin
gi Accession:	4758670
Precursor Mass:	850.7123+3
Delta Mass:	3.168 ppm
XCorr:	40.19

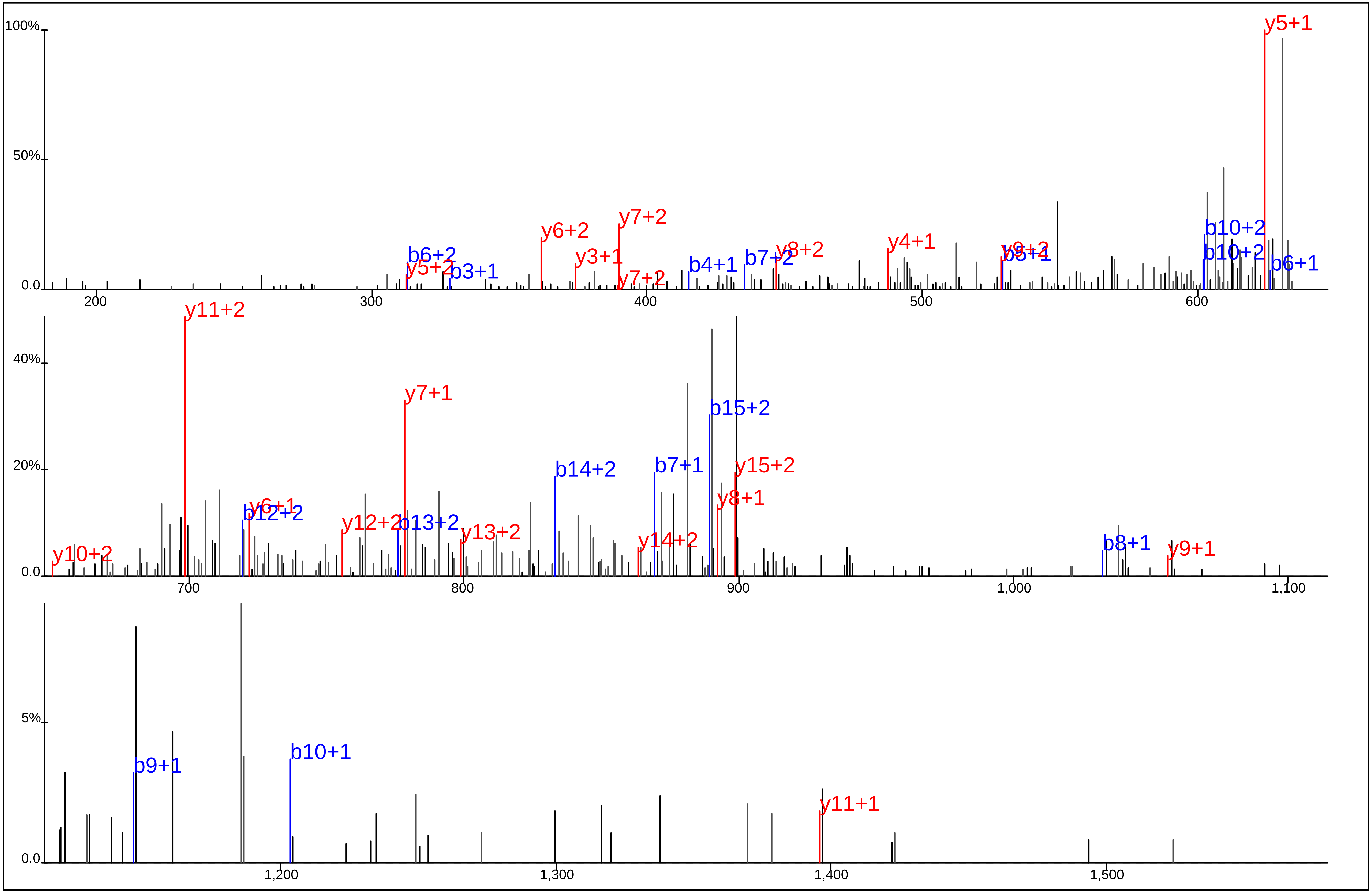

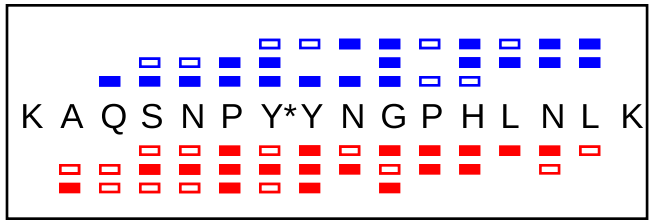

Protein Name: LIM domain only 7
gi Accession: 20521678
Precursor Mass: 641.9772+3
Delta Mass: 2.977 ppm
XCorr: 30.44

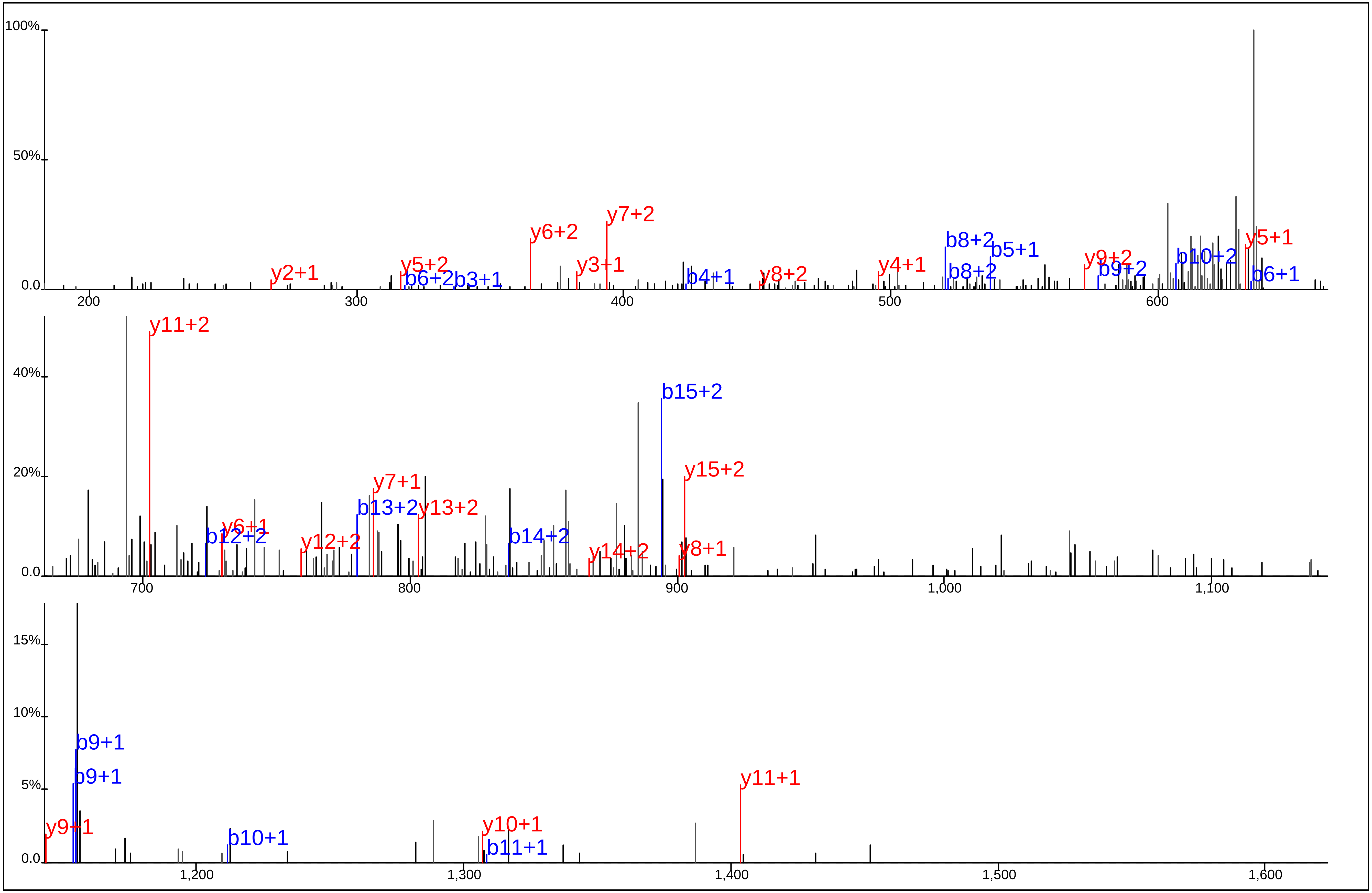

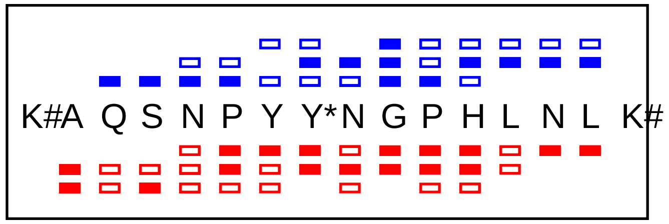

Protein Name:	LIM domain only 7
gi Accession:	20521678
Precursor Mass:	647.3226+3
Delta Mass:	7.056 ppm
XCorr:	22.68

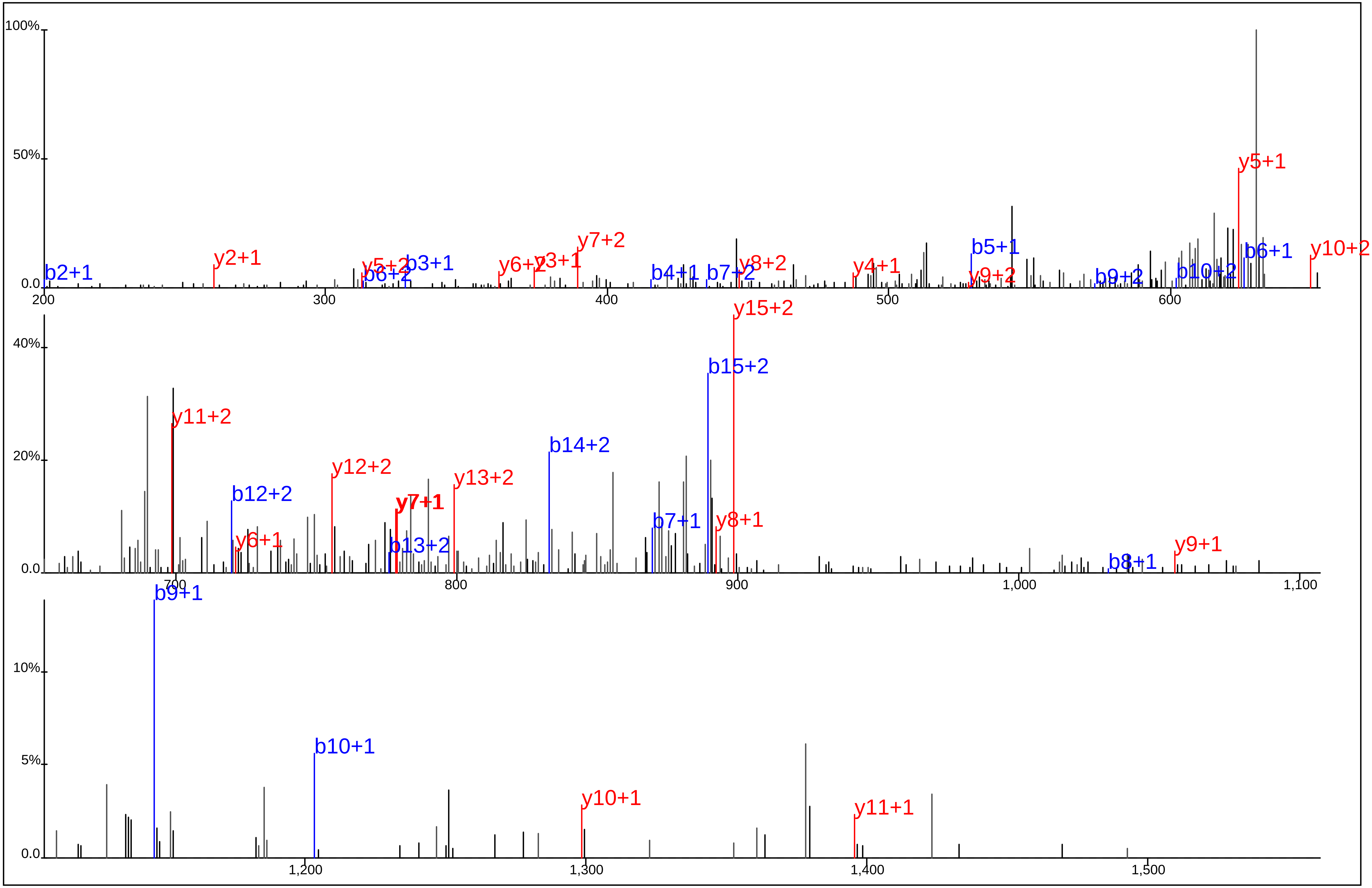

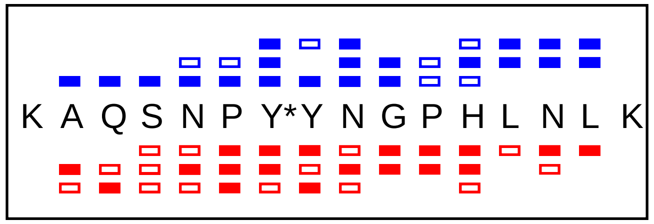

Protein Name:	LIM domain only 7
gi Accession:	20521678
Precursor Mass:	641.9778+3
Delta Mass:	3.928 ppm
XCorr:	33.12

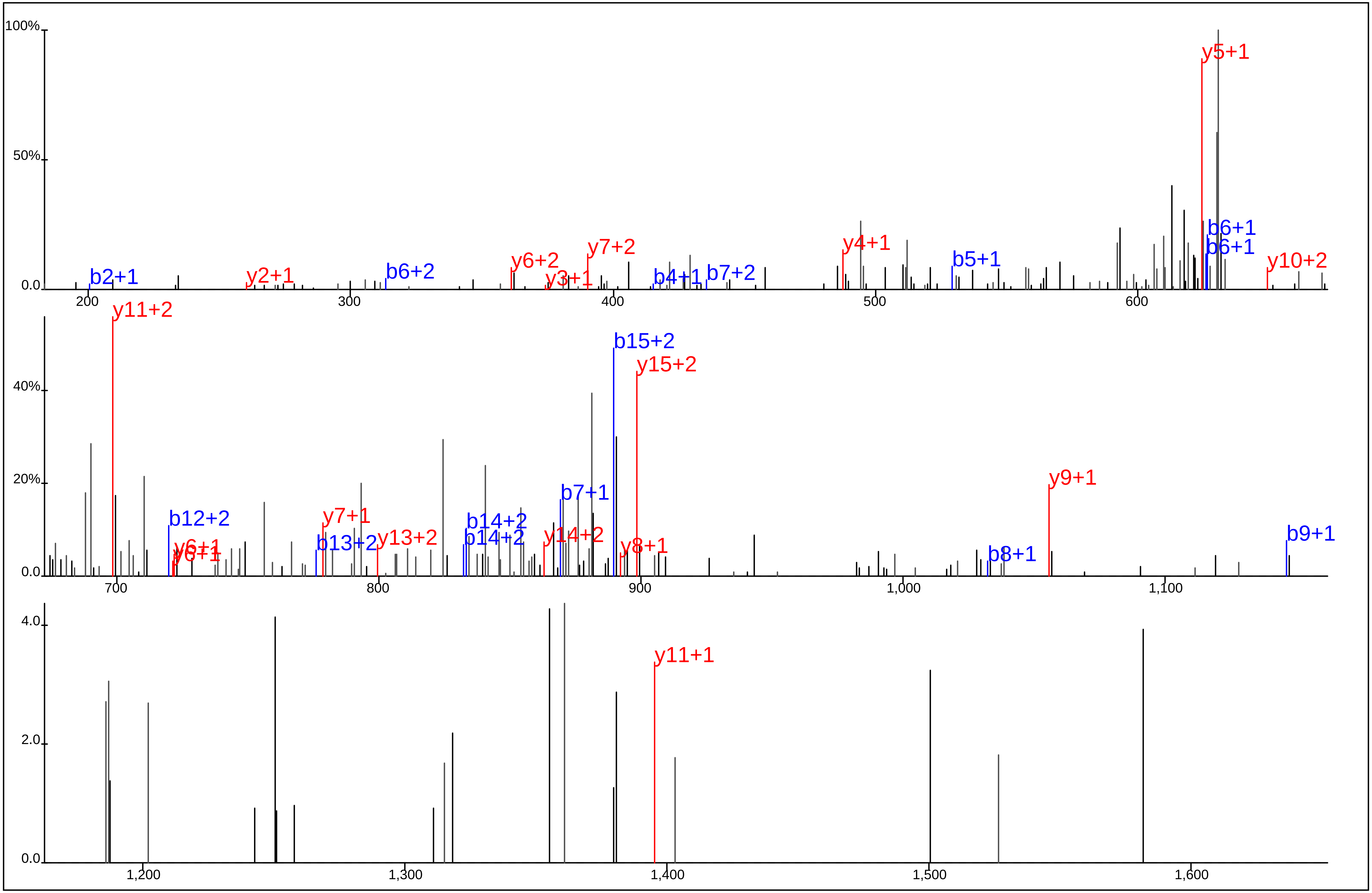

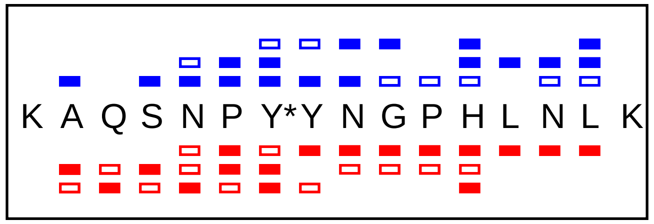

Protein Name:	LIM domain only 7
gi Accession:	20521678
Precursor Mass:	641.9758+3
Delta Mass:	0.883 ppm
XCorr:	15.29

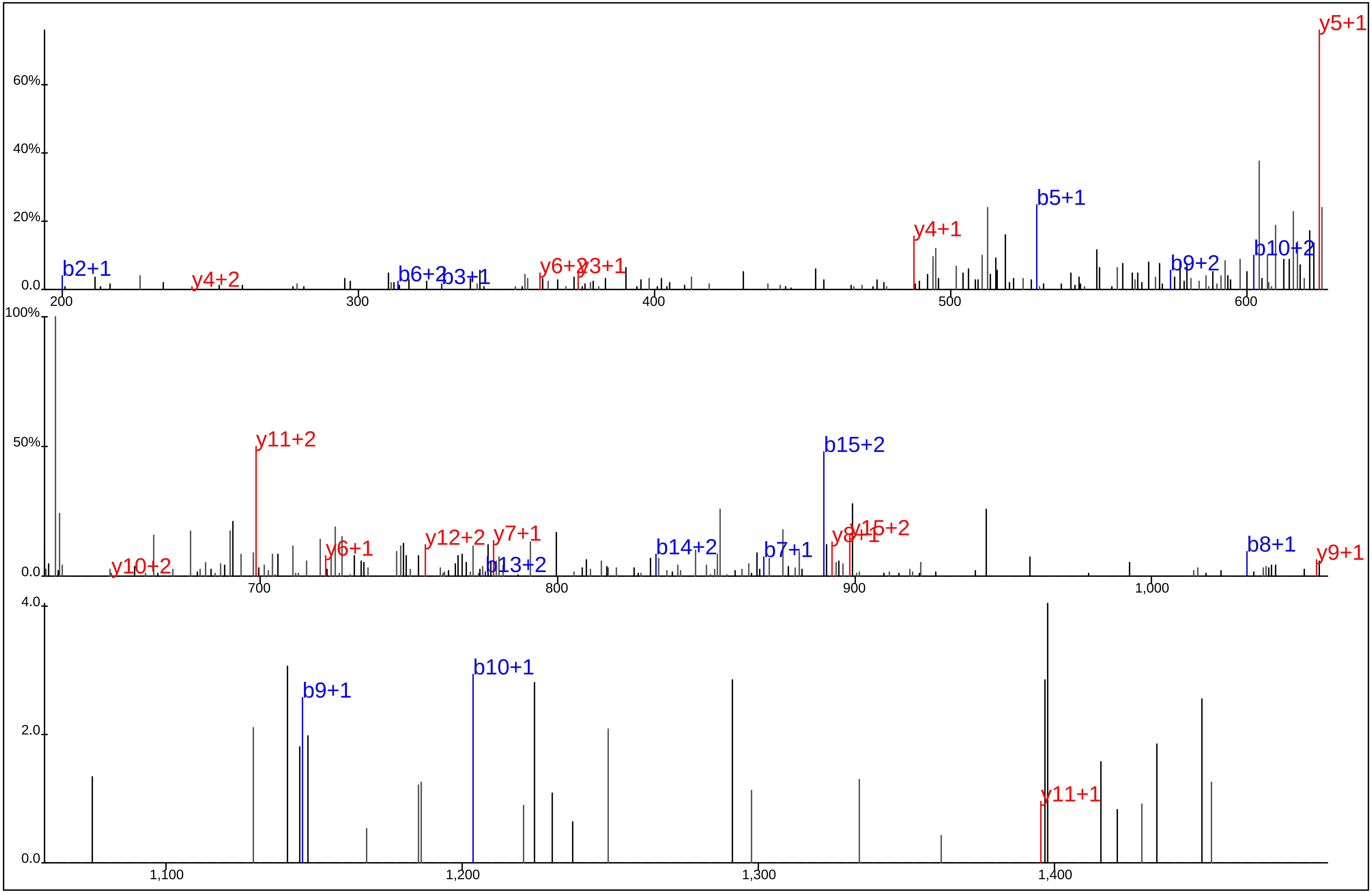

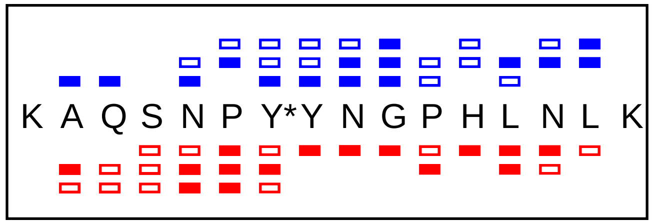

Protein Name:	LIM domain only 7
gi Accession:	20521678
Precursor Mass:	641.9765+3
Delta Mass:	1.928 ppm
XCorr:	19.8

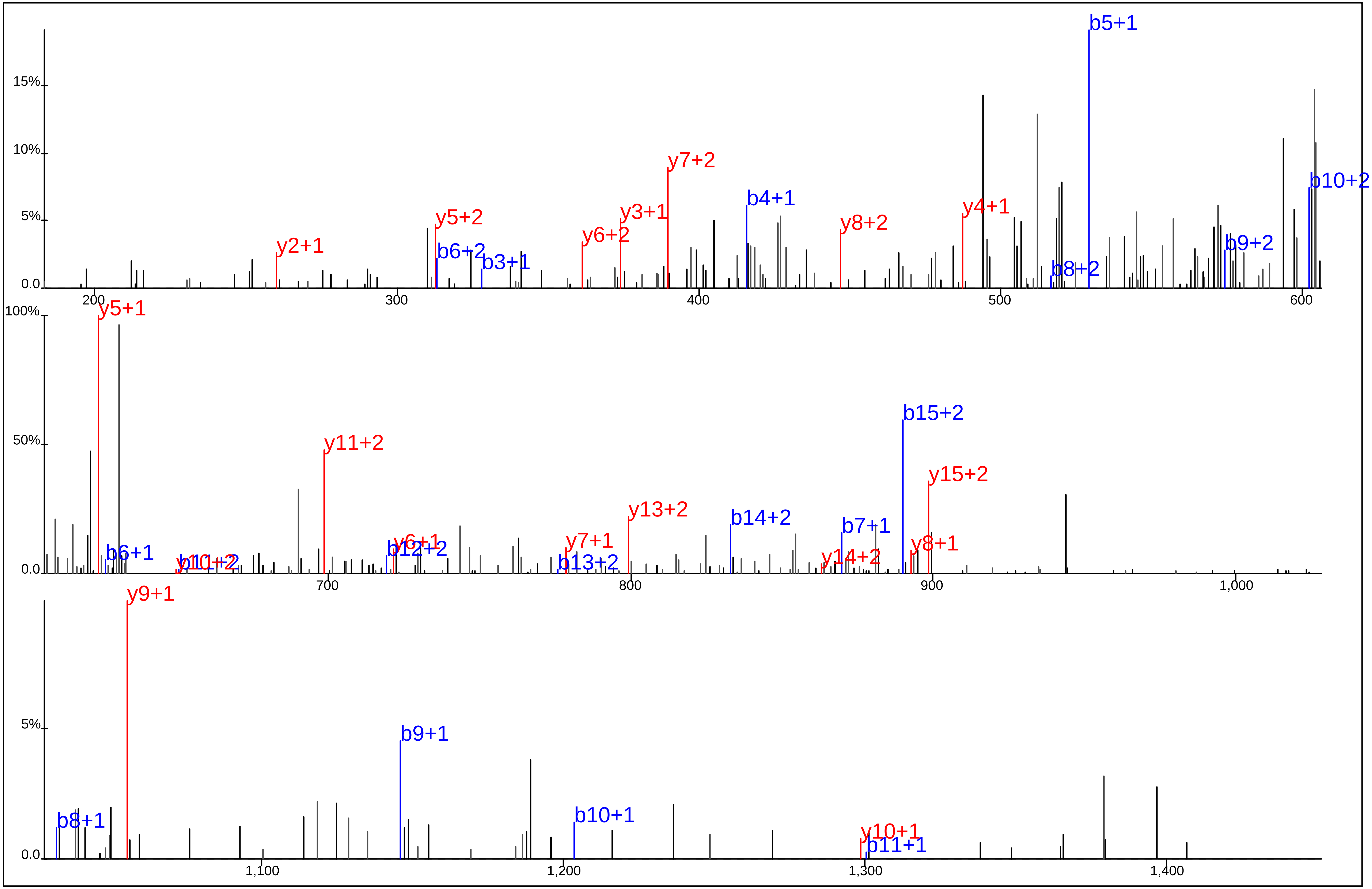

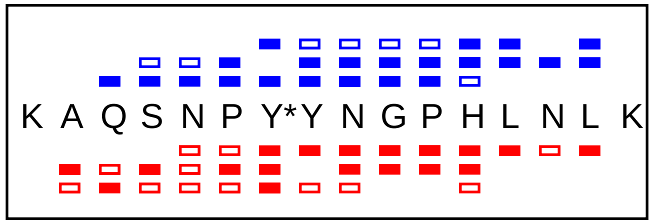

Protein Name: LIM domain only 7
gi Accession: 20521678
Precursor Mass: 641.9761+3
Delta Mass: 1.262 ppm
XCorr: 31.92

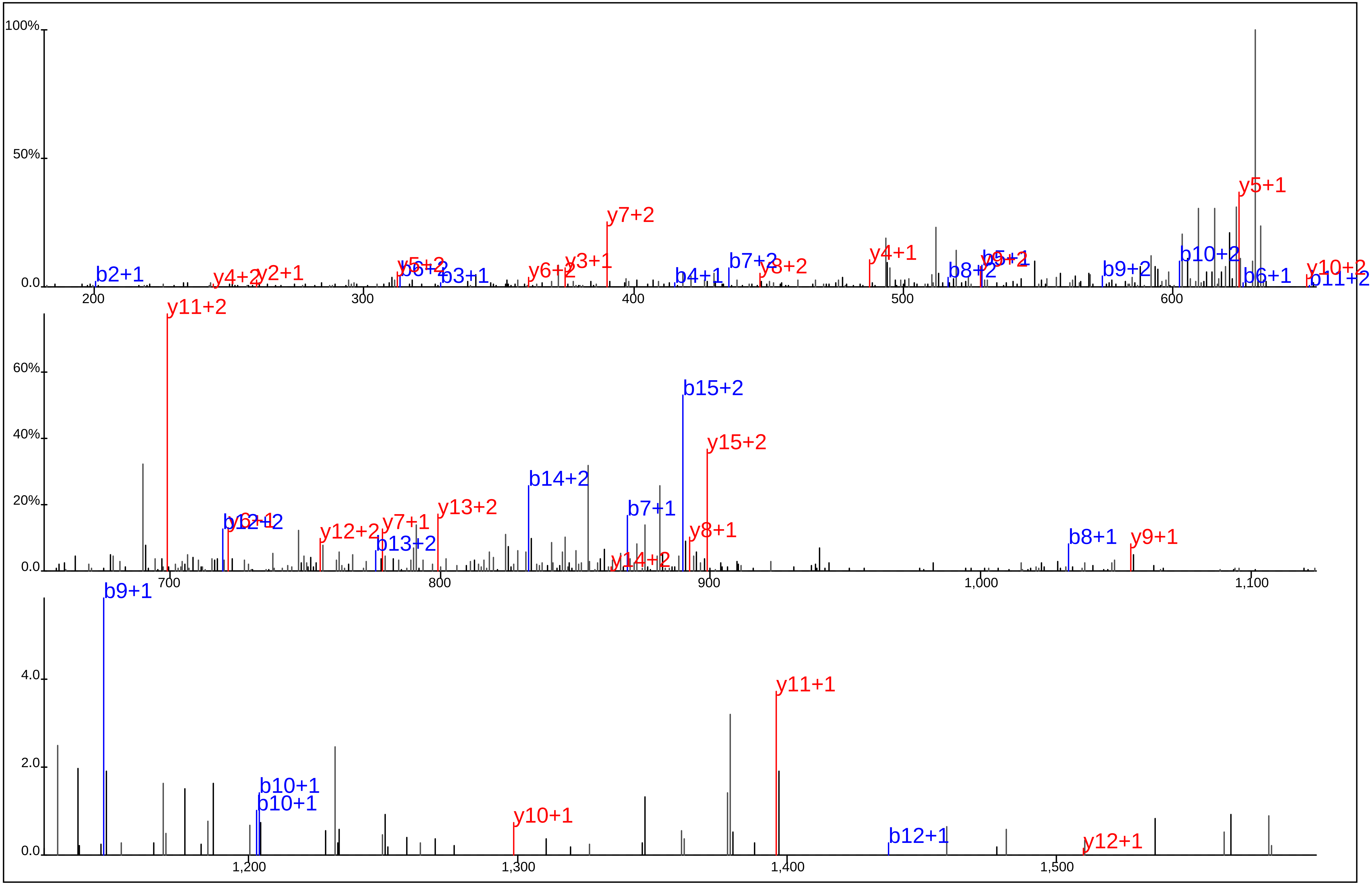

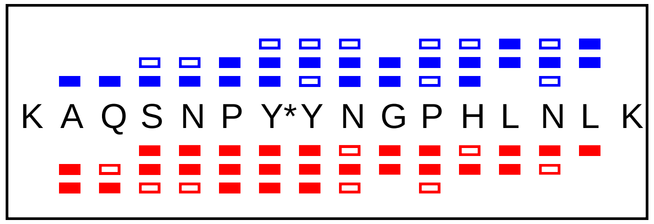

Protein Name: LIM domain only 7
gi Accession: 20521678
Precursor Mass: 641.9758+3
Delta Mass: 0.783 ppm
XCorr: 65.79

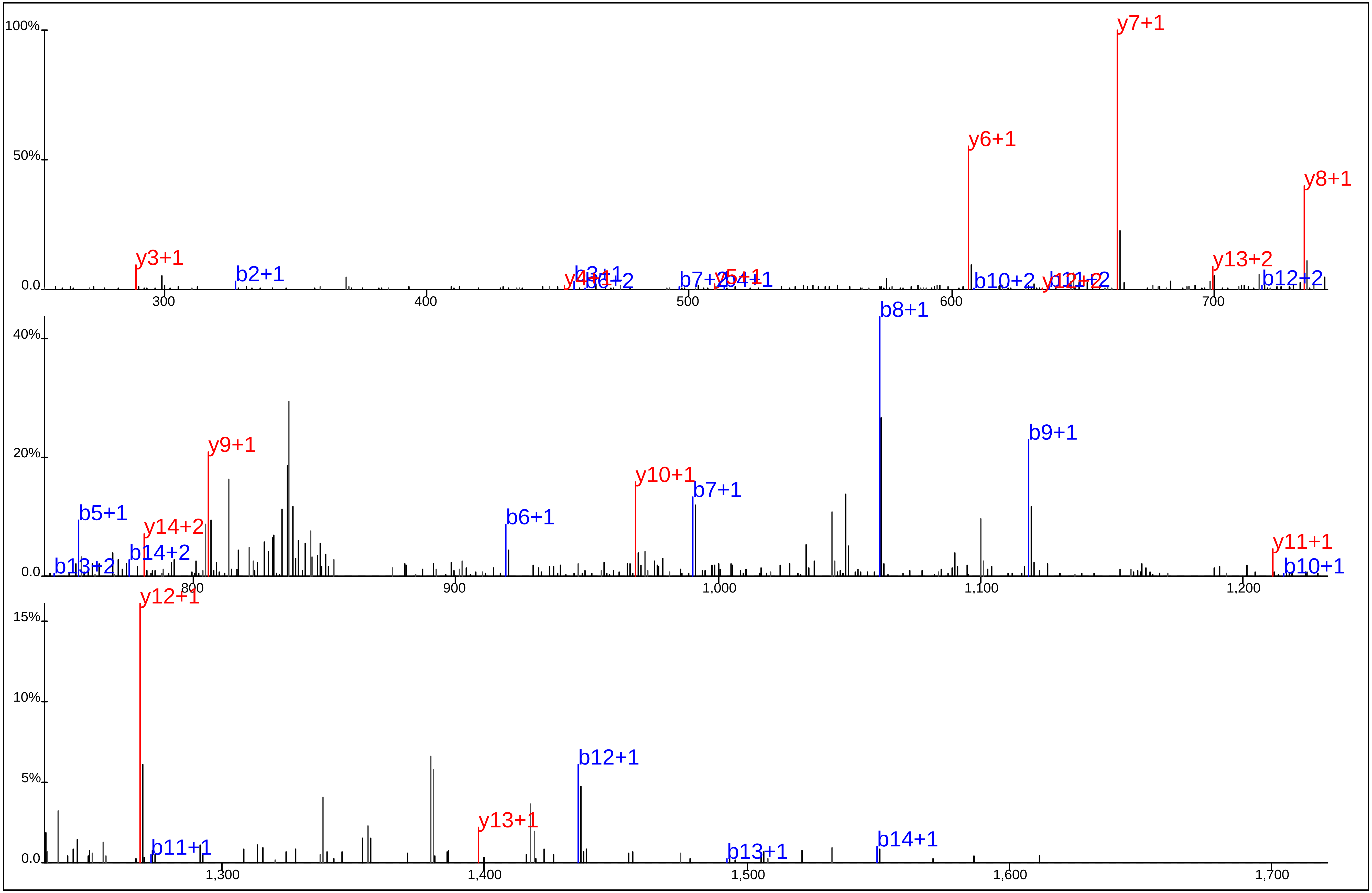

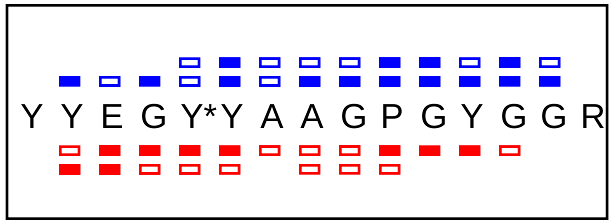

Protein Name:	LPP
gi Accession:	5031887
Precursor Mass:	862.3428+2
Delta Mass:	0.309 ppm
XCorr:	67.94

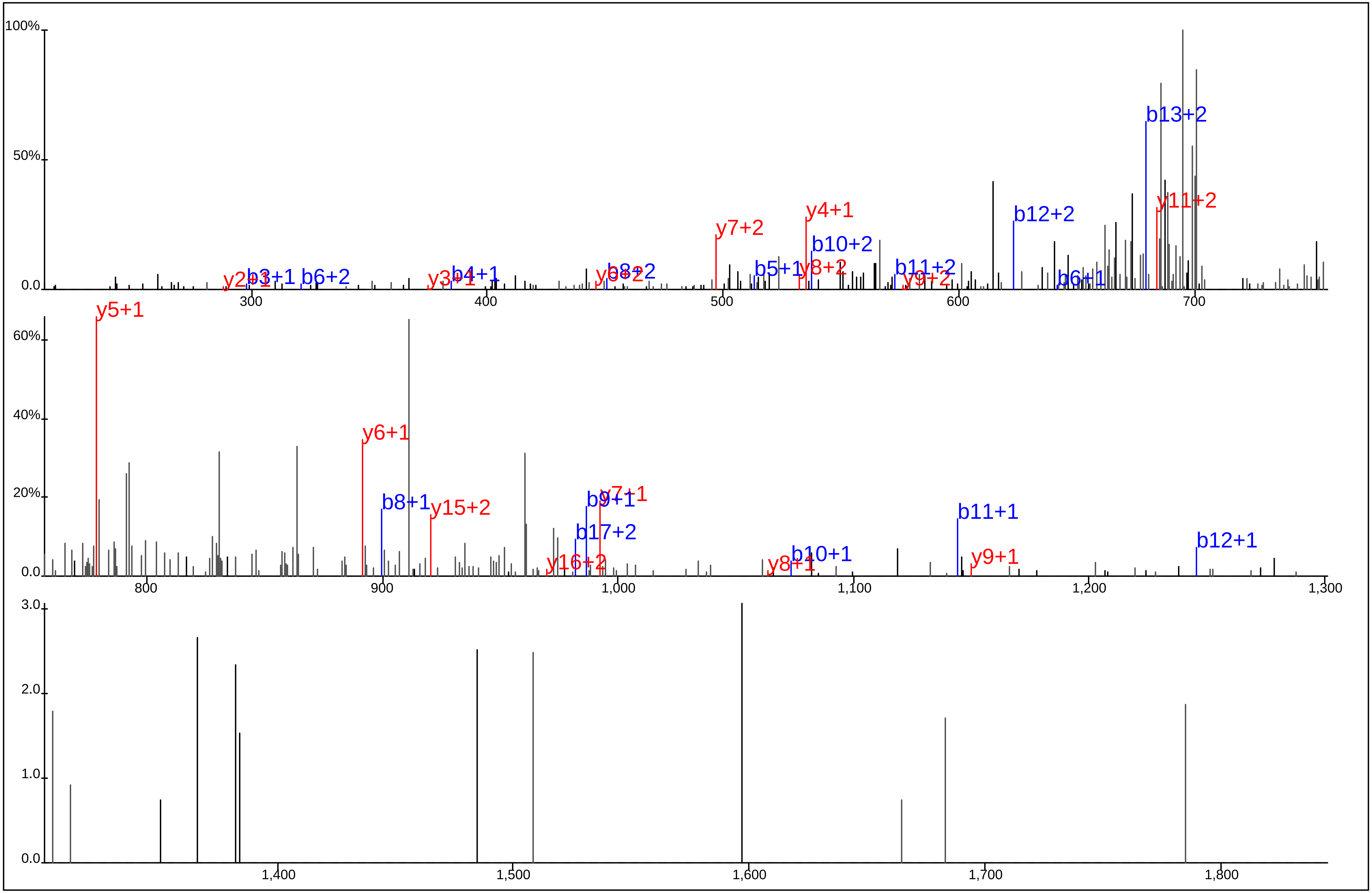

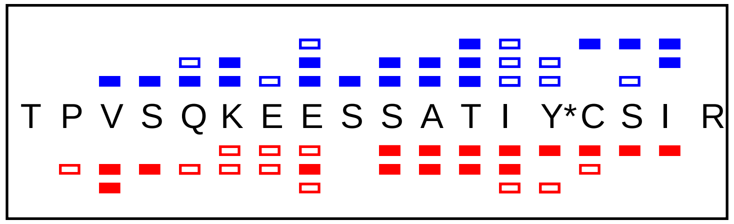

Protein Name:	Lymphocyte antigen 9
gi Accession:	55925578
Precursor Mass:	712.6615+3
Delta Mass:	0.982 ppm
XCorr:	12.68

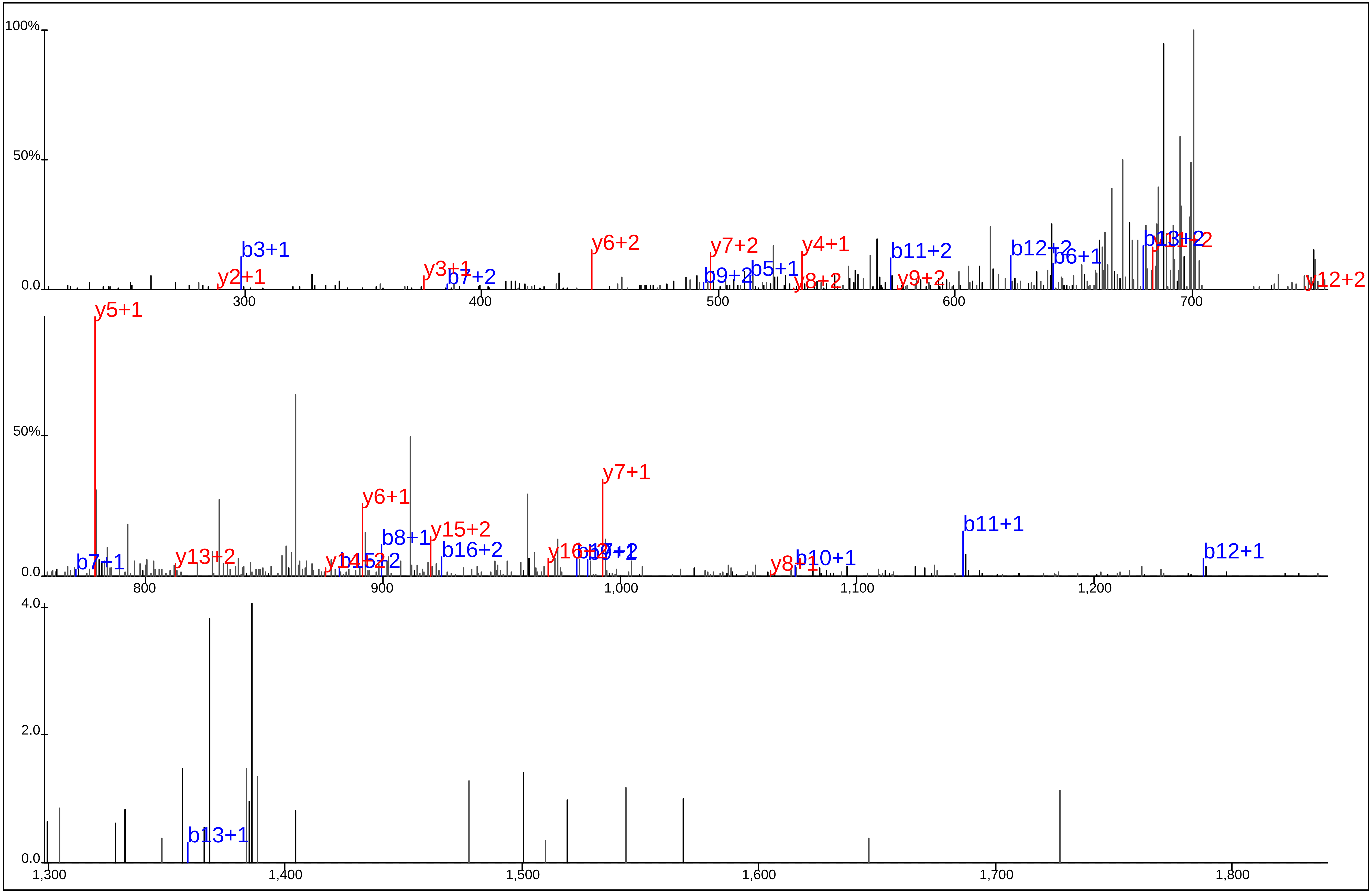

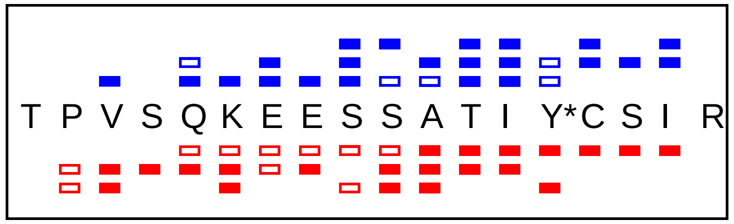

Protein Name: Lymphocyte antigen 9
gi Accession: 55925578
Precursor Mass: 712.6630+3
Delta Mass: 3.042 ppm
XCorr: 16.97

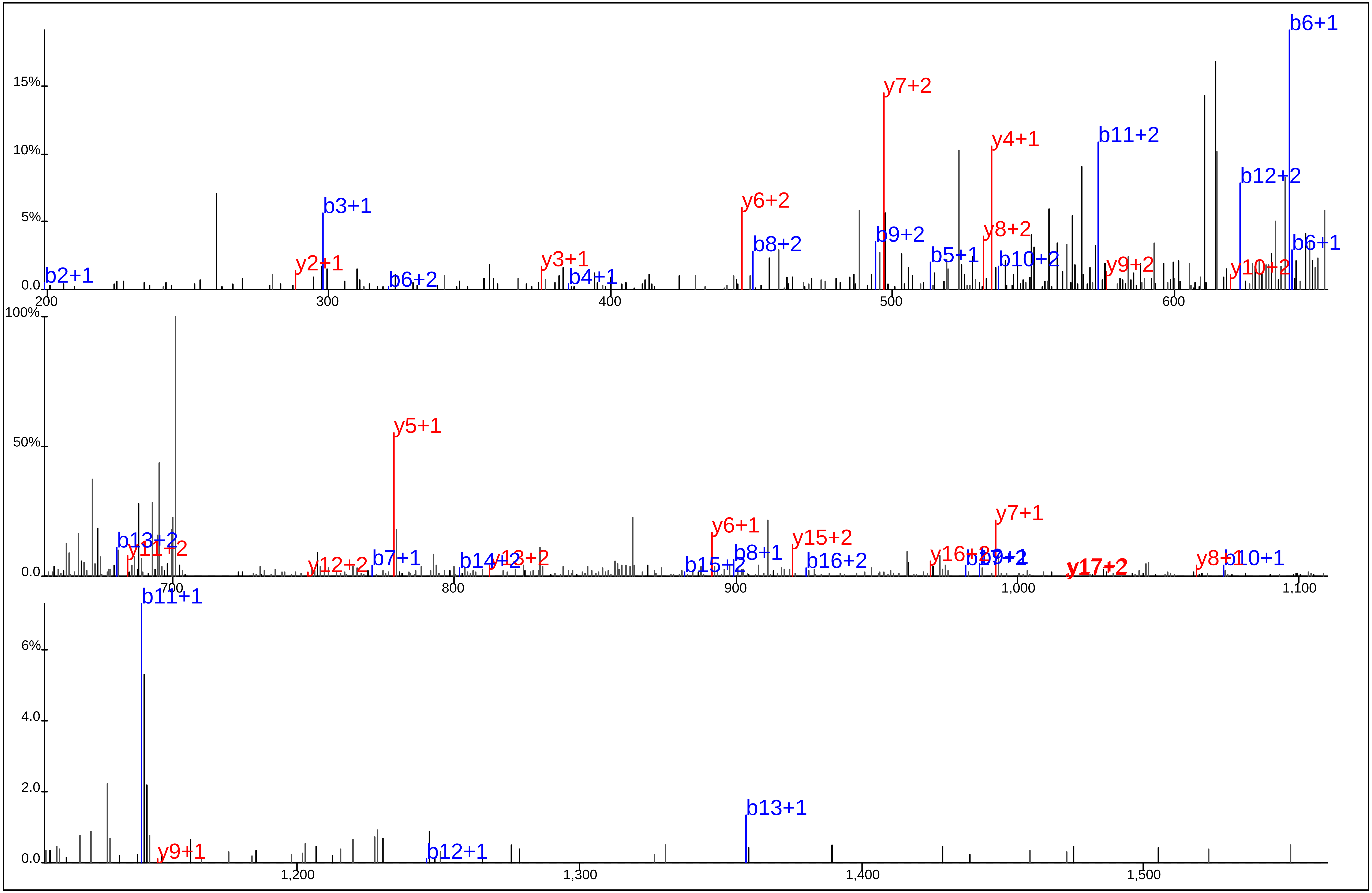

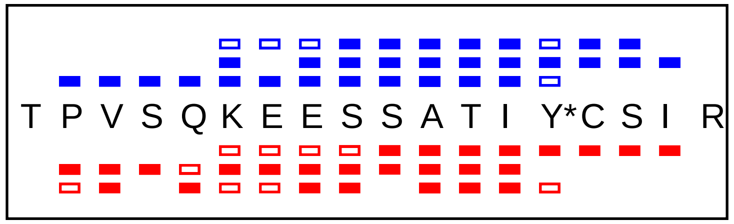

Protein Name: Lymphocyte antigen 9
gi Accession: 55925578
Precursor Mass: 712.6625+3
Delta Mass: 2.354 ppm
XCorr: 23.54

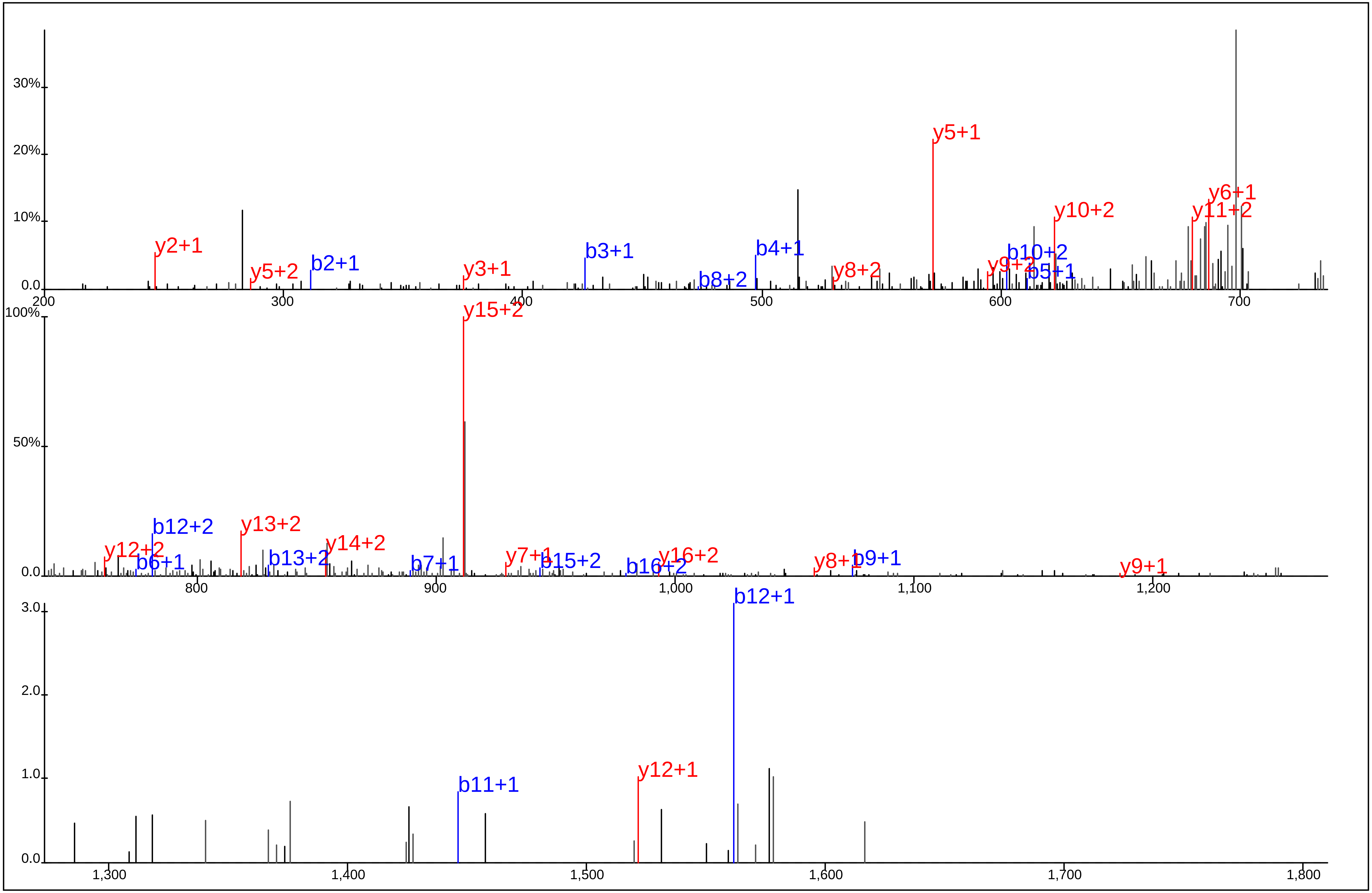

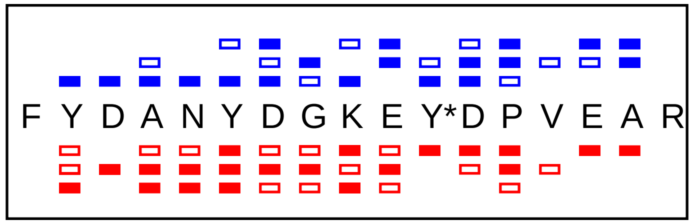

Protein Name:	MAPRE2
gi Accession:	10346135
Precursor Mass:	711.2906+3
Delta Mass:	3.383 ppm
XCorr:	39.68

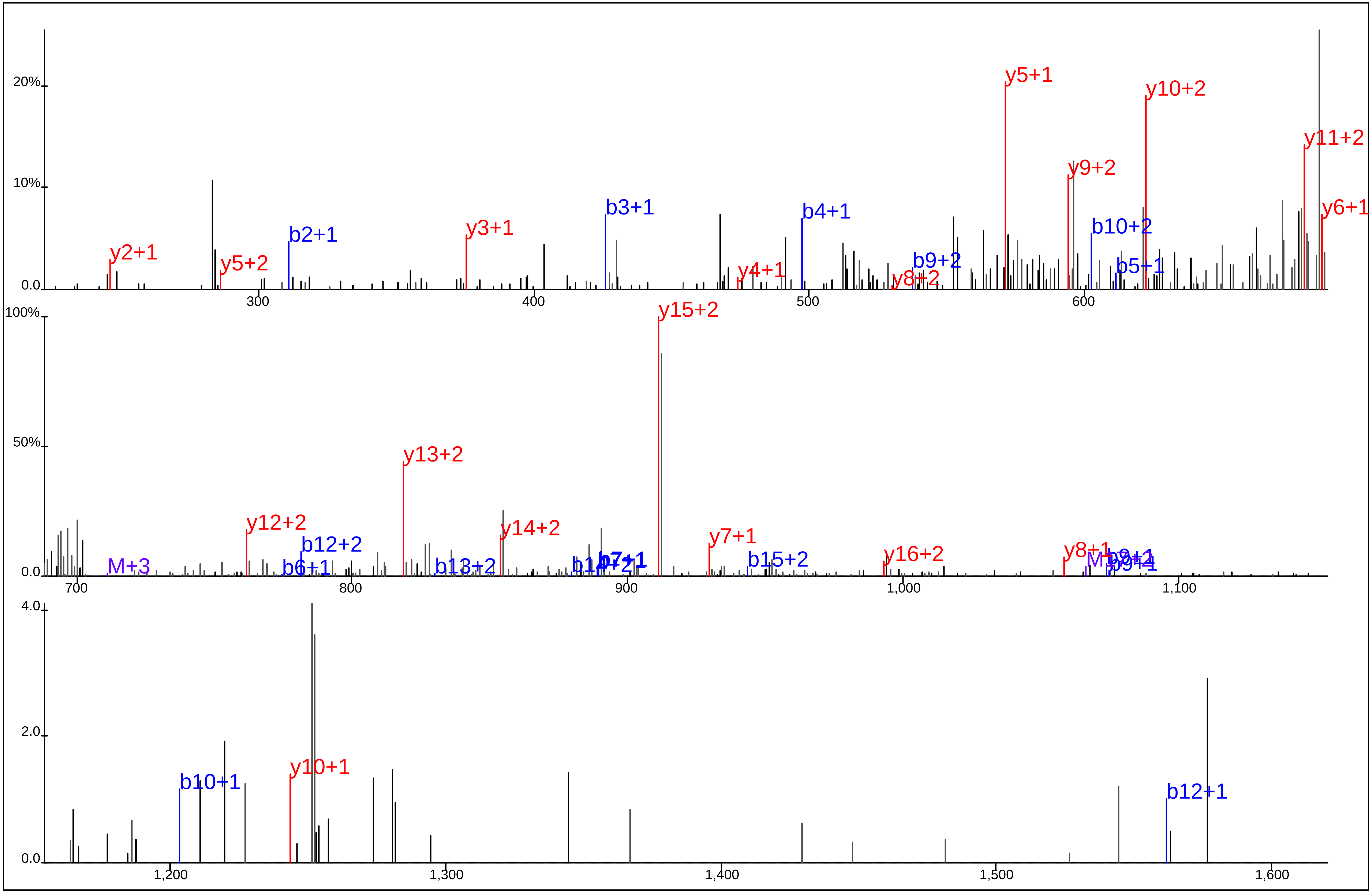

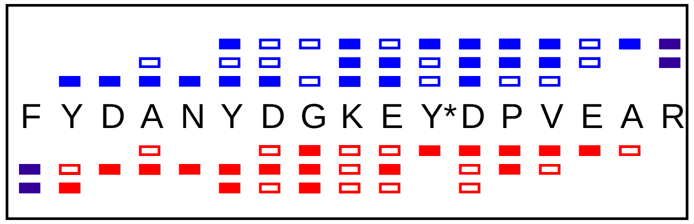

Protein Name: MAPRE2
gi Accession: 10346135
Precursor Mass: 711.2963+3
Delta Mass: 4.605 ppm
XCorr: 30.98

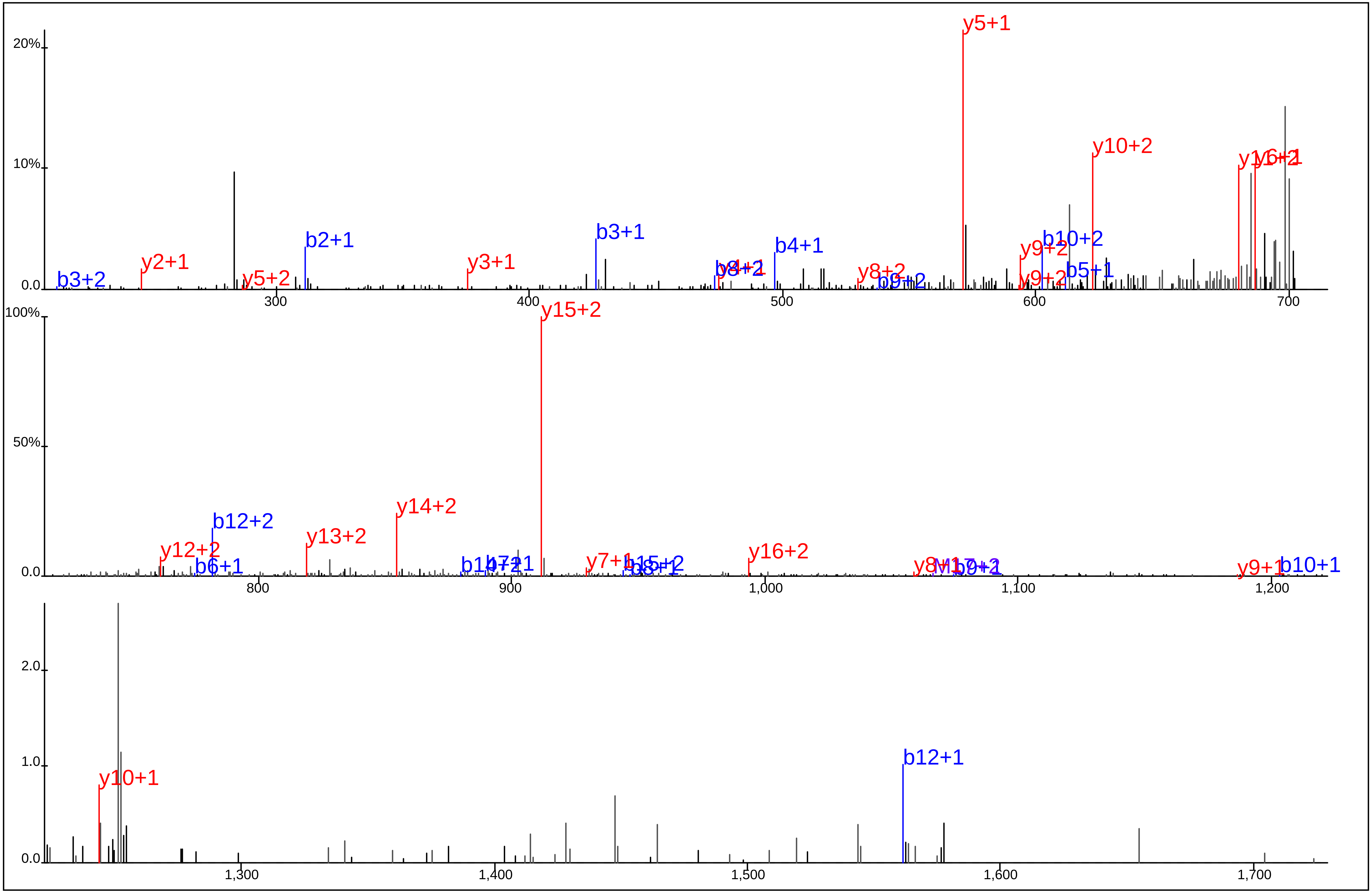

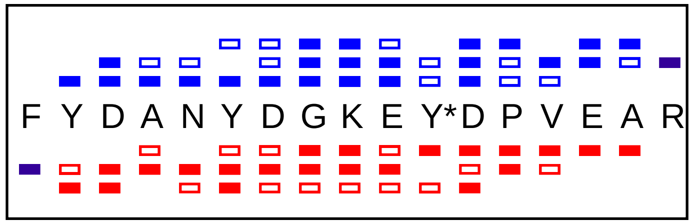

Protein Name: MAPRE2
gi Accession: 10346135
Precursor Mass: 711.2994+3
Delta Mass: 8.986 ppm
XCorr: 44.42

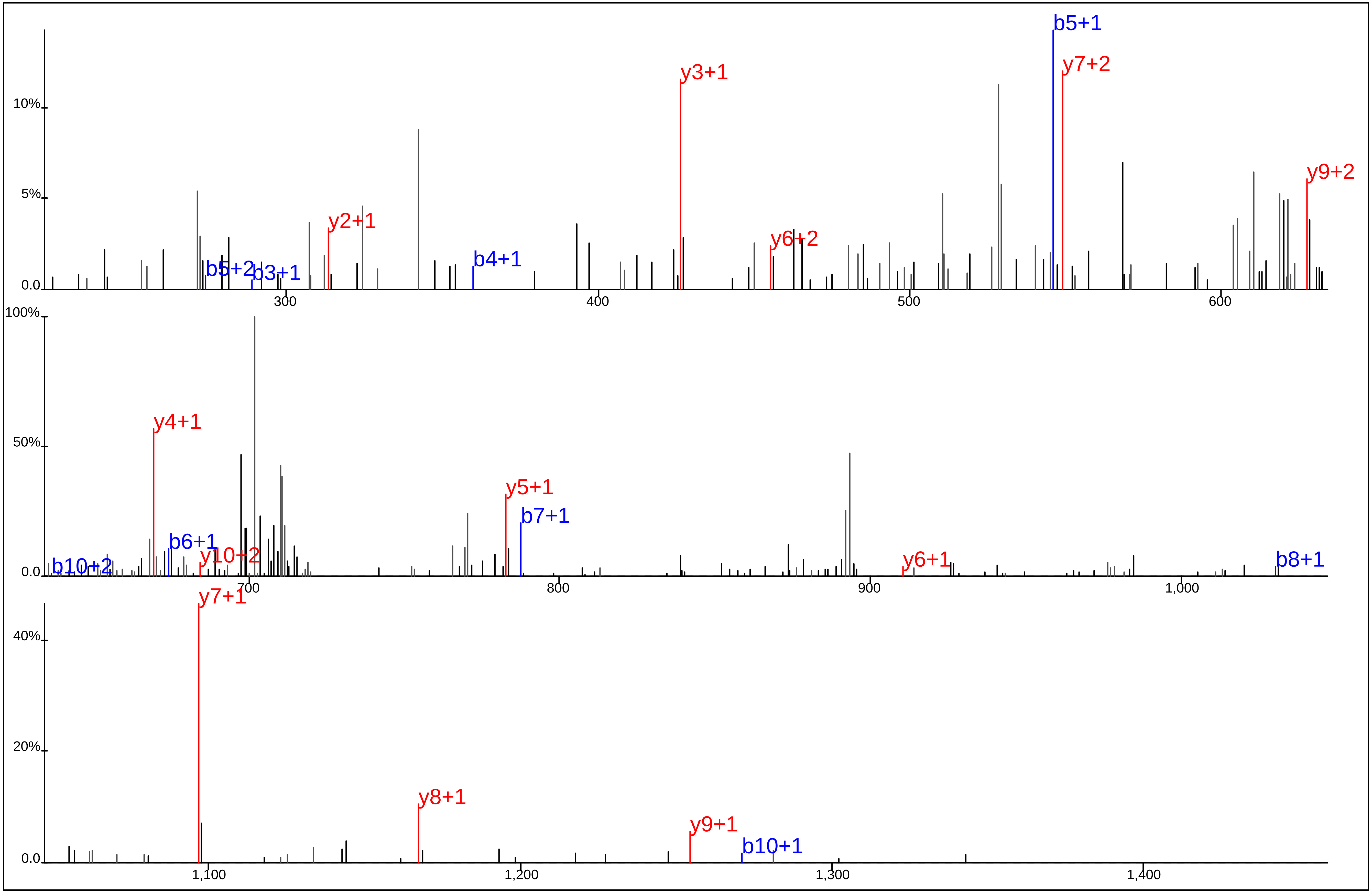

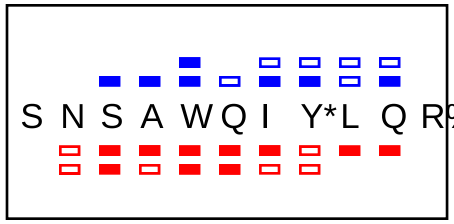

Protein Name: MEKKK6
gi Accession: 29427834
Precursor Mass: 728.3374+2
Delta Mass: 1.366 ppm
XCorr: 29.02

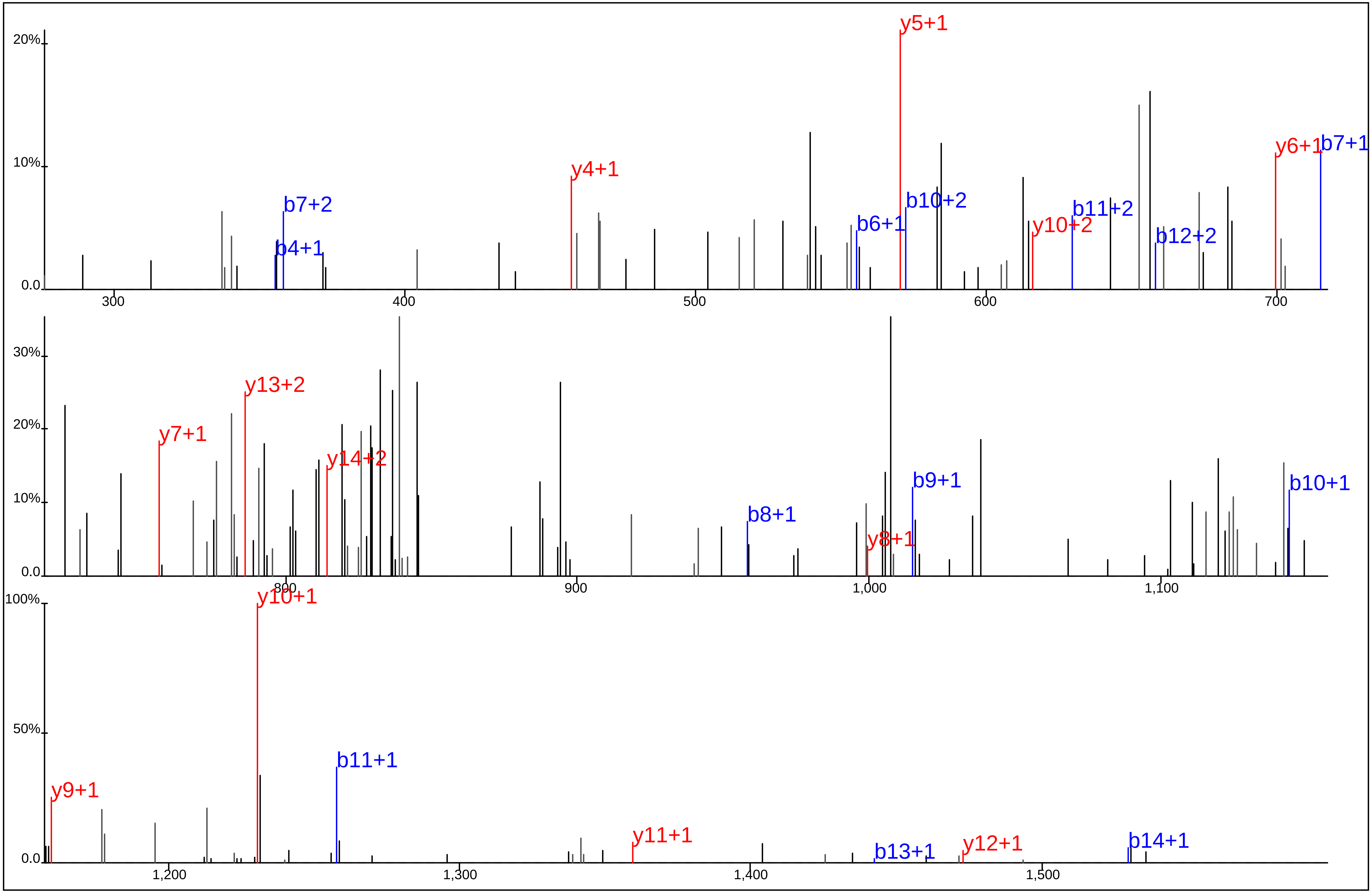

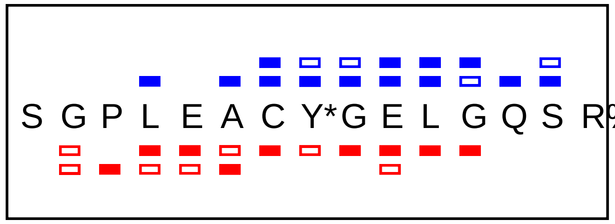

Protein Name: Myotubularin related protein 10
gi Accession: 124028527
Precursor Mass: 857.3614+2
Delta Mass: 1.297 ppm
XCorr: 31.21

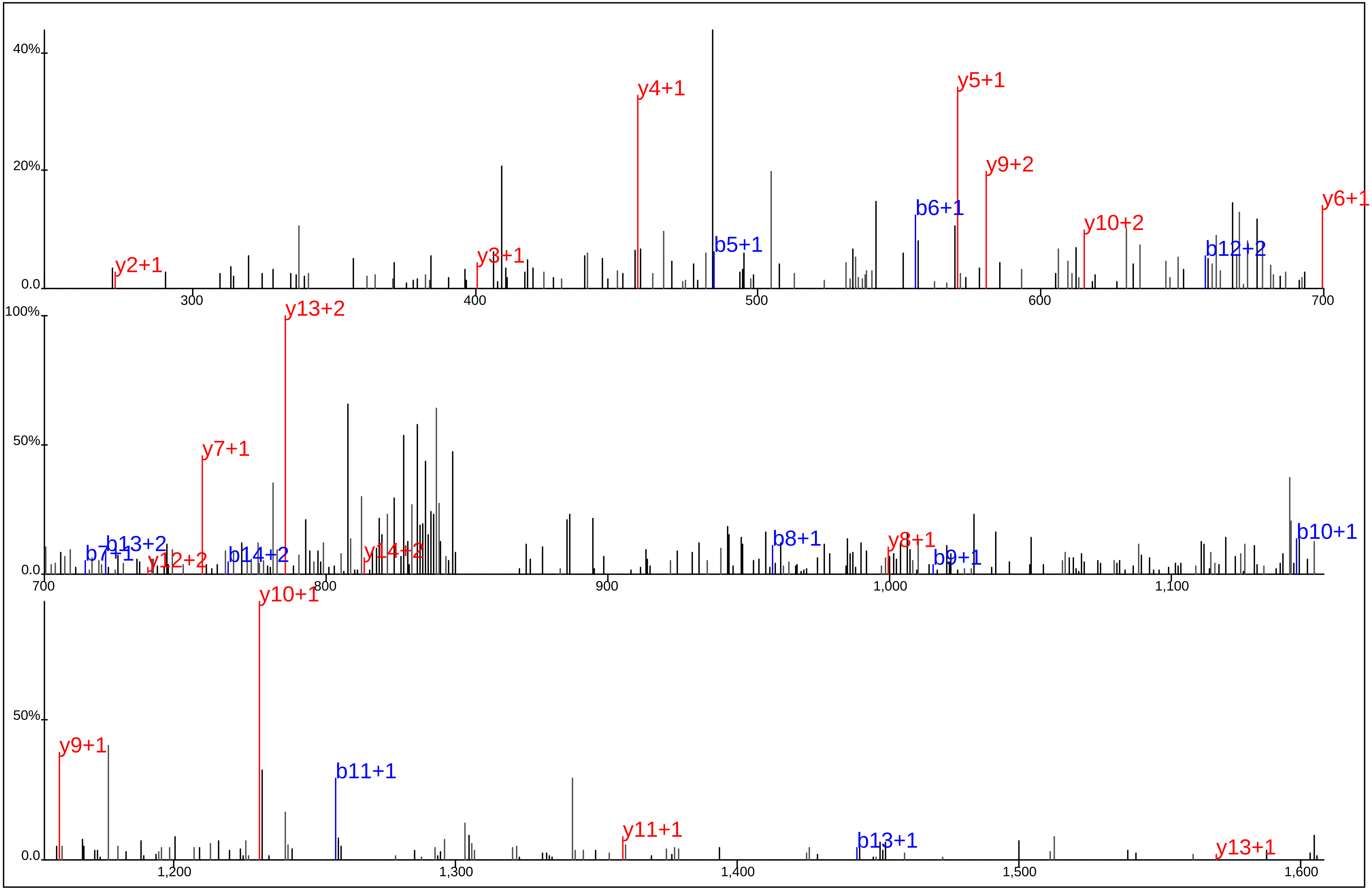

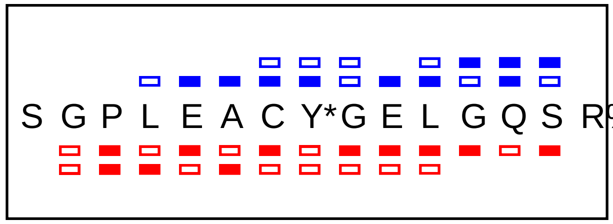

Protein Name:	Myotubularin related protein 10
gi Accession:	124028527
Precursor Mass:	857.3598+2
Delta Mass:	3.146 ppm
XCorr:	22.09

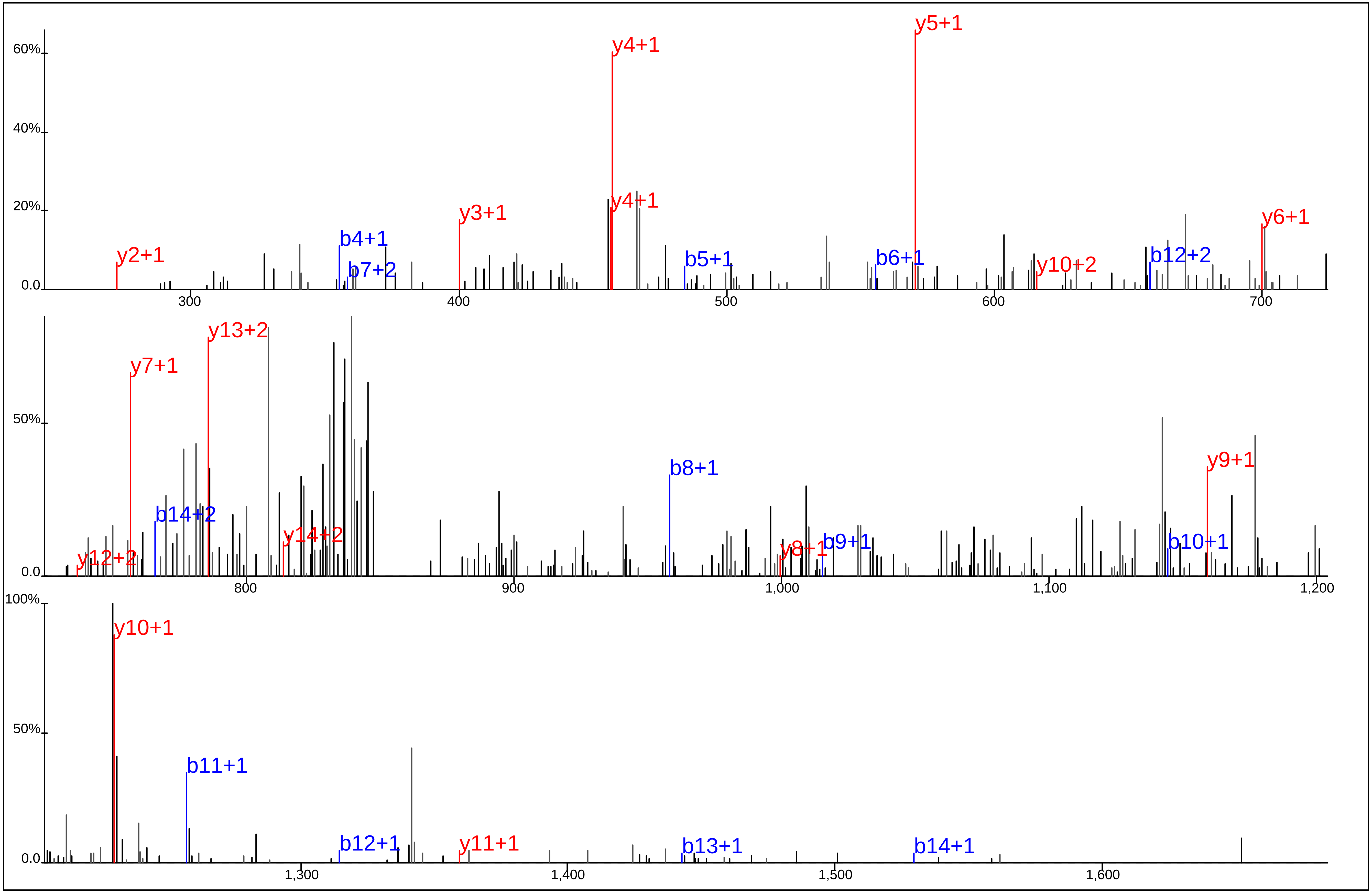

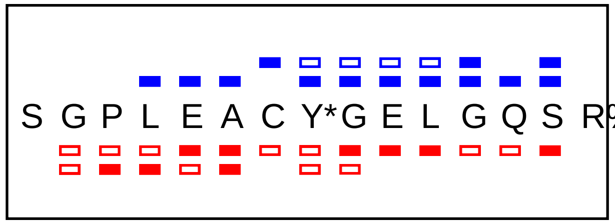

Protein Name: Myotubularin related protein 10

gi Accession: 124028527

Precursor Mass: 857.3616+2

Delta Mass: 1.011 ppm

XCorr: 24.65

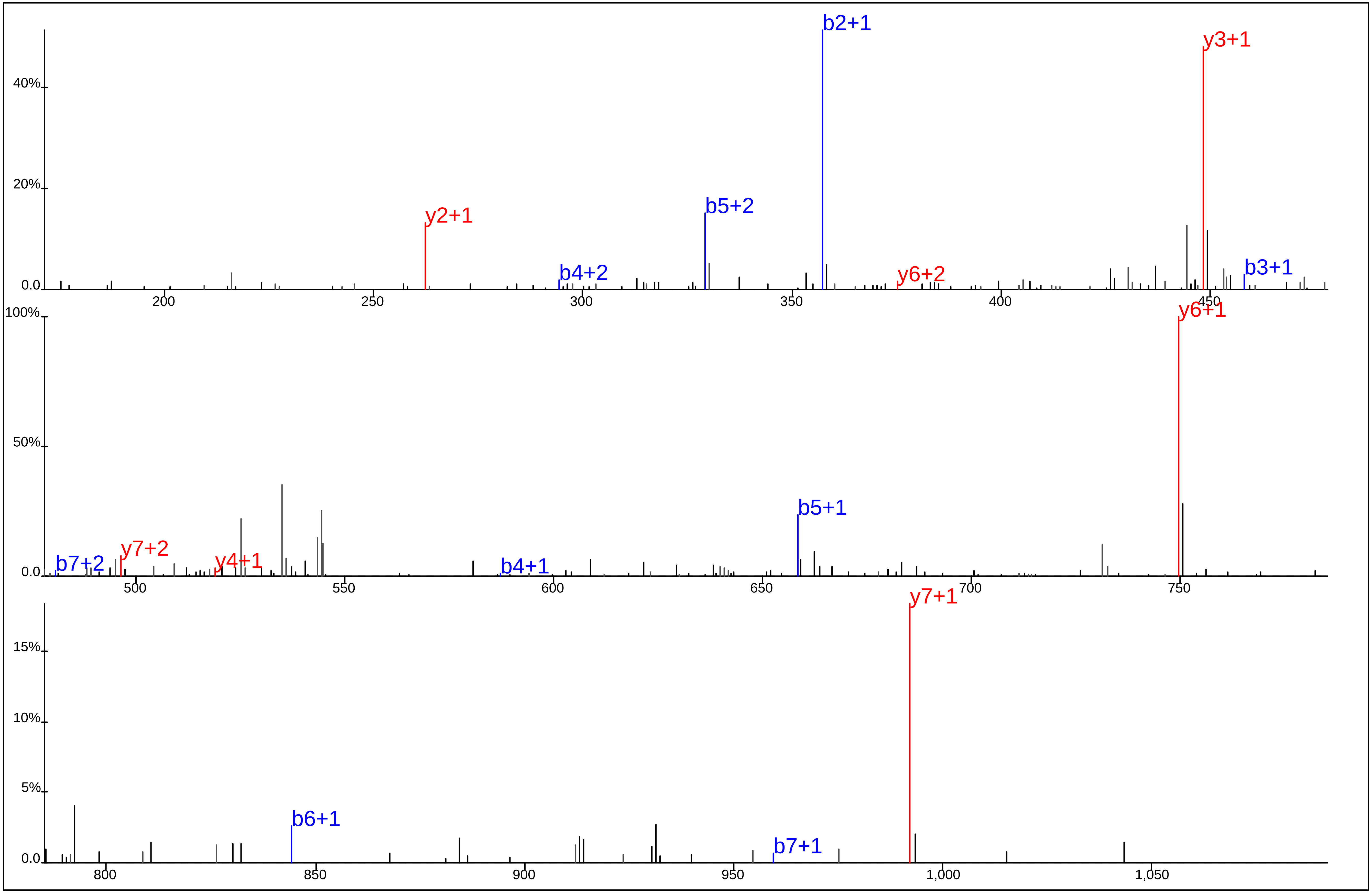

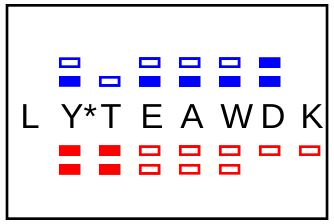

Protein Name: Nebulin
gi Accession: 19856971
Precursor Mass: 553.2411+2
Delta Mass: 13.350 ppm
XCorr: 27.28

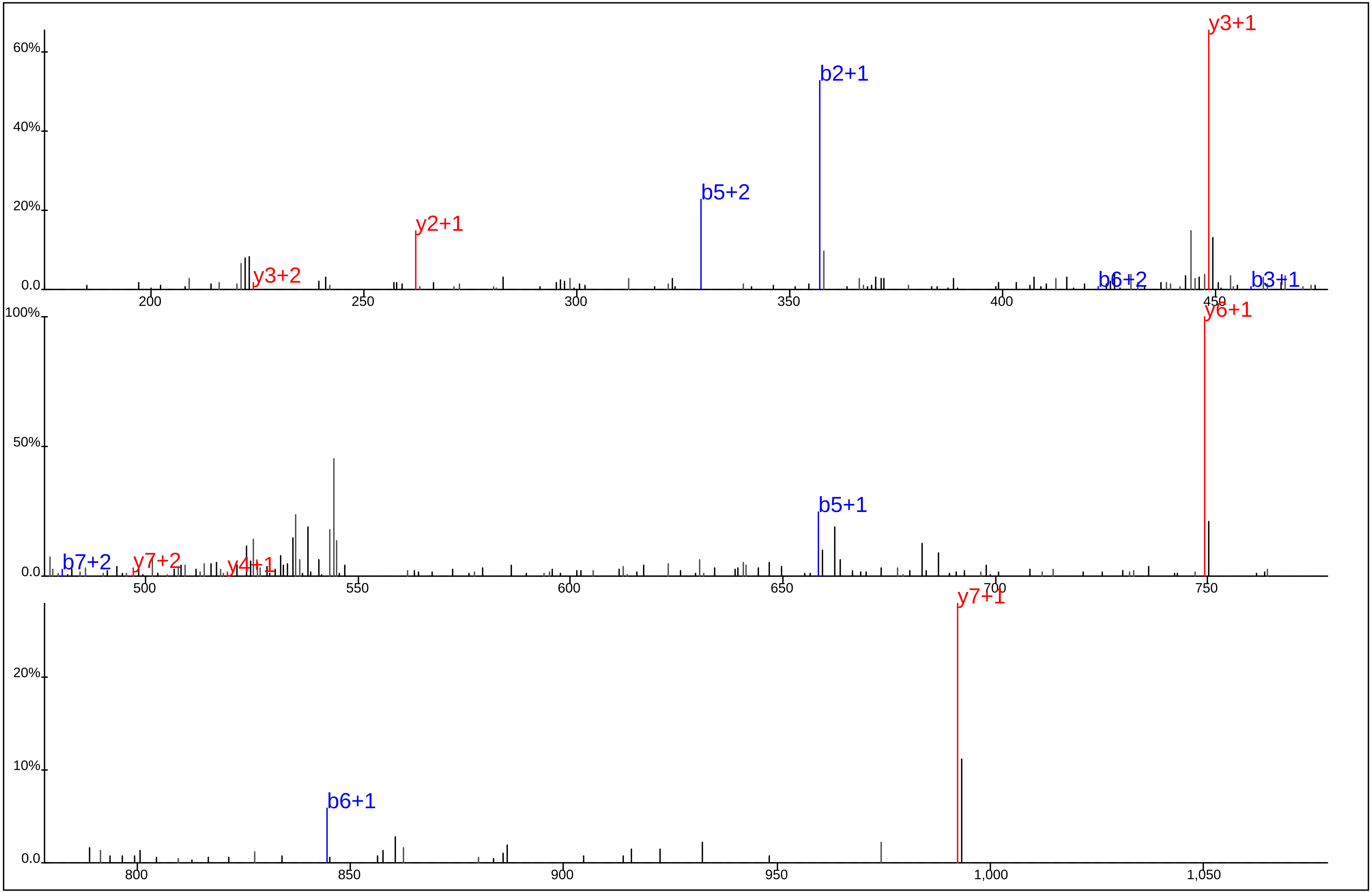

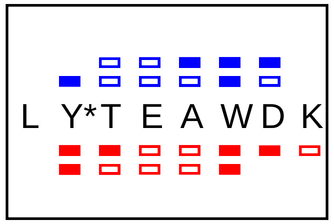

Protein Name: Nebulin
gi Accession: 19856971
Precursor Mass: 553.2424+2
Delta Mass: 15.783 ppm
XCorr: 33.1

626 - Nebulin_3434873.pdf

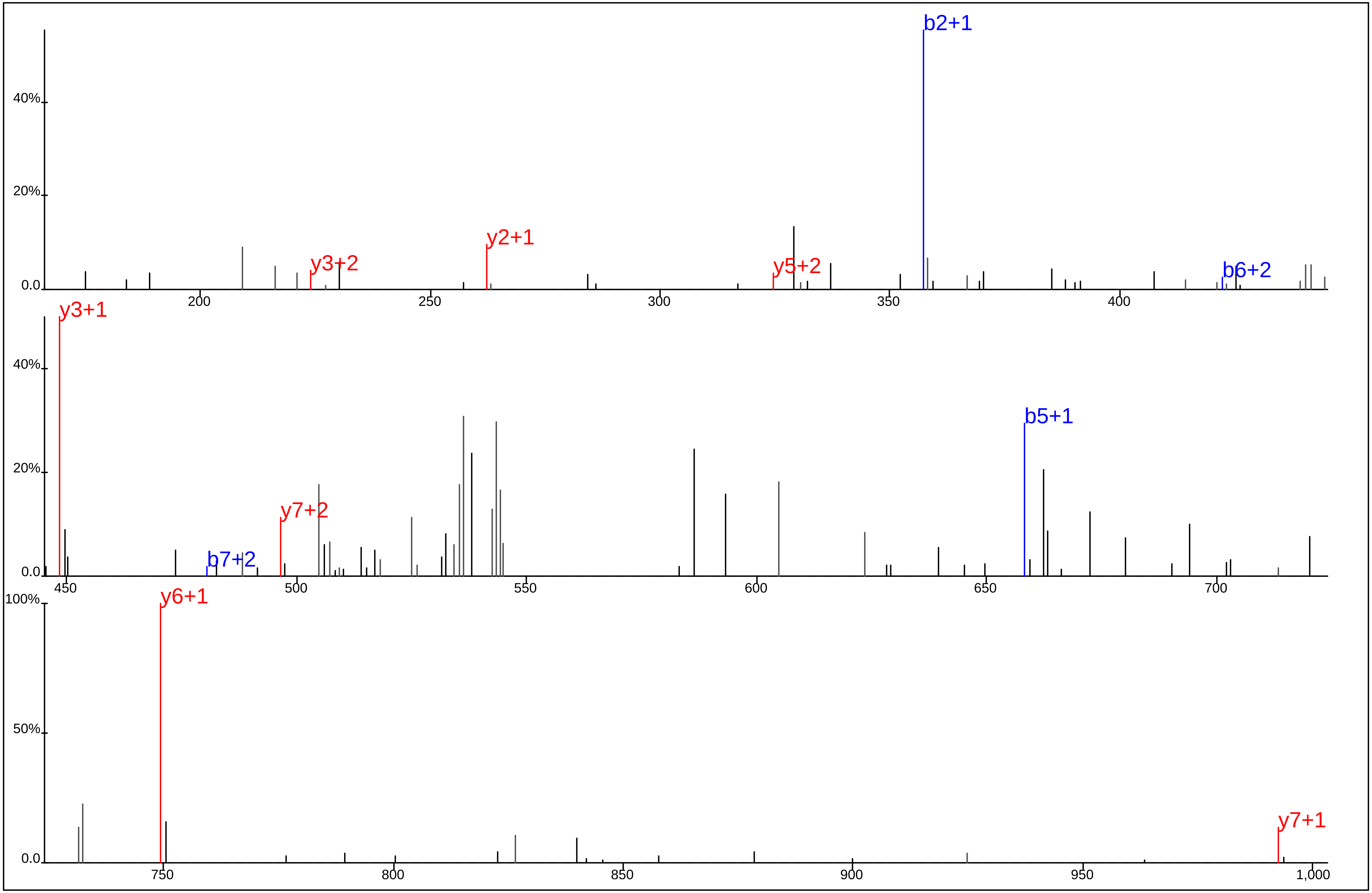

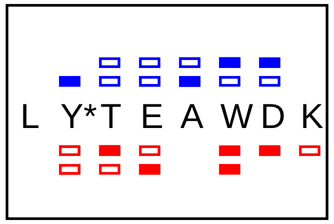

Protein Name: Nebulin
gi Accession: 19856971
Precursor Mass: 553.2430+2
Delta Mass: 16.887 ppm
XCorr: 32.21

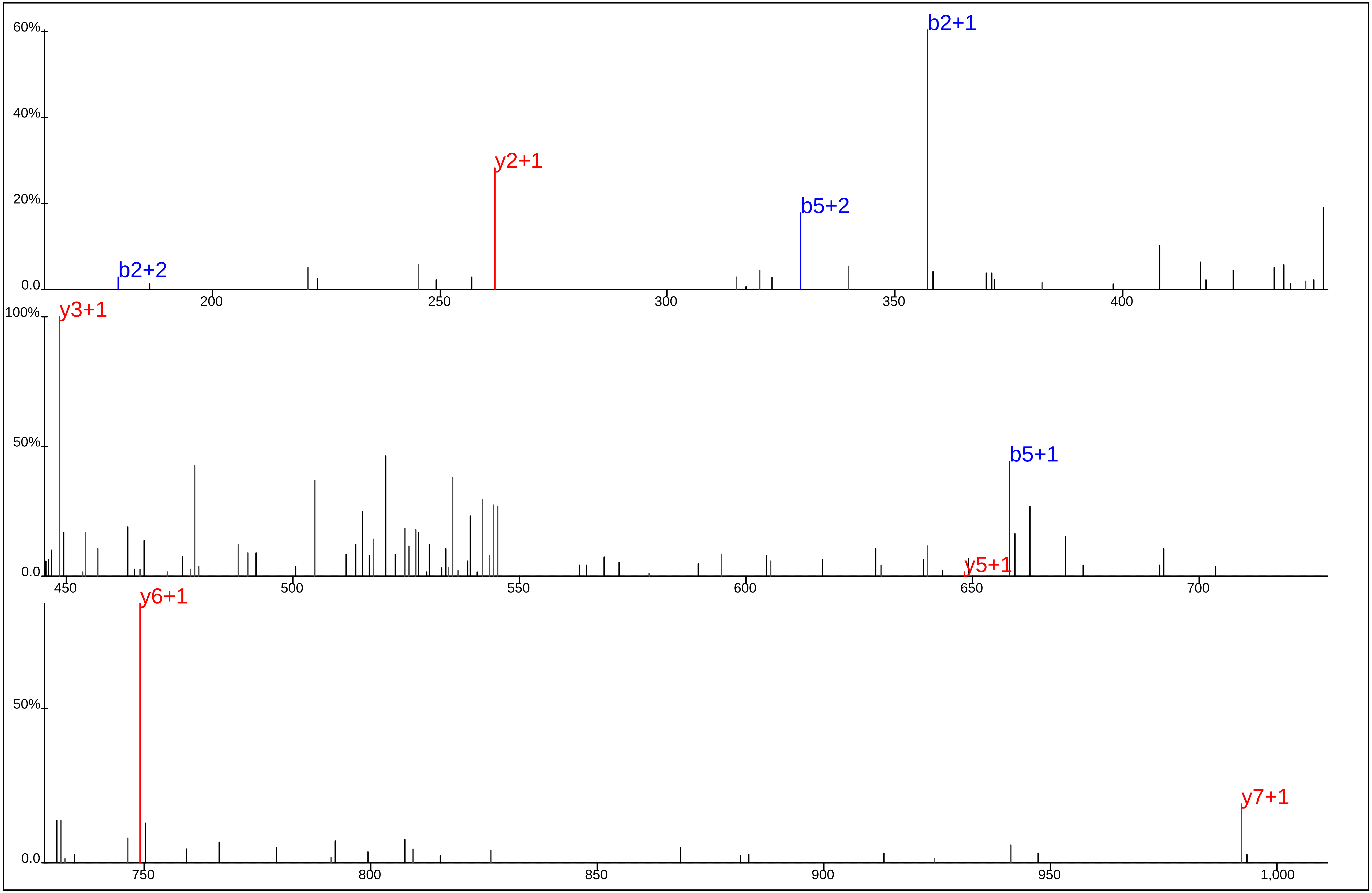

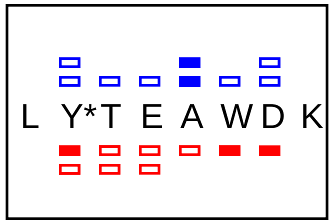

Protein Name: Nebulin
gi Accession: 19856971
Precursor Mass: 553.2419+2
Delta Mass: 14.788 ppm
XCorr: 28.28

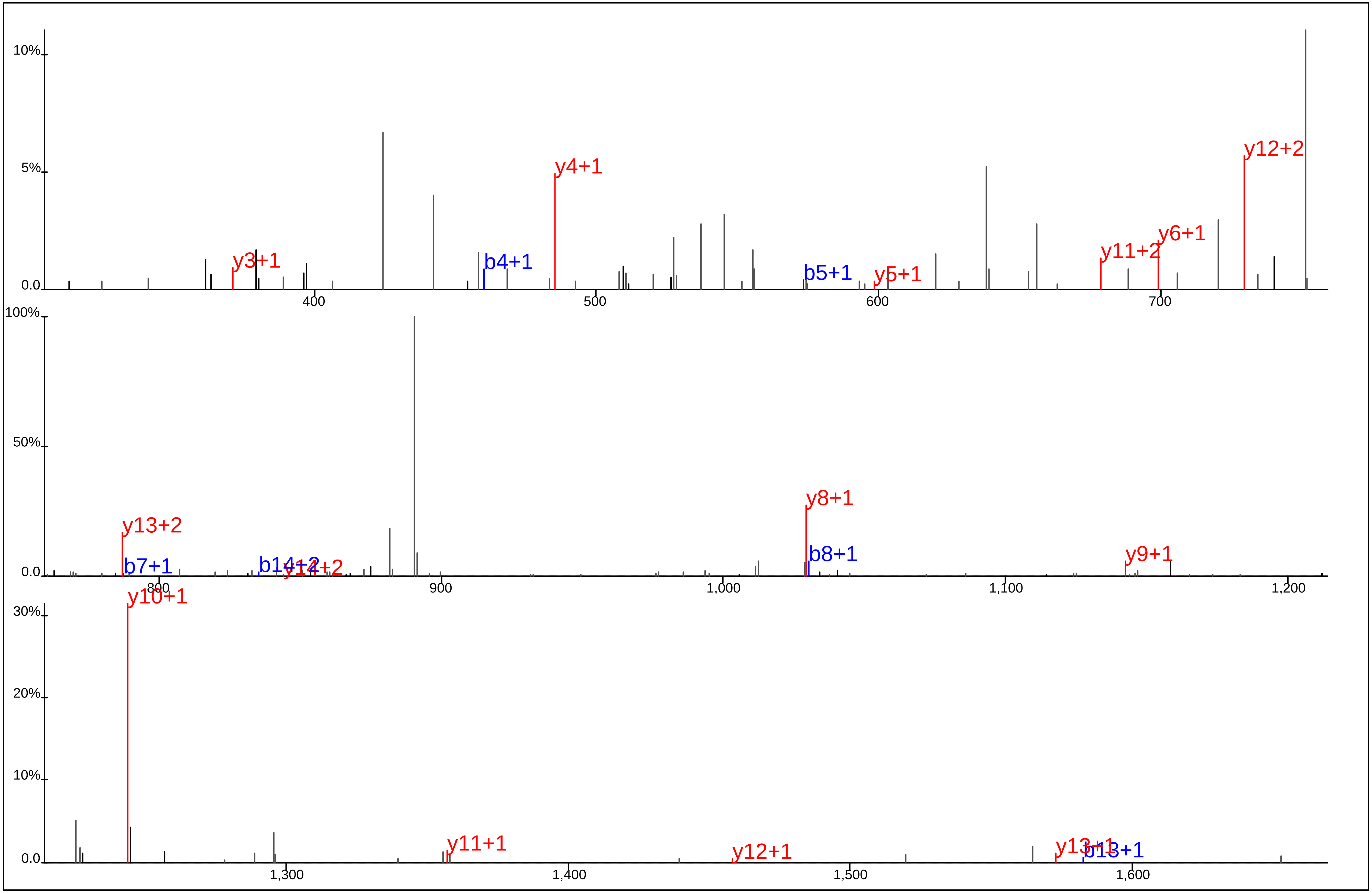

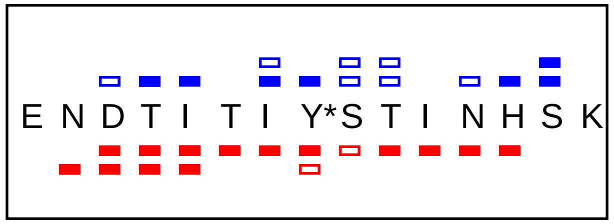

Protein Name: NTBA
gi Accession: 15384843
Precursor Mass: 908.4153+2
Delta Mass: 4.069 ppm
XCorr: 51.91

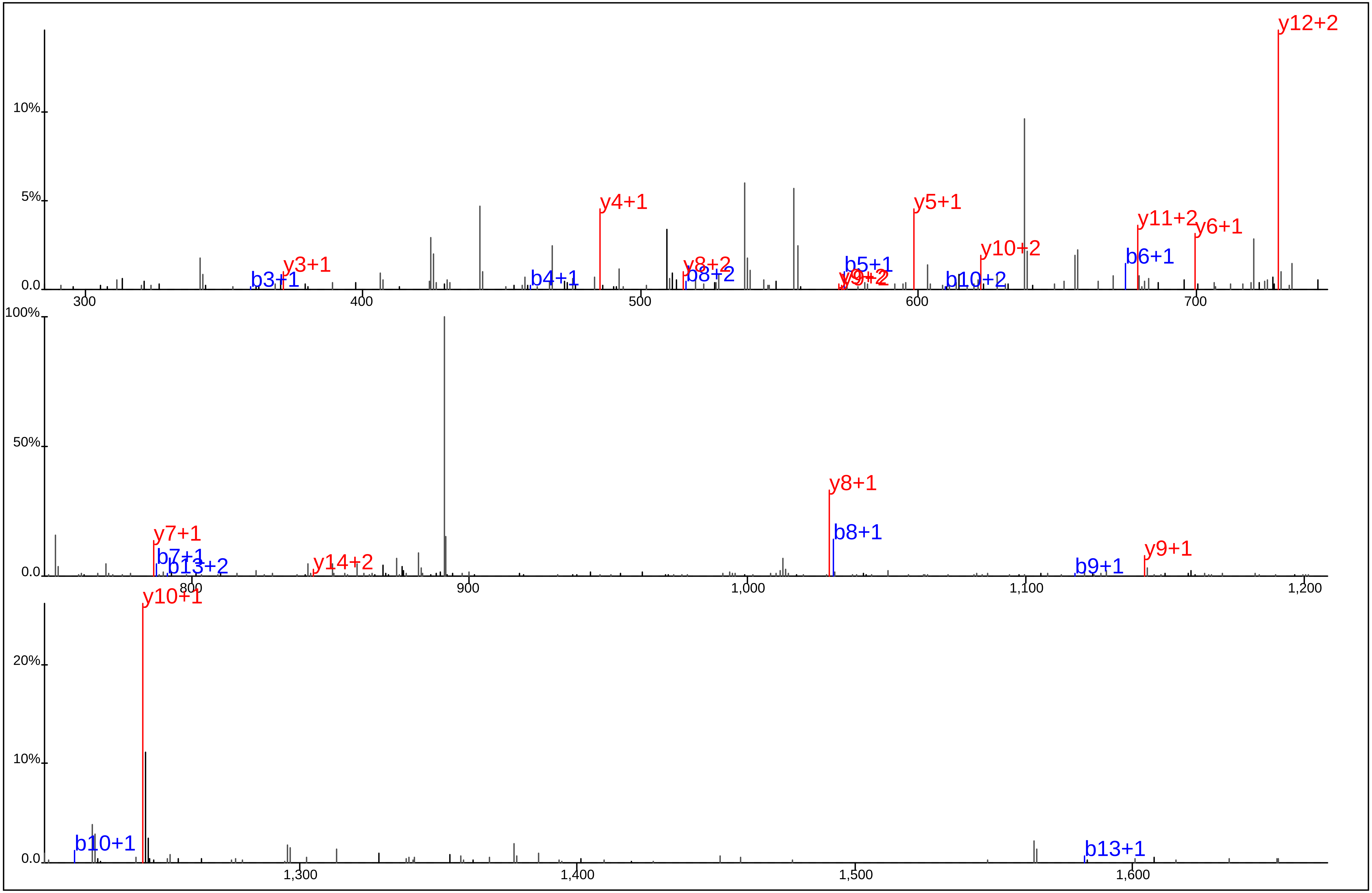

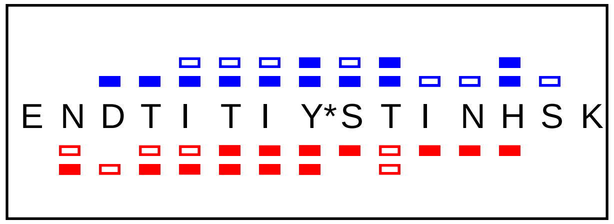

Protein Name:	NTBA
gi Accession:	15384843
Precursor Mass:	908.4108+2
Delta Mass:	0.904 ppm
XCorr:	33.75

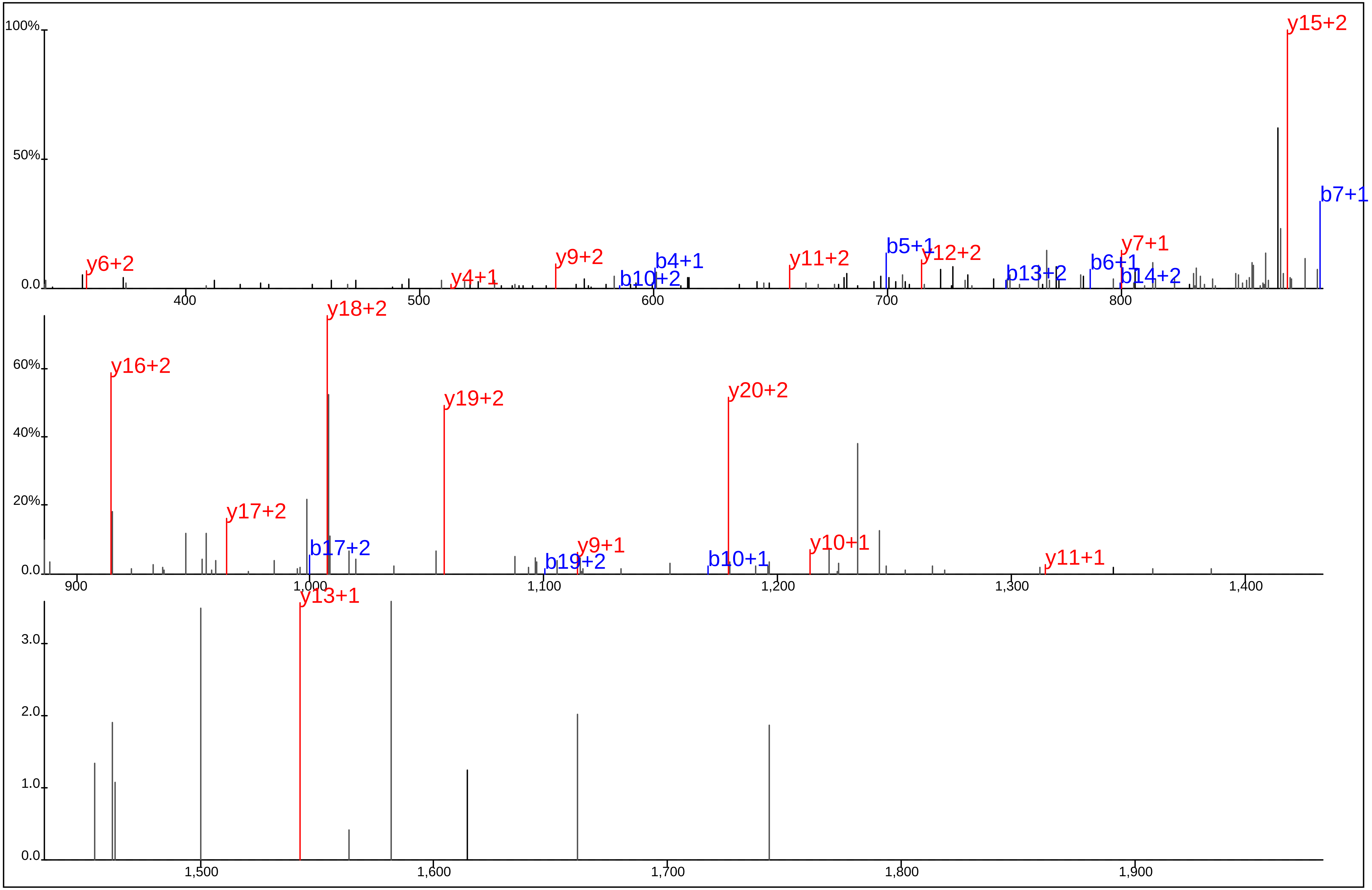

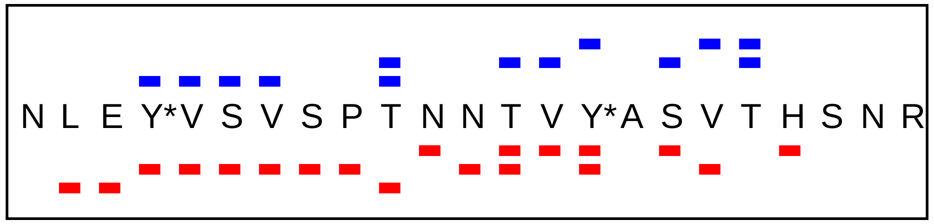

Protein Name:	NTBA
gi Accession:	15384843
Precursor Mass:	904.7297+3
Delta Mass:	1.640 ppm
XCorr:	44.59

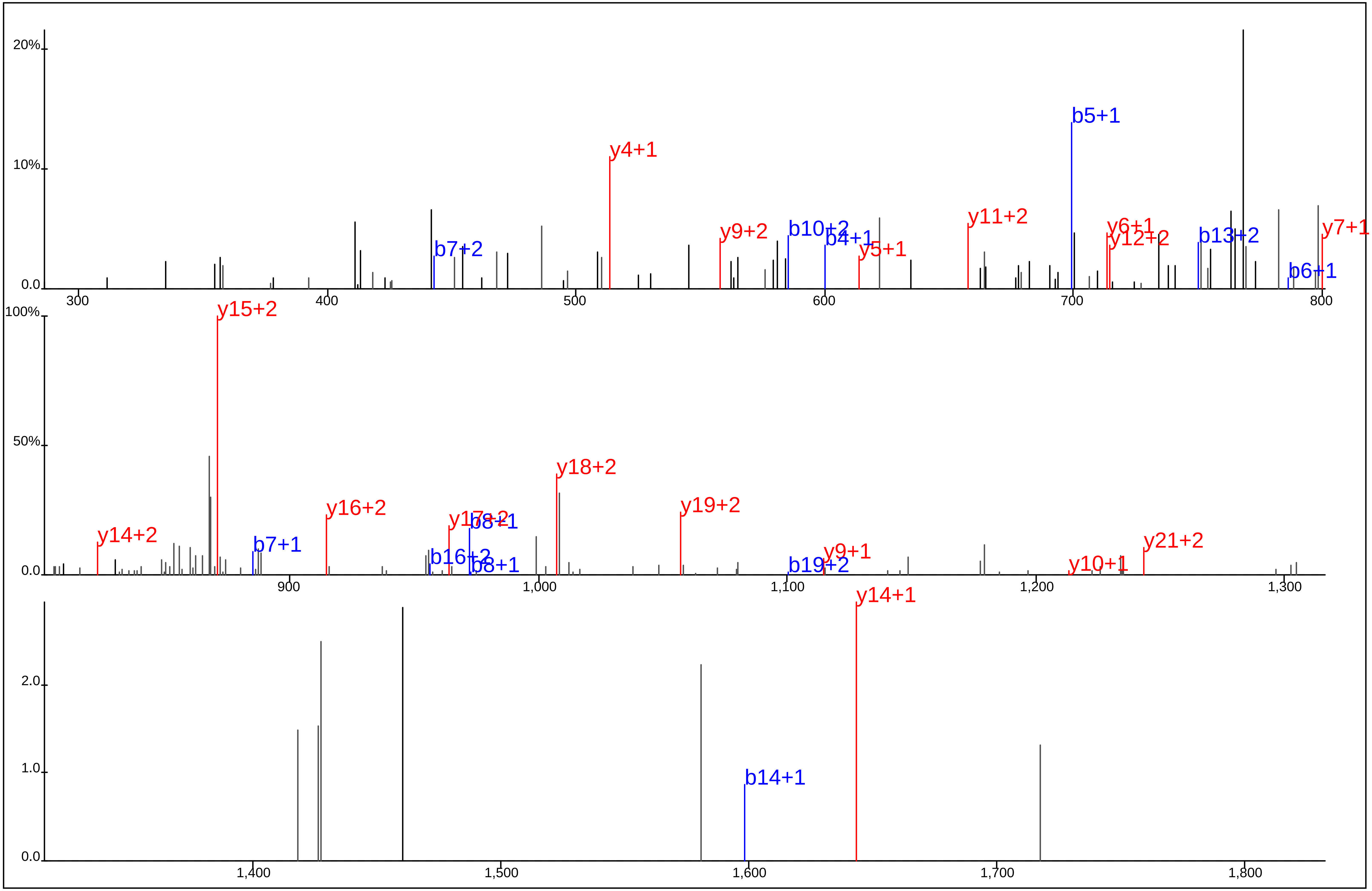

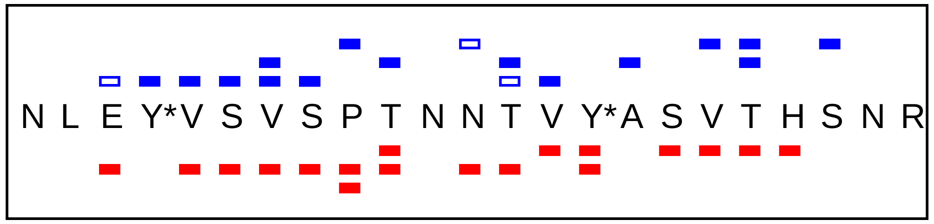

Protein Name: NTBA
gi Accession: 15384843
Precursor Mass: 904.7270+3
Delta Mass: 1.398 ppm
XCorr: 45.05

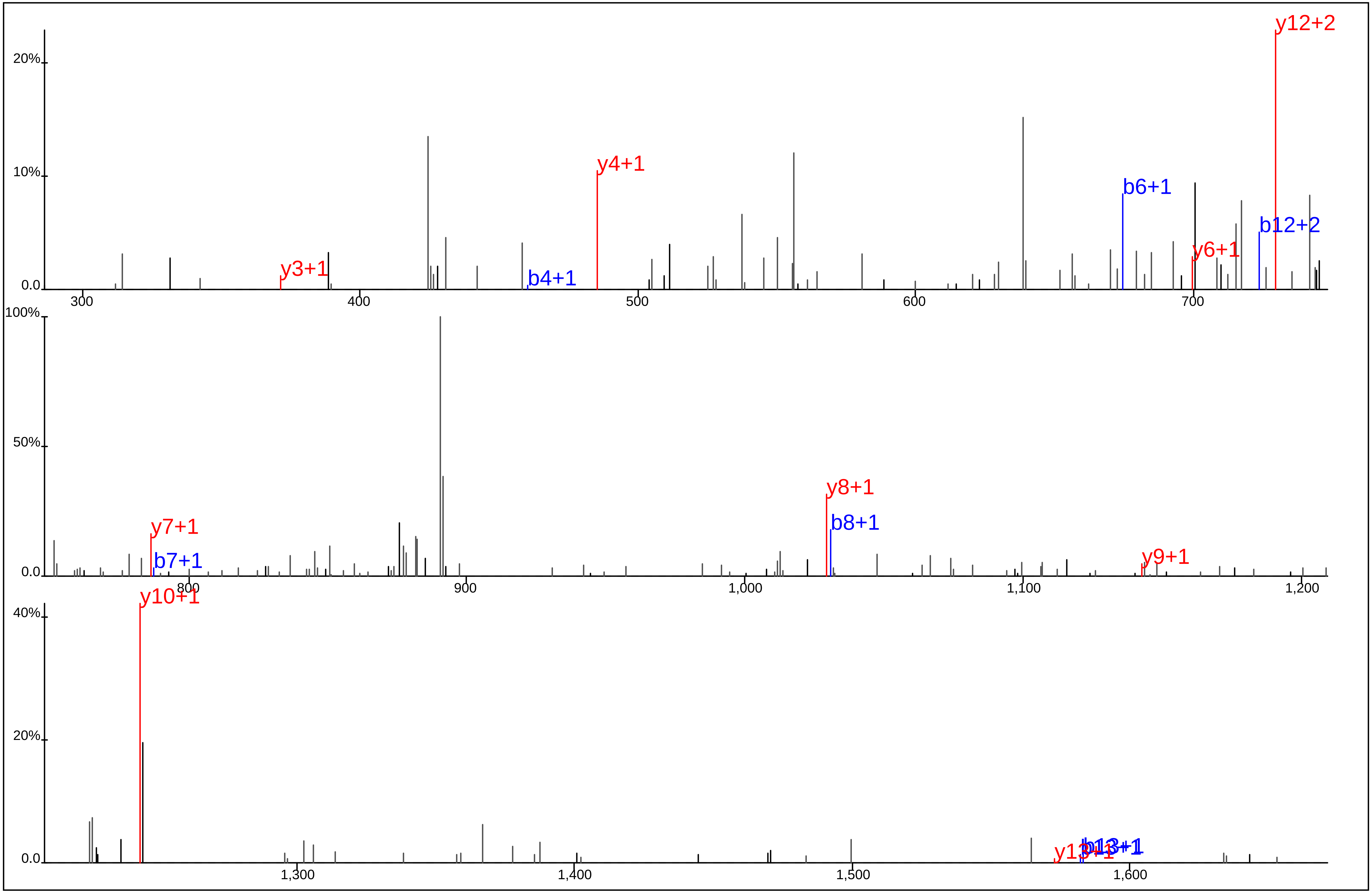

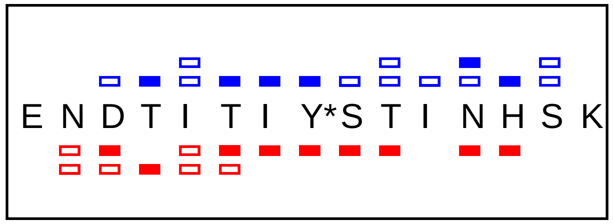

Protein Name: NTBA
gi Accession: 15384843
Precursor Mass: 908.4165+2
Delta Mass: 5.346 ppm
XCorr: 17.38

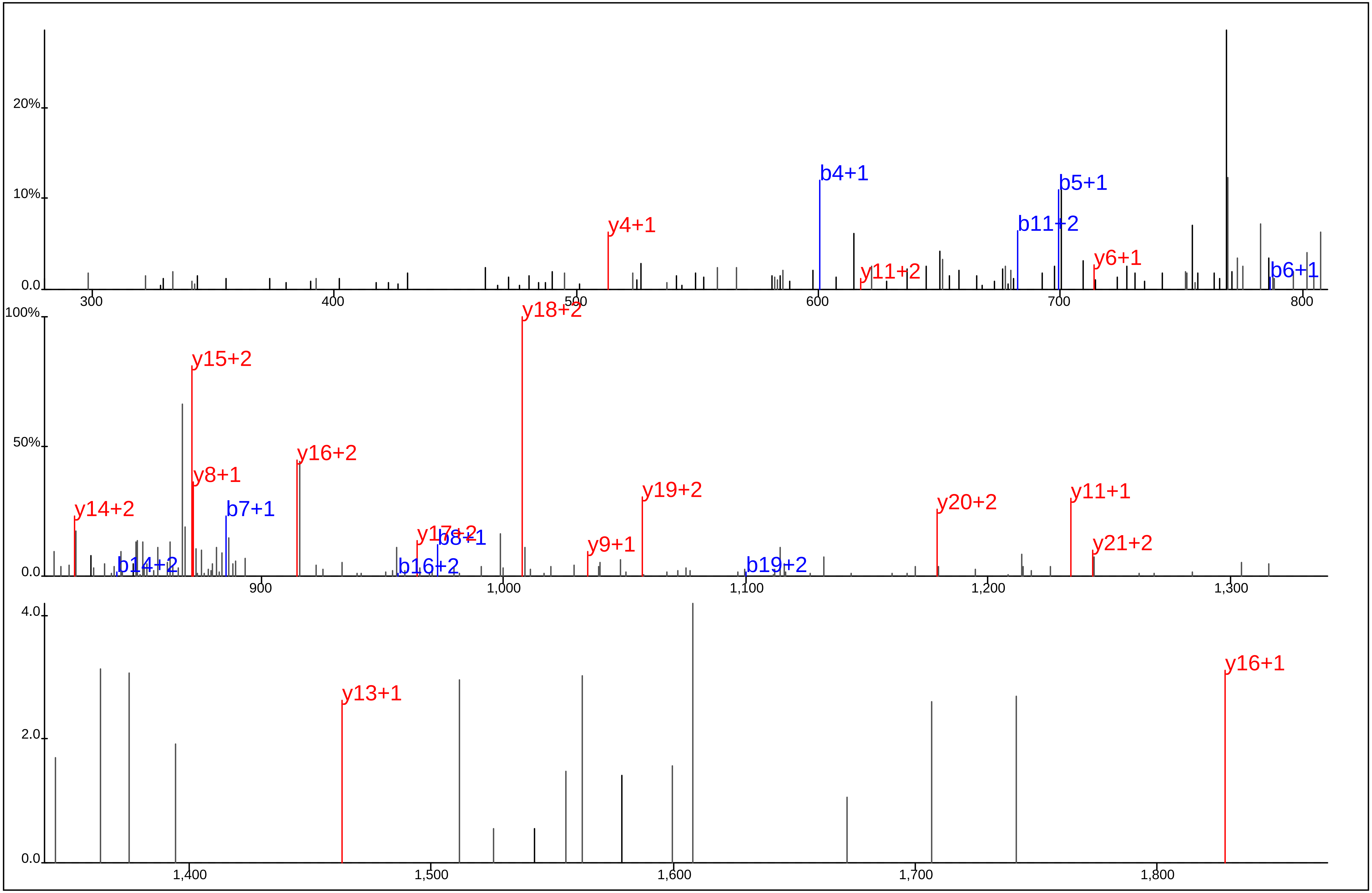

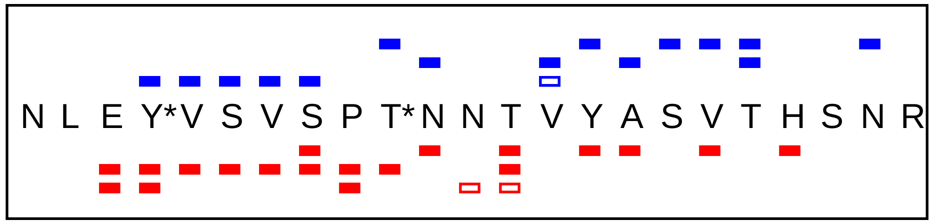

Protein Name:	NTBA
gi Accession:	15384843
Precursor Mass:	904.7325+3
Delta Mass:	4.745 ppm
XCorr:	37.69

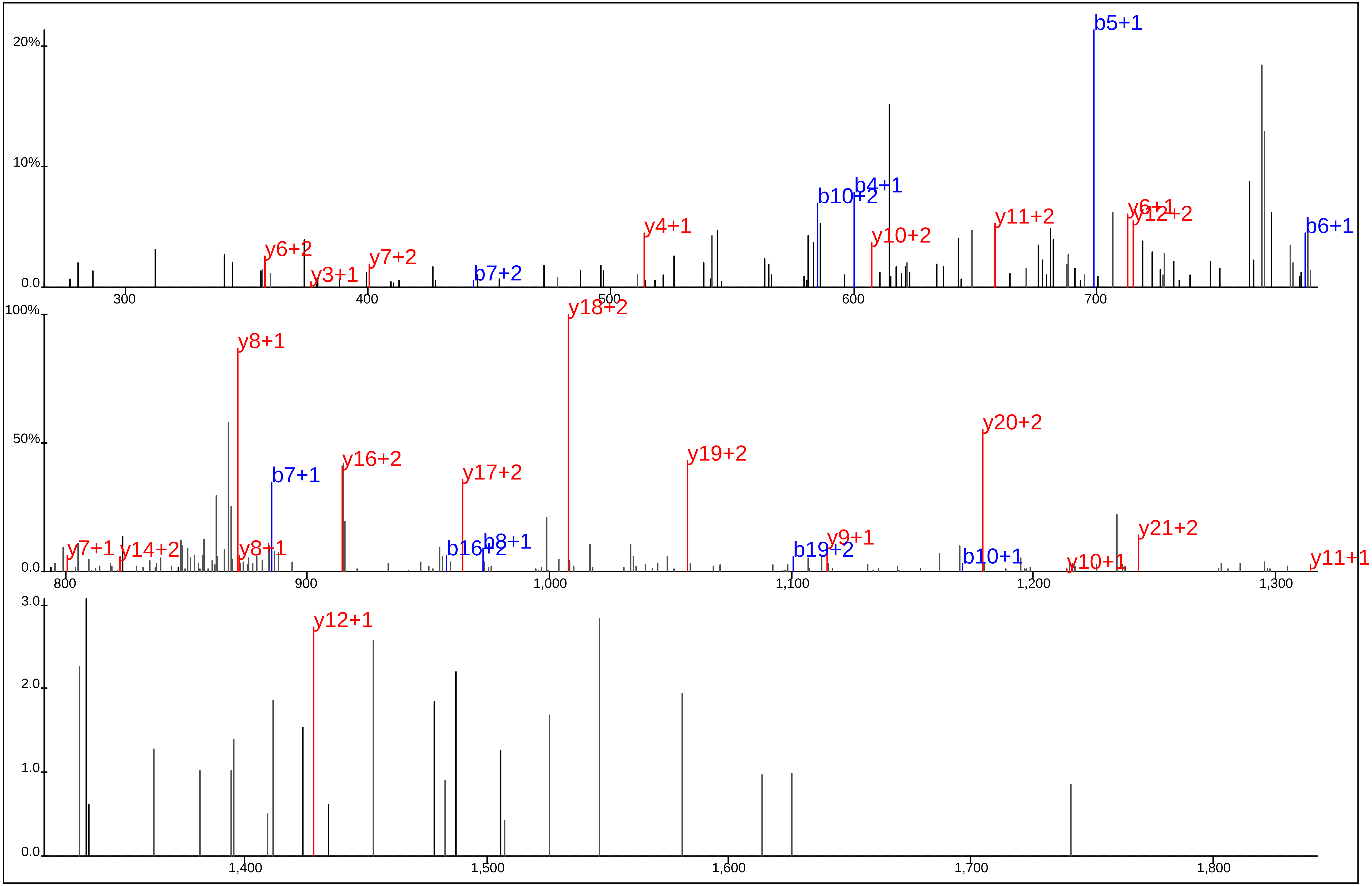

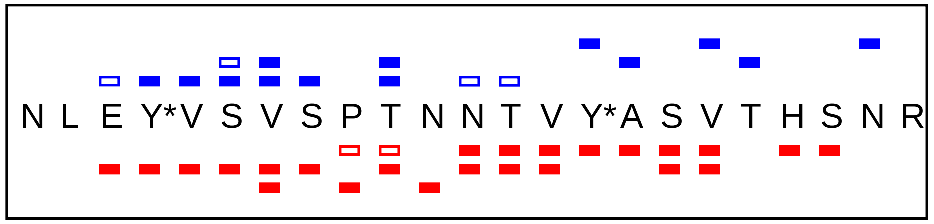

Protein Name: NTBA
gi Accession: 15384843
Precursor Mass: 904.7280+3
Delta Mass: 0.252 ppm
XCorr: 49.13

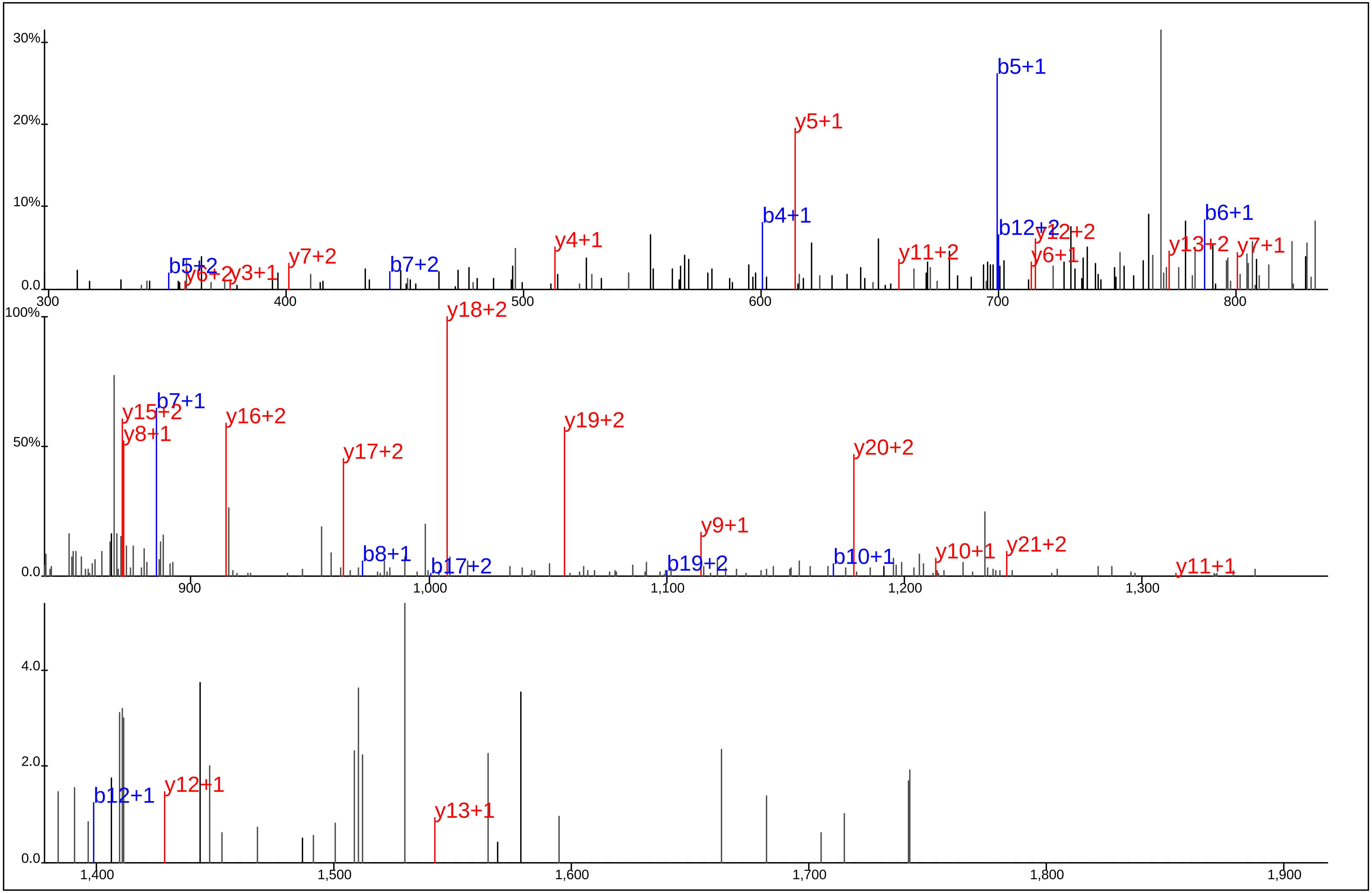

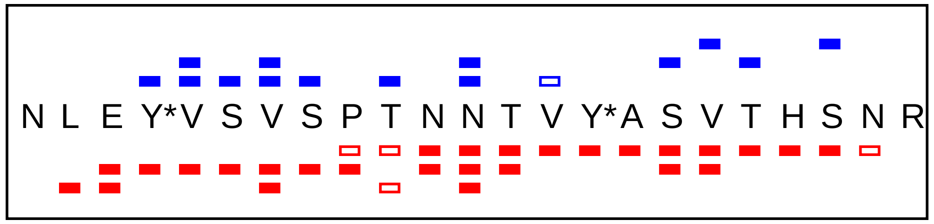

Protein Name:	NTBA
gi Accession:	15384843
Precursor Mass:	904.7315+3
Delta Mass:	3.597 ppm
XCorr:	42.89

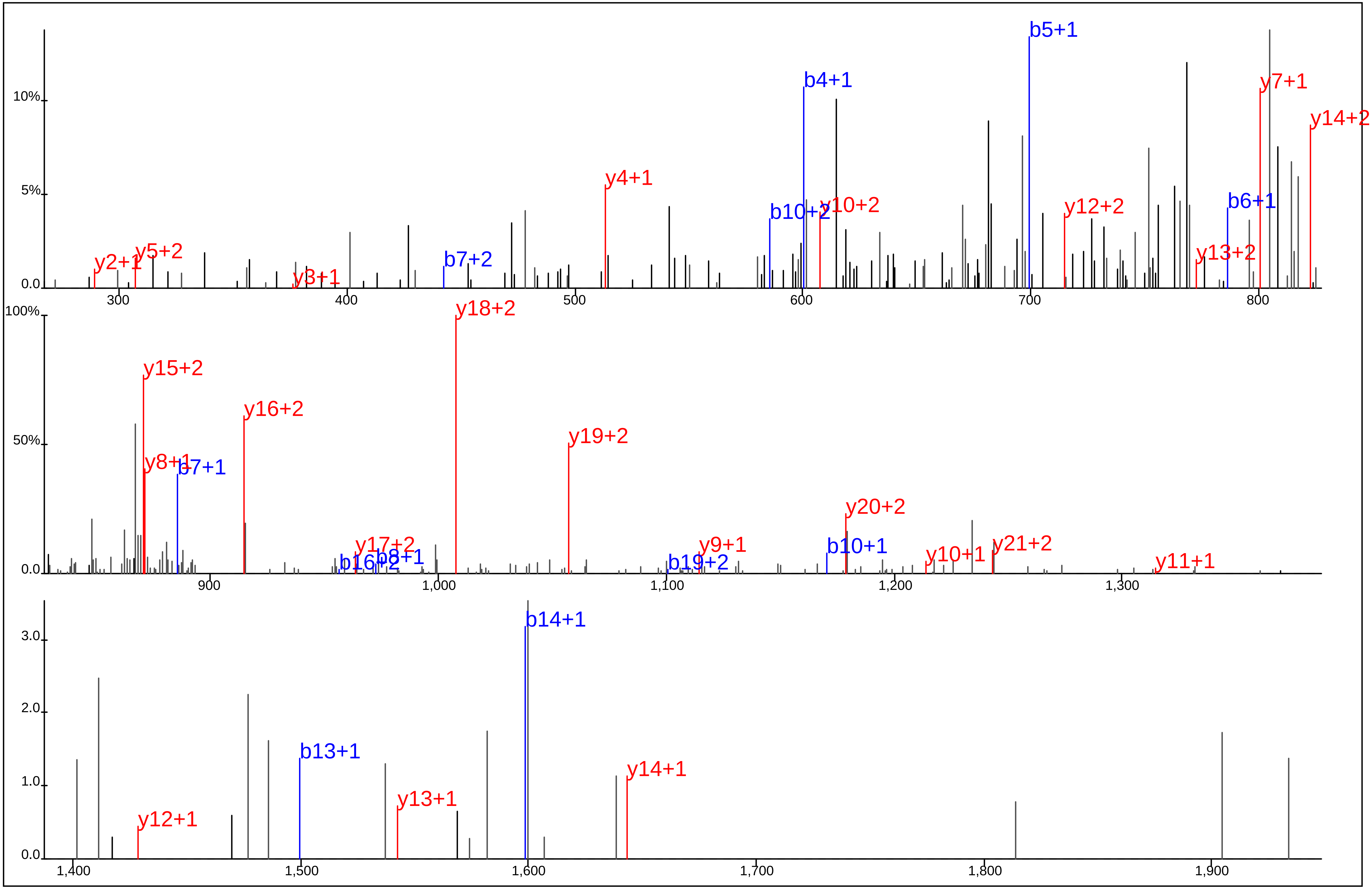

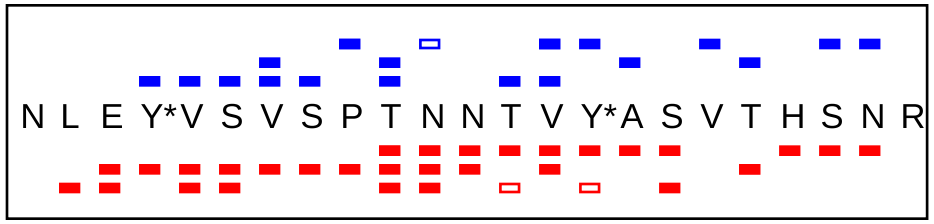

Protein Name:	NTBA
gi Accession:	15384843
Precursor Mass:	904.7294+3
Delta Mass:	1.235 ppm
XCorr:	58.01

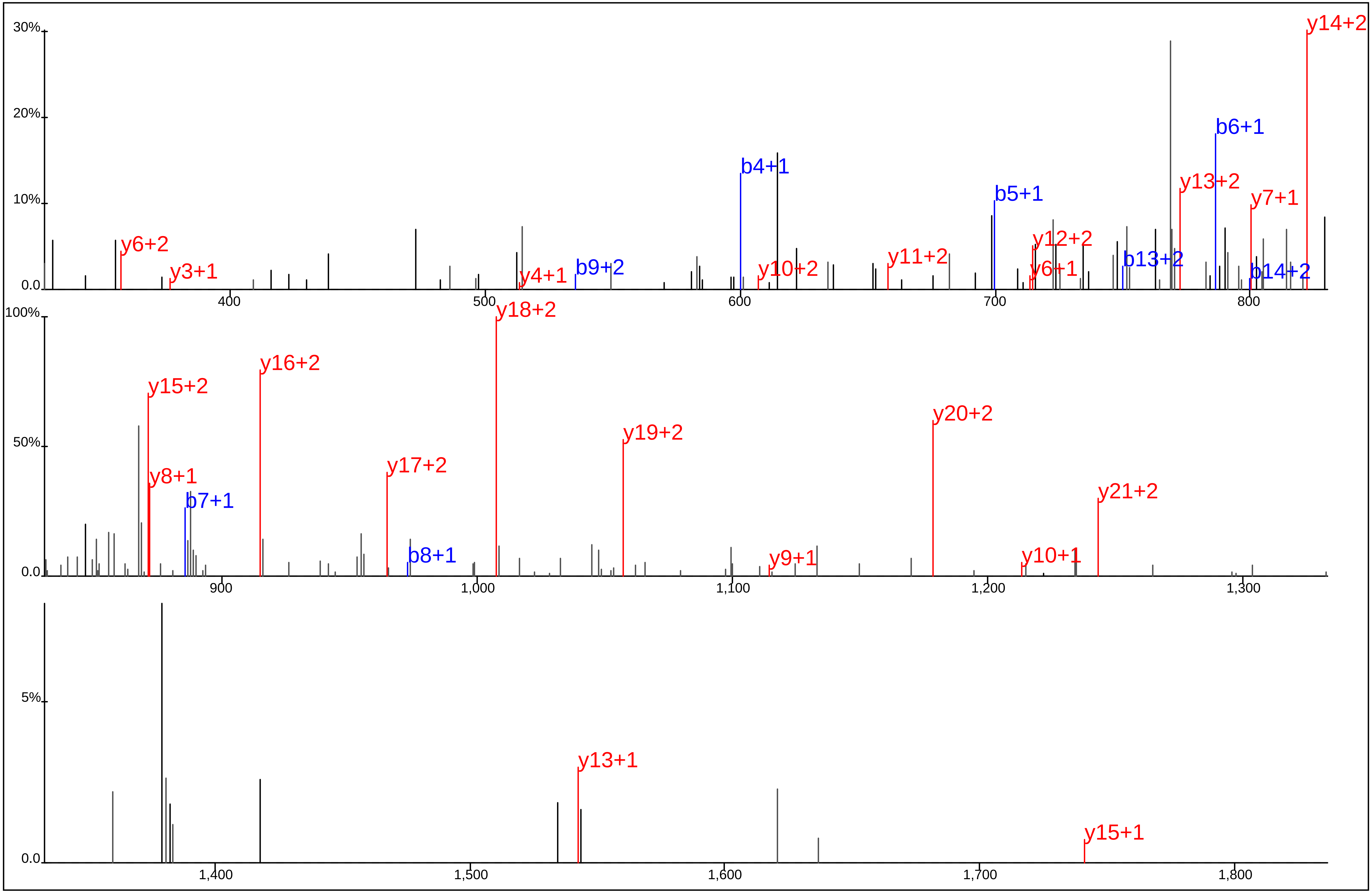

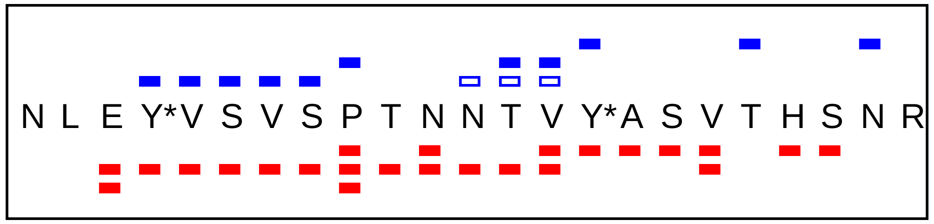

Protein Name:	NTBA
gi Accession:	15384843
Precursor Mass:	904.7308+3
Delta Mass:	2.787 ppm
XCorr:	57.6

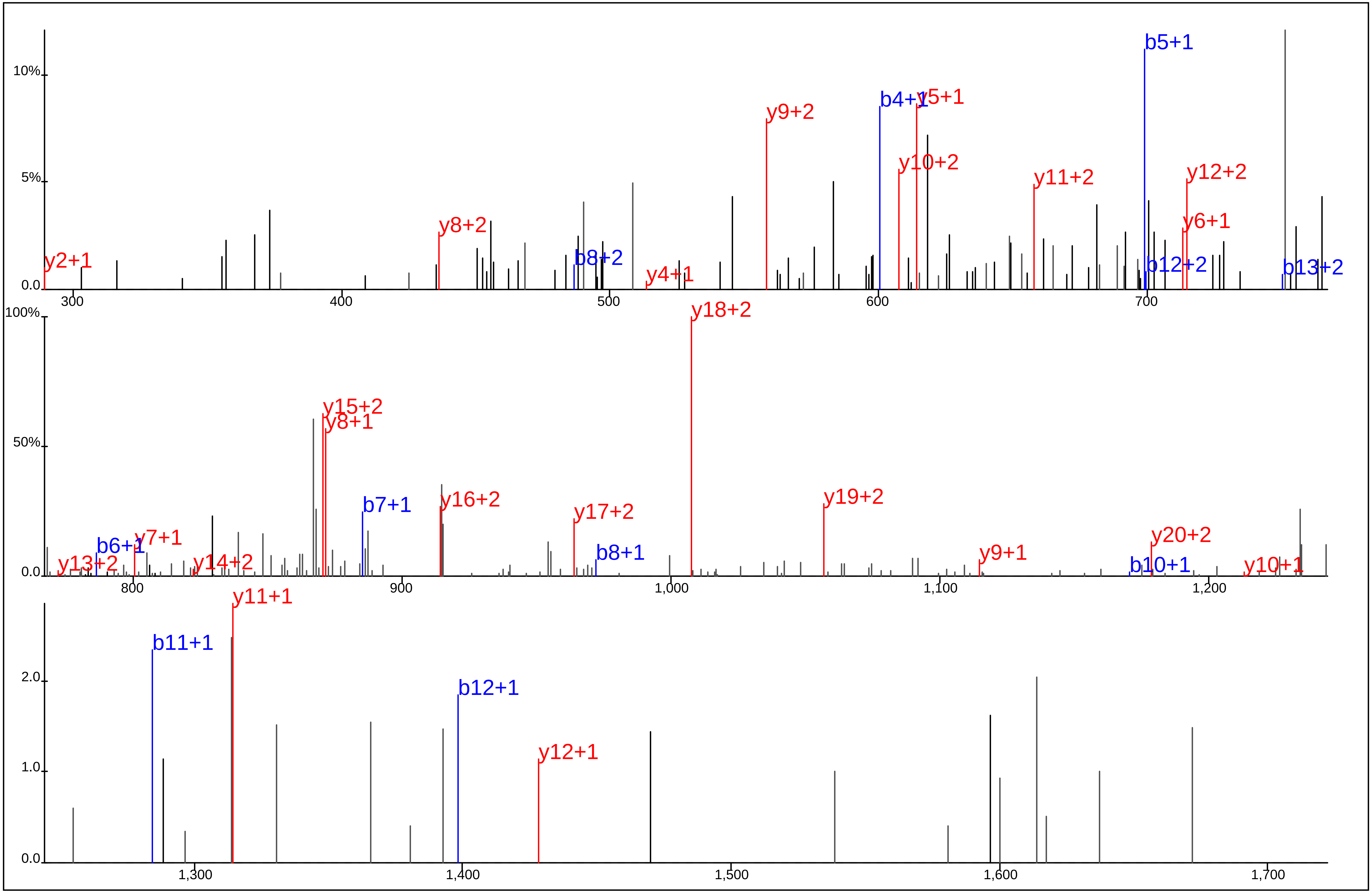

N L E Y*V S V S P T N N T V Y*A S V T H S N R

Protein Name: NTBA
gi Accession: 15384843
Precursor Mass: 904.7315+3
Delta Mass: 3.597 ppm
XCorr: 56.27

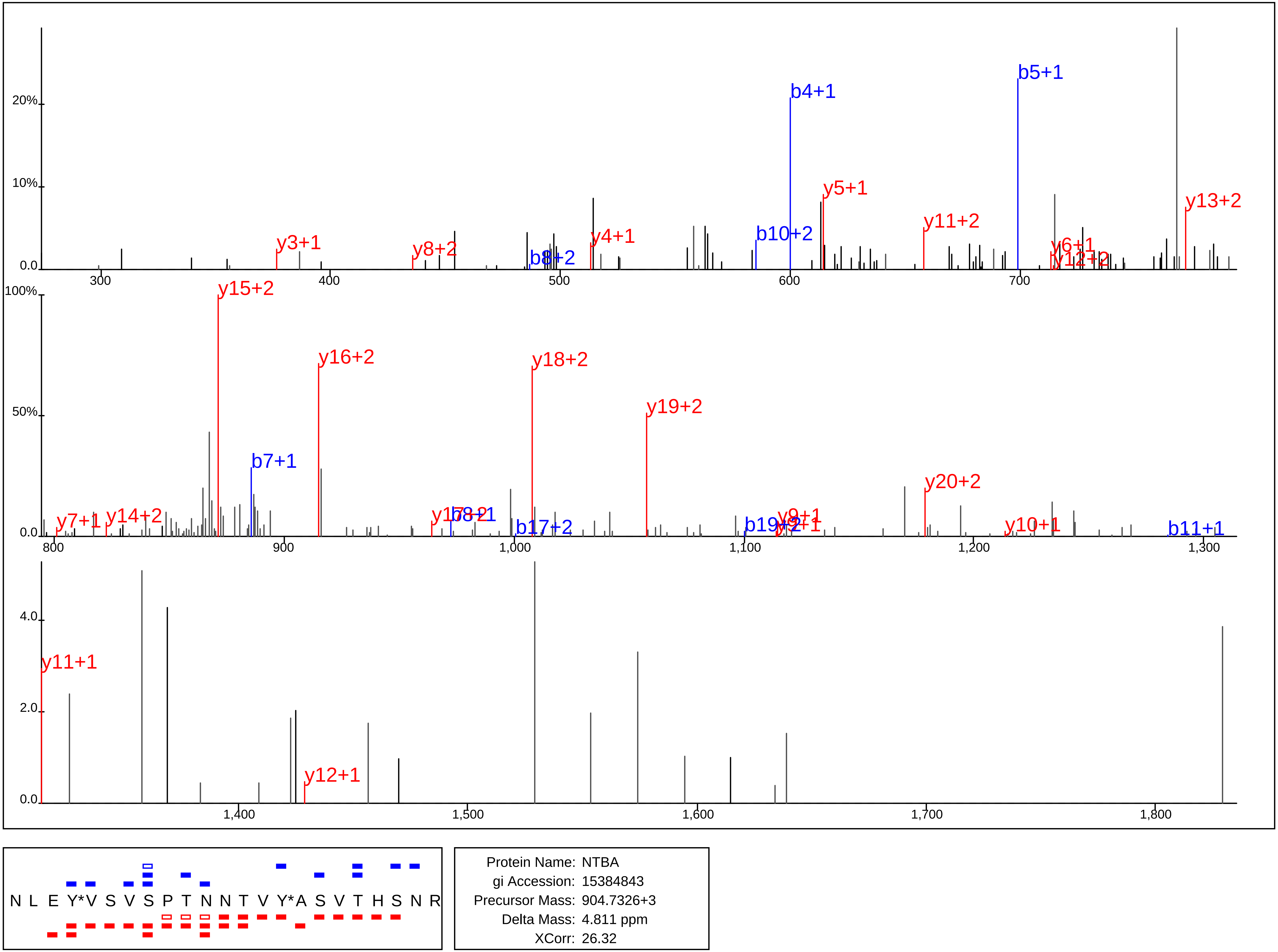

Protein Name: NTBA
gi Accession: 15384843
Precursor Mass: 904.7326+3
Delta Mass: 4.811 ppm
XCorr: 26.32

640 - NTBA_3452167.pdf

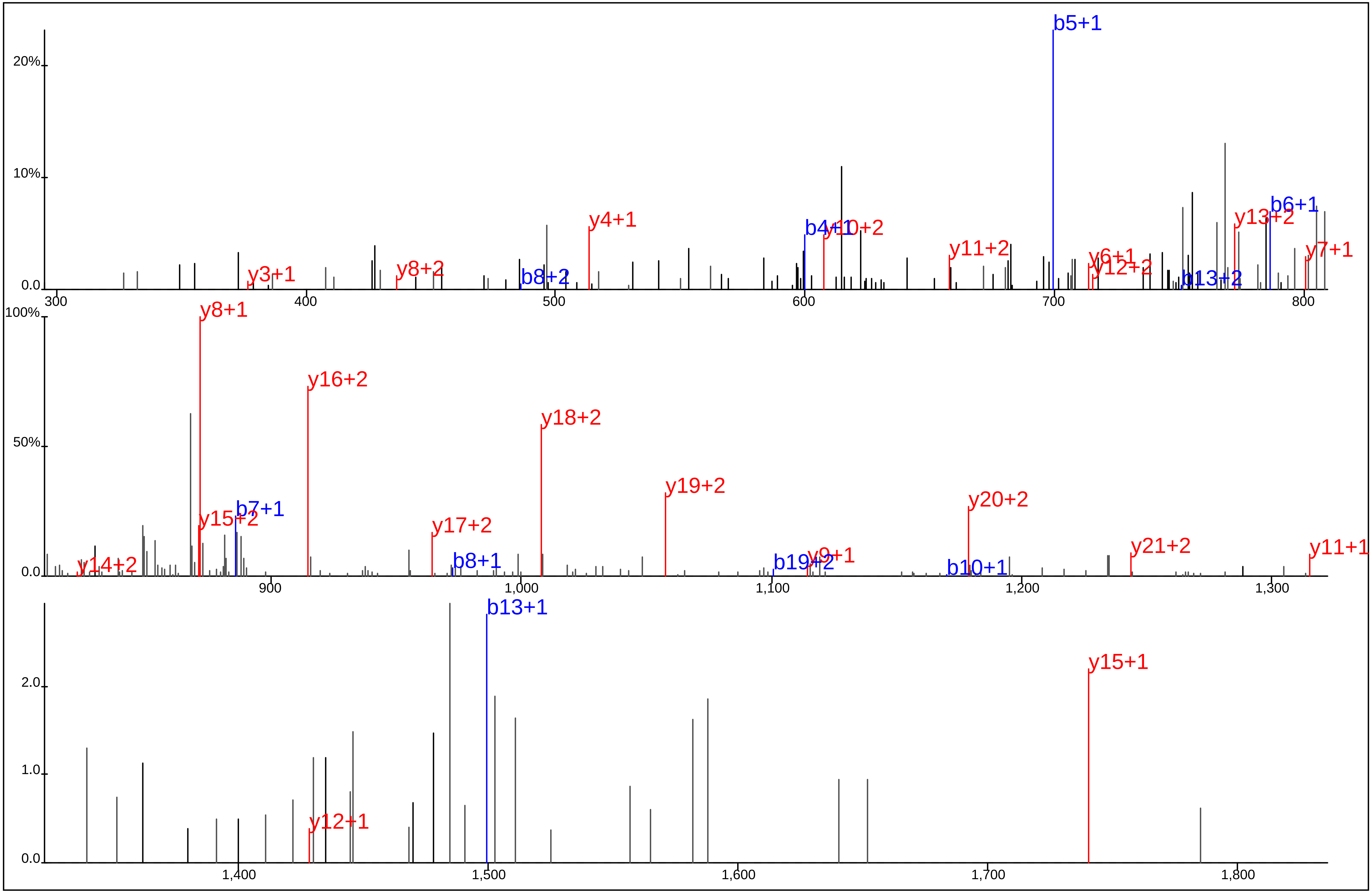

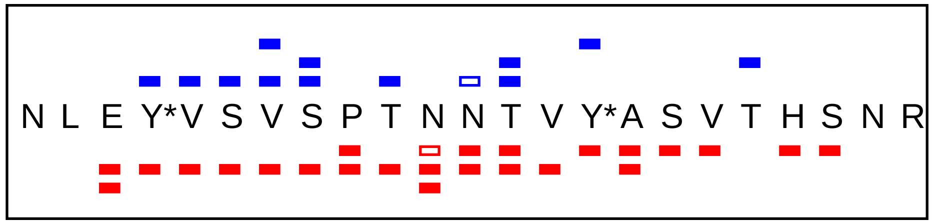

Protein Name: NTBA
gi Accession: 15384843
Precursor Mass: 904.7312+3
Delta Mass: 3.259 ppm
XCorr: 39.91

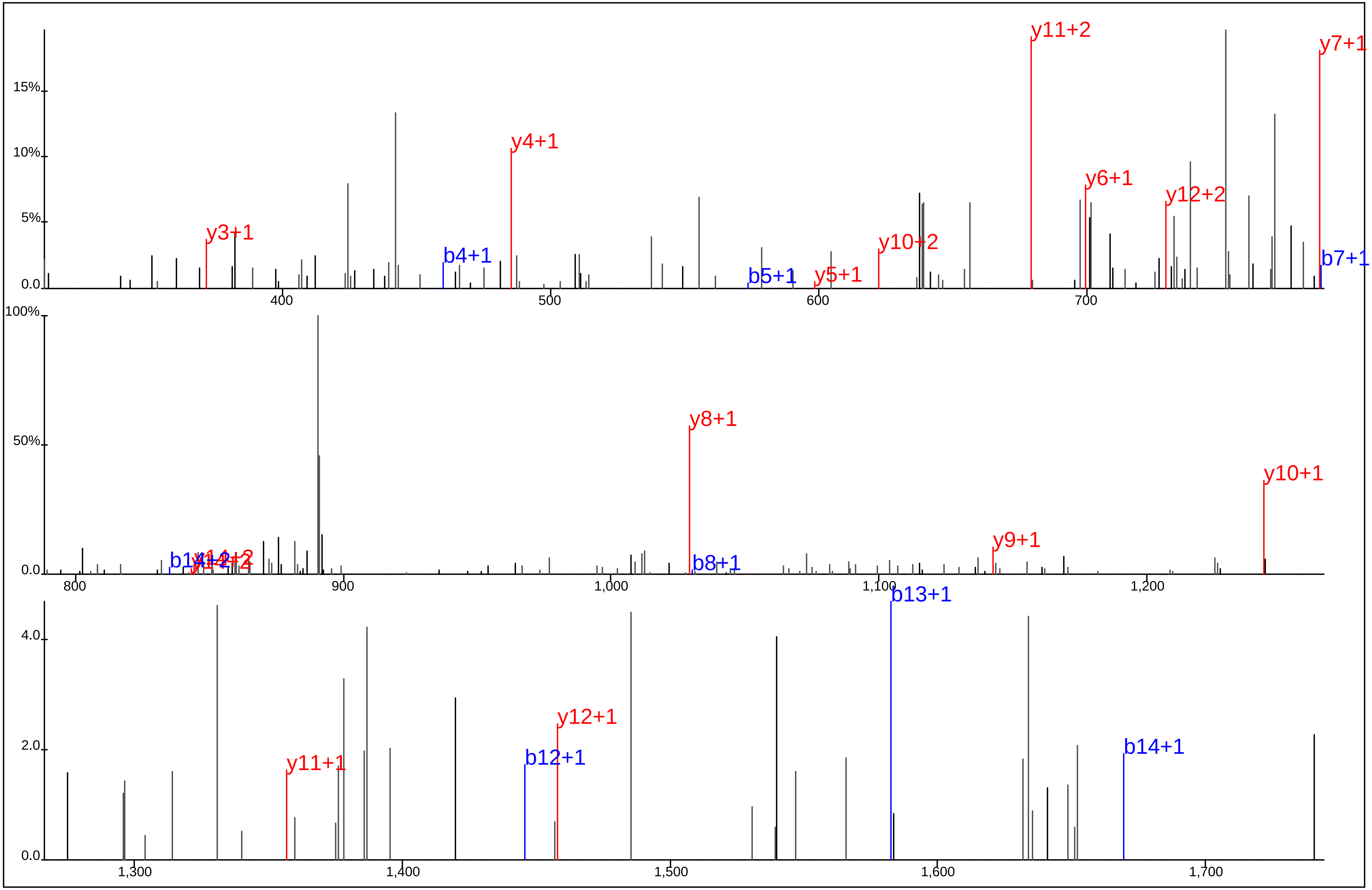

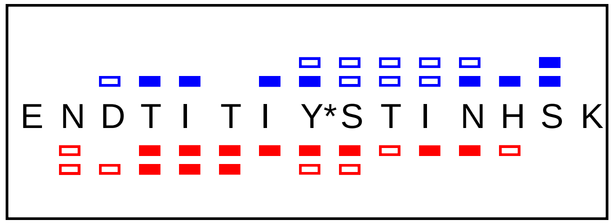

Protein Name: NTBA
gi Accession: 15384843
Precursor Mass: 908.4136+2
Delta Mass: 2.185 ppm
XCorr: 27.13

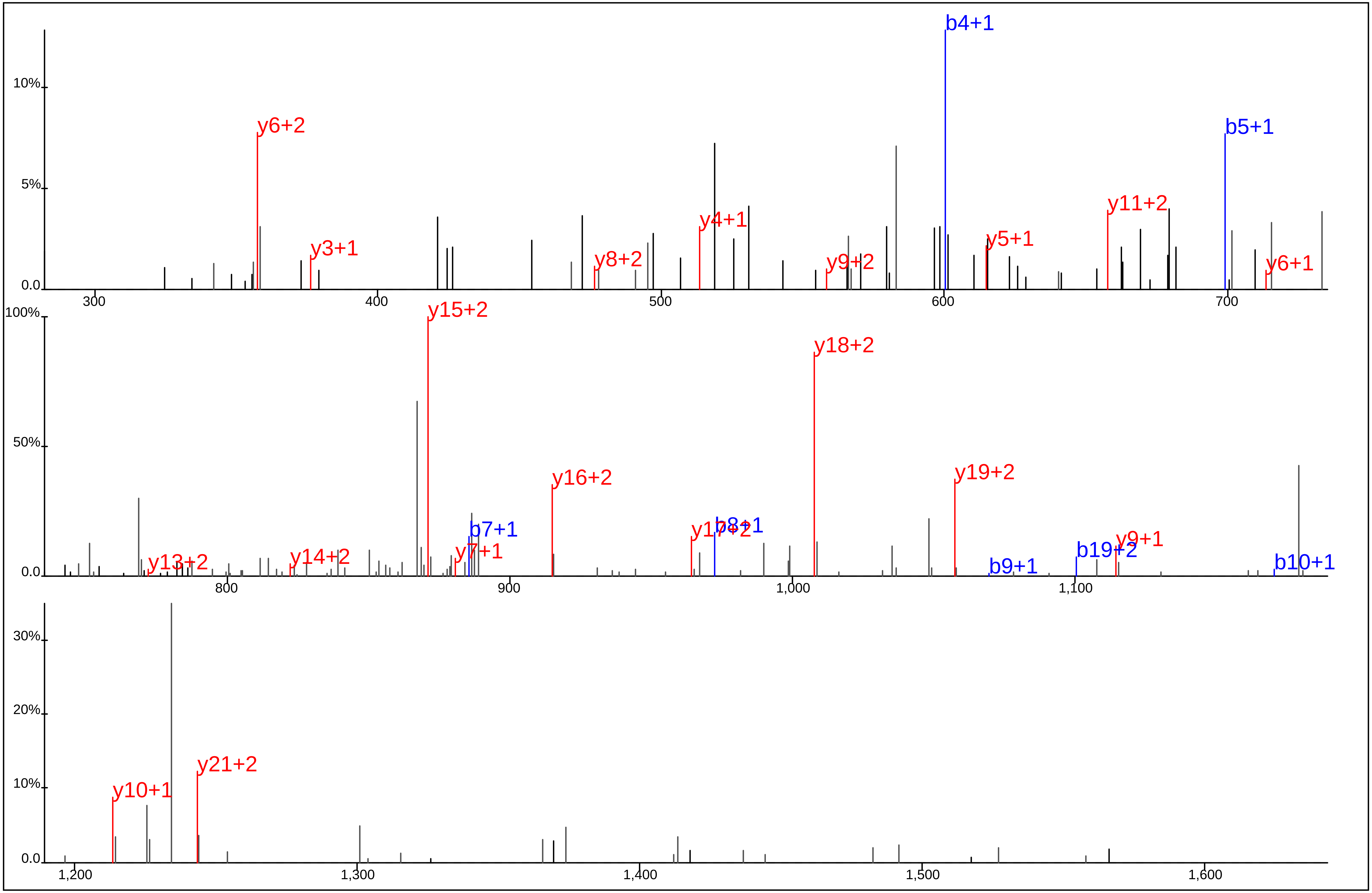

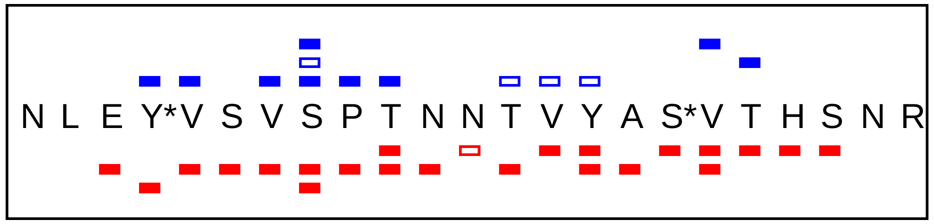

Protein Name:	NTBA
gi Accession:	15384843
Precursor Mass:	904.7255+3
Delta Mass:	3.087 ppm
XCorr:	31.09

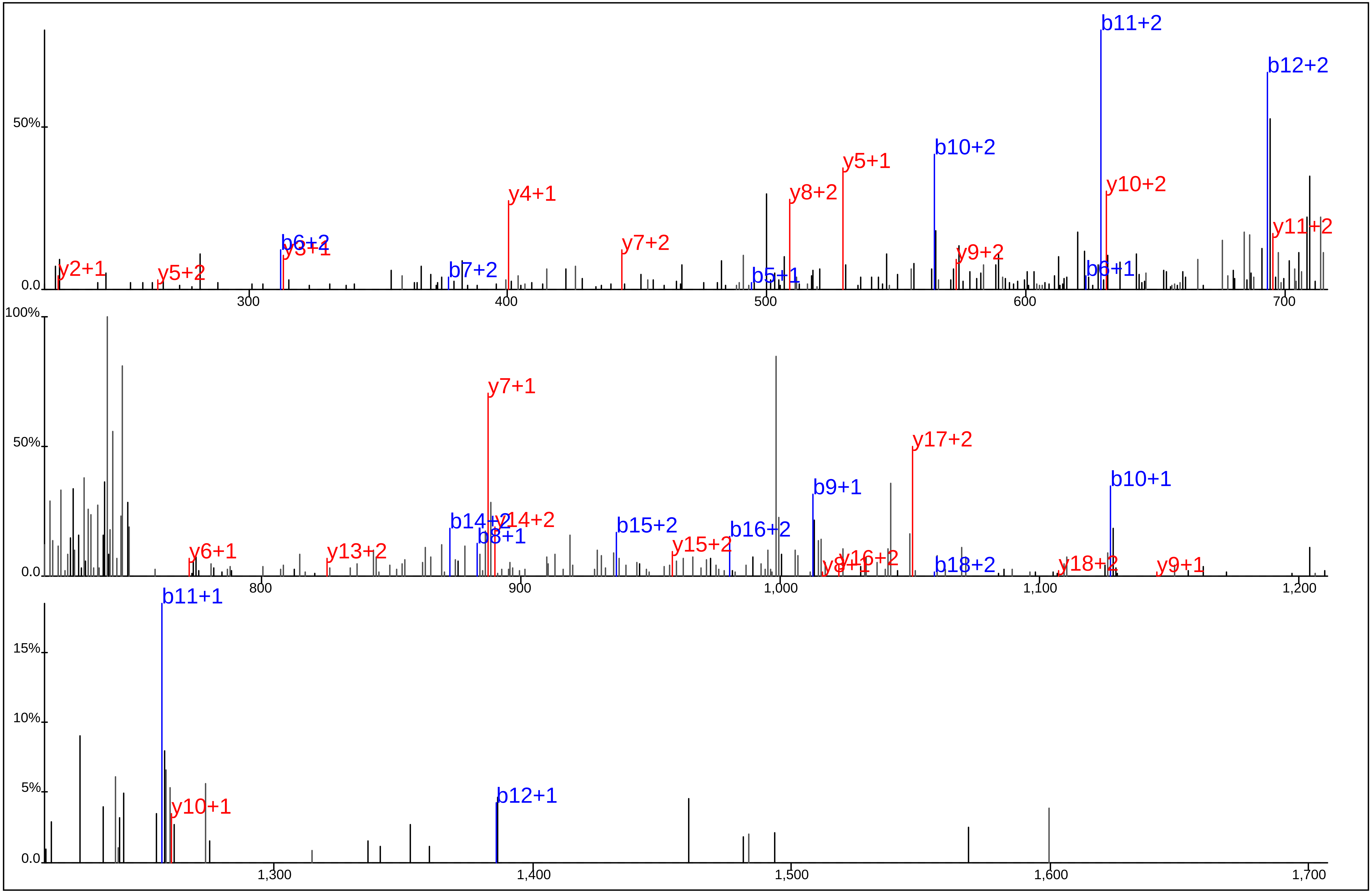

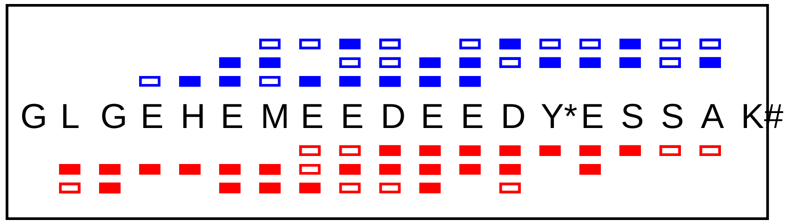

Protein Name:	Oxytocinase
gi Accession:	2209276
Precursor Mass:	757.9517+3
Delta Mass:	1.857 ppm
XCorr:	23.7

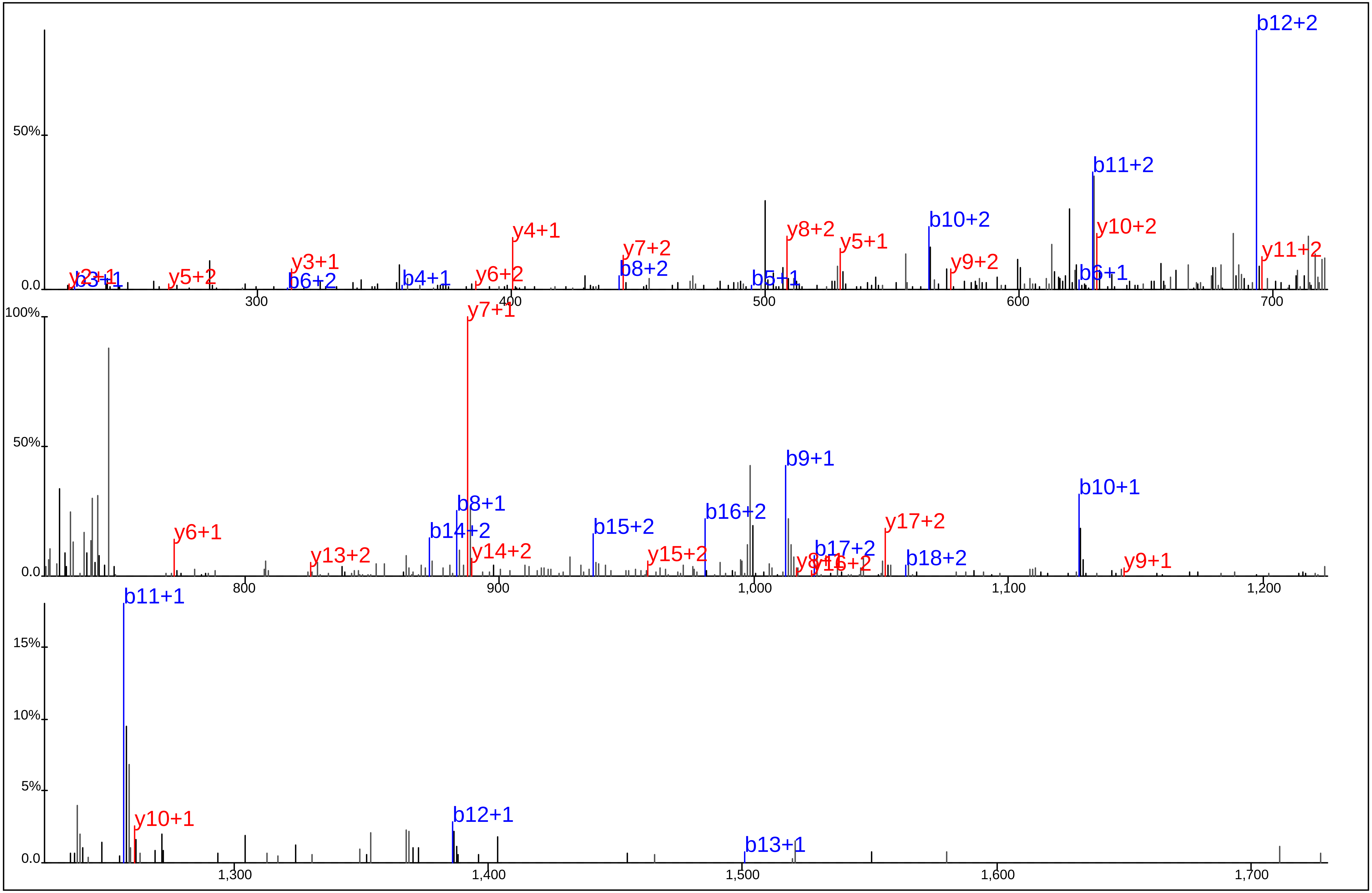

G L G E H E M E E D E E D Y*E S S A K#

Protein Name: Oxytocinase
gi Accession: 2209276
Precursor Mass: 757.9517+3
Delta Mass: 1.857 ppm
XCorr: 41.06

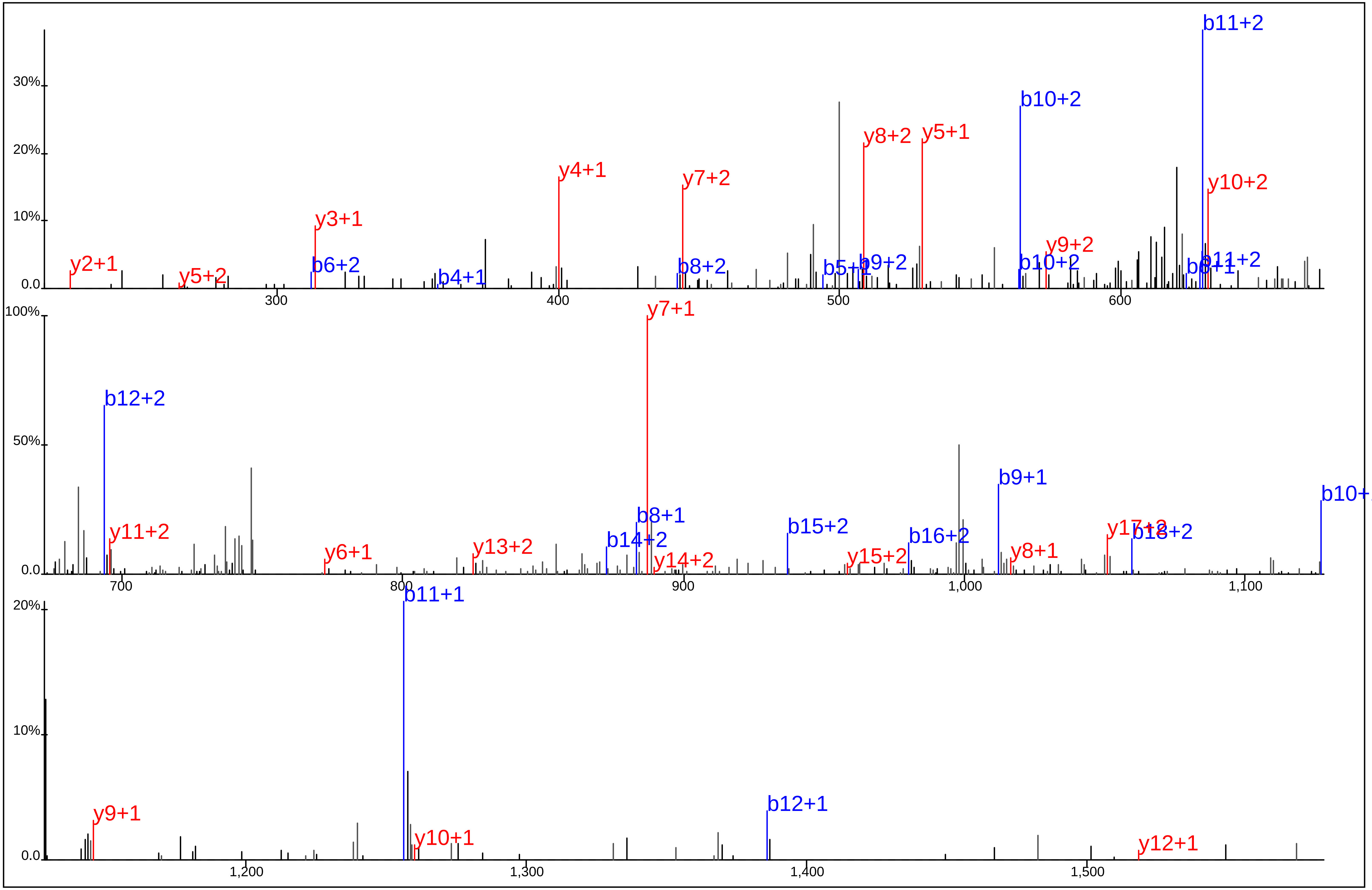

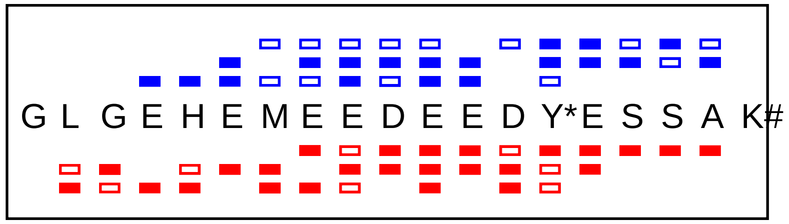

Protein Name: Oxytocinase
gi Accession: 2209276
Precursor Mass: 757.9510+3
Delta Mass: 1.052 ppm
XCorr: 36.24

646 - Oxytocinase_3441577.pdf

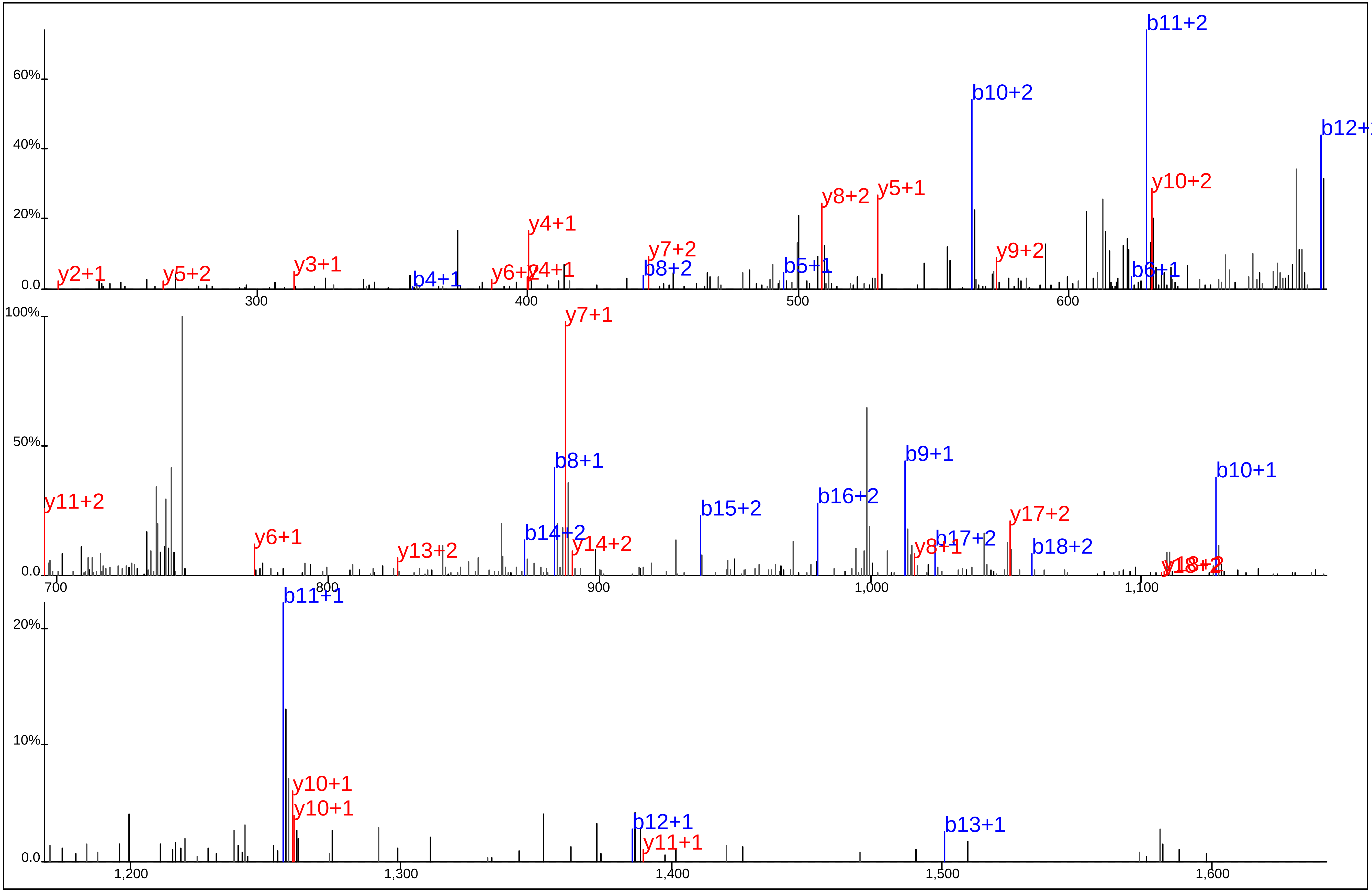

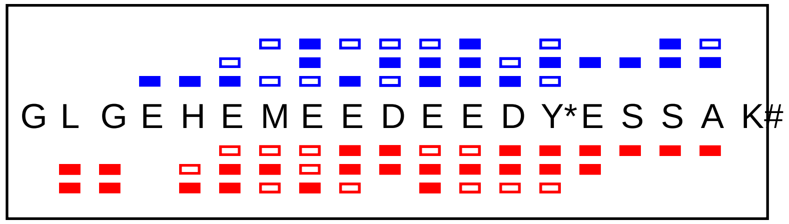

Protein Name: Oxytocinase
gi Accession: 2209276
Precursor Mass: 757.9517+3
Delta Mass: 1.857 ppm
XCorr: 34.79

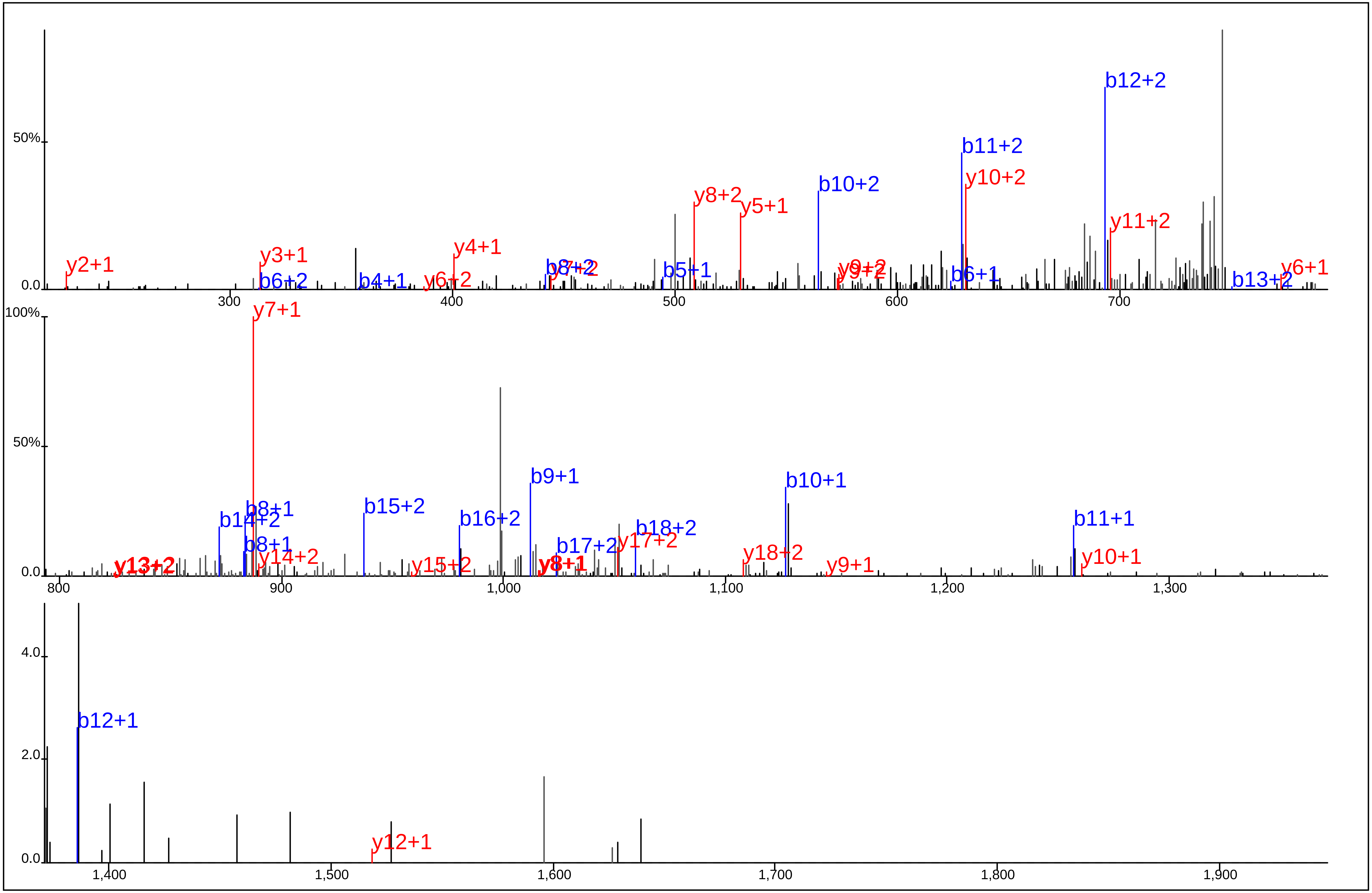

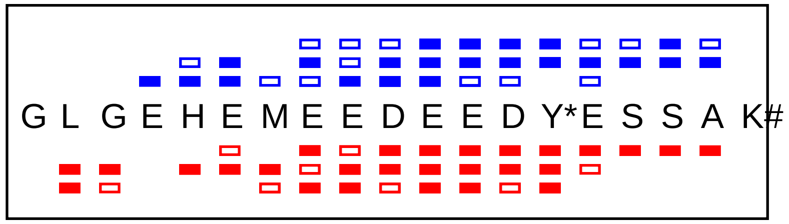

Protein Name: Oxytocinase
gi Accession: 2209276
Precursor Mass: 757.9498+3
Delta Mass: 0.644 ppm
XCorr: 39.57

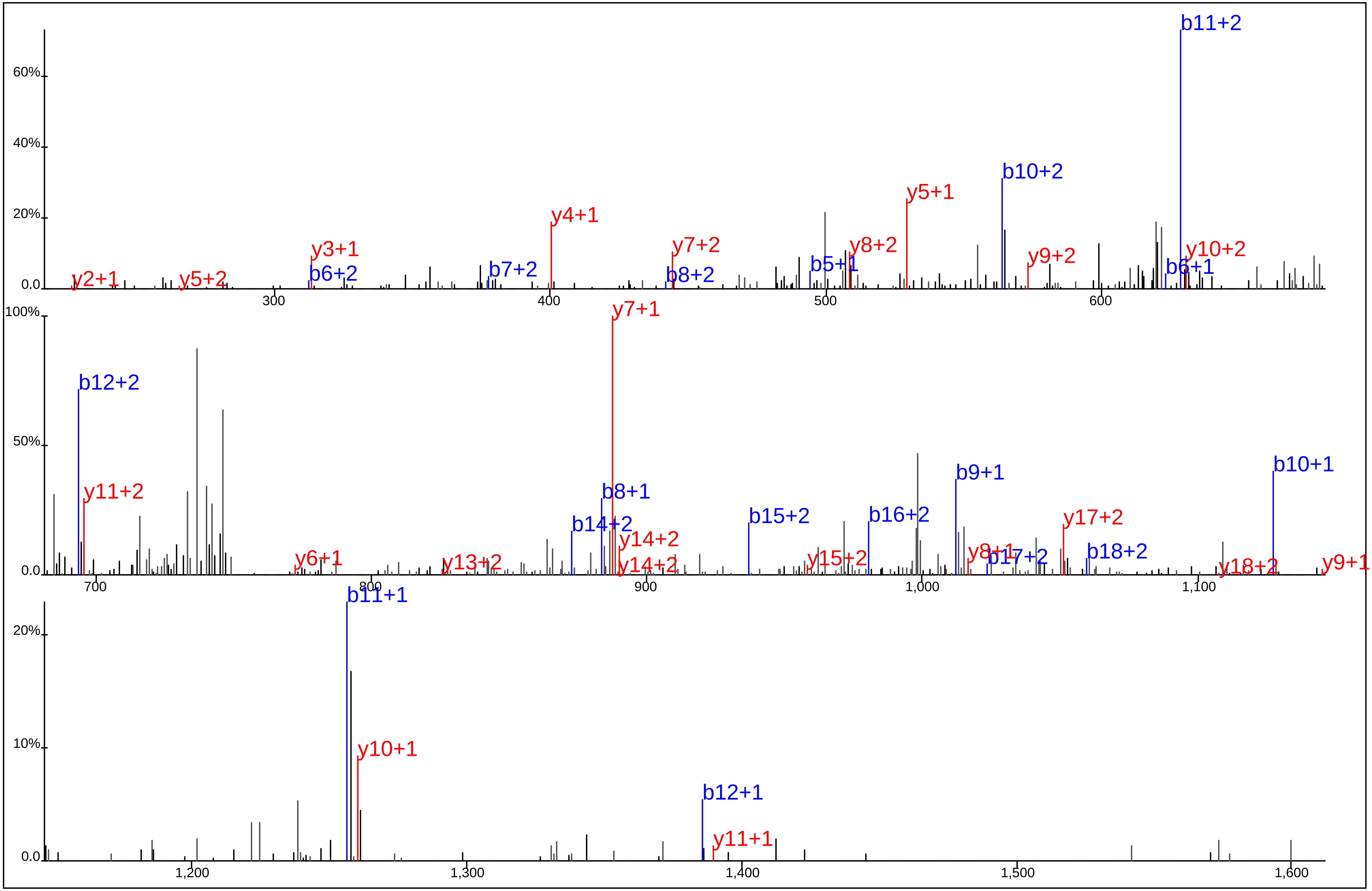

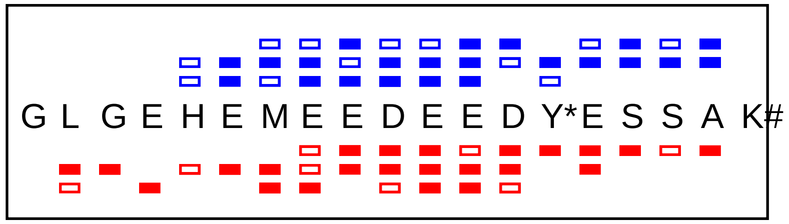

Protein Name: Oxytocinase
gi Accession: 2209276
Precursor Mass: 757.9545+3
Delta Mass: 5.562 ppm
XCorr: 33.76

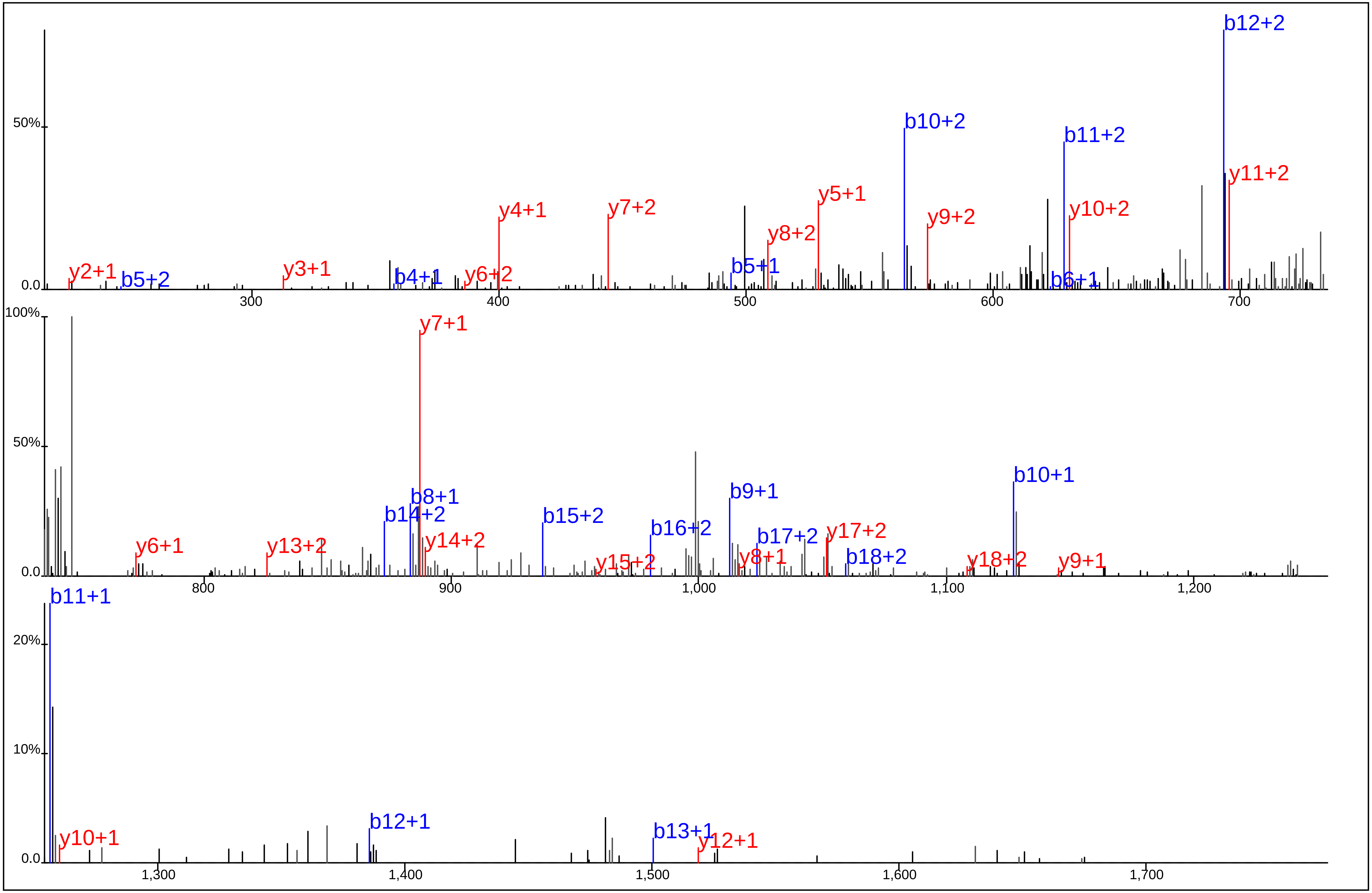

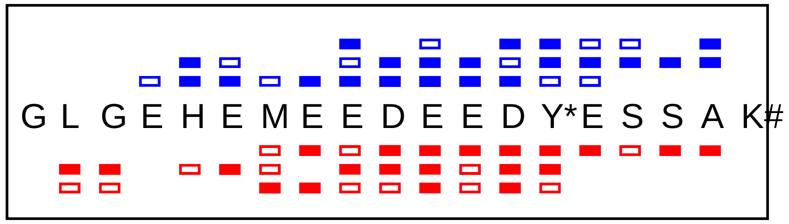

Protein Name: Oxytocinase
gi Accession: 2209276
Precursor Mass: 757.9529+3
Delta Mass: 3.468 ppm
XCorr: 43.18

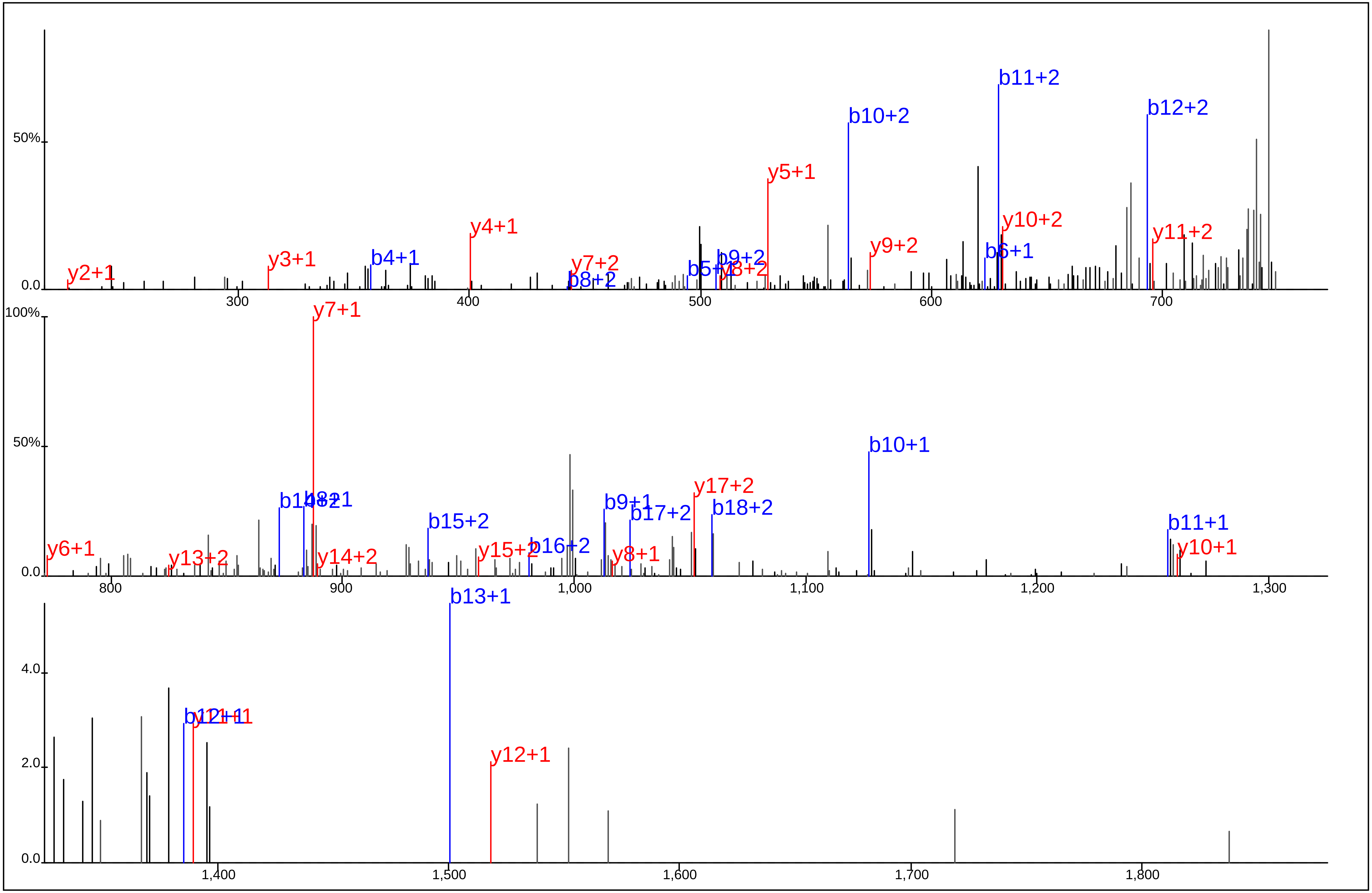

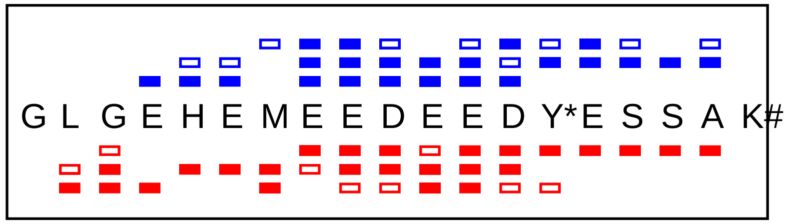

Protein Name: Oxytocinase
gi Accession: 2209276
Precursor Mass: 757.9520+3
Delta Mass: 2.340 ppm
XCorr: 41.55

651 - Oxytocinase_3453746.pdf

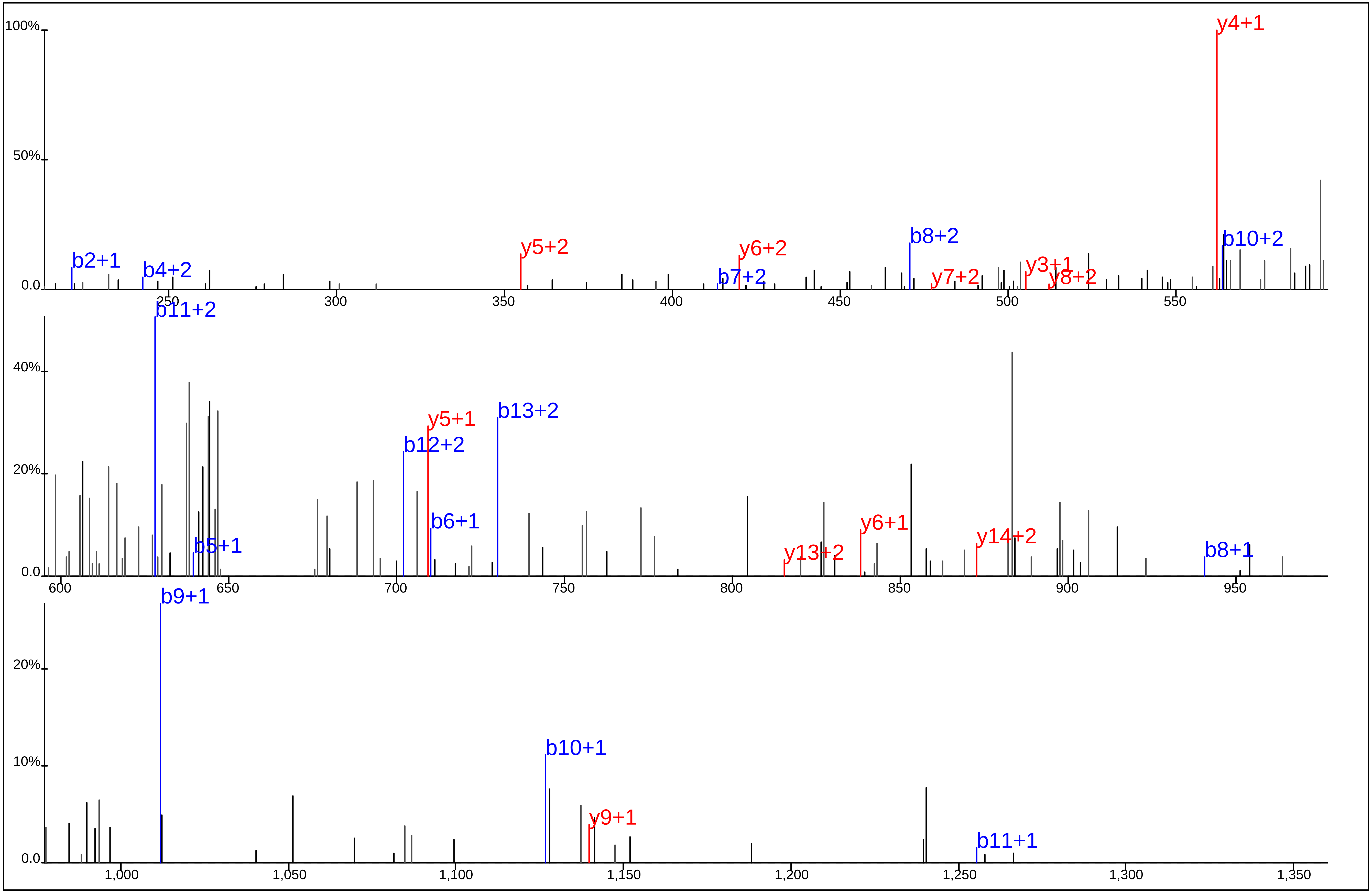

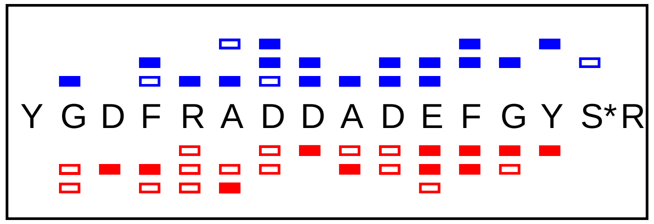

Protein Name: p100 coactivator
gi Accession: 77404397
Precursor Mass: 655.2579+3
Delta Mass: 5.005 ppm
XCorr: 20.59

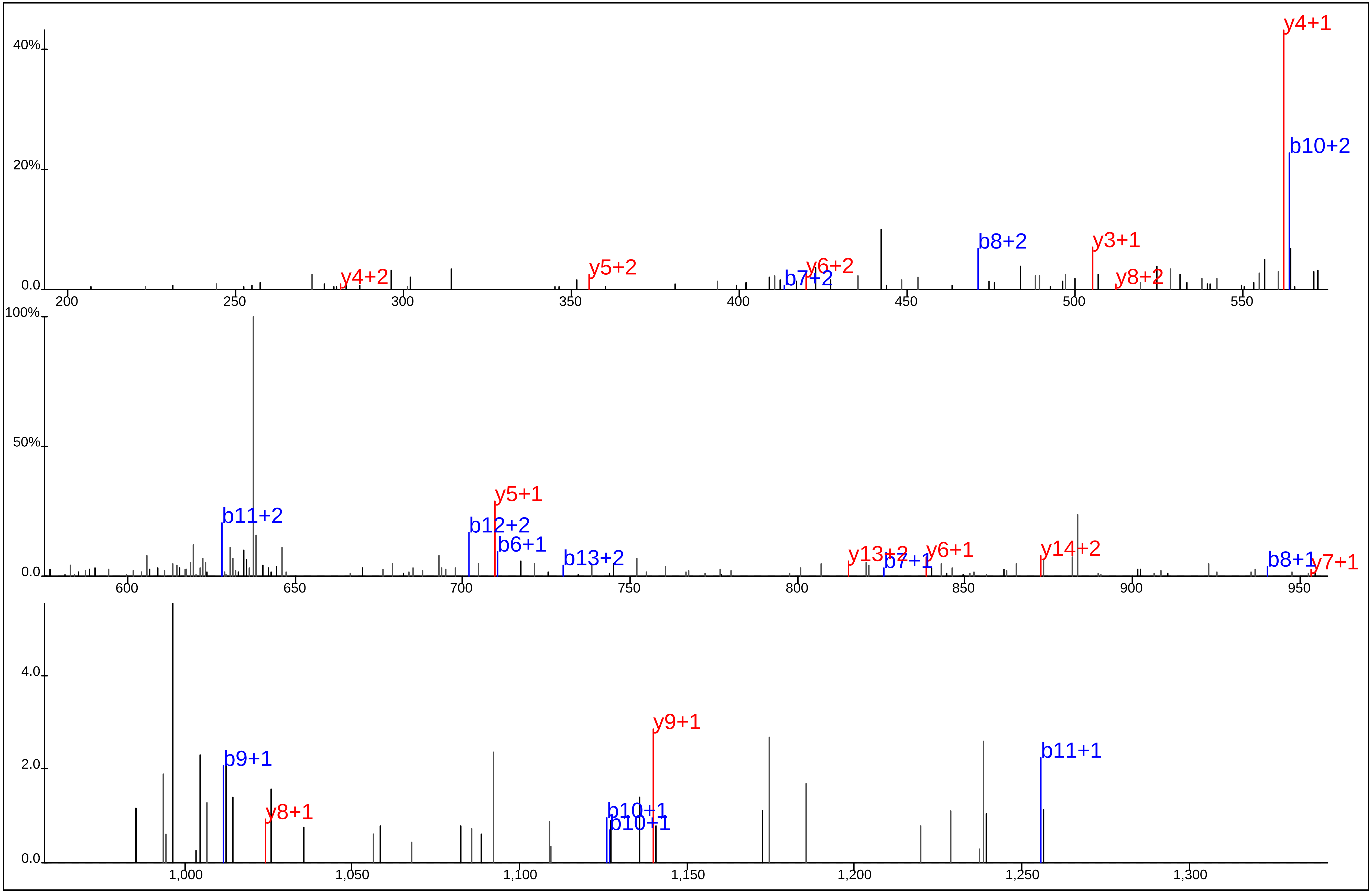

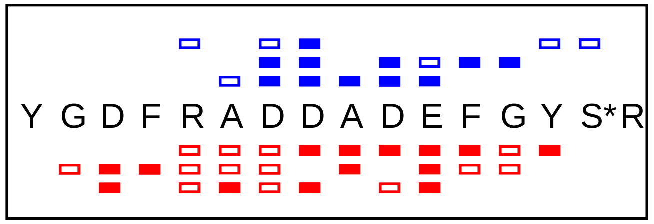

Protein Name:	p100 coactivator
gi Accession:	77404397
Precursor Mass:	655.2560+3
Delta Mass:	2.113 ppm
XCorr:	19.8

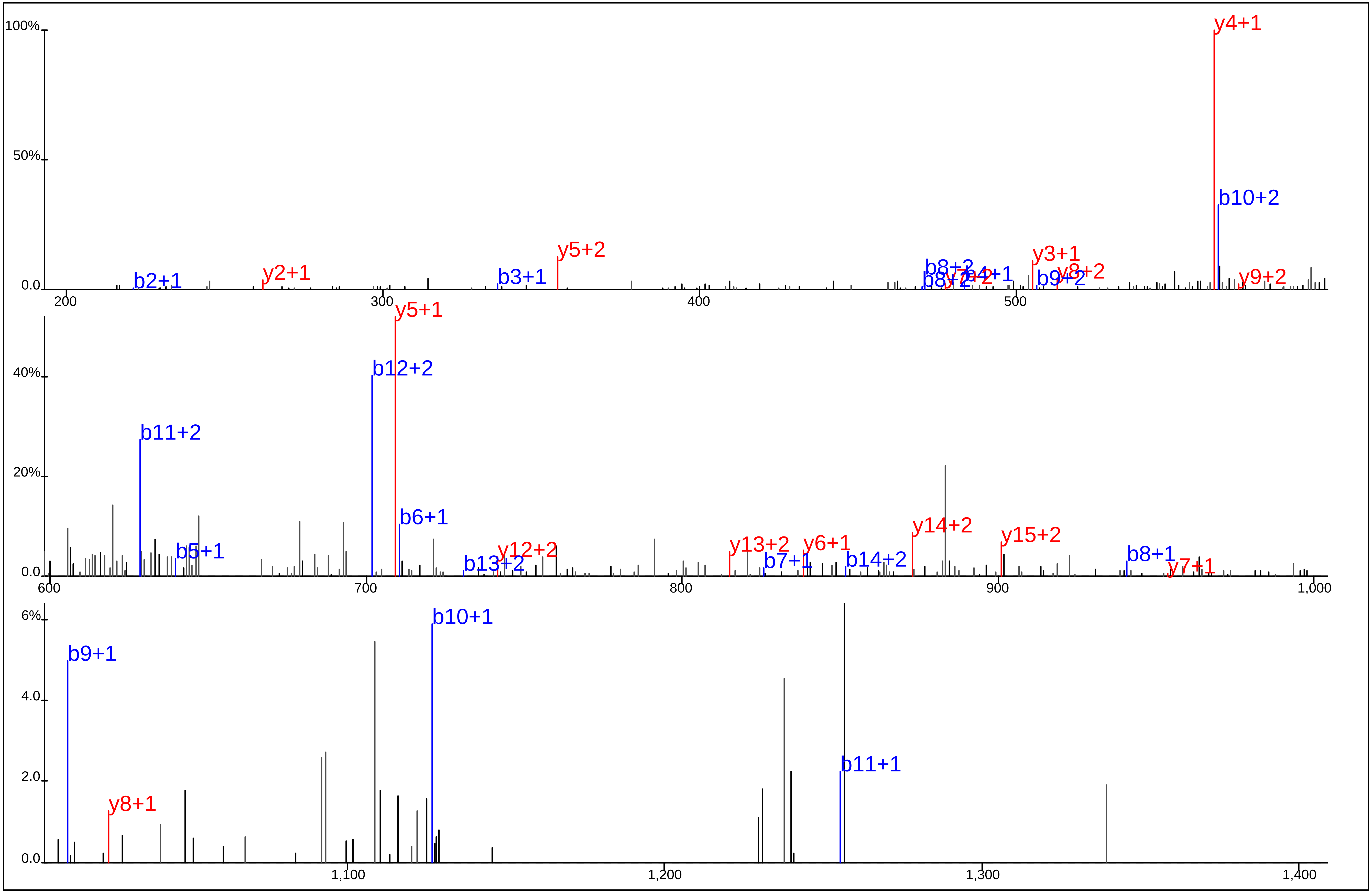

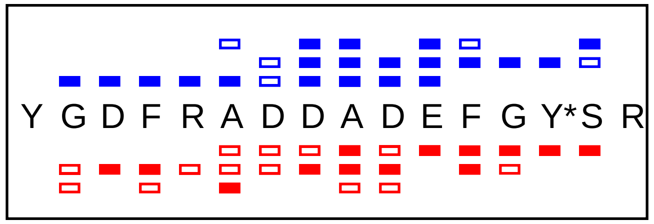

Protein Name: p100 coactivator
gi Accession: 77404397
Precursor Mass: 655.2556+3
Delta Mass: 1.460 ppm
XCorr: 33.32

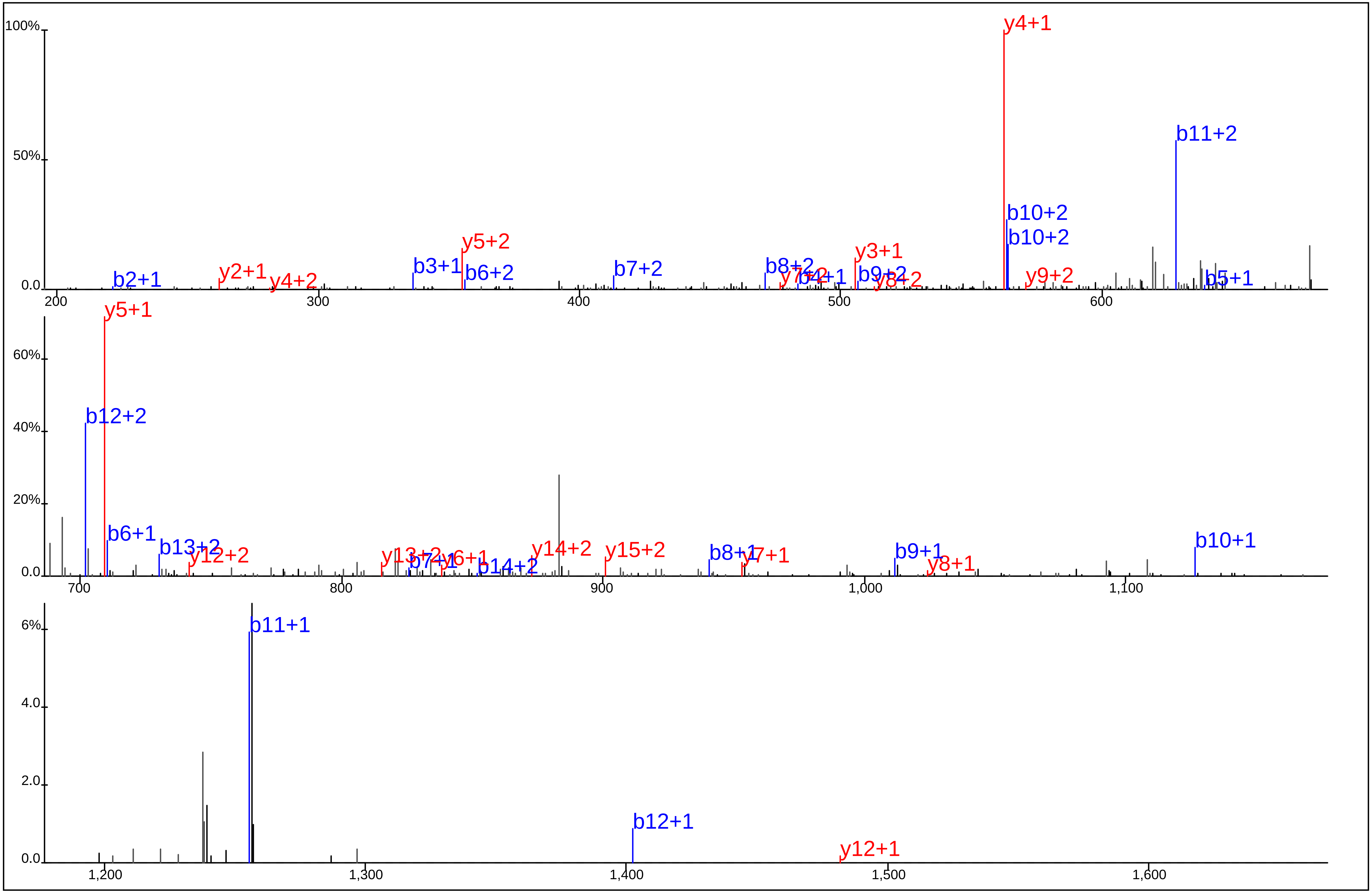

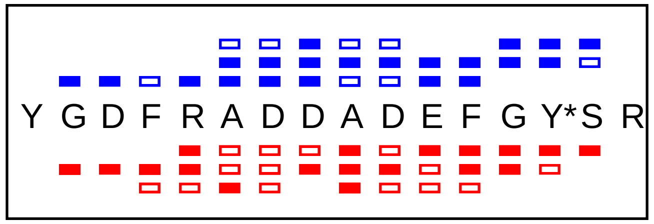

Protein Name:	p100 coactivator
gi Accession:	77404397
Precursor Mass:	655.2578+3
Delta Mass:	4.725 ppm
XCorr:	46.61

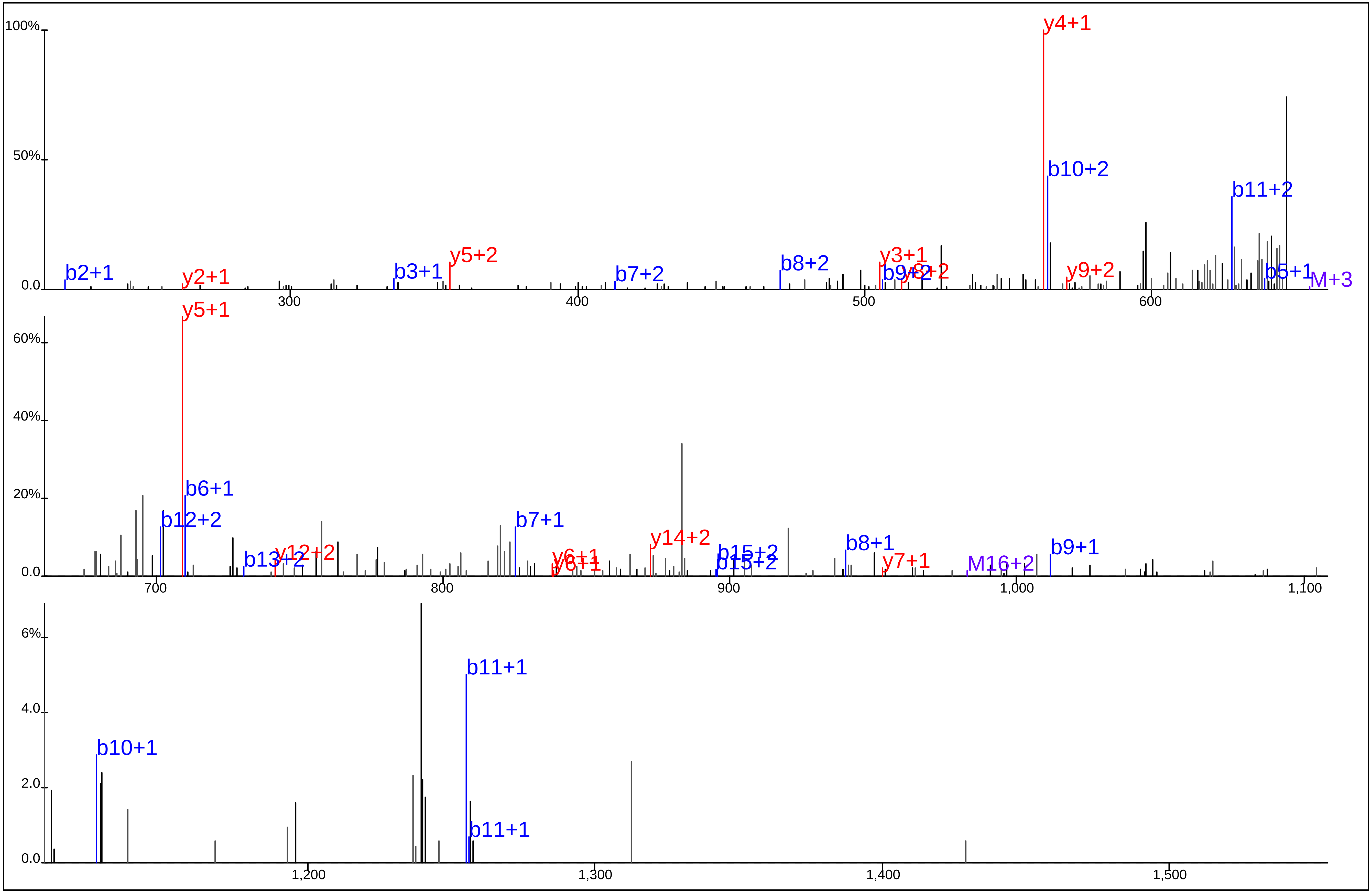

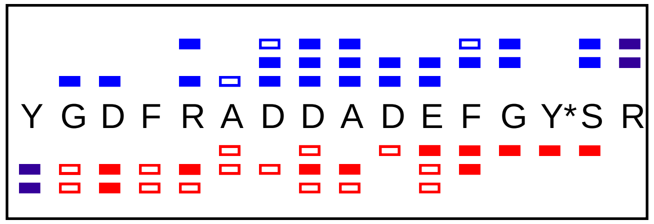

Protein Name:	p100 coactivator
gi Accession:	77404397
Precursor Mass:	655.2559+3
Delta Mass:	1.832 ppm
XCorr:	30.01

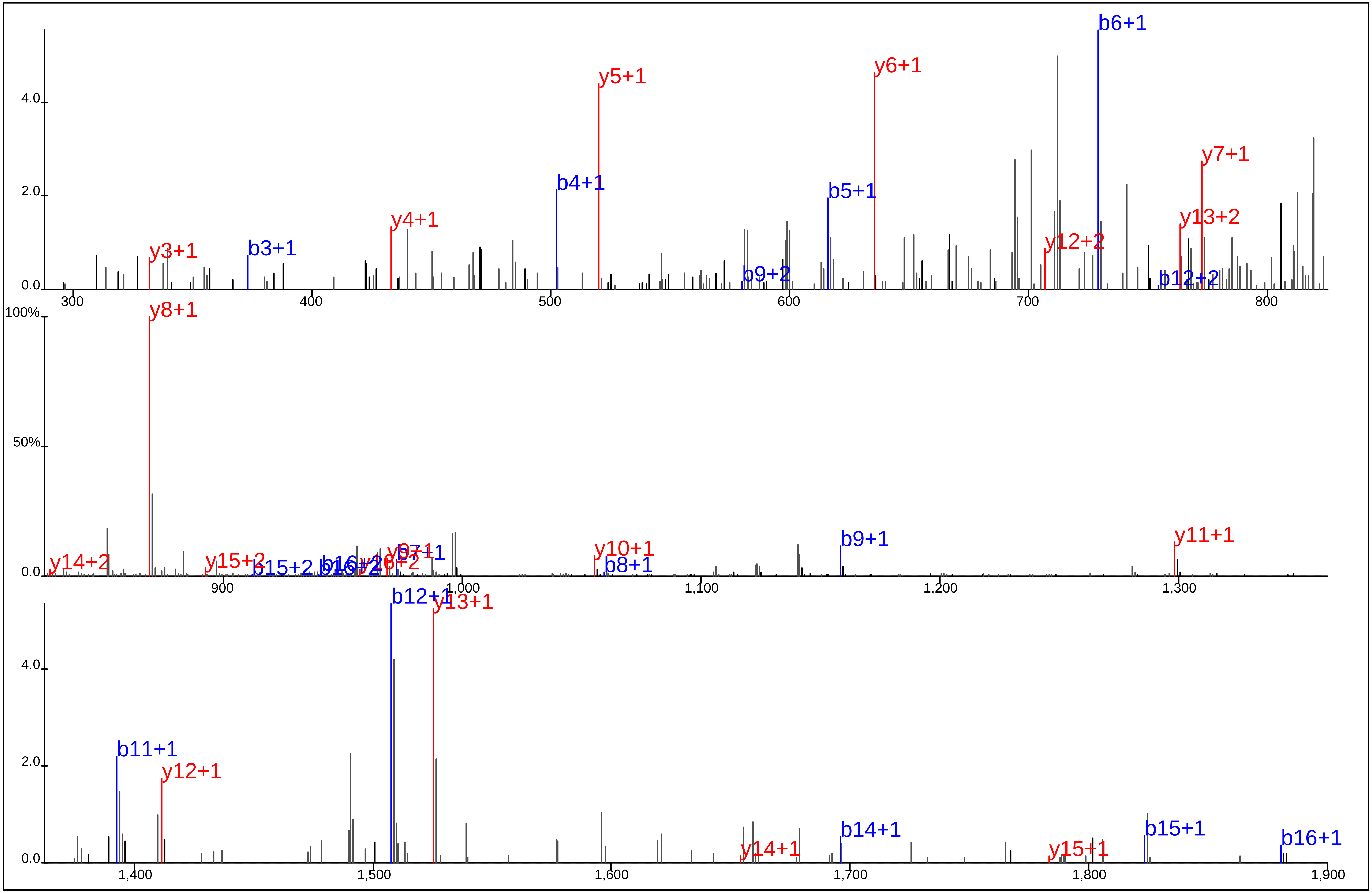

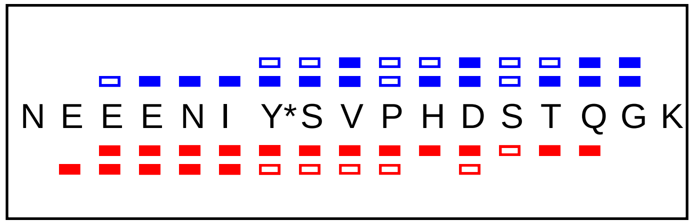

Protein Name: p190 RhoGAP
gi Accession: 150417981
Precursor Mass: 1,013.9269+2
Delta Mass: 3.719 ppm
XCorr: 55.88

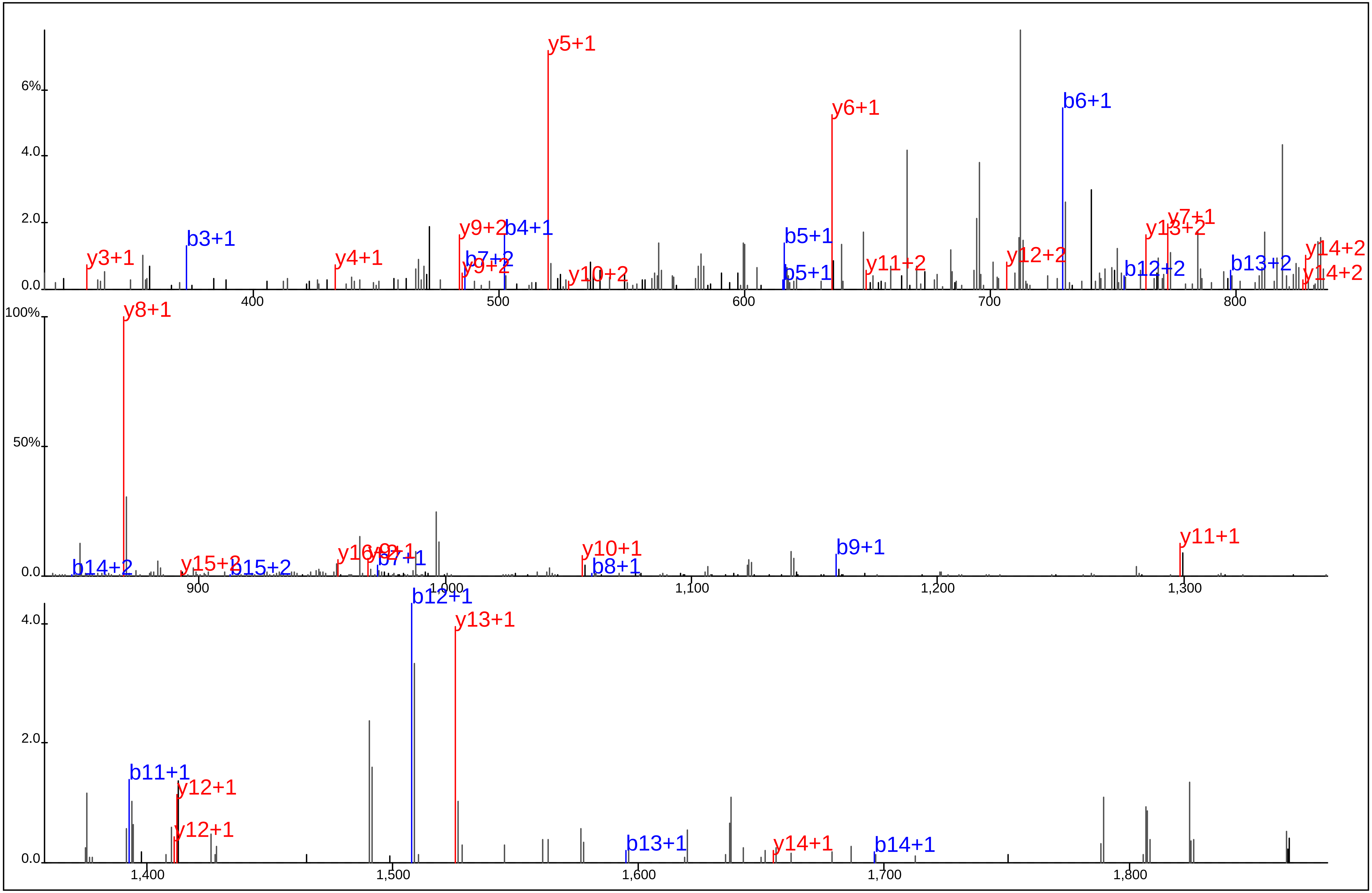

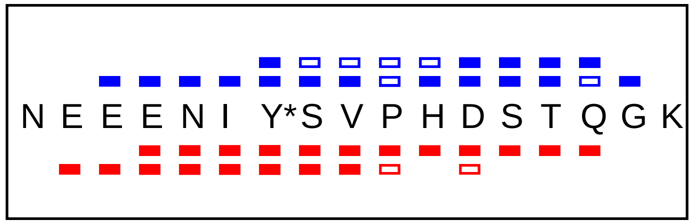

Protein Name: p190 RhoGAP
gi Accession: 150417981
Precursor Mass: 1,013.9238+2
Delta Mass: 0.709 ppm
XCorr: 51.84

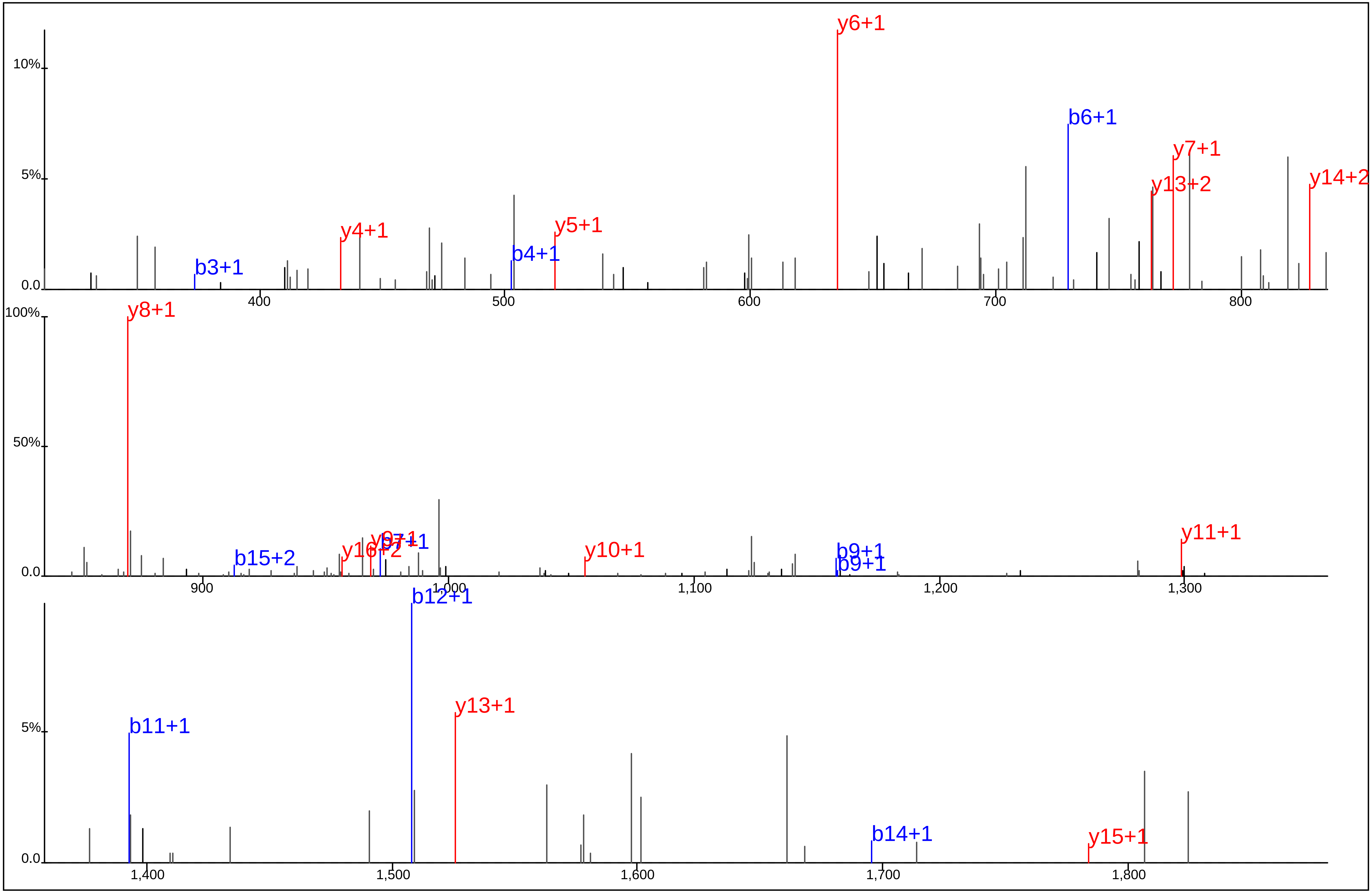

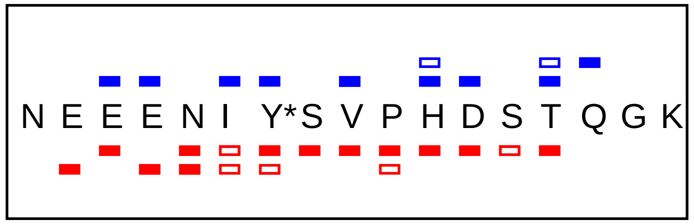

Protein Name:	p190 RhoGAP
gi Accession:	150417981
Precursor Mass:	1,013.9258+2
Delta Mass:	2.698 ppm
XCorr:	48.81

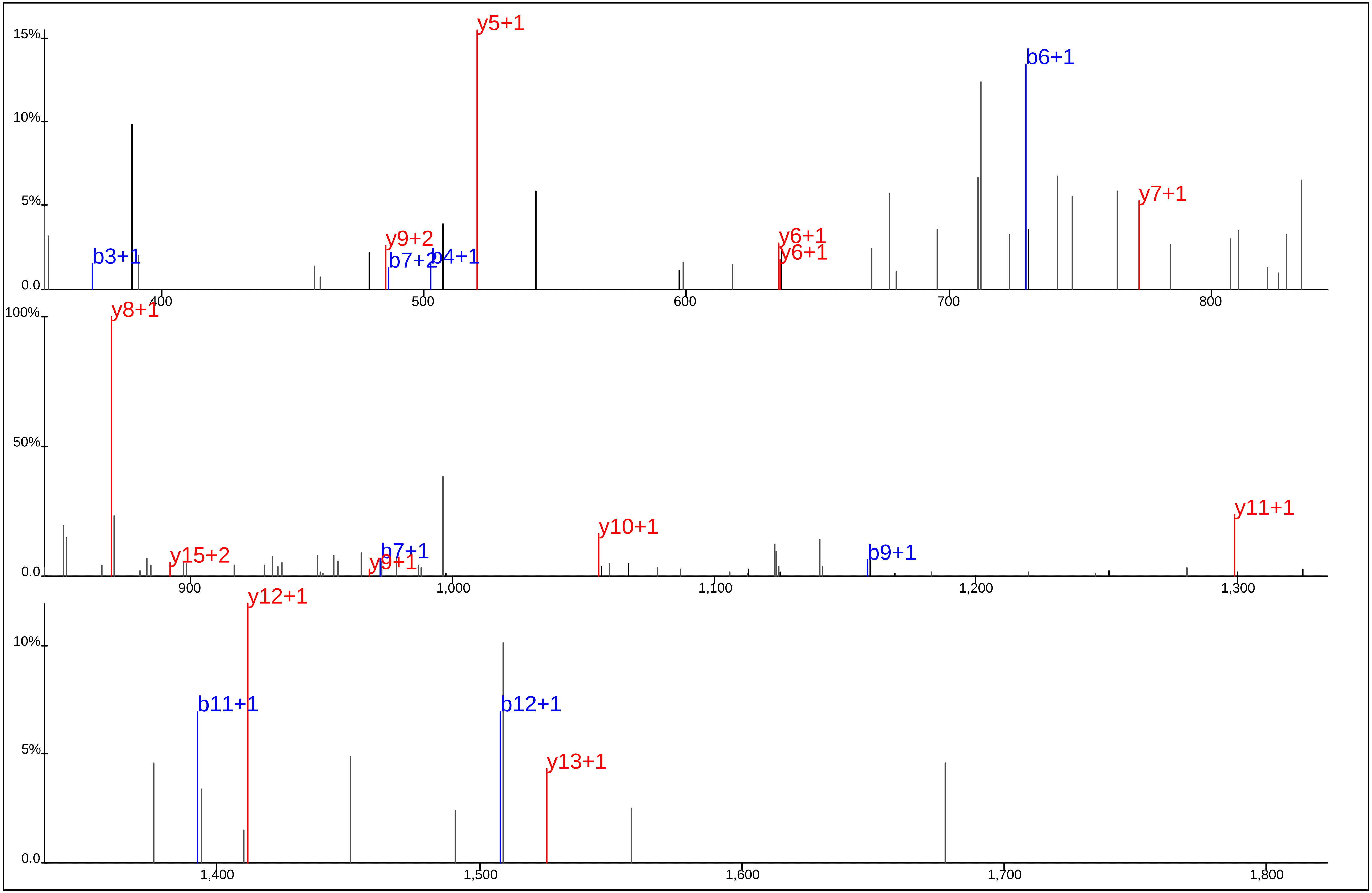

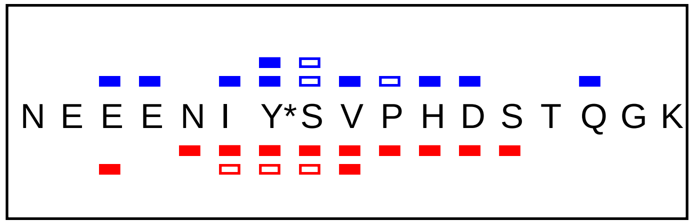

Protein Name: p190 RhoGAP
gi Accession: 150417981
Precursor Mass: 1,013.9278+2
Delta Mass: 4.622 ppm
XCorr: 41.2

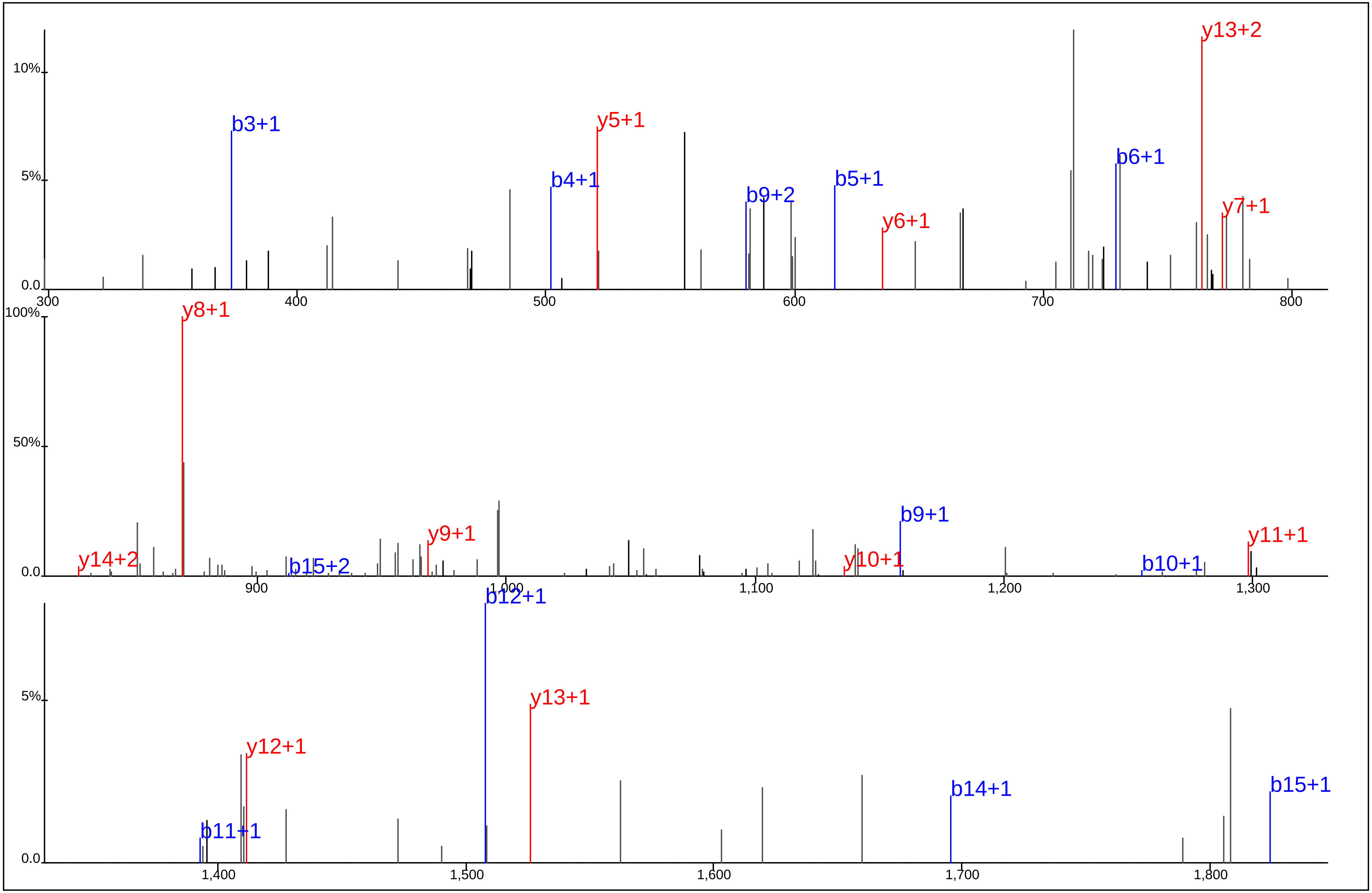

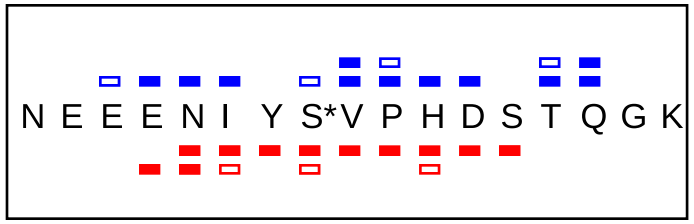

Protein Name: p190 RhoGAP
gi Accession: 150417981
Precursor Mass: 1,013.9256+2
Delta Mass: 2.456 ppm
XCorr: 39.79

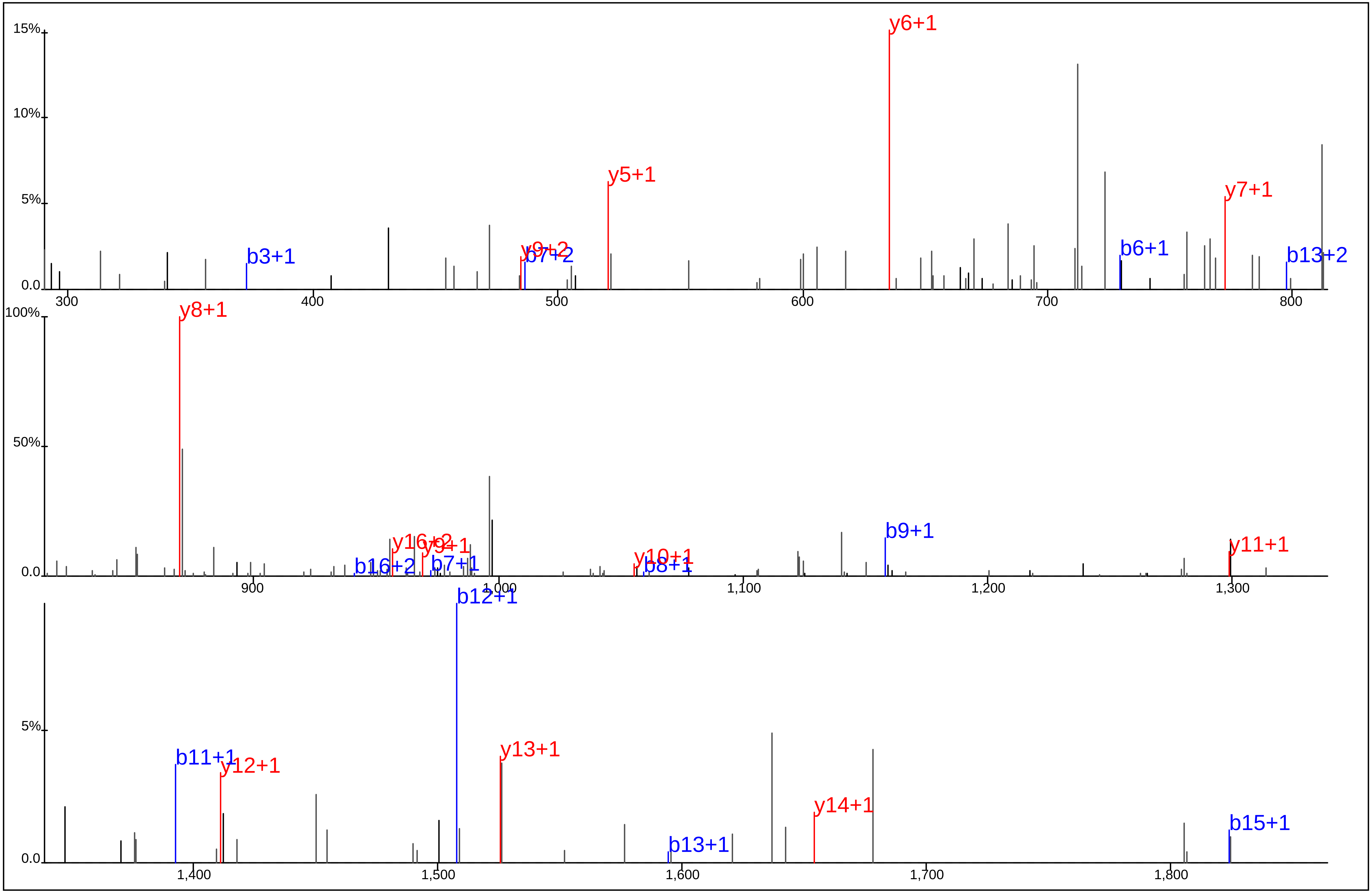

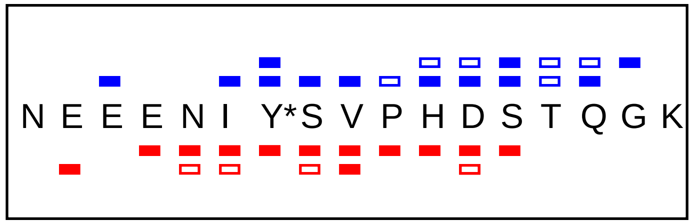

Protein Name:	p190 RhoGAP
gi Accession:	150417981
Precursor Mass:	1,013.9269+2
Delta Mass:	3.783 ppm
XCorr:	44.54

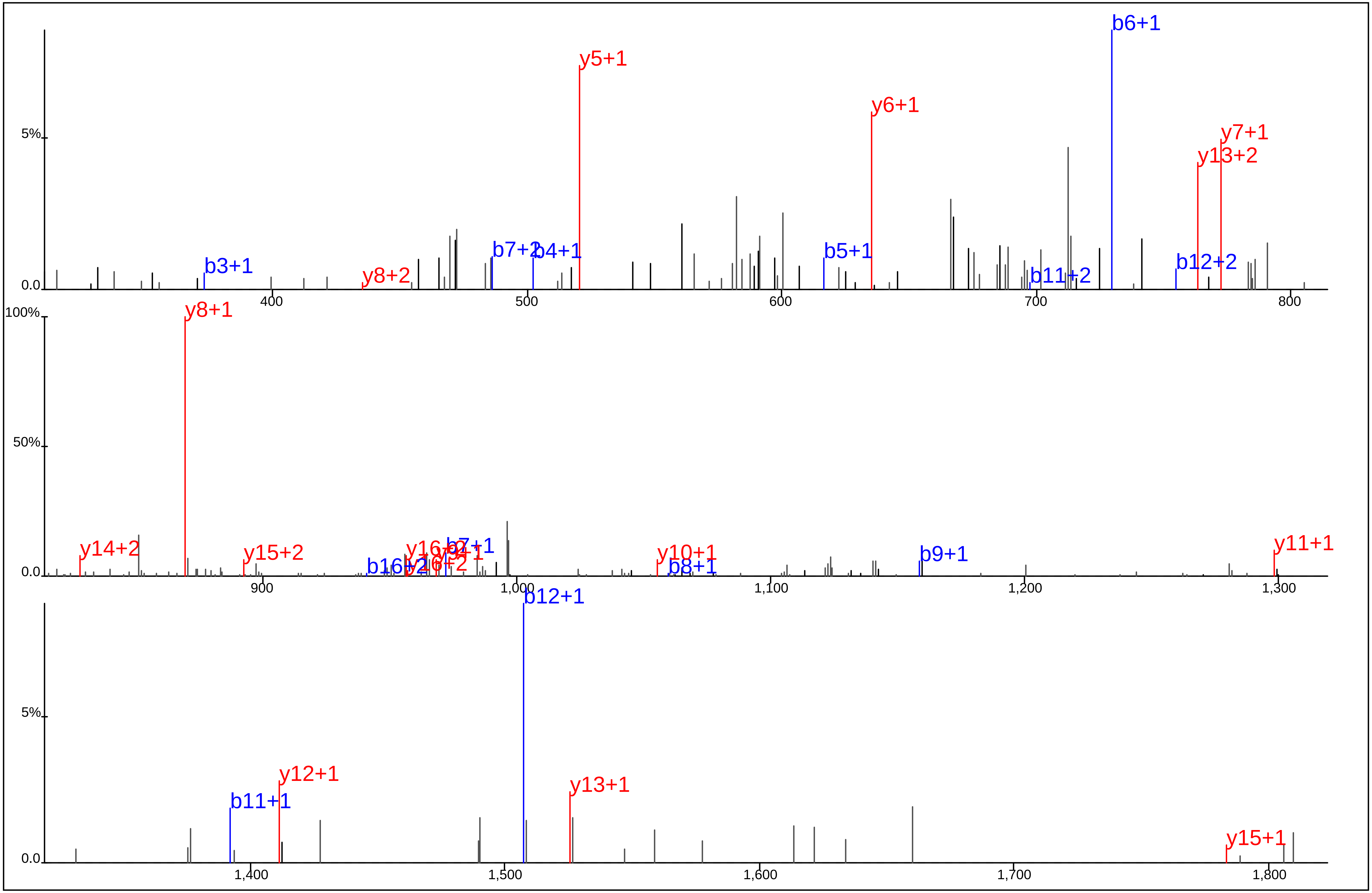

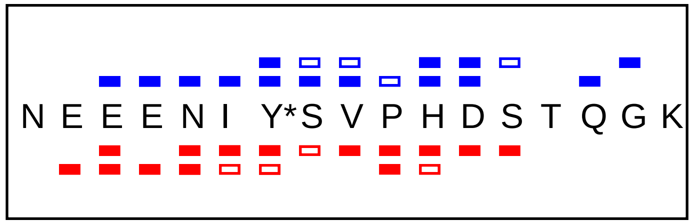

Protein Name: p190 RhoGAP
gi Accession: 150417981
Precursor Mass: 1,013.9283+2
Delta Mass: 5.165 ppm
XCorr: 50.41

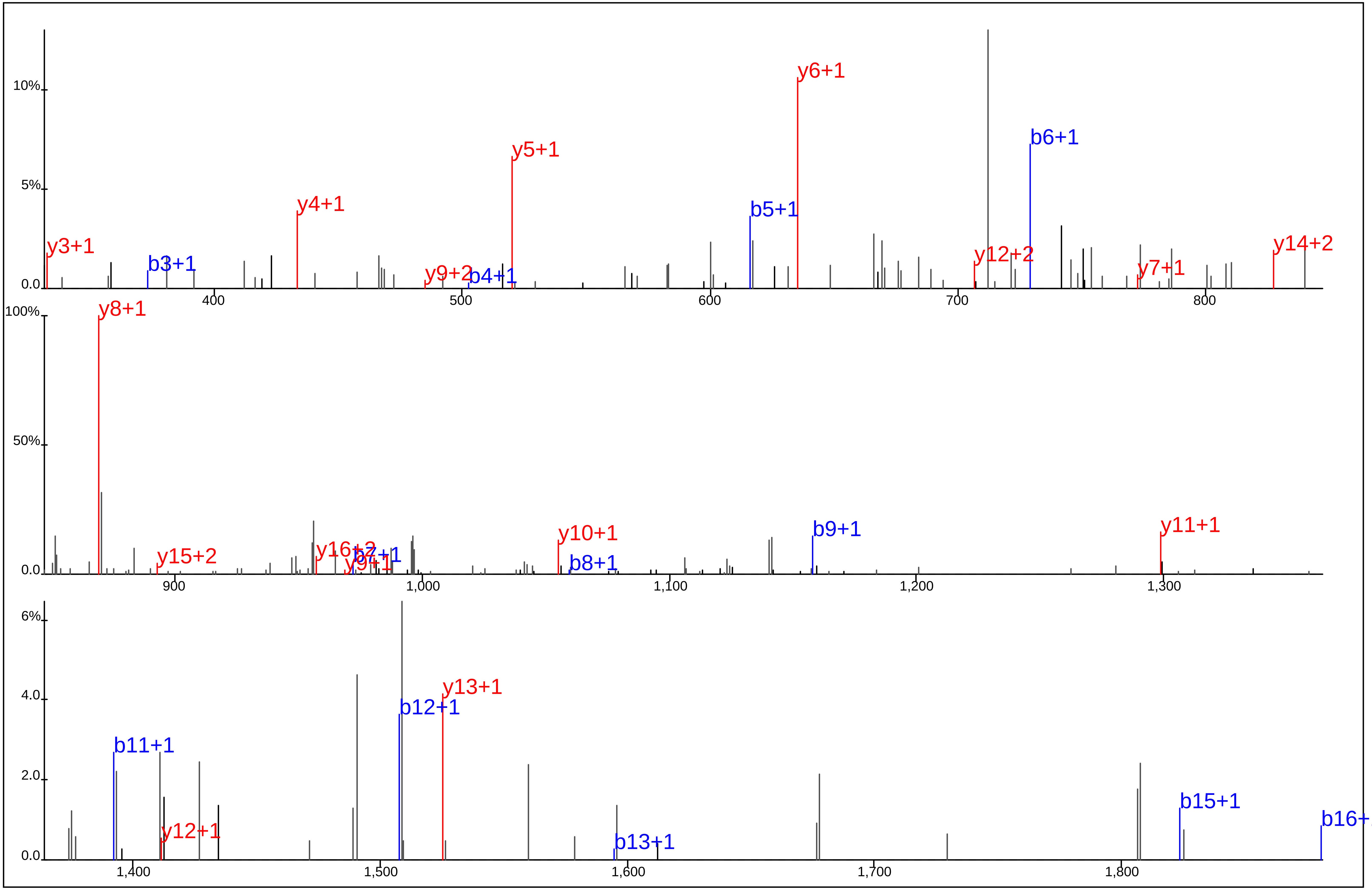

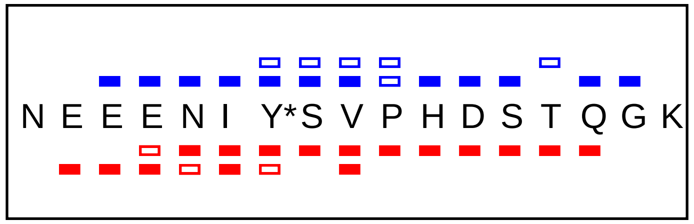

Protein Name: p190 RhoGAP
gi Accession: 150417981
Precursor Mass: 1,013.9265+2
Delta Mass: 3.359 ppm
XCorr: 43.09

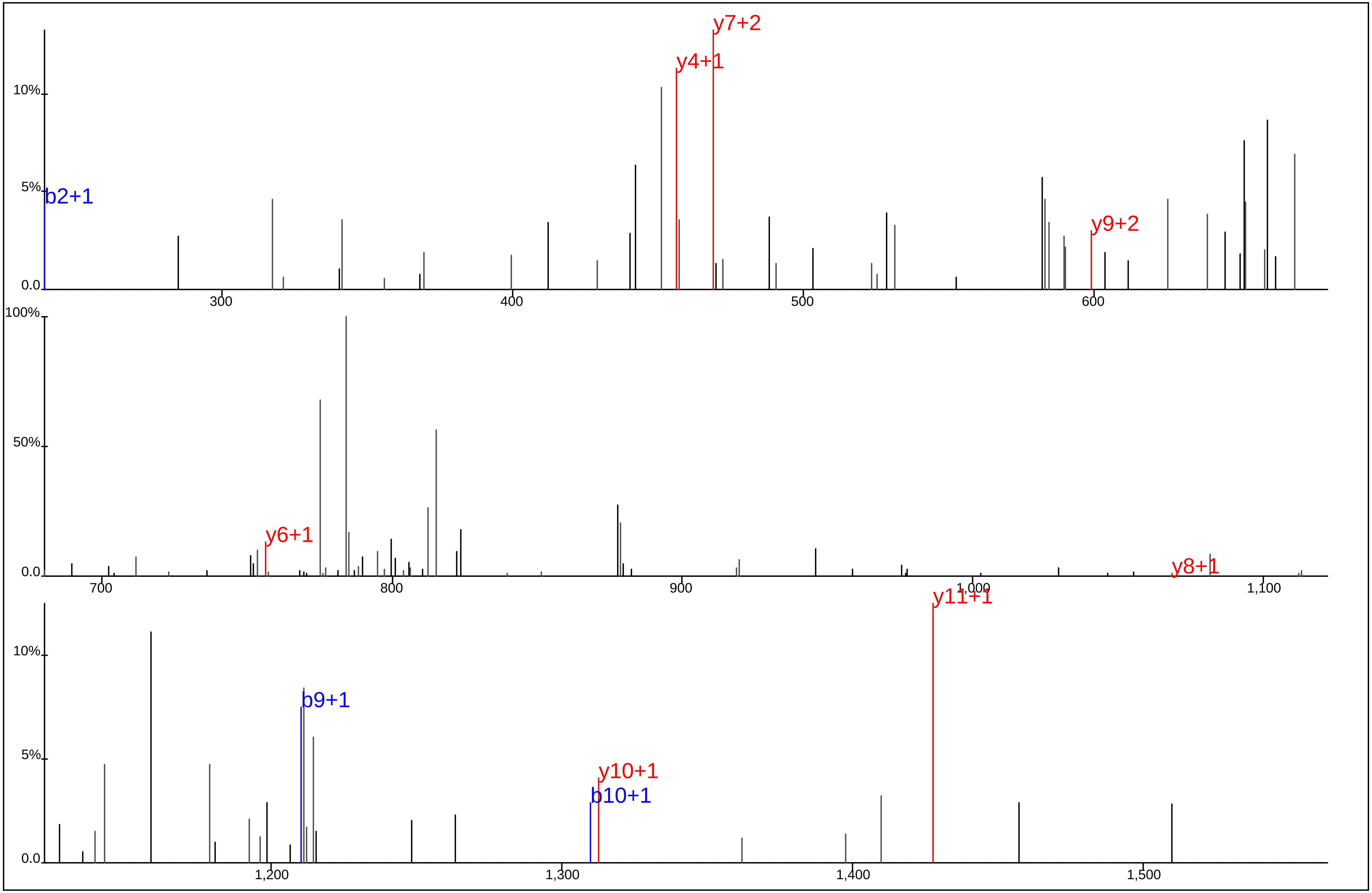

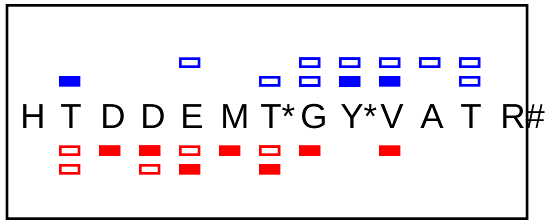

Protein Name: p38
gi Accession: 20986514
Precursor Mass: 833.3024+2
Delta Mass: 5.085 ppm
XCorr: 2.4166

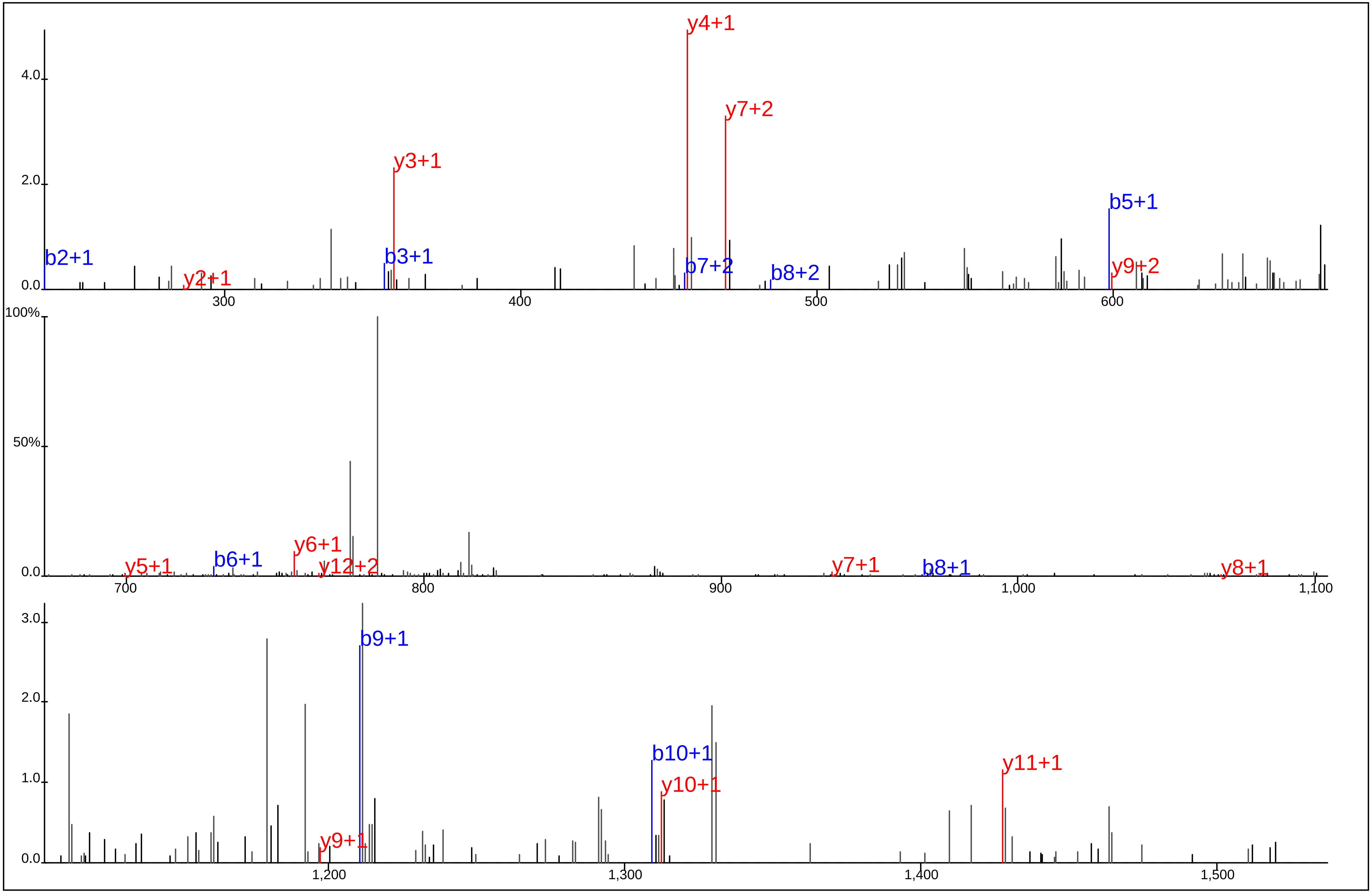

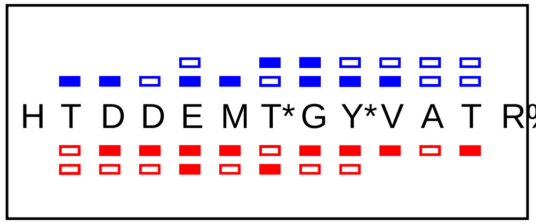

Protein Name:	p38
gi Accession:	20986514
Precursor Mass:	833.3026+2
Delta Mass:	5.306 ppm
XCorr:	38.51

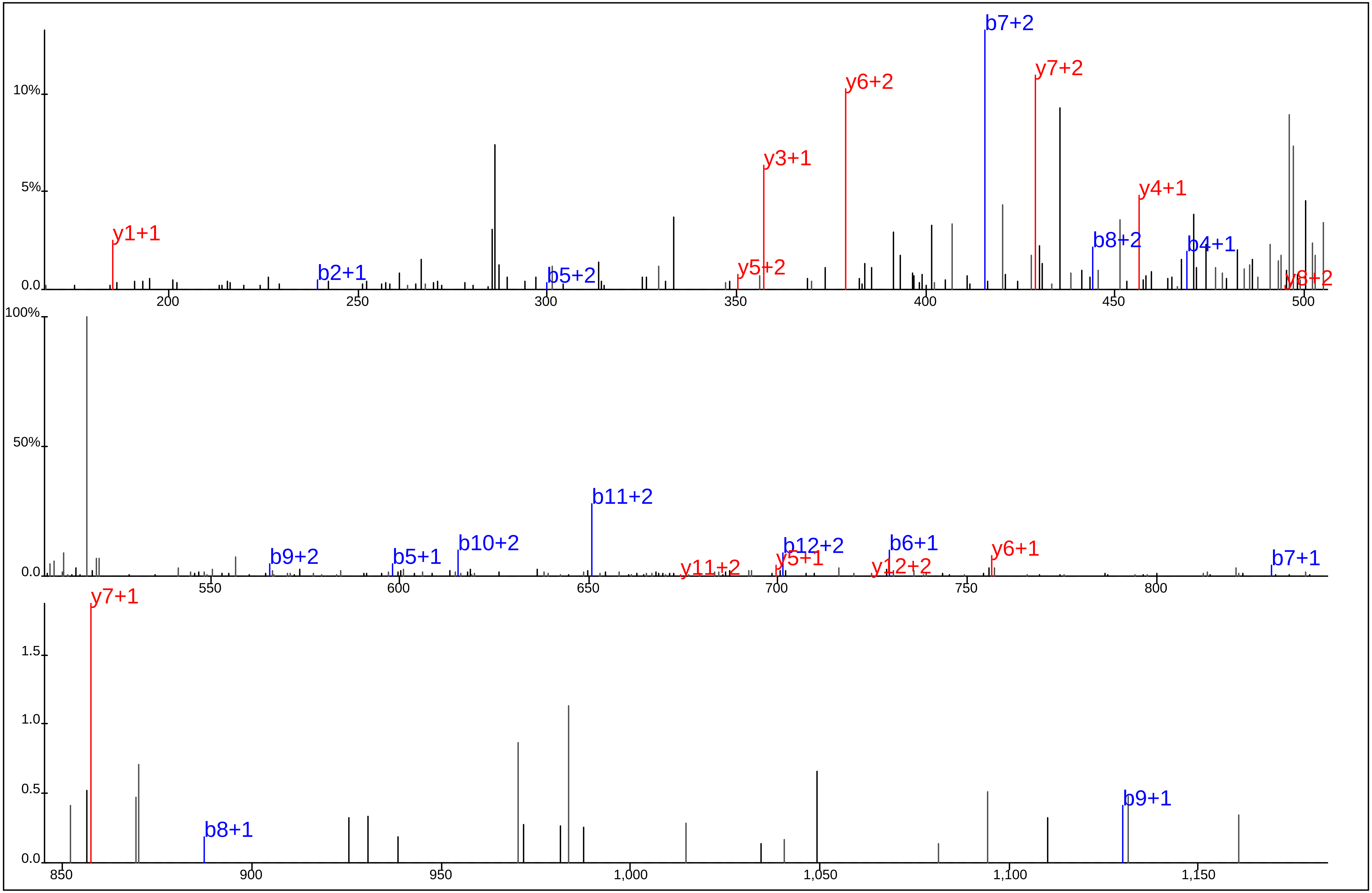

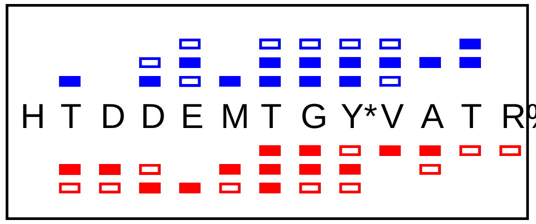

Protein Name: p38
gi Accession: 20986514
Precursor Mass: 529.2141+3
Delta Mass: 3.153 ppm
XCorr: 27.95

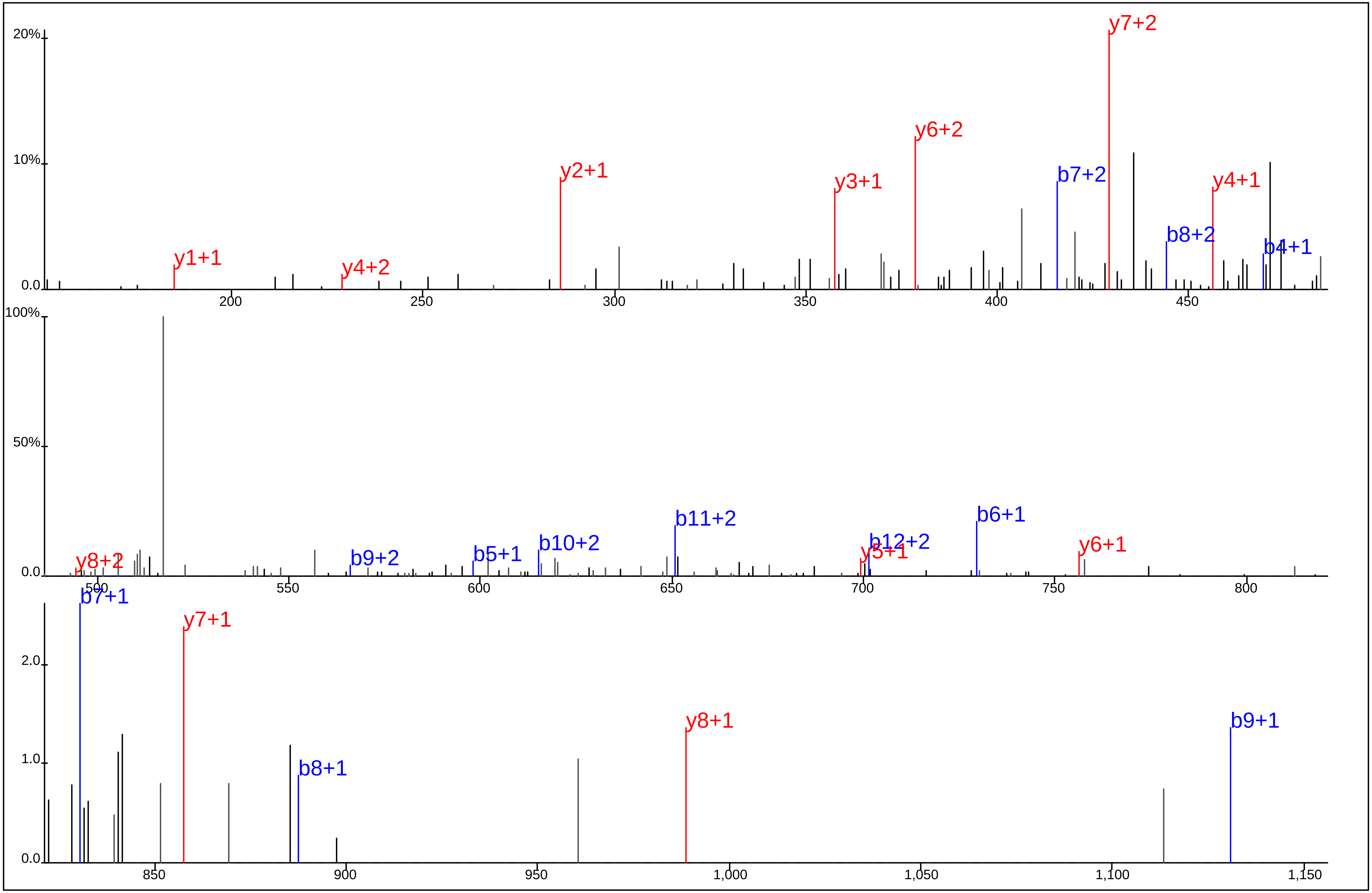

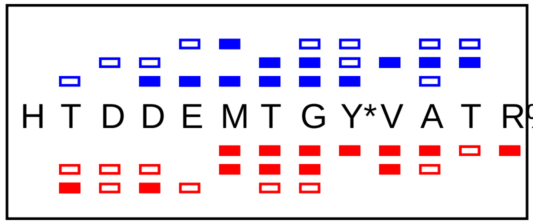

Protein Name: p38
gi Accession: 20986514
Precursor Mass: 529.2133+3
Delta Mass: 1.537 ppm
XCorr: 38.9

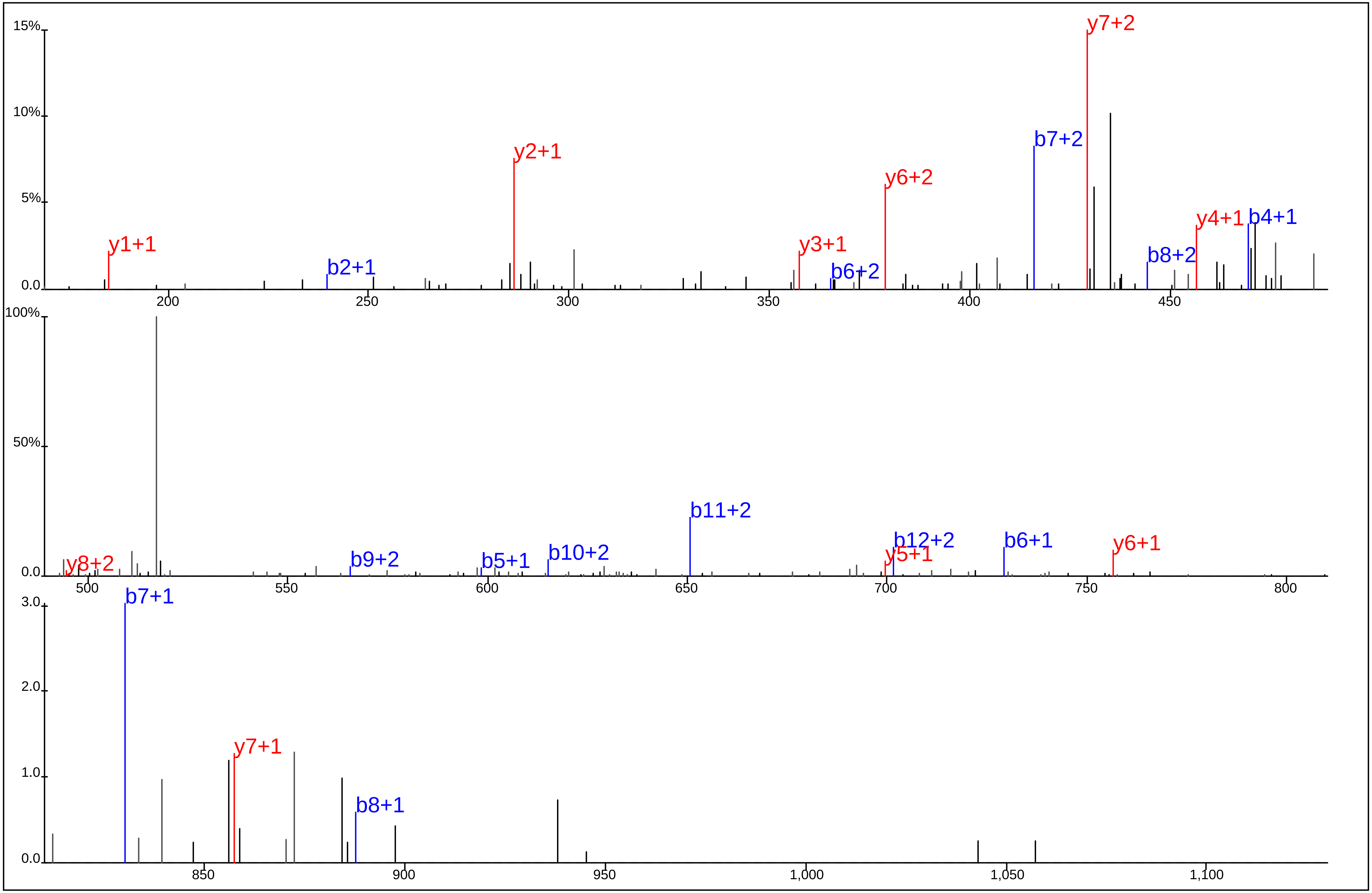

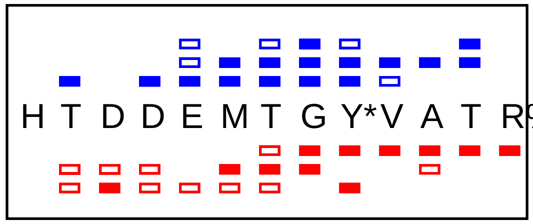

Protein Name: p38
gi Accession: 20986514
Precursor Mass: 529.2169+3
Delta Mass: 8.348 ppm
XCorr: 31.94

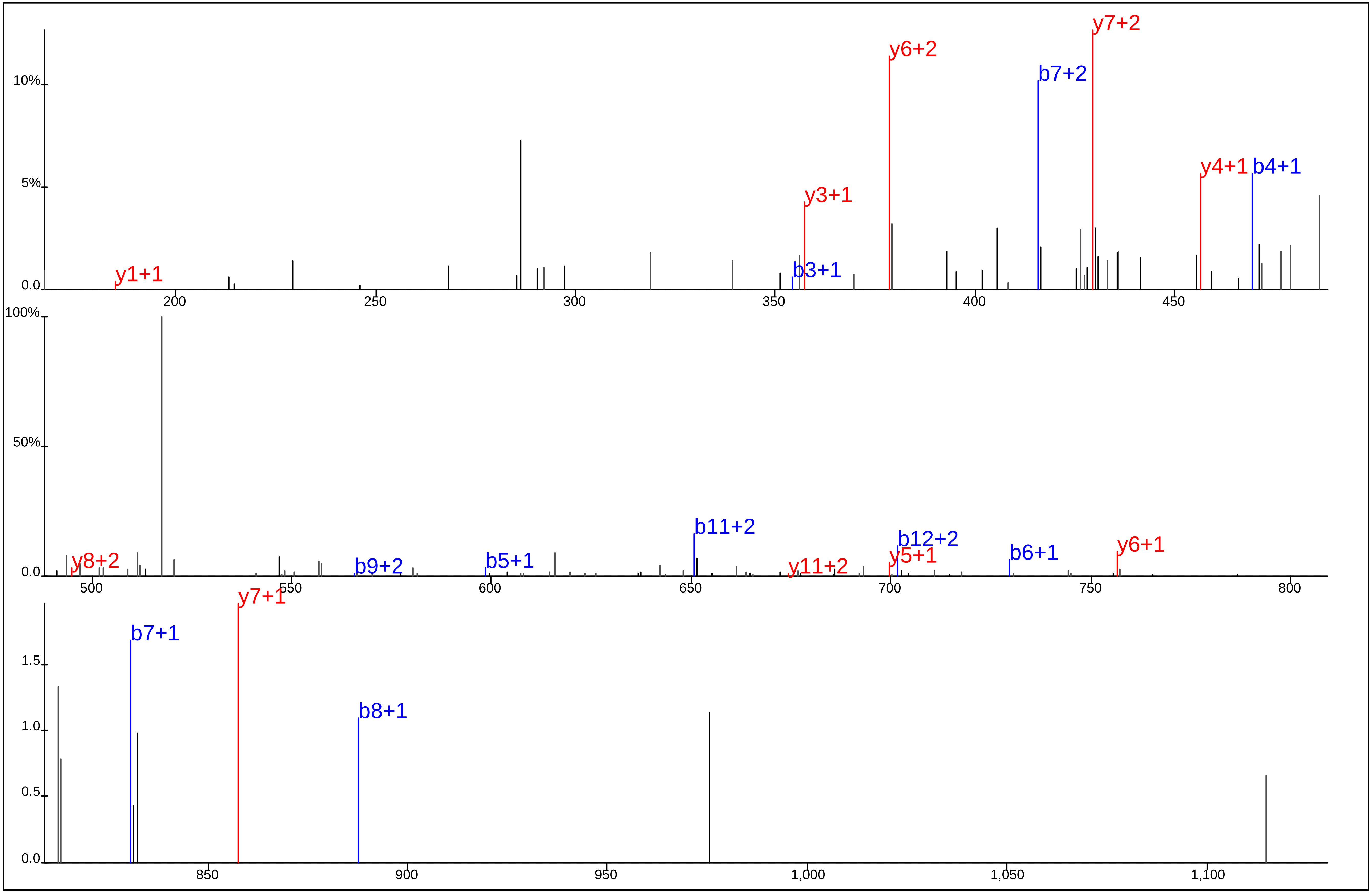

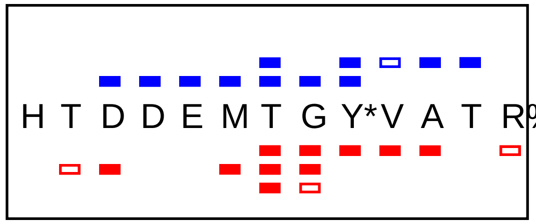

Protein Name: p38
gi Accession: 20986514
Precursor Mass: 529.2137+3
Delta Mass: 2.458 ppm
XCorr: 37.37

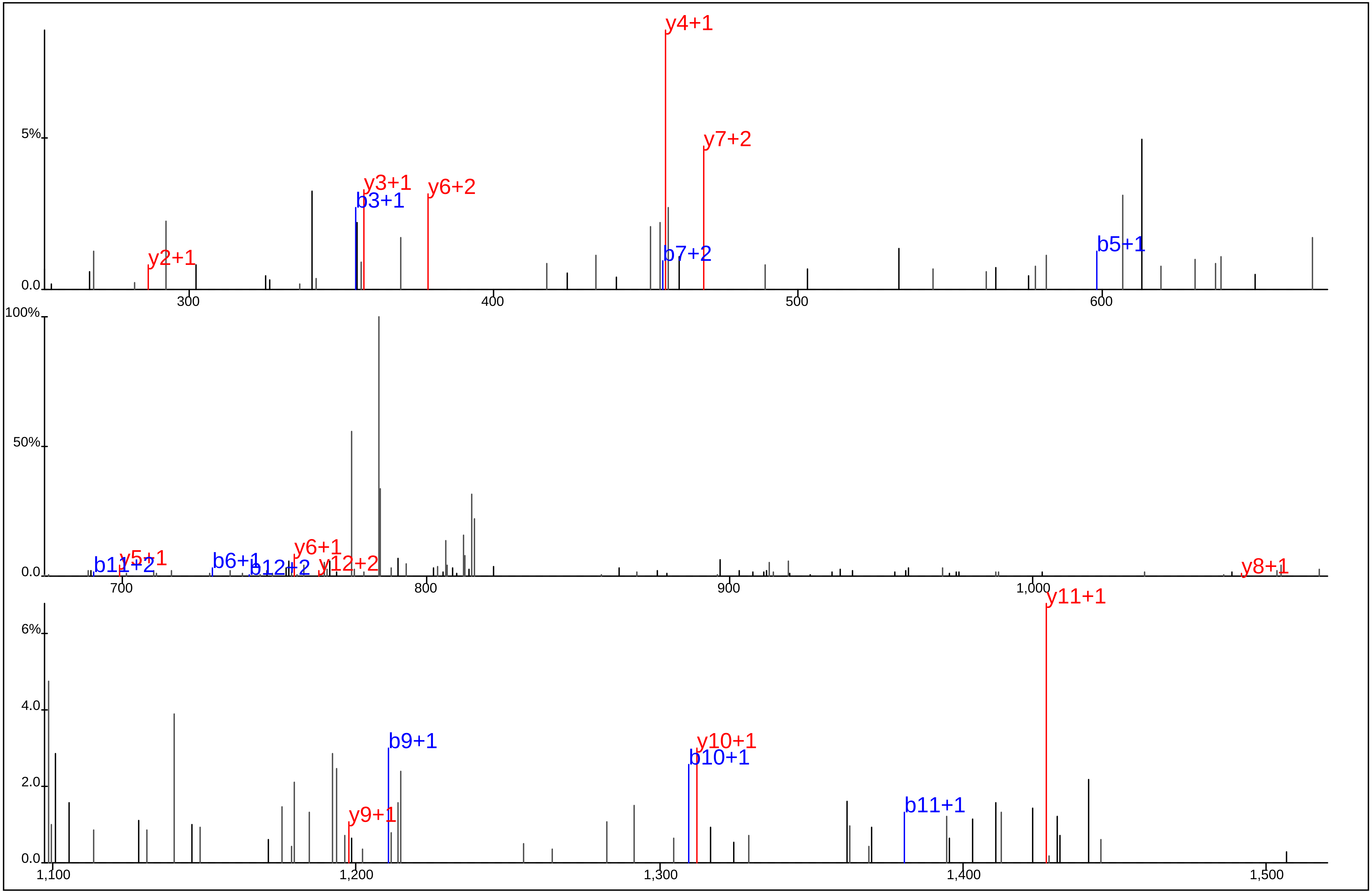

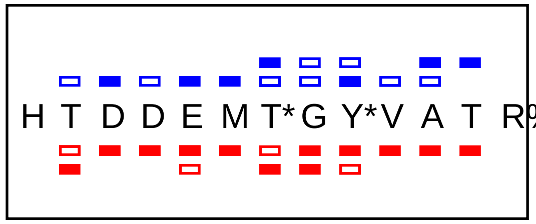

Protein Name: p38
gi Accession: 20986514
Precursor Mass: 833.3027+2
Delta Mass: 5.450 ppm
XCorr: 25.31

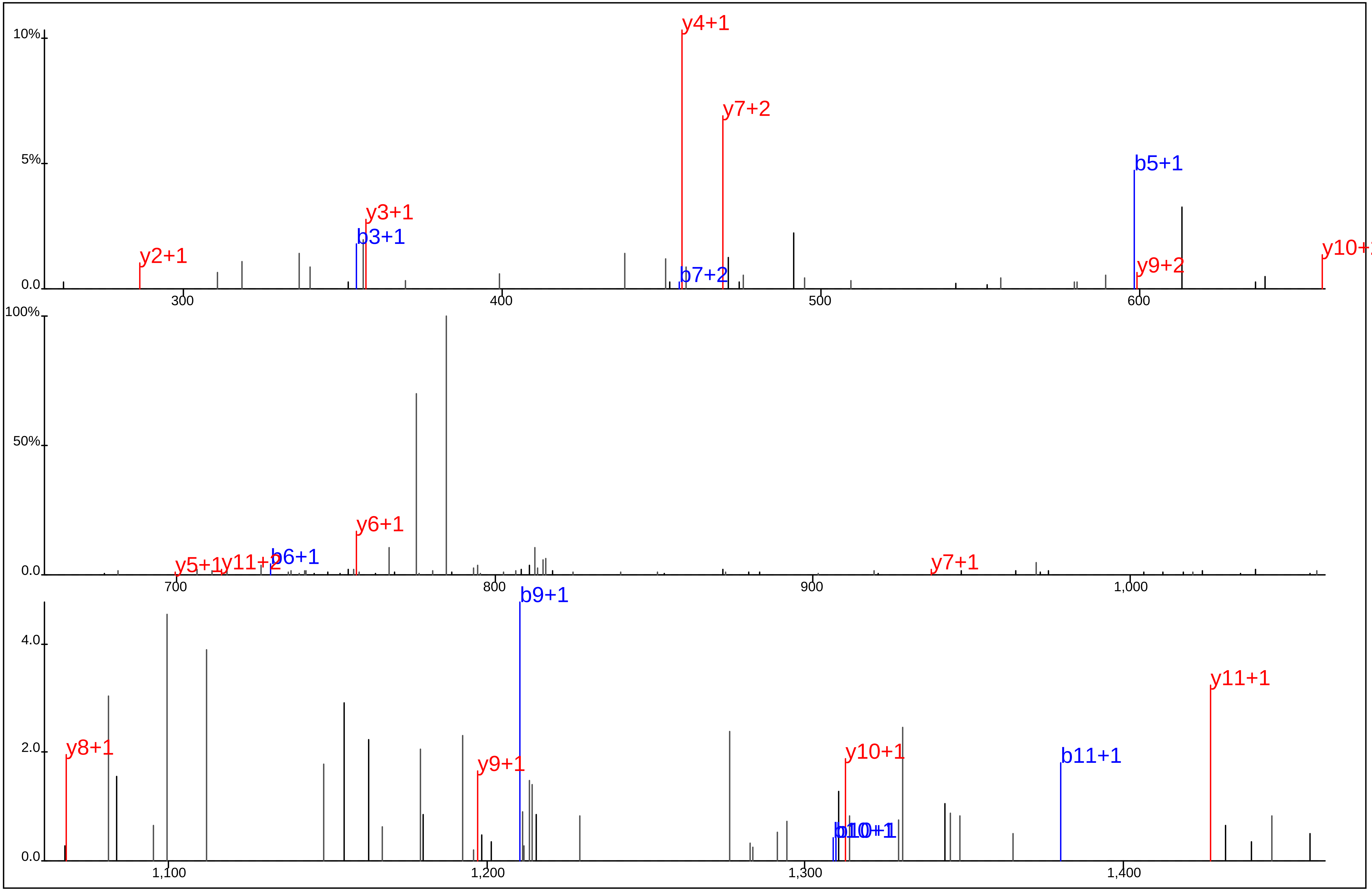

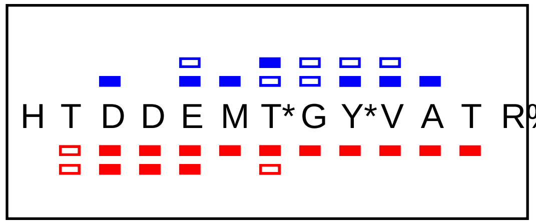

Protein Name: p38
gi Accession: 20986514
Precursor Mass: 833.3033+2
Delta Mass: 6.111 ppm
XCorr: 37.72

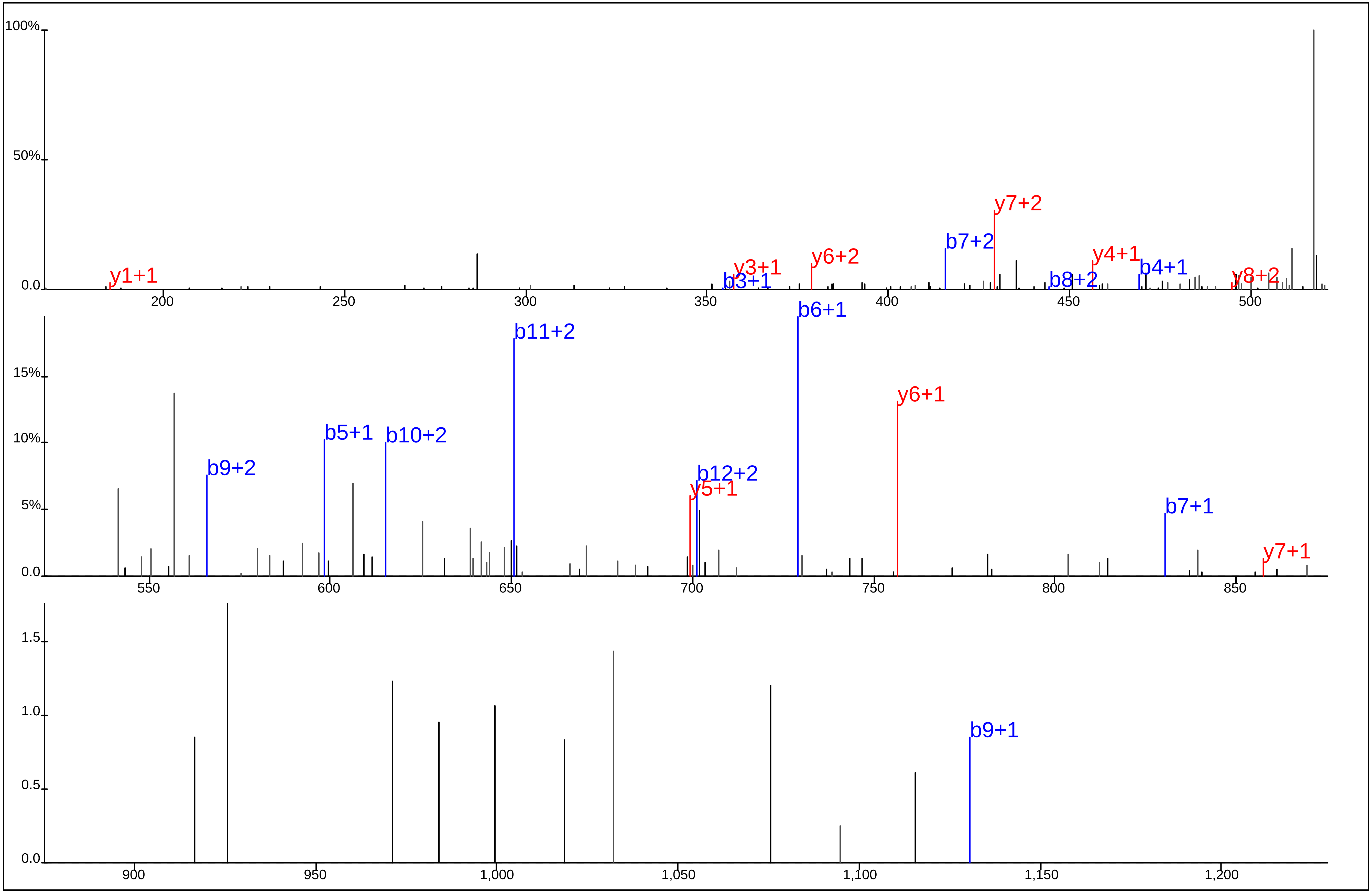

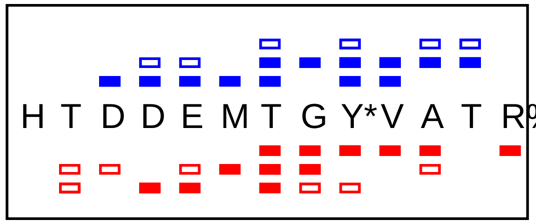

Protein Name: p38
gi Accession: 20986514
Precursor Mass: 529.2147+3
Delta Mass: 4.193 ppm
XCorr: 31.47

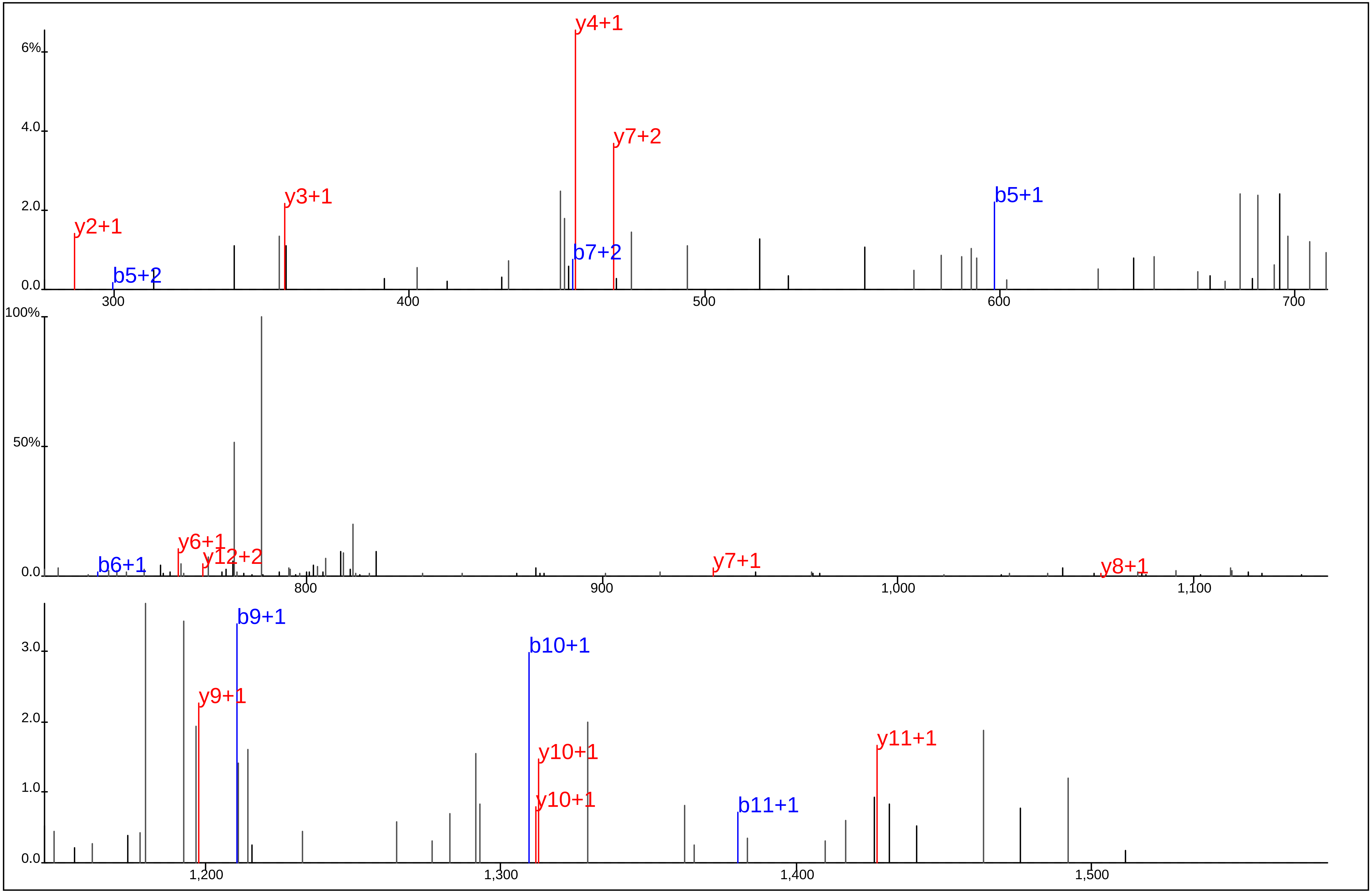

H T D D E M T* G Y* V A T R%

Protein Name: p38
gi Accession: 20986514
Precursor Mass: 833.3044+2
Delta Mass: 7.432 ppm
XCorr: 43.78

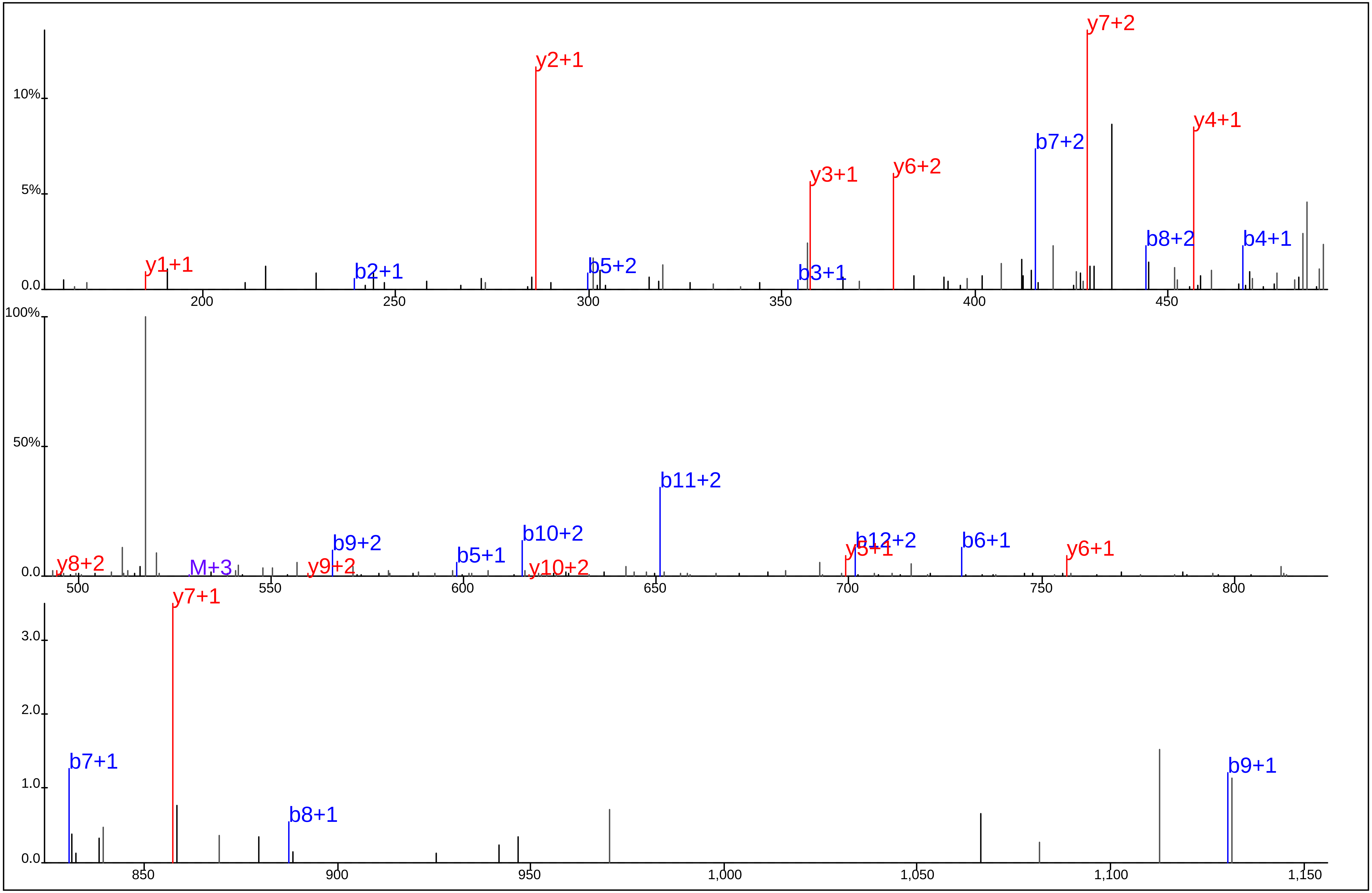

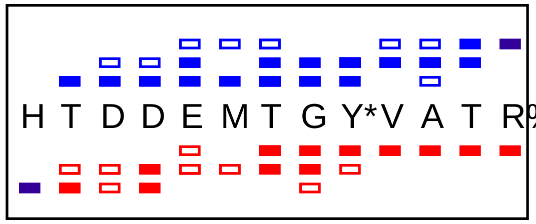

Protein Name:	p38
gi Accession:	20986514
Precursor Mass:	529.2163+3
Delta Mass:	7.308 ppm
XCorr:	38.62

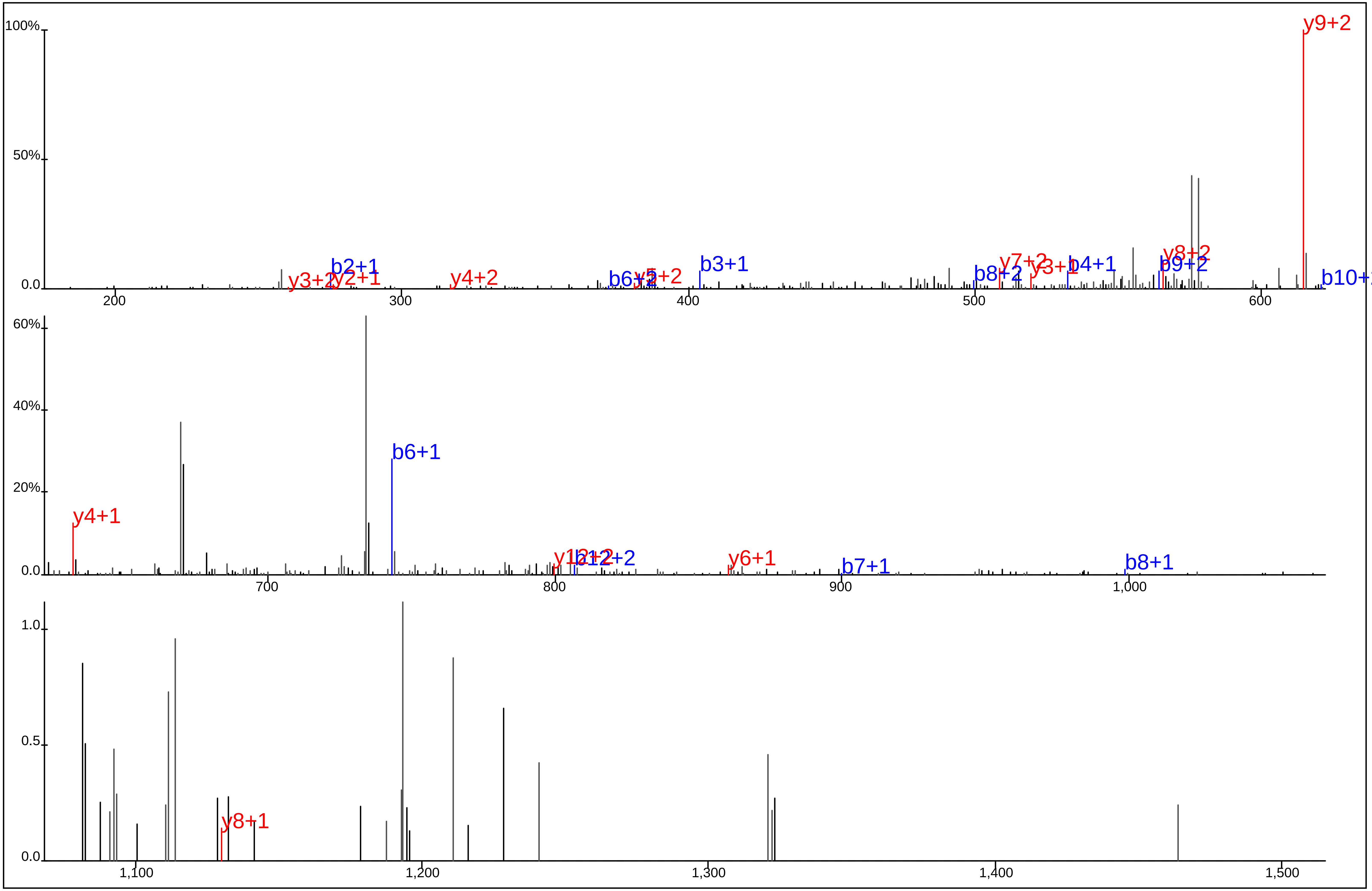

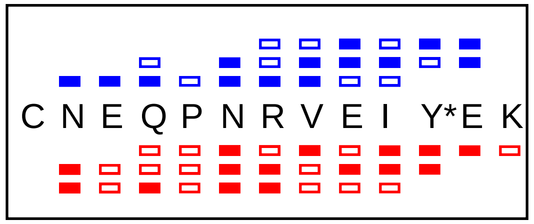

Protein Name:	p53 inducible protein
gi Accession:	559703
Precursor Mass:	586.9229+3
Delta Mass:	1.347 ppm
XCorr:	16.54

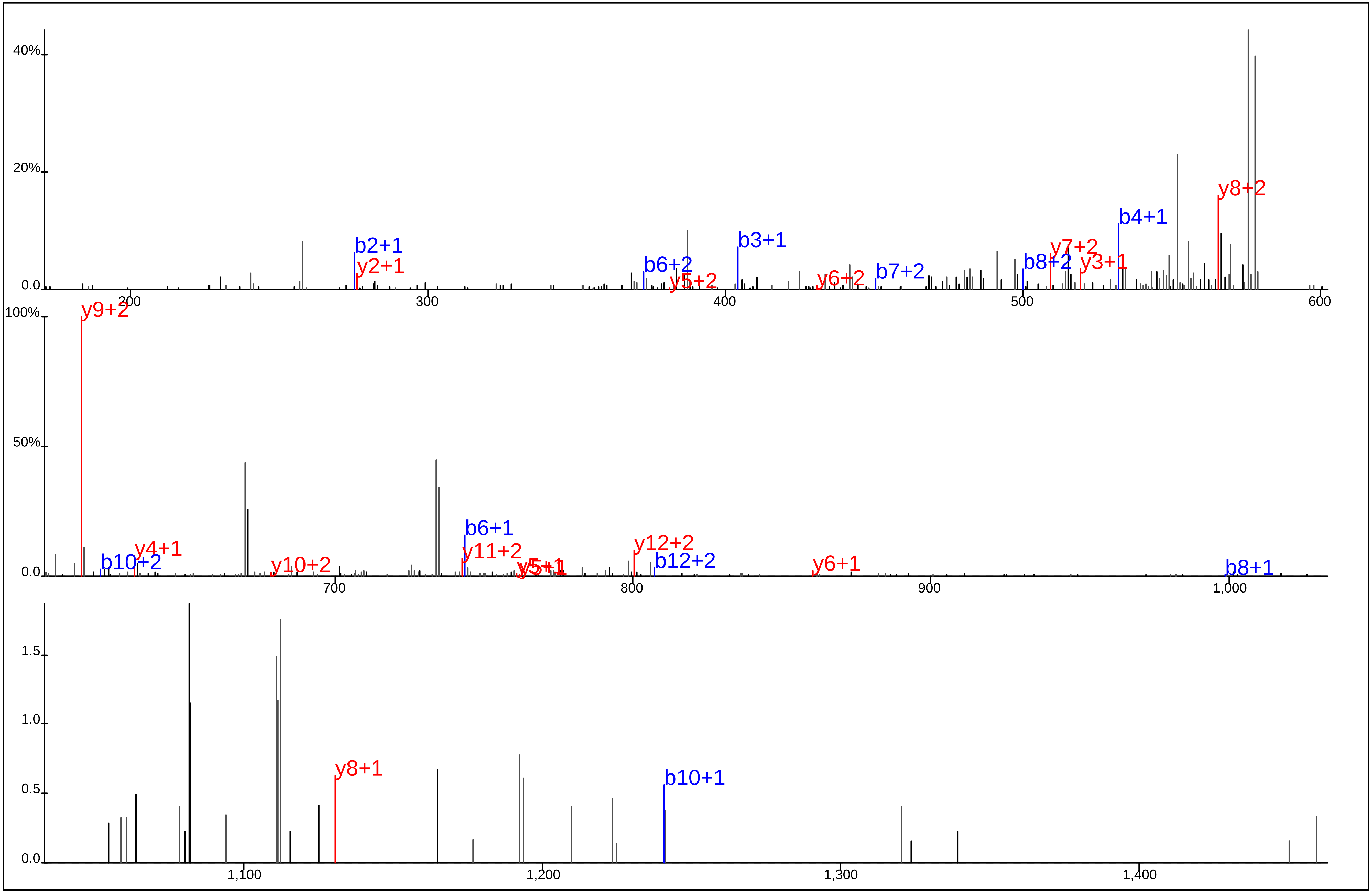

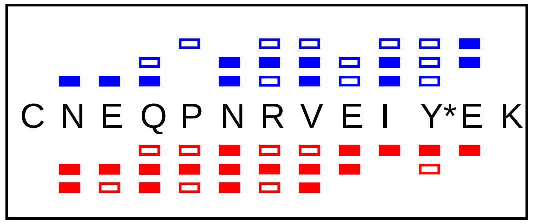

Protein Name: p53 inducible protein
gi Accession: 559703
Precursor Mass: 586.9237+3
Delta Mass: 2.802 ppm
XCorr: 14.62

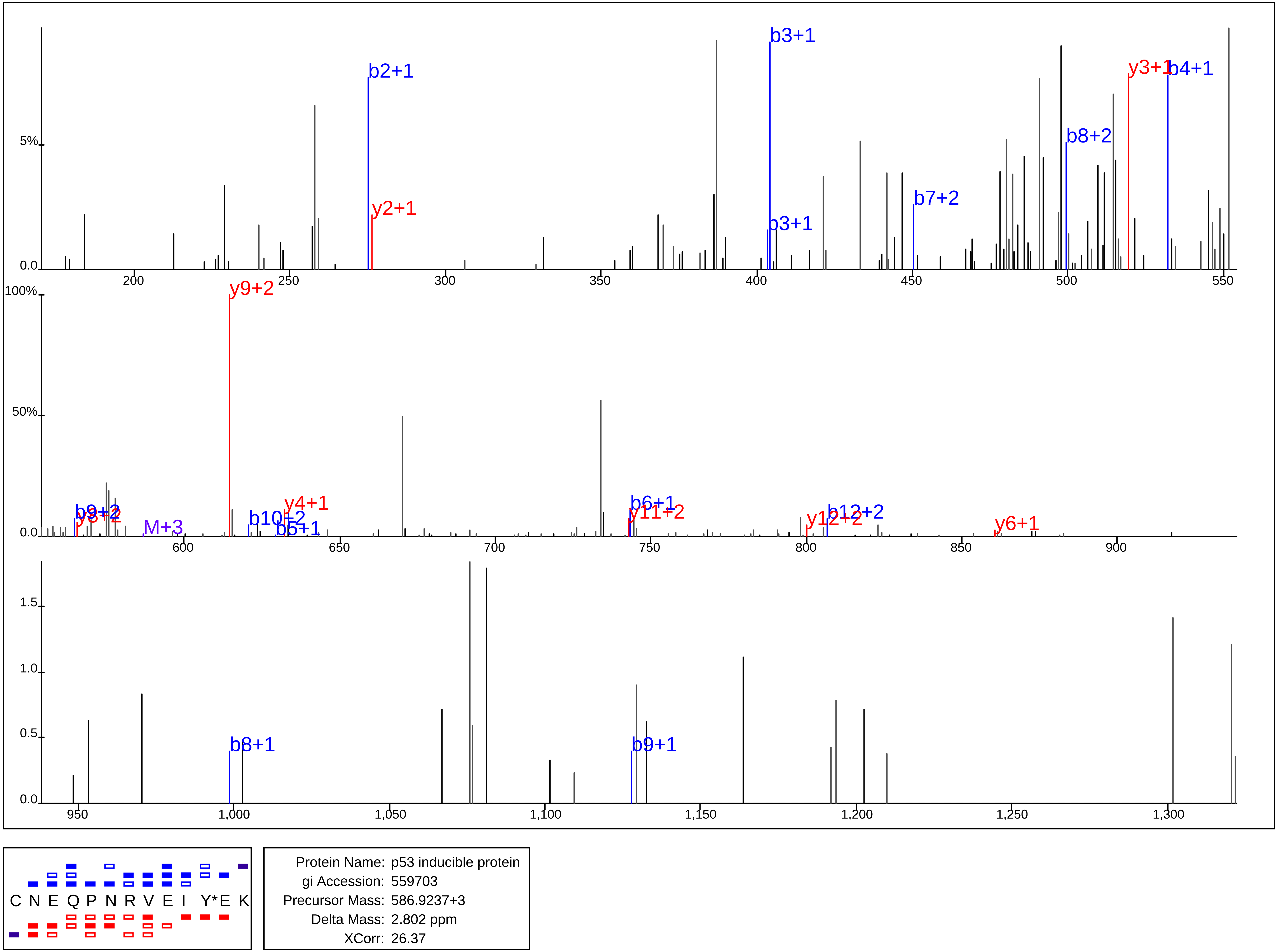

Protein Name:	p53 inducible protein
gi Accession:	559703
Precursor Mass:	586.9237+3
Delta Mass:	2.802 ppm
XCorr:	26.37

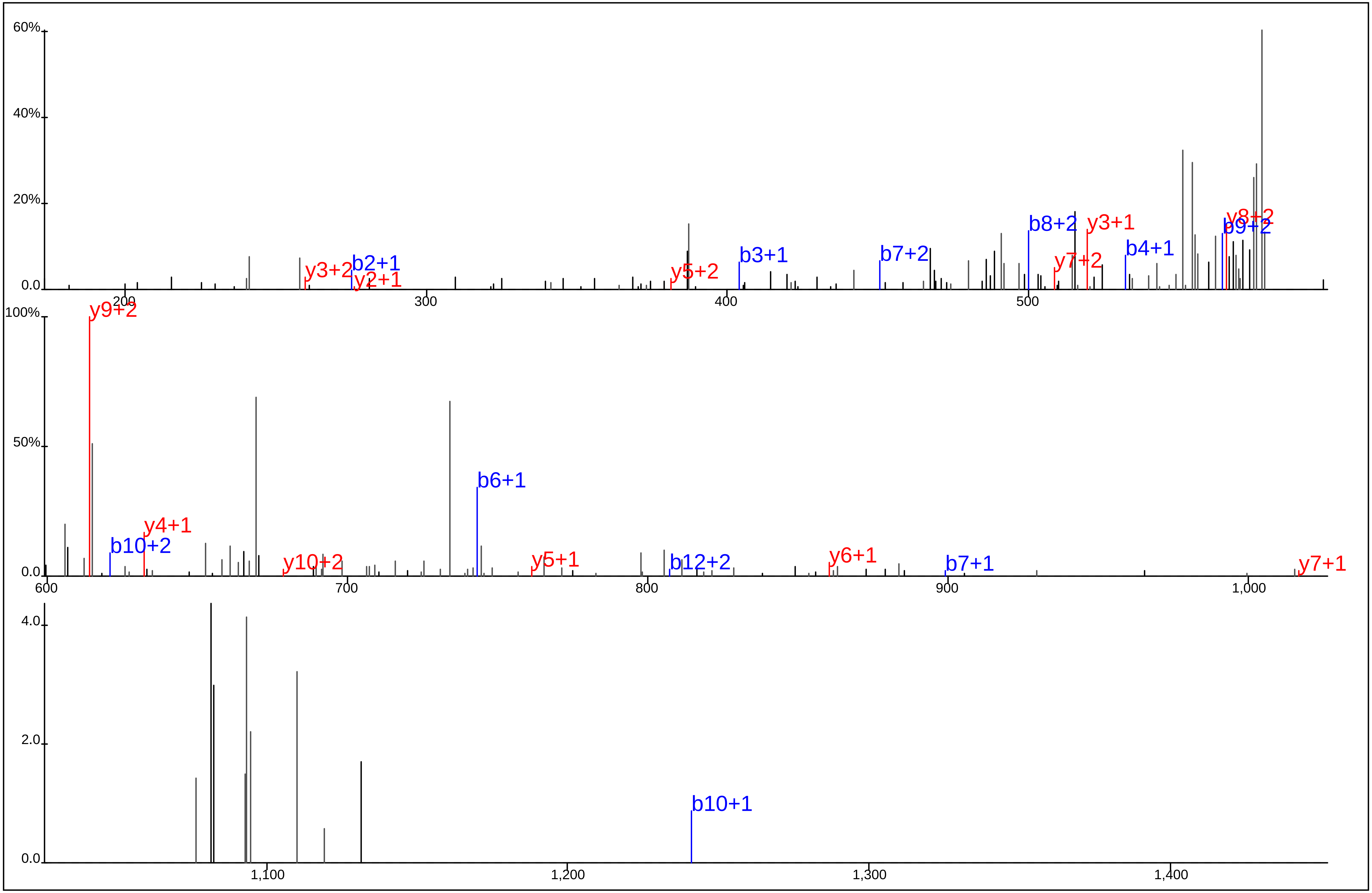

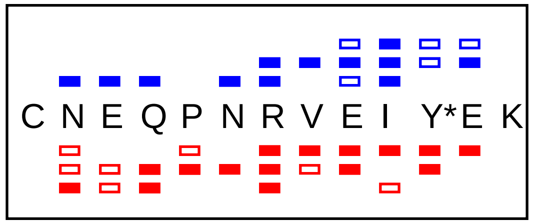

Protein Name:	p53 inducible protein
gi Accession:	559703
Precursor Mass:	586.9244+3
Delta Mass:	3.950 ppm
XCorr:	15.45

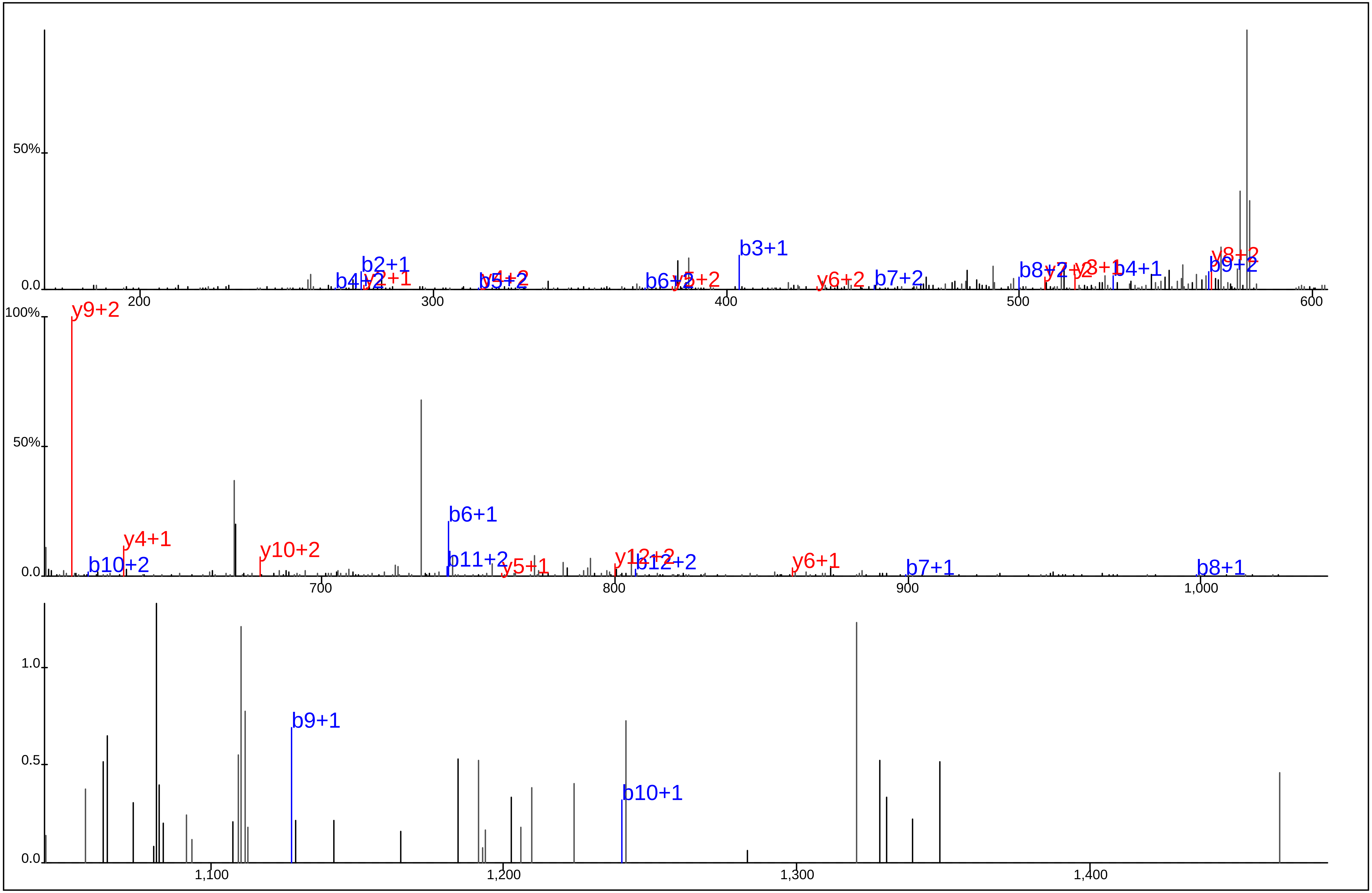

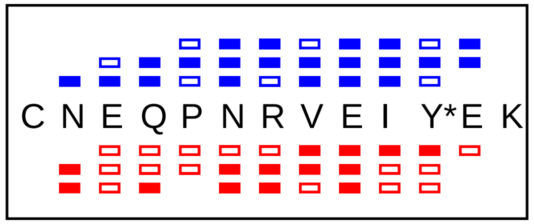

Protein Name: p53 inducible protein
gi Accession: 559703
Precursor Mass: 586.9232+3
Delta Mass: 1.971 ppm
XCorr: 16.46

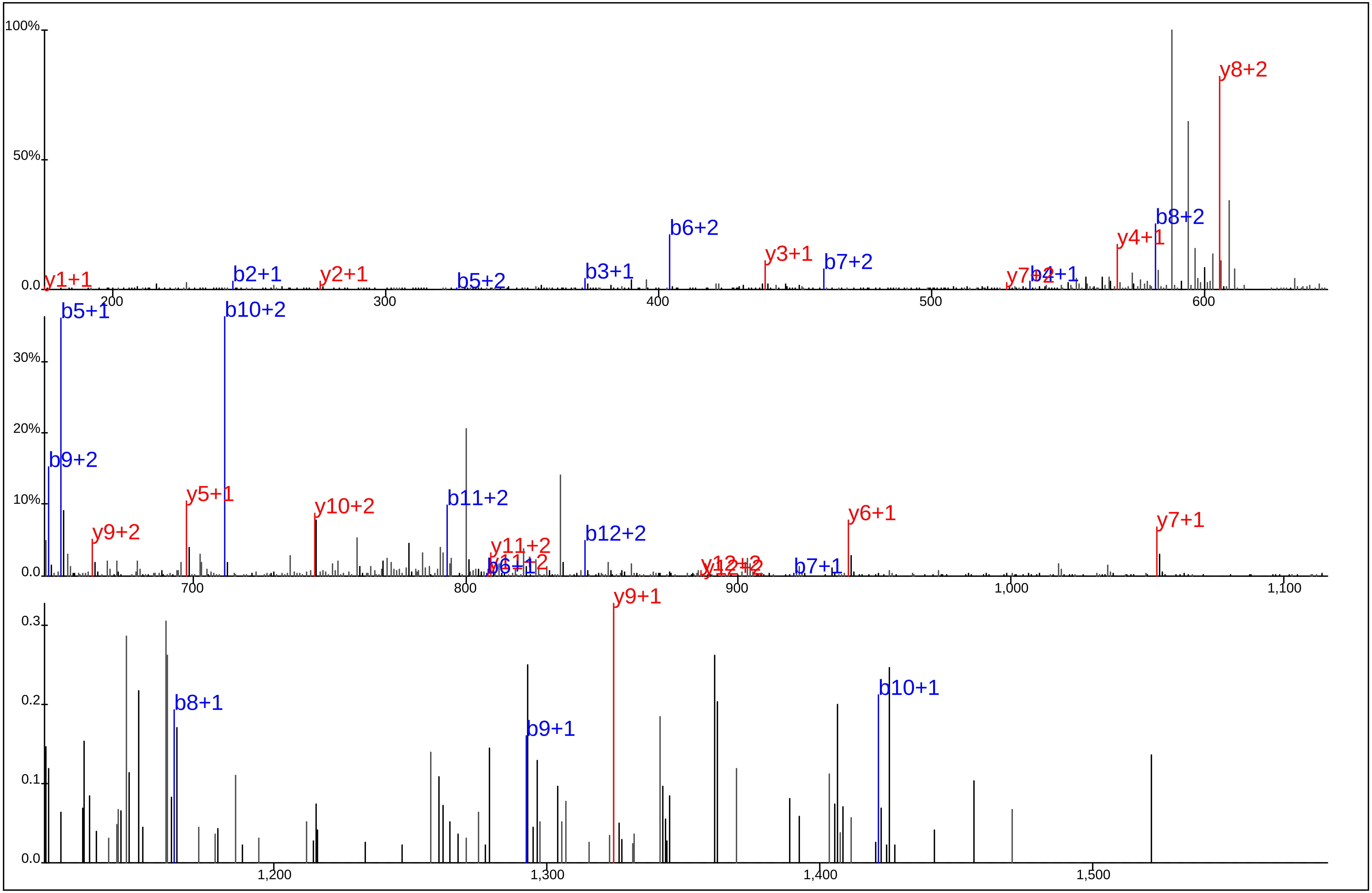

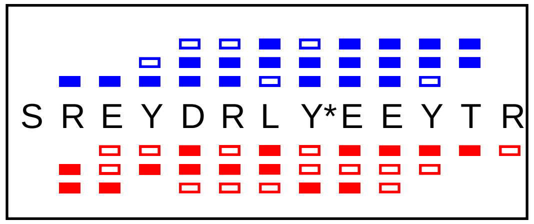

Protein Name: p85 alpha
gi Accession: 32455250
Precursor Mass: 620.6049+3
Delta Mass: 2.301 ppm
XCorr: 37.5

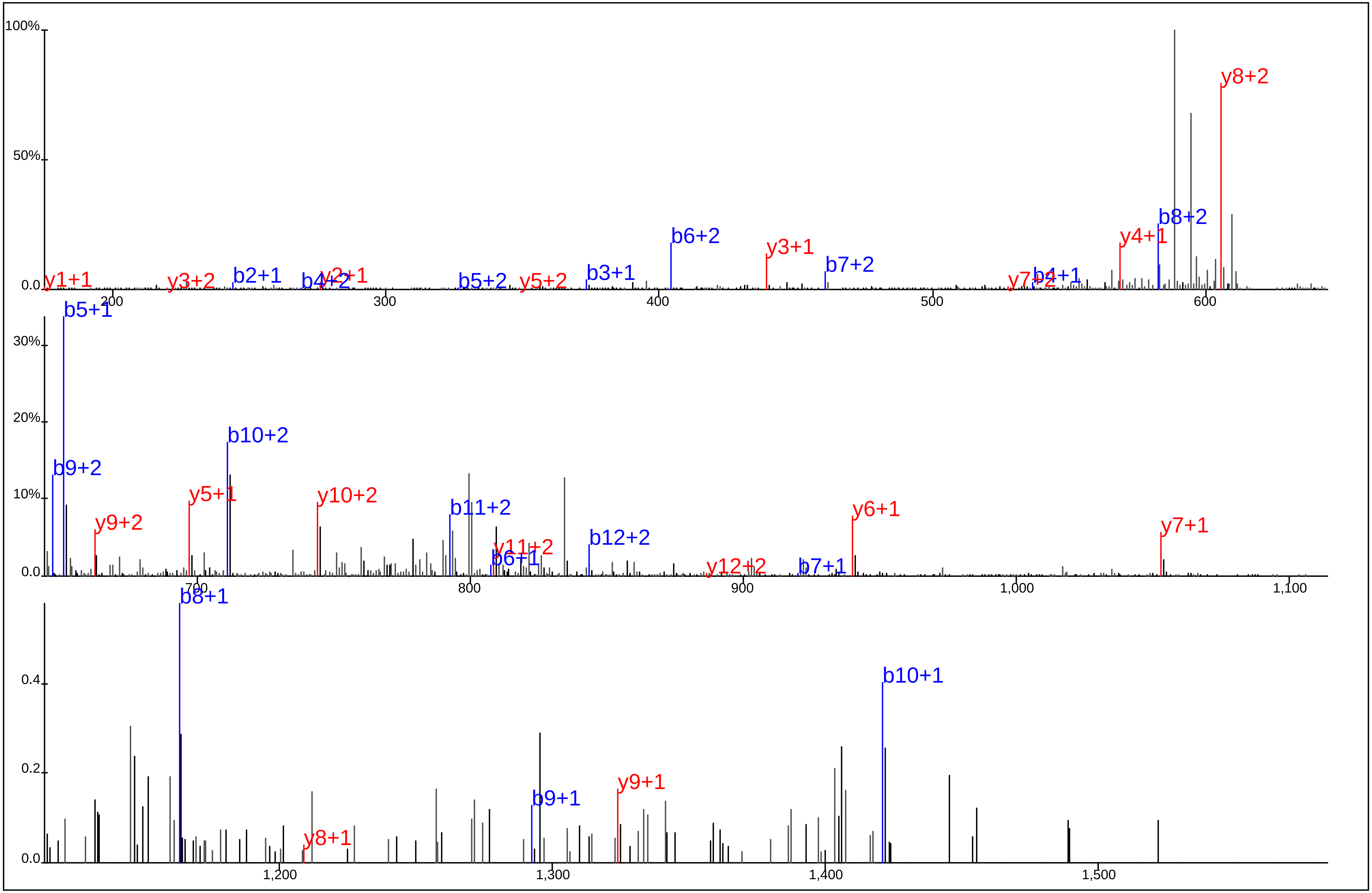

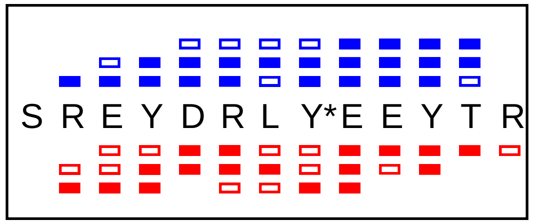

Protein Name: p85 alpha
gi Accession: 32455250
Precursor Mass: 620.6056+3
Delta Mass: 3.382 ppm
XCorr: 38.12

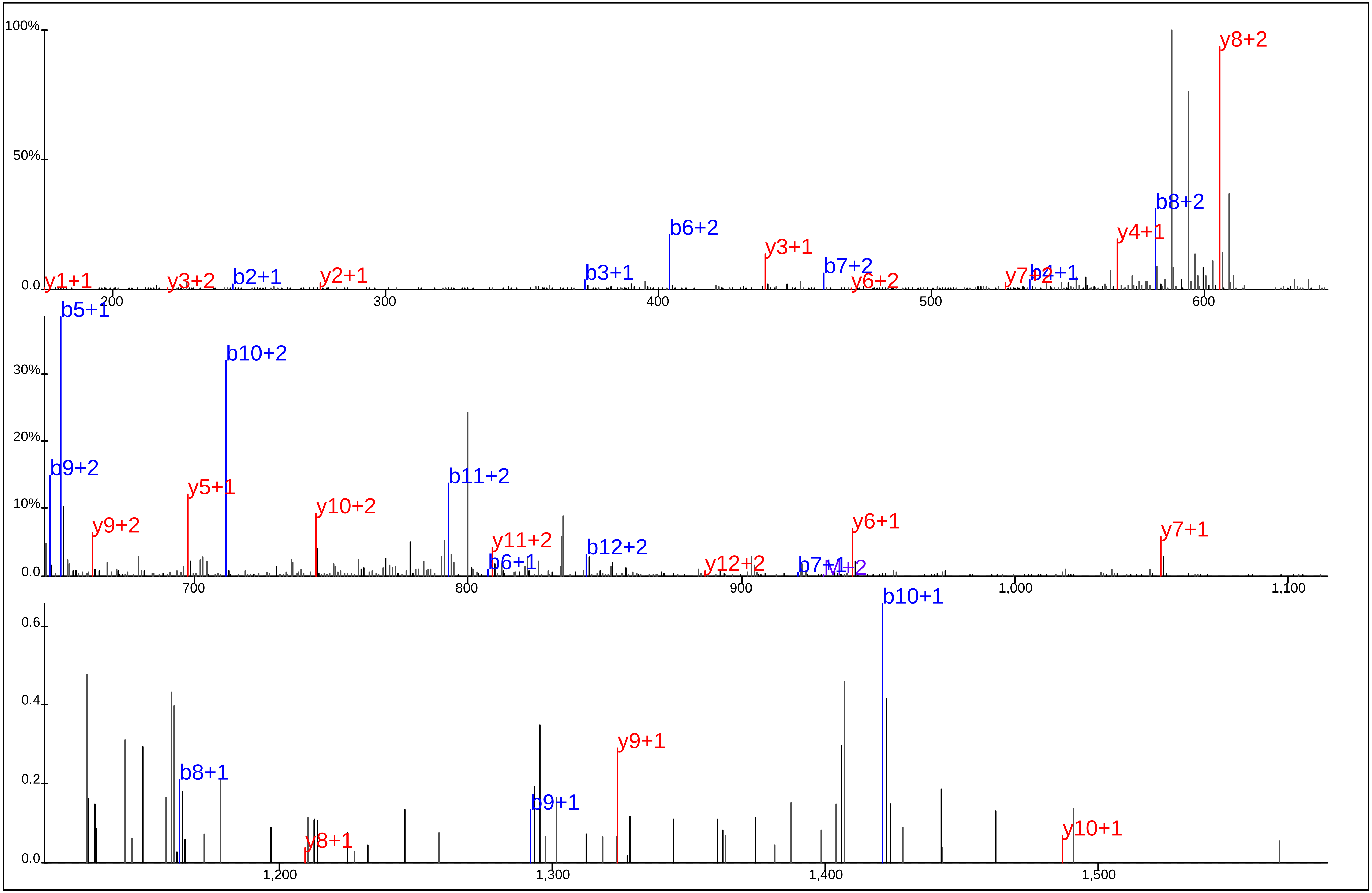

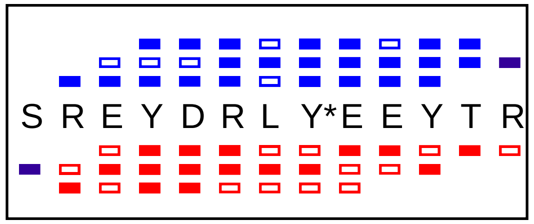

Protein Name: p85 alpha
gi Accession: 32455250
Precursor Mass: 620.6044+3
Delta Mass: 1.510 ppm
XCorr: 47.19

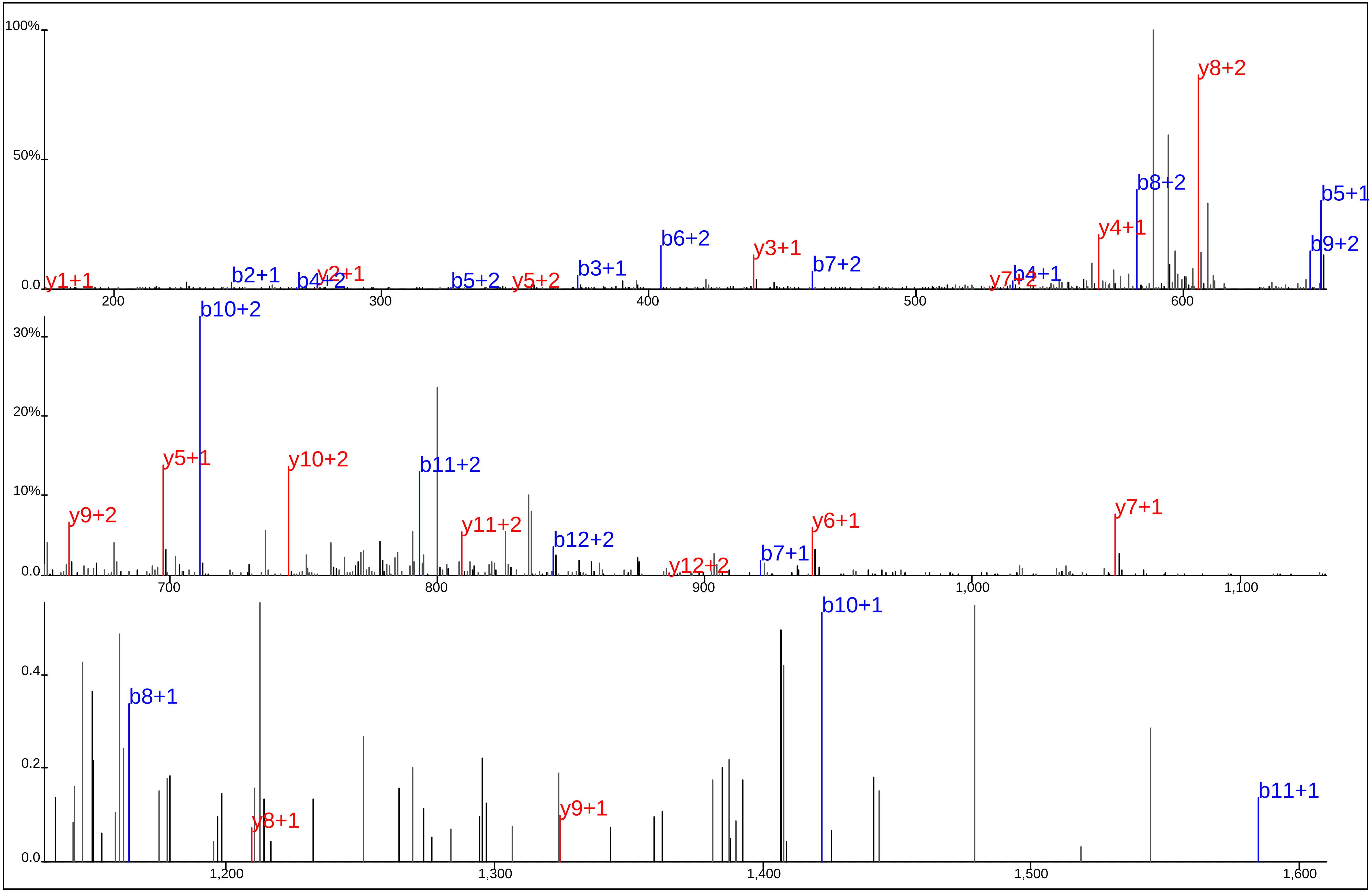

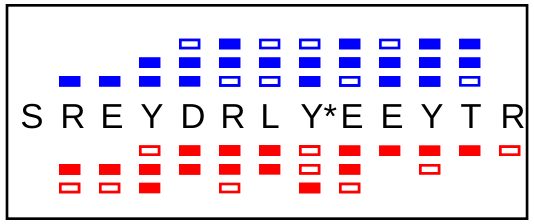

Protein Name: p85 alpha
gi Accession: 32455250
Precursor Mass: 620.6035+3
Delta Mass: 0.031 ppm
XCorr: 33.11

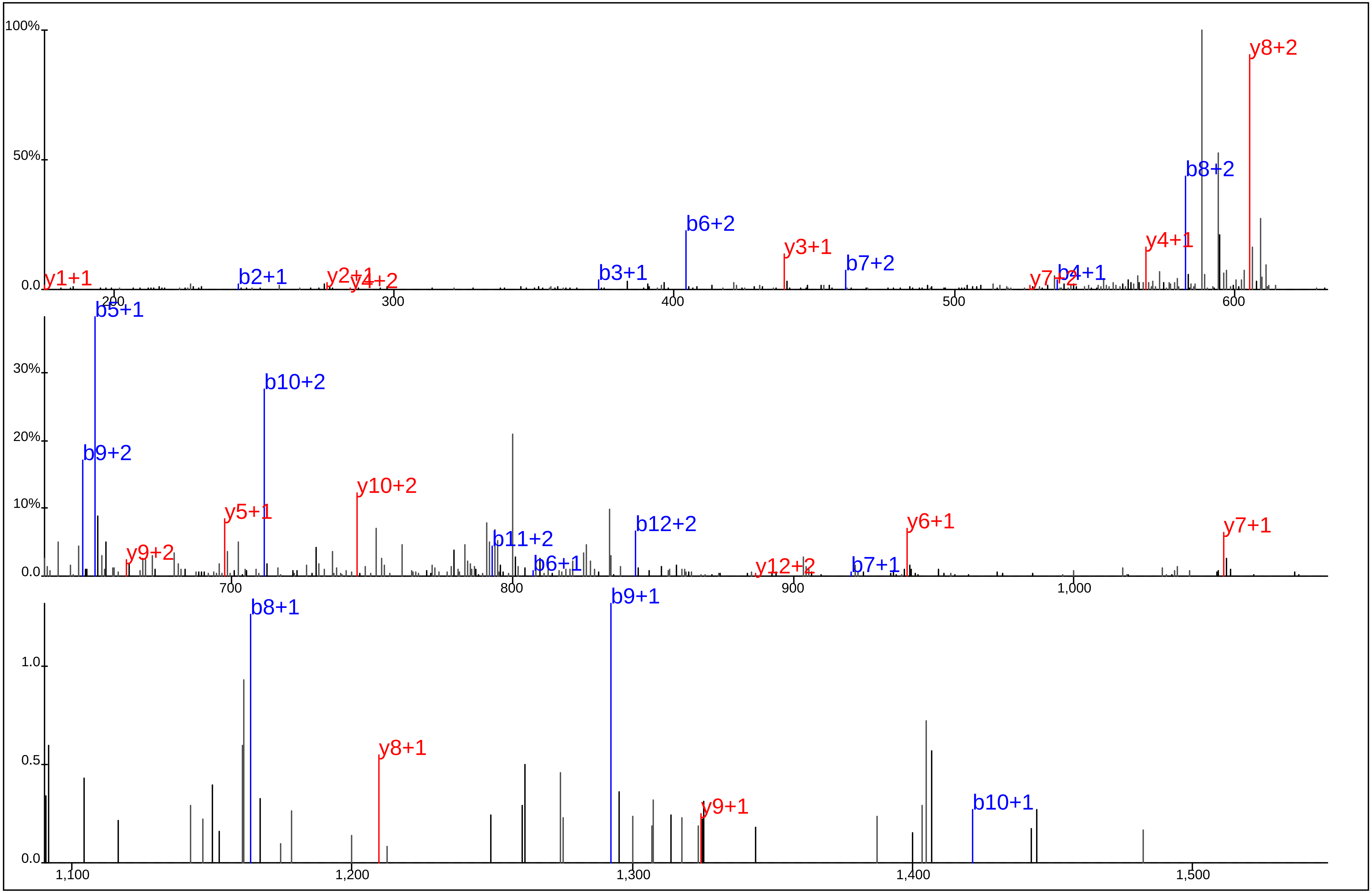

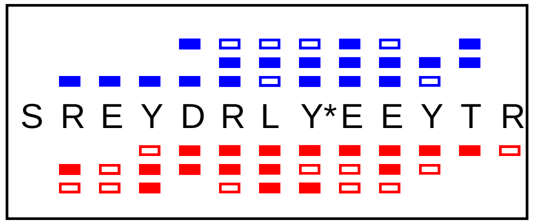

Protein Name: p85 alpha
gi Accession: 32455250
Precursor Mass: 620.6061+3
Delta Mass: 4.269 ppm
XCorr: 41.5

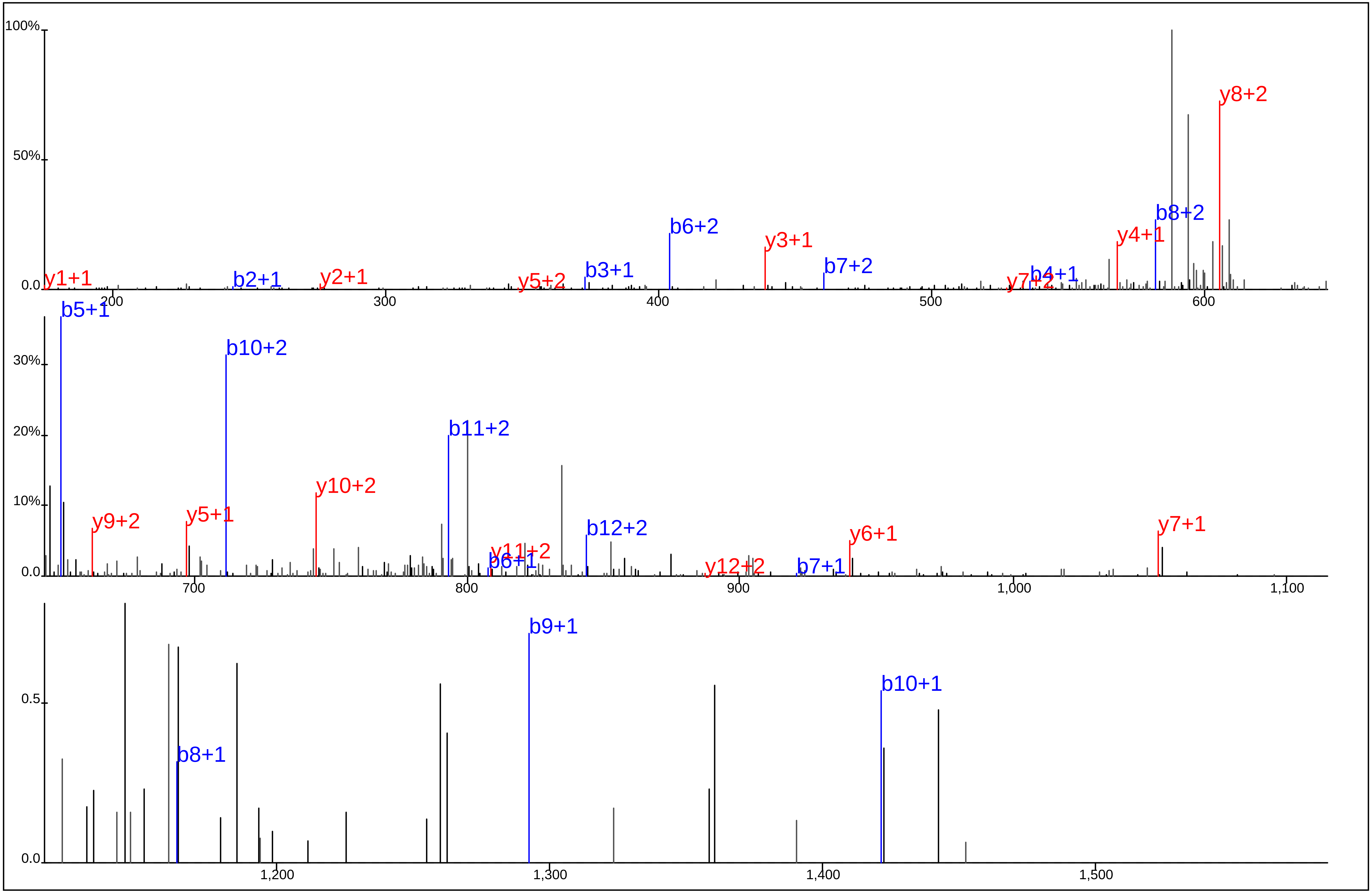

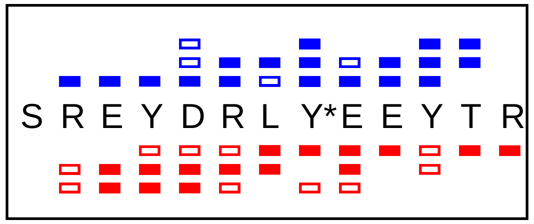

Protein Name: p85 alpha
gi Accession: 32455250
Precursor Mass: 620.6047+3
Delta Mass: 2.004 ppm
XCorr: 30.26

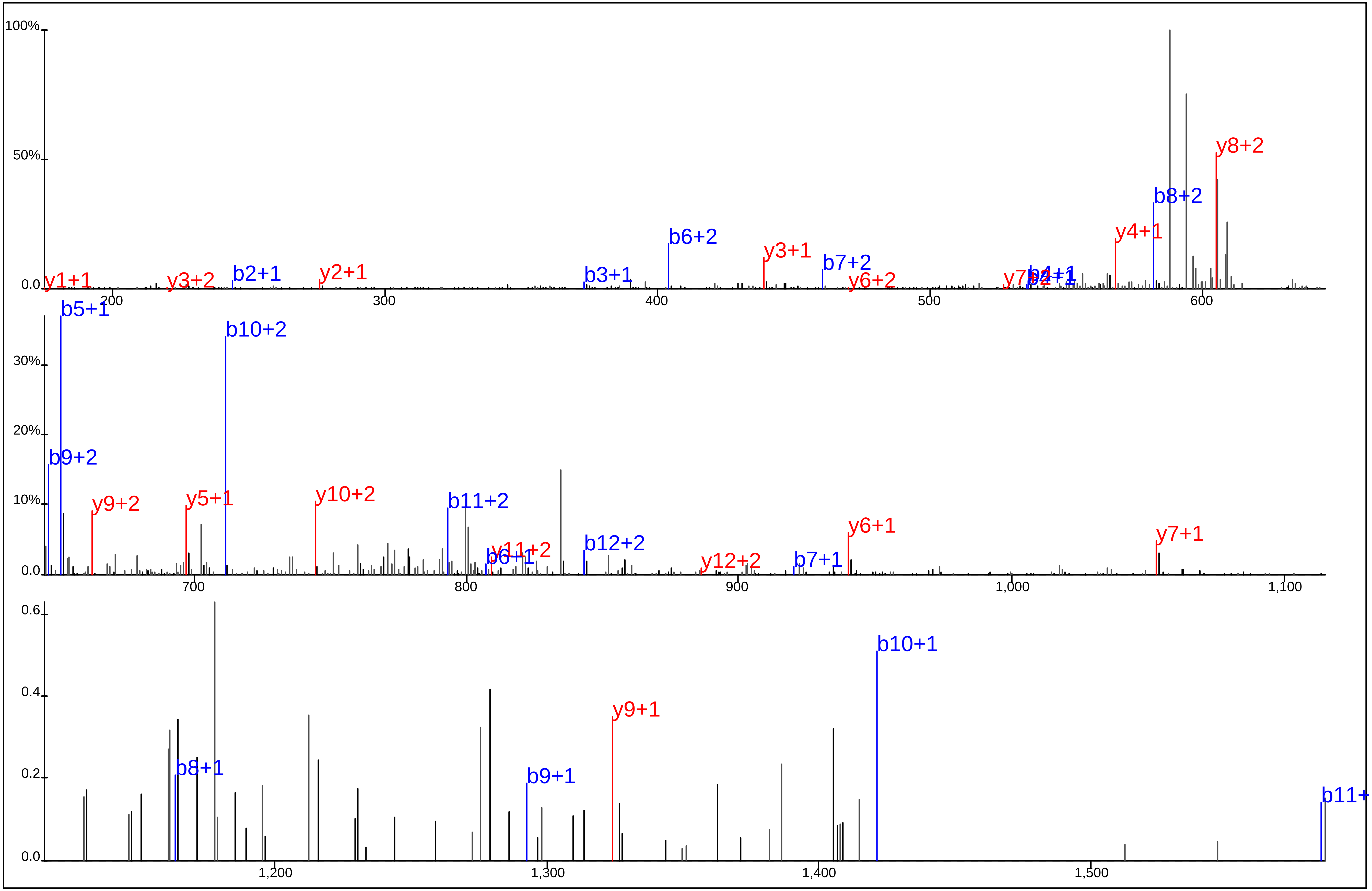

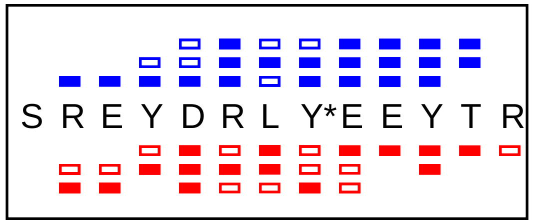

Protein Name:	p85 alpha
gi Accession:	32455250
Precursor Mass:	620.6044+3
Delta Mass:	1.414 ppm
XCorr:	30.5

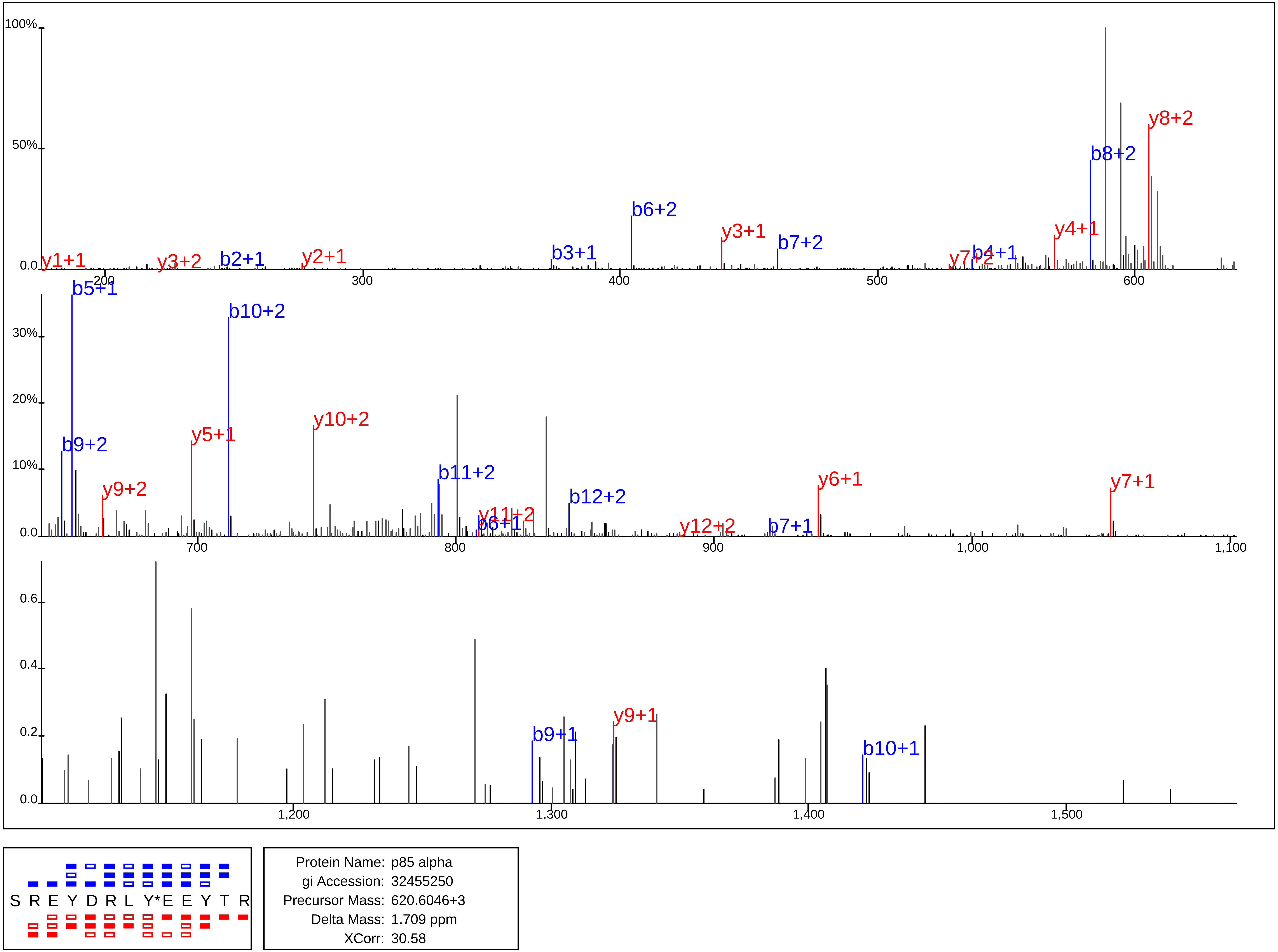

Protein Name: p85 alpha
gi Accession: 32455250
Precursor Mass: 620.6046+3
Delta Mass: 1.709 ppm
XCorr: 30.58

688 - p85alpha_3453245.pdf

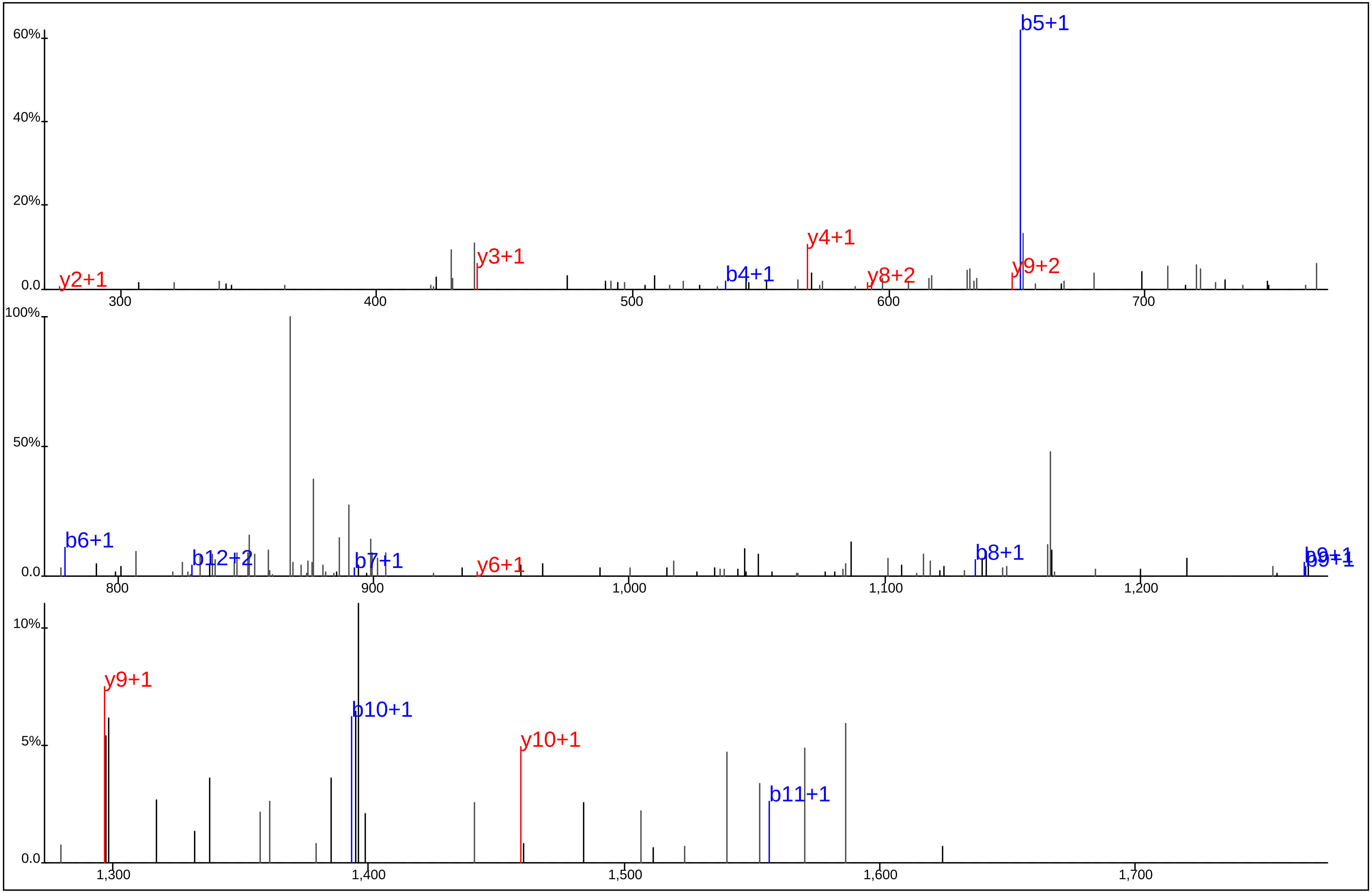

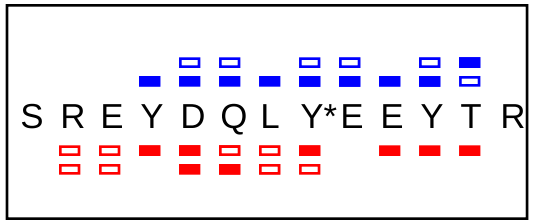

Protein Name: p85 beta
gi Accession: 4826908
Precursor Mass: 916.3834+2
Delta Mass: 3.276 ppm
XCorr: 2.1771

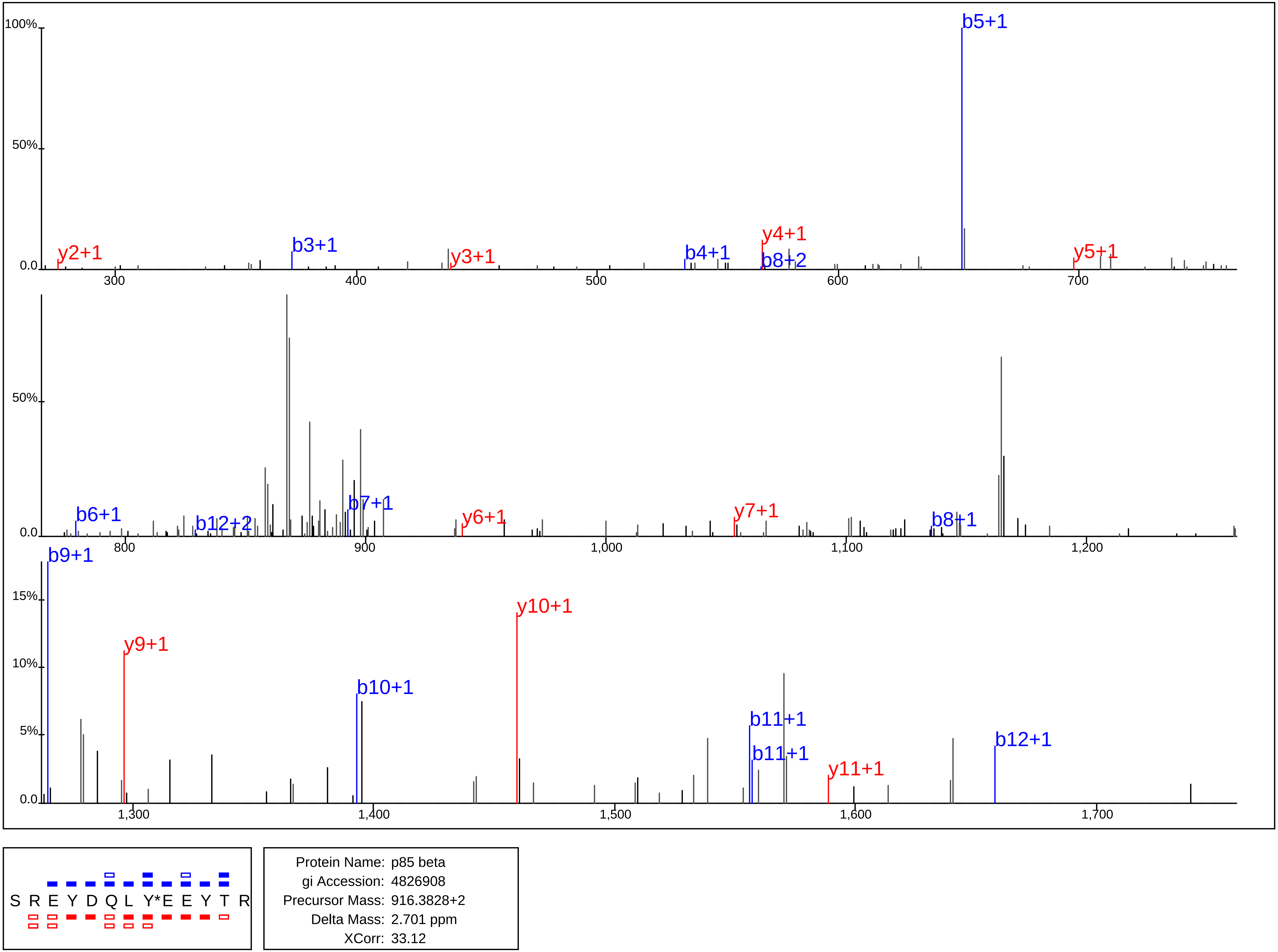

Protein Name: p85 beta
gi Accession: 4826908
Precursor Mass: 916.3828+2
Delta Mass: 2.701 ppm
XCorr: 33.12

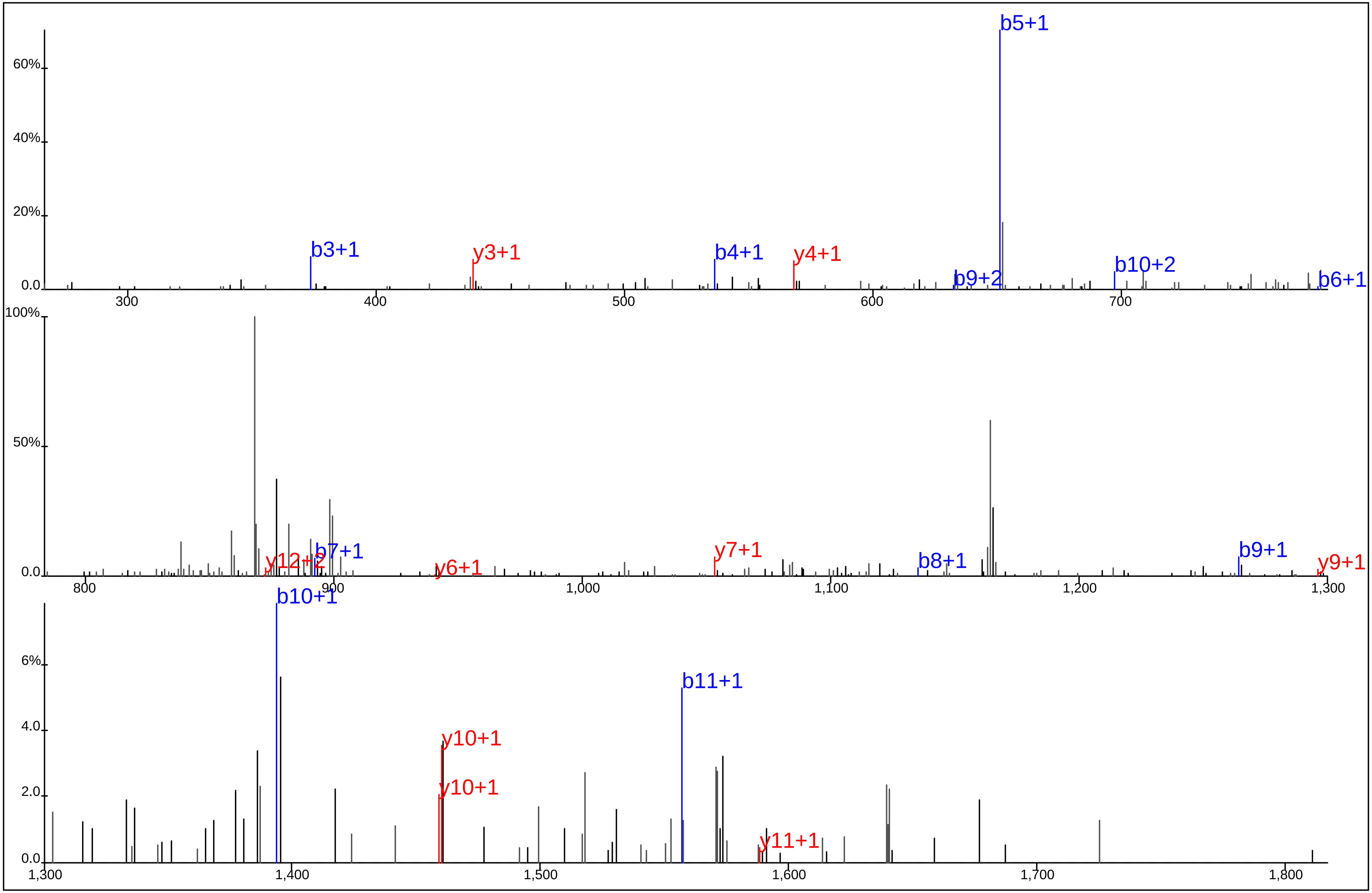

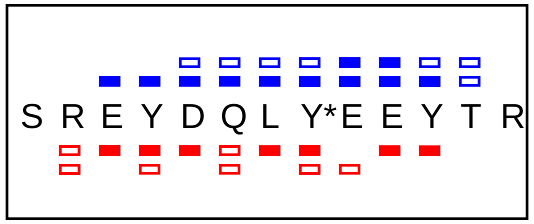

Protein Name:	p85 beta
gi Accession:	4826908
Precursor Mass:	916.3882+2
Delta Mass:	8.630 ppm
XCorr:	29.92

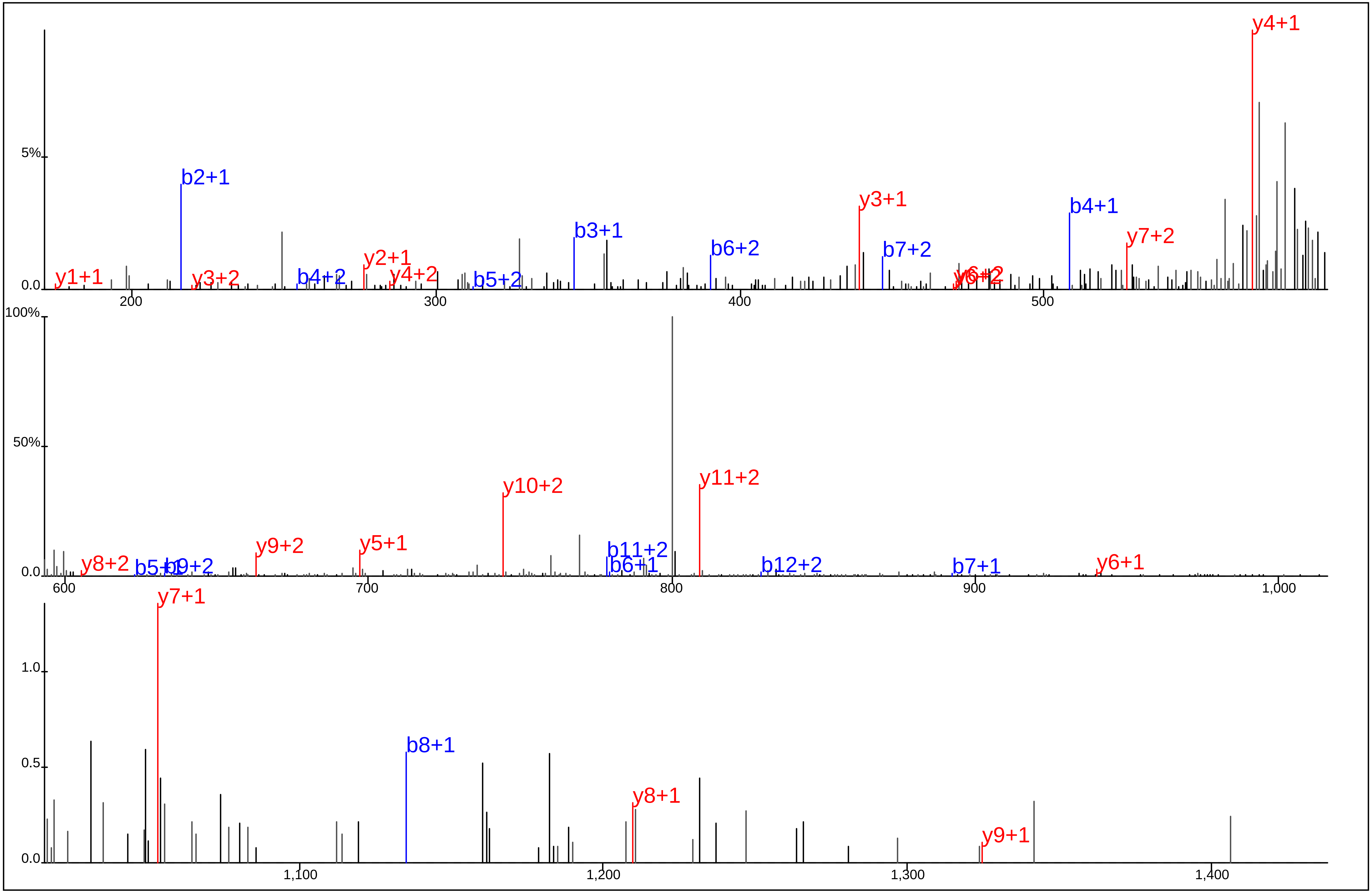

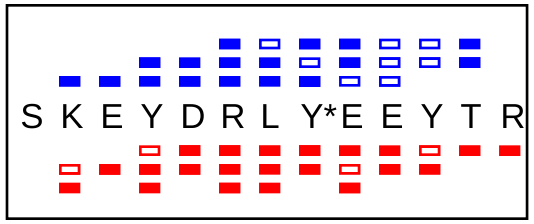

Protein Name: p85 gamma
gi Accession: 12643788
Precursor Mass: 611.2702+3
Delta Mass: 3.422 ppm
XCorr: 34.32

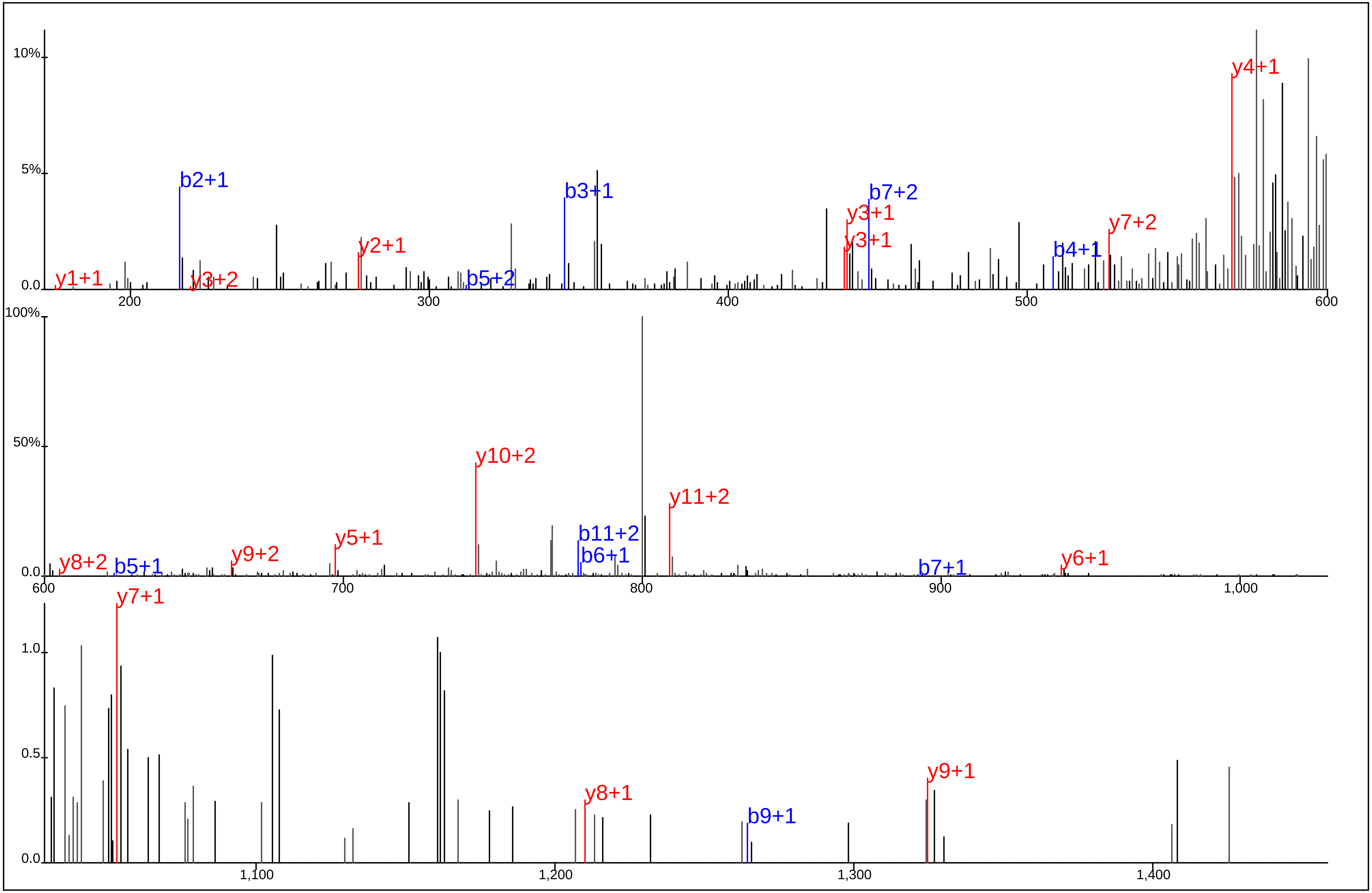

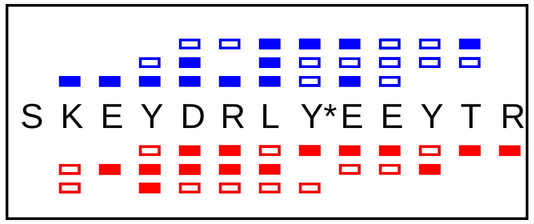

Protein Name: p85 gamma
gi Accession: 12643788
Precursor Mass: 611.2681+3
Delta Mass: 0.073 ppm
XCorr: 24.09

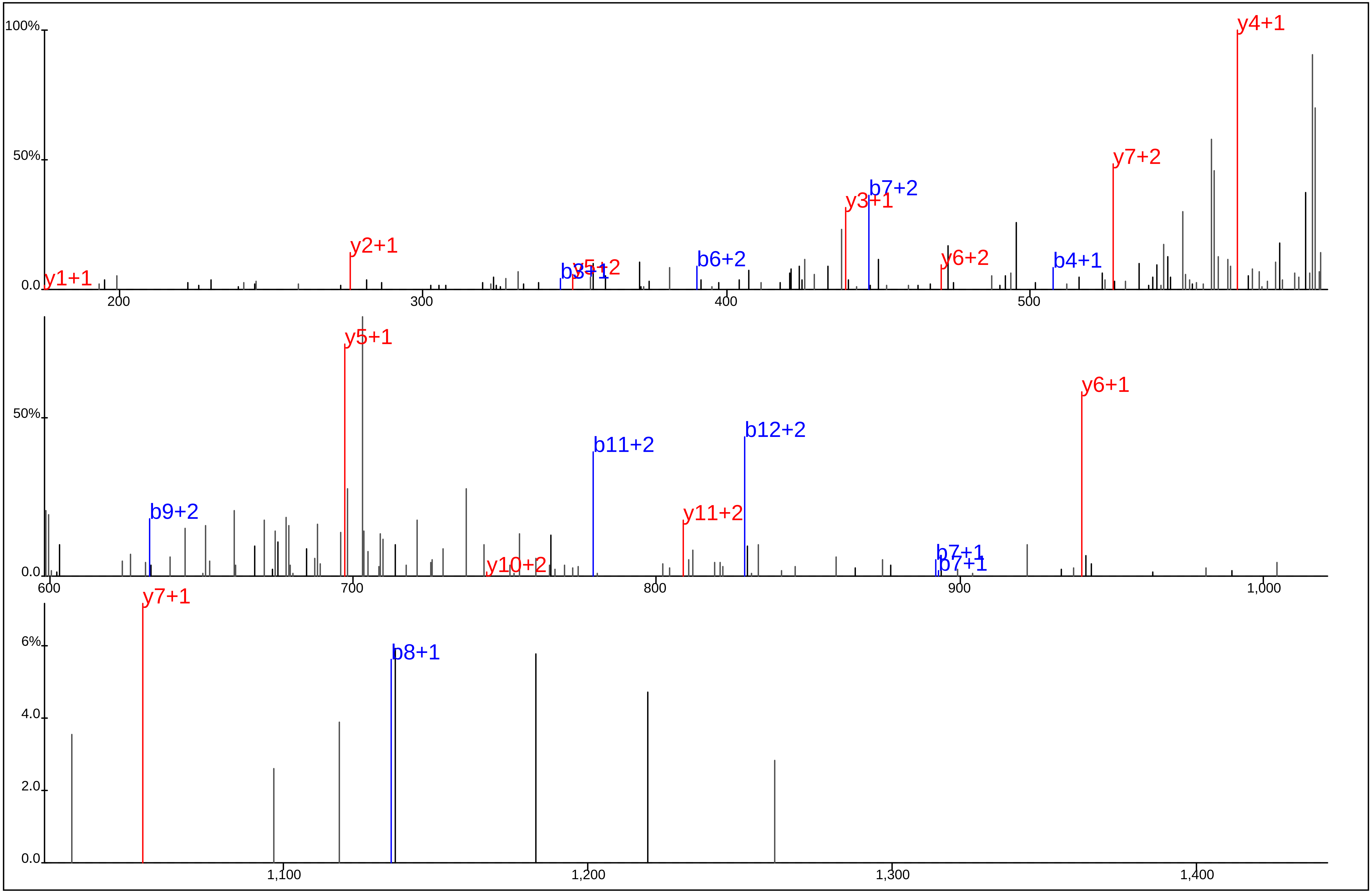

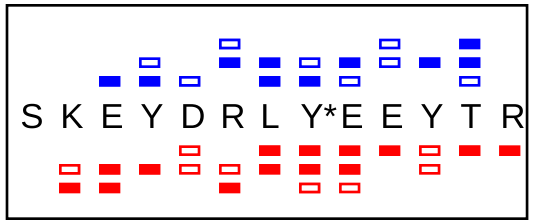

Protein Name:	p85 gamma
gi Accession:	12643788
Precursor Mass:	611.2580+3
Delta Mass:	16.570 ppm
XCorr:	28.54

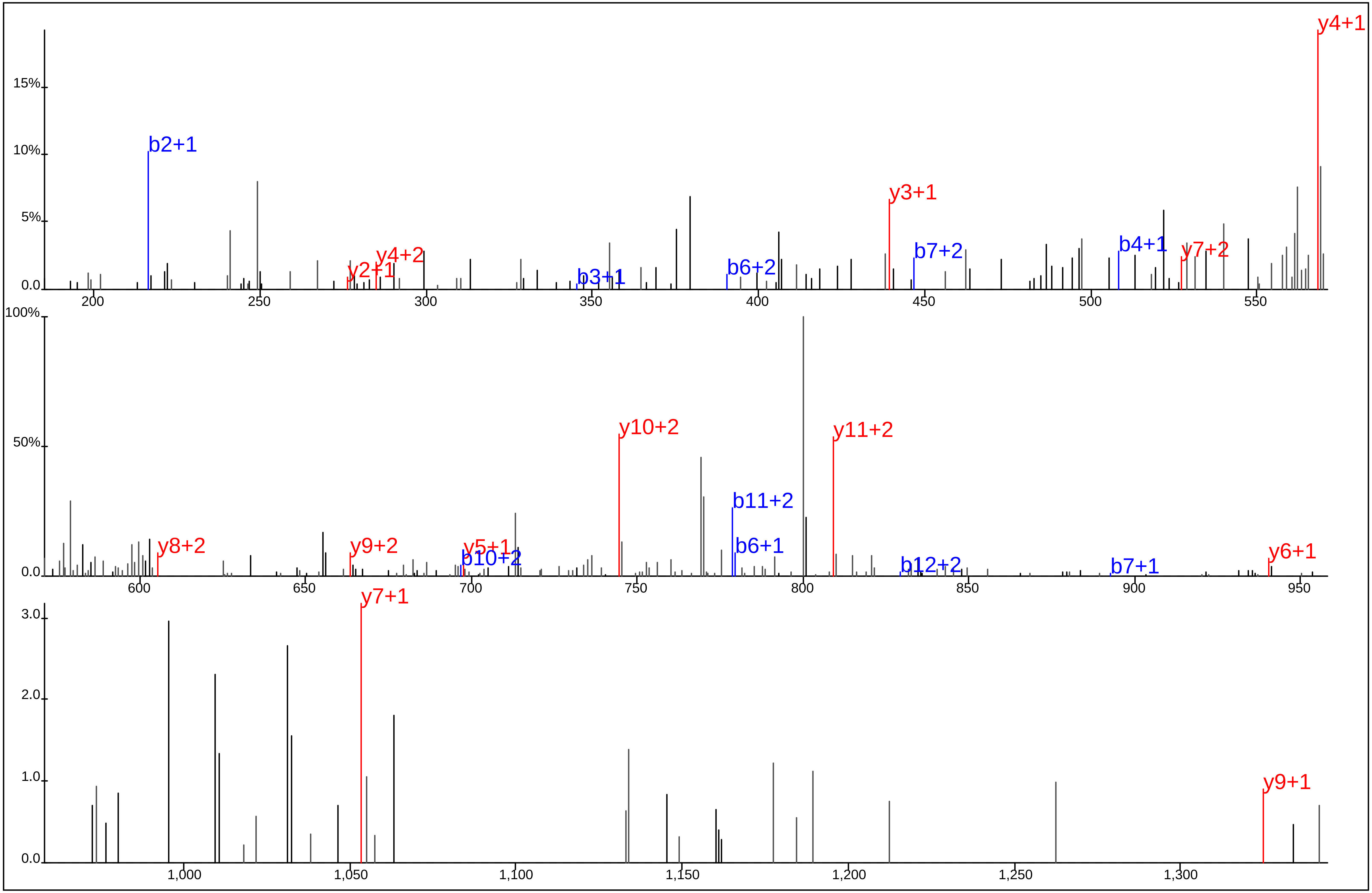

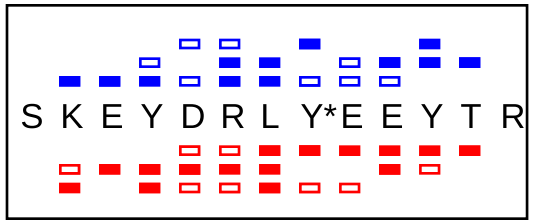

Protein Name:	p85 gamma
gi Accession:	12643788
Precursor Mass:	611.2683+3
Delta Mass:	0.327 ppm
XCorr:	14.1

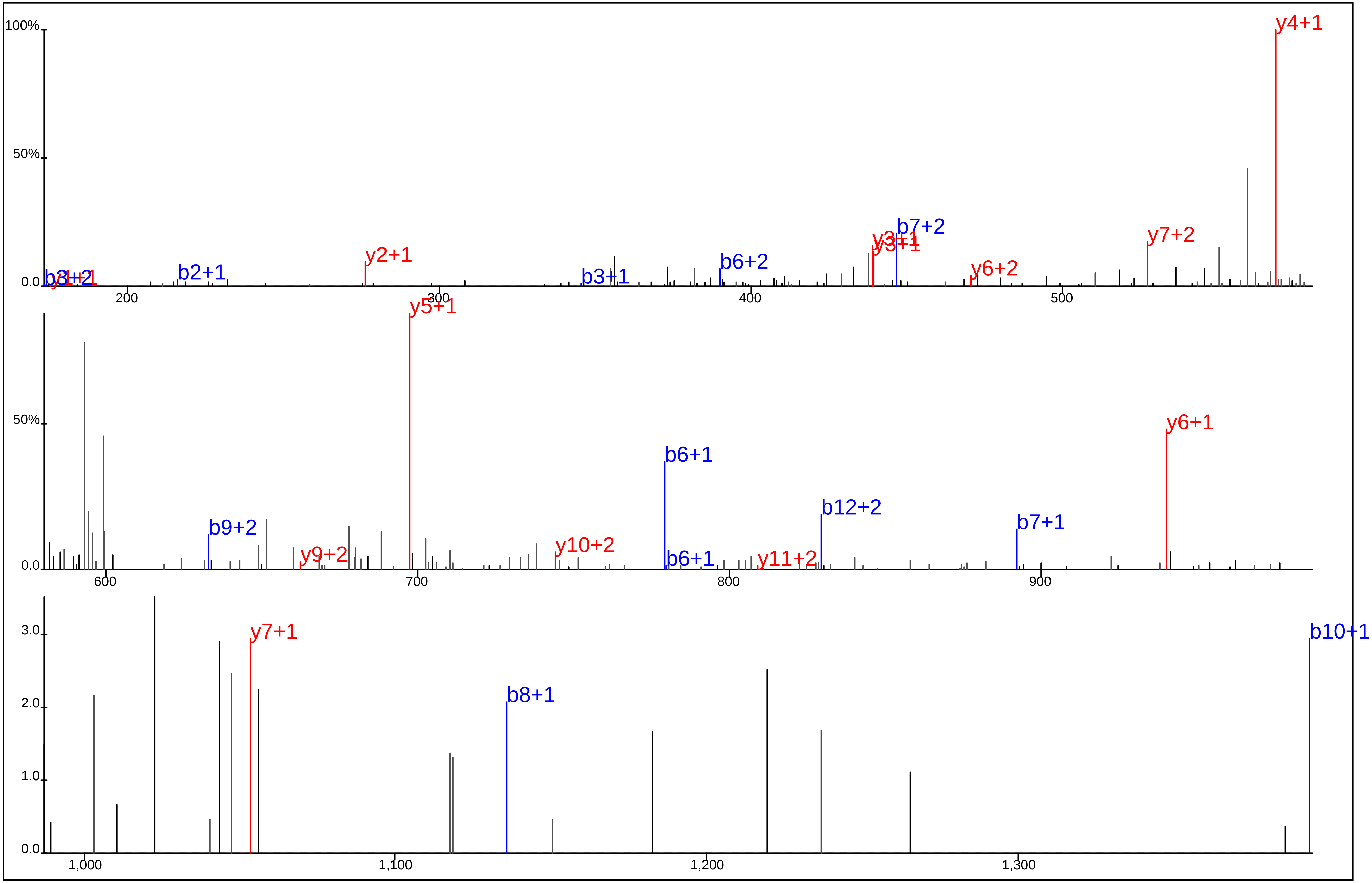

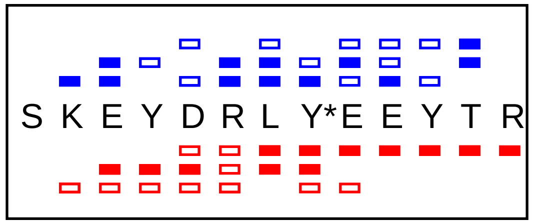

Protein Name:	p85 gamma
gi Accession:	12643788
Precursor Mass:	611.2583+3
Delta Mass:	16.067 ppm
XCorr:	33.12

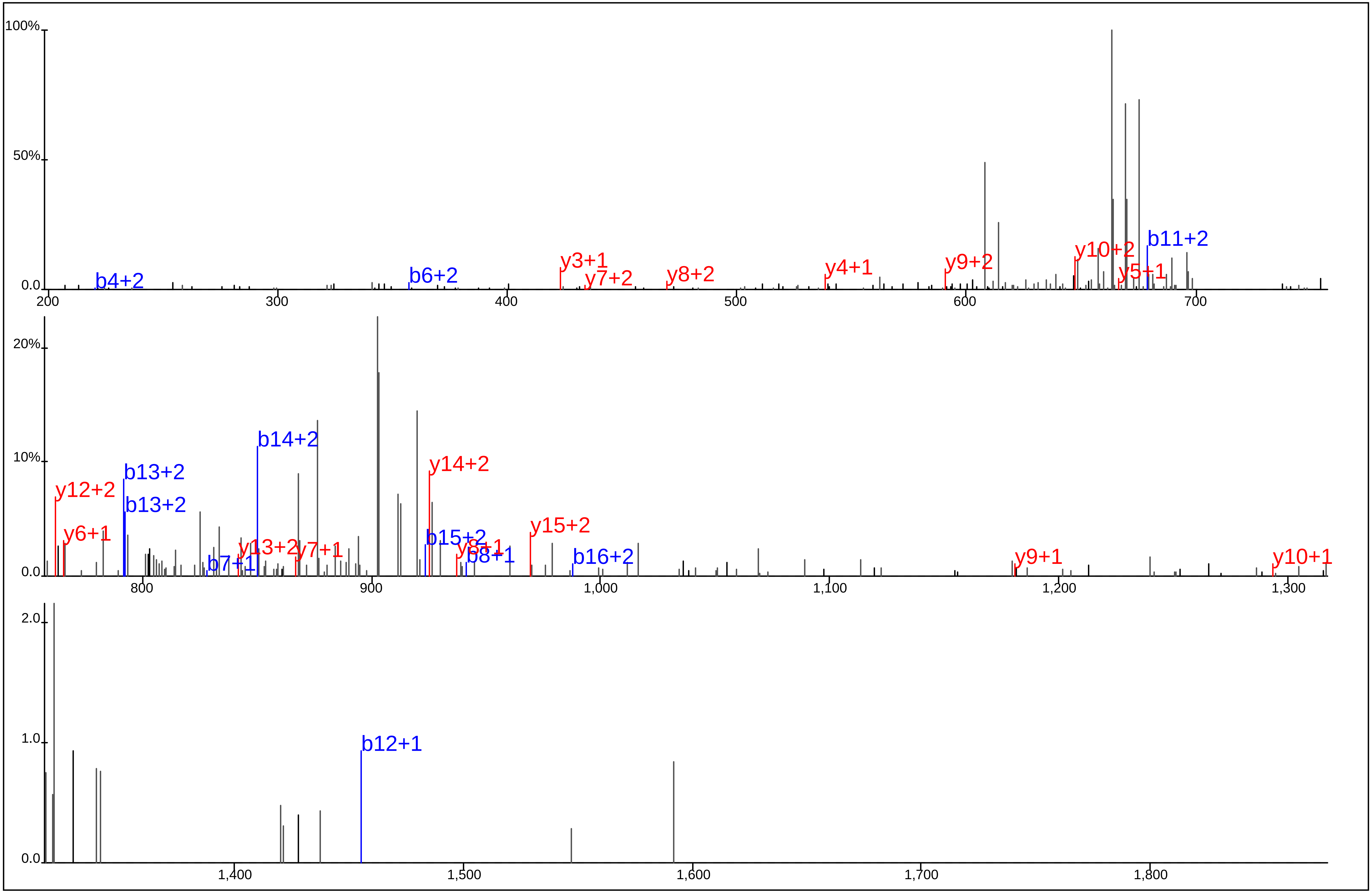

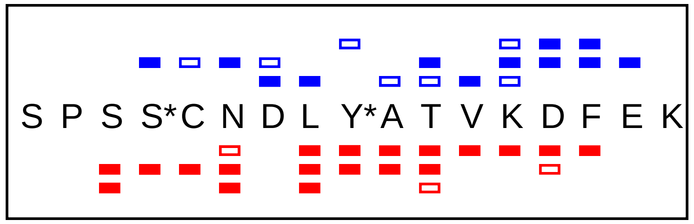

Protein Name: PAG
gi Accession: 7020925
Precursor Mass: 707.6168+3
Delta Mass: 3.480 ppm
XCorr: 2.9444

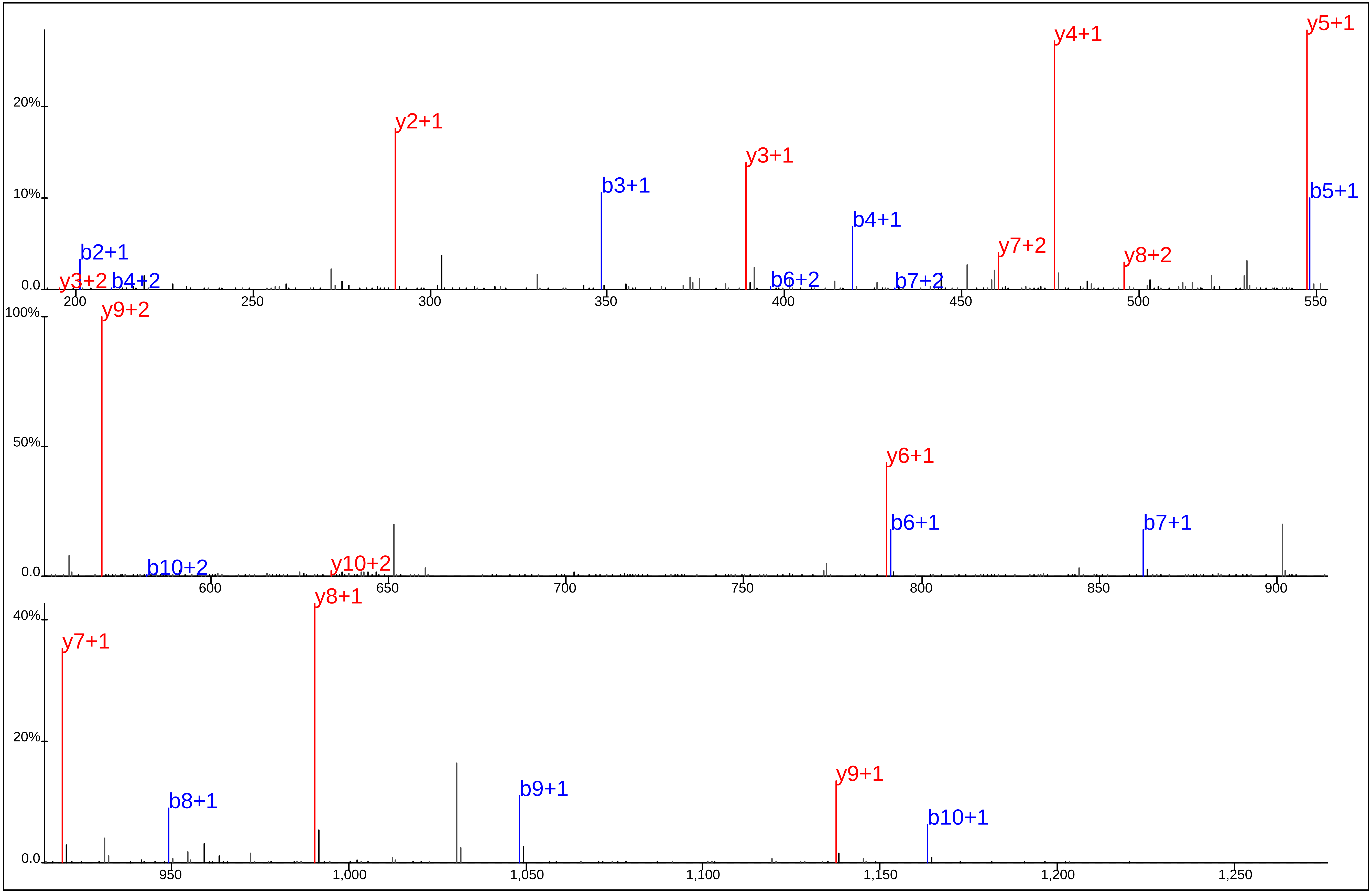

A E F A E Y*A S V D R

Protein Name: PAG
gi Accession: 7020925
Precursor Mass: 669.2740+2
Delta Mass: 0.061 ppm
XCorr: 77.31

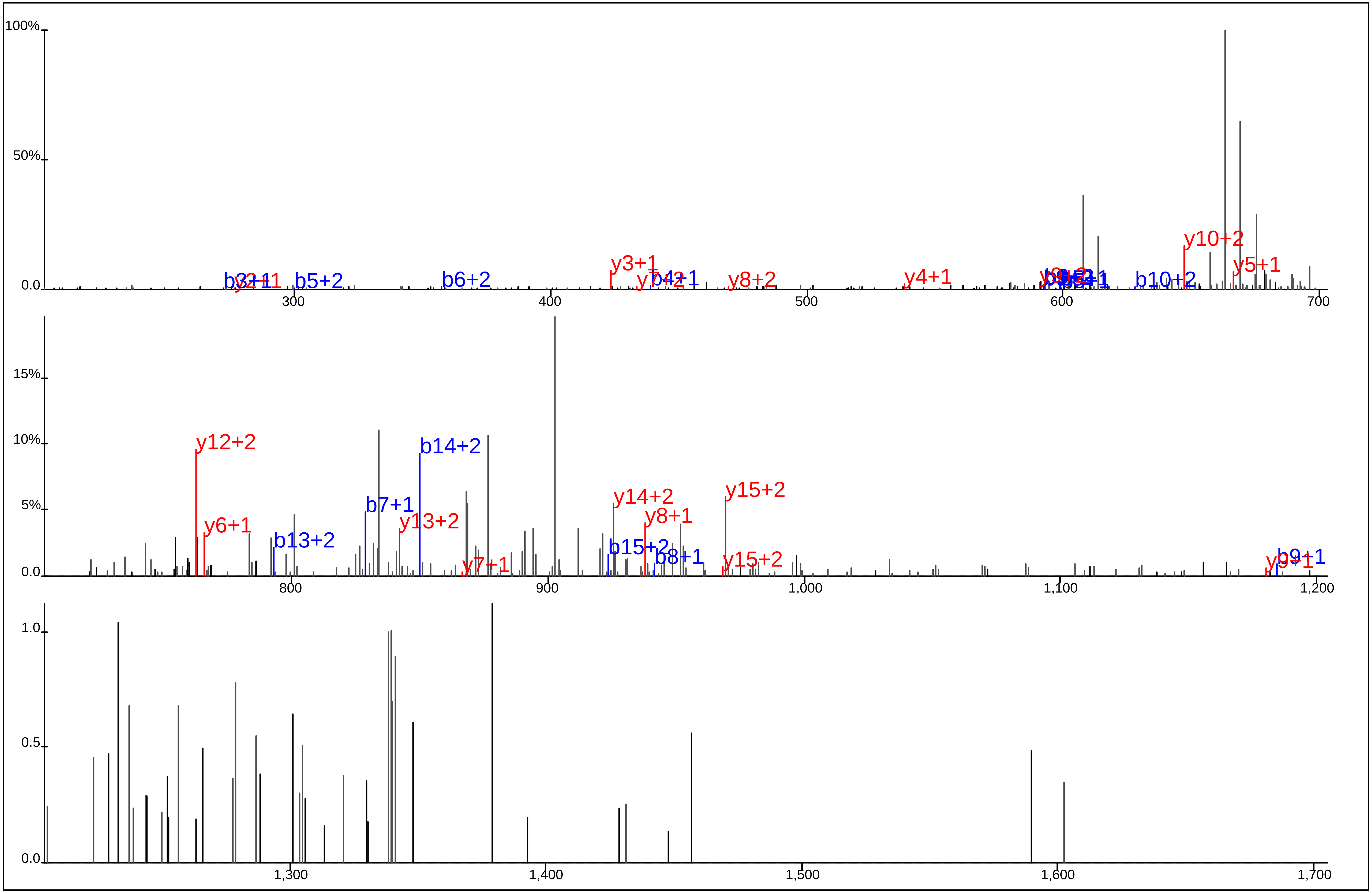

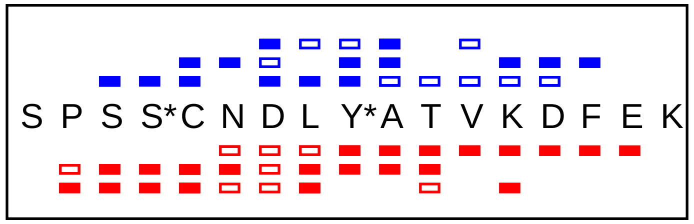

Protein Name: PAG
gi Accession: 7020925
Precursor Mass: 707.6201+3
Delta Mass: 8.156 ppm
XCorr: 16.72

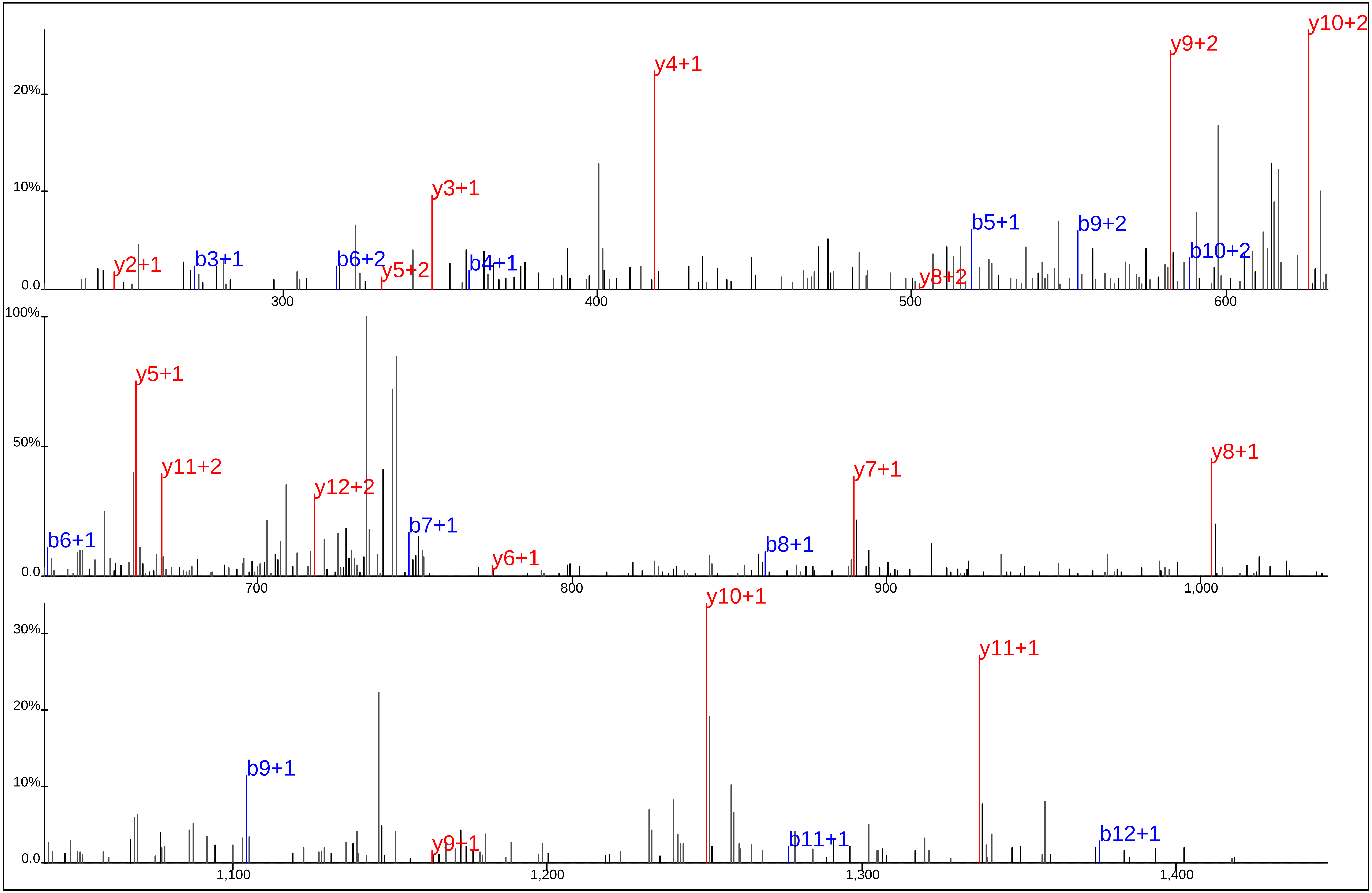

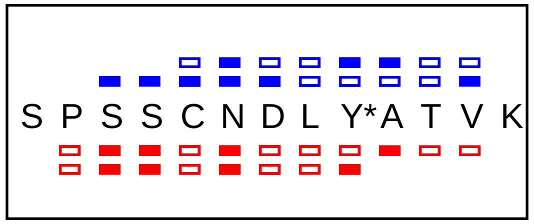

Protein Name: PAG
gi Accession: 7020925
Precursor Mass: 761.3204+2
Delta Mass: 2.963 ppm
XCorr: 38.85

700 - PAG_3433946.pdf

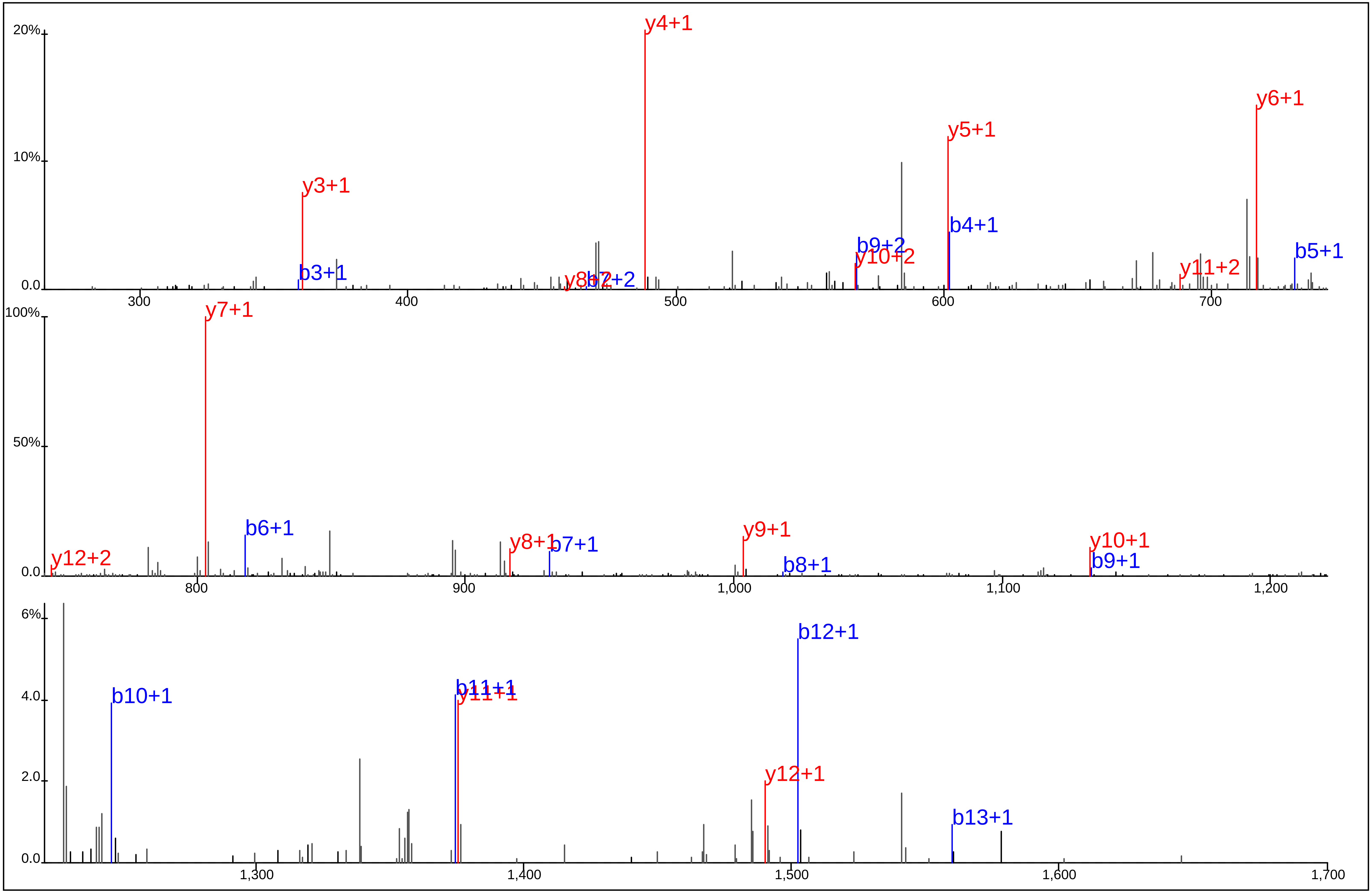

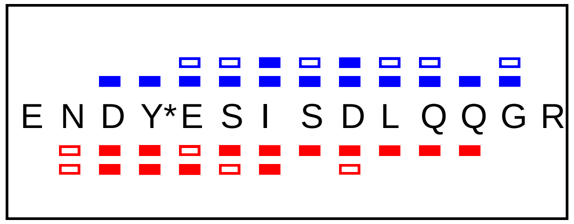

Protein Name: PAG
gi Accession: 7020925
Precursor Mass: 867.3557+2
Delta Mass: 1.527 ppm
XCorr: 72.23

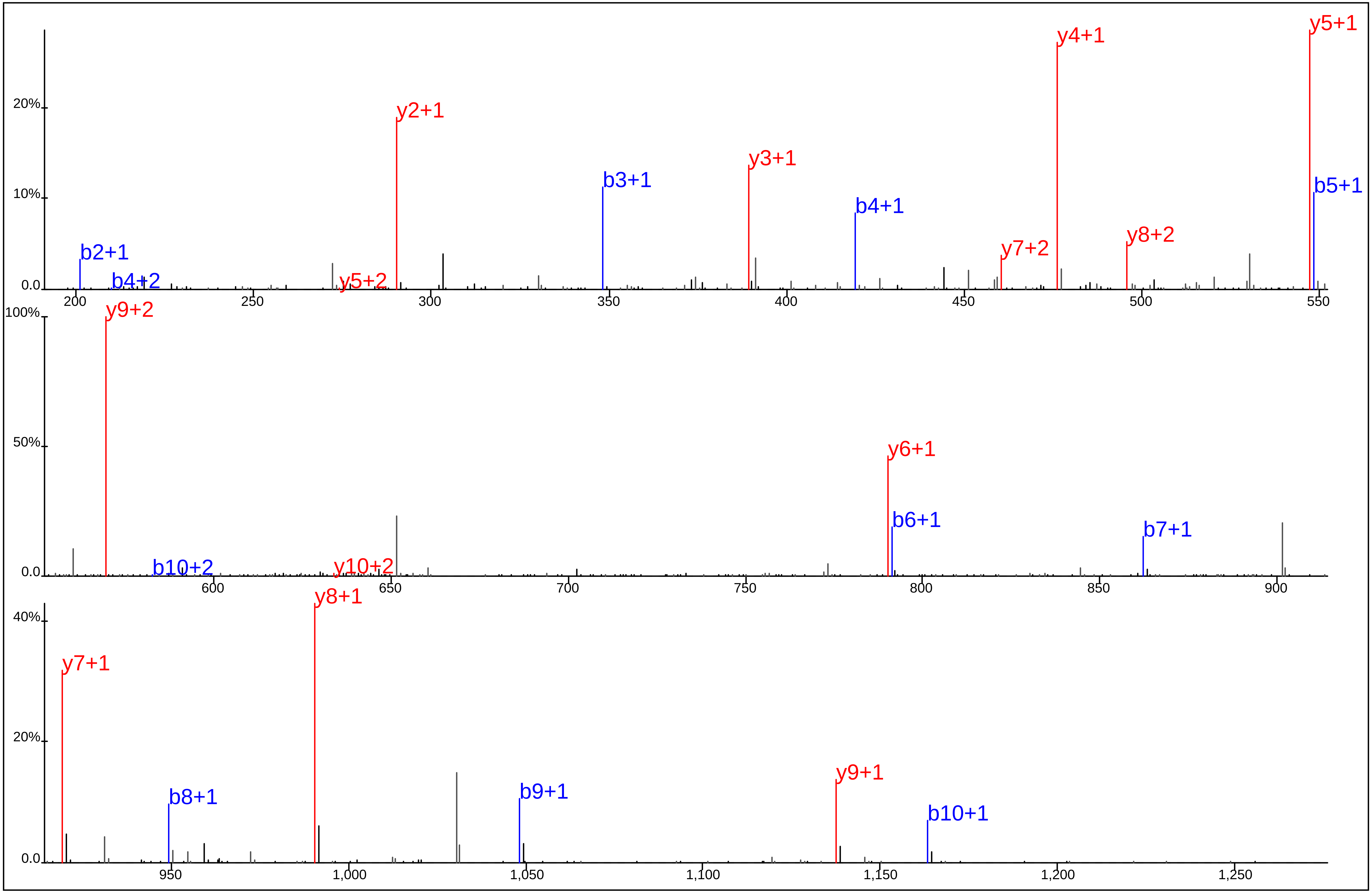

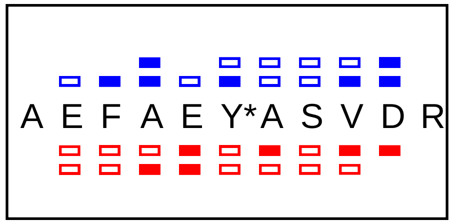

Protein Name: PAG
gi Accession: 7020925
Precursor Mass: 669.2745+2
Delta Mass: 0.574 ppm
XCorr: 81.32

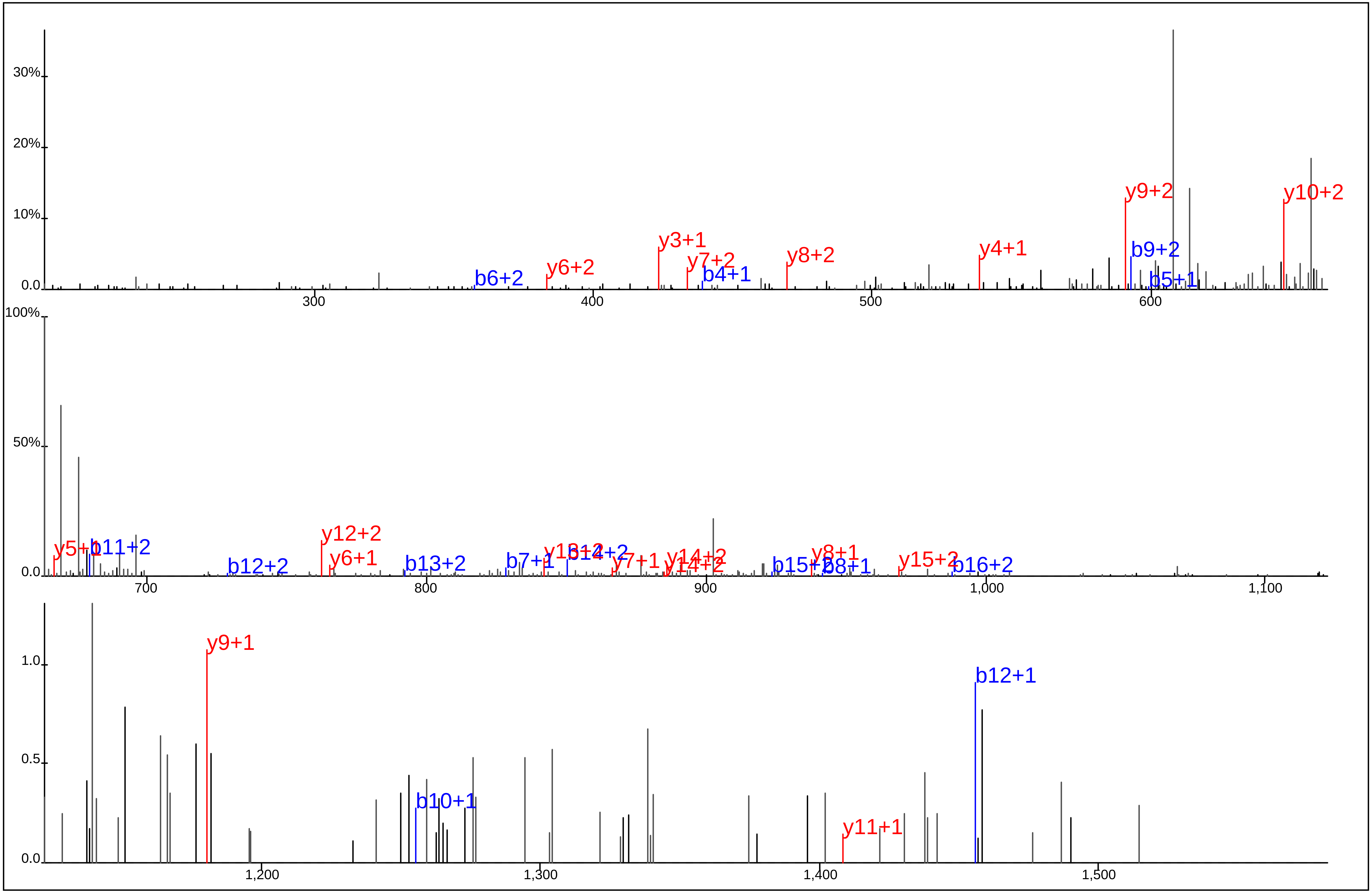

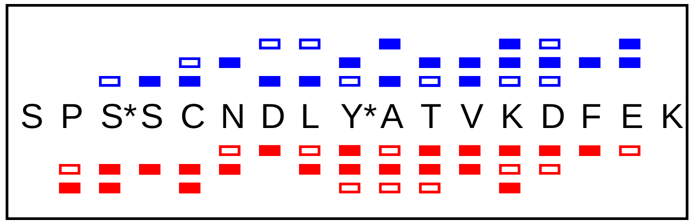

Protein Name:	PAG
gi Accession:	7020925
Precursor Mass:	707.6172+3
Delta Mass:	4.096 ppm
XCorr:	26.71

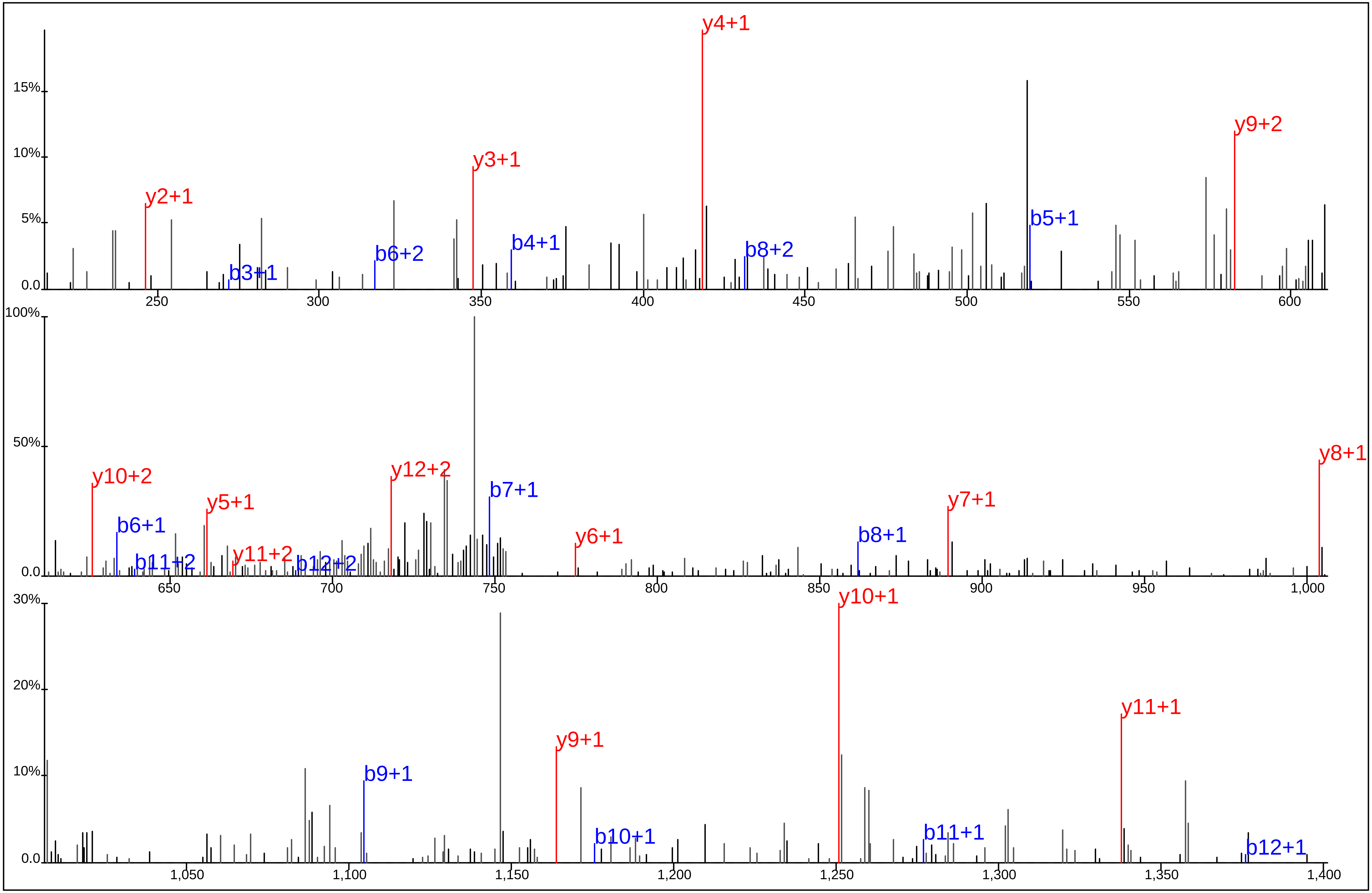

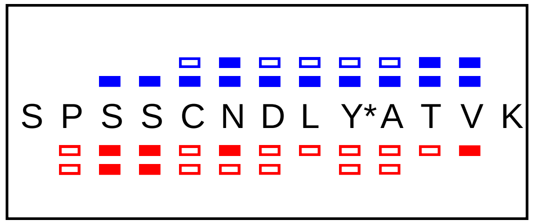

Protein Name: PAG
gi Accession: 7020925
Precursor Mass: 761.3217+2
Delta Mass: 4.566 ppm
XCorr: 61.17

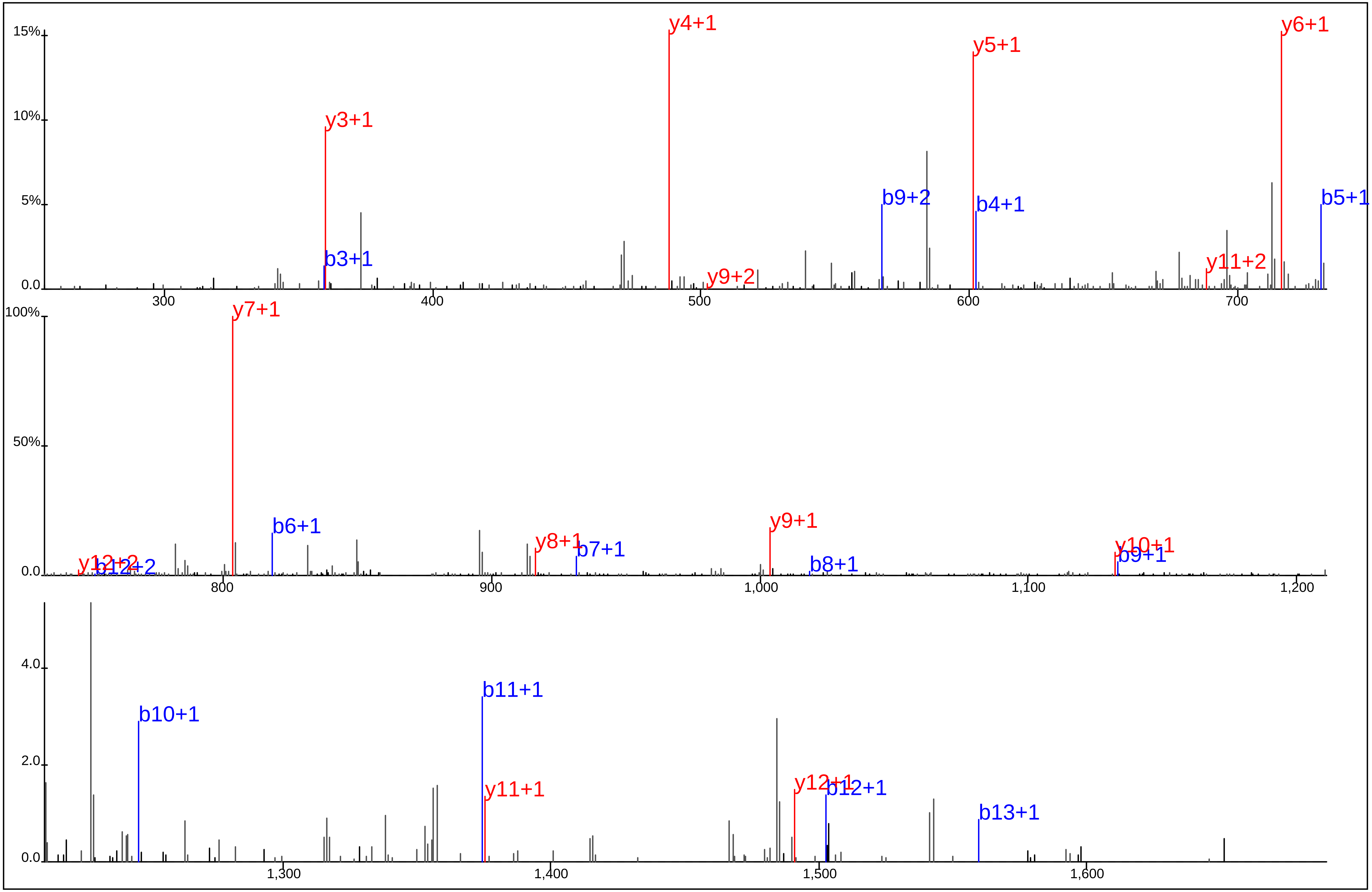

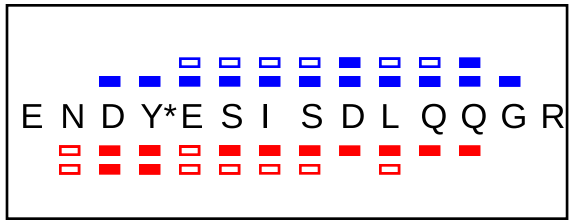

Protein Name: PAG
gi Accession: 7020925
Precursor Mass: 867.3566+2
Delta Mass: 2.583 ppm
XCorr: 71.57

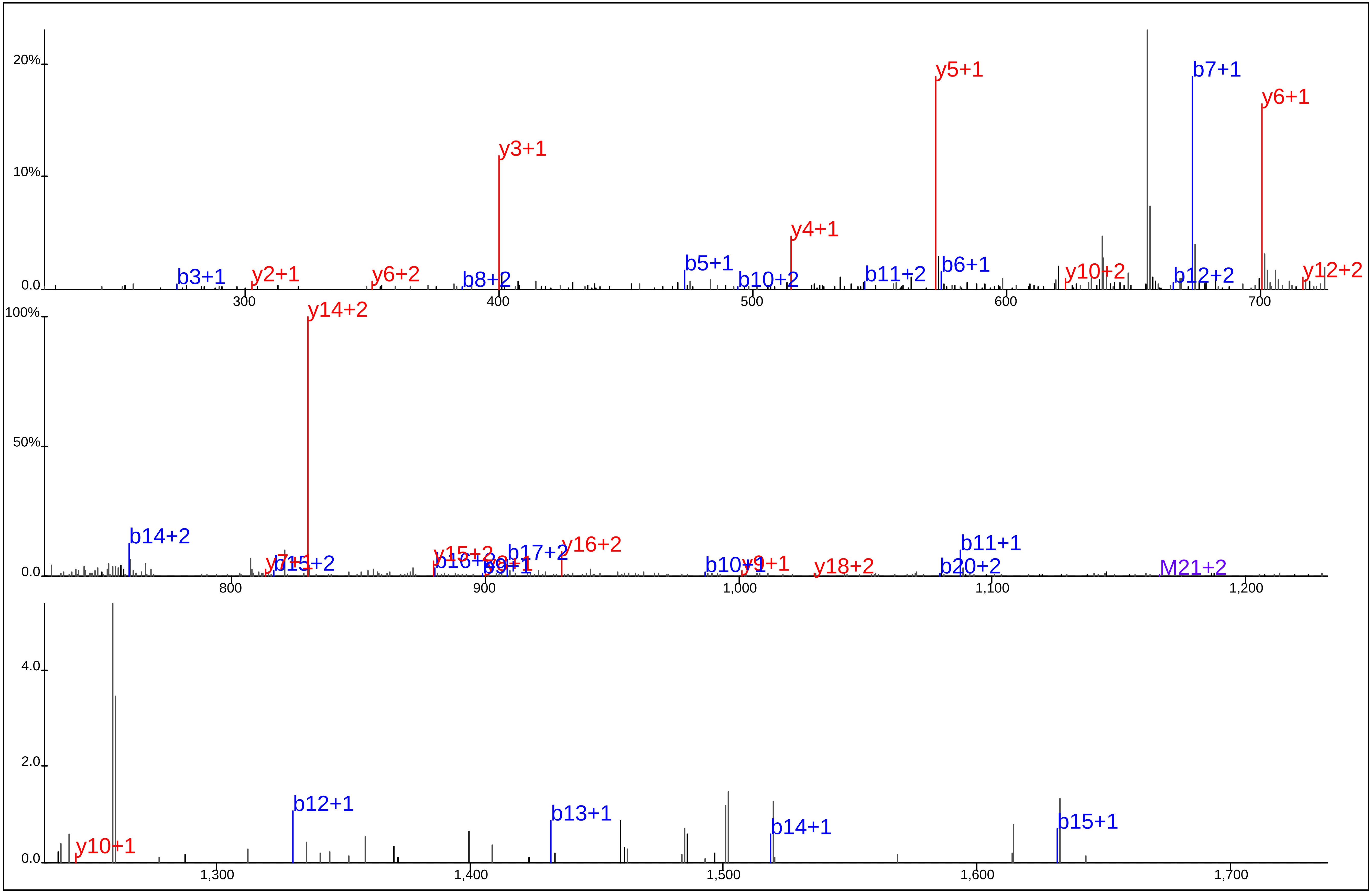

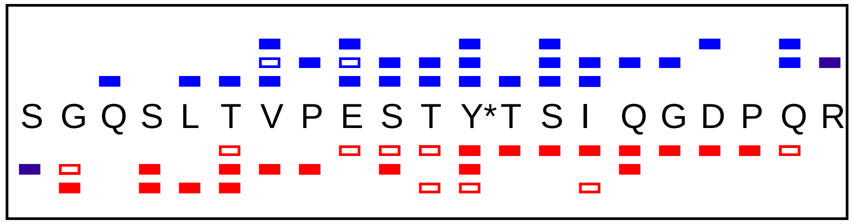

Protein Name:	PAG
gi Accession:	7020925
Precursor Mass:	777.6895+3
Delta Mass:	1.621 ppm
XCorr:	35.28

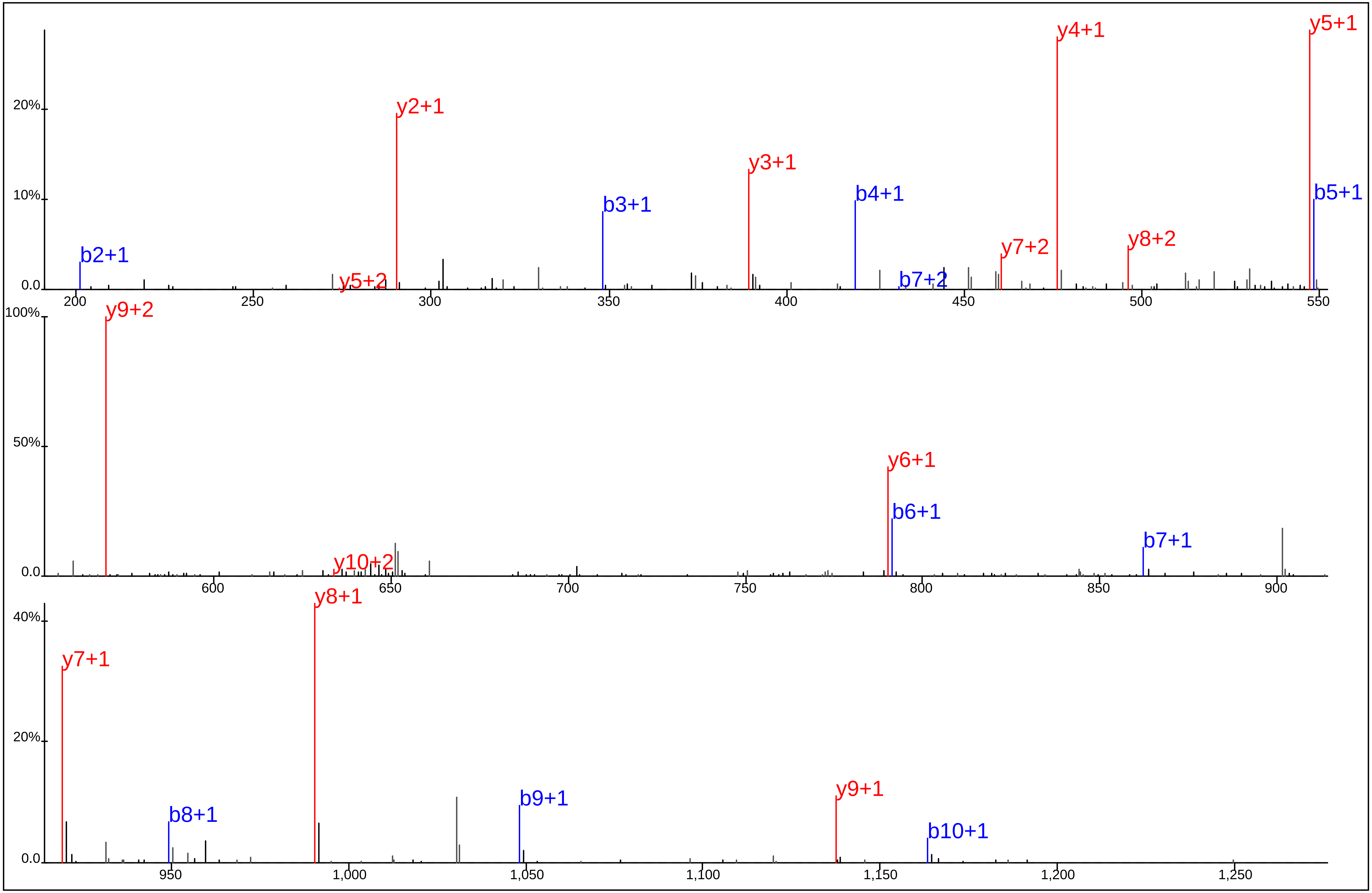

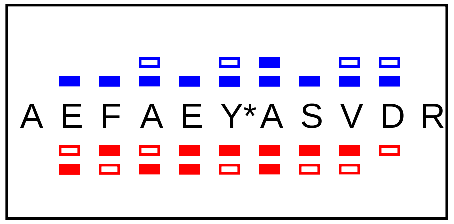

Protein Name: PAG
gi Accession: 7020925
Precursor Mass: 669.2748+2
Delta Mass: 1.127 ppm
XCorr: 77.03

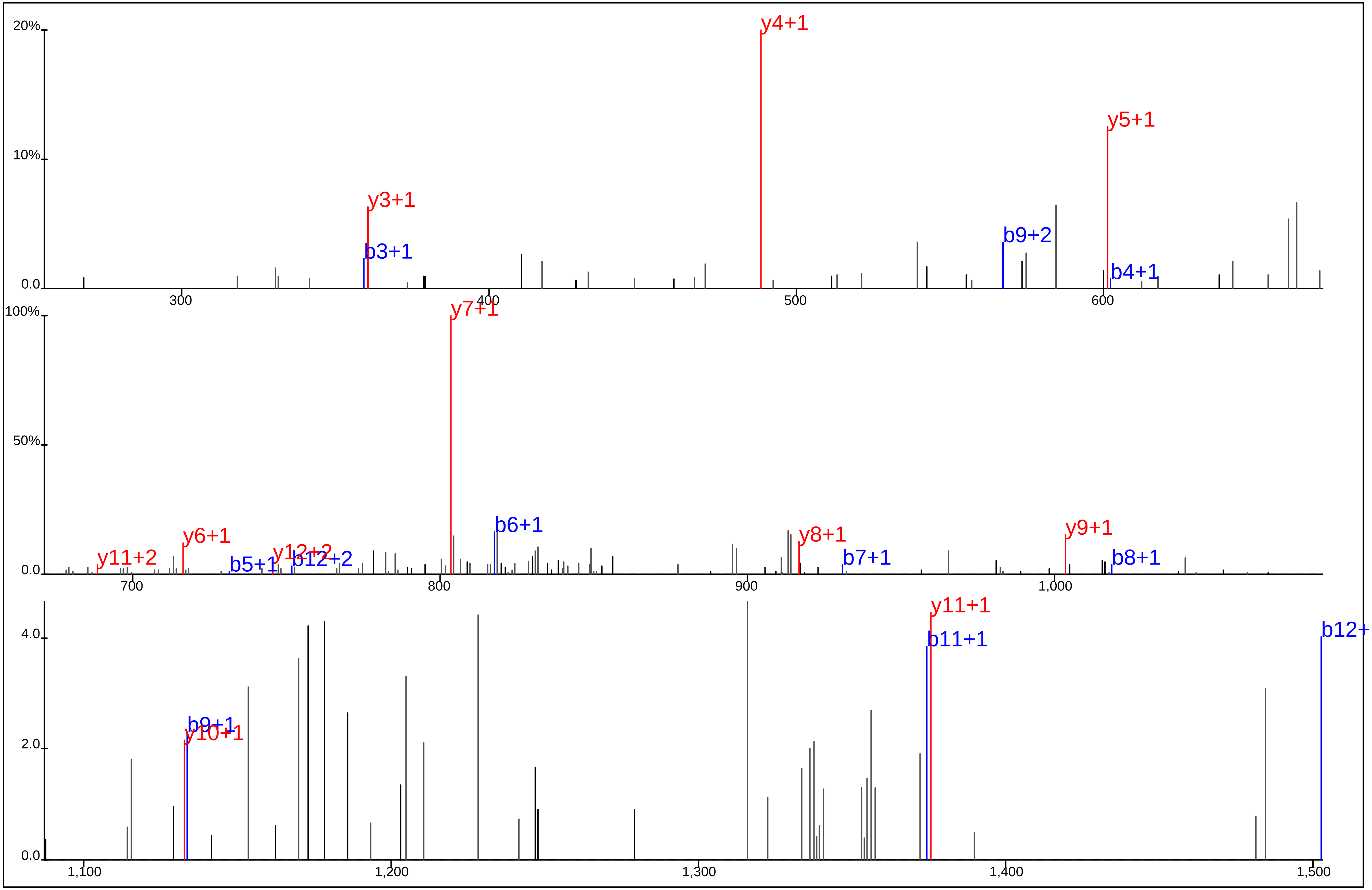

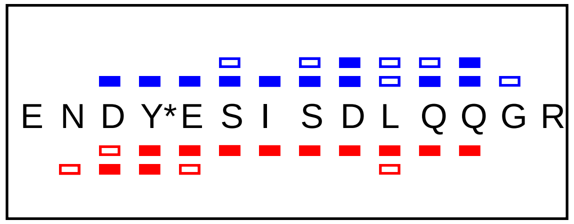

Protein Name:	PAG
gi Accession:	7020925
Precursor Mass:	867.3591+2
Delta Mass:	5.467 ppm
XCorr:	51.08

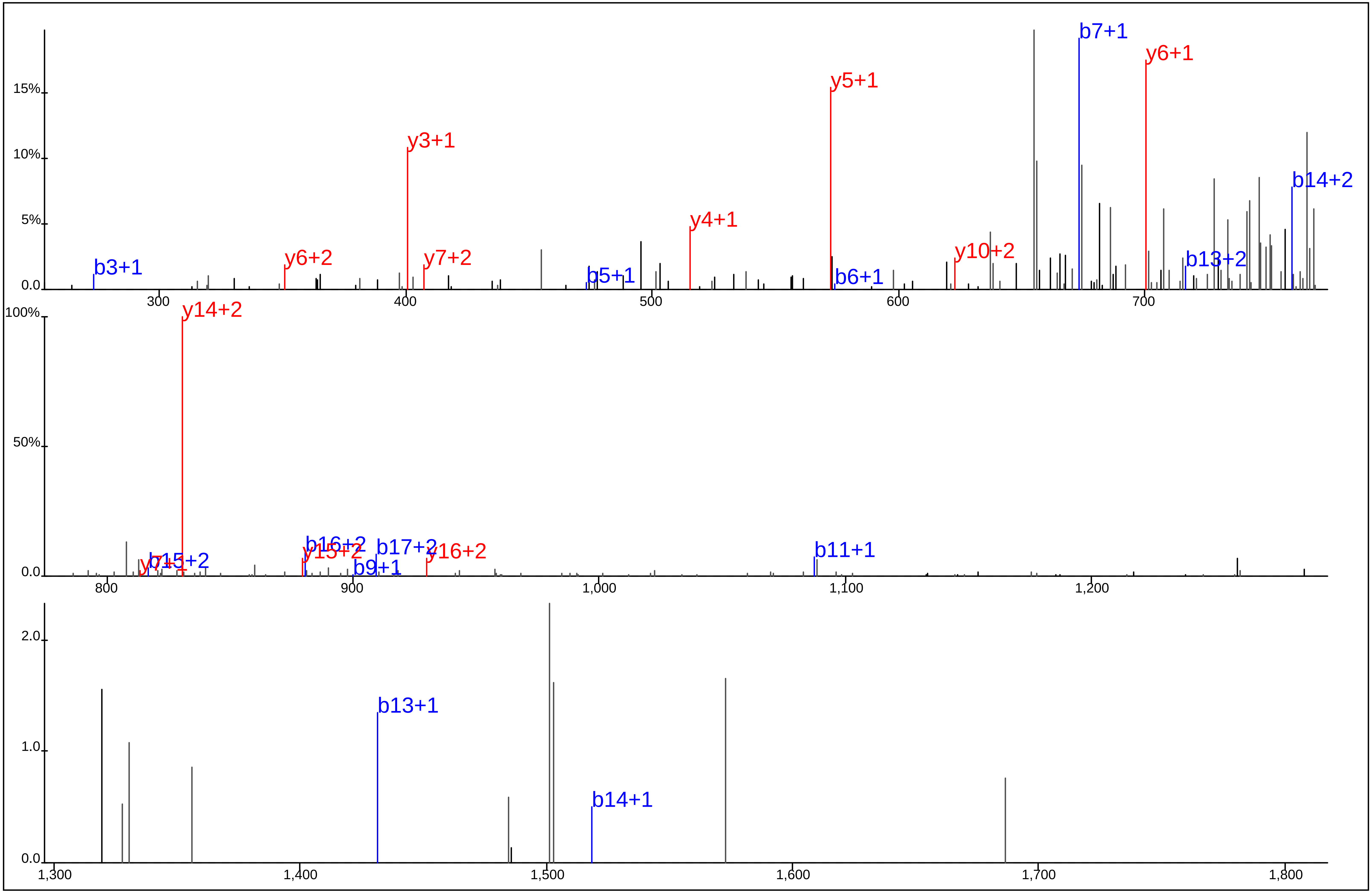

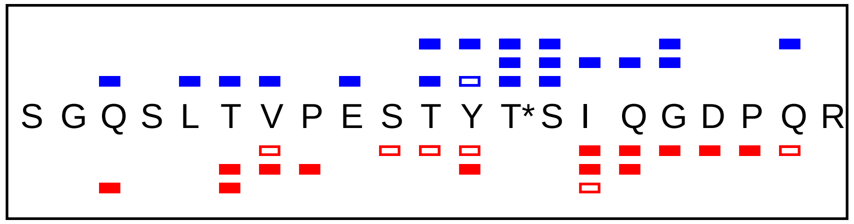

Protein Name: PAG
gi Accession: 7020925
Precursor Mass: 777.6898+3
Delta Mass: 2.011 ppm
XCorr: 28.85

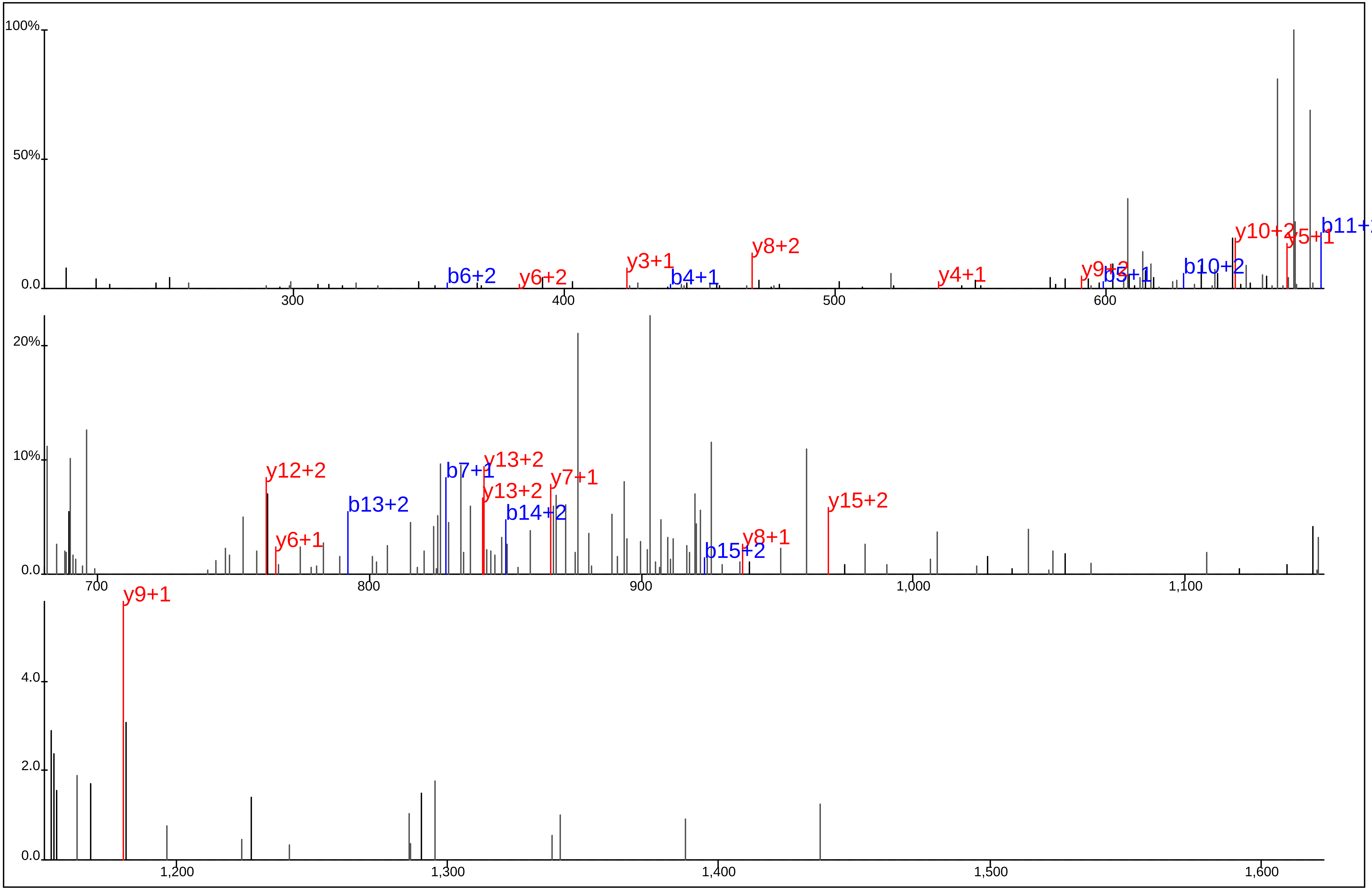

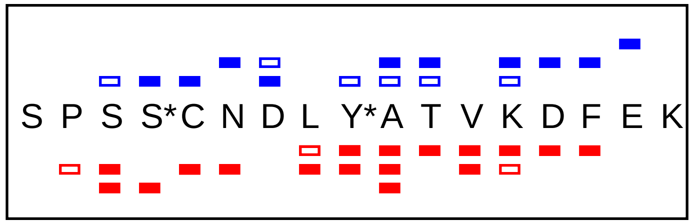

Protein Name: PAG
gi Accession: 7020925
Precursor Mass: 707.6161+3
Delta Mass: 2.540 ppm
XCorr: 14.43

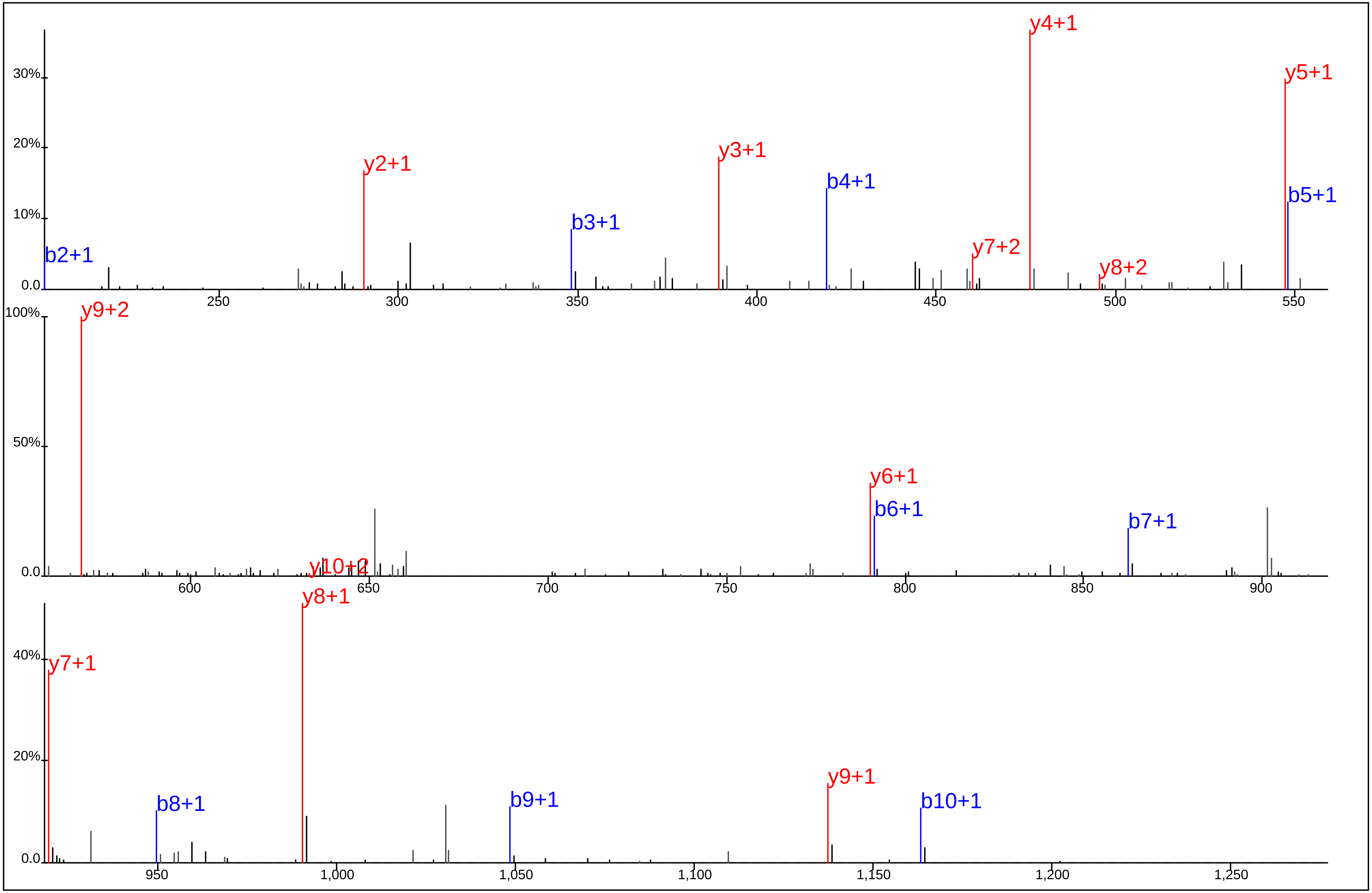

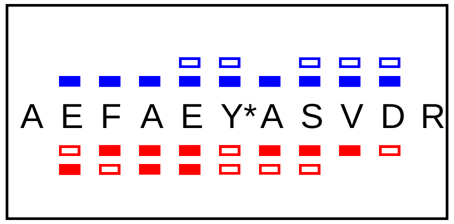

Protein Name: PAG
gi Accession: 7020925
Precursor Mass: 669.2755+2
Delta Mass: 2.129 ppm
XCorr: 73.14

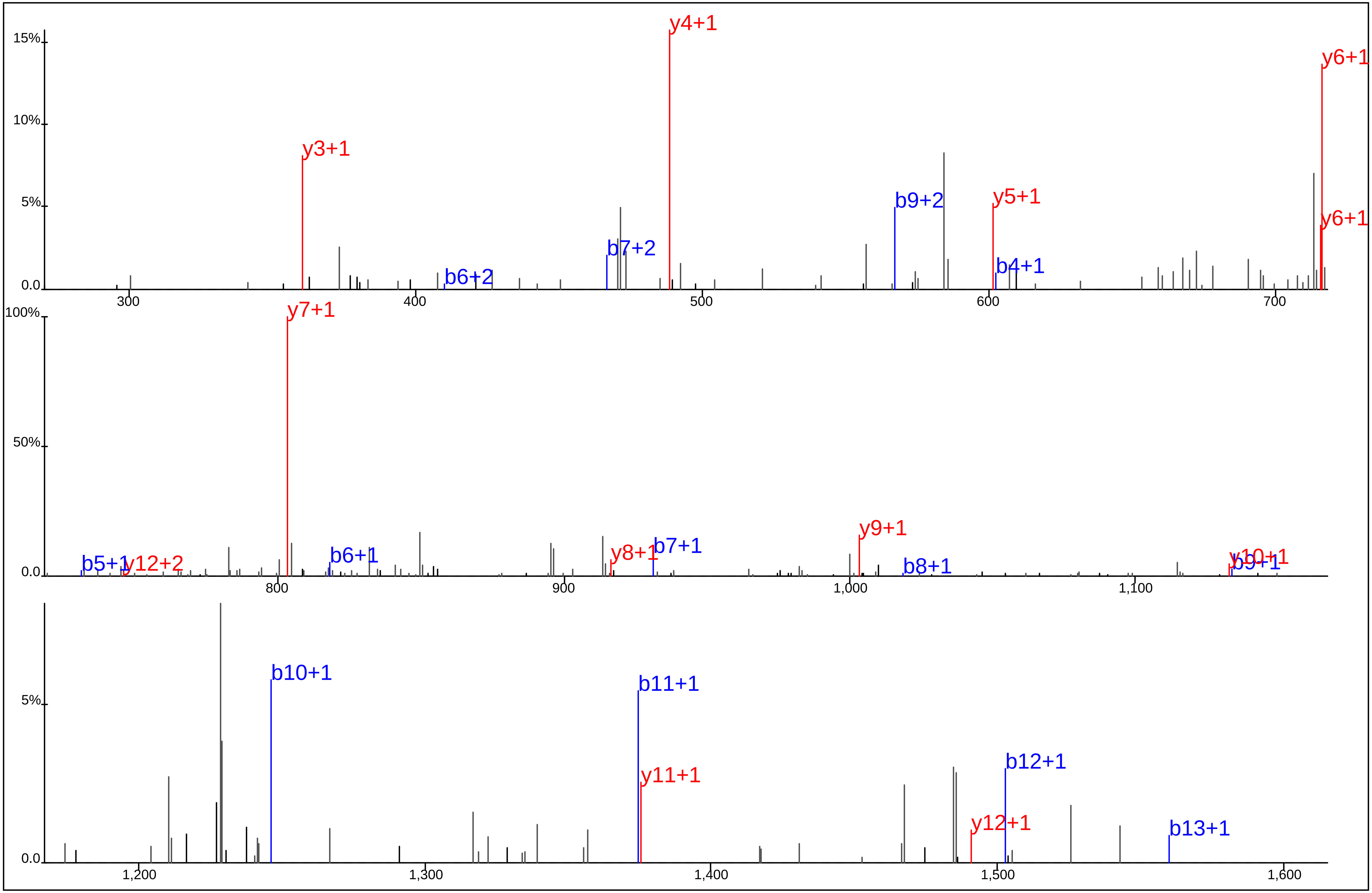

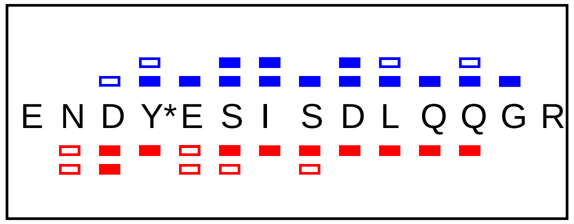

Protein Name: PAG
gi Accession: 7020925
Precursor Mass: 867.3560+2
Delta Mass: 1.879 ppm
XCorr: 60.34

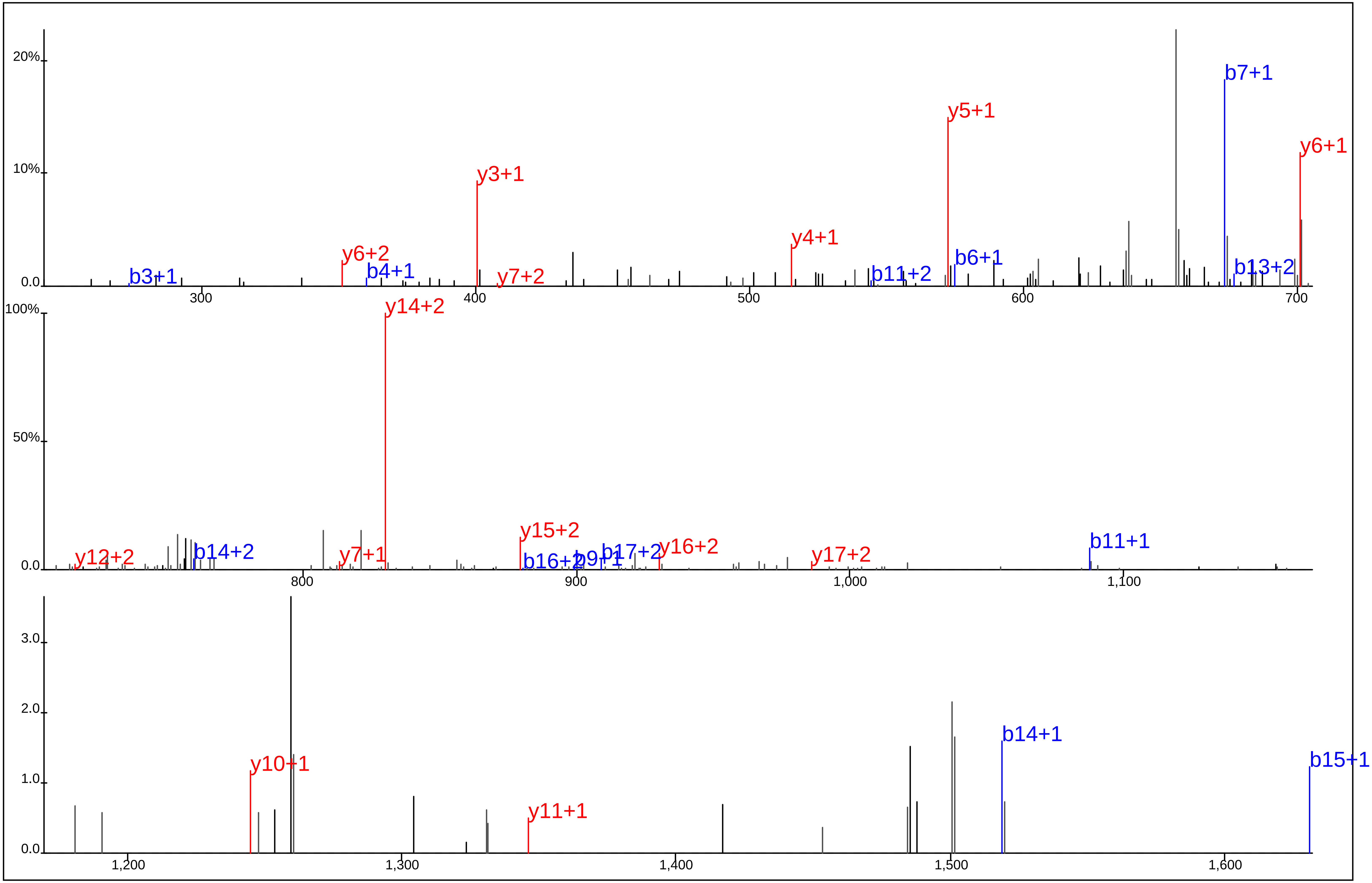

S G Q S L T V P E S T Y T S* I Q G D P Q R

Protein Name:	PAG
gi Accession:	7020925
Precursor Mass:	777.6909+3
Delta Mass:	3.504 ppm
XCorr:	37.97

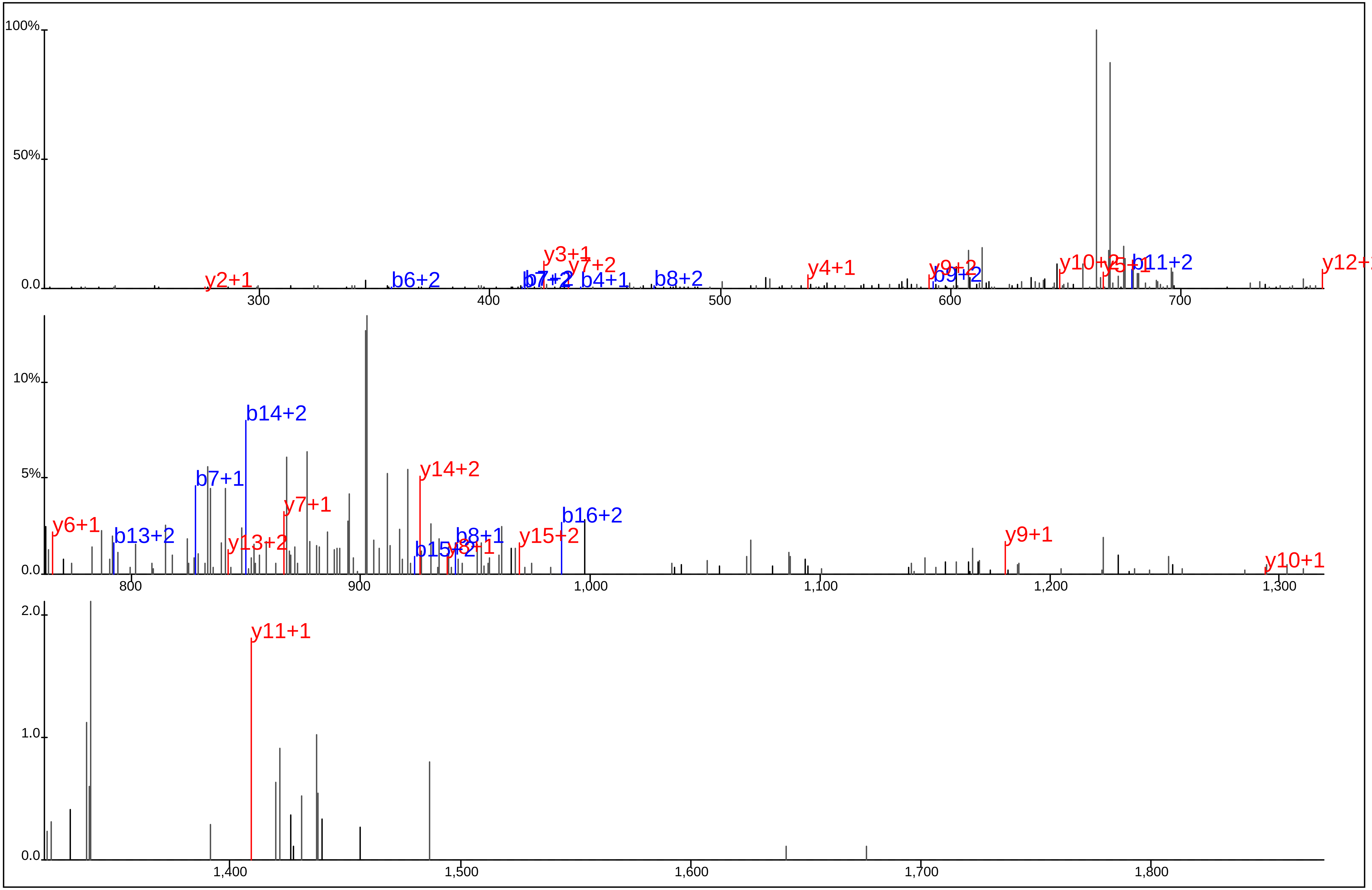

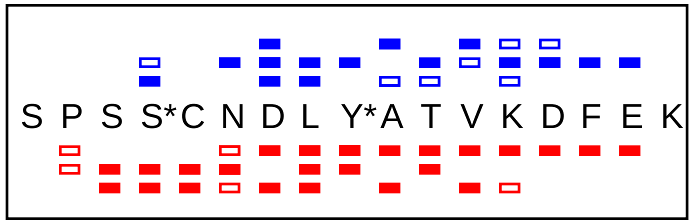

Protein Name:	PAG
gi Accession:	7020925
Precursor Mass:	707.6174+3
Delta Mass:	4.441 ppm
XCorr:	15.58

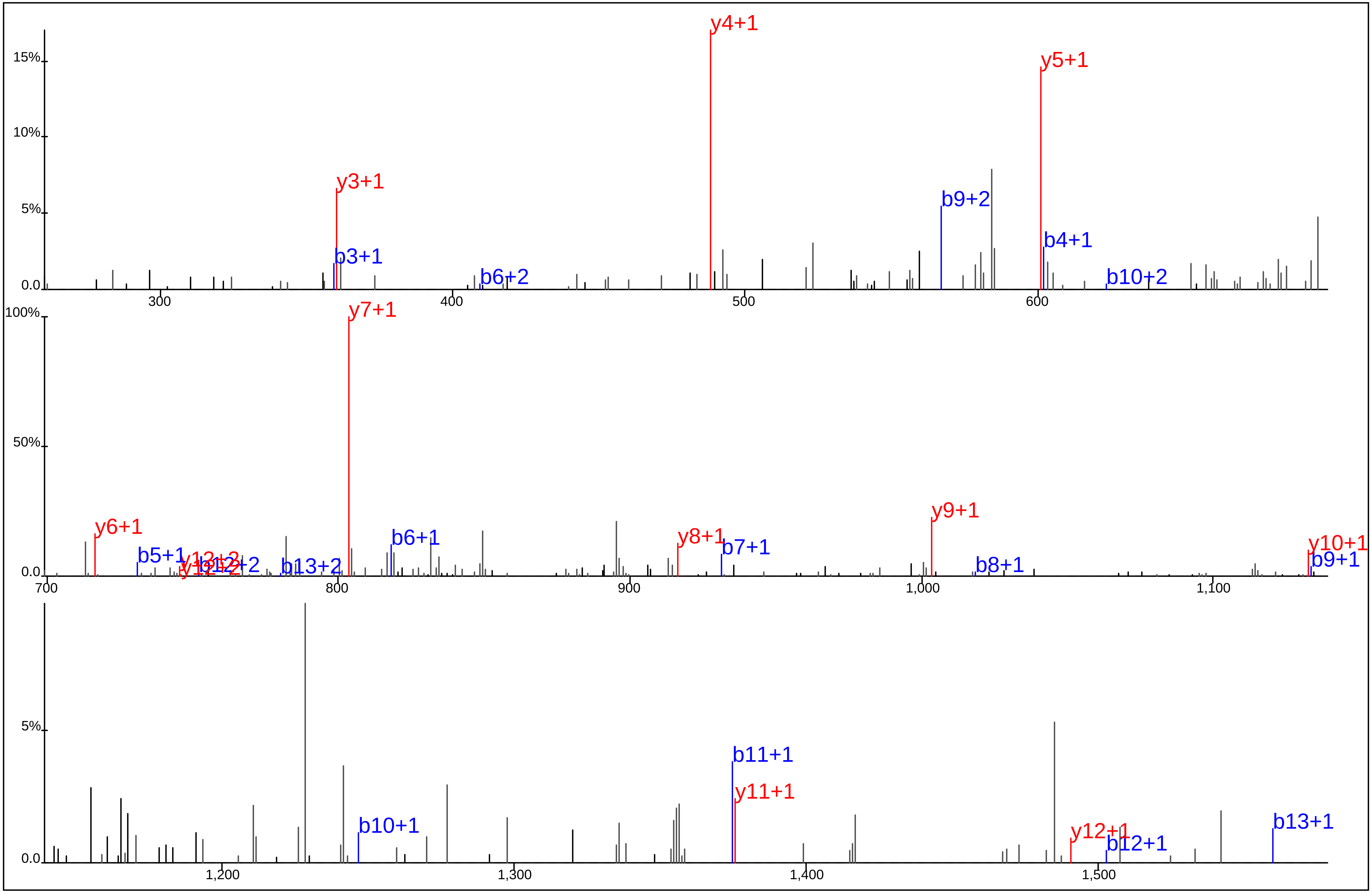

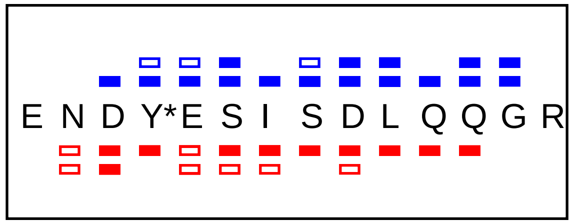

Protein Name:	PAG
gi Accession:	7020925
Precursor Mass:	867.3574+2
Delta Mass:	3.569 ppm
XCorr:	68.71

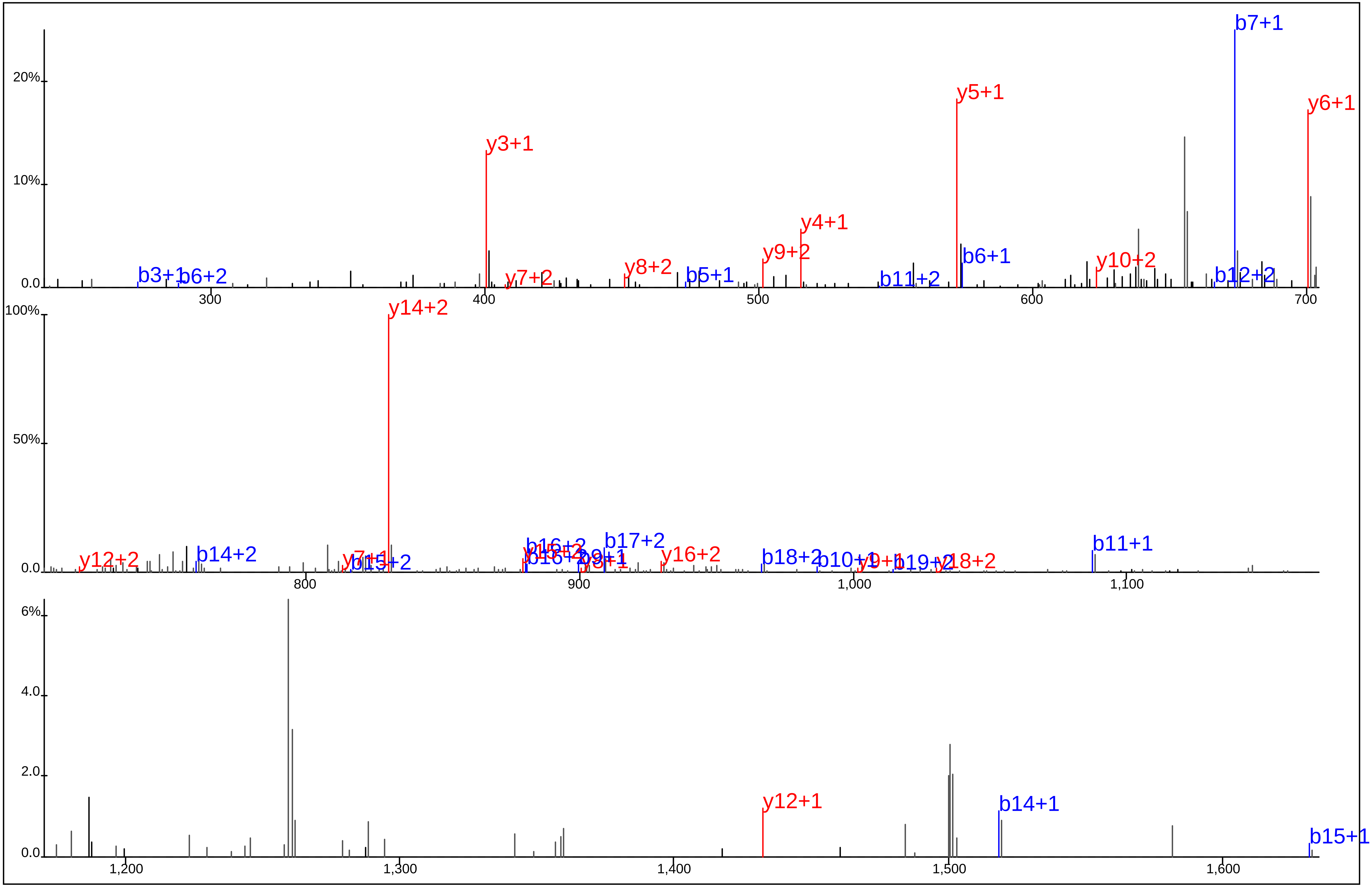

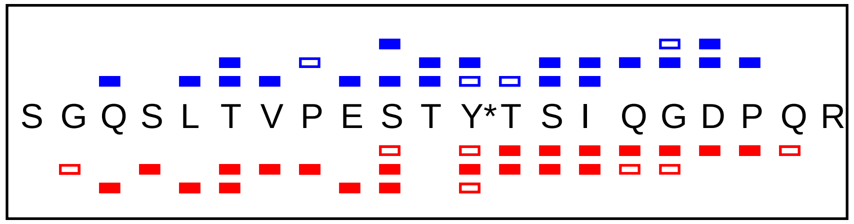

Protein Name:	PAG
gi Accession:	7020925
Precursor Mass:	777.6907+3
Delta Mass:	3.268 ppm
XCorr:	24.64

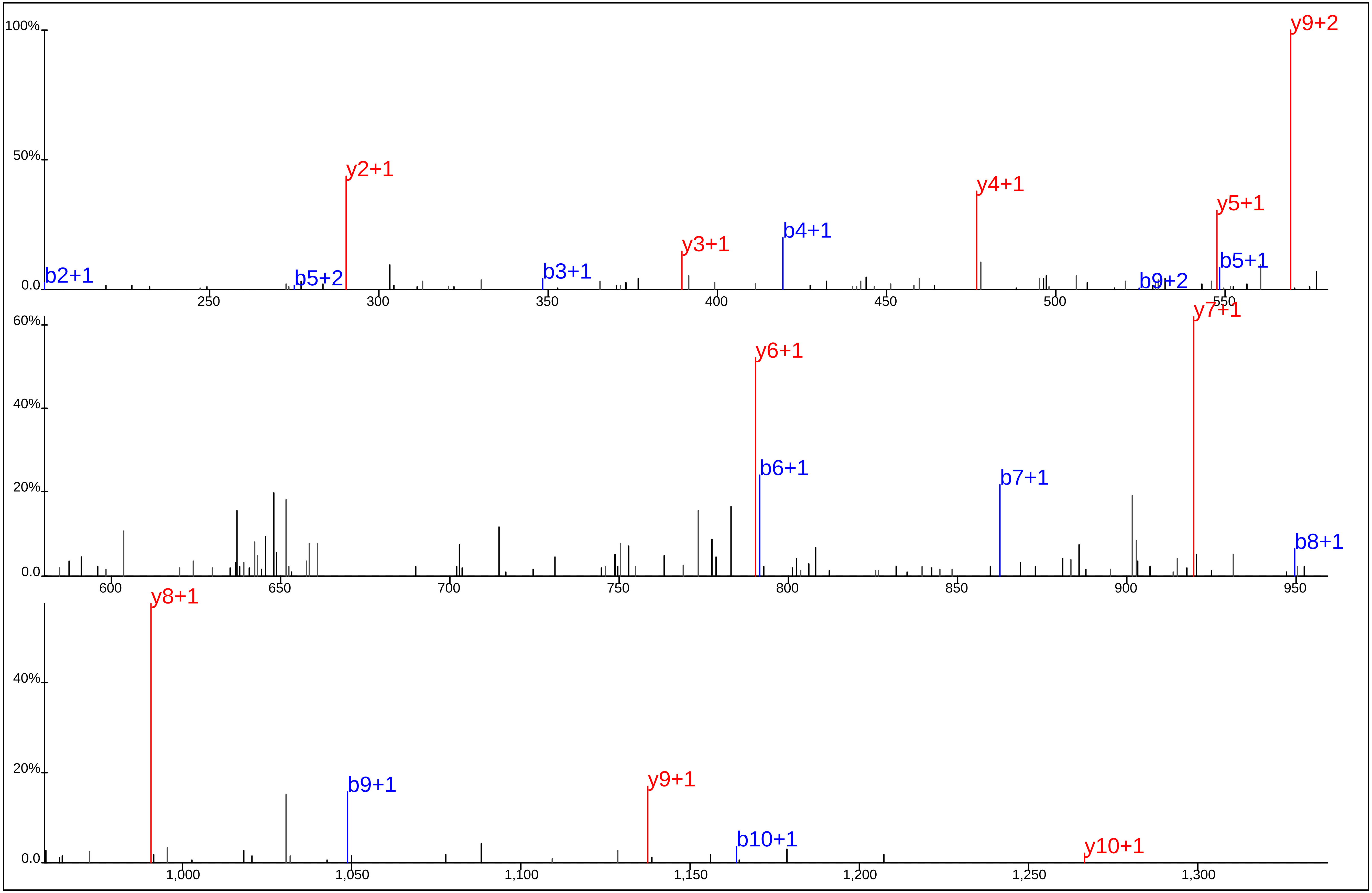

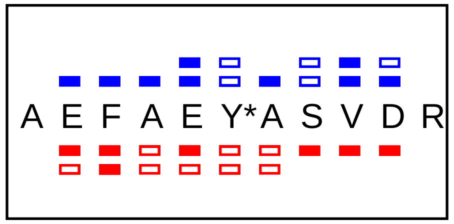

Protein Name: PAG
gi Accession: 7020925
Precursor Mass: 669.2756+2
Delta Mass: 2.219 ppm
XCorr: 76.29

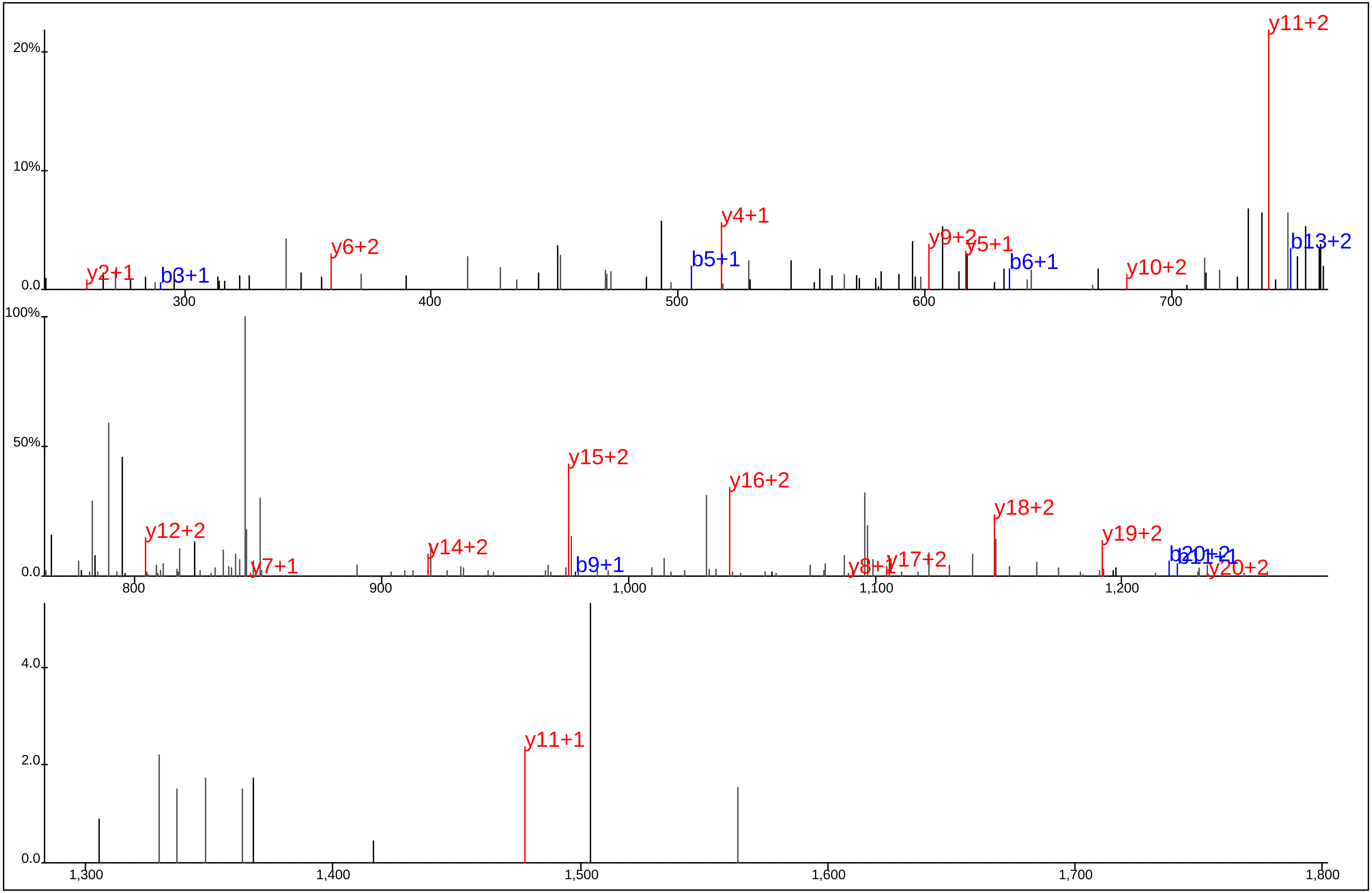

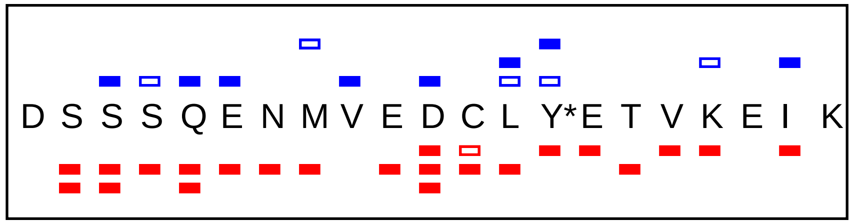

Protein Name: PAG
gi Accession: 7020925
Precursor Mass: 862.0295+3
Delta Mass: 3.456 ppm
XCorr: 24.74

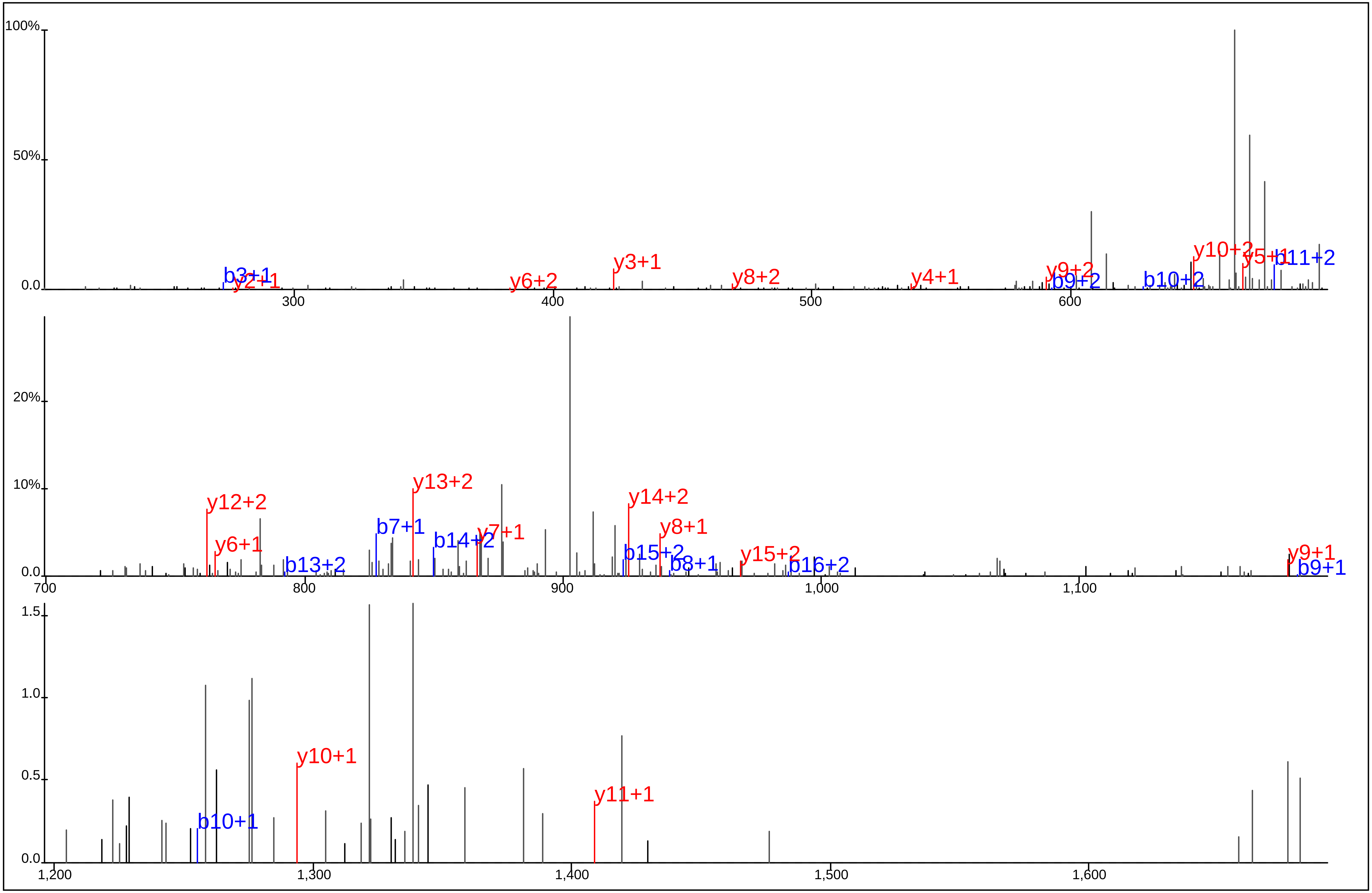

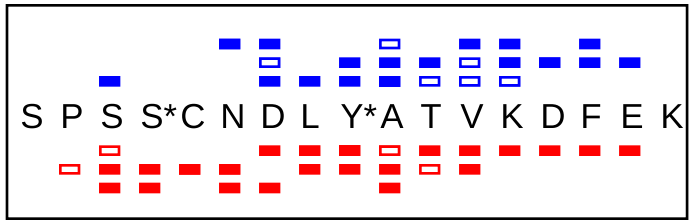

Protein Name: PAG
gi Accession: 7020925
Precursor Mass: 707.6157+3
Delta Mass: 1.937 ppm
XCorr: 32.04

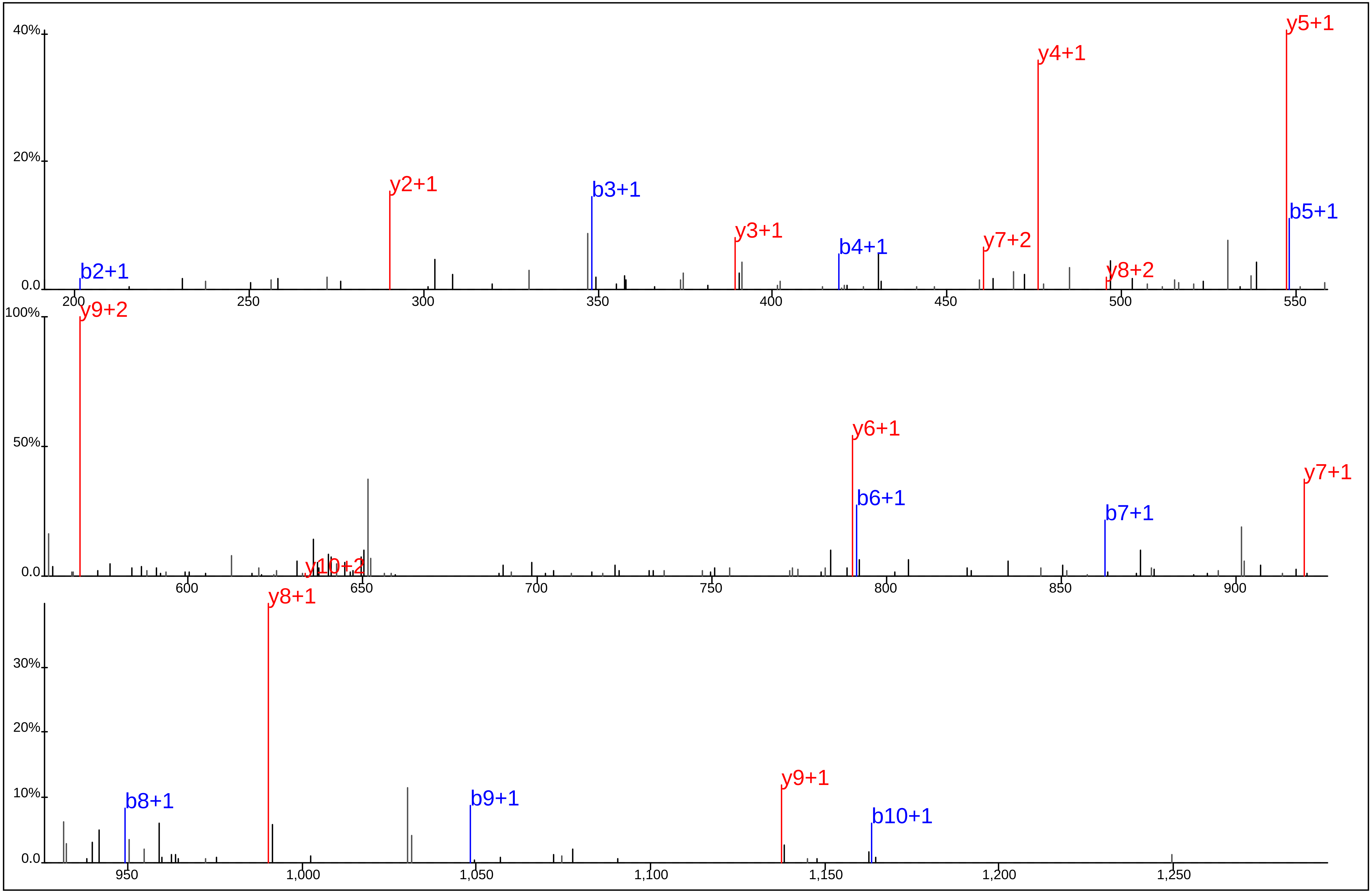

A E F A E Y*A S V D R

Protein Name:	PAG
gi Accession:	7020925
Precursor Mass:	669.2742+2
Delta Mass:	0.215 ppm
XCorr:	61.36

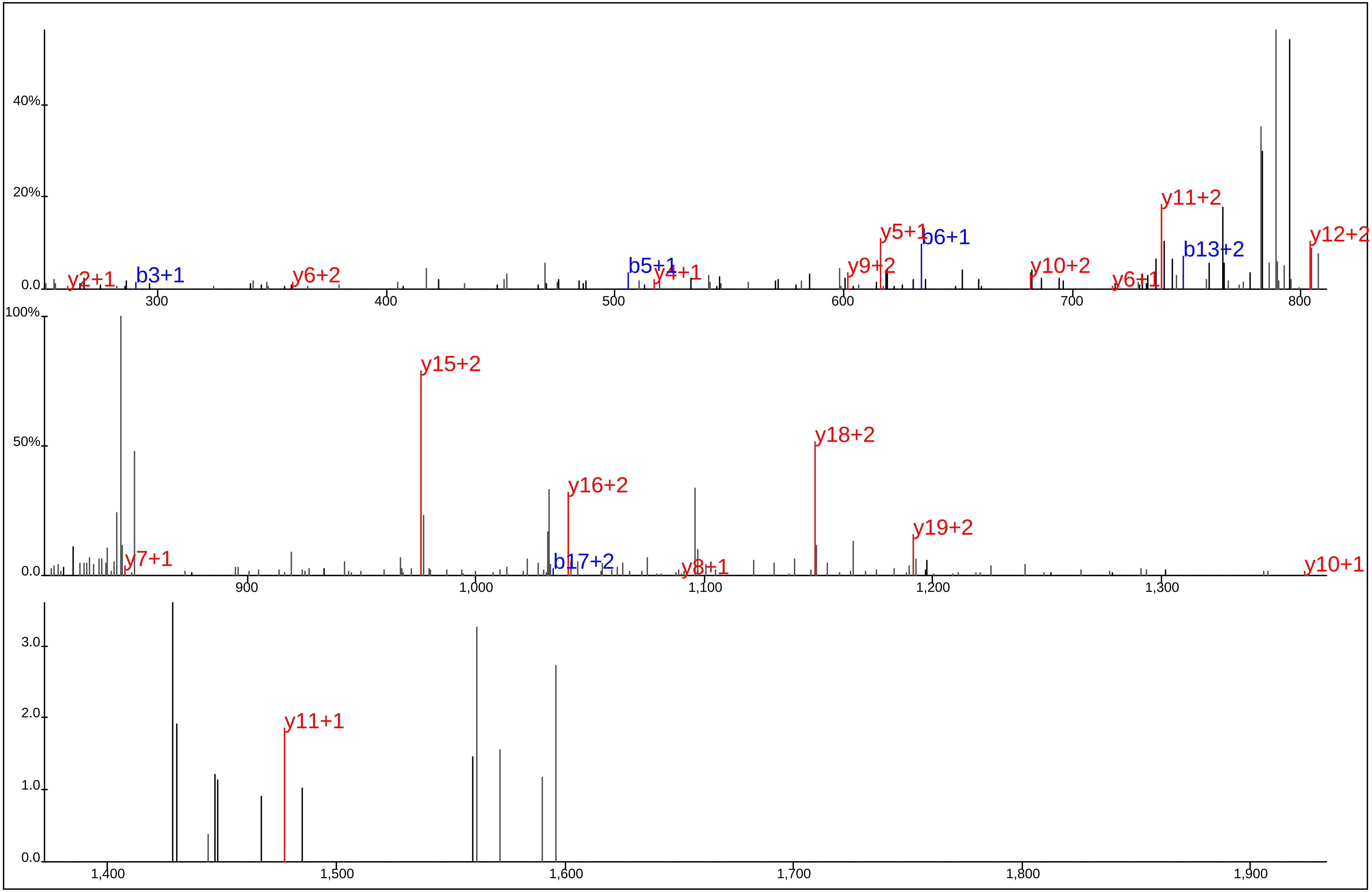

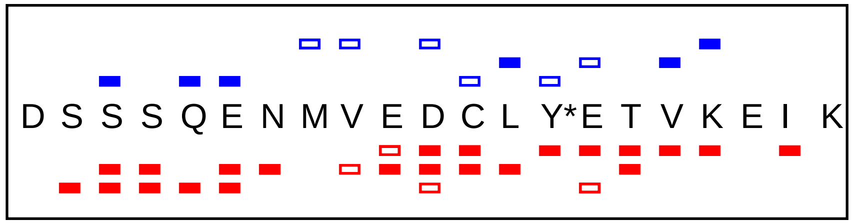

Protein Name: PAG
gi Accession: 7020925
Precursor Mass: 862.0330+3
Delta Mass: 0.584 ppm
XCorr: 16.55

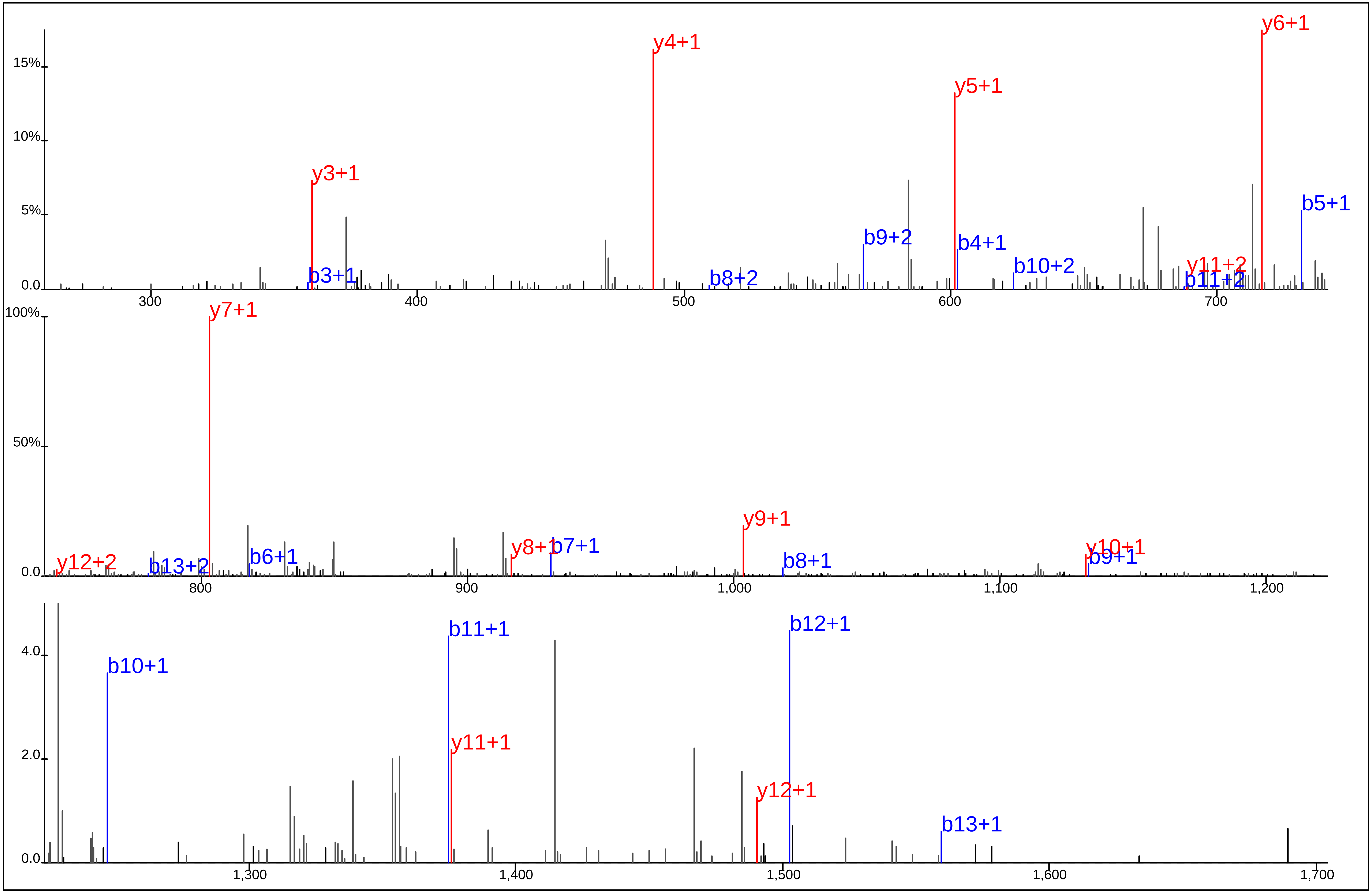

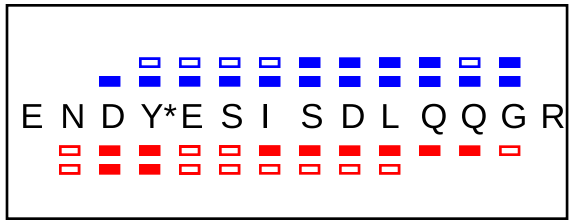

Protein Name: PAG
gi Accession: 7020925
Precursor Mass: 867.3558+2
Delta Mass: 1.666 ppm
XCorr: 57.99

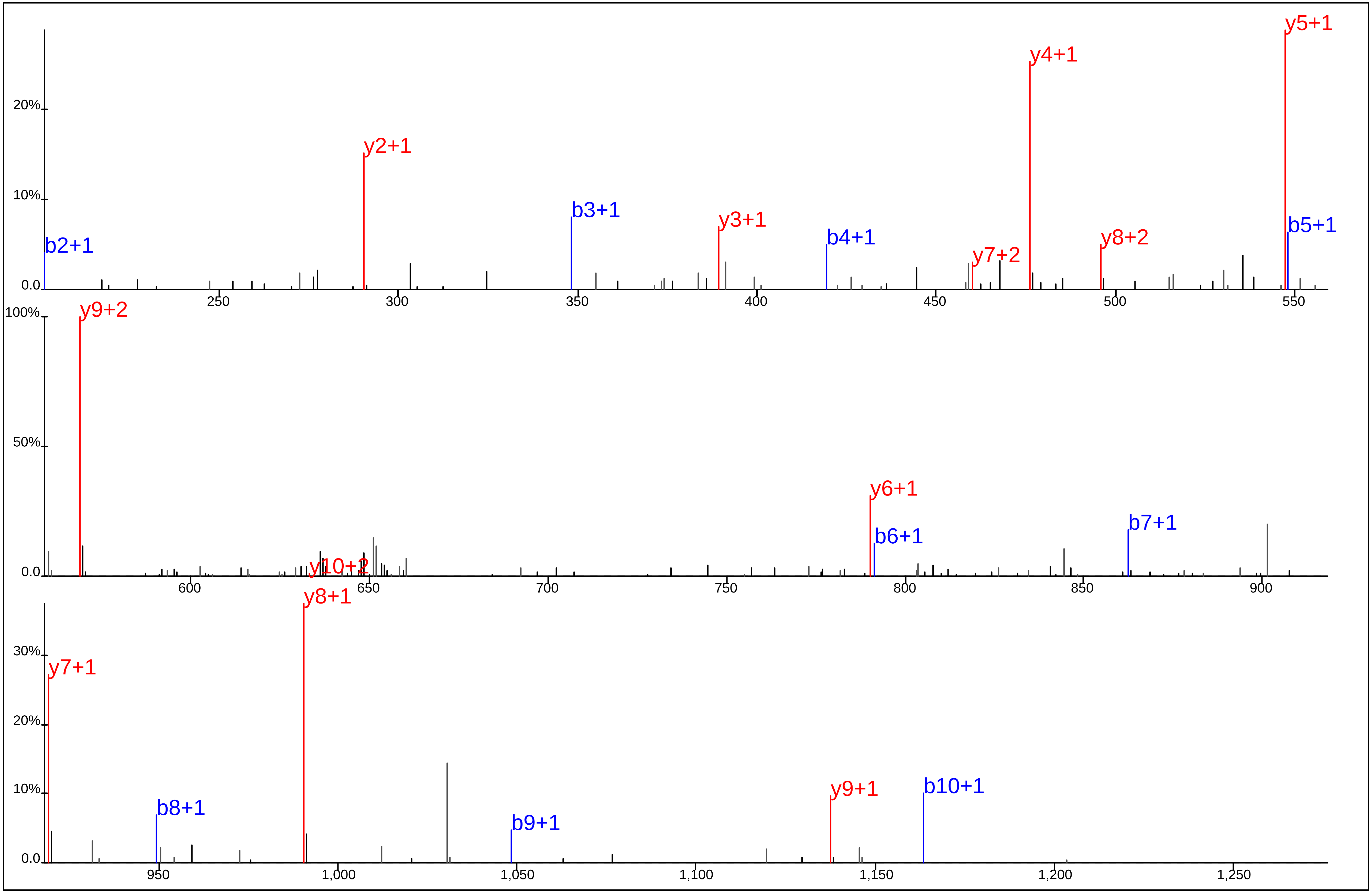

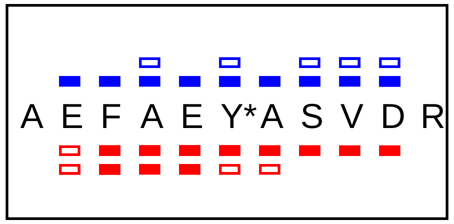

Protein Name: PAG
gi Accession: 7020925
Precursor Mass: 669.2758+2
Delta Mass: 2.585 ppm
XCorr: 76.54

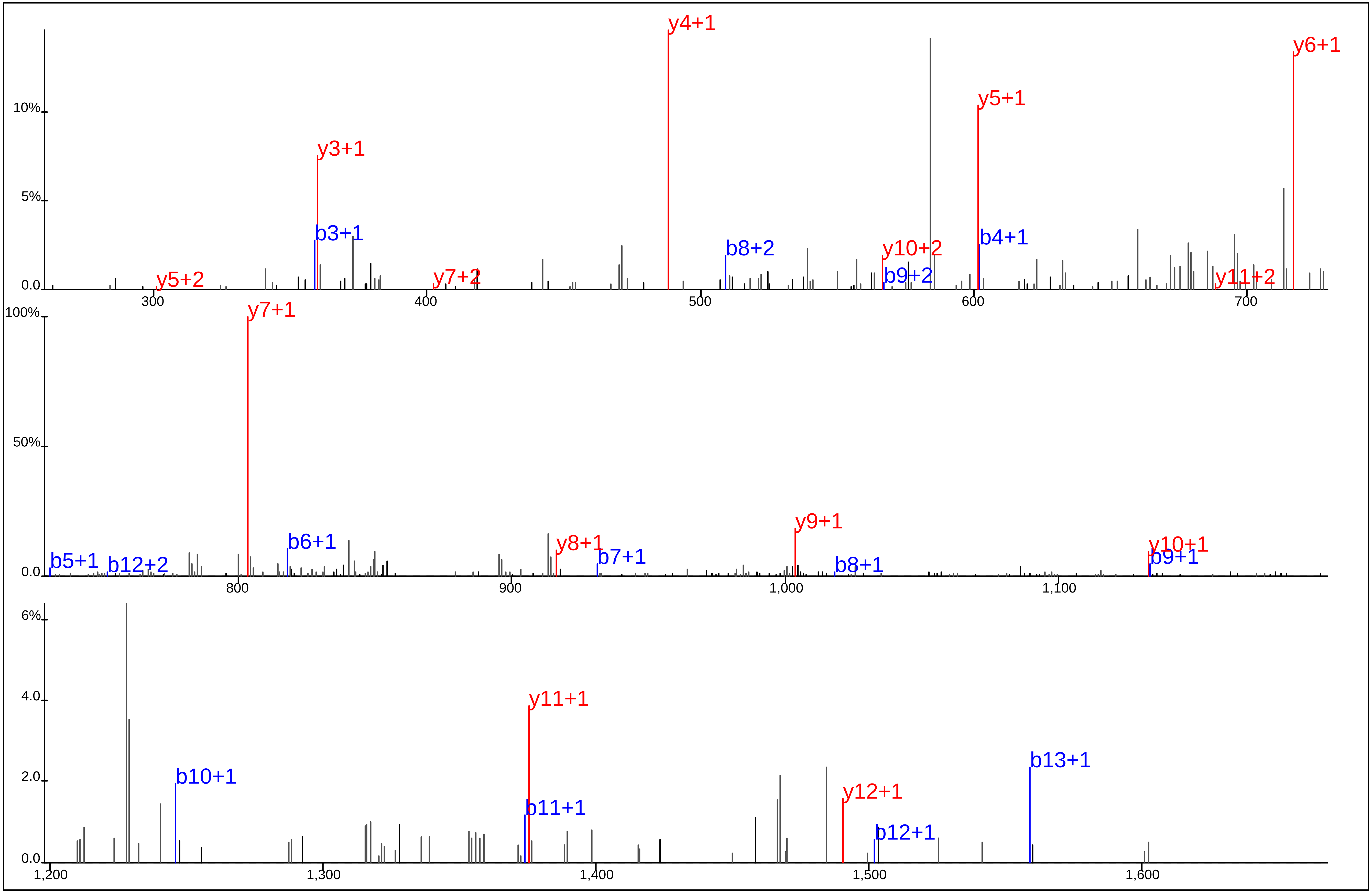

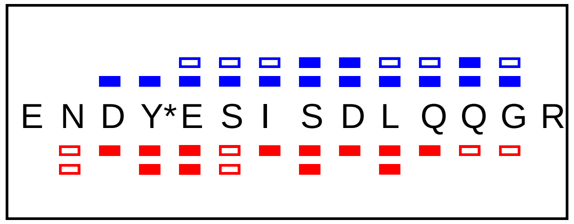

Protein Name: PAG
gi Accession: 7020925
Precursor Mass: 867.3643+2
Delta Mass: 11.454 ppm
XCorr: 67.04

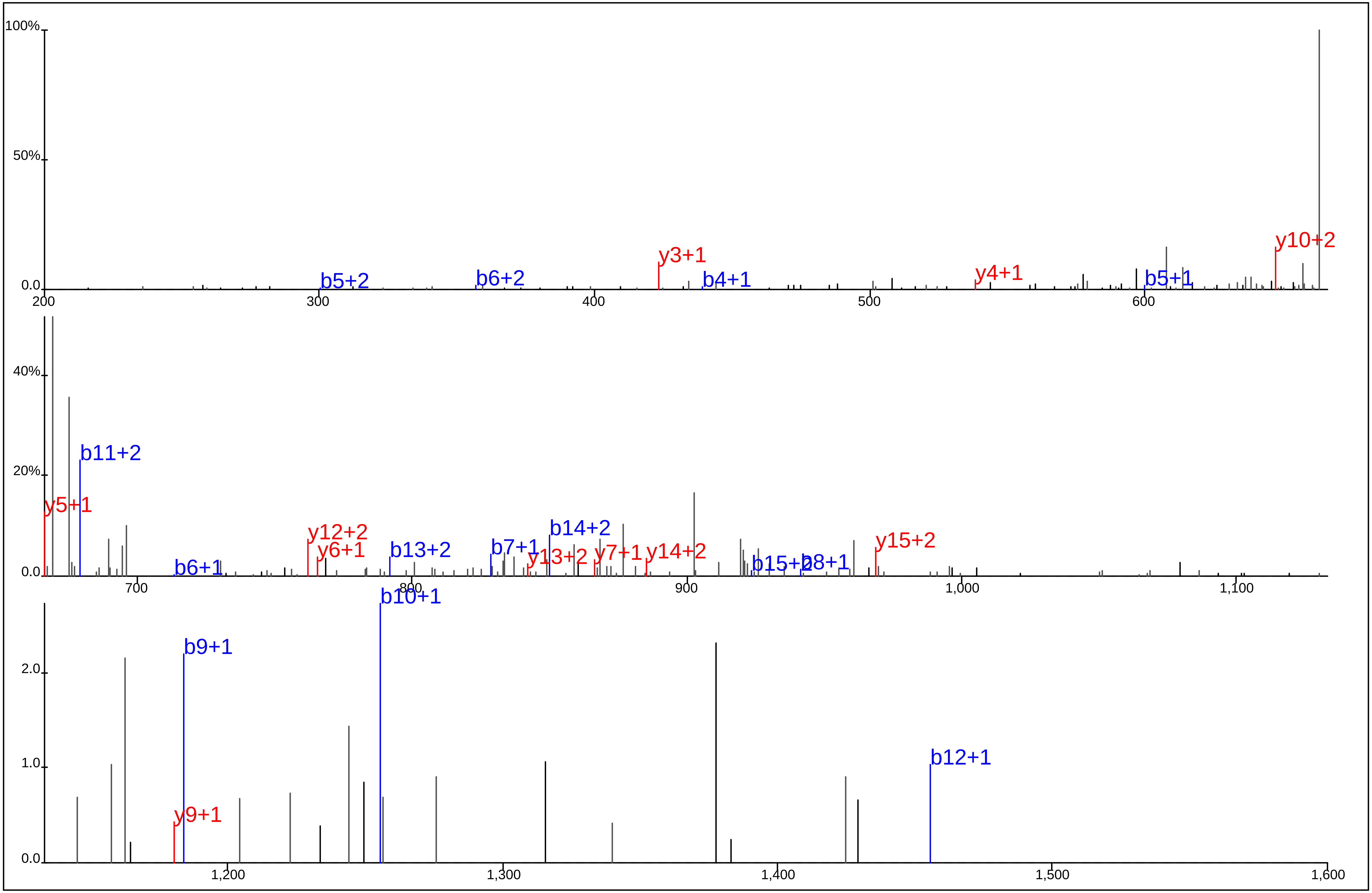

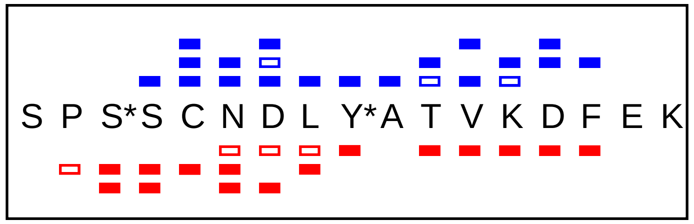

Protein Name: PAG
gi Accession: 7020925
Precursor Mass: 707.6154+3
Delta Mass: 1.507 ppm
XCorr: 23.16

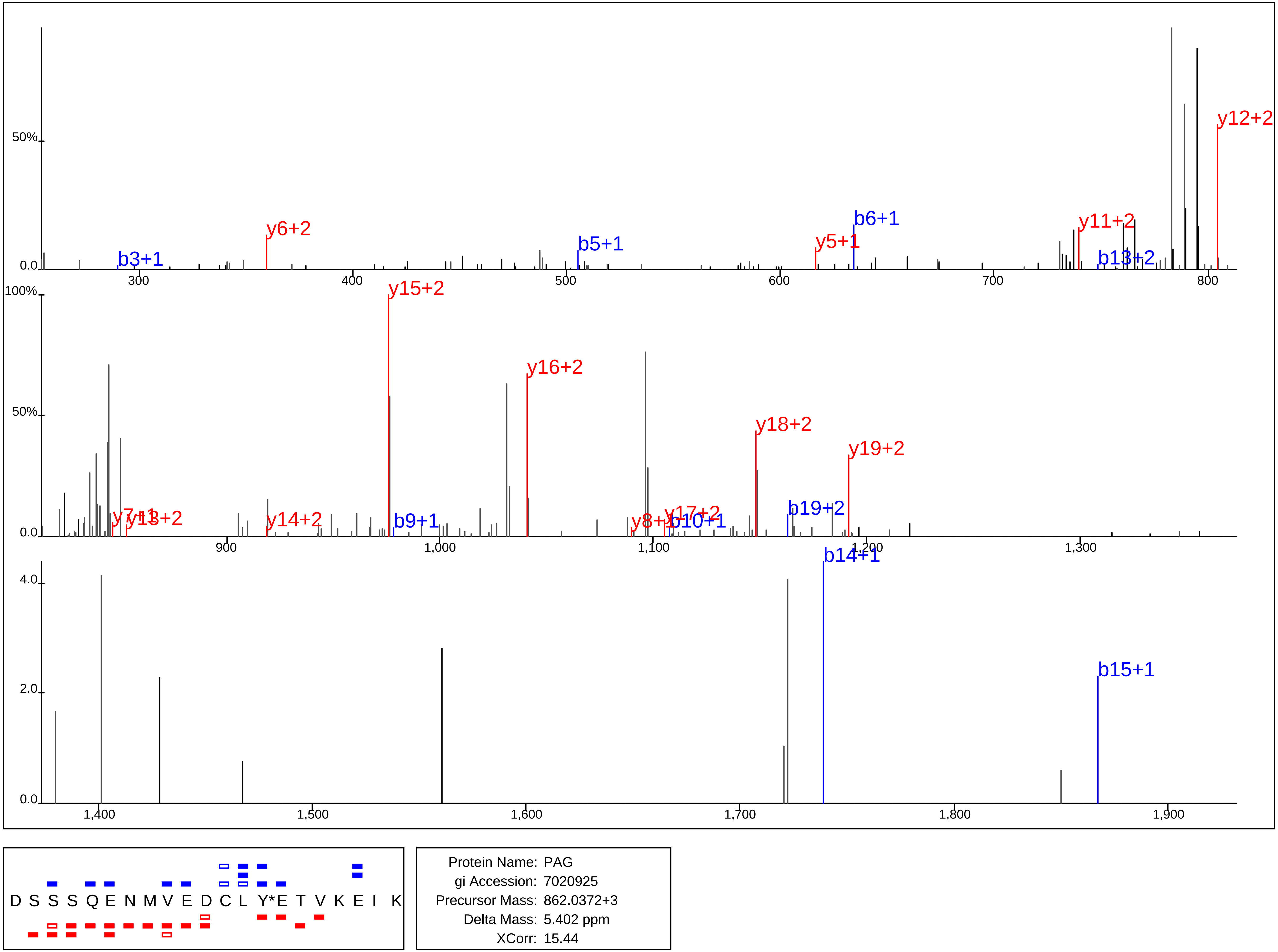

Protein Name: PAG
gi Accession: 7020925
Precursor Mass: 862.0372+3
Delta Mass: 5.402 ppm
XCorr: 15.44

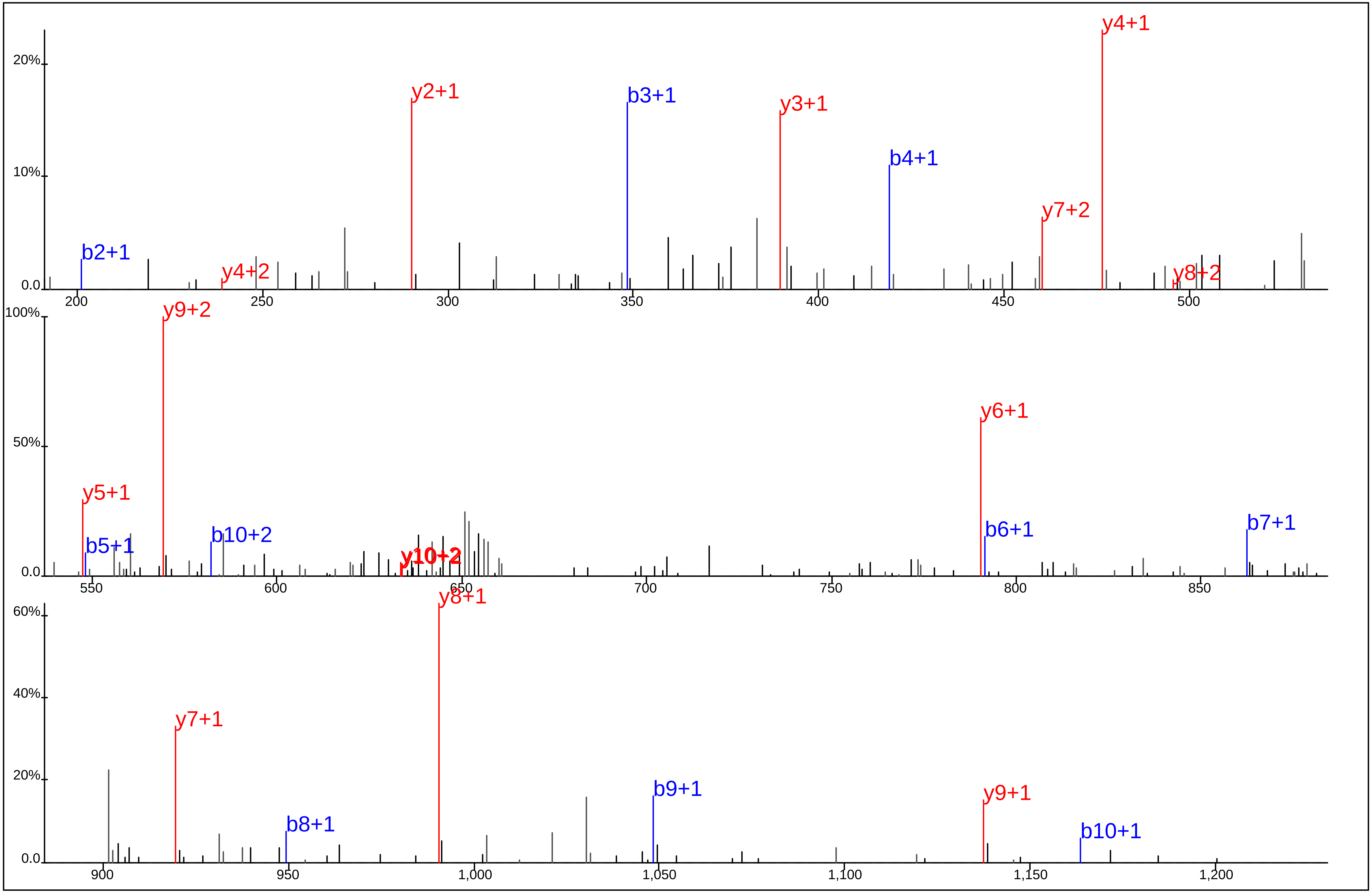

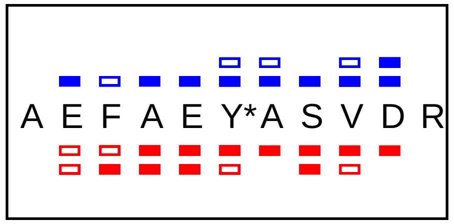

Protein Name:	PAG
gi Accession:	7020925
Precursor Mass:	669.2764+2
Delta Mass:	3.497 ppm
XCorr:	69.4

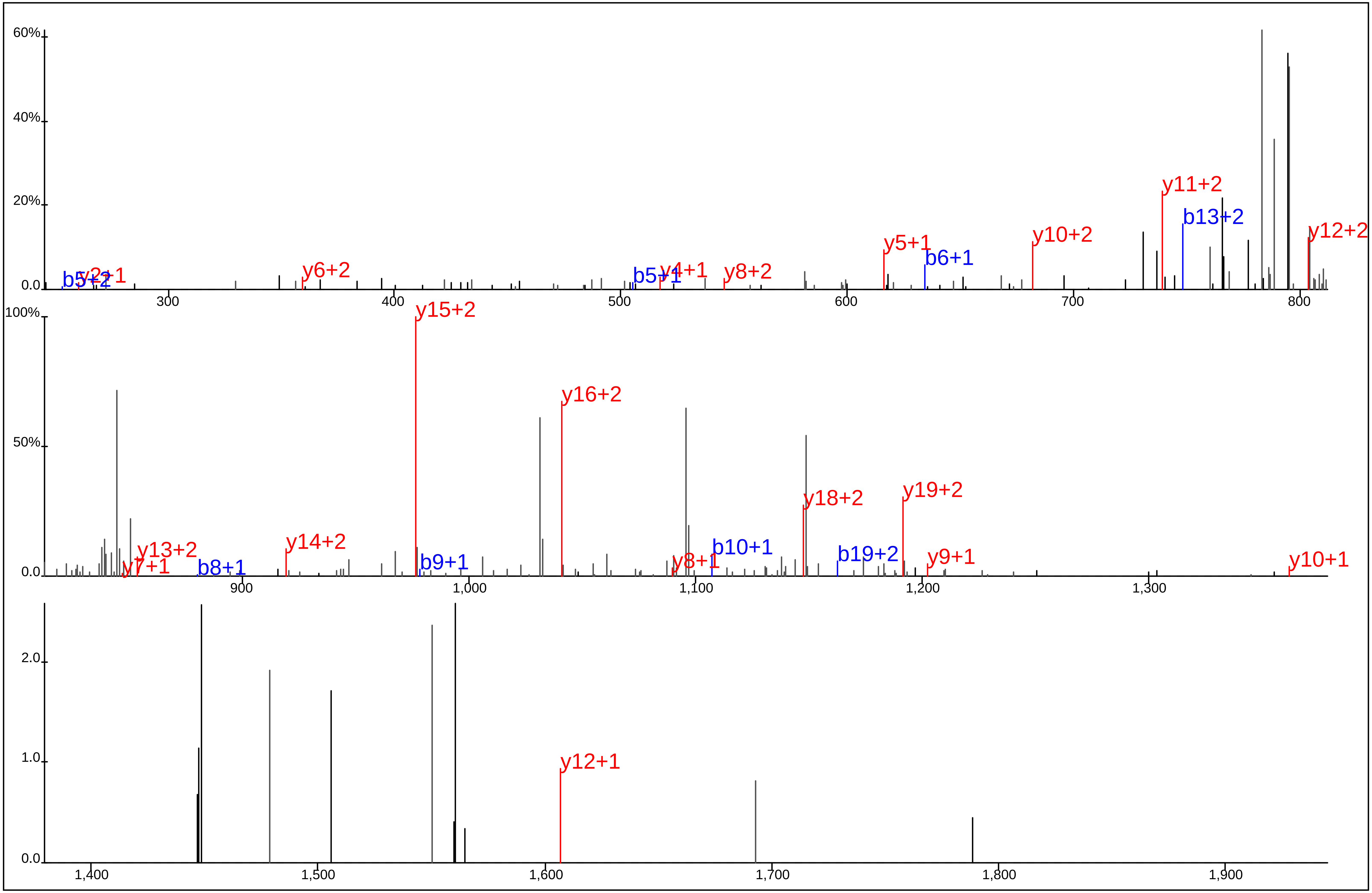

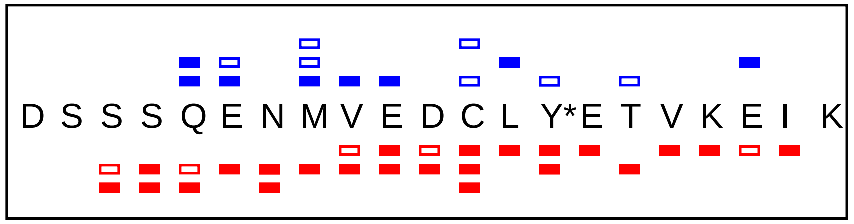

Protein Name:	PAG
gi Accession:	7020925
Precursor Mass:	862.0348+3
Delta Mass:	2.639 ppm
XCorr:	39.12

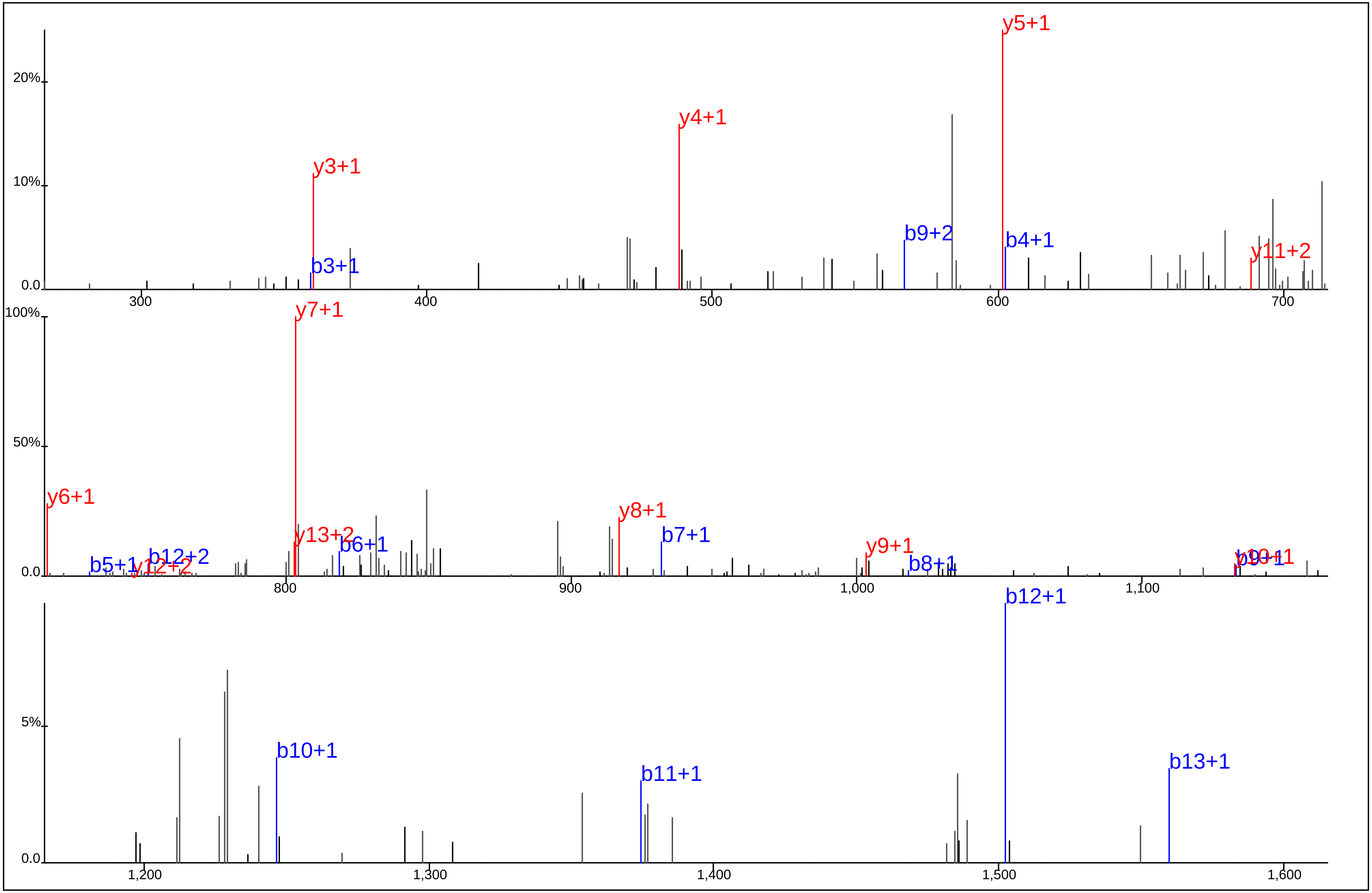

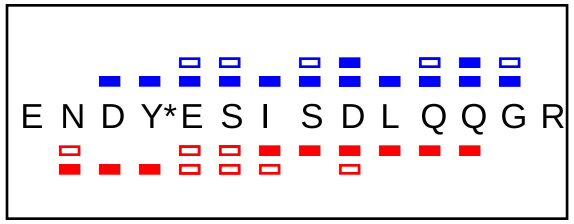

Protein Name: PAG
gi Accession: 7020925
Precursor Mass: 867.3593+2
Delta Mass: 5.680 ppm
XCorr: 54.8

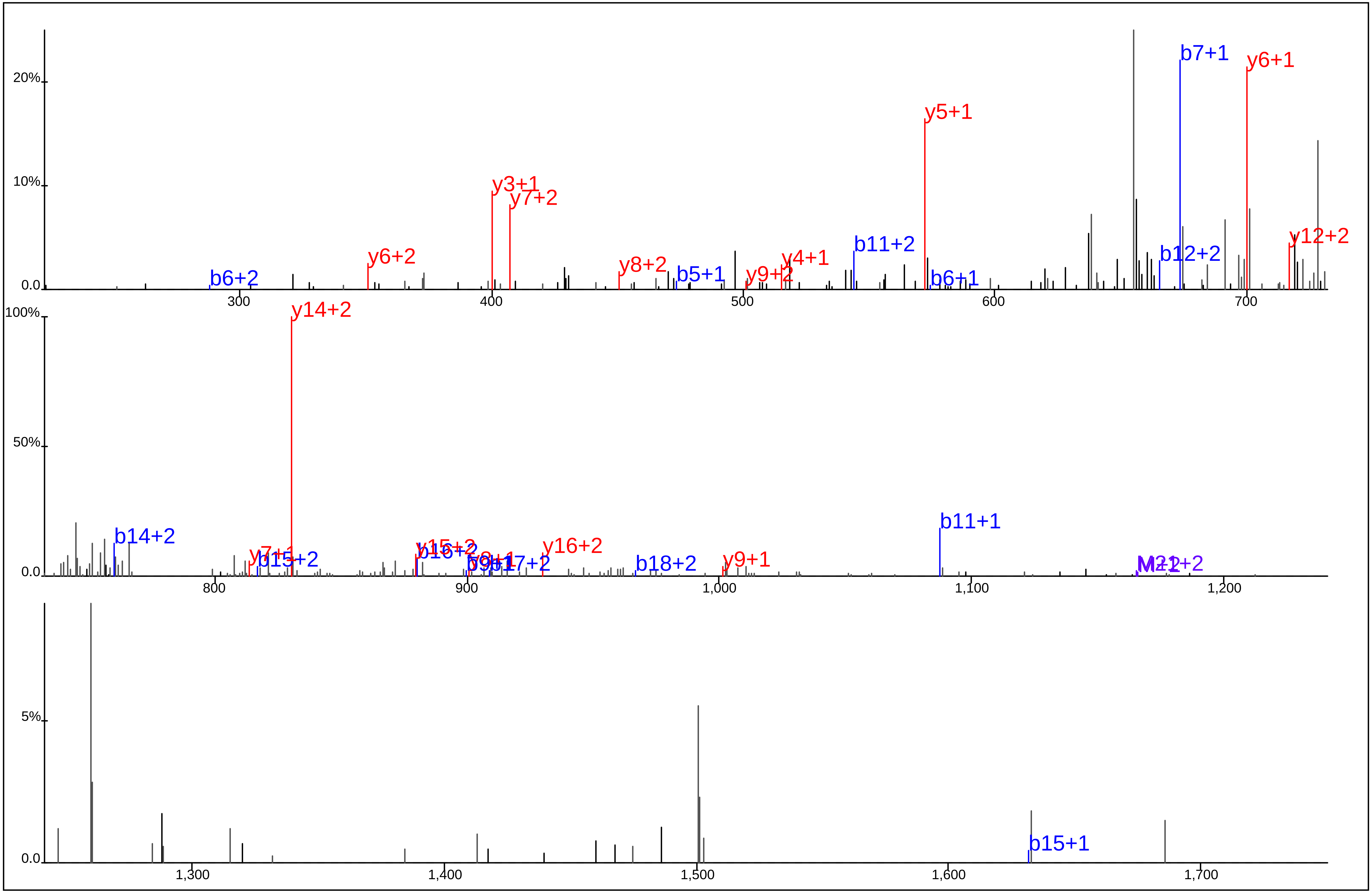

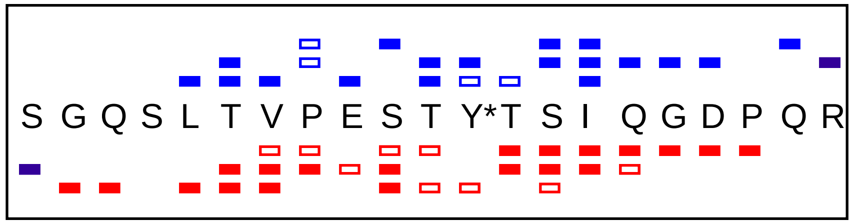

Protein Name:	PAG
gi Accession:	7020925
Precursor Mass:	777.6894+3
Delta Mass:	1.540 ppm
XCorr:	25.17

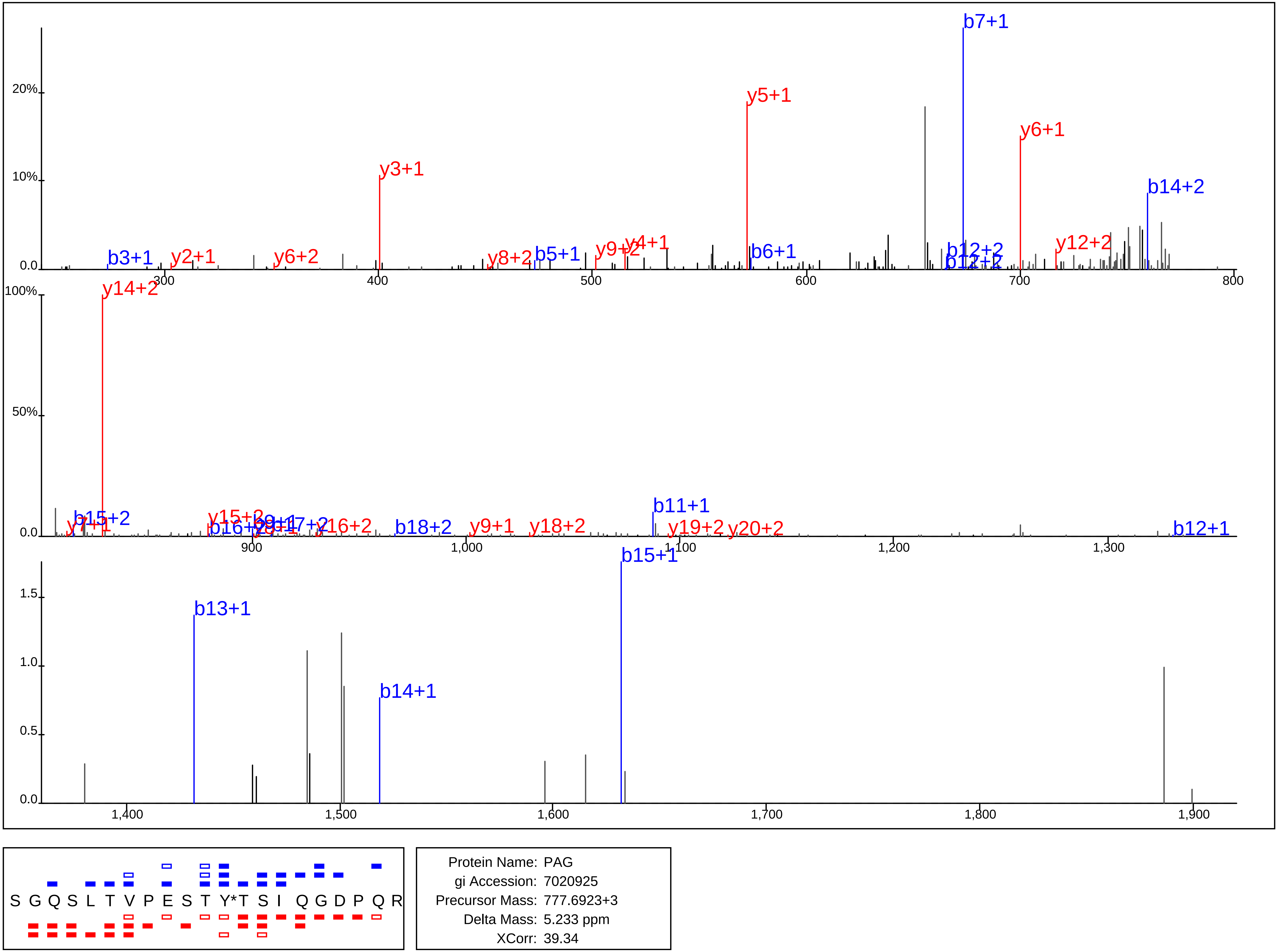

Protein Name: PAG
gi Accession: 7020925
Precursor Mass: 777.6923+3
Delta Mass: 5.233 ppm
XCorr: 39.34

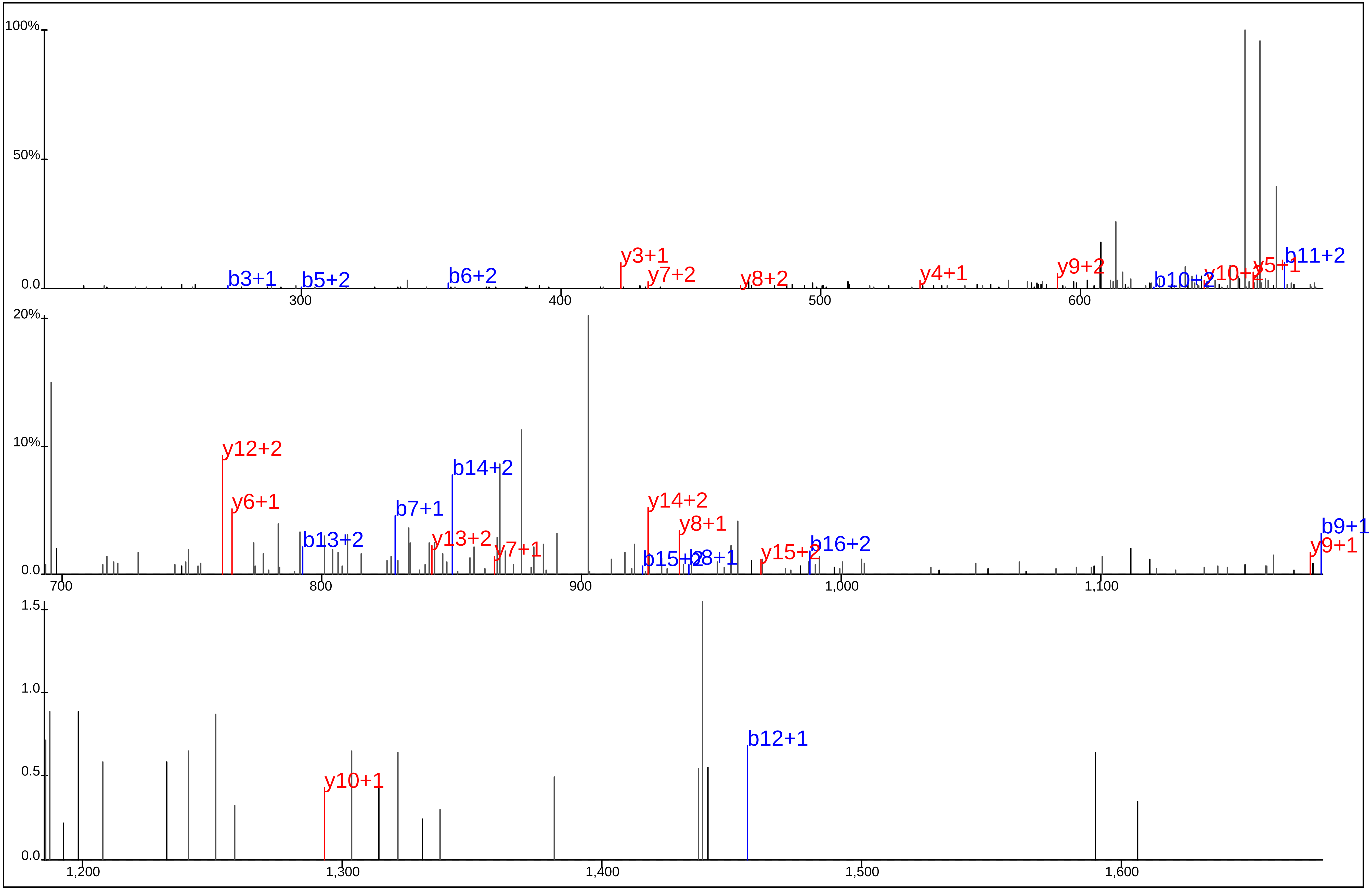

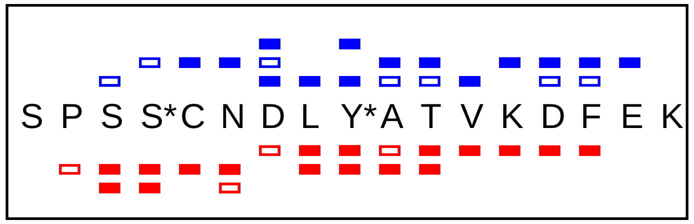

Protein Name:	PAG
gi Accession:	7020925
Precursor Mass:	707.6193+3
Delta Mass:	7.119 ppm
XCorr:	17.4

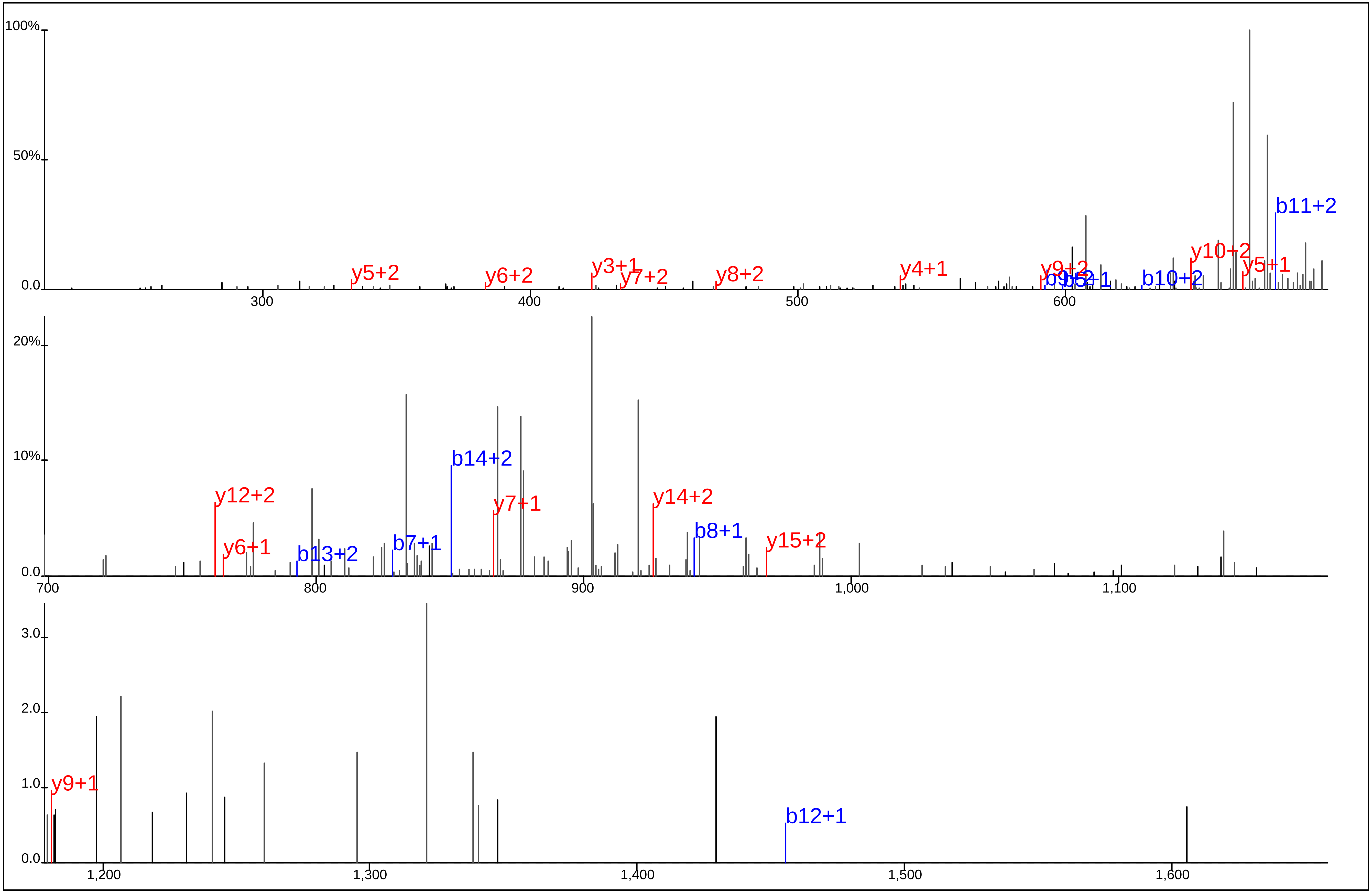

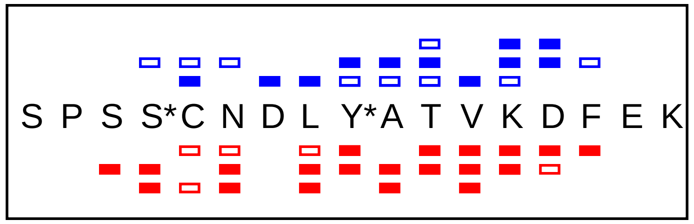

Protein Name: PAG
gi Accession: 7020925
Precursor Mass: 707.6157+3
Delta Mass: 1.937 ppm
XCorr: 12.85

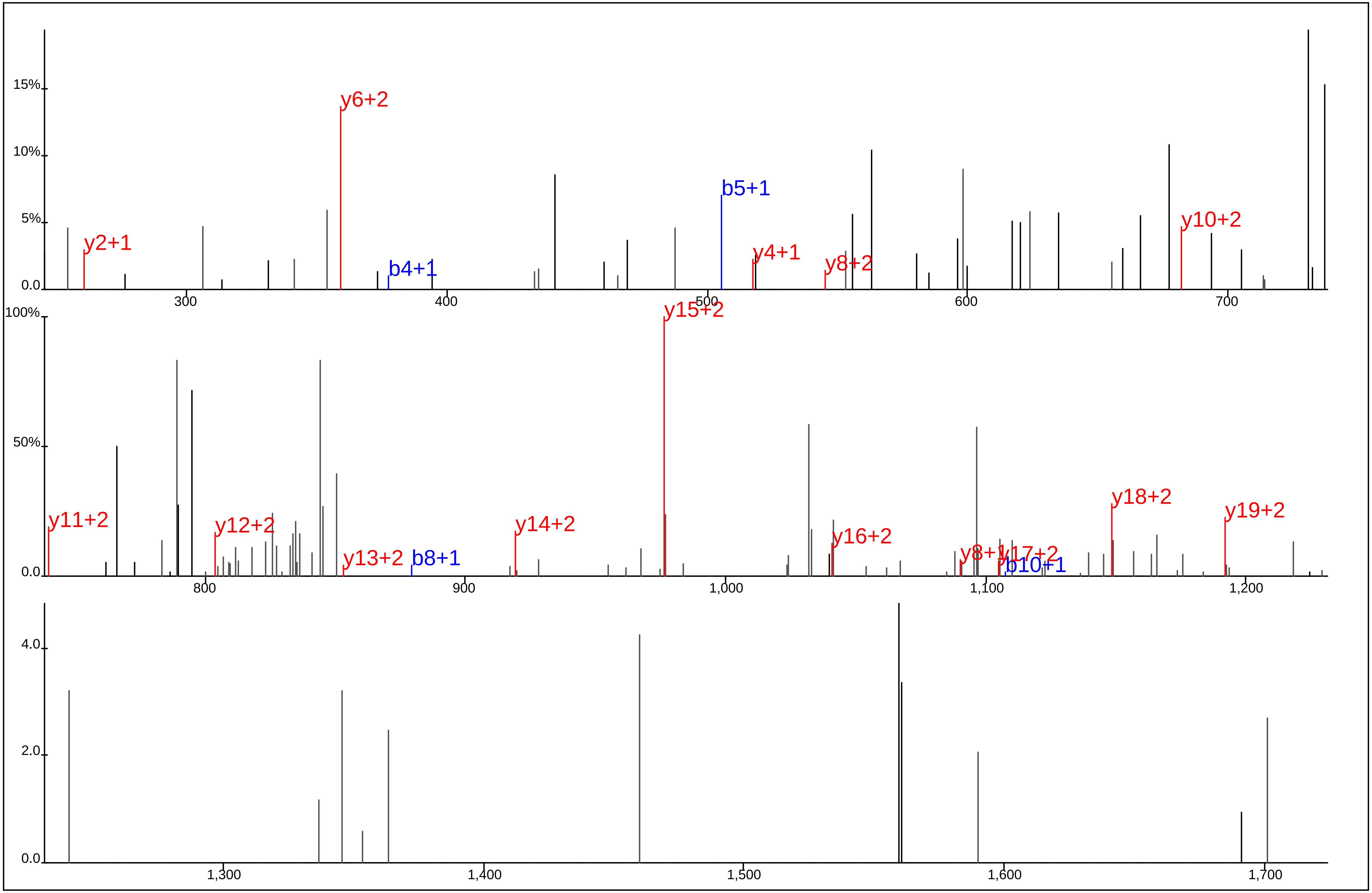

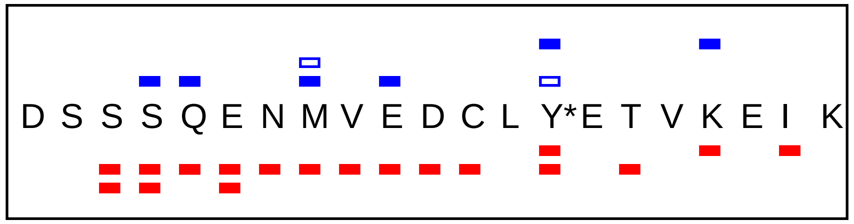

Protein Name: PAG
gi Accession: 7020925
Precursor Mass: 862.0314+3
Delta Mass: 1.258 ppm
XCorr: 23.7

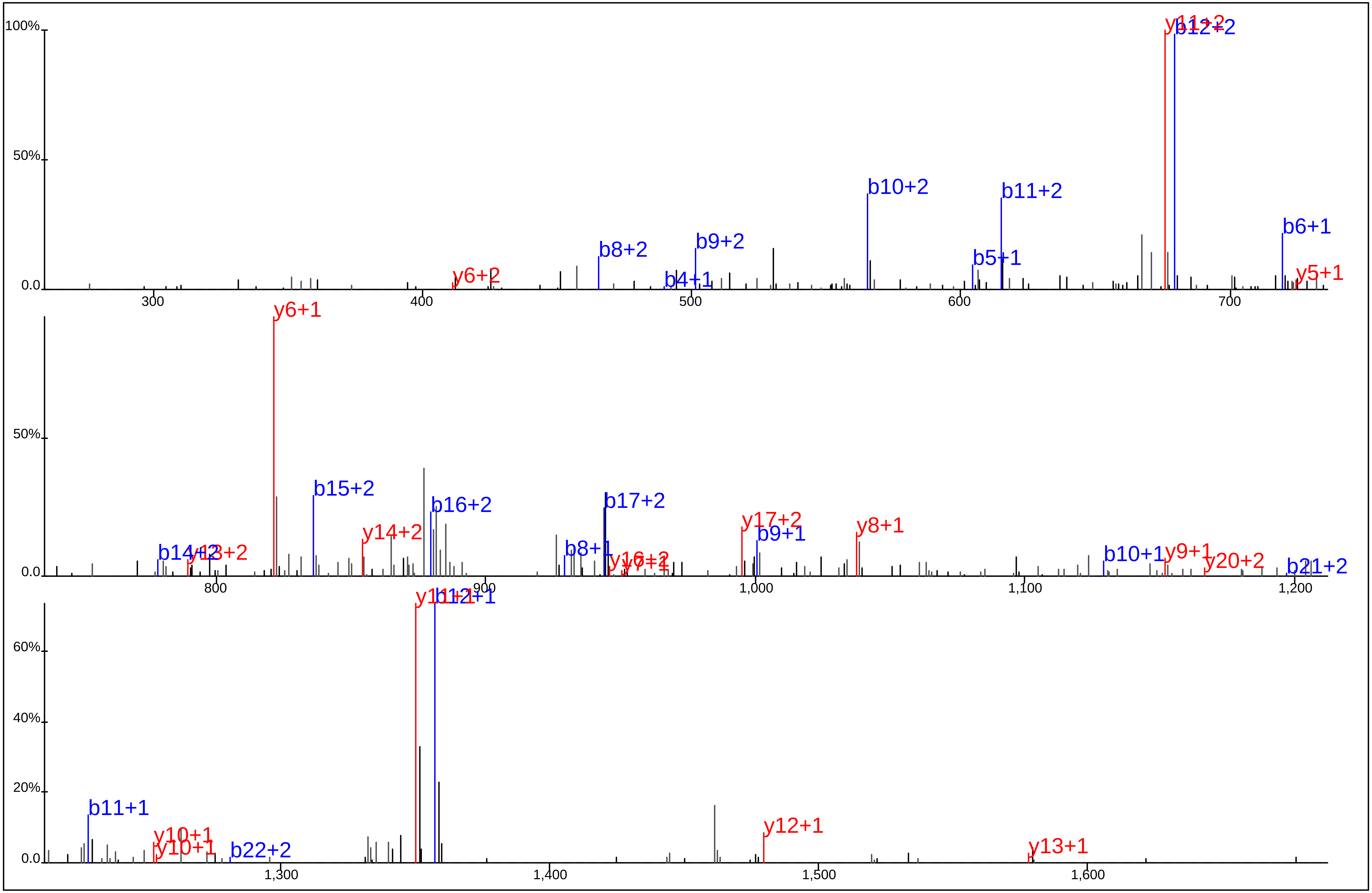

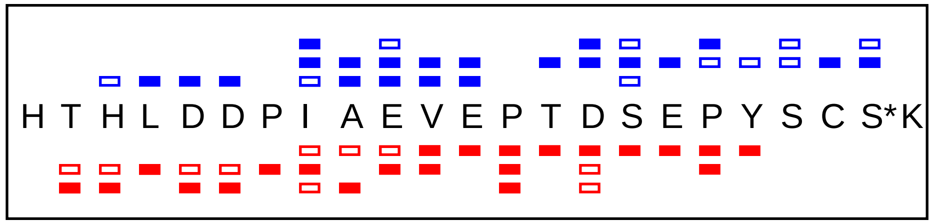

Protein Name: PDZ-GEF2
gi Accession: 34395686
Precursor Mass: 903.0483+3
Delta Mass: 2.718 ppm
XCorr: 32.73

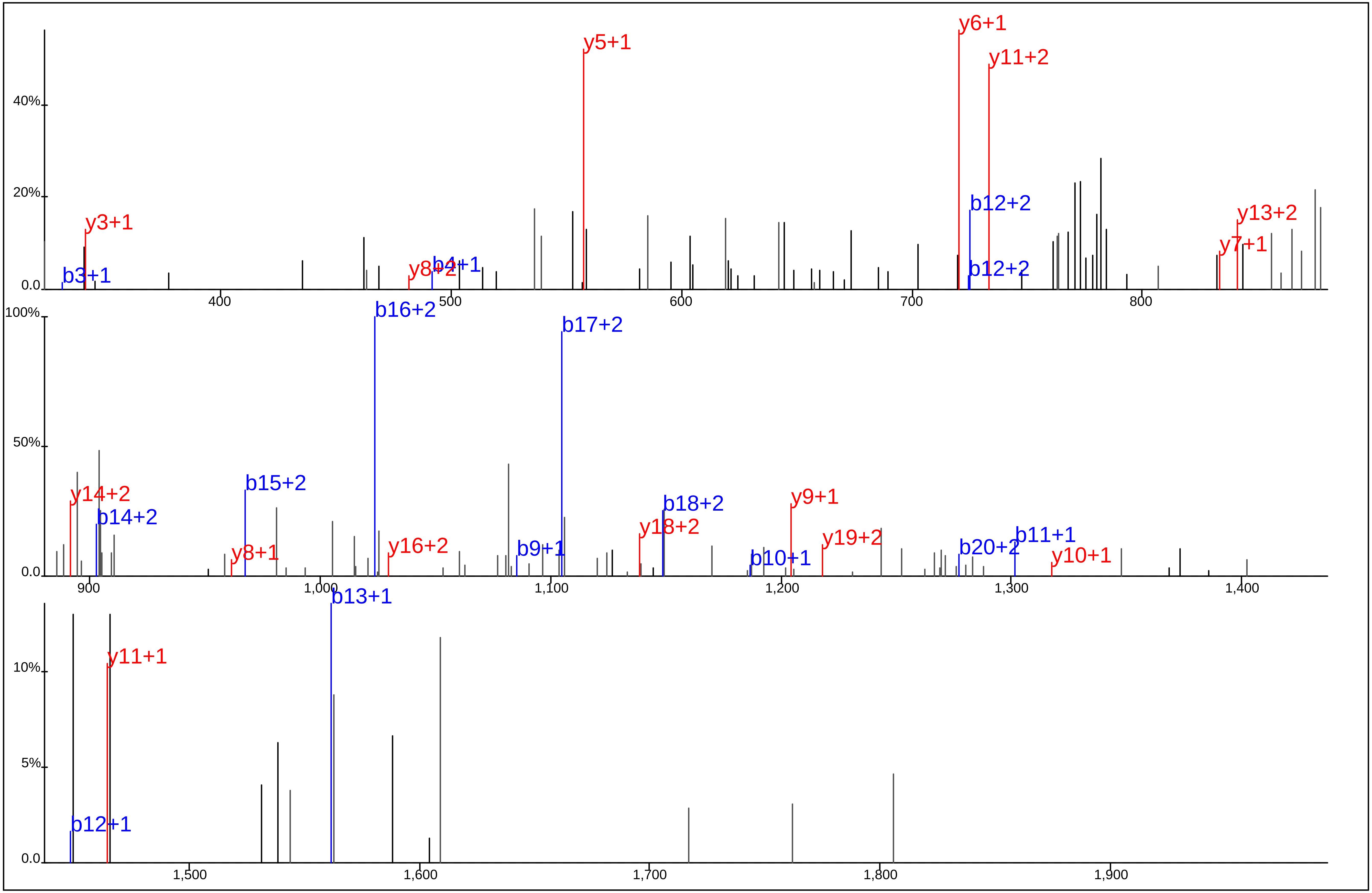

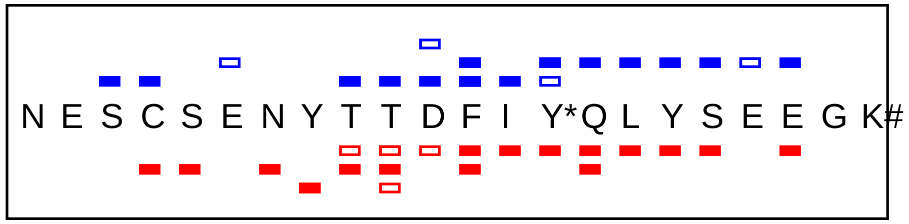

Protein Name:	Phosphofructokinase 1
gi Accession:	11321601
Precursor Mass:	922.3748+3
Delta Mass:	1.656 ppm
XCorr:	26.63

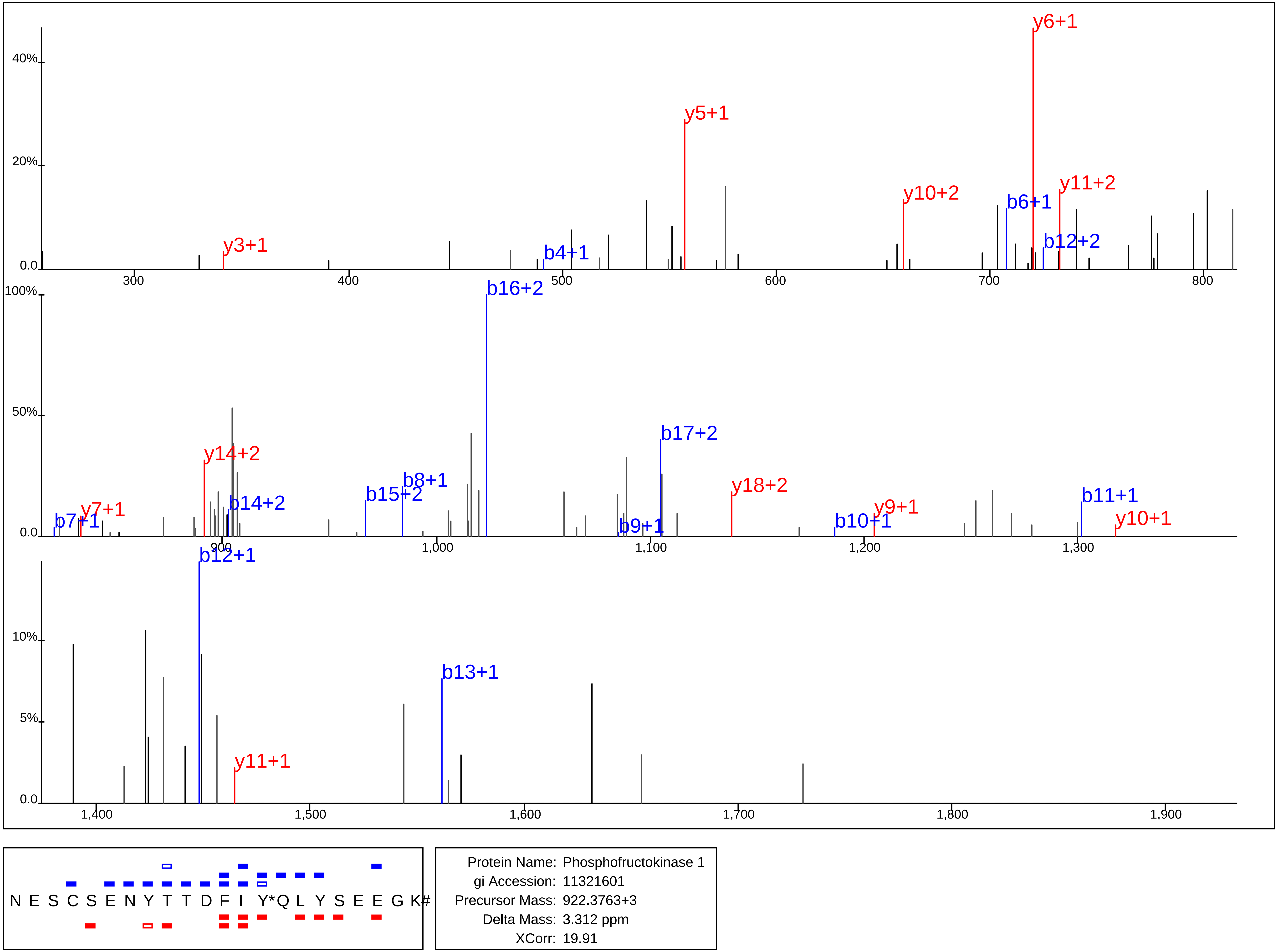

Protein Name: Phosphofructokinase 1
gi Accession: 11321601
Precursor Mass: 922.3763+3
Delta Mass: 3.312 ppm
XCorr: 19.91

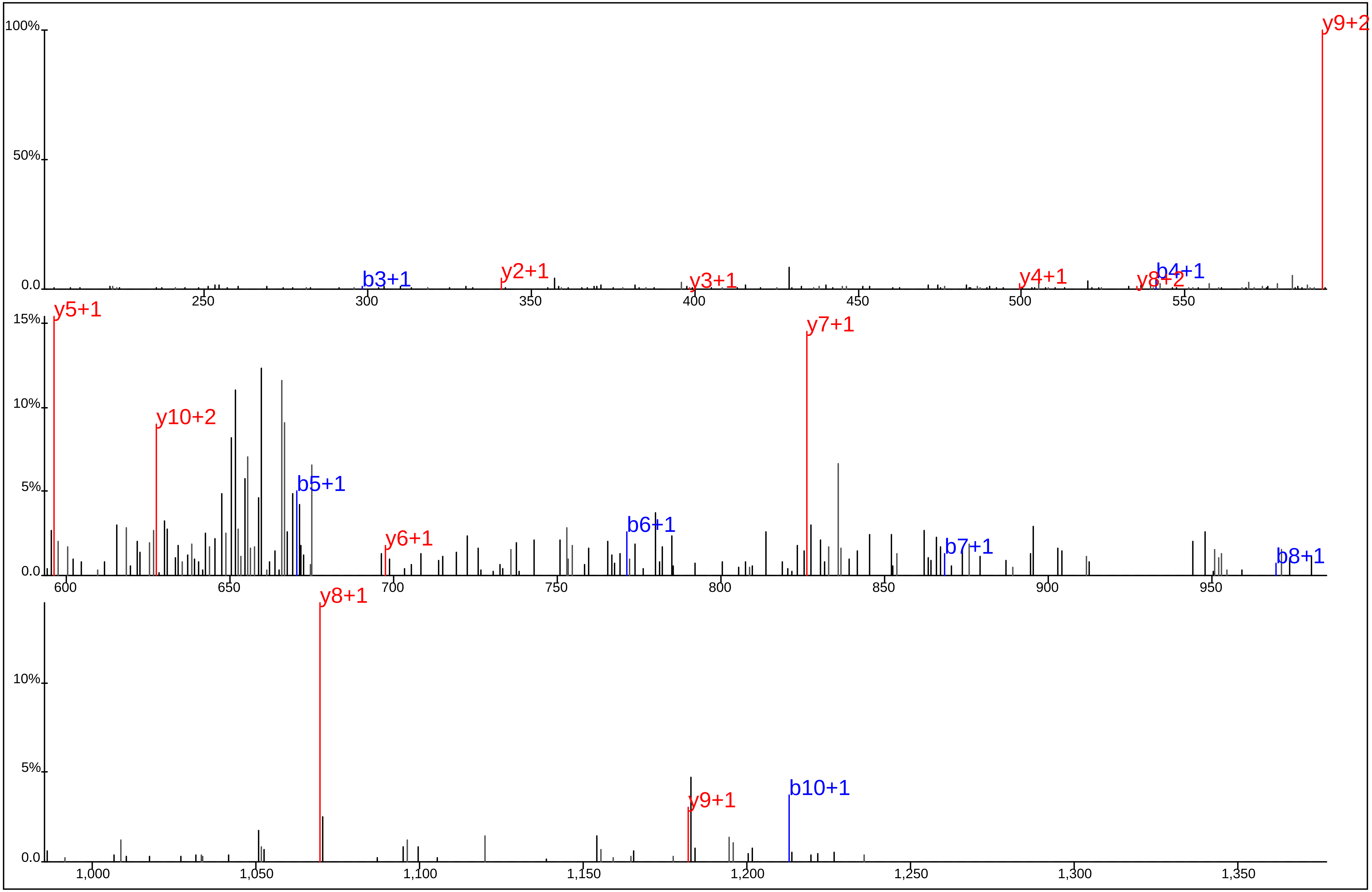

I A L Y*E T P T G W K#

Protein Name:	Phosphoglucomutase 1
gi Accession:	21361621
Precursor Mass:	683.8325+2
Delta Mass:	3.116 ppm
XCorr:	21.43

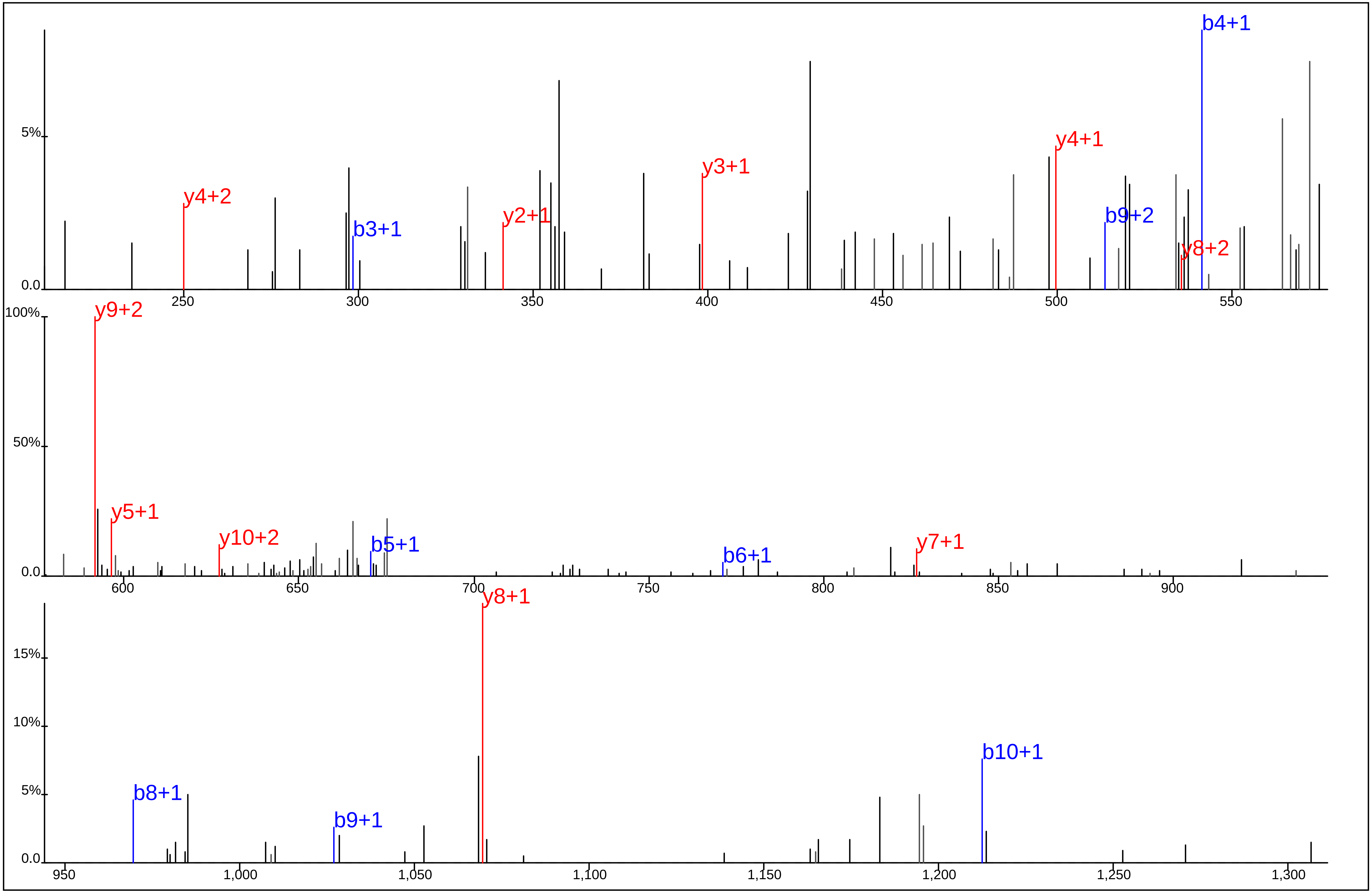

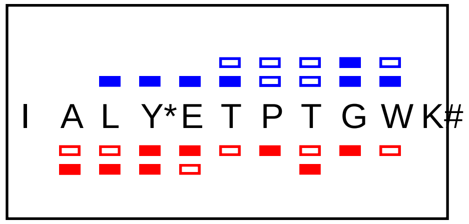

Protein Name:	Phosphoglucomutase 1
gi Accession:	21361621
Precursor Mass:	683.8359+2
Delta Mass:	8.208 ppm
XCorr:	27.94

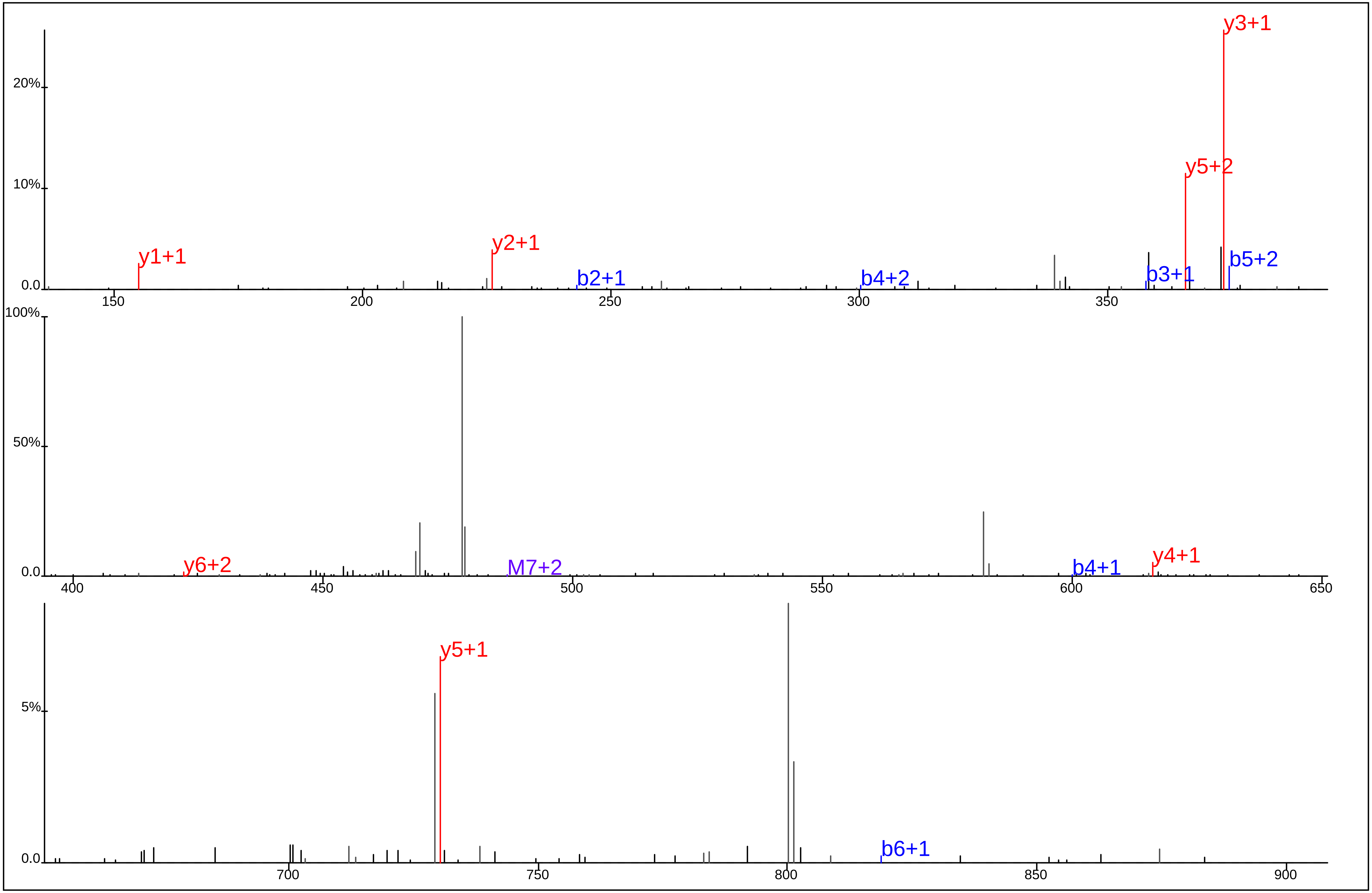

E L N Y*F A K#

Protein Name:	Phosphoglycerate kinase 1
gi Accession:	4505763
Precursor Mass:	486.7203+2
Delta Mass:	1.705 ppm
XCorr:	25.65

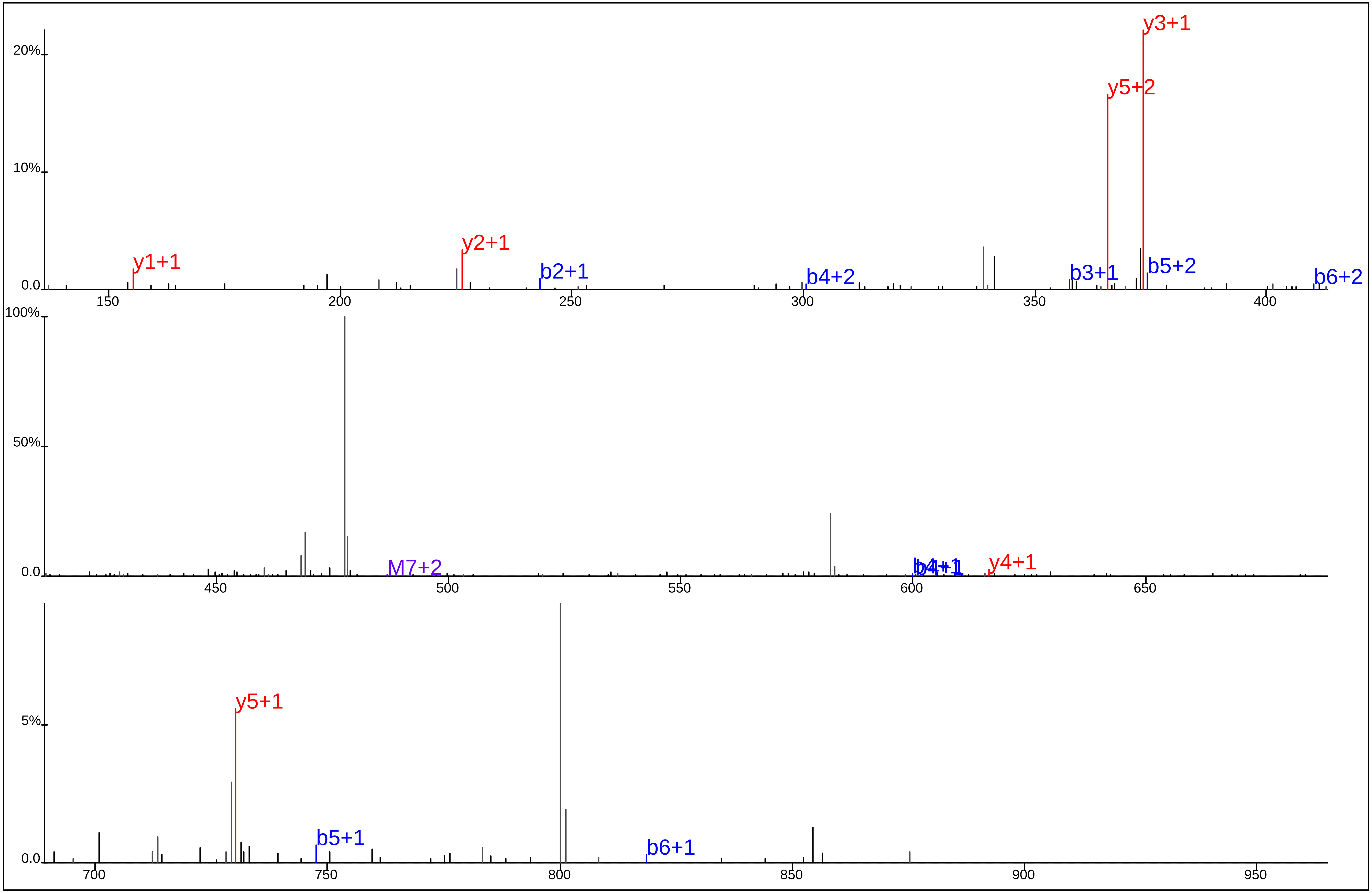

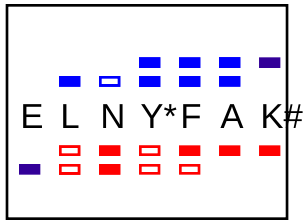

Protein Name:	Phosphoglycerate kinase 1
gi Accession:	4505763
Precursor Mass:	486.7213+2
Delta Mass:	3.649 ppm
XCorr:	38.17

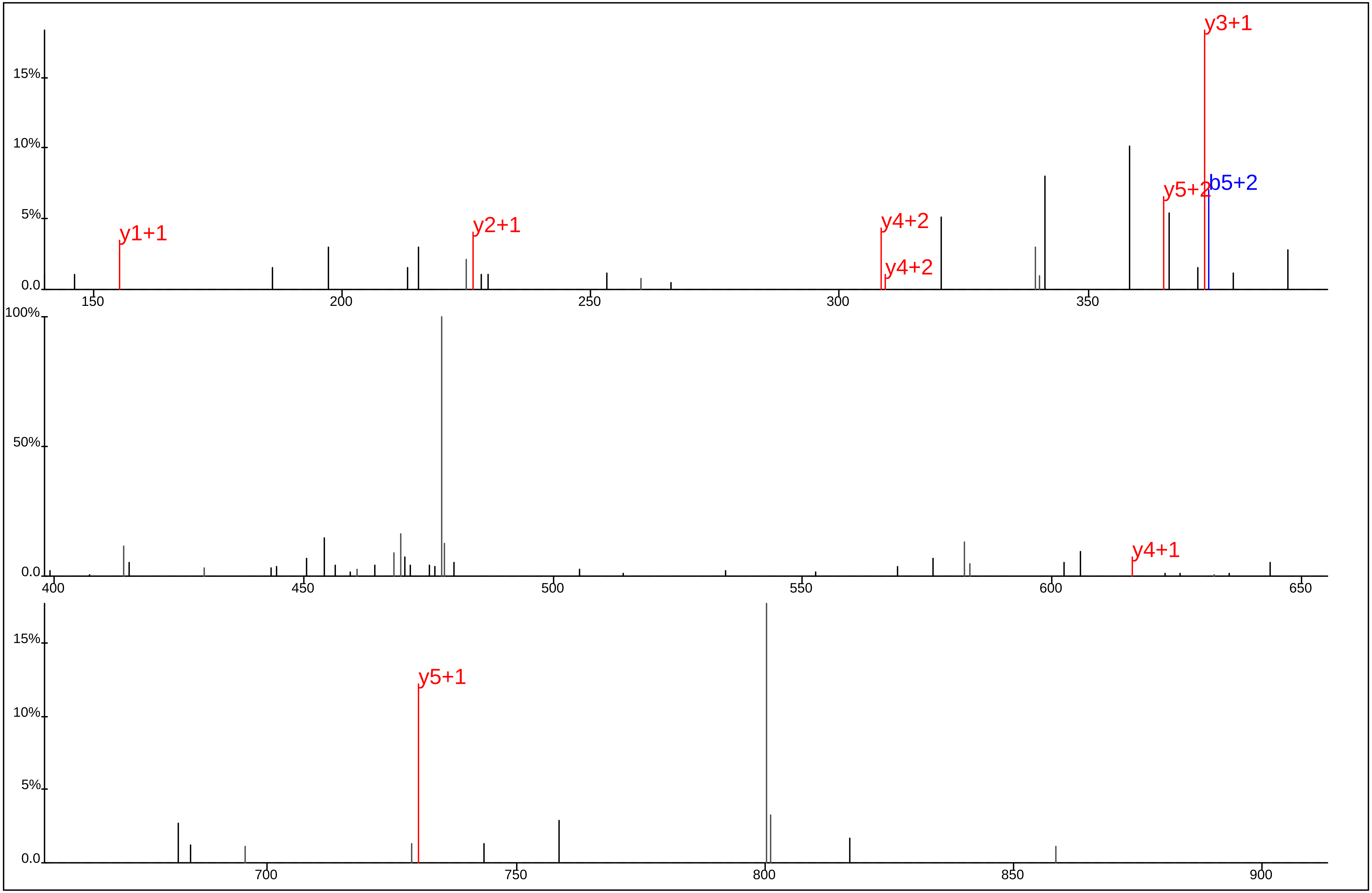

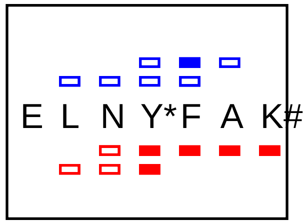

Protein Name:	Phosphoglycerate kinase 1
gi Accession:	4505763
Precursor Mass:	486.7208+2
Delta Mass:	2.579 ppm
XCorr:	19.19

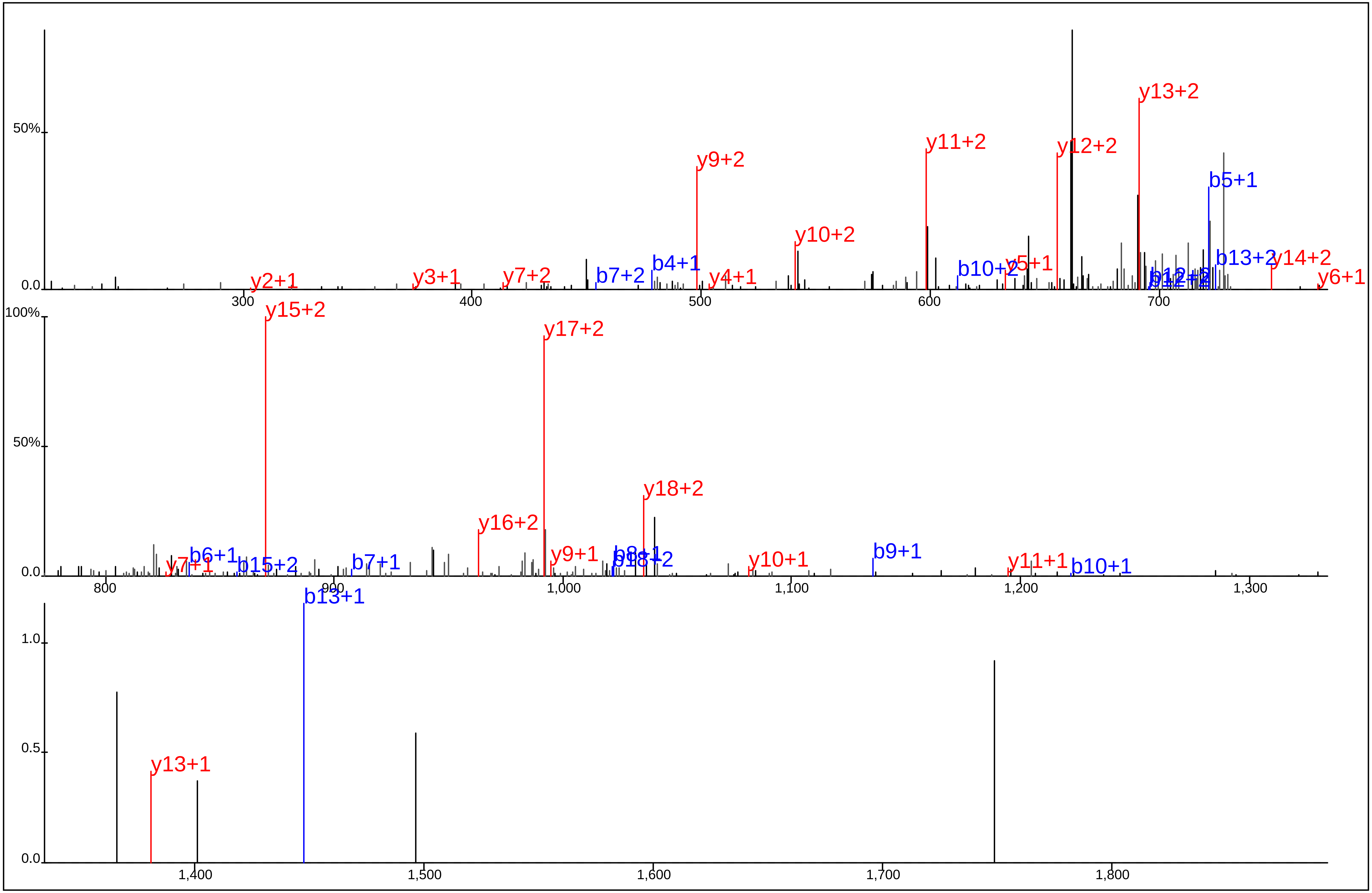

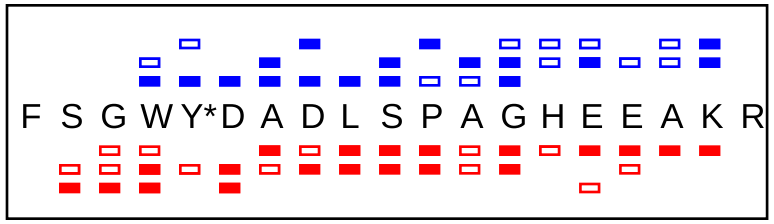

Protein Name:	Phosphoglycerate mutase 1
gi Accession:	4505753
Precursor Mass:	739.3228+3
Delta Mass:	4.196 ppm
XCorr:	32.73

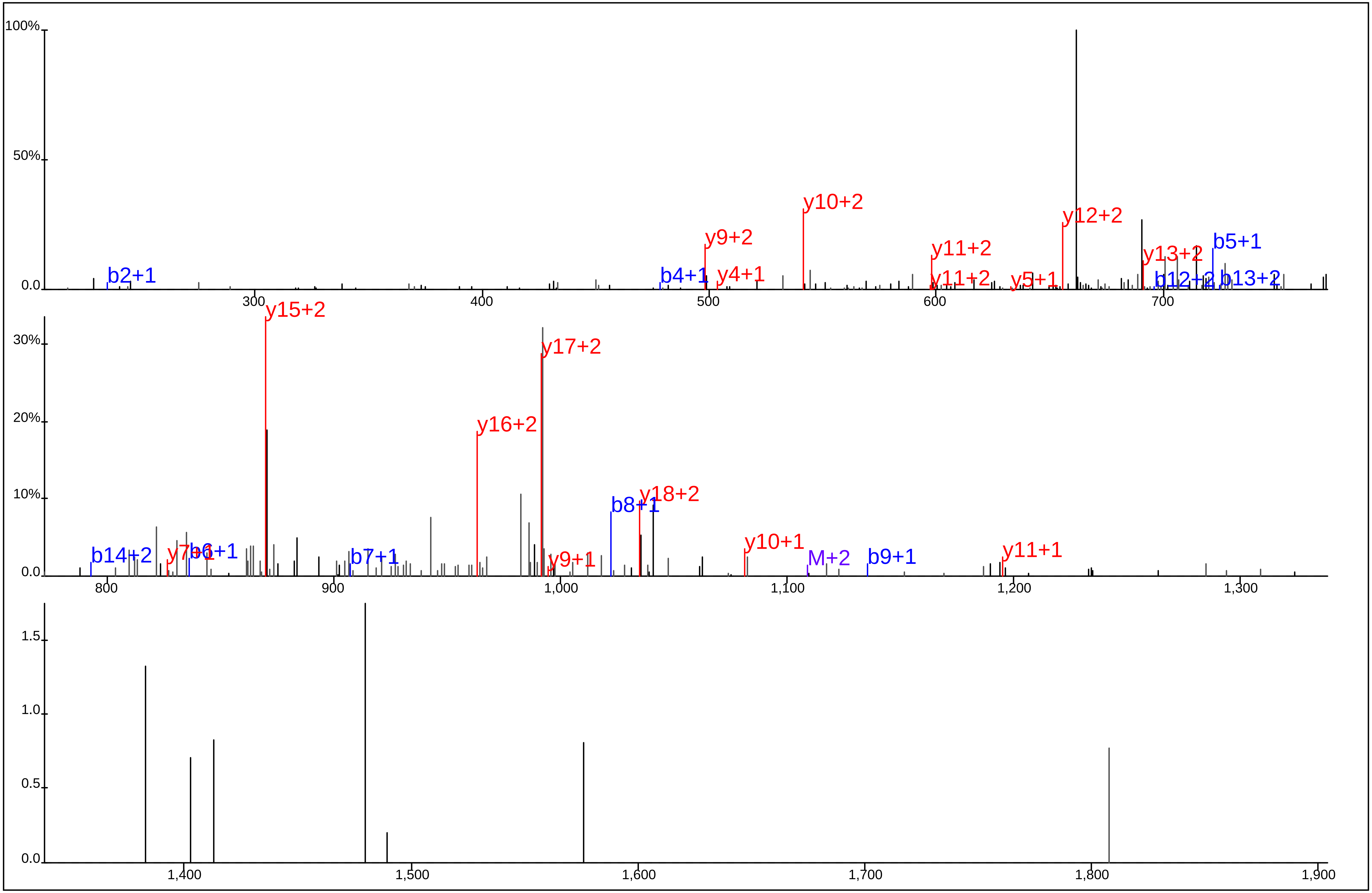

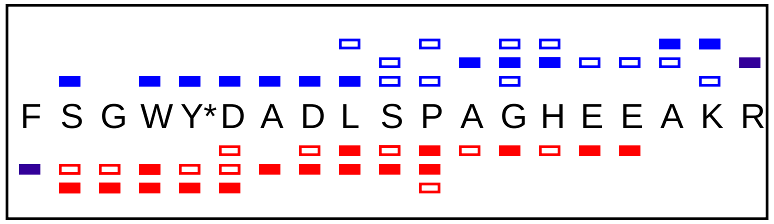

Protein Name:	Phosphoglycerate mutase 1
gi Accession:	4505753
Precursor Mass:	739.3230+3
Delta Mass:	4.525 ppm
XCorr:	27.44

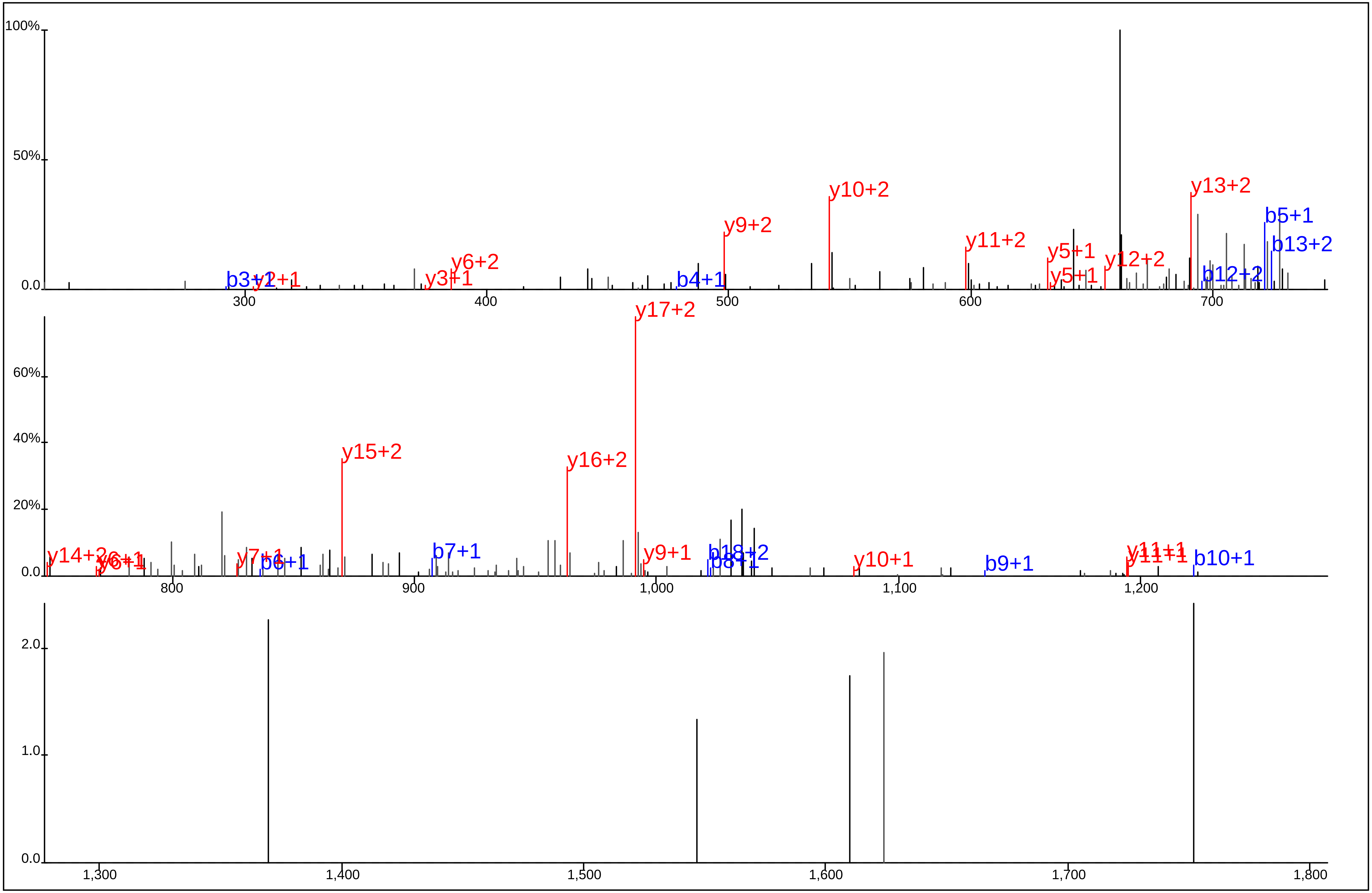

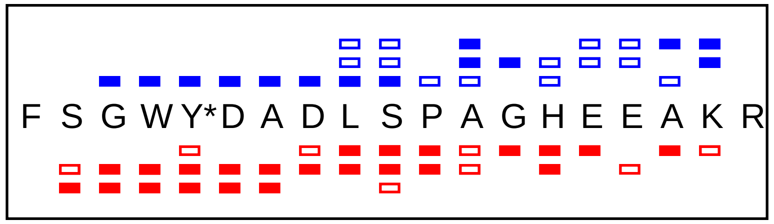

Protein Name: Phosphoglycerate mutase 1
gi Accession: 4505753
Precursor Mass: 739.3218+3
Delta Mass: 2.954 ppm
XCorr: 29.82

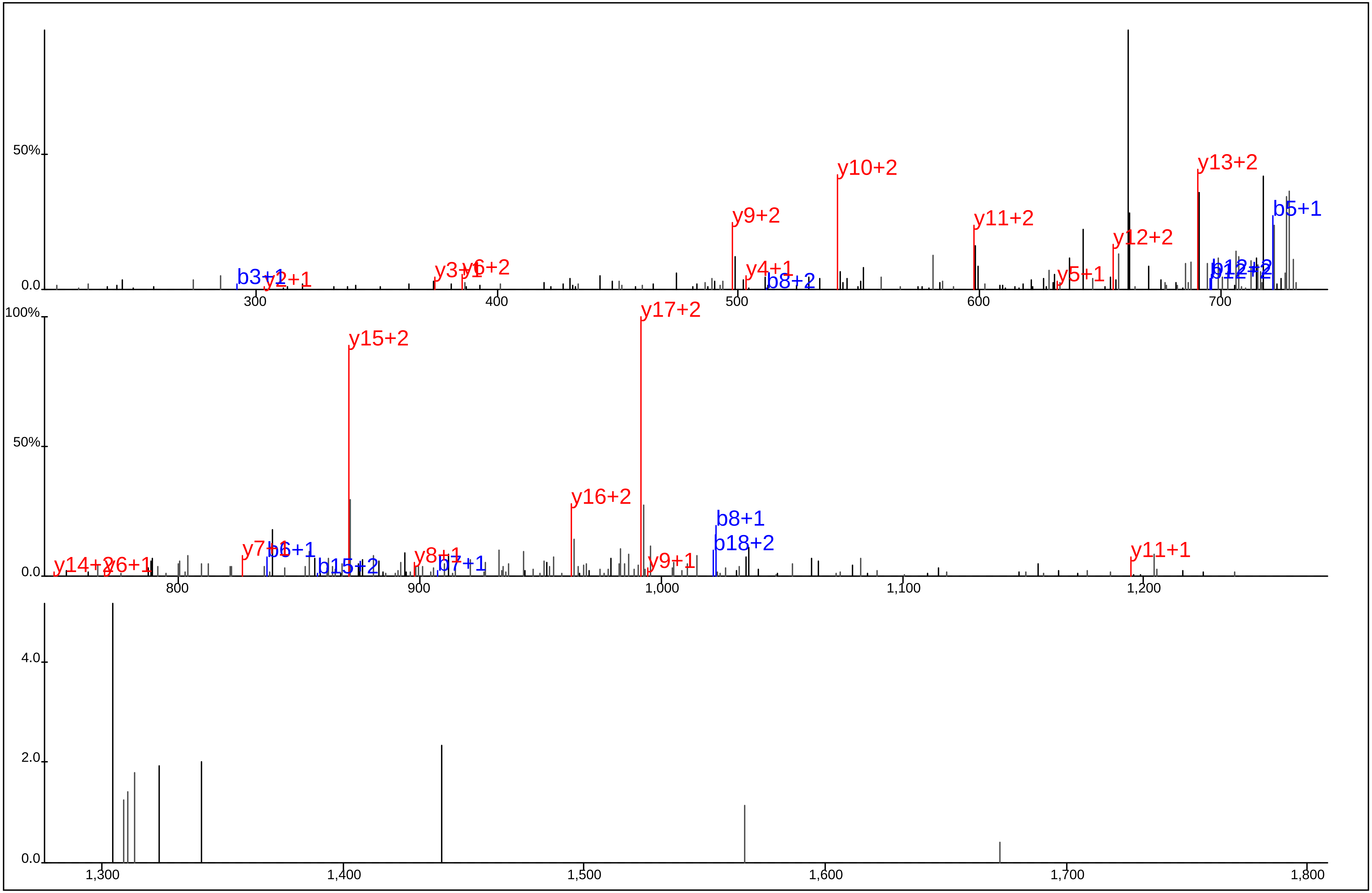

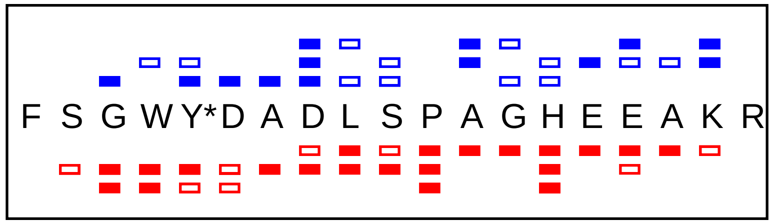

Protein Name: Phosphoglycerate mutase 1
gi Accession: 4505753
Precursor Mass: 739.3221+3
Delta Mass: 3.370 ppm
XCorr: 30.08

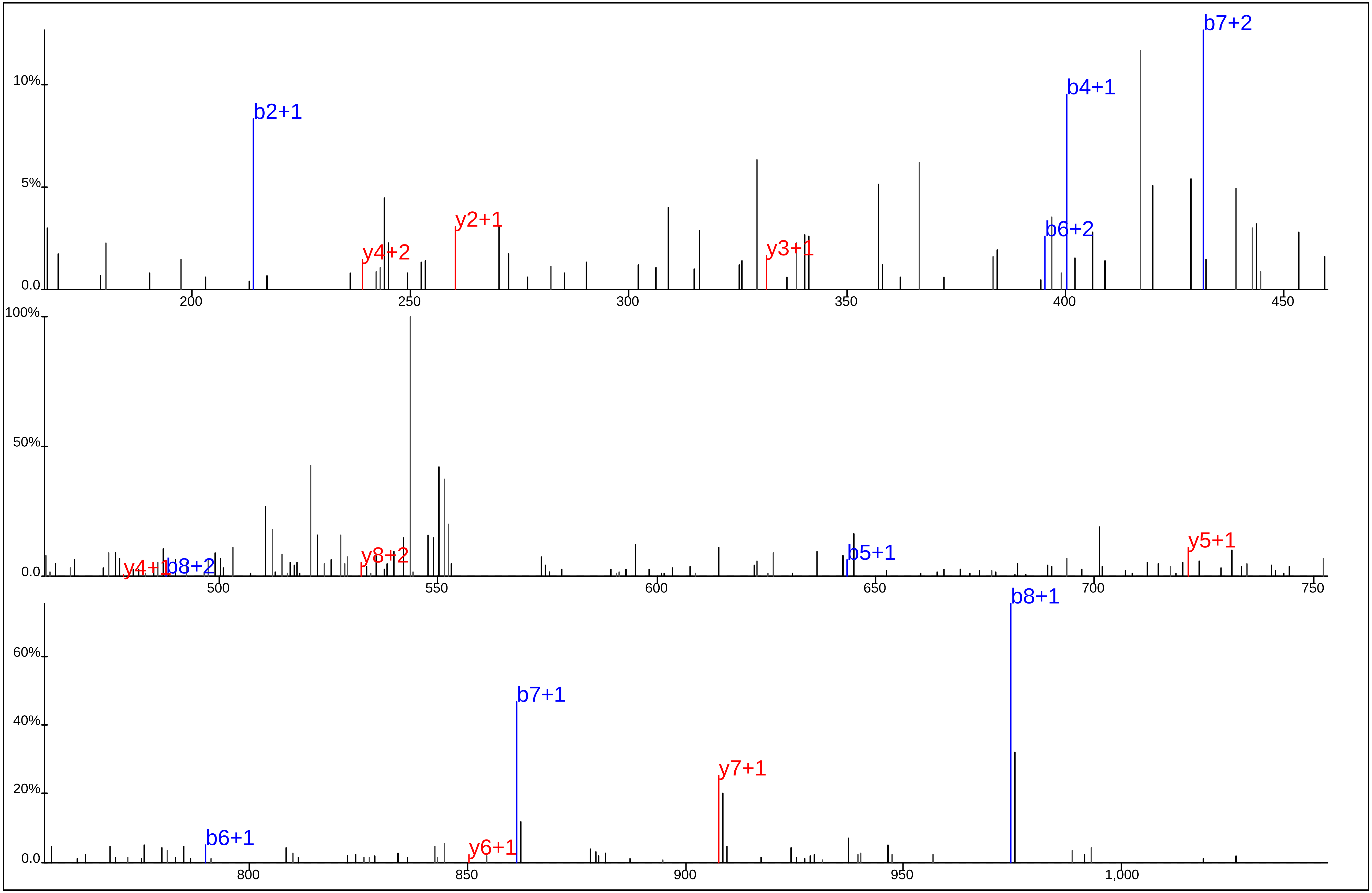

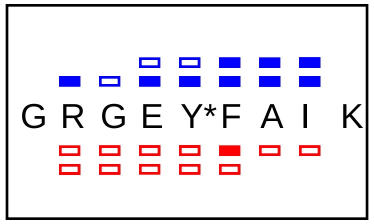

Protein Name: PKC delta
gi Accession: 520587
Precursor Mass: 560.7632+2
Delta Mass: 0.464 ppm
XCorr: 2.3054

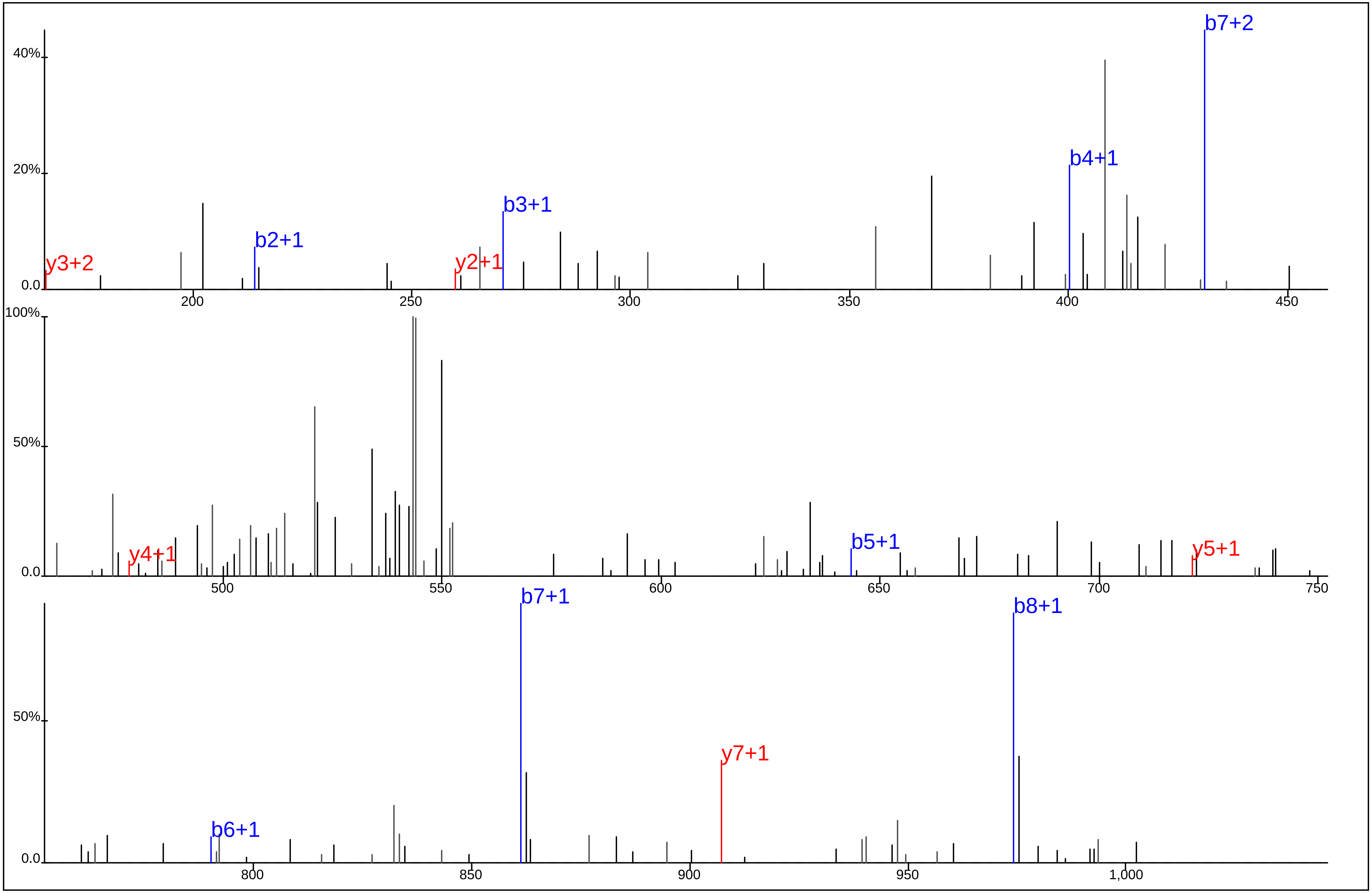

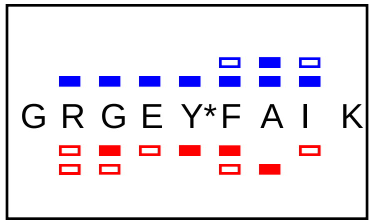

Protein Name: PKC delta
gi Accession: 520587
Precursor Mass: 560.7640+2
Delta Mass: 1.776 ppm
XCorr: 2.2886

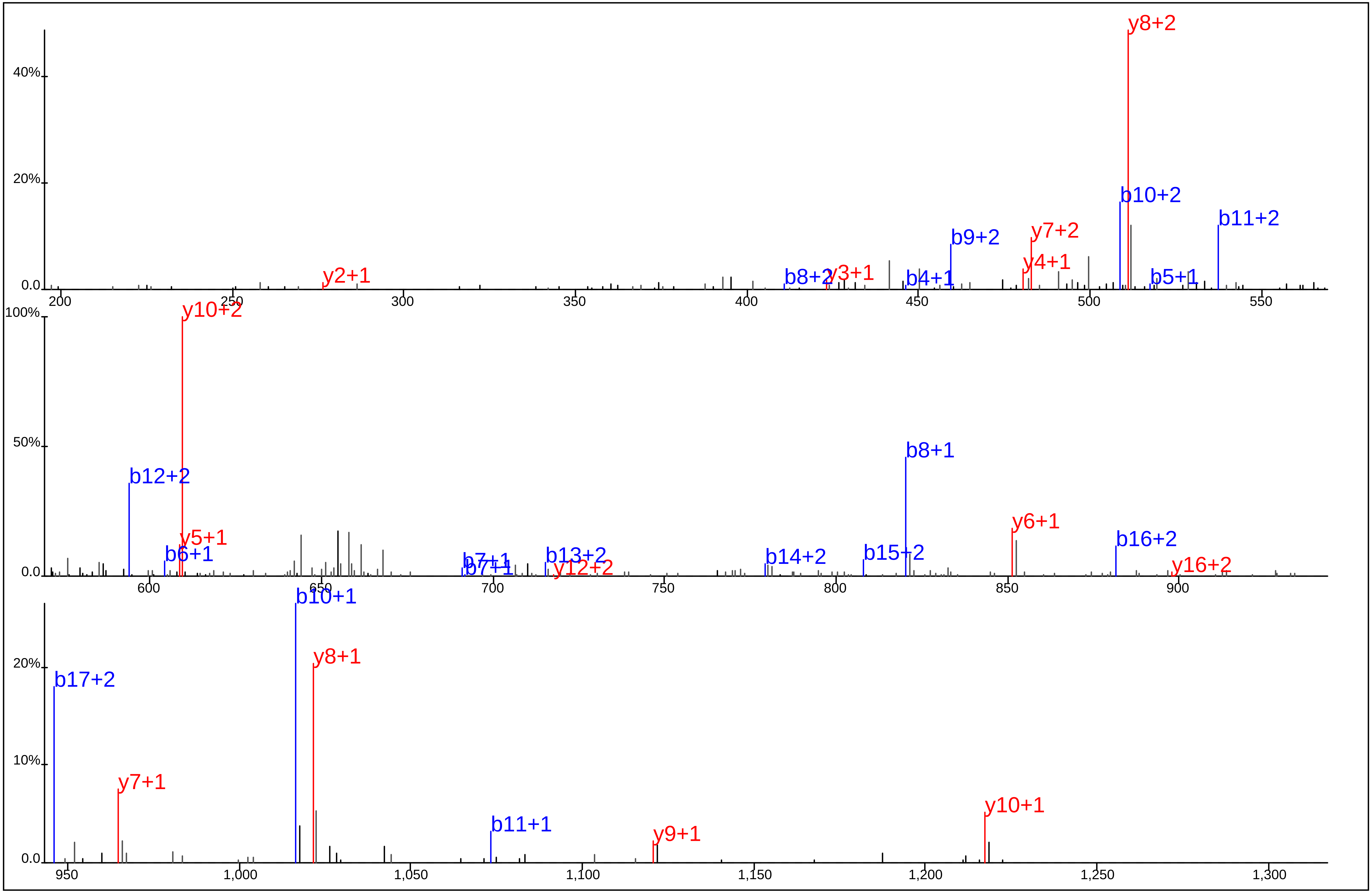

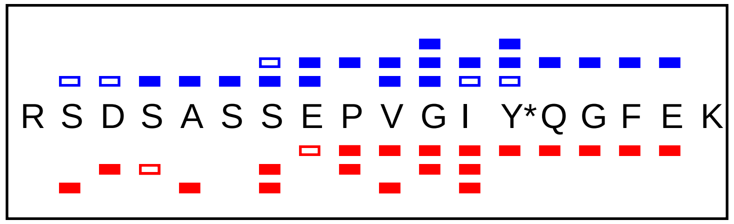

Protein Name:	PKC delta
gi Accession:	520587
Precursor Mass:	679.6400+3
Delta Mass:	4.624 ppm
XCorr:	46.23

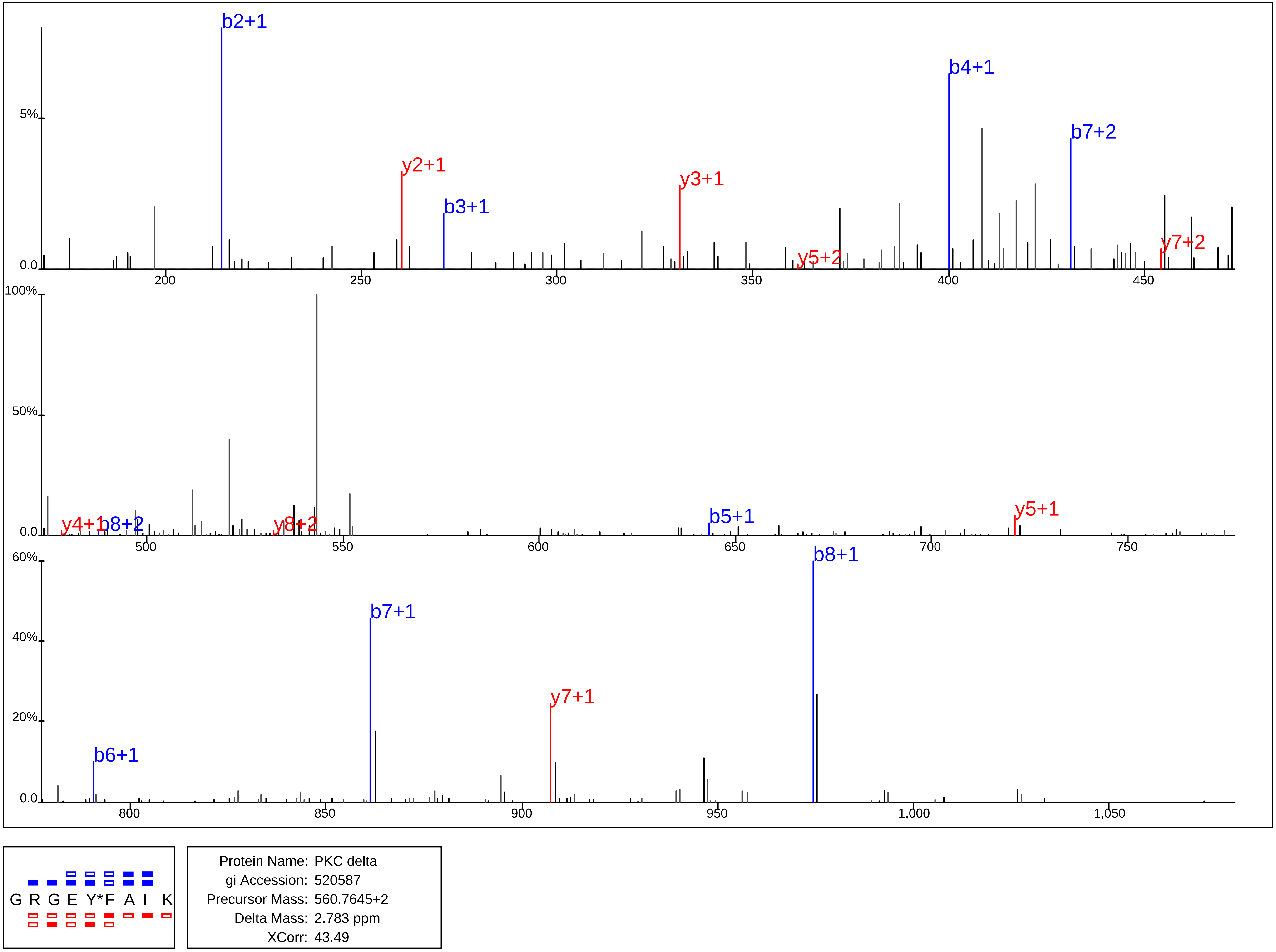

Protein Name: PKC delta
gi Accession: 520587
Precursor Mass: 560.7645+2
Delta Mass: 2.783 ppm
XCorr: 43.49

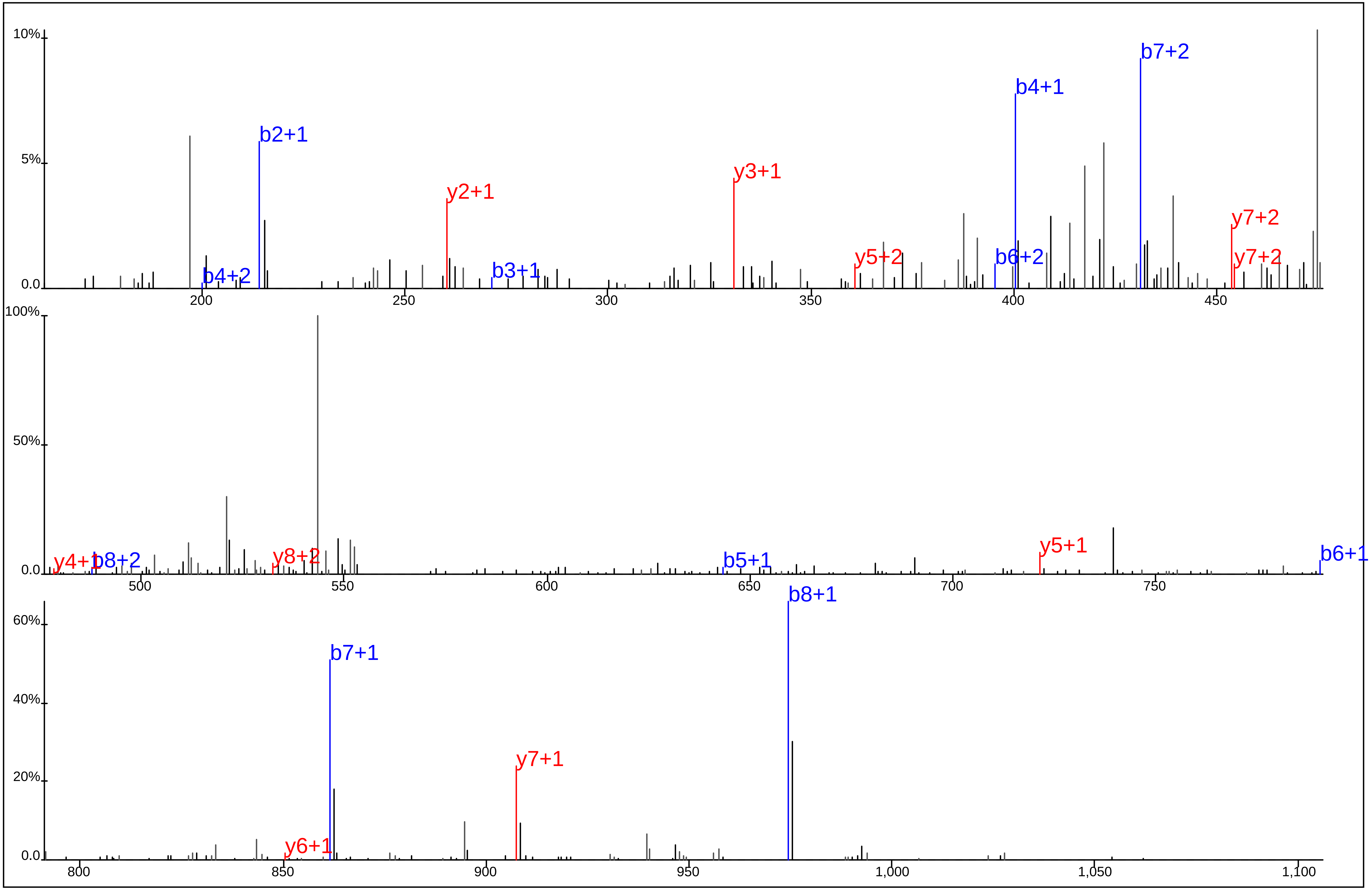

G R G E Y*F A I K

Protein Name: PKC delta
gi Accession: 520587
Precursor Mass: 560.7654+2
Delta Mass: 4.416 ppm
XCorr: 38.8

751 - PKCdelta_3435708.pdf

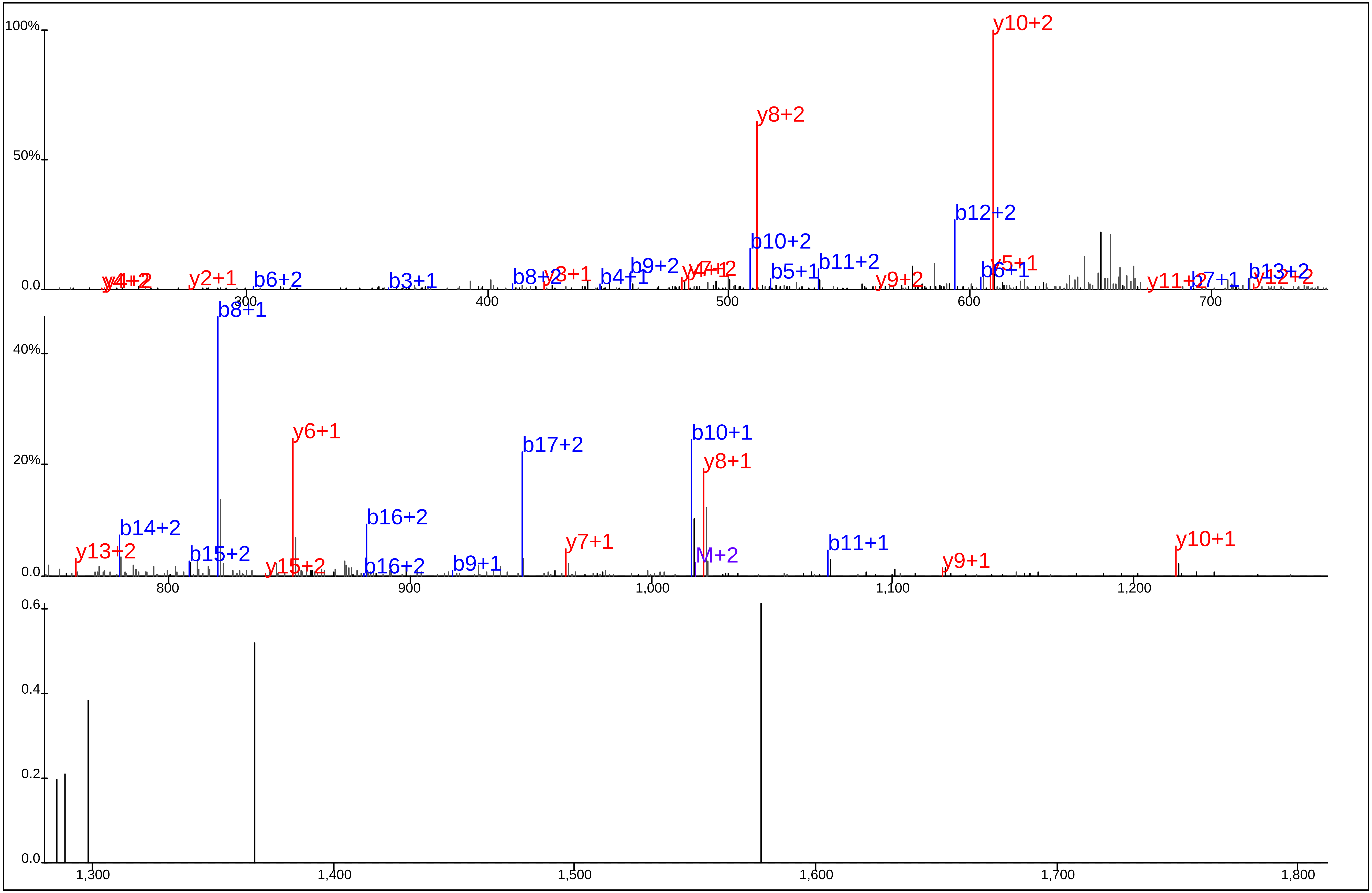

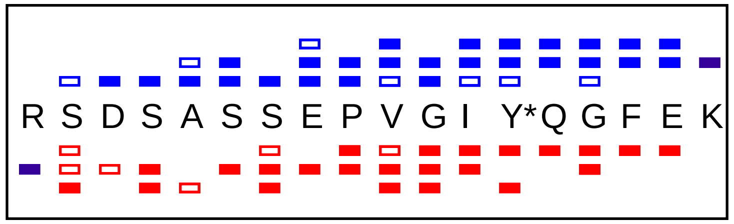

Protein Name: PKC delta
gi Accession: 520587
Precursor Mass: 679.6387+3
Delta Mass: 2.738 ppm
XCorr: 48.82

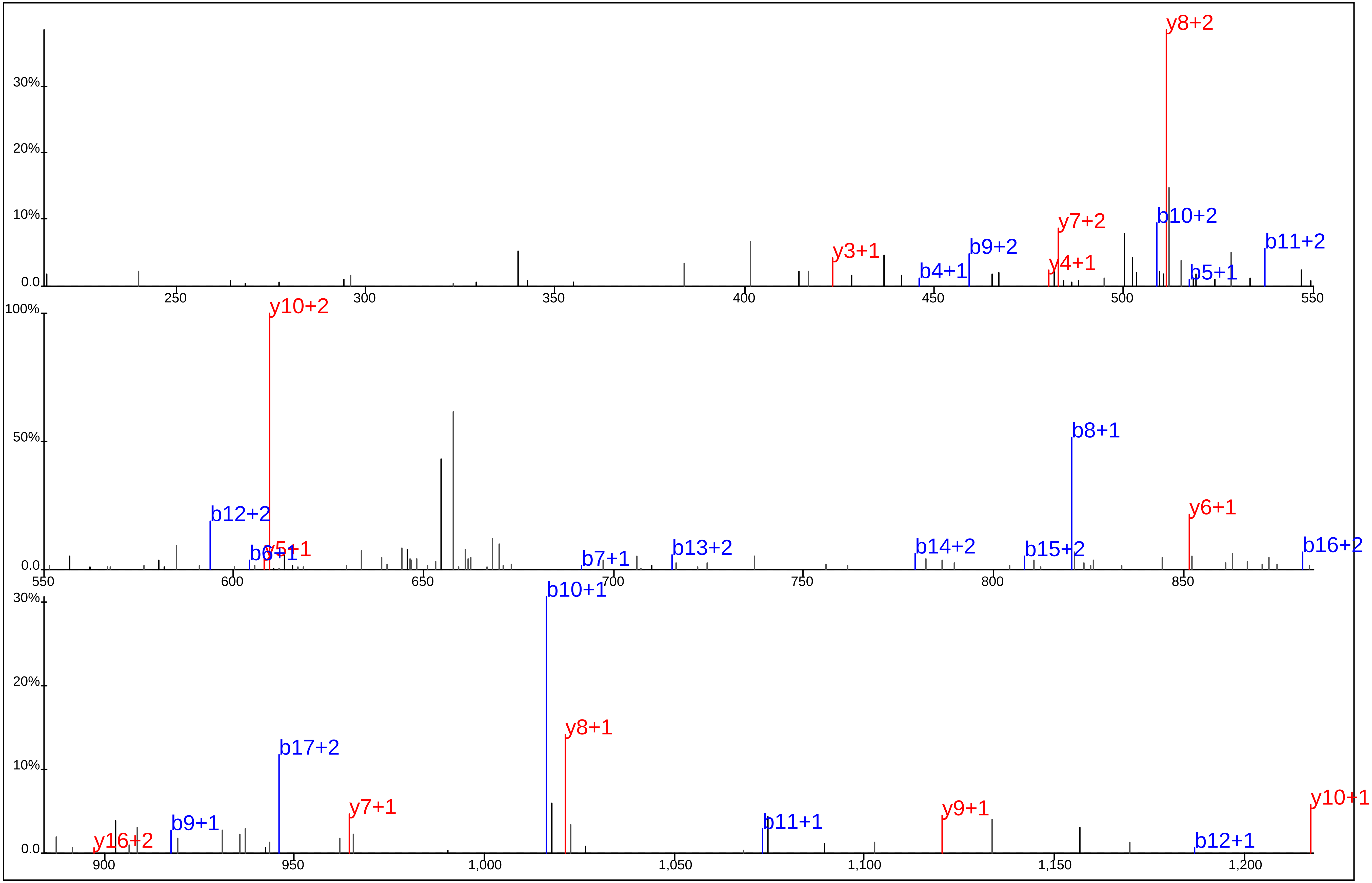

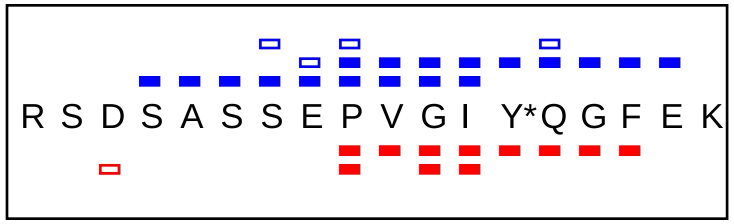

Protein Name: PKC delta
gi Accession: 520587
Precursor Mass: 679.6389+3
Delta Mass: 3.004 ppm
XCorr: 30.63

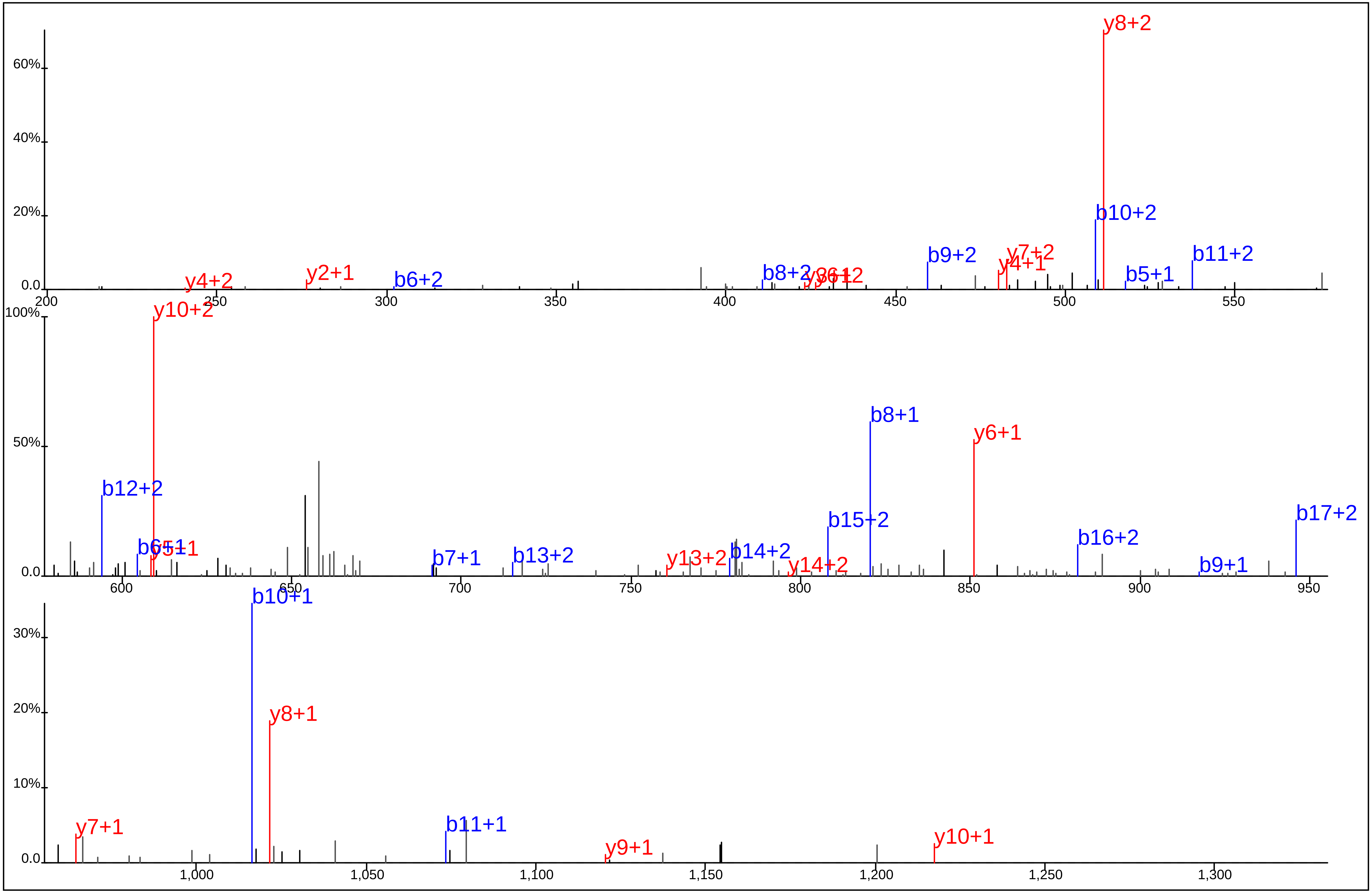

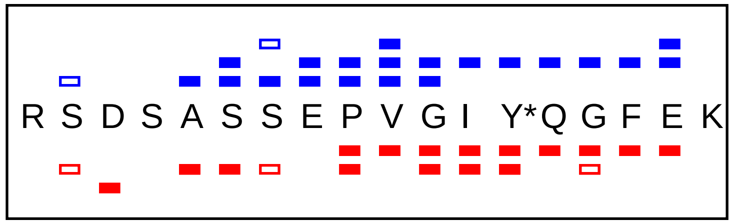

Protein Name: PKC delta
gi Accession: 520587
Precursor Mass: 679.6389+3
Delta Mass: 3.004 ppm
XCorr: 47.73

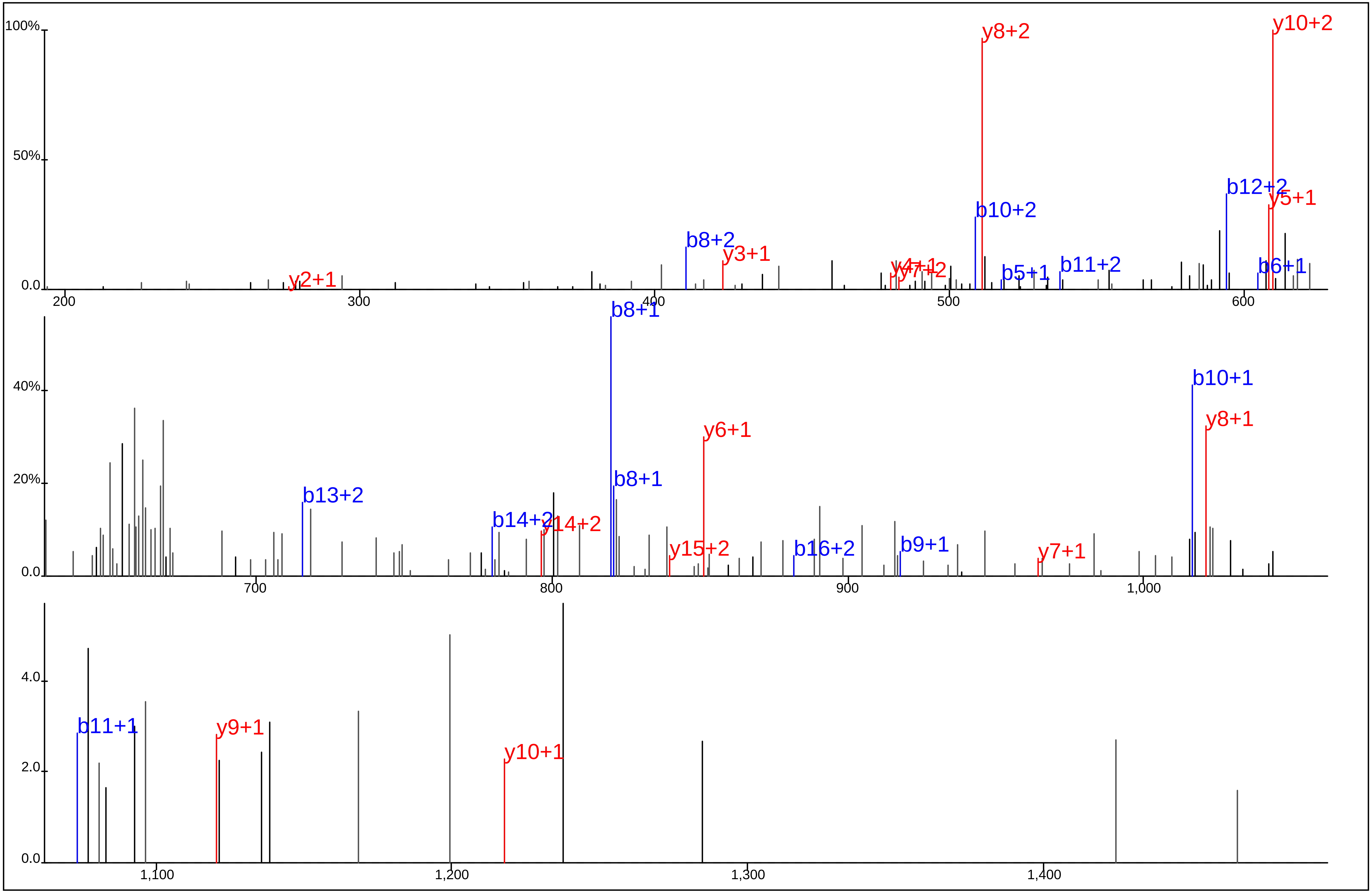

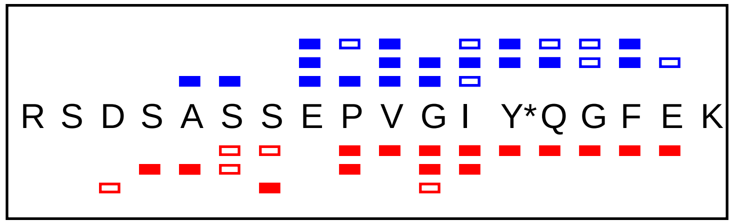

Protein Name: PKC delta
gi Accession: 520587
Precursor Mass: 679.6421+3
Delta Mass: 7.683 ppm
XCorr: 18.05

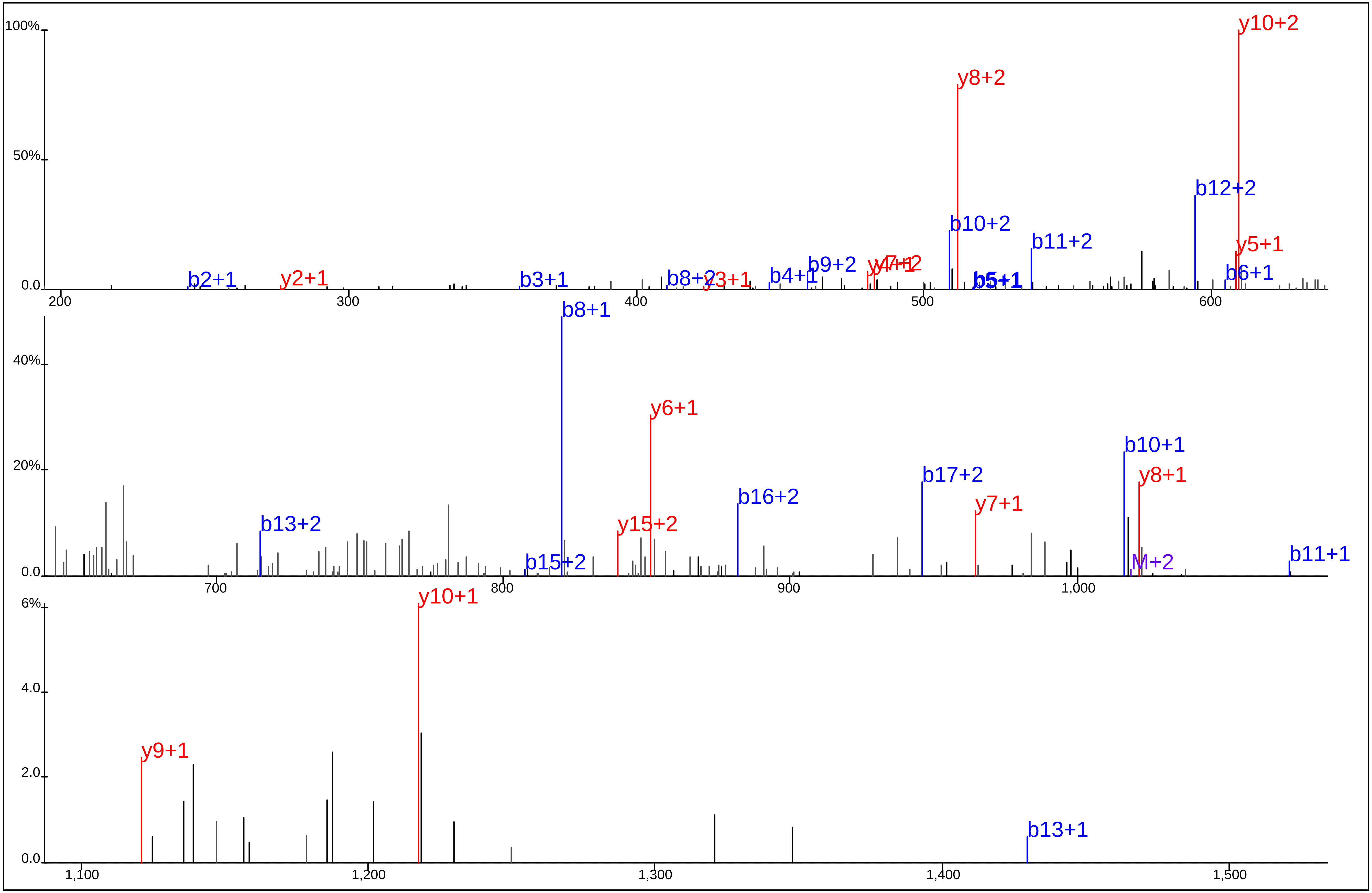

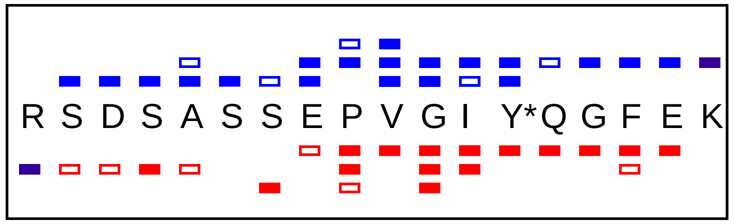

Protein Name: PKC delta
gi Accession: 520587
Precursor Mass: 679.6396+3
Delta Mass: 4.083 ppm
XCorr: 41.46

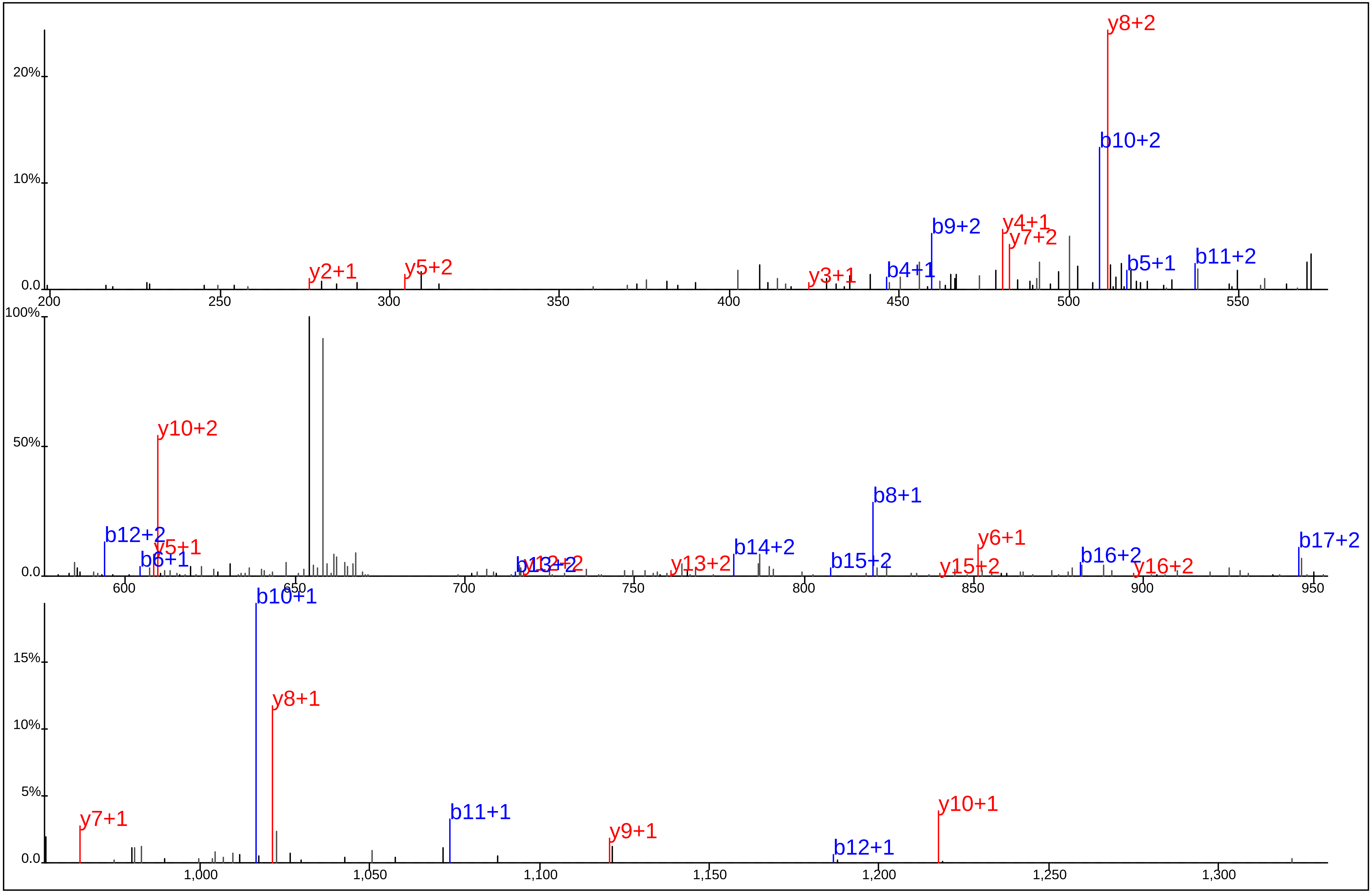

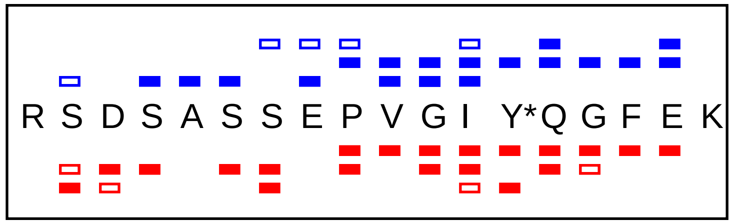

Protein Name: PKC delta
gi Accession: 520587
Precursor Mass: 679.6384+3
Delta Mass: 2.286 ppm
XCorr: 33.69

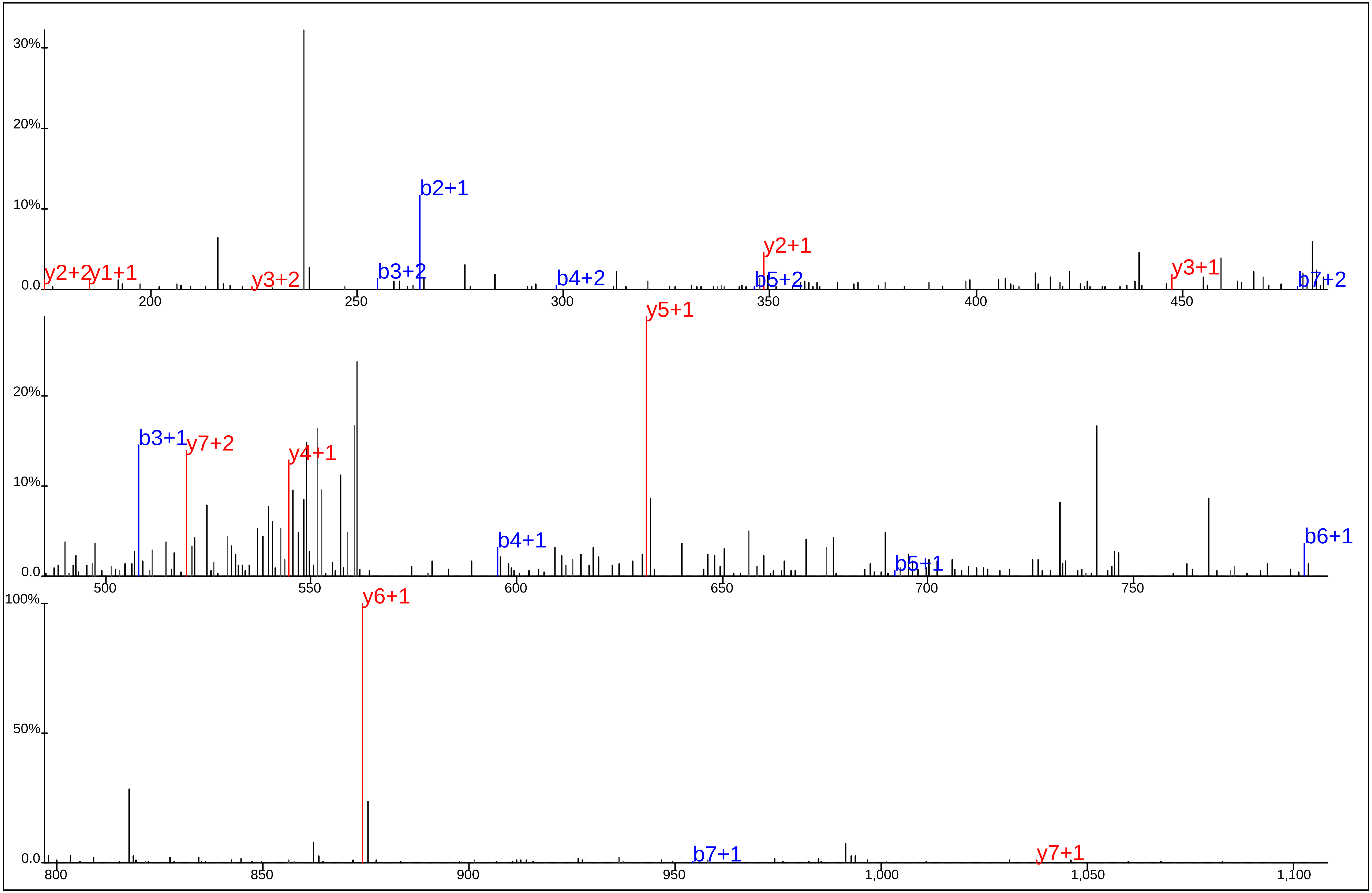

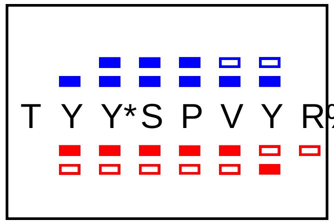

Protein Name: Plakophilin 4
gi Accession: 20139104
Precursor Mass: 569.7484+2
Delta Mass: 4.416 ppm
XCorr: 17.96

758 - Plakophilin4_3435731.pdf

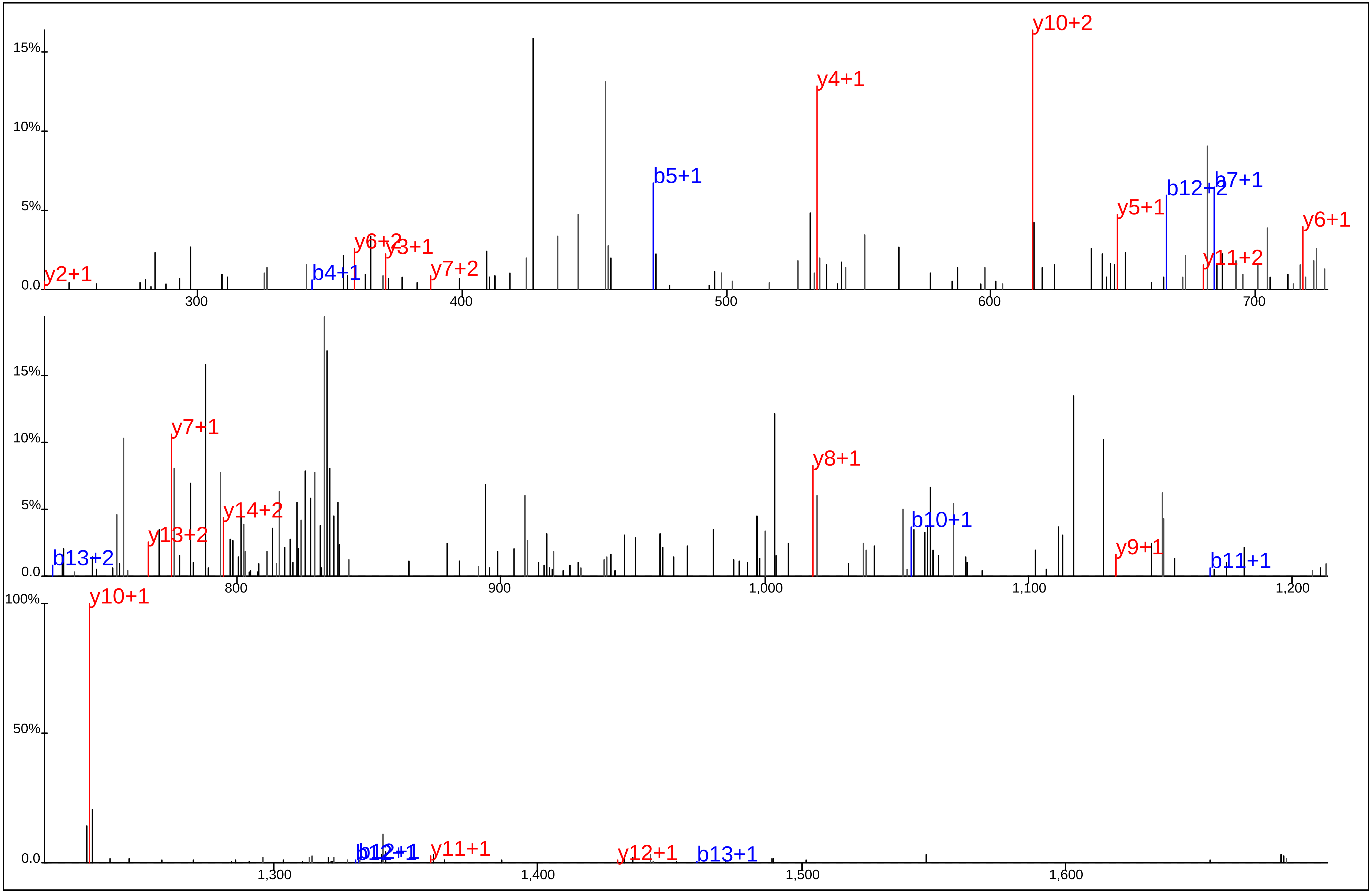

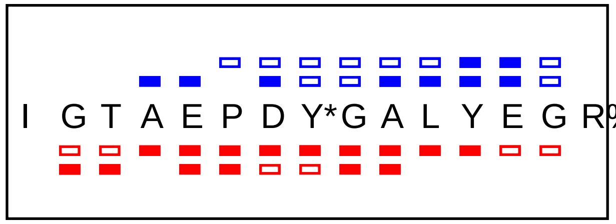

Protein Name: PLC gamma 1
gi Accession: 33598946
Precursor Mass: 851.3807+2
Delta Mass: 8.567 ppm
XCorr: 22.0

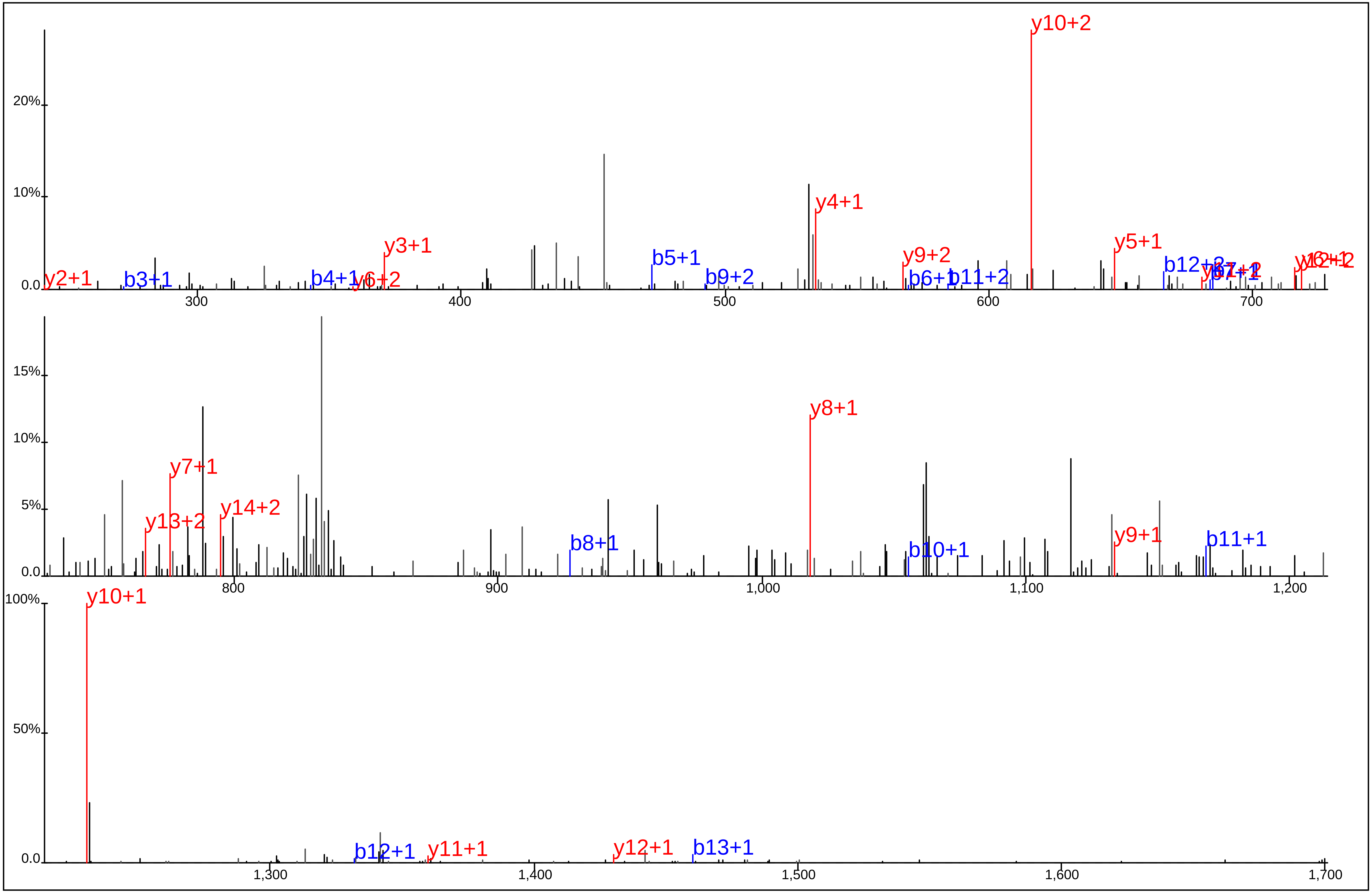

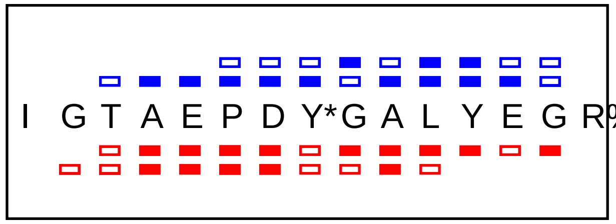

Protein Name: PLC gamma 1
gi Accession: 33598946
Precursor Mass: 851.3776+2
Delta Mass: 4.976 ppm
XCorr: 31.62

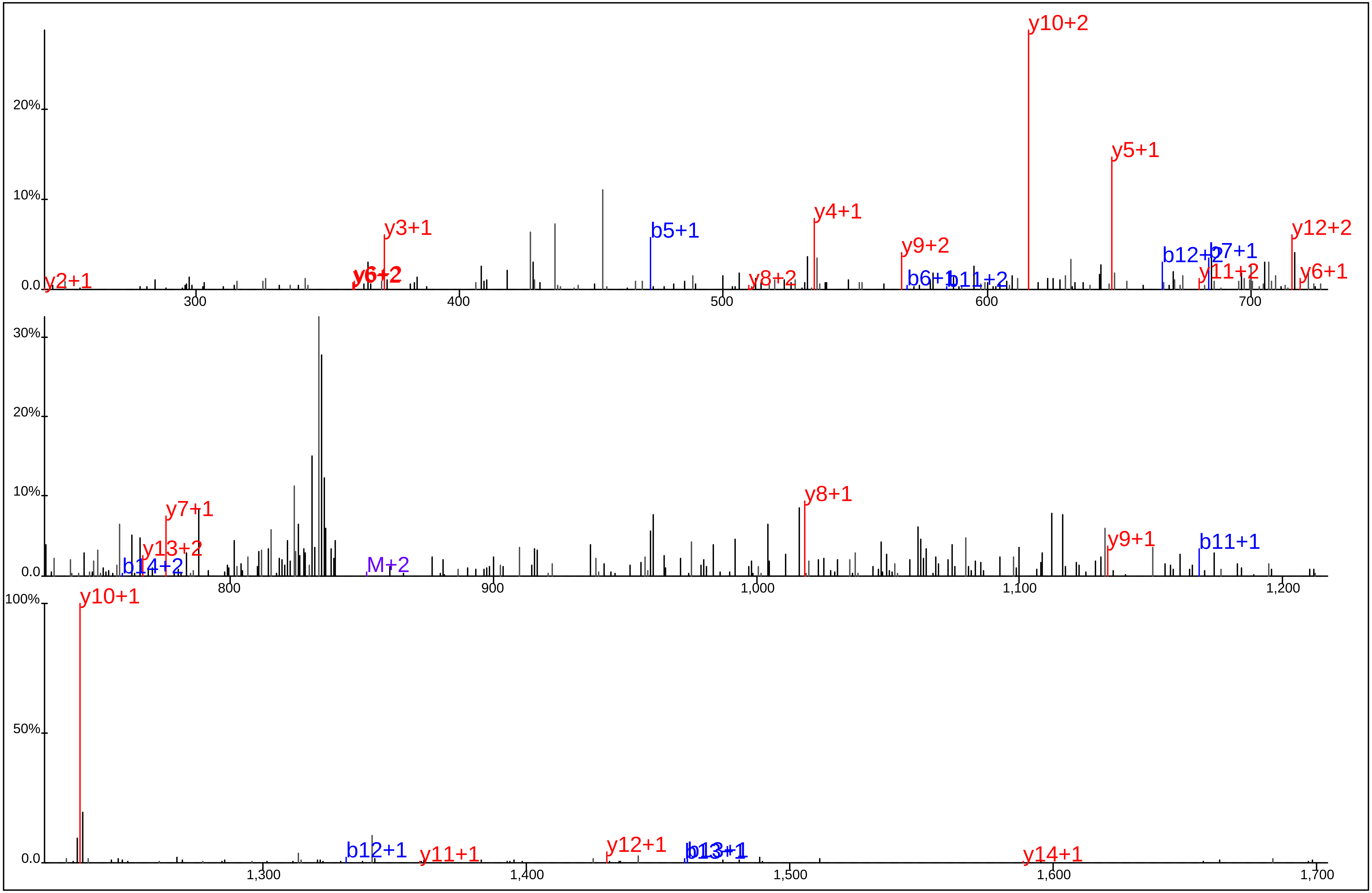

I G T A E P D Y*G A L Y E G R%

Protein Name:	PLC gamma 1
gi Accession:	33598946
Precursor Mass:	851.3720+2
Delta Mass:	1.623 ppm
XCorr:	28.45

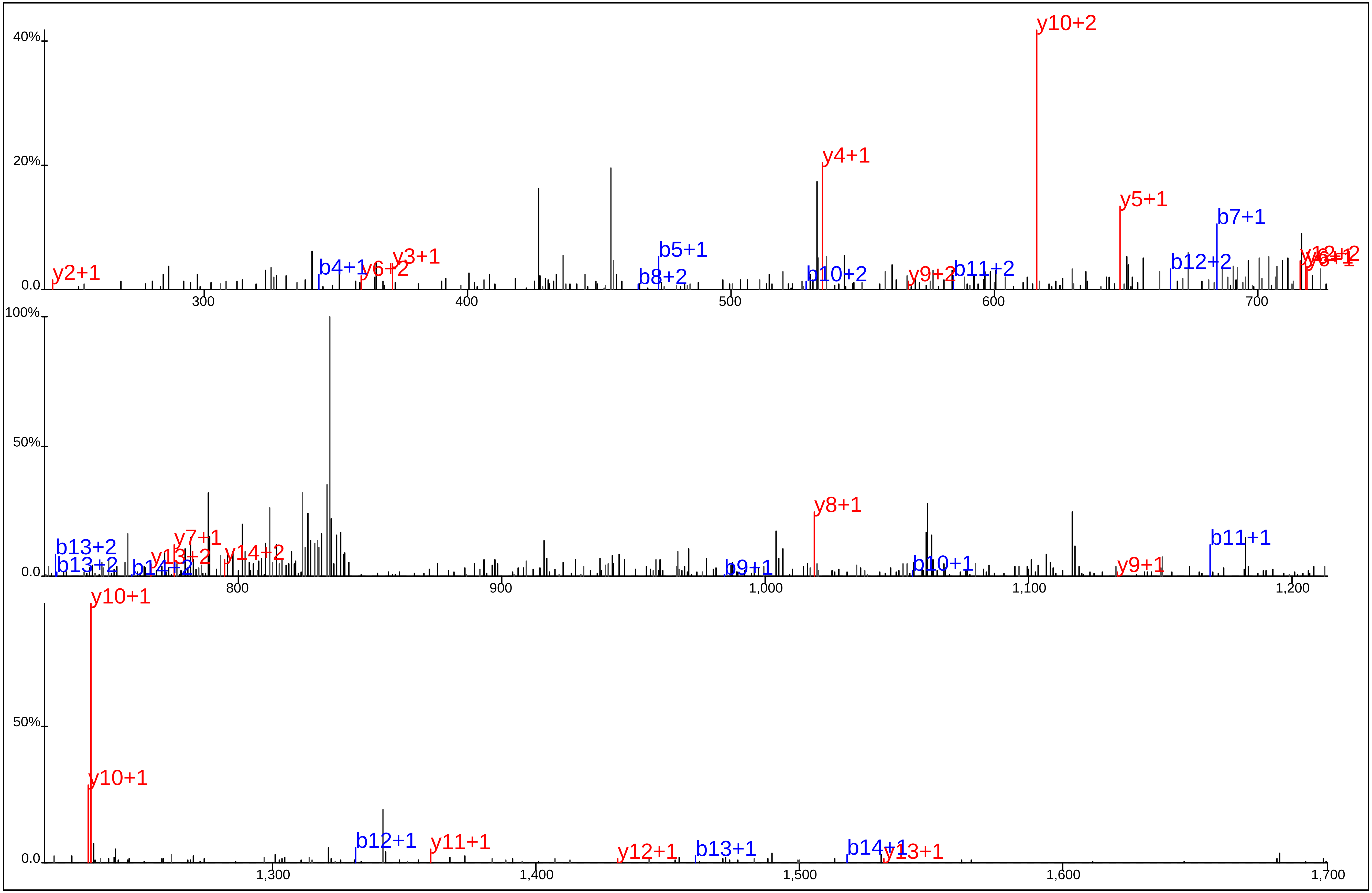

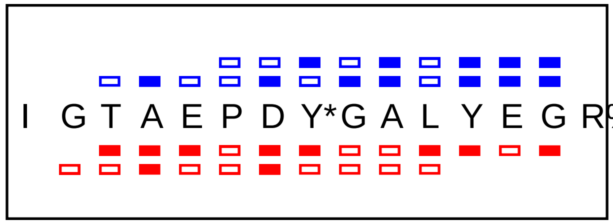

Protein Name: PLC gamma 1
gi Accession: 33598946
Precursor Mass: 851.3795+2
Delta Mass: 7.127 ppm
XCorr: 19.87

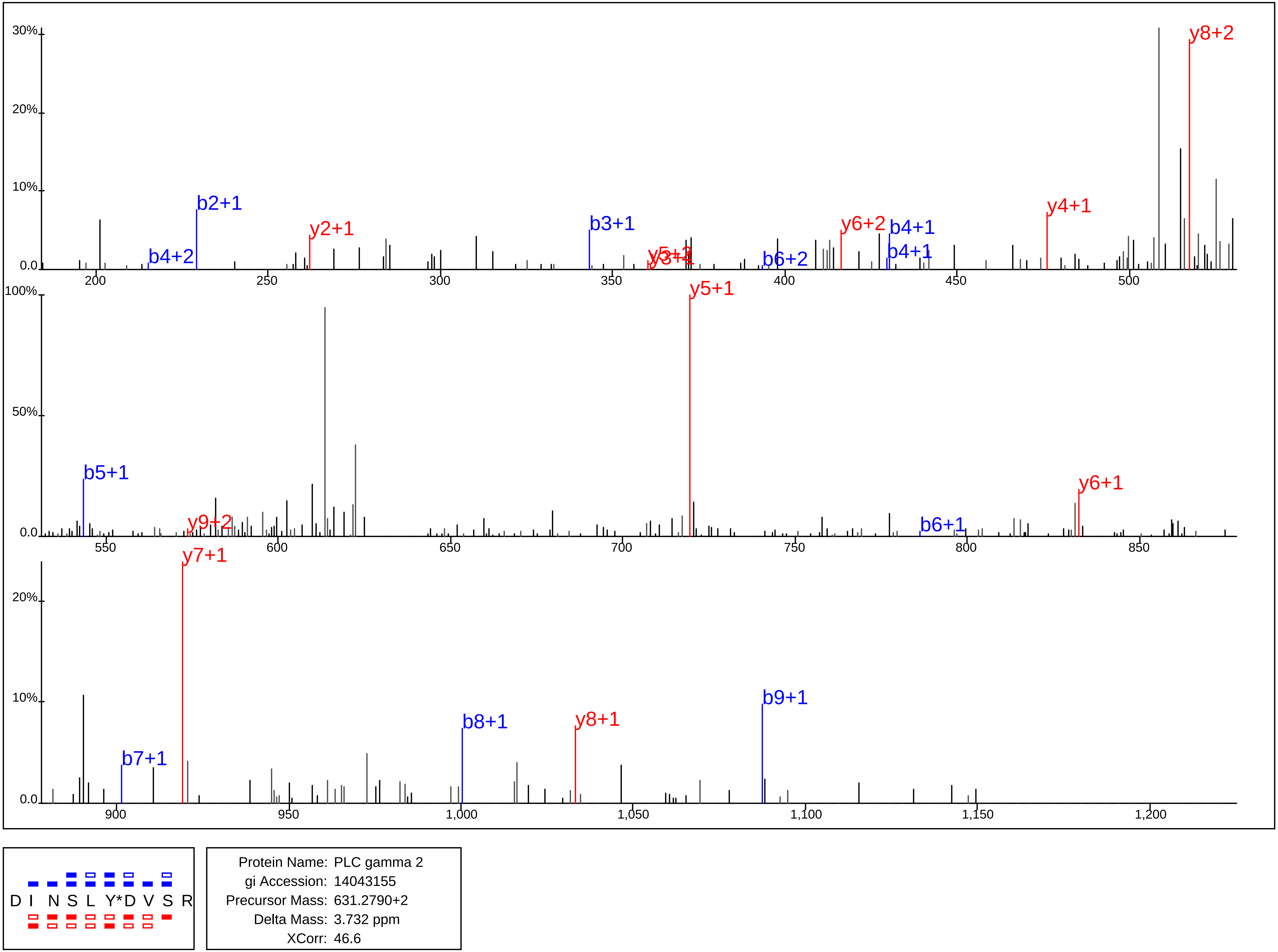

Protein Name: PLC gamma 2
gi Accession: 14043155
Precursor Mass: 631.2790+2
Delta Mass: 3.732 ppm
XCorr: 46.6

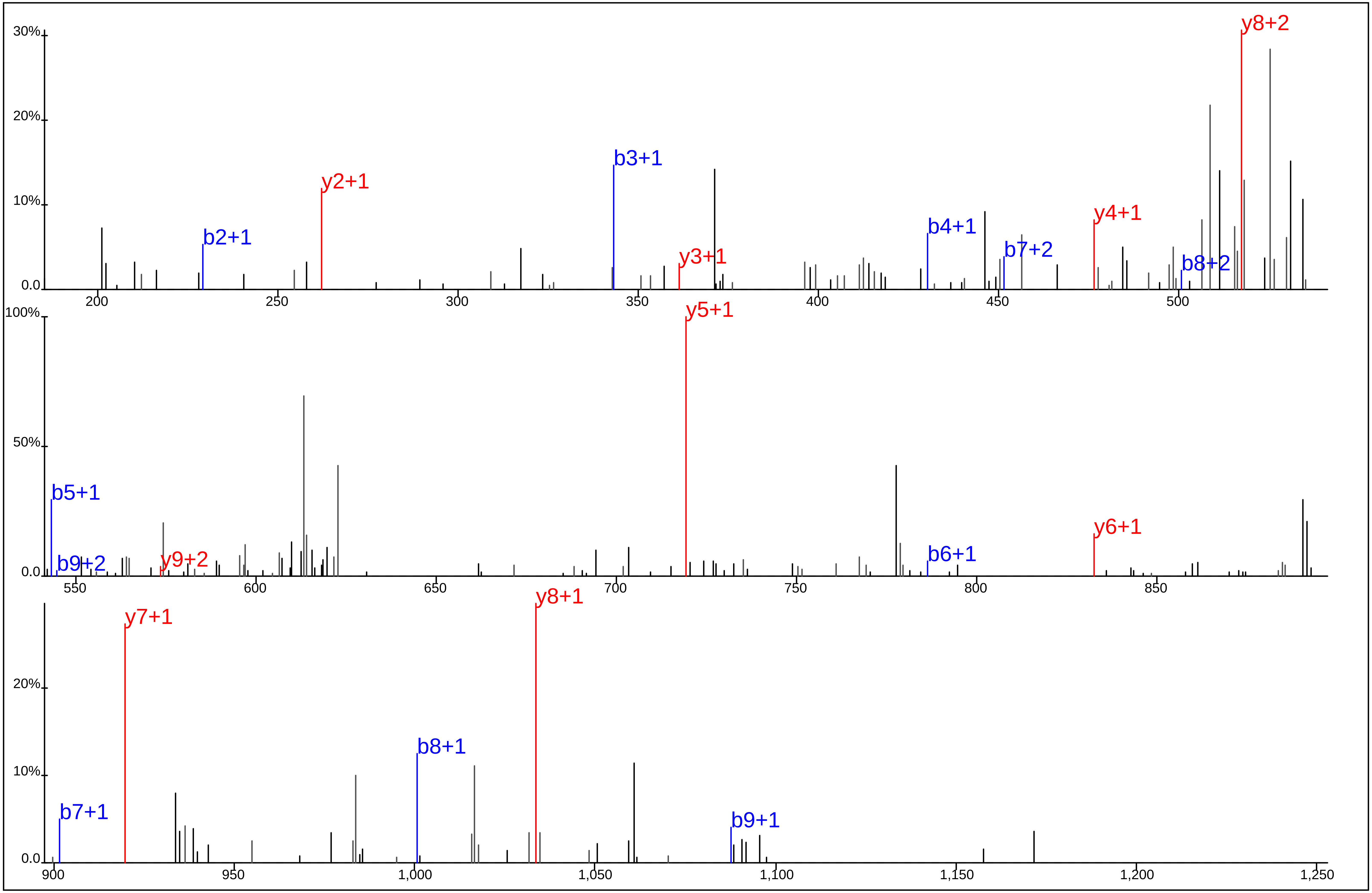

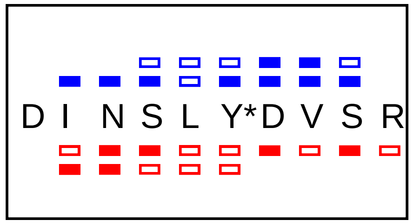

Protein Name: PLC gamma 2
gi Accession: 14043155
Precursor Mass: 631.2806+2
Delta Mass: 6.245 ppm
XCorr: 42.49

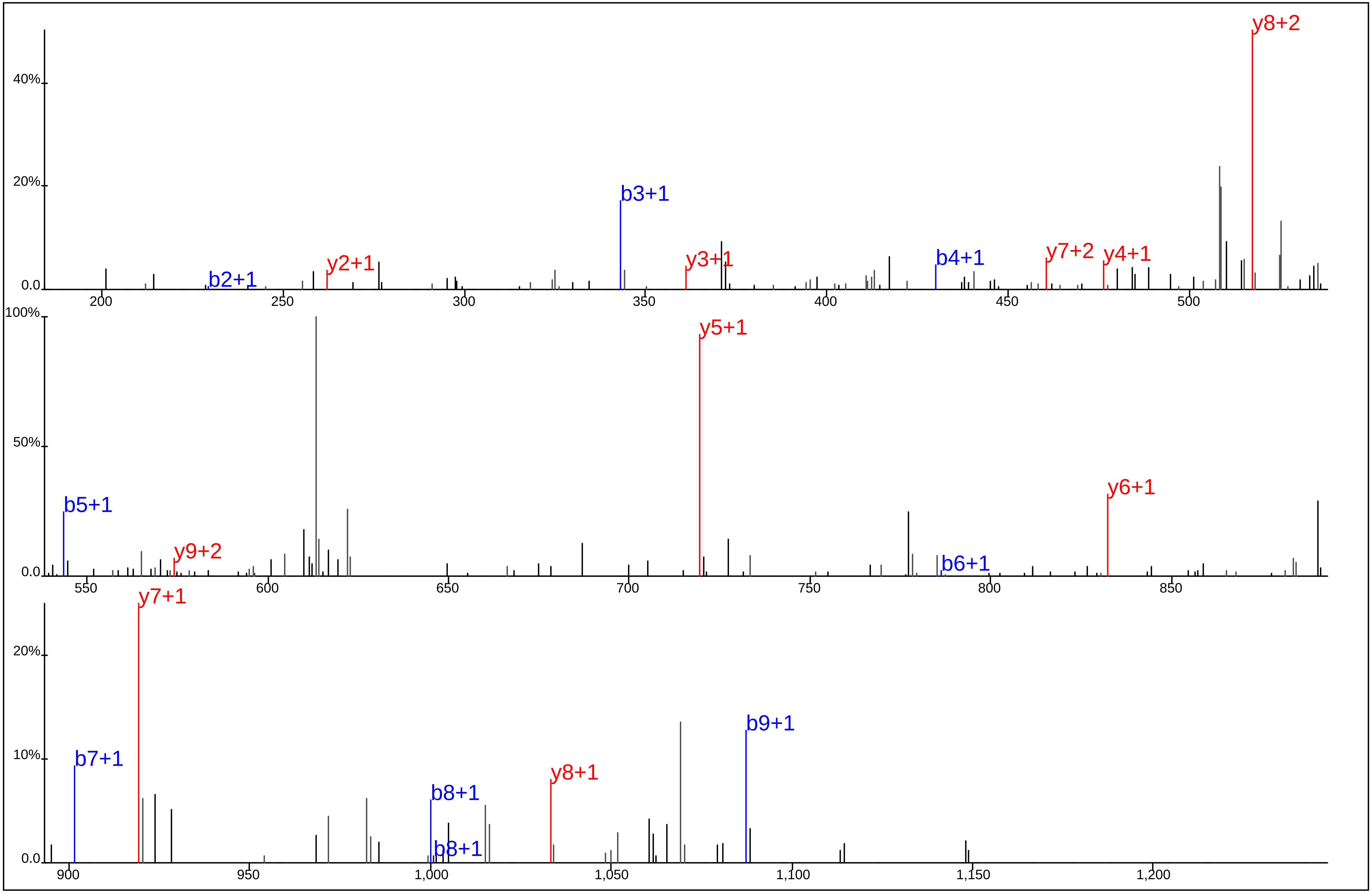

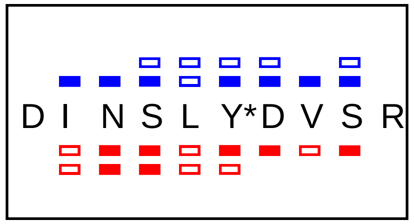

Protein Name: PLC gamma 2
gi Accession: 14043155
Precursor Mass: 631.2797+2
Delta Mass: 4.794 ppm
XCorr: 39.64

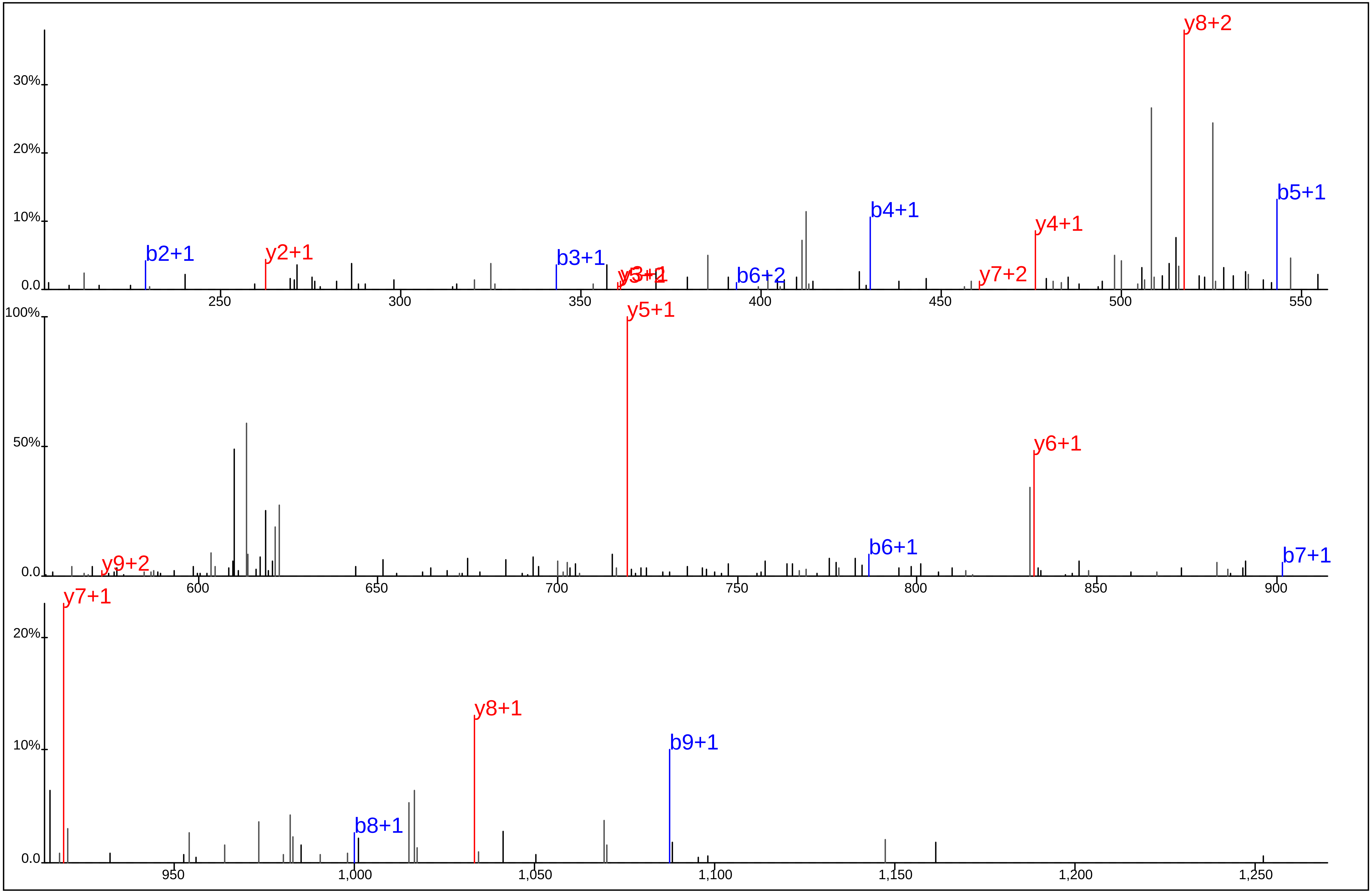

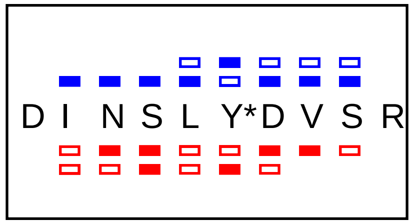

Protein Name: PLC gamma 2
gi Accession: 14043155
Precursor Mass: 631.2781+2
Delta Mass: 2.281 ppm
XCorr: 41.67

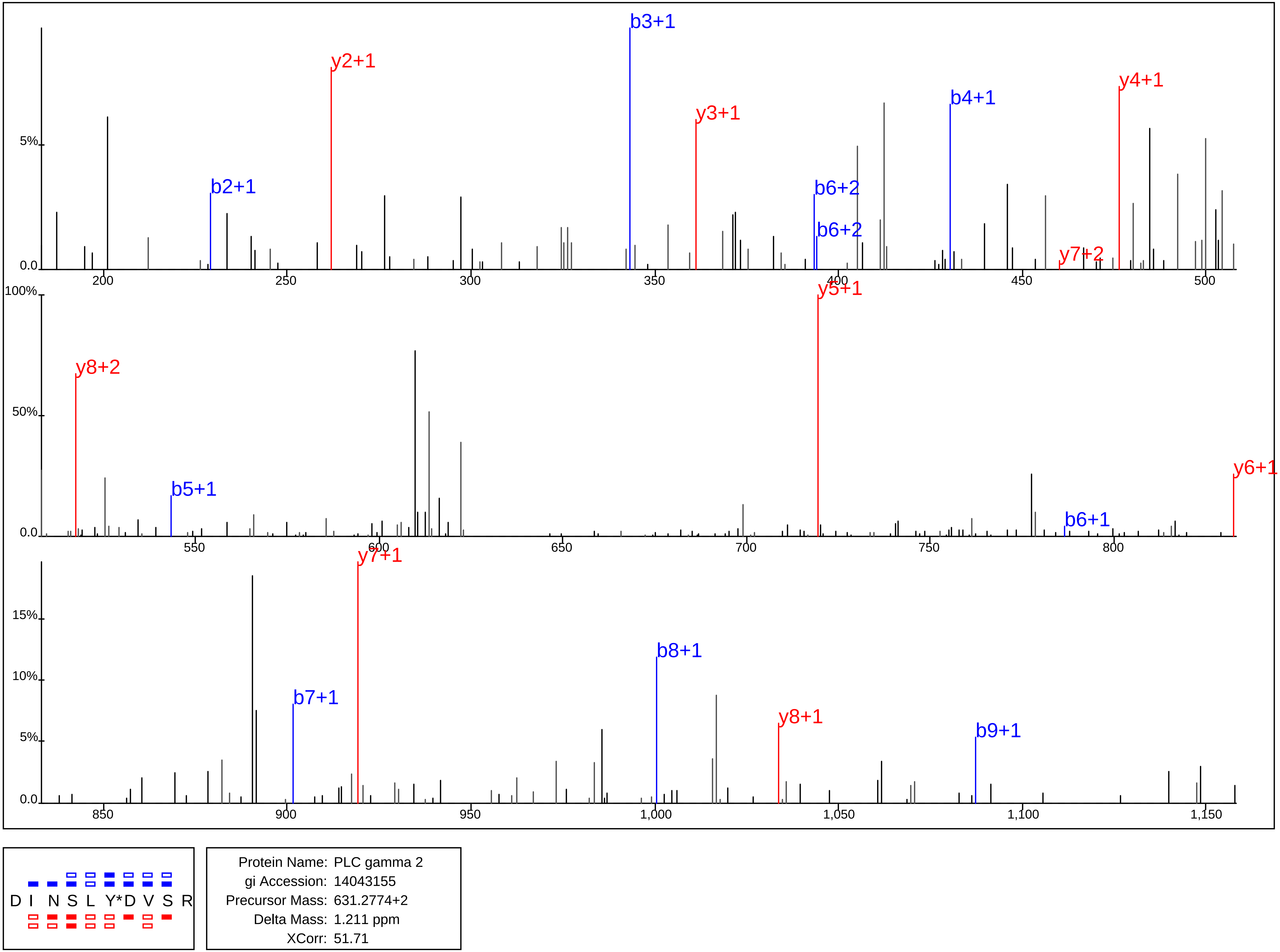

Protein Name: PLC gamma 2
gi Accession: 14043155
Precursor Mass: 631.2774+2
Delta Mass: 1.211 ppm
XCorr: 51.71

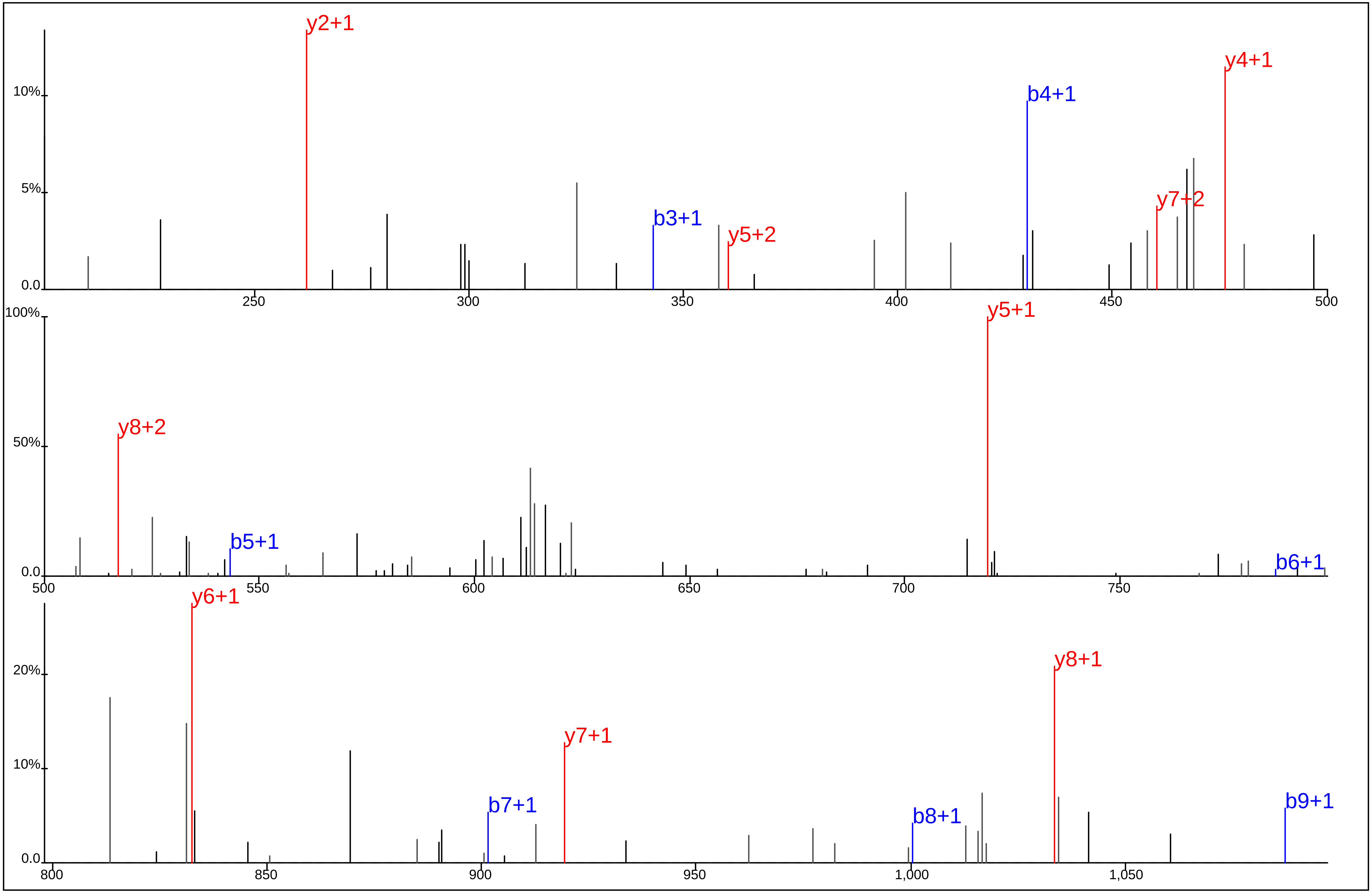

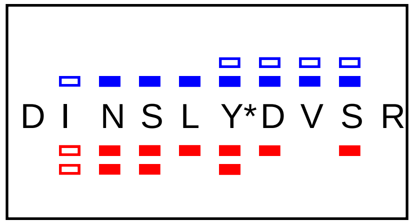

Protein Name: PLC gamma 2
gi Accession: 14043155
Precursor Mass: 631.2792+2
Delta Mass: 4.120 ppm
XCorr: 45.77

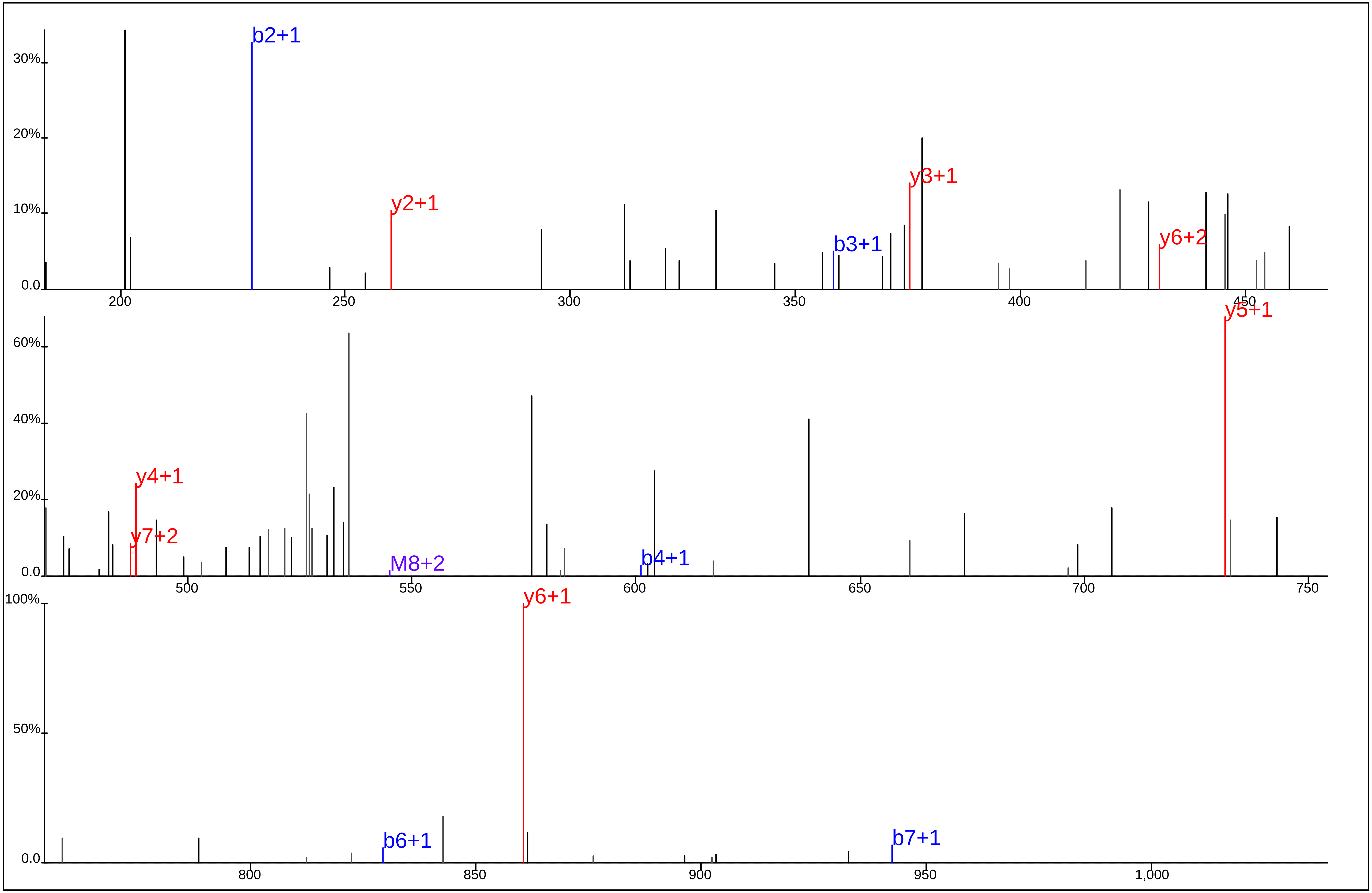

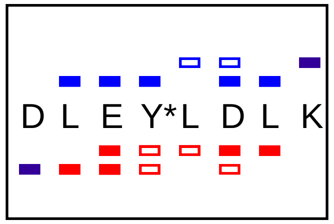

Protein Name: PLEKHA7
gi Accession: 27693901
Precursor Mass: 544.7493+2
Delta Mass: 0.255 ppm
XCorr: 16.44

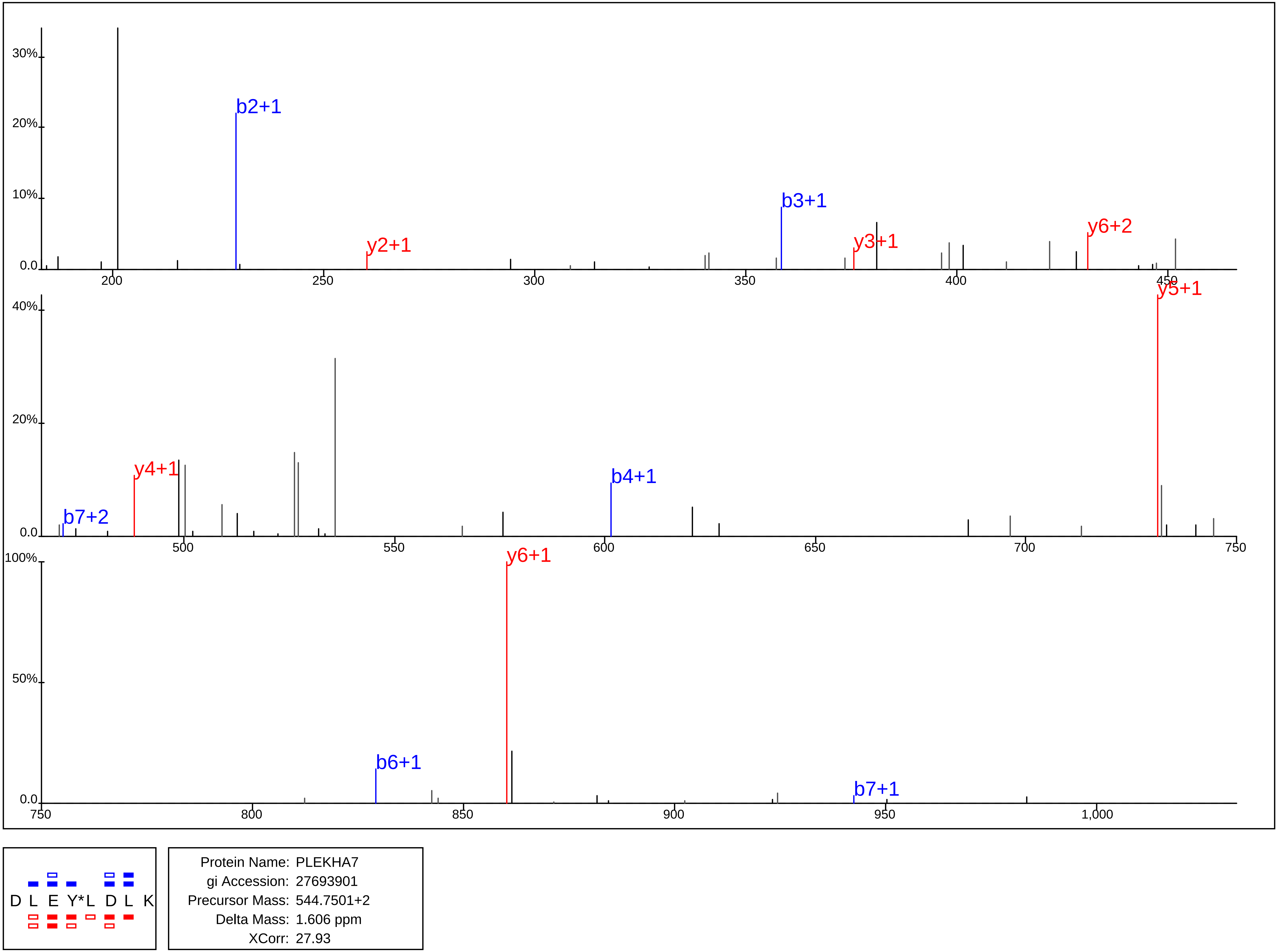

Protein Name: PLEKHA7
gi Accession: 27693901
Precursor Mass: 544.7501+2
Delta Mass: 1.606 ppm
XCorr: 27.93

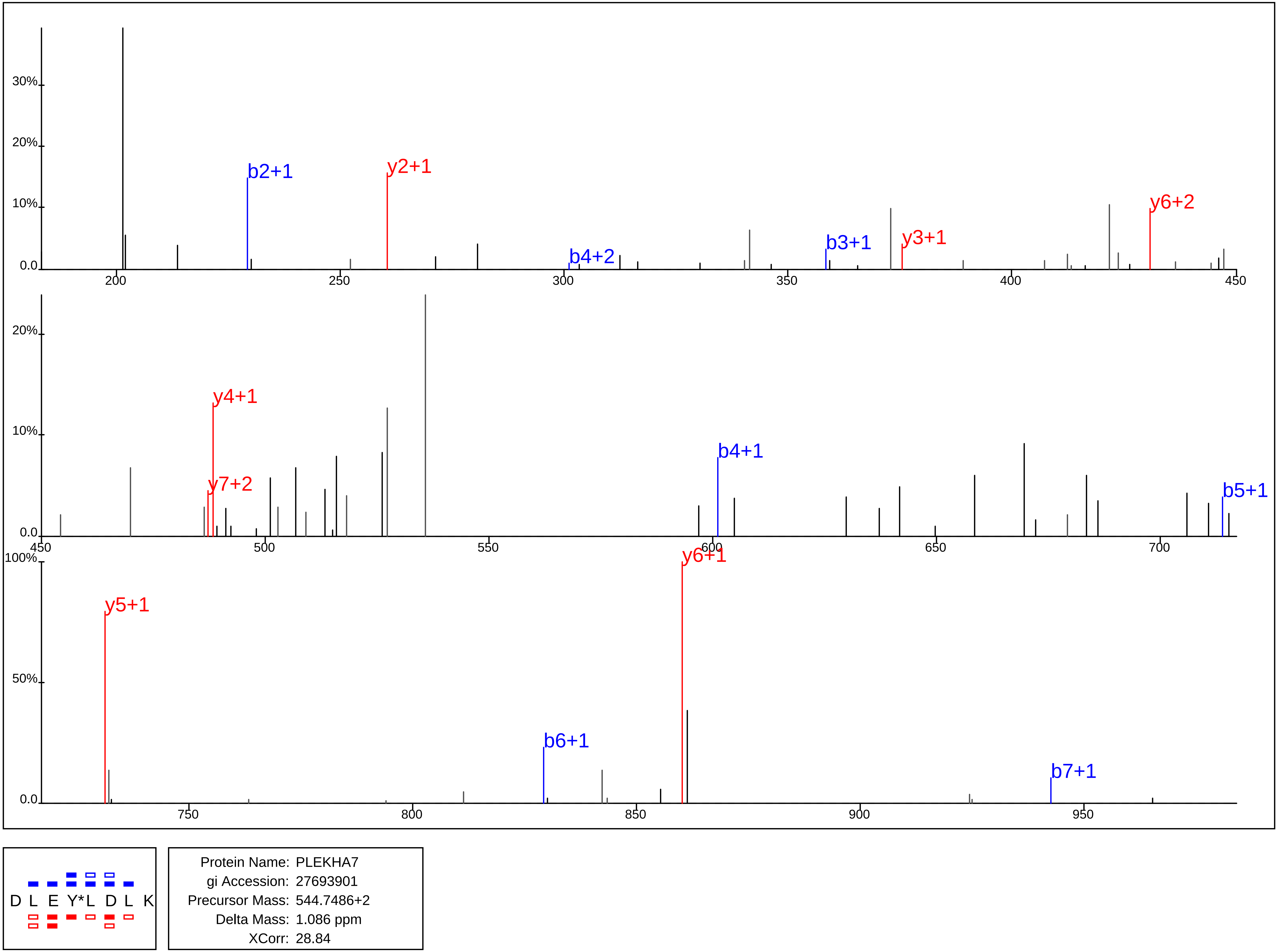

Protein Name: PLEKHA7
gi Accession: 27693901
Precursor Mass: 544.7486+2
Delta Mass: 1.086 ppm
XCorr: 28.84

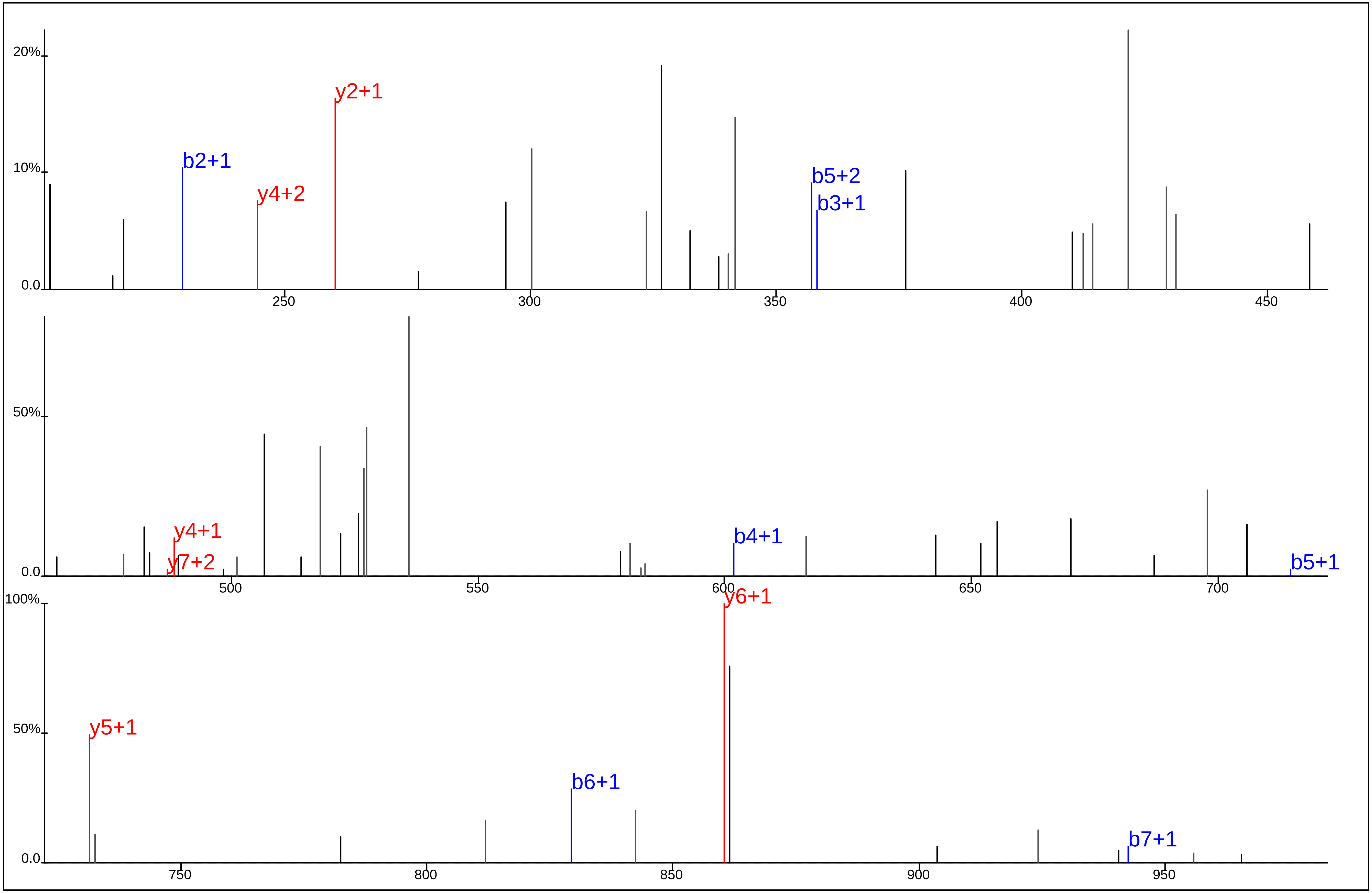

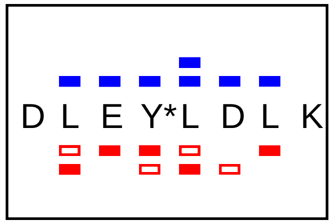

Protein Name: PLEKHA7
gi Accession: 27693901
Precursor Mass: 544.7512+2
Delta Mass: 3.618 ppm
XCorr: 23.02

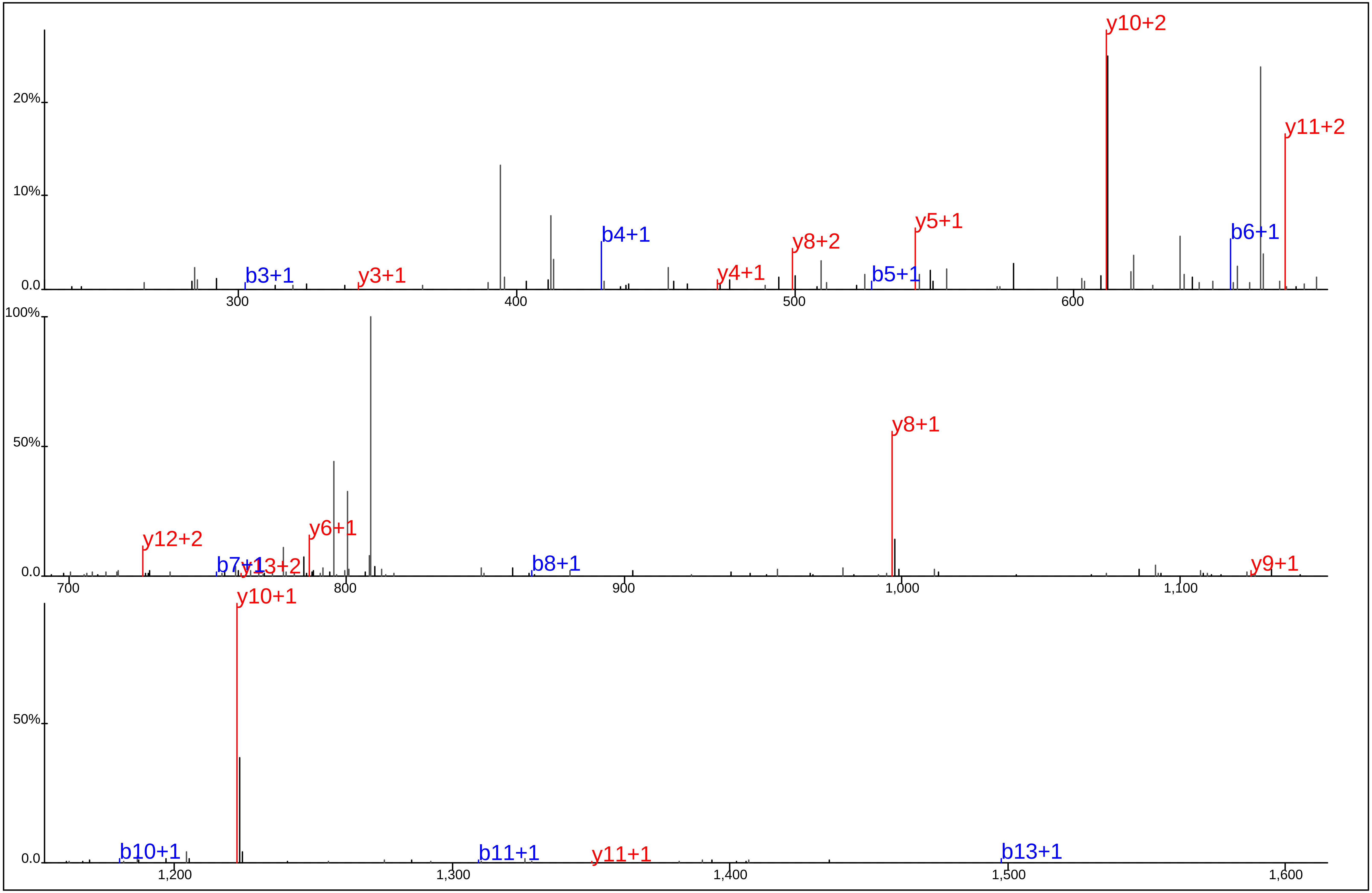

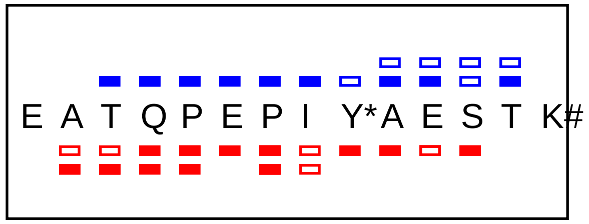

Protein Name: Pragmin
gi Accession: 157909822
Precursor Mass: 826.3767+2
Delta Mass: 7.234 ppm
XCorr: 27.78

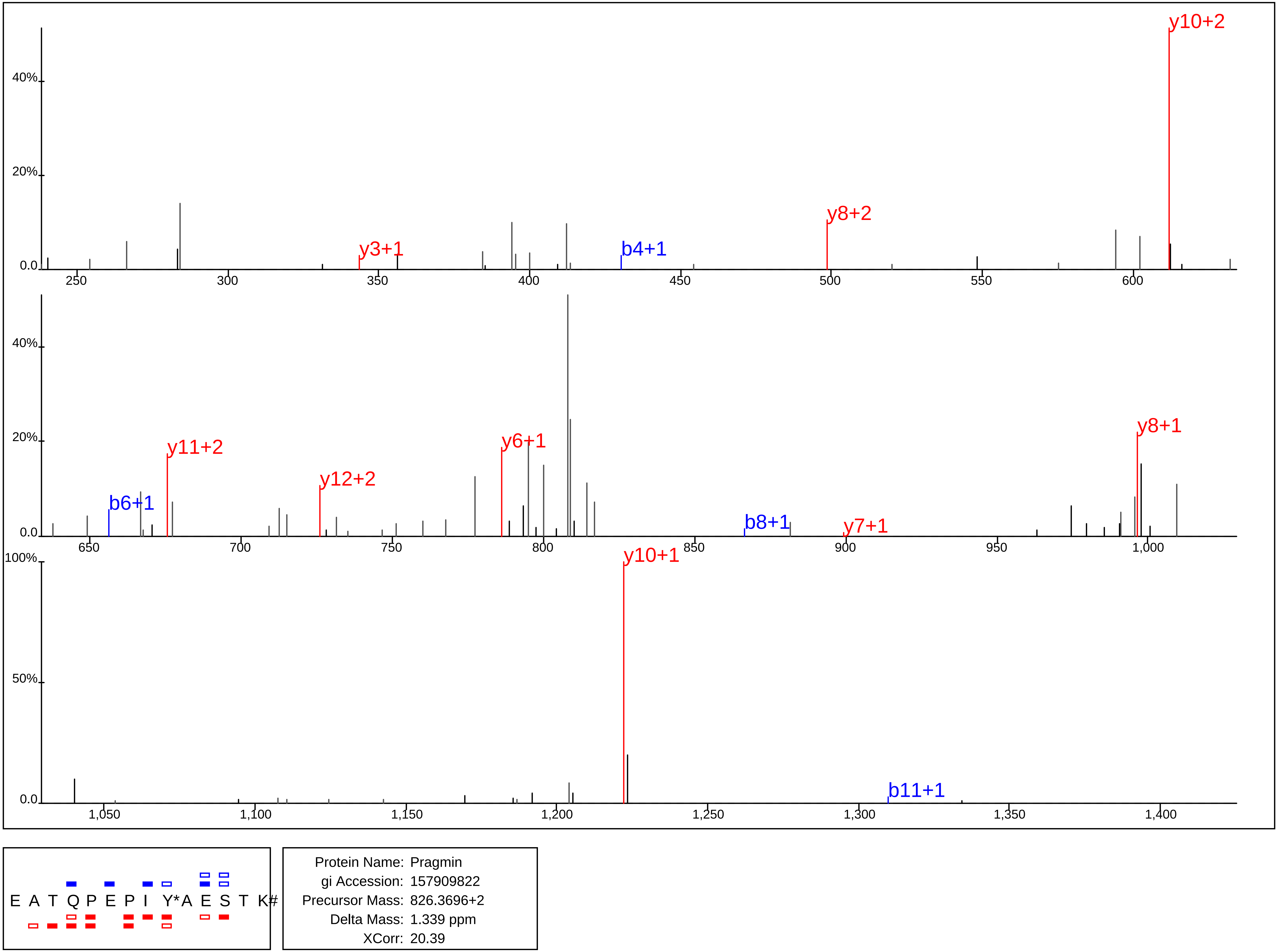

Protein Name: Pragmin
gi Accession: 157909822
Precursor Mass: 826.3696+2
Delta Mass: 1.339 ppm
XCorr: 20.39

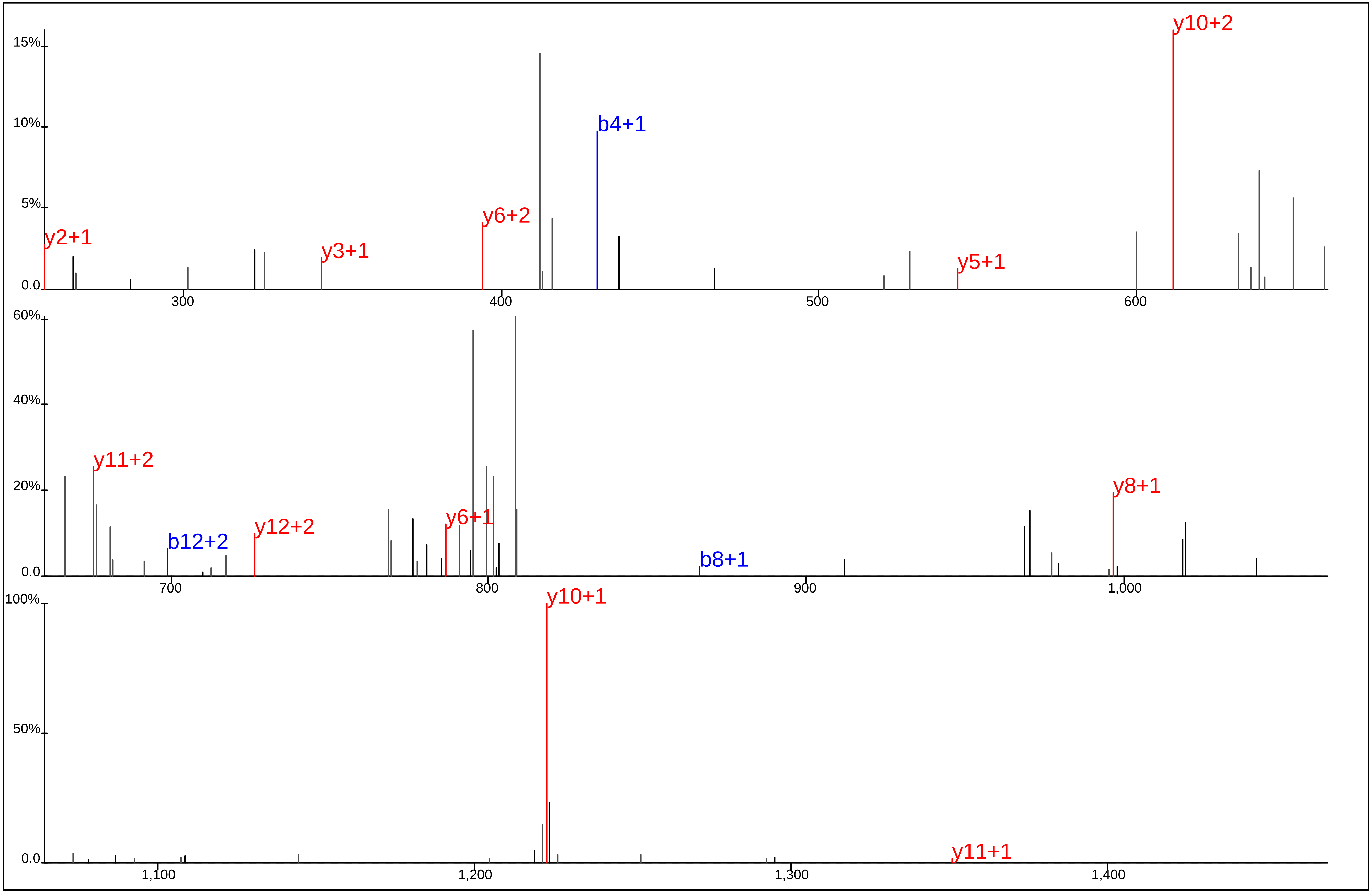

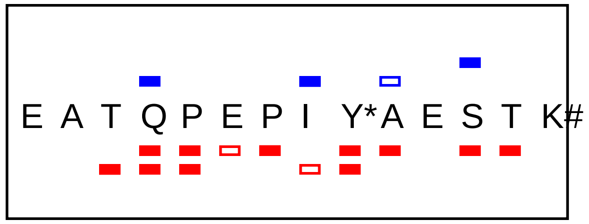

Protein Name: Pragmin
gi Accession: 157909822
Precursor Mass: 826.3690+2
Delta Mass: 2.150 ppm
XCorr: 17.84

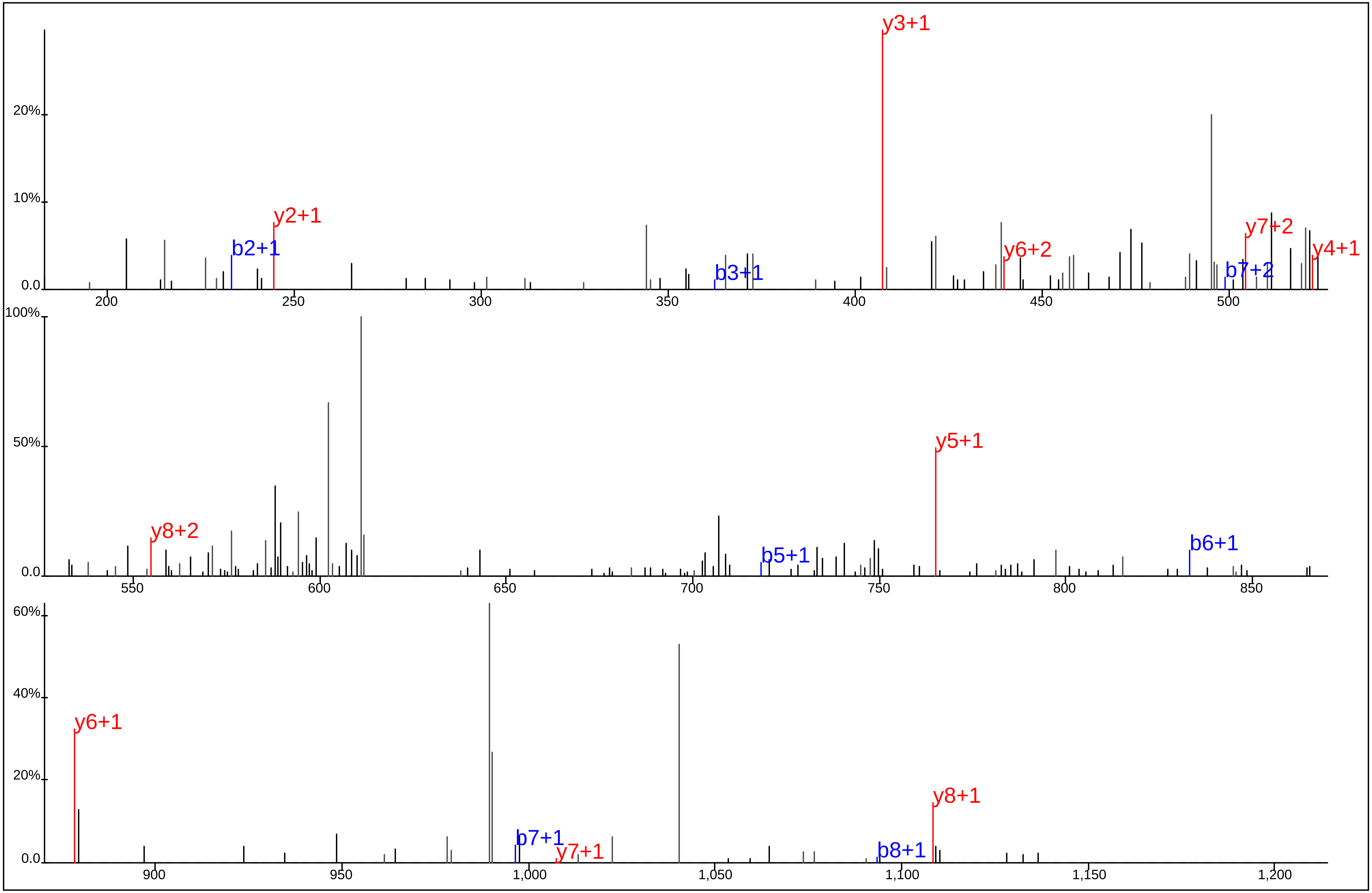

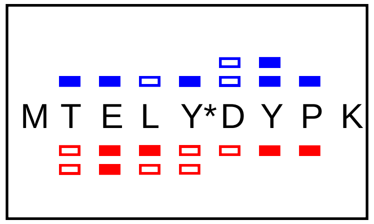

Protein Name: Prolyl endopeptidase
gi Accession: 1346769
Precursor Mass: 620.2563+2
Delta Mass: 4.145 ppm
XCorr: 34.37

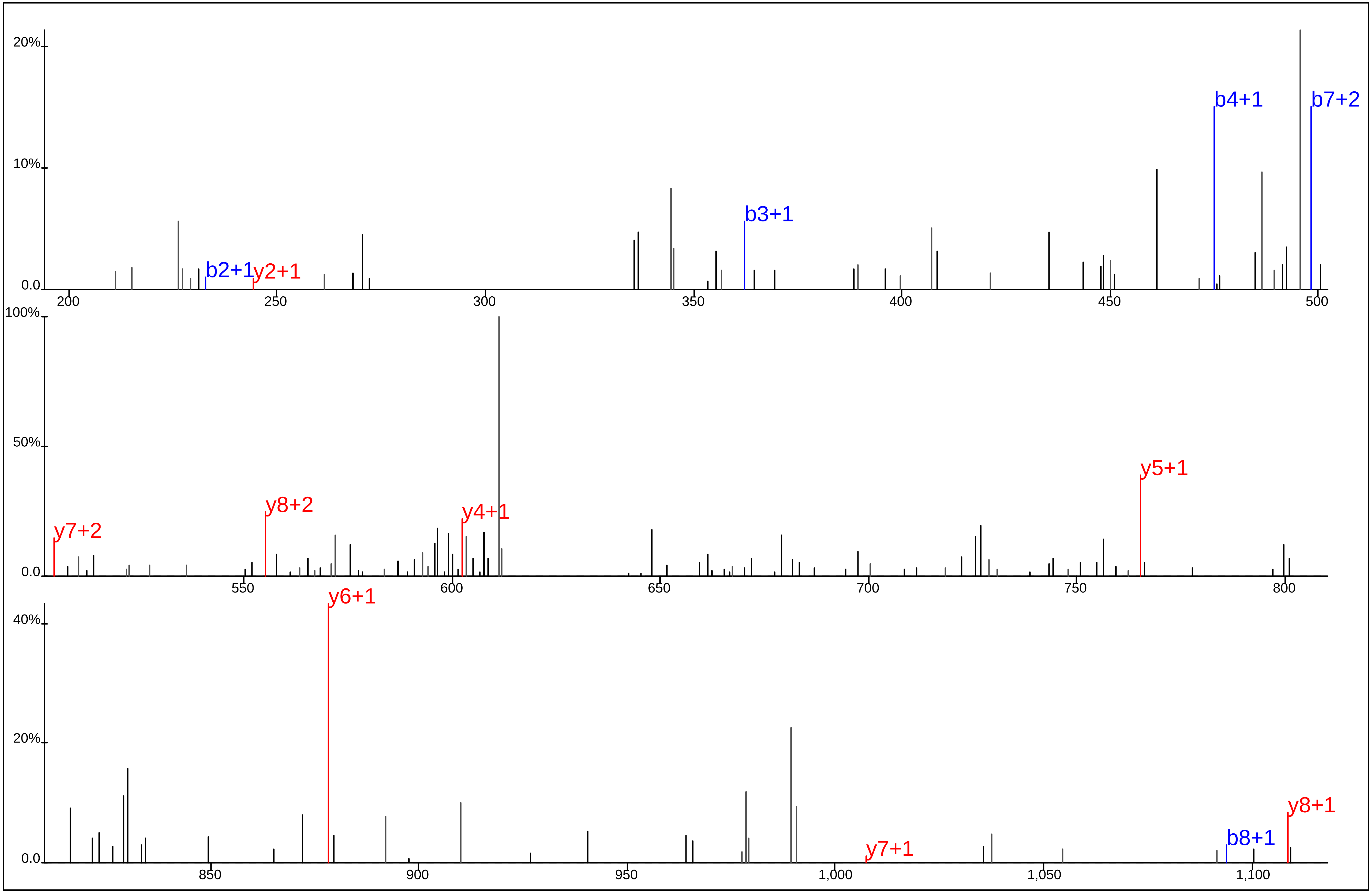

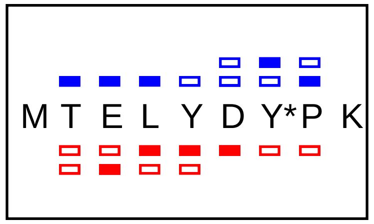

Protein Name: Prolyl endopeptidase
gi Accession: 1346769
Precursor Mass: 620.2567+2
Delta Mass: 4.637 ppm
XCorr: 19.97

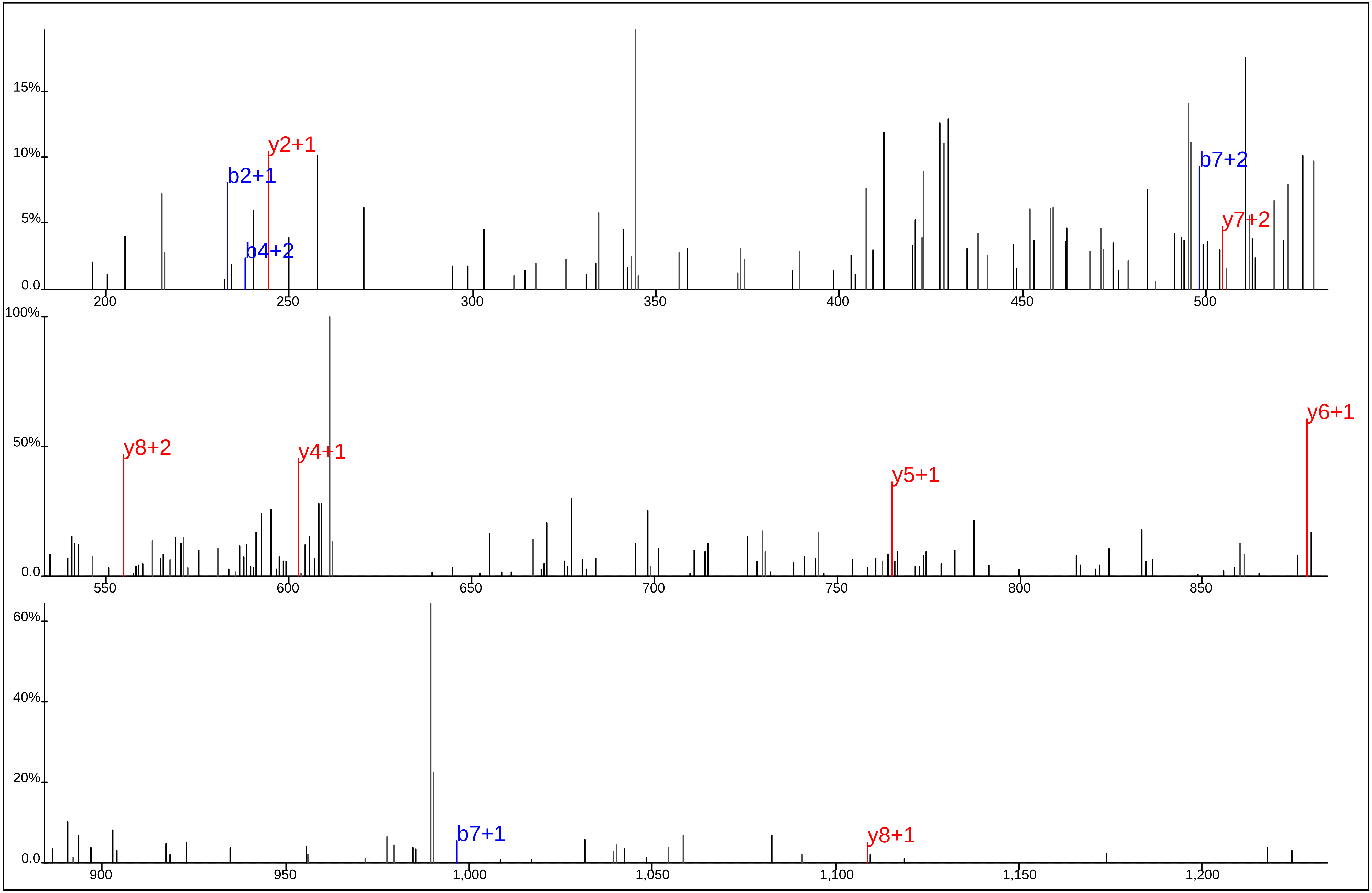

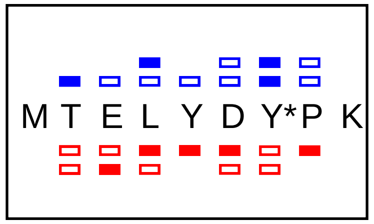

Protein Name:	Prolyl endopeptidase
gi Accession:	1346769
Precursor Mass:	620.2546+2
Delta Mass:	1.386 ppm
XCorr:	21.2

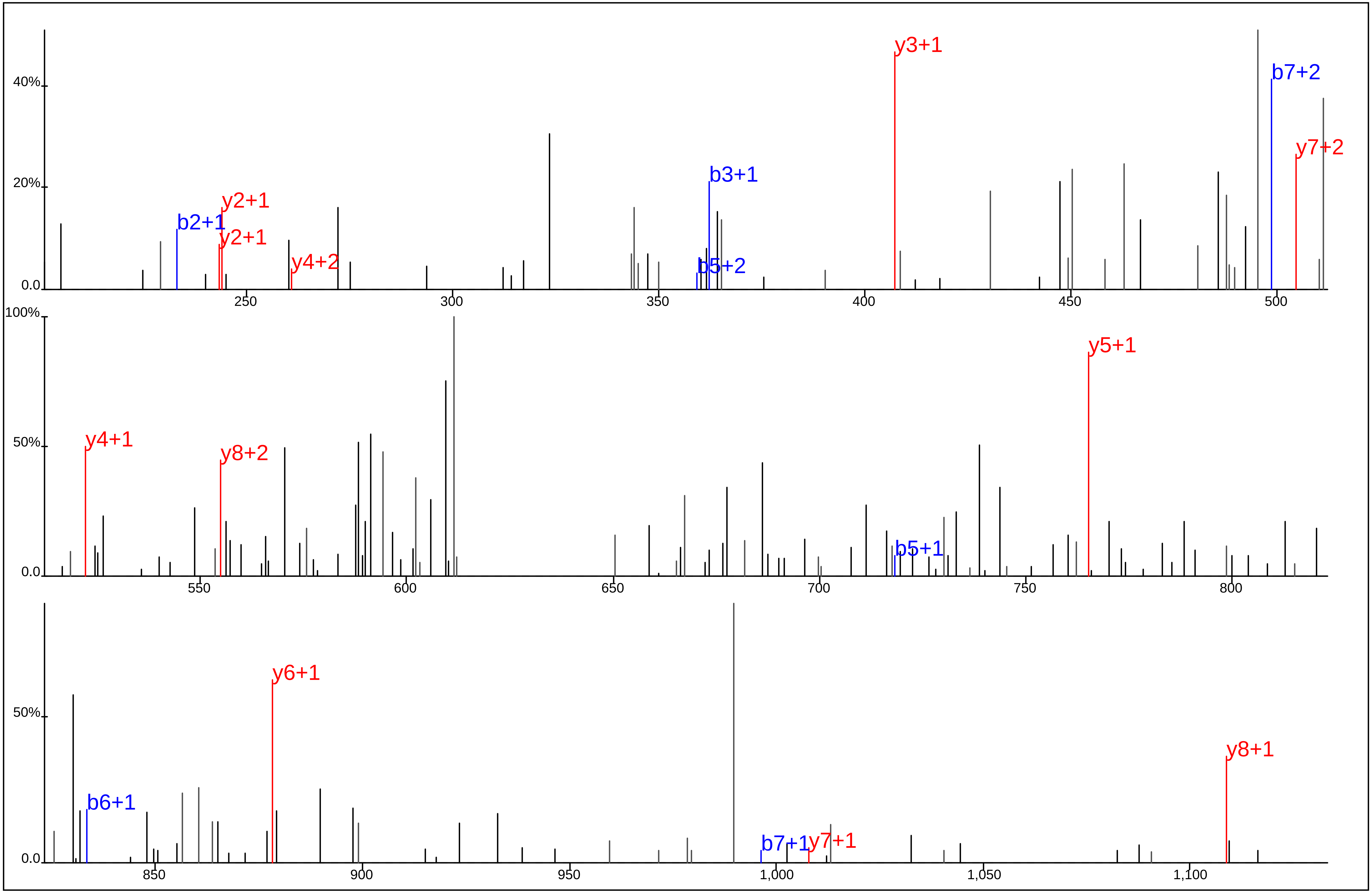

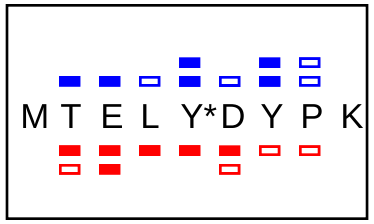

Protein Name: Prolyl endopeptidase
gi Accession: 1346769
Precursor Mass: 620.2591+2
Delta Mass: 8.574 ppm
XCorr: 20.77

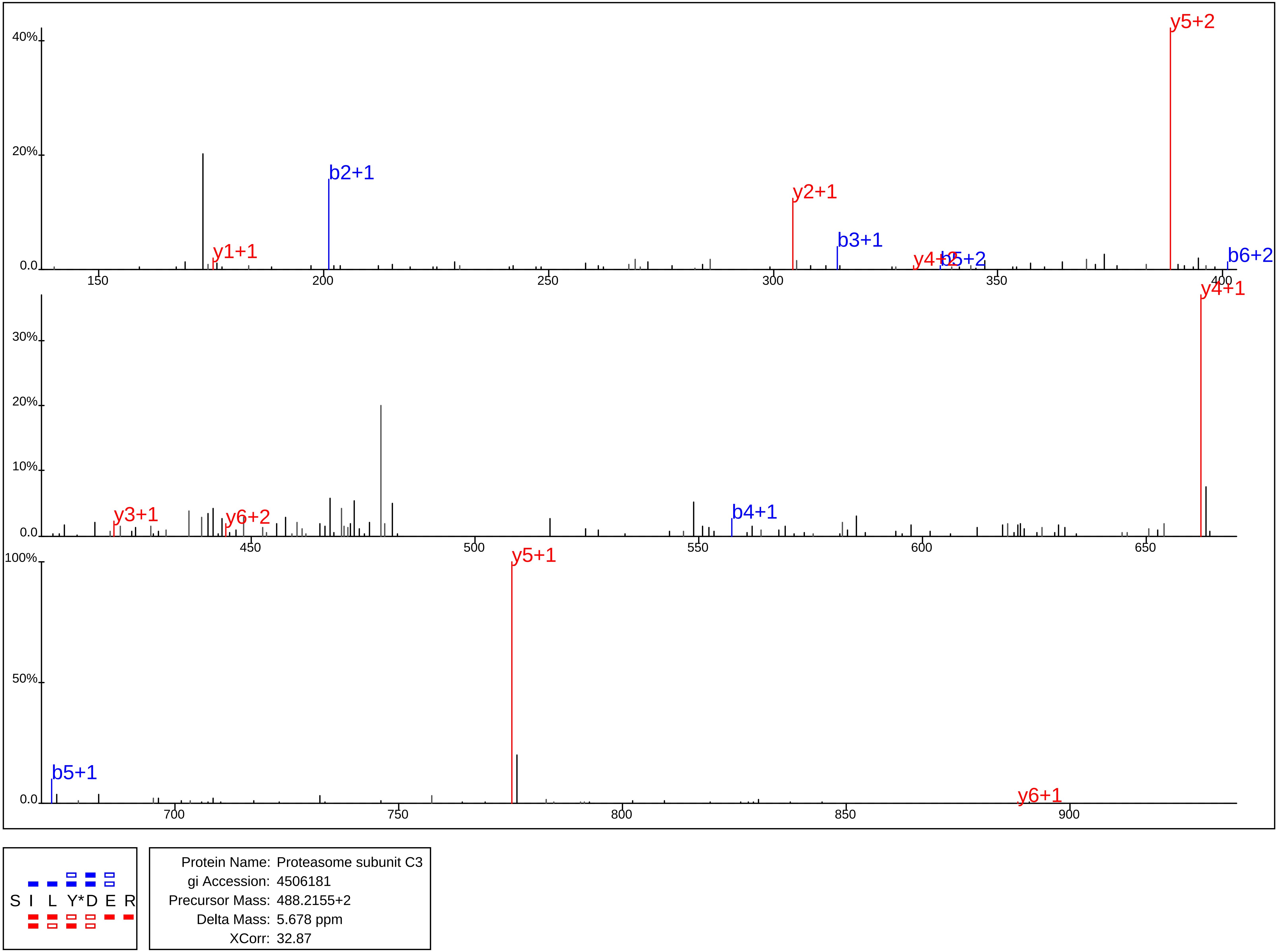

Protein Name:	Proteasome subunit C3
gi Accession:	4506181
Precursor Mass:	488.2155+2
Delta Mass:	5.678 ppm
XCorr:	32.87

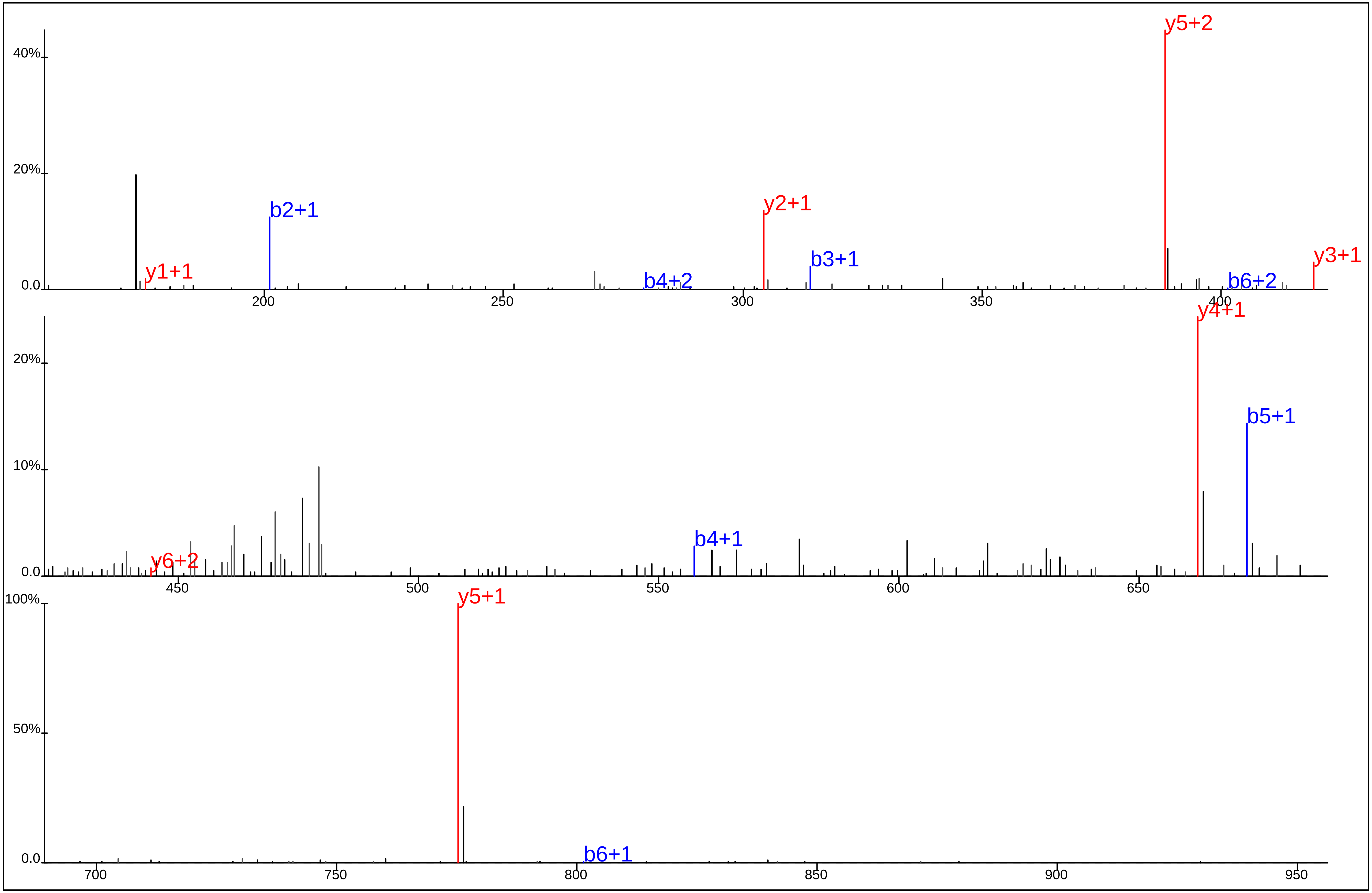

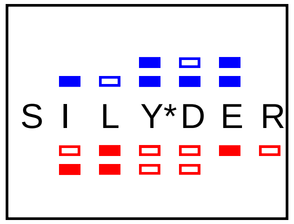

Protein Name: Proteasome subunit C3
gi Accession: 4506181
Precursor Mass: 488.2148+2
Delta Mass: 4.058 ppm
XCorr: 25.44

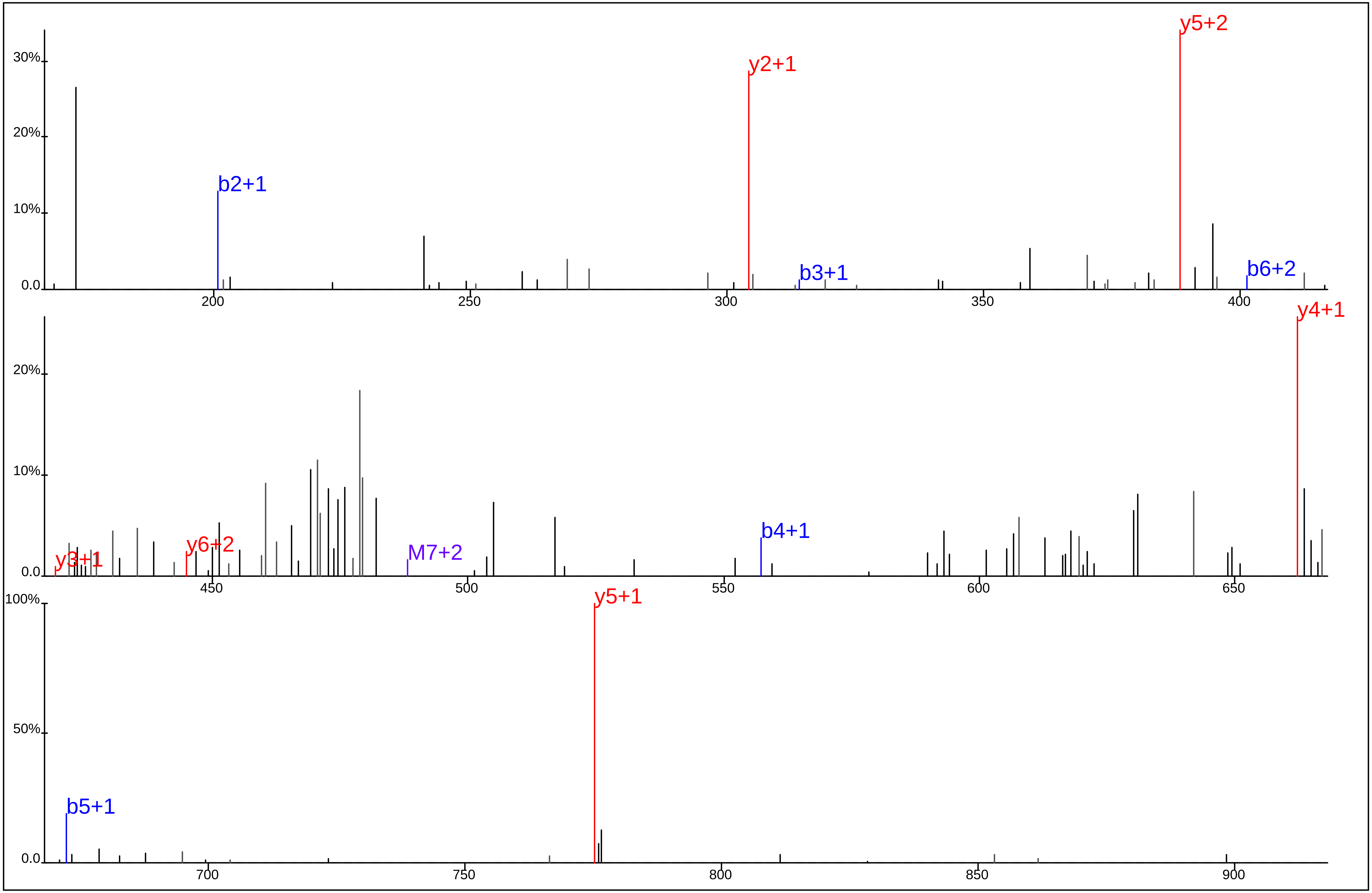

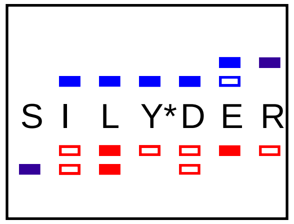

Protein Name: Proteasome subunit C3
gi Accession: 4506181
Precursor Mass: 488.2140+2
Delta Mass: 2.551 ppm
XCorr: 15.06

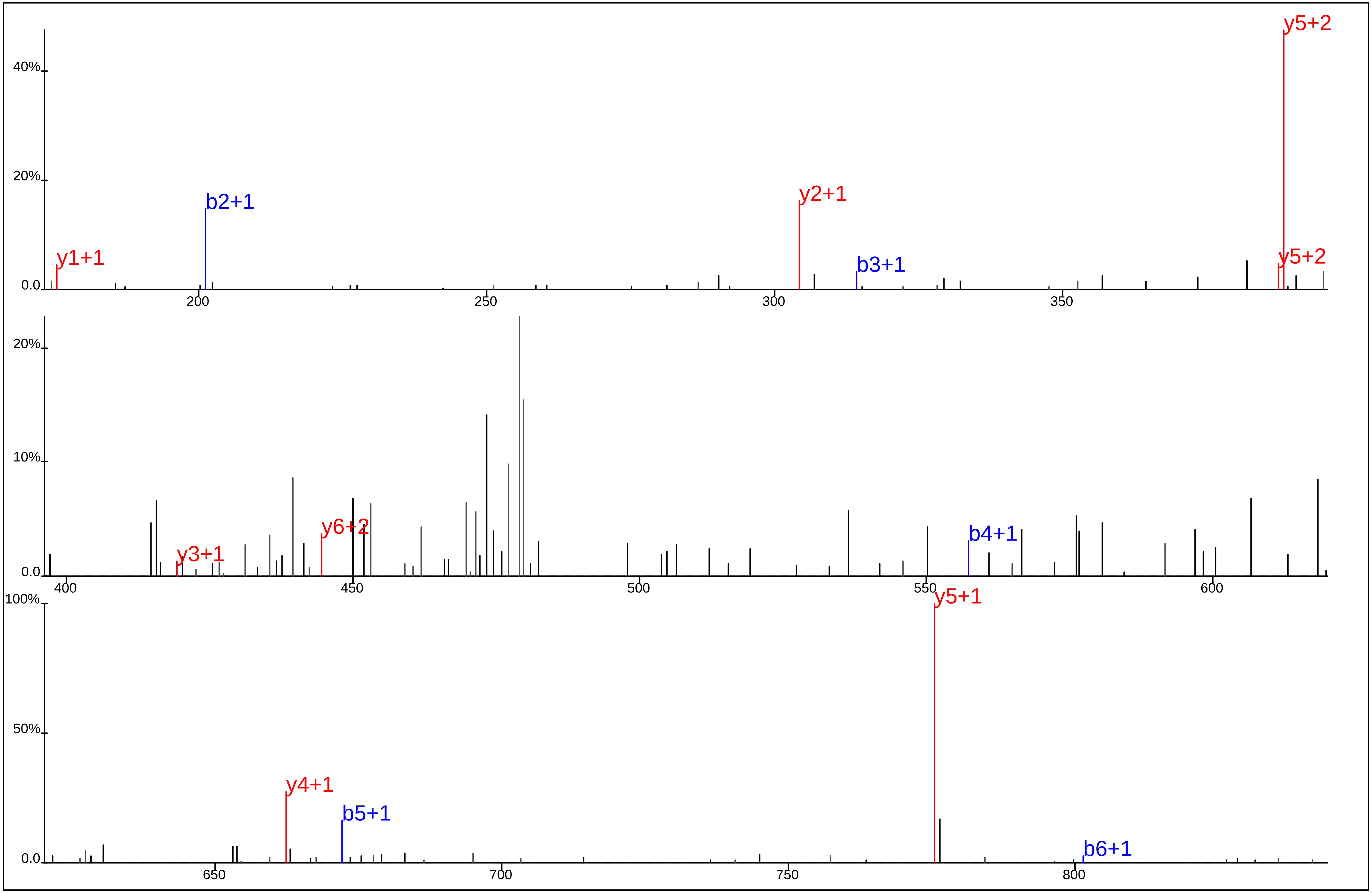

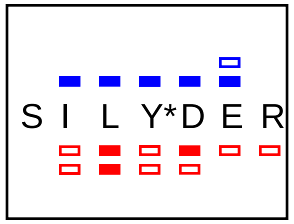

Protein Name:	Proteasome subunit C3
gi Accession:	4506181
Precursor Mass:	488.2141+2
Delta Mass:	2.612 ppm
XCorr:	19.76

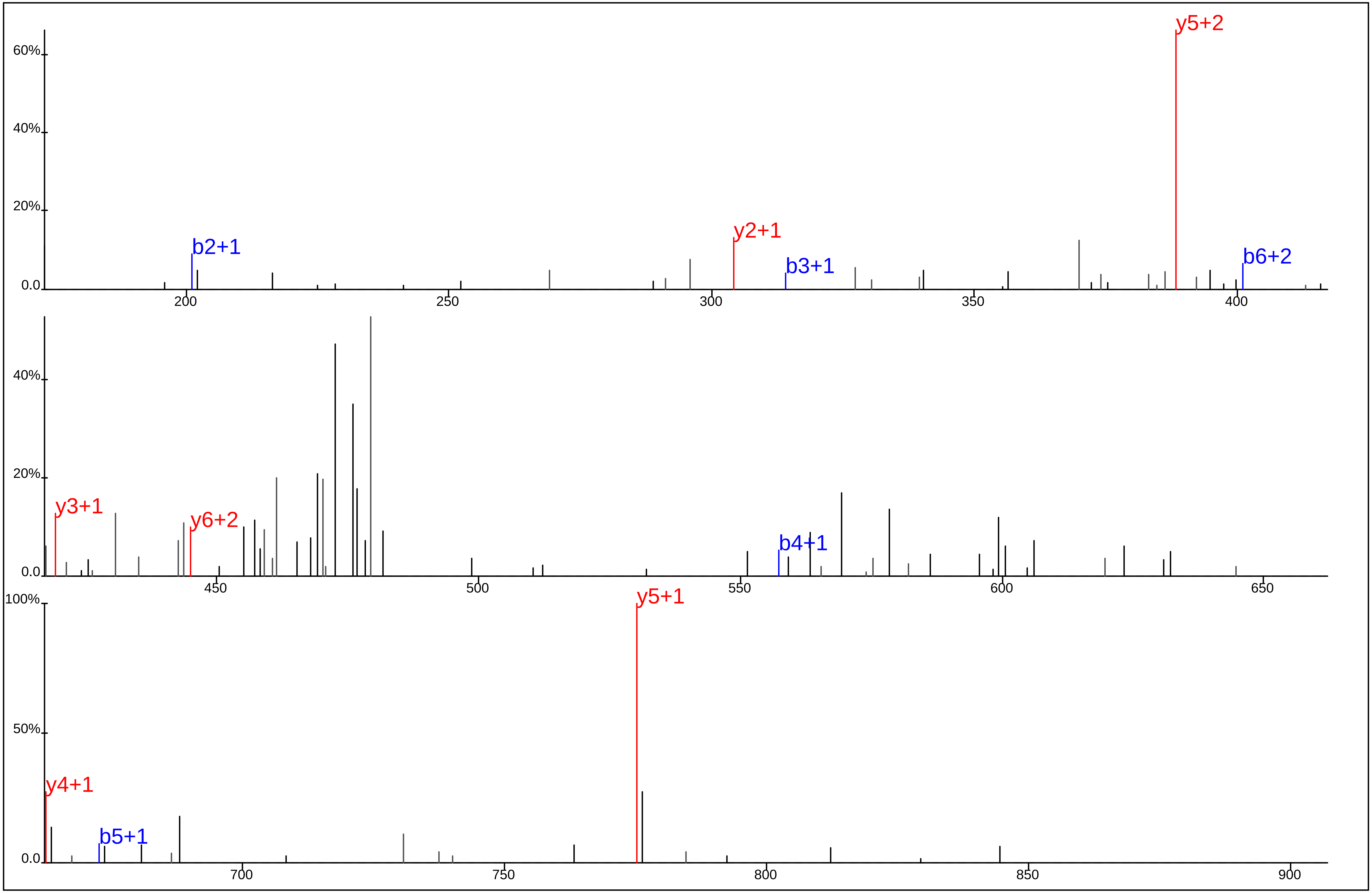

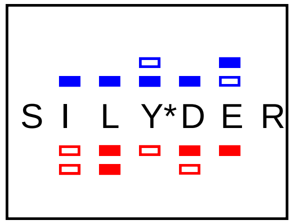

Protein Name:	Proteasome subunit C3
gi Accession:	4506181
Precursor Mass:	488.2148+2
Delta Mass:	4.242 ppm
XCorr:	13.92

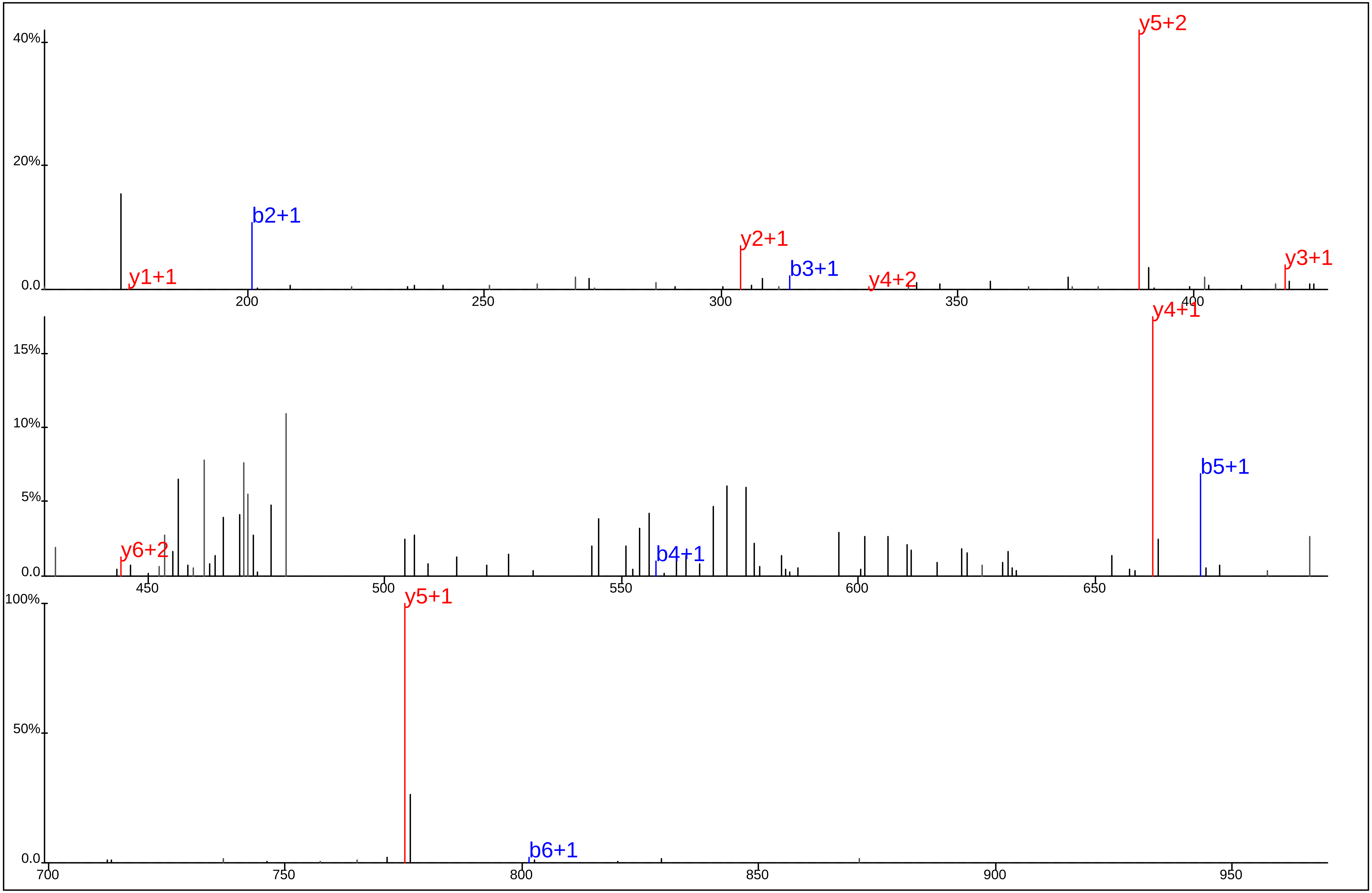

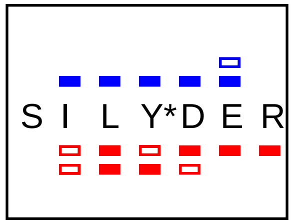

Protein Name: Proteasome subunit C3
gi Accession: 4506181
Precursor Mass: 488.2149+2
Delta Mass: 4.304 ppm
XCorr: 28.62

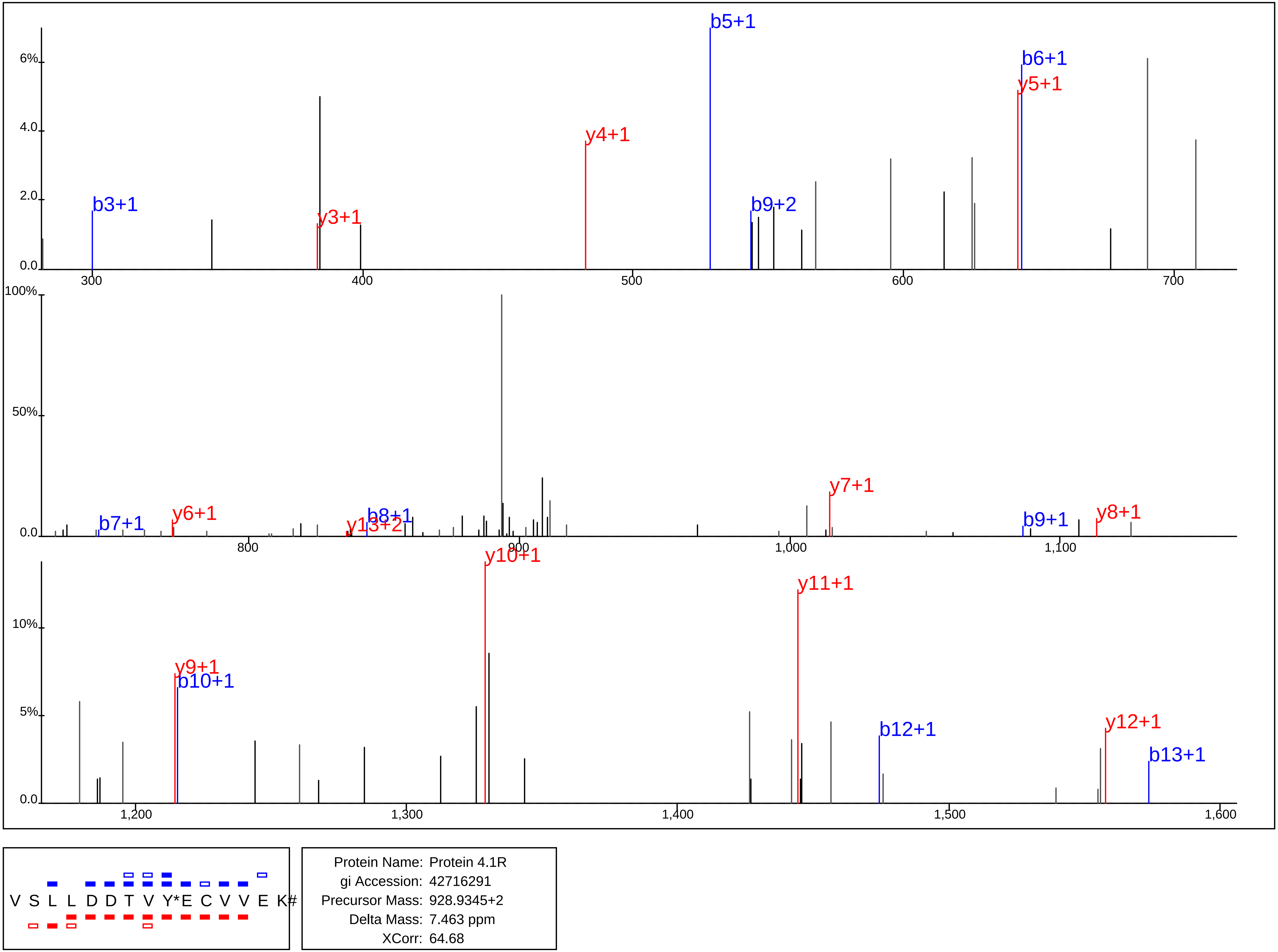

Protein Name: Protein 4.1R
gi Accession: 42716291
Precursor Mass: 928.9345+2
Delta Mass: 7.463 ppm
XCorr: 64.68

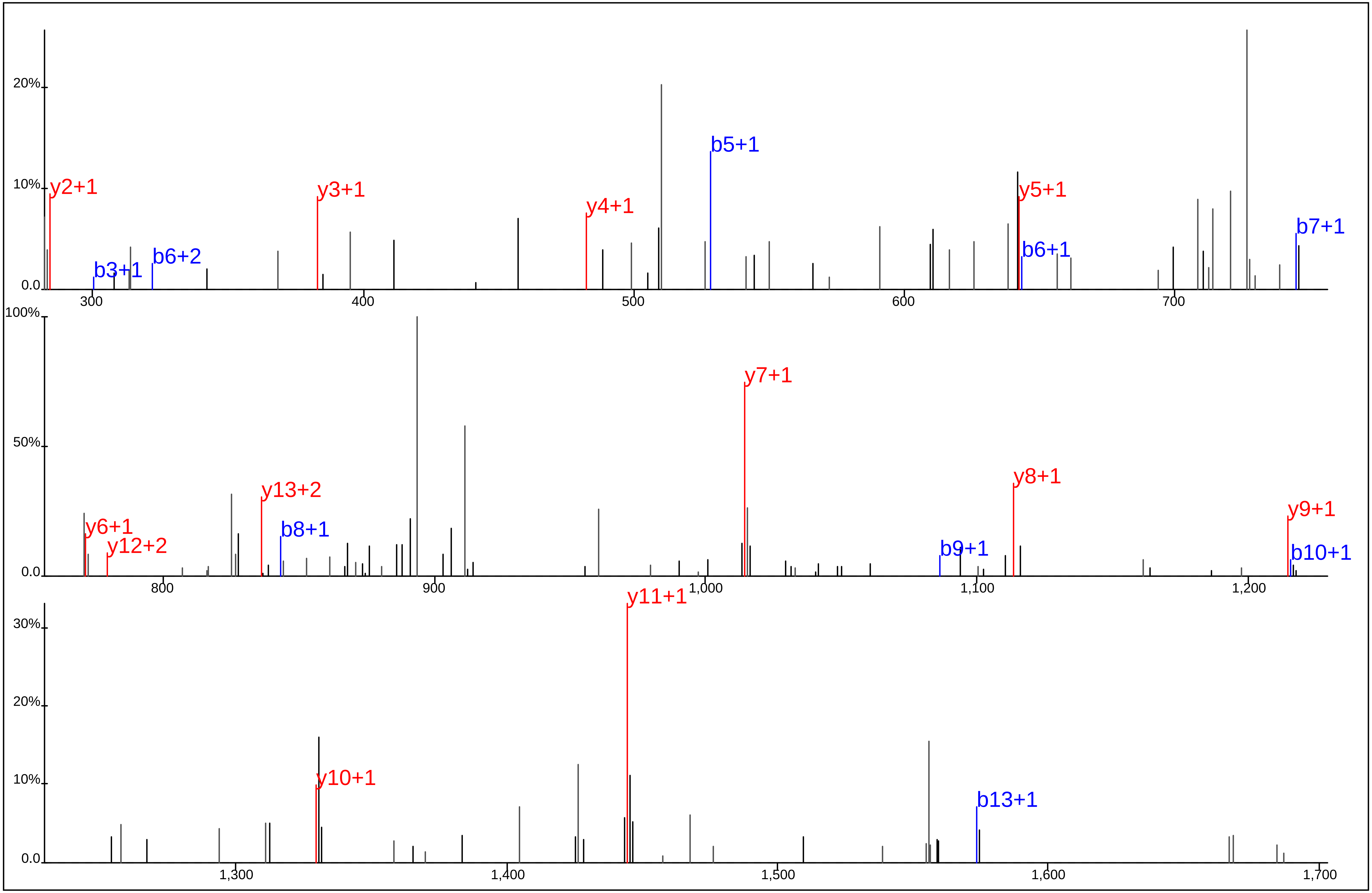

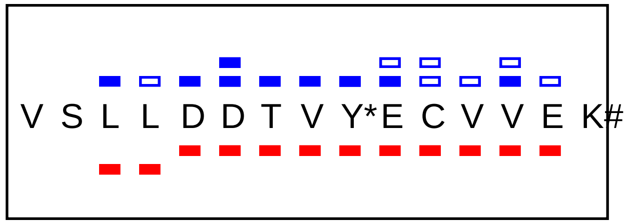

Protein Name: Protein 4.1R
gi Accession: 42716291
Precursor Mass: 928.9327+2
Delta Mass: 5.492 ppm
XCorr: 62.29

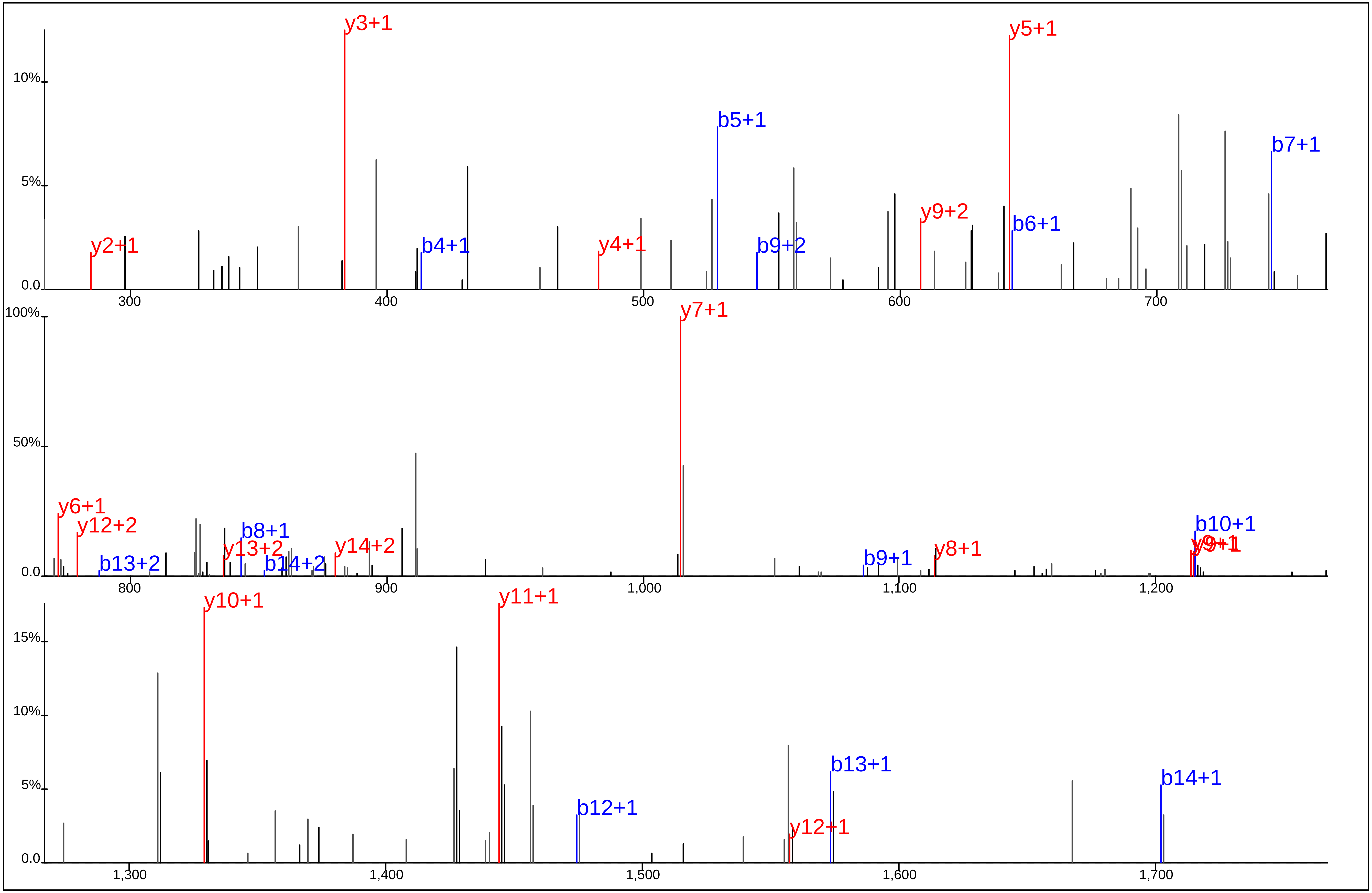

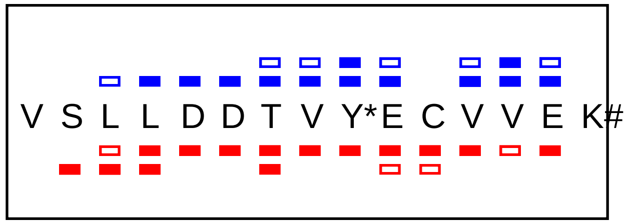

Protein Name: Protein 4.1R
gi Accession: 42716291
Precursor Mass: 928.9300+2
Delta Mass: 2.600 ppm
XCorr: 48.43

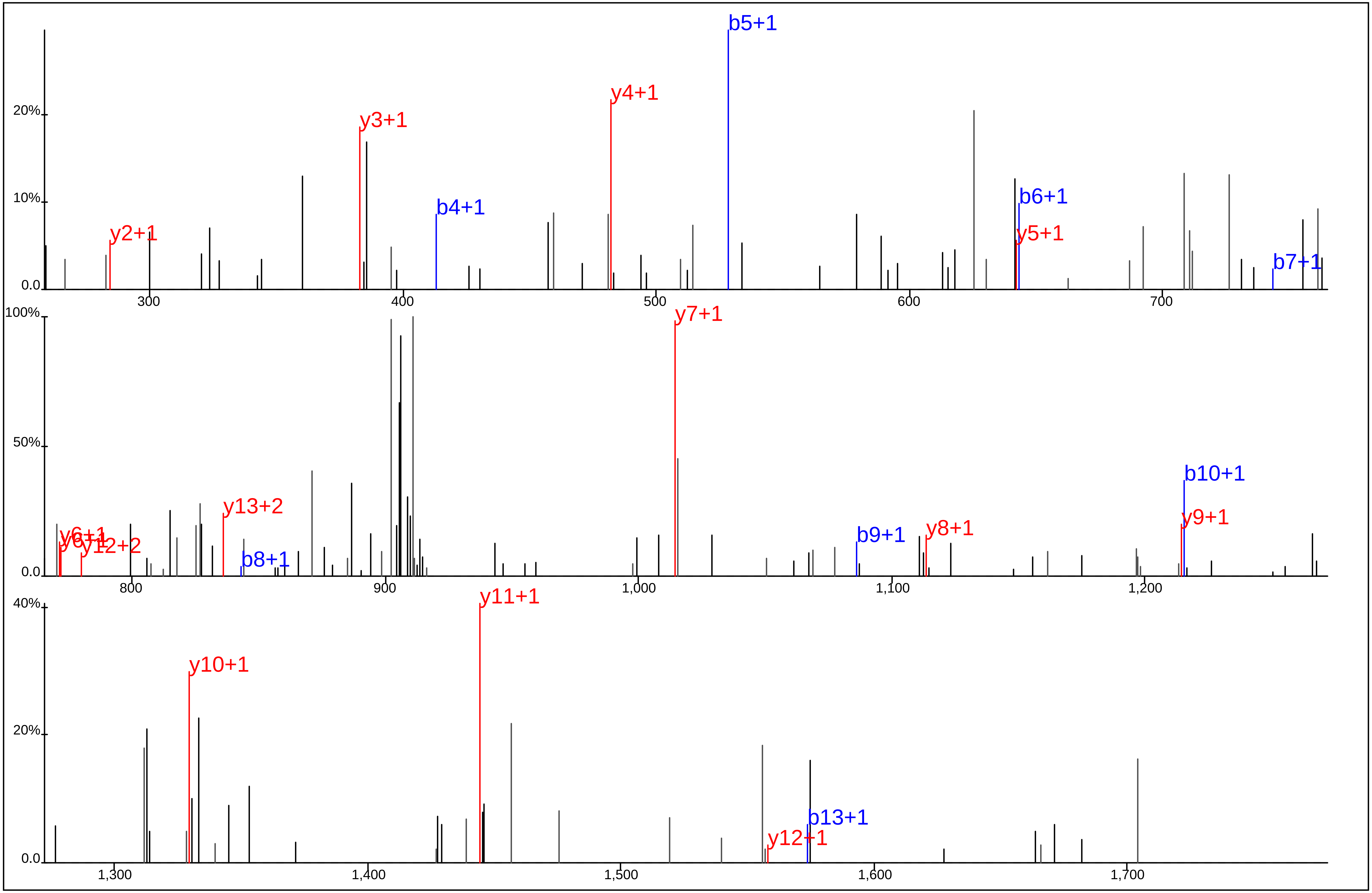

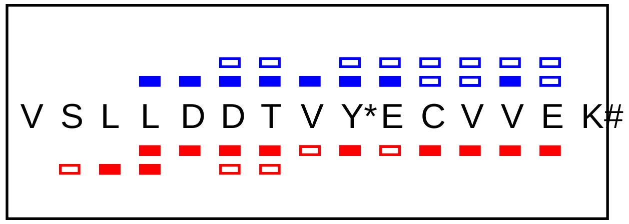

Protein Name: Protein 4.1R
gi Accession: 42716291
Precursor Mass: 928.9376+2
Delta Mass: 10.818 ppm
XCorr: 36.24

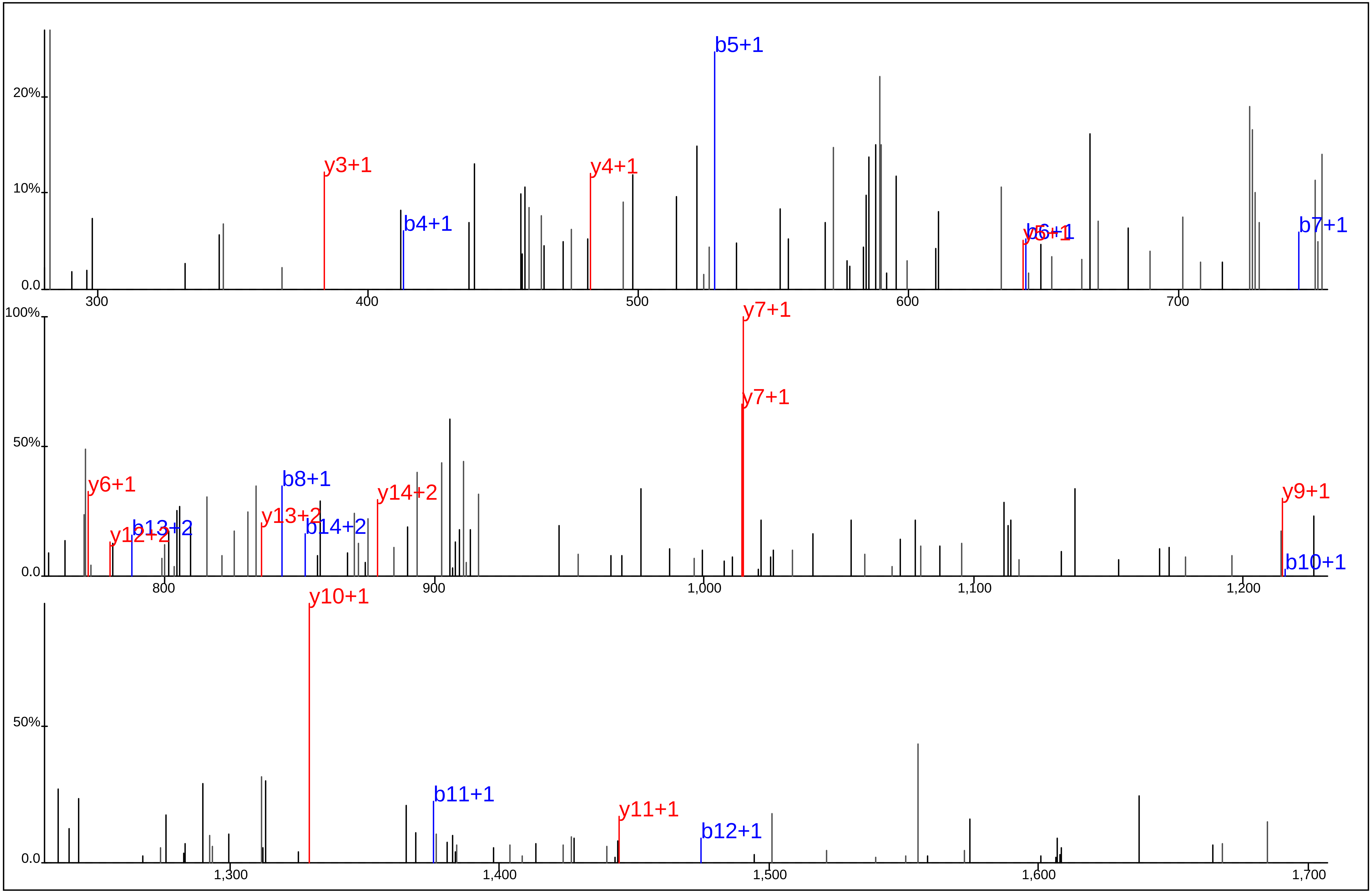

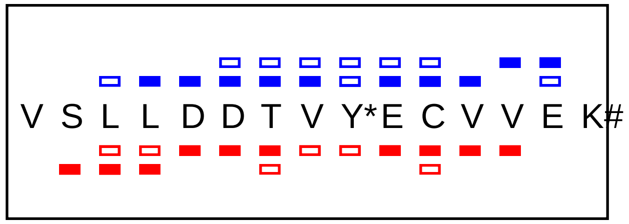

Protein Name: Protein 4.1R
gi Accession: 42716291
Precursor Mass: 928.9358+2
Delta Mass: 8.847 ppm
XCorr: 22.94

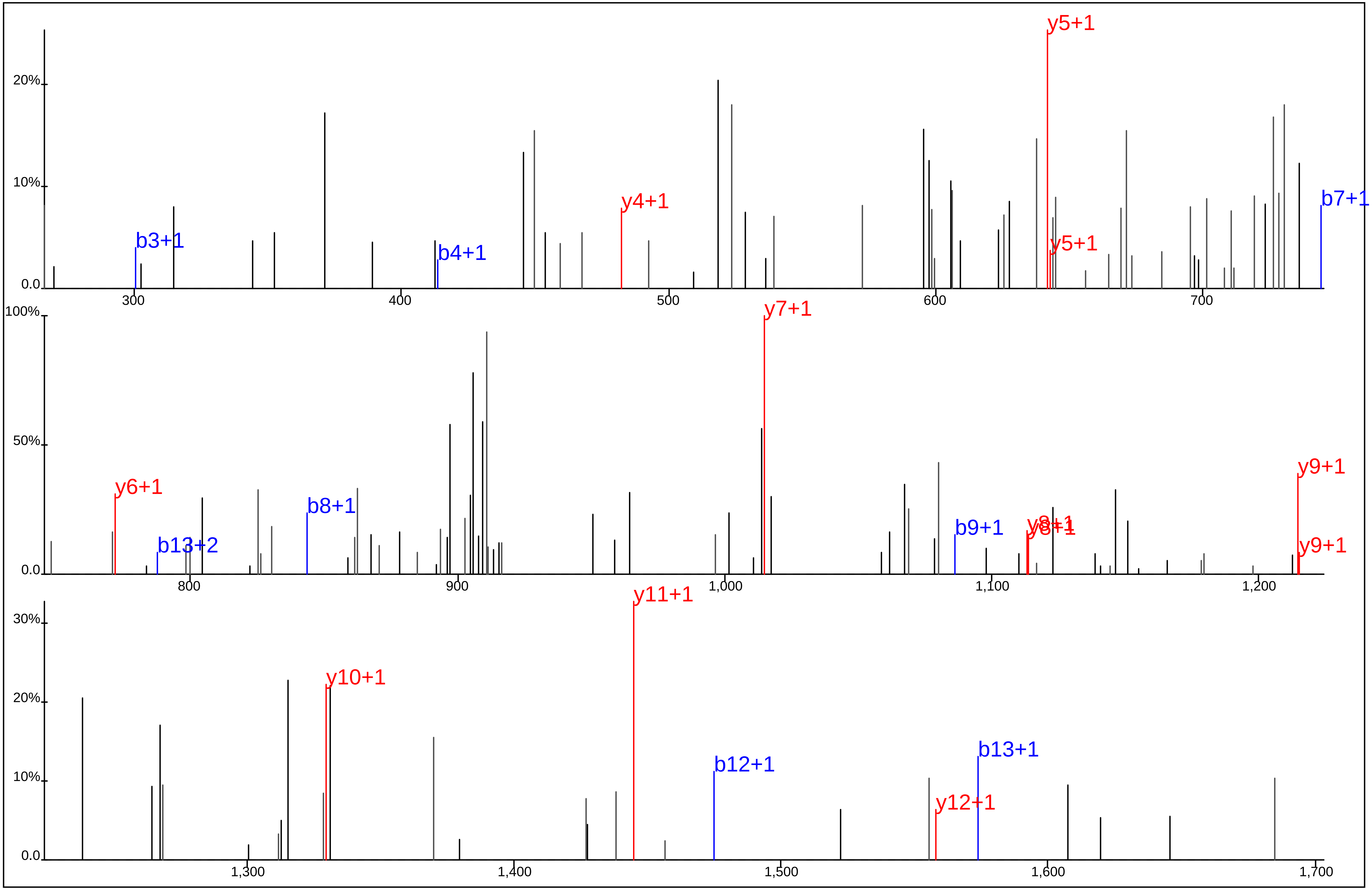

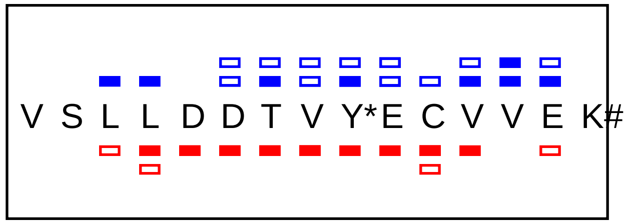

Protein Name: Protein 4.1R
gi Accession: 42716291
Precursor Mass: 928.9272+2
Delta Mass: 0.427 ppm
XCorr: 28.64

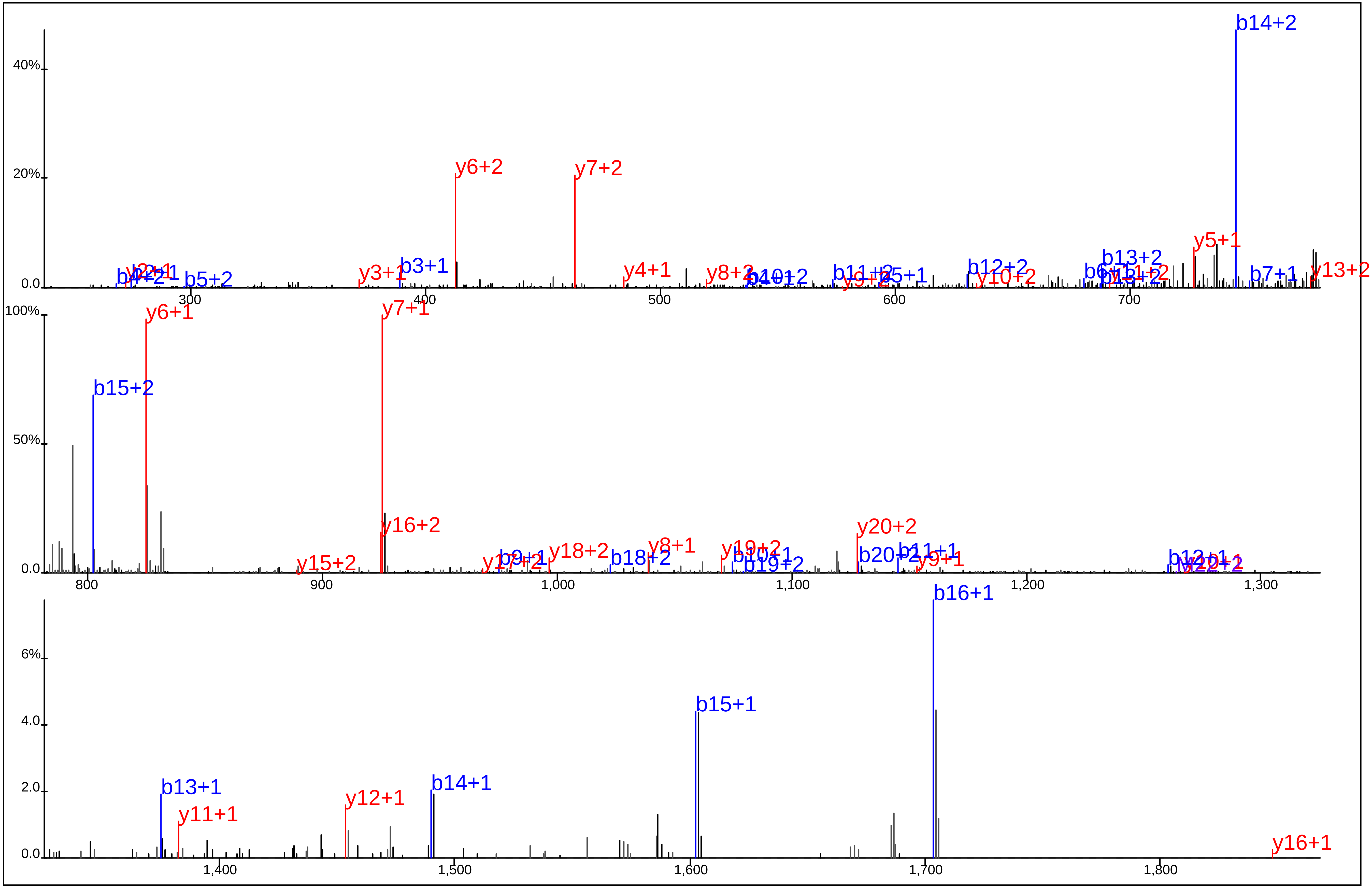

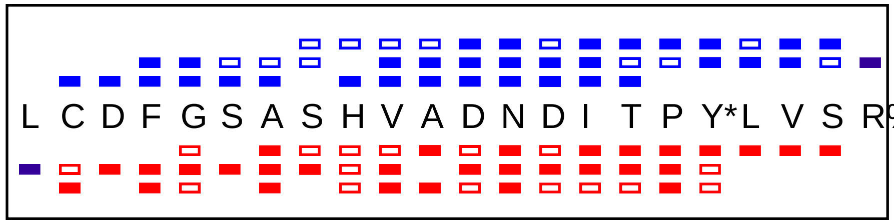

Protein Name:	PRP4 kinase
gi Accession:	3043596
Precursor Mass:	843.0456+3
Delta Mass:	0.941 ppm
XCorr:	67.52

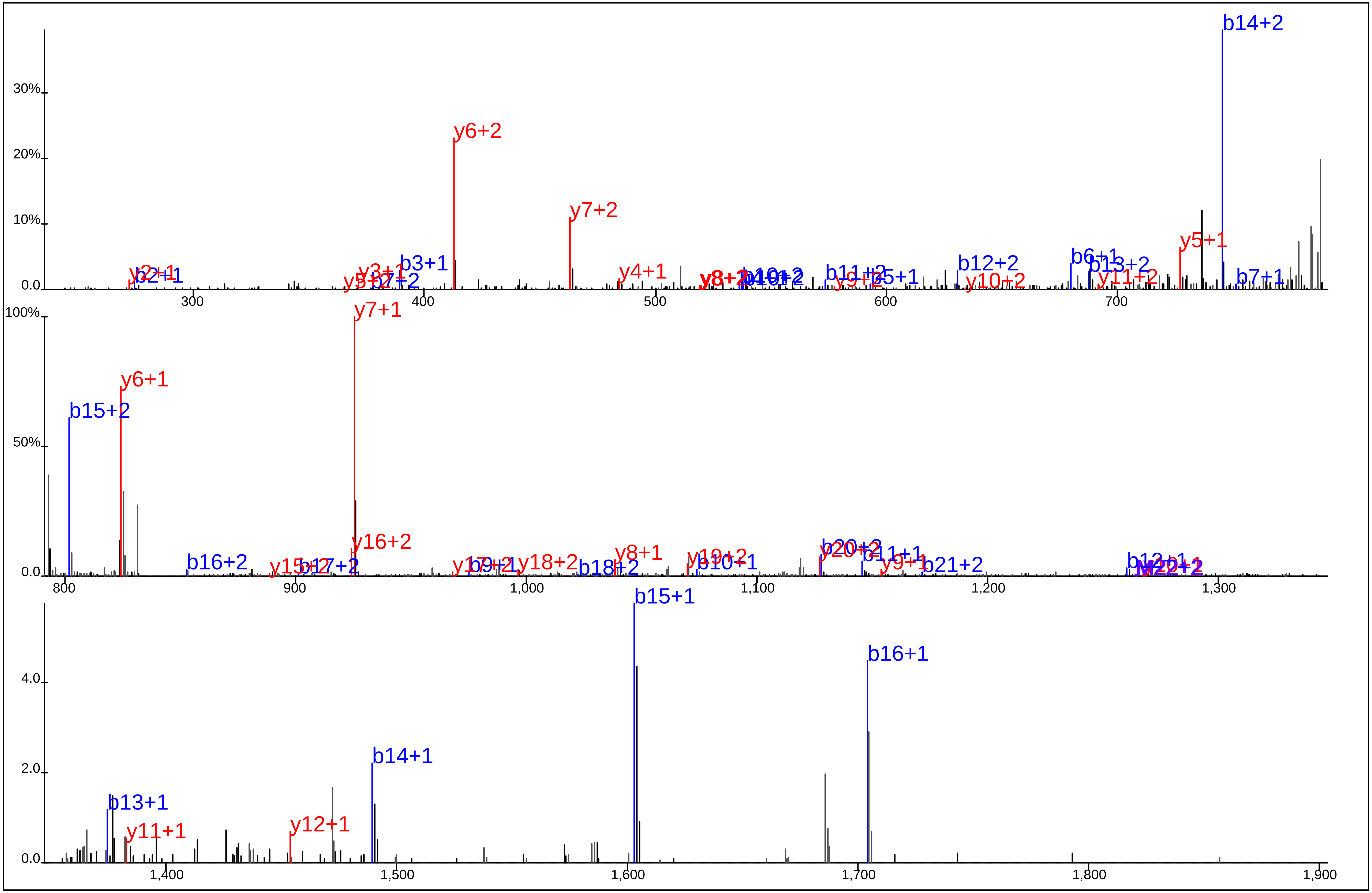

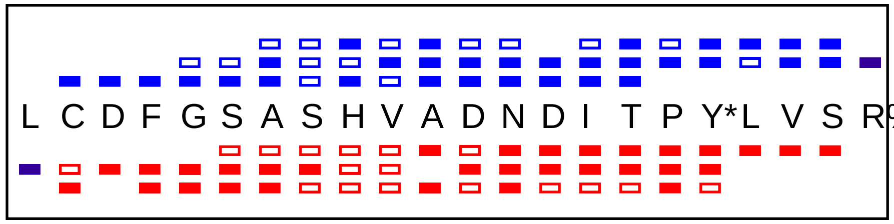

Protein Name: PRP4 kinase
gi Accession: 3043596
Precursor Mass: 843.0478+3
Delta Mass: 3.549 ppm
XCorr: 60.67

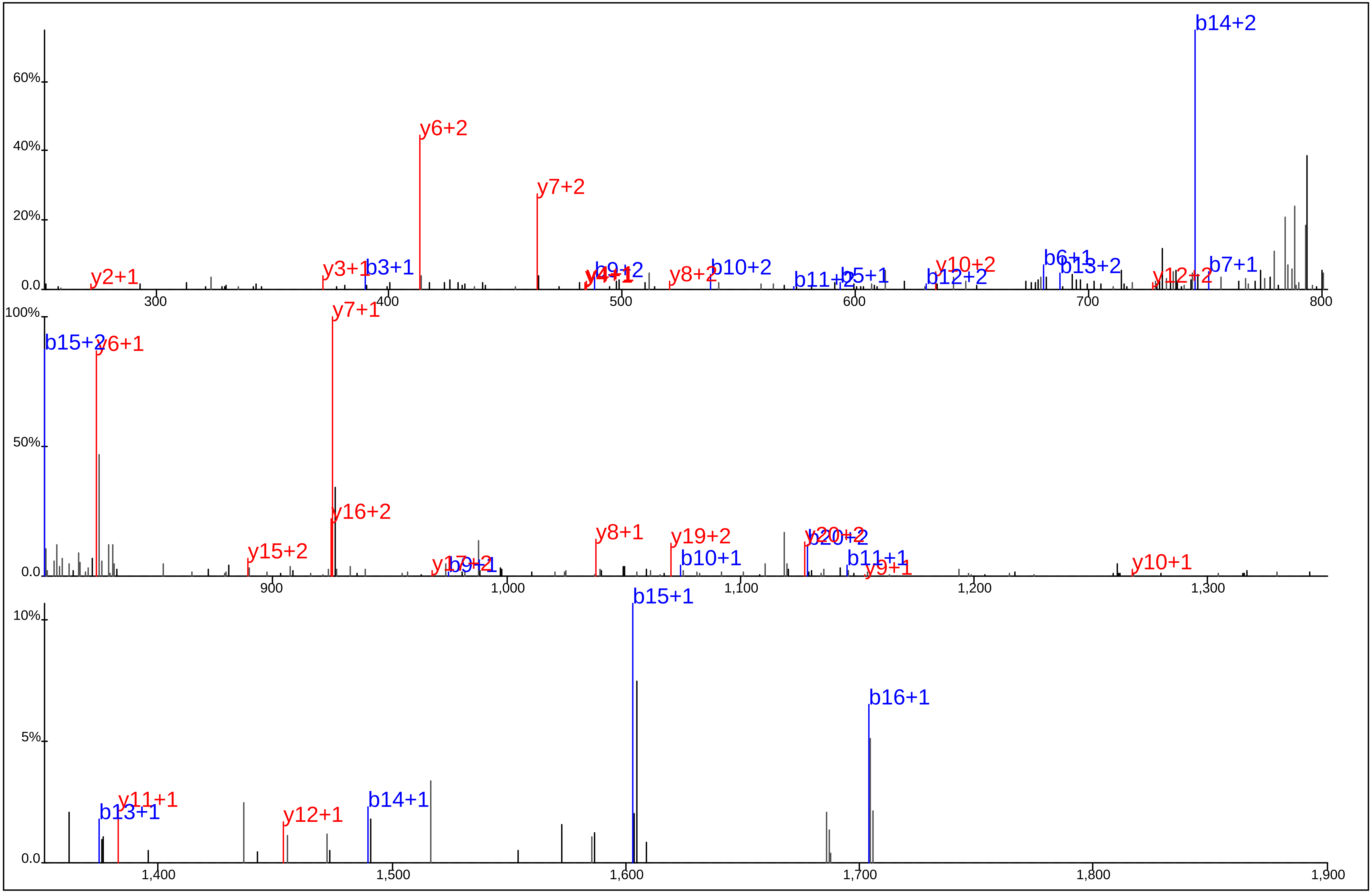

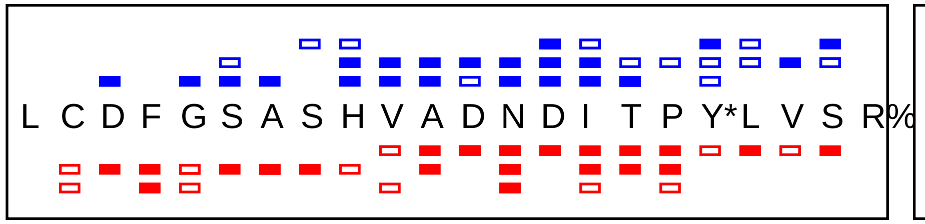

Protein Name: PRP4 kinase
gi Accession: 3043596
Precursor Mass: 843.0500+3
Delta Mass: 6.227 ppm
XCorr: 40.3

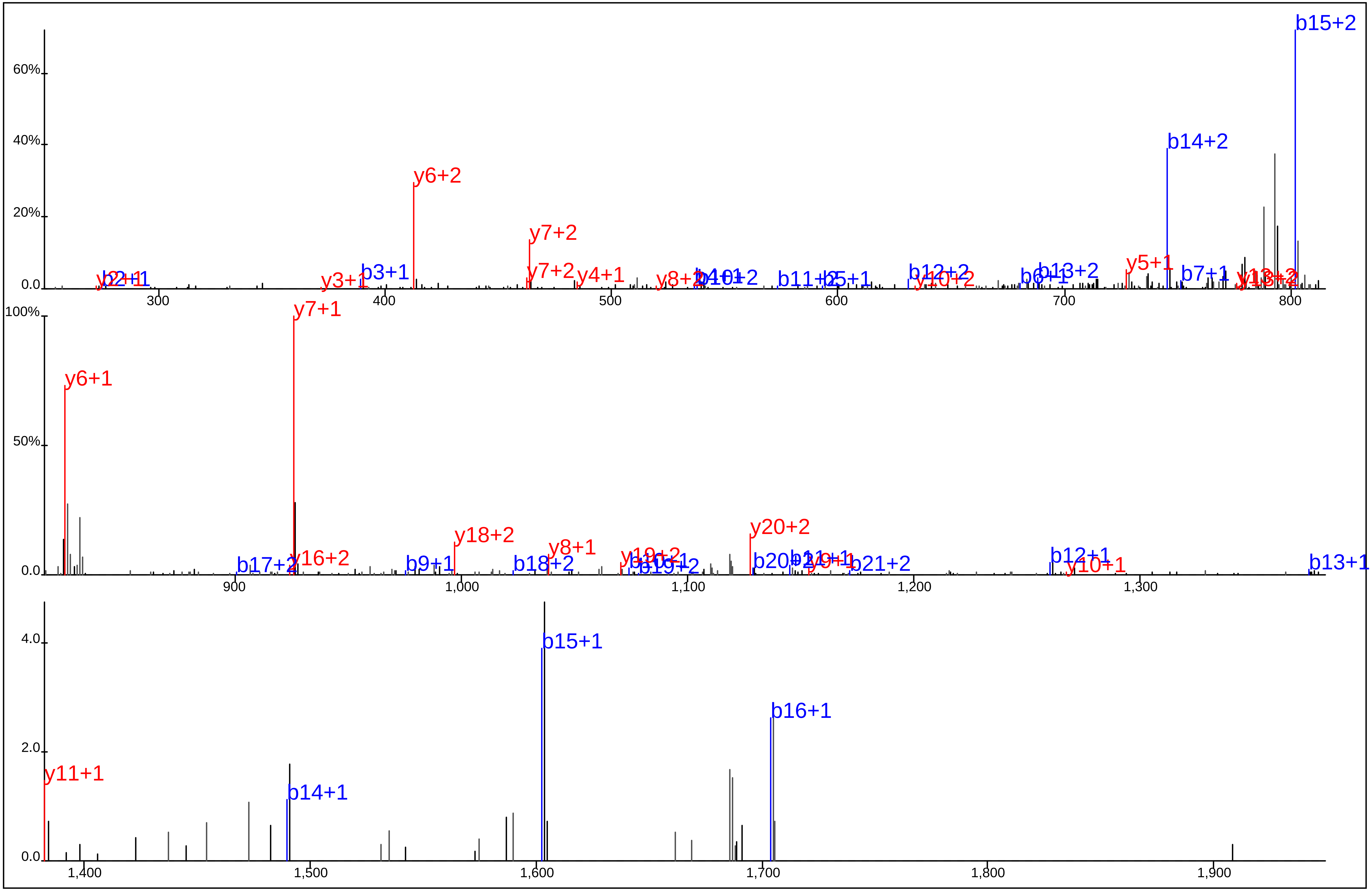

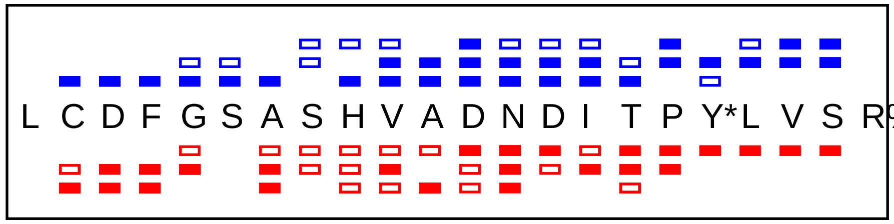

Protein Name: PRP4 kinase
gi Accession: 3043596
Precursor Mass: 843.0464+3
Delta Mass: 1.882 ppm
XCorr: 31.97

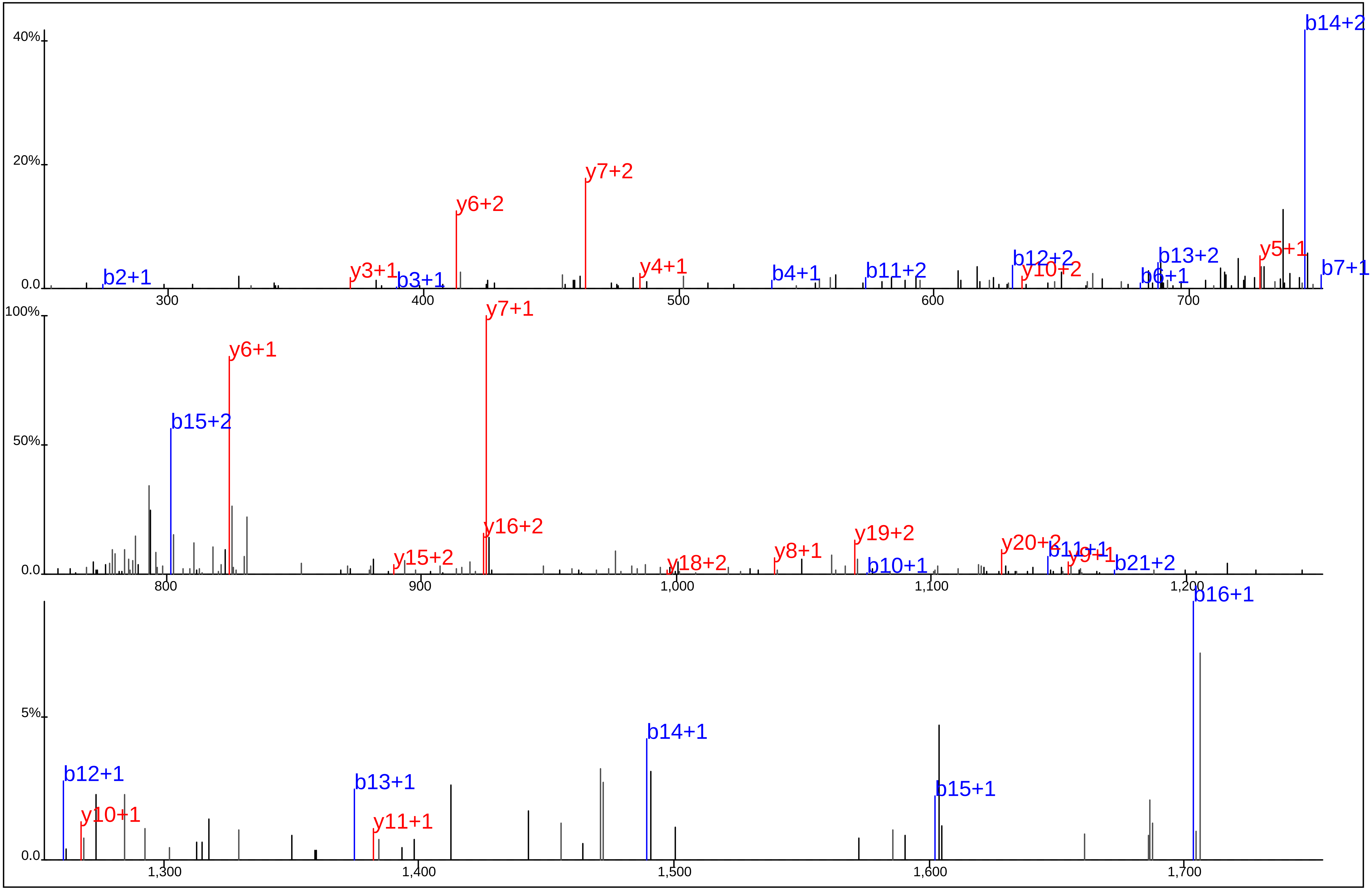

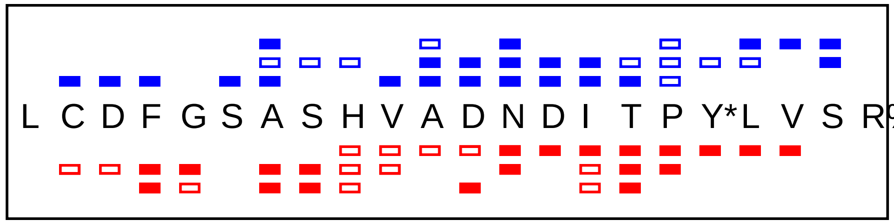

Protein Name: PRP4 kinase
gi Accession: 3043596
Precursor Mass: 843.0492+3
Delta Mass: 5.215 ppm
XCorr: 34.05

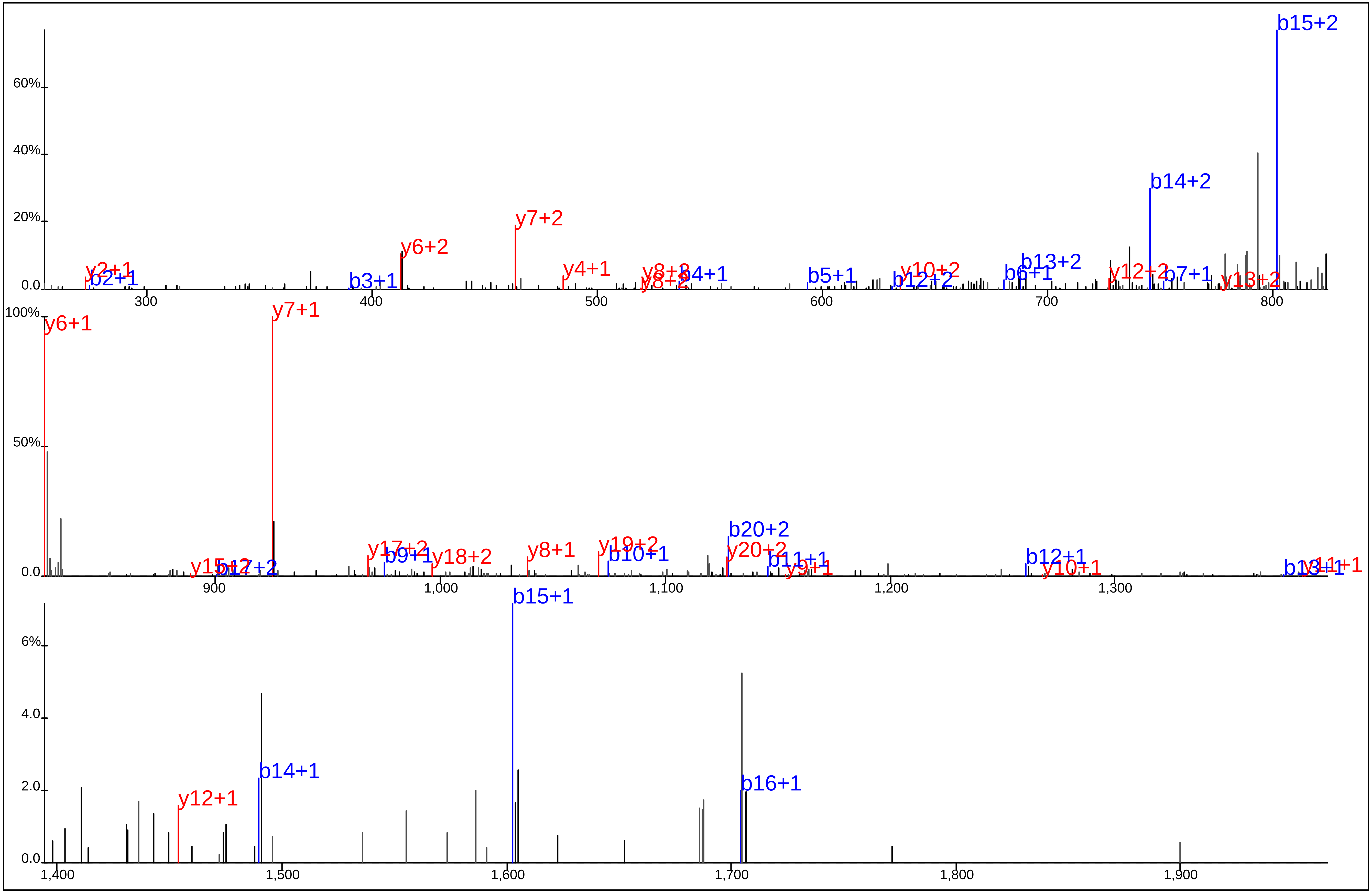

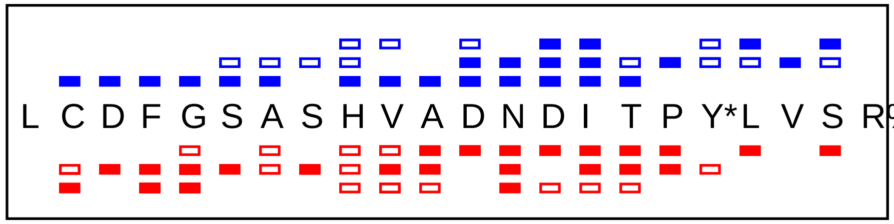

Protein Name: PRP4 kinase
gi Accession: 3043596
Precursor Mass: 843.0477+3
Delta Mass: 3.402 ppm
XCorr: 42.52

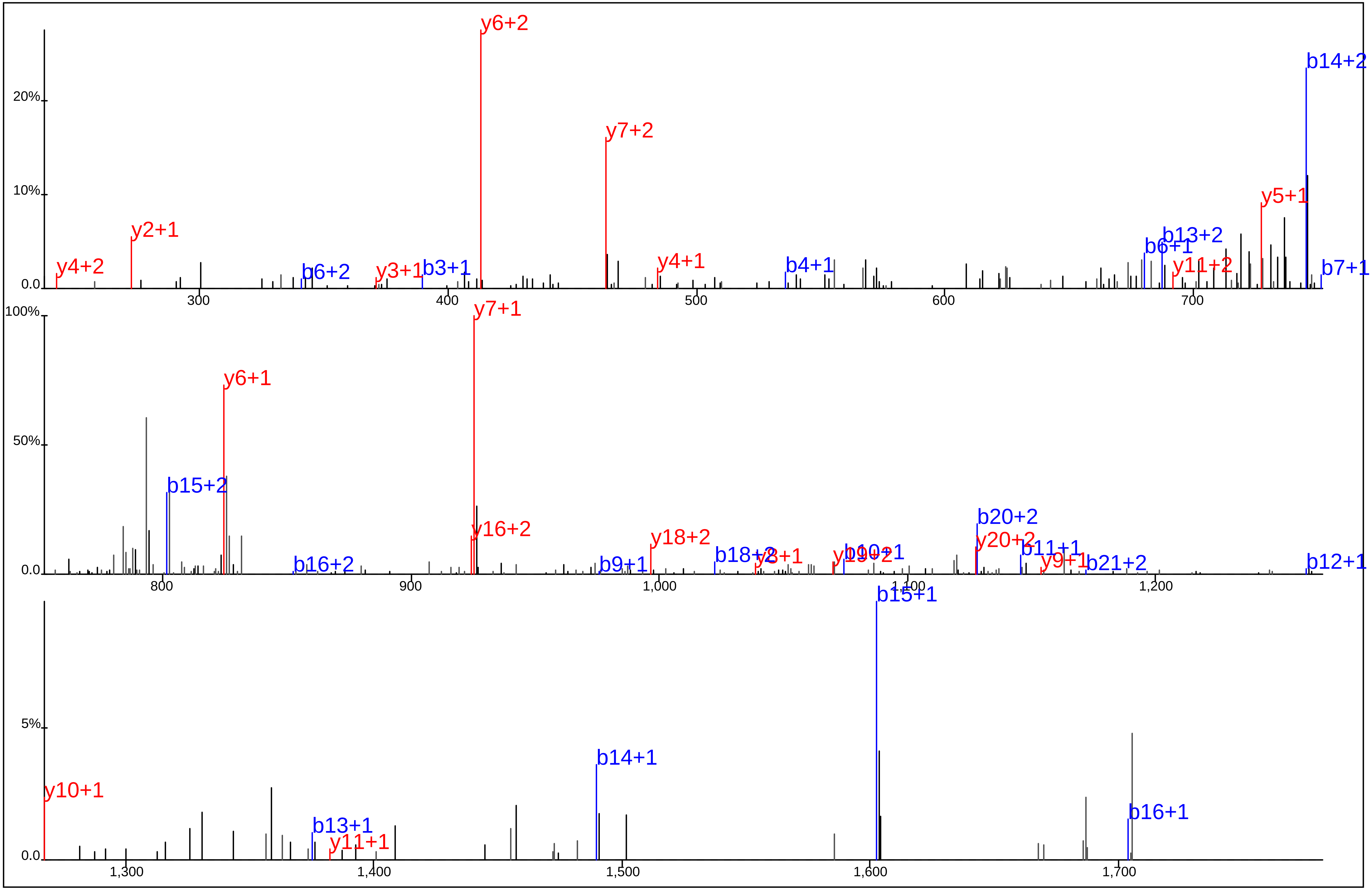

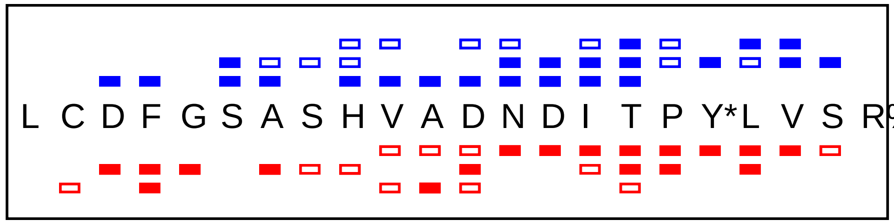

Protein Name:	PRP4 kinase
gi Accession:	3043596
Precursor Mass:	843.0491+3
Delta Mass:	5.143 ppm
XCorr:	41.58

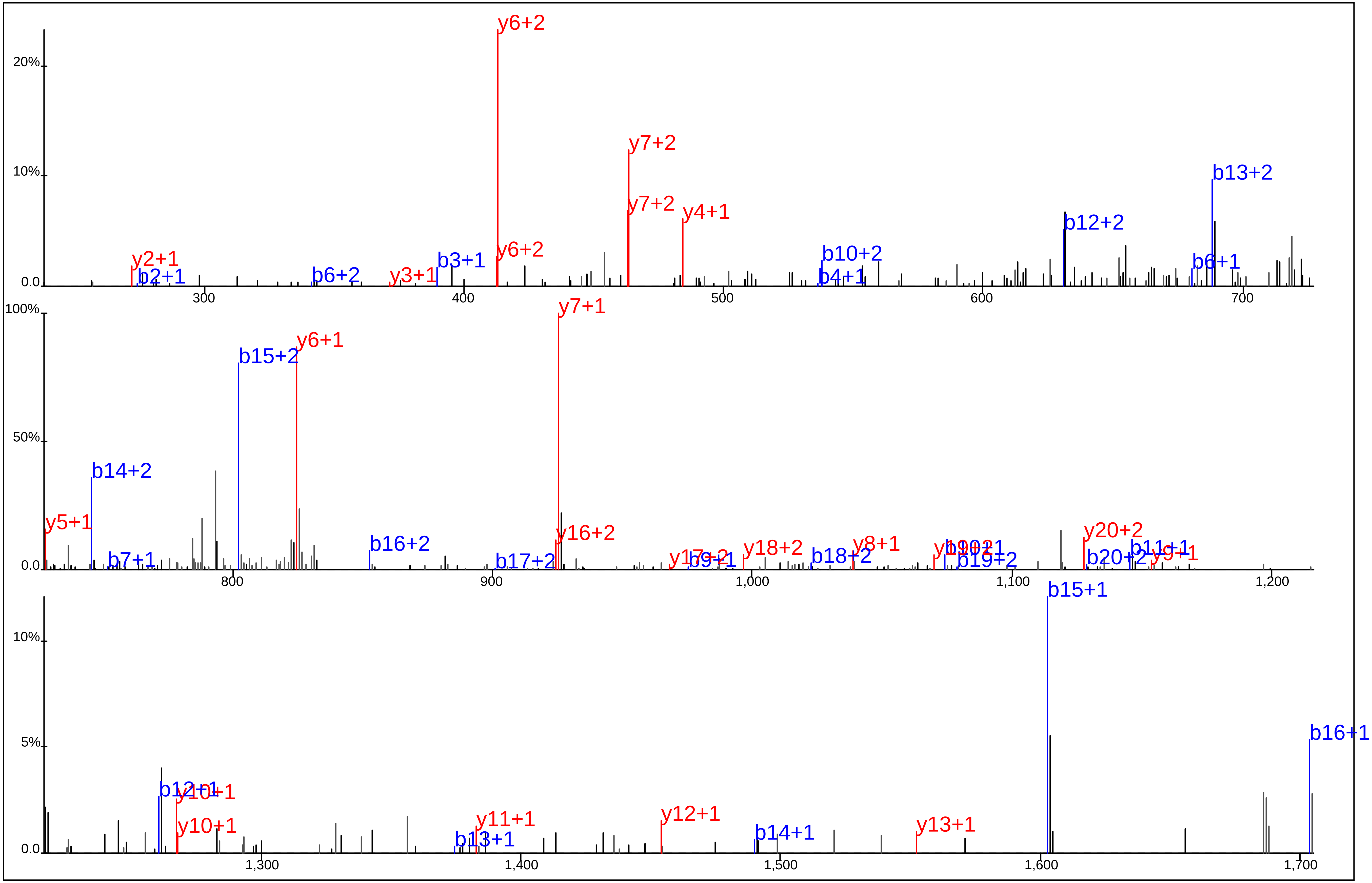

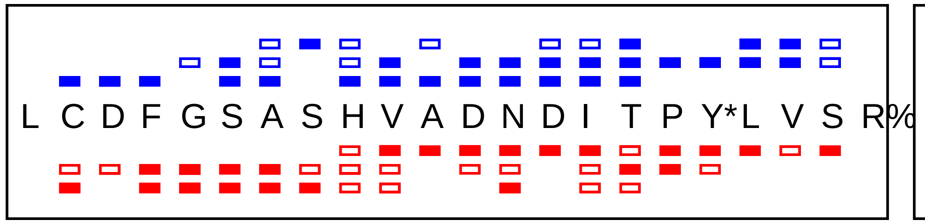

Protein Name: PRP4 kinase
gi Accession: 3043596
Precursor Mass: 843.0460+3
Delta Mass: 1.377 ppm
XCorr: 72.65

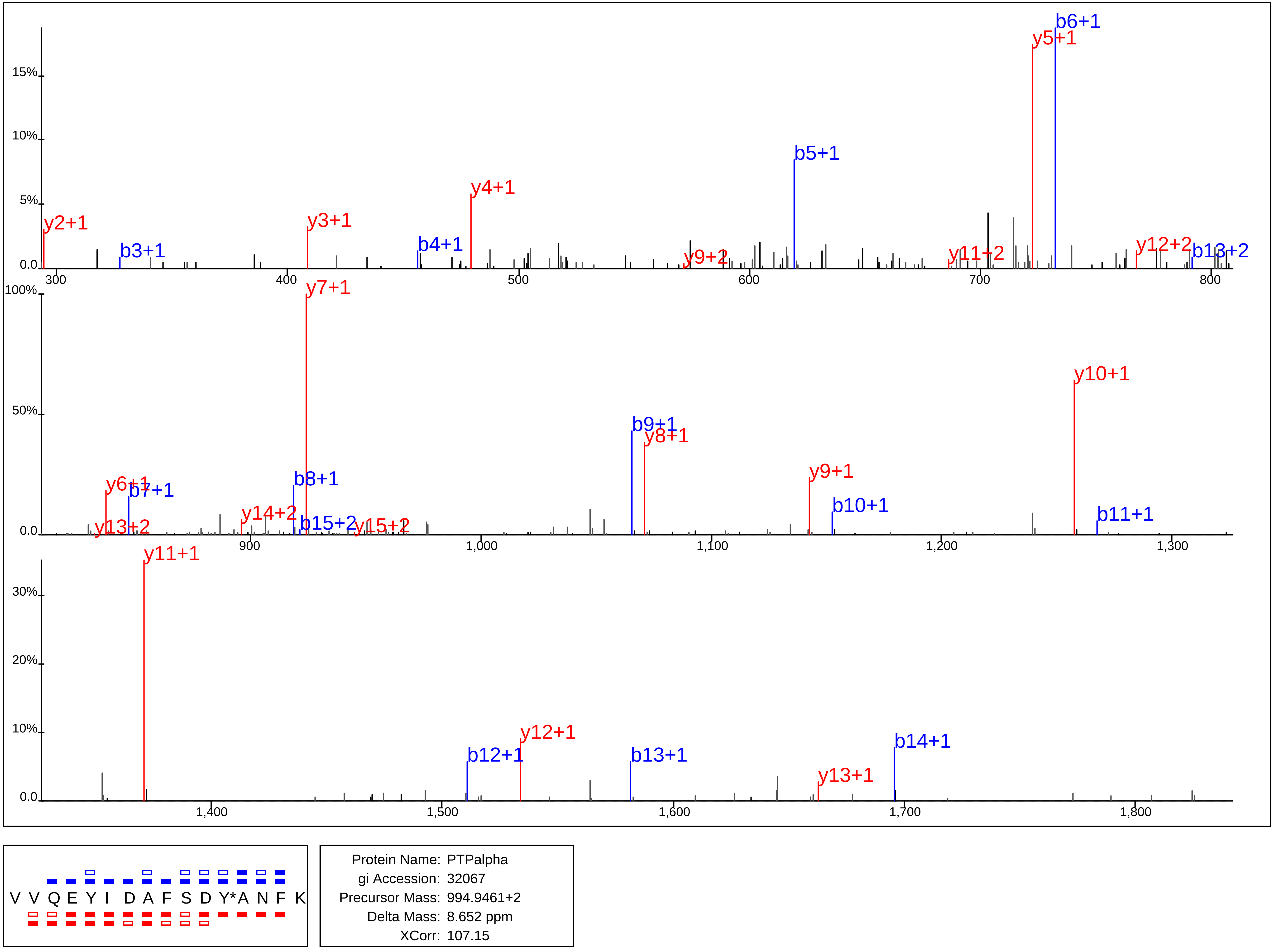

Protein Name:	PTPalpha
gi Accession:	32067
Precursor Mass:	994.9461+2
Delta Mass:	8.652 ppm
XCorr:	107.15

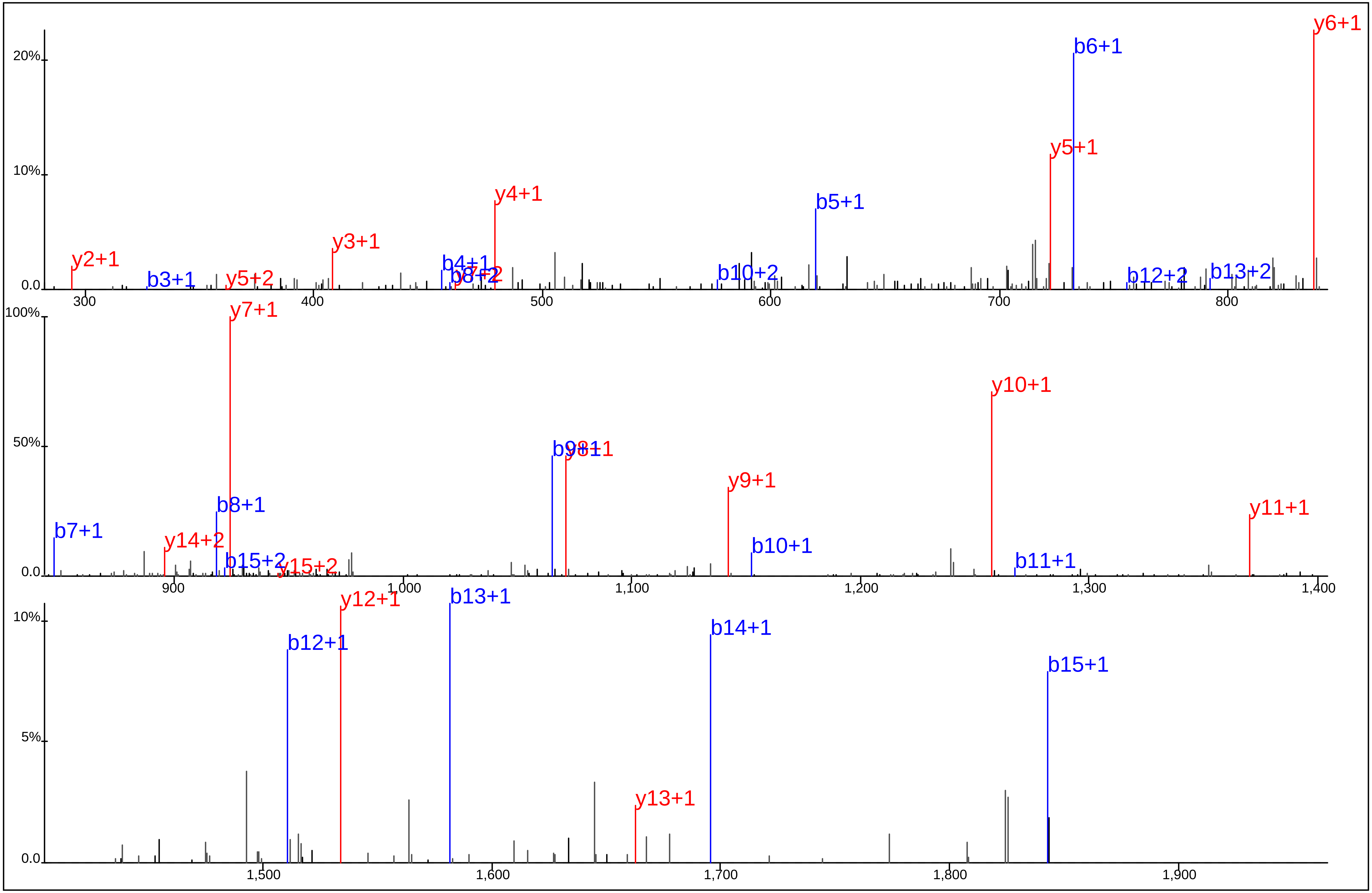

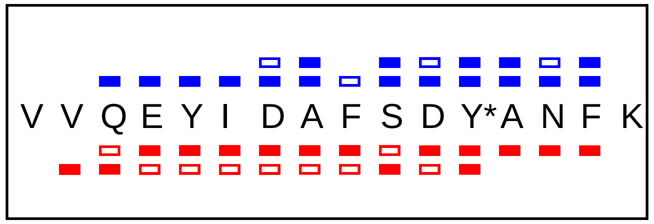

Protein Name: PTPalpha
gi Accession: 32067
Precursor Mass: 994.9412+2
Delta Mass: 3.679 ppm
XCorr: 104.29

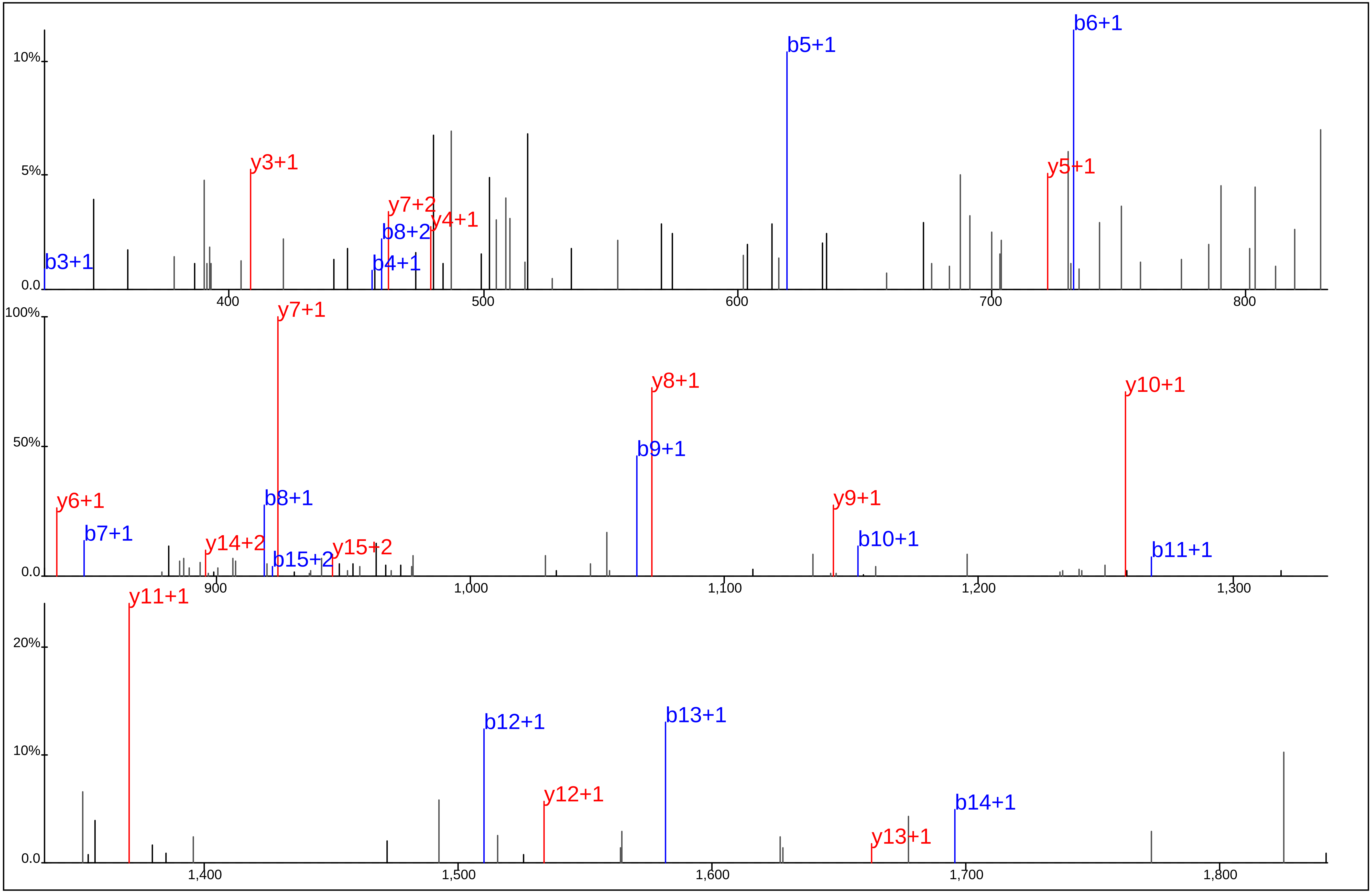

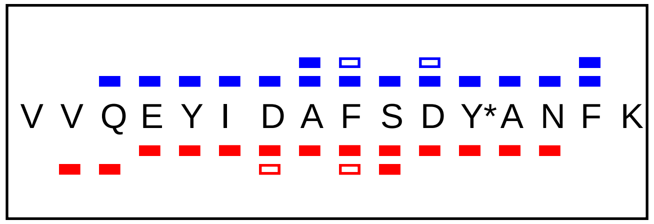

Protein Name:	PTPalpha
gi Accession:	32067
Precursor Mass:	994.9415+2
Delta Mass:	3.986 ppm
XCorr:	79.4

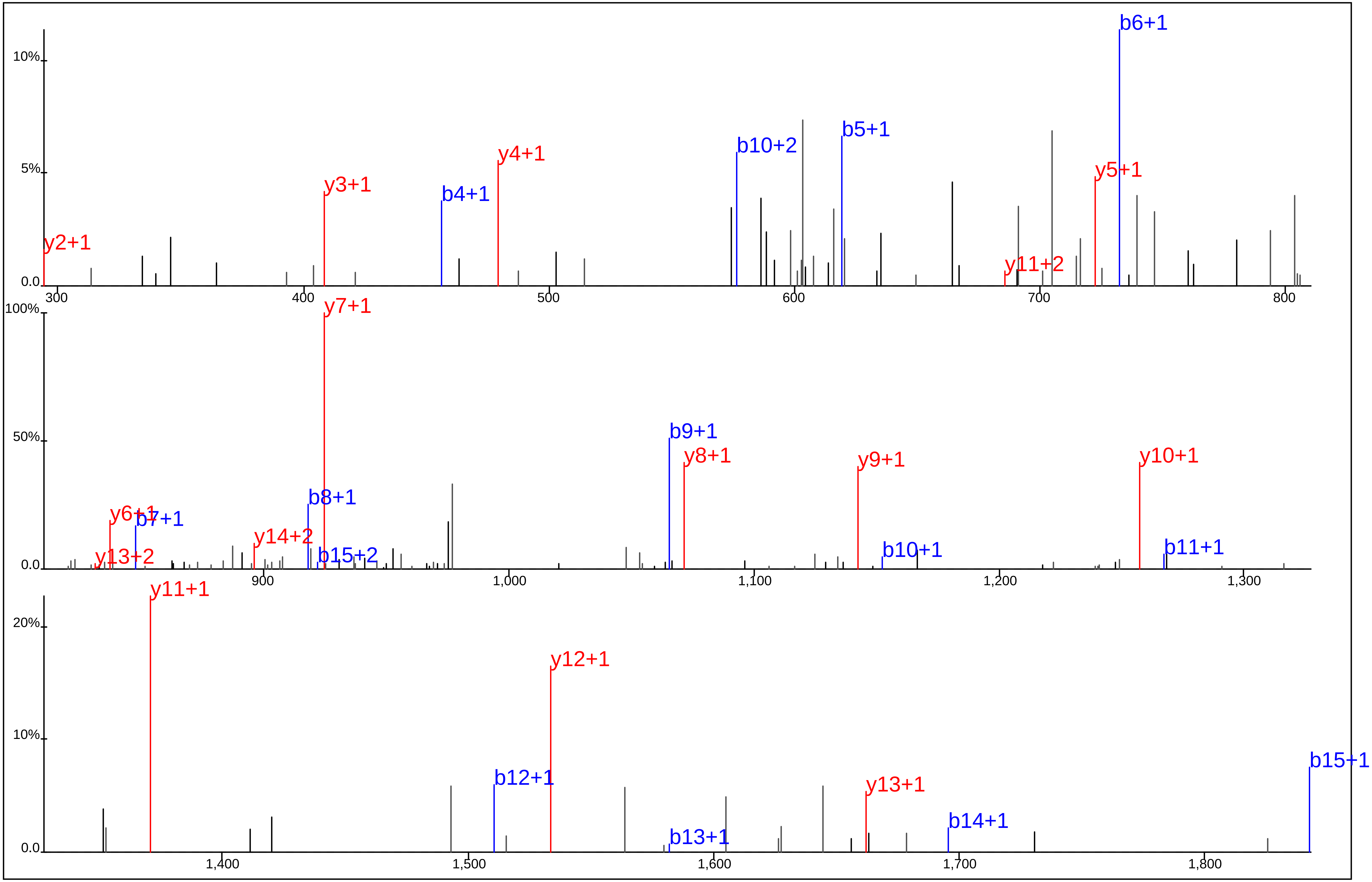

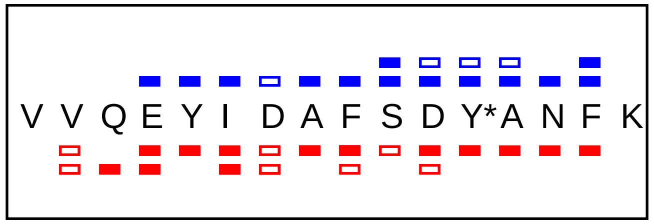

Protein Name: PTPalpha
gi Accession: 32067
Precursor Mass: 994.9426+2
Delta Mass: 5.092 ppm
XCorr: 86.83

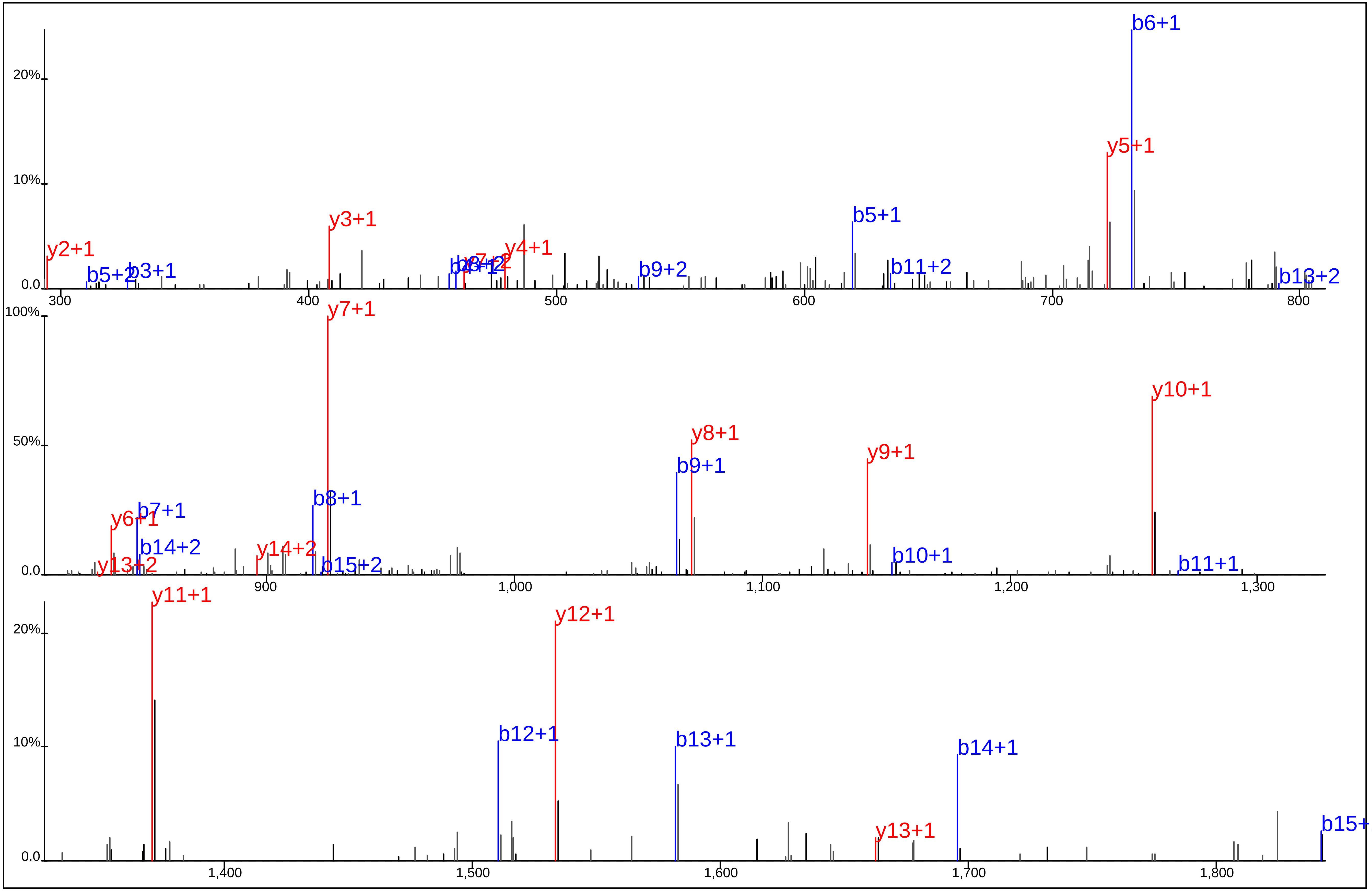

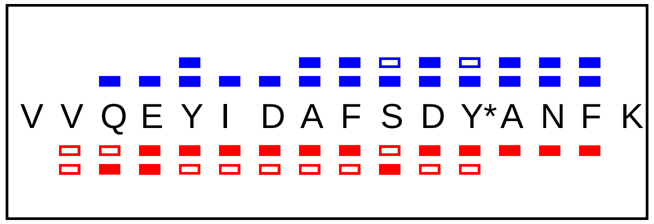

Protein Name:	PTPalpha
gi Accession:	32067
Precursor Mass:	994.9473+2
Delta Mass:	9.814 ppm
XCorr:	70.5

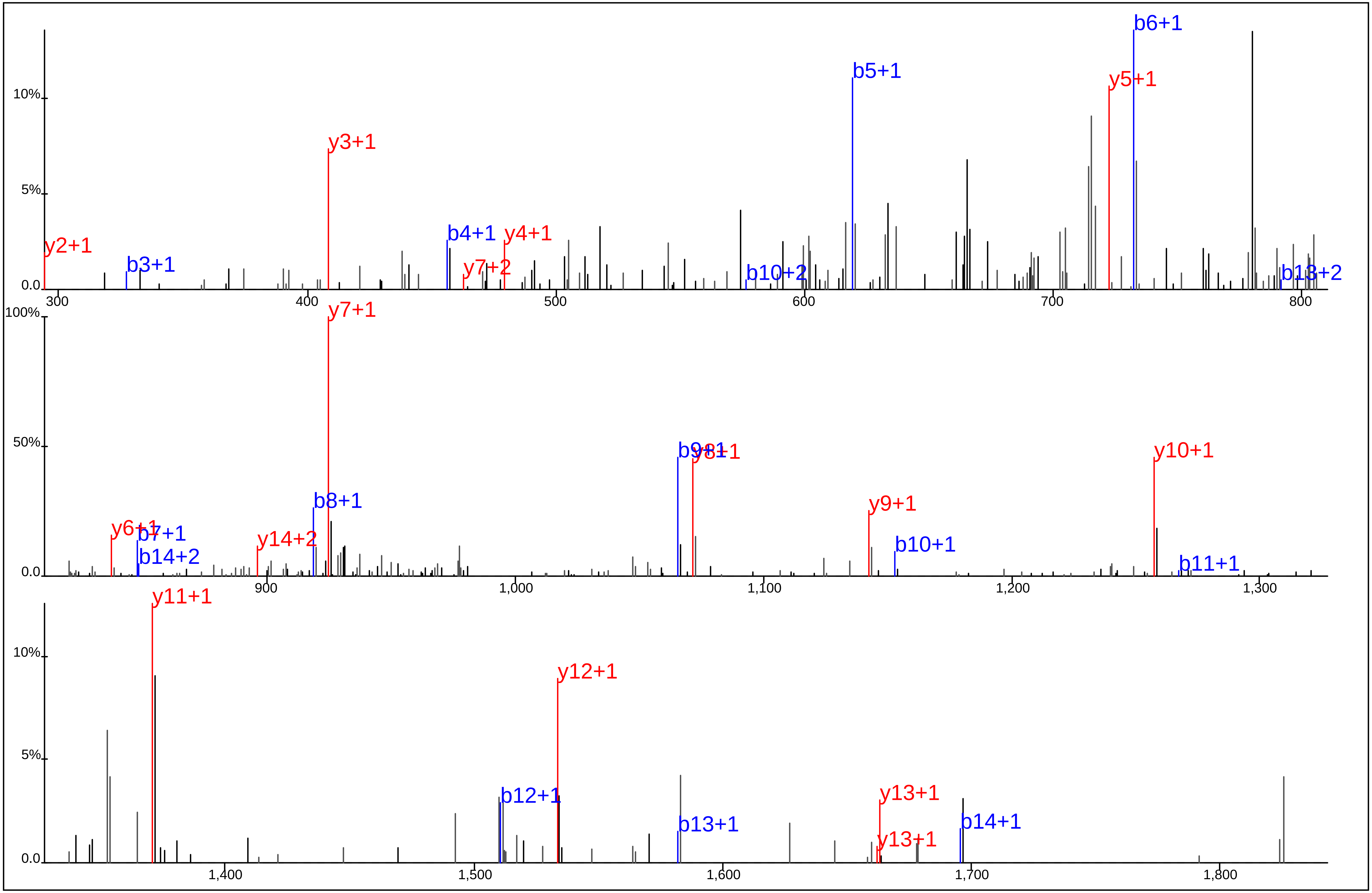

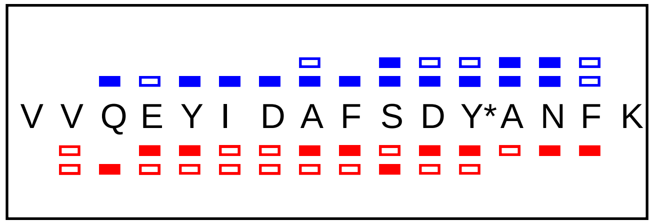

Protein Name: PTPalpha
gi Accession: 32067
Precursor Mass: 994.9418+2
Delta Mass: 4.353 ppm
XCorr: 76.83

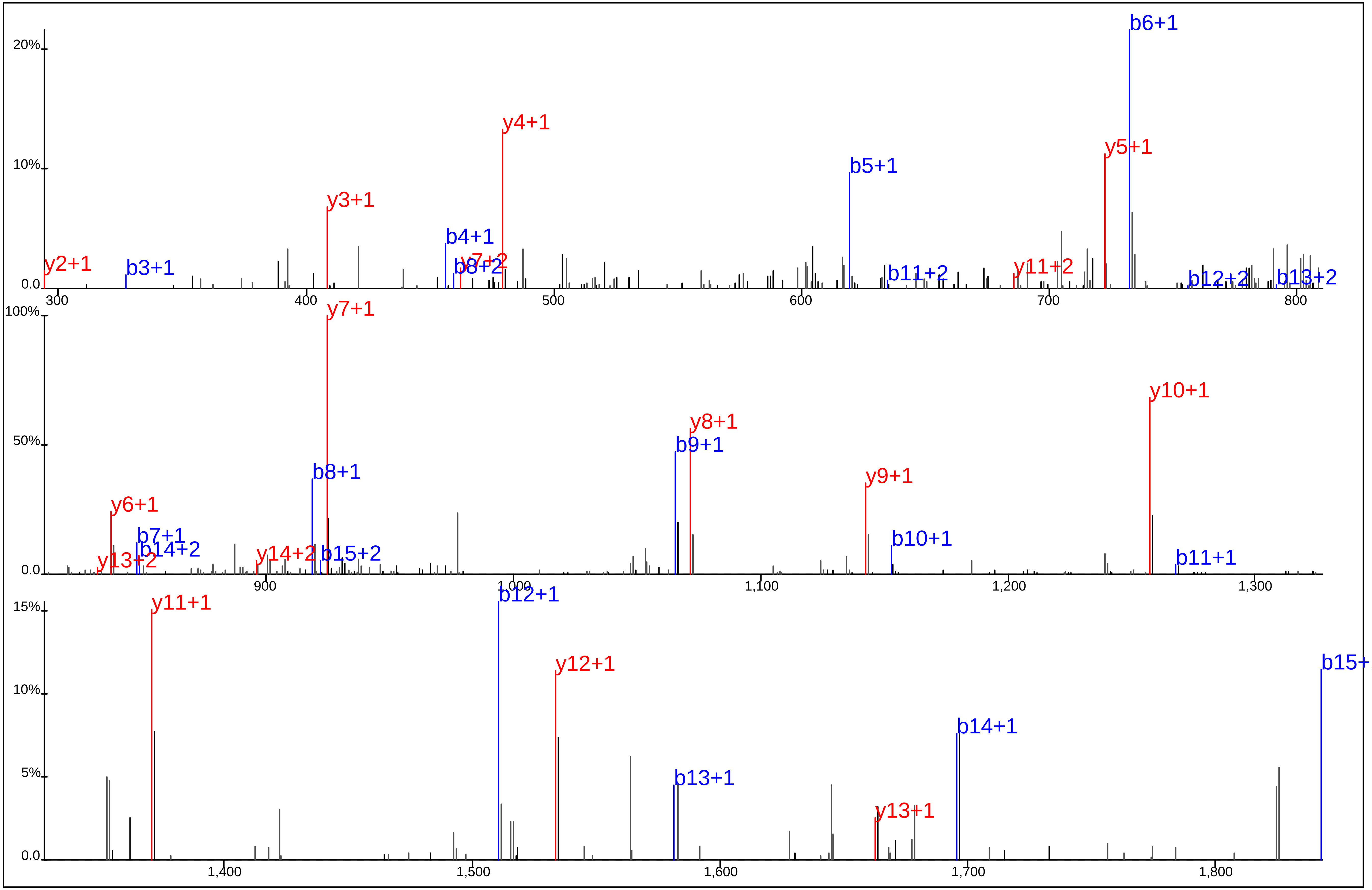

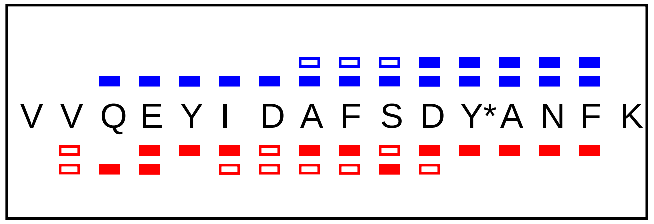

Protein Name: PTPalpha
gi Accession: 32067
Precursor Mass: 994.9417+2
Delta Mass: 4.167 ppm
XCorr: 73.84

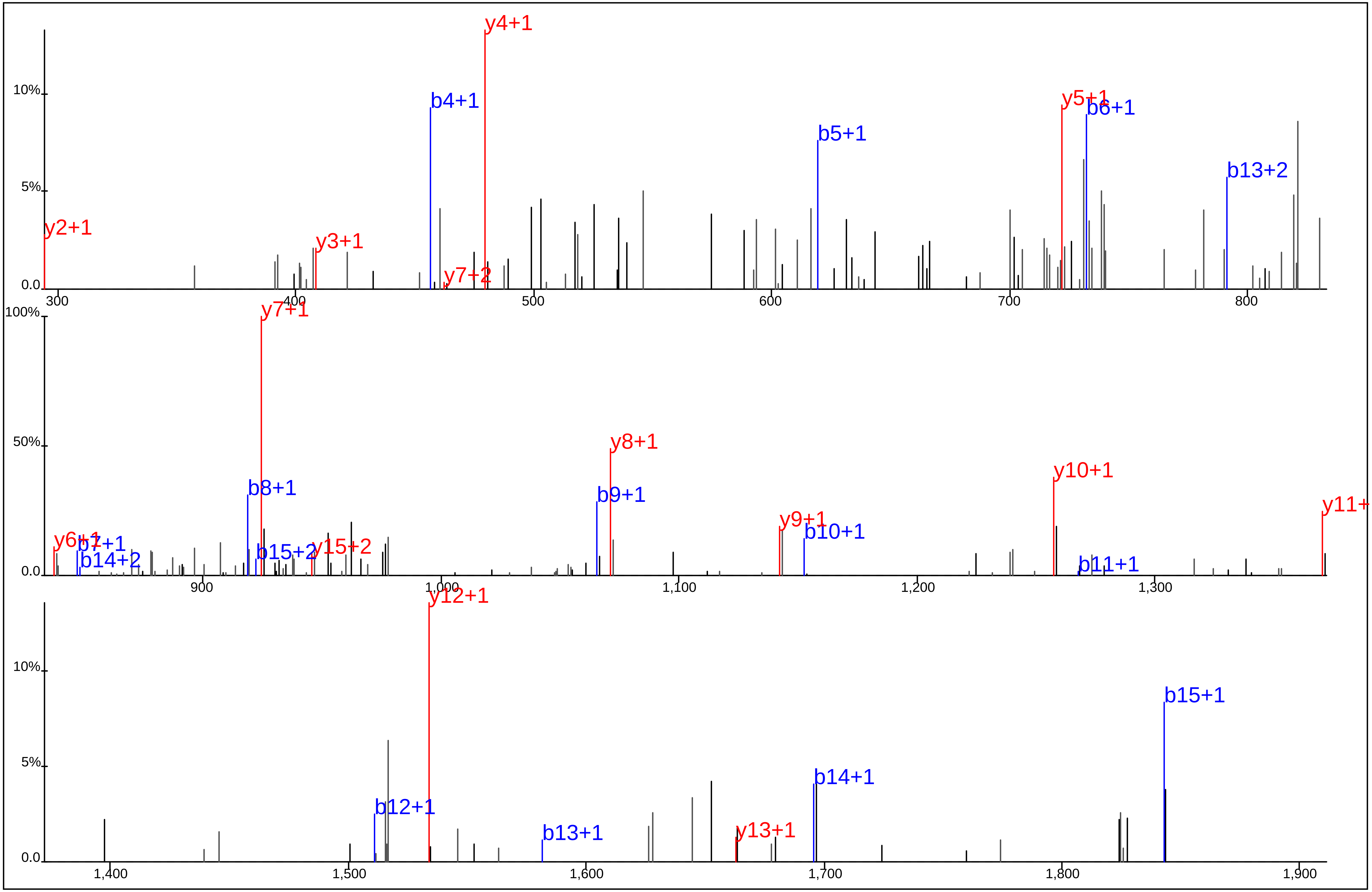

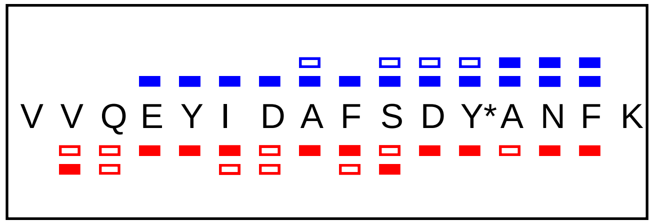

Protein Name:	PTPalpha
gi Accession:	32067
Precursor Mass:	994.9427+2
Delta Mass:	5.273 ppm
XCorr:	78.23

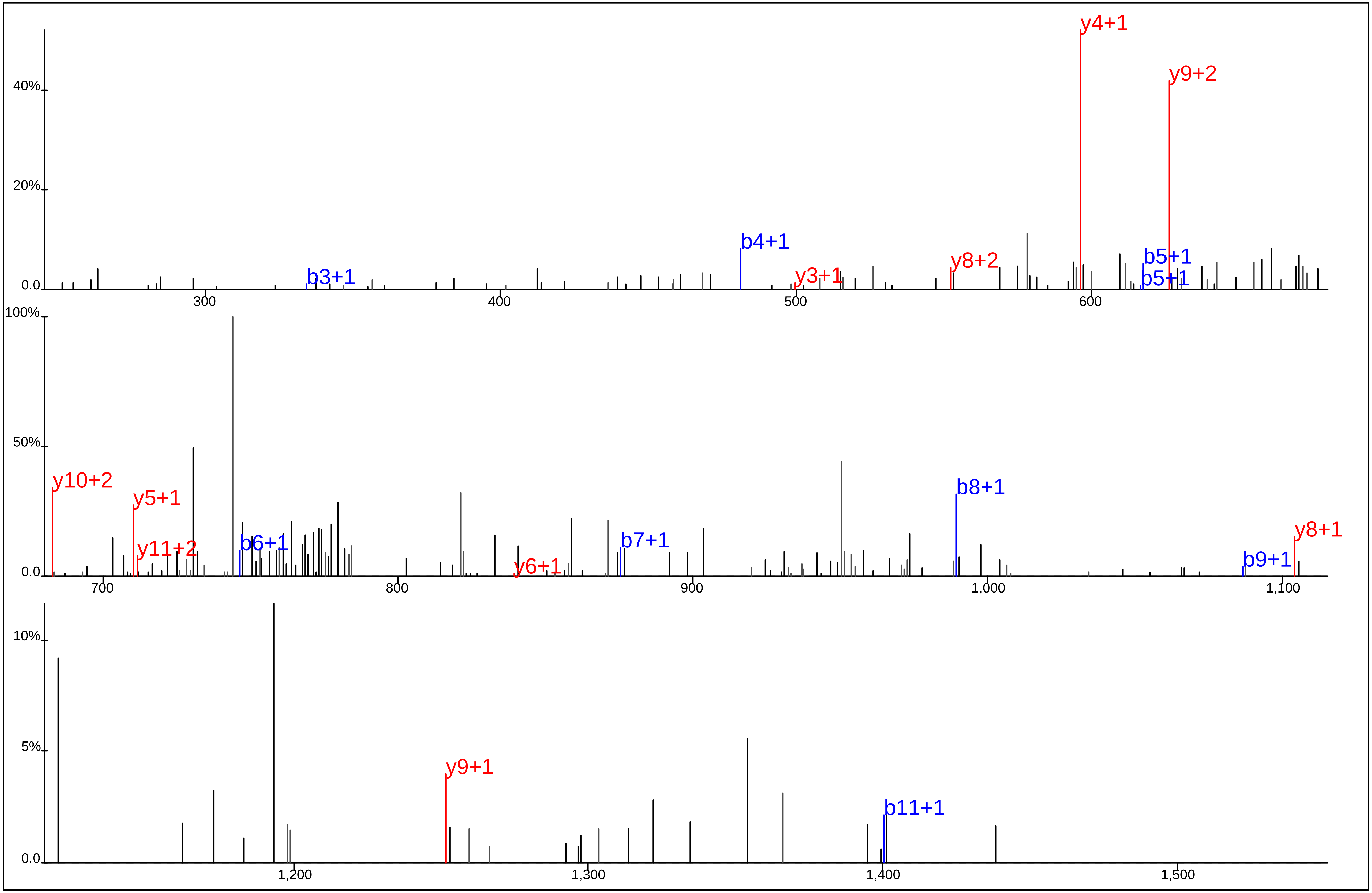

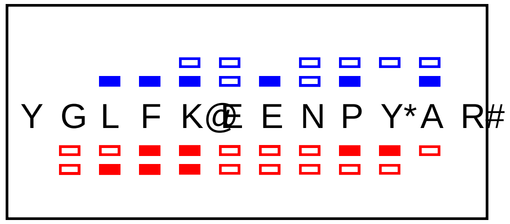

Protein Name:	PTTG binding factor
gi Accession:	4757886
Precursor Mass:	792.8698+2
Delta Mass:	6.897 ppm
XCorr:	2.4431

808 - PTTGbindingfactor_3414608.pdf

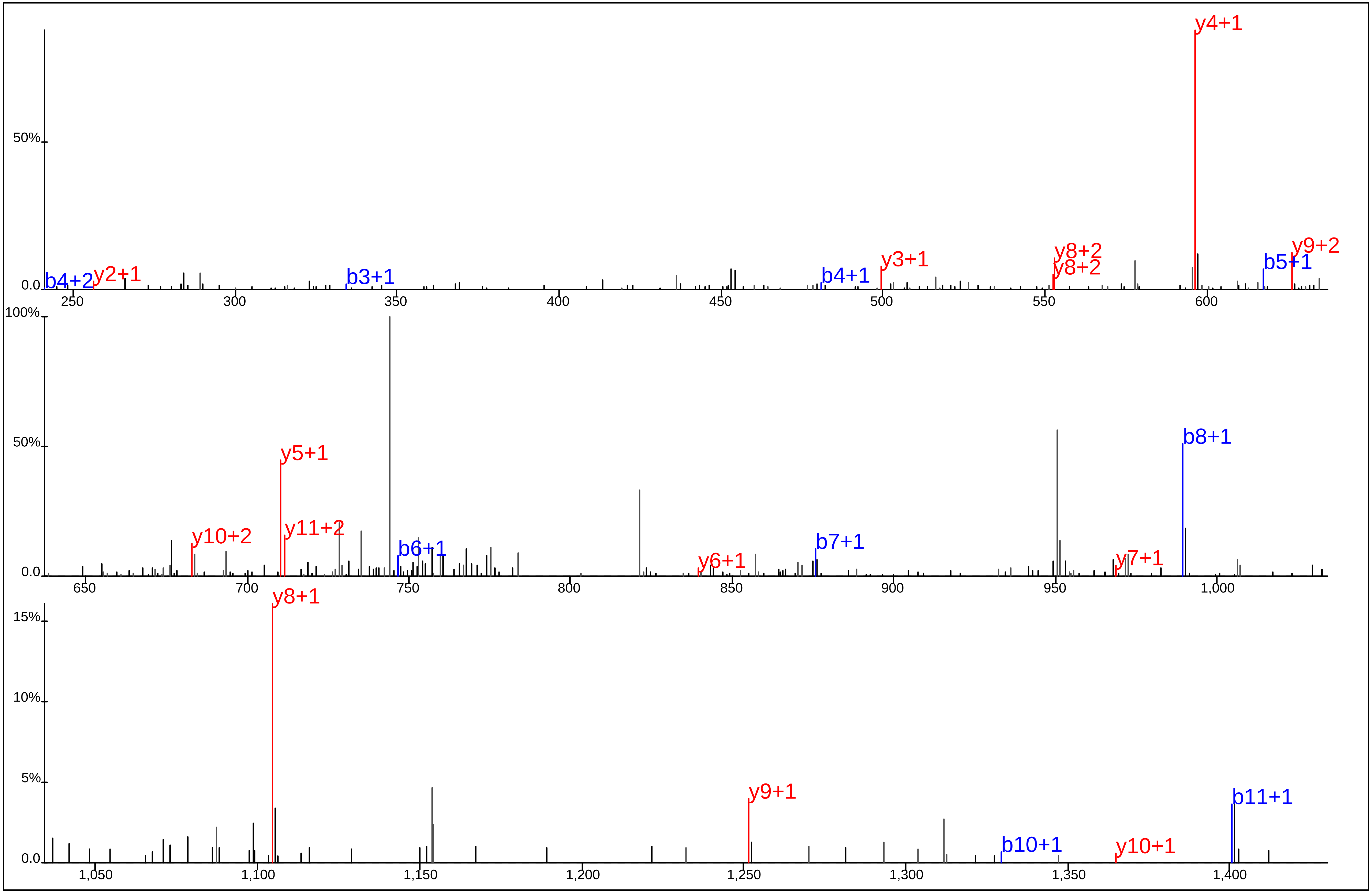

Y G L F K#E E N P Y*A R%

Protein Name: PTTG binding factor
gi Accession: 4757886
Precursor Mass: 792.8666+2
Delta Mass: 2.914 ppm
XCorr: 23.49

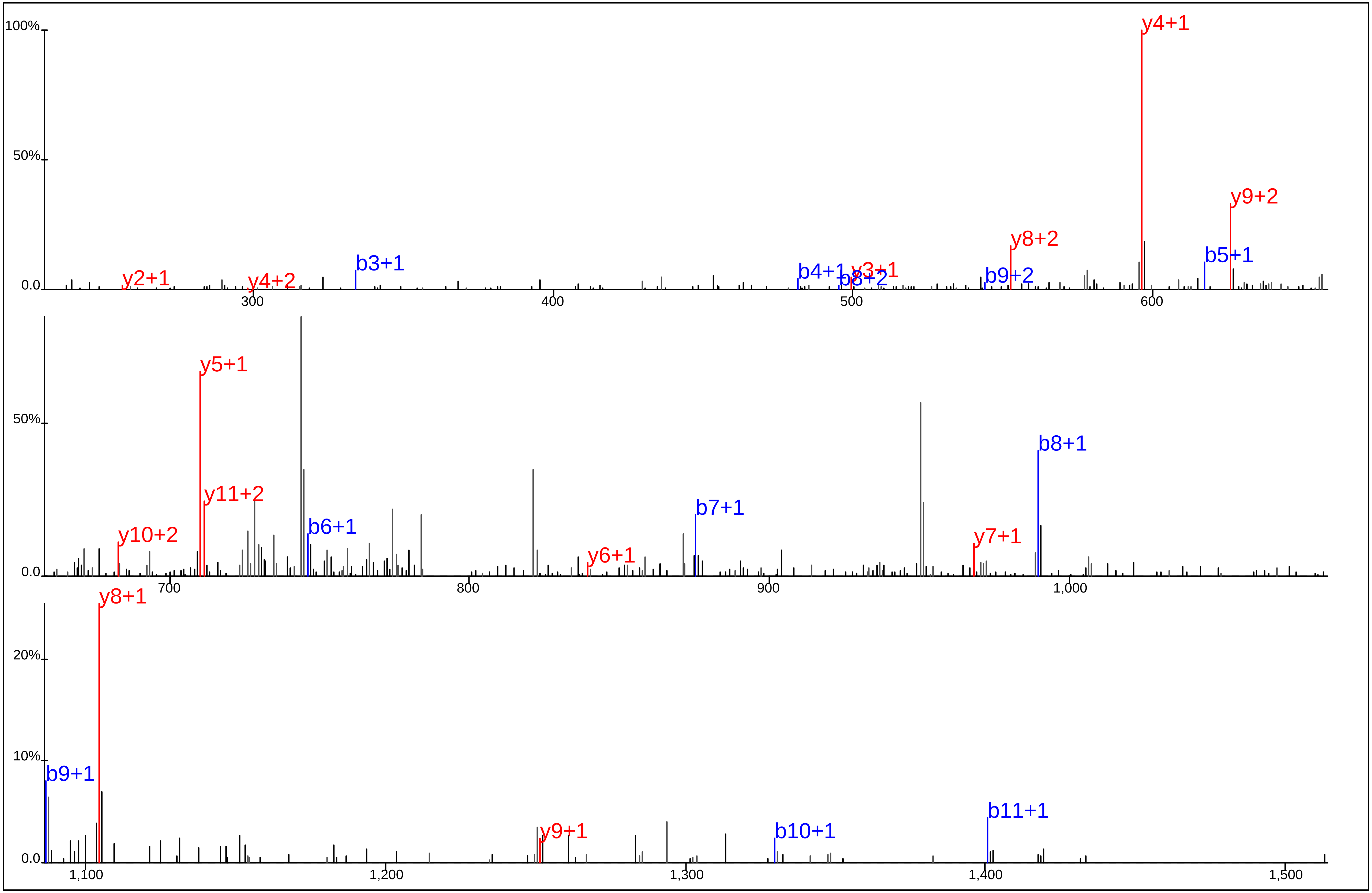

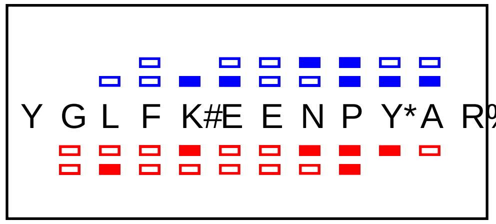

Protein Name: PTTG binding factor
gi Accession: 4757886
Precursor Mass: 792.8670+2
Delta Mass: 3.450 ppm
XCorr: 28.52

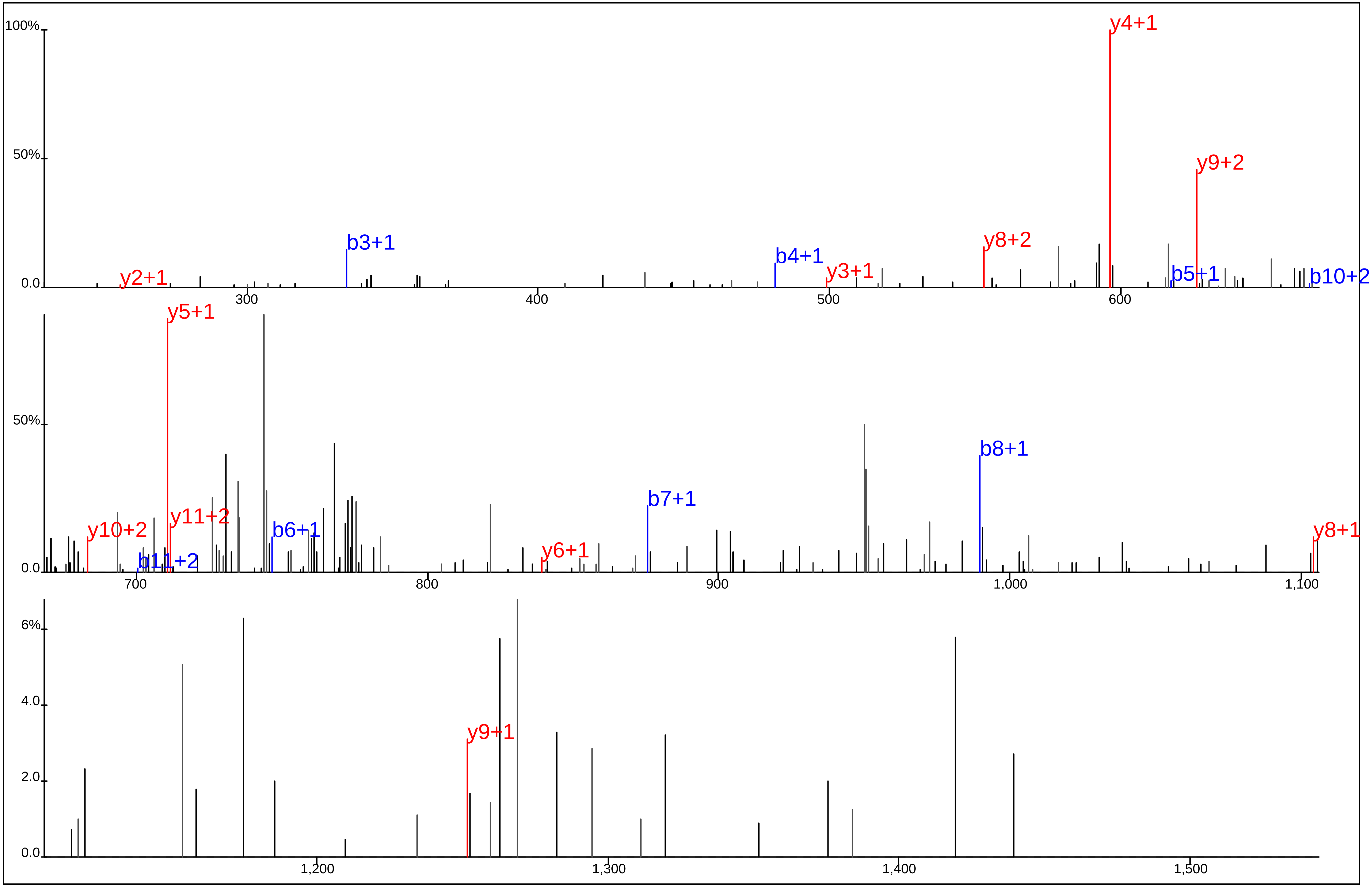

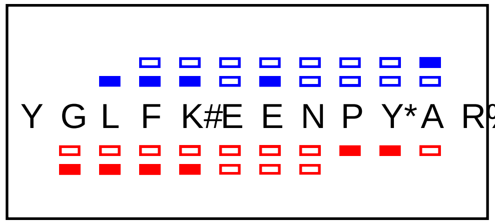

Protein Name: PTTG binding factor
gi Accession: 4757886
Precursor Mass: 792.8685+2
Delta Mass: 5.299 ppm
XCorr: 10.95

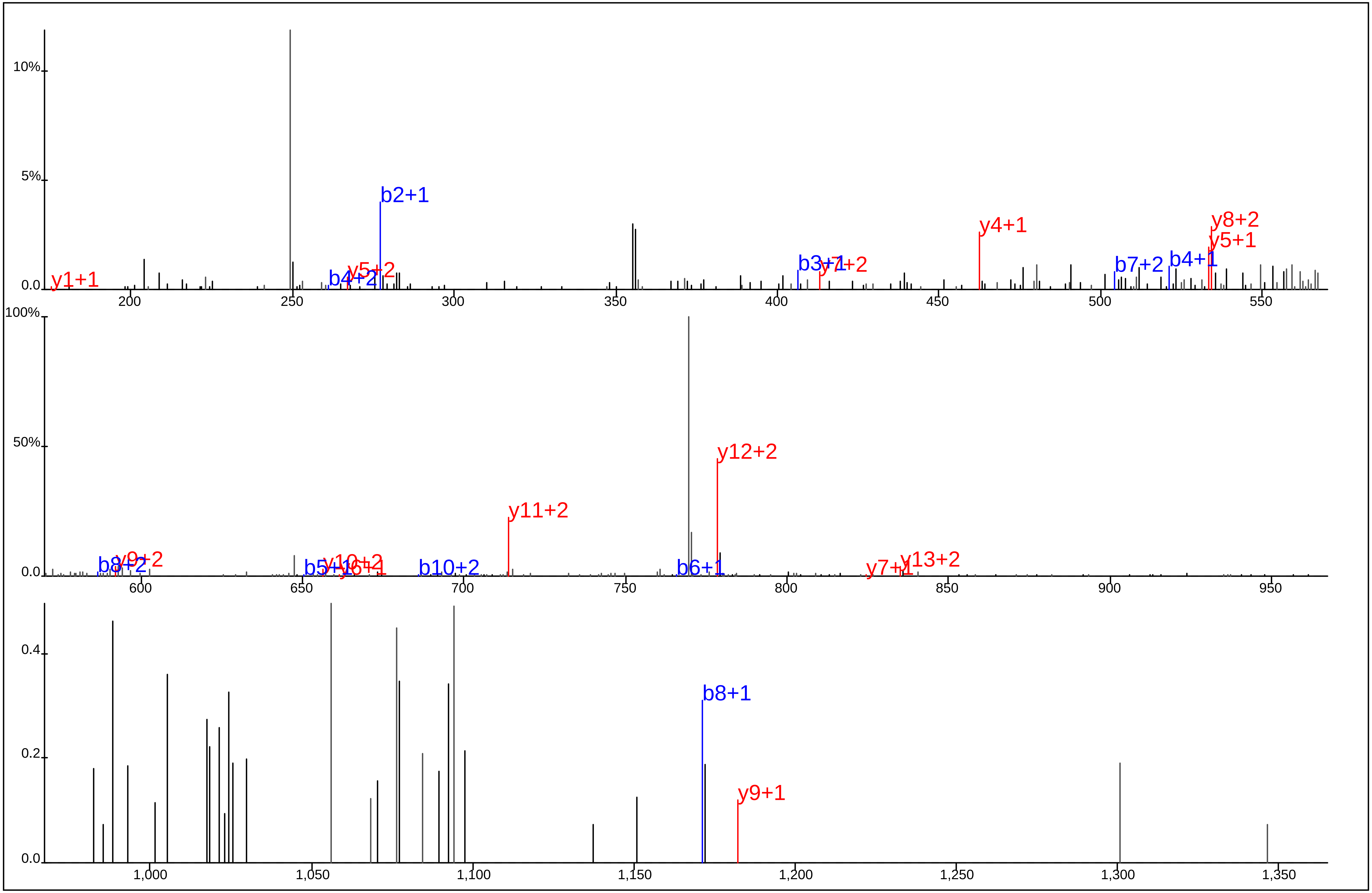

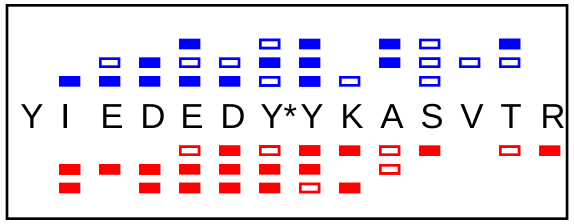

Protein Name: PYK2
gi Accession: 1082034
Precursor Mass: 611.2634+3
Delta Mass: 1.645 ppm
XCorr: 22.85

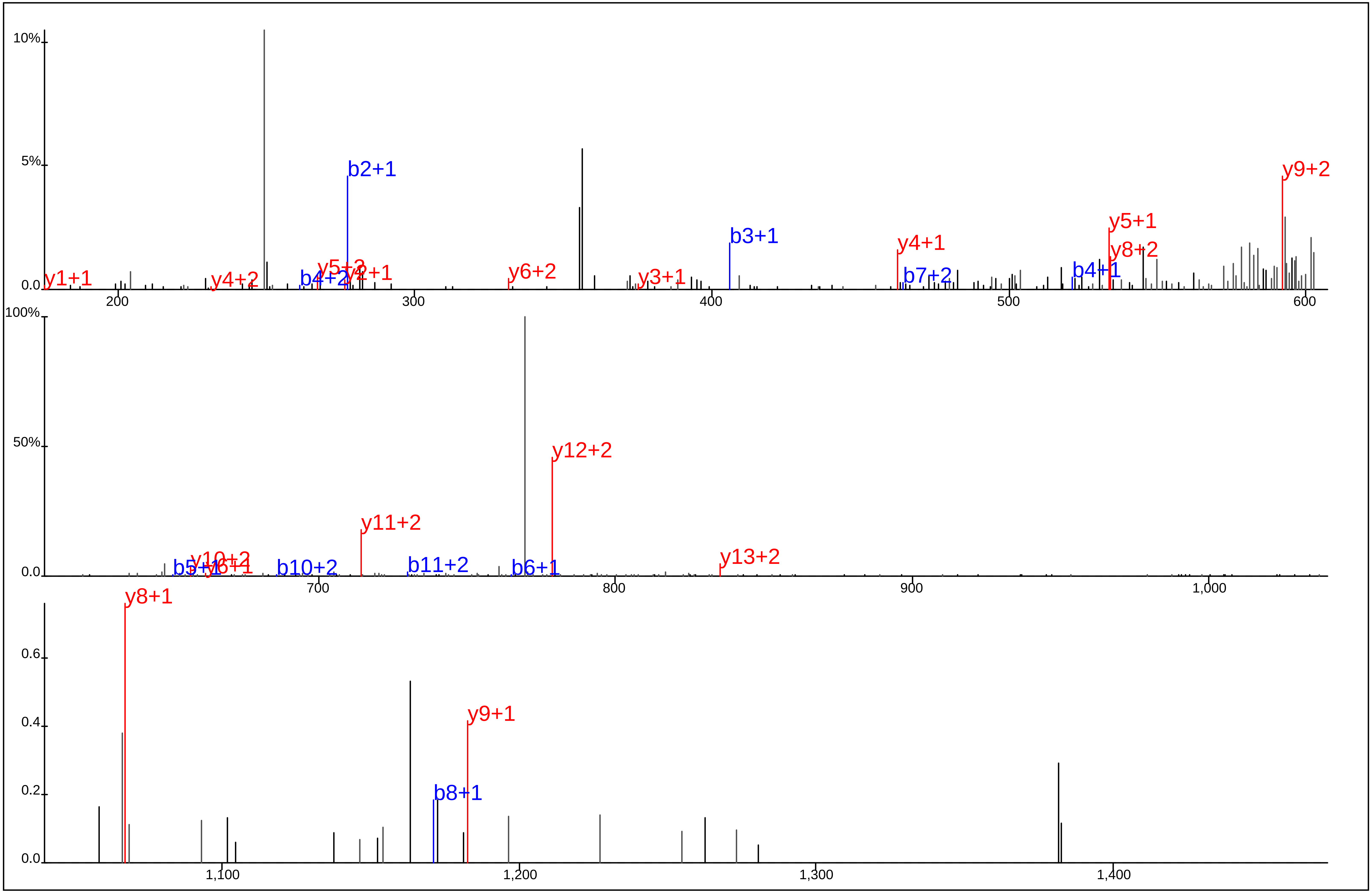

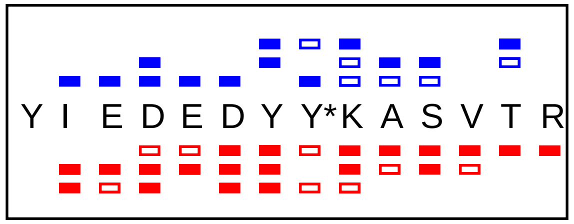

Protein Name: PYK2
gi Accession: 1082034
Precursor Mass: 611.2679+3
Delta Mass: 5.753 ppm
XCorr: 24.78

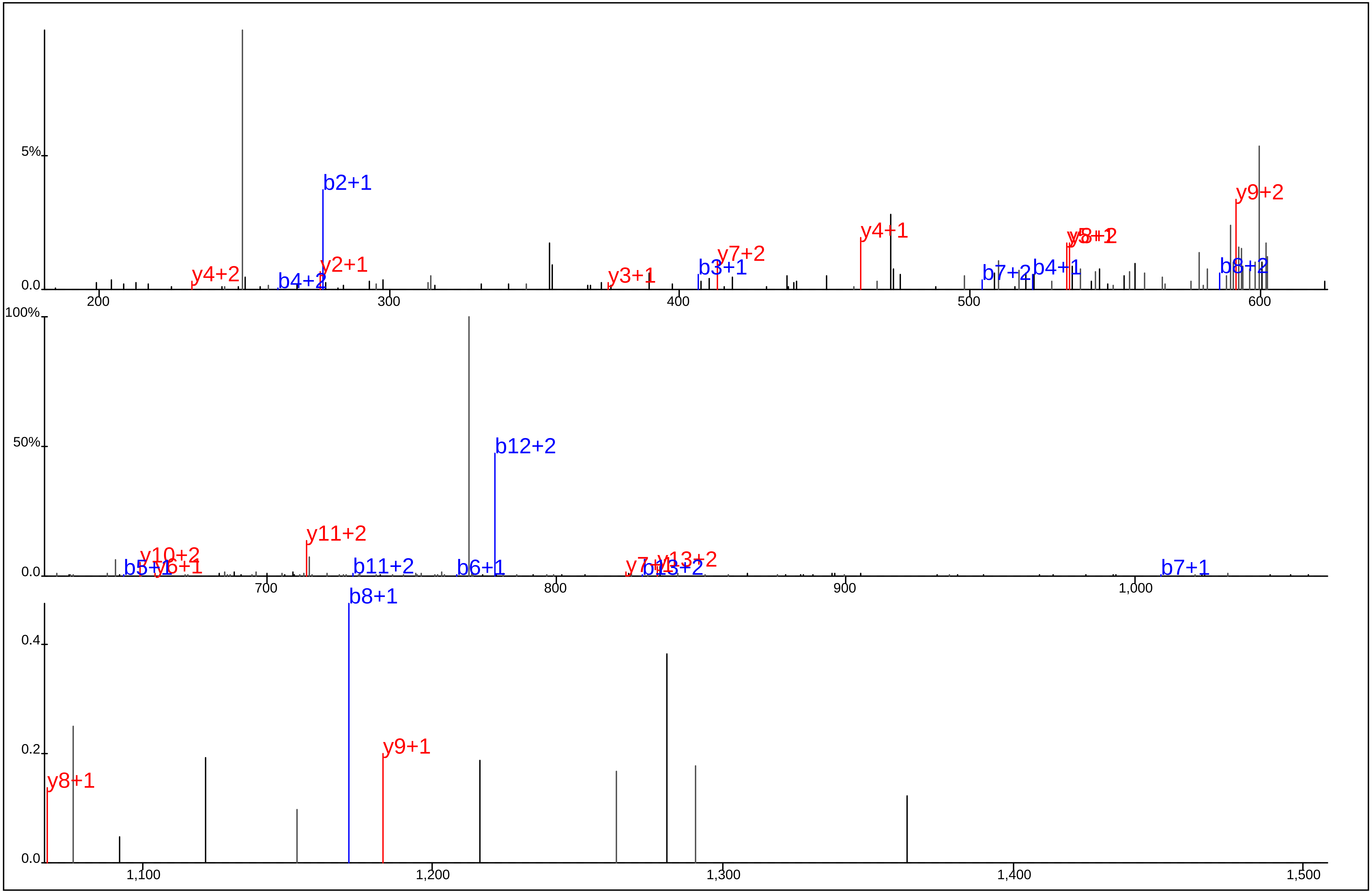

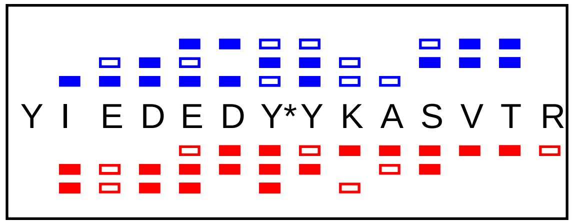

Protein Name:	PYK2
gi Accession:	1082034
Precursor Mass:	611.2664+3
Delta Mass:	3.252 ppm
XCorr:	33.63

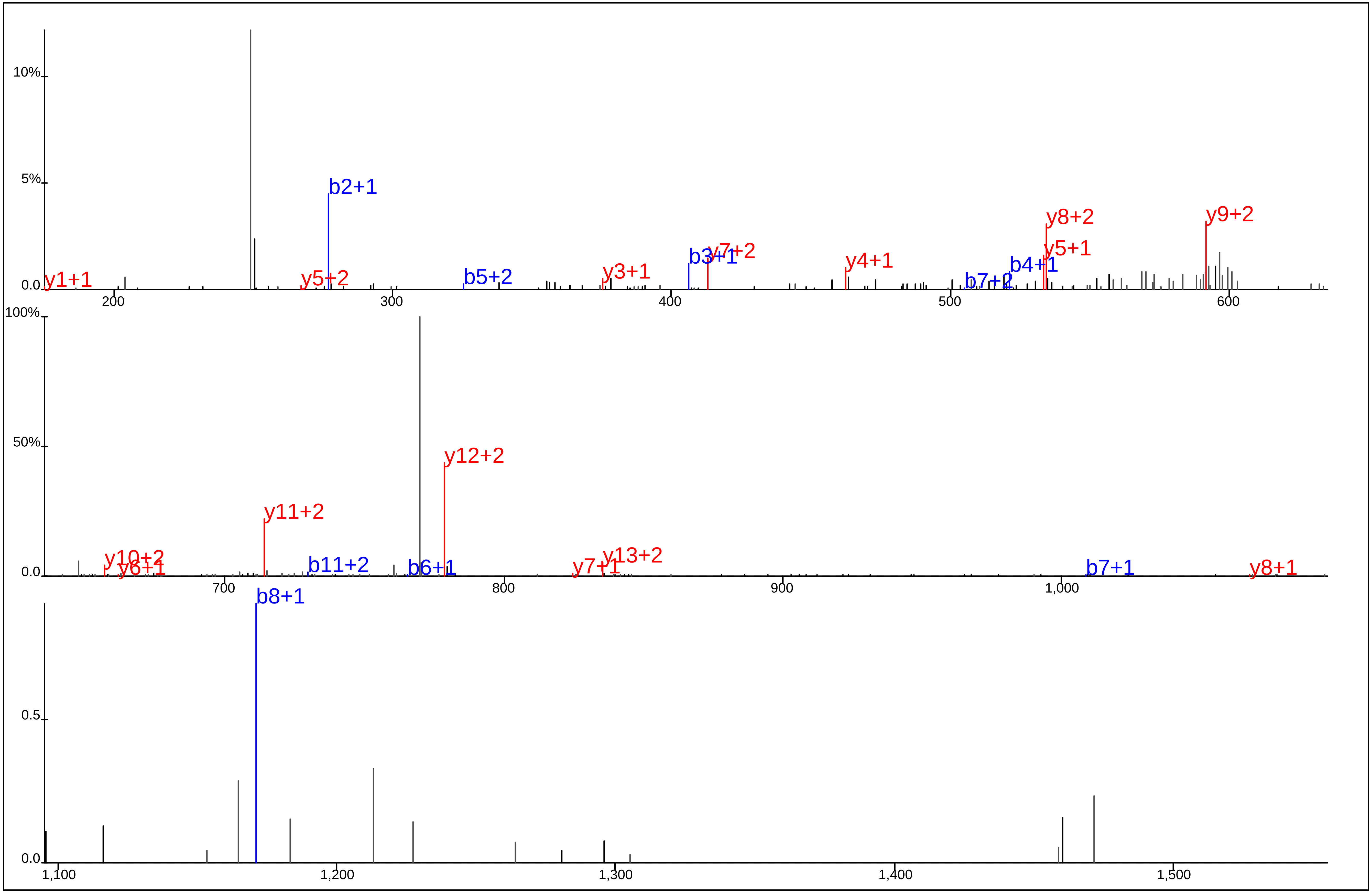

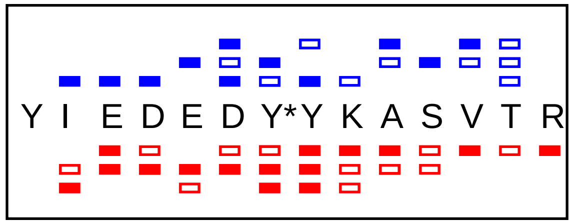

Protein Name: PYK2
gi Accession: 1082034
Precursor Mass: 611.2653+3
Delta Mass: 1.451 ppm
XCorr: 33.77

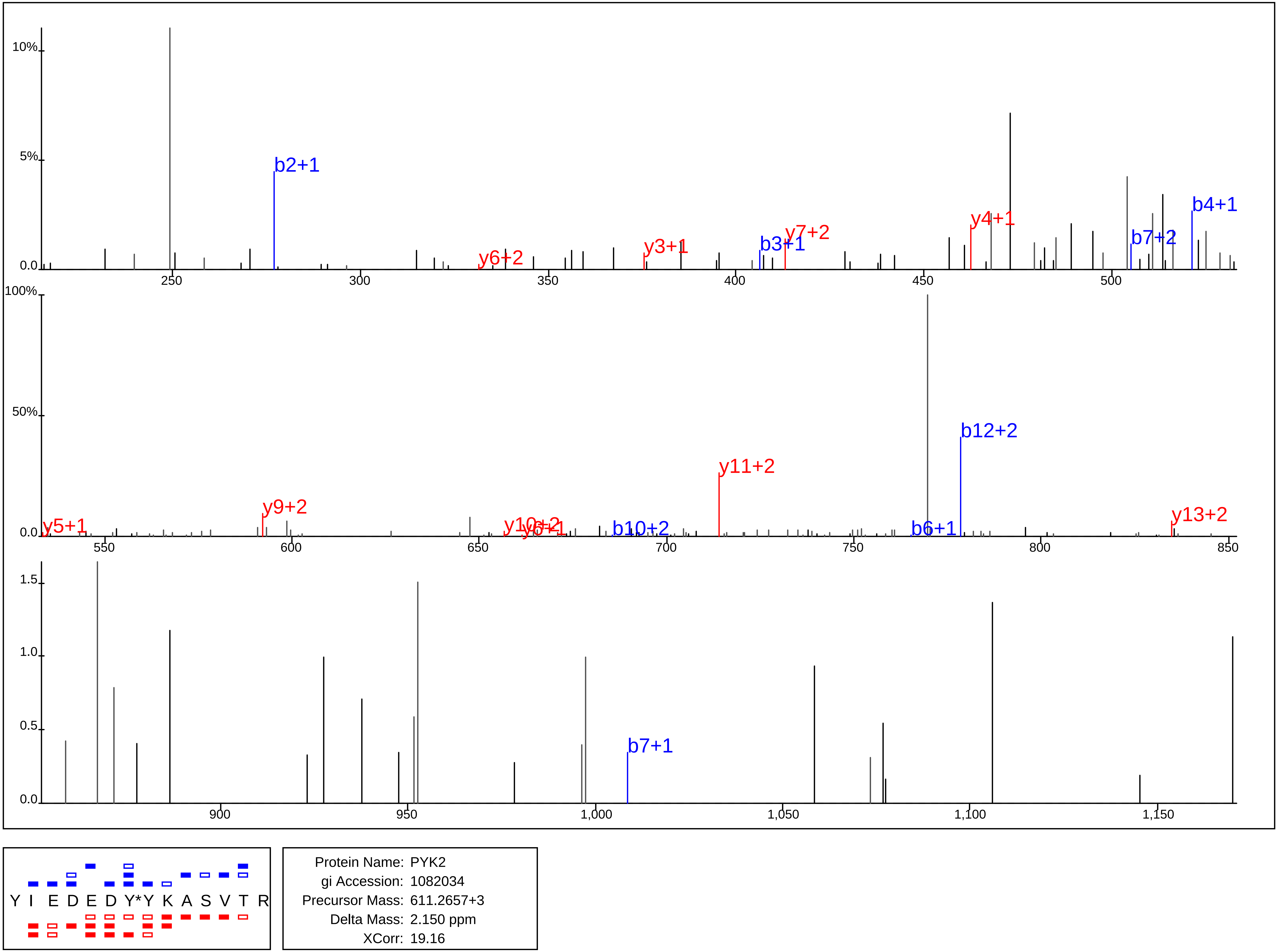

Protein Name: PYK2
gi Accession: 1082034
Precursor Mass: 611.2657+3
Delta Mass: 2.150 ppm
XCorr: 19.16

816 - PYK2_3449179.pdf

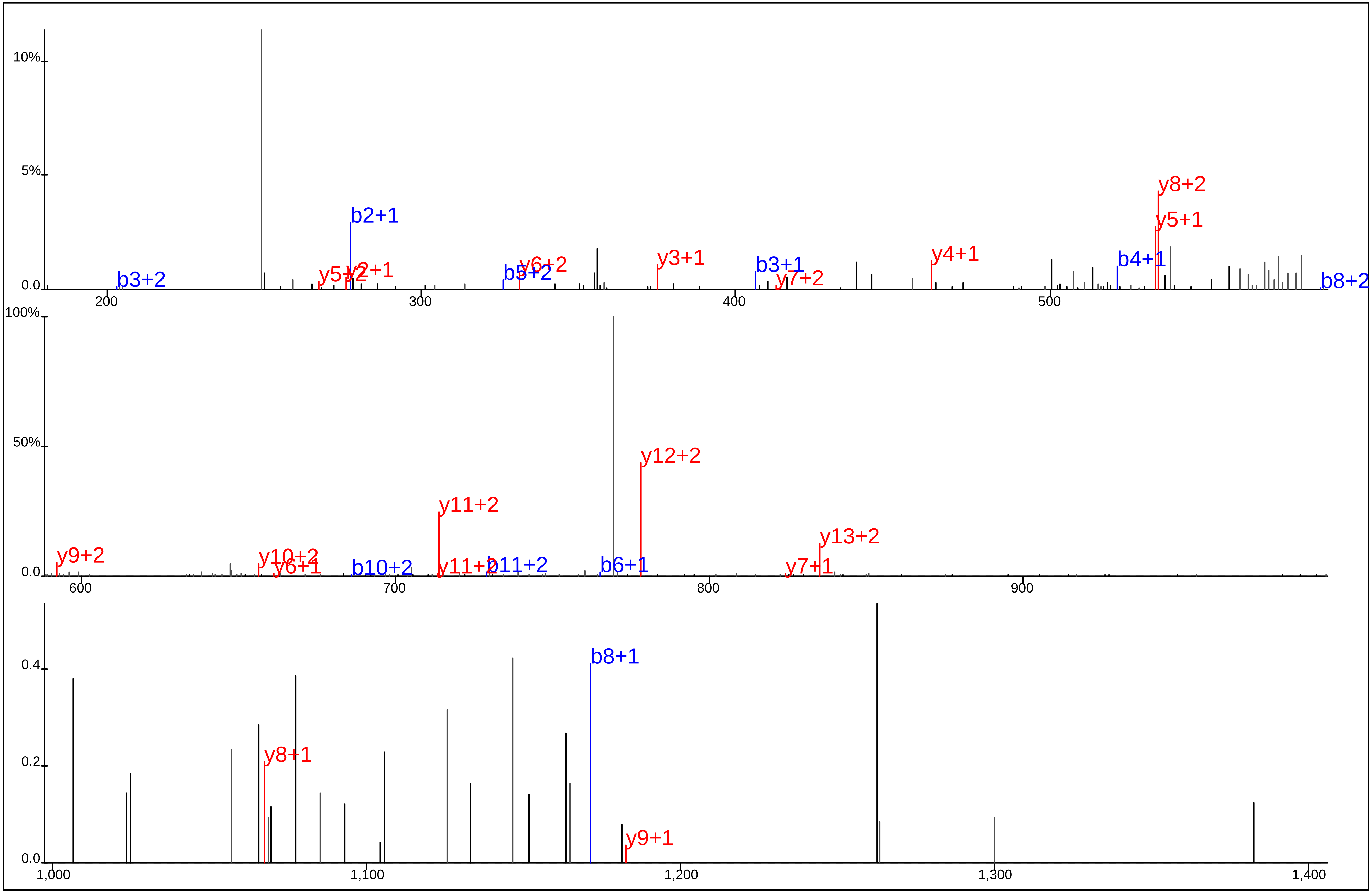

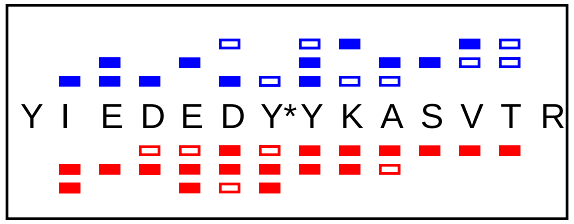

Protein Name: PYK2
gi Accession: 1082034
Precursor Mass: 611.2683+3
Delta Mass: 6.452 ppm
XCorr: 37.6

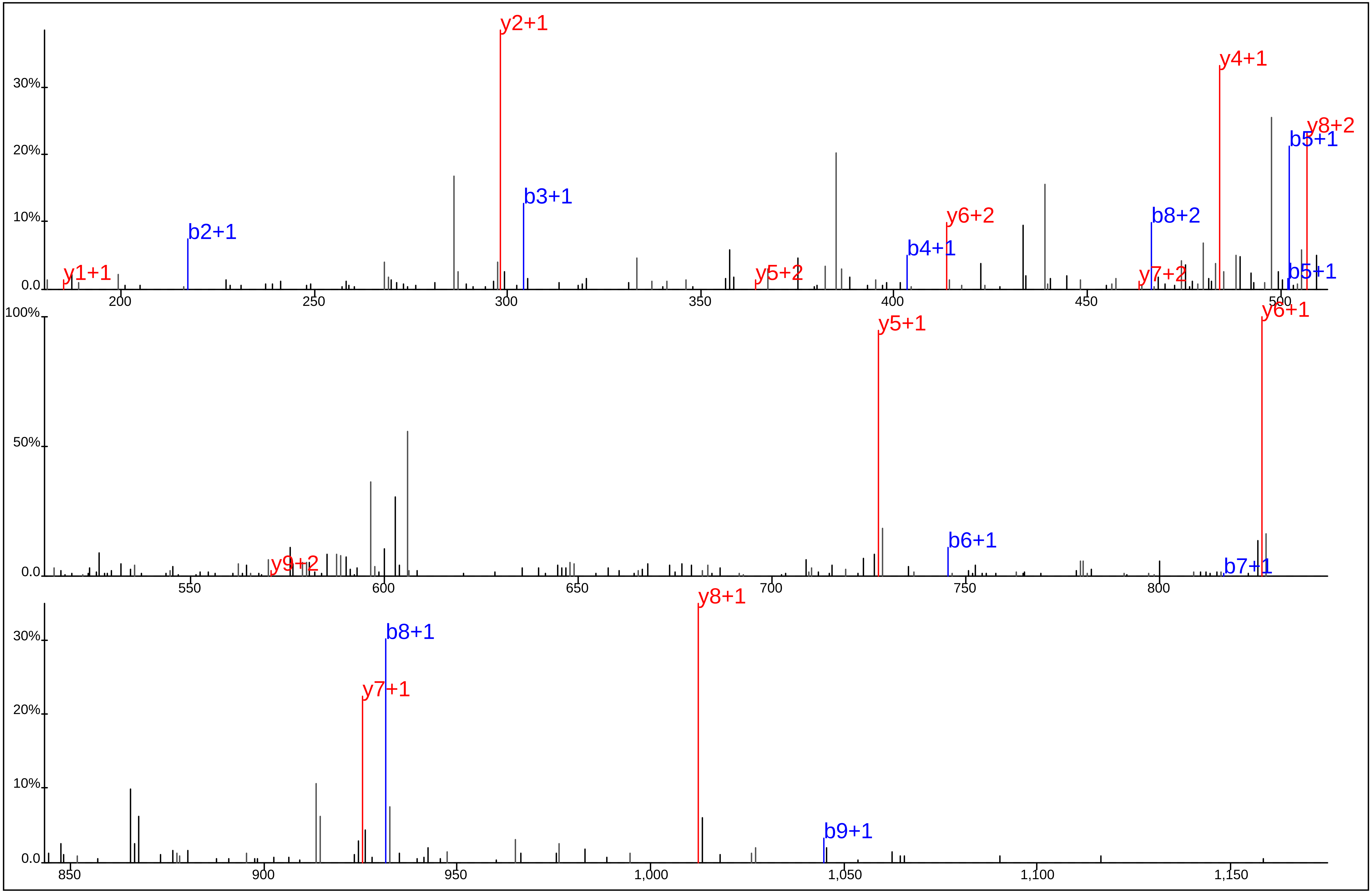

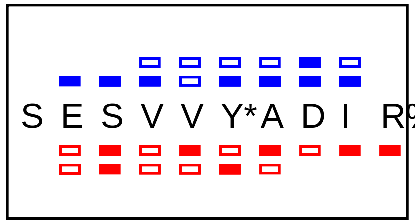

Protein Name: PZR
gi Accession: 5901563
Precursor Mass: 614.7800+2
Delta Mass: 3.515 ppm
XCorr: 46.48

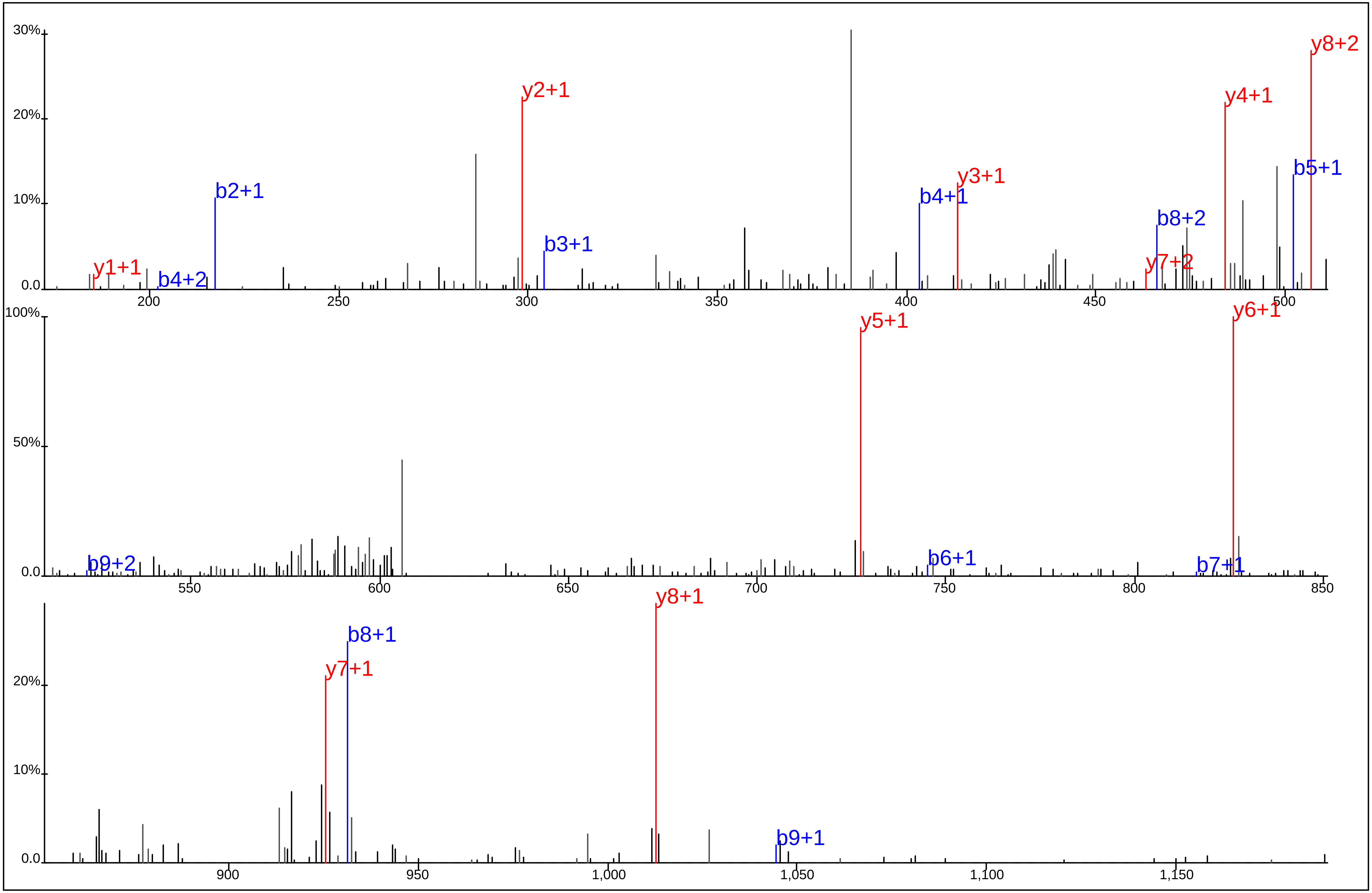

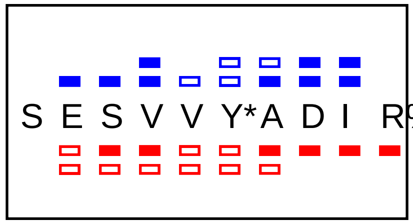

Protein Name: PZR
gi Accession: 5901563
Precursor Mass: 614.7802+2
Delta Mass: 3.816 ppm
XCorr: 46.89

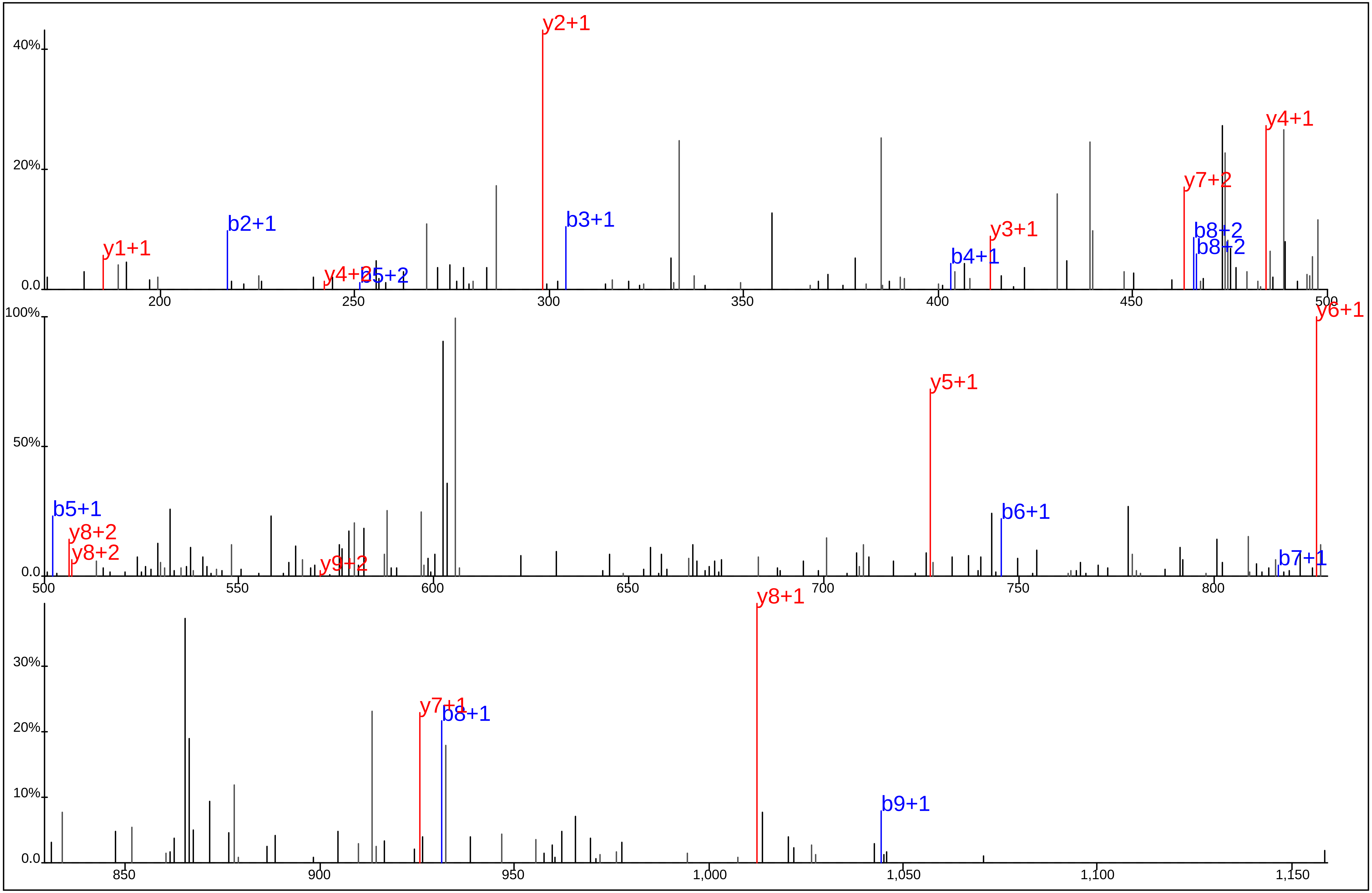

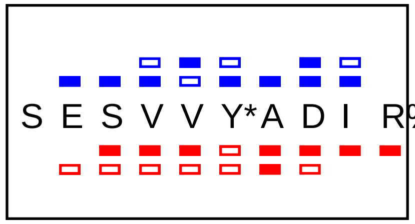

Protein Name: PZR
gi Accession: 5901563
Precursor Mass: 614.7803+2
Delta Mass: 4.011 ppm
XCorr: 39.08

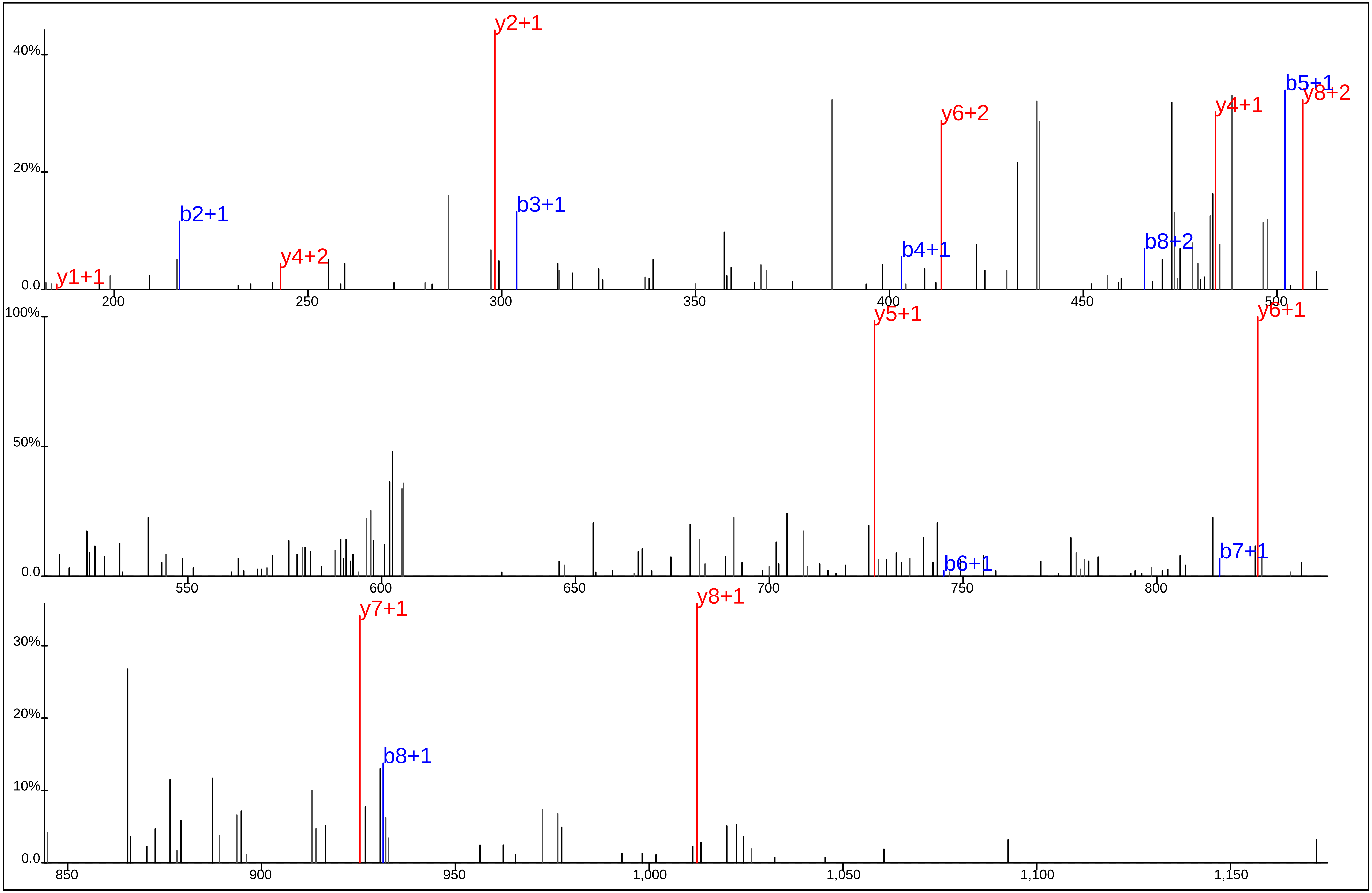

S E S V V Y*A D I R%

Protein Name: PZR
gi Accession: 5901563
Precursor Mass: 614.7784+2
Delta Mass: 0.934 ppm
XCorr: 36.55

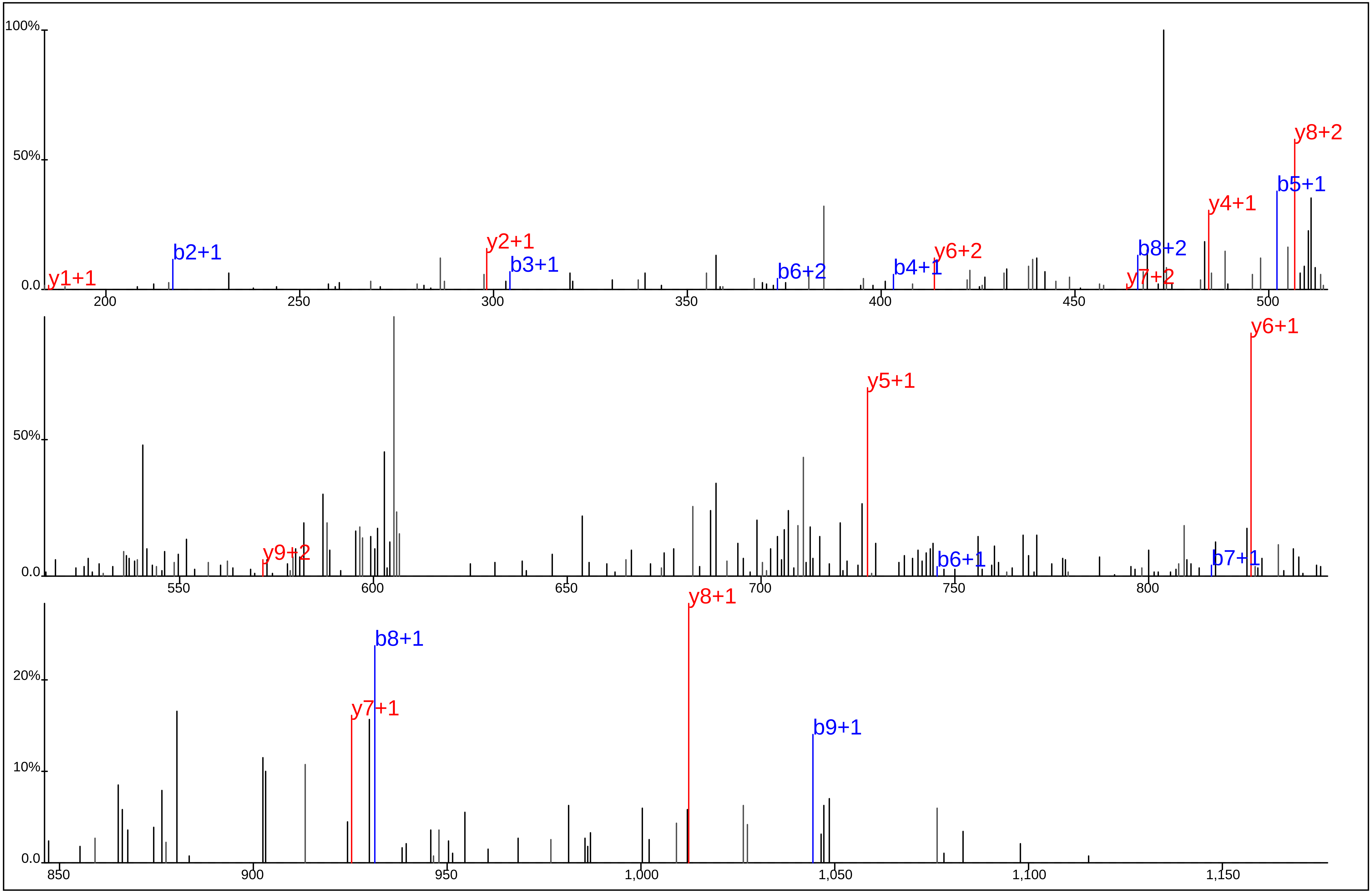

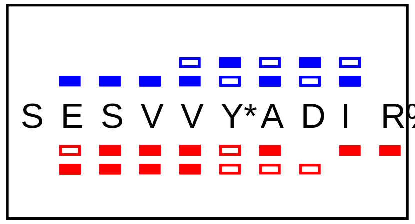

Protein Name: PZR
gi Accession: 5901563
Precursor Mass: 614.7784+2
Delta Mass: 0.934 ppm
XCorr: 24.27

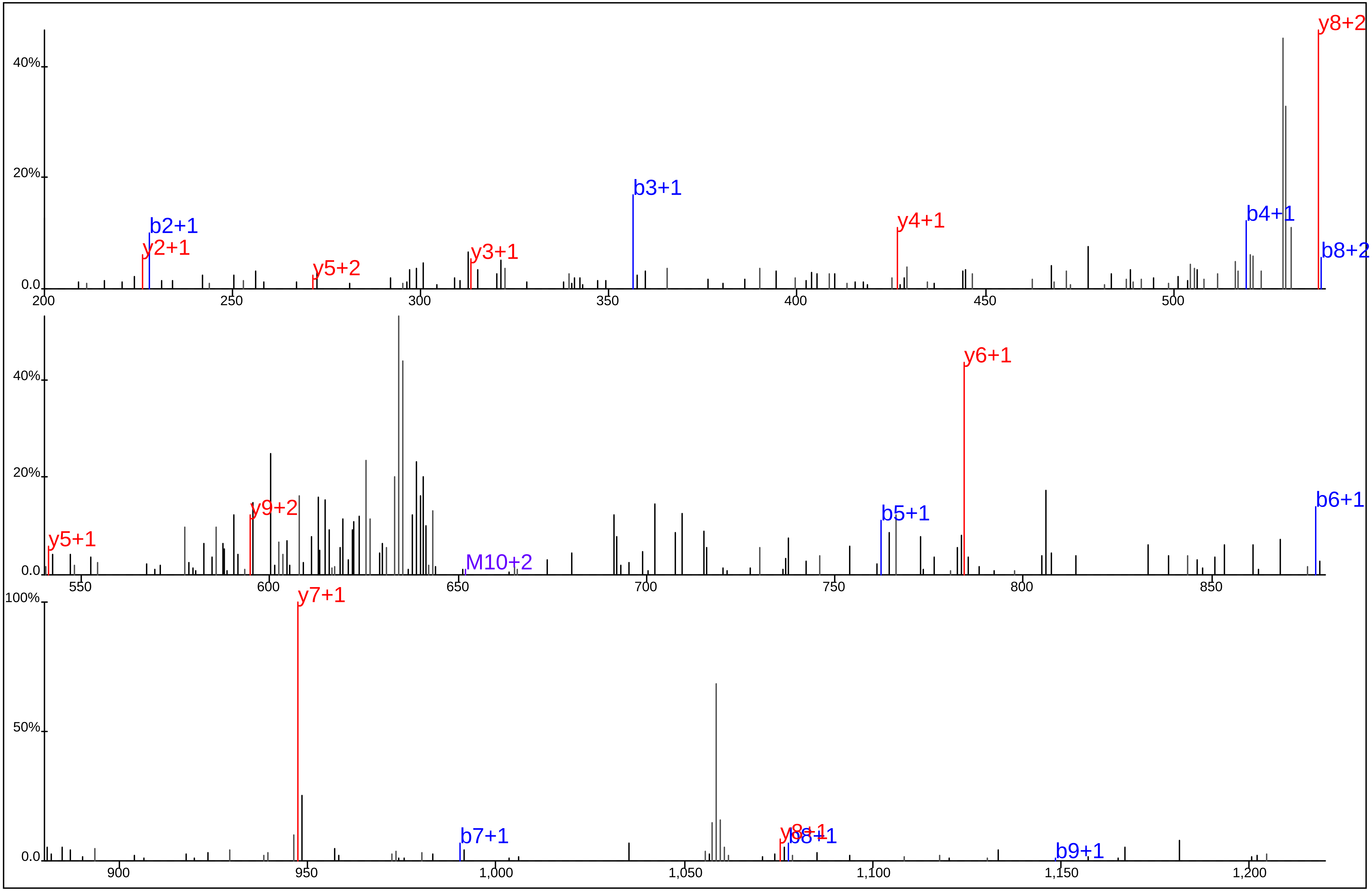

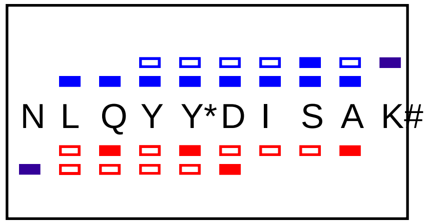

Protein Name: Ran GTPase
gi Accession: 21104432
Precursor Mass: 651.8004+2
Delta Mass: 6.048 ppm
XCorr: 26.47

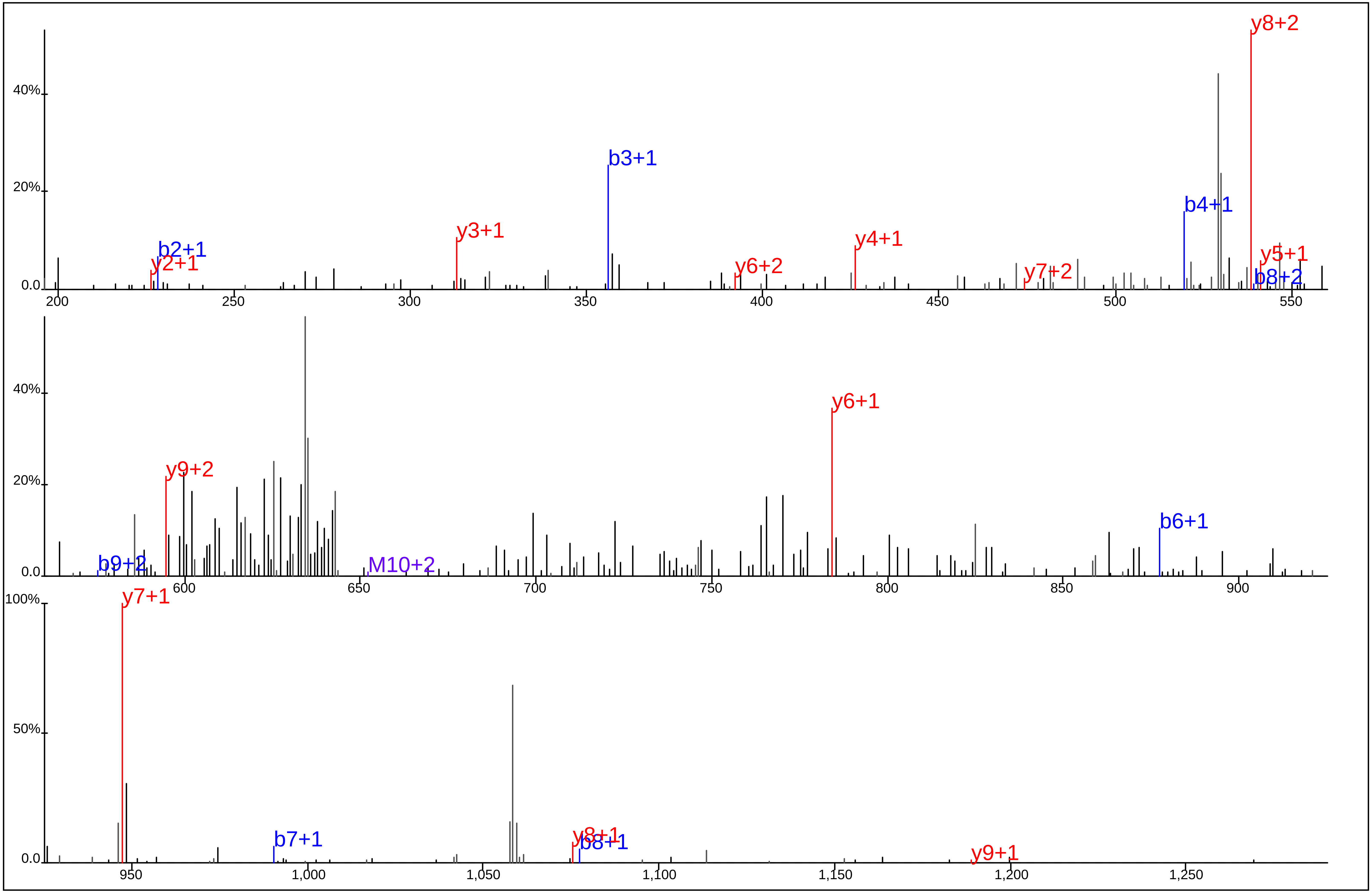

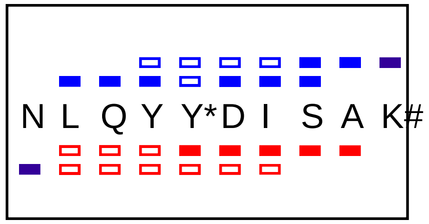

Protein Name: Ran GTPase
gi Accession: 21104432
Precursor Mass: 651.7973+2
Delta Mass: 1.273 ppm
XCorr: 27.02

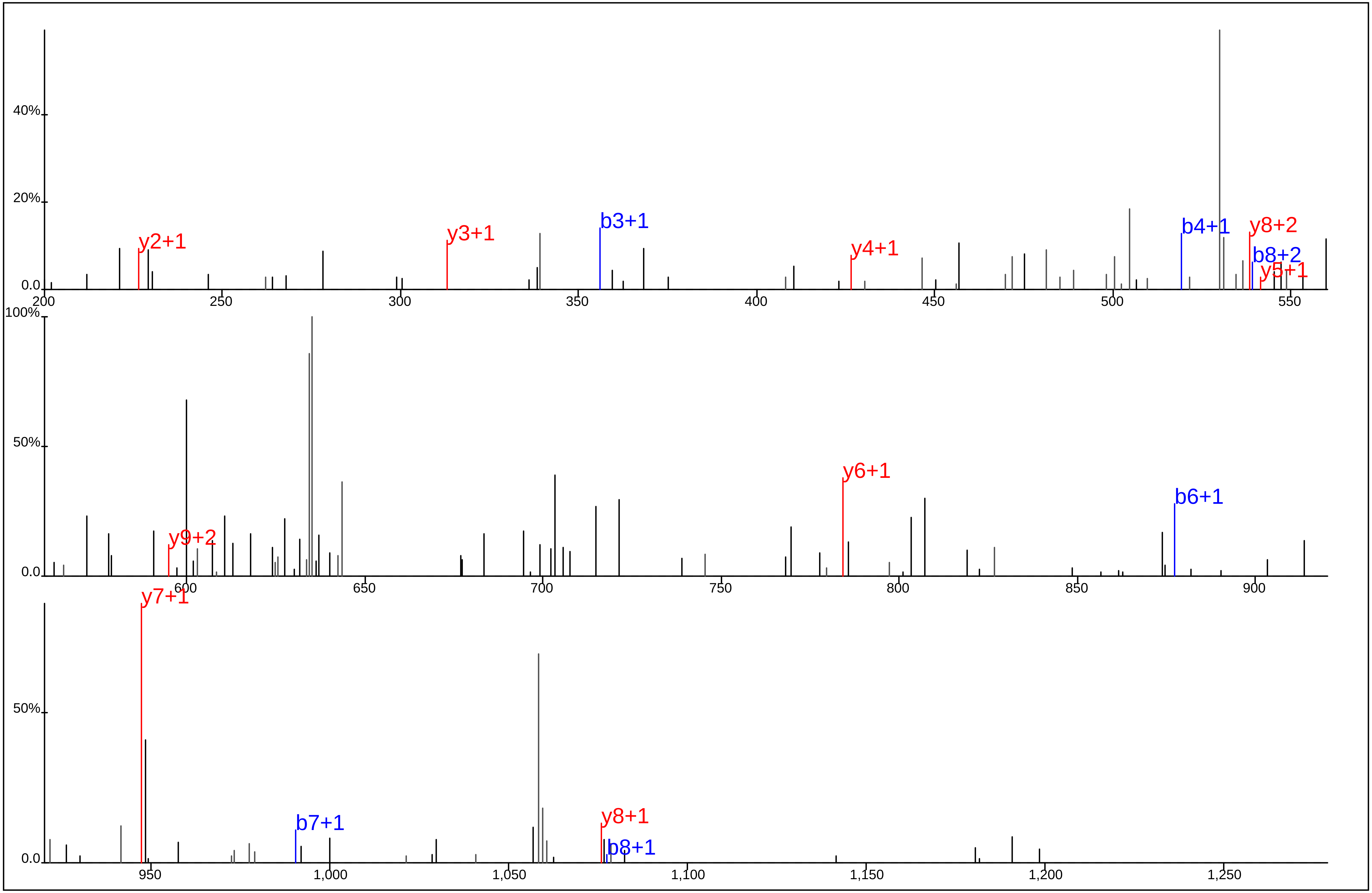

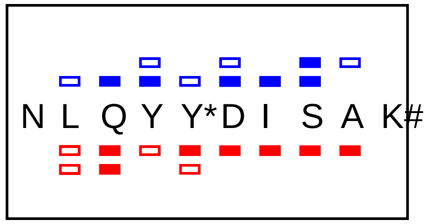

Protein Name: Ran GTPase
gi Accession: 21104432
Precursor Mass: 651.7984+2
Delta Mass: 2.962 ppm
XCorr: 18.93

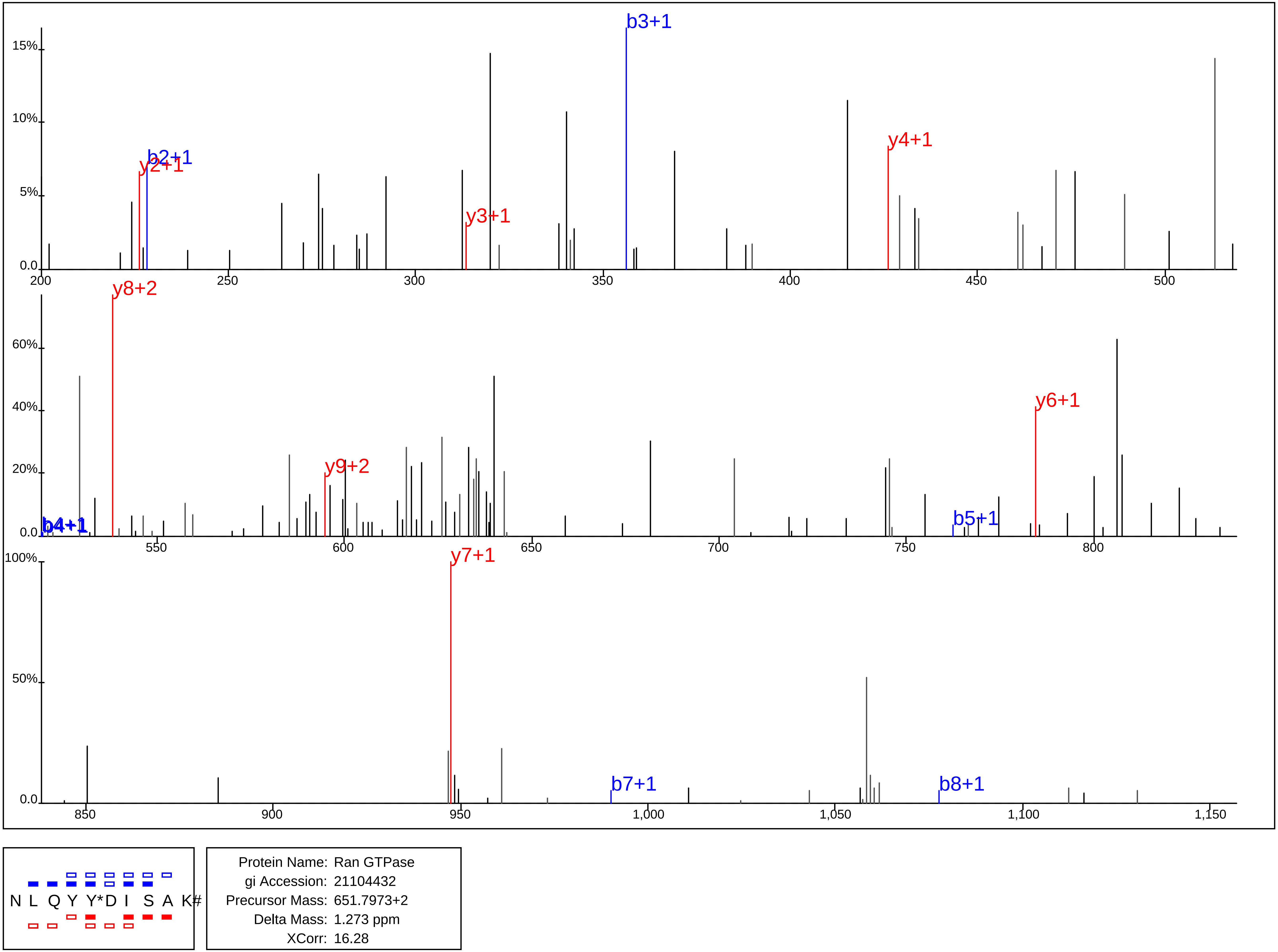

Protein Name: Ran GTPase
gi Accession: 21104432
Precursor Mass: 651.7973+2
Delta Mass: 1.273 ppm
XCorr: 16.28

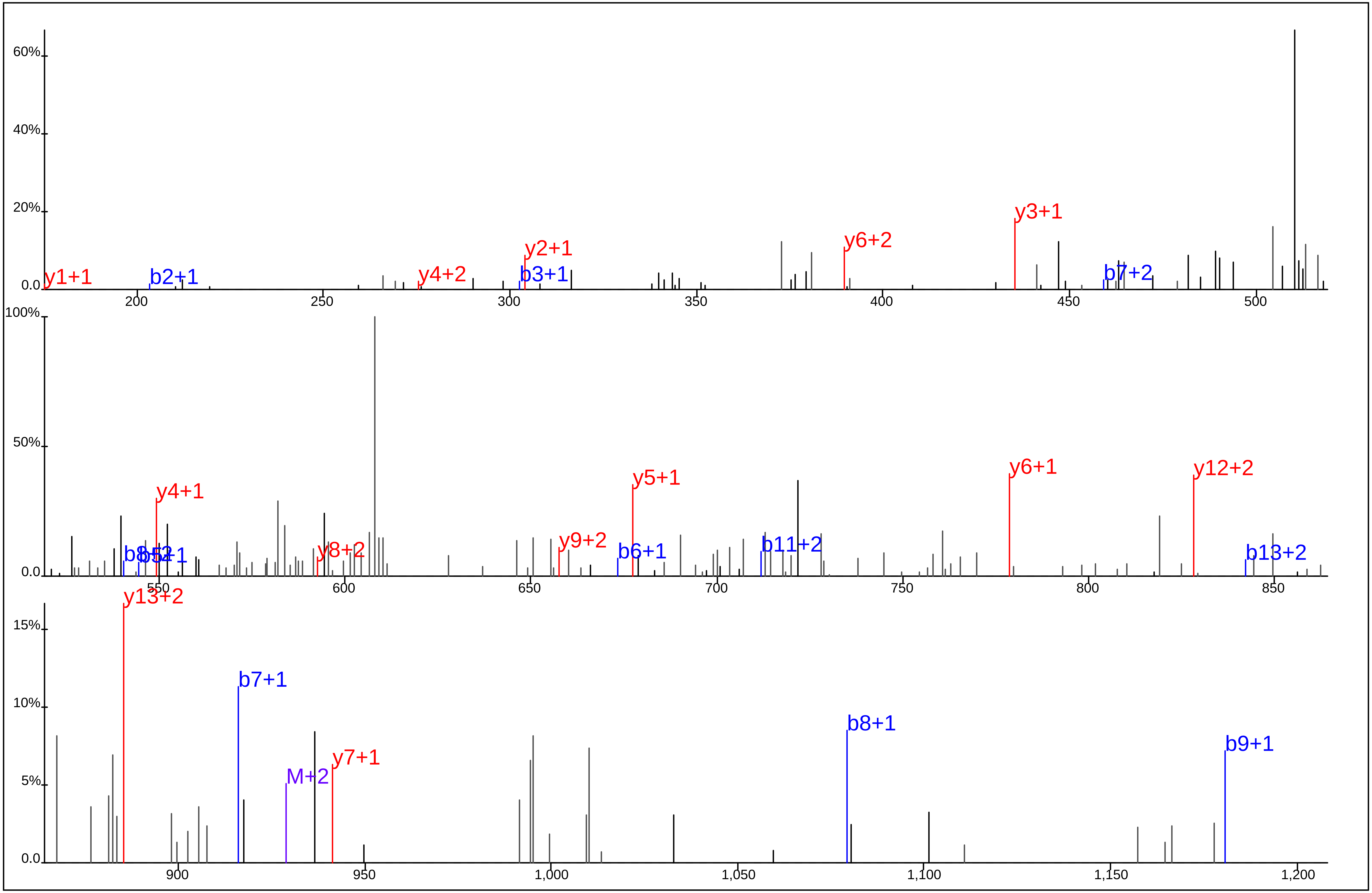

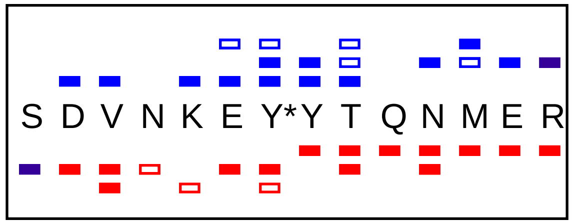

Protein Name: RBM22
gi Accession: 8922328
Precursor Mass: 619.5899+3
Delta Mass: 1.690 ppm
XCorr: 22.91

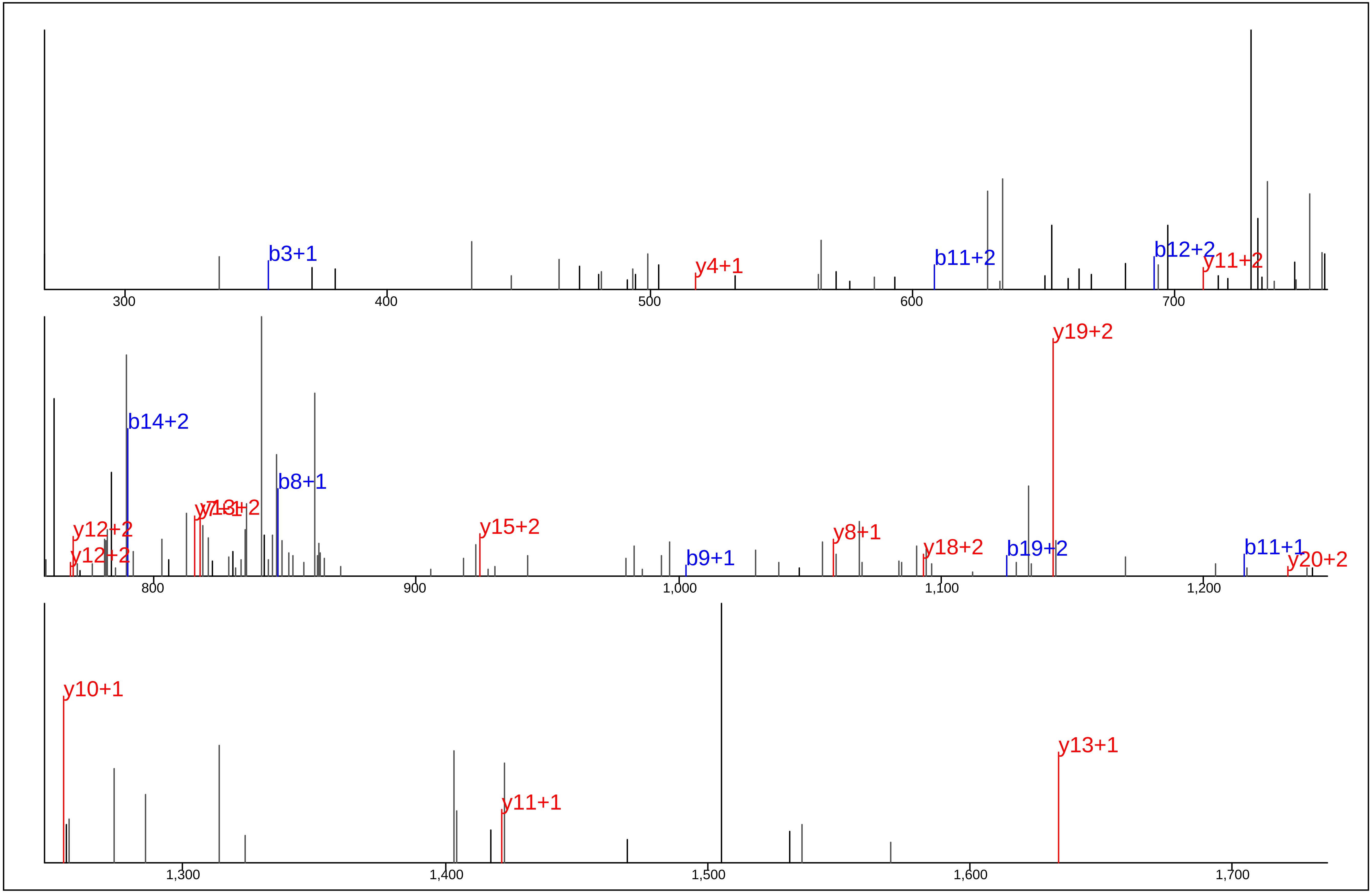

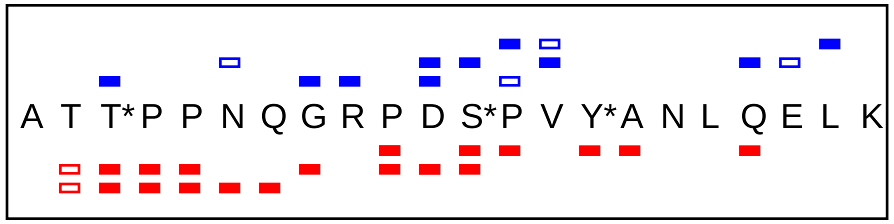

Protein Name: RhoGAP 12
gi Accession: 26986534
Precursor Mass: 879.3859+3
Delta Mass: 8.963 ppm
XCorr: 10.41

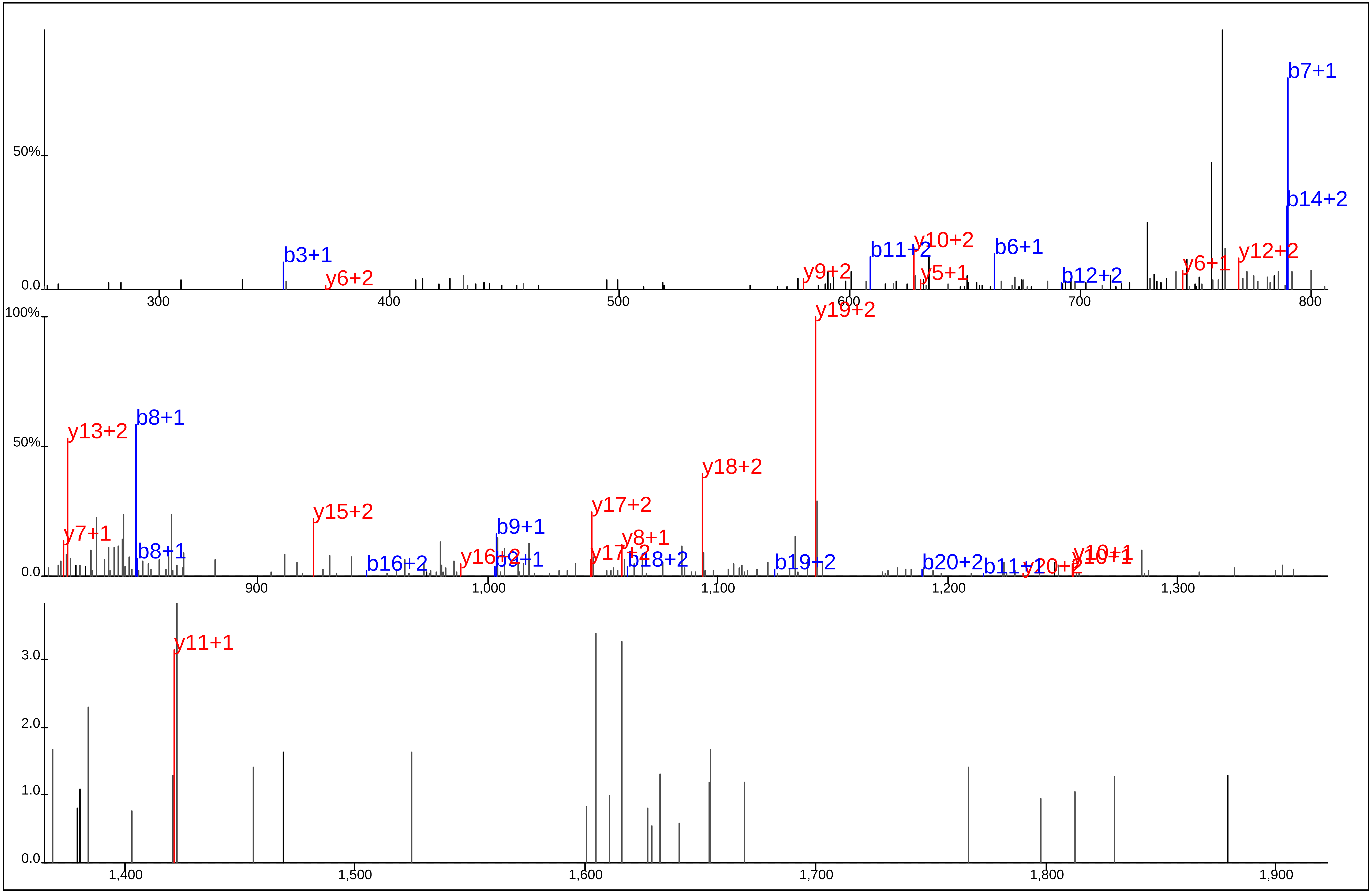

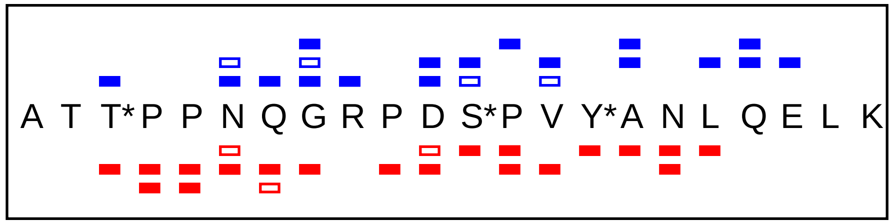

Protein Name: RhoGAP 12
gi Accession: 26986534
Precursor Mass: 879.3848+3
Delta Mass: 7.783 ppm
XCorr: 13.99

829 - RhoGAP12_3435518.pdf

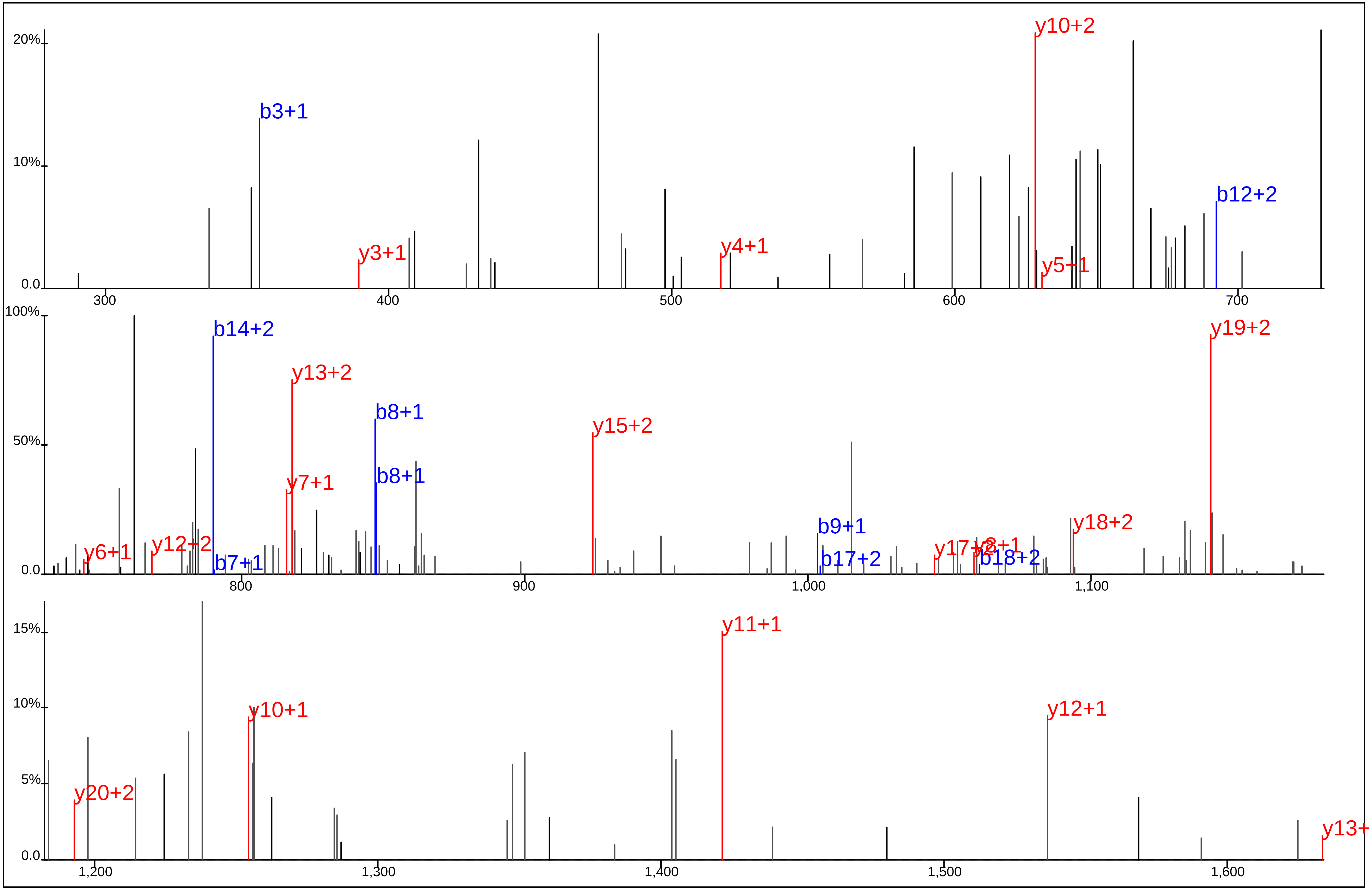

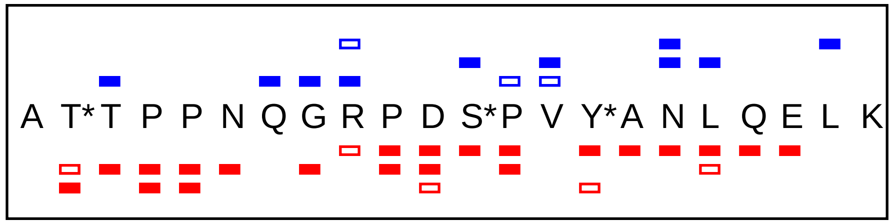

Protein Name: RhoGAP 12
gi Accession: 26986534
Precursor Mass: 879.3845+3
Delta Mass: 7.367 ppm
XCorr: 14.99

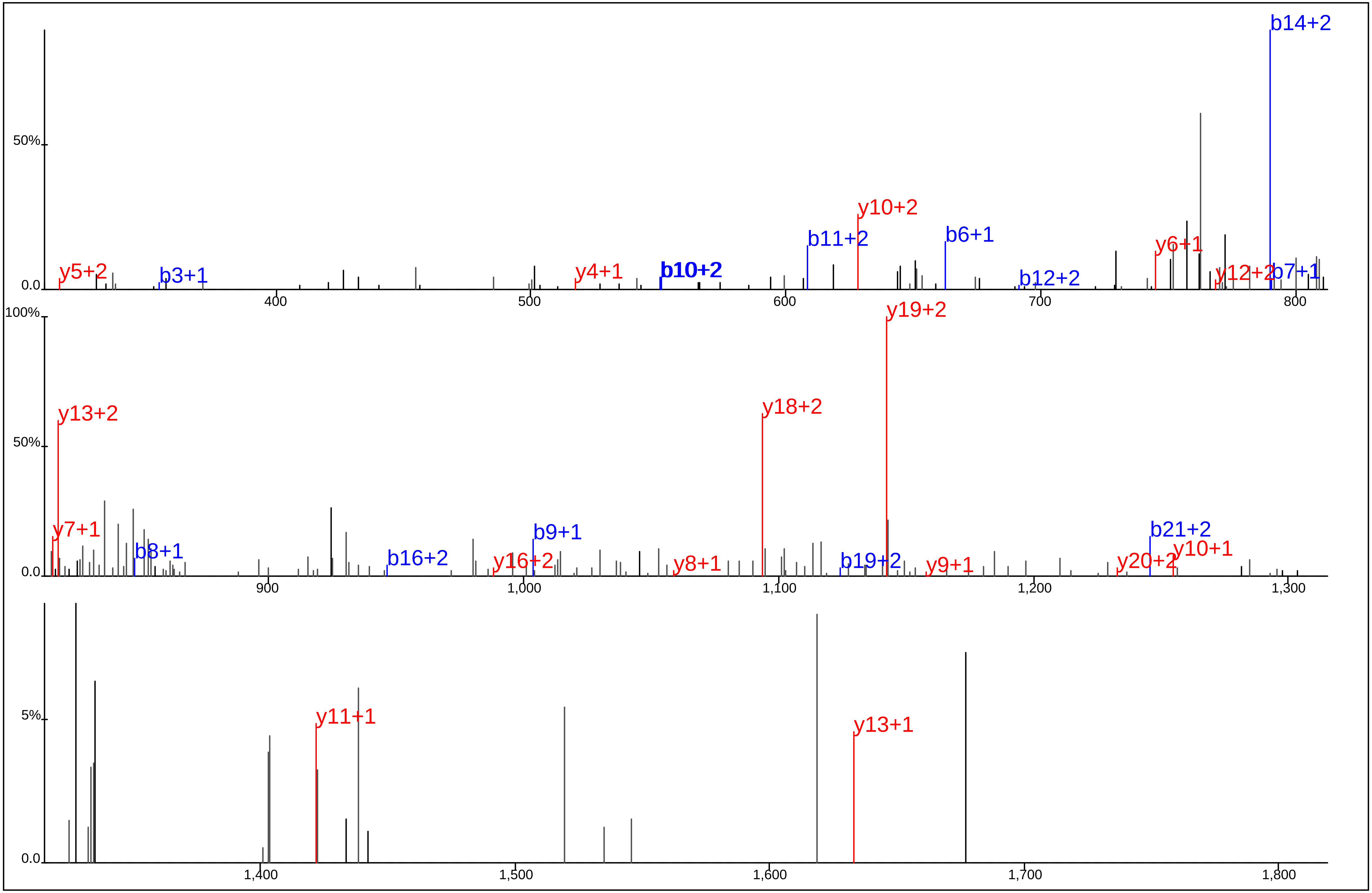

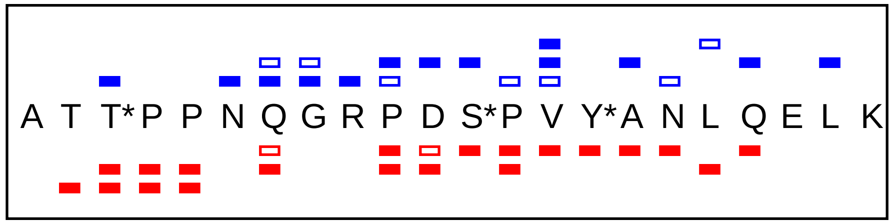

Protein Name: RhoGAP 12
gi Accession: 26986534
Precursor Mass: 879.3817+3
Delta Mass: 4.240 ppm
XCorr: 18.82

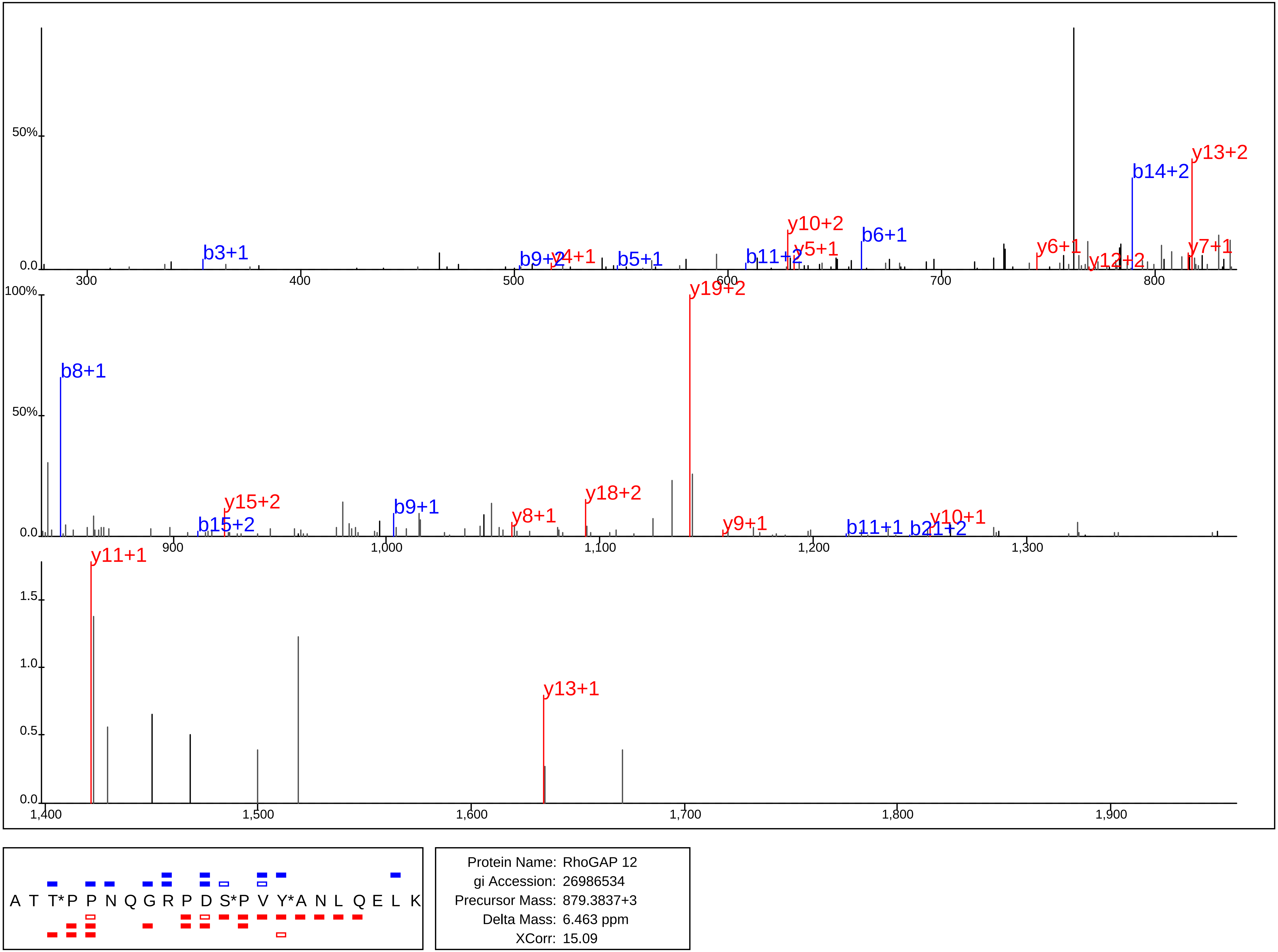

Protein Name: RhoGAP 12
gi Accession: 26986534
Precursor Mass: 879.3837+3
Delta Mass: 6.463 ppm
XCorr: 15.09

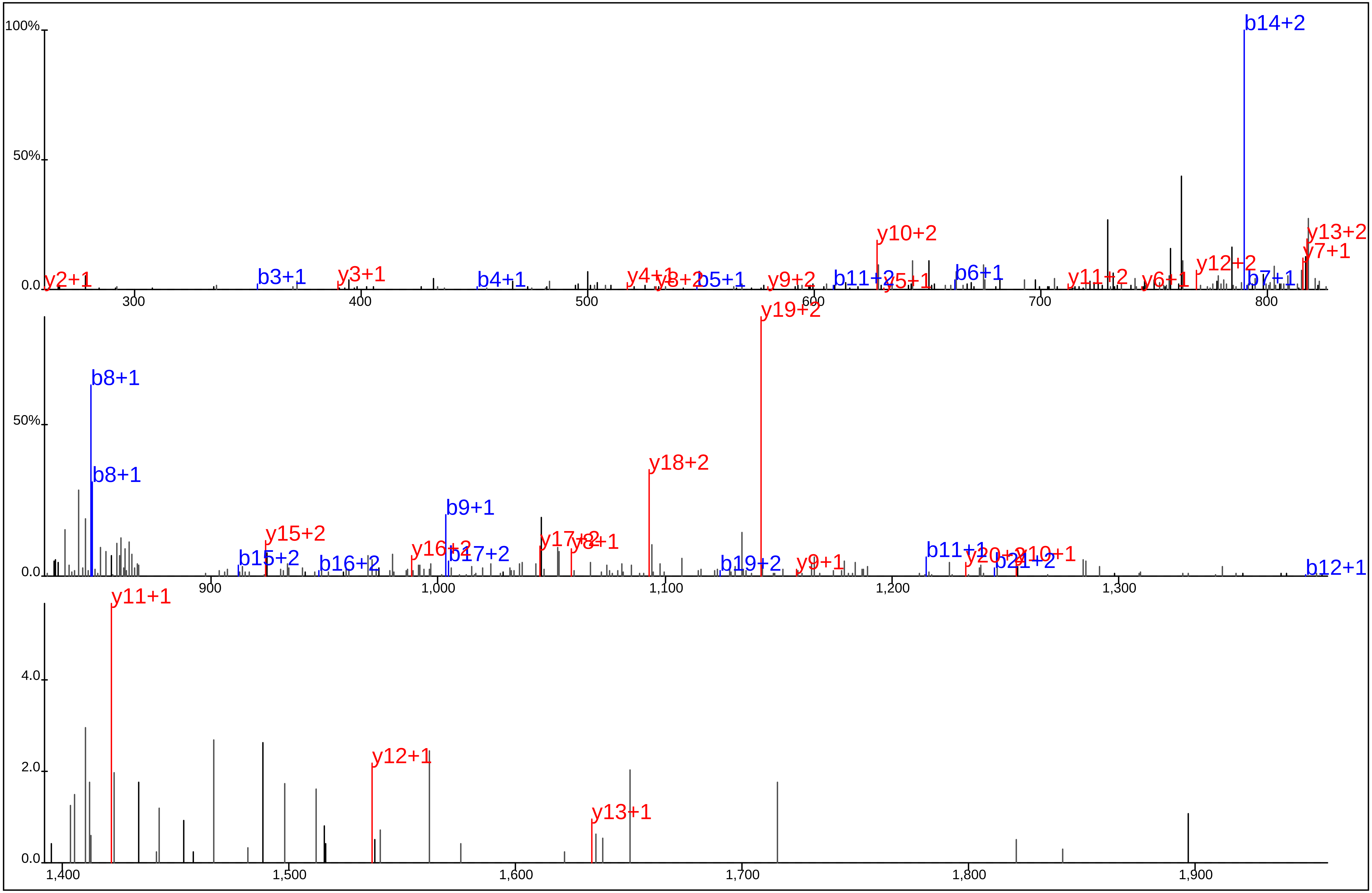

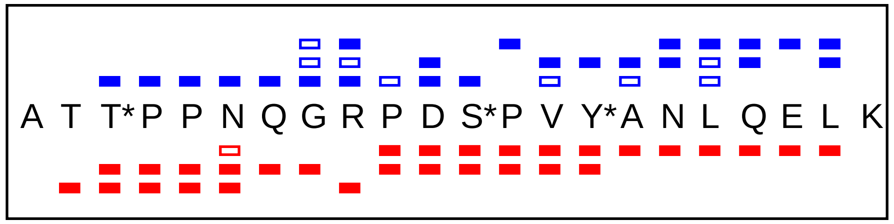

Protein Name: RhoGAP 12
gi Accession: 26986534
Precursor Mass: 879.3813+3
Delta Mass: 3.754 ppm
XCorr: 20.43

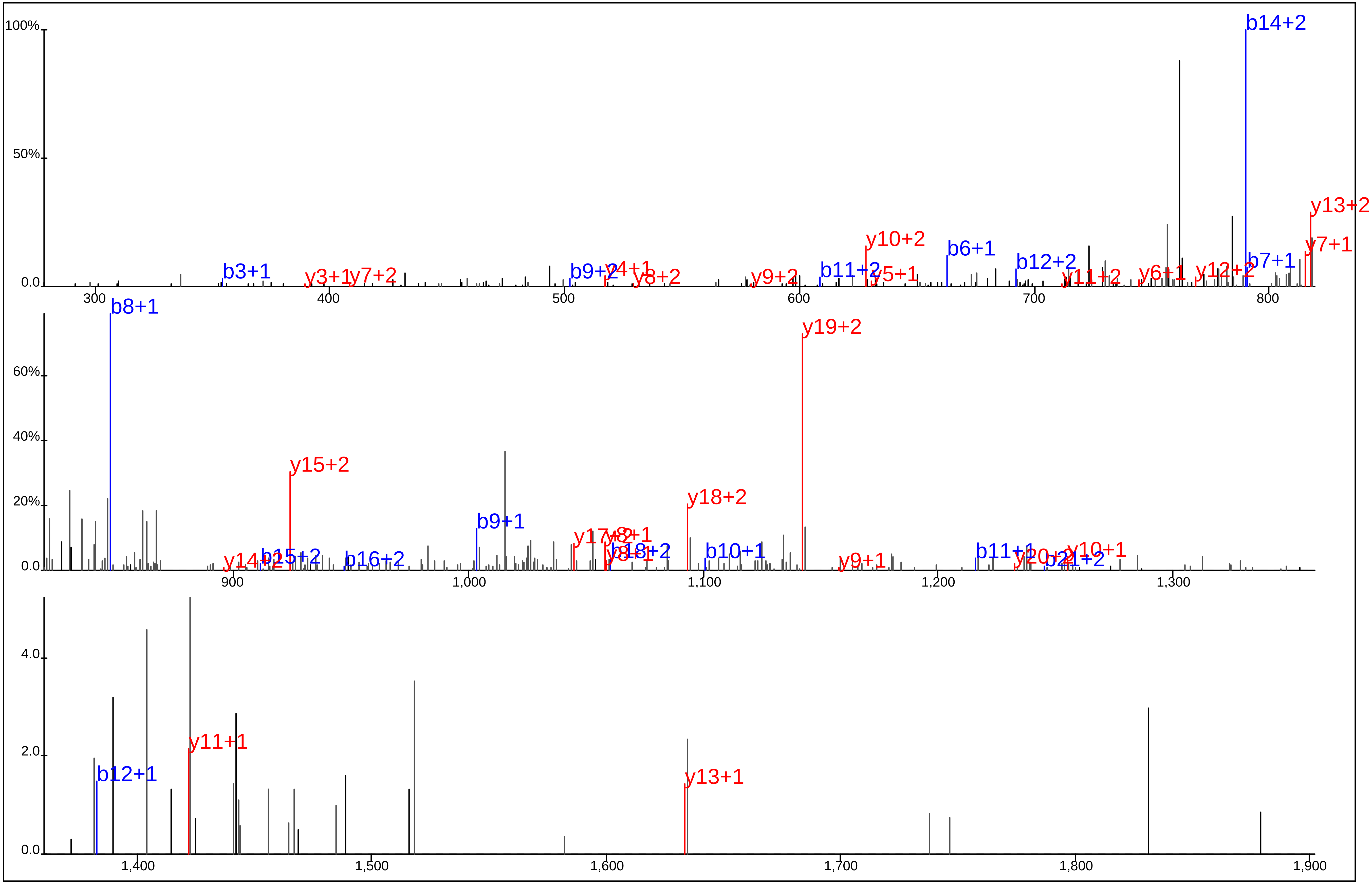

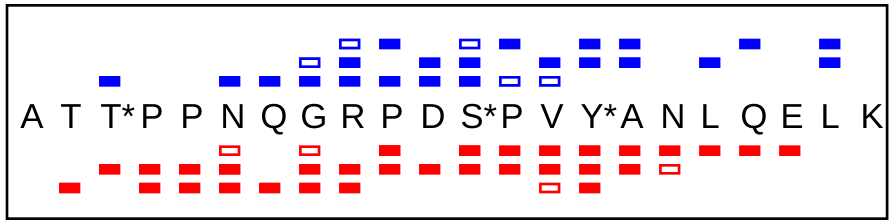

Protein Name: RhoGAP 12
gi Accession: 26986534
Precursor Mass: 879.3707+3
Delta Mass: 8.263 ppm
XCorr: 15.55

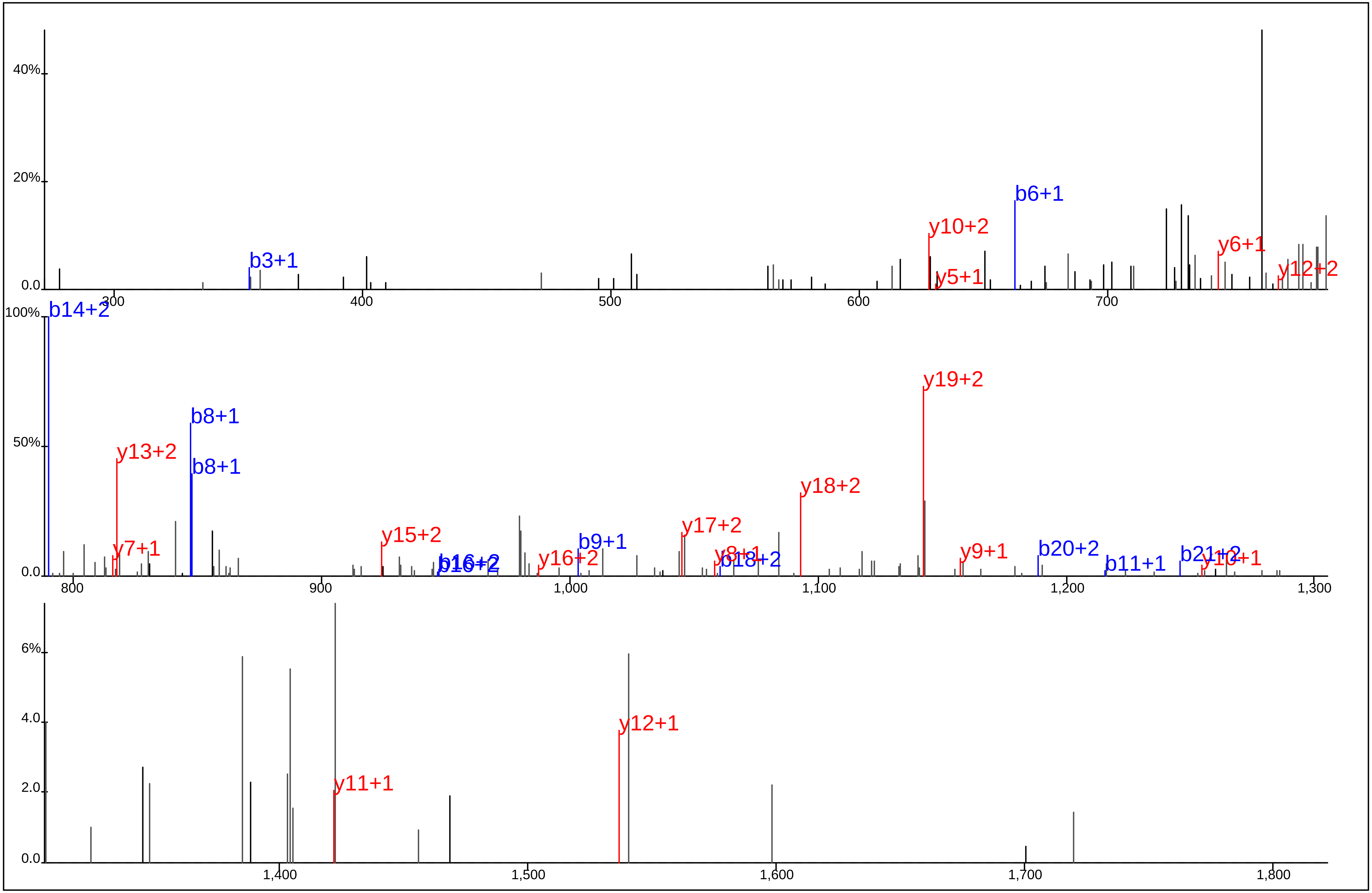

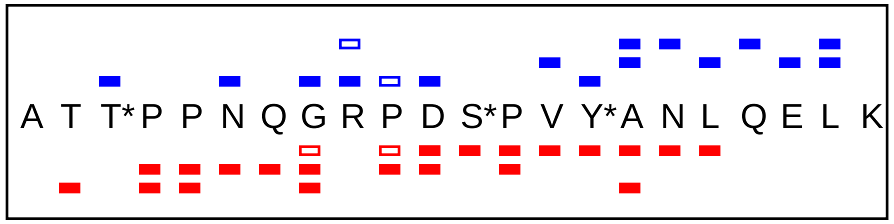

Protein Name: RhoGAP 12
gi Accession: 26986534
Precursor Mass: 879.3904+3
Delta Mass: 14.104 ppm
XCorr: 19.05

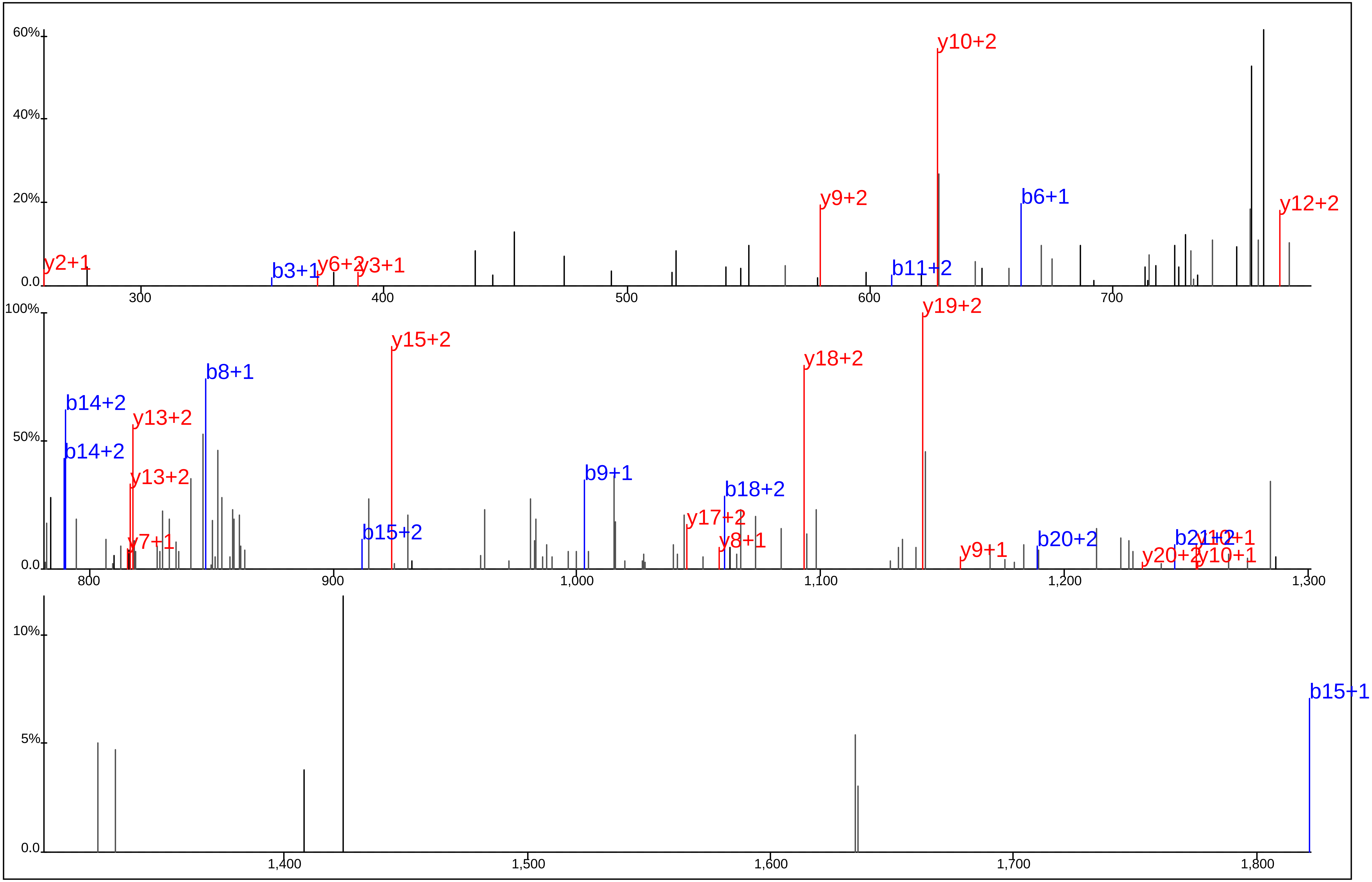

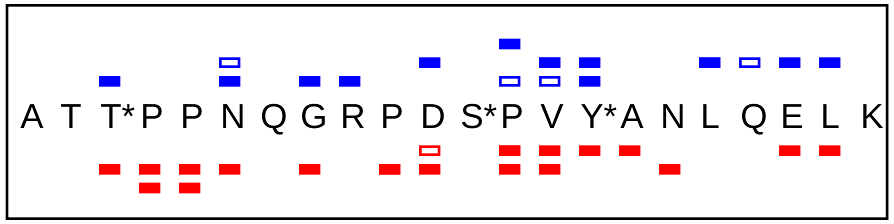

Protein Name:	RhoGAP 12
gi Accession:	26986534
Precursor Mass:	879.3796+3
Delta Mass:	1.808 ppm
XCorr:	25.03

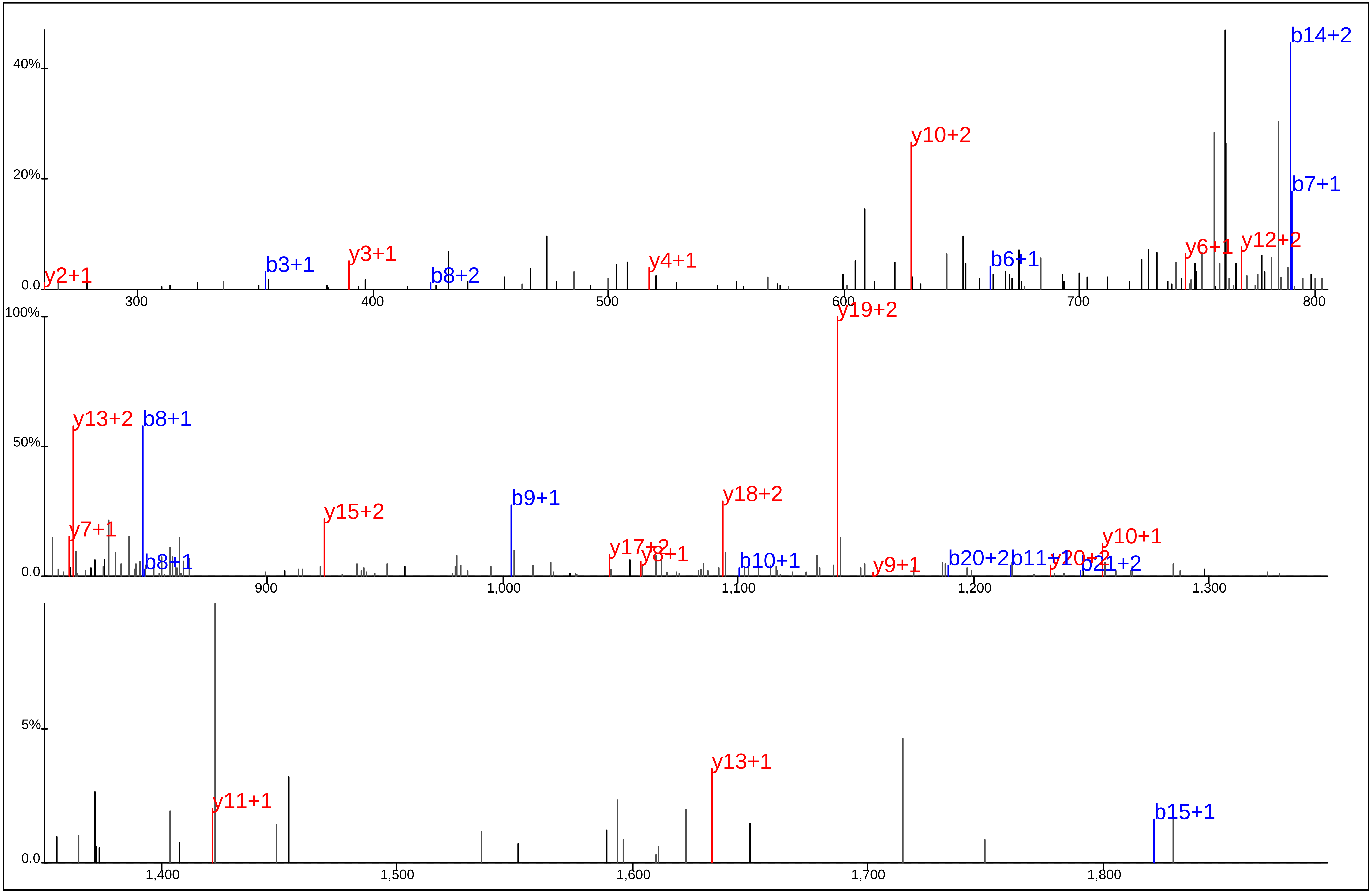

A T T*P P N Q G R P D S*P V Y*A N L Q E L K

Protein Name:	RhoGAP 12
gi Accession:	26986534
Precursor Mass:	879.3810+3
Delta Mass:	3.474 ppm
XCorr:	22.31

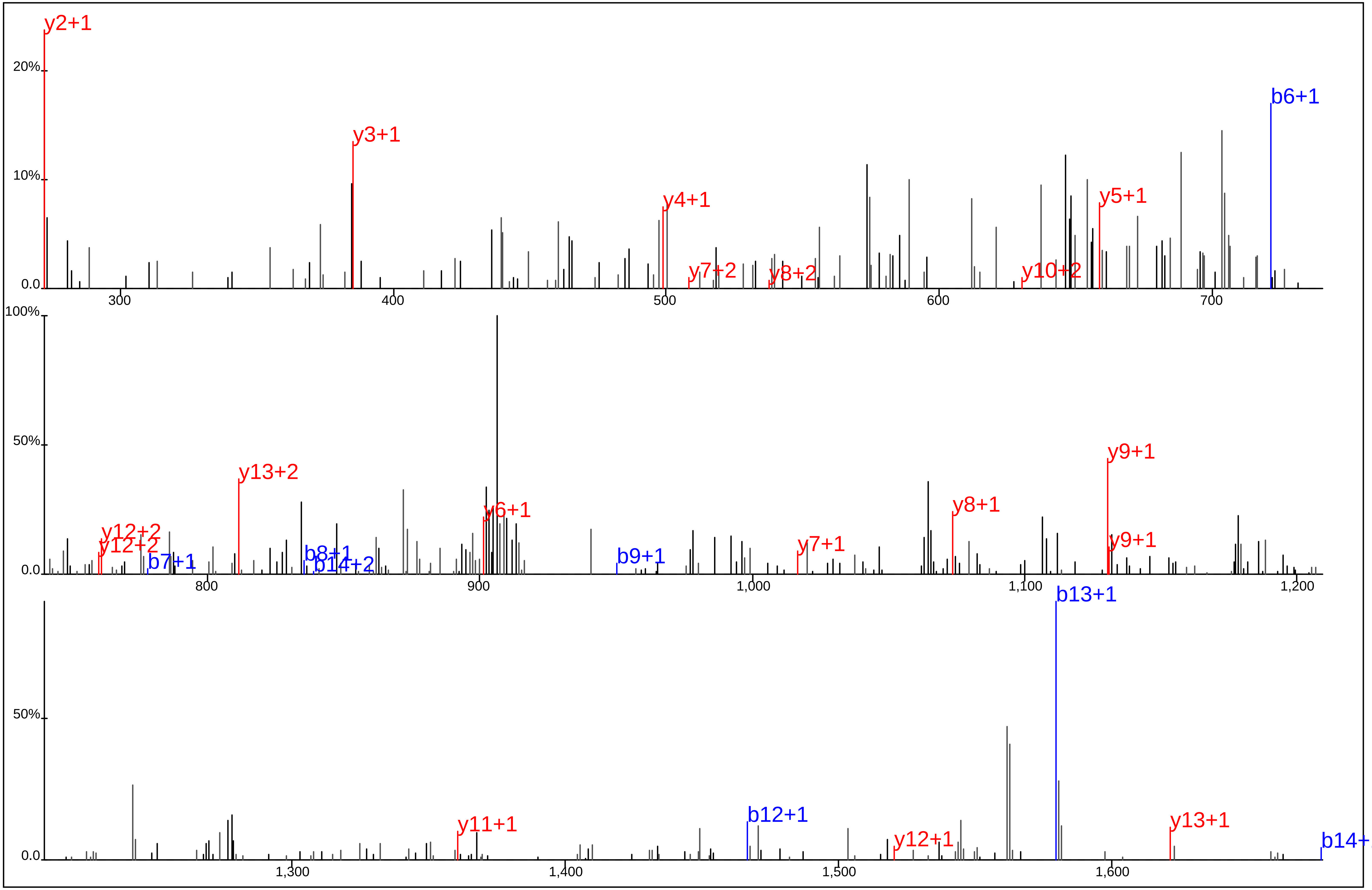

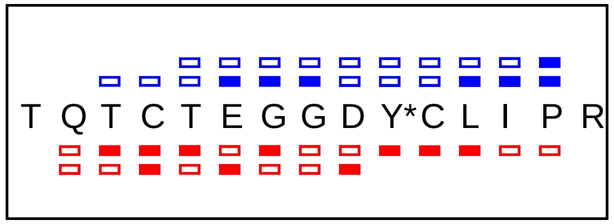

Protein Name:	RhoGAP 30
gi Accession:	47077657
Precursor Mass:	925.8760+2
Delta Mass:	0.044 ppm
XCorr:	22.73

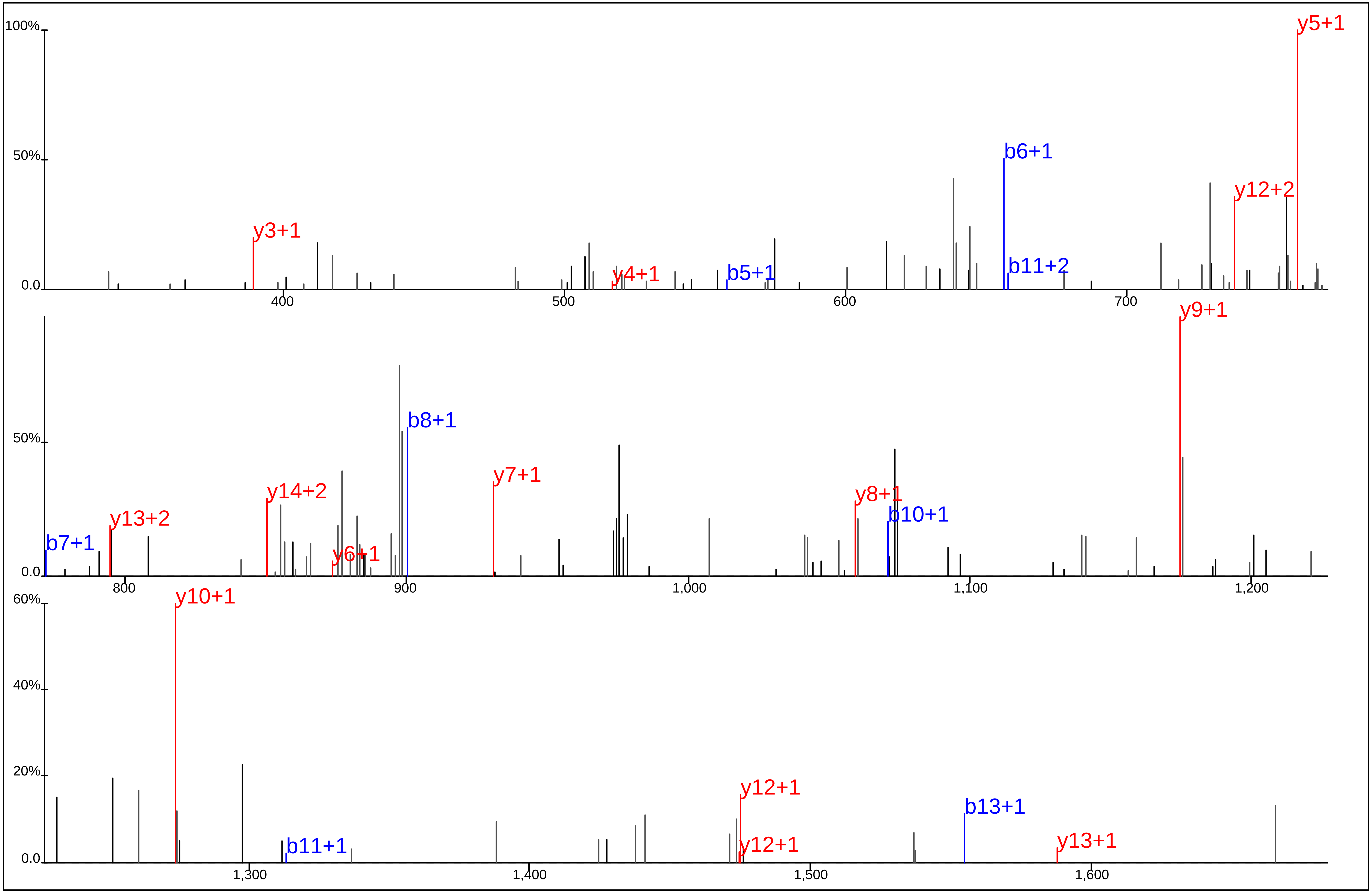

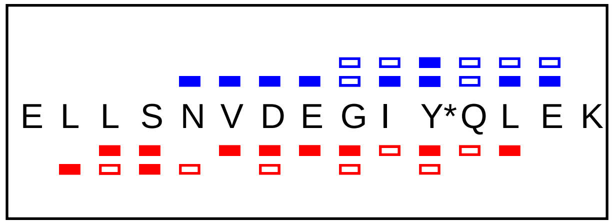

Protein Name: RhoGEF2
gi Accession: 15011974
Precursor Mass: 915.4340+2
Delta Mass: 2.163 ppm
XCorr: 35.88

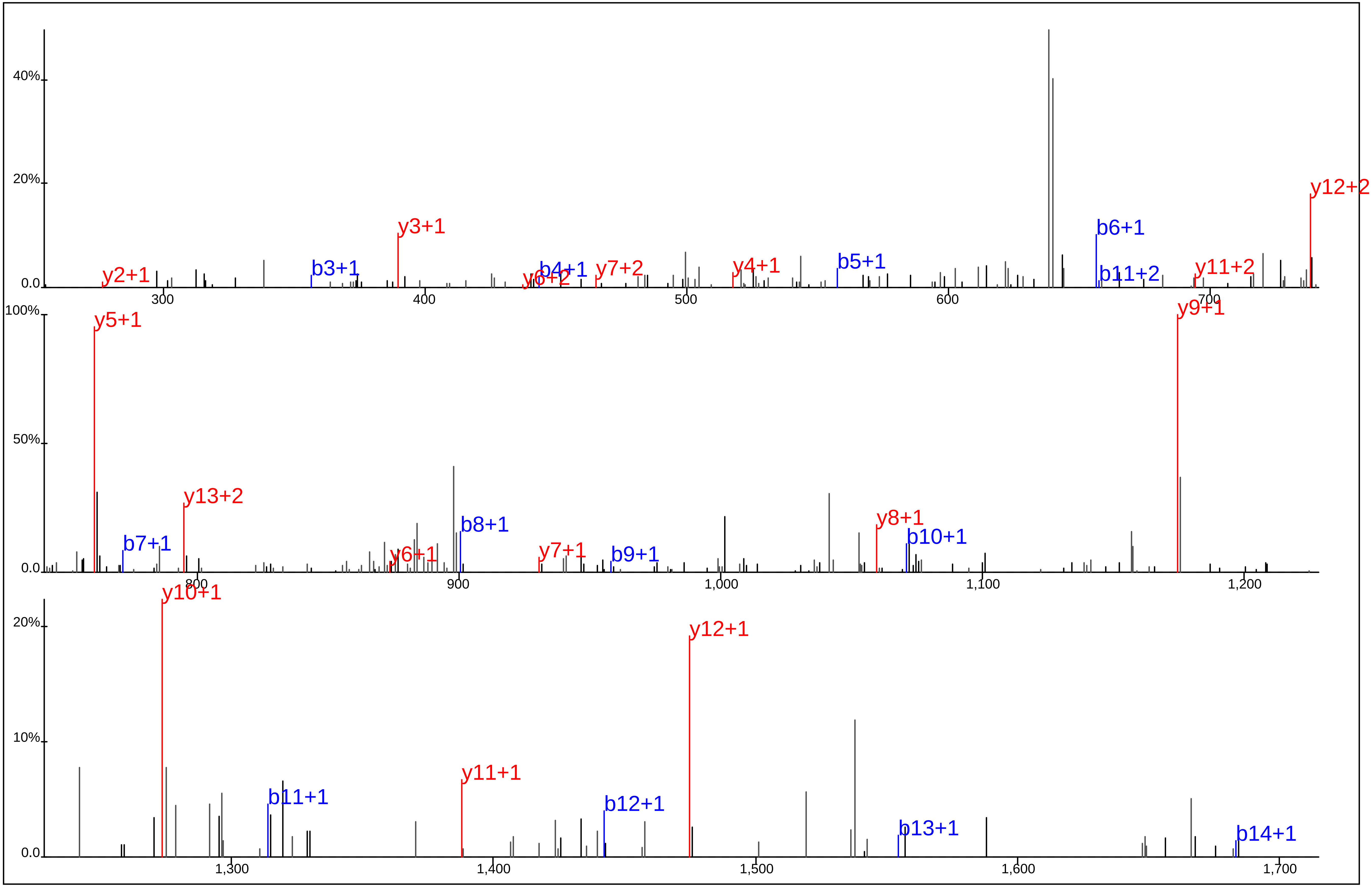

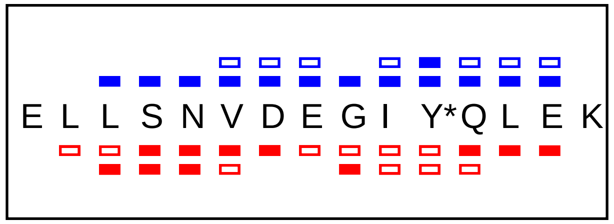

Protein Name:	RhoGEF2
gi Accession:	15011974
Precursor Mass:	915.4363+2
Delta Mass:	4.693 ppm
XCorr:	43.55

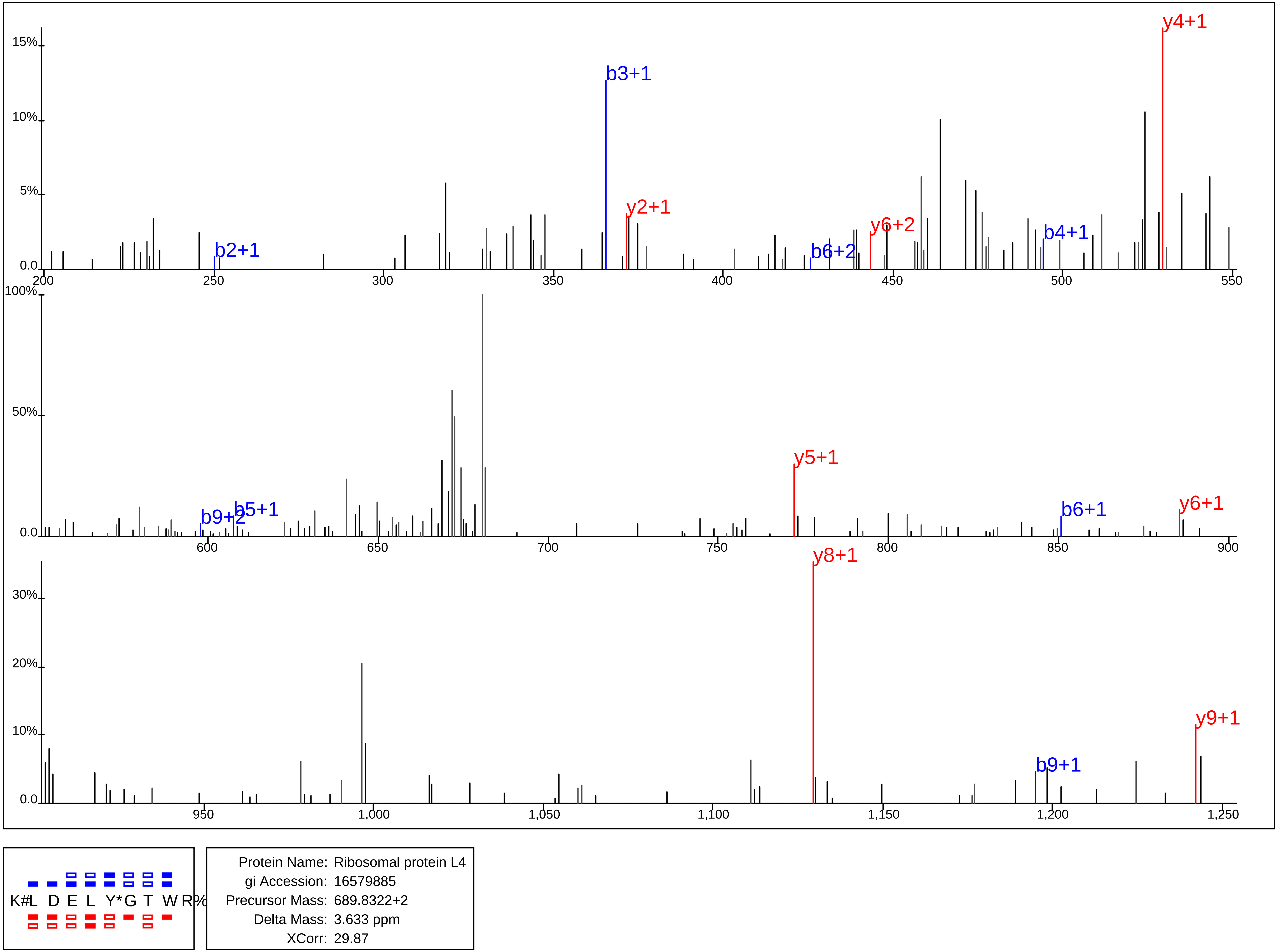

Protein Name: Ribosomal protein L4
gi Accession: 16579885
Precursor Mass: 689.8322+2
Delta Mass: 3.633 ppm
XCorr: 29.87

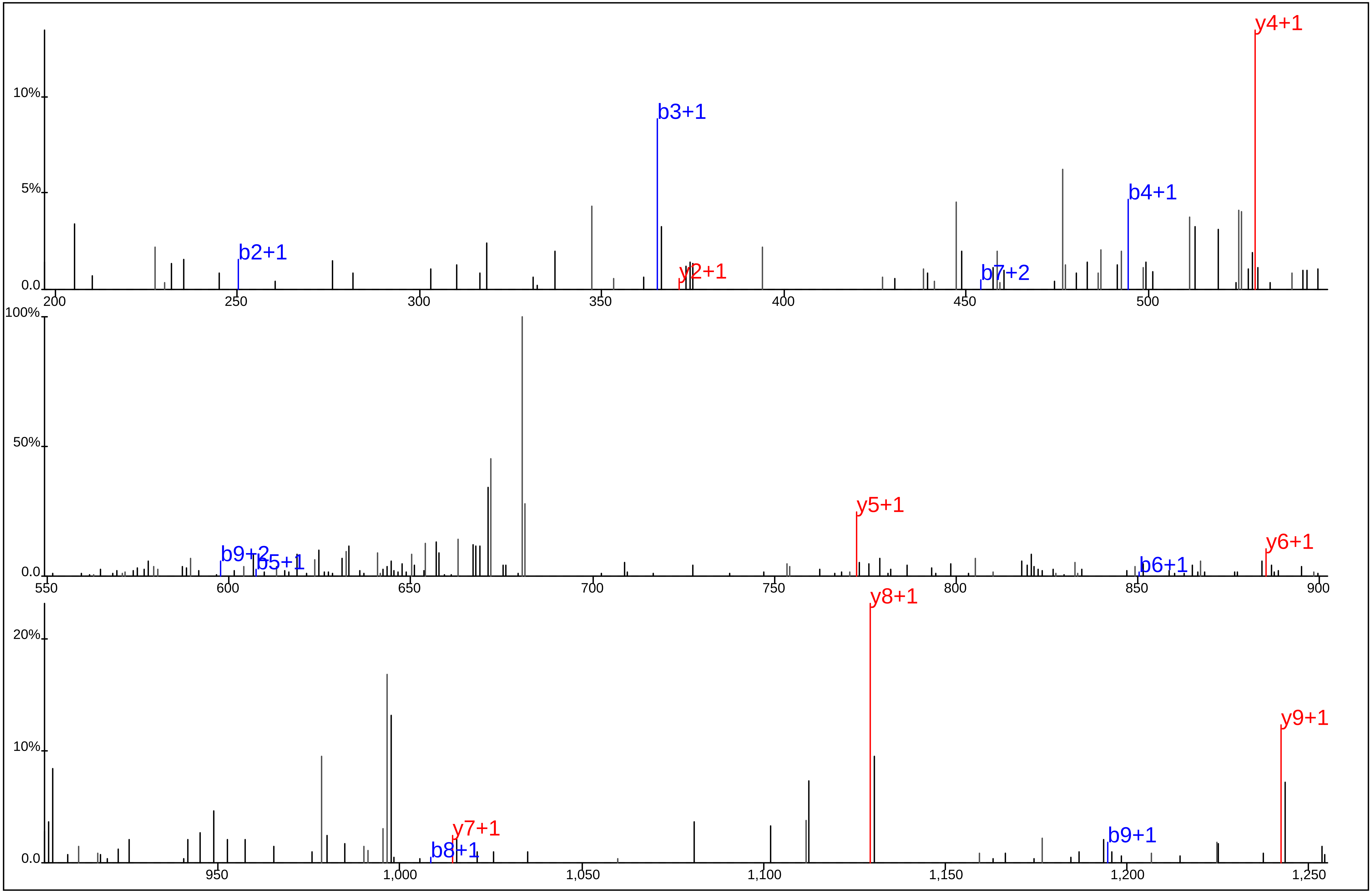

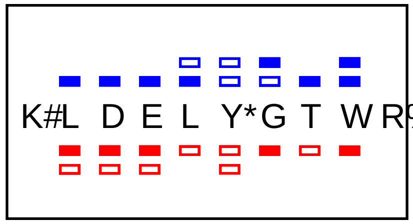

Protein Name:	Ribosomal protein L4
gi Accession:	16579885
Precursor Mass:	689.8326+2
Delta Mass:	4.256 ppm
XCorr:	33.78

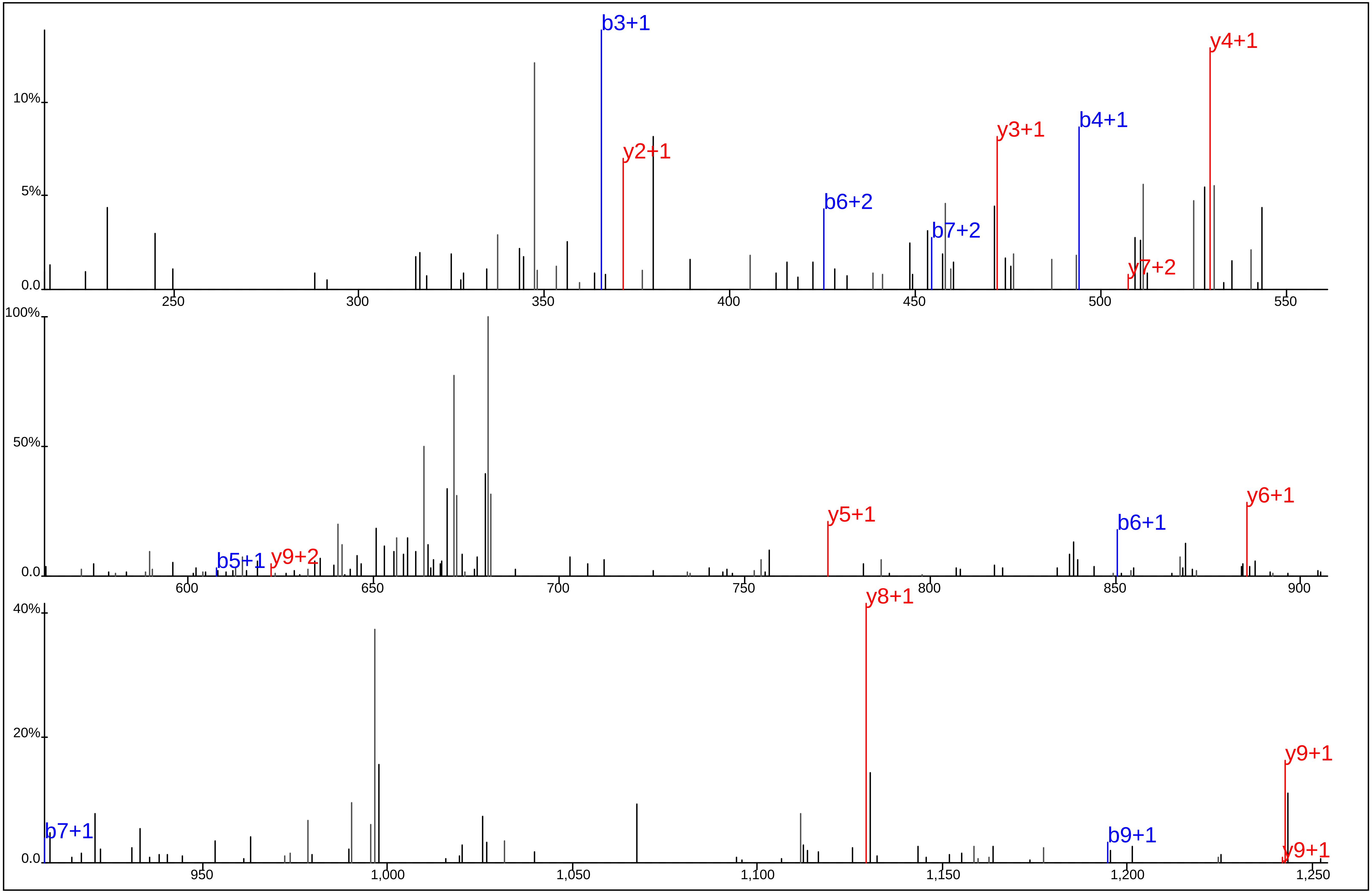

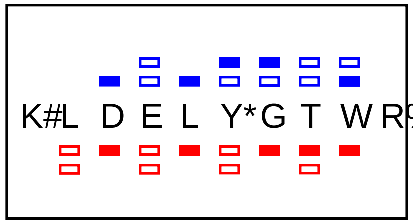

Protein Name: Ribosomal protein L4
gi Accession: 16579885
Precursor Mass: 689.8329+2
Delta Mass: 4.604 ppm
XCorr: 40.58

843 - RibosomalproteinL4_3440789.pdf

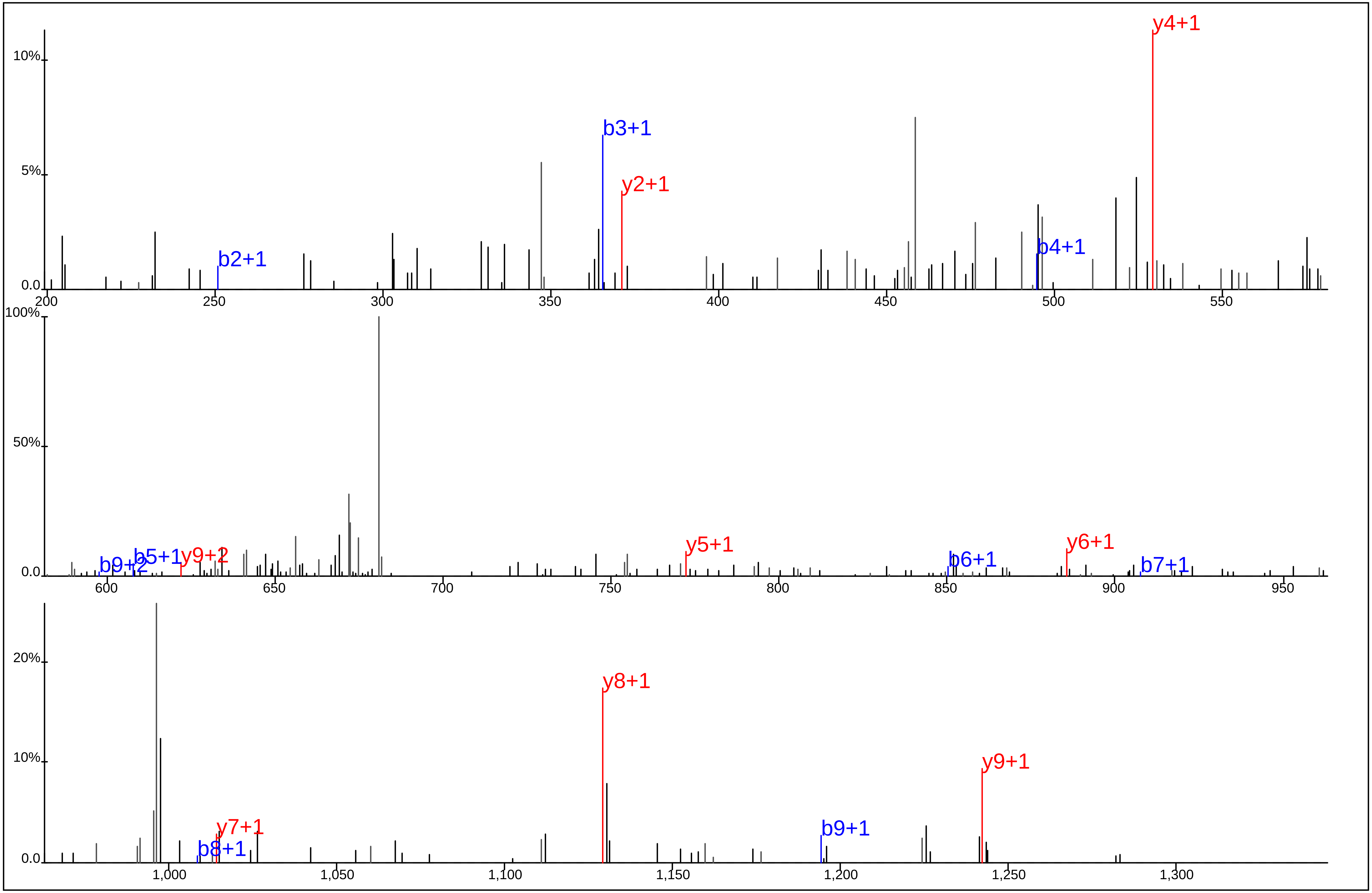

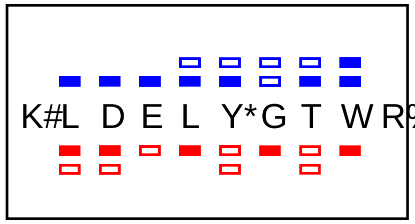

Protein Name:	Ribosomal protein L4
gi Accession:	16579885
Precursor Mass:	689.8311+2
Delta Mass:	2.037 ppm
XCorr:	30.56

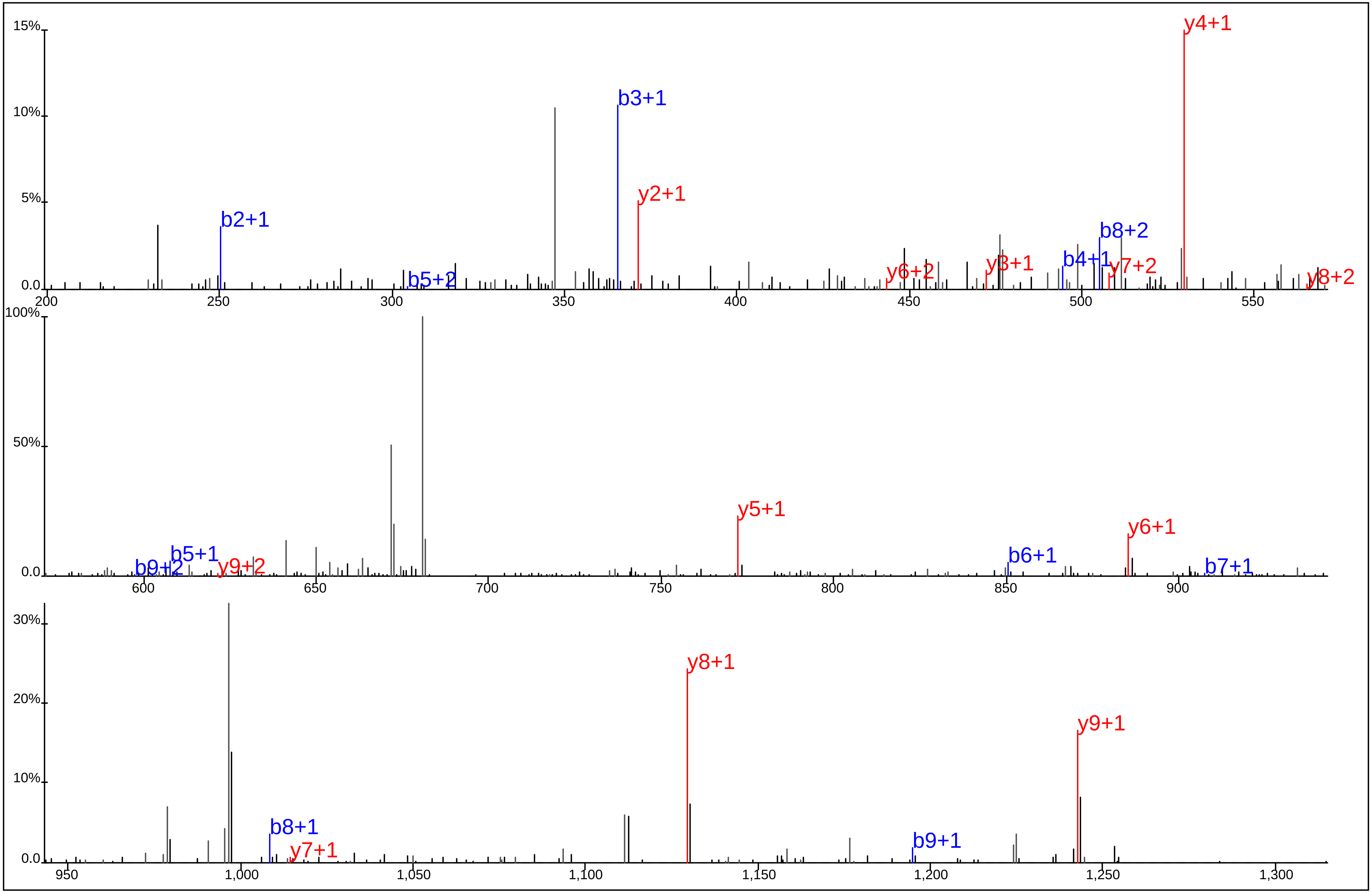

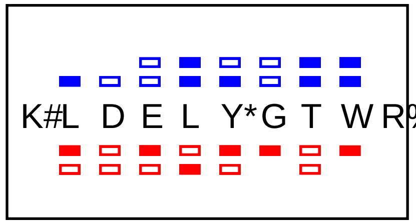

Protein Name: Ribosomal protein L4
gi Accession: 16579885
Precursor Mass: 689.8312+2
Delta Mass: 2.131 ppm
XCorr: 38.0

845 - RibosomalproteinL4_3446901.pdf

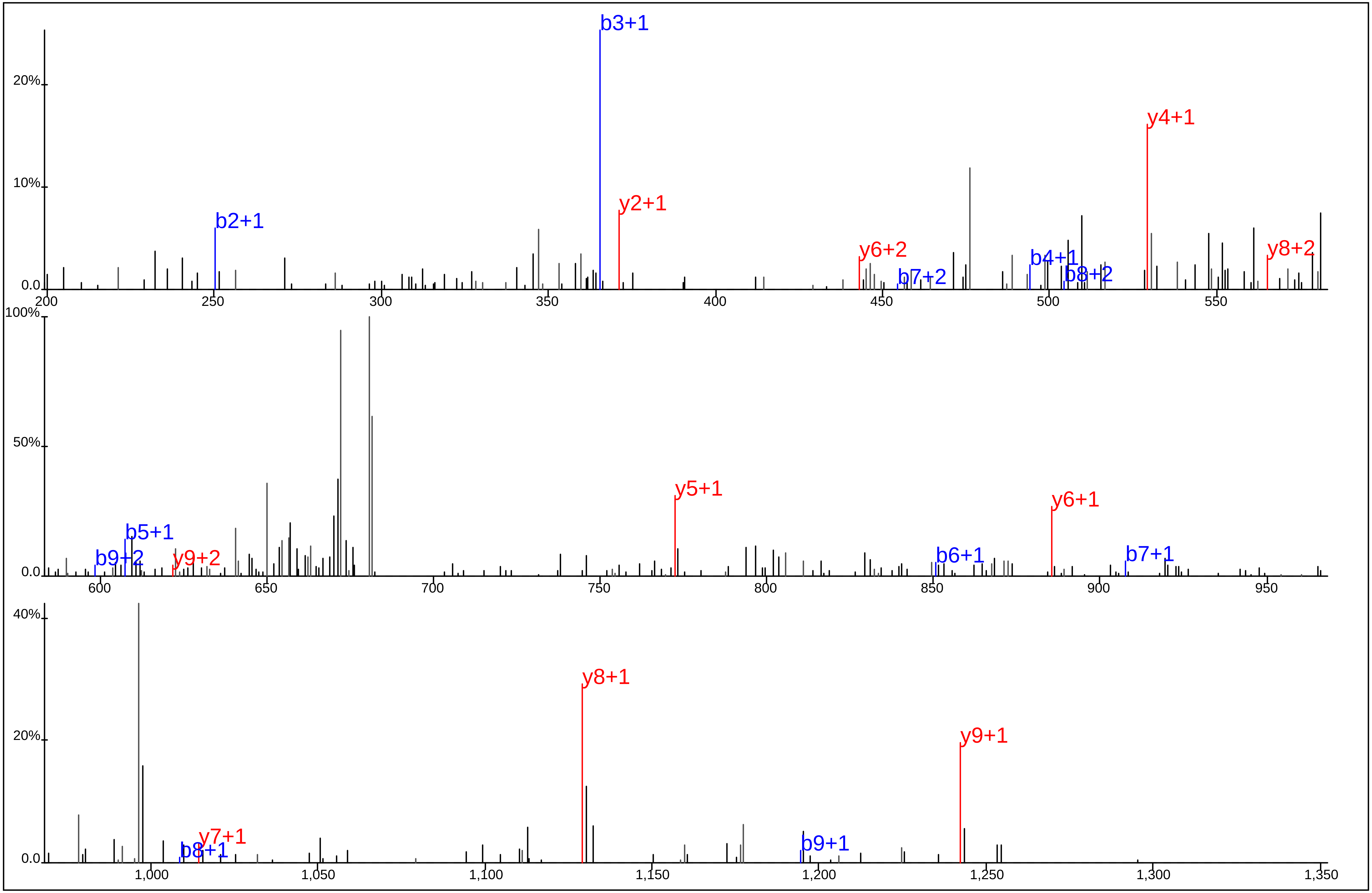

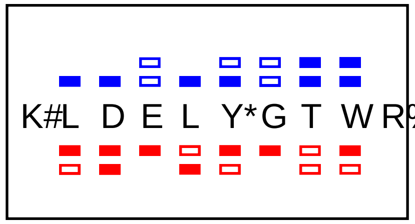

Protein Name:	Ribosomal protein L4
gi Accession:	16579885
Precursor Mass:	689.8318+2
Delta Mass:	3.016 ppm
XCorr:	36.65

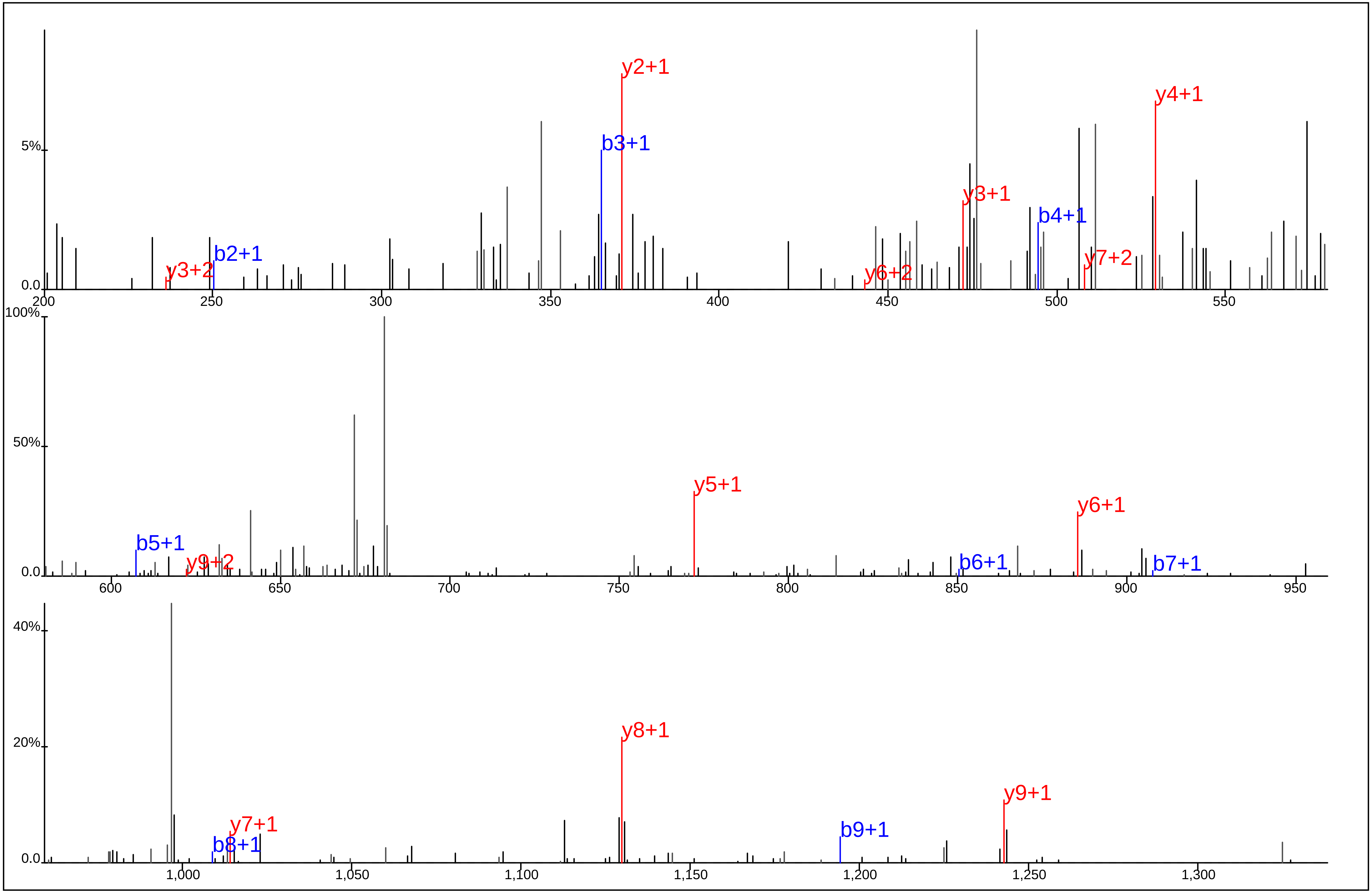

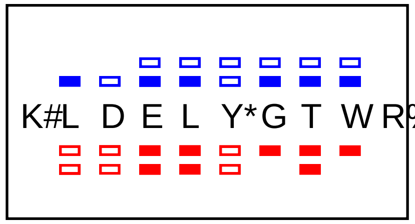

Protein Name: Ribosomal protein L4
gi Accession: 16579885
Precursor Mass: 689.8329+2
Delta Mass: 4.699 ppm
XCorr: 56.31

847 - RibosomalproteinL4_3452087.pdf

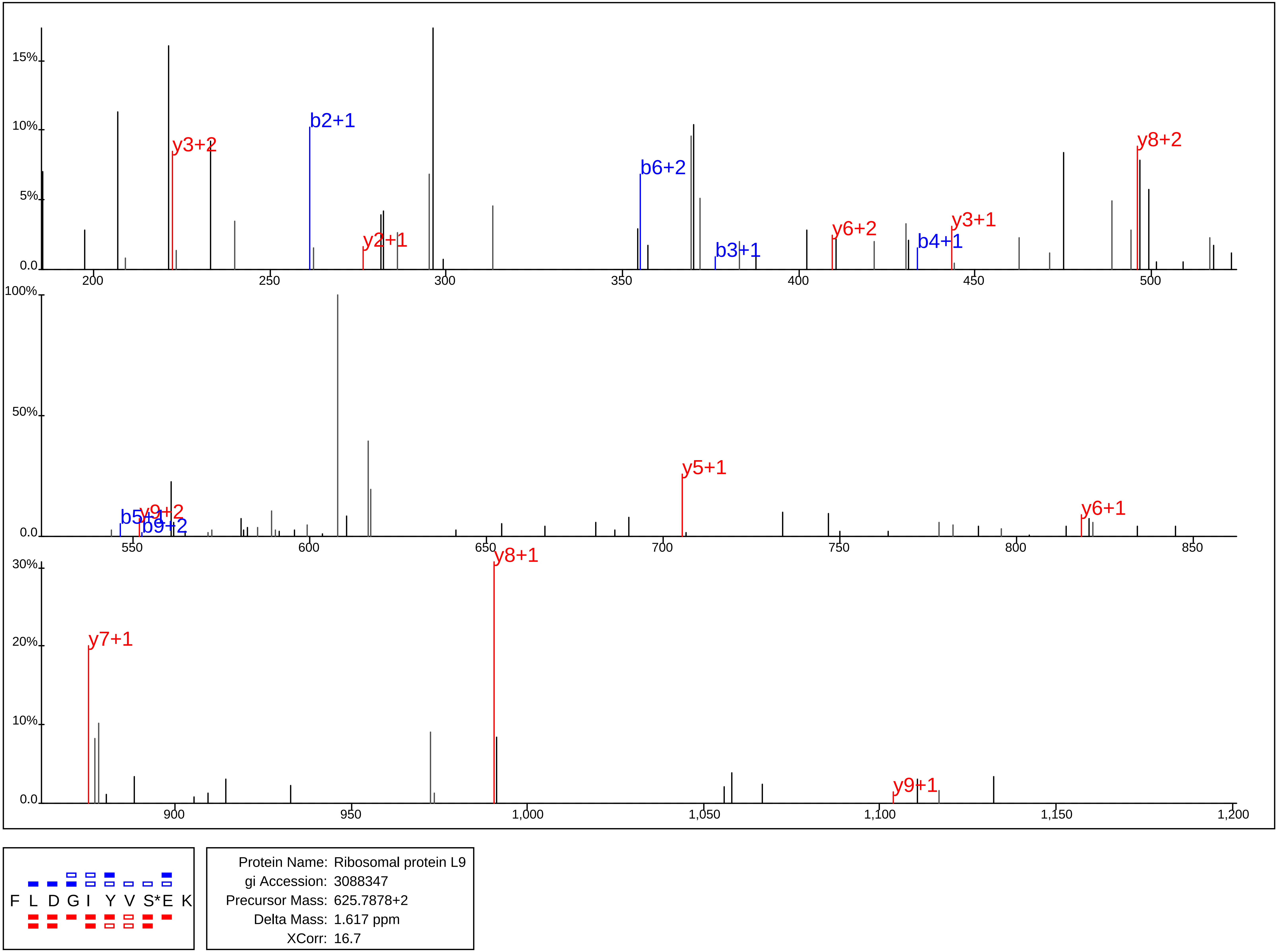

Protein Name: Ribosomal protein L9
gi Accession: 3088347
Precursor Mass: 625.7878+2
Delta Mass: 1.617 ppm
XCorr: 16.7

848 - RibosomalproteinL9_3447833.pdf

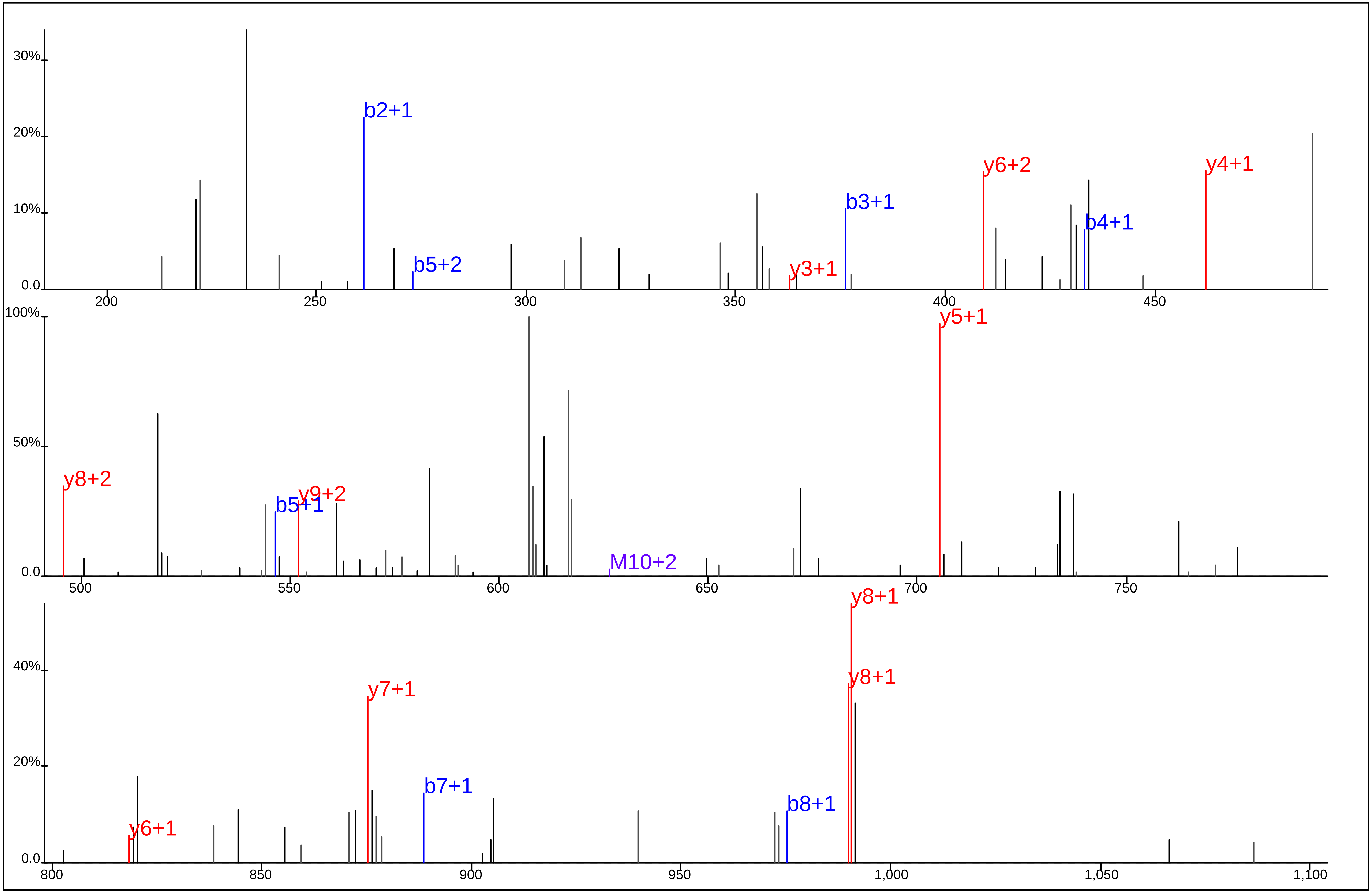

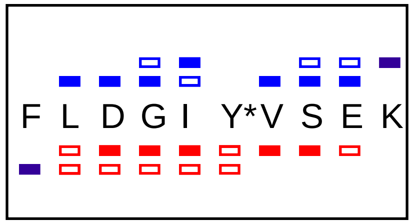

Protein Name:	Ribosomal protein L9
gi Accession:	3088347
Precursor Mass:	625.7848+2
Delta Mass:	6.503 ppm
XCorr:	27.21

849 - RibosomalproteinL9_3448942.pdf

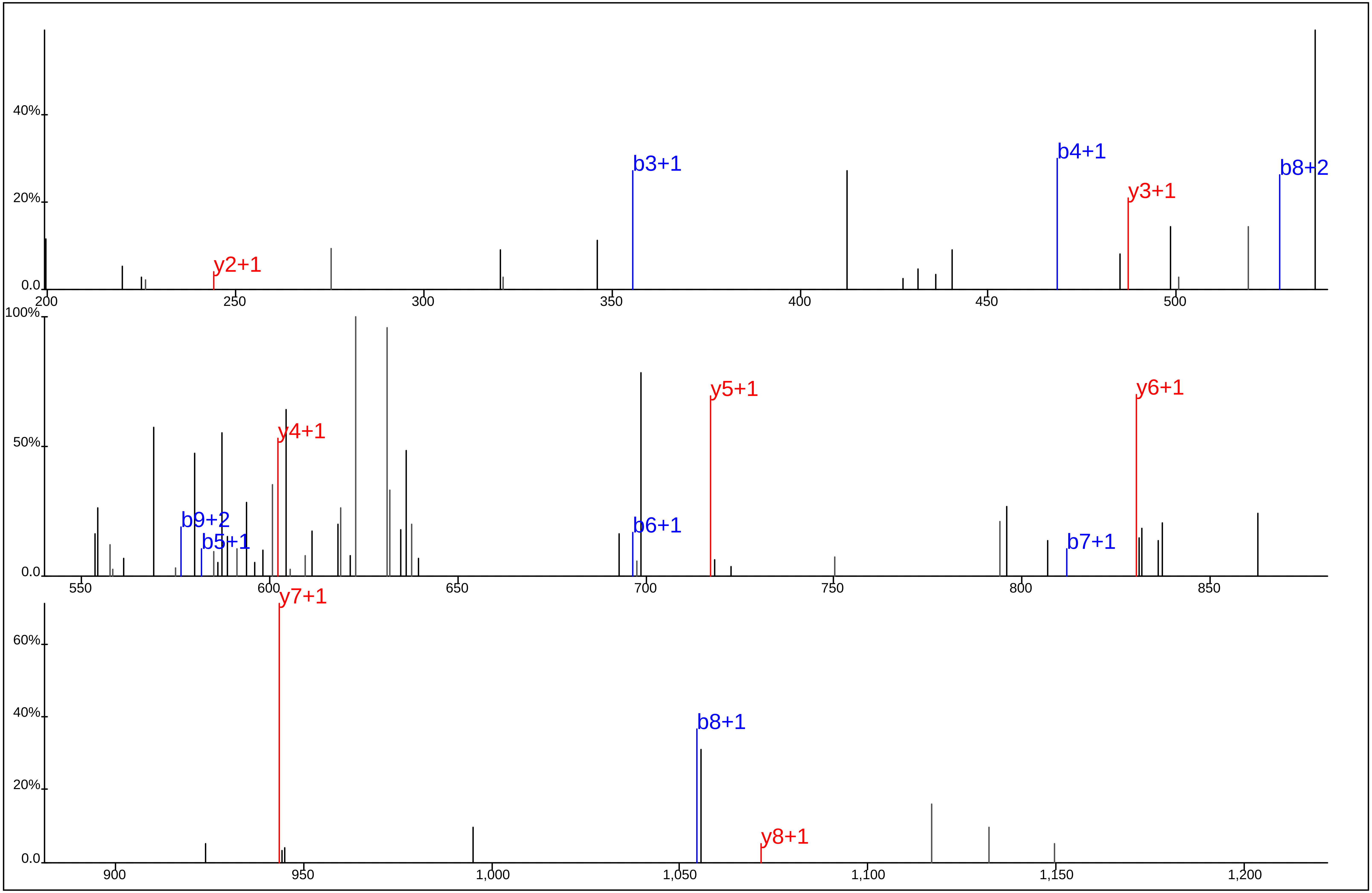

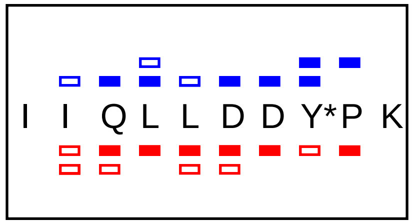

Protein Name: Ribosomal protein P0
gi Accession: 4506667
Precursor Mass: 649.3287+2
Delta Mass: 4.716 ppm
XCorr: 2.2849

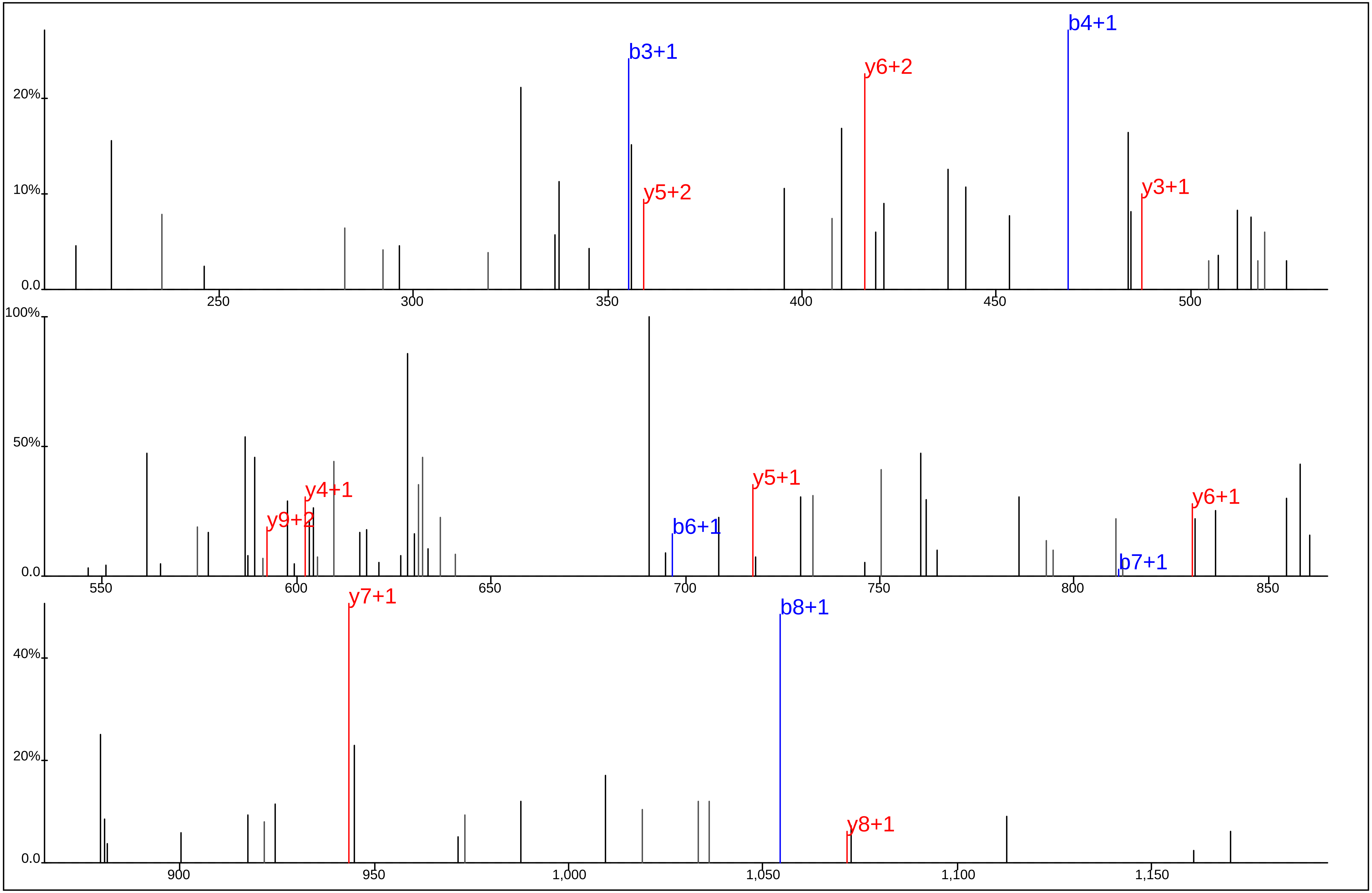

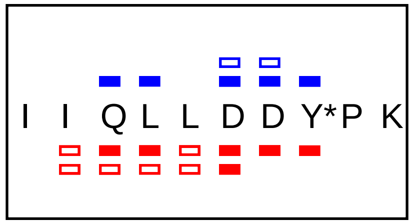

Protein Name:	Ribosomal protein P0
gi Accession:	4506667
Precursor Mass:	649.3258+2
Delta Mass:	0.293 ppm
XCorr:	2.0974

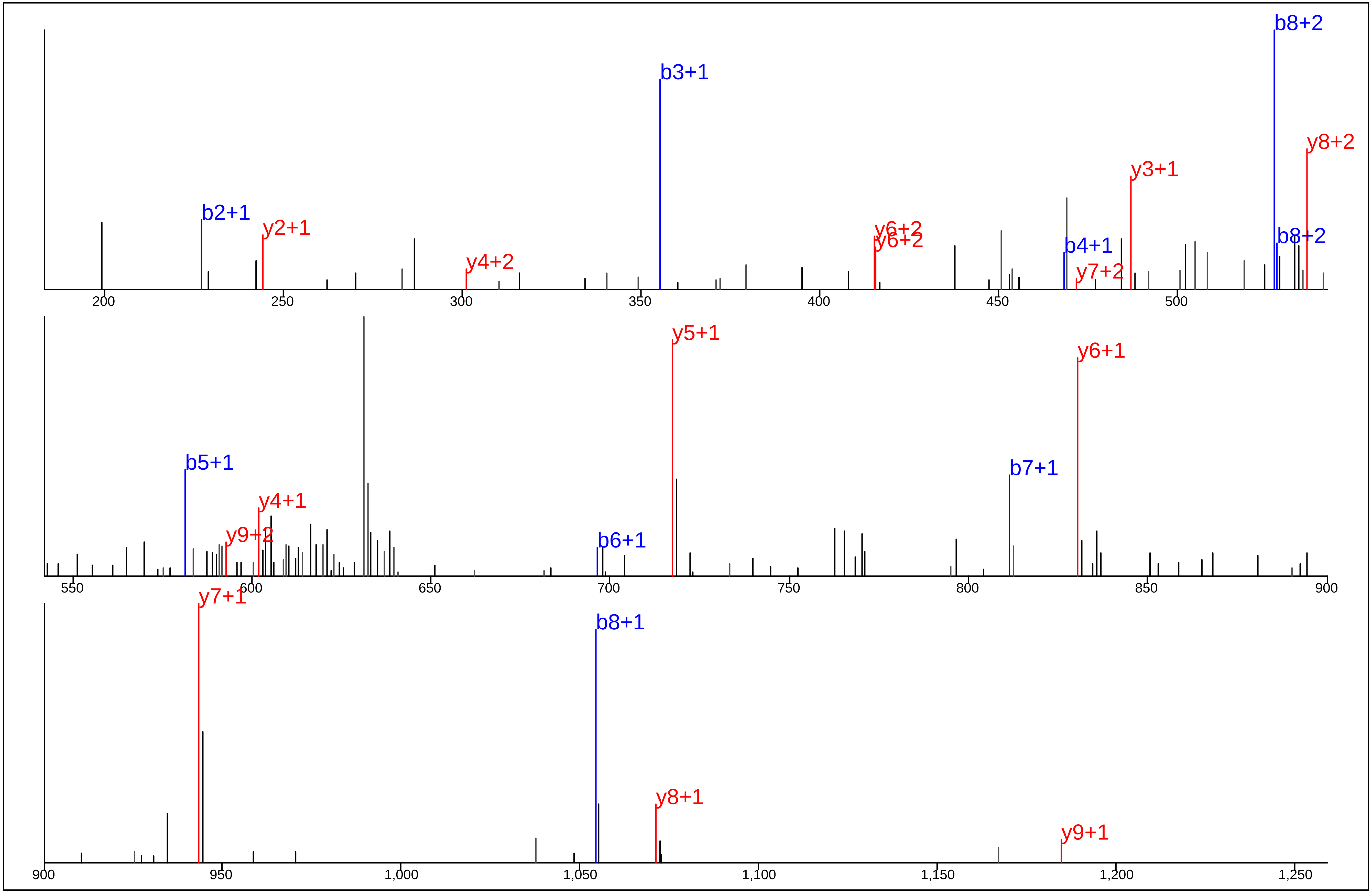

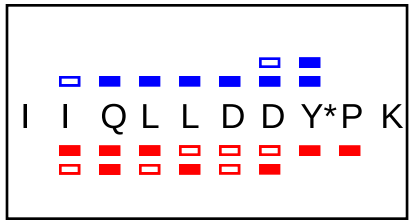

Protein Name: Ribosomal protein P0
gi Accession: 4506667
Precursor Mass: 649.3278+2
Delta Mass: 3.420 ppm
XCorr: 48.44

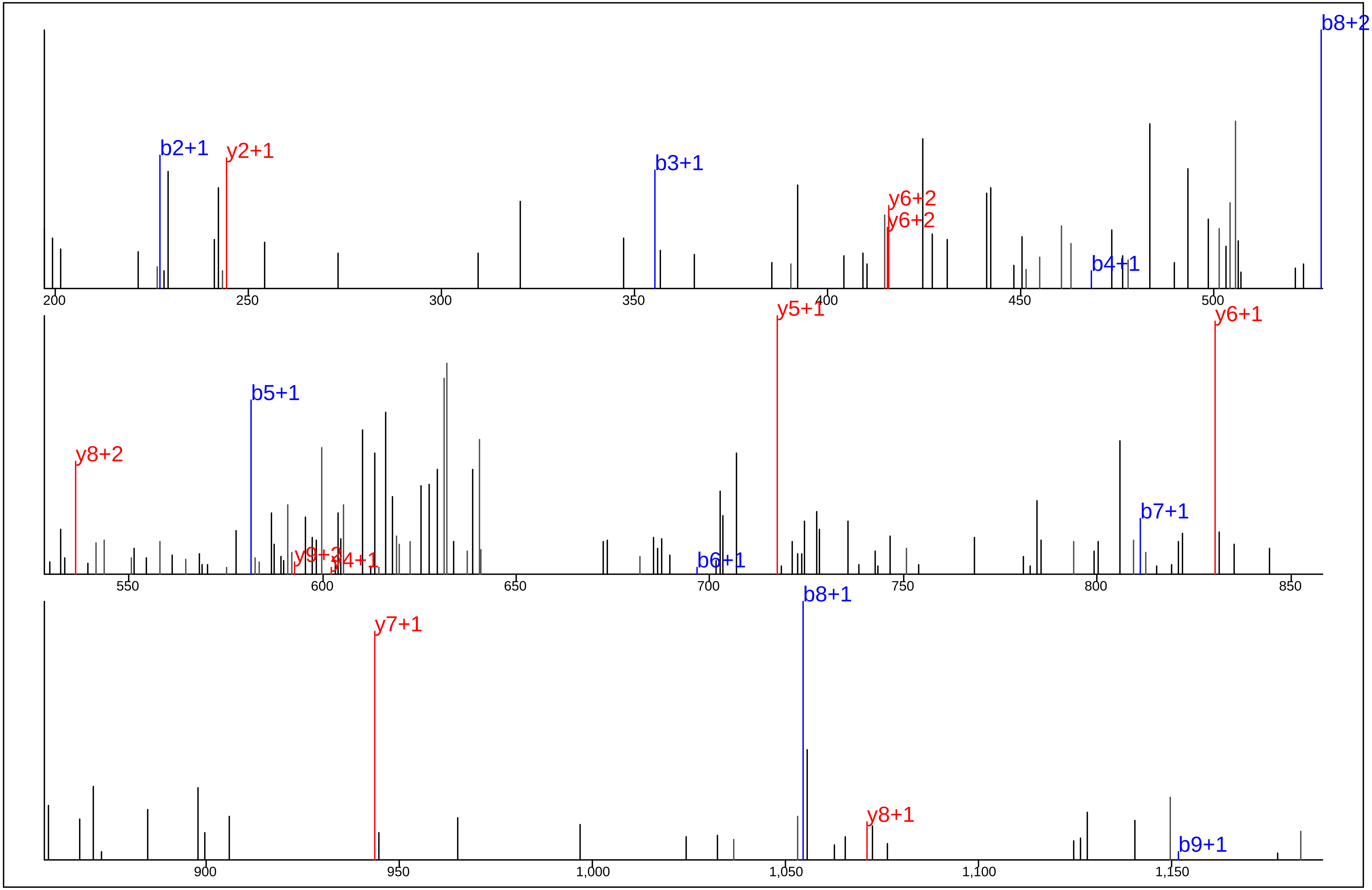

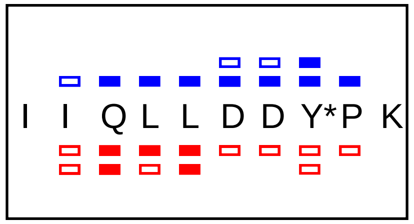

Protein Name:	Ribosomal protein P0
gi Accession:	4506667
Precursor Mass:	649.3303+2
Delta Mass:	7.188 ppm
XCorr:	28.18

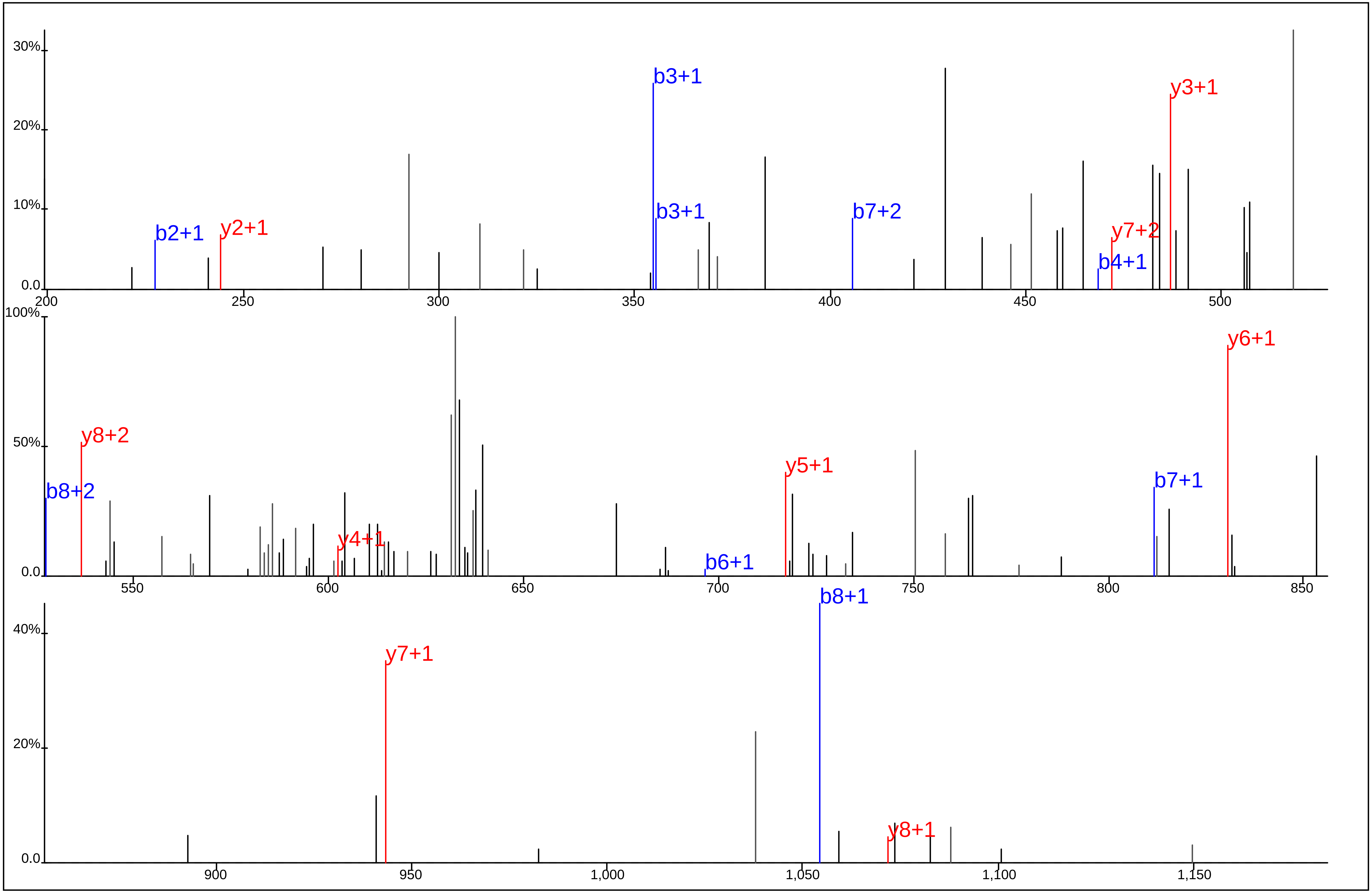

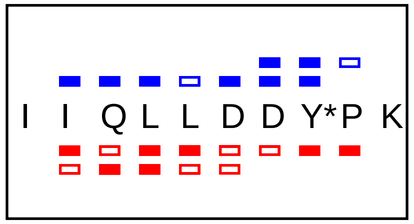

Protein Name:	Ribosomal protein P0
gi Accession:	4506667
Precursor Mass:	649.3313+2
Delta Mass:	8.784 ppm
XCorr:	28.06

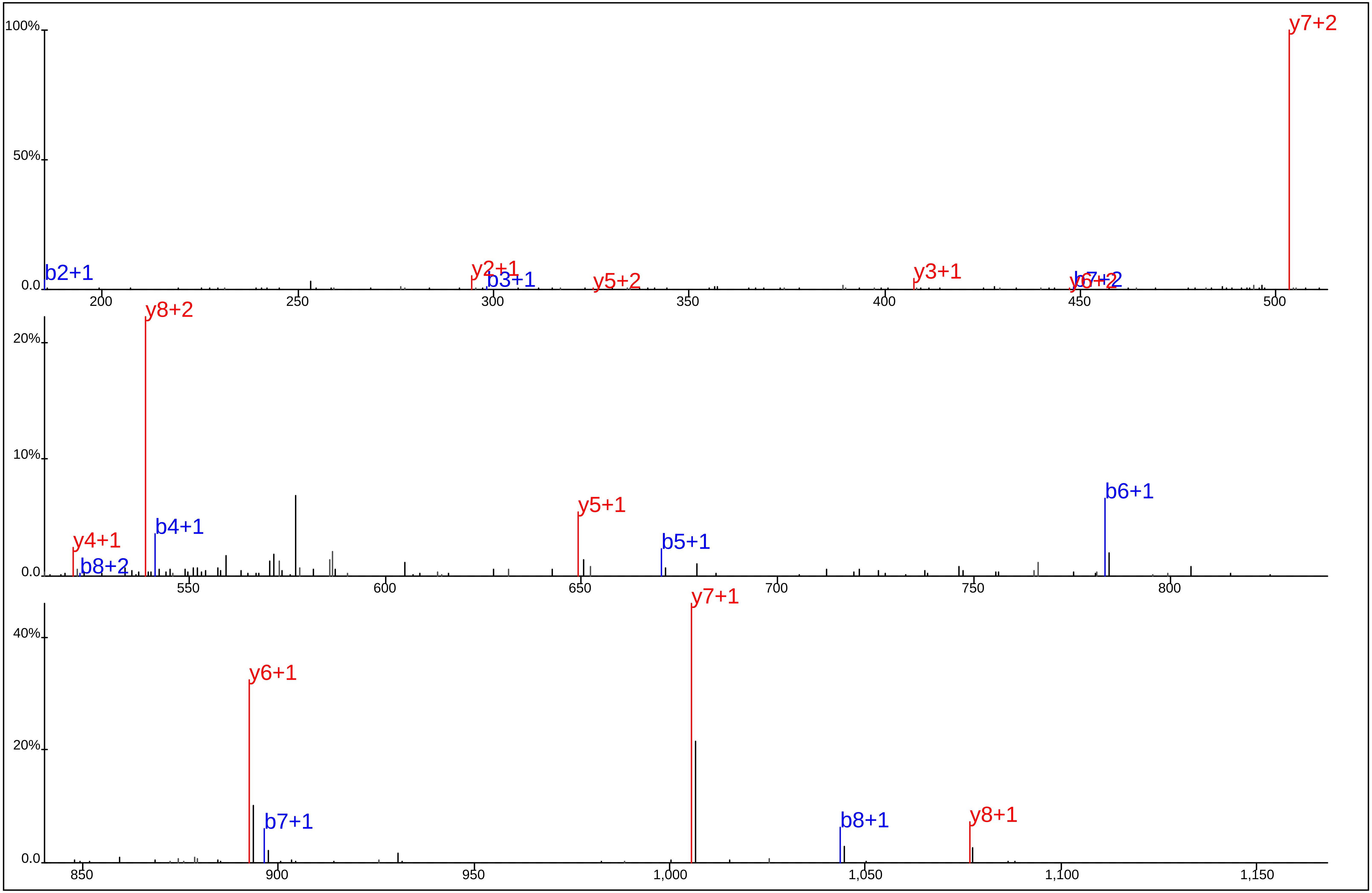

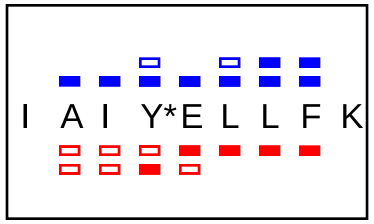

Protein Name:	Ribosomal protein S10
gi Accession:	3088338
Precursor Mass:	595.3193+2
Delta Mass:	3.865 ppm
XCorr:	41.11

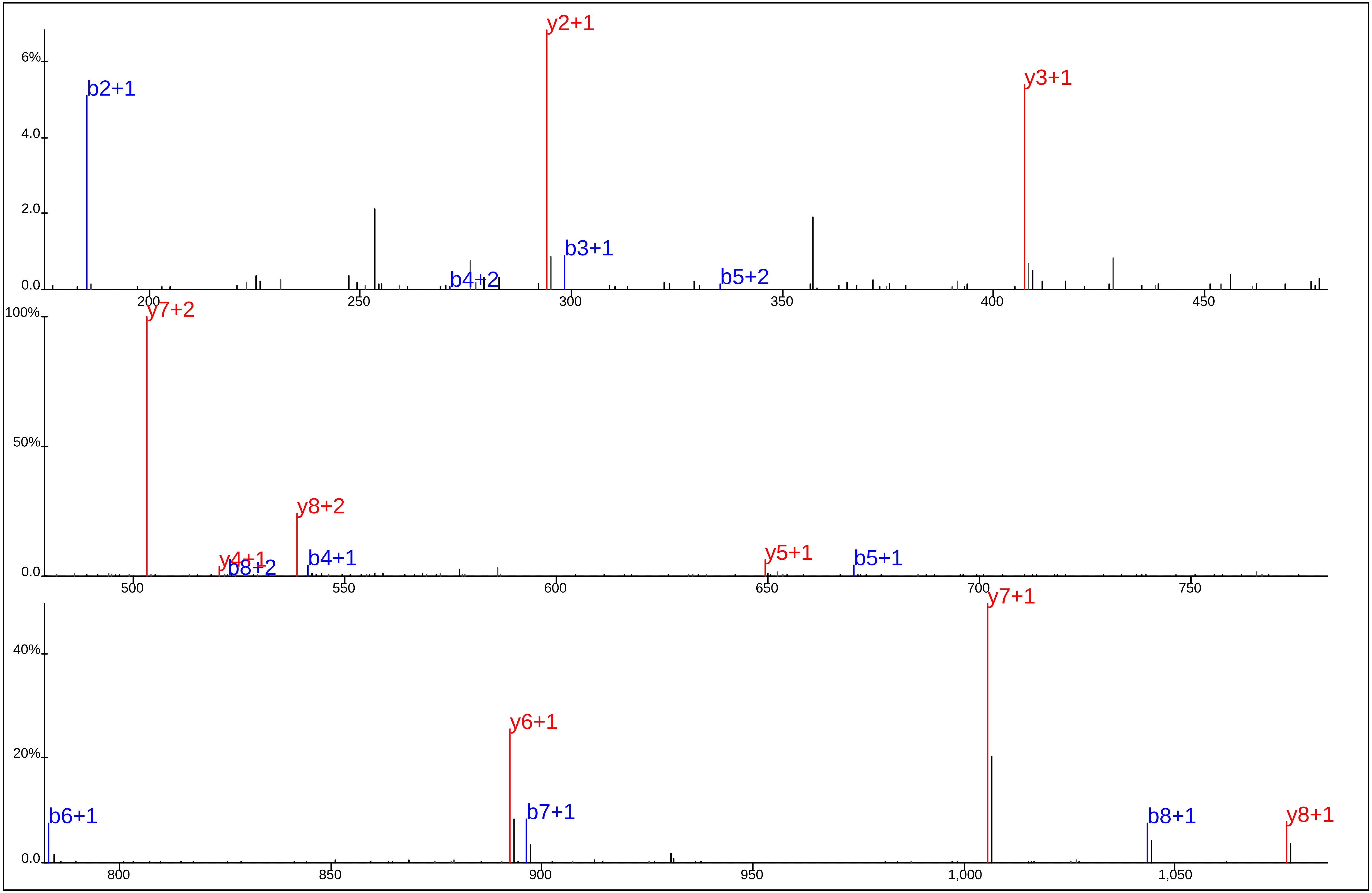

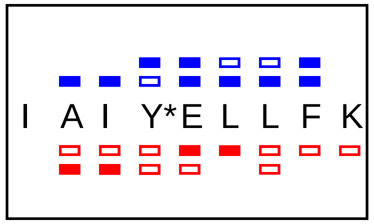

Protein Name: Ribosomal protein S10
gi Accession: 3088338
Precursor Mass: 595.3190+2
Delta Mass: 3.344 ppm
XCorr: 62.87

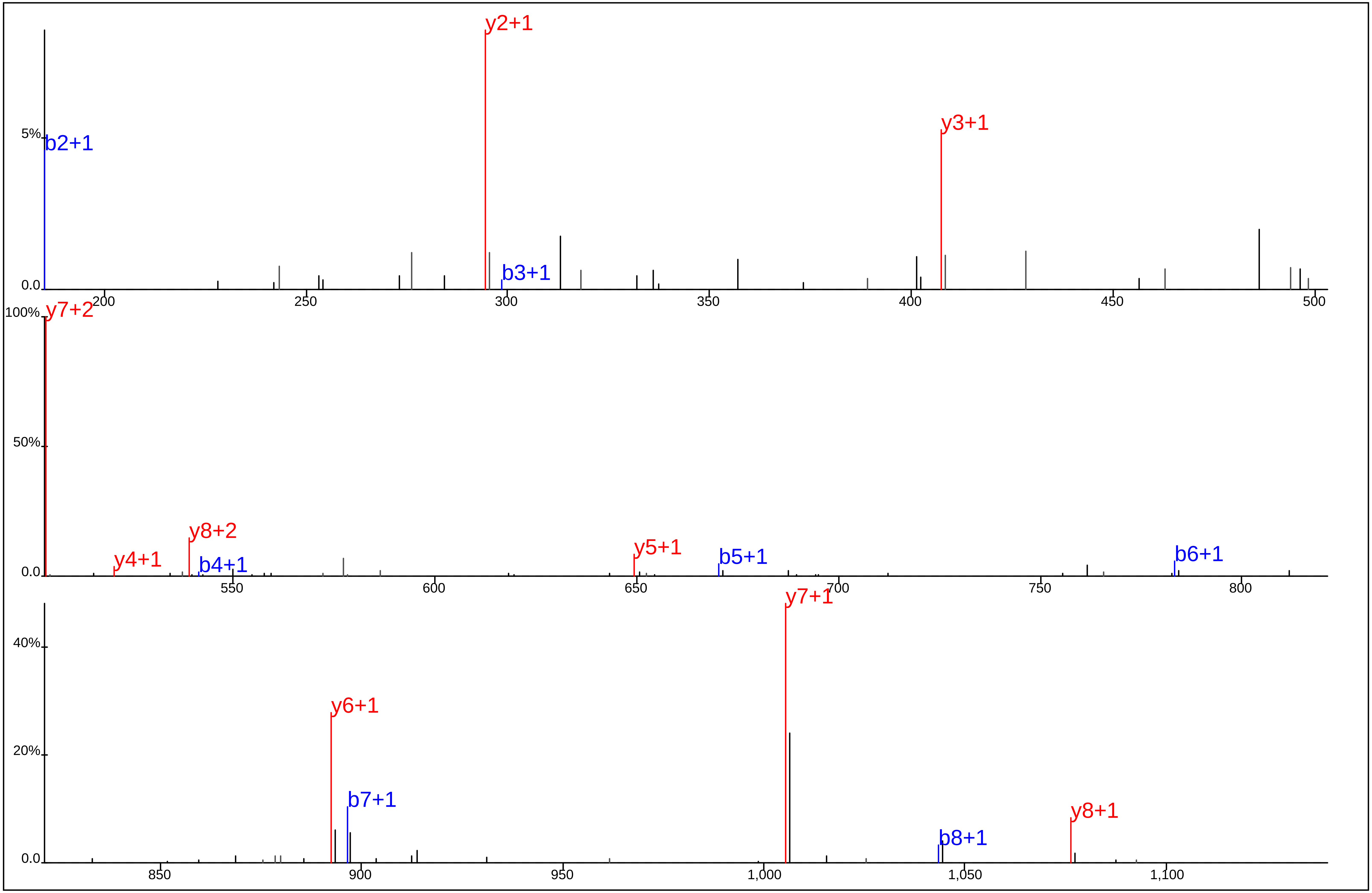

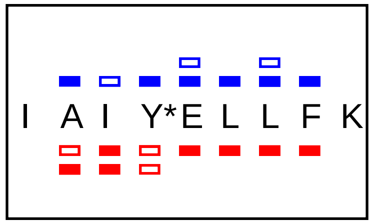

Protein Name:	Ribosomal protein S10
gi Accession:	3088338
Precursor Mass:	595.3190+2
Delta Mass:	3.243 ppm
XCorr:	41.06

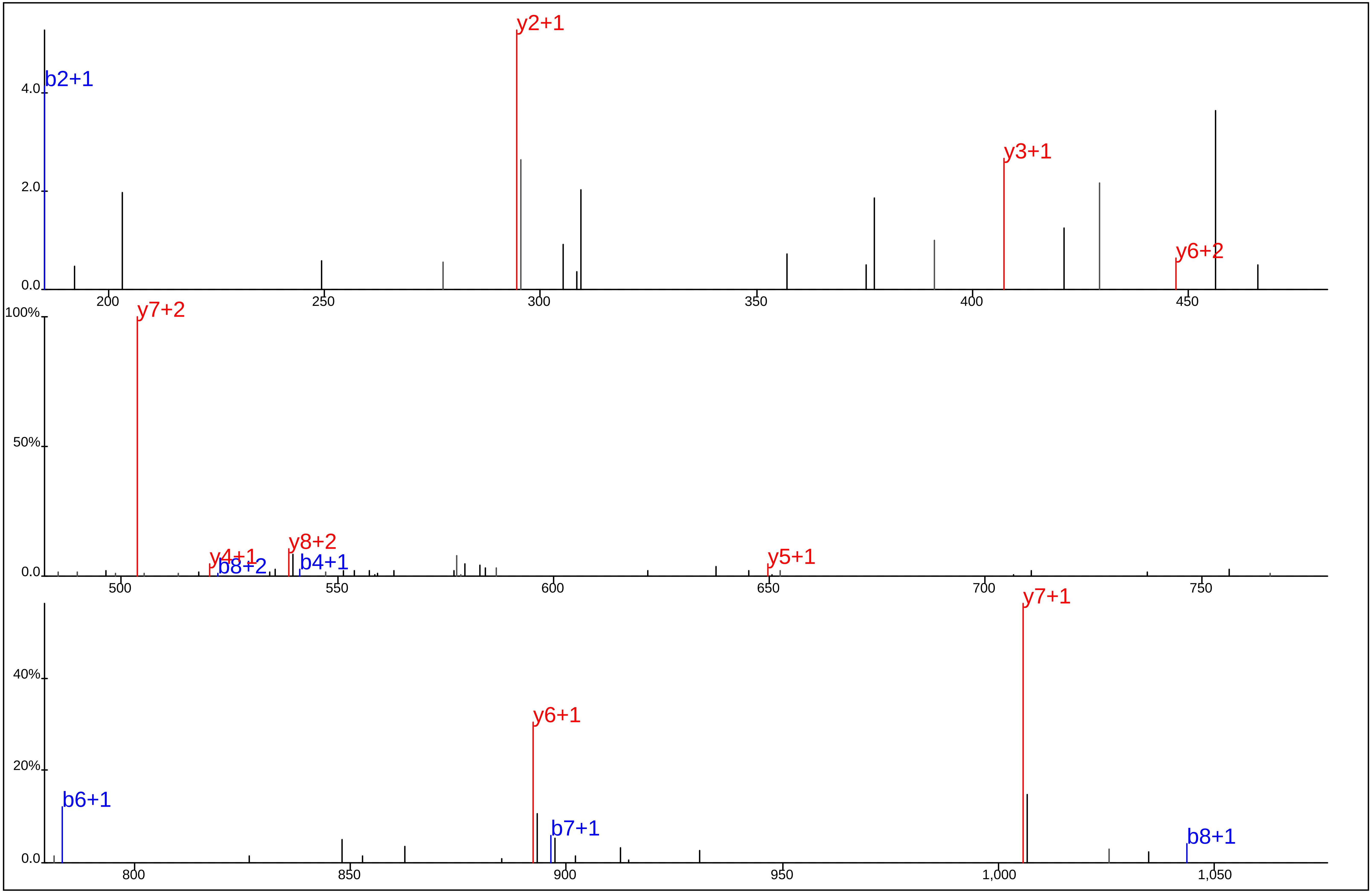

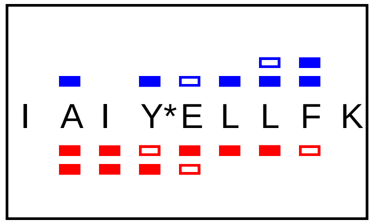

Protein Name: Ribosomal protein S10
gi Accession: 3088338
Precursor Mass: 595.3189+2
Delta Mass: 3.142 ppm
XCorr: 31.92

858 - RibosomalproteinS10_3441477.pdf

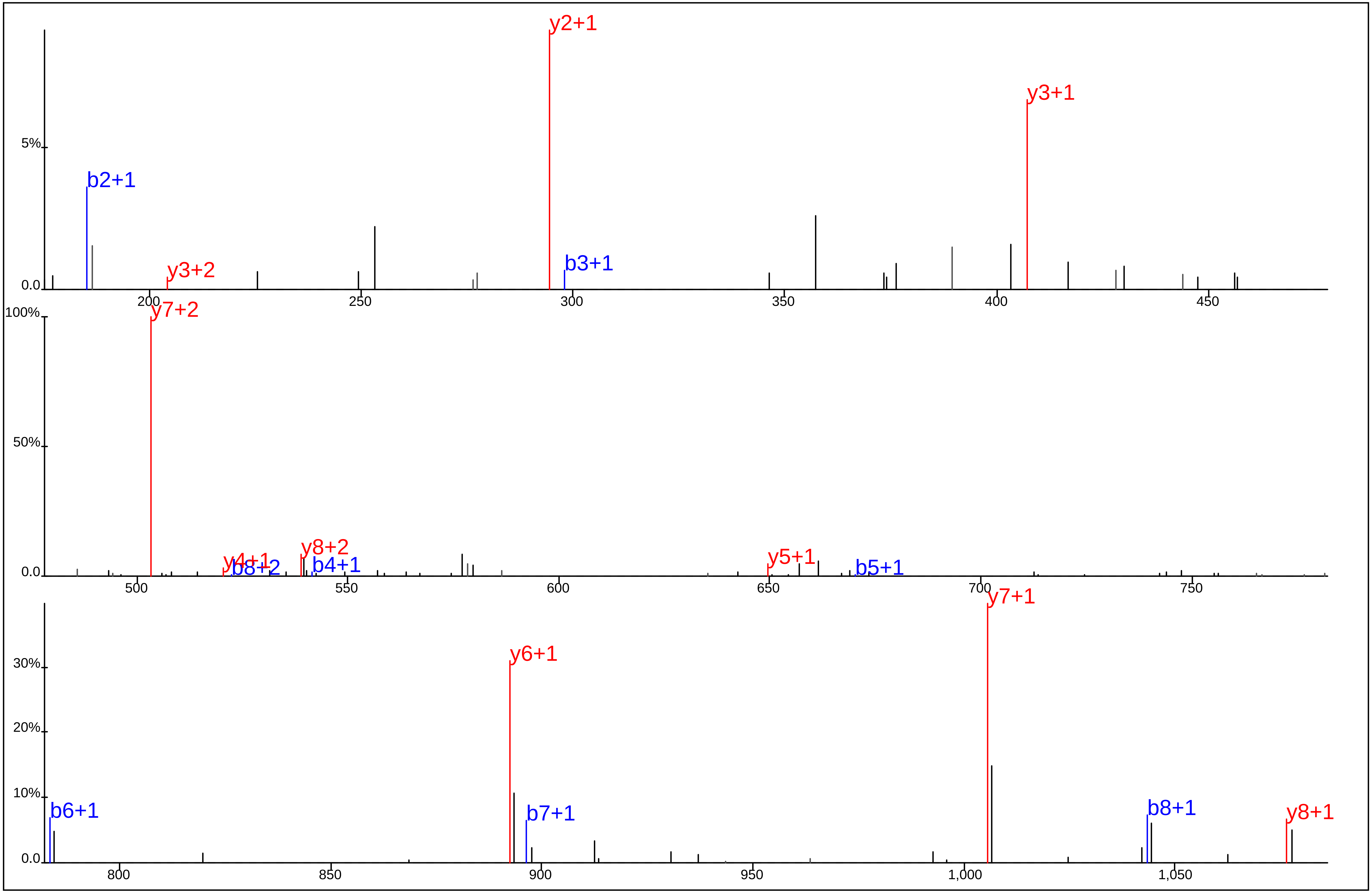

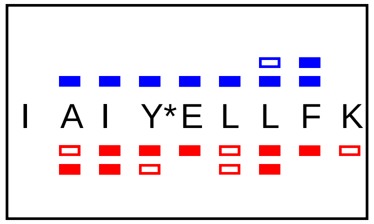

Protein Name:	Ribosomal protein S10
gi Accession:	3088338
Precursor Mass:	595.3197+2
Delta Mass:	4.479 ppm
XCorr:	44.79

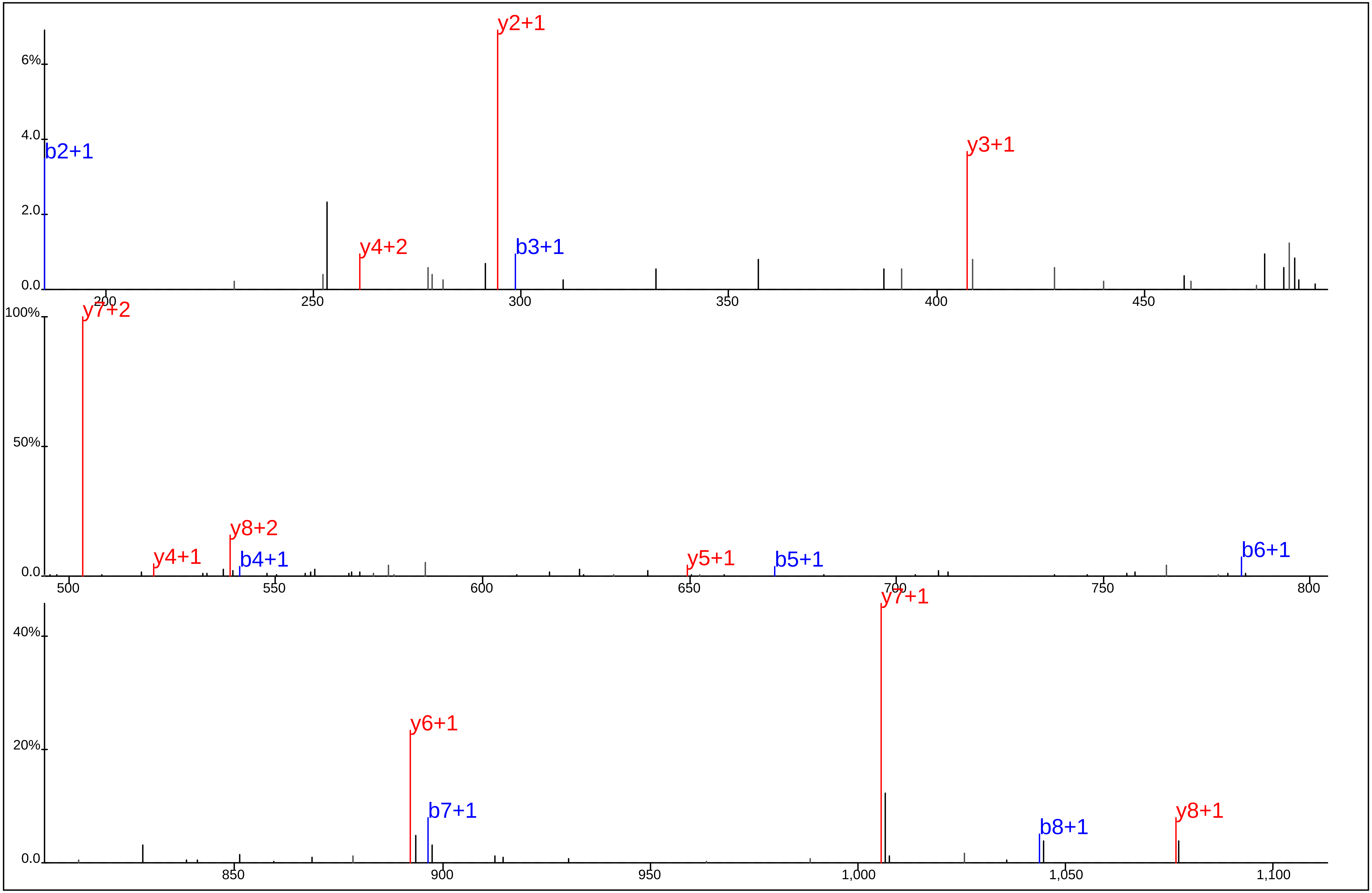

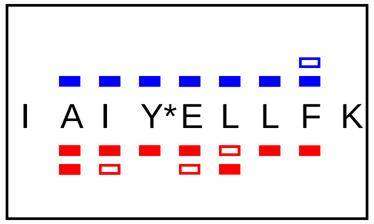

Protein Name:	Ribosomal protein S10
gi Accession:	3088338
Precursor Mass:	595.3200+2
Delta Mass:	4.991 ppm
XCorr:	50.34

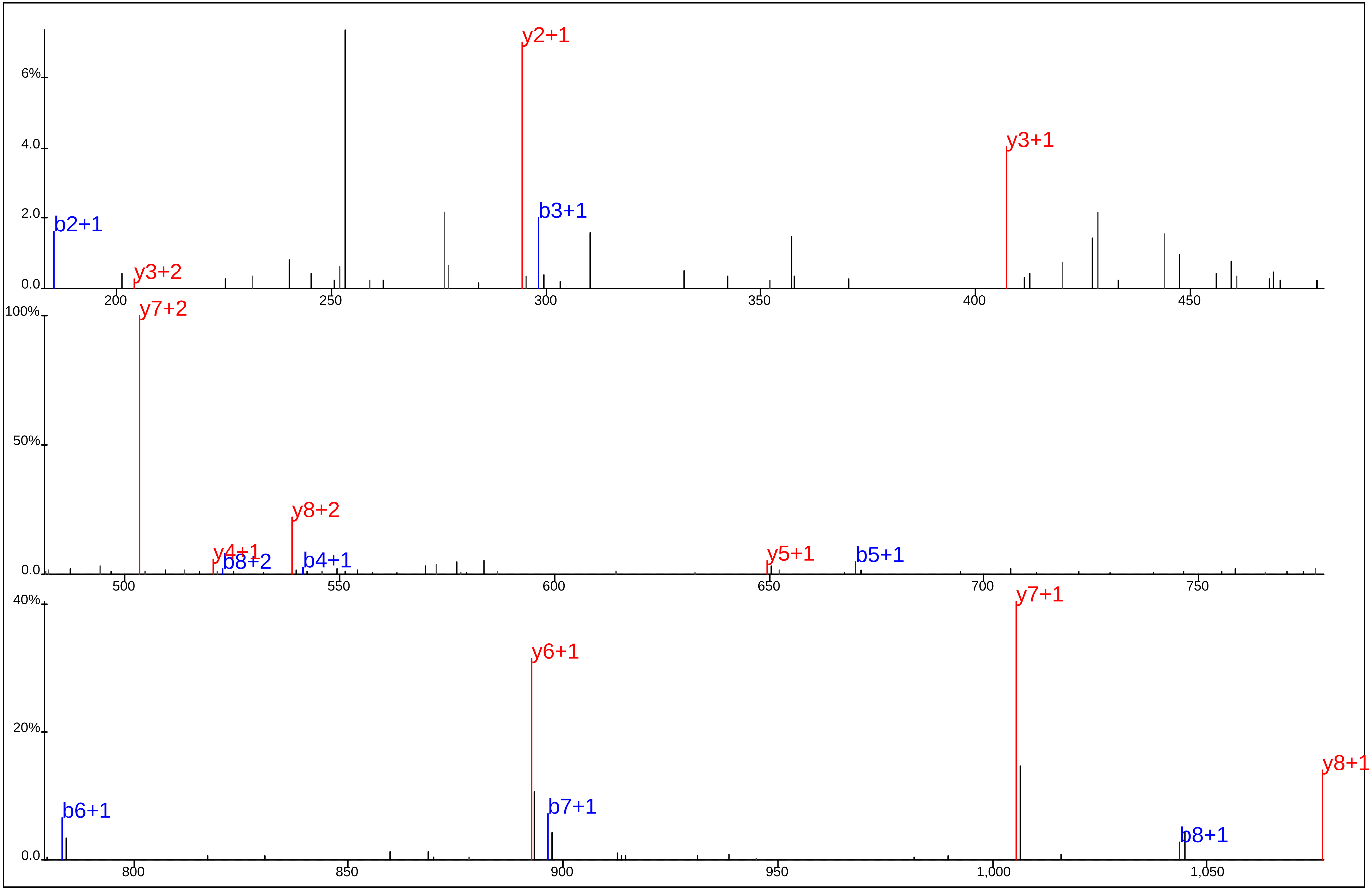

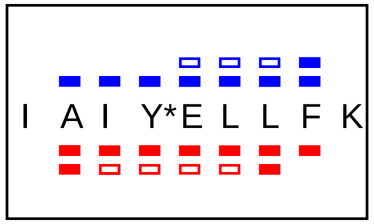

Protein Name:	Ribosomal protein S10
gi Accession:	3088338
Precursor Mass:	595.3203+2
Delta Mass:	5.403 ppm
XCorr:	44.21

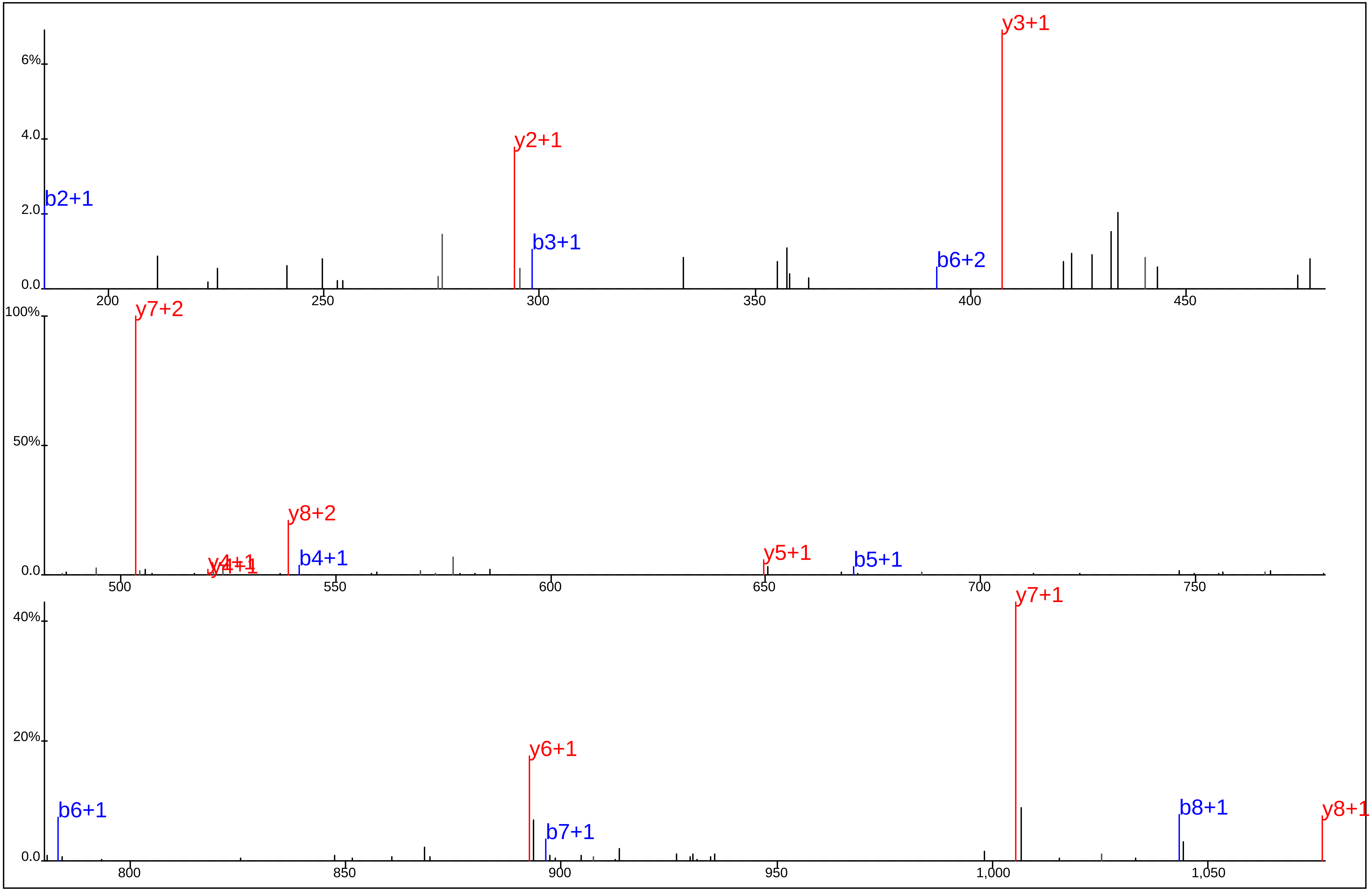

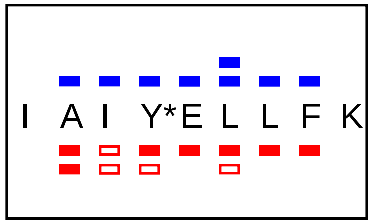

Protein Name: Ribosomal protein S10
gi Accession: 3088338
Precursor Mass: 595.3206+2
Delta Mass: 5.916 ppm
XCorr: 44.8

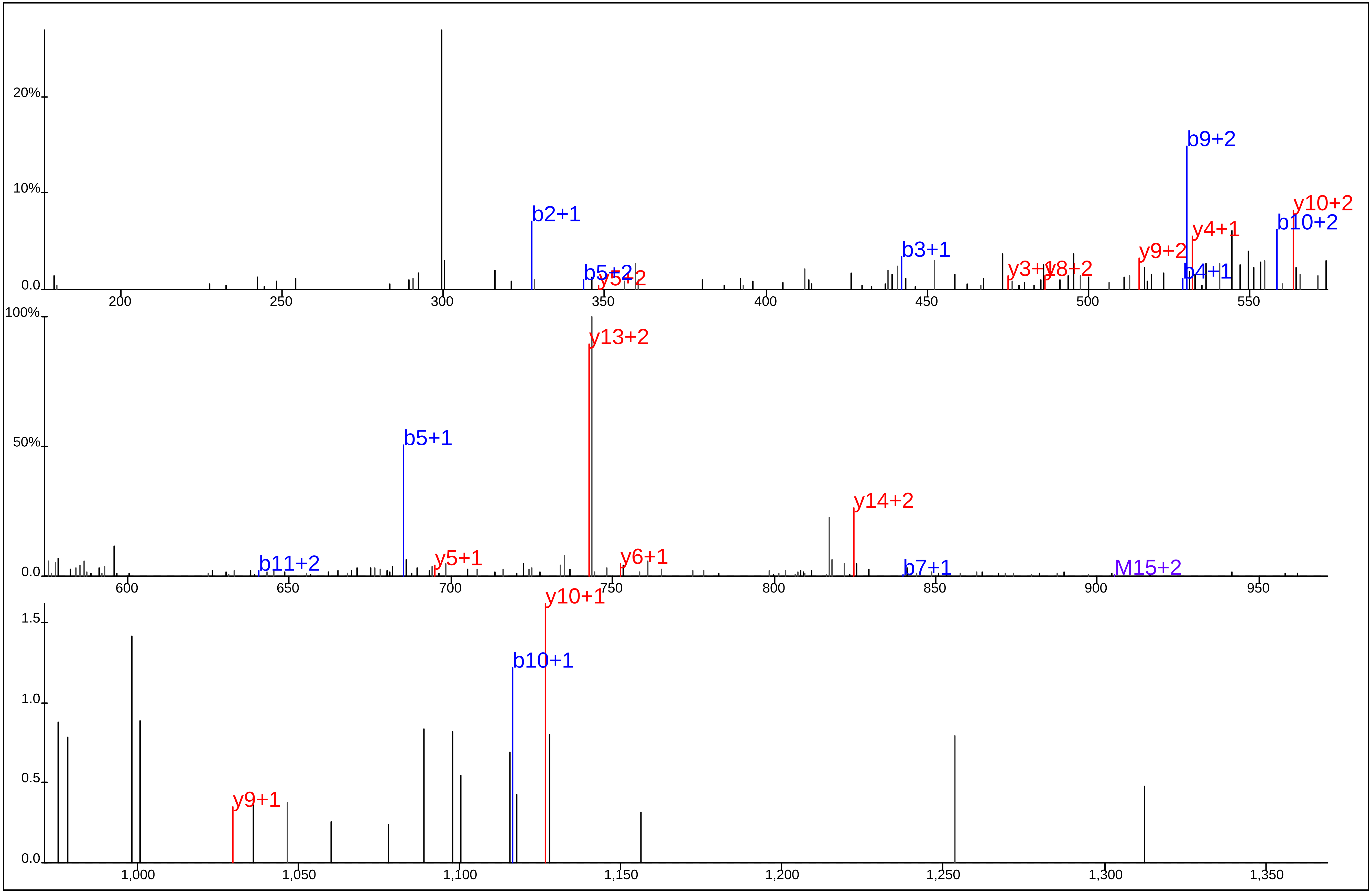

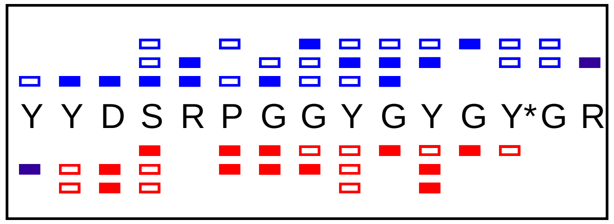

Protein Name:	RNA binding motif protein 3
gi Accession:	5803137
Precursor Mass:	604.2449+3
Delta Mass:	1.116 ppm
XCorr:	2.816

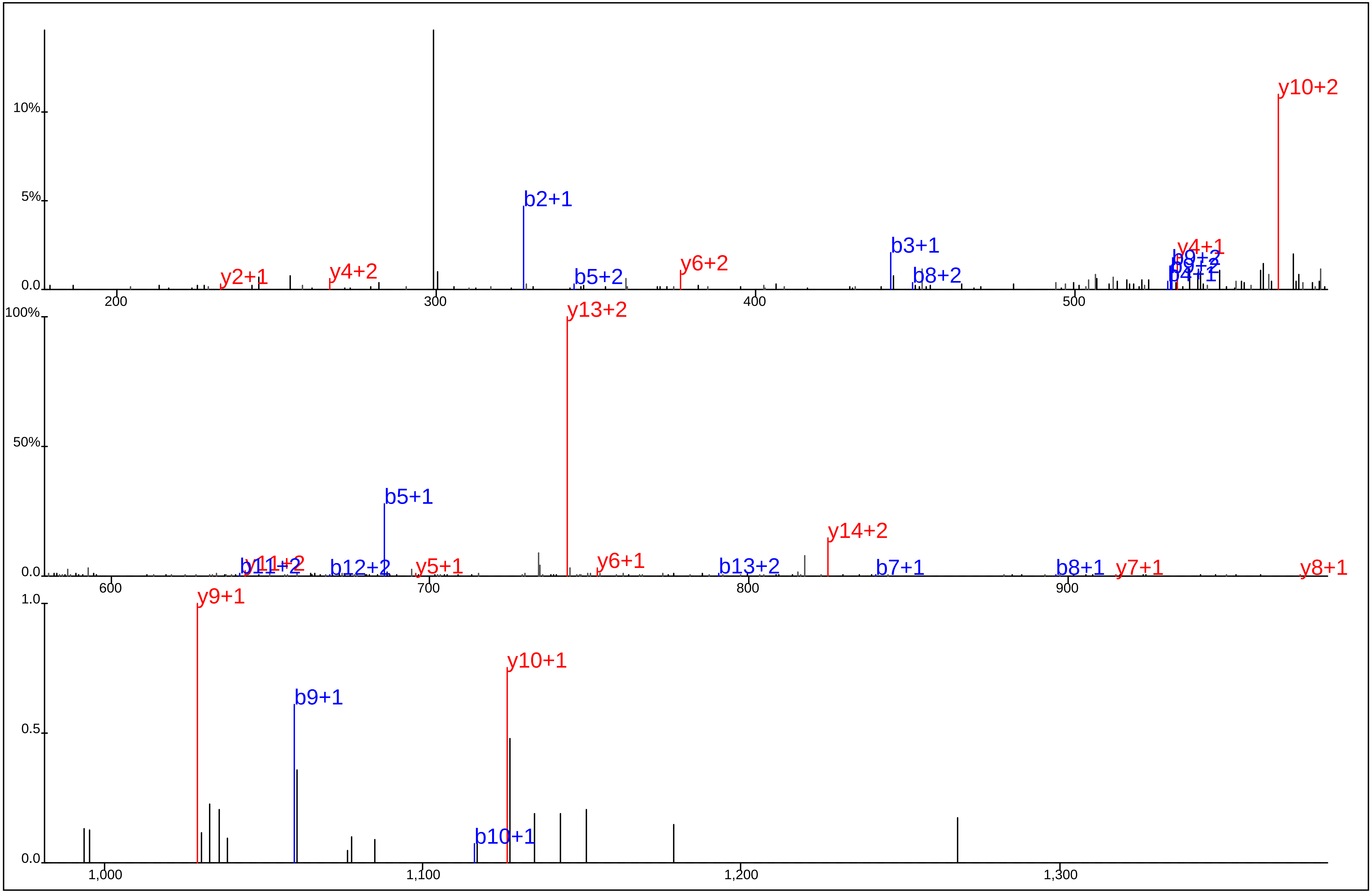

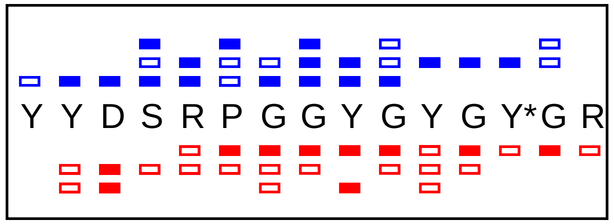

Protein Name:	RNA binding motif protein 3
gi Accession:	5803137
Precursor Mass:	604.2471+3
Delta Mass:	2.534 ppm
XCorr:	33.2

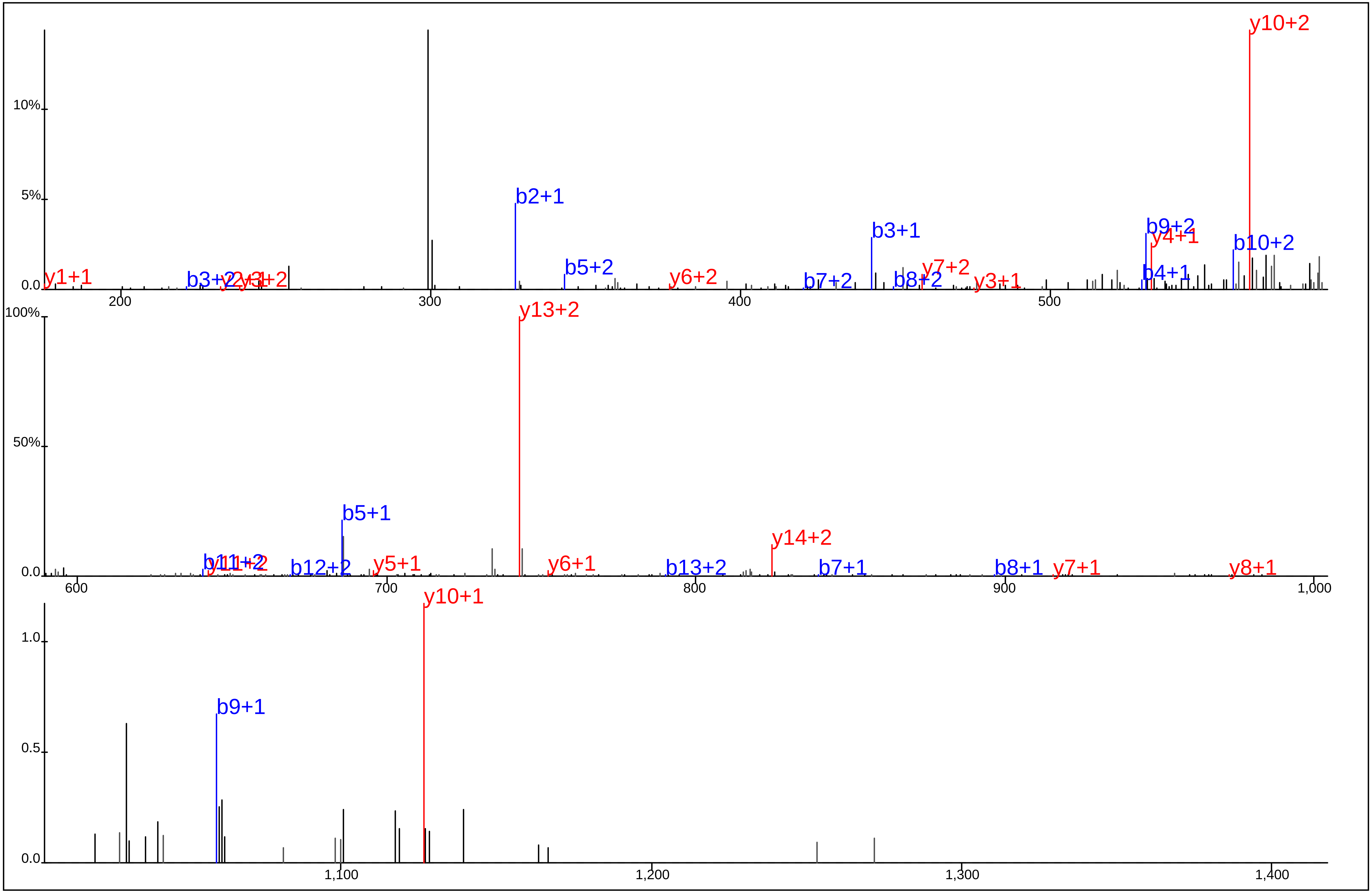

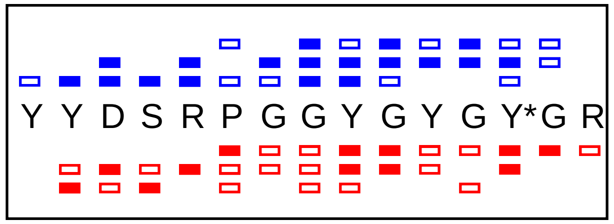

Protein Name:	RNA binding motif protein 3
gi Accession:	5803137
Precursor Mass:	604.2468+3
Delta Mass:	2.130 ppm
XCorr:	27.02

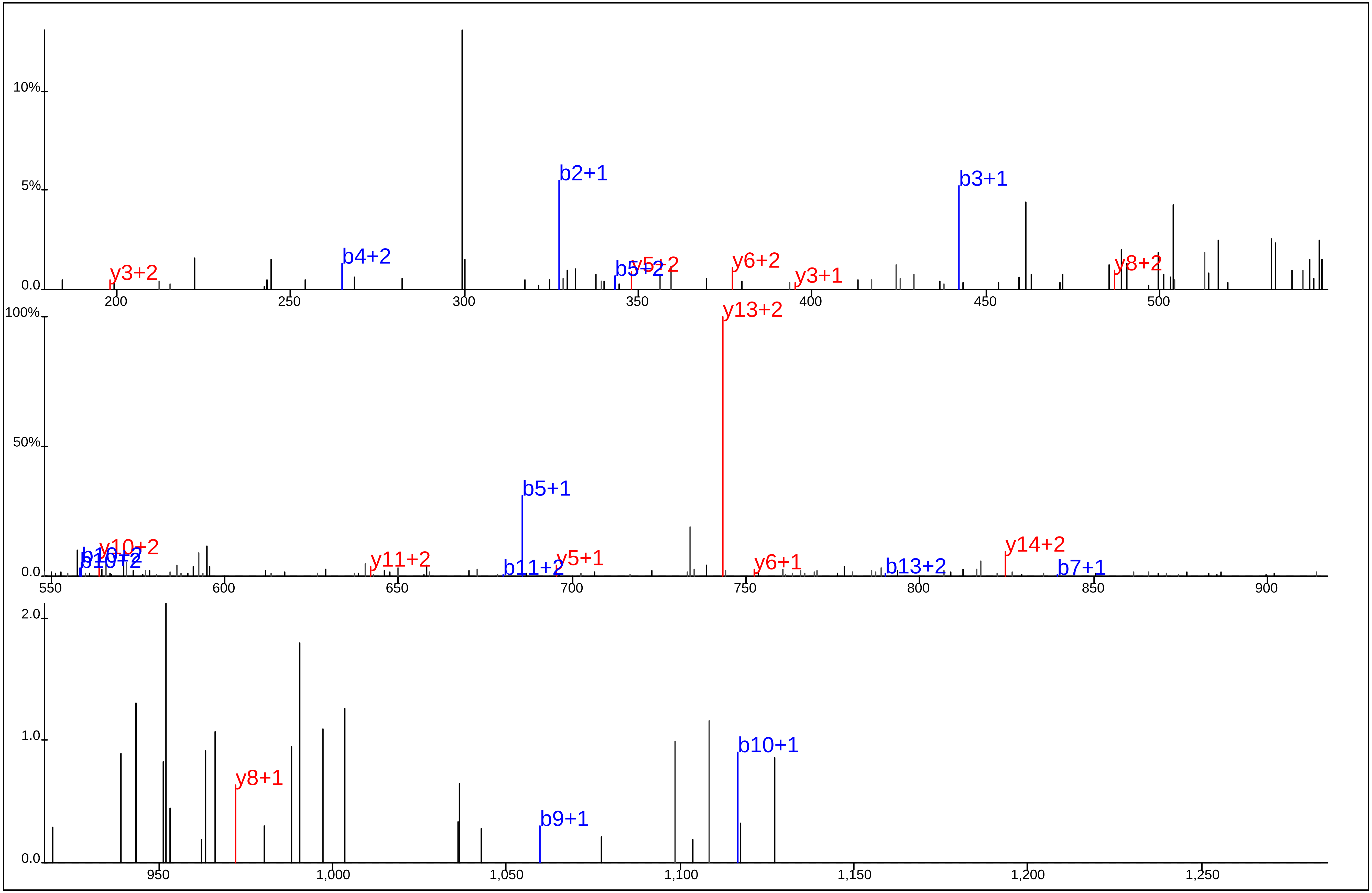

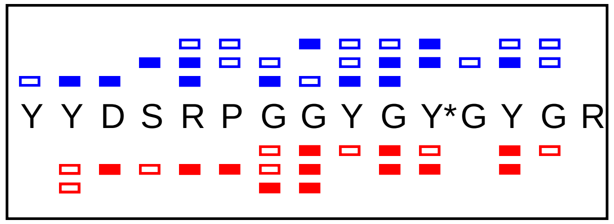

Protein Name: RNA binding motif protein 3
gi Accession: 5803137
Precursor Mass: 604.2498+3
Delta Mass: 6.980 ppm
XCorr: 15.69

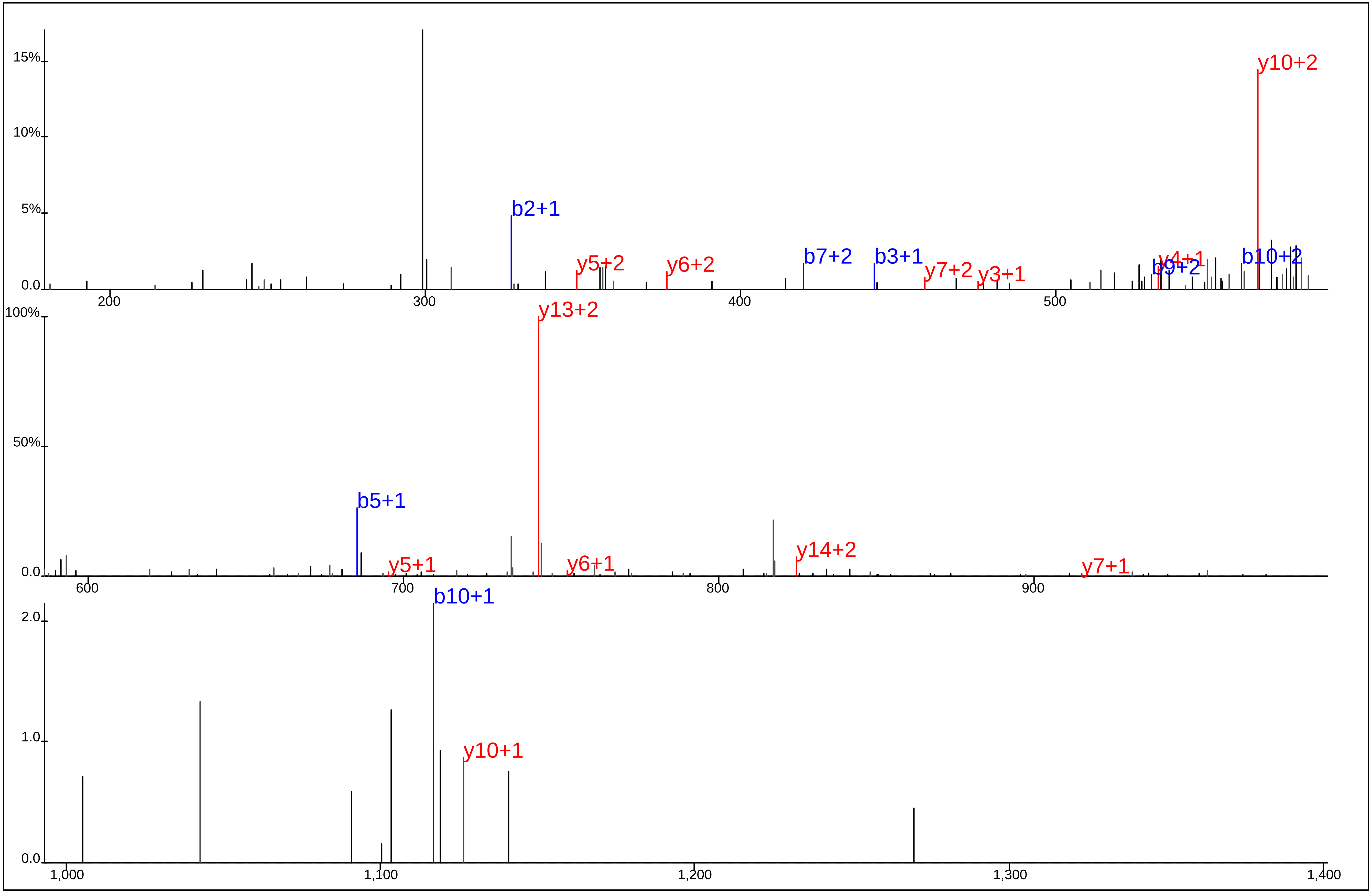

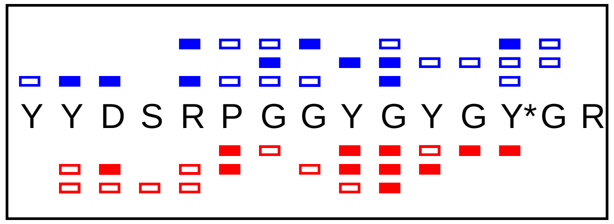

Protein Name: RNA binding motif protein 3
gi Accession: 5803137
Precursor Mass: 604.2460+3
Delta Mass: 0.811 ppm
XCorr: 14.16

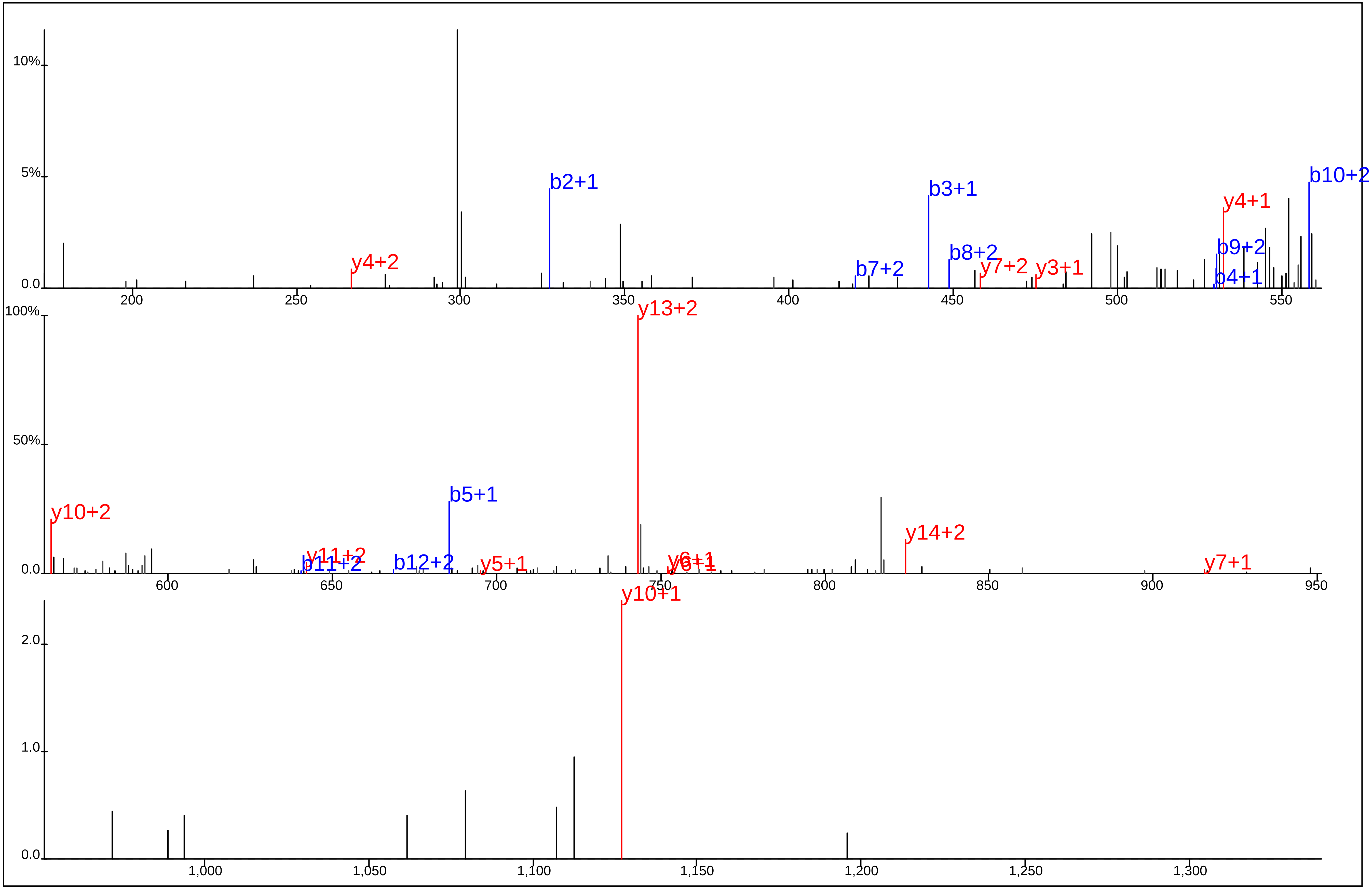

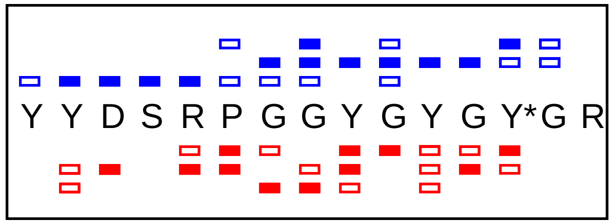

Protein Name: RNA binding motif protein 3
gi Accession: 5803137
Precursor Mass: 604.2426+3
Delta Mass: 4.850 ppm
XCorr: 18.3

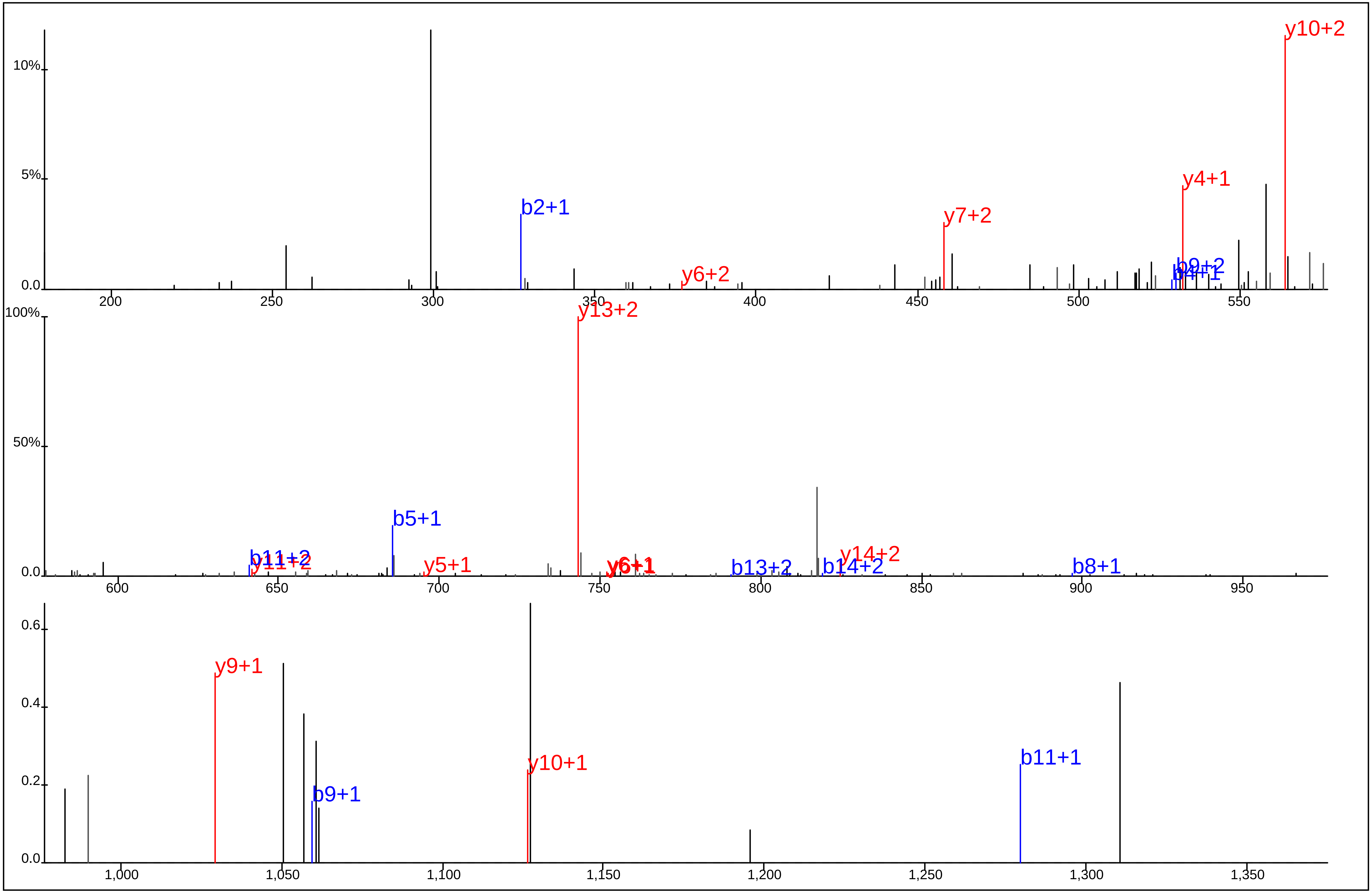

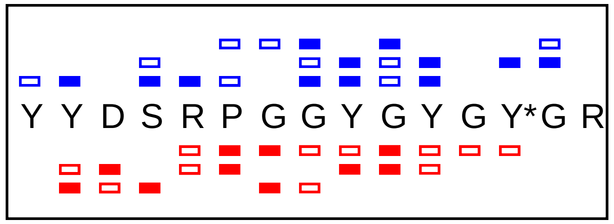

Protein Name:	RNA binding motif protein 3
gi Accession:	5803137
Precursor Mass:	604.2462+3
Delta Mass:	1.114 ppm
XCorr:	22.19

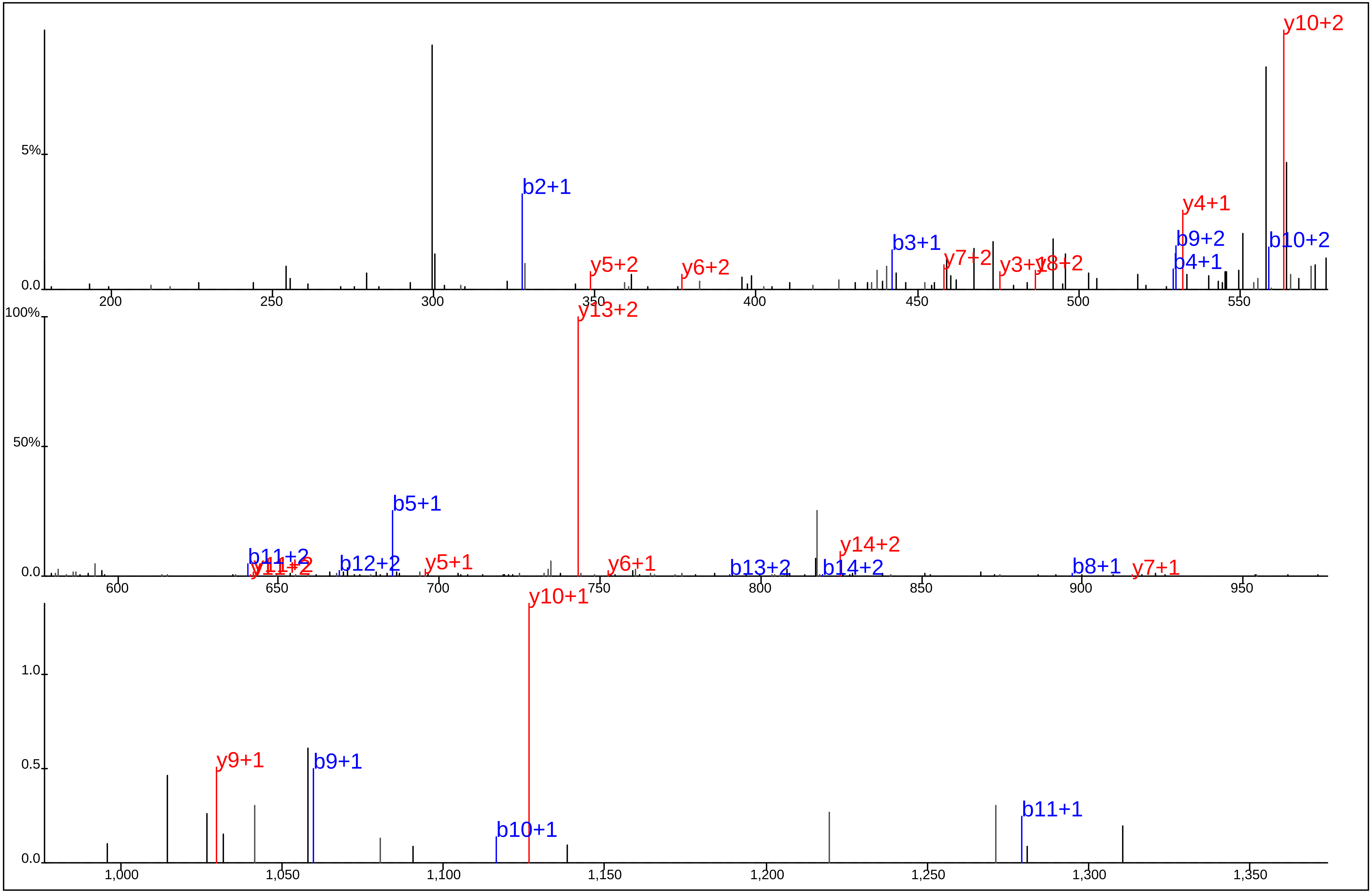

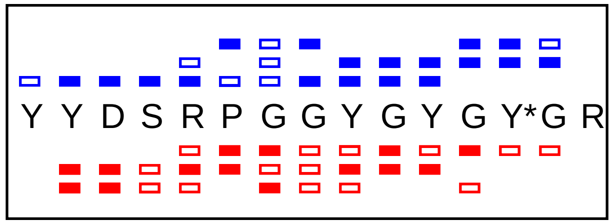

Protein Name:	RNA binding motif protein 3
gi Accession:	5803137
Precursor Mass:	604.2468+3
Delta Mass:	2.130 ppm
XCorr:	26.3

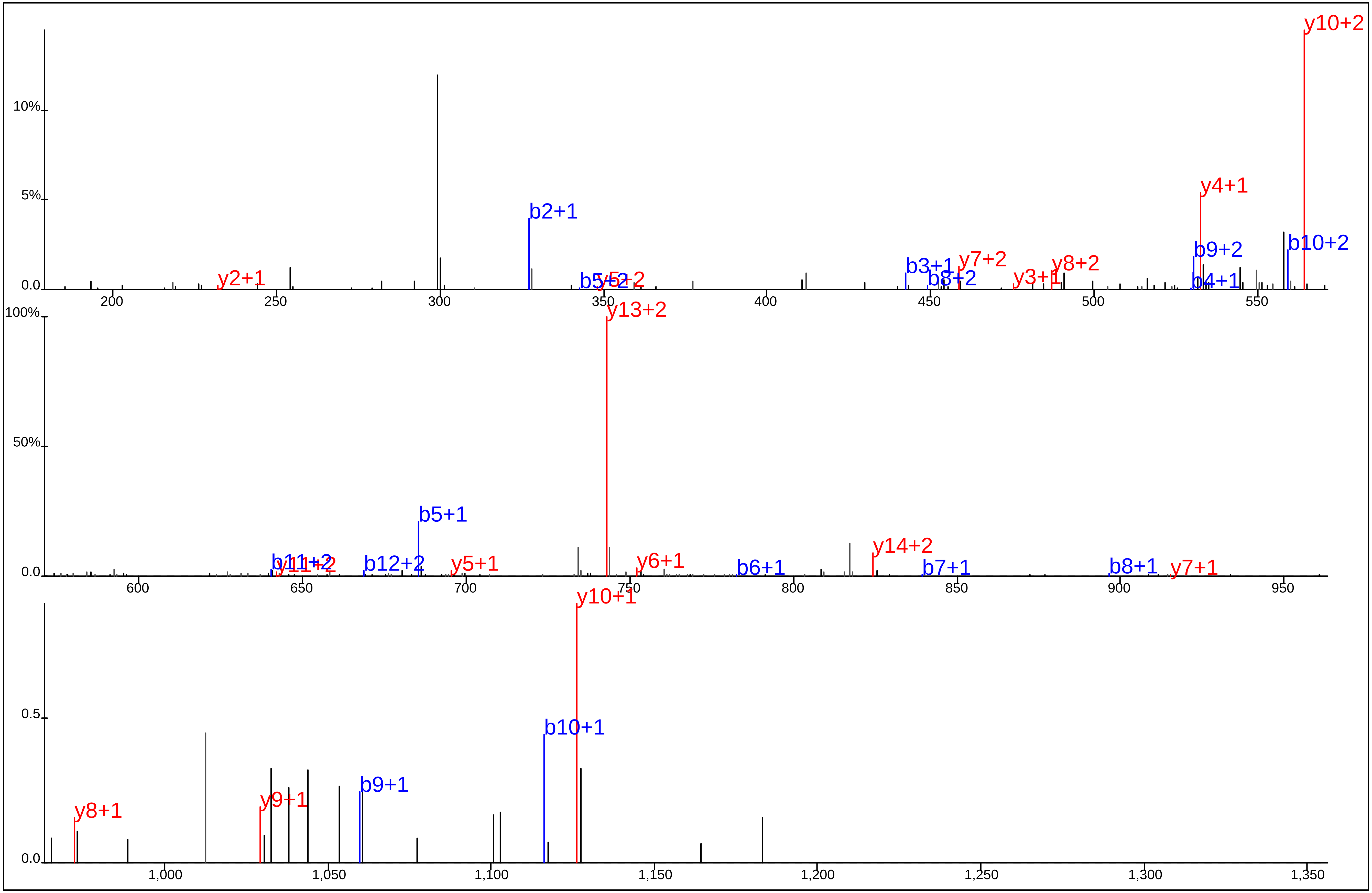

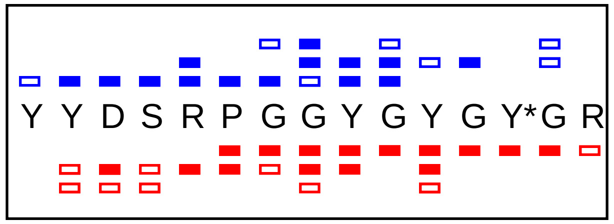

Protein Name:	RNA binding motif protein 3
gi Accession:	5803137
Precursor Mass:	604.2476+3
Delta Mass:	3.446 ppm
XCorr:	36.76

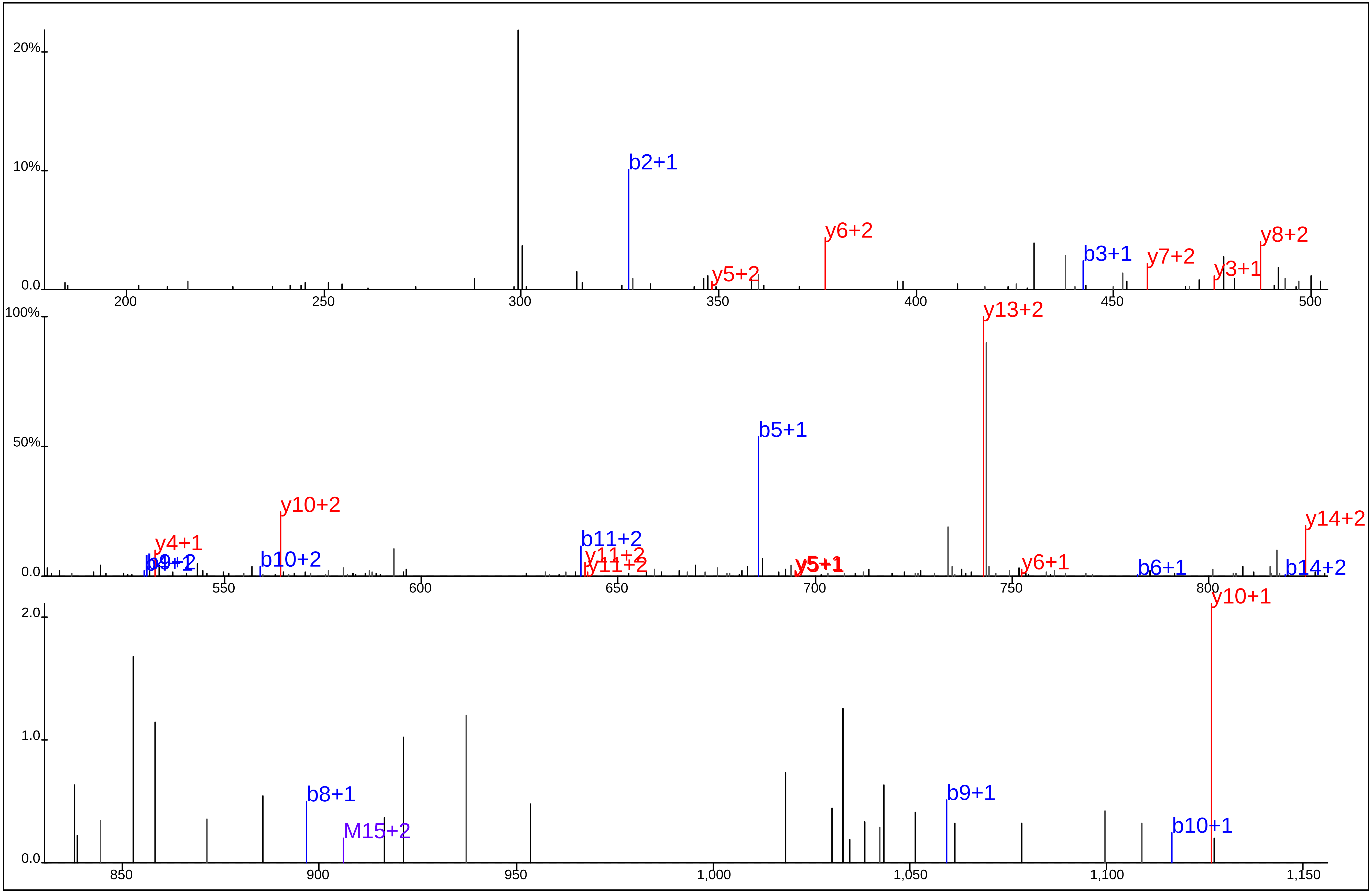

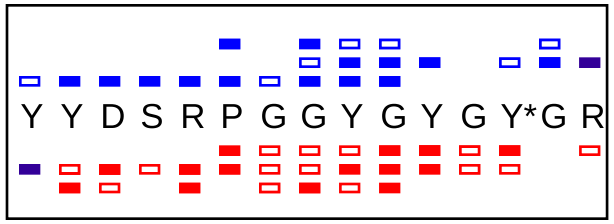

Protein Name:	RNA binding motif protein 3
gi Accession:	5803137
Precursor Mass:	604.2469+3
Delta Mass:	2.229 ppm
XCorr:	29.49

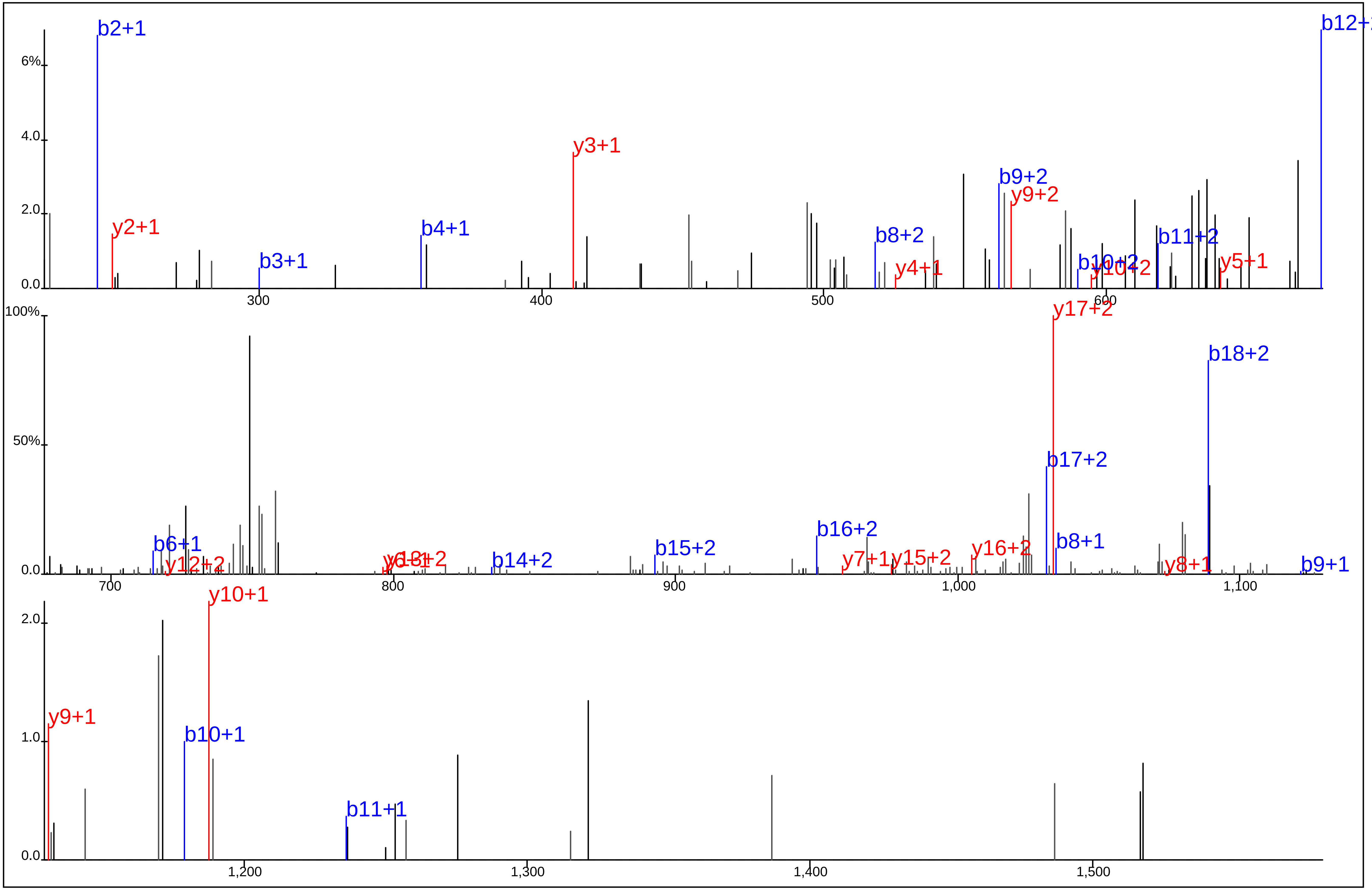

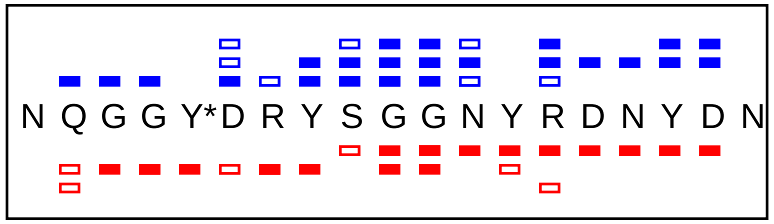

Protein Name: RNA binding motif protein 3
gi Accession: 5803137
Precursor Mass: 769.9658+3
Delta Mass: 6.165 ppm
XCorr: 30.75

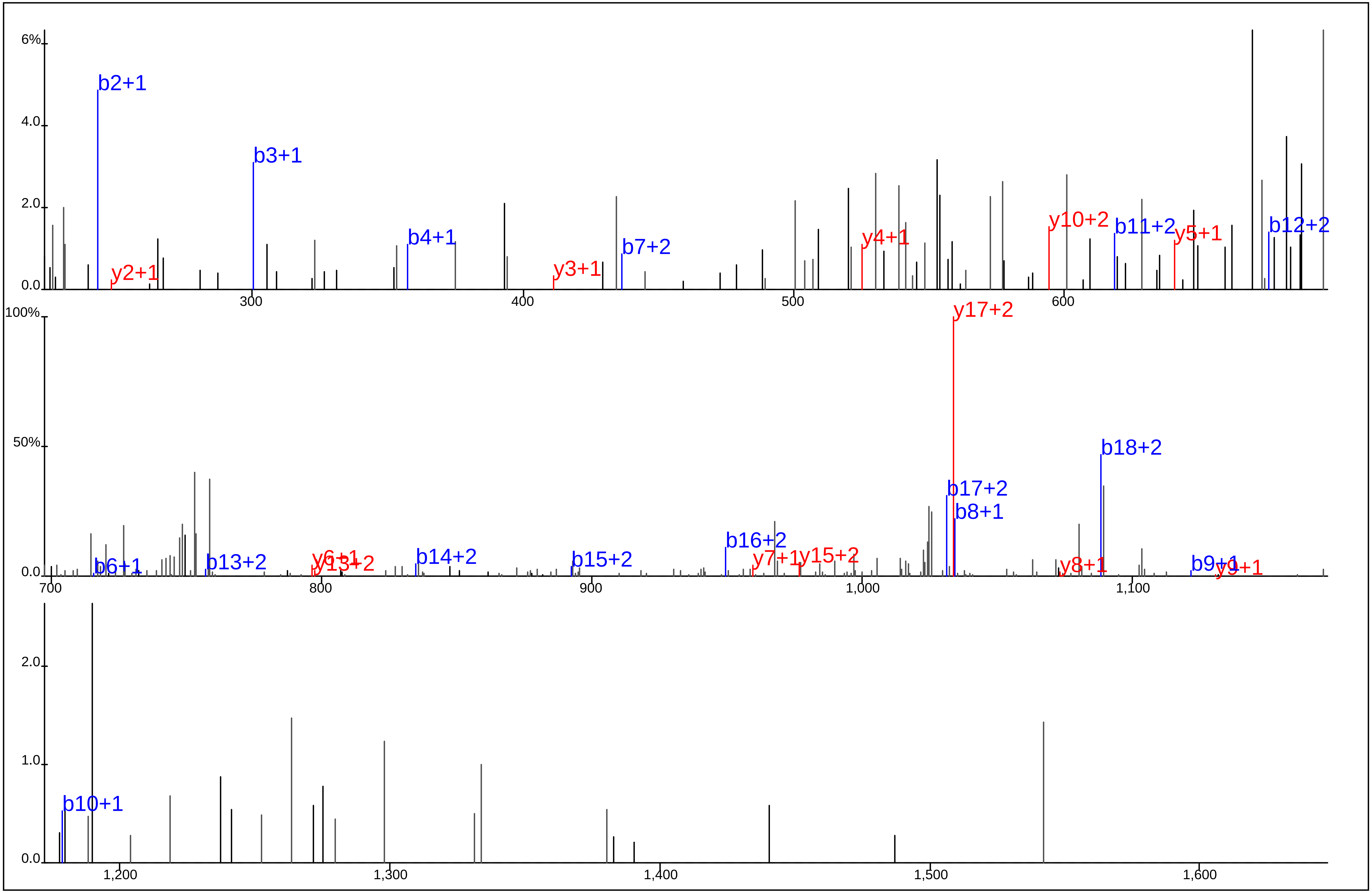

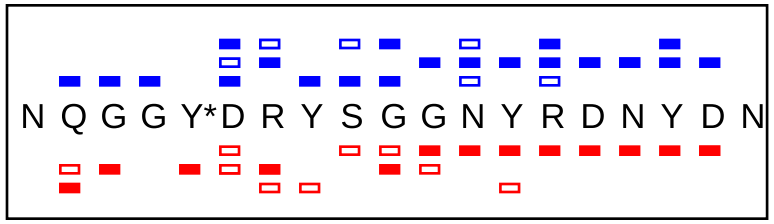

Protein Name:	RNA binding motif protein 3
gi Accession:	5803137
Precursor Mass:	769.9645+3
Delta Mass:	4.496 ppm
XCorr:	12.22

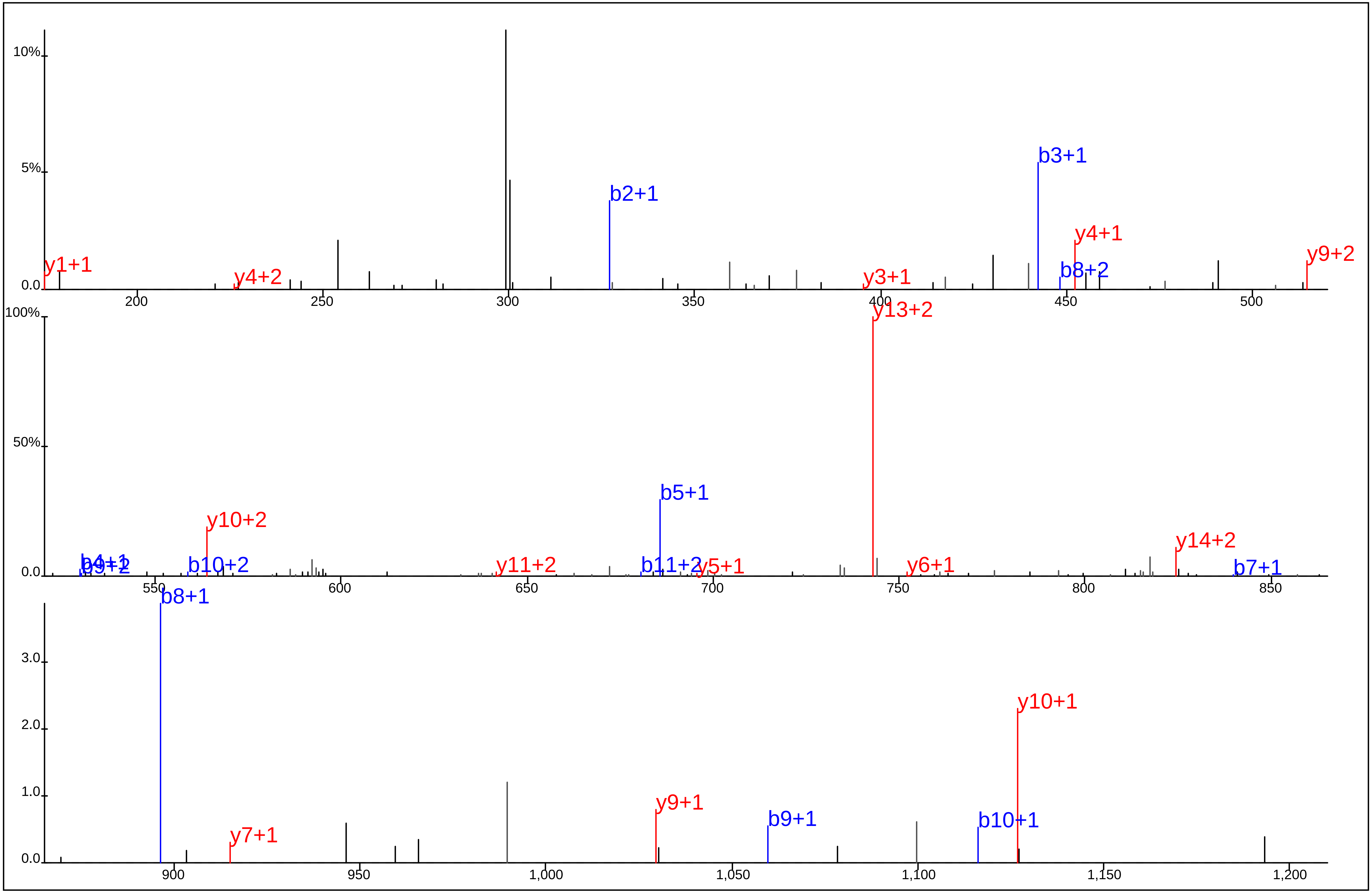

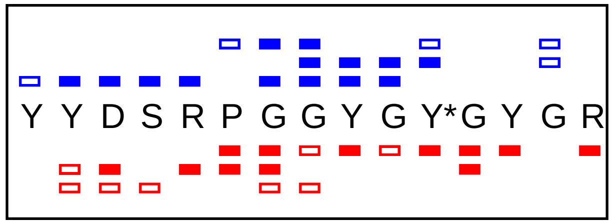

Protein Name:	RNA binding motif protein 3
gi Accession:	5803137
Precursor Mass:	604.2488+3
Delta Mass:	5.467 ppm
XCorr:	32.96

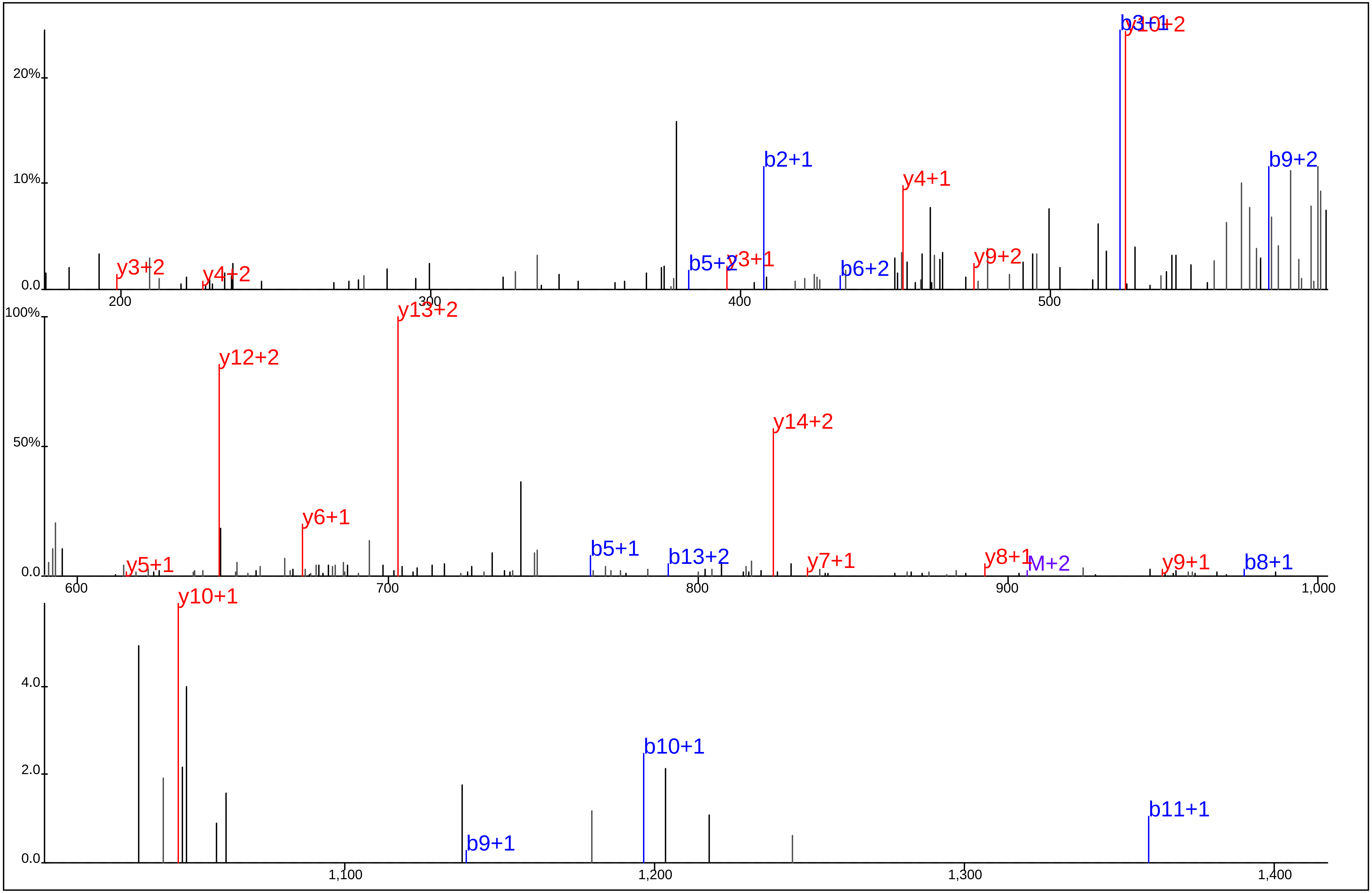

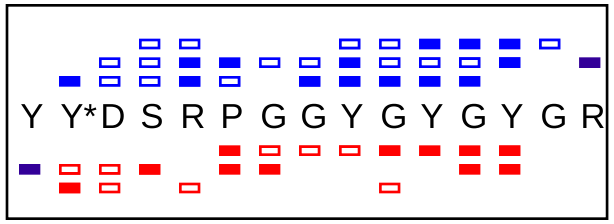

Protein Name:	RNA binding motif protein 3
gi Accession:	5803137
Precursor Mass:	604.2498+3
Delta Mass:	7.084 ppm
XCorr:	37.77

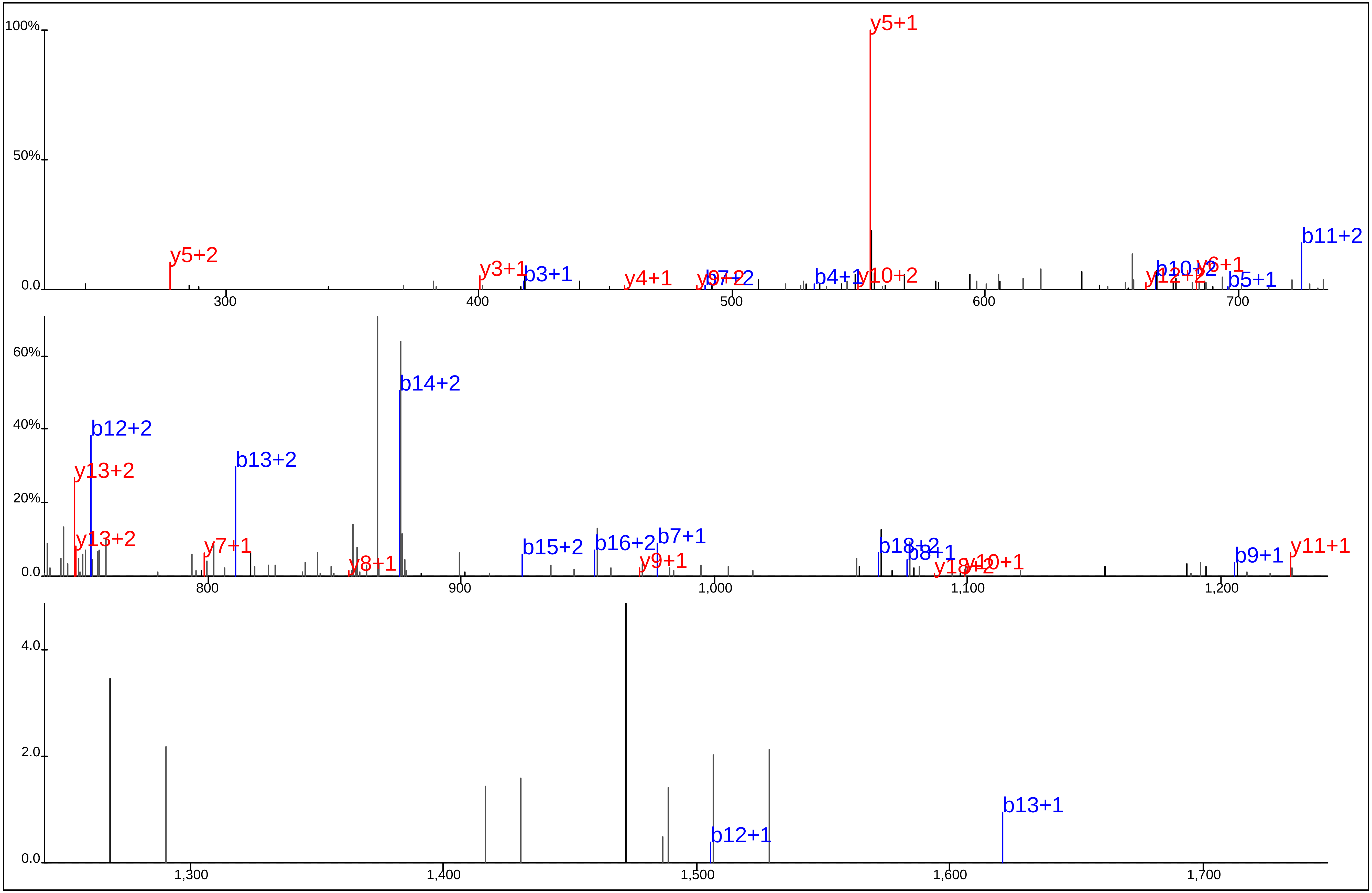

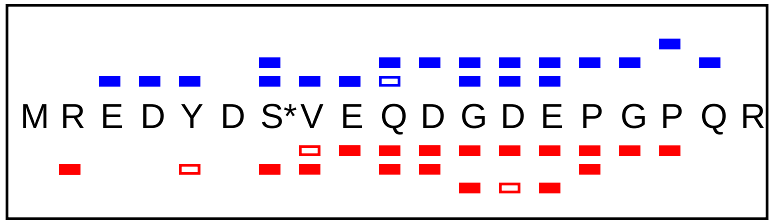

Protein Name: RNA binding motif protein 8
gi Accession: 83320121
Precursor Mass: 768.3060+3
Delta Mass: 5.014 ppm
XCorr: 31.75

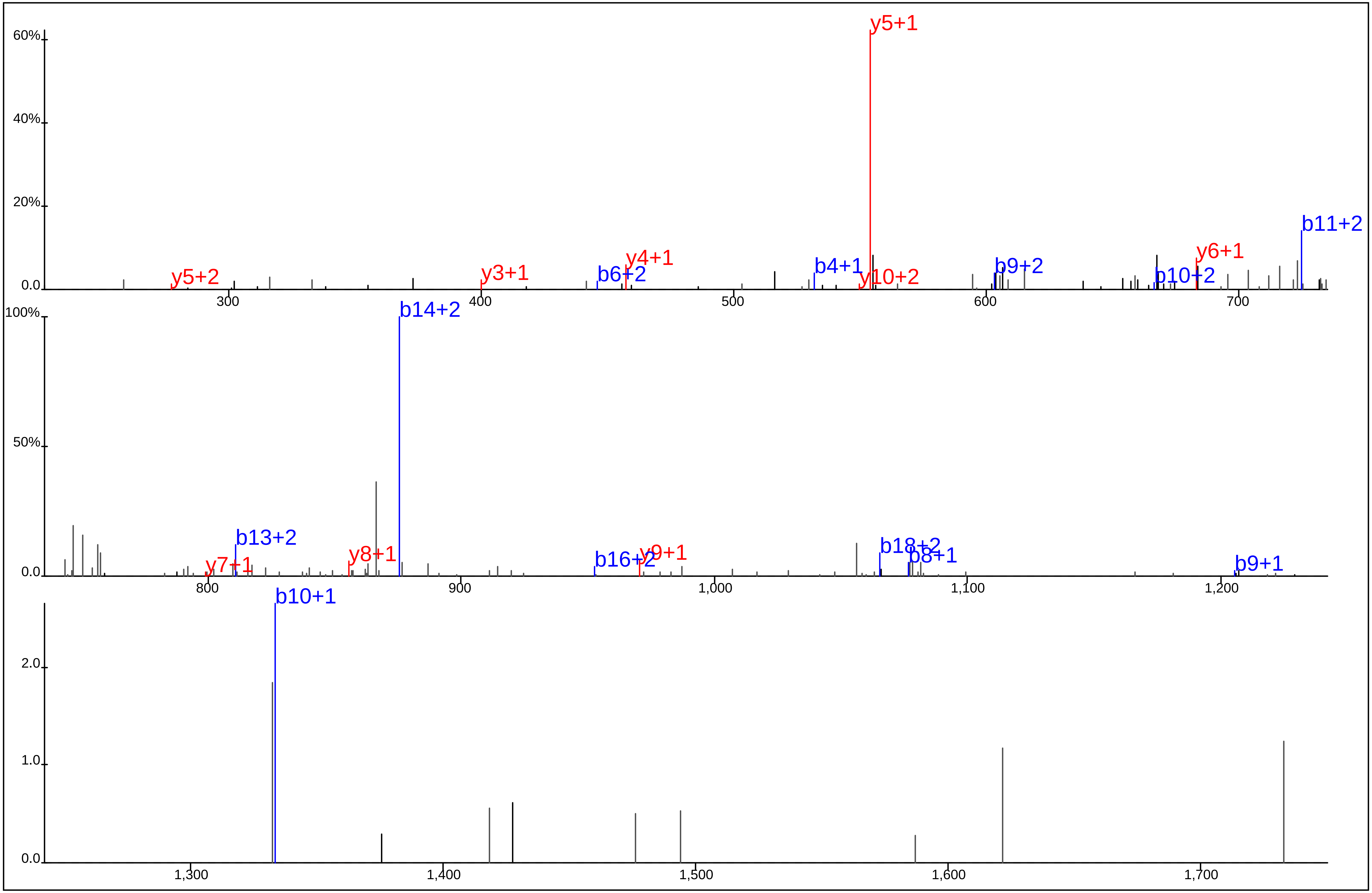

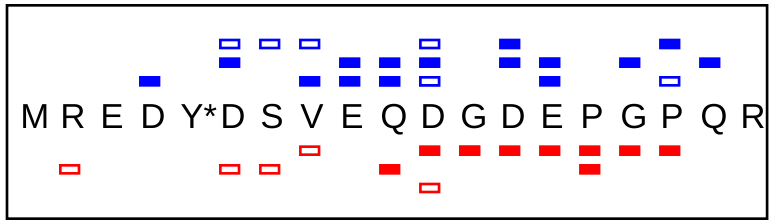

Protein Name:	RNA binding motif protein 8
gi Accession:	83320121
Precursor Mass:	768.3036+3
Delta Mass:	1.914 ppm
XCorr:	25.79

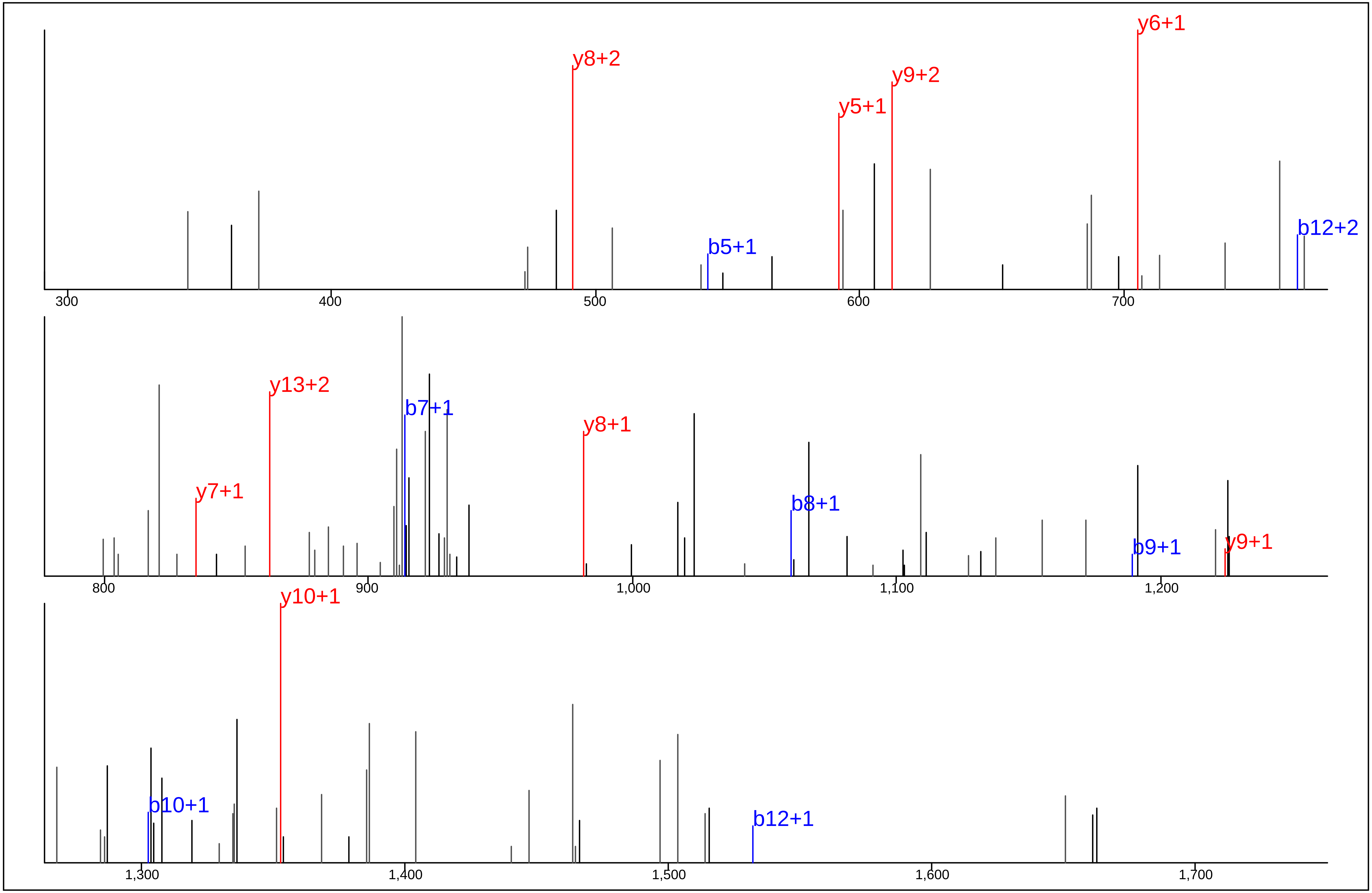

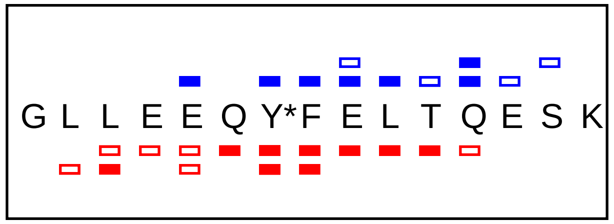

Protein Name: ROCK1
gi Accession: 4885583
Precursor Mass: 947.4275+2
Delta Mass: 2.129 ppm
XCorr: 12.44

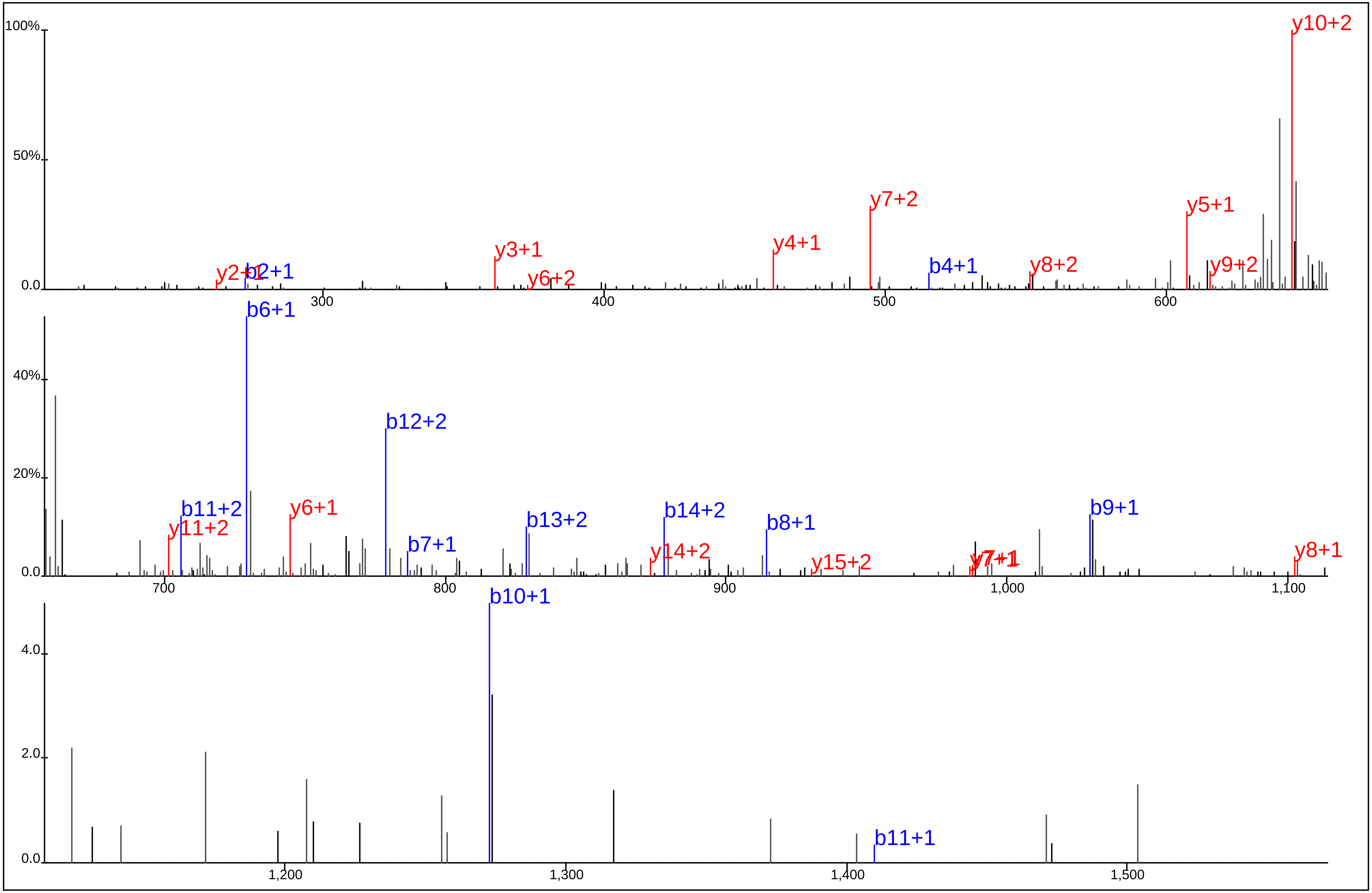

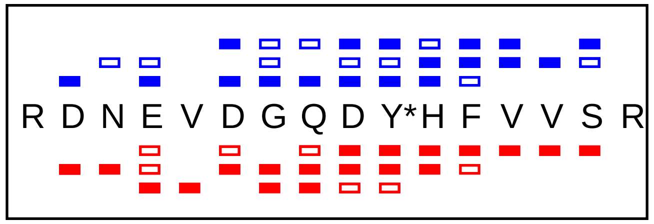

Protein Name: SAP102
gi Accession: 119943106
Precursor Mass: 672.6221+3
Delta Mass: 4.490 ppm
XCorr: 28.73

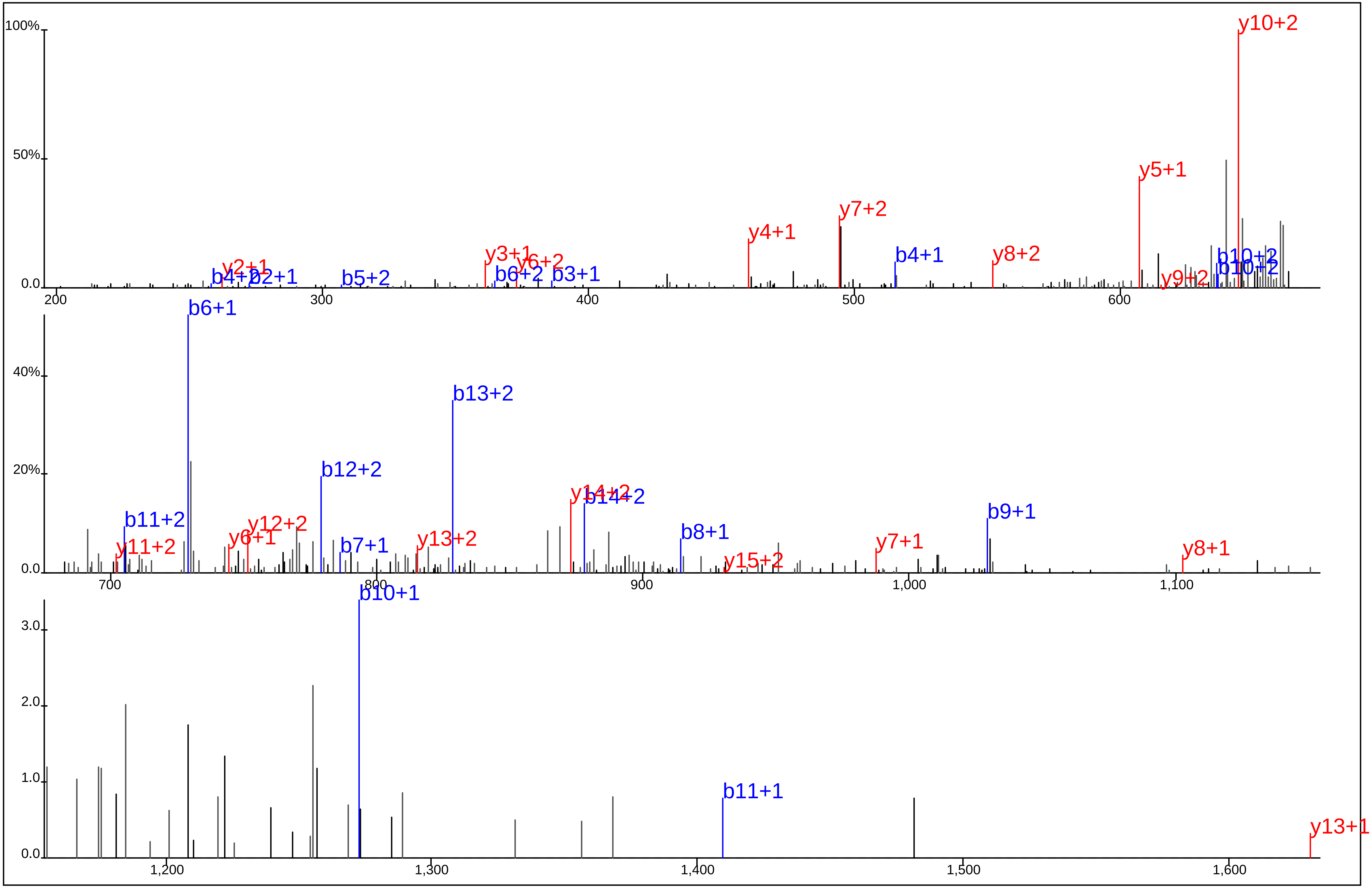

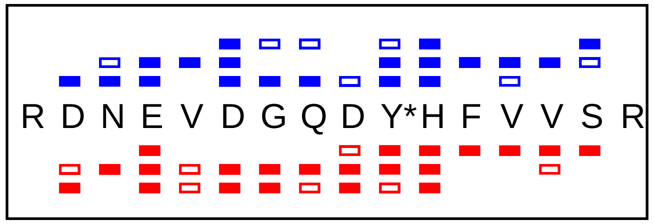

Protein Name: SAP102
gi Accession: 119943106
Precursor Mass: 672.6282+3
Delta Mass: 4.682 ppm
XCorr: 38.85

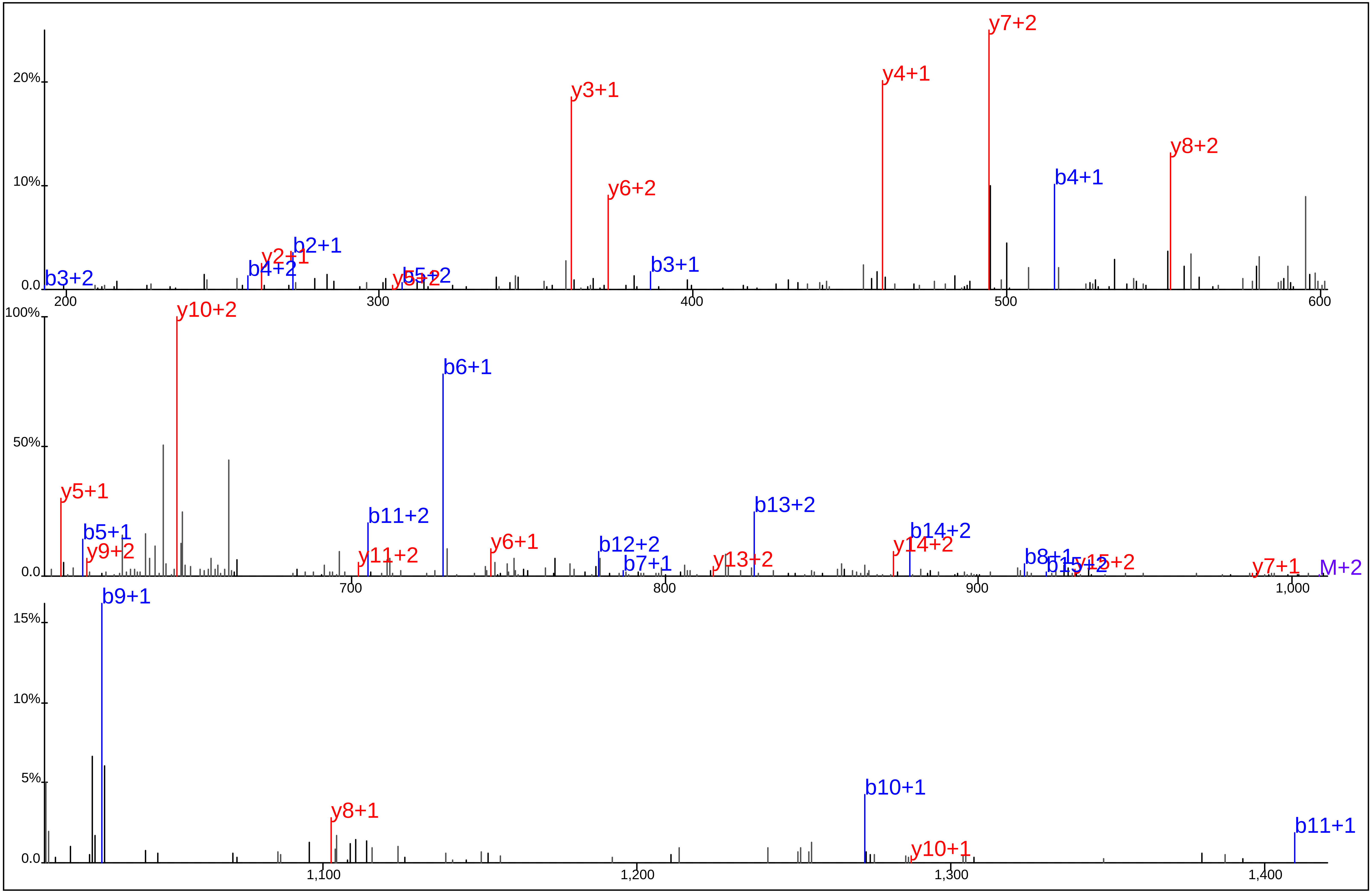

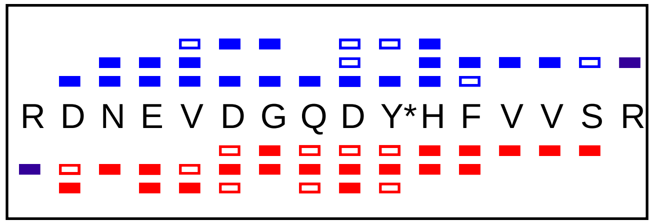

Protein Name: SAP102
gi Accession: 119943106
Precursor Mass: 672.6309+3
Delta Mass: 8.585 ppm
XCorr: 43.9

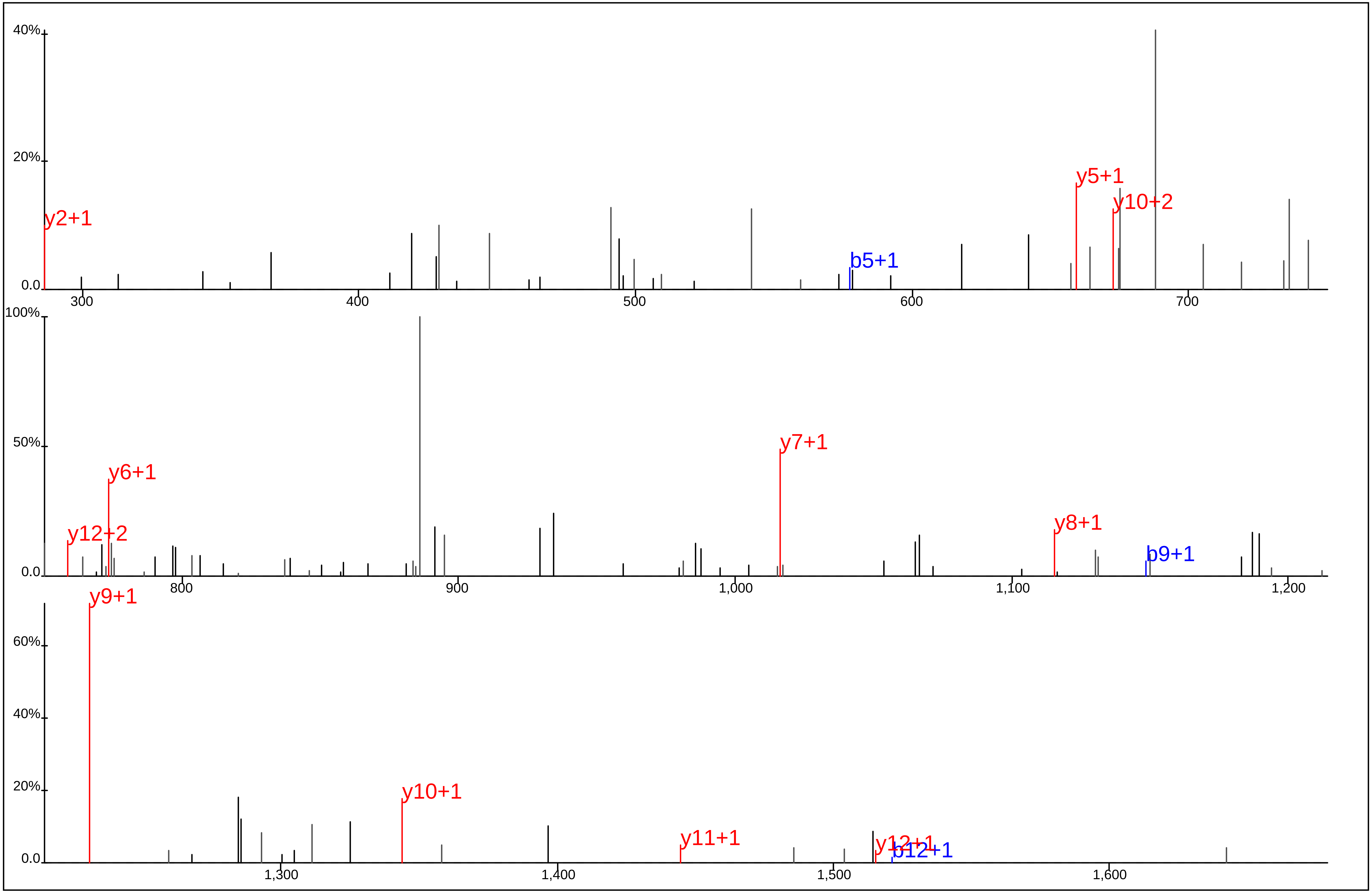

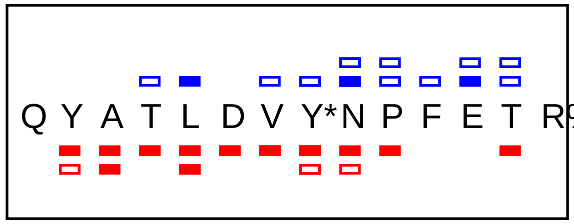

Protein Name: SCAMP3
gi Accession: 16445419
Precursor Mass: 903.9063+2
Delta Mass: 4.421 ppm
XCorr: 39.13

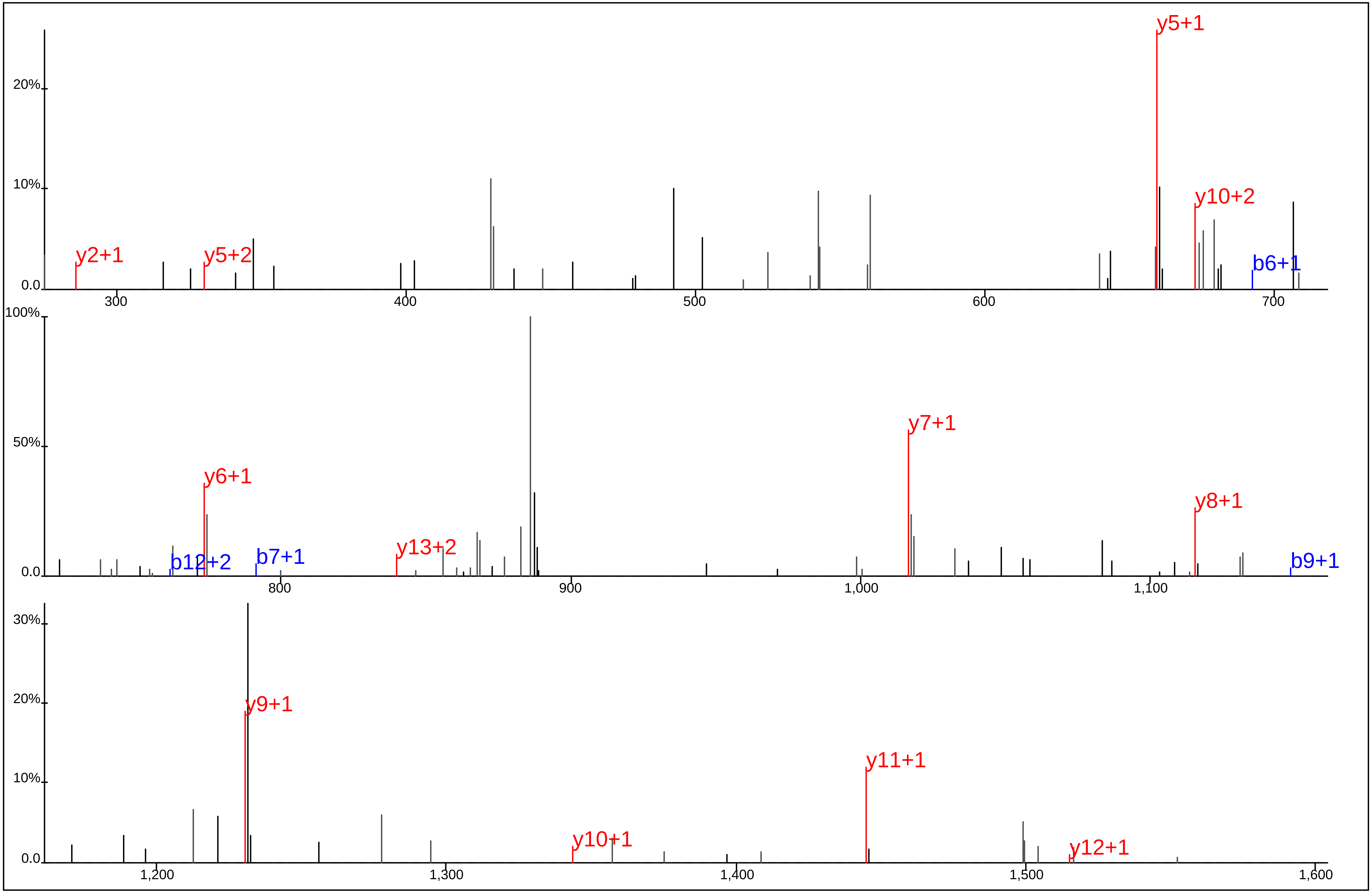

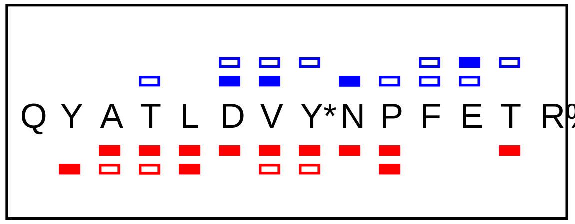

Protein Name:	SCAMP3
gi Accession:	16445419
Precursor Mass:	903.9059+2
Delta Mass:	3.945 ppm
XCorr:	19.35

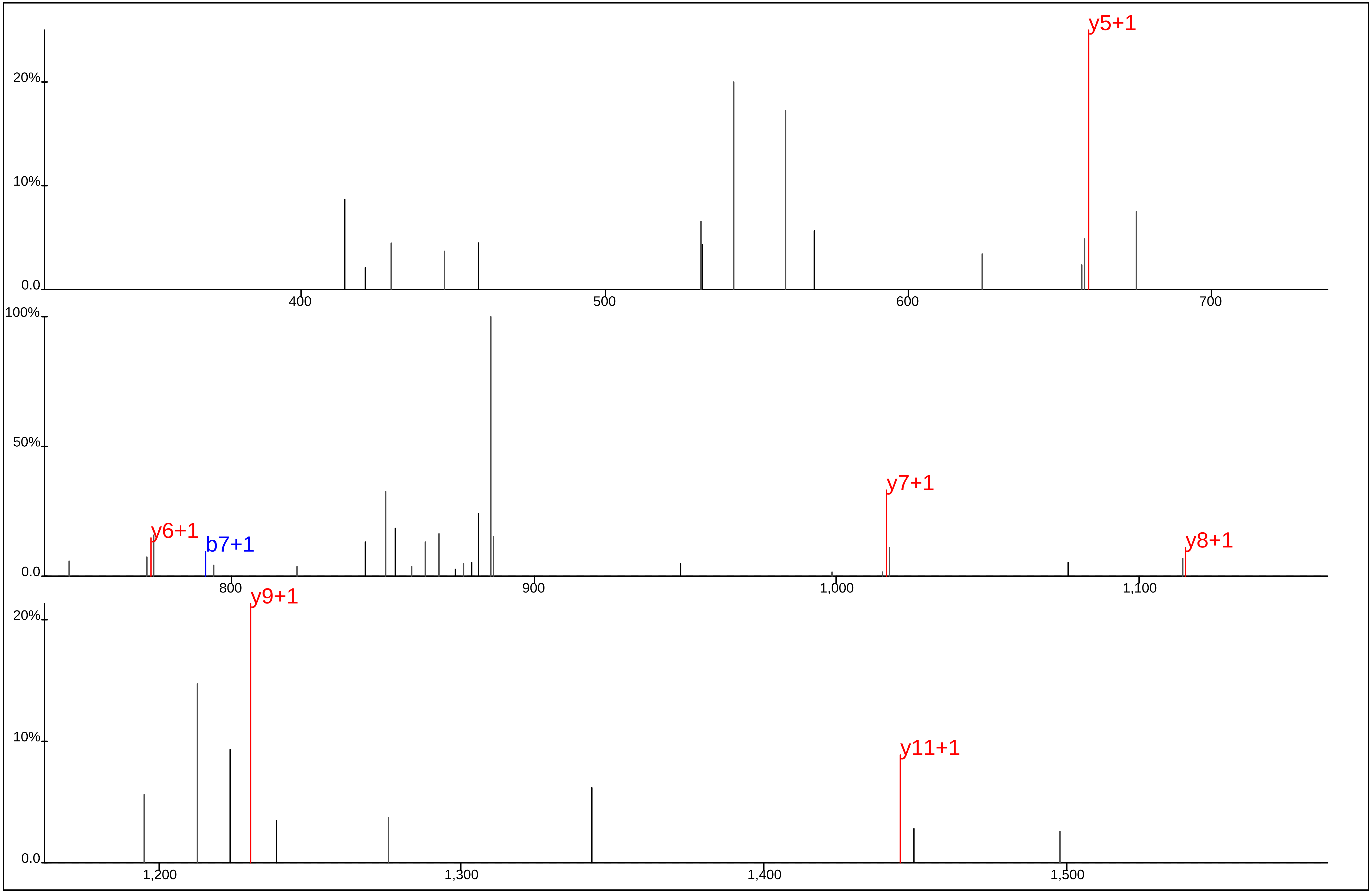

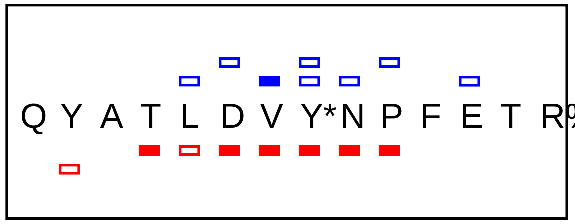

Protein Name: SCAMP3
gi Accession: 16445419
Precursor Mass: 903.9127+2
Delta Mass: 11.511 ppm
XCorr: 17.75

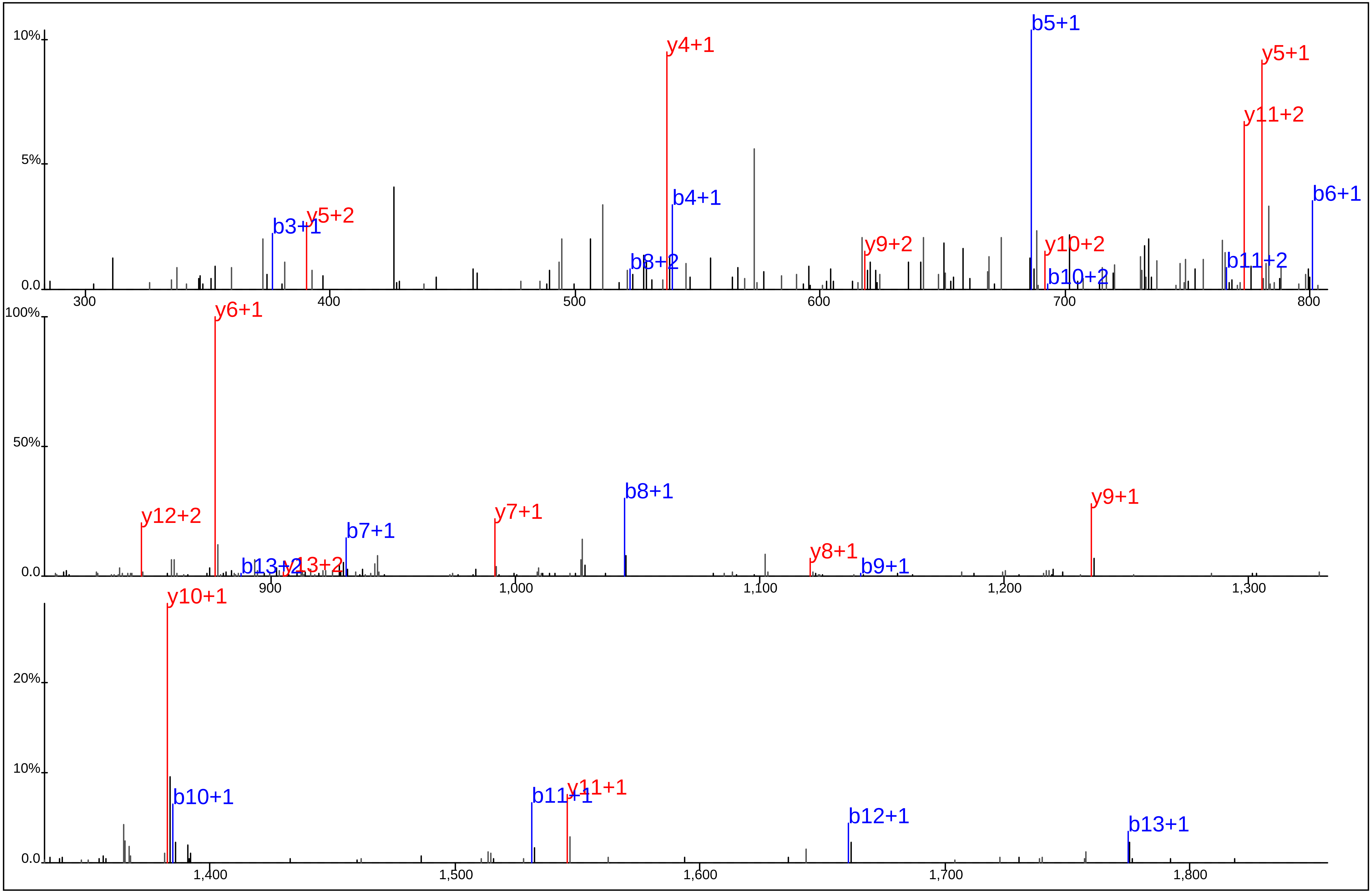

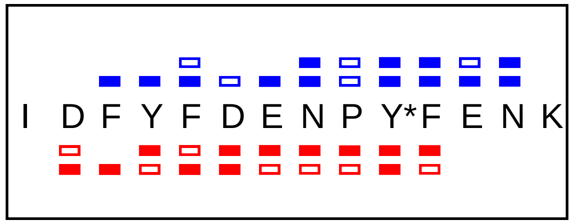

Protein Name: SET protein
gi Accession: 170763498
Precursor Mass: 960.8934+2
Delta Mass: 3.555 ppm
XCorr: 67.61

886 - SETprotein_3434262.pdf

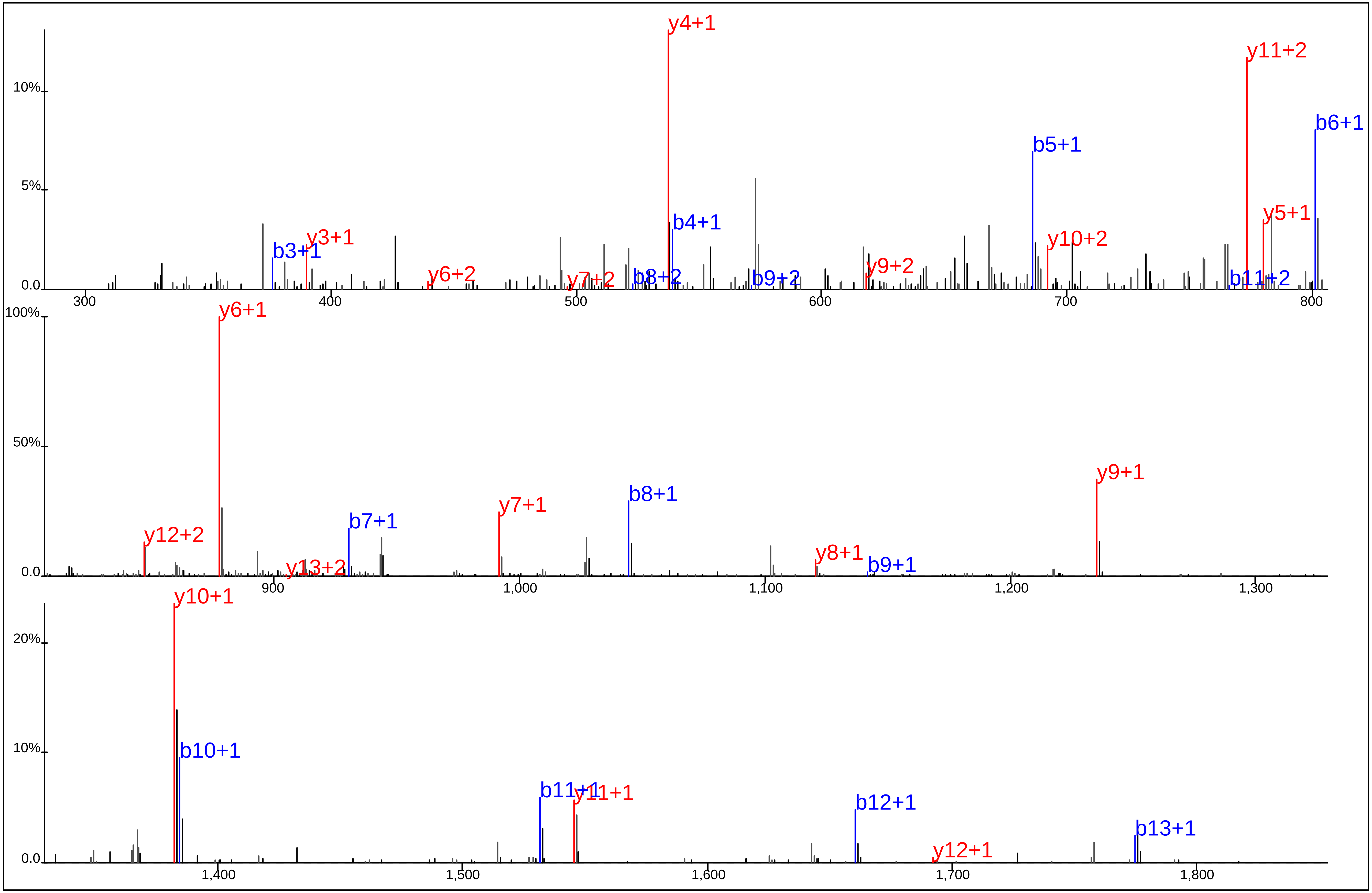

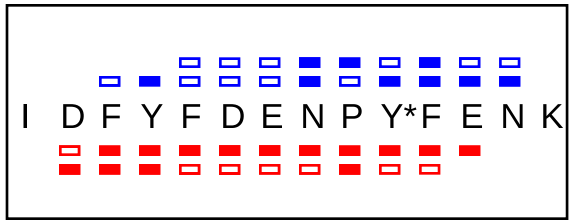

Protein Name: SET protein
gi Accession: 170763498
Precursor Mass: 960.8912+2
Delta Mass: 1.264 ppm
XCorr: 66.92

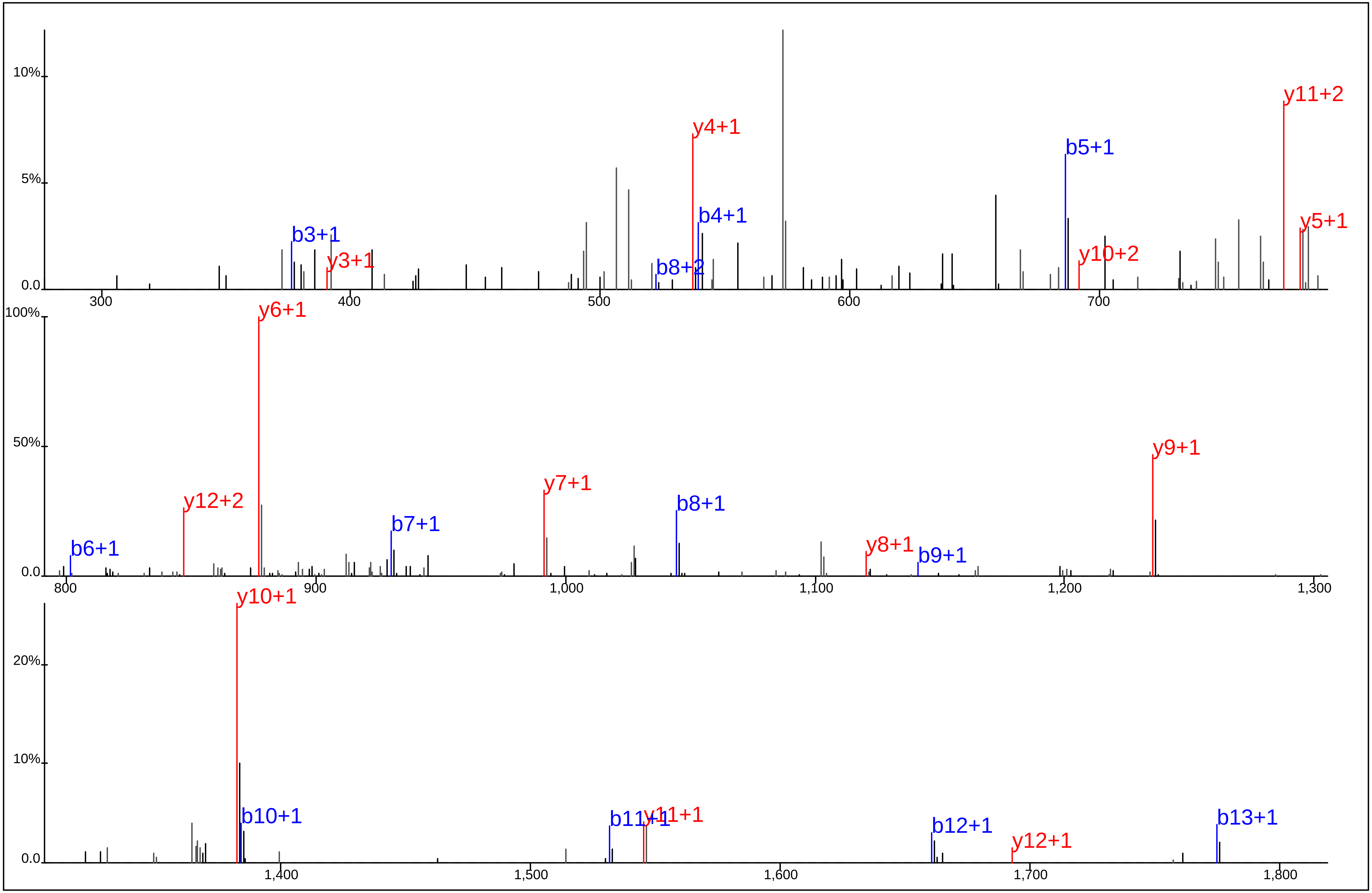

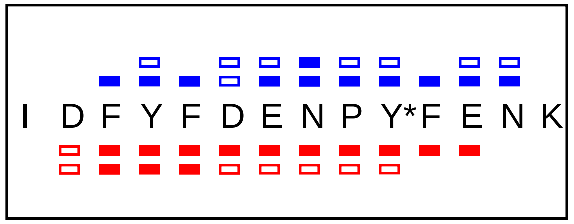

Protein Name: SET protein
gi Accession: 170763498
Precursor Mass: 960.8909+2
Delta Mass: 0.884 ppm
XCorr: 53.23

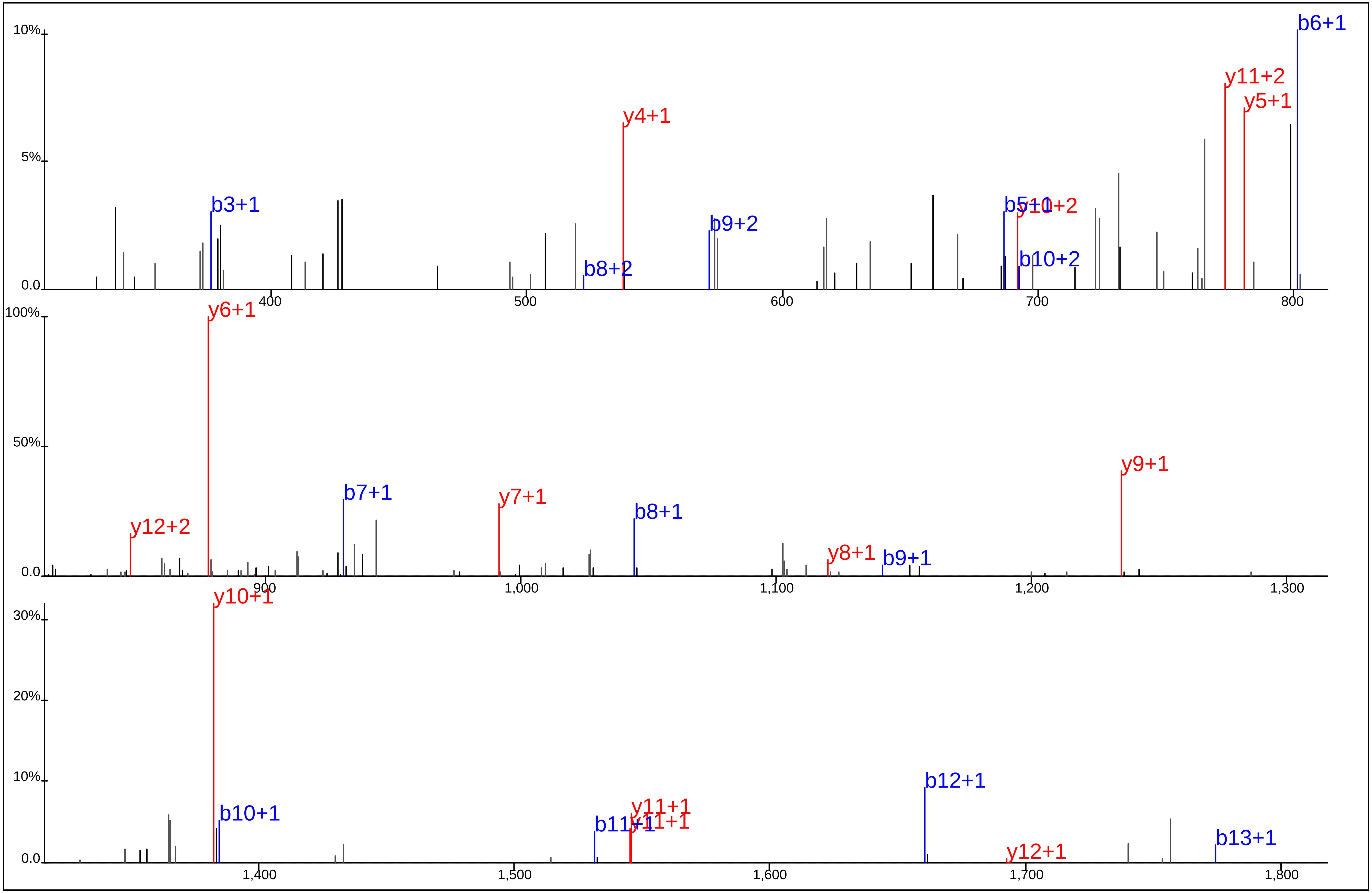

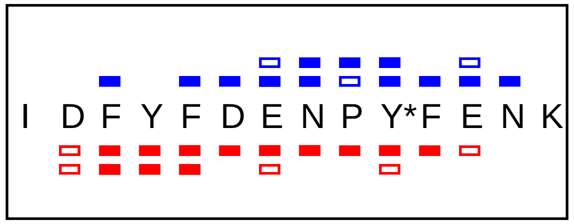

Protein Name: SET protein
gi Accession: 170763498
Precursor Mass: 960.8914+2
Delta Mass: 1.457 ppm
XCorr: 62.05

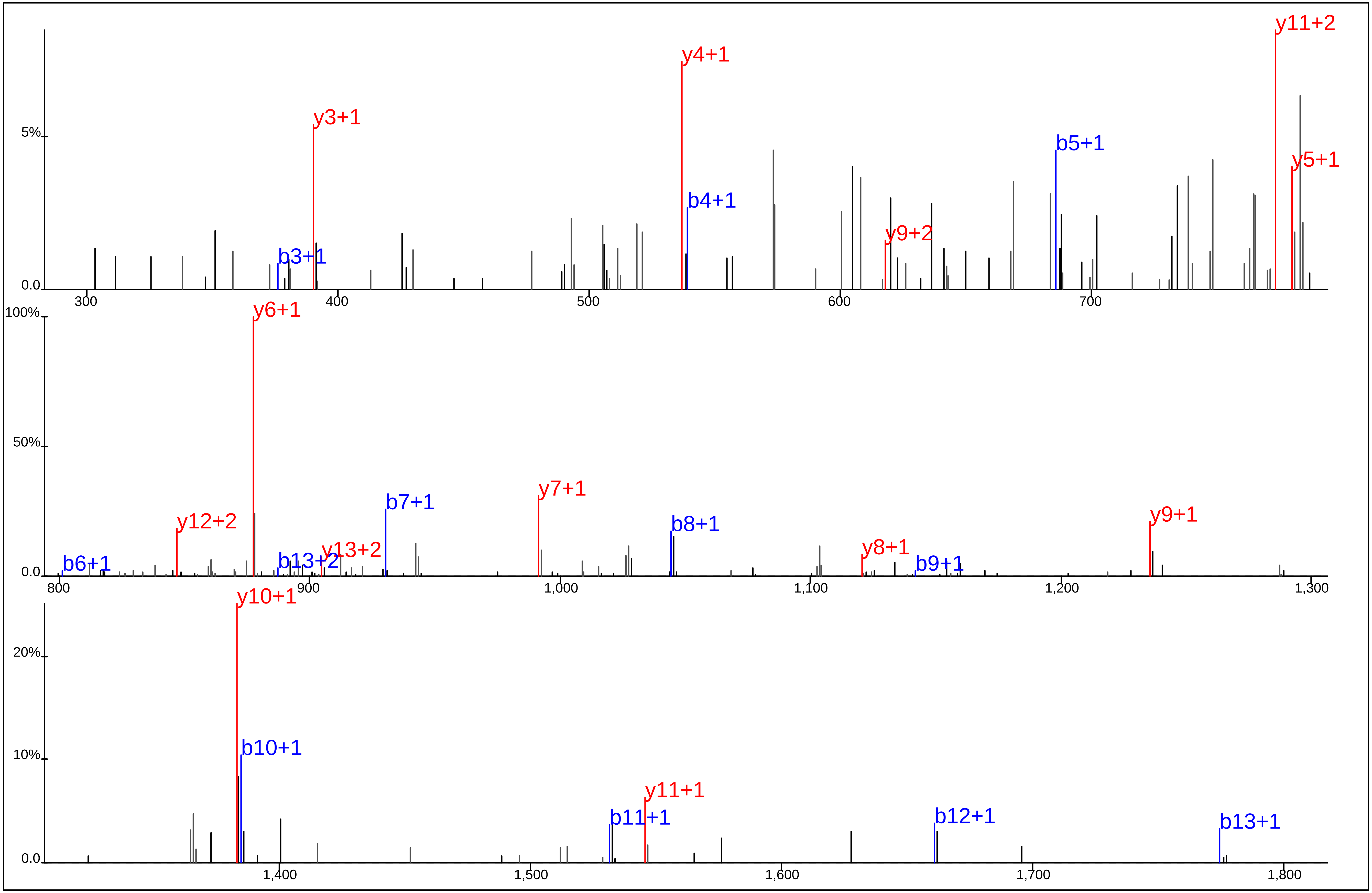

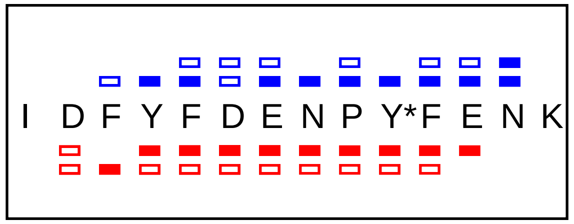

Protein Name:	SET protein
gi Accession:	170763498
Precursor Mass:	960.8906+2
Delta Mass:	0.629 ppm
XCorr:	52.95

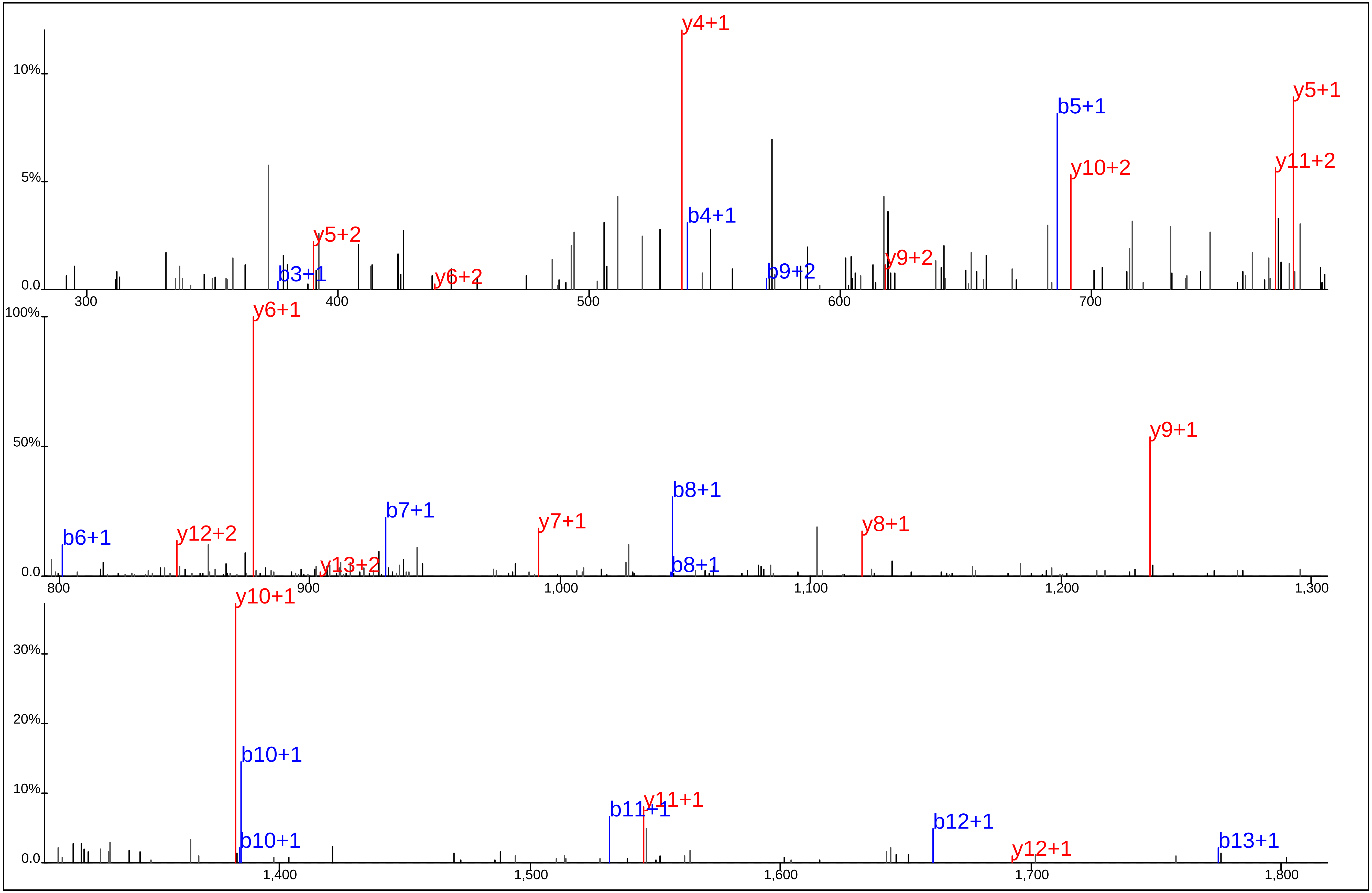

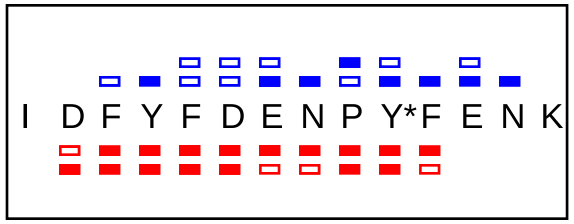

Protein Name: SET protein
gi Accession: 170763498
Precursor Mass: 960.8905+2
Delta Mass: 0.504 ppm
XCorr: 59.04

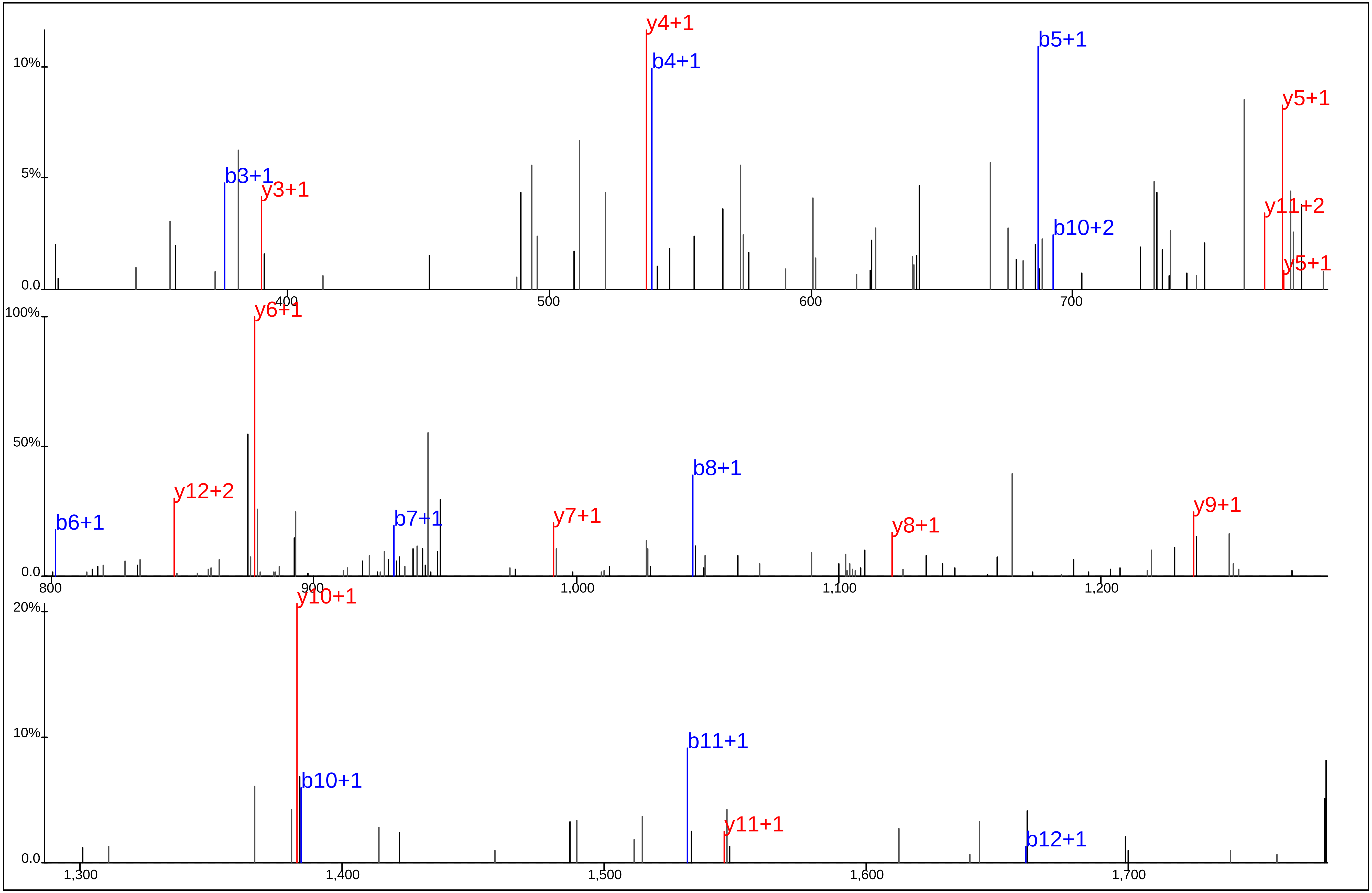

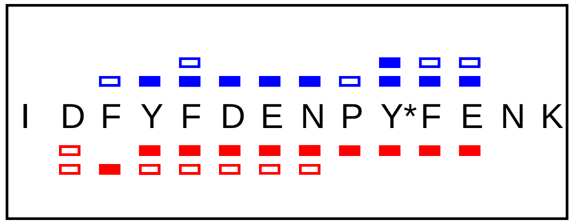

Protein Name: SET protein
gi Accession: 170763498
Precursor Mass: 960.8960+2
Delta Mass: 6.226 ppm
XCorr: 33.16

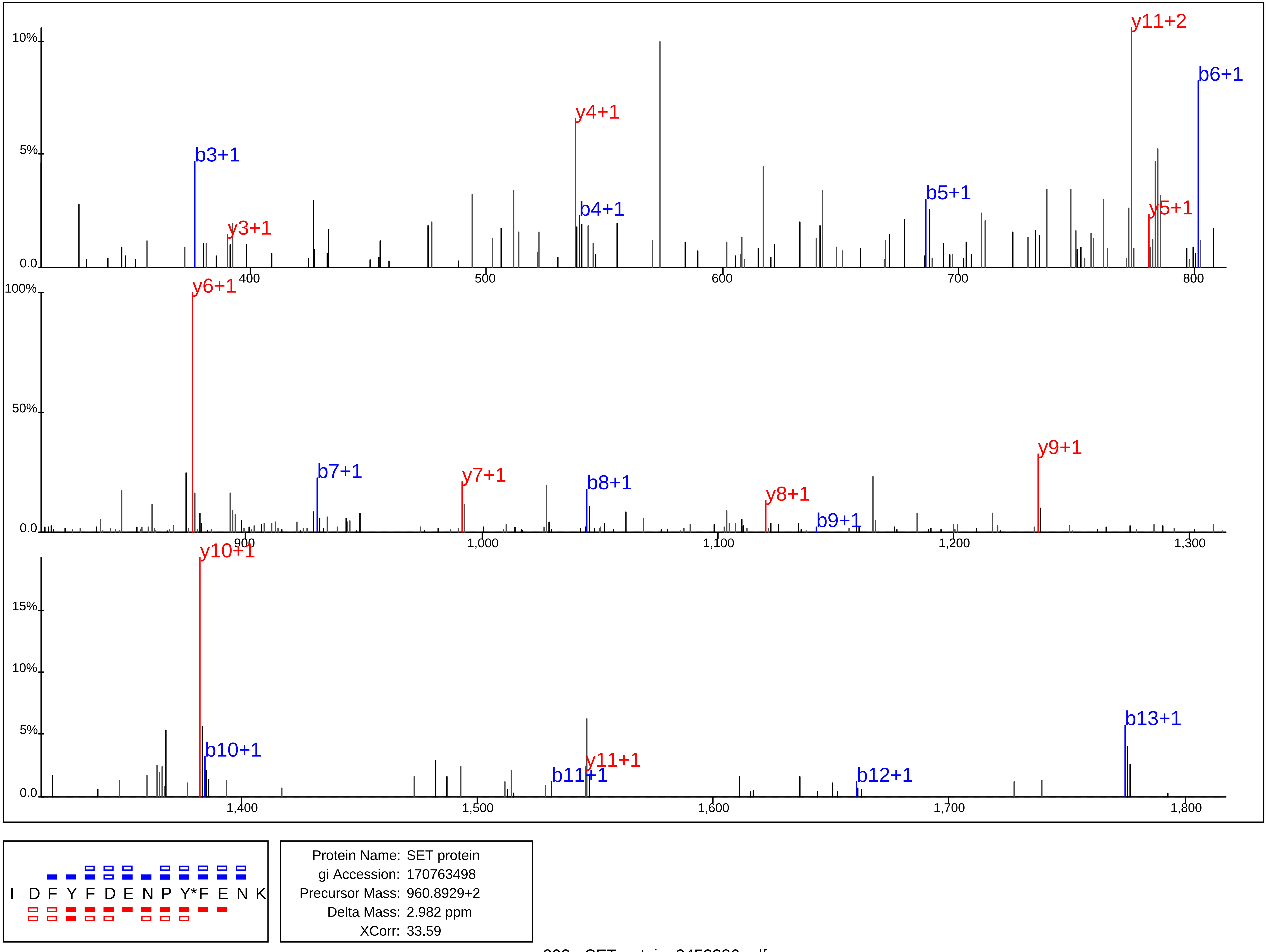

Protein Name:	SET protein
gi Accession:	170763498
Precursor Mass:	960.8929+2
Delta Mass:	2.982 ppm
XCorr:	33.59

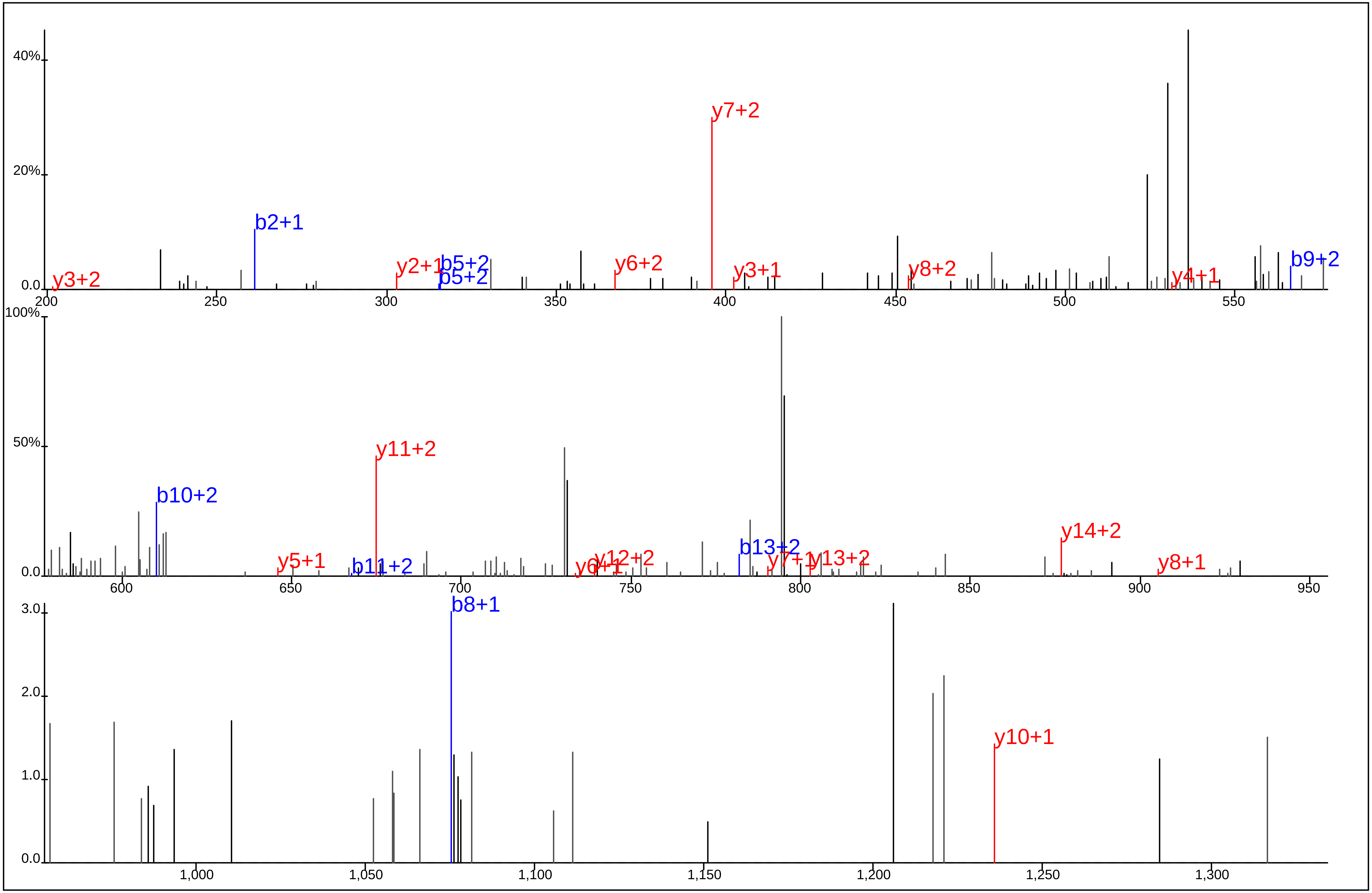

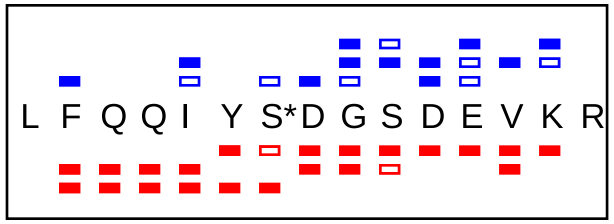

Protein Name:	SGT1
gi Accession:	5730041
Precursor Mass:	622.2893+3
Delta Mass:	3.055 ppm
XCorr:	13.3

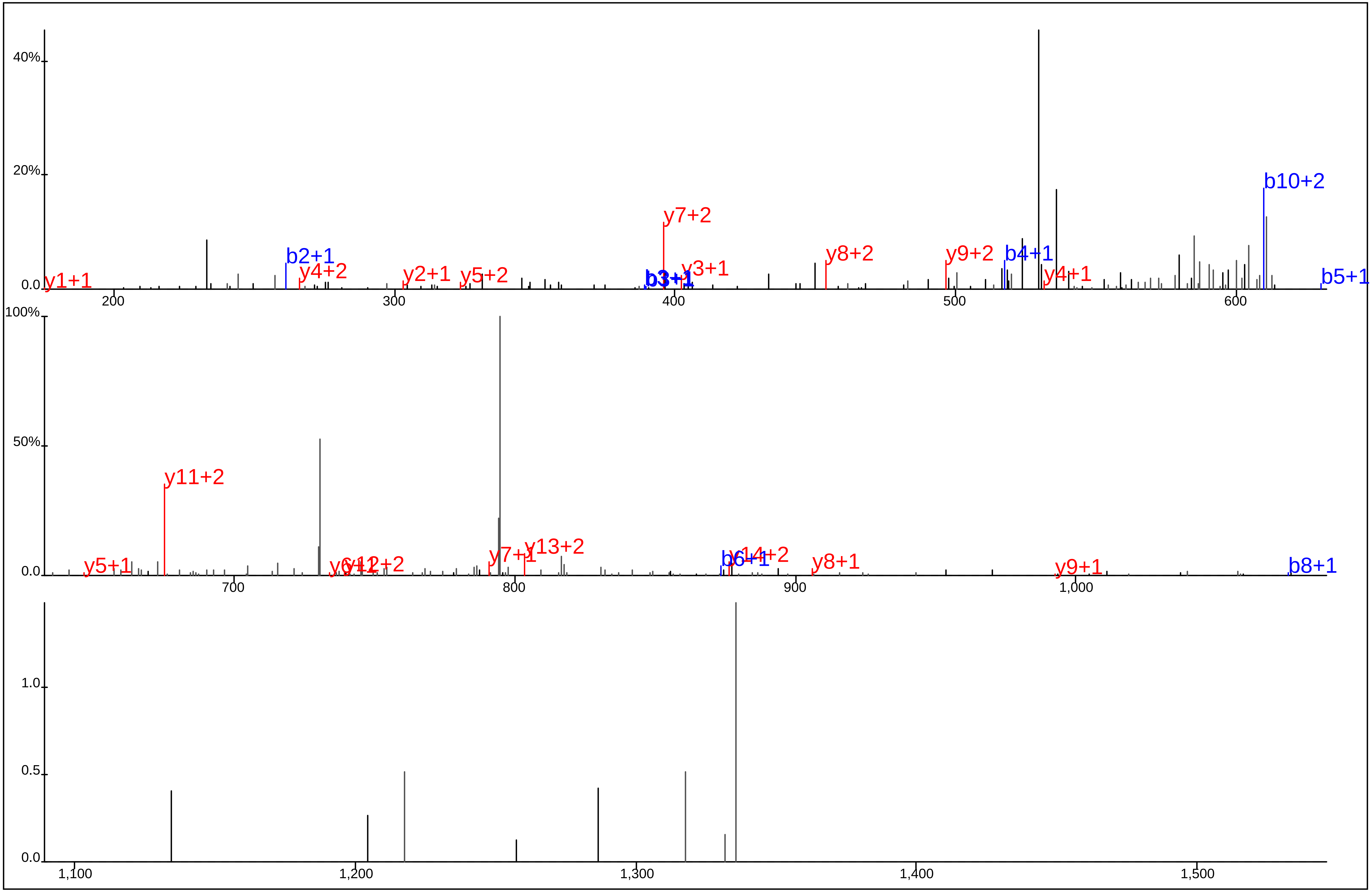

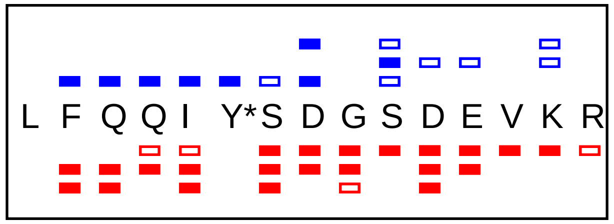

Protein Name: SGT1
gi Accession: 5730041
Precursor Mass: 622.2897+3
Delta Mass: 3.645 ppm
XCorr: 27.18

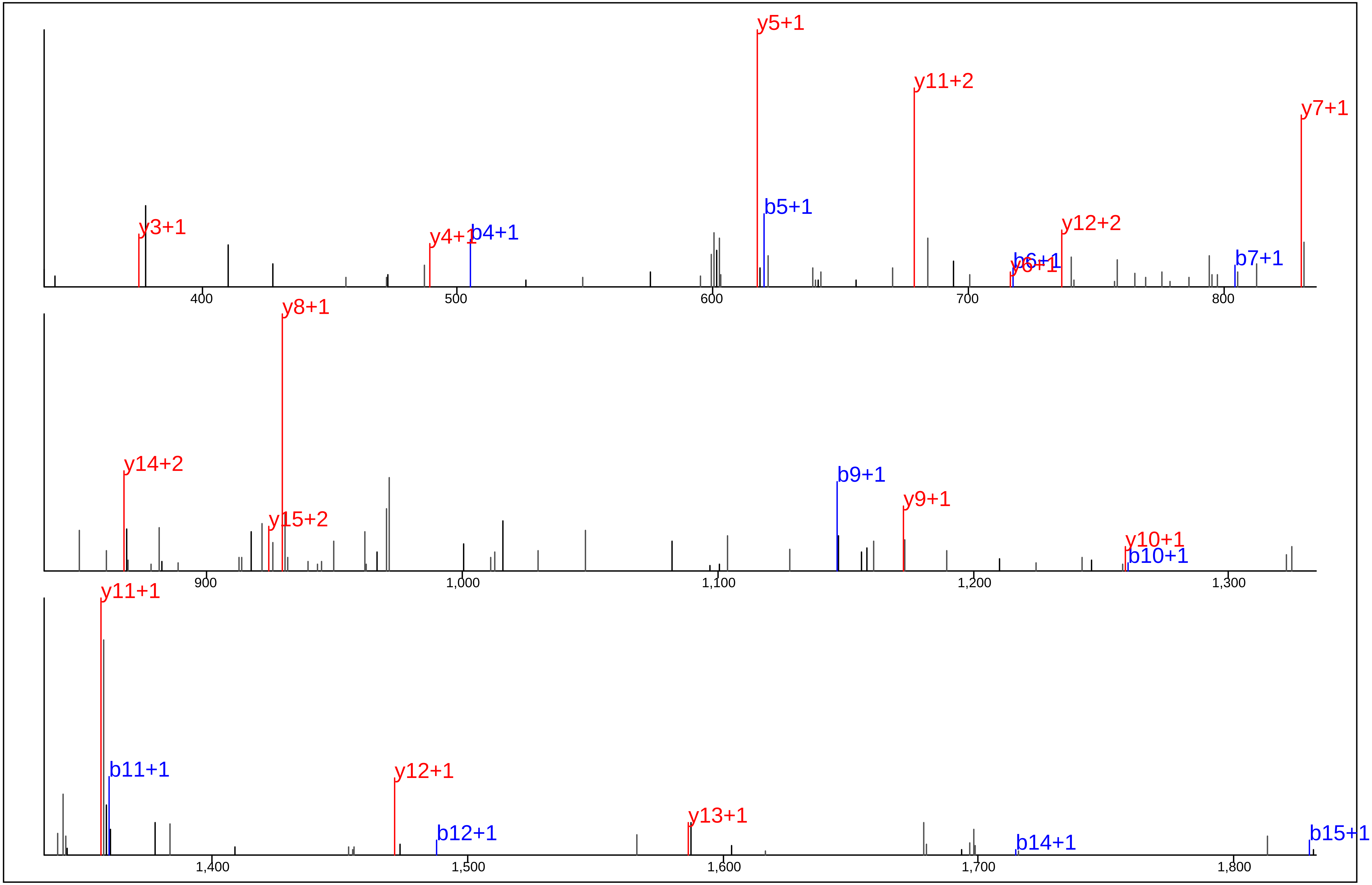

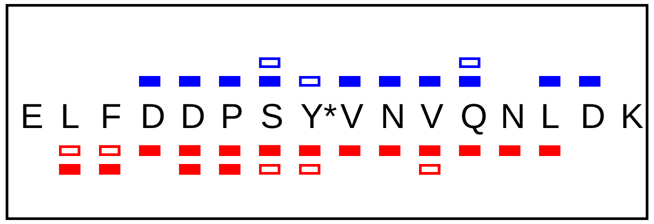

Protein Name: SHC
gi Accession: 32261324
Precursor Mass: 988.4419+2
Delta Mass: 4.073 ppm
XCorr: 72.35

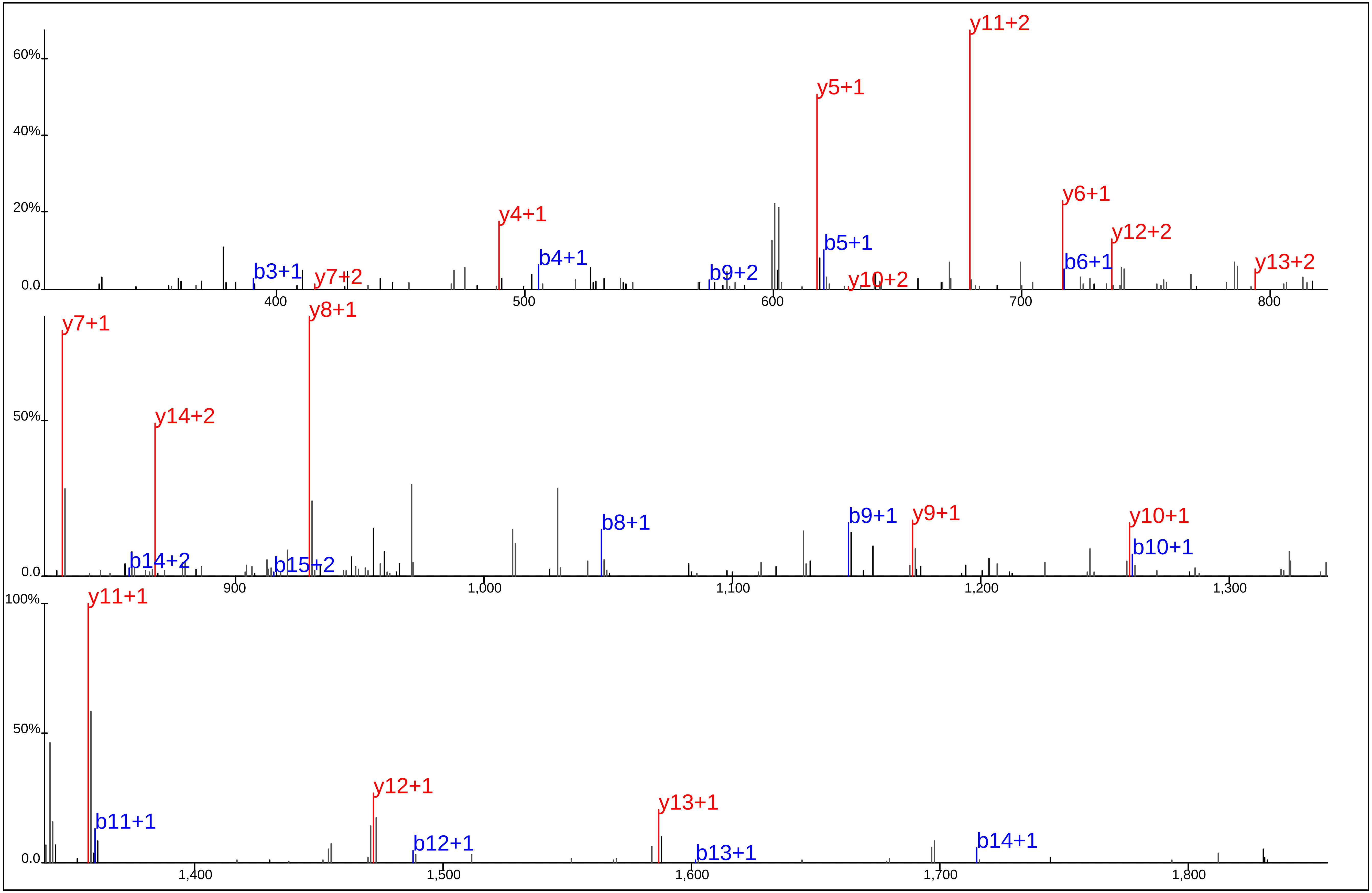

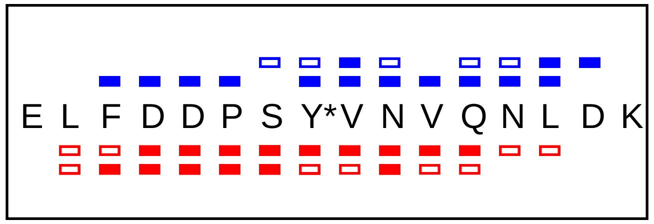

Protein Name: SHC
gi Accession: 32261324
Precursor Mass: 988.4407+2
Delta Mass: 2.838 ppm
XCorr: 75.84

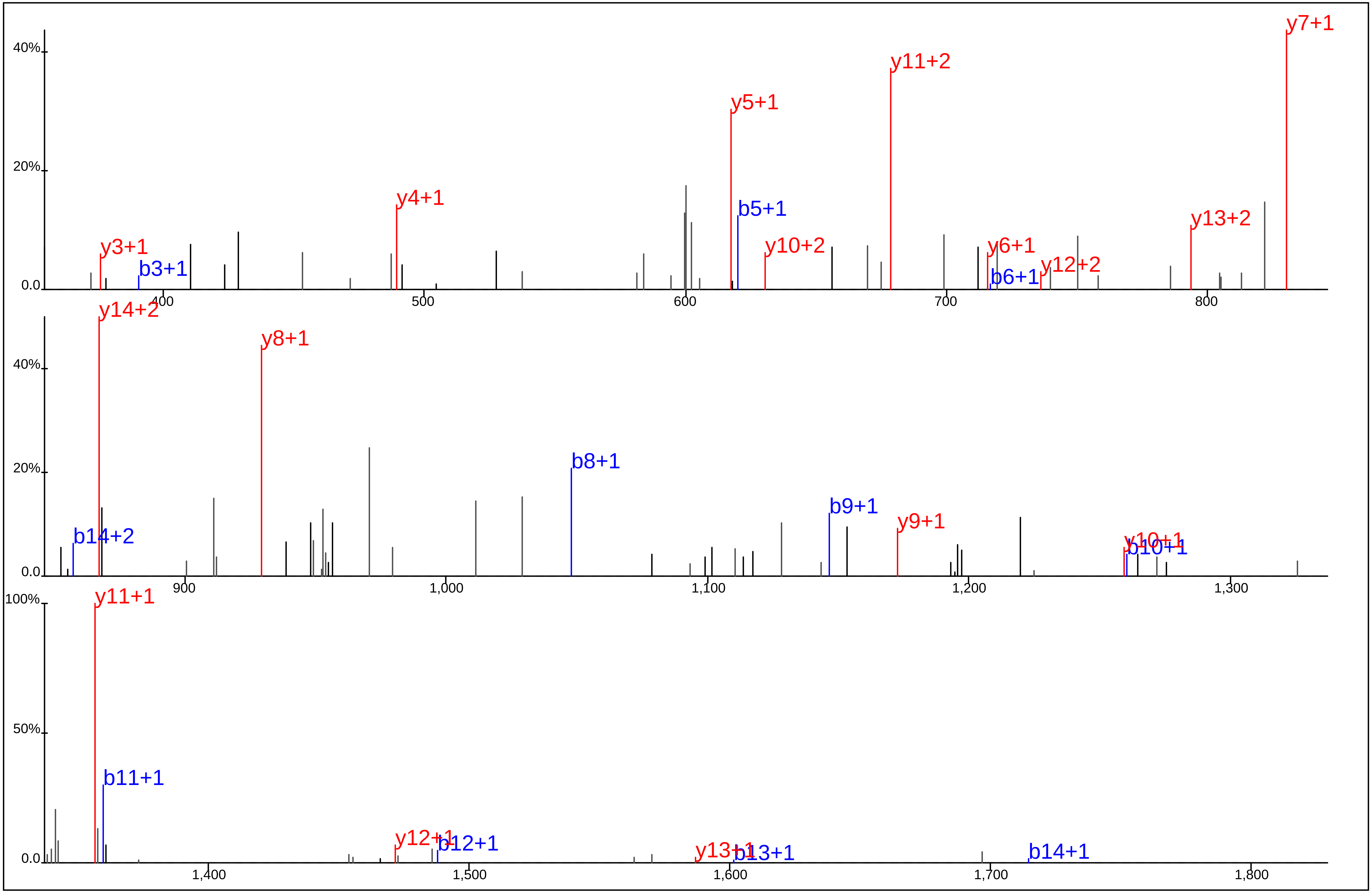

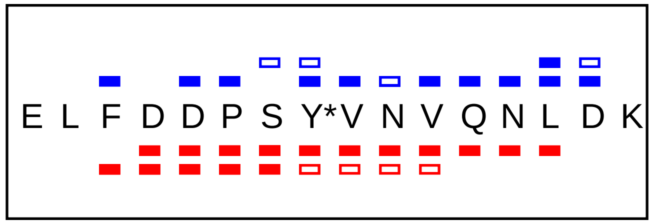

Protein Name: SHC
gi Accession: 32261324
Precursor Mass: 988.4428+2
Delta Mass: 4.999 ppm
XCorr: 61.77

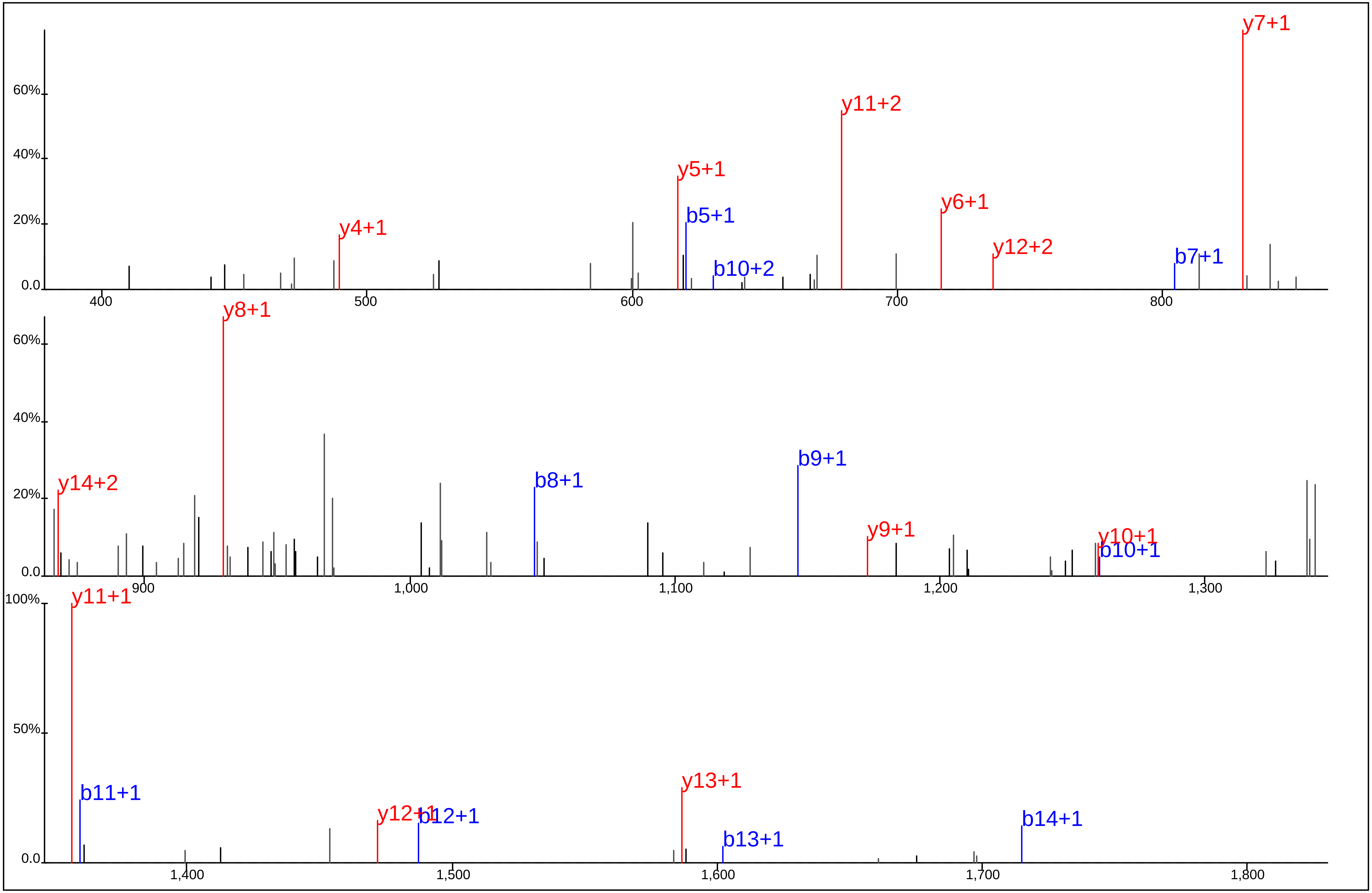

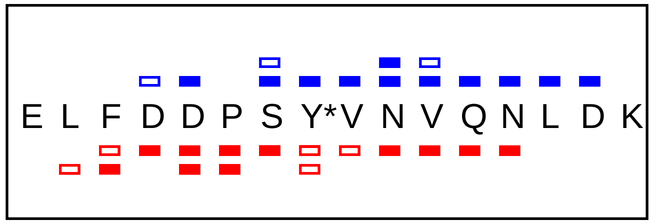

Protein Name: SHC
gi Accession: 32261324
Precursor Mass: 988.4425+2
Delta Mass: 4.691 ppm
XCorr: 77.67

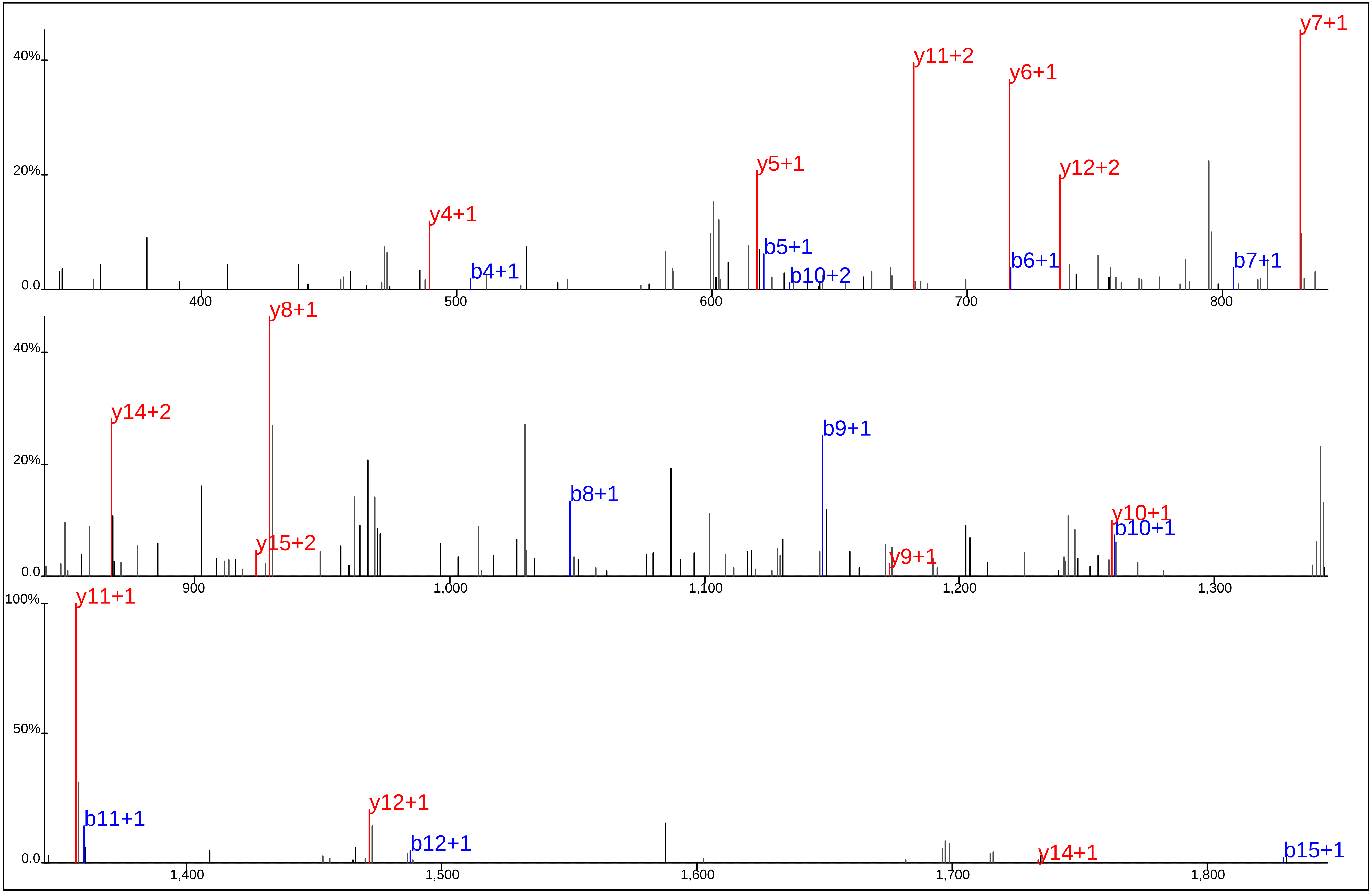

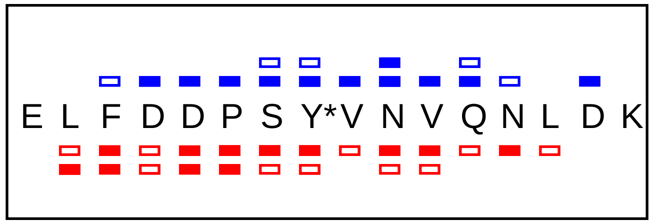

Protein Name: SHC
gi Accession: 32261324
Precursor Mass: 988.4390+2
Delta Mass: 1.173 ppm
XCorr: 43.67

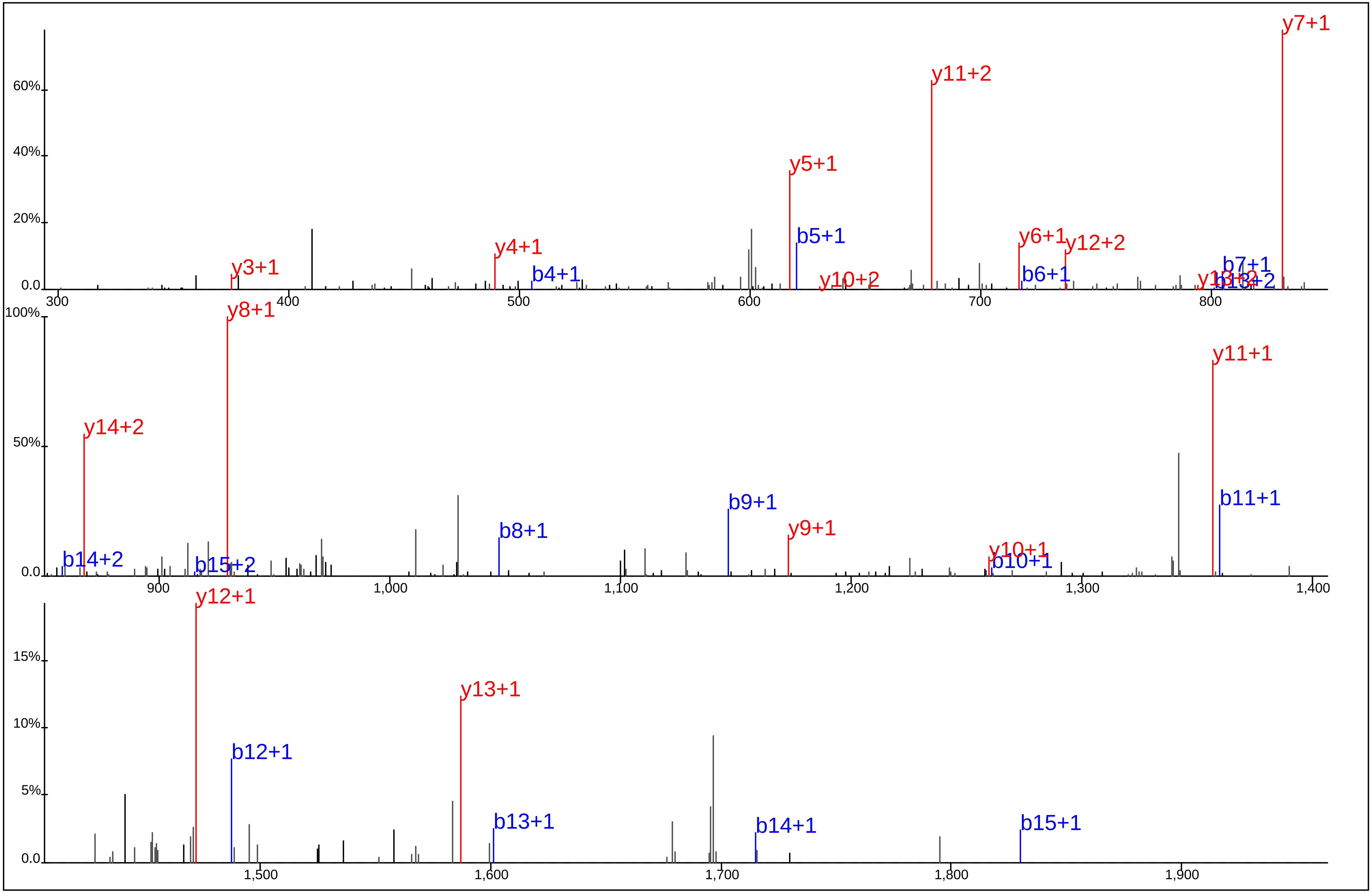

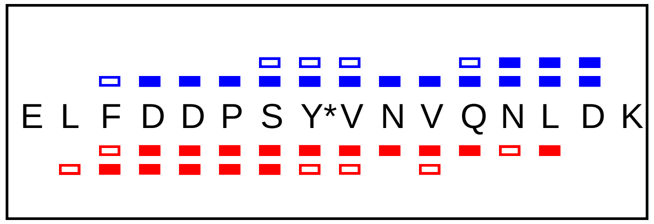

Protein Name: SHC
gi Accession: 32261324
Precursor Mass: 988.4393+2
Delta Mass: 1.416 ppm
XCorr: 83.38

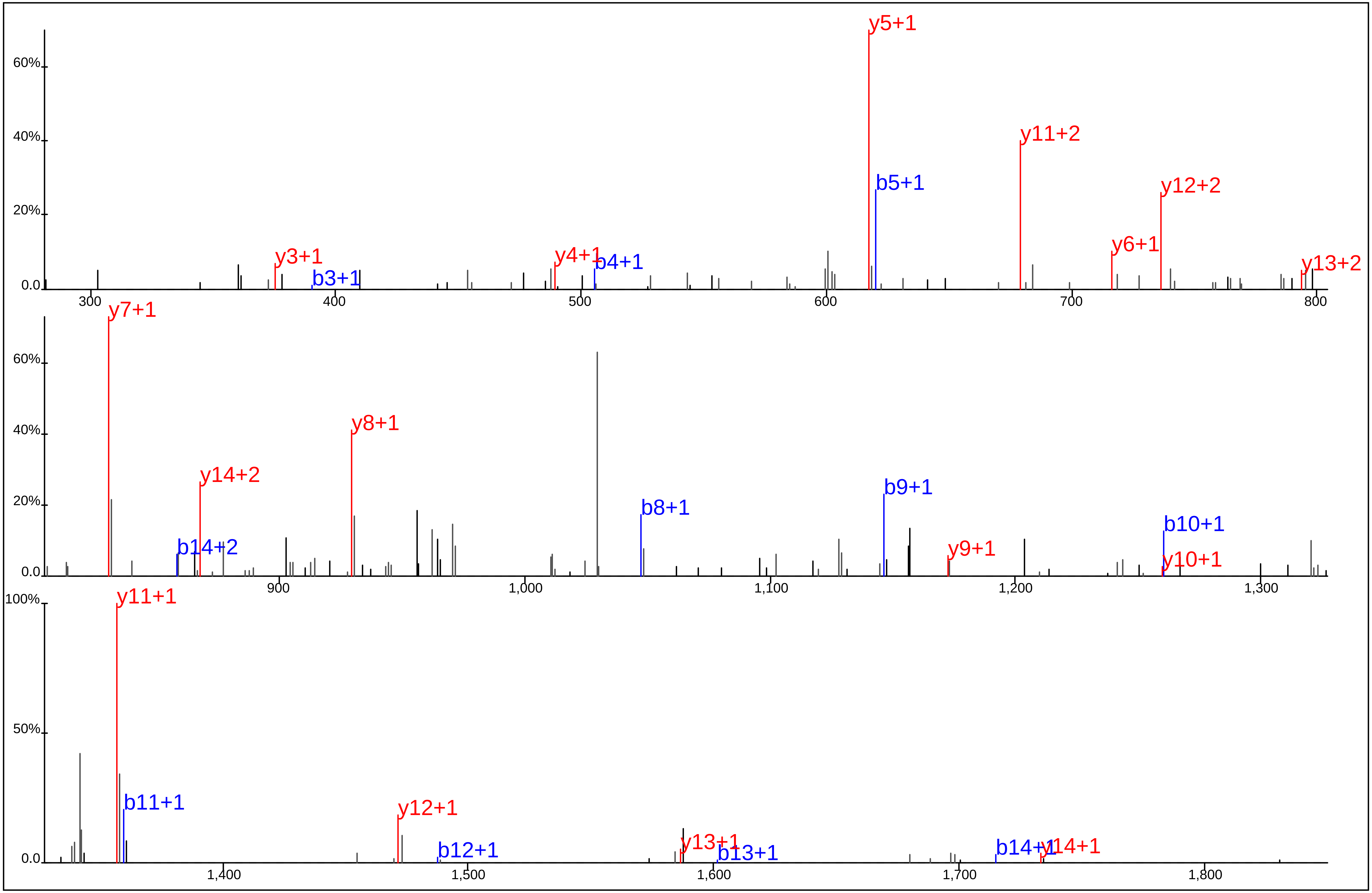

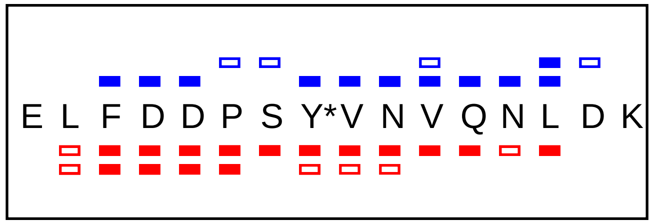

Protein Name: SHC
gi Accession: 32261324
Precursor Mass: 988.4405+2
Delta Mass: 2.651 ppm
XCorr: 68.91

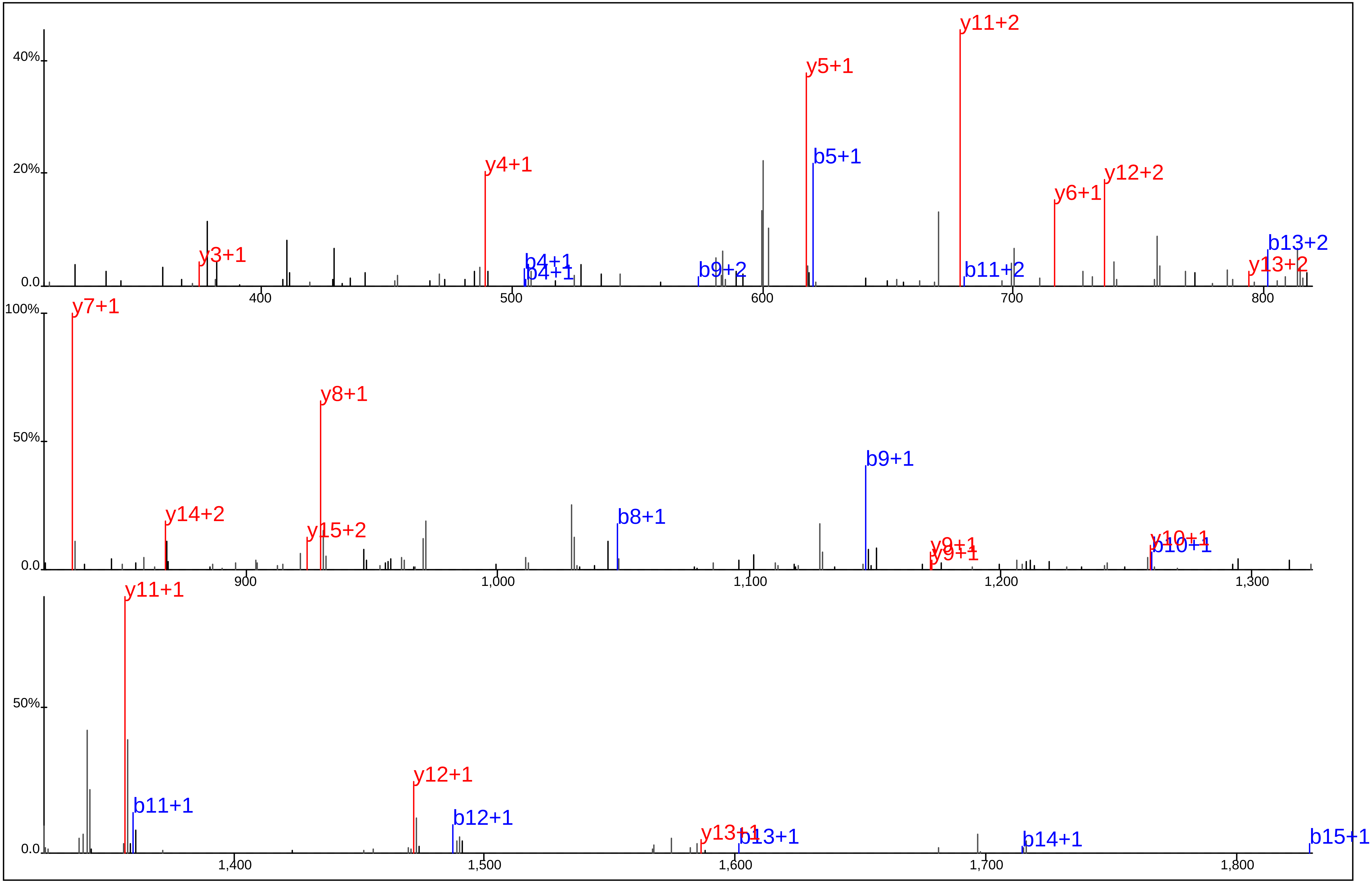

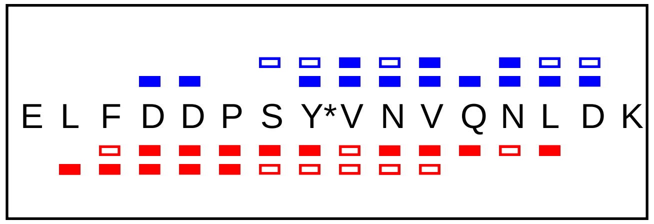

Protein Name: SHC
gi Accession: 32261324
Precursor Mass: 988.4409+2
Delta Mass: 3.086 ppm
XCorr: 67.32

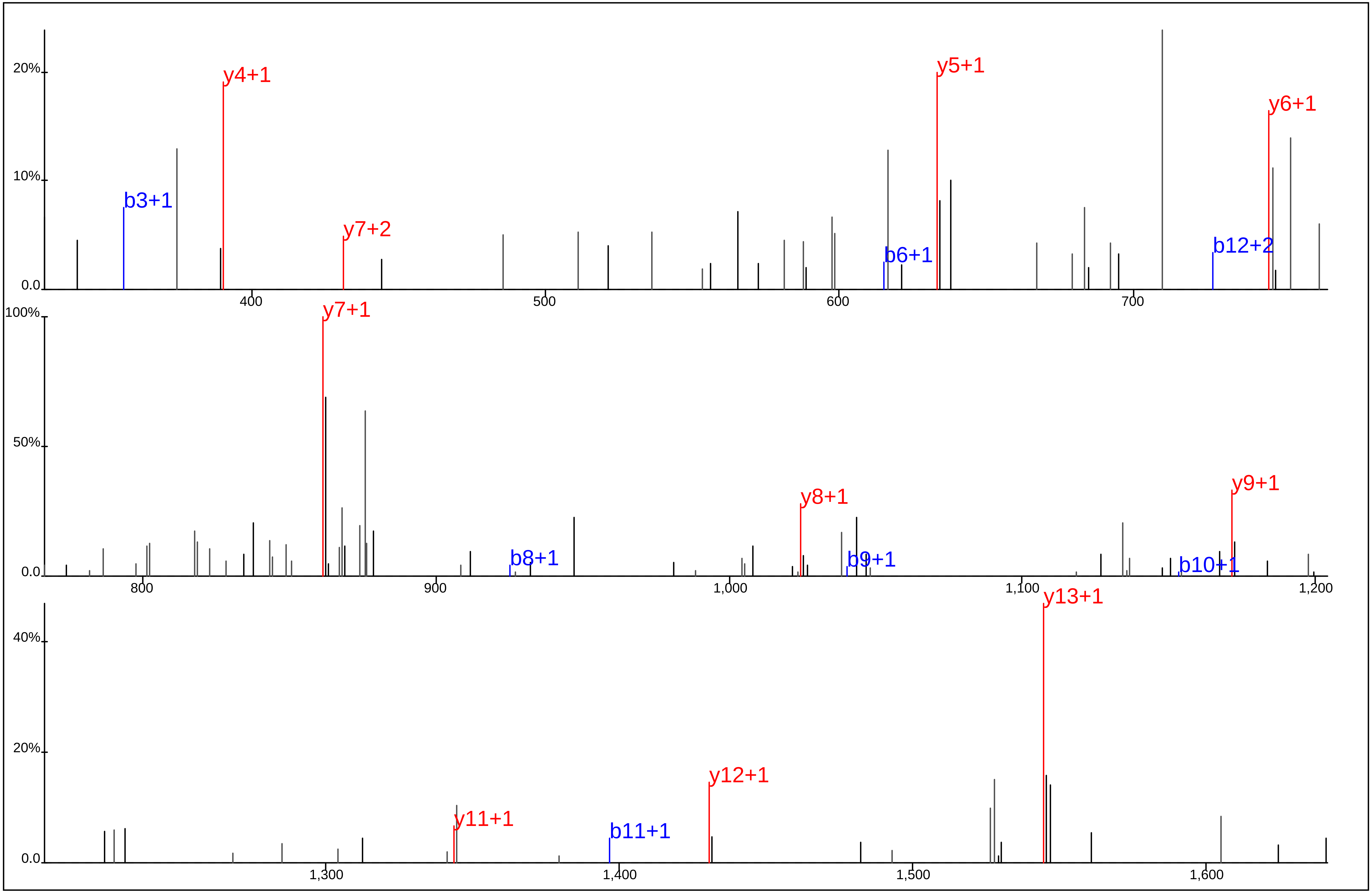

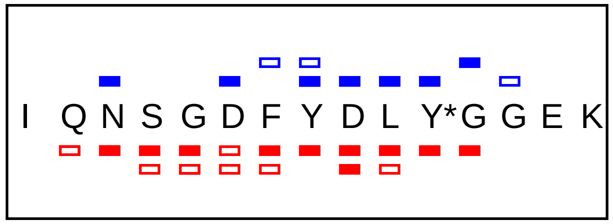

Protein Name: SHP1
gi Accession: 18104991
Precursor Mass: 893.3760+2
Delta Mass: 4.456 ppm
XCorr: 42.42

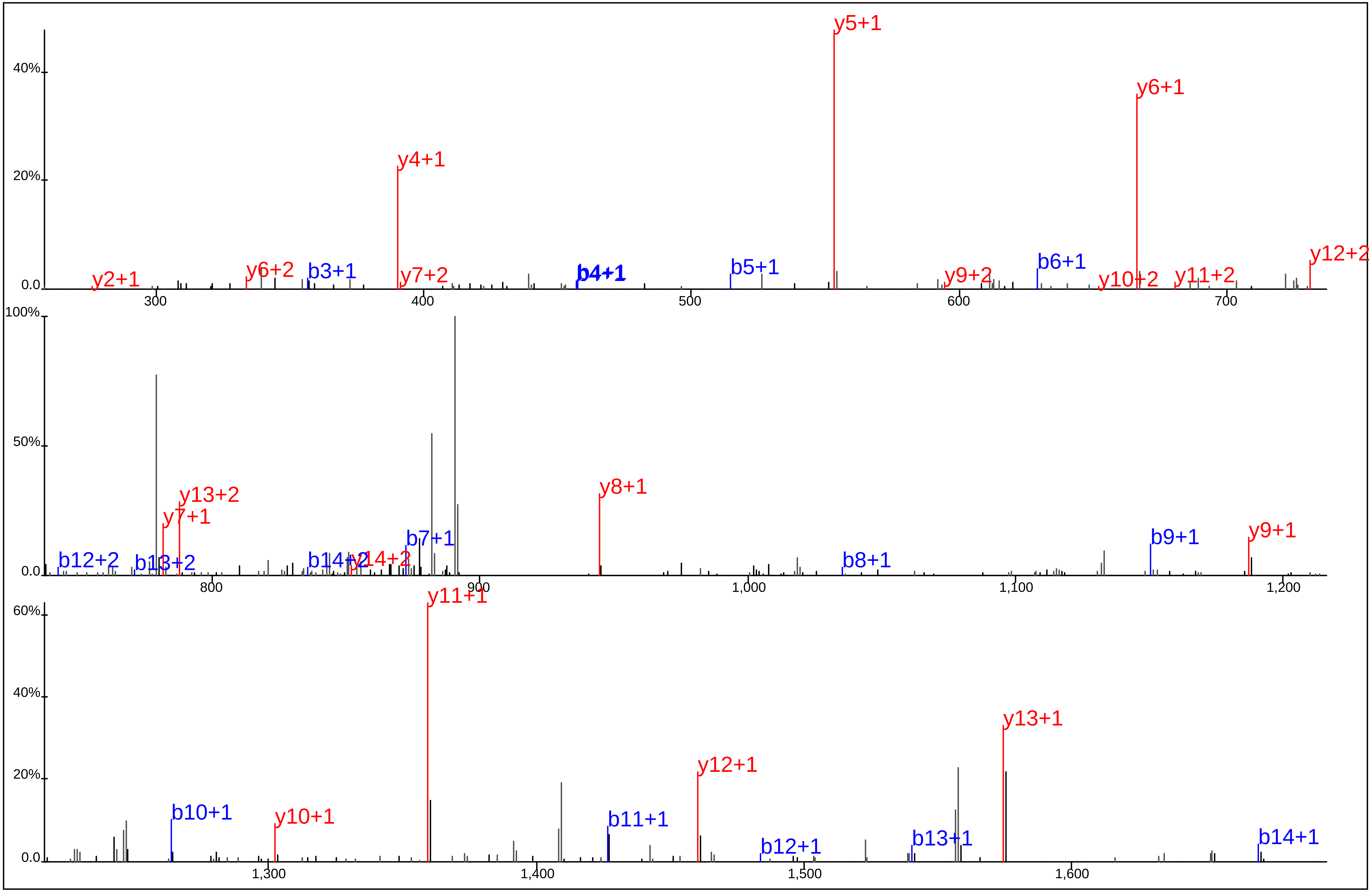

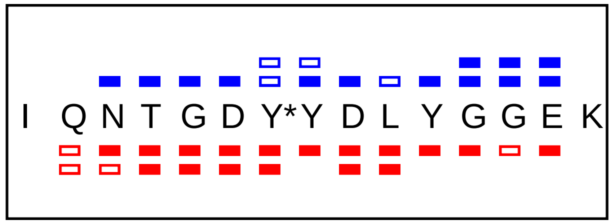

Protein Name: SHP2
gi Accession: 33356177
Precursor Mass: 908.3773+2
Delta Mass: 0.043 ppm
XCorr: 83.97

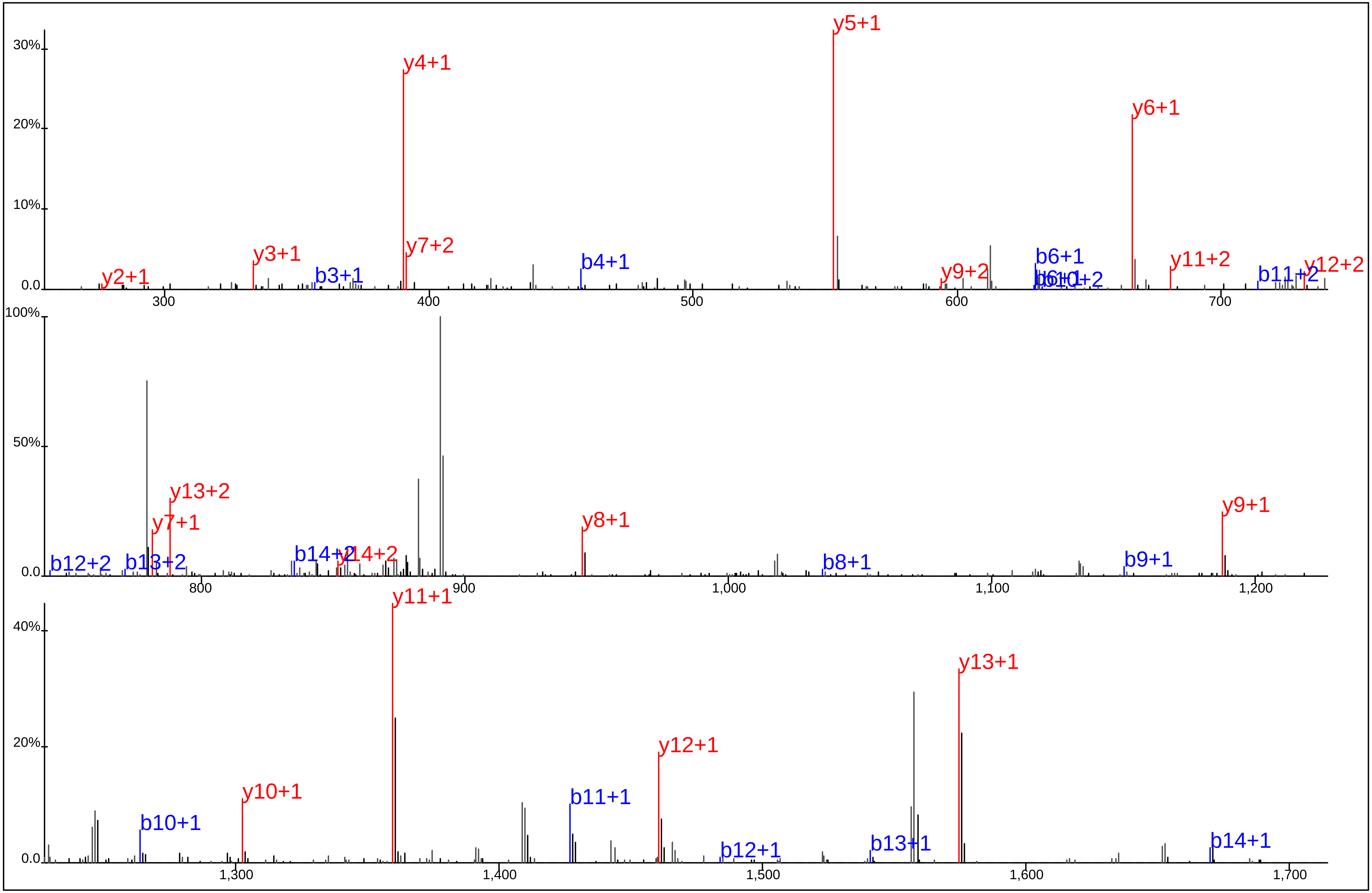

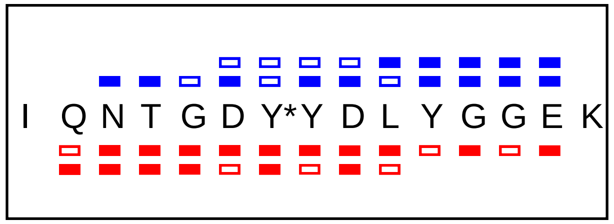

Protein Name:	SHP2
gi Accession:	33356177
Precursor Mass:	908.3777+2
Delta Mass:	0.445 ppm
XCorr:	86.44

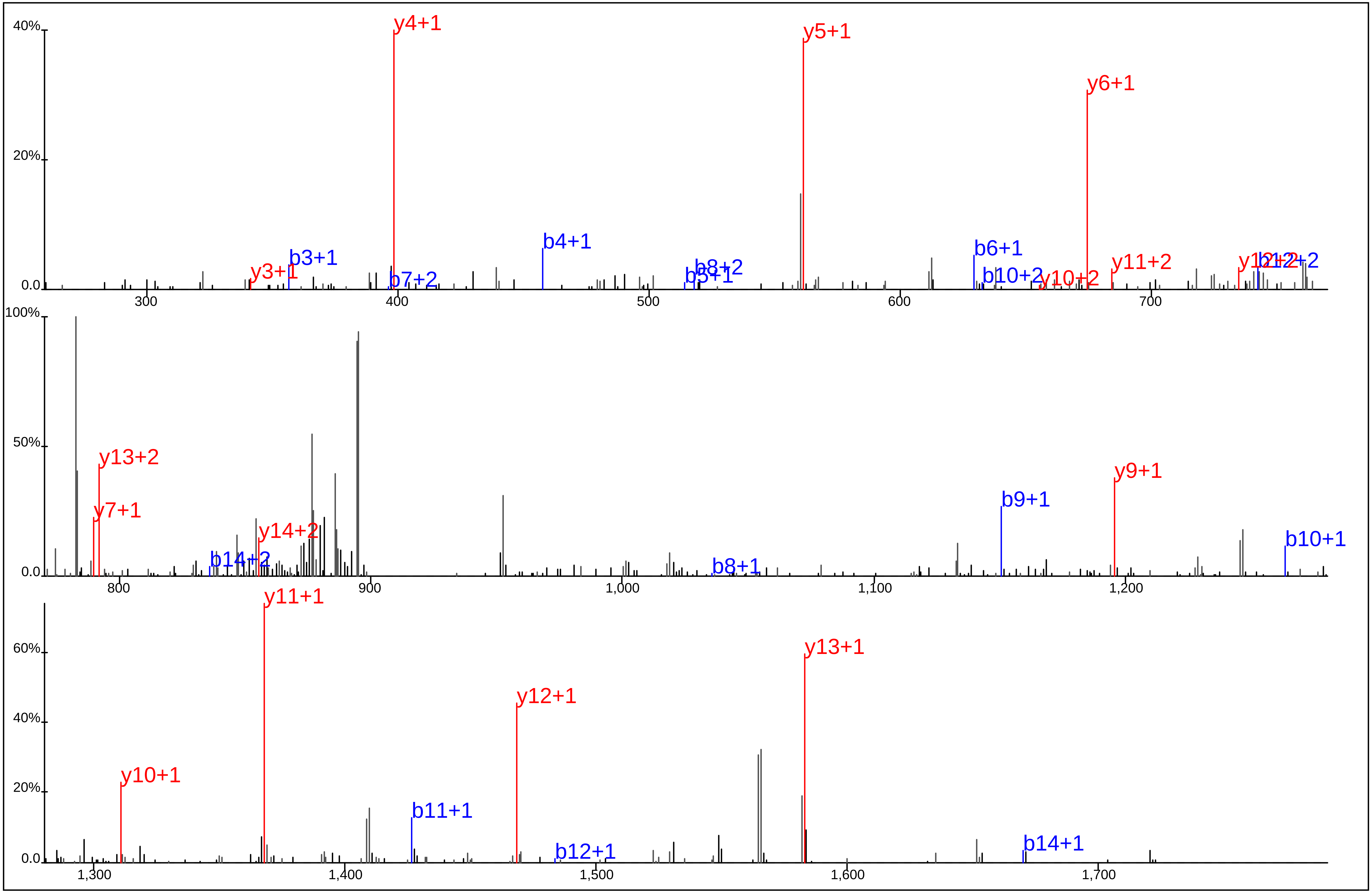

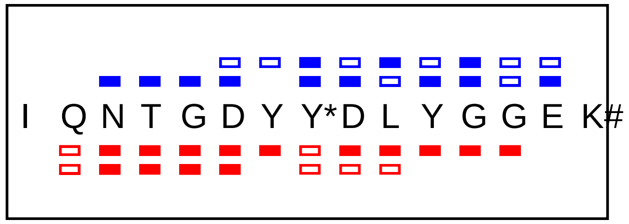

Protein Name:	SHP2
gi Accession:	33356177
Precursor Mass:	912.3885+2
Delta Mass:	4.572 ppm
XCorr:	68.1

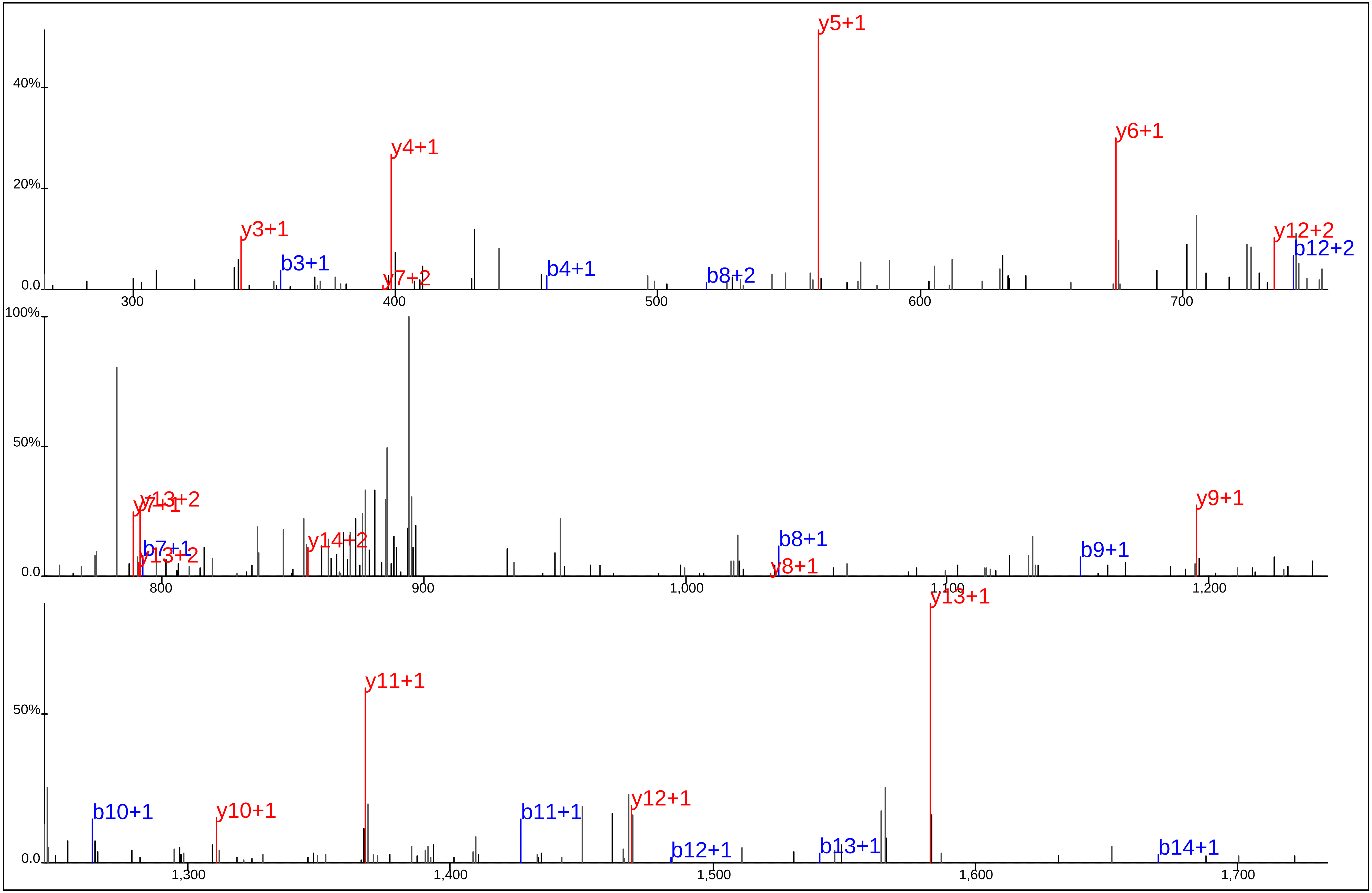

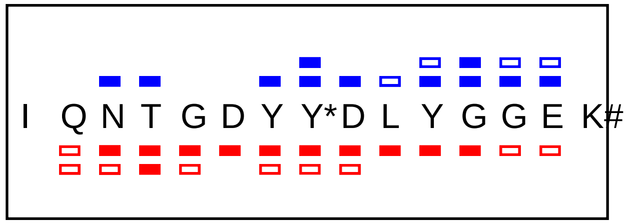

Protein Name:	SHP2
gi Accession:	33356177
Precursor Mass:	912.3857+2
Delta Mass:	1.496 ppm
XCorr:	56.31

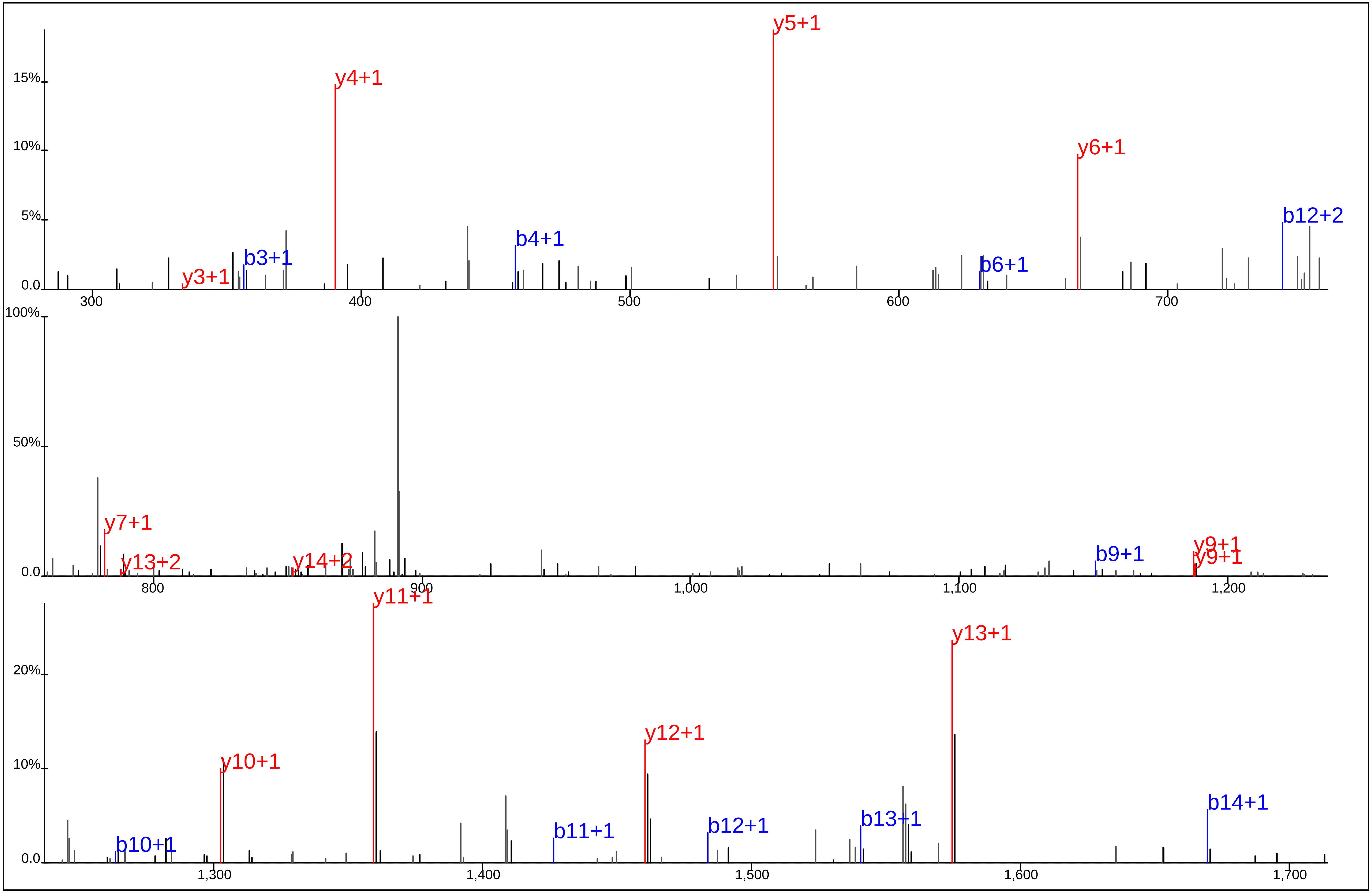

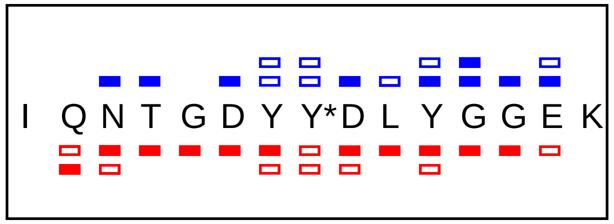

Protein Name: SHP2
gi Accession: 33356177
Precursor Mass: 908.3806+2
Delta Mass: 3.672 ppm
XCorr: 52.72

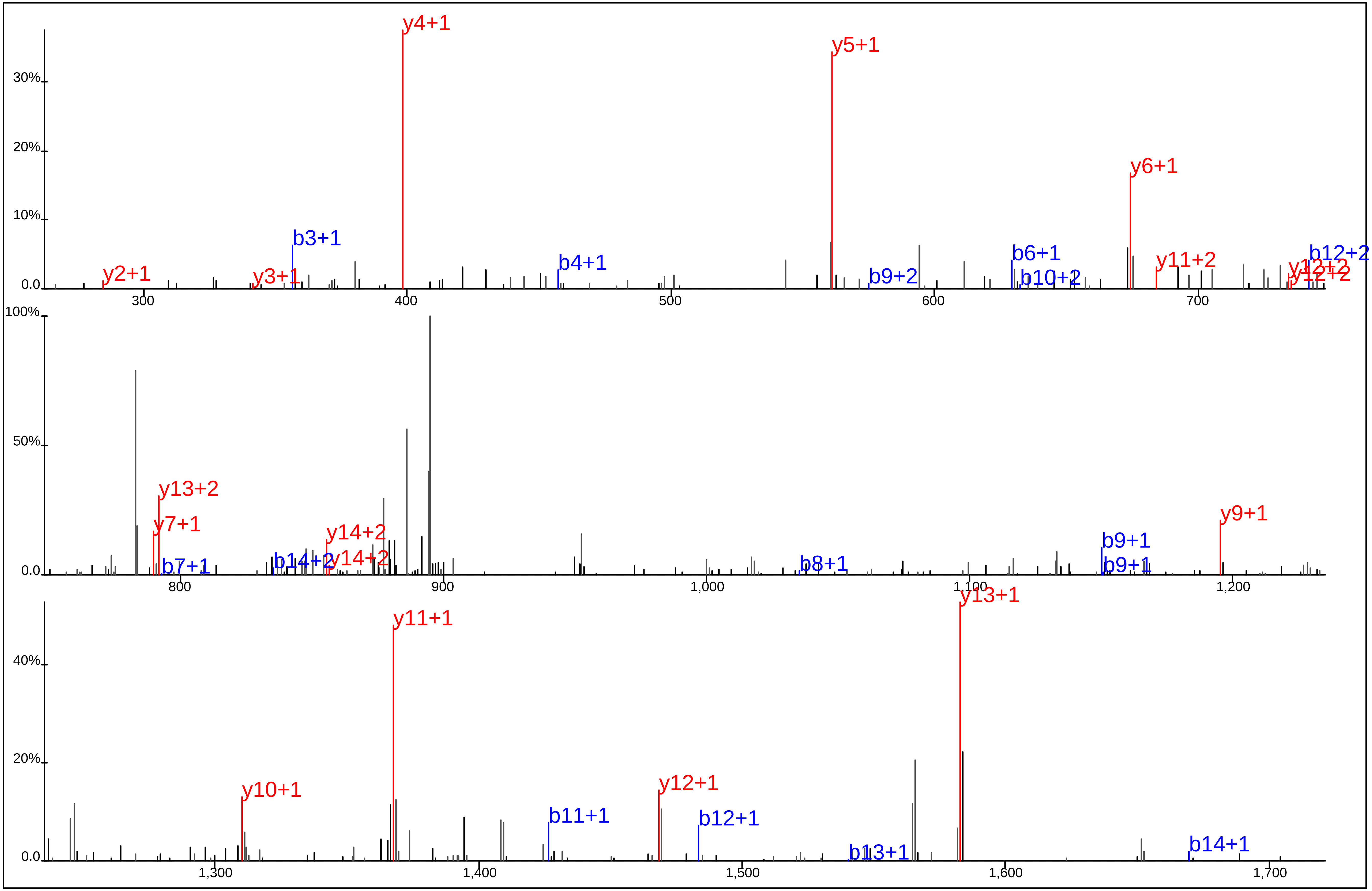

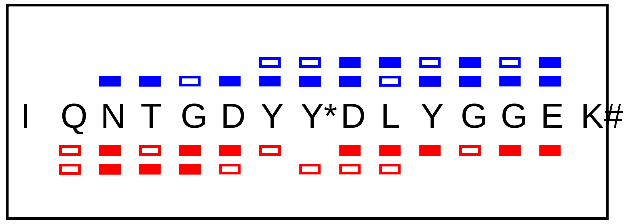

Protein Name: SHP2
gi Accession: 33356177
Precursor Mass: 912.3899+2
Delta Mass: 6.047 ppm
XCorr: 72.5

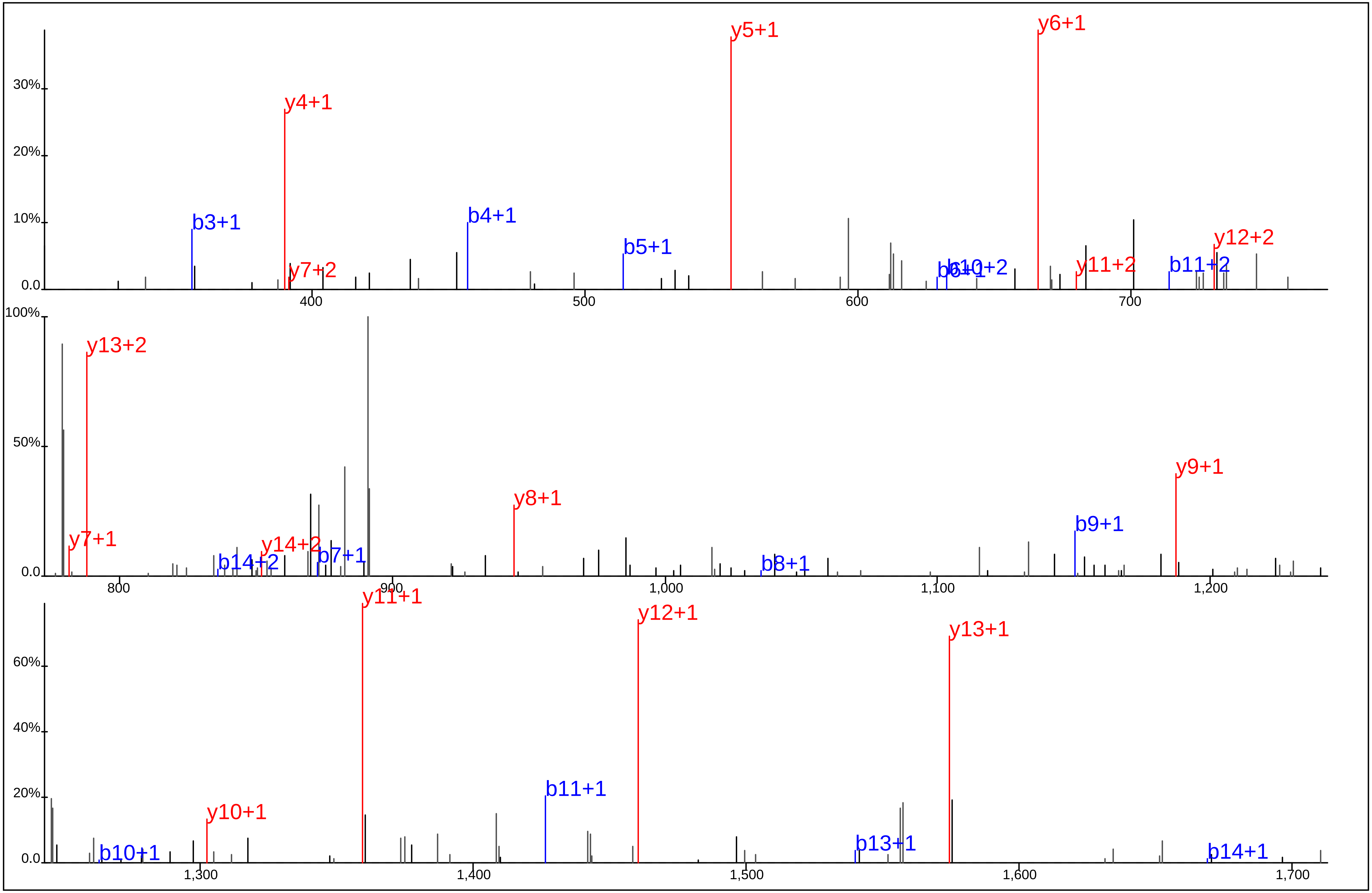

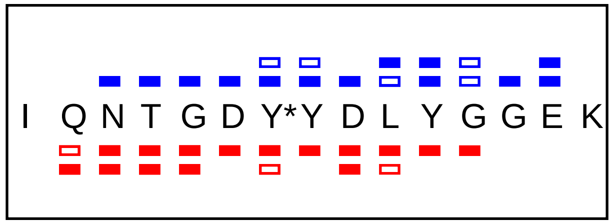

Protein Name: SHP2
gi Accession: 33356177
Precursor Mass: 908.3759+2
Delta Mass: 1.505 ppm
XCorr: 53.73

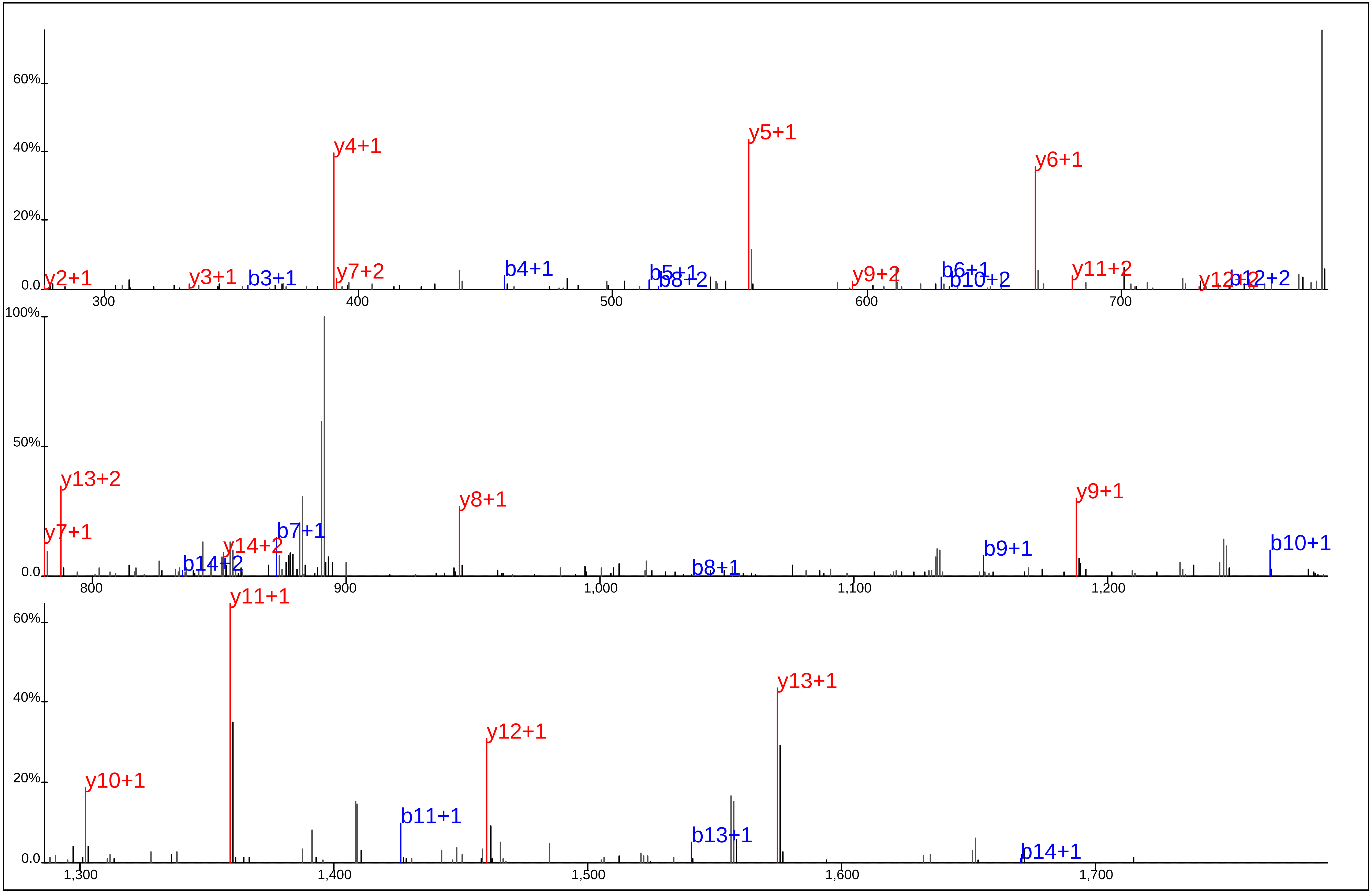

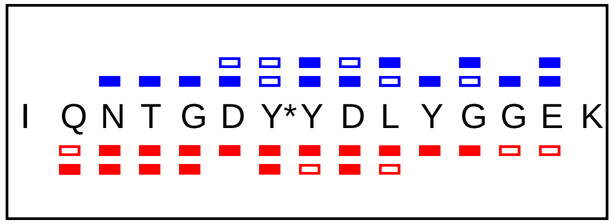

Protein Name:	SHP2
gi Accession:	33356177
Precursor Mass:	908.3782+2
Delta Mass:	0.985 ppm
XCorr:	70.69

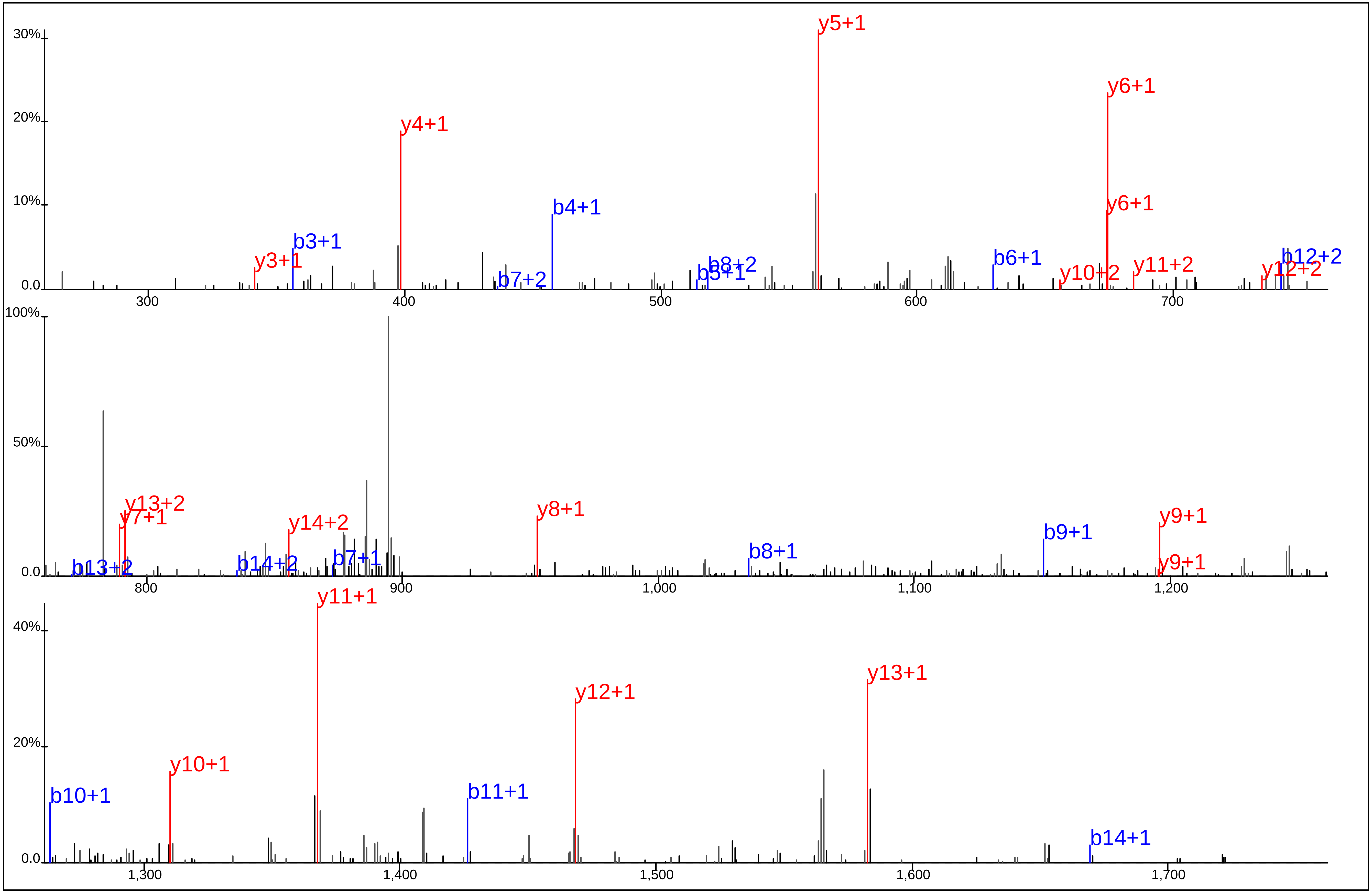

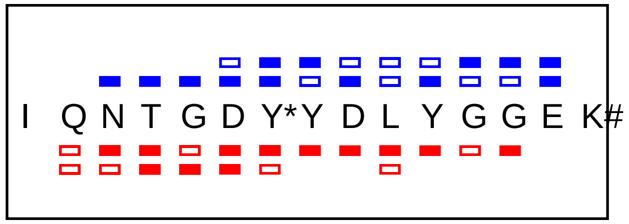

Protein Name:	SHP2
gi Accession:	33356177
Precursor Mass:	912.3856+2
Delta Mass:	1.293 ppm
XCorr:	74.39

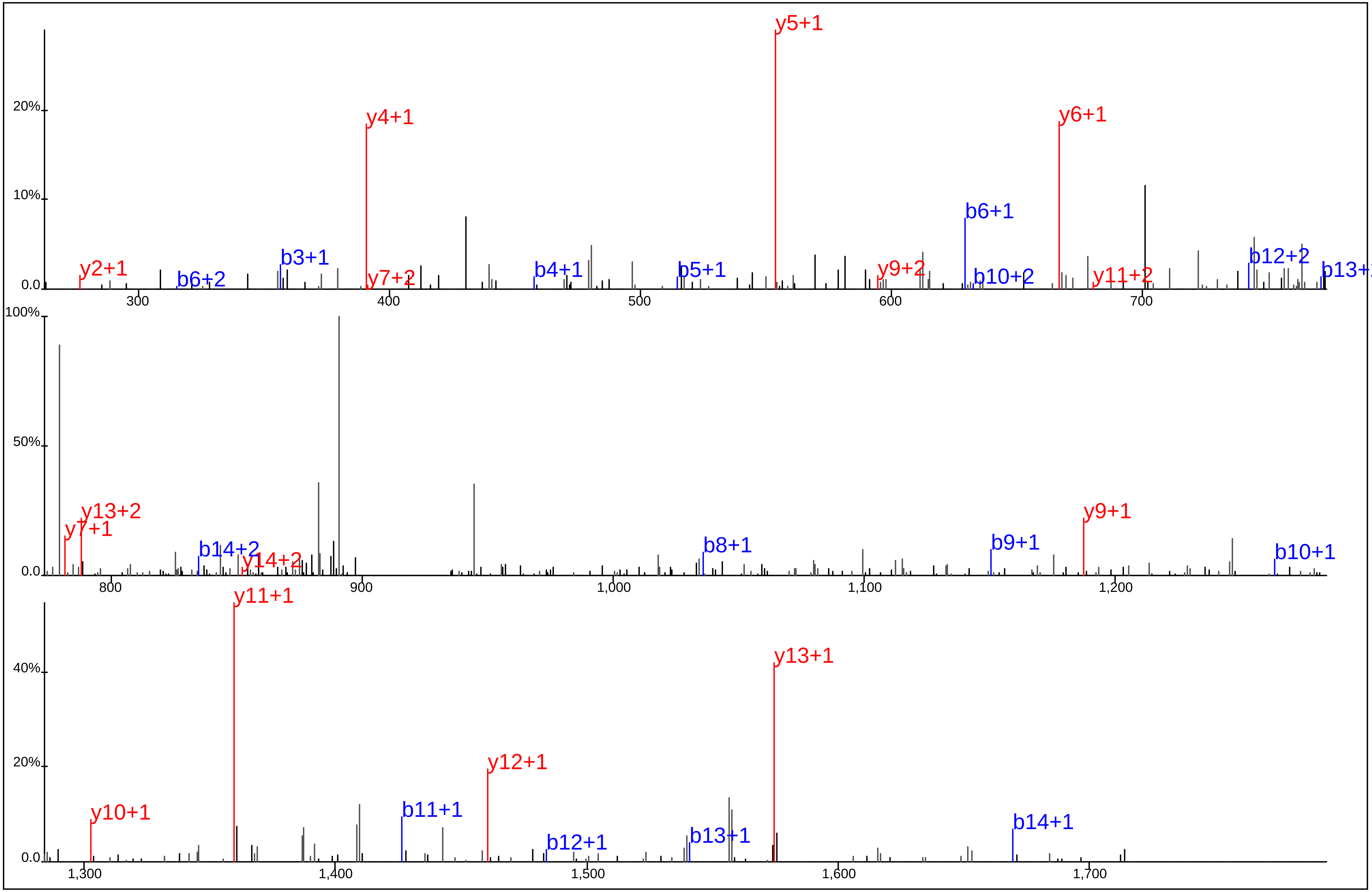

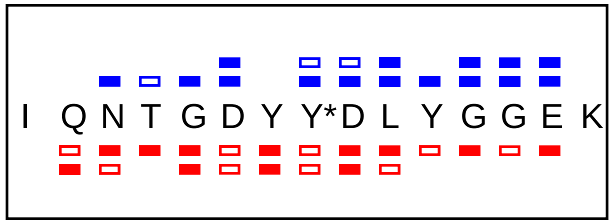

Protein Name: SHP2
gi Accession: 33356177
Precursor Mass: 908.3772+2
Delta Mass: 0.089 ppm
XCorr: 57.89

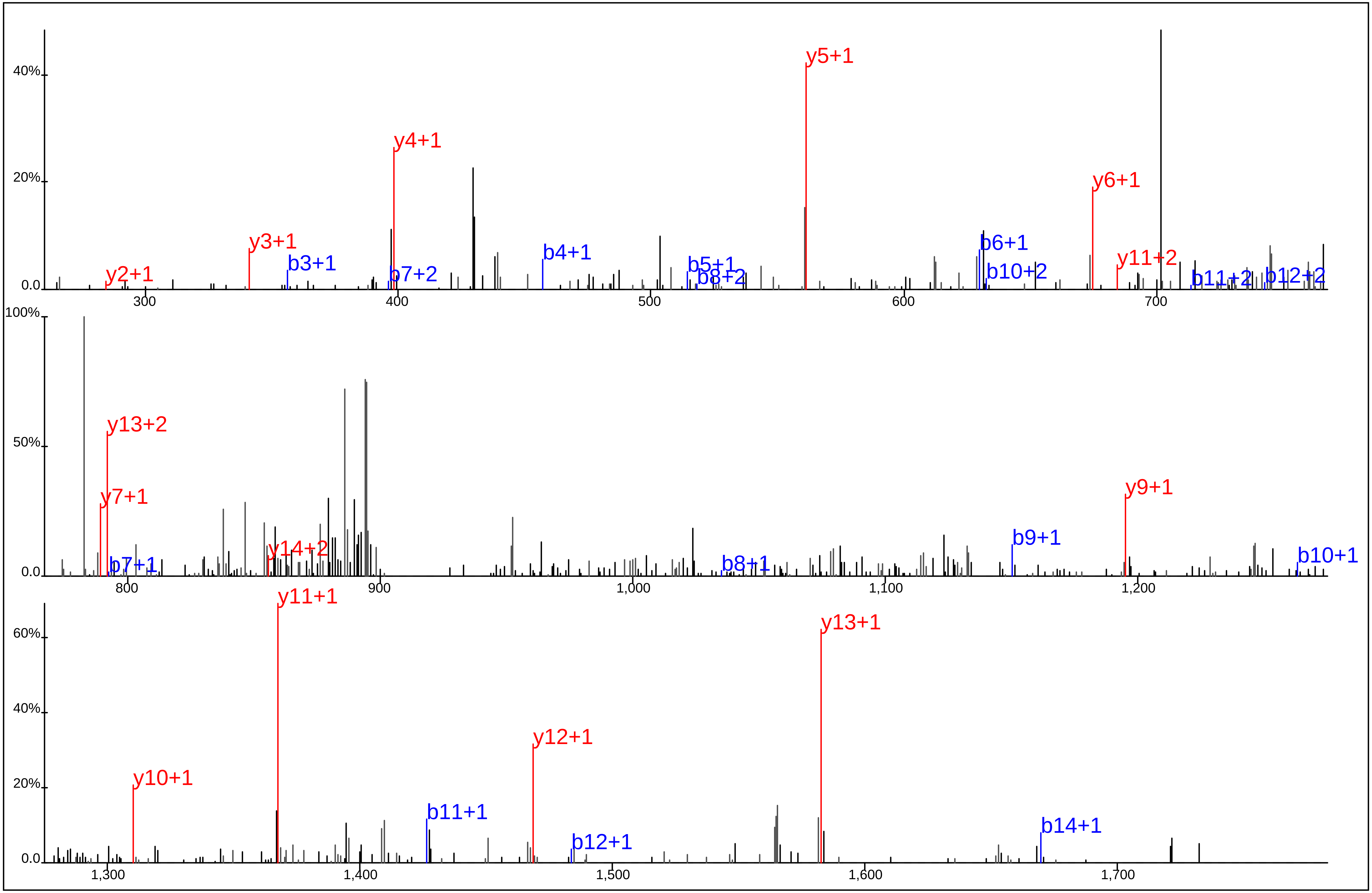

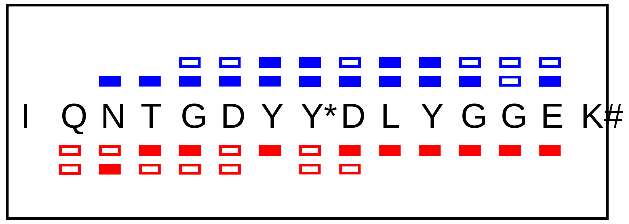

Protein Name: SHP2
gi Accession: 33356177
Precursor Mass: 912.3876+2
Delta Mass: 3.503 ppm
XCorr: 60.45

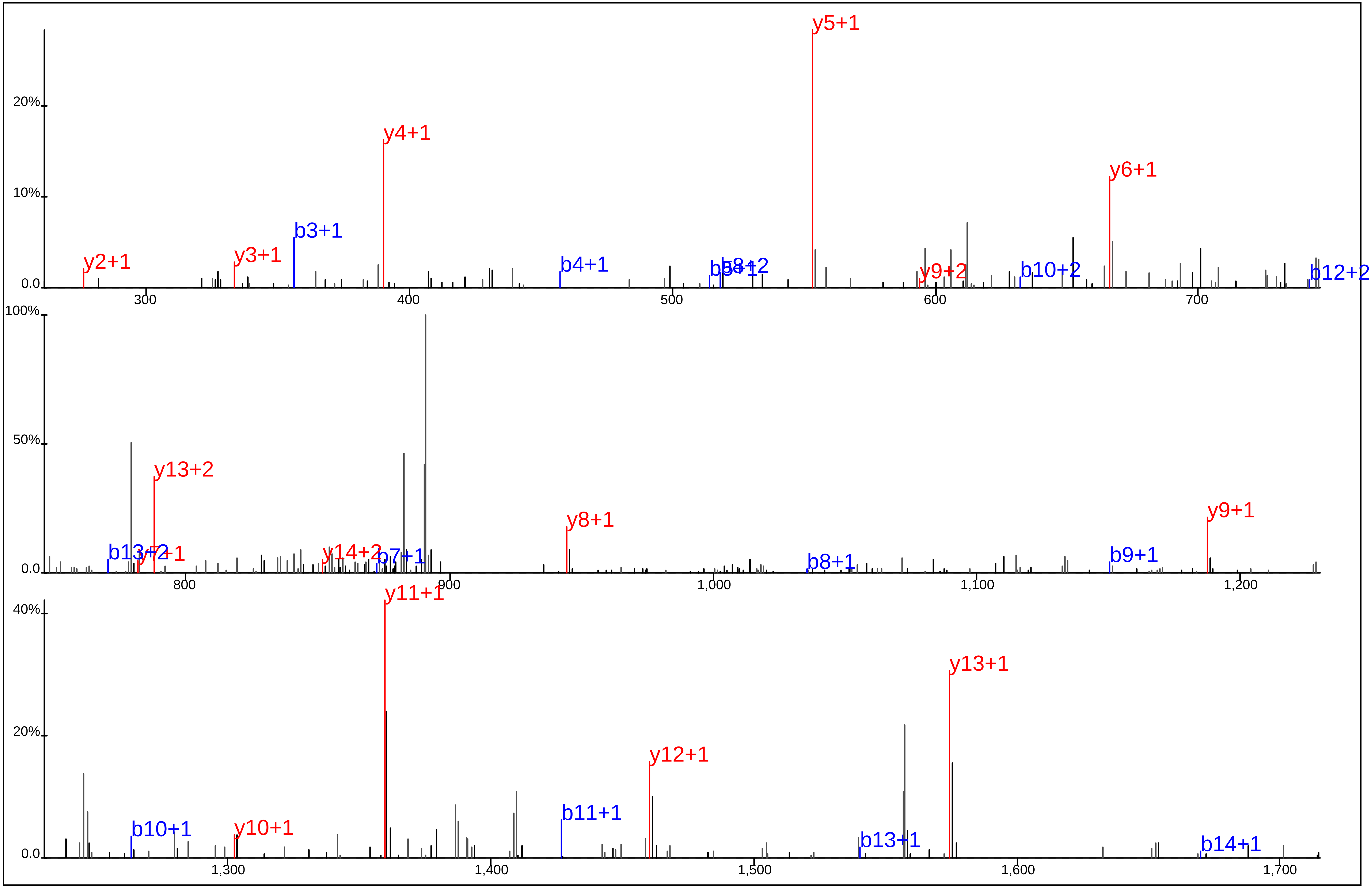

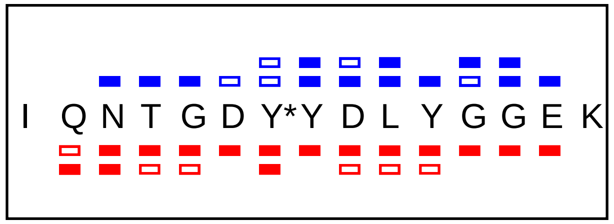

- Protein Name: SHP2
- gi Accession: 33356177
- Precursor Mass: 908.3826+2
- Delta Mass: 5.892 ppm
- XCorr: 48.38

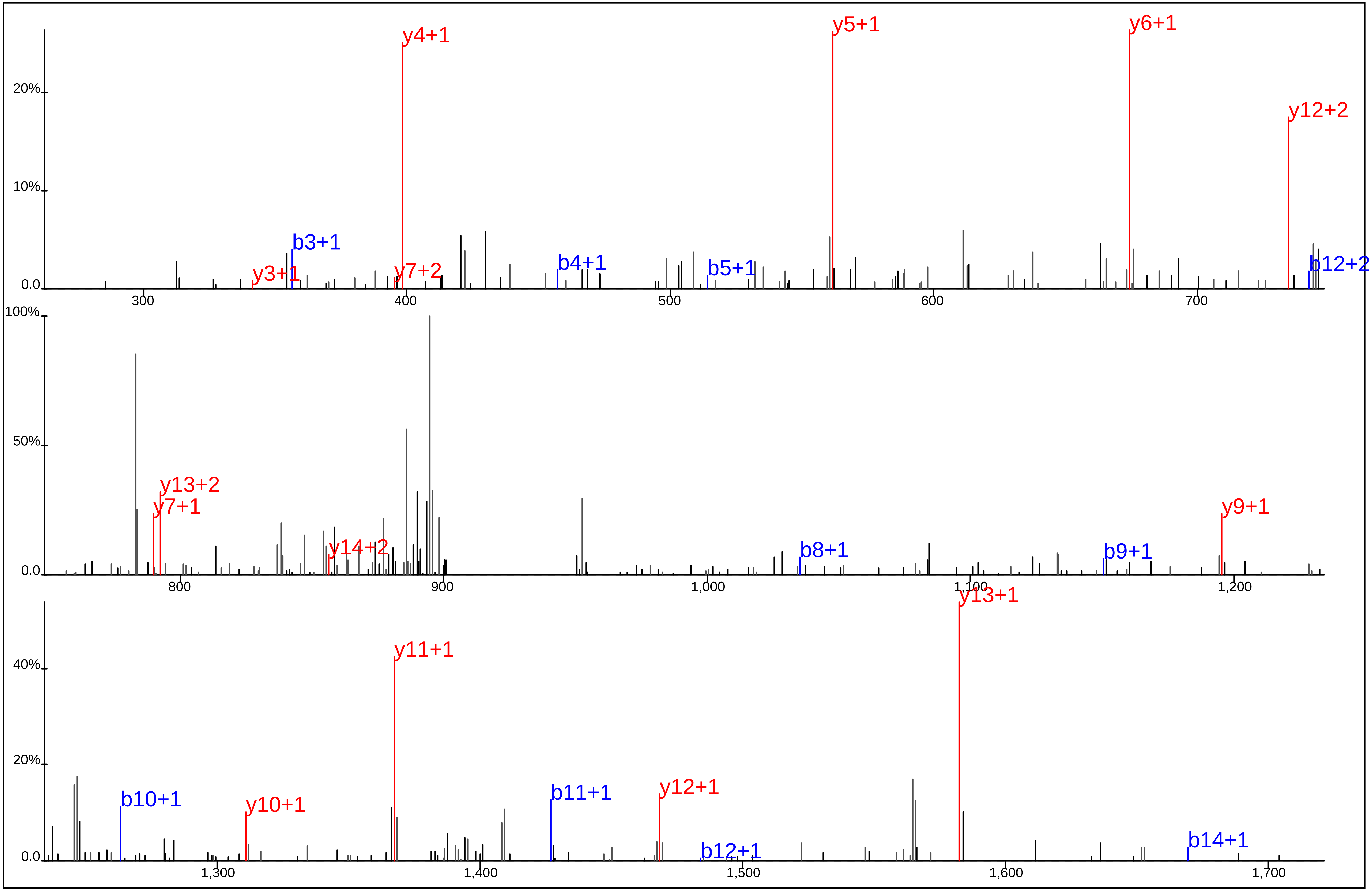

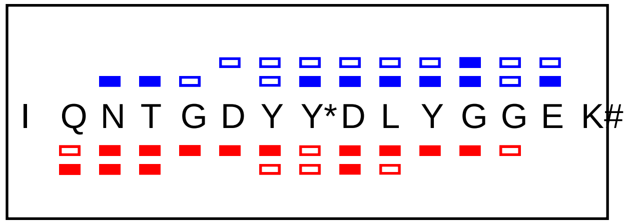

Protein Name:	SHP2
gi Accession:	33356177
Precursor Mass:	912.3886+2
Delta Mass:	4.643 ppm
XCorr:	55.72

917 - SHP2_3448280.pdf

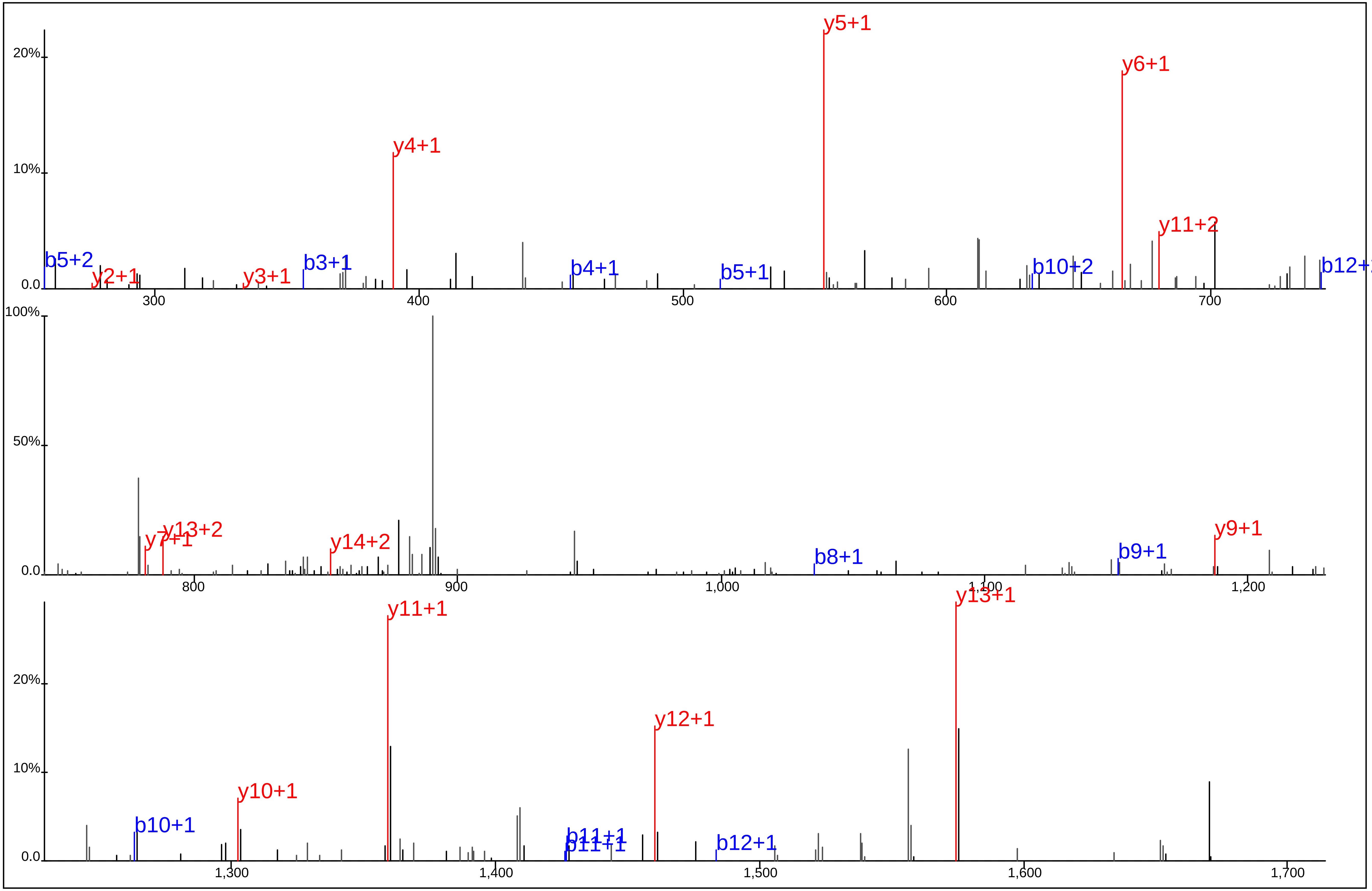

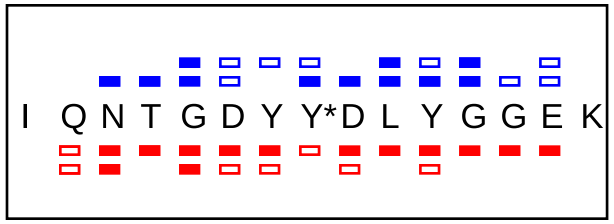

Protein Name: SHP2
gi Accession: 33356177
Precursor Mass: 908.3778+2
Delta Mass: 0.583 ppm
XCorr: 53.54

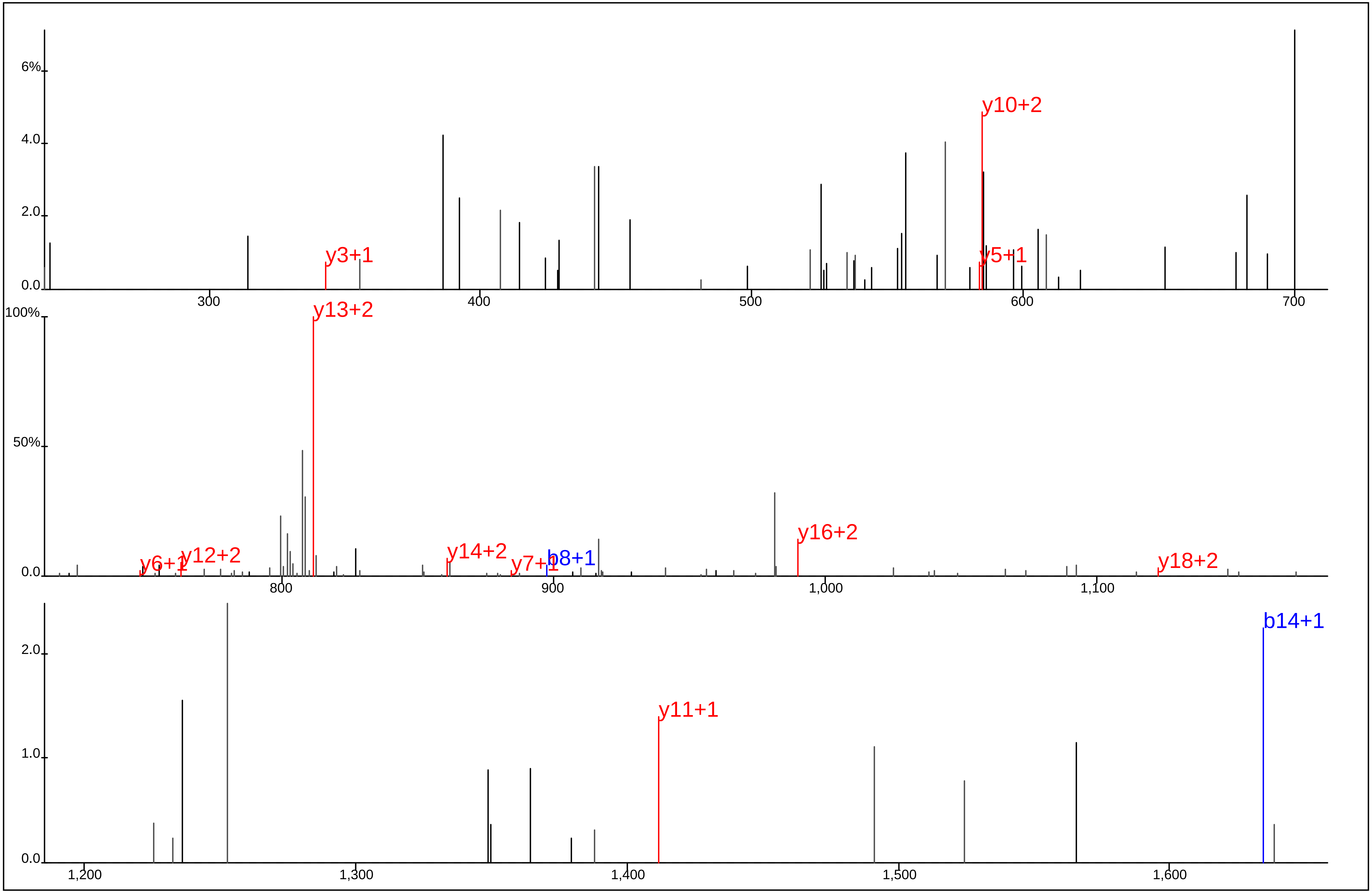

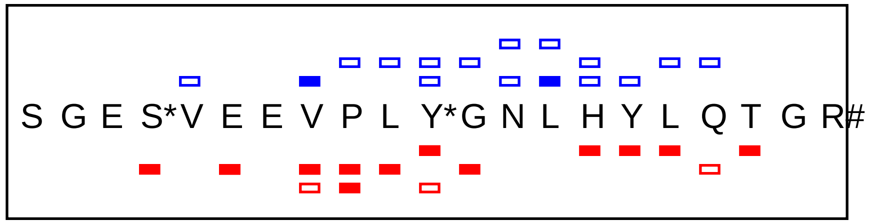

Protein Name: SIT
gi Accession: 7657577
Precursor Mass: 840.0376+3
Delta Mass: 2.224 ppm
XCorr: 3.3797

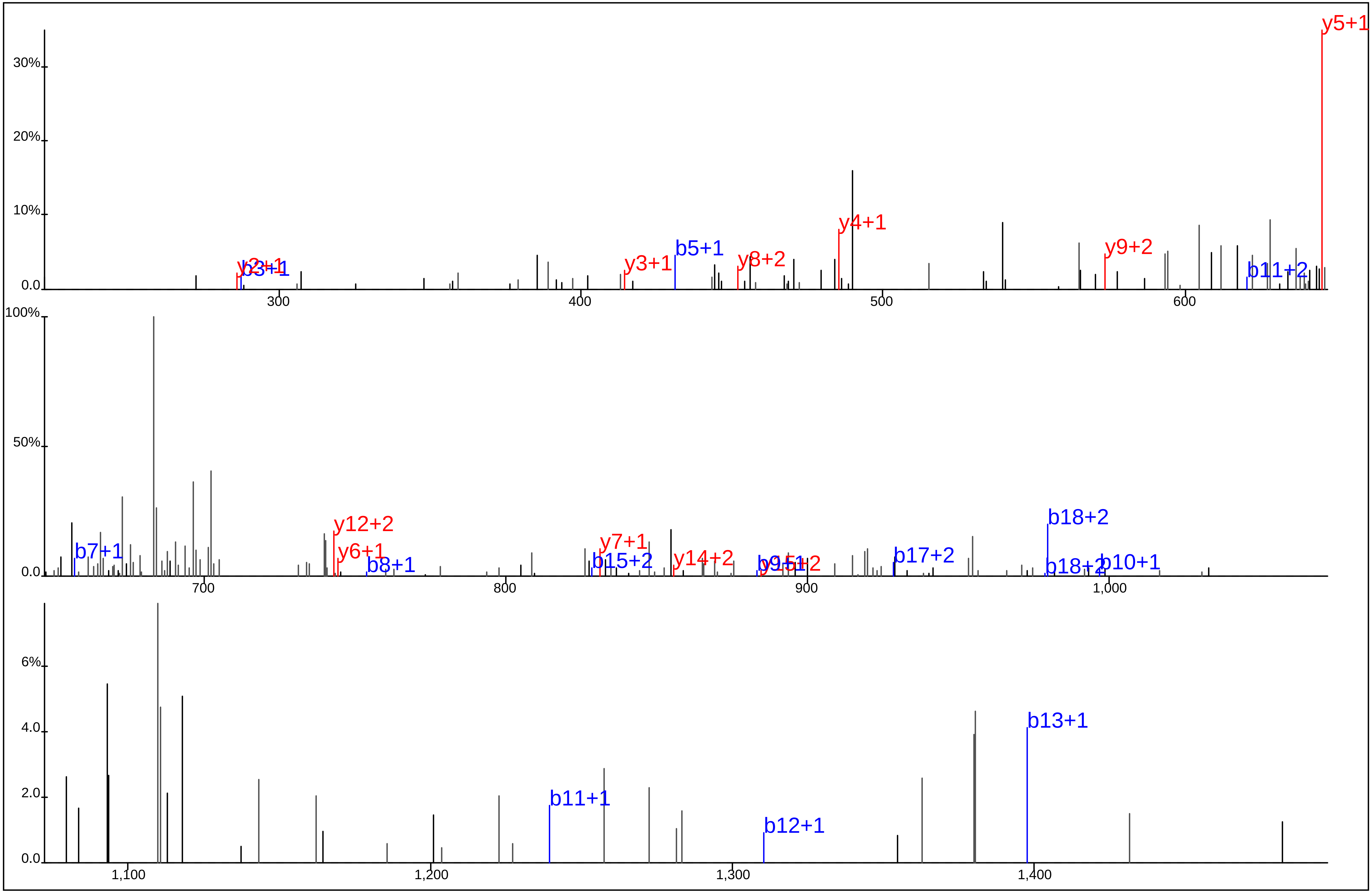

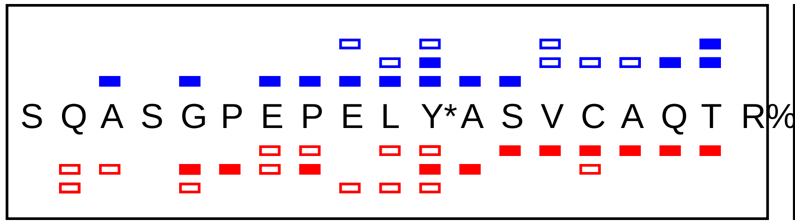

Protein Name: SIT
gi Accession: 7657577
Precursor Mass: 714.3173+3
Delta Mass: 6.067 ppm
XCorr: 14.02

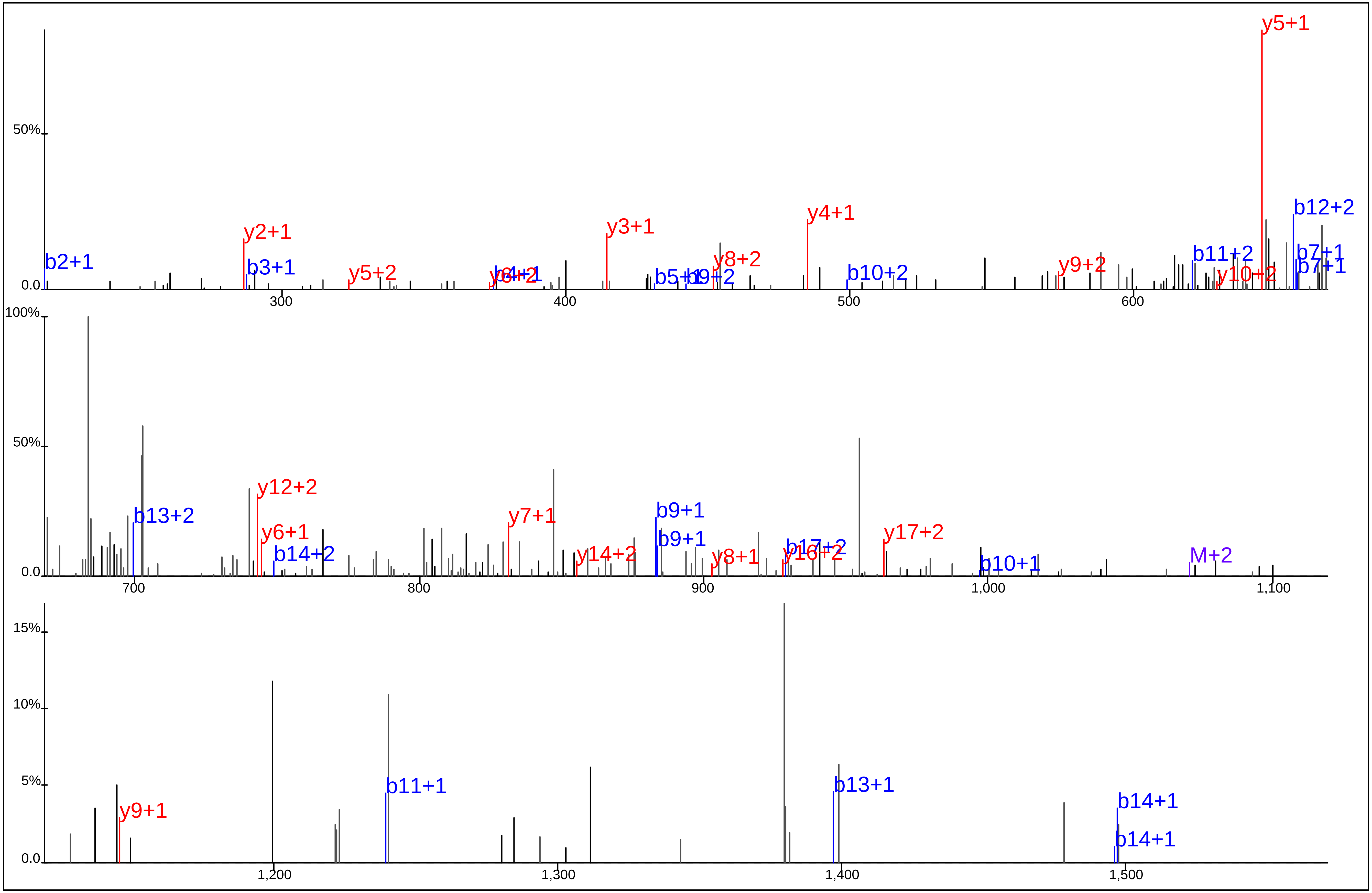

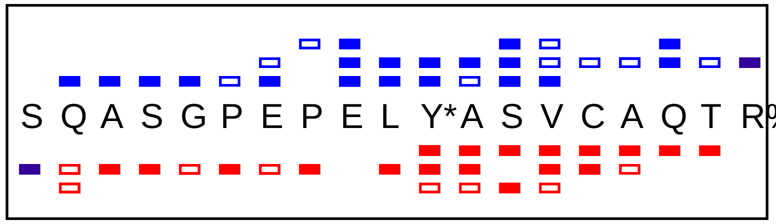

Protein Name: SIT
gi Accession: 7657577
Precursor Mass: 714.3158+3
Delta Mass: 3.927 ppm
XCorr: 26.92

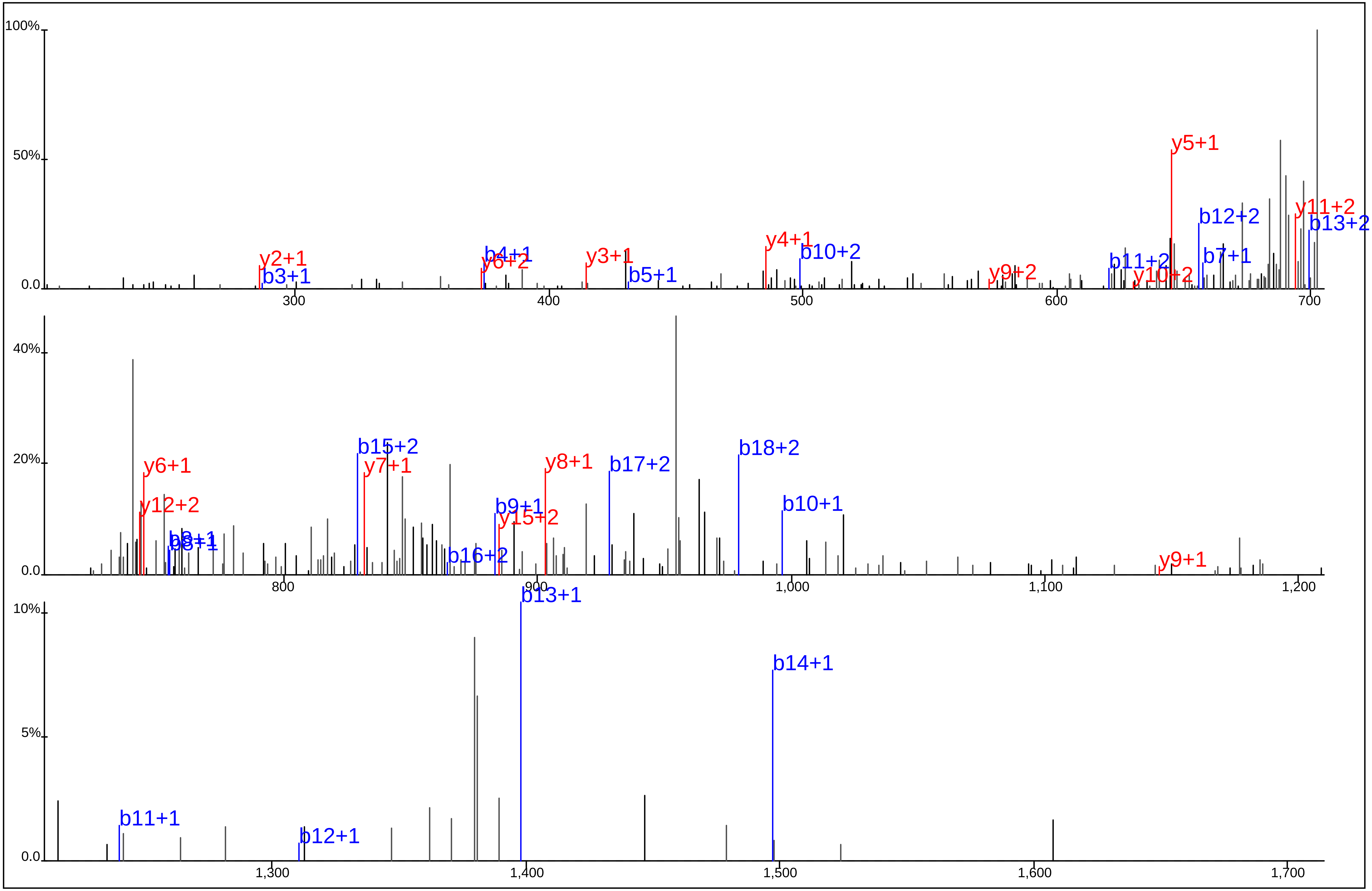

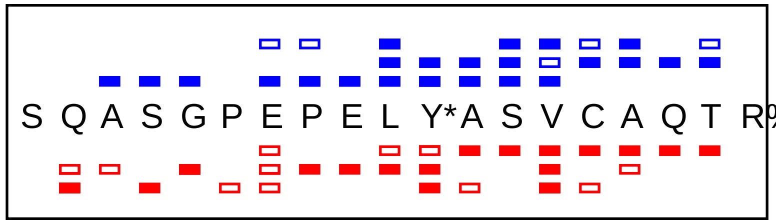

Protein Name: SIT
gi Accession: 7657577
Precursor Mass: 714.3151+3
Delta Mass: 2.904 ppm
XCorr: 24.02

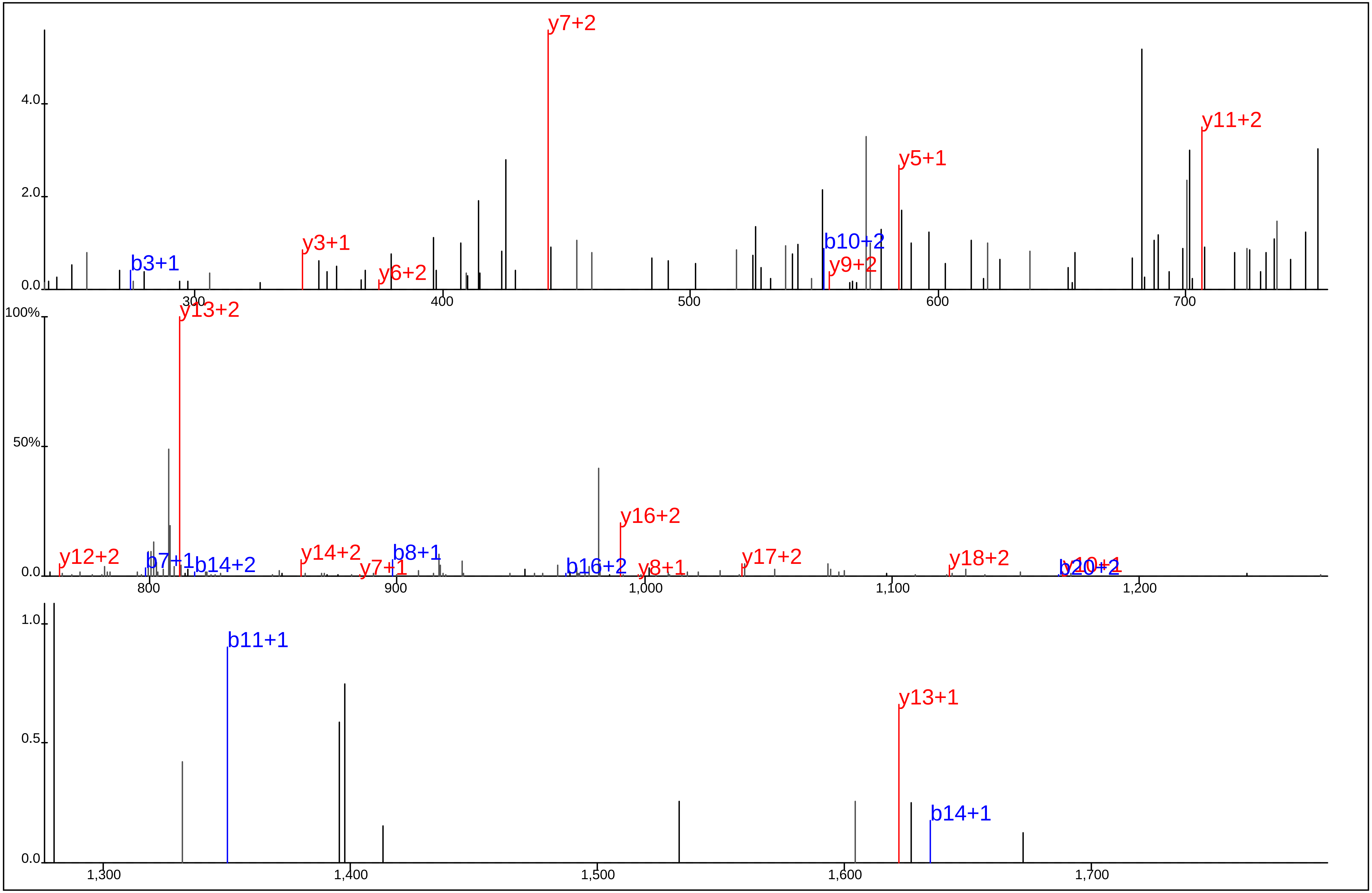

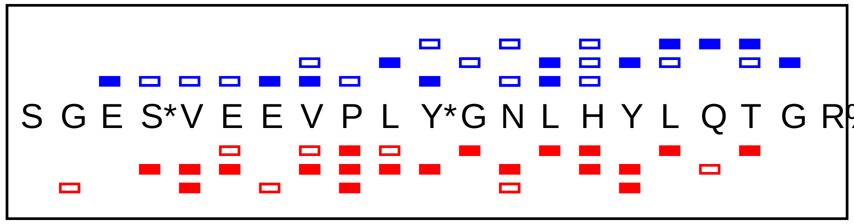

Protein Name:	SIT
gi Accession:	7657577
Precursor Mass:	840.0367+3
Delta Mass:	1.139 ppm
XCorr:	35.06

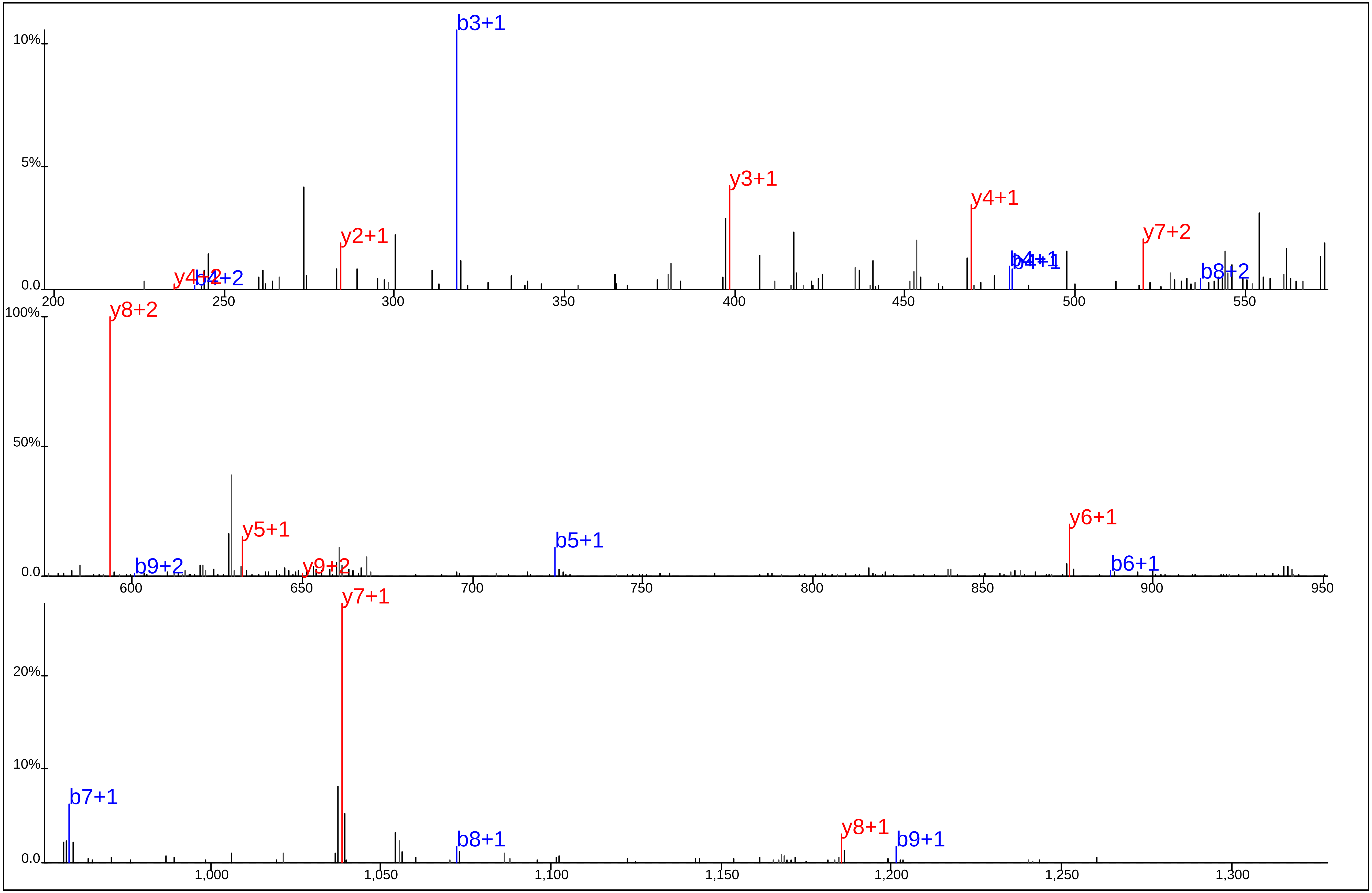

G L F Y Y*Y A N E K#

Protein Name: SKAP55
gi Accession: 114666366
Precursor Mass: 678.2972+2
Delta Mass: 5.325 ppm
XCorr: 32.8

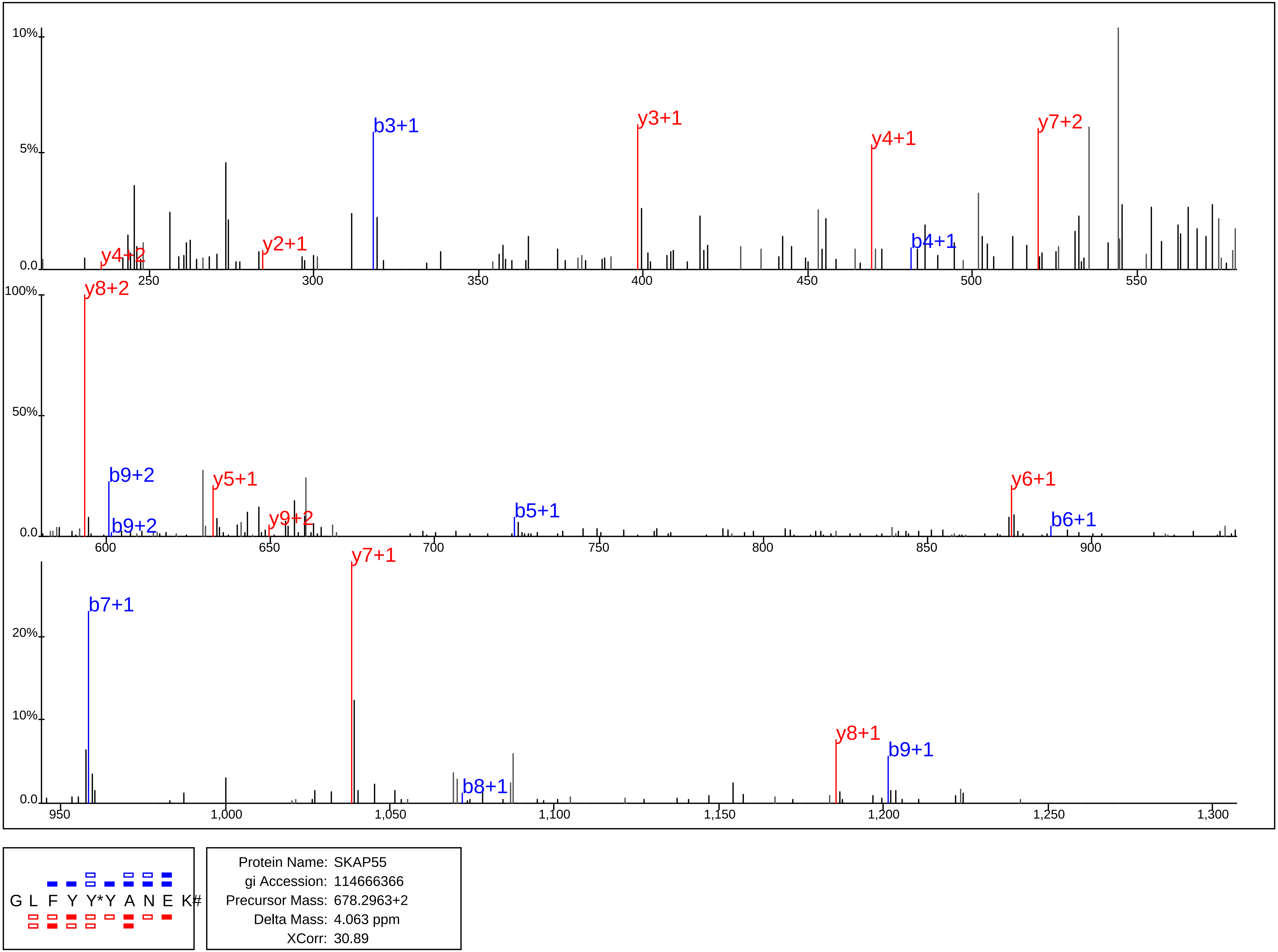

Protein Name: SKAP55
gi Accession: 114666366
Precursor Mass: 678.2963+2
Delta Mass: 4.063 ppm
XCorr: 30.89

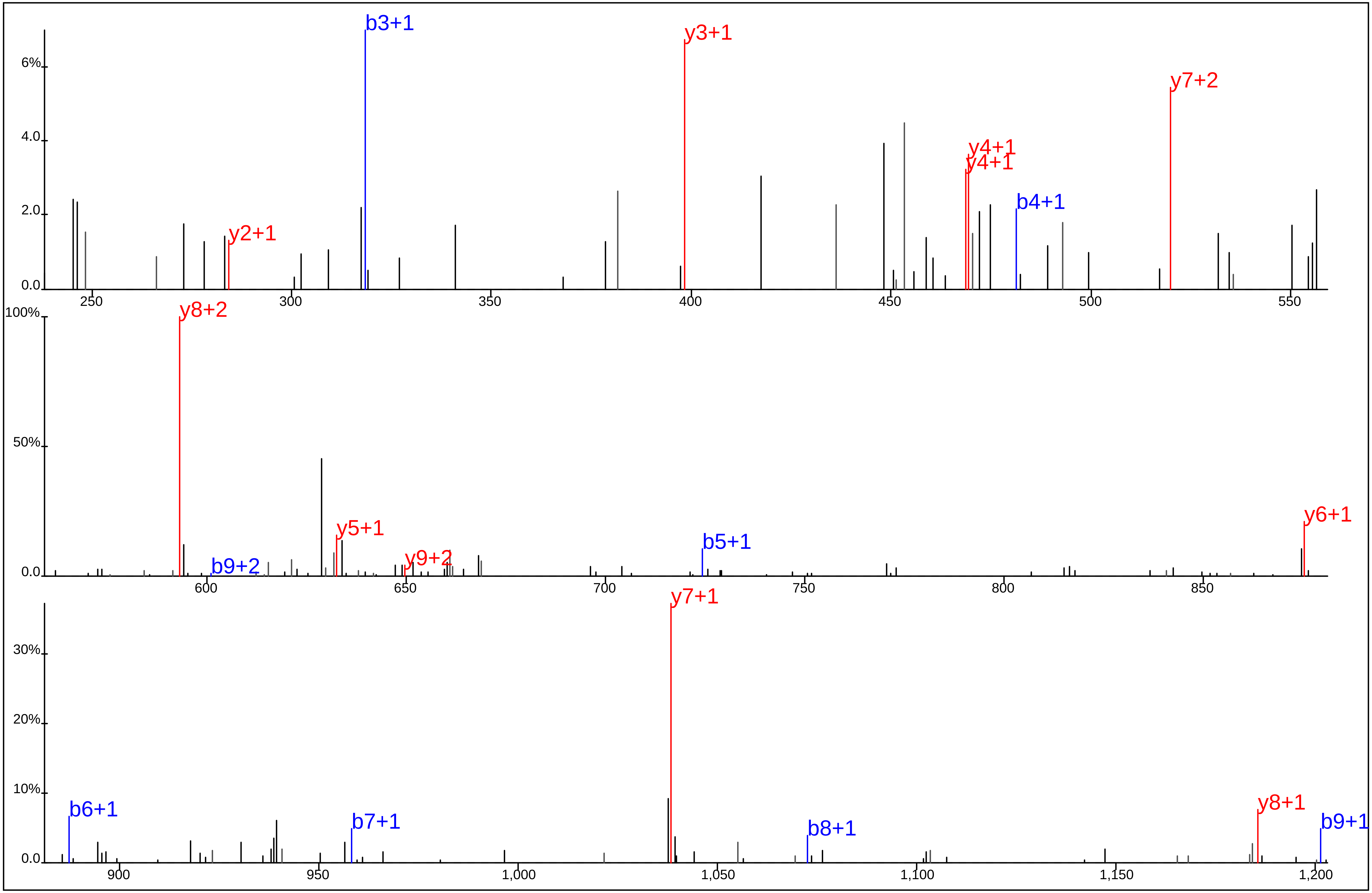

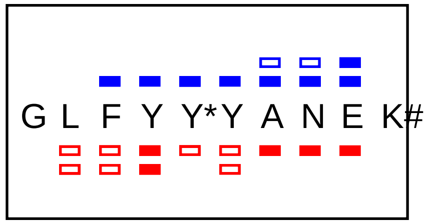

Protein Name: SKAP55
gi Accession: 114666366
Precursor Mass: 678.2963+2
Delta Mass: 3.975 ppm
XCorr: 34.99

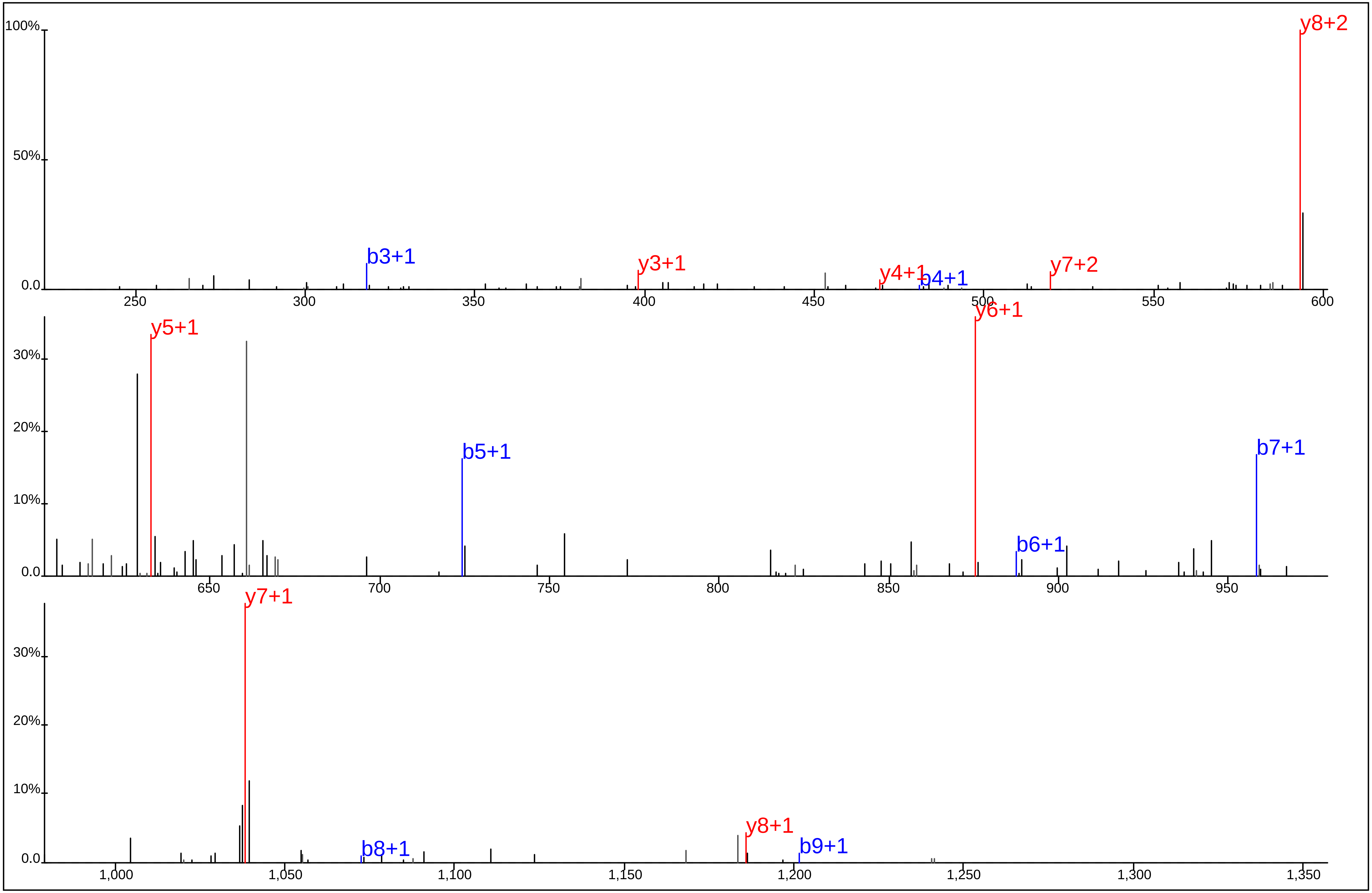

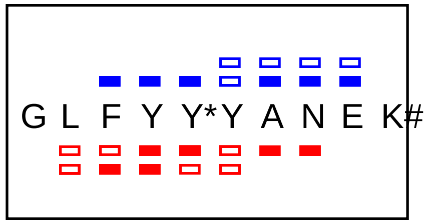

Protein Name: SKAP55
gi Accession: 114666366
Precursor Mass: 678.2964+2
Delta Mass: 4.248 ppm
XCorr: 42.03

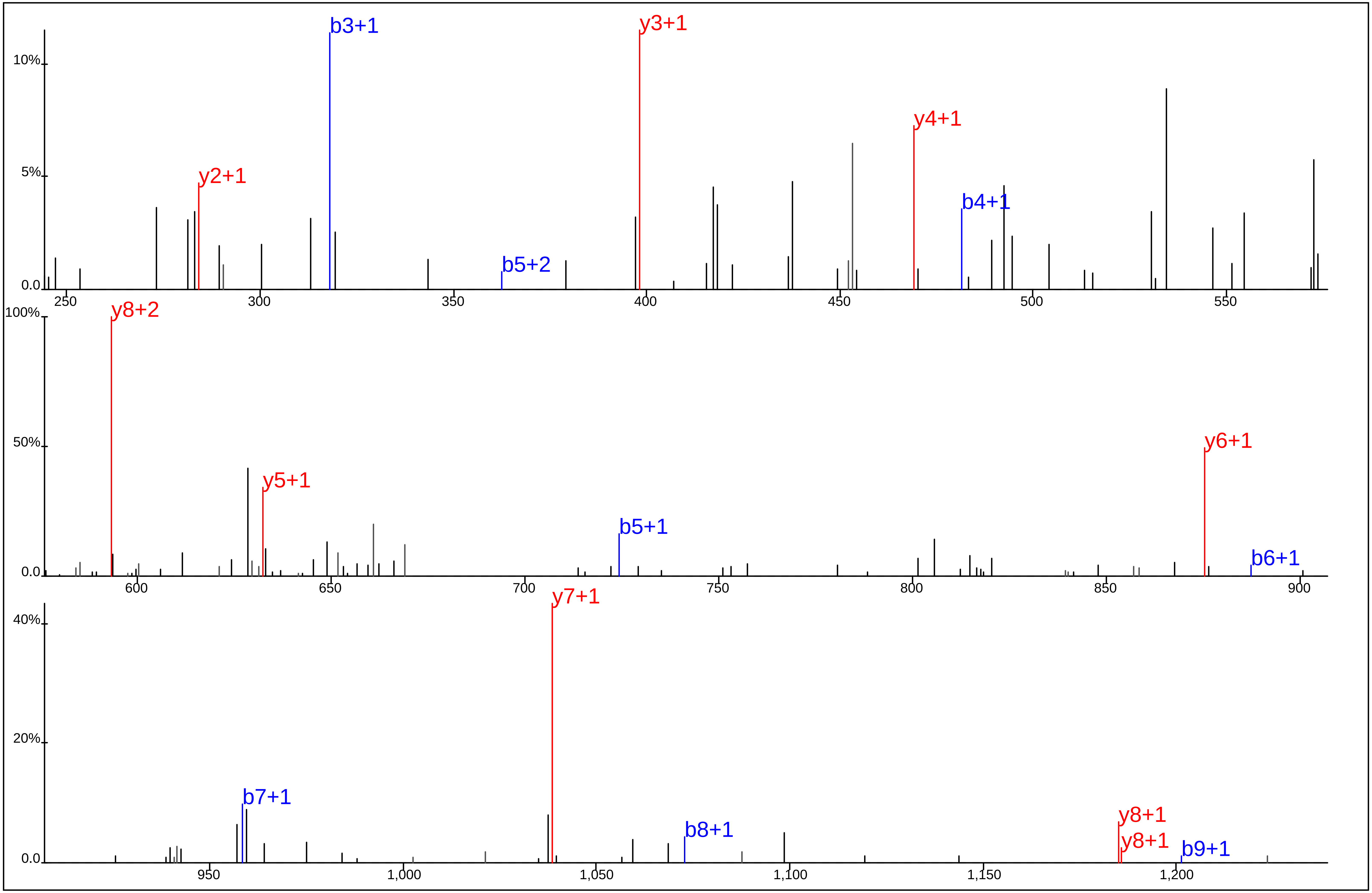

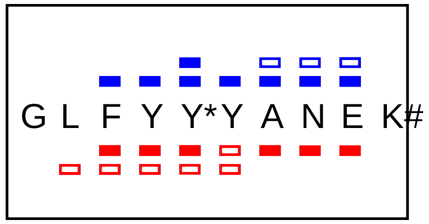

Protein Name: SKAP55
gi Accession: 114666366
Precursor Mass: 678.2967+2
Delta Mass: 4.609 ppm
XCorr: 35.96

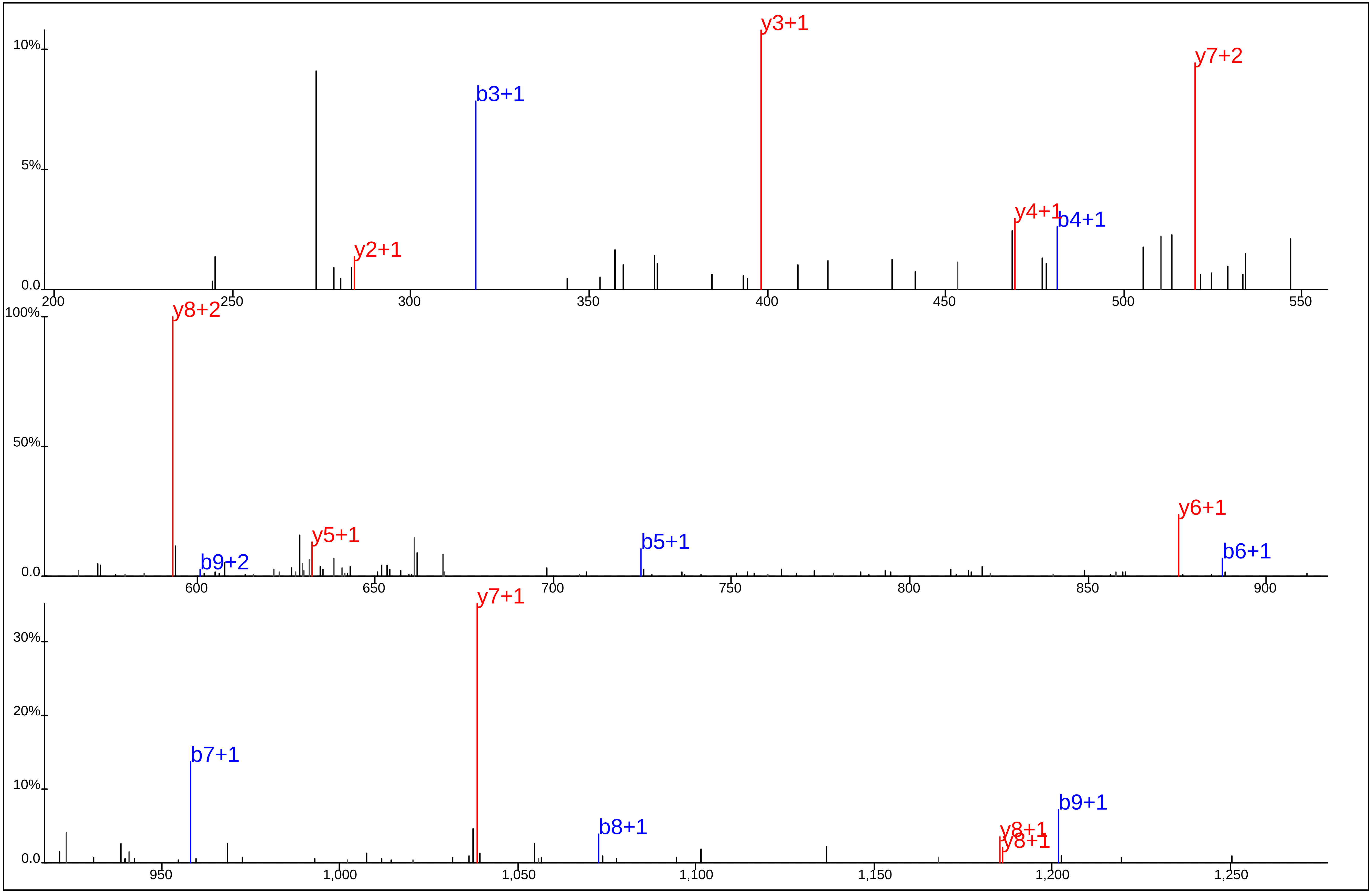

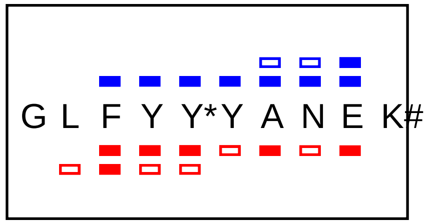

Protein Name:	SKAP55
gi Accession:	114666366
Precursor Mass:	678.2970+2
Delta Mass:	5.059 ppm
XCorr:	43.32

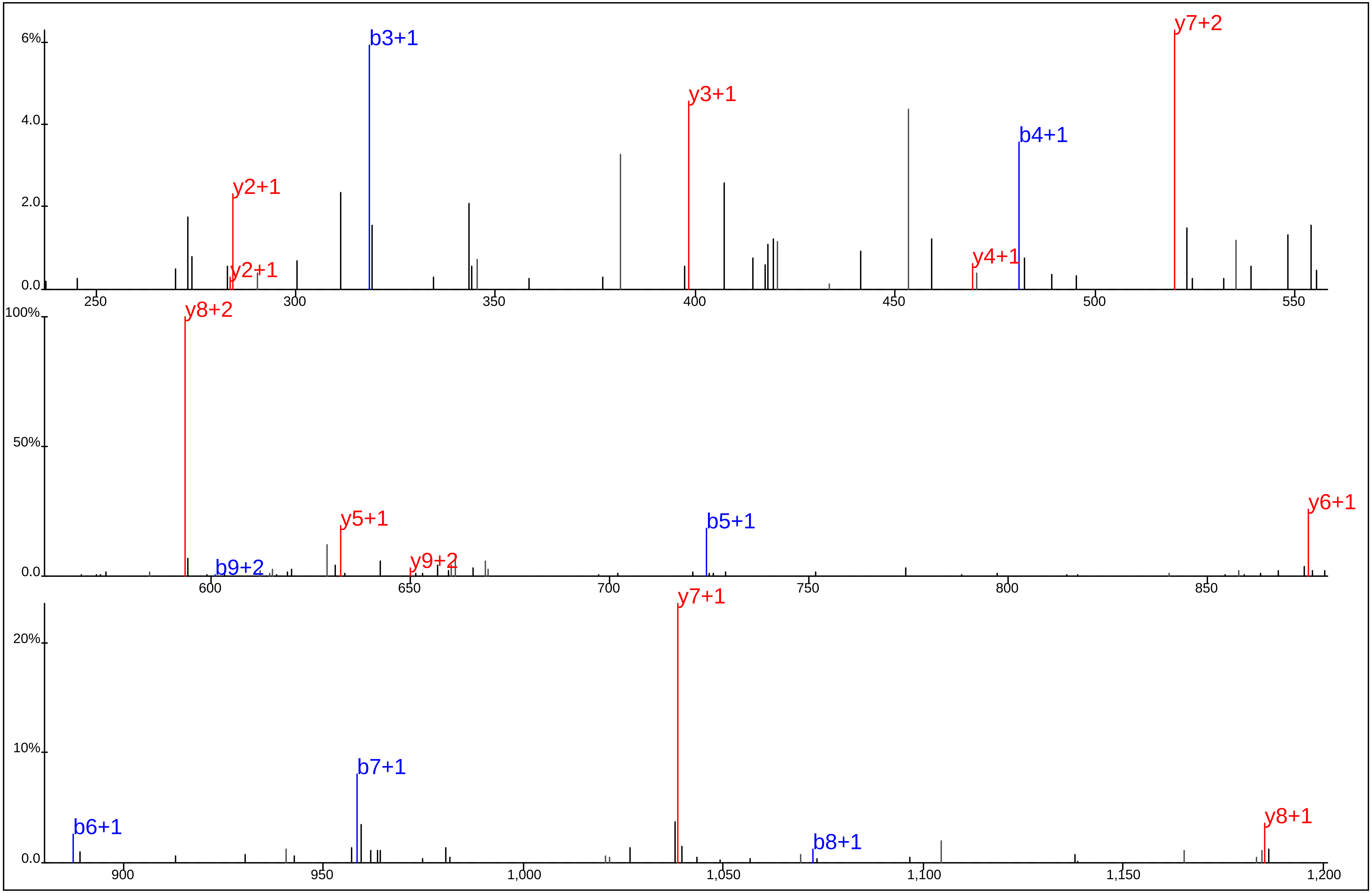

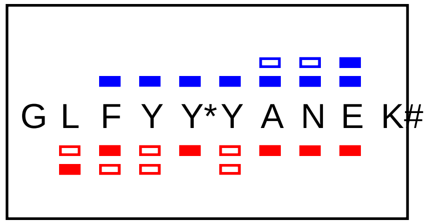

Protein Name: SKAP55
gi Accession: 114666366
Precursor Mass: 678.2980+2
Delta Mass: 6.498 ppm
XCorr: 37.25

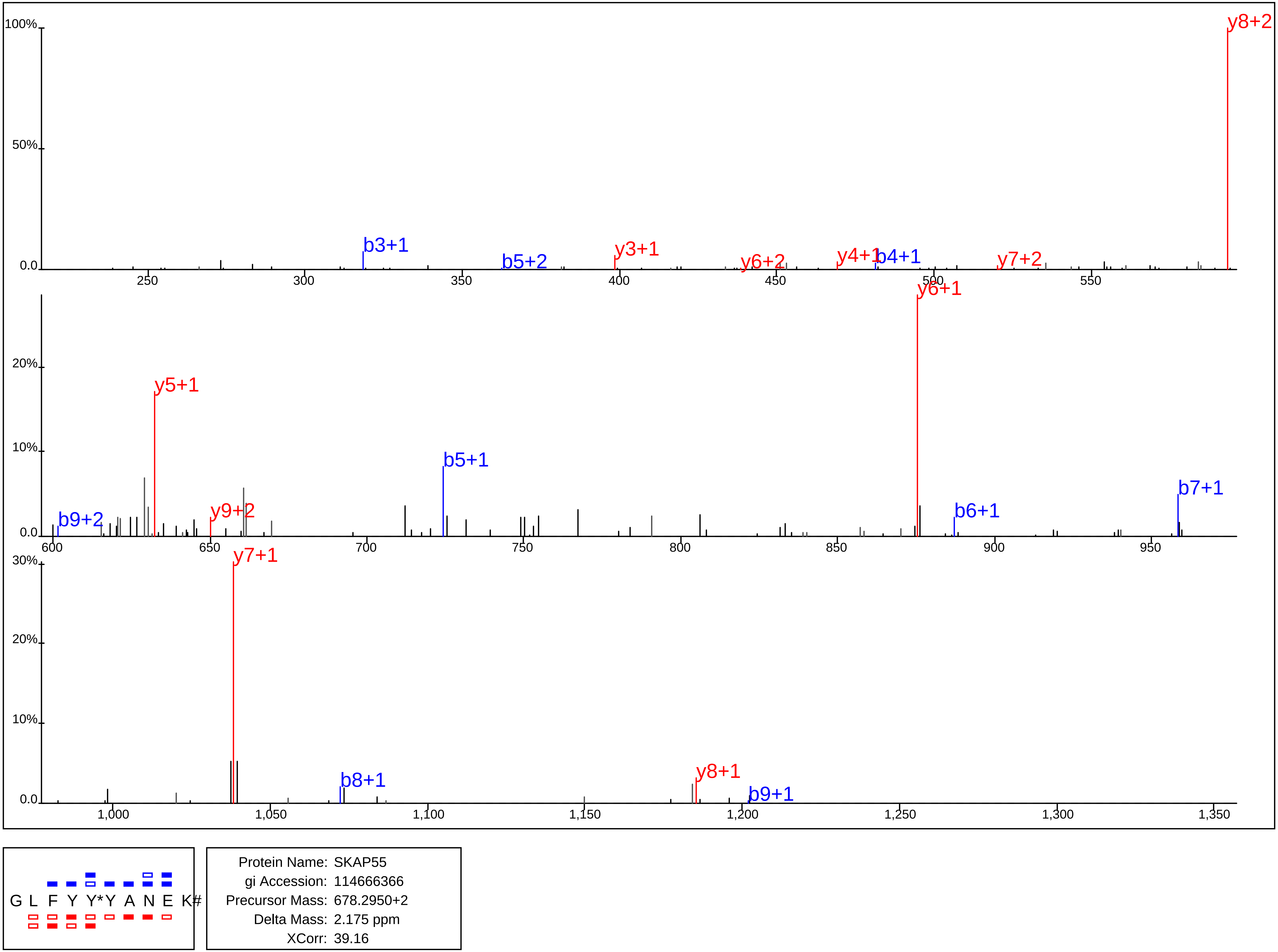

Protein Name: SKAP55
gi Accession: 114666366
Precursor Mass: 678.2950+2
Delta Mass: 2.175 ppm
XCorr: 39.16

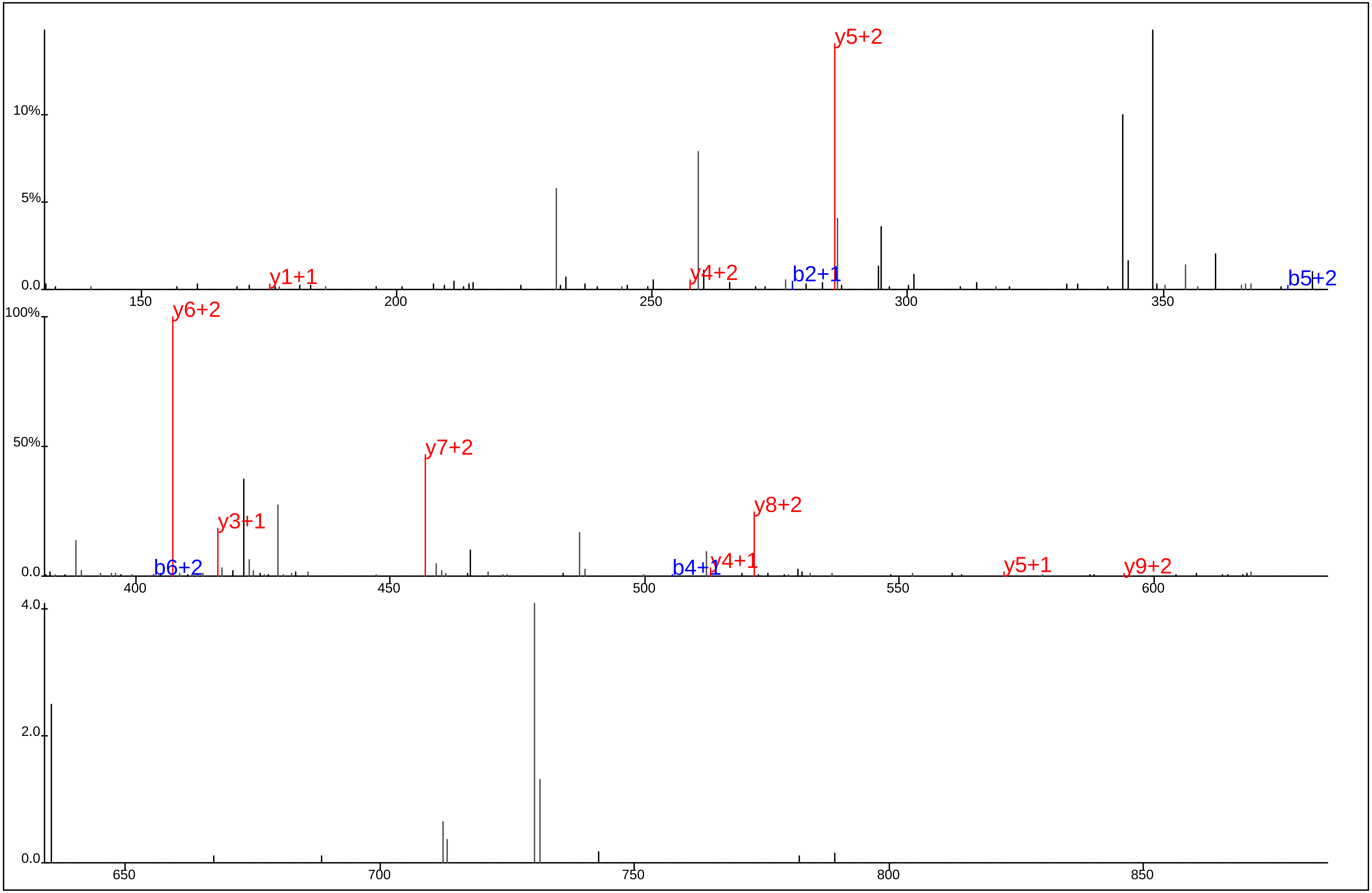

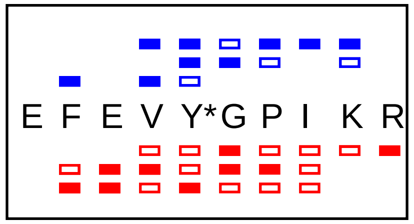

Protein Name: snRNP70
gi Accession: 602021
Precursor Mass: 439.8807+3
Delta Mass: 2.889 ppm
XCorr: 15.96

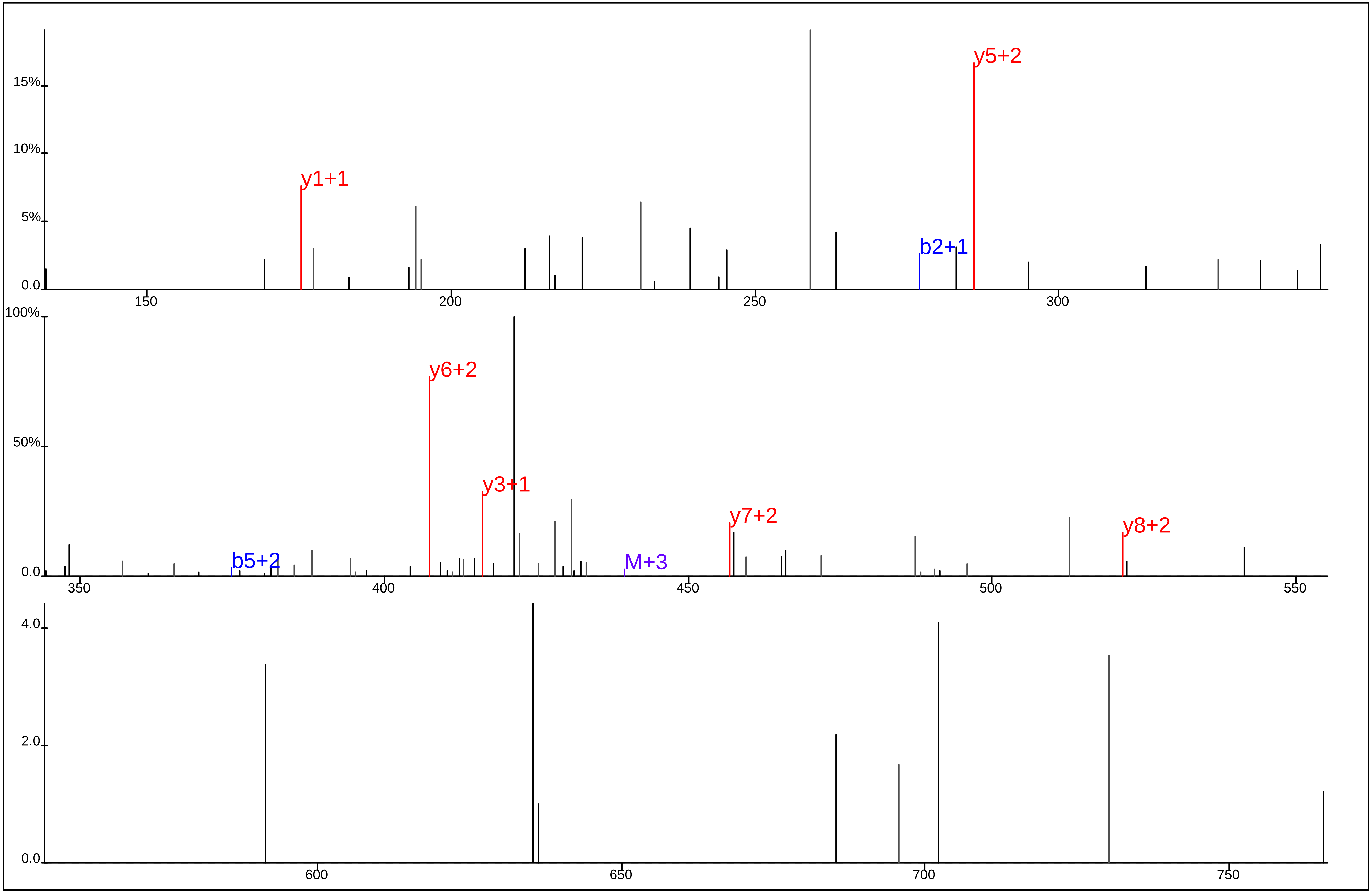

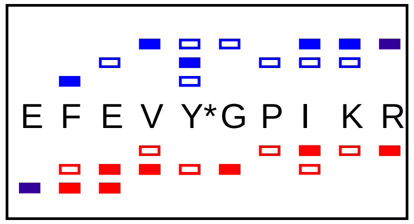

Protein Name: snRNP70
gi Accession: 602021
Precursor Mass: 439.8806+3
Delta Mass: 2.473 ppm
XCorr: 14.17

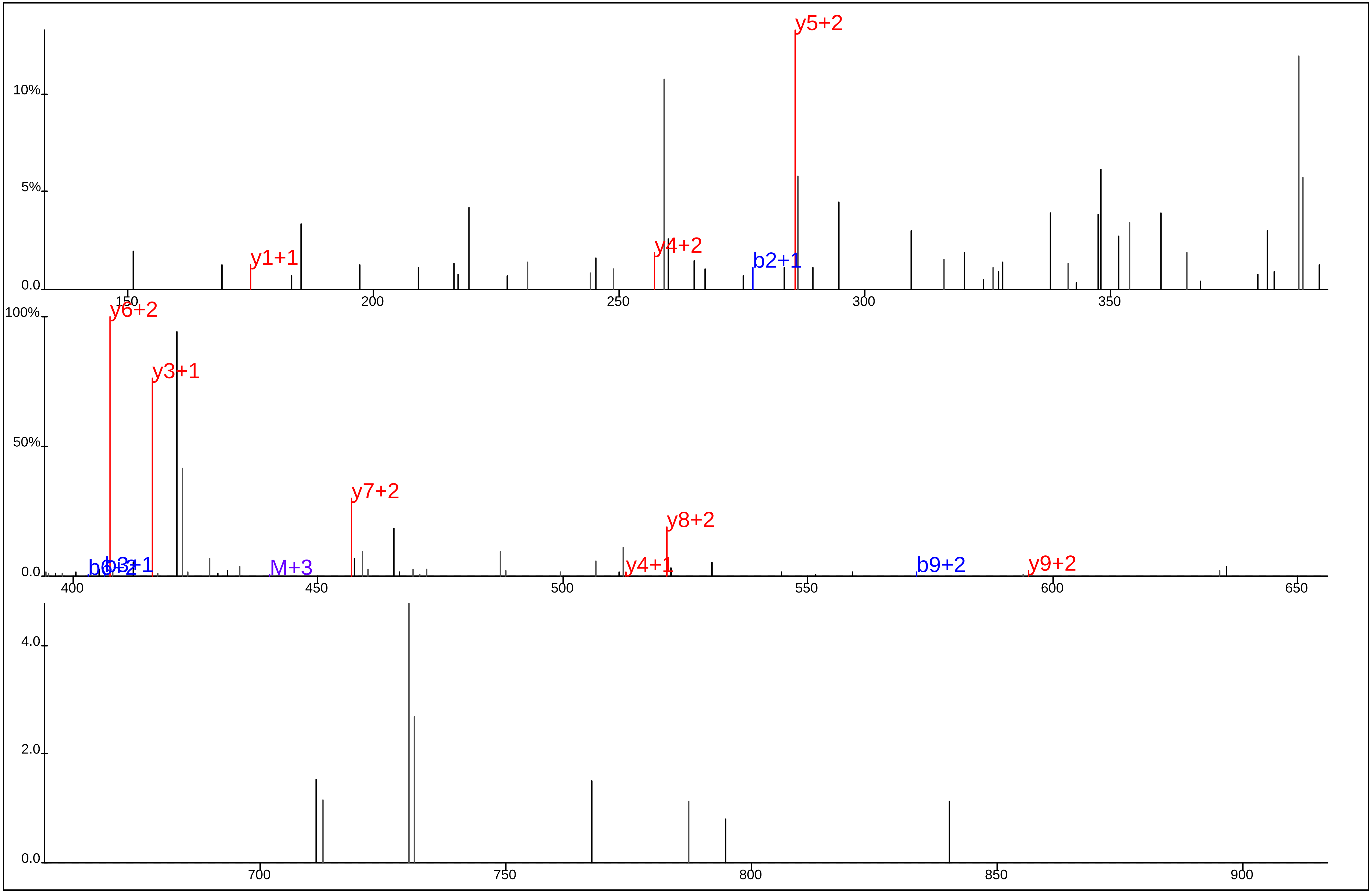

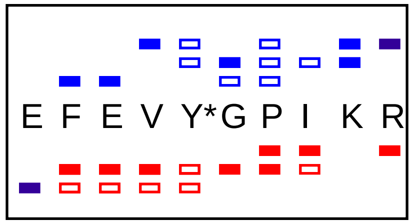

Protein Name: snRNP70
gi Accession: 602021
Precursor Mass: 439.8802+3
Delta Mass: 1.637 ppm
XCorr: 19.48

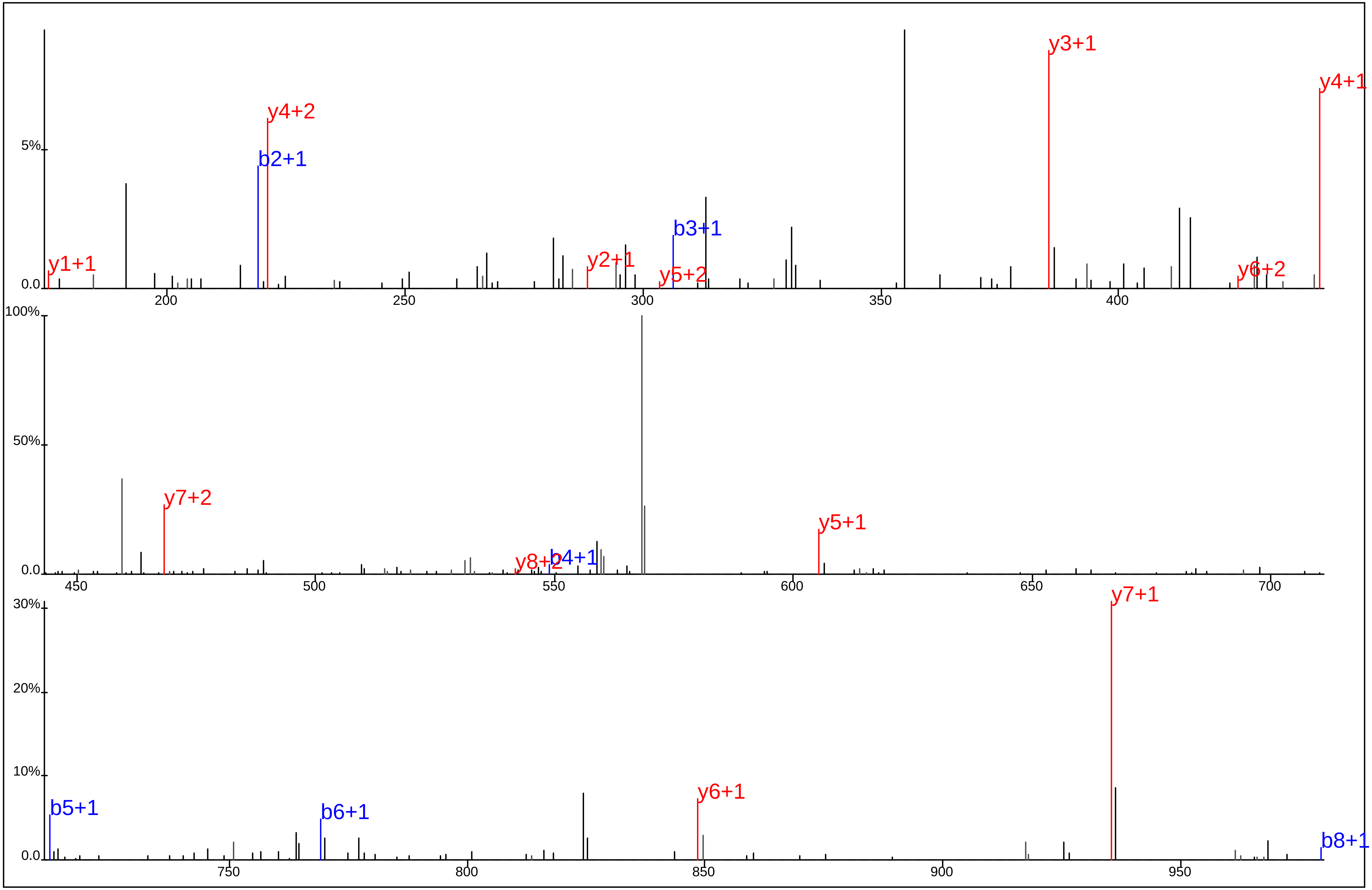

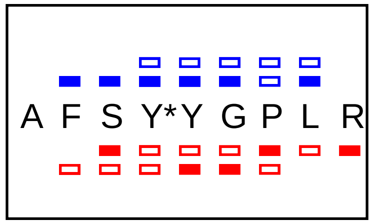

Protein Name: Splicing factor 9G8
gi Accession: 72534660
Precursor Mass: 577.2592+2
Delta Mass: 2.833 ppm
XCorr: 28.7

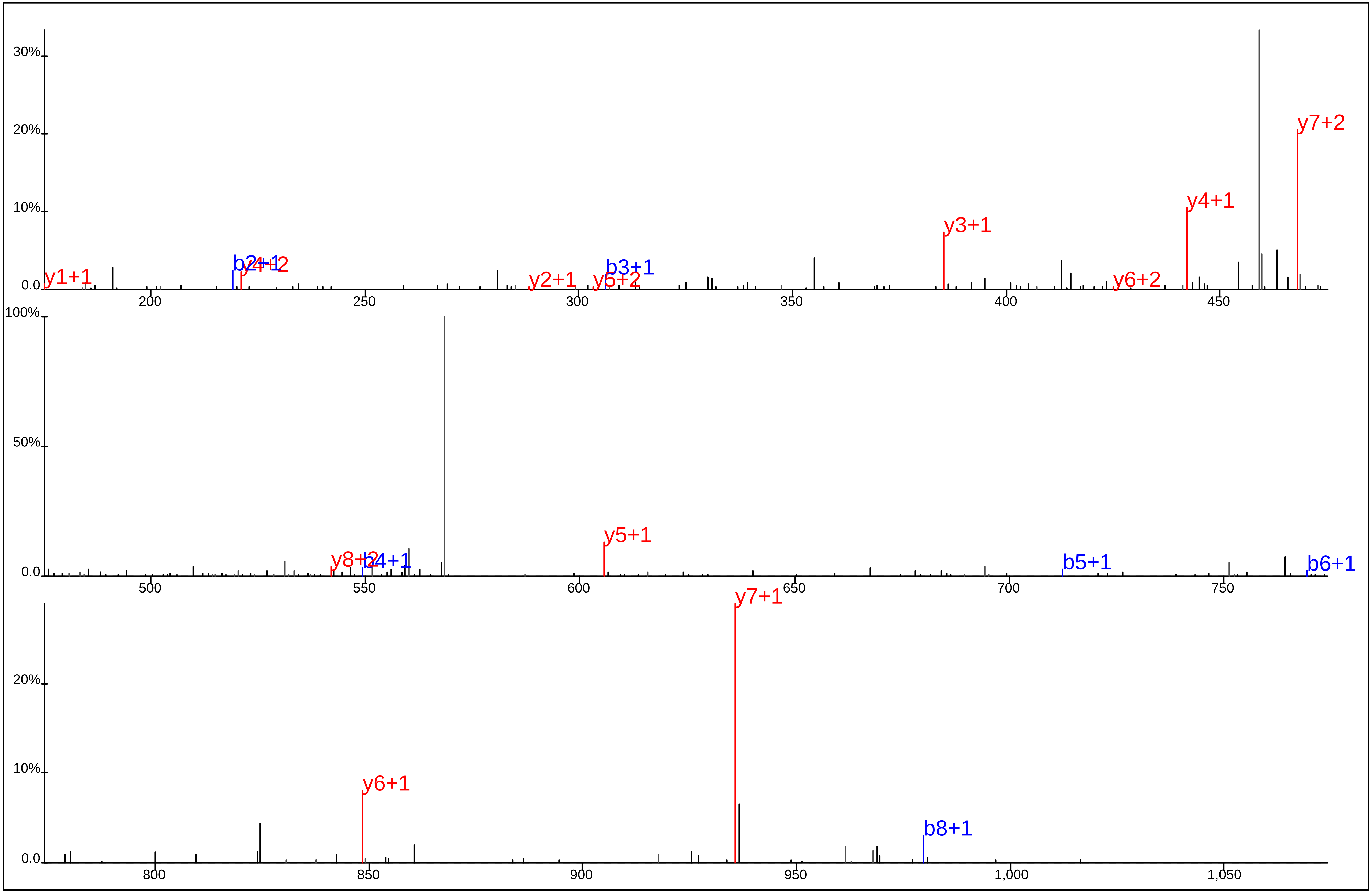

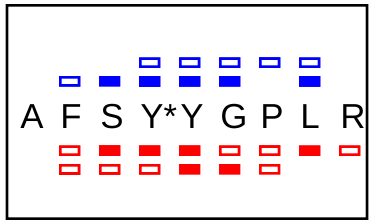

Protein Name:	Splicing factor 9G8
gi Accession:	72534660
Precursor Mass:	577.2595+2
Delta Mass:	3.475 ppm
XCorr:	26.1

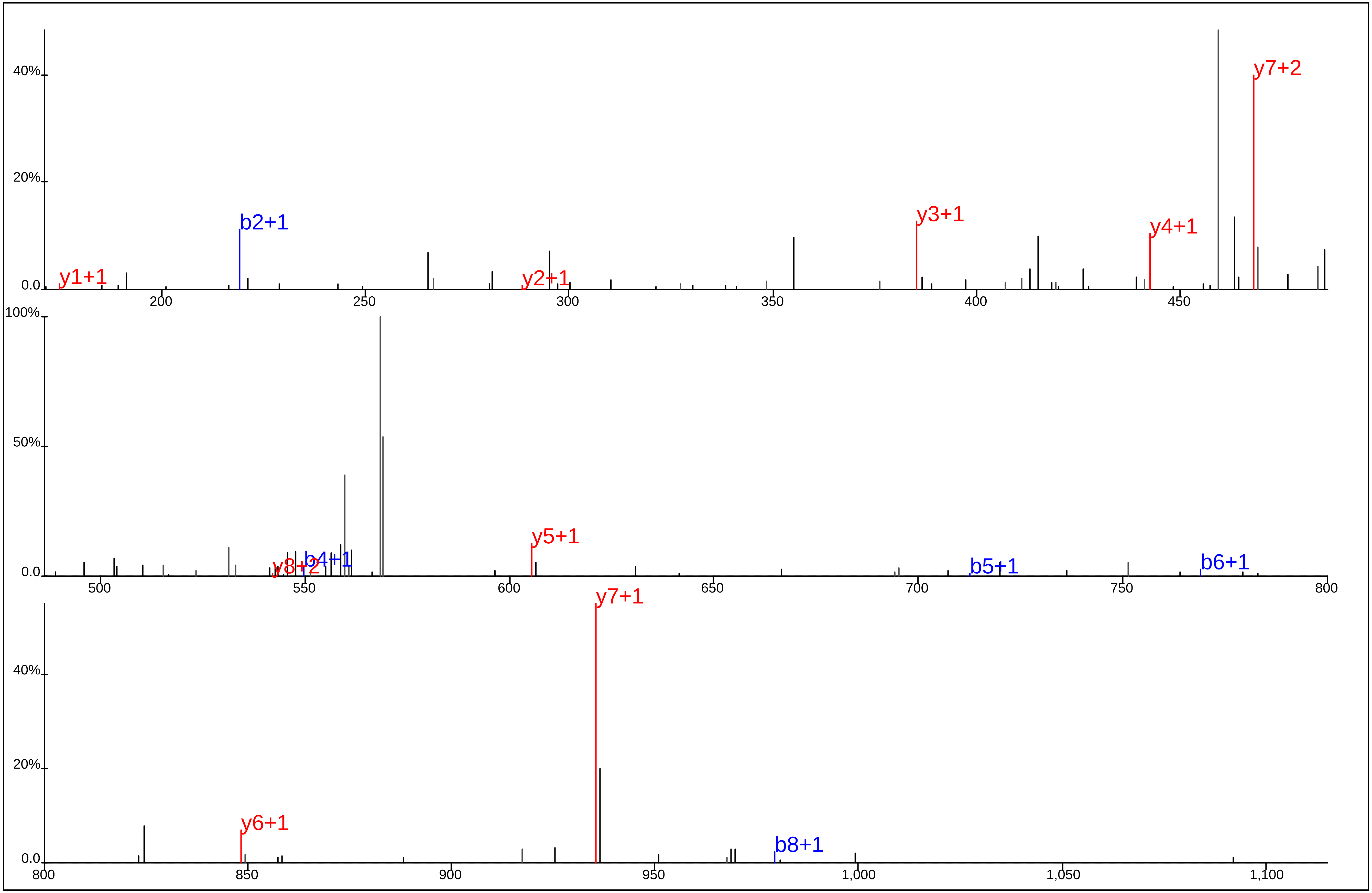

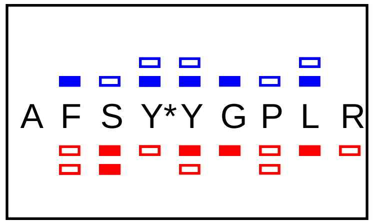

Protein Name:	Splicing factor 9G8
gi Accession:	72534660
Precursor Mass:	577.2598+2
Delta Mass:	4.003 ppm
XCorr:	19.25

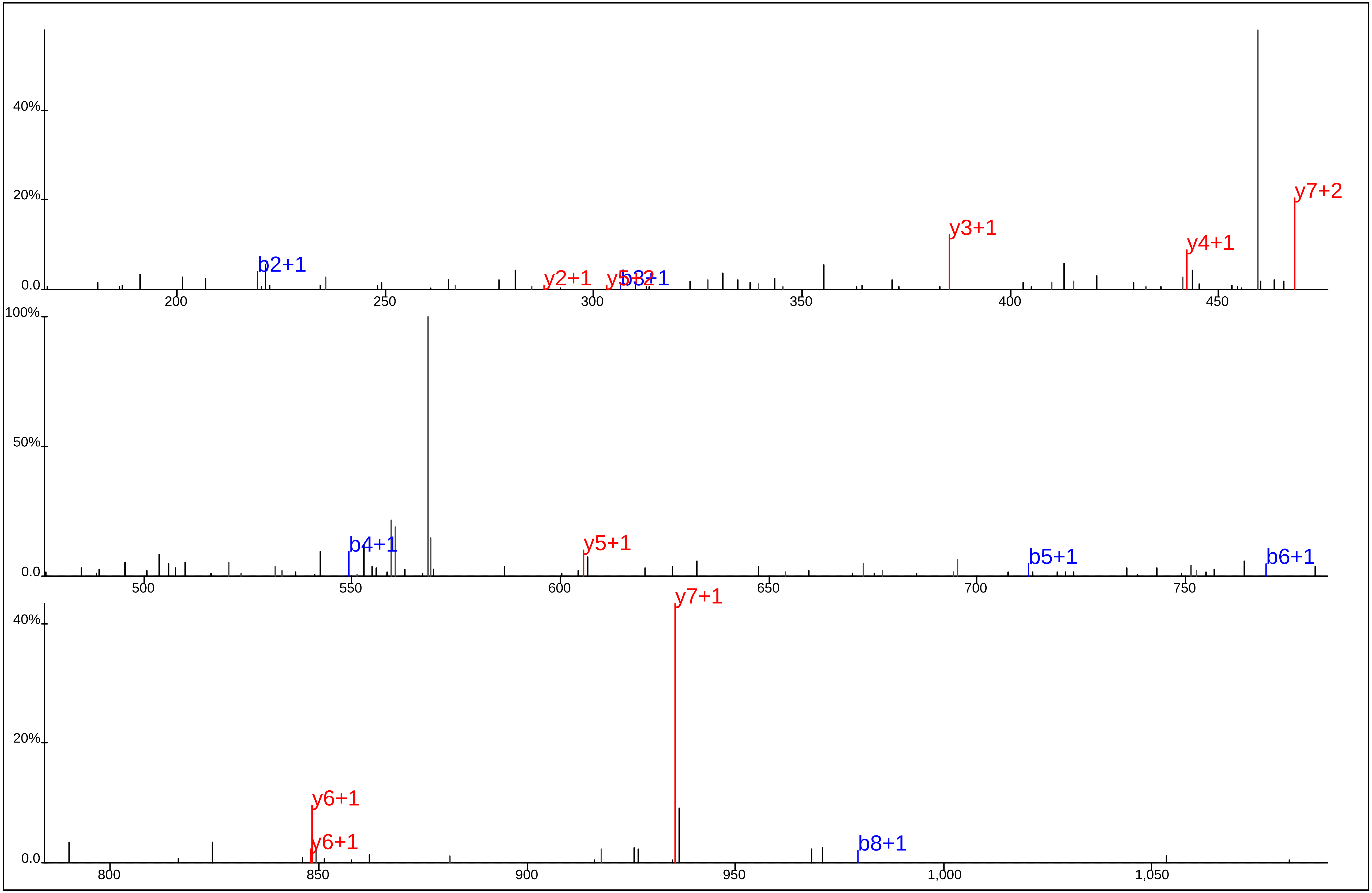

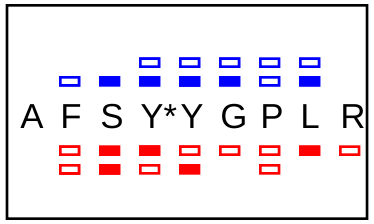

Protein Name:	Splicing factor 9G8
gi Accession:	72534660
Precursor Mass:	577.2594+2
Delta Mass:	3.258 ppm
XCorr:	19.56

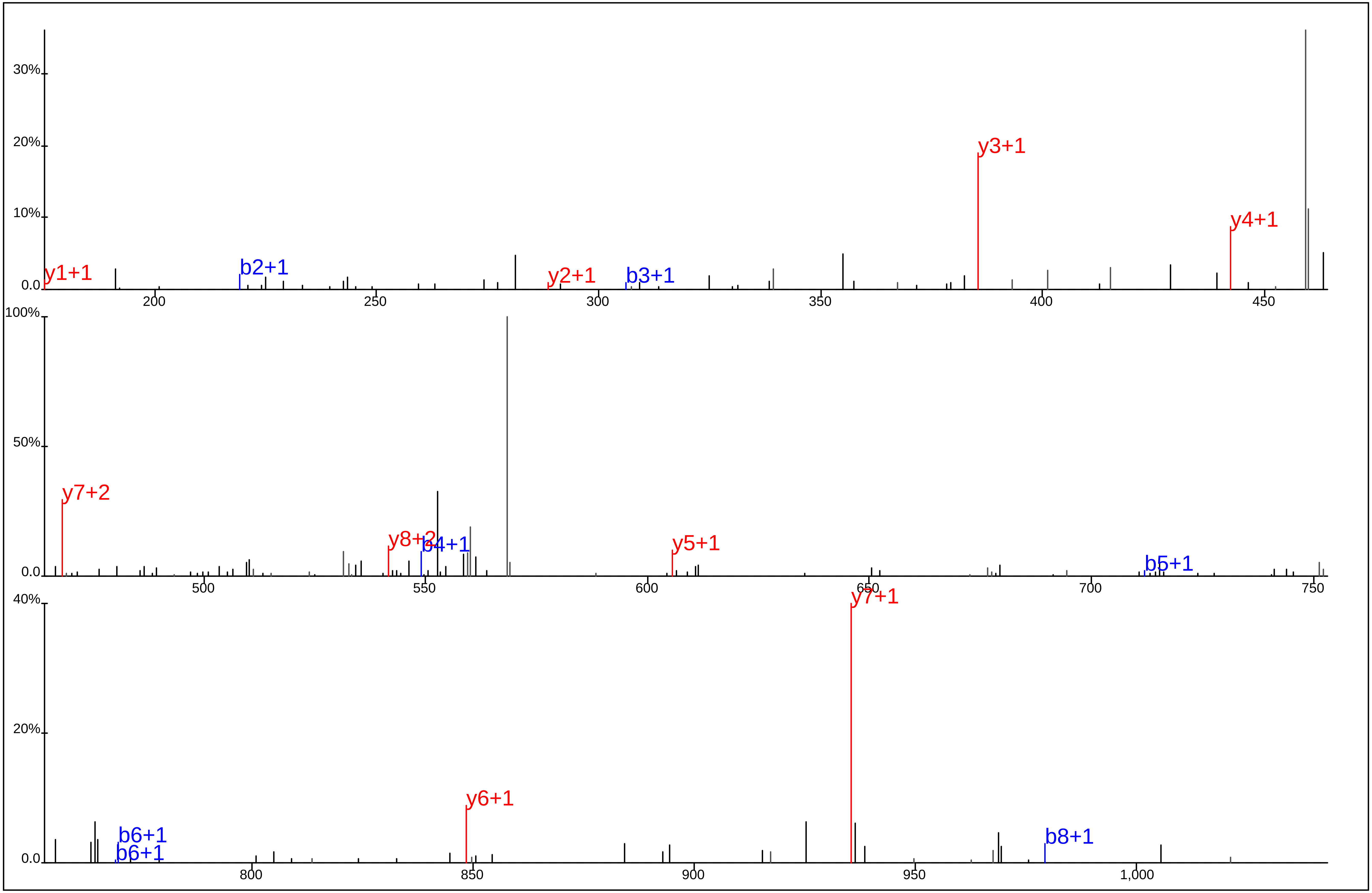

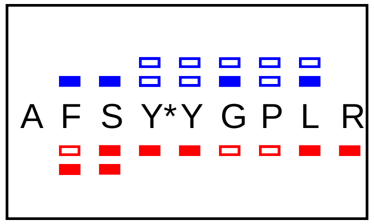

Protein Name:	Splicing factor 9G8
gi Accession:	72534660
Precursor Mass:	577.2589+2
Delta Mass:	2.417 ppm
XCorr:	20.46

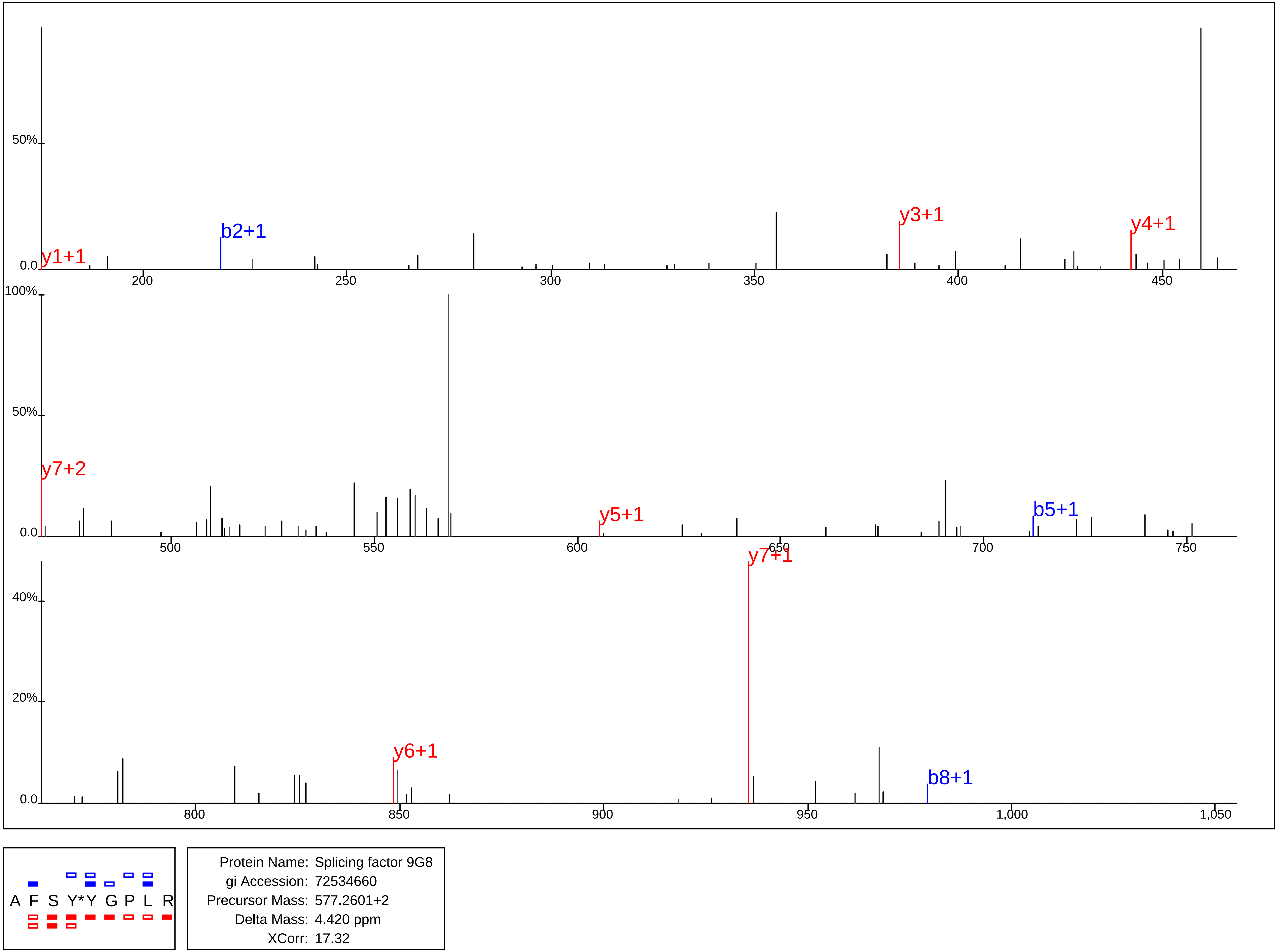

Protein Name:	Splicing factor 9G8
gi Accession:	72534660
Precursor Mass:	577.2601+2
Delta Mass:	4.420 ppm
XCorr:	17.32

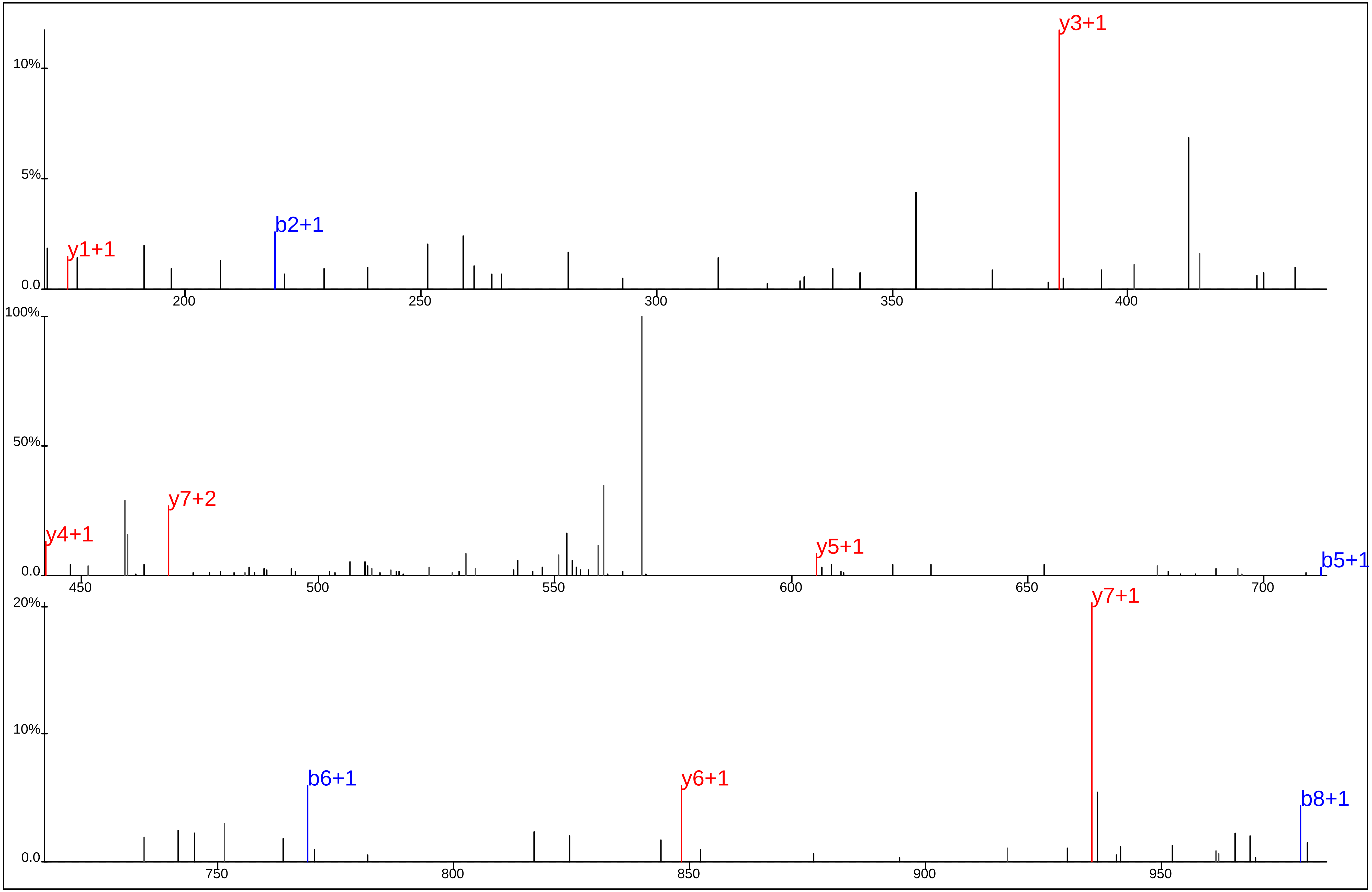

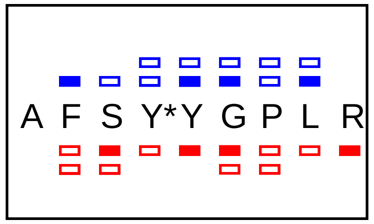

Protein Name:	Splicing factor 9G8
gi Accession:	72534660
Precursor Mass:	577.2643+2
Delta Mass:	11.728 ppm
XCorr:	30.24

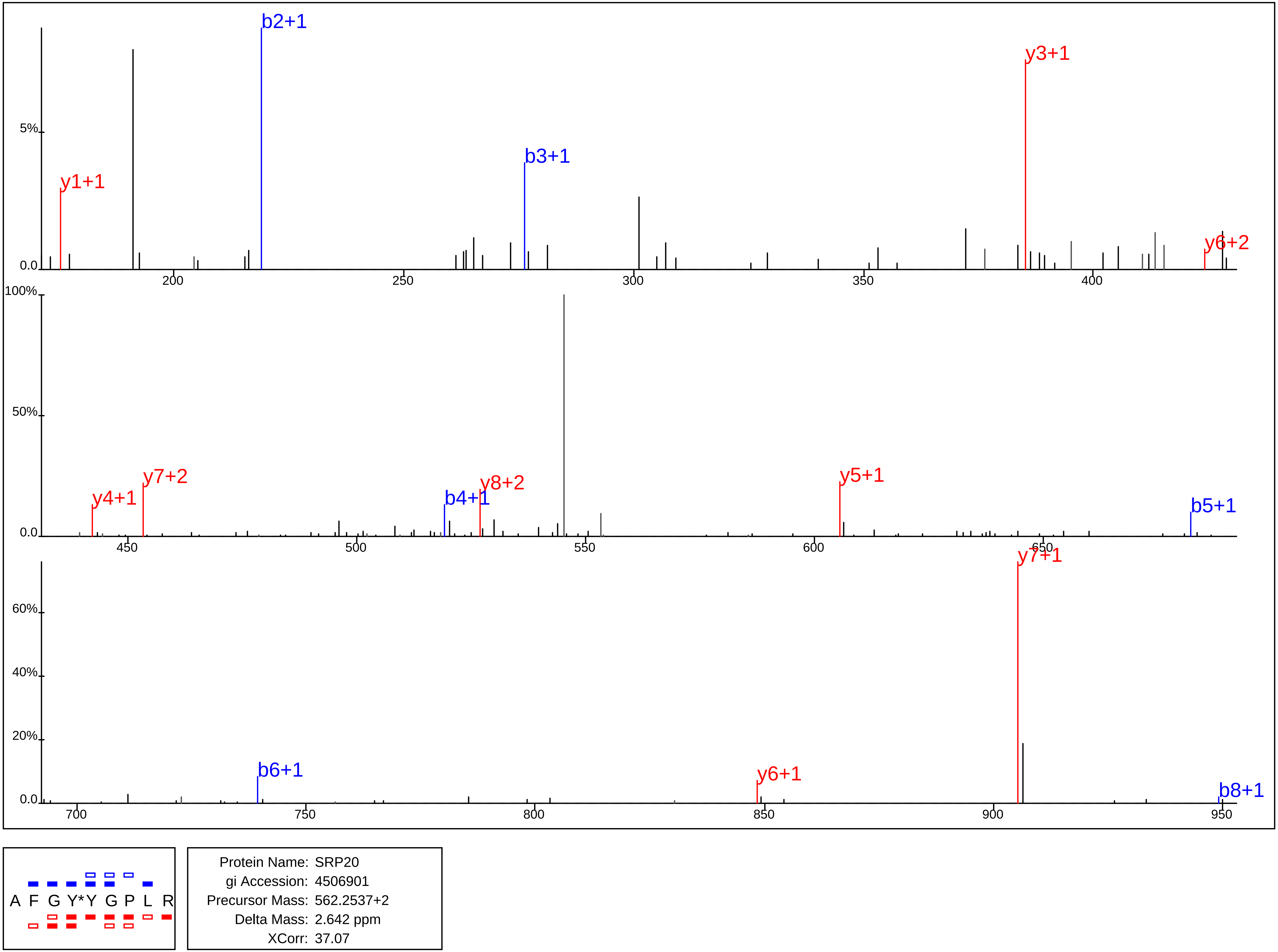

Protein Name: SRP20
gi Accession: 4506901
Precursor Mass: 562.2537+2
Delta Mass: 2.642 ppm
XCorr: 37.07

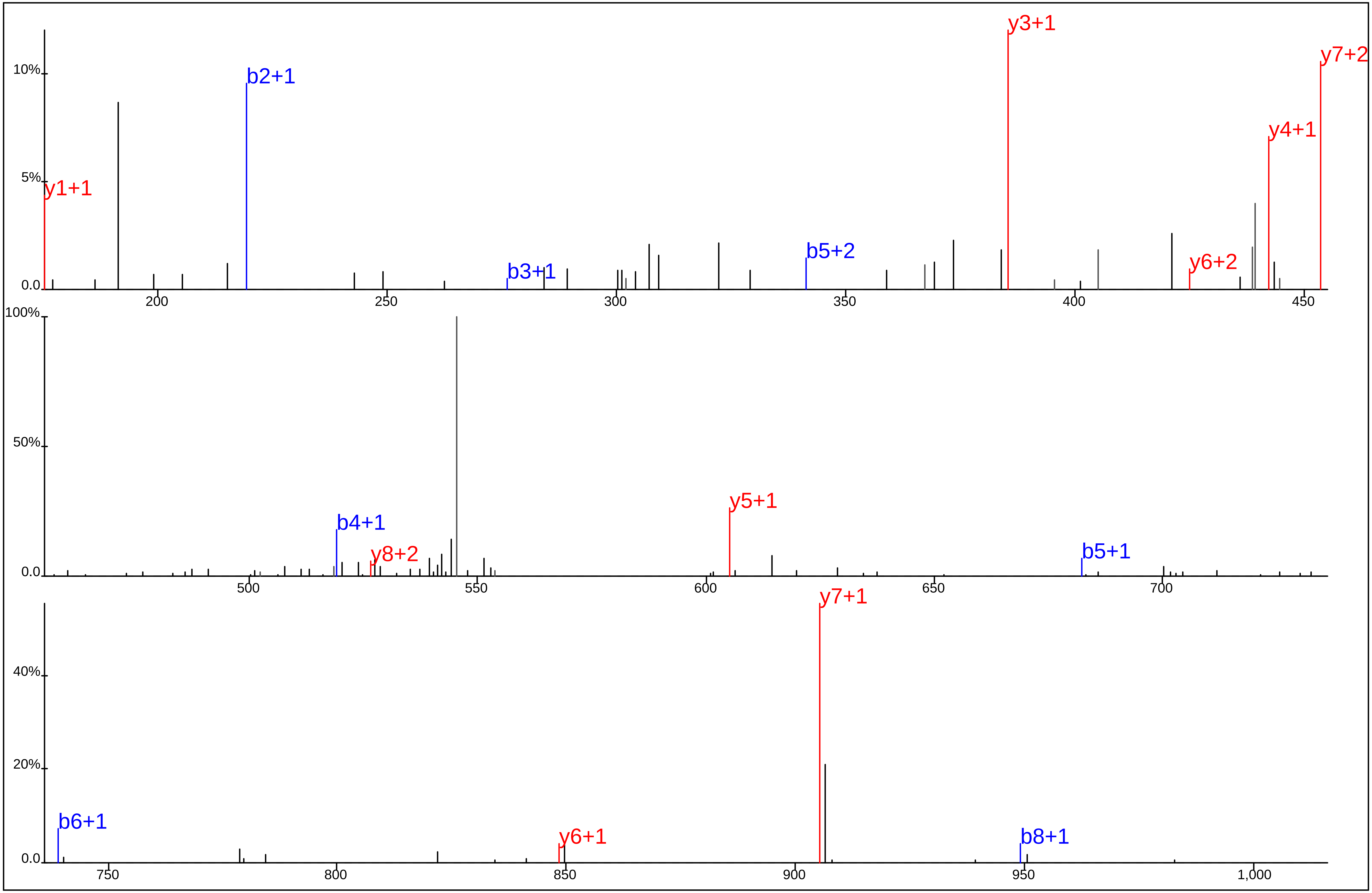

A F G Y*Y G P L R

Protein Name: SRP20
gi Accession: 4506901
Precursor Mass: 562.2524+2
Delta Mass: 0.363 ppm
XCorr: 28.32

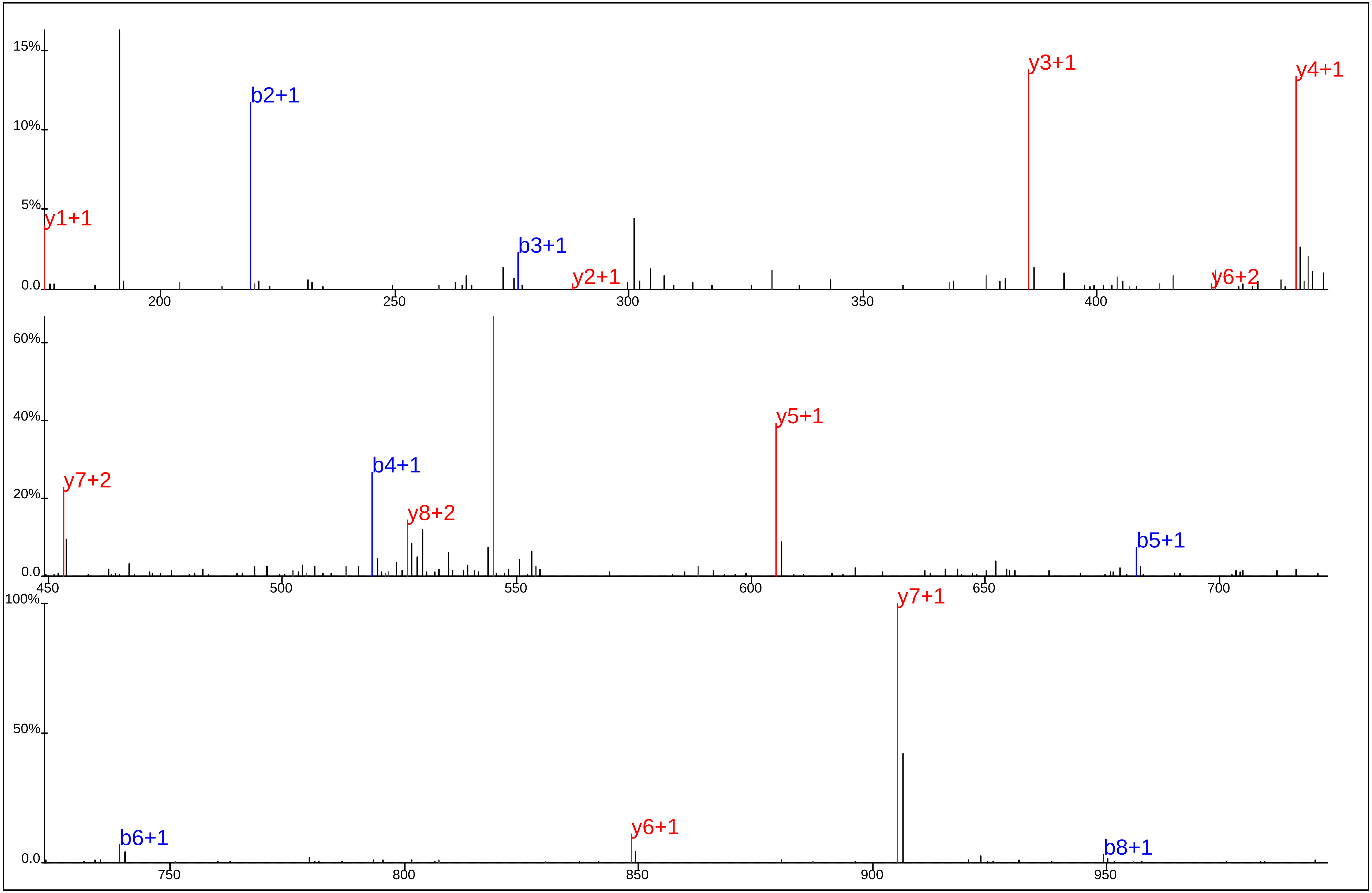

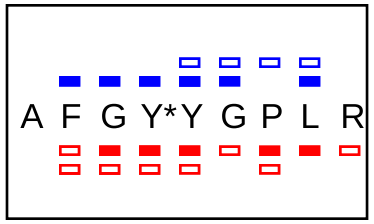

Protein Name: SRP20
gi Accession: 4506901
Precursor Mass: 562.2538+2
Delta Mass: 2.855 ppm
XCorr: 35.73

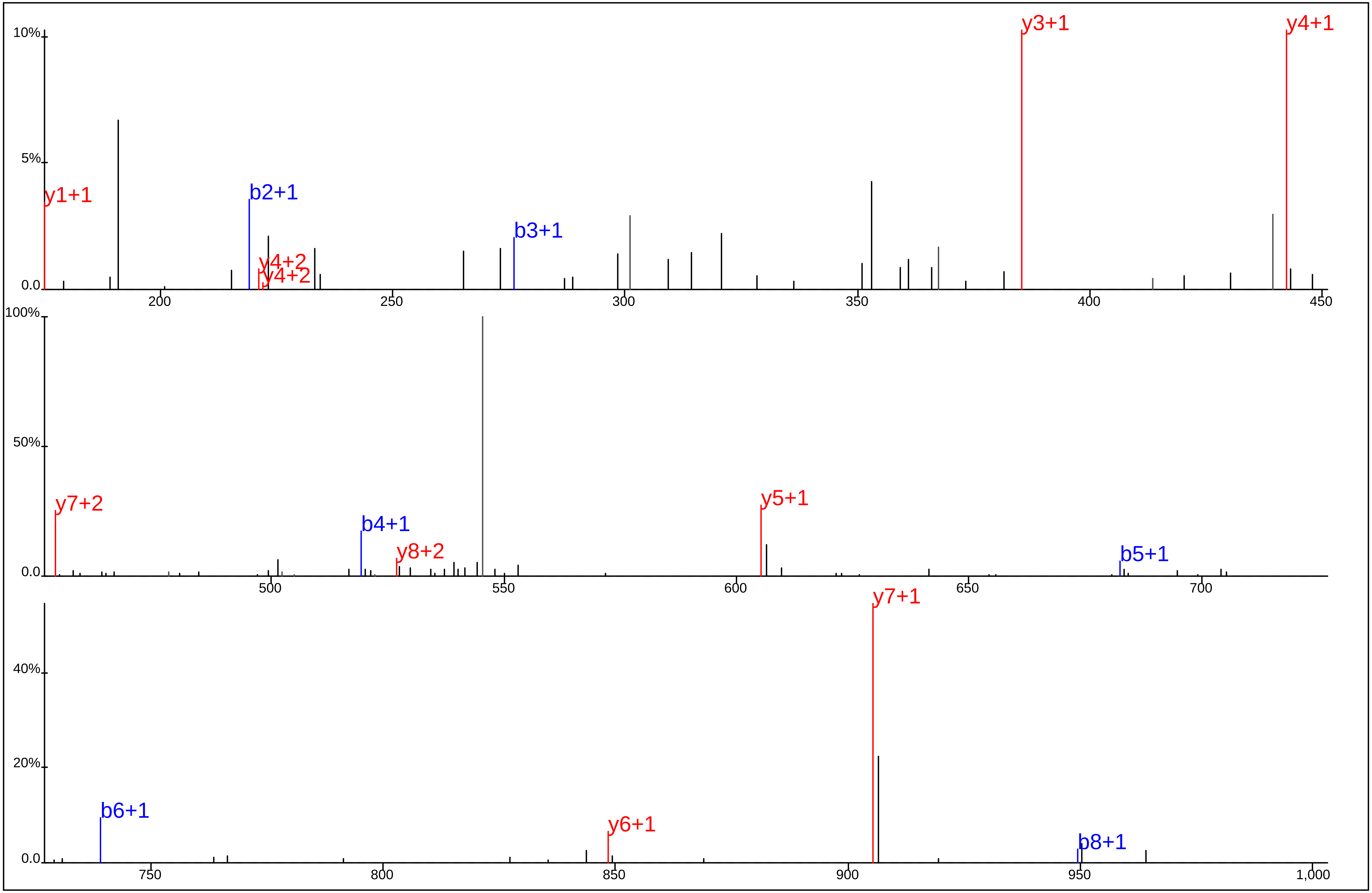

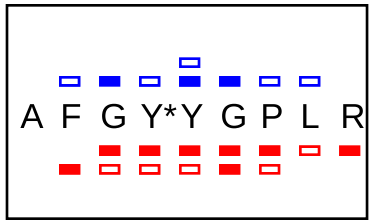

Protein Name: SRP20
gi Accession: 4506901
Precursor Mass: 562.2535+2
Delta Mass: 2.206 ppm
XCorr: 31.54

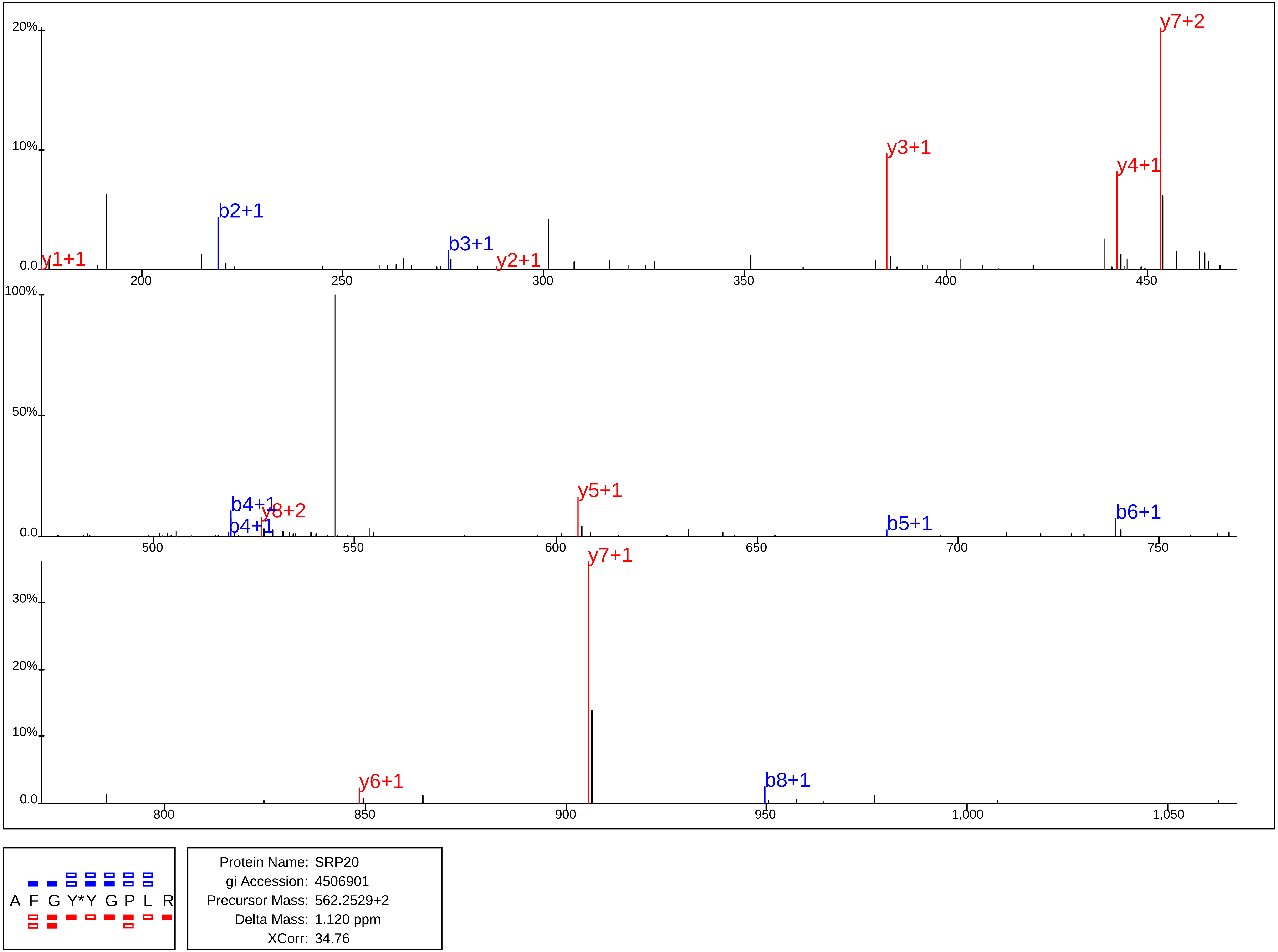

Protein Name: SRP20
gi Accession: 4506901
Precursor Mass: 562.2529+2
Delta Mass: 1.120 ppm
XCorr: 34.76

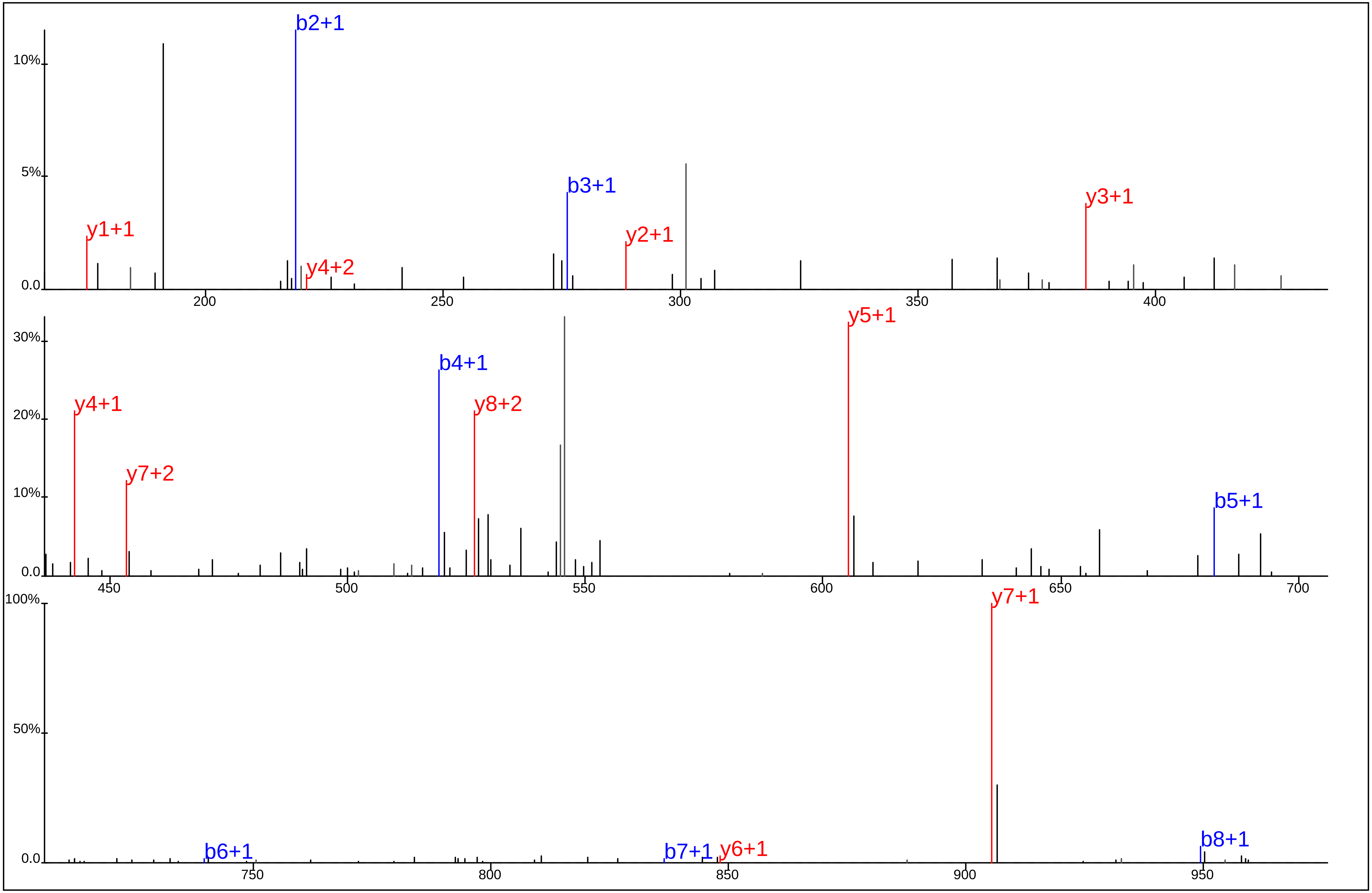

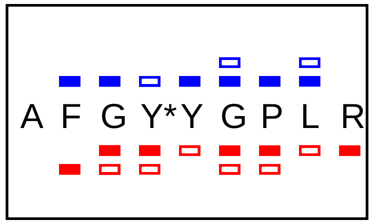

Protein Name:	SRP20
gi Accession:	4506901
Precursor Mass:	562.2521+2
Delta Mass:	0.296 ppm
XCorr:	45.85

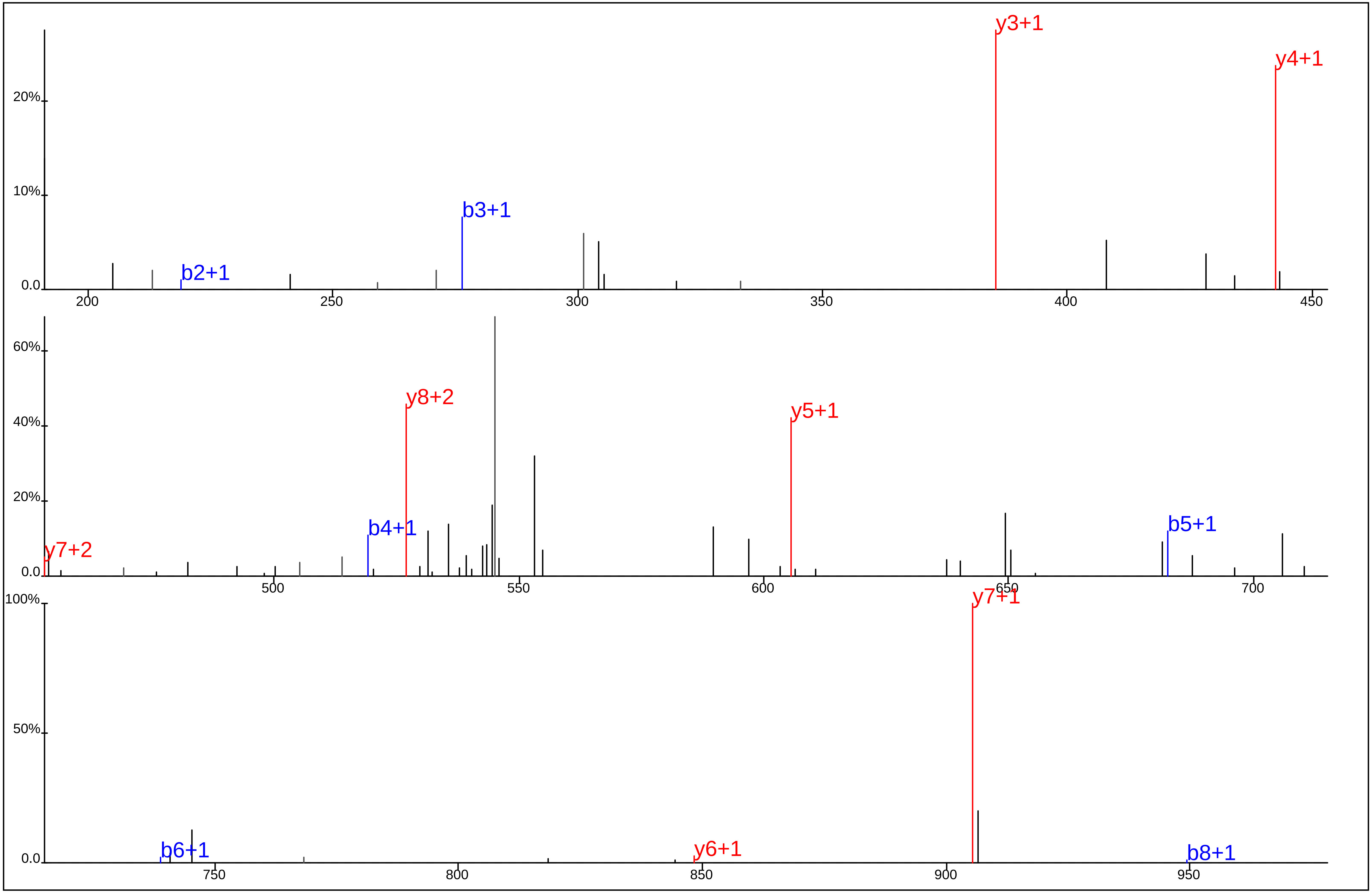

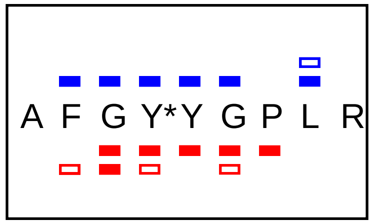

Protein Name: SRP20
gi Accession: 4506901
Precursor Mass: 562.2545+2
Delta Mass: 3.941 ppm
XCorr: 35.22

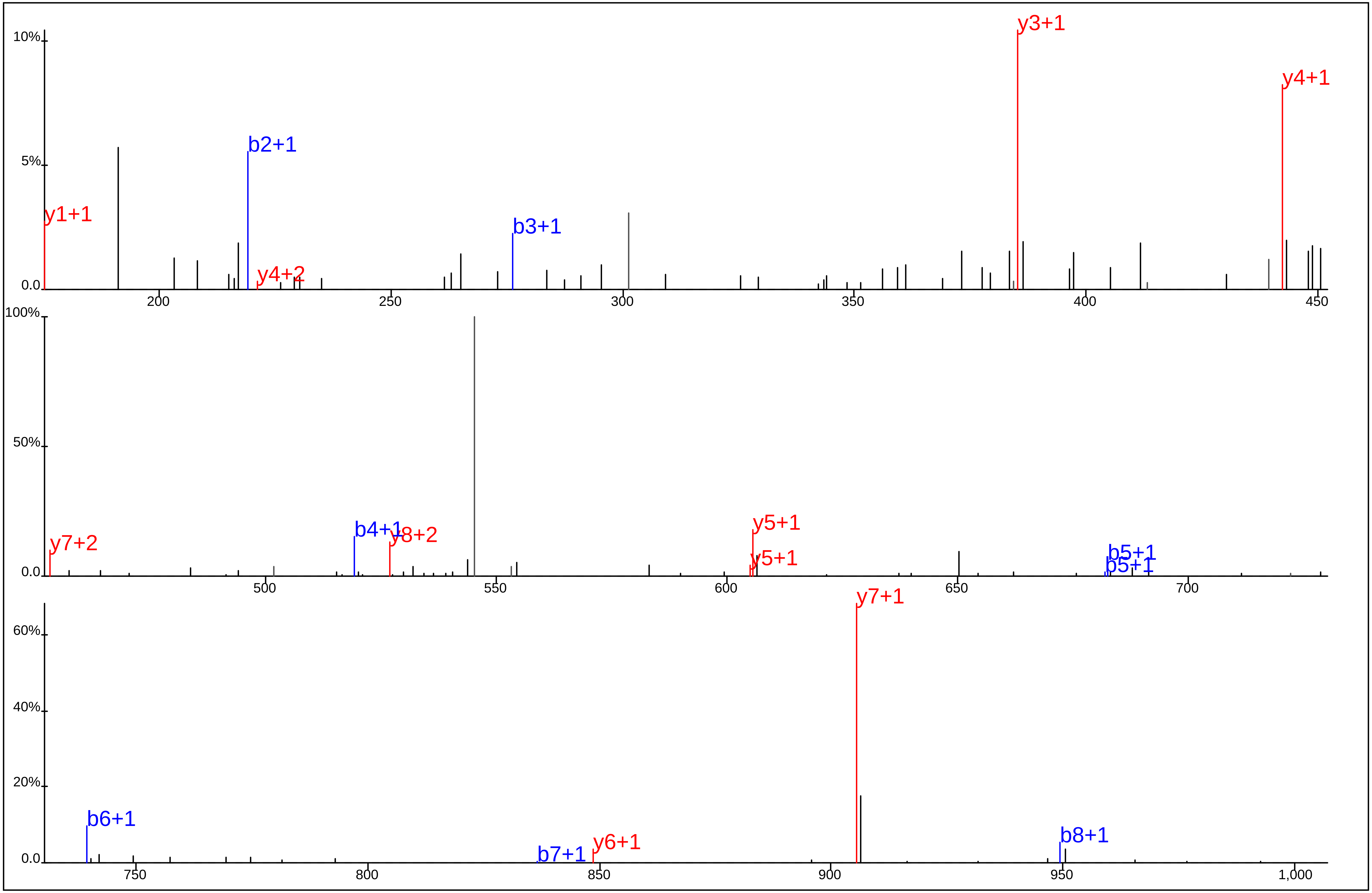

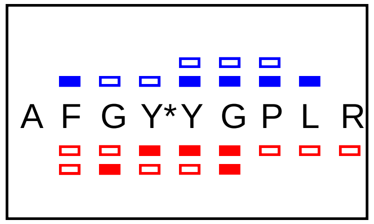

Protein Name: SRP20
gi Accession: 4506901
Precursor Mass: 562.2531+2
Delta Mass: 1.449 ppm
XCorr: 39.99

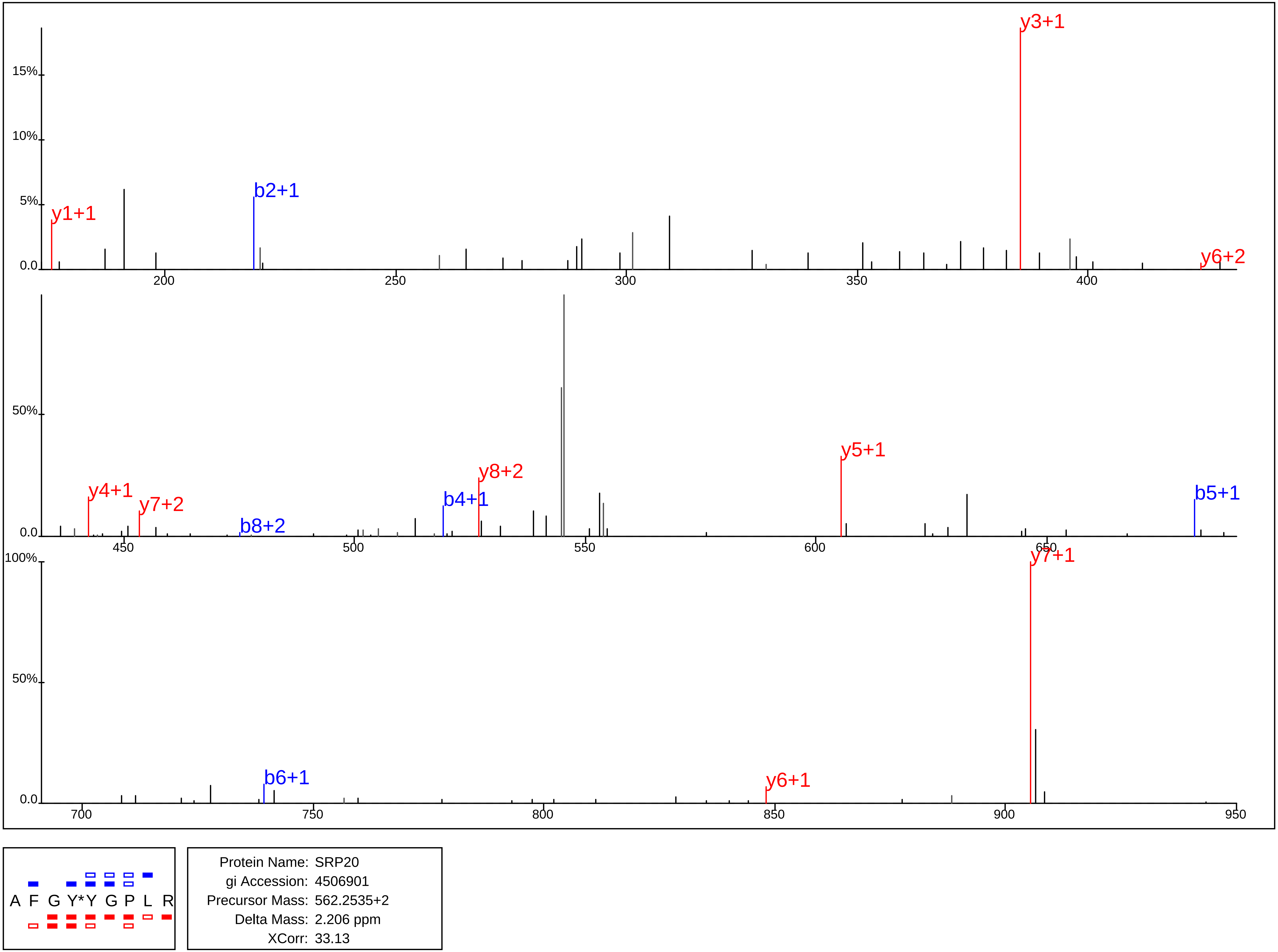

Protein Name: SRP20
gi Accession: 4506901
Precursor Mass: 562.2535+2
Delta Mass: 2.206 ppm
XCorr: 33.13

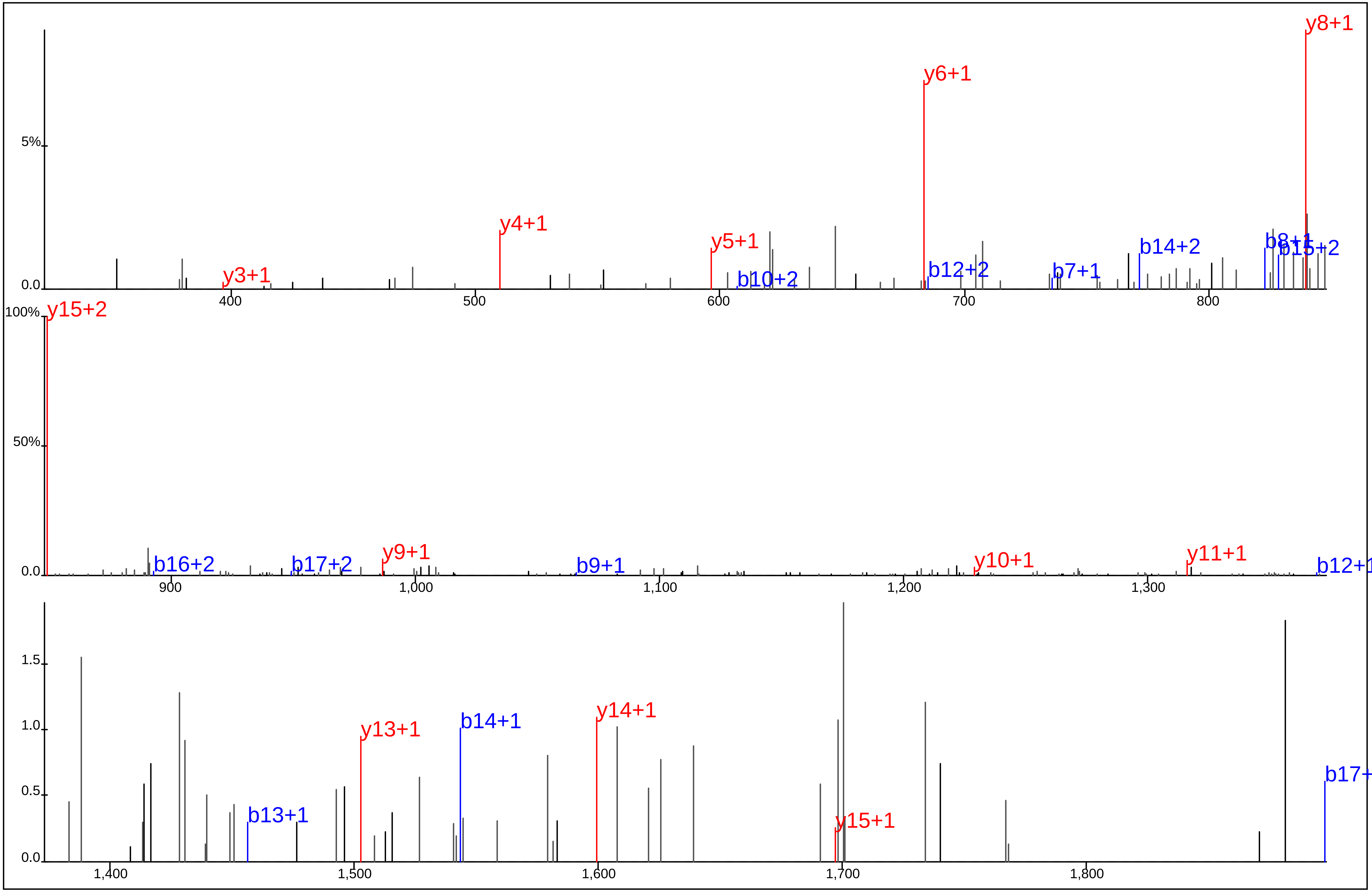

S S*T P P G E S Y*F G V S S L Q L K#

Protein Name: SRRM2
gi Accession: 5821153
Precursor Mass: 1,026.4562+2
Delta Mass: 9.595 ppm
XCorr: 40.17

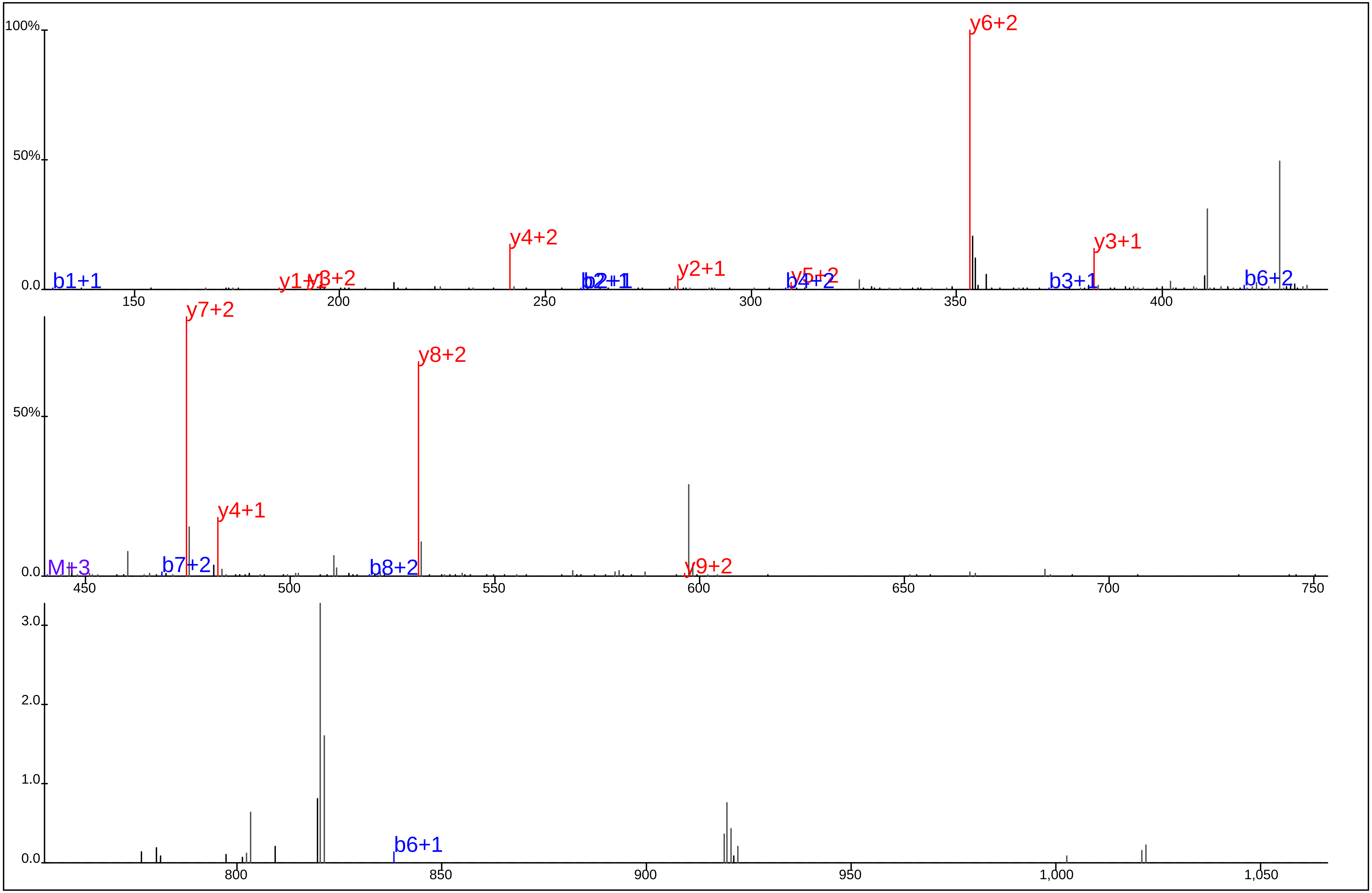

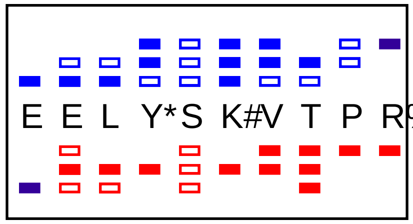

Protein Name: STS1
gi Accession: 18916794
Precursor Mass: 440.5511+3
Delta Mass: 1.863 ppm
XCorr: 26.95

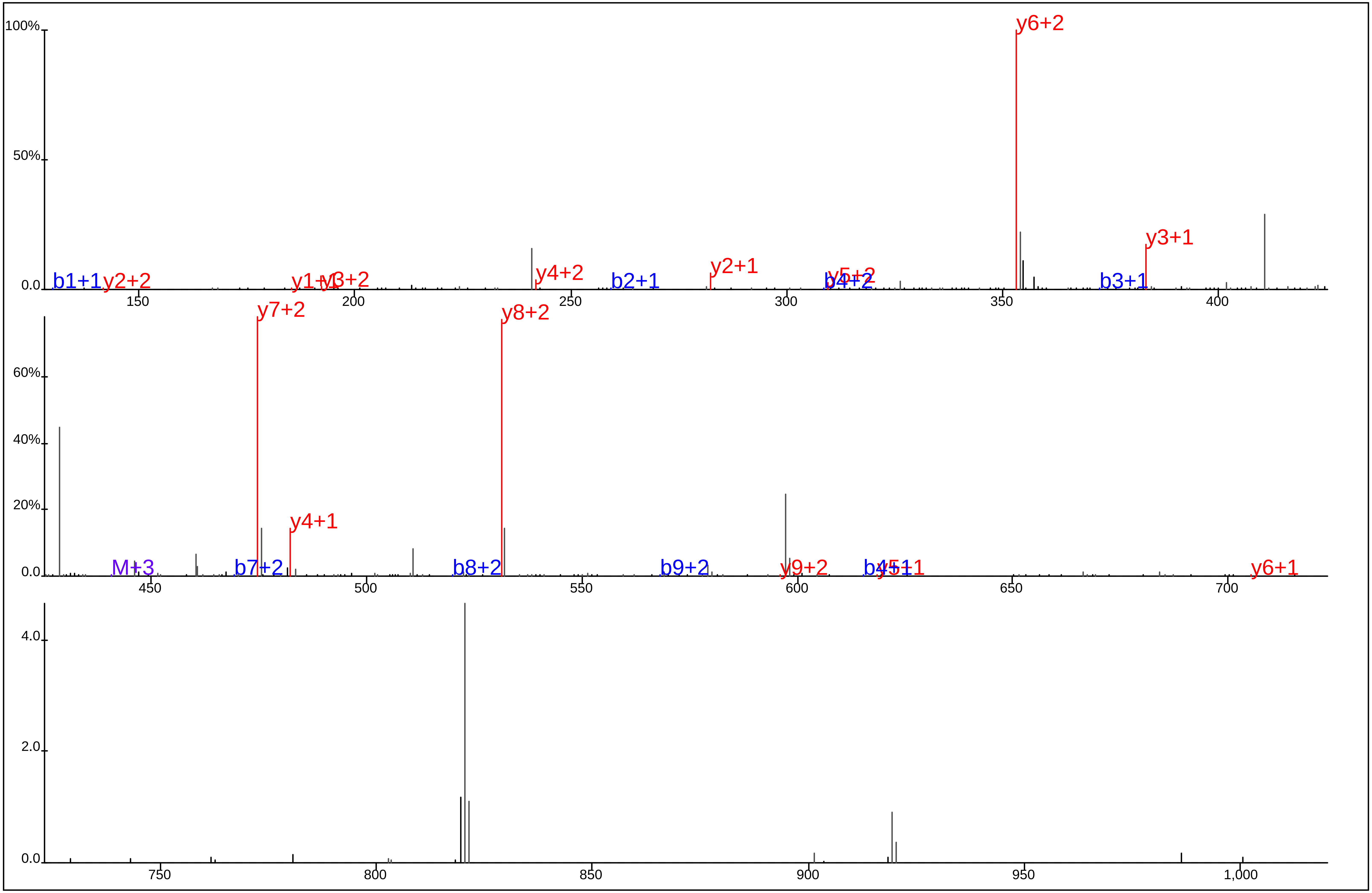

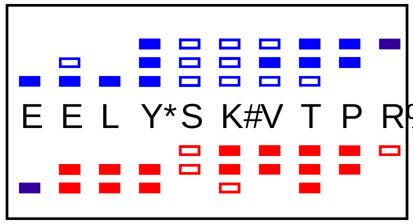

Protein Name: STS1
gi Accession: 18916794
Precursor Mass: 440.5513+3
Delta Mass: 2.424 ppm
XCorr: 15.57

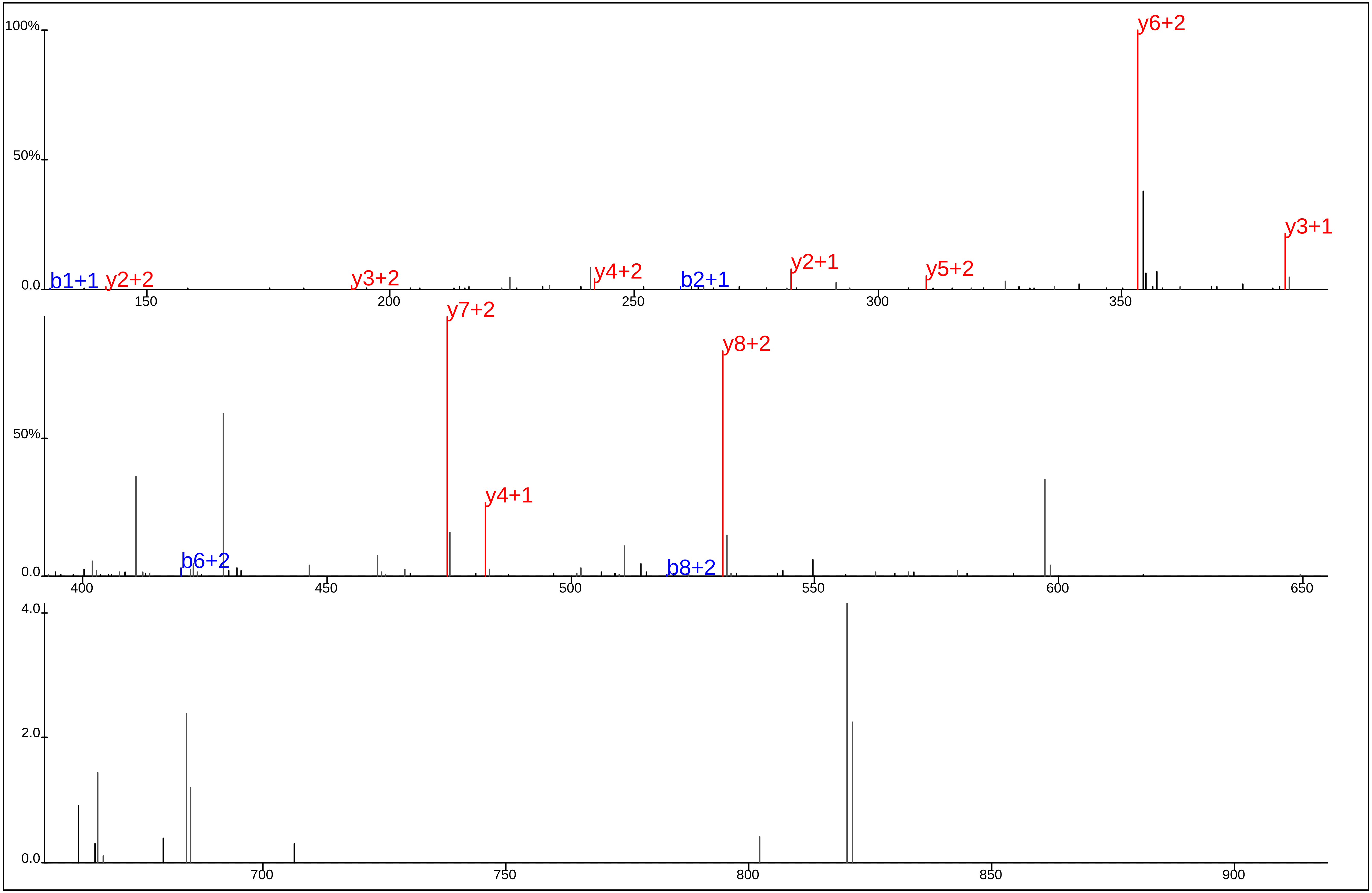

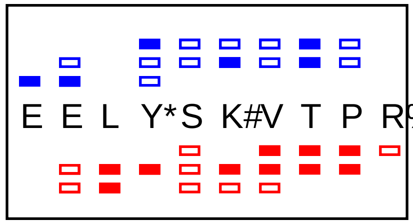

Protein Name: STS1
gi Accession: 18916794
Precursor Mass: 440.5510+3
Delta Mass: 1.794 ppm
XCorr: 27.32

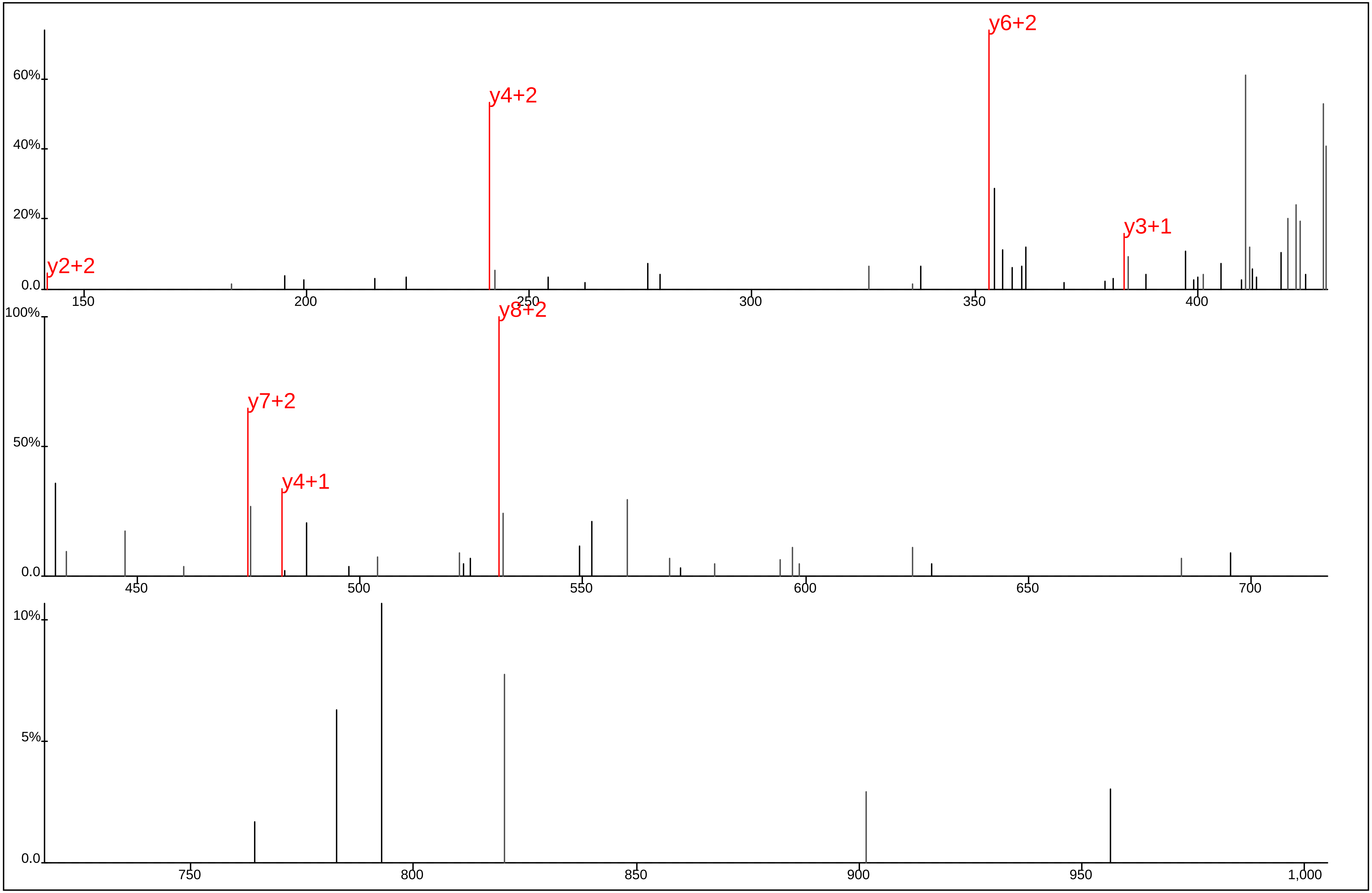

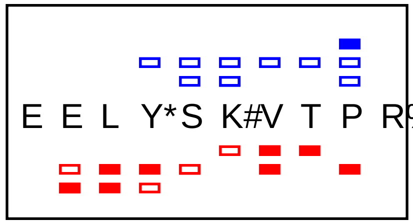

Protein Name: STS1
gi Accession: 18916794
Precursor Mass: 440.5514+3
Delta Mass: 2.697 ppm
XCorr: 17.15

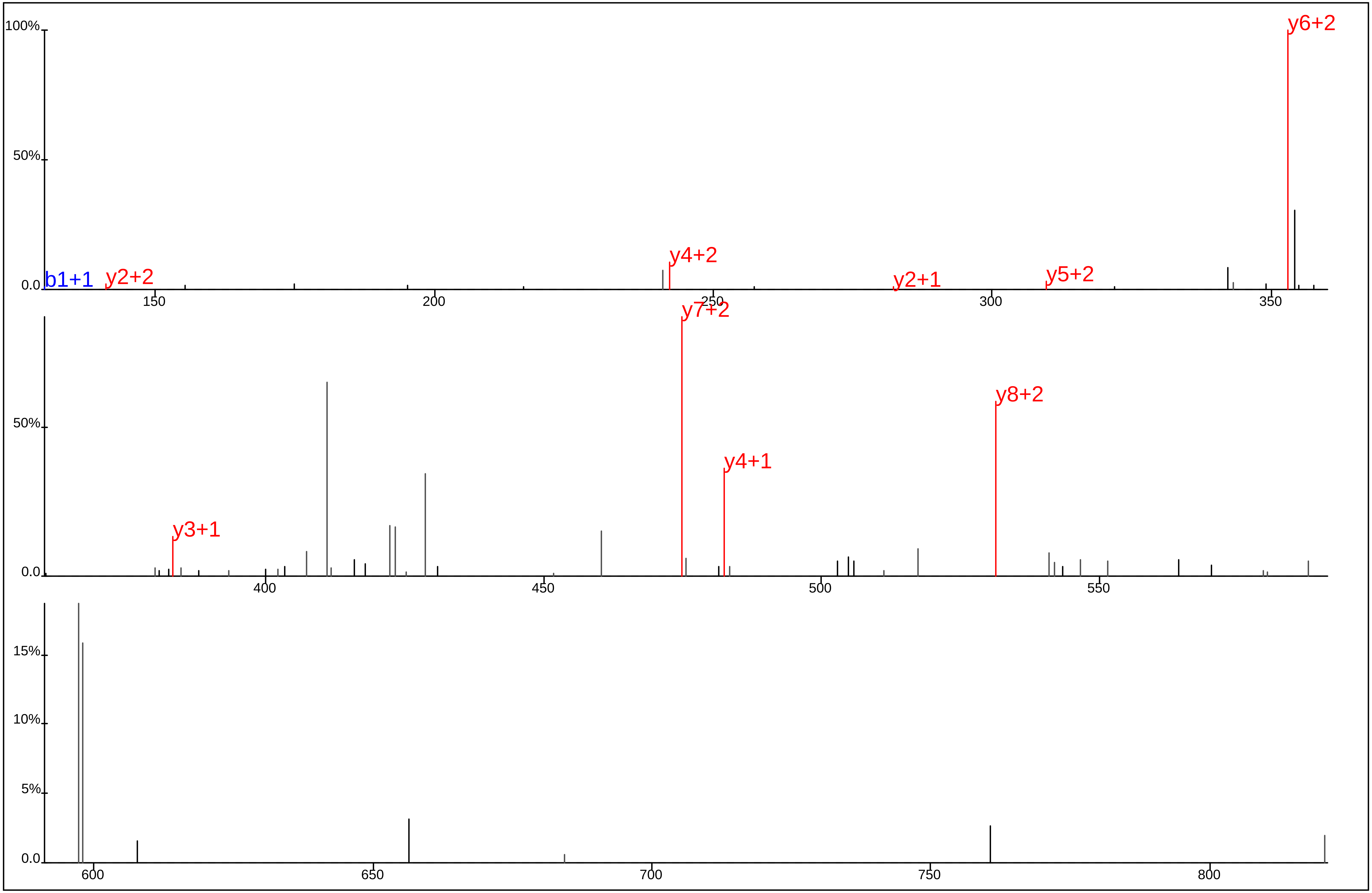

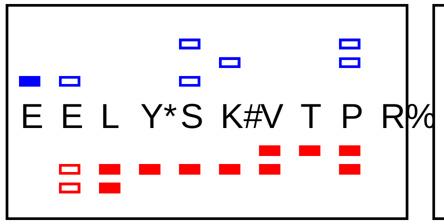

Protein Name: STS1
gi Accession: 18916794
Precursor Mass: 440.5511+3
Delta Mass: 2.006 ppm
XCorr: 37.34

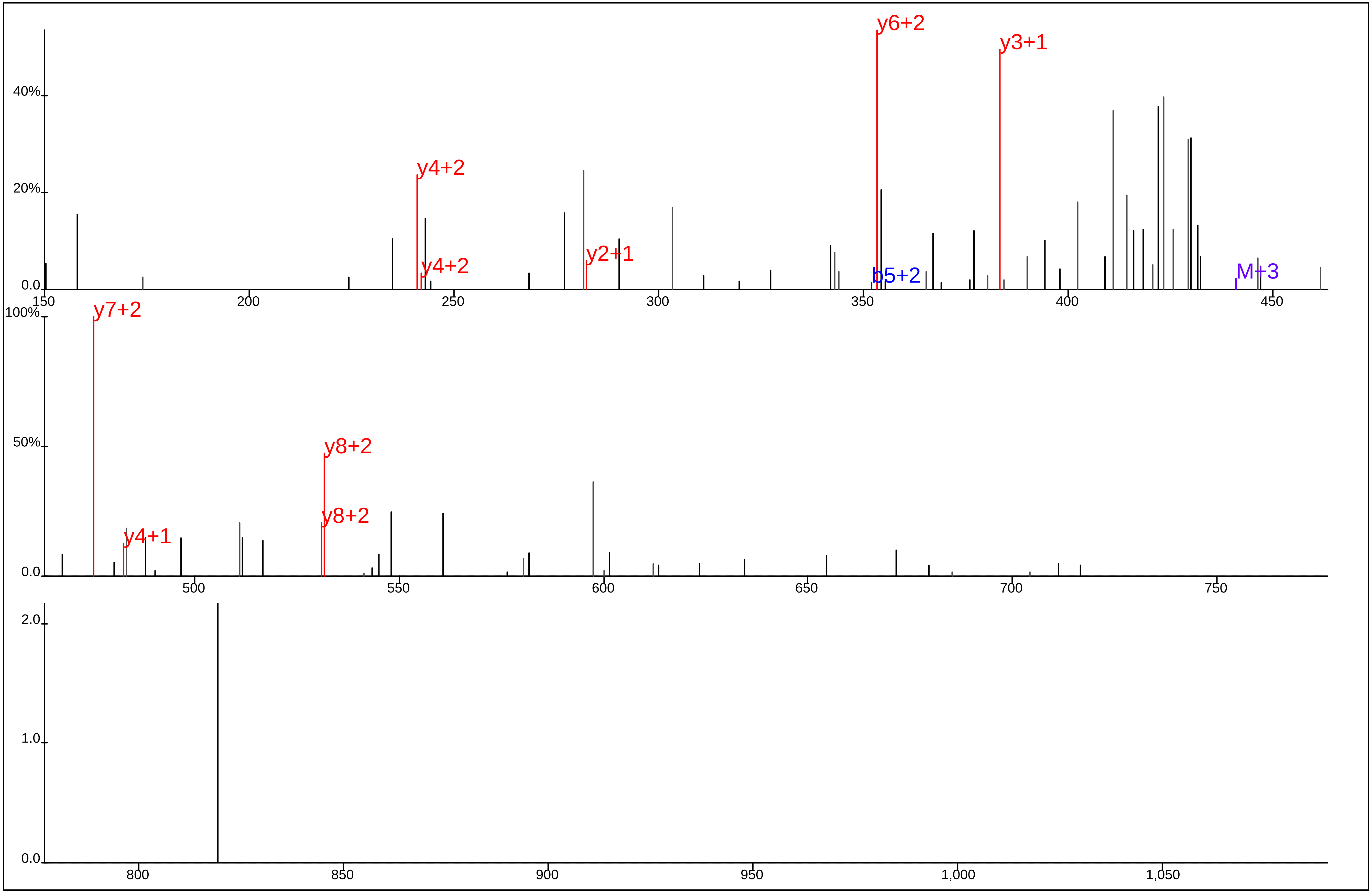

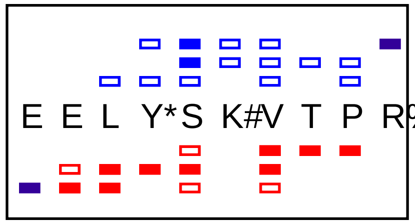

Protein Name:	STS1
gi Accession:	18916794
Precursor Mass:	440.5533+3
Delta Mass:	7.000 ppm
XCorr:	11.56

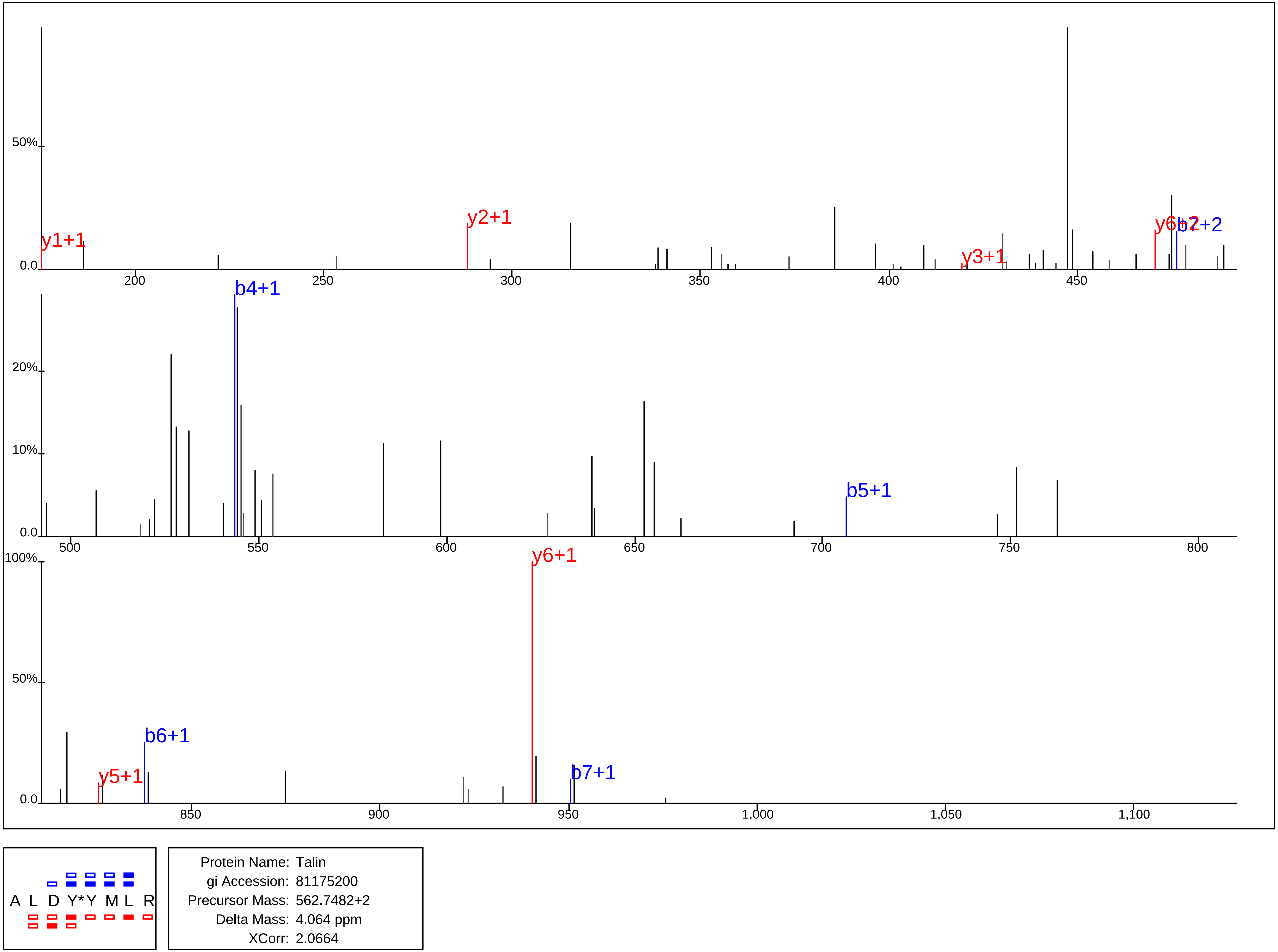

Protein Name:	Talin
gi Accession:	81175200
Precursor Mass:	562.7482+2
Delta Mass:	4.064 ppm
XCorr:	2.0664

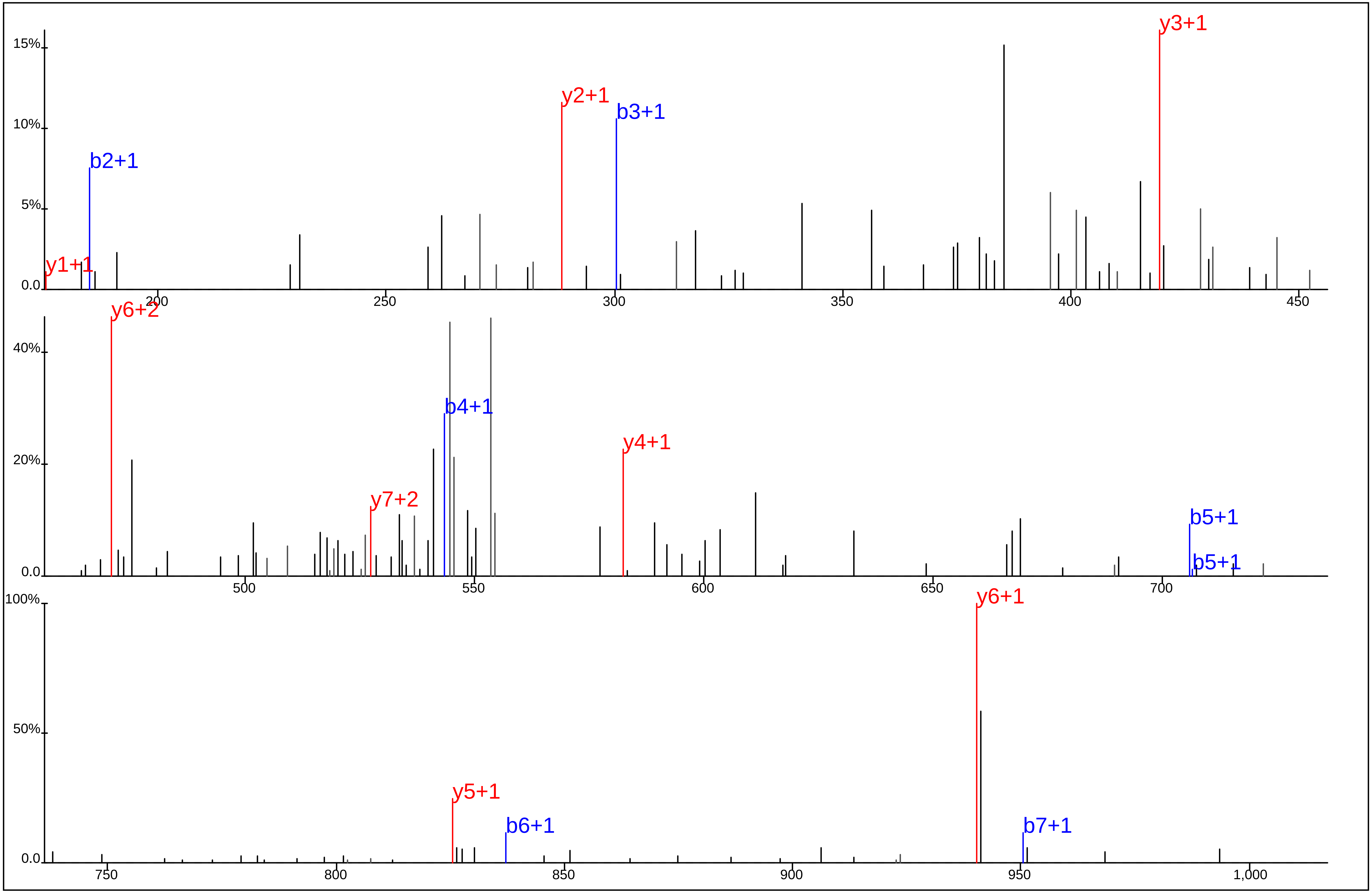

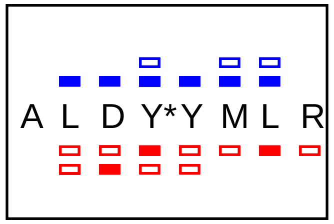

Protein Name: Talin
gi Accession: 81175200
Precursor Mass: 562.7477+2
Delta Mass: 3.235 ppm
XCorr: 32.52

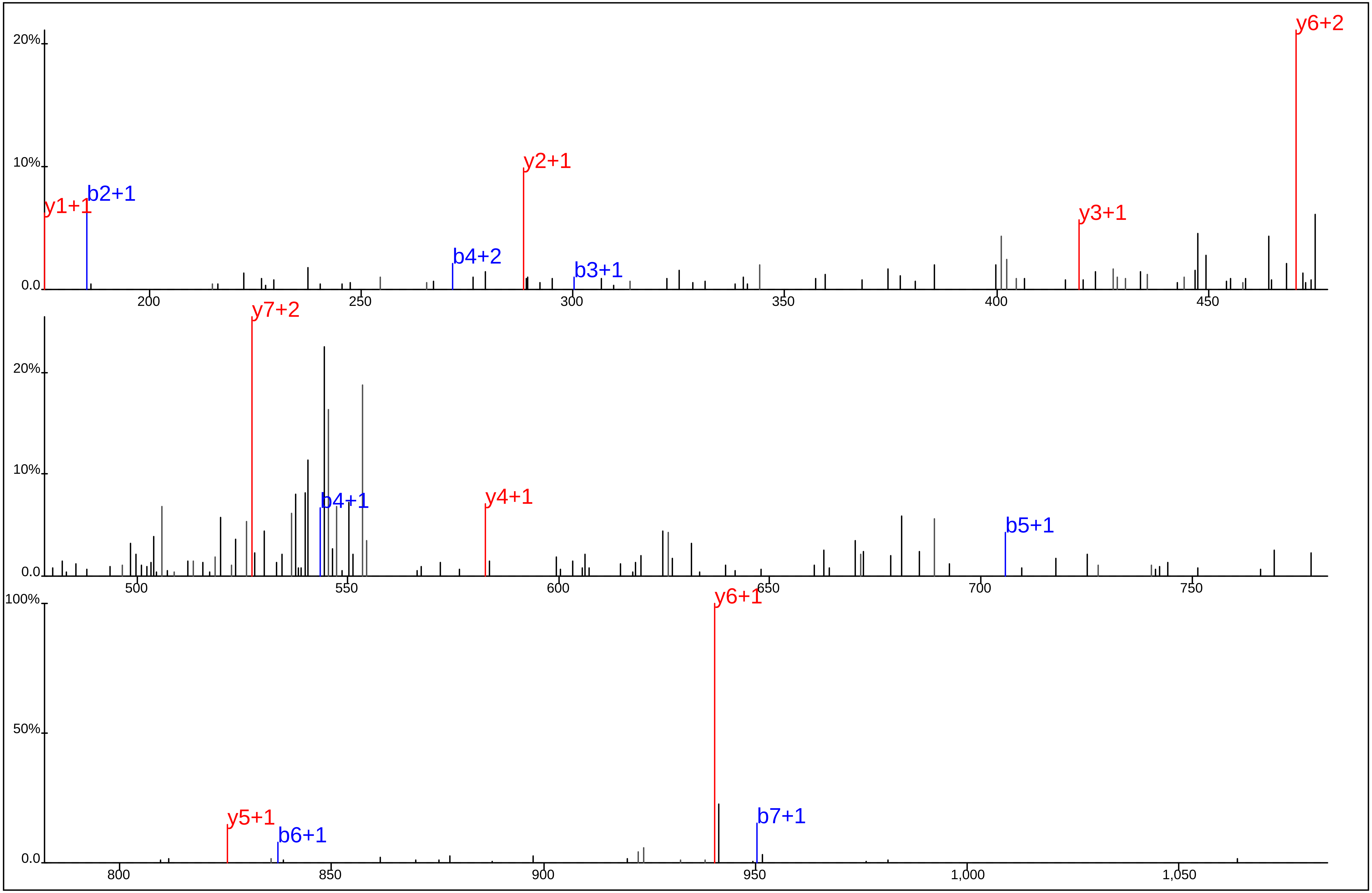

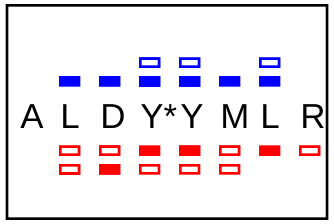

Protein Name: Talin
gi Accession: 81175200
Precursor Mass: 562.7460+2
Delta Mass: 0.194 ppm
XCorr: 35.53

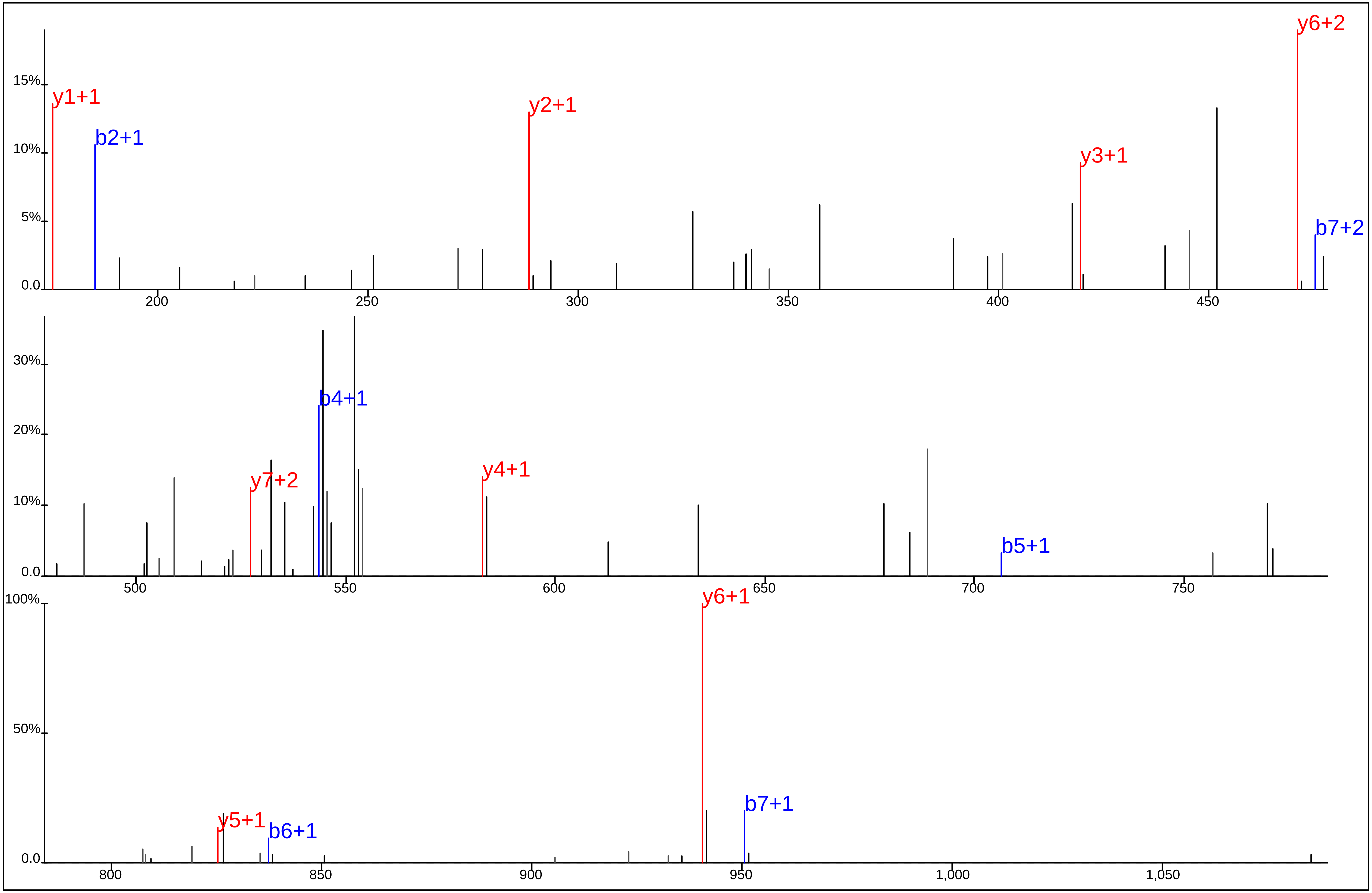

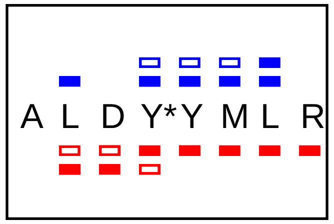

Protein Name: Talin
gi Accession: 81175200
Precursor Mass: 562.7473+2
Delta Mass: 2.364 ppm
XCorr: 34.04

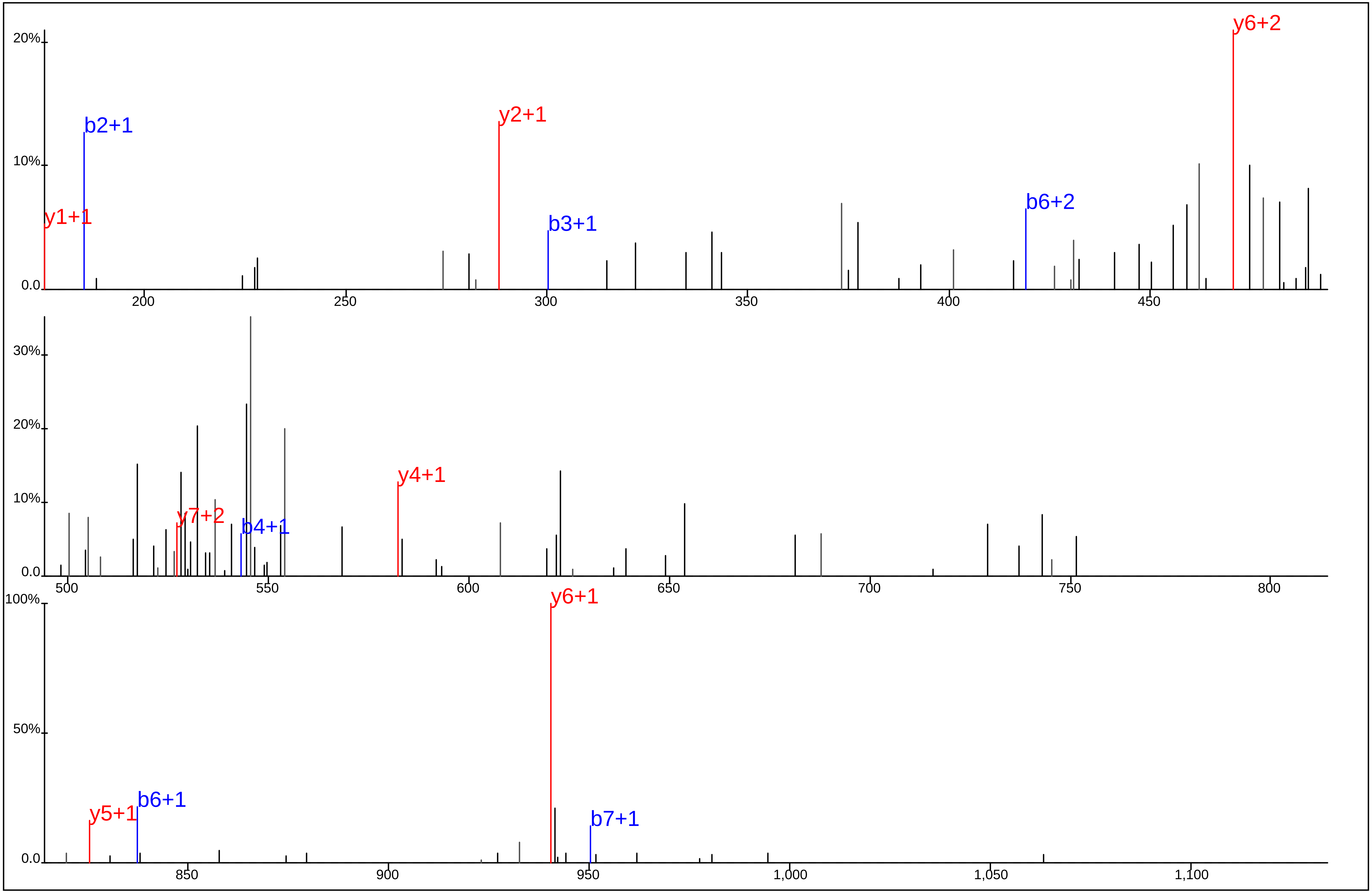

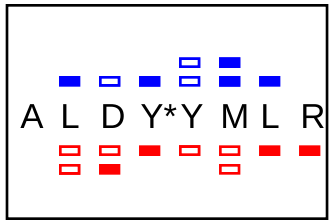

Protein Name: Talin
gi Accession: 81175200
Precursor Mass: 562.7481+2
Delta Mass: 3.884 ppm
XCorr: 27.86

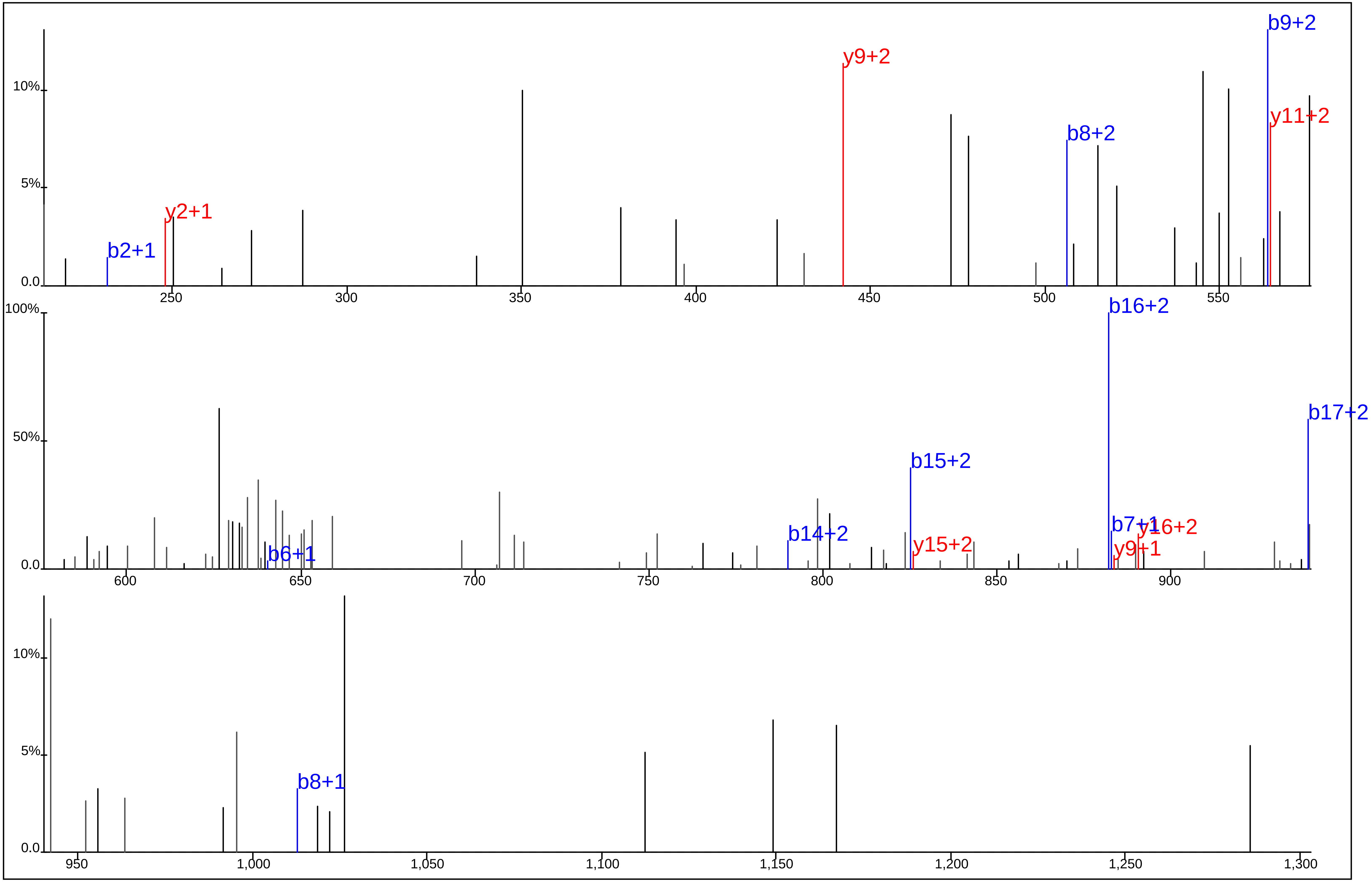

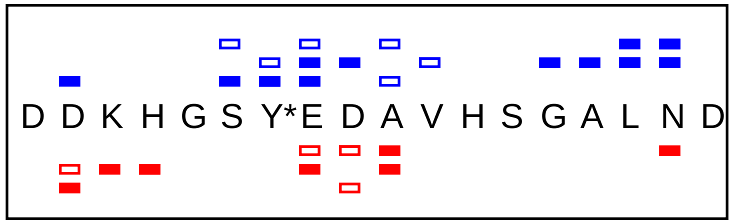

Protein Name: T Complex 1
gi Accession: 57863257
Precursor Mass: 670.6034+3
Delta Mass: 4.164 ppm
XCorr: 11.56

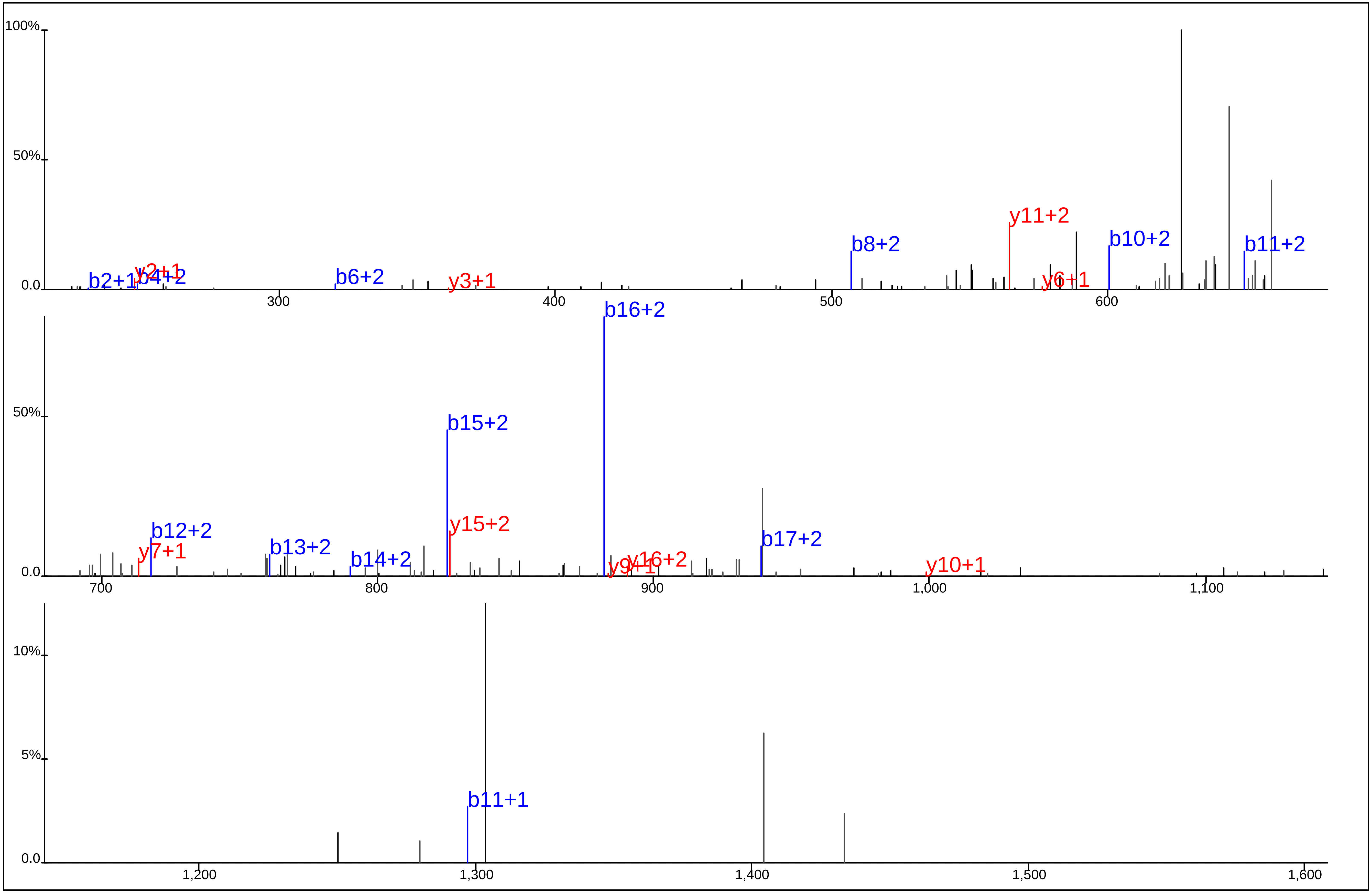

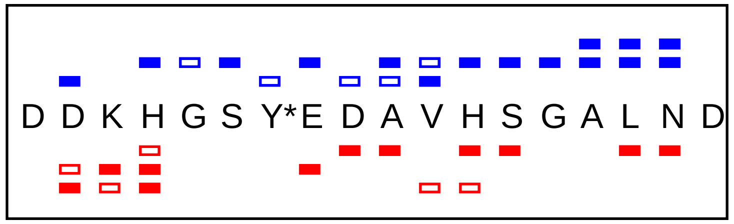

Protein Name: T Complex 1
gi Accession: 57863257
Precursor Mass: 670.6026+3
Delta Mass: 2.980 ppm
XCorr: 29.23

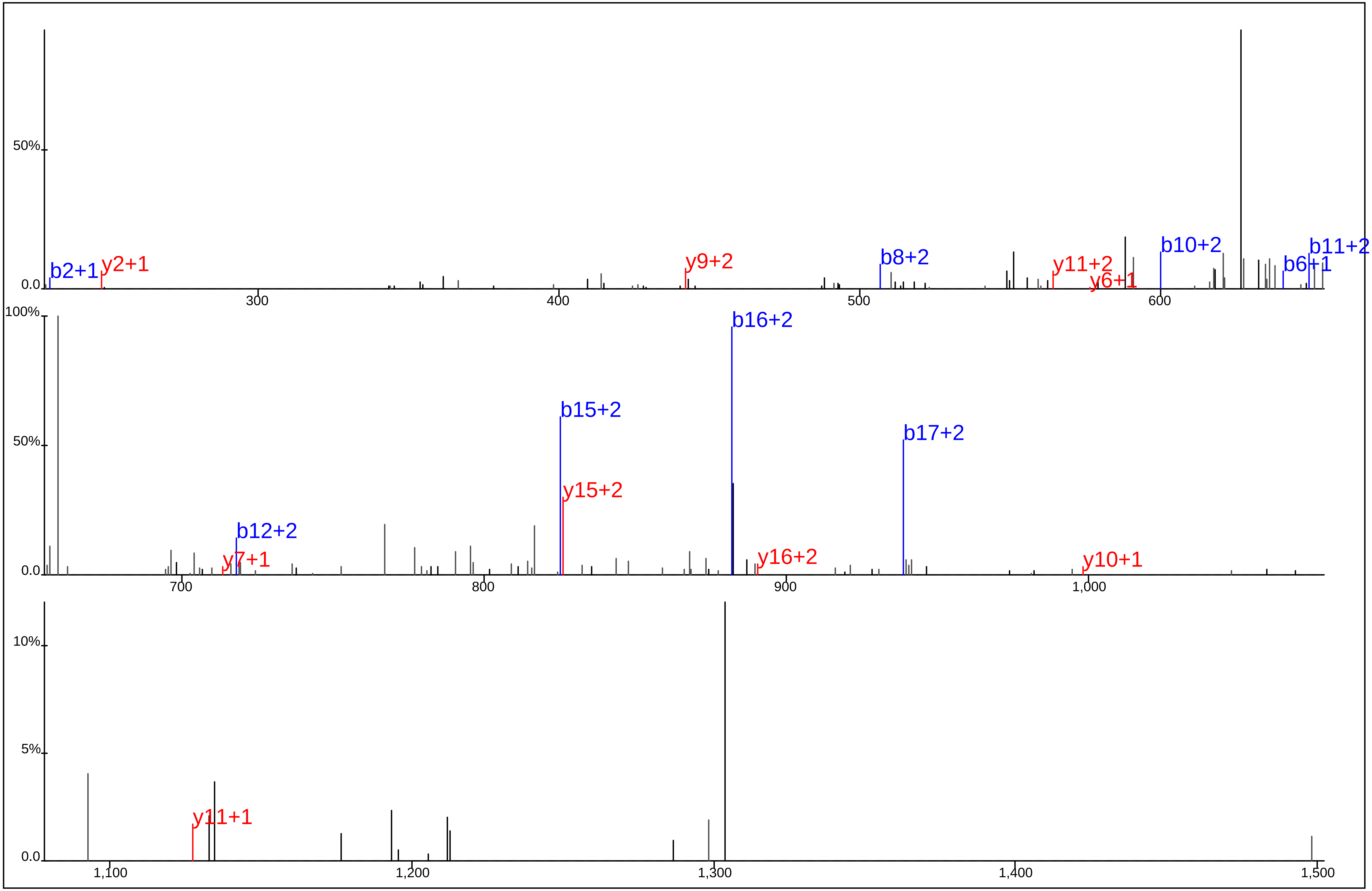

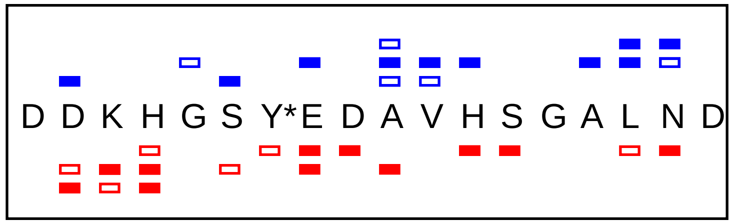

Protein Name: T Complex 1
gi Accession: 57863257
Precursor Mass: 670.6042+3
Delta Mass: 5.437 ppm
XCorr: 40.37

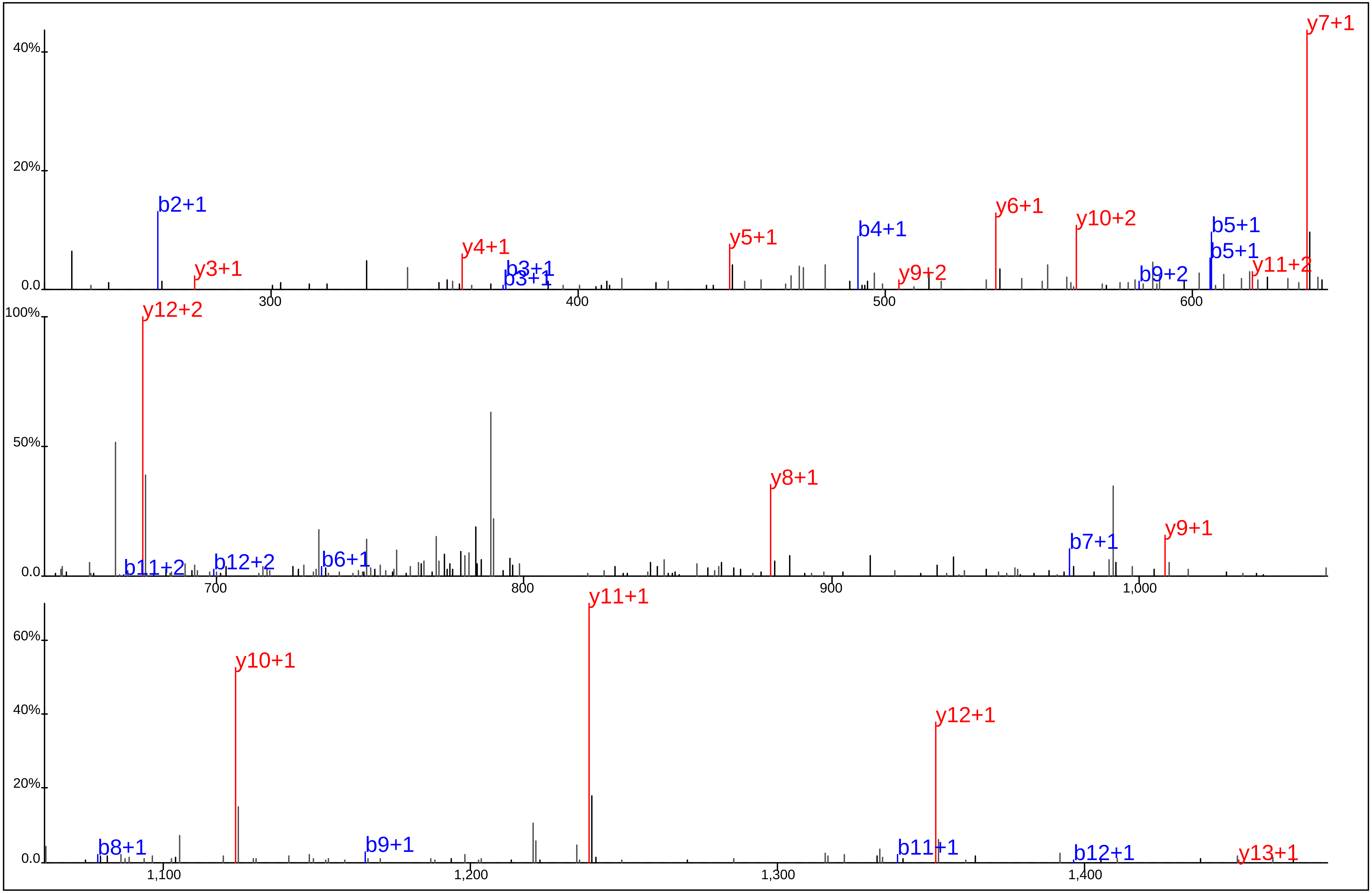

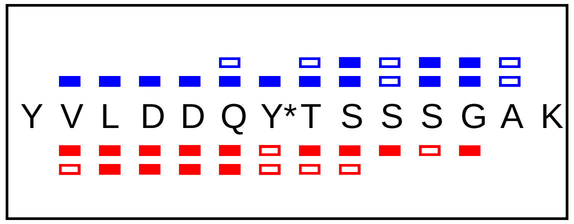

Protein Name: Tec
gi Accession: 130977759
Precursor Mass: 807.3484+2
Delta Mass: 10.187 ppm
XCorr: 76.82

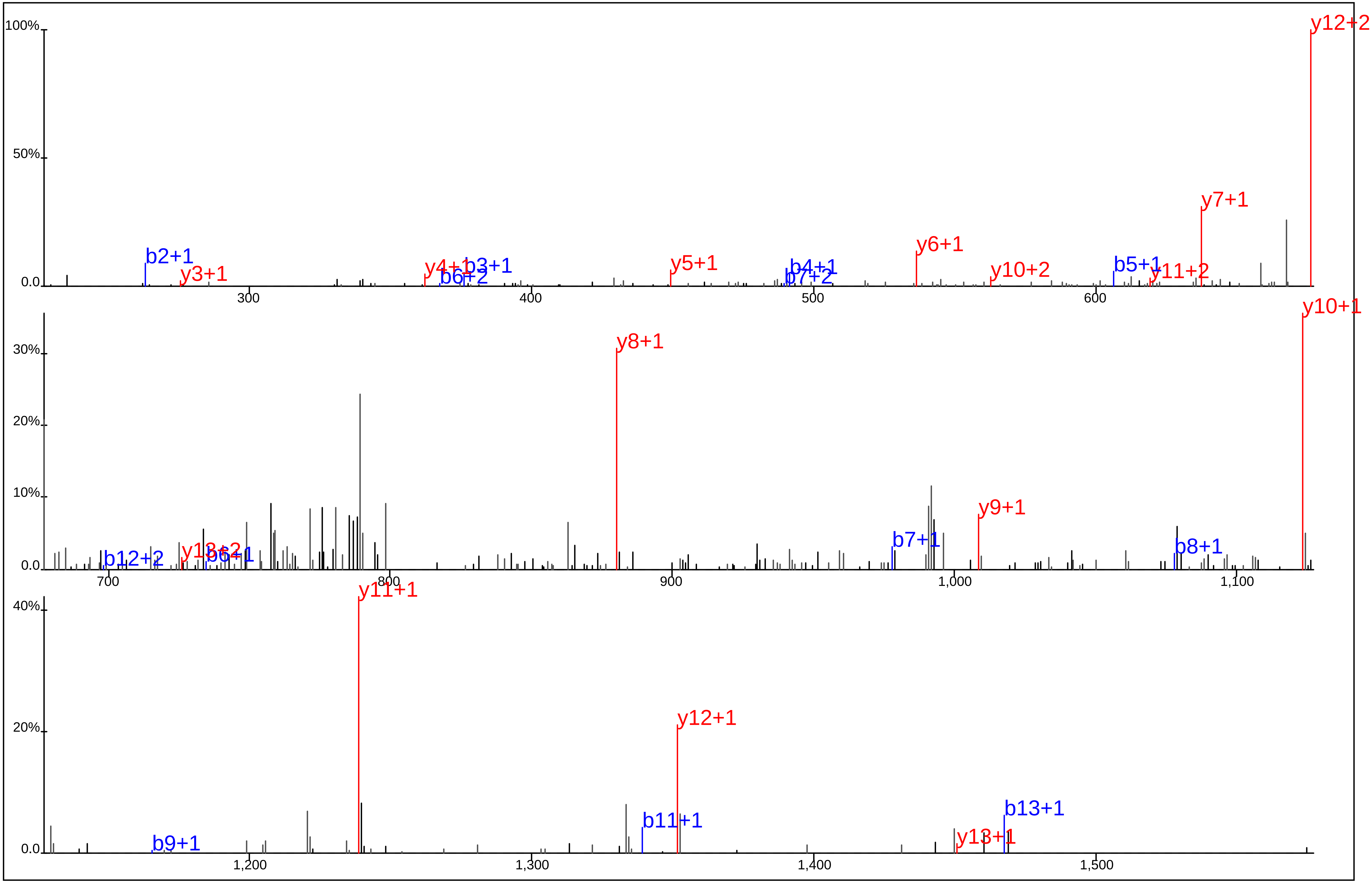

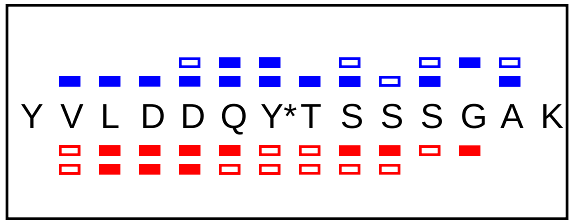

Protein Name: Tec
gi Accession: 130977759
Precursor Mass: 807.3434+2
Delta Mass: 4.058 ppm
XCorr: 73.57

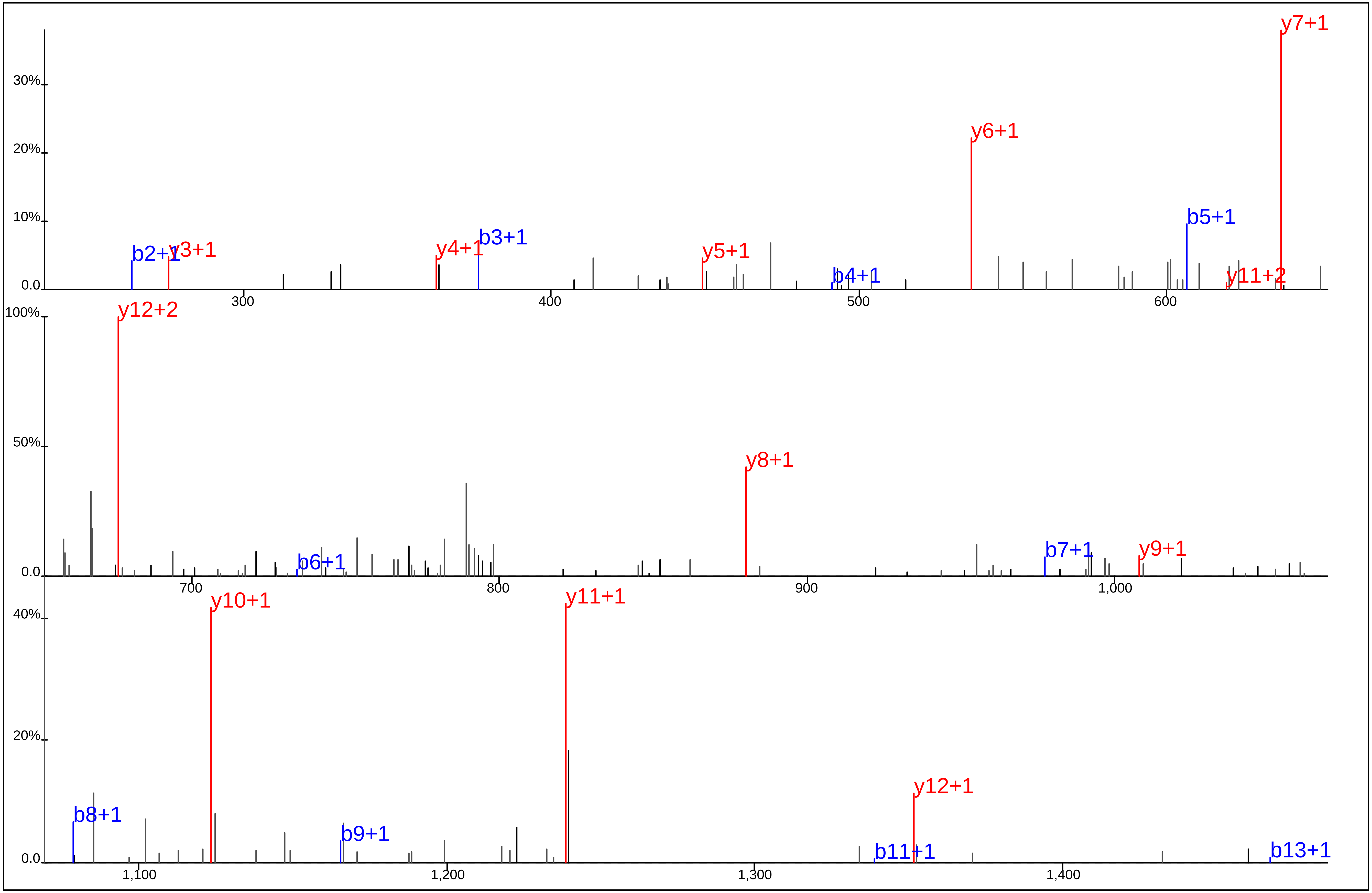

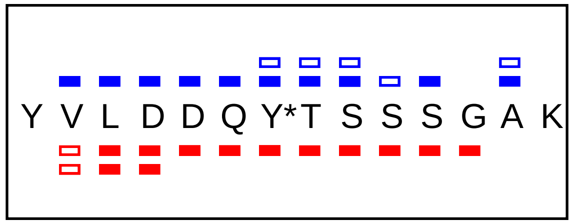

Protein Name: Tec
gi Accession: 130977759
Precursor Mass: 807.3425+2
Delta Mass: 2.849 ppm
XCorr: 57.57

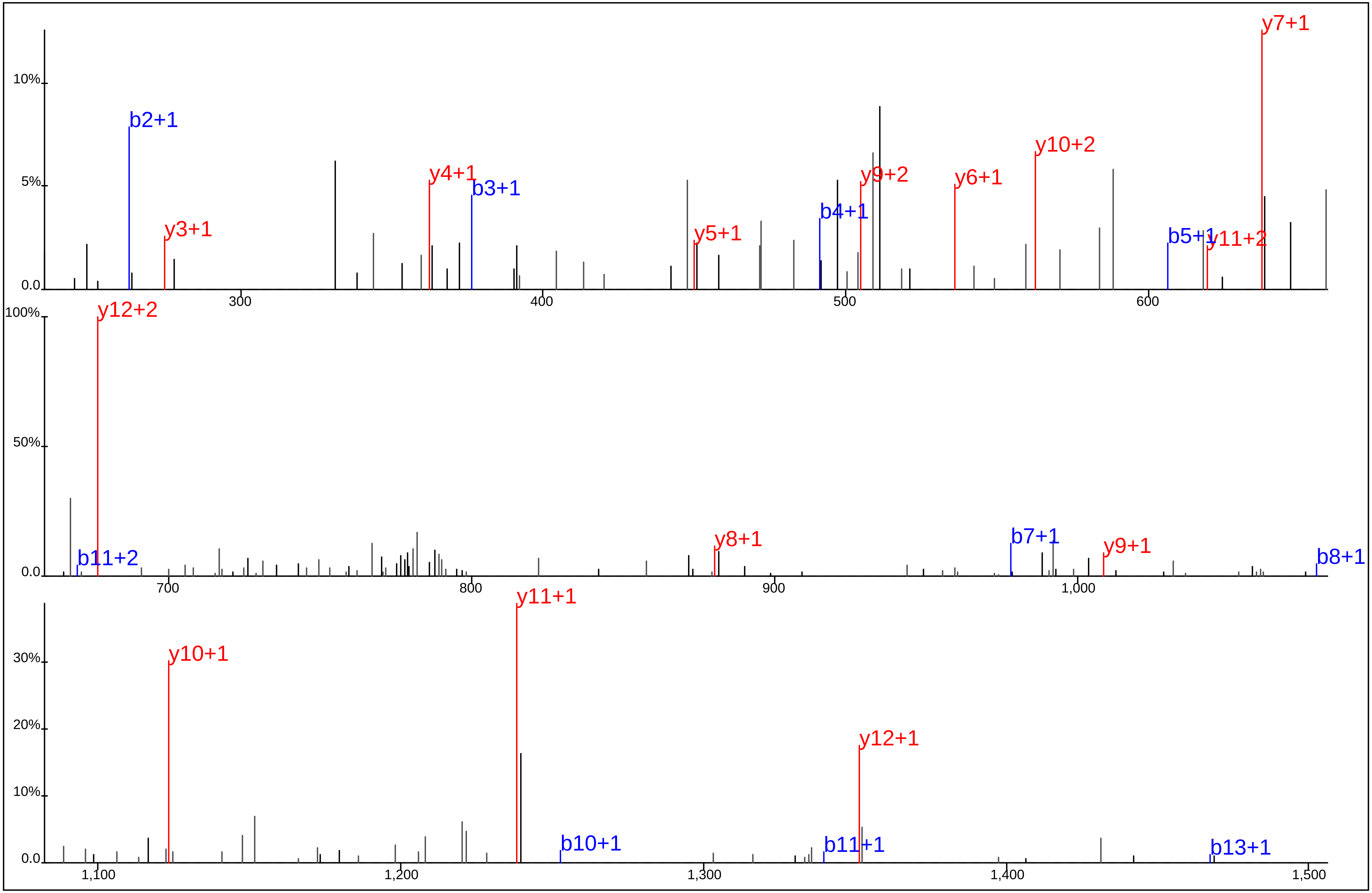

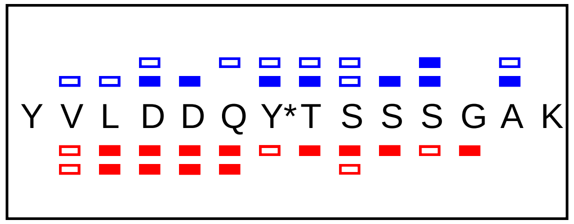

Protein Name:	Tec
gi Accession:	130977759
Precursor Mass:	807.3444+2
Delta Mass:	5.272 ppm
XCorr:	55.86

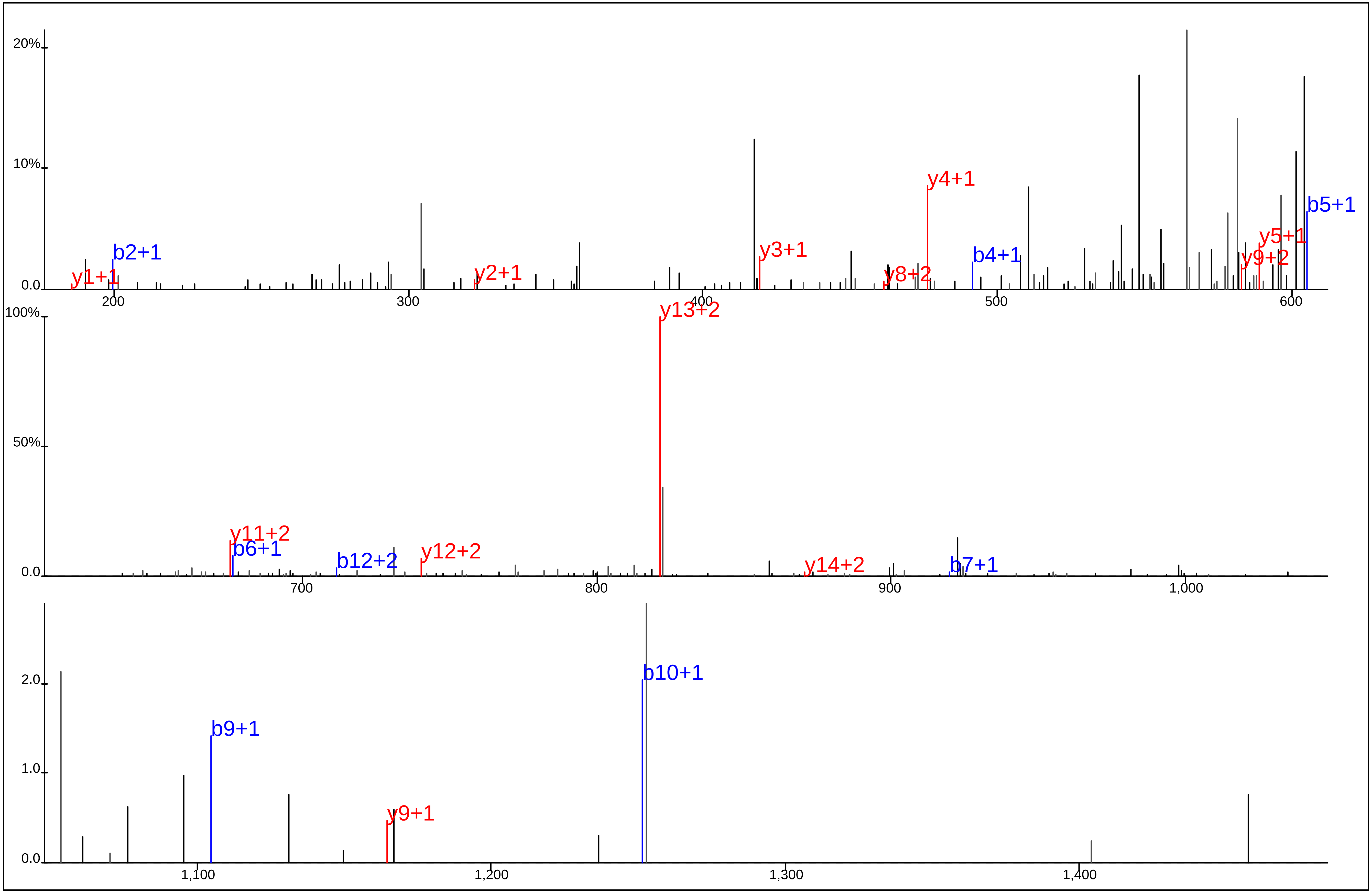

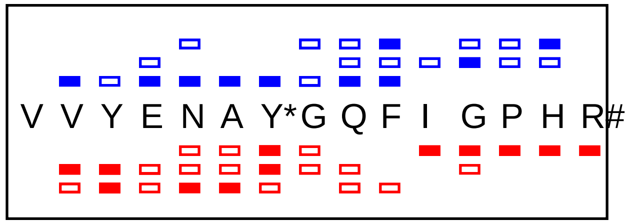

Protein Name:	Thioredoxin reductase 1
gi Accession:	1843434
Precursor Mass:	613.9551+3
Delta Mass:	3.006 ppm
XCorr:	2.6309

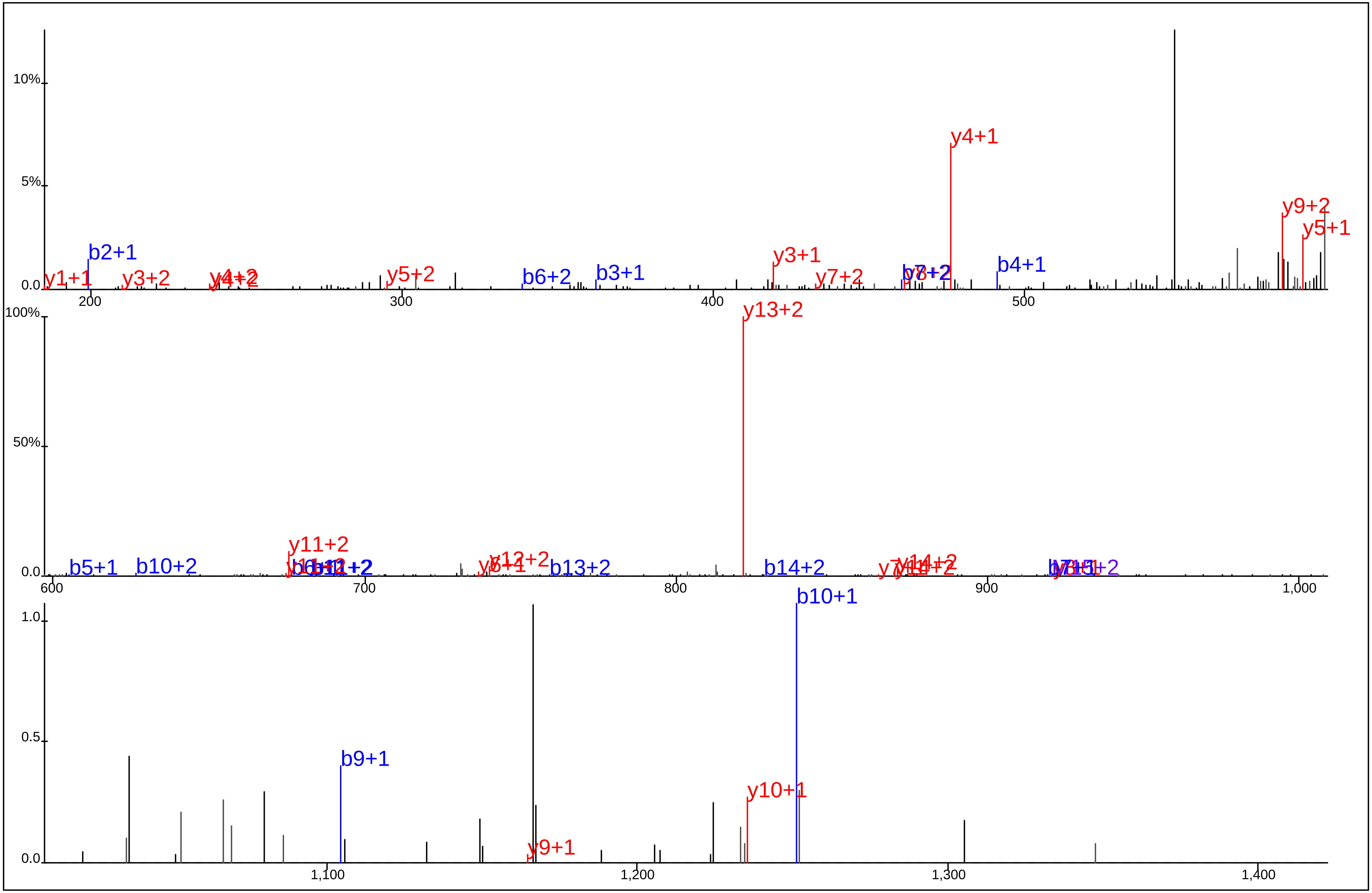

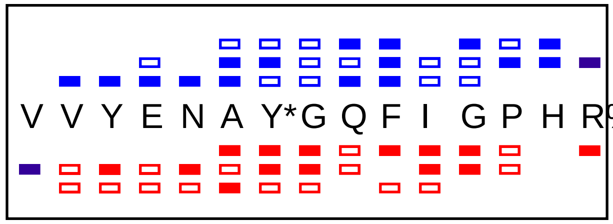

Protein Name:	Thioredoxin reductase 1
gi Accession:	1843434
Precursor Mass:	613.9568+3
Delta Mass:	5.798 ppm
XCorr:	36.74

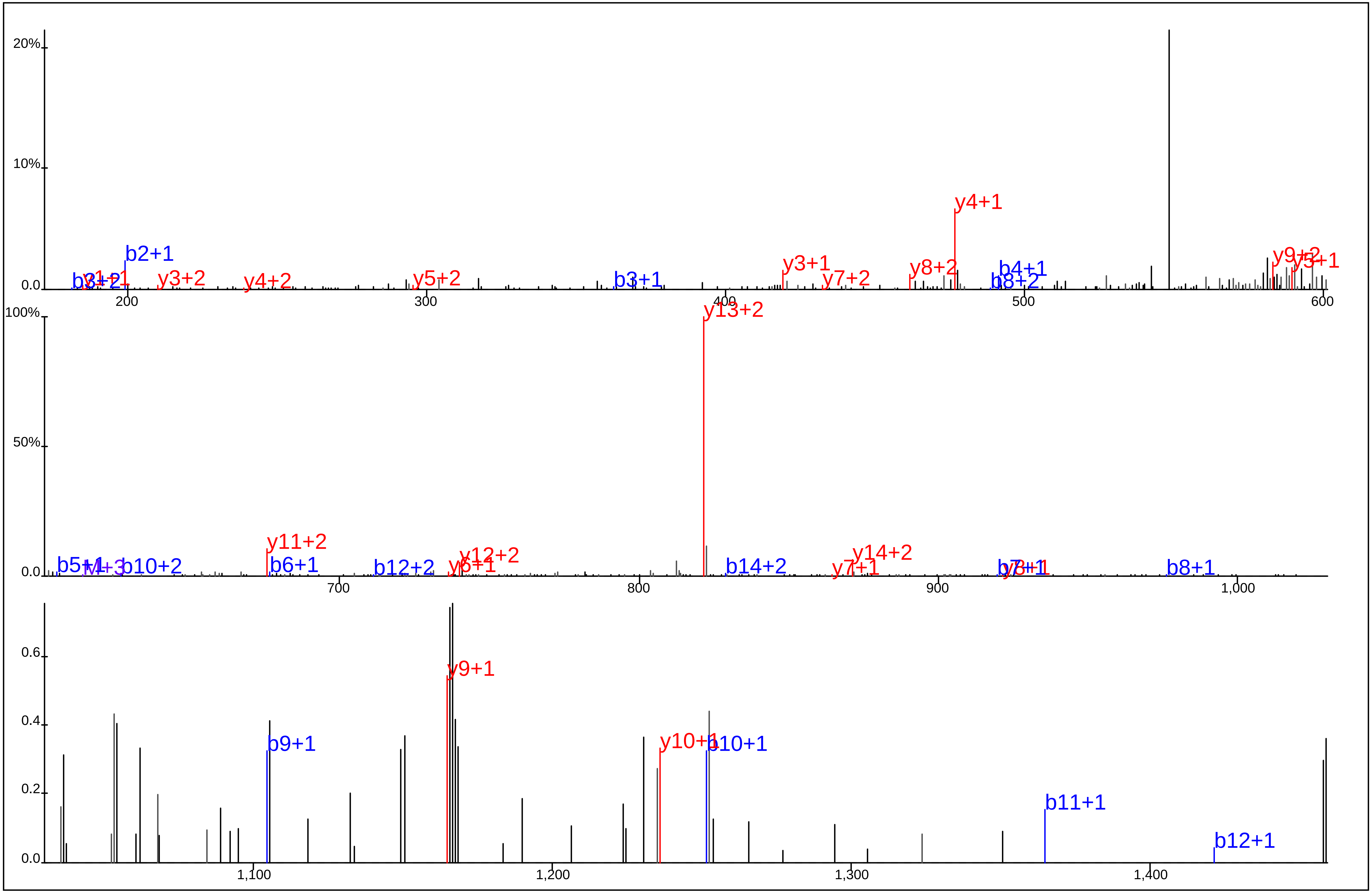

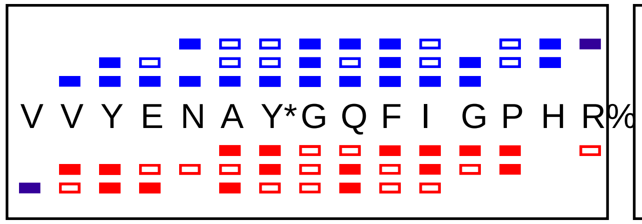

Protein Name:	Thioredoxin reductase 1
gi Accession:	1843434
Precursor Mass:	613.9539+3
Delta Mass:	1.020 ppm
XCorr:	38.86

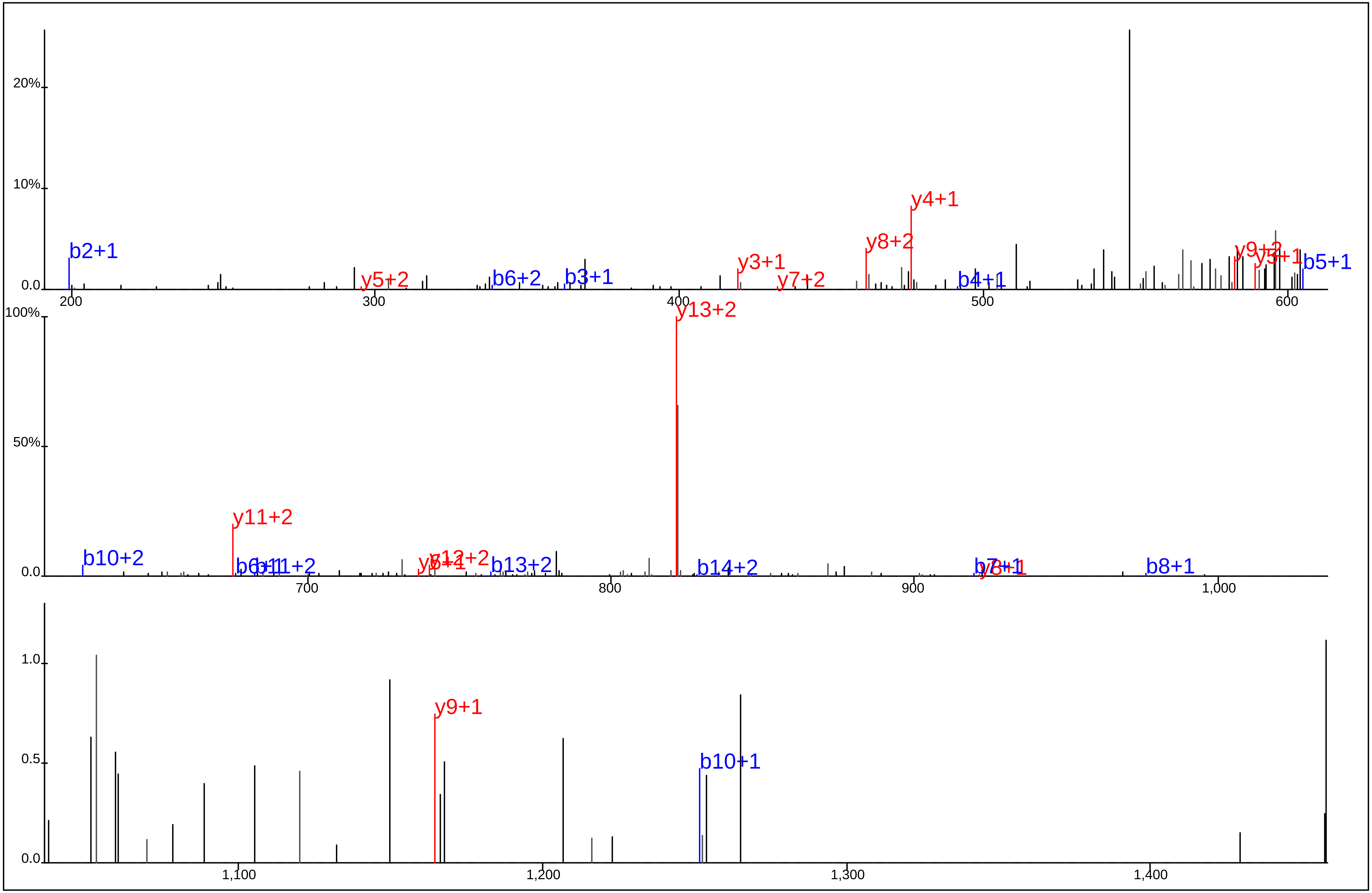

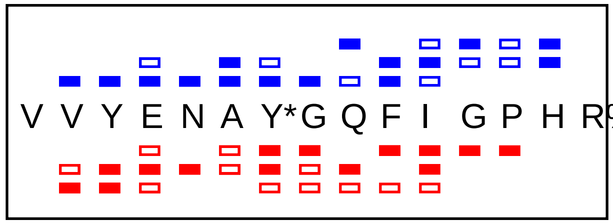

Protein Name:	Thioredoxin reductase 1
gi Accession:	1843434
Precursor Mass:	613.9543+3
Delta Mass:	1.716 ppm
XCorr:	14.14

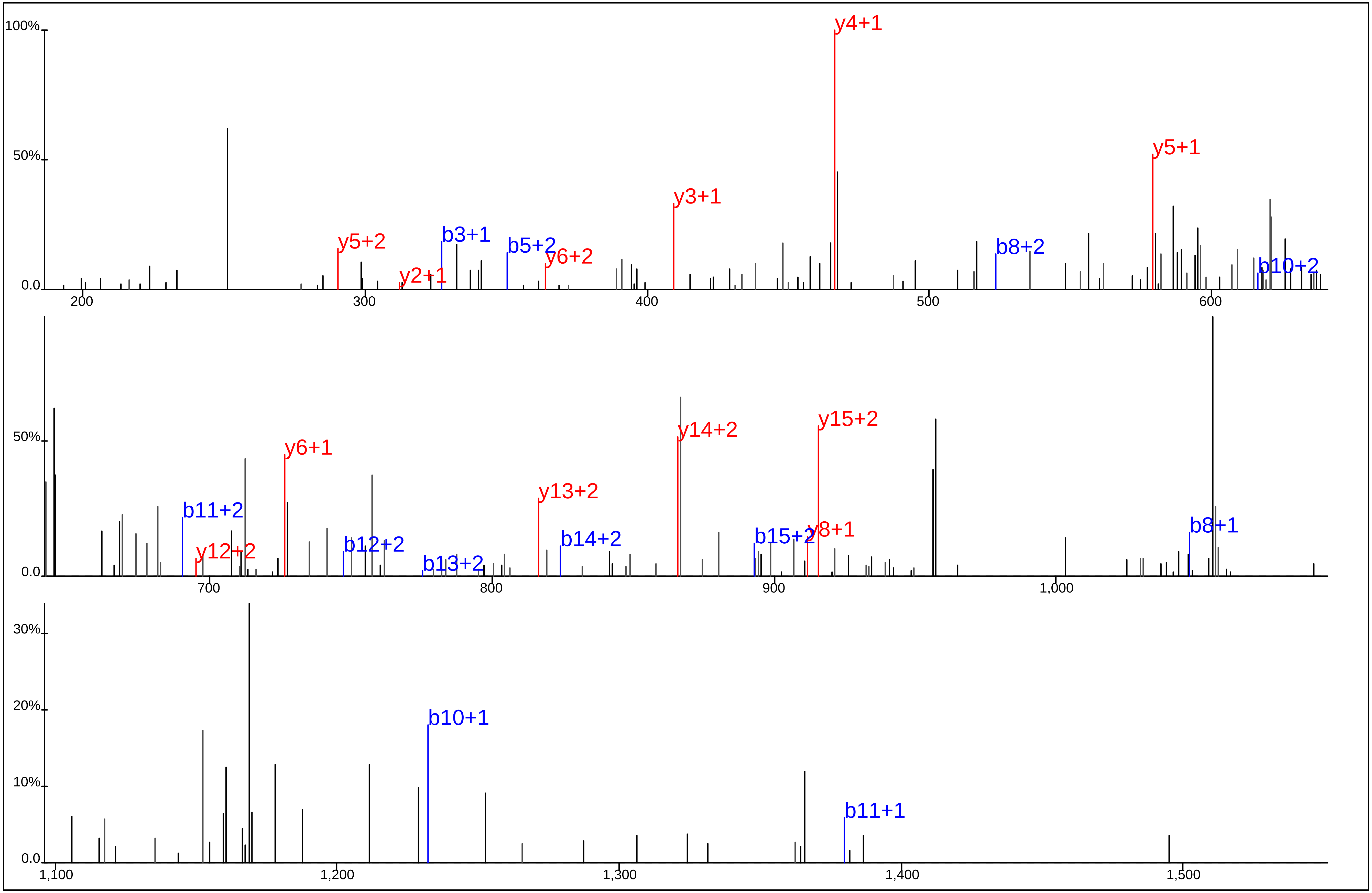

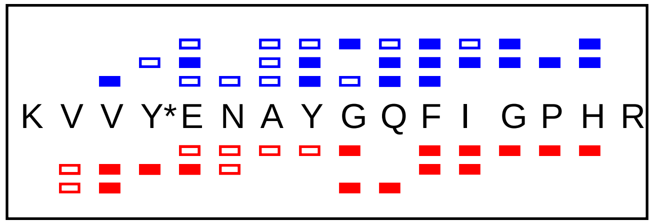

Protein Name:	Thioredoxin reductase 1
gi Accession:	1843434
Precursor Mass:	653.3148+3
Delta Mass:	1.074 ppm
XCorr:	17.66

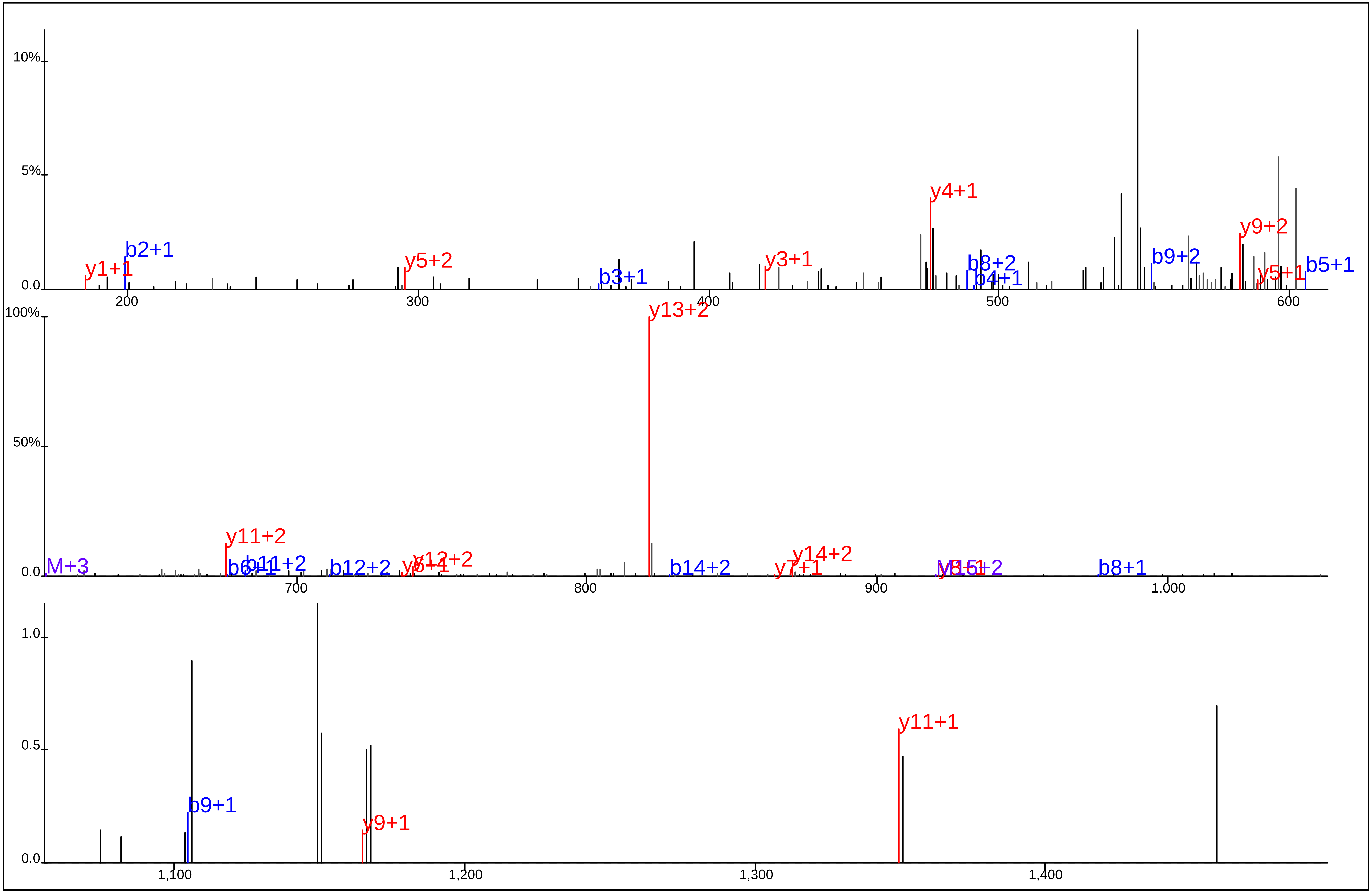

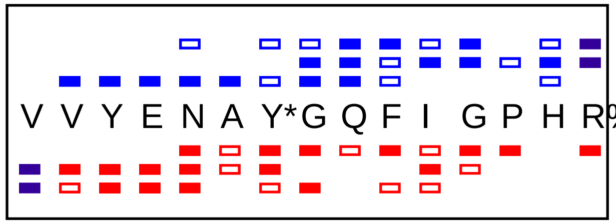

Protein Name:	Thioredoxin reductase 1
gi Accession:	1843434
Precursor Mass:	613.9547+3
Delta Mass:	2.315 ppm
XCorr:	27.12

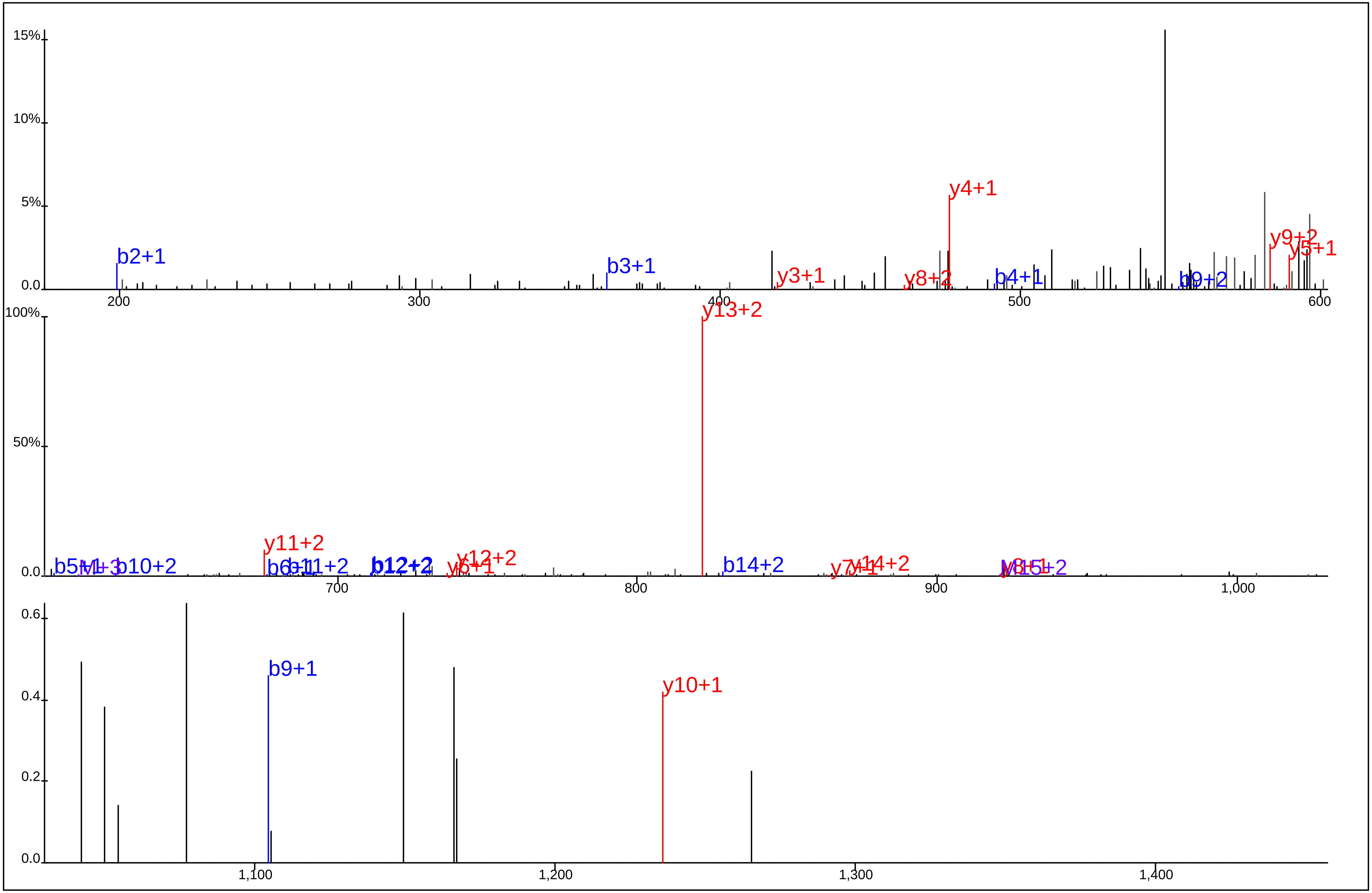

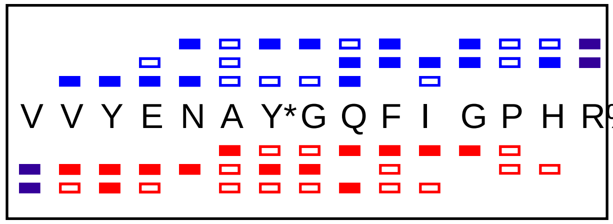

Protein Name: Thioredoxin reductase 1
gi Accession: 1843434
Precursor Mass: 613.9555+3
Delta Mass: 3.608 ppm
XCorr: 17.75

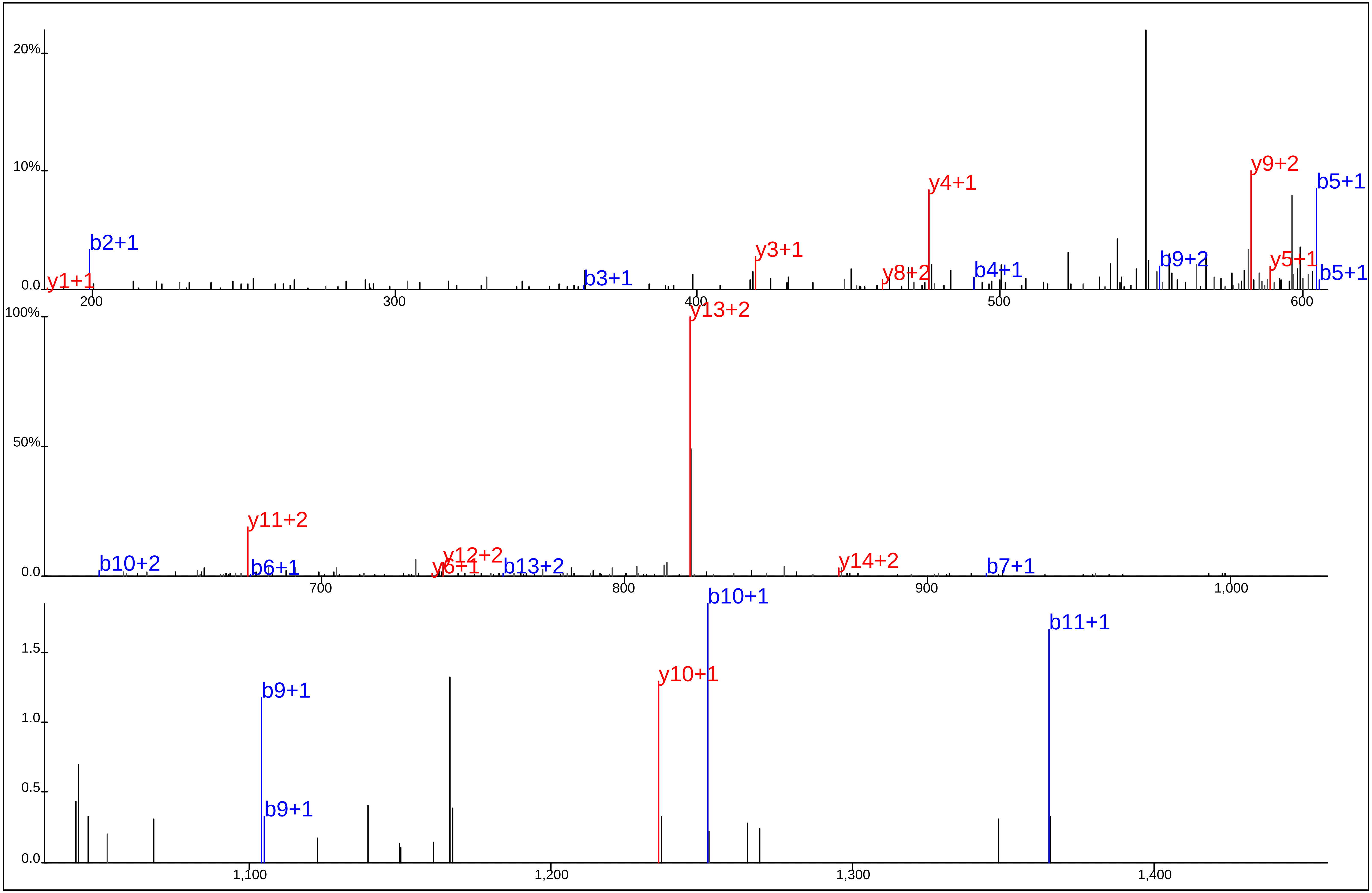

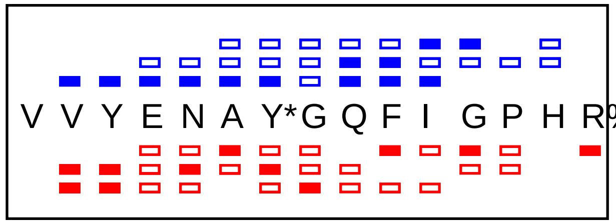

Protein Name:	Thioredoxin reductase 1
gi Accession:	1843434
Precursor Mass:	613.9561+3
Delta Mass:	4.505 ppm
XCorr:	28.89

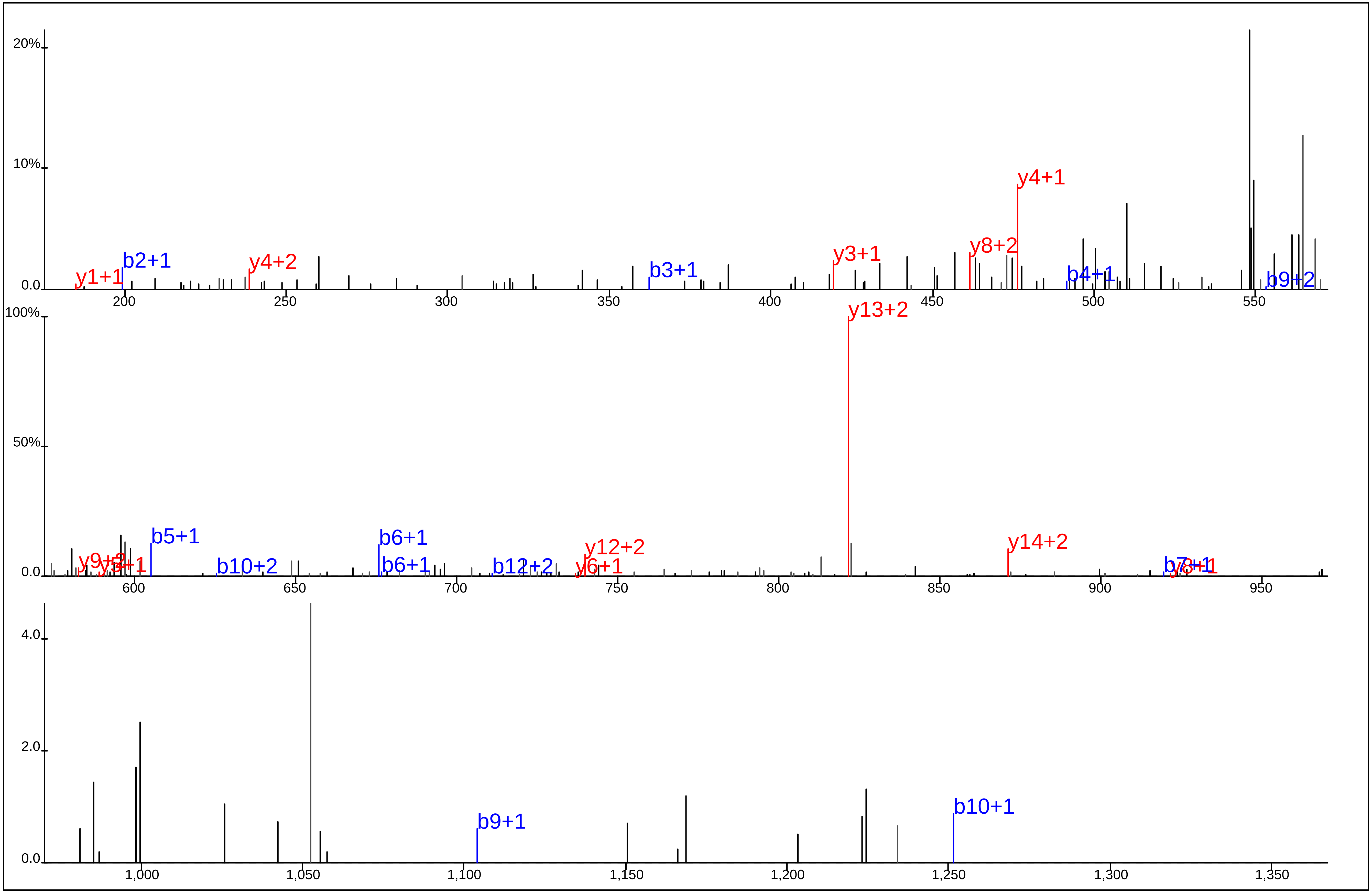

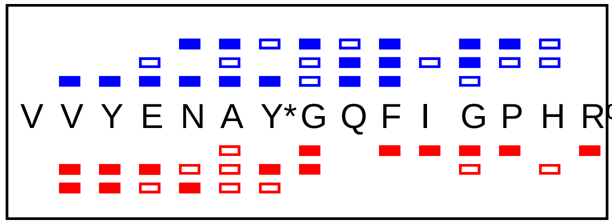

Protein Name:	Thioredoxin reductase 1
gi Accession:	1843434
Precursor Mass:	613.9531+3
Delta Mass:	0.371 ppm
XCorr:	20.14

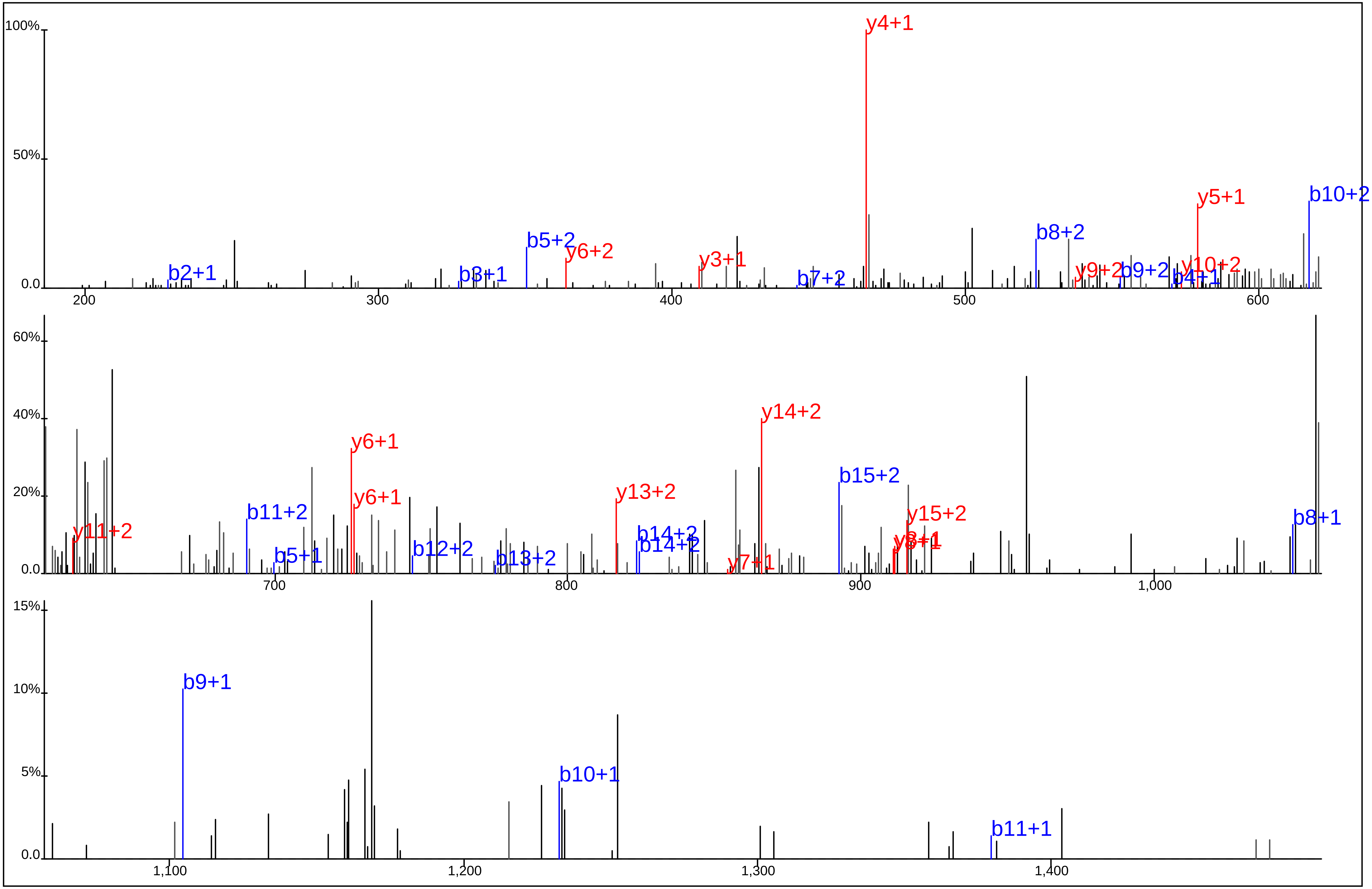

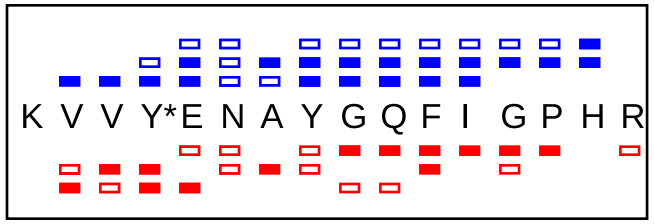

Protein Name:	Thioredoxin reductase 1
gi Accession:	1843434
Precursor Mass:	653.3107+3
Delta Mass:	7.335 ppm
XCorr:	12.2

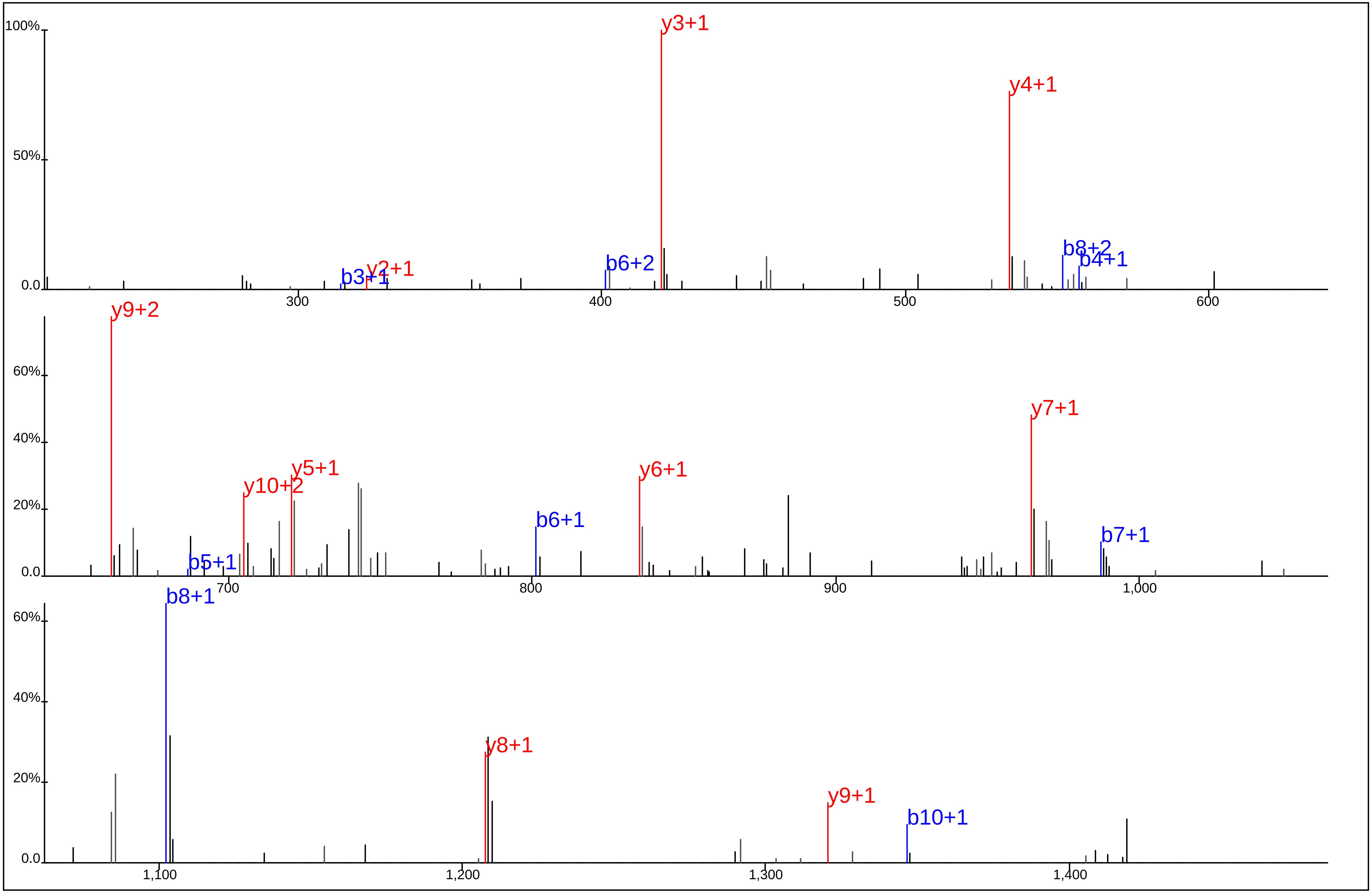

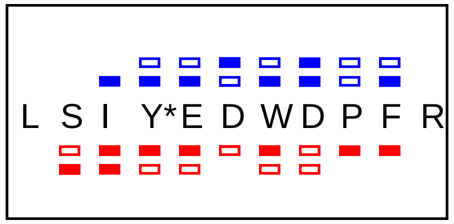

Protein Name: TIAM1
gi Accession: 115583670
Precursor Mass: 760.8259+2
Delta Mass: 0.830 ppm
XCorr: 43.19

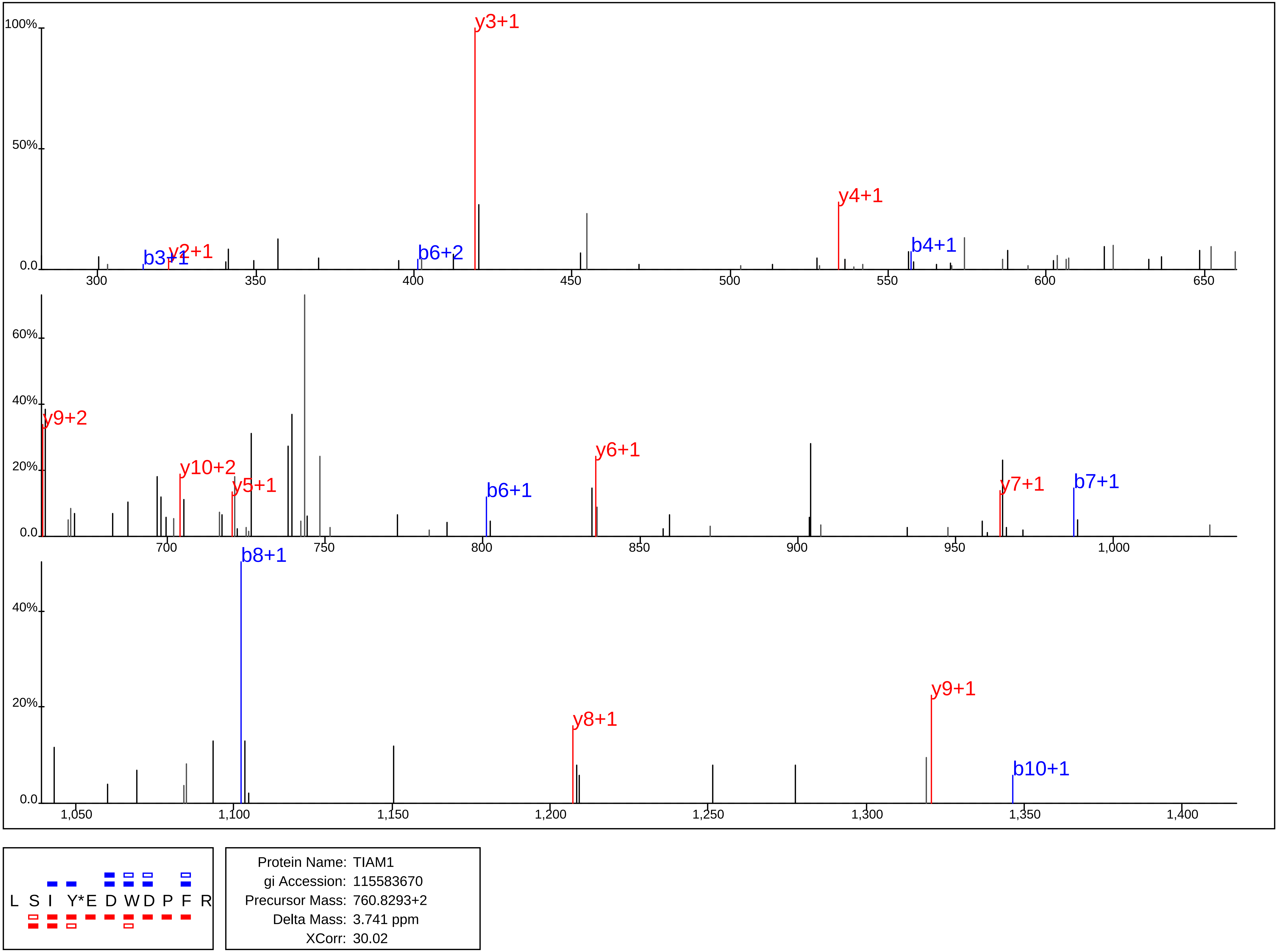

Protein Name: TIAM1

gi Accession: 115583670

Precursor Mass: 760.8293+2

Delta Mass: 3.741 ppm

XCorr: 30.02

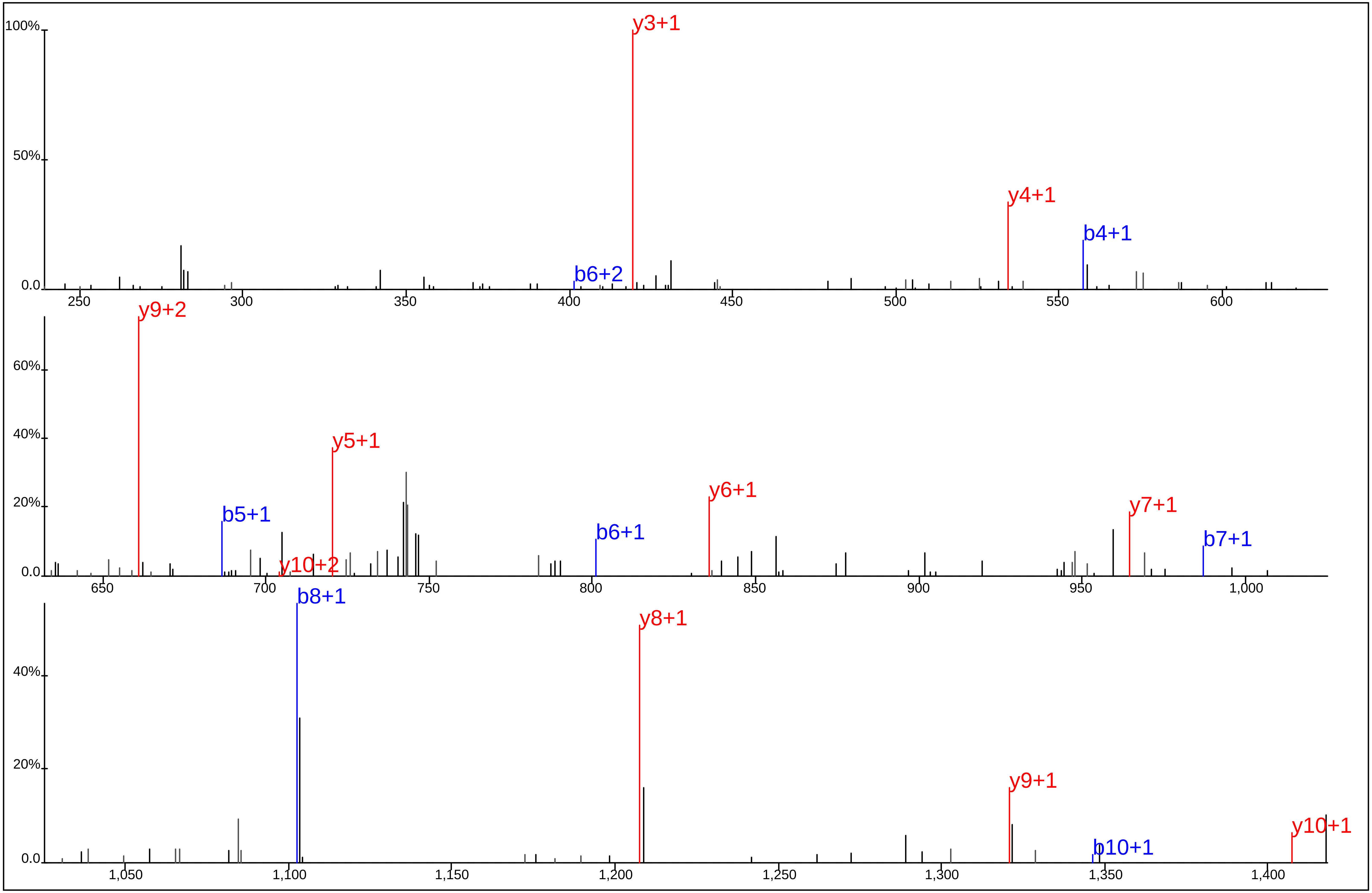

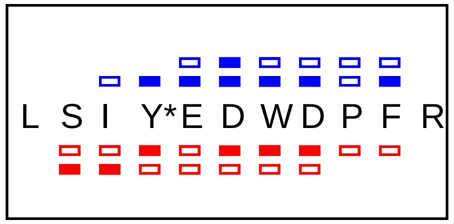

Protein Name:	TIAM1
gi Accession:	115583670
Precursor Mass:	760.8295+2
Delta Mass:	3.984 ppm
XCorr:	45.72

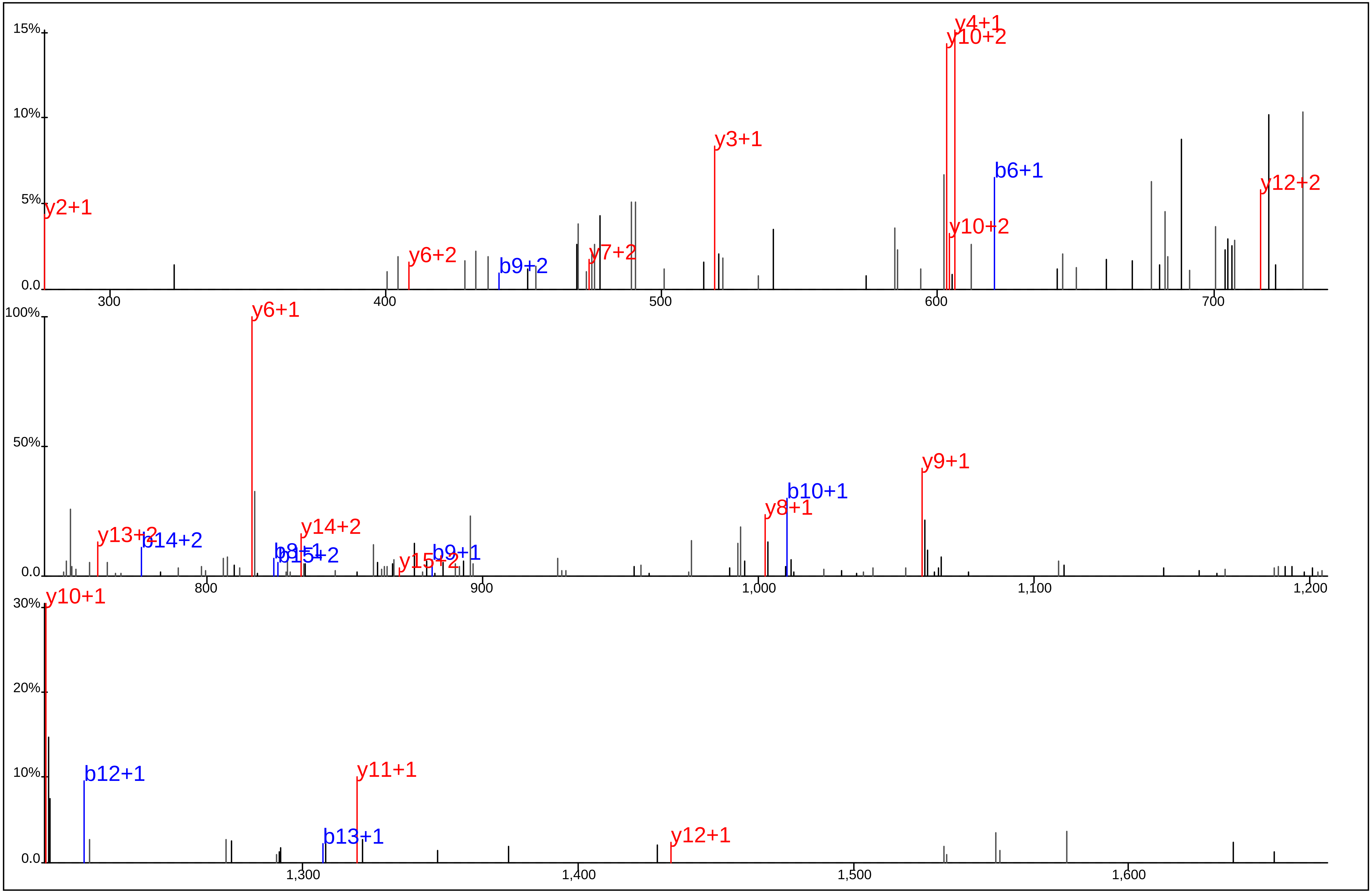

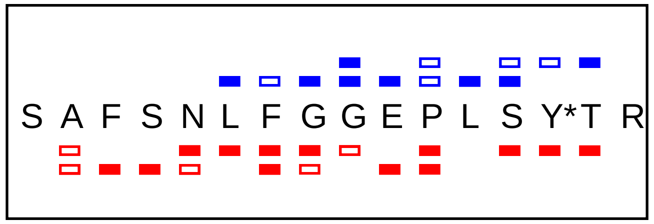

Protein Name:	Transferrin receptor
gi Accession:	37433
Precursor Mass:	913.4155+2
Delta Mass:	4.457 ppm
XCorr:	51.96

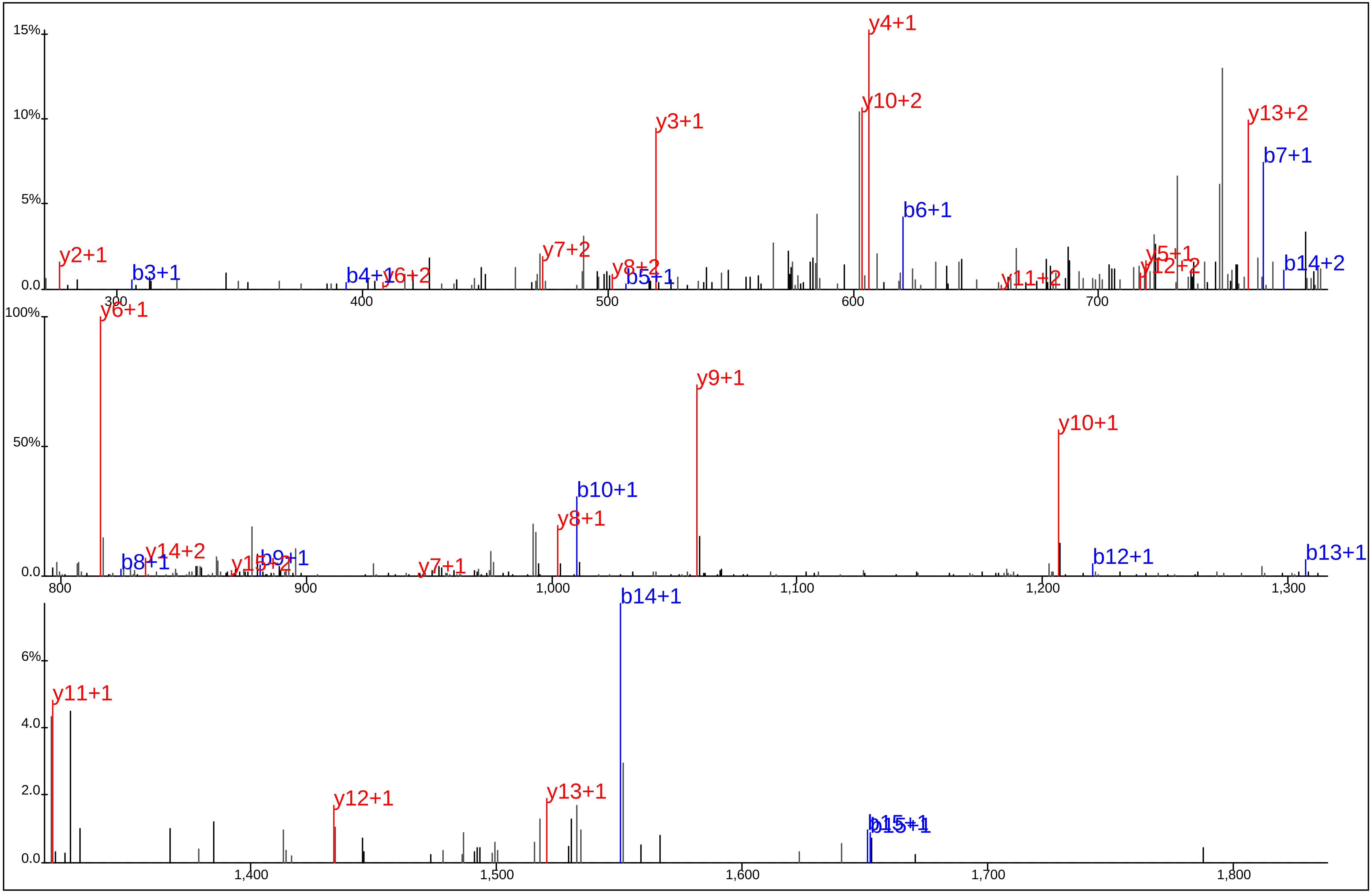

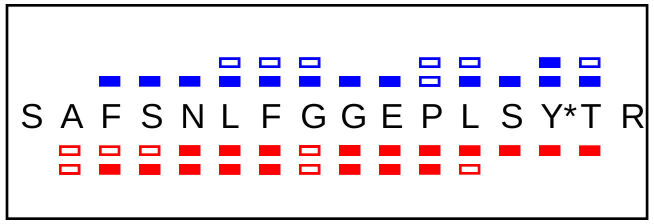

Protein Name:	Transferrin receptor
gi Accession:	37433
Precursor Mass:	913.4132+2
Delta Mass:	1.916 ppm
XCorr:	53.89

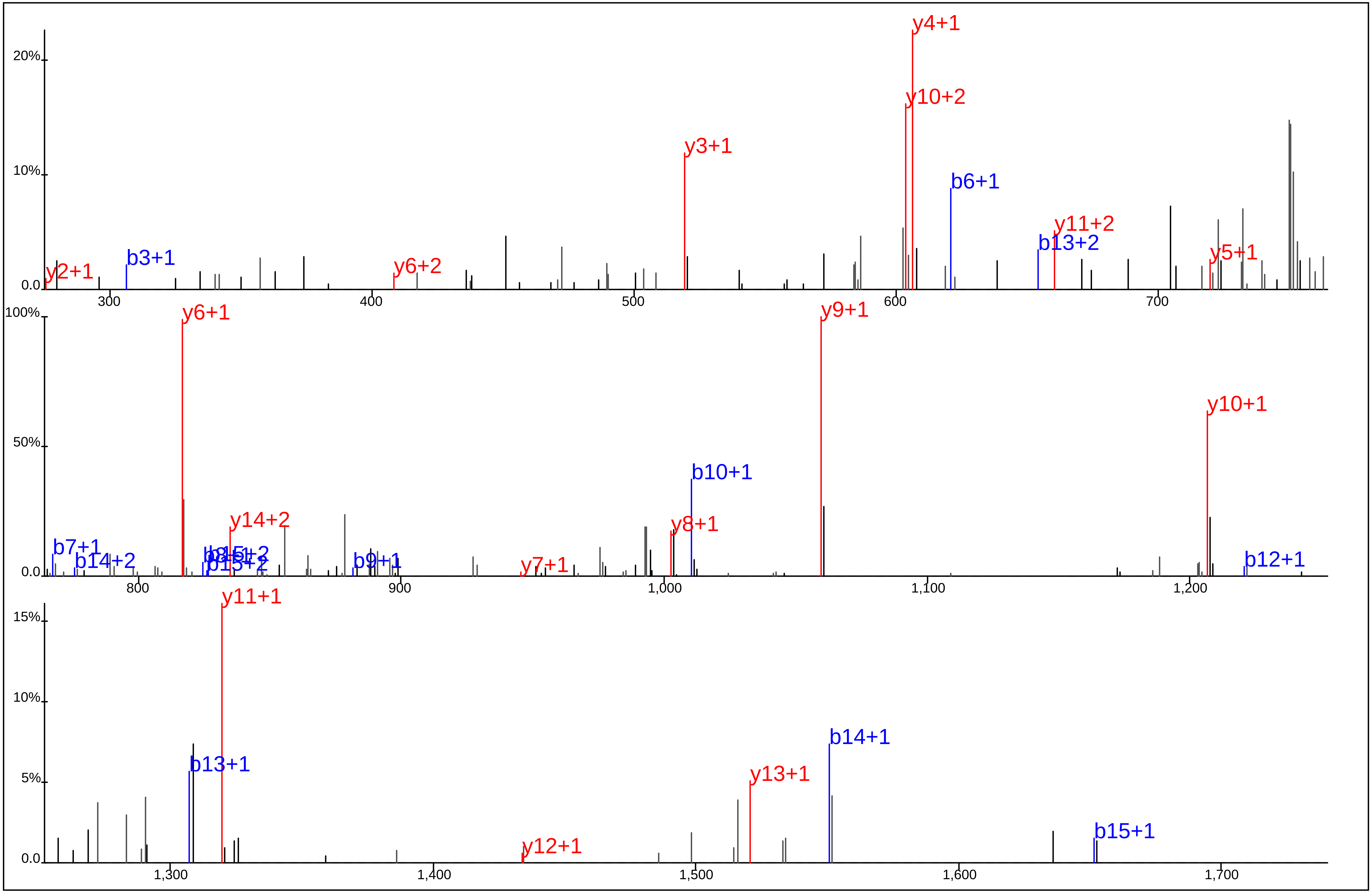

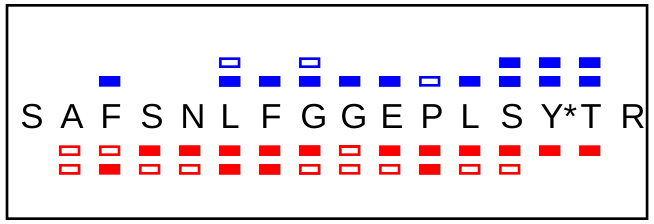

Protein Name:	Transferrin receptor
gi Accession:	37433
Precursor Mass:	913.4124+2
Delta Mass:	1.045 ppm
XCorr:	37.0

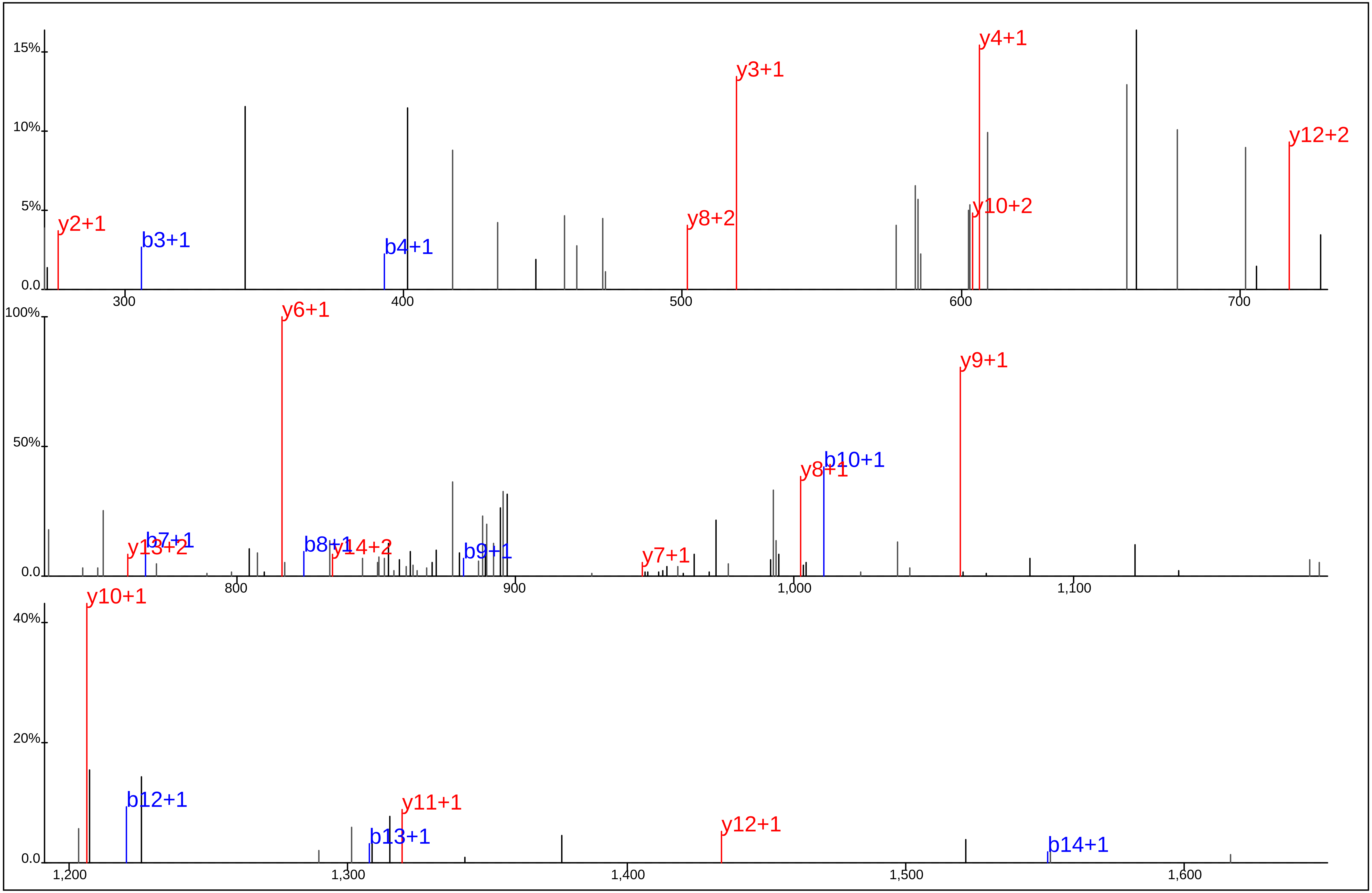

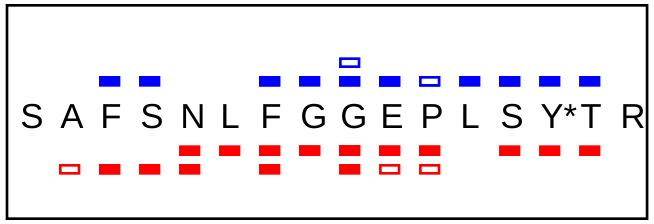

Protein Name: Transferrin receptor
gi Accession: 37433
Precursor Mass: 913.4155+2
Delta Mass: 4.457 ppm
XCorr: 41.36

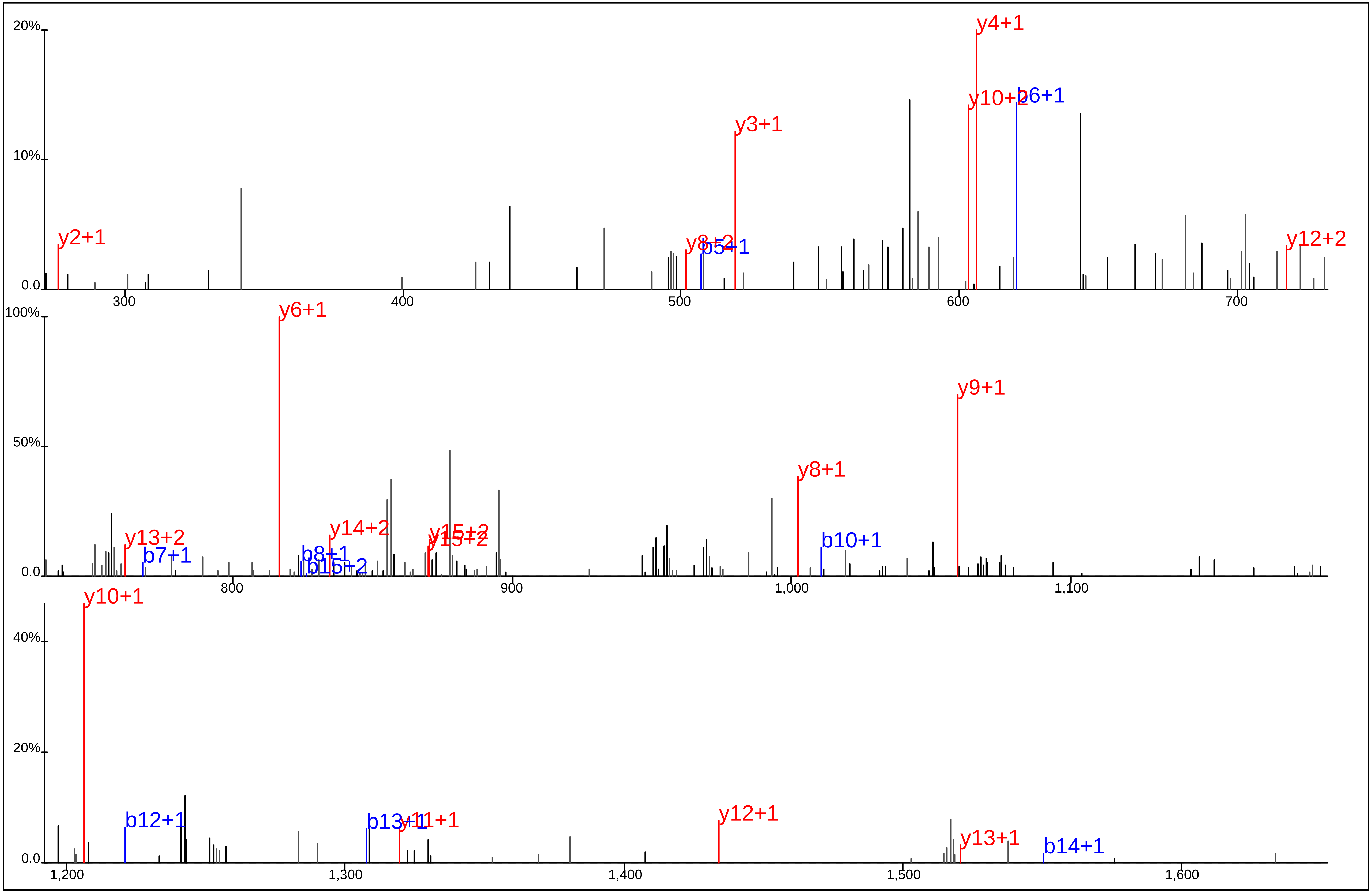

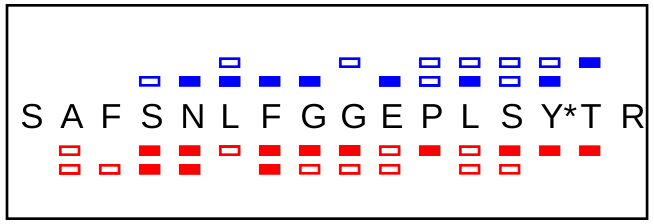

Protein Name:	Transferrin receptor
gi Accession:	37433
Precursor Mass:	913.4113+2
Delta Mass:	0.226 ppm
XCorr:	38.28

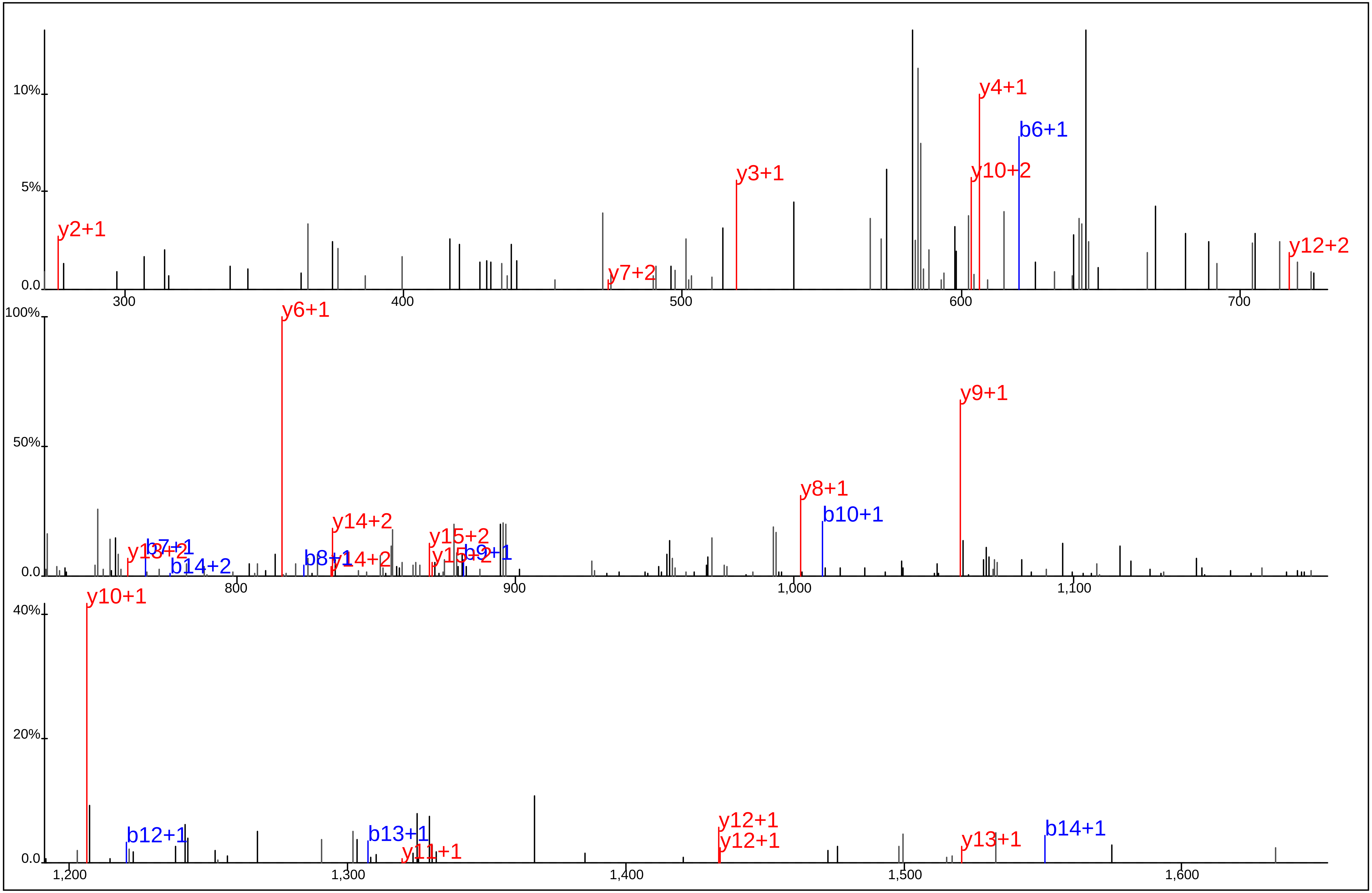

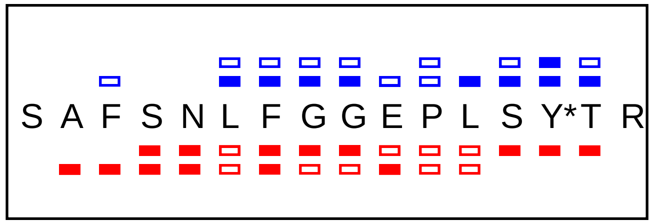

Protein Name: Transferrin receptor
gi Accession: 37433
Precursor Mass: 913.4152+2
Delta Mass: 4.123 ppm
XCorr: 26.64

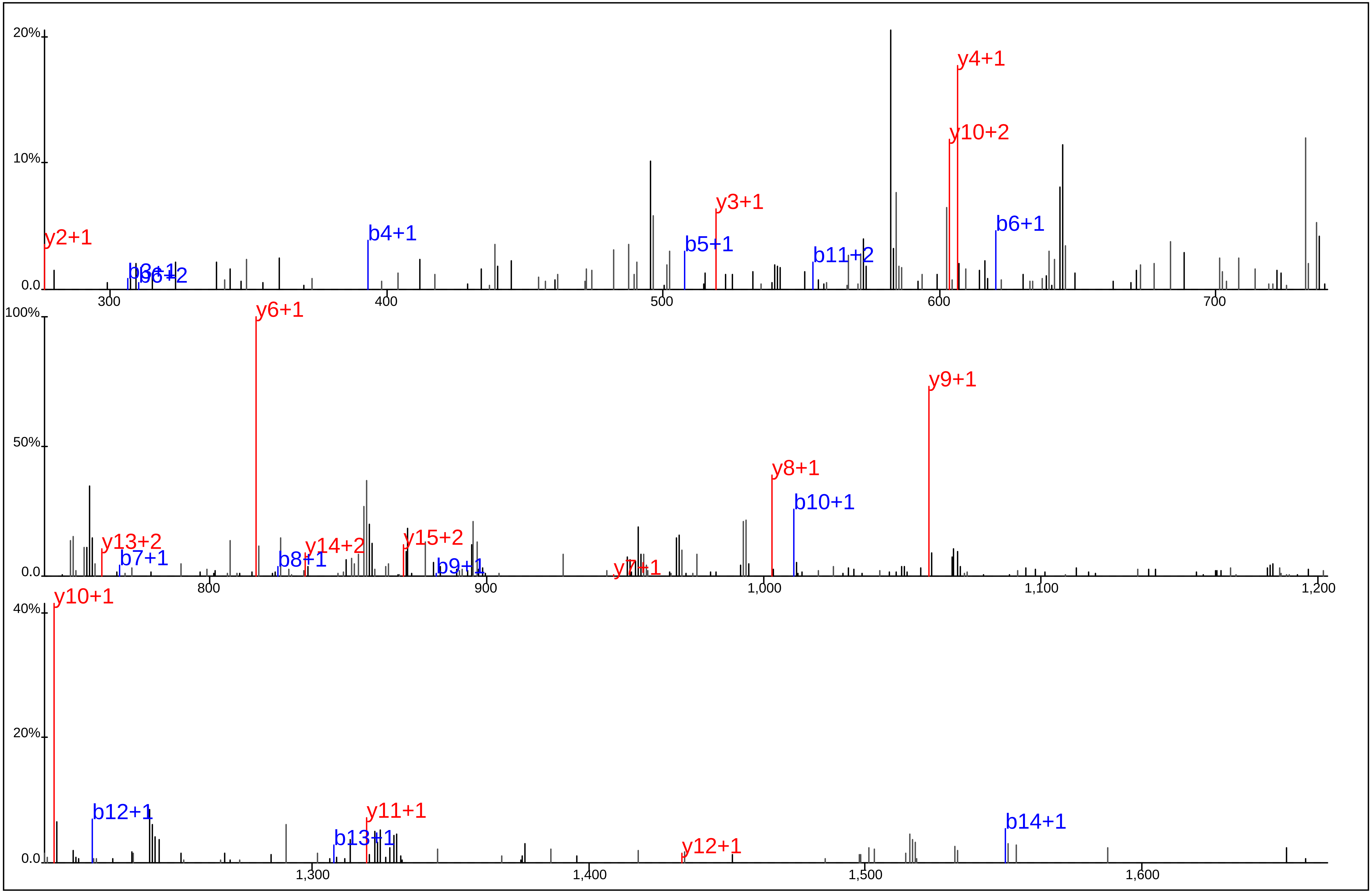

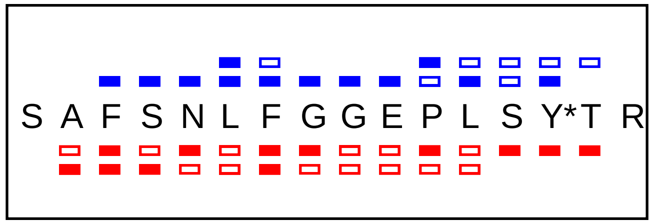

Protein Name:	Transferrin receptor
gi Accession:	37433
Precursor Mass:	913.4159+2
Delta Mass:	4.857 ppm
XCorr:	40.34

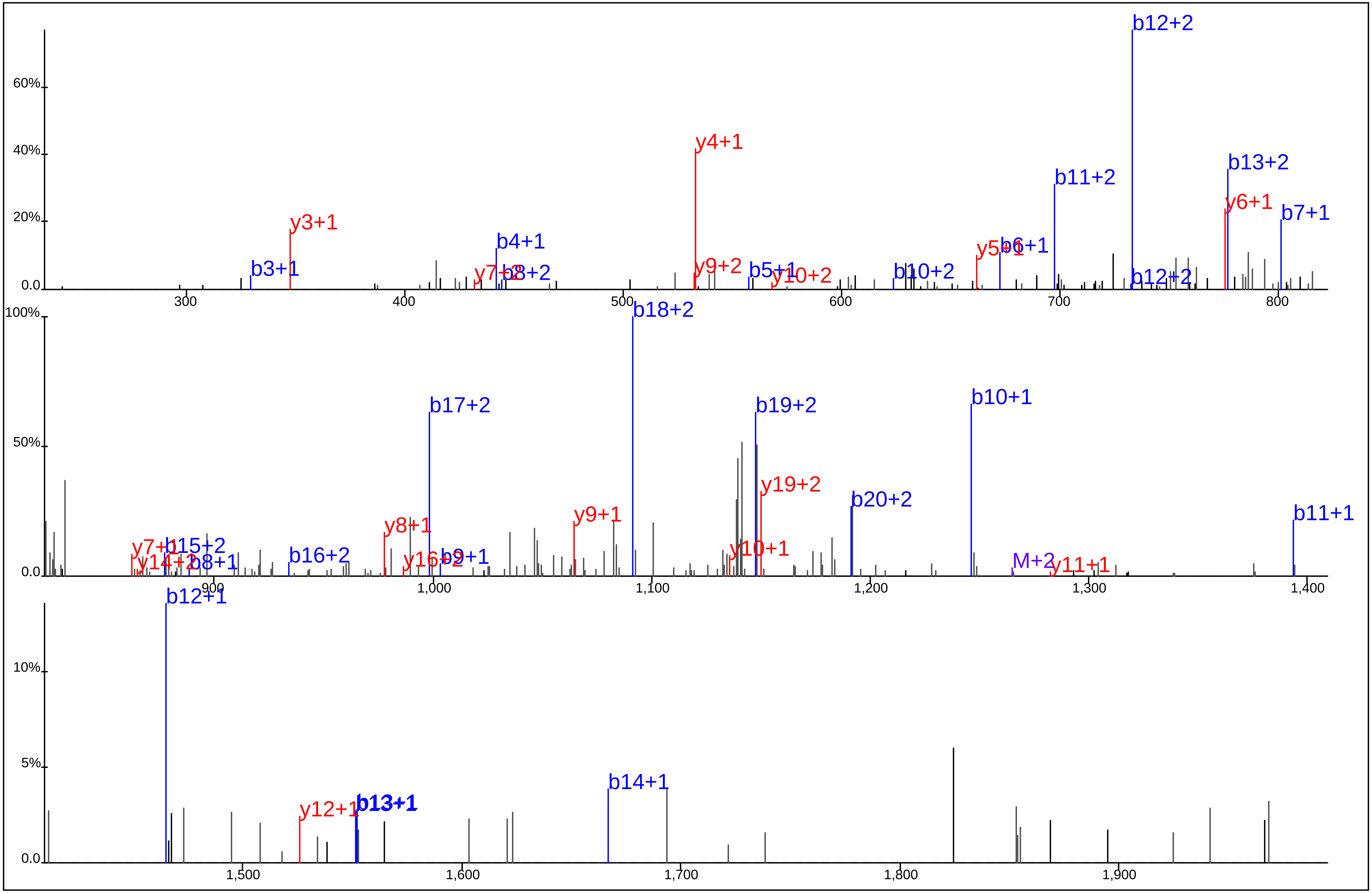

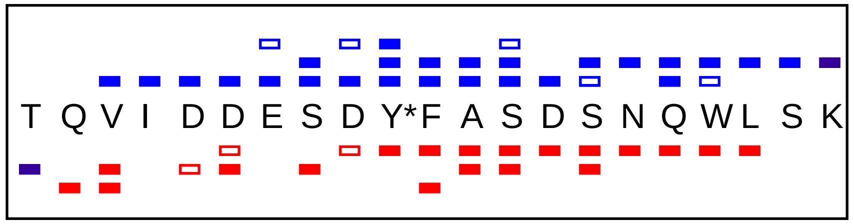

Protein Name:	TRIP4
gi Accession:	6013191
Precursor Mass:	843.3614+3
Delta Mass:	7.724 ppm
XCorr:	58.13

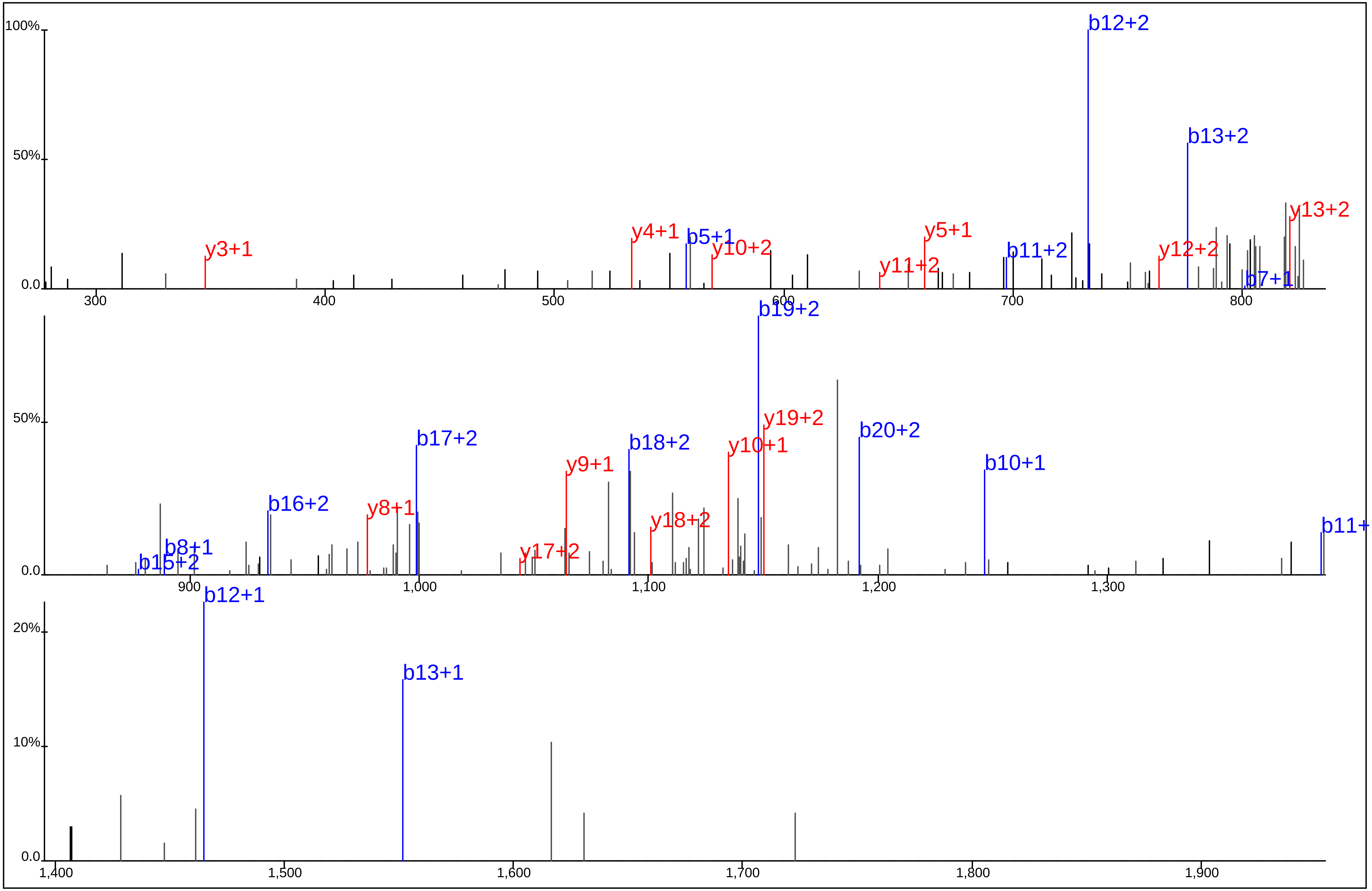

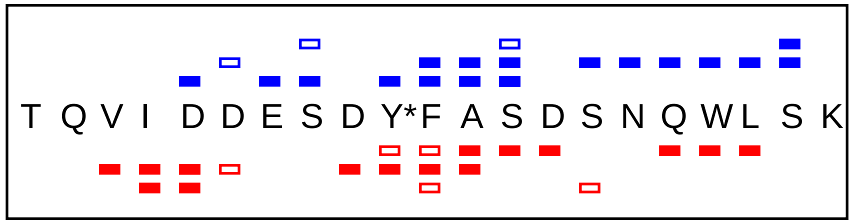

Protein Name: TRIP4
gi Accession: 6013191
Precursor Mass: 843.3543+3
Delta Mass: 0.678 ppm
XCorr: 37.22

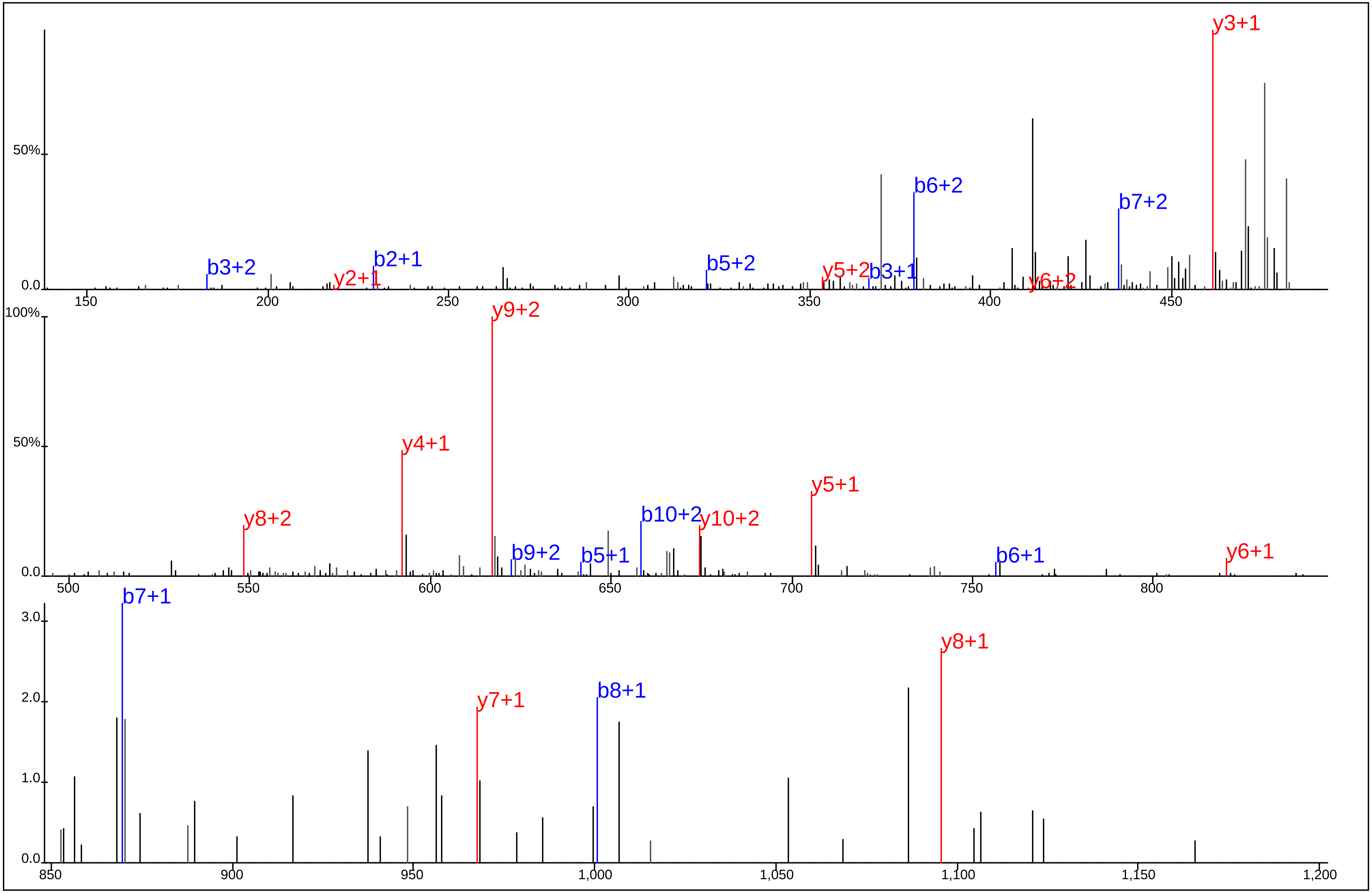

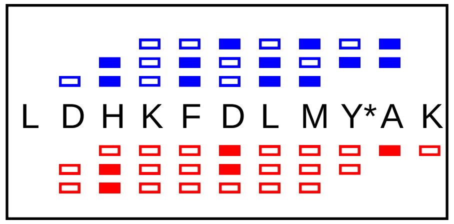

Protein Name: Tubulin alpha
gi Accession: 13376181
Precursor Mass: 487.5612+3
Delta Mass: 3.202 ppm
XCorr: 37.36

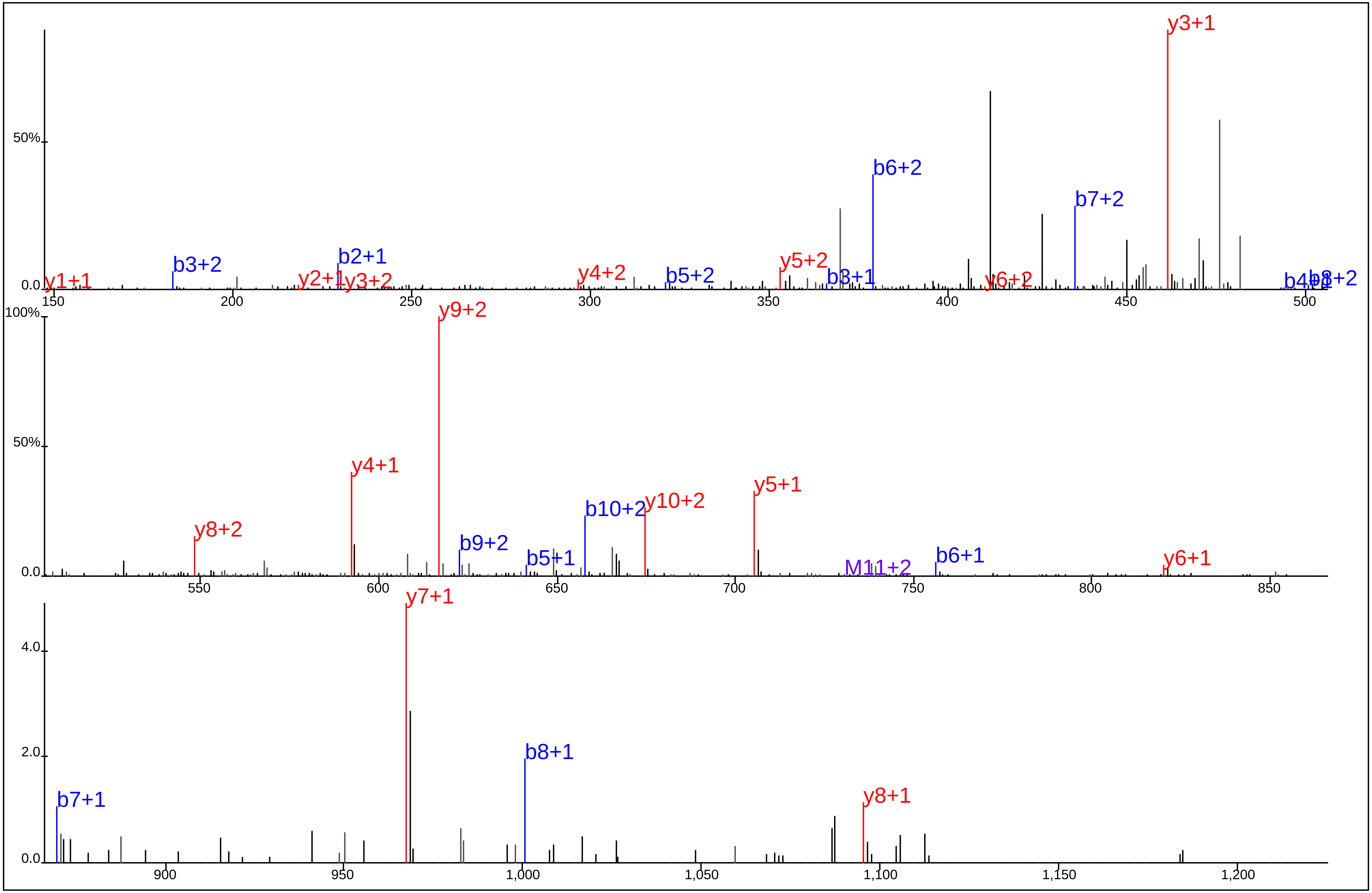

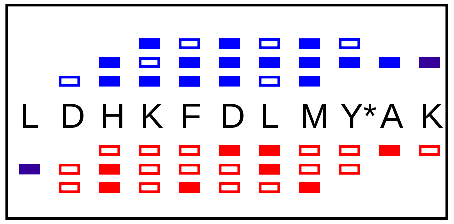

Protein Name: Tubulin alpha
gi Accession: 13376181
Precursor Mass: 487.5601+3
Delta Mass: 1.013 ppm
XCorr: 42.21

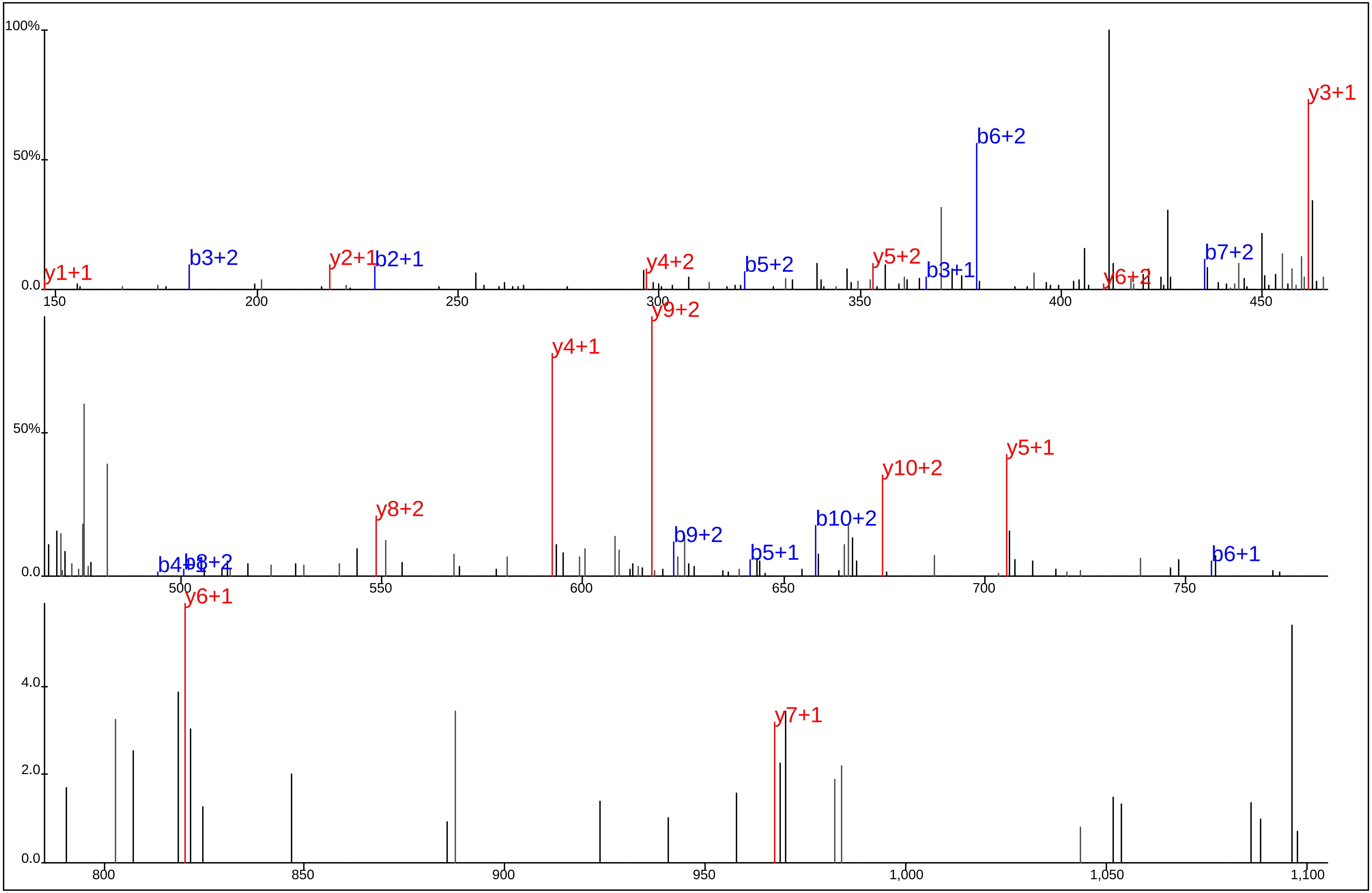

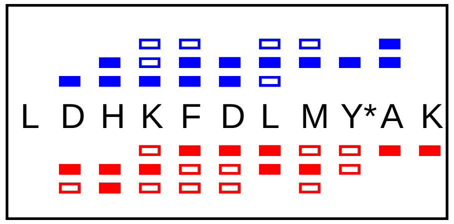

Protein Name: Tubulin alpha
gi Accession: 13376181
Precursor Mass: 487.5606+3
Delta Mass: 2.011 ppm
XCorr: 39.2

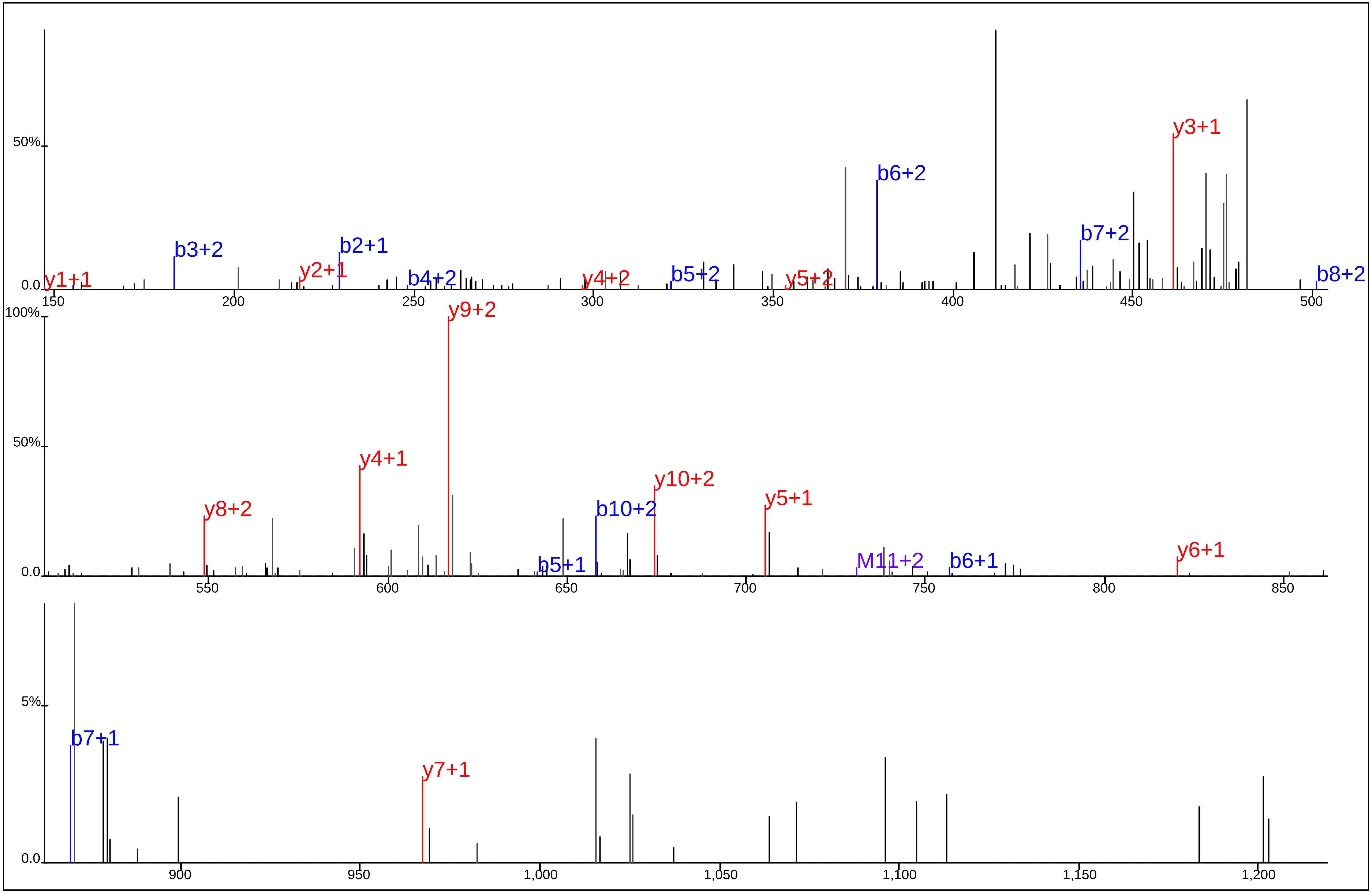

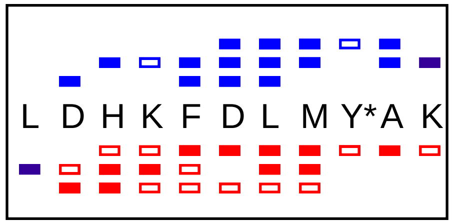

Protein Name: Tubulin alpha
gi Accession: 13376181
Precursor Mass: 487.5605+3
Delta Mass: 1.887 ppm
XCorr: 21.2

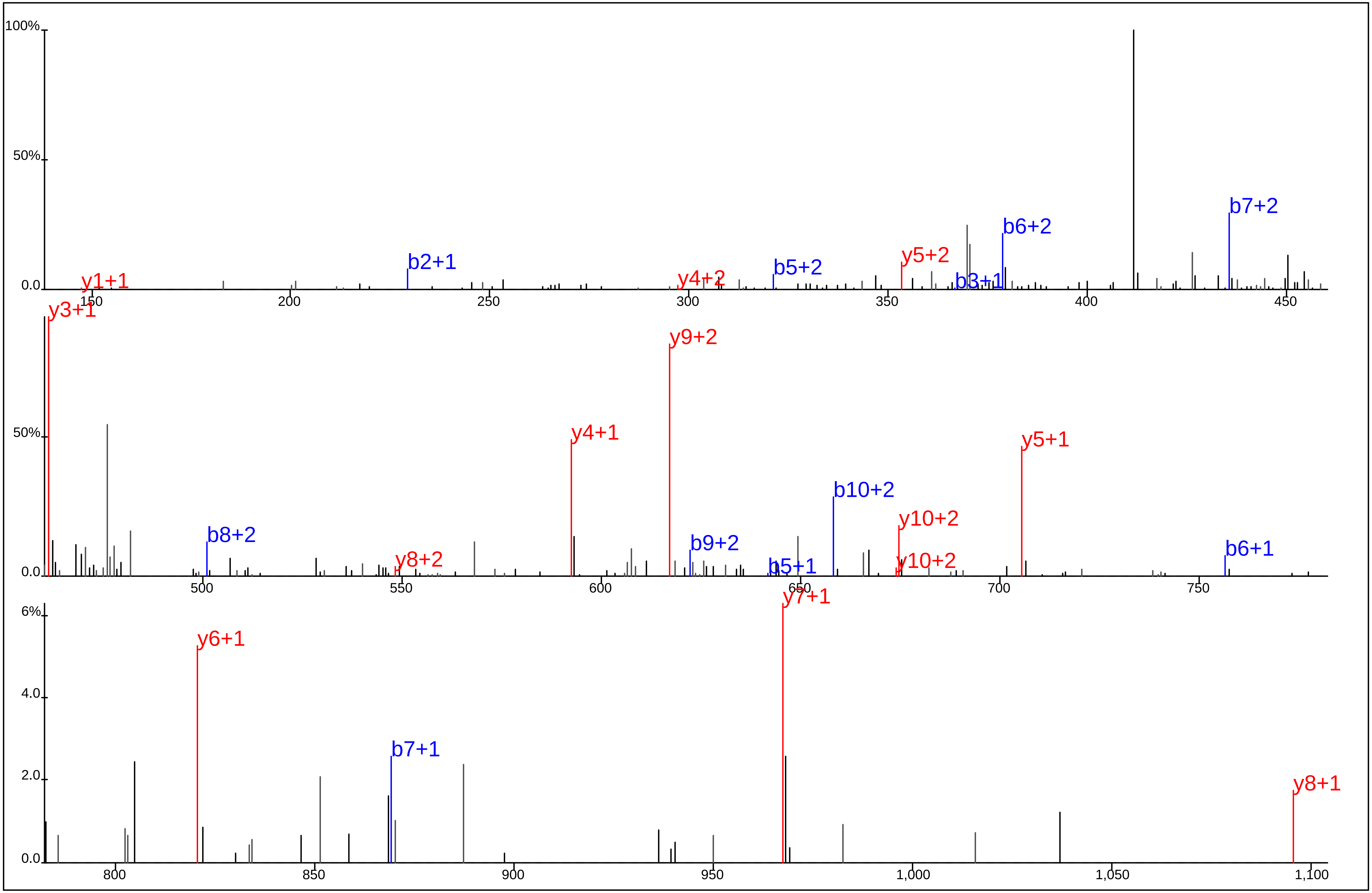

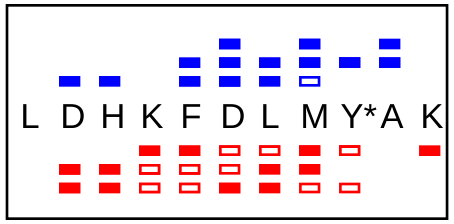

Protein Name: Tubulin alpha
gi Accession: 13376181
Precursor Mass: 487.5605+3
Delta Mass: 1.887 ppm
XCorr: 39.41

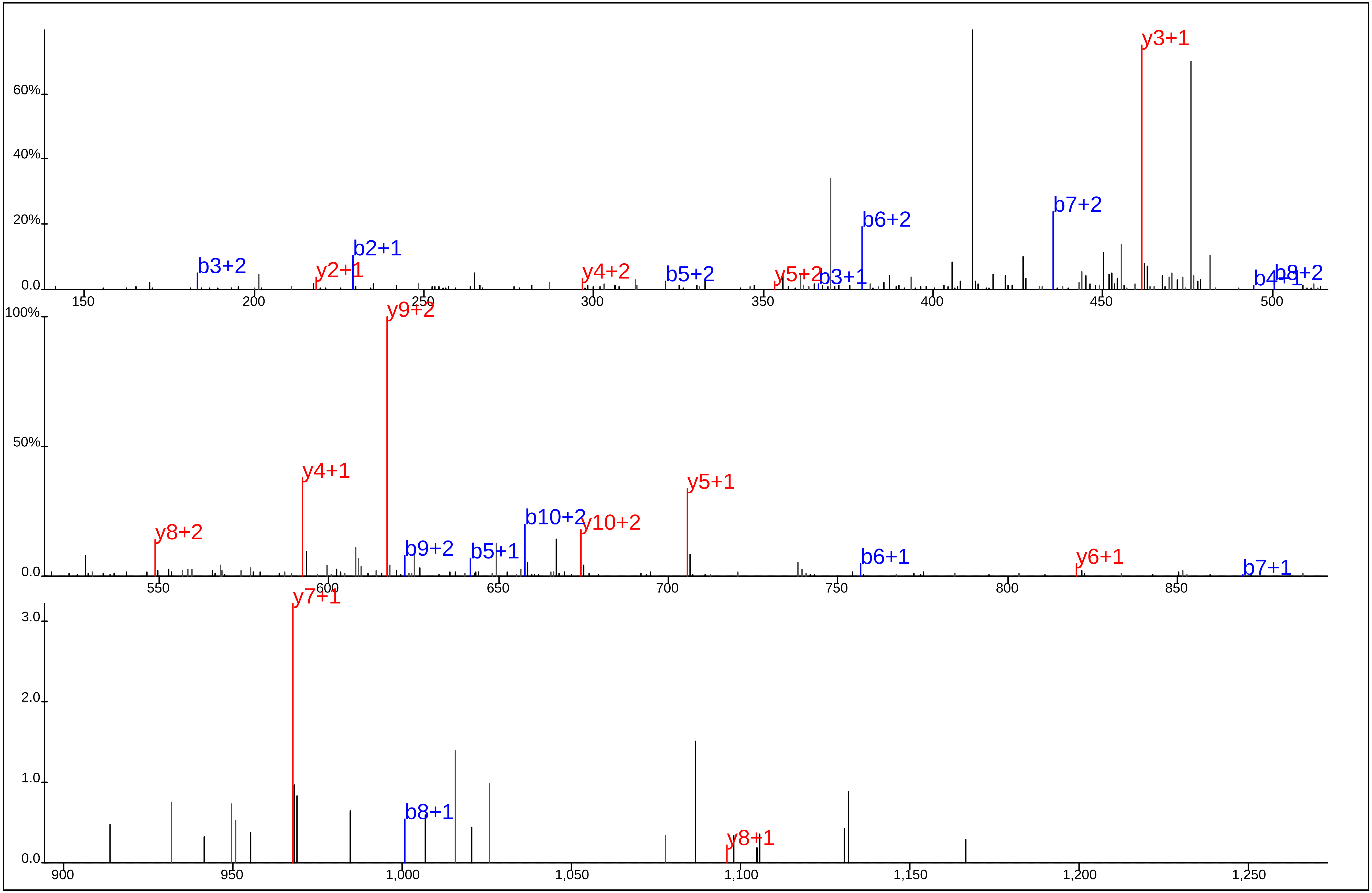

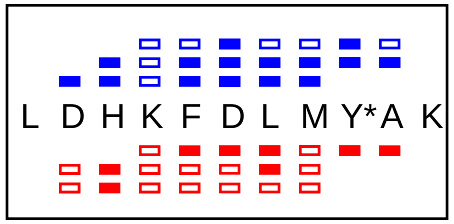

Protein Name: Tubulin alpha
gi Accession: 13376181
Precursor Mass: 487.5602+3
Delta Mass: 1.074 ppm
XCorr: 34.93

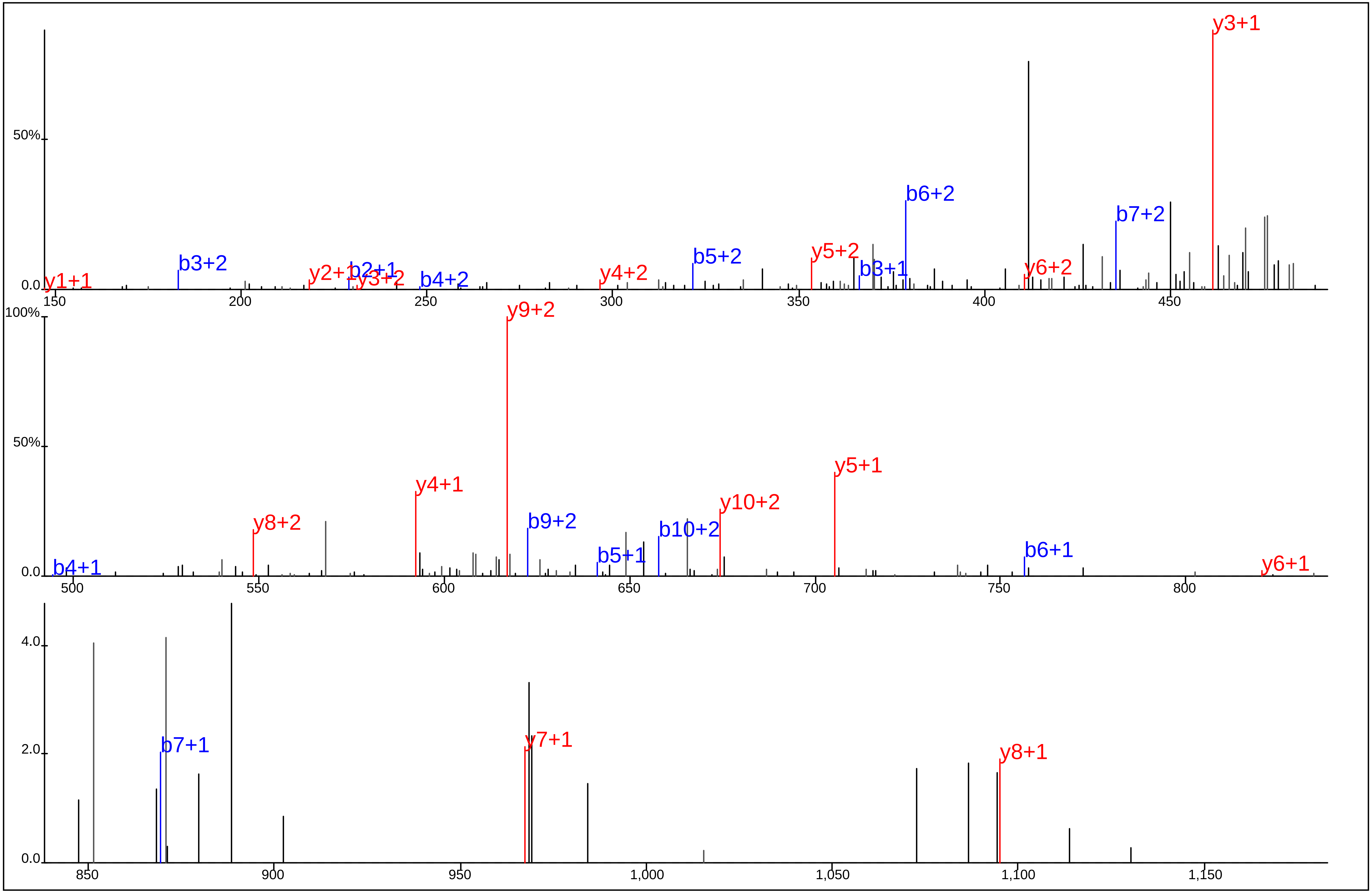

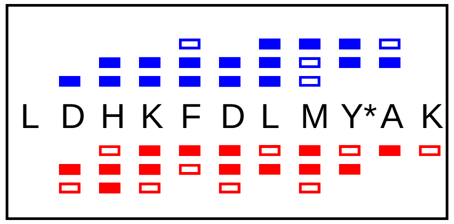

Protein Name: Tubulin alpha
gi Accession: 13376181
Precursor Mass: 487.5611+3
Delta Mass: 2.949 ppm
XCorr: 38.85

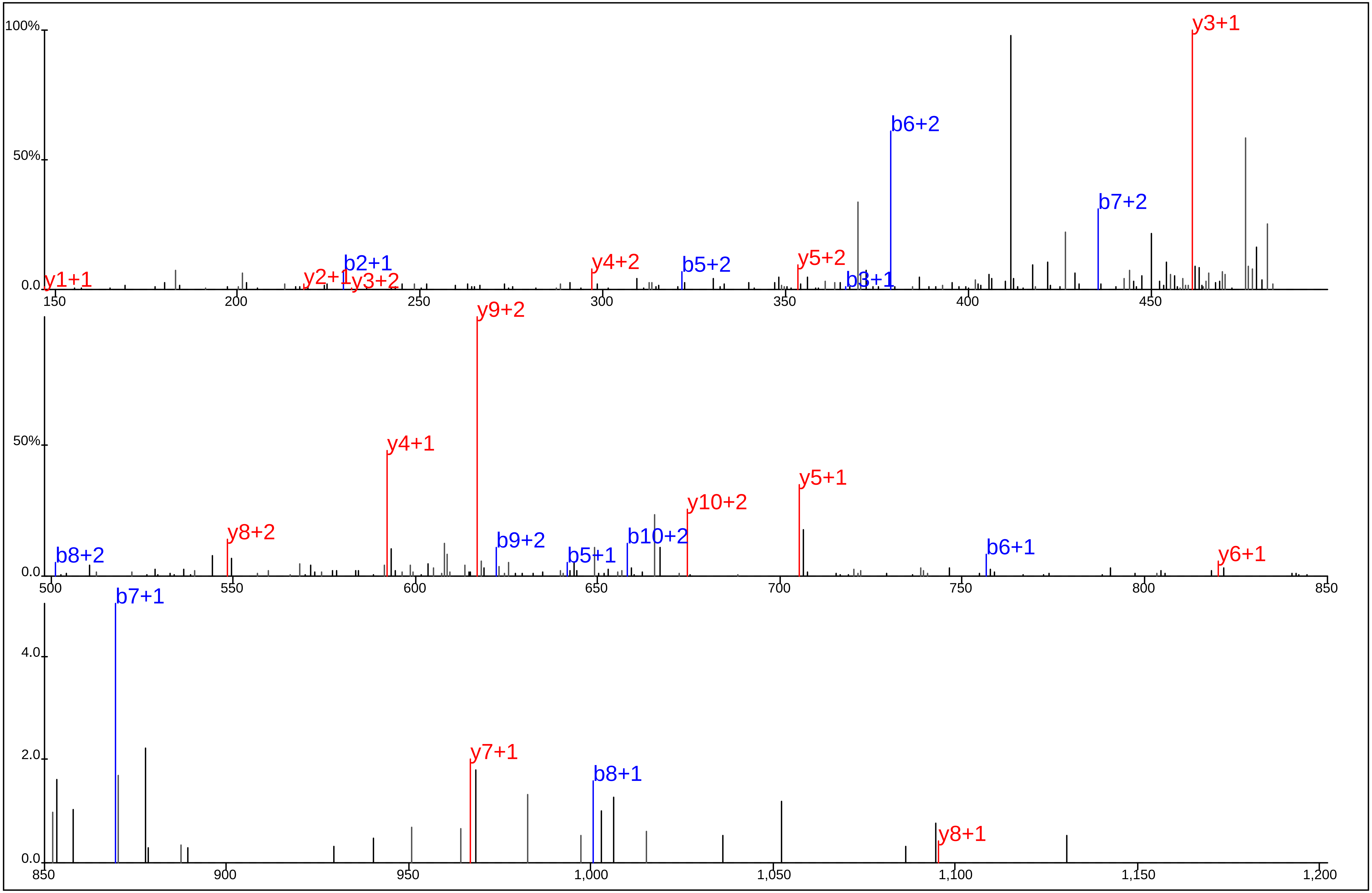

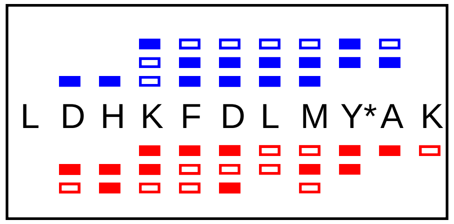

Protein Name: Tubulin alpha
gi Accession: 13376181
Precursor Mass: 487.5633+3
Delta Mass: 7.529 ppm
XCorr: 32.33

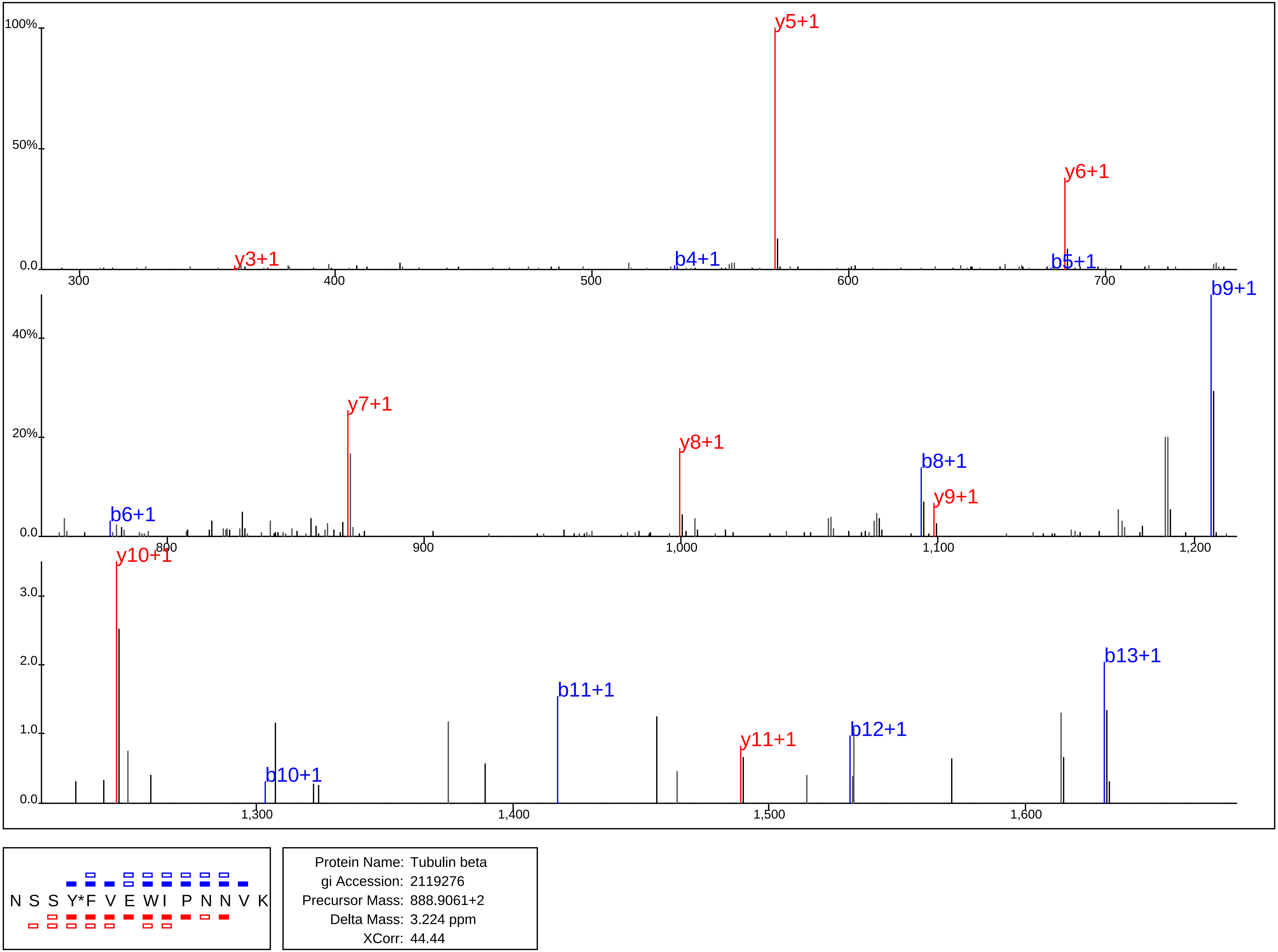

Protein Name: Tubulin beta
gi Accession: 2119276
Precursor Mass: 888.9061+2
Delta Mass: 3.224 ppm
XCorr: 44.44

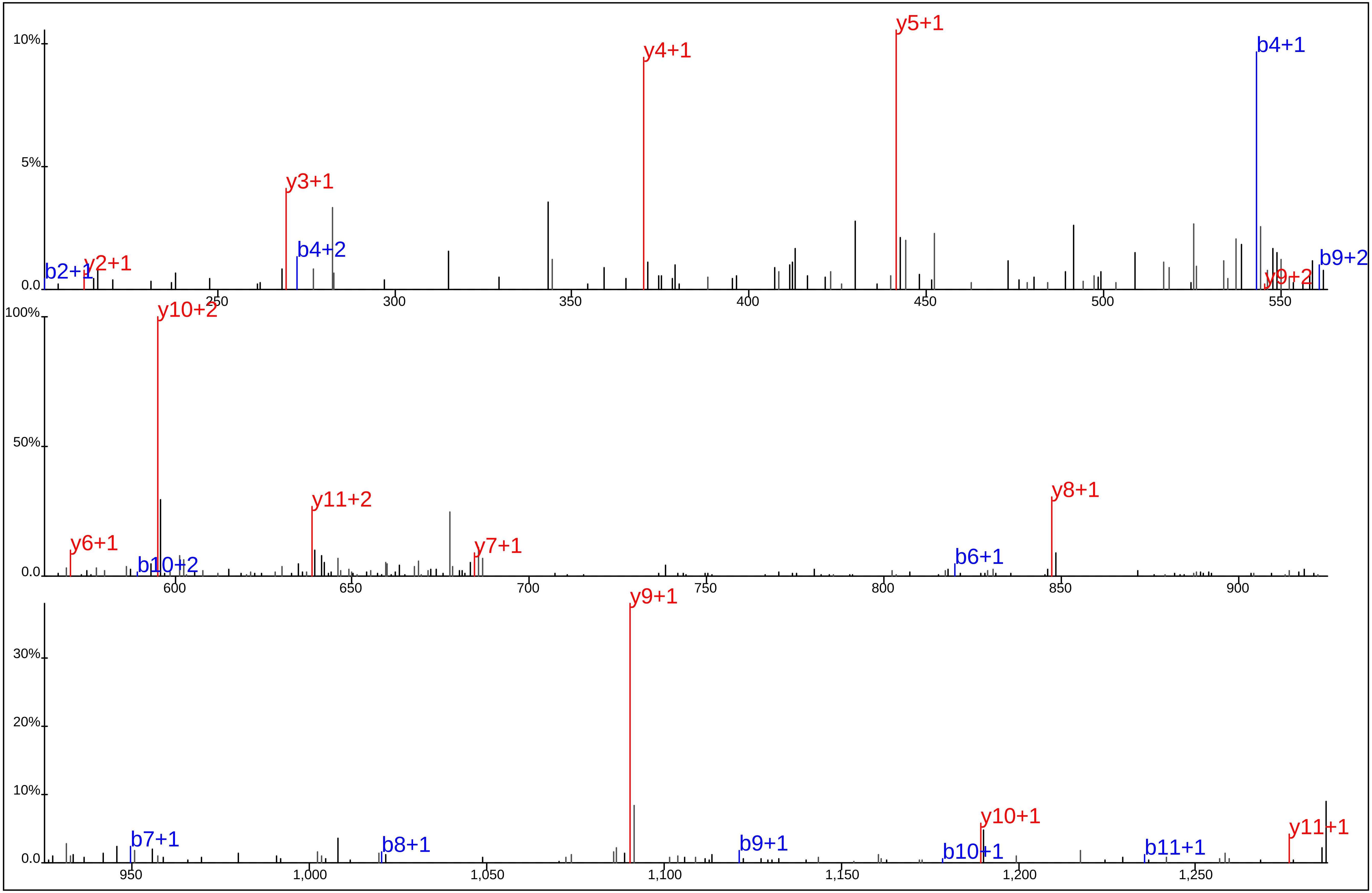

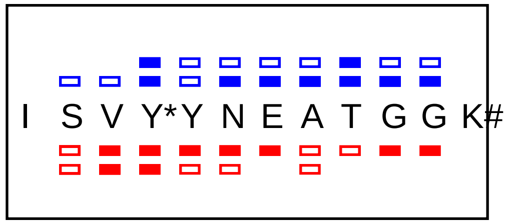

Protein Name:	Tubulin beta
gi Accession:	2119276
Precursor Mass:	695.3134+2
Delta Mass:	1.229 ppm
XCorr:	47.58

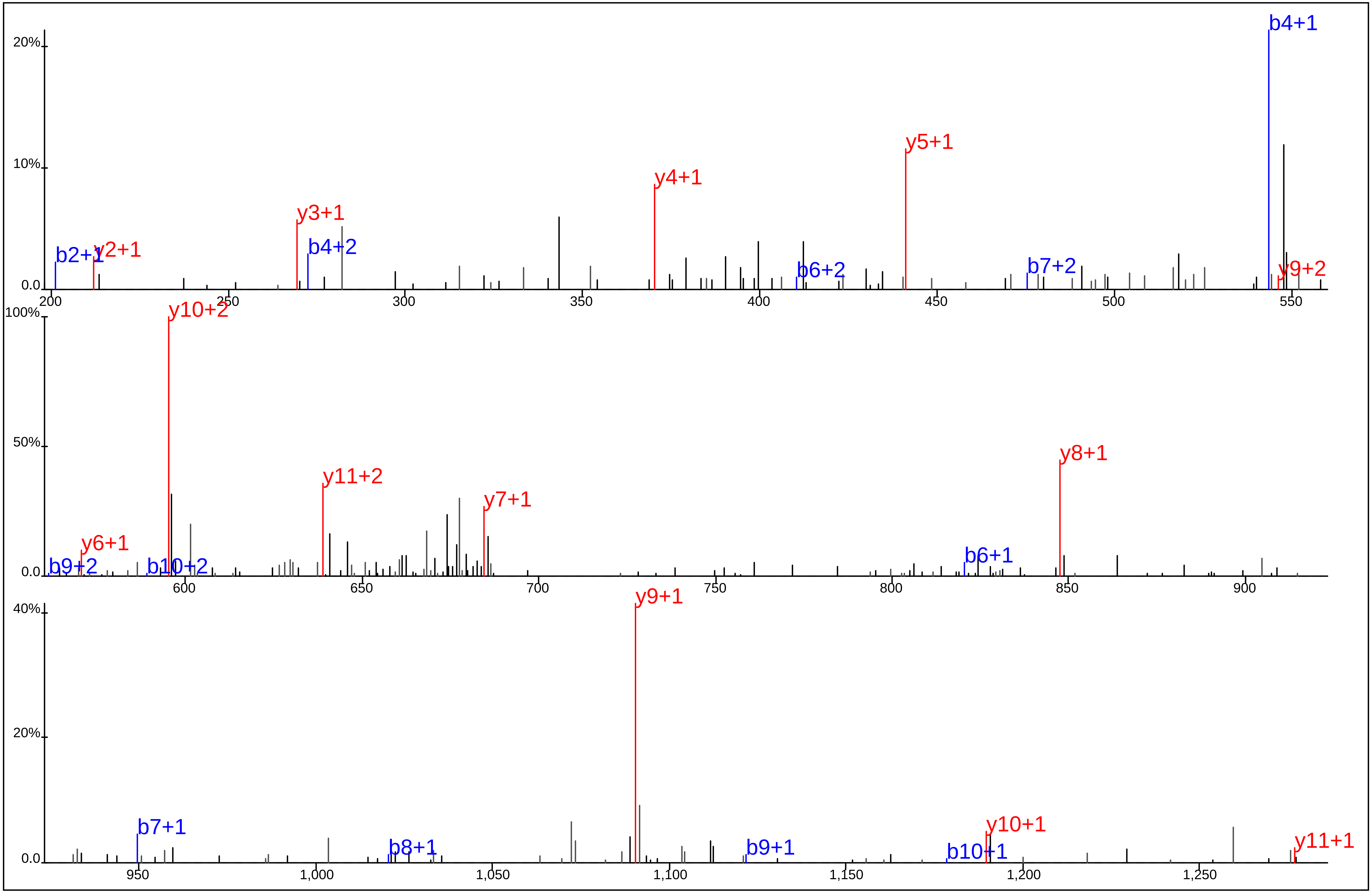

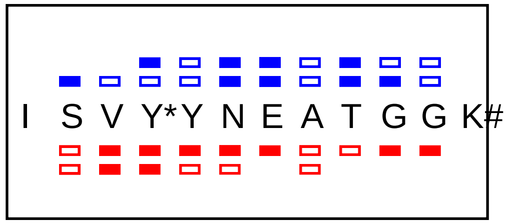

Protein Name: Tubulin beta
gi Accession: 2119276
Precursor Mass: 695.3169+2
Delta Mass: 6.324 ppm
XCorr: 51.11

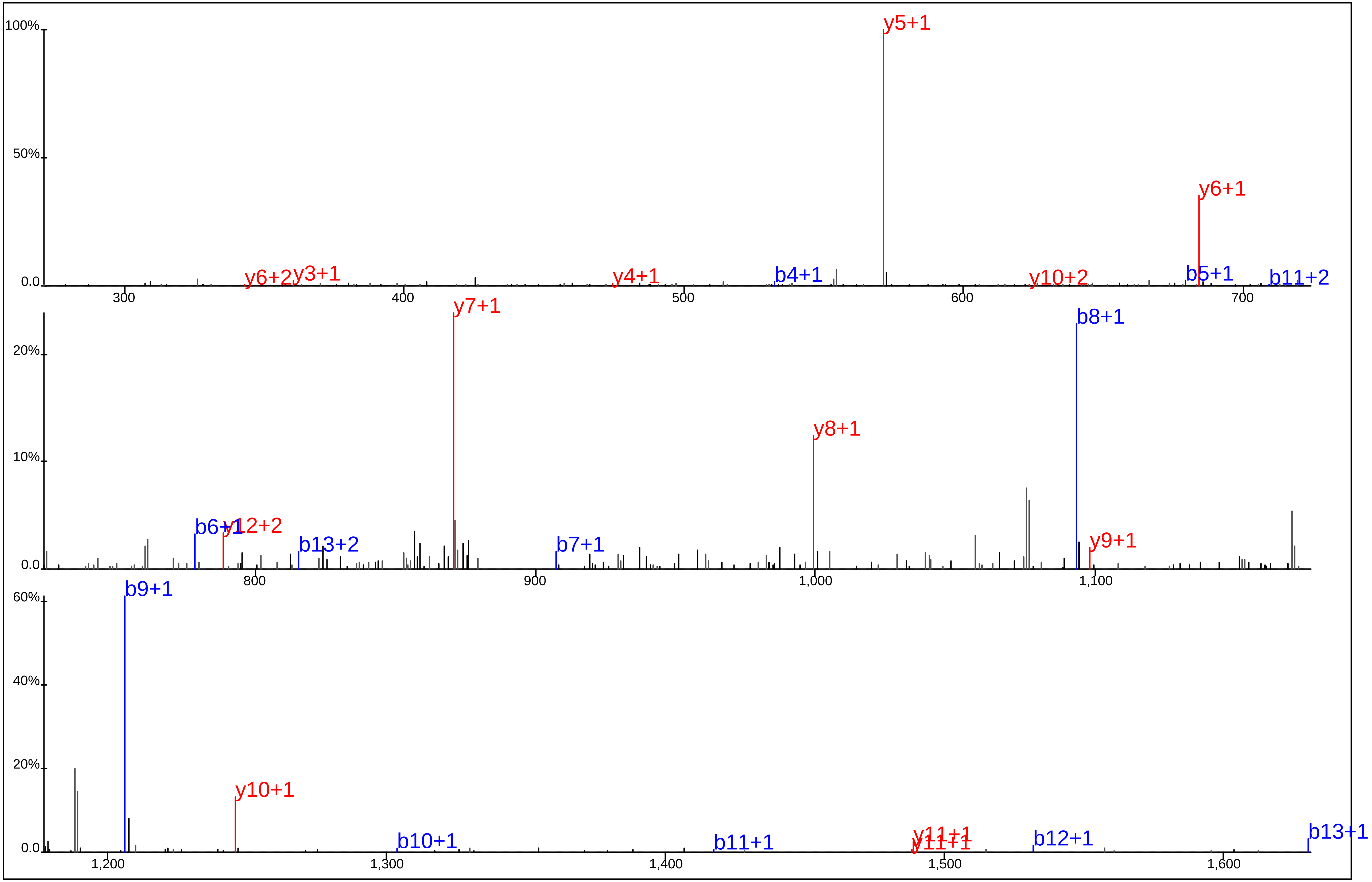

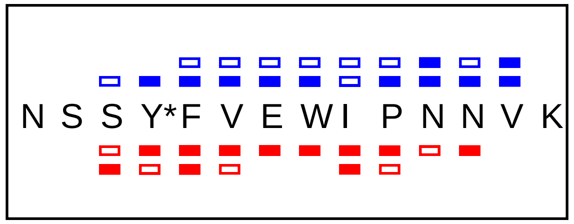

Protein Name: Tubulin beta
gi Accession: 2119276
Precursor Mass: 888.9070+2
Delta Mass: 4.254 ppm
XCorr: 45.71

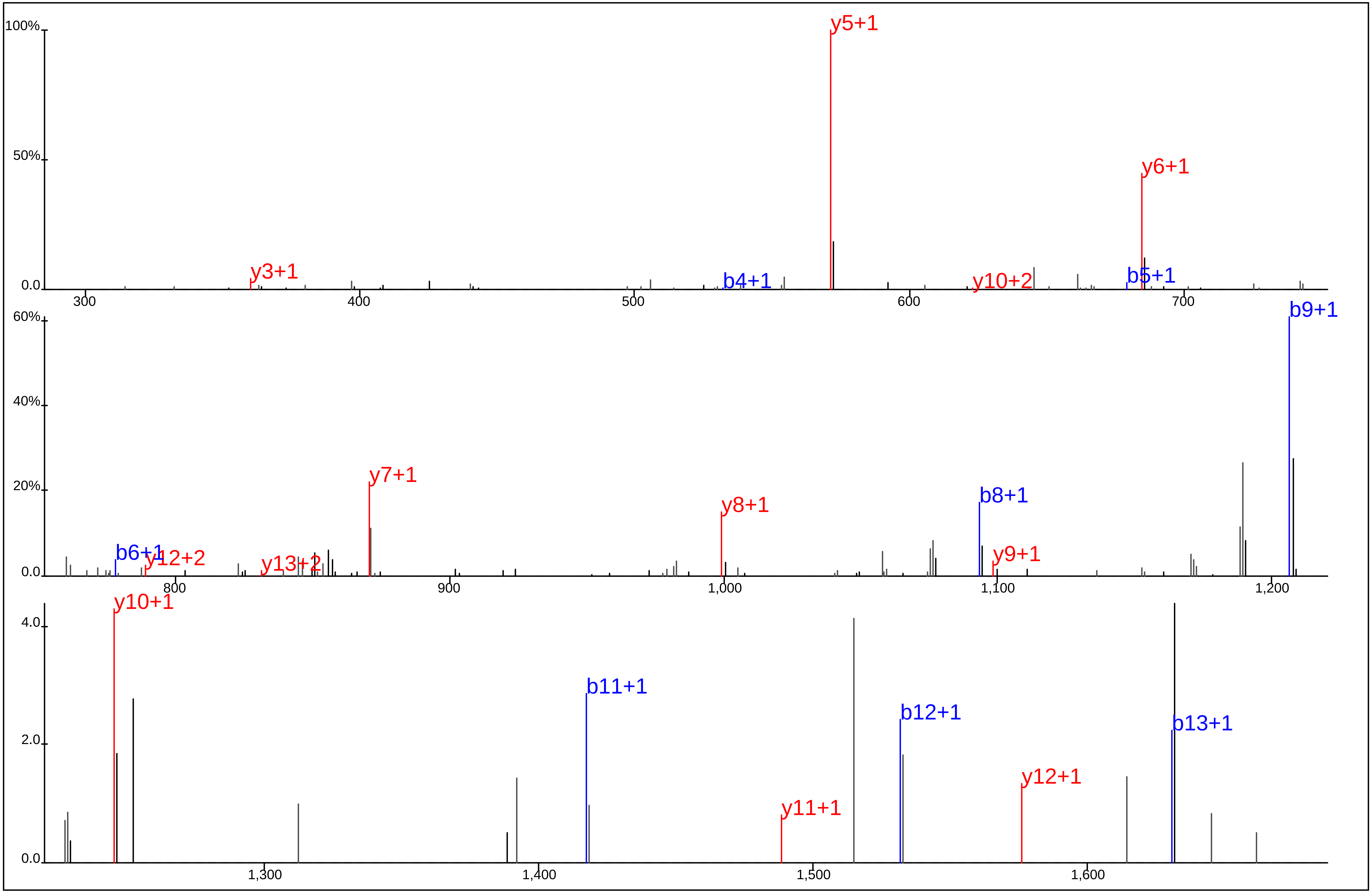

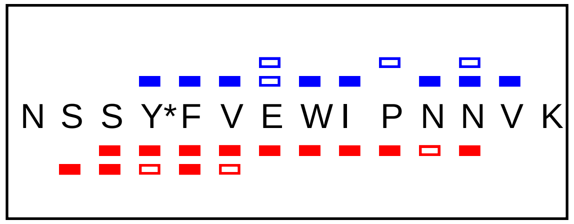

Protein Name: Tubulin beta
gi Accession: 2119276
Precursor Mass: 888.9053+2
Delta Mass: 2.261 ppm
XCorr: 37.16

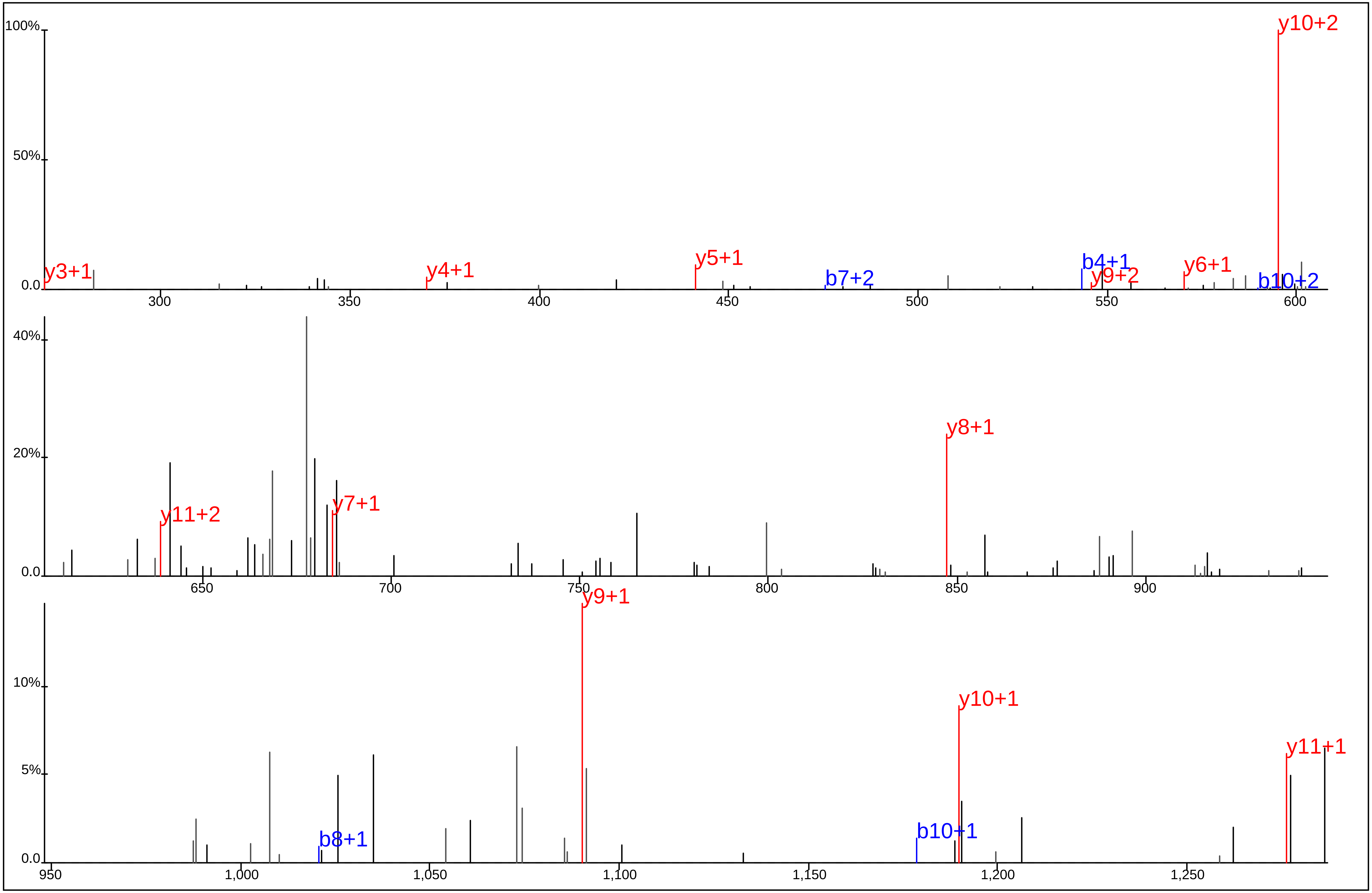

I S V Y*Y N E A T G G K#

Protein Name:	Tubulin beta
gi Accession:	2119276
Precursor Mass:	695.3144+2
Delta Mass:	2.726 ppm
XCorr:	33.26

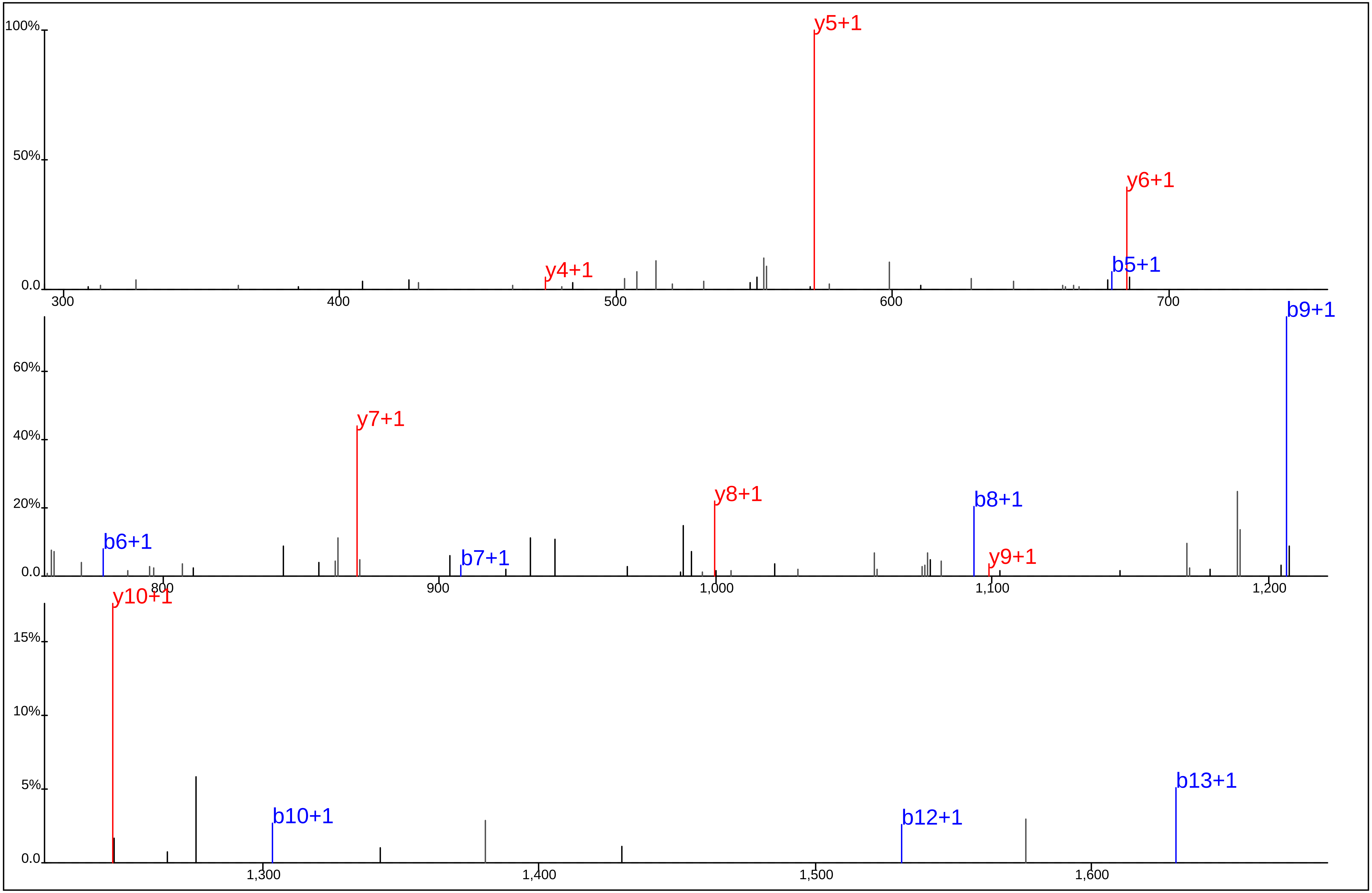

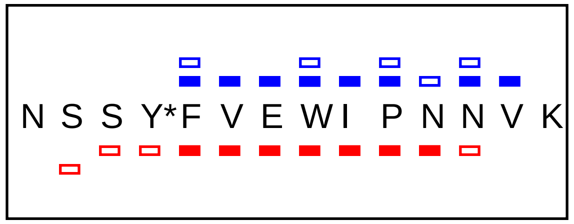

Protein Name:	Tubulin beta
gi Accession:	2119276
Precursor Mass:	888.9062+2
Delta Mass:	3.359 ppm
XCorr:	37.62

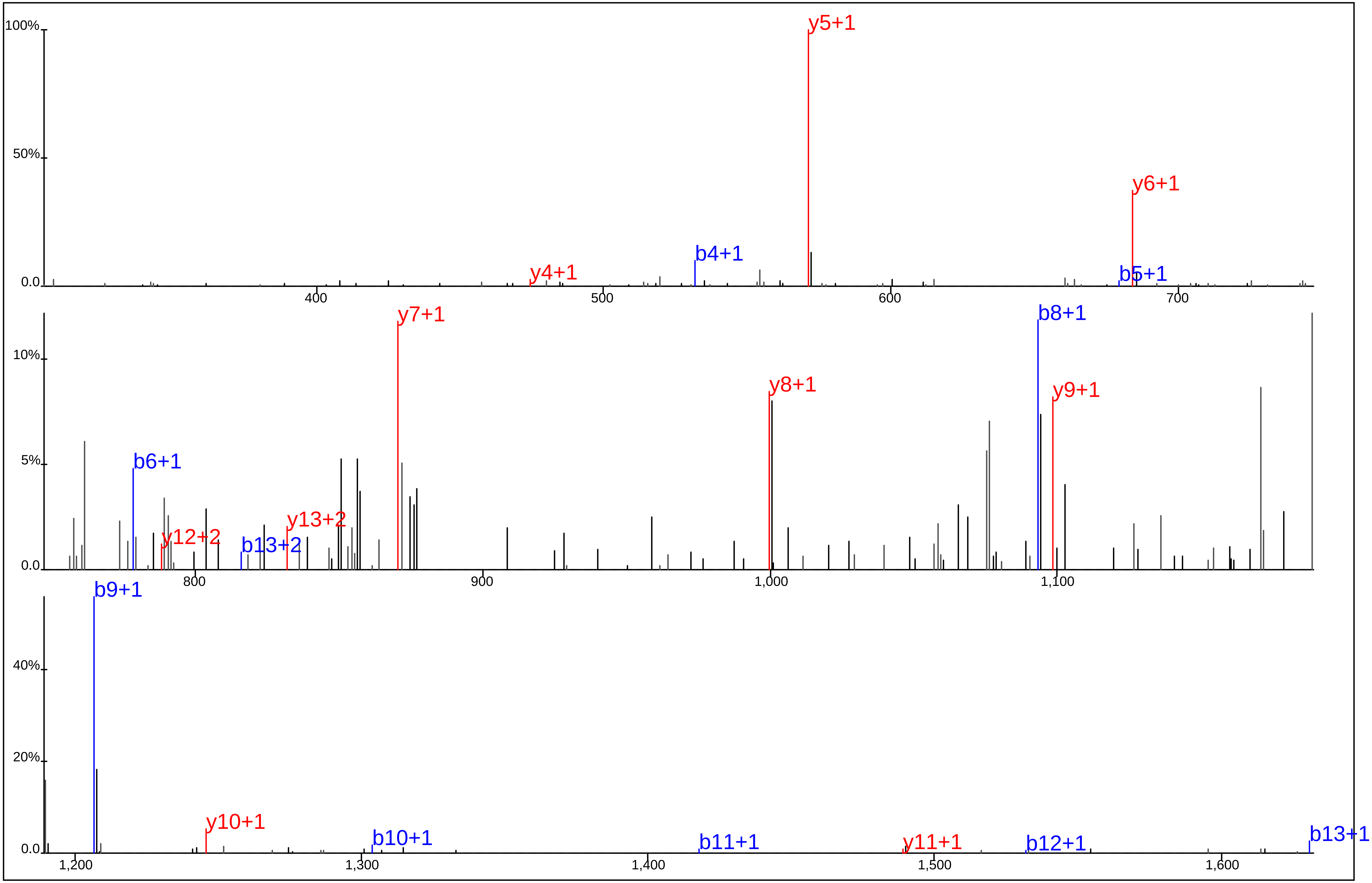

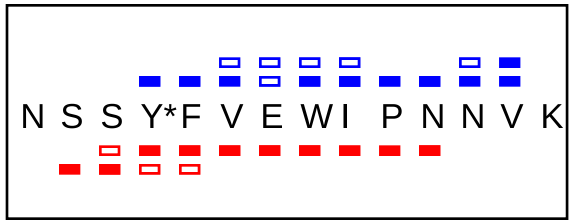

Protein Name:	Tubulin beta
gi Accession:	2119276
Precursor Mass:	888.9024+2
Delta Mass:	0.969 ppm
XCorr:	41.27

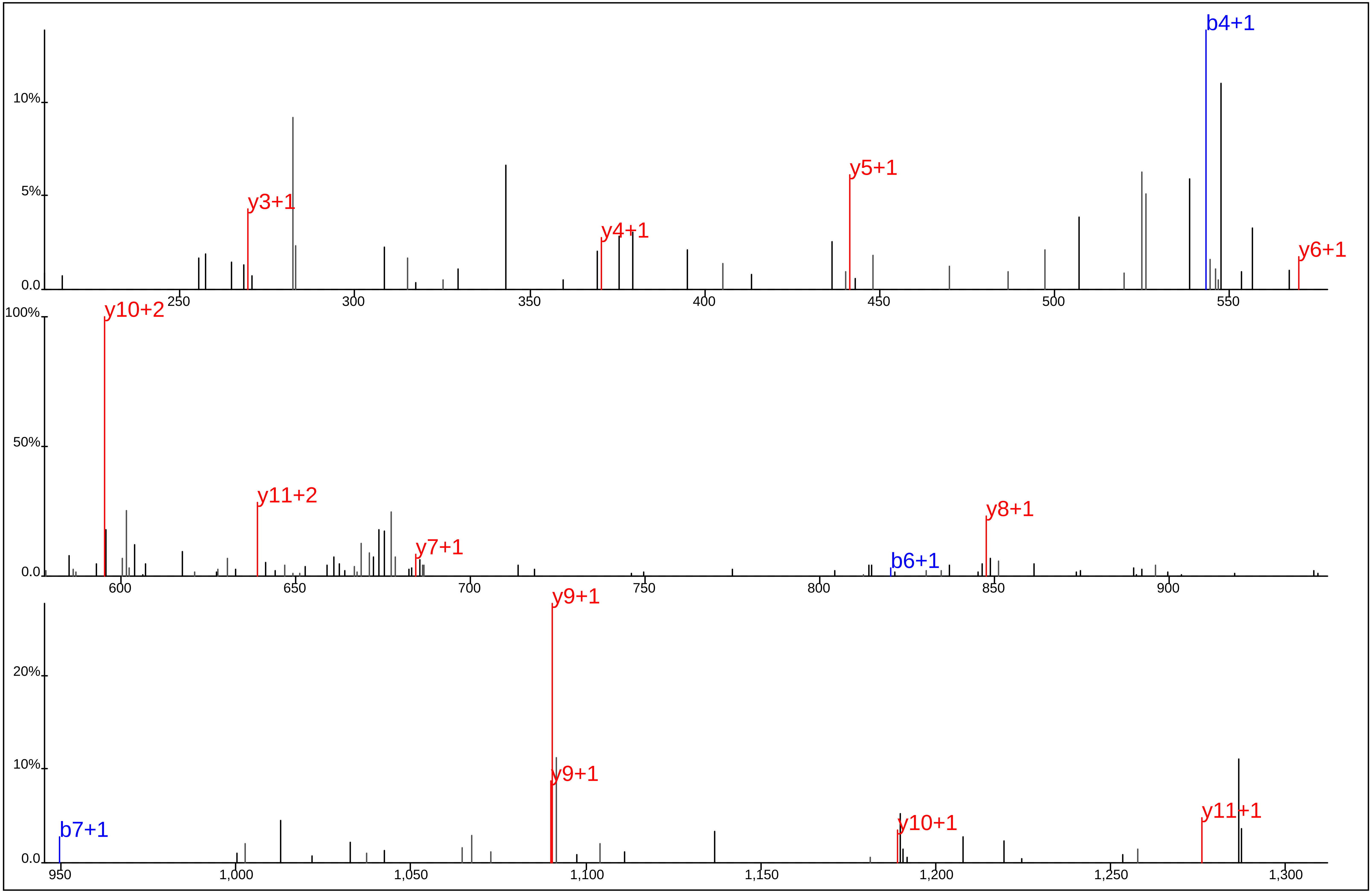

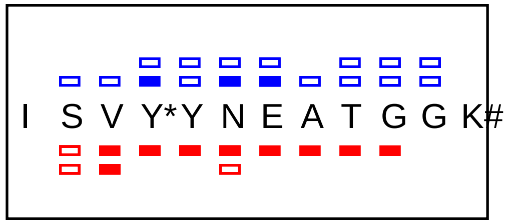

Protein Name: Tubulin beta
gi Accession: 2119276
Precursor Mass: 695.3140+2
Delta Mass: 2.107 ppm
XCorr: 24.05

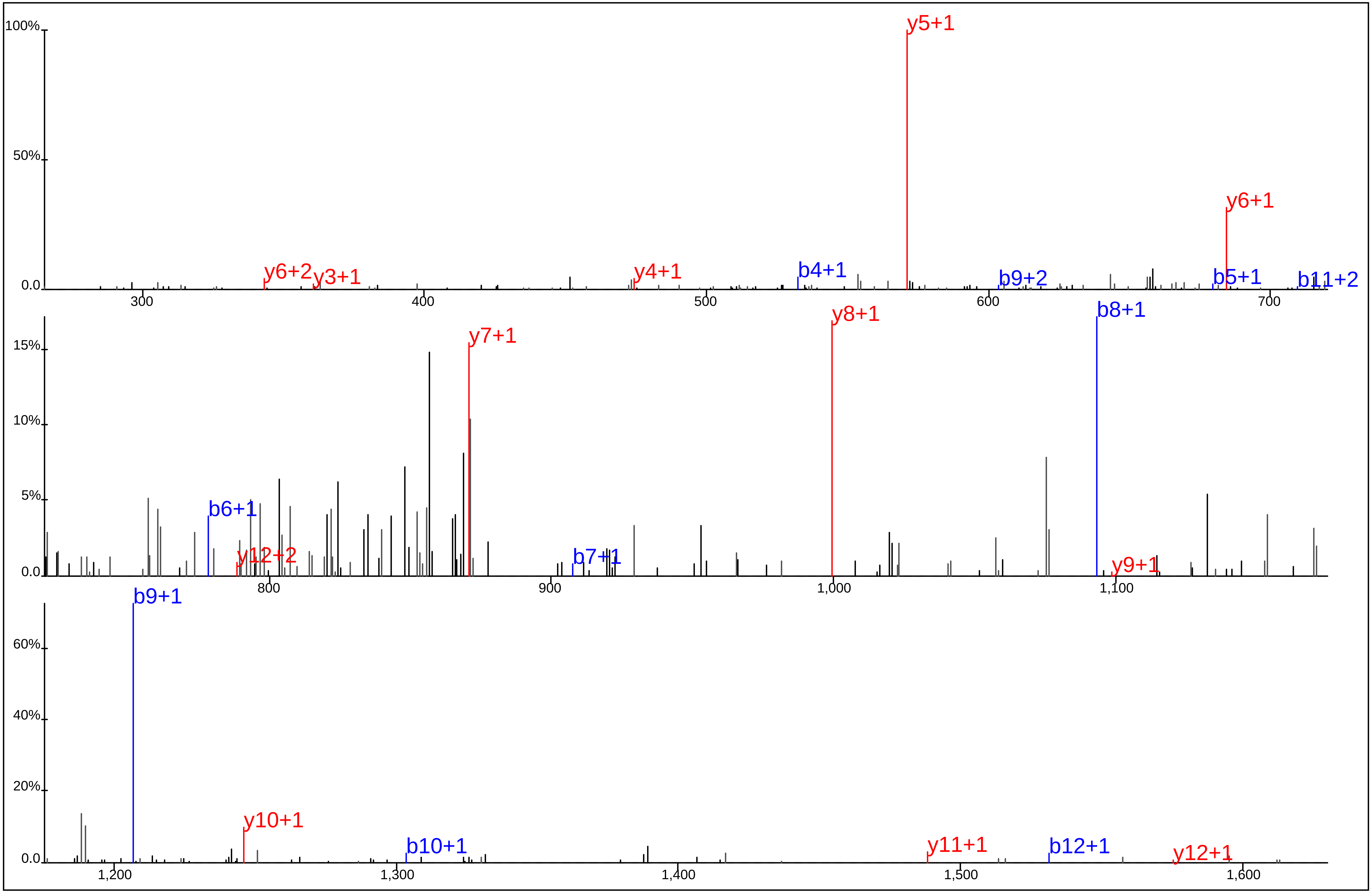

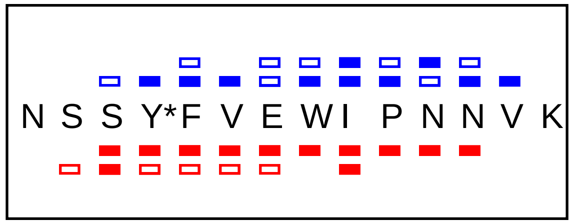

Protein Name:	Tubulin beta
gi Accession:	2119276
Precursor Mass:	888.9148+2
Delta Mass:	12.977 ppm
XCorr:	39.17

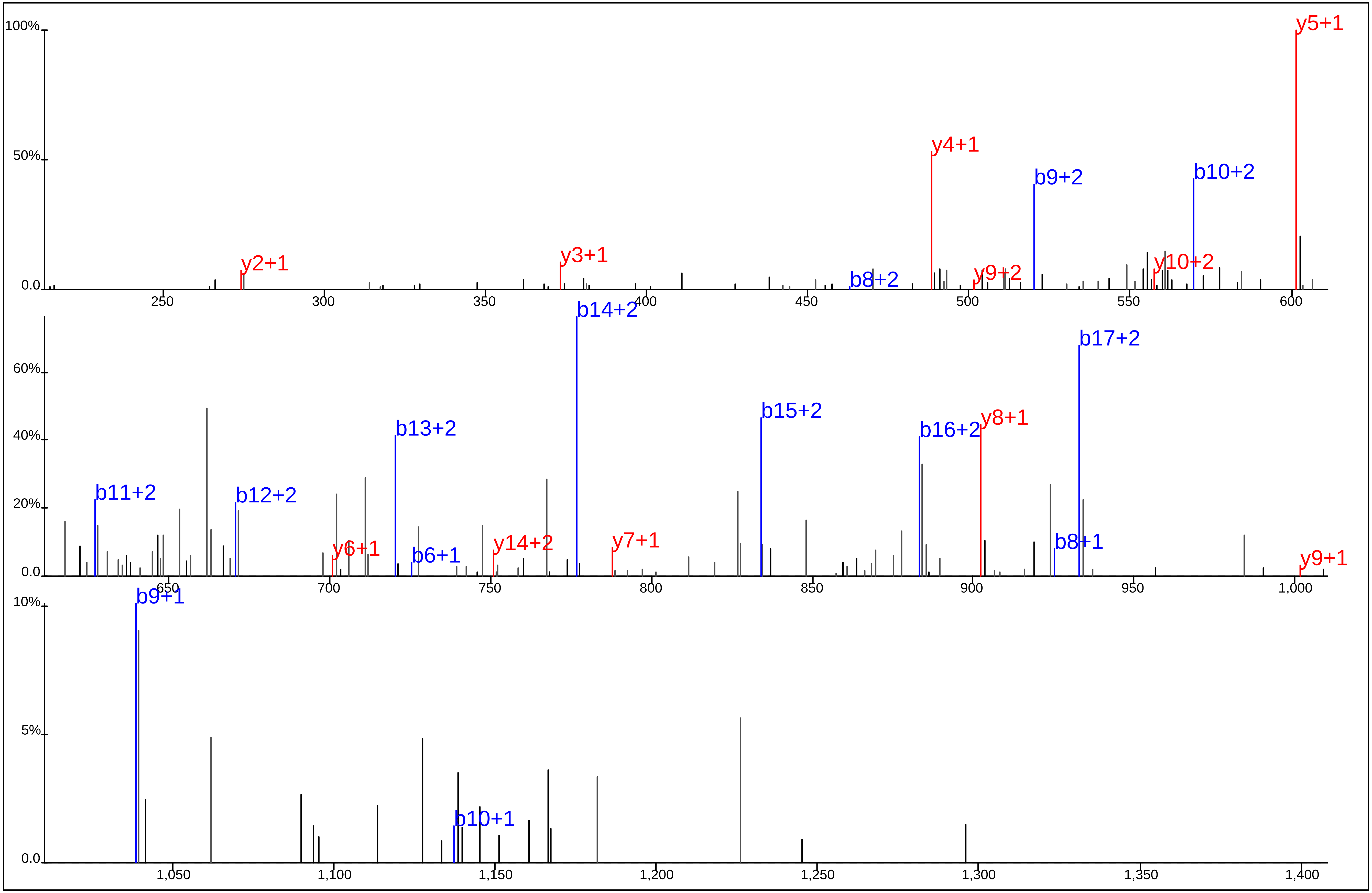

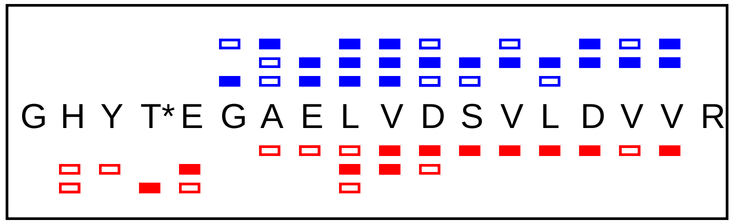

Protein Name:	Tubulin beta
gi Accession:	2119276
Precursor Mass:	680.3248+3
Delta Mass:	5.698 ppm
XCorr:	30.65

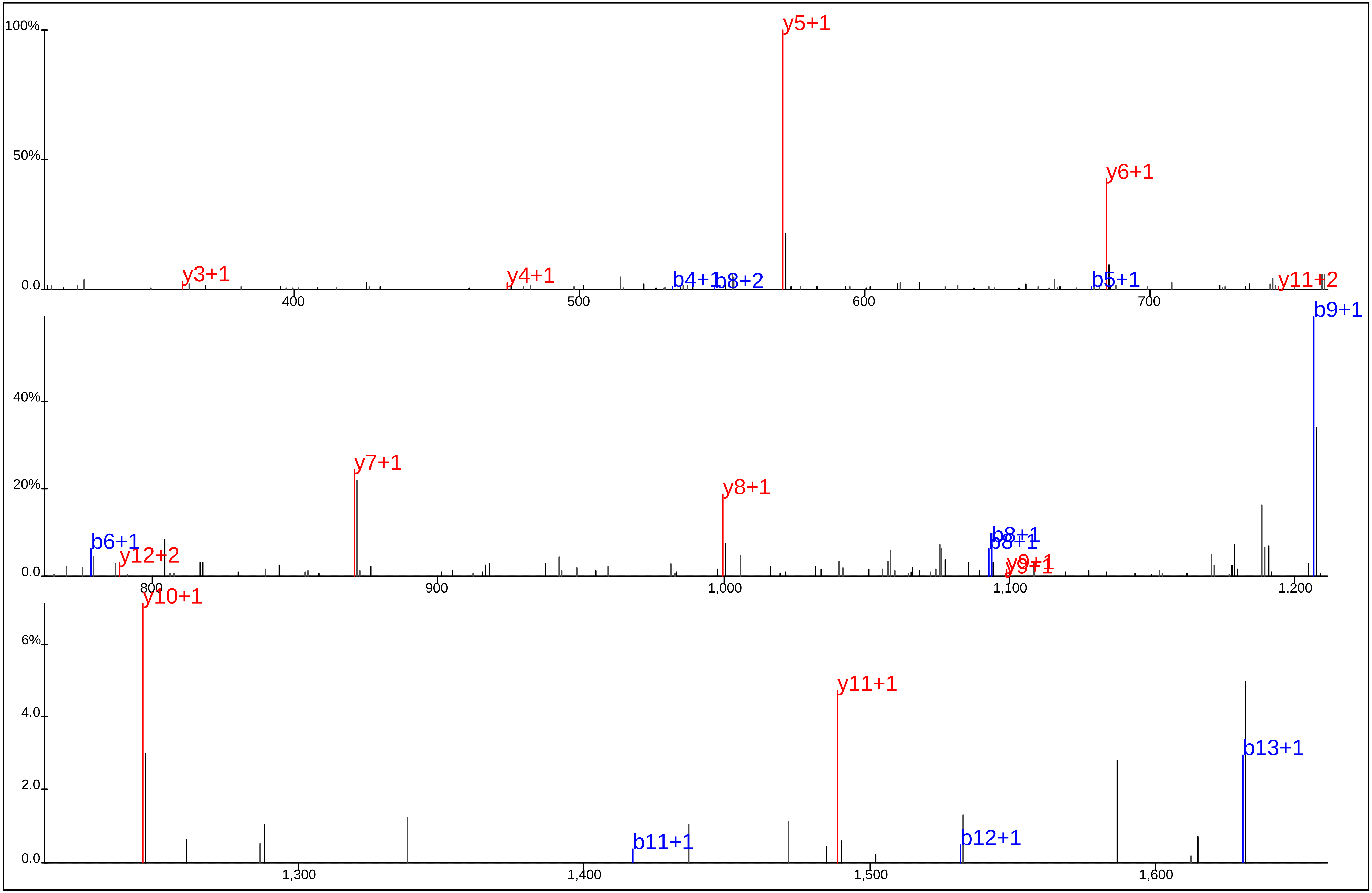

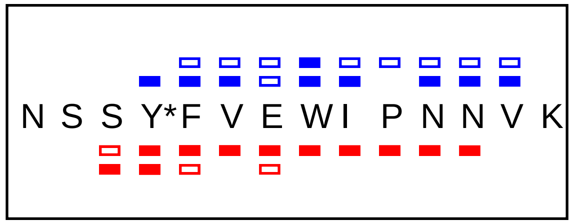

Protein Name: Tubulin beta
gi Accession: 2119276
Precursor Mass: 888.9005+2
Delta Mass: 3.097 ppm
XCorr: 47.6

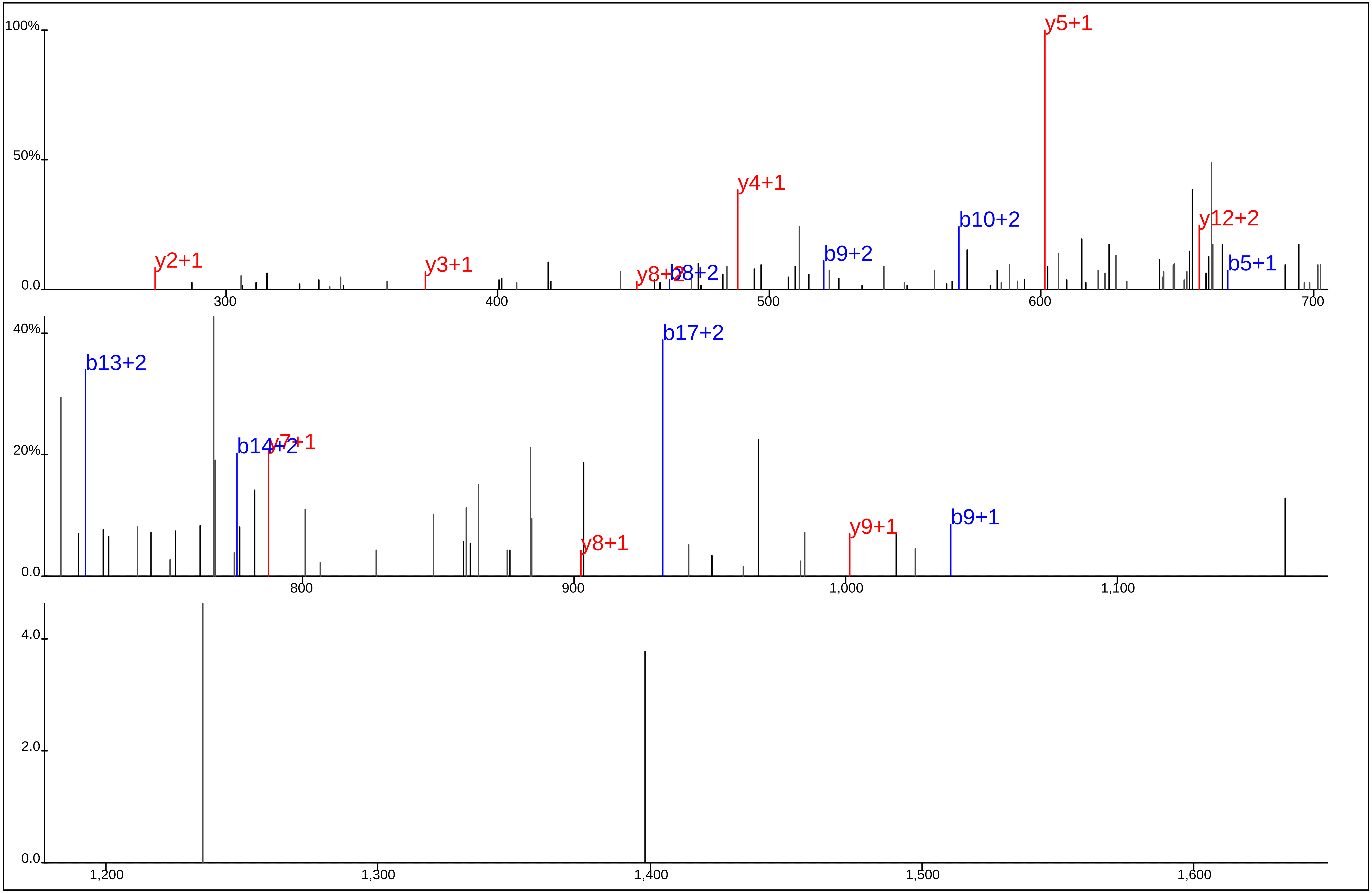

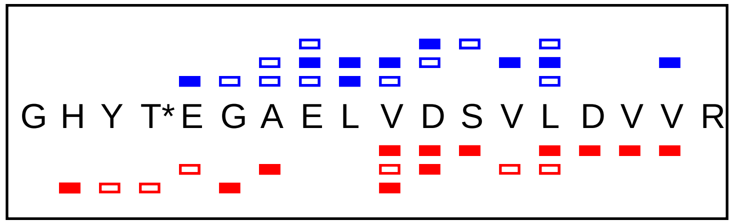

Protein Name: Tubulin beta
gi Accession: 2119276
Precursor Mass: 680.3228+3
Delta Mass: 2.824 ppm
XCorr: 26.68

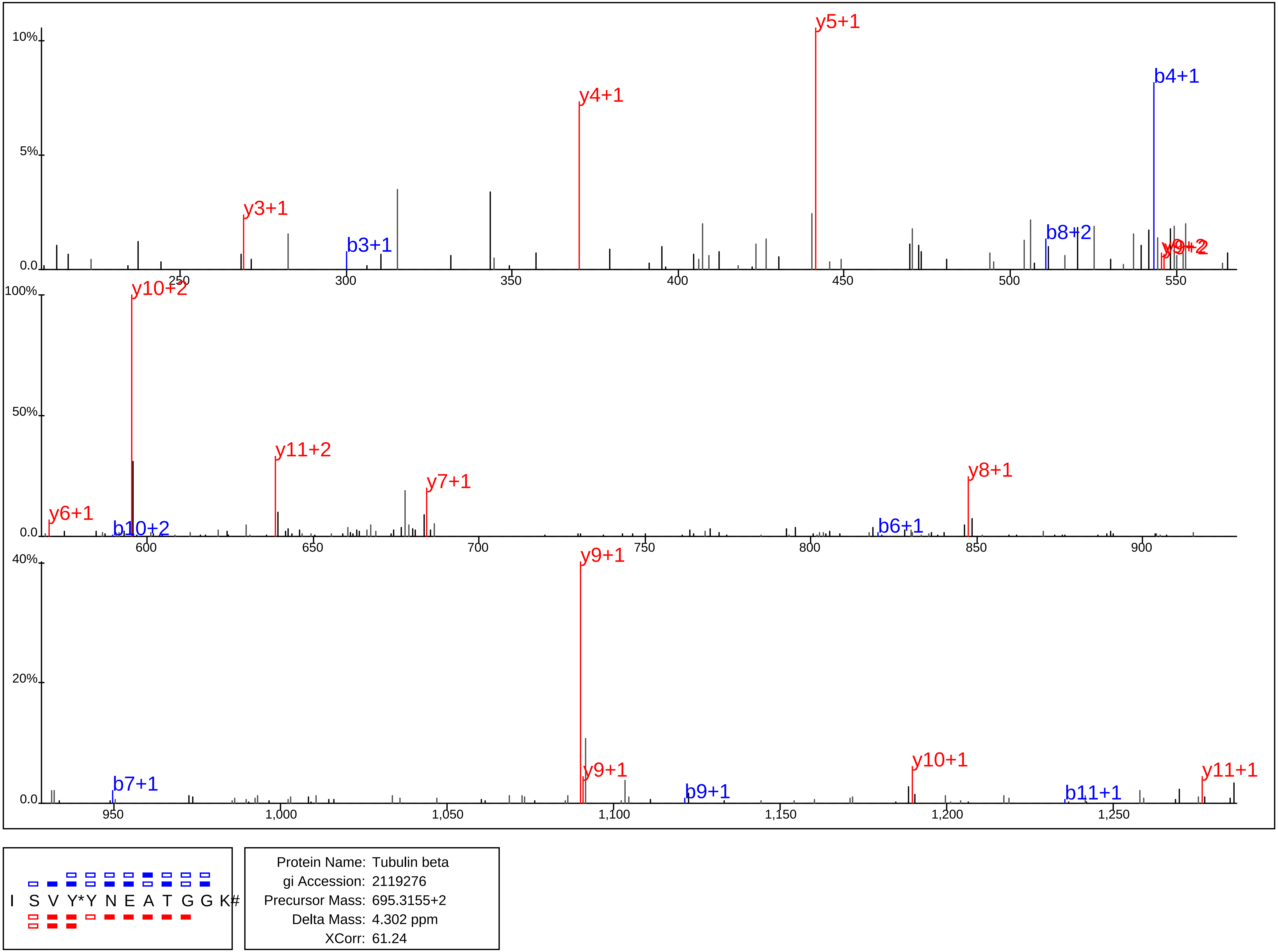

Protein Name:	Tubulin beta
gi Accession:	2119276
Precursor Mass:	695.3155+2
Delta Mass:	4.302 ppm
XCorr:	61.24

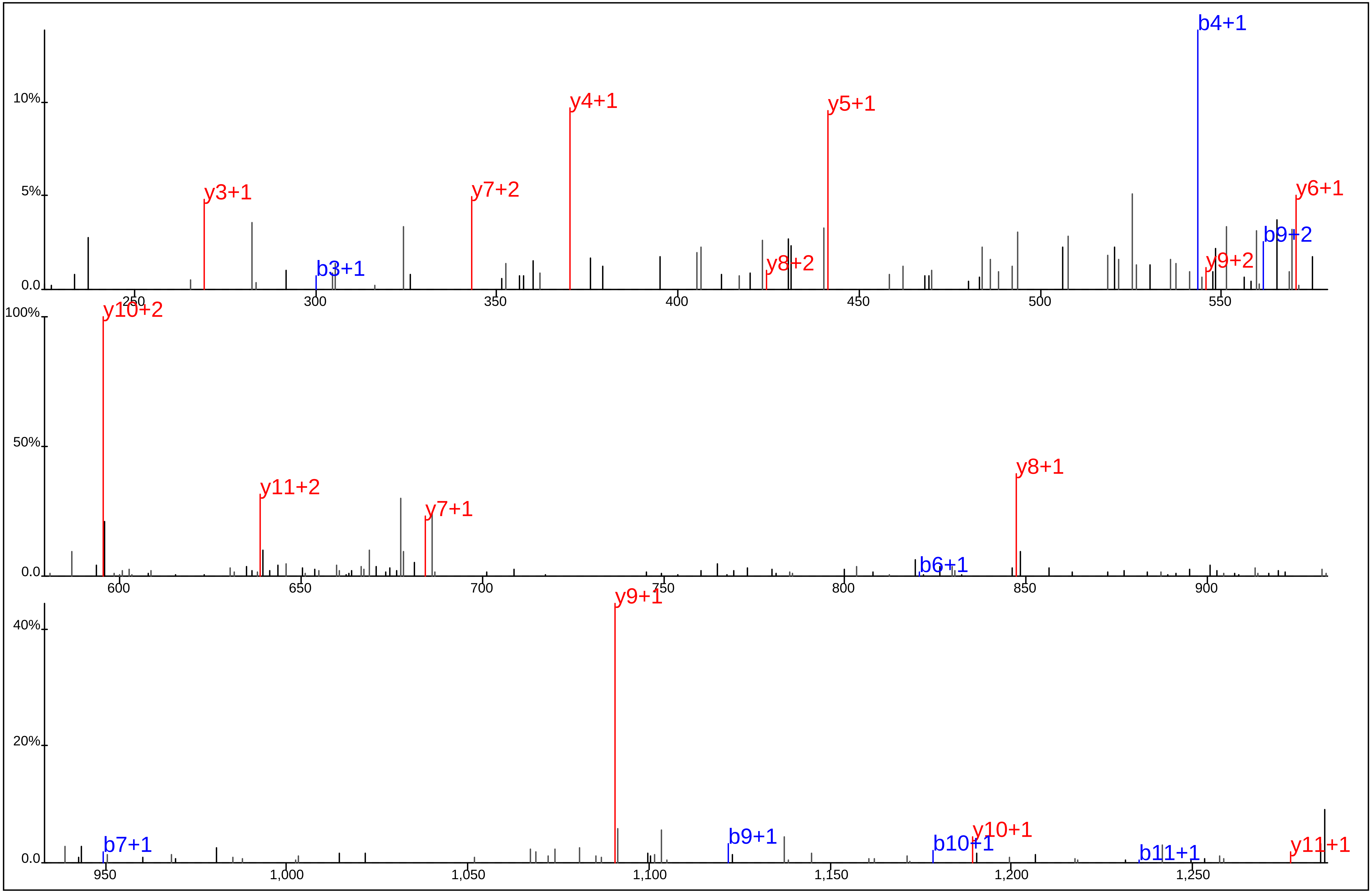

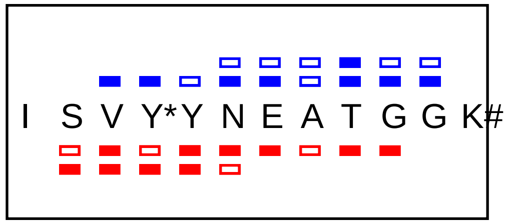

Protein Name: Tubulin beta
gi Accession: 2119276
Precursor Mass: 695.3134+2
Delta Mass: 1.315 ppm
XCorr: 45.02

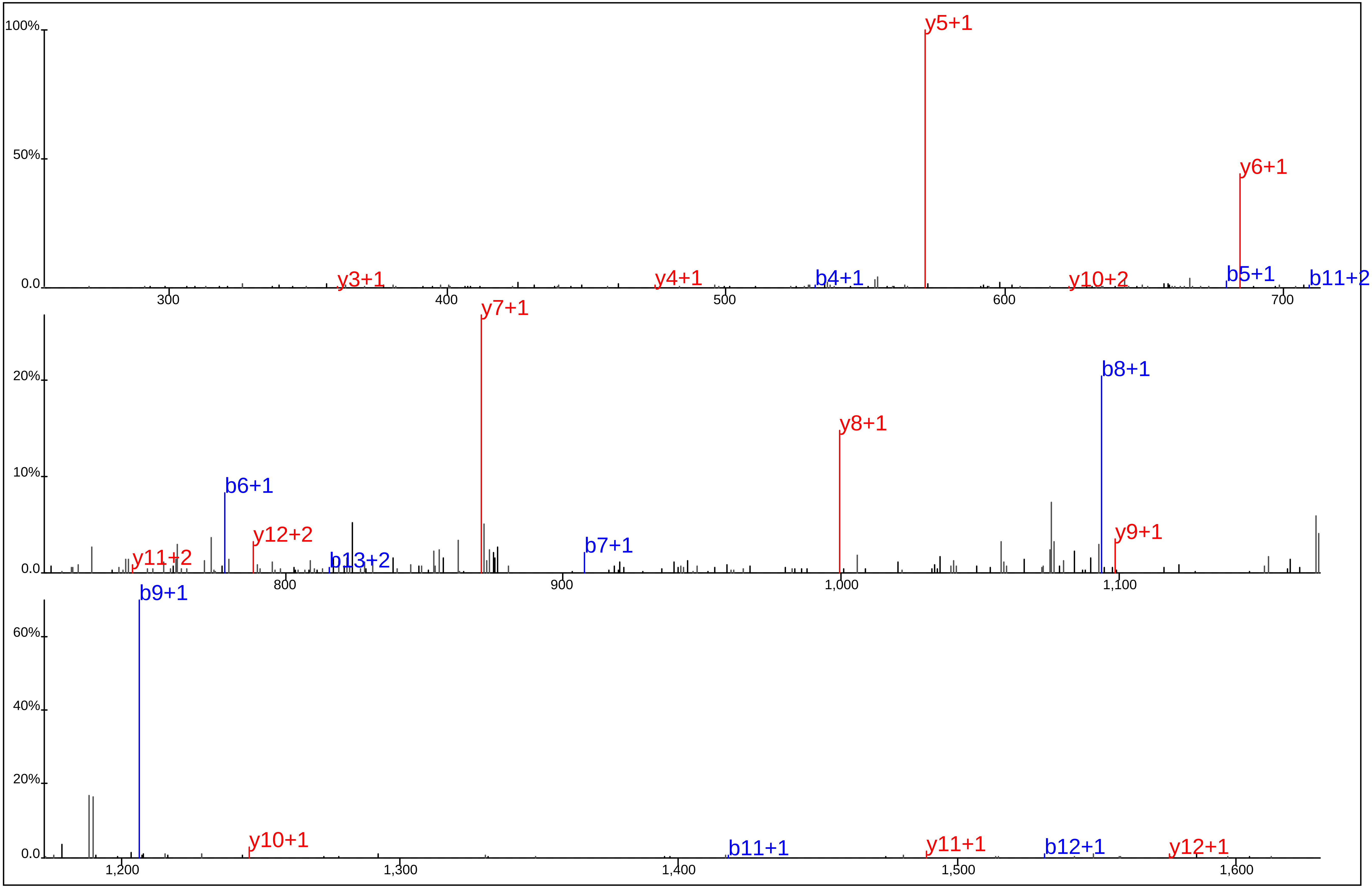

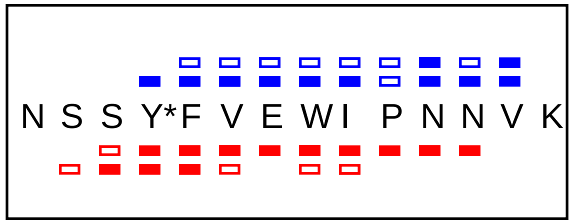

Protein Name: Tubulin beta
gi Accession: 2119276
Precursor Mass: 888.9056+2
Delta Mass: 2.605 ppm
XCorr: 44.83

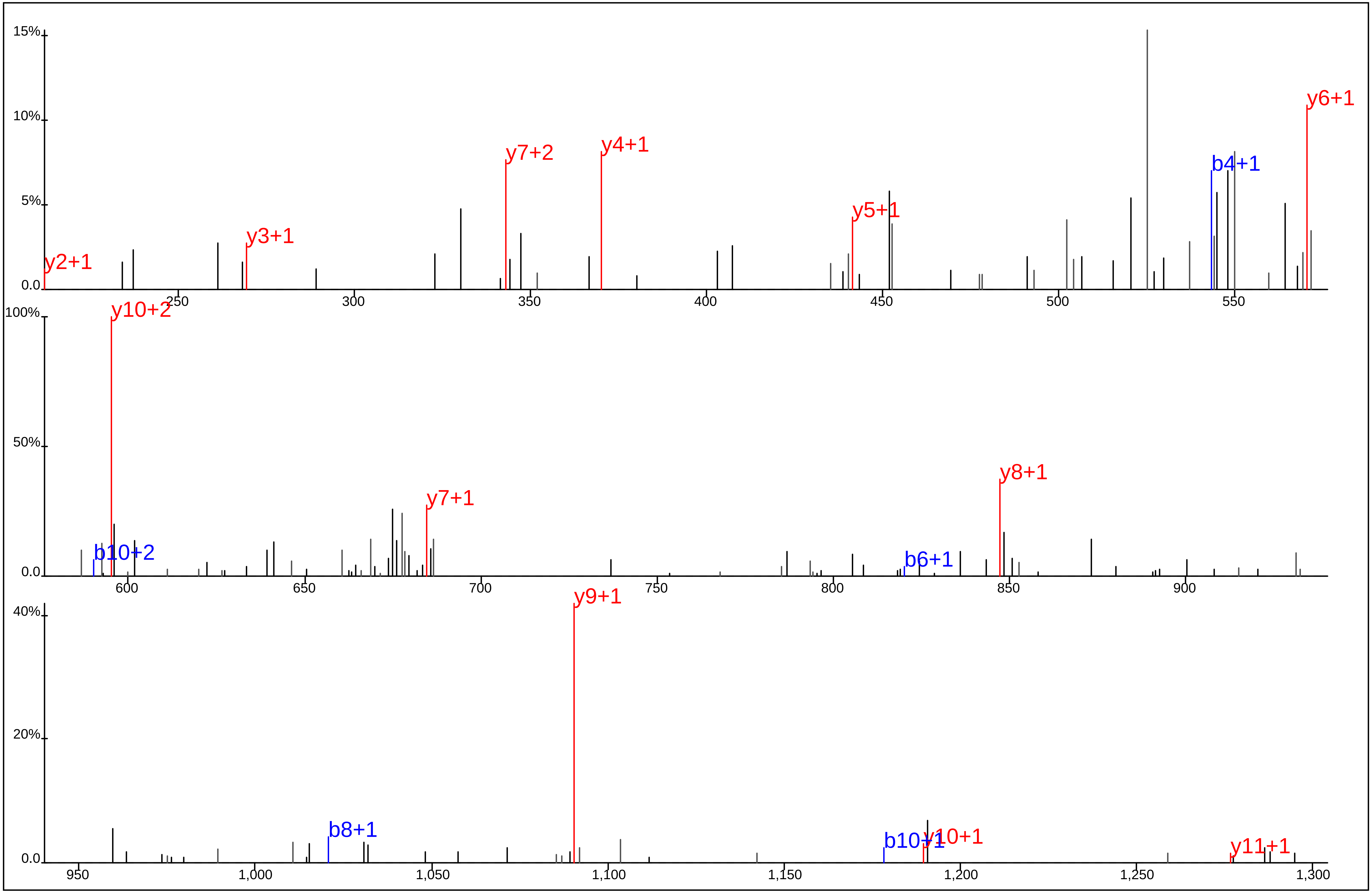

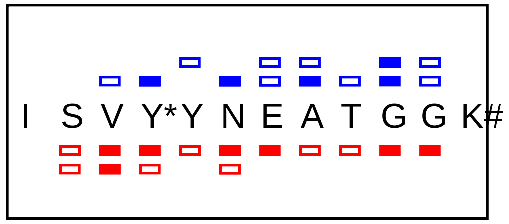

Protein Name: Tubulin beta
gi Accession: 2119276
Precursor Mass: 695.3160+2
Delta Mass: 5.007 ppm
XCorr: 35.17

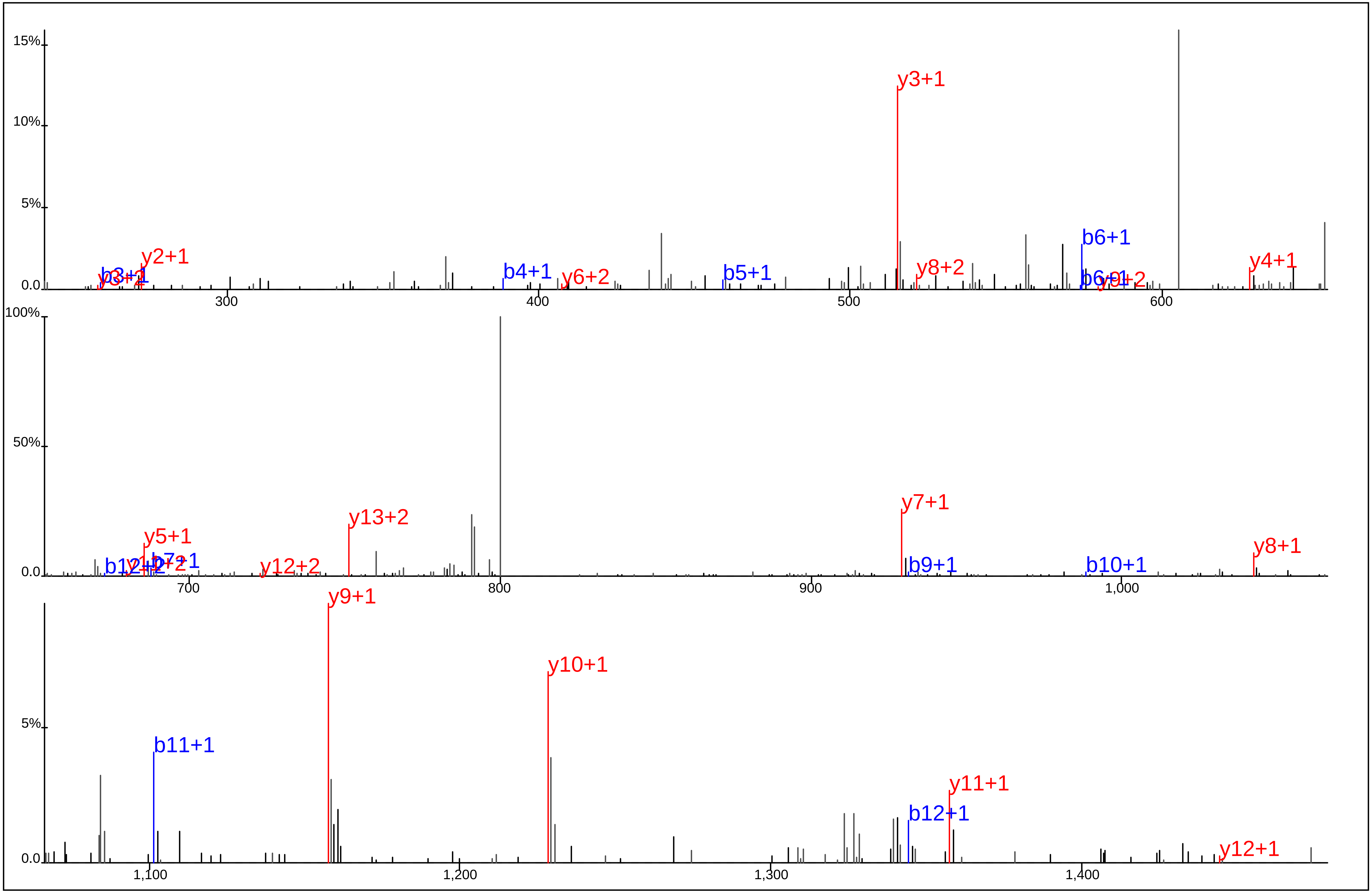

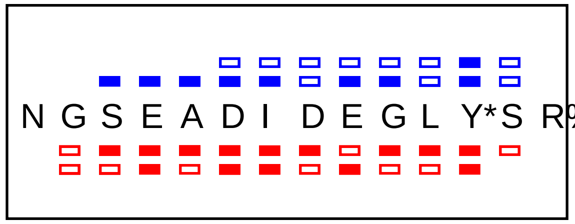

Protein Name: Ubiquitin activating enzyme 1
gi Accession: 23510338
Precursor Mass: 808.3322+2
Delta Mass: 3.762 ppm
XCorr: 55.32

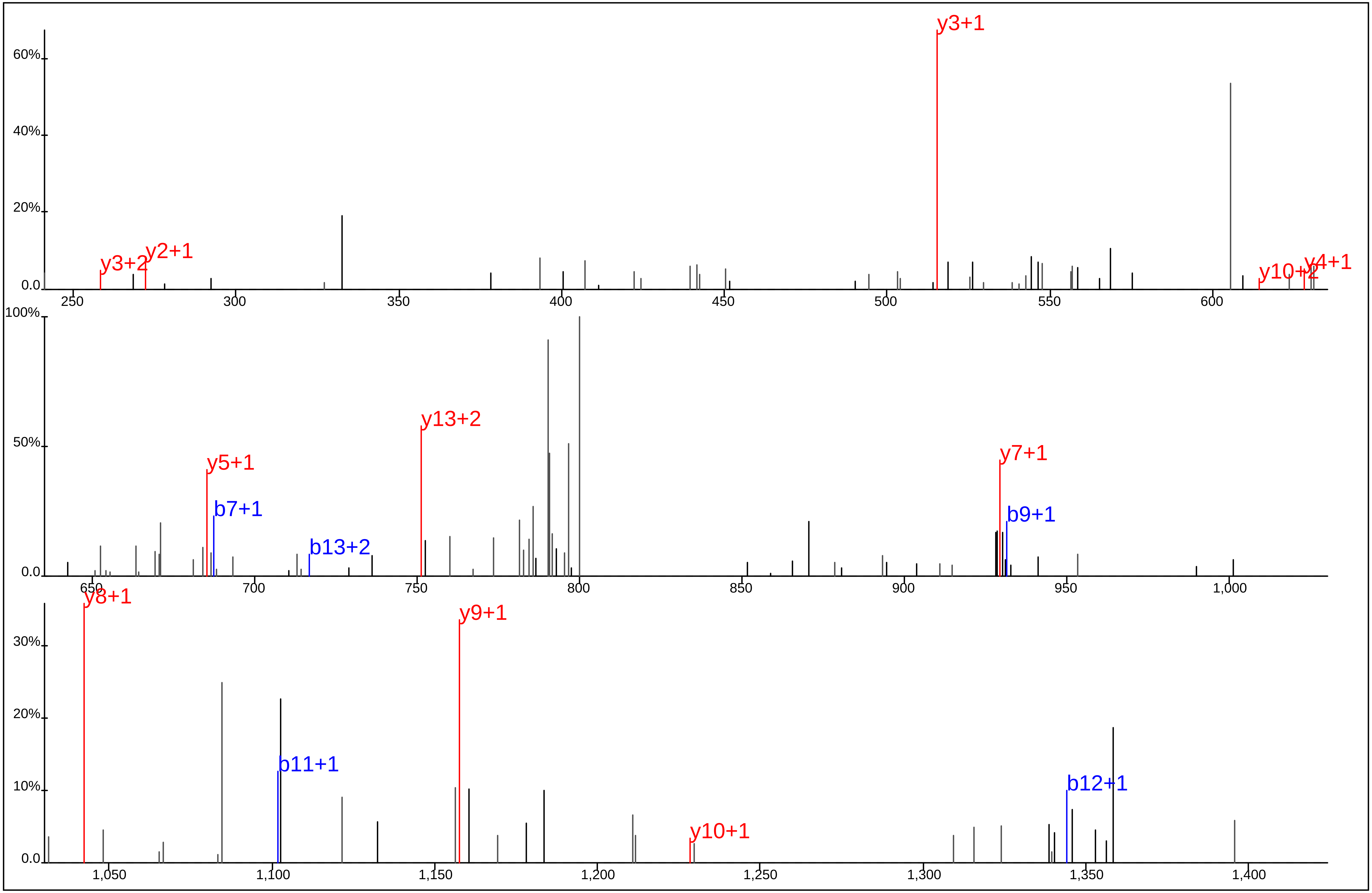

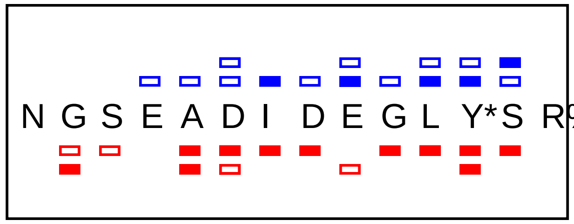

Protein Name:	Ubiquitin activating enzyme 1
gi Accession:	23510338
Precursor Mass:	808.3314+2
Delta Mass:	2.784 ppm
XCorr:	31.63

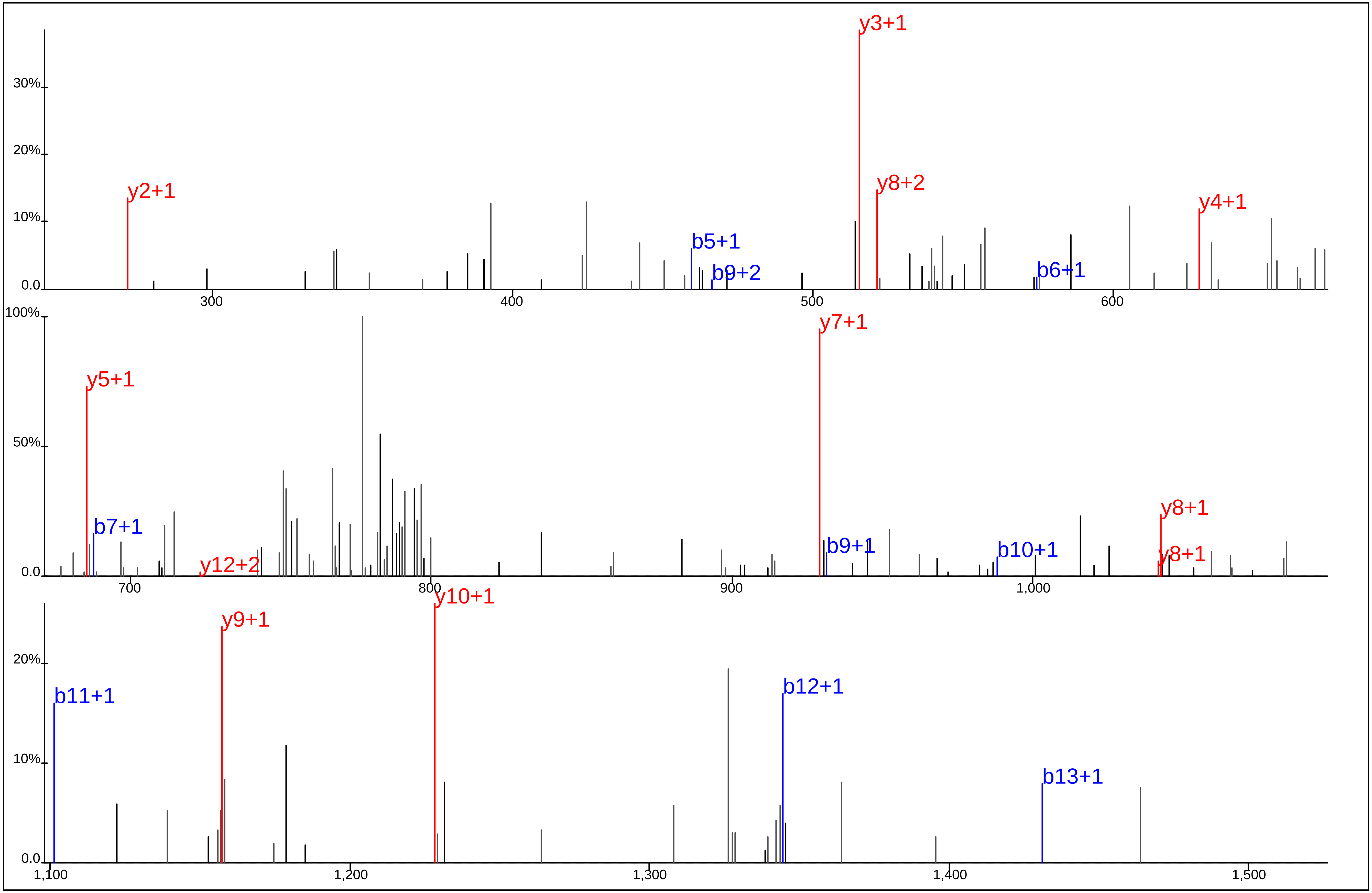

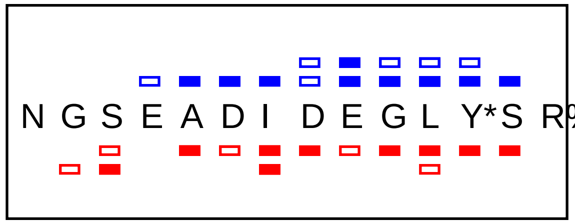

Protein Name: Ubiquitin activating enzyme 1
gi Accession: 23510338
Precursor Mass: 808.3311+2
Delta Mass: 2.326 ppm
XCorr: 47.96

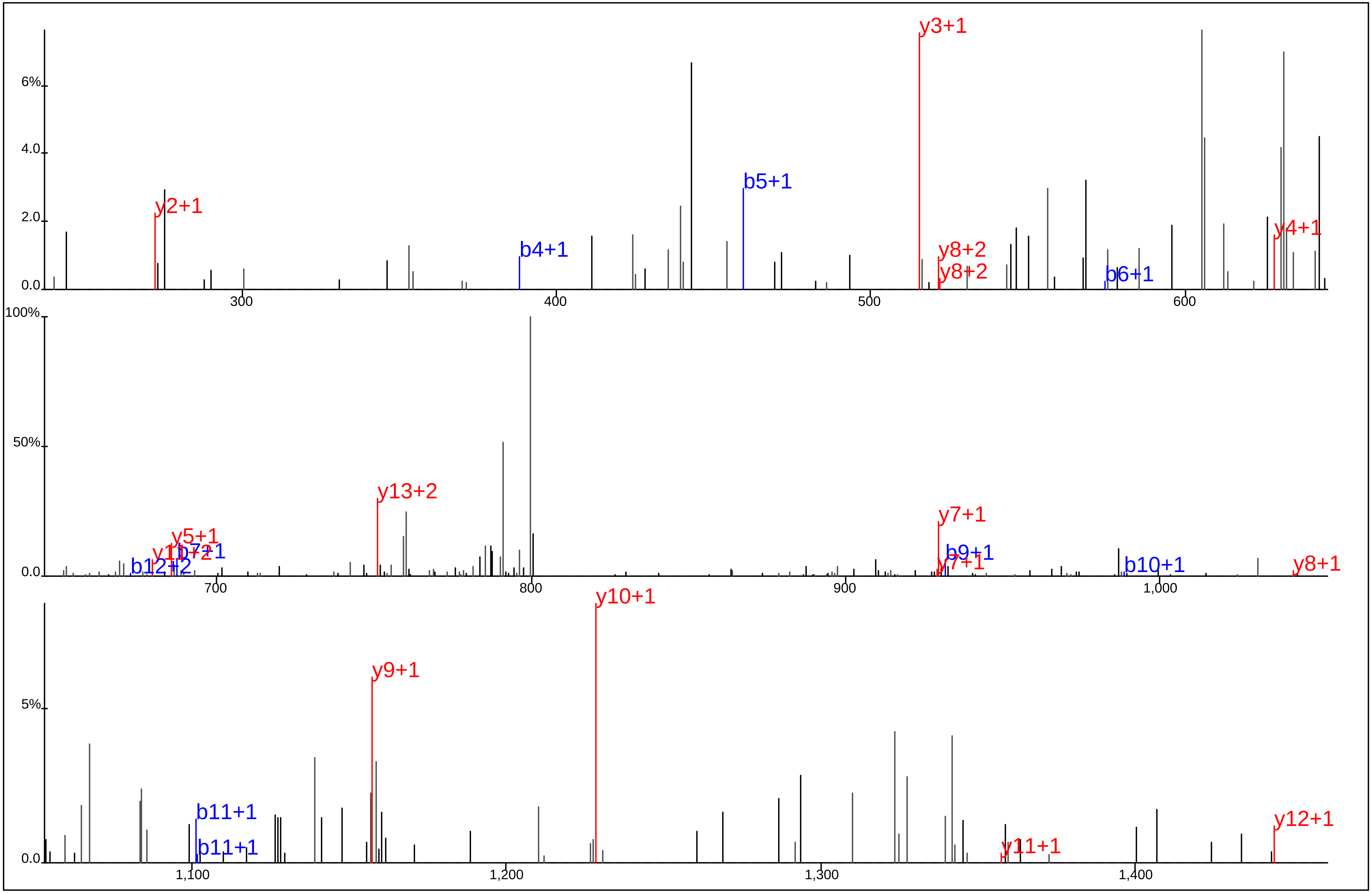

N G S E A D I D E G L Y*S R%

Protein Name:	Ubiquitin activating enzyme 1
gi Accession:	23510338
Precursor Mass:	808.3308+2
Delta Mass:	2.023 ppm
XCorr:	35.9

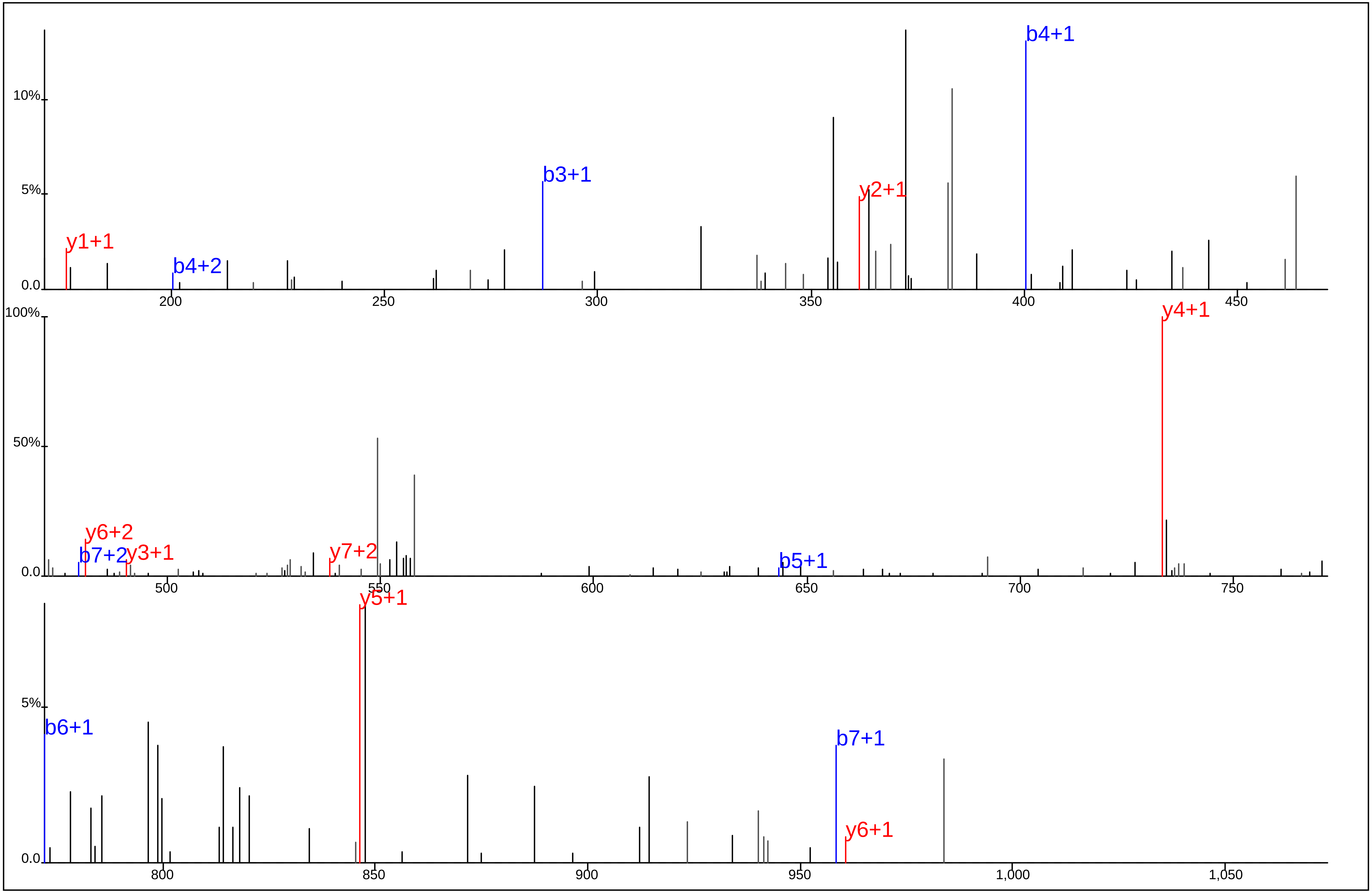

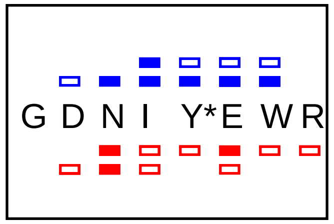

Protein Name: Ubiquitin conjugating enzyme
gi Accession: 22749327
Precursor Mass: 566.7271+2
Delta Mass: 0.804 ppm
XCorr: 2.3723

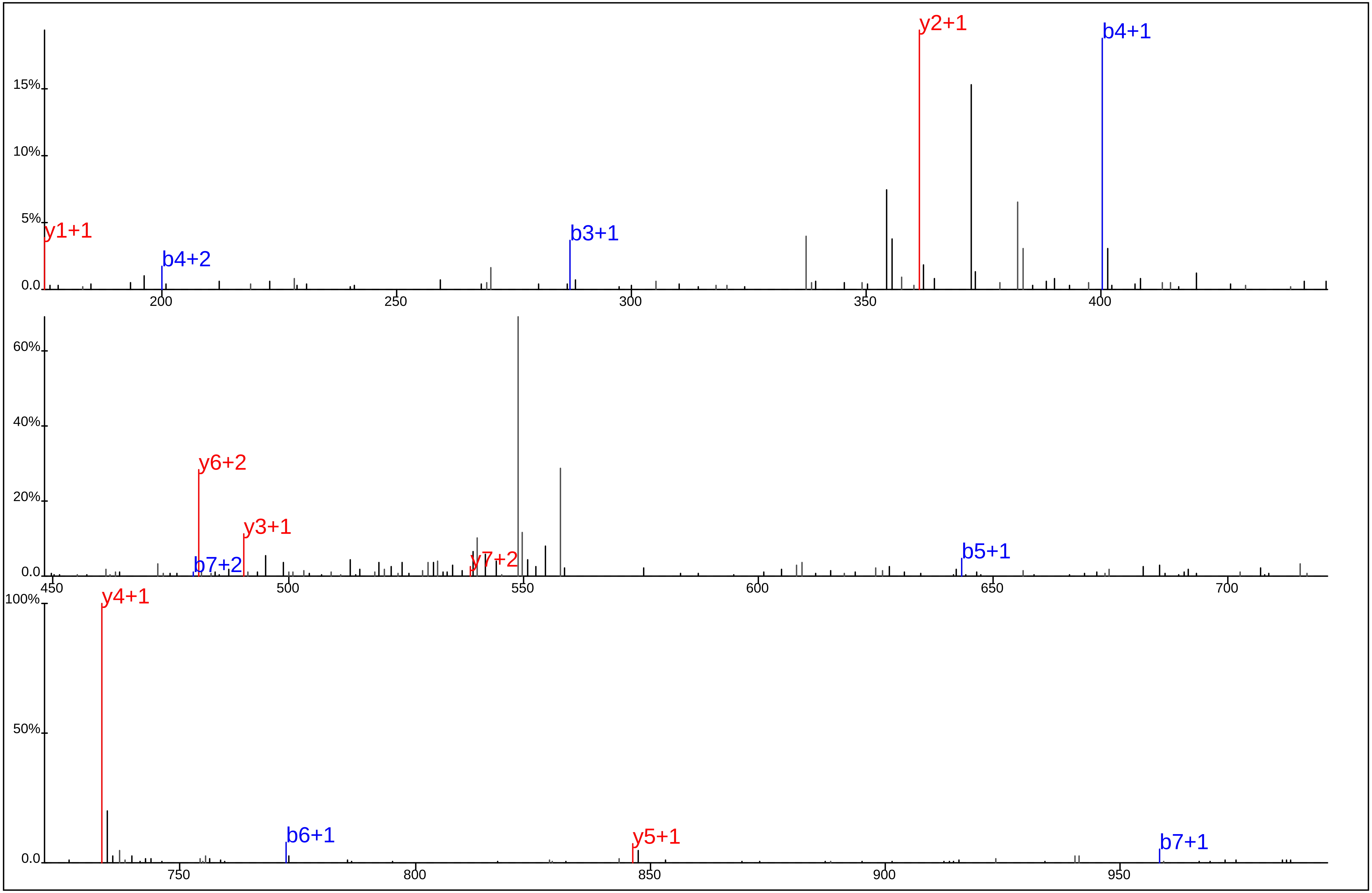

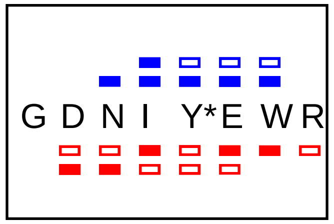

Protein Name: Ubiquitin conjugating enzyme
gi Accession: 22749327
Precursor Mass: 566.7282+2
Delta Mass: 2.868 ppm
XCorr: 34.97

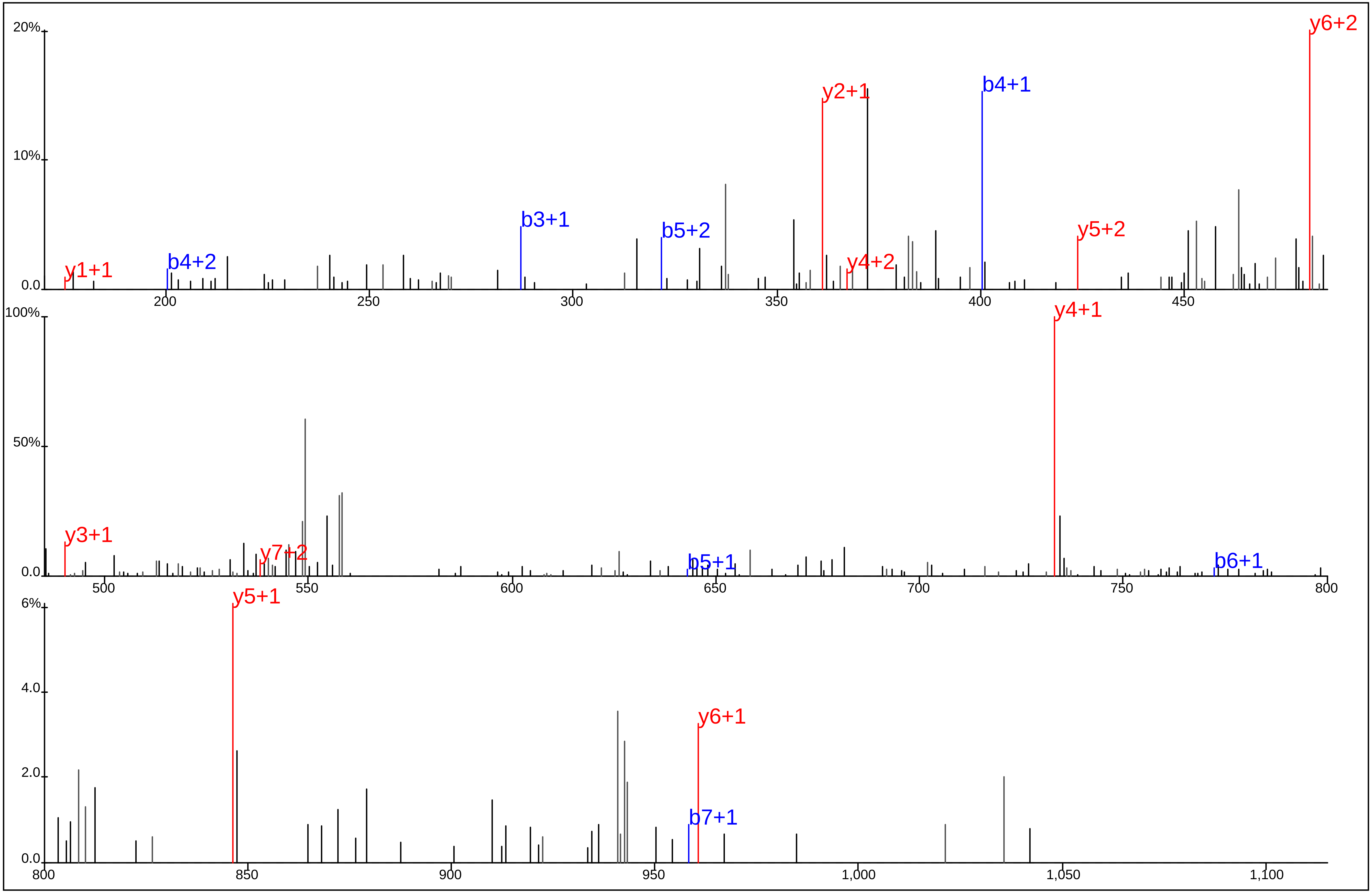

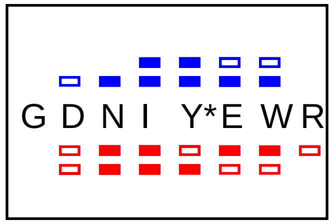

Protein Name: Ubiquitin conjugating enzyme
gi Accession: 22749327
Precursor Mass: 566.7266+2
Delta Mass: 0.037 ppm
XCorr: 14.4

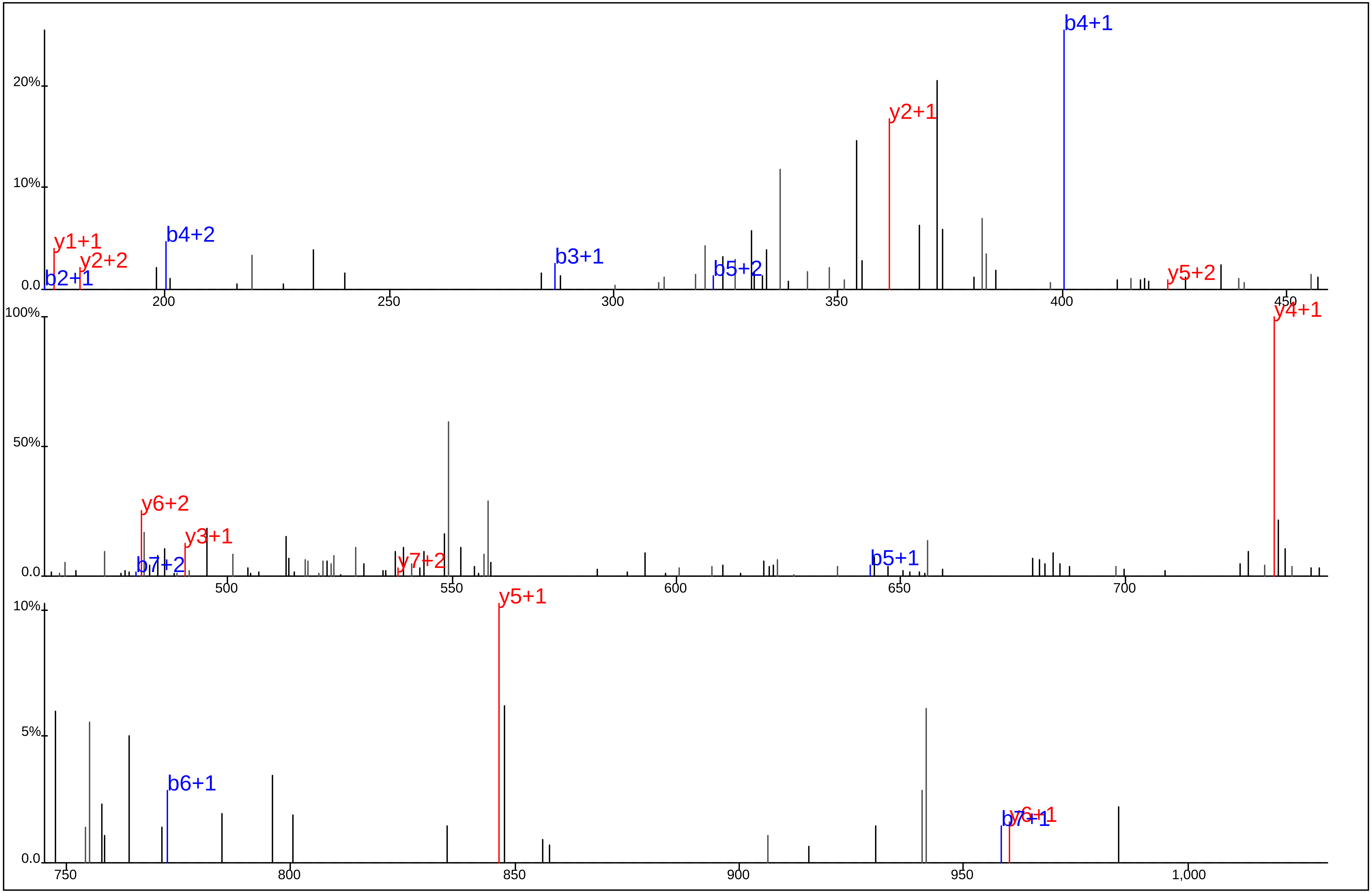

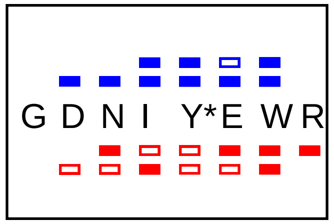

Protein Name:	Ubiquitin conjugating enzyme
gi Accession:	22749327
Precursor Mass:	566.7285+2
Delta Mass:	3.407 ppm
XCorr:	17.62

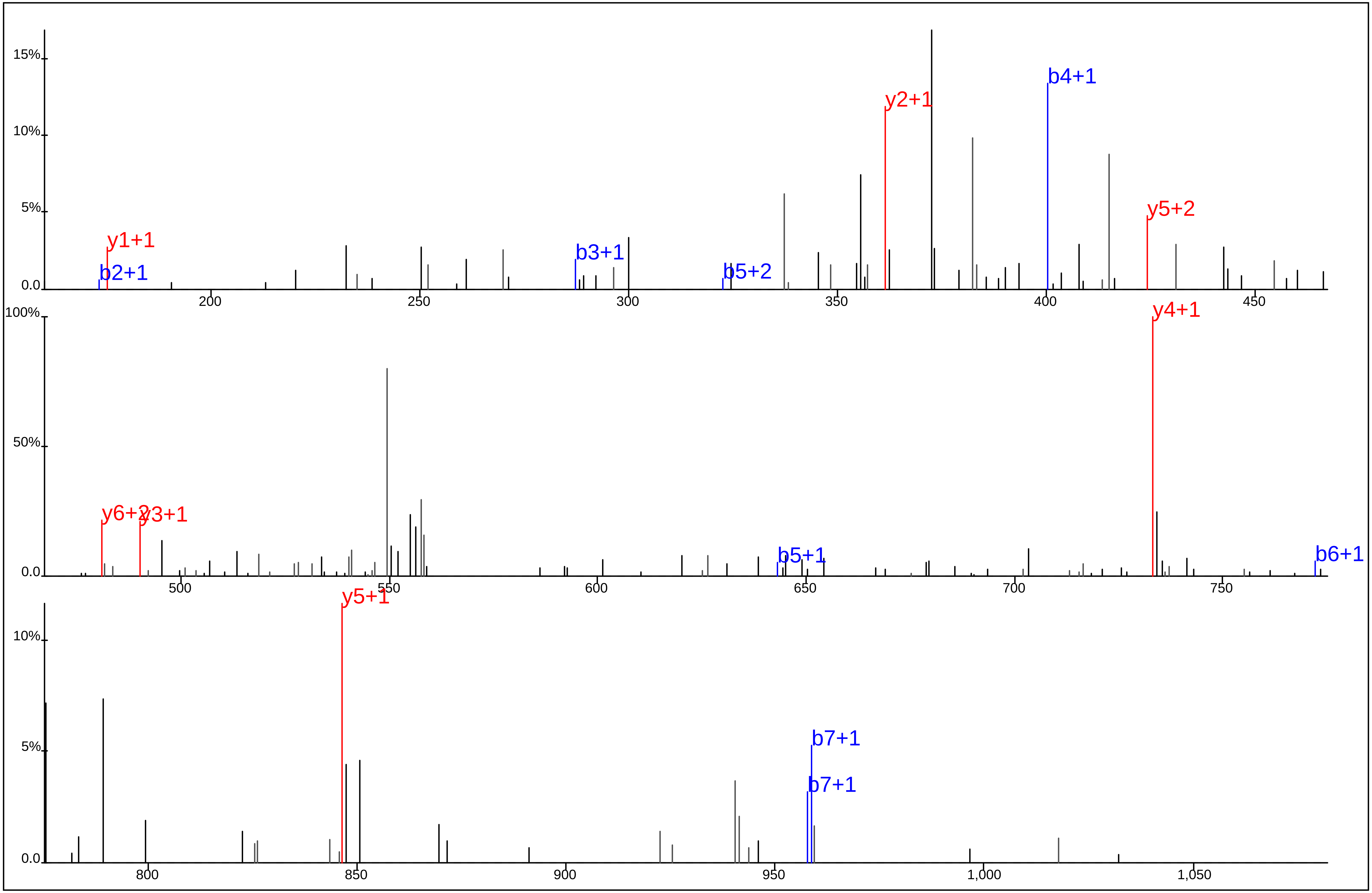

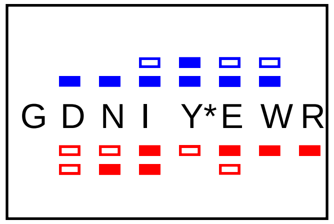

Protein Name: Ubiquitin conjugating enzyme
gi Accession: 22749327
Precursor Mass: 566.7286+2
Delta Mass: 3.628 ppm
XCorr: 17.6

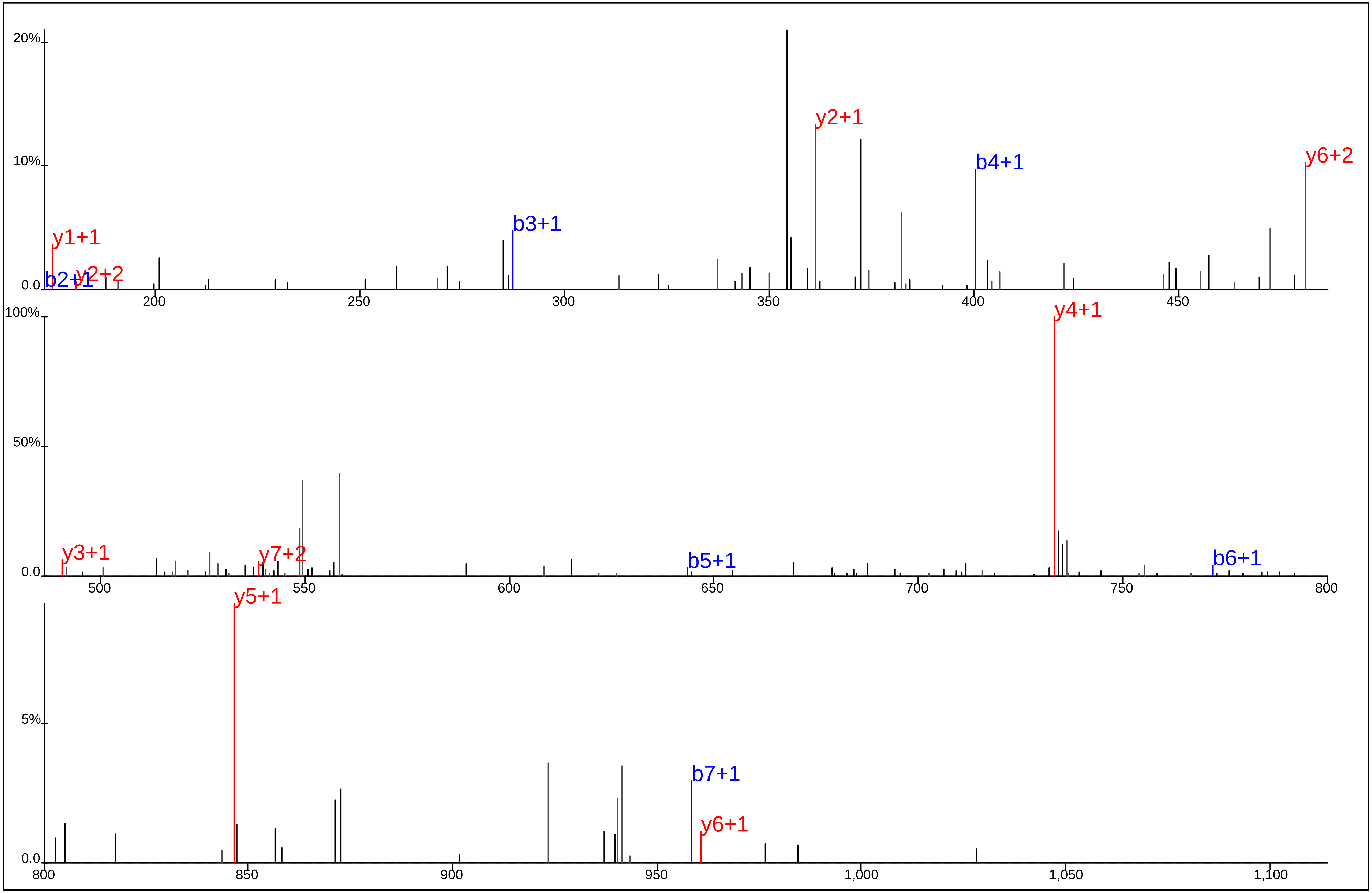

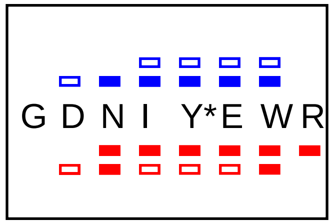

Protein Name: Ubiquitin conjugating enzyme
gi Accession: 22749327
Precursor Mass: 566.7286+2
Delta Mass: 3.522 ppm
XCorr: 19.05

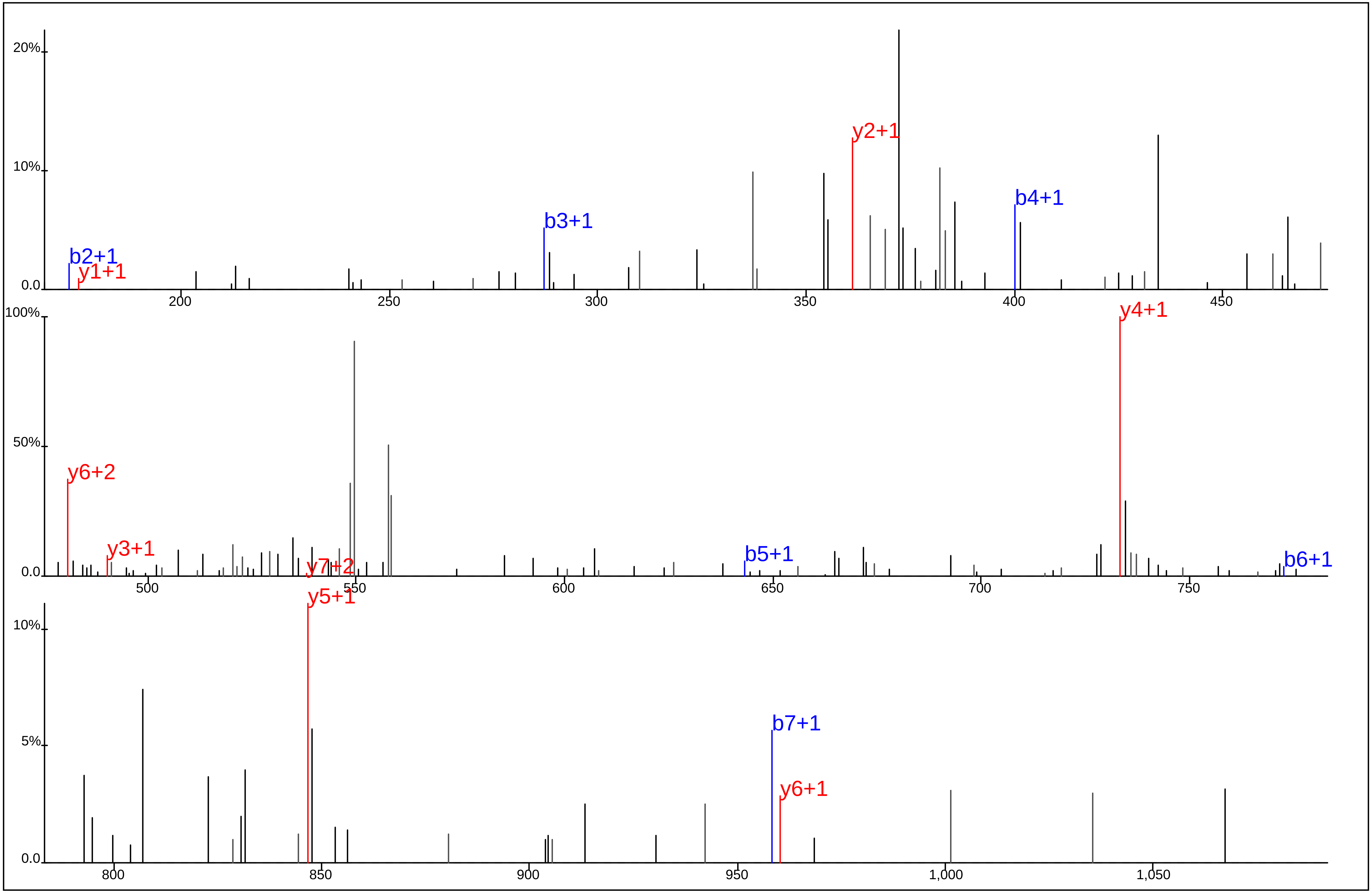

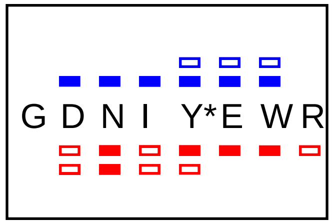

Protein Name: Ubiquitin conjugating enzyme
gi Accession: 22749327
Precursor Mass: 566.7278+2
Delta Mass: 2.118 ppm
XCorr: 17.33

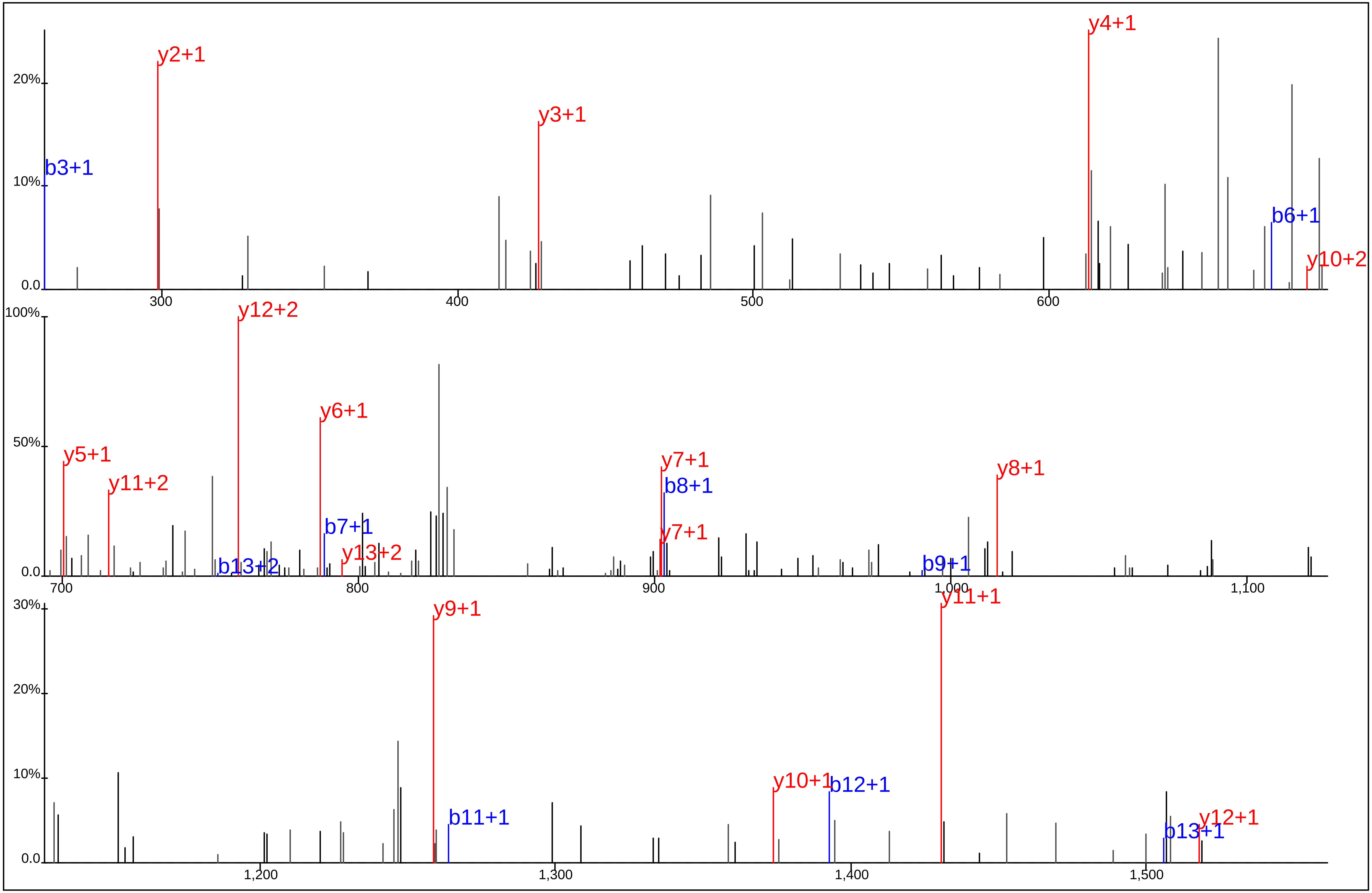

T A S G D Y*I D S S W E L R%

Protein Name: URP2
gi Accession: 41281905
Precursor Mass: 845.3568+2
Delta Mass: 1.833 ppm
XCorr: 66.62

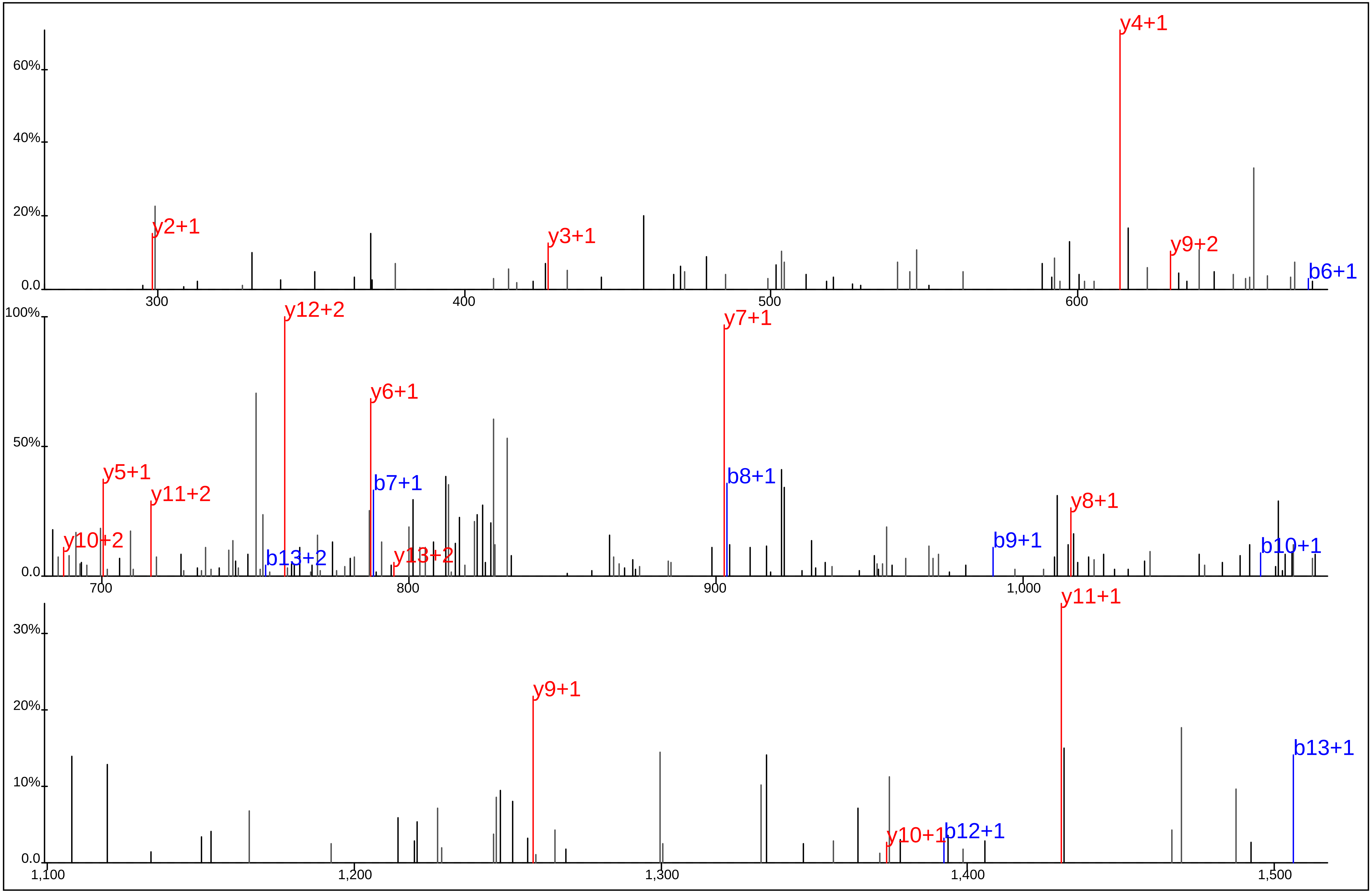

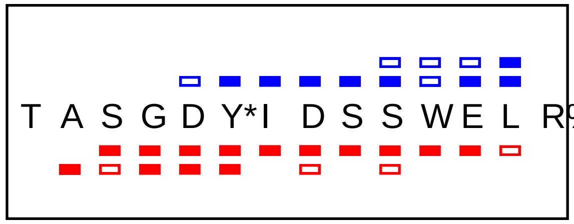

Protein Name:	URP2
gi Accession:	41281905
Precursor Mass:	845.3600+2
Delta Mass:	5.663 ppm
XCorr:	52.72

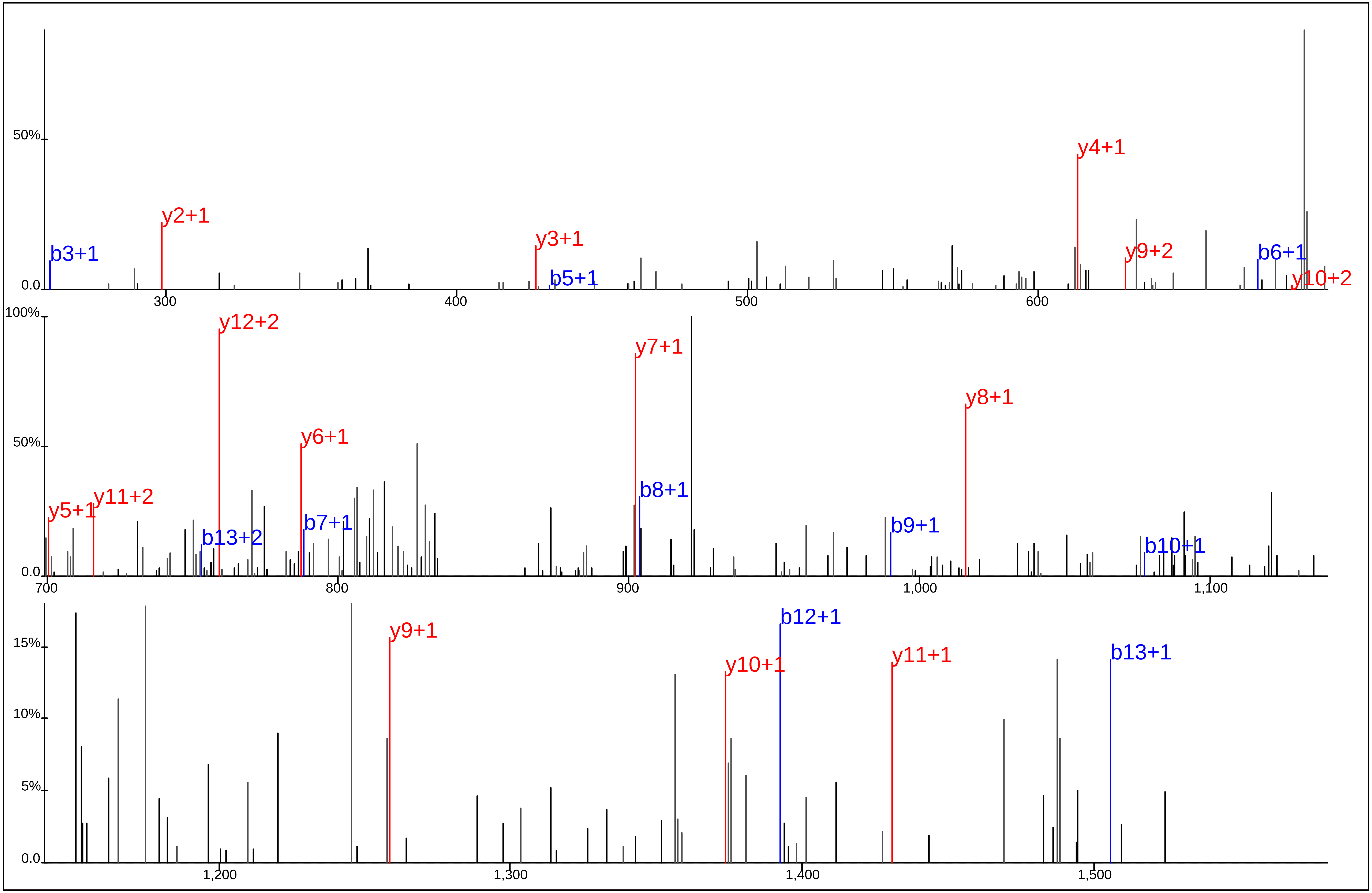

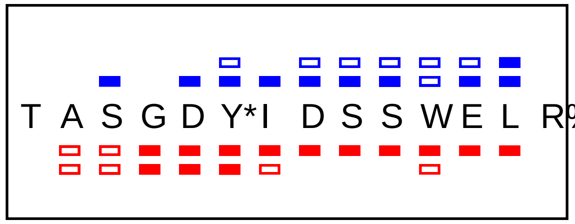

Protein Name: URP2
gi Accession: 41281905
Precursor Mass: 845.3600+2
Delta Mass: 5.663 ppm
XCorr: 46.51

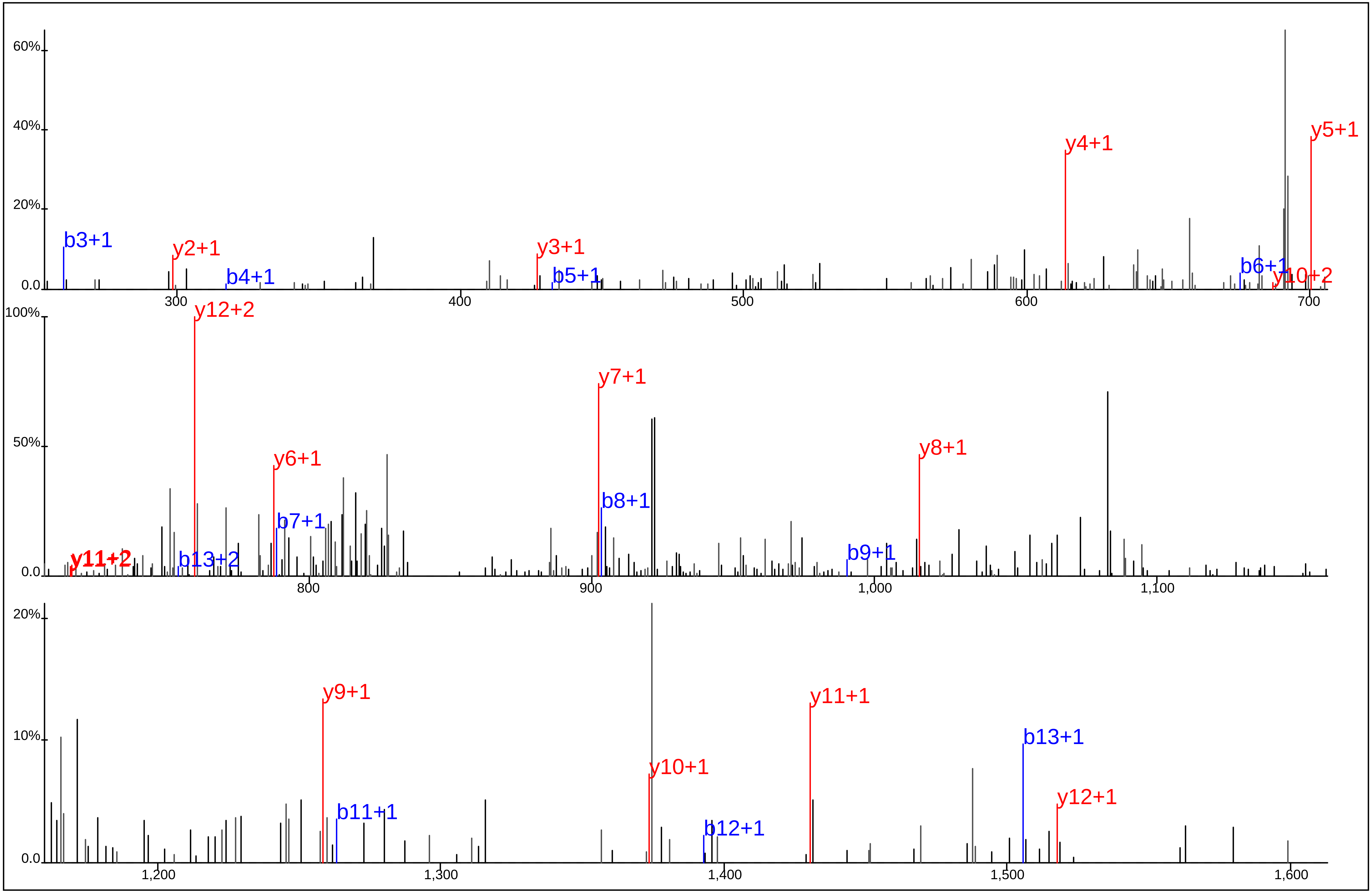

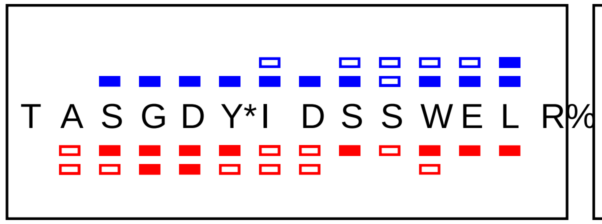

Protein Name: URP2
gi Accession: 41281905
Precursor Mass: 845.3576+2
Delta Mass: 2.845 ppm
XCorr: 59.67

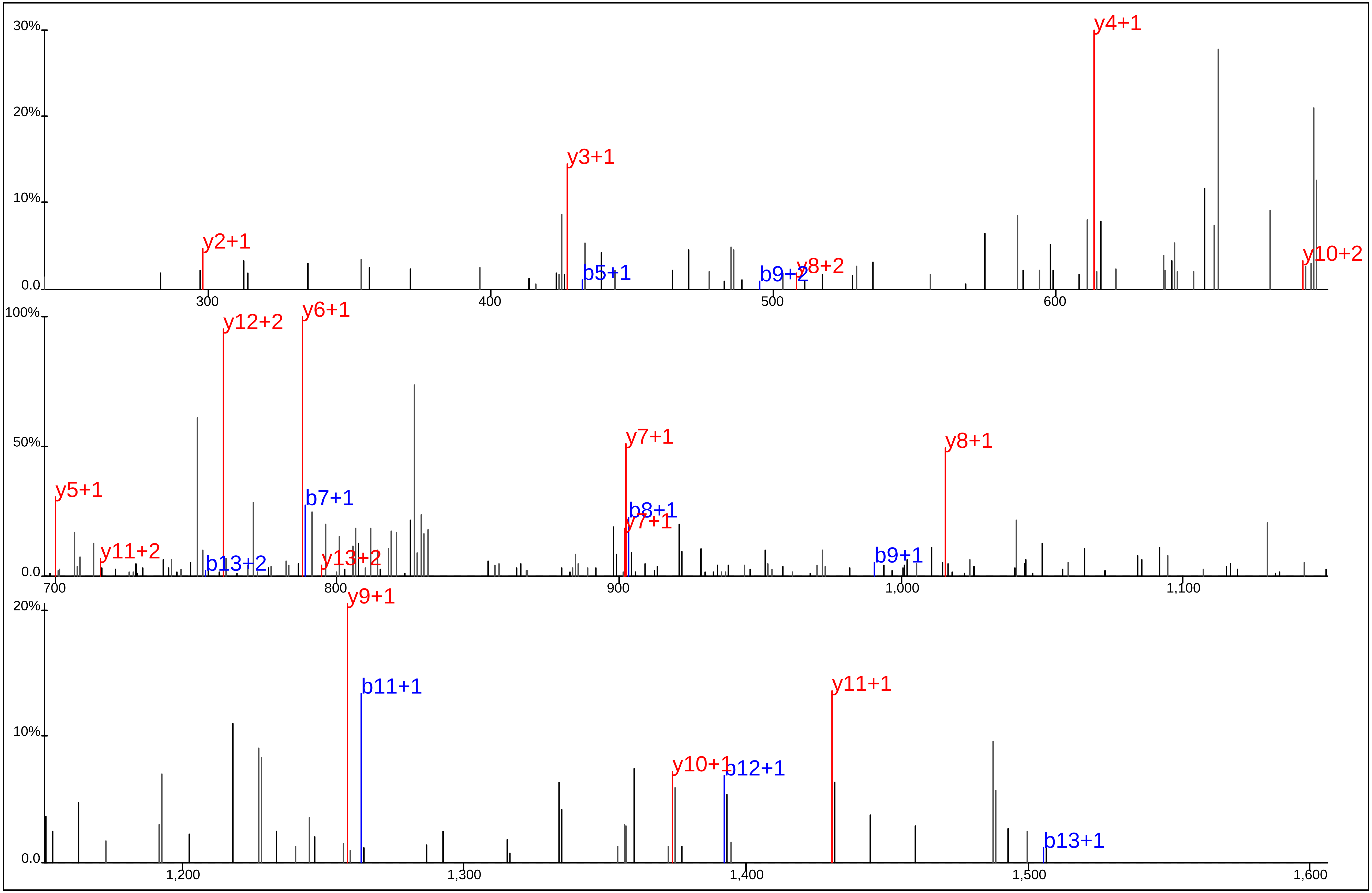

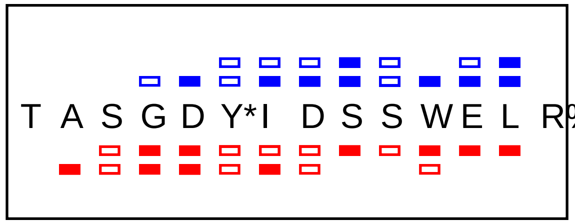

Protein Name:	URP2
gi Accession:	41281905
Precursor Mass:	845.3577+2
Delta Mass:	2.993 ppm
XCorr:	66.48

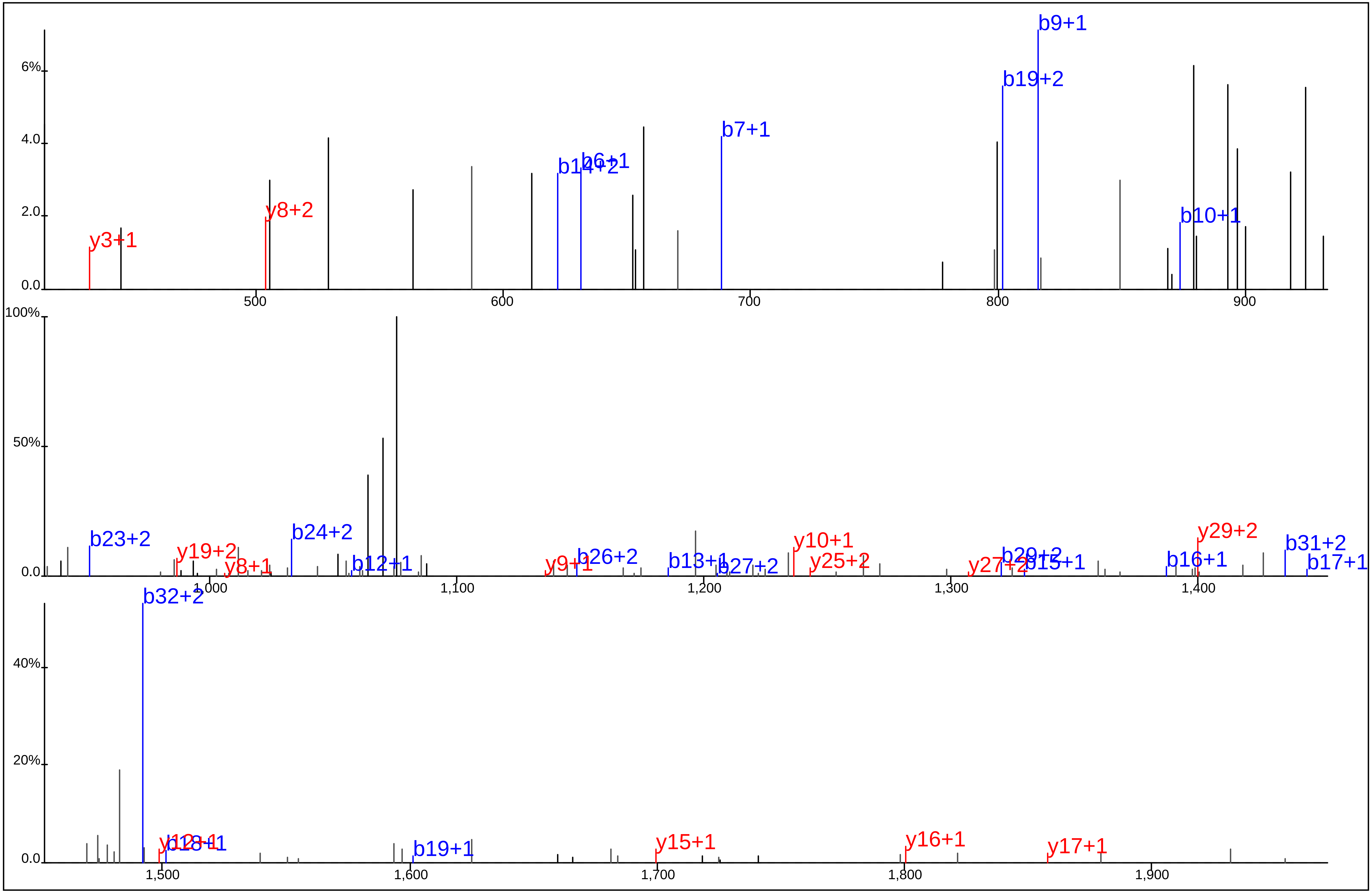

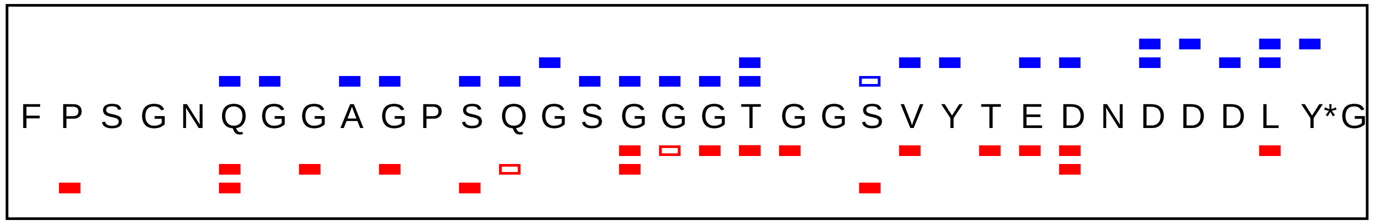

Protein Name:	Valosin containing protein
gi Accession:	6005942
Precursor Mass:	1,100.7711+3
Delta Mass:	3.465 ppm
XCorr:	47.07

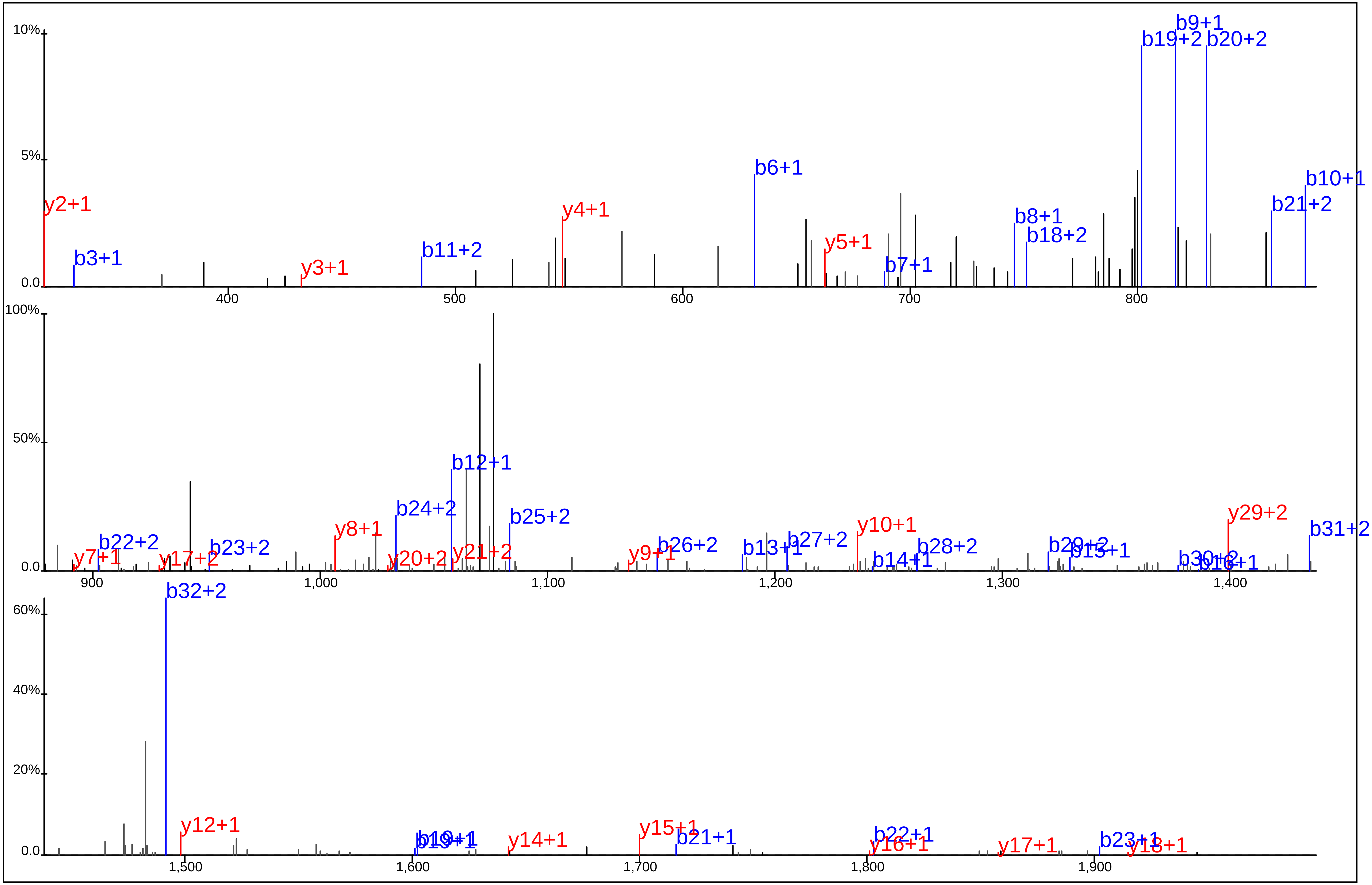

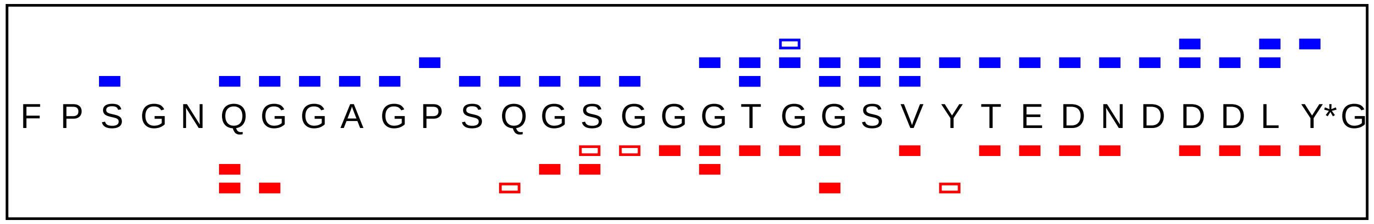

Protein Name:	Valosin containing protein
gi Accession:	6005942
Precursor Mass:	1,100.7733+3
Delta Mass:	5.463 ppm
XCorr:	80.89

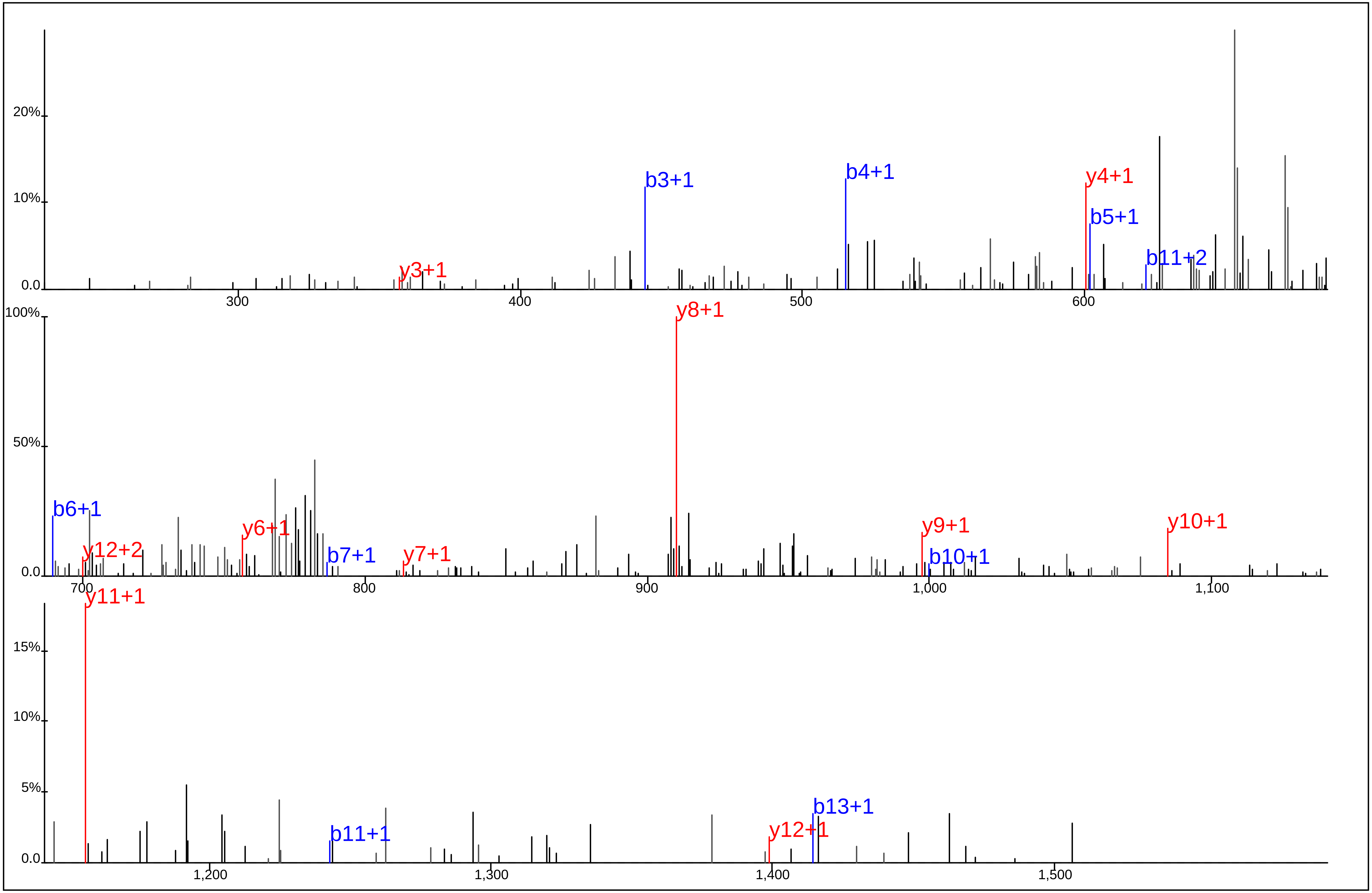

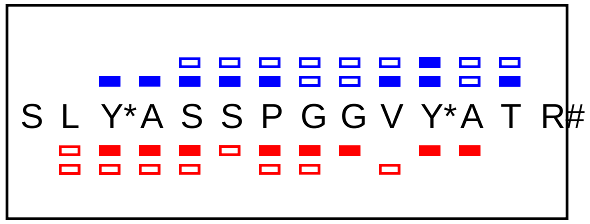

Protein Name: Vimentin
gi Accession: 37848
Precursor Mass: 799.8342+2
Delta Mass: 5.304 ppm
XCorr: 3.3085

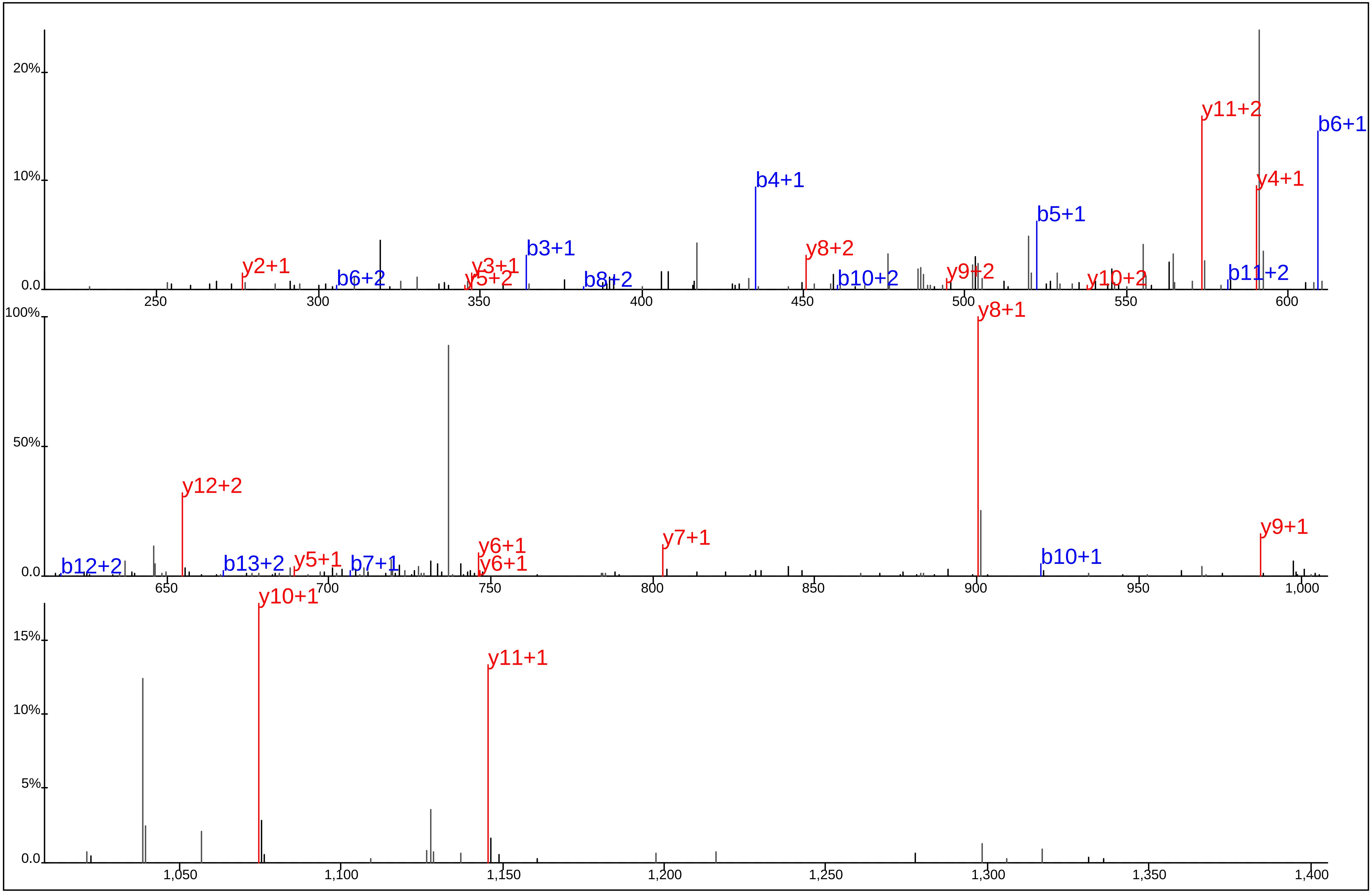

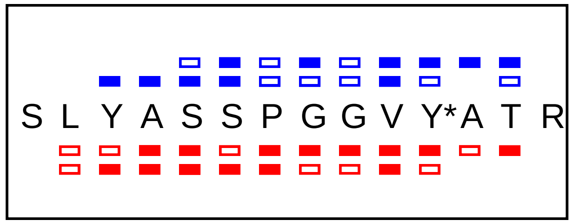

Protein Name: Vimentin
gi Accession: 37848
Precursor Mass: 754.8438+2
Delta Mass: 1.417 ppm
XCorr: 59.45

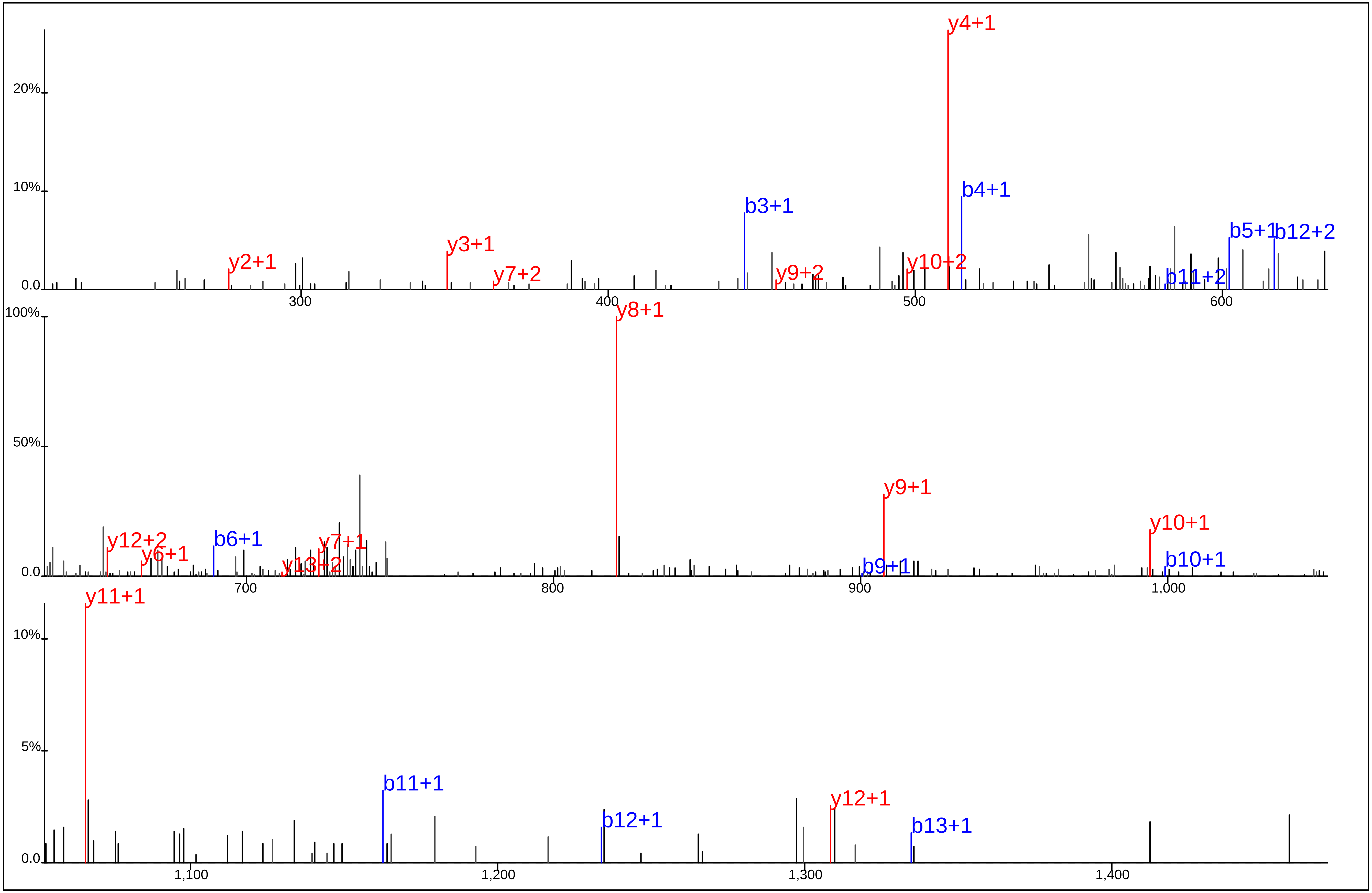

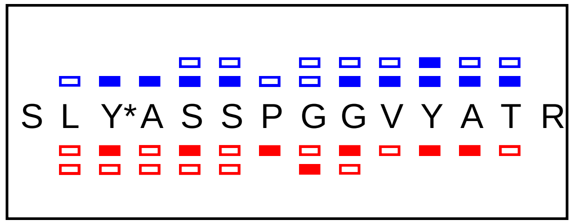

Protein Name: Vimentin
gi Accession: 37848
Precursor Mass: 754.8475+2
Delta Mass: 6.435 ppm
XCorr: 40.61

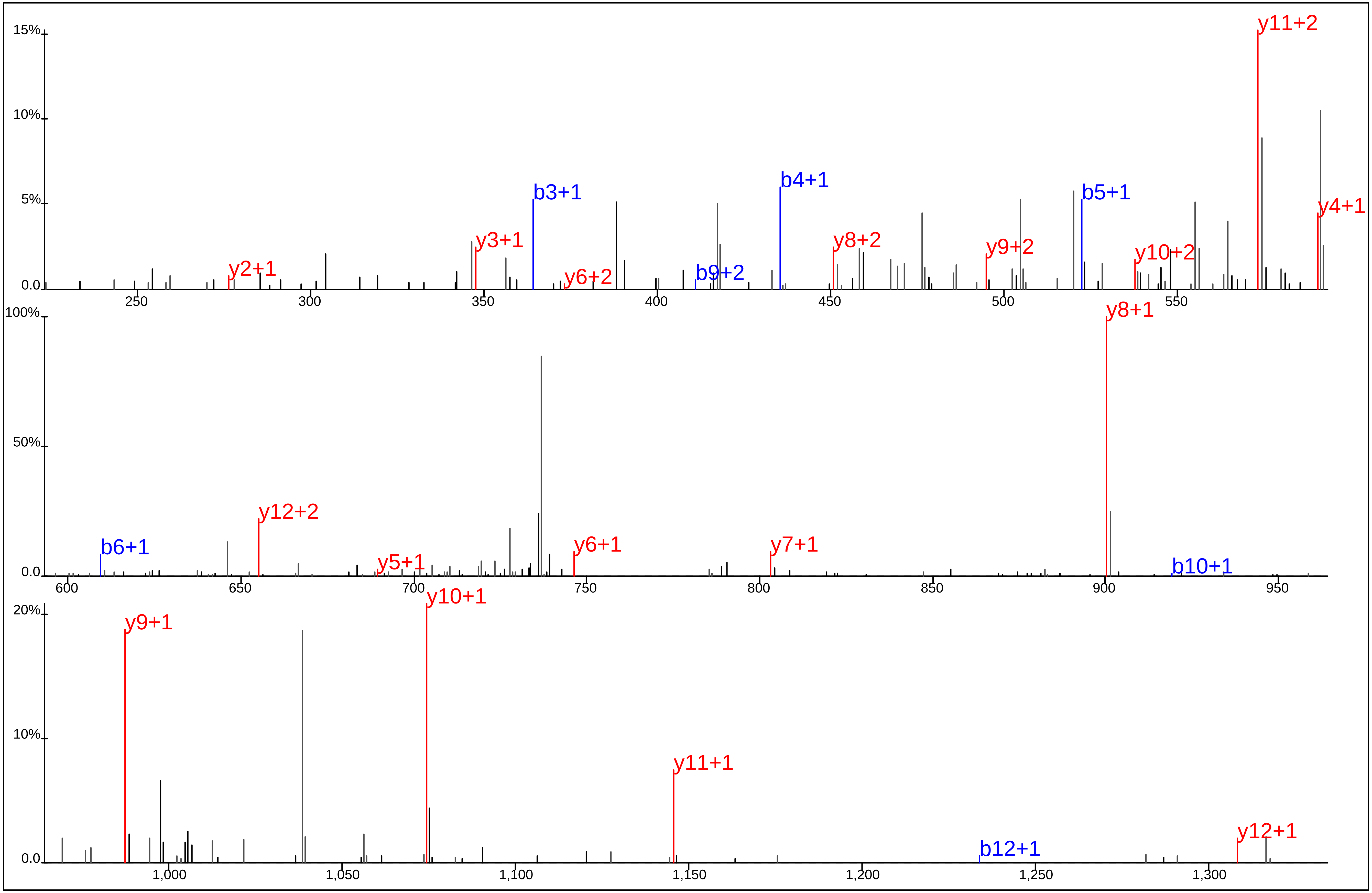

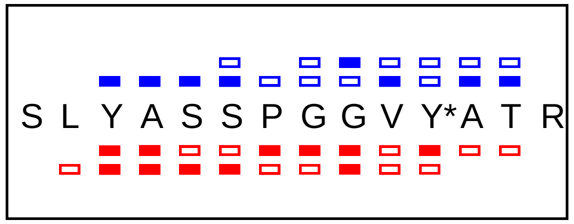

Protein Name: Vimentin
gi Accession: 37848
Precursor Mass: 754.8450+2
Delta Mass: 3.034 ppm
XCorr: 55.98

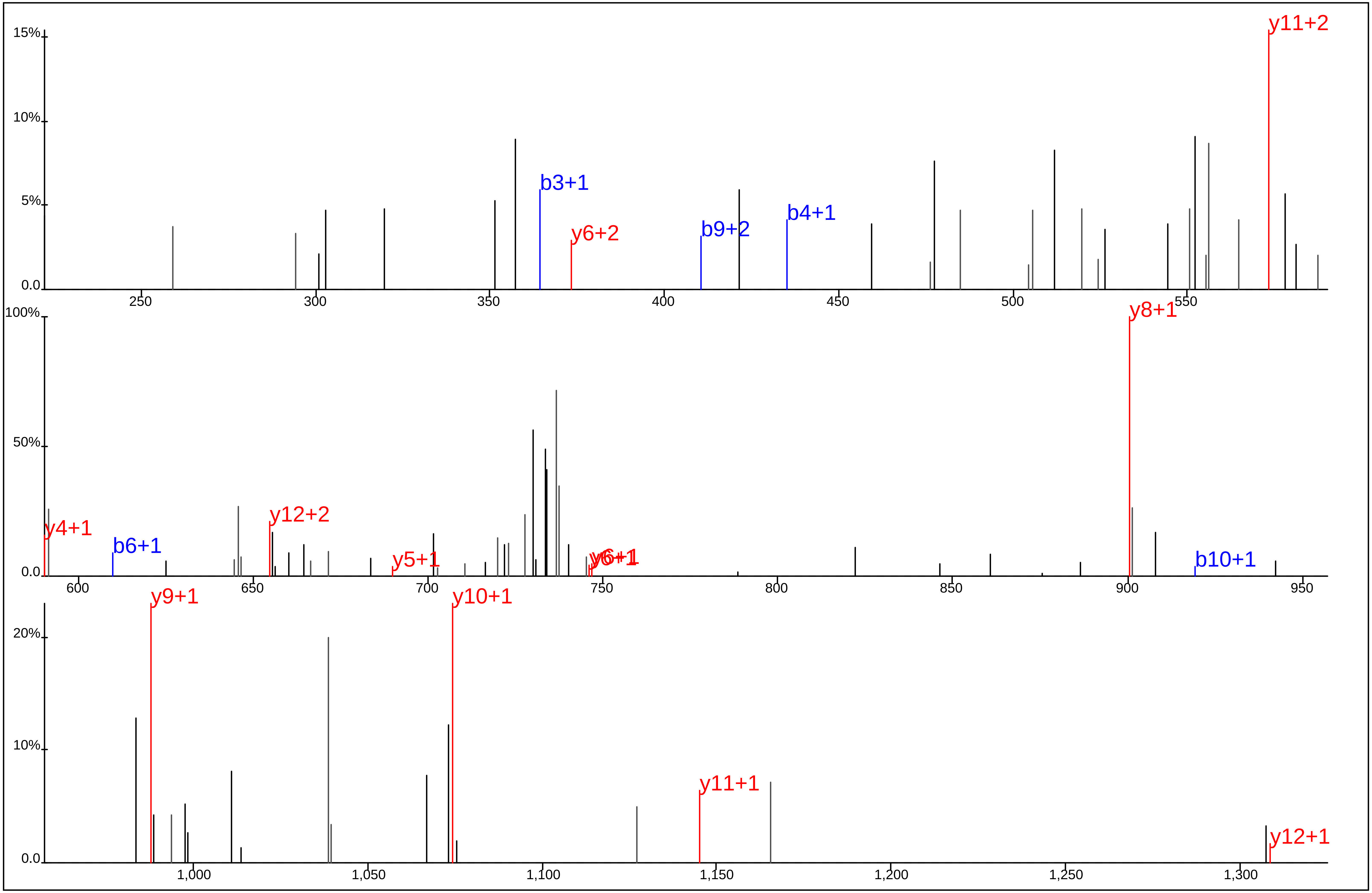

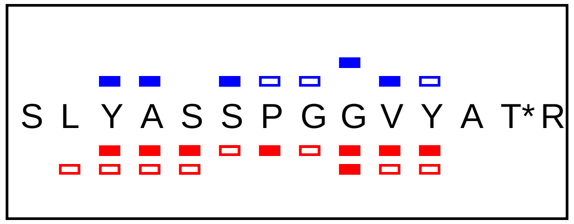

Protein Name: Vimentin
gi Accession: 37848
Precursor Mass: 754.8467+2
Delta Mass: 5.381 ppm
XCorr: 24.55

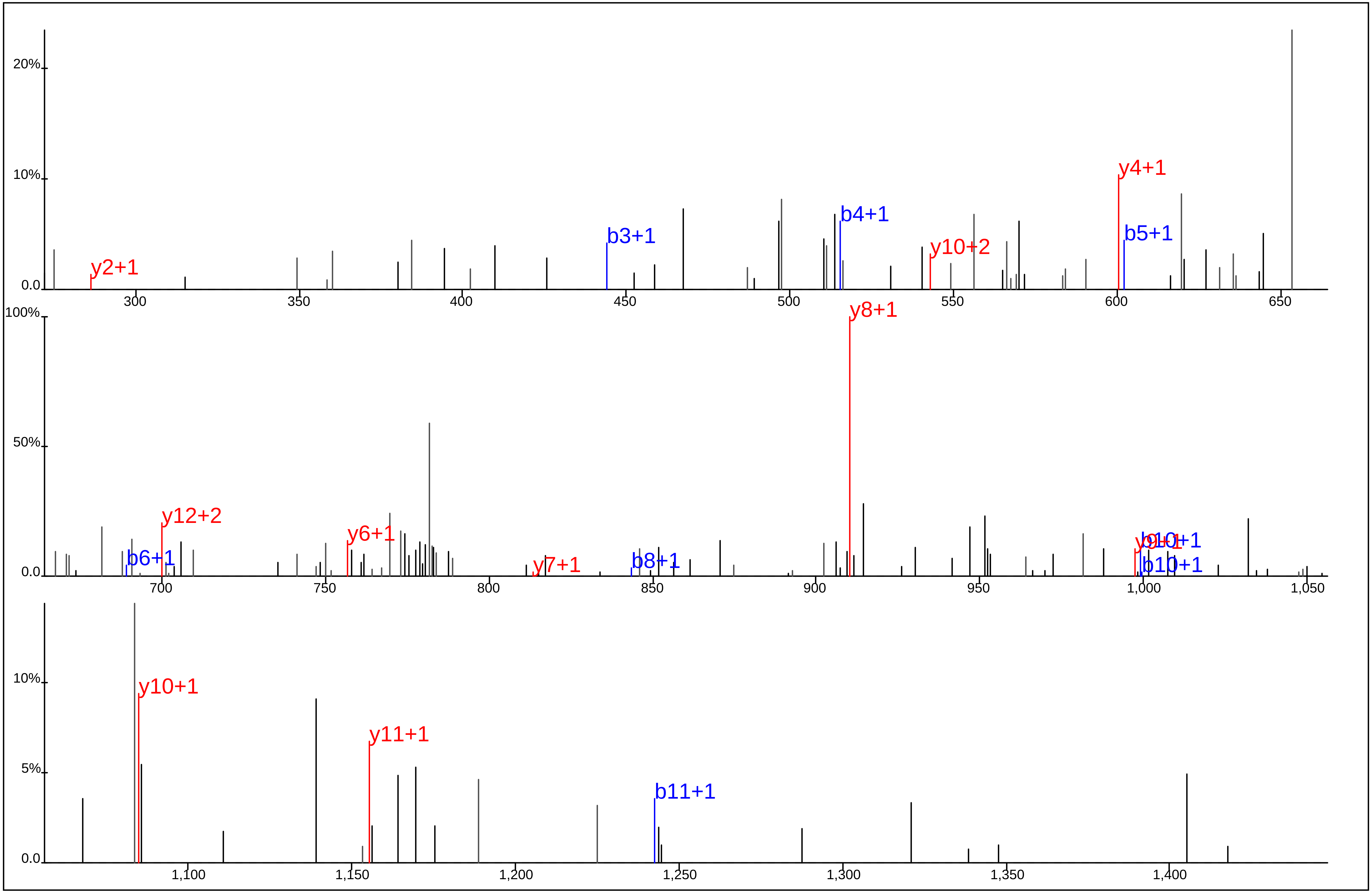

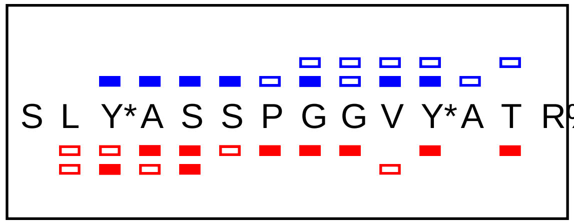

Protein Name: Vimentin
gi Accession: 37848
Precursor Mass: 799.8319+2
Delta Mass: 2.413 ppm
XCorr: 19.2

1040 - Vimentin_3441704.pdf

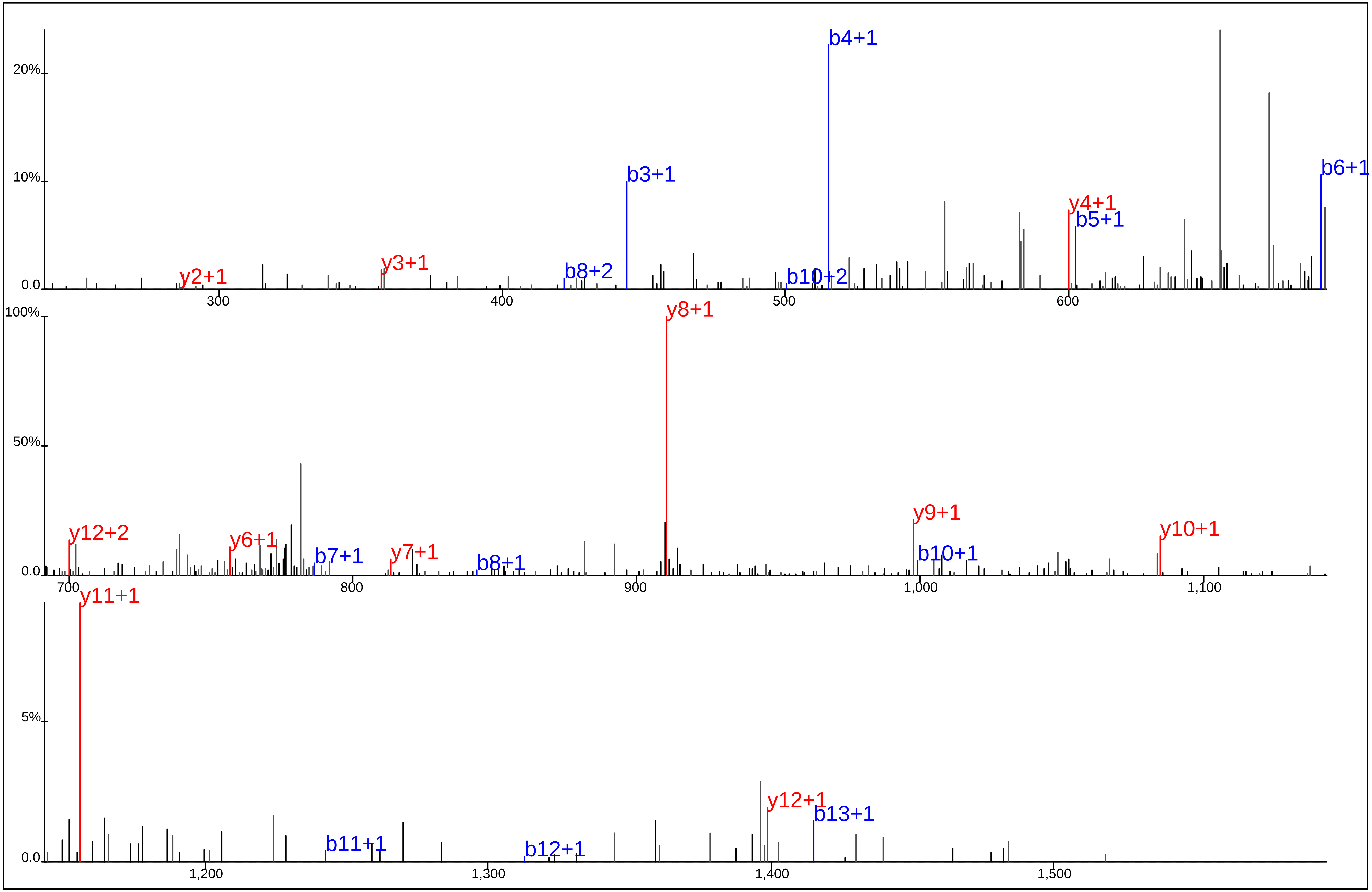

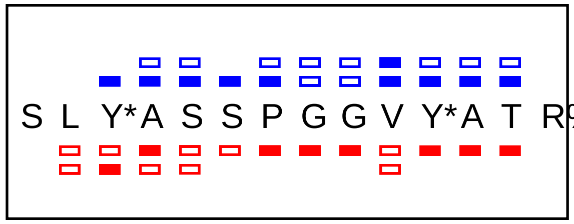

Protein Name: Vimentin
gi Accession: 37848
Precursor Mass: 799.8340+2
Delta Mass: 5.009 ppm
XCorr: 34.23

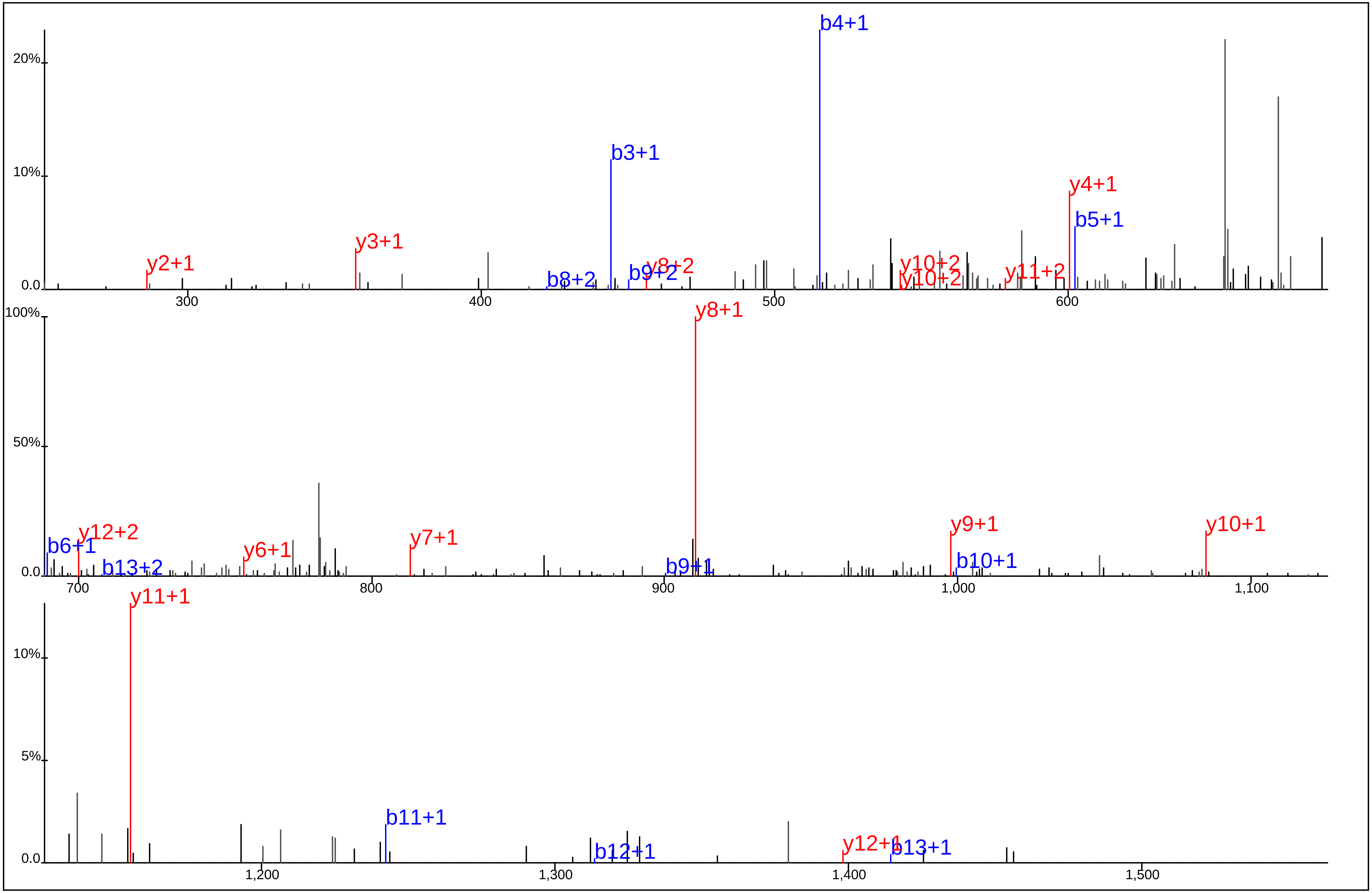

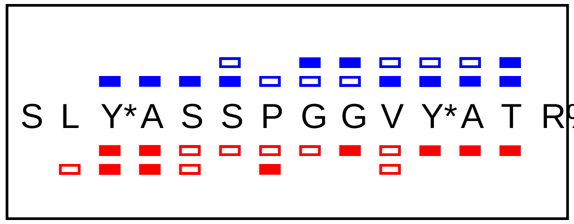

Protein Name: Vimentin
gi Accession: 37848
Precursor Mass: 799.8385+2
Delta Mass: 10.664 ppm
XCorr: 50.24

1042 - Vimentin_3447513.pdf

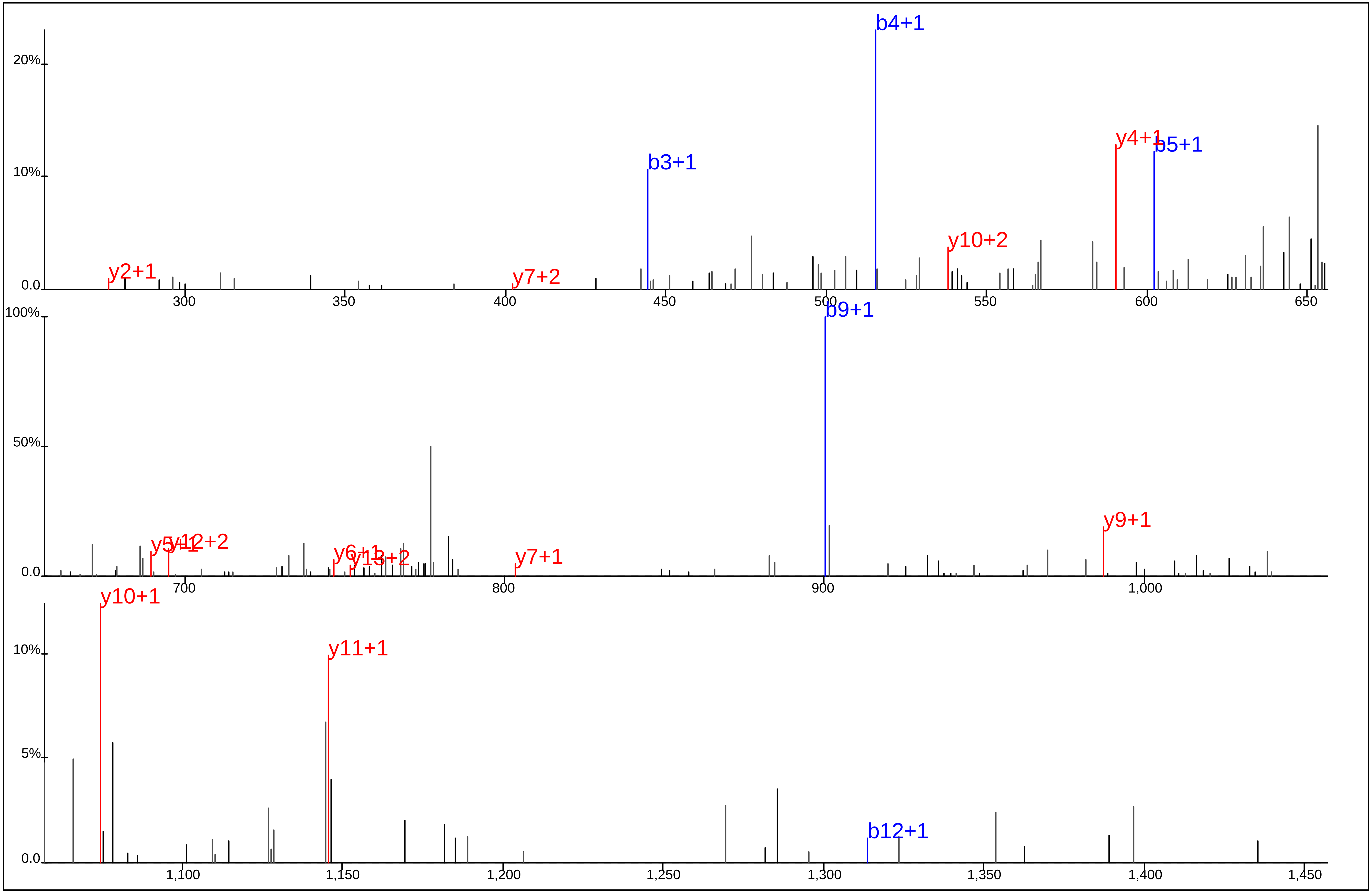

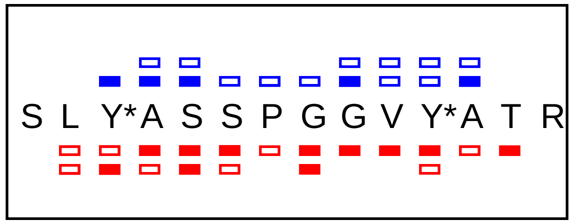

Protein Name: Vimentin
gi Accession: 37848
Precursor Mass: 794.8290+2
Delta Mass: 4.027 ppm
XCorr: 23.06

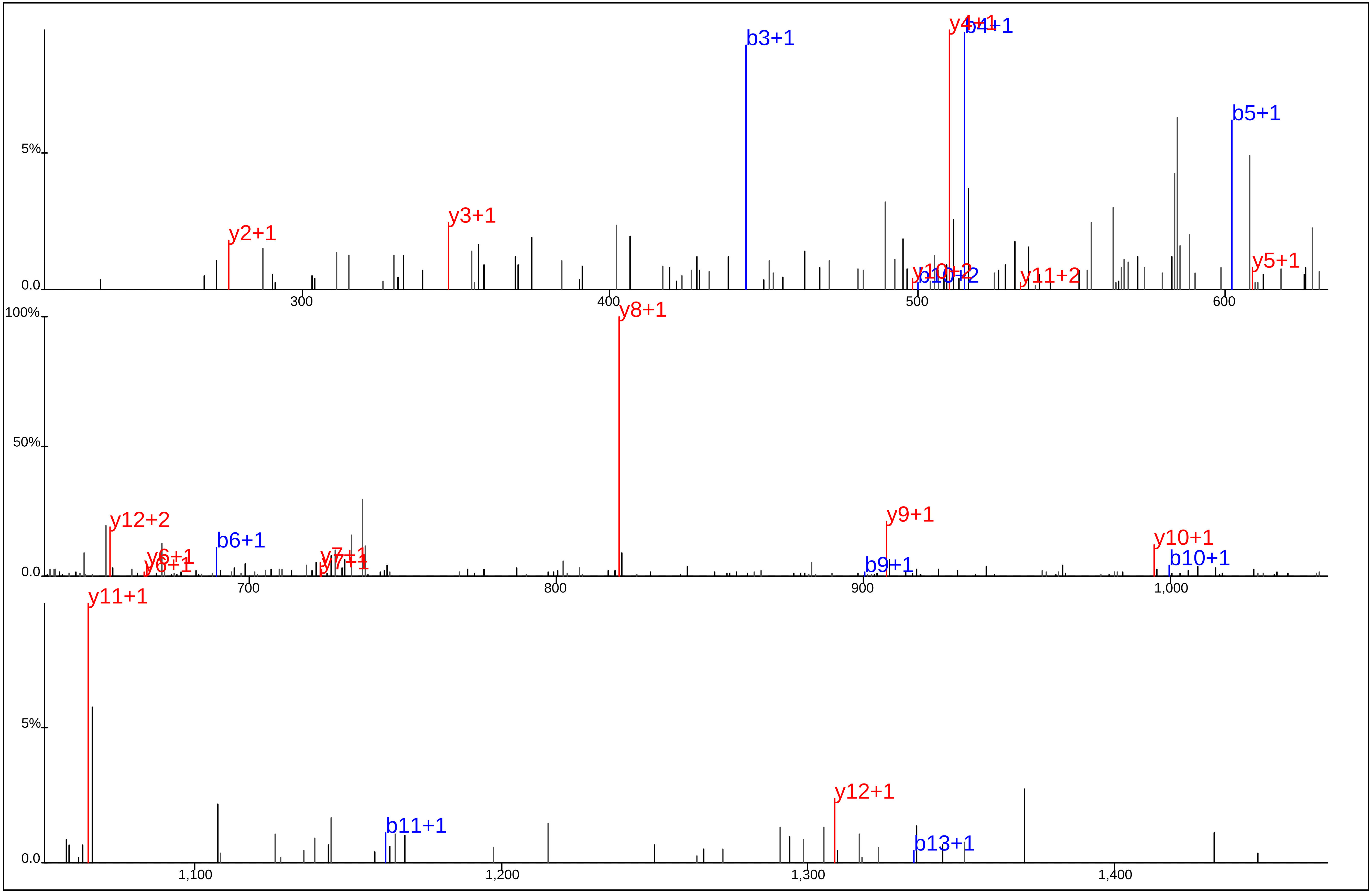

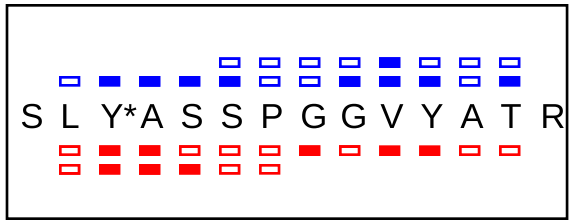

Protein Name: Vimentin
gi Accession: 37848
Precursor Mass: 754.8446+2
Delta Mass: 2.551 ppm
XCorr: 53.24

1044 - Vimentin_3448195.pdf

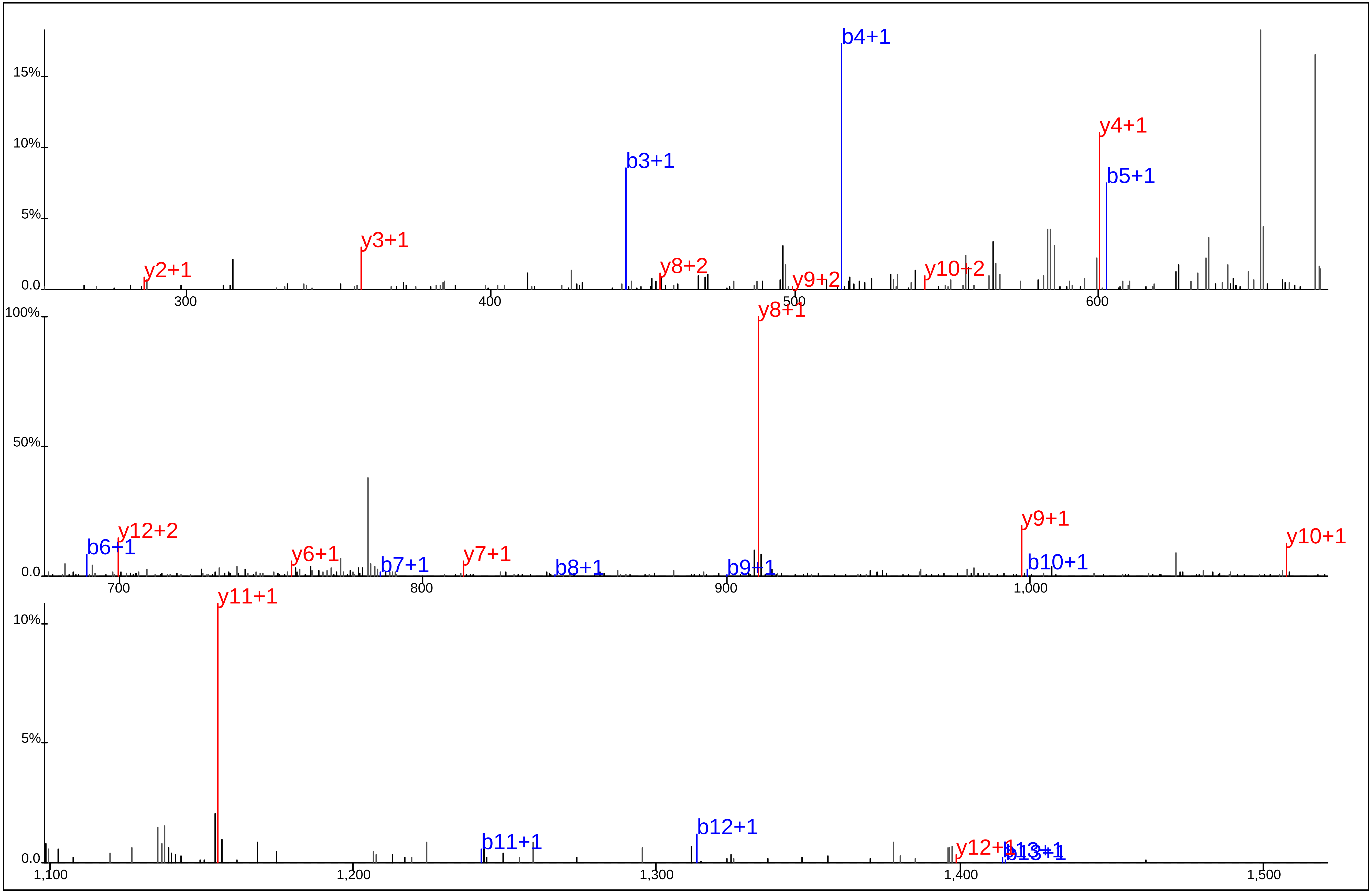

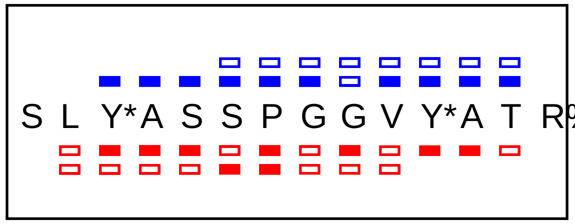

Protein Name: Vimentin
gi Accession: 37848
Precursor Mass: 799.8329+2
Delta Mass: 3.633 ppm
XCorr: 65.82

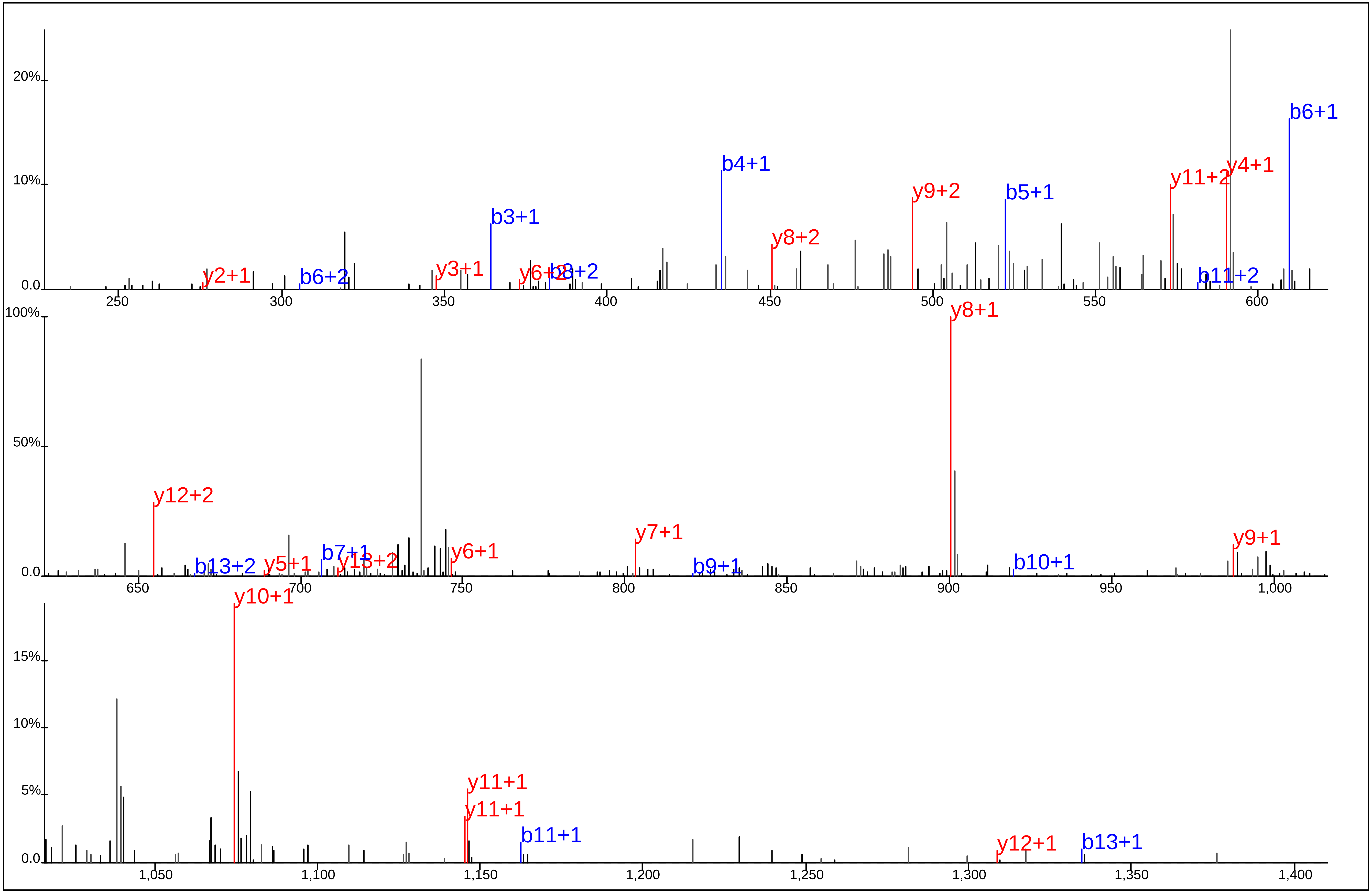

S L Y A S S P G G V Y*A T R

Protein Name: Vimentin
gi Accession: 37848
Precursor Mass: 754.8461+2
Delta Mass: 4.572 ppm
XCorr: 35.03

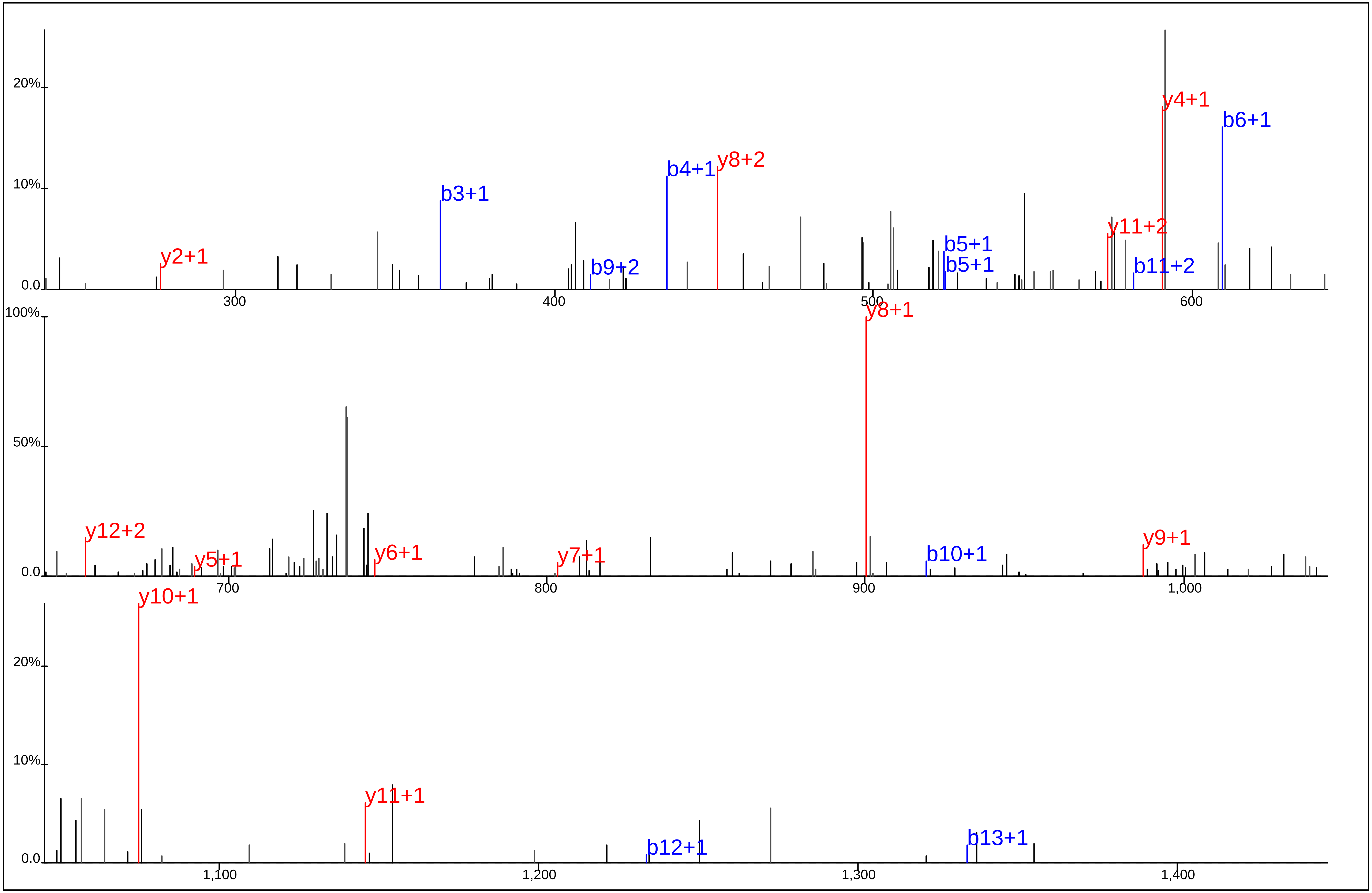

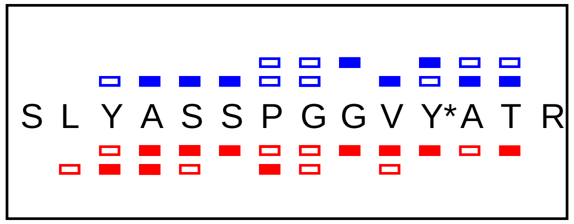

Protein Name: Vimentin
gi Accession: 37848
Precursor Mass: 754.8453+2
Delta Mass: 3.525 ppm
XCorr: 24.37

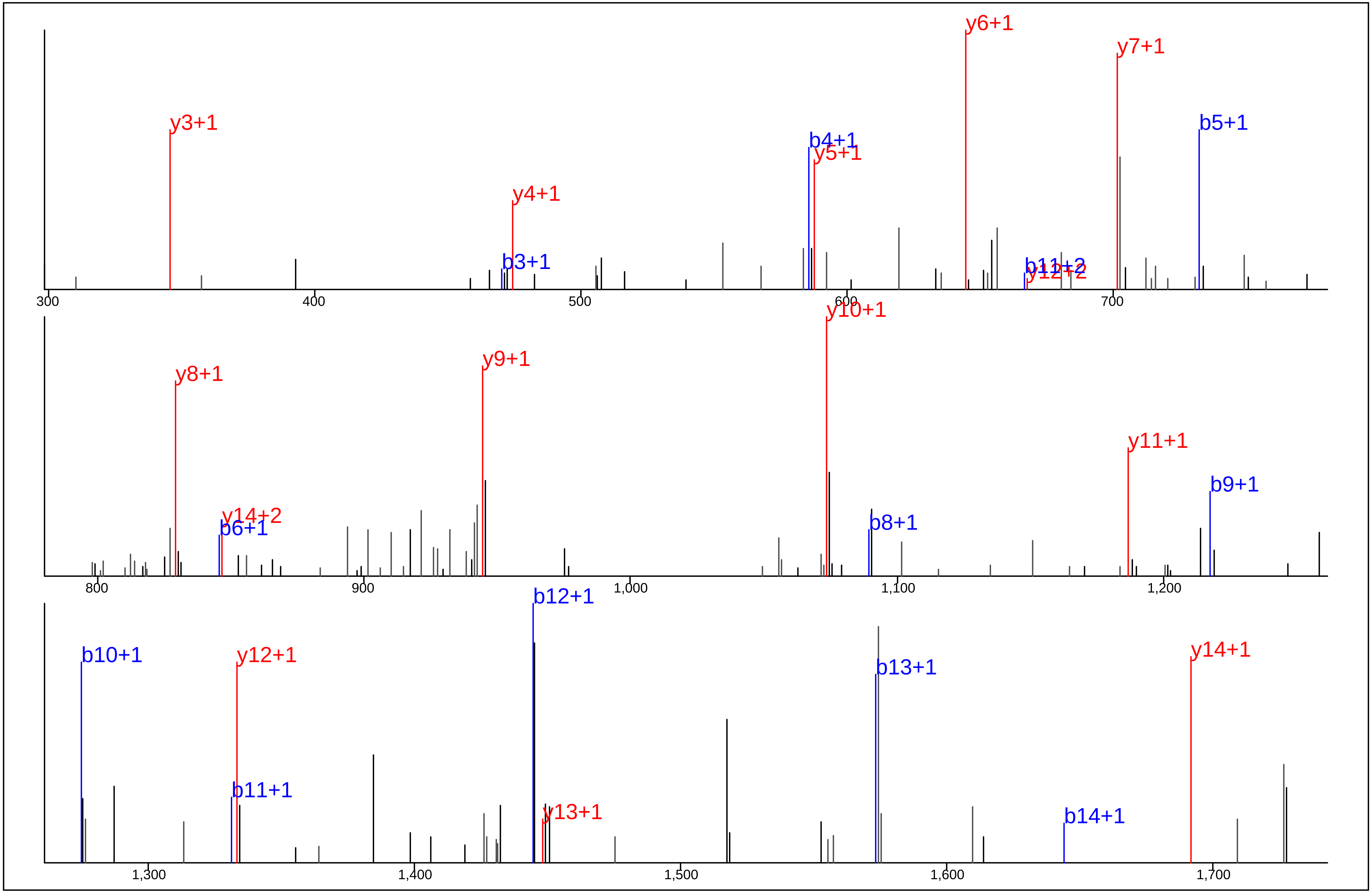

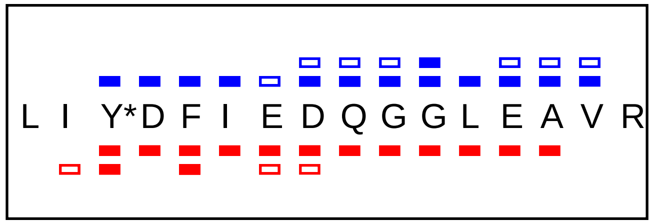

Protein Name:	WASP
gi Accession:	4507909
Precursor Mass:	959.4542+2
Delta Mass:	0.943 ppm
XCorr:	96.33

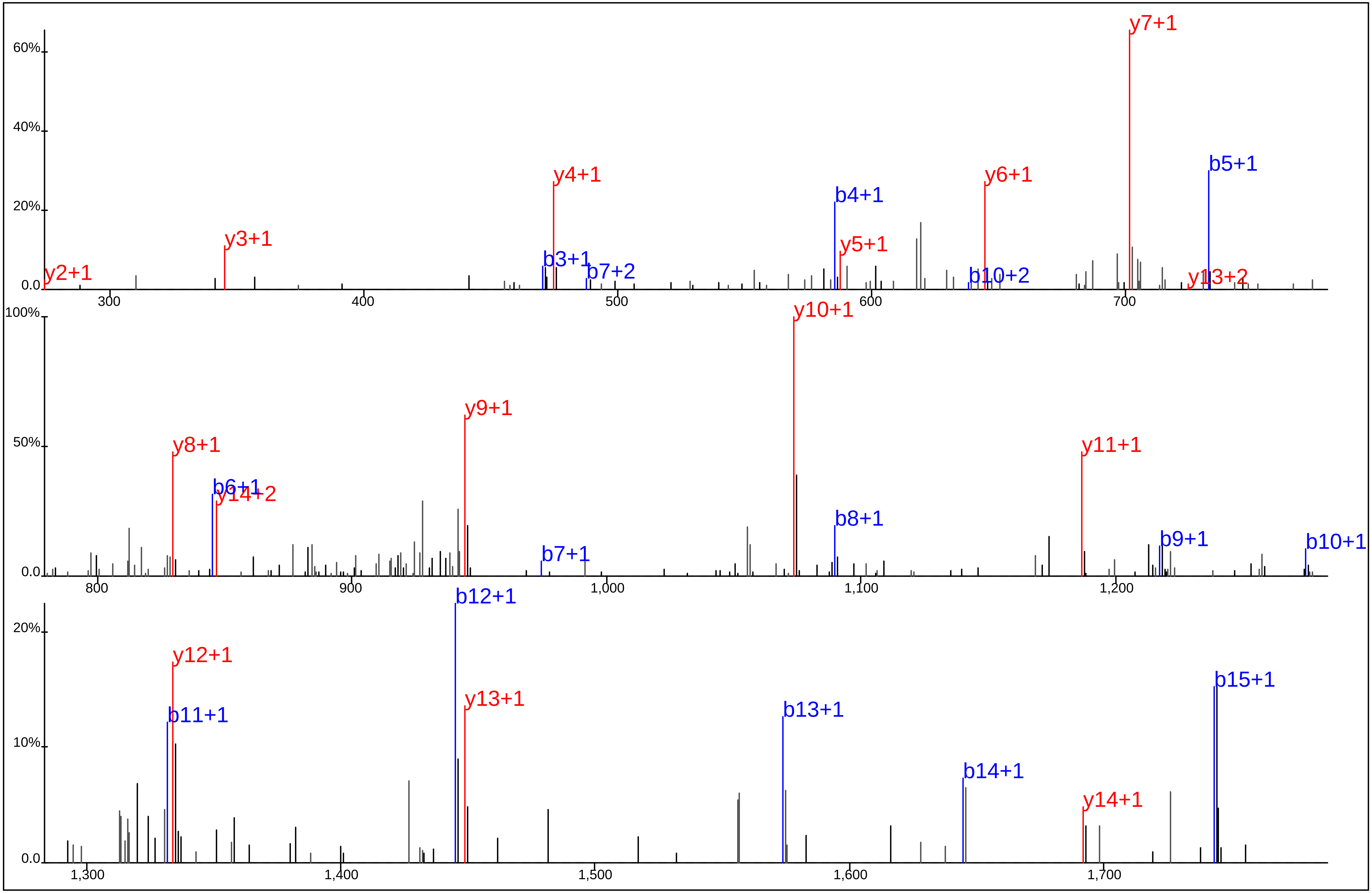

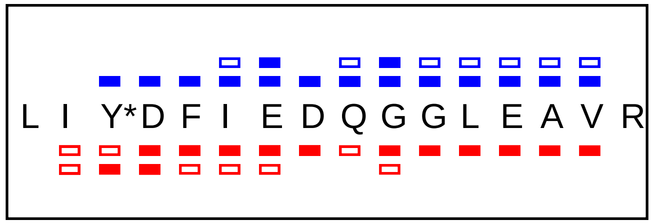

Protein Name:	WASP
gi Accession:	4507909
Precursor Mass:	959.4547+2
Delta Mass:	1.454 ppm
XCorr:	91.08

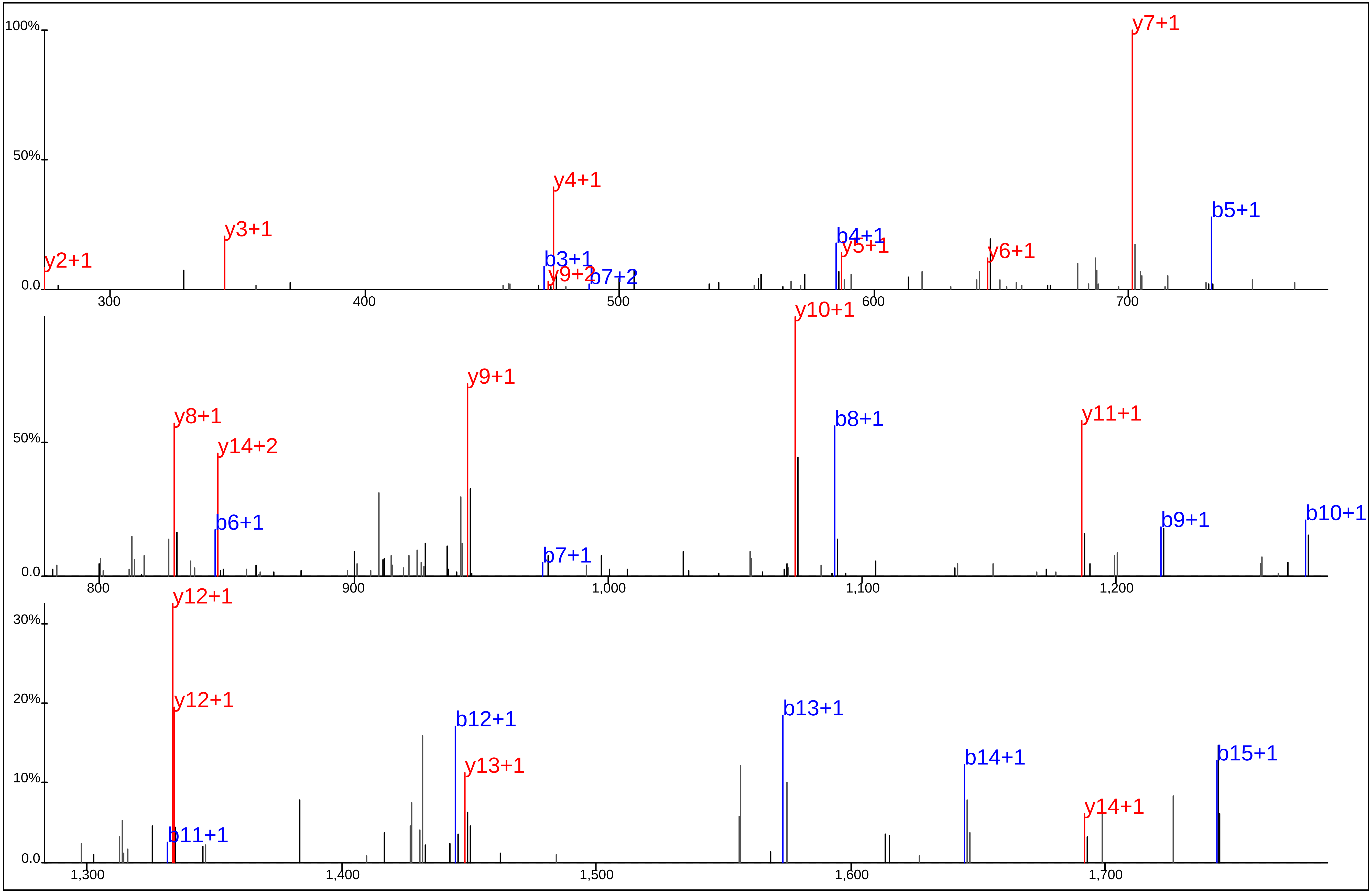

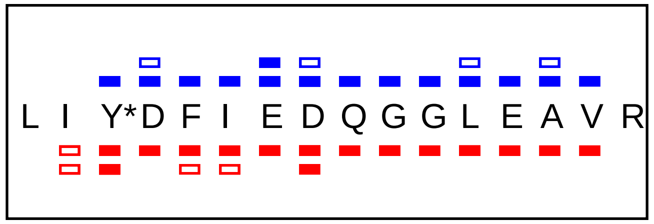

Protein Name: WASP
gi Accession: 4507909
Precursor Mass: 959.4539+2
Delta Mass: 0.625 ppm
XCorr: 82.89

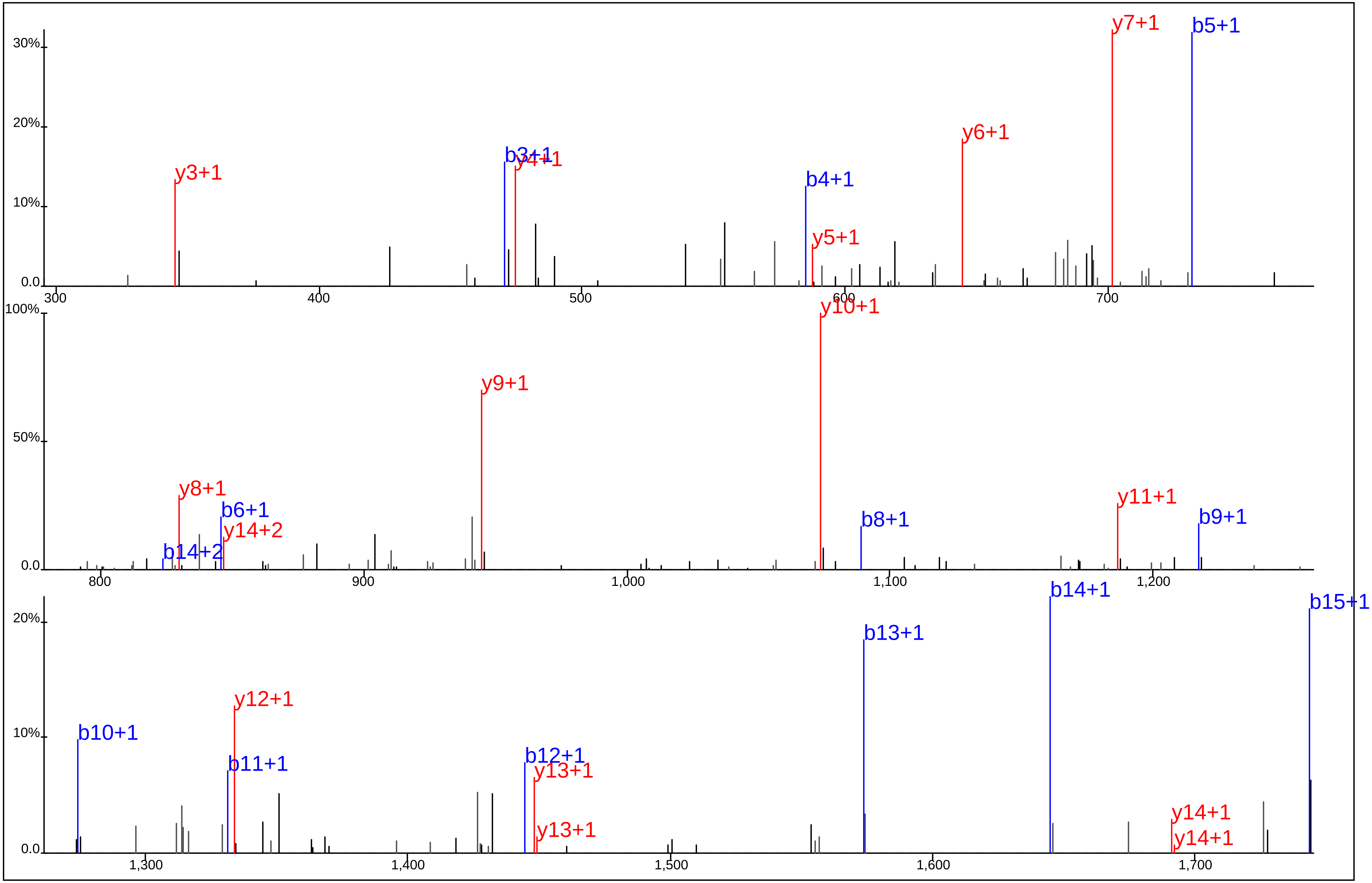

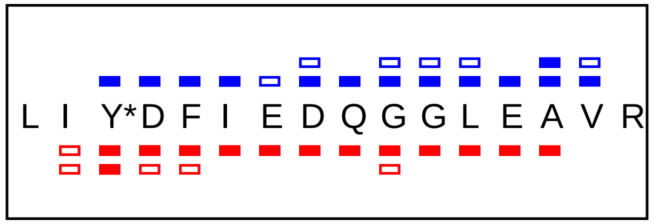

Protein Name: WASP
gi Accession: 4507909
Precursor Mass: 959.4562+2
Delta Mass: 2.981 ppm
XCorr: 118.46

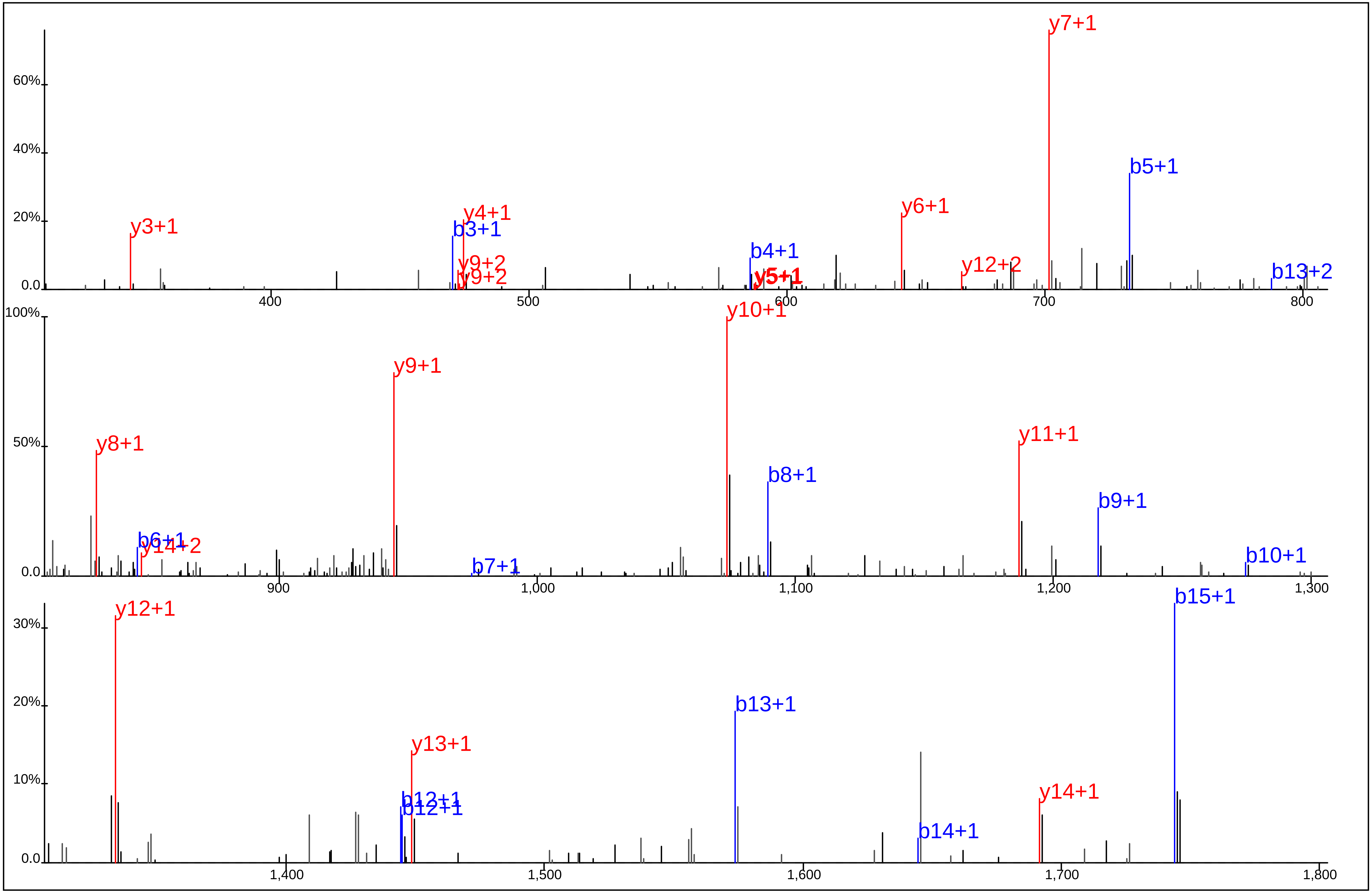

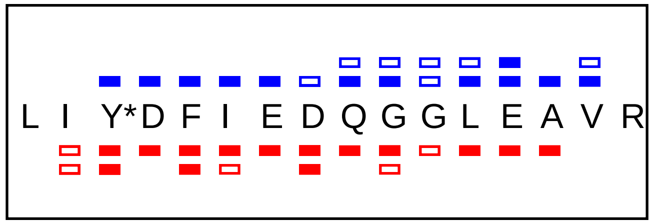

Protein Name: WASP
gi Accession: 4507909
Precursor Mass: 959.4556+2
Delta Mass: 2.345 ppm
XCorr: 84.15

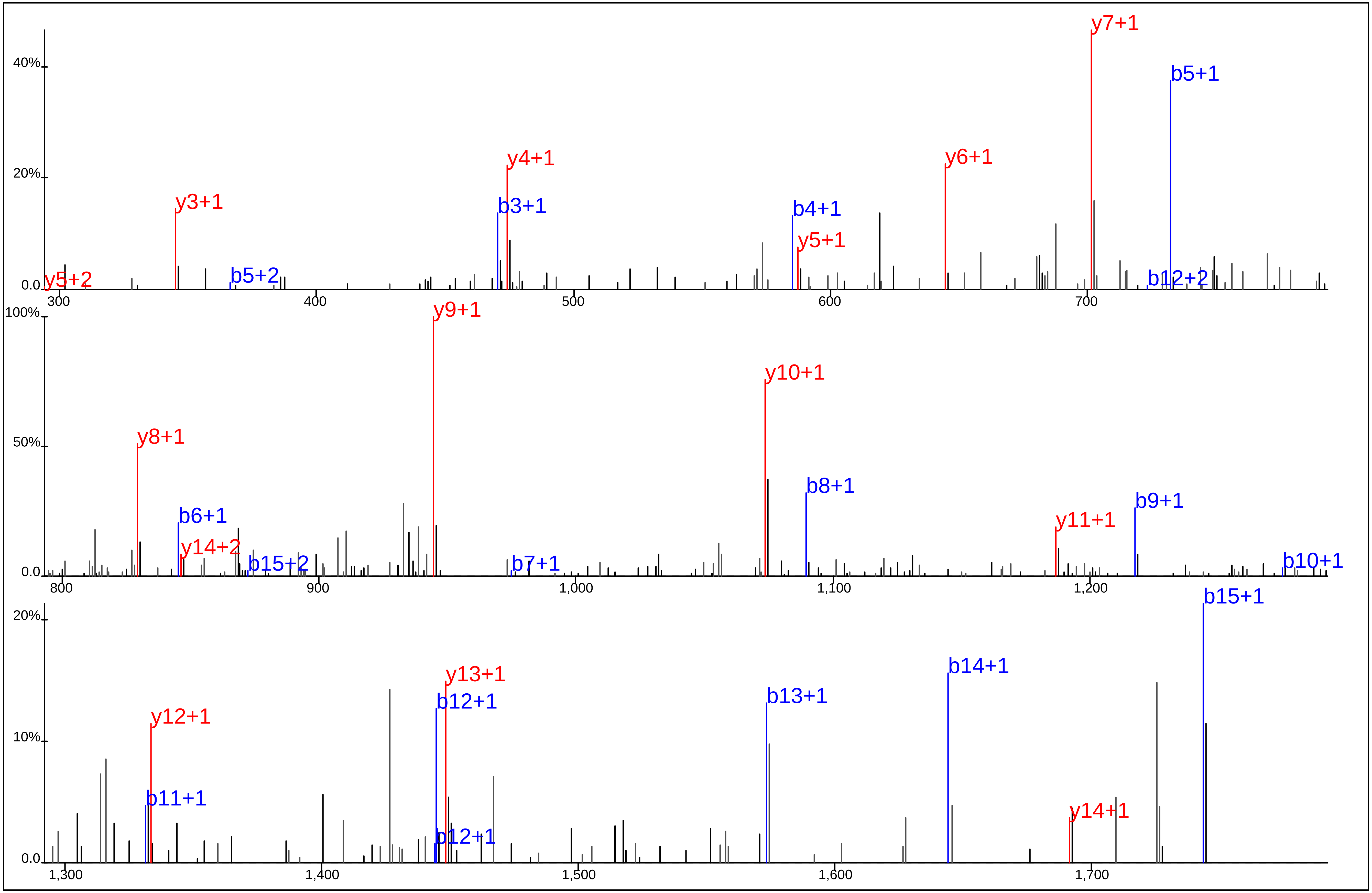

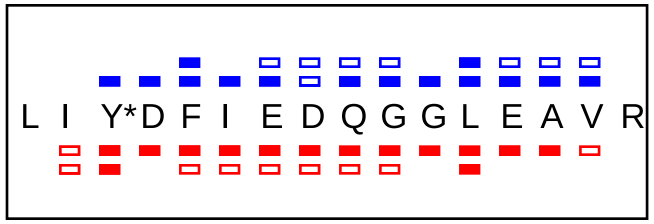

Protein Name: WASP
gi Accession: 4507909
Precursor Mass: 959.4549+2
Delta Mass: 1.641 ppm
XCorr: 102.47

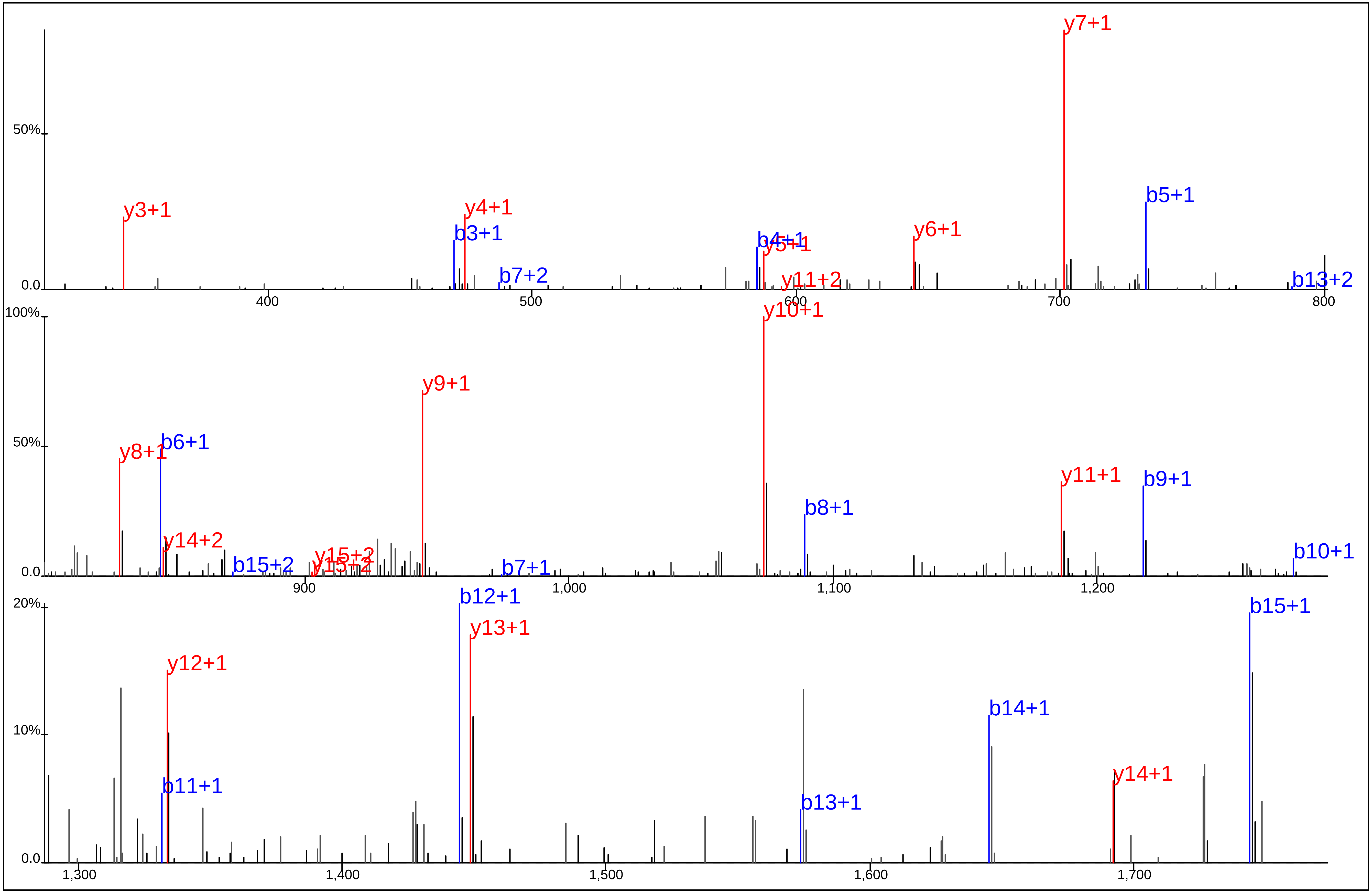

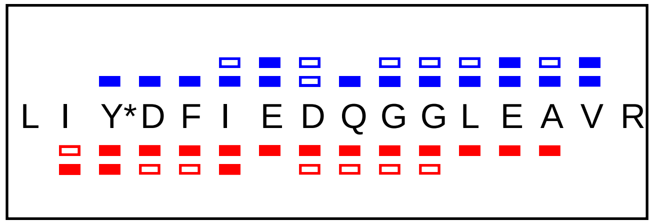

Protein Name:	WASP
gi Accession:	4507909
Precursor Mass:	959.4562+2
Delta Mass:	2.981 ppm
XCorr:	97.39

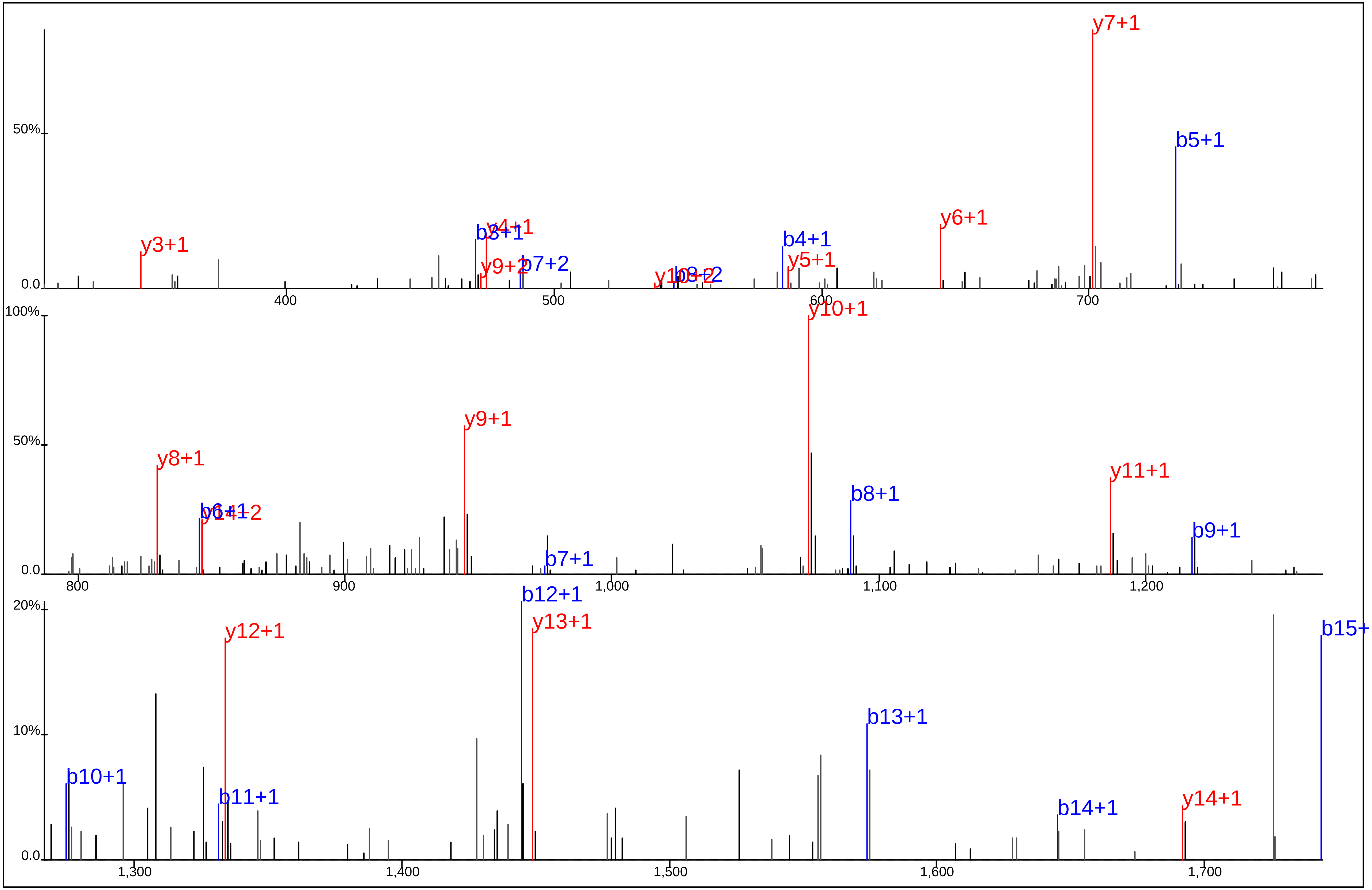

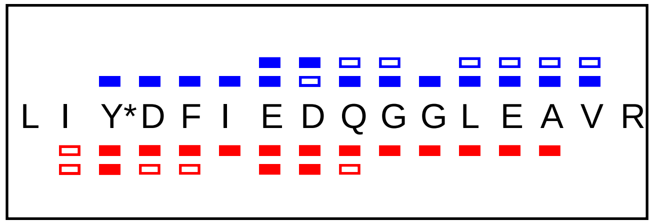

Protein Name: WASP
gi Accession: 4507909
Precursor Mass: 959.4561+2
Delta Mass: 2.851 ppm
XCorr: 102.57

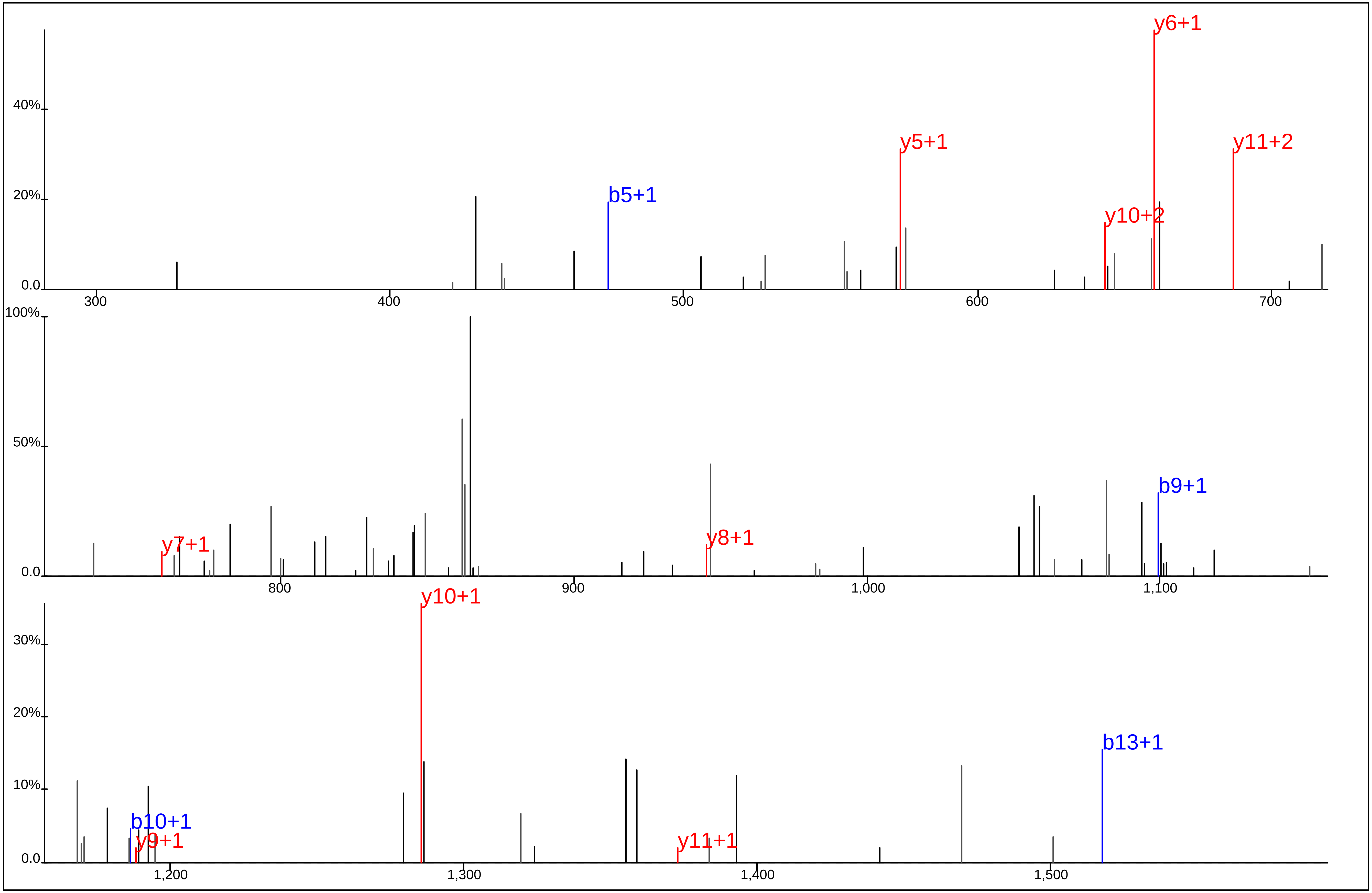

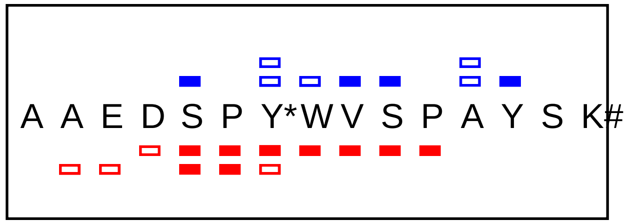

Protein Name: WD repeat domain 70
gi Accession: 12224837
Precursor Mass: 879.8759+2
Delta Mass: 3.202 ppm
XCorr: 12.37

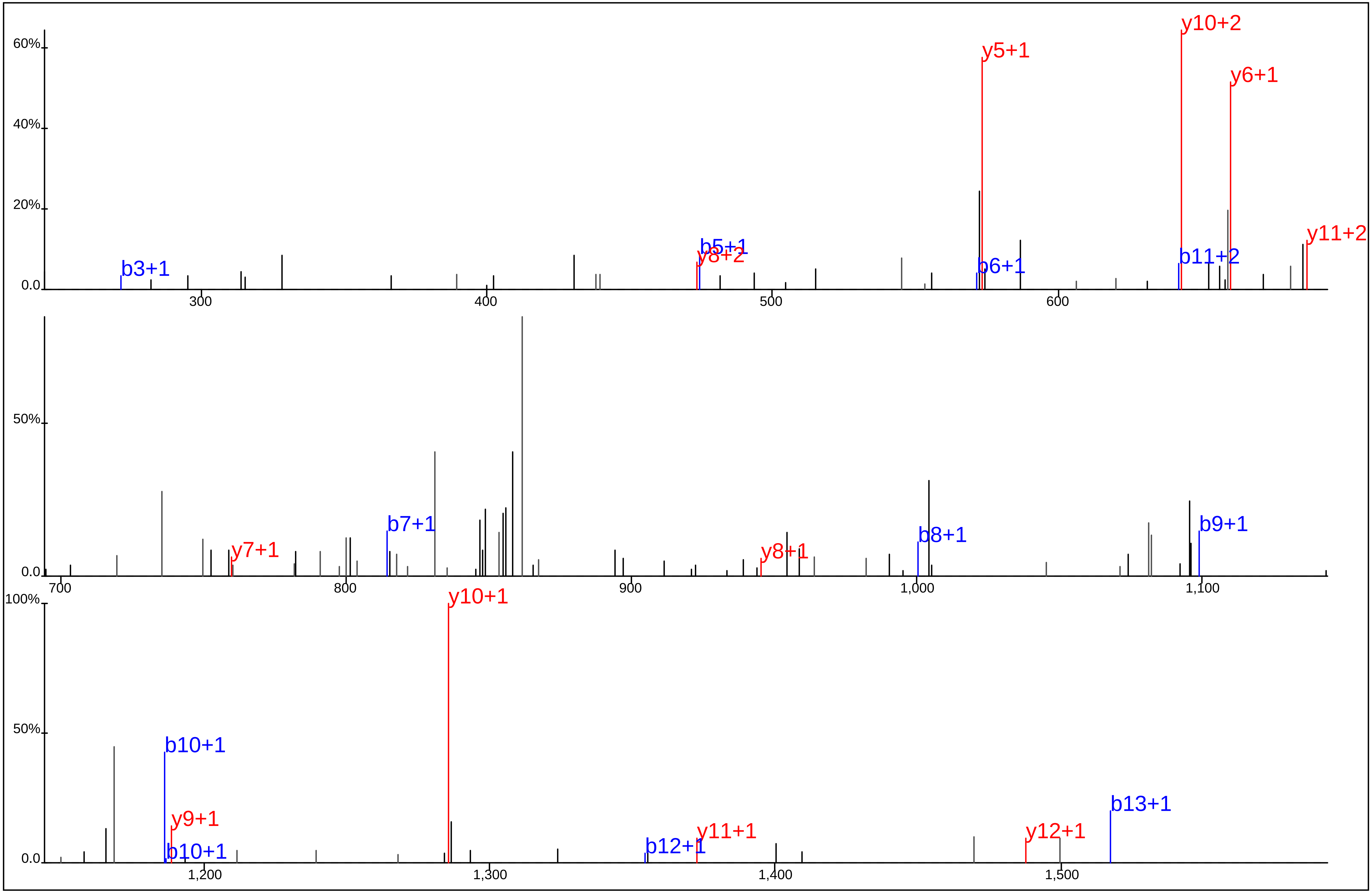

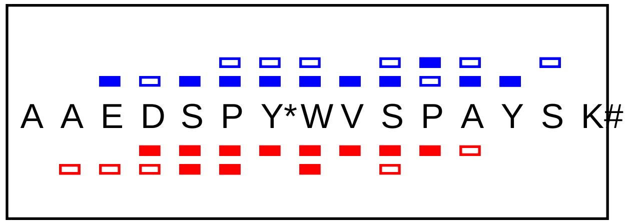

Protein Name: WD repeat domain 70
gi Accession: 12224837
Precursor Mass: 879.8851+2
Delta Mass: 7.282 ppm
XCorr: 33.0

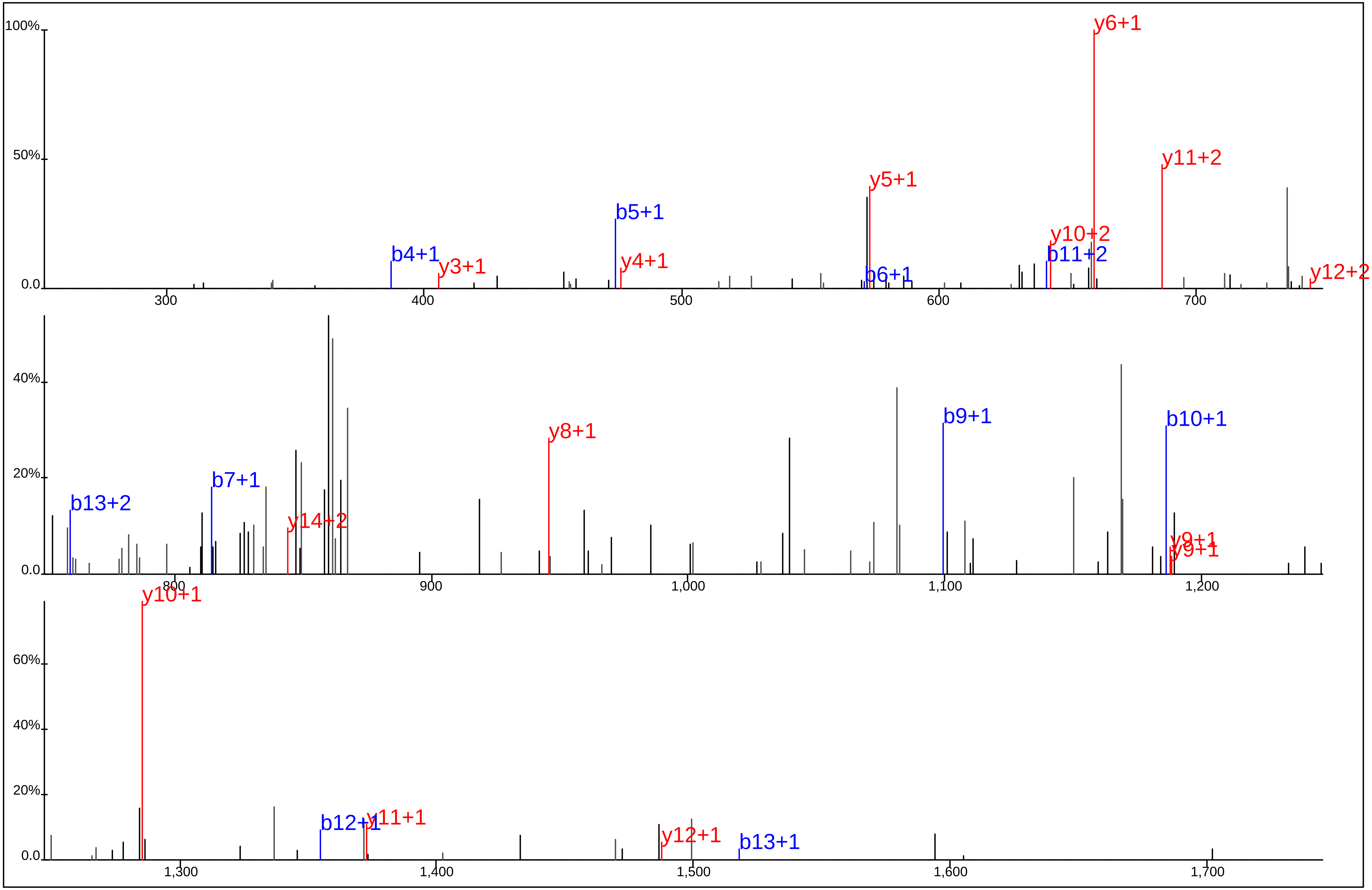

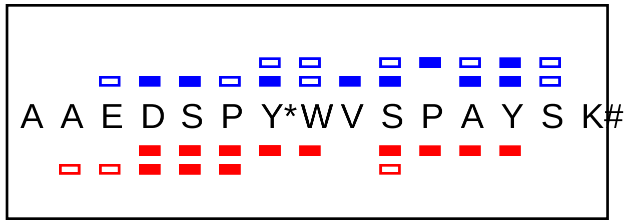

Protein Name: WD repeat domain 70
gi Accession: 12224837
Precursor Mass: 879.8849+2
Delta Mass: 7.004 ppm
XCorr: 34.41

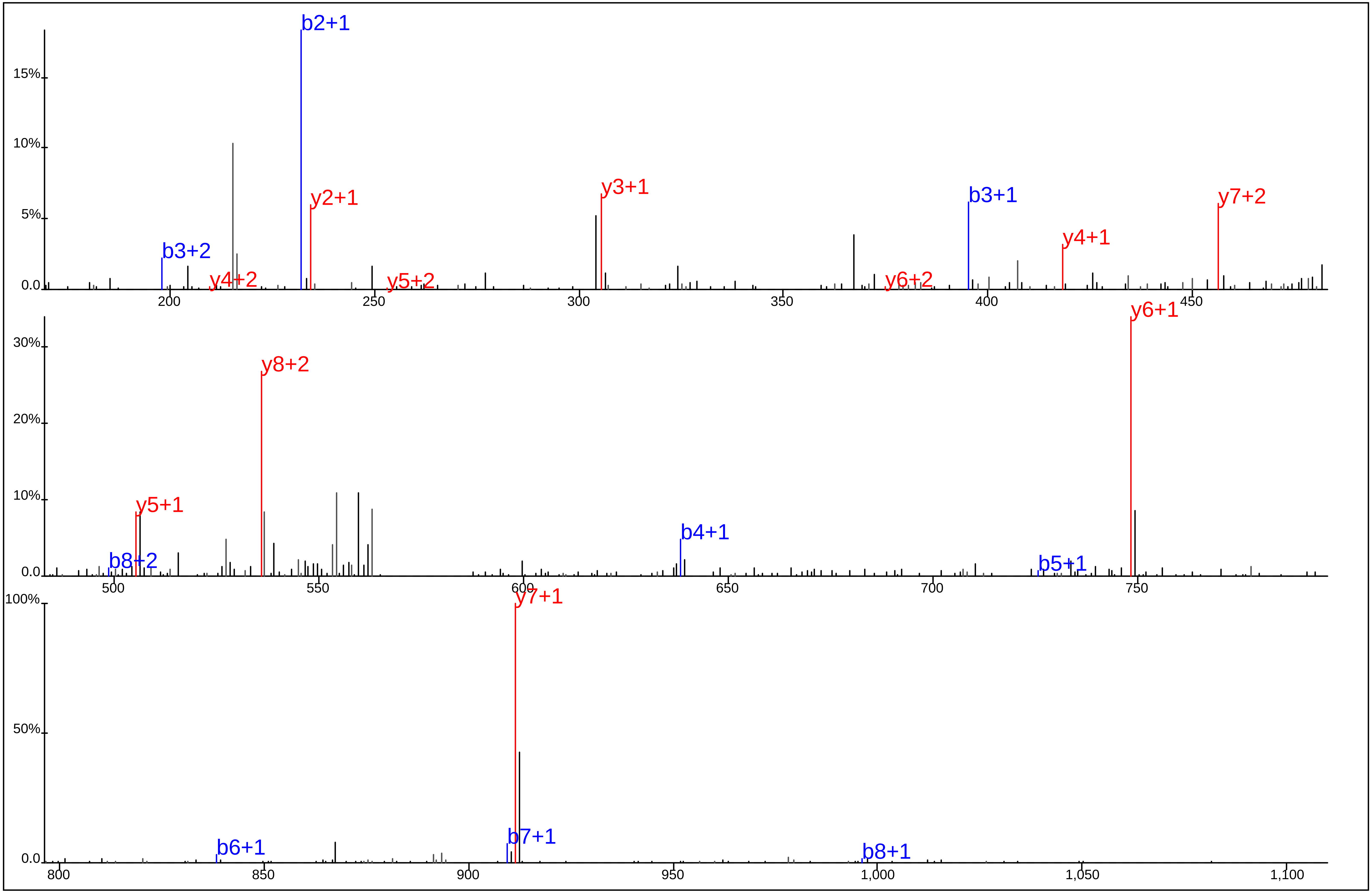

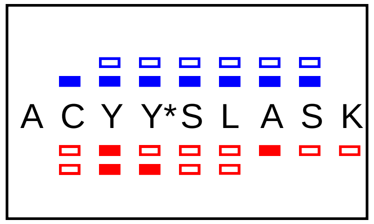

Protein Name: ZAP70
gi Accession: 31455611
Precursor Mass: 571.7337+2
Delta Mass: 1.206 ppm
XCorr: 35.16

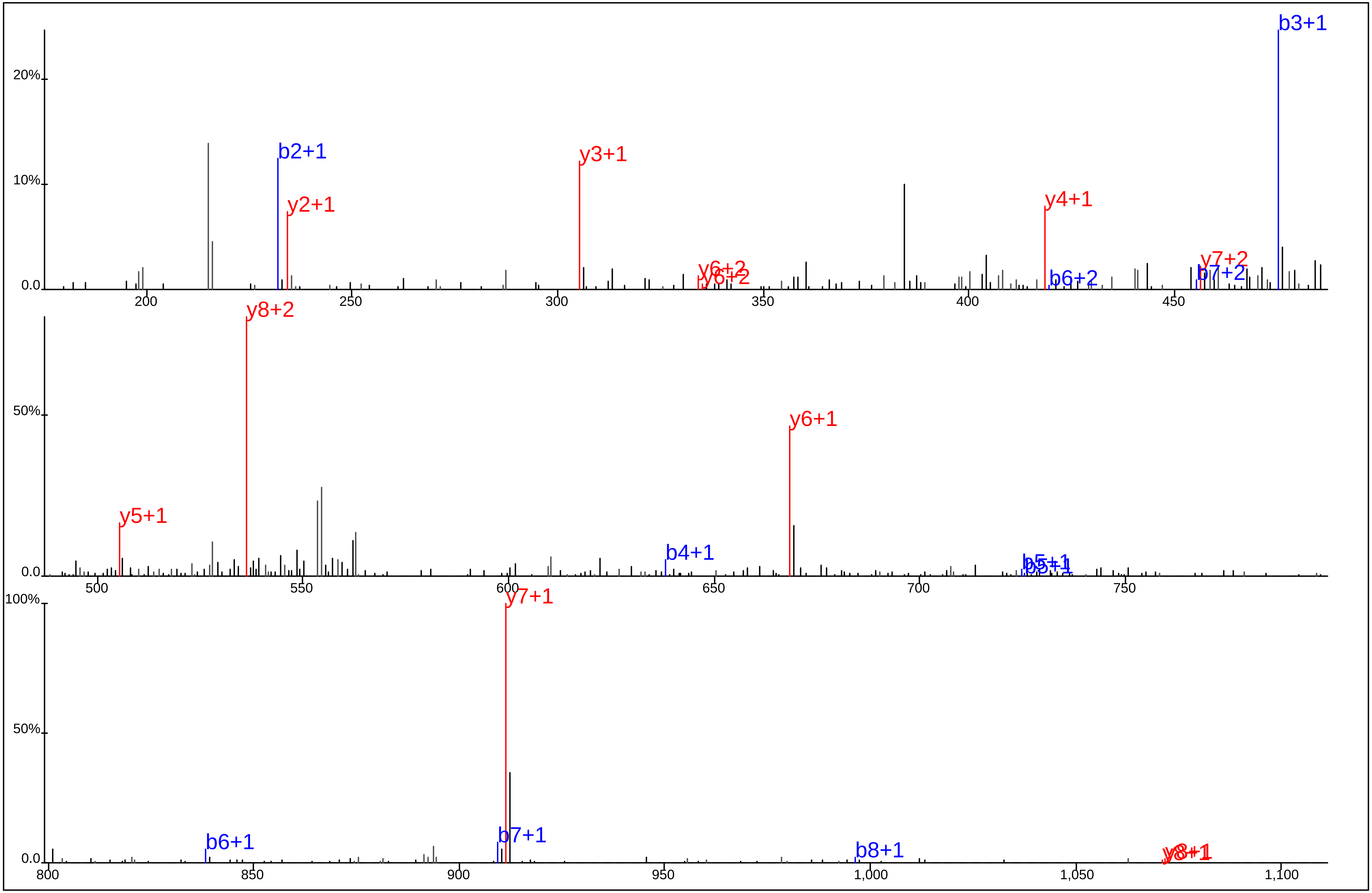

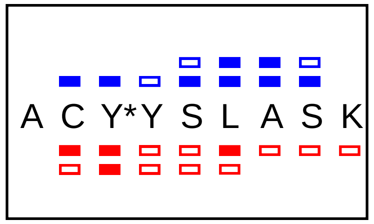

Protein Name: ZAP70
gi Accession: 31455611
Precursor Mass: 571.7338+2
Delta Mass: 1.311 ppm
XCorr: 35.23

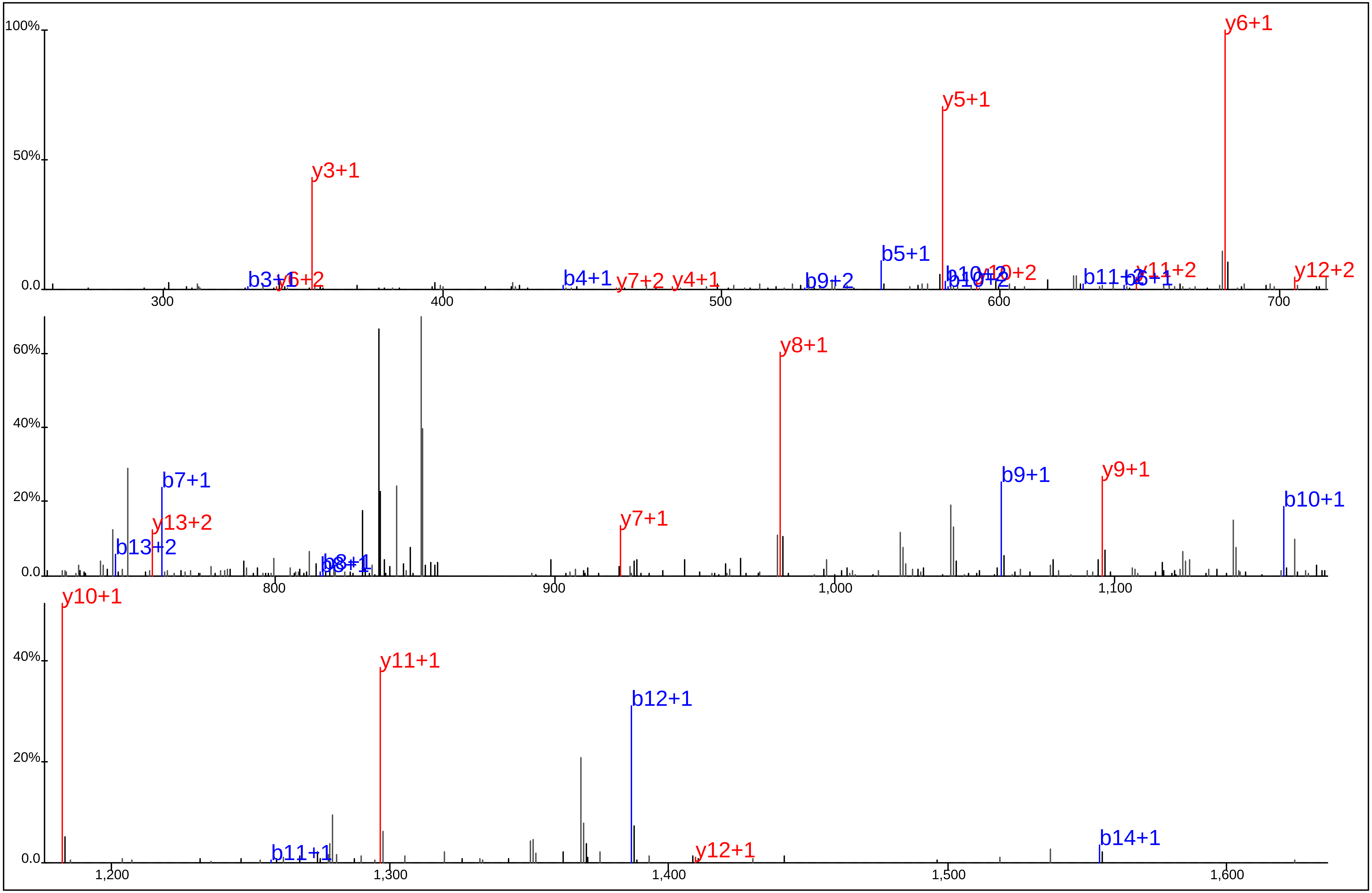

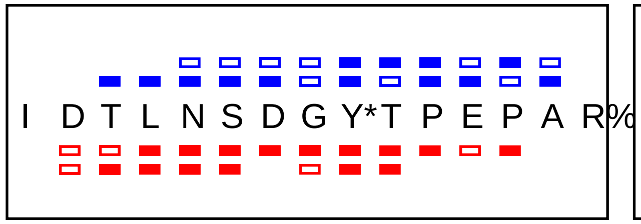

Protein Name: ZAP70
gi Accession: 31455611
Precursor Mass: 869.8841+2
Delta Mass: 2.892 ppm
XCorr: 74.21

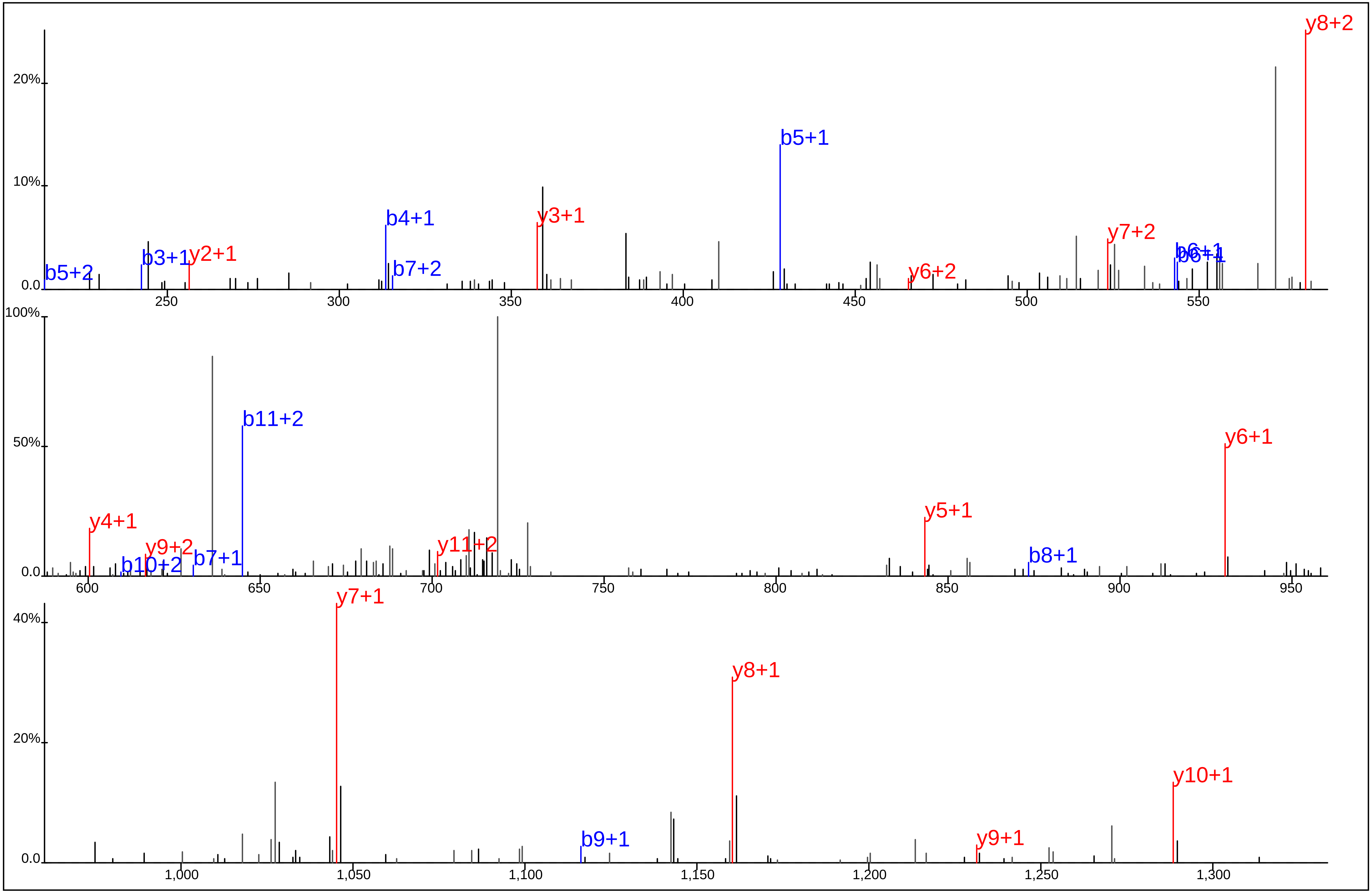

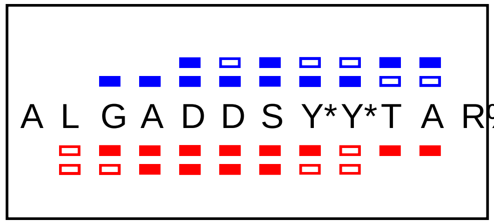

Protein Name: ZAP70
gi Accession: 31455611
Precursor Mass: 736.7722+2
Delta Mass: 0.128 ppm
XCorr: 53.24

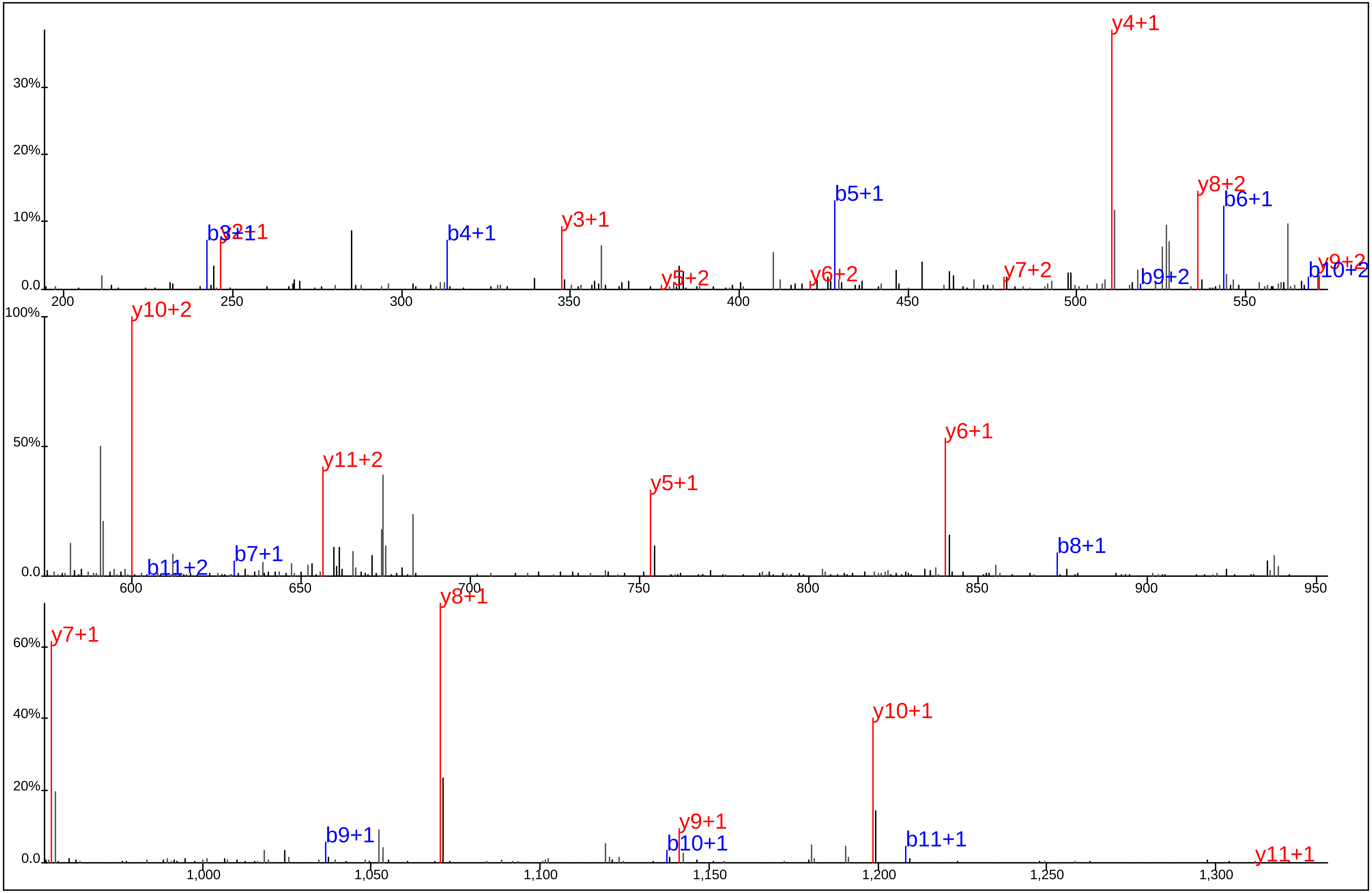

A L G A D D S Y*Y T A R

Protein Name:	ZAP70
gi Accession:	31455611
Precursor Mass:	691.7853+2
Delta Mass:	0.744 ppm
XCorr:	81.16

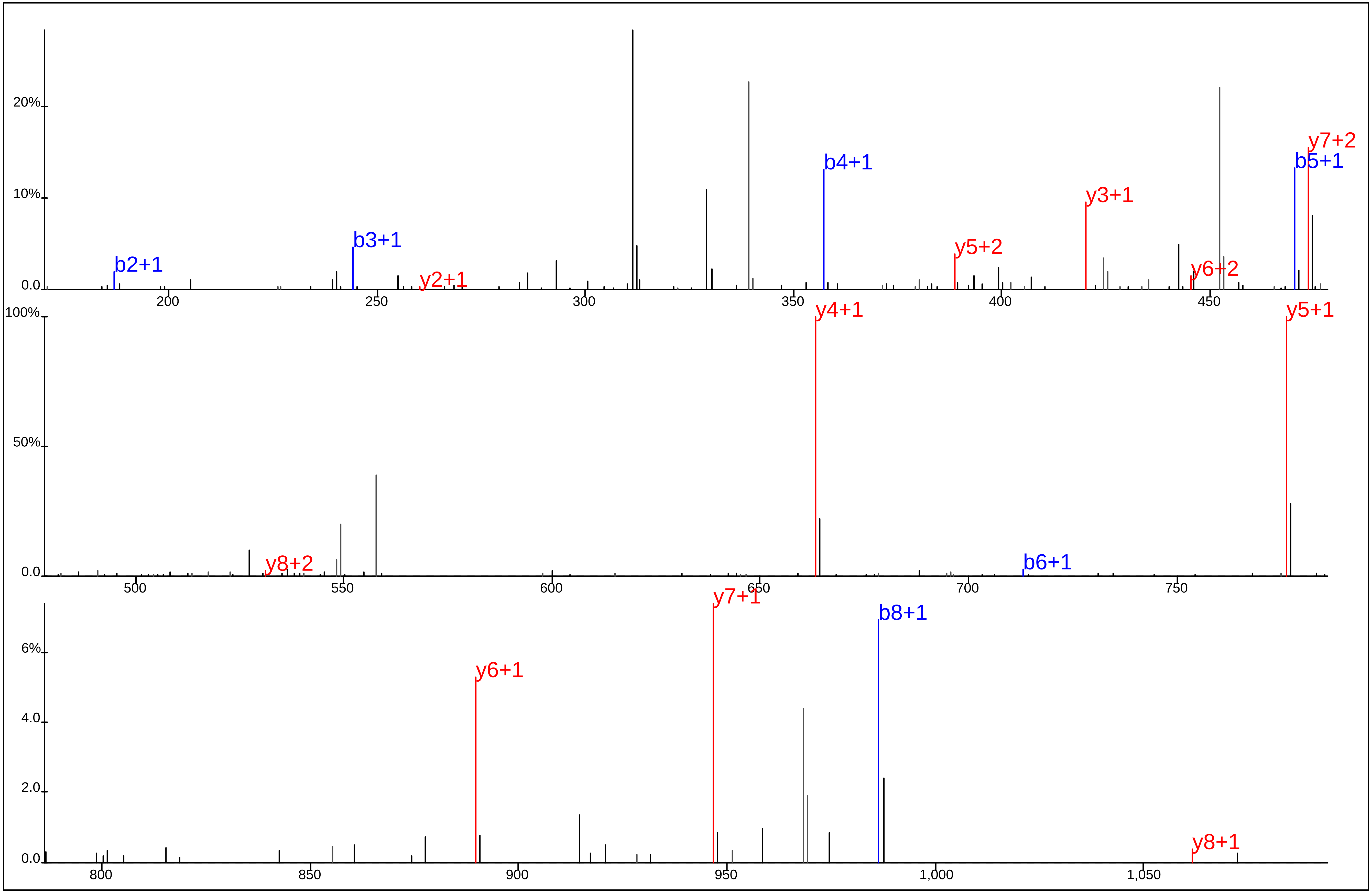

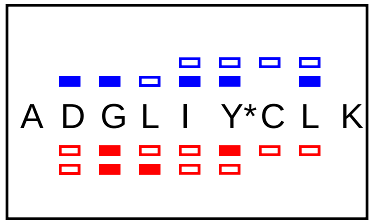

Protein Name: ZAP70
gi Accession: 31455611
Precursor Mass: 566.7620+2
Delta Mass: 5.164 ppm
XCorr: 36.63

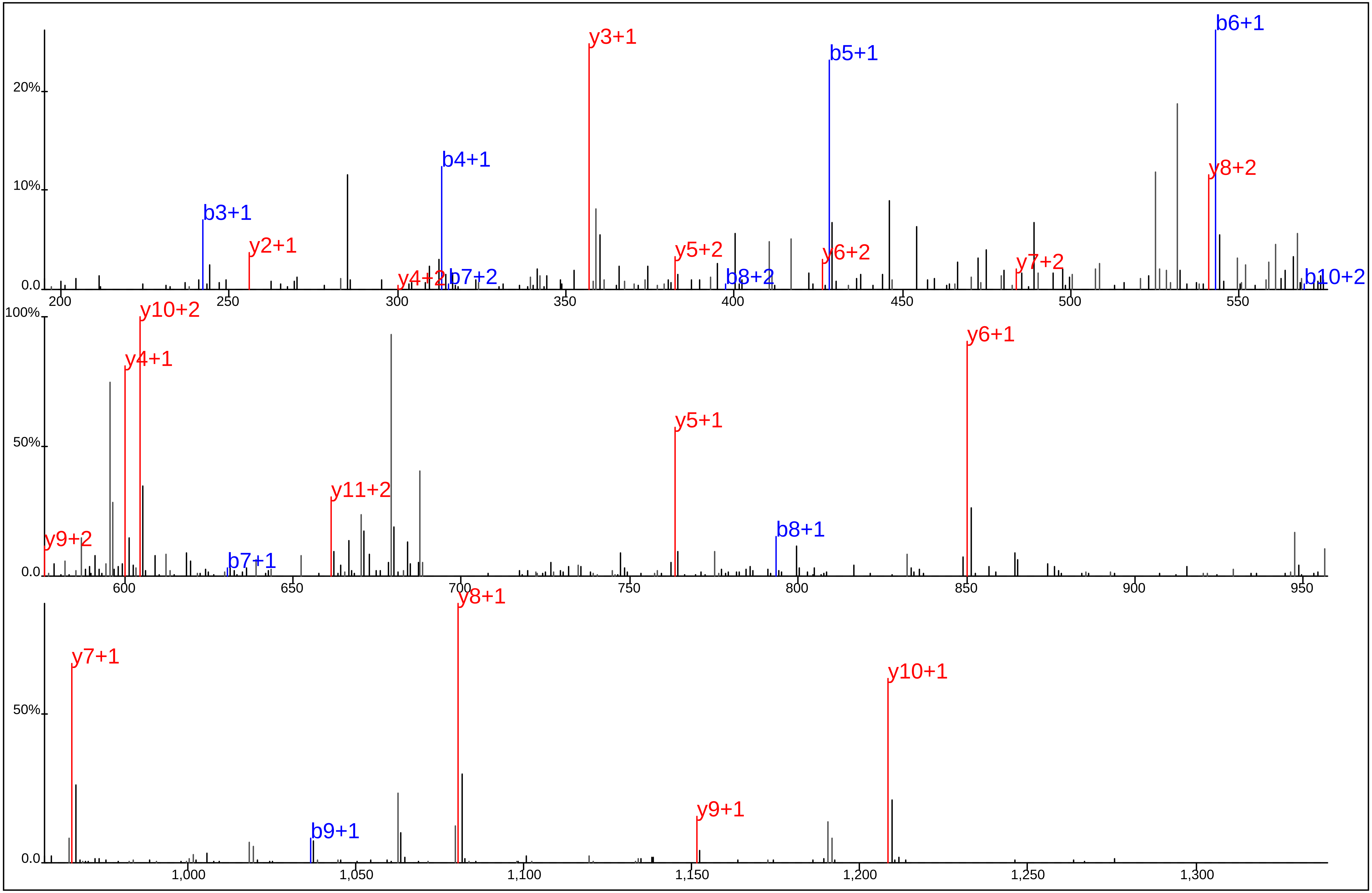

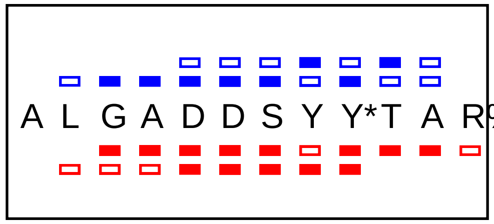

Protein Name:	ZAP70
gi Accession:	31455611
Precursor Mass:	696.7888+2
Delta Mass:	0.203 ppm
XCorr:	61.93

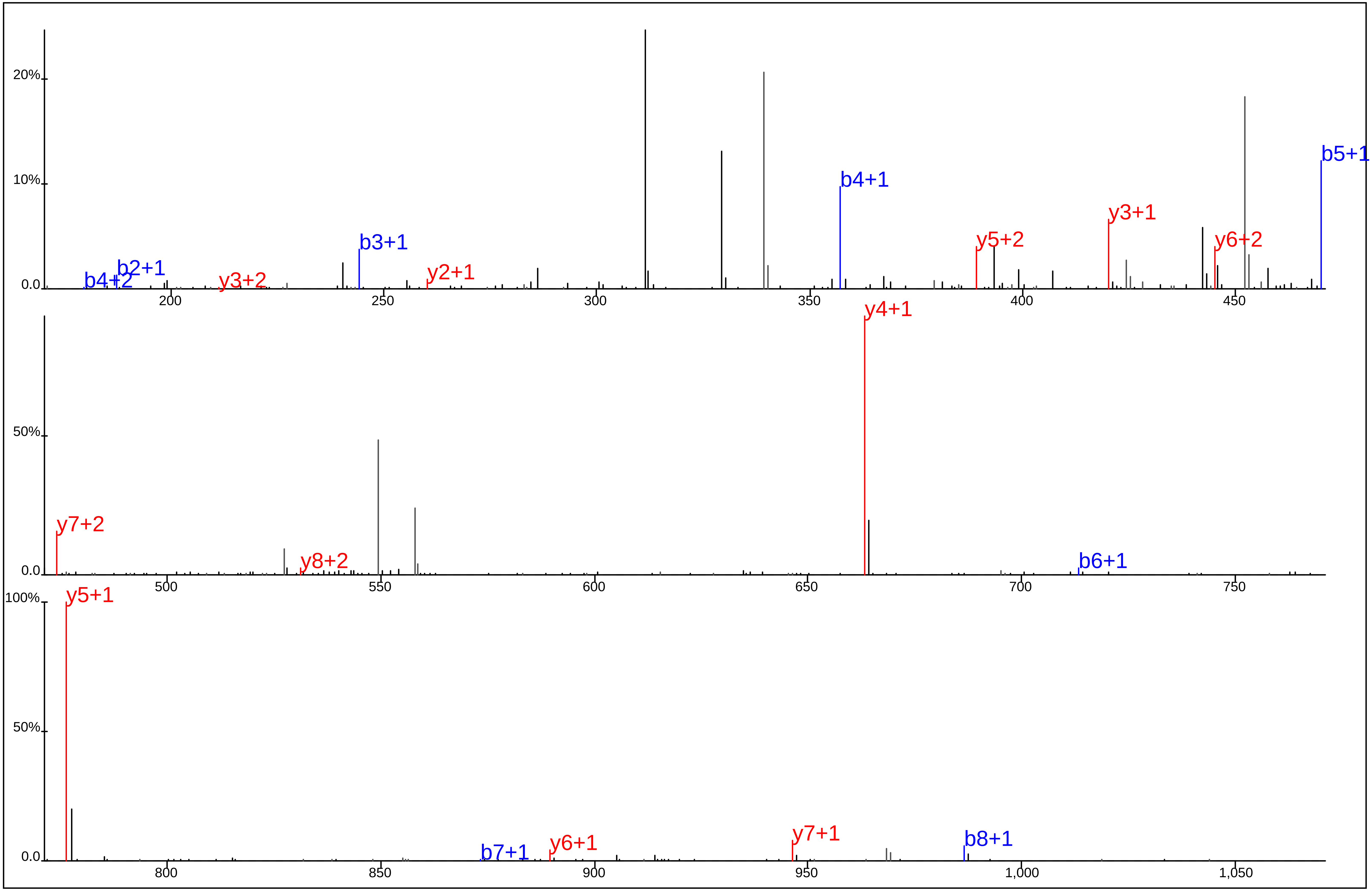

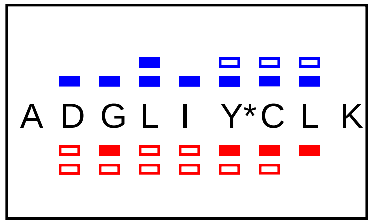

Protein Name: ZAP70
gi Accession: 31455611
Precursor Mass: 566.7606+2
Delta Mass: 2.683 ppm
XCorr: 34.37

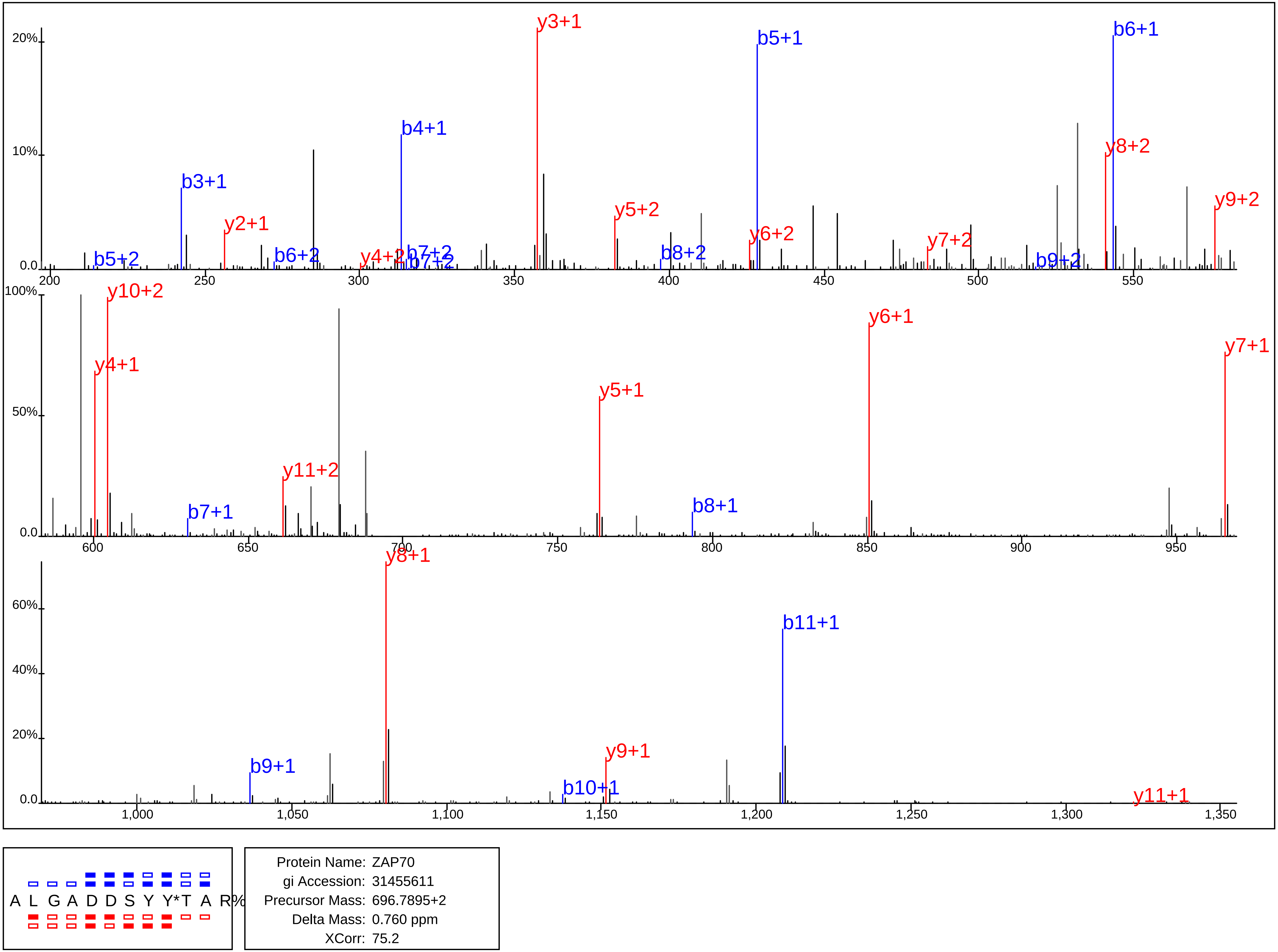

Protein Name: ZAP70
gi Accession: 31455611
Precursor Mass: 696.7895+2
Delta Mass: 0.760 ppm
XCorr: 75.2

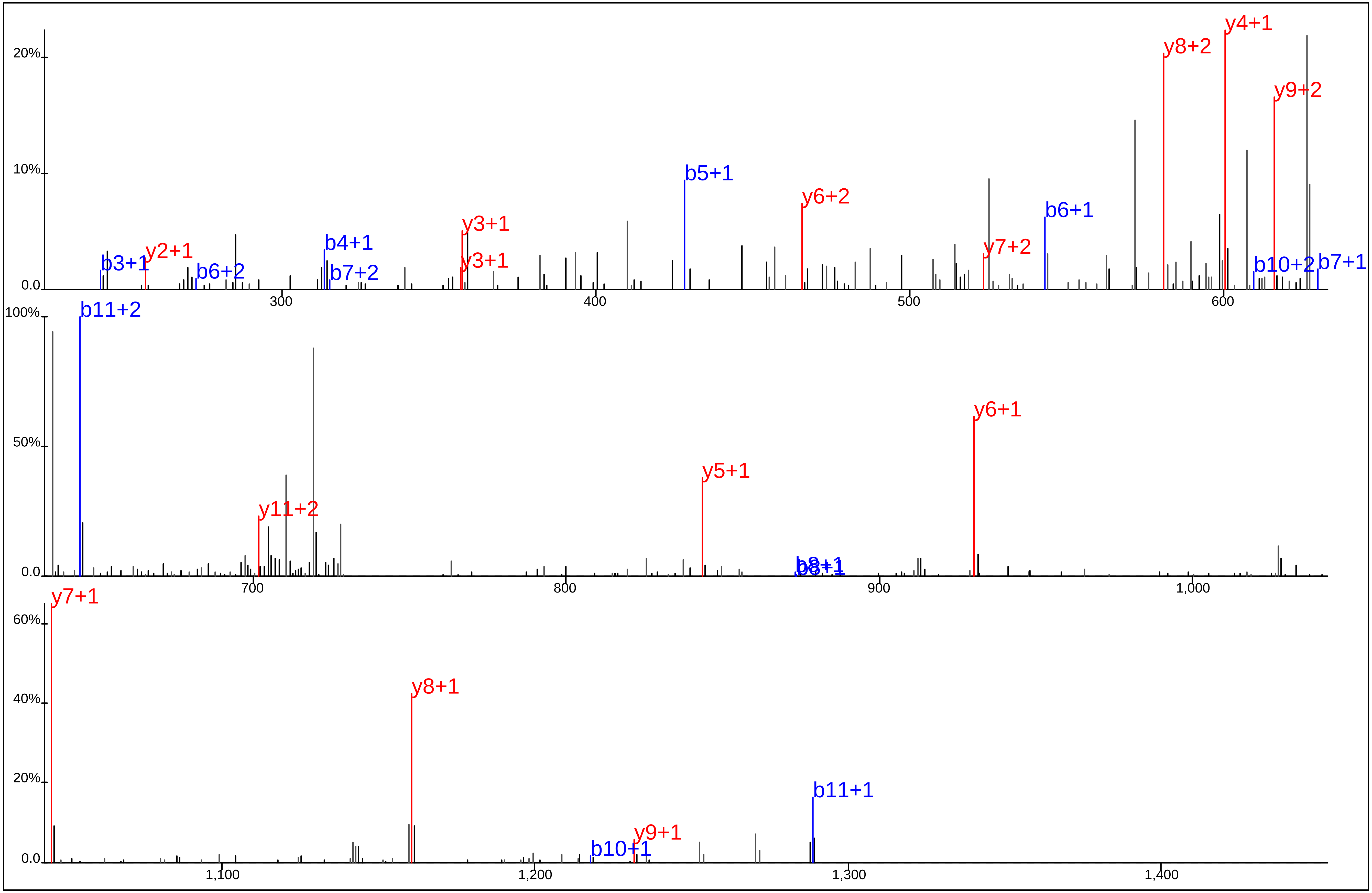

A L G A D D S Y*Y*T A R%

Protein Name: ZAP70
gi Accession: 31455611
Precursor Mass: 736.7754+2
Delta Mass: 4.433 ppm
XCorr: 59.05

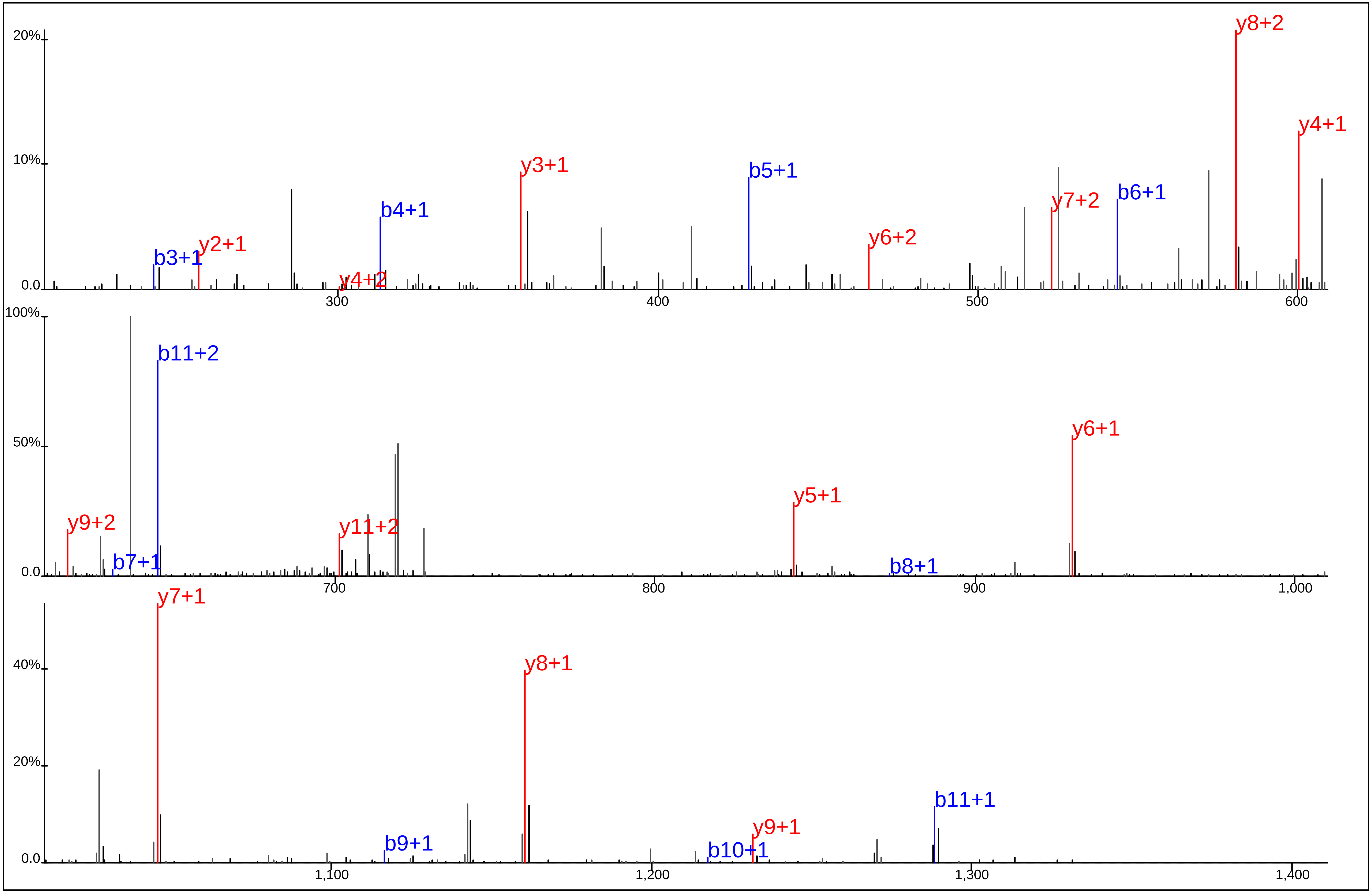

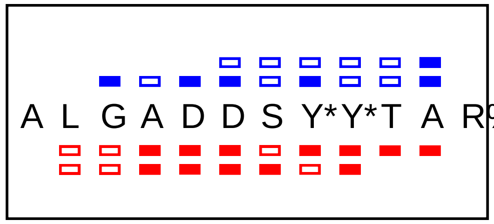

Protein Name: ZAP70
gi Accession: 31455611
Precursor Mass: 736.7737+2
Delta Mass: 2.199 ppm
XCorr: 63.41

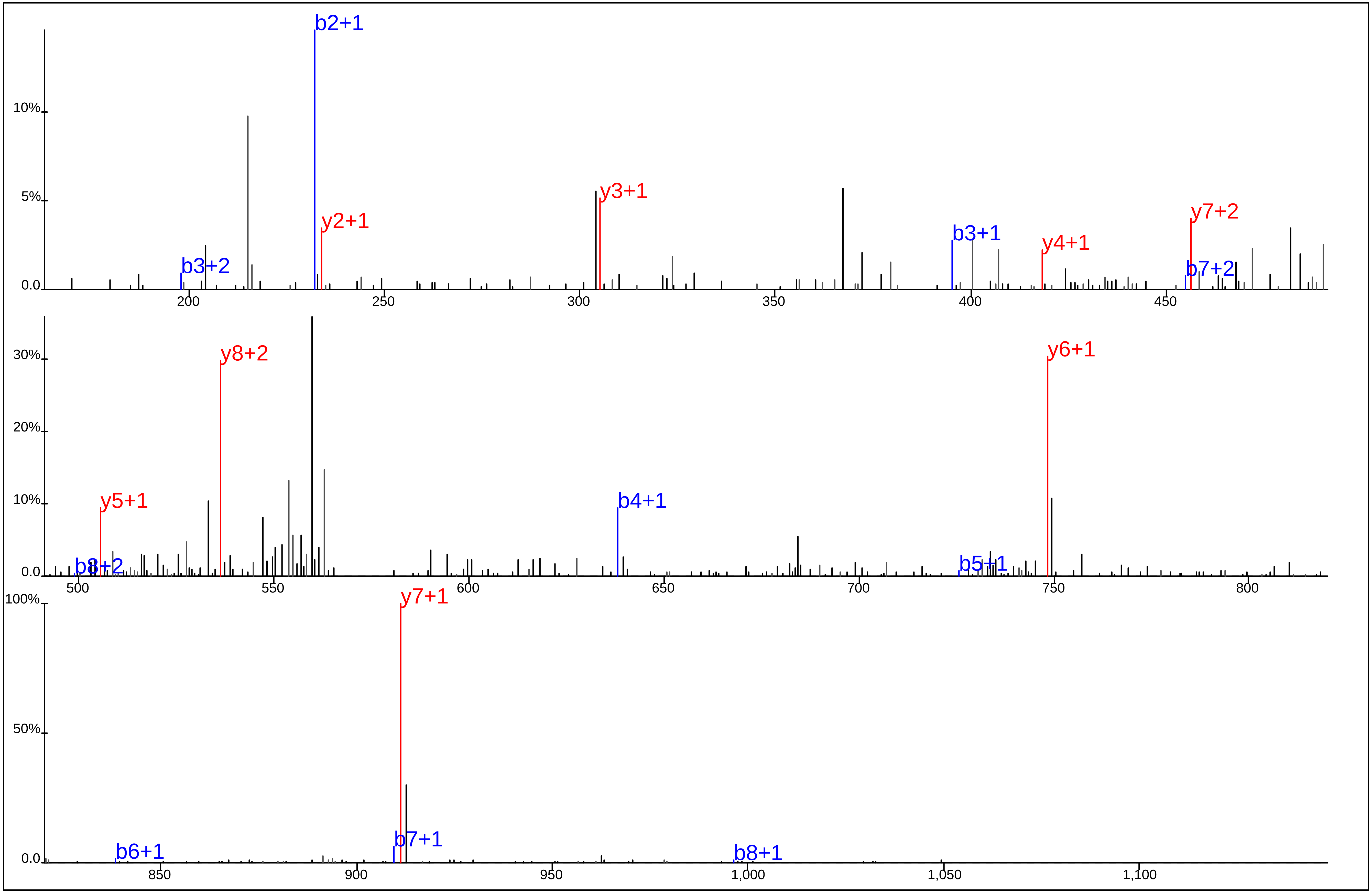

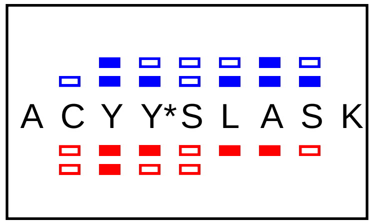

Protein Name:	ZAP70
gi Accession:	31455611
Precursor Mass:	571.7356+2
Delta Mass:	4.515 ppm
XCorr:	32.95

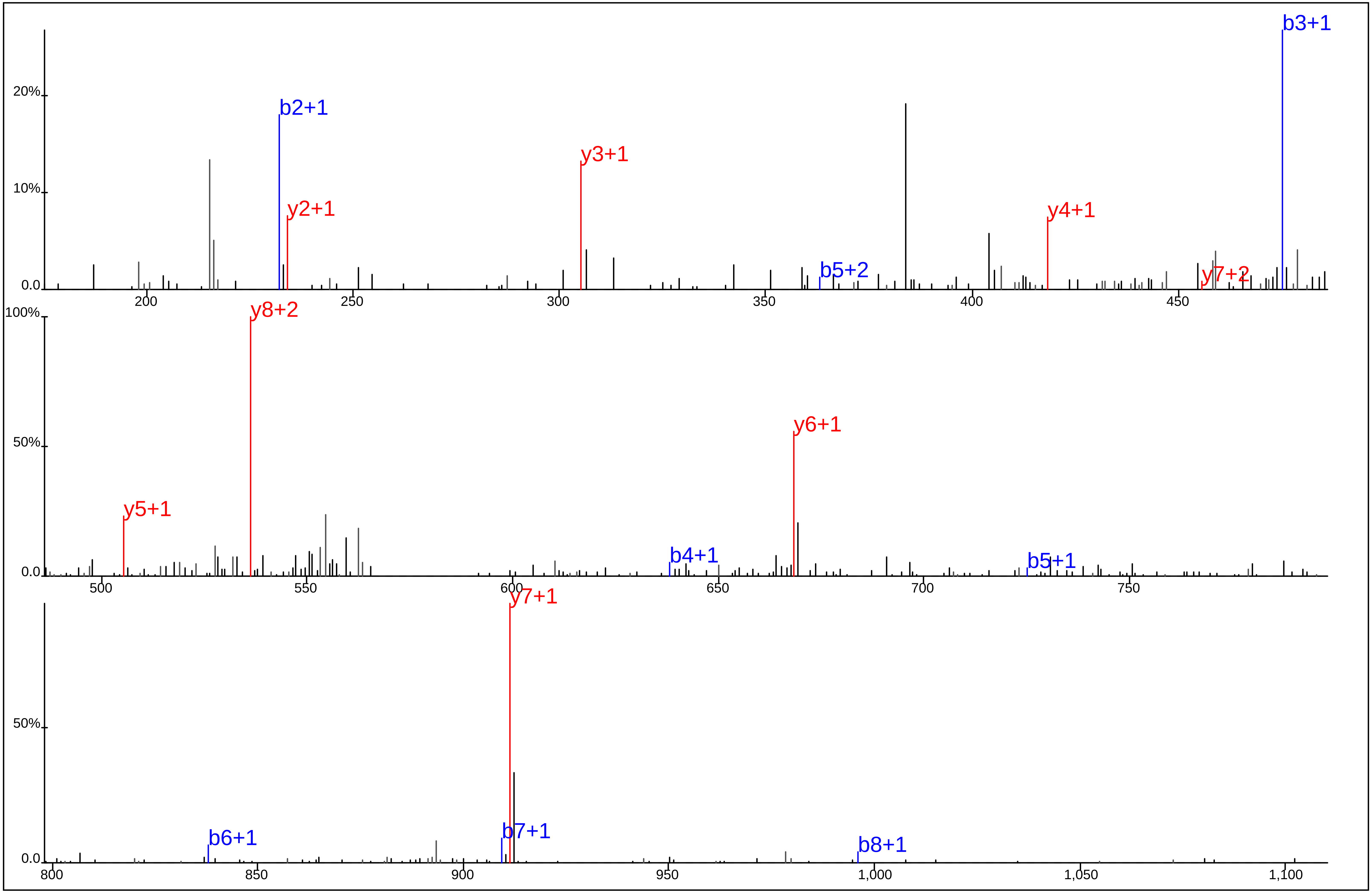

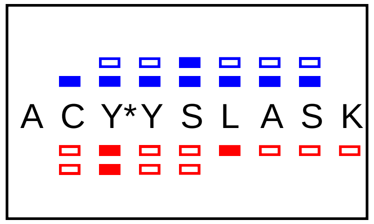

Protein Name: ZAP70
gi Accession: 31455611
Precursor Mass: 571.7346+2
Delta Mass: 2.808 ppm
XCorr: 24.57

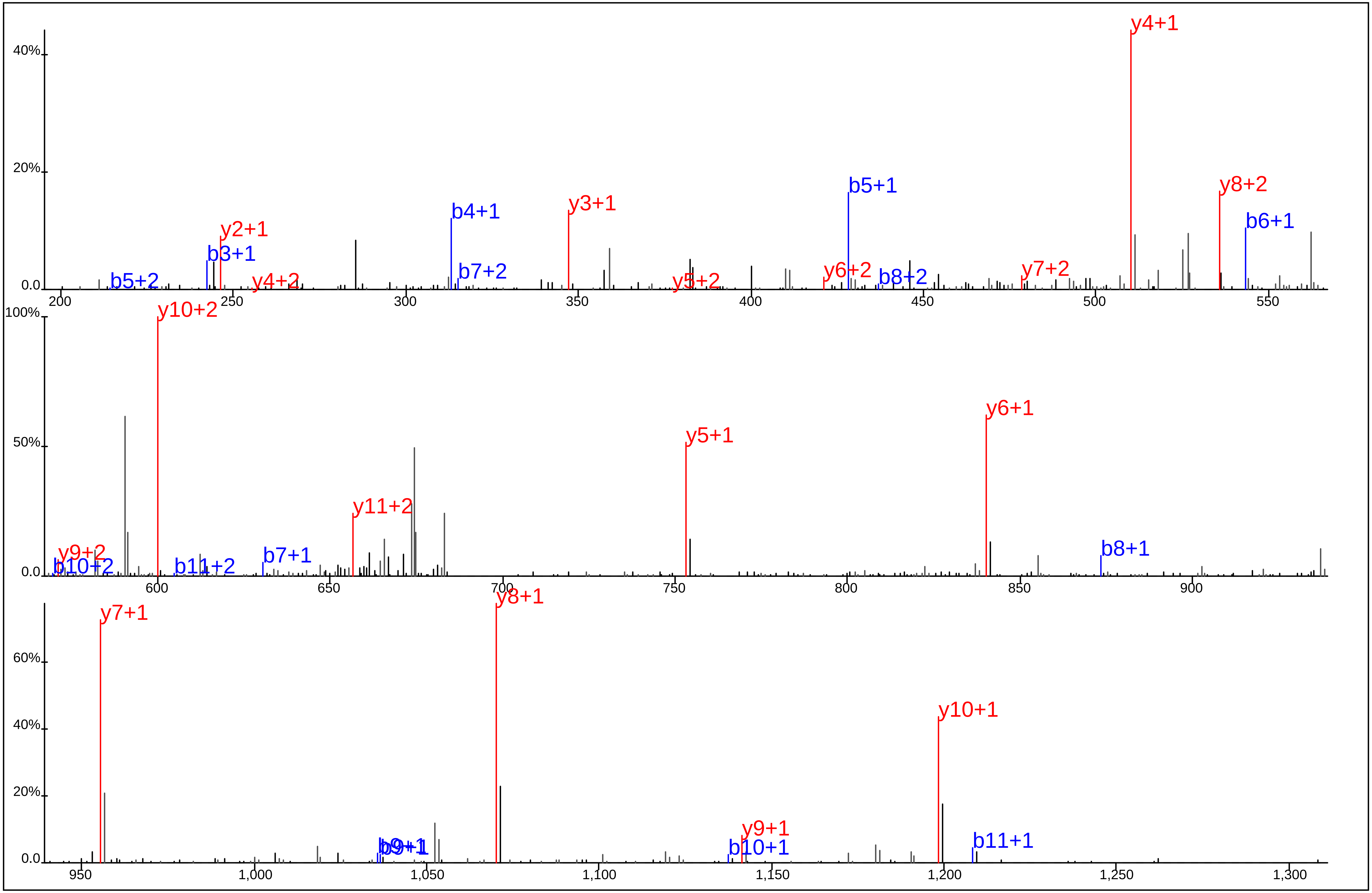

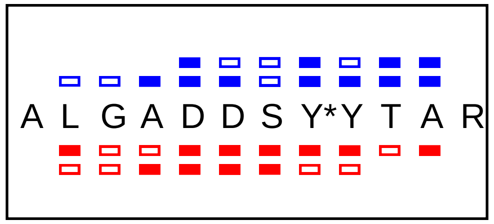

Protein Name: ZAP70
gi Accession: 31455611
Precursor Mass: 691.7875+2
Delta Mass: 3.832 ppm
XCorr: 81.06

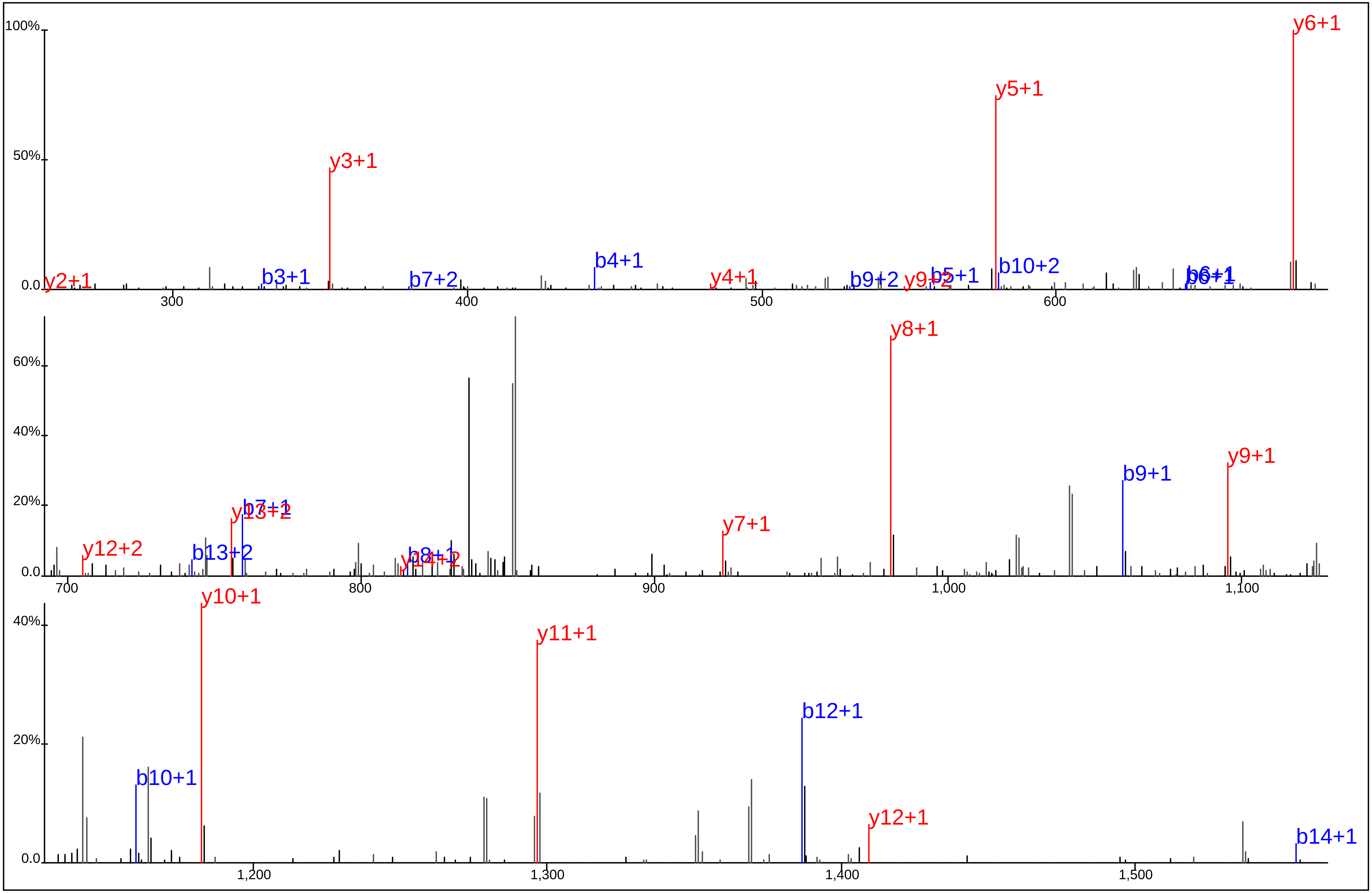

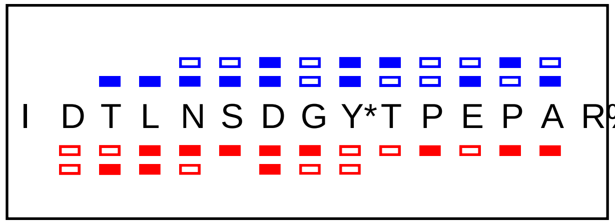

Protein Name: ZAP70
gi Accession: 31455611
Precursor Mass: 869.8817+2
Delta Mass: 0.154 ppm
XCorr: 74.21

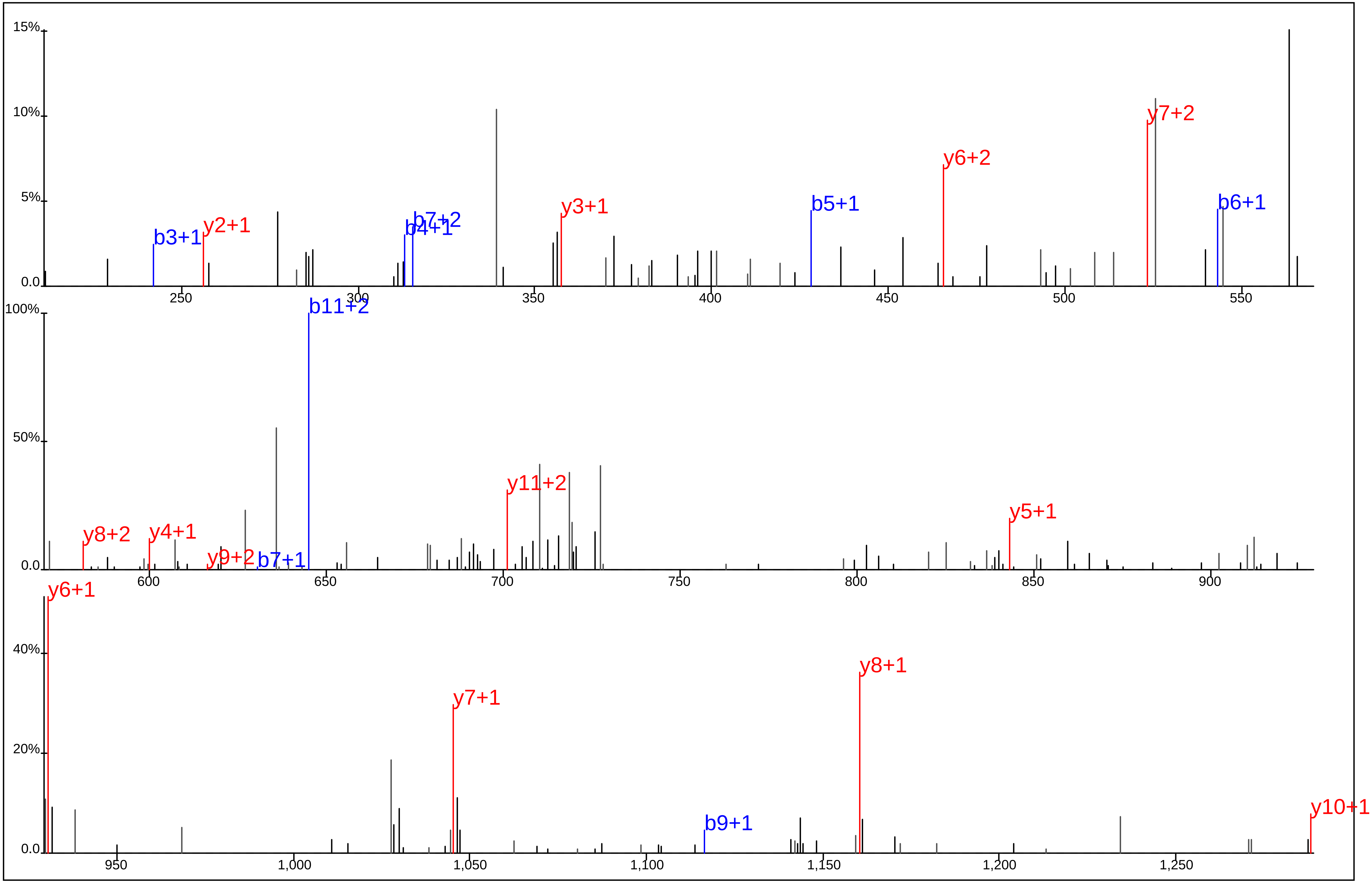

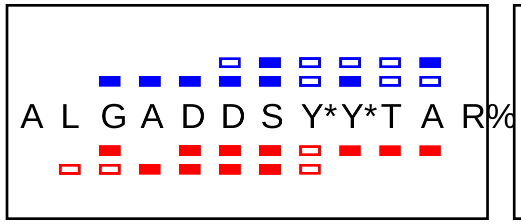

Protein Name: ZAP70
gi Accession: 31455611
Precursor Mass: 736.7736+2
Delta Mass: 1.948 ppm
XCorr: 66.97

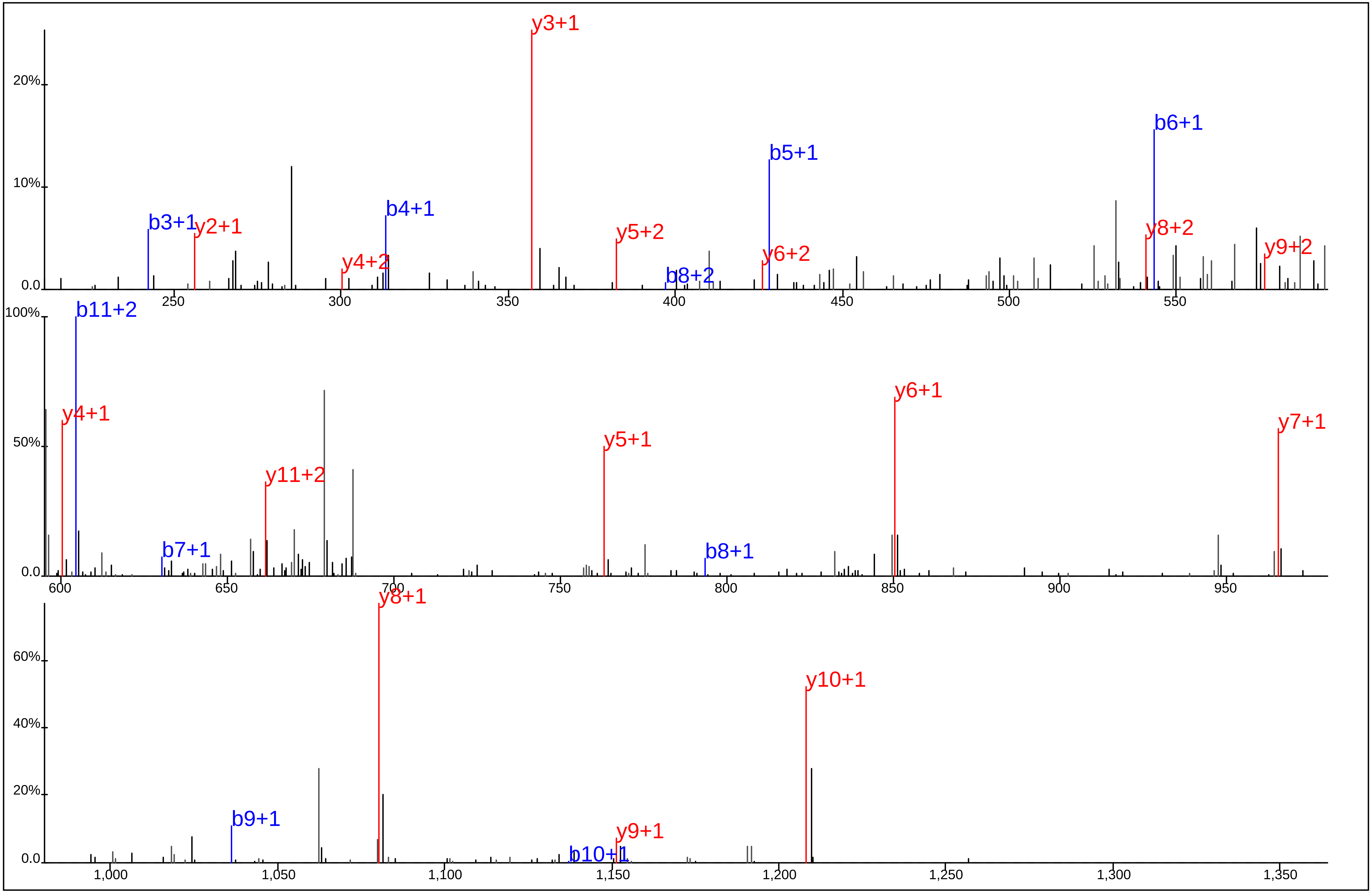

A L G A D D S Y Y*T A R%

Protein Name: ZAP70
gi Accession: 31455611
Precursor Mass: 696.7914+2
Delta Mass: 3.568 ppm
XCorr: 69.9

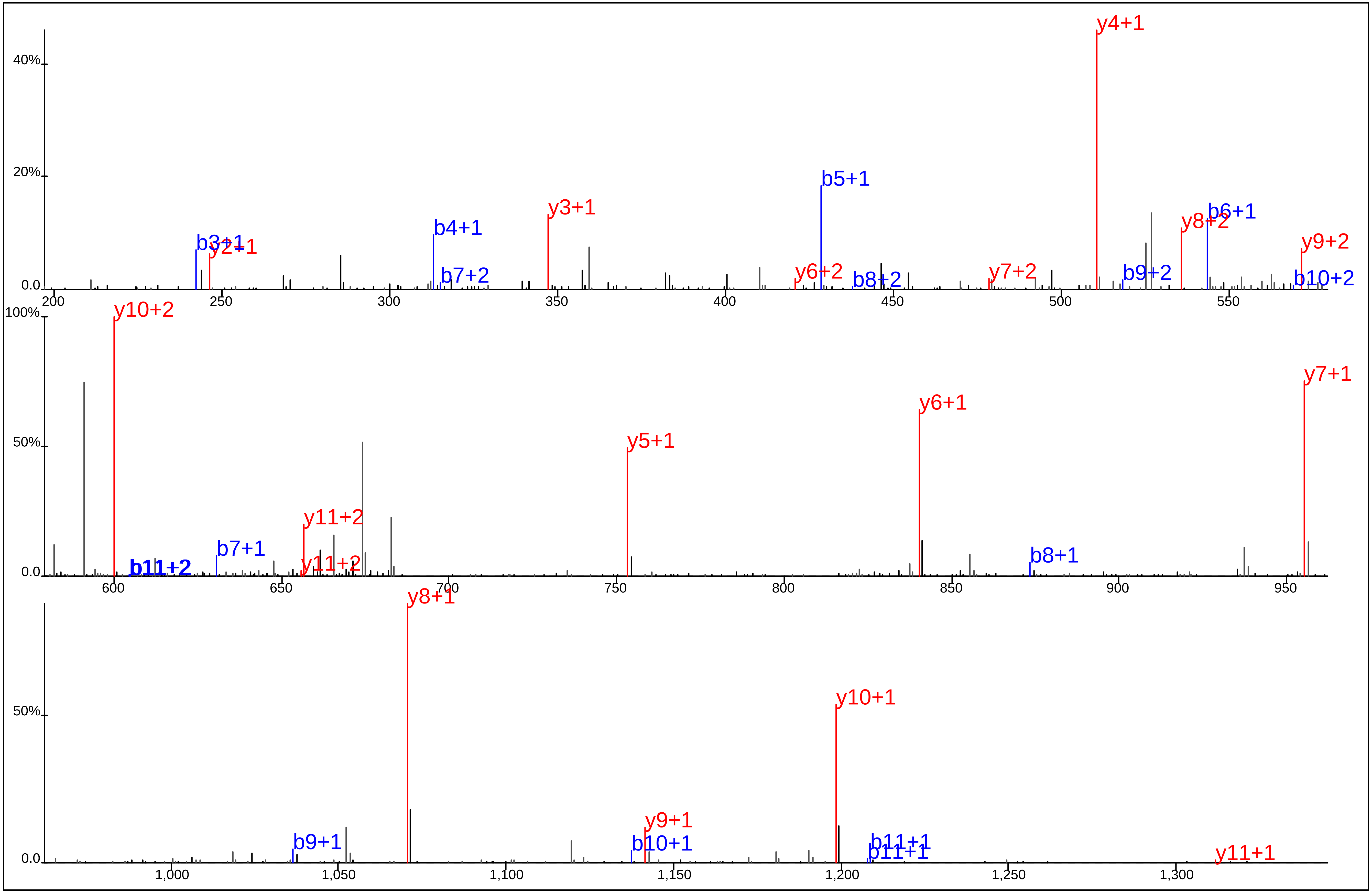

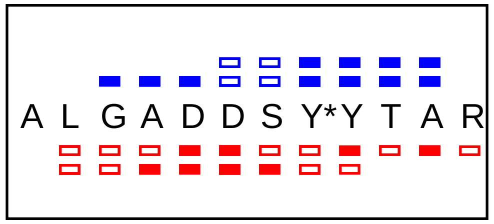

Protein Name: ZAP70
gi Accession: 31455611
Precursor Mass: 691.7851+2
Delta Mass: 0.389 ppm
XCorr: 84.5

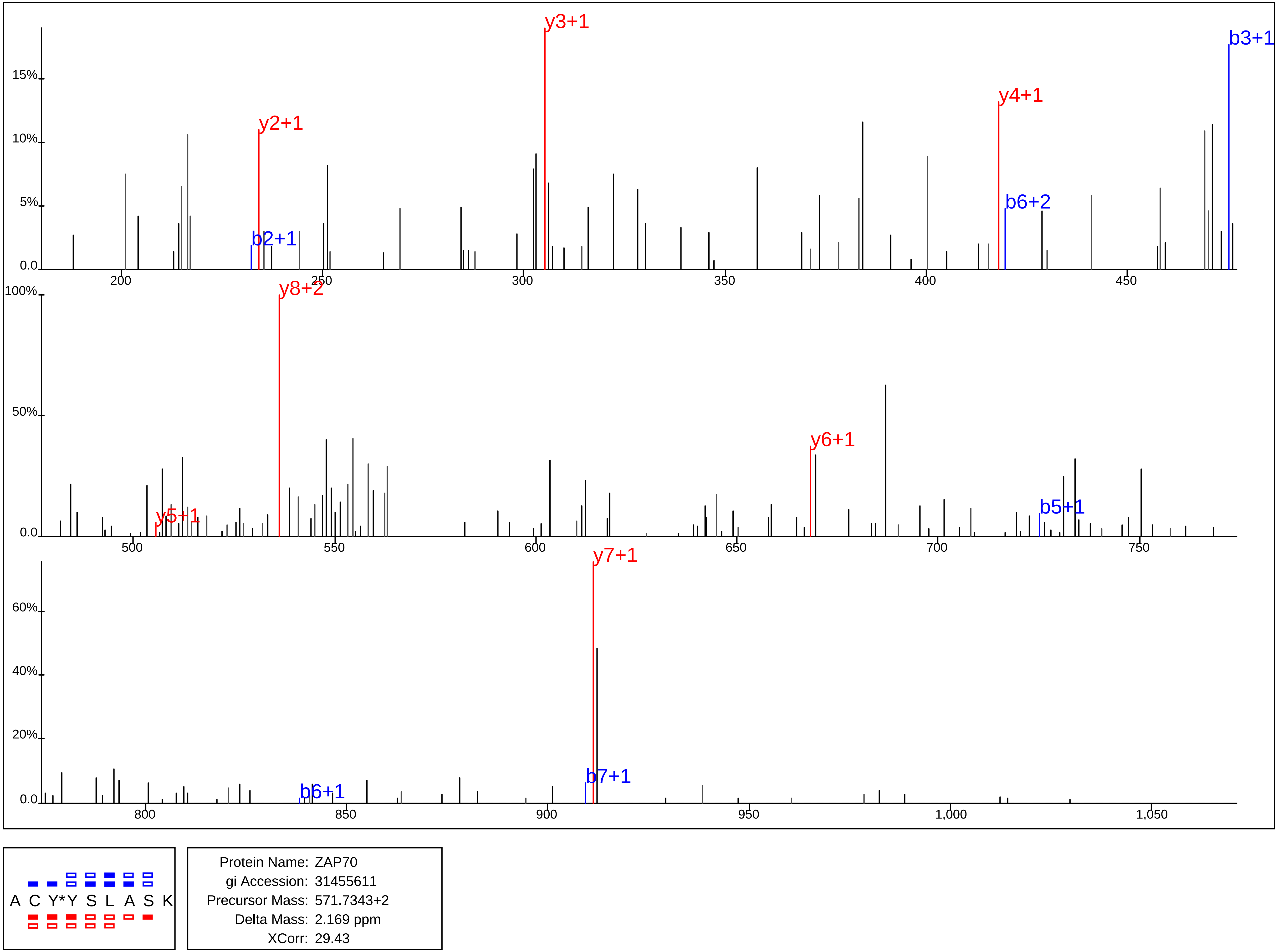

Protein Name: ZAP70
gi Accession: 31455611
Precursor Mass: 571.7343+2
Delta Mass: 2.169 ppm
XCorr: 29.43

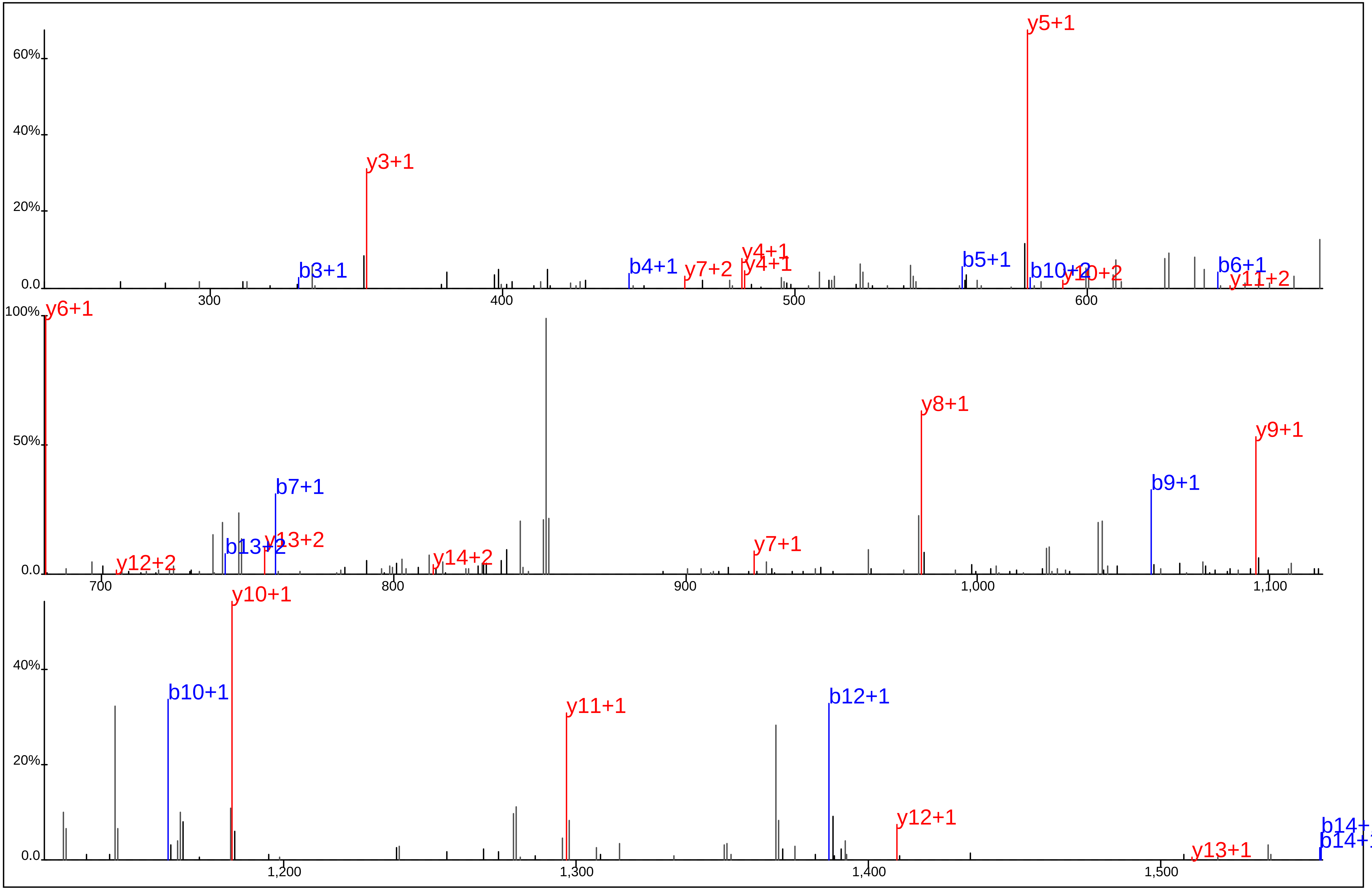

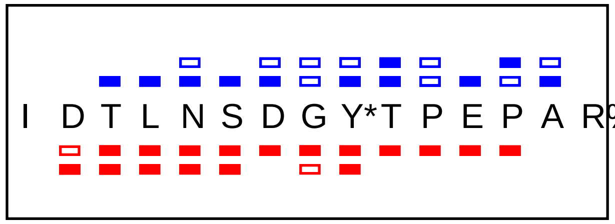

Protein Name: ZAP70
gi Accession: 31455611
Precursor Mass: 869.8839+2
Delta Mass: 2.685 ppm
XCorr: 74.23

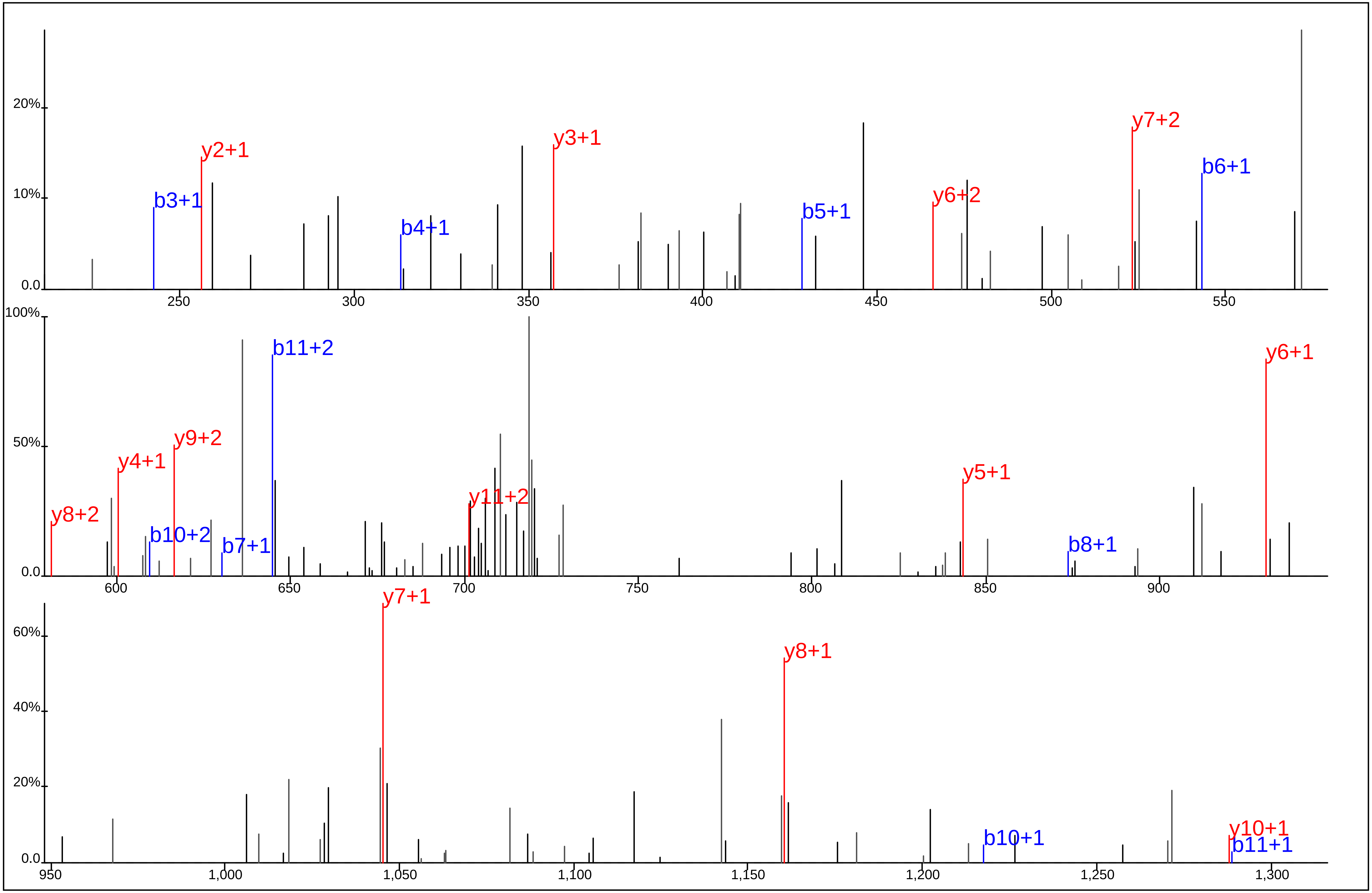

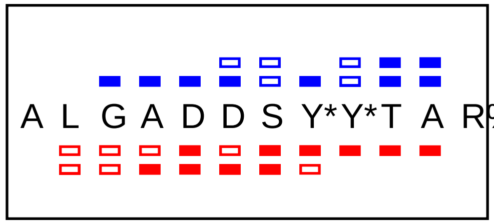

Protein Name: ZAP70
gi Accession: 31455611
Precursor Mass: 736.7745+2
Delta Mass: 3.279 ppm
XCorr: 53.67

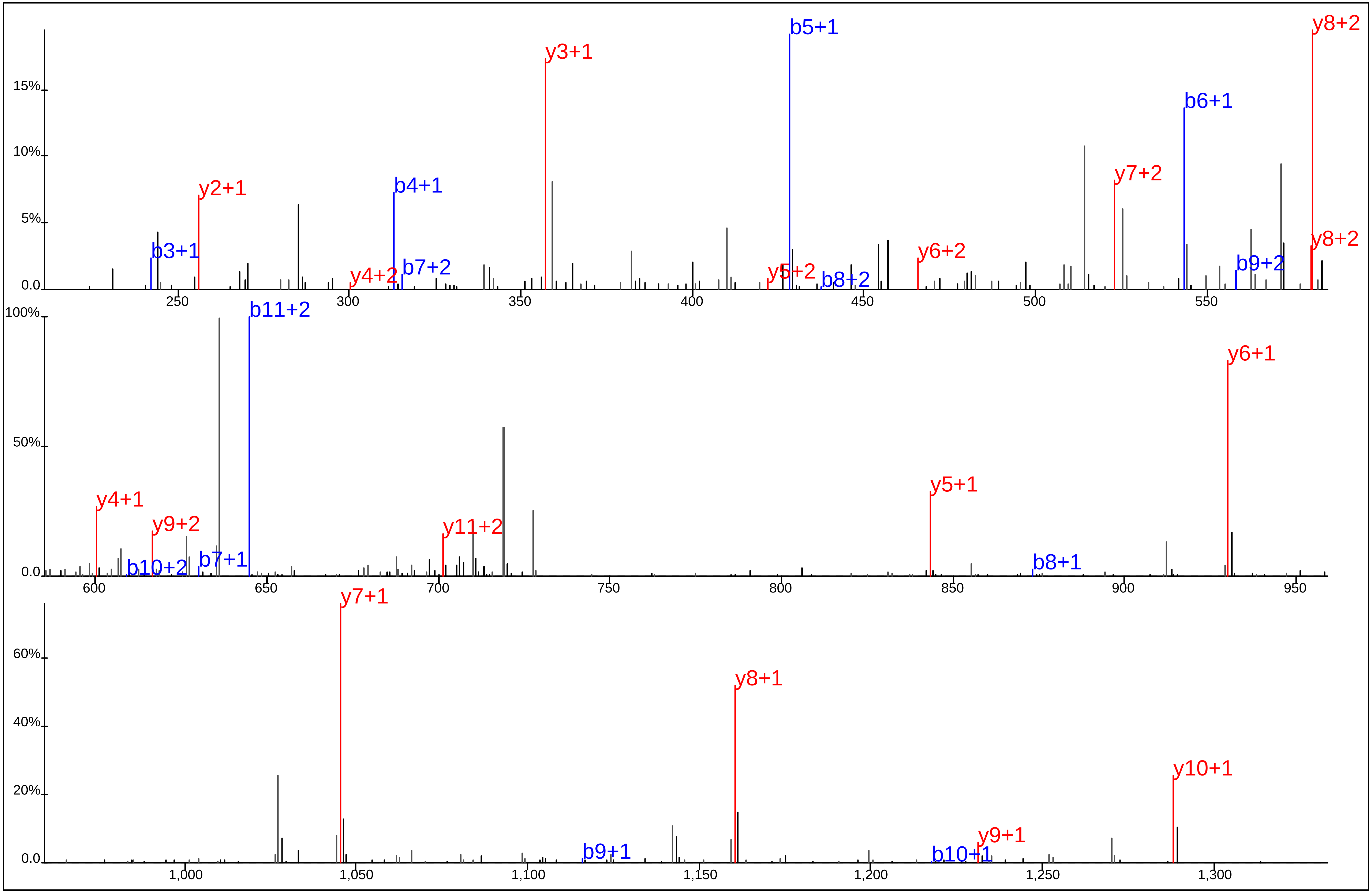

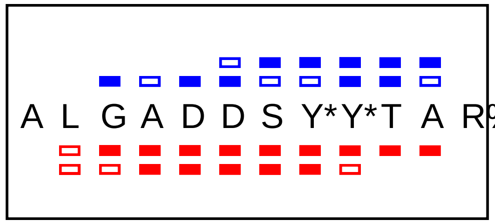

Protein Name: ZAP70
gi Accession: 31455611
Precursor Mass: 736.7734+2
Delta Mass: 1.703 ppm
XCorr: 72.05

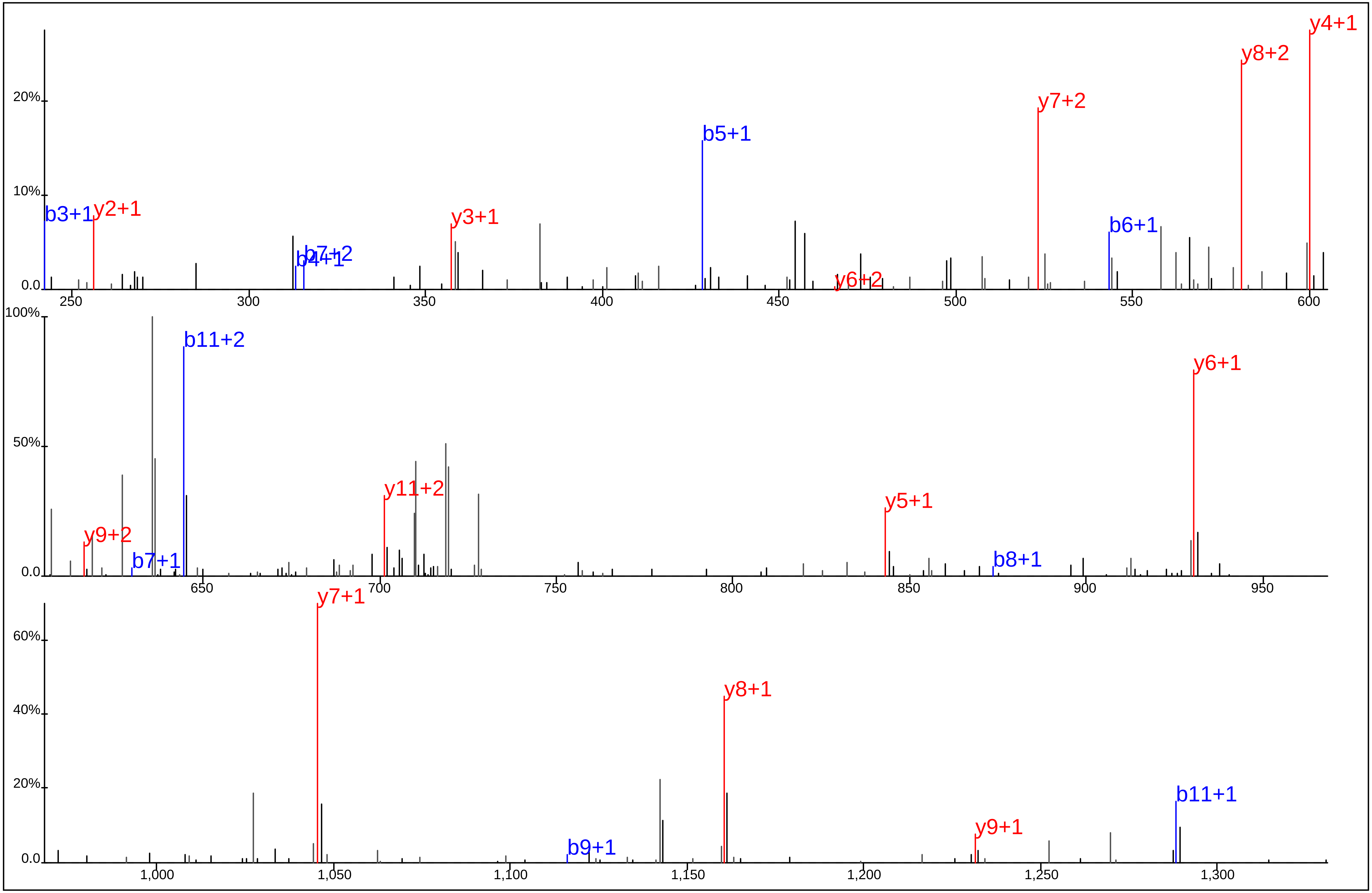

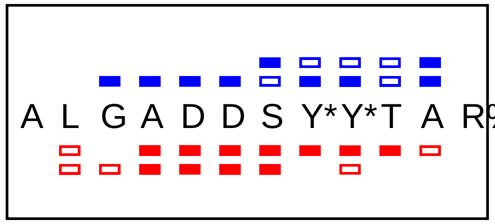

Protein Name: ZAP70
gi Accession: 31455611
Precursor Mass: 736.7729+2
Delta Mass: 1.038 ppm
XCorr: 43.0

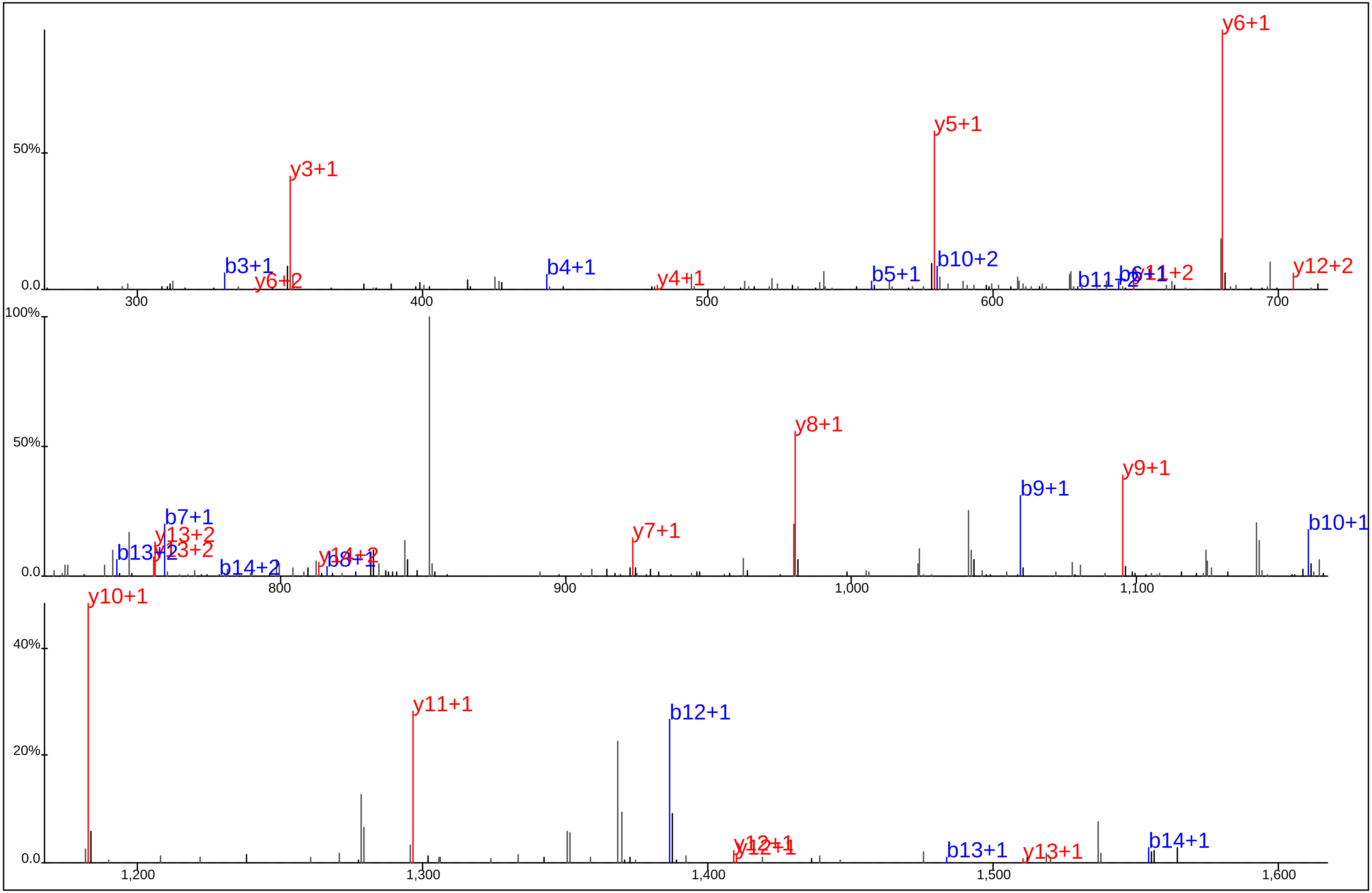

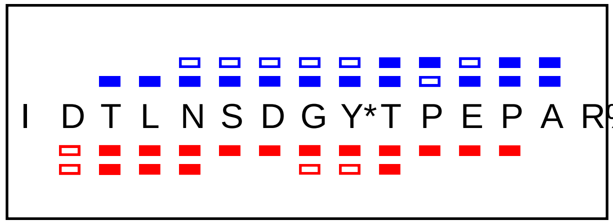

Protein Name: ZAP70
gi Accession: 31455611
Precursor Mass: 869.8829+2
Delta Mass: 1.488 ppm
XCorr: 74.6

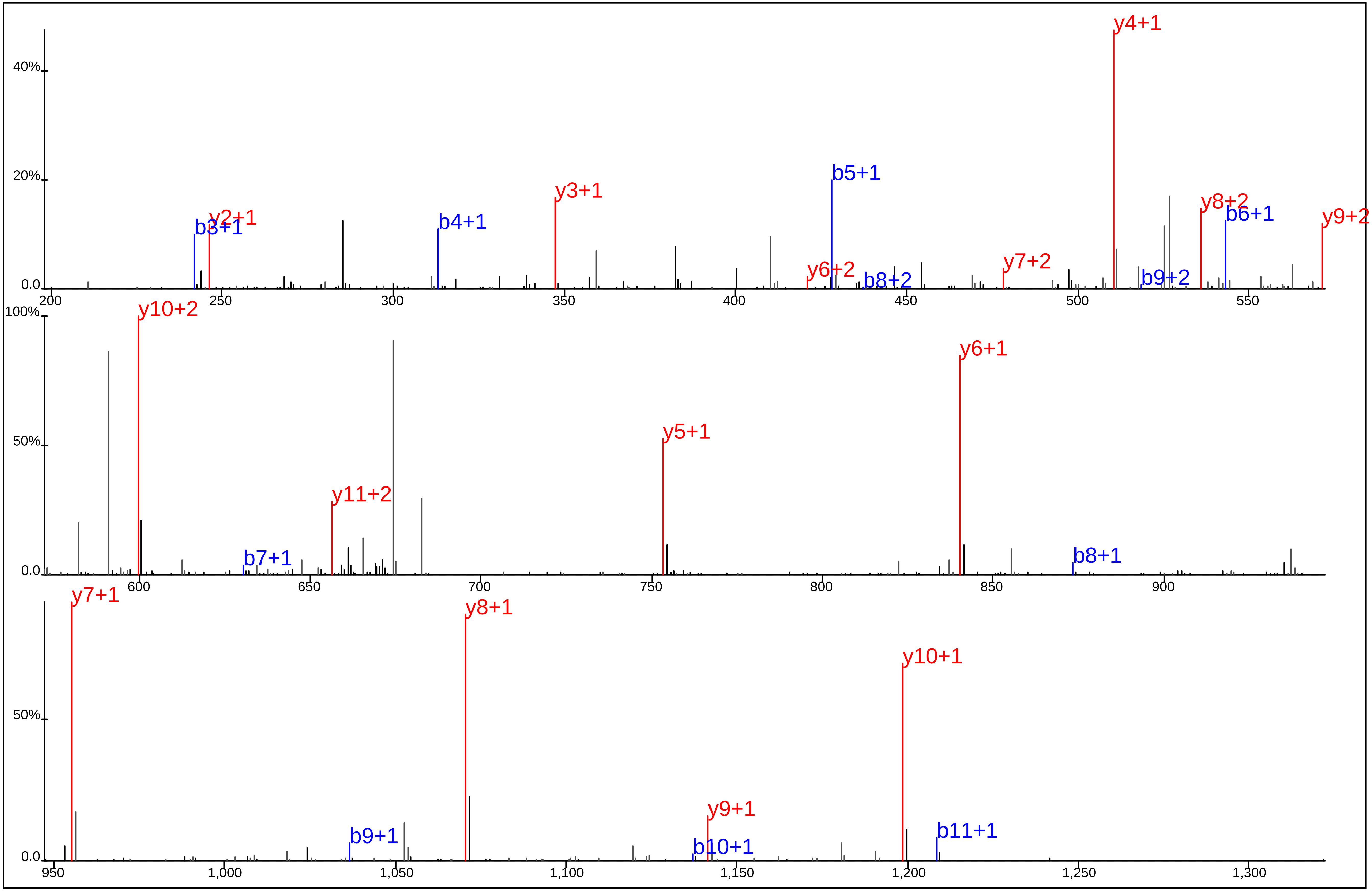

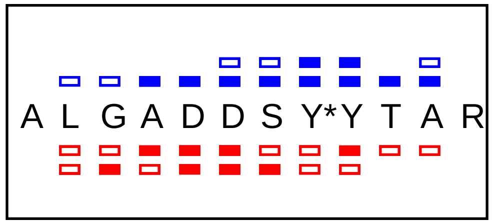

Protein Name: ZAP70
gi Accession: 31455611
Precursor Mass: 691.7846+2
Delta Mass: 0.312 ppm
XCorr: 69.19

1083 - ZAP70_3441611.pdf

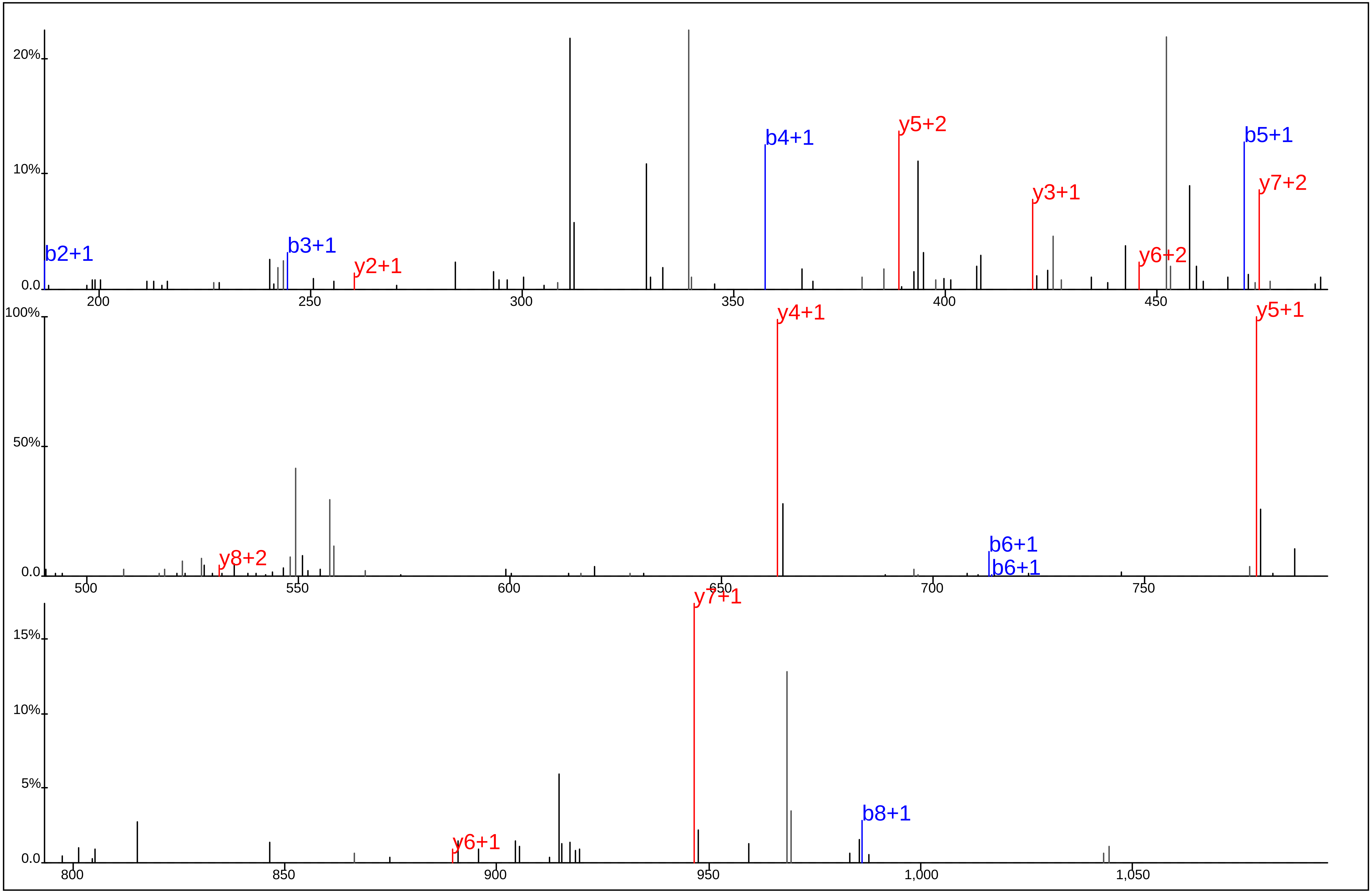

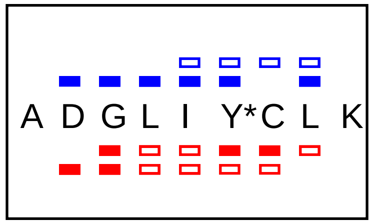

Protein Name: ZAP70
gi Accession: 31455611
Precursor Mass: 566.7625+2
Delta Mass: 6.135 ppm
XCorr: 26.17

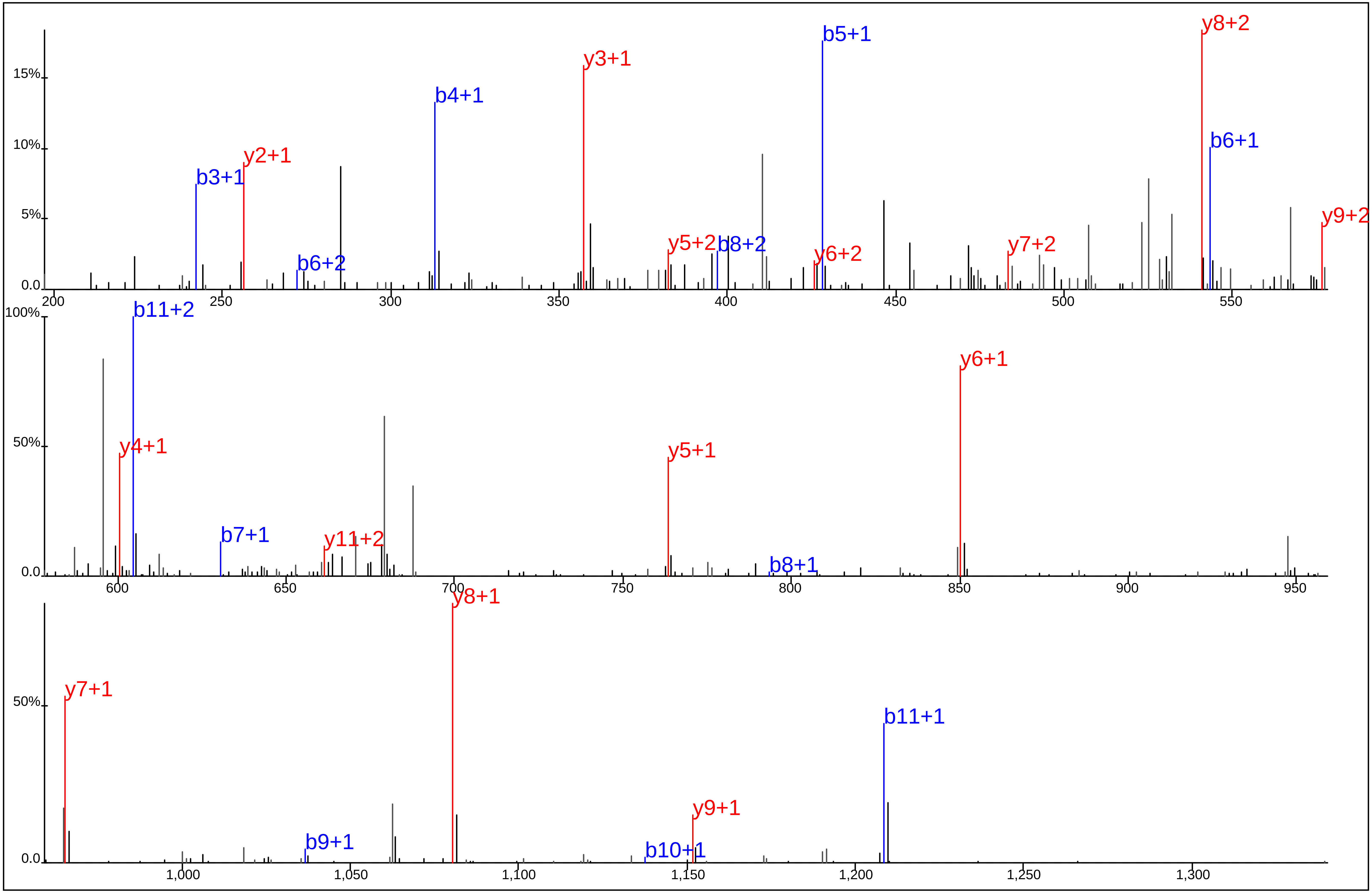

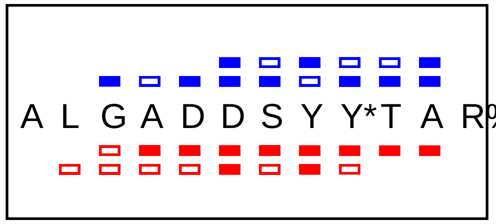

Protein Name: ZAP70
gi Accession: 31455611
Precursor Mass: 696.7901+2
Delta Mass: 1.636 ppm
XCorr: 74.18

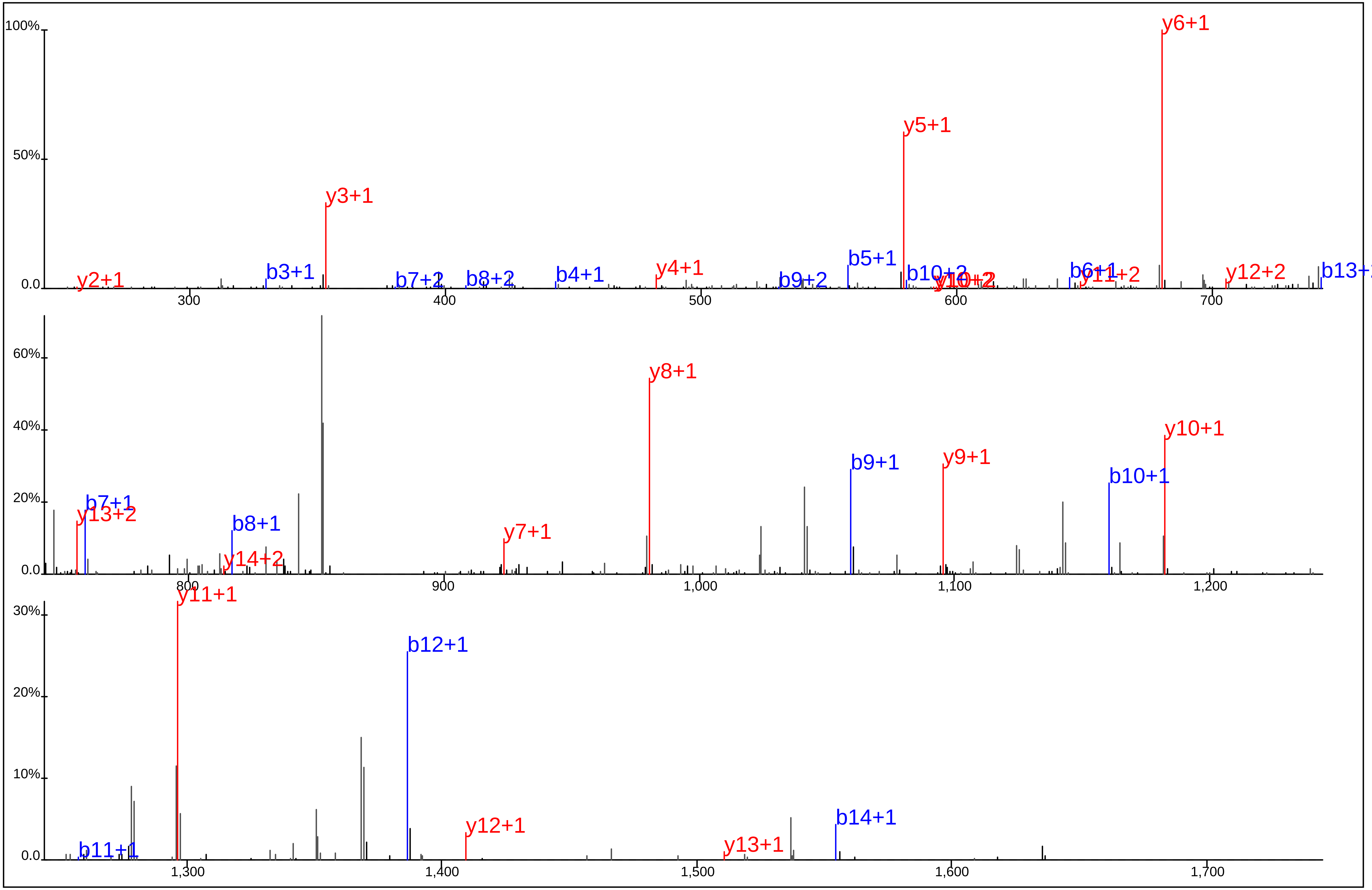

I D T L N S D G Y*T P E P A R%

Protein Name: ZAP70
gi Accession: 31455611
Precursor Mass: 869.8814+2
Delta Mass: 0.197 ppm
XCorr: 80.35

1086 - ZAP70_3444128.pdf

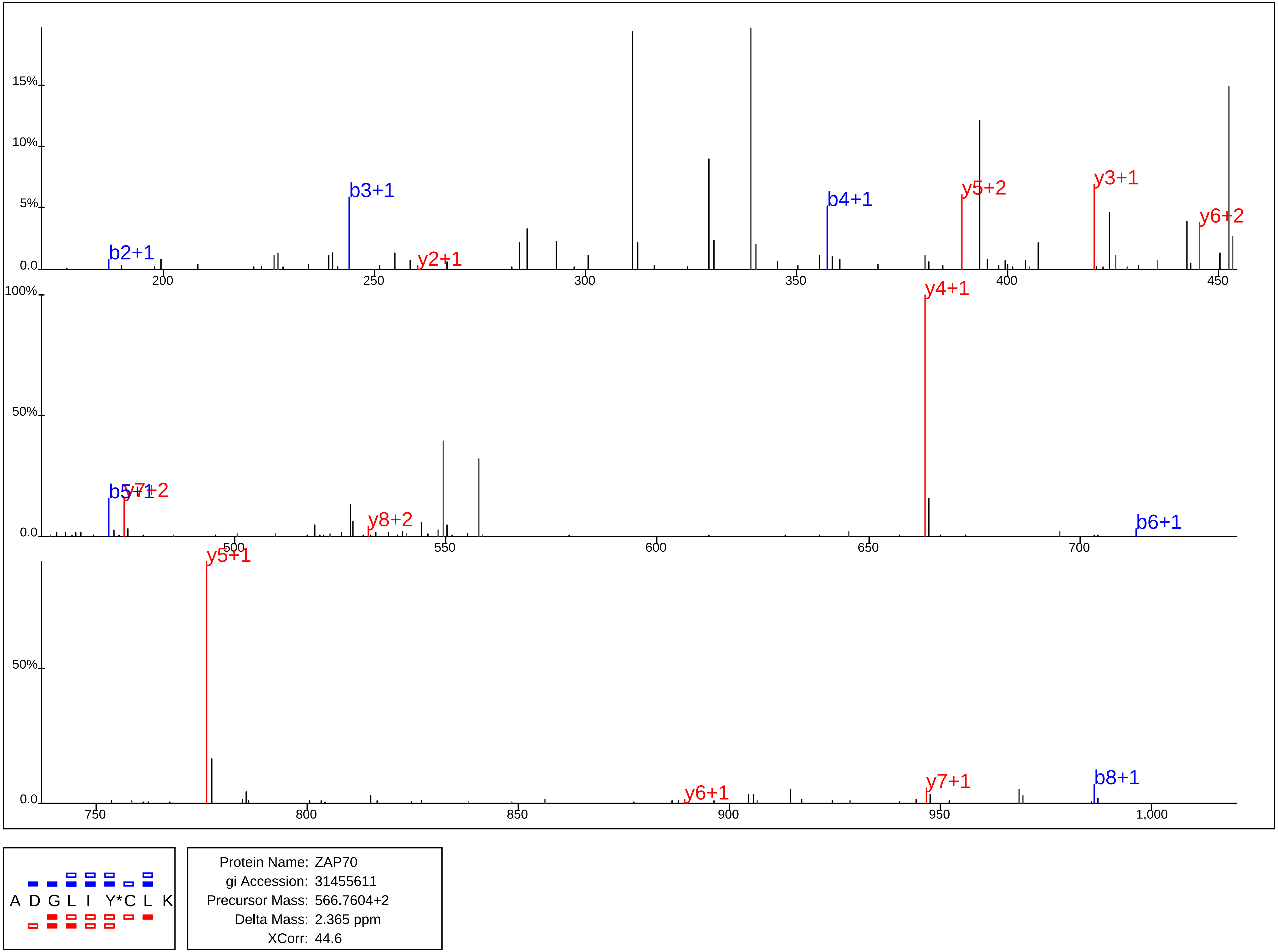

Protein Name: ZAP70
gi Accession: 31455611
Precursor Mass: 566.7604+2
Delta Mass: 2.365 ppm
XCorr: 44.6

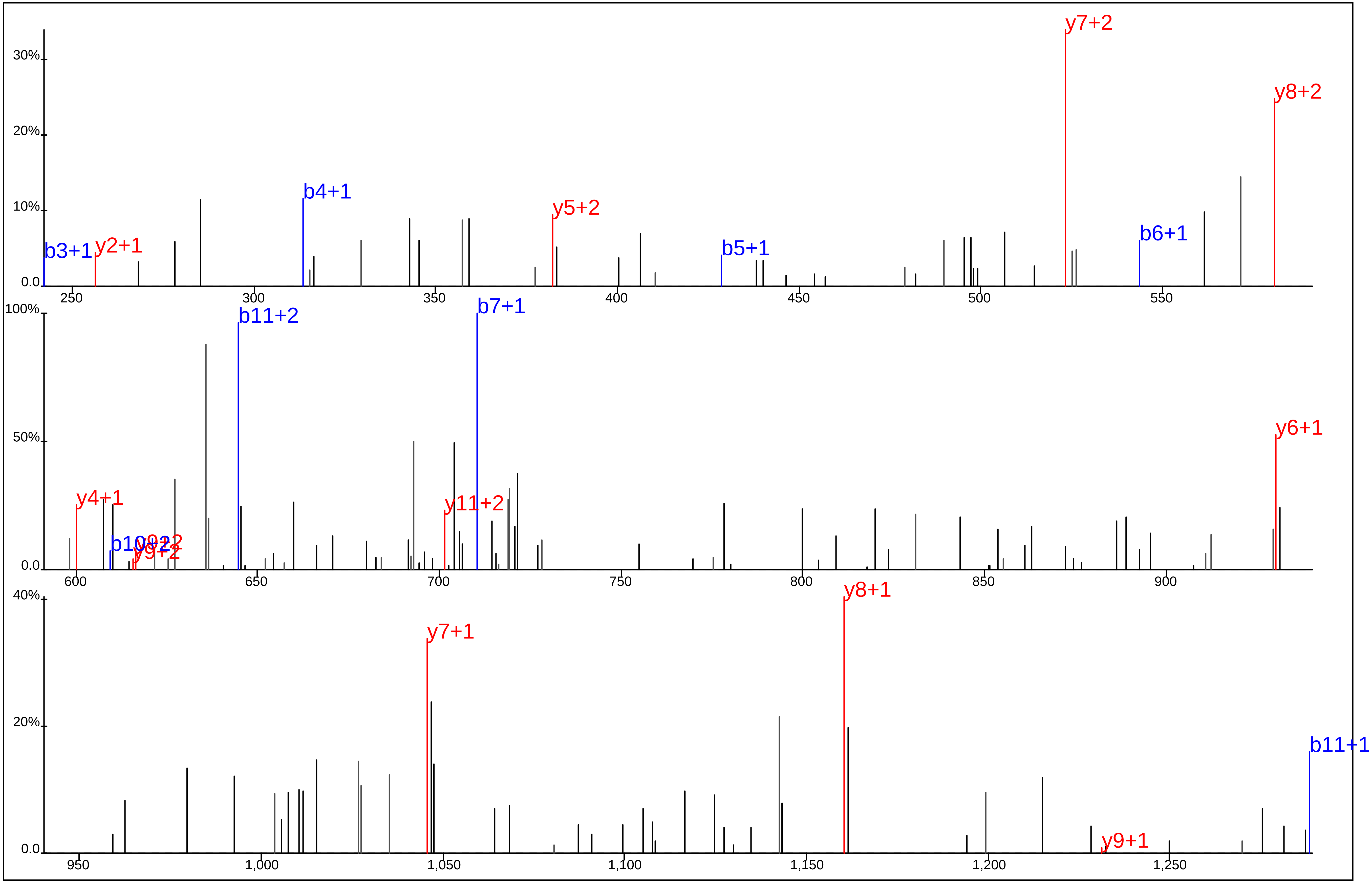

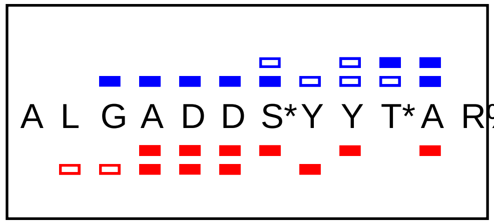

Protein Name: ZAP70
gi Accession: 31455611
Precursor Mass: 736.7721+2
Delta Mass: 0.042 ppm
XCorr: 26.8

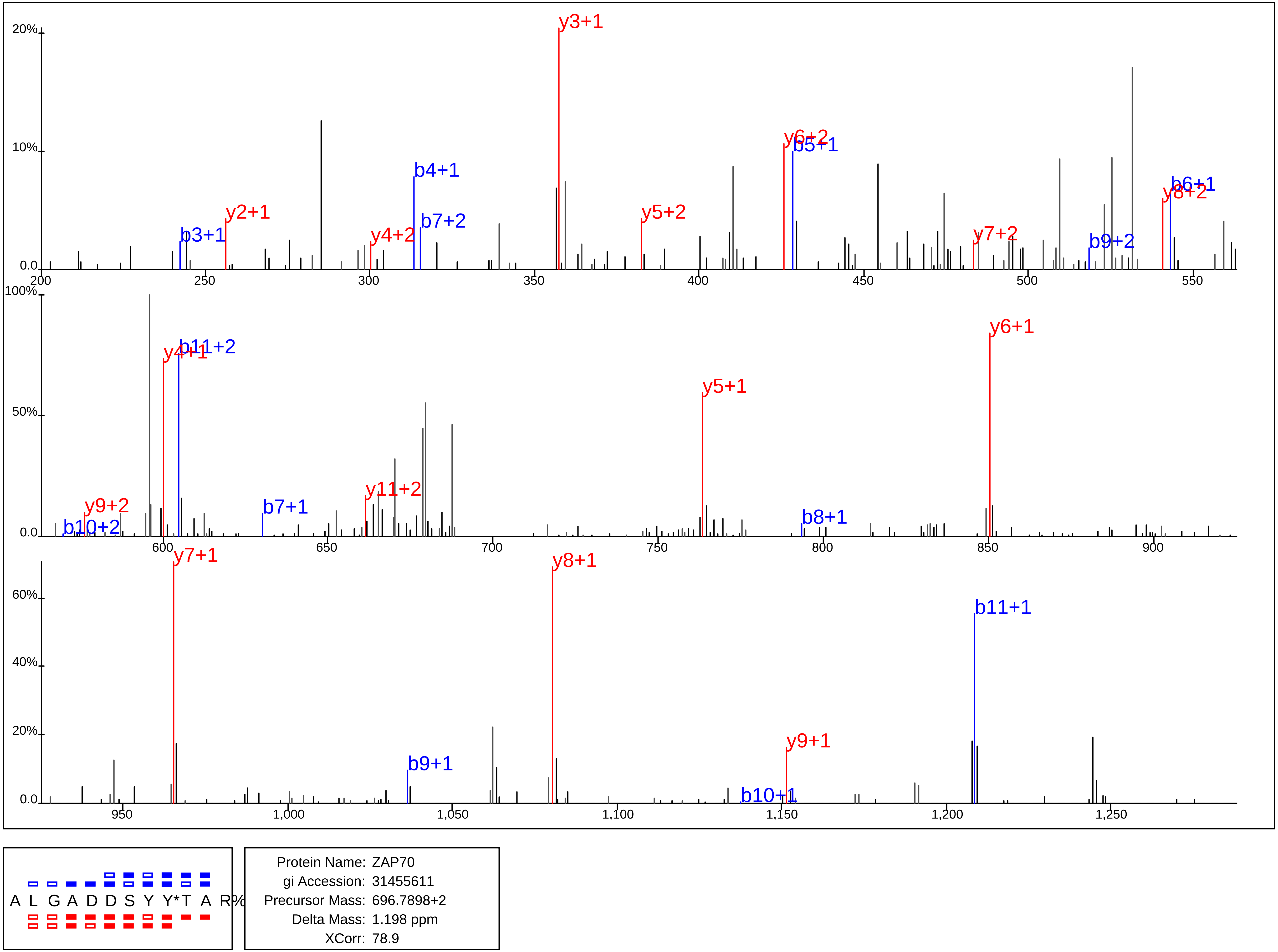

Protein Name: ZAP70
gi Accession: 31455611
Precursor Mass: 696.7898+2
Delta Mass: 1.198 ppm
XCorr: 78.9

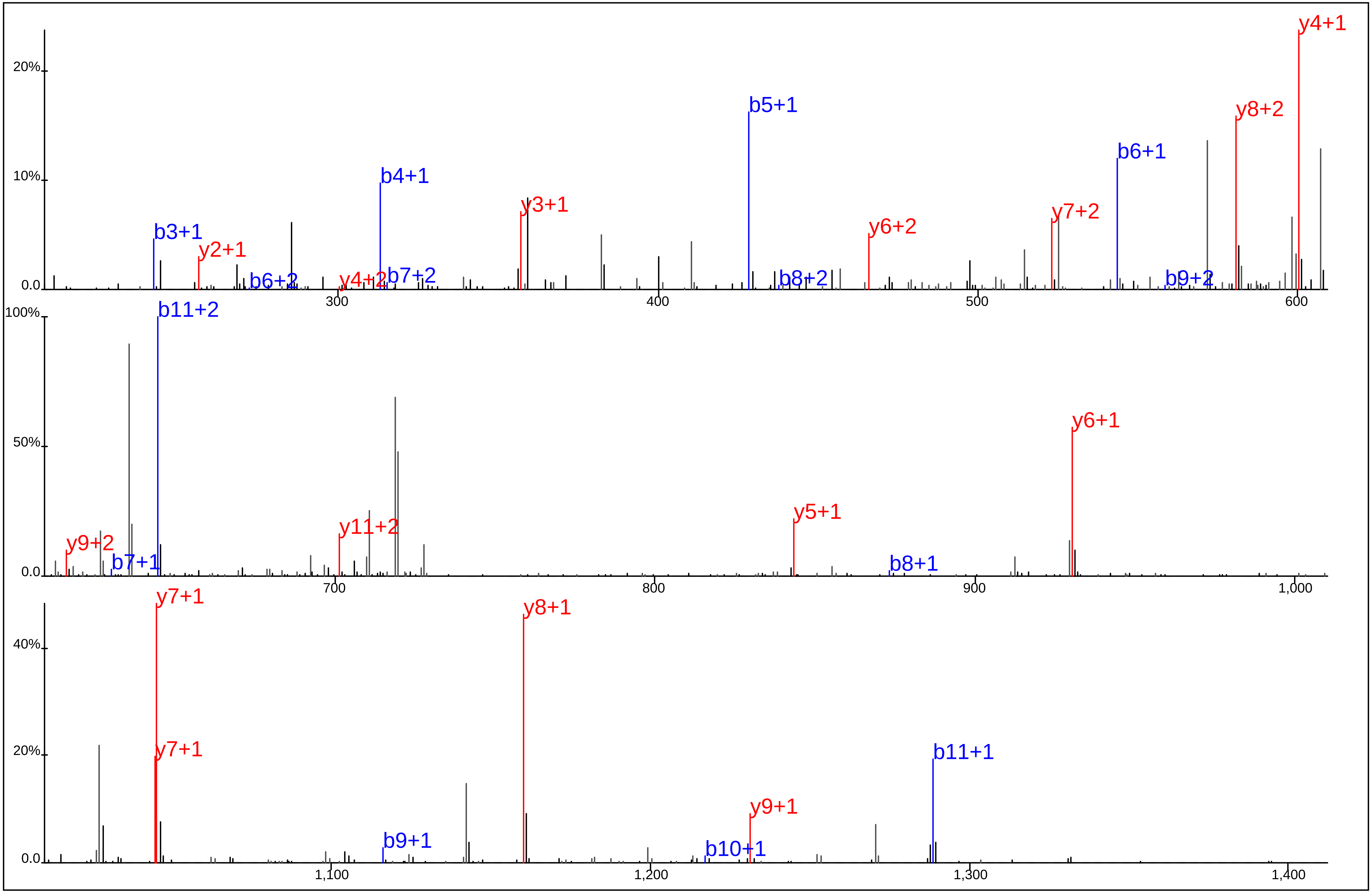

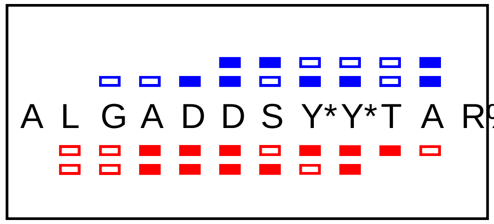

Protein Name: ZAP70
gi Accession: 31455611
Precursor Mass: 736.7717+2
Delta Mass: 0.619 ppm
XCorr: 67.29

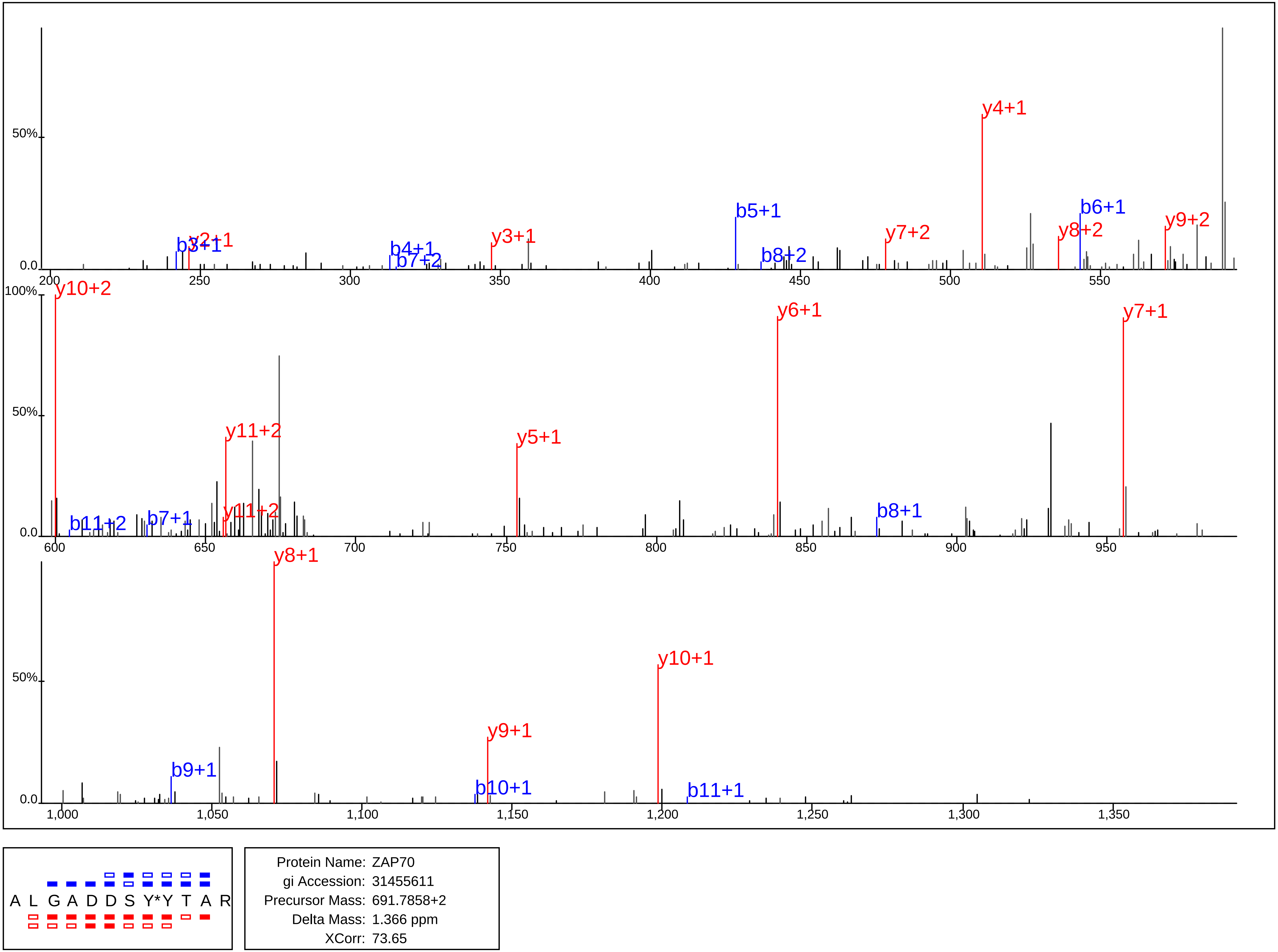

Protein Name: ZAP70
gi Accession: 31455611
Precursor Mass: 691.7858+2
Delta Mass: 1.366 ppm
XCorr: 73.65

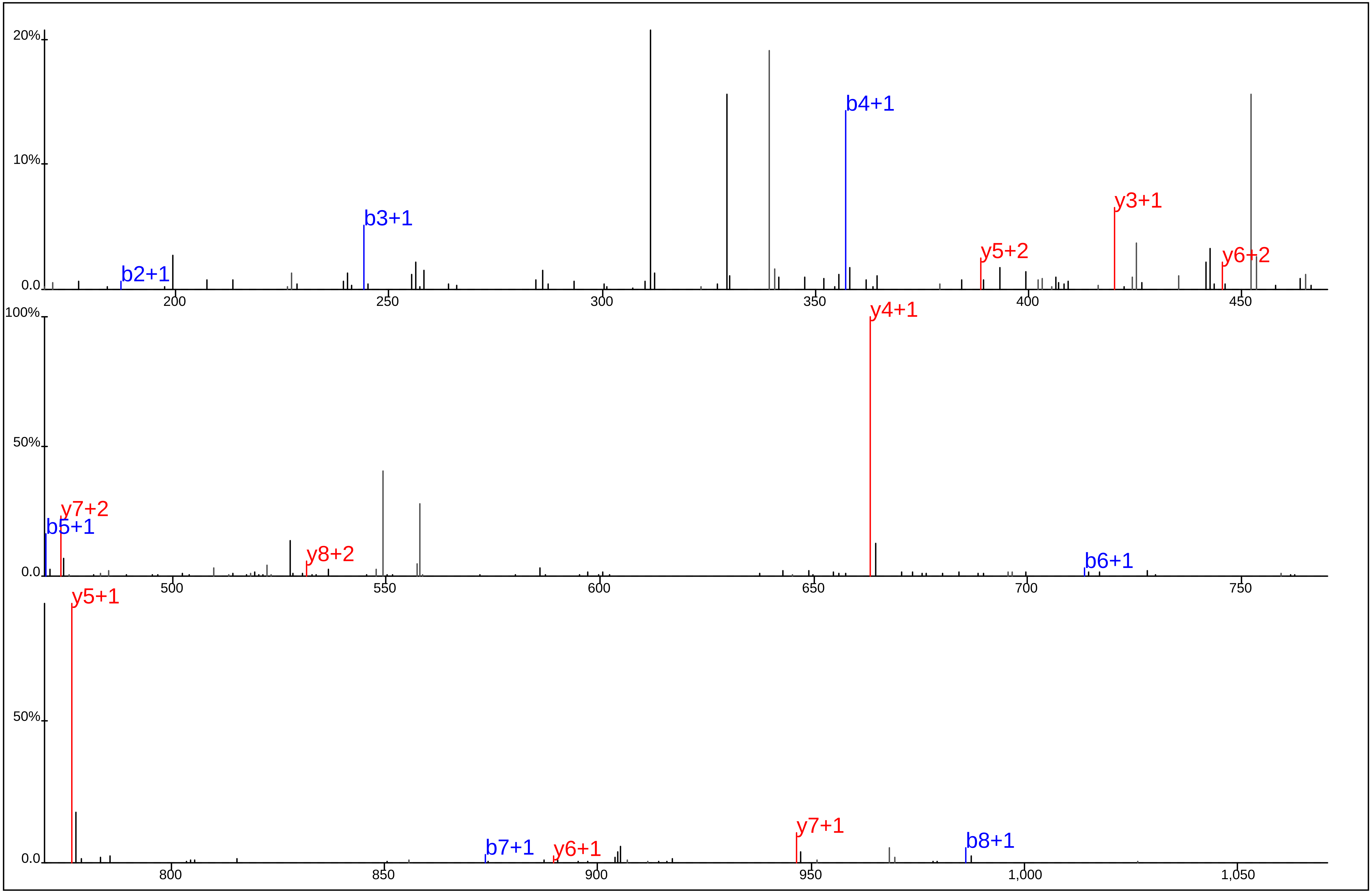

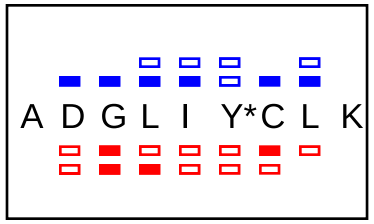

Protein Name: ZAP70
gi Accession: 31455611
Precursor Mass: 566.7604+2
Delta Mass: 2.471 ppm
XCorr: 31.71

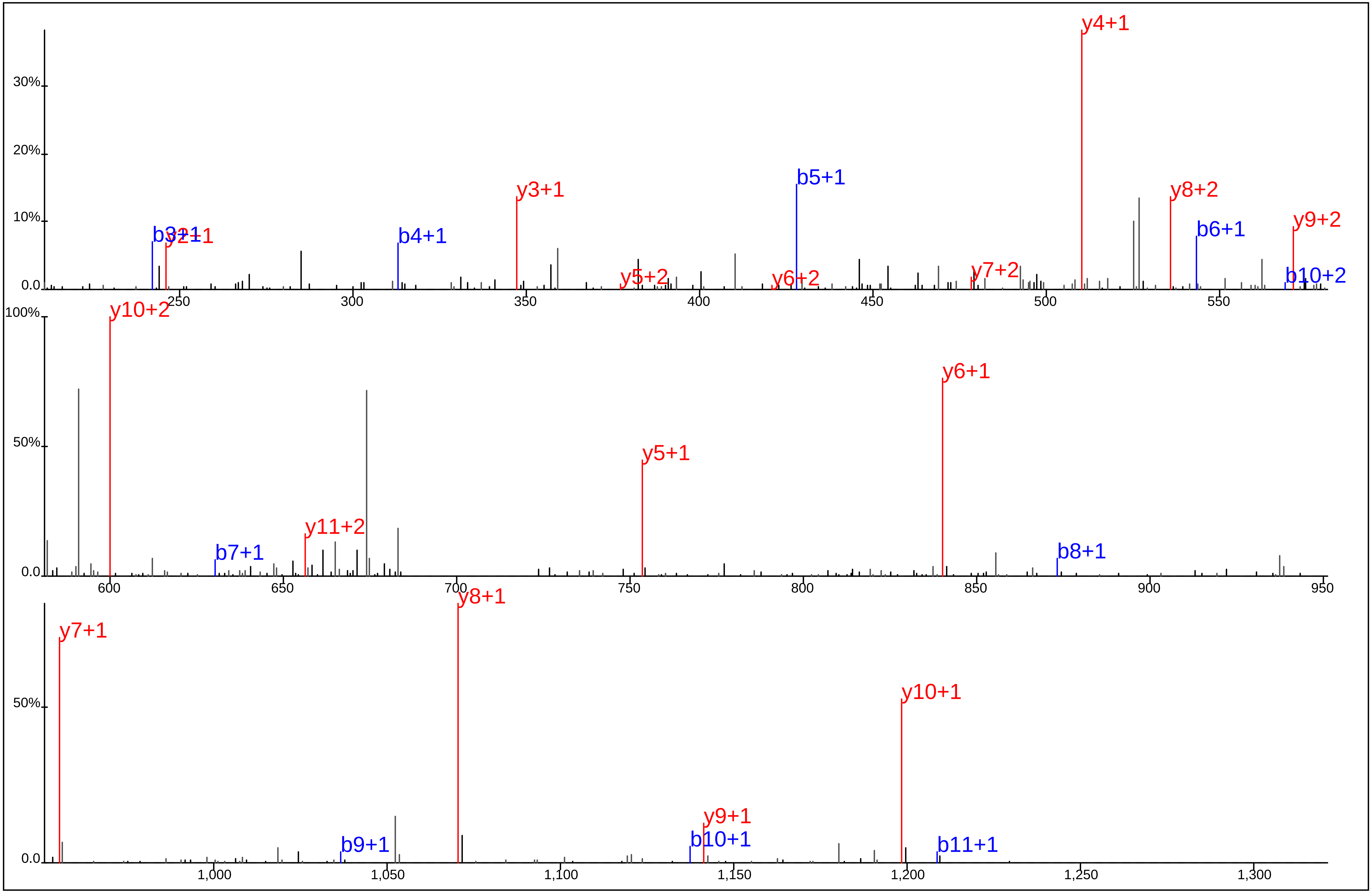

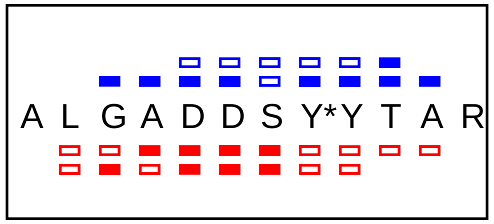

Protein Name: ZAP70
gi Accession: 31455611
Precursor Mass: 691.7852+2
Delta Mass: 0.570 ppm
XCorr: 83.35

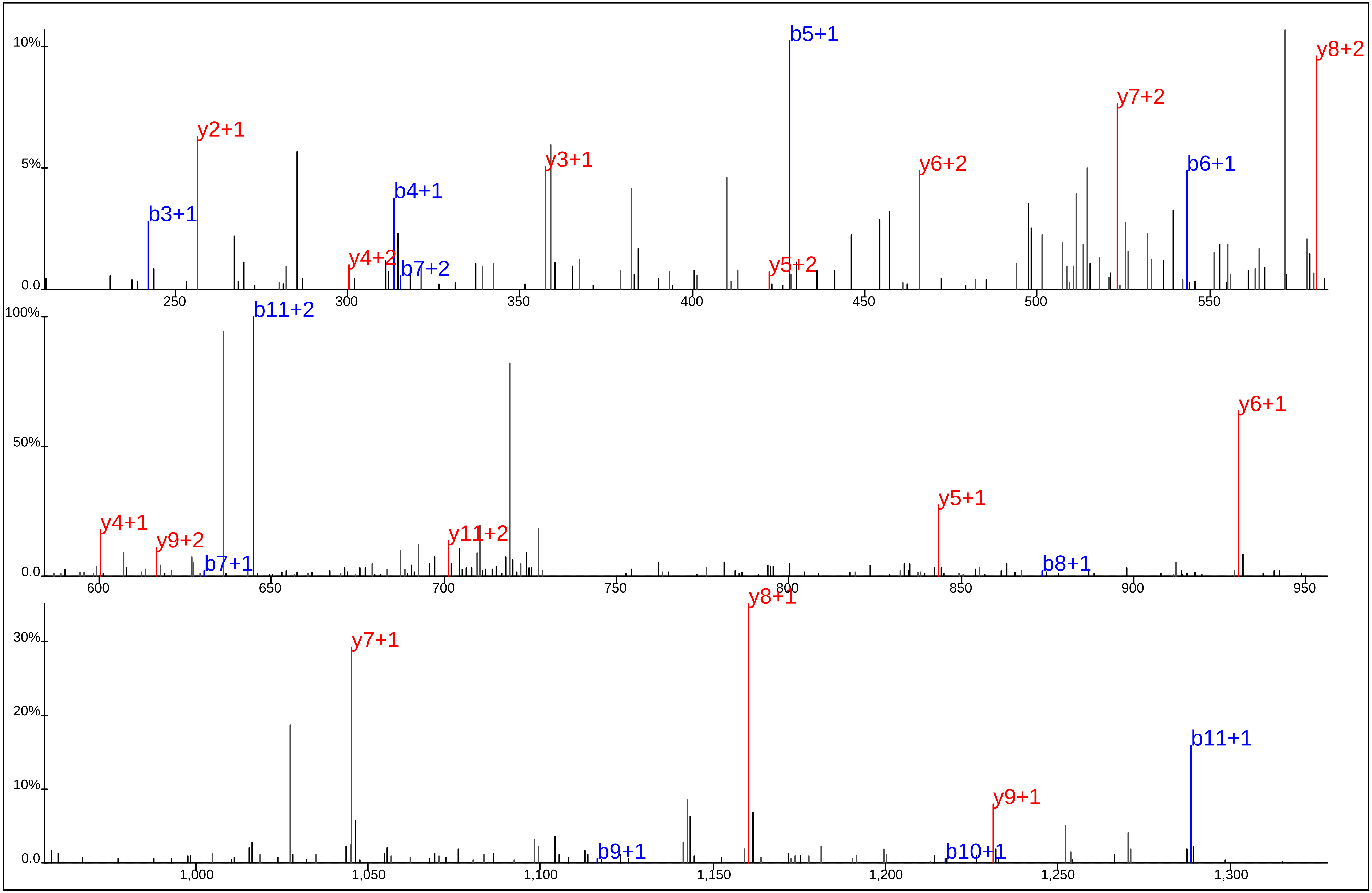

A L G A D D S Y*Y*T A R%

Protein Name: ZAP70
gi Accession: 31455611
Precursor Mass: 736.7730+2
Delta Mass: 1.201 ppm
XCorr: 78.01

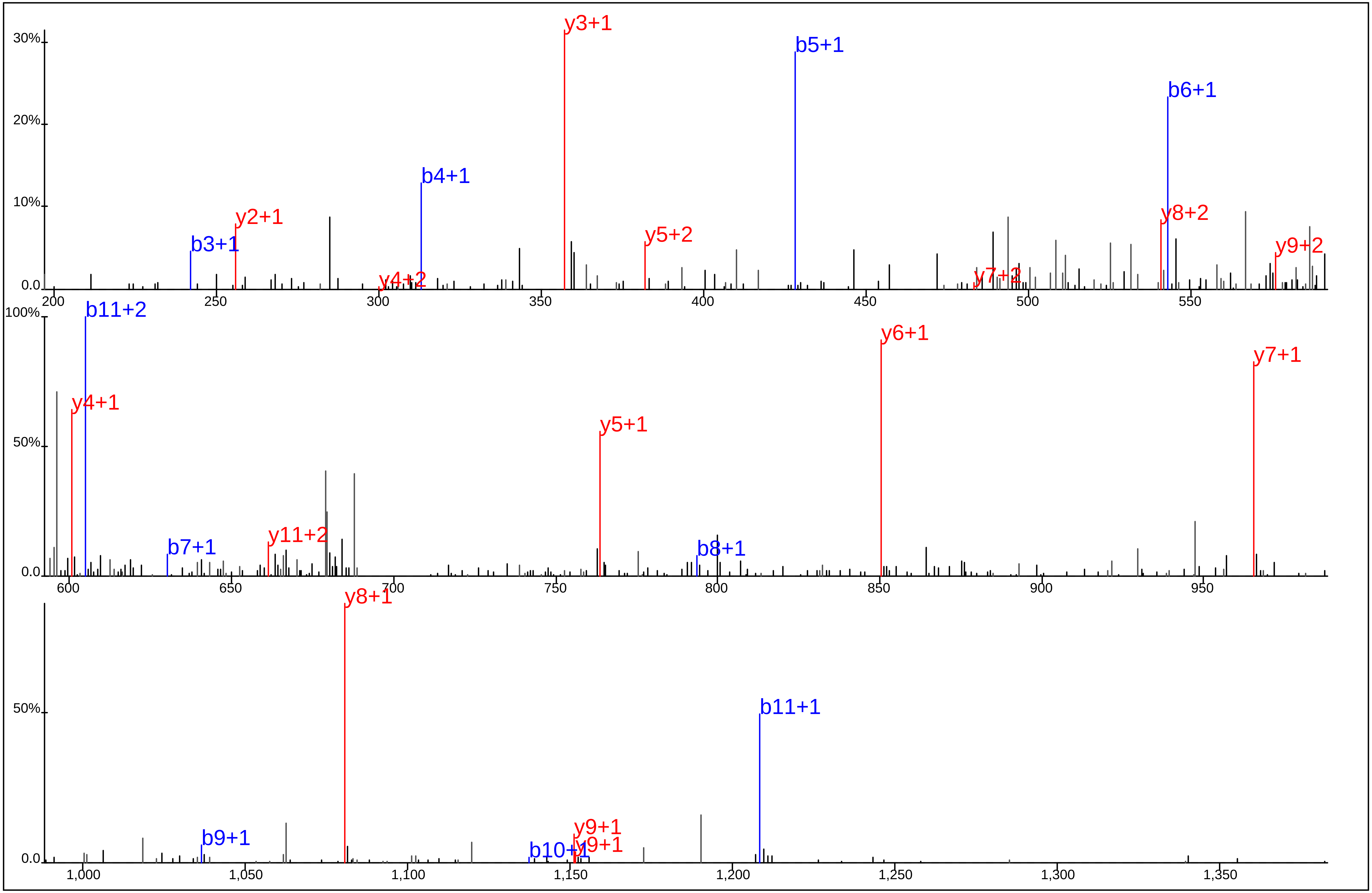

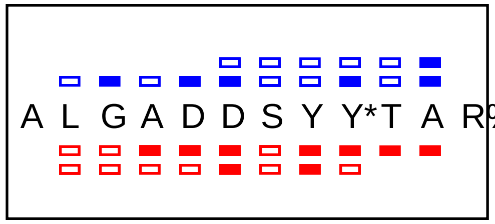

Protein Name: ZAP70
gi Accession: 31455611
Precursor Mass: 696.7906+2
Delta Mass: 2.340 ppm
XCorr: 71.59

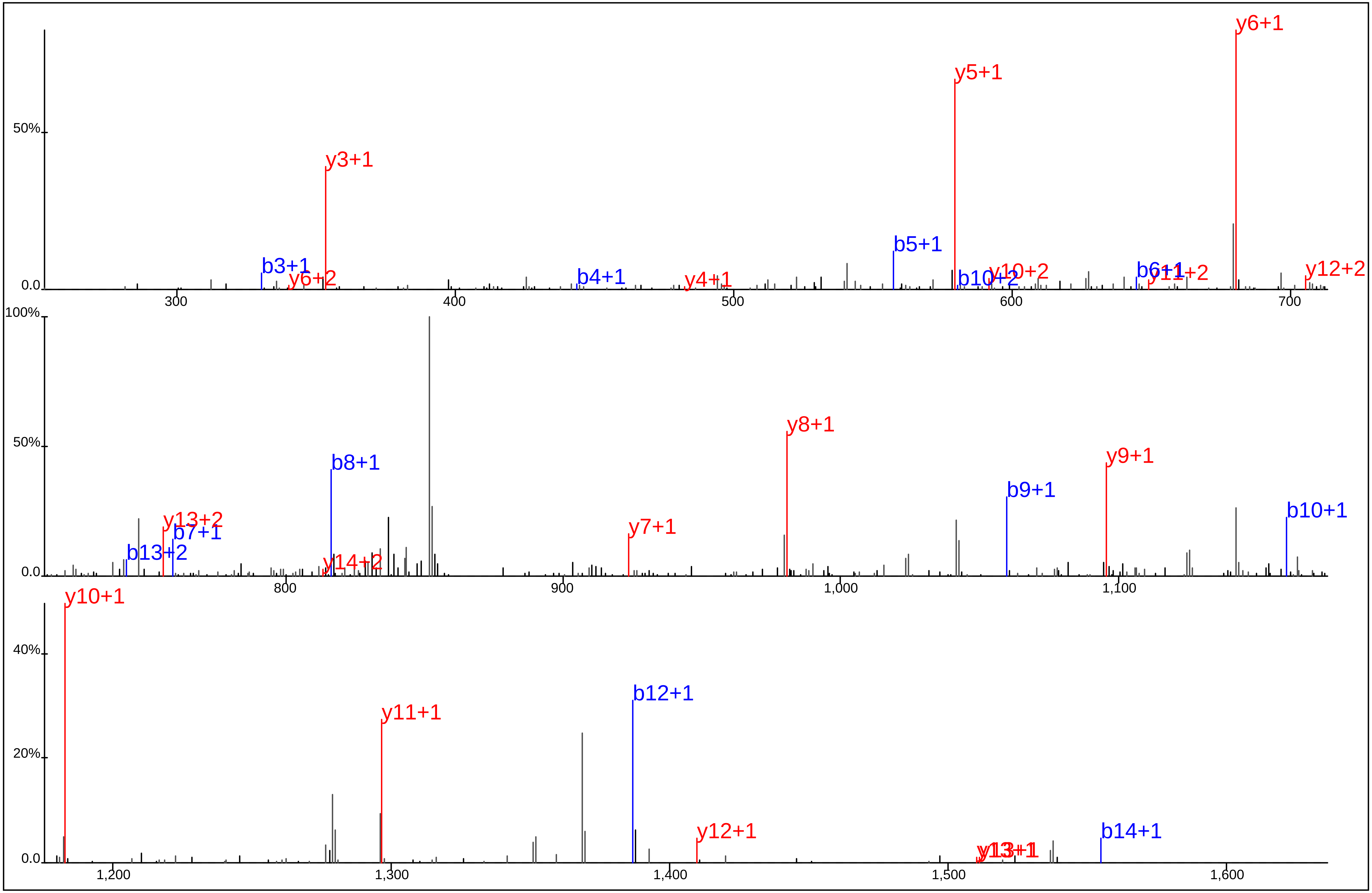

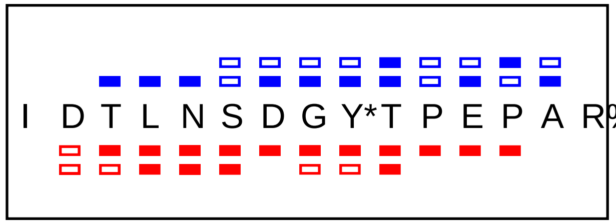

Protein Name: ZAP70
gi Accession: 31455611
Precursor Mass: 869.8851+2
Delta Mass: 4.013 ppm
XCorr: 77.66

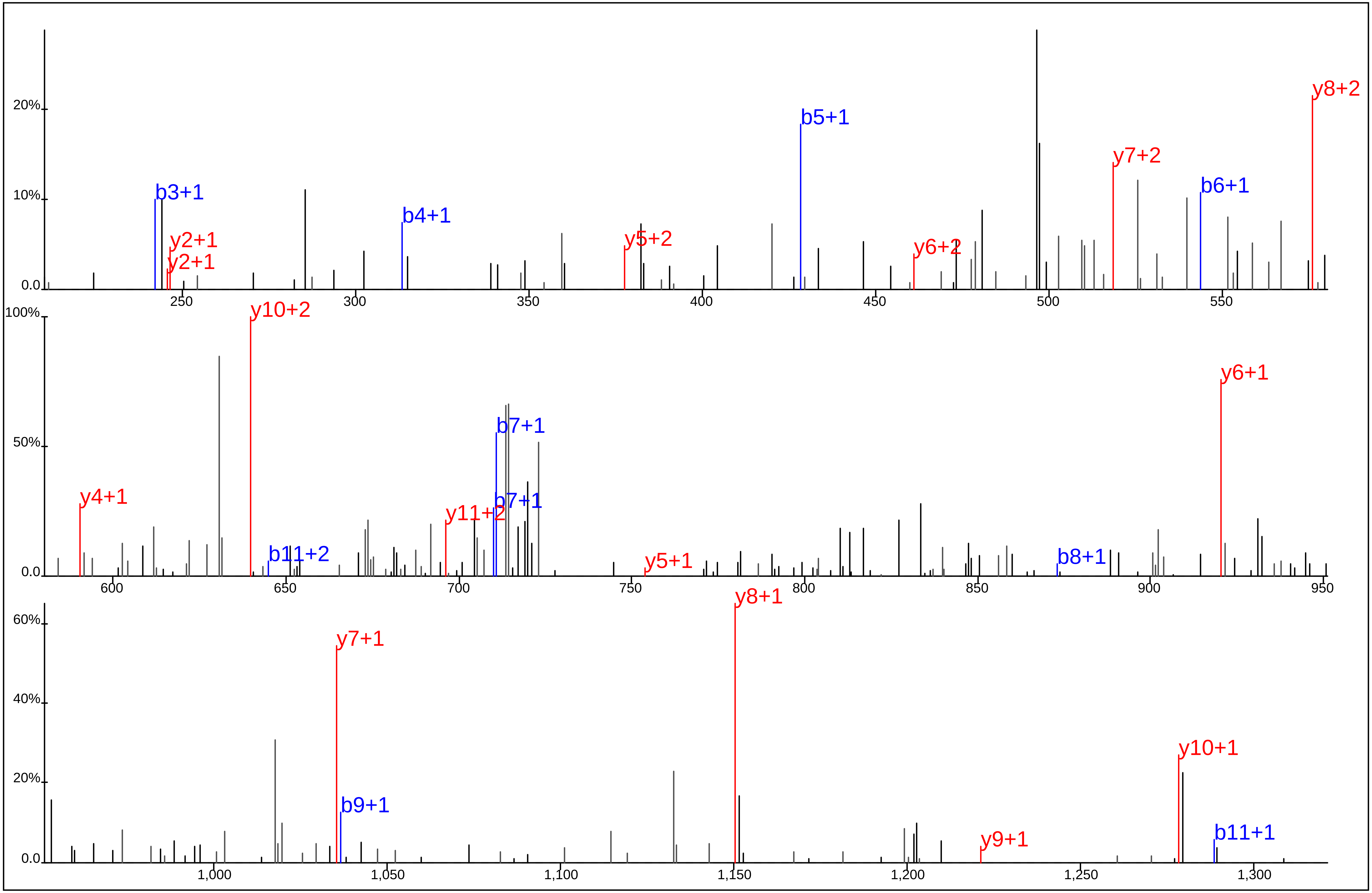

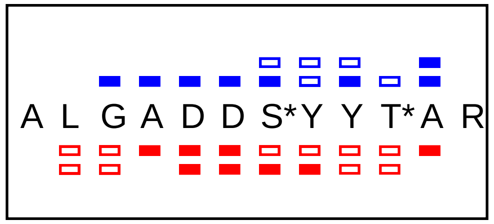

Protein Name: ZAP70
gi Accession: 31455611
Precursor Mass: 731.7704+2
Delta Mass: 3.274 ppm
XCorr: 44.88

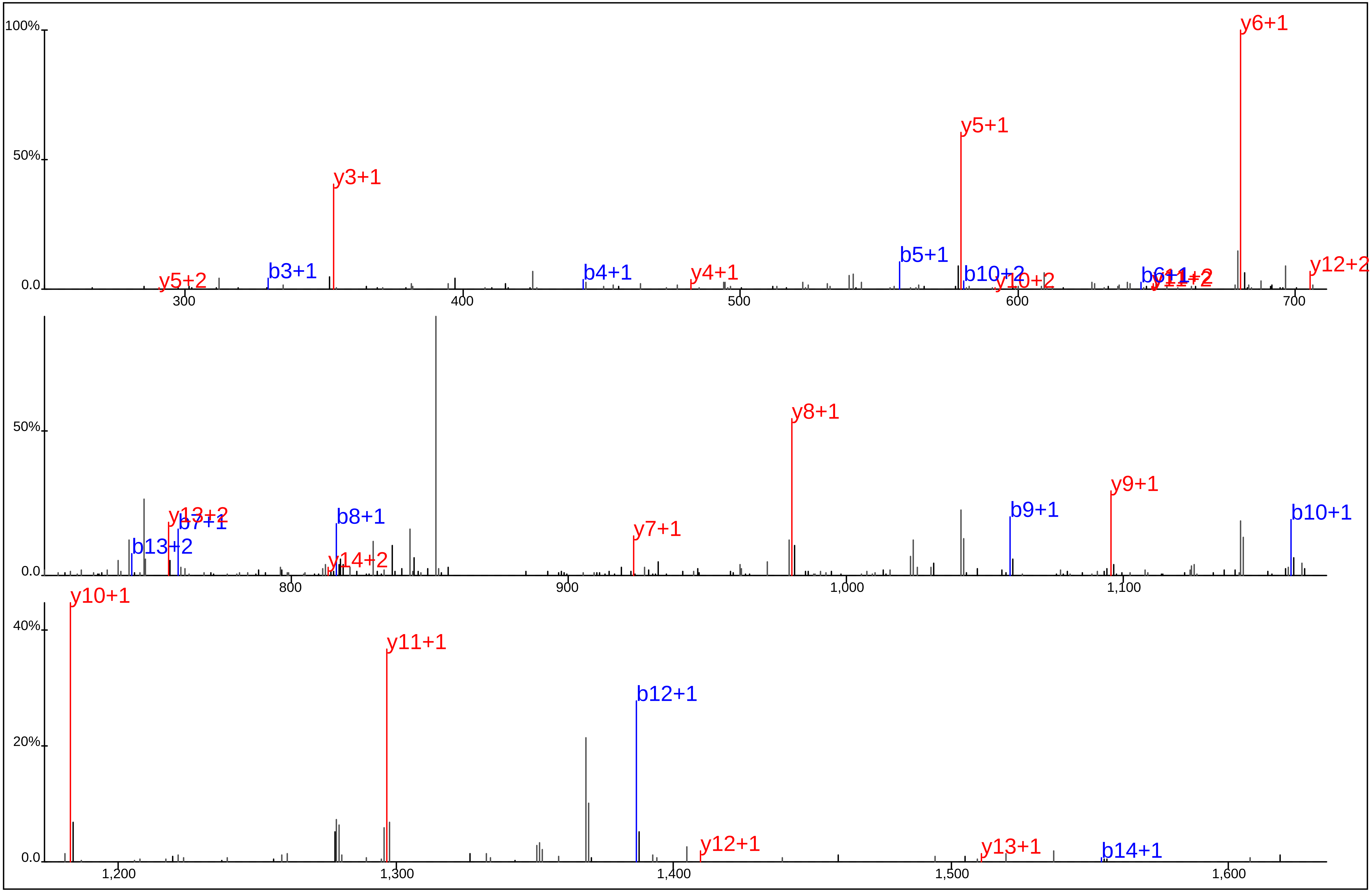

I D T L N S D G Y*T P E P A R%

Protein Name: ZAP70
gi Accession: 31455611
Precursor Mass: 869.8840+2
Delta Mass: 2.823 ppm
XCorr: 75.97

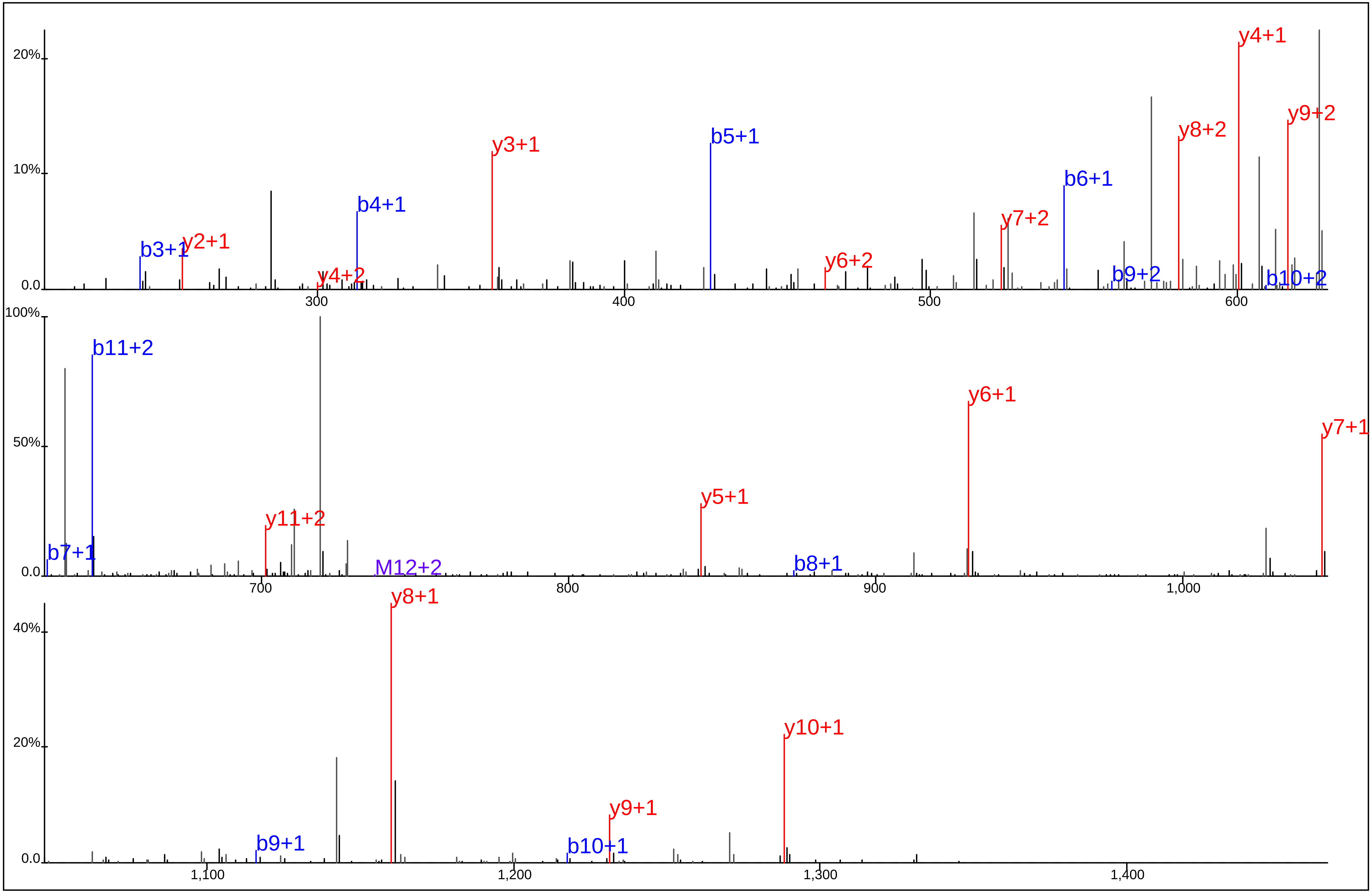

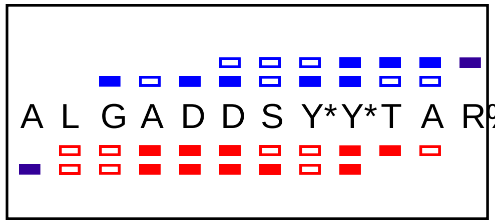

Protein Name: ZAP70
gi Accession: 31455611
Precursor Mass: 736.7739+2
Delta Mass: 2.443 ppm
XCorr: 70.11

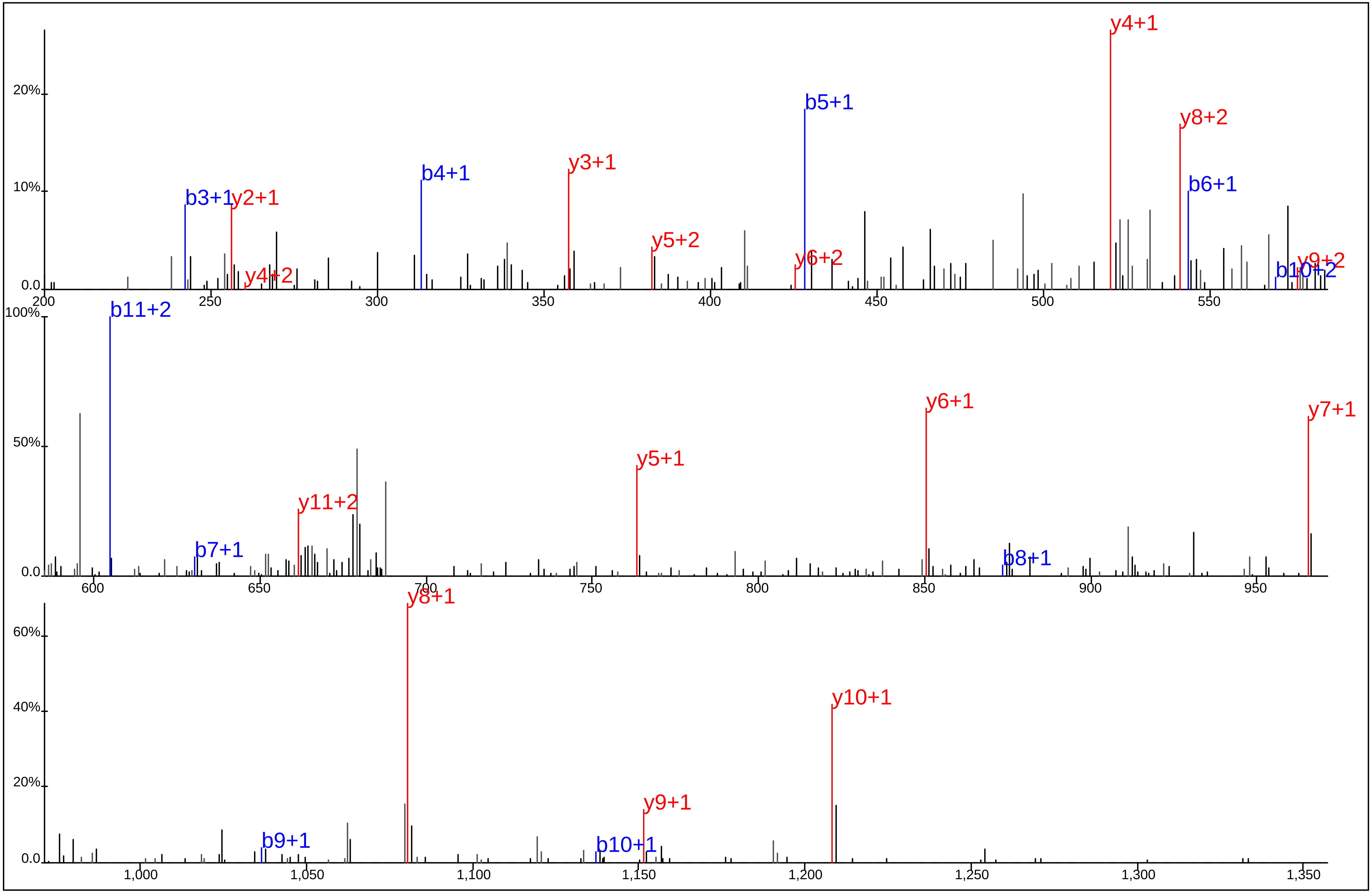

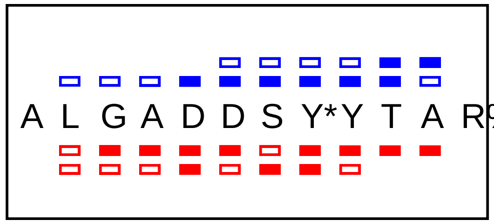

Protein Name: ZAP70
gi Accession: 31455611
Precursor Mass: 696.7920+2
Delta Mass: 4.357 ppm
XCorr: 68.16

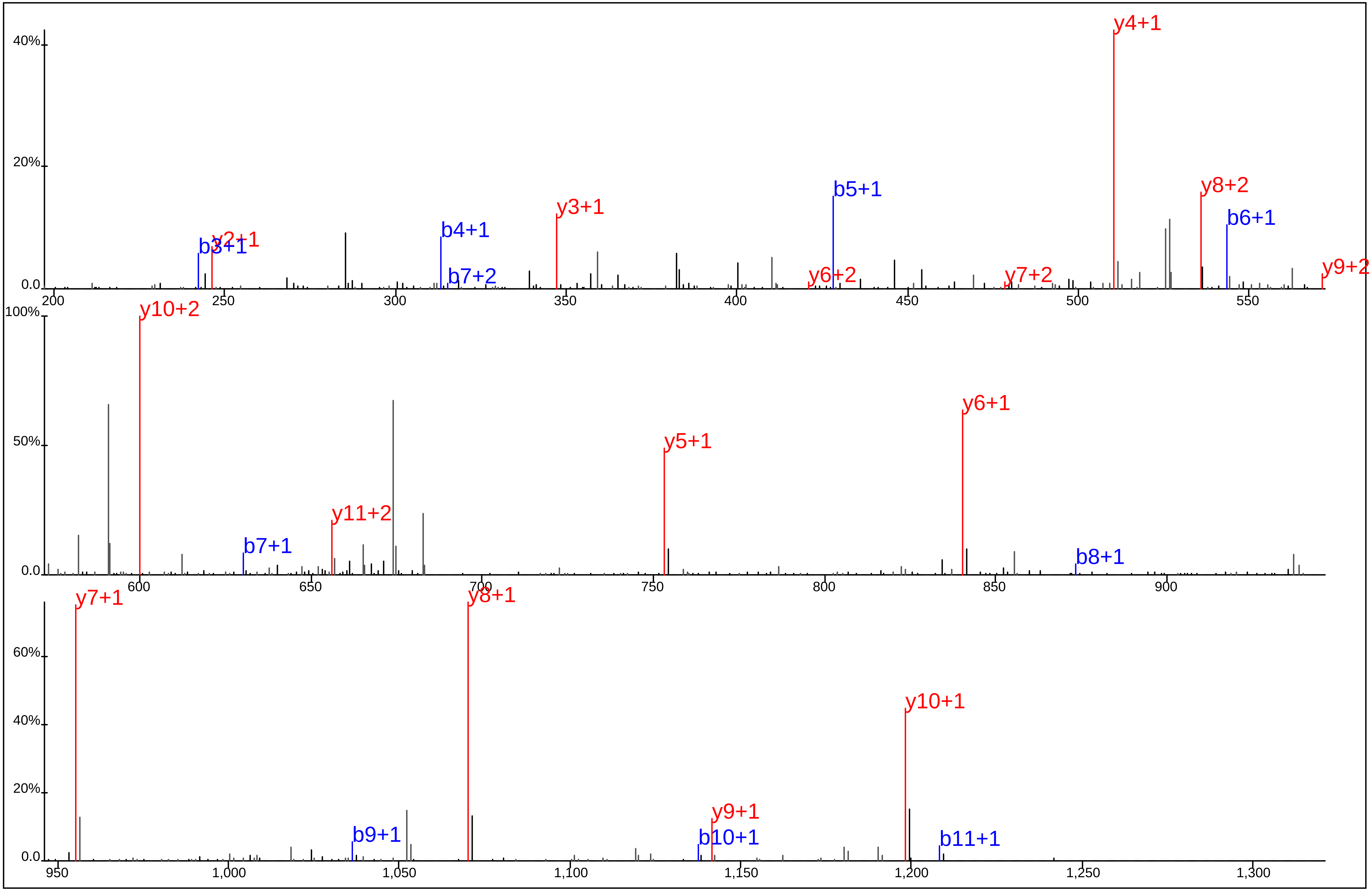

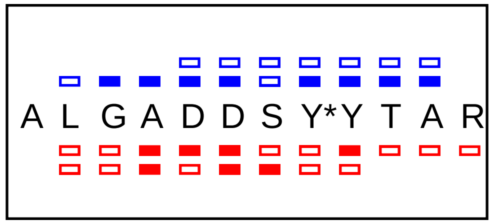

Protein Name: ZAP70
gi Accession: 31455611
Precursor Mass: 691.7852+2
Delta Mass: 0.483 ppm
XCorr: 74.18

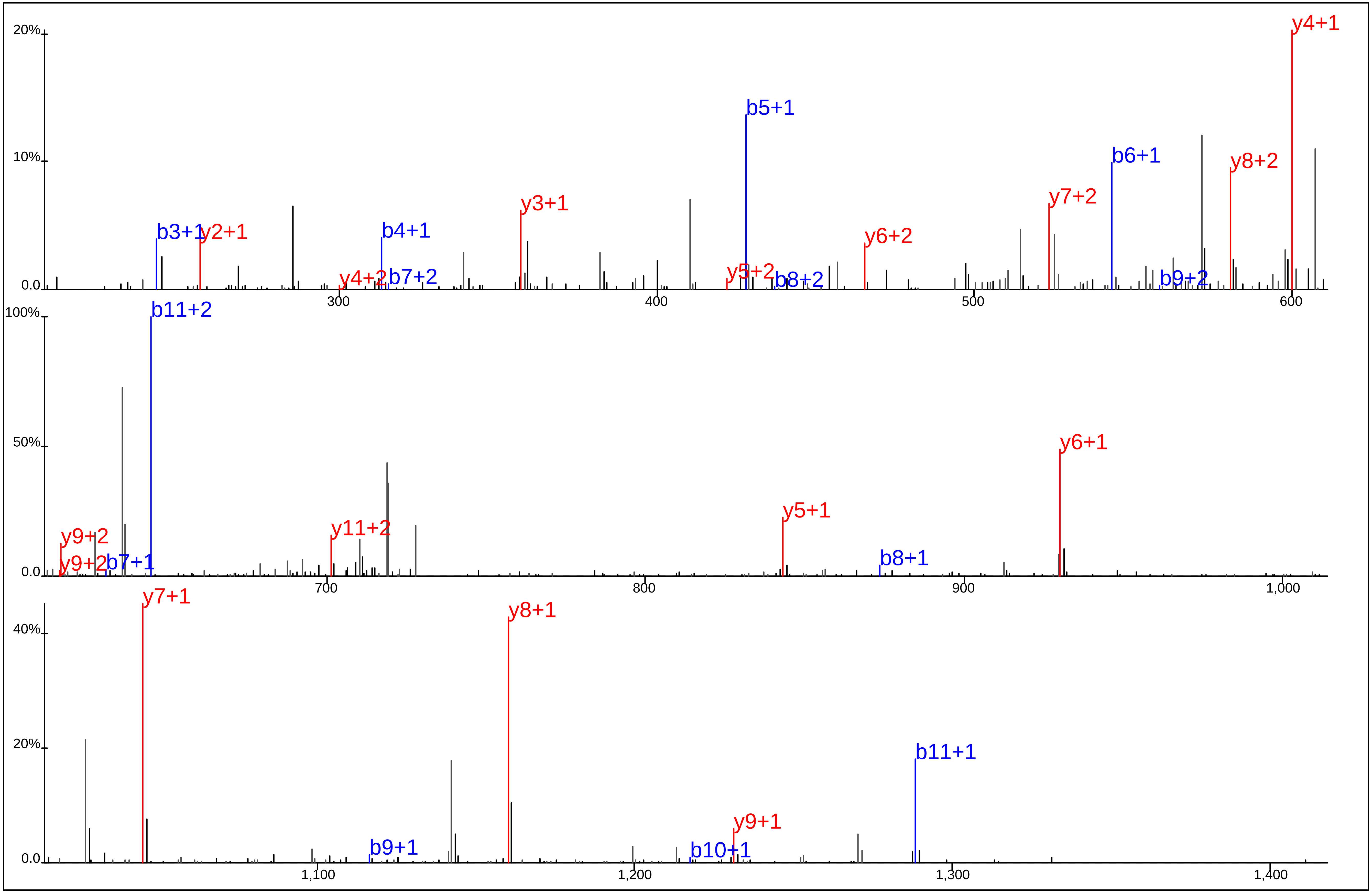

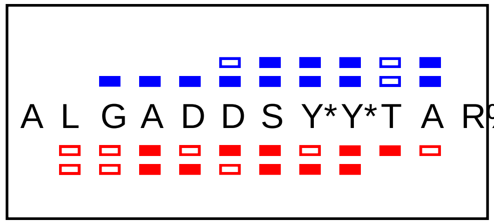

Protein Name: ZAP70
gi Accession: 31455611
Precursor Mass: 736.7750+2
Delta Mass: 3.856 ppm
XCorr: 65.7

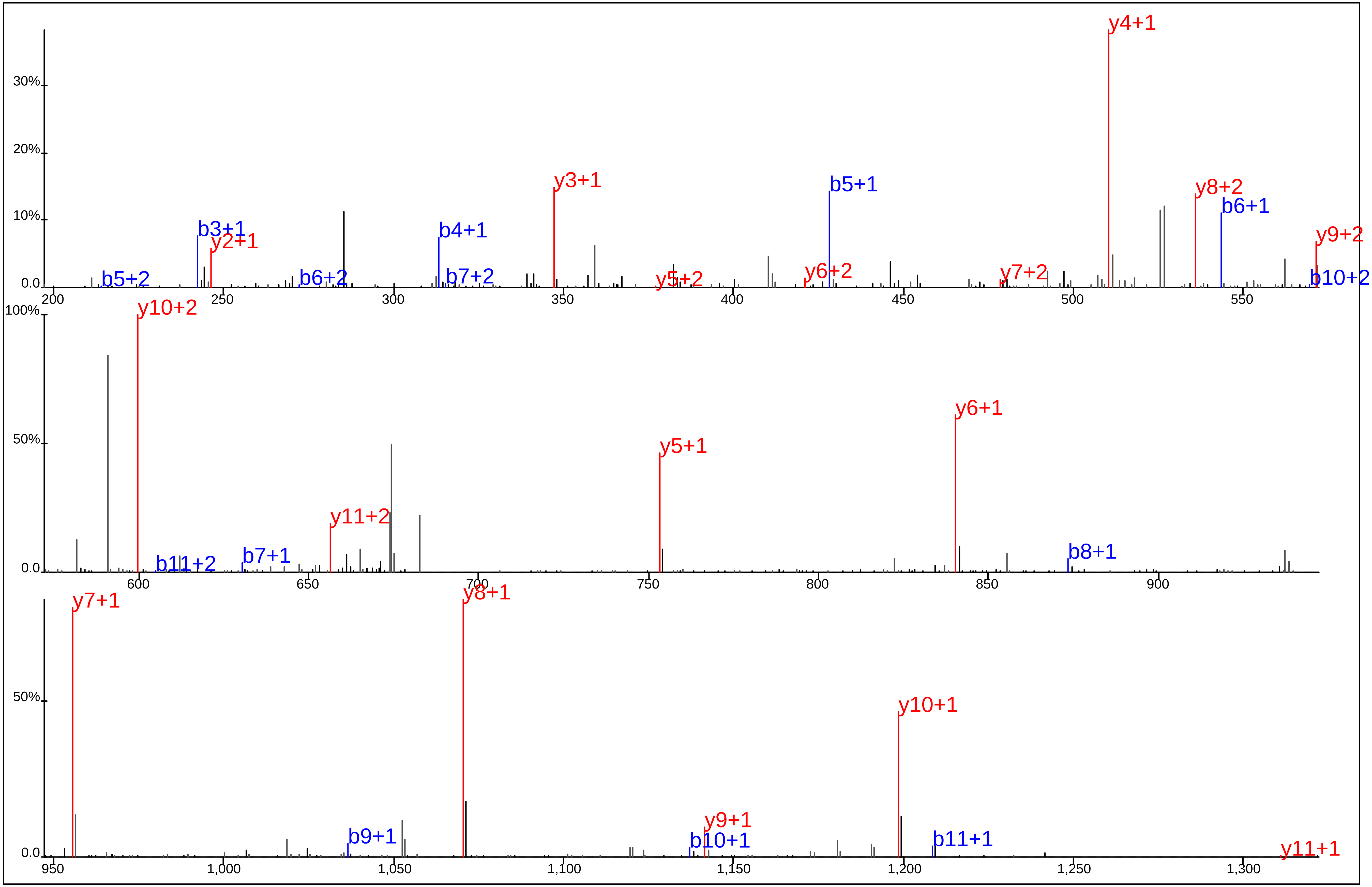

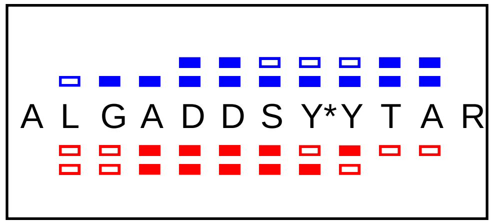

Protein Name: ZAP70
gi Accession: 31455611
Precursor Mass: 691.7852+2
Delta Mass: 0.483 ppm
XCorr: 79.5

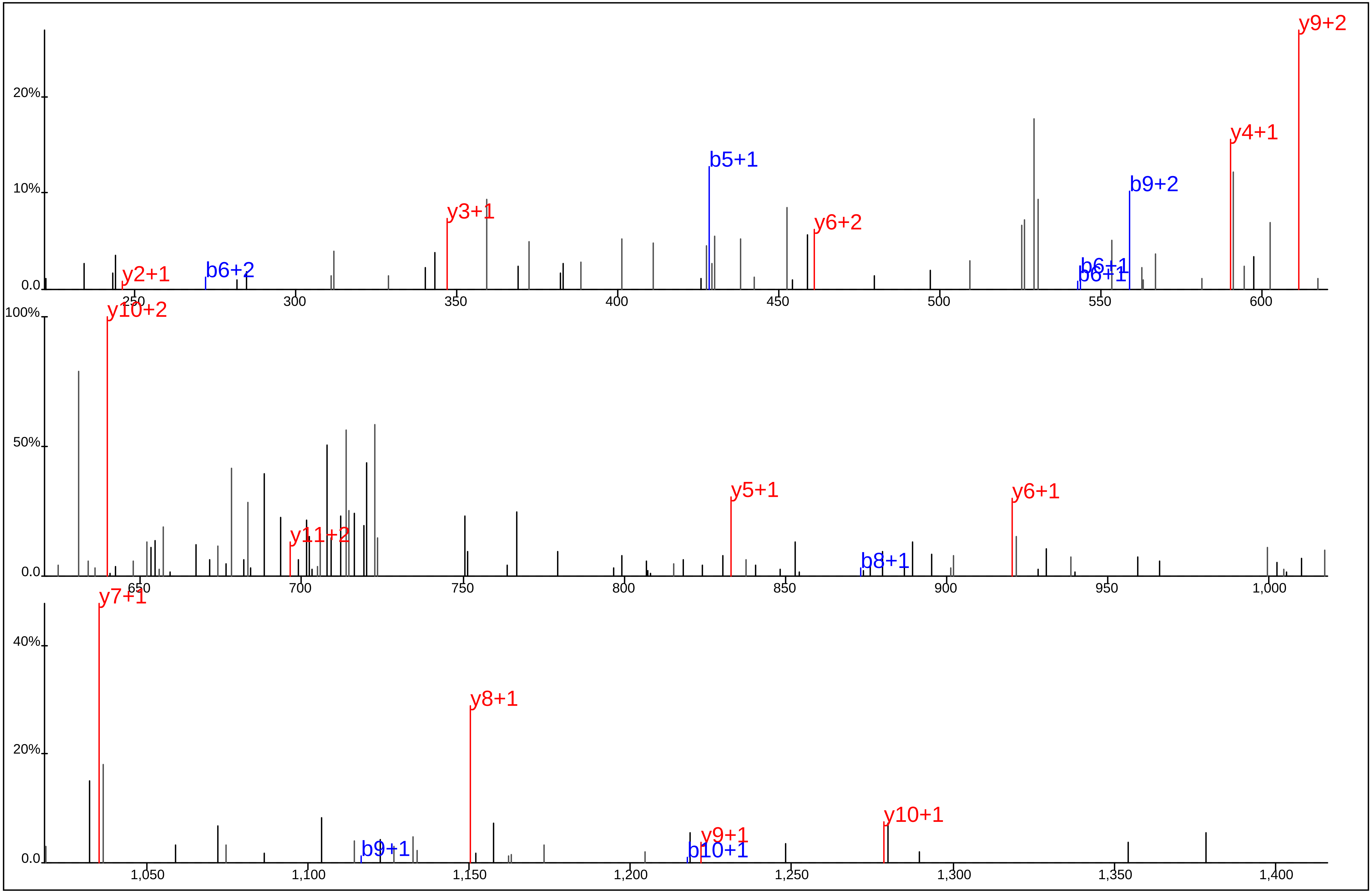

A L G A D D S Y*Y*T A R

Protein Name: ZAP70
gi Accession: 31455611
Precursor Mass: 731.7725+2
Delta Mass: 6.118 ppm
XCorr: 29.37

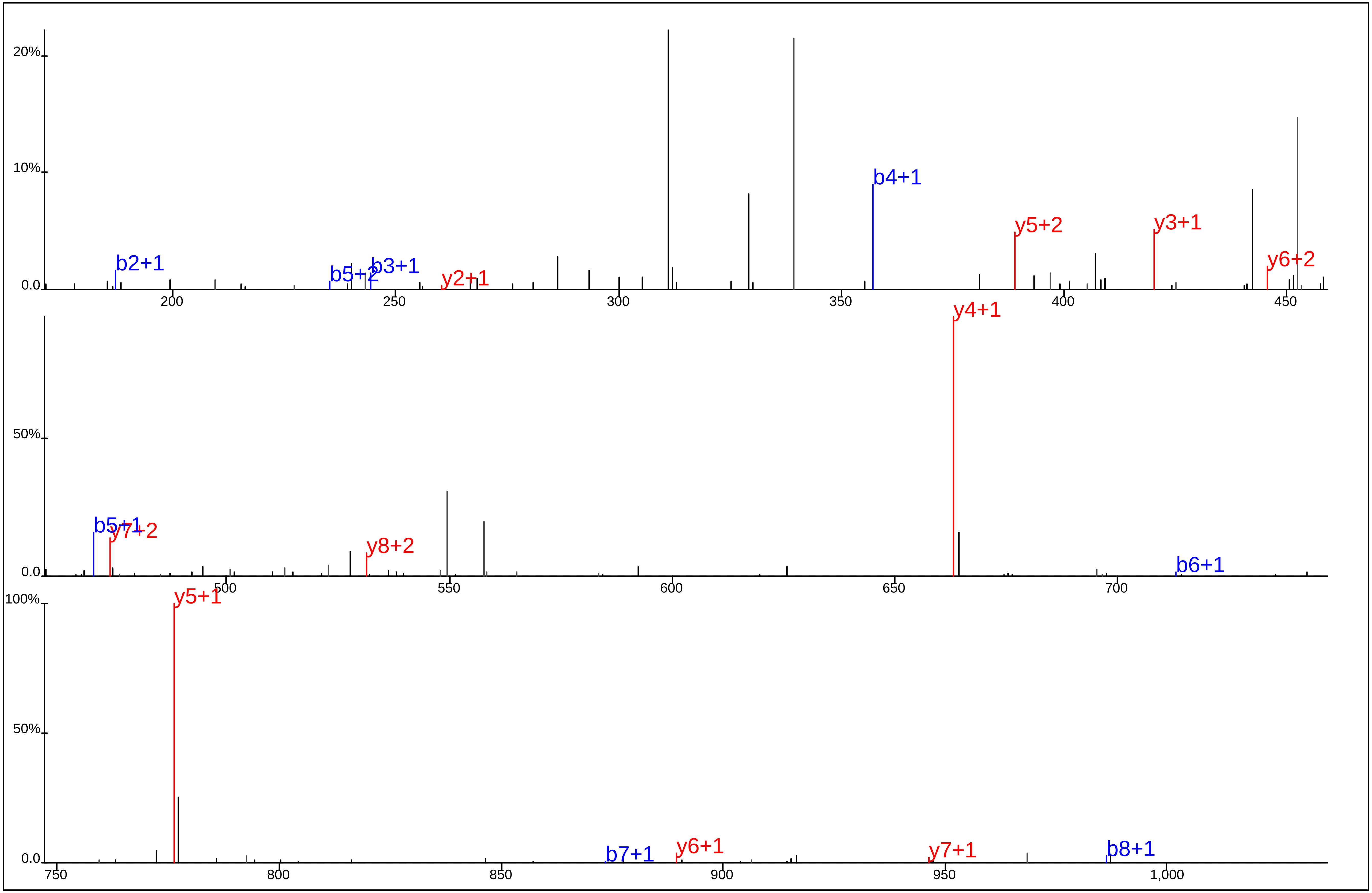

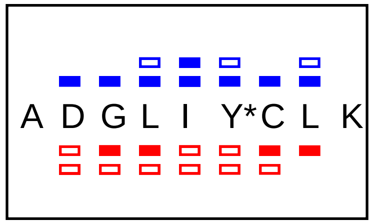

Protein Name: ZAP70
gi Accession: 31455611
Precursor Mass: 566.7603+2
Delta Mass: 2.250 ppm
XCorr: 31.86

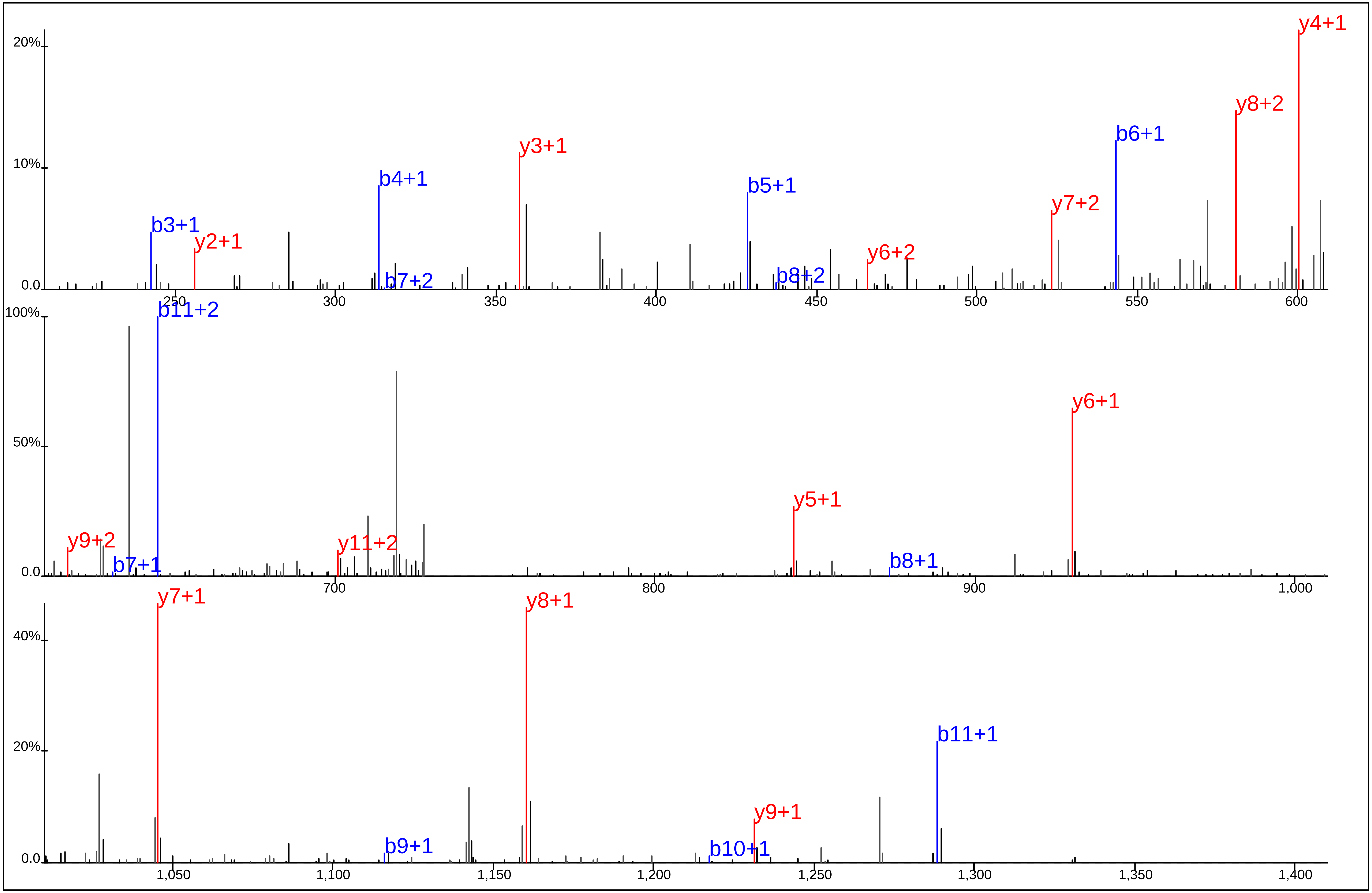

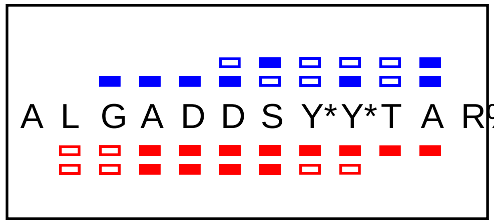

Protein Name: ZAP70
gi Accession: 31455611
Precursor Mass: 736.7729+2
Delta Mass: 1.038 ppm
XCorr: 65.06

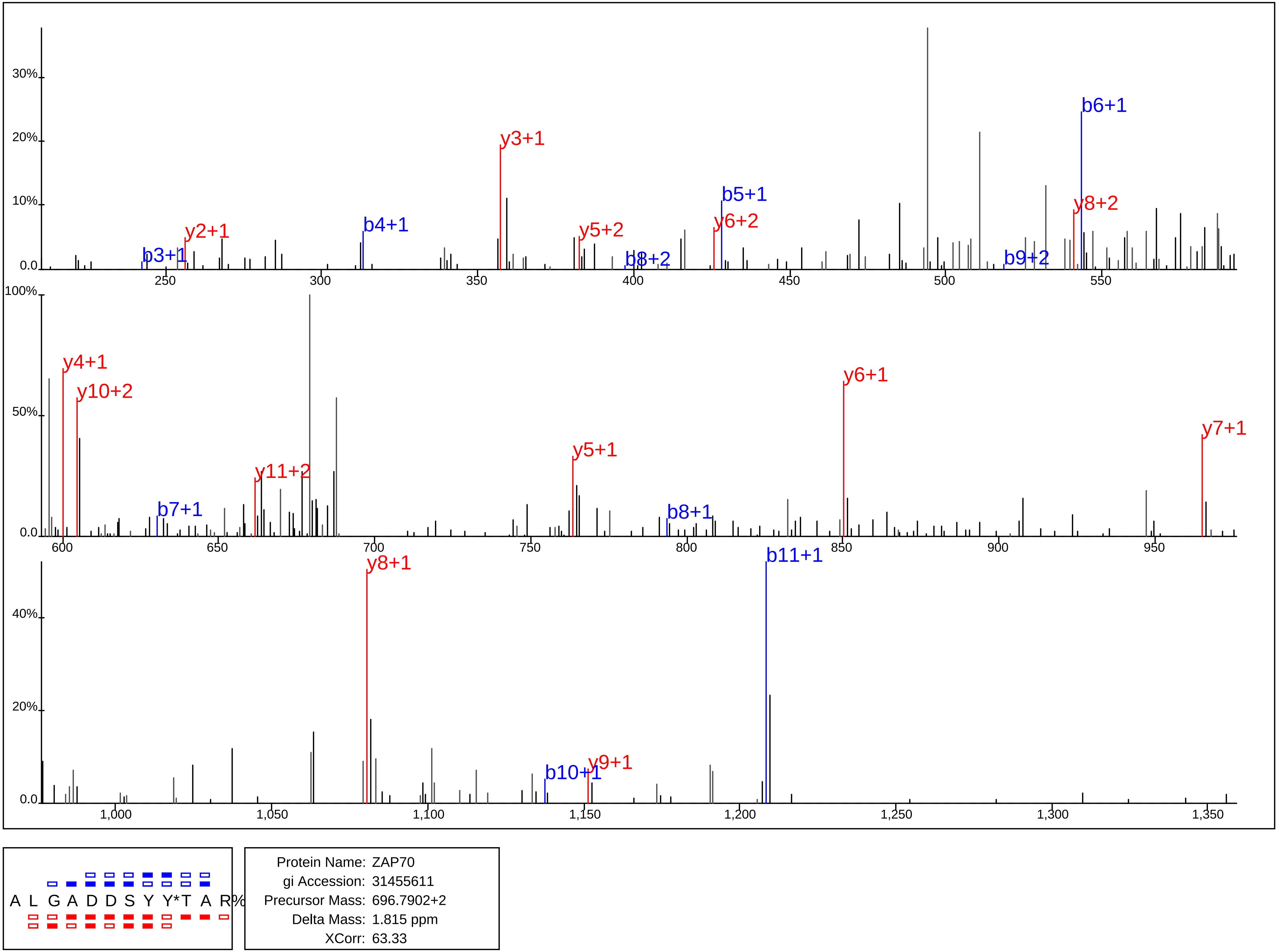

Protein Name: ZAP70
gi Accession: 31455611
Precursor Mass: 696.7902+2
Delta Mass: 1.815 ppm
XCorr: 63.33

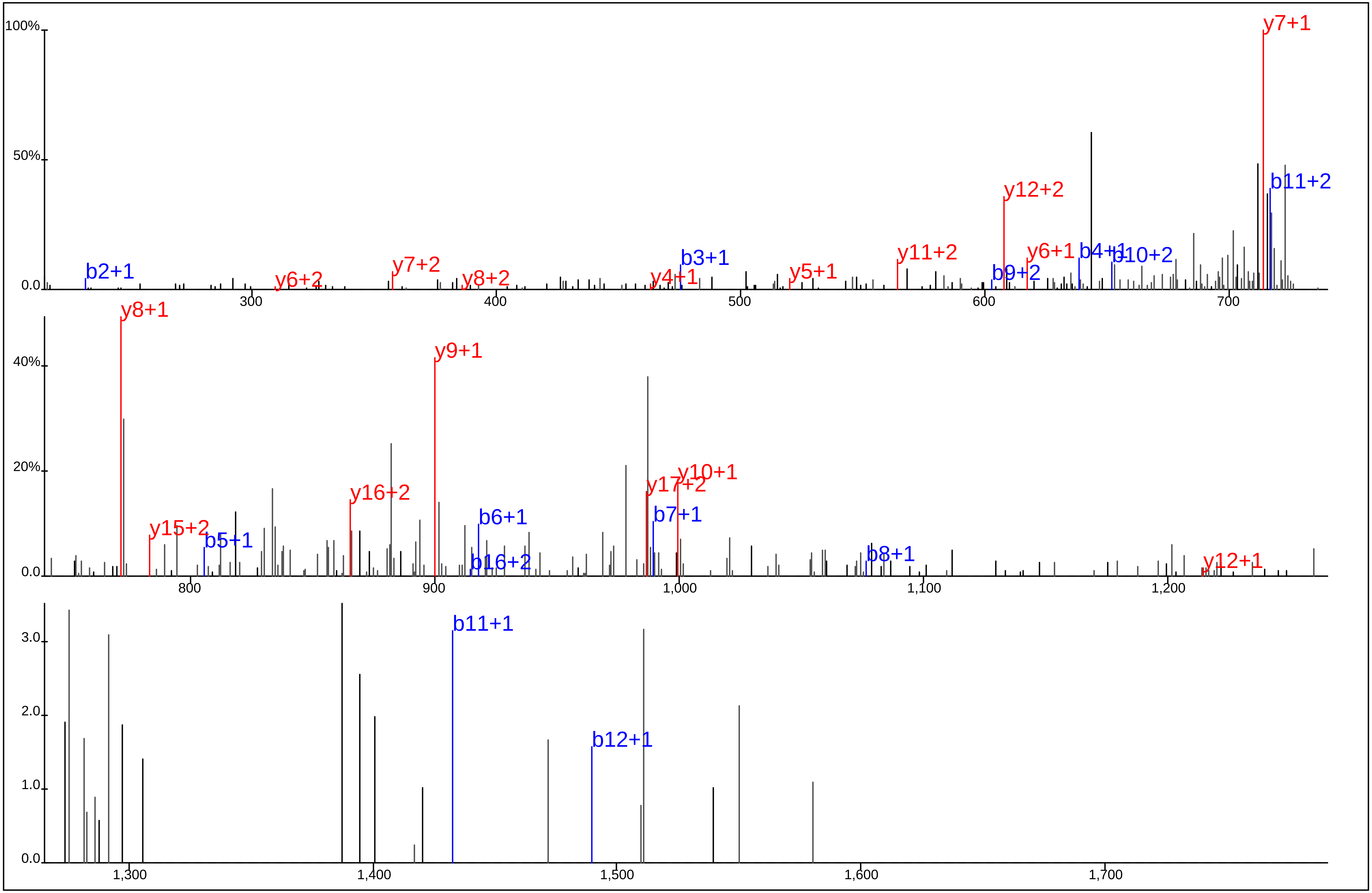

A C Y*Y S*L A S K V E G P P G S T Q K

Protein Name: ZAP70
gi Accession: 31455611
Precursor Mass: 734.9806+3
Delta Mass: 4.276 ppm
XCorr: 23.82

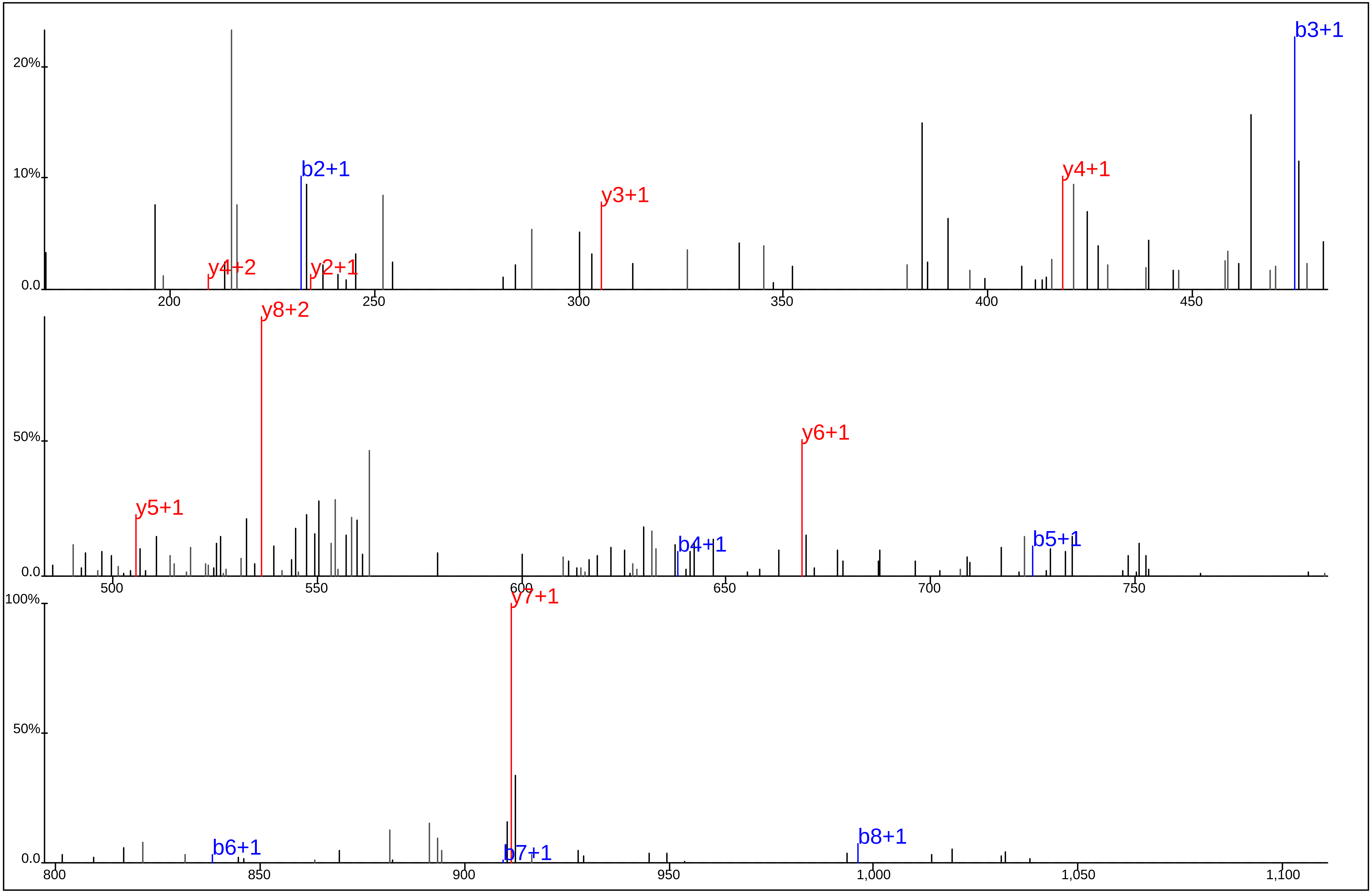

A C Y*Y S L A S K

Protein Name: ZAP70
gi Accession: 31455611
Precursor Mass: 571.7351+2
Delta Mass: 3.666 ppm
XCorr: 13.43

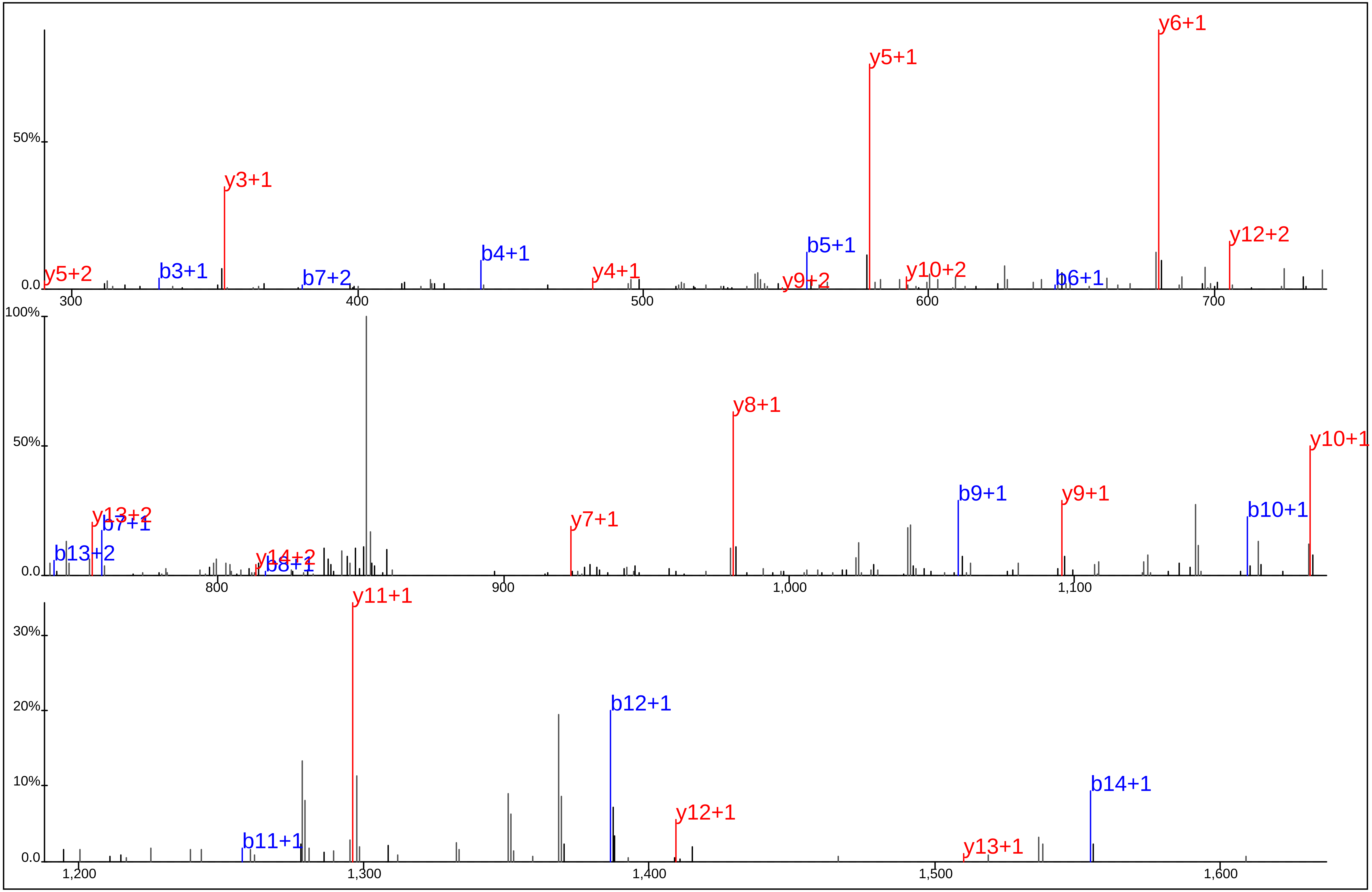

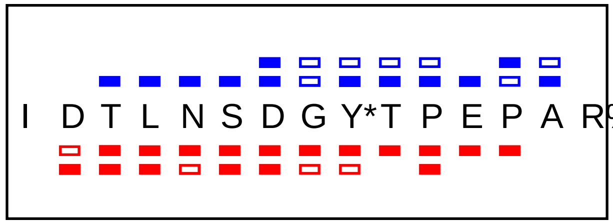

Protein Name: ZAP70
gi Accession: 31455611
Precursor Mass: 869.8835+2
Delta Mass: 2.259 ppm
XCorr: 83.97

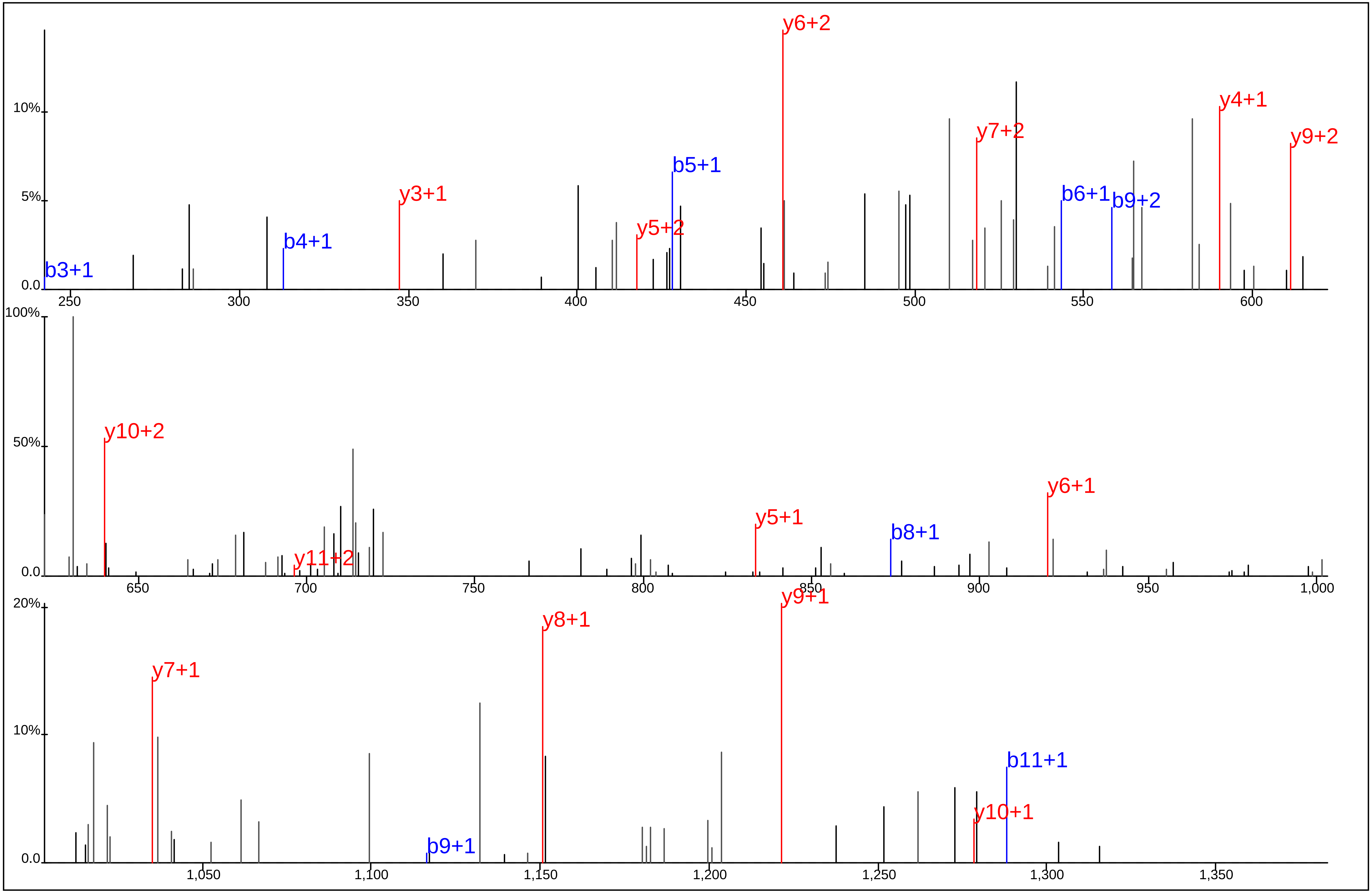

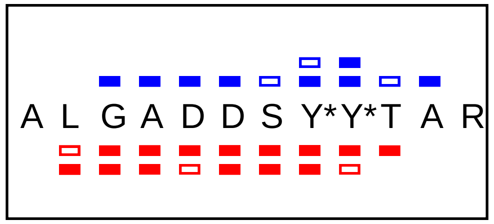

Protein Name: ZAP70
gi Accession: 31455611
Precursor Mass: 731.7758+2
Delta Mass: 10.624 ppm
XCorr: 29.56

1111 - ZAP70_3452616.pdf

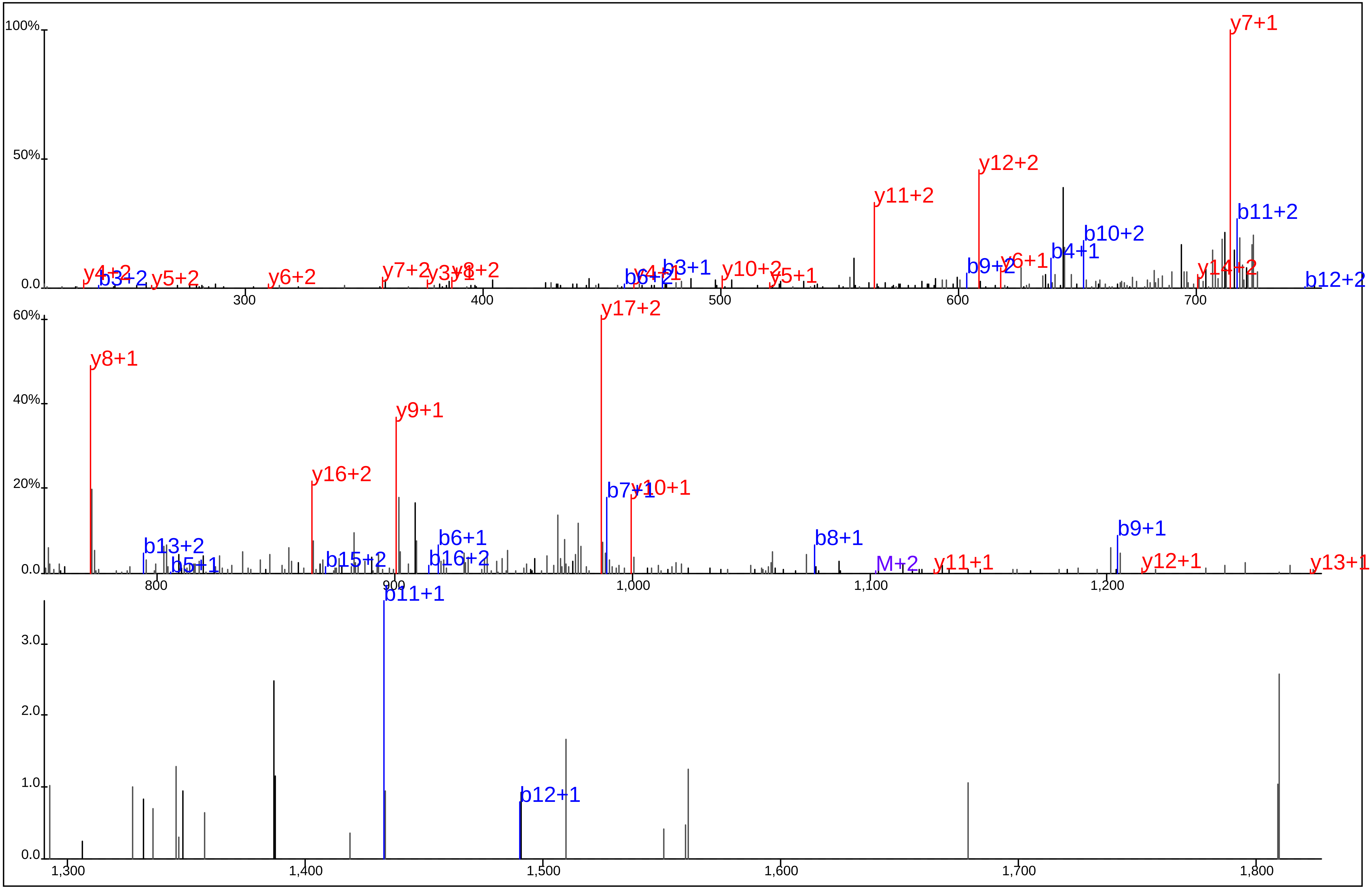

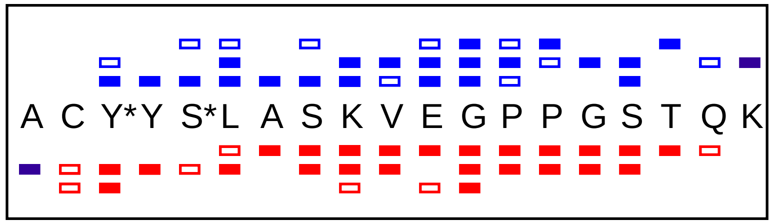

Protein Name: ZAP70
gi Accession: 31455611
Precursor Mass: 734.9777+3
Delta Mass: 0.286 ppm
XCorr: 55.84

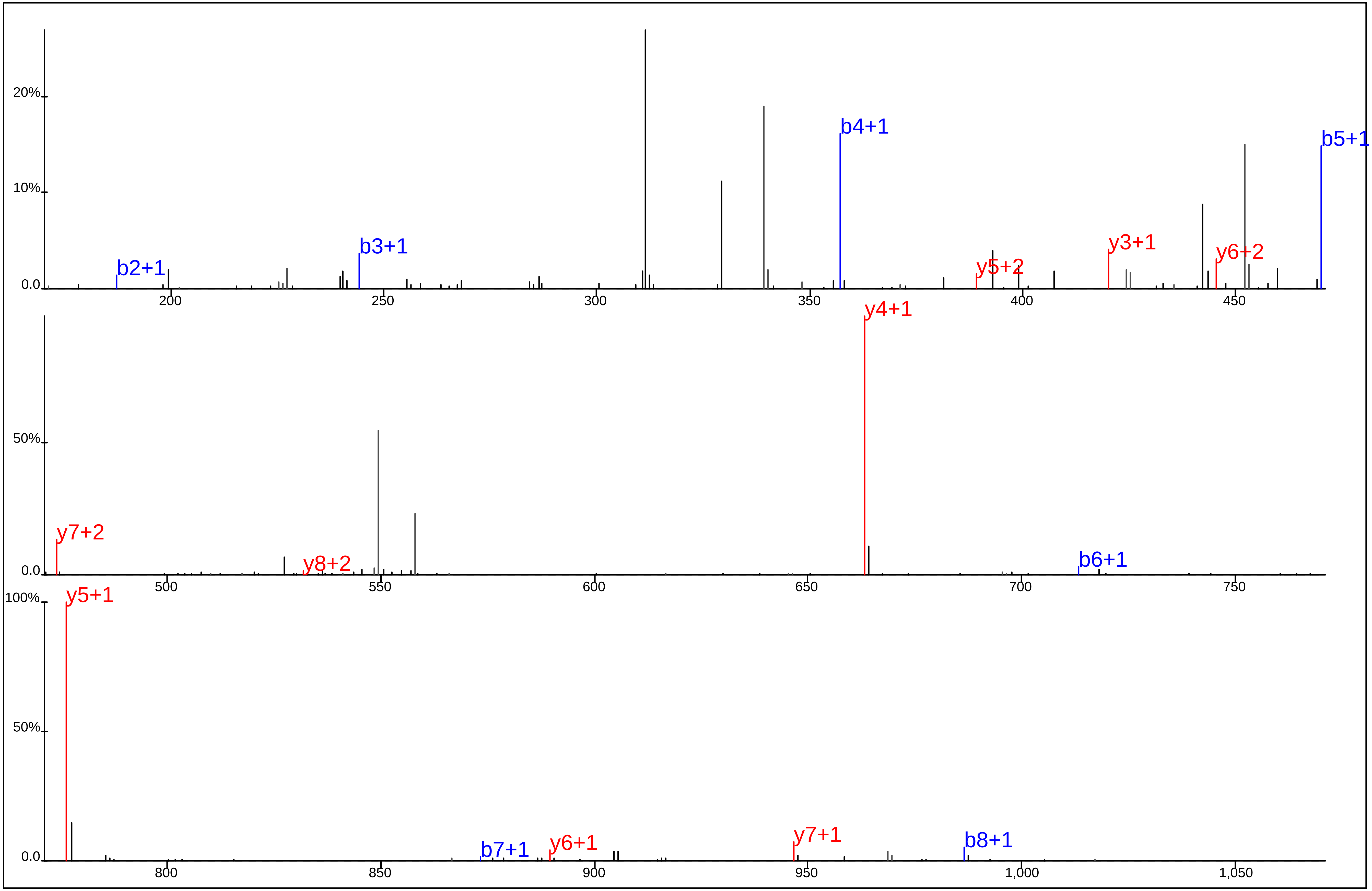

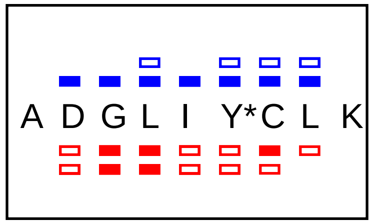

Protein Name: ZAP70
gi Accession: 31455611
Precursor Mass: 566.7616+2
Delta Mass: 4.519 ppm
XCorr: 32.33

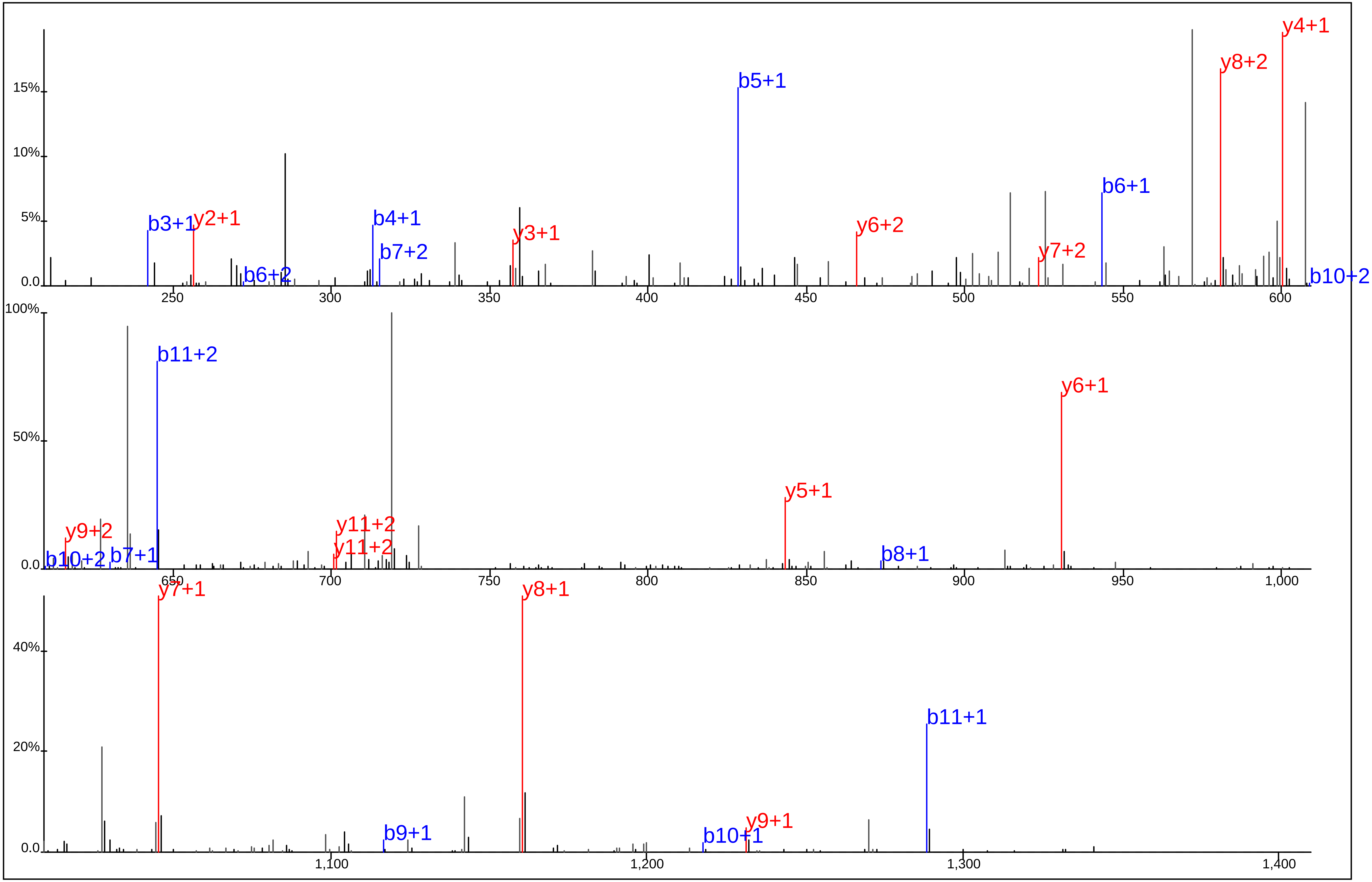

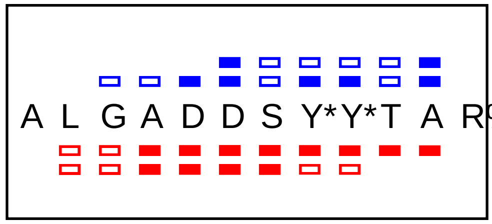

Protein Name: ZAP70
gi Accession: 31455611
Precursor Mass: 736.7742+2
Delta Mass: 2.864 ppm
XCorr: 63.51

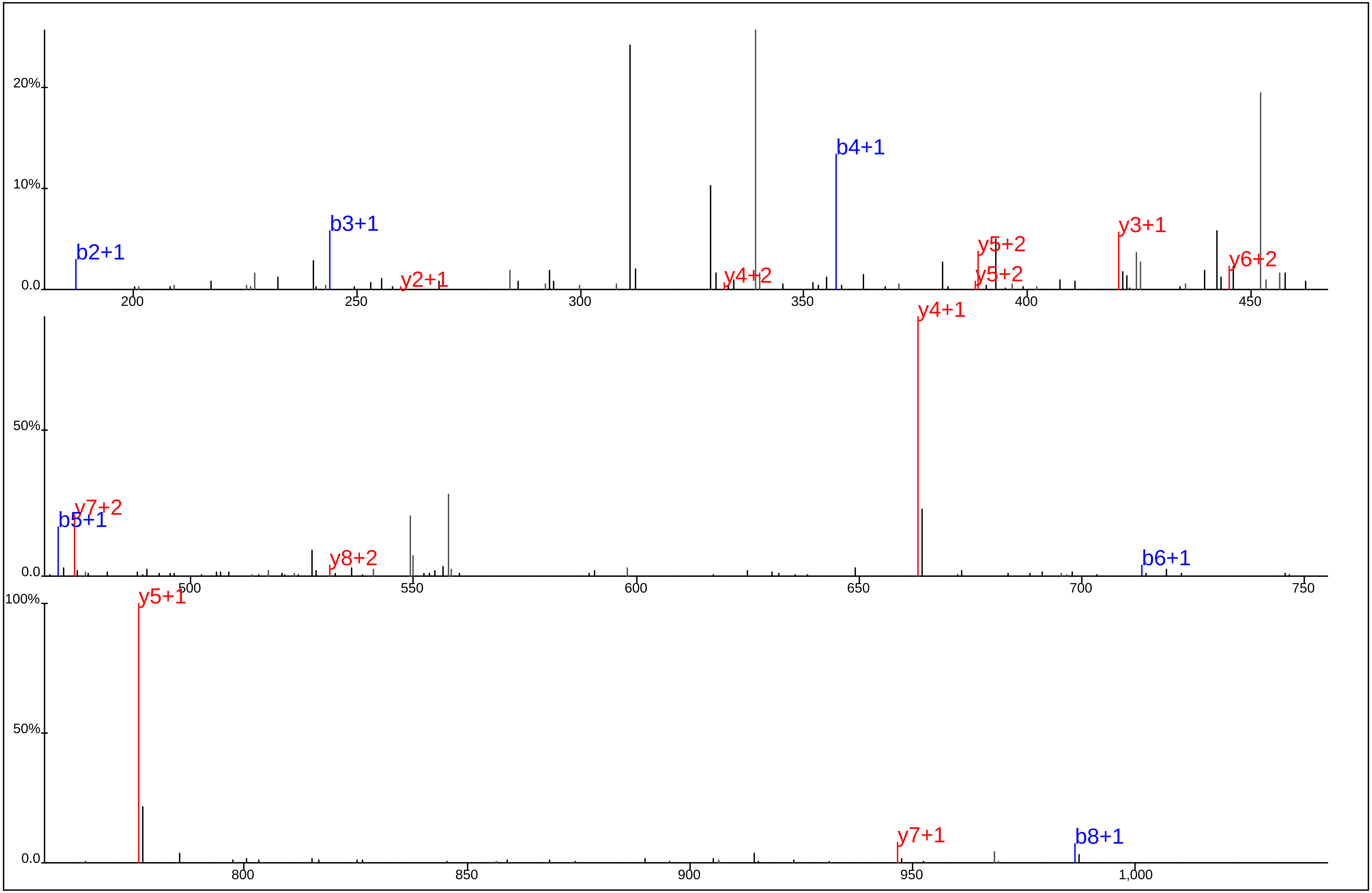

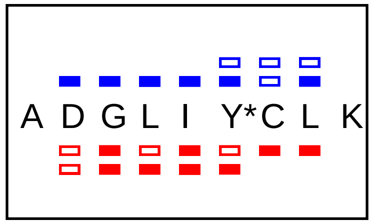

Protein Name: ZAP70
gi Accession: 31455611
Precursor Mass: 566.7612+2
Delta Mass: 3.760 ppm
XCorr: 35.81

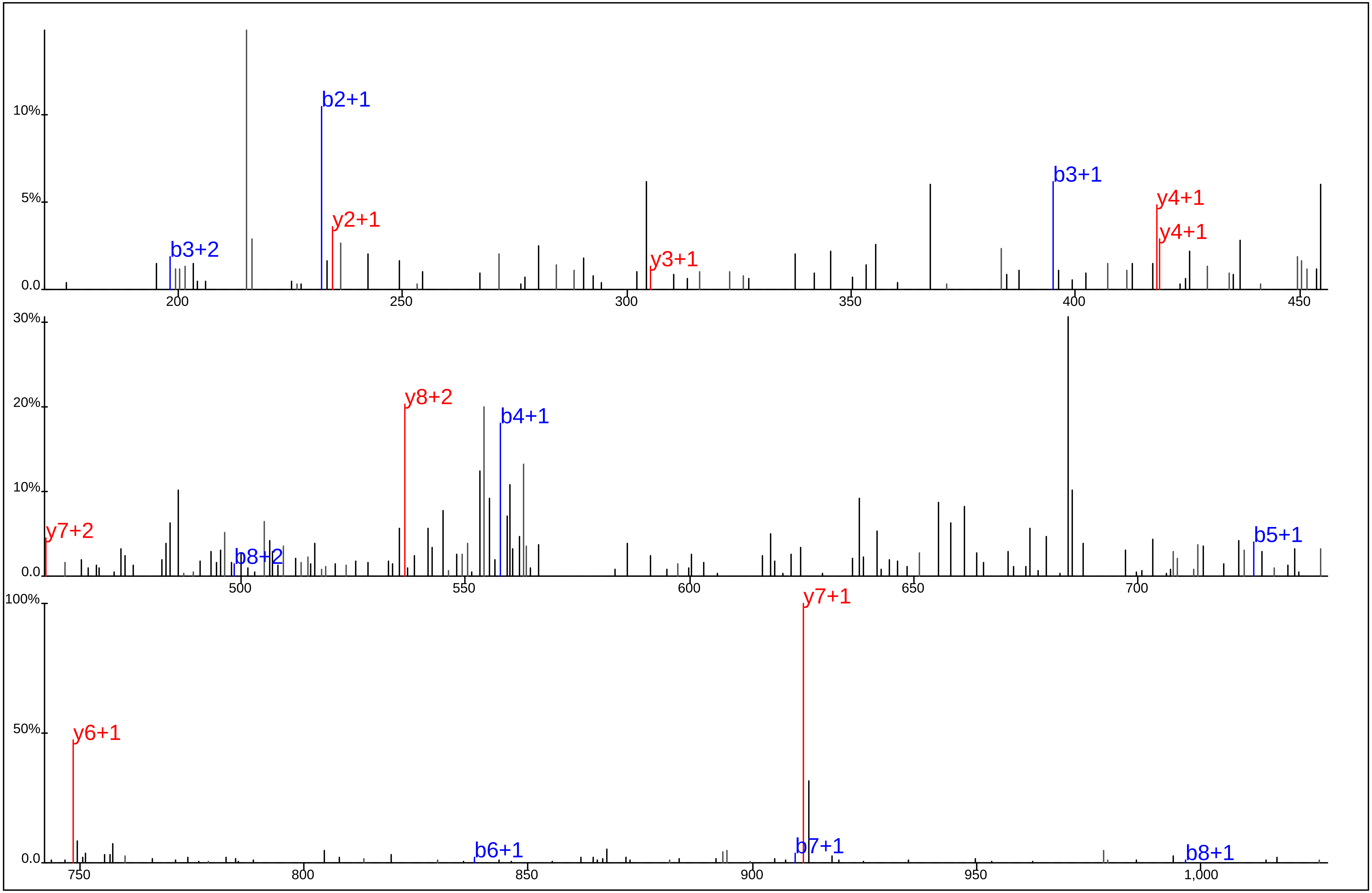

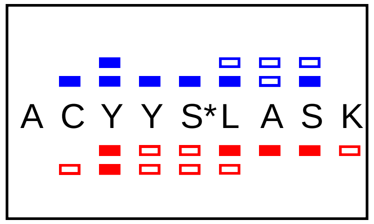

Protein Name:	ZAP70
gi Accession:	31455611
Precursor Mass:	571.7328+2
Delta Mass:	0.396 ppm
XCorr:	16.73

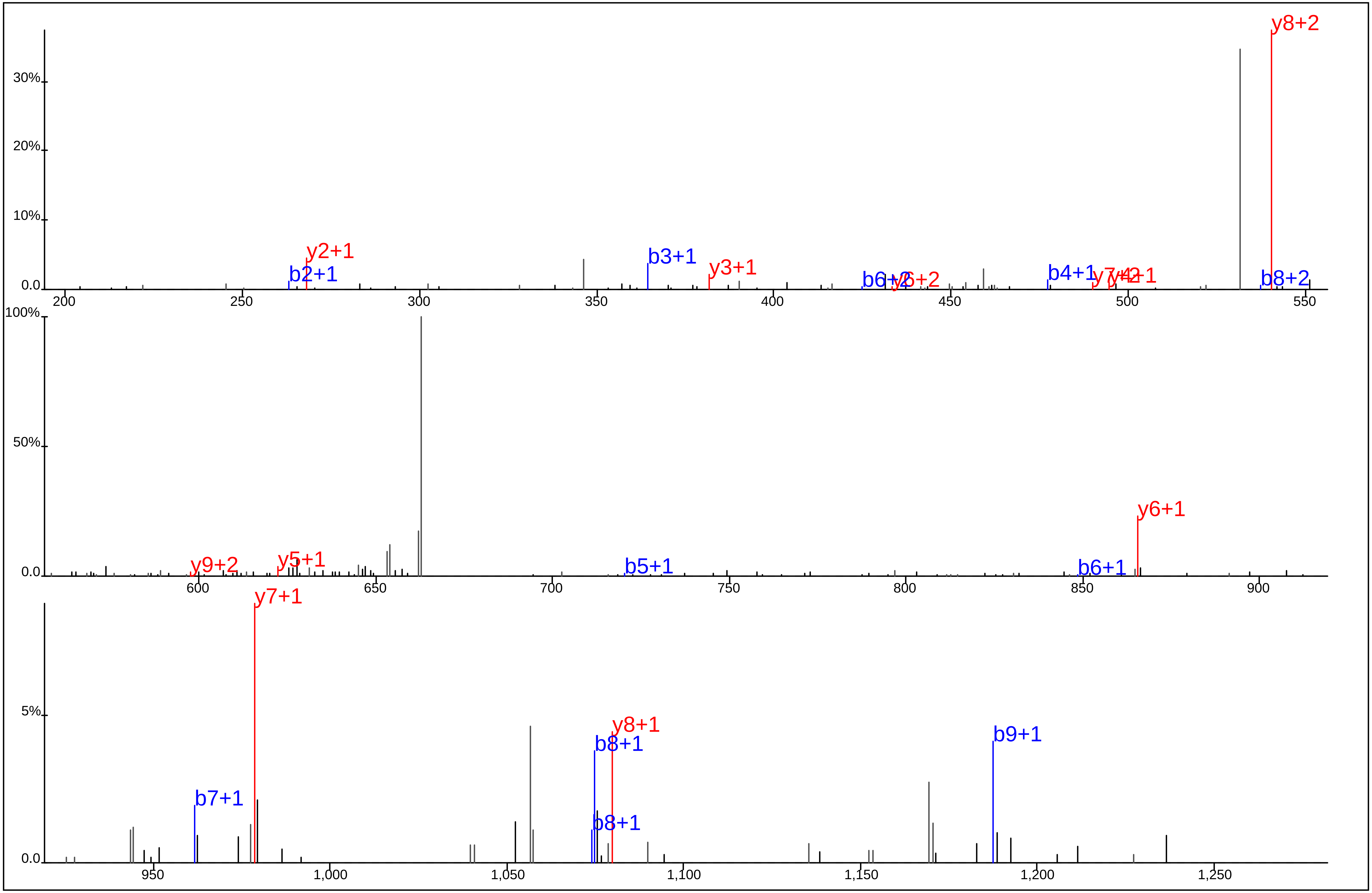

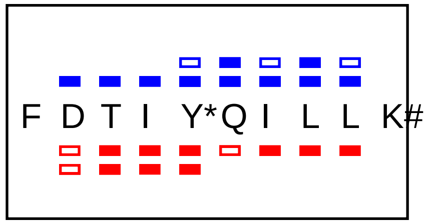

Protein Name:	Zinc finger protein 147
gi Accession:	12585547
Precursor Mass:	671.3539+2
Delta Mass:	4.448 ppm
XCorr:	35.44

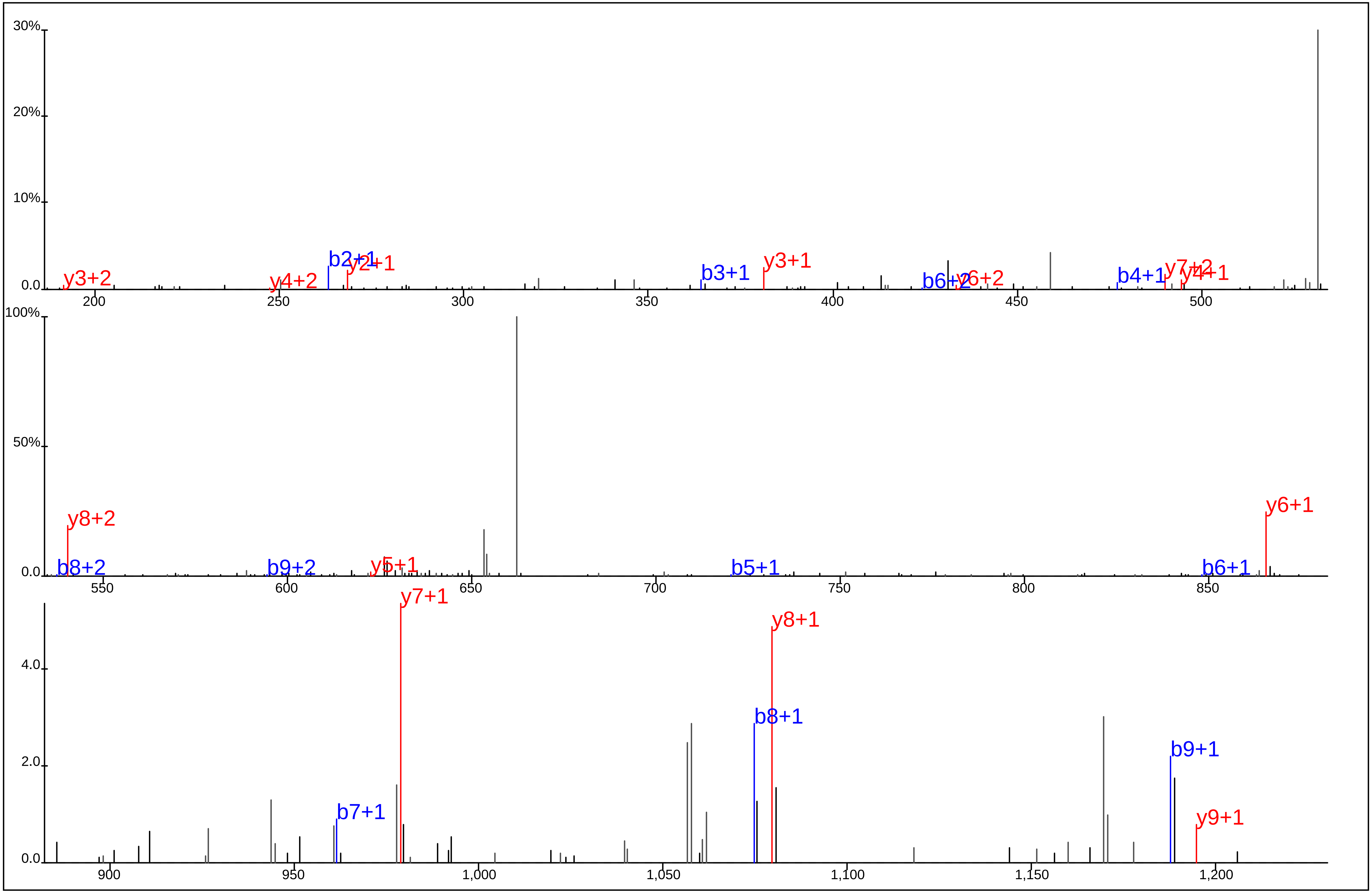

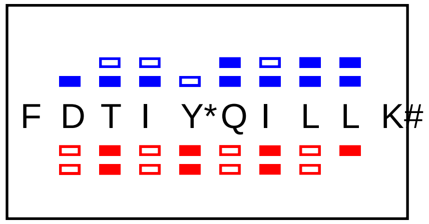

Protein Name: Zinc finger protein 147
gi Accession: 12585547
Precursor Mass: 671.3544+2
Delta Mass: 5.268 ppm
XCorr: 38.24

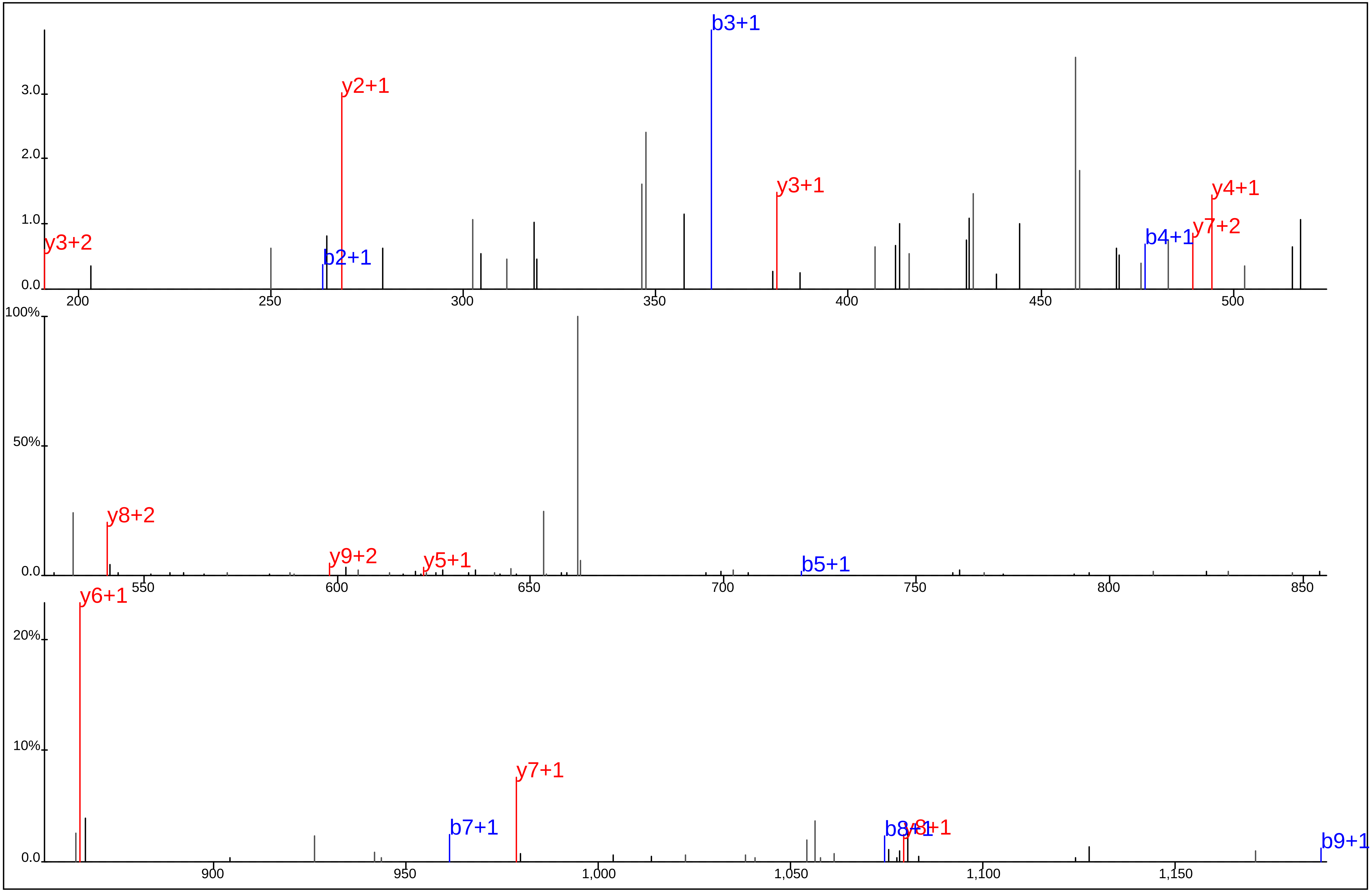

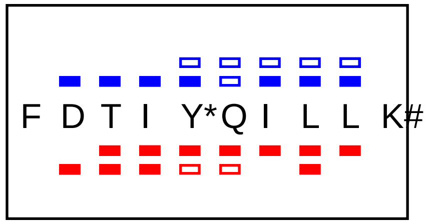

Protein Name: Zinc finger protein 147
gi Accession: 12585547
Precursor Mass: 671.3525+2
Delta Mass: 2.443 ppm
XCorr: 35.82

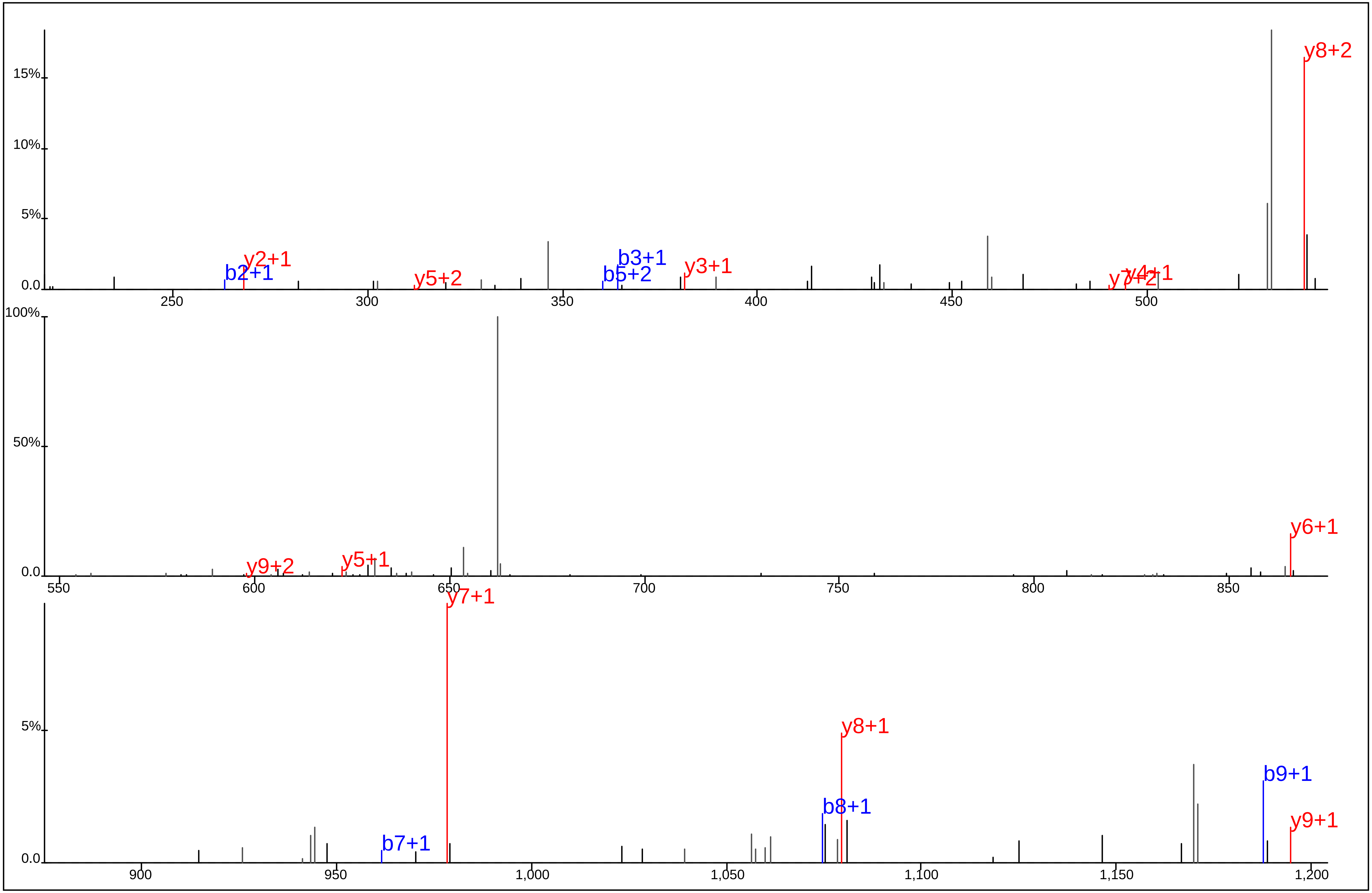

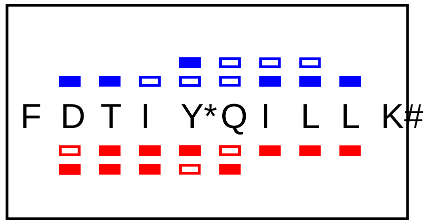

Protein Name:	Zinc finger protein 147
gi Accession:	12585547
Precursor Mass:	671.3534+2
Delta Mass:	3.718 ppm
XCorr:	34.4

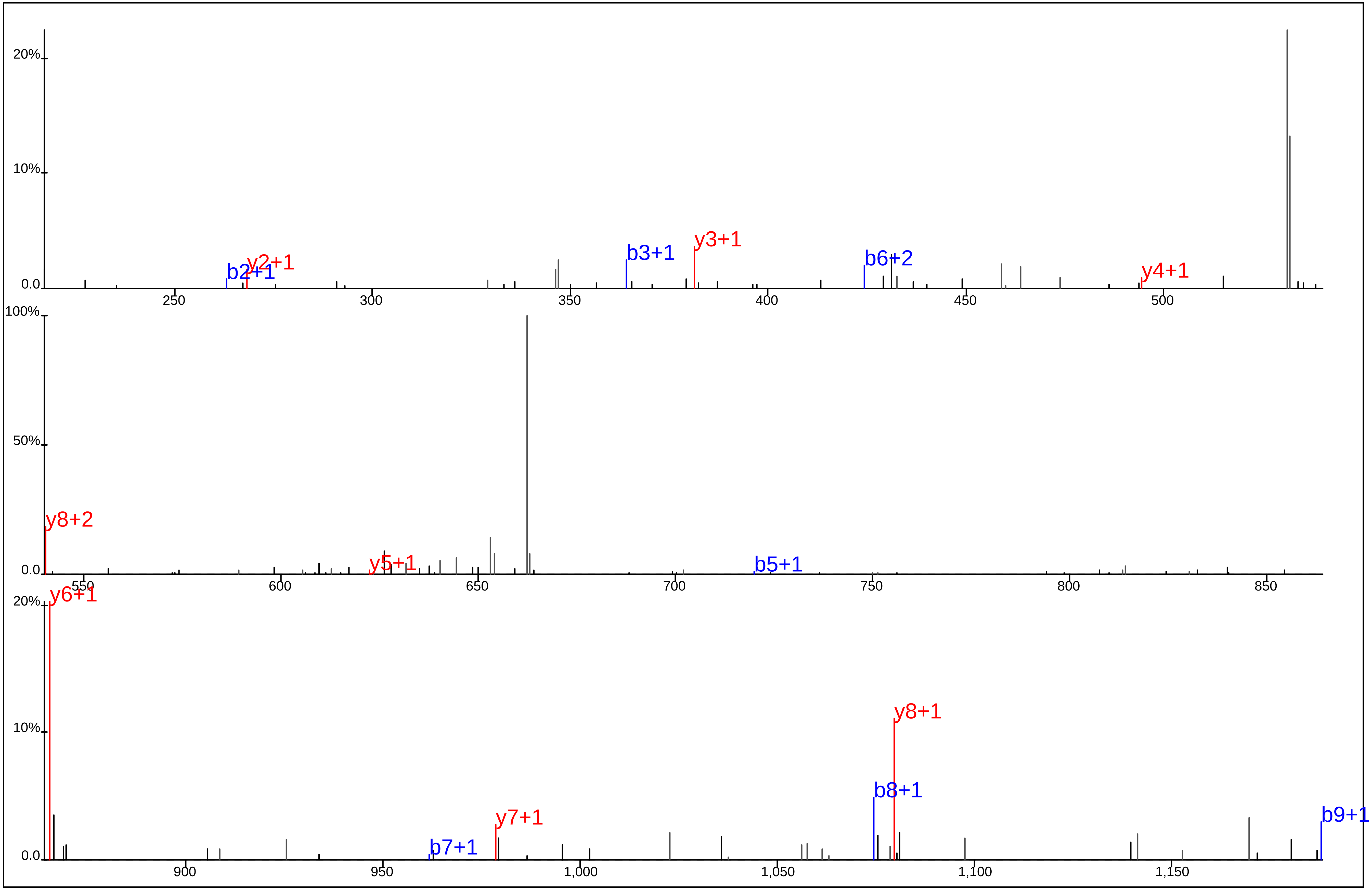

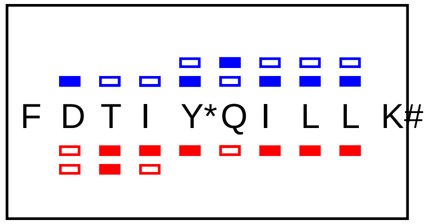

Protein Name: Zinc finger protein 147
gi Accession: 12585547
Precursor Mass: 671.3539+2
Delta Mass: 4.448 ppm
XCorr: 34.87

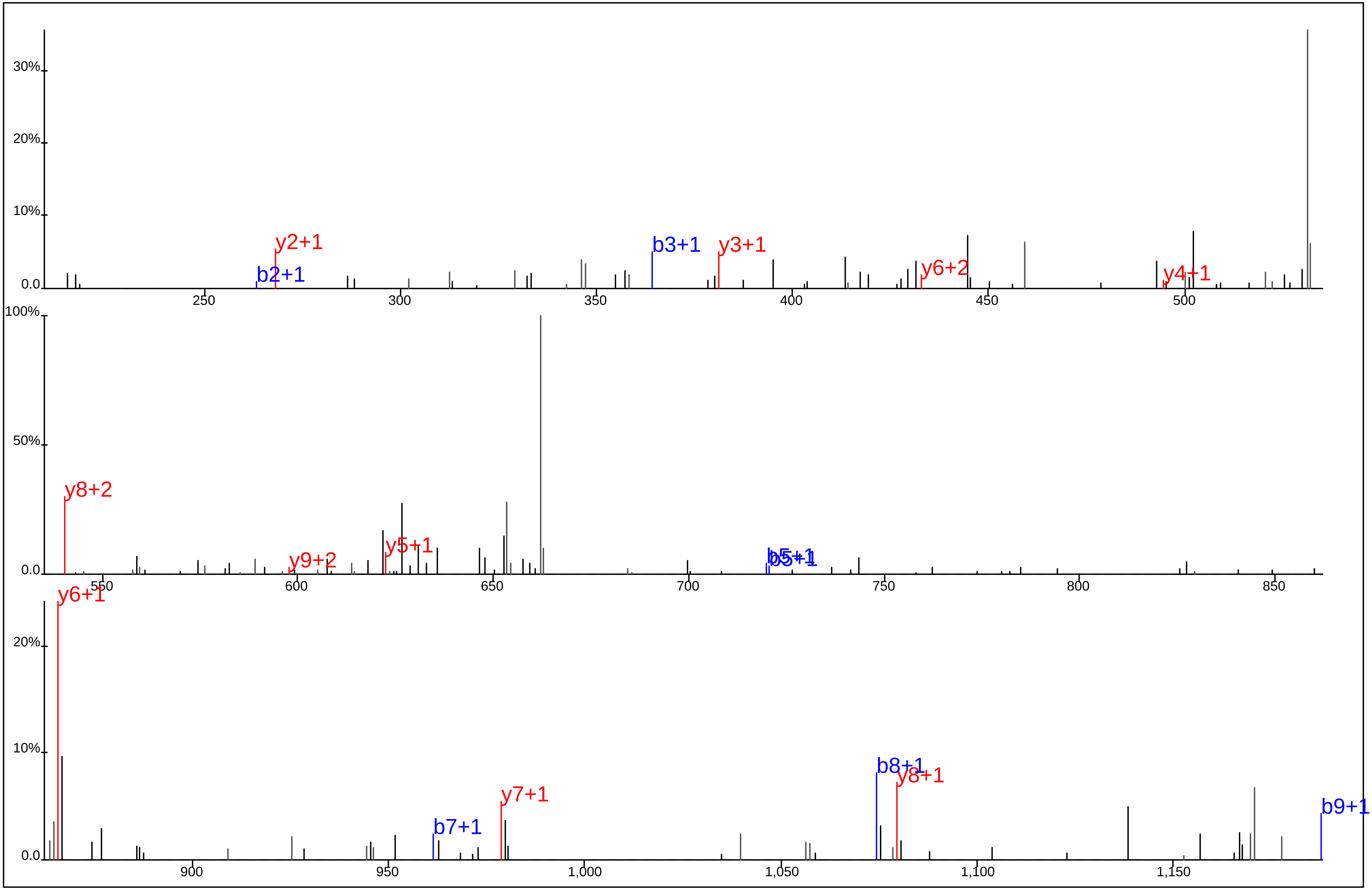

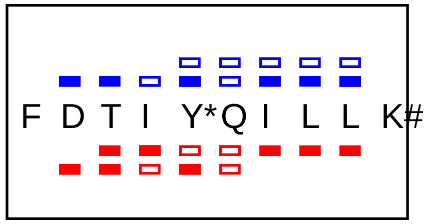

Protein Name: Zinc finger protein 147
gi Accession: 12585547
Precursor Mass: 671.3542+2
Delta Mass: 4.992 ppm
XCorr: 28.86

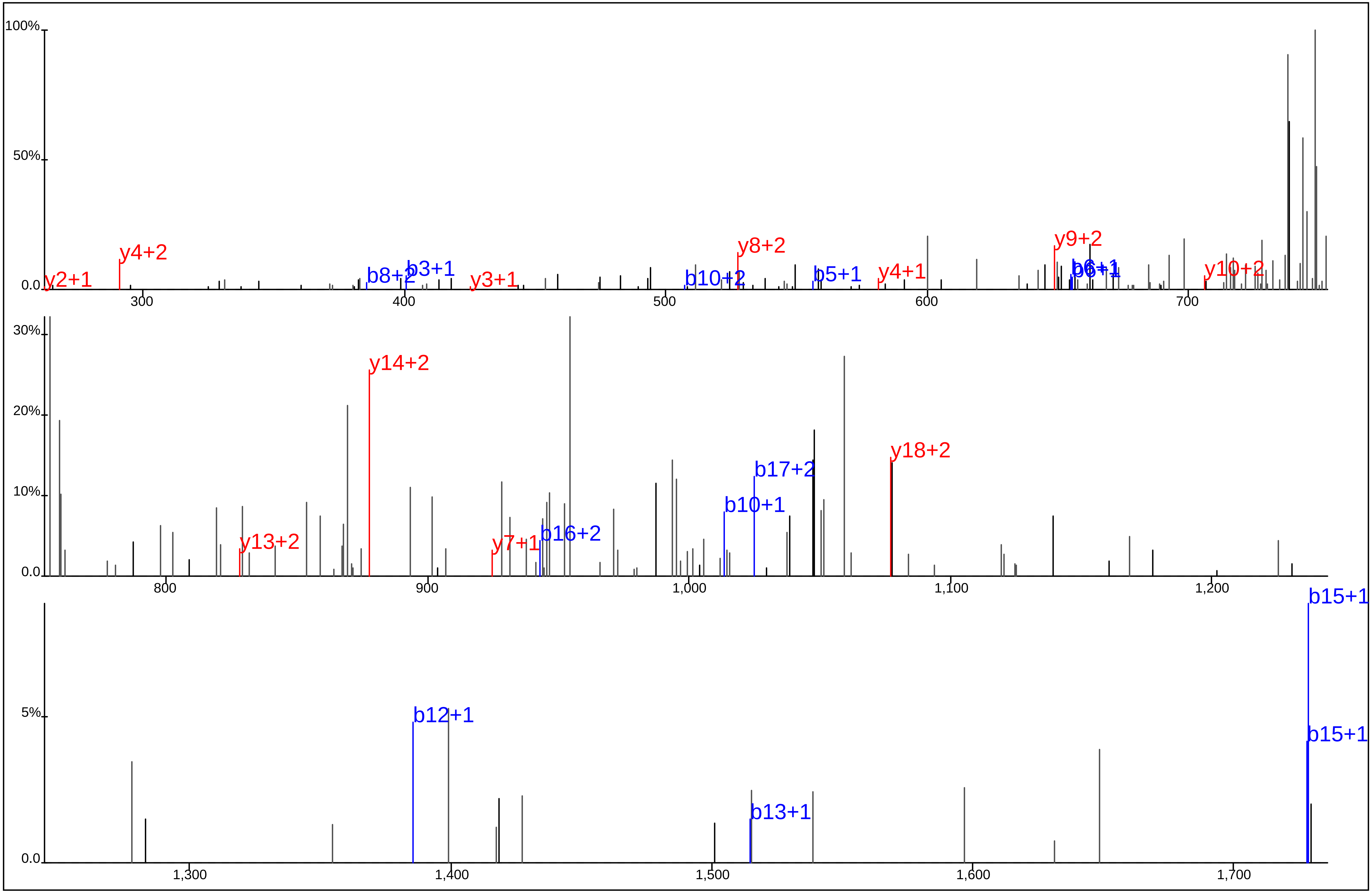

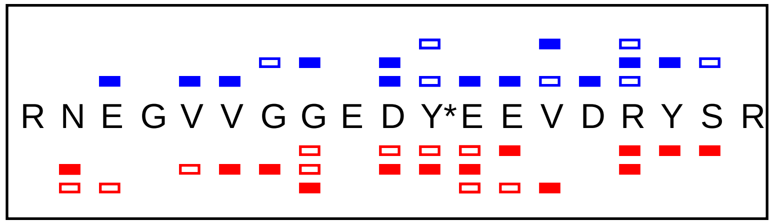

Protein Name: Zinc finger protein 598
gi Accession: 30039694
Precursor Mass: 770.3351+3
Delta Mass: 3.343 ppm
XCorr: 11.47

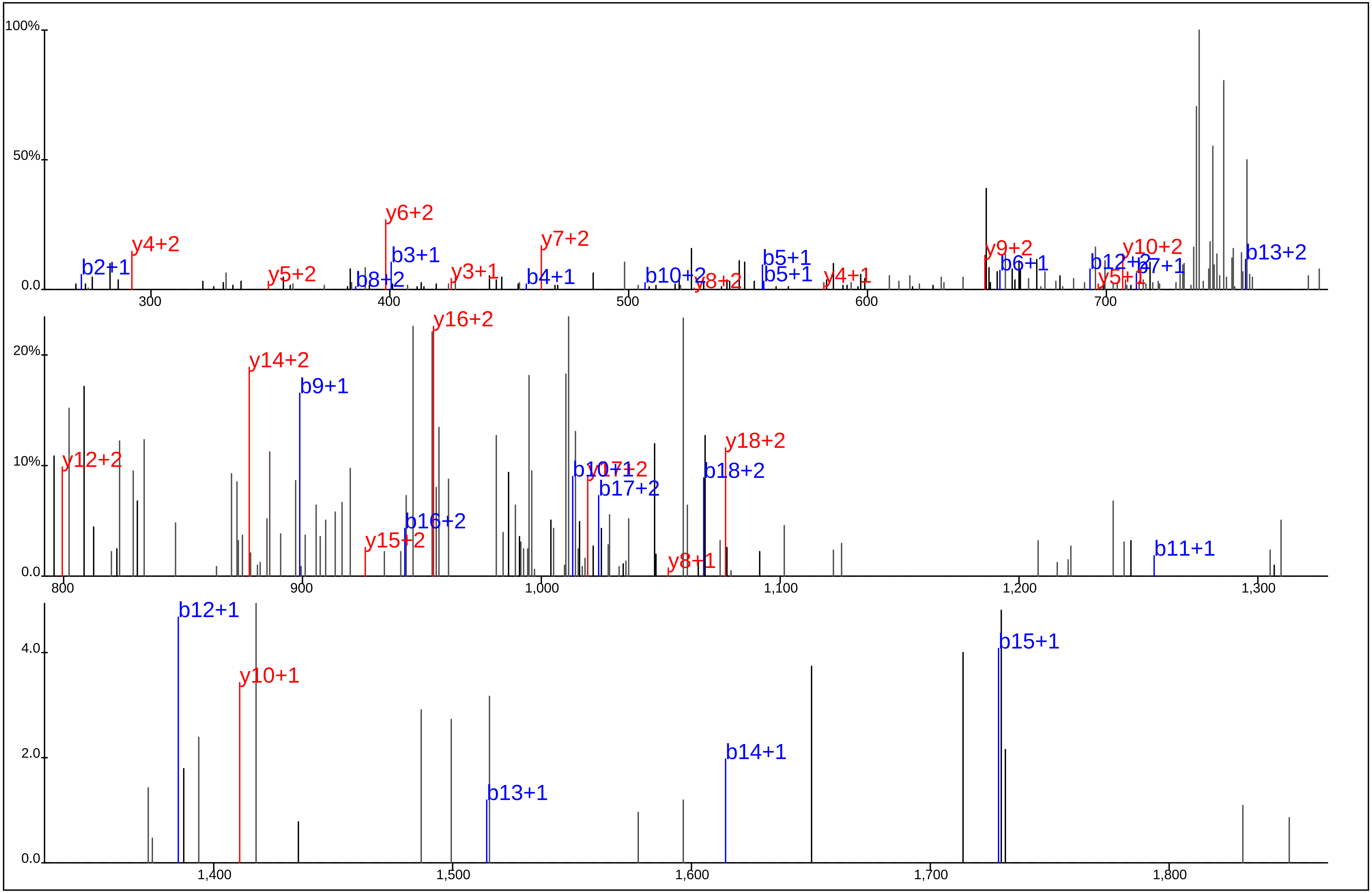

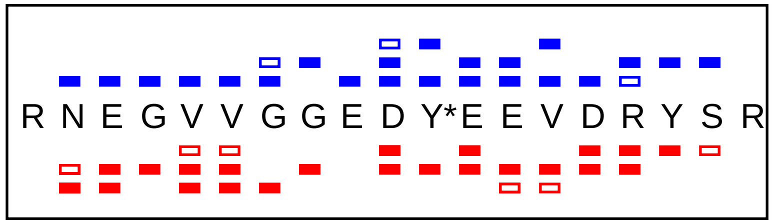

Protein Name:	Zinc finger protein 598
gi Accession:	30039694
Precursor Mass:	770.3360+3
Delta Mass:	4.529 ppm
XCorr:	27.02

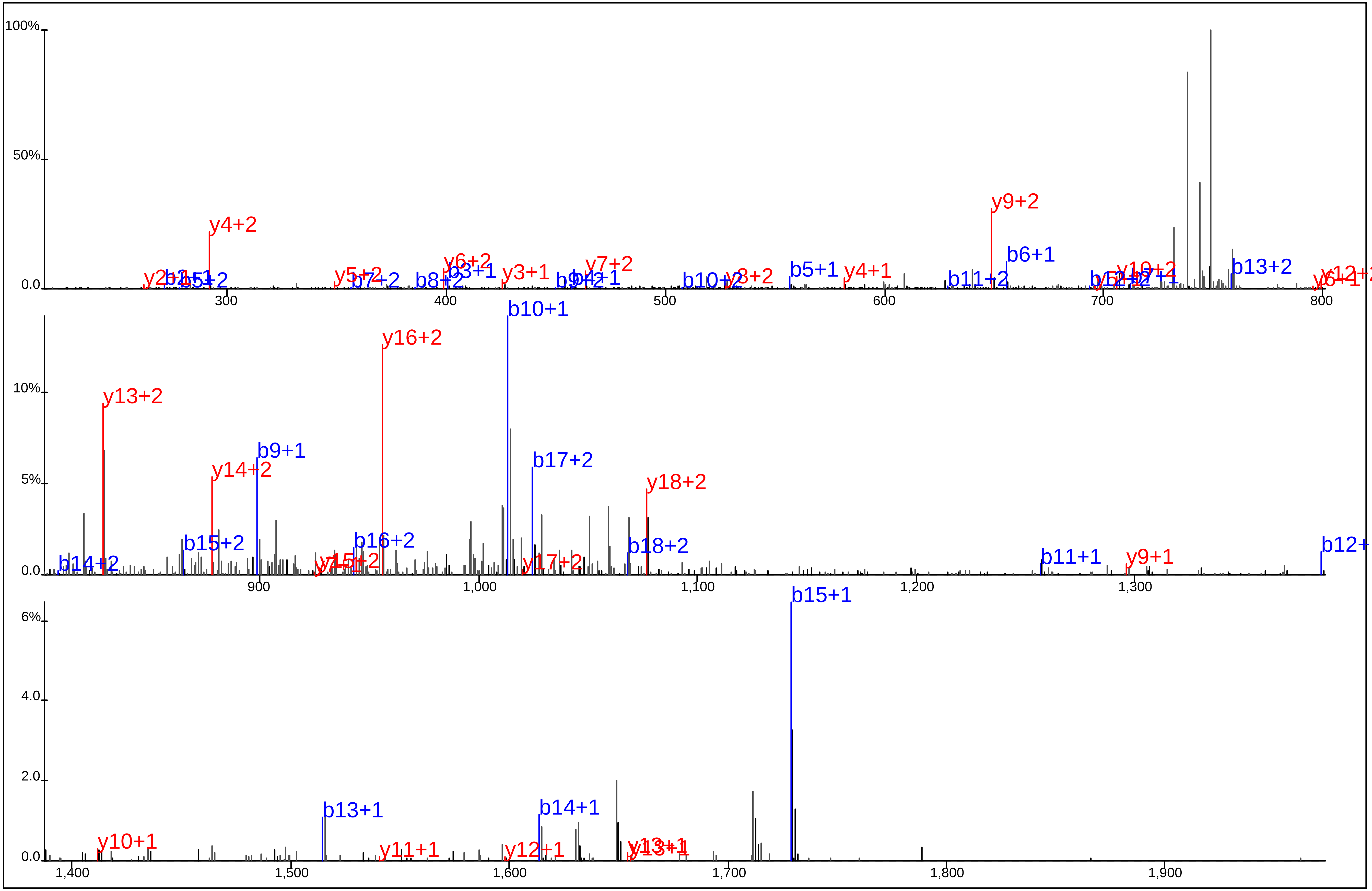

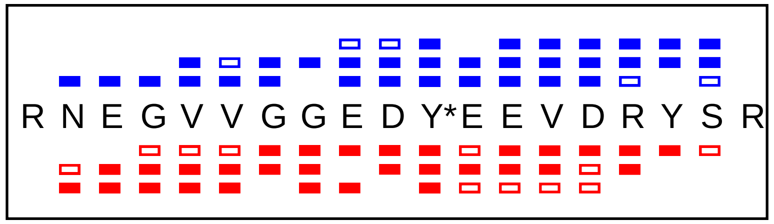

Protein Name: Zinc finger protein 598
gi Accession: 30039694
Precursor Mass: 770.3334+3
Delta Mass: 1.199 ppm
XCorr: 64.22

1125 - Zincfingerprotein598_3446726.pdf

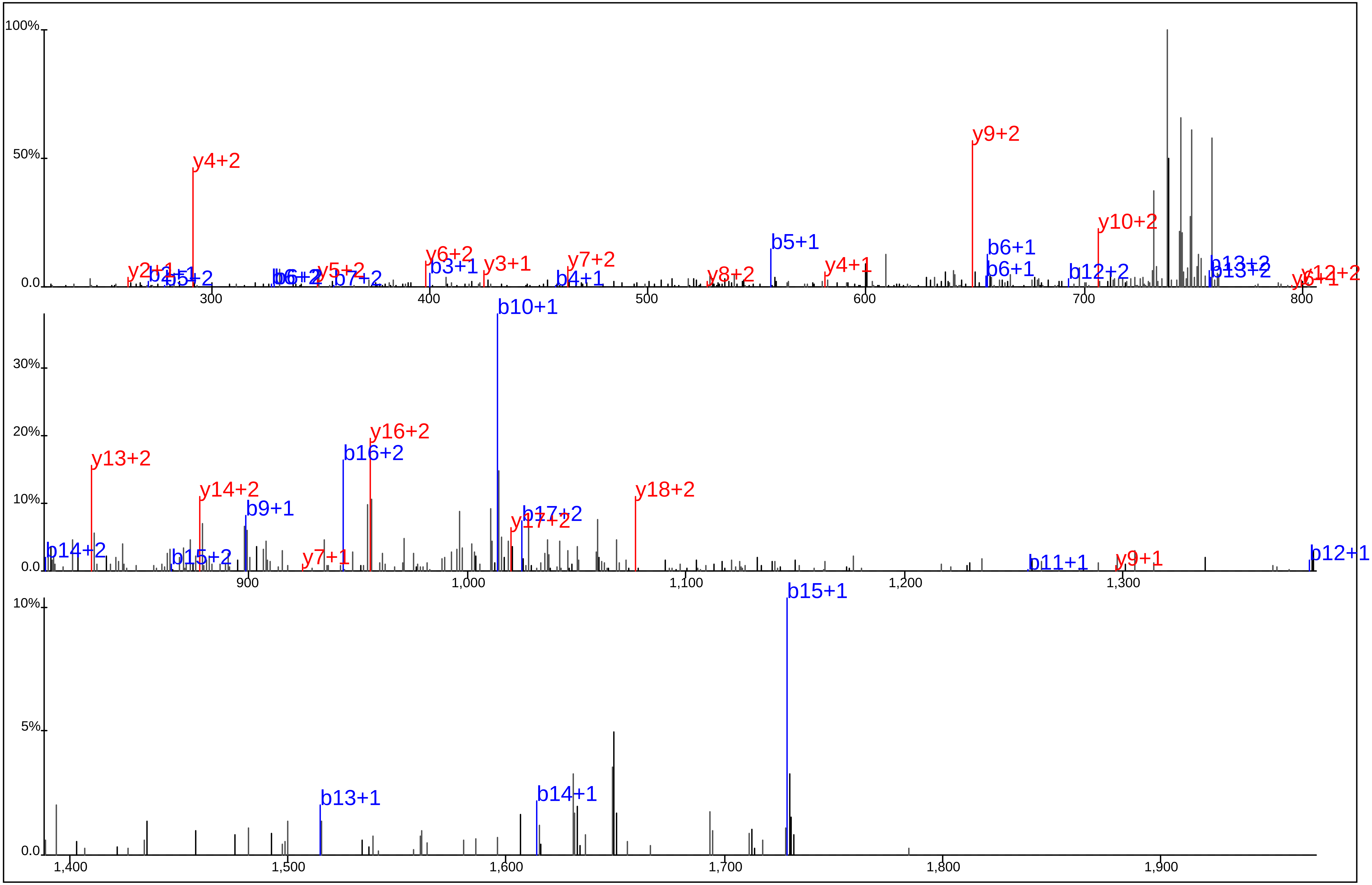

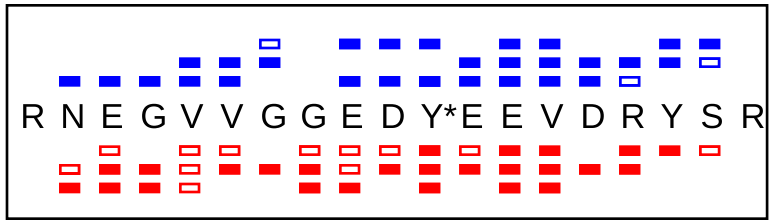

Protein Name: Zinc finger protein 598
gi Accession: 30039694
Precursor Mass: 770.3329+3
Delta Mass: 0.566 ppm
XCorr: 37.44

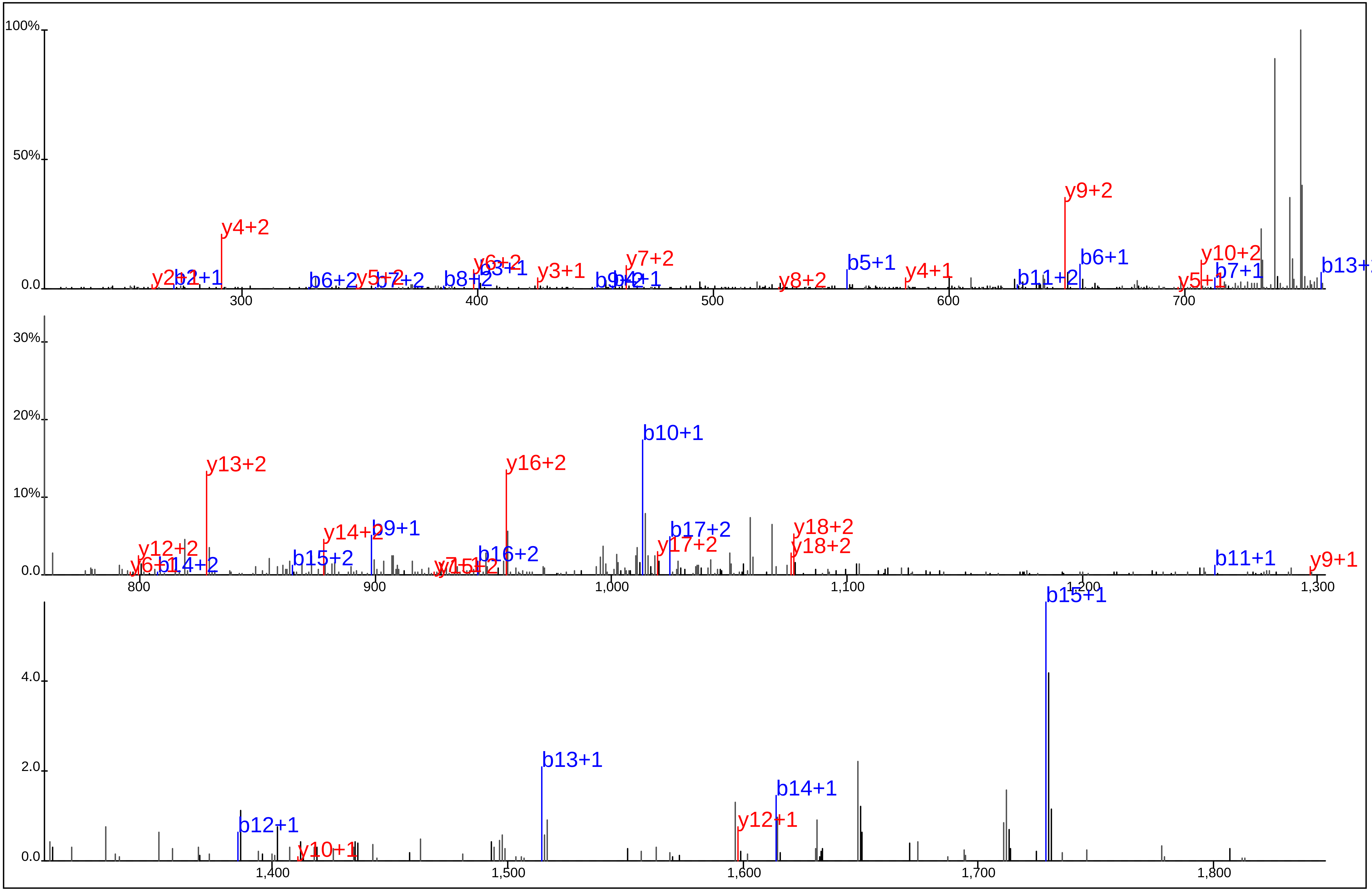

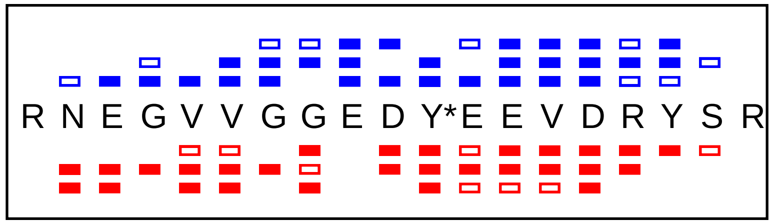

Protein Name:	Zinc finger protein 598
gi Accession:	30039694
Precursor Mass:	770.3340+3
Delta Mass:	1.991 ppm
XCorr:	62.09
