# Supplementary material for: Quantitative Phosphoproteomics Reveals SLP-76 Dependent Regulation of PAG and Src Family Kinases in T Cells
Source: PLoS One. 2012 Oct 11;7(10):e46725. doi: 10.1371/journal.pone.0046725 (PMC3469622; doi:10.1371/journal.pone.0046725)
Supplement: Method S1 — Selection of SILAC labeling conditions. SILAC labeling conditions were tested for both Jurkat T cell lines J14-76-11 (SLP-76 reconstituted) and J14 (SLP-76 deficient) to make sure cells grow and function normally. (PDF) [file pone.0046725.s007.pdf]

Selection of SILAC labeling conditions

Prior to analysis of SLP-76-deficient Jurkat cells, SILAC labeling conditions were tested for both Jurkat T cell lines J14-76-11 and J14 to make sure cells grow and function normally. Briefly, J14-76-11 (SLP-76-reconstituted) cells and J14 (SLP-76-deficient) cells were cultured either in normal medium or in medium containing light version arginine and lysine ($^{12}C_6$, $^{14}N_4$ Arg and $^{12}C_6$, $^{14}N_2$ Lys) and heavy version arginine and lysine ($^{13}C_6$, $^{15}N_4$ Arg and $^{13}C_6$, $^{15}N_2$ Lys) respectively with dialyzed serum for 7 doublings. To prevent from arginine-proline interconversion, different concentrations of arginine were tested for each cell line. Cells were then stimulated, lysed, and cell-derived proteins were trypsin digested into peptides. A list of nonredundant tyrosine phosphorylated peptides for each cell line at a specific cell growth condition was generated as described in methods except that cell lysates from different cell lines were not combined after cell lysis. Concentration of $^{13}C_6$, $^{15}N_4$ Arg at 0.383 mM and $^{13}C_6$, $^{15}N_2$ Lys at 0.219 mM was chosen for the analysis described here because it provides normal cell growth and fully labeled peptides. Furthermore, no conversion of ariginine to proline was observed in the list of 134 nonredundant phosphopeptides generated from cells grown at this condition (data not shown).
